# Author Correction: scRNA-seq in medulloblastoma shows cellular heterogeneity and lineage expansion support resistance to SHH inhibitor therapy

**DOI:** 10.1038/s41467-022-30824-4

**Published:** 2022-05-26

**Authors:** Jennifer Karin Ocasio, Benjamin Babcock, Daniel Malawsky, Seth J. Weir, Lipin Loo, Jeremy M. Simon, Mark J. Zylka, Duhyeong Hwang, Taylor Dismuke, Marina Sokolsky, Elias P. Rosen, Rajeev Vibhakar, Jiao Zhang, Olivier Saulnier, Maria Vladoiu, Ibrahim El-Hamamy, Lincoln D. Stein, Michael D. Taylor, Kyle S. Smith, Paul A. Northcott, Alejandro Colaneri, Kirk Wilhelmsen, Timothy R. Gershon

**Affiliations:** 1 Department of Neurology, University of North Carolina School of Medicine, Chapel Hill, NC 27599 USA; 2 UNC Neuroscience Center, University of North Carolina School of Medicine, Chapel Hill, NC 27599 USA; 3 Department of Genetics, University of North Carolina School of Medicine, Chapel Hill, NC 27599 USA; 4 Department of Cell Biology and Physiology, University of North Carolina School of Medicine, Chapel Hill, NC 27599 USA; 5 Carolina Institute for Developmental Disabilities, University of North Carolina School of Medicine, Chapel Hill, NC 27599 USA; 6 UNC Eshelman School of Pharmacy, University of North Carolina School of Medicine, Chapel Hill, NC 27599 USA; 7 Department of Pediatrics, University of Colorado Anschutz Medical Campus, Aurora, CO USA; 8 Morgan Adams Foundation Pediatric Brain Tumor Research Program, Children’s Hospital Colorado, Aurora, CO USA; 9 Developmental & Stem Cell Biology Program, The Hospital for Sick Children, Toronto, ON M5G 0A4 Canada; 10 The Arthur and Sonia Labatt Brain Tumour Research Centre, The Hospital for Sick Children, Toronto, Ontario M5G 0A4 Canada; 11 Department of Molecular Genetics, University of Toronto, Toronto, ON M5G 0A4 Canada; 12 Program in Computational Biology, Ontario Institute for Cancer Research, Toronto, ON M5G 0A3 Canada; 13 Division of Neurosurgery, The Hospital for Sick Children, Toronto, ON M5S 3E1 Canada; 14 Department of Developmental Neurobiology, St. Jude Children’s Research Hospital, Memphis, TN USA; 15 Renaissance Computing Institute at UNC (RENCI), Chapel Hill, NC 27517 USA; 16 Lineberger Comprehensive Cancer Center, University of North Carolina School of Medicine, Chapel Hill, NC 27599 USA

**Keywords:** Cancer genomics, Cancer therapy, CNS cancer

Correction to: *Nature Communications* 10.1038/s41467-019-13657-6, published online 20 December 2019.

In this article the author name ‘Jennifer Karin Ocasio’ was incorrectly written as ‘Jennifer Ocasio’. The original article has been corrected.

