# Supplementary material for: scRNA-seq in medulloblastoma shows cellular heterogeneity and lineage expansion support resistance to SHH inhibitor therapy
Source: Nat Commun. 2019 Dec 20;10:5829. doi: 10.1038/s41467-019-13657-6 (PMC6925218; doi:10.1038/s41467-019-13657-6)
Supplement: Supplementary file 1 — Supplementary Information [file 41467_2019_13657_MOESM1_ESM.pdf]

Supplementary Figures

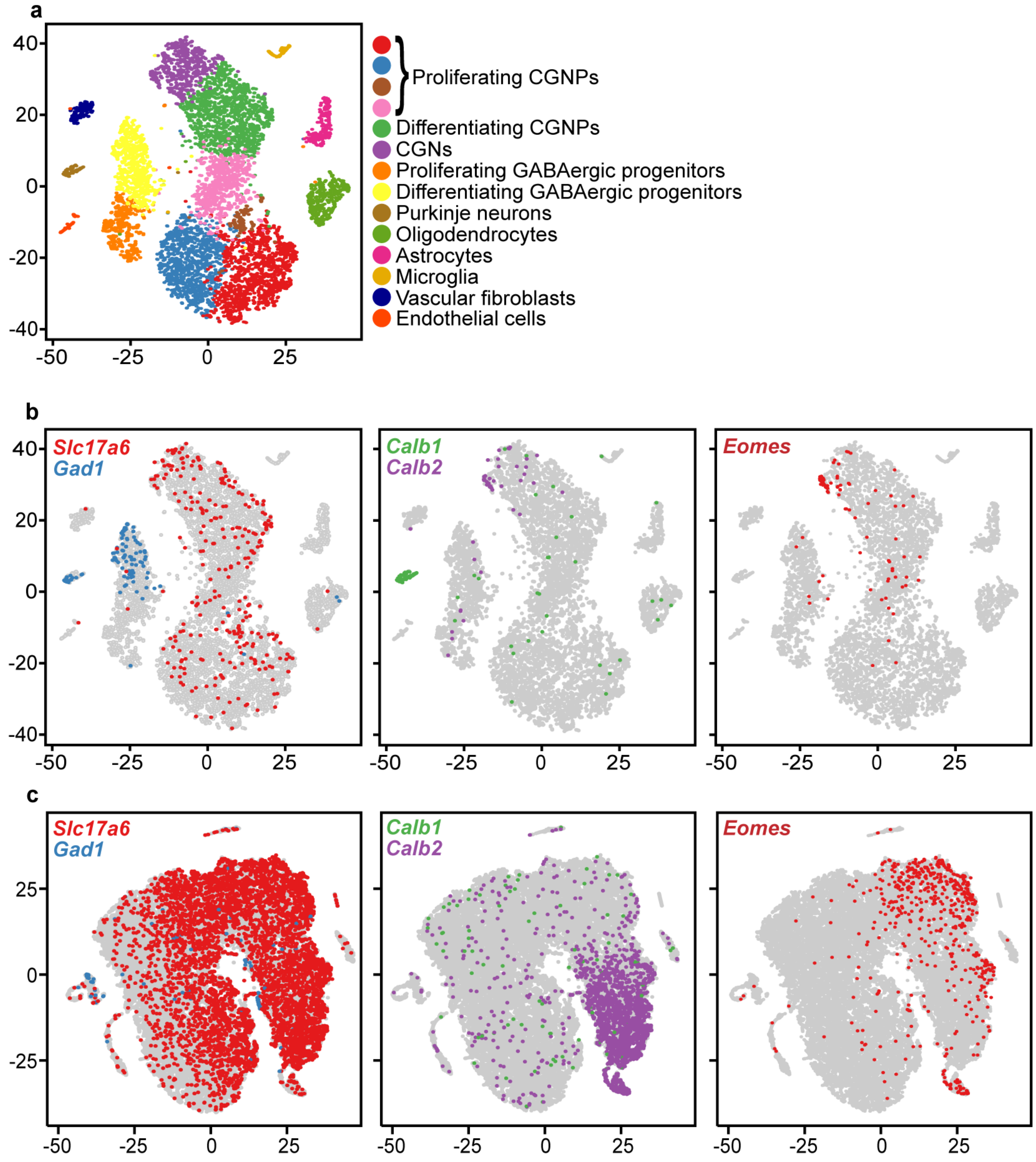

Supplementary Fig. 1: Subclassification of cell types. **a,** Cluster designations in the P7WT t-SNE. **b,c,** neuronal cell types by the expression of indicated marker genes, plotted on **a,** the P7 WT t-SNE and **b**, the t-SNE of vismodegib-treated and control tumors.

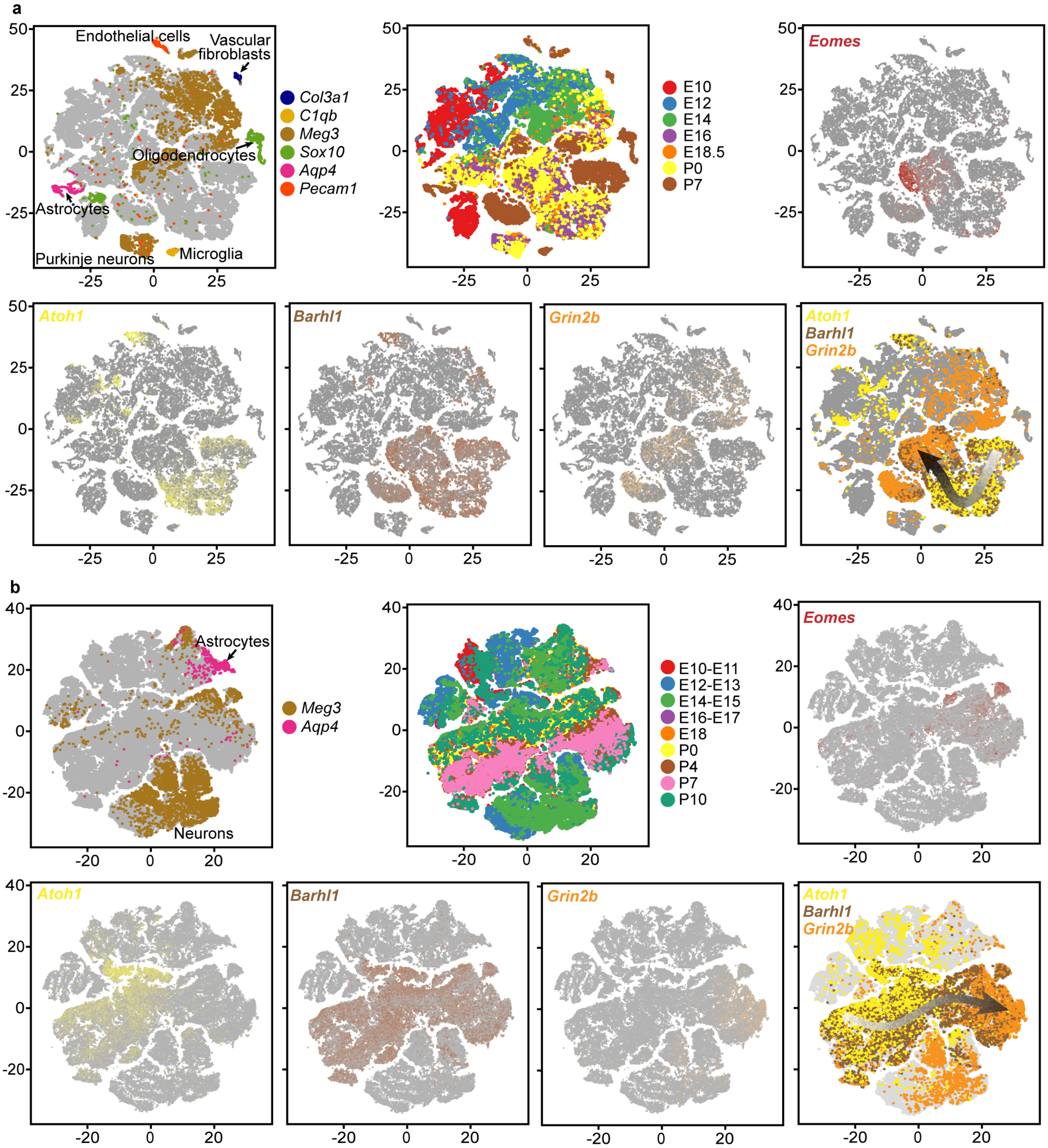

Supplementary Fig. 2: a,b t-SNE projection of WT cells from a, Vladoiu et al 2019 and b, Carter et al 2018, color coded as indicated to show age of developmental age, marker expression and *Atoh1* lineage.

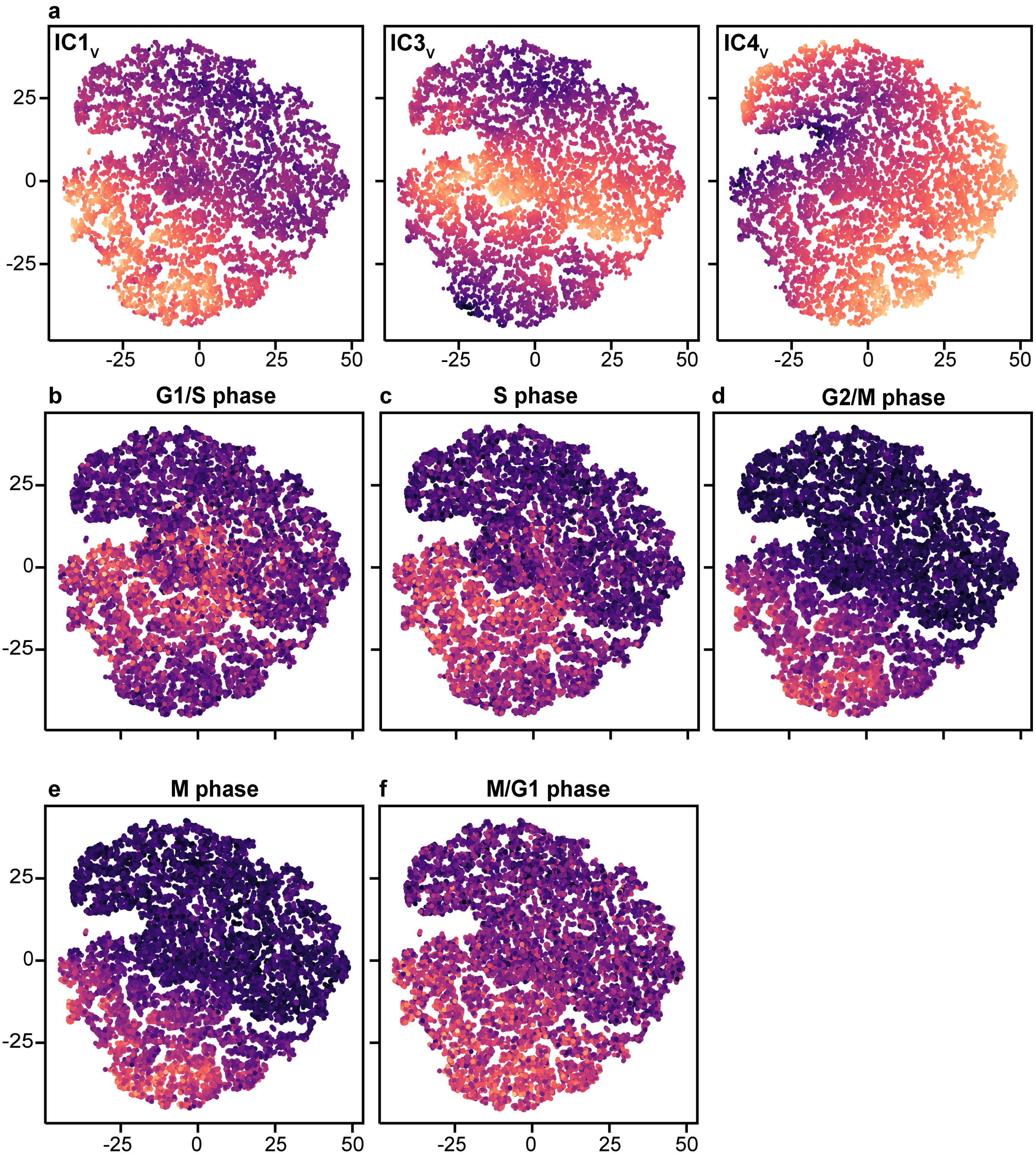

Supplementary Fig. 3: a, ICA-directed t-SNE projection of CGNP-like cells with the indicated ICs color-coded from yellow (high) to blue (low). **b-f,** Cell cycle phase color-coded on the vehicle ICA-directed t-SNE in (a), mapping the genes enriched at the indicated phases of the cell cycle.

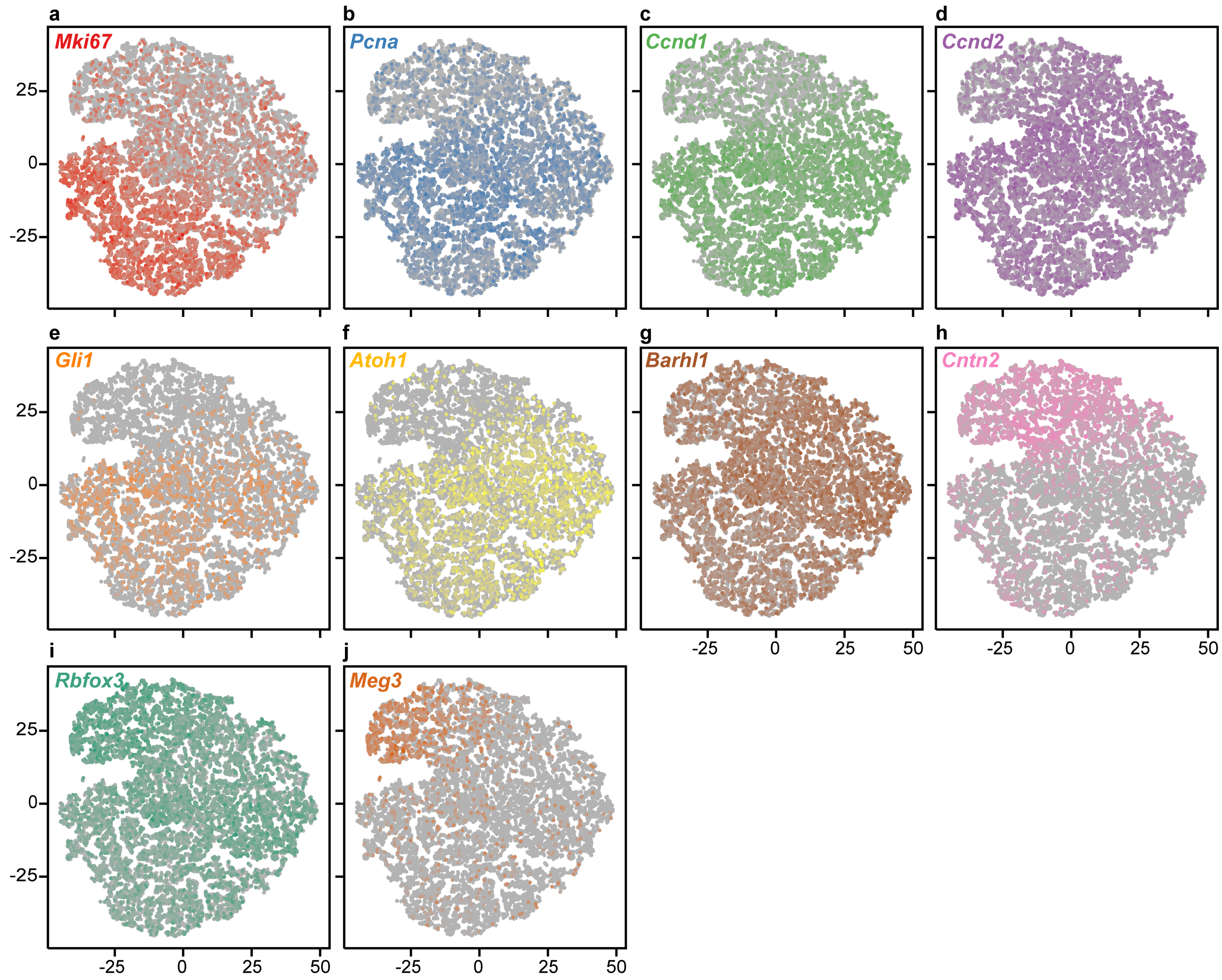

Supplementary Fig. 4: Feature plots of indicated marker genes on the ICA-directed t-SNE projection of CGNP-like cells.

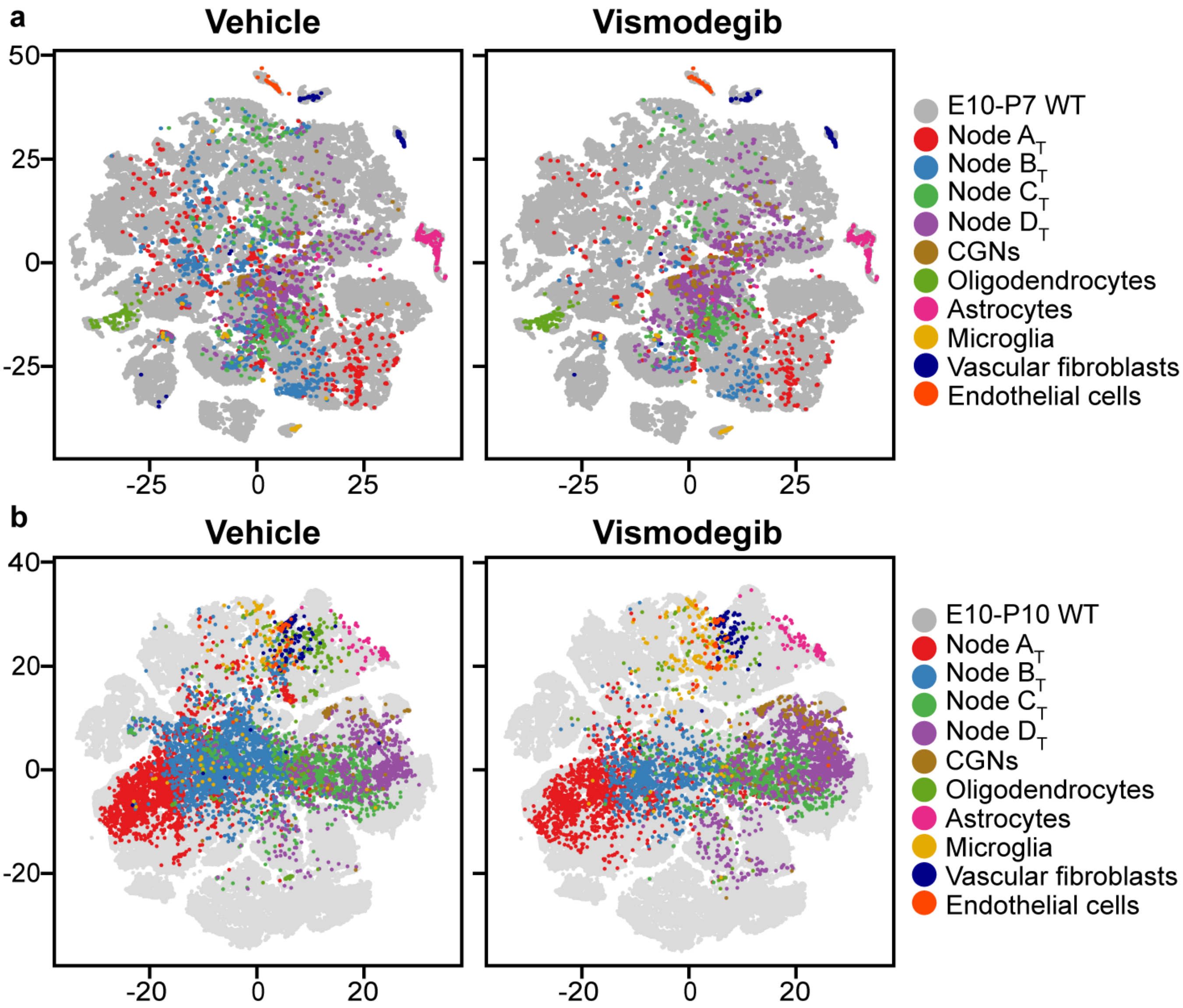

Supplementary Fig. 5: Comparison of vehicle-treated and vismodegib-treated M-Smo tumors, projected into WT datasets. Vismodegib-treated cells of Nodes A_T-D_T mapped to more differentiated regions of the Atoh1 lineage in WT cells from the datasets of **a,** Vladoiu et al. 2019 and **b,** Carter et al. 2018.

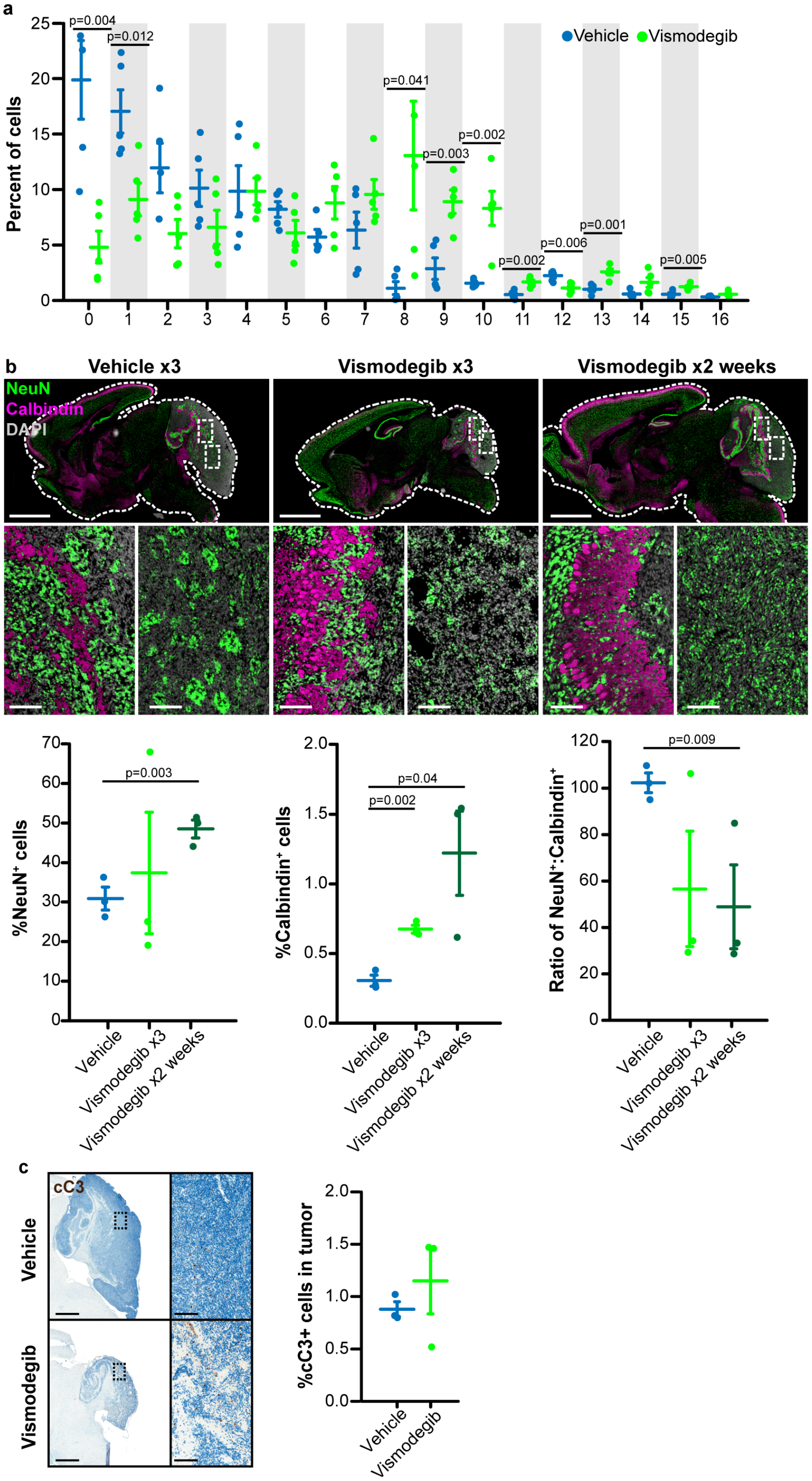

Supplementary Fig. 6: a, Fractional population of each cluster in tumors from vehicle-treated and vismodegib-treated mice, normalized to total number of cells per mouse, formatted as in Fig 6h. **b,** IHC for NeuN and Calbindin, comparing neuronal populations in vehicle-treated

tumors to tumors in mice treated for 3 days or 2 weeks with vismodegib, normalized to the number of Purkinje cells within the section. **c,** IHC for cC3, comparing apoptotic cells in vehicle-treated tumors to tumors in mice treated with vismodegib for 3 days or 2 weeks. In the quantifications, each dot represents an individual replicate animal. Horizontal lines indicate the means, and error bars indicate SEM. Scale bars = 2 mm, except in insets where scale bars = 100 μm.

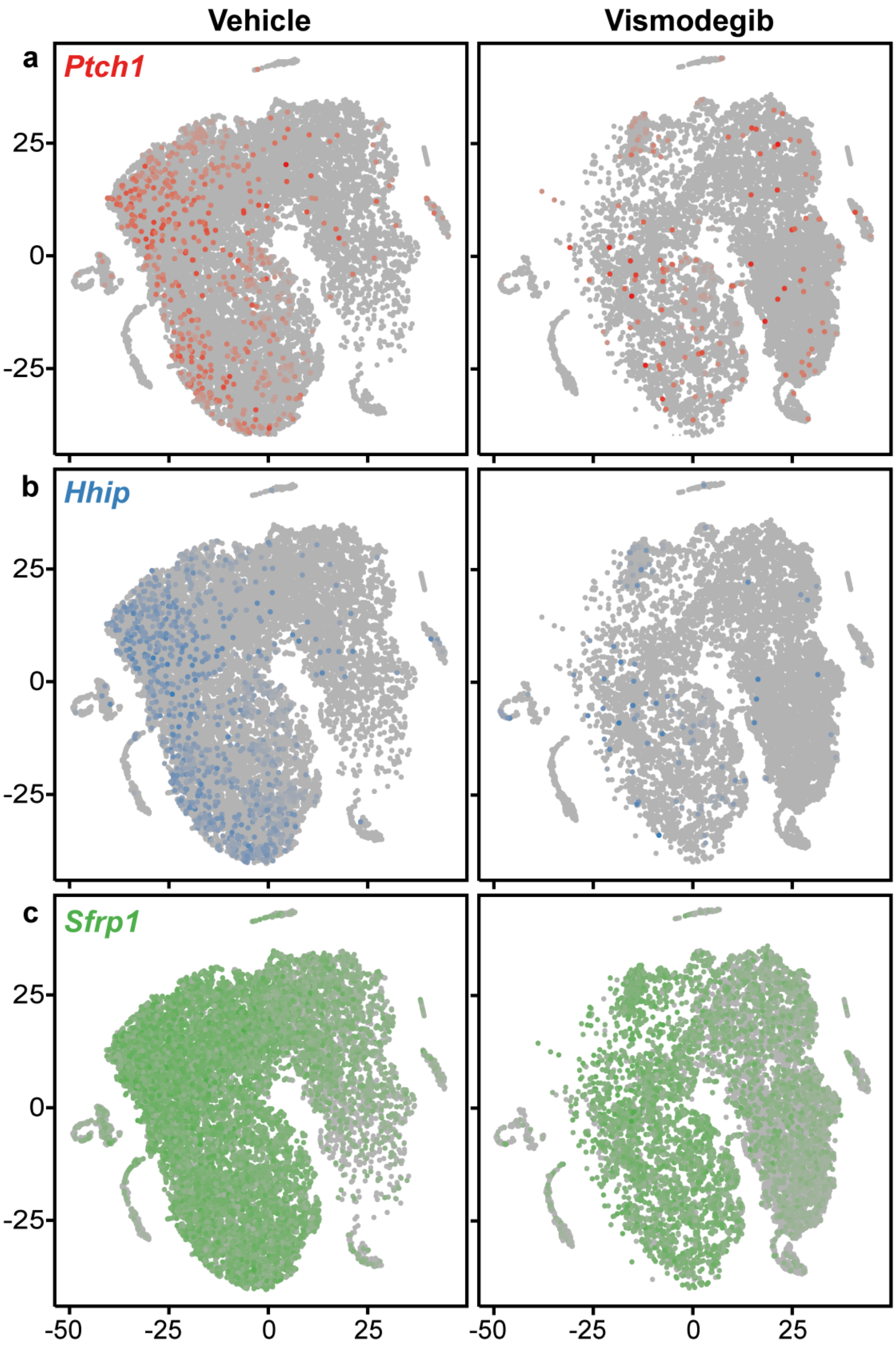

Supplementary Fig. 7: Feature plots of indicated SHH markers on the PCA-directed t-SNE projection of vehicle-treated and vismodegib-treated tumors.

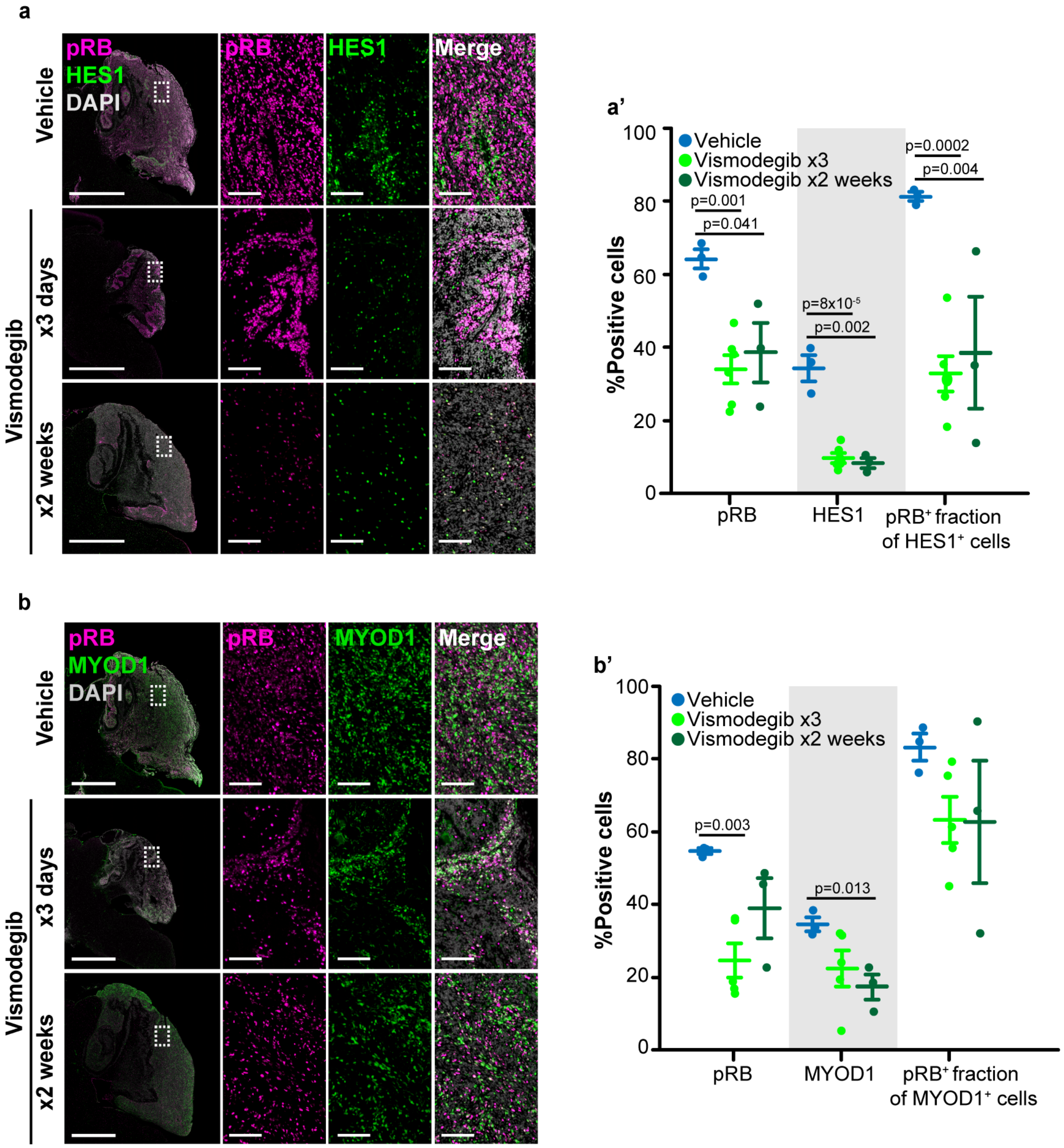

Supplementary Fig. 8: a, b, Medulloblastomas in sagittal hindbrain sections from *M-Smo* mice treated as indicated and stained for (a) HES1 and pRB or (b) MYOD1 and pRB. In the quantifications, each dot represents an individual replicate animal. Horizontal lines indicate the means, and error bars indicate SEM. Scale bars = 2 mm, except insets where scale bars = 100 µm.

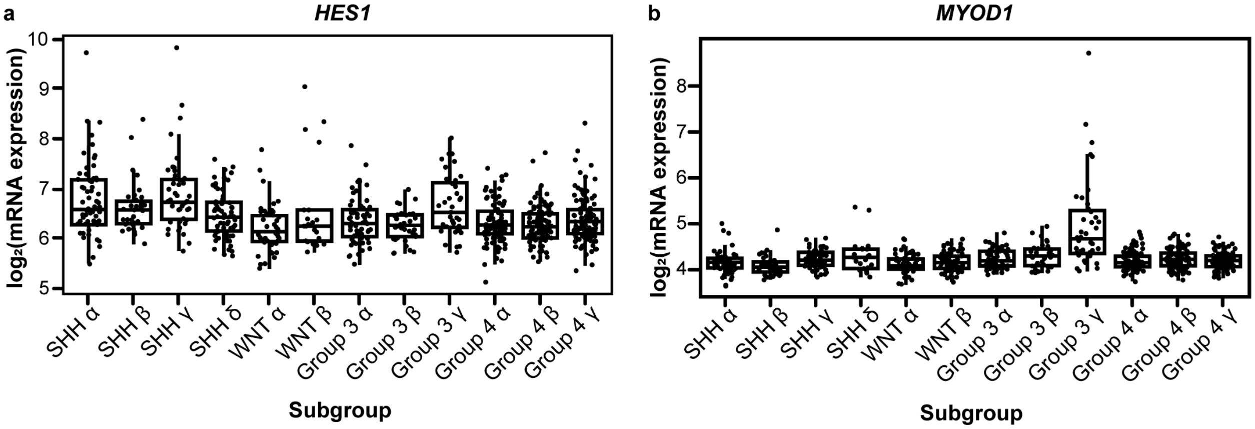

Supplementary Fig. 9: RNA seq data on **a,** *Hes1* and **b,** *Myod1* expression in human medulloblastoma samples, as presented in [44].

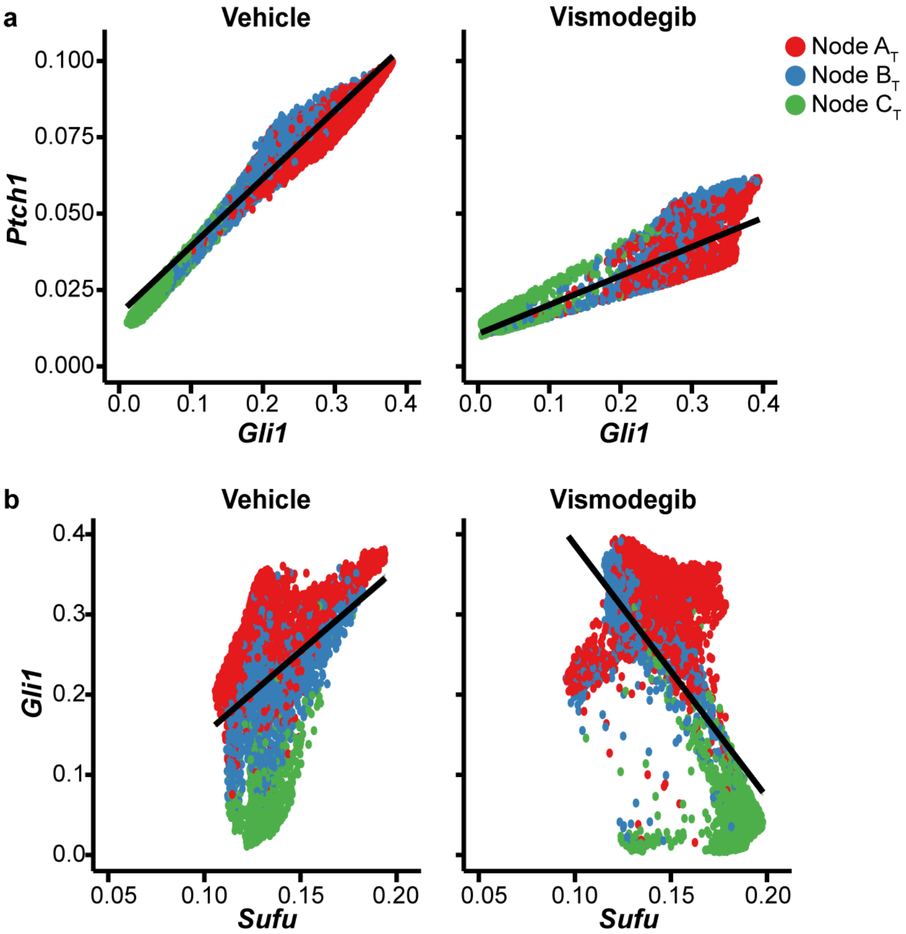

Supplementary Fig. 10 Correlation plot after imputation of data, showing expression of indicated genes as in Fig. 8b, c, with cells of Node D_T removed, and color-coded by Node.

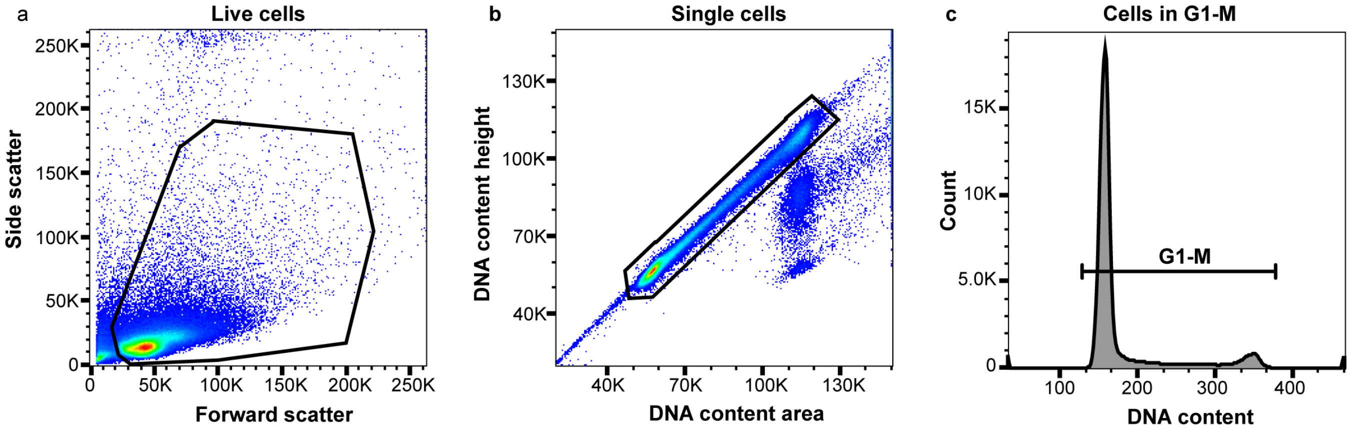

Supplementary Fig. 11 Gating strategy for all flow cytometry studies. Cells are gated first by side scatter over forward scatter to exclude debris, then by DNA dye area over height to include only single cells, and by DNA content to exclude cells with sub-G1 DNA.
