# Supplementary material for: scRNA-seq in medulloblastoma shows cellular heterogeneity and lineage expansion support resistance to SHH inhibitor therapy
Source: Nat Commun. 2019 Dec 20;10:5829. doi: 10.1038/s41467-019-13657-6 (PMC6925218; doi:10.1038/s41467-019-13657-6)
Supplement: Supplementary file 3 — Description of Additional Supplementary Files [file 41467_2019_13657_MOESM3_ESM.pdf]

Description of Additional Supplementary Files

File Name: Supplementary Data 1
Description: cluster-specific differential expression profiles from vehicle-treated tumors.

File Name: Supplementary Data 2
Description: cluster-specific differential expression profiles from WT P7 cerebella.

File Name: Supplementary Data 3
Description: the top 50 genes at the extremes of the 4 ICs from the analysis of vehicle-treated tumors.

File Name: Supplementary Data 4
Description: the set of genes up-regulated in the *Sox2*$^{+}$ population compared to all other cells from vehicle-treated tumors.

File Name: Supplementary Data 5
Description: cluster-specific differential expression profiles from ICA of Sox2^{+} cells from vehicle-treated tumors, showing distinct astrocytic, oligodendrocytic, AP-like, OPC-like and CGNP-like profiles.

File Name: Supplementary Data 6
Description: the top 50 genes at the extremes of the 4 ICs from the analysis of vismodegib-treated and control tumors.

File Name: Supplementary Data 7
Description: the set of genes differentially up-regulated in Cluster 0 in vehicle-treated tumors compared to all other vehicle-treated cells.

File Name: Supplementary Data 8
Description: the set of genes up-regulated in vismodegib-treated cells of Node A_T compared to vehicle-treated cells of Node $B_{T.}$

File Name: Supplementary Data 9
Description: the set of genes expression thresholds used to generate the feature plots.
