# Supplementary material for: scRNA-seq in medulloblastoma shows cellular heterogeneity and lineage expansion support resistance to SHH inhibitor therapy
Source: Nat Commun. 2019 Dec 20;10:5829. doi: 10.1038/s41467-019-13657-6 (PMC6925218; doi:10.1038/s41467-019-13657-6)
Supplement: Supplementary file 4 — Supplementary Data 1 [file 41467_2019_13657_MOESM4_ESM.pdf]

gene	p_val	avg_logFC	pct.Cluster	pct.All_oth	p_val_adj	cluster
2810417H1	0	1.316068	0.836	0.306	0	0
Top2a	0	1.173177	0.881	0.341	0	0
Esco2	0	1.088688	0.522	0.132	0	0
Rrm2	0	0.985892	0.494	0.148	0	0
Pbk	0	0.959796	0.538	0.162	0	0
Dut	0	0.94005	0.762	0.372	0	0
Lig1	0	0.932419	0.678	0.285	0	0
Mki67	0	0.930964	0.782	0.32	0	0
Tyms	0	0.927741	0.51	0.163	0	0
Pcna	0	0.905345	0.679	0.296	0	0
Dek	0	0.901239	0.944	0.645	0	0
Smc2	0	0.891022	0.887	0.48	0	0
Spc25	0	0.886878	0.526	0.168	0	0
Tuba1b	0	0.845357	0.788	0.422	0	0
Cdk1	0	0.819165	0.579	0.183	0	0
Smc4	0	0.777379	0.877	0.507	0	0
H2afx	0	0.761598	0.659	0.267	0	0
Hmgb2	0	0.726051	0.737	0.335	0	0
Ranbp1	0	0.688836	0.883	0.635	0	0
Tubb5	0	0.484792	0.982	0.901	0	0
Birc5	0	0.772736	0.582	0.214	0	0
Hist1h2ak	0	1.000851	0.382	0.097	0	0
Spc24	0	0.859711	0.529	0.187	0	0
Incenp	0	0.81505	0.58	0.217	0	0
Clspn	0	0.907496	0.459	0.138	2.5E-307	0
Anp32b	2.5E-298	0.635838	0.835	0.544	4.1E-294	0
Tk1	2.1E-297	0.840125	0.366	0.086	3.5E-293	0
Anp32e	2.6E-293	0.622143	0.85	0.568	4.4E-289	0
Nasp	3.3E-291	0.609824	0.864	0.597	5.5E-287	0
Prc1	1.7E-290	0.597917	0.537	0.203	2.8E-286	0
Nusap1	1.2E-288	0.735944	0.508	0.173	2.1E-284	0
Atad2	1E-283	0.843581	0.454	0.149	1.7E-279	0
Kif11	3.2E-261	0.749512	0.465	0.156	5.3E-257	0
Ckb	3E-259	-0.74253	0.683	0.831	5E-255	0
Cenph	5.6E-259	0.833841	0.436	0.147	9.4E-255	0
Cenpf	2.6E-254	0.313988	0.624	0.312	4.3E-250	0
Tmsb4x	9.6E-252	-0.59201	0.918	0.954	1.6E-247	0
Neurod1	1.2E-251	-1.14351	0.444	0.549	2E-247	0
Rtn1	3.9E-251	-0.8504	0.544	0.718	6.4E-247	0
H2afv	3.4E-250	0.500301	0.907	0.693	5.6E-246	0
Tpx2	1.1E-242	0.529218	0.543	0.231	1.8E-238	0
Rad51ap1	1.1E-241	0.773908	0.395	0.123	1.9E-237	0
Cdca8	2.5E-234	0.678629	0.527	0.214	4.2E-230	0
Fbxo5	6.5E-226	0.748358	0.377	0.117	1.1E-221	0
Rrm1	4.9E-224	0.730606	0.499	0.214	8.2E-220	0
Prim1	2.1E-221	0.73357	0.504	0.216	3.6E-217	0
Ncapg	4.3E-213	0.736349	0.404	0.141	7.1E-209	0

Hirip3	5.8E-213	0.669457	0.63	0.335	9.6E-209	0
Tmpo	1.4E-212	0.609653	0.696	0.405	2.3E-208	0
Ccna2	1.8E-212	0.641916	0.46	0.175	3.1E-208	0
Dnajc9	1.1E-211	0.662777	0.595	0.302	1.9E-207	0
2700094K1	5.2E-207	0.520978	0.827	0.573	8.7E-203	0
Hnrnpab	1.8E-201	0.421712	0.924	0.795	3E-197	0
Tcf19	6.6E-199	0.681779	0.272	0.065	1.1E-194	0
Hells	2.8E-193	0.670396	0.436	0.17	4.7E-189	0
Dnmt1	3.1E-192	0.655801	0.509	0.233	5.2E-188	0
Aurkb	5.1E-184	0.66616	0.332	0.106	8.5E-180	0
Cntn2	8.5E-184	-1.15995	0.097	0.295	1.4E-179	0
Ezh2	8.1E-182	0.533916	0.791	0.575	1.3E-177	0
Stmn2	2.8E-181	-0.87586	0.432	0.575	4.7E-177	0
Tuba1a	1.6E-178	-0.46629	0.928	0.929	2.6E-174	0
Kif15	6E-177	0.677204	0.381	0.142	1E-172	0
Itm2b	9.2E-172	-0.64751	0.511	0.667	1.5E-167	0
Cdca3	1.7E-171	0.585316	0.435	0.18	2.8E-167	0
Ckap2l	1E-165	0.559954	0.425	0.178	1.7E-161	0
Nrep	6.9E-165	-0.72515	0.421	0.597	1.2E-160	0
Neil3	2.4E-164	0.601079	0.227	0.055	4E-160	0
Fam111a	3.5E-164	0.677465	0.295	0.093	5.9E-160	0
Dtymk	7E-164	0.569994	0.641	0.4	1.2E-159	0
RP23-45G1	2.1E-162	0.525622	0.554	0.283	3.5E-158	0
Usp1	5E-159	0.589321	0.553	0.297	8.4E-155	0
Tipin	9.8E-158	0.611582	0.458	0.216	1.6E-153	0
Fkbp3	2.4E-151	0.414714	0.877	0.719	4E-147	0
Paics	2.4E-147	0.545552	0.615	0.374	4E-143	0
Racgap1	1.2E-146	0.56337	0.458	0.216	2E-142	0
Ran	2.7E-146	0.515043	0.618	0.375	4.6E-142	0
Mxd3	1.5E-145	0.608473	0.285	0.096	2.5E-141	0
Mns1	2.8E-145	0.619786	0.388	0.167	4.6E-141	0
Rad51	4.5E-144	0.579607	0.268	0.087	7.5E-140	0
Mthfd2	1E-142	0.607545	0.225	0.063	1.7E-138	0
Casc5	7.2E-141	0.587302	0.368	0.151	1.2E-136	0
Nucks1	2.3E-139	0.395444	0.831	0.63	3.8E-135	0
Gpm6a	1.4E-138	-0.69926	0.319	0.426	2.3E-134	0
Smc6	1.5E-137	0.513487	0.624	0.384	2.5E-133	0
Tubb3	1.3E-136	-0.77768	0.353	0.497	2.2E-132	0
Apoe	2.9E-136	-1.70742	0.226	0.252	4.9E-132	0
Ndc80	1.5E-133	0.596256	0.258	0.087	2.4E-129	0
Gm10075	1.1E-132	0.467318	0.701	0.479	1.8E-128	0
Kif23	2.5E-132	0.418352	0.391	0.18	4.2E-128	0
Cenpm	9.7E-132	0.586413	0.322	0.131	1.6E-127	0
Chaf1a	3.6E-131	0.540127	0.336	0.136	5.9E-127	0
Gmnn	8.2E-131	0.586264	0.301	0.114	1.4E-126	0
Rpa2	1.6E-130	0.556433	0.378	0.166	2.7E-126	0
Cenpq	2.2E-128	0.58207	0.321	0.129	3.6E-124	0
Cdca5	4.4E-126	0.510457	0.193	0.053	7.4E-122	0

Dpysl3	2.7E-125	-0.83789	0.087	0.244	4.5E-121	0
Map1b	3.8E-125	-0.61401	0.492	0.605	6.3E-121	0
Myod1	3.2E-124	0.630762	0.291	0.112	5.3E-120	0
Rfc4	8.7E-124	0.538371	0.402	0.192	1.5E-119	0
H2afy	7.7E-123	0.431726	0.752	0.561	1.3E-118	0
Cks1b	1.3E-122	0.468484	0.542	0.306	2.2E-118	0
Nuf2	1.7E-119	0.544341	0.29	0.112	2.9E-115	0
Serbp1	5.3E-119	0.27929	0.945	0.854	8.8E-115	0
Supt16	1.1E-118	0.462671	0.669	0.459	1.8E-114	0
Mcm6	2.7E-118	0.482355	0.462	0.24	4.4E-114	0
Tacc3	3.3E-118	0.536945	0.378	0.173	5.4E-114	0
Ncl	3.3E-118	0.280039	0.958	0.884	5.5E-114	0
Stmn4	4.2E-117	-0.78568	0.214	0.365	7.1E-113	0
CRE_RECON	8.8E-117	-0.58966	0.733	0.774	1.5E-112	0
Ccdc34	2.8E-116	0.484259	0.604	0.378	4.7E-112	0
Gng3	4.2E-116	-0.74713	0.17	0.344	7E-112	0
Hnrnpd	2.4E-115	0.418105	0.757	0.582	4E-111	0
Psip1	4E-115	0.382792	0.819	0.662	6.6E-111	0
Bub1	5.6E-115	0.547829	0.273	0.103	9.3E-111	0
Gm9800	6.2E-114	0.438718	0.699	0.503	1E-109	0
Sfrs18	4.9E-113	-0.43778	0.674	0.797	8.1E-109	0
Id2	1E-112	-0.64421	0.343	0.513	1.7E-108	0
Cdc45	2.7E-112	0.504848	0.22	0.071	4.4E-108	0
Ccne2	4.4E-111	0.50717	0.181	0.048	7.3E-107	0
Ncapg2	2.9E-110	0.525774	0.195	0.057	4.9E-106	0
H1f0	4.9E-110	0.442706	0.754	0.59	8.1E-106	0
Cenpk	1.2E-109	0.561176	0.286	0.117	2E-105	0
Kdm5b	2.9E-109	-0.65284	0.053	0.21	4.9E-105	0
Plk4	8.2E-108	0.526556	0.25	0.094	1.4E-103	0
Uhrf1	2.2E-107	0.52968	0.286	0.116	3.6E-103	0
Fen1	2.4E-107	0.500068	0.284	0.114	4E-103	0
Cbx5	4E-105	0.350082	0.806	0.623	6.6E-101	0
Celf4	4.3E-104	-0.73717	0.232	0.337	7.1E-100	0
Kif22	1.1E-103	0.466897	0.311	0.134	1.8E-99	0
Dhfr	8.5E-103	0.496743	0.291	0.121	1.42E-98	0
Hmgn5	5.2E-102	0.475443	0.597	0.391	8.66E-98	0
Miat	7.9E-102	-0.58577	0.435	0.53	1.33E-97	0
Whsc1	8.5E-102	0.435179	0.633	0.432	1.42E-97	0
Cdca2	1.2E-100	0.496685	0.261	0.103	2.01E-96	0
Diap3	2.1E-99	0.515236	0.226	0.083	3.46E-95	0
Ssrp1	4.6E-99	0.385708	0.734	0.546	7.61E-95	0
Ncapd2	8.3E-99	0.509597	0.331	0.157	1.38E-94	0
Melk	5.41E-98	0.483937	0.207	0.072	9.02E-94	0
Pmf1	1.63E-97	0.497401	0.275	0.118	2.72E-93	0
Tubb2a	3.47E-97	-0.71315	0.13	0.267	5.78E-93	0
Prdx4	4.31E-97	0.462145	0.529	0.334	7.19E-93	0
Rangap1	2.89E-96	0.48115	0.417	0.225	4.82E-92	0
Pkmyt1	5.52E-96	0.43257	0.173	0.052	9.21E-92	0

Gap43	8.45E-96	-0.52281	0.543	0.609	1.41E-91	0
Ptma	3.49E-95	0.376443	0.73	0.553	5.82E-91	0
Dlgap4	2.05E-92	-0.59675	0.067	0.21	3.43E-88	0
Sox4	2.47E-92	-0.50502	0.474	0.603	4.12E-88	0
Cst3	5.09E-92	-0.57377	0.513	0.54	8.49E-88	0
Brca1	5.58E-92	0.436487	0.182	0.057	9.31E-88	0
Smc3	5.35E-91	0.371019	0.763	0.608	8.93E-87	0
Rbbp4	2.61E-90	0.396966	0.638	0.453	4.36E-86	0
Atad5	6.22E-90	0.449182	0.282	0.123	1.04E-85	0
BC005764	1.12E-89	-0.65971	0.075	0.206	1.87E-85	0
Hjurp	1.57E-89	0.391874	0.604	0.401	2.62E-85	0
Rad54l	2.03E-89	0.437018	0.2	0.07	3.39E-85	0
D4Wsu53e	2.74E-89	-0.51932	0.333	0.468	4.57E-85	0
Tubb2b	5.95E-89	-0.50961	0.374	0.481	9.93E-85	0
Kif4	1.83E-88	0.482392	0.222	0.086	3.06E-84	0
Ubr7	2.5E-88	0.461877	0.258	0.109	4.17E-84	0
Rbfox3	7.33E-88	-0.52061	0.401	0.511	1.22E-83	0
Elavl4	2.16E-87	-0.61679	0.204	0.36	3.61E-83	0
Topbp1	4.16E-87	0.458623	0.274	0.12	6.94E-83	0
Dcx	4.28E-87	-0.58205	0.26	0.397	7.14E-83	0
H2afz	4.84E-87	0.439809	0.381	0.202	8.07E-83	0
Malat1	1.05E-86	-0.3912	0.919	0.961	1.75E-82	0
Rad21	2.19E-86	0.362625	0.63	0.429	3.65E-82	0
Ankrd12	5.51E-86	-0.67306	0.156	0.297	9.18E-82	0
Zic1	9.24E-86	-0.35192	0.749	0.79	1.54E-81	0
Pdzrn3	1.24E-85	-0.68799	0.047	0.166	2.06E-81	0
Asf1b	1.59E-85	0.395308	0.152	0.044	2.66E-81	0
Cdk5r1	3.1E-85	-0.5783	0.231	0.352	5.18E-81	0
Lmnb1	3.35E-85	0.424386	0.458	0.269	5.58E-81	0
Snrpd1	3.77E-85	0.378294	0.671	0.496	6.29E-81	0
Cltb	7.72E-85	0.442059	0.577	0.393	1.29E-80	0
Ncaph	1.67E-84	0.464576	0.237	0.099	2.79E-80	0
Dtl	3.06E-84	0.44447	0.273	0.121	5.11E-80	0
Nop58	3.4E-84	0.358654	0.735	0.564	5.67E-80	0
Tpm4	7.37E-84	0.431669	0.471	0.287	1.23E-79	0
Hmgn2	1.29E-83	0.449521	0.404	0.227	2.15E-79	0
H1fx	2.37E-83	0.483857	0.388	0.217	3.95E-79	0
Rfc2	2.57E-83	0.450845	0.352	0.183	4.28E-79	0
Hmgb1	4.83E-83	0.414626	0.515	0.328	8.05E-79	0
Ptn	8.18E-83	-0.56269	0.406	0.448	1.36E-78	0
Ccp110	1.55E-82	0.427197	0.397	0.219	2.59E-78	0
Rfc1	3.43E-82	0.427986	0.487	0.3	5.72E-78	0
Mcm2	1.16E-81	0.429263	0.346	0.177	1.94E-77	0
D17H6S56I	2.04E-81	0.431036	0.245	0.105	3.41E-77	0
Hnrnpdl	5.29E-81	0.290467	0.838	0.74	8.82E-77	0
Pa2g4	8.86E-81	0.367214	0.668	0.494	1.48E-76	0
Siva1	2.67E-80	0.441958	0.42	0.243	4.45E-76	0
Banf1	3.83E-80	0.324729	0.809	0.671	6.39E-76	0

Jhdm1d	1.37E-79	-0.5206	0.031	0.143	2.29E-75	0
Calm1	3.74E-79	-0.30024	0.853	0.863	6.24E-75	0
Smc1a	7.03E-79	0.345646	0.754	0.607	1.17E-74	0
Nrxn1	1.48E-78	-0.57659	0.243	0.332	2.46E-74	0
Timeless	2.62E-78	0.403781	0.279	0.129	4.36E-74	0
Thra	8.64E-78	-0.61234	0.119	0.249	1.44E-73	0
Nhlh1	1.39E-77	-0.58471	0.137	0.239	2.32E-73	0
Serinc1	2.12E-77	-0.45818	0.346	0.454	3.53E-73	0
Igfbpl1	2.74E-77	-0.36958	0.623	0.652	4.58E-73	0
Hint1	1.39E-76	0.298585	0.821	0.681	2.32E-72	0
Kif20b	2.82E-76	0.392088	0.323	0.165	4.71E-72	0
Lbr	4.06E-76	0.41808	0.329	0.171	6.78E-72	0
Cenpp	6.37E-76	0.430717	0.22	0.094	1.06E-71	0
Rbbp7	6.88E-76	0.410139	0.465	0.291	1.15E-71	0
Mcm5	7.31E-76	0.420268	0.295	0.144	1.22E-71	0
Alyref	1.08E-75	0.446654	0.33	0.174	1.81E-71	0
Rab3a	3.73E-75	-0.5856	0.061	0.167	6.22E-71	0
Mis18bp1	7.91E-75	0.360972	0.283	0.137	1.32E-70	0
Mrpl18	2.01E-74	0.412785	0.498	0.325	3.35E-70	0
Dctpp1	2.03E-74	0.414077	0.448	0.276	3.38E-70	0
Rpa1	4.6E-74	0.415261	0.215	0.088	7.67E-70	0
Aldoa	6.82E-74	-0.54308	0.14	0.279	1.14E-69	0
Hist1h2ag	1.07E-73	0.374202	0.135	0.041	1.78E-69	0
Acat1	2.71E-73	0.377112	0.586	0.413	4.53E-69	0
Psmc3ip	3.02E-73	0.442424	0.244	0.11	5.04E-69	0
Zfp367	6.07E-73	0.388798	0.191	0.074	1.01E-68	0
Srsf7	6.32E-73	0.357818	0.612	0.437	1.05E-68	0
Sept3	1.15E-72	-0.48271	0.328	0.404	1.92E-68	0
Mcm10	3.09E-72	0.402618	0.153	0.05	5.15E-68	0
Ybx1	4.19E-72	0.275802	0.877	0.768	6.98E-68	0
Mlf1ip	5.05E-72	0.410565	0.165	0.058	8.42E-68	0
Cenpw	7.45E-72	0.436667	0.235	0.107	1.24E-67	0
Zwilch	3.42E-71	0.408096	0.182	0.07	5.71E-67	0
Ctsd	9.99E-71	-0.7552	0.156	0.178	1.67E-66	0
Ccdc25	1.12E-70	0.420223	0.309	0.159	1.87E-66	0
Cdc7	1.22E-70	0.401623	0.289	0.144	2.04E-66	0
Sae1	2.1E-70	0.40214	0.418	0.252	3.51E-66	0
Srsf3	2.48E-70	0.304017	0.763	0.636	4.14E-66	0
Oxct1	2.74E-70	0.342546	0.682	0.528	4.57E-66	0
Tsc22d1	4.66E-70	-0.45622	0.318	0.449	7.78E-66	0
Cdt1	5.65E-70	0.387739	0.235	0.104	9.42E-66	0
Skp2	6.77E-70	0.407053	0.227	0.099	1.13E-65	0
Ina	7.38E-70	-0.47773	0.357	0.46	1.23E-65	0
Mcm3	8.61E-70	0.410063	0.303	0.155	1.44E-65	0
Lsm2	1.06E-69	0.405276	0.361	0.205	1.77E-65	0
Mapt	1.28E-69	-0.626	0.118	0.235	2.14E-65	0
Nrm	1.31E-69	0.412117	0.29	0.146	2.18E-65	0
Mtss1	2.31E-69	-0.54249	0.186	0.285	3.86E-65	0

Pold3	6.84E-69	0.383992	0.264	0.126	1.14E-64	0
Ttc3	7.74E-69	-0.30663	0.798	0.834	1.29E-64	0
Ckap2	2.05E-68	0.393944	0.283	0.14	3.42E-64	0
Cdkn2c	2.55E-68	0.419716	0.254	0.122	4.25E-64	0
Nap1l1	4.56E-68	0.329943	0.652	0.483	7.6E-64	0
Pdgfa	8.32E-68	0.45384	0.385	0.228	1.39E-63	0
Mcm7	1.95E-67	0.337899	0.536	0.36	3.26E-63	0
Slbp	2.55E-67	0.367643	0.169	0.062	4.26E-63	0
Nsl1	5.32E-67	0.369341	0.134	0.043	8.88E-63	0
Pola1	9.55E-67	0.41572	0.199	0.084	1.59E-62	0
Ska1	1.1E-66	0.406677	0.179	0.072	1.83E-62	0
Ctsb	1.11E-66	-0.6489	0.206	0.272	1.85E-62	0
Elavl3	1.87E-66	-0.45509	0.377	0.49	3.11E-62	0
Clic4	2.44E-66	0.399847	0.354	0.203	4.07E-62	0
Gpm6b	4.43E-66	-0.41722	0.407	0.498	7.4E-62	0
Nsmce4a	5.71E-66	0.398546	0.351	0.199	9.53E-62	0
Tmem50a	6.1E-66	-0.46014	0.287	0.427	1.02E-61	0
Psat1	9.9E-66	0.388321	0.501	0.341	1.65E-61	0
Gjc1	1.45E-65	0.401604	0.319	0.172	2.42E-61	0
Myt1	1.73E-65	-0.57561	0.063	0.169	2.89E-61	0
Rif1	3.3E-65	0.373881	0.412	0.249	5.51E-61	0
Snrpb	4E-64	0.30829	0.702	0.548	6.67E-60	0
E2f7	4.08E-64	0.336999	0.112	0.031	6.81E-60	0
Sgol1	4.81E-64	0.39411	0.232	0.107	8.02E-60	0
Ctsl	1.63E-63	-0.50599	0.187	0.273	2.73E-59	0
Atxn7l3b	1.92E-63	-0.33191	0.598	0.687	3.2E-59	0
Ncapd3	2.75E-63	0.385769	0.213	0.093	4.59E-59	0
St18	2.95E-63	-0.55992	0.112	0.205	4.92E-59	0
Barhl1	4.88E-63	-0.40754	0.437	0.537	8.14E-59	0
Blm	5.97E-63	0.371581	0.209	0.091	9.96E-59	0
Syt11	1.67E-62	-0.41383	0.417	0.52	2.78E-58	0
6330403K(	1.95E-62	-0.50943	0.142	0.262	3.26E-58	0
Orc6	2.01E-62	0.380693	0.323	0.177	3.35E-58	0
Mis18a	2.07E-62	0.399261	0.205	0.091	3.46E-58	0
Brca2	2.37E-62	0.381246	0.212	0.094	3.95E-58	0
Foxm1	3.33E-62	0.32852	0.105	0.033	5.55E-58	0
Rad54b	3.47E-62	0.335504	0.138	0.047	5.78E-58	0
Aplp1	5.77E-62	-0.52986	0.069	0.167	9.63E-58	0
Hmgn1	7.96E-62	0.291973	0.743	0.598	1.33E-57	0
Cmc2	8.72E-62	0.389736	0.218	0.1	1.45E-57	0
Pgf	1.13E-61	0.351922	0.148	0.053	1.89E-57	0
Sept4	2.06E-61	-0.55732	0.138	0.243	3.43E-57	0
Csrp2	4.64E-61	0.38997	0.268	0.136	7.74E-57	0
Cep57	5.59E-61	0.379712	0.386	0.233	9.32E-57	0
Phf17	7.76E-61	0.378905	0.179	0.075	1.29E-56	0
Cpe	1.35E-60	-0.40249	0.374	0.471	2.26E-56	0
Nhlh2	1.42E-60	-0.45332	0.355	0.435	2.37E-56	0
Lyar	1.64E-60	0.390964	0.415	0.26	2.74E-56	0

Mxd4	1.99E-60	-0.48852	0.101	0.216	3.31E-56	0
Dscc1	2.72E-60	0.345447	0.128	0.042	4.53E-56	0
Spag5	2.95E-60	0.35624	0.16	0.063	4.92E-56	0
Ttk	4.24E-60	0.369149	0.176	0.073	7.08E-56	0
Rfc3	4.85E-60	0.373626	0.324	0.181	8.08E-56	0
Wdhd1	4.97E-60	0.350946	0.171	0.068	8.29E-56	0
Map2	7.51E-60	-0.40175	0.423	0.504	1.25E-55	0
Rbbp8	8.67E-60	0.392114	0.221	0.103	1.45E-55	0
Nup62	2.05E-59	0.36368	0.282	0.149	3.42E-55	0
Prim2	3.37E-59	0.351439	0.16	0.062	5.63E-55	0
Brd3	5.01E-59	0.326063	0.653	0.508	8.35E-55	0
Chtf18	6.07E-59	0.332624	0.112	0.034	1.01E-54	0
A330076H(	6.45E-59	-0.50548	0.082	0.176	1.08E-54	0
Elmo1	7.62E-59	-0.55718	0.05	0.14	1.27E-54	0
Hpca	8.56E-59	0.380095	0.407	0.252	1.43E-54	0
Trpc4ap	1.52E-58	-0.52372	0.12	0.213	2.53E-54	0
Basp1	1.66E-58	-0.31045	0.672	0.71	2.78E-54	0
Suz12	2.6E-58	0.382319	0.339	0.196	4.34E-54	0
A930011O:	3.14E-58	-0.52778	0.041	0.128	5.24E-54	0
Cklf	5.3E-58	0.397797	0.27	0.145	8.84E-54	0
Ccne1	7.48E-58	0.281974	0.105	0.029	1.25E-53	0
Shcbp1	8.52E-58	0.343088	0.157	0.061	1.42E-53	0
Ptprs	1.15E-57	-0.43899	0.347	0.435	1.92E-53	0
Cadm3	2.47E-57	-0.51375	0.063	0.155	4.12E-53	0
Chd7	9.71E-57	-0.33256	0.587	0.658	1.62E-52	0
Lap3	1.57E-56	0.388298	0.399	0.253	2.63E-52	0
Chgb	2.05E-56	-0.55355	0.167	0.254	3.42E-52	0
Smarcc1	2.18E-56	0.328175	0.565	0.408	3.64E-52	0
Nup85	3.08E-56	0.354116	0.345	0.203	5.13E-52	0
Meg3	5.03E-56	-0.80521	0.032	0.107	8.4E-52	0
Celf2	1.03E-55	-0.33592	0.596	0.645	1.72E-51	0
Nxt1	1.68E-55	0.3647	0.217	0.103	2.81E-51	0
Srsf2	2.3E-55	0.298744	0.682	0.543	3.83E-51	0
Dbf4	2.41E-55	0.334368	0.248	0.125	4.01E-51	0
Dnajc21	2.44E-55	0.34829	0.325	0.187	4.07E-51	0
Gins3	3.63E-55	0.334318	0.141	0.052	6.05E-51	0
Fabp7	3.95E-55	-0.92581	0.101	0.127	6.6E-51	0
Hat1	4.6E-55	0.367612	0.258	0.135	7.68E-51	0
Espl1	4.94E-55	0.342295	0.134	0.051	8.24E-51	0
Vrk1	4.96E-55	0.375042	0.25	0.128	8.27E-51	0
Sqstm1	5.8E-55	-0.43453	0.122	0.246	9.68E-51	0
Mllt11	8.3E-55	-0.49164	0.137	0.233	1.38E-50	0
Syce2	9.2E-55	0.377254	0.162	0.067	1.53E-50	0
Mad2l1	1.27E-54	0.367265	0.216	0.105	2.11E-50	0
Tex14	1.39E-54	-0.53534	0.068	0.16	2.32E-50	0
Apc	5.14E-54	-0.42763	0.349	0.447	8.58E-50	0
Gria2	1.02E-53	-0.38319	0.468	0.531	1.7E-49	0
Arhgap11a	1.03E-53	0.325001	0.276	0.15	1.71E-49	0

E2f2	1.53E-53	0.294245	0.106	0.032	2.55E-49	0
Txn1	2.07E-53	0.286414	0.68	0.537	3.45E-49	0
Lsm3	2.14E-53	0.320423	0.487	0.333	3.57E-49	0
Rad18	2.18E-53	0.354564	0.173	0.074	3.64E-49	0
Wbp5	2.49E-53	0.30062	0.641	0.494	4.15E-49	0
Nop56	2.63E-53	0.329146	0.489	0.336	4.39E-49	0
Baz1b	2.98E-53	0.318104	0.616	0.469	4.97E-49	0
Ska2	3.56E-53	0.346604	0.318	0.183	5.95E-49	0
Cdkn2d	4.59E-53	0.334518	0.26	0.137	7.65E-49	0
Dner	5.75E-53	-0.48098	0.138	0.229	9.6E-49	0
Cenpj	7.25E-53	0.375453	0.24	0.122	1.21E-48	0
2700099C:	1.42E-52	0.345669	0.16	0.068	2.37E-48	0
Slc7a5	2.59E-52	0.366568	0.205	0.097	4.32E-48	0
Brip1	2.65E-52	0.311432	0.105	0.033	4.42E-48	0
Naa50	5.5E-52	0.352823	0.405	0.263	9.17E-48	0
Nsg2	1.46E-51	-0.34687	0.419	0.5	2.43E-47	0
Anln	1.57E-51	0.385277	0.188	0.088	2.62E-47	0
Rbfox2	1.57E-51	-0.44306	0.193	0.3	2.62E-47	0
Cdca4	2.07E-51	0.314088	0.198	0.094	3.45E-47	0
Cenpn	3.5E-51	0.321996	0.139	0.055	5.83E-47	0
Apitd1	4.81E-51	0.344571	0.176	0.079	8.02E-47	0
Dck	6.26E-51	0.350433	0.188	0.086	1.04E-46	0
Chaf1b	8.28E-51	0.359223	0.161	0.069	1.38E-46	0
Insm1	8.4E-51	0.392557	0.404	0.271	1.4E-46	0
Sparcl1	9.42E-51	-0.53508	0.183	0.211	1.57E-46	0
Ypel3	9.52E-51	-0.37414	0.352	0.451	1.59E-46	0
Arpp21	1.02E-50	-0.51671	0.047	0.125	1.7E-46	0
Gsg2	1.05E-50	0.307878	0.109	0.038	1.76E-46	0
Ppp1r14c	8.12E-50	-0.3858	0.227	0.269	1.35E-45	0
Sgol2	1E-49	0.283134	0.252	0.138	1.67E-45	0
Prdx1	2.16E-49	0.272661	0.616	0.465	3.6E-45	0
Tbata	2.78E-49	0.295949	0.464	0.316	4.64E-45	0
Rfc5	2.91E-49	0.339309	0.191	0.091	4.85E-45	0
B3galt2	3.24E-49	-0.45394	0.036	0.109	5.4E-45	0
Kif5a	4.1E-49	-0.45876	0.059	0.141	6.83E-45	0
Ifrd1	9.68E-49	0.346195	0.28	0.158	1.61E-44	0
Snrpe	1.11E-48	0.274417	0.676	0.54	1.85E-44	0
Rab6b	1.14E-48	-0.45116	0.123	0.217	1.9E-44	0
Cdk4	1.95E-48	0.255975	0.743	0.622	3.25E-44	0
Srsf4	2.67E-48	0.328031	0.395	0.257	4.46E-44	0
Pole	2.86E-48	0.299256	0.104	0.035	4.77E-44	0
Cdca7	3.71E-48	0.29906	0.304	0.177	6.18E-44	0
Scg5	6.83E-48	-0.39884	0.254	0.35	1.14E-43	0
Slfn9	9.21E-48	0.338261	0.144	0.059	1.54E-43	0
Kif1b	3.16E-47	-0.34712	0.453	0.533	5.27E-43	0
Rpa3	3.45E-47	0.345801	0.354	0.222	5.76E-43	0
Sema6a	3.96E-47	-0.47106	0.126	0.216	6.61E-43	0
Exo1	5.25E-47	0.282895	0.105	0.035	8.75E-43	0

Arpp19	5.94E-47	0.318042	0.493	0.355	9.91E-43	0
Igsf21	7.59E-47	-0.40531	0.027	0.101	1.27E-42	0
Idh2	1.02E-46	0.317508	0.393	0.256	1.7E-42	0
Ank3	1.09E-46	-0.39146	0.349	0.433	1.82E-42	0
Prnp	1.2E-46	-0.428	0.125	0.222	2.01E-42	0
Gli1	2.91E-46	0.318681	0.19	0.091	4.85E-42	0
Pola2	3.51E-46	0.313849	0.169	0.078	5.86E-42	0
Bub3	4.14E-46	0.327663	0.437	0.299	6.91E-42	0
Rbp4	4.17E-46	0.325083	0.353	0.22	6.96E-42	0
Trim37	4.73E-46	0.348012	0.339	0.213	7.89E-42	0
2700029M	5.06E-46	0.316487	0.404	0.267	8.45E-42	0
Pde1c	6.32E-46	-0.34159	0.357	0.392	1.05E-41	0
Itsn1	6.41E-46	-0.43732	0.168	0.248	1.07E-41	0
Ctsf	1.05E-45	-0.39009	0.034	0.11	1.76E-41	0
Calr	3.69E-45	-0.32106	0.445	0.523	6.15E-41	0
Wdr76	4.64E-45	0.304112	0.152	0.065	7.74E-41	0
Smchd1	6.71E-45	0.345933	0.382	0.252	1.12E-40	0
Kmt2e	7.94E-45	-0.31804	0.503	0.581	1.32E-40	0
Ybx3	1.28E-44	0.320407	0.401	0.268	2.14E-40	0
Pold1	1.34E-44	0.286197	0.157	0.07	2.23E-40	0
Kif5c	1.35E-44	-0.3624	0.343	0.409	2.25E-40	0
Smarca5	2.19E-44	0.295004	0.518	0.379	3.66E-40	0
Rabac1	3.01E-44	-0.4053	0.171	0.274	5.02E-40	0
Trip13	9.64E-44	0.285817	0.151	0.067	1.61E-39	0
Slc25a5	1.43E-43	0.27287	0.621	0.487	2.39E-39	0
Cdk2	2.55E-43	0.302595	0.133	0.055	4.25E-39	0
Ank2	3.75E-43	-0.43679	0.182	0.251	6.26E-39	0
Dnaaf2	3.85E-43	0.325002	0.183	0.089	6.42E-39	0
Pax6	7.31E-43	-0.30391	0.469	0.521	1.22E-38	0
Stil	1.94E-42	0.267099	0.108	0.041	3.23E-38	0
Uncx	2.84E-42	-0.37764	0.256	0.329	4.74E-38	0
Grina	4.75E-42	-0.39184	0.046	0.121	7.93E-38	0
Dpy30	5.28E-42	0.306164	0.408	0.277	8.81E-38	0
Cdk5rap2	5.46E-42	0.327143	0.279	0.164	9.1E-38	0
Ctcf	1.05E-41	0.282006	0.603	0.475	1.75E-37	0
Itm2c	1.17E-41	-0.40367	0.141	0.225	1.96E-37	0
Arl6ip6	1.43E-41	0.277917	0.207	0.109	2.38E-37	0
Larp7	1.46E-41	0.329213	0.418	0.291	2.44E-37	0
Eif4ebp1	2.16E-41	0.349951	0.142	0.066	3.61E-37	0
Bex1	3.31E-41	0.302624	0.326	0.204	5.52E-37	0
Hist1h1e	3.42E-41	0.309234	0.125	0.051	5.71E-37	0
Nmral1	4.11E-41	0.328155	0.281	0.169	6.85E-37	0
Map1lc3b	8.23E-41	-0.35474	0.256	0.345	1.37E-36	0
Chrna3	8.73E-41	-0.42829	0.054	0.13	1.46E-36	0
Trim59	8.89E-41	0.307955	0.261	0.152	1.48E-36	0
Ctps	1.03E-40	0.291454	0.223	0.121	1.72E-36	0
Cnot6	1.38E-40	0.271514	0.506	0.369	2.3E-36	0
Hsd11b2	1.41E-40	0.278144	0.396	0.267	2.35E-36	0

Fignl1	2.19E-40	0.267466	0.13	0.054	3.65E-36	0
Rnaseh2a	2.54E-40	0.322073	0.24	0.136	4.23E-36	0
Naa38	3.36E-40	0.287914	0.421	0.29	5.6E-36	0
Btbd17	3.99E-40	-0.40439	0.092	0.173	6.65E-36	0
Eif4a3	5.32E-40	0.288446	0.465	0.334	8.88E-36	0
Snap25	6.72E-40	-0.39017	0.221	0.293	1.12E-35	0
Tnik	6.92E-40	-0.44991	0.084	0.158	1.15E-35	0
Iqgap2	8.76E-40	0.250279	0.102	0.037	1.46E-35	0
Cep290	8.78E-40	0.318158	0.228	0.126	1.46E-35	0
Snrpf	9.76E-40	0.259965	0.541	0.405	1.63E-35	0
Srsf10	1.42E-39	0.266221	0.441	0.309	2.37E-35	0
S100a16	1.95E-39	-0.42789	0.041	0.105	3.25E-35	0
Bub1b	1.95E-39	0.317566	0.173	0.088	3.25E-35	0
Rnf168	2.02E-39	0.306224	0.305	0.188	3.38E-35	0
Gng2	2.03E-39	-0.33191	0.331	0.408	3.38E-35	0
Ndn	3.05E-39	-0.3838	0.147	0.237	5.09E-35	0
Haus5	3.21E-39	0.26109	0.115	0.046	5.35E-35	0
Kpnb1	3.36E-39	0.296272	0.406	0.28	5.6E-35	0
4930422G(	3.78E-39	0.292257	0.139	0.061	6.31E-35	0
Ipo5	4.24E-39	0.303214	0.222	0.122	7.07E-35	0
1500012F(	4.56E-39	-0.34355	0.322	0.412	7.6E-35	0
Gins2	7.38E-39	0.286139	0.278	0.167	1.23E-34	0
Ldhb	1.26E-38	-0.3562	0.209	0.294	2.1E-34	0
Myt1l	1.98E-38	-0.44489	0.101	0.172	3.31E-34	0
Os9	3.59E-38	-0.37936	0.176	0.265	5.98E-34	0
Nudc	3.73E-38	0.304264	0.383	0.261	6.22E-34	0
Zic4	5.09E-38	-0.32922	0.35	0.431	8.48E-34	0
Ccdc41	8.01E-38	0.329955	0.351	0.235	1.34E-33	0
Gins1	8.49E-38	0.303546	0.191	0.1	1.42E-33	0
Atp6v0b	9.56E-38	-0.35192	0.206	0.278	1.59E-33	0
Lsm6	1.35E-37	0.256246	0.482	0.35	2.26E-33	0
Ptms	1.87E-37	-0.30003	0.406	0.486	3.11E-33	0
Gm11266	3.03E-37	-0.4012	0.079	0.156	5.06E-33	0
Nfatc2ip	3.87E-37	0.29045	0.135	0.061	6.46E-33	0
Klf7	5.21E-37	-0.37524	0.195	0.275	8.7E-33	0
Slc1a3	5.28E-37	-0.45793	0.123	0.158	8.8E-33	0
Casp8ap2	7.01E-37	0.290404	0.343	0.224	1.17E-32	0
Pole3	7.41E-37	0.310283	0.347	0.231	1.24E-32	0
2810008D(	1.07E-36	-0.36404	0.066	0.139	1.78E-32	0
Stag1	1.32E-36	0.287238	0.3	0.188	2.2E-32	0
E2f1	1.36E-36	0.269971	0.229	0.131	2.26E-32	0
Snrpa1	2.6E-36	0.296451	0.431	0.311	4.34E-32	0
Kcnk1	2.7E-36	-0.37798	0.15	0.214	4.5E-32	0
Syncrip	3.04E-36	0.256483	0.596	0.476	5.06E-32	0
A030009H(	4.69E-36	0.281152	0.188	0.099	7.83E-32	0
Pknox1	5.43E-36	0.31494	0.227	0.13	9.05E-32	0
2610203C2	5.64E-36	-0.37017	0.06	0.132	9.41E-32	0
Hprt	5.67E-36	0.287032	0.277	0.17	9.45E-32	0

Snrpd2	7.77E-36	0.259579	0.544	0.421	1.3E-31	0
Zbtb18	1.01E-35	-0.35713	0.203	0.278	1.68E-31	0
Kidins220	1.17E-35	-0.39572	0.078	0.151	1.95E-31	0
Gins4	1.55E-35	0.283914	0.206	0.113	2.59E-31	0
Stub1	1.78E-35	0.258894	0.45	0.326	2.97E-31	0
Nsmce1	1.84E-35	0.294359	0.323	0.21	3.07E-31	0
Mis12	2.83E-35	0.283268	0.167	0.085	4.73E-31	0
Ube2t	3.03E-35	0.264149	0.139	0.065	5.05E-31	0
Gdi1	3.07E-35	-0.32979	0.179	0.239	5.12E-31	0
Bok	3.3E-35	0.291744	0.347	0.232	5.51E-31	0
Phgdh	6.42E-35	0.282371	0.14	0.068	1.07E-30	0
Prr11	9.55E-35	0.250902	0.112	0.048	1.59E-30	0
Tubg1	1.02E-34	0.289911	0.205	0.114	1.7E-30	0
Cntln	1.24E-34	0.336617	0.256	0.156	2.06E-30	0
Mcm4	1.45E-34	0.286677	0.217	0.122	2.41E-30	0
Dnph1	2.49E-34	0.312382	0.191	0.11	4.16E-30	0
Cbfb	3.03E-34	0.291737	0.269	0.166	5.05E-30	0
Lgmn	3.34E-34	-0.38624	0.076	0.127	5.57E-30	0
Uba2	3.48E-34	0.290738	0.35	0.237	5.81E-30	0
Smco4	4.89E-34	0.307782	0.241	0.146	8.15E-30	0
Abhd16a	5.85E-34	-0.36044	0.134	0.2	9.76E-30	0
Snrnp40	6.95E-34	0.260611	0.368	0.252	1.16E-29	0
Azin1	7.7E-34	0.252119	0.292	0.185	1.28E-29	0
Npdc1	7.97E-34	-0.34221	0.199	0.273	1.33E-29	0
Trim28	9.02E-34	0.254885	0.464	0.341	1.5E-29	0
Tagln3	1.18E-33	-0.31821	0.253	0.303	1.96E-29	0
Gm26735	1.2E-33	-0.36845	0.112	0.193	2E-29	0
Bin1	1.23E-33	-0.28	0.393	0.436	2.05E-29	0
Spdl1	1.66E-33	0.289541	0.138	0.067	2.76E-29	0
Vars	2.11E-33	0.259314	0.249	0.15	3.52E-29	0
Rbms1	2.59E-33	0.275987	0.307	0.199	4.32E-29	0
Shmt1	4.66E-33	0.272861	0.171	0.09	7.78E-29	0
Tex30	5.41E-33	0.285216	0.211	0.12	9.02E-29	0
Chd3	6.07E-33	-0.40319	0.085	0.161	1.01E-28	0
Ptges3	6.5E-33	0.285709	0.299	0.195	1.08E-28	0
Aurka	1.15E-32	0.268472	0.165	0.085	1.92E-28	0
Rhno1	1.15E-32	0.279006	0.214	0.123	1.93E-28	0
Sod1	2.37E-32	0.258566	0.538	0.423	3.96E-28	0
Vimp	2.42E-32	-0.34617	0.116	0.203	4.04E-28	0
Mapk8ip1	2.43E-32	-0.35082	0.162	0.239	4.05E-28	0
Ect2	2.53E-32	0.285623	0.161	0.085	4.21E-28	0
Clmp	3.02E-32	-0.36737	0.185	0.253	5.04E-28	0
Set	3.3E-32	0.261716	0.484	0.368	5.51E-28	0
Rcor2	4.3E-32	-0.34416	0.161	0.237	7.17E-28	0
Fam213b	4.85E-32	-0.36298	0.092	0.169	8.08E-28	0
Ncaph2	5.02E-32	0.276362	0.255	0.158	8.38E-28	0
Ncan	5.12E-32	-0.33859	0.047	0.109	8.55E-28	0
Plp1	5.57E-32	-0.76583	0.102	0.116	9.29E-28	0

Soga3	5.97E-32	-0.28166	0.404	0.467	9.97E-28	0
Haus8	6.04E-32	0.251656	0.129	0.06	1.01E-27	0
Apbb1	8.64E-32	-0.34587	0.122	0.179	1.44E-27	0
Tspyl4	1.15E-31	-0.34728	0.074	0.136	1.91E-27	0
Dbn1	1.17E-31	-0.35315	0.08	0.15	1.95E-27	0
Xbp1	1.76E-31	-0.31663	0.066	0.134	2.93E-27	0
Btg2	3.4E-31	-0.37423	0.148	0.219	5.67E-27	0
Clip3	4.39E-31	-0.3116	0.247	0.324	7.33E-27	0
Gnao1	5.05E-31	-0.32343	0.238	0.296	8.42E-27	0
Cdca7l	1.21E-30	0.253624	0.17	0.091	2.02E-26	0
Rab6a	1.23E-30	-0.29955	0.272	0.338	2.05E-26	0
2210016L2	1.26E-30	-0.33927	0.213	0.303	2.1E-26	0
Sox18	1.4E-30	0.254225	0.222	0.133	2.34E-26	0
Pik3r3	1.64E-30	-0.34719	0.084	0.145	2.74E-26	0
Exosc8	1.82E-30	0.277422	0.294	0.193	3.04E-26	0
Ddit4	2.21E-30	-0.32457	0.053	0.115	3.69E-26	0
Mab21l1	2.21E-30	-0.32952	0.173	0.235	3.69E-26	0
Stxbp1	2.48E-30	-0.359	0.109	0.172	4.13E-26	0
Serpinh1	2.7E-30	-0.3329	0.065	0.124	4.5E-26	0
Mbnl2	4.74E-30	-0.33759	0.124	0.189	7.91E-26	0
Prmt2	5.13E-30	-0.30697	0.046	0.103	8.56E-26	0
Cdkn1a	6.15E-30	0.298627	0.153	0.08	1.03E-25	0
Zmynd8	1.18E-29	-0.34142	0.141	0.226	1.96E-25	0
Slc3a2	1.83E-29	0.256928	0.415	0.304	3.06E-25	0
Podxl2	1.86E-29	-0.31966	0.184	0.235	3.11E-25	0
Glce	2.2E-29	-0.34013	0.122	0.187	3.67E-25	0
Pdlim1	3.67E-29	0.254753	0.117	0.056	6.12E-25	0
Nrn1	3.93E-29	-0.36203	0.154	0.234	6.55E-25	0
Sh3bp5	4.14E-29	-0.32958	0.057	0.112	6.9E-25	0
Npc2	6.99E-29	-0.26618	0.408	0.474	1.17E-24	0
Shmt2	9.11E-29	0.250933	0.187	0.106	1.52E-24	0
Cenpl	1.13E-28	0.258138	0.127	0.065	1.89E-24	0
Rtn4	1.15E-28	-0.25338	0.432	0.489	1.91E-24	0
Eid1	1.18E-28	-0.25535	0.45	0.512	1.96E-24	0
Nicn1	1.25E-28	-0.34306	0.144	0.22	2.09E-24	0
Med30	2.02E-28	0.258614	0.285	0.188	3.37E-24	0
Mpp6	3.72E-28	0.293226	0.233	0.147	6.21E-24	0
Cnrip1	4.71E-28	-0.3354	0.133	0.21	7.86E-24	0
Stag2	5.19E-28	0.252621	0.344	0.24	8.66E-24	0
Vim	5.3E-28	-0.31652	0.262	0.31	8.85E-24	0
Gpc2	1.18E-27	-0.30658	0.091	0.148	1.96E-23	0
Rufy3	1.18E-27	-0.26096	0.335	0.378	1.97E-23	0
Sirt2	1.77E-27	-0.35801	0.114	0.163	2.95E-23	0
Srrm4	1.85E-27	-0.30892	0.154	0.203	3.08E-23	0
Kdm6b	2.12E-27	-0.29911	0.053	0.114	3.54E-23	0
Clcn4-2	3.02E-27	-0.30927	0.196	0.261	5.03E-23	0
Timm50	3.07E-27	0.253465	0.32	0.22	5.12E-23	0
Tgfb2	5.68E-27	-0.33922	0.131	0.203	9.47E-23	0

Uchl1	5.93E-27	-0.25782	0.301	0.34	9.89E-23	0
Ogt	6.08E-27	-0.30085	0.16	0.221	1.01E-22	0
Pbdc1	7.71E-27	0.270754	0.284	0.191	1.29E-22	0
Rnmt	1.06E-26	-0.28065	0.297	0.358	1.76E-22	0
Akap8l	1.26E-26	-0.32271	0.103	0.168	2.1E-22	0
Exosc7	1.55E-26	0.254997	0.254	0.165	2.59E-22	0
Prkcb	1.98E-26	-0.27468	0.224	0.261	3.31E-22	0
Pdcd4	2.06E-26	-0.28544	0.264	0.343	3.43E-22	0
Use1	3.45E-26	-0.2939	0.244	0.326	5.76E-22	0
Uchl5	4.46E-26	0.273911	0.27	0.18	7.44E-22	0
Aplp2	1.29E-25	-0.2586	0.287	0.329	2.14E-21	0
Nsg1	1.59E-25	-0.29027	0.234	0.297	2.66E-21	0
Kif20a	3.62E-25	0.252336	0.138	0.077	6.05E-21	0
Pea15a	8.35E-25	-0.32301	0.128	0.185	1.39E-20	0
Tmem66	1.16E-24	-0.28135	0.151	0.208	1.94E-20	0
RP23-32A8	1.19E-24	-0.25921	0.152	0.193	1.98E-20	0
Gabarapl1	1.4E-24	-0.28574	0.167	0.225	2.33E-20	0
Baz2b	1.53E-24	-0.29179	0.304	0.376	2.55E-20	0
Sptan1	1.63E-24	-0.28499	0.132	0.176	2.72E-20	0
Slc22a17	1.69E-24	-0.2916	0.197	0.261	2.82E-20	0
Atp6v1e1	3.31E-24	-0.2748	0.276	0.341	5.52E-20	0
Commd1	4.43E-24	0.252175	0.378	0.283	7.39E-20	0
Hpcal1	4.45E-24	-0.30814	0.061	0.115	7.42E-20	0
Pdrg1	5.3E-24	-0.30277	0.158	0.23	8.84E-20	0
Idh1	5.84E-24	-0.28195	0.096	0.167	9.74E-20	0
Clvs1	6.35E-24	-0.29572	0.087	0.136	1.06E-19	0
Hcfc1r1	6.91E-24	-0.29946	0.139	0.203	1.15E-19	0
H13	7.15E-24	-0.27595	0.19	0.246	1.19E-19	0
Hist3h2a	1.04E-23	-0.30877	0.095	0.152	1.74E-19	0
Ywhag	1.09E-23	-0.29102	0.185	0.251	1.81E-19	0
2410066E1	1.1E-23	-0.28123	0.057	0.106	1.83E-19	0
Tacc2	1.13E-23	-0.3149	0.091	0.15	1.89E-19	0
Ppfia2	1.22E-23	-0.32237	0.071	0.119	2.03E-19	0
Asns	1.32E-23	0.251249	0.138	0.076	2.2E-19	0
2900011O	4.43E-23	-0.29762	0.059	0.112	7.39E-19	0
Ankra2	5.6E-23	-0.28367	0.075	0.128	9.34E-19	0
Gabbr1	6.35E-23	-0.25827	0.109	0.146	1.06E-18	0
Grik2	6.59E-23	-0.2758	0.072	0.107	1.1E-18	0
Cacng4	7.62E-23	-0.30757	0.086	0.104	1.27E-18	0
Gria4	1.04E-22	-0.28703	0.088	0.131	1.74E-18	0
Psap	1.44E-22	-0.30104	0.14	0.192	2.4E-18	0
Bcl7a	1.49E-22	-0.25184	0.279	0.334	2.48E-18	0
Ccdc28b	2.11E-22	-0.29081	0.103	0.173	3.51E-18	0
Igsf8	2.59E-22	-0.27466	0.221	0.278	4.31E-18	0
Hist3h2ba	4.13E-22	-0.28775	0.088	0.147	6.89E-18	0
Flot1	6.23E-22	-0.26755	0.054	0.106	1.04E-17	0
Ing4	1E-21	-0.26043	0.266	0.333	1.67E-17	0
Pnmal2	1.01E-21	-0.2981	0.099	0.155	1.68E-17	0

Klf9	1.08E-21	-0.27719	0.223	0.279	1.8E-17	0
Lrpap1	1.34E-21	-0.26735	0.079	0.124	2.23E-17	0
C130071C(	1.35E-21	-0.26696	0.251	0.318	2.24E-17	0
Pak7	1.88E-21	-0.29205	0.061	0.112	3.14E-17	0
Pik3ip1	3.2E-21	-0.27031	0.073	0.124	5.34E-17	0
Reln	4.23E-21	-0.2859	0.146	0.186	7.05E-17	0
Fbxo32	4.63E-21	-0.27745	0.067	0.126	7.73E-17	0
Agpat4	9.67E-21	-0.26459	0.077	0.125	1.61E-16	0
Pygo1	1.13E-20	-0.29	0.146	0.201	1.88E-16	0
Wdr13	1.19E-20	-0.26282	0.06	0.113	1.99E-16	0
Zfp329	1.53E-20	-0.27238	0.086	0.137	2.55E-16	0
Gm3764	2.73E-20	-0.27418	0.087	0.14	4.55E-16	0
Gramd1b	3.14E-20	-0.28119	0.122	0.178	5.24E-16	0
Prdm8	3.25E-20	-0.25446	0.104	0.13	5.42E-16	0
Bcas1	4.4E-20	-0.29616	0.241	0.29	7.34E-16	0
Dixdc1	5.24E-20	-0.26267	0.195	0.242	8.74E-16	0
4930402H:	5.62E-20	-0.26155	0.073	0.12	9.37E-16	0
Rundc3a	5.87E-20	-0.26625	0.198	0.258	9.79E-16	0
Mkrn1	6.44E-20	-0.26629	0.159	0.218	1.07E-15	0
Mien1	6.98E-20	-0.26595	0.167	0.239	1.16E-15	0
Maged2	7.26E-20	-0.26346	0.123	0.175	1.21E-15	0
Atp6v0e2	1.04E-19	-0.25547	0.202	0.271	1.74E-15	0
Nt5c	1.69E-19	-0.26906	0.132	0.193	2.81E-15	0
Pcmtd2	2.25E-19	-0.26406	0.096	0.152	3.75E-15	0
Tpm1	5.39E-19	-0.27693	0.146	0.199	8.99E-15	0
Mfap4	1.22E-18	-0.26399	0.132	0.167	2.04E-14	0
Sstr2	1.77E-18	-0.27063	0.144	0.199	2.96E-14	0
Kif1a	1.2E-17	-0.26	0.126	0.18	2.01E-13	0
Fyn	1.29E-17	-0.26713	0.188	0.221	2.15E-13	0
Sox5	2.03E-17	-0.25846	0.08	0.126	3.39E-13	0
Slc17a6	3.66E-17	-0.26727	0.114	0.154	6.11E-13	0
Pcdha2	6.6E-17	-0.25903	0.083	0.124	1.1E-12	0
Tmem176b	2.93E-16	-0.25304	0.077	0.115	4.89E-12	0
Mbp	3.42E-16	-0.33672	0.141	0.17	5.71E-12	0
Hes1	5.05E-16	-0.28041	0.123	0.153	8.42E-12	0
Ntm	1.11E-14	-0.25995	0.11	0.152	1.86E-10	0
Akap12	1.37E-14	-0.253	0.08	0.117	2.29E-10	0
Ube2c	0	2.072918	0.908	0.176	0	1
Cenpf	0	1.846262	0.936	0.278	0	1
Cenpa	0	1.757118	0.88	0.16	0	1
Cdc20	0	1.731163	0.795	0.109	0	1
Prc1	0	1.634666	0.817	0.175	0	1
Hmmr	0	1.61588	0.729	0.096	0	1
Cenpe	0	1.587253	0.843	0.204	0	1
Ccnb1	0	1.528323	0.651	0.064	0	1
Tpx2	0	1.520151	0.867	0.196	0	1
Arl6ip1	0	1.495276	0.929	0.494	0	1
Kif23	0	1.475955	0.757	0.135	0	1

Aspm	0	1.437566	0.609	0.074	0	1
Nusap1	0	1.425803	0.765	0.148	0	1
H2afx	0	1.397666	0.809	0.26	0	1
Ckap2l	0	1.371989	0.748	0.14	0	1
Ccnb2	0	1.32937	0.681	0.114	0	1
Sgol2	0	1.328264	0.62	0.089	0	1
Mki67	0	1.326855	0.911	0.319	0	1
Birc5	0	1.325651	0.829	0.192	0	1
Cdk1	0	1.319389	0.751	0.173	0	1
Top2a	0	1.276995	0.912	0.357	0	1
Mis18bp1	0	1.255498	0.615	0.094	0	1
Cdca3	0	1.254135	0.723	0.148	0	1
Cdca8	0	1.234761	0.779	0.189	0	1
Kif20b	0	1.227716	0.633	0.126	0	1
Arhgap11a	0	1.217435	0.612	0.106	0	1
Smc4	0	1.206928	0.966	0.508	0	1
Hmgb2	0	1.199921	0.912	0.325	0	1
Knstrn	0	1.199784	0.652	0.117	0	1
Ccna2	0	1.189292	0.707	0.15	0	1
Incenp	0	1.175681	0.757	0.205	0	1
Spc25	0	1.172452	0.704	0.155	0	1
RP23-45G1	0	1.168539	0.815	0.255	0	1
Casc5	0	1.131691	0.621	0.122	0	1
Kif11	0	1.125204	0.653	0.14	0	1
Fam64a	0	1.09682	0.509	0.06	0	1
Tacc3	0	1.082896	0.653	0.141	0	1
Dlgap5	0	1.038165	0.467	0.06	0	1
Kif2c	0	1.028248	0.461	0.055	0	1
Tubb4b	0	1.0238	0.653	0.18	0	1
Ckap2	0	1.02286	0.55	0.107	0	1
Kif22	0	1.017928	0.552	0.106	0	1
Plk1	0	1.016656	0.442	0.039	0	1
Smc2	0	0.986334	0.944	0.487	0	1
Racgap1	0	0.949777	0.668	0.195	0	1
Cks1b	0	0.947274	0.77	0.282	0	1
Rad21	0	0.93115	0.861	0.403	0	1
C330027C(	0	0.923902	0.47	0.084	0	1
Cdca2	0	0.918777	0.471	0.078	0	1
Ckap5	0	0.917468	0.653	0.209	0	1
Pbk	0	0.914523	0.622	0.164	0	1
Psrc1	0	0.90906	0.384	0.034	0	1
Sgol1	0	0.898585	0.444	0.081	0	1
Ncapg	0	0.896254	0.551	0.129	0	1
Trim59	0	0.895724	0.526	0.117	0	1
Tuba1c	0	0.885594	0.308	0.02	0	1
Nuf2	0	0.88466	0.47	0.092	0	1
Kif15	0	0.883688	0.532	0.129	0	1
Aurka	0	0.882723	0.414	0.052	0	1

Aurkb	0	0.870865	0.478	0.094	0	1
Dbf4	0	0.866254	0.463	0.099	0	1
Cep110	0	0.862971	0.486	0.116	0	1
Gas2l3	0	0.855655	0.338	0.032	0	1
Bora	0	0.849954	0.383	0.05	0	1
Anln	0	0.837519	0.396	0.061	0	1
Ect2	0	0.834308	0.394	0.054	0	1
Nucks1	0	0.817678	0.951	0.62	0	1
Bub1b	0	0.808942	0.394	0.06	0	1
Cep55	0	0.807949	0.349	0.042	0	1
Bub1	0	0.806972	0.439	0.086	0	1
Mxd3	0	0.802157	0.429	0.082	0	1
Spc24	0	0.802042	0.614	0.188	0	1
Cdc25c	0	0.795532	0.334	0.033	0	1
Kif20a	0	0.78636	0.363	0.046	0	1
Tmpo	0	0.779724	0.835	0.396	0	1
Rangap1	0	0.770757	0.616	0.203	0	1
Anp32e	0	0.763169	0.931	0.567	0	1
Sapcd2	0	0.757124	0.343	0.041	0	1
H2afv	0	0.744186	0.967	0.692	0	1
Cks2	0	0.736732	0.325	0.039	0	1
Pif1	0	0.733754	0.28	0.019	0	1
2700094K1	0	0.656612	0.914	0.57	0	1
2810417H1	0	0.500493	0.78	0.335	0	1
Ndc80	0	0.765436	0.397	0.073	0	1
Hjurp	0	0.791773	0.781	0.383	0	1
Troap	0	0.659149	0.269	0.023	0	1
Ccdc34	0	0.710988	0.78	0.361	0	1
Cenpl	0	0.683234	0.312	0.041	2.2E-306	1
Ckb	4.7E-305	-0.80503	0.747	0.817	7.8E-301	1
Nek2	4.4E-304	0.671642	0.272	0.027	7.3E-300	1
Kif4	1E-301	0.741026	0.375	0.069	1.7E-297	1
Ska2	2.3E-295	0.793512	0.526	0.157	3.8E-291	1
Esco2	1.2E-293	0.765743	0.51	0.149	2E-289	1
Ska1	2.8E-287	0.717063	0.334	0.054	4.7E-283	1
Pttg1	2.9E-286	0.953504	0.417	0.099	4.8E-282	1
Nde1	2.5E-281	0.732592	0.386	0.08	4.2E-277	1
Kif18a	3E-278	0.656805	0.288	0.038	5E-274	1
Mns1	7.3E-276	0.748196	0.512	0.158	1.2E-271	1
Neurod1	8.7E-273	-0.97257	0.573	0.526	1.4E-268	1
G2e3	2E-270	0.667291	0.396	0.092	3.4E-266	1
Bub3	2.7E-270	0.769198	0.646	0.273	4.5E-266	1
Spdl1	2.4E-267	0.67627	0.301	0.046	4E-263	1
Arhgef39	8.3E-267	0.524374	0.192	0.01	1.4E-262	1
D17H6S56I	7.3E-265	0.693012	0.39	0.089	1.2E-260	1
Cep89	1.1E-262	0.772978	0.315	0.052	1.8E-258	1
Mad2l1	1.7E-262	0.690544	0.382	0.085	2.8E-258	1
Cdkn2d	4.8E-255	0.683717	0.43	0.117	8E-251	1

Lmnb1	1.1E-251	0.66537	0.621	0.253	1.9E-247	1
Ttk	8.8E-251	0.672544	0.316	0.057	1.5E-246	1
Calm2	2.1E-247	0.441946	0.979	0.859	3.6E-243	1
Fbxo5	1.4E-244	0.698231	0.43	0.12	2.3E-240	1
Tuba1b	2.2E-244	0.597543	0.797	0.435	3.6E-240	1
Hirip3	1.8E-235	0.586835	0.702	0.336	2.9E-231	1
Cdc25b	9.7E-234	0.597593	0.279	0.047	1.6E-229	1
Ncapd2	1.6E-231	0.56485	0.452	0.146	2.6E-227	1
Spag5	8.2E-231	0.563864	0.281	0.049	1.4E-226	1
Hsp90b1	4.3E-230	0.56527	0.893	0.662	7.2E-226	1
Cdkn3	7.6E-230	0.537214	0.201	0.018	1.3E-225	1
Ccdc18	1.5E-228	0.695887	0.349	0.081	2.4E-224	1
Fzr1	2.5E-223	0.586777	0.376	0.101	4.2E-219	1
Banf1	7.4E-219	0.498878	0.917	0.661	1.2E-214	1
Brd8	2.8E-217	0.569725	0.699	0.347	4.7E-213	1
2700099C1	2.9E-217	0.597842	0.285	0.054	4.8E-213	1
Melk	1.1E-215	0.586041	0.304	0.063	1.9E-211	1
Cenpc1	1.8E-210	0.611212	0.4	0.119	3E-206	1
Ccng2	3.8E-207	0.509311	0.469	0.174	6.4E-203	1
Hdgf	5.2E-207	0.540196	0.814	0.49	8.7E-203	1
Hn1	1.8E-206	0.539666	0.82	0.501	3.1E-202	1
Miip	1.9E-206	0.493973	0.239	0.04	3.1E-202	1
Hmgn2	5.1E-205	0.557919	0.533	0.215	8.4E-201	1
Prr11	8.6E-203	0.524612	0.228	0.033	1.4E-198	1
Cenpp	3.7E-200	0.544841	0.327	0.083	6.2E-196	1
Ncaph	2E-198	0.572367	0.339	0.089	3.3E-194	1
Ezh2	3.5E-198	0.494732	0.869	0.572	5.9E-194	1
Rtn1	4.8E-197	-0.57543	0.724	0.685	7.9E-193	1
Nudcd2	2.6E-195	0.554086	0.485	0.185	4.3E-191	1
Ankle1	6.3E-195	0.49199	0.213	0.029	1E-190	1
Kifc1	4E-194	0.490176	0.216	0.031	6.7E-190	1
Pnrc2	6.7E-193	0.539658	0.461	0.17	1.1E-188	1
Nrep	1.8E-191	-0.65915	0.534	0.574	3E-187	1
Cdk5rap2	2.1E-191	0.509896	0.421	0.148	3.6E-187	1
Ska3	3E-191	0.504642	0.241	0.043	5E-187	1
Cdkn2c	1.2E-188	0.523846	0.365	0.111	1.9E-184	1
Usp1	3.5E-187	0.481257	0.615	0.298	5.8E-183	1
Fam83d	4.3E-187	0.367382	0.13	0.006	7.2E-183	1
Ran	1.1E-185	0.491867	0.698	0.372	1.8E-181	1
Kif14	3.4E-185	0.465787	0.183	0.021	5.7E-181	1
Cenpm	4.2E-185	0.535081	0.391	0.128	6.9E-181	1
Gm10075	6.6E-185	0.478256	0.791	0.474	1.1E-180	1
Hmgn5	1.4E-182	0.46901	0.705	0.383	2.3E-178	1
Hyls1	3.5E-182	0.464454	0.211	0.034	5.8E-178	1
Dek	1.7E-181	0.32442	0.92	0.66	2.8E-177	1
Tubb5	5.2E-181	0.334056	0.985	0.904	8.7E-177	1
Lmnb2	8.7E-180	0.51018	0.381	0.126	1.4E-175	1
Igfbpl1	7.2E-178	-0.59005	0.614	0.652	1.2E-173	1

H1fx	7.7E-178	0.553009	0.501	0.207	1.3E-173	1
Espl1	1.6E-176	0.478859	0.227	0.041	2.7E-172	1
Apoe	8E-175	-1.68944	0.287	0.243	1.3E-170	1
Gpm6a	2.1E-173	-0.74136	0.352	0.417	3.5E-169	1
H2afz	4.9E-173	0.507016	0.478	0.195	8.2E-169	1
Rbfox3	1.9E-172	-0.61782	0.453	0.5	3.2E-168	1
Nasp	2E-172	0.309102	0.884	0.604	3.4E-168	1
Sap30	3.1E-172	0.53338	0.348	0.107	5.2E-168	1
Cenpq	4.8E-172	0.541626	0.381	0.128	8E-168	1
Hnrnpa2b1	2.6E-170	0.278302	0.99	0.925	4.4E-166	1
Shcbp1	6.6E-167	0.514049	0.246	0.052	1.1E-162	1
Ube2t	4.6E-164	0.516238	0.245	0.052	7.7E-160	1
Odf2	4.7E-163	0.412526	0.376	0.141	7.9E-159	1
Myod1	4.5E-162	0.602438	0.347	0.111	7.5E-158	1
Cep70	5E-161	0.439341	0.238	0.057	8.4E-157	1
Anp32b	4.1E-158	0.340421	0.838	0.554	6.8E-154	1
Cenph	1.5E-157	0.352342	0.395	0.164	2.6E-153	1
Ddx39	4.6E-157	0.429004	0.399	0.156	7.7E-153	1
Cst3	2.4E-156	-0.31937	0.688	0.513	4E-152	1
Rtkn2	1.5E-155	0.427673	0.174	0.024	2.5E-151	1
Nup37	3.8E-155	0.491625	0.266	0.067	6.3E-151	1
Cntn2	3.2E-153	-1.13207	0.113	0.285	5.4E-149	1
Diap3	1.2E-152	0.501646	0.286	0.08	2.1E-148	1
Pdzrn4	1.6E-152	0.408913	0.334	0.118	2.7E-148	1
Cklf	1.7E-152	0.447276	0.368	0.135	2.8E-148	1
Hmgb3	6.3E-152	0.388098	0.543	0.271	1E-147	1
Gtse1	6.3E-151	0.377562	0.154	0.019	1.1E-146	1
Celf4	2.5E-150	-0.77535	0.275	0.327	4.2E-146	1
Stmn2	8.9E-150	-0.77795	0.49	0.562	1.5E-145	1
Lsm6	1.4E-149	0.43471	0.622	0.335	2.3E-145	1
Plk4	4.3E-149	0.465083	0.302	0.093	7.1E-145	1
Hmgb1	8.6E-149	0.363137	0.598	0.323	1.4E-144	1
Sept3	9E-149	-0.67119	0.332	0.401	1.5E-144	1
Pqlc1	1E-148	0.37323	0.528	0.263	1.7E-144	1
Pmf1	3.2E-148	0.463151	0.337	0.115	5.4E-144	1
Fkbp2	1.6E-147	0.380087	0.563	0.291	2.6E-143	1
Ccnf	2E-146	0.378971	0.163	0.024	3.3E-142	1
Pdgfa	4.9E-146	0.328432	0.465	0.222	8.2E-142	1
Rnaseh2c	5.4E-146	0.41291	0.627	0.345	9E-142	1
Tubb3	6.2E-145	-0.58558	0.484	0.472	1E-140	1
Gap43	1E-144	-0.544	0.613	0.597	1.7E-140	1
Cenpw	2.4E-144	0.428996	0.307	0.101	4.1E-140	1
Gria2	9.1E-144	-0.59009	0.482	0.526	1.5E-139	1
Psat1	1.2E-140	0.381696	0.606	0.332	2E-136	1
Ccnd1	3.3E-140	0.371198	0.765	0.486	5.6E-136	1
Ptn	5.6E-140	-0.25019	0.587	0.42	9.3E-136	1
Dtymk	1.9E-139	0.296584	0.669	0.405	3.2E-135	1
Vbp1	3.5E-139	0.355303	0.546	0.286	5.8E-135	1

Miat	7.5E-139	-0.66806	0.462	0.523	1.2E-134	1
Elavl3	5.5E-138	-0.55092	0.426	0.479	9.1E-134	1
Klf6	2.6E-137	0.418929	0.331	0.121	4.4E-133	1
Rrm1	4.8E-137	0.328319	0.466	0.23	8E-133	1
Barhl1	5.6E-137	-0.41801	0.526	0.521	9.4E-133	1
Rad51ap1	6.4E-137	0.361858	0.349	0.14	1.1E-132	1
Reep4	8E-137	0.325603	0.179	0.039	1.3E-132	1
Cenpk	6.4E-136	0.436851	0.326	0.117	1.1E-131	1
Ccdc77	9.4E-136	0.411107	0.217	0.053	1.6E-131	1
Gas1	1.8E-135	0.441103	0.344	0.129	3E-131	1
Zc3h7a	6.2E-135	0.350447	0.405	0.182	1E-130	1
Map2	9.7E-135	-0.45764	0.491	0.491	1.6E-130	1
Ctcf	1.1E-134	0.348175	0.734	0.461	1.9E-130	1
Syt13	2.9E-134	0.400816	0.296	0.102	4.9E-130	1
Ina	9.9E-134	-0.56319	0.401	0.45	1.6E-129	1
Hmgn1	2.1E-133	0.365036	0.842	0.589	3.6E-129	1
Ubald2	2.4E-132	0.376076	0.253	0.077	4E-128	1
App	2.1E-131	-0.41389	0.569	0.568	3.5E-127	1
Itm2b	4.5E-129	-0.3153	0.711	0.632	7.4E-125	1
Fam110a	3.1E-128	0.271155	0.181	0.049	5.1E-124	1
Gpsm2	1.1E-127	0.372851	0.177	0.036	1.8E-123	1
Cenpt	2.5E-127	0.316901	0.225	0.069	4.2E-123	1
Pcna	2.9E-127	-0.37741	0.376	0.355	4.9E-123	1
Kif1b	3.9E-127	-0.42275	0.518	0.521	6.6E-123	1
Cpe	1.2E-126	-0.36786	0.471	0.453	2E-122	1
Cit	2E-126	0.398064	0.198	0.046	3.3E-122	1
Tubb2b	2.1E-126	-0.33637	0.511	0.457	3.5E-122	1
Ptprs	3.7E-125	-0.58412	0.369	0.428	6.2E-121	1
Cep57	7.4E-125	0.359701	0.46	0.228	1.2E-120	1
Smtn	8.5E-125	0.306344	0.132	0.019	1.4E-120	1
Rbmx	1.3E-124	0.303805	0.523	0.284	2.2E-120	1
Lig1	7.1E-124	-0.34062	0.388	0.342	1.2E-119	1
Dcx	1.2E-123	-0.46187	0.369	0.376	2E-119	1
Lmo4	1.9E-123	0.403292	0.558	0.303	3.2E-119	1
Terf1	3E-123	0.388407	0.214	0.057	5E-119	1
Gsg1l	4.3E-123	0.281165	0.443	0.227	7.3E-119	1
Akirin2	2.5E-122	0.362888	0.382	0.17	4.2E-118	1
Depdc1b	3.1E-122	0.311801	0.123	0.015	5.2E-118	1
Lbr	5.1E-122	0.317342	0.375	0.17	8.5E-118	1
Nhlh2	1.6E-121	-0.44322	0.436	0.421	2.6E-117	1
Arhgap19	2.2E-121	0.326295	0.135	0.019	3.7E-117	1
Cenpv	4E-121	0.268084	0.526	0.299	6.7E-117	1
Ing1	1.5E-120	0.300959	0.352	0.158	2.5E-116	1
Cdca4	7.7E-120	0.401929	0.267	0.088	1.3E-115	1
Bin1	2.2E-119	-0.52492	0.38	0.436	3.7E-115	1
Tgif1	2.4E-119	0.316396	0.238	0.079	4E-115	1
Rrm2	5.1E-119	0.270693	0.373	0.179	8.5E-115	1
Cdca5	7.2E-119	0.382785	0.211	0.055	1.2E-114	1

Ralgps2	1.6E-118	0.32973	0.436	0.217	2.7E-114	1
Lsm4	7.7E-118	0.316134	0.711	0.455	1.3E-113	1
Tdp1	2.9E-117	0.341442	0.246	0.082	4.8E-113	1
Ppp3ca	5E-117	-0.44879	0.37	0.379	8.4E-113	1
Ank3	3.3E-116	-0.38952	0.439	0.417	5.4E-112	1
Chd7	3.6E-116	-0.32601	0.685	0.641	6E-112	1
Sec11c	3.6E-116	0.307803	0.39	0.187	6E-112	1
Basp1	1.1E-115	-0.33652	0.744	0.698	1.8E-111	1
Cenpj	1.6E-115	0.413727	0.306	0.117	2.7E-111	1
Frmd4b	8.3E-115	0.307473	0.274	0.106	1.4E-110	1
Lsm3	1.8E-114	0.281111	0.56	0.329	2.9E-110	1
Ppp2r5c	9.4E-114	0.285537	0.394	0.194	1.6E-109	1
Cenpn	6E-113	0.380645	0.196	0.05	1E-108	1
Pkp4	7.3E-113	0.35155	0.244	0.081	1.2E-108	1
Fam216a	3.4E-112	0.323768	0.337	0.149	5.7E-108	1
Vps36	7.7E-112	0.320013	0.5	0.272	1.3E-107	1
Kif18b	1.9E-111	0.303975	0.127	0.018	3.2E-107	1
Exosc8	2.2E-111	0.321058	0.386	0.184	3.6E-107	1
Clic4	4.2E-111	0.260033	0.396	0.202	7E-107	1
Ncaph2	4.9E-111	0.324136	0.339	0.149	8.2E-107	1
Dnph1	1.3E-110	0.318406	0.269	0.102	2.1E-106	1
Utp3	1.9E-110	0.313796	0.512	0.285	3.2E-106	1
Neil3	3.1E-110	0.372972	0.217	0.063	5.2E-106	1
Prdx1	1E-109	0.254192	0.697	0.459	1.7E-105	1
Ctnnb1	1.3E-109	0.27703	0.59	0.356	2.2E-105	1
Stmn1	1.3E-109	0.260692	0.466	0.257	2.2E-105	1
Celf2	4.1E-109	-0.30342	0.694	0.629	6.9E-105	1
mt-Rnr2	4.3E-109	-0.34002	0.996	0.997	7.1E-105	1
Bok	6.6E-109	0.259357	0.424	0.225	1.1E-104	1
Efs	7.4E-109	0.295817	0.266	0.105	1.2E-104	1
Zfp704	9.4E-109	0.274365	0.436	0.231	1.6E-104	1
Ccdc41	1E-108	0.323704	0.438	0.226	1.7E-104	1
Sae1	1.5E-108	0.31333	0.469	0.251	2.5E-104	1
Ubb	1.7E-108	0.305899	0.754	0.51	2.9E-104	1
Smc1a	2.2E-108	0.35448	0.828	0.601	3.6E-104	1
Chek2	2.9E-108	0.297446	0.154	0.034	4.8E-104	1
Ypel3	4E-108	-0.29785	0.461	0.431	6.7E-104	1
Suv39h2	3.3E-107	0.341825	0.281	0.109	5.6E-103	1
Wapal	1.3E-106	0.261632	0.446	0.244	2.2E-102	1
Ctsd	1.4E-106	-0.76728	0.19	0.172	2.3E-102	1
Ctsb	3.1E-106	-0.57635	0.279	0.259	5.2E-102	1
Cmc2	6.5E-106	0.366298	0.267	0.097	1.1E-101	1
Rfc4	1.2E-105	0.270696	0.394	0.201	2E-101	1
Stmn4	1.4E-105	-0.56964	0.317	0.344	2.4E-101	1
Nrxn1	1.6E-105	-0.518	0.307	0.319	2.7E-101	1
Mrpl51	5.4E-105	0.300458	0.326	0.146	9.1E-101	1
Pcf11	6.4E-105	0.318942	0.34	0.155	1.1E-100	1
Tubb2a	2.6E-104	-0.61418	0.191	0.252	4.3E-100	1

Vrk1	3.6E-104	0.325878	0.3	0.125	6E-100	1
Mis18a	4.9E-104	0.347885	0.251	0.089	8.1E-100	1
Rnaseh2b	5.1E-104	0.258142	0.402	0.212	8.5E-100	1
Cplx2	1E-103	0.264842	0.701	0.465	1.7E-99	1
Tmem138	2E-103	0.251631	0.161	0.044	3.4E-99	1
Uncx	4.1E-103	-0.40489	0.311	0.319	6.8E-99	1
Dynll1	7.7E-103	0.321434	0.794	0.565	1.28E-98	1
Apc	1E-102	-0.33346	0.462	0.427	1.7E-98	1
Trip13	1.1E-102	0.362158	0.209	0.062	1.79E-98	1
Rhno1	1.5E-102	0.285415	0.279	0.117	2.5E-98	1
Sephs1	6.7E-102	0.25933	0.27	0.115	1.12E-97	1
Smco4	1.3E-101	0.301693	0.314	0.139	2.21E-97	1
Mapt	1.7E-101	-0.44818	0.217	0.216	2.88E-97	1
Nfix	5.8E-101	-0.26132	0.719	0.666	9.75E-97	1
Cdk5r1	7.6E-101	-0.43411	0.322	0.335	1.27E-96	1
Hist1h2ak	8E-101	0.434666	0.299	0.12	1.34E-96	1
Kif5c	1.1E-100	-0.45233	0.375	0.401	1.82E-96	1
Eid1	4.9E-100	-0.31136	0.52	0.5	8.18E-96	1
Gnao1	1.1E-99	-0.35714	0.296	0.285	1.76E-95	1
Med30	1.5E-99	0.32304	0.371	0.179	2.47E-95	1
Rdm1	3.1E-99	0.33691	0.176	0.046	5.15E-95	1
Lgals1	5.7E-99	0.355736	0.406	0.202	9.53E-95	1
Ddx11	6.2E-99	0.303387	0.156	0.038	1.03E-94	1
Tmed9	8.1E-99	-0.27425	0.458	0.429	1.35E-94	1
Stil	4.22E-98	0.294112	0.154	0.037	7.03E-94	1
Zwilch	5.94E-98	0.33558	0.215	0.07	9.91E-94	1
Taf5	1.22E-97	0.301317	0.2	0.065	2.03E-93	1
H1f0	1.33E-97	0.255976	0.808	0.588	2.23E-93	1
Hpca	1.48E-97	0.450389	0.468	0.249	2.47E-93	1
Serinc1	1.52E-97	-0.2641	0.476	0.43	2.53E-93	1
Ccdc61	8.03E-97	0.258105	0.141	0.033	1.34E-92	1
Zic4	2.32E-96	-0.26455	0.456	0.412	3.87E-92	1
Asap1	5.29E-96	0.268662	0.328	0.158	8.82E-92	1
2810442I2	6.3E-96	0.334169	0.164	0.041	1.05E-91	1
Dpysl3	6.6E-96	-0.68197	0.144	0.23	1.1E-91	1
Ccp110	8.26E-96	0.252956	0.412	0.224	1.38E-91	1
Hp1bp3	1.02E-95	0.291796	0.778	0.551	1.69E-91	1
Pcnt	2.48E-95	0.31045	0.326	0.152	4.14E-91	1
Chgb	3.01E-95	-0.73654	0.153	0.253	5.02E-91	1
Mcm6	3.5E-95	-0.34849	0.276	0.276	5.84E-91	1
Elavl4	4.36E-95	-0.37268	0.343	0.334	7.27E-91	1
Thra	4.86E-95	-0.61295	0.155	0.239	8.1E-91	1
Hk2	5.93E-95	0.29695	0.321	0.151	9.9E-91	1
Tmsb4x	7.03E-95	-0.3183	0.975	0.944	1.17E-90	1
Mdc1	3.64E-94	0.331181	0.214	0.072	6.07E-90	1
D030056L2	4.77E-94	0.3172	0.285	0.121	7.96E-90	1
5830418K0	7.55E-94	0.295725	0.314	0.146	1.26E-89	1
Kmt2e	9.94E-94	-0.26895	0.608	0.562	1.66E-89	1

Ppp1r14c	2.22E-93	-0.48767	0.238	0.265	3.7E-89	1
CRE_RECON	2.44E-93	-0.4676	0.795	0.764	4.07E-89	1
Aldoa	2.77E-93	-0.58077	0.169	0.27	4.63E-89	1
Gm1673	1.12E-92	-0.31982	0.478	0.472	1.86E-88	1
Sox4	1.29E-92	-0.30139	0.626	0.576	2.16E-88	1
Nudt4	1.45E-92	0.261246	0.373	0.195	2.42E-88	1
Zcwpw1	1.5E-92	0.330886	0.231	0.084	2.5E-88	1
Smarcc2	4E-92	-0.30122	0.378	0.361	6.66E-88	1
Phf17	7.8E-92	0.321729	0.214	0.074	1.3E-87	1
Nin	1.23E-91	0.279815	0.283	0.126	2.05E-87	1
Gng3	1.26E-91	-0.5107	0.277	0.322	2.11E-87	1
Zic1	1.34E-91	-0.25024	0.837	0.776	2.24E-87	1
Iqgap3	1.89E-91	0.280812	0.124	0.024	3.16E-87	1
Aplp2	1.97E-91	-0.3041	0.34	0.32	3.29E-87	1
Gsg2	4.03E-91	0.321375	0.149	0.035	6.73E-87	1
Cntln	4.32E-91	0.317407	0.322	0.15	7.2E-87	1
Nsl1	6.35E-91	0.336566	0.165	0.042	1.06E-86	1
Foxm1	1.23E-90	0.263143	0.135	0.031	2.06E-86	1
D4Wsu53e	1.25E-90	-0.32589	0.457	0.445	2.09E-86	1
Calm3	2.38E-90	0.250239	0.752	0.532	3.98E-86	1
Lhx1	3.38E-90	-0.35759	0.503	0.496	5.65E-86	1
Nsg1	4.43E-90	-0.47085	0.238	0.294	7.39E-86	1
Nmral1	7.16E-90	0.256535	0.332	0.166	1.19E-85	1
Slc1a2	1.09E-89	-0.27014	0.327	0.289	1.82E-85	1
Dclk1	1.33E-89	0.260048	0.598	0.385	2.21E-85	1
Ankrd12	1.47E-88	-0.62428	0.208	0.284	2.44E-84	1
Pde1c	1.03E-87	-0.26879	0.436	0.379	1.72E-83	1
Ift80	6.37E-87	0.256346	0.165	0.05	1.06E-82	1
Cacna2d1	2.07E-86	-0.31184	0.393	0.372	3.45E-82	1
Rbm5	5.5E-86	-0.2521	0.434	0.396	9.18E-82	1
Snap25	2.03E-85	-0.36543	0.283	0.282	3.39E-81	1
E2f5	5.12E-85	0.2524	0.17	0.055	8.55E-81	1
Npdc1	6.37E-85	-0.35979	0.247	0.263	1.06E-80	1
Atp6v0b	2.04E-84	-0.3661	0.251	0.269	3.4E-80	1
Clmp	4.06E-84	-0.38727	0.236	0.243	6.77E-80	1
Nhlh1	9.61E-84	-0.56882	0.159	0.232	1.6E-79	1
Gen1	1.36E-83	0.270219	0.123	0.026	2.27E-79	1
Rnmt	2.19E-83	-0.26148	0.374	0.344	3.65E-79	1
Cntrob	2.77E-83	0.295236	0.159	0.045	4.62E-79	1
Eri2	4.66E-83	0.265552	0.14	0.037	7.77E-79	1
1500012F0	4.9E-83	-0.30166	0.41	0.396	8.17E-79	1
Mtss1	1.39E-82	-0.37609	0.271	0.269	2.31E-78	1
Tex30	1.63E-82	0.258739	0.26	0.117	2.72E-78	1
Sema6a	2.25E-82	-0.46271	0.172	0.206	3.75E-78	1
Cdc27	3.66E-82	0.253355	0.226	0.095	6.1E-78	1
Ank2	4.32E-82	-0.49684	0.209	0.244	7.2E-78	1
Uchl1	1.04E-81	-0.32224	0.339	0.333	1.74E-77	1
Sptbn1	1.37E-81	-0.26918	0.388	0.348	2.29E-77	1

6330403K(	5.65E-81	-0.48275	0.187	0.25	9.42E-77	1
Ttc3	6.18E-80	-0.28533	0.853	0.825	1.03E-75	1
Slc22a17	7.14E-80	-0.28326	0.26	0.249	1.19E-75	1
BC005764	7.3E-80	-0.63278	0.098	0.198	1.22E-75	1
Csrp2	1.14E-79	0.277419	0.291	0.138	1.9E-75	1
Eme1	1.5E-79	0.263218	0.136	0.035	2.5E-75	1
1500016L(	2.06E-79	-0.34947	0.378	0.383	3.43E-75	1
Zbtb18	3.18E-79	-0.28734	0.279	0.264	5.3E-75	1
Dner	8E-79	-0.35364	0.213	0.215	1.34E-74	1
Tbata	2.26E-78	0.253775	0.508	0.315	3.77E-74	1
Mum1l1	2.32E-78	0.301021	0.208	0.077	3.87E-74	1
Mis12	1.42E-77	0.254333	0.206	0.082	2.37E-73	1
Ctsl	2.27E-77	-0.54798	0.199	0.268	3.78E-73	1
Pih1d1	2.45E-77	0.263962	0.303	0.151	4.09E-73	1
Cenpi	2.92E-77	0.25062	0.117	0.026	4.86E-73	1
Map1b	7.92E-77	-0.27531	0.645	0.579	1.32E-72	1
Cep135	9.47E-77	0.252948	0.197	0.077	1.58E-72	1
Mllt11	1.93E-76	-0.50601	0.165	0.226	3.22E-72	1
Map1lc3b	1.52E-75	-0.27479	0.339	0.33	2.54E-71	1
Tk1	2.33E-75	0.298159	0.255	0.113	3.88E-71	1
Rundc3a	2.92E-75	-0.34387	0.236	0.25	4.87E-71	1
Trpc4ap	3.01E-75	-0.42391	0.179	0.201	5.02E-71	1
Nbea	2.61E-74	-0.29856	0.201	0.189	4.35E-70	1
Tagln3	2.67E-74	-0.31396	0.303	0.293	4.45E-70	1
Tbc1d31	2.93E-74	0.261079	0.153	0.047	4.89E-70	1
Oip5	6.19E-74	0.262816	0.123	0.029	1.03E-69	1
Rcor2	1.07E-72	-0.42299	0.181	0.231	1.78E-68	1
Clcn4-2	1E-71	-0.38236	0.225	0.255	1.67E-67	1
Traip	1.28E-71	0.251265	0.128	0.034	2.14E-67	1
2810006K2	1.61E-71	0.254668	0.237	0.106	2.68E-67	1
Prkcb	1.94E-71	-0.36044	0.244	0.257	3.23E-67	1
Itsn1	2.03E-71	-0.38031	0.224	0.237	3.39E-67	1
Sept4	9.66E-71	-0.45429	0.198	0.23	1.61E-66	1
Nktr	4.81E-70	-0.30139	0.382	0.379	8.02E-66	1
Atox1	6.49E-70	-0.29306	0.29	0.293	1.08E-65	1
Pfn2	1.06E-69	-0.28837	0.242	0.241	1.77E-65	1
Mycbp2	4.19E-69	-0.27914	0.361	0.342	6.99E-65	1
Ldhb	1.13E-68	-0.33733	0.259	0.283	1.89E-64	1
Celsr2	2.12E-68	-0.37021	0.212	0.241	3.54E-64	1
Lap3	7.89E-68	0.294613	0.425	0.254	1.32E-63	1
Tmem57	1.54E-67	-0.29701	0.29	0.291	2.57E-63	1
Mapk8ip1	1.56E-67	-0.37526	0.2	0.231	2.6E-63	1
Podxl2	2.08E-67	-0.3326	0.22	0.228	3.47E-63	1
Rab6b	3.62E-67	-0.40295	0.17	0.207	6.03E-63	1
Fabp7	4.65E-67	-0.92439	0.124	0.122	7.76E-63	1
Tnik	7.24E-67	-0.5154	0.095	0.153	1.21E-62	1
Aplp1	9.73E-67	-0.53154	0.083	0.161	1.62E-62	1
Cadm3	1.4E-66	-0.51658	0.081	0.149	2.34E-62	1

Pdzrn3	2.33E-66	-0.63992	0.066	0.159	3.89E-62	1
Itm2c	2.53E-66	-0.35602	0.193	0.214	4.22E-62	1
Igsf8	3.5E-65	-0.38256	0.235	0.274	5.85E-61	1
Cnrip1	4.91E-65	-0.26972	0.202	0.197	8.18E-61	1
C1ql1	1.12E-64	-0.26704	0.286	0.277	1.87E-60	1
Gnl3	1.63E-64	-0.28943	0.294	0.296	2.72E-60	1
Hey1	7.87E-64	-0.3556	0.259	0.274	1.31E-59	1
Adam10	8.07E-64	-0.25081	0.211	0.202	1.35E-59	1
Ntm	9.17E-64	-0.26271	0.158	0.143	1.53E-59	1
Stxbp1	1.88E-63	-0.31589	0.156	0.163	3.13E-59	1
Phf20l1	4.59E-63	-0.29695	0.344	0.339	7.66E-59	1
Dnajc5	1.66E-62	-0.27454	0.251	0.247	2.76E-58	1
Gdi1	4.99E-62	-0.31738	0.214	0.232	8.32E-58	1
A330076H(	6.11E-62	-0.40426	0.13	0.165	1.02E-57	1
Psap	9.16E-62	-0.3344	0.173	0.185	1.53E-57	1
Sowaha	2.56E-61	0.32089	0.284	0.145	4.27E-57	1
Klf7	5.6E-61	-0.32164	0.252	0.263	9.33E-57	1
Slc1a3	3.77E-60	-0.31093	0.188	0.147	6.28E-56	1
Ier2	6.21E-60	-0.36952	0.377	0.382	1.04E-55	1
Chrna3	1.14E-59	-0.52045	0.049	0.128	1.9E-55	1
Tmem66	1.59E-59	-0.25151	0.199	0.199	2.65E-55	1
4631405J1	1.78E-58	0.263081	0.123	0.036	2.97E-54	1
Meg3	3.13E-58	-0.85106	0.034	0.104	5.22E-54	1
Kdm5b	6.65E-58	-0.33358	0.167	0.187	1.11E-53	1
Mxd4	6.89E-58	-0.36985	0.163	0.203	1.15E-53	1
Hcfc1r1	1.22E-57	-0.35299	0.163	0.197	2.03E-53	1
Btg2	2.98E-57	-0.27319	0.219	0.206	4.97E-53	1
Apbb1	3.26E-57	-0.42653	0.124	0.176	5.45E-53	1
Myt1	3.82E-57	-0.48608	0.103	0.159	6.36E-53	1
Sh3gl2	4.51E-57	-0.37683	0.142	0.175	7.51E-53	1
2700089E2	5.88E-57	-0.33074	0.17	0.202	9.8E-53	1
Dlgap4	1.17E-55	-0.33824	0.161	0.191	1.96E-51	1
Abhd16a	2.72E-55	-0.31789	0.175	0.192	4.53E-51	1
Pygo1	2.1E-54	-0.2536	0.2	0.191	3.5E-50	1
Aprt	3.71E-54	-0.4398	0.075	0.15	6.19E-50	1
Hells	4.49E-54	-0.34154	0.197	0.215	7.49E-50	1
Clstn1	6.87E-54	-0.2997	0.195	0.203	1.15E-49	1
Prdm8	2.07E-53	-0.29643	0.121	0.127	3.45E-49	1
Reln	2.09E-53	-0.2703	0.186	0.179	3.48E-49	1
Arpp21	3.16E-53	-0.49916	0.064	0.119	5.28E-49	1
Rab3a	7.01E-53	-0.51453	0.078	0.16	1.17E-48	1
Dbn1	2.03E-52	-0.25281	0.136	0.14	3.39E-48	1
Kidins220	2.23E-52	-0.38866	0.106	0.145	3.71E-48	1
Nt5c	3.38E-52	-0.38405	0.137	0.19	5.64E-48	1
A930011O1	7.13E-52	-0.46745	0.067	0.121	1.19E-47	1
Nrn1	1.15E-51	-0.32175	0.209	0.223	1.91E-47	1
Wdr6	2.27E-51	-0.27906	0.13	0.146	3.78E-47	1
Srrm4	2.68E-51	-0.26004	0.195	0.195	4.47E-47	1

Ung	3.25E-51	-0.41226	0.022	0.102	5.42E-47	1
Pdrg1	4.15E-51	-0.30216	0.199	0.222	6.92E-47	1
Col9a3	5.41E-51	-0.26651	0.176	0.18	9.03E-47	1
Fam213b	9.4E-51	-0.37846	0.119	0.163	1.57E-46	1
Elmo1	2.15E-50	-0.4384	0.091	0.13	3.58E-46	1
Egr1	2.57E-50	-0.31002	0.406	0.381	4.29E-46	1
Fam21	3.22E-50	-0.26064	0.217	0.222	5.37E-46	1
Sh3bgrl3	5.32E-50	-0.28171	0.188	0.2	8.87E-46	1
Kif5a	5.49E-50	-0.41846	0.082	0.134	9.15E-46	1
Lgmn	2.98E-49	-0.27637	0.127	0.117	4.97E-45	1
Zmynd8	5.1E-49	-0.25561	0.21	0.213	8.51E-45	1
Plp1	5.43E-49	-0.78543	0.125	0.112	9.07E-45	1
Pea15a	1.73E-48	-0.33273	0.157	0.178	2.88E-44	1
Mbp	6.34E-47	-0.38539	0.174	0.164	1.06E-42	1
Glce	1E-46	-0.284	0.165	0.178	1.67E-42	1
Btbd17	1.73E-46	-0.25152	0.157	0.16	2.89E-42	1
Gramd1b	3.71E-46	-0.285	0.157	0.171	6.18E-42	1
Jhdm1d	9.39E-46	-0.33721	0.094	0.129	1.57E-41	1
Mcm2	1.27E-44	-0.32156	0.175	0.208	2.11E-40	1
Kif1a	1.58E-44	-0.27322	0.158	0.173	2.64E-40	1
Cacng4	3.33E-44	-0.38239	0.09	0.103	5.56E-40	1
Rnd2	1.77E-43	-0.32832	0.129	0.162	2.95E-39	1
Nrcam	1.78E-43	-0.25522	0.128	0.134	2.97E-39	1
Cadm4	3.37E-43	-0.29163	0.086	0.118	5.61E-39	1
Pkia	3.23E-42	-0.30092	0.112	0.137	5.39E-38	1
Ctsf	4.01E-42	-0.39906	0.04	0.106	6.69E-38	1
Gm17322	6.23E-42	-0.39096	0.09	0.137	1.04E-37	1
Gria4	1.61E-41	-0.31868	0.1	0.128	2.69E-37	1
Gabbr1	7.43E-41	-0.32126	0.11	0.145	1.24E-36	1
Cnpy1	1.6E-40	-0.27965	0.113	0.135	2.67E-36	1
Akap12	3.84E-40	-0.3304	0.088	0.114	6.4E-36	1
Cplx1	4.71E-40	-0.31843	0.112	0.141	7.86E-36	1
Ncan	1.69E-39	-0.28723	0.078	0.102	2.82E-35	1
Ppfia2	1.91E-39	-0.3285	0.089	0.115	3.19E-35	1
Tacc2	3.39E-39	-0.26044	0.132	0.142	5.65E-35	1
Slc17a6	7.26E-39	-0.30282	0.131	0.15	1.21E-34	1
Gamt	1.05E-38	-0.31209	0.139	0.173	1.75E-34	1
Chd3	3.83E-38	-0.38582	0.111	0.154	6.4E-34	1
Mt1	2.21E-37	-0.34304	0.177	0.166	3.69E-33	1
S100a16	6.18E-37	-0.42192	0.049	0.101	1.03E-32	1
Hpcal1	1.66E-36	-0.27125	0.091	0.109	2.76E-32	1
Grina	5.97E-36	-0.37967	0.056	0.117	9.96E-32	1
Malat1	8.89E-36	-0.28584	0.947	0.956	1.48E-31	1
Ppp1r1a	9.5E-36	-0.33944	0.068	0.116	1.58E-31	1
B3galt2	1.74E-35	-0.33764	0.069	0.101	2.9E-31	1
Sbk1	4.49E-35	-0.25203	0.104	0.12	7.49E-31	1
Nenf	5.63E-35	-0.26049	0.118	0.139	9.39E-31	1
Sh3bp5	6.05E-35	-0.30651	0.076	0.107	1.01E-30	1

Grik2	1.23E-34	-0.30208	0.078	0.105	2.06E-30	1
1500011B(	1.71E-34	-0.30908	0.073	0.111	2.86E-30	1
Cdt1	9.15E-34	-0.34131	0.073	0.132	1.53E-29	1
Plcb1	1.97E-33	-0.38156	0.101	0.146	3.29E-29	1
H2-D1	2.08E-33	-0.29456	0.102	0.134	3.47E-29	1
Pmm1	9.35E-31	-0.25803	0.103	0.131	1.56E-26	1
Fos	1.01E-30	-0.28467	0.312	0.303	1.69E-26	1
Nefm	1.66E-30	-0.25027	0.109	0.117	2.76E-26	1
Shd	2.07E-29	-0.25579	0.087	0.112	3.45E-25	1
Cacna1b	2.39E-29	-0.28359	0.071	0.101	3.98E-25	1
2900011O(	8.88E-29	-0.27211	0.081	0.106	1.48E-24	1
Xist	1.81E-26	-0.37979	0.246	0.307	3.01E-22	1
Map7d2	4.87E-26	-0.25337	0.082	0.103	8.12E-22	1
Hes1	0	1.261043	0.432	0.107	0	2
Tubb5	0	-0.73098	0.819	0.928	0	2
Stmn2	0	-1.61873	0.208	0.602	0	2
Neurod1	0	-1.86221	0.203	0.58	0	2
Tuba1a	0	-0.77284	0.852	0.94	0	2
Tubb3	3.6E-300	-1.38389	0.173	0.517	6.1E-296	2
Rpl13a	4.9E-264	0.431994	0.989	0.919	8.2E-260	2
Top2a	1.2E-254	-1.39239	0.18	0.463	2E-250	2
Rps5	1.4E-242	0.389971	0.988	0.949	2.3E-238	2
Rps14	1.4E-240	0.401249	0.989	0.944	2.3E-236	2
Rps9	2.8E-221	0.384842	0.984	0.92	4.7E-217	2
Gnb2l1	1.9E-200	0.427971	0.951	0.86	3.2E-196	2
Egr1	8.8E-199	0.811755	0.639	0.347	1.5E-194	2
Cntn2	4E-198	-1.3293	0.047	0.295	6.7E-194	2
Rpl32	4.7E-196	0.395211	0.963	0.878	7.8E-192	2
Gap43	5.6E-194	-0.92606	0.377	0.631	9.3E-190	2
Draxin	6.2E-192	0.62025	0.766	0.523	1E-187	2
2810417H:	1.7E-189	-1.06542	0.157	0.425	2.9E-185	2
Mki67	1.9E-189	-1.17627	0.166	0.427	3.2E-185	2
Smc4	7.3E-185	-0.90765	0.375	0.594	1.2E-180	2
Gas5	5E-181	0.502751	0.871	0.723	8.3E-177	2
Map1b	5.2E-179	-0.86413	0.368	0.619	8.7E-175	2
Birc5	2.4E-178	-1.01577	0.058	0.304	3.9E-174	2
Cdk1	2.2E-171	-0.98175	0.043	0.276	3.7E-167	2
Tpx2	5.8E-170	-1.07066	0.071	0.311	9.7E-166	2
Rpl8	2.2E-169	0.357531	0.963	0.887	3.7E-165	2
Rps3	4E-168	0.335029	0.976	0.914	6.7E-164	2
Hmgb2	2.1E-167	-0.92668	0.18	0.431	3.5E-163	2
Rplp2	2.2E-166	0.398021	0.928	0.827	3.7E-162	2
Fxyd6	4.7E-165	-0.79179	0.272	0.543	7.8E-161	2
Stmn4	1.9E-164	-1.04704	0.124	0.372	3.1E-160	2
Jun	3.4E-164	0.663199	0.807	0.632	5.7E-160	2
Ube2c	4.4E-164	-1.22928	0.082	0.296	7.4E-160	2
Rplp1	6.5E-162	0.358454	0.963	0.864	1.1E-157	2
Ddah2	5.4E-159	-0.65078	0.509	0.711	9E-155	2

H2afx	1.4E-157	-0.96975	0.141	0.357	2.3E-153	2
Rps19	4.2E-157	0.439092	0.877	0.735	7E-153	2
Miat	2.4E-156	-0.85559	0.281	0.549	4E-152	2
Rtn1	1.9E-155	-0.69137	0.554	0.71	3.2E-151	2
Rpl4	1.3E-154	0.349119	0.959	0.9	2.1E-150	2
Prc1	6E-154	-1.04417	0.068	0.284	1E-149	2
Nusap1	1.2E-153	-0.95595	0.041	0.254	2E-149	2
Cenpf	1.1E-149	-1.12617	0.167	0.39	1.9E-145	2
Cdca8	2.1E-149	-0.87636	0.066	0.293	3.5E-145	2
Spc25	5.2E-148	-0.88238	0.041	0.252	8.6E-144	2
Ckap2l	3.9E-145	-0.87491	0.038	0.243	6.6E-141	2
Ccna2	1.1E-142	-0.79664	0.042	0.247	1.9E-138	2
Rps26	1.6E-142	0.37437	0.935	0.834	2.6E-138	2
Ina	2.3E-142	-0.78905	0.235	0.474	3.8E-138	2
Pbk	5.7E-142	-0.82454	0.042	0.248	9.6E-138	2
Celf4	1.1E-141	-1.0116	0.125	0.348	1.9E-137	2
Rplp0	1.7E-139	0.356464	0.948	0.861	2.8E-135	2
Incenp	2.2E-139	-0.88908	0.079	0.303	3.7E-135	2
Tubb2b	7E-139	-0.72856	0.261	0.494	1.2E-134	2
Eef1a1	7.7E-139	0.360074	0.951	0.874	1.3E-134	2
Rpl22	4.9E-138	0.405326	0.891	0.773	8.1E-134	2
Arl6ip1	9.6E-138	-0.75532	0.401	0.571	1.6E-133	2
Gpm6a	3.9E-137	-0.82579	0.213	0.437	6.5E-133	2
Rps21	1.7E-135	0.377165	0.924	0.82	2.8E-131	2
Sept3	7E-135	-0.80817	0.201	0.42	1.2E-130	2
Rps20	9.8E-130	0.397116	0.893	0.761	1.6E-125	2
Esco2	1.8E-129	-0.79762	0.032	0.218	2.9E-125	2
Cbfa2t3	1E-128	0.597292	0.568	0.378	1.7E-124	2
Barhl1	7.4E-127	0.537521	0.664	0.501	1.2E-122	2
Nrxn1	1.1E-126	-0.88224	0.132	0.345	1.8E-122	2
Cenpa	1.4E-125	-0.97595	0.087	0.275	2.3E-121	2
Sparcl1	6.1E-123	0.542981	0.385	0.181	1E-118	2
Vim	2E-122	0.645278	0.491	0.275	3.3E-118	2
CRE_RECON	6.2E-120	0.577014	0.869	0.753	1E-115	2
Apoe	7.3E-119	-1.25136	0.293	0.242	1.2E-114	2
Spc24	2.2E-118	-0.72063	0.068	0.267	3.7E-114	2
Nhlh2	2.3E-117	-0.74747	0.226	0.451	3.9E-113	2
Cdca3	1.5E-116	-0.77718	0.059	0.245	2.5E-112	2
Kif11	3.2E-116	-0.74175	0.048	0.228	5.3E-112	2
Cdc20	2.3E-114	-0.86208	0.049	0.217	3.9E-110	2
Kif23	3.5E-114	-0.8018	0.054	0.237	5.8E-110	2
Tubb2a	5.2E-113	-0.83104	0.081	0.268	8.7E-109	2
Ier2	3.1E-112	0.638827	0.551	0.357	5.1E-108	2
Ncapg	9.4E-111	-0.66573	0.037	0.204	1.6E-106	2
RP23-45G1	6.5E-110	-0.74641	0.144	0.353	1.1E-105	2
Rps15	1.9E-109	0.355685	0.875	0.775	3.1E-105	2
Elavl3	1.3E-108	-0.64535	0.281	0.5	2.2E-104	2
Smc2	4.3E-108	-0.65659	0.422	0.563	7.1E-104	2

Gpr153	9.3E-108	0.629501	0.35	0.187	1.6E-103	2
Ccnb1	5.4E-107	-0.66401	0.015	0.157	9E-103	2
Ppp1r14c	7.6E-107	-0.77624	0.1	0.285	1.3E-102	2
Mmp14	9.9E-107	0.61328	0.33	0.151	1.7E-102	2
Ccnd1	7.4E-106	0.475991	0.71	0.494	1.2E-101	2
Efhd2	8.9E-105	0.639371	0.255	0.111	1.5E-100	2
Hmmr	1.2E-104	-0.77366	0.035	0.197	1.9E-100	2
Mfap4	1.4E-103	0.680775	0.305	0.14	2.3E-99	2
St18	1.2E-101	-0.76576	0.049	0.211	1.92E-97	2
Btg2	1.8E-101	0.67129	0.341	0.189	3.02E-97	2
Calm2	4E-100	-0.38525	0.81	0.883	6.66E-96	2
Racgap1	4.6E-100	-0.63769	0.091	0.279	7.64E-96	2
Mis18bp1	5.6E-99	-0.62258	0.03	0.179	9.28E-95	2
Tacc3	7.25E-98	-0.62547	0.057	0.228	1.21E-93	2
Rpl39	8.6E-98	0.397209	0.77	0.627	1.44E-93	2
Casc5	1.78E-96	-0.64574	0.045	0.206	2.97E-92	2
Pabpc1	2.77E-95	0.279678	0.942	0.869	4.63E-91	2
Mxd3	2.19E-94	-0.52461	0.014	0.143	3.65E-90	2
Cbx5	4.75E-94	0.410228	0.782	0.634	7.92E-90	2
Hes6	6.91E-93	0.549226	0.37	0.217	1.15E-88	2
Kif15	1.94E-92	-0.6143	0.044	0.2	3.24E-88	2
Rad21	2.38E-91	-0.58686	0.297	0.485	3.97E-87	2
Nuf2	3.67E-91	-0.54913	0.023	0.157	6.12E-87	2
Cdca2	4.25E-91	-0.52019	0.016	0.144	7.09E-87	2
Arhgap11a	5.05E-91	-0.61749	0.04	0.189	8.43E-87	2
Tex14	9.93E-91	-0.70729	0.025	0.162	1.66E-86	2
Chgb	6.88E-90	-0.74234	0.092	0.262	1.15E-85	2
Fam64a	8.75E-90	-0.50689	0.012	0.133	1.46E-85	2
Ezr	9.13E-90	0.483371	0.565	0.419	1.52E-85	2
Sgol2	1.02E-89	-0.62877	0.031	0.174	1.7E-85	2
Nkd1	1.49E-89	0.536379	0.381	0.229	2.48E-85	2
Rpl26	2.46E-89	0.355212	0.802	0.687	4.11E-85	2
Sdpr	2.54E-89	0.680579	0.203	0.07	4.23E-85	2
Cenpe	3.07E-89	-0.81701	0.144	0.306	5.12E-85	2
Kif22	5.95E-89	-0.53467	0.036	0.181	9.93E-85	2
Tmsb10	7.36E-89	-0.44969	0.524	0.684	1.23E-84	2
Gng3	1.02E-88	-0.68555	0.16	0.339	1.7E-84	2
Rrm2	2.35E-88	-0.60831	0.062	0.224	3.93E-84	2
Rangap1	7.46E-88	-0.58795	0.102	0.278	1.24E-83	2
Fstl1	4.24E-87	0.519206	0.314	0.16	7.07E-83	2
Rpl23	4.93E-87	0.404467	0.655	0.521	8.23E-83	2
Sowaha	2.06E-86	0.628132	0.298	0.143	3.43E-82	2
1500012F(	2.32E-86	0.466538	0.532	0.378	3.88E-82	2
Tead2	2.84E-86	0.508972	0.411	0.258	4.74E-82	2
Bub1	3.05E-86	-0.50785	0.019	0.147	5.09E-82	2
Cenph	9.41E-85	-0.5496	0.058	0.213	1.57E-80	2
Rps15a	1.95E-84	0.356221	0.757	0.642	3.26E-80	2
Bin1	2.43E-84	-0.55654	0.268	0.452	4.05E-80	2

Tuba1b	1.38E-83	-0.53135	0.342	0.501	2.31E-79	2
Rps11	2.63E-83	0.324297	0.876	0.763	4.39E-79	2
Aspm	8.48E-83	-0.6139	0.029	0.159	1.41E-78	2
Zmiz1	1.53E-82	0.477814	0.501	0.356	2.55E-78	2
Tagln3	7.09E-82	-0.59424	0.147	0.316	1.18E-77	2
Rpl41	1.35E-81	0.316577	0.871	0.767	2.25E-77	2
Rps24	1.58E-81	0.280537	0.89	0.81	2.63E-77	2
Uchl1	2.18E-81	-0.56088	0.187	0.355	3.64E-77	2
Aurkb	3.28E-81	-0.51038	0.029	0.159	5.48E-77	2
Sept4	4.39E-81	-0.67856	0.092	0.245	7.32E-77	2
Zfp36l1	1.15E-80	0.546731	0.264	0.134	1.92E-76	2
Rad51ap1	1.26E-80	-0.50419	0.043	0.184	2.1E-76	2
Rps18	1.93E-80	0.42962	0.548	0.411	3.22E-76	2
Sptbn1	2.16E-80	-0.56422	0.197	0.376	3.6E-76	2
Mllt11	2.36E-79	-0.64231	0.088	0.237	3.93E-75	2
Basp1	5.8E-79	-0.43501	0.597	0.719	9.68E-75	2
Mns1	1.34E-78	-0.56375	0.067	0.222	2.24E-74	2
Kif20b	1.78E-78	-0.61941	0.06	0.209	2.97E-74	2
Nucks1	4.32E-78	-0.43562	0.557	0.678	7.21E-74	2
Tk1	5.05E-78	-0.50305	0.025	0.146	8.42E-74	2
Atoh1	3.47E-77	0.549799	0.275	0.148	5.79E-73	2
Rps3a1	4.61E-77	0.332122	0.76	0.653	7.7E-73	2
Sgol1	5.72E-77	-0.46235	0.023	0.143	9.54E-73	2
Zeb1	6.78E-77	0.449433	0.502	0.37	1.13E-72	2
Slc17a6	7.72E-77	-0.59721	0.035	0.164	1.29E-72	2
Cdk5r1	7.9E-77	-0.58767	0.184	0.355	1.32E-72	2
Rpl34	1.75E-76	0.336519	0.742	0.639	2.93E-72	2
Podxl2	2.58E-76	-0.57445	0.095	0.246	4.3E-72	2
Ndc80	1.6E-75	-0.4626	0.016	0.128	2.67E-71	2
Calm1	2E-75	-0.35834	0.77	0.875	3.34E-71	2
Dek	3.09E-75	-0.43457	0.608	0.705	5.16E-71	2
Dner	5.18E-74	-0.59651	0.082	0.234	8.65E-70	2
Slc1a2	6.01E-74	0.502729	0.4	0.278	1E-69	2
Ckap2	1.4E-73	-0.51359	0.045	0.18	2.34E-69	2
Sox9	3.16E-73	0.506209	0.376	0.237	5.27E-69	2
Dlgap5	7.57E-73	-0.47058	0.016	0.125	1.26E-68	2
2410006H1	1.39E-72	0.418678	0.577	0.442	2.31E-68	2
Map2	1.02E-71	-0.47722	0.345	0.513	1.69E-67	2
Prkcb	2.03E-71	-0.56922	0.116	0.276	3.39E-67	2
Dut	2.07E-71	-0.52687	0.294	0.455	3.45E-67	2
Sfrp1	2.49E-71	0.323731	0.904	0.798	4.15E-67	2
Knstrn	8.4E-71	-0.56384	0.064	0.203	1.4E-66	2
D430041D	1.1E-70	0.417627	0.613	0.474	1.83E-66	2
Rps2	1.28E-70	0.372808	0.559	0.433	2.14E-66	2
Sox4	2.11E-70	0.429735	0.669	0.57	3.53E-66	2
Rps10	3E-70	0.330507	0.726	0.623	5.01E-66	2
Ctsd	3.21E-70	-0.49302	0.188	0.172	5.35E-66	2
Npm1	3.49E-70	0.329718	0.756	0.63	5.82E-66	2

Serpinh1	4.3E-70	0.444169	0.224	0.099	7.18E-66	2
Ntrk3	6.24E-70	0.495932	0.248	0.141	1.04E-65	2
Kif5c	7.36E-70	-0.51333	0.246	0.42	1.23E-65	2
Fbxo5	8.03E-70	-0.50247	0.046	0.175	1.34E-65	2
Rab3a	1.18E-69	-0.60074	0.041	0.165	1.97E-65	2
Hn1	1.19E-69	-0.44497	0.396	0.562	1.98E-65	2
Ccnb2	5.33E-69	-0.57955	0.065	0.204	8.89E-65	2
Kif2c	6.24E-69	-0.41476	0.016	0.12	1.04E-64	2
Sema6a	9.11E-69	-0.58215	0.085	0.219	1.52E-64	2
Gsg1l	1.47E-68	0.451209	0.366	0.239	2.45E-64	2
Rnd3	2.02E-68	0.419768	0.541	0.415	3.36E-64	2
Tyms	4.19E-68	-0.52448	0.094	0.236	6.99E-64	2
Rufy3	3.43E-67	-0.47889	0.223	0.393	5.71E-63	2
Plk1	4.74E-67	-0.3754	0.009	0.102	7.91E-63	2
Rpl35a	1.29E-66	0.330015	0.672	0.555	2.14E-62	2
Ckap5	1.3E-66	-0.51757	0.129	0.285	2.16E-62	2
Clspn	1.33E-66	-0.52122	0.067	0.207	2.22E-62	2
Pdlim3	1.44E-66	0.515936	0.141	0.05	2.39E-62	2
Ska1	1.98E-66	-0.36344	0.009	0.101	3.3E-62	2
Eno1	2.32E-66	0.42177	0.326	0.215	3.86E-62	2
Cdkn2d	3.98E-66	-0.46139	0.048	0.173	6.64E-62	2
Dbf4	4.25E-66	-0.45714	0.04	0.16	7.09E-62	2
Anp32e	4.77E-66	-0.40269	0.531	0.625	7.96E-62	2
Calm3	6.64E-66	-0.40395	0.403	0.583	1.11E-61	2
Id2	6.95E-66	0.433211	0.61	0.468	1.16E-61	2
Pou3f2	9.4E-66	0.45534	0.377	0.261	1.57E-61	2
Whsc1	1.37E-65	-0.42947	0.299	0.488	2.28E-61	2
Cxcr4	3.21E-65	0.468997	0.178	0.078	5.35E-61	2
Ank3	3.3E-65	-0.48242	0.281	0.44	5.5E-61	2
Nnat	1.51E-64	-0.45104	0.604	0.725	2.51E-60	2
Celf2	1.67E-64	-0.39459	0.54	0.652	2.79E-60	2
Rpl14	2.41E-64	0.289093	0.78	0.684	4.03E-60	2
Srrm3	4.47E-64	-0.52926	0.109	0.258	7.46E-60	2
Rps25	4.95E-64	0.36457	0.594	0.463	8.26E-60	2
Gas6	5.82E-64	0.520571	0.172	0.067	9.7E-60	2
Lmnb1	3.3E-63	-0.46475	0.156	0.32	5.5E-59	2
Apc	3.61E-63	-0.47386	0.288	0.452	6.01E-59	2
Hist1h2ak	4.12E-63	-0.55807	0.042	0.157	6.88E-59	2
Eef1b2	6.39E-63	0.300235	0.812	0.72	1.07E-58	2
Kif4	1.44E-62	-0.4017	0.02	0.12	2.4E-58	2
Tmpo	1.56E-62	-0.46484	0.318	0.471	2.61E-58	2
Cdh20	2.64E-62	0.437227	0.3	0.188	4.4E-58	2
Eif3f	5.47E-62	0.280694	0.708	0.619	9.12E-58	2
Fos	6.69E-62	0.575783	0.427	0.286	1.12E-57	2
Klc1	7.92E-62	-0.42399	0.217	0.382	1.32E-57	2
D17H6S56I	9.81E-62	-0.38798	0.032	0.141	1.64E-57	2
Cenpm	1.96E-61	-0.43123	0.053	0.177	3.28E-57	2
E130114P1	2.24E-61	0.378999	0.693	0.568	3.73E-57	2

Ttk	3.05E-61	-0.35423	0.012	0.101	5.08E-57	2
Ect2	3.68E-61	-0.35408	0.016	0.109	6.14E-57	2
Tgfb2	1.14E-60	0.499615	0.291	0.177	1.91E-56	2
Myt1	2.22E-60	-0.55225	0.049	0.167	3.7E-56	2
Aurka	8.95E-60	-0.35175	0.017	0.11	1.49E-55	2
Hjurp	1.08E-59	-0.45696	0.29	0.454	1.8E-55	2
Mtss1	4.65E-59	-0.52965	0.154	0.286	7.75E-55	2
Anln	4.97E-59	-0.38375	0.02	0.116	8.29E-55	2
Stmn1	6.98E-59	-0.42861	0.15	0.303	1.16E-54	2
Mapt	8.68E-59	-0.58636	0.112	0.231	1.45E-54	2
Angptl2	1.17E-58	0.427441	0.189	0.105	1.96E-54	2
C330027C(	1.2E-58	-0.43143	0.037	0.147	2.01E-54	2
Loxl1	4.49E-58	0.425359	0.162	0.079	7.49E-54	2
Cenpq	7.72E-58	-0.43372	0.055	0.175	1.29E-53	2
Fnbp1l	1.59E-57	-0.42187	0.317	0.473	2.65E-53	2
Gria2	2.35E-57	-0.4486	0.389	0.54	3.92E-53	2
Atad2	2.57E-57	-0.47753	0.084	0.214	4.29E-53	2
Myt1l	3.57E-57	-0.55585	0.059	0.175	5.96E-53	2
Bub1b	4.08E-57	-0.37578	0.02	0.114	6.8E-53	2
Sema7a	5.37E-57	0.436996	0.246	0.142	8.96E-53	2
Dhx32	1.1E-56	0.431186	0.288	0.187	1.84E-52	2
Ncapd2	1.16E-56	-0.40546	0.075	0.2	1.93E-52	2
Eef2	1.3E-56	0.273142	0.793	0.705	2.17E-52	2
2700094K:	1.61E-56	-0.38573	0.501	0.63	2.68E-52	2
Elmo1	2.48E-56	-0.55537	0.036	0.138	4.14E-52	2
Cacng5	3.16E-56	0.430156	0.116	0.04	5.26E-52	2
Nhlh1	4.03E-56	-0.5327	0.112	0.239	6.72E-52	2
Rrm1	4.65E-56	-0.43772	0.133	0.278	7.75E-52	2
Dpysl3	6.83E-56	-0.58544	0.107	0.235	1.14E-51	2
Diap3	7.19E-56	-0.37843	0.023	0.118	1.2E-51	2
Mad2l1	7.99E-56	-0.36693	0.033	0.136	1.33E-51	2
Myc	1.7E-54	0.427146	0.177	0.089	2.84E-50	2
Ezh2	2.43E-54	-0.39681	0.488	0.627	4.06E-50	2
Olfm1	4.15E-54	-0.46352	0.091	0.214	6.92E-50	2
Ctsb	1.4E-53	-0.53958	0.194	0.271	2.34E-49	2
Lig1	4.79E-53	-0.47527	0.216	0.367	8E-49	2
Crmp1	6.88E-53	-0.35311	0.548	0.685	1.15E-48	2
Melk	1.67E-52	-0.32165	0.018	0.105	2.79E-48	2
Rpl37	2.33E-52	0.322799	0.579	0.492	3.88E-48	2
Plk4	2.46E-52	-0.36958	0.033	0.132	4.11E-48	2
Nt5dc2	1.66E-51	-0.41308	0.123	0.253	2.77E-47	2
Hirip3	1.78E-51	-0.43969	0.254	0.401	2.96E-47	2
Pdlim4	2E-51	0.437778	0.146	0.065	3.33E-47	2
Ank2	2.06E-51	-0.52983	0.135	0.255	3.44E-47	2
Clmp	2.51E-51	-0.47382	0.124	0.259	4.18E-47	2
Ska2	3.11E-51	-0.41976	0.093	0.22	5.2E-47	2
Gm1673	4.83E-51	-0.36456	0.339	0.492	8.06E-47	2
Fam111a	5.55E-51	-0.42198	0.039	0.138	9.26E-47	2

Ncor2	8.24E-51	0.405477	0.247	0.157	1.37E-46	2
Cenpk	8.53E-51	-0.40855	0.049	0.158	1.42E-46	2
Ccng2	1.65E-50	-0.40706	0.107	0.227	2.76E-46	2
Mdk	2.51E-50	0.38629	0.491	0.363	4.18E-46	2
Meg3	2.91E-50	-0.80969	0.028	0.105	4.86E-46	2
Fabp7	3.52E-50	-0.85158	0.102	0.126	5.87E-46	2
Stxbp1	6.17E-50	-0.47319	0.068	0.176	1.03E-45	2
Rpl7	8.92E-50	0.300363	0.583	0.498	1.49E-45	2
Rpl18a	9.75E-50	0.339325	0.488	0.387	1.63E-45	2
H2afv	4.72E-49	-0.27889	0.705	0.731	7.87E-45	2
Myod1	8.07E-49	-0.44213	0.052	0.154	1.35E-44	2
Prmt8	1.06E-48	0.409738	0.361	0.259	1.76E-44	2
Pde1c	2.37E-48	-0.45755	0.274	0.403	3.95E-44	2
Frmd4a	4.07E-48	-0.38046	0.185	0.326	6.78E-44	2
B3galt2	1.22E-47	-0.4619	0.023	0.108	2.03E-43	2
Gm11478	5.08E-47	0.369722	0.293	0.203	8.48E-43	2
Itsn1	7.6E-47	-0.47563	0.129	0.251	1.27E-42	2
Sept11	7.94E-47	-0.37787	0.2	0.339	1.32E-42	2
Plcb1	9.01E-47	-0.49165	0.052	0.153	1.5E-42	2
Stmn3	9.22E-47	-0.3284	0.511	0.637	1.54E-42	2
Mroh2a	1.86E-46	-0.48143	0.037	0.124	3.1E-42	2
Rfc4	2.98E-46	-0.35288	0.116	0.242	4.98E-42	2
Zic1	3.44E-46	-0.2822	0.739	0.79	5.73E-42	2
Ppp2r2c	5.22E-46	-0.33674	0.376	0.523	8.71E-42	2
Ier5	1.01E-45	0.363927	0.474	0.382	1.68E-41	2
Ccnd2	1.03E-45	-0.35152	0.591	0.672	1.71E-41	2
Sparc	2.87E-45	-0.38238	0.103	0.082	4.79E-41	2
Dll3	3.21E-45	0.418401	0.135	0.055	5.36E-41	2
Atp6v0e	4.03E-45	0.306703	0.48	0.397	6.72E-41	2
Elavl4	4.52E-45	-0.42498	0.216	0.352	7.53E-41	2
Ckb	5.02E-45	-0.27135	0.779	0.812	8.38E-41	2
Rprml	6.4E-45	0.434024	0.145	0.071	1.07E-40	2
Gm10260	7.78E-45	0.34339	0.309	0.215	1.3E-40	2
Fosb	2.32E-44	0.472003	0.243	0.143	3.88E-40	2
Sfrp2	2.99E-44	0.351632	0.206	0.139	4.98E-40	2
Pax6	3.44E-44	-0.31996	0.384	0.531	5.73E-40	2
Kcnk1	3.58E-44	-0.42151	0.104	0.219	5.97E-40	2
Nrn1	4E-44	-0.4483	0.116	0.236	6.68E-40	2
Cenpp	5.34E-44	-0.31224	0.039	0.125	8.9E-40	2
Gstm5	5.7E-44	0.334759	0.399	0.311	9.5E-40	2
Cst3	6.91E-44	-0.28619	0.53	0.536	1.15E-39	2
Ramp2	7.33E-44	0.369392	0.194	0.108	1.22E-39	2
A930011O	9.14E-44	-0.47517	0.037	0.126	1.52E-39	2
Rbfox1	1.33E-43	-0.38315	0.023	0.102	2.22E-39	2
Rpl18	1.36E-43	0.294998	0.367	0.295	2.27E-39	2
Ppic	1.57E-43	0.372271	0.327	0.226	2.62E-39	2
Ptn	1.69E-43	-0.46587	0.347	0.455	2.81E-39	2
Pdgfra	1.86E-43	-0.41173	0.086	0.195	3.11E-39	2

Fzd1	2.15E-43	0.356118	0.128	0.059	3.58E-39	2
Nasp	3.77E-43	-0.33471	0.541	0.654	6.29E-39	2
Uncx	8.56E-43	-0.36391	0.209	0.333	1.43E-38	2
Cog7	1.09E-42	-0.3261	0.46	0.602	1.82E-38	2
Tspan13	1.14E-42	-0.36879	0.109	0.221	1.9E-38	2
Aplp1	1.53E-42	-0.45502	0.066	0.163	2.56E-38	2
Gpx8	2.36E-42	0.340036	0.115	0.044	3.94E-38	2
Ptprg	3.25E-42	0.390894	0.209	0.133	5.42E-38	2
Trpc4ap	3.43E-42	-0.44499	0.118	0.209	5.72E-38	2
Ncam1	4.44E-42	-0.30824	0.265	0.404	7.4E-38	2
Psip1	5.14E-42	-0.2935	0.581	0.703	8.57E-38	2
Pcna	5.45E-42	-0.40541	0.254	0.373	9.09E-38	2
Dcx	6.67E-42	-0.386	0.266	0.391	1.11E-37	2
H2afz	9.13E-42	-0.30174	0.128	0.246	1.52E-37	2
Rpl37a	1.17E-41	0.275185	0.546	0.456	1.95E-37	2
Trim59	1.4E-41	-0.3593	0.078	0.182	2.34E-37	2
H1fx	2.31E-41	-0.36637	0.139	0.26	3.86E-37	2
BC005764	5.47E-41	-0.46458	0.094	0.199	9.13E-37	2
Eef1g	6.17E-41	0.294202	0.403	0.324	1.03E-36	2
1700025G(	6.28E-41	-0.37196	0.159	0.278	1.05E-36	2
Rad51	7.69E-41	-0.29549	0.04	0.127	1.28E-36	2
Pea15a	8.41E-41	-0.42026	0.091	0.188	1.4E-36	2
Lbr	5.11E-40	-0.29419	0.102	0.21	8.52E-36	2
2810055G:	5.87E-40	0.377753	0.246	0.159	9.79E-36	2
Ccdc34	6.99E-40	-0.37281	0.296	0.431	1.17E-35	2
Srebf1	1.15E-39	0.358102	0.422	0.321	1.93E-35	2
Gm17322	1.47E-39	0.431446	0.207	0.12	2.45E-35	2
Dnmt1	2.04E-39	-0.34014	0.169	0.293	3.4E-35	2
Ncaph	2.21E-39	-0.29969	0.045	0.132	3.68E-35	2
Gmnn	2.38E-39	-0.30795	0.062	0.156	3.96E-35	2
Cdc7	3.13E-39	-0.2671	0.084	0.179	5.22E-35	2
Hadh	4.16E-39	0.310689	0.13	0.065	6.95E-35	2
Gm13826	4.23E-39	0.279614	0.377	0.302	7.06E-35	2
Clvs1	4.97E-39	-0.38193	0.049	0.14	8.29E-35	2
Cenpw	5.2E-39	-0.31527	0.051	0.138	8.67E-35	2
Fabp5	5.53E-39	-0.30692	0.29	0.419	9.22E-35	2
Cenpj	6.56E-39	-0.33734	0.061	0.153	1.09E-34	2
Wdr89	7.89E-39	0.294355	0.269	0.206	1.32E-34	2
Gnai2	8.46E-39	0.282191	0.508	0.412	1.41E-34	2
Mis18a	1.17E-38	-0.28652	0.04	0.12	1.96E-34	2
Npc2	1.4E-38	0.273586	0.533	0.453	2.33E-34	2
Cdc45	3.37E-38	-0.28681	0.029	0.104	5.62E-34	2
Lhx1	5.61E-38	-0.35472	0.395	0.512	9.36E-34	2
Fen1	6.71E-38	-0.30731	0.062	0.153	1.12E-33	2
Smpd2	7.99E-38	0.34375	0.224	0.157	1.33E-33	2
Cks1b	8.92E-38	-0.38091	0.241	0.359	1.49E-33	2
Irs1	9.11E-38	0.355053	0.178	0.11	1.52E-33	2
Mrpl52	9.66E-38	0.279892	0.498	0.423	1.61E-33	2

Atp1b3	1.06E-37	-0.3395	0.226	0.353	1.78E-33	2
Cdk6	1.51E-37	0.320233	0.304	0.234	2.52E-33	2
Nrep	2.17E-37	-0.28975	0.502	0.579	3.62E-33	2
Rps7	2.34E-37	0.275072	0.461	0.39	3.91E-33	2
Snhg1	2.44E-37	0.292291	0.441	0.345	4.07E-33	2
Ppp3ca	2.47E-37	-0.3211	0.281	0.392	4.12E-33	2
Gltscr2	3.74E-37	0.292233	0.411	0.331	6.24E-33	2
Olig2	4.37E-37	0.355428	0.148	0.067	7.29E-33	2
Sox1	5.02E-37	0.347445	0.118	0.063	8.38E-33	2
Pmf1	5.76E-37	-0.268	0.066	0.154	9.61E-33	2
Brd8	6.53E-37	-0.30042	0.279	0.408	1.09E-32	2
Lrig3	8.75E-37	0.335538	0.228	0.161	1.46E-32	2
Rftn2	1E-36	0.332015	0.184	0.114	1.67E-32	2
App	3.29E-36	-0.27855	0.474	0.582	5.49E-32	2
1110038B1	5.66E-36	0.278179	0.352	0.29	9.43E-32	2
Vps37b	6.22E-36	0.270747	0.533	0.461	1.04E-31	2
Dnajc9	9.22E-36	-0.32888	0.238	0.365	1.54E-31	2
Gm17750	9.76E-36	-0.30432	0.267	0.381	1.63E-31	2
Ptch2	1.07E-35	0.347775	0.204	0.144	1.78E-31	2
Tubb4b	1.18E-35	-0.34946	0.142	0.254	1.96E-31	2
2410004N0	1.22E-35	0.316239	0.245	0.175	2.03E-31	2
Bub3	1.59E-35	-0.35028	0.215	0.336	2.65E-31	2
Dusp5	1.61E-35	0.411373	0.152	0.078	2.69E-31	2
Nes	1.65E-35	0.38769	0.152	0.076	2.75E-31	2
Snhg6	2.26E-35	0.308223	0.324	0.249	3.77E-31	2
Usp46	2.34E-35	-0.30035	0.143	0.249	3.9E-31	2
Cacng4	5.19E-35	-0.4742	0.042	0.11	8.66E-31	2
Rps28	6.44E-35	0.285335	0.264	0.194	1.07E-30	2
Rtn4	1.29E-34	-0.28619	0.375	0.495	2.16E-30	2
2610203C2	1.63E-34	0.356328	0.187	0.111	2.71E-30	2
Cadm3	1.89E-34	-0.42318	0.068	0.151	3.15E-30	2
Kcnip3	2.01E-34	-0.34043	0.049	0.13	3.35E-30	2
Trim37	2.24E-34	-0.29722	0.14	0.246	3.73E-30	2
Cplx1	2.3E-34	-0.3408	0.063	0.149	3.84E-30	2
Rpl22l1	4.02E-34	0.261418	0.401	0.343	6.71E-30	2
Eif3e	4.7E-34	0.257228	0.439	0.373	7.85E-30	2
Ttc9b	4.91E-34	-0.29122	0.107	0.207	8.19E-30	2
Hspe1	4.97E-34	0.250649	0.408	0.33	8.29E-30	2
Use1	5.7E-34	0.281535	0.377	0.304	9.51E-30	2
Rps8	9.16E-34	0.250192	0.459	0.387	1.53E-29	2
Whrn	9.55E-34	0.320973	0.127	0.066	1.59E-29	2
Fyn	1.34E-33	-0.347	0.143	0.226	2.23E-29	2
Igdcc4	1.36E-33	0.34476	0.15	0.086	2.26E-29	2
Srgap2	1.36E-33	-0.31563	0.06	0.142	2.27E-29	2
Mgll	1.52E-33	-0.34583	0.062	0.143	2.54E-29	2
Hsd11b2	2.11E-33	0.341329	0.359	0.277	3.52E-29	2
Dync1i2	2.94E-33	-0.26925	0.471	0.582	4.9E-29	2
Tle1	3.93E-33	0.287161	0.281	0.217	6.56E-29	2

Hells	5.37E-33	-0.37317	0.12	0.226	8.97E-29	2
A030009H(	6.39E-33	-0.29149	0.048	0.123	1.07E-28	2
Fkbp7	7.27E-33	0.307957	0.114	0.06	1.21E-28	2
Nrcam	7.39E-33	-0.35833	0.06	0.144	1.23E-28	2
Prim1	8.1E-33	-0.32593	0.173	0.275	1.35E-28	2
Naca	1.03E-32	0.250629	0.528	0.455	1.72E-28	2
Nfyb	1.34E-32	-0.26584	0.148	0.247	2.23E-28	2
Gm11223	1.46E-32	-0.41105	0.153	0.221	2.43E-28	2
Bora	1.48E-32	-0.25186	0.033	0.101	2.47E-28	2
Rab6b	1.51E-32	-0.35857	0.115	0.215	2.52E-28	2
Pkia	1.62E-32	-0.31816	0.061	0.144	2.71E-28	2
Myl12a	1.7E-32	0.26633	0.396	0.318	2.83E-28	2
Nme4	2.3E-32	0.315236	0.177	0.109	3.84E-28	2
Pdzrn4	2.51E-32	0.341426	0.19	0.139	4.19E-28	2
Tead1	3.68E-32	0.335645	0.225	0.166	6.14E-28	2
Nek7	3.69E-32	0.306186	0.169	0.109	6.15E-28	2
Nav2	3.69E-32	-0.2775	0.042	0.114	6.16E-28	2
Usp1	4.23E-32	-0.30304	0.239	0.353	7.06E-28	2
Odf2	4.89E-32	-0.29293	0.091	0.182	8.15E-28	2
Gdpd1	4.99E-32	-0.26696	0.156	0.255	8.33E-28	2
Tceal8	5.48E-32	0.271882	0.294	0.238	9.14E-28	2
Pnrc1	6.77E-32	0.307539	0.336	0.261	1.13E-27	2
Syt11	8.22E-32	-0.27926	0.405	0.518	1.37E-27	2
Nsg2	9.37E-32	-0.26639	0.392	0.501	1.56E-27	2
Azin1	1.01E-31	-0.26751	0.118	0.215	1.68E-27	2
Kif5a	1.05E-31	-0.37819	0.061	0.137	1.75E-27	2
Chaf1a	1.07E-31	-0.28295	0.088	0.18	1.79E-27	2
Apbb1	1.08E-31	-0.36257	0.096	0.18	1.8E-27	2
Smc6	3.04E-31	-0.28112	0.31	0.438	5.07E-27	2
Lmnb2	3.53E-31	-0.2635	0.085	0.169	5.88E-27	2
Snord104	3.68E-31	0.316449	0.215	0.143	6.14E-27	2
Lpin2	4.41E-31	0.311471	0.224	0.173	7.36E-27	2
H2afy2	4.58E-31	-0.26275	0.244	0.351	7.64E-27	2
Cadm1	5.91E-31	0.280091	0.446	0.382	9.86E-27	2
Mab21l1	6.3E-31	-0.32388	0.141	0.237	1.05E-26	2
Crip2	7.62E-31	-0.29639	0.15	0.253	1.27E-26	2
Elavl2	8.22E-31	-0.28168	0.184	0.282	1.37E-26	2
Hsp90aa1	1.01E-30	-0.25105	0.521	0.63	1.68E-26	2
Strbp	1.16E-30	-0.26764	0.25	0.361	1.93E-26	2
Dynll2	1.43E-30	-0.26586	0.201	0.309	2.38E-26	2
Nsg1	1.59E-30	-0.31169	0.207	0.299	2.65E-26	2
2810004N:	1.77E-30	0.258556	0.308	0.255	2.95E-26	2
Rbp4	2.08E-30	0.326464	0.306	0.232	3.48E-26	2
Prdm8	2.47E-30	-0.37386	0.061	0.136	4.13E-26	2
Gnl3	3.61E-30	0.287086	0.363	0.286	6.01E-26	2
Sh3bp5	6.05E-30	-0.32585	0.043	0.112	1.01E-25	2
Gsto1	7E-30	0.312774	0.189	0.119	1.17E-25	2
Rab13	7.69E-30	0.311509	0.135	0.077	1.28E-25	2

Ap1s2	1.17E-29	-0.28763	0.085	0.171	1.94E-25	2
Rbfox2	1.33E-29	-0.31677	0.201	0.295	2.22E-25	2
Ndrg2	1.39E-29	0.250522	0.233	0.177	2.32E-25	2
Igsf3	1.91E-29	-0.25524	0.067	0.142	3.19E-25	2
Arpp21	2.37E-29	-0.41338	0.053	0.121	3.96E-25	2
Sacs	2.66E-29	0.303685	0.239	0.183	4.44E-25	2
Actn1	3.68E-29	0.280818	0.107	0.057	6.14E-25	2
Acat1	4.11E-29	-0.2518	0.35	0.454	6.85E-25	2
Chd3	4.47E-29	-0.37621	0.077	0.159	7.46E-25	2
2900011O(	4.58E-29	-0.33888	0.045	0.112	7.64E-25	2
Dhfr	6.42E-29	-0.25138	0.077	0.158	1.07E-24	2
Rcn3	7.91E-29	0.297768	0.143	0.079	1.32E-24	2
Wdr60	9.21E-29	0.325665	0.173	0.11	1.54E-24	2
Lima1	9.76E-29	0.324628	0.182	0.113	1.63E-24	2
Plp1	1.06E-28	-0.6432	0.111	0.115	1.76E-24	2
Uhrf1	1.08E-28	-0.28555	0.074	0.153	1.8E-24	2
Tipin	1.29E-28	-0.253	0.168	0.267	2.16E-24	2
Oxct1	1.64E-28	-0.25851	0.448	0.567	2.74E-24	2
Boc	1.86E-28	0.291384	0.201	0.149	3.09E-24	2
Hey1	2.07E-28	0.282539	0.336	0.263	3.45E-24	2
Trps1	3.91E-28	0.282635	0.136	0.084	6.52E-24	2
Brd7	6.08E-28	0.265386	0.366	0.299	1.01E-23	2
Pcdha2	7.13E-28	-0.31235	0.057	0.127	1.19E-23	2
Rcn1	7.32E-28	0.255217	0.287	0.225	1.22E-23	2
Nexn	8.66E-28	0.408115	0.127	0.069	1.44E-23	2
Stx4a	9.89E-28	0.294692	0.21	0.146	1.65E-23	2
Akap12	1.08E-27	-0.35706	0.048	0.12	1.8E-23	2
Epb4.1	1.67E-27	-0.25247	0.147	0.241	2.78E-23	2
Srrm4	1.71E-27	-0.29025	0.123	0.205	2.85E-23	2
Dnmt3a	1.82E-27	0.279461	0.255	0.195	3.03E-23	2
Tnik	1.91E-27	-0.36893	0.079	0.156	3.18E-23	2
Hip1r	4.64E-27	-0.31218	0.067	0.142	7.74E-23	2
Gm26735	6.05E-27	0.332872	0.241	0.172	1.01E-22	2
Plxnb2	6.16E-27	-0.26937	0.098	0.181	1.03E-22	2
Tcf19	6.47E-27	-0.28452	0.04	0.106	1.08E-22	2
Btbd11	8.63E-27	0.272998	0.108	0.063	1.44E-22	2
Chrna3	9E-27	-0.35423	0.054	0.127	1.5E-22	2
Kif21a	1.04E-26	0.280007	0.407	0.336	1.74E-22	2
Gpatch4	1.21E-26	0.280061	0.234	0.171	2.01E-22	2
Islr2	1.29E-26	0.296011	0.23	0.172	2.15E-22	2
Cep170	1.48E-26	-0.27829	0.227	0.316	2.46E-22	2
Adamts1	1.83E-26	0.306263	0.187	0.149	3.05E-22	2
Mapk8ip1	2.54E-26	-0.28892	0.152	0.238	4.23E-22	2
Cmtm3	2.75E-26	0.254894	0.163	0.11	4.59E-22	2
Prnp	4.98E-26	-0.27239	0.135	0.217	8.31E-22	2
Etfb	6.77E-26	0.25215	0.228	0.178	1.13E-21	2
Arhgef7	1.1E-25	-0.26994	0.085	0.158	1.84E-21	2
Dpysl5	1.46E-25	-0.25624	0.04	0.103	2.43E-21	2

Hddc2	6.11E-25	0.270812	0.141	0.086	1.02E-20	2
Cttnbp2	7.71E-25	0.310605	0.136	0.075	1.29E-20	2
1500011K1	1.13E-24	0.26062	0.2	0.142	1.89E-20	2
Rrp15	1.31E-24	0.263845	0.218	0.167	2.18E-20	2
Tshz2	1.47E-24	0.264807	0.373	0.311	2.44E-20	2
Glce	2.21E-24	-0.29391	0.109	0.186	3.69E-20	2
Sstr2	2.68E-24	-0.27606	0.121	0.2	4.47E-20	2
Zfp462	2.76E-24	0.269918	0.199	0.15	4.61E-20	2
Wwc1	3.42E-24	0.261929	0.114	0.062	5.71E-20	2
Cd1d1	5.73E-24	0.258923	0.128	0.082	9.56E-20	2
Robo2	6.38E-24	0.305642	0.206	0.143	1.06E-19	2
Hmgcs1	1.07E-23	-0.28522	0.091	0.16	1.79E-19	2
C77370	1.81E-23	-0.26199	0.047	0.109	3.01E-19	2
Pfn2	2.11E-23	-0.2585	0.17	0.251	3.51E-19	2
Nbea	5.68E-23	-0.30191	0.12	0.2	9.48E-19	2
Dusp6	5.73E-23	0.280317	0.135	0.086	9.56E-19	2
Ifitm2	6.97E-23	0.253031	0.163	0.107	1.16E-18	2
Eif4e3	8.83E-23	-0.27678	0.07	0.139	1.47E-18	2
Dusp1	1.35E-22	0.256943	0.197	0.158	2.25E-18	2
Pnmal2	1.69E-22	-0.26296	0.084	0.155	2.83E-18	2
S100a16	2.66E-22	-0.35207	0.046	0.101	4.43E-18	2
Abhd16a	5.9E-22	-0.2633	0.13	0.198	9.84E-18	2
Adcyap1r1	8.05E-22	0.286005	0.125	0.077	1.34E-17	2
Kcnh7	1.02E-21	0.250401	0.102	0.06	1.7E-17	2
Tspyl4	1.19E-21	-0.27157	0.072	0.134	1.98E-17	2
Gstm1	1.19E-21	0.256785	0.131	0.074	1.98E-17	2
Dbn1	2.84E-21	-0.27903	0.083	0.147	4.73E-17	2
Lama5	4.53E-21	0.279239	0.118	0.074	7.56E-17	2
Gria4	7.14E-21	-0.26162	0.071	0.132	1.19E-16	2
Pak7	9.68E-21	-0.27244	0.053	0.112	1.61E-16	2
Gria3	1.69E-20	0.264831	0.132	0.083	2.82E-16	2
Mt2	1.75E-20	0.29998	0.143	0.084	2.92E-16	2
Mbp	6.6E-17	-0.26584	0.13	0.17	1.1E-12	2
Xist	4.18E-14	0.257118	0.369	0.289	6.98E-10	2
Top2a	3.2E-188	-1.1085	0.255	0.452	5.3E-184	3
Mki67	3.4E-159	-1.02468	0.224	0.419	5.7E-155	3
Prc1	2.3E-147	-1.04044	0.078	0.282	3.8E-143	3
Smc4	1.1E-144	-0.68255	0.487	0.578	1.9E-140	3
Arl6ip1	4.8E-144	-0.77501	0.429	0.567	8.1E-140	3
CRE_RECOM	2.4E-141	0.556051	0.902	0.748	4.1E-137	3
Neurod1	1.3E-140	-0.86048	0.502	0.537	2.2E-136	3
Nusap1	5.1E-134	-0.913	0.06	0.251	8.5E-130	3
Ube2c	1.8E-133	-1.11333	0.116	0.291	3E-129	3
Birc5	3.7E-133	-0.88352	0.101	0.298	6.2E-129	3
Cenpf	8.5E-132	-1.05015	0.206	0.385	1.4E-127	3
Hmgb2	3.7E-129	-0.70661	0.278	0.417	6.1E-125	3
H2afx	6.6E-127	-0.83717	0.194	0.35	1.1E-122	3
Tpx2	8.2E-127	-0.91427	0.115	0.305	1.4E-122	3

Cdk1	1.1E-118	-0.83694	0.086	0.27	1.9E-114	3
Kif23	7.9E-117	-0.83504	0.06	0.236	1.3E-112	3
Sfrp1	1.6E-114	0.353816	0.926	0.795	2.7E-110	3
Ckap2l	6.8E-114	-0.80812	0.063	0.24	1.1E-109	3
Spc25	7.7E-114	-0.79569	0.07	0.248	1.3E-109	3
Hmmr	5.8E-112	-0.83283	0.037	0.196	9.8E-108	3
Cdca8	4.6E-111	-0.78183	0.105	0.287	7.7E-107	3
Igfbpl1	3.4E-110	0.356657	0.839	0.619	5.6E-106	3
Barhl1	1.1E-107	0.447027	0.724	0.492	1.8E-103	3
Ccnd2	3.9E-102	0.423265	0.812	0.64	6.57E-98	3
Cdc20	6.1E-102	-0.83765	0.061	0.215	1.02E-97	3
Hey1	6.6E-100	0.582245	0.459	0.245	1.11E-95	3
Cdca3	4.6E-99	-0.72589	0.073	0.243	7.64E-95	3
Cenpa	7.25E-98	-0.87952	0.108	0.272	1.21E-93	3
Kif11	4.84E-97	-0.69922	0.066	0.226	8.07E-93	3
Ptn	6.28E-96	-0.80887	0.307	0.461	1.05E-91	3
Gm17322	1.63E-94	0.576028	0.284	0.109	2.73E-90	3
Srebf1	1.17E-93	0.519959	0.522	0.306	1.95E-89	3
Incenp	1.6E-93	-0.73549	0.125	0.296	2.68E-89	3
Apoe	7.67E-93	-1.67653	0.211	0.254	1.28E-88	3
Slc29a1	2.18E-92	0.437477	0.725	0.537	3.64E-88	3
Smc2	2.31E-91	-0.50672	0.495	0.552	3.86E-87	3
Ccna2	1.85E-90	-0.66366	0.081	0.241	3.08E-86	3
Pbk	1.06E-89	-0.68541	0.082	0.242	1.77E-85	3
Cenpe	1.2E-85	-0.81211	0.155	0.304	2E-81	3
Ccnb1	1.69E-85	-0.62383	0.029	0.155	2.83E-81	3
Rtn1	2.68E-84	-0.39616	0.707	0.687	4.47E-80	3
Rps26	5.07E-83	0.28112	0.921	0.836	8.46E-79	3
Calm2	1.56E-82	-0.31611	0.862	0.876	2.61E-78	3
Itm2b	3.05E-82	-0.46707	0.572	0.652	5.09E-78	3
Nucks1	2.52E-81	-0.35782	0.638	0.666	4.2E-77	3
Sgol2	5.96E-79	-0.63452	0.045	0.172	9.94E-75	3
Casc5	1.54E-76	-0.61548	0.066	0.203	2.57E-72	3
Tacc3	3.83E-75	-0.59797	0.08	0.224	6.38E-71	3
Lhx1	4.72E-75	0.390517	0.673	0.471	7.87E-71	3
Esco2	2.95E-74	-0.64417	0.075	0.212	4.93E-70	3
Miat	1.52E-73	0.251892	0.689	0.49	2.53E-69	3
Aurkb	5.72E-71	-0.50822	0.039	0.157	9.53E-67	3
Draxin	3.06E-70	0.355775	0.718	0.53	5.1E-66	3
Arhgap11a	7.88E-69	-0.58421	0.059	0.186	1.31E-64	3
Rad21	2.91E-68	-0.43706	0.385	0.472	4.86E-64	3
Ncapg	6.81E-67	-0.55514	0.072	0.199	1.14E-62	3
Ccnb2	1.18E-66	-0.6081	0.07	0.203	1.97E-62	3
Fam210b	1.39E-66	0.435937	0.474	0.29	2.33E-62	3
Mis18bp1	2.46E-66	-0.55936	0.055	0.176	4.1E-62	3
Fam64a	4.61E-66	-0.4649	0.024	0.131	7.68E-62	3
Kif22	7.08E-66	-0.51657	0.053	0.178	1.18E-61	3
2810417H	2.13E-65	-0.58268	0.275	0.408	3.56E-61	3

Celf4	5.1E-65	-0.63742	0.243	0.331	8.5E-61	3
Cdca2	3.74E-64	-0.46081	0.034	0.142	6.24E-60	3
C1ql1	4.64E-63	0.426009	0.431	0.256	7.74E-59	3
Spc24	2.25E-62	-0.54291	0.135	0.257	3.75E-58	3
Gpm6a	8.25E-62	-0.49889	0.348	0.418	1.38E-57	3
Cst3	2.72E-61	-0.53512	0.514	0.538	4.53E-57	3
Ung	1.31E-60	0.266394	0.188	0.078	2.19E-56	3
Gamt	8.54E-60	0.42531	0.3	0.15	1.42E-55	3
Aspm	2.26E-59	-0.56338	0.049	0.156	3.77E-55	3
Cntn2	2.84E-59	-0.59068	0.21	0.271	4.74E-55	3
Kif15	5.88E-59	-0.51244	0.071	0.196	9.81E-55	3
Ndc80	1.32E-58	-0.42754	0.027	0.127	2.2E-54	3
Tubb5	1.93E-58	-0.25953	0.914	0.914	3.21E-54	3
Nrxn1	2.82E-58	-0.58139	0.23	0.33	4.71E-54	3
Cd63	1.06E-57	0.253388	0.871	0.738	1.77E-53	3
Hsd11b2	1.12E-57	0.379493	0.437	0.266	1.87E-53	3
Kif2c	1.98E-57	-0.40859	0.023	0.119	3.3E-53	3
RP23-45G1	1.45E-56	-0.49828	0.229	0.34	2.41E-52	3
Crmp1	1.86E-56	0.28582	0.8	0.648	3.11E-52	3
Rassf4	4.13E-56	0.389802	0.524	0.354	6.9E-52	3
Bub1	5.96E-56	-0.4368	0.039	0.144	9.94E-52	3
Knstrn	6.13E-56	-0.52344	0.084	0.2	1.02E-51	3
Meg3	1.68E-54	-0.85289	0.025	0.105	2.8E-50	3
Ckap5	1.2E-53	-0.49428	0.163	0.28	2E-49	3
Plk1	5.16E-53	-0.36027	0.017	0.101	8.6E-49	3
Mxd3	8.45E-53	-0.42533	0.039	0.139	1.41E-48	3
Cdkn2d	3.7E-52	-0.45242	0.059	0.171	6.18E-48	3
Gap43	9.74E-52	-0.42461	0.551	0.606	1.63E-47	3
Pde1c	2E-51	0.284336	0.535	0.365	3.33E-47	3
Cbfa2t3	4.83E-51	0.322096	0.55	0.381	8.06E-47	3
Mroh2a	5.51E-51	0.50356	0.213	0.099	9.19E-47	3
Ckap2	2.31E-50	-0.46846	0.067	0.177	3.86E-46	3
1500016L(	2.47E-50	0.309471	0.531	0.361	4.12E-46	3
Islr2	2.64E-49	0.401111	0.299	0.162	4.41E-45	3
Dpysl4	3.23E-49	0.303208	0.634	0.47	5.38E-45	3
Sparcl1	3.74E-49	-0.74603	0.127	0.218	6.24E-45	3
Nuf2	4.18E-48	-0.42116	0.052	0.153	6.96E-44	3
Selm	8.81E-48	0.363302	0.291	0.157	1.47E-43	3
Bub1b	1.74E-47	-0.3601	0.028	0.113	2.9E-43	3
Mdk	2.15E-47	0.393503	0.511	0.36	3.59E-43	3
Racgap1	4.37E-47	-0.45388	0.162	0.269	7.28E-43	3
Map1b	1.42E-44	-0.25161	0.614	0.583	2.37E-40	3
Sept3	1.44E-44	-0.31647	0.383	0.394	2.4E-40	3
Ctsd	3.16E-44	-0.68129	0.161	0.176	5.28E-40	3
Dpysl3	2.32E-43	-0.52864	0.139	0.231	3.86E-39	3
Fabp7	1.02E-42	-0.91367	0.098	0.126	1.69E-38	3
Ppp1r14c	3.34E-42	-0.47577	0.186	0.273	5.56E-38	3
Tmpo	5.62E-42	-0.25722	0.432	0.454	9.37E-38	3

Fbxo5	1.69E-41	-0.42443	0.076	0.171	2.82E-37	3
Kif20b	2.87E-41	-0.48073	0.102	0.203	4.79E-37	3
Sgol1	4.72E-41	-0.38211	0.052	0.138	7.87E-37	3
Mapt	6.12E-41	-0.51441	0.142	0.227	1.02E-36	3
Elmo1	7.78E-41	-0.51101	0.052	0.136	1.3E-36	3
Rangap1	1.54E-40	-0.41776	0.153	0.271	2.56E-36	3
Tubb2a	2.07E-40	-0.48704	0.171	0.255	3.46E-36	3
Kif4	2.66E-40	-0.35392	0.037	0.118	4.45E-36	3
Hist1h2ak	8.04E-40	-0.46528	0.068	0.153	1.34E-35	3
Dek	8.23E-40	-0.26711	0.679	0.695	1.37E-35	3
Dlgap5	1.01E-39	-0.37359	0.04	0.122	1.68E-35	3
Tuba1b	2.35E-39	-0.27525	0.451	0.485	3.91E-35	3
Vim	4.1E-39	-0.45586	0.225	0.313	6.84E-35	3
Rcor2	5.34E-38	0.293865	0.338	0.208	8.91E-34	3
Aurka	1.39E-37	-0.30856	0.03	0.108	2.32E-33	3
Ccm2	2.58E-37	0.323865	0.336	0.208	4.3E-33	3
Anln	9.79E-37	-0.35006	0.037	0.114	1.63E-32	3
Mycn	1.07E-36	0.293365	0.486	0.345	1.79E-32	3
Rpl35a	1.29E-36	0.250089	0.681	0.554	2.15E-32	3
Ect2	1.62E-36	-0.3195	0.033	0.107	2.7E-32	3
Dbf4	2.19E-36	-0.36651	0.066	0.157	3.65E-32	3
Hn1	8.74E-36	-0.26303	0.504	0.547	1.46E-31	3
Slc1a2	2.32E-35	0.313007	0.411	0.277	3.87E-31	3
Mcm2	3.27E-35	0.266236	0.309	0.189	5.46E-31	3
C330027C(	5.42E-35	-0.36362	0.06	0.143	9.04E-31	3
Ctsb	2E-34	-0.47853	0.237	0.265	3.34E-30	3
Rnd3	6.27E-34	0.27164	0.552	0.413	1.05E-29	3
Mtss1	6.72E-34	-0.40275	0.212	0.277	1.12E-29	3
Eln	2.12E-33	0.311763	0.136	0.059	3.53E-29	3
Tk1	2.41E-33	-0.36475	0.065	0.14	4.02E-29	3
Cacng2	2.9E-32	0.28592	0.453	0.321	4.84E-28	3
Trpc4ap	2.91E-32	-0.4334	0.133	0.207	4.85E-28	3
Arpp21	6.28E-31	-0.43903	0.062	0.12	1.05E-26	3
Melk	6.5E-31	-0.29236	0.037	0.102	1.08E-26	3
Gnl3	7.13E-31	0.250335	0.406	0.279	1.19E-26	3
Fxyd6	2.11E-30	-0.26404	0.477	0.513	3.52E-26	3
Tubb4b	2.12E-30	-0.36632	0.165	0.251	3.54E-26	3
Ncapd2	2.55E-30	-0.34467	0.104	0.196	4.25E-26	3
Plp1	4.36E-30	-0.66904	0.124	0.113	7.27E-26	3
Sema7a	4.76E-30	0.288328	0.244	0.142	7.95E-26	3
D17H6S56I	3.22E-29	-0.32434	0.064	0.136	5.37E-25	3
Ccng2	3.53E-29	-0.32533	0.123	0.224	5.9E-25	3
2310022B(	5.68E-29	0.282484	0.35	0.233	9.48E-25	3
Cks1b	6.51E-29	-0.27682	0.303	0.35	1.09E-24	3
Atp2b1	6.81E-29	-0.30004	0.514	0.579	1.14E-24	3
Hspa5	1.69E-28	0.272277	0.643	0.536	2.82E-24	3
Stmn4	2.67E-28	-0.32123	0.323	0.344	4.45E-24	3
Atoh1	6.02E-28	0.276614	0.25	0.151	1E-23	3

Egr1	6.54E-28	-0.40557	0.318	0.393	1.09E-23	3
Ccdc34	1.35E-27	-0.29246	0.365	0.421	2.25E-23	3
Cacng4	7.54E-27	-0.4543	0.052	0.109	1.26E-22	3
Mad2l1	2.48E-26	-0.29528	0.062	0.132	4.14E-22	3
St18	4.45E-26	-0.3935	0.135	0.198	7.43E-22	3
Ank2	6.74E-26	-0.39511	0.178	0.249	1.12E-21	3
Cenpq	2.58E-25	-0.32747	0.093	0.169	4.3E-21	3
B2m	3.4E-25	-0.29623	0.216	0.233	5.66E-21	3
Hes1	7.74E-25	-0.43248	0.091	0.156	1.29E-20	3
Tprn	7.9E-25	0.297772	0.314	0.213	1.32E-20	3
Diap3	9.1E-25	-0.28793	0.049	0.114	1.52E-20	3
Mns1	1.87E-24	-0.34041	0.132	0.213	3.12E-20	3
Prdm8	2.28E-24	-0.36977	0.07	0.134	3.8E-20	3
Dner	2.36E-24	-0.35932	0.159	0.222	3.93E-20	3
Klf6	2.41E-23	-0.3387	0.095	0.156	4.03E-19	3
Trim59	5.99E-23	-0.29661	0.105	0.178	9.99E-19	3
Gse1	7.33E-23	0.256229	0.322	0.224	1.22E-18	3
Nek6	1.38E-21	0.271366	0.215	0.133	2.3E-17	3
Pttg1	5.8E-21	-0.32874	0.082	0.147	9.68E-17	3
Sptbn1	1.18E-20	-0.30859	0.289	0.362	1.96E-16	3
Sema6a	7.22E-20	-0.31002	0.162	0.207	1.2E-15	3
Rrm2	1.47E-19	-0.28698	0.157	0.21	2.45E-15	3
Ska2	3.45E-19	-0.28259	0.141	0.213	5.75E-15	3
Thra	3.81E-19	-0.31864	0.18	0.236	6.35E-15	3
Add3	3.83E-19	-0.2951	0.055	0.111	6.39E-15	3
Kcnk1	1.23E-18	-0.31734	0.151	0.212	2.04E-14	3
Cenph	2.31E-18	-0.30026	0.135	0.202	3.86E-14	3
Tspan7	2.74E-18	-0.30625	0.064	0.111	4.58E-14	3
H1fx	3.56E-18	-0.26479	0.195	0.252	5.93E-14	3
Mt1	7.47E-18	-0.34237	0.149	0.17	1.25E-13	3
Slc1a3	1.86E-17	-0.38313	0.123	0.157	3.11E-13	3
Cenpw	4.37E-17	-0.25482	0.078	0.134	7.29E-13	3
G2e3	4.69E-17	-0.25068	0.079	0.138	7.82E-13	3
Rad51ap1	6.78E-17	-0.27406	0.112	0.174	1.13E-12	3
Rab3a	2.25E-15	-0.29238	0.106	0.156	3.76E-11	3
Gnao1	2.88E-15	-0.26631	0.233	0.295	4.81E-11	3
Odf2	4.56E-15	-0.25691	0.121	0.178	7.61E-11	3
Stxbp1	6.19E-15	-0.27237	0.123	0.168	1.03E-10	3
Cep110	2.12E-14	-0.25884	0.111	0.17	3.53E-10	3
Aplp1	3.01E-14	-0.27705	0.111	0.157	5.01E-10	3
Lgals1	4.88E-14	-0.26941	0.197	0.233	8.15E-10	3
A930011O	8.02E-14	-0.26791	0.086	0.118	1.34E-09	3
Myt1l	1.77E-12	-0.26985	0.127	0.165	2.95E-08	3
Fyn	2.34E-12	-0.25698	0.184	0.22	3.9E-08	3
Pea15a	1.58E-11	-0.25107	0.134	0.182	2.63E-07	3
Fos	5.16E-09	-0.258	0.265	0.31	8.6E-05	3
Neurod1	6E-221	-1.5216	0.256	0.564	1E-216	4
Pcna	1.4E-186	0.751477	0.583	0.332	2.4E-182	4

Lig1	2.5E-181	0.798601	0.592	0.32	4.1E-177	4
Mcm6	1.6E-180	0.815715	0.504	0.249	2.6E-176	4
Tuba1a	9.1E-175	-0.62696	0.861	0.937	1.5E-170	4
Stmn2	1.3E-158	-1.07653	0.321	0.579	2.1E-154	4
Ung	1.1E-148	0.781064	0.276	0.071	1.9E-144	4
Hells	5.2E-147	0.811418	0.428	0.188	8.7E-143	4
Rtn1	6.6E-143	-0.7894	0.486	0.713	1.1E-138	4
Arl6ip1	4E-140	-0.9151	0.327	0.575	6.6E-136	4
Cntn2	5.2E-140	-1.23377	0.053	0.287	8.7E-136	4
Mcm3	2.9E-136	0.758386	0.376	0.157	4.8E-132	4
Tubb3	1.8E-122	-0.94536	0.262	0.498	3.1E-118	4
Ube2c	1.1E-119	-1.18307	0.086	0.29	1.8E-115	4
Tmsb4x	1.4E-119	-0.50638	0.895	0.954	2.3E-115	4
Gpm6a	5.9E-116	-0.88191	0.184	0.434	9.9E-112	4
Map1b	2.2E-112	-0.75516	0.376	0.611	3.7E-108	4
Rpa2	1.1E-111	0.675926	0.373	0.18	1.9E-107	4
Mcm2	8.5E-110	0.678645	0.384	0.184	1.4E-105	4
Gap43	8E-109	-0.75512	0.409	0.621	1.3E-104	4
Cenpa	4.4E-106	-1.00428	0.072	0.272	7.4E-102	4
Ranbp1	1.1E-104	0.42937	0.782	0.663	1.9E-100	4
Calm2	3.2E-104	-0.44477	0.763	0.886	5.3E-100	4
Rplp1	6.8E-103	0.338164	0.94	0.869	1.13E-98	4
Dut	6.9E-102	0.536558	0.611	0.414	1.16E-97	4
Itm2b	2.2E-101	-0.61615	0.452	0.664	3.74E-97	4
Rps5	1.1E-100	0.289953	0.982	0.95	1.82E-96	4
Ccnd1	1.01E-96	0.497873	0.696	0.502	1.68E-92	4
Tbata	3.08E-90	0.617945	0.509	0.32	5.13E-86	4
Nasp	3.25E-89	0.407497	0.749	0.627	5.42E-85	4
Dek	1.65E-87	0.369456	0.83	0.677	2.74E-83	4
Stmn4	1.4E-86	-0.80615	0.152	0.363	2.34E-82	4
Cenpf	2.03E-86	-0.93748	0.207	0.38	3.39E-82	4
Rpl8	3.09E-86	0.303553	0.943	0.892	5.16E-82	4
Cltb	8.85E-85	0.516543	0.539	0.409	1.48E-80	4
Tpx2	8.79E-84	-0.82623	0.122	0.299	1.47E-79	4
Srebf1	1.25E-83	0.587363	0.498	0.315	2.08E-79	4
Nrxn1	1.36E-83	-0.80574	0.139	0.338	2.27E-79	4
Cdt1	9.9E-83	0.602432	0.266	0.109	1.65E-78	4
Eef1b2	2.56E-81	0.361351	0.821	0.722	4.27E-77	4
Cenpe	5.53E-80	-0.8837	0.124	0.303	9.22E-76	4
Siva1	1.24E-78	0.548767	0.416	0.255	2.06E-74	4
Rpl13a	9.87E-78	0.27087	0.96	0.924	1.65E-73	4
Mcm5	1.26E-77	0.571198	0.314	0.151	2.1E-73	4
Zic1	5.23E-77	-0.42641	0.655	0.798	8.72E-73	4
Cdca7	1.62E-76	0.573048	0.337	0.181	2.7E-72	4
Tubb2a	2.17E-75	-0.73203	0.085	0.263	3.62E-71	4
Npm1	4.49E-75	0.370127	0.742	0.635	7.48E-71	4
Ptn	4.58E-74	-0.748	0.279	0.46	7.63E-70	4
Tubb2b	5.3E-73	-0.59025	0.278	0.485	8.84E-69	4

Mtss1	6.27E-73	-0.66778	0.104	0.288	1.05E-68	4
Uhrf1	1.93E-72	0.553951	0.271	0.129	3.23E-68	4
Kif23	2.95E-72	-0.73075	0.078	0.229	4.92E-68	4
Ddah2	1.41E-71	-0.47294	0.525	0.704	2.35E-67	4
Rps3	1.6E-71	0.267907	0.955	0.918	2.67E-67	4
Apoe	3.19E-71	-1.49766	0.232	0.25	5.32E-67	4
Cdk5r1	8.54E-71	-0.60972	0.156	0.353	1.42E-66	4
Paics	8.9E-71	0.445471	0.52	0.401	1.48E-66	4
Hn1	2.28E-70	-0.49526	0.351	0.563	3.8E-66	4
Dpysl3	3.63E-70	-0.73743	0.073	0.236	6.05E-66	4
Anp32b	4.14E-69	0.383948	0.701	0.578	6.91E-65	4
Rplp2	3.94E-68	0.28599	0.898	0.833	6.58E-64	4
Mcm4	1.89E-66	0.540185	0.261	0.123	3.15E-62	4
Gng3	2.41E-66	-0.65814	0.154	0.335	4.02E-62	4
Mcm7	2.51E-66	0.43399	0.5	0.376	4.18E-62	4
Nrep	5.87E-66	-0.53362	0.43	0.585	9.79E-62	4
Elavl4	7.3E-66	-0.6163	0.165	0.354	1.22E-61	4
Cdc20	1.65E-65	-0.74076	0.069	0.21	2.76E-61	4
Hmmr	1.99E-65	-0.71105	0.054	0.19	3.32E-61	4
Nhlh2	8.04E-65	-0.5907	0.246	0.443	1.34E-60	4
Celf4	8.66E-65	-0.7606	0.169	0.337	1.44E-60	4
Malat1	3.09E-64	-0.4047	0.927	0.958	5.15E-60	4
Rps20	4.14E-64	0.299883	0.869	0.767	6.91E-60	4
Prc1	4.86E-64	-0.74394	0.13	0.271	8.11E-60	4
Cdc6	1.26E-63	0.458113	0.139	0.037	2.1E-59	4
Chaf1b	4.94E-63	0.496572	0.194	0.071	8.24E-59	4
Ckb	5.84E-63	-0.41076	0.728	0.817	9.75E-59	4
Nhlh1	2.98E-62	-0.66034	0.084	0.239	4.96E-58	4
Dhfr	4.1E-62	0.522699	0.271	0.134	6.83E-58	4
Ccnb2	4.2E-62	-0.63718	0.06	0.201	7E-58	4
Dtl	4.28E-62	0.526118	0.263	0.132	7.14E-58	4
Rplp0	5.09E-62	0.270336	0.909	0.868	8.5E-58	4
Apc	1.67E-61	-0.51887	0.254	0.451	2.79E-57	4
St18	3.21E-61	-0.67491	0.063	0.205	5.35E-57	4
Serinc1	5.08E-61	-0.48125	0.26	0.456	8.47E-57	4
Gnb2l1	1.63E-60	0.263739	0.913	0.867	2.71E-56	4
Elmo1	2.73E-60	-0.64057	0.023	0.137	4.56E-56	4
Ccnb1	8.31E-60	-0.58084	0.035	0.151	1.39E-55	4
Nusap1	1.62E-59	-0.67238	0.099	0.241	2.7E-55	4
Mapt	1.85E-59	-0.68074	0.083	0.231	3.09E-55	4
Serbp1	2E-59	0.268093	0.904	0.865	3.34E-55	4
Tex14	8.45E-59	-0.62751	0.034	0.158	1.41E-54	4
Ccnd2	9.04E-59	0.383668	0.774	0.649	1.51E-54	4
Rps21	6.44E-58	0.280608	0.883	0.827	1.07E-53	4
Mllt11	1.02E-57	-0.60783	0.084	0.233	1.7E-53	4
Rps19	1.43E-57	0.307787	0.829	0.745	2.39E-53	4
Sfrp1	3.4E-57	0.298867	0.895	0.802	5.66E-53	4
Ccng2	6.72E-57	-0.49698	0.079	0.227	1.12E-52	4

Tagln3	1.15E-56	-0.53744	0.142	0.312	1.92E-52	4
Nop58	9.34E-56	0.35484	0.679	0.581	1.56E-51	4
Gria2	3.64E-55	-0.49604	0.366	0.538	6.07E-51	4
H3f3b	7.59E-55	-0.27652	0.837	0.914	1.27E-50	4
Sema6a	1.97E-54	-0.58956	0.076	0.216	3.28E-50	4
Calm1	3.81E-54	-0.32882	0.772	0.872	6.35E-50	4
Rpl14	3.85E-54	0.300185	0.752	0.69	6.41E-50	4
Ckap2l	7.89E-54	-0.60604	0.094	0.231	1.32E-49	4
Rpl41	2.12E-53	0.276487	0.842	0.773	3.54E-49	4
Pdzrn3	4.35E-53	-0.62198	0.04	0.159	7.26E-49	4
Dner	7.7E-53	-0.56343	0.085	0.229	1.28E-48	4
Celf2	1.1E-52	-0.41016	0.491	0.654	1.83E-48	4
Rab3a	1.34E-52	-0.55412	0.042	0.162	2.23E-48	4
Dctpp1	3.32E-52	0.415499	0.4	0.293	5.54E-48	4
Cdca3	3.49E-52	-0.57932	0.1	0.235	5.82E-48	4
Sept3	4.67E-52	-0.54631	0.256	0.408	7.8E-48	4
Nap1l1	5.13E-52	0.333718	0.576	0.503	8.56E-48	4
Clspn	1.13E-51	0.505234	0.311	0.175	1.88E-47	4
Nhp2	1.82E-51	0.392145	0.463	0.36	3.04E-47	4
Map2	2.34E-51	-0.4559	0.333	0.509	3.9E-47	4
Spc25	7.42E-51	-0.58835	0.108	0.238	1.24E-46	4
Aplp1	7.57E-51	-0.54108	0.044	0.163	1.26E-46	4
Ppp1r14c	9.55E-51	-0.59962	0.131	0.277	1.59E-46	4
H2afx	7.15E-50	-0.53657	0.244	0.34	1.19E-45	4
Cbx5	1.5E-48	0.296458	0.729	0.644	2.5E-44	4
Basp1	2.3E-48	-0.36066	0.59	0.717	3.83E-44	4
Myt1	4.66E-48	-0.53546	0.048	0.164	7.78E-44	4
Rps11	1.48E-47	0.258588	0.828	0.771	2.47E-43	4
Snhg1	3.25E-47	0.397242	0.445	0.347	5.42E-43	4
Prmt8	5.65E-47	0.446983	0.377	0.26	9.43E-43	4
Cadm3	7.03E-47	-0.53846	0.042	0.152	1.17E-42	4
Sptbn1	7.42E-47	-0.498	0.208	0.37	1.24E-42	4
Hat1	8.44E-47	0.451077	0.257	0.143	1.41E-42	4
Nolc1	9.2E-47	0.380631	0.482	0.4	1.53E-42	4
Tsc22d1	1.11E-46	-0.36085	0.271	0.446	1.84E-42	4
Syt11	5.05E-46	-0.3987	0.351	0.522	8.42E-42	4
Hsd11b2	6.49E-46	0.452769	0.392	0.275	1.08E-41	4
Mif	6.93E-46	0.365252	0.426	0.348	1.16E-41	4
E2f1	1.04E-45	0.469065	0.241	0.136	1.73E-41	4
Ckap5	2.05E-45	-0.49247	0.135	0.28	3.42E-41	4
Atp5e	7.32E-45	0.26816	0.696	0.647	1.22E-40	4
Dcx	2.12E-44	-0.45823	0.233	0.392	3.54E-40	4
Tcf19	1.18E-43	0.401611	0.198	0.087	1.97E-39	4
H2afy	1.19E-43	0.276782	0.645	0.585	1.99E-39	4
Birc5	4.73E-43	-0.5355	0.168	0.285	7.9E-39	4
Itsn1	4.93E-43	-0.50843	0.113	0.249	8.22E-39	4
Fam64a	6.18E-43	-0.40709	0.031	0.127	1.03E-38	4
Timeless	1.27E-42	0.443538	0.243	0.143	2.12E-38	4

Ina	3.27E-42	-0.45752	0.31	0.459	5.45E-38	4
Rfc3	5.95E-42	0.416544	0.3	0.193	9.93E-38	4
Trpc4ap	6.71E-42	-0.53207	0.089	0.21	1.12E-37	4
Chaf1a	7.37E-42	0.433613	0.25	0.159	1.23E-37	4
Pkm	1.06E-41	0.309824	0.484	0.423	1.77E-37	4
Ptms	1.07E-41	-0.38242	0.324	0.49	1.79E-37	4
Aspm	1.1E-41	-0.51418	0.053	0.153	1.83E-37	4
Nop10	1.26E-41	0.296615	0.528	0.476	2.11E-37	4
Cst3	1.55E-41	-0.50783	0.453	0.545	2.59E-37	4
Cdkn2d	1.73E-41	-0.43864	0.058	0.168	2.89E-37	4
Tipin	4.43E-41	0.399772	0.339	0.245	7.39E-37	4
Tnik	2.27E-40	-0.47507	0.051	0.157	3.78E-36	4
Dnajc9	9.2E-40	0.380766	0.442	0.339	1.53E-35	4
Pdgfa	2.12E-39	0.443763	0.349	0.242	3.53E-35	4
Top2a	1.05E-38	-0.38963	0.409	0.429	1.76E-34	4
Ankrd12	1.31E-38	-0.49283	0.151	0.288	2.18E-34	4
Chgb	1.83E-38	-0.56253	0.132	0.253	3.06E-34	4
B3galt2	2.37E-38	-0.44233	0.023	0.106	3.95E-34	4
Ubb	3.58E-38	-0.31537	0.396	0.558	5.96E-34	4
Mki67	4.24E-38	-0.47696	0.33	0.402	7.07E-34	4
Tyms	5.41E-38	0.405756	0.322	0.207	9.02E-34	4
Rfc2	6.1E-38	0.401487	0.301	0.2	1.02E-33	4
Rps3a1	7.03E-38	0.265508	0.724	0.66	1.17E-33	4
Gmnn	1.95E-37	0.40516	0.235	0.134	3.26E-33	4
Cdk1	2.76E-37	-0.47985	0.167	0.255	4.6E-33	4
Nop56	3.45E-37	0.357591	0.451	0.35	5.76E-33	4
Gpm6b	7.63E-37	-0.35087	0.342	0.5	1.27E-32	4
Ssrp1	1.15E-36	0.268233	0.634	0.569	1.93E-32	4
Chd3	2.94E-36	-0.4472	0.057	0.159	4.91E-32	4
Pax6	3.43E-36	-0.33368	0.378	0.528	5.73E-32	4
Prim1	4.08E-36	0.390315	0.361	0.251	6.81E-32	4
Nsg2	5.8E-36	-0.34087	0.349	0.503	9.68E-32	4
Dtymk	6.61E-36	0.309798	0.516	0.43	1.1E-31	4
Srsf7	8.22E-36	0.271837	0.509	0.46	1.37E-31	4
Rps25	9.58E-36	0.295093	0.535	0.473	1.6E-31	4
Dync1i2	1.43E-35	-0.2966	0.427	0.584	2.39E-31	4
Rtn4	1.55E-35	-0.32717	0.341	0.496	2.59E-31	4
Hspd1	2E-35	0.302955	0.421	0.358	3.34E-31	4
Cbfa2t3	2.96E-35	0.34115	0.474	0.394	4.93E-31	4
Kcnk1	3.06E-35	-0.43574	0.1	0.216	5.1E-31	4
Smc4	4.49E-35	-0.26075	0.562	0.567	7.5E-31	4
Arpp21	5.14E-35	-0.49981	0.035	0.121	8.57E-31	4
Hist3h2a	1.12E-34	-0.4165	0.054	0.153	1.86E-30	4
Pde1c	1.14E-34	-0.41602	0.259	0.401	1.91E-30	4
Shmt1	1.33E-34	0.370696	0.172	0.095	2.21E-30	4
Hey1	1.36E-34	0.39634	0.355	0.263	2.26E-30	4
Slc25a5	1.51E-34	0.257096	0.551	0.503	2.52E-30	4
H1f0	1.69E-34	-0.31251	0.488	0.631	2.82E-30	4

Rif1	2.45E-34	0.377779	0.354	0.266	4.09E-30	4
Akirin2	2.46E-34	-0.31157	0.096	0.209	4.1E-30	4
Rps2	3.5E-34	0.269592	0.487	0.445	5.83E-30	4
Idh2	7.21E-34	0.328396	0.339	0.271	1.2E-29	4
A330076H(	7.27E-34	-0.41624	0.068	0.171	1.21E-29	4
Arhgap11a	7.58E-34	-0.44334	0.078	0.181	1.26E-29	4
Rpa1	1.12E-33	0.373884	0.18	0.1	1.86E-29	4
A930011O:	1.61E-33	-0.46436	0.037	0.123	2.68E-29	4
Elavl3	5.53E-33	-0.36739	0.346	0.487	9.22E-29	4
Bola2	9.25E-33	0.307743	0.448	0.391	1.54E-28	4
Ctsb	9.77E-33	-0.57573	0.182	0.271	1.63E-28	4
Rbfox2	9.87E-33	-0.385	0.168	0.296	1.65E-28	4
Ank2	1.11E-32	-0.47164	0.133	0.252	1.85E-28	4
Snrpd1	1.41E-32	0.263324	0.573	0.518	2.35E-28	4
D4Wsu53e	1.6E-32	-0.32181	0.314	0.462	2.67E-28	4
Mis18bp1	1.86E-32	-0.43974	0.074	0.17	3.1E-28	4
Thra	1.93E-32	-0.45981	0.125	0.24	3.22E-28	4
Myt1l	2.63E-32	-0.4891	0.073	0.171	4.38E-28	4
Brd8	3.46E-32	-0.3219	0.263	0.406	5.77E-28	4
Sox9	3.58E-32	0.389317	0.339	0.245	5.97E-28	4
Rbp4	5.88E-32	0.395281	0.326	0.232	9.81E-28	4
Dnmt1	9.33E-32	0.361567	0.366	0.267	1.56E-27	4
Meg3	1.14E-31	-0.74175	0.035	0.102	1.91E-27	4
D430041D	1.49E-31	0.299075	0.551	0.485	2.48E-27	4
Ccm2	1.58E-31	0.373402	0.301	0.216	2.64E-27	4
Aurka	2.08E-31	-0.29713	0.029	0.106	3.46E-27	4
Prkcb	2.11E-31	-0.32914	0.146	0.268	3.52E-27	4
Gli1	2.66E-31	0.373018	0.166	0.101	4.44E-27	4
Ccne2	3.64E-31	0.367912	0.14	0.061	6.08E-27	4
Prnp	3.84E-31	-0.33664	0.107	0.218	6.41E-27	4
Supt16	4.05E-31	0.252431	0.527	0.489	6.76E-27	4
Cdca8	5.57E-31	-0.4473	0.166	0.275	9.29E-27	4
Prdx4	6.52E-31	0.308128	0.428	0.358	1.09E-26	4
Dkc1	6.9E-31	0.325401	0.397	0.325	1.15E-26	4
Rad21	6.91E-31	-0.35925	0.345	0.475	1.15E-26	4
Calm3	7.52E-31	-0.26054	0.428	0.575	1.25E-26	4
Fxyd6	1.01E-30	-0.31999	0.394	0.522	1.69E-26	4
Kdm5b	1.07E-30	-0.4097	0.09	0.195	1.79E-26	4
Fam111a	1.14E-30	0.40234	0.204	0.117	1.9E-26	4
Kif11	1.42E-30	-0.41946	0.108	0.217	2.37E-26	4
Zfp367	1.89E-30	0.346967	0.166	0.084	3.16E-26	4
Fnbp1l	2.04E-30	-0.32639	0.327	0.467	3.4E-26	4
Gins2	2.11E-30	0.350172	0.246	0.178	3.52E-26	4
Afap1	8.82E-30	-0.30231	0.068	0.161	1.47E-25	4
Cpe	9.66E-30	-0.31724	0.333	0.469	1.61E-25	4
Klf6	1.18E-29	-0.35421	0.064	0.158	1.97E-25	4
Sparcl1	1.18E-29	-0.56932	0.147	0.214	1.98E-25	4
Sept4	1.31E-29	-0.41604	0.126	0.238	2.19E-25	4

Tead2	1.79E-29	0.326593	0.348	0.269	2.98E-25	4
Srm	2.44E-29	0.361648	0.266	0.183	4.07E-25	4
Cdkn1b	4.13E-29	-0.29282	0.34	0.481	6.88E-25	4
Tmsb10	4.57E-29	-0.26282	0.546	0.677	7.62E-25	4
Kif5c	7.08E-29	-0.36139	0.279	0.412	1.18E-24	4
Dscc1	9.32E-29	0.320945	0.117	0.048	1.55E-24	4
Dlgap5	9.61E-29	-0.32263	0.041	0.12	1.6E-24	4
Rab6a	1.01E-28	-0.29737	0.212	0.341	1.69E-24	4
Uncx	1.44E-28	-0.35058	0.204	0.331	2.4E-24	4
Ank3	1.64E-28	-0.35531	0.301	0.433	2.74E-24	4
Gnl3	2.32E-28	0.335954	0.362	0.288	3.87E-24	4
Soga3	2.37E-28	-0.28149	0.332	0.471	3.95E-24	4
C1qbp	2.41E-28	0.276094	0.42	0.372	4.01E-24	4
Abhd16a	2.52E-28	-0.3234	0.097	0.2	4.2E-24	4
RP23-45G1	3.01E-28	-0.40659	0.227	0.338	5.02E-24	4
Atp6v0b	3.37E-28	-0.34676	0.161	0.279	5.61E-24	4
Btbd17	3.63E-28	-0.35414	0.074	0.17	6.06E-24	4
Rnf5	4.1E-28	-0.26864	0.155	0.269	6.84E-24	4
1110038B1	4.98E-28	0.302554	0.352	0.292	8.3E-24	4
Kif1b	6.34E-28	-0.29206	0.397	0.535	1.06E-23	4
Baz1a	7.84E-28	0.363957	0.224	0.159	1.31E-23	4
Prdm8	1.06E-27	-0.40143	0.051	0.135	1.76E-23	4
Eef1d	1.22E-27	0.265101	0.389	0.345	2.04E-23	4
Gsg1l	1.24E-27	0.332498	0.316	0.248	2.06E-23	4
Atad5	1.62E-27	0.368995	0.219	0.14	2.71E-23	4
Slc29a1	1.71E-27	0.270306	0.624	0.554	2.86E-23	4
Nol4	2.26E-27	-0.27	0.087	0.181	3.77E-23	4
Pafah1b1	4.4E-27	-0.26355	0.355	0.487	7.34E-23	4
Dbn1	4.81E-27	-0.329	0.062	0.148	8.03E-23	4
Rbm5	5.19E-27	-0.27068	0.284	0.414	8.65E-23	4
Klf7	5.26E-27	-0.36923	0.159	0.273	8.78E-23	4
Slc17a6	6.48E-27	-0.32711	0.068	0.157	1.08E-22	4
Mab21l1	7.84E-27	-0.34038	0.127	0.236	1.31E-22	4
Pola2	8.64E-27	0.336607	0.157	0.085	1.44E-22	4
Atad2	9.5E-27	0.341407	0.276	0.189	1.58E-22	4
Cdc45	1.1E-26	0.319746	0.16	0.087	1.84E-22	4
Gdi1	1.28E-26	-0.34171	0.131	0.241	2.14E-22	4
Mms22l	1.76E-26	0.307865	0.12	0.056	2.94E-22	4
Idh1	2.39E-26	-0.27124	0.077	0.165	3.99E-22	4
Rufy3	3.01E-26	-0.31021	0.257	0.384	5.02E-22	4
Boc	3.82E-26	0.338519	0.21	0.149	6.37E-22	4
Kif5a	4.2E-26	-0.3474	0.054	0.136	7.01E-22	4
Msh6	4.76E-26	0.332706	0.151	0.081	7.93E-22	4
Tacc3	5.48E-26	-0.36472	0.117	0.216	9.14E-22	4
Kif2c	9.18E-26	-0.31524	0.042	0.114	1.53E-21	4
Bin1	9.24E-26	-0.31134	0.315	0.442	1.54E-21	4
Polr2f	9.59E-26	0.274738	0.426	0.368	1.6E-21	4
Rangap1	1.16E-25	-0.30954	0.155	0.267	1.93E-21	4

Pnmal2	1.59E-25	-0.32063	0.068	0.155	2.66E-21	4
Mdk	1.97E-25	0.308897	0.454	0.37	3.29E-21	4
Slfn9	2.3E-25	0.33184	0.138	0.066	3.83E-21	4
Uchl1	2.43E-25	-0.33888	0.226	0.347	4.05E-21	4
Gng2	2.77E-25	-0.28	0.281	0.409	4.62E-21	4
Gdpd1	2.98E-25	-0.29639	0.145	0.254	4.96E-21	4
Stxbp1	3.54E-25	-0.35626	0.08	0.171	5.91E-21	4
2810417H1	5.42E-25	0.274176	0.477	0.381	9.04E-21	4
Rab6b	6.27E-25	-0.37207	0.113	0.212	1.05E-20	4
Knstrn	6.31E-25	-0.36765	0.1	0.195	1.05E-20	4
Fam210b	6.6E-25	0.3155	0.386	0.305	1.1E-20	4
Grina	9.11E-25	-0.32803	0.042	0.116	1.52E-20	4
Hsp90b1	1.26E-24	-0.2601	0.602	0.702	2.11E-20	4
Mbnl2	1.28E-24	-0.31967	0.094	0.188	2.14E-20	4
Arhgef2	1.85E-24	-0.2609	0.182	0.293	3.09E-20	4
Mcm10	2.08E-24	0.316349	0.12	0.061	3.46E-20	4
Nnat	2.7E-24	-0.29129	0.647	0.717	4.5E-20	4
Mxd4	3.03E-24	-0.25713	0.114	0.207	5.06E-20	4
Wdr76	3.79E-24	0.31815	0.14	0.072	6.33E-20	4
Jhdm1d	4.3E-24	-0.26367	0.057	0.132	7.17E-20	4
Hist3h2ba	4.49E-24	-0.31319	0.063	0.146	7.5E-20	4
Polr2h	5.21E-24	0.286938	0.27	0.219	8.7E-20	4
Fen1	5.38E-24	0.324672	0.204	0.134	8.98E-20	4
Usp1	9.32E-24	0.300551	0.415	0.329	1.55E-19	4
Sgol2	1.74E-23	-0.38018	0.083	0.165	2.91E-19	4
Pqlc1	2.05E-23	0.292634	0.347	0.291	3.43E-19	4
Rfc4	2.14E-23	0.312171	0.295	0.218	3.56E-19	4
Ppat	2.55E-23	0.322125	0.169	0.101	4.25E-19	4
Ect2	3.9E-23	-0.25502	0.037	0.104	6.51E-19	4
Kif22	8.77E-23	-0.32964	0.085	0.171	1.46E-18	4
Npdc1	9.54E-23	-0.30483	0.166	0.272	1.59E-18	4
Rpa3	1.27E-22	0.289297	0.296	0.237	2.11E-18	4
Casp8ap2	1.29E-22	0.324374	0.314	0.235	2.15E-18	4
Mapk8ip1	1.46E-22	-0.25318	0.139	0.237	2.43E-18	4
Rfc1	1.65E-22	0.302268	0.396	0.323	2.75E-18	4
Os9	2E-22	-0.27485	0.158	0.261	3.33E-18	4
Ptch2	2.12E-22	0.332096	0.215	0.145	3.53E-18	4
Miat	2.52E-22	-0.31986	0.416	0.527	4.2E-18	4
Podxl2	2.91E-22	-0.34639	0.14	0.237	4.85E-18	4
Hmgb2	3.53E-22	-0.25101	0.372	0.402	5.89E-18	4
Kif20b	4.06E-22	-0.36494	0.108	0.199	6.77E-18	4
Pold1	4.6E-22	0.293512	0.135	0.079	7.68E-18	4
Wdhd1	6.47E-22	0.311858	0.143	0.077	1.08E-17	4
Chrna3	6.47E-22	-0.32684	0.052	0.125	1.08E-17	4
Sox4	7.3E-22	-0.25263	0.481	0.594	1.22E-17	4
Fyn	9.06E-22	-0.33518	0.135	0.225	1.51E-17	4
Dtx1	9.11E-22	0.313463	0.216	0.159	1.52E-17	4
Fabp7	9.29E-22	-0.84447	0.084	0.127	1.55E-17	4

Taf1d	1.5E-21	0.272587	0.387	0.336	2.5E-17	4
Ldha	1.89E-21	0.28452	0.281	0.226	3.15E-17	4
Mthfd1	2.15E-21	0.288843	0.168	0.109	3.59E-17	4
Ipo5	2.23E-21	0.290883	0.189	0.132	3.72E-17	4
Rrm1	2.27E-21	0.299361	0.329	0.252	3.79E-17	4
Apitd1	2.32E-21	0.304361	0.149	0.088	3.87E-17	4
Incenp	2.41E-21	-0.37787	0.2	0.283	4.02E-17	4
Cenph	2.64E-21	0.318388	0.263	0.185	4.41E-17	4
Cdca7l	3.26E-21	0.297587	0.16	0.098	5.44E-17	4
Gnao1	3.49E-21	-0.31232	0.193	0.298	5.83E-17	4
Ctsd	3.49E-21	-0.60303	0.14	0.178	5.83E-17	4
Klc1	5.24E-21	-0.27027	0.259	0.373	8.74E-17	4
Clip3	7.17E-21	-0.26873	0.215	0.322	1.2E-16	4
Id2	7.56E-21	-0.27685	0.389	0.497	1.26E-16	4
Dclk1	7.78E-21	0.263268	0.466	0.406	1.3E-16	4
Ncam1	8.88E-21	-0.25509	0.283	0.398	1.48E-16	4
Pik3r3	8.89E-21	-0.26988	0.068	0.143	1.48E-16	4
Mcmbp	1.09E-20	0.27734	0.258	0.211	1.82E-16	4
BC005764	1.11E-20	-0.36636	0.111	0.194	1.86E-16	4
Apbb1	1.44E-20	-0.28743	0.094	0.178	2.41E-16	4
Clvs1	1.66E-20	-0.31657	0.062	0.136	2.76E-16	4
Pea15a	1.71E-20	-0.35412	0.1	0.184	2.86E-16	4
Racgap1	2.18E-20	-0.32507	0.168	0.265	3.63E-16	4
2900011O(	2.66E-20	-0.30826	0.044	0.11	4.43E-16	4
Rad54l	3.13E-20	0.262606	0.124	0.087	5.22E-16	4
Casc5	3.14E-20	-0.33263	0.108	0.195	5.24E-16	4
Tspyl4	3.7E-20	-0.28913	0.061	0.134	6.18E-16	4
Lrpap1	4.21E-20	-0.28237	0.054	0.124	7.03E-16	4
Stx7	5.9E-20	-0.26542	0.053	0.123	9.85E-16	4
Gins1	6.95E-20	0.286919	0.164	0.109	1.16E-15	4
Nuf2	7.43E-20	-0.29697	0.074	0.148	1.24E-15	4
Rad51	8.47E-20	0.28882	0.176	0.109	1.41E-15	4
Pkia	8.51E-20	-0.30751	0.067	0.141	1.42E-15	4
Lyar	9.82E-20	0.266367	0.323	0.281	1.64E-15	4
Zbtb18	1.17E-19	-0.314	0.178	0.276	1.95E-15	4
Atp6v1e1	1.28E-19	-0.26305	0.234	0.342	2.14E-15	4
Srrm4	2E-19	-0.3131	0.116	0.204	3.33E-15	4
Chek1	2.36E-19	0.270973	0.119	0.067	3.94E-15	4
Srgap2	2.38E-19	-0.27574	0.068	0.139	3.97E-15	4
Lap3	3.12E-19	0.279007	0.338	0.269	5.21E-15	4
Rnf168	3.28E-19	0.292706	0.268	0.2	5.48E-15	4
Clasp2	3.51E-19	-0.27084	0.094	0.175	5.86E-15	4
Rprml	6.51E-19	0.31782	0.134	0.074	1.09E-14	4
Snap25	6.57E-19	-0.28266	0.195	0.292	1.1E-14	4
Snhg4	7.04E-19	0.295527	0.142	0.083	1.17E-14	4
Pcdha2	8.14E-19	-0.32366	0.057	0.125	1.36E-14	4
Ablim2	8.73E-19	0.282619	0.117	0.071	1.46E-14	4
1700001O2	1.19E-18	0.271291	0.155	0.105	1.99E-14	4

Cep170	1.57E-18	-0.26989	0.213	0.315	2.62E-14	4
Thsd7a	2.84E-18	-0.2607	0.065	0.134	4.73E-14	4
Bok	4.62E-18	0.262856	0.301	0.245	7.71E-14	4
Gstp1	4.9E-18	0.259756	0.193	0.151	8.17E-14	4
Cacng4	5.31E-18	-0.41143	0.053	0.107	8.86E-14	4
Rnaseh2a	5.63E-18	0.280876	0.212	0.146	9.4E-14	4
Rrm2	7.96E-18	0.25315	0.272	0.196	1.33E-13	4
Gart	1.18E-17	0.255319	0.205	0.161	1.97E-13	4
Mycn	1.52E-17	0.25194	0.406	0.358	2.53E-13	4
4930422G(	1.89E-17	0.285263	0.115	0.069	3.15E-13	4
Ak2	3.23E-17	0.252003	0.255	0.208	5.39E-13	4
Kif4	5.65E-17	-0.25223	0.052	0.114	9.43E-13	4
Nexn	7.47E-17	0.348049	0.124	0.071	1.25E-12	4
Syce2	9.13E-17	0.274117	0.134	0.077	1.52E-12	4
Glce	1.01E-16	-0.26317	0.107	0.184	1.69E-12	4
Aplp2	1.19E-16	-0.26378	0.236	0.333	1.99E-12	4
Gria4	1.22E-16	-0.27449	0.065	0.131	2.04E-12	4
Nrn1	1.29E-16	-0.28188	0.144	0.23	2.15E-12	4
Ccna2	2.18E-16	-0.30795	0.157	0.228	3.64E-12	4
Gnaq	2.24E-16	-0.27172	0.201	0.294	3.74E-12	4
4930402H:	2.73E-16	-0.25017	0.057	0.119	4.56E-12	4
Gm11266	4.88E-16	-0.29169	0.081	0.151	8.13E-12	4
Noc2l	5.06E-16	0.261719	0.158	0.116	8.44E-12	4
Trmt6	5.73E-16	0.257801	0.217	0.169	9.56E-12	4
Psmc3ip	6.3E-16	0.261664	0.174	0.127	1.05E-11	4
Lrig3	7.09E-16	0.250204	0.208	0.165	1.18E-11	4
Gsr	1.3E-15	0.257721	0.137	0.084	2.17E-11	4
Nrcam	1.36E-15	-0.27105	0.074	0.141	2.27E-11	4
Sema7a	2.81E-15	0.260803	0.194	0.151	4.69E-11	4
Xist	3.09E-15	0.315576	0.357	0.292	5.15E-11	4
Plcb1	3.71E-15	-0.32703	0.082	0.147	6.19E-11	4
Gpr180	3.72E-15	0.252705	0.122	0.075	6.2E-11	4
Kidins220	4.07E-15	-0.27199	0.079	0.146	6.78E-11	4
Lpin2	7.77E-15	0.254263	0.215	0.176	1.3E-10	4
Pold3	9.06E-15	0.253656	0.194	0.143	1.51E-10	4
Ntrk3	1.28E-14	0.256418	0.197	0.15	2.13E-10	4
Ppfia2	1.83E-14	-0.25738	0.059	0.118	3.05E-10	4
Angptl2	4.88E-14	0.254525	0.157	0.111	8.14E-10	4
Rad51ap1	9.06E-14	0.263844	0.223	0.16	1.51E-09	4
Msh2	9.63E-14	0.253962	0.193	0.137	1.61E-09	4
Aprt	3.72E-13	0.25268	0.184	0.136	6.2E-09	4
Clmp	5.39E-13	-0.29105	0.176	0.249	8.99E-09	4
Gm17322	6.93E-13	0.264103	0.179	0.125	1.16E-08	4
Fos	8.41E-13	-0.32651	0.239	0.312	1.4E-08	4
Lgmn	2.12E-12	-0.26455	0.075	0.124	3.54E-08	4
Kif15	4.4E-12	-0.25398	0.121	0.187	7.34E-08	4
Tspan7	7.08E-11	-0.25817	0.063	0.11	1.18E-06	4
Esco2	1.66E-09	-0.25332	0.147	0.2	2.77E-05	4

Gm11223	7.37E-09	-0.25185	0.166	0.218	0.000123	4
Plp1	1.23E-06	-0.59581	0.093	0.116	0.020558	4
Tubb3	0	1.22049	0.866	0.43	0	5
Pde1c	0	1.112396	0.684	0.353	0	5
Cntn2	0	1.082691	0.674	0.218	0	5
Miat	0	1.021771	0.838	0.479	0	5
Nnat	0	0.971628	0.84	0.695	0	5
Igfbpl1	0	0.932525	0.902	0.619	0	5
Stmn2	0	0.759197	0.898	0.514	0	5
Ckb	0	0.719046	0.953	0.792	0	5
Tuba1a	0	0.576307	0.986	0.923	0	5
Nhlh2	1.1E-278	0.933906	0.737	0.388	1.8E-274	5
Ccnd1	1.7E-257	-1.26846	0.173	0.56	2.8E-253	5
Nhlh1	5.1E-234	1.005761	0.511	0.191	8.4E-230	5
Ddah2	3.1E-232	0.6786	0.865	0.666	5.1E-228	5
Neurod1	2.7E-228	0.669993	0.823	0.5	4.5E-224	5
Smc2	1E-223	-1.21808	0.252	0.577	1.7E-219	5
Lhx1	6.9E-213	0.782629	0.711	0.473	1.2E-208	5
Dek	1.9E-212	-0.9256	0.427	0.722	3.1E-208	5
Elavl3	1.6E-210	0.753833	0.725	0.444	2.6E-206	5
Top2a	7.1E-198	-1.41035	0.156	0.457	1.2E-193	5
Gap43	6.9E-196	0.707671	0.831	0.573	1.1E-191	5
Mki67	9.9E-193	-1.36822	0.126	0.424	1.7E-188	5
Chrna3	3.1E-183	0.943557	0.347	0.093	5.2E-179	5
Pdzrn3	6E-183	0.981202	0.392	0.12	1E-178	5
2810417H1	1.5E-174	-1.14366	0.113	0.422	2.6E-170	5
Hmgb2	9.6E-172	-1.08004	0.127	0.43	1.6E-167	5
Nrn1	2.6E-162	0.857539	0.444	0.196	4.3E-158	5
Elavl4	4.7E-161	0.783818	0.57	0.309	7.9E-157	5
Sept4	6.2E-160	0.881072	0.459	0.2	1E-155	5
Rtn1	1.2E-152	0.502994	0.867	0.67	2E-148	5
Birc5	7.7E-152	-1.03929	0.046	0.298	1.3E-147	5
Anp32b	3E-151	-0.78655	0.338	0.618	5E-147	5
Cdk1	1.9E-147	-1.00744	0.033	0.27	3.1E-143	5
Tpx2	4.4E-146	-1.10144	0.055	0.306	7.3E-142	5
Cenpf	8.4E-146	-1.26896	0.124	0.388	1.4E-141	5
Bin1	2.3E-143	0.671946	0.636	0.406	3.8E-139	5
Map2	2.9E-140	0.652983	0.676	0.471	4.9E-136	5
Nfix	1.9E-132	0.507098	0.8	0.659	3.2E-128	5
Basp1	4.1E-131	0.508337	0.824	0.69	6.8E-127	5
Nfib	8E-130	0.412706	0.982	0.928	1.3E-125	5
Cbx5	6E-129	-0.65329	0.411	0.679	1E-124	5
Tuba1b	6.8E-128	-0.78933	0.237	0.508	1.1E-123	5
Nusap1	7.6E-128	-0.96995	0.034	0.248	1.3E-123	5
Clmp	1.1E-127	0.782523	0.435	0.22	1.8E-123	5
Pbk	1.3E-127	-0.86631	0.031	0.243	2.2E-123	5
Cdca3	3.1E-127	-0.88981	0.031	0.242	5.1E-123	5
Ccna2	1E-122	-0.82416	0.033	0.242	1.7E-118	5

Cdca8	3.2E-122	-0.90837	0.063	0.286	5.4E-118	5
Pdgfa	1.2E-121	-0.86401	0.053	0.275	2E-117	5
H2afx	2.6E-121	-0.99332	0.12	0.353	4.4E-117	5
Ina	7.7E-121	0.58801	0.632	0.422	1.3E-116	5
Spc24	1.9E-119	-0.79394	0.046	0.264	3.2E-115	5
Smc4	3.4E-119	-0.81951	0.376	0.587	5.7E-115	5
Dut	4.1E-119	-0.80661	0.198	0.461	6.9E-115	5
Nasp	3.5E-117	-0.65792	0.426	0.663	5.8E-113	5
Kif11	1.1E-114	-0.81955	0.029	0.225	1.8E-110	5
Mllt11	1.6E-114	0.685243	0.427	0.195	2.6E-110	5
Spc25	2.8E-113	-0.84294	0.04	0.246	4.7E-109	5
Egr1	9.4E-112	-0.90487	0.153	0.409	1.6E-107	5
Cenpa	5.2E-111	-1.04045	0.067	0.272	8.7E-107	5
Prc1	9.1E-111	-0.9907	0.066	0.277	1.5E-106	5
Map1b	1.5E-110	0.510269	0.761	0.568	2.5E-106	5
Tex14	7.9E-110	0.796892	0.325	0.125	1.3E-105	5
Tmpo	4.7E-109	-0.7263	0.22	0.477	7.8E-105	5
Ube2c	1.3E-108	-1.12292	0.085	0.289	2.2E-104	5
Cenpe	3.9E-108	-0.99982	0.085	0.307	6.6E-104	5
Crmp1	5.2E-103	0.430557	0.771	0.656	8.7E-99	5
Incenp	8.1E-103	-0.81586	0.079	0.296	1.35E-98	5
Uncx	1E-102	0.63589	0.494	0.298	1.7E-98	5
Dbi	1.4E-102	-0.75418	0.249	0.487	2.39E-98	5
1500016L(	3.1E-102	0.608465	0.553	0.364	5.11E-98	5
Ank3	5.5E-102	0.593574	0.573	0.403	9.25E-98	5
Abhd16a	1.8E-101	0.703297	0.349	0.172	3.05E-97	5
Ranbp1	2.9E-101	-0.57649	0.504	0.694	4.83E-97	5
Stmn4	1.5E-100	0.607168	0.547	0.318	2.55E-96	5
Esco2	2.2E-100	-0.76417	0.031	0.213	3.69E-96	5
Pcna	6.8E-99	-0.77401	0.155	0.38	1.14E-94	5
Mcm6	2.95E-97	-0.7507	0.087	0.296	4.92E-93	5
Srrm3	1.11E-96	0.668844	0.396	0.222	1.85E-92	5
Kif23	3.63E-96	-0.8291	0.048	0.232	6.06E-92	5
Rrm2	2.89E-95	-0.71311	0.04	0.222	4.82E-91	5
Tcf4	3.3E-95	0.345296	0.956	0.921	5.5E-91	5
Kif15	3.79E-95	-0.70583	0.028	0.197	6.32E-91	5
Ckap2l	3.25E-94	-0.73744	0.048	0.236	5.43E-90	5
Cog7	3.61E-94	0.47458	0.714	0.569	6.02E-90	5
Ncapg	4.65E-94	-0.6615	0.029	0.2	7.76E-90	5
Kif5c	8.37E-94	0.555664	0.566	0.379	1.4E-89	5
Gpr153	1.2E-93	-0.67798	0.043	0.226	2.01E-89	5
H2afv	2.02E-93	-0.52968	0.575	0.744	3.37E-89	5
Zic1	3.93E-91	0.35447	0.873	0.774	6.55E-87	5
Apc	3.29E-90	0.547263	0.584	0.414	5.49E-86	5
Cks1b	6.73E-90	-0.68754	0.145	0.366	1.12E-85	5
Tubb2b	1.8E-89	0.514991	0.62	0.447	3E-85	5
Syt11	2.1E-89	0.517753	0.632	0.49	3.5E-85	5
Ran	1.41E-88	-0.61956	0.207	0.436	2.35E-84	5

Usp1	2.54E-88	-0.65145	0.138	0.36	4.24E-84	5
Cxxc5	3.33E-88	0.438578	0.633	0.533	5.55E-84	5
Casc5	1.29E-87	-0.67883	0.036	0.202	2.16E-83	5
Cdc20	2.16E-87	-0.83539	0.044	0.213	3.6E-83	5
2700094K1	1.21E-85	-0.54741	0.417	0.635	2.03E-81	5
Ccnb2	3.33E-85	-0.74465	0.039	0.203	5.55E-81	5
Pqlc1	3.27E-84	-0.64994	0.111	0.317	5.45E-80	5
Tmsb4x	6.58E-84	0.278621	0.975	0.945	1.1E-79	5
Nrep	9.07E-84	0.374406	0.718	0.553	1.51E-79	5
Knstrn	1.27E-83	-0.66515	0.037	0.202	2.11E-79	5
Myt1	5.47E-83	0.677411	0.312	0.134	9.12E-79	5
Rad51ap1	1.89E-82	-0.58442	0.028	0.182	3.16E-78	5
Rplp1	2.98E-82	-0.38273	0.789	0.886	4.98E-78	5
RP23-45G1	8.91E-82	-0.71155	0.145	0.347	1.49E-77	5
Hmmr	1.04E-81	-0.76071	0.034	0.192	1.74E-77	5
Nucks1	1.36E-81	-0.51808	0.488	0.682	2.27E-77	5
Lig1	4.46E-81	-0.69848	0.155	0.369	7.44E-77	5
Clspn	5.09E-81	-0.67351	0.041	0.206	8.49E-77	5
Ckap2	5.31E-81	-0.61473	0.027	0.178	8.85E-77	5
Tyms	9.02E-81	-0.66558	0.06	0.236	1.5E-76	5
Cenph	1.98E-80	-0.64482	0.044	0.21	3.3E-76	5
Ptprs	7.89E-80	0.566405	0.554	0.406	1.32E-75	5
Ccnb1	4.31E-79	-0.64197	0.017	0.153	7.19E-75	5
Hirip3	1.25E-78	-0.63265	0.189	0.404	2.08E-74	5
Dtymk	6.52E-78	-0.58284	0.242	0.46	1.09E-73	5
Dcx	2.19E-77	0.541583	0.527	0.359	3.66E-73	5
Tacc3	4.44E-77	-0.63882	0.055	0.223	7.41E-73	5
Aurkb	5.46E-77	-0.55238	0.019	0.156	9.11E-73	5
Hes1	6.28E-77	-0.77028	0.024	0.162	1.05E-72	5
Dnajc9	7E-77	-0.57834	0.159	0.37	1.17E-72	5
Thsd7a	1.05E-76	0.656938	0.246	0.114	1.75E-72	5
Nuf2	3.62E-76	-0.54752	0.019	0.154	6.04E-72	5
Fbxo5	7.46E-76	-0.57991	0.028	0.174	1.24E-71	5
Zfp36l1	1.74E-75	-0.57579	0.024	0.164	2.9E-71	5
Gng3	2.75E-75	0.536351	0.482	0.298	4.59E-71	5
Cltb	3.33E-75	-0.60254	0.229	0.444	5.55E-71	5
Kif20b	4.31E-75	-0.6754	0.047	0.206	7.19E-71	5
Sgol2	9.35E-75	-0.62486	0.027	0.17	1.56E-70	5
1700025G(	5.28E-74	0.546187	0.371	0.251	8.8E-70	5
Mis18bp1	9.41E-74	-0.56834	0.031	0.175	1.57E-69	5
Cdca2	1.06E-73	-0.5041	0.014	0.141	1.76E-69	5
Rbp4	1.14E-73	-0.62377	0.081	0.259	1.89E-69	5
Serbp1	1.2E-73	-0.35639	0.766	0.88	2.01E-69	5
Gas1	3.24E-73	-0.55547	0.028	0.171	5.4E-69	5
Tagln3	4.47E-73	0.54526	0.447	0.278	7.46E-69	5
Npm1	5.42E-73	-0.47062	0.471	0.665	9.03E-69	5
Nkd1	5.67E-73	-0.58149	0.086	0.266	9.46E-69	5
Sparcl1	6E-73	-0.9525	0.067	0.222	1E-68	5

Slc17a6	6.98E-73	0.639821	0.288	0.132	1.16E-68	5
Nsg2	8.68E-73	0.431388	0.595	0.475	1.45E-68	5
Prim1	1.56E-72	-0.59013	0.097	0.281	2.61E-68	5
Sstr2	2.14E-72	0.584503	0.328	0.175	3.57E-68	5
Cdk5r1	4.4E-72	0.525557	0.489	0.316	7.34E-68	5
Cenpm	8.4E-72	-0.53778	0.032	0.176	1.4E-67	5
Anp32e	1.01E-71	-0.5128	0.443	0.632	1.69E-67	5
Tbata	1.36E-71	-0.68218	0.167	0.358	2.27E-67	5
Rcor2	2.03E-71	0.561002	0.364	0.209	3.38E-67	5
Dclk1	7.28E-71	-0.53049	0.219	0.433	1.21E-66	5
Gm10075	1.13E-70	-0.48923	0.321	0.536	1.88E-66	5
Chgb	1.32E-70	0.569606	0.404	0.222	2.2E-66	5
BC005764	2.57E-70	0.543267	0.349	0.167	4.29E-66	5
Podxl2	2.84E-69	0.597794	0.363	0.212	4.73E-65	5
Kif22	3.22E-69	-0.53342	0.034	0.177	5.37E-65	5
CRE_RECON	4.1E-69	0.382962	0.849	0.759	6.83E-65	5
Mxd3	4.26E-69	-0.48052	0.016	0.139	7.1E-65	5
Chrnb4	5.91E-69	0.460817	0.105	0.017	9.86E-65	5
Apoe	5.95E-69	-1.59515	0.192	0.255	9.92E-65	5
C1ql1	8.14E-69	0.519264	0.405	0.264	1.36E-64	5
Gnai2	9.11E-69	-0.53761	0.238	0.445	1.52E-64	5
Atad2	1.04E-68	-0.58678	0.056	0.213	1.74E-64	5
Gmnn	3.57E-68	-0.52278	0.025	0.157	5.95E-64	5
Ndc80	2.12E-67	-0.47564	0.011	0.126	3.54E-63	5
Aspm	3.45E-67	-0.60151	0.025	0.155	5.75E-63	5
Mapk8ip1	4.44E-67	0.544837	0.359	0.212	7.41E-63	5
Tk1	4.77E-67	-0.51428	0.019	0.143	7.95E-63	5
Bub1	2.43E-66	-0.48021	0.02	0.143	4.06E-62	5
Sgol1	9.53E-66	-0.44818	0.019	0.139	1.59E-61	5
Rab6b	3.34E-65	0.558907	0.331	0.188	5.56E-61	5
Hells	3.9E-65	-0.57508	0.069	0.229	6.5E-61	5
Tmsb10	2.38E-64	0.339015	0.727	0.656	3.98E-60	5
Marcksl1	3.23E-64	0.309482	0.769	0.716	5.38E-60	5
Hmgn5	4.31E-64	-0.57848	0.247	0.443	7.19E-60	5
Gsg1l	4.39E-64	-0.57039	0.101	0.272	7.33E-60	5
Mns1	4.84E-64	-0.59325	0.064	0.218	8.08E-60	5
Ccdc34	8.43E-64	-0.50985	0.231	0.435	1.41E-59	5
Mcm3	8.95E-64	-0.5523	0.049	0.194	1.49E-59	5
Sfrp2	3.49E-63	-0.47089	0.031	0.161	5.82E-59	5
Txn1	5.23E-63	-0.44214	0.382	0.579	8.73E-59	5
Rpa2	5.84E-63	-0.5438	0.063	0.215	9.74E-59	5
Arhgap11a	5.92E-63	-0.58286	0.044	0.184	9.88E-59	5
Lap3	1.08E-62	-0.56023	0.12	0.293	1.81E-58	5
Kif4	2.22E-62	-0.4085	0.012	0.118	3.7E-58	5
Cenpq	5.63E-62	-0.49198	0.038	0.173	9.39E-58	5
Pmf1	8.81E-62	-0.46696	0.029	0.155	1.47E-57	5
Hint1	1.49E-61	-0.36581	0.534	0.722	2.48E-57	5
D17H6S56I	2.17E-61	-0.45194	0.021	0.139	3.62E-57	5

D430041D	8.7E-61	-0.46438	0.312	0.511	1.45E-56	5
Rrm1	1.94E-60	-0.50041	0.109	0.276	3.24E-56	5
Kif2c	4.83E-60	-0.41973	0.012	0.117	8.06E-56	5
Nop58	5.08E-60	-0.43716	0.416	0.611	8.48E-56	5
Gm2694	1.61E-59	-0.49554	0.189	0.377	2.68E-55	5
Rab3a	3.03E-59	0.505157	0.287	0.134	5.05E-55	5
Prdx1	2.71E-58	-0.47125	0.326	0.507	4.51E-54	5
Cenpk	5.43E-58	-0.49513	0.032	0.156	9.05E-54	5
Angptl2	1.04E-57	-0.44703	0.018	0.127	1.74E-53	5
C330027C(	1.09E-57	-0.46226	0.027	0.145	1.82E-53	5
A330076H(	3.8E-57	0.54879	0.288	0.147	6.33E-53	5
Cdca7	5.01E-57	-0.50124	0.067	0.212	8.35E-53	5
Elavl2	2.22E-56	0.483999	0.364	0.259	3.71E-52	5
Paics	2.26E-56	-0.46683	0.242	0.432	3.78E-52	5
Bub1b	7.51E-56	-0.37393	0.013	0.112	1.25E-51	5
Cplx2	9.24E-56	-0.43212	0.319	0.514	1.54E-51	5
Dlgap5	2.71E-55	-0.4184	0.018	0.122	4.51E-51	5
Mt1	3.1E-55	-0.71731	0.052	0.181	5.17E-51	5
Chaf1a	5.49E-55	-0.49929	0.05	0.181	9.15E-51	5
Arl6ip1	1.03E-54	-0.50881	0.451	0.56	1.72E-50	5
Fam64a	1.16E-54	-0.4225	0.021	0.128	1.93E-50	5
Prdx4	1.32E-54	-0.39243	0.203	0.383	2.2E-50	5
Mcm5	1.6E-54	-0.50342	0.051	0.181	2.67E-50	5
Sema6a	6.82E-54	0.544012	0.317	0.189	1.14E-49	5
Afap1	8.32E-54	0.508988	0.249	0.141	1.39E-49	5
Klc1	9.72E-54	0.416666	0.454	0.351	1.62E-49	5
Rufy3	2.04E-53	0.412827	0.477	0.359	3.41E-49	5
App	2.61E-53	0.363541	0.642	0.56	4.35E-49	5
Dkc1	2.74E-53	-0.42622	0.176	0.35	4.57E-49	5
Hsbp1	2.81E-53	0.322909	0.645	0.588	4.69E-49	5
Mad2l1	3.59E-53	-0.39696	0.024	0.134	5.99E-49	5
Lmnb1	1.84E-52	-0.4503	0.148	0.316	3.07E-48	5
Ttc3	2.52E-52	0.264889	0.875	0.824	4.2E-48	5
Tipin	2.71E-52	-0.471	0.115	0.27	4.51E-48	5
Mex3a	2.75E-52	0.381022	0.541	0.454	4.58E-48	5
A930011O:	3.66E-52	0.553466	0.231	0.102	6.11E-48	5
Hk2	4.83E-52	-0.44549	0.055	0.185	8.06E-48	5
Plk4	4.96E-52	-0.39377	0.024	0.13	8.28E-48	5
Nap1l1	8.21E-52	-0.38533	0.34	0.529	1.37E-47	5
Kmt2e	8.66E-52	0.344528	0.619	0.563	1.44E-47	5
Aurka	9.61E-52	-0.36503	0.013	0.108	1.6E-47	5
Sptbn1	1.2E-51	0.457581	0.454	0.342	2E-47	5
Barhl1	1.45E-51	0.323189	0.611	0.511	2.41E-47	5
Ncl	2.61E-51	-0.29078	0.82	0.904	4.35E-47	5
Melk	2.72E-51	-0.35618	0.011	0.103	4.54E-47	5
Cnbp	3.27E-51	-0.32794	0.602	0.755	5.46E-47	5
Igsf8	3.64E-51	0.468564	0.382	0.256	6.08E-47	5
Cdkn1b	5.05E-51	0.346748	0.538	0.459	8.43E-47	5

Chd4	8.05E-51	0.261688	0.8	0.766	1.34E-46	5
Cenpp	1.17E-50	-0.37107	0.021	0.124	1.95E-46	5
Itsn1	1.77E-50	0.506454	0.346	0.223	2.95E-46	5
Rangap1	2.51E-50	-0.4762	0.116	0.271	4.18E-46	5
E130114P1	2.68E-50	0.361156	0.675	0.574	4.48E-46	5
Dpysl4	3.48E-50	0.374863	0.571	0.481	5.81E-46	5
Hrk	6.43E-50	0.575011	0.138	0.051	1.07E-45	5
Ect2	1.16E-49	-0.3532	0.014	0.107	1.93E-45	5
Stmn3	1.31E-49	0.314863	0.685	0.614	2.18E-45	5
Gm17750	2.62E-49	0.42854	0.45	0.357	4.37E-45	5
Cd63	2.66E-49	0.258636	0.795	0.75	4.44E-45	5
Nt5dc2	2.7E-49	0.455305	0.331	0.226	4.51E-45	5
H2afz	2.87E-49	-0.40623	0.099	0.246	4.79E-45	5
Tubb4b	3.22E-49	-0.47426	0.106	0.255	5.37E-45	5
Hmgb1	5.59E-49	-0.37573	0.203	0.375	9.33E-45	5
Trim59	5.71E-49	-0.42964	0.056	0.182	9.52E-45	5
B2m	7.87E-49	-0.58151	0.108	0.245	1.31E-44	5
Atp2b1	8.06E-49	-0.37053	0.407	0.589	1.34E-44	5
Cklf	8.35E-49	-0.42042	0.053	0.177	1.39E-44	5
Dbf4	8.99E-49	-0.39631	0.042	0.157	1.5E-44	5
Sowaha	9.75E-49	-0.57678	0.054	0.174	1.63E-44	5
Sox9	1.05E-48	-0.50697	0.123	0.269	1.75E-44	5
Rbfox2	1.5E-48	0.428514	0.382	0.272	2.5E-44	5
Rfc4	1.53E-48	-0.39492	0.098	0.24	2.55E-44	5
Ppib	1.62E-48	0.284647	0.74	0.696	2.7E-44	5
Ncaph	5.83E-48	-0.38583	0.027	0.131	9.73E-44	5
Gdpd1	6.17E-48	0.426672	0.325	0.234	1.03E-43	5
Uhrf1	6.81E-48	-0.43078	0.041	0.155	1.14E-43	5
Pax6	7.98E-48	0.375605	0.598	0.503	1.33E-43	5
Fkbp3	1.12E-47	-0.34014	0.624	0.758	1.87E-43	5
Siva1	1.3E-47	-0.44251	0.136	0.287	2.16E-43	5
Gria2	1.72E-47	0.353702	0.633	0.508	2.87E-43	5
Gsk3b	2.8E-47	0.328693	0.551	0.493	4.67E-43	5
Hmgn2	2.9E-47	-0.44671	0.123	0.27	4.84E-43	5
Celf2	3.75E-47	0.282608	0.716	0.629	6.26E-43	5
Fam111a	8.68E-47	-0.46342	0.031	0.136	1.45E-42	5
Ebf3	2.65E-46	0.456208	0.289	0.193	4.41E-42	5
Anln	3.43E-46	-0.39617	0.019	0.113	5.72E-42	5
Snrpb	3.75E-46	-0.33868	0.411	0.591	6.26E-42	5
Arhgef2	7.87E-46	0.397688	0.351	0.274	1.31E-41	5
Hjurp	9.54E-46	-0.43325	0.28	0.451	1.59E-41	5
Mcm7	1.23E-45	-0.3573	0.237	0.405	2.04E-41	5
Supt16	1.28E-45	-0.35666	0.332	0.511	2.14E-41	5
Rif1	1.33E-45	-0.43894	0.138	0.291	2.22E-41	5
Draxin	1.82E-45	-0.37628	0.399	0.571	3.04E-41	5
Fth1	2.62E-45	-0.33364	0.579	0.722	4.38E-41	5
Fos	3.19E-45	-0.60188	0.166	0.319	5.32E-41	5
Rbbp7	1.8E-44	-0.36259	0.176	0.335	3E-40	5

Frmd4b	2.81E-44	-0.35823	0.035	0.137	4.69E-40	5
Rad21	3.15E-44	-0.43069	0.313	0.478	5.25E-40	5
Vim	3.49E-44	-0.4995	0.165	0.317	5.82E-40	5
Dixdc1	1.43E-43	0.444086	0.318	0.225	2.39E-39	5
Slc7a5	1.71E-43	-0.41338	0.028	0.124	2.86E-39	5
Jun	1.95E-43	-0.38608	0.526	0.668	3.26E-39	5
Rad51	1.97E-43	-0.37745	0.028	0.126	3.29E-39	5
Hpca	2.45E-43	-0.50769	0.146	0.291	4.08E-39	5
Slc3a2	2.55E-43	-0.37386	0.181	0.338	4.25E-39	5
Lrig3	2.85E-43	-0.3992	0.063	0.181	4.76E-39	5
Ppp2r2c	4E-43	0.341706	0.586	0.495	6.68E-39	5
Nde1	6.34E-43	-0.35798	0.03	0.129	1.06E-38	5
Racgap1	7.69E-43	-0.42991	0.126	0.269	1.28E-38	5
Atp1b3	9.34E-43	0.368309	0.407	0.329	1.56E-38	5
Pard6a	1.05E-42	0.450288	0.165	0.083	1.76E-38	5
Rtn4	1.8E-42	0.320706	0.531	0.474	3E-38	5
Fosb	4.53E-42	-0.55858	0.054	0.167	7.56E-38	5
Rpl41	4.63E-42	-0.27331	0.669	0.792	7.73E-38	5
Dtl	5.51E-42	-0.40463	0.047	0.156	9.19E-38	5
Ddr1	6.89E-42	0.443372	0.212	0.126	1.15E-37	5
Banp	6.92E-42	0.452952	0.204	0.129	1.16E-37	5
Mif	7.8E-42	-0.38266	0.218	0.372	1.3E-37	5
Myod1	8.03E-42	-0.43377	0.045	0.151	1.34E-37	5
B3galt2	1.56E-41	0.480192	0.196	0.086	2.61E-37	5
Hnrnpd	3.89E-41	-0.31464	0.469	0.626	6.5E-37	5
Smco4	5.26E-41	-0.40479	0.059	0.172	8.77E-37	5
Sept8	6.25E-41	-0.34779	0.04	0.139	1.04E-36	5
Pabpc1	6.37E-41	-0.2646	0.818	0.885	1.06E-36	5
Diap3	8.7E-41	-0.36731	0.024	0.115	1.45E-36	5
Smc6	9.75E-41	-0.32164	0.273	0.439	1.63E-36	5
Ybx1	1.04E-40	-0.27432	0.671	0.798	1.73E-36	5
Hist1h2ak	1.05E-40	-0.50225	0.048	0.153	1.76E-36	5
Topbp1	1.07E-40	-0.35157	0.05	0.155	1.78E-36	5
Mcm2	1.72E-40	-0.44805	0.091	0.217	2.87E-36	5
Dnmt1	1.79E-40	-0.41716	0.147	0.292	2.98E-36	5
Brd7	2.04E-40	-0.32095	0.176	0.322	3.4E-36	5
Soga3	2.48E-40	0.326335	0.518	0.45	4.13E-36	5
Nop56	2.63E-40	-0.3837	0.219	0.376	4.39E-36	5
Schip1	3.28E-40	0.423941	0.273	0.172	5.46E-36	5
Rps19	8.83E-40	-0.28633	0.638	0.766	1.47E-35	5
Hsd11b2	9.99E-40	-0.46655	0.159	0.302	1.67E-35	5
Ncapd2	1.41E-39	-0.35409	0.078	0.196	2.35E-35	5
Clic1	1.65E-39	0.351735	0.211	0.168	2.75E-35	5
Idh2	1.85E-39	-0.3449	0.15	0.292	3.08E-35	5
Lmcd1	2.11E-39	0.438063	0.12	0.055	3.53E-35	5
Hat1	4.01E-39	-0.36696	0.057	0.166	6.7E-35	5
Eef1d	4.46E-39	-0.29533	0.214	0.364	7.44E-35	5
2610203C2	6.32E-39	0.467796	0.204	0.111	1.05E-34	5

Itm2b	7.87E-39	0.290858	0.699	0.636	1.31E-34	5
Rps25	1.02E-38	-0.31641	0.338	0.495	1.7E-34	5
Cbfa2t3	1.7E-38	-0.34824	0.271	0.417	2.84E-34	5
Pkp4	1.83E-38	-0.31455	0.025	0.111	3.06E-34	5
Tead2	2.01E-38	-0.39738	0.155	0.291	3.36E-34	5
Hmgn3	3.53E-38	-0.37052	0.146	0.283	5.88E-34	5
Fstl1	3.62E-38	-0.42503	0.077	0.191	6.04E-34	5
Lsm2	4.54E-38	-0.36599	0.114	0.243	7.58E-34	5
Banf1	7.33E-38	-0.31049	0.573	0.707	1.22E-33	5
Gli1	8.55E-38	-0.36979	0.028	0.116	1.43E-33	5
Rplp2	1.4E-37	-0.25481	0.756	0.849	2.34E-33	5
Aldoa	1.65E-37	0.396724	0.351	0.247	2.74E-33	5
Lsm6	1.72E-37	-0.32095	0.237	0.386	2.86E-33	5
S100a16	2.85E-37	0.464767	0.175	0.085	4.75E-33	5
Chst15	6.96E-37	0.41892	0.155	0.069	1.16E-32	5
Gdi1	1.08E-36	0.385416	0.311	0.22	1.8E-32	5
Dhfr	1.33E-36	-0.40086	0.055	0.158	2.21E-32	5
Hip1r	1.95E-36	0.440875	0.208	0.124	3.25E-32	5
Cenpw	2.13E-36	-0.33075	0.042	0.137	3.55E-32	5
Exosc8	2.35E-36	-0.25767	0.107	0.221	3.91E-32	5
6330403K(	3E-36	0.408593	0.325	0.233	5E-32	5
Cald1	3.3E-36	0.270662	0.588	0.529	5.51E-32	5
Klf7	3.49E-36	0.398829	0.348	0.252	5.81E-32	5
Rpl22	4.37E-36	-0.26384	0.682	0.799	7.29E-32	5
Cep110	4.91E-36	-0.35699	0.067	0.173	8.19E-32	5
Dctpp1	5.79E-36	-0.36447	0.179	0.318	9.66E-32	5
Pa2g4	1.53E-35	-0.29086	0.38	0.538	2.55E-31	5
Clic4	2.44E-35	-0.32063	0.116	0.239	4.07E-31	5
Lmo4	2.53E-35	-0.32406	0.206	0.349	4.22E-31	5
St18	2.73E-35	0.466654	0.283	0.18	4.56E-31	5
Atp6v0e	3.77E-35	0.280844	0.458	0.402	6.29E-31	5
Nudcd2	4.25E-35	-0.29128	0.118	0.235	7.09E-31	5
Cd24a	5.74E-35	0.261819	0.684	0.628	9.57E-31	5
Dnph1	6.21E-35	-0.33472	0.04	0.132	1.04E-30	5
Gadd45a	8.84E-35	0.424826	0.159	0.08	1.47E-30	5
Ezh2	8.84E-35	-0.34312	0.493	0.622	1.47E-30	5
Skp2	9.37E-35	-0.26933	0.041	0.128	1.56E-30	5
Snrpd1	1.1E-34	-0.30484	0.384	0.539	1.84E-30	5
Necab3	1.62E-34	0.408631	0.123	0.045	2.7E-30	5
Lgals1	1.64E-34	-0.45279	0.121	0.24	2.74E-30	5
Serinc1	1.72E-34	0.27309	0.476	0.432	2.87E-30	5
Naa50	1.8E-34	-0.26984	0.168	0.298	3E-30	5
Shmt1	1.83E-34	-0.29964	0.028	0.111	3.06E-30	5
Scg3	2.28E-34	0.290693	0.485	0.442	3.81E-30	5
Snhg1	2.3E-34	-0.27136	0.234	0.37	3.84E-30	5
Rfc2	2.59E-34	-0.33732	0.103	0.222	4.33E-30	5
Dctn3	3.14E-34	0.287711	0.407	0.364	5.24E-30	5
Isoc1	3.34E-34	-0.33167	0.081	0.191	5.57E-30	5

Pttg1	3.5E-34	-0.39975	0.053	0.149	5.84E-30	5
Sept3	3.68E-34	0.298468	0.505	0.38	6.14E-30	5
Ccdc41	4.24E-34	-0.29077	0.142	0.266	7.08E-30	5
Cdca7l	5.55E-34	-0.32992	0.029	0.112	9.26E-30	5
H13	8E-34	0.356496	0.303	0.23	1.33E-29	5
Srsf7	8.69E-34	-0.31062	0.327	0.48	1.45E-29	5
Nolc1	9.02E-34	-0.27837	0.277	0.423	1.51E-29	5
Rpl22l1	1.11E-33	-0.30263	0.224	0.365	1.85E-29	5
Hdgf	1.11E-33	-0.31073	0.399	0.546	1.85E-29	5
Slc25a5	1.32E-33	-0.25556	0.385	0.522	2.2E-29	5
Cdt1	1.38E-33	-0.35774	0.042	0.134	2.3E-29	5
Tpm4	1.68E-33	-0.27976	0.196	0.33	2.8E-29	5
Mab21l2	2.32E-33	0.42302	0.169	0.096	3.87E-29	5
Nsmce4a	2.36E-33	-0.2693	0.12	0.235	3.94E-29	5
Hes6	2.49E-33	-0.32495	0.128	0.248	4.16E-29	5
Cbfb	2.9E-33	-0.2893	0.087	0.193	4.84E-29	5
BC031181	2.95E-33	0.330578	0.32	0.254	4.92E-29	5
Fen1	3.33E-33	-0.33068	0.054	0.151	5.55E-29	5
Mrpl42	3.57E-33	-0.30006	0.196	0.331	5.95E-29	5
Rnmt	3.89E-33	0.328393	0.405	0.342	6.48E-29	5
Lmnb2	4.73E-33	-0.27271	0.069	0.168	7.89E-29	5
Ctsd	4.89E-33	-0.71205	0.119	0.181	8.16E-29	5
Spop	5.06E-33	-0.29539	0.194	0.328	8.43E-29	5
Irs1	5.08E-33	-0.31348	0.04	0.127	8.48E-29	5
Snrpf	6.05E-33	-0.31195	0.295	0.441	1.01E-28	5
Add3	7.91E-33	-0.35273	0.03	0.112	1.32E-28	5
Rfc1	7.97E-33	-0.30375	0.207	0.344	1.33E-28	5
G3bp1	8.14E-33	-0.26628	0.206	0.341	1.36E-28	5
Btbd17	9.75E-33	0.41377	0.241	0.151	1.63E-28	5
Sri	1.39E-32	-0.35544	0.072	0.176	2.33E-28	5
Rpl14	1.72E-32	-0.26056	0.578	0.71	2.87E-28	5
Fabp7	2E-32	-0.8872	0.09	0.126	3.34E-28	5
Ckap5	2.02E-32	-0.34638	0.151	0.278	3.36E-28	5
Carhsp1	2.31E-32	0.326858	0.307	0.247	3.85E-28	5
Timeless	3.11E-32	-0.31706	0.065	0.163	5.19E-28	5
Lyar	3.16E-32	-0.28516	0.171	0.298	5.28E-28	5
Lbr	4.02E-32	-0.30207	0.098	0.207	6.7E-28	5
Usp46	4.15E-32	0.338737	0.286	0.23	6.91E-28	5
Rps20	4.91E-32	-0.25482	0.693	0.787	8.2E-28	5
Zic4	4.92E-32	0.312271	0.479	0.411	8.21E-28	5
Tcf19	5.11E-32	-0.34106	0.027	0.106	8.53E-28	5
Gng2	1.19E-31	0.279684	0.44	0.391	1.99E-27	5
Cdc45	1.56E-31	-0.30252	0.025	0.102	2.6E-27	5
Cep57	2.94E-31	-0.27298	0.149	0.269	4.91E-27	5
Kif1b	3.38E-31	0.252237	0.575	0.514	5.65E-27	5
Plk3	3.4E-31	0.385503	0.142	0.065	5.68E-27	5
Fbxo32	3.57E-31	0.411584	0.191	0.108	5.95E-27	5
Pou3f2	3.68E-31	-0.31591	0.164	0.288	6.14E-27	5

Cacna2d1	4.05E-31	0.322518	0.434	0.368	6.75E-27	5
Ptn	4.09E-31	-0.35823	0.346	0.452	6.82E-27	5
Nr3c1	4.85E-31	-0.33526	0.045	0.134	8.09E-27	5
Rundc3a	5.52E-31	0.326848	0.31	0.241	9.21E-27	5
Sae1	9.56E-31	-0.28147	0.167	0.291	1.6E-26	5
Ntm	1.08E-30	-0.40477	0.061	0.154	1.8E-26	5
Aplp1	1.48E-30	0.4011	0.247	0.14	2.47E-26	5
Pfn1	1.67E-30	-0.26917	0.384	0.524	2.78E-26	5
Atoh1	1.91E-30	-0.37227	0.073	0.174	3.19E-26	5
Mis12	2.02E-30	-0.27373	0.029	0.106	3.37E-26	5
Cmtm3	2.07E-30	-0.29377	0.042	0.125	3.45E-26	5
Ctnnb1	3.07E-30	-0.26026	0.262	0.4	5.12E-26	5
Ddx39	3.11E-30	-0.26542	0.095	0.197	5.19E-26	5
Dlgap4	4.24E-30	0.379449	0.271	0.178	7.07E-26	5
Ier2	4.45E-30	-0.34866	0.262	0.395	7.42E-26	5
D4Wsu53e	4.98E-30	0.286767	0.501	0.44	8.31E-26	5
Fam213b	5.05E-30	0.402951	0.234	0.148	8.43E-26	5
Clip3	5.12E-30	0.328302	0.387	0.303	8.54E-26	5
Psat1	5.93E-30	-0.32583	0.245	0.38	9.9E-26	5
Gpc2	6.48E-30	0.391044	0.215	0.131	1.08E-25	5
Tubb2a	7.36E-30	0.376487	0.348	0.233	1.23E-25	5
Suz12	8.73E-30	-0.29266	0.121	0.229	1.46E-25	5
Acot7	9.51E-30	-0.3183	0.098	0.205	1.59E-25	5
Mtap	1E-29	-0.3041	0.027	0.102	1.67E-25	5
Mcm4	1.36E-29	-0.34752	0.055	0.147	2.26E-25	5
Uchl1	1.39E-29	0.316081	0.405	0.326	2.33E-25	5
Pik3r3	1.47E-29	0.398409	0.212	0.127	2.45E-25	5
Csrp2	1.95E-29	-0.33891	0.07	0.167	3.26E-25	5
Cadm1	2.07E-29	-0.30148	0.267	0.404	3.45E-25	5
Ska2	2.82E-29	-0.31497	0.106	0.215	4.7E-25	5
Usp22	2.83E-29	0.263959	0.4	0.356	4.71E-25	5
Syt13	3.02E-29	-0.32526	0.049	0.135	5.04E-25	5
Gm11266	3.15E-29	0.400002	0.214	0.136	5.25E-25	5
Nup62	3.43E-29	-0.26248	0.083	0.18	5.72E-25	5
H2afy	4.74E-29	-0.25985	0.471	0.605	7.9E-25	5
Dbn1	5.95E-29	0.389115	0.217	0.131	9.92E-25	5
Cerk	7.54E-29	-0.29794	0.067	0.16	1.26E-24	5
Cntln	8.38E-29	-0.31822	0.083	0.182	1.4E-24	5
Rpa1	9.01E-29	-0.28276	0.037	0.117	1.5E-24	5
Ssrp1	1.01E-28	-0.27295	0.454	0.589	1.69E-24	5
Hist3h2a	1.04E-28	0.392822	0.209	0.136	1.73E-24	5
Adamts1	1.28E-28	-0.35952	0.072	0.163	2.13E-24	5
Ccdc18	1.29E-28	-0.29752	0.042	0.123	2.15E-24	5
Hsp90b1	1.6E-28	-0.28523	0.588	0.703	2.67E-24	5
Boc	1.83E-28	-0.32679	0.069	0.165	3.05E-24	5
Kdm5b	2.5E-28	0.374656	0.274	0.175	4.17E-24	5
Igsf21	2.63E-28	0.406112	0.16	0.081	4.39E-24	5
Ybx3	5.08E-28	-0.26837	0.183	0.301	8.47E-24	5

Fnbp1l	7.53E-28	0.270849	0.496	0.448	1.26E-23	5
Pola1	9.32E-28	-0.29808	0.034	0.11	1.55E-23	5
Smpd2	9.47E-28	-0.30331	0.079	0.175	1.58E-23	5
Cst3	1.43E-27	-0.3382	0.487	0.541	2.39E-23	5
Nrm	1.72E-27	-0.27767	0.084	0.178	2.88E-23	5
Alyref	1.76E-27	-0.28309	0.106	0.209	2.94E-23	5
Mphosph1(	2.24E-27	-0.27772	0.132	0.242	3.73E-23	5
Mrpl18	2.43E-27	-0.25713	0.239	0.366	4.05E-23	5
Nmral1	2.99E-27	-0.25323	0.099	0.197	4.98E-23	5
Ptch2	3.31E-27	-0.27344	0.071	0.161	5.52E-23	5
Bub3	3.38E-27	-0.26403	0.211	0.333	5.64E-23	5
Id2	4.83E-27	-0.31329	0.368	0.499	8.06E-23	5
C530008M	5.03E-27	0.321011	0.318	0.262	8.39E-23	5
Nin	6.66E-27	-0.28102	0.067	0.155	1.11E-22	5
Clvs1	6.7E-27	0.374856	0.2	0.121	1.12E-22	5
Prkcb	7.78E-27	0.349457	0.331	0.247	1.3E-22	5
Ramp2	7.98E-27	-0.3359	0.047	0.127	1.33E-22	5
Rad50	8.68E-27	-0.31995	0.08	0.175	1.45E-22	5
Gpr56	9.3E-27	0.325378	0.277	0.216	1.55E-22	5
Hmgn1	9.72E-27	-0.25108	0.503	0.634	1.62E-22	5
Nudc	1.05E-26	-0.28541	0.175	0.292	1.75E-22	5
Ddx21	1.21E-26	-0.26983	0.215	0.334	2.02E-22	5
Vrk1	1.56E-26	-0.25271	0.069	0.156	2.6E-22	5
Casp8ap2	1.8E-26	-0.26249	0.146	0.254	3E-22	5
Rrbp1	2.07E-26	-0.32641	0.099	0.198	3.46E-22	5
Stx4a	2.14E-26	-0.29162	0.072	0.163	3.56E-22	5
Gramd1a	2.25E-26	0.317452	0.211	0.16	3.76E-22	5
Enox2	2.85E-26	0.33394	0.108	0.05	4.75E-22	5
Ube2d1	3.14E-26	0.269611	0.261	0.224	5.24E-22	5
Dda1	3.28E-26	0.255322	0.282	0.249	5.47E-22	5
H1fx	3.71E-26	-0.31901	0.144	0.256	6.2E-22	5
Ccdc50	5.44E-26	0.257455	0.363	0.322	9.07E-22	5
Mab21l1	6.05E-26	0.33332	0.285	0.218	1.01E-21	5
Ldha	8.29E-26	-0.27908	0.136	0.242	1.38E-21	5
Mmp14	1.57E-25	-0.29242	0.09	0.183	2.62E-21	5
Pbdc1	1.63E-25	-0.27671	0.115	0.216	2.73E-21	5
Fibp	1.66E-25	0.294196	0.217	0.175	2.76E-21	5
Zfp423	1.74E-25	0.346361	0.143	0.077	2.91E-21	5
Smarcd1	3.05E-25	0.271666	0.302	0.267	5.08E-21	5
Atp6v1e1	3.12E-25	0.277636	0.375	0.326	5.21E-21	5
Smc5	3.19E-25	-0.26427	0.159	0.268	5.33E-21	5
Stxbp1	3.64E-25	0.362057	0.235	0.154	6.07E-21	5
Nsg1	5.85E-25	0.295323	0.34	0.281	9.75E-21	5
Tprn	6.08E-25	0.354284	0.302	0.218	1.01E-20	5
Frmd4a	7.39E-25	0.302081	0.36	0.303	1.23E-20	5
Srebf1	7.57E-25	-0.27526	0.231	0.345	1.26E-20	5
Mpp6	1E-24	-0.26552	0.081	0.17	1.67E-20	5
Elovl6	1.06E-24	0.289013	0.322	0.273	1.77E-20	5

Myt1l	1.8E-24	0.387753	0.226	0.153	3E-20	5
Apitd1	3.26E-24	-0.26915	0.033	0.101	5.44E-20	5
Scg5	4.42E-24	0.267183	0.381	0.329	7.37E-20	5
Bcl2l1	4.96E-24	0.332537	0.134	0.083	8.28E-20	5
Fam115a	5.51E-24	0.250171	0.396	0.345	9.2E-20	5
Strbp	5.77E-24	0.275776	0.394	0.341	9.62E-20	5
Map6	5.95E-24	0.347186	0.141	0.072	9.93E-20	5
Mien1	9.06E-24	0.283017	0.264	0.223	1.51E-19	5
Pvrl3	9.1E-24	-0.28638	0.053	0.129	1.52E-19	5
Snx5	9.87E-24	-0.28509	0.084	0.172	1.65E-19	5
Pak7	1.4E-23	0.368187	0.169	0.097	2.34E-19	5
Kif5a	1.44E-23	0.358826	0.203	0.119	2.4E-19	5
Lpin2	1.63E-23	-0.30414	0.097	0.189	2.71E-19	5
Baz2b	1.69E-23	0.26741	0.389	0.361	2.82E-19	5
Maged2	3.18E-23	0.31876	0.221	0.16	5.3E-19	5
Clcn4-2	3.21E-23	0.296634	0.306	0.245	5.36E-19	5
Map1lc3b	6.52E-23	0.250013	0.375	0.326	1.09E-18	5
Cdc7	9.19E-23	-0.25187	0.092	0.176	1.53E-18	5
Arid3a	1.44E-22	0.338305	0.154	0.089	2.39E-18	5
Psmc3ip	1.44E-22	-0.27975	0.06	0.139	2.4E-18	5
Rock2	1.95E-22	-0.27625	0.126	0.221	3.24E-18	5
Ccp110	2.34E-22	-0.26003	0.155	0.258	3.91E-18	5
Brca2	2.49E-22	-0.25544	0.049	0.12	4.16E-18	5
Fam107b	2.68E-22	0.326412	0.153	0.096	4.47E-18	5
Atad5	3.07E-22	-0.28797	0.073	0.157	5.12E-18	5
Dner	3.42E-22	0.311229	0.289	0.206	5.71E-18	5
Glul	4.21E-22	-0.28264	0.125	0.209	7.01E-18	5
Ppp2r2b	4.52E-22	0.348576	0.137	0.078	7.54E-18	5
Trpc4ap	5.18E-22	0.364527	0.259	0.191	8.64E-18	5
Chd3	6.45E-22	0.36467	0.212	0.142	1.08E-17	5
Meis1	6.57E-22	0.254038	0.428	0.366	1.1E-17	5
Rnf165	6.8E-22	0.320363	0.22	0.167	1.13E-17	5
Tmx4	8.82E-22	0.299391	0.225	0.172	1.47E-17	5
Npdc1	9.42E-22	0.28002	0.311	0.256	1.57E-17	5
Ppat	9.52E-22	-0.26545	0.045	0.115	1.59E-17	5
Klhl7	1.01E-21	0.261665	0.196	0.161	1.68E-17	5
Brsk2	1.07E-21	0.340581	0.137	0.082	1.79E-17	5
Oraov1	1.78E-21	-0.26536	0.054	0.128	2.96E-17	5
Efhd2	5.57E-21	-0.2753	0.063	0.137	9.29E-17	5
St8sia3	8.2E-21	0.273787	0.295	0.245	1.37E-16	5
Nol4	1.02E-20	0.286032	0.218	0.166	1.7E-16	5
Zic5	1.1E-20	0.287148	0.191	0.157	1.84E-16	5
Nav2	1.33E-20	0.311714	0.147	0.1	2.22E-16	5
Grb2	1.56E-20	0.270414	0.29	0.244	2.6E-16	5
Pdgfra	1.61E-20	0.30918	0.247	0.174	2.68E-16	5
Rnf122	1.8E-20	0.301205	0.145	0.097	3E-16	5
Sbk1	2.68E-20	0.317888	0.167	0.113	4.47E-16	5
Srgap2	2.81E-20	0.273662	0.162	0.129	4.69E-16	5

Srrm4	2.9E-20	0.318049	0.255	0.188	4.83E-16	5
Cadm3	3.38E-20	0.327447	0.209	0.133	5.64E-16	5
Scrt1	3.97E-20	0.301815	0.104	0.053	6.61E-16	5
Dcc	4.08E-20	0.298012	0.106	0.058	6.81E-16	5
Nova2	4.44E-20	0.303455	0.105	0.053	7.4E-16	5
Acd	4.6E-20	0.291794	0.207	0.156	7.67E-16	5
Dusp8	5.6E-20	0.314857	0.132	0.072	9.34E-16	5
Smpd3	5.71E-20	0.270189	0.191	0.158	9.52E-16	5
Tmem57	6.53E-20	0.252155	0.337	0.285	1.09E-15	5
Tspan7	7.03E-20	-0.29528	0.048	0.112	1.17E-15	5
Atp6v0b	7.25E-20	0.270238	0.314	0.262	1.21E-15	5
Smoc1	9.7E-20	-0.25714	0.067	0.141	1.62E-15	5
Fam20c	9.72E-20	0.290111	0.106	0.055	1.62E-15	5
Dst	1.22E-19	0.264463	0.307	0.262	2.04E-15	5
9330159F1	1.66E-19	0.261806	0.207	0.164	2.76E-15	5
Smarcd2	1.68E-19	0.305127	0.144	0.092	2.8E-15	5
Baz1a	1.73E-19	-0.28994	0.092	0.174	2.89E-15	5
Bzrap1	1.73E-19	0.328653	0.107	0.056	2.89E-15	5
Nek7	1.84E-19	-0.26349	0.054	0.123	3.07E-15	5
Sox11	2.68E-19	0.263384	0.149	0.111	4.46E-15	5
Plcb1	2.7E-19	0.354972	0.193	0.135	4.51E-15	5
Mical1	3.04E-19	-0.2677	0.061	0.132	5.07E-15	5
Myc	3.85E-19	-0.28278	0.044	0.106	6.42E-15	5
Klf6	3.99E-19	-0.25319	0.081	0.155	6.66E-15	5
Mapk10	4.23E-19	0.292528	0.15	0.093	7.06E-15	5
Slc1a3	5.05E-19	-0.36853	0.098	0.158	8.42E-15	5
Adk	7.67E-19	-0.253	0.094	0.173	1.28E-14	5
Ywhag	1.21E-18	0.251381	0.277	0.236	2.03E-14	5
Eif4e3	1.35E-18	0.303758	0.183	0.124	2.25E-14	5
Gamt	1.44E-18	0.286632	0.218	0.164	2.4E-14	5
Ldb1	1.53E-18	0.279801	0.228	0.179	2.55E-14	5
Scmh1	2.26E-18	0.275801	0.114	0.073	3.77E-14	5
Pea15a	3.62E-18	0.293016	0.227	0.17	6.04E-14	5
Trak1	4.02E-18	0.281036	0.124	0.085	6.71E-14	5
Tmeff1	4.66E-18	0.284291	0.201	0.143	7.77E-14	5
Grina	7.22E-18	0.308549	0.16	0.103	1.2E-13	5
Atl1	7.98E-18	0.275249	0.132	0.099	1.33E-13	5
Fam57b	1.86E-17	0.284148	0.24	0.179	3.11E-13	5
Tmem178b	3.34E-17	0.275365	0.111	0.065	5.57E-13	5
Tubb4a	6.42E-17	0.275867	0.107	0.062	1.07E-12	5
Snapc3	7.97E-17	0.257924	0.164	0.12	1.33E-12	5
Atat1	9E-17	0.272553	0.149	0.098	1.5E-12	5
Celsr2	1.02E-16	0.260844	0.277	0.233	1.71E-12	5
Prmt2	1.17E-16	0.265181	0.139	0.089	1.94E-12	5
Clybl	1.35E-16	0.279182	0.16	0.111	2.25E-12	5
Rnasel	1.53E-16	0.307269	0.125	0.067	2.55E-12	5
Csrp1	1.64E-16	0.25011	0.124	0.075	2.73E-12	5
Nrcam	2.53E-16	0.278715	0.167	0.13	4.22E-12	5

Lingo1	3.77E-16	0.300203	0.145	0.089	6.3E-12	5
Gramd1b	5.2E-16	0.260534	0.202	0.165	8.67E-12	5
Shd	2.67E-15	0.25684	0.144	0.105	4.46E-11	5
Gdap1	3.54E-15	0.25212	0.24	0.194	5.91E-11	5
Mroh2a	3.58E-15	0.299622	0.162	0.108	5.97E-11	5
Reln	4.39E-15	0.251364	0.211	0.176	7.32E-11	5
Dpysl3	1.97E-14	0.284824	0.284	0.212	3.29E-10	5
Itga7	2.19E-14	0.262453	0.136	0.085	3.65E-10	5
Trafd1	3.21E-14	0.251379	0.156	0.113	5.35E-10	5
Tmem2	5.66E-14	0.254173	0.116	0.08	9.44E-10	5
Maml3	1.06E-13	0.253326	0.138	0.096	1.76E-09	5
Apbb1	1.45E-13	0.256484	0.214	0.165	2.42E-09	5
2700081O	2.18E-13	0.251801	0.148	0.106	3.64E-09	5
Ncan	3.94E-13	0.255271	0.132	0.095	6.57E-09	5
Ralgds	4.77E-13	0.255746	0.139	0.098	7.95E-09	5
Jhdm1d	1.53E-12	0.255539	0.177	0.119	2.55E-08	5
Plp1	3.01E-06	-0.39867	0.108	0.115	0.050195	5
Celf4	0	1.681048	0.82	0.267	0	6
Neurod1	0	1.600624	0.968	0.486	0	6
Meg3	0	1.526734	0.406	0.062	0	6
Mapt	0	1.506963	0.635	0.171	0	6
Gpm6a	0	1.47765	0.834	0.364	0	6
Arpp21	0	1.477205	0.47	0.075	0	6
Stmn2	0	1.300621	0.949	0.51	0	6
Nrxn1	0	1.281652	0.676	0.28	0	6
Neurod2	0	1.278677	0.348	0.037	0	6
Dpysl3	0	1.273969	0.582	0.181	0	6
Tubb2a	0	1.271607	0.612	0.206	0	6
Stmn4	0	1.262127	0.702	0.303	0	6
Sept3	0	1.2473	0.756	0.354	0	6
Rtn1	0	1.233829	0.959	0.661	0	6
Ank2	0	1.227259	0.562	0.206	0	6
Ppp1r14c	0	1.219707	0.604	0.226	0	6
Thra	0	1.213681	0.564	0.193	0	6
Mtss1	0	1.129457	0.592	0.235	0	6
Nrep	0	1.129283	0.85	0.539	0	6
Map1b	0	0.958603	0.836	0.561	0	6
Gria2	0	0.951727	0.773	0.494	0	6
Celf2	0	0.945906	0.834	0.617	0	6
Atp2b1	0	0.929486	0.743	0.553	0	6
Gap43	0	0.871443	0.855	0.572	0	6
Zic1	0	0.84991	0.951	0.766	0	6
Calm1	0	0.718997	0.941	0.853	0	6
Tuba1a	0	0.703571	0.975	0.924	0	6
Rpl4	0	-0.77334	0.706	0.929	0	6
Rps9	0	-0.82149	0.723	0.95	0	6
Pabpc1	0	-0.8372	0.613	0.906	0	6
Rps5	0	-0.843	0.81	0.969	0	6

Rpl32	0	-0.84343	0.635	0.916	0	6
Rpl8	0	-0.87321	0.648	0.923	0	6
Rps3	0	-0.94	0.669	0.948	0	6
Rps26	0	-0.9783	0.528	0.881	0	6
Rpl13a	0	-0.98557	0.7	0.952	0	6
Rps20	0	-0.99186	0.393	0.818	0	6
Rplp1	0	-1.06185	0.548	0.911	0	6
Rps19	0	-1.06274	0.359	0.795	0	6
Sfrp1	0	-1.19615	0.46	0.849	0	6
Fxyd6	0	0.893414	0.732	0.485	0	6
Rplp2	0	-0.87182	0.531	0.873	0	6
Rbfox3	0	0.93189	0.755	0.466	0	6
Pcp4	0	1.45529	0.291	0.027	0	6
Rps14	0	-0.6641	0.828	0.963	0	6
Gnb2l1	0	-0.80992	0.604	0.9	0	6
Car10	0	1.208347	0.265	0.016	0	6
Rpl22	1.1E-302	-0.90257	0.436	0.825	1.9E-298	6
Ccnd1	4.2E-300	-1.38791	0.124	0.564	7E-296	6
Malat1	8E-299	0.699127	0.988	0.951	1.3E-294	6
Rps11	3.6E-296	-0.9069	0.426	0.814	6.1E-292	6
Jph4	3.1E-289	1.21574	0.32	0.042	5.1E-285	6
Ttc3	4.8E-285	0.644091	0.915	0.819	8.1E-281	6
L1cam	1.8E-284	1.143773	0.291	0.027	3E-280	6
Ccnd2	7E-281	-1.1272	0.283	0.702	1.2E-276	6
Elmo1	5.5E-280	1.199653	0.414	0.095	9.2E-276	6
Eef1a1	7E-279	-0.755	0.664	0.907	1.2E-274	6
Rplp0	3.2E-277	-0.75921	0.624	0.898	5.4E-273	6
Cd63	6.7E-277	-0.89341	0.403	0.792	1.1E-272	6
Gng3	1.2E-273	1.008	0.611	0.285	2.1E-269	6
Sez6	8.3E-273	1.139778	0.278	0.032	1.4E-268	6
Rps21	1.1E-271	-0.78411	0.531	0.865	1.9E-267	6
Tubb2b	3.5E-267	0.848069	0.686	0.441	5.8E-263	6
Smc2	1E-261	-1.33624	0.177	0.584	1.7E-257	6
Rpl41	3.7E-260	-0.80707	0.447	0.815	6.2E-256	6
Npm1	1.1E-259	-0.95699	0.266	0.686	1.9E-255	6
Cntn2	1.5E-257	0.974909	0.613	0.226	2.4E-253	6
Plxna2	1.7E-251	1.116784	0.292	0.046	2.8E-247	6
Prdx1	2.2E-251	-1.08103	0.122	0.528	3.6E-247	6
Ankrd12	2.3E-247	1.054716	0.539	0.246	3.8E-243	6
Anp32b	7.1E-246	-1.01507	0.218	0.63	1.2E-241	6
Ranbp1	1.6E-243	-0.9577	0.324	0.712	2.8E-239	6
Dner	1E-241	1.027152	0.484	0.186	1.7E-237	6
H2afv	9.9E-241	-0.92199	0.414	0.76	1.6E-236	6
Cdk5r1	1.1E-240	0.917934	0.579	0.307	1.9E-236	6
Tmsb4x	4.5E-239	0.536699	0.978	0.945	7.5E-235	6
Rps15	2.1E-233	-0.7477	0.481	0.82	3.5E-229	6
Gnao1	1.5E-226	0.920883	0.516	0.262	2.5E-222	6
Ina	2.1E-223	0.82175	0.664	0.42	3.6E-219	6

Cdk4	7.1E-223	-0.83202	0.285	0.679	1.2E-218	6
Cadps2	2.5E-221	1.082316	0.255	0.041	4.2E-217	6
Rpl14	3.6E-221	-0.81552	0.363	0.732	6E-217	6
Basp1	3.3E-220	0.638136	0.834	0.69	5.5E-216	6
Kcnk1	4.9E-220	0.977438	0.446	0.179	8.2E-216	6
Dusp26	2.6E-219	1.107952	0.275	0.042	4.4E-215	6
Eef1b2	6E-219	-0.77705	0.408	0.766	1E-214	6
Anp32e	1.4E-218	-0.95828	0.268	0.649	2.3E-214	6
Rps24	2.5E-218	-0.69842	0.553	0.848	4.1E-214	6
Rps3a1	1.6E-217	-0.81257	0.323	0.703	2.7E-213	6
Zfpm2	1.7E-217	0.952978	0.224	0.023	2.8E-213	6
Draxin	5.2E-217	-0.94861	0.201	0.592	8.7E-213	6
2810417H1	5.1E-216	-1.31717	0.077	0.424	8.5E-212	6
Smc4	1.2E-215	-1.1743	0.24	0.601	2E-211	6
Mdk	9.1E-215	-1.20161	0.065	0.412	1.5E-210	6
Uchl1	8.3E-211	0.808399	0.52	0.314	1.4E-206	6
Nbea	1.8E-210	0.988685	0.405	0.167	3.1E-206	6
Srebf1	1.1E-208	-1.14223	0.041	0.364	1.9E-204	6
Cadm2	3.8E-208	1.062379	0.232	0.028	6.3E-204	6
Camk2d	8.7E-207	1.005594	0.272	0.053	1.5E-202	6
Sv2a	3.9E-206	1.028613	0.285	0.049	6.6E-202	6
Hmgb2	1.3E-205	-1.18737	0.088	0.432	2.2E-201	6
Rpl39	1.4E-205	-0.81583	0.309	0.681	2.3E-201	6
Dbi	3.3E-205	-1.06785	0.137	0.497	5.5E-201	6
E130114P1	2.4E-204	-0.88582	0.241	0.621	4E-200	6
Dcx	2.5E-204	0.825671	0.596	0.352	4.2E-200	6
Mki67	1.8E-198	-1.36623	0.093	0.426	3.1E-194	6
Gnai2	8.6E-195	-0.89614	0.109	0.458	1.4E-190	6
Zbtb18	3E-193	0.839701	0.468	0.245	4.9E-189	6
Snca	2.2E-192	1.097697	0.232	0.041	3.6E-188	6
Stmn3	3.2E-191	0.612499	0.741	0.609	5.3E-187	6
Kif1b	3.4E-191	0.67987	0.662	0.505	5.7E-187	6
Cks1b	7.7E-191	-1.02207	0.057	0.374	1.3E-186	6
Phf20l1	2.5E-190	0.816142	0.507	0.322	4.2E-186	6
Prkcb	1.5E-189	0.864421	0.481	0.232	2.6E-185	6
Ppp3ca	1.1E-188	0.781925	0.552	0.359	1.8E-184	6
Rab3a	1.8E-188	0.955667	0.383	0.125	3E-184	6
Rnd3	1.6E-187	-0.91672	0.119	0.464	2.7E-183	6
Grin2b	3.5E-187	0.941035	0.227	0.028	5.8E-183	6
Cadm3	1.3E-186	0.961913	0.366	0.117	2.1E-182	6
Zfand5	1.5E-186	0.71254	0.545	0.401	2.5E-182	6
Tspan4	4.4E-186	0.993488	0.255	0.045	7.3E-182	6
Kcna1	5.8E-186	0.876431	0.17	0.011	9.7E-182	6
Tnik	2.5E-185	1.003695	0.375	0.122	4.1E-181	6
Aplp1	3.6E-185	0.944273	0.379	0.127	6E-181	6
Rps15a	2.9E-184	-0.73508	0.341	0.69	4.8E-180	6
Sh3gl2	8.8E-183	0.916092	0.37	0.15	1.5E-178	6
Psat1	2.7E-182	-0.90777	0.077	0.398	4.5E-178	6

2900079G:	7.8E-182	0.924724	0.218	0.03	1.3E-177	6
Prdm8	5.8E-179	0.967077	0.33	0.105	9.6E-175	6
Chd7	2.5E-178	0.589073	0.748	0.636	4.1E-174	6
Gm12022	2.9E-178	0.938392	0.161	0.011	4.9E-174	6
Hmgn5	3.4E-177	-0.98167	0.124	0.456	5.7E-173	6
Epb4.1l1	7E-177	0.956329	0.287	0.07	1.2E-172	6
Nop58	2.8E-176	-0.75416	0.27	0.626	4.6E-172	6
Camk4	3.9E-175	0.88215	0.204	0.026	6.6E-171	6
Fkbp3	9.1E-173	-0.70387	0.469	0.774	1.5E-168	6
Cygb	2.2E-172	0.921562	0.219	0.033	3.7E-168	6
Rps27l	2.9E-172	-0.75322	0.181	0.524	4.9E-168	6
Marcks	8.5E-172	0.447699	0.95	0.912	1.4E-167	6
Ndst3	1.3E-171	0.885374	0.181	0.018	2.2E-167	6
Top2a	2.2E-171	-1.29109	0.158	0.456	3.7E-167	6
Prkce	2.8E-170	0.901459	0.228	0.047	4.7E-166	6
Dek	5E-169	-0.80682	0.43	0.721	8.4E-165	6
Lrrtm2	6.2E-167	0.904795	0.212	0.034	1E-162	6
Tead2	1.3E-166	-0.88383	0.034	0.303	2.1E-162	6
Anks1b	7.2E-166	0.916079	0.238	0.045	1.2E-161	6
Mcm6	1.4E-164	-0.96639	0.034	0.301	2.3E-160	6
Grin1	2.7E-164	0.822336	0.182	0.02	4.5E-160	6
Id2	3.1E-164	0.726093	0.631	0.47	5.2E-160	6
Rps2	3.7E-164	-0.77698	0.151	0.481	6.2E-160	6
Ybx1	6.6E-164	-0.61225	0.533	0.812	1.1E-159	6
Serinc1	9.7E-164	0.643066	0.571	0.422	1.6E-159	6
Ephb1	3.4E-163	0.848843	0.191	0.024	5.7E-159	6
Cpe	5.2E-162	0.646449	0.594	0.441	8.7E-158	6
Ryr2	6.3E-162	0.810638	0.141	0.008	1.1E-157	6
Tbata	3.7E-161	-1.05813	0.075	0.367	6.1E-157	6
Dut	3.2E-160	-0.89203	0.147	0.465	5.3E-156	6
Pax6	9.8E-159	0.621098	0.669	0.496	1.6E-154	6
Hnrnpab	6.1E-158	-0.58107	0.589	0.839	1E-153	6
Pcsk1n	4E-157	0.884894	0.275	0.063	6.7E-153	6
Nasp	1.2E-156	-0.7634	0.356	0.67	2E-152	6
Nhp2	1.6E-156	-0.81926	0.097	0.399	2.7E-152	6
C1ql1	1.8E-156	-0.92184	0.039	0.304	3E-152	6
Rpl26	3E-156	-0.62128	0.418	0.731	5E-152	6
Rpl35a	3.3E-156	-0.68476	0.27	0.602	5.5E-152	6
Sema6a	5.4E-155	0.845591	0.399	0.181	9.1E-151	6
Synpr	2.3E-154	0.834779	0.184	0.027	3.8E-150	6
Pcna	3.2E-154	-0.96517	0.094	0.385	5.4E-150	6
Ran	7.1E-154	-0.78475	0.131	0.443	1.2E-149	6
Tpx2	3.1E-153	-1.0794	0.043	0.306	5.2E-149	6
Rbp4	3.7E-153	-0.94432	0.022	0.264	6.1E-149	6
Gsg1l	8.5E-153	-0.8827	0.029	0.279	1.4E-148	6
Stxbp1	2.9E-152	0.836162	0.343	0.143	4.8E-148	6
D3Bwg056	3.7E-152	0.868784	0.159	0.017	6.2E-148	6
Ppp1r14b	6E-151	-0.79228	0.124	0.425	1E-146	6

Birc5	1.3E-150	-1.01906	0.041	0.298	2.2E-146	6
Snrpf	4.4E-150	-0.7187	0.149	0.456	7.4E-146	6
Tmpo	3.7E-149	-0.79786	0.165	0.482	6.1E-145	6
Pvrl3	5.3E-149	0.820722	0.261	0.107	8.8E-145	6
Mllt11	5.8E-149	0.823514	0.407	0.198	9.7E-145	6
Cplx1	1.9E-147	0.844142	0.309	0.12	3.1E-143	6
Cdk1	2.7E-145	-0.9792	0.029	0.269	4.4E-141	6
Hey1	5.3E-145	-0.92383	0.043	0.297	8.9E-141	6
Tuba1b	1E-144	-0.81301	0.202	0.51	1.7E-140	6
Pkia	3E-144	0.821844	0.299	0.116	5.1E-140	6
Porcn	4.2E-144	0.85843	0.25	0.065	7E-140	6
Gabra2	6.1E-144	0.671117	0.117	0.005	1E-139	6
Apbb1	1.9E-143	0.826596	0.349	0.151	3.1E-139	6
Rps25	2.6E-143	-0.68404	0.197	0.509	4.3E-139	6
D4Wsu53e	5.9E-143	0.615566	0.567	0.434	9.8E-139	6
Rps10	8.2E-143	-0.5932	0.353	0.666	1.4E-138	6
Rbfox1	1E-142	0.832238	0.255	0.075	1.7E-138	6
Prdx6	2.4E-142	-0.79543	0.078	0.352	4E-138	6
Kif5c	3.9E-142	0.671191	0.556	0.381	6.4E-138	6
Elavl3	6E-142	0.583408	0.641	0.454	9.9E-138	6
Cdca8	9.1E-142	-0.94944	0.041	0.288	1.5E-137	6
RP23-45G1	6E-141	-0.93395	0.08	0.353	1E-136	6
Rpl34	3.3E-140	-0.59068	0.371	0.682	5.4E-136	6
Shfm1	3.4E-140	-0.57461	0.41	0.715	5.6E-136	6
Grik2	4.4E-140	0.852205	0.267	0.084	7.4E-136	6
Ncam1	1.4E-139	0.609011	0.504	0.374	2.3E-135	6
CRE_RECON	2.3E-139	-0.81461	0.582	0.788	3.8E-135	6
Fnbp1l	1.6E-138	0.629933	0.583	0.439	2.6E-134	6
Atp6v0e	3.6E-138	-0.67559	0.146	0.435	6.1E-134	6
Hmgn1	9.8E-138	-0.59273	0.337	0.651	1.6E-133	6
Cenpf	1E-137	-1.22334	0.119	0.388	1.7E-133	6
Dtymk	3.5E-137	-0.73143	0.168	0.467	5.9E-133	6
Eid1	4.5E-137	0.549669	0.604	0.492	7.5E-133	6
Ier5	3.9E-136	-0.728	0.135	0.421	6.5E-132	6
Ube2c	1E-135	-1.23607	0.055	0.291	1.7E-131	6
2700094K1	1.5E-135	-0.69282	0.349	0.642	2.5E-131	6
Tomm7	1.7E-135	-0.6366	0.218	0.527	2.8E-131	6
Cadm1	2.2E-135	-0.69715	0.131	0.418	3.7E-131	6
Grm1	5.8E-135	0.725783	0.125	0.008	9.8E-131	6
Hsd11b2	1E-134	-0.84051	0.059	0.311	1.7E-130	6
Syp	2.9E-134	0.818852	0.22	0.052	4.8E-130	6
St18	8E-133	0.771877	0.388	0.169	1.3E-128	6
Dctpp1	1.3E-132	-0.7319	0.072	0.328	2.1E-128	6
Spc24	1.5E-132	-0.84792	0.034	0.264	2.4E-128	6
Slc25a5	3E-132	-0.64757	0.235	0.537	4.9E-128	6
Cenpa	7.5E-132	-1.12318	0.044	0.274	1.3E-127	6
Paics	8.1E-131	-0.70446	0.152	0.441	1.4E-126	6
Hirip3	2.5E-130	-0.79544	0.127	0.409	4.2E-126	6

Racgap1	4.1E-130	-0.80896	0.043	0.278	6.8E-126	6
Itsn1	4.6E-130	0.752756	0.4	0.218	7.6E-126	6
H2afy	7.7E-130	-0.60153	0.317	0.62	1.3E-125	6
Myl12a	2.8E-129	-0.6808	0.092	0.353	4.6E-125	6
Trpc4ap	4.5E-129	0.783476	0.354	0.181	7.4E-125	6
Rpl23	5.5E-129	-0.59645	0.265	0.567	9.1E-125	6
Gnaq	6E-129	0.681579	0.423	0.27	1E-124	6
Tagln3	7.7E-128	0.704389	0.463	0.277	1.3E-123	6
Cog7	1.5E-127	-0.56675	0.311	0.613	2.5E-123	6
Pbk	6.4E-127	-0.84884	0.027	0.243	1.1E-122	6
Nap1l1	2.1E-126	-0.58951	0.242	0.539	3.4E-122	6
Prmt8	5.2E-126	-0.80026	0.055	0.295	8.7E-122	6
H2afx	6.2E-126	-0.9783	0.101	0.354	1E-121	6
Lsm6	2.9E-125	-0.66069	0.126	0.398	4.9E-121	6
Dpysl4	8.8E-125	-0.65199	0.229	0.518	1.5E-120	6
Kcnk2	9.3E-125	0.75398	0.162	0.027	1.6E-120	6
Kif5a	1.2E-124	0.793041	0.3	0.109	1.9E-120	6
BC005764	1.9E-124	0.801856	0.37	0.166	3.1E-120	6
Hook1	3.7E-124	0.81783	0.194	0.048	6.2E-120	6
Sox9	5.5E-124	-0.82094	0.046	0.277	9.2E-120	6
Cenpe	1.2E-123	-1.07951	0.068	0.308	2.1E-119	6
Nsg1	1.4E-123	0.6496	0.43	0.272	2.4E-119	6
Rufy3	3.5E-123	0.616171	0.501	0.357	5.9E-119	6
Cdca3	5.3E-123	-0.86316	0.029	0.242	8.8E-119	6
Siva1	1.1E-122	-0.70574	0.059	0.294	1.8E-118	6
Rps18	1.3E-122	-0.63356	0.174	0.455	2.2E-118	6
Ppic	1.5E-122	-0.74615	0.039	0.26	2.5E-118	6
Pcdha2	1.7E-122	0.803583	0.268	0.102	2.8E-118	6
Fam210b	4.4E-122	-0.72955	0.086	0.337	7.3E-118	6
Sox4	7.9E-122	0.51011	0.676	0.572	1.3E-117	6
Hnrnpm	9.6E-122	-0.49133	0.487	0.761	1.6E-117	6
Scg5	5.3E-121	0.648024	0.476	0.32	8.8E-117	6
Ptms	6.4E-121	0.503308	0.55	0.465	1.1E-116	6
Pqlc1	7.9E-121	-0.73375	0.077	0.32	1.3E-116	6
Nfib	1.3E-120	0.404053	0.968	0.929	2.2E-116	6
Slc29a1	2.3E-120	-0.61952	0.303	0.589	3.8E-116	6
Gm10075	6.7E-120	-0.63007	0.253	0.542	1.1E-115	6
Itm2b	9.5E-120	0.463483	0.73	0.633	1.6E-115	6
Srcin1	1E-119	0.775579	0.198	0.038	1.7E-115	6
Cnbp	1.6E-119	-0.51584	0.506	0.764	2.6E-115	6
Mycbp2	1.8E-119	0.643273	0.479	0.33	3E-115	6
Itgb1	6.3E-119	-0.56524	0.129	0.387	1.1E-114	6
Apex1	8.5E-119	-0.54231	0.198	0.478	1.4E-114	6
Rpl18a	1E-118	-0.55625	0.161	0.425	1.7E-114	6
Prc1	3.7E-118	-0.98539	0.053	0.278	6.1E-114	6
Fam63b	5.8E-118	0.788748	0.228	0.073	9.6E-114	6
Gpr153	7.9E-118	-0.76531	0.025	0.227	1.3E-113	6
Hdgf	9.1E-118	-0.58445	0.271	0.559	1.5E-113	6

Sfrs18	9.8E-118	0.463504	0.842	0.771	1.6E-113	6
Prim1	1E-117	-0.71414	0.057	0.284	1.7E-113	6
Ncl	1.1E-117	-0.44777	0.765	0.91	1.9E-113	6
Apc	3.6E-117	0.600706	0.576	0.416	6E-113	6
Snap25	4.7E-117	0.639864	0.42	0.267	7.8E-113	6
Hmgn2	7.7E-117	-0.69312	0.052	0.277	1.3E-112	6
Mcm2	1.2E-116	-0.75502	0.024	0.223	2E-112	6
Prdx4	1.6E-116	-0.6461	0.13	0.39	2.7E-112	6
Ube2ql1	1.9E-116	0.64656	0.129	0.013	3.2E-112	6
Lsm4	2.3E-116	-0.55855	0.229	0.515	3.9E-112	6
Sh3bp5	4.2E-116	0.758916	0.248	0.088	7E-112	6
Bola2	6.1E-116	-0.59314	0.154	0.422	1E-111	6
Serbp1	1E-115	-0.45043	0.719	0.885	1.7E-111	6
Camta1	1.8E-115	0.544315	0.501	0.398	2.9E-111	6
Chgb	2.5E-115	0.740026	0.434	0.22	4.1E-111	6
Ckb	2.7E-115	0.401495	0.864	0.802	4.5E-111	6
Galr1	4.9E-115	0.731137	0.183	0.033	8.1E-111	6
Nusap1	6.2E-115	-0.89386	0.036	0.247	1E-110	6
Trp53	6.9E-115	-0.59463	0.084	0.32	1.2E-110	6
Pdgfa	9.2E-115	-0.7731	0.052	0.274	1.5E-110	6
Banf1	1.5E-114	-0.54816	0.459	0.718	2.5E-110	6
Srsf7	2E-114	-0.56204	0.214	0.491	3.3E-110	6
Ccna2	2.1E-114	-0.77617	0.034	0.241	3.5E-110	6
Npdc1	8.8E-114	0.641558	0.4	0.246	1.5E-109	6
Nefl	1.2E-113	0.752985	0.197	0.043	2E-109	6
Snrpd2	1.3E-113	-0.58551	0.193	0.467	2.2E-109	6
Cbx5	6.2E-113	-0.57536	0.41	0.678	1E-108	6
Kcnc1	6.5E-113	0.757448	0.231	0.074	1.1E-108	6
Eif3f	7.2E-113	-0.4903	0.376	0.657	1.2E-108	6
Ckap2l	1.4E-112	-0.84651	0.034	0.237	2.4E-108	6
Ccdc34	1.8E-112	-0.65592	0.171	0.44	3E-108	6
Aplp2	2.6E-112	0.605111	0.436	0.311	4.4E-108	6
Snrpe	2.9E-112	-0.51596	0.305	0.589	4.8E-108	6
Atp5e	3.5E-112	-0.49697	0.404	0.678	5.8E-108	6
Gng2	4E-112	0.529557	0.486	0.386	6.7E-108	6
Ifi203	5.7E-112	0.619227	0.109	0.008	9.4E-108	6
Cdh20	6E-112	-0.70535	0.026	0.221	9.9E-108	6
Cdca7	6.2E-112	-0.70736	0.024	0.216	1E-107	6
Cenpv	1.8E-111	-0.5958	0.108	0.352	3.1E-107	6
Myt1l	2.1E-111	0.80302	0.326	0.143	3.4E-107	6
5830416P1	2.7E-111	0.657807	0.121	0.012	4.5E-107	6
Nop10	6.4E-111	-0.5447	0.232	0.508	1.1E-106	6
Map1lc3a	7.8E-111	0.580832	0.373	0.265	1.3E-106	6
Kif11	8.4E-111	-0.75382	0.028	0.224	1.4E-106	6
Rpl22l1	1.2E-110	-0.63307	0.122	0.375	2.1E-106	6
Fut9	1.3E-110	0.680094	0.334	0.199	2.2E-106	6
Eef1d	2.1E-110	-0.56567	0.128	0.373	3.6E-106	6
Naca	2.4E-110	-0.52101	0.22	0.49	4.1E-106	6

Nnat	2.4E-110	-0.61887	0.493	0.732	4.1E-106	6
Ntm	7.4E-110	0.780692	0.27	0.132	1.2E-105	6
Snrpb	8.6E-110	-0.49759	0.322	0.599	1.4E-105	6
Lyar	1.1E-109	-0.67701	0.077	0.307	1.9E-105	6
Scaper	1.6E-109	0.751165	0.243	0.096	2.7E-105	6
Lig1	3.2E-109	-0.79942	0.125	0.371	5.3E-105	6
Hes6	3.2E-109	-0.67	0.048	0.256	5.3E-105	6
Gdi1	3.9E-109	0.634422	0.354	0.216	6.5E-105	6
Tpm4	4.3E-109	-0.62059	0.099	0.339	7.1E-105	6
Chd3	8.2E-109	0.750146	0.311	0.132	1.4E-104	6
Lap3	1.2E-108	-0.73943	0.072	0.298	2E-104	6
Commd1	1.6E-108	-0.60786	0.089	0.32	2.7E-104	6
Mcm7	2.5E-108	-0.56695	0.16	0.413	4.1E-104	6
Scg2	2.6E-108	0.767522	0.163	0.04	4.4E-104	6
Nsg2	3.8E-108	0.49598	0.596	0.475	6.3E-104	6
Dnajc9	4.9E-108	-0.61651	0.125	0.373	8.3E-104	6
Tox3	2.2E-107	-0.61725	0.123	0.369	3.6E-103	6
Ezr	2.2E-107	-0.58341	0.2	0.463	3.7E-103	6
Bok	2.4E-107	-0.64173	0.058	0.271	4.1E-103	6
Ndrg4	3.7E-107	0.719564	0.174	0.038	6.2E-103	6
Lgals1	5.9E-107	-0.84019	0.044	0.248	9.9E-103	6
Hnrnpu	6.3E-107	-0.41885	0.706	0.882	1.1E-102	6
Arhgef7	1.5E-106	0.687884	0.281	0.135	2.5E-102	6
Sv2b	1.9E-106	0.706955	0.231	0.088	3.2E-102	6
Mycn	2E-106	-0.63529	0.138	0.387	3.3E-102	6
Mbnl2	2.3E-106	0.674539	0.304	0.165	3.8E-102	6
Dync1i2	3.5E-106	0.43924	0.626	0.562	5.8E-102	6
Mns1	4E-106	-0.71938	0.03	0.221	6.7E-102	6
Rrm1	5.2E-106	-0.64016	0.066	0.28	8.7E-102	6
Rpl7	5.7E-106	-0.49783	0.265	0.535	9.5E-102	6
Casc5	6.1E-106	-0.72653	0.022	0.203	1E-101	6
Rrm2	6.3E-106	-0.71312	0.03	0.222	1.1E-101	6
mt-Nd1	7.2E-106	-0.39074	0.827	0.937	1.2E-101	6
Spc25	7.6E-106	-0.80956	0.042	0.244	1.3E-101	6
Incenp	1.8E-105	-0.82037	0.072	0.296	3E-101	6
Zeb1	1.8E-105	-0.56893	0.16	0.411	3.1E-101	6
C1qbp	2.1E-105	-0.51486	0.155	0.401	3.4E-101	6
Tyms	1.7E-104	-0.71673	0.039	0.237	2.9E-100	6
Clspn	1.8E-104	-0.71529	0.025	0.207	3E-100	6
Pkm	2.8E-104	-0.51511	0.201	0.453	4.7E-100	6
Ly6h	3.3E-104	0.611236	0.117	0.012	5.6E-100	6
Knstrn	5.8E-104	-0.71271	0.022	0.202	9.7E-100	6
Ssrp1	2E-103	-0.49348	0.327	0.602	3.4E-99	6
Pcbp1	2.6E-103	-0.44512	0.347	0.619	4.3E-99	6
Spock2	9.7E-103	0.727951	0.198	0.051	1.63E-98	6
Atp6v0b	1.6E-102	0.59942	0.385	0.254	2.62E-98	6
Tacc3	3.7E-102	-0.73309	0.034	0.224	6.16E-98	6
Pgm2l1	8.2E-102	0.744209	0.228	0.083	1.37E-97	6

Sub1	1.2E-101	-0.38879	0.345	0.614	2E-97	6
Tipin	3.8E-101	-0.58056	0.067	0.274	6.41E-97	6
Snrpg	4.2E-101	-0.55381	0.122	0.357	7.04E-97	6
Nfix	4.7E-101	0.385097	0.722	0.668	7.9E-97	6
Tmsb10	6.9E-101	0.385897	0.705	0.659	1.15E-96	6
Zwint	7.9E-101	0.52802	0.366	0.283	1.32E-96	6
Park7	8.6E-101	-0.45127	0.307	0.571	1.43E-96	6
Rgs17	1.1E-100	0.70589	0.158	0.034	1.83E-96	6
B2m	1.1E-100	-0.79958	0.05	0.25	1.9E-96	6
Runx1t1	1.3E-100	0.651416	0.115	0.013	2.23E-96	6
Mpp3	1.6E-100	0.686254	0.159	0.036	2.62E-96	6
Rnasel	2.1E-100	0.769725	0.213	0.057	3.53E-96	6
Sphkap	2.9E-100	0.704755	0.164	0.035	4.8E-96	6
Smarcc2	3.3E-100	0.519529	0.451	0.354	5.56E-96	6
Atp1a3	5.4E-100	0.616799	0.137	0.021	8.94E-96	6
Tshz2	6E-100	-0.61945	0.111	0.341	1.01E-95	6
Camk2b	8E-100	0.727301	0.228	0.076	1.33E-95	6
Rps4x	9.4E-100	-0.55714	0.095	0.316	1.57E-95	6
Gm9800	9.8E-100	-0.52206	0.3	0.559	1.63E-95	6
Tnrc6c	1E-99	0.680292	0.298	0.165	1.66E-95	6
Cdc20	1E-99	-0.87353	0.031	0.213	1.73E-95	6
Snrpd1	2.4E-99	-0.44405	0.29	0.549	4.08E-95	6
Nrxn2	3.1E-99	0.717582	0.228	0.075	5.13E-95	6
Ctnnb1	3.8E-99	-0.47838	0.168	0.409	6.36E-95	6
Adcy1	1.24E-98	0.635039	0.122	0.015	2.07E-94	6
Atp6v1e1	1.6E-98	0.528915	0.423	0.321	2.67E-94	6
Cklf	1.85E-98	-0.59351	0.016	0.181	3.08E-94	6
Rnaseh2c	2.68E-98	-0.49355	0.162	0.404	4.46E-94	6
Smarcc1	3.31E-98	-0.53106	0.206	0.457	5.52E-94	6
Sptan1	5.38E-98	0.681	0.298	0.155	8.97E-94	6
Esco2	6.85E-98	-0.74759	0.03	0.212	1.14E-93	6
Gm1673	9.53E-98	0.471046	0.563	0.463	1.59E-93	6
Nrxn3	1.21E-97	0.71711	0.142	0.025	2.02E-93	6
Rbfox2	1.29E-97	0.598117	0.404	0.27	2.15E-93	6
Nmral1	2.06E-97	-0.62008	0.026	0.204	3.44E-93	6
Pfn1	2.89E-97	-0.46512	0.277	0.535	4.83E-93	6
Rpl37	3.07E-97	-0.51436	0.274	0.527	5.12E-93	6
Pdia6	3.15E-97	-0.53138	0.132	0.362	5.26E-93	6
Hmmr	5.76E-97	-0.82902	0.022	0.192	9.6E-93	6
Rab6a	7.24E-97	0.509694	0.412	0.319	1.21E-92	6
Ccnb2	7.74E-97	-0.76671	0.027	0.203	1.29E-92	6
Prnp	7.74E-97	0.621217	0.321	0.194	1.29E-92	6
Hspe1	1.5E-96	-0.5229	0.132	0.362	2.5E-92	6
Idh2	1.79E-96	-0.54834	0.088	0.298	2.99E-92	6
Rpa2	2.13E-96	-0.64427	0.035	0.217	3.55E-92	6
Vps37b	4.89E-96	-0.43492	0.245	0.494	8.15E-92	6
Adh5	9.42E-96	-0.4411	0.191	0.427	1.57E-91	6
A930011O	1.16E-95	0.762191	0.265	0.098	1.93E-91	6

Luc7l3	1.35E-95	0.410229	0.788	0.743	2.25E-91	6
Hells	1.39E-95	-0.78048	0.043	0.231	2.32E-91	6
Abracl	1.49E-95	-0.58823	0.101	0.323	2.48E-91	6
Rps8	2.21E-95	-0.5213	0.175	0.42	3.68E-91	6
Zmat4	2.26E-95	0.565178	0.102	0.01	3.76E-91	6
2810004N1	2.51E-95	-0.57685	0.077	0.281	4.19E-91	6
Eif3i	3.04E-95	-0.48123	0.234	0.486	5.07E-91	6
BC029214	5.59E-95	0.663485	0.212	0.08	9.32E-91	6
Cenph	1.24E-94	-0.62082	0.033	0.21	2.06E-90	6
Dkc1	1.69E-94	-0.58643	0.125	0.354	2.83E-90	6
Btbd3	2.73E-94	0.651997	0.188	0.08	4.55E-90	6
Ptma	3.09E-94	-0.46589	0.344	0.606	5.16E-90	6
Ndufa2	4.09E-94	-0.39962	0.371	0.627	6.82E-90	6
Rpl14-ps1	5.26E-94	-0.48418	0.097	0.303	8.77E-90	6
Pou3f2	6.41E-94	-0.62391	0.085	0.296	1.07E-89	6
Ptprs	1.53E-93	0.542418	0.53	0.409	2.55E-89	6
Rsl1d1	1.61E-93	-0.40856	0.264	0.513	2.69E-89	6
Snrpd3	3.41E-93	-0.43856	0.301	0.559	5.68E-89	6
Rps7	3.94E-93	-0.4778	0.185	0.422	6.58E-89	6
Ndufa12	8.67E-93	-0.46941	0.204	0.444	1.45E-88	6
Rtn4	9.12E-93	0.418	0.525	0.475	1.52E-88	6
Uba52	9.39E-93	-0.52434	0.118	0.337	1.57E-88	6
Hmgb1	1.18E-92	-0.49975	0.15	0.38	1.97E-88	6
Slc3a2	1.3E-92	-0.49437	0.126	0.343	2.16E-88	6
Ppfia2	1.45E-92	0.730462	0.249	0.097	2.42E-88	6
Rfc4	2.43E-92	-0.5347	0.057	0.244	4.05E-88	6
Calm2	3.35E-92	0.335424	0.914	0.87	5.58E-88	6
Cct3	3.52E-92	-0.412	0.273	0.518	5.88E-88	6
Txn1	5.07E-92	-0.41306	0.33	0.584	8.47E-88	6
Mif	5.52E-92	-0.53304	0.147	0.378	9.21E-88	6
Add3	7.33E-92	0.685049	0.209	0.092	1.22E-87	6
Usp1	9.19E-92	-0.57076	0.132	0.36	1.53E-87	6
Smco4	9.33E-92	-0.57441	0.018	0.176	1.56E-87	6
Cntn1	1.98E-91	0.641964	0.171	0.038	3.31E-87	6
Gmnn	4.59E-91	-0.58199	0.011	0.158	7.66E-87	6
Mllt4	9.78E-91	0.536383	0.464	0.352	1.63E-86	6
Ddx39b	1.27E-90	-0.42217	0.228	0.467	2.11E-86	6
Tcp1	1.33E-90	-0.43954	0.241	0.486	2.21E-86	6
Kidins220	1.42E-90	0.685129	0.27	0.126	2.38E-86	6
Elavl4	1.45E-90	0.555018	0.479	0.32	2.42E-86	6
Adam10	1.64E-90	0.56219	0.283	0.194	2.73E-86	6
Cbfa2t3	1.82E-90	-0.54588	0.189	0.425	3.04E-86	6
Mrpl52	2.43E-90	-0.41476	0.219	0.455	4.06E-86	6
Map2	2.89E-90	0.447154	0.586	0.481	4.83E-86	6
Homer2	3.2E-90	-0.47333	0.075	0.264	5.34E-86	6
Tacc2	7.56E-90	0.690934	0.267	0.127	1.26E-85	6
Gm8292	7.72E-90	-0.52548	0.161	0.391	1.29E-85	6
Pnmal2	8.68E-90	0.672206	0.273	0.133	1.45E-85	6

Kif23	8.95E-90	-0.75929	0.048	0.231	1.49E-85	6
Srsf6	9.36E-90	-0.42482	0.23	0.468	1.56E-85	6
Chchd2	9.73E-90	-0.38501	0.426	0.677	1.62E-85	6
Calb2	1.18E-89	0.654732	0.108	0.013	1.98E-85	6
Csnk1e	1.42E-89	0.442108	0.43	0.381	2.37E-85	6
G3bp1	2.07E-89	-0.47066	0.13	0.348	3.45E-85	6
Psma4	2.83E-89	-0.39483	0.206	0.435	4.72E-85	6
Supt16	3.24E-89	-0.49549	0.266	0.517	5.4E-85	6
Snhg1	3.89E-89	-0.5289	0.152	0.379	6.49E-85	6
Sod1	5.46E-89	-0.4264	0.227	0.464	9.11E-85	6
Cep170	5.63E-89	0.54806	0.391	0.296	9.38E-85	6
Hjurp	5.66E-89	-0.58729	0.218	0.456	9.45E-85	6
Adrbk2	7.95E-89	0.67244	0.22	0.087	1.33E-84	6
mt-Rnr2	8.58E-89	0.30634	0.996	0.997	1.43E-84	6
Mrpl13	1.1E-88	-0.47357	0.118	0.327	1.83E-84	6
Mab21l1	1.19E-88	0.601806	0.334	0.213	1.98E-84	6
Klf7	1.29E-88	0.594765	0.38	0.249	2.15E-84	6
Atoh1	1.74E-88	-0.64159	0.021	0.179	2.9E-84	6
B3galt2	1.89E-88	0.712027	0.225	0.084	3.15E-84	6
Nudt3	3.17E-88	0.507442	0.325	0.24	5.28E-84	6
Ezh2	3.96E-88	-0.52826	0.387	0.633	6.61E-84	6
Ssbp3	3.98E-88	0.605554	0.275	0.155	6.64E-84	6
Lrig3	4.8E-88	-0.60008	0.024	0.185	8.01E-84	6
Cplx2	6.63E-88	0.462545	0.545	0.49	1.11E-83	6
Mcm3	8.12E-88	-0.61622	0.029	0.195	1.35E-83	6
Slc1a2	8.83E-88	-0.58842	0.105	0.314	1.47E-83	6
Fgf13	1.44E-87	0.603815	0.151	0.042	2.41E-83	6
Ncapg	3.38E-87	-0.64198	0.031	0.199	5.64E-83	6
1810009A1	5.56E-87	-0.50654	0.083	0.278	9.28E-83	6
Nudcd2	6.22E-87	-0.52103	0.059	0.24	1.04E-82	6
Asap1	1.1E-86	-0.60206	0.029	0.196	1.84E-82	6
Tmem145	1.43E-86	0.64448	0.154	0.039	2.38E-82	6
Smim11	1.93E-86	-0.47098	0.097	0.295	3.21E-82	6
Rab6b	2.84E-86	0.611021	0.335	0.188	4.73E-82	6
Mmp24	3.06E-86	0.657124	0.165	0.041	5.1E-82	6
Larp7	3.14E-86	-0.46884	0.125	0.331	5.23E-82	6
Cnrip1	3.67E-86	0.584028	0.297	0.187	6.13E-82	6
Timp3	4.23E-86	0.729589	0.194	0.067	7.06E-82	6
Reep2	4.69E-86	0.663111	0.177	0.056	7.82E-82	6
Cdc42	6.94E-86	0.345346	0.529	0.513	1.16E-81	6
Rpl37a	1.25E-85	-0.45045	0.253	0.491	2.09E-81	6
Tubb4b	1.34E-85	-0.58272	0.069	0.258	2.24E-81	6
Chaf1a	2.81E-85	-0.55338	0.026	0.183	4.68E-81	6
Gas1	3.34E-85	-0.59022	0.019	0.171	5.58E-81	6
2900011O0	3.48E-85	0.659659	0.226	0.09	5.8E-81	6
Gar1	3.6E-85	-0.55218	0.049	0.225	6E-81	6
Fam115a	3.99E-85	0.45646	0.4	0.345	6.65E-81	6
Tspyl4	4.09E-85	0.65625	0.242	0.114	6.82E-81	6

Mtch2	7.41E-85	0.44937	0.35	0.288	1.24E-80	6
Ndn	8.04E-85	0.523925	0.308	0.214	1.34E-80	6
Smarca5	8.86E-85	-0.45248	0.196	0.423	1.48E-80	6
Ank3	1.12E-84	0.494675	0.503	0.411	1.87E-80	6
Cadps	1.14E-84	0.645844	0.177	0.061	1.9E-80	6
Klc1	1.85E-84	0.489133	0.463	0.35	3.09E-80	6
Rpl6	2.87E-84	-0.46616	0.11	0.31	4.79E-80	6
Ppib	4.39E-84	-0.3332	0.488	0.723	7.31E-80	6
Grina	5.36E-84	0.608502	0.234	0.096	8.95E-80	6
Ptn	6.94E-84	0.316548	0.557	0.429	1.16E-79	6
Cenpm	8.38E-84	-0.56007	0.022	0.176	1.4E-79	6
Hpcal1	1.28E-83	0.661221	0.215	0.095	2.14E-79	6
Npc2	1.47E-83	-0.43076	0.254	0.486	2.46E-79	6
Atxn7l3b	1.53E-83	0.319819	0.679	0.672	2.55E-79	6
Soga3	1.65E-83	0.427023	0.501	0.452	2.76E-79	6
Tubb3	1.72E-83	0.369454	0.626	0.458	2.86E-79	6
Arpp19	3.5E-83	-0.37376	0.185	0.397	5.84E-79	6
Cald1	3.89E-83	-0.38626	0.316	0.558	6.48E-79	6
Srsf3	4.4E-83	-0.3882	0.438	0.679	7.33E-79	6
Trim59	4.44E-83	-0.52295	0.029	0.184	7.41E-79	6
Unc5c	4.56E-83	0.60465	0.101	0.015	7.6E-79	6
Sowaha	5.43E-83	-0.7027	0.023	0.177	9.05E-79	6
Cct2	6.13E-83	-0.36459	0.311	0.546	1.02E-78	6
Gm13826	6.61E-83	-0.48002	0.127	0.331	1.1E-78	6
Uhrf1	7.32E-83	-0.54856	0.015	0.157	1.22E-78	6
Smpd2	1.1E-82	-0.55714	0.026	0.18	1.83E-78	6
Rpl18	1.16E-82	-0.49359	0.118	0.324	1.93E-78	6
Isoc1	2.46E-82	-0.51935	0.036	0.196	4.11E-78	6
Peli2	2.64E-82	0.569404	0.262	0.154	4.4E-78	6
Aurkb	3.3E-82	-0.55912	0.014	0.156	5.5E-78	6
Pa2g4	3.75E-82	-0.38883	0.308	0.545	6.25E-78	6
Psmb2	3.87E-82	-0.30337	0.235	0.448	6.46E-78	6
Islr2	4.67E-82	-0.59599	0.033	0.195	7.79E-78	6
Bcl11a	6.91E-82	-0.50852	0.054	0.226	1.15E-77	6
Hnrnpd	8.09E-82	-0.43264	0.397	0.633	1.35E-77	6
Mcm5	8.43E-82	-0.56682	0.027	0.183	1.41E-77	6
Kif15	9.22E-82	-0.59531	0.035	0.195	1.54E-77	6
Hnrnpc	1.01E-81	-0.36454	0.454	0.689	1.68E-77	6
Pde1c	1.04E-81	-0.57754	0.192	0.407	1.73E-77	6
Reln	1.86E-81	0.653409	0.292	0.168	3.11E-77	6
App	1.9E-81	0.378618	0.627	0.562	3.17E-77	6
Clcn3	2.07E-81	0.498953	0.341	0.265	3.46E-77	6
Cnn3	4.34E-81	-0.35971	0.238	0.457	7.24E-77	6
Sox18	4.78E-81	-0.58088	0.017	0.161	7.97E-77	6
Srrm2	8.51E-81	0.3105	0.729	0.732	1.42E-76	6
2210016L2	8.53E-81	0.501466	0.366	0.28	1.42E-76	6
Tulp4	8.67E-81	0.55194	0.298	0.194	1.45E-76	6
Rpa3	1.13E-80	-0.51242	0.076	0.261	1.89E-76	6

Nolc1	1.31E-80	-0.43797	0.204	0.43	2.19E-76	6
Prrt1	1.38E-80	0.533915	0.105	0.013	2.3E-76	6
Sep15	1.44E-80	-0.36545	0.297	0.526	2.4E-76	6
Pdzrn4	1.54E-80	-0.61614	0.016	0.159	2.56E-76	6
Ywhag	2.14E-80	0.510962	0.325	0.231	3.58E-76	6
Rbm8a	3.49E-80	-0.33404	0.265	0.486	5.82E-76	6
Ckap2	3.56E-80	-0.58905	0.026	0.177	5.94E-76	6
Clcn4-2	1.04E-79	0.547613	0.35	0.24	1.74E-75	6
Pja2	1.89E-79	0.481278	0.3	0.227	3.15E-75	6
Cd200	2.06E-79	0.613042	0.147	0.034	3.44E-75	6
Nicn1	2.74E-79	0.586008	0.313	0.197	4.57E-75	6
Adamts1	4.8E-79	-0.5962	0.022	0.168	8.01E-75	6
H2afz	5.04E-79	-0.46996	0.072	0.248	8.4E-75	6
Pmf1	5.75E-79	-0.47519	0.018	0.156	9.59E-75	6
Rbbp7	9.56E-79	-0.39456	0.141	0.338	1.6E-74	6
Fstl1	9.61E-79	-0.59129	0.036	0.195	1.6E-74	6
Sfrp2	9.72E-79	-0.53223	0.019	0.161	1.62E-74	6
Gm10260	1.15E-78	-0.48651	0.069	0.244	1.91E-74	6
Ubxn1	1.98E-78	-0.31834	0.28	0.499	3.3E-74	6
Naa38	2.16E-78	-0.3627	0.139	0.329	3.61E-74	6
Cst3	2.45E-78	-0.58797	0.356	0.554	4.08E-74	6
Pak1	2.57E-78	0.629777	0.171	0.047	4.29E-74	6
Grb2	3.55E-78	0.460525	0.303	0.243	5.93E-74	6
Ddx21	3.68E-78	-0.49069	0.138	0.341	6.15E-74	6
Cirbp	6.63E-78	0.431229	0.408	0.349	1.11E-73	6
Rtn3	6.67E-78	0.34864	0.496	0.474	1.11E-73	6
Eef1g	8.7E-78	-0.4317	0.15	0.354	1.45E-73	6
Sez6l2	9.09E-78	0.596146	0.129	0.027	1.52E-73	6
Uqcrq	1.04E-77	-0.33888	0.343	0.572	1.73E-73	6
Snrpb2	1.17E-77	-0.33151	0.204	0.403	1.95E-73	6
Tcf25	1.47E-77	0.319367	0.603	0.602	2.44E-73	6
Baz1a	1.48E-77	-0.56731	0.029	0.181	2.47E-73	6
Hivep2	1.99E-77	0.605193	0.151	0.044	3.32E-73	6
Eif5a	2.07E-77	-0.40206	0.209	0.423	3.46E-73	6
Kifap3	2.23E-77	0.514188	0.362	0.263	3.72E-73	6
Nucks1	2.27E-77	-0.46215	0.475	0.682	3.79E-73	6
Clasp2	2.81E-77	0.574073	0.264	0.157	4.7E-73	6
Kif20b	2.87E-77	-0.64958	0.042	0.206	4.78E-73	6
Egr1	3.75E-77	0.591485	0.474	0.374	6.26E-73	6
Syt1	6.6E-77	0.601873	0.124	0.023	1.1E-72	6
Mrpl17	7.29E-77	-0.42743	0.095	0.277	1.22E-72	6
Nop56	8.32E-77	-0.39854	0.174	0.38	1.39E-72	6
Marcksl1	9.41E-77	0.306226	0.743	0.719	1.57E-72	6
Sae1	9.47E-77	-0.44606	0.108	0.296	1.58E-72	6
Snrpa1	1.06E-76	-0.39191	0.151	0.349	1.78E-72	6
Emg1	1.09E-76	-0.40104	0.138	0.333	1.81E-72	6
Cacna1a	1.12E-76	0.622491	0.178	0.061	1.88E-72	6
Glce	1.53E-76	0.594007	0.281	0.165	2.55E-72	6

Sgol2	1.65E-76	-0.60959	0.025	0.17	2.76E-72	6
Mbd3	2.05E-76	-0.43516	0.119	0.309	3.42E-72	6
Atp5b	3.03E-76	-0.3262	0.51	0.734	5.05E-72	6
Kmt2e	3.46E-76	0.335348	0.582	0.567	5.78E-72	6
Ccnb1	3.82E-76	-0.61115	0.016	0.152	6.37E-72	6
Polr2f	3.88E-76	-0.38322	0.187	0.394	6.47E-72	6
Rpl29	5.82E-76	-0.44678	0.094	0.274	9.71E-72	6
Gm11541	7.31E-76	-0.53803	0.012	0.14	1.22E-71	6
Cenpq	1.41E-75	-0.5098	0.029	0.174	2.34E-71	6
Ndufa3	1.46E-75	-0.26963	0.307	0.517	2.43E-71	6
Mrpl42	1.73E-75	-0.44967	0.139	0.337	2.88E-71	6
2810025M	1.78E-75	-0.48753	0.033	0.181	2.97E-71	6
Eny2	3.24E-75	-0.37553	0.2	0.407	5.41E-71	6
Lman1	3.74E-75	-0.41798	0.147	0.343	6.24E-71	6
Rpl38	4.71E-75	-0.3978	0.197	0.402	7.85E-71	6
Set	4.97E-75	-0.38185	0.2	0.407	8.29E-71	6
Pafah1b1	5.19E-75	0.352216	0.496	0.472	8.65E-71	6
Wbp5	5.26E-75	-0.29384	0.32	0.539	8.77E-71	6
Erc1	9.07E-75	0.595695	0.202	0.101	1.51E-70	6
Snap47	9.48E-75	0.539054	0.228	0.131	1.58E-70	6
Mphosph1(	1.03E-74	-0.49077	0.075	0.247	1.72E-70	6
Eif3k	1.23E-74	-0.32281	0.327	0.541	2.04E-70	6
Gng5	1.25E-74	-0.48414	0.031	0.179	2.09E-70	6
Eif3g	1.28E-74	-0.31542	0.219	0.421	2.14E-70	6
Clic4	1.75E-74	-0.46387	0.072	0.243	2.93E-70	6
Chchd1	1.95E-74	-0.30735	0.221	0.419	3.25E-70	6
Sparcl1	2.53E-74	-0.90344	0.059	0.222	4.22E-70	6
Arhgap11a	2.66E-74	-0.60093	0.033	0.185	4.44E-70	6
2010107E(	2.69E-74	-0.30046	0.374	0.592	4.49E-70	6
Ntrk3	2.79E-74	-0.57538	0.025	0.169	4.65E-70	6
Cct5	2.8E-74	-0.31517	0.283	0.497	4.68E-70	6
Nuf2	3.58E-74	-0.50678	0.019	0.153	5.98E-70	6
Hes1	3.99E-74	-0.74664	0.022	0.161	6.66E-70	6
Bub1	4E-74	-0.50398	0.014	0.143	6.67E-70	6
Eif3d	4.03E-74	-0.29474	0.225	0.424	6.73E-70	6
Pbdc1	5.16E-74	-0.47607	0.058	0.221	8.61E-70	6
Rpl13	6.35E-74	-0.46897	0.134	0.331	1.06E-69	6
Golim4	8.49E-74	-0.50011	0.075	0.249	1.42E-69	6
2310044G	9.29E-74	0.554957	0.132	0.046	1.55E-69	6
Ypel3	9.74E-74	0.343066	0.458	0.432	1.62E-69	6
Cacna2d1	1.2E-73	0.479842	0.451	0.366	2.01E-69	6
Apoe	1.24E-73	-1.64336	0.145	0.259	2.08E-69	6
Gas5	1.26E-73	-0.36695	0.556	0.761	2.1E-69	6
Alcam	1.3E-73	-0.54376	0.015	0.147	2.17E-69	6
Rnmt	1.42E-73	0.435756	0.406	0.342	2.37E-69	6
Mmp14	1.44E-73	-0.53483	0.038	0.188	2.4E-69	6
Rbm5	1.78E-73	0.398368	0.449	0.396	2.98E-69	6
Gabbr1	2.24E-73	0.568074	0.239	0.13	3.73E-69	6

Hspa5	2.31E-73	-0.42986	0.353	0.571	3.86E-69	6
Bccip	2.91E-73	-0.3681	0.142	0.328	4.85E-69	6
D17H6S56I	3.18E-73	-0.50111	0.012	0.139	5.31E-69	6
Dpm3	3.5E-73	-0.42869	0.105	0.287	5.83E-69	6
Cdk6	3.56E-73	-0.47599	0.085	0.259	5.95E-69	6
Zfp36l1	4.14E-73	-0.55444	0.024	0.164	6.91E-69	6
Mt1	4.33E-73	-0.79779	0.033	0.182	7.23E-69	6
Uqcr11	4.79E-73	-0.2688	0.296	0.501	7.99E-69	6
Ccl27a	5.88E-73	0.569473	0.131	0.034	9.82E-69	6
Ssr3	8.38E-73	-0.31798	0.242	0.444	1.4E-68	6
Arhgap20	9.4E-73	0.61834	0.152	0.043	1.57E-68	6
Sec11c	1.14E-72	-0.50939	0.062	0.228	1.9E-68	6
Polr2h	1.46E-72	-0.40826	0.079	0.24	2.44E-68	6
Uqcrh	1.76E-72	-0.29999	0.463	0.677	2.94E-68	6
Hspd1	1.89E-72	-0.40267	0.181	0.384	3.14E-68	6
Rps16	2.04E-72	-0.45759	0.062	0.224	3.4E-68	6
Mis18bp1	2.83E-72	-0.55167	0.03	0.174	4.72E-68	6
Tceal5	3.5E-72	0.537182	0.116	0.023	5.83E-68	6
Scd2	3.67E-72	-0.41326	0.131	0.314	6.13E-68	6
Fbxo5	5.43E-72	-0.51198	0.03	0.173	9.06E-68	6
Rbbp4	6.23E-72	-0.34429	0.287	0.504	1.04E-67	6
Rps6ka3	7.85E-72	0.562291	0.142	0.055	1.31E-67	6
Psmb6	8.31E-72	-0.26039	0.419	0.631	1.39E-67	6
Atrx	1.23E-71	0.32122	0.748	0.739	2.05E-67	6
Ndufb6	1.26E-71	-0.3644	0.2	0.398	2.11E-67	6
Eif4a1	2.12E-71	-0.3696	0.292	0.504	3.54E-67	6
Irf2bpl	2.89E-71	0.538688	0.126	0.031	4.81E-67	6
2410066E1	3.14E-71	0.584116	0.199	0.087	5.23E-67	6
Myod1	3.79E-71	-0.6028	0.02	0.154	6.32E-67	6
Wdr89	4.01E-71	-0.43719	0.068	0.229	6.69E-67	6
Lsm3	4.86E-71	-0.36858	0.181	0.377	8.1E-67	6
Serpini1	5.19E-71	0.603353	0.162	0.054	8.66E-67	6
Slc25a3	6.85E-71	-0.28158	0.383	0.593	1.14E-66	6
Sptbn1	7.12E-71	0.47309	0.459	0.342	1.19E-66	6
Acot7	8.4E-71	-0.44038	0.055	0.209	1.4E-66	6
Pole3	9.09E-71	-0.38139	0.098	0.266	1.52E-66	6
Cdk14	9.82E-71	0.534934	0.176	0.096	1.64E-66	6
Kcnj3	1.1E-70	0.597457	0.167	0.058	1.83E-66	6
Mxd3	1.15E-70	-0.47052	0.014	0.138	1.92E-66	6
Thoc7	1.19E-70	-0.27812	0.332	0.541	1.99E-66	6
Ndufa7	1.62E-70	-0.25489	0.267	0.458	2.71E-66	6
Ebna1bp2	2.99E-70	-0.39332	0.146	0.333	4.98E-66	6
Btf3	3.87E-70	-0.31509	0.182	0.366	6.45E-66	6
Laptm4a	4.22E-70	-0.30696	0.36	0.574	7.04E-66	6
Sema7a	6.1E-70	-0.5302	0.028	0.169	1.02E-65	6
Cxcl12	7.08E-70	0.410777	0.129	0.035	1.18E-65	6
Cnih4	7.38E-70	-0.38281	0.089	0.25	1.23E-65	6
Ntrk2	8.17E-70	0.6185	0.191	0.072	1.36E-65	6

Cacna1b	8.57E-70	0.601923	0.19	0.087	1.43E-65	6
Nup62	8.74E-70	-0.41905	0.041	0.184	1.46E-65	6
Igfbpl1	9.95E-70	-0.42993	0.48	0.665	1.66E-65	6
2010107G:	1.3E-69	0.516222	0.111	0.019	2.17E-65	6
Nktr	1.76E-69	0.410923	0.417	0.376	2.93E-65	6
Fam168a	1.99E-69	0.452542	0.344	0.281	3.32E-65	6
Akap9	2.04E-69	0.386671	0.531	0.497	3.41E-65	6
Lin7a	2.08E-69	0.592999	0.145	0.042	3.47E-65	6
Hspa9	2.62E-69	-0.32529	0.199	0.39	4.38E-65	6
Parp1	3.37E-69	-0.32744	0.152	0.33	5.62E-65	6
Srgap2	4.76E-69	0.559731	0.215	0.123	7.94E-65	6
Tspan3	7.08E-69	-0.33241	0.248	0.447	1.18E-64	6
Spcs2	8.25E-69	-0.25349	0.253	0.441	1.38E-64	6
Serping1	9.94E-69	0.545769	0.119	0.024	1.66E-64	6
Hint1	9.98E-69	-0.3411	0.518	0.723	1.66E-64	6
Fundc2	1.21E-68	-0.39055	0.114	0.287	2.02E-64	6
Nek6	1.37E-68	-0.49362	0.024	0.156	2.29E-64	6
Gtpbp4	1.66E-68	-0.45557	0.116	0.299	2.76E-64	6
Eno1	1.85E-68	-0.42487	0.082	0.245	3.09E-64	6
Srsf9	1.87E-68	-0.38084	0.089	0.252	3.12E-64	6
Minos1	2.25E-68	-0.32803	0.373	0.589	3.75E-64	6
Fmnl2	2.42E-68	0.515285	0.231	0.149	4.03E-64	6
Gpm6b	2.75E-68	0.359253	0.531	0.479	4.59E-64	6
Optn	2.78E-68	0.571854	0.147	0.047	4.64E-64	6
Dbn1	3.25E-68	0.555765	0.241	0.128	5.43E-64	6
Cpne3	3.93E-68	-0.45259	0.06	0.211	6.56E-64	6
mt-Cytb	3.98E-68	-0.26984	0.853	0.947	6.65E-64	6
Mak16	4.08E-68	-0.41191	0.079	0.24	6.8E-64	6
Rad51ap1	4.16E-68	-0.49383	0.038	0.18	6.94E-64	6
Lsamp	5.14E-68	0.653683	0.184	0.066	8.57E-64	6
Actl6a	5.24E-68	-0.35677	0.085	0.241	8.74E-64	6
Fam162a	5.32E-68	-0.39533	0.132	0.313	8.88E-64	6
Mybbp1a	5.53E-68	-0.36698	0.101	0.265	9.23E-64	6
Gm17322	5.73E-68	-0.61516	0.017	0.143	9.56E-64	6
Sf3b5	8.52E-68	-0.38063	0.157	0.345	1.42E-63	6
mt-Nd2	9.15E-68	-0.2591	0.449	0.658	1.53E-63	6
Pdgfra	9.56E-68	-0.54087	0.047	0.195	1.59E-63	6
Lsm2	1.12E-67	-0.36635	0.088	0.245	1.87E-63	6
Tcf3	1.13E-67	-0.4317	0.078	0.241	1.89E-63	6
Cct8	1.23E-67	-0.25447	0.27	0.459	2.06E-63	6
2310022B(	1.29E-67	-0.42004	0.096	0.264	2.16E-63	6
Ndufb9	1.44E-67	-0.25011	0.355	0.559	2.4E-63	6
1110038B:	1.57E-67	-0.38754	0.138	0.315	2.61E-63	6
Dnph1	1.58E-67	-0.45322	0.014	0.134	2.64E-63	6
Atad2	1.65E-67	-0.52699	0.057	0.213	2.74E-63	6
Gstm5	2.28E-67	-0.37691	0.155	0.34	3.81E-63	6
Gjc1	2.95E-67	-0.44037	0.058	0.21	4.92E-63	6
Syncrip	3.29E-67	-0.30521	0.307	0.515	5.49E-63	6

Snap91	3.76E-67	0.553895	0.138	0.04	6.27E-63	6
Sgol1	4.3E-67	-0.45374	0.017	0.139	7.18E-63	6
Igsf21	5.36E-67	0.574589	0.202	0.077	8.94E-63	6
9330159F1	5.52E-67	0.525487	0.254	0.159	9.21E-63	6
Serp2	6.41E-67	0.55664	0.177	0.077	1.07E-62	6
Magoh	7.08E-67	-0.34282	0.167	0.35	1.18E-62	6
Mrpl18	7.94E-67	-0.37239	0.179	0.372	1.32E-62	6
Tk1	9.47E-67	-0.47404	0.019	0.143	1.58E-62	6
Mad2l1	1.13E-66	-0.41524	0.016	0.134	1.89E-62	6
Ostc	1.15E-66	-0.37371	0.122	0.29	1.92E-62	6
Cox7b	1.4E-66	-0.31009	0.355	0.568	2.34E-62	6
Map3k12	1.59E-66	0.543186	0.182	0.089	2.65E-62	6
Smarca2	1.67E-66	0.433804	0.34	0.279	2.78E-62	6
Rb1cc1	1.75E-66	0.494107	0.296	0.227	2.92E-62	6
C330027C(	1.77E-66	-0.46058	0.02	0.145	2.95E-62	6
Eif3e	1.78E-66	-0.25139	0.224	0.398	2.97E-62	6
Ybx3	2.36E-66	-0.43079	0.127	0.307	3.94E-62	6
Hmg20b	2.53E-66	-0.39212	0.08	0.237	4.22E-62	6
Uncx	2.65E-66	0.457903	0.411	0.308	4.42E-62	6
Kif2c	3.32E-66	-0.44339	0.008	0.117	5.53E-62	6
Ect2	3.4E-66	-0.42184	0.004	0.107	5.67E-62	6
Manf	3.68E-66	-0.35704	0.164	0.344	6.13E-62	6
Ndufc2	4.43E-66	-0.32567	0.418	0.631	7.39E-62	6
Kcnd2	4.48E-66	0.555052	0.102	0.018	7.48E-62	6
Csrp2	4.89E-66	-0.45186	0.034	0.171	8.16E-62	6
Hat1	5.82E-66	-0.4288	0.035	0.167	9.71E-62	6
Ppp3cb	8.18E-66	0.366271	0.334	0.307	1.36E-61	6
Trim2	9.98E-66	0.470426	0.35	0.275	1.66E-61	6
Pdrg1	1.01E-65	0.472869	0.291	0.211	1.68E-61	6
Insm1	1.01E-65	-0.49336	0.131	0.309	1.68E-61	6
Nde1	1.05E-65	-0.43806	0.013	0.13	1.75E-61	6
Cox6c	1.35E-65	-0.28571	0.531	0.738	2.25E-61	6
Ramp2	1.35E-65	-0.51444	0.014	0.13	2.26E-61	6
Tmem256	1.67E-65	-0.26481	0.319	0.51	2.78E-61	6
Myl12b	1.88E-65	0.305186	0.387	0.365	3.13E-61	6
Cnksr2	2.25E-65	0.562434	0.19	0.098	3.75E-61	6
Gltscr2	2.26E-65	-0.28652	0.184	0.358	3.77E-61	6
Gli1	2.3E-65	-0.47155	0.008	0.118	3.84E-61	6
Nsmce1	2.95E-65	-0.34229	0.09	0.243	4.93E-61	6
Slc4a4	3.13E-65	0.388053	0.119	0.032	5.22E-61	6
Canx	3.32E-65	-0.30105	0.405	0.615	5.54E-61	6
Srrm4	3.48E-65	0.536766	0.29	0.185	5.8E-61	6
Bcas1	3.87E-65	-0.51306	0.122	0.299	6.46E-61	6
Pitpnc1	4.46E-65	0.553041	0.163	0.065	7.44E-61	6
AI854517	4.49E-65	-0.44347	0.057	0.203	7.48E-61	6
Actl6b	4.91E-65	0.544288	0.148	0.045	8.2E-61	6
Adk	5.2E-65	-0.4412	0.039	0.179	8.68E-61	6
Phf5a	8.6E-65	-0.27644	0.177	0.347	1.43E-60	6

Mrps14	1.01E-64	-0.35097	0.094	0.251	1.68E-60	6
Bub3	1.07E-64	-0.42279	0.154	0.338	1.79E-60	6
Tmem57	1.12E-64	0.407126	0.336	0.286	1.87E-60	6
Vgll4	1.34E-64	-0.38955	0.036	0.165	2.24E-60	6
Plcb1	1.34E-64	0.608327	0.247	0.129	2.24E-60	6
Ddx17	1.61E-64	0.339488	0.399	0.379	2.69E-60	6
Gria4	2.67E-64	0.576345	0.227	0.113	4.45E-60	6
Atxn10	3.08E-64	0.287259	0.372	0.369	5.15E-60	6
Zeb2	3.69E-64	-0.40466	0.101	0.264	6.16E-60	6
Aspm	3.79E-64	-0.58745	0.025	0.155	6.33E-60	6
Naa10	7.85E-64	-0.36074	0.104	0.264	1.31E-59	6
Cltb	8.97E-64	-0.48296	0.247	0.441	1.5E-59	6
Cenpp	9.38E-64	-0.40225	0.013	0.125	1.57E-59	6
Macf1	9.7E-64	0.488334	0.266	0.204	1.62E-59	6
Atp5a1	1.04E-63	-0.2551	0.537	0.737	1.73E-59	6
Zfp521	1.2E-63	0.570463	0.147	0.057	2E-59	6
Mettl9	1.35E-63	-0.29122	0.151	0.316	2.26E-59	6
Tia1	1.44E-63	0.269906	0.538	0.553	2.41E-59	6
Psmc4	1.45E-63	-0.29701	0.169	0.337	2.42E-59	6
Psma7	2.26E-63	-0.27252	0.611	0.799	3.77E-59	6
Ivns1abp	2.47E-63	-0.31927	0.244	0.433	4.12E-59	6
1700001O	3.04E-63	-0.42672	0.011	0.12	5.06E-59	6
Dhx32	3.17E-63	-0.42739	0.065	0.214	5.29E-59	6
Lrpap1	3.21E-63	0.519858	0.191	0.109	5.35E-59	6
Ubb	3.66E-63	0.26091	0.526	0.543	6.1E-59	6
Tspan13	3.78E-63	0.445599	0.265	0.201	6.3E-59	6
Map1lc3b	5.16E-63	0.363184	0.367	0.327	8.61E-59	6
Ndufv3	6.99E-63	0.344604	0.314	0.296	1.17E-58	6
Ppa1	7.52E-63	-0.36427	0.086	0.235	1.25E-58	6
Rftn2	7.79E-63	-0.43303	0.018	0.134	1.3E-58	6
Etfa	8.27E-63	-0.35624	0.107	0.269	1.38E-58	6
Lmnb1	1.03E-62	-0.41932	0.138	0.317	1.71E-58	6
Cdca2	1.26E-62	-0.41765	0.022	0.139	2.1E-58	6
Myt1	1.27E-62	0.609426	0.28	0.139	2.12E-58	6
Gabarapl1	1.37E-62	0.429443	0.276	0.209	2.28E-58	6
2410015M	1.73E-62	-0.29813	0.206	0.377	2.88E-58	6
Cnpy2	1.81E-62	-0.30562	0.185	0.359	3.02E-58	6
Tsc22d4	1.85E-62	-0.51567	0.042	0.179	3.09E-58	6
Ift74	1.94E-62	-0.42225	0.084	0.239	3.24E-58	6
Utp3	2.09E-62	-0.2513	0.168	0.33	3.48E-58	6
Cnr1	2.14E-62	0.566578	0.142	0.049	3.57E-58	6
Pttg1	2.44E-62	-0.54208	0.025	0.151	4.08E-58	6
Gpx1	2.71E-62	-0.35753	0.21	0.4	4.51E-58	6
Kbtbd11	2.76E-62	0.531032	0.157	0.08	4.6E-58	6
Luc7l2	3.99E-62	0.275701	0.497	0.494	6.65E-58	6
Ncor2	4.4E-62	-0.43176	0.044	0.181	7.35E-58	6
Tle1	4.67E-62	-0.36543	0.089	0.24	7.79E-58	6
Shmt1	4.83E-62	-0.37567	0.01	0.113	8.05E-58	6

Mad2l2	4.98E-62	-0.40134	0.112	0.278	8.3E-58	6
Cfdp1	5.26E-62	-0.2552	0.315	0.502	8.78E-58	6
Npepps	7.08E-62	0.300029	0.324	0.317	1.18E-57	6
Eif3h	7.18E-62	-0.27908	0.317	0.511	1.2E-57	6
Dtl	8.72E-62	-0.44638	0.03	0.157	1.45E-57	6
Rad51	9.08E-62	-0.42656	0.014	0.127	1.51E-57	6
Ccdc88a	9.78E-62	0.297936	0.469	0.483	1.63E-57	6
Hsp90b1	1.18E-61	-0.39731	0.526	0.709	1.96E-57	6
Mrpl15	1.4E-61	-0.3259	0.144	0.31	2.33E-57	6
Alyref	1.94E-61	-0.3669	0.07	0.213	3.23E-57	6
Gse1	2.38E-61	-0.41867	0.094	0.252	3.97E-57	6
Ywhab	3.88E-61	0.255567	0.601	0.607	6.47E-57	6
Ncor1	4.77E-61	0.252653	0.541	0.557	7.96E-57	6
RP23-32A8	6.07E-61	0.450808	0.242	0.181	1.01E-56	6
Fkbp2	6.12E-61	-0.26107	0.177	0.342	1.02E-56	6
Ccdc41	6.36E-61	-0.3726	0.108	0.269	1.06E-56	6
P4hb	7.67E-61	-0.27027	0.144	0.293	1.28E-56	6
Rpl3	1.11E-60	-0.42295	0.074	0.226	1.85E-56	6
Efs	1.17E-60	-0.4215	0.02	0.136	1.95E-56	6
Rpl35	1.38E-60	-0.33958	0.098	0.248	2.31E-56	6
Kif22	1.46E-60	-0.48749	0.041	0.175	2.44E-56	6
Dpy30	1.47E-60	-0.27858	0.154	0.313	2.46E-56	6
Pafah1b3	1.56E-60	0.412491	0.244	0.184	2.59E-56	6
Carhsp1	1.63E-60	-0.31571	0.118	0.268	2.71E-56	6
Gatsl2	1.63E-60	0.555128	0.175	0.082	2.72E-56	6
Gnl3	1.7E-60	-0.37981	0.14	0.312	2.83E-56	6
Syt13	1.94E-60	-0.46776	0.02	0.138	3.23E-56	6
Mfap4	2.02E-60	-0.61185	0.039	0.174	3.37E-56	6
Rpl36al	2.16E-60	-0.3536	0.145	0.314	3.61E-56	6
Msra	2.64E-60	0.488648	0.166	0.081	4.4E-56	6
Psmb1	2.72E-60	-0.29496	0.497	0.699	4.54E-56	6
Kdelr2	2.84E-60	-0.35987	0.074	0.219	4.74E-56	6
Eif3b	3.25E-60	-0.31112	0.093	0.233	5.42E-56	6
Polr1c	3.52E-60	-0.34958	0.088	0.232	5.87E-56	6
Dnajc2	3.78E-60	-0.32599	0.171	0.345	6.3E-56	6
Clk1	4.02E-60	0.332031	0.362	0.337	6.7E-56	6
Pja1	4.32E-60	0.458429	0.237	0.166	7.21E-56	6
Ddx39	4.51E-60	-0.37701	0.061	0.2	7.52E-56	6
Cib2	4.69E-60	0.492226	0.102	0.025	7.83E-56	6
Smim18	5.38E-60	0.534353	0.133	0.04	8.98E-56	6
Ccm2	6.67E-60	-0.44136	0.085	0.239	1.11E-55	6
Hmgn3	7.88E-60	-0.32514	0.128	0.284	1.31E-55	6
Gins2	8.11E-60	-0.44678	0.055	0.198	1.35E-55	6
Psmg4	9.9E-60	-0.32104	0.081	0.22	1.65E-55	6
Cep57	1.03E-59	-0.26217	0.128	0.271	1.72E-55	6
Plk4	1.15E-59	-0.42532	0.017	0.13	1.92E-55	6
Cenpk	1.16E-59	-0.46764	0.031	0.156	1.93E-55	6
Gm11478	1.29E-59	-0.41144	0.079	0.229	2.14E-55	6

Srsf2	1.47E-59	-0.25491	0.388	0.584	2.45E-55	6
Rcc2	1.62E-59	-0.39611	0.057	0.191	2.69E-55	6
Smoc1	2.57E-59	-0.46032	0.025	0.146	4.29E-55	6
E2f1	2.99E-59	-0.46981	0.032	0.159	4.98E-55	6
Rif1	3.31E-59	-0.37511	0.126	0.291	5.52E-55	6
Ndufs6	3.48E-59	-0.30391	0.206	0.383	5.81E-55	6
Dync1li2	4.4E-59	0.38497	0.297	0.255	7.35E-55	6
Ssna1	4.45E-59	-0.33683	0.125	0.282	7.42E-55	6
Utp14a	4.56E-59	-0.36491	0.099	0.246	7.61E-55	6
Cd9	5.76E-59	-0.25752	0.319	0.502	9.6E-55	6
Sobp	5.86E-59	0.503725	0.207	0.119	9.77E-55	6
Akap12	6.13E-59	0.577179	0.2	0.102	1.02E-54	6
BC018507	6.22E-59	0.50071	0.188	0.115	1.04E-54	6
Arl6ip1	6.35E-59	-0.39312	0.461	0.559	1.06E-54	6
Mical1	8.87E-59	-0.43497	0.021	0.136	1.48E-54	6
Barhl1	9.11E-59	-0.38044	0.344	0.54	1.52E-54	6
Nek7	9.33E-59	-0.43331	0.016	0.127	1.56E-54	6
Tpt1	9.52E-59	-0.31953	0.118	0.271	1.59E-54	6
Cbln1	9.58E-59	0.51539	0.162	0.082	1.6E-54	6
Ak2	9.87E-59	-0.3172	0.085	0.226	1.65E-54	6
Fscn1	1.03E-58	-0.38033	0.075	0.216	1.71E-54	6
Nrcam	1.05E-58	0.518413	0.224	0.124	1.75E-54	6
Mroh2a	1.17E-58	-0.57583	0.015	0.124	1.96E-54	6
Fxr1	1.39E-58	-0.27295	0.123	0.269	2.32E-54	6
Cdh4	1.42E-58	-0.46772	0.034	0.157	2.36E-54	6
Vars	1.55E-58	-0.357	0.05	0.178	2.58E-54	6
Ndc80	1.68E-58	-0.42075	0.016	0.125	2.8E-54	6
Scrn1	1.82E-58	0.519503	0.124	0.042	3.03E-54	6
Dcakd	1.86E-58	-0.28777	0.154	0.311	3.1E-54	6
Pura	2.77E-58	0.373116	0.313	0.283	4.62E-54	6
Gm9843	2.88E-58	-0.33401	0.098	0.241	4.8E-54	6
Taf1d	2.88E-58	-0.34253	0.185	0.358	4.81E-54	6
Mvd	2.91E-58	0.511938	0.142	0.052	4.85E-54	6
Nptn	2.96E-58	0.398899	0.233	0.183	4.93E-54	6
Cdc45	3.07E-58	-0.37616	0.007	0.104	5.12E-54	6
Snrnp40	3.76E-58	-0.28769	0.132	0.285	6.28E-54	6
Tmem107	4.19E-58	-0.38524	0.024	0.137	6.99E-54	6
Mrpl33	5.11E-58	-0.2965	0.141	0.297	8.53E-54	6
Rrs1	5.9E-58	-0.40389	0.062	0.202	9.85E-54	6
Naa50	6.43E-58	-0.37366	0.135	0.301	1.07E-53	6
Mrfap1	6.46E-58	-0.26307	0.243	0.42	1.08E-53	6
Romo1	6.47E-58	-0.27108	0.267	0.447	1.08E-53	6
Parp6	7.1E-58	0.500821	0.201	0.117	1.18E-53	6
2610203C2	1.03E-57	-0.50159	0.018	0.131	1.71E-53	6
Fzd2	1.07E-57	-0.43441	0.043	0.173	1.78E-53	6
Prkacb	1.2E-57	0.41897	0.237	0.181	2.01E-53	6
Ifitm2	1.55E-57	-0.40778	0.016	0.124	2.58E-53	6
Slirp	1.8E-57	-0.27943	0.198	0.366	2.99E-53	6

Rpl30	2.13E-57	-0.36352	0.122	0.283	3.55E-53	6
Ndufs8	2.43E-57	-0.29155	0.195	0.364	4.06E-53	6
Clip3	2.44E-57	0.37616	0.36	0.306	4.07E-53	6
Ptch2	2.51E-57	-0.45329	0.037	0.164	4.18E-53	6
Dnajc8	3.53E-57	-0.25013	0.272	0.451	5.89E-53	6
Prmt5	3.88E-57	-0.30829	0.124	0.27	6.48E-53	6
Ldha	4.04E-57	-0.39362	0.095	0.246	6.75E-53	6
Boc	4.07E-57	-0.40394	0.042	0.167	6.78E-53	6
Odf2	4.09E-57	0.424477	0.218	0.165	6.83E-53	6
Coq7	4.73E-57	-0.34739	0.08	0.22	7.88E-53	6
2700029M	6.14E-57	-0.25243	0.152	0.304	1.02E-52	6
Fth1	6.24E-57	-0.3115	0.548	0.725	1.04E-52	6
Napb	6.81E-57	0.52382	0.148	0.053	1.14E-52	6
Pcbp2	7.62E-57	-0.26023	0.445	0.639	1.27E-52	6
Dnajc15	8.09E-57	-0.41627	0.024	0.139	1.35E-52	6
Cenpw	9.07E-57	-0.35386	0.027	0.138	1.51E-52	6
Rnaseh2b	9.1E-57	-0.34227	0.102	0.25	1.52E-52	6
Dlgap5	9.47E-57	-0.45016	0.015	0.122	1.58E-52	6
Rwdd3	1.59E-56	0.544817	0.155	0.067	2.65E-52	6
Dctn2	1.83E-56	0.313557	0.338	0.318	3.05E-52	6
Gprin1	2.1E-56	0.507119	0.163	0.084	3.5E-52	6
Pnrc2	2.31E-56	-0.35312	0.081	0.221	3.86E-52	6
Syn2	2.36E-56	0.552012	0.161	0.077	3.93E-52	6
Fubp1	2.79E-56	-0.26316	0.397	0.584	4.66E-52	6
Lims1	2.98E-56	-0.35848	0.062	0.195	4.97E-52	6
Tm7sf2	3.46E-56	0.483266	0.103	0.026	5.77E-52	6
Hist1h2ak	3.62E-56	-0.5446	0.033	0.154	6.04E-52	6
Chga	3.68E-56	0.506606	0.161	0.077	6.13E-52	6
Laptm4b	3.96E-56	-0.41923	0.032	0.153	6.61E-52	6
Reep5	4.25E-56	0.439636	0.205	0.13	7.08E-52	6
Cmtm3	4.32E-56	-0.41247	0.018	0.127	7.21E-52	6
Bhlhe22	4.56E-56	0.526197	0.167	0.069	7.61E-52	6
Mrpl21	4.6E-56	-0.28576	0.143	0.294	7.67E-52	6
1700025G	4.61E-56	-0.34939	0.125	0.278	7.7E-52	6
Dhfr	4.94E-56	-0.4435	0.036	0.16	8.24E-52	6
Klf9	5.07E-56	0.3996	0.323	0.265	8.46E-52	6
Exosc8	5.26E-56	-0.29103	0.089	0.222	8.77E-52	6
Cask	5.92E-56	0.521176	0.182	0.101	9.88E-52	6
Ppp2r3a	6.07E-56	0.529424	0.176	0.085	1.01E-51	6
Gsk3b	6.08E-56	0.272165	0.503	0.499	1.01E-51	6
Cers4	6.2E-56	0.496488	0.128	0.053	1.03E-51	6
Sh3bgrl	6.7E-56	-0.28125	0.186	0.353	1.12E-51	6
Atp6v1a	7.96E-56	0.307846	0.298	0.286	1.33E-51	6
Tsc22d1	8.06E-56	0.306992	0.456	0.425	1.34E-51	6
Nptxr	1.01E-55	0.459084	0.102	0.027	1.68E-51	6
Brd7	1.21E-55	-0.28502	0.168	0.322	2.02E-51	6
Ing4	1.48E-55	0.311684	0.342	0.32	2.48E-51	6
Eci2	1.5E-55	-0.3109	0.12	0.263	2.51E-51	6

Casp3	1.86E-55	0.315417	0.248	0.232	3.1E-51	6
Syt11	2.06E-55	0.339597	0.558	0.498	3.44E-51	6
Serp1	2.14E-55	-0.35913	0.088	0.231	3.56E-51	6
Ncaph	2.35E-55	-0.40383	0.021	0.131	3.91E-51	6
Peg3	2.5E-55	0.376246	0.32	0.28	4.17E-51	6
Rps28	3.05E-55	-0.38709	0.075	0.216	5.1E-51	6
Sec61b	3.57E-55	-0.2847	0.182	0.344	5.95E-51	6
Rfc1	4.8E-55	-0.26507	0.185	0.345	8E-51	6
Mapk8ip1	5.04E-55	0.451842	0.305	0.219	8.4E-51	6
Map7d1	5.26E-55	0.347634	0.265	0.239	8.77E-51	6
Nol4	5.67E-55	0.445255	0.23	0.165	9.46E-51	6
Kat6b	5.95E-55	0.484903	0.223	0.149	9.92E-51	6
Mrps28	6.34E-55	-0.37172	0.054	0.181	1.06E-50	6
Cdkn2c	6.36E-55	-0.36992	0.036	0.154	1.06E-50	6
Hist3h2ba	7.77E-55	0.486093	0.215	0.129	1.3E-50	6
Gspt1	8.17E-55	-0.32847	0.135	0.292	1.36E-50	6
Bin1	9.37E-55	0.349647	0.484	0.423	1.56E-50	6
Cd81	1.01E-54	-0.25261	0.308	0.478	1.69E-50	6
Cdc42se1	1.28E-54	-0.28692	0.059	0.176	2.14E-50	6
Rabac1	1.38E-54	0.371754	0.302	0.253	2.31E-50	6
Tgif1	1.46E-54	-0.38197	0.011	0.108	2.44E-50	6
Napg	1.52E-54	0.49338	0.196	0.121	2.54E-50	6
Itm2c	1.73E-54	0.435354	0.276	0.205	2.88E-50	6
Lbr	2.04E-54	-0.33393	0.074	0.209	3.4E-50	6
Fkbp4	2.35E-54	-0.27178	0.257	0.43	3.93E-50	6
Fam212b	2.46E-54	-0.35406	0.093	0.236	4.1E-50	6
Rab3c	3.14E-54	0.489305	0.219	0.14	5.24E-50	6
Mcm4	3.15E-54	-0.42708	0.032	0.149	5.26E-50	6
Tead1	3.25E-54	-0.38508	0.057	0.186	5.42E-50	6
Pgrmc2	3.27E-54	-0.34594	0.074	0.209	5.46E-50	6
Stxbp5l	3.49E-54	0.531565	0.126	0.044	5.82E-50	6
Efhd2	3.51E-54	-0.47448	0.026	0.14	5.86E-50	6
Amph	3.68E-54	0.499601	0.166	0.082	6.15E-50	6
Angptl2	4.24E-54	-0.44554	0.018	0.126	7.08E-50	6
Paxbp1	4.27E-54	0.376542	0.305	0.254	7.12E-50	6
Dnmt1	4.27E-54	-0.32976	0.138	0.292	7.13E-50	6
Nrm	4.53E-54	-0.29968	0.061	0.18	7.55E-50	6
Rrp15	4.58E-54	-0.37025	0.058	0.186	7.64E-50	6
Mki67ip	5.82E-54	-0.27673	0.117	0.257	9.7E-50	6
Gab1	6.1E-54	0.508755	0.148	0.063	1.02E-49	6
Usp22	6.19E-54	0.254318	0.359	0.361	1.03E-49	6
Brix1	6.76E-54	-0.29999	0.085	0.218	1.13E-49	6
Cxxc5	7.08E-54	-0.26895	0.377	0.561	1.18E-49	6
Tceal8	7.48E-54	-0.26256	0.123	0.258	1.25E-49	6
Gpatch4	7.7E-54	-0.39208	0.061	0.192	1.28E-49	6
Pard3	7.81E-54	-0.40172	0.025	0.137	1.3E-49	6
Fen1	8.3E-54	-0.37371	0.036	0.152	1.38E-49	6
Topbp1	9.64E-54	-0.35171	0.041	0.156	1.61E-49	6

Bub1b	1.01E-53	-0.37124	0.013	0.111	1.69E-49	6
Atp6v1b2	1.08E-53	0.422523	0.221	0.165	1.8E-49	6
Os9	1.27E-53	0.383955	0.297	0.246	2.12E-49	6
Adarb1	1.37E-53	0.505707	0.112	0.033	2.28E-49	6
Ube2e3	1.58E-53	0.250841	0.409	0.417	2.64E-49	6
Dnajc5	1.74E-53	0.37649	0.284	0.243	2.91E-49	6
Lmo4	1.9E-53	-0.29728	0.19	0.351	3.17E-49	6
Nudc	2.01E-53	-0.29033	0.144	0.295	3.35E-49	6
Timeless	2.7E-53	-0.38418	0.044	0.165	4.51E-49	6
Cnih2	2.99E-53	0.45393	0.101	0.026	4.98E-49	6
Atp1b3	3.26E-53	0.302552	0.349	0.335	5.43E-49	6
Cep110	3.98E-53	-0.41335	0.049	0.175	6.64E-49	6
Tcf19	5.27E-53	-0.36225	0.013	0.107	8.79E-49	6
Fam111a	5.72E-53	-0.40773	0.026	0.136	9.54E-49	6
Mpp6	5.87E-53	-0.36391	0.05	0.173	9.79E-49	6
Dpysl2	6.98E-53	0.359848	0.279	0.245	1.16E-48	6
Atp6ap1	7.3E-53	0.402784	0.188	0.134	1.22E-48	6
Nop16	8.17E-53	-0.38484	0.057	0.186	1.36E-48	6
Mxd4	8.49E-53	0.420342	0.261	0.191	1.42E-48	6
Rufy2	1.03E-52	0.421968	0.202	0.153	1.72E-48	6
Clvs1	1.19E-52	0.511701	0.219	0.119	1.99E-48	6
Hk2	1.21E-52	-0.28083	0.068	0.184	2.03E-48	6
Imp3	1.47E-52	-0.27016	0.124	0.26	2.46E-48	6
Nin	1.49E-52	-0.43636	0.037	0.157	2.48E-48	6
Eif3a	1.51E-52	-0.25393	0.522	0.706	2.52E-48	6
Micu3	1.58E-52	0.496811	0.164	0.071	2.63E-48	6
Kif4	1.88E-52	-0.39117	0.016	0.117	3.13E-48	6
Myc	2E-52	-0.40621	0.013	0.109	3.34E-48	6
Rassf4	2.18E-52	-0.33651	0.227	0.392	3.64E-48	6
Rpn2	2.35E-52	-0.25403	0.111	0.239	3.93E-48	6
Ap3d1	2.56E-52	0.427151	0.264	0.197	4.26E-48	6
Tmem151b	2.85E-52	0.496213	0.116	0.036	4.76E-48	6
Rnaseh2a	2.96E-52	-0.353	0.046	0.164	4.93E-48	6
Gpr56	2.99E-52	-0.34433	0.095	0.236	4.99E-48	6
Efr3b	3E-52	0.481571	0.117	0.042	5.01E-48	6
Ska2	3.32E-52	-0.35184	0.083	0.217	5.54E-48	6
Nsmce4a	3.69E-52	-0.25055	0.108	0.235	6.15E-48	6
Rps24-ps3	4.47E-52	-0.30603	0.067	0.191	7.46E-48	6
Cbfb	7.24E-52	-0.33575	0.069	0.195	1.21E-47	6
Kif1a	7.65E-52	0.436664	0.236	0.164	1.28E-47	6
Srm	9.86E-52	-0.36047	0.074	0.204	1.64E-47	6
2610001J0	1.13E-51	-0.28981	0.079	0.206	1.89E-47	6
Ctnna1	1.53E-51	-0.33144	0.048	0.164	2.55E-47	6
Ncapd2	1.6E-51	-0.26646	0.077	0.196	2.67E-47	6
Gm10076	1.65E-51	-0.35458	0.033	0.144	2.75E-47	6
Gprasp1	1.76E-51	0.398013	0.248	0.202	2.94E-47	6
Psmc3ip	2.65E-51	-0.37635	0.032	0.142	4.41E-47	6
Celf5	2.66E-51	0.497636	0.135	0.048	4.44E-47	6

H1f0	3.58E-51	-0.25019	0.449	0.634	5.96E-47	6
Clic1	5.19E-51	-0.34464	0.061	0.184	8.65E-47	6
Rpf2	5.47E-51	-0.33254	0.082	0.214	9.12E-47	6
Taok3	6.63E-51	0.526539	0.173	0.078	1.11E-46	6
Dnm1l	6.78E-51	0.307858	0.305	0.29	1.13E-46	6
Ndrg3	7.08E-51	0.449229	0.188	0.108	1.18E-46	6
Lhx1	7.53E-51	-0.34415	0.335	0.514	1.26E-46	6
Suclg1	8.21E-51	-0.26563	0.142	0.286	1.37E-46	6
Sf3a3	9.08E-51	-0.25105	0.149	0.294	1.52E-46	6
Tspan6	1.17E-50	-0.25333	0.124	0.258	1.95E-46	6
Irs1	1.31E-50	-0.40255	0.023	0.128	2.18E-46	6
Smpd3	1.54E-50	0.44204	0.233	0.153	2.58E-46	6
Cisd2	1.86E-50	-0.25217	0.131	0.27	3.1E-46	6
Anln	2.3E-50	-0.35454	0.018	0.113	3.83E-46	6
Lta4h	2.84E-50	-0.30448	0.115	0.256	4.74E-46	6
Uba1	3.19E-50	0.285921	0.28	0.27	5.32E-46	6
1700020I1	3.87E-50	0.284072	0.284	0.273	6.46E-46	6
Cerk	4.16E-50	-0.35102	0.046	0.162	6.95E-46	6
Ift27	4.63E-50	-0.29026	0.119	0.255	7.73E-46	6
Add1	4.95E-50	0.375235	0.206	0.159	8.25E-46	6
Melk	5.42E-50	-0.36134	0.011	0.103	9.03E-46	6
Lsmd1	5.64E-50	-0.30096	0.099	0.232	9.4E-46	6
Ctps	5.98E-50	-0.34629	0.037	0.148	9.98E-46	6
Tcof1	6.33E-50	-0.33904	0.058	0.179	1.06E-45	6
Eml4	8.59E-50	-0.28597	0.092	0.221	1.43E-45	6
Prkd3	9.02E-50	-0.40871	0.038	0.153	1.5E-45	6
Abcc5	1.05E-49	0.410759	0.165	0.118	1.75E-45	6
Phactr1	1.05E-49	0.379345	0.223	0.179	1.75E-45	6
Samm50	1.11E-49	-0.28099	0.091	0.215	1.85E-45	6
Serf1	1.46E-49	0.284428	0.285	0.28	2.44E-45	6
Ankra2	1.47E-49	0.435989	0.171	0.115	2.46E-45	6
Ier2	1.93E-49	0.319763	0.377	0.382	3.21E-45	6
Dbf4	1.97E-49	-0.35137	0.043	0.156	3.28E-45	6
Rad21	2.05E-49	-0.33638	0.299	0.478	3.42E-45	6
Snx32	2.32E-49	0.45748	0.104	0.03	3.86E-45	6
Rassf3	2.33E-49	-0.41064	0.035	0.149	3.88E-45	6
Dlgap4	2.55E-49	0.458497	0.266	0.179	4.26E-45	6
Strbp	3.04E-49	0.27767	0.354	0.346	5.07E-45	6
Zic2	3.33E-49	0.412987	0.174	0.115	5.55E-45	6
C530008M	3.45E-49	-0.32853	0.135	0.282	5.75E-45	6
Tspan5	3.67E-49	0.368525	0.233	0.192	6.12E-45	6
Vamp2	3.91E-49	0.444138	0.127	0.067	6.52E-45	6
Ftsj3	5.12E-49	-0.30491	0.089	0.218	8.54E-45	6
Zfp57	5.66E-49	0.472556	0.14	0.059	9.44E-45	6
Cdca7l	6.54E-49	-0.3623	0.016	0.113	1.09E-44	6
Mum1l1	8.17E-49	-0.3646	0.012	0.103	1.36E-44	6
Rabgap1l	8.18E-49	0.512309	0.137	0.054	1.36E-44	6
Sema4g	1.32E-48	0.482969	0.101	0.031	2.2E-44	6

Lman2	1.44E-48	-0.26913	0.047	0.149	2.41E-44	6
Ip6k1	1.75E-48	0.380311	0.222	0.171	2.93E-44	6
Zmynd8	2.07E-48	0.360255	0.239	0.21	3.45E-44	6
Rcn1	2.2E-48	-0.28913	0.111	0.246	3.67E-44	6
Csrnp3	2.69E-48	0.474602	0.147	0.076	4.49E-44	6
Suv39h2	3.06E-48	-0.28987	0.039	0.14	5.1E-44	6
Ung	3.33E-48	-0.41382	0.011	0.101	5.55E-44	6
Mrps17	4.68E-48	-0.26994	0.125	0.259	7.81E-44	6
Mndal	4.86E-48	0.466431	0.101	0.032	8.11E-44	6
Mrpl12	7.04E-48	-0.31175	0.089	0.216	1.17E-43	6
D19Bwg13	7.59E-48	-0.25202	0.148	0.286	1.27E-43	6
Hnrnpf	7.73E-48	-0.32227	0.031	0.135	1.29E-43	6
Hmgb3	7.88E-48	-0.25927	0.174	0.32	1.31E-43	6
Eif2s1	8.78E-48	-0.28208	0.085	0.208	1.46E-43	6
Dclk1	1.02E-47	-0.28097	0.268	0.427	1.71E-43	6
Prdx5	1.12E-47	0.297941	0.282	0.262	1.87E-43	6
Nme2	1.14E-47	-0.36506	0.016	0.11	1.9E-43	6
Vrk1	1.29E-47	-0.33804	0.047	0.158	2.16E-43	6
Man1c1	1.56E-47	-0.3804	0.031	0.136	2.6E-43	6
Mlf2	1.66E-47	0.285304	0.296	0.277	2.78E-43	6
Dgkd	1.8E-47	0.426375	0.157	0.101	3E-43	6
Olfm1	2.35E-47	0.39676	0.25	0.193	3.92E-43	6
Hpca	2.44E-47	-0.43545	0.14	0.291	4.07E-43	6
Gm26735	2.61E-47	0.370573	0.218	0.176	4.35E-43	6
Nfyb	4.02E-47	0.339451	0.263	0.231	6.7E-43	6
Hprt	4.04E-47	-0.26112	0.081	0.199	6.74E-43	6
Dph3	4.47E-47	-0.28436	0.096	0.222	7.46E-43	6
Plekho2	5.52E-47	-0.36176	0.013	0.102	9.21E-43	6
Diap3	6.4E-47	-0.34758	0.02	0.115	1.07E-42	6
Jam3	7.47E-47	-0.34661	0.038	0.145	1.25E-42	6
2610017I0	8.54E-47	-0.32779	0.089	0.216	1.42E-42	6
Zfp367	1.04E-46	-0.32737	0.014	0.101	1.74E-42	6
Pdia4	1.34E-46	-0.2664	0.088	0.207	2.24E-42	6
Pold2	1.35E-46	-0.34	0.024	0.123	2.25E-42	6
Rars	1.77E-46	-0.29716	0.077	0.195	2.95E-42	6
Fam96a	1.82E-46	-0.30748	0.068	0.185	3.04E-42	6
Cd164	2.02E-46	-0.31412	0.03	0.129	3.37E-42	6
Gpatch8	2.11E-46	0.309236	0.298	0.278	3.52E-42	6
Serpinh1	2.54E-46	-0.39788	0.026	0.124	4.24E-42	6
Aurka	2.97E-46	-0.31879	0.017	0.107	4.95E-42	6
Atp6v0e2	3.37E-46	0.29915	0.281	0.257	5.62E-42	6
Dixdc1	3.5E-46	0.359798	0.265	0.231	5.83E-42	6
Chchd3	3.67E-46	-0.29917	0.074	0.195	6.13E-42	6
Lin7c	3.91E-46	0.325815	0.251	0.214	6.52E-42	6
Baiap2	4.34E-46	0.435034	0.104	0.045	7.24E-42	6
Lphn3	4.81E-46	0.469793	0.127	0.054	8.03E-42	6
Gabrg2	5.35E-46	0.443691	0.118	0.042	8.93E-42	6
Aig1	7.25E-46	0.443893	0.156	0.089	1.21E-41	6

Evl	7.33E-46	0.358697	0.22	0.173	1.22E-41	6
Atcay	7.89E-46	0.448127	0.133	0.058	1.32E-41	6
Mapre2	8.13E-46	0.371012	0.21	0.162	1.36E-41	6
Map6	9.39E-46	0.470714	0.157	0.071	1.57E-41	6
Snx5	1.07E-45	-0.34374	0.057	0.174	1.78E-41	6
Agtpbp1	1.11E-45	0.433057	0.152	0.096	1.86E-41	6
Sqstm1	1.48E-45	0.298278	0.245	0.225	2.48E-41	6
Apc2	1.76E-45	0.45719	0.118	0.036	2.94E-41	6
Rbbp8	1.95E-45	-0.32048	0.032	0.131	3.26E-41	6
Smap1	1.95E-45	0.290147	0.23	0.222	3.26E-41	6
Pea15a	1.98E-45	0.417347	0.257	0.167	3.3E-41	6
Ndufaf2	2.39E-45	-0.31691	0.09	0.214	3.99E-41	6
Dusp8	2.46E-45	0.48535	0.158	0.07	4.1E-41	6
Jarid2	2.49E-45	0.342296	0.207	0.175	4.16E-41	6
Zfp148	2.5E-45	0.259757	0.281	0.284	4.17E-41	6
Wdr12	2.79E-45	-0.28704	0.072	0.186	4.66E-41	6
1810037I1	3.21E-45	-0.29343	0.087	0.208	5.36E-41	6
Gtf3c6	4.63E-45	-0.35307	0.084	0.212	7.72E-41	6
Abi2	5.23E-45	0.291381	0.2	0.181	8.72E-41	6
Gcsh	5.24E-45	-0.31465	0.046	0.152	8.74E-41	6
Pom121	5.28E-45	-0.28518	0.075	0.19	8.81E-41	6
Cdc42se2	6.66E-45	-0.28383	0.119	0.251	1.11E-40	6
Eef1e1	7.52E-45	0.281691	0.194	0.18	1.25E-40	6
Bcar1	7.99E-45	-0.27256	0.087	0.203	1.33E-40	6
Snrpn	8.05E-45	0.35094	0.19	0.154	1.34E-40	6
Smc5	9.01E-45	-0.27632	0.135	0.27	1.5E-40	6
Mkrn1	9.16E-45	0.313751	0.231	0.206	1.53E-40	6
Gng12	1.03E-44	-0.33306	0.031	0.13	1.71E-40	6
Klhl29	1.04E-44	0.452983	0.102	0.029	1.74E-40	6
Ptprd	1.13E-44	0.310339	0.357	0.338	1.88E-40	6
Cd63-ps	1.18E-44	-0.3236	0.031	0.128	1.97E-40	6
Bod1l	1.2E-44	0.250253	0.367	0.379	2.01E-40	6
Exosc7	1.3E-44	-0.25903	0.077	0.19	2.18E-40	6
Plekha1	1.7E-44	0.437962	0.121	0.054	2.84E-40	6
Zcchc18	1.81E-44	0.406395	0.222	0.164	3.03E-40	6
Gins1	1.84E-44	-0.33458	0.027	0.124	3.07E-40	6
Rfc3	1.99E-44	-0.29648	0.092	0.216	3.32E-40	6
Mtap	2.94E-44	-0.33917	0.015	0.103	4.9E-40	6
Atp6v0a1	3.21E-44	0.450604	0.167	0.088	5.36E-40	6
Gm6472	3.6E-44	-0.29249	0.066	0.177	6.01E-40	6
Mdga1	3.63E-44	-0.4037	0.038	0.145	6.06E-40	6
Elavl1	4.97E-44	-0.30339	0.081	0.201	8.29E-40	6
Tmem132a	5.25E-44	0.410506	0.157	0.096	8.76E-40	6
Hcfc1r1	5.3E-44	0.345302	0.233	0.188	8.84E-40	6
Gart	5.51E-44	-0.26624	0.068	0.176	9.19E-40	6
Cmc2	6.23E-44	-0.34017	0.029	0.128	1.04E-39	6
Synj1	7.23E-44	0.432059	0.146	0.072	1.21E-39	6
Zfp277	7.39E-44	-0.29451	0.041	0.141	1.23E-39	6

Etfb	9.04E-44	-0.3272	0.078	0.195	1.51E-39	6
Cdc42bpa	9.73E-44	0.39254	0.183	0.132	1.62E-39	6
Ipo5	1.07E-43	-0.31762	0.044	0.148	1.79E-39	6
Mpdz	1.23E-43	-0.31163	0.043	0.144	2.06E-39	6
Ptbp1	1.29E-43	-0.3357	0.019	0.109	2.16E-39	6
Strip1	1.38E-43	0.366358	0.19	0.149	2.31E-39	6
Riok3	1.64E-43	0.374349	0.17	0.126	2.74E-39	6
Erbb4	1.99E-43	0.452146	0.114	0.039	3.31E-39	6
Mest	2E-43	0.426689	0.145	0.077	3.34E-39	6
Znrd1	2.21E-43	-0.26018	0.102	0.223	3.68E-39	6
Cntln	2.39E-43	-0.35152	0.067	0.183	3.99E-39	6
Cenpj	2.97E-43	-0.35831	0.045	0.151	4.95E-39	6
Snx1	3.03E-43	-0.27337	0.07	0.178	5.06E-39	6
Mycl	3.11E-43	-0.35142	0.042	0.147	5.19E-39	6
Rpl27a	3.32E-43	-0.25031	0.088	0.198	5.54E-39	6
Rp9	3.44E-43	0.250266	0.239	0.242	5.73E-39	6
Zranb1	3.75E-43	0.336951	0.238	0.205	6.25E-39	6
Hist3h2a	4.08E-43	0.443069	0.218	0.135	6.8E-39	6
Pkn2	4.13E-43	-0.28328	0.098	0.219	6.88E-39	6
Mbd6	4.27E-43	0.428141	0.136	0.076	7.13E-39	6
Ppa2	4.42E-43	-0.31525	0.055	0.165	7.38E-39	6
Snhg6	5.15E-43	-0.25492	0.142	0.27	8.59E-39	6
Mapk8ip2	5.95E-43	0.401298	0.193	0.137	9.93E-39	6
Hn1	8.16E-43	0.264293	0.558	0.539	1.36E-38	6
Trafd1	9.33E-43	0.402459	0.17	0.112	1.56E-38	6
Gnl3l	9.34E-43	0.324933	0.225	0.184	1.56E-38	6
Arhgef2	1.22E-42	0.286053	0.305	0.279	2.03E-38	6
Rabep1	1.31E-42	0.2969	0.243	0.234	2.18E-38	6
Ninj1	1.52E-42	-0.33751	0.019	0.106	2.54E-38	6
Mlec	1.57E-42	-0.28339	0.064	0.172	2.62E-38	6
Smchd1	1.76E-42	-0.25961	0.152	0.285	2.94E-38	6
Slc17a6	1.76E-42	0.460741	0.235	0.138	2.94E-38	6
Skp2	2.28E-42	-0.25752	0.036	0.128	3.8E-38	6
Igdcc4	2.45E-42	-0.37309	0.016	0.103	4.08E-38	6
Ccnl2	3.04E-42	0.252762	0.328	0.319	5.08E-38	6
Dpysl5	3.39E-42	0.433512	0.16	0.088	5.65E-38	6
Ppp2r2b	3.46E-42	0.497816	0.168	0.075	5.76E-38	6
Wbp2	3.62E-42	0.297943	0.213	0.193	6.04E-38	6
Arhgap21	4.07E-42	0.366566	0.197	0.16	6.79E-38	6
Zfp608	4.23E-42	0.294482	0.2	0.183	7.06E-38	6
Slc7a5	4.53E-42	-0.34388	0.031	0.123	7.55E-38	6
Ptprg	4.6E-42	-0.32328	0.048	0.152	7.67E-38	6
Cdt1	5.62E-42	-0.36409	0.035	0.134	9.38E-38	6
Stx7	5.93E-42	0.407868	0.18	0.109	9.9E-38	6
Prpf40b	6.93E-42	0.293968	0.16	0.144	1.16E-37	6
Dars	8.29E-42	-0.2724	0.062	0.167	1.38E-37	6
Tspyl1	8.36E-42	0.360949	0.192	0.141	1.39E-37	6
Arhgef25	9.43E-42	0.455095	0.144	0.072	1.57E-37	6

Tprn	1.05E-41	0.359238	0.287	0.22	1.76E-37	6
Sap30	1.12E-41	-0.29211	0.046	0.147	1.86E-37	6
Ncdn	1.29E-41	0.381482	0.117	0.061	2.14E-37	6
Ptges3	1.46E-41	-0.25564	0.104	0.223	2.43E-37	6
Fam57b	1.66E-41	0.32079	0.217	0.182	2.77E-37	6
Scamp1	1.7E-41	0.447923	0.137	0.065	2.84E-37	6
C77370	2.02E-41	0.451507	0.157	0.095	3.38E-37	6
Ldhb	2.08E-41	0.320121	0.32	0.276	3.46E-37	6
Idh1	2.15E-41	0.371664	0.202	0.151	3.58E-37	6
Fam64a	2.2E-41	-0.367	0.03	0.127	3.66E-37	6
Asrgl1	2.35E-41	-0.33337	0.043	0.143	3.92E-37	6
C130071C(	2.49E-41	0.289995	0.335	0.305	4.16E-37	6
Ogt	3.41E-41	0.297044	0.233	0.209	5.69E-37	6
Rpl7a	3.74E-41	-0.30588	0.055	0.16	6.23E-37	6
Ppid	4.91E-41	-0.25759	0.097	0.213	8.18E-37	6
Cxadr	5.68E-41	0.427834	0.145	0.073	9.47E-37	6
Phf3	5.75E-41	0.257025	0.267	0.27	9.59E-37	6
Zcwpw1	8.37E-41	-0.33963	0.022	0.111	1.4E-36	6
Ypel5	9.59E-41	0.363774	0.157	0.11	1.6E-36	6
Gphn	1.03E-40	0.329688	0.164	0.138	1.72E-36	6
Col9a3	1.08E-40	0.283113	0.193	0.178	1.8E-36	6
Hid1	1.18E-40	0.430702	0.123	0.048	1.97E-36	6
Tex9	1.34E-40	-0.2992	0.055	0.157	2.23E-36	6
Camsap2	1.42E-40	0.410427	0.184	0.127	2.36E-36	6
Nhlh2	1.99E-40	0.284041	0.473	0.417	3.33E-36	6
Atp9a	2E-40	0.454513	0.136	0.061	3.34E-36	6
Sept6	2.27E-40	0.397106	0.124	0.069	3.78E-36	6
Rev3l	2.35E-40	0.410111	0.153	0.091	3.92E-36	6
Robo2	2.44E-40	0.393426	0.199	0.146	4.07E-36	6
Cnpy1	3.86E-40	0.41353	0.19	0.126	6.44E-36	6
Gm13092	4.47E-40	-0.26914	0.027	0.112	7.46E-36	6
Gdpd1	4.71E-40	0.253857	0.252	0.242	7.85E-36	6
Rps16-ps2	5.04E-40	-0.3039	0.049	0.149	8.41E-36	6
Wdr13	5.16E-40	0.422509	0.167	0.098	8.61E-36	6
Abtb1	6.38E-40	0.406521	0.139	0.078	1.06E-35	6
Cdca4	6.95E-40	-0.2723	0.03	0.119	1.16E-35	6
Ssbp2	6.99E-40	-0.3054	0.039	0.135	1.17E-35	6
Itgb3bp	7.65E-40	-0.33674	0.023	0.112	1.28E-35	6
Mis18a	8.38E-40	-0.30794	0.028	0.118	1.4E-35	6
Fam171a2	8.75E-40	0.414513	0.112	0.049	1.46E-35	6
Pfn2	9.14E-40	0.305844	0.272	0.238	1.52E-35	6
Mrpl36	1.01E-39	-0.27983	0.07	0.175	1.68E-35	6
Klhdc3	1.05E-39	0.265328	0.193	0.184	1.75E-35	6
Ensa	1.16E-39	0.261318	0.23	0.227	1.93E-35	6
Nudt21	1.16E-39	-0.25729	0.047	0.142	1.93E-35	6
Actr1a	1.28E-39	0.278137	0.191	0.175	2.14E-35	6
Pabpn1	1.31E-39	0.256393	0.2	0.193	2.19E-35	6
Osbpl1a	1.39E-39	0.405079	0.107	0.054	2.32E-35	6

Arl6ip6	1.58E-39	-0.29715	0.039	0.134	2.63E-35	6
Pick1	1.69E-39	0.385706	0.177	0.122	2.81E-35	6
Agap1	1.8E-39	0.363163	0.172	0.127	3E-35	6
Actr2	1.95E-39	0.28653	0.217	0.195	3.25E-35	6
Polb	2E-39	0.329958	0.201	0.162	3.33E-35	6
Fh1	2.24E-39	-0.25892	0.085	0.193	3.74E-35	6
Pak7	3.04E-39	0.442458	0.171	0.097	5.07E-35	6
Rpa1	3.35E-39	-0.2877	0.029	0.117	5.59E-35	6
Mapk10	3.74E-39	0.401455	0.153	0.093	6.23E-35	6
Fam171b	3.92E-39	0.309796	0.25	0.221	6.54E-35	6
Mgst3	4.93E-39	0.43131	0.163	0.088	8.22E-35	6
A830010M	4.95E-39	0.433092	0.102	0.041	8.26E-35	6
Igsf3	5.28E-39	0.362022	0.174	0.128	8.8E-35	6
Snx10	5.4E-39	0.36013	0.186	0.142	9.01E-35	6
Nae1	6.49E-39	0.261774	0.231	0.223	1.08E-34	6
Xrcc5	6.59E-39	-0.32581	0.031	0.124	1.1E-34	6
Ubl3	6.61E-39	0.269533	0.217	0.208	1.1E-34	6
Mthfd1	8.14E-39	-0.29618	0.033	0.124	1.36E-34	6
Zfp292	8.86E-39	0.290392	0.356	0.34	1.48E-34	6
Ankrd46	1.06E-38	0.328325	0.193	0.157	1.77E-34	6
Mef2a	1.06E-38	0.350794	0.174	0.135	1.77E-34	6
Ctsz	1.28E-38	-0.36872	0.047	0.146	2.14E-34	6
Pik3r2	1.81E-38	0.431215	0.147	0.074	3.01E-34	6
Eftud2	2.46E-38	-0.2571	0.083	0.192	4.11E-34	6
Prr13	2.85E-38	0.387802	0.142	0.084	4.75E-34	6
Dnaaf2	2.85E-38	-0.30481	0.026	0.113	4.75E-34	6
2510002D	2.86E-38	0.413148	0.115	0.043	4.76E-34	6
Slc22a17	3.34E-38	0.291589	0.277	0.248	5.58E-34	6
Pfkm	3.87E-38	0.395173	0.122	0.06	6.46E-34	6
Ttc4	4.2E-38	0.277483	0.192	0.178	7E-34	6
Cdk5rap2	4.21E-38	-0.27328	0.084	0.193	7.02E-34	6
Rangap1	4.82E-38	-0.28587	0.142	0.268	8.04E-34	6
Slc1a3	4.95E-38	-0.496	0.068	0.161	8.25E-34	6
Ubr7	5.56E-38	-0.28088	0.048	0.142	9.28E-34	6
Sept8	5.84E-38	-0.33936	0.041	0.138	9.73E-34	6
Ccdc58	7.01E-38	-0.29071	0.046	0.14	1.17E-33	6
Vegfb	7.49E-38	-0.27087	0.037	0.127	1.25E-33	6
Rusc1	1.23E-37	0.405466	0.108	0.05	2.05E-33	6
Ppm1l	1.66E-37	0.409224	0.149	0.089	2.78E-33	6
Zfp318	1.71E-37	0.279899	0.185	0.174	2.85E-33	6
Ccdc18	1.81E-37	-0.26429	0.038	0.123	3.02E-33	6
Apitd1	1.99E-37	-0.28314	0.022	0.102	3.32E-33	6
Scamp2	2.53E-37	-0.27501	0.051	0.144	4.22E-33	6
Snord104	2.82E-37	-0.27201	0.062	0.161	4.71E-33	6
Sgip1	3.16E-37	0.446089	0.129	0.057	5.26E-33	6
Arl8a	4.28E-37	0.386086	0.156	0.089	7.15E-33	6
Alg2	4.48E-37	0.370227	0.145	0.095	7.48E-33	6
Rpl3-ps1	5.03E-37	-0.2692	0.026	0.108	8.4E-33	6

Lamp2	5.74E-37	-0.29863	0.051	0.146	9.58E-33	6
Clstn1	6.44E-37	0.332667	0.244	0.198	1.07E-32	6
Ctxn1	6.5E-37	0.301596	0.197	0.167	1.08E-32	6
Ctnna2	6.89E-37	0.363165	0.134	0.081	1.15E-32	6
Odc1	7.02E-37	-0.29998	0.025	0.109	1.17E-32	6
BC034090	7.5E-37	-0.31542	0.044	0.136	1.25E-32	6
Nme4	8.88E-37	-0.28372	0.038	0.126	1.48E-32	6
Vcan	9.94E-37	-0.30432	0.045	0.138	1.66E-32	6
Pola1	1.1E-36	-0.33495	0.025	0.111	1.84E-32	6
Rhebl1	1.82E-36	0.303738	0.126	0.103	3.04E-32	6
Gsto1	1.91E-36	-0.31497	0.044	0.137	3.18E-32	6
Ctnnd2	1.91E-36	0.37321	0.139	0.094	3.19E-32	6
Grik5	2.32E-36	0.370997	0.15	0.105	3.87E-32	6
Mrps15	2.68E-36	-0.25147	0.125	0.241	4.47E-32	6
Lpgat1	2.78E-36	0.302202	0.185	0.165	4.64E-32	6
Fbxl15	3.06E-36	0.354318	0.128	0.081	5.1E-32	6
Shd	3.42E-36	0.380968	0.165	0.103	5.7E-32	6
Lima1	4.72E-36	-0.29986	0.042	0.13	7.88E-32	6
Brca2	5.85E-36	-0.25956	0.038	0.121	9.76E-32	6
A630007B(	6.02E-36	0.288295	0.224	0.203	1E-31	6
Tmem30a	6.73E-36	0.253664	0.185	0.178	1.12E-31	6
Herc1	7.22E-36	0.253017	0.192	0.186	1.2E-31	6
Pcgf2	9.03E-36	0.393061	0.139	0.08	1.51E-31	6
Samd14	9.04E-36	0.363263	0.112	0.073	1.51E-31	6
Tmem237	1.16E-35	-0.29035	0.046	0.139	1.93E-31	6
Mtch1	1.2E-35	0.252582	0.233	0.221	2E-31	6
Zc2hc1a	1.22E-35	0.367578	0.144	0.089	2.04E-31	6
Ndrg2	1.28E-35	-0.28704	0.088	0.194	2.13E-31	6
4930402H:	1.28E-35	0.375507	0.16	0.108	2.13E-31	6
Jakmip2	1.67E-35	0.36655	0.206	0.151	2.79E-31	6
Nedd4l	1.8E-35	0.343391	0.168	0.124	3E-31	6
Lphn2	2.23E-35	0.374526	0.118	0.065	3.72E-31	6
Trappc3	2.3E-35	0.302034	0.124	0.098	3.84E-31	6
Tom1l1	3.98E-35	-0.28055	0.029	0.111	6.63E-31	6
Klhl13	4.1E-35	-0.28812	0.03	0.112	6.85E-31	6
Hcfc2	6.7E-35	0.384447	0.118	0.064	1.12E-30	6
H1fx	7.9E-35	-0.25835	0.141	0.256	1.32E-30	6
Gpc2	8.38E-35	0.351965	0.182	0.135	1.4E-30	6
Akap8l	8.83E-35	0.357256	0.2	0.153	1.47E-30	6
Stau2	9.46E-35	0.331347	0.192	0.145	1.58E-30	6
A330076H(	9.61E-35	0.448003	0.238	0.152	1.6E-30	6
Prmt2	9.86E-35	0.398412	0.145	0.089	1.64E-30	6
Pcmtd1	1.04E-34	0.323858	0.158	0.122	1.74E-30	6
Cdk5rap3	1.59E-34	-0.2614	0.098	0.202	2.64E-30	6
Rps23	1.66E-34	-0.26847	0.026	0.105	2.77E-30	6
Rrp1b	1.84E-34	-0.28871	0.051	0.144	3.07E-30	6
Tmem63b	2.08E-34	0.379769	0.131	0.075	3.46E-30	6
Gamt	2.82E-34	-0.32027	0.075	0.179	4.7E-30	6

Necab3	2.92E-34	0.407591	0.125	0.046	4.87E-30	6
Cspp1	2.97E-34	0.266443	0.251	0.237	4.96E-30	6
Mau2	3.26E-34	0.314922	0.179	0.145	5.44E-30	6
Ctsd	4.32E-34	-0.47113	0.142	0.178	7.2E-30	6
Syngr1	4.33E-34	0.363626	0.144	0.087	7.23E-30	6
Smarca1	4.57E-34	0.42459	0.108	0.051	7.62E-30	6
Araf	4.68E-34	0.306238	0.168	0.134	7.81E-30	6
Orc2	5.64E-34	-0.27548	0.067	0.164	9.4E-30	6
Shc2	7.6E-34	-0.2908	0.024	0.101	1.27E-29	6
Mpped2	8.05E-34	0.338273	0.163	0.122	1.34E-29	6
Rps27a	8.9E-34	-0.26048	0.032	0.113	1.48E-29	6
Zfp536	9.34E-34	0.312481	0.135	0.107	1.56E-29	6
Tram1l1	9.6E-34	0.35864	0.129	0.081	1.6E-29	6
Pkig	1.24E-33	-0.27614	0.048	0.136	2.07E-29	6
Pkp4	1.95E-33	0.254161	0.107	0.102	3.25E-29	6
Pip5k1c	2.14E-33	0.38204	0.131	0.077	3.57E-29	6
Rnpc3	2.2E-33	0.329296	0.134	0.1	3.67E-29	6
Pik3ca	3.08E-33	0.252491	0.165	0.162	5.13E-29	6
Podxl2	3.2E-33	0.310234	0.28	0.221	5.33E-29	6
Fbxo21	3.44E-33	0.322921	0.131	0.086	5.74E-29	6
Oraov1	3.53E-33	-0.27485	0.042	0.129	5.89E-29	6
Kdm5b	3.99E-33	0.35114	0.231	0.18	6.65E-29	6
Hmgcs1	4.11E-33	0.348271	0.192	0.147	6.85E-29	6
Stox2	5.63E-33	0.255993	0.209	0.197	9.4E-29	6
Fos	8.74E-33	0.428674	0.364	0.298	1.46E-28	6
Lpin2	8.92E-33	-0.29831	0.085	0.19	1.49E-28	6
6330403K(	9.91E-33	0.3017	0.287	0.238	1.65E-28	6
Cyfip2	1.05E-32	0.364023	0.111	0.047	1.75E-28	6
Tmem178	1.13E-32	0.292153	0.214	0.183	1.88E-28	6
Chd9	1.44E-32	0.35393	0.155	0.115	2.41E-28	6
Senp7	1.54E-32	0.306599	0.135	0.104	2.57E-28	6
Scn8a	1.57E-32	0.340721	0.172	0.133	2.62E-28	6
Plcb4	1.58E-32	0.368873	0.184	0.134	2.63E-28	6
Tpi1	1.63E-32	0.329255	0.16	0.117	2.72E-28	6
Fam184a	1.68E-32	0.387853	0.109	0.062	2.8E-28	6
Wdr60	1.74E-32	-0.28503	0.043	0.126	2.9E-28	6
1500011B(	2.32E-32	0.372751	0.158	0.101	3.87E-28	6
Blcap	2.48E-32	0.379654	0.132	0.073	4.14E-28	6
Wasf2	2.58E-32	-0.27485	0.039	0.121	4.3E-28	6
Gdap1	4.11E-32	0.25796	0.215	0.197	6.86E-28	6
Dennd2a	4.81E-32	-0.26649	0.037	0.114	8.02E-28	6
Fabp7	5.09E-32	-0.8378	0.082	0.127	8.49E-28	6
Tspan7	6.41E-32	0.348342	0.133	0.102	1.07E-27	6
Agpat4	7.73E-32	0.33917	0.16	0.113	1.29E-27	6
Bdh1	9.14E-32	0.330692	0.117	0.066	1.53E-27	6
Gm5124	1.05E-31	0.412367	0.121	0.06	1.76E-27	6
Sox5	1.12E-31	0.343792	0.149	0.116	1.87E-27	6
Vkorc1	1.66E-31	-0.25162	0.038	0.118	2.77E-27	6

Socs7	1.68E-31	0.288041	0.117	0.094	2.79E-27	6
Phf21b	2.13E-31	-0.25261	0.052	0.135	3.55E-27	6
Peo1	2.4E-31	-0.26583	0.047	0.132	4E-27	6
Elovl4	2.5E-31	0.254237	0.125	0.108	4.17E-27	6
Tmx4	2.92E-31	0.267107	0.2	0.175	4.87E-27	6
Mapk9	2.92E-31	0.367859	0.119	0.072	4.88E-27	6
Rnf165	3.39E-31	0.309096	0.205	0.169	5.65E-27	6
Tro	4.15E-31	0.298918	0.153	0.123	6.93E-27	6
Usp33	4.91E-31	0.299783	0.175	0.145	8.2E-27	6
Gramd1a	5.3E-31	0.251215	0.178	0.163	8.84E-27	6
Tmem176b	6.3E-31	-0.30879	0.037	0.116	1.05E-26	6
Nefm	7.28E-31	0.39558	0.156	0.112	1.21E-26	6
Armcx1	1.45E-30	0.364911	0.131	0.078	2.42E-26	6
Map9	3.53E-30	0.320317	0.18	0.144	5.89E-26	6
Fbxo11	3.67E-30	0.292219	0.177	0.152	6.11E-26	6
Socs2	3.72E-30	0.299852	0.165	0.135	6.21E-26	6
Rundc3a	4.66E-30	0.255247	0.273	0.246	7.78E-26	6
Sqle	4.72E-30	0.262163	0.135	0.119	7.88E-26	6
Tmem35	6.32E-30	0.282329	0.129	0.1	1.05E-25	6
Rnf219	7.18E-30	-0.25533	0.063	0.148	1.2E-25	6
D430019H	7.47E-30	0.367458	0.108	0.051	1.25E-25	6
Ltbp3	8.9E-30	-0.28769	0.044	0.125	1.48E-25	6
Gdap1l1	1.13E-29	0.322087	0.119	0.079	1.89E-25	6
Tsga10	1.41E-29	0.329546	0.104	0.058	2.35E-25	6
Atat1	1.44E-29	0.25918	0.123	0.101	2.41E-25	6
Necap1	1.49E-29	0.332623	0.105	0.066	2.49E-25	6
Tln1	1.69E-29	0.295249	0.182	0.146	2.82E-25	6
Prkx	1.85E-29	0.313311	0.144	0.109	3.09E-25	6
Enho	2.08E-29	0.366981	0.105	0.043	3.47E-25	6
2810008D	2.08E-29	0.298162	0.158	0.124	3.47E-25	6
Hyi	2.1E-29	0.27259	0.101	0.073	3.5E-25	6
Rps13	2.64E-29	-0.25937	0.029	0.103	4.4E-25	6
Celsr2	2.65E-29	0.269536	0.27	0.234	4.41E-25	6
Gabrb3	3.4E-29	0.33081	0.137	0.098	5.68E-25	6
Trio	6.9E-29	0.251092	0.141	0.123	1.15E-24	6
Ophn1	7.94E-29	0.330226	0.13	0.079	1.32E-24	6
Cd99l2	8.41E-29	0.310841	0.131	0.095	1.4E-24	6
Slc25a27	8.74E-29	0.297177	0.141	0.106	1.46E-24	6
Vopp1	2.09E-28	0.303479	0.152	0.115	3.48E-24	6
Rbm33	2.21E-28	0.26869	0.148	0.128	3.69E-24	6
Reep1	2.94E-28	0.315942	0.119	0.078	4.91E-24	6
Camsap1	3.3E-28	0.337551	0.135	0.098	5.5E-24	6
Flot1	4.28E-28	0.33003	0.141	0.093	7.14E-24	6
Pmvk	1.02E-27	0.281707	0.124	0.091	1.7E-23	6
Phyhipl	1.25E-27	0.318104	0.107	0.057	2.09E-23	6
Tbce	1.26E-27	0.301472	0.109	0.075	2.1E-23	6
Arhgef9	1.3E-27	0.329012	0.127	0.084	2.17E-23	6
Fnbp1	1.41E-27	0.344014	0.122	0.068	2.35E-23	6

Nipsnap1	1.92E-27	0.304873	0.167	0.132	3.21E-23	6
Kif3c	2.25E-27	0.34222	0.115	0.058	3.74E-23	6
Zfp329	2.78E-27	0.274521	0.154	0.126	4.64E-23	6
4632415L(	2.99E-27	0.29621	0.133	0.112	4.99E-23	6
Ddhd2	2.99E-27	0.261631	0.152	0.127	4.99E-23	6
Lingo1	7.28E-27	0.339346	0.142	0.089	1.21E-22	6
Fbxo9	1.09E-26	0.270888	0.15	0.125	1.82E-22	6
Nsf	1.52E-26	0.269913	0.107	0.083	2.54E-22	6
Narf	2.05E-26	0.269407	0.141	0.114	3.41E-22	6
2510009E(	3.76E-26	0.272519	0.117	0.094	6.27E-22	6
Chfr	5.96E-26	0.266831	0.118	0.097	9.94E-22	6
Myo9a	6.21E-26	0.275707	0.18	0.155	1.04E-21	6
Xpr1	6.63E-26	0.32534	0.129	0.088	1.11E-21	6
Tspyl2	1.79E-25	0.322442	0.105	0.065	2.99E-21	6
Pygo1	2.13E-25	0.25051	0.207	0.19	3.55E-21	6
Map1a	2.16E-25	0.349753	0.122	0.068	3.61E-21	6
Dcaf6	2.35E-25	0.307889	0.112	0.077	3.92E-21	6
Asxl3	3.57E-25	0.34073	0.101	0.059	5.95E-21	6
Nova2	4.12E-25	0.316737	0.102	0.053	6.88E-21	6
Zdhhc17	1.02E-24	0.283131	0.107	0.074	1.71E-20	6
Bmyc	1.5E-24	0.25545	0.12	0.098	2.5E-20	6
Kcnq2	1.64E-24	0.332941	0.112	0.068	2.73E-20	6
Nenf	2.91E-24	0.260237	0.158	0.134	4.86E-20	6
Tmem127	4.2E-24	0.265842	0.117	0.092	7E-20	6
Eml5	5.58E-24	0.325226	0.124	0.089	9.31E-20	6
Dip2b	5.68E-24	0.281327	0.102	0.077	9.47E-20	6
Tsc1	5.99E-24	0.281204	0.138	0.1	9.99E-20	6
Thsd7a	7.5E-24	-0.25554	0.061	0.134	1.25E-19	6
Zfyve27	8.25E-24	0.319383	0.101	0.058	1.38E-19	6
Ctsf	8.39E-24	0.317864	0.143	0.093	1.4E-19	6
4933427D:	1.14E-23	0.320089	0.114	0.073	1.91E-19	6
Ntn4	1.23E-23	0.327207	0.115	0.078	2.05E-19	6
Csnk1g1	1.52E-23	0.315417	0.115	0.073	2.54E-19	6
Ppp1r1a	2.1E-23	0.30916	0.153	0.105	3.5E-19	6
Casd1	3.27E-23	0.295436	0.117	0.079	5.45E-19	6
Gm10036	3.66E-23	-0.28836	0.099	0.184	6.11E-19	6
Mcoln1	5.76E-23	0.257423	0.101	0.073	9.61E-19	6
Dirc2	5.81E-23	0.258759	0.117	0.093	9.69E-19	6
RP23-199B	6.9E-23	0.311207	0.102	0.058	1.15E-18	6
Pik3r3	1.6E-22	0.297617	0.172	0.132	2.67E-18	6
Lztr1	1.72E-22	0.260745	0.13	0.097	2.86E-18	6
Ttc28	1.9E-22	0.258575	0.139	0.121	3.17E-18	6
Dctn1	2.39E-22	0.269982	0.11	0.079	3.99E-18	6
Dmxl2	4.08E-22	0.312288	0.118	0.07	6.81E-18	6
Egfr	4.54E-22	0.282884	0.112	0.08	7.56E-18	6
Meis3	6.63E-22	0.270368	0.109	0.073	1.11E-17	6
Vezt	1.16E-21	0.263457	0.116	0.09	1.93E-17	6
Mgll	1.66E-21	0.252646	0.152	0.131	2.76E-17	6

Gm3764	6.64E-21	0.274845	0.173	0.127	1.11E-16	6
Sbk1	7.51E-21	0.30824	0.16	0.114	1.25E-16	6
BC068157	1.82E-20	0.296712	0.117	0.077	3.03E-16	6
Tceal3	7.07E-20	0.2568	0.135	0.108	1.18E-15	6
Limk2	9.57E-19	0.264917	0.101	0.067	1.6E-14	6
Zer1	1.16E-18	0.291841	0.102	0.064	1.94E-14	6
Brsk2	4.04E-17	0.259645	0.117	0.084	6.73E-13	6
Plp1	1.17E-14	-0.49416	0.091	0.117	1.96E-10	6
Cntn2	0	1.570952	0.891	0.22	0	7
Neurod1	0	1.446811	0.987	0.501	0	7
Tubb3	0	1.295784	0.941	0.442	0	7
Gap43	0	1.170999	0.954	0.574	0	7
Stmn2	0	1.049003	0.966	0.524	0	7
Map1b	0	1.014594	0.937	0.563	0	7
Rtn1	0	0.962461	0.969	0.671	0	7
Tuba1a	0	0.958779	0.996	0.924	0	7
Ckb	0	0.860872	0.986	0.795	0	7
Tmsb4x	0	0.742296	0.997	0.945	0	7
Nhlh2	1.3E-305	1.083243	0.838	0.394	2.2E-301	7
Miat	1.1E-295	1.006084	0.902	0.489	1.9E-291	7
Tubb2b	4.5E-266	0.931847	0.832	0.439	7.5E-262	7
Basp1	4.9E-264	0.814525	0.928	0.688	8.1E-260	7
Dpysl3	1.9E-260	1.086915	0.657	0.189	3.1E-256	7
Nhlh1	6E-255	1.12979	0.638	0.194	1E-250	7
Tex14	3.4E-244	1.221127	0.522	0.119	5.6E-240	7
St18	1.2E-243	1.199817	0.582	0.163	2E-239	7
Ina	3.4E-239	0.94216	0.809	0.418	5.7E-235	7
Trpc4ap	6.1E-239	1.140128	0.579	0.172	1E-234	7
Stmn4	1.2E-237	0.98023	0.762	0.312	2E-233	7
Pdzrn3	2.1E-226	1.129845	0.529	0.12	3.5E-222	7
Sept3	1.4E-222	0.933506	0.767	0.366	2.4E-218	7
Ccnd1	2.6E-222	-1.52516	0.109	0.55	4.4E-218	7
Rplp1	3E-216	-0.77794	0.728	0.887	5E-212	7
Rpl13a	1E-207	-0.6829	0.851	0.933	1.7E-203	7
Zic1	6.9E-205	0.639432	0.955	0.772	1.2E-200	7
Sept4	3.2E-203	1.010626	0.606	0.2	5.3E-199	7
Map2	7.5E-201	0.859892	0.799	0.47	1.2E-196	7
Ppp1r14c	1E-197	0.956563	0.631	0.236	1.7E-193	7
Gpm6a	2.1E-197	0.789711	0.778	0.383	3.5E-193	7
Tubb2a	1.5E-192	0.95499	0.617	0.219	2.4E-188	7
Mtss1	2E-192	0.976951	0.627	0.244	3.4E-188	7
Podxl2	2.2E-192	0.997219	0.577	0.203	3.7E-188	7
BC005764	2.5E-188	1.015133	0.541	0.161	4.1E-184	7
Itm2b	1.3E-187	0.713548	0.878	0.626	2.2E-183	7
Nrep	1E-184	0.673215	0.863	0.549	1.7E-180	7
Rpl8	4.9E-184	-0.66726	0.784	0.905	8.1E-180	7
Igfbpl1	1.7E-182	0.704795	0.898	0.63	2.8E-178	7
Ank3	7.9E-180	0.864273	0.738	0.398	1.3E-175	7

Draxin	8.9E-180	-1.20902	0.18	0.58	1.5E-175	7
Celf4	1.2E-177	0.81366	0.69	0.294	2.1E-173	7
Nrxn1	1.8E-177	0.896213	0.666	0.294	3E-173	7
Dek	3.7E-175	-1.0548	0.42	0.712	6.2E-171	7
Cdk5r1	1.2E-174	0.858148	0.683	0.309	2E-170	7
Chgb	1.7E-174	0.920469	0.599	0.216	2.8E-170	7
Ddah2	8.9E-173	0.670651	0.895	0.671	1.5E-168	7
Smc2	4.2E-170	-1.34528	0.229	0.567	7E-166	7
2810417H1	5.4E-163	-1.43923	0.062	0.414	9E-159	7
Elavl4	3.9E-162	0.824263	0.68	0.311	6.5E-158	7
Dcx	4.7E-161	0.809233	0.694	0.353	7.9E-157	7
Gng3	3.3E-160	0.859299	0.665	0.292	5.5E-156	7
Clmp	1.8E-157	0.922035	0.561	0.22	3E-153	7
Apc	6.8E-157	0.801646	0.729	0.411	1.1E-152	7
Nfib	8.4E-156	0.500777	0.987	0.929	1.4E-151	7
Celf2	1.6E-155	0.679	0.859	0.622	2.7E-151	7
Anp32b	6.4E-155	-1.0462	0.255	0.613	1.1E-150	7
Myt1	1.9E-154	0.910057	0.461	0.131	3.1E-150	7
Sema6a	1.1E-151	0.892873	0.523	0.18	1.8E-147	7
Rab3a	2.2E-149	0.841153	0.458	0.128	3.7E-145	7
H2afv	6.6E-148	-0.82725	0.539	0.74	1.1E-143	7
Rplp2	2.5E-146	-0.67546	0.672	0.851	4.1E-142	7
Elmo1	1E-145	0.897637	0.412	0.106	1.7E-141	7
Bin1	7.8E-145	0.753586	0.728	0.408	1.3E-140	7
Sfrp1	1.6E-144	-0.73484	0.712	0.818	2.7E-140	7
Myt1l	7.8E-141	0.936797	0.454	0.14	1.3E-136	7
Rps5	8.7E-140	-0.5248	0.923	0.956	1.4E-135	7
Ranbp1	1.1E-139	-0.88845	0.425	0.692	1.9E-135	7
Pabpc1	2E-138	-0.60068	0.775	0.885	3.3E-134	7
Malat1	2.4E-138	0.437144	0.995	0.952	4.1E-134	7
Rpl4	1.8E-136	-0.54842	0.843	0.912	3E-132	7
A930011O1	3.3E-135	0.896445	0.381	0.096	5.4E-131	7
Rps19	3.9E-135	-0.74288	0.538	0.768	6.5E-131	7
Mki67	2.5E-131	-1.40036	0.12	0.413	4.2E-127	7
Top2a	2.7E-128	-1.37343	0.173	0.445	4.4E-124	7
Srebf1	3E-128	-1.12807	0.053	0.353	5E-124	7
Npm1	1.1E-127	-0.84074	0.385	0.664	1.9E-123	7
Hmgb2	3.6E-126	-1.16373	0.113	0.419	6E-122	7
Itsn1	3.5E-125	0.811863	0.521	0.216	5.9E-121	7
Aplp1	4.4E-125	0.779452	0.429	0.132	7.3E-121	7
Rps26	2.6E-124	-0.60682	0.74	0.854	4.4E-120	7
Gria2	4.5E-124	0.643784	0.795	0.502	7.4E-120	7
Dut	5.6E-124	-1.07529	0.141	0.455	9.3E-120	7
Cadm3	5.9E-124	0.756885	0.401	0.123	9.8E-120	7
Prdx1	6.2E-124	-0.94905	0.177	0.511	1E-119	7
Dbi	5.2E-123	-1.08405	0.165	0.483	8.7E-119	7
Pax6	8.4E-123	0.636459	0.764	0.495	1.4E-118	7
Cadm1	1.2E-122	-0.99608	0.092	0.411	1.9E-118	7

Elavl3	1.6E-122	0.653861	0.75	0.453	2.7E-118	7
Mllt11	1.3E-119	0.763257	0.502	0.198	2.1E-115	7
Sptbn1	3.5E-117	0.780071	0.604	0.336	5.8E-113	7
Rps20	4.6E-116	-0.66185	0.588	0.791	7.7E-112	7
Rpl32	1.4E-115	-0.53999	0.796	0.895	2.3E-111	7
Anp32e	2.1E-115	-0.86662	0.356	0.631	3.5E-111	7
2700094K1	1.5E-114	-0.83985	0.367	0.631	2.4E-110	7
B3galt2	1.1E-110	0.765323	0.323	0.082	1.8E-106	7
Rbfox3	2.2E-110	0.592004	0.753	0.476	3.7E-106	7
Nasp	4.2E-110	-0.80213	0.418	0.655	7E-106	7
Tnik	3.5E-109	0.770833	0.391	0.129	5.9E-105	7
Tcf4	4E-108	0.413047	0.974	0.921	6.7E-104	7
Rps11	1.9E-106	-0.61375	0.618	0.788	3.1E-102	7
Pcna	2.5E-106	-1.02547	0.096	0.376	4.1E-102	7
Gnai2	2.3E-105	-0.92282	0.149	0.443	3.8E-101	7
Kif5c	2.3E-105	0.665983	0.66	0.38	3.8E-101	7
Birc5	8.9E-105	-1.04982	0.036	0.289	1.5E-100	7
Chrna3	1E-104	0.755772	0.352	0.102	1.7E-100	7
Uncx	1.6E-104	0.719972	0.583	0.299	2.7E-100	7
Dner	2.6E-104	0.750035	0.48	0.196	4.3E-100	7
Galr1	5E-104	0.741285	0.221	0.035	8.3E-100	7
Tmsb10	6.1E-104	0.518463	0.84	0.651	1E-99	7
Ttc3	7.8E-104	0.450847	0.941	0.821	1.3E-99	7
Mcm6	7.1E-103	-0.96883	0.04	0.292	1.19E-98	7
Rpl41	5.3E-102	-0.59324	0.611	0.792	8.86E-98	7
Ube2c	1.4E-101	-1.33995	0.053	0.284	2.27E-97	7
Hsd11b2	2.5E-101	-0.98681	0.048	0.304	4.12E-97	7
Rpl22	6E-101	-0.59414	0.616	0.799	1.01E-96	7
App	9.5E-101	0.578816	0.779	0.553	1.59E-96	7
Rps3	1.4E-100	-0.44577	0.882	0.924	2.36E-96	7
Gnb2l1	1E-99	-0.50205	0.786	0.877	1.73E-95	7
Tuba1b	2.4E-99	-0.86441	0.245	0.497	3.95E-95	7
Rufy3	7.5E-99	0.655483	0.62	0.354	1.25E-94	7
Rbp4	1.87E-98	-0.93892	0.026	0.256	3.12E-94	7
Fkbp3	4.58E-98	-0.64928	0.562	0.757	7.64E-94	7
Tpx2	6.74E-98	-1.12545	0.053	0.297	1.12E-93	7
Smc4	4.04E-97	-0.90211	0.379	0.579	6.75E-93	7
Gsg1l	1.55E-96	-0.86826	0.032	0.27	2.58E-92	7
Syt11	2.47E-95	0.588101	0.731	0.488	4.12E-91	7
Btbd17	3.61E-95	0.738595	0.392	0.144	6.03E-91	7
Pdgfa	7.04E-95	-0.95148	0.034	0.268	1.17E-90	7
Igsf21	1.02E-94	0.711875	0.292	0.075	1.71E-90	7
Epb4.1l1	1.23E-94	0.764956	0.292	0.077	2.05E-90	7
Cenpf	1.13E-92	-1.24655	0.146	0.377	1.89E-88	7
Rab6b	1.7E-92	0.677508	0.438	0.186	2.84E-88	7
Ncl	3.46E-92	-0.46812	0.82	0.901	5.77E-88	7
Srrm4	6.48E-92	0.72539	0.429	0.179	1.08E-87	7
Cnbp	7.58E-92	-0.58319	0.56	0.752	1.26E-87	7

Hist3h2a	8.03E-92	0.743654	0.361	0.128	1.34E-87	7
Cks1b	8.85E-92	-0.88085	0.095	0.361	1.48E-87	7
Cdca8	9.47E-91	-0.96587	0.047	0.279	1.58E-86	7
Ptprs	9.7E-91	0.623822	0.659	0.404	1.62E-86	7
Eef1b2	1.18E-90	-0.60822	0.546	0.745	1.97E-86	7
Rbfox2	7.06E-90	0.6561	0.53	0.266	1.18E-85	7
Serbp1	1.73E-89	-0.47115	0.781	0.875	2.88E-85	7
Pnoc	1.76E-89	0.71296	0.124	0.009	2.93E-85	7
Hmgn5	3.42E-89	-0.88467	0.163	0.442	5.71E-85	7
Cdk1	4.03E-88	-0.96422	0.038	0.261	6.73E-84	7
Rps9	4.06E-88	-0.42313	0.887	0.931	6.77E-84	7
Eef1a1	1.67E-87	-0.46955	0.809	0.889	2.79E-83	7
Rplp0	1.05E-86	-0.46791	0.805	0.877	1.75E-82	7
H2afx	1.54E-86	-1.02939	0.109	0.345	2.58E-82	7
Spc24	3.55E-86	-0.84347	0.035	0.256	5.92E-82	7
Tubb5	6.3E-85	0.285711	0.986	0.909	1.05E-80	7
Ank2	8.23E-85	0.670172	0.48	0.223	1.37E-80	7
Plcb1	1.15E-84	0.760409	0.35	0.126	1.91E-80	7
Nfix	3.53E-84	0.457383	0.82	0.663	5.88E-80	7
Pbk	5.8E-84	-0.8635	0.027	0.235	9.68E-80	7
Gpr153	2.93E-83	-0.80533	0.021	0.221	4.89E-79	7
Ppp2r2b	4.21E-83	0.714642	0.265	0.071	7.02E-79	7
Glce	1.09E-82	0.700021	0.393	0.161	1.81E-78	7
Ankrd12	1.94E-82	0.661672	0.515	0.258	3.23E-78	7
Apbb1	5.36E-82	0.638865	0.39	0.155	8.94E-78	7
Prdm8	1.15E-81	0.725905	0.325	0.112	1.91E-77	7
Tagln3	1.21E-81	0.602037	0.532	0.278	2.02E-77	7
Kcnk1	2.4E-81	0.676385	0.427	0.189	4E-77	7
Kif5a	4.95E-81	0.69079	0.334	0.114	8.25E-77	7
Rnasel	3.54E-80	0.639677	0.244	0.061	5.9E-76	7
Cenpa	4.06E-80	-1.09263	0.056	0.265	6.78E-76	7
Mif	8.56E-80	-0.76534	0.123	0.372	1.43E-75	7
Prkcb	1.11E-79	0.599484	0.489	0.239	1.86E-75	7
Chd7	1.4E-79	0.47374	0.817	0.635	2.33E-75	7
Gpc2	4.08E-79	0.66641	0.341	0.125	6.81E-75	7
Stmn3	4.74E-79	0.462495	0.782	0.61	7.9E-75	7
RP23-45G1	1.53E-78	-0.88832	0.116	0.341	2.56E-74	7
Pqlc1	1.69E-78	-0.80101	0.079	0.312	2.83E-74	7
Stxbp1	1.7E-78	0.656678	0.376	0.147	2.83E-74	7
Paics	1.94E-78	-0.7735	0.173	0.429	3.24E-74	7
Cdca3	7.05E-78	-0.84838	0.032	0.234	1.18E-73	7
Hirip3	7.69E-78	-0.80837	0.144	0.399	1.28E-73	7
Ccna2	1.55E-77	-0.80441	0.032	0.234	2.59E-73	7
Serinc1	1.79E-77	0.544657	0.643	0.422	2.99E-73	7
Nop58	3.08E-77	-0.6809	0.363	0.607	5.14E-73	7
Hells	5.24E-77	-0.84222	0.029	0.225	8.73E-73	7
Elavl2	1.24E-76	0.651808	0.481	0.255	2.07E-72	7
Apc2	1.74E-76	0.589629	0.182	0.034	2.9E-72	7

Pde1c	5.84E-75	0.599703	0.599	0.372	9.75E-71	7
Rrm2	6.48E-75	-0.76316	0.025	0.216	1.08E-70	7
Fnbp1l	3.98E-74	0.565368	0.647	0.44	6.63E-70	7
Incenp	4.69E-74	-0.89617	0.075	0.288	7.83E-70	7
Mab21l1	6.66E-74	0.656777	0.433	0.21	1.11E-69	7
Rps3a1	6.83E-74	-0.5674	0.487	0.679	1.14E-69	7
Nusap1	8.43E-74	-0.91166	0.041	0.239	1.41E-69	7
Rpl14	1.64E-73	-0.55538	0.513	0.709	2.73E-69	7
Rps15	3.58E-73	-0.48909	0.649	0.797	5.97E-69	7
Ybx1	7.66E-73	-0.48011	0.665	0.794	1.28E-68	7
Dctpp1	1.71E-72	-0.73364	0.09	0.319	2.85E-68	7
Nsg1	2.42E-72	0.598701	0.501	0.273	4.03E-68	7
Gdpd5	1.15E-71	0.538493	0.154	0.025	1.92E-67	7
Nsg2	1.24E-71	0.515161	0.673	0.474	2.07E-67	7
Kif1b	1.26E-71	0.492114	0.707	0.508	2.11E-67	7
Klc1	1.31E-71	0.570979	0.563	0.347	2.19E-67	7
Cbx5	1.38E-71	-0.59926	0.476	0.665	2.3E-67	7
Esco2	1.7E-71	-0.81935	0.025	0.206	2.84E-67	7
Hes6	2.81E-71	-0.75253	0.049	0.249	4.68E-67	7
Ran	4.7E-71	-0.70817	0.187	0.429	7.84E-67	7
Cenpe	5.57E-71	-1.03811	0.088	0.299	9.29E-67	7
Mapk8ip1	1.6E-70	0.575093	0.441	0.212	2.67E-66	7
Txn1	2.09E-70	-0.63665	0.336	0.575	3.48E-66	7
Rps21	5.67E-70	-0.45921	0.715	0.841	9.45E-66	7
Sox9	5.8E-70	-0.76935	0.06	0.268	9.67E-66	7
Marcksl1	1.37E-69	0.404165	0.835	0.714	2.28E-65	7
Tmpo	1.6E-69	-0.72796	0.232	0.467	2.66E-65	7
Mcm3	1.82E-69	-0.71218	0.019	0.19	3.04E-65	7
Spc25	4.02E-69	-0.8198	0.046	0.237	6.7E-65	7
Tyms	7.5E-69	-0.74226	0.042	0.231	1.25E-64	7
Knstrn	3.26E-68	-0.73921	0.023	0.196	5.45E-64	7
Usp1	4.17E-68	-0.73775	0.123	0.353	6.95E-64	7
Kif11	4.37E-68	-0.77126	0.034	0.217	7.28E-64	7
Lig1	4.43E-68	-0.85883	0.139	0.362	7.4E-64	7
D4Wsu53e	6.92E-68	0.513331	0.66	0.432	1.15E-63	7
Calm2	7.55E-68	0.327095	0.946	0.869	1.26E-63	7
Gng2	5.3E-67	0.527749	0.578	0.383	8.84E-63	7
Ckap2l	5.38E-67	-0.80067	0.04	0.229	8.98E-63	7
Gdi1	5.47E-67	0.533921	0.428	0.216	9.13E-63	7
Ppm1h	7.66E-67	0.538311	0.151	0.026	1.28E-62	7
Nucks1	8.83E-67	-0.58384	0.496	0.674	1.47E-62	7
Srgap3	9.16E-67	0.526998	0.16	0.031	1.53E-62	7
Akap12	1.79E-66	0.645214	0.282	0.099	2.98E-62	7
Cdca7	2.01E-66	-0.68832	0.03	0.209	3.34E-62	7
Chd3	5.58E-66	0.610918	0.345	0.135	9.31E-62	7
Dtymk	6.02E-66	-0.69486	0.218	0.454	1E-61	7
Tbata	9.46E-66	-0.84347	0.136	0.353	1.58E-61	7
Sobp	9.97E-66	0.628529	0.295	0.116	1.66E-61	7

L1cam	1.02E-65	0.522424	0.186	0.044	1.7E-61	7
Clspn	1.54E-65	-0.71781	0.026	0.2	2.57E-61	7
Dbn1	2.31E-65	0.626603	0.323	0.126	3.85E-61	7
A330076H(	4.95E-65	0.615784	0.352	0.147	8.26E-61	7
Mdk	1.25E-64	-0.79399	0.171	0.393	2.08E-60	7
Tmem2	3.02E-64	0.617896	0.236	0.073	5.04E-60	7
Rnmt	3.52E-64	0.54628	0.537	0.335	5.87E-60	7
Cenph	4.32E-64	-0.71127	0.031	0.204	7.21E-60	7
Nrcam	5.75E-64	0.642079	0.309	0.122	9.59E-60	7
Smco4	9E-64	-0.58731	0.015	0.171	1.5E-59	7
Ptn	1.39E-63	0.319582	0.65	0.427	2.33E-59	7
Prim1	1.87E-63	-0.70296	0.071	0.276	3.11E-59	7
Ccnb2	3.46E-63	-0.77656	0.028	0.197	5.78E-59	7
Clvs1	8.14E-63	0.599908	0.298	0.117	1.36E-58	7
Lsm4	9.83E-63	-0.60551	0.269	0.502	1.64E-58	7
Kif23	1.29E-62	-0.81549	0.044	0.225	2.14E-58	7
Ncapg	1.47E-62	-0.69231	0.026	0.194	2.46E-58	7
Porcn	1.74E-62	0.566587	0.237	0.072	2.91E-58	7
Prc1	3.37E-62	-0.89898	0.077	0.269	5.63E-58	7
Hmgn2	5.44E-62	-0.67603	0.07	0.268	9.08E-58	7
Rps14	6.12E-62	-0.34802	0.918	0.952	1.02E-57	7
Abhd16a	9.3E-62	0.558114	0.376	0.177	1.55E-57	7
Ccdc34	1.61E-61	-0.69227	0.194	0.429	2.69E-57	7
Banf1	1.91E-61	-0.51092	0.53	0.704	3.19E-57	7
Klf7	2.1E-61	0.571617	0.457	0.248	3.51E-57	7
Fam210b	2.37E-61	-0.71248	0.115	0.327	3.95E-57	7
Grin2b	2.39E-61	0.523891	0.173	0.039	3.99E-57	7
Gmnn	4.95E-61	-0.57729	0.01	0.154	8.26E-57	7
Plcd1	5.5E-61	0.473168	0.154	0.032	9.17E-57	7
Gnao1	1.09E-60	0.526583	0.486	0.273	1.81E-56	7
Pak7	1.37E-60	0.563142	0.263	0.093	2.29E-56	7
Nhp2	1.61E-60	-0.63941	0.16	0.385	2.68E-56	7
Cbfa2t3	3.38E-60	-0.68029	0.204	0.416	5.63E-56	7
Psat1	6.82E-60	-0.65046	0.159	0.381	1.14E-55	7
Tacc3	7.27E-60	-0.69626	0.043	0.217	1.21E-55	7
mt-Nd1	7.59E-60	-0.35661	0.863	0.931	1.27E-55	7
Cep170	8.6E-60	0.564287	0.493	0.292	1.43E-55	7
Atoh1	9.15E-60	-0.69063	0.021	0.174	1.53E-55	7
Tead2	1.42E-59	-0.67616	0.091	0.29	2.36E-55	7
Gas1	1.56E-59	-0.60112	0.016	0.166	2.61E-55	7
Sfrp2	2.7E-59	-0.56566	0.012	0.157	4.5E-55	7
Rpa2	4.05E-59	-0.63927	0.038	0.211	6.76E-55	7
B2m	8.43E-59	-0.7596	0.064	0.242	1.41E-54	7
Siva1	1.87E-58	-0.66432	0.084	0.284	3.12E-54	7
Uchl1	1.9E-58	0.54767	0.526	0.321	3.17E-54	7
Hmmr	4.71E-58	-0.76517	0.027	0.186	7.85E-54	7
Igsf8	5.48E-58	0.527143	0.461	0.256	9.14E-54	7
Necab3	6.33E-58	0.518719	0.181	0.044	1.06E-53	7

Hey1	6.55E-58	-0.75475	0.092	0.285	1.09E-53	7
Isoc1	7.32E-58	-0.58583	0.03	0.191	1.22E-53	7
Soga3	8.45E-58	0.473073	0.622	0.446	1.41E-53	7
Cltb	1.26E-57	-0.69733	0.238	0.435	2.11E-53	7
Hist3h2ba	1.58E-57	0.580174	0.302	0.126	2.63E-53	7
Cenpm	3.69E-57	-0.57019	0.02	0.171	6.16E-53	7
Slc25a5	5.57E-57	-0.59099	0.311	0.522	9.3E-53	7
Mns1	6.07E-57	-0.68034	0.043	0.214	1.01E-52	7
Pmf1	6.55E-57	-0.54124	0.012	0.152	1.09E-52	7
Smarcc2	8.32E-57	0.503754	0.532	0.351	1.39E-52	7
Npdc1	1.98E-56	0.523935	0.446	0.249	3.3E-52	7
Aurkb	2.08E-56	-0.55512	0.012	0.151	3.47E-52	7
Rtn4	2.71E-56	0.442842	0.648	0.469	4.52E-52	7
Hes1	4.09E-56	-0.7801	0.018	0.157	6.83E-52	7
Lap3	6.9E-56	-0.68084	0.093	0.289	1.15E-51	7
Marcks	8.61E-56	0.279865	0.96	0.913	1.44E-51	7
Ppfia2	1.36E-55	0.5906	0.271	0.101	2.27E-51	7
Rnd3	1.63E-55	-0.62143	0.226	0.445	2.72E-51	7
Map6	2.15E-55	0.521353	0.22	0.07	3.59E-51	7
Gm10075	2.6E-55	-0.57765	0.321	0.528	4.34E-51	7
Apoe	2.62E-55	-1.71558	0.173	0.254	4.38E-51	7
Bhlhe22	7.81E-55	0.577724	0.214	0.069	1.3E-50	7
Rrm1	8.19E-55	-0.63252	0.083	0.272	1.37E-50	7
Mis18bp1	1.27E-54	-0.63863	0.022	0.17	2.12E-50	7
Gria4	1.59E-54	0.543613	0.28	0.114	2.66E-50	7
Zfp36l1	1.84E-54	-0.59747	0.019	0.159	3.07E-50	7
Dync1i2	1.91E-54	0.439778	0.71	0.558	3.19E-50	7
Cdc20	2.03E-54	-0.80075	0.044	0.206	3.39E-50	7
Frmd4a	2.81E-54	0.514888	0.487	0.296	4.69E-50	7
Slc17a6	3E-54	0.566713	0.318	0.136	5.01E-50	7
Dnajc9	3.66E-54	-0.62415	0.151	0.363	6.11E-50	7
Gm17322	3.86E-54	-0.6572	0.011	0.139	6.44E-50	7
Mmp24	3.86E-54	0.478577	0.173	0.045	6.44E-50	7
Ccnb1	4.05E-54	-0.61578	0.013	0.148	6.75E-50	7
Sparcl1	4.72E-54	-0.9959	0.055	0.217	7.87E-50	7
Rps25	7.08E-54	-0.55883	0.278	0.493	1.18E-49	7
Hpca	7.88E-54	-0.727	0.099	0.289	1.31E-49	7
Nap1l1	1.16E-53	-0.5598	0.33	0.523	1.93E-49	7
Ckap2	1.51E-53	-0.60767	0.025	0.172	2.51E-49	7
Serpini1	1.55E-53	0.470913	0.191	0.055	2.59E-49	7
Hn1	1.82E-53	0.440187	0.715	0.529	3.04E-49	7
Tk1	1.99E-53	-0.54382	0.01	0.139	3.33E-49	7
Gadd45a	2.38E-53	0.603299	0.228	0.079	3.97E-49	7
Dclk1	8.37E-53	-0.64165	0.214	0.425	1.4E-48	7
Kmt2e	9.27E-53	0.416064	0.715	0.558	1.55E-48	7
Cpe	3.54E-52	0.451465	0.632	0.443	5.91E-48	7
Smim18	4.92E-52	0.482533	0.162	0.041	8.21E-48	7
Dkc1	5.83E-52	-0.58649	0.141	0.345	9.72E-48	7

Rpl35a	1.19E-51	-0.50022	0.391	0.582	1.98E-47	7
Dpysl5	1.23E-51	0.53292	0.234	0.085	2.06E-47	7
Casc5	1.37E-51	-0.65671	0.039	0.196	2.28E-47	7
Hjurp	1.45E-51	-0.63022	0.249	0.446	2.42E-47	7
Cacna2d1	1.58E-51	0.51131	0.552	0.362	2.64E-47	7
Mllt4	1.62E-51	0.487716	0.532	0.351	2.71E-47	7
Rps24	3.14E-51	-0.38436	0.724	0.827	5.24E-47	7
Fbxo5	4.79E-51	-0.56218	0.025	0.168	7.99E-47	7
Mt1	5.47E-51	-0.84324	0.033	0.177	9.12E-47	7
Jhdm1d	6.64E-51	0.550924	0.277	0.114	1.11E-46	7
Rpl22l1	6.72E-51	-0.6083	0.165	0.363	1.12E-46	7
Rad51ap1	8.56E-51	-0.56674	0.031	0.176	1.43E-46	7
Uhrf1	9.74E-51	-0.54373	0.018	0.152	1.63E-46	7
Samd12	1.4E-50	0.434347	0.134	0.027	2.33E-46	7
Mcm5	2.14E-50	-0.57832	0.031	0.177	3.58E-46	7
Kdm5b	2.49E-50	0.540538	0.352	0.173	4.15E-46	7
Fth1	2.67E-50	-0.48066	0.567	0.718	4.46E-46	7
Mcm2	3.73E-50	-0.60632	0.051	0.215	6.22E-46	7
Slc3a2	3.97E-50	-0.55268	0.137	0.335	6.63E-46	7
Bok	5.81E-50	-0.6017	0.084	0.262	9.68E-46	7
Hmgn1	7.02E-50	-0.47985	0.468	0.632	1.17E-45	7
Cenpk	7.76E-50	-0.55925	0.018	0.153	1.29E-45	7
Prdx4	1.09E-49	-0.59759	0.18	0.378	1.82E-45	7
Kif15	1.45E-49	-0.61269	0.038	0.19	2.41E-45	7
Prmt8	1.78E-49	-0.62833	0.102	0.283	2.97E-45	7
Mapt	3.42E-49	0.467916	0.388	0.204	5.7E-45	7
Myod1	4.33E-49	-0.60602	0.018	0.149	7.22E-45	7
Gsk3b	4.9E-49	0.425062	0.64	0.489	8.17E-45	7
Aspm	5.31E-49	-0.63808	0.02	0.151	8.87E-45	7
Lhx1	6.16E-49	0.407335	0.67	0.485	1.03E-44	7
Acot7	7.13E-49	-0.57786	0.051	0.204	1.19E-44	7
Cd24a	7.61E-49	0.38177	0.778	0.624	1.27E-44	7
S100a16	7.83E-49	0.519398	0.238	0.084	1.31E-44	7
Kif20b	1.13E-48	-0.65512	0.046	0.2	1.88E-44	7
D17H6S56I	1.67E-48	-0.49332	0.012	0.135	2.79E-44	7
Tipin	2.37E-48	-0.56432	0.088	0.266	3.95E-44	7
Thra	3.21E-48	0.460915	0.401	0.217	5.36E-44	7
Sox4	3.58E-48	0.412588	0.733	0.572	5.98E-44	7
Lsm6	5.16E-48	-0.55704	0.179	0.385	8.61E-44	7
Pa2g4	5.36E-48	-0.51837	0.33	0.535	8.94E-44	7
Kidins220	6.62E-48	0.560996	0.296	0.129	1.1E-43	7
Atad2	6.93E-48	-0.59122	0.052	0.208	1.16E-43	7
Arl6ip1	7.82E-48	-0.44703	0.513	0.552	1.3E-43	7
Hdgf	1.6E-47	-0.51776	0.353	0.544	2.67E-43	7
Hint1	1.98E-47	-0.42665	0.56	0.713	3.3E-43	7
Nrn1	2.01E-47	0.521587	0.392	0.209	3.36E-43	7
Nav2	2.35E-47	0.549455	0.241	0.095	3.92E-43	7
2410066E1	3.52E-47	0.497778	0.224	0.089	5.87E-43	7

Ebf3	4.46E-47	0.512393	0.367	0.191	7.44E-43	7
Neurod2	5.66E-47	0.471541	0.187	0.059	9.44E-43	7
Sgol2	9.14E-47	-0.62182	0.028	0.165	1.52E-42	7
Cklf	1.21E-46	-0.5271	0.032	0.174	2.01E-42	7
Rps2	1.29E-46	-0.53365	0.267	0.462	2.15E-42	7
Cdca2	1.7E-46	-0.50483	0.015	0.136	2.84E-42	7
Kif21b	1.86E-46	0.471725	0.159	0.044	3.11E-42	7
Kif22	1.88E-46	-0.5324	0.032	0.171	3.14E-42	7
Prdx6	1.94E-46	-0.56709	0.148	0.338	3.24E-42	7
Scg5	3.68E-46	0.474458	0.499	0.323	6.14E-42	7
Arhgap11a	4.58E-46	-0.61089	0.037	0.179	7.63E-42	7
Rpl39	5.31E-46	-0.44283	0.513	0.655	8.86E-42	7
Nuf2	6.02E-46	-0.5126	0.02	0.149	1E-41	7
Dlgap4	7.6E-46	0.502638	0.351	0.176	1.27E-41	7
Clip3	1.09E-45	0.474512	0.469	0.3	1.81E-41	7
Lgals1	5.28E-45	-0.71723	0.083	0.238	8.81E-41	7
Nmral1	7.29E-45	-0.50791	0.047	0.196	1.22E-40	7
Ppp3ca	8.56E-45	0.448201	0.553	0.366	1.43E-40	7
H2afy	9.06E-45	-0.46857	0.439	0.602	1.51E-40	7
Rps15a	9.76E-45	-0.41468	0.537	0.665	1.63E-40	7
Sv2a	1.17E-44	0.422955	0.188	0.064	1.96E-40	7
Dusp14	1.24E-44	0.470043	0.147	0.039	2.06E-40	7
Jph4	1.28E-44	0.388586	0.181	0.061	2.14E-40	7
Actl6b	1.51E-44	0.436855	0.165	0.048	2.52E-40	7
Pou3f2	1.65E-44	-0.56447	0.113	0.287	2.76E-40	7
Serping1	1.76E-44	0.42295	0.123	0.027	2.93E-40	7
Sowaha	1.81E-44	-0.67269	0.037	0.171	3.02E-40	7
Ntrk3	3.53E-44	-0.53267	0.031	0.163	5.88E-40	7
Mxd3	3.68E-44	-0.49551	0.017	0.134	6.14E-40	7
Atp1b3	3.77E-44	0.468401	0.496	0.326	6.29E-40	7
Tubb4b	4.57E-44	-0.58011	0.086	0.25	7.62E-40	7
Cenpp	6.15E-44	-0.41366	0.011	0.121	1.03E-39	7
Sfrs18	6.89E-44	0.332349	0.877	0.771	1.15E-39	7
Racgap1	8.56E-44	-0.5941	0.1	0.266	1.43E-39	7
Afap1	1.14E-43	0.517694	0.3	0.142	1.89E-39	7
Tmem57	1.18E-43	0.482253	0.447	0.28	1.97E-39	7
Dusp8	1.44E-43	0.45069	0.201	0.07	2.39E-39	7
Scd2	1.83E-43	-0.58969	0.13	0.308	3.05E-39	7
Homer2	1.98E-43	-0.52659	0.088	0.257	3.3E-39	7
Nop10	2.72E-43	-0.50003	0.296	0.494	4.53E-39	7
Dtl	2.83E-43	-0.50879	0.025	0.153	4.72E-39	7
Stxbp5l	3.95E-43	0.46681	0.155	0.045	6.59E-39	7
Boc	6.17E-43	-0.54256	0.032	0.164	1.03E-38	7
Cenpq	8.18E-43	-0.50366	0.035	0.168	1.36E-38	7
Adamts1	9.54E-43	-0.58797	0.033	0.163	1.59E-38	7
Grina	1.3E-42	0.492209	0.245	0.099	2.16E-38	7
Klc2	1.47E-42	0.450131	0.13	0.032	2.46E-38	7
Cnih2	1.6E-42	0.378912	0.119	0.028	2.67E-38	7

Lrig3	1.94E-42	-0.5218	0.04	0.179	3.24E-38	7
Gli1	2.31E-42	-0.45635	0.009	0.114	3.85E-38	7
Smpd2	2.59E-42	-0.52564	0.038	0.174	4.32E-38	7
Hsp90b1	2.99E-42	-0.42782	0.596	0.698	4.99E-38	7
Smpd3	2.99E-42	0.515046	0.308	0.151	4.99E-38	7
Tomm7	4.75E-42	-0.48307	0.323	0.509	7.93E-38	7
Rps27l	4.88E-42	-0.49553	0.313	0.504	8.15E-38	7
Rps10	5.01E-42	-0.41019	0.499	0.645	8.36E-38	7
Zfp521	5.11E-42	0.484526	0.176	0.058	8.52E-38	7
Hist1h2ak	6.71E-42	-0.59234	0.026	0.151	1.12E-37	7
Mcm7	8.88E-42	-0.49434	0.205	0.401	1.48E-37	7
Hnrnpu	1.3E-41	-0.29076	0.834	0.867	2.16E-37	7
Arhgef2	2.06E-41	0.457662	0.435	0.271	3.44E-37	7
Shmt1	2.23E-41	-0.40286	0.008	0.109	3.73E-37	7
Chaf1a	2.31E-41	-0.51809	0.04	0.177	3.86E-37	7
Snrpb	2.71E-41	-0.45787	0.414	0.584	4.52E-37	7
Bub1	2.8E-41	-0.45861	0.021	0.138	4.66E-37	7
Nefl	4.21E-41	0.52266	0.166	0.051	7.03E-37	7
Mex3b	4.76E-41	0.50802	0.243	0.107	7.93E-37	7
Gpm6b	5.18E-41	0.348337	0.635	0.473	8.63E-37	7
Cnpy1	6.14E-41	0.502223	0.264	0.124	1.02E-36	7
Erbb4	7.2E-41	0.496223	0.144	0.039	1.2E-36	7
Clcn4-2	8.54E-41	0.448881	0.406	0.24	1.42E-36	7
2900079G	1.53E-40	0.424319	0.148	0.042	2.56E-36	7
Snrpf	1.97E-40	-0.50239	0.251	0.439	3.28E-36	7
Dnph1	2.74E-40	-0.43307	0.018	0.13	4.57E-36	7
Cacna1b	3.11E-40	0.46495	0.223	0.088	5.19E-36	7
Myl12a	4.85E-40	-0.49773	0.166	0.339	8.09E-36	7
Rab6a	6.57E-40	0.406292	0.475	0.318	1.1E-35	7
Slc29a1	6.6E-40	-0.46578	0.421	0.571	1.1E-35	7
Syt13	6.86E-40	-0.48705	0.019	0.134	1.14E-35	7
Sox11	8.66E-40	0.470626	0.24	0.106	1.44E-35	7
Timp3	2.41E-39	0.487917	0.187	0.071	4.01E-35	7
Smoc1	3.1E-39	-0.49746	0.025	0.141	5.18E-35	7
Nop56	6.89E-39	-0.49929	0.192	0.372	1.15E-34	7
Ndc80	7.04E-39	-0.4194	0.015	0.121	1.18E-34	7
Pgm2l1	7.15E-39	0.394071	0.215	0.089	1.19E-34	7
Rps18	1.3E-38	-0.47776	0.263	0.44	2.17E-34	7
Cdkn1b	1.38E-38	0.375683	0.6	0.458	2.3E-34	7
Mad2l1	1.44E-38	-0.40307	0.019	0.13	2.41E-34	7
Mcm4	1.83E-38	-0.44967	0.027	0.145	3.05E-34	7
Ier5	3.27E-38	-0.51257	0.233	0.405	5.45E-34	7
Nnat	6.01E-38	0.440246	0.801	0.703	1E-33	7
Rfc4	6.74E-38	-0.50575	0.085	0.236	1.12E-33	7
Eif5a	1.22E-37	-0.46138	0.237	0.414	2.03E-33	7
Anln	1.43E-37	-0.40923	0.011	0.11	2.39E-33	7
Zbtb18	1.54E-37	0.446075	0.414	0.256	2.57E-33	7
Cdh20	1.71E-37	-0.47792	0.068	0.211	2.86E-33	7

Mad2l2	1.8E-37	-0.49794	0.113	0.272	3E-33	7
Lyar	1.94E-37	-0.54629	0.13	0.296	3.23E-33	7
Tcf19	1.95E-37	-0.42542	0.009	0.104	3.26E-33	7
Ncam1	2.53E-37	0.405862	0.529	0.377	4.22E-33	7
Ect2	4.35E-37	-0.39042	0.01	0.104	7.26E-33	7
Snhg1	5.07E-37	-0.47894	0.201	0.368	8.45E-33	7
H2afz	5.13E-37	-0.4479	0.088	0.241	8.56E-33	7
Ttyh2	6.27E-37	0.413546	0.123	0.033	1.05E-32	7
2900011O(	7.22E-37	0.432017	0.222	0.095	1.21E-32	7
Csrp2	7.37E-37	-0.44656	0.041	0.165	1.23E-32	7
Tubb4a	7.7E-37	0.399959	0.169	0.06	1.28E-32	7
Eno1	7.81E-37	-0.47872	0.093	0.238	1.3E-32	7
Sept8	7.98E-37	-0.47185	0.025	0.136	1.33E-32	7
Angptl2	8.06E-37	-0.44503	0.018	0.122	1.34E-32	7
Ramp2	1.03E-36	-0.49953	0.019	0.126	1.72E-32	7
Ncaph	1.23E-36	-0.38859	0.021	0.127	2.05E-32	7
Fstl1	1.41E-36	-0.53612	0.055	0.188	2.35E-32	7
Zfp423	1.99E-36	0.478289	0.19	0.076	3.32E-32	7
Ptms	2.09E-36	0.358563	0.591	0.465	3.49E-32	7
Tspyl4	2.17E-36	0.434451	0.25	0.118	3.62E-32	7
Fam213b	2.81E-36	0.482458	0.294	0.148	4.69E-32	7
Mmp14	2.88E-36	-0.54047	0.052	0.182	4.81E-32	7
Ybx3	3.03E-36	-0.50396	0.138	0.3	5.05E-32	7
Kif4	4.2E-36	-0.3937	0.015	0.114	7E-32	7
C330027C(	5.78E-36	-0.44317	0.027	0.14	9.64E-32	7
Cyfip2	5.83E-36	0.401152	0.149	0.046	9.73E-32	7
Hmgb1	6.54E-36	-0.44366	0.197	0.369	1.09E-31	7
Slc7a5	8.34E-36	-0.45803	0.018	0.121	1.39E-31	7
Gm11541	1.08E-35	-0.47901	0.026	0.134	1.81E-31	7
Atp6v1e1	1.25E-35	0.396069	0.471	0.321	2.09E-31	7
Bola2	1.27E-35	-0.46034	0.238	0.408	2.12E-31	7
Pkm	1.33E-35	-0.45056	0.259	0.441	2.22E-31	7
Gm1673	1.39E-35	0.361506	0.616	0.463	2.32E-31	7
Grik2	1.46E-35	0.444723	0.219	0.093	2.44E-31	7
Hat1	1.54E-35	-0.44774	0.04	0.163	2.57E-31	7
Darc	1.94E-35	0.359751	0.114	0.029	3.24E-31	7
9330159F1	2.29E-35	0.434202	0.299	0.159	3.82E-31	7
Pdrg1	2.57E-35	0.428487	0.359	0.209	4.29E-31	7
Trp53inp2	2.7E-35	0.43432	0.145	0.046	4.51E-31	7
E130309F1	3.68E-35	0.35797	0.116	0.029	6.14E-31	7
Atp5e	3.81E-35	-0.37679	0.522	0.661	6.35E-31	7
6330403K(	4E-35	0.432755	0.387	0.232	6.68E-31	7
Cenpw	7.81E-35	-0.40059	0.025	0.134	1.3E-30	7
Rcor2	8.17E-35	0.434686	0.373	0.214	1.36E-30	7
1500016L(	8.42E-35	0.368981	0.525	0.373	1.4E-30	7
Reep2	9.9E-35	0.377615	0.162	0.061	1.65E-30	7
Baz2b	1.28E-34	0.42262	0.51	0.354	2.14E-30	7
Ntm	1.68E-34	-0.5633	0.038	0.152	2.81E-30	7

Naa50	1.79E-34	-0.46096	0.137	0.296	2.99E-30	7
Sgol1	1.85E-34	-0.44681	0.027	0.134	3.08E-30	7
Lingo1	3.09E-34	0.466074	0.206	0.087	5.15E-30	7
Hk2	3.24E-34	-0.47018	0.053	0.181	5.41E-30	7
Pgm2	3.4E-34	0.380394	0.111	0.028	5.67E-30	7
Rad51	3.52E-34	-0.40788	0.022	0.122	5.87E-30	7
Ctsd	3.97E-34	-0.64261	0.145	0.176	6.62E-30	7
2810025M	4.21E-34	-0.42994	0.05	0.175	7.02E-30	7
Ndufc2	4.23E-34	-0.39644	0.471	0.62	7.06E-30	7
Kif2c	4.43E-34	-0.39147	0.016	0.113	7.39E-30	7
Eef1d	4.64E-34	-0.45406	0.194	0.36	7.74E-30	7
Cdh4	5.78E-34	-0.46551	0.037	0.153	9.64E-30	7
Os9	6.53E-34	0.445819	0.389	0.241	1.09E-29	7
Selm	6.62E-34	-0.5232	0.055	0.182	1.1E-29	7
Cenpv	8.53E-34	-0.45263	0.176	0.339	1.42E-29	7
Celf3	9.25E-34	0.367816	0.121	0.035	1.54E-29	7
Nfasc	9.31E-34	0.341668	0.112	0.03	1.55E-29	7
Dnmt1	1.03E-33	-0.50359	0.13	0.287	1.72E-29	7
Dpysl4	1.17E-33	-0.45302	0.327	0.502	1.95E-29	7
Phf20l1	1.56E-33	0.403973	0.468	0.331	2.6E-29	7
Hnrnpab	1.68E-33	-0.29504	0.765	0.819	2.8E-29	7
Snrpd3	1.68E-33	-0.40226	0.371	0.546	2.81E-29	7
Gdpd1	1.77E-33	0.365885	0.376	0.234	2.95E-29	7
Klhl29	2.01E-33	0.384604	0.114	0.031	3.35E-29	7
Fmnl2	2.19E-33	0.460619	0.271	0.149	3.65E-29	7
Ephb1	2.51E-33	0.411272	0.123	0.035	4.19E-29	7
Lrpap1	2.85E-33	0.410757	0.223	0.109	4.75E-29	7
Pea15a	3.28E-33	0.36701	0.3	0.167	5.46E-29	7
Ssrp1	5.61E-33	-0.40192	0.417	0.587	9.36E-29	7
Cmtm3	6E-33	-0.39946	0.023	0.124	1E-28	7
Snrpa1	6.92E-33	-0.44735	0.182	0.34	1.15E-28	7
Fam111a	6.97E-33	-0.46018	0.026	0.132	1.16E-28	7
Mvd	7.02E-33	0.394735	0.152	0.054	1.17E-28	7
Scmh1	8.72E-33	0.405626	0.173	0.071	1.45E-28	7
Dhfr	9.45E-33	-0.46024	0.039	0.156	1.58E-28	7
Ccnd2	1.22E-32	-0.42119	0.56	0.668	2.04E-28	7
Psmc3ip	1.33E-32	-0.39979	0.031	0.138	2.23E-28	7
Kifap3	1.37E-32	0.386753	0.398	0.264	2.29E-28	7
Ldha	1.47E-32	-0.46505	0.099	0.241	2.45E-28	7
Rbm5	1.73E-32	0.359339	0.534	0.392	2.88E-28	7
Fam115a	1.75E-32	0.368596	0.465	0.342	2.92E-28	7
Bub1b	1.8E-32	-0.35811	0.016	0.108	3.01E-28	7
Rpl26	1.87E-32	-0.33377	0.617	0.707	3.13E-28	7
Srgap2	2.18E-32	0.365547	0.245	0.124	3.63E-28	7
Ezr	2.44E-32	-0.43797	0.288	0.448	4.07E-28	7
Atp6v0b	2.65E-32	0.396011	0.398	0.258	4.42E-28	7
Parp6	2.75E-32	0.422899	0.236	0.117	4.59E-28	7
D430041D	3.05E-32	-0.4267	0.345	0.502	5.09E-28	7

Plk4	4.16E-32	-0.38171	0.025	0.126	6.93E-28	7
Shf	4.34E-32	0.365016	0.131	0.04	7.24E-28	7
Shfm1	4.56E-32	-0.35276	0.558	0.694	7.61E-28	7
Pfn1	4.94E-32	-0.40571	0.361	0.521	8.24E-28	7
Dbf4	5.31E-32	-0.42777	0.04	0.152	8.86E-28	7
Srcin1	5.36E-32	0.364705	0.141	0.048	8.94E-28	7
Scrt1	5.7E-32	0.397643	0.152	0.052	9.51E-28	7
Sphkap	8.64E-32	0.360442	0.131	0.041	1.44E-27	7
Pnmal2	9.68E-32	0.454965	0.264	0.138	1.62E-27	7
Timeless	1.04E-31	-0.46127	0.045	0.161	1.74E-27	7
Rpa3	1.08E-31	-0.44685	0.108	0.253	1.8E-27	7
Celsr2	1.19E-31	0.390732	0.37	0.228	1.99E-27	7
Mycbp2	1.33E-31	0.416924	0.48	0.335	2.22E-27	7
Pik3r3	1.62E-31	0.437871	0.251	0.128	2.7E-27	7
Cdca7l	2.3E-31	-0.36728	0.017	0.11	3.84E-27	7
Nde1	2.75E-31	-0.38867	0.025	0.125	4.58E-27	7
Nek7	3.43E-31	-0.37847	0.024	0.123	5.73E-27	7
Ldhb	3.71E-31	0.403827	0.408	0.271	6.19E-27	7
Srsf7	4.94E-31	-0.40243	0.306	0.476	8.24E-27	7
Diap3	5.73E-31	-0.38116	0.019	0.112	9.55E-27	7
Ywhag	5.8E-31	0.37385	0.357	0.232	9.67E-27	7
Dlgap5	6.67E-31	-0.40332	0.021	0.118	1.11E-26	7
Rbbp7	8.56E-31	-0.37764	0.177	0.328	1.43E-26	7
Adk	1.58E-30	-0.40591	0.053	0.173	2.63E-26	7
Islr2	1.58E-30	-0.47127	0.063	0.187	2.64E-26	7
Pttg1	1.77E-30	-0.50763	0.039	0.146	2.95E-26	7
Snrpg	1.9E-30	-0.41643	0.187	0.345	3.17E-26	7
Idh2	2.15E-30	-0.42252	0.139	0.288	3.58E-26	7
1700001O	4.19E-30	-0.37213	0.021	0.116	6.98E-26	7
Pdzrn4	4.86E-30	-0.47014	0.042	0.153	8.1E-26	7
Gm11223	5.86E-30	0.528078	0.321	0.205	9.77E-26	7
Ubl3	5.89E-30	0.385786	0.321	0.201	9.83E-26	7
Cdt1	5.92E-30	-0.3997	0.03	0.131	9.88E-26	7
Ssbp3	6.98E-30	0.363365	0.284	0.159	1.16E-25	7
Nkd1	1.05E-29	-0.41999	0.119	0.257	1.75E-25	7
Ypel4	1.23E-29	0.339921	0.117	0.035	2.06E-25	7
Cerk	1.37E-29	-0.43515	0.046	0.158	2.28E-25	7
Tulp4	1.64E-29	0.414223	0.328	0.196	2.74E-25	7
Tacc2	2.17E-29	0.419202	0.259	0.132	3.62E-25	7
Sez6l2	2.41E-29	0.353831	0.11	0.032	4.02E-25	7
Syp	2.61E-29	0.347977	0.16	0.062	4.36E-25	7
Fam64a	2.66E-29	-0.37713	0.028	0.124	4.44E-25	7
Map1lc3a	3.38E-29	0.349072	0.391	0.268	5.63E-25	7
Nova2	3.42E-29	0.360977	0.144	0.052	5.71E-25	7
Gnl3	3.5E-29	-0.41379	0.158	0.305	5.83E-25	7
Ccm2	3.82E-29	-0.48143	0.102	0.233	6.37E-25	7
Rpl23	4.97E-29	-0.38576	0.404	0.547	8.29E-25	7
Idh1	5.28E-29	0.410732	0.267	0.148	8.81E-25	7

Pcsk1n	6.11E-29	0.326426	0.18	0.077	1.02E-24	7
Rps7	6.69E-29	-0.40352	0.259	0.409	1.12E-24	7
Trim59	6.99E-29	-0.4255	0.06	0.177	1.17E-24	7
Csrnp3	8.42E-29	0.411483	0.18	0.076	1.4E-24	7
Ctnna2	8.89E-29	0.374516	0.175	0.08	1.48E-24	7
Wbp5	9.72E-29	-0.36824	0.376	0.527	1.62E-24	7
Cpne3	1.21E-28	-0.42074	0.079	0.205	2.01E-24	7
Atat1	1.23E-28	0.397815	0.207	0.096	2.05E-24	7
Tpm4	1.41E-28	-0.42318	0.18	0.326	2.36E-24	7
Ppp1r14b	1.41E-28	-0.41144	0.253	0.406	2.36E-24	7
Pdia6	1.41E-28	-0.39476	0.201	0.349	2.36E-24	7
Arpp21	2.02E-28	0.330077	0.215	0.105	3.36E-24	7
Clybl	3.13E-28	0.42486	0.22	0.109	5.21E-24	7
Sh3kbp1	3.65E-28	0.417109	0.194	0.09	6.08E-24	7
Snrpd1	4.39E-28	-0.37958	0.388	0.533	7.33E-24	7
Dixdc1	4.57E-28	0.38448	0.347	0.227	7.63E-24	7
Nt5dc2	4.75E-28	0.393137	0.35	0.229	7.92E-24	7
Kcnj3	6.57E-28	0.335341	0.154	0.063	1.1E-23	7
Sbk1	6.96E-28	0.395832	0.227	0.111	1.16E-23	7
Nicn1	7.42E-28	0.384921	0.329	0.199	1.24E-23	7
Gm20033	7.78E-28	0.389477	0.125	0.04	1.3E-23	7
Exosc7	8.5E-28	-0.34717	0.07	0.187	1.42E-23	7
Eid1	8.78E-28	0.304573	0.62	0.494	1.46E-23	7
Cst3	9.91E-28	-0.39152	0.508	0.537	1.65E-23	7
Map1lc3b	1.09E-27	0.345417	0.452	0.322	1.82E-23	7
Rnaseh2b	1.35E-27	-0.37839	0.116	0.245	2.25E-23	7
Ndrg2	1.37E-27	-0.45833	0.073	0.192	2.29E-23	7
Fkbp2	1.41E-27	-0.39161	0.189	0.335	2.35E-23	7
Nolc1	1.84E-27	-0.4114	0.263	0.418	3.07E-23	7
Rnaseh2c	1.93E-27	-0.39934	0.24	0.39	3.23E-23	7
Rpl18a	2E-27	-0.39757	0.257	0.41	3.34E-23	7
Aurka	2.15E-27	-0.33603	0.018	0.104	3.59E-23	7
Hspe1	2.16E-27	-0.39181	0.201	0.35	3.6E-23	7
Ephb2	2.25E-27	0.41757	0.174	0.074	3.75E-23	7
Rltpr	2.34E-27	0.325674	0.109	0.034	3.91E-23	7
Nudcd2	2.94E-27	-0.39698	0.101	0.232	4.91E-23	7
Gar1	3.38E-27	-0.35746	0.093	0.216	5.63E-23	7
Akap9	3.41E-27	0.337523	0.599	0.493	5.68E-23	7
Trp53	4.04E-27	-0.38228	0.164	0.307	6.74E-23	7
Spop	4.91E-27	-0.42692	0.18	0.324	8.19E-23	7
Hivep2	5.09E-27	0.335928	0.131	0.049	8.49E-23	7
Ubash3b	5.79E-27	0.39697	0.171	0.071	9.66E-23	7
Cct3	5.96E-27	-0.36349	0.351	0.504	9.95E-23	7
Pbdc1	6.18E-27	-0.38637	0.089	0.214	1.03E-22	7
Srsf3	7.09E-27	-0.3079	0.569	0.662	1.18E-22	7
Pcdha2	7.26E-27	0.403493	0.222	0.111	1.21E-22	7
Atxn10	7.91E-27	0.289905	0.464	0.363	1.32E-22	7
Lpin2	8.32E-27	-0.42202	0.076	0.187	1.39E-22	7

Irs1	1.05E-26	-0.36668	0.032	0.124	1.75E-22	7
Nova1	1.09E-26	0.374925	0.165	0.076	1.82E-22	7
Brsk2	1.22E-26	0.413429	0.18	0.081	2.03E-22	7
Ccl27a	1.27E-26	0.349701	0.116	0.038	2.12E-22	7
Clic4	1.28E-26	-0.3558	0.108	0.235	2.13E-22	7
Ndufa12	1.61E-26	-0.37733	0.286	0.43	2.69E-22	7
Nup62	1.69E-26	-0.3555	0.067	0.178	2.81E-22	7
Ube2d1	1.74E-26	0.348578	0.331	0.22	2.9E-22	7
Pcbp1	1.75E-26	-0.34744	0.475	0.601	2.92E-22	7
Srrm3	1.84E-26	0.378875	0.37	0.231	3.07E-22	7
Snrpd2	1.9E-26	-0.36231	0.313	0.449	3.17E-22	7
Cog7	2E-26	0.298379	0.71	0.575	3.33E-22	7
Slc22a17	2.14E-26	0.363575	0.37	0.243	3.56E-22	7
Mapk8ip2	2.23E-26	0.379241	0.24	0.136	3.73E-22	7
Sema7a	2.55E-26	-0.40318	0.054	0.162	4.25E-22	7
Gnaq	2.81E-26	0.358604	0.399	0.276	4.69E-22	7
Naa38	2.92E-26	-0.3818	0.182	0.32	4.88E-22	7
Nktr	2.95E-26	0.339622	0.489	0.372	4.91E-22	7
Cdk6	3.64E-26	-0.40331	0.123	0.251	6.06E-22	7
Usp22	4.59E-26	0.289863	0.451	0.354	7.66E-22	7
Ttc28	4.97E-26	0.419775	0.222	0.116	8.29E-22	7
Alyref	5.88E-26	-0.39538	0.088	0.207	9.8E-22	7
Dmxl2	6.92E-26	0.371036	0.161	0.068	1.15E-21	7
Cplx2	7.37E-26	-0.37031	0.353	0.505	1.23E-21	7
Srsf2	9.24E-26	-0.35386	0.441	0.574	1.54E-21	7
Bex1	9.7E-26	-0.4076	0.105	0.232	1.62E-21	7
Ppic	1.02E-25	-0.40958	0.123	0.247	1.7E-21	7
Chst15	1.64E-25	0.345906	0.165	0.072	2.74E-21	7
Rsl1d1	1.65E-25	-0.35564	0.351	0.499	2.75E-21	7
Efr3b	1.8E-25	0.357433	0.125	0.044	3E-21	7
Hmgn3	2.16E-25	-0.37356	0.142	0.278	3.6E-21	7
Myc	2.29E-25	-0.36638	0.022	0.105	3.82E-21	7
Fam110a	2.66E-25	0.335213	0.137	0.061	4.44E-21	7
Gramd1a	3.22E-25	0.354006	0.275	0.157	5.38E-21	7
Gramd1b	3.39E-25	0.346637	0.268	0.162	5.66E-21	7
Tnrc6c	3.56E-25	0.358595	0.281	0.171	5.95E-21	7
Tmeff1	3.61E-25	0.38586	0.254	0.142	6.01E-21	7
Pik3r2	4.23E-25	0.346252	0.166	0.076	7.05E-21	7
Eny2	4.26E-25	-0.39484	0.25	0.396	7.11E-21	7
Topbp1	4.42E-25	-0.35158	0.05	0.151	7.38E-21	7
Sri	4.63E-25	-0.38682	0.067	0.172	7.72E-21	7
Cntln	6.11E-25	-0.43338	0.067	0.179	1.02E-20	7
Lzts1	6.23E-25	0.328606	0.109	0.036	1.04E-20	7
Ncor2	6.35E-25	-0.3918	0.066	0.175	1.06E-20	7
Tmem107	7.34E-25	-0.35965	0.038	0.132	1.22E-20	7
Rrs1	7.7E-25	-0.37398	0.08	0.196	1.29E-20	7
Rps4x	7.74E-25	-0.40074	0.168	0.304	1.29E-20	7
Paxbp1	8.13E-25	0.369203	0.356	0.252	1.36E-20	7

Snrnp40	8.33E-25	-0.32524	0.15	0.279	1.39E-20	7
Vrk1	8.88E-25	-0.36721	0.055	0.154	1.48E-20	7
Dcps	9.39E-25	-0.32375	0.027	0.114	1.57E-20	7
Rnaseh2a	9.62E-25	-0.36016	0.057	0.159	1.6E-20	7
Mrpl42	1.04E-24	-0.3319	0.185	0.327	1.73E-20	7
Larp7	1.05E-24	-0.38623	0.181	0.32	1.75E-20	7
Supt16	1.09E-24	-0.38682	0.365	0.502	1.82E-20	7
Ccp110	1.27E-24	-0.37542	0.131	0.256	2.12E-20	7
Cask	1.29E-24	0.392271	0.198	0.103	2.15E-20	7
Sae1	1.3E-24	-0.35523	0.155	0.287	2.17E-20	7
Nedd4l	1.47E-24	0.365183	0.224	0.121	2.45E-20	7
Ptch2	1.57E-24	-0.38939	0.057	0.158	2.62E-20	7
Peli2	1.63E-24	0.35696	0.264	0.157	2.72E-20	7
Vegfb	1.72E-24	-0.31312	0.034	0.124	2.87E-20	7
Asxl3	1.74E-24	0.36565	0.141	0.058	2.9E-20	7
Ap1s2	1.76E-24	0.389121	0.255	0.153	2.93E-20	7
Fdft1	1.82E-24	0.360518	0.173	0.095	3.04E-20	7
Tshz2	2.2E-24	-0.4145	0.186	0.328	3.68E-20	7
Rusc1	2.72E-24	0.29547	0.13	0.05	4.53E-20	7
Smim11	3.03E-24	-0.38682	0.153	0.284	5.05E-20	7
Rtn3	3.27E-24	0.296043	0.565	0.47	5.45E-20	7
Cbfb	3.46E-24	-0.33802	0.083	0.19	5.76E-20	7
Skp2	3.52E-24	-0.34249	0.036	0.125	5.87E-20	7
Scrn1	3.52E-24	0.30702	0.123	0.044	5.88E-20	7
Nol7	4.09E-24	-0.31781	0.438	0.578	6.83E-20	7
Rad21	4.2E-24	-0.38467	0.356	0.468	7.01E-20	7
Eif3i	4.28E-24	-0.35697	0.328	0.471	7.15E-20	7
Fen1	4.98E-24	-0.3387	0.049	0.148	8.3E-20	7
Sstr2	5.02E-24	0.401743	0.303	0.183	8.37E-20	7
Mroh2a	7.03E-24	-0.47307	0.036	0.119	1.17E-19	7
Ing4	7.74E-24	0.314171	0.421	0.315	1.29E-19	7
Uqcrq	8.49E-24	-0.32311	0.419	0.559	1.42E-19	7
RP23-32A8	8.53E-24	0.357839	0.291	0.179	1.42E-19	7
Rpl7	8.72E-24	-0.32921	0.372	0.518	1.45E-19	7
Pkia	9.37E-24	0.352773	0.238	0.126	1.56E-19	7
Mphosph1(	9.54E-24	-0.36706	0.119	0.239	1.59E-19	7
Trp53i11	1.03E-23	0.352585	0.299	0.194	1.72E-19	7
Rfc5	1.07E-23	-0.31354	0.028	0.113	1.79E-19	7
C1qbp	1.13E-23	-0.37073	0.244	0.386	1.89E-19	7
Cnih4	1.13E-23	-0.33297	0.122	0.242	1.89E-19	7
Lmnb1	1.16E-23	-0.36167	0.183	0.308	1.94E-19	7
Baz1a	1.25E-23	-0.40975	0.066	0.173	2.08E-19	7
G3bp1	1.39E-23	-0.3596	0.194	0.337	2.33E-19	7
Hmgcs1	1.43E-23	0.412153	0.241	0.145	2.39E-19	7
Ndufa2	1.48E-23	-0.32409	0.491	0.61	2.47E-19	7
Clasp2	1.72E-23	0.357489	0.264	0.16	2.87E-19	7
Atl1	1.83E-23	0.367011	0.193	0.096	3.05E-19	7
Gm10260	1.99E-23	-0.37502	0.118	0.235	3.33E-19	7

Ppp2r2c	2.01E-23	0.278388	0.618	0.497	3.36E-19	7
Rabep1	2.11E-23	0.348077	0.336	0.228	3.52E-19	7
Strbp	2.21E-23	0.322971	0.445	0.34	3.68E-19	7
Sod1	2.52E-23	-0.36194	0.31	0.45	4.21E-19	7
Spag9	2.69E-23	0.284682	0.41	0.299	4.49E-19	7
Sox18	2.82E-23	-0.4002	0.054	0.153	4.71E-19	7
Prkd3	2.95E-23	-0.31418	0.052	0.148	4.91E-19	7
Cplx1	3.05E-23	0.33826	0.235	0.131	5.08E-19	7
Id2	3.85E-23	0.341482	0.581	0.479	6.42E-19	7
March1	3.88E-23	0.346219	0.107	0.039	6.47E-19	7
Dpm3	4.19E-23	-0.34481	0.158	0.277	6.98E-19	7
Alcam	4.43E-23	-0.36512	0.046	0.14	7.39E-19	7
Naca	4.59E-23	-0.34745	0.332	0.474	7.65E-19	7
Mex3a	4.73E-23	0.294955	0.561	0.456	7.89E-19	7
Zic2	4.87E-23	0.345867	0.212	0.114	8.13E-19	7
Slco5a1	5.24E-23	0.379732	0.134	0.055	8.74E-19	7
Vim	6.6E-23	-0.45496	0.191	0.31	1.1E-18	7
Cnrip1	6.68E-23	0.307668	0.301	0.19	1.11E-18	7
Gm11266	6.73E-23	0.397062	0.245	0.137	1.12E-18	7
Rpl18	7.3E-23	-0.35263	0.18	0.313	1.22E-18	7
Gm9800	7.68E-23	-0.32669	0.405	0.543	1.28E-18	7
Rif1	8.58E-23	-0.38568	0.152	0.284	1.43E-18	7
Atp5b	9E-23	-0.26824	0.639	0.717	1.5E-18	7
Rb1cc1	9.06E-23	0.353418	0.333	0.227	1.51E-18	7
Etfb	9.65E-23	-0.3857	0.086	0.191	1.61E-18	7
RP23-199B	9.96E-23	0.332709	0.139	0.057	1.66E-18	7
Ska2	1.1E-22	-0.34097	0.103	0.211	1.83E-18	7
Etfa	1.21E-22	-0.37345	0.138	0.261	2.02E-18	7
Meis1	1.26E-22	0.285571	0.478	0.365	2.1E-18	7
Mak16	1.28E-22	-0.31687	0.12	0.232	2.14E-18	7
Shd	1.47E-22	0.325272	0.201	0.103	2.45E-18	7
Klf9	1.49E-22	0.348586	0.367	0.264	2.48E-18	7
Rps16	1.62E-22	-0.3342	0.104	0.216	2.71E-18	7
Reln	1.66E-22	0.390619	0.291	0.172	2.77E-18	7
Vgll4	2.05E-22	-0.33279	0.063	0.159	3.42E-18	7
Pak1	2.07E-22	0.282603	0.13	0.054	3.45E-18	7
Clk1	2.14E-22	0.302487	0.433	0.333	3.57E-18	7
Mical1	2.23E-22	-0.36016	0.042	0.131	3.72E-18	7
Mybbp1a	2.3E-22	-0.36253	0.133	0.257	3.84E-18	7
Ift27	2.75E-22	-0.33432	0.136	0.25	4.59E-18	7
Stx7	2.97E-22	0.332176	0.197	0.11	4.96E-18	7
Mthfd1	3.05E-22	-0.31597	0.035	0.121	5.08E-18	7
Park7	5.59E-22	-0.32593	0.418	0.554	9.33E-18	7
Mbd3	5.64E-22	-0.30893	0.168	0.299	9.41E-18	7
Npepps	5.85E-22	0.27201	0.412	0.311	9.76E-18	7
Sdc3	7.08E-22	0.298459	0.153	0.072	1.18E-17	7
Hspd1	7.15E-22	-0.33825	0.249	0.372	1.19E-17	7
Acd	7.35E-22	0.363257	0.252	0.155	1.23E-17	7

4930402H:	7.82E-22	0.383837	0.201	0.107	1.3E-17	7
Mpdz	8.43E-22	-0.34117	0.048	0.141	1.41E-17	7
Ninj1	1.29E-21	-0.3214	0.025	0.102	2.14E-17	7
Tcp1	1.38E-21	-0.3359	0.337	0.471	2.3E-17	7
Asrgl1	1.55E-21	-0.35422	0.049	0.139	2.58E-17	7
Gabbr1	1.78E-21	0.347276	0.242	0.133	2.97E-17	7
Exosc8	1.96E-21	-0.36798	0.105	0.217	3.27E-17	7
Dpysl2	1.98E-21	0.315729	0.349	0.242	3.3E-17	7
Riok3	2.07E-21	0.357124	0.221	0.124	3.46E-17	7
Cd81	2.14E-21	-0.32847	0.353	0.469	3.57E-17	7
Cdkn2c	2.25E-21	-0.291	0.061	0.148	3.75E-17	7
Ddx21	2.31E-21	-0.36236	0.198	0.33	3.85E-17	7
Mat2a	2.42E-21	-0.3099	0.285	0.428	4.03E-17	7
Scn8a	2.49E-21	0.340237	0.233	0.13	4.15E-17	7
Synj1	2.69E-21	0.368143	0.154	0.074	4.49E-17	7
Bicd1	2.99E-21	0.379129	0.243	0.146	4.99E-17	7
Ctxn1	3.05E-21	0.321972	0.254	0.164	5.09E-17	7
Dhx32	3.18E-21	-0.3669	0.099	0.207	5.3E-17	7
Bzrap1	3.65E-21	0.304838	0.131	0.056	6.09E-17	7
Prmt2	3.93E-21	0.354876	0.179	0.088	6.56E-17	7
Nxt1	4.08E-21	-0.29141	0.041	0.127	6.8E-17	7
Ncaph2	4.1E-21	-0.32761	0.079	0.18	6.84E-17	7
Brca2	4.2E-21	-0.32849	0.035	0.118	7.01E-17	7
Rfc1	4.22E-21	-0.39956	0.209	0.338	7.03E-17	7
Eif4e3	4.31E-21	0.301973	0.21	0.125	7.19E-17	7
Fabp7	4.32E-21	-0.93865	0.075	0.126	7.21E-17	7
Zfpm2	5.32E-21	0.283189	0.105	0.038	8.88E-17	7
Ssb	5.58E-21	-0.2757	0.612	0.709	9.31E-17	7
Ipo5	5.61E-21	-0.29853	0.054	0.144	9.35E-17	7
Sh3bp5	5.81E-21	0.348448	0.191	0.097	9.69E-17	7
Arpc5	6.39E-21	0.267855	0.404	0.315	1.07E-16	7
Hnrnpm	6.95E-21	-0.26325	0.647	0.741	1.16E-16	7
Pfn2	7.64E-21	0.267889	0.329	0.235	1.27E-16	7
Ankrd13a	7.68E-21	0.319447	0.141	0.06	1.28E-16	7
Cadps	7.94E-21	0.357183	0.149	0.067	1.32E-16	7
Tub	9.86E-21	0.271467	0.102	0.039	1.64E-16	7
Arid3a	1.05E-20	0.330543	0.176	0.09	1.76E-16	7
Scg3	1.08E-20	0.302058	0.545	0.439	1.8E-16	7
Kcnq2	1.1E-20	0.294315	0.147	0.067	1.84E-16	7
Gng12	1.19E-20	-0.29834	0.041	0.126	1.98E-16	7
Etf1	1.23E-20	-0.30241	0.156	0.268	2.06E-16	7
Tcf3	1.24E-20	-0.32099	0.131	0.232	2.07E-16	7
Rpl37	1.26E-20	-0.27066	0.422	0.509	2.1E-16	7
Naa10	1.36E-20	-0.32658	0.144	0.256	2.27E-16	7
1810009A:	1.38E-20	-0.344	0.148	0.267	2.3E-16	7
Gm10076	1.42E-20	-0.2676	0.053	0.139	2.37E-16	7
Tgif1	1.65E-20	-0.29363	0.027	0.104	2.76E-16	7
Celf5	1.73E-20	0.345234	0.125	0.051	2.89E-16	7

Atcay	2.42E-20	0.313992	0.138	0.06	4.03E-16	7
Hnrnpd	2.6E-20	-0.2993	0.518	0.616	4.35E-16	7
Bdh1	2.76E-20	0.303756	0.144	0.066	4.6E-16	7
Srsf1	4.32E-20	-0.28949	0.202	0.302	7.21E-16	7
Kdm1a	4.41E-20	0.294918	0.467	0.357	7.35E-16	7
Atad5	4.49E-20	-0.33519	0.062	0.154	7.5E-16	7
Bccip	4.78E-20	-0.32449	0.191	0.318	7.97E-16	7
Ssr1	5.16E-20	-0.30039	0.18	0.296	8.6E-16	7
Itgb1	5.39E-20	-0.30058	0.238	0.371	8.99E-16	7
Vars	5.57E-20	-0.33118	0.074	0.172	9.29E-16	7
Rnf165	6.04E-20	0.322494	0.272	0.165	1.01E-15	7
Hip1r	6.12E-20	0.311086	0.228	0.126	1.02E-15	7
Ddx39	6.29E-20	-0.32929	0.091	0.193	1.05E-15	7
Alg2	6.41E-20	0.301713	0.173	0.094	1.07E-15	7
Mpp6	7.16E-20	-0.28582	0.074	0.167	1.19E-15	7
Cep120	8.02E-20	0.280031	0.13	0.064	1.34E-15	7
Nefm	8.11E-20	0.41262	0.196	0.11	1.35E-15	7
Mrpl52	8.54E-20	-0.27839	0.31	0.44	1.42E-15	7
Rpl38	8.85E-20	-0.3131	0.26	0.391	1.48E-15	7
Lsm2	9.38E-20	-0.28584	0.129	0.237	1.56E-15	7
Pafah1b1	9.5E-20	0.281358	0.541	0.469	1.58E-15	7
Gart	1E-19	-0.32348	0.075	0.172	1.67E-15	7
Sfxn1	1.05E-19	-0.36041	0.145	0.257	1.75E-15	7
Cdk4	1.12E-19	-0.27052	0.57	0.646	1.87E-15	7
Mtmr4	1.14E-19	0.311432	0.111	0.051	1.91E-15	7
Rps8	1.15E-19	-0.32052	0.282	0.404	1.92E-15	7
Mycn	1.18E-19	-0.35731	0.257	0.371	1.96E-15	7
Srm	1.2E-19	-0.32849	0.103	0.197	2E-15	7
Pdap1	1.29E-19	-0.27117	0.588	0.689	2.15E-15	7
Dnmt3a	1.4E-19	-0.31202	0.102	0.21	2.33E-15	7
Stx4a	1.51E-19	-0.31904	0.069	0.16	2.52E-15	7
Rwdd3	1.51E-19	0.335184	0.15	0.071	2.52E-15	7
Trafd1	1.62E-19	0.291662	0.2	0.112	2.71E-15	7
Dctn2	1.82E-19	0.297702	0.413	0.314	3.03E-15	7
Eif3a	1.83E-19	-0.28538	0.594	0.695	3.05E-15	7
Rpl14-ps1	1.88E-19	-0.3288	0.172	0.291	3.13E-15	7
Rpl34	1.95E-19	-0.25618	0.565	0.658	3.25E-15	7
Pkp4	1.99E-19	-0.31821	0.031	0.107	3.31E-15	7
Ssr3	2.06E-19	-0.31074	0.311	0.432	3.44E-15	7
Mapk10	2.12E-19	0.266493	0.172	0.093	3.53E-15	7
Rai1	2.15E-19	-0.3217	0.071	0.165	3.58E-15	7
Nme2	2.39E-19	-0.29541	0.031	0.106	3.98E-15	7
Ifitm2	2.75E-19	-0.32471	0.039	0.119	4.58E-15	7
H2afy2	3.29E-19	0.299399	0.426	0.331	5.49E-15	7
Lrrtm2	3.45E-19	0.34313	0.117	0.046	5.75E-15	7
Fundc2	3.83E-19	-0.2971	0.165	0.277	6.39E-15	7
Gm11478	3.91E-19	-0.33477	0.116	0.221	6.52E-15	7
Thsd7a	4.57E-19	0.374501	0.208	0.121	7.63E-15	7

Daam1	4.81E-19	0.315968	0.264	0.178	8.03E-15	7
Mrpl33	5.84E-19	-0.30827	0.177	0.289	9.75E-15	7
Rcn1	6.24E-19	-0.2842	0.13	0.24	1.04E-14	7
Ccdc18	6.52E-19	-0.29337	0.041	0.12	1.09E-14	7
Ppa1	6.58E-19	-0.29606	0.128	0.227	1.1E-14	7
Rpl3	7.58E-19	-0.34052	0.114	0.218	1.26E-14	7
Ptbp1	7.78E-19	-0.26521	0.032	0.105	1.3E-14	7
Abracl	8.32E-19	-0.34215	0.194	0.309	1.39E-14	7
Rpl29	8.55E-19	-0.28591	0.158	0.264	1.43E-14	7
Scaper	8.62E-19	0.337007	0.184	0.105	1.44E-14	7
Clic1	9.29E-19	0.313639	0.251	0.167	1.55E-14	7
Add2	9.64E-19	0.275766	0.129	0.063	1.61E-14	7
Lmnb2	9.87E-19	-0.29174	0.071	0.164	1.65E-14	7
Fxyd6	9.96E-19	0.264784	0.595	0.502	1.66E-14	7
Jmjd1c	1.12E-18	0.304342	0.265	0.18	1.86E-14	7
Sdha	1.24E-18	-0.29166	0.141	0.248	2.07E-14	7
Ctnnb1	1.24E-18	-0.32156	0.267	0.394	2.08E-14	7
Cdk5rap2	1.54E-18	-0.30349	0.091	0.189	2.57E-14	7
Onecut2	1.55E-18	0.330945	0.116	0.046	2.59E-14	7
Tmem63b	1.58E-18	0.325621	0.154	0.075	2.63E-14	7
Uba52	1.6E-18	-0.30148	0.203	0.324	2.67E-14	7
2810004N	1.7E-18	-0.34399	0.152	0.269	2.83E-14	7
Sema6c	1.71E-18	0.272147	0.125	0.056	2.85E-14	7
Cyth2	1.81E-18	0.278154	0.358	0.267	3.02E-14	7
Snx5	1.97E-18	-0.27733	0.076	0.169	3.28E-14	7
Rpl6	1.97E-18	-0.3253	0.181	0.298	3.29E-14	7
Smarcd2	2.27E-18	0.341048	0.18	0.091	3.79E-14	7
Brd7	2.6E-18	-0.33381	0.196	0.315	4.34E-14	7
Exosc1	2.6E-18	-0.32473	0.079	0.168	4.34E-14	7
Nin	2.8E-18	-0.28533	0.064	0.151	4.67E-14	7
Klf13	3.15E-18	-0.30565	0.14	0.243	5.25E-14	7
Arhgef7	3.17E-18	0.323437	0.236	0.143	5.29E-14	7
Cep110	3.88E-18	-0.35926	0.076	0.169	6.47E-14	7
Set	3.98E-18	-0.30471	0.277	0.394	6.63E-14	7
Tpt1	3.99E-18	-0.30257	0.158	0.263	6.66E-14	7
Sptan1	4.06E-18	0.279923	0.257	0.163	6.77E-14	7
Dars	4.16E-18	-0.28001	0.074	0.163	6.94E-14	7
Asap1	4.9E-18	-0.30999	0.095	0.185	8.18E-14	7
Grb2	4.98E-18	0.283006	0.327	0.243	8.31E-14	7
Pola1	5.17E-18	-0.2585	0.039	0.107	8.63E-14	7
Hpcal1	5.22E-18	0.32334	0.187	0.101	8.71E-14	7
Hcfc1r1	5.46E-18	0.313801	0.277	0.187	9.11E-14	7
Pygo1	5.54E-18	0.325486	0.285	0.185	9.25E-14	7
Gabarapl1	5.6E-18	0.29803	0.309	0.209	9.34E-14	7
Smc6	5.78E-18	-0.33645	0.306	0.43	9.64E-14	7
Nol4	5.87E-18	0.274543	0.257	0.165	9.78E-14	7
Cep57	5.9E-18	-0.28122	0.155	0.264	9.85E-14	7
Cox7b	6.31E-18	-0.28995	0.433	0.556	1.05E-13	7

Ccdc41	6.48E-18	-0.33422	0.151	0.26	1.08E-13	7
Sox5	6.6E-18	0.332838	0.196	0.114	1.1E-13	7
BC029214	6.89E-18	0.312958	0.171	0.087	1.15E-13	7
Mrpl17	6.9E-18	-0.27327	0.165	0.266	1.15E-13	7
Rfc2	7.62E-18	-0.31116	0.118	0.217	1.27E-13	7
Enox2	7.63E-18	0.345176	0.118	0.052	1.27E-13	7
Atp6v0a1	7.96E-18	0.337228	0.171	0.091	1.33E-13	7
Nrm	8.74E-18	-0.31274	0.081	0.175	1.46E-13	7
Rbm8a	9.13E-18	-0.2931	0.363	0.472	1.52E-13	7
Ppil1	9.33E-18	-0.26342	0.049	0.128	1.56E-13	7
Dst	9.58E-18	0.317212	0.358	0.26	1.6E-13	7
Rrp15	9.58E-18	-0.28675	0.085	0.18	1.6E-13	7
Wsb1	1E-17	0.303573	0.334	0.24	1.67E-13	7
Kcnc1	1.1E-17	0.316716	0.167	0.084	1.84E-13	7
Mrpl13	1.21E-17	-0.31907	0.197	0.315	2.02E-13	7
Rbm3	1.24E-17	-0.27786	0.05	0.128	2.07E-13	7
Sv2b	1.3E-17	0.317471	0.18	0.097	2.17E-13	7
Cited2	1.32E-17	0.354734	0.232	0.146	2.2E-13	7
Lsm3	1.34E-17	-0.27243	0.243	0.366	2.24E-13	7
Pold3	1.35E-17	-0.28436	0.069	0.154	2.25E-13	7
Gjc1	1.35E-17	-0.31331	0.105	0.202	2.25E-13	7
Atp5d	1.37E-17	-0.25052	0.464	0.593	2.29E-13	7
Evl	1.37E-17	0.301444	0.265	0.172	2.29E-13	7
Polr2i	1.46E-17	-0.29854	0.219	0.323	2.44E-13	7
Rpl30	1.5E-17	-0.26547	0.17	0.275	2.5E-13	7
Nr3c1	1.51E-17	-0.30967	0.049	0.13	2.52E-13	7
Snrpe	1.54E-17	-0.26518	0.468	0.569	2.56E-13	7
Tspan7	1.74E-17	-0.36173	0.046	0.109	2.91E-13	7
Epc1	1.76E-17	0.31306	0.305	0.227	2.94E-13	7
Glo1	1.77E-17	-0.2836	0.04	0.112	2.95E-13	7
St7	1.8E-17	0.294563	0.159	0.085	3.01E-13	7
Aig1	1.92E-17	0.328902	0.17	0.091	3.2E-13	7
Srsf10	2.01E-17	-0.28033	0.226	0.337	3.36E-13	7
Laptm4b	2.02E-17	-0.2522	0.067	0.146	3.37E-13	7
Sf3b5	2.24E-17	-0.28001	0.212	0.335	3.74E-13	7
Arl8a	2.25E-17	0.306741	0.171	0.091	3.76E-13	7
Mrfap1	2.28E-17	-0.28171	0.286	0.411	3.8E-13	7
Dtx1	2.45E-17	-0.32867	0.08	0.171	4.08E-13	7
Tram1	2.55E-17	-0.26445	0.053	0.133	4.25E-13	7
Egfr	2.73E-17	0.318929	0.149	0.079	4.55E-13	7
Pold2	2.81E-17	-0.26946	0.044	0.118	4.69E-13	7
Zcwpw1	3.02E-17	-0.26056	0.036	0.107	5.03E-13	7
Ak2	3.56E-17	-0.28869	0.119	0.219	5.93E-13	7
Mycl	3.6E-17	-0.27832	0.06	0.142	6.01E-13	7
Celsr3	3.97E-17	0.291383	0.195	0.118	6.62E-13	7
Meis3	4.06E-17	0.302481	0.15	0.072	6.78E-13	7
Epb4.1	4.21E-17	0.305669	0.307	0.223	7.02E-13	7
Ankra2	4.45E-17	0.291079	0.187	0.115	7.42E-13	7

Uqcr10	4.78E-17	-0.27606	0.317	0.441	7.98E-13	7
Rpl37a	5.77E-17	-0.26701	0.363	0.475	9.63E-13	7
Sep15	5.81E-17	-0.25408	0.412	0.511	9.7E-13	7
1500011B(	6.03E-17	0.341082	0.187	0.101	1.01E-12	7
Ssbp2	6.33E-17	-0.25612	0.052	0.131	1.06E-12	7
Maged2	6.59E-17	0.269255	0.247	0.161	1.1E-12	7
Prnp	6.67E-17	0.255155	0.292	0.2	1.11E-12	7
Zfp292	6.7E-17	0.293938	0.434	0.335	1.12E-12	7
Nop16	7.16E-17	-0.27448	0.092	0.179	1.19E-12	7
Sec11c	7.55E-17	-0.34287	0.116	0.219	1.26E-12	7
Lman1	8.11E-17	-0.30922	0.216	0.332	1.35E-12	7
Pard6a	8.54E-17	0.262175	0.162	0.087	1.42E-12	7
Timm13	8.64E-17	-0.27232	0.347	0.461	1.44E-12	7
Kif3c	8.7E-17	0.265688	0.123	0.059	1.45E-12	7
Sqle	9.1E-17	0.296447	0.181	0.116	1.52E-12	7
Taf1d	9.81E-17	-0.28838	0.23	0.349	1.64E-12	7
Hprt	1.04E-16	-0.26576	0.103	0.193	1.74E-12	7
Nacad	1.09E-16	0.301788	0.105	0.042	1.82E-12	7
Brix1	1.11E-16	-0.27065	0.116	0.211	1.85E-12	7
Rbbp8	1.16E-16	-0.25813	0.052	0.127	1.94E-12	7
Gsto1	1.19E-16	-0.31189	0.055	0.133	1.98E-12	7
A430005L1	1.22E-16	-0.26407	0.045	0.12	2.03E-12	7
Slc7a1	1.23E-16	-0.2813	0.033	0.103	2.05E-12	7
Flot1	1.31E-16	0.281596	0.166	0.093	2.18E-12	7
Peli1	1.31E-16	0.305322	0.28	0.196	2.19E-12	7
Rnf122	1.32E-16	0.319529	0.17	0.097	2.19E-12	7
Apex1	1.37E-16	-0.28677	0.348	0.458	2.28E-12	7
Nfyb	1.44E-16	0.288677	0.313	0.229	2.4E-12	7
1110038B1	1.49E-16	-0.29152	0.198	0.305	2.49E-12	7
Gm8292	1.59E-16	-0.25391	0.281	0.375	2.65E-12	7
St8sia3	1.65E-16	0.276095	0.339	0.244	2.76E-12	7
Rnf168	1.79E-16	-0.29781	0.118	0.213	2.98E-12	7
H1fx	1.8E-16	-0.32894	0.158	0.251	2.99E-12	7
Arpp19	1.82E-16	-0.27426	0.264	0.385	3.04E-12	7
Pole4	1.82E-16	-0.25639	0.069	0.149	3.04E-12	7
Pard6g	1.84E-16	0.317969	0.172	0.1	3.07E-12	7
Dync1li2	1.86E-16	0.256146	0.341	0.254	3.11E-12	7
Slc1a2	1.96E-16	-0.31899	0.204	0.3	3.28E-12	7
Commd1	1.97E-16	-0.28856	0.197	0.305	3.29E-12	7
Mrps28	2.13E-16	-0.27313	0.086	0.175	3.55E-12	7
Nipbl	2.14E-16	0.252718	0.567	0.487	3.58E-12	7
Prmt5	2.28E-16	-0.29477	0.156	0.263	3.79E-12	7
Rpa1	2.44E-16	-0.27452	0.041	0.113	4.08E-12	7
Gm17750	2.57E-16	0.266802	0.457	0.36	4.29E-12	7
Prkx	2.88E-16	0.302978	0.185	0.107	4.81E-12	7
Imp3	2.94E-16	-0.28006	0.151	0.253	4.9E-12	7
Tspyl1	2.96E-16	0.281318	0.223	0.14	4.94E-12	7
Cnksr2	2.98E-16	0.328531	0.175	0.102	4.97E-12	7

Armcx1	3E-16	0.328998	0.149	0.079	5E-12	7
Zfp57	3.1E-16	0.252022	0.126	0.063	5.18E-12	7
Vezt	3.23E-16	0.312008	0.16	0.088	5.38E-12	7
Nudc	3.88E-16	-0.26762	0.18	0.287	6.47E-12	7
Nsmce1	4.57E-16	-0.26765	0.142	0.234	7.62E-12	7
Dirc2	5.13E-16	0.289058	0.159	0.091	8.56E-12	7
Rcc2	5.26E-16	-0.25188	0.095	0.184	8.77E-12	7
Lims1	5.28E-16	-0.26041	0.099	0.188	8.81E-12	7
Ift74	5.74E-16	-0.29364	0.132	0.231	9.58E-12	7
Map7d1	5.98E-16	0.284423	0.313	0.236	9.97E-12	7
Rftn2	6.08E-16	-0.31599	0.054	0.128	1.01E-11	7
Acat1	6.3E-16	-0.26487	0.349	0.447	1.05E-11	7
Hspa5	6.39E-16	-0.29797	0.464	0.556	1.07E-11	7
Mgat5b	7.71E-16	0.290673	0.135	0.068	1.29E-11	7
Ppat	7.8E-16	-0.25103	0.041	0.112	1.3E-11	7
Atp6v0e2	7.91E-16	0.268001	0.338	0.254	1.32E-11	7
Mkrn1	8.46E-16	0.293032	0.283	0.203	1.41E-11	7
Aff4	8.5E-16	0.289352	0.292	0.211	1.42E-11	7
Map3k12	8.53E-16	0.292381	0.165	0.093	1.42E-11	7
Gm5620	9.34E-16	0.261245	0.154	0.097	1.56E-11	7
Blcap	9.39E-16	0.292197	0.144	0.074	1.57E-11	7
Tmem178	1.03E-15	0.31572	0.266	0.18	1.71E-11	7
Eif3b	1.05E-15	-0.27429	0.132	0.226	1.75E-11	7
Stau2	1.08E-15	0.30037	0.218	0.145	1.79E-11	7
Nme1	1.31E-15	-0.25617	0.425	0.515	2.19E-11	7
Srsf9	1.51E-15	-0.28031	0.145	0.242	2.52E-11	7
Rfc3	1.59E-15	-0.26416	0.116	0.21	2.65E-11	7
Ezh2	1.7E-15	-0.26805	0.546	0.614	2.84E-11	7
Serf1	1.87E-15	0.28401	0.362	0.275	3.12E-11	7
Gab1	2.05E-15	0.269172	0.13	0.067	3.42E-11	7
Setbp1	2.09E-15	0.320931	0.239	0.154	3.48E-11	7
Cenpj	2.24E-15	-0.26101	0.068	0.146	3.73E-11	7
Rassf3	2.35E-15	-0.29195	0.065	0.143	3.92E-11	7
Brd3	2.5E-15	-0.27484	0.436	0.538	4.17E-11	7
Bub3	2.55E-15	-0.30003	0.232	0.327	4.25E-11	7
Gins2	2.59E-15	-0.28621	0.106	0.19	4.32E-11	7
Cog1	2.68E-15	0.272153	0.18	0.11	4.47E-11	7
Hnrnpl	2.71E-15	-0.25679	0.257	0.37	4.53E-11	7
Btf3	2.76E-15	-0.25774	0.248	0.356	4.61E-11	7
Zfp608	2.77E-15	0.273226	0.255	0.18	4.62E-11	7
Pafah1b3	2.8E-15	0.267254	0.249	0.186	4.67E-11	7
Stx6	3.03E-15	-0.25119	0.084	0.166	5.05E-11	7
Zfp157	3.14E-15	0.297363	0.165	0.098	5.23E-11	7
Jam3	3.53E-15	-0.25594	0.062	0.14	5.89E-11	7
1810037I1	3.77E-15	-0.25044	0.118	0.201	6.29E-11	7
Fam162a	3.82E-15	-0.28509	0.197	0.303	6.38E-11	7
Canx	3.95E-15	-0.2587	0.504	0.602	6.59E-11	7
Mrps14	4.36E-15	-0.26747	0.144	0.242	7.27E-11	7

Lsmd1	4.62E-15	-0.28455	0.133	0.226	7.71E-11	7
Ccdc88a	4.99E-15	0.252417	0.563	0.476	8.33E-11	7
1500011K1	5.1E-15	-0.30614	0.074	0.154	8.51E-11	7
Rab3c	5.27E-15	0.272999	0.225	0.142	8.79E-11	7
Psmg4	5.35E-15	-0.25375	0.119	0.213	8.92E-11	7
Med30	5.61E-15	-0.26888	0.117	0.209	9.35E-11	7
Eef1g	5.81E-15	-0.25347	0.243	0.341	9.69E-11	7
Jun	5.98E-15	-0.30008	0.595	0.658	9.97E-11	7
C1ql1	6.4E-15	-0.30036	0.182	0.285	1.07E-10	7
Tmem176b	6.84E-15	-0.34652	0.049	0.113	1.14E-10	7
Edil3	9.54E-15	0.280227	0.102	0.046	1.59E-10	7
Ntrk2	1.08E-14	0.297921	0.148	0.079	1.8E-10	7
Eif1ax	1.18E-14	-0.251	0.294	0.404	1.97E-10	7
Glrx5	1.22E-14	-0.25653	0.266	0.367	2.04E-10	7
Pabpn1	1.22E-14	0.282461	0.265	0.189	2.04E-10	7
Gcsh	1.31E-14	-0.25784	0.072	0.146	2.18E-10	7
Ppp2r3a	1.31E-14	0.256253	0.155	0.089	2.18E-10	7
Pick1	1.42E-14	0.267485	0.195	0.123	2.37E-10	7
Dnajc2	1.43E-14	-0.28561	0.235	0.335	2.39E-10	7
Mrpl18	1.5E-14	-0.25844	0.246	0.361	2.51E-10	7
Tsc22d4	1.55E-14	-0.29579	0.094	0.171	2.58E-10	7
Jakmip2	1.59E-14	0.2817	0.233	0.151	2.65E-10	7
Nhsl1	1.64E-14	0.26973	0.11	0.053	2.73E-10	7
Emg1	1.93E-14	-0.27095	0.218	0.321	3.22E-10	7
Gm13092	2.12E-14	-0.25054	0.041	0.108	3.53E-10	7
Ftsj3	2.21E-14	-0.29804	0.123	0.211	3.68E-10	7
Jarid2	2.35E-14	0.310973	0.25	0.174	3.91E-10	7
Chchd1	2.58E-14	-0.26017	0.296	0.407	4.3E-10	7
Rexo2	2.65E-14	-0.2818	0.099	0.186	4.42E-10	7
Trim28	2.66E-14	-0.26971	0.257	0.368	4.44E-10	7
Aes	2.87E-14	0.26472	0.29	0.206	4.79E-10	7
Nbea	3.2E-14	0.290934	0.269	0.185	5.34E-10	7
Neurod6	3.21E-14	0.293645	0.248	0.166	5.35E-10	7
Mpped2	3.31E-14	0.272241	0.197	0.121	5.52E-10	7
Zc2hc1a	3.99E-14	0.283076	0.158	0.09	6.65E-10	7
Lsm14a	4.44E-14	-0.27093	0.083	0.165	7.4E-10	7
Atxn7l2	5E-14	0.262688	0.158	0.098	8.34E-10	7
Lmo4	5.17E-14	-0.28	0.247	0.341	8.62E-10	7
Trp53bp1	5.17E-14	0.257382	0.195	0.125	8.62E-10	7
Gnl3l	5.34E-14	0.251744	0.242	0.184	8.9E-10	7
Gcc2	5.48E-14	0.257793	0.236	0.166	9.13E-10	7
Polr2h	5.65E-14	-0.27498	0.142	0.23	9.42E-10	7
2700029M	6.01E-14	-0.27859	0.194	0.296	1E-09	7
Actl6a	6.18E-14	-0.25575	0.141	0.232	1.03E-09	7
Map9	6.51E-14	0.290588	0.22	0.142	1.09E-09	7
Ubr7	6.63E-14	-0.26915	0.062	0.137	1.11E-09	7
Hcfc2	8.2E-14	0.251964	0.125	0.065	1.37E-09	7
Zfp462	8.33E-14	-0.27913	0.082	0.161	1.39E-09	7

Mgst3	8.74E-14	0.254255	0.155	0.091	1.46E-09	7
Timm10	9.47E-14	-0.28401	0.07	0.144	1.58E-09	7
Plp1	9.86E-14	-0.57058	0.113	0.114	1.65E-09	7
Fam168a	9.92E-14	0.250634	0.357	0.282	1.65E-09	7
Pja2	1.06E-13	0.262697	0.292	0.23	1.77E-09	7
Fbxo11	1.18E-13	0.26802	0.231	0.149	1.97E-09	7
Golm1	1.24E-13	0.303232	0.259	0.183	2.07E-09	7
Tln1	1.24E-13	0.269587	0.21	0.145	2.07E-09	7
Tmx4	1.42E-13	0.277607	0.246	0.172	2.38E-09	7
Ola1	1.54E-13	-0.26707	0.145	0.234	2.57E-09	7
Rps28	1.81E-13	-0.27198	0.118	0.209	3.02E-09	7
Isy1	2E-13	-0.27605	0.074	0.146	3.34E-09	7
Polr1c	2.08E-13	-0.26671	0.135	0.224	3.47E-09	7
Leprotl1	2.5E-13	0.262585	0.234	0.175	4.16E-09	7
Xrcc1	2.5E-13	-0.25101	0.046	0.114	4.18E-09	7
Suv420h1	2.63E-13	0.250746	0.196	0.139	4.39E-09	7
Dpy30	2.65E-13	-0.26129	0.206	0.304	4.42E-09	7
Vopp1	2.66E-13	0.275416	0.181	0.114	4.44E-09	7
Exosc5	2.73E-13	-0.25496	0.045	0.108	4.56E-09	7
Enox1	2.91E-13	0.261776	0.101	0.052	4.85E-09	7
4933427D1	3.37E-13	0.270893	0.13	0.073	5.62E-09	7
Rnf220	3.64E-13	-0.25053	0.163	0.24	6.07E-09	7
Uchl5	3.65E-13	-0.25994	0.116	0.2	6.09E-09	7
Mgea5	4.05E-13	0.276018	0.303	0.226	6.75E-09	7
Polb	4.68E-13	0.268817	0.229	0.161	7.8E-09	7
Limk2	4.87E-13	0.252883	0.124	0.066	8.13E-09	7
Ctps	4.89E-13	-0.2527	0.069	0.142	8.16E-09	7
Rere	5.47E-13	0.253044	0.216	0.146	9.12E-09	7
Mfap4	5.56E-13	-0.2943	0.086	0.166	9.28E-09	7
Lima1	5.69E-13	-0.25971	0.056	0.126	9.48E-09	7
Egr1	6.17E-13	-0.32321	0.284	0.391	1.03E-08	7
Wasf2	6.42E-13	-0.25868	0.051	0.117	1.07E-08	7
Reep1	6.88E-13	0.25223	0.134	0.078	1.15E-08	7
Gpatch8	6.91E-13	0.270523	0.354	0.275	1.15E-08	7
Hook3	7.06E-13	0.271814	0.371	0.294	1.18E-08	7
Akap8l	7.92E-13	0.256654	0.223	0.153	1.32E-08	7
Ctsf	9.19E-13	0.278282	0.162	0.094	1.53E-08	7
Mettl9	9.25E-13	-0.25847	0.207	0.306	1.54E-08	7
Tom1l1	9.26E-13	-0.25393	0.043	0.107	1.54E-08	7
Oraov1	1E-12	-0.25462	0.056	0.125	1.67E-08	7
Mxd4	1.2E-12	0.267643	0.282	0.192	2.01E-08	7
Rsbn1	1.57E-12	0.258434	0.244	0.178	2.62E-08	7
Jak1	1.64E-12	0.286371	0.185	0.124	2.73E-08	7
Gatsl2	1.85E-12	0.261561	0.152	0.086	3.08E-08	7
Msl3	2.16E-12	-0.2551	0.069	0.139	3.6E-08	7
Ccng2	2.47E-12	0.259321	0.289	0.206	4.11E-08	7
Nr2f2	2.54E-12	0.261916	0.135	0.075	4.24E-08	7
Banp	2.73E-12	0.255518	0.195	0.133	4.55E-08	7

Suz12	3.38E-12	-0.25455	0.142	0.224	5.63E-08	7
Vps28	3.49E-12	0.250251	0.311	0.24	5.82E-08	7
Mapre2	3.52E-12	0.26889	0.23	0.162	5.87E-08	7
Fam107b	3.71E-12	0.267703	0.158	0.098	6.19E-08	7
Efhd2	3.87E-12	-0.27047	0.071	0.133	6.46E-08	7
Slc1a3	3.98E-12	-0.31585	0.12	0.155	6.64E-08	7
Zfp711	4.09E-12	0.283671	0.139	0.085	6.81E-08	7
Plk3	4.85E-12	0.279517	0.125	0.069	8.1E-08	7
Gm26735	7.54E-12	0.267549	0.251	0.175	1.26E-07	7
Psmg2	8.28E-12	-0.25223	0.085	0.156	1.38E-07	7
2810006K2	1.04E-11	-0.26424	0.061	0.127	1.74E-07	7
Hid1	1.42E-11	0.269626	0.108	0.052	2.38E-07	7
Abcc5	1.58E-11	0.257514	0.182	0.119	2.64E-07	7
Dpf2	1.66E-11	0.275481	0.166	0.107	2.76E-07	7
Phactr3	2.36E-11	0.258522	0.129	0.07	3.93E-07	7
Myo9a	2.69E-11	0.266631	0.217	0.153	4.48E-07	7
Mapk9	3.8E-11	0.256698	0.127	0.073	6.34E-07	7
Zmiz2	5.05E-11	0.262817	0.139	0.081	8.42E-07	7
Zbtb38	1.59E-10	0.252129	0.103	0.056	2.65E-06	7
Serpinh1	7.79E-09	-0.26586	0.063	0.119	0.00013	7
Cenpa	2.4E-214	1.116561	0.778	0.228	4.1E-210	8
Cenpe	6.1E-144	0.99569	0.718	0.266	1E-139	8
Ccnb2	6.7E-135	1.021999	0.574	0.169	1.1E-130	8
Hsp90b1	1.4E-118	0.709507	0.891	0.683	2.3E-114	8
Tpx2	1.6E-116	0.785592	0.682	0.263	2.6E-112	8
Cenpf	1.1E-115	0.59148	0.759	0.345	1.9E-111	8
Cdc20	6.4E-112	0.847283	0.559	0.18	1.1E-107	8
Hmgb2	1.17E-93	0.664128	0.755	0.384	1.95E-89	8
Mki67	3.08E-90	0.526943	0.749	0.379	5.14E-86	8
H2afv	1.83E-83	0.495295	0.937	0.718	3.05E-79	8
Knstrn	2.92E-79	0.759751	0.484	0.172	4.88E-75	8
Nucks1	3.57E-76	0.544433	0.885	0.653	5.96E-72	8
RP23-45G1	1.69E-75	0.63242	0.652	0.312	2.81E-71	8
2700094K1	6.21E-69	0.510579	0.868	0.602	1.04E-64	8
Cdca8	5.29E-64	0.501086	0.549	0.252	8.83E-60	8
Dynll1	3.52E-59	0.52002	0.811	0.584	5.87E-55	8
Pttg1	1.6E-56	0.697937	0.368	0.129	2.66E-52	8
Cdca3	8.17E-54	0.546854	0.477	0.21	1.36E-49	8
Tubb5	1.69E-53	-0.41696	0.883	0.916	2.81E-49	8
Hdgf	3.13E-53	0.505268	0.778	0.521	5.21E-49	8
Anp32e	7.13E-52	0.432466	0.844	0.603	1.19E-47	8
Hnrnpa2b1	2.14E-51	0.293846	0.985	0.931	3.57E-47	8
Neurod1	2.3E-50	-0.88444	0.487	0.534	3.83E-46	8
Fstl1	2.36E-50	0.593858	0.414	0.17	3.94E-46	8
Rad21	1.85E-49	0.493069	0.712	0.45	3.09E-45	8
Malat1	1.36E-48	-0.48931	0.953	0.955	2.27E-44	8
Gm11266	3.67E-47	0.644547	0.353	0.134	6.13E-43	8
CRE_RECON	1.24E-46	0.549354	0.9	0.762	2.07E-42	8

Gria2	4.58E-42	-0.72788	0.353	0.528	7.64E-38	8
Ckb	5.86E-42	-0.51914	0.756	0.81	9.78E-38	8
Hmgn5	1.08E-40	0.487592	0.66	0.414	1.8E-36	8
Birc5	4.14E-40	0.341249	0.489	0.264	6.9E-36	8
Nrep	8.25E-39	-0.51897	0.526	0.571	1.38E-34	8
C330027C(	1.08E-38	0.552835	0.315	0.125	1.8E-34	8
Cep89	1.86E-38	0.525874	0.243	0.079	3.1E-34	8
Banf1	6.9E-38	0.296283	0.888	0.685	1.15E-33	8
Miat	1.52E-37	-0.7381	0.353	0.522	2.54E-33	8
Sowaha	4.05E-37	0.669154	0.341	0.155	6.76E-33	8
Apoe	2.81E-36	-1.58536	0.237	0.249	4.69E-32	8
Gm10075	3.78E-36	0.395513	0.733	0.505	6.31E-32	8
Rtn1	1.95E-35	-0.42445	0.698	0.69	3.25E-31	8
Lmnb1	2.33E-35	0.399097	0.519	0.29	3.89E-31	8
Hmgn2	3.86E-35	0.471901	0.466	0.246	6.44E-31	8
Ccdc34	1.37E-34	0.393551	0.64	0.404	2.28E-30	8
Celf4	2.42E-34	-0.82066	0.195	0.325	4.04E-30	8
Sept3	1.14E-33	-0.64131	0.288	0.397	1.89E-29	8
Hes6	5.61E-33	0.415088	0.437	0.227	9.35E-29	8
Racgap1	1.35E-32	0.447799	0.46	0.246	2.25E-28	8
Top1	5.67E-32	0.369792	0.829	0.655	9.45E-28	8
Tacc3	8.7E-31	0.404489	0.392	0.198	1.45E-26	8
Tubb2a	1.03E-29	-0.64232	0.159	0.248	1.72E-25	8
Dlgap5	1.05E-29	0.437515	0.26	0.105	1.75E-25	8
Tubb4b	2.75E-29	0.420333	0.43	0.231	4.59E-25	8
Gpm6a	3.51E-29	-0.57553	0.334	0.412	5.85E-25	8
Basp1	4.77E-29	-0.43481	0.635	0.707	7.96E-25	8
Gap43	9.02E-29	-0.48325	0.565	0.6	1.51E-24	8
Kif23	3.27E-28	0.375146	0.391	0.206	5.46E-24	8
Arhgap11a	4.38E-28	0.379475	0.334	0.163	7.3E-24	8
Shfm1	1.07E-27	0.33146	0.83	0.679	1.78E-23	8
Stmn2	1.61E-27	-0.57397	0.501	0.555	2.69E-23	8
Hist1h2bc	3E-27	0.445621	0.133	0.034	5.01E-23	8
Cep110	1.27E-26	0.456088	0.323	0.156	2.12E-22	8
Zic1	4.09E-26	-0.28722	0.801	0.783	6.82E-22	8
Fabp7	4.1E-26	-0.77477	0.143	0.122	6.84E-22	8
Cacna2d1	4.3E-26	-0.51592	0.283	0.379	7.18E-22	8
Pcna	6.65E-26	-0.45748	0.302	0.36	1.11E-21	8
Bin1	8.54E-26	-0.51428	0.323	0.434	1.42E-21	8
Tubb3	1.06E-25	-0.49557	0.441	0.475	1.76E-21	8
Hmgn1	1.2E-25	0.296247	0.794	0.614	1.99E-21	8
App	1.53E-25	-0.39888	0.511	0.571	2.55E-21	8
Cdc25c	1.7E-25	0.390503	0.189	0.066	2.83E-21	8
Ezr	1.93E-25	0.346246	0.629	0.429	3.22E-21	8
Paip2	2.32E-25	0.317155	0.733	0.54	3.87E-21	8
Tubb2b	2.96E-25	-0.39559	0.43	0.466	4.93E-21	8
Hpca	3.85E-25	0.433733	0.457	0.269	6.42E-21	8
Lig1	4E-25	-0.54146	0.267	0.351	6.67E-21	8

Cks1b	4.38E-25	0.333643	0.532	0.336	7.3E-21	8
Smim11	4.84E-25	0.434394	0.447	0.269	8.07E-21	8
Vim	5.54E-25	0.368914	0.487	0.294	9.24E-21	8
Cntn2	5.77E-25	-0.73495	0.168	0.267	9.63E-21	8
Syt13	6.91E-25	0.46327	0.268	0.12	1.15E-20	8
Hmmr	1.13E-24	0.275694	0.318	0.17	1.88E-20	8
Celf2	3.34E-24	-0.38368	0.6	0.639	5.57E-20	8
Cdkn3	4.2E-24	0.338821	0.132	0.037	7.01E-20	8
Top2a	8.23E-24	-0.30306	0.47	0.425	1.37E-19	8
Ctsb	1.09E-23	-0.5324	0.249	0.262	1.82E-19	8
Cbx1	2.28E-23	0.287739	0.829	0.678	3.8E-19	8
Lgals1	2.37E-23	0.422637	0.395	0.221	3.95E-19	8
Ckap5	2.53E-23	0.402341	0.438	0.258	4.21E-19	8
Mns1	2.86E-23	0.360717	0.36	0.196	4.77E-19	8
Hnrnpm	3.64E-23	0.266534	0.871	0.729	6.07E-19	8
Rbfox3	6.04E-23	-0.48586	0.399	0.498	1.01E-18	8
Pde1c	6.93E-23	-0.48303	0.323	0.389	1.16E-18	8
Dpysl3	1.27E-22	-0.4968	0.175	0.221	2.12E-18	8
Hmgb3	1.29E-22	0.348723	0.481	0.298	2.16E-18	8
Rps27l	1.66E-22	0.261878	0.673	0.484	2.76E-18	8
Ptprs	5.65E-22	-0.51476	0.323	0.425	9.43E-18	8
Igfbpl1	8.29E-22	-0.401	0.592	0.65	1.38E-17	8
Ctnnb1	8.56E-22	0.319102	0.565	0.378	1.43E-17	8
Gng3	9.02E-22	-0.43262	0.278	0.318	1.5E-17	8
Xist	1.23E-21	-0.63147	0.205	0.303	2.06E-17	8
Ran	2.55E-21	0.286069	0.59	0.406	4.26E-17	8
Vbp1	2.9E-21	0.343885	0.491	0.312	4.83E-17	8
Mdh1	1.2E-20	0.295263	0.627	0.444	2E-16	8
Hnrnpc	1.98E-20	0.255885	0.815	0.66	3.3E-16	8
Efhd2	2.1E-20	0.345184	0.257	0.124	3.5E-16	8
Pdia6	2.15E-20	0.272173	0.505	0.333	3.59E-16	8
Aspm	2.46E-20	0.335217	0.272	0.137	4.11E-16	8
Ppp1r14b	2.66E-20	0.328205	0.567	0.389	4.43E-16	8
Rangap1	2.89E-20	0.277106	0.41	0.249	4.81E-16	8
Ppp1r14c	3.29E-20	-0.52493	0.185	0.265	5.48E-16	8
Ank3	5.89E-20	-0.36488	0.387	0.421	9.83E-16	8
H2afz	1.07E-19	0.332022	0.379	0.224	1.79E-15	8
Thra	2.38E-19	-0.39704	0.195	0.23	3.97E-15	8
Nfib	2.95E-19	-0.26535	0.943	0.933	4.91E-15	8
Fam64a	7.71E-19	0.311901	0.235	0.112	1.29E-14	8
Sep15	1.11E-18	0.282288	0.667	0.497	1.85E-14	8
Stmn4	1.52E-18	-0.4938	0.283	0.343	2.53E-14	8
Nde1	1.73E-18	0.368329	0.236	0.114	2.89E-14	8
Lyar	1.96E-18	0.313101	0.441	0.278	3.27E-14	8
Pdlim3	4.19E-18	0.294183	0.15	0.057	7E-14	8
Otx2	5.02E-18	0.352077	0.36	0.21	8.37E-14	8
Gnai2	5.61E-18	0.310626	0.585	0.417	9.36E-14	8
Ctsd	5.87E-18	-0.59071	0.183	0.174	9.78E-14	8

Mad2l1	6.08E-18	0.318985	0.241	0.118	1.01E-13	8
Hmgn3	6.31E-18	0.326805	0.418	0.263	1.05E-13	8
Map2	7.74E-18	-0.34781	0.456	0.493	1.29E-13	8
Kif1b	7.96E-18	-0.38227	0.439	0.524	1.33E-13	8
Bub1b	1.03E-17	0.288985	0.21	0.097	1.72E-13	8
Cct5	2.21E-17	0.268361	0.636	0.469	3.68E-13	8
Tax1bp1	2.42E-17	0.269726	0.728	0.571	4.03E-13	8
BC005764	2.67E-17	-0.52167	0.102	0.189	4.45E-13	8
Ppp2r5c	3.07E-17	0.324397	0.357	0.213	5.12E-13	8
Lsm4	4.78E-17	0.25443	0.643	0.481	7.97E-13	8
Nrxn1	5.37E-17	-0.47383	0.26	0.32	8.96E-13	8
Gas6	5.43E-17	0.353283	0.179	0.076	9.05E-13	8
Sh3gl2	5.56E-17	-0.49417	0.077	0.175	9.28E-13	8
Cd81	5.92E-17	-0.35617	0.398	0.464	9.88E-13	8
Pcf11	7.04E-17	0.334018	0.309	0.173	1.17E-12	8
Mapt	9.5E-17	-0.51392	0.15	0.219	1.58E-12	8
Cenpv	1E-16	0.3094	0.478	0.322	1.67E-12	8
Ralgps2	3.16E-16	0.305135	0.385	0.239	5.27E-12	8
Nudcd2	3.24E-16	0.337781	0.36	0.217	5.41E-12	8
Psmc1	5.11E-16	0.280806	0.565	0.412	8.52E-12	8
Sh3bgrl	6.15E-16	0.277161	0.487	0.33	1.03E-11	8
Atp6v1e1	8.44E-16	-0.38452	0.261	0.334	1.41E-11	8
Mtss1	8.83E-16	-0.375	0.236	0.27	1.47E-11	8
Ank2	1.02E-15	-0.5167	0.154	0.244	1.7E-11	8
1700123O	1.16E-15	0.258068	0.251	0.135	1.94E-11	8
Cbx5	1.31E-15	-0.2773	0.615	0.654	2.19E-11	8
Tra2b	1.82E-15	0.292312	0.54	0.398	3.03E-11	8
Mphosph1	2.23E-15	0.331515	0.365	0.225	3.72E-11	8
Cox7b	2.39E-15	0.262662	0.69	0.542	3.98E-11	8
Sec11c	2.9E-15	0.337722	0.342	0.207	4.84E-11	8
Hmgb1	3.31E-15	0.271631	0.505	0.352	5.53E-11	8
Clspn	4.62E-15	-0.45008	0.112	0.193	7.7E-11	8
Aplp1	4.86E-15	-0.46085	0.081	0.154	8.1E-11	8
Myt1l	5.07E-15	-0.48424	0.096	0.163	8.45E-11	8
Pqlc1	5.15E-15	0.27329	0.437	0.291	8.59E-11	8
Smarcc2	5.6E-15	-0.41179	0.265	0.367	9.34E-11	8
Cks2	6.11E-15	0.26906	0.164	0.071	1.02E-10	8
Trpc4ap	8.79E-15	-0.3317	0.179	0.199	1.47E-10	8
Myt1	1.12E-14	-0.45432	0.093	0.155	1.87E-10	8
Gadd45g	1.23E-14	0.348189	0.167	0.076	2.06E-10	8
Zfc3h1	1.51E-14	0.274913	0.178	0.084	2.51E-10	8
Spop	1.55E-14	0.254647	0.453	0.309	2.58E-10	8
Sept4	1.58E-14	-0.43264	0.173	0.228	2.63E-10	8
Kif5c	2.18E-14	-0.35881	0.348	0.4	3.63E-10	8
Cenpw	2.74E-14	0.267397	0.23	0.123	4.57E-10	8
Zeb1	3.8E-14	-0.29147	0.354	0.388	6.33E-10	8
Chgb	4.17E-14	-0.49651	0.175	0.243	6.95E-10	8
Ubb	4.85E-14	-0.27326	0.489	0.543	8.09E-10	8

Bzw1	4.95E-14	0.275215	0.5	0.353	8.25E-10	8
Magoh	5.42E-14	0.256018	0.472	0.326	9.03E-10	8
Nfia	7E-14	-0.25548	0.752	0.774	1.17E-09	8
Slc35b1	8.21E-14	0.289878	0.429	0.287	1.37E-09	8
Odc1	8.74E-14	0.314961	0.194	0.097	1.46E-09	8
Grik2	1.12E-13	-0.39808	0.043	0.104	1.87E-09	8
Ect2	1.16E-13	0.277686	0.191	0.093	1.94E-09	8
Taf7	1.89E-13	0.319471	0.237	0.128	3.16E-09	8
Ift74	3.1E-13	0.296346	0.346	0.219	5.17E-09	8
Kif15	3.14E-13	0.276679	0.292	0.175	5.24E-09	8
Prkcb	3.97E-13	-0.36319	0.208	0.258	6.62E-09	8
Elavl4	4.02E-13	-0.33344	0.302	0.336	6.7E-09	8
D4Wsu53e	4.59E-13	-0.27364	0.422	0.447	7.66E-09	8
Gnao1	4.92E-13	-0.36276	0.236	0.289	8.2E-09	8
Ankrd12	5.59E-13	-0.42489	0.22	0.277	9.33E-09	8
Rufy3	7.3E-13	-0.278	0.348	0.372	1.22E-08	8
Tnik	1.21E-12	-0.46324	0.075	0.149	2.02E-08	8
Apc	1.64E-12	-0.28812	0.407	0.432	2.73E-08	8
Gsk3b	1.75E-12	-0.30922	0.431	0.502	2.91E-08	8
Mapk8ip1	1.93E-12	-0.25635	0.212	0.228	3.22E-08	8
Rab6b	1.96E-12	-0.38068	0.143	0.205	3.26E-08	8
Cog7	2.56E-12	-0.27343	0.543	0.586	4.28E-08	8
Chd3	2.57E-12	-0.31427	0.127	0.15	4.29E-08	8
Csrp2	3.03E-12	0.261054	0.261	0.153	5.05E-08	8
Tagln3	3.86E-12	-0.40726	0.212	0.298	6.44E-08	8
Tcf19	4.75E-12	-0.32921	0.035	0.101	7.93E-08	8
Nbea	4.82E-12	-0.42855	0.115	0.193	8.05E-08	8
Dcx	5.35E-12	-0.25899	0.368	0.376	8.93E-08	8
Nsg1	5.36E-12	-0.28017	0.26	0.288	8.94E-08	8
Cadm3	6.82E-12	-0.40132	0.086	0.143	1.14E-07	8
Ckap2	8.92E-12	0.278571	0.264	0.158	1.49E-07	8
Akap6	1.15E-11	-0.34906	0.158	0.221	1.92E-07	8
Atp2b1	1.24E-11	-0.27729	0.542	0.572	2.06E-07	8
Klc1	1.57E-11	-0.28382	0.325	0.363	2.61E-07	8
Chd7	1.66E-11	-0.26269	0.606	0.649	2.77E-07	8
Rbbp6	1.68E-11	0.261378	0.557	0.427	2.8E-07	8
Gas1	2.04E-11	0.275971	0.257	0.152	3.4E-07	8
Pak7	2.1E-11	-0.36098	0.051	0.106	3.51E-07	8
Phf20l1	2.35E-11	-0.35354	0.274	0.343	3.93E-07	8
Itsn1	2.47E-11	-0.32743	0.201	0.237	4.11E-07	8
Ccdc77	2.72E-11	0.257958	0.148	0.07	4.53E-07	8
Ina	4.01E-11	-0.25917	0.43	0.444	6.7E-07	8
Sema6a	5.37E-11	-0.37008	0.148	0.204	8.96E-07	8
Grina	6.11E-11	-0.30992	0.07	0.111	1.02E-06	8
Ppp3ca	6.75E-11	-0.34626	0.306	0.381	1.13E-06	8
Ptprd	7.08E-11	-0.32574	0.28	0.342	1.18E-06	8
Jam3	8.14E-11	0.262282	0.228	0.131	1.36E-06	8
Hist3h2a	9.14E-11	-0.36026	0.088	0.146	1.52E-06	8

Cnpy1	1.06E-10	-0.36524	0.066	0.135	1.76E-06	8
Gdi1	1.06E-10	-0.30627	0.185	0.231	1.77E-06	8
Cenpp	1.14E-10	0.296656	0.198	0.11	1.9E-06	8
Elmo1	1.35E-10	-0.4395	0.061	0.128	2.25E-06	8
Gm8203	1.38E-10	0.252836	0.151	0.074	2.3E-06	8
Fam111a	1.47E-10	-0.35521	0.065	0.128	2.46E-06	8
Cacng4	2.61E-10	-0.34133	0.082	0.102	4.35E-06	8
Chrna3	2.73E-10	-0.32142	0.082	0.119	4.55E-06	8
Kifap3	3.75E-10	-0.29265	0.232	0.274	6.25E-06	8
2810008D(	3.81E-10	0.263104	0.214	0.123	6.36E-06	8
Apbb1	3.92E-10	-0.32461	0.127	0.172	6.53E-06	8
Arpp21	4.01E-10	-0.4261	0.055	0.115	6.68E-06	8
Tacc2	4.01E-10	-0.33413	0.094	0.143	6.69E-06	8
Slc1a2	4.54E-10	-0.28891	0.26	0.295	7.57E-06	8
Ypel3	4.59E-10	-0.27183	0.38	0.437	7.65E-06	8
Itm2c	5.06E-10	-0.27106	0.187	0.212	8.43E-06	8
Zwint	5.1E-10	-0.3342	0.214	0.294	8.5E-06	8
Pea15a	5.78E-10	-0.35007	0.128	0.178	9.64E-06	8
Mum1l1	7.65E-10	0.254852	0.171	0.091	1.28E-05	8
Celsr2	7.79E-10	-0.30666	0.193	0.239	1.3E-05	8
Stxbp1	8.09E-10	-0.34805	0.113	0.164	1.35E-05	8
Mycbp2	9.02E-10	-0.31142	0.288	0.347	1.5E-05	8
Fyn	1.01E-09	-0.2698	0.202	0.216	1.69E-05	8
Gnaq	1.24E-09	-0.28372	0.247	0.286	2.07E-05	8
Aldoa	1.37E-09	-0.29001	0.216	0.259	2.28E-05	8
2810474O(	1.39E-09	0.252654	0.346	0.236	2.32E-05	8
Atp6v0b	1.6E-09	-0.3225	0.205	0.27	2.68E-05	8
Ppfia2	1.75E-09	-0.37177	0.055	0.114	2.92E-05	8
Lmnb2	2E-09	0.269229	0.249	0.154	3.33E-05	8
Maml3	2.42E-09	-0.30997	0.047	0.102	4.04E-05	8
Hes1	2.43E-09	0.298096	0.237	0.144	4.06E-05	8
A330076H(	2.53E-09	-0.3662	0.1	0.163	4.22E-05	8
Slc17a6	2.53E-09	-0.33746	0.104	0.15	4.22E-05	8
Cnksr2	2.56E-09	-0.30432	0.067	0.109	4.27E-05	8
Zmynd8	3.47E-09	-0.32009	0.16	0.215	5.79E-05	8
Pkia	4.31E-09	-0.30368	0.094	0.135	7.18E-05	8
Srgap2	4.72E-09	-0.2814	0.1	0.133	7.88E-05	8
Hcfc1r1	5.26E-09	-0.2954	0.146	0.194	8.78E-05	8
Esco2	5.37E-09	-0.3307	0.15	0.196	8.96E-05	8
Erc1	5.63E-09	-0.31423	0.066	0.112	9.39E-05	8
Hells	7.33E-09	-0.27528	0.187	0.214	0.000122	8
Srrm3	7.72E-09	-0.25146	0.222	0.24	0.000129	8
Zmiz1	7.85E-09	-0.2615	0.33	0.377	0.000131	8
Psap	8.54E-09	-0.27323	0.158	0.185	0.000142	8
Snap25	9.08E-09	-0.318	0.235	0.284	0.000151	8
Ppp1r1a	1.21E-08	-0.30857	0.062	0.112	0.000201	8
Gria4	1.25E-08	-0.33363	0.069	0.127	0.000209	8
Dnmt1	1.53E-08	-0.31626	0.209	0.28	0.000255	8

Reln	1.58E-08	-0.32975	0.136	0.182	0.000264	8
Mllt11	1.64E-08	-0.2533	0.199	0.219	0.000274	8
Kmt2c	1.81E-08	-0.31824	0.22	0.298	0.000302	8
Akap9	2.21E-08	-0.25234	0.47	0.501	0.000369	8
Eif4g3	2.24E-08	-0.26129	0.33	0.386	0.000374	8
Fmnl2	2.29E-08	-0.31026	0.105	0.159	0.000382	8
Pdzrn3	2.96E-08	-0.36634	0.101	0.149	0.000494	8
Prdm8	3.11E-08	-0.26762	0.104	0.127	0.000519	8
Rab3a	3.31E-08	-0.31122	0.102	0.152	0.000552	8
Plp1	3.74E-08	-0.6897	0.109	0.114	0.000624	8
Mgll	4.55E-08	-0.32441	0.077	0.135	0.000758	8
Dner	4.69E-08	-0.27221	0.189	0.216	0.000783	8
Ntrk3	4.84E-08	-0.27308	0.113	0.157	0.000807	8
Ncan	5.14E-08	-0.27693	0.062	0.101	0.000857	8
Aplp2	5.62E-08	-0.25298	0.286	0.324	0.000937	8
Gabbr1	6.71E-08	-0.26554	0.106	0.142	0.001119	8
Gphn	7.3E-08	-0.29322	0.089	0.143	0.001217	8
Vcan	9.08E-08	-0.28537	0.09	0.131	0.001515	8
Kif5a	1.01E-07	-0.33505	0.075	0.13	0.001679	8
Agap1	1.04E-07	-0.29977	0.081	0.134	0.001728	8
Mllt4	1.06E-07	-0.26945	0.307	0.365	0.001774	8
Klf9	1.15E-07	-0.27493	0.228	0.272	0.001913	8
Podxl2	1.2E-07	-0.33342	0.162	0.23	0.002001	8
Stag2	1.21E-07	-0.26963	0.21	0.258	0.002019	8
Clcn4-2	1.32E-07	-0.27683	0.21	0.253	0.002199	8
Ddx26b	1.49E-07	-0.25677	0.094	0.131	0.002482	8
Plcb1	1.81E-07	-0.33305	0.096	0.143	0.003011	8
Clasp2	1.9E-07	-0.29933	0.113	0.169	0.003175	8
Mllt3	1.98E-07	-0.26696	0.179	0.228	0.003298	8
Scaper	1.99E-07	-0.26036	0.084	0.111	0.00332	8
Zcchc18	2.15E-07	-0.25269	0.139	0.171	0.00359	8
Sv2b	3.02E-07	-0.2543	0.069	0.104	0.00503	8
Rere	3.62E-07	-0.2923	0.09	0.153	0.006039	8
Fut9	4.26E-07	-0.283	0.167	0.213	0.007107	8
Cplx1	5.28E-07	-0.26078	0.106	0.139	0.008802	8
Kidins220	5.28E-07	-0.30286	0.096	0.142	0.008809	8
St18	5.66E-07	-0.32712	0.144	0.192	0.009439	8
Trio	7.22E-07	-0.28201	0.071	0.127	0.01205	8
Aprt	7.86E-07	-0.26862	0.093	0.143	0.013104	8
Aurkb	7.88E-07	-0.25129	0.094	0.145	0.013146	8
Tspyl4	9.48E-07	-0.26559	0.081	0.128	0.015805	8
Adrbk2	1.21E-06	-0.26499	0.067	0.101	0.020117	8
Nrcam	1.25E-06	-0.29047	0.089	0.136	0.020917	8
Phactr1	1.3E-06	-0.28237	0.137	0.185	0.021612	8
Fam213b	1.41E-06	-0.28835	0.111	0.159	0.02357	8
Rabgap1	1.8E-06	-0.2793	0.17	0.226	0.029954	8
Sox5	2.16E-06	-0.27874	0.075	0.121	0.036098	8
Stau2	2.56E-06	-0.27151	0.104	0.152	0.042681	8

Abhd16a	2.95E-06	-0.27746	0.139	0.192	0.0492	8
Scn8a	3.8E-06	-0.256	0.097	0.139	0.06335	8
Mt1	4.48E-06	-0.36813	0.139	0.169	0.074754	8
Shd	5.48E-06	-0.25308	0.069	0.111	0.091402	8
Rad51ap1	6.03E-06	-0.26424	0.115	0.169	0.100659	8
Ntm	1.49E-05	-0.28015	0.105	0.147	0.248532	8
Olig1	0	2.902829	0.937	0.059	0	9
Serpine2	0	2.715137	0.844	0.038	0	9
Cspg5	0	2.683292	0.693	0.013	0	9
3110035E1	0	2.3893	0.713	0.007	0	9
Cacng4	0	2.361371	0.808	0.085	0	9
Fabp7	0	2.295238	0.827	0.106	0	9
Ptprz1	0	2.209687	0.698	0.021	0	9
Scrg1	0	2.182568	0.703	0.009	0	9
Cntn1	0	2.16389	0.732	0.035	0	9
Gpr17	0	2.022637	0.526	0.003	0	9
Gpr37l1	0	1.973763	0.684	0.015	0	9
Pllp	0	1.780244	0.538	0.003	0	9
Sox10	0	1.68034	0.526	0.002	0	9
Olig2	0	2.021847	0.754	0.062	0	9
Plp1	0	3.538733	0.326	0.109	2.7E-305	9
Sulf2	2E-284	1.741819	0.533	0.01	3.3E-280	9
Ptpre	1E-283	1.653078	0.496	0.005	1.7E-279	9
Bcas1	9.5E-276	1.863839	0.672	0.273	1.6E-271	9
S100a13	5.4E-271	2.01217	0.535	0.019	9E-267	9
Sirt2	6.6E-269	2.064177	0.55	0.146	1.1E-264	9
Enpp2	1.2E-267	1.804524	0.572	0.019	2E-263	9
Cnp	2.4E-262	2.339049	0.484	0.023	4E-258	9
Bcan	4.1E-262	1.879737	0.655	0.038	6.8E-258	9
Ramp1	3.6E-257	1.715161	0.545	0.015	6E-253	9
Fyn	1E-242	1.869148	0.603	0.206	1.7E-238	9
S100a1	9.2E-240	1.832083	0.482	0.015	1.5E-235	9
Rgcc	2.1E-233	1.551647	0.428	0.006	3.6E-229	9
Ugt8a	5.4E-233	1.57135	0.428	0.006	9.1E-229	9
Slc35f1	1.5E-228	1.450607	0.431	0.007	2.5E-224	9
Ppfibp1	4.5E-223	1.602462	0.501	0.021	7.6E-219	9
Cspg4	2.7E-214	1.630552	0.433	0.01	4.5E-210	9
Nfib	9.5E-213	-1.50827	0.54	0.942	1.6E-208	9
Pdgfra	8.2E-201	1.670338	0.62	0.171	1.4E-196	9
Gjc3	8.3E-198	1.458354	0.375	0.006	1.4E-193	9
Gatm	1.1E-195	1.469929	0.489	0.024	1.8E-191	9
Ednrb	2.8E-188	1.529893	0.457	0.019	4.7E-184	9
Tpm1	7E-186	1.557406	0.674	0.179	1.2E-181	9
Pcsk1n	7.5E-184	1.527661	0.633	0.071	1.2E-179	9
Nap1l5	2.8E-179	1.553163	0.584	0.069	4.6E-175	9
Itpr2	1.4E-177	1.547662	0.423	0.02	2.3E-173	9
Epn2	4.8E-173	1.522306	0.572	0.072	8E-169	9
Tagln2	7.1E-173	1.571526	0.462	0.034	1.2E-168	9

Nkx2-2	4.2E-171	1.180123	0.299	0.002	7E-167	9
B3gat2	2.2E-170	1.300348	0.365	0.01	3.6E-166	9
Plekhb1	2.5E-168	1.189842	0.314	0.004	4.2E-164	9
Lsamp	3.4E-168	1.501843	0.579	0.065	5.7E-164	9
Qpct	5.2E-166	1.311559	0.377	0.013	8.6E-162	9
Sfrp1	1.2E-165	-1.74955	0.304	0.823	2.1E-161	9
Mfsd2a	5.1E-163	1.088409	0.309	0.004	8.6E-159	9
Dnm3	7.1E-162	1.32497	0.406	0.017	1.2E-157	9
Omg	1.1E-161	1.158875	0.307	0.004	1.9E-157	9
Gria3	5.7E-160	1.522734	0.55	0.078	9.5E-156	9
Mbp	2.7E-157	2.073282	0.326	0.161	4.4E-153	9
Zic1	2.9E-154	-1.65544	0.219	0.797	4.9E-150	9
Ptn	1.8E-153	1.267744	0.818	0.432	3E-149	9
Tsc22d4	1.4E-152	1.461678	0.625	0.155	2.4E-148	9
Sox6	3E-152	1.142568	0.29	0.004	5.1E-148	9
Phlda1	9.7E-152	1.310419	0.384	0.017	1.6E-147	9
Tmem100	1.6E-150	1.127033	0.282	0.004	2.7E-146	9
Asrgl1	4.9E-148	1.354862	0.608	0.122	8.1E-144	9
Spon1	1.4E-144	1.13373	0.311	0.007	2.4E-140	9
Cd9	4E-143	1.15447	0.701	0.479	6.6E-139	9
Ddah1	5.8E-143	1.306043	0.409	0.03	9.7E-139	9
CRE_RECON	1.3E-142	-2.05969	0.248	0.78	2.2E-138	9
Cdo1	2.3E-141	1.287764	0.341	0.013	3.9E-137	9
Lims2	1.5E-140	1.020915	0.248	0.002	2.4E-136	9
Ncald	5E-140	1.369376	0.633	0.154	8.3E-136	9
Hsp90ab1	2.2E-136	-0.57099	0.99	0.998	3.6E-132	9
Susd4	2.4E-134	1.012502	0.292	0.007	4E-130	9
Ascl1	3.4E-134	0.948014	0.234	0.002	5.7E-130	9
Tril	4E-134	1.126869	0.363	0.018	6.7E-130	9
Ncam2	2.9E-133	1.098209	0.311	0.01	4.8E-129	9
Cyp2j6	5.8E-128	1.098664	0.309	0.011	9.7E-124	9
Tmem176b	4.4E-127	1.364364	0.555	0.098	7.3E-123	9
Igfbpl1	6.3E-126	-1.85801	0.112	0.66	1E-121	9
Cd24a	1.4E-124	-1.62233	0.095	0.646	2.3E-120	9
Sox2	4.4E-123	1.198093	0.416	0.039	7.3E-119	9
Neu4	4.8E-122	0.863523	0.197	0.001	8.1E-118	9
Kcnj10	5.7E-121	1.128784	0.333	0.018	9.5E-117	9
2810468N	7.4E-120	1.115626	0.309	0.015	1.2E-115	9
Dbi	8.9E-120	1.094424	0.798	0.455	1.5E-115	9
Kctd4	2.6E-119	0.738452	0.195	0.001	4.4E-115	9
Klhl5	2.7E-119	1.159013	0.382	0.031	4.5E-115	9
Ttyh1	4.1E-119	1.160428	0.358	0.024	6.9E-115	9
S100a16	8.7E-119	1.437618	0.499	0.085	1.4E-114	9
Cadm2	1.6E-118	1.20264	0.418	0.039	2.7E-114	9
Dmrtb1	5.2E-117	0.718697	0.187	0.001	8.7E-113	9
Pcdh17	4.1E-116	1.213113	0.341	0.021	6.8E-112	9
Lrrc4c	7.5E-116	0.958672	0.241	0.005	1.2E-111	9
Sox8	6.8E-114	1.208148	0.331	0.022	1.1E-109	9

Cmtm5	6.6E-113	0.904539	0.287	0.011	1.1E-108	9
Slc1a1	6.8E-113	0.980495	0.285	0.011	1.1E-108	9
Rlbp1	8.1E-113	1.116926	0.241	0.006	1.3E-108	9
Afap1l2	1.9E-112	0.828839	0.221	0.003	3.1E-108	9
Zcchc24	5.8E-110	1.089812	0.324	0.021	9.6E-106	9
Sept7	1.9E-109	0.927384	0.818	0.583	3.1E-105	9
Vcan	1.9E-108	1.281638	0.513	0.12	3.2E-104	9
Opcml	3.3E-108	1.016942	0.29	0.013	5.5E-104	9
Anks1b	1.8E-107	1.218667	0.44	0.055	2.9E-103	9
Enc1	5.6E-106	1.012164	0.255	0.009	9.4E-102	9
Bmp4	2.1E-105	1.293061	0.187	0.002	3.5E-101	9
Atp1a2	5.4E-105	1.072214	0.404	0.05	9E-101	9
Ostf1	7.6E-105	1.219191	0.411	0.055	1.3E-100	9
Car8	1.7E-103	0.901617	0.219	0.005	2.9E-99	9
Mmp15	2.7E-103	1.008322	0.258	0.011	4.5E-99	9
S100b	2.8E-103	1.346371	0.358	0.033	4.6E-99	9
Rprm	2.1E-102	1.113938	0.326	0.027	3.58E-98	9
Nxph1	7E-102	0.863208	0.231	0.006	1.17E-97	9
Ppap2b	1.6E-101	1.03696	0.365	0.035	2.71E-97	9
Pxdc1	1.6E-99	0.732621	0.204	0.004	2.66E-95	9
3632451O(	3.12E-97	1.004365	0.333	0.028	5.2E-93	9
Pou3f1	3.6E-97	0.825435	0.175	0.002	6.01E-93	9
Gal3st1	1.04E-96	0.576701	0.151	0	1.74E-92	9
Degs1	1.51E-96	1.114284	0.423	0.068	2.51E-92	9
Brinp3	2.06E-96	0.8185	0.221	0.006	3.43E-92	9
Gfra1	3.72E-96	0.840487	0.214	0.005	6.21E-92	9
Matn4	4.62E-96	0.792718	0.185	0.002	7.7E-92	9
Tnr	1.41E-95	0.612111	0.165	0.001	2.35E-91	9
Kcnd2	1.49E-95	1.088778	0.297	0.019	2.49E-91	9
Sema5a	1.51E-95	0.79634	0.185	0.002	2.52E-91	9
Tspan3	7.19E-93	0.925656	0.766	0.42	1.2E-88	9
Scd2	1.88E-92	1.059481	0.667	0.288	3.14E-88	9
Arsb	1.14E-91	1.001949	0.277	0.019	1.9E-87	9
Tmem255b	1.46E-91	0.702145	0.151	0.001	2.44E-87	9
Fa2h	1E-90	0.731765	0.163	0.001	1.67E-86	9
Tubb5	5.08E-90	-0.83457	0.766	0.918	8.47E-86	9
Anp32a	1.01E-89	-0.90282	0.586	0.842	1.68E-85	9
Sox2ot	4.7E-89	0.774647	0.168	0.002	7.83E-85	9
Pcdh10	7.39E-89	0.860649	0.226	0.009	1.23E-84	9
Tspan7	5.48E-88	1.093166	0.479	0.096	9.15E-84	9
Enpp6	1.42E-87	0.927245	0.158	0.002	2.38E-83	9
AW04773C	2.31E-87	1.079007	0.265	0.018	3.85E-83	9
Sema5b	2.95E-87	0.774638	0.19	0.004	4.91E-83	9
Dscam	5.15E-87	0.899838	0.246	0.012	8.6E-83	9
Timp4	9.08E-87	0.87051	0.238	0.011	1.51E-82	9
Sh3d19	5.22E-86	0.953938	0.311	0.029	8.71E-82	9
Cdh13	4.53E-85	1.007015	0.343	0.04	7.55E-81	9
H3f3b	7.66E-85	-0.75099	0.742	0.91	1.28E-80	9

Barhl1	1.75E-84	-1.45972	0.085	0.532	2.91E-80	9
Megf11	2.02E-84	0.811024	0.197	0.006	3.37E-80	9
Cd81	2.47E-84	0.895775	0.776	0.454	4.12E-80	9
1810041L1	4.3E-84	0.717513	0.18	0.003	7.18E-80	9
Tmem132b	1.47E-83	0.607691	0.144	0.001	2.45E-79	9
Il1rap	3.53E-82	0.851516	0.226	0.011	5.89E-78	9
Phyhipl	3.65E-81	1.151075	0.333	0.055	6.09E-77	9
Slc6a1	5.73E-81	0.826085	0.273	0.021	9.56E-77	9
Gm2a	6.79E-81	0.995252	0.333	0.041	1.13E-76	9
Arl4a	8.4E-81	1.100842	0.367	0.057	1.4E-76	9
Grm5	9.04E-81	0.904329	0.202	0.007	1.51E-76	9
Cldn11	1.64E-80	1.458065	0.168	0.031	2.74E-76	9
Draxin	8.6E-80	-1.40598	0.134	0.564	1.43E-75	9
Meg3	1.6E-79	0.658537	0.416	0.087	2.67E-75	9
Resp18	4.09E-79	0.756287	0.165	0.003	6.82E-75	9
Kcnip3	1.61E-78	1.14894	0.401	0.113	2.68E-74	9
Tns3	4.35E-78	0.751655	0.187	0.006	7.25E-74	9
Lypd1	2.4E-77	0.71224	0.156	0.002	4.01E-73	9
Cog7	2.55E-77	-1.29887	0.165	0.594	4.25E-73	9
Gltp	3.49E-76	1.058131	0.37	0.076	5.82E-72	9
Sema3d	4.97E-76	0.66779	0.151	0.002	8.29E-72	9
Crmp1	1.39E-75	-1.16331	0.268	0.677	2.32E-71	9
Vstm2b	1.66E-75	0.600189	0.136	0.001	2.77E-71	9
Scn3a	3.61E-75	0.839347	0.217	0.011	6.03E-71	9
Clu	4.08E-75	0.816275	0.229	0.015	6.81E-71	9
Gsn	4.19E-74	0.959593	0.195	0.009	6.98E-70	9
Calm2	5.08E-74	-0.79872	0.662	0.879	8.47E-70	9
Tspan2	6.77E-74	0.967811	0.207	0.011	1.13E-69	9
Ddah2	9.33E-74	-1.1595	0.316	0.694	1.56E-69	9
Slc22a3	1.55E-73	0.566328	0.117	0	2.58E-69	9
Slc29a1	1.69E-73	-1.28067	0.151	0.571	2.81E-69	9
Nlgn3	2.68E-73	0.877928	0.263	0.022	4.47E-69	9
Luzp2	4.08E-73	0.803838	0.219	0.012	6.81E-69	9
Adam9	1.46E-72	0.940095	0.328	0.046	2.43E-68	9
Pik3r1	2.52E-72	0.995204	0.436	0.094	4.2E-68	9
Adora1	5.85E-72	0.76297	0.19	0.008	9.75E-68	9
Dab1	7.34E-72	0.958017	0.268	0.027	1.22E-67	9
Stk32a	1.09E-71	0.602171	0.144	0.002	1.81E-67	9
Tmem88b	1.23E-70	0.662955	0.148	0.003	2.05E-66	9
Pax6	2.17E-70	-1.3041	0.114	0.522	3.62E-66	9
Phactr3	5.33E-70	0.982201	0.375	0.066	8.88E-66	9
Bricd5	7.36E-70	0.543058	0.127	0.001	1.23E-65	9
Hip1	1.41E-69	1.019847	0.44	0.109	2.35E-65	9
Lhx1	2.77E-69	-1.39688	0.114	0.506	4.63E-65	9
Sh3bp4	2.89E-69	0.824641	0.241	0.019	4.82E-65	9
Gnb4	1.05E-68	0.994874	0.411	0.097	1.75E-64	9
Tmsb10	1.92E-68	-1.07693	0.311	0.672	3.2E-64	9
Tgfa	2.71E-68	0.572772	0.146	0.003	4.52E-64	9

Slc22a23	3.2E-68	0.707932	0.175	0.007	5.34E-64	9
Ezr	3.76E-68	-1.25187	0.061	0.446	6.27E-64	9
Lrp1	1.03E-67	0.928696	0.309	0.041	1.72E-63	9
Ptprt	2.12E-67	0.675528	0.148	0.003	3.54E-63	9
Dlgap1	2.7E-67	0.96484	0.353	0.06	4.51E-63	9
Chst11	3.2E-67	0.721958	0.187	0.009	5.33E-63	9
Nfia	3.41E-67	-0.92785	0.474	0.781	5.69E-63	9
C1ql2	5.58E-67	0.489329	0.112	0	9.3E-63	9
Slc22a17	9.37E-67	0.92949	0.601	0.242	1.56E-62	9
Syt11	1.05E-66	0.786482	0.798	0.497	1.75E-62	9
Calr	1.29E-66	0.790858	0.779	0.504	2.16E-62	9
Alcam	1.74E-66	0.99064	0.462	0.126	2.9E-62	9
Cd63	1.74E-66	-0.91223	0.47	0.762	2.91E-62	9
Dcaf12l1	2.8E-66	0.552358	0.134	0.002	4.67E-62	9
Rap1gap	9.1E-66	0.650601	0.178	0.007	1.52E-61	9
Stmn2	1.22E-65	-1.55578	0.178	0.561	2.03E-61	9
Zfp365	3.16E-65	0.70768	0.19	0.01	5.27E-61	9
Brinp1	2.47E-64	0.764199	0.248	0.024	4.12E-60	9
G0s2	2.79E-64	0.596495	0.148	0.004	4.66E-60	9
Slc38a3	2.91E-63	0.841924	0.217	0.017	4.86E-59	9
Rab31	3.83E-63	0.896056	0.311	0.049	6.38E-59	9
Tmsb4x	1.02E-62	-0.73114	0.893	0.95	1.71E-58	9
Gng12	1.29E-62	0.943342	0.416	0.113	2.15E-58	9
B3gat1	5.24E-62	0.839778	0.217	0.02	8.74E-58	9
S100a6	1.27E-61	0.837266	0.214	0.017	2.12E-57	9
Ppp1r16b	1.4E-61	0.553641	0.131	0.002	2.33E-57	9
Sstr1	2.06E-61	0.443619	0.107	0.001	3.44E-57	9
Fbxo7	2.29E-61	0.71901	0.234	0.022	3.83E-57	9
Neurod1	5.88E-61	-1.73922	0.182	0.541	9.8E-57	9
Deb1	1.32E-60	0.935502	0.474	0.16	2.2E-56	9
Lrrtm1	1.39E-60	0.622283	0.136	0.003	2.32E-56	9
Pmp22	3.12E-60	0.791342	0.195	0.013	5.2E-56	9
Rbfox3	6.26E-60	-1.25069	0.124	0.502	1.04E-55	9
D430041D	8.47E-60	-1.18139	0.129	0.5	1.41E-55	9
Pfn2	1.85E-59	0.938212	0.487	0.235	3.08E-55	9
Kank1	2.76E-59	0.786209	0.248	0.03	4.61E-55	9
Pcdh11x	3.17E-59	0.620996	0.158	0.006	5.29E-55	9
Pcdh9	5.31E-59	0.83911	0.268	0.035	8.86E-55	9
Nfasc	7.14E-59	1.077608	0.238	0.031	1.19E-54	9
Ehd3	7.26E-59	0.774727	0.241	0.025	1.21E-54	9
Emid1	9.99E-59	0.725579	0.153	0.006	1.67E-54	9
Npas3	1.63E-58	0.828341	0.226	0.022	2.71E-54	9
Eps8	1.84E-58	0.783694	0.243	0.027	3.07E-54	9
Il18	2.52E-58	0.625886	0.178	0.01	4.21E-54	9
Zic4	5.09E-58	-1.16447	0.068	0.426	8.49E-54	9
Dock9	5.94E-58	0.716185	0.185	0.012	9.9E-54	9
Nrep	9.04E-58	-1.1965	0.217	0.577	1.51E-53	9
E130114P1	9.48E-58	-1.11412	0.226	0.593	1.58E-53	9

Pgp	1.24E-57	0.928919	0.445	0.143	2.06E-53	9
4833424O:	3.42E-57	0.855244	0.273	0.038	5.7E-53	9
Timp2	1.87E-56	0.679184	0.209	0.021	3.11E-52	9
Rnd3	4.46E-56	-1.19061	0.092	0.439	7.43E-52	9
Trio	8.59E-56	0.916559	0.414	0.118	1.43E-51	9
Pid1	1.18E-55	0.817495	0.27	0.038	1.97E-51	9
Gpm6b	1.7E-55	0.734407	0.766	0.477	2.84E-51	9
Sash1	2.37E-55	0.867221	0.234	0.03	3.95E-51	9
Rhoc	6.25E-55	0.75768	0.217	0.023	1.04E-50	9
Chn2	1.04E-54	0.984828	0.17	0.01	1.73E-50	9
Tm7sf3	1.44E-54	0.79058	0.316	0.059	2.4E-50	9
Igfbp3	5.03E-54	0.654362	0.158	0.009	8.38E-50	9
H2afv	6.14E-54	-0.88568	0.462	0.734	1.02E-49	9
Canx	7.95E-54	0.635116	0.766	0.591	1.33E-49	9
Pabpc1	9.85E-54	-0.58289	0.81	0.88	1.64E-49	9
Sox21	1.1E-53	0.531438	0.129	0.004	1.83E-49	9
Spry4	1.55E-53	0.593801	0.124	0.003	2.59E-49	9
Nhlh2	1.67E-53	-1.27773	0.09	0.43	2.78E-49	9
Mmd2	1.72E-53	0.744708	0.253	0.034	2.86E-49	9
2900011O(	2.07E-53	0.862781	0.392	0.096	3.45E-49	9
Wscd1	2.31E-53	0.794545	0.309	0.057	3.86E-49	9
Spry1	3.06E-53	0.694832	0.161	0.01	5.1E-49	9
Slitrk3	3.17E-53	0.575796	0.163	0.01	5.28E-49	9
Dpp6	1.66E-52	0.69033	0.212	0.022	2.77E-48	9
Mpzl1	1.77E-52	0.93742	0.37	0.139	2.96E-48	9
Pde4b	2.09E-52	0.821063	0.275	0.045	3.49E-48	9
Nfix	3.13E-52	-0.88148	0.336	0.681	5.23E-48	9
Cyp2j9	3.99E-52	0.50089	0.124	0.003	6.65E-48	9
Cplx2	4.95E-52	-1.12346	0.158	0.503	8.26E-48	9
1500016L(	7.73E-52	-1.17504	0.066	0.39	1.29E-47	9
Neto1	8.5E-52	0.486329	0.107	0.002	1.42E-47	9
Mmp16	1.41E-50	0.916933	0.326	0.082	2.34E-46	9
Chadl	2.51E-50	0.501684	0.129	0.005	4.18E-46	9
Epb4.1l2	3.15E-50	0.946384	0.26	0.089	5.25E-46	9
Lrrfip1	5.49E-50	0.798891	0.253	0.039	9.16E-46	9
Dusp26	9.38E-50	0.720113	0.299	0.059	1.56E-45	9
Plk2	1.85E-49	0.654596	0.153	0.009	3.09E-45	9
Col16a1	2.04E-49	0.482271	0.112	0.003	3.4E-45	9
Flrt1	2.63E-49	0.614241	0.139	0.006	4.39E-45	9
Cbr3	2.93E-49	0.538776	0.134	0.006	4.89E-45	9
Ppapdc1a	3.76E-49	0.510941	0.122	0.004	6.27E-45	9
Cbfa2t3	4.65E-49	-1.09203	0.083	0.41	7.75E-45	9
Cacng2	5.7E-49	-1.07277	0.041	0.345	9.5E-45	9
A930009A:	1.55E-48	0.699037	0.202	0.022	2.58E-44	9
Tbata	2.03E-48	-1.19778	0.044	0.346	3.39E-44	9
Hnrnpab	7.1E-48	-0.6316	0.667	0.819	1.18E-43	9
S100a4	7.23E-48	0.522493	0.109	0.003	1.21E-43	9
Chpt1	1.15E-47	0.777299	0.285	0.054	1.91E-43	9

Rpl13a	1.72E-47	-0.52912	0.876	0.929	2.87E-43	9
Cbx1	2.01E-47	-0.77577	0.438	0.691	3.36E-43	9
Sdc3	2.3E-47	0.855747	0.319	0.071	3.83E-43	9
Mapt	2.9E-47	0.719896	0.53	0.208	4.83E-43	9
Ntrk2	4.95E-47	0.765434	0.336	0.077	8.26E-43	9
Arhgdig	5.55E-47	0.64192	0.173	0.015	9.26E-43	9
Ptma	1.04E-46	-0.87641	0.282	0.588	1.73E-42	9
Cadm4	1.2E-46	0.768847	0.387	0.107	2E-42	9
Sgk1	1.59E-46	0.849421	0.209	0.03	2.65E-42	9
Cacna2d1	1.65E-46	-1.11565	0.075	0.382	2.76E-42	9
Rps5	1.87E-46	-0.5021	0.92	0.954	3.12E-42	9
Gria2	2.01E-46	0.631393	0.793	0.514	3.35E-42	9
H1f0	5.61E-46	-0.89817	0.319	0.623	9.36E-42	9
Tmem176a	1.34E-45	0.600168	0.158	0.012	2.24E-41	9
Fam3c	4.03E-45	0.801133	0.314	0.079	6.72E-41	9
5730559C1	1.96E-44	0.386859	0.102	0.002	3.27E-40	9
Abhd12	2.5E-44	0.723013	0.345	0.087	4.17E-40	9
Tubb3	2.53E-44	-1.22473	0.173	0.481	4.21E-40	9
Rtkn	2.72E-44	0.594006	0.187	0.02	4.54E-40	9
Zdhhc2	3.17E-44	0.711959	0.204	0.028	5.3E-40	9
Mt3	4.35E-44	0.643622	0.19	0.021	7.26E-40	9
Sh3gl3	1.05E-43	0.689899	0.234	0.037	1.76E-39	9
Plekha2	1.44E-43	0.484316	0.107	0.003	2.41E-39	9
Fam210b	1.75E-43	-1.0502	0.044	0.32	2.92E-39	9
Celf2	3.32E-43	-0.89488	0.345	0.644	5.54E-39	9
Uncx	3.93E-43	-1.10051	0.056	0.324	6.56E-39	9
Pea15a	4.98E-43	0.818402	0.457	0.169	8.3E-39	9
Lphn3	6.26E-43	0.699702	0.277	0.056	1.04E-38	9
Dpysl4	7.09E-43	-0.94603	0.173	0.498	1.18E-38	9
Mycn	9.39E-43	-1.00175	0.073	0.37	1.57E-38	9
Hsd11b2	1.05E-42	-1.07733	0.032	0.293	1.74E-38	9
Nrxn2	1.19E-42	0.763707	0.333	0.084	1.98E-38	9
Rplp0	1.46E-42	-0.52777	0.781	0.874	2.44E-38	9
Cp	2.54E-42	0.565436	0.139	0.011	4.23E-38	9
Ppp2r2c	4.12E-42	-0.93275	0.2	0.512	6.87E-38	9
Acox1	5.41E-42	0.644079	0.265	0.052	9.02E-38	9
Cav2	5.62E-42	0.49265	0.124	0.007	9.38E-38	9
Cdh11	7.05E-42	0.597248	0.151	0.012	1.18E-37	9
Cyfip2	8.8E-42	0.778214	0.255	0.048	1.47E-37	9
Hsd17b12	1.17E-41	0.77392	0.46	0.181	1.96E-37	9
Hmgcs1	1.37E-41	0.834108	0.409	0.145	2.29E-37	9
Dusp15	1.84E-41	0.48768	0.134	0.008	3.07E-37	9
Smc2	3.07E-41	-1.02038	0.304	0.551	5.12E-37	9
Rps9	4.59E-41	-0.42893	0.915	0.929	7.65E-37	9
Rps3	4.59E-41	-0.4591	0.876	0.923	7.66E-37	9
Rpl4	5.94E-41	-0.48219	0.864	0.909	9.91E-37	9
Tspan6	7.15E-41	0.751788	0.494	0.24	1.19E-36	9
Gm2694	1.07E-40	-1.00008	0.078	0.365	1.78E-36	9

Marc2	1.2E-40	0.776901	0.299	0.078	2E-36	9
Scamp2	1.31E-40	0.780661	0.382	0.129	2.19E-36	9
Kcnd3	3.87E-40	0.624066	0.151	0.013	6.46E-36	9
Tead2	8.71E-40	-0.88743	0.029	0.283	1.45E-35	9
Prkcq	9.61E-40	0.737772	0.255	0.058	1.6E-35	9
Slitrk2	2.76E-39	0.474447	0.109	0.005	4.61E-35	9
Lnx1	3.42E-39	0.700487	0.173	0.022	5.7E-35	9
Cdh10	3.57E-39	0.45686	0.134	0.011	5.95E-35	9
Taf9b	4.68E-39	0.473275	0.139	0.011	7.8E-35	9
Rassf4	5.04E-39	-0.98464	0.1	0.382	8.4E-35	9
Fbn2	9.25E-39	0.568455	0.117	0.007	1.54E-34	9
Tmem255a	1.16E-38	0.499796	0.105	0.005	1.94E-34	9
Nacc2	1.19E-38	0.593432	0.153	0.015	1.98E-34	9
Limch1	1.32E-38	0.627561	0.173	0.02	2.2E-34	9
Ier5	1.43E-38	-0.91646	0.105	0.401	2.38E-34	9
Ccnd2	1.77E-38	-0.83697	0.421	0.667	2.96E-34	9
Necab2	6.94E-38	0.412913	0.114	0.006	1.16E-33	9
Cav1	1.24E-37	0.570529	0.148	0.016	2.07E-33	9
Eid1	1.52E-37	0.59676	0.757	0.496	2.53E-33	9
Hepacam	1.76E-37	0.527152	0.117	0.007	2.94E-33	9
Dock10	2.03E-37	0.536506	0.144	0.013	3.39E-33	9
Spock2	2.63E-37	0.629287	0.265	0.06	4.39E-33	9
Srebf1	3.07E-37	-1.0181	0.078	0.34	5.11E-33	9
Epha4	3.35E-37	0.474668	0.127	0.009	5.59E-33	9
Mex3a	4.25E-37	-0.88642	0.173	0.47	7.09E-33	9
Tmem9b	7.68E-37	0.680873	0.35	0.106	1.28E-32	9
Npc1	9.95E-37	0.650125	0.214	0.038	1.66E-32	9
Lrrtm3	1.23E-36	0.483468	0.146	0.015	2.05E-32	9
Pde1c	1.56E-36	-1.0028	0.112	0.393	2.59E-32	9
Wipf1	1.74E-36	0.59279	0.175	0.024	2.91E-32	9
Rev3l	2.71E-36	0.803003	0.307	0.092	4.52E-32	9
Ildr2	3.11E-36	0.442827	0.107	0.005	5.19E-32	9
Gabra3	4.34E-36	0.580239	0.144	0.015	7.23E-32	9
Ppp2r2b	5.03E-36	0.659206	0.297	0.079	8.4E-32	9
Hbegf	6.19E-36	0.627009	0.156	0.017	1.03E-31	9
Dynlt3	6.39E-36	0.618279	0.224	0.042	1.07E-31	9
Ptpro	8.13E-36	0.431548	0.136	0.013	1.36E-31	9
Hsp90b1	9.7E-36	0.525504	0.793	0.689	1.62E-31	9
Insm1	1.01E-35	-0.94472	0.049	0.298	1.69E-31	9
Camk1	1.19E-35	0.692909	0.241	0.052	1.99E-31	9
Mgll	1.46E-35	0.798101	0.358	0.127	2.44E-31	9
Lbh	2.78E-35	0.732149	0.27	0.069	4.63E-31	9
Pcdh7	3.48E-35	0.465329	0.134	0.012	5.81E-31	9
Taok3	3.67E-35	0.670958	0.302	0.082	6.12E-31	9
Sv2a	4.2E-35	0.5312	0.263	0.067	7E-31	9
Nasp	5.55E-35	-0.7042	0.453	0.644	9.26E-31	9
Sapcd2	6.21E-35	0.729546	0.234	0.076	1.04E-30	9
Cd302	1.47E-34	0.699815	0.231	0.048	2.45E-30	9

Gria4	1.65E-34	0.733891	0.36	0.119	2.75E-30	9
Tln2	1.69E-34	0.528344	0.151	0.017	2.82E-30	9
Kif21a	1.81E-34	-0.9683	0.102	0.351	3.02E-30	9
Cpne8	1.87E-34	0.486688	0.127	0.011	3.12E-30	9
Kazn	3.02E-34	0.512821	0.148	0.018	5.04E-30	9
Cryab	3.19E-34	0.710688	0.153	0.019	5.32E-30	9
Miat	3.59E-34	-0.9868	0.265	0.521	5.99E-30	9
Ppic	3.68E-34	-0.83773	0.024	0.244	6.14E-30	9
Mif4gd	4.48E-34	0.551546	0.18	0.027	7.48E-30	9
Rps14	5.58E-34	-0.40936	0.922	0.951	9.31E-30	9
Nim1	7.42E-34	0.519366	0.136	0.013	1.24E-29	9
Shc4	8.24E-34	0.641022	0.19	0.033	1.37E-29	9
Hpca	1.19E-33	-0.96474	0.049	0.282	1.99E-29	9
Lamp1	1.62E-33	0.649078	0.569	0.33	2.7E-29	9
Dynll2	1.71E-33	0.678332	0.409	0.293	2.86E-29	9
Apoe	2.05E-33	-0.92752	0.401	0.245	3.42E-29	9
Lrrc4	3.15E-33	0.370363	0.105	0.007	5.26E-29	9
Gsg1l	3.87E-33	-0.86744	0.036	0.26	6.46E-29	9
Arxes1	4.25E-33	0.624393	0.253	0.06	7.09E-29	9
Smc4	4.7E-33	-0.85978	0.372	0.571	7.84E-29	9
Fermt2	5.99E-33	0.642028	0.467	0.198	1E-28	9
Pdlim5	7.55E-33	0.451753	0.127	0.011	1.26E-28	9
Gpt2	8.83E-33	0.64847	0.251	0.062	1.47E-28	9
Shisa4	1.01E-32	0.645938	0.224	0.048	1.68E-28	9
Trib2	1.11E-32	0.691439	0.282	0.083	1.85E-28	9
Vimp	1.21E-32	0.726993	0.414	0.184	2.02E-28	9
Slc44a1	1.28E-32	0.799496	0.282	0.099	2.14E-28	9
Metrn	1.4E-32	0.522139	0.148	0.018	2.33E-28	9
Rps24	1.59E-32	-0.50326	0.691	0.823	2.66E-28	9
Chd5	1.75E-32	0.523063	0.102	0.006	2.92E-28	9
Caskin2	1.99E-32	0.472316	0.105	0.007	3.31E-28	9
Tmbim6	2.23E-32	0.649564	0.574	0.318	3.72E-28	9
Rps20	2.23E-32	-0.54431	0.633	0.781	3.72E-28	9
Hmgb2	3.51E-32	-0.91368	0.161	0.405	5.86E-28	9
Lrch3	4E-32	0.647632	0.273	0.072	6.67E-28	9
Aldoc	7.18E-32	0.538487	0.17	0.029	1.2E-27	9
Meis1	7.32E-32	-0.86326	0.124	0.378	1.22E-27	9
Arvcf	9.94E-32	0.536418	0.124	0.011	1.66E-27	9
Cd200	1.05E-31	0.619207	0.207	0.041	1.76E-27	9
Evi5l	1.92E-31	0.64046	0.197	0.037	3.2E-27	9
Gpx3	1.94E-31	0.49399	0.109	0.008	3.23E-27	9
Cdk6	2.56E-31	-0.7884	0.032	0.248	4.28E-27	9
Gyg	3E-31	0.410777	0.105	0.007	5.01E-27	9
Cks1b	3.03E-31	-0.88387	0.109	0.349	5.05E-27	9
Grin3a	3.43E-31	0.667237	0.231	0.056	5.72E-27	9
Tspan13	3.75E-31	0.70626	0.431	0.201	6.26E-27	9
Syt16	5.2E-31	0.487534	0.119	0.011	8.68E-27	9
Eif1b	6.53E-31	0.621412	0.564	0.33	1.09E-26	9

Sec11c	8.1E-31	0.693732	0.433	0.207	1.35E-26	9
Rps26	1.01E-30	-0.464	0.779	0.848	1.68E-26	9
Dbndd2	1.1E-30	0.569435	0.153	0.021	1.83E-26	9
Fgf9	1.22E-30	-0.8237	0.044	0.265	2.04E-26	9
Gucy1a3	1.95E-30	0.49542	0.105	0.008	3.25E-26	9
Tcf4	2.03E-30	-0.41947	0.876	0.926	3.38E-26	9
Fchsd2	2.04E-30	0.588132	0.18	0.031	3.4E-26	9
Add3	7.26E-30	0.632865	0.311	0.099	1.21E-25	9
Stmn1	9.92E-30	-0.7751	0.066	0.289	1.65E-25	9
Spry2	1.46E-29	0.64614	0.202	0.045	2.43E-25	9
Cntn2	1.73E-29	-1.18667	0.061	0.268	2.88E-25	9
Gng3	1.76E-29	0.453568	0.589	0.31	2.94E-25	9
Mtap	2.02E-29	0.67475	0.27	0.09	3.37E-25	9
Chd7	3.51E-29	-0.66467	0.423	0.652	5.85E-25	9
Rbp4	4.05E-29	-0.85666	0.039	0.246	6.76E-25	9
Gm11223	6.55E-29	-1.02055	0.027	0.217	1.09E-24	9
Gm17750	6.98E-29	-0.83553	0.122	0.372	1.16E-24	9
Otx2	7.51E-29	-0.79478	0.034	0.221	1.25E-24	9
Elovl7	7.73E-29	0.523377	0.117	0.012	1.29E-24	9
Gm9800	1.01E-28	-0.66764	0.316	0.539	1.68E-24	9
Dtd1	1.02E-28	0.61222	0.265	0.075	1.7E-24	9
Clmp	1.32E-28	-0.90641	0.041	0.247	2.21E-24	9
Arxes2	1.51E-28	0.650438	0.285	0.094	2.52E-24	9
Fez1	1.59E-28	0.611032	0.562	0.327	2.65E-24	9
Nenf	2.2E-28	0.592765	0.358	0.131	3.66E-24	9
Fam49b	2.43E-28	0.587196	0.275	0.08	4.05E-24	9
Mfap2	3.01E-28	0.560861	0.197	0.042	5.02E-24	9
Mageh1	3.42E-28	0.639204	0.321	0.115	5.71E-24	9
Kif5c	4.13E-28	-0.84137	0.173	0.403	6.89E-24	9
Arl2bp	4.52E-28	0.659995	0.433	0.212	7.54E-24	9
Lmbrd1	6.03E-28	0.560983	0.246	0.066	1.01E-23	9
Itgb8	6.2E-28	0.541333	0.131	0.018	1.03E-23	9
Polg	6.27E-28	0.627513	0.209	0.05	1.05E-23	9
Ly6h	6.93E-28	0.455127	0.136	0.02	1.16E-23	9
2810417H1	8.54E-28	-0.90626	0.165	0.396	1.42E-23	9
Jam2	9.58E-28	0.524382	0.168	0.03	1.6E-23	9
Ggct	9.62E-28	0.557336	0.175	0.033	1.6E-23	9
Cxxc5	1.26E-27	-0.69794	0.302	0.549	2.1E-23	9
Arhgap31	1.45E-27	0.578298	0.18	0.036	2.43E-23	9
Tub	1.55E-27	0.58256	0.19	0.039	2.59E-23	9
Hnrnpu	2.07E-27	-0.38717	0.808	0.866	3.45E-23	9
Atpif1	3.02E-27	-0.45741	0.689	0.818	5.03E-23	9
1810037I1	3.06E-27	0.600248	0.418	0.191	5.1E-23	9
Scamp5	3.48E-27	0.56364	0.229	0.058	5.8E-23	9
Limd1	3.66E-27	0.64631	0.234	0.067	6.1E-23	9
Lrrn3	3.88E-27	0.515168	0.146	0.022	6.48E-23	9
Tceal3	4.3E-27	0.669733	0.302	0.106	7.17E-23	9
Rgs7bp	4.37E-27	0.550228	0.153	0.025	7.28E-23	9

Gjc1	4.62E-27	-0.69597	0.019	0.2	7.7E-23	9
Amz1	5.57E-27	0.3705	0.119	0.014	9.29E-23	9
C1ql3	5.86E-27	0.620348	0.212	0.054	9.77E-23	9
Apba2	8.84E-27	-0.73603	0.175	0.419	1.47E-22	9
Vapa	9.44E-27	0.574059	0.557	0.334	1.58E-22	9
Nhlh1	1.21E-26	-0.88467	0.039	0.227	2.02E-22	9
Grid1	1.26E-26	0.440367	0.107	0.01	2.1E-22	9
Hmgn1	1.54E-26	-0.5765	0.411	0.626	2.57E-22	9
Rpl22	1.86E-26	-0.4305	0.713	0.789	3.11E-22	9
2700094K1	2.33E-26	-0.54124	0.448	0.617	3.88E-22	9
A230050P2	2.43E-26	0.403258	0.117	0.013	4.05E-22	9
Mcm7	2.74E-26	-0.71672	0.151	0.394	4.57E-22	9
Sox9	2.88E-26	-0.81041	0.056	0.259	4.8E-22	9
Mmp2	2.94E-26	0.47978	0.114	0.014	4.91E-22	9
Fkbp3	3.26E-26	-0.51555	0.62	0.748	5.44E-22	9
Dek	3.31E-26	-0.52545	0.623	0.695	5.53E-22	9
BC005764	3.97E-26	-0.8471	0.022	0.189	6.63E-22	9
Rin2	5.66E-26	0.501014	0.129	0.018	9.44E-22	9
Fam213a	6.23E-26	0.652585	0.263	0.084	1.04E-21	9
Rab33a	8.73E-26	0.682829	0.19	0.048	1.46E-21	9
Mt1	1.19E-25	0.552136	0.384	0.163	1.99E-21	9
Reep5	1.48E-25	0.631518	0.336	0.133	2.46E-21	9
Ebf3	1.61E-25	-0.74692	0.027	0.207	2.69E-21	9
Rap2a	1.77E-25	0.689569	0.353	0.165	2.95E-21	9
Nrxn1	2.97E-25	0.482791	0.572	0.312	4.95E-21	9
Mab21l1	4.35E-25	-0.75717	0.041	0.229	7.26E-21	9
Tle1	6.38E-25	-0.72175	0.056	0.229	1.06E-20	9
Selk	6.49E-25	0.524541	0.628	0.39	1.08E-20	9
Bcl11a	1.06E-24	-0.6713	0.034	0.214	1.76E-20	9
Prmt8	1.21E-24	-0.74374	0.068	0.277	2.02E-20	9
Rasl11a	1.33E-24	0.594698	0.131	0.027	2.21E-20	9
Phldb1	1.45E-24	0.583683	0.192	0.047	2.42E-20	9
Wasf1	1.95E-24	0.607926	0.294	0.105	3.25E-20	9
Sox4	2.17E-24	-0.68012	0.389	0.587	3.62E-20	9
Crip2	2.8E-24	-0.75609	0.051	0.244	4.67E-20	9
Psap	2.98E-24	0.462209	0.399	0.179	4.97E-20	9
Ezh2	3.19E-24	-0.58838	0.423	0.614	5.33E-20	9
Abtb2	3.4E-24	0.377446	0.107	0.013	5.67E-20	9
Lgi3	3.88E-24	0.463041	0.119	0.016	6.47E-20	9
Hap1	4.8E-24	0.531623	0.158	0.032	8E-20	9
Tmco3	4.88E-24	0.425028	0.161	0.032	8.14E-20	9
Dhcr24	5.23E-24	0.565539	0.226	0.065	8.72E-20	9
Dynll1	7.07E-24	0.454924	0.691	0.591	1.18E-19	9
Tmem191c	7.09E-24	0.456666	0.144	0.025	1.18E-19	9
Sstr2	9.45E-24	-0.75101	0.027	0.194	1.58E-19	9
Rplp2	1.02E-23	-0.38259	0.8	0.841	1.71E-19	9
Fnta	1.21E-23	0.580407	0.328	0.134	2.01E-19	9
Vps37b	1.22E-23	-0.67028	0.251	0.475	2.04E-19	9

Ugdh	1.23E-23	0.621276	0.26	0.092	2.06E-19	9
Rab14	1.7E-23	0.511397	0.616	0.407	2.83E-19	9
Ranbp1	2.14E-23	-0.49776	0.591	0.677	3.57E-19	9
Spag9	2.46E-23	0.56124	0.526	0.301	4.11E-19	9
Dmd	2.89E-23	0.514702	0.136	0.023	4.82E-19	9
Malat1	2.9E-23	0.416888	0.956	0.955	4.83E-19	9
Serbp1	2.98E-23	-0.35142	0.832	0.87	4.98E-19	9
Ccnd1	3.5E-23	0.544797	0.659	0.518	5.85E-19	9
Abcg1	4.99E-23	0.40808	0.127	0.022	8.33E-19	9
Mllt4	5E-23	-0.70273	0.141	0.368	8.34E-19	9
H2afy	5.19E-23	-0.58078	0.387	0.596	8.65E-19	9
Rcor2	5.27E-23	-0.74304	0.051	0.229	8.79E-19	9
Car11	6.36E-23	0.441839	0.122	0.018	1.06E-18	9
Gnb2l1	6.57E-23	-0.37828	0.815	0.873	1.1E-18	9
Ppt1	7.81E-23	0.498887	0.268	0.09	1.3E-18	9
Ust	9.04E-23	0.414105	0.153	0.031	1.51E-18	9
Fnbp1l	1.01E-22	-0.71196	0.241	0.458	1.68E-18	9
Hk2	1.04E-22	-0.63673	0.019	0.176	1.74E-18	9
Itm2c	1.31E-22	0.545749	0.431	0.206	2.19E-18	9
Rps19	1.44E-22	-0.44659	0.655	0.756	2.4E-18	9
Hey1	1.5E-22	-0.79171	0.085	0.277	2.5E-18	9
Hirip3	1.71E-22	-0.68716	0.165	0.387	2.86E-18	9
Fam63b	1.71E-22	0.492747	0.253	0.083	2.86E-18	9
Snx22	1.75E-22	0.48757	0.185	0.045	2.91E-18	9
Usp24	1.95E-22	0.56806	0.146	0.031	3.26E-18	9
Slc17a6	2.03E-22	-0.73013	0.012	0.151	3.39E-18	9
Eef2	2.1E-22	-0.47159	0.564	0.719	3.5E-18	9
Map3k1	2.21E-22	-0.69452	0.158	0.375	3.69E-18	9
Pqlc1	2.74E-22	-0.69656	0.097	0.302	4.56E-18	9
Gnal	2.95E-22	0.43767	0.112	0.015	4.93E-18	9
Atcay	2.99E-22	0.535031	0.217	0.062	4.98E-18	9
Ddx5	3.01E-22	-0.41803	0.689	0.818	5.02E-18	9
Dut	3.39E-22	-0.70187	0.217	0.44	5.66E-18	9
Laptm4b	3.7E-22	0.582831	0.319	0.137	6.18E-18	9
Stxbp3a	4.18E-22	0.432233	0.117	0.018	6.97E-18	9
Gpm6a	4.28E-22	0.331417	0.635	0.403	7.15E-18	9
Papss1	4.95E-22	0.557226	0.336	0.142	8.26E-18	9
Abcd3	5.13E-22	0.590896	0.375	0.177	8.56E-18	9
Taldo1	5.31E-22	0.550592	0.484	0.317	8.86E-18	9
Irs2	7.62E-22	0.524785	0.209	0.06	1.27E-17	9
Fip1l1	1.03E-21	0.557064	0.509	0.304	1.72E-17	9
Cald1	1.04E-21	-0.6505	0.348	0.54	1.74E-17	9
Srrm4	1.06E-21	-0.70561	0.034	0.199	1.77E-17	9
Abracl	1.06E-21	-0.6354	0.1	0.306	1.77E-17	9
Tubb2a	1.09E-21	0.356941	0.455	0.24	1.82E-17	9
Aplp2	1.13E-21	0.540926	0.54	0.318	1.88E-17	9
Cxcl14	1.39E-21	0.564754	0.158	0.038	2.31E-17	9
Dpysl3	1.43E-21	0.384578	0.418	0.214	2.38E-17	9

Myl12a	1.62E-21	-0.68215	0.151	0.332	2.7E-17	9
Abhd2	1.63E-21	0.534987	0.136	0.029	2.72E-17	9
Gng2	1.75E-21	-0.59929	0.173	0.401	2.93E-17	9
Hjurp	2.81E-21	-0.68124	0.241	0.438	4.68E-17	9
Zbtb18	2.85E-21	-0.72315	0.08	0.27	4.75E-17	9
Nkd1	3.98E-21	-0.65986	0.068	0.252	6.64E-17	9
Abhd6	4.62E-21	0.411365	0.156	0.034	7.71E-17	9
Cbx5	4.75E-21	-0.51823	0.513	0.656	7.93E-17	9
Acap3	5.69E-21	0.485751	0.178	0.044	9.49E-17	9
Hmgcl	6.35E-21	0.516191	0.229	0.074	1.06E-16	9
Itga9	7.29E-21	0.485902	0.109	0.017	1.22E-16	9
Sept2	8.76E-21	0.536881	0.414	0.217	1.46E-16	9
Lrrtm2	9.11E-21	0.358286	0.17	0.048	1.52E-16	9
Eef1b2	1.09E-20	-0.4696	0.584	0.736	1.82E-16	9
Scn2a1	1.09E-20	0.361661	0.102	0.015	1.83E-16	9
Traf4	1.45E-20	0.511219	0.241	0.08	2.42E-16	9
Tceal5	1.62E-20	0.479927	0.144	0.029	2.7E-16	9
Txndc15	1.73E-20	0.583783	0.299	0.126	2.88E-16	9
Celf4	1.9E-20	-0.8704	0.146	0.324	3.16E-16	9
Atoh1	2.05E-20	-0.66957	0.024	0.167	3.42E-16	9
Pcdhga9	2.68E-20	0.485235	0.606	0.415	4.47E-16	9
Tanc2	2.77E-20	0.415007	0.102	0.014	4.62E-16	9
Heg1	2.97E-20	-0.62938	0.022	0.16	4.96E-16	9
Lcorl	3.28E-20	0.611241	0.248	0.092	5.46E-16	9
Mki67	4.36E-20	-0.74587	0.2	0.399	7.28E-16	9
Fzd2	4.69E-20	-0.5889	0.019	0.164	7.82E-16	9
Arl6ip1	4.79E-20	0.507516	0.747	0.545	7.99E-16	9
Reep1	5.25E-20	0.417738	0.231	0.078	8.76E-16	9
Islr2	5.82E-20	-0.6293	0.029	0.183	9.71E-16	9
Kcna1	5.96E-20	0.312669	0.107	0.025	9.94E-16	9
Ccng2	6.89E-20	-0.64948	0.054	0.215	1.15E-15	9
Reep3	7.32E-20	0.519069	0.465	0.251	1.22E-15	9
Map1b	7.34E-20	-0.5918	0.37	0.592	1.22E-15	9
Orai1	7.41E-20	0.440459	0.119	0.021	1.24E-15	9
Maged1	8.68E-20	0.473888	0.62	0.424	1.45E-15	9
Wdr1	1.94E-19	0.525718	0.238	0.084	3.24E-15	9
Aatk	2.44E-19	0.360834	0.129	0.026	4.07E-15	9
Rnmt	2.46E-19	-0.67572	0.168	0.352	4.1E-15	9
Spred1	3.17E-19	0.526916	0.268	0.104	5.28E-15	9
Rsu1	4.15E-19	0.509492	0.287	0.114	6.92E-15	9
Thra	4.8E-19	0.510165	0.428	0.224	8E-15	9
Anp32b	4.99E-19	-0.49819	0.457	0.593	8.33E-15	9
Npm1	6.32E-19	-0.44846	0.53	0.649	1.05E-14	9
Nr3c1	6.34E-19	0.52973	0.292	0.121	1.06E-14	9
Rbms1	6.64E-19	-0.59514	0.054	0.22	1.11E-14	9
Gaa	7.94E-19	0.374799	0.124	0.025	1.32E-14	9
Ctnnd2	8.63E-19	0.610349	0.236	0.095	1.44E-14	9
Lgals1	8.91E-19	-0.75326	0.066	0.232	1.49E-14	9

Nipa1	8.99E-19	0.381766	0.127	0.025	1.5E-14	9
Tmbim4	9.12E-19	0.503312	0.321	0.14	1.52E-14	9
RP23-45G1	9.45E-19	-0.61639	0.131	0.331	1.58E-14	9
Ptch2	9.67E-19	-0.57594	0.022	0.155	1.61E-14	9
Hnrnpm	1.02E-18	-0.41832	0.625	0.737	1.7E-14	9
Hnrnpd	1.34E-18	-0.48763	0.465	0.613	2.24E-14	9
Ralb	1.42E-18	0.376214	0.105	0.016	2.37E-14	9
Mdk	1.46E-18	-0.69937	0.185	0.384	2.43E-14	9
Gde1	1.86E-18	0.462767	0.316	0.137	3.1E-14	9
Atp6v0b	2.12E-18	0.478063	0.467	0.262	3.54E-14	9
Fstl1	2.66E-18	-0.64343	0.039	0.183	4.43E-14	9
Mfge8	3.03E-18	0.446705	0.139	0.031	5.05E-14	9
Ndn	3.18E-18	0.504736	0.418	0.218	5.3E-14	9
Srsf3	4.25E-18	-0.43413	0.521	0.659	7.09E-14	9
Serinc5	4.74E-18	0.513097	0.18	0.058	7.91E-14	9
Rpl32	4.79E-18	-0.31413	0.861	0.89	7.98E-14	9
Paip2	5.73E-18	-0.52079	0.392	0.552	9.56E-14	9
Zfand5	5.8E-18	-0.6209	0.243	0.419	9.67E-14	9
Sept8	6.4E-18	0.50951	0.292	0.125	1.07E-13	9
Zdbf2	6.52E-18	-0.52984	0.017	0.136	1.09E-13	9
Top2a	6.63E-18	-0.76614	0.255	0.431	1.11E-13	9
Snx25	7.68E-18	0.439285	0.107	0.02	1.28E-13	9
Lima1	7.95E-18	0.510712	0.285	0.118	1.33E-13	9
Ypel3	9.09E-18	-0.59327	0.263	0.439	1.52E-13	9
Cdkn1b	9.18E-18	-0.55689	0.282	0.471	1.53E-13	9
Boc	9.96E-18	-0.57733	0.024	0.158	1.66E-13	9
Chd4	1.05E-17	-0.4238	0.647	0.772	1.76E-13	9
Tmpo	1.11E-17	-0.59241	0.263	0.456	1.86E-13	9
Atp1b1	1.12E-17	0.527434	0.173	0.049	1.87E-13	9
Emc7	1.14E-17	0.531935	0.307	0.14	1.91E-13	9
Celsr2	1.47E-17	-0.65945	0.083	0.241	2.46E-13	9
Smc1a	1.53E-17	-0.45813	0.479	0.634	2.55E-13	9
Snrpf	1.57E-17	-0.55636	0.26	0.431	2.62E-13	9
Glrb	1.57E-17	0.45548	0.148	0.037	2.62E-13	9
Pcna	1.76E-17	-0.64536	0.221	0.361	2.94E-13	9
Spc24	1.8E-17	-0.60927	0.078	0.246	3E-13	9
Rps21	1.81E-17	-0.34276	0.776	0.834	3.02E-13	9
Chrna3	1.85E-17	-0.61572	0.01	0.12	3.08E-13	9
Nckap1	2.04E-17	0.526718	0.292	0.127	3.4E-13	9
Nrxn3	2.09E-17	0.449107	0.139	0.034	3.49E-13	9
Zic5	2.18E-17	-0.58045	0.027	0.163	3.64E-13	9
Gse1	2.2E-17	-0.62033	0.073	0.24	3.67E-13	9
Hmgb3	2.59E-17	-0.58241	0.134	0.31	4.32E-13	9
Nap1l3	2.59E-17	0.307541	0.102	0.02	4.33E-13	9
Osbpl1a	3.54E-17	0.489059	0.182	0.056	5.91E-13	9
Gtl3	3.62E-17	0.534084	0.316	0.181	6.04E-13	9
Reln	3.89E-17	-0.68366	0.041	0.183	6.49E-13	9
H3f3a	3.93E-17	-0.56535	0.207	0.385	6.56E-13	9

Ncl	4.75E-17	-0.28877	0.876	0.896	7.92E-13	9
BC034090	4.79E-17	-0.52859	0.012	0.13	7.99E-13	9
Nell2	4.8E-17	0.554417	0.265	0.129	8E-13	9
Hmgn5	4.84E-17	-0.6379	0.253	0.428	8.08E-13	9
Cuedc1	5.1E-17	0.547858	0.236	0.092	8.5E-13	9
Stat3	5.8E-17	0.422301	0.151	0.039	9.67E-13	9
Gm3764	5.81E-17	0.541731	0.287	0.127	9.7E-13	9
Trp53i11	6.35E-17	-0.60581	0.056	0.204	1.06E-12	9
Tex14	6.9E-17	-0.70109	0.024	0.148	1.15E-12	9
Syt13	7.89E-17	-0.50111	0.012	0.129	1.32E-12	9
Tcf7l2	8.26E-17	0.325981	0.114	0.023	1.38E-12	9
Hdgf	8.6E-17	-0.49321	0.36	0.536	1.44E-12	9
Frmd4b	9.85E-17	-0.50999	0.015	0.13	1.64E-12	9
Sez6l	1.06E-16	0.41613	0.185	0.058	1.77E-12	9
Selt	1.08E-16	0.488873	0.236	0.091	1.8E-12	9
Rps11	1.14E-16	-0.35517	0.689	0.779	1.9E-12	9
Tmem63b	1.53E-16	0.408533	0.214	0.077	2.55E-12	9
Mfap4	1.84E-16	-0.6601	0.032	0.164	3.07E-12	9
Ndrg2	1.95E-16	0.511341	0.355	0.18	3.26E-12	9
8430419L(	1.98E-16	0.410238	0.192	0.063	3.3E-12	9
Prox1	1.99E-16	-0.54804	0.071	0.232	3.32E-12	9
Dhx32	2.22E-16	-0.59303	0.063	0.203	3.71E-12	9
Rrbp1	2.26E-16	0.52189	0.363	0.184	3.76E-12	9
Lhfpl2	2.57E-16	0.346572	0.117	0.025	4.29E-12	9
Akap6	2.67E-16	-0.60881	0.071	0.222	4.46E-12	9
Arpc5	2.77E-16	0.476743	0.496	0.317	4.63E-12	9
Tuba1b	2.82E-16	-0.51167	0.348	0.484	4.7E-12	9
Efhd2	2.96E-16	-0.52226	0.015	0.132	4.93E-12	9
Tmem30a	3.13E-16	0.4905	0.35	0.175	5.22E-12	9
Slc35b2	3.31E-16	0.483103	0.353	0.177	5.52E-12	9
Sox18	3.44E-16	-0.55277	0.024	0.15	5.75E-12	9
Dhrs7	4.09E-16	0.475125	0.197	0.068	6.83E-12	9
Mcm6	4.18E-16	-0.63011	0.112	0.279	6.98E-12	9
Klhl13	4.22E-16	0.523088	0.241	0.1	7.03E-12	9
Hdac2	4.29E-16	-0.4547	0.358	0.519	7.16E-12	9
Cenpf	4.39E-16	-0.57863	0.197	0.366	7.32E-12	9
Gpr153	4.47E-16	-0.5744	0.058	0.212	7.45E-12	9
Tpm4	4.54E-16	-0.58133	0.146	0.321	7.58E-12	9
Abhd4	4.77E-16	0.401289	0.153	0.042	7.96E-12	9
Robo2	4.88E-16	-0.57407	0.027	0.154	8.13E-12	9
Dner	4.91E-16	0.479065	0.394	0.21	8.19E-12	9
Cttnbp2	4.94E-16	0.488306	0.219	0.08	8.24E-12	9
Ppp2r2a	5.2E-16	0.486914	0.418	0.239	8.68E-12	9
Agrn	5.96E-16	0.415252	0.202	0.072	9.94E-12	9
Rab10	5.97E-16	0.433926	0.47	0.288	9.95E-12	9
Btg2	6.57E-16	-0.66962	0.068	0.211	1.1E-11	9
Coro2b	6.69E-16	-0.56697	0.044	0.177	1.12E-11	9
Cyb5	7.22E-16	0.478356	0.36	0.183	1.2E-11	9

Zdhhc14	7.28E-16	0.357244	0.141	0.038	1.21E-11	9
Chst2	7.43E-16	0.455738	0.119	0.03	1.24E-11	9
Itgav	7.66E-16	0.516539	0.17	0.058	1.28E-11	9
Nova1	8.16E-16	0.491287	0.214	0.079	1.36E-11	9
Zic2	9.18E-16	-0.46957	0.012	0.123	1.53E-11	9
Lrig3	1.01E-15	-0.51656	0.036	0.173	1.68E-11	9
Btbd17	1.06E-15	-0.60355	0.039	0.163	1.76E-11	9
Birc5	1.11E-15	-0.50776	0.114	0.277	1.85E-11	9
Rest	1.13E-15	0.38659	0.105	0.021	1.88E-11	9
Gm11541	1.14E-15	-0.50734	0.017	0.13	1.9E-11	9
Notch1	1.26E-15	0.4231	0.102	0.021	2.1E-11	9
Snrpe	1.31E-15	-0.46272	0.418	0.565	2.19E-11	9
Tsn	1.31E-15	-0.46286	0.414	0.593	2.19E-11	9
Sel1l	1.57E-15	0.392385	0.209	0.079	2.61E-11	9
Aplp1	1.57E-15	0.491528	0.314	0.147	2.62E-11	9
Myod1	1.7E-15	-0.57271	0.024	0.144	2.84E-11	9
Tmem59	1.84E-15	0.4176	0.579	0.387	3.07E-11	9
Atxn7l3b	1.93E-15	0.323664	0.818	0.669	3.22E-11	9
Wbp5	2.18E-15	-0.49651	0.353	0.522	3.64E-11	9
Fubp1	2.28E-15	-0.46826	0.416	0.57	3.8E-11	9
Atp1b2	2.31E-15	0.413753	0.153	0.045	3.85E-11	9
Pigyl	2.46E-15	0.436951	0.304	0.14	4.1E-11	9
Hn1	2.65E-15	-0.48711	0.392	0.545	4.42E-11	9
Tgif1	2.84E-15	-0.40724	0.005	0.101	4.74E-11	9
Dtna	2.88E-15	0.350401	0.122	0.029	4.8E-11	9
Mdga1	3.22E-15	-0.50495	0.022	0.138	5.38E-11	9
Siva1	3.29E-15	-0.57142	0.114	0.275	5.49E-11	9
Htatsf1	3.31E-15	-0.52836	0.353	0.502	5.53E-11	9
Sec62	3.37E-15	0.386726	0.594	0.401	5.62E-11	9
Neurod6	3.39E-15	-0.59801	0.046	0.175	5.66E-11	9
Hprt	4.03E-15	-0.51855	0.066	0.19	6.73E-11	9
Ube2b	4.13E-15	-0.48701	0.358	0.524	6.88E-11	9
Nmnat2	4.15E-15	0.434084	0.122	0.029	6.93E-11	9
Snx3	4.82E-15	0.433323	0.518	0.338	8.04E-11	9
Fam19a5	5.08E-15	0.365899	0.107	0.022	8.47E-11	9
Pak4	5.37E-15	0.437338	0.127	0.036	8.95E-11	9
Gm11266	5.39E-15	-0.5897	0.027	0.146	8.99E-11	9
Rabgap1l	5.39E-15	0.434351	0.178	0.059	9E-11	9
Acsl3	5.45E-15	0.517122	0.277	0.143	9.08E-11	9
Elavl2	5.53E-15	-0.60269	0.109	0.273	9.22E-11	9
Baz2b	5.71E-15	-0.56469	0.182	0.369	9.52E-11	9
Tmem107	5.81E-15	-0.45601	0.017	0.129	9.68E-11	9
Tpx2	5.86E-15	-0.55019	0.124	0.285	9.77E-11	9
Bpgm	6.22E-15	0.379381	0.19	0.066	1.04E-10	9
Nedd4	6.4E-15	-0.36992	0.572	0.685	1.07E-10	9
Ccdc134	6.93E-15	0.363829	0.124	0.03	1.16E-10	9
Gm17322	7.14E-15	-0.57562	0.019	0.133	1.19E-10	9
Serpinh1	7.59E-15	-0.51569	0.015	0.117	1.27E-10	9

Incenp	7.86E-15	-0.63164	0.117	0.278	1.31E-10	9
Gpc2	1E-14	-0.54181	0.029	0.142	1.68E-10	9
Phip	1.01E-14	-0.52449	0.277	0.463	1.69E-10	9
B2m	1.16E-14	0.400017	0.399	0.227	1.94E-10	9
Ccdc107	1.26E-14	0.45129	0.187	0.069	2.1E-10	9
Ier3	1.45E-14	0.41956	0.127	0.032	2.42E-10	9
Cdk4	1.45E-14	-0.38242	0.526	0.644	2.42E-10	9
Sept3	1.46E-14	-0.60839	0.265	0.395	2.44E-10	9
Cdk1	1.5E-14	-0.54147	0.092	0.25	2.51E-10	9
Specc1	1.53E-14	0.437209	0.153	0.046	2.55E-10	9
Prex1	1.59E-14	0.457301	0.175	0.06	2.66E-10	9
Mtus1	1.69E-14	-0.49236	0.019	0.129	2.83E-10	9
Nlgn1	2.06E-14	0.411774	0.105	0.022	3.43E-10	9
Hmgn2	2.08E-14	-0.55407	0.107	0.259	3.47E-10	9
Ccna2	2.1E-14	-0.56051	0.075	0.224	3.5E-10	9
Tox3	2.2E-14	-0.53431	0.173	0.349	3.66E-10	9
Sar1b	2.27E-14	0.469945	0.36	0.194	3.78E-10	9
Gm10075	2.32E-14	-0.45359	0.392	0.517	3.87E-10	9
Cdc37l1	2.45E-14	0.497987	0.248	0.11	4.08E-10	9
Fam69b	2.74E-14	0.356994	0.144	0.041	4.57E-10	9
Rpl8	2.9E-14	-0.26729	0.881	0.897	4.84E-10	9
Cdk14	2.93E-14	0.3422	0.231	0.101	4.88E-10	9
Sema7a	3.14E-14	-0.50799	0.034	0.158	5.24E-10	9
Ccnd3	3.37E-14	0.474515	0.195	0.079	5.62E-10	9
Fam53b	3.4E-14	-0.49899	0.029	0.138	5.67E-10	9
Foxp1	3.46E-14	-0.52763	0.044	0.175	5.77E-10	9
Pbk	3.64E-14	-0.5277	0.075	0.225	6.08E-10	9
Apex1	3.91E-14	-0.46607	0.277	0.455	6.53E-10	9
Baz1b	4.05E-14	-0.49514	0.328	0.496	6.75E-10	9
Abat	4.56E-14	0.251106	0.109	0.028	7.61E-10	9
Map1a	4.79E-14	0.454408	0.192	0.07	7.99E-10	9
Angptl2	5.28E-14	-0.44402	0.015	0.118	8.8E-10	9
Smco4	5.37E-14	-0.48329	0.039	0.164	8.95E-10	9
Tspyl4	5.82E-14	0.350766	0.268	0.123	9.7E-10	9
Ctsl	5.92E-14	0.399564	0.438	0.255	9.87E-10	9
Bok	6.85E-14	-0.53087	0.1	0.254	1.14E-09	9
Kif3a	6.96E-14	0.461323	0.477	0.312	1.16E-09	9
Timp3	6.97E-14	0.354662	0.195	0.076	1.16E-09	9
Rdh5	7.25E-14	-0.37705	0.022	0.128	1.21E-09	9
Aldh9a1	7.59E-14	0.421757	0.163	0.053	1.27E-09	9
Snx18	7.68E-14	0.351659	0.136	0.038	1.28E-09	9
Ssrp1	8.15E-14	-0.40993	0.465	0.579	1.36E-09	9
Tmem50b	8.18E-14	0.260772	0.109	0.027	1.36E-09	9
Basp1	8.21E-14	-0.44145	0.577	0.707	1.37E-09	9
Mtf2	9.19E-14	-0.50094	0.2	0.361	1.53E-09	9
Prc1	9.72E-14	-0.62963	0.107	0.26	1.62E-09	9
Banf1	9.77E-14	-0.32732	0.596	0.696	1.63E-09	9
Lmnb1	1.02E-13	-0.52578	0.144	0.303	1.7E-09	9

Prnp	1.03E-13	0.426194	0.375	0.202	1.72E-09	9
Ina	1.04E-13	-0.5394	0.304	0.447	1.73E-09	9
Rab11a	1.12E-13	0.422485	0.445	0.273	1.88E-09	9
Ube2e3	1.14E-13	-0.49358	0.253	0.42	1.9E-09	9
Dpy19l1	1.14E-13	0.43795	0.178	0.063	1.9E-09	9
Slc25a4	1.22E-13	0.282927	0.878	0.805	2.03E-09	9
Adcyap1r1	1.28E-13	0.411109	0.204	0.08	2.13E-09	9
Rassf3	1.34E-13	-0.4837	0.027	0.14	2.24E-09	9
C530008M	1.36E-13	-0.54359	0.114	0.271	2.26E-09	9
Mroh2a	1.4E-13	-0.57379	0.017	0.115	2.34E-09	9
Ifitm2	1.61E-13	-0.40747	0.015	0.116	2.68E-09	9
Bcap29	1.73E-13	0.45456	0.255	0.119	2.89E-09	9
Smyd2	1.77E-13	0.473454	0.221	0.097	2.96E-09	9
Fam168a	1.89E-13	0.371835	0.462	0.283	3.15E-09	9
Spc25	1.89E-13	-0.58617	0.083	0.228	3.15E-09	9
Mrps7	1.91E-13	0.474015	0.282	0.148	3.18E-09	9
Hells	1.97E-13	-0.59499	0.075	0.216	3.29E-09	9
Rpl35a	2.23E-13	-0.41795	0.431	0.573	3.72E-09	9
Lta4h	2.33E-13	-0.52667	0.114	0.245	3.89E-09	9
Sgcb	2.37E-13	0.450885	0.248	0.112	3.95E-09	9
Atp5j	2.45E-13	-0.34638	0.603	0.725	4.08E-09	9
Tubb4a	2.52E-13	0.46616	0.178	0.064	4.21E-09	9
Atraid	2.8E-13	0.437546	0.389	0.222	4.68E-09	9
Map4k4	3.17E-13	-0.50128	0.28	0.453	5.29E-09	9
Ccdc88a	3.26E-13	0.403949	0.633	0.478	5.44E-09	9
Cdca7	3.36E-13	-0.4955	0.063	0.201	5.6E-09	9
Ccdc34	3.79E-13	-0.52098	0.263	0.418	6.33E-09	9
Ilf2	3.81E-13	-0.43042	0.409	0.544	6.36E-09	9
Hipk2	3.86E-13	0.309469	0.141	0.045	6.44E-09	9
Elf1	4.12E-13	0.355246	0.156	0.051	6.88E-09	9
Ppp2r3a	4.34E-13	0.467679	0.219	0.09	7.24E-09	9
Lmo1	4.37E-13	0.396394	0.127	0.037	7.3E-09	9
Ier2	4.61E-13	-0.57904	0.221	0.385	7.69E-09	9
Fkbp15	5.12E-13	0.431834	0.107	0.032	8.54E-09	9
Carhsp1	5.63E-13	-0.53492	0.109	0.257	9.39E-09	9
Pdia6	5.64E-13	0.401589	0.494	0.336	9.4E-09	9
Dad1	5.7E-13	0.414056	0.504	0.339	9.51E-09	9
Tk1	5.76E-13	-0.43628	0.024	0.133	9.6E-09	9
Acbd5	5.79E-13	0.489502	0.238	0.106	9.65E-09	9
Cyp51	6.09E-13	0.456637	0.282	0.137	1.02E-08	9
Spcs2	6.38E-13	0.392788	0.564	0.42	1.06E-08	9
Hnrnpa0	6.53E-13	-0.48686	0.299	0.46	1.09E-08	9
H2afz	6.6E-13	-0.46443	0.088	0.234	1.1E-08	9
Idh2	6.68E-13	-0.52353	0.134	0.282	1.11E-08	9
Snrpg	6.71E-13	-0.49452	0.178	0.338	1.12E-08	9
H1fx	6.8E-13	-0.50153	0.102	0.248	1.13E-08	9
Cltb	6.9E-13	-0.48034	0.336	0.425	1.15E-08	9
Ebpl	7.06E-13	0.312795	0.207	0.086	1.18E-08	9

Sept11	7.24E-13	-0.50702	0.168	0.325	1.21E-08	9
Tspan12	7.47E-13	0.340618	0.114	0.03	1.25E-08	9
Cnpy1	8.45E-13	-0.51004	0.029	0.135	1.41E-08	9
Ick	8.49E-13	0.46453	0.282	0.143	1.42E-08	9
Pcmtd1	8.89E-13	0.380555	0.263	0.122	1.48E-08	9
2310022B(	8.96E-13	-0.50623	0.102	0.251	1.49E-08	9
Dhcr7	9.37E-13	0.365067	0.151	0.049	1.56E-08	9
Asah1	9.48E-13	0.3865	0.17	0.06	1.58E-08	9
Ypel1	9.49E-13	-0.46272	0.051	0.177	1.58E-08	9
Elavl3	1.01E-12	-0.49882	0.353	0.475	1.68E-08	9
Myt1	1.07E-12	0.359334	0.294	0.149	1.78E-08	9
Sdf2l1	1.14E-12	0.397941	0.148	0.049	1.9E-08	9
Cenph	1.16E-12	-0.51002	0.063	0.196	1.93E-08	9
Pdia3	1.18E-12	0.425236	0.45	0.292	1.97E-08	9
4833439L1	1.19E-12	0.387187	0.299	0.151	1.98E-08	9
Itgb1	1.23E-12	0.395644	0.53	0.358	2.05E-08	9
E2f1	1.25E-12	-0.49041	0.036	0.149	2.08E-08	9
Rbfox2	1.27E-12	-0.5391	0.131	0.286	2.11E-08	9
Rrm2	1.3E-12	-0.4712	0.073	0.207	2.17E-08	9
Magee1	1.31E-12	0.417904	0.144	0.046	2.18E-08	9
Igsf21	1.43E-12	0.293999	0.197	0.086	2.38E-08	9
Camta1	1.45E-12	-0.4685	0.243	0.411	2.42E-08	9
Ptms	1.47E-12	-0.46989	0.341	0.476	2.45E-08	9
Pcyt1b	1.56E-12	0.451338	0.148	0.049	2.61E-08	9
Cdc42se1	1.65E-12	0.417414	0.302	0.162	2.75E-08	9
Snx1	1.73E-12	0.44256	0.307	0.165	2.89E-08	9
Zeb2	1.77E-12	0.45844	0.367	0.246	2.95E-08	9
Tmem9	1.79E-12	0.413029	0.236	0.106	2.99E-08	9
Os9	1.86E-12	0.449513	0.406	0.247	3.1E-08	9
Kif23	2.14E-12	-0.54305	0.078	0.217	3.56E-08	9
Socs2	2.16E-12	-0.48968	0.032	0.141	3.6E-08	9
Fndc4	2.2E-12	0.453347	0.248	0.122	3.68E-08	9
Cask	2.31E-12	0.446061	0.238	0.106	3.86E-08	9
Cyld	2.43E-12	0.443226	0.139	0.047	4.05E-08	9
Dusp6	2.44E-12	0.467298	0.192	0.09	4.06E-08	9
Pbrm1	2.48E-12	-0.45191	0.377	0.534	4.13E-08	9
Tmcc3	2.58E-12	0.419274	0.207	0.088	4.31E-08	9
Plxnb2	2.65E-12	-0.49962	0.054	0.173	4.42E-08	9
Tyms	2.71E-12	-0.54124	0.092	0.221	4.52E-08	9
Pgm2	2.84E-12	0.31903	0.114	0.031	4.74E-08	9
Iffo1	2.92E-12	0.309213	0.136	0.042	4.88E-08	9
Cdca8	2.95E-12	-0.38003	0.122	0.267	4.92E-08	9
Cdca2	3.12E-12	-0.33248	0.029	0.13	5.2E-08	9
Elovl1	3.2E-12	0.31826	0.117	0.033	5.34E-08	9
Unc50	3.24E-12	0.389129	0.26	0.122	5.4E-08	9
Tppp3	3.29E-12	-0.46379	0.044	0.16	5.48E-08	9
Syncrip	3.36E-12	-0.44507	0.348	0.499	5.6E-08	9
Cklf	3.39E-12	-0.4715	0.051	0.168	5.65E-08	9

Igsf3	3.4E-12	-0.48593	0.034	0.135	5.66E-08	9
Ncapg	3.42E-12	-0.5079	0.061	0.186	5.7E-08	9
Jam3	3.55E-12	0.397714	0.273	0.131	5.92E-08	9
Mcm3	3.6E-12	-0.51708	0.058	0.182	6E-08	9
Ostm1	3.61E-12	0.302549	0.139	0.045	6.02E-08	9
Tgfb2	3.72E-12	-0.54181	0.066	0.194	6.2E-08	9
Cited2	3.75E-12	-0.48501	0.044	0.155	6.26E-08	9
Mpc2	3.92E-12	0.426479	0.448	0.293	6.53E-08	9
Ccdc18	4.37E-12	-0.31758	0.027	0.117	7.29E-08	9
Fam69a	4.5E-12	0.358039	0.109	0.029	7.51E-08	9
Snrpb	4.6E-12	-0.38311	0.45	0.576	7.68E-08	9
Sacs	4.78E-12	-0.51586	0.068	0.193	7.98E-08	9
Dcakd	4.82E-12	-0.48795	0.153	0.299	8.03E-08	9
Racgap1	4.83E-12	-0.46442	0.109	0.259	8.06E-08	9
Snx27	4.93E-12	0.396815	0.297	0.15	8.22E-08	9
Smdt1	4.96E-12	0.368006	0.516	0.351	8.28E-08	9
A930011O	5.13E-12	-0.5644	0.027	0.117	8.56E-08	9
Sult4a1	5.51E-12	-0.50528	0.092	0.225	9.19E-08	9
Adrbk2	5.96E-12	-0.43843	0.015	0.102	9.94E-08	9
Map7d2	6.2E-12	0.410737	0.224	0.097	1.03E-07	9
Nedd4l	6.2E-12	-0.46889	0.039	0.13	1.03E-07	9
Rnaseh2c	6.26E-12	-0.45537	0.231	0.384	1.04E-07	9
2510003E(	6.87E-12	0.456643	0.229	0.104	1.15E-07	9
Psip1	7.25E-12	-0.34161	0.584	0.69	1.21E-07	9
Fam171b	7.48E-12	0.458564	0.363	0.22	1.25E-07	9
Ccdc28b	7.61E-12	0.438281	0.299	0.159	1.27E-07	9
Rasa3	8.16E-12	0.301178	0.122	0.037	1.36E-07	9
Pdap1	9.1E-12	-0.34456	0.572	0.685	1.52E-07	9
Fktn	9.14E-12	0.400074	0.238	0.108	1.52E-07	9
Sv2b	9.44E-12	-0.43229	0.015	0.104	1.57E-07	9
Clcn3	9.46E-12	0.394015	0.433	0.269	1.58E-07	9
Tacc2	9.5E-12	0.443444	0.273	0.138	1.58E-07	9
Clspn	9.93E-12	-0.53579	0.066	0.192	1.66E-07	9
Dtl	1.01E-11	-0.46966	0.039	0.148	1.68E-07	9
Csrp2	1.04E-11	-0.45525	0.046	0.16	1.73E-07	9
Ube2c	1.09E-11	-0.62387	0.131	0.272	1.82E-07	9
2810055G	1.11E-11	-0.50137	0.056	0.173	1.85E-07	9
Mab21l2	1.12E-11	-0.44592	0.015	0.106	1.86E-07	9
Tcf12	1.16E-11	0.358068	0.418	0.251	1.94E-07	9
Rad51ap1	1.19E-11	-0.42651	0.051	0.169	1.98E-07	9
Dcx	1.24E-11	-0.5231	0.234	0.379	2.07E-07	9
Rps15	1.3E-11	-0.28169	0.74	0.789	2.17E-07	9
Smarca1	1.31E-11	0.321966	0.148	0.054	2.19E-07	9
Tmeff2	1.36E-11	0.425513	0.163	0.063	2.27E-07	9
Chd3	1.42E-11	0.474085	0.275	0.146	2.37E-07	9
Cenpe	1.54E-11	-0.6131	0.139	0.289	2.57E-07	9
Snhg5	1.59E-11	-0.48799	0.236	0.384	2.66E-07	9
Kif11	1.64E-11	-0.29213	0.09	0.208	2.74E-07	9

Slc6a6	1.66E-11	0.345576	0.131	0.042	2.77E-07	9
Mxd3	1.7E-11	-0.41034	0.027	0.129	2.83E-07	9
St18	1.71E-11	-0.59453	0.095	0.192	2.85E-07	9
Arhgef2	2.01E-11	-0.52301	0.148	0.285	3.36E-07	9
Sc4mol	2.02E-11	0.437106	0.202	0.089	3.37E-07	9
Comt	2.04E-11	0.41004	0.231	0.106	3.4E-07	9
Atad2	2.09E-11	-0.48961	0.075	0.201	3.48E-07	9
Gphn	2.19E-11	0.342714	0.275	0.137	3.65E-07	9
Fbxo8	2.19E-11	0.338739	0.131	0.041	3.66E-07	9
Pdzrn3	2.23E-11	-0.60785	0.041	0.149	3.72E-07	9
Otud7b	2.35E-11	0.365377	0.153	0.054	3.92E-07	9
Ccm2	2.54E-11	-0.45153	0.097	0.227	4.24E-07	9
Rpl14	2.56E-11	-0.28736	0.652	0.698	4.28E-07	9
Btg1	2.92E-11	-0.46536	0.063	0.186	4.86E-07	9
Lss	3.42E-11	0.29128	0.136	0.047	5.7E-07	9
Tceal6	3.43E-11	0.309229	0.136	0.046	5.73E-07	9
Setd8	3.45E-11	-0.48974	0.127	0.264	5.75E-07	9
Tmem66	3.46E-11	0.387256	0.348	0.195	5.78E-07	9
Gli1	3.89E-11	-0.34213	0.019	0.109	6.49E-07	9
D17Wsu10	3.96E-11	0.380186	0.367	0.212	6.6E-07	9
Kif1a	4.09E-11	0.371458	0.314	0.168	6.82E-07	9
Cers2	4.14E-11	0.407154	0.234	0.111	6.91E-07	9
Efcab14	4.57E-11	0.378089	0.185	0.075	7.63E-07	9
Tmem5	4.61E-11	0.369245	0.19	0.079	7.69E-07	9
Twf1	4.68E-11	0.35623	0.268	0.133	7.8E-07	9
Evi5	4.72E-11	0.454168	0.26	0.139	7.88E-07	9
Mmp14	5.17E-11	-0.44212	0.058	0.177	8.62E-07	9
BC029722	5.58E-11	0.336483	0.114	0.033	9.31E-07	9
Esco2	5.68E-11	-0.55154	0.08	0.197	9.47E-07	9
Cdca3	5.79E-11	-0.35831	0.095	0.224	9.65E-07	9
Rpl39	5.81E-11	-0.32339	0.574	0.647	9.69E-07	9
Myh10	6.05E-11	-0.49118	0.18	0.327	1.01E-06	9
Tbc1d12	6.26E-11	0.293605	0.136	0.046	1.04E-06	9
Serinc1	6.61E-11	0.325178	0.599	0.432	1.1E-06	9
Pnrc1	7.1E-11	-0.46463	0.134	0.273	1.18E-06	9
Ccp110	7.1E-11	-0.48575	0.117	0.251	1.19E-06	9
Pop5	7.4E-11	0.376388	0.217	0.097	1.24E-06	9
Pex2	8.17E-11	0.385588	0.217	0.098	1.36E-06	9
Psma7	8.39E-11	-0.30711	0.686	0.783	1.4E-06	9
Slc35a5	8.4E-11	0.256121	0.102	0.029	1.4E-06	9
Frmd4a	8.69E-11	-0.29026	0.17	0.312	1.45E-06	9
Nusap1	8.95E-11	-0.55727	0.1	0.23	1.49E-06	9
Hnrnph1	9.17E-11	-0.36033	0.501	0.631	1.53E-06	9
Tead1	9.33E-11	-0.42429	0.058	0.176	1.56E-06	9
Marcksl1	9.43E-11	-0.32426	0.63	0.724	1.57E-06	9
Samd8	9.73E-11	0.370074	0.185	0.077	1.62E-06	9
Atp9a	9.97E-11	0.323714	0.158	0.066	1.66E-06	9
Rab8b	1E-10	0.321637	0.139	0.047	1.67E-06	9

Vps28	1.05E-10	0.378534	0.397	0.241	1.76E-06	9
Agpat4	1.06E-10	0.371376	0.238	0.114	1.77E-06	9
Clybl	1.07E-10	-0.39435	0.024	0.118	1.78E-06	9
Ncaph	1.1E-10	-0.30912	0.029	0.123	1.84E-06	9
Snrpb2	1.11E-10	-0.44437	0.238	0.387	1.85E-06	9
Stk39	1.11E-10	0.299325	0.122	0.041	1.86E-06	9
Tbc1d16	1.12E-10	-0.47606	0.078	0.199	1.86E-06	9
Uhrf1	1.21E-10	-0.38556	0.041	0.146	2.02E-06	9
Rap1gds1	1.22E-10	0.276583	0.131	0.044	2.04E-06	9
Snca	1.26E-10	0.269807	0.146	0.058	2.1E-06	9
Cacnb4	1.26E-10	0.347462	0.109	0.032	2.11E-06	9
Nap1l1	1.28E-10	-0.37174	0.394	0.513	2.13E-06	9
Chchd2	1.29E-10	-0.32319	0.557	0.655	2.15E-06	9
Kif15	1.3E-10	-0.44515	0.063	0.183	2.17E-06	9
P4ha1	1.32E-10	0.338825	0.156	0.059	2.2E-06	9
Rps25	1.33E-10	-0.41574	0.355	0.482	2.22E-06	9
Itm2b	1.37E-10	0.279227	0.764	0.639	2.29E-06	9
Rps15a	1.48E-10	-0.32312	0.577	0.659	2.47E-06	9
Ccdc47	1.53E-10	0.380856	0.394	0.241	2.55E-06	9
Chaf1a	1.63E-10	-0.40601	0.056	0.171	2.73E-06	9
Jun	1.87E-10	-0.43844	0.54	0.657	3.11E-06	9
Ap2a1	1.87E-10	0.297057	0.102	0.028	3.12E-06	9
Blvra	1.89E-10	0.297887	0.117	0.036	3.15E-06	9
Lig1	1.97E-10	-0.51425	0.207	0.351	3.29E-06	9
Nt5dc2	1.98E-10	-0.48598	0.112	0.24	3.3E-06	9
Gabbr1	2.03E-10	0.430602	0.263	0.137	3.39E-06	9
Mllt3	2.07E-10	-0.48109	0.131	0.228	3.45E-06	9
Itsn1	2.07E-10	-0.53792	0.129	0.238	3.45E-06	9
Dhx9	2.21E-10	-0.39372	0.333	0.466	3.68E-06	9
Sdc2	2.28E-10	0.370749	0.144	0.052	3.8E-06	9
Cdc20	2.33E-10	-0.4653	0.078	0.199	3.88E-06	9
H2afx	2.38E-10	-0.5454	0.217	0.333	3.96E-06	9
Tcp11l2	2.52E-10	0.277471	0.127	0.045	4.2E-06	9
Rpl26	2.68E-10	-0.31772	0.603	0.703	4.47E-06	9
Sep15	2.71E-10	0.32399	0.65	0.501	4.51E-06	9
Msi1	2.76E-10	-0.38393	0.029	0.112	4.6E-06	9
Vamp3	2.78E-10	0.332438	0.182	0.076	4.63E-06	9
Tmed5	2.87E-10	0.395286	0.236	0.123	4.79E-06	9
Usp1	2.94E-10	-0.41302	0.195	0.342	4.9E-06	9
Hpcal1	2.97E-10	-0.45557	0.024	0.108	4.96E-06	9
Tmem147	3.04E-10	0.398274	0.394	0.258	5.08E-06	9
Vldlr	3.54E-10	0.338844	0.153	0.058	5.9E-06	9
Arhgef9	3.56E-10	0.399808	0.195	0.086	5.93E-06	9
Rufy3	3.59E-10	-0.45858	0.263	0.374	5.98E-06	9
Dirc2	4.13E-10	0.34196	0.204	0.092	6.88E-06	9
Ssbp3	4.65E-10	-0.47439	0.068	0.169	7.76E-06	9
Ltbp3	4.94E-10	-0.40099	0.032	0.12	8.24E-06	9
Dock7	4.99E-10	-0.40676	0.027	0.115	8.33E-06	9

Pou3f2	5.22E-10	-0.4573	0.141	0.279	8.7E-06	9
Epb4.1	5.4E-10	-0.44676	0.102	0.232	9E-06	9
Fam155a	5.54E-10	0.435631	0.221	0.113	9.24E-06	9
H2-D1	5.59E-10	0.292406	0.246	0.127	9.32E-06	9
Rad21	5.67E-10	-0.35949	0.358	0.464	9.46E-06	9
Eif4e3	5.85E-10	-0.44436	0.036	0.133	9.76E-06	9
Casp3	6.18E-10	-0.4588	0.122	0.236	1.03E-05	9
Ppp1r18	6.63E-10	0.28869	0.153	0.061	1.11E-05	9
March1	6.98E-10	0.304541	0.122	0.041	1.16E-05	9
Atp6v0e2	7.01E-10	0.358044	0.404	0.256	1.17E-05	9
Dll3	7.39E-10	0.402131	0.158	0.063	1.23E-05	9
Rbm8a	7.66E-10	-0.39465	0.336	0.468	1.28E-05	9
Cdk5r1	7.71E-10	-0.50588	0.207	0.336	1.29E-05	9
Dnajc9	7.72E-10	-0.43925	0.204	0.353	1.29E-05	9
Tmem246	7.78E-10	0.360488	0.182	0.079	1.3E-05	9
Tshz2	8.2E-10	-0.43644	0.178	0.323	1.37E-05	9
Rnf165	8.34E-10	-0.44552	0.066	0.175	1.39E-05	9
Astn1	8.35E-10	0.32197	0.114	0.036	1.39E-05	9
Trp53	8.77E-10	-0.42594	0.161	0.301	1.46E-05	9
Lactb	9.74E-10	0.306588	0.107	0.032	1.62E-05	9
Ncan	1E-09	0.362365	0.207	0.096	1.67E-05	9
Dctpp1	1.06E-09	-0.428	0.195	0.306	1.77E-05	9
Hadha	1.07E-09	0.382751	0.28	0.152	1.78E-05	9
Glud1	1.12E-09	0.394344	0.26	0.138	1.87E-05	9
Etfb	1.12E-09	0.353198	0.319	0.181	1.87E-05	9
Ndfip1	1.13E-09	0.350944	0.178	0.077	1.88E-05	9
Sept4	1.13E-09	-0.37005	0.107	0.229	1.88E-05	9
Mrpl34	1.13E-09	-0.44881	0.182	0.29	1.89E-05	9
Setbp1	1.16E-09	-0.4517	0.058	0.162	1.93E-05	9
Trim59	1.17E-09	-0.39056	0.061	0.172	1.95E-05	9
Ybx3	1.38E-09	-0.40573	0.163	0.293	2.3E-05	9
Arl8a	1.42E-09	0.296203	0.2	0.093	2.37E-05	9
Mllt11	1.61E-09	-0.46302	0.148	0.22	2.69E-05	9
Ccdc41	1.69E-09	-0.42876	0.129	0.256	2.82E-05	9
Celsr3	1.7E-09	-0.41094	0.034	0.125	2.83E-05	9
Dbt	1.85E-09	0.359588	0.195	0.091	3.09E-05	9
Dusp1	1.9E-09	-0.47263	0.061	0.166	3.17E-05	9
Klhdc2	1.91E-09	-0.4066	0.19	0.33	3.19E-05	9
Mid1ip1	1.97E-09	0.29217	0.134	0.049	3.28E-05	9
Hmgb1	2.03E-09	-0.39654	0.229	0.361	3.39E-05	9
Ctsz	2.13E-09	-0.47228	0.044	0.138	3.55E-05	9
Sptssa	2.28E-09	0.371836	0.382	0.243	3.8E-05	9
Hsp90aa1	2.29E-09	-0.3187	0.518	0.619	3.82E-05	9
Capns1	2.36E-09	0.304635	0.367	0.224	3.94E-05	9
2700089E2	2.42E-09	0.352526	0.331	0.195	4.03E-05	9
Fam173a	2.53E-09	0.354914	0.275	0.15	4.22E-05	9
Casc4	2.63E-09	0.35999	0.27	0.144	4.38E-05	9
Lgalsl	2.69E-09	0.360358	0.195	0.09	4.48E-05	9

Rhbdd2	2.73E-09	0.334495	0.148	0.058	4.55E-05	9
Jagn1	2.97E-09	0.345841	0.258	0.136	4.96E-05	9
Mvk	3.01E-09	0.329357	0.151	0.06	5.02E-05	9
Nuf2	3.03E-09	-0.36939	0.044	0.143	5.05E-05	9
Tex30	3.11E-09	-0.32643	0.041	0.137	5.19E-05	9
Gabarapl1	3.13E-09	0.368664	0.348	0.213	5.21E-05	9
Lap3	3.13E-09	-0.46826	0.151	0.279	5.22E-05	9
Ctnna1	3.18E-09	-0.40394	0.056	0.155	5.3E-05	9
Lmf1	3.2E-09	0.283392	0.102	0.032	5.33E-05	9
Man2a2	3.36E-09	0.307883	0.122	0.042	5.6E-05	9
Clvs1	3.46E-09	-0.44991	0.046	0.131	5.77E-05	9
Zfp462	3.51E-09	0.384024	0.277	0.153	5.85E-05	9
Rap2b	3.51E-09	0.380243	0.265	0.145	5.86E-05	9
Nceh1	3.81E-09	0.297718	0.105	0.033	6.36E-05	9
Sgol1	3.87E-09	-0.38007	0.036	0.13	6.45E-05	9
Eif3f	3.87E-09	-0.31494	0.526	0.633	6.46E-05	9
Hmmr	3.94E-09	-0.396	0.071	0.179	6.58E-05	9
Prim1	4.11E-09	-0.45059	0.139	0.265	6.85E-05	9
Kif20b	4.12E-09	-0.48866	0.08	0.193	6.86E-05	9
Ttc3	4.49E-09	-0.27131	0.779	0.83	7.48E-05	9
Map4k5	4.67E-09	0.303213	0.151	0.06	7.79E-05	9
Smpd3	4.76E-09	-0.45392	0.061	0.163	7.94E-05	9
Mcm5	4.89E-09	-0.38164	0.066	0.17	8.16E-05	9
Strbp	5.14E-09	-0.43657	0.236	0.349	8.58E-05	9
Ctcf	5.24E-09	-0.38019	0.363	0.498	8.73E-05	9
Mad2l1	5.63E-09	-0.34173	0.034	0.125	9.4E-05	9
Casc5	5.86E-09	-0.45896	0.078	0.188	9.78E-05	9
Podxl2	5.9E-09	-0.48663	0.119	0.23	9.84E-05	9
Camsap2	6.1E-09	0.373133	0.248	0.13	0.000102	9
Cox7a2	6.28E-09	-0.27228	0.65	0.713	0.000105	9
Ppm1l	6.42E-09	0.294037	0.195	0.093	0.000107	9
Usp16	6.55E-09	0.3865	0.375	0.238	0.000109	9
Slc48a1	7.01E-09	0.374645	0.144	0.058	0.000117	9
Rph3a	7.32E-09	0.312902	0.107	0.038	0.000122	9
Abi2	7.66E-09	0.369696	0.307	0.179	0.000128	9
Ncapd2	7.87E-09	-0.36451	0.08	0.187	0.000131	9
Gpsm1	8.1E-09	-0.43233	0.073	0.178	0.000135	9
Nucks1	8.18E-09	-0.26693	0.601	0.664	0.000136	9
Ddost	8.54E-09	0.350202	0.399	0.266	0.000142	9
Prpf40a	8.89E-09	-0.35203	0.467	0.573	0.000148	9
Parp6	9.31E-09	-0.41819	0.044	0.127	0.000155	9
Tcerg1	9.46E-09	-0.39028	0.338	0.459	0.000158	9
Magoh	1.02E-08	-0.40212	0.214	0.335	0.00017	9
Desi1	1.06E-08	0.38395	0.187	0.091	0.000177	9
Grik5	1.09E-08	0.333008	0.217	0.107	0.000182	9
Ifnar2	1.11E-08	0.293464	0.129	0.048	0.000186	9
1700001O	1.14E-08	-0.34726	0.029	0.112	0.000189	9
Kmt2e	1.16E-08	-0.35114	0.47	0.57	0.000194	9

Glce	1.17E-08	-0.45923	0.075	0.179	0.000195	9
Src	1.19E-08	0.316962	0.102	0.033	0.000198	9
Ank3	1.24E-08	-0.3751	0.358	0.421	0.000207	9
Ccnb2	1.25E-08	-0.37061	0.083	0.189	0.000209	9
G6pc3	1.26E-08	0.27342	0.112	0.038	0.00021	9
Ndc80	1.26E-08	-0.37015	0.032	0.116	0.00021	9
Mum1	1.28E-08	-0.41068	0.071	0.171	0.000213	9
Stard3nl	1.3E-08	0.291572	0.224	0.114	0.000217	9
Pcdha2	1.34E-08	0.263769	0.221	0.115	0.000223	9
Gins2	1.4E-08	-0.35278	0.078	0.187	0.000233	9
Slc50a1	1.41E-08	0.331264	0.178	0.08	0.000235	9
Sri	1.42E-08	0.283624	0.285	0.163	0.000237	9
Nop58	1.48E-08	-0.3115	0.53	0.593	0.000247	9
Sqle	1.5E-08	0.296715	0.229	0.118	0.00025	9
Ankrd46	1.5E-08	0.373821	0.275	0.157	0.000251	9
Epb4.1l3	1.5E-08	0.389631	0.209	0.111	0.000251	9
Impad1	1.53E-08	0.360925	0.348	0.22	0.000255	9
Dtymk	1.58E-08	-0.39138	0.326	0.441	0.000264	9
Kif22	1.62E-08	-0.39195	0.063	0.165	0.000269	9
Wls	1.62E-08	0.336422	0.18	0.085	0.00027	9
Srrt	1.66E-08	-0.39192	0.314	0.426	0.000277	9
Rpl34	1.69E-08	-0.30119	0.564	0.654	0.000282	9
Fam13c	1.73E-08	0.35472	0.144	0.06	0.000288	9
Tprn	1.76E-08	-0.45294	0.119	0.229	0.000293	9
Ankrd11	1.79E-08	0.341348	0.586	0.47	0.000298	9
Pmf1	1.81E-08	-0.37327	0.054	0.145	0.000302	9
Epdr1	1.87E-08	0.322969	0.141	0.057	0.000312	9
Atxn7l2	1.9E-08	-0.36525	0.024	0.104	0.000316	9
Ufl1	1.91E-08	0.361345	0.209	0.104	0.000319	9
Prps1	1.97E-08	-0.36494	0.051	0.144	0.000329	9
Phf21b	2E-08	-0.3787	0.041	0.129	0.000333	9
Gnai1	2.06E-08	0.389175	0.192	0.097	0.000343	9
Rab6a	2.08E-08	0.339804	0.465	0.324	0.000346	9
Atrx	2.11E-08	-0.29595	0.64	0.742	0.000352	9
Atp8a1	2.15E-08	0.303819	0.163	0.071	0.000359	9
Tmem106b	2.17E-08	0.313571	0.161	0.07	0.000363	9
Sh3bgrl	2.23E-08	-0.38994	0.207	0.34	0.000372	9
Ankrd32	2.25E-08	-0.43722	0.085	0.185	0.000375	9
Tmem101	2.27E-08	0.304839	0.165	0.074	0.000379	9
Hist1h2ak	2.28E-08	-0.42413	0.061	0.145	0.00038	9
Zfp191	2.3E-08	0.338273	0.345	0.214	0.000384	9
Clic1	2.31E-08	-0.39445	0.071	0.175	0.000385	9
Afap1	2.35E-08	-0.43054	0.061	0.154	0.000391	9
Pcbp1	2.39E-08	-0.25172	0.557	0.594	0.000399	9
Ly6e	2.44E-08	-0.37731	0.156	0.284	0.000407	9
Nrn1	2.48E-08	-0.46375	0.119	0.223	0.000413	9
Tmem50a	2.5E-08	0.250893	0.538	0.402	0.000417	9
Rpl18a	2.51E-08	-0.39088	0.275	0.403	0.000419	9

Usp46	2.54E-08	-0.41792	0.122	0.238	0.000424	9
Tom1l1	2.56E-08	-0.3512	0.027	0.105	0.000427	9
Rpn2	2.57E-08	0.348909	0.35	0.224	0.000428	9
Pknox1	2.59E-08	-0.41107	0.058	0.147	0.000432	9
Zswim6	2.6E-08	0.292487	0.131	0.051	0.000434	9
Preb	2.61E-08	0.342082	0.265	0.152	0.000435	9
Homer2	2.65E-08	-0.39345	0.129	0.249	0.000442	9
Cenpa	2.68E-08	-0.52985	0.134	0.254	0.000447	9
Elovl6	2.92E-08	0.374066	0.399	0.275	0.000488	9
Flna	2.98E-08	-0.41219	0.061	0.154	0.000497	9
Ndufa8	2.99E-08	0.354568	0.443	0.327	0.000499	9
Mapre2	3E-08	0.33613	0.287	0.164	0.000501	9
Spats2l	3.31E-08	0.322906	0.119	0.044	0.000552	9
Rabac1	3.34E-08	0.290229	0.394	0.254	0.000558	9
Paqr4	3.65E-08	0.303792	0.122	0.045	0.000608	9
Cenpk	3.76E-08	-0.33739	0.054	0.146	0.000627	9
Slc15a2	3.78E-08	0.333271	0.107	0.037	0.00063	9
Tm2d2	3.96E-08	0.309493	0.382	0.246	0.000661	9
1500011K1	4.15E-08	0.341703	0.251	0.147	0.000692	9
Ank2	4.19E-08	-0.44186	0.182	0.241	0.000699	9
Cep290	4.24E-08	-0.34833	0.054	0.145	0.000708	9
Fjx1	4.48E-08	0.280166	0.105	0.036	0.000747	9
Rpa2	4.59E-08	-0.40949	0.097	0.202	0.000766	9
Vmp1	4.63E-08	0.302889	0.221	0.114	0.000771	9
Prkar2b	4.63E-08	0.296215	0.156	0.067	0.000773	9
Fabp5	4.68E-08	-0.34413	0.273	0.406	0.000781	9
Pura	4.8E-08	0.34002	0.421	0.283	0.000801	9
Napa	4.88E-08	-0.39084	0.236	0.352	0.000815	9
Smim15	4.91E-08	0.321536	0.226	0.118	0.000819	9
Cecr2	4.95E-08	-0.3802	0.044	0.131	0.000825	9
Dkc1	5.08E-08	-0.28993	0.217	0.335	0.000848	9
Extl2	5.11E-08	0.296893	0.131	0.052	0.000853	9
Prdx5	5.15E-08	0.275292	0.399	0.261	0.000859	9
Ddx25	5.24E-08	0.269398	0.102	0.034	0.000874	9
Larp7	5.33E-08	-0.39747	0.19	0.314	0.000889	9
Prdx4	5.41E-08	-0.38343	0.241	0.368	0.000902	9
Pgrmc2	5.57E-08	0.351604	0.302	0.193	0.000929	9
Emc2	5.58E-08	0.316062	0.29	0.168	0.000931	9
Anp32e	5.71E-08	-0.29695	0.562	0.614	0.000952	9
Dtd2	5.71E-08	0.261351	0.105	0.036	0.000953	9
Cct2	6.02E-08	-0.32274	0.426	0.526	0.001004	9
Cebpg	6.09E-08	-0.38778	0.09	0.195	0.001015	9
Nup62	6.21E-08	-0.30428	0.078	0.173	0.001035	9
Ptpra	6.25E-08	0.302816	0.365	0.231	0.001042	9
Eif1	6.59E-08	-0.2692	0.528	0.583	0.001099	9
Tpp1	6.77E-08	0.258519	0.148	0.064	0.001128	9
Rfc1	6.8E-08	-0.39518	0.209	0.333	0.001135	9
Pepd	6.94E-08	0.276226	0.112	0.04	0.001158	9

Tbca	7.03E-08	-0.35108	0.287	0.423	0.001173	9
Tmem167	7.04E-08	0.358277	0.38	0.27	0.001175	9
Ago3	7.24E-08	0.283928	0.165	0.075	0.001207	9
Zfand6	7.48E-08	-0.38707	0.219	0.315	0.001248	9
Eif3a	7.53E-08	-0.2896	0.618	0.69	0.001256	9
Rnf5	8.5E-08	0.333086	0.384	0.254	0.001418	9
Lmcd1	8.55E-08	0.269718	0.141	0.06	0.001426	9
Ctnnal1	8.65E-08	0.277308	0.124	0.048	0.001442	9
Kif13a	8.92E-08	0.346248	0.119	0.047	0.001488	9
Krcc1	9.23E-08	0.306284	0.173	0.081	0.00154	9
Ankmy2	9.25E-08	0.306776	0.109	0.04	0.001542	9
Smim13	9.25E-08	0.263054	0.139	0.058	0.001543	9
Aprt	9.33E-08	0.260997	0.246	0.138	0.001556	9
Rif1	9.43E-08	-0.42579	0.168	0.278	0.001573	9
Tceal1	9.44E-08	0.316458	0.158	0.071	0.001574	9
Uba52	9.76E-08	-0.37062	0.207	0.319	0.001628	9
Uqcrb	9.77E-08	0.360567	0.336	0.217	0.00163	9
0610007P1	9.8E-08	0.361605	0.265	0.159	0.001635	9
Smc3	9.92E-08	-0.29025	0.526	0.635	0.001655	9
Aurkb	1.02E-07	-0.35467	0.054	0.145	0.001696	9
Knstrn	1.02E-07	-0.37577	0.088	0.188	0.001701	9
Lrrn1	1.03E-07	0.371783	0.202	0.111	0.001717	9
C130071C(	1.03E-07	-0.37927	0.19	0.31	0.001724	9
Maged2	1.06E-07	0.309158	0.282	0.164	0.001762	9
Znhit6	1.06E-07	0.363768	0.219	0.117	0.001773	9
Elovl5	1.07E-07	0.353935	0.214	0.116	0.001793	9
Samd4b	1.11E-07	0.28583	0.156	0.069	0.00185	9
Ate1	1.12E-07	0.356154	0.231	0.127	0.001868	9
Midn	1.13E-07	0.338031	0.236	0.13	0.001887	9
Ivns1abp	1.2E-07	-0.35265	0.29	0.418	0.001996	9
Rpl23	1.2E-07	-0.32185	0.445	0.54	0.002001	9
Leprot	1.21E-07	0.351003	0.217	0.115	0.002021	9
Tia1	1.21E-07	-0.31415	0.472	0.553	0.002025	9
Sox11	1.22E-07	-0.36061	0.036	0.116	0.002037	9
Gm8292	1.23E-07	-0.37303	0.255	0.371	0.002058	9
Pak1	1.25E-07	0.293206	0.136	0.057	0.002093	9
Gpx7	1.28E-07	0.262929	0.141	0.061	0.002135	9
B3gnt1	1.3E-07	0.320757	0.141	0.06	0.002171	9
Myo6	1.3E-07	0.284004	0.122	0.047	0.002173	9
Gmnn	1.35E-07	-0.30555	0.056	0.146	0.002254	9
Pfdn4	1.37E-07	-0.38801	0.204	0.307	0.002286	9
Clptm1l	1.47E-07	0.334835	0.221	0.12	0.002445	9
Hmgn3	1.47E-07	0.338649	0.394	0.267	0.002448	9
Mboat2	1.47E-07	0.266793	0.119	0.046	0.00246	9
Eif3k	1.51E-07	-0.33127	0.401	0.523	0.002524	9
1500012F(	1.51E-07	-0.37584	0.29	0.4	0.002526	9
Snap25	1.53E-07	-0.42401	0.17	0.284	0.002547	9
Hes6	1.59E-07	-0.41527	0.139	0.238	0.002644	9

Ccnh	1.65E-07	0.346844	0.251	0.147	0.002757	9
Pmvk	1.73E-07	0.264728	0.185	0.092	0.002882	9
Cpe	1.77E-07	0.295957	0.579	0.453	0.002959	9
Rnf208	1.78E-07	0.259677	0.161	0.075	0.002968	9
Cd63-ps	1.79E-07	-0.32151	0.039	0.12	0.002987	9
Tmem18	1.82E-07	0.329952	0.112	0.044	0.003039	9
Nlk	1.84E-07	0.266709	0.178	0.086	0.003077	9
Ramp2	1.85E-07	0.279835	0.219	0.117	0.003092	9
Cdca7l	1.89E-07	-0.30716	0.029	0.106	0.003145	9
Hdhd2	1.9E-07	0.26439	0.217	0.115	0.003177	9
Myo5a	1.91E-07	0.35909	0.302	0.188	0.00319	9
Mtdh	1.91E-07	0.257948	0.667	0.546	0.003192	9
Ddx26b	1.94E-07	0.357758	0.224	0.127	0.003234	9
Ptprd	1.94E-07	-0.37939	0.214	0.343	0.003241	9
Syne2	1.95E-07	-0.42432	0.122	0.222	0.00325	9
Nsg2	2.02E-07	-0.34961	0.367	0.49	0.003362	9
Fbxo5	2.03E-07	-0.33489	0.066	0.161	0.003384	9
Sgce	2.03E-07	0.306139	0.2	0.101	0.003394	9
Rab9	2.09E-07	0.328444	0.204	0.107	0.003494	9
Supt16	2.11E-07	-0.3463	0.387	0.495	0.003522	9
Asf1a	2.16E-07	-0.3719	0.163	0.282	0.0036	9
Rnf13	2.22E-07	0.320348	0.165	0.079	0.00371	9
Secisbp2l	2.23E-07	0.378116	0.255	0.152	0.003721	9
Chgb	2.23E-07	-0.49602	0.136	0.243	0.003721	9
Baz1a	2.24E-07	-0.39858	0.088	0.168	0.003734	9
Magt1	2.26E-07	0.301493	0.131	0.055	0.003777	9
Cdc42se2	2.3E-07	0.344	0.35	0.236	0.00383	9
Arpc1a	2.3E-07	0.295659	0.443	0.362	0.003842	9
Klf7	2.31E-07	-0.4	0.192	0.263	0.003846	9
Tm9sf3	2.31E-07	0.324709	0.47	0.341	0.003853	9
Atp6v0e	2.37E-07	0.29743	0.506	0.405	0.003948	9
Golm1	2.43E-07	-0.40205	0.092	0.19	0.004047	9
Sbf2	2.44E-07	0.32062	0.168	0.08	0.004072	9
Hsd17b4	2.45E-07	0.298729	0.243	0.137	0.004079	9
Ubl3	2.5E-07	0.320362	0.328	0.206	0.004177	9
Pole3	2.54E-07	-0.3822	0.141	0.252	0.004231	9
Myt1l	2.62E-07	-0.47454	0.08	0.162	0.004375	9
Atp13a3	2.64E-07	0.29109	0.122	0.052	0.004399	9
Usp22	2.64E-07	-0.35151	0.27	0.363	0.004407	9
Rab5c	2.66E-07	0.284614	0.19	0.095	0.004443	9
Deaf1	2.67E-07	0.272014	0.175	0.085	0.004457	9
Rad51	2.84E-07	-0.2868	0.039	0.118	0.004739	9
Psph	2.85E-07	0.340222	0.151	0.072	0.004755	9
Tfdp2	2.95E-07	-0.3897	0.073	0.162	0.004924	9
Dnajb11	3.01E-07	0.327827	0.27	0.16	0.005027	9
Soga3	3.23E-07	-0.35123	0.37	0.459	0.005382	9
Sptlc1	3.28E-07	0.30216	0.119	0.049	0.005476	9
Ddx3x	3.35E-07	-0.34129	0.353	0.469	0.005592	9

Tor1aip2	3.39E-07	0.287639	0.18	0.088	0.005651	9
1700025G(	3.5E-07	0.346307	0.38	0.26	0.005842	9
Purb	3.51E-07	-0.2919	0.491	0.55	0.00586	9
Rgs12	3.62E-07	-0.37199	0.061	0.148	0.006046	9
Atp2a2	3.68E-07	0.30385	0.319	0.199	0.006143	9
Cldn25	3.71E-07	0.296575	0.353	0.229	0.006186	9
Rap1a	3.74E-07	0.29418	0.221	0.121	0.006244	9
Sppl2a	3.83E-07	0.309198	0.217	0.117	0.006385	9
Fam184a	3.9E-07	0.276254	0.144	0.065	0.006511	9
Mis18bp1	3.93E-07	-0.37859	0.068	0.163	0.006552	9
Dclk1	3.96E-07	-0.38614	0.299	0.414	0.006612	9
Smarca5	4.12E-07	-0.32976	0.314	0.403	0.006874	9
Ap3m1	4.2E-07	0.279344	0.144	0.065	0.007012	9
Khdrbs1	4.23E-07	-0.30758	0.382	0.496	0.007054	9
Rfc2	4.3E-07	-0.35565	0.114	0.212	0.007171	9
Ndrg3	4.37E-07	0.288638	0.212	0.113	0.007296	9
Mia3	4.5E-07	0.300685	0.365	0.242	0.00751	9
Cenpj	4.53E-07	-0.30481	0.058	0.143	0.007552	9
Rnaseh2a	4.59E-07	-0.35259	0.071	0.154	0.007657	9
Arhgap11a	4.59E-07	-0.35922	0.075	0.172	0.00766	9
Suv39h2	4.85E-07	-0.32735	0.049	0.133	0.008088	9
Ndufa13	4.92E-07	0.262359	0.652	0.53	0.008202	9
Nf1	5E-07	0.294691	0.148	0.067	0.008341	9
Zcchc18	5.2E-07	0.279572	0.28	0.167	0.008675	9
Mpv17	5.35E-07	0.277154	0.185	0.093	0.008924	9
Fcho2	5.35E-07	0.320278	0.156	0.075	0.008925	9
Gpbp1	5.37E-07	-0.35339	0.268	0.358	0.008957	9
Cenpw	5.39E-07	-0.25639	0.049	0.129	0.008986	9
Chga	5.48E-07	0.309762	0.17	0.083	0.00914	9
Snx4	5.68E-07	0.295036	0.287	0.173	0.009472	9
Srrm3	5.76E-07	-0.40065	0.136	0.242	0.0096	9
Hnrnpk	5.91E-07	-0.25428	0.56	0.637	0.00985	9
Prdm8	5.91E-07	-0.42739	0.056	0.128	0.009863	9
Cdk2ap1	5.96E-07	-0.29521	0.032	0.101	0.009949	9
Smap1	6.14E-07	0.280877	0.343	0.22	0.010238	9
Sdhb	6.23E-07	0.299867	0.513	0.397	0.010389	9
Mns1	6.27E-07	-0.37143	0.102	0.205	0.01046	9
Ehbp1	6.34E-07	-0.37665	0.061	0.138	0.01057	9
Cpox	6.37E-07	0.291534	0.151	0.071	0.010629	9
Jund	6.4E-07	-0.36064	0.229	0.354	0.010668	9
Elavl4	6.56E-07	-0.40204	0.221	0.338	0.010939	9
Nfkbib	6.58E-07	0.316113	0.158	0.075	0.010982	9
Lrrc42	6.75E-07	0.314936	0.136	0.062	0.011256	9
Arrdc3	6.87E-07	-0.31431	0.034	0.105	0.01146	9
Sppl2b	6.94E-07	0.323953	0.114	0.055	0.011569	9
Ica1	7.22E-07	0.262924	0.114	0.046	0.012037	9
Npc2	7.69E-07	-0.34204	0.358	0.466	0.012825	9
Dennd5a	8.06E-07	0.258929	0.192	0.1	0.013451	9

Fasn	8.28E-07	0.304044	0.238	0.135	0.013817	9
Psmb10	8.33E-07	0.288495	0.136	0.06	0.013887	9
Mbip	8.62E-07	0.267236	0.146	0.068	0.014385	9
Rpl7	8.85E-07	-0.29796	0.433	0.511	0.014768	9
Nptn	8.87E-07	0.291698	0.299	0.185	0.014796	9
Kbtbd11	8.89E-07	0.318704	0.168	0.085	0.014824	9
Nfyb	9.07E-07	-0.38228	0.134	0.237	0.015134	9
Rbbp4	9.07E-07	-0.30444	0.377	0.486	0.015136	9
Taf15	9.11E-07	-0.36749	0.129	0.224	0.01519	9
Dbf4	9.5E-07	-0.30483	0.061	0.147	0.01585	9
Agl	9.59E-07	0.298228	0.102	0.039	0.015992	9
Cdca4	9.92E-07	-0.27208	0.036	0.112	0.016551	9
Bub1b	1.06E-06	-0.26917	0.032	0.104	0.01767	9
Limd2	1.07E-06	-0.28528	0.063	0.136	0.017842	9
Pcnt	1.07E-06	-0.38077	0.085	0.176	0.017928	9
Bub1	1.09E-06	-0.27666	0.051	0.132	0.018175	9
Timeless	1.15E-06	-0.32442	0.066	0.155	0.019157	9
Mlf2	1.15E-06	0.306109	0.397	0.276	0.019197	9
Maml3	1.16E-06	-0.27204	0.032	0.102	0.019377	9
Fam107b	1.24E-06	-0.30525	0.034	0.104	0.020725	9
Emc3	1.26E-06	0.266971	0.251	0.146	0.021033	9
Tmed10	1.3E-06	0.295527	0.311	0.201	0.02168	9
Akap12	1.33E-06	0.334738	0.197	0.109	0.022255	9
Stmn4	1.35E-06	-0.27055	0.341	0.341	0.02248	9
2310061I0	1.42E-06	0.310904	0.148	0.07	0.02364	9
Fen1	1.49E-06	-0.34536	0.066	0.143	0.024774	9
Rpl37	1.57E-06	-0.30797	0.406	0.506	0.026128	9
Chtop	1.7E-06	0.308862	0.343	0.246	0.028388	9
Tmem33	1.76E-06	0.297324	0.243	0.142	0.029274	9
Pccb	1.76E-06	0.316531	0.207	0.116	0.029341	9
Nol4	1.76E-06	-0.37061	0.083	0.173	0.029393	9
BC004004	1.78E-06	0.30108	0.217	0.12	0.029729	9
Atp2b1	1.92E-06	-0.29061	0.533	0.572	0.032039	9
Zfp91	1.98E-06	-0.29942	0.394	0.491	0.03309	9
Ten1	2.04E-06	0.290648	0.253	0.149	0.03399	9
Glo1	2.06E-06	0.304357	0.192	0.105	0.034369	9
Itsn2	2.07E-06	0.295953	0.168	0.084	0.034595	9
Arhgdia	2.09E-06	0.276379	0.319	0.205	0.034891	9
Ska2	2.11E-06	-0.33555	0.139	0.206	0.03519	9
Banp	2.16E-06	-0.33776	0.08	0.138	0.036032	9
Golph3	2.31E-06	0.319634	0.139	0.072	0.03854	9
Tor1b	2.34E-06	0.296902	0.226	0.129	0.039013	9
Palm	2.4E-06	-0.31211	0.08	0.14	0.039993	9
Gm26735	2.42E-06	-0.39038	0.095	0.182	0.040334	9
Ubtf	2.44E-06	-0.3144	0.355	0.454	0.040684	9
Ap1s2	2.54E-06	-0.33441	0.073	0.162	0.042332	9
Slc3a2	2.57E-06	-0.29943	0.27	0.323	0.042905	9
Ythdc1	2.66E-06	-0.31016	0.355	0.479	0.044379	9

Whsc1	2.69E-06	-0.28436	0.401	0.465	0.044872	9
5830418K(	2.74E-06	-0.29764	0.083	0.17	0.045739	9
Maoa	2.85E-06	0.273915	0.139	0.066	0.0476	9
Snhg1	2.96E-06	-0.32954	0.248	0.359	0.049392	9
Cep78	2.97E-06	-0.3461	0.066	0.142	0.049567	9
Lsm3	3.01E-06	-0.32549	0.268	0.36	0.050198	9
9330159F1	3.03E-06	-0.35154	0.08	0.17	0.050526	9
Frrs1l	3.12E-06	0.335649	0.294	0.188	0.051967	9
Brpf1	3.16E-06	-0.27789	0.061	0.142	0.052673	9
Hibadh	3.18E-06	0.271519	0.165	0.083	0.05303	9
Blm	3.23E-06	-0.28359	0.039	0.112	0.053855	9
Ndufa7	3.25E-06	0.281648	0.545	0.437	0.054283	9
Arhgap20	3.26E-06	0.26941	0.117	0.052	0.054351	9
Smarcc1	3.31E-06	-0.31329	0.326	0.436	0.055187	9
Ptbp1	3.31E-06	-0.28494	0.034	0.102	0.055271	9
Polr2h	3.33E-06	-0.29321	0.139	0.227	0.055494	9
Slc25a1	3.36E-06	0.300114	0.129	0.059	0.056035	9
Rora	3.36E-06	0.270958	0.119	0.053	0.056072	9
Nfic	3.37E-06	-0.36281	0.158	0.263	0.056175	9
Fam57b	3.37E-06	-0.35907	0.095	0.187	0.056213	9
Hmgcr	3.42E-06	0.326803	0.217	0.127	0.056978	9
Ckap2l	3.48E-06	-0.38181	0.122	0.219	0.058115	9
Bnip3l	3.53E-06	-0.34594	0.088	0.165	0.05883	9
Tipin	3.62E-06	-0.34019	0.151	0.257	0.060314	9
Ddhd1	3.7E-06	0.296818	0.119	0.054	0.061746	9
Sdf4	3.7E-06	0.289483	0.328	0.22	0.061784	9
Apc	3.74E-06	-0.35695	0.35	0.433	0.062319	9
Ncdn	3.79E-06	0.252417	0.139	0.065	0.063213	9
1810009A1	3.83E-06	-0.36612	0.17	0.261	0.063806	9
Cadm3	3.84E-06	-0.31355	0.112	0.141	0.064048	9
Tbl1x	3.85E-06	-0.31569	0.212	0.33	0.064255	9
Zeb1	3.89E-06	-0.33352	0.273	0.39	0.064883	9
Pbx3	3.93E-06	-0.27945	0.051	0.128	0.065602	9
Lrpap1	3.94E-06	0.288124	0.207	0.115	0.06567	9
Dbn1	4.21E-06	-0.37164	0.073	0.141	0.070212	9
Btbd9	4.22E-06	0.304064	0.195	0.107	0.070372	9
Gpr180	4.35E-06	0.270576	0.148	0.079	0.072501	9
Psma4	4.4E-06	-0.31608	0.324	0.415	0.073394	9
Cers4	4.49E-06	0.303221	0.129	0.058	0.074897	9
Kifap3	4.54E-06	0.308445	0.382	0.27	0.075777	9
Vrk1	4.65E-06	-0.28782	0.066	0.149	0.07756	9
Cnn3	4.7E-06	-0.29595	0.331	0.439	0.078343	9
Gins1	4.87E-06	-0.2635	0.044	0.116	0.081264	9
Scp2	4.95E-06	0.27609	0.146	0.071	0.08255	9
Vkorc1	4.97E-06	0.284264	0.195	0.108	0.082884	9
Eif3j1	5.35E-06	0.269658	0.153	0.076	0.089191	9
Vapb	5.58E-06	0.256799	0.17	0.091	0.093015	9
Cenpb	5.63E-06	-0.32762	0.117	0.211	0.093918	9

Eny2	5.64E-06	-0.29062	0.326	0.388	0.094	9
Rpn1	5.72E-06	0.302025	0.292	0.19	0.095417	9
Galnt1	5.79E-06	0.317782	0.18	0.102	0.096549	9
Saysd1	5.8E-06	0.323309	0.102	0.051	0.096683	9
Trib1	5.81E-06	0.278567	0.109	0.057	0.096846	9
Rpa3	5.85E-06	-0.35322	0.153	0.246	0.097506	9
Xpo1	5.86E-06	-0.3279	0.158	0.229	0.097702	9
Bag1	5.91E-06	-0.30656	0.229	0.347	0.098571	9
Stxbp1	6.02E-06	-0.39356	0.088	0.164	0.100404	9
Kif4	6.04E-06	-0.2597	0.039	0.109	0.100754	9
Rac1	6.1E-06	0.287482	0.426	0.344	0.101716	9
Hmg20b	6.49E-06	-0.34925	0.134	0.224	0.108269	9
Rai1	6.59E-06	-0.35246	0.08	0.161	0.109911	9
Tsc22d1	6.78E-06	0.258474	0.543	0.425	0.113106	9
N6amt1	6.96E-06	0.282966	0.18	0.096	0.116087	9
Tram1l1	7E-06	0.257004	0.163	0.084	0.116794	9
Pa2g4	7.07E-06	-0.25769	0.44	0.524	0.117906	9
Mis18a	7.09E-06	-0.28813	0.051	0.111	0.118312	9
Fkbp2	7.1E-06	0.281173	0.436	0.323	0.118388	9
Srp14	7.12E-06	0.254607	0.477	0.354	0.118795	9
Agfg1	7.16E-06	0.276793	0.129	0.06	0.119347	9
Lmnb2	7.29E-06	-0.32777	0.078	0.16	0.121628	9
Dazap1	7.8E-06	-0.31483	0.17	0.274	0.130071	9
Cdk5rap3	7.85E-06	-0.3284	0.105	0.194	0.130946	9
Creb1	7.96E-06	-0.29845	0.131	0.224	0.132781	9
Crebbp	8.02E-06	-0.3414	0.131	0.225	0.133759	9
Scfd1	8.04E-06	0.266148	0.212	0.121	0.134102	9
Mier3	8.07E-06	-0.31612	0.051	0.121	0.134666	9
Rrm1	8.12E-06	-0.30314	0.158	0.262	0.135427	9
Ift27	8.16E-06	-0.29678	0.144	0.245	0.136169	9
Pdgfa	8.19E-06	-0.38639	0.165	0.255	0.136676	9
Pim3	8.33E-06	0.267584	0.165	0.085	0.138965	9
Mesdc2	8.34E-06	0.317826	0.282	0.204	0.139104	9
Ktn1	8.44E-06	0.268709	0.543	0.457	0.140746	9
Zfp292	8.61E-06	-0.36413	0.238	0.344	0.143696	9
Tdp2	8.67E-06	-0.30315	0.075	0.151	0.144569	9
D030056L2	8.81E-06	-0.28366	0.063	0.144	0.146893	9
Pafah1b2	8.89E-06	-0.32583	0.158	0.257	0.148207	9
Ndufaf7	9E-06	0.27804	0.18	0.098	0.150049	9
Smc6	9.28E-06	-0.31736	0.311	0.425	0.154756	9
Arl6ip6	1.02E-05	-0.30304	0.063	0.126	0.169643	9
Acap2	1.05E-05	0.295605	0.141	0.07	0.175314	9
Snrnp40	1.11E-05	-0.2886	0.195	0.272	0.185623	9
Paics	1.15E-05	-0.25245	0.302	0.416	0.192432	9
Agap1	1.16E-05	0.279289	0.221	0.129	0.193054	9
Wwp1	1.2E-05	0.284044	0.156	0.082	0.200713	9
Cdkn1c	1.24E-05	0.279061	0.129	0.061	0.206733	9
Ift46	1.3E-05	-0.31337	0.058	0.118	0.21633	9

Cspp1	1.32E-05	-0.30177	0.153	0.24	0.219788	9
Tkt	1.33E-05	0.279049	0.304	0.201	0.221596	9
Nipbl	1.35E-05	-0.31078	0.404	0.494	0.224959	9
Grik2	1.35E-05	-0.32935	0.058	0.102	0.22512	9
Hddc2	1.39E-05	0.275267	0.17	0.091	0.231742	9
Ptpla	1.39E-05	0.26225	0.151	0.077	0.232106	9
Srsf7	1.4E-05	-0.26465	0.382	0.467	0.232721	9
Set	1.4E-05	-0.27717	0.302	0.389	0.233109	9
Dhx15	1.41E-05	-0.27466	0.311	0.419	0.235621	9
Gnptg	1.44E-05	0.285906	0.26	0.164	0.239622	9
Mcm2	1.44E-05	-0.31631	0.119	0.206	0.23984	9
Tmed9	1.46E-05	-0.2507	0.392	0.434	0.242799	9
Cyth2	1.46E-05	-0.31771	0.204	0.274	0.244145	9
Rbm3	1.5E-05	-0.26193	0.061	0.125	0.249397	9
Mrpl13	1.53E-05	-0.32311	0.217	0.309	0.254836	9
Tacc3	1.53E-05	-0.34427	0.117	0.208	0.255156	9
Sh3gl2	1.58E-05	-0.35553	0.102	0.173	0.262863	9
Snx5	1.65E-05	0.268263	0.258	0.161	0.275189	9
Txnrd1	1.65E-05	-0.30634	0.297	0.39	0.275717	9
Litaf	1.66E-05	0.28194	0.131	0.067	0.277278	9
Caprin1	1.68E-05	-0.26703	0.309	0.395	0.279996	9
5830428H:	1.74E-05	0.259774	0.129	0.063	0.289682	9
Uggt2	1.74E-05	0.30109	0.122	0.059	0.290098	9
Glg1	1.74E-05	0.26855	0.324	0.216	0.290948	9
Nnat	1.77E-05	-0.28235	0.698	0.71	0.295056	9
Gnao1	1.79E-05	-0.31172	0.241	0.288	0.298064	9
Rps28	1.79E-05	-0.2994	0.129	0.205	0.298807	9
Fbxo32	1.84E-05	-0.34373	0.054	0.118	0.30619	9
Rfc4	1.87E-05	-0.28151	0.134	0.228	0.311728	9
1110065P:	1.9E-05	0.272173	0.2	0.116	0.317629	9
Commd6	1.92E-05	0.312051	0.263	0.177	0.319998	9
2410006H:	1.93E-05	-0.30884	0.36	0.461	0.322256	9
Cdv3	1.94E-05	0.316691	0.231	0.156	0.32284	9
Ggh	2.02E-05	0.287769	0.18	0.122	0.336489	9
2810474O:	2.13E-05	-0.32296	0.144	0.243	0.355409	9
Akap11	2.13E-05	0.264709	0.229	0.137	0.355893	9
Pak2	2.19E-05	0.250756	0.409	0.298	0.365845	9
Ccnl1	2.21E-05	-0.27692	0.294	0.37	0.369078	9
Grlf1	2.26E-05	0.271503	0.175	0.096	0.377332	9
Tardbp	2.29E-05	-0.25845	0.399	0.484	0.382291	9
Uimc1	2.33E-05	-0.31488	0.061	0.134	0.389065	9
Serf1	2.37E-05	-0.31426	0.187	0.283	0.395977	9
Scamp3	2.44E-05	0.289799	0.204	0.12	0.406205	9
Mt2	2.52E-05	0.323502	0.163	0.09	0.420642	9
Acp2	2.7E-05	0.261527	0.168	0.091	0.450068	9
Sod2	2.7E-05	0.257882	0.27	0.173	0.45079	9
Crot	2.73E-05	0.285351	0.156	0.087	0.454691	9
Zc3h13	2.78E-05	-0.31629	0.37	0.464	0.463087	9

Ap3b2	2.91E-05	-0.27089	0.117	0.204	0.485325	9
Psme4	2.94E-05	-0.30215	0.19	0.286	0.490356	9
Hsph1	2.96E-05	-0.32916	0.141	0.229	0.492916	9
Brd3	3.04E-05	-0.25601	0.455	0.533	0.507266	9
Psma1	3.17E-05	-0.26999	0.355	0.446	0.528144	9
Tmem57	3.17E-05	-0.33861	0.207	0.293	0.529424	9
Stard4	3.21E-05	-0.31536	0.073	0.142	0.534651	9
Pdzrn4	3.21E-05	0.302048	0.182	0.145	0.536025	9
Hnrnph3	3.23E-05	-0.27397	0.338	0.428	0.538528	9
Rps7	3.23E-05	-0.28972	0.316	0.401	0.53918	9
Ing4	3.27E-05	-0.30785	0.226	0.324	0.54469	9
Cep57	3.4E-05	-0.31425	0.173	0.259	0.5678	9
Tbcb	3.44E-05	0.270975	0.416	0.308	0.573469	9
Man1c1	3.5E-05	-0.30761	0.058	0.127	0.583353	9
Dnajc21	3.56E-05	-0.31693	0.136	0.211	0.594102	9
1110038B1	3.77E-05	-0.30298	0.204	0.3	0.629653	9
Mllt10	3.87E-05	-0.32019	0.202	0.288	0.644847	9
Ensa	3.87E-05	-0.32281	0.144	0.229	0.645836	9
Ptp4a2	4.06E-05	0.264729	0.436	0.339	0.676478	9
Tshz1	4.14E-05	0.299164	0.224	0.139	0.690912	9
Lpcat1	4.29E-05	0.277696	0.219	0.133	0.715985	9
Rfc5	4.29E-05	-0.27177	0.051	0.109	0.716226	9
Ssbp2	4.37E-05	-0.25652	0.056	0.127	0.729409	9
Pttg1	4.39E-05	-0.32046	0.066	0.141	0.732243	9
Arid2	4.45E-05	-0.32525	0.151	0.243	0.741578	9
2700049A(	4.62E-05	-0.27849	0.056	0.123	0.769819	9
Rab7	4.65E-05	0.257842	0.277	0.181	0.776416	9
Psmd6	4.7E-05	0.267049	0.397	0.306	0.783608	9
Ldb1	4.78E-05	-0.33875	0.107	0.186	0.796963	9
Naa38	4.98E-05	-0.2731	0.217	0.313	0.830318	9
Vps37a	5.06E-05	0.258598	0.112	0.057	0.843895	9
Arl6ip4	5.16E-05	-0.32942	0.151	0.238	0.860216	9
Phf20l1	5.2E-05	-0.335	0.26	0.342	0.86731	9
Zfp706	5.25E-05	-0.28785	0.275	0.365	0.875736	9
Phactr1	5.28E-05	0.25168	0.277	0.181	0.879946	9
Atad5	5.35E-05	-0.32063	0.078	0.15	0.892833	9
Med19	5.69E-05	-0.31507	0.238	0.319	0.948525	9
Faim	5.71E-05	0.307449	0.241	0.167	0.952349	9
Zmiz1	5.74E-05	-0.31355	0.285	0.377	0.957934	9
Ccni	5.95E-05	-0.32068	0.139	0.224	0.993145	9
Irs1	6.06E-05	-0.2952	0.058	0.12	1	9
Stk11	6.23E-05	-0.30384	0.151	0.231	1	9
Plcg1	6.59E-05	-0.2612	0.109	0.162	1	9
Zfp827	6.83E-05	0.261414	0.114	0.055	1	9
Klhl24	6.96E-05	-0.31543	0.075	0.14	1	9
Gtf2f2	7.07E-05	0.268501	0.19	0.115	1	9
Anapc5	7.11E-05	-0.29914	0.243	0.335	1	9
Psmc3ip	7.24E-05	-0.29425	0.066	0.133	1	9

Lbr	7.41E-05	-0.29735	0.117	0.198	1	9
Sgol2	7.62E-05	-0.29206	0.08	0.158	1	9
Myl12b	7.75E-05	-0.28245	0.29	0.369	1	9
Cirbp	7.97E-05	-0.28102	0.297	0.356	1	9
Git2	8.02E-05	-0.29051	0.056	0.111	1	9
Xpr1	8.39E-05	0.253131	0.163	0.09	1	9
Jarid2	8.69E-05	-0.31333	0.102	0.18	1	9
Lphn1	8.73E-05	0.251933	0.19	0.111	1	9
Agpat6	8.81E-05	0.307954	0.192	0.129	1	9
Sesn3	8.9E-05	0.259572	0.102	0.049	1	9
Dync2h1	8.92E-05	-0.30681	0.054	0.117	1	9
Plekha1	9.05E-05	0.293319	0.112	0.059	1	9
Bcar1	9.27E-05	-0.31376	0.117	0.194	1	9
Shmt2	9.63E-05	-0.26546	0.054	0.121	1	9
Gtf2e2	9.87E-05	-0.29567	0.105	0.179	1	9
Ywhaz	0.0001	-0.2739	0.294	0.363	1	9
Slc38a1	0.000105	-0.27041	0.175	0.272	1	9
Rbmx	0.000106	-0.2927	0.229	0.317	1	9
Tro	0.000108	0.295851	0.202	0.124	1	9
Ptprs	0.000109	-0.31251	0.326	0.423	1	9
Dnajc2	0.00011	-0.27668	0.251	0.33	1	9
Rfc3	0.000113	-0.27886	0.122	0.206	1	9
Armc10	0.000116	-0.27263	0.049	0.11	1	9
Mapk8ip1	0.000116	-0.29666	0.148	0.229	1	9
Ube2g2	0.000126	-0.28541	0.061	0.126	1	9
Blmh	0.000127	-0.27634	0.221	0.319	1	9
Evl	0.000128	-0.29895	0.1	0.18	1	9
Sumo3	0.000131	-0.29798	0.217	0.3	1	9
Ppp1r1a	0.000131	-0.25641	0.068	0.111	1	9
Pttg1ip	0.000132	0.266433	0.148	0.084	1	9
Shmt1	0.000132	-0.256	0.049	0.104	1	9
Cplx1	0.000136	-0.29018	0.068	0.139	1	9
Zfr	0.000137	0.263851	0.421	0.352	1	9
Dnajc1	0.000139	0.258625	0.219	0.138	1	9
Rps4x	0.000139	-0.28612	0.209	0.297	1	9
Scaf11	0.00014	-0.2696	0.292	0.375	1	9
Ccdc53	0.000142	0.255527	0.131	0.071	1	9
Klc1	0.000142	-0.28261	0.304	0.362	1	9
Cdh20	0.000143	-0.28395	0.129	0.204	1	9
Kdm1a	0.000153	-0.28633	0.275	0.366	1	9
Pcbp4	0.000155	-0.27504	0.243	0.326	1	9
Dnmt1	0.000155	-0.2715	0.187	0.279	1	9
Picalm	0.000156	0.260415	0.219	0.139	1	9
Zfp422	0.000158	-0.28585	0.161	0.247	1	9
Nsmce4a	0.00016	-0.28396	0.139	0.225	1	9
Sclt1	0.00016	-0.26957	0.063	0.128	1	9
Wasl	0.000163	0.269153	0.311	0.234	1	9
Ctbp2	0.000167	-0.28014	0.131	0.217	1	9

D19Bwg13	0.000167	-0.30229	0.187	0.275	1	9
Ddx39b	0.000173	-0.25758	0.353	0.446	1	9
Prkar2a	0.000182	0.260314	0.212	0.133	1	9
Tchp	0.000185	-0.28671	0.068	0.118	1	9
Scg3	0.000186	-0.27777	0.372	0.448	1	9
Herpud1	0.000188	-0.29047	0.073	0.137	1	9
Brd7	0.000191	-0.29518	0.231	0.309	1	9
Polr1c	0.000193	-0.269	0.163	0.219	1	9
Ulk1	0.000198	-0.27616	0.051	0.105	1	9
Pebp1	0.000199	0.267677	0.319	0.24	1	9
Gltscr2	0.000204	-0.28378	0.26	0.343	1	9
Cnksr2	0.00021	-0.28796	0.049	0.108	1	9
Nes	0.000219	0.260666	0.148	0.085	1	9
Zfp36l1	0.000248	0.284109	0.209	0.149	1	9
Fam64a	0.000255	-0.26966	0.056	0.119	1	9
Dpy30	0.000259	-0.28026	0.212	0.3	1	9
Arfrp1	0.000262	0.267593	0.187	0.116	1	9
Dock1	0.000264	0.273651	0.146	0.089	1	9
Pcsk2	0.000265	0.256565	0.158	0.1	1	9
Kcnk1	0.000269	-0.32958	0.134	0.206	1	9
Sass6	0.00027	-0.2599	0.061	0.127	1	9
Klhl7	0.000273	-0.28019	0.097	0.166	1	9
Casp8ap2	0.000276	-0.29244	0.158	0.245	1	9
Ankrd49	0.000279	0.274557	0.156	0.094	1	9
Lyar	0.000284	-0.27725	0.195	0.287	1	9
Rnf220	0.000298	-0.2977	0.158	0.237	1	9
Mad2l2	0.0003	-0.28924	0.187	0.264	1	9
Ccdc23	0.000301	0.259369	0.285	0.196	1	9
Rab3c	0.000312	-0.29248	0.08	0.149	1	9
2700081O	0.000318	-0.29247	0.054	0.112	1	9
Poc1b	0.000323	-0.25725	0.071	0.128	1	9
Nmral1	0.000334	-0.26832	0.112	0.189	1	9
Gm13826	0.000337	-0.27011	0.241	0.313	1	9
Tmco1	0.000342	0.257398	0.324	0.239	1	9
Gm11478	0.000373	-0.27793	0.136	0.216	1	9
Zdhhc20	0.00038	0.272714	0.217	0.143	1	9
Fkbp4	0.00038	-0.2511	0.333	0.416	1	9
Ano6	0.000392	0.281484	0.146	0.089	1	9
Nfu1	0.000398	0.257039	0.204	0.129	1	9
Iqgap1	0.000405	-0.26999	0.068	0.133	1	9
Frg1	0.000409	-0.27049	0.238	0.3	1	9
Lin7c	0.000411	-0.28384	0.139	0.219	1	9
Sowaha	0.000411	-0.31807	0.122	0.163	1	9
Snord104	0.00042	-0.28562	0.097	0.153	1	9
Klf13	0.000426	-0.28048	0.165	0.238	1	9
Pou3f3	0.000426	0.272703	0.238	0.17	1	9
Polr2c	0.000441	-0.27201	0.127	0.203	1	9
Celf1	0.000465	-0.26603	0.304	0.381	1	9

Rbbp8	0.000469	-0.26559	0.063	0.123	1	9
2610203C2	0.00047	-0.29347	0.061	0.122	1	9
Mpp6	0.000474	0.258367	0.234	0.159	1	9
Tiparp	0.000478	-0.28266	0.097	0.148	1	9
Rpf1	0.00048	-0.27864	0.119	0.195	1	9
Mxi1	0.000513	-0.28389	0.131	0.192	1	9
Tbpl1	0.00052	-0.28169	0.119	0.188	1	9
Rundc3a	0.000556	-0.27464	0.19	0.25	1	9
Emd	0.000558	-0.25018	0.119	0.183	1	9
Rraga	0.000559	0.259258	0.246	0.171	1	9
Ube2d1	0.000604	-0.25089	0.163	0.229	1	9
Sf3a3	0.000613	-0.25379	0.204	0.282	1	9
Cotl1	0.000624	0.273435	0.217	0.152	1	9
6330403K0	0.000704	-0.29612	0.178	0.244	1	9
Nkain4	0.000713	0.279002	0.192	0.127	1	9
R3hcc1	0.000752	-0.278	0.102	0.16	1	9
Ndufaf2	0.000771	0.254905	0.28	0.2	1	9
Oraov1	0.0008	0.259294	0.175	0.119	1	9
Ncor2	0.000847	-0.27309	0.102	0.17	1	9
Smad4	0.000867	-0.27844	0.085	0.145	1	9
Phc2	0.00088	-0.281	0.144	0.201	1	9
Mapk3	0.000884	-0.25184	0.063	0.113	1	9
Dgkz	0.000899	-0.26304	0.054	0.105	1	9
Nfkbia	0.000923	-0.27437	0.122	0.172	1	9
Klf9	0.000928	-0.28865	0.195	0.272	1	9
Pcyt1a	0.000941	0.270106	0.156	0.097	1	9
Aspm	0.000998	-0.29869	0.08	0.144	1	9
Rpl6	0.001004	-0.25618	0.212	0.292	1	9
Suz12	0.001083	-0.25327	0.144	0.22	1	9
Slc12a2	0.00109	0.264845	0.168	0.109	1	9
Elp3	0.001121	-0.25563	0.083	0.138	1	9
Cyb5r3	0.001144	-0.27123	0.151	0.2	1	9
Mrpl48	0.001343	0.255332	0.192	0.136	1	9
1700020I1	0.00168	-0.25605	0.212	0.275	1	9
Smchd1	0.001984	-0.25992	0.204	0.274	1	9
A330076H0	0.002068	-0.29632	0.1	0.162	1	9
Tagln3	0.00216	-0.26951	0.234	0.296	1	9
Zfp704	0.002202	-0.25921	0.202	0.258	1	9
Nemf	0.002425	-0.25923	0.268	0.34	1	9
Srgap2	0.002486	-0.2628	0.085	0.133	1	9
Pnpla8	0.002495	-0.25606	0.178	0.232	1	9
Esf1	0.002567	-0.25808	0.328	0.393	1	9
Atp1b3	0.002959	-0.25236	0.263	0.338	1	9
Acd	0.003346	-0.25558	0.109	0.162	1	9
Rab6b	0.003645	-0.25224	0.161	0.203	1	9
Hes1	0.00447	-0.29902	0.107	0.149	1	9
Kdm5a	0.004514	-0.25489	0.202	0.257	1	9
Pde4dip	0.004597	-0.25173	0.158	0.222	1	9

A030009H(	0.00475	-0.25105	0.066	0.115	1	9
Plcb4	0.006991	-0.25339	0.088	0.14	1	9
Apoe	0	4.814817	0.983	0.241	0	10
Ctsd	0	4.346427	0.971	0.166	0	10
C1qb	0	4.200489	0.977	0.009	0	10
Lyz2	0	4.183327	0.943	0.009	0	10
C1qc	0	3.891818	0.954	0.006	0	10
Sepp1	0	3.835728	0.96	0.046	0	10
Hexb	0	3.773298	0.908	0.023	0	10
Tyrobp	0	3.736191	0.971	0.008	0	10
Ctsb	0	3.67162	0.966	0.254	0	10
Ctss	0	3.593406	0.937	0.004	0	10
Cst3	0	3.303421	0.989	0.531	0	10
Fcrls	0	3.268441	0.902	0.004	0	10
Fcer1g	0	3.17988	0.902	0.004	0	10
C1qa	0	3.03047	0.897	0.003	0	10
Aif1	0	3.006934	0.885	0.003	0	10
Grn	0	2.897274	0.856	0.078	0	10
B2m	0	2.870834	0.948	0.224	0	10
Lgmn	0	2.849949	0.908	0.111	0	10
Ctsz	0	2.652496	0.92	0.128	0	10
Trem2	0	3.083342	0.856	0.003	0	10
Laptm5	0	2.910531	0.862	0.003	0	10
Ly86	6.6E-301	2.683389	0.851	0.004	1.1E-296	10
Csf1r	3.8E-294	2.643149	0.776	0.001	6.3E-290	10
Cx3cr1	2E-275	2.722999	0.77	0.003	3.4E-271	10
Cd68	1.1E-266	2.566251	0.787	0.006	1.9E-262	10
Hsp90ab1	1.3E-263	-1.12379	0.931	0.998	2.1E-259	10
Igf1	1.7E-255	2.292626	0.753	0.003	2.8E-251	10
Fth1	2.9E-255	1.734292	0.989	0.705	4.8E-251	10
Fcgr3	3E-255	2.270977	0.707	0.001	5.1E-251	10
Ctsa	3.5E-245	2.417381	0.805	0.117	5.9E-241	10
Rnase4	1E-240	2.575893	0.753	0.014	1.7E-236	10
Timp2	7.9E-236	2.740073	0.799	0.018	1.3E-231	10
Cyba	6.8E-233	2.400059	0.782	0.022	1.1E-228	10
Hexa	1.2E-230	2.444486	0.81	0.058	2E-226	10
Mpeg1	1.9E-228	2.267285	0.661	0.002	3.2E-224	10
Rgs10	2.9E-219	2.166143	0.747	0.009	4.9E-215	10
Creg1	1.2E-218	2.478473	0.799	0.051	2E-214	10
Ctsl	3.5E-214	2.30494	0.822	0.254	5.9E-210	10
AF251705	3E-204	1.748915	0.58	0.001	5E-200	10
Hpgds	1.7E-200	1.804674	0.603	0.002	2.9E-196	10
Emr1	6.2E-200	1.6905	0.552	0	1E-195	10
Ptpn18	1.4E-198	1.87344	0.598	0.002	2.3E-194	10
Unc93b1	1.8E-198	2.070428	0.655	0.006	3E-194	10
Fyb	2.8E-198	1.842407	0.609	0.002	4.7E-194	10
P2ry12	3.2E-196	2.101146	0.563	0.001	5.3E-192	10
Lamp1	6.9E-190	1.85854	0.885	0.33	1.1E-185	10

Cd53	1.7E-186	1.798367	0.54	0.001	2.9E-182	10
Maf	1.7E-185	2.340153	0.672	0.013	2.9E-181	10
Gpr34	4.9E-184	1.798368	0.534	0.001	8.2E-180	10
C3ar1	7.1E-177	1.722745	0.517	0.001	1.2E-172	10
Vamp8	8.1E-176	1.846939	0.609	0.007	1.3E-171	10
Sat1	2.3E-175	2.181892	0.787	0.059	3.8E-171	10
Psap	4E-173	2.082532	0.782	0.178	6.7E-169	10
Cyth4	5.6E-173	1.580755	0.517	0.001	9.3E-169	10
Cd52	1.2E-170	1.913042	0.489	0.001	2E-166	10
Tmsb4x	2E-170	1.452509	1	0.948	3.4E-166	10
Pld4	2E-166	1.537548	0.483	0.001	3.3E-162	10
Arpc1b	1.4E-165	1.953874	0.713	0.03	2.3E-161	10
Ctsc	1.7E-165	1.841566	0.54	0.003	2.9E-161	10
Npc2	8.2E-164	1.60084	0.954	0.458	1.4E-159	10
Arhgdib	3.5E-163	1.695537	0.54	0.003	5.8E-159	10
Rac2	3.3E-162	1.437899	0.489	0.001	5.5E-158	10
Abca1	5.1E-162	2.095325	0.649	0.017	8.6E-158	10
Ctsh	5.5E-161	1.864346	0.586	0.006	9.2E-157	10
Sirpa	1.1E-156	2.014017	0.695	0.031	1.8E-152	10
Ltc4s	4.9E-155	1.561183	0.454	0.001	8.2E-151	10
Man2b1	2.7E-153	1.940246	0.672	0.027	4.5E-149	10
Stab1	3.2E-151	1.992318	0.523	0.004	5.3E-147	10
Pycard	6E-151	1.802954	0.54	0.006	9.9E-147	10
Itgb5	1E-150	1.568442	0.517	0.004	1.7E-146	10
Lpcat2	1.5E-149	1.480897	0.489	0.002	2.6E-145	10
Evi2a	9.6E-148	1.502647	0.483	0.002	1.6E-143	10
Ccl3	2.9E-147	2.329239	0.431	0.001	4.8E-143	10
Cd84	3.6E-147	1.188706	0.42	0	6E-143	10
Apbb1ip	6.4E-147	1.539618	0.511	0.004	1.1E-142	10
Ms4a7	1.1E-144	2.34827	0.431	0.001	1.9E-140	10
Ms4a6c	6.4E-144	1.597501	0.425	0.001	1.1E-139	10
Ncf1	2.2E-139	1.314925	0.425	0.001	3.7E-135	10
H2-K1	3.9E-139	1.768939	0.546	0.009	6.4E-135	10
Apoc1	4.4E-138	2.369226	0.443	0.003	7.4E-134	10
Itm2b	4.4E-137	1.484552	0.966	0.639	7.4E-133	10
Irf8	1.7E-136	1.455471	0.506	0.006	2.8E-132	10
Bst2	2.7E-134	1.448648	0.42	0.001	4.6E-130	10
Serinc3	7.9E-134	2.009635	0.747	0.113	1.3E-129	10
Lair1	9.7E-133	1.323846	0.391	0	1.6E-128	10
Anxa3	5.8E-130	1.462236	0.448	0.003	9.7E-126	10
Abhd12	8E-130	1.84069	0.718	0.086	1.3E-125	10
Fam105a	6.2E-129	1.499624	0.517	0.008	1E-124	10
Cd300a	1.6E-128	1.27143	0.397	0.001	2.6E-124	10
Pf4	1.2E-126	2.640508	0.391	0.002	2E-122	10
Mertk	3E-126	1.527668	0.437	0.003	4.9E-122	10
Abcg1	9.6E-124	1.767135	0.58	0.018	1.6E-119	10
Mt1	3.4E-123	1.986689	0.851	0.161	5.7E-119	10
Rgs2	3.7E-123	1.916484	0.638	0.046	6.1E-119	10

Spi1	5.3E-123	1.198803	0.362	0	8.8E-119	10
Npl	2.4E-121	1.255541	0.425	0.003	3.9E-117	10
4632428N(	7.5E-121	1.355061	0.408	0.002	1.3E-116	10
Fcgr1	1.4E-120	1.109739	0.356	0	2.3E-116	10
Tnfaip8l2	1.6E-118	1.089487	0.351	0	2.6E-114	10
Cotl1	9.8E-118	1.71375	0.787	0.147	1.6E-113	10
Lcp1	2.4E-116	1.291431	0.379	0.002	4E-112	10
Tuba1a	5.6E-116	-1.94209	0.402	0.934	9.3E-112	10
Sh3bgrl3	1E-115	1.714074	0.776	0.192	1.7E-111	10
Cd86	2E-115	1.037691	0.339	0	3.3E-111	10
Syngr1	4.2E-114	1.84939	0.69	0.087	6.9E-110	10
Mrc1	5.3E-114	1.980449	0.368	0.002	8.8E-110	10
Selplg	7.6E-114	1.248164	0.328	0	1.3E-109	10
Bin2	1.1E-113	1.181839	0.333	0	1.8E-109	10
Nfib	4.3E-113	-1.72788	0.454	0.938	7.1E-109	10
Psmb8	4.3E-112	1.258633	0.397	0.003	7.2E-108	10
Cd37	1.6E-111	1.136944	0.351	0.001	2.6E-107	10
Fgd2	4.8E-110	1.041012	0.328	0	8E-106	10
Anxa5	4E-108	1.607152	0.511	0.015	6.7E-104	10
Plek	2E-107	1.416354	0.443	0.007	3.4E-103	10
Ptpn6	3.3E-107	0.98173	0.328	0.001	5.5E-103	10
Gpx3	6.8E-107	1.306953	0.437	0.006	1.1E-102	10
Gns	2.7E-106	1.710641	0.632	0.064	4.6E-102	10
Cryba4	9.9E-106	1.200686	0.316	0	1.7E-101	10
Spp1	3.8E-105	2.858392	0.408	0.007	6.3E-101	10
Clec7a	2.6E-103	1.342418	0.328	0.001	4.4E-99	10
Plin2	1.4E-101	1.681296	0.563	0.047	2.4E-97	10
Ptgs1	8.2E-100	1.217195	0.305	0.001	1.36E-95	10
Ccl4	1.9E-99	2.314333	0.305	0.001	3.18E-95	10
Ms4a6d	2.2E-99	1.024471	0.305	0.001	3.66E-95	10
Hmha1	5.4E-99	0.975048	0.305	0.001	8.99E-95	10
Inpp5d	2.31E-98	1.042058	0.299	0	3.85E-94	10
Tbxas1	1.17E-96	0.96283	0.293	0	1.95E-92	10
P2ry13	2.25E-96	0.968719	0.282	0	3.75E-92	10
Scamp2	4.95E-96	1.638507	0.667	0.13	8.26E-92	10
Pld3	5.97E-95	1.6171	0.598	0.052	9.96E-91	10
Il6ra	8.45E-95	1.113025	0.328	0.002	1.41E-90	10
Ccr5	1.25E-94	0.930527	0.27	0	2.09E-90	10
Ms4a6b	2.19E-94	1.208261	0.276	0	3.65E-90	10
Rasgrp3	2.49E-94	1.083078	0.333	0.002	4.15E-90	10
Zfp36	2.56E-94	1.661074	0.379	0.009	4.27E-90	10
Wfdc17	2.88E-94	1.357068	0.293	0.001	4.81E-90	10
Ccl12	3.16E-94	1.582229	0.287	0	5.27E-90	10
Gusb	3.54E-93	1.571421	0.655	0.075	5.9E-89	10
Fermt3	1.14E-92	0.988281	0.322	0.002	1.9E-88	10
Tmem86a	2.36E-92	1.383675	0.466	0.015	3.93E-88	10
Cst7	7.3E-91	0.989011	0.27	0	1.22E-86	10
H2-D1	1.27E-90	1.624674	0.69	0.124	2.12E-86	10

Plxdc2	2.82E-90	1.333726	0.471	0.016	4.7E-86	10
Cstb	6.8E-89	1.616443	0.667	0.111	1.13E-84	10
Cybb	2.41E-88	1.253868	0.287	0.001	4.02E-84	10
Slc11a1	4.24E-88	1.012472	0.282	0.001	7.08E-84	10
Rgs1	6.2E-88	1.114283	0.259	0	1.03E-83	10
Lgals9	6.3E-88	1.206463	0.362	0.005	1.05E-83	10
Mylip	2.4E-87	1.244245	0.397	0.009	4E-83	10
Tpp1	3.11E-87	1.5222	0.592	0.061	5.18E-83	10
Arl4c	3.21E-87	1.548412	0.523	0.036	5.36E-83	10
Nckap1l	7.56E-87	0.934822	0.282	0.001	1.26E-82	10
Tcf4	2.67E-86	-1.32887	0.586	0.928	4.45E-82	10
Hpgd	4.78E-86	1.259704	0.305	0.002	7.98E-82	10
Myo1f	7.46E-86	0.822446	0.259	0	1.24E-81	10
Trf	9.93E-86	1.495332	0.534	0.032	1.66E-81	10
Sparc	1.66E-85	2.222616	0.626	0.079	2.76E-81	10
Serpine2	2.49E-85	1.763444	0.598	0.051	4.15E-81	10
Syk	2.57E-85	0.844177	0.276	0.001	4.29E-81	10
Abcc3	4.81E-85	0.927273	0.264	0	8.02E-81	10
Slc7a7	1.08E-84	0.941007	0.305	0.002	1.8E-80	10
Tlr7	5.71E-84	0.862914	0.241	0	9.53E-80	10
Snx5	6.11E-84	1.455335	0.724	0.157	1.02E-79	10
Lpl	3.81E-83	1.742082	0.466	0.054	6.36E-79	10
Sfrp1	6.02E-83	-1.90556	0.305	0.816	1E-78	10
Cd83	6.95E-83	1.180944	0.316	0.003	1.16E-78	10
Olfml3	1.23E-82	1.019316	0.351	0.006	2.05E-78	10
Ehd4	2.61E-82	1.319316	0.431	0.016	4.36E-78	10
Rab3il1	2.96E-82	1.029572	0.322	0.003	4.93E-78	10
Bcl2a1b	3.61E-82	0.776961	0.236	0	6.02E-78	10
Ccl6	5.16E-82	1.039264	0.259	0.001	8.61E-78	10
Nrp1	6.65E-82	1.650663	0.529	0.039	1.11E-77	10
Dcxr	8.85E-82	1.159529	0.385	0.009	1.48E-77	10
Siglech	9.62E-82	1.154252	0.247	0	1.6E-77	10
Mef2c	1.12E-81	1.519509	0.586	0.063	1.88E-77	10
Ucp2	2.19E-81	0.900385	0.299	0.002	3.65E-77	10
Clta	5.8E-81	1.135093	0.902	0.561	9.68E-77	10
Ctse	7.99E-81	0.902731	0.241	0	1.33E-76	10
Tcn2	1.21E-79	1.198016	0.425	0.015	2.02E-75	10
Blnk	3.32E-79	0.830744	0.247	0	5.54E-75	10
Mafb	7.02E-79	1.476866	0.529	0.046	1.17E-74	10
Il10ra	1.07E-78	0.784683	0.241	0	1.79E-74	10
Entpd1	3.58E-78	0.969145	0.287	0.002	5.97E-74	10
Adap2	3.89E-78	0.85303	0.27	0.002	6.49E-74	10
Gngt2	4.24E-78	0.781823	0.253	0.001	7.08E-74	10
Tmem37	5.93E-78	0.981126	0.322	0.004	9.88E-74	10
Gpx1	8.9E-78	1.269495	0.856	0.377	1.48E-73	10
H2-DMa	1.15E-77	1.056484	0.299	0.003	1.92E-73	10
Srgn	1.54E-77	1.107863	0.293	0.003	2.57E-73	10
Lgals3	2.4E-77	1.562207	0.287	0.003	4E-73	10

Havcr2	1.39E-76	0.801369	0.236	0	2.31E-72	10
Arhgap30	3.37E-76	0.777714	0.23	0	5.62E-72	10
Uap1l1	1.21E-75	1.161177	0.328	0.006	2.01E-71	10
Hcls1	2.7E-75	0.833003	0.23	0	4.51E-71	10
Lrp1	3.41E-75	1.468828	0.523	0.042	5.69E-71	10
Lyn	3.8E-75	1.054687	0.339	0.007	6.34E-71	10
Slc6a6	7.28E-75	1.220914	0.523	0.039	1.22E-70	10
Klhl6	8.91E-75	0.787209	0.236	0.001	1.49E-70	10
Ighm	9.42E-75	0.839053	0.23	0	1.57E-70	10
Il10rb	1.36E-74	0.936815	0.293	0.003	2.26E-70	10
Lyl1	3.79E-74	0.73833	0.236	0.001	6.32E-70	10
Slco2b1	4.95E-74	0.842274	0.27	0.002	8.26E-70	10
Emp3	1.56E-73	1.189786	0.333	0.007	2.6E-69	10
Hnrnpa2b1	2.85E-73	-0.96059	0.776	0.935	4.75E-69	10
Lipa	3.82E-73	1.268173	0.46	0.027	6.37E-69	10
Slc15a3	1E-72	1.013447	0.299	0.004	1.67E-68	10
Lgals3bp	3.4E-72	0.75696	0.27	0.002	5.66E-68	10
Ptprc	6.11E-72	0.715259	0.224	0	1.02E-67	10
Renbp	8.11E-72	0.926175	0.293	0.004	1.35E-67	10
Lamp2	1.37E-71	1.403454	0.661	0.132	2.28E-67	10
Itgb2	1.57E-71	0.728327	0.236	0.001	2.61E-67	10
F11r	2.04E-71	0.794546	0.259	0.002	3.41E-67	10
Tmem176a	3.03E-71	1.258184	0.368	0.012	5.05E-67	10
Slc40a1	3.21E-71	0.997329	0.282	0.003	5.36E-67	10
Dhrs3	4.97E-71	1.099167	0.408	0.018	8.29E-67	10
Cd180	1.42E-70	0.756206	0.218	0	2.36E-66	10
Tlr13	3.56E-70	0.76279	0.207	0	5.94E-66	10
Folr2	7.45E-70	0.899963	0.201	0	1.24E-65	10
Actb	9.14E-70	0.783232	0.989	0.979	1.52E-65	10
Rhog	1.47E-69	1.217808	0.437	0.026	2.45E-65	10
Ifi30	1.56E-69	1.205064	0.362	0.012	2.6E-65	10
Ccl2	2.05E-69	1.696644	0.224	0.001	3.42E-65	10
Dnase2a	2.84E-69	1.070569	0.356	0.011	4.74E-65	10
Cd36	2.09E-68	1.155608	0.218	0.001	3.48E-64	10
Fcgr2b	5.19E-68	0.921757	0.201	0	8.66E-64	10
Zic1	1.11E-67	-1.73558	0.224	0.789	1.86E-63	10
Arsb	3.36E-67	1.220963	0.408	0.021	5.61E-63	10
Ctla2b	1.23E-66	0.83871	0.224	0.001	2.04E-62	10
Lst1	2.03E-66	0.935487	0.218	0.001	3.38E-62	10
Sgk1	2.58E-66	1.417306	0.425	0.03	4.31E-62	10
Apobec1	1.04E-65	1.261451	0.339	0.015	1.73E-61	10
Vav1	1.22E-65	0.757643	0.195	0	2.04E-61	10
Lcp2	2.59E-65	0.803554	0.241	0.002	4.32E-61	10
Ifi27	8.59E-64	1.092514	0.339	0.012	1.43E-59	10
Cxcl16	6.76E-63	1.084095	0.247	0.003	1.13E-58	10
Gna15	7.36E-63	0.644752	0.184	0	1.23E-58	10
Prkcd	1.04E-62	0.905052	0.27	0.004	1.73E-58	10
Tcirg1	3.12E-62	1.091366	0.305	0.008	5.2E-58	10

Dock2	3.13E-62	0.572524	0.19	0	5.22E-58	10
Axl	3.25E-62	0.914351	0.276	0.005	5.42E-58	10
Rps6ka1	3.63E-62	0.84837	0.276	0.005	6.05E-58	10
Ostf1	3.93E-62	1.272783	0.523	0.058	6.56E-58	10
Sla	1.15E-61	0.650184	0.19	0	1.91E-57	10
Camk1	1.46E-61	1.224117	0.5	0.052	2.43E-57	10
Gmfg	1.08E-60	0.687954	0.178	0	1.8E-56	10
Ncf2	1.35E-60	0.683465	0.19	0	2.25E-56	10
Arhgap25	1.98E-60	0.675455	0.201	0.001	3.3E-56	10
CRE_RECON	2.87E-60	-2.03151	0.264	0.773	4.79E-56	10
Nrros	9.02E-60	0.852558	0.23	0.002	1.5E-55	10
Lgals1	2E-59	1.451918	0.684	0.224	3.34E-55	10
Gpr183	5.41E-59	0.721485	0.172	0	9.02E-55	10
Msr1	6.05E-59	0.805726	0.178	0	1.01E-54	10
Pfn1	6.15E-59	1.028051	0.845	0.507	1.03E-54	10
Pla2g15	6.2E-59	0.957774	0.328	0.012	1.03E-54	10
Fli1	1.66E-58	0.801023	0.247	0.003	2.77E-54	10
Cfh	2.47E-58	0.804007	0.247	0.005	4.12E-54	10
KCTD12	2.65E-58	1.217132	0.333	0.015	4.41E-54	10
Cd14	4.6E-58	0.923852	0.178	0	7.67E-54	10
Itga6	7.29E-58	0.92821	0.31	0.01	1.22E-53	10
Susd3	1.27E-57	0.63476	0.195	0.001	2.12E-53	10
Igsf6	1.29E-57	0.732473	0.195	0.001	2.16E-53	10
Arpc2	1.4E-57	1.058863	0.868	0.504	2.34E-53	10
Rrbp1	2.18E-57	1.287909	0.695	0.183	3.64E-53	10
Rin2	4.23E-57	1.122036	0.345	0.017	7.06E-53	10
P2ry6	9.71E-57	0.596618	0.167	0	1.62E-52	10
Gcnt1	1.1E-56	0.713286	0.19	0.001	1.83E-52	10
Pon3	2.88E-56	0.837918	0.241	0.004	4.81E-52	10
Tubb5	4.82E-56	-1.05358	0.672	0.917	8.04E-52	10
Pmepa1	5E-56	1.190488	0.368	0.024	8.34E-52	10
Ang	6.39E-56	0.834019	0.213	0.002	1.07E-51	10
Tpd52	7.68E-56	1.141828	0.402	0.032	1.28E-51	10
Dock8	9.23E-56	0.592593	0.178	0	1.54E-51	10
Ptplad2	1.13E-55	0.841141	0.253	0.005	1.88E-51	10
Tmem106a	1.52E-55	0.800552	0.195	0.001	2.54E-51	10
Fuca2	2E-55	0.669825	0.27	0.007	3.34E-51	10
Slc37a2	9.73E-55	0.729608	0.201	0.001	1.62E-50	10
Tifab	1.01E-54	0.557158	0.161	0	1.69E-50	10
Lilrb4	1.92E-54	0.644538	0.172	0	3.21E-50	10
Abi3	3.47E-54	0.61167	0.167	0	5.78E-50	10
Tlr2	5.69E-54	0.721825	0.195	0.001	9.49E-50	10
Runx1	7.19E-54	0.636287	0.19	0.001	1.2E-49	10
Hk3	7.73E-54	0.619512	0.161	0	1.29E-49	10
Atf3	2.4E-53	1.41582	0.259	0.015	4E-49	10
Ecscr	2.82E-53	0.851732	0.224	0.003	4.7E-49	10
Rab32	4.74E-53	0.799372	0.224	0.003	7.91E-49	10
Erp29	5.93E-53	1.129853	0.741	0.275	9.9E-49	10

Efhd2	1.05E-52	1.206858	0.615	0.125	1.75E-48	10
H2-DMb1	1.22E-52	0.587007	0.149	0	2.03E-48	10
Anp32a	1.82E-52	-1.08202	0.523	0.839	3.04E-48	10
Tnfrsf1b	1.95E-52	0.605315	0.161	0	3.26E-48	10
Cd24a	2.03E-52	-1.65974	0.098	0.639	3.39E-48	10
Ikzf1	2.67E-52	0.547983	0.161	0	4.45E-48	10
Kcnk6	2.68E-52	0.567293	0.161	0	4.48E-48	10
Pbxip1	4.74E-52	0.973614	0.31	0.014	7.9E-48	10
Slc9a9	5.35E-52	0.756102	0.241	0.005	8.93E-48	10
Tmem176b	5.9E-52	1.363595	0.534	0.105	9.84E-48	10
Slc7a8	6.08E-52	0.647451	0.218	0.003	1.01E-47	10
Klf2	2.21E-51	1.518505	0.293	0.015	3.68E-47	10
Parvg	3.71E-51	0.635106	0.19	0.001	6.19E-47	10
Csf2rb	4.4E-51	0.502833	0.161	0	7.34E-47	10
Soat1	9.41E-51	0.884795	0.328	0.018	1.57E-46	10
Tnfrsf11a	9.97E-50	0.584595	0.161	0	1.66E-45	10
Skap2	1.49E-49	0.703042	0.213	0.003	2.48E-45	10
Scarb2	2.31E-49	0.951757	0.397	0.036	3.86E-45	10
Pmp22	3.12E-49	1.00154	0.305	0.015	5.21E-45	10
A630001G	4.27E-49	0.509845	0.155	0	7.13E-45	10
Crmp1	6.02E-49	-1.45188	0.132	0.673	1E-44	10
Gpr65	6.16E-49	0.685366	0.155	0	1.03E-44	10
Tspo	8.72E-49	0.86311	0.293	0.013	1.45E-44	10
Ntpcr	8.75E-49	0.958001	0.339	0.022	1.46E-44	10
Asah1	1.34E-48	1.167098	0.443	0.059	2.24E-44	10
Igfbpl1	1.55E-48	-1.71632	0.132	0.652	2.58E-44	10
Csf3r	1.65E-48	0.551939	0.138	0	2.75E-44	10
Hhex	1.69E-48	0.481684	0.155	0.001	2.82E-44	10
Plcg2	2.31E-48	0.499273	0.161	0.001	3.86E-44	10
Fes	3.29E-48	0.713122	0.184	0.001	5.49E-44	10
Nnat	3.54E-48	-1.7137	0.236	0.714	5.91E-44	10
Neat1	7.21E-48	1.005386	0.287	0.013	1.2E-43	10
Rasal3	9.59E-48	0.448252	0.144	0	1.6E-43	10
Stxbp2	2.31E-47	0.572806	0.178	0.002	3.85E-43	10
Ncl	4.15E-47	-0.81852	0.753	0.897	6.93E-43	10
Ebi3	1.13E-46	0.5744	0.155	0	1.88E-42	10
Tgfbr2	1.35E-46	0.708825	0.236	0.006	2.25E-42	10
Gas6	1.46E-46	1.247118	0.483	0.077	2.44E-42	10
Ddah2	2.3E-46	-1.49583	0.207	0.69	3.83E-42	10
Epb4.1l2	6.41E-46	1.137184	0.506	0.088	1.07E-41	10
Rtn1	6.86E-46	-1.67499	0.207	0.695	1.14E-41	10
Marcksl1	1.01E-45	-1.25486	0.241	0.726	1.69E-41	10
Pnpla7	1.05E-45	0.791221	0.253	0.009	1.75E-41	10
Nfix	1.56E-45	-1.39198	0.155	0.678	2.6E-41	10
Fxyd5	1.64E-45	0.70738	0.184	0.002	2.73E-41	10
Alox5ap	2.25E-45	0.712815	0.161	0.001	3.75E-41	10
Ttc3	3.5E-45	-1.13627	0.466	0.832	5.84E-41	10
Nedd4	4.58E-45	-1.21569	0.195	0.688	7.65E-41	10

Ppfia4	5.94E-45	0.823072	0.247	0.01	9.91E-41	10
Npc1	6.46E-45	0.949508	0.385	0.038	1.08E-40	10
Fam49b	2.08E-44	1.169659	0.471	0.081	3.47E-40	10
Tnf	2.17E-44	0.524096	0.126	0	3.61E-40	10
Nfia	2.91E-44	-1.26854	0.414	0.777	4.85E-40	10
Fblim1	3.25E-44	0.572516	0.19	0.004	5.42E-40	10
AB124611	3.93E-44	0.604719	0.144	0	6.56E-40	10
Lmo2	4.98E-44	0.9536	0.362	0.033	8.3E-40	10
Glul	6.92E-44	1.172296	0.626	0.196	1.15E-39	10
Ccl7	8.09E-44	1.170523	0.138	0	1.35E-39	10
Shisa5	9.63E-44	0.826851	0.316	0.022	1.61E-39	10
H3f3b	1.07E-43	-0.78174	0.77	0.908	1.79E-39	10
Hck	1.6E-43	0.724101	0.213	0.006	2.66E-39	10
Cd4	1.68E-43	0.741182	0.155	0.001	2.8E-39	10
Hvcn1	2.34E-43	0.557638	0.149	0.001	3.9E-39	10
Gpr157	2.72E-43	0.467595	0.149	0.001	4.54E-39	10
Daglb	3.18E-43	0.932092	0.368	0.036	5.3E-39	10
Plin3	3.79E-43	0.74313	0.218	0.006	6.32E-39	10
Icam1	4.86E-43	0.530453	0.167	0.001	8.11E-39	10
0610031J0	8.86E-43	0.894298	0.431	0.058	1.48E-38	10
Ccl9	9.4E-43	0.892458	0.132	0	1.57E-38	10
Glipr1	1.24E-42	0.53508	0.161	0.001	2.06E-38	10
Tgfbr1	1.4E-42	1.085456	0.414	0.058	2.33E-38	10
Ccnd2	1.76E-42	-1.52458	0.213	0.666	2.94E-38	10
Slc43a2	5.39E-42	0.972185	0.305	0.021	8.99E-38	10
Atp13a2	6.17E-42	0.982759	0.414	0.053	1.03E-37	10
Psip1	6.33E-42	-1.29522	0.224	0.692	1.06E-37	10
Cd33	9.52E-42	0.494704	0.138	0	1.59E-37	10
Aldh2	1.27E-41	0.860624	0.31	0.023	2.12E-37	10
Stmn3	1.86E-41	-1.39694	0.155	0.626	3.1E-37	10
Syngr2	2.09E-41	0.862073	0.293	0.019	3.48E-37	10
Npnt	2.1E-41	0.539588	0.167	0.002	3.5E-37	10
Psmb9	3.56E-41	0.526647	0.144	0.001	5.94E-37	10
Ncf4	3.56E-41	0.37286	0.115	0	5.95E-37	10
Tmem140	5.6E-41	0.440305	0.161	0.002	9.35E-37	10
Arhgap9	9.08E-41	0.451742	0.132	0	1.51E-36	10
H2afv	2.02E-40	-1.09388	0.466	0.73	3.37E-36	10
Gba	2.05E-40	0.975612	0.408	0.054	3.42E-36	10
Tmsb10	2.17E-40	-1.35713	0.207	0.668	3.61E-36	10
Ifngr1	2.32E-40	0.977026	0.339	0.034	3.87E-36	10
Pik3cg	2.39E-40	0.459616	0.126	0	3.98E-36	10
Slfn2	2.81E-40	0.701264	0.138	0.001	4.69E-36	10
Nfam1	2.84E-40	0.457507	0.115	0	4.74E-36	10
Cbx5	2.99E-40	-1.32191	0.195	0.657	4.98E-36	10
Litaf	4.28E-40	0.958016	0.431	0.065	7.13E-36	10
Slamf9	4.8E-40	0.453134	0.121	0	8.01E-36	10
Tapbp	5.35E-40	0.736138	0.264	0.015	8.92E-36	10
Hnrnpu	6.04E-40	-0.7401	0.741	0.866	1.01E-35	10

Cd48	9.16E-40	0.437766	0.121	0	1.53E-35	10
Ptafr	1.66E-39	0.423003	0.121	0	2.77E-35	10
Trim30a	2.04E-39	0.529594	0.126	0	3.41E-35	10
Tnfaip3	2.65E-39	0.591812	0.149	0.001	4.43E-35	10
Clec5a	3.07E-39	0.407831	0.109	0	5.12E-35	10
Hebp1	6.55E-39	0.694763	0.195	0.005	1.09E-34	10
Ifngr2	1.52E-38	0.827667	0.287	0.021	2.54E-34	10
Itgal	1.91E-38	0.418838	0.121	0	3.18E-34	10
Hnrnpab	2.64E-38	-0.87395	0.552	0.818	4.4E-34	10
C5ar1	2.7E-38	0.563896	0.115	0	4.51E-34	10
Gap43	3.89E-38	-1.66089	0.144	0.603	6.48E-34	10
Hpse	4.35E-38	0.362458	0.132	0.001	7.26E-34	10
Coro1a	4.75E-38	0.720831	0.218	0.009	7.92E-34	10
Adipor1	1.62E-37	1.02597	0.546	0.136	2.7E-33	10
Tmem119	1.74E-37	0.63081	0.144	0.001	2.9E-33	10
Atox1	1.74E-37	0.965041	0.718	0.288	2.9E-33	10
Fam26f	2.85E-37	0.513614	0.126	0.001	4.75E-33	10
Csf2ra	3.29E-37	0.80512	0.27	0.018	5.49E-33	10
Slc29a3	3.69E-37	0.671083	0.19	0.005	6.16E-33	10
Man1a	3.87E-37	0.567345	0.184	0.005	6.46E-33	10
Nagpa	3.99E-37	0.806392	0.299	0.025	6.66E-33	10
Chst1	6.16E-37	0.482478	0.161	0.003	1.03E-32	10
Fkbp3	7.31E-37	-1.00383	0.42	0.748	1.22E-32	10
Sipa1	8.16E-37	0.598392	0.207	0.008	1.36E-32	10
Cyfip1	1.41E-36	1.024207	0.506	0.117	2.36E-32	10
Cmtm6	1.73E-36	0.804698	0.276	0.021	2.89E-32	10
P2rx4	2.85E-36	0.728821	0.224	0.011	4.76E-32	10
Fam212a	4.36E-36	0.552472	0.144	0.001	7.28E-32	10
Tnfrsf13b	5.87E-36	0.392125	0.126	0.001	9.78E-32	10
Gatm	6.42E-36	1.058913	0.31	0.032	1.07E-31	10
Gpr137b	6.72E-36	0.848013	0.236	0.014	1.12E-31	10
Stard8	8.04E-36	0.701382	0.155	0.002	1.34E-31	10
Gaa	8.86E-36	0.66786	0.276	0.025	1.48E-31	10
Itgam	1.18E-35	0.923044	0.247	0.017	1.96E-31	10
Sdcbp	1.26E-35	1.053076	0.563	0.187	2.09E-31	10
Hnrnpdl	1.41E-35	-0.94826	0.489	0.758	2.34E-31	10
E130114P1	1.69E-35	-1.40388	0.149	0.589	2.81E-31	10
Anxa2	1.98E-35	0.895639	0.218	0.01	3.3E-31	10
Rassf5	2.38E-35	0.373896	0.121	0.001	3.97E-31	10
Eva1a	2.39E-35	0.567238	0.178	0.005	3.98E-31	10
Wwp2	2.52E-35	0.950398	0.316	0.033	4.21E-31	10
Rbm47	5E-35	0.416182	0.115	0	8.34E-31	10
Bex2	5.1E-35	-1.26383	0.121	0.575	8.51E-31	10
Serbp1	9.22E-35	-0.6753	0.776	0.87	1.54E-30	10
Cog7	1.09E-34	-1.32646	0.172	0.588	1.81E-30	10
Fcgrt	1.3E-34	1.031171	0.253	0.025	2.18E-30	10
Ifnar2	1.58E-34	0.863827	0.356	0.047	2.63E-30	10
Tm6sf1	1.99E-34	0.81071	0.236	0.015	3.33E-30	10

Ralb	3.1E-34	0.755003	0.247	0.016	5.16E-30	10
Capzb	3.98E-34	0.907455	0.713	0.346	6.63E-30	10
S100a1	4.48E-34	0.809759	0.27	0.024	7.47E-30	10
Adssl1	5.33E-34	0.398622	0.126	0.001	8.89E-30	10
Stmn2	6.23E-34	-1.80363	0.167	0.556	1.04E-29	10
Rnh1	7.87E-34	0.904434	0.379	0.059	1.31E-29	10
Colec12	8.44E-34	0.709676	0.184	0.007	1.41E-29	10
Cpe	9.1E-34	-1.41521	0.052	0.459	1.52E-29	10
Clec4n	1.12E-33	0.587855	0.103	0	1.86E-29	10
Slc25a45	1.89E-33	0.425997	0.115	0	3.15E-29	10
Cd38	2.19E-33	0.631397	0.132	0.001	3.66E-29	10
Luc7l3	2.22E-33	-1.03774	0.42	0.751	3.71E-29	10
Smagp	2.32E-33	0.481406	0.121	0.001	3.87E-29	10
Junb	2.68E-33	1.124764	0.276	0.032	4.47E-29	10
Pla2g7	4.44E-33	0.826437	0.241	0.018	7.41E-29	10
Sdc4	5.22E-33	0.658856	0.19	0.007	8.71E-29	10
Msn	6.39E-33	1.007566	0.425	0.089	1.07E-28	10
Pax6	6.42E-33	-1.40033	0.098	0.517	1.07E-28	10
Paqr7	6.54E-33	0.559172	0.167	0.004	1.09E-28	10
Nceh1	9.5E-33	0.849318	0.299	0.032	1.58E-28	10
Gm17750	1.04E-32	-1.39623	0.017	0.37	1.74E-28	10
Gdi2	1.35E-32	0.86992	0.718	0.373	2.25E-28	10
Matr3	1.36E-32	-0.99822	0.414	0.709	2.27E-28	10
Fbxw4	2.02E-32	0.657308	0.253	0.021	3.38E-28	10
Lhfpl2	2.68E-32	0.755573	0.27	0.025	4.47E-28	10
Lhx1	2.7E-32	-1.48691	0.098	0.501	4.5E-28	10
Cd302	2.88E-32	0.919664	0.351	0.05	4.81E-28	10
Gm26532	4E-32	0.688892	0.115	0.001	6.68E-28	10
Gm2a	4.51E-32	0.855565	0.333	0.045	7.52E-28	10
Msrb1	5.67E-32	1.005505	0.345	0.058	9.46E-28	10
Cbx1	8.64E-32	-1.0113	0.368	0.688	1.44E-27	10
Snx20	9.16E-32	0.364344	0.132	0.002	1.53E-27	10
Nrp2	9.65E-32	0.483733	0.155	0.004	1.61E-27	10
Cald1	1.44E-31	-1.33946	0.126	0.539	2.4E-27	10
Card9	2.17E-31	0.410544	0.103	0	3.62E-27	10
Necap2	2.75E-31	0.741652	0.322	0.042	4.59E-27	10
6330416G	3.15E-31	0.552669	0.184	0.007	5.25E-27	10
Banf1	3.22E-31	-0.91686	0.448	0.696	5.37E-27	10
Pros1	3.95E-31	0.735194	0.253	0.021	6.59E-27	10
AI662270	4.43E-31	0.348385	0.103	0.001	7.4E-27	10
Cnn3	4.7E-31	-1.2974	0.052	0.44	7.83E-27	10
Akr1a1	5.39E-31	0.748959	0.845	0.583	8.99E-27	10
Il4ra	5.46E-31	0.726368	0.218	0.014	9.11E-27	10
Blvrb	6.09E-31	0.974121	0.339	0.057	1.02E-26	10
Acss1	6.79E-31	0.411922	0.149	0.004	1.13E-26	10
Mex3a	7.18E-31	-1.30901	0.063	0.467	1.2E-26	10
Ccrl2	7.23E-31	0.509752	0.115	0.001	1.21E-26	10
Stat6	7.96E-31	0.386547	0.121	0.001	1.33E-26	10

Ngfrap1	8.56E-31	-1.01185	0.287	0.661	1.43E-26	10
Barhl1	9.15E-31	-1.31835	0.109	0.525	1.53E-26	10
Fam46c	1.02E-30	0.705465	0.161	0.005	1.69E-26	10
Soga3	1.44E-30	-1.29166	0.069	0.461	2.4E-26	10
Gria2	1.52E-30	-1.44366	0.121	0.525	2.53E-26	10
Plod1	1.7E-30	0.728858	0.253	0.022	2.84E-26	10
S1pr1	2.32E-30	0.610148	0.178	0.007	3.86E-26	10
Cela1	2.47E-30	0.619188	0.144	0.003	4.13E-26	10
Htatip2	2.87E-30	0.480153	0.132	0.002	4.79E-26	10
Slc38a6	3.99E-30	0.633985	0.207	0.013	6.66E-26	10
Cryl1	4.05E-30	0.674919	0.184	0.008	6.76E-26	10
Adam15	4.26E-30	0.631141	0.172	0.006	7.11E-26	10
Cebpa	4.4E-30	0.494484	0.161	0.005	7.34E-26	10
Map1b	4.49E-30	-1.42764	0.201	0.591	7.49E-26	10
Tubb3	4.79E-30	-1.66353	0.098	0.478	7.99E-26	10
Tnfrsf1a	4.82E-30	0.544812	0.241	0.023	8.04E-26	10
Rtn4rl1	5.29E-30	0.46947	0.126	0.002	8.82E-26	10
Edem1	6.42E-30	0.885805	0.253	0.028	1.07E-25	10
Pon2	6.47E-30	0.775749	0.299	0.036	1.08E-25	10
Bmp2k	6.93E-30	0.730788	0.253	0.023	1.16E-25	10
Cndp2	7.45E-30	0.901179	0.368	0.064	1.24E-25	10
Elavl3	8.19E-30	-1.40228	0.086	0.476	1.37E-25	10
Xlr	8.48E-30	0.377492	0.109	0.001	1.41E-25	10
Adcy7	1.53E-29	0.499492	0.161	0.005	2.55E-25	10
Cd9	2.25E-29	0.804719	0.736	0.482	3.76E-25	10
H1f0	2.28E-29	-1.19044	0.253	0.619	3.81E-25	10
Gpnmb	2.97E-29	1.028207	0.126	0.003	4.96E-25	10
Ier3	3.14E-29	1.092635	0.259	0.032	5.24E-25	10
Tmem50a	4.96E-29	0.774253	0.764	0.401	8.28E-25	10
Smim1	6.25E-29	0.539642	0.144	0.003	1.04E-24	10
Rcsd1	7.64E-29	0.611773	0.132	0.003	1.27E-24	10
Scg3	8.13E-29	-1.27665	0.075	0.45	1.36E-24	10
Naglu	9.27E-29	0.529209	0.172	0.007	1.55E-24	10
Hnrnph1	9.5E-29	-0.94973	0.339	0.631	1.59E-24	10
Arap1	9.58E-29	0.305013	0.121	0.002	1.6E-24	10
Klf6	1.18E-28	1.121697	0.402	0.145	1.96E-24	10
Grap	1.28E-28	0.435355	0.126	0.003	2.14E-24	10
Miat	1.44E-28	-1.39102	0.121	0.519	2.4E-24	10
Ftl1	2.05E-28	0.611982	0.293	0.04	3.41E-24	10
Gabarap	2.05E-28	0.793808	0.741	0.441	3.42E-24	10
Sfrs18	2.62E-28	-0.85968	0.534	0.78	4.37E-24	10
Calm2	2.64E-28	-0.68832	0.759	0.875	4.41E-24	10
Dpp7	2.86E-28	0.52749	0.213	0.019	4.77E-24	10
Nrep	2.88E-28	-1.26237	0.167	0.573	4.81E-24	10
Vwa5a	2.96E-28	0.66298	0.201	0.013	4.94E-24	10
Vcam1	3.14E-28	0.821537	0.149	0.006	5.24E-24	10
Sfpq	3.44E-28	-0.90824	0.385	0.673	5.73E-24	10
Tspan4	3.95E-28	0.563029	0.322	0.063	6.59E-24	10

Ppp2r2c	5.8E-28	-1.18706	0.115	0.508	9.67E-24	10
Draxin	6.72E-28	-1.21974	0.161	0.558	1.12E-23	10
Myh9	7.15E-28	0.76889	0.247	0.025	1.19E-23	10
C2	1.12E-27	0.499102	0.109	0.001	1.86E-23	10
Atrx	1.23E-27	-0.82185	0.466	0.743	2.05E-23	10
Capza2	1.25E-27	0.808651	0.707	0.357	2.09E-23	10
Ptbp3	1.26E-27	0.919402	0.431	0.107	2.11E-23	10
Fxyd6	1.27E-27	-1.25733	0.126	0.512	2.13E-23	10
Slc46a3	1.6E-27	0.450516	0.126	0.002	2.67E-23	10
Chd4	2.72E-27	-0.82869	0.477	0.772	4.53E-23	10
Kcnk13	3.14E-27	0.351889	0.144	0.006	5.24E-23	10
Plbd2	3.43E-27	0.665852	0.276	0.035	5.72E-23	10
Sqrdl	3.93E-27	0.336002	0.103	0.001	6.56E-23	10
Rps5	4.91E-27	-0.51004	0.954	0.954	8.18E-23	10
Ezh2	5.82E-27	-1.08437	0.259	0.613	9.7E-23	10
S100a13	6.11E-27	0.697321	0.247	0.028	1.02E-22	10
Cpd	6.5E-27	0.774749	0.345	0.06	1.08E-22	10
Akap13	7.01E-27	0.889074	0.356	0.069	1.17E-22	10
Acin1	7.08E-27	-0.90459	0.431	0.726	1.18E-22	10
Nhlh2	7.09E-27	-1.38033	0.057	0.426	1.18E-22	10
Smc3	7.65E-27	-1.06096	0.282	0.636	1.28E-22	10
Sft2d2	1.11E-26	0.469562	0.132	0.003	1.85E-22	10
P4ha1	1.21E-26	0.65133	0.333	0.058	2.01E-22	10
Nsg2	1.87E-26	-1.18755	0.109	0.491	3.13E-22	10
Srsf3	2E-26	-0.86756	0.379	0.659	3.33E-22	10
Ilf2	2.05E-26	-1.09361	0.155	0.545	3.42E-22	10
Tubb2b	2.06E-26	-1.2887	0.115	0.468	3.43E-22	10
Lpcat3	2.17E-26	0.668933	0.287	0.041	3.61E-22	10
Rnd3	2.18E-26	-1.24863	0.075	0.434	3.64E-22	10
P2rx7	2.33E-26	0.607528	0.155	0.006	3.88E-22	10
Hist1h1c	2.68E-26	0.855287	0.247	0.031	4.46E-22	10
Nasp	2.68E-26	-0.90341	0.356	0.643	4.47E-22	10
1700003F1	3.25E-26	0.422844	0.103	0.001	5.41E-22	10
Nfkbid	3.38E-26	0.63595	0.138	0.004	5.65E-22	10
AI413582	3.42E-26	0.619785	0.247	0.029	5.71E-22	10
Elovl1	3.78E-26	0.648628	0.264	0.033	6.31E-22	10
Map2	3.93E-26	-1.24374	0.115	0.495	6.56E-22	10
Rnf130	5.66E-26	0.89489	0.42	0.109	9.44E-22	10
Fam46a	5.96E-26	0.608735	0.138	0.004	9.95E-22	10
Tia1	7.36E-26	-1.06338	0.207	0.555	1.23E-21	10
Cebpb	9.38E-26	0.634329	0.155	0.007	1.57E-21	10
Neurod1	9.87E-26	-1.77492	0.218	0.535	1.65E-21	10
Irf5	1.07E-25	0.584927	0.155	0.006	1.78E-21	10
Ndufa4	1.2E-25	-0.6594	0.621	0.803	2E-21	10
0610040J0	1.26E-25	0.421461	0.121	0.002	2.11E-21	10
Dpysl4	1.35E-25	-1.19266	0.121	0.494	2.24E-21	10
Cap1	1.59E-25	0.815018	0.402	0.093	2.66E-21	10
1700017B0	1.74E-25	0.515805	0.149	0.006	2.91E-21	10

Ranbp1	1.99E-25	-0.88184	0.454	0.677	3.31E-21	10
Pik3cd	2.27E-25	0.518005	0.138	0.005	3.78E-21	10
Rapsn	3.36E-25	0.487003	0.103	0.001	5.6E-21	10
Pcdhga9	3.51E-25	-1.17303	0.092	0.423	5.86E-21	10
Speg	3.63E-25	0.311446	0.109	0.002	6.05E-21	10
Abca9	4.5E-25	0.545202	0.138	0.004	7.51E-21	10
Man2b2	5.1E-25	0.33771	0.115	0.002	8.5E-21	10
Hdac2	5.54E-25	-1.07238	0.172	0.518	9.25E-21	10
Hfe	5.85E-25	0.470326	0.121	0.003	9.76E-21	10
Chd7	5.96E-25	-1.04075	0.322	0.65	9.93E-21	10
Cltc	6.41E-25	0.846838	0.489	0.148	1.07E-20	10
Tor3a	6.47E-25	0.522277	0.155	0.007	1.08E-20	10
Hsp90aa1	7.95E-25	-0.91435	0.264	0.62	1.33E-20	10
BC028528	8.11E-25	0.510072	0.144	0.006	1.35E-20	10
Picalm	8.58E-25	0.842672	0.471	0.138	1.43E-20	10
Ank3	9.22E-25	-1.28634	0.075	0.423	1.54E-20	10
Golm1	9.79E-25	0.917689	0.506	0.185	1.63E-20	10
Gpm6a	1.12E-24	-1.37477	0.063	0.412	1.87E-20	10
Pnn	1.14E-24	-0.70672	0.477	0.724	1.91E-20	10
Gpsm3	1.22E-24	0.422175	0.109	0.002	2.03E-20	10
Crybb1	1.22E-24	0.480254	0.103	0.001	2.04E-20	10
Anp32e	1.28E-24	-1.01024	0.322	0.616	2.14E-20	10
Ninj1	1.65E-24	0.826624	0.397	0.094	2.75E-20	10
Prdx2	1.93E-24	-0.8276	0.385	0.682	3.23E-20	10
Tfpi	2.15E-24	0.376293	0.144	0.007	3.58E-20	10
Tmem141	2.18E-24	0.452674	0.132	0.004	3.64E-20	10
Clic1	2.23E-24	0.81588	0.517	0.169	3.71E-20	10
Crip1	2.3E-24	0.978182	0.172	0.012	3.83E-20	10
Ppt1	2.31E-24	0.819023	0.391	0.091	3.86E-20	10
Atxn7l3b	3.19E-24	-0.90795	0.374	0.675	5.31E-20	10
Gm13476	3.19E-24	0.697281	0.23	0.026	5.33E-20	10
Ncam1	3.78E-24	-1.05194	0.052	0.39	6.31E-20	10
D430041D	4.39E-24	-1.09294	0.126	0.495	7.33E-20	10
Wdr1	4.51E-24	0.782904	0.368	0.085	7.53E-20	10
1500016L(	4.86E-24	-1.22969	0.052	0.386	8.1E-20	10
Rab11fip5	5.36E-24	0.575261	0.195	0.017	8.94E-20	10
H2-T23	6.18E-24	0.667294	0.23	0.026	1.03E-19	10
Smc1a	7.38E-24	-0.90138	0.328	0.633	1.23E-19	10
Pde1c	7.69E-24	-1.3371	0.069	0.39	1.28E-19	10
Trim47	7.71E-24	0.444806	0.109	0.002	1.29E-19	10
Hnrnpm	9.78E-24	-0.6188	0.58	0.736	1.63E-19	10
Fubp1	1.06E-23	-0.99212	0.276	0.569	1.76E-19	10
Pcm1	1.14E-23	-1.04997	0.184	0.525	1.9E-19	10
Ubtd1	1.15E-23	0.621488	0.132	0.004	1.91E-19	10
Syt11	1.18E-23	-1.13584	0.144	0.508	1.97E-19	10
Serpinb6a	1.24E-23	0.823081	0.305	0.056	2.07E-19	10
Slc29a1	1.36E-23	-1.0269	0.259	0.564	2.26E-19	10
Ramp1	1.38E-23	0.732959	0.224	0.025	2.31E-19	10

Ly6e	2.34E-23	0.858605	0.592	0.278	3.9E-19	10
Gmpr	2.39E-23	0.424992	0.161	0.011	3.99E-19	10
Zic4	2.59E-23	-1.10498	0.08	0.421	4.31E-19	10
Dse	3.13E-23	0.528682	0.161	0.009	5.22E-19	10
Dtx4	3.47E-23	0.392573	0.138	0.006	5.79E-19	10
Tnfrsf21	3.76E-23	0.678143	0.316	0.061	6.27E-19	10
Mfsd1	4.26E-23	0.761298	0.414	0.108	7.1E-19	10
Ckb	4.74E-23	-0.88865	0.609	0.81	7.91E-19	10
Itm2c	6.12E-23	0.840479	0.552	0.208	1.02E-18	10
Gpm6b	7.22E-23	-1.09045	0.126	0.487	1.2E-18	10
Gm6977	7.52E-23	0.518979	0.23	0.03	1.25E-18	10
Cnbp	7.94E-23	-0.68573	0.563	0.741	1.32E-18	10
Mycn	8.76E-23	-1.15987	0.052	0.366	1.46E-18	10
Gnao1	9.23E-23	-1.11379	0.011	0.29	1.54E-18	10
Pnp	1.05E-22	0.568587	0.195	0.018	1.76E-18	10
Tec	1.1E-22	0.43943	0.138	0.006	1.84E-18	10
Snrnp70	1.19E-22	-0.76945	0.471	0.695	1.98E-18	10
Prune2	1.44E-22	0.55476	0.149	0.008	2.4E-18	10
Dcx	1.44E-22	-1.23929	0.057	0.379	2.4E-18	10
Itpripl2	1.49E-22	0.352443	0.115	0.003	2.48E-18	10
Pfkfb3	1.52E-22	0.645534	0.201	0.019	2.53E-18	10
Dek	1.6E-22	-0.73111	0.592	0.694	2.67E-18	10
Twf2	2.04E-22	0.640194	0.299	0.054	3.4E-18	10
Il18	2.52E-22	0.544096	0.172	0.013	4.21E-18	10
Tmed3	2.76E-22	0.742462	0.351	0.08	4.61E-18	10
Tox3	2.82E-22	-1.05039	0.034	0.348	4.7E-18	10
Cplx2	2.96E-22	-1.08269	0.161	0.498	4.94E-18	10
Comt	3E-22	0.837586	0.397	0.106	5.01E-18	10
Cpt1a	3.74E-22	0.485338	0.178	0.016	6.23E-18	10
Adrb2	4.52E-22	0.599615	0.103	0.003	7.54E-18	10
Mfsd12	4.54E-22	0.51817	0.178	0.016	7.58E-18	10
Scoc	6.16E-22	0.700495	0.345	0.076	1.03E-17	10
Malat1	6.5E-22	-0.25216	0.874	0.955	1.08E-17	10
Arpc5	6.98E-22	0.80697	0.621	0.318	1.16E-17	10
Dynlt3	7.39E-22	0.648028	0.27	0.044	1.23E-17	10
Sept3	7.56E-22	-1.26572	0.075	0.395	1.26E-17	10
Slc16a6	7.67E-22	0.678432	0.259	0.039	1.28E-17	10
Hnrnpr	7.92E-22	-0.94012	0.264	0.547	1.32E-17	10
Apba2	8.85E-22	-1.04858	0.08	0.417	1.48E-17	10
Hmgn1	9.36E-22	-0.63692	0.454	0.623	1.56E-17	10
Ddx5	9.87E-22	-0.61123	0.661	0.817	1.65E-17	10
Casp8	1.15E-21	0.337318	0.109	0.003	1.91E-17	10
Adam17	1.16E-21	0.752485	0.27	0.047	1.94E-17	10
Mt2	1.33E-21	0.792155	0.368	0.089	2.22E-17	10
Nop58	1.34E-21	-0.94219	0.293	0.594	2.24E-17	10
Pdk1	1.77E-21	0.474931	0.132	0.006	2.96E-17	10
Ptma	1.79E-21	-0.87172	0.287	0.584	2.98E-17	10
Parp9	1.97E-21	0.468704	0.144	0.008	3.29E-17	10

Slc46a1	2.1E-21	0.305033	0.132	0.009	3.5E-17	10
Limd2	2.15E-21	0.851197	0.408	0.132	3.58E-17	10
Srrm2	2.3E-21	-0.71838	0.5	0.734	3.83E-17	10
Ssb	2.38E-21	-0.71886	0.483	0.705	3.96E-17	10
Tanc2	2.51E-21	0.457349	0.161	0.014	4.18E-17	10
Hnrnpd	3.03E-21	-0.90107	0.328	0.613	5.06E-17	10
Edem2	3.11E-21	0.603956	0.264	0.044	5.19E-17	10
Scg5	3.17E-21	-1.11391	0.04	0.337	5.28E-17	10
Fnbp1l	3.43E-21	-1.13943	0.144	0.456	5.72E-17	10
Cln3	3.92E-21	0.709589	0.236	0.034	6.54E-17	10
Brd3	3.99E-21	-0.96248	0.27	0.534	6.66E-17	10
Kif5c	5.63E-21	-1.17642	0.092	0.401	9.38E-17	10
Abhd6	5.99E-21	0.541733	0.236	0.035	1E-16	10
Rbfox3	8.12E-21	-1.14111	0.172	0.497	1.35E-16	10
Nfe2l2	1E-20	0.767799	0.339	0.081	1.67E-16	10
Isyna1	1.21E-20	0.377536	0.138	0.009	2.02E-16	10
Celf4	1.22E-20	-1.41355	0.034	0.323	2.03E-16	10
Rnf128	1.24E-20	0.361244	0.121	0.005	2.07E-16	10
Plekha2	1.41E-20	0.427172	0.121	0.005	2.35E-16	10
Tns3	1.87E-20	0.56854	0.144	0.008	3.11E-16	10
Bhlhe41	1.88E-20	0.600235	0.149	0.011	3.13E-16	10
Nfkbia	1.91E-20	0.94298	0.368	0.169	3.18E-16	10
1110001A1	1.95E-20	0.826243	0.356	0.094	3.24E-16	10
Usp2	1.98E-20	0.420677	0.149	0.011	3.3E-16	10
Tmem135	2.01E-20	0.541486	0.218	0.029	3.35E-16	10
Galns	2.08E-20	0.398041	0.121	0.005	3.47E-16	10
Map3k8	2.11E-20	0.458493	0.115	0.004	3.52E-16	10
Sulf2	2.3E-20	0.554594	0.184	0.02	3.84E-16	10
Smpdl3a	2.44E-20	0.413352	0.144	0.01	4.08E-16	10
Vps37b	2.71E-20	-1.00876	0.167	0.473	4.52E-16	10
Gas5	2.76E-20	-0.70043	0.511	0.744	4.6E-16	10
Anxa4	3.27E-20	0.66195	0.241	0.039	5.45E-16	10
Hnrnpc	3.29E-20	-0.6843	0.448	0.669	5.48E-16	10
Atp6v0b	3.39E-20	0.765162	0.586	0.264	5.65E-16	10
Arpc4	3.49E-20	0.727473	0.46	0.152	5.83E-16	10
Manba	3.54E-20	0.373012	0.115	0.004	5.9E-16	10
As3mt	4.5E-20	0.488518	0.155	0.012	7.5E-16	10
Bin1	4.77E-20	0.683053	0.736	0.426	7.95E-16	10
Gm10075	5.15E-20	-0.94702	0.213	0.517	8.59E-16	10
Cdk4	5.24E-20	-0.7404	0.42	0.644	8.74E-16	10
Blvra	5.52E-20	0.713835	0.236	0.036	9.21E-16	10
Mef2a	5.65E-20	0.698246	0.437	0.135	9.43E-16	10
Fuca1	5.87E-20	0.775771	0.443	0.144	9.79E-16	10
Slc25a4	5.97E-20	-0.56001	0.713	0.808	9.96E-16	10
Sgpl1	6.38E-20	0.735497	0.448	0.145	1.06E-15	10
Rps26	6.42E-20	-0.49836	0.816	0.847	1.07E-15	10
Pkib	6.9E-20	0.394129	0.155	0.018	1.15E-15	10
Capg	7.02E-20	0.525473	0.109	0.004	1.17E-15	10

Skp1a	7.5E-20	-0.87626	0.282	0.584	1.25E-15	10
Ina	7.53E-20	-1.13318	0.138	0.446	1.26E-15	10
Dnajb14	9.09E-20	0.679435	0.31	0.067	1.52E-15	10
Gm9800	9.24E-20	-0.80652	0.247	0.537	1.54E-15	10
Uncx	9.44E-20	-1.10702	0.034	0.32	1.57E-15	10
Nap1l1	1.39E-19	-0.8581	0.213	0.513	2.32E-15	10
Leprot	1.4E-19	0.580419	0.391	0.115	2.33E-15	10
Cdkn1b	1.44E-19	-0.95449	0.155	0.47	2.41E-15	10
Commd9	1.91E-19	0.763469	0.328	0.081	3.18E-15	10
Prdx5	1.96E-19	0.745597	0.58	0.261	3.26E-15	10
Cacna2d1	1.99E-19	-1.10102	0.075	0.378	3.31E-15	10
Csf1	2.01E-19	0.620752	0.132	0.008	3.35E-15	10
Atp6ap2	2.27E-19	0.730992	0.494	0.181	3.79E-15	10
Camta1	2.55E-19	-0.99956	0.115	0.41	4.26E-15	10
Rasa4	2.64E-19	0.335446	0.109	0.006	4.4E-15	10
Nacc2	2.8E-19	0.437617	0.167	0.017	4.67E-15	10
Snrpd1	2.95E-19	-0.88119	0.259	0.526	4.92E-15	10
M6pr	3.04E-19	0.715191	0.379	0.106	5.08E-15	10
Fez1	3.17E-19	-1.02075	0.052	0.335	5.28E-15	10
Elk3	3.31E-19	0.485934	0.178	0.019	5.51E-15	10
Sall1	3.34E-19	0.423251	0.144	0.01	5.57E-15	10
Ptn	3.4E-19	-1.28553	0.149	0.444	5.67E-15	10
Amdhd2	3.49E-19	0.661387	0.247	0.042	5.82E-15	10
Pla2g16	4.28E-19	0.590855	0.126	0.007	7.15E-15	10
Bclaf1	4.84E-19	-0.80631	0.368	0.585	8.07E-15	10
Rassf2	5.12E-19	0.472847	0.161	0.014	8.54E-15	10
Uchl1	5.23E-19	-0.99852	0.046	0.337	8.72E-15	10
Plekho1	6.38E-19	0.581356	0.264	0.05	1.06E-14	10
Mllt4	7.22E-19	-0.96059	0.069	0.365	1.2E-14	10
Rnf13	7.65E-19	0.617666	0.322	0.078	1.28E-14	10
Cib1	7.82E-19	0.701437	0.276	0.055	1.3E-14	10
Orai1	8.14E-19	0.50587	0.184	0.021	1.36E-14	10
Sec14l1	8.31E-19	0.550317	0.259	0.05	1.39E-14	10
Arhgap17	8.69E-19	0.623854	0.333	0.083	1.45E-14	10
Aga	8.81E-19	0.504311	0.218	0.034	1.47E-14	10
Smc4	9.15E-19	-1.0371	0.328	0.569	1.53E-14	10
H2afy2	1.24E-18	-0.99011	0.057	0.34	2.06E-14	10
Hist1h2bc	1.26E-18	0.763551	0.224	0.036	2.1E-14	10
Hn1	1.28E-18	-0.88048	0.27	0.544	2.13E-14	10
Rap2a	1.33E-18	0.695423	0.471	0.166	2.22E-14	10
Ubc	1.34E-18	0.730528	0.563	0.357	2.24E-14	10
Gla	1.35E-18	0.4866	0.19	0.023	2.24E-14	10
Hnrnpa0	1.36E-18	-0.90294	0.218	0.459	2.26E-14	10
Supt16	1.61E-18	-0.84766	0.27	0.495	2.69E-14	10
Gyg	1.63E-18	0.522653	0.132	0.008	2.71E-14	10
Insm1	1.93E-18	-1.03969	0.029	0.295	3.22E-14	10
Pepd	1.95E-18	0.689756	0.236	0.04	3.26E-14	10
Top2b	2.31E-18	-0.97768	0.167	0.416	3.86E-14	10

Fam134b	2.49E-18	0.459269	0.207	0.032	4.16E-14	10
Cltb	2.55E-18	-1.0548	0.172	0.425	4.25E-14	10
H2afj	2.59E-18	0.664191	0.517	0.202	4.32E-14	10
Krcc1	2.73E-18	0.716141	0.322	0.08	4.55E-14	10
Cd81	2.82E-18	0.637214	0.569	0.46	4.7E-14	10
Khk	2.9E-18	0.290472	0.126	0.01	4.83E-14	10
Rpl4	3.09E-18	-0.50295	0.822	0.909	5.15E-14	10
Acox3	3.28E-18	0.58835	0.167	0.017	5.48E-14	10
Tagln3	3.48E-18	-1.07522	0.034	0.297	5.81E-14	10
Rufy3	3.93E-18	-1.04314	0.103	0.374	6.56E-14	10
Glb1	4.06E-18	0.624553	0.201	0.028	6.78E-14	10
Cd34	4.1E-18	0.412982	0.103	0.004	6.84E-14	10
Mdk	4.21E-18	-1.14157	0.098	0.382	7.02E-14	10
Snx3	4.5E-18	0.690093	0.632	0.339	7.51E-14	10
Dynll1	5.11E-18	-0.79198	0.333	0.596	8.53E-14	10
Nfkbiz	5.53E-18	0.579217	0.138	0.011	9.23E-14	10
Arhgdia	5.7E-18	0.763632	0.477	0.205	9.5E-14	10
Zfhx3	5.7E-18	0.679415	0.236	0.042	9.51E-14	10
Srsf11	5.82E-18	-0.61843	0.466	0.691	9.7E-14	10
Top1	5.94E-18	-0.74665	0.454	0.665	9.9E-14	10
Celf2	6.13E-18	-0.82469	0.466	0.639	1.02E-13	10
Hmgn5	6.37E-18	-1.00667	0.138	0.427	1.06E-13	10
Smc2	7.48E-18	-0.94706	0.287	0.547	1.25E-13	10
Hdgf	7.74E-18	-0.87224	0.264	0.534	1.29E-13	10
Mgat4a	8.23E-18	0.425348	0.132	0.009	1.37E-13	10
Rnpep	9.59E-18	0.593328	0.241	0.043	1.6E-13	10
Wasf2	1.01E-17	0.696541	0.368	0.11	1.69E-13	10
Hdgfrp3	1.06E-17	-0.96694	0.063	0.34	1.76E-13	10
Tmem9b	1.06E-17	0.642027	0.374	0.109	1.77E-13	10
Gsdmd	1.07E-17	0.366644	0.167	0.023	1.78E-13	10
Atp6v1c1	1.09E-17	0.636819	0.368	0.106	1.83E-13	10
Rragc	1.42E-17	0.615724	0.259	0.051	2.37E-13	10
Ptov1	1.47E-17	-0.85802	0.224	0.486	2.46E-13	10
Hnrnph3	1.6E-17	-0.90739	0.149	0.429	2.67E-13	10
Kmt2e	1.86E-17	-0.83779	0.299	0.571	3.1E-13	10
Tshz2	1.99E-17	-1.00632	0.057	0.322	3.33E-13	10
Il16	2.29E-17	0.261841	0.103	0.01	3.82E-13	10
Thoc7	2.37E-17	-0.77508	0.282	0.523	3.95E-13	10
Tpi1	2.49E-17	0.658447	0.385	0.118	4.16E-13	10
Mpp1	2.5E-17	0.621014	0.213	0.033	4.18E-13	10
Cfdp1	2.75E-17	-0.84893	0.264	0.486	4.59E-13	10
Pdap1	3.06E-17	-0.66131	0.454	0.685	5.1E-13	10
Dclk1	3.13E-17	-0.96117	0.126	0.415	5.21E-13	10
Stmn4	3.25E-17	-1.23216	0.069	0.344	5.42E-13	10
Rad21	3.41E-17	-0.93557	0.19	0.464	5.68E-13	10
Htatsf1	3.49E-17	-0.89456	0.201	0.501	5.82E-13	10
Pip4k2a	3.63E-17	0.566951	0.236	0.044	6.06E-13	10
Hsd11b2	3.71E-17	-1.02104	0.034	0.29	6.18E-13	10

Rap1b	3.89E-17	0.6845	0.402	0.131	6.5E-13	10
Snhg5	4.12E-17	-0.85136	0.092	0.384	6.88E-13	10
Eif4g2	4.15E-17	-0.59179	0.506	0.707	6.92E-13	10
Ppp1r18	4.36E-17	0.612529	0.276	0.061	7.27E-13	10
Fam115a	4.37E-17	-0.98941	0.092	0.353	7.29E-13	10
Gmip	4.93E-17	0.43074	0.144	0.015	8.23E-13	10
Nhlrc3	4.95E-17	0.40161	0.138	0.012	8.26E-13	10
Tpst2	5.94E-17	0.651132	0.299	0.072	9.91E-13	10
Il18bp	6.13E-17	0.627292	0.121	0.008	1.02E-12	10
Sox4	7.04E-17	-0.88242	0.333	0.585	1.17E-12	10
Ezr	8.17E-17	-0.95584	0.167	0.44	1.36E-12	10
Tsc22d1	8.54E-17	-0.92895	0.138	0.43	1.43E-12	10
Dtnbp1	8.91E-17	0.616632	0.339	0.094	1.49E-12	10
Hmgb3	9.18E-17	-0.89664	0.052	0.308	1.53E-12	10
Irf9	9.6E-17	0.390775	0.144	0.015	1.6E-12	10
Clip3	9.65E-17	-0.91994	0.052	0.314	1.61E-12	10
Ccnd1	1.03E-16	-0.88295	0.305	0.524	1.73E-12	10
Tbata	1.05E-16	-1.12605	0.08	0.342	1.75E-12	10
Kif21a	1.08E-16	-1.03318	0.08	0.348	1.8E-12	10
Sh3glb1	1.1E-16	0.645685	0.655	0.363	1.83E-12	10
Cacng2	1.12E-16	-0.96275	0.069	0.341	1.88E-12	10
Anp32b	1.19E-16	-0.59814	0.431	0.592	1.98E-12	10
Rdx	1.25E-16	-0.74545	0.379	0.605	2.09E-12	10
Fus	1.4E-16	-0.52814	0.569	0.738	2.34E-12	10
Trim2	1.53E-16	-0.97008	0.034	0.285	2.55E-12	10
Elavl4	1.66E-16	-1.09428	0.086	0.337	2.76E-12	10
Sdf2l1	1.82E-16	0.615938	0.247	0.049	3.04E-12	10
Strbp	2.06E-16	-0.96553	0.08	0.349	3.43E-12	10
Ssrp1	2.14E-16	-0.76997	0.305	0.579	3.58E-12	10
Cyb5r1	2.47E-16	0.490219	0.178	0.024	4.12E-12	10
Gnai2	2.56E-16	0.573791	0.73	0.421	4.26E-12	10
Zeb1	2.61E-16	-0.95709	0.109	0.39	4.36E-12	10
Akap9	3.04E-16	-0.97473	0.241	0.503	5.07E-12	10
Mvp	3.08E-16	0.300892	0.103	0.007	5.14E-12	10
Tep1	3.15E-16	0.396358	0.109	0.006	5.25E-12	10
Ddx26b	3.43E-16	0.814493	0.356	0.127	5.72E-12	10
Bri3	3.58E-16	0.68409	0.305	0.085	5.98E-12	10
Tor1aip1	3.69E-16	0.565923	0.322	0.089	6.15E-12	10
Basp1	3.94E-16	-0.73066	0.529	0.705	6.57E-12	10
C1ql1	4.08E-16	-0.93828	0.034	0.281	6.81E-12	10
Gng3	4.2E-16	-1.08432	0.057	0.319	7E-12	10
Kif1b	4.96E-16	-0.90632	0.241	0.523	8.28E-12	10
Dpysl3	5.22E-16	-1.08459	0.011	0.221	8.71E-12	10
Nucks1	5.23E-16	-0.6268	0.517	0.664	8.72E-12	10
Dram2	5.41E-16	0.571793	0.305	0.085	9.03E-12	10
Meis1	5.99E-16	-0.92834	0.098	0.375	1E-11	10
Tram1	6.2E-16	0.619758	0.385	0.125	1.03E-11	10
Arid4b	6.3E-16	-0.91908	0.161	0.442	1.05E-11	10

Elf1	6.87E-16	0.60598	0.247	0.051	1.15E-11	10
Sash1	7.37E-16	0.445223	0.195	0.033	1.23E-11	10
Rundc3a	8.02E-16	-0.93663	0.023	0.25	1.34E-11	10
Pde3b	9.17E-16	0.642993	0.305	0.083	1.53E-11	10
Rbfox2	9.5E-16	-0.94744	0.04	0.285	1.59E-11	10
Ttyh2	1.2E-15	0.447115	0.195	0.037	2.01E-11	10
Ktn1	1.31E-15	-0.81916	0.184	0.461	2.19E-11	10
Crlf2	1.39E-15	0.438464	0.144	0.014	2.32E-11	10
Gsn	1.42E-15	0.469741	0.132	0.012	2.37E-11	10
Ahi1	1.47E-15	-0.962	0.057	0.309	2.45E-11	10
Prcp	1.54E-15	0.551621	0.207	0.035	2.57E-11	10
Myh10	1.59E-15	-0.9183	0.075	0.326	2.65E-11	10
Ptgr2	1.67E-15	0.436725	0.161	0.021	2.78E-11	10
Fam210b	1.91E-15	-0.94634	0.063	0.316	3.18E-11	10
Elavl2	2.18E-15	-0.92906	0.034	0.272	3.63E-11	10
Capns1	2.32E-15	0.630443	0.517	0.224	3.86E-11	10
Cd164	2.33E-15	0.664897	0.362	0.117	3.88E-11	10
Sstr2	2.37E-15	-0.81703	0.011	0.192	3.95E-11	10
Pcbp2	2.69E-15	-0.57002	0.448	0.622	4.49E-11	10
Rab31	3.07E-15	0.503346	0.241	0.053	5.13E-11	10
2700094K1	3.24E-15	-0.65432	0.425	0.615	5.41E-11	10
Tmbim6	3.39E-15	0.626203	0.603	0.321	5.66E-11	10
Srebf1	3.57E-15	-0.99437	0.086	0.336	5.95E-11	10
Bzw2	3.62E-15	-0.83258	0.218	0.491	6.04E-11	10
Hpca	3.63E-15	-0.72905	0.046	0.279	6.06E-11	10
Cox7a2	3.63E-15	-0.42797	0.626	0.713	6.06E-11	10
Slc36a1	3.86E-15	0.42629	0.138	0.015	6.44E-11	10
Aldoa	4.25E-15	0.646652	0.54	0.254	7.08E-11	10
Abcd2	4.73E-15	0.478875	0.31	0.092	7.89E-11	10
Ppp1r14c	5.05E-15	-1.04261	0.04	0.264	8.42E-11	10
Eif2ak2	5.1E-15	0.517364	0.121	0.01	8.5E-11	10
Atpif1	5.39E-15	-0.48981	0.713	0.816	8.98E-11	10
Thra	6.18E-15	-0.91457	0.057	0.23	1.03E-10	10
Metap2	7.05E-15	-0.68952	0.351	0.599	1.18E-10	10
Nrxn1	8.59E-15	-1.1183	0.08	0.32	1.43E-10	10
Fnip2	8.59E-15	0.469531	0.144	0.015	1.43E-10	10
Lactb	8.72E-15	0.544466	0.19	0.033	1.45E-10	10
Pdlim4	9.44E-15	0.578685	0.282	0.074	1.57E-10	10
Arrdc1	9.91E-15	0.412967	0.115	0.009	1.65E-10	10
Hey1	1E-14	-0.97374	0.057	0.275	1.67E-10	10
Pcbp4	1.01E-14	-0.83232	0.069	0.327	1.68E-10	10
6330403K0	1.06E-14	-0.93091	0.034	0.245	1.77E-10	10
Gm5617	1.08E-14	0.588657	0.253	0.058	1.79E-10	10
Tcerg1	1.15E-14	-0.81665	0.224	0.459	1.92E-10	10
Lamtor1	1.23E-14	0.668592	0.46	0.196	2.06E-10	10
Dusp3	1.25E-14	0.439665	0.172	0.027	2.09E-10	10
Mfsd11	1.36E-14	0.582188	0.236	0.05	2.27E-10	10
Maged1	1.37E-14	-0.84582	0.184	0.431	2.29E-10	10

Vps18	1.51E-14	0.404194	0.172	0.028	2.51E-10	10
Orai3	1.59E-14	0.348749	0.103	0.006	2.66E-10	10
Prim1	1.71E-14	-0.8758	0.069	0.264	2.86E-10	10
Ppib	1.72E-14	-0.49885	0.638	0.701	2.86E-10	10
Tecr	1.83E-14	-0.69293	0.351	0.531	3.05E-10	10
Tmem219	1.84E-14	0.495835	0.276	0.073	3.06E-10	10
Rrp1	1.89E-14	-0.63697	0.448	0.607	3.16E-10	10
Tacc1	2.03E-14	0.607571	0.27	0.068	3.38E-10	10
H2afy	2.18E-14	-0.61298	0.42	0.593	3.63E-10	10
Kif3a	2.36E-14	-0.89172	0.098	0.318	3.93E-10	10
Ptprs	2.55E-14	-0.96336	0.161	0.423	4.25E-10	10
Txnrd1	2.8E-14	-0.83316	0.126	0.391	4.66E-10	10
Mcfd2	2.8E-14	0.586677	0.293	0.081	4.67E-10	10
Ncor1	3.03E-14	-0.72448	0.351	0.557	5.05E-10	10
Map3k1	3.27E-14	-0.85851	0.149	0.372	5.46E-10	10
Smap2	3.33E-14	0.680373	0.356	0.12	5.56E-10	10
Podxl2	3.53E-14	-0.90706	0.023	0.229	5.89E-10	10
Tsn	3.54E-14	-0.6538	0.379	0.591	5.91E-10	10
Snrpf	3.68E-14	-0.8036	0.19	0.429	6.14E-10	10
Clmp	3.72E-14	-0.94746	0.034	0.244	6.2E-10	10
Magt1	3.75E-14	0.565465	0.241	0.055	6.25E-10	10
Galc	3.85E-14	0.537319	0.207	0.039	6.42E-10	10
Hmgb2	3.92E-14	-0.93593	0.167	0.402	6.54E-10	10
Eva1b	4.05E-14	0.316976	0.103	0.009	6.75E-10	10
Safb	4.2E-14	-0.77272	0.287	0.505	7E-10	10
Nap1l4	4.33E-14	-0.75042	0.287	0.492	7.22E-10	10
Whsc1	4.45E-14	-0.82851	0.213	0.466	7.42E-10	10
Rnmt	4.85E-14	-0.89486	0.098	0.351	8.08E-10	10
Tmem179b	5.02E-14	0.646353	0.224	0.05	8.37E-10	10
Wipf1	5.03E-14	0.494436	0.172	0.027	8.38E-10	10
Acp2	5.51E-14	0.34912	0.276	0.091	9.19E-10	10
Tmpo	5.67E-14	-0.76903	0.224	0.454	9.45E-10	10
Hmgn3	6.08E-14	-0.70193	0.046	0.272	1.01E-09	10
Nagk	7.4E-14	0.598715	0.207	0.041	1.23E-09	10
Tgfb2	7.77E-14	-0.79221	0.017	0.193	1.3E-09	10
Apc	7.97E-14	-0.92723	0.19	0.434	1.33E-09	10
Atp6v0e2	8.37E-14	-0.83428	0.046	0.262	1.4E-09	10
Map4k4	8.94E-14	-0.79745	0.241	0.451	1.49E-09	10
Cyp4f13	9.28E-14	0.3667	0.121	0.011	1.55E-09	10
mt-Cytb	9.75E-14	-0.39177	0.891	0.938	1.63E-09	10
Gng12	1.01E-13	0.677335	0.339	0.118	1.68E-09	10
Cfl2	1.02E-13	-0.76747	0.149	0.411	1.7E-09	10
Degs1	1.05E-13	0.591964	0.276	0.074	1.74E-09	10
Ppic	1.09E-13	-0.81806	0.04	0.241	1.82E-09	10
Snrpe	1.11E-13	-0.63736	0.362	0.564	1.85E-09	10
Ctcf	1.11E-13	-0.78129	0.276	0.497	1.85E-09	10
Pfn2	1.13E-13	-0.85241	0.034	0.243	1.88E-09	10
Ccdc88a	1.16E-13	-0.84978	0.247	0.484	1.94E-09	10

Slc12a2	1.16E-13	0.614232	0.333	0.108	1.94E-09	10
Nkd1	1.2E-13	-0.8506	0.04	0.25	2.01E-09	10
Gm2694	1.23E-13	-0.88842	0.109	0.361	2.06E-09	10
Arrb2	1.32E-13	0.649204	0.333	0.116	2.2E-09	10
Pcbp1	1.47E-13	-0.55999	0.466	0.594	2.46E-09	10
Cd63	1.47E-13	0.440558	0.868	0.754	2.46E-09	10
Dnaja1	1.57E-13	-0.79764	0.161	0.376	2.62E-09	10
Itpripl1	1.6E-13	0.323409	0.115	0.011	2.68E-09	10
Dynlrb1	1.7E-13	-0.60996	0.443	0.58	2.83E-09	10
Pou3f2	1.72E-13	-0.87323	0.052	0.278	2.87E-09	10
Ncald	1.77E-13	-0.71454	0.011	0.167	2.95E-09	10
Rps3a1	1.82E-13	-0.46392	0.586	0.667	3.04E-09	10
Pald1	1.85E-13	0.492782	0.264	0.072	3.09E-09	10
Fkbp4	1.86E-13	-0.75167	0.19	0.416	3.11E-09	10
Mcm7	1.97E-13	-0.77054	0.138	0.391	3.28E-09	10
Tln1	2.21E-13	0.647675	0.391	0.147	3.69E-09	10
St3gal6	2.26E-13	0.394802	0.109	0.009	3.77E-09	10
Lrp10	2.31E-13	0.392535	0.19	0.039	3.85E-09	10
Sf3b2	2.38E-13	-0.57728	0.483	0.626	3.97E-09	10
Nsg1	2.43E-13	-0.8818	0.069	0.289	4.05E-09	10
Bex1	2.61E-13	-0.75869	0.023	0.225	4.36E-09	10
Tsc22d4	2.66E-13	0.538735	0.414	0.163	4.43E-09	10
Usp22	2.66E-13	-0.81736	0.126	0.363	4.44E-09	10
Rbp4	2.66E-13	-0.89257	0.04	0.243	4.44E-09	10
Fgf9	3.02E-13	-0.82196	0.04	0.262	5.03E-09	10
Prmt8	3.05E-13	-0.84326	0.052	0.274	5.09E-09	10
Tmem173	3.05E-13	0.514545	0.126	0.015	5.09E-09	10
Tuba1c	3.18E-13	0.5483	0.23	0.055	5.31E-09	10
Mab21l1	3.35E-13	-0.83153	0.023	0.227	5.6E-09	10
Btbd17	3.76E-13	-0.7464	0	0.161	6.27E-09	10
Cdk5rap2	3.97E-13	-0.74642	0.006	0.185	6.62E-09	10
Flcn	3.98E-13	0.487708	0.19	0.036	6.64E-09	10
Kdm1a	4E-13	-0.72059	0.115	0.366	6.67E-09	10
Atp6ap1	4.18E-13	0.47144	0.368	0.137	6.97E-09	10
Hjurp	4.45E-13	-0.8314	0.213	0.436	7.42E-09	10
Serp1	4.51E-13	0.54847	0.471	0.214	7.52E-09	10
Hirip3	4.63E-13	-0.83422	0.155	0.384	7.72E-09	10
Nhlh1	4.66E-13	-0.94798	0.034	0.225	7.78E-09	10
Nop56	4.72E-13	-0.81451	0.115	0.363	7.87E-09	10
Sppl2a	4.96E-13	0.589102	0.333	0.117	8.27E-09	10
Srrm4	5.08E-13	-0.83689	0.017	0.197	8.47E-09	10
1110007C(	5.19E-13	0.427851	0.207	0.049	8.66E-09	10
Adcy3	5.33E-13	0.297847	0.132	0.019	8.88E-09	10
Pou2f2	5.48E-13	0.438202	0.155	0.027	9.14E-09	10
Snx18	5.99E-13	0.482639	0.195	0.039	9.99E-09	10
Srrt	6.05E-13	-0.8028	0.184	0.426	1.01E-08	10
Mapk14	6.31E-13	0.513976	0.247	0.068	1.05E-08	10
Wwp1	6.47E-13	0.692068	0.276	0.082	1.08E-08	10

Sord	6.48E-13	0.434005	0.161	0.025	1.08E-08	10
2310022B(	6.61E-13	-0.80678	0.046	0.25	1.1E-08	10
Luc7l	7.33E-13	-0.79162	0.167	0.362	1.22E-08	10
Zfp710	7.67E-13	0.35287	0.109	0.01	1.28E-08	10
Tead2	7.98E-13	-0.80961	0.057	0.28	1.33E-08	10
Cdk5r1	8.24E-13	-0.92042	0.115	0.335	1.37E-08	10
Ptprd	8.43E-13	-0.7649	0.092	0.342	1.41E-08	10
Chgb	9.44E-13	-1.01354	0.034	0.242	1.57E-08	10
Mki67	9.5E-13	-0.94524	0.178	0.396	1.58E-08	10
Cdh20	9.63E-13	-0.71761	0.017	0.204	1.61E-08	10
Rsrc2	9.79E-13	-0.57292	0.316	0.549	1.63E-08	10
Fam171b	9.79E-13	-0.80812	0.034	0.225	1.63E-08	10
Tpm3	1E-12	0.562404	0.586	0.313	1.68E-08	10
Ywhae	1.11E-12	-0.45003	0.644	0.727	1.85E-08	10
Grina	1.12E-12	0.290871	0.276	0.107	1.87E-08	10
Hp1bp3	1.12E-12	-0.58578	0.425	0.582	1.87E-08	10
Actr3	1.14E-12	0.598998	0.339	0.126	1.9E-08	10
Prpf4b	1.15E-12	-0.74713	0.27	0.473	1.92E-08	10
Psma7	1.18E-12	-0.46651	0.644	0.783	1.96E-08	10
Arhgap19	1.22E-12	0.49928	0.172	0.033	2.04E-08	10
Adam9	1.22E-12	0.562341	0.218	0.051	2.04E-08	10
Klhdc2	1.29E-12	-0.80618	0.103	0.329	2.14E-08	10
Scpep1	1.3E-12	0.423522	0.218	0.052	2.17E-08	10
Cbfa2t3	1.31E-12	-0.82489	0.161	0.405	2.18E-08	10
Itfg3	1.36E-12	0.466183	0.126	0.016	2.26E-08	10
AI854517	1.36E-12	-0.67904	0.011	0.19	2.27E-08	10
Cct6a	1.43E-12	-0.69325	0.276	0.488	2.39E-08	10
Psmb10	1.65E-12	0.493556	0.236	0.06	2.75E-08	10
Khdrbs1	1.74E-12	-0.69305	0.293	0.495	2.91E-08	10
Paip2	1.88E-12	-0.67266	0.328	0.551	3.13E-08	10
Fut9	1.88E-12	-0.82811	0.023	0.213	3.14E-08	10
Cntn2	1.98E-12	-1.16946	0.069	0.265	3.3E-08	10
Tmed5	2.03E-12	0.524966	0.345	0.124	3.39E-08	10
Dut	2.39E-12	-0.7571	0.201	0.437	3.98E-08	10
Smarca4	2.47E-12	-0.71495	0.27	0.507	4.12E-08	10
Set	2.51E-12	-0.7176	0.178	0.389	4.18E-08	10
Zbtb18	2.51E-12	-0.8161	0.098	0.268	4.18E-08	10
Tmem66	2.9E-12	0.481411	0.448	0.196	4.84E-08	10
Sf3b1	2.92E-12	-0.51142	0.546	0.669	4.87E-08	10
Gbp7	2.92E-12	0.472139	0.103	0.009	4.87E-08	10
Ap3b2	3.17E-12	-0.72259	0.017	0.204	5.29E-08	10
Dap	3.17E-12	0.359949	0.195	0.047	5.29E-08	10
Zc3h13	3.18E-12	-0.79837	0.253	0.464	5.3E-08	10
Pde1b	3.3E-12	0.387512	0.132	0.019	5.51E-08	10
Rcn1	3.46E-12	-0.74923	0.034	0.235	5.76E-08	10
Trp53i11	3.58E-12	-0.64563	0.023	0.203	5.97E-08	10
Sept11	3.62E-12	-0.81435	0.092	0.324	6.04E-08	10
Dixdc1	3.79E-12	-0.82381	0.063	0.236	6.32E-08	10

Rbmx	3.92E-12	-0.74523	0.092	0.317	6.55E-08	10
Dgkz	3.96E-12	0.433338	0.299	0.102	6.6E-08	10
Ubtf	4E-12	-0.74256	0.236	0.454	6.68E-08	10
Npdc1	4.07E-12	-0.81651	0.052	0.263	6.79E-08	10
Mllt3	4.48E-12	-0.80439	0.034	0.227	7.48E-08	10
Syncrip	4.5E-12	-0.65781	0.305	0.497	7.5E-08	10
Slc1a2	4.59E-12	-0.87271	0.08	0.296	7.66E-08	10
Gng5	5.48E-12	0.601959	0.391	0.162	9.13E-08	10
Bcl11a	5.86E-12	-0.71746	0.023	0.211	9.77E-08	10
Elovl6	5.96E-12	-0.8041	0.069	0.28	9.95E-08	10
Apex1	6.05E-12	-0.61529	0.207	0.453	1.01E-07	10
Npm1	6.31E-12	-0.47026	0.534	0.647	1.05E-07	10
Bcas1	6.33E-12	-0.89678	0.069	0.284	1.06E-07	10
Akap6	6.38E-12	-0.75236	0.029	0.22	1.06E-07	10
1110004F1	6.53E-12	-0.62186	0.195	0.425	1.09E-07	10
Slc45a4	6.59E-12	0.498719	0.138	0.021	1.1E-07	10
Scarb1	6.61E-12	0.321465	0.167	0.035	1.1E-07	10
Snx2	7.05E-12	0.622358	0.276	0.094	1.18E-07	10
Sptbn1	7.29E-12	-0.8688	0.132	0.355	1.22E-07	10
Mast3	7.49E-12	0.365157	0.109	0.011	1.25E-07	10
C130071C(	7.51E-12	-0.67483	0.08	0.31	1.25E-07	10
Neu1	8.58E-12	0.514984	0.195	0.045	1.43E-07	10
March1	8.6E-12	0.323891	0.172	0.042	1.43E-07	10
Ythdc1	8.89E-12	-0.71088	0.224	0.479	1.48E-07	10
Dkc1	9.14E-12	-0.74735	0.103	0.335	1.52E-07	10
Sypl	9.31E-12	0.534854	0.253	0.072	1.55E-07	10
Pbrm1	9.76E-12	-0.64394	0.356	0.532	1.63E-07	10
Slc35c2	1.08E-11	0.473358	0.178	0.036	1.8E-07	10
Atp6v1f	1.13E-11	0.567816	0.552	0.298	1.88E-07	10
Ddx46	1.18E-11	-0.76581	0.218	0.444	1.97E-07	10
Hnrnpk	1.23E-11	-0.55261	0.46	0.637	2.06E-07	10
Arl3	1.26E-11	-0.6442	0.126	0.367	2.1E-07	10
Tspan6	1.26E-11	-0.6598	0.046	0.248	2.11E-07	10
Stx7	1.42E-11	0.469644	0.316	0.114	2.37E-07	10
Cirbp	1.43E-11	-0.77949	0.149	0.357	2.39E-07	10
Dner	1.44E-11	-0.86527	0.034	0.216	2.41E-07	10
RP23-45G1	1.57E-11	-0.88023	0.109	0.329	2.62E-07	10
Myo9b	1.6E-11	0.384861	0.19	0.046	2.67E-07	10
Cflar	1.6E-11	0.535095	0.207	0.049	2.67E-07	10
Cnot6	1.68E-11	-0.71453	0.167	0.393	2.8E-07	10
Sdf4	1.7E-11	0.521055	0.471	0.22	2.83E-07	10
Csnk1a1	1.82E-11	-0.4968	0.511	0.668	3.03E-07	10
Sacs	1.85E-11	-0.73974	0.029	0.192	3.09E-07	10
Aim2	1.86E-11	0.388569	0.109	0.012	3.1E-07	10
Cln5	1.93E-11	0.417428	0.172	0.034	3.22E-07	10
Rplp0	1.95E-11	-0.33451	0.874	0.872	3.25E-07	10
Gstm5	1.98E-11	-0.75406	0.138	0.324	3.3E-07	10
Eny2	2.02E-11	-0.57089	0.167	0.389	3.37E-07	10

Islr2	2.1E-11	-0.72645	0.017	0.181	3.5E-07	10
Dbnl	2.12E-11	0.551946	0.299	0.108	3.54E-07	10
Pigk	2.2E-11	0.463364	0.195	0.044	3.67E-07	10
Rogdi	2.32E-11	0.578448	0.213	0.062	3.86E-07	10
Top2a	2.49E-11	-0.96145	0.241	0.429	4.15E-07	10
Plcb4	2.5E-11	-0.68544	0	0.14	4.18E-07	10
Homer2	2.6E-11	-0.75349	0.057	0.248	4.34E-07	10
Lmnb1	2.65E-11	-0.74684	0.086	0.302	4.43E-07	10
Ccar1	2.84E-11	-0.53315	0.408	0.573	4.73E-07	10
Slc35f6	2.98E-11	0.297084	0.121	0.018	4.97E-07	10
Mitf	2.98E-11	0.370414	0.103	0.01	4.97E-07	10
Akr1b10	3.08E-11	0.45471	0.167	0.033	5.14E-07	10
Prrc2c	3.1E-11	-0.56749	0.368	0.554	5.18E-07	10
Dock10	3.11E-11	0.404495	0.121	0.015	5.18E-07	10
Cenpv	3.11E-11	-0.73558	0.103	0.33	5.18E-07	10
Aldh9a1	3.14E-11	0.464301	0.213	0.054	5.24E-07	10
Ank	3.22E-11	0.550805	0.213	0.054	5.37E-07	10
Col27a1	3.32E-11	0.322084	0.109	0.013	5.53E-07	10
H2afx	3.36E-11	-0.87105	0.155	0.332	5.61E-07	10
Gsk3b	3.47E-11	-0.68128	0.253	0.502	5.79E-07	10
P4hb	3.65E-11	0.55812	0.523	0.276	6.09E-07	10
Dnm2	3.81E-11	0.439067	0.236	0.069	6.35E-07	10
2810417H	4.01E-11	-0.86373	0.201	0.393	6.7E-07	10
Rab8b	4.05E-11	0.484211	0.201	0.048	6.75E-07	10
Gm8292	4.07E-11	-0.72718	0.161	0.371	6.79E-07	10
Aplp1	4.27E-11	-0.7217	0.023	0.152	7.12E-07	10
Dusp6	4.29E-11	0.601004	0.276	0.091	7.16E-07	10
1700025G	4.37E-11	-0.77877	0.069	0.265	7.29E-07	10
Sult4a1	4.43E-11	-0.72204	0.034	0.224	7.38E-07	10
Rap2b	4.54E-11	0.639425	0.328	0.146	7.58E-07	10
Ldhb	4.98E-11	0.614752	0.443	0.279	8.31E-07	10
Mfap4	5.02E-11	-0.74375	0.011	0.163	8.38E-07	10
Usp1	5.13E-11	-0.77726	0.115	0.34	8.55E-07	10
Clstn1	5.16E-11	-0.59763	0.121	0.203	8.6E-07	10
Atp6v1g1	5.23E-11	0.533572	0.437	0.208	8.73E-07	10
Nrn1	5.33E-11	-0.80907	0.052	0.223	8.88E-07	10
Zfp326	5.51E-11	-0.71295	0.19	0.411	9.2E-07	10
Snrnp40	5.71E-11	-0.64156	0.069	0.272	9.53E-07	10
Pdgfra	5.81E-11	-0.78989	0.017	0.183	9.69E-07	10
Ubl3	5.99E-11	0.477385	0.443	0.206	1E-06	10
Tcp1	6.2E-11	-0.54524	0.333	0.464	1.03E-06	10
Ostm1	6.25E-11	0.530017	0.195	0.046	1.04E-06	10
Mroh1	6.49E-11	0.289697	0.103	0.013	1.08E-06	10
Rbm25	6.53E-11	-0.46675	0.684	0.813	1.09E-06	10
Bcl7a	6.61E-11	-0.7727	0.115	0.327	1.1E-06	10
Ebf3	6.8E-11	-0.75347	0.029	0.204	1.13E-06	10
Peg3	6.81E-11	-0.81264	0.08	0.286	1.14E-06	10
Ilf3	7.05E-11	-0.64971	0.172	0.378	1.18E-06	10

Zc3hav1	7.22E-11	0.363203	0.109	0.012	1.2E-06	10
H1fx	7.25E-11	-0.73931	0.052	0.246	1.21E-06	10
Slc17a6	7.47E-11	-0.72075	0.011	0.149	1.25E-06	10
Derl1	7.54E-11	0.397316	0.264	0.093	1.26E-06	10
Apbb1	7.55E-11	-0.72318	0.023	0.171	1.26E-06	10
Cln8	7.63E-11	0.378003	0.19	0.047	1.27E-06	10
Mapt	7.65E-11	-0.91602	0.046	0.218	1.28E-06	10
Rsf1	7.65E-11	-0.809	0.144	0.34	1.28E-06	10
Stmn1	7.72E-11	-0.7325	0.086	0.286	1.29E-06	10
Rplp1	7.92E-11	0.331179	0.96	0.876	1.32E-06	10
Prr13	8.03E-11	0.494714	0.27	0.087	1.34E-06	10
Rbm8a	8.55E-11	-0.61905	0.299	0.467	1.43E-06	10
Rbm39	8.85E-11	-0.39026	0.701	0.793	1.48E-06	10
Naga	9.49E-11	0.38294	0.161	0.032	1.58E-06	10
Ltbr	9.54E-11	0.33298	0.115	0.015	1.59E-06	10
Siva1	9.61E-11	-0.75985	0.086	0.273	1.6E-06	10
Wbp5	9.99E-11	-0.625	0.333	0.52	1.67E-06	10
Brk1	1.02E-10	0.527456	0.603	0.373	1.7E-06	10
Rhob	1.03E-10	0.641688	0.305	0.137	1.72E-06	10
Purb	1.04E-10	-0.64722	0.356	0.551	1.73E-06	10
Actr2	1.09E-10	0.589931	0.42	0.195	1.82E-06	10
Rhoc	1.13E-10	0.371294	0.144	0.026	1.88E-06	10
Cxxc5	1.14E-10	-0.64365	0.356	0.545	1.9E-06	10
Tle1	1.16E-10	-0.70772	0.04	0.227	1.93E-06	10
Dnajc9	1.19E-10	-0.75925	0.155	0.351	1.99E-06	10
Eif1ax	1.2E-10	-0.62841	0.224	0.398	2.01E-06	10
Prdx1	1.24E-10	0.503018	0.695	0.487	2.07E-06	10
Fam168a	1.25E-10	-0.69089	0.155	0.288	2.08E-06	10
Mapk8ip2	1.28E-10	-0.63026	0.011	0.144	2.14E-06	10
Baz1b	1.33E-10	-0.62823	0.316	0.494	2.21E-06	10
Mcur1	1.33E-10	0.448191	0.184	0.042	2.22E-06	10
Vasp	1.35E-10	0.465443	0.23	0.065	2.25E-06	10
Cenpe	1.36E-10	-1.02095	0.092	0.287	2.27E-06	10
St8sia3	1.4E-10	-0.74389	0.063	0.252	2.34E-06	10
Kif2a	1.4E-10	-0.73661	0.08	0.252	2.34E-06	10
Plod3	1.44E-10	0.331877	0.213	0.064	2.39E-06	10
Nek6	1.44E-10	0.550667	0.339	0.142	2.4E-06	10
Ophn1	1.5E-10	0.486638	0.259	0.082	2.5E-06	10
Nr2f1	1.51E-10	-0.64789	0.029	0.205	2.52E-06	10
Pkn1	1.6E-10	0.439706	0.184	0.043	2.68E-06	10
Nol7	1.81E-10	-0.55493	0.391	0.57	3.02E-06	10
Wls	1.86E-10	0.483985	0.264	0.085	3.1E-06	10
Sept7	1.87E-10	-0.52911	0.448	0.59	3.13E-06	10
Pgd	1.9E-10	0.541528	0.253	0.081	3.17E-06	10
Ddx42	1.9E-10	-0.68568	0.213	0.384	3.18E-06	10
Reep5	2.02E-10	0.440681	0.339	0.136	3.38E-06	10
Rab5c	2.07E-10	0.539033	0.282	0.095	3.44E-06	10
Rnd2	2.08E-10	-0.68299	0.017	0.159	3.46E-06	10

Cct2	2.14E-10	-0.58111	0.351	0.525	3.57E-06	10
Ube2e3	2.18E-10	-0.66719	0.23	0.418	3.64E-06	10
Bok	2.2E-10	-0.72065	0.063	0.252	3.67E-06	10
Mid1ip1	2.4E-10	0.407904	0.195	0.05	4E-06	10
Satb1	2.41E-10	-0.71537	0.034	0.211	4.02E-06	10
2510039O	2.41E-10	0.262511	0.138	0.03	4.02E-06	10
Sowaha	2.42E-10	-0.78403	0.011	0.164	4.04E-06	10
Tmod2	2.55E-10	-0.72889	0.034	0.208	4.25E-06	10
Kcnk1	2.56E-10	-0.7935	0.034	0.206	4.26E-06	10
Chd6	2.66E-10	-0.75245	0.109	0.306	4.44E-06	10
Nfkb1	2.67E-10	0.312269	0.132	0.024	4.45E-06	10
Ppcdc	2.68E-10	0.347358	0.144	0.028	4.48E-06	10
Coro2b	2.69E-10	-0.66671	0.017	0.175	4.49E-06	10
Cetn3	2.7E-10	-0.62827	0.316	0.488	4.51E-06	10
Colgalt1	2.78E-10	0.377141	0.305	0.119	4.63E-06	10
Ssh2	2.83E-10	0.513578	0.184	0.043	4.72E-06	10
Srrm1	2.95E-10	-0.50526	0.46	0.612	4.93E-06	10
Otx2	2.98E-10	-0.741	0.04	0.218	4.97E-06	10
Tspyl4	3.02E-10	-0.61717	0	0.128	5.04E-06	10
Snrpb	3.18E-10	-0.51604	0.414	0.574	5.3E-06	10
Srrm3	3.34E-10	-0.79814	0.052	0.241	5.57E-06	10
Gm3764	3.4E-10	-0.48111	0.006	0.132	5.67E-06	10
Rps19	3.61E-10	-0.30247	0.759	0.753	6.02E-06	10
Rps9	3.79E-10	-0.25082	0.925	0.929	6.33E-06	10
Ssr4	3.79E-10	0.52609	0.598	0.374	6.33E-06	10
Heg1	3.87E-10	-0.64026	0.023	0.158	6.45E-06	10
Fbxo6	3.99E-10	0.411023	0.132	0.022	6.65E-06	10
Ptprg	3.99E-10	-0.46307	0.011	0.143	6.66E-06	10
Ttc9b	4.03E-10	-0.67275	0.029	0.196	6.71E-06	10
Sema6a	4.07E-10	-0.74251	0.034	0.203	6.79E-06	10
Pak3	4.26E-10	-0.75001	0.034	0.207	7.1E-06	10
Ehbp1l1	4.29E-10	0.369577	0.126	0.021	7.16E-06	10
Coa5	4.31E-10	0.48256	0.195	0.05	7.19E-06	10
Atp5b	4.39E-10	-0.35646	0.678	0.713	7.33E-06	10
Crip2	4.42E-10	-0.75887	0.052	0.241	7.38E-06	10
Cks1b	4.64E-10	-0.68665	0.138	0.346	7.73E-06	10
Tmem178	4.74E-10	-0.67883	0.023	0.187	7.91E-06	10
Dusp1	5.07E-10	0.652305	0.345	0.161	8.46E-06	10
Matk	5.09E-10	0.40302	0.103	0.012	8.49E-06	10
Adrbk1	5.8E-10	0.385434	0.19	0.053	9.68E-06	10
Sltm	5.9E-10	-0.64232	0.345	0.533	9.84E-06	10
Thoc2	5.93E-10	-0.62318	0.316	0.449	9.89E-06	10
Pa2g4	6E-10	-0.53266	0.356	0.524	1E-05	10
Prc1	6.62E-10	-0.84029	0.109	0.258	1.1E-05	10
Ccser2	6.7E-10	-0.72911	0.086	0.26	1.12E-05	10
2610017I0	6.82E-10	-0.68469	0.04	0.205	1.14E-05	10
Dtymk	6.89E-10	-0.67471	0.23	0.441	1.15E-05	10
St18	6.96E-10	-0.82981	0.029	0.192	1.16E-05	10

Klc1	7.1E-10	-0.70089	0.138	0.363	1.18E-05	10
Fermt2	8.06E-10	-0.68448	0.034	0.206	1.34E-05	10
Sox9	8.48E-10	-0.70065	0.063	0.257	1.41E-05	10
Tex14	8.72E-10	-0.75308	0.023	0.146	1.46E-05	10
Aprt	8.82E-10	0.590923	0.333	0.139	1.47E-05	10
BC005764	8.88E-10	-0.76873	0.029	0.187	1.48E-05	10
Ralgps2	8.9E-10	-0.72808	0.063	0.247	1.48E-05	10
Ptpn1	8.97E-10	0.535663	0.362	0.159	1.5E-05	10
Rtf1	9.2E-10	-0.62132	0.345	0.513	1.53E-05	10
Rbbp6	9.41E-10	-0.47958	0.322	0.433	1.57E-05	10
B4galt1	9.69E-10	0.393402	0.149	0.029	1.62E-05	10
Snrpn	9.76E-10	-0.62486	0.017	0.159	1.63E-05	10
Camk2d	1.03E-09	0.257657	0.201	0.072	1.71E-05	10
Ppp3cb	1.05E-09	-0.70968	0.132	0.311	1.74E-05	10
Atp5o	1.05E-09	-0.43865	0.454	0.588	1.75E-05	10
Usp46	1.06E-09	-0.6963	0.086	0.237	1.77E-05	10
Slc23a2	1.1E-09	0.276443	0.115	0.019	1.83E-05	10
Lpp	1.13E-09	0.39183	0.126	0.022	1.89E-05	10
Pacsin2	1.14E-09	0.453134	0.126	0.022	1.9E-05	10
St6galnac4	1.15E-09	0.321615	0.138	0.032	1.92E-05	10
Fam57b	1.16E-09	-0.67348	0.04	0.187	1.94E-05	10
Ubash3b	1.21E-09	0.371087	0.23	0.076	2.01E-05	10
Txnip	1.22E-09	0.560362	0.287	0.105	2.03E-05	10
Fstl1	1.27E-09	-0.68293	0.023	0.181	2.11E-05	10
Cdk6	1.29E-09	-0.70419	0.075	0.244	2.16E-05	10
Kif11	1.3E-09	-0.7426	0.04	0.207	2.17E-05	10
Med19	1.31E-09	-0.63113	0.132	0.319	2.19E-05	10
Itgav	1.32E-09	0.316329	0.195	0.059	2.2E-05	10
Ccp110	1.37E-09	-0.69417	0.063	0.25	2.28E-05	10
Rab6b	1.41E-09	-0.74223	0.052	0.204	2.34E-05	10
Rab7	1.46E-09	0.525241	0.397	0.181	2.44E-05	10
Igfbp4	1.53E-09	0.626596	0.115	0.018	2.55E-05	10
Stat3	1.57E-09	0.417641	0.167	0.041	2.63E-05	10
Kmt2a	1.59E-09	-0.58152	0.23	0.331	2.66E-05	10
Pdcd4	1.6E-09	-0.72368	0.138	0.333	2.67E-05	10
9330159F1	1.65E-09	-0.67739	0.029	0.17	2.74E-05	10
Zfp704	1.65E-09	-0.72802	0.086	0.259	2.75E-05	10
Ube2b	1.66E-09	-0.54661	0.362	0.522	2.77E-05	10
Snap25	1.68E-09	-0.73639	0.092	0.284	2.8E-05	10
Rnf20	1.75E-09	-0.71194	0.115	0.29	2.92E-05	10
Srpk2	1.8E-09	-0.61044	0.276	0.431	3E-05	10
Fez2	1.9E-09	0.375021	0.161	0.036	3.17E-05	10
Cadm3	1.9E-09	-0.69651	0.017	0.142	3.18E-05	10
Sept4	1.91E-09	-0.84672	0.057	0.228	3.19E-05	10
Rab2a	1.92E-09	-0.5974	0.287	0.432	3.2E-05	10
Kifap3	1.94E-09	-0.73631	0.092	0.274	3.24E-05	10
Nucb1	1.94E-09	0.418705	0.23	0.073	3.24E-05	10
Myef2	1.96E-09	-0.56089	0.138	0.34	3.27E-05	10

Chrna3	1.96E-09	-0.62122	0.011	0.119	3.27E-05	10
Atoh1	1.97E-09	-0.68142	0.023	0.165	3.28E-05	10
Marc2	1.98E-09	0.350459	0.236	0.082	3.31E-05	10
Amz1	1.99E-09	0.345356	0.109	0.015	3.33E-05	10
Smarcc1	2.02E-09	-0.64869	0.23	0.435	3.37E-05	10
Bnip2	2.11E-09	0.515889	0.408	0.215	3.52E-05	10
Nudt14	2.15E-09	0.303286	0.115	0.018	3.58E-05	10
Tpm4	2.34E-09	-0.65572	0.155	0.318	3.9E-05	10
Fam212b	2.34E-09	-0.69164	0.052	0.224	3.9E-05	10
Trim37	2.36E-09	-0.69734	0.075	0.234	3.94E-05	10
Dtx1	2.52E-09	-0.63939	0.017	0.167	4.21E-05	10
Tmed10	2.53E-09	0.518165	0.414	0.202	4.23E-05	10
Olfm1	2.54E-09	-0.74227	0.04	0.2	4.23E-05	10
Sh3gl2	2.58E-09	-0.683	0.034	0.172	4.31E-05	10
Pdzrn3	2.6E-09	-0.80616	0.011	0.148	4.34E-05	10
1110001J0	2.62E-09	-0.67322	0.057	0.239	4.38E-05	10
Slc25a10	2.65E-09	0.258236	0.126	0.026	4.42E-05	10
Rcor2	2.66E-09	-0.72175	0.057	0.226	4.44E-05	10
Dhx36	2.71E-09	-0.68237	0.155	0.372	4.52E-05	10
Nolc1	2.74E-09	-0.63031	0.236	0.41	4.57E-05	10
Gltp	2.74E-09	0.309553	0.23	0.082	4.58E-05	10
Prex1	2.77E-09	0.267162	0.19	0.062	4.61E-05	10
Fnip1	2.8E-09	0.273116	0.207	0.071	4.67E-05	10
Snrpd3	2.84E-09	-0.53661	0.356	0.536	4.73E-05	10
Vezf1	2.87E-09	-0.65744	0.155	0.343	4.79E-05	10
Arglu1	2.96E-09	-0.50522	0.374	0.559	4.94E-05	10
Dcakd	2.98E-09	-0.63262	0.098	0.298	4.97E-05	10
Mpdz	2.98E-09	-0.5792	0.006	0.136	4.97E-05	10
Fmnl3	3.14E-09	0.340326	0.132	0.025	5.24E-05	10
Taok3	3.3E-09	0.431218	0.247	0.086	5.51E-05	10
Sec11c	3.32E-09	0.477896	0.431	0.21	5.54E-05	10
Ldha	3.46E-09	0.359081	0.431	0.229	5.77E-05	10
Mdga1	3.46E-09	-0.55053	0.017	0.136	5.77E-05	10
Sema7a	3.52E-09	-0.50419	0.017	0.157	5.87E-05	10
Arpc3	3.6E-09	0.482865	0.328	0.14	6E-05	10
Ubb	3.65E-09	-0.51675	0.42	0.542	6.09E-05	10
Camk1d	3.78E-09	0.364152	0.115	0.022	6.3E-05	10
Gpr153	3.82E-09	-0.68537	0.04	0.21	6.36E-05	10
Cr1l	3.93E-09	0.291514	0.167	0.045	6.55E-05	10
Rab11a	3.93E-09	0.489966	0.5	0.275	6.56E-05	10
Os9	3.93E-09	0.342668	0.466	0.248	6.56E-05	10
Gna12	4.01E-09	0.297216	0.224	0.081	6.69E-05	10
Acat1	4.02E-09	-0.62128	0.253	0.442	6.7E-05	10
Gsg1l	4.11E-09	-0.65712	0.075	0.257	6.86E-05	10
Casc4	4.19E-09	-0.58783	0.011	0.149	7E-05	10
Slc12a9	4.2E-09	0.452901	0.167	0.042	7E-05	10
Cdkn1a	4.21E-09	0.747417	0.167	0.091	7.03E-05	10
Gjc1	4.25E-09	-0.6269	0.034	0.197	7.1E-05	10

Nemf	4.27E-09	-0.6515	0.213	0.34	7.12E-05	10
Atp1b3	4.27E-09	0.43096	0.569	0.334	7.13E-05	10
R3hdm1	4.32E-09	-0.62455	0.115	0.299	7.2E-05	10
Cant1	4.32E-09	0.260345	0.138	0.034	7.2E-05	10
Zfp422	4.33E-09	-0.63805	0.086	0.247	7.23E-05	10
Tmeff1	4.36E-09	-0.581	0.011	0.15	7.26E-05	10
Cdc5l	4.45E-09	-0.65255	0.207	0.402	7.42E-05	10
Chtop	4.51E-09	-0.59033	0.121	0.25	7.52E-05	10
Bnip3	4.53E-09	0.37375	0.132	0.025	7.55E-05	10
Sirt2	4.59E-09	0.400736	0.351	0.153	7.65E-05	10
Smox	4.67E-09	0.365908	0.207	0.064	7.79E-05	10
Bach1	4.97E-09	0.316803	0.224	0.079	8.29E-05	10
Smc6	5.01E-09	-0.63319	0.23	0.424	8.36E-05	10
Mllt11	5.07E-09	-0.68099	0.046	0.22	8.46E-05	10
Cmtm3	5.17E-09	0.442072	0.287	0.115	8.62E-05	10
H3f3a	5.21E-09	-0.60977	0.218	0.382	8.68E-05	10
Tbc1d12	5.27E-09	0.401392	0.178	0.047	8.79E-05	10
Ppfia2	5.4E-09	-0.61743	0	0.113	9E-05	10
Pcbd2	5.57E-09	0.376078	0.155	0.035	9.3E-05	10
Zfp462	5.61E-09	-0.5931	0.017	0.157	9.36E-05	10
Rassf4	5.63E-09	-0.6133	0.247	0.377	9.39E-05	10
Polr2h	5.65E-09	-0.61185	0.057	0.226	9.42E-05	10
Tex9	5.72E-09	-0.59149	0.017	0.149	9.55E-05	10
Igsf3	5.95E-09	-0.58937	0.006	0.134	9.92E-05	10
Smpd3	5.97E-09	-0.65564	0.023	0.162	9.95E-05	10
Nell2	5.97E-09	-0.56346	0.006	0.133	9.96E-05	10
Ndn	6.04E-09	-0.68846	0.057	0.224	0.000101	10
Man1c1	6.08E-09	0.39594	0.305	0.124	0.000101	10
Epc2	6.13E-09	-0.67543	0.092	0.251	0.000102	10
Tro	6.57E-09	-0.54364	0.023	0.127	0.00011	10
Bcap31	6.65E-09	0.443628	0.374	0.172	0.000111	10
Snx6	6.69E-09	0.452777	0.569	0.366	0.000112	10
Prkcb	6.88E-09	-0.75341	0.092	0.257	0.000115	10
Tprn	6.9E-09	-0.69014	0.052	0.228	0.000115	10
Setbp1	7.11E-09	-0.62919	0.017	0.161	0.000119	10
Klf9	7.22E-09	-0.66845	0.08	0.272	0.00012	10
Sars	7.22E-09	-0.62024	0.241	0.422	0.00012	10
Eid1	7.24E-09	-0.59602	0.339	0.504	0.000121	10
Zcchc18	7.28E-09	-0.62724	0.023	0.171	0.000121	10
Rsl1d1	7.4E-09	-0.59425	0.293	0.491	0.000123	10
1700001O	7.64E-09	-0.46516	0	0.111	0.000127	10
Pqlc1	7.84E-09	-0.68373	0.103	0.299	0.000131	10
Stox2	7.86E-09	-0.66922	0.04	0.2	0.000131	10
Elovl4	8.06E-09	-0.49901	0	0.111	0.000134	10
Dhrs1	9.02E-09	0.349531	0.247	0.091	0.00015	10
Rhoa	9.14E-09	0.460407	0.328	0.14	0.000153	10
Cyc1	9.28E-09	-0.5484	0.276	0.414	0.000155	10
Gm11266	9.45E-09	-0.65412	0.023	0.145	0.000158	10

Gas1	9.85E-09	-0.59415	0.017	0.158	0.000164	10
Zic2	9.88E-09	-0.48374	0.011	0.121	0.000165	10
Ank2	1.02E-08	-0.81277	0.069	0.242	0.00017	10
Sub1	1.06E-08	-0.42841	0.494	0.589	0.000176	10
Coro1b	1.06E-08	0.446093	0.454	0.236	0.000177	10
Cct8	1.08E-08	-0.45526	0.299	0.442	0.00018	10
Rnf187	1.08E-08	-0.57169	0.23	0.422	0.00018	10
Carhsp1	1.08E-08	-0.64785	0.086	0.255	0.000181	10
Mknk1	1.11E-08	0.379972	0.138	0.033	0.000185	10
Neurod6	1.14E-08	-0.66892	0.029	0.173	0.00019	10
App	1.17E-08	-0.53604	0.448	0.569	0.000195	10
Rnf144a	1.2E-08	-0.48689	0.023	0.161	0.0002	10
Rgl2	1.22E-08	0.381521	0.282	0.113	0.000203	10
Setd8	1.22E-08	-0.65607	0.08	0.263	0.000203	10
Tardbp	1.24E-08	-0.57692	0.293	0.484	0.000207	10
Myo5a	1.3E-08	0.523932	0.391	0.189	0.000216	10
Phip	1.31E-08	-0.58916	0.276	0.461	0.000219	10
Smarcd1	1.34E-08	-0.50472	0.086	0.273	0.000223	10
Akirin1	1.34E-08	0.368648	0.144	0.031	0.000224	10
Mphosph8	1.38E-08	-0.62847	0.23	0.366	0.00023	10
Ndufa5	1.38E-08	-0.55347	0.322	0.454	0.000231	10
A330076H(	1.41E-08	-0.69129	0.023	0.162	0.000235	10
Tpx2	1.53E-08	-0.82658	0.115	0.283	0.000255	10
Mfng	1.57E-08	0.309893	0.126	0.028	0.000262	10
Rbm5	1.57E-08	-0.59293	0.276	0.402	0.000262	10
Sh2b3	1.59E-08	0.351927	0.103	0.016	0.000265	10
Snx8	1.6E-08	0.401623	0.126	0.024	0.000266	10
Hmgn2	1.63E-08	-0.65346	0.086	0.257	0.000272	10
Clspn	1.65E-08	-0.6574	0.034	0.191	0.000274	10
Cdc42	1.65E-08	0.361284	0.741	0.513	0.000275	10
Cpeb4	1.68E-08	0.298533	0.161	0.045	0.000281	10
Fut8	1.77E-08	-0.62832	0.057	0.229	0.000295	10
Pold2	1.79E-08	-0.44001	0.011	0.114	0.000299	10
Efs	1.82E-08	-0.47906	0.017	0.126	0.000303	10
Myt1	1.88E-08	-0.72997	0.017	0.154	0.000313	10
Gdpd1	1.88E-08	-0.65797	0.069	0.245	0.000313	10
Amer2	1.96E-08	-0.48256	0.023	0.134	0.000326	10
Slc22a17	2.03E-08	-0.6843	0.086	0.252	0.000339	10
Ran	2.13E-08	-0.57879	0.259	0.415	0.000356	10
Pbx1	2.18E-08	-0.62248	0.034	0.188	0.000363	10
Syne2	2.19E-08	-0.63218	0.052	0.221	0.000365	10
Pygo1	2.25E-08	-0.70092	0.04	0.193	0.000375	10
Ewsr1	2.28E-08	-0.47578	0.293	0.393	0.00038	10
Rab3c	2.3E-08	-0.62353	0.017	0.149	0.000384	10
Scly	2.3E-08	0.297457	0.121	0.023	0.000384	10
Tmem57	2.36E-08	-0.68126	0.126	0.292	0.000394	10
Calm3	2.41E-08	-0.45435	0.471	0.561	0.000402	10
Asf1a	2.42E-08	-0.63109	0.098	0.281	0.000404	10

Itsn2	2.45E-08	0.464681	0.236	0.084	0.000409	10
Ccdc34	2.46E-08	-0.63489	0.23	0.416	0.000411	10
Spc25	2.61E-08	-0.69661	0.092	0.226	0.000435	10
Fkbp1a	2.66E-08	-0.55615	0.264	0.454	0.000444	10
Vat1	2.67E-08	0.299357	0.161	0.046	0.000445	10
C530008M	2.78E-08	-0.6486	0.086	0.27	0.000464	10
Fam133b	2.96E-08	-0.6764	0.144	0.309	0.000494	10
Fkbp15	2.98E-08	0.351489	0.138	0.032	0.000496	10
Tppp3	2.99E-08	-0.54049	0.023	0.159	0.000498	10
Map7d2	3.01E-08	-0.5089	0.011	0.101	0.000503	10
Cttnbp2nl	3.03E-08	0.431866	0.201	0.066	0.000506	10
Mtss1	3.09E-08	-0.76299	0.115	0.27	0.000515	10
Snhg1	3.12E-08	-0.59342	0.178	0.359	0.00052	10
Nptn	3.14E-08	0.451976	0.385	0.186	0.000524	10
Slc39a6	3.18E-08	-0.62186	0.069	0.2	0.000531	10
Brd8	3.25E-08	-0.61164	0.236	0.393	0.000542	10
Epc1	3.33E-08	-0.59654	0.115	0.233	0.000555	10
Srsf10	3.44E-08	-0.60396	0.19	0.331	0.000573	10
U2surp	3.49E-08	-0.49483	0.333	0.507	0.000582	10
Atp5a1	3.52E-08	-0.33506	0.672	0.718	0.000588	10
Fam98b	3.71E-08	-0.62103	0.138	0.337	0.000618	10
Armcx4	3.72E-08	-0.48772	0.011	0.114	0.000621	10
Fmr1	3.77E-08	-0.52867	0.109	0.301	0.000629	10
Magoh	3.86E-08	-0.57292	0.155	0.334	0.000644	10
Daam1	4.04E-08	-0.60093	0.034	0.185	0.000674	10
Tnik	4.1E-08	-0.71148	0.023	0.147	0.000683	10
Eef1a1	4.11E-08	-0.28298	0.902	0.884	0.000685	10
Ten1	4.28E-08	0.406395	0.333	0.15	0.000715	10
Rbfa	4.31E-08	0.302874	0.155	0.042	0.000719	10
Celsr2	4.34E-08	-0.66633	0.069	0.239	0.000724	10
Tspan14	4.35E-08	0.294135	0.155	0.043	0.000726	10
Sft2d1	4.39E-08	0.324437	0.115	0.021	0.000732	10
Sf3a3	4.49E-08	-0.60735	0.144	0.282	0.000749	10
Etfb	4.52E-08	0.445123	0.379	0.182	0.000754	10
Gdap1	4.67E-08	-0.65911	0.052	0.2	0.000778	10
Wdr26	4.68E-08	0.395781	0.563	0.337	0.000781	10
Mns1	4.89E-08	-0.6657	0.052	0.204	0.000816	10
Wdr6	5.04E-08	-0.582	0.023	0.145	0.00084	10
Zwint	5.22E-08	-0.65177	0.126	0.293	0.000871	10
Eprs	5.28E-08	-0.55892	0.316	0.493	0.000881	10
Spcs2	5.46E-08	0.443488	0.621	0.421	0.000911	10
Pltp	5.57E-08	0.381545	0.126	0.026	0.00093	10
Cecr2	5.6E-08	-0.53942	0.011	0.131	0.000934	10
Cdca3	5.73E-08	-0.6539	0.069	0.223	0.000956	10
Gde1	5.75E-08	0.416433	0.316	0.14	0.000958	10
Bub3	5.88E-08	-0.52008	0.218	0.322	0.000981	10
Arf3	5.96E-08	0.389837	0.195	0.061	0.000994	10
Ybx3	6.05E-08	-0.61389	0.109	0.291	0.001009	10

Cul3	6.15E-08	-0.50798	0.075	0.163	0.001026	10
Srsf7	6.18E-08	-0.5151	0.305	0.466	0.001031	10
Hip1r	6.22E-08	-0.59373	0.011	0.134	0.001038	10
Senp6	6.34E-08	-0.63127	0.218	0.39	0.001058	10
Mrps5	6.39E-08	-0.64802	0.103	0.274	0.001066	10
Cwc27	6.45E-08	-0.62778	0.034	0.17	0.001075	10
Epb4.1l3	6.53E-08	0.307073	0.264	0.111	0.00109	10
Cenpf	6.62E-08	-0.80922	0.207	0.363	0.001105	10
Usp34	6.81E-08	-0.61838	0.161	0.291	0.001137	10
Grb2	6.82E-08	0.391205	0.448	0.246	0.001138	10
Cct7	6.86E-08	-0.54054	0.305	0.486	0.001144	10
Specc1	6.87E-08	0.432496	0.172	0.048	0.001145	10
Csrp2	6.91E-08	-0.50499	0.023	0.159	0.001153	10
Ndufa12	7.06E-08	-0.50093	0.241	0.423	0.001178	10
Tmem160	7.25E-08	0.372378	0.437	0.232	0.001209	10
Prpf40a	7.25E-08	-0.50245	0.391	0.573	0.001209	10
Boc	7.36E-08	-0.58853	0.023	0.157	0.001228	10
Lamtor4	7.41E-08	0.424853	0.293	0.126	0.001236	10
Spats2	7.44E-08	-0.57055	0.034	0.183	0.001241	10
Cct5	7.63E-08	-0.41713	0.368	0.478	0.001272	10
2900011O(	7.69E-08	-0.40292	0.006	0.104	0.001283	10
Tcf25	7.88E-08	-0.44161	0.494	0.603	0.001315	10
Plcb1	7.94E-08	-0.64984	0.023	0.142	0.001324	10
Cplx1	7.96E-08	-0.60763	0.023	0.139	0.001328	10
Rp2h	7.98E-08	0.455777	0.161	0.045	0.001331	10
Rcan1	8.18E-08	0.521229	0.103	0.035	0.001365	10
Atp5j	8.21E-08	-0.40039	0.615	0.723	0.001369	10
A630007B(	8.24E-08	-0.68307	0.075	0.206	0.001374	10
Naa38	8.28E-08	-0.51096	0.121	0.313	0.001382	10
Gpbp1	8.34E-08	-0.60175	0.178	0.358	0.001391	10
Mum1	8.55E-08	-0.5642	0.029	0.17	0.001427	10
Sox11	8.68E-08	-0.47342	0.011	0.116	0.001447	10
Ehmt1	8.75E-08	-0.55507	0.103	0.253	0.00146	10
Zfp637	8.77E-08	-0.53224	0.086	0.258	0.001463	10
Gprasp1	8.86E-08	-0.62818	0.052	0.208	0.001478	10
Hsd17b11	8.86E-08	0.312829	0.184	0.061	0.001479	10
Ddx39b	8.87E-08	-0.42976	0.328	0.445	0.001479	10
Slc25a23	8.96E-08	-0.44653	0.029	0.108	0.001494	10
Spc24	9E-08	-0.60094	0.08	0.243	0.001502	10
Ift74	9.11E-08	-0.63	0.098	0.226	0.001519	10
Csnk1e	9.19E-08	-0.40929	0.345	0.386	0.001533	10
Snrpa1	9.43E-08	-0.59239	0.19	0.332	0.001573	10
Zfp91	9.99E-08	-0.48265	0.362	0.49	0.001666	10
Rsbn1l	1.01E-07	-0.66543	0.155	0.313	0.001684	10
Eif3h	1.01E-07	-0.4541	0.385	0.494	0.001685	10
Chmp6	1.06E-07	0.410142	0.236	0.087	0.00177	10
Phf14	1.09E-07	-0.60987	0.224	0.376	0.001826	10
Eif1	1.13E-07	-0.43346	0.477	0.583	0.001883	10

Cox20	1.18E-07	-0.50592	0.201	0.286	0.001971	10
Vav2	1.19E-07	0.368215	0.155	0.042	0.001978	10
Tceal8	1.21E-07	-0.58427	0.109	0.246	0.002022	10
Ppp3ca	1.23E-07	-0.6114	0.259	0.379	0.002046	10
Rcn2	1.25E-07	-0.59078	0.126	0.282	0.002077	10
Hist3h2ba	1.25E-07	-0.46304	0.017	0.138	0.002078	10
Bicd1	1.29E-07	-0.6186	0.023	0.154	0.002145	10
Tcf3	1.3E-07	-0.50195	0.069	0.227	0.002165	10
Coro7	1.33E-07	0.267697	0.109	0.023	0.002213	10
Nkain4	1.33E-07	-0.56056	0.011	0.129	0.002214	10
Ddx6	1.33E-07	-0.4671	0.362	0.493	0.002215	10
Mbnl1	1.34E-07	0.414819	0.328	0.149	0.00223	10
Trim59	1.41E-07	-0.5725	0.034	0.17	0.002353	10
Rap1a	1.42E-07	0.413273	0.287	0.122	0.002369	10
Upf3b	1.43E-07	-0.58181	0.184	0.321	0.002387	10
Gabrb3	1.45E-07	-0.41708	0.011	0.103	0.002413	10
Zcchc11	1.45E-07	-0.58213	0.138	0.322	0.002414	10
Snap23	1.49E-07	0.305803	0.149	0.04	0.00248	10
Fbxo32	1.51E-07	-0.55308	0.006	0.117	0.002515	10
Atp6v1a	1.51E-07	0.459618	0.489	0.286	0.002521	10
Bmyc	1.51E-07	0.296027	0.241	0.099	0.002524	10
Srsf2	1.52E-07	-0.37335	0.431	0.567	0.002534	10
Mtf2	1.52E-07	-0.60504	0.172	0.359	0.00254	10
Thrap3	1.55E-07	-0.57292	0.224	0.389	0.002581	10
Casp3	1.57E-07	-0.60364	0.08	0.235	0.002627	10
Eif3e	1.58E-07	-0.48565	0.23	0.383	0.002639	10
Extl3	1.59E-07	0.374945	0.184	0.058	0.002654	10
Trappc6a	1.6E-07	0.300216	0.144	0.04	0.002662	10
Rfc4	1.68E-07	-0.59903	0.069	0.227	0.00281	10
Zfp428	1.72E-07	-0.5241	0.029	0.168	0.002869	10
Rgs19	1.74E-07	0.300432	0.161	0.047	0.002904	10
Cct3	1.78E-07	-0.49803	0.322	0.496	0.002967	10
Cdh2	1.8E-07	-0.51053	0.023	0.154	0.00301	10
Zc3h15	1.87E-07	-0.57138	0.207	0.367	0.003125	10
Hmgb1	1.93E-07	-0.56674	0.19	0.36	0.003225	10
Cnot7	1.95E-07	-0.59146	0.08	0.217	0.003261	10
Gm11541	2.02E-07	-0.52201	0.011	0.128	0.003365	10
Fam174a	2.05E-07	0.42382	0.19	0.063	0.003418	10
Ccng1	2.09E-07	0.415939	0.149	0.038	0.003486	10
Hgsnat	2.1E-07	0.423997	0.155	0.043	0.003495	10
Rap1gds1	2.1E-07	0.365354	0.155	0.045	0.003505	10
Smarca5	2.15E-07	-0.52135	0.236	0.403	0.003592	10
Surf4	2.2E-07	0.33833	0.264	0.117	0.003672	10
Prpf38b	2.3E-07	-0.52114	0.368	0.496	0.003843	10
Naa10	2.35E-07	-0.5893	0.092	0.25	0.003913	10
Tubb2a	2.38E-07	-0.6338	0.161	0.245	0.003965	10
Ube3a	2.41E-07	-0.57333	0.224	0.351	0.004013	10
Kif1a	2.43E-07	-0.61213	0.04	0.172	0.004045	10

Maf1	2.46E-07	-0.53754	0.213	0.332	0.004095	10
Phf21b	2.47E-07	-0.52488	0.011	0.129	0.004113	10
Ddx17	2.48E-07	-0.53088	0.276	0.382	0.004143	10
Ap1b1	2.61E-07	0.397834	0.161	0.045	0.004346	10
Frrs1l	2.75E-07	-0.63305	0.052	0.192	0.00458	10
Lmbrd1	2.78E-07	0.264213	0.184	0.069	0.004643	10
Fam111a	2.81E-07	0.349463	0.282	0.124	0.004691	10
Cox6c	2.82E-07	-0.29783	0.69	0.719	0.004703	10
Egr2	2.84E-07	0.510525	0.132	0.046	0.004744	10
Tmem14c	2.88E-07	0.403001	0.374	0.186	0.004797	10
Fam3c	2.91E-07	0.254294	0.207	0.083	0.00485	10
Eef2	2.98E-07	-0.37334	0.598	0.717	0.004972	10
Cttn	3E-07	-0.46334	0.006	0.115	0.005	10
Fbxo5	3.04E-07	-0.46512	0.034	0.16	0.005063	10
Bcar1	3.04E-07	-0.55068	0.046	0.193	0.005067	10
Timeless	3.08E-07	-0.55616	0.029	0.154	0.005137	10
Odf2	3.11E-07	-0.44991	0.103	0.171	0.005192	10
Eif3d	3.16E-07	-0.49128	0.287	0.406	0.005273	10
BC005561	3.22E-07	-0.63127	0.149	0.291	0.005367	10
G3bp2	3.23E-07	-0.38157	0.471	0.552	0.005388	10
Lrig3	3.23E-07	-0.54357	0.034	0.171	0.005391	10
Reln	3.35E-07	-0.66064	0.04	0.181	0.005582	10
Eif3c	3.38E-07	-0.39902	0.483	0.603	0.005634	10
Hes6	3.4E-07	-0.60122	0.115	0.237	0.005676	10
Sh3kbp1	3.41E-07	0.340357	0.236	0.095	0.00568	10
Phpt1	3.43E-07	-0.53861	0.092	0.203	0.005718	10
Zfp354c	3.44E-07	-0.52647	0.011	0.126	0.00573	10
Lmnb2	3.57E-07	-0.52521	0.034	0.159	0.005959	10
Rtn3	3.76E-07	-0.43573	0.397	0.477	0.006275	10
Mapk8ip1	3.76E-07	-0.59442	0.069	0.228	0.006278	10
Rab6a	3.78E-07	-0.54865	0.155	0.329	0.0063	10
Zmynd11	3.87E-07	-0.51952	0.305	0.461	0.006449	10
Smarcb1	3.88E-07	-0.5218	0.213	0.324	0.00647	10
Pdzrn4	3.88E-07	-0.56539	0.023	0.147	0.006473	10
Sfrp2	3.91E-07	-0.51569	0.023	0.149	0.006523	10
Zfp292	3.94E-07	-0.64264	0.178	0.343	0.00657	10
Gpatch8	4.01E-07	-0.61526	0.138	0.282	0.006681	10
Lrrn1	4.12E-07	-0.47731	0.011	0.114	0.006871	10
Jakmip2	4.19E-07	-0.5921	0.029	0.158	0.00699	10
Serf1	4.33E-07	-0.61043	0.132	0.282	0.007225	10
Prox1	4.41E-07	-0.49403	0.109	0.23	0.00736	10
Tubg1	4.42E-07	-0.45852	0.034	0.13	0.007367	10
Ier5	4.42E-07	-0.57132	0.241	0.395	0.007374	10
Xrn2	4.56E-07	-0.5017	0.293	0.474	0.007608	10
Nop10	4.63E-07	-0.44478	0.356	0.483	0.007723	10
Stk4	4.67E-07	0.404347	0.218	0.085	0.007796	10
Ckap4	4.69E-07	-0.56904	0.149	0.291	0.007817	10
Rhobtb3	4.75E-07	-0.55646	0.04	0.165	0.007927	10

Sox5	4.77E-07	-0.52962	0.017	0.12	0.007959	10
Fndc4	4.79E-07	-0.47607	0.023	0.126	0.007989	10
Ckap2l	4.81E-07	-0.53517	0.075	0.219	0.008022	10
Lmo4	4.87E-07	-0.55948	0.155	0.337	0.008117	10
Ift43	4.87E-07	-0.46397	0.017	0.128	0.008125	10
Bod1	5E-07	-0.56634	0.046	0.178	0.008344	10
Orc6	5.01E-07	-0.55202	0.052	0.202	0.008364	10
Kat6b	5.02E-07	-0.58957	0.029	0.158	0.008377	10
Prpf6	5.03E-07	-0.60644	0.092	0.233	0.008391	10
Lyar	5.16E-07	-0.61894	0.132	0.287	0.008613	10
Dpy19l4	5.17E-07	0.267641	0.155	0.05	0.008617	10
Pou3f3	5.19E-07	-0.57299	0.04	0.173	0.008661	10
Nsd1	5.2E-07	-0.57386	0.178	0.341	0.008669	10
Casp8ap2	5.34E-07	-0.63736	0.098	0.245	0.008913	10
Rnf166	5.36E-07	0.404669	0.19	0.066	0.008946	10
Snrpd2	5.61E-07	-0.46386	0.282	0.442	0.009365	10
Rab3ip	5.7E-07	-0.43297	0.011	0.101	0.009513	10
Nrcam	5.73E-07	-0.56901	0.023	0.135	0.009558	10
Rufy2	5.75E-07	-0.51044	0.029	0.159	0.009584	10
Samhd1	5.76E-07	0.448401	0.161	0.052	0.009605	10
Cacng4	5.88E-07	-0.65955	0.006	0.102	0.009809	10
Atp6v1b2	5.93E-07	0.271768	0.328	0.169	0.009891	10
Ulk2	6.18E-07	0.295395	0.201	0.077	0.010316	10
Mpped2	6.2E-07	-0.49133	0.017	0.127	0.010349	10
Tacc2	6.25E-07	-0.58721	0.034	0.142	0.010426	10
Arpc1a	6.25E-07	-0.48744	0.264	0.364	0.010429	10
Pttg1ip	6.29E-07	0.419123	0.213	0.084	0.010487	10
Luc7l2	6.47E-07	-0.48889	0.339	0.496	0.01079	10
Lrpap1	6.48E-07	0.410677	0.27	0.115	0.010811	10
Srp19	6.52E-07	-0.37998	0.316	0.354	0.010876	10
Ptpn11	6.56E-07	-0.57043	0.144	0.271	0.01094	10
Pitpnc1	6.58E-07	0.34879	0.201	0.073	0.010972	10
Tfrc	6.63E-07	-0.57321	0.046	0.17	0.011051	10
Gpr85	6.63E-07	-0.56248	0.034	0.165	0.011065	10
Scn8a	6.67E-07	-0.54048	0.023	0.138	0.011118	10
Son	6.7E-07	-0.31655	0.753	0.788	0.011175	10
Tmem87b	6.7E-07	0.281398	0.23	0.097	0.01118	10
Mad2l2	6.77E-07	-0.55493	0.115	0.263	0.011299	10
Rcbtb2	6.83E-07	0.464255	0.316	0.148	0.011398	10
Nfyb	6.86E-07	-0.58895	0.098	0.236	0.011434	10
Clptm1l	6.98E-07	0.380353	0.276	0.121	0.011639	10
Atp6v0a1	7.27E-07	0.399738	0.236	0.094	0.012127	10
Nono	7.3E-07	-0.5046	0.115	0.22	0.012173	10
Fasn	7.32E-07	-0.50252	0.034	0.138	0.012217	10
Col9a3	7.43E-07	-0.60317	0.046	0.181	0.01239	10
Eif3m	7.58E-07	-0.50244	0.195	0.302	0.012643	10
Wasf1	7.66E-07	-0.45498	0.011	0.111	0.01278	10
Zfp664	7.74E-07	-0.56848	0.08	0.23	0.012914	10

Npepl1	7.75E-07	0.311105	0.126	0.033	0.01293	10
Rpn2	7.9E-07	0.405345	0.408	0.225	0.013179	10
Ska2	7.9E-07	-0.55484	0.057	0.206	0.013179	10
Pcyox1	7.91E-07	0.296943	0.207	0.082	0.013192	10
Stxbp1	7.96E-07	-0.62667	0.04	0.163	0.013277	10
Tubb4b	8.02E-07	-0.56697	0.121	0.241	0.013379	10
Mrpl20	8.16E-07	-0.44228	0.23	0.311	0.013607	10
Nuak1	8.24E-07	0.314476	0.132	0.034	0.013751	10
Ube2k	8.32E-07	-0.50186	0.328	0.452	0.013884	10
Mycl	8.34E-07	-0.50471	0.023	0.138	0.013913	10
Vps26a	8.4E-07	0.443437	0.282	0.13	0.014011	10
Polr2i	8.41E-07	-0.58103	0.167	0.318	0.01403	10
Calr	8.5E-07	0.392349	0.684	0.509	0.014174	10
Ankrd12	8.76E-07	-0.30988	0.305	0.274	0.01462	10
Brwd1	8.8E-07	-0.55418	0.08	0.194	0.014673	10
Cdk11b	8.95E-07	-0.36472	0.356	0.512	0.014931	10
Ntm	9.02E-07	-0.62622	0.034	0.146	0.01505	10
Hscb	9.11E-07	0.474837	0.155	0.059	0.015196	10
Zdbf2	9.16E-07	-0.40703	0.023	0.134	0.015279	10
Csde1	9.17E-07	-0.3906	0.356	0.437	0.015288	10
Ppp1r14b	9.23E-07	-0.49772	0.224	0.398	0.015389	10
Serpinh1	9.41E-07	-0.50271	0.011	0.116	0.015694	10
Cdc123	9.45E-07	-0.55753	0.172	0.314	0.01577	10
Abcf1	9.64E-07	-0.51037	0.328	0.458	0.016081	10
Cdca8	9.79E-07	-0.61414	0.144	0.265	0.016336	10
Gse1	9.91E-07	-0.58517	0.08	0.238	0.016528	10
Rbx1	9.98E-07	-0.40302	0.351	0.497	0.016651	10
Plgrkt	9.99E-07	0.383076	0.374	0.193	0.016656	10
Ppp2r5e	1E-06	-0.54717	0.109	0.221	0.016679	10
Srek1	1E-06	-0.54665	0.241	0.384	0.016745	10
Spag9	1.01E-06	-0.58769	0.155	0.308	0.016799	10
Prps2	1.04E-06	0.328425	0.167	0.052	0.017307	10
Rpa3	1.04E-06	-0.5759	0.092	0.245	0.017349	10
Rps15a	1.05E-06	-0.28451	0.609	0.657	0.0175	10
Cnksr2	1.05E-06	-0.51447	0.006	0.108	0.017569	10
Tbc1d20	1.12E-06	0.266025	0.218	0.094	0.018692	10
Trove2	1.12E-06	-0.59009	0.057	0.187	0.018719	10
Trp53	1.13E-06	-0.52741	0.155	0.299	0.018869	10
Schip1	1.15E-06	-0.57711	0.052	0.183	0.019121	10
Pnmal2	1.16E-06	-0.52461	0.029	0.147	0.019294	10
Arl8b	1.16E-06	0.293108	0.328	0.166	0.01936	10
Polr2f	1.19E-06	-0.52873	0.236	0.376	0.019773	10
Gnl3l	1.22E-06	-0.56239	0.052	0.19	0.020298	10
Pafah1b3	1.22E-06	-0.54887	0.063	0.191	0.020331	10
Gm11223	1.23E-06	-0.7231	0.075	0.214	0.020476	10
Mcl1	1.28E-06	0.476534	0.362	0.21	0.021406	10
Tfdp2	1.34E-06	-0.54777	0.034	0.162	0.022304	10
Atp8a1	1.34E-06	0.404251	0.201	0.072	0.022342	10

Jak1	1.35E-06	0.279683	0.264	0.126	0.022475	10
Ccdc41	1.35E-06	-0.60842	0.115	0.255	0.022507	10
Pdcd11	1.35E-06	-0.53681	0.04	0.165	0.022562	10
Tax1bp1	1.37E-06	-0.43968	0.408	0.58	0.022849	10
Ywhaq	1.38E-06	-0.52328	0.184	0.361	0.022961	10
Slc15a4	1.38E-06	0.338062	0.126	0.035	0.023052	10
Myt1l	1.38E-06	-0.66521	0.034	0.162	0.023087	10
Hes1	1.44E-06	-0.71258	0.04	0.149	0.023939	10
N4bp2	1.44E-06	-0.44279	0.057	0.132	0.024057	10
Hmox2	1.47E-06	0.404818	0.305	0.142	0.024599	10
Morc3	1.5E-06	0.257974	0.224	0.096	0.025046	10
Tuba1b	1.5E-06	-0.30323	0.414	0.481	0.025062	10
Ptbp2	1.54E-06	-0.57236	0.086	0.221	0.025696	10
Rps14	1.58E-06	-0.26222	0.937	0.95	0.026308	10
Ccdc104	1.58E-06	-0.58705	0.149	0.315	0.026334	10
Lman2	1.59E-06	0.286955	0.287	0.137	0.026506	10
Cdc7	1.6E-06	-0.52621	0.04	0.169	0.026722	10
Wdr82	1.61E-06	-0.49585	0.075	0.174	0.026798	10
Ddx1	1.62E-06	-0.46738	0.282	0.389	0.026972	10
E330009J0	1.62E-06	-0.47337	0.023	0.117	0.027104	10
Dnajc3	1.65E-06	0.388171	0.339	0.175	0.027579	10
Fos	1.7E-06	0.600628	0.454	0.303	0.028333	10
Tet1	1.72E-06	-0.47373	0.023	0.141	0.028676	10
Gtf2h5	1.75E-06	-0.3817	0.287	0.44	0.029214	10
Trp53bp1	1.76E-06	-0.48095	0.017	0.13	0.029335	10
Gm1673	1.77E-06	-0.46483	0.379	0.474	0.029556	10
Pfdn4	1.78E-06	-0.55636	0.144	0.306	0.029637	10
Tex264	1.78E-06	0.384035	0.19	0.067	0.029691	10
Epm2aip1	1.8E-06	-0.43807	0.011	0.106	0.030053	10
Ssx2ip	1.82E-06	-0.46811	0.04	0.131	0.03036	10
Ift27	1.84E-06	-0.53535	0.086	0.244	0.030646	10
Clvs1	1.85E-06	-0.53236	0.017	0.13	0.030797	10
Ptch2	1.87E-06	-0.5043	0.029	0.153	0.031218	10
Aagab	1.88E-06	0.292901	0.178	0.063	0.031305	10
Tead1	1.88E-06	-0.56725	0.052	0.174	0.031356	10
Nob1	1.93E-06	-0.48571	0.04	0.171	0.032137	10
Zic5	1.94E-06	-0.53756	0.034	0.161	0.032356	10
Magohb	1.94E-06	-0.50894	0.034	0.156	0.032434	10
Ptpn12	2E-06	-0.4556	0.017	0.124	0.033443	10
Man2a2	2.07E-06	0.25064	0.138	0.043	0.034522	10
Smarcad1	2.11E-06	-0.51253	0.069	0.169	0.035218	10
Atl1	2.12E-06	-0.482	0.006	0.103	0.035407	10
Smarcc2	2.13E-06	-0.52327	0.213	0.364	0.035467	10
Rnf7	2.16E-06	0.333162	0.374	0.207	0.036075	10
Rexo2	2.21E-06	0.491988	0.322	0.179	0.036846	10
Stard3nl	2.21E-06	0.406591	0.264	0.116	0.036895	10
Selm	2.23E-06	-0.58781	0.057	0.175	0.03723	10
Acot7	2.23E-06	-0.49807	0.057	0.196	0.037245	10

Racgap1	2.27E-06	-0.5508	0.098	0.257	0.037864	10
Mxd3	2.29E-06	-0.4559	0.017	0.127	0.038122	10
Cc2d1b	2.31E-06	0.32517	0.138	0.04	0.038456	10
Gtf2i	2.31E-06	-0.47102	0.253	0.389	0.038614	10
Clip1	2.32E-06	0.404222	0.23	0.092	0.038768	10
Nbea	2.33E-06	-0.63201	0.063	0.191	0.038909	10
Tgoln1	2.37E-06	0.301998	0.201	0.082	0.039578	10
Ndufb9	2.4E-06	-0.30538	0.5	0.539	0.040091	10
Eps15	2.43E-06	0.323114	0.172	0.059	0.040573	10
Ski	2.46E-06	0.355751	0.19	0.068	0.04095	10
Flywch1	2.46E-06	-0.46732	0.017	0.129	0.040977	10
Celsr3	2.48E-06	-0.51291	0.017	0.124	0.041351	10
Prnp	2.48E-06	-0.57163	0.063	0.208	0.041448	10
Map9	2.49E-06	-0.52748	0.029	0.148	0.041507	10
Ndufs4	2.5E-06	-0.44144	0.276	0.366	0.041764	10
Esf1	2.53E-06	-0.51664	0.236	0.393	0.04225	10
Gpsm1	2.55E-06	-0.54781	0.046	0.177	0.042523	10
Cdca7	2.57E-06	-0.48662	0.057	0.199	0.042931	10
Pde4dip	2.59E-06	-0.52058	0.086	0.222	0.043138	10
Prdm8	2.59E-06	-0.57844	0.023	0.127	0.04323	10
Rac1	2.61E-06	0.380554	0.54	0.344	0.043527	10
Dnajc2	2.62E-06	-0.48846	0.224	0.329	0.043711	10
Ggh	2.62E-06	0.333992	0.264	0.122	0.043745	10
Glce	2.64E-06	-0.59217	0.057	0.177	0.043981	10
Ndufb2	2.67E-06	-0.30859	0.351	0.367	0.044496	10
Kif20b	2.75E-06	-0.62917	0.075	0.191	0.045886	10
Klhl7	2.77E-06	-0.52692	0.046	0.165	0.046204	10
Cisd1	2.78E-06	-0.49979	0.23	0.343	0.046346	10
Rest	2.79E-06	0.350353	0.103	0.022	0.04658	10
Stard4	2.86E-06	-0.42567	0.029	0.141	0.0477	10
Appl1	2.95E-06	-0.55302	0.075	0.21	0.049236	10
Cdk2ap1	2.97E-06	-0.40723	0.006	0.101	0.049608	10
Ndrg2	3.04E-06	-0.5689	0.052	0.186	0.050789	10
Tcf12	3.07E-06	-0.55954	0.138	0.256	0.051273	10
Nckap1	3.11E-06	-0.50414	0.029	0.132	0.051847	10
Robo2	3.11E-06	-0.55016	0.04	0.152	0.05189	10
Mdh2	3.13E-06	-0.41475	0.402	0.529	0.05214	10
2210018M	3.24E-06	-0.37978	0.046	0.105	0.054012	10
Arpp21	3.24E-06	-0.59901	0.011	0.114	0.054084	10
Pgrmc1	3.26E-06	-0.55805	0.138	0.296	0.054298	10
Birc5	3.36E-06	-0.63885	0.138	0.274	0.055981	10
Ube2d1	3.37E-06	-0.54055	0.098	0.229	0.056173	10
Strn3	3.47E-06	-0.50042	0.144	0.314	0.057831	10
1110037F(	3.52E-06	-0.41086	0.023	0.105	0.058689	10
Hsp90b1	3.52E-06	0.290135	0.851	0.69	0.058726	10
Dnajc8	3.56E-06	-0.40347	0.322	0.435	0.059417	10
Ckap5	3.6E-06	-0.36875	0.138	0.266	0.060021	10
Emg1	3.63E-06	-0.45711	0.19	0.316	0.060485	10

Nans	3.63E-06	0.346917	0.241	0.106	0.060618	10
Chchd2	3.7E-06	-0.30339	0.609	0.653	0.061685	10
Cyth2	3.74E-06	-0.55016	0.138	0.274	0.062457	10
Lpar6	3.79E-06	0.325969	0.184	0.071	0.063157	10
Atad5	3.8E-06	-0.51247	0.046	0.149	0.063327	10
Ctr9	3.81E-06	-0.54946	0.109	0.243	0.063613	10
Adam10	3.83E-06	0.365758	0.374	0.201	0.063846	10
Pds5b	3.87E-06	-0.40425	0.126	0.273	0.064622	10
Oat	3.97E-06	0.38525	0.19	0.069	0.066261	10
Mob1a	3.99E-06	0.340989	0.161	0.052	0.066611	10
Ctbp1	4.03E-06	-0.49104	0.236	0.395	0.067184	10
Kif22	4.04E-06	-0.42408	0.046	0.164	0.067449	10
Clpp	4.06E-06	-0.53516	0.121	0.239	0.067723	10
Morf4l2	4.18E-06	-0.52428	0.132	0.273	0.069647	10
Ppp1r1a	4.21E-06	-0.45277	0.011	0.111	0.070305	10
Dctpp1	4.22E-06	-0.53478	0.149	0.305	0.070338	10
Gar1	4.23E-06	-0.51749	0.069	0.209	0.070554	10
Ube2e1	4.23E-06	-0.45548	0.144	0.228	0.070565	10
Cdca7l	4.24E-06	-0.3381	0.011	0.105	0.070719	10
Plk4	4.25E-06	-0.43008	0.017	0.12	0.070952	10
Vps36	4.28E-06	-0.4216	0.207	0.302	0.071355	10
Cdh4	4.29E-06	-0.50409	0.029	0.147	0.07154	10
Txn1	4.33E-06	-0.3878	0.466	0.561	0.072217	10
Ndufc2	4.35E-06	-0.35979	0.511	0.611	0.072572	10
Chmp3	4.43E-06	0.341508	0.264	0.127	0.07386	10
Creb1	4.46E-06	-0.5018	0.132	0.223	0.07442	10
Nedd4l	4.48E-06	-0.48616	0.029	0.129	0.074723	10
Naca	4.58E-06	-0.4201	0.351	0.466	0.076411	10
Nhp2	4.61E-06	-0.43118	0.264	0.371	0.07692	10
Zfml	4.71E-06	-0.54739	0.19	0.313	0.07849	10
D030056L2	4.81E-06	-0.46956	0.034	0.143	0.080311	10
Eif5a	5.01E-06	-0.33714	0.328	0.403	0.08355	10
Smdt1	5.12E-06	0.312077	0.54	0.353	0.085433	10
Cfl1	5.19E-06	0.413861	0.529	0.376	0.086628	10
Ift81	5.23E-06	-0.46612	0.029	0.147	0.087209	10
Tk2	5.34E-06	0.257471	0.126	0.038	0.089143	10
Sumf1	5.44E-06	0.379338	0.126	0.034	0.090698	10
Zmiz1	5.45E-06	-0.43664	0.305	0.375	0.090834	10
Cnpy1	5.46E-06	-0.5041	0.023	0.134	0.091049	10
Itpa	5.5E-06	-0.45584	0.121	0.212	0.091766	10
Acot13	5.55E-06	0.449074	0.253	0.112	0.092638	10
Appbp2	5.6E-06	-0.48626	0.098	0.2	0.093412	10
Pip5k1a	5.6E-06	-0.41451	0.046	0.118	0.093444	10
Esco2	5.89E-06	-0.60319	0.063	0.196	0.098328	10
Mpzl1	5.91E-06	-0.47956	0.052	0.145	0.098631	10
Gtf2a2	5.94E-06	-0.38794	0.282	0.384	0.099151	10
Vbp1	6.04E-06	-0.42003	0.155	0.321	0.100735	10
Nipa2	6.06E-06	0.345701	0.184	0.067	0.101121	10

Mageh1	6.11E-06	-0.43281	0.017	0.12	0.101941	10
Mcm6	6.13E-06	-0.56539	0.121	0.277	0.102214	10
Dhx9	6.17E-06	-0.40333	0.362	0.464	0.102915	10
Cuta	6.26E-06	0.403162	0.46	0.294	0.104449	10
Thsd7a	6.39E-06	-0.54085	0.023	0.128	0.106641	10
Hint1	6.44E-06	-0.31682	0.638	0.704	0.107477	10
Rbbp4	6.58E-06	-0.35206	0.414	0.484	0.109708	10
Fam181b	6.6E-06	-0.46847	0.029	0.143	0.110056	10
Srsf5	6.6E-06	-0.35901	0.368	0.427	0.110143	10
Nicn1	6.66E-06	-0.5868	0.075	0.209	0.111127	10
Smarce1	6.82E-06	-0.5215	0.069	0.205	0.113723	10
Gm17322	6.89E-06	-0.56201	0.034	0.132	0.114893	10
Psmc6	6.92E-06	-0.48597	0.213	0.341	0.115342	10
Afap1	6.94E-06	-0.5199	0.034	0.153	0.11578	10
Sbk1	7.05E-06	-0.49721	0.017	0.119	0.117662	10
Dbn1	7.21E-06	-0.53518	0.034	0.14	0.12031	10
Polr2m	7.24E-06	-0.39351	0.138	0.245	0.120729	10
Polr2b	7.25E-06	-0.51026	0.057	0.179	0.12099	10
Mgst1	7.27E-06	0.361779	0.121	0.032	0.121255	10
Lsm14b	7.43E-06	-0.49615	0.046	0.154	0.123951	10
Nefm	7.65E-06	-0.48237	0.017	0.117	0.127586	10
Rnf165	7.67E-06	-0.5473	0.052	0.173	0.127868	10
Cdc16	7.68E-06	-0.5134	0.155	0.283	0.128032	10
Pdia3	7.69E-06	0.35113	0.471	0.294	0.128292	10
Ndnl2	7.71E-06	-0.41814	0.046	0.13	0.128541	10
Vopp1	7.9E-06	-0.42574	0.017	0.12	0.131844	10
Gkap1	7.94E-06	-0.53171	0.092	0.221	0.132363	10
Txnrd2	7.94E-06	0.291516	0.103	0.024	0.132395	10
Nol4	8.04E-06	-0.51606	0.046	0.172	0.134174	10
Brd2	8.07E-06	-0.48151	0.253	0.396	0.13458	10
2210016L2	8.1E-06	-0.55769	0.161	0.29	0.135104	10
Taldo1	8.41E-06	0.340564	0.5	0.319	0.140205	10
Ubqln2	8.41E-06	-0.5393	0.092	0.215	0.140263	10
Dph3	8.51E-06	-0.50953	0.098	0.211	0.141898	10
Kcnip3	8.54E-06	-0.48082	0.017	0.121	0.14249	10
Zranb2	8.7E-06	-0.47491	0.264	0.407	0.145037	10
Mab21l2	8.89E-06	-0.49428	0.011	0.105	0.148309	10
Mfsd5	8.91E-06	0.335828	0.126	0.035	0.148547	10
G2e3	8.91E-06	-0.4411	0.023	0.132	0.148563	10
Yipf1	9E-06	0.297289	0.201	0.082	0.150082	10
Psma5	9.06E-06	-0.47608	0.201	0.322	0.151151	10
Bptf	9.27E-06	-0.45729	0.293	0.374	0.154707	10
Tipin	9.35E-06	-0.52979	0.138	0.256	0.156004	10
Tex30	9.37E-06	-0.45018	0.029	0.136	0.156335	10
Gm9843	9.42E-06	0.255057	0.385	0.225	0.15712	10
Got1	9.49E-06	0.317779	0.155	0.052	0.158328	10
Ech1	9.56E-06	0.403844	0.247	0.113	0.159516	10
Kdm5b	9.69E-06	-0.5905	0.069	0.186	0.1616	10

Ebna1bp2	9.78E-06	-0.50439	0.161	0.316	0.163182	10
Mllt10	9.79E-06	-0.53501	0.155	0.287	0.163334	10
Pafah1b1	9.85E-06	-0.37149	0.414	0.474	0.164343	10
Ndufv2	9.93E-06	-0.30569	0.402	0.443	0.165593	10
Tmem35	9.96E-06	-0.42968	0.017	0.104	0.166186	10
Anapc5	1.01E-05	-0.45867	0.224	0.334	0.169035	10
Eif4a1	1.02E-05	-0.38687	0.345	0.485	0.170324	10
Grik2	1.03E-05	-0.48342	0.011	0.102	0.17231	10
Gamt	1.04E-05	-0.53056	0.069	0.17	0.173417	10
Mycbp2	1.05E-05	-0.55275	0.224	0.346	0.174345	10
Impad1	1.05E-05	-0.53956	0.092	0.224	0.17571	10
Vcp	1.06E-05	-0.4148	0.236	0.305	0.177048	10
Ifnar1	1.07E-05	0.37033	0.218	0.095	0.178807	10
Agps	1.09E-05	0.261849	0.144	0.05	0.181711	10
Rnps1	1.09E-05	-0.43678	0.029	0.138	0.181939	10
Rnpc3	1.1E-05	-0.43256	0.023	0.104	0.183084	10
Tbl1x	1.1E-05	-0.36728	0.282	0.328	0.183556	10
Ercc1	1.12E-05	-0.46408	0.04	0.137	0.186638	10
Pdcd6ip	1.13E-05	0.390763	0.224	0.094	0.187756	10
Rab11b	1.13E-05	-0.36622	0.098	0.153	0.188617	10
Zfp36l1	1.14E-05	0.462898	0.293	0.149	0.190752	10
Fzd2	1.16E-05	-0.49829	0.046	0.162	0.193178	10
Ykt6	1.16E-05	0.41677	0.247	0.111	0.193496	10
Mex3b	1.18E-05	-0.46696	0.023	0.116	0.196244	10
Vcan	1.19E-05	-0.48003	0.023	0.13	0.19822	10
Prdx6	1.2E-05	-0.51011	0.184	0.327	0.199989	10
Ndufs8	1.2E-05	0.253935	0.511	0.346	0.200065	10
Stip1	1.24E-05	-0.43777	0.155	0.304	0.20648	10
Ndufv3	1.24E-05	0.271744	0.471	0.296	0.206676	10
Cacybp	1.26E-05	-0.37424	0.23	0.357	0.209494	10
Cdipt	1.27E-05	0.26264	0.213	0.095	0.211909	10
Hmgcl	1.29E-05	0.422509	0.195	0.077	0.21495	10
Ccna2	1.32E-05	-0.47476	0.08	0.222	0.219964	10
2610203C2	1.35E-05	-0.49342	0.023	0.121	0.224864	10
Fam92a	1.36E-05	-0.36067	0.069	0.199	0.226274	10
Osbpl11	1.37E-05	0.340815	0.161	0.055	0.228971	10
Stoml2	1.38E-05	-0.48062	0.155	0.263	0.229445	10
Snw1	1.39E-05	-0.39928	0.345	0.432	0.232255	10
Phactr1	1.4E-05	-0.55421	0.063	0.184	0.233216	10
Cdkn2c	1.41E-05	-0.45182	0.034	0.144	0.235736	10
Pja1	1.42E-05	-0.50408	0.052	0.174	0.237563	10
Tmem30a	1.43E-05	0.324989	0.333	0.177	0.238029	10
Parp6	1.43E-05	-0.48066	0.023	0.126	0.239058	10
Smarca2	1.44E-05	-0.54744	0.167	0.286	0.240418	10
Ube2e2	1.45E-05	-0.42821	0.023	0.126	0.241561	10
Prpf4	1.49E-05	-0.39434	0.08	0.14	0.247799	10
Rab14	1.52E-05	0.324727	0.598	0.41	0.253751	10
Smco4	1.53E-05	-0.35545	0.046	0.162	0.254796	10

Rpf2	1.55E-05	-0.51381	0.086	0.203	0.259369	10
Bcas2	1.56E-05	-0.36847	0.31	0.406	0.260671	10
Pbk	1.57E-05	-0.57296	0.092	0.223	0.261674	10
Mical1	1.58E-05	-0.33992	0.086	0.126	0.263987	10
Ncapd2	1.59E-05	-0.48655	0.057	0.186	0.265371	10
Neo1	1.59E-05	-0.41068	0.023	0.107	0.265599	10
Rnaset2a	1.62E-05	0.263703	0.144	0.048	0.269421	10
Map1lc3a	1.63E-05	-0.46721	0.19	0.276	0.271432	10
Srsf1	1.65E-05	-0.50982	0.155	0.297	0.275283	10
Tiprl	1.65E-05	-0.48291	0.075	0.205	0.275571	10
Ywhaz	1.7E-05	-0.39544	0.282	0.363	0.282864	10
Xist	1.7E-05	-0.72365	0.172	0.3	0.283	10
Abi2	1.7E-05	-0.49704	0.069	0.184	0.283967	10
Tmem107	1.72E-05	-0.42644	0.023	0.127	0.286981	10
Pabpn1	1.73E-05	-0.50565	0.086	0.195	0.288064	10
Cspp1	1.73E-05	-0.55196	0.098	0.239	0.288672	10
Lig1	1.73E-05	-0.51185	0.259	0.349	0.288996	10
Vma21	1.74E-05	-0.44329	0.034	0.137	0.290752	10
Myod1	1.75E-05	-0.51846	0.034	0.142	0.29226	10
Rbmx2	1.76E-05	-0.44529	0.023	0.115	0.293077	10
Tspan5	1.76E-05	-0.51887	0.069	0.197	0.29424	10
Bcl2l13	1.79E-05	-0.38665	0.052	0.115	0.297823	10
Stx6	1.8E-05	-0.41436	0.092	0.161	0.301017	10
Ap2s1	1.81E-05	0.2807	0.437	0.274	0.301126	10
Trpc2	1.81E-05	-0.44971	0.029	0.135	0.301707	10
Cenpm	1.81E-05	-0.44278	0.046	0.163	0.30269	10
Ctxn1	1.83E-05	-0.4869	0.052	0.171	0.305738	10
BC034090	1.85E-05	-0.48282	0.029	0.128	0.30805	10
Ryk	1.85E-05	-0.48086	0.034	0.144	0.308185	10
Agpat4	1.86E-05	-0.47392	0.023	0.118	0.310567	10
Amer1	1.87E-05	-0.44546	0.017	0.108	0.312267	10
Tmem256	1.88E-05	0.360341	0.649	0.49	0.314161	10
Tiparp	1.92E-05	-0.44367	0.075	0.147	0.319442	10
Ddr1	1.92E-05	-0.49907	0.04	0.136	0.320175	10
Lbr	1.94E-05	-0.43257	0.075	0.197	0.323427	10
Nudt3	1.96E-05	-0.43545	0.172	0.249	0.326672	10
Smc5	1.99E-05	-0.5373	0.121	0.259	0.331984	10
Pik3r3	2E-05	-0.52272	0.04	0.136	0.332882	10
Tmem251	2E-05	0.420846	0.155	0.057	0.333206	10
Ikzf5	2E-05	-0.38269	0.034	0.145	0.334208	10
Klf13	2.01E-05	-0.46859	0.103	0.237	0.335301	10
Prmt5	2.03E-05	-0.50792	0.126	0.257	0.337986	10
Pard3	2.03E-05	-0.41131	0.023	0.127	0.338101	10
Elp3	2.08E-05	-0.42549	0.057	0.137	0.347258	10
Pole3	2.09E-05	-0.46351	0.161	0.25	0.348007	10
Ilkap	2.09E-05	-0.42356	0.121	0.231	0.348426	10
Arl6	2.1E-05	-0.41172	0.023	0.115	0.350636	10
Mgea5	2.1E-05	-0.49431	0.138	0.232	0.350929	10

Ankrd32	2.12E-05	-0.5103	0.069	0.184	0.352811	10
Ankrd11	2.14E-05	-0.4353	0.368	0.474	0.356837	10
Psmc3ip	2.14E-05	-0.44341	0.04	0.132	0.3569	10
Phf6	2.15E-05	-0.44388	0.052	0.174	0.359061	10
Shmt2	2.23E-05	-0.38353	0.023	0.12	0.37123	10
Rnf122	2.26E-05	-0.40886	0.011	0.103	0.37659	10
Rrm1	2.26E-05	-0.49917	0.149	0.261	0.377772	10
Nipsnap1	2.27E-05	-0.45799	0.029	0.137	0.378161	10
Ssu72	2.37E-05	0.322751	0.385	0.221	0.395826	10
Zfp580	2.39E-05	-0.41685	0.023	0.123	0.399168	10
Abi1	2.41E-05	0.390811	0.287	0.143	0.402314	10
Clic4	2.42E-05	-0.48494	0.092	0.228	0.40307	10
Caprin1	2.42E-05	-0.36843	0.316	0.394	0.403896	10
Ypel1	2.45E-05	-0.47801	0.052	0.175	0.408028	10
Dis3	2.45E-05	-0.39197	0.023	0.125	0.408715	10
Golim4	2.46E-05	-0.54363	0.109	0.233	0.410957	10
Ywhag	2.48E-05	-0.46232	0.161	0.241	0.413935	10
Usp10	2.49E-05	-0.49273	0.075	0.187	0.415181	10
Ccnb2	2.49E-05	-0.50381	0.063	0.188	0.415233	10
Nusap1	2.52E-05	-0.60699	0.092	0.228	0.420272	10
Ndufa10	2.53E-05	-0.35698	0.322	0.372	0.421813	10
Pmpcb	2.54E-05	-0.41624	0.144	0.218	0.424298	10
Fyn	2.62E-05	-0.54604	0.132	0.216	0.43644	10
Lta4h	2.63E-05	-0.46987	0.126	0.243	0.439349	10
Pitpna	2.65E-05	0.334124	0.264	0.13	0.44126	10
Ppp2ca	2.65E-05	-0.29753	0.345	0.376	0.441728	10
Zfp266	2.66E-05	-0.4766	0.063	0.168	0.443942	10
Rnf168	2.69E-05	-0.48741	0.115	0.208	0.447883	10
Spop	2.71E-05	-0.47971	0.207	0.316	0.45169	10
Arf4	2.71E-05	-0.43592	0.287	0.405	0.452016	10
C77370	2.75E-05	-0.45048	0.011	0.102	0.45793	10
Cdc20	2.78E-05	-0.6375	0.075	0.197	0.463022	10
Gpatch4	2.82E-05	-0.45031	0.109	0.18	0.469767	10
Knstrn	2.84E-05	-0.53004	0.063	0.186	0.474052	10
Erdr1	2.87E-05	-0.49151	0.046	0.162	0.478305	10
Las1l	2.87E-05	-0.47105	0.121	0.256	0.479439	10
Znrf2	2.89E-05	0.267895	0.201	0.087	0.482462	10
Slc25a27	2.96E-05	-0.44066	0.017	0.11	0.494152	10
Hnrnpul2	3.04E-05	-0.47052	0.161	0.258	0.506272	10
Phf3	3.05E-05	-0.52295	0.126	0.271	0.508222	10
Cenpa	3.05E-05	-0.65551	0.132	0.253	0.508297	10
Lsm6	3.05E-05	-0.40784	0.27	0.372	0.508858	10
Tdp2	3.07E-05	-0.36987	0.04	0.151	0.511415	10
Npepps	3.08E-05	-0.41416	0.241	0.318	0.513155	10
Lhfpl4	3.08E-05	-0.41177	0.017	0.108	0.513836	10
Slc25a5	3.16E-05	0.305736	0.672	0.507	0.527694	10
Cep57	3.2E-05	-0.494	0.115	0.259	0.53338	10
Sri	3.23E-05	0.383196	0.31	0.164	0.538559	10

Ptrhd1	3.26E-05	0.434085	0.178	0.074	0.54417	10
Golga4	3.33E-05	-0.45776	0.259	0.325	0.555748	10
Hspa5	3.38E-05	0.319364	0.718	0.548	0.563361	10
Ppp1cb	3.42E-05	-0.42264	0.207	0.292	0.571205	10
Tecpr1	3.43E-05	0.342865	0.19	0.076	0.57286	10
Ube2j1	3.45E-05	0.361667	0.195	0.079	0.575232	10
Mphosph6	3.45E-05	-0.38732	0.075	0.141	0.575551	10
Id2	3.46E-05	-0.50055	0.356	0.487	0.577936	10
Aspm	3.49E-05	-0.52719	0.046	0.143	0.582125	10
Nt5c3	3.55E-05	-0.45999	0.046	0.15	0.592426	10
Twf1	3.55E-05	0.380109	0.27	0.135	0.592729	10
Jmy	3.58E-05	-0.50763	0.063	0.187	0.597422	10
Tulp4	3.59E-05	-0.51281	0.098	0.205	0.598102	10
Mff	3.59E-05	-0.45356	0.172	0.285	0.599027	10
Stard3	3.72E-05	0.350246	0.126	0.045	0.620661	10
Ccdc25	3.77E-05	-0.36563	0.069	0.184	0.628154	10
Psme4	3.77E-05	-0.49109	0.138	0.285	0.628938	10
Dync1i2	3.78E-05	-0.30893	0.534	0.568	0.631035	10
Smim15	3.78E-05	0.446352	0.236	0.119	0.631193	10
Snrpb2	3.79E-05	-0.32343	0.339	0.384	0.631571	10
Zfp451	3.83E-05	-0.48712	0.057	0.171	0.639549	10
Zcrb1	3.87E-05	-0.45157	0.236	0.372	0.645085	10
Bcap29	3.87E-05	0.402448	0.247	0.121	0.645634	10
Sqle	3.87E-05	-0.447	0.029	0.121	0.646069	10
Smchd1	3.89E-05	-0.51282	0.155	0.274	0.64932	10
Cbx3	3.93E-05	-0.38452	0.138	0.275	0.655359	10
Sec62	3.95E-05	-0.30078	0.385	0.406	0.659287	10
Gpr56	3.97E-05	-0.43666	0.149	0.223	0.661622	10
Ube2c	3.99E-05	-0.68689	0.149	0.27	0.66519	10
Polr3k	4.04E-05	-0.49106	0.132	0.272	0.673199	10
Eif4a3	4.07E-05	-0.25275	0.333	0.355	0.679406	10
Papola	4.08E-05	-0.41117	0.293	0.402	0.680331	10
Prpf19	4.1E-05	-0.45737	0.19	0.307	0.683683	10
Sox18	4.1E-05	-0.4883	0.04	0.148	0.683958	10
Scd2	4.1E-05	0.346264	0.46	0.295	0.683966	10
Cnep1r1	4.11E-05	-0.2875	0.103	0.132	0.684752	10
Rbm6	4.14E-05	-0.44845	0.138	0.224	0.690534	10
Mis18bp1	4.18E-05	-0.39802	0.063	0.161	0.698014	10
Aurkb	4.23E-05	-0.36405	0.04	0.144	0.705193	10
G3bp1	4.23E-05	-0.40429	0.23	0.328	0.706104	10
Mcm2	4.24E-05	-0.47552	0.075	0.205	0.707718	10
Gnb4	4.29E-05	-0.41961	0.017	0.105	0.716096	10
Phb2	4.34E-05	-0.3994	0.259	0.333	0.723913	10
Deb1	4.36E-05	-0.44093	0.063	0.168	0.727728	10
A930011O	4.4E-05	-0.55888	0.023	0.115	0.734389	10
Zfp131	4.41E-05	-0.4963	0.086	0.202	0.734759	10
Rab3a	4.42E-05	-0.5623	0.046	0.151	0.738024	10
Ccdc28b	4.43E-05	-0.49703	0.057	0.163	0.738658	10

Trip11	4.5E-05	-0.50317	0.063	0.154	0.750675	10
Paxbp1	4.52E-05	-0.46032	0.144	0.26	0.753357	10
Prmt1	4.54E-05	-0.47071	0.092	0.225	0.757227	10
Nktr	4.55E-05	-0.50527	0.247	0.381	0.759368	10
5031439G(	4.57E-05	0.280495	0.126	0.041	0.761639	10
Mettl9	4.6E-05	-0.44439	0.19	0.301	0.766915	10
Cul5	4.63E-05	-0.49505	0.167	0.316	0.771678	10
Eif4g1	4.66E-05	-0.47098	0.236	0.353	0.776455	10
Cep78	4.68E-05	-0.43598	0.034	0.141	0.780256	10
Eif3g	4.69E-05	-0.39326	0.31	0.403	0.781662	10
Iws1	4.73E-05	-0.49156	0.109	0.216	0.789722	10
D19Bwg13	4.77E-05	-0.45479	0.178	0.274	0.796186	10
Mak16	4.82E-05	-0.4896	0.098	0.226	0.804426	10
Fnbp1	4.9E-05	0.296689	0.172	0.072	0.817117	10
Tpr	5.01E-05	-0.35844	0.454	0.554	0.835719	10
Stau2	5.04E-05	-0.48815	0.057	0.151	0.841363	10
Cd63-ps	5.06E-05	0.297539	0.241	0.117	0.843766	10
Fhl1	5.08E-05	-0.40442	0.017	0.106	0.846867	10
4921524J1	5.08E-05	-0.45614	0.04	0.15	0.846991	10
Clcn4-2	5.11E-05	-0.50795	0.144	0.252	0.852391	10
Igsf8	5.25E-05	-0.4988	0.132	0.27	0.876075	10
Irs1	5.29E-05	-0.37954	0.023	0.119	0.882206	10
Dync2h1	5.29E-05	-0.4523	0.023	0.116	0.882411	10
Bzw1	5.33E-05	-0.36229	0.264	0.36	0.888549	10
Sh3bp2	5.36E-05	0.280976	0.126	0.041	0.894153	10
Irf2bp2	5.37E-05	0.381191	0.213	0.093	0.896003	10
Bex4	5.4E-05	-0.40751	0.029	0.128	0.899973	10
Jund	5.46E-05	-0.48848	0.207	0.353	0.911048	10
Fabp7	5.51E-05	-0.73492	0.149	0.122	0.919781	10
Ppil4	5.53E-05	-0.50804	0.115	0.25	0.923164	10
Sobp	5.54E-05	-0.47377	0.029	0.128	0.924557	10
Hspa9	5.6E-05	-0.43296	0.236	0.373	0.933882	10
Cyb5	5.62E-05	0.254921	0.328	0.185	0.938091	10
Gtl3	5.67E-05	-0.43825	0.098	0.185	0.945471	10
Clybl	5.73E-05	-0.4561	0.029	0.117	0.955798	10
Pdxp	5.8E-05	-0.42037	0.023	0.114	0.968018	10
Nup62	5.81E-05	-0.39829	0.098	0.171	0.968848	10
Kif5a	5.98E-05	-0.47003	0.029	0.129	0.998186	10
Palm	6.06E-05	-0.42758	0.034	0.14	1	10
Acat2	6.15E-05	-0.46952	0.069	0.183	1	10
Zranb1	6.18E-05	-0.48775	0.092	0.209	1	10
Dzip3	6.21E-05	-0.47348	0.063	0.168	1	10
Klf7	6.31E-05	-0.49491	0.144	0.263	1	10
Pomp	6.42E-05	0.303133	0.58	0.412	1	10
Ctdspl2	6.44E-05	-0.41887	0.04	0.141	1	10
Snap47	6.44E-05	-0.44557	0.057	0.141	1	10
Prpf40b	6.5E-05	-0.44085	0.04	0.147	1	10
Pura	6.55E-05	-0.44383	0.195	0.287	1	10

Zfp148	6.58E-05	-0.50176	0.172	0.285	1	10
Uhrf1	6.6E-05	-0.42543	0.04	0.144	1	10
Hsbp1	6.68E-05	-0.34471	0.489	0.595	1	10
Myl12a	6.79E-05	0.339522	0.477	0.327	1	10
Usp12	6.8E-05	0.279819	0.109	0.031	1	10
Cpsf6	6.85E-05	-0.41757	0.149	0.292	1	10
Ttc4	6.86E-05	-0.47549	0.08	0.18	1	10
Sart3	6.89E-05	-0.47472	0.126	0.226	1	10
Acaa2	6.97E-05	0.330253	0.115	0.034	1	10
Tm9sf3	7.05E-05	0.360993	0.506	0.342	1	10
Tra2a	7.06E-05	-0.45157	0.253	0.393	1	10
Dync1li2	7.07E-05	-0.47546	0.161	0.26	1	10
Dtl	7.08E-05	-0.40793	0.04	0.146	1	10
Csnk2b	7.13E-05	-0.39888	0.126	0.2	1	10
Ahsa2	7.18E-05	-0.44455	0.109	0.207	1	10
Cfp	7.43E-05	0.445411	0.109	0.042	1	10
Jarid2	7.45E-05	-0.48417	0.063	0.18	1	10
Spen	7.57E-05	-0.4711	0.121	0.203	1	10
Srsf6	7.69E-05	-0.30044	0.374	0.446	1	10
Dnajb11	7.7E-05	0.302334	0.299	0.161	1	10
Vdac2	7.78E-05	0.257082	0.511	0.347	1	10
Cdc42bpa	7.94E-05	-0.47888	0.046	0.137	1	10
Ap3b1	8.02E-05	0.320255	0.247	0.121	1	10
Lsm3	8.08E-05	-0.3863	0.241	0.359	1	10
Chmp4b	8.35E-05	0.349558	0.305	0.213	1	10
Ick	8.37E-05	-0.40684	0.04	0.147	1	10
Gna13	8.52E-05	0.28004	0.247	0.127	1	10
Psmd7	8.61E-05	-0.35598	0.385	0.48	1	10
Srpk1	8.71E-05	-0.45443	0.149	0.247	1	10
Tpm1	8.73E-05	-0.51598	0.098	0.191	1	10
Sfswap	8.84E-05	-0.44686	0.155	0.24	1	10
Nt5dc2	8.87E-05	-0.4986	0.109	0.238	1	10
Sipa1l2	9.02E-05	0.296948	0.149	0.056	1	10
Rsu1	9.3E-05	0.34214	0.23	0.117	1	10
Mocs2	9.33E-05	-0.30038	0.172	0.195	1	10
Akap8	9.35E-05	-0.3783	0.172	0.233	1	10
Dot1l	9.41E-05	-0.49111	0.149	0.28	1	10
Fam216a	9.6E-05	-0.40328	0.057	0.174	1	10
Ntrk3	9.65E-05	-0.44999	0.046	0.156	1	10
Prps1	9.71E-05	-0.41431	0.04	0.143	1	10
Tra2b	9.72E-05	-0.39279	0.31	0.405	1	10
Tob1	9.73E-05	-0.46334	0.052	0.155	1	10
Slc39a10	9.74E-05	-0.46971	0.08	0.195	1	10
Bod1l	9.74E-05	-0.41921	0.316	0.379	1	10
5830418K(	9.76E-05	-0.47727	0.063	0.169	1	10
Paics	9.79E-05	-0.40455	0.305	0.414	1	10
Bcat1	9.9E-05	-0.42133	0.029	0.121	1	10
Tomm70a	9.91E-05	-0.42169	0.155	0.229	1	10

Tceal3	0.000101	-0.43282	0.023	0.111	1	10
Mapre1	0.000101	-0.3116	0.42	0.518	1	10
Ash1l	0.000102	-0.5183	0.149	0.278	1	10
Sv2b	0.000103	-0.4308	0.017	0.103	1	10
Uqcrfs1	0.000103	-0.38275	0.282	0.388	1	10
Zeb2	0.000104	0.40521	0.374	0.247	1	10
Stx4a	0.000105	0.339111	0.276	0.153	1	10
Rbmxl1	0.000105	-0.34023	0.098	0.153	1	10
Phf5a	0.000106	-0.40238	0.201	0.332	1	10
Cenpk	0.000107	-0.44062	0.04	0.145	1	10
Kidins220	0.000107	-0.34012	0.115	0.14	1	10
Krr1	0.000107	-0.43371	0.052	0.152	1	10
Smoc1	0.000109	-0.42254	0.034	0.135	1	10
Cadm4	0.000111	-0.40936	0.023	0.114	1	10
Btf3	0.000111	-0.39643	0.253	0.35	1	10
Arhgef2	0.000111	-0.34788	0.247	0.282	1	10
Sarnp	0.000111	-0.46468	0.115	0.239	1	10
Tada2a	0.000113	-0.36782	0.017	0.101	1	10
Actl6a	0.000118	-0.43784	0.109	0.227	1	10
Gm13092	0.00012	-0.34009	0.034	0.104	1	10
Vars	0.000121	-0.41066	0.08	0.167	1	10
Nek7	0.000122	0.2845	0.236	0.115	1	10
Fbxl5	0.000127	-0.29373	0.086	0.118	1	10
Scp2	0.000129	0.300088	0.172	0.072	1	10
Nav2	0.00013	-0.38001	0.046	0.105	1	10
Fam98a	0.00013	-0.44005	0.052	0.146	1	10
Ndufb11	0.000131	-0.2951	0.414	0.462	1	10
Hsph1	0.000131	-0.47558	0.103	0.228	1	10
Grik5	0.000131	-0.40487	0.023	0.11	1	10
Kif15	0.000133	-0.45364	0.063	0.181	1	10
Lasp1	0.000133	0.251724	0.195	0.09	1	10
1110051M	0.000135	-0.3832	0.023	0.101	1	10
Lphn1	0.000135	-0.40175	0.023	0.114	1	10
Mga	0.000136	-0.47084	0.092	0.191	1	10
Pkia	0.000136	-0.47954	0.04	0.134	1	10
Nedd8	0.000138	-0.2613	0.523	0.542	1	10
Rb1cc1	0.000138	-0.4937	0.144	0.235	1	10
Rbm26	0.00014	-0.47433	0.178	0.298	1	10
Agpat3	0.000142	0.257804	0.247	0.128	1	10
Lias	0.000142	-0.35567	0.098	0.151	1	10
Dhx15	0.000143	-0.3887	0.316	0.417	1	10
Chkb	0.000143	-0.35421	0.103	0.157	1	10
Rhno1	0.000144	-0.41167	0.04	0.139	1	10
Sfr1	0.000144	-0.39117	0.27	0.395	1	10
Angptl2	0.000146	-0.38578	0.034	0.117	1	10
Gna11	0.000146	-0.43475	0.04	0.138	1	10
Txndc17	0.000146	0.288488	0.362	0.241	1	10
Hnrnpl	0.000146	-0.33059	0.316	0.363	1	10

Gphn	0.000148	-0.44842	0.052	0.142	1	10
Gabbr1	0.000148	-0.45678	0.052	0.141	1	10
Hprt	0.000148	0.298932	0.328	0.186	1	10
Tmem132a	0.000148	-0.38513	0.017	0.103	1	10
Cep290	0.000148	-0.4955	0.057	0.143	1	10
Mroh2a	0.000152	-0.52487	0.029	0.114	1	10
Cenpj	0.000154	-0.39597	0.046	0.142	1	10
Asrgl1	0.000155	-0.44619	0.04	0.134	1	10
Psmd14	0.000155	-0.2999	0.27	0.351	1	10
2810004N1	0.000155	-0.4718	0.149	0.263	1	10
Psmc1	0.000156	-0.41119	0.276	0.42	1	10
Slc38a1	0.000158	-0.4671	0.144	0.271	1	10
Snrpg	0.000159	-0.4353	0.213	0.336	1	10
Klc4	0.000159	0.275105	0.149	0.058	1	10
Vamp3	0.000161	0.296565	0.178	0.078	1	10
Dnm1l	0.000161	-0.47207	0.172	0.293	1	10
Pet100	0.000162	0.25003	0.316	0.186	1	10
Tbrg1	0.000162	-0.47107	0.115	0.243	1	10
Celf1	0.000165	-0.39465	0.293	0.38	1	10
Shd	0.000173	-0.42499	0.029	0.11	1	10
Dazap1	0.000175	-0.45889	0.149	0.273	1	10
U2af1l4	0.000175	-0.42558	0.115	0.196	1	10
Taok1	0.000176	-0.33159	0.19	0.225	1	10
2410089E0	0.000176	-0.43356	0.04	0.131	1	10
Ddx23	0.000177	-0.32701	0.098	0.207	1	10
Eif3i	0.000179	-0.25378	0.431	0.462	1	10
Brcc3	0.00018	-0.43283	0.063	0.176	1	10
Fbxw7	0.00018	-0.42319	0.046	0.12	1	10
Atxn7l2	0.00018	-0.39682	0.029	0.103	1	10
Prdm2	0.000181	-0.42572	0.04	0.133	1	10
Dctn2	0.000181	-0.29293	0.299	0.321	1	10
Kif23	0.000182	-0.49933	0.092	0.215	1	10
Eif3f	0.000187	-0.28816	0.575	0.631	1	10
Ttc14	0.00019	-0.47439	0.155	0.262	1	10
Acadl	0.000191	0.280207	0.155	0.063	1	10
Ccnl2	0.000192	-0.33953	0.276	0.321	1	10
Erc1	0.000192	-0.43641	0.04	0.111	1	10
Pvrl3	0.000193	-0.42094	0.029	0.123	1	10
Trim33	0.000193	-0.44878	0.052	0.15	1	10
Irak1bp1	0.000196	-0.39915	0.04	0.14	1	10
Ankhd1	0.000198	-0.37264	0.08	0.182	1	10
Fam155a	0.000199	-0.3687	0.029	0.116	1	10
Mrps30	0.000199	-0.26434	0.075	0.107	1	10
Qdpr	0.0002	-0.37558	0.207	0.268	1	10
Dnmt1	0.0002	-0.43973	0.149	0.279	1	10
Cct4	0.000201	-0.30241	0.322	0.382	1	10
Cyp51	0.000201	-0.40011	0.04	0.142	1	10
Dnaja2	0.000201	-0.27412	0.253	0.334	1	10

Rps6kb1	0.000202	-0.43179	0.167	0.279	1	10
Chaf1a	0.000203	-0.3812	0.069	0.169	1	10
Arl2bp	0.000204	-0.41204	0.092	0.218	1	10
Utp3	0.000205	-0.34898	0.195	0.315	1	10
Rps7	0.000207	-0.3791	0.299	0.4	1	10
Hnrnpll	0.000207	-0.40291	0.092	0.171	1	10
Slc3a2	0.00021	0.298928	0.483	0.321	1	10
5430416N(	0.000212	-0.40183	0.04	0.141	1	10
Mrps22	0.000213	-0.40656	0.052	0.134	1	10
Stk25	0.000214	-0.43403	0.098	0.185	1	10
Adk	0.000215	-0.38726	0.057	0.166	1	10
Trappc4	0.000215	-0.41427	0.149	0.225	1	10
Mrpl11	0.000216	-0.44022	0.132	0.238	1	10
Mat2a	0.000216	-0.39209	0.282	0.42	1	10
Gtf2f1	0.000218	-0.4191	0.236	0.344	1	10
H2-Ke2	0.000225	-0.38705	0.103	0.196	1	10
Nabp2	0.000227	-0.38031	0.069	0.162	1	10
Uhrf2	0.000227	-0.41267	0.069	0.151	1	10
Ankrd46	0.000234	-0.43021	0.075	0.161	1	10
Rftn1	0.000234	0.354569	0.195	0.088	1	10
Zfp536	0.000234	-0.38604	0.023	0.111	1	10
Psmc4	0.000238	-0.41229	0.207	0.322	1	10
Rdh5	0.000238	-0.40499	0.034	0.126	1	10
Sptssa	0.000239	0.306777	0.351	0.246	1	10
Agtpbp1	0.00024	-0.40648	0.023	0.102	1	10
Ccdc66	0.000242	-0.40485	0.063	0.173	1	10
Rab1	0.000243	0.278358	0.511	0.36	1	10
Tbc1d16	0.000243	-0.32455	0.161	0.196	1	10
Nipbl	0.000244	-0.34116	0.437	0.492	1	10
Cox7c	0.000247	-0.27888	0.241	0.284	1	10
Kpnb1	0.000248	-0.31239	0.259	0.3	1	10
1700021F(	0.000249	-0.37964	0.075	0.141	1	10
Zmym5	0.000251	-0.473	0.126	0.233	1	10
Pcdha2	0.000251	-0.41416	0.029	0.119	1	10
Sparcl1	0.000254	-0.7628	0.098	0.208	1	10
Zfp512	0.000255	-0.40613	0.029	0.119	1	10
Cask	0.000256	-0.43579	0.034	0.11	1	10
Rpl36	0.000257	-0.36391	0.029	0.109	1	10
Frmd4a	0.000258	-0.38587	0.247	0.309	1	10
Rad51ap1	0.000259	-0.30378	0.063	0.167	1	10
Wdr12	0.000261	-0.37909	0.098	0.176	1	10
Acsl3	0.000262	-0.40011	0.046	0.147	1	10
Stub1	0.000263	-0.32462	0.276	0.347	1	10
Alas1	0.000264	0.26194	0.144	0.056	1	10
0610009D(	0.000268	-0.25612	0.305	0.384	1	10
Copg1	0.000268	-0.36462	0.092	0.153	1	10
Zfp260	0.000273	-0.44822	0.092	0.206	1	10
Gdi1	0.000277	-0.47604	0.115	0.231	1	10

Nudcd3	0.000285	-0.35912	0.121	0.17	1	10
Rangap1	0.000286	-0.42892	0.161	0.257	1	10
Mki67ip	0.000288	-0.37527	0.115	0.245	1	10
Srsf4	0.000288	-0.37672	0.149	0.28	1	10
Hs6st1	0.00029	0.344255	0.115	0.039	1	10
Gripap1	0.00029	-0.44118	0.075	0.189	1	10
Snrnp27	0.000291	-0.40693	0.207	0.311	1	10
Rheb	0.000291	-0.33378	0.213	0.274	1	10
Tial1	0.000294	-0.28967	0.316	0.348	1	10
Uri1	0.000296	-0.34681	0.115	0.21	1	10
Socs2	0.000296	-0.40328	0.04	0.139	1	10
Pdrg1	0.000297	-0.43977	0.138	0.22	1	10
Vps4a	0.000299	-0.34733	0.075	0.137	1	10
Arid2	0.0003	-0.42529	0.132	0.242	1	10
Cntln	0.0003	-0.46009	0.063	0.173	1	10
Sf1	0.000301	-0.4018	0.155	0.237	1	10
Pik3ip1	0.000301	-0.32121	0.034	0.117	1	10
Rgs12	0.000302	-0.44071	0.052	0.147	1	10
Clcn3	0.000303	-0.34308	0.201	0.273	1	10
Tbca	0.000304	-0.29557	0.362	0.42	1	10
Fopnl	0.000306	-0.38328	0.086	0.203	1	10
2700049A(	0.000309	-0.42046	0.04	0.122	1	10
Pnrc2	0.000309	-0.35679	0.149	0.208	1	10
Rrs1	0.00031	-0.41625	0.086	0.19	1	10
Ndufs2	0.000315	-0.27567	0.322	0.369	1	10
Cdca2	0.000315	-0.38574	0.034	0.129	1	10
Dnajc7	0.000317	-0.32927	0.236	0.281	1	10
Zfr	0.000318	-0.41012	0.259	0.354	1	10
Ppil1	0.000321	-0.31998	0.04	0.124	1	10
Rufy1	0.000323	0.292333	0.138	0.052	1	10
Tmem70	0.000325	-0.31247	0.069	0.119	1	10
Ccnb1	0.000331	-0.47213	0.046	0.14	1	10
Pard6g	0.000333	-0.38753	0.023	0.105	1	10
Dhx40	0.000334	-0.40827	0.063	0.148	1	10
R3hdm2	0.000337	-0.38727	0.092	0.168	1	10
Brd9	0.000337	-0.4405	0.109	0.214	1	10
Msi2	0.000339	-0.3923	0.075	0.188	1	10
Tex261	0.00034	0.252429	0.224	0.114	1	10
Rabgap1	0.000346	-0.47372	0.121	0.225	1	10
Zfp608	0.000346	-0.46806	0.08	0.186	1	10
Sae1	0.000349	-0.388	0.19	0.279	1	10
Eif4g3	0.000351	-0.30015	0.351	0.384	1	10
Hmmr	0.000351	-0.51918	0.069	0.177	1	10
Cdk14	0.000352	-0.37934	0.023	0.104	1	10
Maged2	0.000354	-0.42986	0.08	0.167	1	10
Syt13	0.000355	-0.35818	0.034	0.128	1	10
Hmgcs1	0.000355	-0.46382	0.057	0.152	1	10
Jam3	0.000357	-0.3327	0.04	0.136	1	10

Rnf220	0.000367	-0.42289	0.149	0.236	1	10
Tmem55b	0.000368	0.317459	0.224	0.111	1	10
Rps8	0.000368	-0.31223	0.31	0.397	1	10
Ccdc59	0.000373	-0.39363	0.213	0.314	1	10
Gmps	0.000375	-0.3397	0.092	0.211	1	10
Eef1g	0.000377	-0.36384	0.259	0.335	1	10
Ppp1ca	0.00038	-0.30276	0.42	0.483	1	10
Rftn2	0.000382	-0.37125	0.04	0.124	1	10
Otub1	0.000382	-0.26735	0.138	0.161	1	10
Bms1	0.000386	-0.26778	0.098	0.183	1	10
Wdr61	0.000388	-0.39759	0.103	0.181	1	10
Rbm4b	0.000389	-0.40772	0.046	0.141	1	10
Rcor3	0.00039	-0.35734	0.04	0.107	1	10
Ing1	0.000396	-0.41208	0.103	0.184	1	10
Rbm22	0.000398	-0.39389	0.126	0.223	1	10
A830080D(	0.000398	-0.38898	0.029	0.114	1	10
Rrm2	0.0004	-0.39249	0.092	0.205	1	10
B9d2	0.000402	-0.31688	0.052	0.101	1	10
Pafah1b2	0.000403	-0.39826	0.155	0.255	1	10
Snapc3	0.000403	-0.37688	0.057	0.125	1	10
Nisch	0.000404	-0.37816	0.23	0.301	1	10
Aimp1	0.000406	-0.39281	0.195	0.28	1	10
Vmp1	0.000407	0.283674	0.224	0.115	1	10
Tdp1	0.000407	-0.32605	0.023	0.103	1	10
Ssbp3	0.000407	-0.433	0.063	0.168	1	10
Ctbp2	0.000408	-0.42112	0.126	0.215	1	10
Dusp11	0.00041	-0.25491	0.19	0.201	1	10
Slc35b1	0.00041	-0.35973	0.224	0.294	1	10
Trim28	0.000416	-0.36213	0.287	0.362	1	10
Scaper	0.000416	-0.44324	0.034	0.111	1	10
Gria4	0.000418	-0.44935	0.046	0.125	1	10
Ddx55	0.000421	-0.43269	0.069	0.166	1	10
Rnaseh2c	0.000422	-0.4168	0.253	0.382	1	10
Anapc15	0.000423	0.259839	0.172	0.079	1	10
Gli1	0.000424	-0.37059	0.034	0.108	1	10
Tnrc6a	0.000426	-0.42725	0.213	0.3	1	10
Actr6	0.000426	-0.37718	0.029	0.108	1	10
Rtn2	0.000428	-0.38219	0.029	0.116	1	10
Ssr3	0.00043	-0.32639	0.362	0.425	1	10
Naa15	0.000432	-0.38747	0.276	0.392	1	10
Mzt1	0.000435	-0.40066	0.057	0.152	1	10
Odc1	0.000436	-0.34262	0.023	0.101	1	10
Cnot2	0.000445	-0.43147	0.092	0.186	1	10
0610007P1	0.000445	-0.38478	0.063	0.163	1	10
Acd	0.000448	-0.44017	0.063	0.162	1	10
Taf2	0.000455	-0.3553	0.046	0.105	1	10
Iscu	0.000456	0.261957	0.167	0.073	1	10
Cenpq	0.000458	-0.40278	0.063	0.161	1	10

Taf7	0.000465	-0.40221	0.057	0.133	1	10
Meis3	0.000466	0.309808	0.172	0.076	1	10
Ntan1	0.000469	-0.42288	0.092	0.187	1	10
Ssbp2	0.000471	-0.38511	0.04	0.126	1	10
Rbbp8	0.000487	-0.34773	0.04	0.123	1	10
Dhx32	0.000488	-0.42795	0.098	0.201	1	10
Fcho2	0.00049	0.309974	0.172	0.076	1	10
Ckap2	0.000495	-0.43764	0.075	0.164	1	10
Ubqln1	0.0005	-0.34066	0.247	0.305	1	10
Rnf146	0.000501	-0.38697	0.046	0.144	1	10
Lancl2	0.000504	-0.36929	0.029	0.105	1	10
Phf20l1	0.000505	-0.45621	0.236	0.341	1	10
Nsmce4a	0.000511	-0.43155	0.109	0.224	1	10
Dlgap5	0.000513	-0.3085	0.029	0.112	1	10
Trio	0.000514	-0.37513	0.063	0.125	1	10
1700020I1	0.000516	-0.35305	0.201	0.275	1	10
Nxf1	0.000521	-0.41027	0.098	0.186	1	10
Atad3a	0.000521	-0.29255	0.092	0.126	1	10
Gabarapl1	0.000525	-0.43983	0.103	0.217	1	10
Dvl1	0.000525	-0.3007	0.092	0.126	1	10
Ogt	0.000528	-0.33924	0.167	0.211	1	10
Rpap3	0.000528	-0.42762	0.052	0.145	1	10
Usp11	0.000533	-0.2966	0.023	0.101	1	10
Mrfap1	0.000544	-0.31762	0.293	0.404	1	10
Mta1	0.000544	-0.34631	0.046	0.104	1	10
Abt1	0.000546	-0.35572	0.04	0.127	1	10
Fdft1	0.000547	-0.3503	0.023	0.101	1	10
Gm5914	0.000548	-0.36429	0.04	0.112	1	10
Sqstm1	0.000555	0.255006	0.356	0.225	1	10
Cenpc1	0.000557	-0.41842	0.057	0.156	1	10
Lsm4	0.000563	-0.34223	0.402	0.488	1	10
Exosc1	0.000565	-0.3916	0.086	0.163	1	10
Baz2b	0.000567	-0.38977	0.282	0.365	1	10
Vps16	0.000575	-0.26084	0.069	0.101	1	10
Wbp11	0.000582	-0.37657	0.218	0.297	1	10
Ddx3x	0.000582	-0.25981	0.437	0.466	1	10
Emc6	0.00059	-0.27402	0.23	0.276	1	10
Zfp207	0.000592	-0.37468	0.282	0.37	1	10
Hist3h2a	0.000593	-0.41485	0.08	0.144	1	10
Imp3	0.000594	-0.42059	0.132	0.248	1	10
Ash2l	0.000599	-0.39393	0.126	0.216	1	10
Fip1l1	0.0006	-0.40845	0.201	0.309	1	10
Zkscan1	0.000601	-0.41296	0.052	0.152	1	10
Sh3bp5	0.000608	0.262053	0.201	0.102	1	10
Park7	0.00061	-0.32136	0.466	0.546	1	10
Cdc26	0.000611	-0.38308	0.063	0.144	1	10
Tceb2	0.000616	-0.27489	0.466	0.514	1	10
Sec61a2	0.000619	-0.38389	0.034	0.116	1	10

Bmpr1a	0.000629	-0.32761	0.023	0.103	1	10
Pja2	0.00063	-0.44614	0.132	0.235	1	10
Gm26735	0.00063	-0.43381	0.075	0.181	1	10
Hmgxb4	0.000633	-0.40252	0.057	0.155	1	10
Ift57	0.000635	-0.34749	0.023	0.103	1	10
Zc3h7b	0.000646	-0.30631	0.086	0.128	1	10
Ddit4	0.000646	-0.37405	0.046	0.105	1	10
Psma6	0.000648	-0.27765	0.391	0.452	1	10
Nop14	0.000656	-0.39392	0.132	0.218	1	10
Igf1r	0.000657	-0.36776	0.04	0.114	1	10
Srm	0.000657	-0.41296	0.109	0.192	1	10
Anapc11	0.000662	-0.3197	0.241	0.329	1	10
Rai1	0.000669	-0.38595	0.063	0.16	1	10
Ptges3	0.000671	-0.38737	0.126	0.212	1	10
2810008D(	0.000672	-0.28124	0.103	0.127	1	10
Epb4.1	0.000701	-0.40439	0.109	0.23	1	10
Cox5b	0.000704	-0.27748	0.31	0.357	1	10
Phf20	0.000715	-0.44348	0.121	0.221	1	10
Ep400	0.000735	-0.41079	0.08	0.181	1	10
Lrrc45	0.000746	-0.33134	0.034	0.102	1	10
Cstf3	0.000753	-0.37926	0.052	0.14	1	10
Pebp1	0.00076	-0.36517	0.138	0.243	1	10
Gng2	0.000766	-0.33887	0.31	0.396	1	10
Raf1	0.000769	-0.41585	0.086	0.187	1	10
Ccdc47	0.000789	-0.25307	0.236	0.244	1	10
Nip7	0.000797	-0.37725	0.086	0.163	1	10
Ctnnbl1	0.000797	-0.41849	0.086	0.185	1	10
Dnttip2	0.000807	-0.43967	0.201	0.325	1	10
Zfp386	0.000809	-0.40168	0.052	0.139	1	10
Ndc80	0.000812	-0.26415	0.034	0.115	1	10
Chchd1	0.000818	-0.35092	0.328	0.401	1	10
Sbno1	0.000826	-0.39865	0.236	0.352	1	10
Ndufv1	0.000828	-0.40554	0.132	0.237	1	10
0610011F(	0.00083	-0.36557	0.063	0.127	1	10
Zfand5	0.000836	-0.33771	0.299	0.416	1	10
Mier3	0.000837	-0.34771	0.034	0.12	1	10
Gga1	0.000838	0.259432	0.115	0.044	1	10
Dhx30	0.00084	-0.37807	0.04	0.124	1	10
Kif2c	0.000841	-0.35155	0.029	0.108	1	10
Ociad1	0.000843	-0.27645	0.322	0.358	1	10
Gatad2b	0.000858	-0.34477	0.034	0.121	1	10
R3hcc1	0.00086	-0.38379	0.063	0.16	1	10
Elp2	0.000861	-0.37165	0.172	0.243	1	10
Cops5	0.000869	-0.40949	0.126	0.223	1	10
Uba1	0.00087	-0.38303	0.195	0.272	1	10
Wnk1	0.000876	0.344417	0.305	0.182	1	10
Hells	0.000877	-0.41303	0.115	0.214	1	10
Vimp	0.000891	0.307251	0.316	0.188	1	10

5730455P1	0.000892	-0.40246	0.052	0.128	1	10
Hspd1	0.000895	-0.36142	0.253	0.365	1	10
Slc7a5	0.000899	-0.37219	0.034	0.115	1	10
Hdac3	0.000906	-0.37818	0.075	0.153	1	10
Papss1	0.00091	-0.38824	0.063	0.147	1	10
Ccar2	0.000928	-0.35414	0.029	0.103	1	10
Usp3	0.000929	-0.3772	0.075	0.142	1	10
Rps18	0.000938	-0.34768	0.322	0.429	1	10
Pfdn5	0.000953	0.288135	0.644	0.519	1	10
Med11	0.000973	0.356453	0.121	0.048	1	10
Nae1	0.000975	-0.42095	0.109	0.225	1	10
1500012F0	0.000979	-0.34193	0.322	0.399	1	10
Cby1	0.000981	-0.35972	0.052	0.127	1	10
Alcam	0.000986	-0.31397	0.046	0.135	1	10
Nudc	0.000993	-0.40456	0.161	0.282	1	10
Xrn1	0.000998	-0.38568	0.034	0.111	1	10
Cdc27	0.001	-0.29333	0.069	0.112	1	10
Topors	0.001004	-0.39492	0.098	0.183	1	10
Elp4	0.001012	-0.32517	0.046	0.103	1	10
Bap1	0.001012	-0.3582	0.029	0.109	1	10
Pick1	0.001024	-0.3815	0.057	0.128	1	10
Tsen34	0.001029	-0.41087	0.126	0.234	1	10
Txndc9	0.001038	-0.40167	0.121	0.212	1	10
Upf2	0.001043	-0.42434	0.075	0.155	1	10
Mbtd1	0.001044	-0.40922	0.132	0.215	1	10
Nup85	0.001047	-0.37847	0.109	0.227	1	10
Tom1l1	0.001047	-0.33768	0.04	0.104	1	10
Gsto1	0.001054	0.304583	0.236	0.127	1	10
Mark3	0.001055	-0.41365	0.126	0.236	1	10
Ctps	0.001058	-0.35297	0.046	0.138	1	10
Ccdc18	0.001058	-0.35942	0.034	0.116	1	10
Wdr43	0.00106	-0.3903	0.138	0.242	1	10
Psme1	0.001081	0.288624	0.287	0.172	1	10
2410006H1	0.001083	-0.37243	0.356	0.46	1	10
Cep170	0.001083	-0.41143	0.213	0.306	1	10
Eif4h	0.001109	-0.30022	0.391	0.471	1	10
Ostc	0.001111	-0.33539	0.201	0.274	1	10
Incenp	0.001118	-0.40282	0.184	0.276	1	10
Ensa	0.001128	-0.36619	0.155	0.228	1	10
Ube2g1	0.001131	-0.28879	0.057	0.144	1	10
Pdhb	0.001137	-0.3635	0.109	0.182	1	10
Pin1	0.001146	-0.39463	0.121	0.238	1	10
Pcnt	0.001165	-0.40283	0.086	0.175	1	10
March7	0.001165	-0.31259	0.195	0.239	1	10
Myl6	0.001166	0.282794	0.328	0.201	1	10
Sfxn1	0.00117	-0.38608	0.172	0.25	1	10
2700081O1	0.001184	-0.389	0.046	0.111	1	10
Gars	0.001188	-0.39832	0.126	0.228	1	10

Bfar	0.001188	-0.41097	0.115	0.192	1	10
Ip6k2	0.001199	-0.39916	0.04	0.122	1	10
Tspan3	0.001199	-0.29325	0.339	0.429	1	10
Strap	0.001215	-0.38778	0.19	0.309	1	10
4632415L(	0.00122	-0.36894	0.034	0.115	1	10
Tmed9	0.001234	-0.28046	0.391	0.433	1	10
C1qbp	0.001235	-0.35958	0.282	0.378	1	10
Ltbp3	0.001237	-0.3804	0.034	0.118	1	10
Galk2	0.001242	0.257862	0.126	0.052	1	10
Emc10	0.001248	-0.30861	0.241	0.316	1	10
Mrpl13	0.001248	-0.28687	0.218	0.308	1	10
Knop1	0.001249	-0.41884	0.178	0.272	1	10
Sclt1	0.001254	-0.37681	0.04	0.127	1	10
Xpo1	0.00126	-0.35865	0.144	0.228	1	10
Mnat1	0.001262	-0.31878	0.046	0.105	1	10
Gnl3	0.001268	-0.41505	0.19	0.296	1	10
Ppa1	0.001269	-0.36918	0.121	0.222	1	10
Atf4	0.001271	-0.32203	0.299	0.397	1	10
Ccndbp1	0.001296	-0.28643	0.08	0.117	1	10
Ppp2r1a	0.001311	-0.29727	0.172	0.214	1	10
Mau2	0.001311	-0.28675	0.121	0.149	1	10
Wdr33	0.001312	-0.36735	0.092	0.196	1	10
Ddx56	0.001333	-0.32379	0.04	0.102	1	10
Napg	0.001353	-0.36046	0.075	0.129	1	10
Eif2a	0.001362	-0.40804	0.126	0.224	1	10
Rpa2	0.001364	-0.41979	0.109	0.2	1	10
Slc35a1	0.001365	-0.37094	0.052	0.125	1	10
Gnb2	0.001382	-0.26854	0.443	0.484	1	10
Zmat2	0.001386	-0.29546	0.333	0.441	1	10
Sms	0.001396	-0.38678	0.057	0.137	1	10
Ammecr1l	0.001404	-0.31794	0.075	0.123	1	10
Uba2	0.001416	-0.35532	0.155	0.256	1	10
Ftsj3	0.00143	-0.39748	0.103	0.207	1	10
Gzf1	0.00144	-0.30486	0.034	0.115	1	10
Adamts1	0.001454	-0.41874	0.063	0.155	1	10
Fosb	0.001456	0.472266	0.259	0.154	1	10
Sgol1	0.001486	-0.36472	0.046	0.128	1	10
Anln	0.001503	-0.28543	0.029	0.105	1	10
Cops8	0.001511	-0.38583	0.149	0.241	1	10
Aftph	0.001521	0.262163	0.213	0.118	1	10
Parp1	0.001523	-0.38766	0.19	0.314	1	10
Erbb2ip	0.00154	0.27079	0.259	0.173	1	10
Psma4	0.001548	-0.32976	0.305	0.414	1	10
Taf1	0.001564	-0.38512	0.109	0.202	1	10
Ist1	0.001567	-0.30719	0.149	0.201	1	10
Polr1c	0.001572	-0.28902	0.132	0.219	1	10
Fmnl2	0.00158	-0.40812	0.08	0.158	1	10
Cdc40	0.00158	-0.39081	0.098	0.17	1	10

Poglut1	0.00159	-0.36772	0.057	0.131	1	10
Tmed4	0.001596	-0.2938	0.149	0.189	1	10
Bbip1	0.001603	-0.27033	0.201	0.231	1	10
Suv39h2	0.001609	-0.36252	0.046	0.132	1	10
Dck	0.001611	-0.31303	0.029	0.103	1	10
Timm50	0.001617	-0.34242	0.155	0.237	1	10
Cpsf7	0.001634	-0.37574	0.08	0.184	1	10
Tsnax	0.00165	-0.37916	0.115	0.191	1	10
Tk1	0.001667	-0.3427	0.046	0.132	1	10
1110038B1	0.001673	-0.38744	0.201	0.299	1	10
Hectd1	0.001676	-0.40119	0.121	0.203	1	10
Mmadhc	0.001683	-0.3573	0.161	0.233	1	10
Cherp	0.001685	-0.31964	0.063	0.121	1	10
Mettl14	0.001687	-0.36177	0.052	0.123	1	10
Frg1	0.001692	-0.3566	0.213	0.299	1	10
Snord104	0.001703	-0.40509	0.069	0.153	1	10
Phax	0.001705	-0.35421	0.276	0.348	1	10
Twistnb	0.001721	-0.37919	0.069	0.151	1	10
Inpp5f	0.001727	-0.35304	0.052	0.113	1	10
Taf1d	0.001741	-0.39267	0.241	0.342	1	10
Zcwpw1	0.001765	-0.27531	0.029	0.104	1	10
1500011B0	0.001772	-0.36679	0.04	0.107	1	10
4930402H1	0.00178	-0.30284	0.034	0.113	1	10
Ncor2	0.0018	-0.40239	0.08	0.169	1	10
Setd5	0.001822	-0.39	0.132	0.248	1	10
Sac3d1	0.001824	-0.26721	0.069	0.107	1	10
Snap29	0.001824	0.25179	0.155	0.073	1	10
Vegfb	0.001832	0.253028	0.213	0.117	1	10
Sumo1	0.001865	-0.38313	0.126	0.218	1	10
Nme1	0.001865	-0.32658	0.42	0.51	1	10
Tshz1	0.001869	-0.32025	0.075	0.142	1	10
Sepw1	0.001876	-0.37876	0.195	0.301	1	10
Ppid	0.001897	-0.38098	0.103	0.202	1	10
Chic2	0.001898	-0.26944	0.121	0.15	1	10
Ubp1	0.00191	-0.39415	0.08	0.17	1	10
Larp7	0.001915	-0.28461	0.207	0.312	1	10
Tmem184c	0.001923	-0.35599	0.04	0.123	1	10
Cxx1b	0.001945	-0.36218	0.04	0.121	1	10
Abl1	0.001956	0.256939	0.144	0.064	1	10
Rsbn1	0.001963	-0.3941	0.109	0.183	1	10
Fam168b	0.001965	-0.29112	0.195	0.241	1	10
Fnbp4	0.001987	-0.3619	0.161	0.277	1	10
Usp7	0.001989	-0.39632	0.161	0.265	1	10
Ccnt2	0.001995	-0.33401	0.086	0.135	1	10
Casc5	0.002003	-0.41634	0.086	0.187	1	10
Arid4a	0.002023	-0.34947	0.161	0.284	1	10
Fam107b	0.002024	-0.34025	0.04	0.103	1	10
Ndufaf2	0.002039	-0.30754	0.098	0.203	1	10

Mbd3	0.002055	-0.32931	0.218	0.291	1	10
Psme2	0.002058	0.319962	0.201	0.103	1	10
Isoc1	0.002063	-0.38077	0.098	0.181	1	10
Rab24	0.002069	-0.2904	0.178	0.215	1	10
Akap12	0.002104	-0.40229	0.034	0.112	1	10
Arid1b	0.002111	-0.26532	0.075	0.103	1	10
Stk16	0.002113	-0.31245	0.092	0.169	1	10
Uchl3	0.002147	-0.36962	0.092	0.187	1	10
Tnrc6b	0.002163	-0.30716	0.167	0.248	1	10
1110038F1	0.002167	-0.38361	0.109	0.192	1	10
Qser1	0.002179	-0.35358	0.046	0.128	1	10
Eftud2	0.0022	-0.35927	0.109	0.183	1	10
Rexo1	0.002214	-0.32944	0.144	0.212	1	10
Lrp11	0.002215	-0.29697	0.029	0.101	1	10
Aff4	0.002311	-0.3982	0.144	0.217	1	10
Asun	0.002342	-0.34475	0.034	0.107	1	10
Supt6	0.002345	-0.39289	0.086	0.158	1	10
H2afz	0.002346	-0.35355	0.132	0.232	1	10
Commd1	0.002349	-0.31425	0.218	0.299	1	10
l7Rn6	0.002353	-0.34237	0.115	0.201	1	10
Mrpl30	0.002366	-0.30797	0.236	0.309	1	10
C330027C(	0.00239	-0.36874	0.057	0.134	1	10
Kdm6b	0.002406	-0.35608	0.034	0.105	1	10
Cenpp	0.002411	-0.31568	0.04	0.115	1	10
Rpf1	0.002471	-0.38004	0.115	0.194	1	10
Lrrfip1	0.002473	0.268487	0.109	0.043	1	10
Thoc3	0.002484	-0.31519	0.069	0.159	1	10
Ddx18	0.002485	-0.28887	0.086	0.127	1	10
Palld	0.002492	0.311649	0.103	0.04	1	10
Man2a1	0.002495	0.295816	0.109	0.041	1	10
Ercc5	0.002567	-0.33977	0.063	0.112	1	10
Tomm40	0.002589	-0.28117	0.126	0.166	1	10
Pmm1	0.002611	-0.35215	0.057	0.128	1	10
Thumpd1	0.002612	-0.37849	0.086	0.166	1	10
Ogfrl1	0.002618	0.264679	0.167	0.081	1	10
Oraov1	0.002618	-0.29502	0.04	0.121	1	10
Pum1	0.002619	-0.38525	0.161	0.277	1	10
Zcchc7	0.002628	-0.30582	0.115	0.151	1	10
Ubxn6	0.002699	-0.33358	0.109	0.168	1	10
Rif1	0.002709	-0.38354	0.178	0.276	1	10
Ehbp1	0.002773	-0.36558	0.057	0.137	1	10
Senp2	0.002793	-0.31439	0.052	0.115	1	10
Hspa8	0.002799	-0.26118	0.351	0.416	1	10
Cpsf3l	0.002799	-0.26604	0.063	0.11	1	10
Trnt1	0.0028	-0.29747	0.069	0.153	1	10
Pik3ca	0.002819	-0.36123	0.103	0.163	1	10
Dcaf15	0.002858	-0.26899	0.132	0.161	1	10
Hmgcr	0.00286	-0.34822	0.046	0.13	1	10

Kdm5a	0.002911	-0.40268	0.167	0.257	1	10
Nin	0.002957	-0.3764	0.075	0.146	1	10
Csnk2a1	0.002973	-0.35966	0.132	0.245	1	10
Hnrnph2	0.002975	-0.32579	0.08	0.144	1	10
Cdc23	0.00299	-0.34501	0.052	0.135	1	10
Sod1	0.002993	-0.2865	0.374	0.442	1	10
Bhlhb9	0.002998	-0.32236	0.04	0.117	1	10
Vps29	0.003006	0.301316	0.328	0.225	1	10
Tcea1	0.003047	-0.32804	0.057	0.137	1	10
H13	0.003052	-0.31756	0.19	0.238	1	10
Bmi1	0.003053	-0.32748	0.057	0.147	1	10
Msi1	0.003082	-0.30948	0.034	0.111	1	10
Lin7c	0.003159	-0.36359	0.138	0.218	1	10
Ccdc127	0.003167	-0.29386	0.115	0.208	1	10
Rcc2	0.003173	-0.33578	0.109	0.179	1	10
Ntmt1	0.003176	-0.3329	0.057	0.144	1	10
Bola1	0.003195	-0.37662	0.086	0.173	1	10
Wbp1	0.003267	-0.36085	0.063	0.149	1	10
Dohh	0.003296	-0.34193	0.098	0.169	1	10
Bccip	0.003334	-0.2698	0.247	0.311	1	10
Mad2l1	0.003356	-0.30901	0.046	0.124	1	10
Kmt2c	0.003356	-0.35695	0.201	0.296	1	10
Ppp2r2d	0.003368	-0.33738	0.121	0.203	1	10
Mapk7	0.003378	-0.31941	0.034	0.106	1	10
Zfp68	0.003401	-0.35161	0.069	0.14	1	10
Wbp4	0.003417	-0.33219	0.224	0.276	1	10
Cops3	0.003428	-0.36275	0.172	0.251	1	10
Cdk1	0.00347	-0.39385	0.144	0.247	1	10
Gtpbp4	0.003476	-0.34687	0.184	0.282	1	10
Rpl27	0.00348	-0.30164	0.034	0.107	1	10
Dstn	0.003551	-0.31049	0.224	0.331	1	10
Ndufs7	0.003552	-0.30951	0.264	0.335	1	10
Anapc16	0.003592	-0.36226	0.161	0.234	1	10
Pbdc1	0.003601	-0.31378	0.155	0.206	1	10
Ndufa1	0.003608	0.29777	0.466	0.341	1	10
Maml3	0.00361	-0.32186	0.034	0.101	1	10
Ppp1r12a	0.003652	-0.37085	0.172	0.251	1	10
Fbxw2	0.003657	-0.35629	0.086	0.16	1	10
Foxp1	0.003661	-0.38825	0.092	0.172	1	10
Ube2z	0.003663	0.267452	0.126	0.057	1	10
Zfp322a	0.003752	-0.35133	0.063	0.123	1	10
Wapal	0.003785	-0.29524	0.201	0.271	1	10
Sugt1	0.003818	-0.33845	0.167	0.232	1	10
Lars	0.003857	-0.34555	0.115	0.218	1	10
Cdk12	0.003875	-0.30283	0.126	0.166	1	10
Mdh1	0.003877	-0.28183	0.374	0.453	1	10
Blmh	0.003897	-0.29949	0.241	0.317	1	10
F2r	0.003959	-0.31837	0.04	0.105	1	10

Mrpl38	0.00396	-0.26328	0.034	0.107	1	10
Ube2r2	0.003964	-0.27144	0.264	0.317	1	10
Ranbp3	0.004096	-0.3213	0.109	0.172	1	10
Paf1	0.004098	-0.32539	0.103	0.171	1	10
Med4	0.004136	-0.33585	0.092	0.155	1	10
Hars	0.004147	-0.25866	0.184	0.211	1	10
Cbr1	0.004268	-0.32346	0.08	0.149	1	10
Mrps6	0.004274	-0.31676	0.063	0.152	1	10
Immt	0.004279	-0.25391	0.224	0.251	1	10
Brca2	0.004309	-0.34503	0.052	0.114	1	10
Carkd	0.004309	-0.30209	0.178	0.225	1	10
Wasl	0.004319	-0.38764	0.138	0.237	1	10
Ylpm1	0.004327	-0.35768	0.155	0.242	1	10
Pqbp1	0.004333	-0.36185	0.178	0.264	1	10
Casc3	0.004366	-0.31922	0.063	0.13	1	10
Eif4b	0.004397	-0.32515	0.184	0.276	1	10
Sssca1	0.004418	-0.34159	0.132	0.203	1	10
Cdv3	0.004423	-0.32336	0.069	0.159	1	10
Arhgef7	0.004464	-0.32867	0.103	0.149	1	10
Copb2	0.004509	-0.29061	0.167	0.218	1	10
Cog1	0.004572	-0.26968	0.04	0.115	1	10
Lcmt1	0.004573	-0.34579	0.086	0.169	1	10
Tmem183a	0.004597	-0.35197	0.092	0.173	1	10
Ppat	0.004668	-0.30802	0.034	0.108	1	10
Rbm17	0.004681	-0.28005	0.316	0.39	1	10
Ccdc174	0.004694	-0.38041	0.121	0.205	1	10
St3gal5	0.00471	-0.30277	0.121	0.194	1	10
Rfc1	0.004723	-0.26321	0.241	0.331	1	10
Chmp7	0.004736	-0.33818	0.063	0.129	1	10
Yme1l1	0.004854	-0.28161	0.172	0.222	1	10
Midn	0.004869	-0.33098	0.075	0.133	1	10
Mphosph1(	0.004937	-0.3863	0.138	0.232	1	10
Rtca	0.004951	-0.34403	0.057	0.129	1	10
Hook3	0.004997	-0.38278	0.195	0.3	1	10
Rwdd1	0.005031	-0.34193	0.167	0.277	1	10
Mecp2	0.005065	-0.34855	0.046	0.121	1	10
Abhd17b	0.005091	-0.33907	0.08	0.143	1	10
Prdx4	0.005097	-0.26776	0.287	0.366	1	10
Rnf11	0.005121	-0.34337	0.086	0.148	1	10
Srp72	0.005124	-0.26288	0.259	0.322	1	10
Senp1	0.005131	-0.35196	0.063	0.136	1	10
Hk2	0.00515	0.256128	0.264	0.172	1	10
Cuedc2	0.005179	-0.34651	0.293	0.388	1	10
Arhgap21	0.005227	-0.3732	0.075	0.165	1	10
Ubfd1	0.005258	-0.26295	0.075	0.11	1	10
A430005L1	0.005306	-0.2793	0.057	0.116	1	10
Sp3	0.005306	-0.25926	0.075	0.163	1	10
Mpnd	0.005355	-0.35374	0.098	0.172	1	10

Commd4	0.005423	0.26344	0.368	0.249	1	10
Rrn3	0.00544	-0.26632	0.046	0.12	1	10
Pnpla8	0.005458	-0.34373	0.149	0.232	1	10
Fen1	0.00546	-0.31389	0.086	0.142	1	10
Parl	0.005474	-0.32332	0.063	0.124	1	10
Mrpl21	0.005478	-0.32665	0.19	0.281	1	10
2610524H	0.005493	-0.33426	0.063	0.127	1	10
Rhebl1	0.005509	-0.27754	0.034	0.106	1	10
Gm12696	0.005532	-0.2979	0.057	0.133	1	10
Smim8	0.005543	-0.29998	0.034	0.106	1	10
Cdc25a	0.005544	-0.33568	0.046	0.119	1	10
Ncapd3	0.00558	-0.26829	0.04	0.113	1	10
Clasp1	0.00561	-0.30455	0.04	0.115	1	10
Dennd2a	0.005633	-0.32137	0.04	0.108	1	10
Gle1	0.005642	-0.28377	0.057	0.14	1	10
Lrrcc1	0.005688	-0.33778	0.098	0.152	1	10
Chd1	0.005699	-0.34383	0.109	0.21	1	10
Klhl13	0.005723	-0.26667	0.034	0.104	1	10
Ralgds	0.005776	-0.3378	0.034	0.103	1	10
Orc2	0.005789	-0.32382	0.092	0.156	1	10
Psmd11	0.005793	-0.34035	0.23	0.321	1	10
Yeats4	0.005914	-0.31237	0.207	0.264	1	10
Dus3l	0.005962	-0.3129	0.08	0.131	1	10
Hist1h2ak	0.006112	-0.41279	0.069	0.143	1	10
Ankrd10	0.006125	-0.32541	0.103	0.159	1	10
Dnajc21	0.00615	-0.33996	0.138	0.21	1	10
Xrcc5	0.006151	-0.29686	0.04	0.115	1	10
Pelp1	0.006176	-0.27491	0.069	0.112	1	10
Ap4s1	0.006272	-0.28771	0.057	0.104	1	10
Wipi2	0.006313	-0.2925	0.075	0.12	1	10
Nmral1	0.006314	-0.29335	0.103	0.188	1	10
Zfp191	0.006373	-0.34155	0.126	0.218	1	10
Rad23b	0.006436	-0.28084	0.155	0.202	1	10
Ramp2	0.00645	-0.35835	0.046	0.12	1	10
Lsm2	0.006493	-0.32805	0.126	0.231	1	10
Eif4e3	0.006544	-0.33633	0.052	0.131	1	10
Spin1	0.006567	-0.31409	0.052	0.119	1	10
Dnal4	0.006579	-0.31316	0.046	0.11	1	10
Smpd2	0.006658	-0.34799	0.086	0.166	1	10
Rfk	0.006686	-0.27336	0.098	0.139	1	10
Gtf2h2	0.006687	0.30009	0.167	0.092	1	10
Mrpl47	0.006703	-0.33062	0.086	0.147	1	10
Vps72	0.006765	-0.29662	0.075	0.154	1	10
Ssna1	0.006792	-0.33835	0.19	0.267	1	10
Myo10	0.006821	-0.33523	0.046	0.122	1	10
Suv420h1	0.006824	-0.32969	0.092	0.143	1	10
Rab11fip2	0.006944	-0.34128	0.046	0.107	1	10
Psmb3	0.006984	-0.32078	0.184	0.259	1	10

Ccdc58	0.00702	-0.30433	0.057	0.132	1	10
Peli2	0.007071	-0.28048	0.08	0.165	1	10
Smek2	0.007098	-0.2654	0.195	0.223	1	10
Slc38a2	0.007147	-0.36246	0.138	0.221	1	10
Eef1e1	0.007239	-0.34484	0.109	0.182	1	10
Med31	0.007268	-0.33473	0.063	0.135	1	10
Mysm1	0.007295	-0.34503	0.086	0.145	1	10
Pdgfa	0.007321	-0.32579	0.178	0.254	1	10
Lap3	0.007337	0.278039	0.397	0.275	1	10
Akap11	0.00751	-0.29406	0.103	0.14	1	10
Scaf11	0.007548	-0.26112	0.316	0.373	1	10
Mrps12	0.007607	-0.32511	0.103	0.19	1	10
Ccng2	0.007657	-0.33414	0.144	0.212	1	10
2810055G1	0.007686	-0.27512	0.144	0.17	1	10
Zfp318	0.007725	-0.37741	0.103	0.176	1	10
Sgce	0.00777	0.256458	0.172	0.103	1	10
Chd3	0.007859	-0.37299	0.092	0.149	1	10
Zfp281	0.007867	-0.26834	0.103	0.137	1	10
Yeats2	0.007878	-0.33211	0.046	0.115	1	10
Tmem11	0.007883	-0.28259	0.075	0.149	1	10
Elovl5	0.007945	-0.28334	0.069	0.119	1	10
Brd7	0.008016	-0.29328	0.23	0.308	1	10
Zrsr2	0.008061	-0.30798	0.161	0.236	1	10
Ppan	0.008079	-0.31607	0.08	0.14	1	10
Smek1	0.008136	-0.28566	0.132	0.179	1	10
Ing4	0.008159	-0.30248	0.264	0.323	1	10
Topbp1	0.008208	-0.34197	0.069	0.146	1	10
Ptms	0.008262	-0.26095	0.42	0.474	1	10
Cul4a	0.00827	-0.31507	0.046	0.111	1	10
Ftsj1	0.008291	-0.28898	0.052	0.104	1	10
Pdha1	0.008312	-0.32353	0.092	0.163	1	10
Uqcrc2	0.008382	-0.29113	0.287	0.357	1	10
Pgrmc2	0.008464	-0.28963	0.126	0.197	1	10
Rps27a	0.00851	-0.28716	0.052	0.106	1	10
Bcl10	0.008595	0.273549	0.167	0.086	1	10
Emd	0.008613	-0.31953	0.115	0.182	1	10
Ubxn2a	0.008678	-0.35772	0.08	0.163	1	10
Banp	0.008722	-0.34944	0.057	0.137	1	10
Ptpra	0.008725	0.263155	0.333	0.234	1	10
Kars	0.008731	-0.29877	0.138	0.195	1	10
Chka	0.008777	-0.33265	0.098	0.159	1	10
Tpgs2	0.008798	-0.29283	0.052	0.104	1	10
Txn2	0.008801	-0.2893	0.149	0.199	1	10
Tfg	0.008841	-0.27337	0.086	0.15	1	10
Zfp329	0.008864	-0.35561	0.057	0.129	1	10
Itsn1	0.008903	-0.39158	0.155	0.236	1	10
E2f1	0.008984	-0.3179	0.092	0.147	1	10
Pitpnb	0.009093	-0.27252	0.172	0.213	1	10

Nosip	0.009104	-0.27251	0.132	0.168	1	10
Isca2	0.009155	-0.31957	0.103	0.162	1	10
Rfc5	0.009186	-0.27602	0.04	0.108	1	10
Rps19bp1	0.009222	-0.34205	0.092	0.175	1	10
Ints7	0.009235	-0.30727	0.063	0.117	1	10
Msantd3	0.009248	-0.29183	0.04	0.112	1	10
Krt10	0.009279	-0.32714	0.08	0.162	1	10
Zcchc9	0.009289	-0.30244	0.052	0.111	1	10
Tars	0.009291	-0.33381	0.092	0.178	1	10
Ncaph2	0.009336	-0.3212	0.103	0.174	1	10
Mprip	0.0094	-0.34537	0.086	0.152	1	10
Nuf2	0.009427	-0.26706	0.063	0.141	1	10
Sgol2	0.009541	-0.37247	0.075	0.157	1	10
Utp14a	0.009551	-0.32394	0.172	0.233	1	10
Ikbkap	0.009572	-0.32928	0.063	0.127	1	10
Herc1	0.009608	-0.27073	0.155	0.186	1	10
Usp5	0.009673	-0.2904	0.092	0.14	1	10
Zfp945	0.009674	-0.27728	0.046	0.111	1	10
1700123O:	0.009719	-0.28629	0.086	0.141	1	10
Med1	0.009727	-0.33222	0.098	0.173	1	10
Tnrc6c	0.009776	-0.31404	0.092	0.179	1	10
Gm10260	0.009934	-0.25087	0.19	0.228	1	10
Pop4	0.009943	-0.29395	0.08	0.162	1	10
Fabp7	0	4.487123	0.992	0.117	0	11
Slc1a3	0	3.661779	0.992	0.147	0	11
Dbi	0	2.747635	1	0.459	0	11
Apoe	0	3.60396	1	0.243	0	11
Aqp4	0	3.971161	0.967	0.019	8.7E-305	11
Cst3	1.3E-300	2.722055	1	0.532	2.2E-296	11
Sparcl1	2.3E-268	3.171955	1	0.201	3.8E-264	11
Slc4a4	3.1E-261	3.342439	0.95	0.034	5.2E-257	11
Mt1	2.5E-243	3.104502	0.933	0.163	4.1E-239	11
Aldoc	5.6E-237	3.162611	0.933	0.026	9.3E-233	11
Atp1a2	1.2E-231	3.352355	1	0.052	2.1E-227	11
Glul	5.2E-229	2.490691	0.933	0.196	8.6E-225	11
Ttyh1	9.8E-222	2.888883	0.958	0.025	1.6E-217	11
Pla2g7	9.9E-220	2.958166	0.883	0.014	1.7E-215	11
Ednrb	9.5E-209	2.995227	0.95	0.023	1.6E-204	11
Mlc1	4E-198	2.232735	0.833	0.004	6.6E-194	11
Ppap2b	4.1E-188	2.780529	0.908	0.037	6.8E-184	11
Clu	1.3E-182	2.67564	0.833	0.014	2.2E-178	11
Id3	4.2E-178	2.983439	0.858	0.026	6.9E-174	11
Car2	1.8E-175	2.62709	0.767	0.012	3E-171	11
Agt	1.7E-171	2.425021	0.792	0.01	2.9E-167	11
Gpr37l1	2.3E-167	2.597701	0.892	0.025	3.9E-163	11
Sparc	1.5E-166	2.946163	0.975	0.079	2.5E-162	11
Acsbg1	9.6E-165	2.01798	0.742	0.005	1.6E-160	11
Timp4	2.7E-161	2.138963	0.8	0.011	4.5E-157	11

Gpm6b	4.7E-157	1.952047	0.95	0.481	7.8E-153	11
Aldh1l1	7.3E-154	1.937856	0.675	0.004	1.2E-149	11
Ptn	2.3E-148	2.074195	0.975	0.438	3.9E-144	11
Tril	7.6E-147	2.168631	0.8	0.02	1.3E-142	11
Kcnj10	4.1E-146	2.508106	0.767	0.02	6.9E-142	11
Mt2	1.4E-145	2.542942	0.858	0.086	2.3E-141	11
Mt3	7E-144	2.186024	0.783	0.02	1.2E-139	11
Slc25a18	6.5E-142	1.679426	0.658	0.004	1.1E-137	11
Igfbp2	2E-141	2.590301	0.758	0.015	3.3E-137	11
Cd81	5.3E-136	1.70931	0.975	0.458	8.8E-132	11
Mmd2	3.7E-135	2.056276	0.833	0.034	6.2E-131	11
Tnc	1.7E-134	1.870529	0.567	0.002	2.9E-130	11
Serpine2	5.9E-134	2.387718	0.908	0.051	9.9E-130	11
Hsp90ab1	2E-133	-1.01903	0.975	0.998	3.3E-129	11
Ramp1	3.2E-132	2.052332	0.8	0.022	5.3E-128	11
Bcan	2.6E-131	2.223818	0.892	0.046	4.4E-127	11
Hepacam	7.8E-129	1.628469	0.633	0.005	1.3E-124	11
Cmtm5	1.3E-128	1.857032	0.717	0.013	2.2E-124	11
Scd2	1.1E-127	1.916921	0.908	0.292	1.8E-123	11
Id4	2.8E-127	1.801043	0.667	0.008	4.7E-123	11
Ptprz1	1.1E-125	2.27019	0.825	0.031	1.8E-121	11
Atp1b2	1.4E-125	2.089395	0.825	0.042	2.4E-121	11
Gria1	2.3E-121	1.677636	0.625	0.007	3.8E-117	11
Tspan7	2.3E-118	2.103693	0.9	0.1	3.9E-114	11
Lcat	1.1E-115	1.660266	0.608	0.007	1.9E-111	11
Npy	1.6E-111	1.954262	0.492	0.002	2.6E-107	11
S100b	2E-109	2.125688	0.767	0.035	3.4E-105	11
Pea15a	7E-108	1.989219	0.875	0.171	1.2E-103	11
Pla2g16	1E-106	1.358423	0.542	0.005	1.7E-102	11
Nid1	1.1E-105	1.739732	0.642	0.014	1.8E-101	11
Chchd10	7.6E-105	1.923461	0.717	0.029	1.3E-100	11
Hopx	6.3E-104	1.636641	0.525	0.005	1E-99	11
Sepp1	2.1E-103	1.142915	0.758	0.05	3.5E-99	11
S1pr1	3.7E-103	1.459845	0.533	0.005	6.1E-99	11
Cspg5	7.2E-102	1.55544	0.667	0.024	1.2E-97	11
Paqr8	1.1E-99	1.399768	0.542	0.007	1.79E-95	11
Gja1	3.29E-97	1.865783	0.583	0.016	5.49E-93	11
Htra1	5.11E-97	1.65892	0.558	0.011	8.52E-93	11
Gstm1	2.97E-93	1.90743	0.717	0.077	4.96E-89	11
Mfge8	1.44E-92	1.780226	0.65	0.029	2.41E-88	11
Lxn	5.39E-91	1.79505	0.708	0.048	8.99E-87	11
Adora1	1.52E-90	1.367967	0.525	0.008	2.54E-86	11
Tmem176a	3.31E-90	1.498	0.558	0.012	5.53E-86	11
Ncan	1.78E-88	1.817064	0.8	0.094	2.98E-84	11
Lsamp	2.03E-88	1.774628	0.808	0.072	3.39E-84	11
Sdc4	3.03E-88	1.273792	0.483	0.006	5.05E-84	11
Cryab	1.56E-87	1.586036	0.6	0.018	2.59E-83	11
Emid1	1.57E-86	1.485912	0.483	0.006	2.62E-82	11

Gfap	3.74E-86	1.843872	0.558	0.026	6.24E-82	11
Ntrk2	3.09E-85	1.745939	0.8	0.078	5.15E-81	11
Ifitm3	3.26E-85	1.431923	0.517	0.01	5.45E-81	11
Casp12	1.11E-84	1.07462	0.375	0.001	1.86E-80	11
Scrg1	2.03E-84	1.523916	0.6	0.021	3.39E-80	11
F3	4.04E-84	1.348014	0.45	0.005	6.74E-80	11
AW04773C	1.52E-83	1.573359	0.592	0.02	2.54E-79	11
Tsc22d4	3.88E-83	1.731387	0.8	0.161	6.46E-79	11
Tmem176b	4.66E-83	1.727081	0.817	0.104	7.78E-79	11
Lrig1	9.66E-81	1.366865	0.533	0.014	1.61E-76	11
Metrn	2.3E-79	1.31347	0.558	0.017	3.83E-75	11
Plat	4.07E-79	1.412362	0.558	0.018	6.8E-75	11
S100a1	3.38E-78	1.443275	0.575	0.022	5.63E-74	11
Slc9a3r1	1.05E-77	1.119058	0.492	0.01	1.76E-73	11
Hsd11b1	1.44E-77	1.608851	0.383	0.003	2.39E-73	11
Glud1	2.29E-76	1.603206	0.792	0.137	3.82E-72	11
Ccdc80	3.41E-76	1.299297	0.433	0.006	5.68E-72	11
Luzp2	3.82E-76	1.355863	0.517	0.014	6.38E-72	11
Gabrb1	4.24E-76	0.839911	0.342	0.001	7.07E-72	11
Hes5	1.27E-75	1.366471	0.408	0.004	2.11E-71	11
Slc6a1	1.27E-75	1.486908	0.575	0.023	2.12E-71	11
Prss23	6.14E-75	1.181293	0.367	0.002	1.02E-70	11
Atp1b1	1.56E-74	1.780926	0.658	0.047	2.61E-70	11
Chl1	3.36E-74	1.237599	0.483	0.012	5.6E-70	11
Fgfr3	3.13E-73	0.956566	0.342	0.001	5.23E-69	11
Kcnj16	4.1E-73	1.093049	0.408	0.005	6.84E-69	11
Cyp26b1	2.3E-72	1.141274	0.35	0.002	3.83E-68	11
Rgs5	2.53E-72	1.837958	0.442	0.01	4.21E-68	11
Gjb6	7.99E-71	1.279179	0.35	0.002	1.33E-66	11
Rps5	2.28E-70	-1.07626	0.9	0.954	3.81E-66	11
Limch1	2.76E-70	1.322433	0.533	0.02	4.6E-66	11
Lpar1	4.23E-70	1.065741	0.408	0.006	7.05E-66	11
Sfxn5	2.7E-69	1.172592	0.483	0.014	4.5E-65	11
Lamp1	3.25E-69	1.380261	0.908	0.332	5.42E-65	11
Npas3	1.73E-68	1.328612	0.542	0.023	2.89E-64	11
Slc39a12	2.18E-68	0.929573	0.267	0	3.64E-64	11
Tubb5	3.55E-68	-1.50652	0.583	0.916	5.92E-64	11
Vcam1	4.62E-68	0.852246	0.367	0.005	7.7E-64	11
Ndrg2	1.7E-67	1.610995	0.783	0.18	2.83E-63	11
Cnp	1.01E-66	1.024991	0.525	0.03	1.69E-62	11
Sfrp1	1.81E-66	-1.98514	0.367	0.814	3.01E-62	11
Prex2	2.36E-66	1.231327	0.442	0.01	3.93E-62	11
Gpnmb	6.66E-66	1.009489	0.325	0.002	1.11E-61	11
Emp2	1.03E-65	1.067347	0.35	0.003	1.72E-61	11
Vim	1.19E-65	1.565345	0.883	0.298	1.99E-61	11
Fxyd1	1.31E-65	1.133874	0.433	0.01	2.19E-61	11
Sox2	3.4E-65	1.286099	0.617	0.044	5.67E-61	11
Pbxip1	3.46E-65	1.112927	0.458	0.014	5.77E-61	11

Slc38a3	3.58E-65	1.259258	0.483	0.019	5.97E-61	11
Ddah1	9.63E-65	1.525051	0.567	0.036	1.61E-60	11
Tlcd1	3.61E-64	1.396683	0.5	0.024	6.02E-60	11
Thbs3	6.75E-64	0.925905	0.383	0.007	1.13E-59	11
Tspan3	2.9E-63	1.273433	0.908	0.425	4.83E-59	11
Gm3764	2.86E-62	1.540259	0.75	0.127	4.76E-58	11
Cacng4	6.89E-62	1.550115	0.733	0.097	1.15E-57	11
Slc7a10	1.24E-61	1.1454	0.325	0.003	2.07E-57	11
Nfib	1.65E-61	-1.38318	0.75	0.934	2.76E-57	11
Tmem47	2.58E-61	1.443324	0.617	0.06	4.3E-57	11
Atp13a4	4.81E-61	0.954861	0.258	0	8.02E-57	11
Smpdl3a	5.71E-61	0.97563	0.392	0.008	9.52E-57	11
Shisa9	6.13E-61	1.103283	0.35	0.004	1.02E-56	11
Gsta4	2.06E-60	0.835913	0.367	0.009	3.43E-56	11
Ckb	2.17E-60	1.047331	0.975	0.807	3.62E-56	11
S100a13	4.95E-60	1.151793	0.5	0.027	8.26E-56	11
Pdpn	5.12E-60	0.999744	0.383	0.008	8.54E-56	11
H2-K1	6.52E-60	1.20719	0.417	0.011	1.09E-55	11
Dkk3	2.19E-59	0.891622	0.367	0.007	3.65E-55	11
Gjc3	1.39E-58	1.30045	0.417	0.012	2.31E-54	11
Slc13a3	3.71E-58	1.010419	0.283	0.001	6.2E-54	11
Mgst1	7.7E-58	1.246139	0.517	0.03	1.28E-53	11
Myo6	1.62E-57	1.386721	0.575	0.046	2.71E-53	11
Itm2b	1.73E-57	1.136482	0.95	0.64	2.88E-53	11
Daam2	5.67E-57	0.833172	0.283	0.002	9.46E-53	11
S100a10	1.81E-56	1.300522	0.483	0.026	3.02E-52	11
Cp	5.77E-56	0.803019	0.358	0.011	9.62E-52	11
Slc1a2	3.38E-55	1.414454	0.842	0.29	5.63E-51	11
Cd44	2.39E-54	0.922518	0.425	0.017	3.98E-50	11
Asrgl1	2.64E-54	1.47834	0.692	0.129	4.4E-50	11
Cybrd1	2.97E-54	0.727112	0.267	0.002	4.96E-50	11
Spon1	3.88E-54	0.896136	0.375	0.012	6.47E-50	11
Gucy1a3	5.57E-54	0.705285	0.333	0.008	9.29E-50	11
Eps8	1.37E-53	1.073652	0.475	0.029	2.28E-49	11
Omg	1.6E-53	1.017662	0.358	0.009	2.67E-49	11
Gmpr	6.71E-53	0.996467	0.367	0.01	1.12E-48	11
Gabrg1	9.41E-53	0.725307	0.283	0.003	1.57E-48	11
Acadl	9.66E-53	1.293429	0.592	0.06	1.61E-48	11
Ncl	1.69E-52	-1.14745	0.692	0.897	2.81E-48	11
Itga6	2.46E-52	1.032512	0.375	0.011	4.1E-48	11
Acot1	3.56E-52	1.316372	0.508	0.037	5.95E-48	11
S100a16	6.45E-52	1.326587	0.667	0.09	1.08E-47	11
Cyp2j9	1.33E-51	0.811837	0.3	0.004	2.21E-47	11
Psap	2.59E-51	1.287652	0.808	0.18	4.32E-47	11
Slc6a11	5.03E-51	1.397292	0.375	0.016	8.38E-47	11
S100a6	1.76E-50	1.232833	0.417	0.019	2.93E-46	11
Fjx1	2.61E-50	1.077441	0.492	0.034	4.36E-46	11
Cd302	5.35E-50	1.101574	0.542	0.049	8.92E-46	11

Itm2c	6.88E-50	1.31453	0.808	0.207	1.15E-45	11
Rps9	8.32E-50	-1.01793	0.808	0.929	1.39E-45	11
Me1	1.19E-49	0.727732	0.3	0.006	1.99E-45	11
Ctsl	1.38E-49	1.281309	0.808	0.256	2.3E-45	11
Arhgef26	2.71E-49	0.932825	0.392	0.016	4.53E-45	11
Gpd1	3.76E-49	0.964006	0.325	0.007	6.27E-45	11
Hnrnpa2b1	4.88E-49	-0.78254	0.917	0.933	8.14E-45	11
B2m	1.1E-48	1.355697	0.783	0.227	1.84E-44	11
Tjp2	1.61E-48	1.083463	0.533	0.048	2.69E-44	11
Sat1	1.75E-48	1.475812	0.558	0.063	2.92E-44	11
Pcp4l1	2.19E-48	1.009802	0.275	0.003	3.65E-44	11
Chst2	2.9E-48	1.031999	0.458	0.029	4.83E-44	11
Rfx4	4.27E-48	0.656145	0.242	0.002	7.13E-44	11
Wnt7a	4.49E-48	0.744739	0.233	0.002	7.49E-44	11
Zcchc24	7.98E-48	0.954675	0.425	0.025	1.33E-43	11
Slc6a9	8.62E-48	0.899412	0.317	0.007	1.44E-43	11
Rpl13a	1.3E-47	-0.91156	0.9	0.928	2.17E-43	11
Pcdh10	1.88E-47	0.751402	0.342	0.012	3.13E-43	11
Tuba1a	3.77E-47	-1.24833	0.8	0.93	6.3E-43	11
Cldn10	5.07E-47	0.948538	0.242	0.002	8.45E-43	11
Timp3	7.21E-47	1.083699	0.592	0.075	1.2E-42	11
1700084C(	1.26E-46	0.565965	0.208	0.001	2.11E-42	11
Pcx	7.24E-46	1.145676	0.542	0.057	1.21E-41	11
Vamp8	8.89E-46	0.631842	0.3	0.011	1.48E-41	11
Degs1	9.88E-46	1.143506	0.583	0.072	1.65E-41	11
Abhd3	1.34E-45	0.843973	0.275	0.004	2.24E-41	11
Cntn1	2.91E-45	0.757002	0.442	0.048	4.86E-41	11
Adcyap1r1	4.05E-45	1.124791	0.6	0.079	6.76E-41	11
Jam2	6.26E-45	1.013521	0.442	0.03	1.04E-40	11
Abca1	6.78E-45	0.843142	0.367	0.021	1.13E-40	11
Cntnap2	7.59E-45	0.838133	0.3	0.007	1.27E-40	11
H3f3b	7.75E-45	-1.01495	0.692	0.908	1.29E-40	11
Rps26	8.44E-45	-1.12802	0.65	0.848	1.41E-40	11
Abat	1.03E-44	0.911563	0.417	0.027	1.72E-40	11
Sorl1	3.4E-44	1.025859	0.392	0.02	5.68E-40	11
Timp1	4.51E-44	0.641705	0.242	0.002	7.52E-40	11
Igfbp4	5.37E-44	1.197953	0.367	0.016	8.96E-40	11
Cpq	7.59E-44	0.7864	0.283	0.006	1.27E-39	11
Rps3	1.4E-43	-0.85716	0.858	0.922	2.34E-39	11
Pamr1	1.63E-43	0.579933	0.208	0.001	2.71E-39	11
Gabra4	1.78E-43	0.68915	0.325	0.012	2.97E-39	11
Naaa	2.55E-43	0.792765	0.258	0.004	4.25E-39	11
Dbx2	2.91E-43	0.898909	0.25	0.003	4.85E-39	11
Adrb1	2.96E-43	0.549832	0.2	0.001	4.94E-39	11
Nrarp	2.98E-43	0.818294	0.283	0.006	4.97E-39	11
Lyn	3.35E-43	0.609828	0.283	0.008	5.58E-39	11
Phyhipl	3.42E-43	0.93544	0.517	0.059	5.71E-39	11
Sox21	5.2E-43	0.636743	0.258	0.005	8.68E-39	11

1810037I1	5.41E-43	1.211006	0.75	0.192	9.02E-39	11
Rpl4	1.43E-42	-0.91481	0.817	0.908	2.39E-38	11
Slc14a1	1.87E-42	0.582641	0.192	0.001	3.13E-38	11
Rplp0	2.9E-42	-1.06302	0.7	0.873	4.83E-38	11
Adcy8	2.96E-42	0.560647	0.2	0.001	4.93E-38	11
Gpx8	4.21E-42	0.971002	0.492	0.05	7.02E-38	11
Plcd4	5.43E-42	0.699704	0.242	0.003	9.05E-38	11
Slc35f1	6.87E-42	0.699488	0.317	0.015	1.15E-37	11
CRE_RECON	7.22E-42	-1.97418	0.408	0.77	1.2E-37	11
S100a11	2.41E-41	0.689103	0.325	0.014	4.02E-37	11
Hnrnpab	3.57E-41	-1.07304	0.608	0.817	5.96E-37	11
Calm2	7E-41	-1.03211	0.7	0.875	1.17E-36	11
Rhod	7.5E-41	0.674856	0.225	0.002	1.25E-36	11
Gatm	7.82E-41	0.896289	0.408	0.032	1.3E-36	11
Pax3	8.97E-41	0.681753	0.267	0.008	1.5E-36	11
Anp32a	1.01E-40	-0.94376	0.65	0.837	1.69E-36	11
Cryl1	1.28E-40	0.920167	0.292	0.008	2.13E-36	11
Ifi27	1.74E-40	0.777033	0.317	0.013	2.91E-36	11
Epha4	2.68E-40	0.943771	0.3	0.01	4.47E-36	11
Rpl32	3.34E-40	-0.88969	0.792	0.89	5.57E-36	11
Paqr7	4.4E-40	0.776223	0.25	0.004	7.34E-36	11
Igsf11	1.15E-39	0.673481	0.242	0.004	1.92E-35	11
Prdx6	4.39E-39	1.121172	0.792	0.322	7.32E-35	11
Trpm3	7.82E-39	0.730845	0.283	0.009	1.31E-34	11
Dhrs1	8.59E-39	1.112595	0.575	0.089	1.43E-34	11
Aldh2	9.35E-39	0.839198	0.367	0.023	1.56E-34	11
Gdpd2	1.08E-38	0.476478	0.175	0.001	1.8E-34	11
Slc1a4	1.13E-38	1.152839	0.475	0.054	1.88E-34	11
Rps14	1.23E-38	-0.80219	0.875	0.95	2.05E-34	11
Gpr126	1.25E-38	0.532854	0.225	0.004	2.09E-34	11
Slc13a5	1.64E-38	0.65426	0.225	0.003	2.74E-34	11
Grm3	2.67E-38	0.48499	0.192	0.001	4.45E-34	11
Il33	2.86E-38	0.508958	0.217	0.004	4.77E-34	11
Fam213a	4.19E-38	1.042314	0.558	0.085	7E-34	11
Hnrnpu	4.79E-38	-0.84368	0.767	0.866	7.99E-34	11
Ctso	8.36E-38	0.714465	0.308	0.014	1.39E-33	11
Rab31	9.27E-38	0.996694	0.475	0.052	1.55E-33	11
Angpt1	1.12E-37	1.051309	0.45	0.044	1.87E-33	11
Bmpr1b	1.22E-37	0.621797	0.233	0.004	2.03E-33	11
Rgl1	1.26E-37	0.738454	0.258	0.006	2.1E-33	11
Efhd1	3.75E-37	0.694174	0.358	0.028	6.26E-33	11
Kcnn2	4.74E-37	0.870841	0.283	0.01	7.91E-33	11
Nacc2	5.89E-37	0.838336	0.325	0.016	9.82E-33	11
Arap2	6.04E-37	0.727129	0.267	0.007	1.01E-32	11
Sash1	6.47E-37	0.900441	0.392	0.032	1.08E-32	11
Hbegf	6.67E-37	0.806329	0.325	0.018	1.11E-32	11
Abhd4	1.07E-36	0.916345	0.433	0.042	1.79E-32	11
BC064078	1.27E-36	0.499765	0.167	0.001	2.12E-32	11

2810459M	1.3E-36	0.497489	0.192	0.002	2.16E-32	11
Cml1	1.65E-36	0.824487	0.292	0.011	2.76E-32	11
Igfbpl1	2.55E-36	-1.77332	0.133	0.651	4.26E-32	11
Mro	4.19E-36	0.503887	0.2	0.003	6.98E-32	11
Basp1	6.3E-36	-1.61492	0.233	0.707	1.05E-31	11
Add3	8.45E-36	0.896525	0.567	0.1	1.41E-31	11
Tmem37	1.34E-35	0.726999	0.242	0.006	2.23E-31	11
Itih5	2.15E-35	0.297736	0.217	0.013	3.59E-31	11
Rarres2	2.18E-35	0.722638	0.225	0.005	3.64E-31	11
Fgfr1	2.66E-35	1.015383	0.525	0.077	4.44E-31	11
Pon2	3.05E-35	0.848243	0.4	0.036	5.09E-31	11
Gria4	3.4E-35	1.066269	0.617	0.121	5.67E-31	11
Sncg	3.79E-35	0.774427	0.158	0	6.33E-31	11
Nbl1	4.1E-35	0.768994	0.342	0.025	6.84E-31	11
Sirpa	4.43E-35	0.894516	0.392	0.035	7.38E-31	11
Rplp2	2.19E-34	-0.95874	0.675	0.841	3.66E-30	11
Rrbp1	2.79E-34	1.113137	0.7	0.185	4.66E-30	11
Rps21	2.99E-34	-0.90085	0.692	0.834	4.98E-30	11
Serbp1	3.26E-34	-0.77376	0.8	0.869	5.44E-30	11
Nwd1	3.85E-34	0.431018	0.158	0.001	6.42E-30	11
Paqr4	4.99E-34	0.899915	0.417	0.045	8.33E-30	11
0610040J0	5.29E-34	0.410991	0.183	0.002	8.83E-30	11
Dtna	8.73E-34	0.677553	0.333	0.029	1.46E-29	11
Slc12a4	1.76E-33	0.821716	0.375	0.033	2.94E-29	11
Hnrnpm	1.86E-33	-0.98957	0.55	0.736	3.09E-29	11
Epas1	2.39E-33	0.635291	0.233	0.007	3.98E-29	11
Agrn	2.64E-33	0.850442	0.483	0.073	4.4E-29	11
Angptl4	3.97E-33	0.635878	0.233	0.007	6.62E-29	11
Aldh1a1	4.59E-33	0.579997	0.208	0.005	7.66E-29	11
Fam20a	4.74E-33	0.551387	0.167	0.001	7.9E-29	11
Tpp1	5.07E-33	0.861574	0.467	0.064	8.45E-29	11
Rhoc	1E-32	0.85531	0.342	0.025	1.68E-28	11
Gnb2l1	1.14E-32	-0.85773	0.742	0.872	1.89E-28	11
Clrn1	1.15E-32	0.385904	0.167	0.003	1.93E-28	11
Id1	1.2E-32	1.13495	0.483	0.07	2E-28	11
Crmp1	1.39E-32	-1.43244	0.242	0.67	2.32E-28	11
Chpt1	1.76E-32	0.998849	0.45	0.057	2.93E-28	11
Adam11	2.1E-32	0.45975	0.242	0.016	3.5E-28	11
Ephx1	2.75E-32	0.663092	0.225	0.006	4.58E-28	11
Pabpc1	2.87E-32	-0.84186	0.792	0.879	4.78E-28	11
Tmsb10	3.62E-32	-1.46137	0.217	0.666	6.03E-28	11
Btbd17	4.88E-32	1.101906	0.642	0.156	8.14E-28	11
Slc27a1	5.11E-32	0.862994	0.408	0.045	8.53E-28	11
Elovl5	5.22E-32	1.022123	0.575	0.116	8.7E-28	11
Itih3	6.06E-32	1.067321	0.208	0.007	1.01E-27	11
Nfasc	6.07E-32	0.915421	0.358	0.033	1.01E-27	11
Slc7a2	6.3E-32	0.542316	0.217	0.005	1.05E-27	11
Cbr3	8.09E-32	0.567078	0.225	0.007	1.35E-27	11

Egfl6	8.3E-32	0.590232	0.167	0.001	1.39E-27	11
Gnb4	9.66E-32	0.928136	0.55	0.102	1.61E-27	11
Acox1	1.01E-31	0.644474	0.4	0.054	1.69E-27	11
Asah1	1.09E-31	0.865396	0.45	0.06	1.81E-27	11
Pmm1	1.09E-31	0.901684	0.592	0.124	1.83E-27	11
Sfpq	1.12E-31	-1.09035	0.425	0.671	1.87E-27	11
Stat3	1.15E-31	0.701725	0.367	0.04	1.92E-27	11
Aspa	1.15E-31	0.458629	0.158	0.001	1.92E-27	11
Cog7	1.37E-31	-1.58595	0.15	0.587	2.29E-27	11
Cd9	2.09E-31	0.923999	0.867	0.482	3.49E-27	11
Tmem56	2.11E-31	0.448177	0.217	0.009	3.52E-27	11
Prrx1	2.65E-31	0.558475	0.2	0.005	4.42E-27	11
Tmem198b	2.69E-31	0.876562	0.358	0.033	4.49E-27	11
Sema6d	2.91E-31	0.641251	0.325	0.03	4.86E-27	11
Tmem9b	3.28E-31	0.82726	0.55	0.109	5.47E-27	11
Slc25a33	3.43E-31	0.769934	0.392	0.044	5.72E-27	11
Fam181a	3.73E-31	0.337538	0.125	0	6.22E-27	11
Tspan12	4.56E-31	0.717053	0.333	0.03	7.6E-27	11
Abi3bp	4.75E-31	0.400547	0.15	0.001	7.93E-27	11
Emc7	4.85E-31	0.787374	0.608	0.14	8.09E-27	11
Nfia	7.73E-31	-1.1623	0.533	0.775	1.29E-26	11
Dlgap1	1.02E-30	0.734957	0.425	0.064	1.7E-26	11
Ctsd	1.45E-30	0.416835	0.633	0.171	2.42E-26	11
Pld2	1.52E-30	0.423948	0.158	0.001	2.53E-26	11
Gpld1	1.61E-30	0.760826	0.183	0.003	2.69E-26	11
Slc22a4	1.66E-30	0.487199	0.192	0.005	2.76E-26	11
Grid2	1.76E-30	0.676646	0.258	0.012	2.94E-26	11
Ddx5	1.9E-30	-0.889	0.625	0.817	3.16E-26	11
Gpr123	1.94E-30	0.498079	0.175	0.002	3.24E-26	11
Scrn1	2.23E-30	0.66413	0.367	0.047	3.72E-26	11
Gde1	2.29E-30	0.908998	0.608	0.138	3.81E-26	11
Tpi1	2.3E-30	0.890126	0.567	0.118	3.83E-26	11
Hnrnpc	3.08E-30	-0.78939	0.583	0.667	5.14E-26	11
AI464131	3.15E-30	0.493995	0.167	0.002	5.26E-26	11
Mrps6	3.23E-30	1.055396	0.608	0.148	5.4E-26	11
Cd24a	4.08E-30	-1.23699	0.167	0.637	6.81E-26	11
Cyp2d22	4.59E-30	0.428118	0.158	0.001	7.65E-26	11
Scg3	5.27E-30	0.943681	0.858	0.443	8.8E-26	11
Stk32a	6.52E-30	0.620729	0.2	0.004	1.09E-25	11
Rnf13	6.72E-30	0.687641	0.458	0.078	1.12E-25	11
Cpxm1	7.16E-30	0.553182	0.242	0.013	1.19E-25	11
Ptpra	7.24E-30	0.939625	0.733	0.231	1.21E-25	11
Clec3b	1.26E-29	0.479718	0.167	0.002	2.11E-25	11
Pmp22	1.34E-29	0.72239	0.275	0.016	2.23E-25	11
Rps24	1.73E-29	-0.796	0.725	0.821	2.88E-25	11
Wnt3	1.93E-29	0.68477	0.217	0.007	3.23E-25	11
Pth1r	1.98E-29	0.534086	0.192	0.005	3.3E-25	11
Slc43a3	2.34E-29	0.55925	0.2	0.004	3.91E-25	11

Irx2	2.41E-29	0.596973	0.225	0.008	4.03E-25	11
Rnf182	2.6E-29	0.540331	0.217	0.009	4.34E-25	11
Rxrg	2.8E-29	0.404899	0.133	0.001	4.66E-25	11
Ranbp1	2.85E-29	-0.94129	0.575	0.676	4.75E-25	11
Dtx4	2.94E-29	0.333162	0.183	0.006	4.9E-25	11
Atpif1	2.94E-29	-0.82462	0.683	0.816	4.9E-25	11
Rps19	2.99E-29	-0.89958	0.617	0.754	4.99E-25	11
Vwa1	3.21E-29	0.409177	0.2	0.01	5.36E-25	11
Fbxo2	3.62E-29	0.788075	0.258	0.012	6.03E-25	11
Itpr2	3.83E-29	0.639735	0.3	0.028	6.39E-25	11
Elmo2	4.22E-29	0.720408	0.375	0.045	7.04E-25	11
Cdh13	4.41E-29	0.754306	0.375	0.044	7.36E-25	11
Hnrnph1	4.69E-29	-1.10037	0.358	0.63	7.82E-25	11
Mcc	5E-29	0.51177	0.175	0.002	8.34E-25	11
Eps15	6.37E-29	0.906856	0.425	0.057	1.06E-24	11
Hsdl2	6.43E-29	0.826833	0.4	0.051	1.07E-24	11
Ampd3	6.47E-29	0.539818	0.167	0.002	1.08E-24	11
Msx2	9.42E-29	0.32673	0.117	0	1.57E-24	11
Akr1b10	1.03E-28	0.693137	0.333	0.032	1.72E-24	11
Lix1l	1.24E-28	0.417837	0.192	0.006	2.07E-24	11
Gng12	1.42E-28	1.025686	0.542	0.118	2.37E-24	11
Ppp1r1a	1.52E-28	0.926534	0.533	0.107	2.54E-24	11
Gabbr2	2.02E-28	0.663146	0.25	0.017	3.37E-24	11
Rps15	2.07E-28	-0.77464	0.7	0.788	3.45E-24	11
Ppargc1a	2.27E-28	0.537728	0.192	0.005	3.79E-24	11
Osbpl1a	2.31E-28	0.757448	0.4	0.056	3.86E-24	11
Sept4	3.68E-28	1.137812	0.667	0.223	6.14E-24	11
Mertk	3.81E-28	0.518121	0.192	0.006	6.35E-24	11
Npm1	6.14E-28	-1.0502	0.433	0.647	1.02E-23	11
Cpne2	1.18E-27	0.54559	0.2	0.006	1.97E-23	11
Axl	1.25E-27	0.545018	0.2	0.006	2.08E-23	11
Spry2	1.28E-27	0.876537	0.383	0.046	2.14E-23	11
Tnfrsf19	1.6E-27	0.442299	0.175	0.004	2.67E-23	11
Tst	1.6E-27	0.541151	0.25	0.015	2.67E-23	11
Sez6l	1.86E-27	0.811222	0.4	0.059	3.11E-23	11
Gaa	2.13E-27	0.550902	0.275	0.025	3.56E-23	11
Rgs7bp	2.16E-27	0.634627	0.283	0.026	3.61E-23	11
Grina	2.45E-27	0.530227	0.458	0.106	4.09E-23	11
H2afv	2.5E-27	-0.9077	0.617	0.728	4.16E-23	11
Cystm1	2.51E-27	0.620463	0.317	0.036	4.19E-23	11
AI413582	2.97E-27	0.527713	0.283	0.029	4.95E-23	11
Frem2	4.02E-27	0.439348	0.15	0.002	6.7E-23	11
Cbx1	4.88E-27	-0.99309	0.442	0.686	8.15E-23	11
Amot	5.67E-27	0.597145	0.25	0.014	9.46E-23	11
Pltp	6.61E-27	0.597612	0.283	0.026	1.1E-22	11
Serpina3n	6.82E-27	0.354477	0.117	0	1.14E-22	11
Chd7	7.41E-27	-1.22625	0.35	0.649	1.24E-22	11
Gramd3	8.29E-27	0.513596	0.192	0.005	1.38E-22	11

Marc2	1.1E-26	0.850347	0.458	0.081	1.84E-22	11
Timp2	1.21E-26	0.492395	0.25	0.024	2.03E-22	11
Gm2a	1.29E-26	0.706584	0.358	0.046	2.15E-22	11
Fuca2	1.38E-26	0.41644	0.2	0.008	2.3E-22	11
Zic1	1.38E-26	-0.90151	0.658	0.784	2.3E-22	11
Rps20	1.42E-26	-0.82737	0.658	0.779	2.36E-22	11
Cgrrf1	1.44E-26	0.592359	0.358	0.052	2.4E-22	11
Hist1h2bc	1.5E-26	0.841025	0.333	0.036	2.5E-22	11
Trim9	1.5E-26	0.61913	0.242	0.015	2.5E-22	11
Arxes2	1.54E-26	0.925548	0.475	0.096	2.58E-22	11
Bhlhe40	1.61E-26	0.339015	0.15	0.002	2.69E-22	11
Fth1	1.64E-26	0.692477	0.917	0.707	2.74E-22	11
Pfkm	1.75E-26	0.822608	0.417	0.064	2.92E-22	11
Ddah2	1.89E-26	-1.15557	0.425	0.687	3.16E-22	11
Hrsp12	1.9E-26	0.60667	0.333	0.041	3.17E-22	11
Ctsb	1.97E-26	0.565573	0.725	0.258	3.28E-22	11
Tmbim6	2.22E-26	0.930684	0.767	0.321	3.71E-22	11
Ubtd1	2.49E-26	0.37234	0.167	0.005	4.15E-22	11
Lrrc4c	2.6E-26	0.404585	0.183	0.009	4.34E-22	11
Cyr61	2.79E-26	0.891374	0.267	0.017	4.66E-22	11
Nfix	2.94E-26	-1.07167	0.45	0.674	4.91E-22	11
Nop58	3.41E-26	-1.18708	0.308	0.593	5.69E-22	11
Tmem229a	3.5E-26	0.893979	0.383	0.053	5.83E-22	11
Slc29a1	3.96E-26	-1.42432	0.142	0.564	6.61E-22	11
Mfn1	4.35E-26	0.564397	0.308	0.036	7.25E-22	11
C030005K(	5.03E-26	0.524524	0.125	0.001	8.39E-22	11
Elovl2	5.52E-26	0.824211	0.333	0.036	9.21E-22	11
Nebl	5.92E-26	0.491421	0.175	0.006	9.87E-22	11
Trf	6.27E-26	0.785474	0.325	0.035	1.05E-21	11
Wwc1	6.79E-26	0.691379	0.408	0.067	1.13E-21	11
Cgref1	1.11E-25	0.285591	0.125	0.001	1.85E-21	11
Plce1	1.15E-25	0.537573	0.192	0.006	1.91E-21	11
Cadm2	1.16E-25	0.373624	0.275	0.046	1.94E-21	11
Ecm2	1.24E-25	0.515309	0.158	0.002	2.07E-21	11
Oat	1.27E-25	0.803692	0.417	0.068	2.12E-21	11
Mpp6	1.32E-25	0.942675	0.592	0.158	2.21E-21	11
Phgdh	1.44E-25	0.908174	0.442	0.077	2.4E-21	11
Wls	1.51E-25	0.980992	0.458	0.084	2.53E-21	11
Arpp21	1.61E-25	0.28574	0.383	0.111	2.69E-21	11
Psip1	1.77E-25	-0.785	0.558	0.688	2.94E-21	11
Agtrap	2.32E-25	0.533094	0.183	0.006	3.87E-21	11
Lmbrd1	3.59E-25	0.615129	0.392	0.068	5.98E-21	11
Fkbp10	3.67E-25	0.418405	0.175	0.007	6.13E-21	11
Sec14l2	3.92E-25	0.428433	0.158	0.003	6.54E-21	11
Snrpd1	3.94E-25	-0.7167	0.475	0.524	6.57E-21	11
Map1b	4.06E-25	-1.52253	0.258	0.589	6.77E-21	11
Prr5l	4.27E-25	0.492362	0.158	0.003	7.13E-21	11
Dek	4.43E-25	-1.22145	0.4	0.695	7.39E-21	11

Ldha	4.79E-25	0.941638	0.667	0.228	7.99E-21	11
Acsl6	5.15E-25	0.767244	0.3	0.028	8.58E-21	11
Hn1	5.37E-25	-1.13201	0.3	0.543	8.96E-21	11
Dbndd2	5.43E-25	0.581074	0.258	0.023	9.06E-21	11
Slc15a2	5.66E-25	0.603481	0.308	0.037	9.45E-21	11
Acss1	5.77E-25	0.567609	0.175	0.004	9.63E-21	11
Rps11	6.14E-25	-0.8065	0.625	0.778	1.02E-20	11
Notch1	6.66E-25	0.585862	0.258	0.021	1.11E-20	11
Cd151	8.4E-25	0.600411	0.242	0.016	1.4E-20	11
Npl	1.09E-24	0.443607	0.175	0.006	1.81E-20	11
Anxa5	1.13E-24	0.465806	0.225	0.018	1.89E-20	11
Col1a2	1.14E-24	0.329557	0.183	0.013	1.9E-20	11
Smad9	1.21E-24	0.339007	0.142	0.003	2.01E-20	11
Sned1	1.92E-24	0.870454	0.267	0.022	3.2E-20	11
Itgb8	2.07E-24	0.589179	0.25	0.019	3.45E-20	11
Tmie	2.27E-24	0.461934	0.133	0.001	3.78E-20	11
Gas5	2.39E-24	-0.8617	0.592	0.743	3.98E-20	11
Draxin	2.48E-24	-1.27965	0.233	0.556	4.14E-20	11
Fus	2.48E-24	-0.74216	0.617	0.737	4.14E-20	11
Bmpr1a	2.55E-24	0.77236	0.475	0.1	4.26E-20	11
Matr3	2.8E-24	-0.80005	0.6	0.707	4.67E-20	11
Rarres1	2.81E-24	0.343518	0.117	0.001	4.69E-20	11
Stmn3	3.27E-24	-1.23946	0.258	0.624	5.45E-20	11
Spry1	3.32E-24	0.50206	0.208	0.012	5.54E-20	11
Oaf	3.36E-24	0.303554	0.142	0.003	5.6E-20	11
Cxcl14	3.5E-24	0.916996	0.333	0.039	5.84E-20	11
Itgb5	4.09E-24	0.420658	0.175	0.008	6.82E-20	11
Cbs	5.16E-24	0.515939	0.142	0.002	8.6E-20	11
Eif3a	6.18E-24	-0.82253	0.542	0.689	1.03E-19	11
4931406C(	7.22E-24	0.460685	0.292	0.044	1.2E-19	11
Smc2	7.33E-24	-1.34054	0.308	0.546	1.22E-19	11
Banf1	8.57E-24	-0.70835	0.633	0.694	1.43E-19	11
Dbp	9.4E-24	0.653376	0.317	0.038	1.57E-19	11
Rgma	9.87E-24	0.65199	0.333	0.044	1.65E-19	11
Entpd2	1.14E-23	0.452978	0.133	0.001	1.9E-19	11
Lysmd2	1.26E-23	0.697729	0.4	0.069	2.11E-19	11
Tex264	1.28E-23	0.623079	0.375	0.066	2.13E-19	11
Appl2	1.34E-23	0.784392	0.525	0.128	2.24E-19	11
4930402H:	1.38E-23	0.652174	0.467	0.11	2.31E-19	11
Cck	1.52E-23	0.771518	0.133	0.001	2.54E-19	11
Reep5	1.53E-23	0.824177	0.533	0.135	2.55E-19	11
Olfm2	1.55E-23	0.328108	0.133	0.003	2.58E-19	11
Pgm2	1.79E-23	0.571766	0.275	0.031	2.98E-19	11
Chd4	1.83E-23	-0.78302	0.675	0.77	3.05E-19	11
Anp32b	2.41E-23	-1.0979	0.342	0.592	4.01E-19	11
Eef1a1	2.46E-23	-0.732	0.783	0.884	4.11E-19	11
Klhl5	2.7E-23	0.674629	0.308	0.037	4.5E-19	11
Hnrnpdl	2.94E-23	-0.8311	0.575	0.757	4.9E-19	11

Capns1	2.98E-23	0.866879	0.65	0.224	4.98E-19	11
Cdh22	3.05E-23	0.563306	0.192	0.008	5.09E-19	11
Cdk4	3.13E-23	-0.90462	0.425	0.643	5.22E-19	11
Ptma	3.88E-23	-1.09604	0.258	0.583	6.47E-19	11
Usp24	3.98E-23	0.397377	0.25	0.032	6.64E-19	11
Mras	4.02E-23	0.70615	0.35	0.051	6.71E-19	11
Rpl23	4.07E-23	-0.8248	0.425	0.539	6.78E-19	11
Etv4	4.15E-23	0.435785	0.158	0.005	6.92E-19	11
Mmp14	4.4E-23	0.813094	0.592	0.171	7.33E-19	11
Slc7a11	5.31E-23	0.69008	0.15	0.003	8.86E-19	11
Il6st	5.45E-23	0.50269	0.292	0.038	9.1E-19	11
Thrsp	6.39E-23	0.968434	0.292	0.031	1.07E-18	11
Clip1	6.66E-23	0.722035	0.442	0.091	1.11E-18	11
Aldoa	6.69E-23	0.847035	0.7	0.254	1.12E-18	11
Abcd3	1E-22	0.854822	0.6	0.179	1.67E-18	11
Smc3	1.04E-22	-0.83833	0.517	0.634	1.74E-18	11
Them4	1.05E-22	0.795676	0.342	0.049	1.76E-18	11
Hsd17b12	1.15E-22	0.89424	0.6	0.185	1.92E-18	11
Plec	1.23E-22	0.391759	0.183	0.01	2.06E-18	11
Neurod1	1.31E-22	-2.02268	0.175	0.535	2.19E-18	11
Lamb2	1.41E-22	0.64064	0.2	0.01	2.36E-18	11
Serpinh1	1.48E-22	0.669651	0.475	0.112	2.47E-18	11
Cyp4v3	1.49E-22	0.32228	0.133	0.002	2.48E-18	11
Sybu	1.49E-22	0.338257	0.142	0.004	2.49E-18	11
St3gal6	1.54E-22	0.456173	0.183	0.008	2.56E-18	11
Eef2	1.59E-22	-0.79202	0.558	0.717	2.65E-18	11
Srsf3	1.61E-22	-0.82492	0.483	0.657	2.69E-18	11
Mtss1l	1.86E-22	0.538387	0.25	0.025	3.1E-18	11
Lfng	1.92E-22	0.667051	0.283	0.029	3.21E-18	11
Phyh	1.99E-22	0.745105	0.375	0.062	3.32E-18	11
Rpl8	2E-22	-0.61499	0.875	0.897	3.34E-18	11
Epdr1	2.09E-22	0.5815	0.342	0.057	3.49E-18	11
Kcne1l	2.1E-22	0.821587	0.342	0.047	3.51E-18	11
Cib1	2.23E-22	0.709416	0.358	0.055	3.71E-18	11
Stom	2.38E-22	0.427199	0.158	0.004	3.96E-18	11
Kank1	2.55E-22	0.589001	0.283	0.033	4.26E-18	11
Ldhb	2.59E-22	0.767517	0.725	0.277	4.32E-18	11
Slc30a10	3.14E-22	0.695566	0.367	0.06	5.23E-18	11
Nasp	3.14E-22	-1.0707	0.392	0.641	5.24E-18	11
Gsap	3.41E-22	0.376165	0.167	0.007	5.69E-18	11
Ccnd2	3.58E-22	-1.13743	0.442	0.663	5.97E-18	11
Atp6v0b	3.67E-22	0.508201	0.683	0.264	6.12E-18	11
Ccdc24	4.9E-22	0.40889	0.142	0.003	8.17E-18	11
Syt15	4.95E-22	0.309178	0.108	0.001	8.26E-18	11
Hip1	5.01E-22	0.675689	0.475	0.115	8.35E-18	11
Pgpep1	5.42E-22	0.331052	0.167	0.009	9.03E-18	11
Ttyh3	6.13E-22	0.451578	0.442	0.121	1.02E-17	11
Ttc3	6.2E-22	-0.80088	0.717	0.829	1.03E-17	11

Hdhd2	7.98E-22	0.568715	0.458	0.115	1.33E-17	11
Tapbp	8.33E-22	0.298727	0.183	0.016	1.39E-17	11
Ybx1	8.74E-22	-0.7042	0.675	0.786	1.46E-17	11
Hexa	9.74E-22	0.423051	0.325	0.063	1.62E-17	11
Sox4	1.04E-21	-1.1509	0.35	0.584	1.74E-17	11
Rpl34	1.17E-21	-0.76183	0.517	0.653	1.95E-17	11
Parp3	1.19E-21	0.391035	0.125	0.002	1.98E-17	11
Dmd	1.2E-21	0.558143	0.25	0.024	2E-17	11
Adamts5	1.23E-21	0.407539	0.15	0.004	2.04E-17	11
Tagln2	1.26E-21	0.585005	0.3	0.043	2.1E-17	11
Lrp1	1.62E-21	0.718281	0.317	0.045	2.71E-17	11
Pdap1	1.72E-21	-0.72195	0.567	0.683	2.87E-17	11
Dynlt3	1.77E-21	0.735203	0.325	0.044	2.95E-17	11
Necap2	1.8E-21	0.550042	0.3	0.043	3E-17	11
Mex3a	2.3E-21	-1.32273	0.108	0.465	3.83E-17	11
Smc1a	2.44E-21	-0.84018	0.483	0.631	4.07E-17	11
Acin1	2.51E-21	-0.78315	0.542	0.724	4.18E-17	11
Elavl3	2.59E-21	-1.39429	0.075	0.475	4.32E-17	11
C4b	2.6E-21	0.475909	0.117	0.001	4.34E-17	11
Slc20a2	2.63E-21	0.663083	0.317	0.045	4.38E-17	11
Ech1	2.63E-21	0.781783	0.467	0.112	4.39E-17	11
Colgalt2	3.37E-21	0.281563	0.108	0.001	5.62E-17	11
Rasl11a	3.48E-21	0.860199	0.267	0.028	5.81E-17	11
Nnat	3.74E-21	-0.94641	0.442	0.711	6.24E-17	11
Rbm25	4.1E-21	-0.73694	0.692	0.813	6.83E-17	11
Hdac2	4.33E-21	-1.04557	0.267	0.517	7.22E-17	11
Tns3	5.28E-21	0.330815	0.158	0.009	8.81E-17	11
Il18	5.63E-21	0.597581	0.208	0.013	9.39E-17	11
Gm9800	6.81E-21	-1.05846	0.267	0.536	1.14E-16	11
Barhl1	6.86E-21	-1.32606	0.15	0.524	1.14E-16	11
Mboat2	8.7E-21	0.620501	0.317	0.046	1.45E-16	11
Fgfr2	8.85E-21	0.452963	0.192	0.011	1.48E-16	11
Fkbp11	9.61E-21	0.560188	0.192	0.01	1.6E-16	11
Marcks	1.02E-20	-0.58647	0.858	0.916	1.69E-16	11
Phlda1	1.05E-20	0.609393	0.242	0.024	1.75E-16	11
Prex1	1.17E-20	0.734929	0.358	0.061	1.95E-16	11
Proca1	1.39E-20	0.377268	0.117	0.001	2.32E-16	11
Pygb	1.78E-20	0.587247	0.225	0.017	2.97E-16	11
Rpl22	1.79E-20	-0.72366	0.667	0.789	2.98E-16	11
Ezh2	1.9E-20	-1.10911	0.325	0.611	3.16E-16	11
Pdgfrl	2.44E-20	0.380717	0.15	0.007	4.07E-16	11
Rplp1	2.48E-20	-0.54389	0.892	0.876	4.14E-16	11
Plvap	2.61E-20	0.288822	0.117	0.002	4.35E-16	11
Nkain4	2.69E-20	0.834282	0.492	0.126	4.49E-16	11
Plin3	2.7E-20	0.366893	0.158	0.007	4.5E-16	11
Celf2	2.75E-20	-0.94158	0.5	0.638	4.59E-16	11
Hpgd	2.96E-20	0.441409	0.142	0.004	4.93E-16	11
Lpl	3.15E-20	0.71153	0.342	0.056	5.26E-16	11

Fkbp3	3.26E-20	-0.70867	0.683	0.745	5.44E-16	11
Col5a3	3.29E-20	0.39049	0.125	0.002	5.49E-16	11
Shisa4	3.63E-20	0.48806	0.292	0.051	6.05E-16	11
Crot	3.74E-20	0.66187	0.4	0.086	6.24E-16	11
Fubp1	5.1E-20	-0.9004	0.383	0.567	8.51E-16	11
Rpl14	5.17E-20	-0.63367	0.617	0.697	8.63E-16	11
Orai1	5.2E-20	0.518877	0.225	0.022	8.67E-16	11
Smc4	5.6E-20	-1.06402	0.425	0.567	9.34E-16	11
Pdlim5	5.82E-20	0.456581	0.192	0.013	9.71E-16	11
Fgf1	5.82E-20	0.257397	0.108	0.001	9.71E-16	11
Gstk1	6.94E-20	0.456615	0.167	0.007	1.16E-15	11
Spred1	7.38E-20	0.505714	0.417	0.106	1.23E-15	11
Rpl26	7.65E-20	-0.67948	0.6	0.702	1.28E-15	11
Trib2	8E-20	0.71419	0.408	0.086	1.34E-15	11
Fads2	8.67E-20	0.533145	0.217	0.017	1.45E-15	11
Nrep	8.77E-20	-1.17181	0.342	0.571	1.46E-15	11
Megf10	9.12E-20	0.490365	0.167	0.007	1.52E-15	11
Kdelr3	1.02E-19	0.346646	0.15	0.008	1.7E-15	11
Tcf4	1.15E-19	-0.65234	0.85	0.925	1.91E-15	11
Spock3	1.16E-19	0.288935	0.117	0.002	1.94E-15	11
Lamp2	1.24E-19	0.812445	0.5	0.135	2.06E-15	11
Cdh10	1.33E-19	0.265662	0.15	0.013	2.22E-15	11
Rbfox3	1.42E-19	-1.34961	0.125	0.496	2.36E-15	11
Mfap3l	1.46E-19	0.315713	0.133	0.004	2.43E-15	11
Cpeb4	1.6E-19	0.410186	0.267	0.045	2.67E-15	11
Rora	1.75E-19	0.454538	0.292	0.053	2.92E-15	11
Gm10075	1.85E-19	-0.81844	0.367	0.515	3.09E-15	11
Grm5	1.88E-19	0.689155	0.183	0.01	3.13E-15	11
Tmem33	1.94E-19	0.612229	0.492	0.142	3.23E-15	11
Lhx1	1.98E-19	-1.33587	0.158	0.499	3.3E-15	11
Cap2	1.99E-19	0.363188	0.158	0.009	3.32E-15	11
Grin3a	2.38E-19	0.77303	0.342	0.058	3.98E-15	11
Hsp90aa1	2.41E-19	-0.81958	0.433	0.617	4.02E-15	11
Ccnd1	2.91E-19	-1.13225	0.308	0.523	4.85E-15	11
Ptchd4	3.55E-19	0.349452	0.158	0.011	5.92E-15	11
Abhd6	3.71E-19	0.569309	0.267	0.035	6.18E-15	11
Gas7	4.78E-19	0.258032	0.125	0.006	7.98E-15	11
Nrcam	4.8E-19	0.721468	0.483	0.131	8E-15	11
Sirt2	5.09E-19	0.488895	0.492	0.153	8.49E-15	11
Bclaf1	5.2E-19	-0.99485	0.317	0.584	8.68E-15	11
KCTD12	5.38E-19	0.484532	0.2	0.017	8.98E-15	11
Ahcyl1	5.62E-19	0.672969	0.525	0.157	9.37E-15	11
Sdc3	5.9E-19	0.437768	0.325	0.075	9.84E-15	11
Pigs	6.32E-19	0.578413	0.233	0.027	1.05E-14	11
Plekhb1	6.77E-19	0.490045	0.167	0.01	1.13E-14	11
Sgcb	6.88E-19	0.315564	0.367	0.113	1.15E-14	11
Golph3	7.69E-19	0.606212	0.358	0.071	1.28E-14	11
Galc	7.82E-19	0.525882	0.275	0.039	1.3E-14	11

Lrp4	7.97E-19	0.391285	0.15	0.006	1.33E-14	11
Stmn2	8.04E-19	-1.40315	0.325	0.554	1.34E-14	11
Rpl39	8.52E-19	-0.73479	0.533	0.646	1.42E-14	11
Cdo1	8.54E-19	0.458178	0.2	0.02	1.42E-14	11
Vimp	8.56E-19	0.780476	0.567	0.187	1.43E-14	11
Fads1	8.59E-19	0.567713	0.408	0.103	1.43E-14	11
Fam195a	9.35E-19	0.435894	0.167	0.01	1.56E-14	11
Fuca1	9.72E-19	0.666522	0.5	0.145	1.62E-14	11
Gm266	9.87E-19	0.339067	0.125	0.003	1.65E-14	11
Srsf11	1.16E-18	-0.73735	0.5	0.69	1.94E-14	11
Hnrnpd	1.21E-18	-0.85789	0.375	0.612	2.01E-14	11
Aco2	1.21E-18	0.719047	0.742	0.335	2.02E-14	11
Tcn2	1.25E-18	0.309819	0.175	0.018	2.08E-14	11
Rasa3	1.26E-18	0.524392	0.258	0.037	2.1E-14	11
Tmem106b	1.37E-18	0.602269	0.35	0.07	2.28E-14	11
Rps3a1	1.41E-18	-0.7532	0.525	0.667	2.36E-14	11
Slc35e4	1.43E-18	0.47918	0.2	0.016	2.39E-14	11
Rab7l1	1.55E-18	0.501202	0.192	0.013	2.59E-14	11
Sypl	1.57E-18	0.590042	0.358	0.072	2.61E-14	11
Hdgf	1.57E-18	-0.76413	0.408	0.532	2.63E-14	11
Nfkbiz	1.73E-18	0.444025	0.175	0.011	2.88E-14	11
Atrx	1.96E-18	-0.77687	0.608	0.741	3.27E-14	11
Srrm2	1.98E-18	-0.50777	0.717	0.732	3.3E-14	11
Tnfaip6	2.26E-18	0.286364	0.125	0.004	3.77E-14	11
Efemp2	2.28E-18	0.47616	0.192	0.015	3.81E-14	11
Nucks1	2.59E-18	-0.64926	0.617	0.663	4.32E-14	11
Laptm4b	2.74E-18	0.624339	0.483	0.139	4.56E-14	11
4933407L2	3.08E-18	0.393543	0.125	0.003	5.13E-14	11
Miat	3.15E-18	-1.41625	0.192	0.517	5.25E-14	11
Decr1	3.59E-18	0.538944	0.342	0.07	5.98E-14	11
Pnn	3.6E-18	-0.73616	0.508	0.723	6.01E-14	11
Fabp5	3.7E-18	0.797304	0.733	0.4	6.17E-14	11
Enho	3.71E-18	0.341331	0.242	0.048	6.19E-14	11
Igfbp3	3.9E-18	0.66424	0.183	0.011	6.5E-14	11
Camk1	4.09E-18	0.359319	0.267	0.055	6.83E-14	11
Tubb3	4.13E-18	-1.48966	0.117	0.476	6.89E-14	11
Suclg2	4.13E-18	0.382178	0.208	0.025	6.89E-14	11
Fermt2	4.17E-18	0.712277	0.583	0.202	6.96E-14	11
Eya1	4.37E-18	0.37185	0.125	0.003	7.29E-14	11
Rgcc	4.49E-18	0.521482	0.183	0.015	7.49E-14	11
Rhoj	4.67E-18	0.525191	0.242	0.03	7.79E-14	11
Uqcr10	4.69E-18	0.703645	0.783	0.431	7.82E-14	11
D430041D	4.7E-18	-1.22014	0.142	0.494	7.84E-14	11
Ctnnd2	4.71E-18	0.551662	0.392	0.096	7.85E-14	11
Cox14	4.91E-18	0.71274	0.717	0.313	8.18E-14	11
Itgav	5.14E-18	0.504496	0.308	0.058	8.58E-14	11
Samd4	5.18E-18	0.555298	0.258	0.036	8.64E-14	11
Cdh11	6.05E-18	0.542927	0.192	0.014	1.01E-13	11

Mid1ip1	6.4E-18	0.651885	0.308	0.049	1.07E-13	11
Fuom	7E-18	0.490075	0.233	0.03	1.17E-13	11
BC026585	7.03E-18	0.322264	0.117	0.002	1.17E-13	11
Rlbp1	7.47E-18	0.457988	0.158	0.011	1.25E-13	11
Renbp	8.9E-18	0.374729	0.142	0.006	1.49E-13	11
Adcy2	9.39E-18	0.53932	0.133	0.004	1.57E-13	11
Nckap5	1.03E-17	0.278182	0.108	0.002	1.71E-13	11
Snrpb	1.05E-17	-0.65308	0.5	0.573	1.75E-13	11
Cct6a	1.06E-17	-0.7766	0.35	0.487	1.77E-13	11
Lrp10	1.15E-17	0.544461	0.258	0.039	1.91E-13	11
Lpcat1	1.16E-17	0.568722	0.458	0.133	1.94E-13	11
Marcksl1	1.19E-17	-0.7179	0.6	0.723	1.98E-13	11
Cacng5	1.34E-17	0.599453	0.292	0.048	2.23E-13	11
Agpat5	1.37E-17	0.706389	0.558	0.197	2.29E-13	11
Usp2	1.44E-17	0.325827	0.158	0.011	2.4E-13	11
Hp1bp3	1.65E-17	-0.67762	0.517	0.58	2.74E-13	11
Car10	1.66E-17	0.47244	0.233	0.039	2.77E-13	11
Ilf2	1.68E-17	-0.91267	0.325	0.542	2.81E-13	11
Rps15a	1.69E-17	-0.70806	0.533	0.658	2.82E-13	11
Itpkb	1.8E-17	0.477147	0.158	0.008	3.01E-13	11
Zfyve21	1.9E-17	0.509292	0.292	0.051	3.18E-13	11
Gng11	1.97E-17	0.352377	0.158	0.014	3.28E-13	11
Acadm	2.05E-17	0.463822	0.35	0.085	3.42E-13	11
Atraid	2.08E-17	0.531326	0.592	0.224	3.46E-13	11
Gm15417	2.11E-17	0.361303	0.133	0.006	3.53E-13	11
H1f0	2.14E-17	-1.06896	0.317	0.618	3.57E-13	11
Cbfa2t3	2.22E-17	-1.21638	0.1	0.404	3.7E-13	11
Tmem66	2.35E-17	0.657766	0.567	0.196	3.93E-13	11
Tmed5	2.38E-17	0.582852	0.442	0.124	3.98E-13	11
Rnh1	2.41E-17	0.545295	0.308	0.06	4.02E-13	11
Rhbdf1	2.43E-17	0.294488	0.125	0.005	4.06E-13	11
Mfhas1	2.57E-17	0.592218	0.333	0.067	4.29E-13	11
Pde4b	2.61E-17	0.577206	0.292	0.049	4.35E-13	11
Safb	2.62E-17	-0.83983	0.342	0.504	4.37E-13	11
Luc7l3	2.82E-17	-0.61592	0.642	0.748	4.71E-13	11
Ppp1r14b	2.96E-17	-0.91078	0.225	0.398	4.93E-13	11
Uap1l1	3.22E-17	0.393798	0.15	0.008	5.38E-13	11
Slc5a3	3.55E-17	0.419924	0.175	0.013	5.92E-13	11
Ank3	3.71E-17	-1.23753	0.142	0.422	6.19E-13	11
Tspan15	3.85E-17	0.332213	0.15	0.009	6.42E-13	11
Rabac1	4.09E-17	0.622488	0.642	0.255	6.82E-13	11
Bzw2	4.3E-17	-0.92091	0.283	0.489	7.17E-13	11
Anp32e	4.31E-17	-0.67448	0.525	0.613	7.18E-13	11
Soat1	4.54E-17	0.573976	0.208	0.019	7.57E-13	11
Nudt19	4.62E-17	0.440514	0.392	0.11	7.7E-13	11
Ptplb	4.71E-17	0.772131	0.433	0.113	7.86E-13	11
Gap43	4.71E-17	-1.18354	0.375	0.6	7.86E-13	11
Ina	4.81E-17	-1.26126	0.117	0.446	8.03E-13	11

Malat1	4.84E-17	0.561335	0.958	0.955	8.07E-13	11
Cyp4f13	5.05E-17	0.354657	0.158	0.011	8.42E-13	11
Eva1a	5.44E-17	0.486176	0.142	0.005	9.07E-13	11
Ilvbl	5.46E-17	0.292738	0.15	0.012	9.11E-13	11
Acadvl	5.49E-17	0.564407	0.4	0.104	9.15E-13	11
Ssbp4	5.49E-17	0.415851	0.283	0.059	9.16E-13	11
Ptplad1	5.74E-17	0.6536	0.633	0.248	9.58E-13	11
Sdf2	5.74E-17	0.480252	0.558	0.212	9.58E-13	11
Bex2	6.18E-17	-0.91762	0.308	0.572	1.03E-12	11
Vamp5	6.22E-17	0.377492	0.117	0.003	1.04E-12	11
Pcdh17	6.32E-17	0.497559	0.217	0.027	1.05E-12	11
Slc35c2	6.44E-17	0.653125	0.258	0.036	1.07E-12	11
Enpp5	6.74E-17	0.319309	0.183	0.026	1.12E-12	11
Mxra8	6.85E-17	0.414904	0.192	0.021	1.14E-12	11
Fam181b	7.18E-17	0.715944	0.475	0.14	1.2E-12	11
S1pr3	7.36E-17	0.476764	0.125	0.004	1.23E-12	11
Ngfrap1	7.45E-17	-0.68671	0.525	0.658	1.24E-12	11
Cplx2	7.73E-17	-1.19277	0.183	0.497	1.29E-12	11
Syt12	7.89E-17	0.511401	0.25	0.035	1.32E-12	11
1110065P2	8.09E-17	0.326852	0.367	0.116	1.35E-12	11
Npc1	8.96E-17	0.416194	0.242	0.04	1.49E-12	11
Hmgn1	9.38E-17	-0.69396	0.475	0.622	1.56E-12	11
Rbbp4	9.67E-17	-0.62138	0.408	0.484	1.61E-12	11
Cnbp	1.01E-16	-0.53588	0.717	0.74	1.68E-12	11
Rps10	1.09E-16	-0.72224	0.5	0.637	1.82E-12	11
Gpd2	1.11E-16	0.727036	0.342	0.07	1.86E-12	11
Mamdc2	1.12E-16	0.405103	0.2	0.02	1.87E-12	11
Eno1	1.15E-16	0.612006	0.6	0.226	1.92E-12	11
Cyp2j6	1.17E-16	0.351259	0.167	0.017	1.95E-12	11
Iqsec1	1.19E-16	0.371378	0.167	0.014	1.98E-12	11
Ctsc	1.26E-16	0.278353	0.125	0.008	2.1E-12	11
Rasa2	1.28E-16	0.567859	0.325	0.071	2.14E-12	11
Magt1	1.35E-16	0.49049	0.292	0.055	2.25E-12	11
Arhgef12	1.38E-16	0.406047	0.4	0.123	2.29E-12	11
Syngr1	1.39E-16	0.482392	0.358	0.091	2.32E-12	11
Rdh14	1.4E-16	0.355777	0.208	0.029	2.33E-12	11
Galnt18	1.45E-16	0.276789	0.142	0.01	2.42E-12	11
Slitrk2	1.52E-16	0.324598	0.133	0.007	2.54E-12	11
Kcnip3	1.54E-16	0.55127	0.417	0.118	2.57E-12	11
Rhoq	1.62E-16	0.529787	0.283	0.049	2.71E-12	11
1110001A1	1.68E-16	0.275491	0.317	0.095	2.8E-12	11
Acot13	1.88E-16	0.589672	0.408	0.111	3.13E-12	11
Haghl	1.88E-16	0.519753	0.358	0.088	3.14E-12	11
Gstt1	1.91E-16	0.503508	0.258	0.042	3.19E-12	11
Skap2	2.02E-16	0.461308	0.125	0.004	3.37E-12	11
Cyba	2.13E-16	0.330704	0.192	0.028	3.55E-12	11
Ugp2	2.32E-16	0.465014	0.35	0.088	3.87E-12	11
Chst7	2.4E-16	0.260915	0.117	0.005	4.01E-12	11

Nhlh2	2.46E-16	-1.30021	0.133	0.425	4.1E-12	11
Gabbr1	2.61E-16	0.461952	0.425	0.138	4.35E-12	11
Hnrnpa0	2.87E-16	-0.8703	0.292	0.457	4.79E-12	11
Baz1b	2.88E-16	-0.89531	0.317	0.494	4.81E-12	11
Hirip3	2.93E-16	-1.12472	0.133	0.384	4.89E-12	11
Ddt	3.13E-16	0.568962	0.392	0.103	5.22E-12	11
Hmgb2	3.18E-16	-1.03469	0.208	0.401	5.31E-12	11
Tprgl	3.26E-16	0.42103	0.4	0.122	5.44E-12	11
Tuba1b	3.3E-16	-0.7983	0.342	0.482	5.51E-12	11
Cdkn1b	3.41E-16	-0.70413	0.375	0.467	5.68E-12	11
Naga	3.47E-16	0.376662	0.217	0.032	5.78E-12	11
Cacng2	3.53E-16	-1.15214	0.05	0.34	5.89E-12	11
Fzd9	3.55E-16	0.450916	0.108	0.002	5.92E-12	11
As3mt	3.58E-16	0.425401	0.167	0.012	5.98E-12	11
mt-Nd2	3.65E-16	0.642838	0.867	0.637	6.09E-12	11
Srrm1	3.68E-16	-0.70344	0.458	0.612	6.14E-12	11
Alpl	3.72E-16	0.373227	0.108	0.002	6.2E-12	11
Ccar1	3.92E-16	-0.86328	0.358	0.573	6.54E-12	11
Metap2	4.07E-16	-0.71279	0.408	0.597	6.8E-12	11
H2afy	4.24E-16	-0.59815	0.542	0.592	7.07E-12	11
Vcl	4.31E-16	0.40331	0.175	0.016	7.19E-12	11
Fcgrt	4.43E-16	0.481331	0.208	0.026	7.39E-12	11
Glo1	4.69E-16	0.649508	0.408	0.105	7.82E-12	11
2700094K1	5.34E-16	-0.68808	0.517	0.614	8.91E-12	11
Ctsh	5.62E-16	0.280022	0.133	0.011	9.38E-12	11
Phlpp1	5.93E-16	0.285553	0.175	0.022	9.89E-12	11
Ccdc107	6.22E-16	0.589119	0.325	0.07	1.04E-11	11
Pttg1ip	6.28E-16	0.607424	0.358	0.084	1.05E-11	11
Epn2	6.47E-16	0.318184	0.3	0.082	1.08E-11	11
Nsg2	6.63E-16	-1.04155	0.167	0.489	1.11E-11	11
Eif3c	7.56E-16	-0.57074	0.533	0.603	1.26E-11	11
Dclk1	8.31E-16	0.700038	0.775	0.409	1.39E-11	11
Dpysl4	9.28E-16	-1.03222	0.217	0.492	1.55E-11	11
Nim1	1.11E-15	0.452651	0.175	0.015	1.85E-11	11
Stk25	1.16E-15	-0.27788	0.25	0.183	1.93E-11	11
Sf3b1	1.25E-15	-0.65123	0.542	0.669	2.08E-11	11
Creg1	1.36E-15	0.329562	0.258	0.057	2.26E-11	11
Ppp2r2c	1.5E-15	-0.98817	0.242	0.506	2.49E-11	11
Dag1	1.5E-15	0.442066	0.233	0.036	2.5E-11	11
Sec11c	1.52E-15	0.603833	0.55	0.21	2.53E-11	11
Junb	1.53E-15	0.740181	0.242	0.033	2.56E-11	11
Eif4g2	1.53E-15	-0.59717	0.6	0.705	2.56E-11	11
Ap3m2	1.62E-15	0.284422	0.225	0.049	2.7E-11	11
Tulp3	1.75E-15	0.404255	0.208	0.03	2.92E-11	11
Cpt1a	1.88E-15	0.445891	0.175	0.016	3.14E-11	11
Extl3	1.97E-15	0.268427	0.242	0.058	3.28E-11	11
Aldh6a1	2.06E-15	0.366923	0.175	0.02	3.44E-11	11
Cnn3	2.1E-15	0.651235	0.758	0.434	3.51E-11	11

Csf1	2.24E-15	0.329181	0.133	0.008	3.74E-11	11
Acyp2	2.25E-15	0.517768	0.3	0.062	3.76E-11	11
Hibadh	2.3E-15	0.465401	0.333	0.084	3.84E-11	11
Tmem147	2.37E-15	0.306114	0.567	0.259	3.96E-11	11
Tmem50b	2.39E-15	0.404103	0.2	0.027	3.99E-11	11
Usp6nl	2.47E-15	0.377276	0.208	0.031	4.13E-11	11
Luc7l2	2.51E-15	-0.76616	0.35	0.496	4.19E-11	11
Nap1l1	2.54E-15	-0.5613	0.475	0.511	4.23E-11	11
Ppt1	2.55E-15	0.611885	0.367	0.092	4.26E-11	11
Cyfip1	2.58E-15	0.496687	0.4	0.118	4.3E-11	11
Klf15	2.64E-15	0.344389	0.108	0.003	4.41E-11	11
Lgalsl	2.65E-15	0.351692	0.317	0.091	4.41E-11	11
Dut	2.87E-15	-1.08361	0.192	0.436	4.79E-11	11
Rnd3	2.93E-15	-1.11397	0.133	0.433	4.89E-11	11
Aebp1	3.25E-15	0.329905	0.133	0.008	5.42E-11	11
Rcn2	3.46E-15	0.626335	0.625	0.278	5.77E-11	11
Sft2d2	3.6E-15	0.267823	0.108	0.004	6E-11	11
Etfb	3.7E-15	0.576165	0.517	0.182	6.18E-11	11
Tmem38a	3.76E-15	0.335271	0.175	0.02	6.28E-11	11
Lrpap1	4.14E-15	0.280444	0.342	0.115	6.91E-11	11
Dab1	4.32E-15	0.448919	0.217	0.032	7.21E-11	11
Cald1	4.34E-15	-0.9543	0.325	0.537	7.25E-11	11
Ncor1	4.42E-15	-0.69366	0.467	0.556	7.38E-11	11
Ctsa	4.46E-15	0.291635	0.367	0.122	7.43E-11	11
Nr3c1	4.46E-15	0.561049	0.408	0.123	7.44E-11	11
Trip6	4.89E-15	0.327658	0.183	0.024	8.16E-11	11
Mtss1	4.99E-15	-0.90127	0.15	0.27	8.32E-11	11
2410006H	5.59E-15	-0.65278	0.4	0.459	9.33E-11	11
Ralb	5.78E-15	0.408365	0.175	0.017	9.64E-11	11
H2afx	5.82E-15	-0.95981	0.217	0.331	9.71E-11	11
Vegfa	6.02E-15	0.479891	0.208	0.025	1E-10	11
Erp29	6.12E-15	0.367346	0.6	0.278	1.02E-10	11
Txndc15	6.75E-15	0.389871	0.392	0.129	1.13E-10	11
Fam173a	6.76E-15	0.534386	0.45	0.151	1.13E-10	11
Zfp36l1	6.81E-15	0.340952	0.408	0.149	1.14E-10	11
Fam69a	6.87E-15	0.402409	0.208	0.03	1.15E-10	11
Fam167a	7.03E-15	0.287015	0.117	0.005	1.17E-10	11
Gna13	7.09E-15	0.509696	0.408	0.126	1.18E-10	11
Slc33a1	7.35E-15	0.343484	0.192	0.028	1.23E-10	11
Tnfrsf21	7.43E-15	0.333989	0.258	0.062	1.24E-10	11
Nedd4	7.43E-15	-0.61873	0.583	0.683	1.24E-10	11
Gadd45g	8.04E-15	0.669934	0.342	0.078	1.34E-10	11
Shisa5	8.74E-15	0.46143	0.2	0.024	1.46E-10	11
Cd200	8.77E-15	0.431251	0.225	0.044	1.46E-10	11
Capn2	8.94E-15	0.49069	0.342	0.088	1.49E-10	11
Prpf40a	9.25E-15	-0.67944	0.45	0.572	1.54E-10	11
Eif3f	9.65E-15	-0.55712	0.583	0.63	1.61E-10	11
Pex11a	1.01E-14	0.339382	0.133	0.008	1.68E-10	11

Fam210b	1.03E-14	-0.92006	0.15	0.314	1.71E-10	11
Klhl13	1.05E-14	0.58468	0.375	0.102	1.76E-10	11
Gpm6a	1.06E-14	0.450743	0.758	0.406	1.78E-10	11
Tmem30a	1.08E-14	0.391262	0.467	0.177	1.81E-10	11
Tgfb2	1.12E-14	0.33496	0.467	0.19	1.87E-10	11
Rnf215	1.13E-14	0.421791	0.275	0.061	1.88E-10	11
Tmpo	1.16E-14	-0.98032	0.208	0.453	1.94E-10	11
Srsf2	1.16E-14	-0.61159	0.467	0.566	1.94E-10	11
Crip1	1.18E-14	0.37639	0.15	0.012	1.97E-10	11
Hmgcs1	1.2E-14	0.619094	0.45	0.149	2.01E-10	11
Naprt1	1.24E-14	0.367747	0.125	0.006	2.07E-10	11
Gas2l1	1.27E-14	0.372917	0.125	0.006	2.11E-10	11
Rgs20	1.31E-14	0.472132	0.342	0.089	2.18E-10	11
0610031J0	1.34E-14	0.540641	0.292	0.06	2.23E-10	11
1110054M	1.34E-14	0.511358	0.142	0.011	2.24E-10	11
Sept9	1.36E-14	0.482923	0.317	0.077	2.27E-10	11
Dcxr	1.4E-14	0.462229	0.158	0.012	2.33E-10	11
Scd1	1.44E-14	0.569958	0.133	0.009	2.41E-10	11
Mxd4	1.45E-14	0.285983	0.45	0.196	2.42E-10	11
Top2a	1.47E-14	-1.30861	0.217	0.429	2.45E-10	11
Ggh	1.5E-14	0.551729	0.408	0.121	2.5E-10	11
Olig1	1.54E-14	0.934613	0.333	0.078	2.57E-10	11
Hsd17b10	1.58E-14	0.301088	0.417	0.163	2.64E-10	11
Anxa7	1.58E-14	0.29718	0.133	0.01	2.64E-10	11
Pcdhga9	1.63E-14	0.506248	0.783	0.417	2.71E-10	11
Eva1b	1.72E-14	0.382209	0.133	0.009	2.86E-10	11
Fam49a	1.72E-14	0.411071	0.233	0.043	2.86E-10	11
Wipi1	1.75E-14	0.521961	0.225	0.032	2.93E-10	11
Pcbp1	1.8E-14	-0.51052	0.567	0.593	3.01E-10	11
Dcx	1.85E-14	-1.19805	0.083	0.377	3.09E-10	11
Sept3	1.91E-14	-1.23348	0.117	0.394	3.19E-10	11
1500009L1	1.93E-14	0.437619	0.225	0.037	3.21E-10	11
Ntsr2	1.94E-14	0.609528	0.125	0.007	3.24E-10	11
Gm5607	2.15E-14	0.562112	0.133	0.007	3.59E-10	11
Arl6ip1	2.18E-14	0.593517	0.858	0.547	3.63E-10	11
Tmbim4	2.25E-14	0.455604	0.417	0.142	3.75E-10	11
Stt3b	2.29E-14	0.50334	0.533	0.209	3.81E-10	11
Sltm	2.29E-14	-0.64884	0.467	0.532	3.82E-10	11
Mcm7	2.52E-14	-0.97307	0.142	0.39	4.2E-10	11
Eef1b2	2.58E-14	-0.54124	0.692	0.732	4.31E-10	11
Tacc1	2.72E-14	0.435116	0.292	0.068	4.55E-10	11
Unc50	2.78E-14	0.455058	0.392	0.123	4.64E-10	11
Fnbp1l	3.25E-14	-1.01709	0.225	0.455	5.41E-10	11
Ptpmt1	3.49E-14	0.389689	0.358	0.11	5.82E-10	11
Pcm1	3.79E-14	-0.73432	0.417	0.522	6.33E-10	11
G3bp2	3.86E-14	-0.67719	0.45	0.552	6.44E-10	11
Ergic3	4.18E-14	0.302096	0.508	0.226	6.98E-10	11
Agpat3	4.27E-14	0.534483	0.408	0.128	7.12E-10	11

Syncrip	4.41E-14	-0.59304	0.425	0.496	7.35E-10	11
Tia1	4.59E-14	-0.8107	0.358	0.553	7.65E-10	11
Fat1	4.95E-14	0.255494	0.167	0.025	8.26E-10	11
Smox	4.97E-14	0.509532	0.292	0.064	8.29E-10	11
Ssrp1	5.03E-14	-0.70253	0.45	0.577	8.39E-10	11
Prnp	5.03E-14	0.618737	0.525	0.204	8.39E-10	11
Acsl3	5.08E-14	0.649275	0.442	0.144	8.47E-10	11
2700060E(	5.1E-14	-0.40997	0.383	0.352	8.51E-10	11
Vcan	5.54E-14	0.527983	0.408	0.127	9.24E-10	11
Dnajc3	5.62E-14	0.59648	0.492	0.174	9.37E-10	11
H3f3a	5.75E-14	-0.82116	0.2	0.382	9.59E-10	11
Eny2	6.26E-14	-0.59191	0.333	0.387	1.04E-09	11
Plk2	6.41E-14	0.504296	0.15	0.012	1.07E-09	11
Sox9	6.41E-14	0.598569	0.6	0.252	1.07E-09	11
Rpl37a	6.49E-14	-0.58925	0.4	0.468	1.08E-09	11
Fam63b	6.86E-14	0.319819	0.292	0.086	1.14E-09	11
Ufl1	7.19E-14	0.496891	0.367	0.105	1.2E-09	11
Cenpf	7.19E-14	-1.30798	0.2	0.363	1.2E-09	11
Syt11	8.04E-14	0.527924	0.842	0.502	1.34E-09	11
Sf3b2	8.62E-14	-0.47215	0.6	0.625	1.44E-09	11
Lhfp	8.9E-14	0.341871	0.15	0.018	1.49E-09	11
Hnrnpr	9.04E-14	-0.69482	0.383	0.546	1.51E-09	11
Irx1	9.25E-14	0.294663	0.108	0.005	1.54E-09	11
Ndufc2	9.98E-14	0.597969	0.8	0.609	1.66E-09	11
Ppp2r5a	1E-13	0.477062	0.258	0.049	1.67E-09	11
Hadha	1.01E-13	0.550759	0.442	0.153	1.68E-09	11
Zc3h13	1.17E-13	-0.91347	0.283	0.463	1.95E-09	11
Kif5c	1.19E-13	-1.09802	0.133	0.4	1.98E-09	11
Ier5	1.23E-13	-0.97531	0.125	0.395	2.05E-09	11
Cxxc5	1.28E-13	-0.83791	0.292	0.545	2.14E-09	11
Snta1	1.31E-13	0.302718	0.133	0.01	2.18E-09	11
Zfp36	1.33E-13	0.312864	0.133	0.011	2.22E-09	11
H2afj	1.34E-13	0.387246	0.492	0.203	2.24E-09	11
Phip	1.37E-13	-0.93046	0.242	0.461	2.29E-09	11
Grn	1.49E-13	0.329105	0.3	0.085	2.49E-09	11
Rpl41	1.5E-13	-0.59703	0.65	0.781	2.5E-09	11
Maf	1.56E-13	0.302731	0.15	0.018	2.61E-09	11
Etfa	1.57E-13	0.556582	0.592	0.251	2.62E-09	11
Abca3	1.59E-13	0.333773	0.2	0.037	2.65E-09	11
Gng2	1.6E-13	-1.03115	0.133	0.397	2.67E-09	11
Cyb5	1.6E-13	0.553756	0.5	0.185	2.67E-09	11
Os9	1.7E-13	0.356533	0.55	0.249	2.84E-09	11
Tm7sf2	1.76E-13	0.274698	0.175	0.032	2.94E-09	11
Top1	1.91E-13	-0.57093	0.583	0.663	3.18E-09	11
Btbd6	1.94E-13	0.353776	0.167	0.021	3.23E-09	11
Ikbip	1.94E-13	0.479874	0.217	0.035	3.23E-09	11
Lgmn	1.94E-13	0.368837	0.367	0.117	3.24E-09	11
Anxa2	1.99E-13	0.447714	0.142	0.011	3.32E-09	11

Lifr	2.05E-13	0.364217	0.117	0.005	3.41E-09	11
Gyg	2.06E-13	0.280034	0.125	0.009	3.43E-09	11
Neat1	2.1E-13	0.565021	0.167	0.015	3.5E-09	11
Smpd1	2.12E-13	0.448855	0.217	0.038	3.53E-09	11
8430419L(	2.17E-13	0.348044	0.258	0.065	3.62E-09	11
Eif4a1	2.19E-13	-0.70333	0.333	0.485	3.65E-09	11
2810417H:	2.27E-13	-1.10464	0.183	0.392	3.78E-09	11
Efemp1	2.27E-13	0.251278	0.108	0.006	3.79E-09	11
Mir22hg	2.49E-13	0.295273	0.108	0.005	4.16E-09	11
Akap9	2.61E-13	-0.77523	0.408	0.501	4.35E-09	11
Camta1	2.74E-13	-0.85855	0.233	0.409	4.56E-09	11
Dolk	2.75E-13	0.300756	0.192	0.034	4.58E-09	11
Col9a3	2.78E-13	0.549127	0.475	0.178	4.64E-09	11
Gpr56	2.98E-13	0.586144	0.525	0.22	4.97E-09	11
Galk1	3.04E-13	0.281113	0.217	0.049	5.07E-09	11
Gpi1	3.09E-13	0.371117	0.375	0.129	5.15E-09	11
Snrpf	3.24E-13	-0.78877	0.242	0.428	5.41E-09	11
Tox3	3.25E-13	-0.99432	0.083	0.347	5.42E-09	11
Cpped1	3.3E-13	0.537899	0.275	0.059	5.5E-09	11
Surf4	3.51E-13	0.51787	0.375	0.117	5.85E-09	11
Xrn2	3.56E-13	-0.8091	0.308	0.474	5.93E-09	11
Psma7	3.89E-13	-0.49251	0.725	0.782	6.5E-09	11
Rfc1	4E-13	-0.80909	0.2	0.331	6.67E-09	11
Emc3	4.05E-13	0.52576	0.425	0.147	6.75E-09	11
Kifc3	4.19E-13	0.332455	0.108	0.005	6.99E-09	11
Pnp	4.21E-13	0.319338	0.158	0.019	7.02E-09	11
Acaa2	4.27E-13	0.490249	0.217	0.034	7.12E-09	11
Rps2	4.33E-13	-0.54265	0.408	0.449	7.23E-09	11
Cct3	4.46E-13	-0.49323	0.475	0.495	7.45E-09	11
Ptgr2	4.52E-13	0.446053	0.175	0.021	7.55E-09	11
Serhl	4.68E-13	0.339134	0.108	0.004	7.81E-09	11
C230037L1	5.08E-13	0.315806	0.108	0.006	8.47E-09	11
Htatsf1	5.3E-13	-0.82323	0.317	0.5	8.84E-09	11
D3Bwg056	5.56E-13	0.31682	0.167	0.029	9.28E-09	11
Tmtc2	5.84E-13	0.331945	0.183	0.032	9.74E-09	11
Scamp2	6.17E-13	0.511208	0.408	0.133	1.03E-08	11
Cox7a1	6.57E-13	0.312938	0.108	0.005	1.1E-08	11
Hjurp	6.71E-13	-1.01682	0.175	0.435	1.12E-08	11
Ginm1	7.13E-13	0.395992	0.292	0.08	1.19E-08	11
Rab10	7.27E-13	0.313688	0.592	0.291	1.21E-08	11
Rsl1d1	7.43E-13	-0.56493	0.433	0.49	1.24E-08	11
Gsk3b	7.98E-13	-0.4438	0.508	0.499	1.33E-08	11
Irgm1	8.05E-13	0.264139	0.15	0.019	1.34E-08	11
Cyhr1	8.51E-13	0.530574	0.333	0.093	1.42E-08	11
Srsf6	8.53E-13	-0.70186	0.292	0.446	1.42E-08	11
Ddrgk1	8.56E-13	0.400152	0.45	0.186	1.43E-08	11
Mki67	8.64E-13	-1.22708	0.175	0.396	1.44E-08	11
Cenpe	8.81E-13	-1.28786	0.075	0.287	1.47E-08	11

Gm2694	9.13E-13	-1.05264	0.083	0.36	1.52E-08	11
Ccdc141	9.42E-13	0.413648	0.125	0.008	1.57E-08	11
Cotl1	9.45E-13	0.45253	0.425	0.151	1.58E-08	11
Cables1	9.54E-13	0.319609	0.117	0.008	1.59E-08	11
Cbx5	9.58E-13	-0.55075	0.55	0.653	1.6E-08	11
Srsf7	9.82E-13	-0.80733	0.242	0.466	1.64E-08	11
Prkcdbp	1.06E-12	0.546051	0.333	0.088	1.77E-08	11
Tubb2a	1.06E-12	0.337212	0.525	0.243	1.77E-08	11
Tmem205	1.1E-12	0.33809	0.25	0.064	1.83E-08	11
Orai3	1.12E-12	0.257216	0.108	0.007	1.86E-08	11
Uqcrb	1.18E-12	0.466803	0.525	0.218	1.97E-08	11
Naca	1.24E-12	-0.61443	0.367	0.465	2.07E-08	11
Lrrc8a	1.25E-12	0.287752	0.167	0.027	2.09E-08	11
Ctsf	1.26E-12	0.277616	0.267	0.097	2.1E-08	11
Pa2g4	1.26E-12	-0.61738	0.408	0.523	2.11E-08	11
Desi1	1.29E-12	0.259064	0.283	0.092	2.15E-08	11
Sccpdh	1.3E-12	0.299515	0.275	0.082	2.16E-08	11
Preb	1.3E-12	0.392764	0.408	0.153	2.17E-08	11
Ntm	1.31E-12	0.439603	0.408	0.143	2.19E-08	11
Efhd2	1.33E-12	0.359555	0.367	0.128	2.22E-08	11
Laptm4a	1.36E-12	0.521174	0.85	0.552	2.28E-08	11
Specc1	1.38E-12	0.449681	0.242	0.048	2.3E-08	11
Slc41a1	1.39E-12	0.550794	0.225	0.038	2.32E-08	11
Tpr	1.42E-12	-0.5854	0.467	0.553	2.37E-08	11
Echs1	1.46E-12	0.523206	0.483	0.185	2.44E-08	11
Ctcf	1.48E-12	-0.7616	0.342	0.496	2.47E-08	11
Lrrc1	1.49E-12	0.412519	0.175	0.022	2.49E-08	11
Tshz2	1.5E-12	-0.81123	0.183	0.32	2.51E-08	11
Snrpd3	1.55E-12	-0.47536	0.5	0.535	2.59E-08	11
Soga3	1.57E-12	-0.88617	0.258	0.458	2.63E-08	11
Casc4	1.6E-12	0.275636	0.375	0.146	2.68E-08	11
Sec14l1	1.64E-12	0.390996	0.233	0.051	2.74E-08	11
Khdrbs1	1.69E-12	-0.63047	0.383	0.494	2.81E-08	11
E130114P1	1.73E-12	-0.68581	0.467	0.585	2.89E-08	11
Ndufa1	1.74E-12	0.423901	0.667	0.34	2.9E-08	11
Rnase4	1.83E-12	0.33207	0.15	0.021	3.05E-08	11
Eif2ak2	1.84E-12	0.259361	0.117	0.011	3.07E-08	11
Pbrm1	1.84E-12	-0.66294	0.425	0.531	3.07E-08	11
Rpl35a	1.91E-12	-0.54607	0.483	0.571	3.18E-08	11
Hcfc1r1	2E-12	0.29125	0.442	0.191	3.34E-08	11
Cyth2	2.05E-12	-0.61142	0.2	0.273	3.42E-08	11
Tmod2	2.12E-12	0.596337	0.517	0.205	3.54E-08	11
Slc7a4	2.14E-12	0.285376	0.108	0.007	3.57E-08	11
Nckap1	2.15E-12	0.253954	0.342	0.129	3.58E-08	11
Rufy3	2.21E-12	-0.91394	0.167	0.373	3.68E-08	11
Ptms	2.3E-12	-0.59831	0.4	0.474	3.84E-08	11
Ssb	2.32E-12	-0.52034	0.642	0.704	3.86E-08	11
Pigyl	2.34E-12	0.287846	0.367	0.142	3.9E-08	11

St8sia3	2.46E-12	-0.95077	0.017	0.252	4.1E-08	11
Ypel3	2.77E-12	-0.76926	0.292	0.436	4.63E-08	11
Bcl2	2.87E-12	0.263004	0.175	0.031	4.79E-08	11
Snrpe	2.88E-12	-0.71659	0.383	0.563	4.81E-08	11
Fmn2	2.92E-12	0.425555	0.217	0.04	4.87E-08	11
Slc25a4	2.98E-12	0.42552	0.933	0.806	4.97E-08	11
Snrnp27	3.06E-12	-0.37547	0.35	0.31	5.1E-08	11
Snx5	3.06E-12	0.402563	0.425	0.161	5.11E-08	11
Dhx9	3.14E-12	-0.7529	0.292	0.464	5.25E-08	11
Tmem100	3.17E-12	0.388843	0.125	0.009	5.29E-08	11
Rps7	3.18E-12	-0.6694	0.283	0.4	5.3E-08	11
Gng5	3.2E-12	0.349594	0.417	0.163	5.34E-08	11
Impact	3.26E-12	0.363053	0.225	0.05	5.43E-08	11
Rorb	3.3E-12	0.344626	0.158	0.02	5.5E-08	11
Pccb	3.52E-12	0.478595	0.367	0.116	5.87E-08	11
Klhdc8b	3.53E-12	0.294563	0.15	0.022	5.88E-08	11
D17Wsu10	3.82E-12	0.556301	0.508	0.213	6.37E-08	11
Vldlr	3.97E-12	0.303108	0.233	0.059	6.61E-08	11
Tsn	3.99E-12	-0.58122	0.483	0.589	6.66E-08	11
Pmvk	4E-12	0.272036	0.275	0.093	6.67E-08	11
Fahd2a	4.23E-12	0.268855	0.208	0.051	7.06E-08	11
Pam	4.47E-12	0.428452	0.283	0.078	7.46E-08	11
Ak1	4.47E-12	0.323949	0.117	0.009	7.46E-08	11
Sdhc	4.49E-12	0.542926	0.592	0.267	7.48E-08	11
Plekhh2	4.51E-12	0.303345	0.125	0.011	7.52E-08	11
Itfg1	4.57E-12	0.358454	0.292	0.085	7.62E-08	11
Tmem9	4.59E-12	0.440472	0.342	0.108	7.66E-08	11
Stard3	4.74E-12	0.254248	0.192	0.045	7.91E-08	11
Rbm8a	4.87E-12	-0.48386	0.408	0.465	8.13E-08	11
Fbxo44	4.93E-12	0.451079	0.2	0.035	8.23E-08	11
Surf1	4.97E-12	0.344968	0.317	0.103	8.29E-08	11
Srp72	5.4E-12	-0.6006	0.258	0.322	9.01E-08	11
Adam17	5.49E-12	0.364579	0.225	0.048	9.16E-08	11
Uncx	5.68E-12	-1.03829	0.092	0.319	9.48E-08	11
Tardbp	5.8E-12	-0.57444	0.408	0.483	9.67E-08	11
Rdh10	5.99E-12	0.448896	0.183	0.026	1E-07	11
Wwtr1	6.01E-12	0.318821	0.125	0.011	1E-07	11
Nucb1	6.19E-12	0.417473	0.275	0.073	1.03E-07	11
Nipa1	6.28E-12	0.318289	0.167	0.026	1.05E-07	11
Trp53	6.48E-12	-0.63076	0.2	0.299	1.08E-07	11
Tbata	6.58E-12	-1.14019	0.075	0.341	1.1E-07	11
Efr3a	6.64E-12	0.260726	0.283	0.093	1.11E-07	11
Smarca5	7.11E-12	-0.82706	0.217	0.402	1.19E-07	11
mt-Rnr2	7.12E-12	-0.34824	1	0.997	1.19E-07	11
Hint1	7.15E-12	-0.4386	0.7	0.703	1.19E-07	11
Psmb10	7.19E-12	0.411149	0.25	0.061	1.2E-07	11
Smc6	7.42E-12	-0.61933	0.342	0.423	1.24E-07	11
Rps25	7.45E-12	-0.57428	0.392	0.48	1.24E-07	11

Lmnb1	8E-12	-0.91893	0.075	0.301	1.33E-07	11
Eif5b	8.17E-12	-0.63344	0.508	0.595	1.36E-07	11
Serf1	8.2E-12	-0.51861	0.258	0.281	1.37E-07	11
Kpnb1	8.34E-12	-0.43749	0.3	0.3	1.39E-07	11
Wnt5a	8.48E-12	0.253074	0.108	0.008	1.41E-07	11
Pqbp1	8.55E-12	-0.30815	0.317	0.263	1.43E-07	11
Ccm2	8.6E-12	-0.62061	0.15	0.225	1.43E-07	11
Ube2b	8.61E-12	-0.55571	0.442	0.521	1.44E-07	11
Hsd11b2	8.68E-12	-0.98279	0.033	0.289	1.45E-07	11
Bphl	8.68E-12	0.366795	0.233	0.058	1.45E-07	11
Smarca4	9.11E-12	-0.70575	0.342	0.506	1.52E-07	11
Tnfrsf1a	9.39E-12	0.341822	0.167	0.024	1.57E-07	11
Polr3h	9.63E-12	0.361007	0.308	0.098	1.61E-07	11
Anxa6	1.02E-11	0.273205	0.225	0.06	1.7E-07	11
Ptprg	1.06E-11	0.38185	0.383	0.141	1.78E-07	11
Commd6	1.08E-11	0.283017	0.4	0.178	1.8E-07	11
Hpca	1.08E-11	-1.06142	0.042	0.278	1.8E-07	11
Adam9	1.09E-11	0.341332	0.225	0.051	1.81E-07	11
Vps36	1.13E-11	-0.52921	0.275	0.301	1.88E-07	11
G0s2	1.19E-11	0.324757	0.108	0.006	1.99E-07	11
Pxdc1	1.19E-11	0.339677	0.108	0.007	1.99E-07	11
Selk	1.19E-11	0.445676	0.7	0.393	1.99E-07	11
Rgs2	1.2E-11	0.482965	0.233	0.05	2.01E-07	11
Zfand5	1.22E-11	-0.53778	0.392	0.415	2.04E-07	11
Nrbp2	1.26E-11	0.488497	0.175	0.028	2.11E-07	11
Rap2a	1.27E-11	0.264575	0.4	0.168	2.12E-07	11
Mycn	1.29E-11	-0.96394	0.125	0.365	2.16E-07	11
Hnrnph3	1.34E-11	-0.7234	0.267	0.427	2.23E-07	11
Gas6	1.34E-11	0.286367	0.258	0.079	2.24E-07	11
Psmc1	1.34E-11	-0.36793	0.458	0.418	2.24E-07	11
Gabarapl1	1.39E-11	0.283433	0.467	0.214	2.31E-07	11
Ccnd3	1.39E-11	0.380685	0.283	0.081	2.32E-07	11
Klhl25	1.42E-11	0.260258	0.117	0.01	2.37E-07	11
0610011F0	1.44E-11	0.341774	0.342	0.125	2.4E-07	11
Ptprf	1.53E-11	0.435288	0.317	0.096	2.56E-07	11
Tusc3	1.56E-11	0.299411	0.367	0.146	2.6E-07	11
Bhlhe41	1.58E-11	0.268356	0.125	0.011	2.63E-07	11
Fkbp1a	1.58E-11	-0.50118	0.392	0.452	2.64E-07	11
Ly6e	1.63E-11	-0.95604	0.042	0.283	2.72E-07	11
Hnrnpk	1.64E-11	-0.52598	0.533	0.636	2.74E-07	11
Aard	1.74E-11	0.299192	0.158	0.023	2.9E-07	11
Trappc3	1.75E-11	0.347222	0.308	0.099	2.93E-07	11
Eif3h	1.79E-11	-0.39119	0.483	0.493	2.99E-07	11
Mbd3	1.79E-11	-0.26385	0.358	0.29	2.99E-07	11
Ahcyl2	1.83E-11	0.309397	0.158	0.025	3.05E-07	11
Pigc	1.84E-11	0.269938	0.167	0.031	3.07E-07	11
Rest	1.85E-11	0.268133	0.15	0.022	3.09E-07	11
Ddhd1	1.89E-11	0.415833	0.242	0.055	3.15E-07	11

Slc25a29	1.94E-11	0.411784	0.125	0.009	3.23E-07	11
Cacna2d1	1.99E-11	-1.01156	0.1	0.376	3.32E-07	11
Amer2	2.19E-11	0.335275	0.35	0.132	3.65E-07	11
Tcerg1	2.27E-11	-0.80213	0.267	0.458	3.79E-07	11
Hlf	2.39E-11	0.451703	0.158	0.018	3.98E-07	11
Usp22	2.4E-11	-0.70813	0.233	0.362	4E-07	11
Rab7	2.44E-11	0.467255	0.458	0.181	4.07E-07	11
Pitpnc1	2.45E-11	0.337221	0.258	0.073	4.09E-07	11
Pls3	2.69E-11	0.513579	0.275	0.07	4.49E-07	11
Camk2n1	2.8E-11	0.362274	0.275	0.084	4.67E-07	11
Acbd5	2.8E-11	0.552133	0.342	0.107	4.68E-07	11
Ppp2r1a	2.86E-11	-0.25564	0.275	0.213	4.76E-07	11
Hmgn3	2.91E-11	0.429797	0.567	0.267	4.86E-07	11
Csad	3.05E-11	0.452826	0.267	0.067	5.08E-07	11
Csde1	3.09E-11	-0.5728	0.358	0.436	5.15E-07	11
Rpl37	3.18E-11	-0.54954	0.408	0.504	5.3E-07	11
Shfm1	3.24E-11	-0.47069	0.633	0.686	5.41E-07	11
Paip2	3.28E-11	-0.65227	0.417	0.549	5.46E-07	11
Sod2	3.29E-11	0.399542	0.433	0.173	5.48E-07	11
Pax6	3.34E-11	-0.79174	0.342	0.514	5.57E-07	11
Ddx39b	3.38E-11	-0.63867	0.317	0.445	5.64E-07	11
Rdx	3.39E-11	-0.32237	0.667	0.603	5.65E-07	11
Brd3	3.39E-11	-0.80561	0.317	0.533	5.65E-07	11
Nup93	3.48E-11	0.313173	0.233	0.059	5.8E-07	11
Gpc4	3.48E-11	0.337227	0.117	0.008	5.81E-07	11
Prim1	3.64E-11	-0.84133	0.1	0.264	6.08E-07	11
Derl2	3.91E-11	0.342322	0.25	0.069	6.53E-07	11
mt-Nd4	4E-11	0.467627	0.925	0.775	6.66E-07	11
Bicc1	4E-11	0.260172	0.108	0.009	6.67E-07	11
Prmt8	4.01E-11	-0.95574	0.05	0.273	6.69E-07	11
Fam102b	4.1E-11	0.381667	0.208	0.042	6.84E-07	11
Comt	4.1E-11	0.416682	0.333	0.107	6.84E-07	11
Fos	4.12E-11	0.991542	0.525	0.303	6.87E-07	11
Gab2	4.47E-11	0.349747	0.125	0.013	7.45E-07	11
Ncam2	4.66E-11	0.303514	0.133	0.016	7.77E-07	11
Erlec1	4.76E-11	0.363269	0.375	0.143	7.93E-07	11
Zfp326	4.95E-11	-0.79497	0.233	0.41	8.26E-07	11
Mtf2	5.02E-11	-0.84777	0.183	0.358	8.37E-07	11
E2f5	5.24E-11	0.474072	0.275	0.068	8.74E-07	11
Sfxn1	5.34E-11	0.529306	0.542	0.248	8.91E-07	11
Vhl	5.36E-11	0.30148	0.217	0.055	8.94E-07	11
Cfdp1	5.38E-11	-0.59429	0.392	0.484	8.98E-07	11
Lactb	5.41E-11	0.268932	0.167	0.033	9.03E-07	11
Cct7	5.42E-11	-0.42484	0.467	0.484	9.04E-07	11
Smarcc1	5.6E-11	-0.84685	0.217	0.435	9.34E-07	11
Arl8a	5.61E-11	0.389364	0.3	0.094	9.36E-07	11
C1galt1c1	5.63E-11	0.324476	0.175	0.031	9.4E-07	11
Plxnb1	5.65E-11	0.37593	0.233	0.058	9.42E-07	11

Eif1	5.68E-11	-0.41823	0.558	0.582	9.48E-07	11
Apba2	5.89E-11	-0.55328	0.358	0.414	9.82E-07	11
1500012F(	6.03E-11	-0.85023	0.217	0.399	1.01E-06	11
Sstr2	6.03E-11	-0.82994	0.017	0.192	1.01E-06	11
Rbp4	6.07E-11	-0.93573	0.042	0.243	1.01E-06	11
Snw1	6.21E-11	-0.53209	0.375	0.431	1.04E-06	11
Aldh7a1	6.33E-11	0.44112	0.25	0.065	1.06E-06	11
Aplp2	6.33E-11	0.461311	0.633	0.321	1.06E-06	11
Nudt2	6.4E-11	0.31814	0.242	0.069	1.07E-06	11
Klc1	6.47E-11	-0.31058	0.433	0.36	1.08E-06	11
Sema6a	6.58E-11	0.262518	0.433	0.2	1.1E-06	11
Stmn4	6.59E-11	-1.14369	0.108	0.343	1.1E-06	11
Gnptg	6.74E-11	0.308245	0.392	0.165	1.13E-06	11
Macf1	6.85E-11	0.485196	0.483	0.208	1.14E-06	11
Tmem184c	6.86E-11	0.250669	0.317	0.121	1.14E-06	11
Tmem14c	6.92E-11	0.327005	0.433	0.186	1.15E-06	11
Ddx46	7.01E-11	-0.77919	0.267	0.443	1.17E-06	11
C530008M	7.04E-11	-0.86	0.092	0.269	1.17E-06	11
Usp1	7.19E-11	-0.81746	0.175	0.339	1.2E-06	11
Hspa5	7.21E-11	0.332094	0.85	0.548	1.2E-06	11
Kmt2e	7.35E-11	-0.72922	0.408	0.569	1.23E-06	11
Rprm	7.37E-11	0.316679	0.175	0.032	1.23E-06	11
Anapc5	7.41E-11	-0.57118	0.258	0.334	1.24E-06	11
Scp2	7.43E-11	0.359192	0.258	0.072	1.24E-06	11
Trps1	7.45E-11	0.491174	0.308	0.089	1.24E-06	11
Pigt	7.57E-11	0.262043	0.15	0.024	1.26E-06	11
Ube2e3	7.57E-11	-0.74172	0.233	0.417	1.26E-06	11
Slc25a1	7.78E-11	0.275952	0.217	0.059	1.3E-06	11
Sec62	7.86E-11	0.525074	0.717	0.403	1.31E-06	11
B4galt4	8.42E-11	0.368292	0.167	0.025	1.4E-06	11
Arsk	8.79E-11	0.328297	0.167	0.027	1.47E-06	11
Tmem246	8.85E-11	0.319462	0.267	0.08	1.48E-06	11
Cltb	8.88E-11	-0.82189	0.267	0.424	1.48E-06	11
Ddx42	8.97E-11	-0.66827	0.275	0.383	1.5E-06	11
Gusb	9.2E-11	0.390583	0.275	0.079	1.53E-06	11
Nhp2	9.2E-11	-0.46632	0.358	0.37	1.53E-06	11
Prdx5	9.36E-11	0.426502	0.55	0.262	1.56E-06	11
Celf4	9.36E-11	-1.20719	0.067	0.322	1.56E-06	11
Etv5	9.44E-11	0.260582	0.158	0.03	1.57E-06	11
Uqcrc1	9.89E-11	0.407982	0.592	0.292	1.65E-06	11
Map3k1	1.05E-10	-0.89003	0.142	0.371	1.76E-06	11
Gpt2	1.1E-10	0.546544	0.267	0.065	1.84E-06	11
Sall2	1.12E-10	0.366579	0.208	0.048	1.86E-06	11
Mmp15	1.16E-10	0.251939	0.125	0.016	1.93E-06	11
Sspn	1.16E-10	0.270493	0.125	0.014	1.94E-06	11
Incenp	1.18E-10	-1.00029	0.083	0.276	1.97E-06	11
Ndufa11	1.23E-10	0.458767	0.625	0.329	2.04E-06	11
Caprin1	1.28E-10	-0.59	0.308	0.394	2.14E-06	11

Rad21	1.29E-10	-0.62579	0.342	0.462	2.15E-06	11
Tagln3	1.3E-10	0.445124	0.592	0.293	2.17E-06	11
Cetn3	1.31E-10	-0.55902	0.417	0.487	2.18E-06	11
Snrpg	1.33E-10	-0.64431	0.208	0.335	2.22E-06	11
Nol7	1.33E-10	-0.44891	0.55	0.569	2.22E-06	11
Sumo3	1.34E-10	-0.63517	0.2	0.298	2.23E-06	11
Apex1	1.35E-10	-0.79365	0.242	0.452	2.25E-06	11
Nolc1	1.37E-10	-0.75982	0.25	0.409	2.29E-06	11
Smn1	1.41E-10	-0.37797	0.15	0.159	2.35E-06	11
Rbmx	1.45E-10	-0.74714	0.158	0.316	2.41E-06	11
Itgb1bp1	1.47E-10	0.278921	0.225	0.062	2.45E-06	11
Ghr	1.5E-10	0.260892	0.142	0.021	2.51E-06	11
Rtn3	1.51E-10	0.423224	0.783	0.474	2.52E-06	11
BC031181	1.56E-10	0.374981	0.542	0.259	2.6E-06	11
Mboat7	1.56E-10	0.552974	0.2	0.038	2.61E-06	11
Polr3k	1.57E-10	-0.5955	0.192	0.271	2.62E-06	11
Sox18	1.58E-10	-0.63018	0.017	0.148	2.63E-06	11
Rbfox2	1.6E-10	-0.93248	0.075	0.284	2.66E-06	11
Srp68	1.66E-10	-0.25772	0.125	0.108	2.76E-06	11
Smim13	1.68E-10	0.264576	0.217	0.058	2.81E-06	11
Rassf4	1.73E-10	-0.90273	0.142	0.378	2.89E-06	11
Pcdh9	1.74E-10	0.427559	0.2	0.039	2.9E-06	11
Pdlim3	1.75E-10	0.398216	0.233	0.06	2.92E-06	11
Tmed1	1.77E-10	0.384684	0.317	0.103	2.95E-06	11
Rpl7	1.81E-10	-0.33354	0.55	0.509	3.02E-06	11
Sys1	1.84E-10	0.448252	0.442	0.195	3.07E-06	11
Pcbp4	1.88E-10	-0.88897	0.092	0.326	3.13E-06	11
Jtb	1.92E-10	0.388197	0.483	0.217	3.19E-06	11
Metrnl	1.97E-10	0.288119	0.108	0.009	3.29E-06	11
Trib1	1.98E-10	0.252302	0.208	0.057	3.3E-06	11
Ndp	2.1E-10	0.352854	0.133	0.014	3.5E-06	11
Snhg5	2.17E-10	-0.88894	0.133	0.382	3.62E-06	11
Wbp5	2.2E-10	-0.37672	0.517	0.518	3.66E-06	11
Sub1	2.21E-10	-0.39516	0.567	0.588	3.69E-06	11
Dhrs4	2.35E-10	0.365413	0.2	0.045	3.92E-06	11
Ide	2.38E-10	0.410554	0.325	0.112	3.97E-06	11
Epha5	2.38E-10	0.355694	0.133	0.015	3.97E-06	11
Tcp1	2.39E-10	-0.5275	0.392	0.463	3.99E-06	11
Elavl4	2.55E-10	-0.99264	0.133	0.336	4.26E-06	11
Stk17b	2.62E-10	0.264111	0.225	0.064	4.37E-06	11
Zmiz1	2.69E-10	-0.59051	0.308	0.375	4.48E-06	11
Snn	2.69E-10	0.469056	0.35	0.126	4.48E-06	11
Wscd1	2.76E-10	0.352198	0.233	0.062	4.6E-06	11
Cox8a	2.84E-10	0.448874	0.833	0.699	4.73E-06	11
Hsd17b4	2.88E-10	0.48441	0.383	0.138	4.81E-06	11
Ankrd11	2.91E-10	-0.62315	0.392	0.473	4.85E-06	11
Pex2	2.97E-10	0.46287	0.3	0.099	4.95E-06	11
Prpsap1	2.98E-10	0.346998	0.408	0.172	4.97E-06	11

Thrap3	3.12E-10	-0.60764	0.267	0.388	5.2E-06	11
Krcc1	3.23E-10	0.343512	0.267	0.082	5.38E-06	11
Snap25	3.3E-10	-0.96254	0.067	0.283	5.5E-06	11
Gm8292	3.34E-10	-0.60684	0.258	0.369	5.57E-06	11
Slc50a1	3.36E-10	0.314912	0.258	0.081	5.61E-06	11
Pcbp2	3.53E-10	-0.37016	0.6	0.621	5.9E-06	11
Maoa	3.57E-10	0.347539	0.242	0.066	5.95E-06	11
Cirbp	3.59E-10	-0.64625	0.258	0.355	5.98E-06	11
Erp44	3.63E-10	0.285759	0.3	0.107	6.05E-06	11
Cdk1	3.64E-10	-0.74328	0.15	0.247	6.08E-06	11
Bai1	3.76E-10	0.457442	0.15	0.021	6.27E-06	11
Mlf2	3.81E-10	0.270913	0.533	0.277	6.36E-06	11
Nkd1	3.82E-10	-0.8606	0.042	0.249	6.38E-06	11
Leprel4	3.86E-10	0.354852	0.167	0.028	6.44E-06	11
Vmp1	3.92E-10	0.36858	0.317	0.115	6.54E-06	11
Cox6b2	4.04E-10	0.425547	0.133	0.014	6.74E-06	11
Rnmt	4.25E-10	-0.8304	0.167	0.349	7.09E-06	11
Evi5	4.44E-10	0.541866	0.392	0.14	7.4E-06	11
Hmgb1	4.44E-10	-0.70445	0.217	0.359	7.4E-06	11
Dscr3	4.55E-10	0.36362	0.25	0.071	7.59E-06	11
Zmat2	4.59E-10	-0.60643	0.35	0.44	7.65E-06	11
Fndc5	4.6E-10	0.285536	0.125	0.017	7.67E-06	11
Clptm1	4.61E-10	0.267068	0.208	0.059	7.69E-06	11
Fkbp2	4.72E-10	0.261059	0.575	0.324	7.86E-06	11
Phf5a	4.81E-10	-0.40144	0.325	0.331	8.02E-06	11
Zeb1	4.84E-10	-0.77128	0.217	0.388	8.07E-06	11
Zwint	5.03E-10	-0.26769	0.358	0.291	8.4E-06	11
Eif3k	5.16E-10	-0.4592	0.492	0.52	8.62E-06	11
Eif4h	5.25E-10	-0.40868	0.45	0.471	8.76E-06	11
Kctd5	5.28E-10	0.353961	0.308	0.106	8.8E-06	11
Hspa8	5.43E-10	-0.52493	0.342	0.415	9.06E-06	11
Abracl	5.62E-10	-0.791	0.125	0.303	9.37E-06	11
Lig1	5.87E-10	-1.02909	0.125	0.349	9.8E-06	11
Clcn3	5.98E-10	0.414201	0.55	0.271	9.98E-06	11
Grhpr	6.05E-10	0.410981	0.133	0.015	1.01E-05	11
Tm2d2	6.28E-10	0.263319	0.492	0.248	1.05E-05	11
1500016L(	6.3E-10	-0.90932	0.142	0.384	1.05E-05	11
Hdgfrp3	6.35E-10	-0.78493	0.167	0.339	1.06E-05	11
Ubtf	6.65E-10	-0.70334	0.3	0.453	1.11E-05	11
Samd8	6.89E-10	0.381647	0.25	0.078	1.15E-05	11
Rab9	6.92E-10	0.294682	0.3	0.108	1.15E-05	11
Dpy19l3	6.96E-10	0.296824	0.142	0.023	1.16E-05	11
Gpx1	6.97E-10	-0.37894	0.408	0.382	1.16E-05	11
Cpe	7.11E-10	0.589229	0.7	0.454	1.19E-05	11
Dhrs7	7.39E-10	0.302778	0.233	0.07	1.23E-05	11
Ran	7.44E-10	-0.53177	0.308	0.414	1.24E-05	11
Slc35f5	7.51E-10	0.281731	0.158	0.031	1.25E-05	11
Rtn1	7.65E-10	-0.71082	0.617	0.69	1.28E-05	11

Scg5	7.89E-10	-0.85158	0.108	0.336	1.32E-05	11
Fut9	7.93E-10	0.512269	0.483	0.21	1.32E-05	11
Cks1b	7.95E-10	-0.86229	0.142	0.345	1.33E-05	11
Txnrd1	8.26E-10	-0.65013	0.267	0.389	1.38E-05	11
Psmd4	8.32E-10	-0.35216	0.425	0.393	1.39E-05	11
Aldh9a1	8.36E-10	0.379913	0.217	0.055	1.39E-05	11
Nop56	8.49E-10	-0.81478	0.158	0.362	1.42E-05	11
Mrpl13	8.53E-10	-0.27102	0.367	0.307	1.42E-05	11
Ddx6	8.53E-10	-0.63559	0.358	0.493	1.42E-05	11
Rps8	8.91E-10	-0.62207	0.275	0.397	1.49E-05	11
Vdac1	9.27E-10	0.377692	0.575	0.3	1.55E-05	11
Lamtor4	9.32E-10	0.263909	0.317	0.126	1.55E-05	11
Smim15	9.51E-10	0.338227	0.317	0.119	1.59E-05	11
Aga	9.52E-10	0.27381	0.167	0.035	1.59E-05	11
Whsc1	1.01E-09	-0.64473	0.342	0.465	1.68E-05	11
Tmem59	1.04E-09	0.430548	0.692	0.39	1.74E-05	11
Cldn25	1.06E-09	0.274361	0.45	0.23	1.76E-05	11
Eif3e	1.07E-09	-0.66323	0.25	0.382	1.79E-05	11
Nme1	1.17E-09	-0.48229	0.442	0.509	1.95E-05	11
Yif1a	1.18E-09	0.291026	0.392	0.177	1.96E-05	11
Mcm6	1.18E-09	-0.90148	0.075	0.277	1.97E-05	11
Bmyc	1.19E-09	0.262921	0.275	0.099	1.98E-05	11
Sh3glb2	1.26E-09	0.349014	0.258	0.083	2.1E-05	11
Sbds	1.28E-09	0.274454	0.333	0.135	2.13E-05	11
Vegfb	1.28E-09	0.366699	0.325	0.117	2.14E-05	11
Rhoa	1.28E-09	0.371098	0.358	0.14	2.14E-05	11
Pcna	1.3E-09	-0.68614	0.267	0.358	2.17E-05	11
Sugt1	1.32E-09	-0.3999	0.225	0.231	2.21E-05	11
Hmgb3	1.34E-09	-0.83304	0.092	0.307	2.24E-05	11
Vps37b	1.35E-09	-0.57903	0.383	0.471	2.26E-05	11
Map2	1.37E-09	-0.41514	0.533	0.491	2.29E-05	11
Cit	1.39E-09	0.275458	0.225	0.064	2.32E-05	11
Rpe	1.42E-09	0.257099	0.192	0.05	2.37E-05	11
Cct5	1.44E-09	-0.3826	0.475	0.476	2.4E-05	11
Fam212b	1.54E-09	-0.48215	0.192	0.223	2.57E-05	11
Tmx2	1.54E-09	0.288663	0.292	0.107	2.57E-05	11
Acaa1a	1.61E-09	0.423723	0.308	0.102	2.69E-05	11
U2surp	1.64E-09	-0.43156	0.492	0.505	2.74E-05	11
Pde1c	1.65E-09	-0.88809	0.225	0.388	2.76E-05	11
Gbp7	1.66E-09	0.297168	0.108	0.009	2.77E-05	11
2610507B1	1.67E-09	0.374915	0.417	0.179	2.78E-05	11
C1ql1	1.68E-09	-0.86744	0.083	0.28	2.8E-05	11
Supt16	1.73E-09	-0.59439	0.375	0.494	2.89E-05	11
Sdc2	1.79E-09	0.423436	0.225	0.053	2.98E-05	11
Eprs	1.79E-09	-0.52567	0.433	0.491	2.99E-05	11
Sept2	1.81E-09	0.261186	0.45	0.22	3.02E-05	11
Rpl18a	1.87E-09	-0.64911	0.25	0.401	3.12E-05	11
Slc35f6	1.89E-09	0.334449	0.133	0.018	3.15E-05	11

Suv39h2	1.94E-09	-0.48829	0.042	0.131	3.24E-05	11
Arl2	2.06E-09	0.252244	0.275	0.102	3.44E-05	11
Suclg1	2.06E-09	0.331726	0.533	0.27	3.44E-05	11
Cs	2.1E-09	0.476825	0.5	0.226	3.51E-05	11
Per3	2.12E-09	0.268936	0.117	0.015	3.53E-05	11
Birc5	2.12E-09	-0.88105	0.117	0.274	3.53E-05	11
Ik	2.14E-09	-0.42224	0.425	0.465	3.57E-05	11
Pros1	2.25E-09	0.289759	0.142	0.023	3.76E-05	11
Thoc7	2.26E-09	-0.54028	0.417	0.522	3.76E-05	11
Cachd1	2.26E-09	0.298679	0.15	0.026	3.77E-05	11
Adh5	2.26E-09	-0.38573	0.417	0.404	3.78E-05	11
Ssfa2	2.32E-09	0.270405	0.133	0.02	3.87E-05	11
Slc48a1	2.34E-09	0.325758	0.217	0.059	3.9E-05	11
Gng3	2.36E-09	-0.99387	0.1	0.318	3.94E-05	11
Gse1	2.36E-09	-0.84586	0.042	0.238	3.94E-05	11
Hadh	2.37E-09	0.406416	0.258	0.072	3.96E-05	11
Cdca8	2.43E-09	-0.86863	0.1	0.265	4.05E-05	11
Cdip1	2.46E-09	0.275258	0.267	0.101	4.11E-05	11
2700029M	2.52E-09	-0.46654	0.258	0.289	4.21E-05	11
Dusp6	2.53E-09	0.500199	0.3	0.091	4.21E-05	11
Skp1a	2.54E-09	-0.37518	0.592	0.581	4.24E-05	11
Neurod6	2.57E-09	-0.73346	0.025	0.173	4.29E-05	11
Fip1l1	2.69E-09	-0.64477	0.208	0.309	4.49E-05	11
Ubc	2.7E-09	0.290055	0.625	0.357	4.5E-05	11
Smim20	2.8E-09	0.404131	0.208	0.048	4.66E-05	11
Phb2	2.84E-09	-0.2828	0.383	0.332	4.74E-05	11
Rpl14-ps1	3.07E-09	-0.58392	0.183	0.284	5.13E-05	11
Actb	3.27E-09	-0.27013	1	0.978	5.45E-05	11
Snrnp70	3.42E-09	-0.4374	0.617	0.694	5.71E-05	11
Arpc1a	3.43E-09	-0.39834	0.367	0.363	5.73E-05	11
Nars	3.45E-09	-0.36338	0.475	0.441	5.76E-05	11
D10Jhu81e	3.46E-09	0.298636	0.242	0.074	5.77E-05	11
Hspb6	3.46E-09	0.294085	0.108	0.011	5.78E-05	11
Tm7sf3	3.52E-09	0.335677	0.225	0.064	5.88E-05	11
Rsrc2	3.8E-09	-0.55673	0.458	0.548	6.34E-05	11
H2afy2	3.9E-09	-0.83097	0.108	0.339	6.5E-05	11
Prpf38b	4E-09	-0.48177	0.467	0.495	6.67E-05	11
Vbp1	4.12E-09	-0.40416	0.308	0.319	6.88E-05	11
Sptssa	4.14E-09	0.284022	0.475	0.245	6.91E-05	11
Uqcc2	4.23E-09	0.312165	0.575	0.31	7.05E-05	11
Ncald	4.26E-09	0.322964	0.375	0.164	7.11E-05	11
Dirc2	4.3E-09	0.376221	0.258	0.094	7.17E-05	11
Gnai2	4.31E-09	0.300145	0.7	0.422	7.18E-05	11
Srsf10	4.38E-09	-0.65658	0.2	0.331	7.3E-05	11
Ophn1	4.4E-09	0.300255	0.25	0.083	7.33E-05	11
Arfip1	4.43E-09	0.296321	0.267	0.091	7.39E-05	11
Tra2b	4.66E-09	-0.69668	0.25	0.405	7.78E-05	11
Emg1	4.75E-09	-0.41382	0.3	0.315	7.92E-05	11

Gpx7	4.83E-09	0.422669	0.225	0.061	8.06E-05	11
Mad2l2	4.89E-09	-0.5476	0.192	0.262	8.16E-05	11
Meis1	4.93E-09	-0.6096	0.283	0.373	8.22E-05	11
Lbh	4.98E-09	0.410337	0.242	0.072	8.31E-05	11
Gdpd1	5.01E-09	-0.65143	0.133	0.244	8.36E-05	11
Nudt4	5.08E-09	0.512452	0.458	0.216	8.47E-05	11
Ghitm	5.17E-09	0.301552	0.533	0.282	8.62E-05	11
Kazn	5.2E-09	0.340719	0.133	0.02	8.67E-05	11
Eif3b	5.34E-09	-0.44284	0.192	0.22	8.91E-05	11
Pigp	5.35E-09	0.252303	0.308	0.127	8.92E-05	11
Lsm6	5.47E-09	0.338922	0.642	0.37	9.13E-05	11
Lactb2	5.69E-09	0.301135	0.117	0.013	9.49E-05	11
Scaf11	5.73E-09	-0.51641	0.317	0.373	9.55E-05	11
Prr13	5.76E-09	0.26131	0.25	0.088	9.6E-05	11
Spag9	5.94E-09	0.2619	0.542	0.305	9.91E-05	11
Asf1a	6.01E-09	-0.44125	0.233	0.28	0.0001	11
Gm11223	6.08E-09	-1.03965	0.025	0.214	0.000101	11
Kif11	6.09E-09	-0.7293	0.083	0.206	0.000102	11
Neto2	6.18E-09	0.329378	0.242	0.073	0.000103	11
Ing4	6.21E-09	-0.52783	0.267	0.323	0.000104	11
Krtcap2	6.26E-09	0.35259	0.75	0.466	0.000104	11
Thoc2	6.46E-09	-0.70109	0.3	0.449	0.000108	11
Csnk1e	6.66E-09	-0.6722	0.258	0.386	0.000111	11
1110008F1	6.73E-09	0.499611	0.483	0.227	0.000112	11
Cdc42se1	6.8E-09	0.303829	0.375	0.164	0.000113	11
Srrm4	6.94E-09	-0.80577	0.033	0.196	0.000116	11
Myt1	7.09E-09	-0.75568	0.033	0.153	0.000118	11
Rpn2	7.53E-09	0.368681	0.475	0.225	0.000126	11
Tead2	7.65E-09	-0.65011	0.158	0.278	0.000128	11
Esf1	7.71E-09	-0.55093	0.35	0.392	0.000129	11
Ythdc1	8.07E-09	-0.63958	0.325	0.477	0.000135	11
Mdk	8.19E-09	-0.38744	0.425	0.379	0.000137	11
Cluh	8.34E-09	0.256429	0.175	0.046	0.000139	11
Hsp90b1	8.37E-09	0.387133	0.917	0.69	0.00014	11
Nipbl	8.37E-09	-0.44943	0.458	0.492	0.00014	11
Pfkl	8.44E-09	0.336846	0.117	0.013	0.000141	11
Ano6	8.81E-09	0.363045	0.267	0.089	0.000147	11
Pak2	8.82E-09	-0.30226	0.342	0.3	0.000147	11
Tmem5	8.88E-09	0.320648	0.25	0.08	0.000148	11
Sar1b	9.11E-09	0.418076	0.442	0.196	0.000152	11
Cntn2	9.17E-09	-1.15407	0.092	0.264	0.000153	11
Pnkd	9.22E-09	0.259985	0.217	0.067	0.000154	11
Id2	9.26E-09	0.36239	0.733	0.484	0.000155	11
Rbm17	9.31E-09	-0.5489	0.308	0.39	0.000155	11
Tmed9	9.42E-09	-0.27794	0.475	0.433	0.000157	11
0610012G(	9.64E-09	0.250991	0.325	0.145	0.000161	11
Insm1	9.68E-09	-0.88004	0.075	0.294	0.000161	11
Slc35b2	9.7E-09	0.284574	0.392	0.18	0.000162	11

Cct2	9.72E-09	-0.55038	0.392	0.524	0.000162	11
Prrc2b	9.74E-09	-0.4227	0.208	0.222	0.000163	11
Srgap1	9.97E-09	0.403404	0.133	0.017	0.000166	11
Atp1b3	1.01E-08	-0.25579	0.408	0.336	0.000168	11
0610009L1	1.04E-08	0.309658	0.117	0.015	0.000173	11
N4bp2l2	1.06E-08	-0.53898	0.2	0.261	0.000176	11
Arl8b	1.09E-08	0.250724	0.35	0.166	0.000181	11
Psat1	1.09E-08	-0.30565	0.4	0.367	0.000182	11
Brd2	1.11E-08	-0.41032	0.375	0.395	0.000185	11
Arsb	1.11E-08	0.39831	0.15	0.024	0.000186	11
Srsf5	1.12E-08	-0.42345	0.4	0.426	0.000187	11
Tle1	1.16E-08	-0.46275	0.192	0.226	0.000194	11
H2-D1	1.16E-08	0.408088	0.333	0.128	0.000194	11
Bcl7a	1.17E-08	-0.77435	0.142	0.326	0.000195	11
Brd8	1.21E-08	-0.65899	0.258	0.392	0.000202	11
Cdc5l	1.22E-08	-0.67876	0.25	0.402	0.000203	11
Cadm4	1.22E-08	0.251782	0.275	0.112	0.000204	11
Pdgfra	1.23E-08	-0.83155	0.008	0.182	0.000204	11
Abcf1	1.25E-08	-0.31408	0.4	0.457	0.000208	11
Lrrc42	1.3E-08	0.273019	0.208	0.062	0.000216	11
Set	1.31E-08	-0.73482	0.192	0.388	0.000218	11
Klf4	1.33E-08	0.504602	0.142	0.023	0.000221	11
Pde6d	1.34E-08	0.317085	0.325	0.128	0.000224	11
Celf1	1.35E-08	-0.67618	0.242	0.38	0.000225	11
Rpn1	1.39E-08	0.483347	0.425	0.19	0.000232	11
Dkc1	1.46E-08	-0.75657	0.125	0.334	0.000243	11
Akr1e1	1.48E-08	0.338897	0.267	0.092	0.000246	11
Ube2q1	1.51E-08	-0.31959	0.208	0.191	0.000252	11
Ctage5	1.54E-08	-0.58744	0.192	0.267	0.000258	11
Fam115a	1.55E-08	-0.37824	0.367	0.35	0.000259	11
Clmp	1.57E-08	-0.90107	0.042	0.243	0.000262	11
Pou3f3	1.62E-08	0.310949	0.383	0.17	0.000271	11
Tmbim1	1.67E-08	0.272746	0.108	0.014	0.000279	11
Arhgap31	1.68E-08	0.254143	0.158	0.038	0.00028	11
Prkcb	1.68E-08	-0.87437	0.067	0.257	0.00028	11
Hells	1.71E-08	-0.74897	0.092	0.213	0.000286	11
Sars	1.73E-08	-0.38381	0.425	0.42	0.000288	11
Fadd	1.75E-08	0.297943	0.108	0.012	0.000293	11
Sc5d	1.8E-08	0.510428	0.233	0.059	0.000301	11
Rdh11	1.84E-08	0.317789	0.233	0.079	0.000307	11
Bola2	1.85E-08	-0.44819	0.358	0.397	0.000309	11
Dync1i2	1.85E-08	-0.2898	0.617	0.567	0.000309	11
Dixdc1	1.87E-08	-0.81801	0.042	0.236	0.000311	11
Fam98b	1.87E-08	-0.34728	0.35	0.335	0.000313	11
Selt	1.91E-08	0.305826	0.258	0.094	0.000319	11
Cdc42ep4	1.96E-08	0.325327	0.192	0.047	0.000327	11
Srrt	1.96E-08	-0.73729	0.225	0.425	0.000328	11
Klf13	1.99E-08	-0.73046	0.058	0.237	0.000331	11

Fkbp4	1.99E-08	-0.52995	0.333	0.414	0.000332	11
Ndufa9	2E-08	0.287903	0.425	0.208	0.000334	11
Dzip1l	2.04E-08	0.37072	0.142	0.023	0.00034	11
Stmn1	2.05E-08	-0.7432	0.108	0.285	0.000342	11
Gm13826	2.07E-08	-0.53805	0.233	0.312	0.000345	11
Ctbp1	2.07E-08	-0.43069	0.35	0.394	0.000346	11
Gm6472	2.12E-08	-0.34442	0.158	0.167	0.000354	11
Pja2	2.14E-08	-0.26727	0.283	0.234	0.000358	11
Slc16a1	2.15E-08	0.260693	0.167	0.037	0.000359	11
Iars2	2.15E-08	0.35887	0.258	0.083	0.000359	11
Cenpa	2.16E-08	-0.72262	0.175	0.252	0.00036	11
Snhg1	2.2E-08	-0.55445	0.283	0.357	0.000366	11
Rab2a	2.2E-08	-0.30067	0.475	0.43	0.000367	11
Ier3	2.21E-08	0.255613	0.142	0.034	0.000368	11
Eif2s2	2.22E-08	-0.44048	0.308	0.33	0.00037	11
Rhbdd2	2.23E-08	0.264722	0.2	0.059	0.000372	11
Derl1	2.24E-08	0.394032	0.275	0.093	0.000374	11
Plscr4	2.33E-08	0.300797	0.15	0.027	0.000389	11
Stxbp3a	2.36E-08	0.258579	0.125	0.019	0.000394	11
Sox8	2.37E-08	0.276071	0.142	0.029	0.000395	11
Rbm39	2.37E-08	-0.42646	0.717	0.793	0.000396	11
Ncam1	2.39E-08	-0.39229	0.392	0.387	0.000398	11
Cyp51	2.42E-08	0.456414	0.35	0.139	0.000403	11
Tprn	2.46E-08	-0.8295	0.05	0.227	0.000411	11
Atp2b1	2.47E-08	-0.38491	0.617	0.571	0.000411	11
Cox6c	2.53E-08	0.433999	0.867	0.717	0.000422	11
Zc3h15	2.67E-08	-0.48423	0.317	0.366	0.000446	11
Uqcrq	2.74E-08	0.428531	0.8	0.548	0.000457	11
Zbtb18	2.75E-08	-0.83066	0.067	0.267	0.000459	11
Ogfr	3E-08	-0.35114	0.167	0.17	0.000501	11
mt-Nd1	3.02E-08	0.379696	0.992	0.926	0.000504	11
Chchd1	3.03E-08	-0.33386	0.425	0.4	0.000506	11
Siva1	3.05E-08	-0.66619	0.142	0.272	0.000509	11
Arhgap11a	3.06E-08	-0.76513	0.008	0.171	0.00051	11
Nop10	3.09E-08	-0.44492	0.45	0.482	0.000515	11
Lsm4	3.16E-08	-0.43236	0.442	0.488	0.000526	11
Bin1	3.16E-08	-0.80336	0.225	0.43	0.000526	11
Slc39a1	3.33E-08	0.356387	0.167	0.034	0.000556	11
Prmt5	3.34E-08	-0.36506	0.258	0.256	0.000557	11
Nap1l5	3.52E-08	0.391796	0.25	0.079	0.000586	11
Lbr	3.56E-08	-0.66739	0.05	0.197	0.000594	11
Csnk1a1	3.64E-08	-0.30463	0.7	0.666	0.000606	11
Adamts1	3.64E-08	0.654289	0.342	0.153	0.000607	11
Stk40	3.78E-08	0.378526	0.15	0.035	0.00063	11
Gdap1	3.82E-08	-0.73406	0.05	0.199	0.000637	11
Atp5g3	3.86E-08	0.436677	0.775	0.515	0.000644	11
Zfp292	3.95E-08	-0.83318	0.15	0.343	0.000659	11
Ppp1ca	3.96E-08	-0.36945	0.475	0.483	0.00066	11

Eid1	4.05E-08	-0.42446	0.492	0.502	0.000676	11
Idh1	4.05E-08	0.308213	0.35	0.155	0.000676	11
Blvrb	4.08E-08	0.32676	0.208	0.058	0.00068	11
Gm2000	4.08E-08	-0.4243	0.058	0.129	0.000681	11
Cenpv	4.13E-08	-0.6387	0.192	0.329	0.000689	11
Mphosph8	4.25E-08	-0.37483	0.375	0.365	0.000709	11
Ndufa13	4.28E-08	0.493159	0.75	0.531	0.000713	11
Erlin2	4.74E-08	0.338317	0.183	0.045	0.000791	11
Ctnnb1	4.76E-08	0.34123	0.633	0.384	0.000795	11
Pfdn4	4.77E-08	-0.59447	0.208	0.305	0.000796	11
Mfsd1	4.84E-08	0.267617	0.275	0.11	0.000808	11
Papola	4.84E-08	-0.3033	0.442	0.401	0.000808	11
Dnajc9	4.84E-08	-0.64446	0.225	0.35	0.000808	11
Fmr1	4.92E-08	-0.36011	0.267	0.299	0.000821	11
Rnft1	4.92E-08	0.262934	0.158	0.036	0.000821	11
Tpx2	4.93E-08	-0.75511	0.175	0.282	0.000822	11
Rnf20	4.95E-08	-0.7018	0.15	0.289	0.000825	11
Cdk5r1	4.98E-08	-0.83589	0.133	0.334	0.000831	11
Klf2	4.99E-08	0.402446	0.125	0.017	0.000833	11
Napa	5.05E-08	-0.3186	0.367	0.349	0.000842	11
Ccdc55	5.1E-08	-0.50809	0.233	0.267	0.000851	11
Rtf1	5.11E-08	-0.65865	0.358	0.513	0.000853	11
Ralbp1	5.22E-08	-0.44101	0.3	0.322	0.00087	11
Fkbp9	5.22E-08	0.316532	0.15	0.029	0.00087	11
Polr2m	5.32E-08	-0.35987	0.242	0.244	0.000888	11
Gtf3a	5.34E-08	-0.44512	0.15	0.194	0.00089	11
Prpf4b	5.41E-08	-0.55311	0.383	0.471	0.000902	11
Canx	5.45E-08	0.433273	0.817	0.594	0.000908	11
Ash1l	5.49E-08	-0.26032	0.333	0.277	0.000916	11
Cox20	5.59E-08	-0.38115	0.283	0.285	0.000932	11
Hnrnpul2	5.81E-08	-0.53391	0.192	0.257	0.00097	11
Ywhaz	6.03E-08	-0.45081	0.308	0.362	0.001006	11
Arhgef2	6.19E-08	-0.68951	0.158	0.283	0.001032	11
Fgf9	6.22E-08	-0.78094	0.075	0.261	0.001037	11
Amfr	6.23E-08	0.472596	0.242	0.073	0.001039	11
Aes	6.25E-08	-0.40264	0.192	0.212	0.001042	11
Rbbp7	6.34E-08	-0.28919	0.35	0.319	0.001058	11
Parp1	6.43E-08	-0.66178	0.167	0.314	0.001073	11
Atp6v0e2	6.61E-08	0.271754	0.483	0.258	0.001103	11
Ddx21	6.66E-08	-0.71321	0.183	0.323	0.001111	11
Bcas2	6.76E-08	-0.44438	0.367	0.405	0.001128	11
Srebf1	6.77E-08	-0.7037	0.225	0.334	0.00113	11
Kif2a	6.78E-08	-0.53496	0.183	0.251	0.001131	11
Fam32a	6.8E-08	-0.59669	0.175	0.269	0.001135	11
Gbas	6.88E-08	0.383922	0.333	0.131	0.001148	11
Tpm4	6.91E-08	-0.62444	0.2	0.317	0.001153	11
Apc	7.12E-08	-0.48459	0.425	0.431	0.001188	11
Nudc	7.27E-08	-0.54172	0.183	0.281	0.001212	11

Mrfap1	7.29E-08	-0.33992	0.408	0.403	0.001216	11
Myef2	7.36E-08	-0.51592	0.267	0.338	0.001228	11
Dad1	7.54E-08	0.389096	0.608	0.341	0.001258	11
Vamp3	7.62E-08	0.320391	0.233	0.078	0.001272	11
Cct8	7.73E-08	-0.43315	0.383	0.441	0.00129	11
Arsa	7.93E-08	0.295366	0.117	0.017	0.001323	11
Top2b	8.11E-08	-0.5713	0.325	0.415	0.001353	11
Nktr	8.23E-08	-0.49182	0.35	0.38	0.001373	11
Ccdc50	8.52E-08	-0.53233	0.242	0.326	0.001421	11
Spc25	8.71E-08	-0.54165	0.175	0.225	0.001452	11
Ncln	8.86E-08	0.254127	0.233	0.1	0.001478	11
Rab24	9.1E-08	-0.32848	0.225	0.214	0.001518	11
Ubb	9.45E-08	-0.40248	0.517	0.541	0.001576	11
Mia3	9.67E-08	-0.28017	0.283	0.244	0.001613	11
Lpgat1	9.7E-08	-0.63568	0.008	0.168	0.001619	11
Tceal3	9.78E-08	0.553343	0.3	0.109	0.001631	11
Fam168a	9.81E-08	0.33524	0.525	0.285	0.001636	11
Dusp3	9.89E-08	0.31365	0.142	0.028	0.001649	11
Atoh1	9.94E-08	-0.63143	0.058	0.164	0.001658	11
Kif21a	9.97E-08	-0.31471	0.383	0.345	0.001663	11
Fam213b	1.02E-07	-0.45337	0.125	0.157	0.001693	11
Golga4	1.04E-07	-0.69171	0.217	0.325	0.001727	11
Gar1	1.04E-07	-0.67785	0.05	0.209	0.001734	11
Pak3	1.05E-07	-0.68835	0.092	0.206	0.00175	11
Mocs2	1.06E-07	0.280693	0.4	0.193	0.001761	11
Mfsd11	1.06E-07	0.282858	0.175	0.051	0.001764	11
Casc5	1.07E-07	-0.72612	0.05	0.187	0.001785	11
Dennd5a	1.07E-07	0.281757	0.267	0.101	0.001789	11
Gucy1b3	1.11E-07	0.340704	0.167	0.037	0.001844	11
Zranb2	1.18E-07	-0.33645	0.425	0.406	0.001962	11
Cfl2	1.22E-07	-0.32401	0.417	0.408	0.00203	11
Ppp3ca	1.24E-07	-0.41008	0.383	0.378	0.002064	11
Dctpp1	1.27E-07	-0.62045	0.183	0.305	0.002121	11
C1qbp	1.29E-07	-0.32046	0.392	0.377	0.002156	11
Sac3d1	1.29E-07	-0.35164	0.067	0.107	0.002158	11
Idh3a	1.29E-07	0.299473	0.358	0.161	0.002159	11
Rpl6	1.31E-07	-0.63548	0.158	0.291	0.002182	11
Smim11	1.31E-07	-0.29117	0.308	0.276	0.002183	11
Gpr153	1.32E-07	-0.70493	0.075	0.209	0.002202	11
March7	1.33E-07	-0.35356	0.233	0.238	0.002211	11
Aimp1	1.34E-07	-0.51573	0.217	0.279	0.002229	11
Rexo2	1.34E-07	0.384359	0.4	0.179	0.002234	11
Rab34	1.35E-07	0.372528	0.3	0.116	0.002245	11
Supt4a	1.37E-07	-0.26958	0.167	0.154	0.002287	11
Cwc15	1.4E-07	-0.27368	0.425	0.398	0.002328	11
Itsn1	1.41E-07	-0.41678	0.242	0.235	0.002347	11
Kdm5b	1.43E-07	-0.44109	0.167	0.185	0.002382	11
Zic4	1.45E-07	-0.67755	0.25	0.419	0.002414	11

Mut	1.49E-07	0.31466	0.192	0.053	0.002488	11
Pqlc1	1.49E-07	-0.73397	0.142	0.298	0.00249	11
Zcchc11	1.52E-07	-0.75111	0.15	0.322	0.00254	11
Prdx1	1.54E-07	0.334135	0.75	0.488	0.00257	11
Ddx3x	1.55E-07	-0.42771	0.408	0.467	0.002578	11
Chgb	1.55E-07	-0.96721	0.067	0.241	0.002582	11
Elavl2	1.61E-07	-0.79081	0.067	0.271	0.002682	11
Bex1	1.61E-07	-0.6462	0.108	0.224	0.002686	11
Ndufa12	1.61E-07	0.335641	0.65	0.419	0.002688	11
Setd5	1.61E-07	-0.41361	0.225	0.247	0.002693	11
A630007B(	1.62E-07	-0.60374	0.133	0.206	0.002706	11
Kif3a	1.64E-07	-0.45783	0.275	0.316	0.002729	11
Gapvd1	1.64E-07	-0.33849	0.242	0.23	0.00274	11
C130071C(	1.65E-07	-0.50657	0.25	0.308	0.002753	11
Cyth3	1.67E-07	0.493673	0.208	0.055	0.002778	11
Zfp422	1.72E-07	-0.55916	0.15	0.246	0.002872	11
Tmem150a	1.74E-07	0.336138	0.117	0.017	0.002903	11
Rps4x	1.77E-07	-0.4874	0.233	0.295	0.002947	11
Cited2	1.87E-07	-0.55658	0.075	0.152	0.003117	11
Pdgfa	1.91E-07	-0.80642	0.1	0.254	0.003185	11
Podxl2	1.91E-07	-0.8008	0.05	0.228	0.003189	11
Ebf3	1.93E-07	-0.7165	0.058	0.203	0.003222	11
Rrp1	1.93E-07	-0.49442	0.517	0.606	0.003225	11
RP23-45G1	1.93E-07	-0.55557	0.25	0.327	0.003227	11
Myl12b	1.98E-07	-0.39131	0.342	0.367	0.003303	11
Ndufs8	2.04E-07	0.406334	0.608	0.346	0.003402	11
Mns1	2.05E-07	-0.7349	0.05	0.204	0.003414	11
Ncor2	2.08E-07	-0.62766	0.042	0.169	0.003473	11
Gtf2a2	2.09E-07	-0.32547	0.367	0.383	0.003491	11
Rnf122	2.14E-07	-0.32647	0.075	0.102	0.003563	11
Paics	2.16E-07	-0.32551	0.433	0.413	0.003605	11
Magoh	2.17E-07	-0.5329	0.25	0.333	0.003617	11
Sfr1	2.17E-07	-0.30222	0.408	0.394	0.003618	11
Ccp110	2.23E-07	-0.67612	0.108	0.249	0.003717	11
P4ha1	2.24E-07	0.34603	0.208	0.06	0.003737	11
1110038B1	2.32E-07	-0.6402	0.167	0.299	0.003867	11
Gpr19	2.33E-07	0.287819	0.208	0.066	0.003887	11
Alcam	2.37E-07	0.36563	0.317	0.133	0.003953	11
Cebpz	2.4E-07	-0.45072	0.267	0.305	0.003997	11
Hmgxb4	2.41E-07	-0.48509	0.092	0.155	0.004028	11
Ckap2l	2.42E-07	-0.53002	0.175	0.218	0.004033	11
Gcsh	2.47E-07	0.397859	0.342	0.14	0.004119	11
Dctn3	2.48E-07	-0.37099	0.367	0.368	0.004145	11
Psmd7	2.52E-07	-0.38902	0.458	0.479	0.004199	11
Esco2	2.58E-07	-0.76163	0.058	0.195	0.004308	11
Rcor2	2.72E-07	-0.74103	0.067	0.226	0.00453	11
Ctxn1	2.77E-07	-0.61637	0.042	0.171	0.004618	11
Txnl4a	2.77E-07	-0.33274	0.192	0.194	0.004623	11

Cacng7	2.78E-07	0.279298	0.183	0.051	0.004638	11
Dazap1	2.79E-07	-0.63728	0.142	0.273	0.004661	11
Fam58b	2.81E-07	0.286489	0.158	0.04	0.004684	11
Ewsr1	2.85E-07	-0.47603	0.325	0.392	0.004761	11
Lgr4	2.86E-07	0.260136	0.108	0.017	0.004775	11
Smarcd1	2.89E-07	-0.52004	0.2	0.271	0.004824	11
Itpa	2.91E-07	-0.27852	0.233	0.21	0.004848	11
Acat1	2.95E-07	-0.31516	0.45	0.44	0.004914	11
Srrm3	2.95E-07	-0.72658	0.117	0.24	0.004914	11
Lsm3	2.97E-07	-0.65518	0.2	0.359	0.004952	11
Txn1	2.98E-07	-0.38373	0.533	0.56	0.004964	11
Bcar1	2.98E-07	-0.61933	0.075	0.193	0.004973	11
Hmgn2	3E-07	-0.66456	0.108	0.257	0.005011	11
Phf14	3.01E-07	-0.55452	0.258	0.375	0.005026	11
Btf3	3.04E-07	-0.28808	0.375	0.349	0.005073	11
Gsg1l	3.08E-07	-0.73269	0.058	0.256	0.005129	11
Casc3	3.14E-07	-0.27845	0.133	0.129	0.00524	11
Nap1l4	3.16E-07	-0.35397	0.475	0.49	0.005266	11
Gltscr2	3.28E-07	-0.54728	0.25	0.342	0.005465	11
Actr1a	3.3E-07	-0.2886	0.192	0.177	0.0055	11
Dnmt1	3.31E-07	-0.73777	0.1	0.278	0.005523	11
BC005764	3.35E-07	-0.79981	0.042	0.186	0.005583	11
Pkm	3.41E-07	-0.2818	0.458	0.429	0.005693	11
Srpk2	3.42E-07	-0.35252	0.433	0.429	0.005708	11
Mllt11	3.49E-07	-0.75381	0.092	0.219	0.005829	11
Rpl13	3.5E-07	-0.36668	0.292	0.313	0.005831	11
Med19	3.58E-07	-0.53293	0.242	0.317	0.005969	11
Ndufa3	3.6E-07	0.327945	0.75	0.495	0.006008	11
Polr2h	3.62E-07	-0.35933	0.217	0.225	0.00603	11
Zcrb1	3.67E-07	-0.40049	0.342	0.371	0.006121	11
Frrs1l	3.69E-07	0.362033	0.4	0.189	0.006147	11
St18	4.02E-07	-0.79367	0.058	0.191	0.006712	11
Strn3	4.21E-07	-0.33555	0.325	0.312	0.007015	11
Bcl11a	4.24E-07	-0.6702	0.033	0.211	0.00707	11
Ahsa1	4.3E-07	-0.26337	0.275	0.249	0.007179	11
Snrpb2	4.31E-07	-0.55418	0.267	0.385	0.007186	11
Fam214b	4.37E-07	0.273968	0.125	0.025	0.007282	11
Utp14a	4.39E-07	-0.55944	0.158	0.233	0.007322	11
Psma1	4.45E-07	-0.42874	0.392	0.444	0.00743	11
Bptf	4.52E-07	-0.53077	0.308	0.374	0.007543	11
Slc16a2	4.54E-07	0.28264	0.2	0.068	0.007581	11
Flcn	4.68E-07	0.333479	0.158	0.036	0.0078	11
Sumo1	4.68E-07	-0.41301	0.175	0.218	0.007813	11
Gars	4.76E-07	-0.42918	0.192	0.227	0.007941	11
Ssna1	4.81E-07	-0.29865	0.283	0.266	0.00803	11
Pfdn1	5.09E-07	0.281026	0.508	0.297	0.008492	11
Pds5b	5.11E-07	-0.50456	0.208	0.272	0.008524	11
Hmgn5	5.12E-07	-0.69012	0.258	0.425	0.008539	11

Purb	5.19E-07	-0.28274	0.592	0.548	0.008663	11
Atp2a2	5.27E-07	0.275891	0.392	0.2	0.008785	11
Mab21l1	5.31E-07	-0.76222	0.067	0.226	0.008864	11
Nsmce4a	5.35E-07	-0.53539	0.133	0.224	0.008929	11
Arid4b	5.39E-07	-0.67639	0.275	0.44	0.008985	11
Calr	5.48E-07	0.330739	0.75	0.509	0.009136	11
Bcap31	5.58E-07	0.403271	0.383	0.172	0.009309	11
Glce	5.58E-07	-0.7233	0.025	0.177	0.00931	11
Rpl38	5.6E-07	-0.42994	0.342	0.382	0.00934	11
Psmd12	5.76E-07	-0.25774	0.417	0.372	0.009599	11
Acer3	6.07E-07	0.422279	0.192	0.051	0.010123	11
Trpc4ap	6.08E-07	-0.27192	0.25	0.197	0.010138	11
Tpm3	6.08E-07	-0.30614	0.317	0.315	0.010145	11
Tmco3	6.09E-07	0.300011	0.15	0.034	0.010163	11
Tmem18	6.1E-07	0.273889	0.167	0.045	0.010178	11
Apoa1bp	6.11E-07	0.255558	0.367	0.196	0.010195	11
Zmynd8	6.13E-07	-0.65016	0.1	0.213	0.010225	11
Ank	6.2E-07	0.508256	0.2	0.054	0.010334	11
Denr	6.29E-07	-0.50458	0.258	0.346	0.010489	11
2410089E(	6.29E-07	-0.40967	0.092	0.13	0.01049	11
Idh2	6.44E-07	-0.28211	0.3	0.278	0.01075	11
Spag5	6.54E-07	0.447021	0.225	0.078	0.010903	11
Cep57	6.63E-07	-0.60717	0.15	0.258	0.011065	11
Hes1	6.64E-07	0.438998	0.342	0.147	0.011081	11
Snrnp40	6.64E-07	-0.53329	0.175	0.271	0.011082	11
Bpnt1	6.67E-07	0.336594	0.167	0.051	0.011127	11
Igsf8	6.68E-07	-0.45185	0.233	0.269	0.011143	11
Fam168b	6.73E-07	-0.42834	0.208	0.241	0.011218	11
Rnaseh2c	6.75E-07	-0.25415	0.408	0.381	0.01126	11
Akirin2	6.77E-07	-0.34194	0.192	0.197	0.011295	11
Lpp	6.79E-07	0.316558	0.125	0.022	0.011318	11
Fgd6	7.06E-07	0.254194	0.2	0.067	0.011777	11
Dpysl2	7.27E-07	-0.37368	0.242	0.248	0.012132	11
Fmnl2	7.46E-07	0.307469	0.342	0.156	0.012443	11
Myod1	7.5E-07	-0.65724	0.008	0.142	0.01251	11
Dtymk	7.64E-07	-0.46018	0.375	0.439	0.012736	11
Eef1e1	7.81E-07	-0.3813	0.158	0.181	0.013025	11
Ubap2l	7.83E-07	-0.54347	0.217	0.317	0.013058	11
Bok	8.03E-07	-0.66435	0.083	0.252	0.013394	11
Clip3	8.11E-07	-0.54321	0.233	0.312	0.013533	11
mt-Cytb	8.32E-07	0.335259	0.967	0.938	0.013874	11
Rpl3	8.35E-07	-0.43807	0.158	0.212	0.013925	11
Pdcd4	8.38E-07	-0.39056	0.317	0.331	0.013973	11
Dhx15	8.47E-07	-0.4426	0.358	0.417	0.014121	11
Nmral1	8.73E-07	-0.61218	0.05	0.188	0.014569	11
Cnot6	8.78E-07	-0.53336	0.3	0.392	0.014647	11
Ppic	9.01E-07	-0.38475	0.225	0.239	0.015033	11
Atg3	9.05E-07	0.371221	0.383	0.178	0.015094	11

Ten1	9.12E-07	0.301303	0.325	0.151	0.015218	11
Rbm4b	9.2E-07	-0.52448	0.042	0.141	0.015344	11
Eif3g	9.24E-07	-0.4024	0.367	0.402	0.015414	11
Kras	9.28E-07	-0.32454	0.258	0.251	0.015475	11
Btd	9.36E-07	0.327439	0.117	0.018	0.015619	11
Cyc1	9.44E-07	0.402899	0.658	0.41	0.015738	11
Ift27	9.68E-07	-0.39364	0.217	0.242	0.016144	11
Actr6	9.69E-07	-0.30356	0.092	0.107	0.016158	11
Mrpl21	9.75E-07	-0.32126	0.292	0.28	0.016271	11
4933426M	9.81E-07	0.359156	0.175	0.046	0.016356	11
Baz2b	9.9E-07	-0.46625	0.333	0.364	0.016519	11
Sidt2	1.01E-06	0.334469	0.167	0.045	0.016835	11
Cuedc2	1.04E-06	-0.35061	0.367	0.387	0.017425	11
Tnrc6a	1.08E-06	-0.29203	0.325	0.299	0.017943	11
Psph	1.08E-06	0.372688	0.225	0.073	0.017976	11
1110004F1	1.09E-06	-0.47428	0.35	0.423	0.018114	11
Ivns1abp	1.09E-06	-0.42184	0.367	0.415	0.018256	11
Ppig	1.1E-06	-0.44482	0.442	0.486	0.018364	11
Sucla2	1.11E-06	0.3288	0.317	0.146	0.018447	11
Spcs2	1.13E-06	0.313005	0.667	0.422	0.018822	11
Mphosph9	1.14E-06	-0.5953	0.025	0.148	0.019038	11
Naa38	1.16E-06	-0.38184	0.275	0.311	0.019361	11
Chd8	1.18E-06	-0.46404	0.15	0.201	0.019601	11
Dtl	1.18E-06	-0.59547	0.017	0.146	0.019612	11
Uchl1	1.18E-06	-0.57784	0.242	0.335	0.019658	11
Sult4a1	1.2E-06	-0.68604	0.067	0.223	0.01999	11
Cadm1	1.21E-06	-0.59936	0.225	0.391	0.02013	11
Rnf165	1.21E-06	-0.67037	0.033	0.173	0.020134	11
Adipor1	1.22E-06	0.29805	0.308	0.138	0.02029	11
Ift81	1.22E-06	-0.3428	0.133	0.146	0.020311	11
Olfm1	1.23E-06	-0.62318	0.108	0.199	0.020454	11
Epb4.1	1.24E-06	-0.68508	0.083	0.23	0.020706	11
Slc22a17	1.26E-06	0.336741	0.45	0.249	0.021072	11
Cdk11b	1.28E-06	-0.41349	0.433	0.511	0.021292	11
Il11ra1	1.28E-06	0.312814	0.183	0.056	0.021361	11
Lsm2	1.29E-06	-0.37794	0.175	0.23	0.021565	11
Tacc2	1.3E-06	-0.67187	0.008	0.142	0.021648	11
Nptn	1.3E-06	0.372498	0.392	0.186	0.021735	11
Ppp1r14c	1.34E-06	-0.42557	0.267	0.262	0.022327	11
Ylpm1	1.35E-06	-0.40126	0.225	0.241	0.022493	11
Uqcrfs1	1.36E-06	0.264568	0.608	0.386	0.022684	11
Snx13	1.36E-06	0.257176	0.158	0.044	0.022708	11
Larp7	1.39E-06	-0.63291	0.175	0.312	0.023135	11
Gm10260	1.42E-06	-0.42684	0.183	0.228	0.023637	11
Rpl29	1.42E-06	-0.30865	0.225	0.257	0.023754	11
Nhsl1	1.43E-06	0.358865	0.183	0.055	0.023802	11
Cpsf2	1.47E-06	-0.32559	0.242	0.236	0.024482	11
Hook3	1.49E-06	-0.33257	0.317	0.299	0.024881	11

Ypel1	1.5E-06	-0.62915	0.042	0.175	0.025091	11
Nsd1	1.55E-06	-0.39597	0.325	0.339	0.025871	11
Tnrc6c	1.56E-06	-0.50394	0.125	0.178	0.026055	11
Rab33b	1.57E-06	0.26254	0.15	0.038	0.026231	11
Kmt2a	1.58E-06	-0.56936	0.242	0.33	0.026429	11
Fhl1	1.62E-06	0.253799	0.25	0.104	0.027072	11
Myl12a	1.62E-06	-0.27884	0.358	0.328	0.027096	11
Myt1l	1.66E-06	-0.77194	0.017	0.162	0.027692	11
Ilf3	1.69E-06	-0.61669	0.208	0.377	0.028121	11
Arl6ip4	1.69E-06	-0.25199	0.275	0.236	0.028177	11
Eif4g1	1.71E-06	-0.29093	0.367	0.351	0.028477	11
Sept7	1.71E-06	0.284388	0.817	0.587	0.028511	11
Smpd3	1.74E-06	-0.62022	0.05	0.162	0.029054	11
Rbm26	1.77E-06	-0.50262	0.225	0.297	0.02958	11
Carhsp1	1.79E-06	-0.49554	0.192	0.254	0.029852	11
Rif1	1.82E-06	-0.55523	0.192	0.276	0.030294	11
Nup85	1.88E-06	-0.56348	0.117	0.226	0.031442	11
Bzw1	1.9E-06	-0.31278	0.367	0.359	0.031656	11
Tprkb	1.9E-06	0.273118	0.283	0.125	0.031671	11
Ttc9b	1.9E-06	-0.64881	0.033	0.195	0.031702	11
Trim28	1.94E-06	-0.64399	0.183	0.362	0.032354	11
Atp5d	1.94E-06	0.395042	0.783	0.583	0.03242	11
Ezr	1.95E-06	-0.35667	0.442	0.437	0.032476	11
Nup62	1.96E-06	-0.57663	0.042	0.171	0.032747	11
Ska2	1.96E-06	-0.59206	0.092	0.205	0.032753	11
Rb1cc1	1.96E-06	-0.33276	0.25	0.234	0.032768	11
A930011O	1.97E-06	-0.6674	0	0.115	0.032836	11
Cbx3	2.02E-06	-0.53643	0.167	0.274	0.033664	11
Sh3gl2	2.02E-06	-0.44177	0.142	0.171	0.033715	11
Tmem57	2.08E-06	-0.66912	0.125	0.292	0.034619	11
Csrp2	2.17E-06	-0.54937	0.042	0.158	0.03616	11
Gtpbp4	2.18E-06	-0.54431	0.2	0.282	0.036323	11
Senp6	2.22E-06	-0.38054	0.383	0.389	0.037054	11
Cd320	2.24E-06	0.257724	0.225	0.085	0.037355	11
Mki67ip	2.27E-06	-0.47334	0.192	0.244	0.037793	11
Smdt1	2.27E-06	0.381609	0.592	0.353	0.037793	11
Ube2c	2.28E-06	-0.85827	0.183	0.269	0.038032	11
Pfdn2	2.4E-06	-0.58195	0.183	0.31	0.040024	11
Rpf2	2.42E-06	-0.56113	0.1	0.202	0.040373	11
Sobp	2.43E-06	-0.40257	0.1	0.128	0.04057	11
Mpv17l2	2.46E-06	0.346554	0.208	0.071	0.04105	11
Golim4	2.46E-06	-0.47646	0.183	0.233	0.041101	11
Mpc2	2.48E-06	0.256507	0.508	0.296	0.041348	11
Tra2a	2.48E-06	-0.40616	0.317	0.392	0.041392	11
Sf3a3	2.48E-06	-0.68003	0.108	0.282	0.041441	11
Hspa4	2.53E-06	-0.34785	0.35	0.368	0.04222	11
5430416N	2.59E-06	-0.59999	0.008	0.141	0.043143	11
Orc6	2.61E-06	-0.54881	0.108	0.201	0.043481	11

Ube2a	2.68E-06	0.397779	0.35	0.161	0.044757	11
Sema7a	2.72E-06	-0.61067	0.017	0.156	0.045302	11
Utp3	2.73E-06	-0.52757	0.225	0.315	0.045477	11
Clspn	2.77E-06	-0.72811	0.042	0.19	0.046164	11
Usp3	2.81E-06	-0.55296	0.033	0.142	0.046793	11
B230219D:	2.85E-06	-0.37316	0.3	0.321	0.047605	11
Zic5	2.86E-06	-0.44995	0.117	0.16	0.047734	11
Gspt1	2.87E-06	-0.28247	0.292	0.277	0.047836	11
Dcakd	2.92E-06	-0.58605	0.167	0.297	0.048649	11
Dhrs3	2.92E-06	0.253258	0.108	0.021	0.048741	11
Crnkl1	3E-06	-0.476	0.117	0.186	0.050012	11
Myh10	3.01E-06	-0.68184	0.133	0.325	0.050135	11
Tbpl1	3.04E-06	-0.52053	0.1	0.187	0.050644	11
Ank2	3.06E-06	0.280579	0.442	0.238	0.050989	11
Bub3	3.06E-06	-0.38142	0.25	0.321	0.051004	11
Srsf9	3.09E-06	-0.25745	0.258	0.236	0.051487	11
Slc17a6	3.1E-06	-0.66596	0.042	0.148	0.051726	11
Rpl18	3.15E-06	-0.51787	0.2	0.305	0.052604	11
Rfc3	3.17E-06	-0.58871	0.092	0.205	0.052886	11
Pole3	3.21E-06	-0.40406	0.217	0.25	0.053521	11
Dnajc2	3.26E-06	-0.59836	0.208	0.329	0.054298	11
Dnajc21	3.27E-06	-0.65739	0.092	0.21	0.054498	11
Prdx2	3.3E-06	-0.27748	0.708	0.679	0.054976	11
Wapal	3.31E-06	-0.43365	0.225	0.27	0.055242	11
Usp7	3.33E-06	-0.48022	0.2	0.264	0.055545	11
Cenpm	3.36E-06	-0.51398	0.067	0.162	0.056036	11
Fam50a	3.41E-06	-0.29846	0.25	0.239	0.05682	11
Mdga1	3.45E-06	-0.59613	0.008	0.136	0.057604	11
Trappc4	3.46E-06	-0.26461	0.25	0.224	0.05764	11
Nemf	3.51E-06	-0.5151	0.275	0.339	0.058544	11
Gm561	3.54E-06	0.251193	0.242	0.112	0.059008	11
Peg3	3.57E-06	-0.66636	0.158	0.285	0.059527	11
Cln3	3.69E-06	0.337824	0.142	0.035	0.061549	11
Wbp11	3.71E-06	-0.39633	0.25	0.296	0.061829	11
Fez1	3.71E-06	0.270005	0.533	0.331	0.061892	11
Plscr1	3.85E-06	0.284324	0.125	0.024	0.064225	11
Hnrnpa3	3.85E-06	-0.46911	0.075	0.159	0.064225	11
Mllt10	3.88E-06	-0.33824	0.292	0.286	0.064743	11
Twf1	3.88E-06	0.251795	0.283	0.135	0.064798	11
Smarca2	3.93E-06	-0.36845	0.267	0.285	0.065559	11
Eif5a	4E-06	-0.31976	0.392	0.403	0.066793	11
Fopnl	4.02E-06	-0.35528	0.183	0.202	0.066977	11
Rps18	4.09E-06	-0.48545	0.333	0.429	0.068181	11
Nop14	4.1E-06	-0.30924	0.233	0.217	0.068448	11
Slc3a2	4.11E-06	0.261071	0.533	0.321	0.068535	11
Mfap4	4.16E-06	-0.71715	0.033	0.162	0.069333	11
Snrnp25	4.17E-06	-0.2729	0.125	0.126	0.069612	11
Ep400	4.19E-06	-0.44153	0.125	0.18	0.069914	11

Mycbp2	4.34E-06	-0.39332	0.342	0.345	0.072378	11
Ppp1r7	4.36E-06	-0.43799	0.15	0.198	0.072753	11
Cep170	4.38E-06	-0.6795	0.158	0.306	0.072992	11
Jakmip2	4.42E-06	-0.30349	0.167	0.156	0.073678	11
Rnf187	4.45E-06	-0.41663	0.333	0.421	0.074165	11
Wwp1	4.54E-06	0.316579	0.225	0.083	0.075779	11
Zfp318	4.56E-06	-0.34246	0.175	0.176	0.076124	11
Polr2c	4.61E-06	-0.41823	0.15	0.202	0.076882	11
Nfkbia	4.64E-06	0.318637	0.35	0.17	0.077474	11
Lap3	4.7E-06	-0.47473	0.225	0.276	0.078316	11
Dnajc8	4.71E-06	-0.26006	0.458	0.434	0.078525	11
Rsbn1l	4.71E-06	-0.41322	0.283	0.312	0.078636	11
Psmd14	4.8E-06	-0.28528	0.35	0.35	0.080145	11
Coro2b	4.81E-06	-0.36529	0.15	0.174	0.080246	11
Calm1	4.84E-06	-0.30097	0.883	0.861	0.080766	11
Safb2	4.96E-06	-0.56257	0.217	0.314	0.082661	11
Myo10	5E-06	0.46489	0.275	0.12	0.083331	11
Prdm8	5E-06	-0.60616	0.025	0.127	0.083448	11
Polr2f	5.12E-06	-0.32602	0.358	0.374	0.085441	11
Dhx36	5.12E-06	-0.3665	0.358	0.37	0.085445	11
Tsg101	5.15E-06	-0.32574	0.258	0.264	0.085968	11
Cops2	5.25E-06	-0.33364	0.217	0.226	0.087547	11
Gtf2f1	5.38E-06	-0.5299	0.25	0.344	0.089752	11
Nop16	5.41E-06	-0.46779	0.1	0.174	0.090219	11
Rps16	5.42E-06	-0.40823	0.167	0.209	0.090381	11
Zfml	5.47E-06	-0.44125	0.275	0.312	0.091177	11
Stau2	5.47E-06	-0.40177	0.117	0.15	0.091191	11
Abr	5.5E-06	0.327481	0.242	0.098	0.091801	11
Rpa2	5.53E-06	-0.58357	0.092	0.2	0.092314	11
Ikzf5	5.57E-06	-0.34295	0.117	0.144	0.09287	11
Tmem178	5.57E-06	-0.64163	0.033	0.187	0.092919	11
Gm26735	5.59E-06	-0.56817	0.092	0.181	0.093244	11
Nhlh1	5.76E-06	-0.78134	0.067	0.224	0.09601	11
Mrpl30	5.8E-06	-0.3734	0.283	0.308	0.096685	11
Tubgcp2	5.8E-06	-0.29608	0.092	0.117	0.096695	11
H2afz	6.04E-06	-0.44261	0.15	0.231	0.100701	11
Mcm2	6.07E-06	-0.58863	0.1	0.205	0.10129	11
Dstn	6.21E-06	-0.40887	0.275	0.331	0.103614	11
Zfp704	6.22E-06	-0.56588	0.15	0.258	0.103727	11
Pde7a	6.23E-06	-0.37943	0.067	0.112	0.103966	11
Tubb2b	6.32E-06	-0.36647	0.467	0.464	0.105404	11
Eif3l	6.33E-06	-0.32653	0.283	0.302	0.105597	11
Tcf3	6.33E-06	-0.57017	0.108	0.226	0.105628	11
Tmem192	6.69E-06	0.269235	0.125	0.031	0.111523	11
2610001J0	6.72E-06	0.257244	0.367	0.192	0.112109	11
Mgrn1	7.06E-06	0.307751	0.175	0.053	0.117714	11
Mcl1	7.11E-06	-0.25383	0.233	0.211	0.118588	11
Mapk10	7.14E-06	0.349072	0.242	0.098	0.11914	11

Sacs	7.17E-06	-0.59914	0.083	0.191	0.119592	11
Rbbp6	7.31E-06	-0.50473	0.342	0.433	0.121849	11
Gm5617	7.31E-06	0.461033	0.183	0.059	0.121986	11
Dnaja1	7.43E-06	-0.3313	0.367	0.374	0.123969	11
Pdzrn4	7.54E-06	-0.39014	0.125	0.146	0.125789	11
Smap1	7.58E-06	0.255327	0.4	0.222	0.126517	11
Nt5dc2	7.72E-06	-0.63534	0.1	0.238	0.128851	11
Ptprs	7.73E-06	-0.33324	0.458	0.42	0.128901	11
Phf20l1	7.79E-06	-0.59718	0.225	0.341	0.129876	11
Ccng2	7.81E-06	-0.61373	0.083	0.212	0.1302	11
Mapk8ip1	7.88E-06	-0.45368	0.183	0.227	0.131423	11
Nsg1	8E-06	-0.64711	0.142	0.288	0.133485	11
Mak16	8.04E-06	-0.56134	0.125	0.225	0.134113	11
Chrna3	8.39E-06	-0.60662	0.017	0.119	0.139995	11
Nadk2	8.53E-06	0.341889	0.142	0.032	0.142273	11
2900011O	8.53E-06	-0.54581	0	0.104	0.142306	11
Sep15	8.62E-06	0.374448	0.725	0.503	0.143775	11
Trim2	8.99E-06	-0.40201	0.267	0.282	0.149936	11
Nt5c3	8.99E-06	-0.36158	0.117	0.149	0.149977	11
Smarcd3	8.99E-06	0.276781	0.183	0.061	0.150028	11
Fam155a	9.2E-06	0.375127	0.275	0.114	0.153478	11
Rpl30	9.31E-06	-0.40095	0.217	0.268	0.155208	11
Prc1	9.4E-06	-0.78154	0.133	0.257	0.156852	11
Eif4e3	9.52E-06	-0.54327	0.033	0.131	0.158842	11
Smek1	9.54E-06	-0.40238	0.133	0.179	0.159162	11
Cpsf6	9.62E-06	-0.38939	0.258	0.291	0.160379	11
Reln	9.65E-06	-0.70082	0.042	0.181	0.160907	11
Chd1	9.65E-06	-0.53854	0.125	0.209	0.160983	11
Snrpa1	9.74E-06	-0.47185	0.258	0.331	0.162475	11
Txndc9	9.85E-06	-0.28282	0.217	0.211	0.16426	11
Nin	9.91E-06	-0.50121	0.017	0.147	0.165334	11
Hibch	9.92E-06	0.322555	0.142	0.033	0.165546	11
Ralgps2	1.01E-05	-0.62825	0.117	0.246	0.167949	11
Mapre1	1.01E-05	-0.28969	0.525	0.517	0.168136	11
Bcas1	1.01E-05	-0.7157	0.15	0.283	0.16819	11
R3hdm1	1.01E-05	-0.64195	0.142	0.298	0.168826	11
Hars	1.01E-05	-0.37129	0.183	0.211	0.169139	11
Srsf4	1.06E-05	-0.36585	0.233	0.279	0.176099	11
Stag1	1.07E-05	-0.56704	0.1	0.207	0.178398	11
Msantd3	1.08E-05	-0.25088	0.108	0.111	0.180588	11
Pgrmc1	1.09E-05	0.318471	0.508	0.293	0.181821	11
Nicn1	1.09E-05	-0.39566	0.183	0.208	0.182029	11
Birc6	1.1E-05	-0.25953	0.183	0.168	0.183483	11
Nrm	1.12E-05	-0.50007	0.075	0.17	0.187235	11
Rbms1	1.15E-05	-0.48417	0.15	0.216	0.192118	11
Lpcat3	1.16E-05	0.390003	0.158	0.042	0.19323	11
Imp3	1.17E-05	-0.4478	0.183	0.247	0.19473	11
Prox1	1.18E-05	-0.66301	0.075	0.23	0.196895	11

Ercc5	1.21E-05	-0.28718	0.108	0.112	0.202393	11
Smg6	1.21E-05	-0.45454	0.058	0.126	0.202573	11
Vps29	1.22E-05	0.360127	0.425	0.225	0.204188	11
Tsc22d1	1.23E-05	-0.52102	0.308	0.428	0.20571	11
Spc24	1.29E-05	-0.67131	0.092	0.243	0.215455	11
Kcnd2	1.32E-05	0.284388	0.108	0.025	0.219709	11
Bnip3l	1.34E-05	-0.33153	0.15	0.164	0.224222	11
Dnttip2	1.35E-05	-0.48381	0.258	0.324	0.225247	11
Polr1c	1.36E-05	-0.44331	0.158	0.218	0.226603	11
Ccnl1	1.38E-05	-0.41334	0.317	0.368	0.230386	11
Homer2	1.38E-05	-0.56836	0.108	0.247	0.23078	11
Psmc6	1.39E-05	-0.38721	0.3	0.34	0.231846	11
Vps4b	1.42E-05	-0.31455	0.158	0.171	0.236853	11
Arf1	1.46E-05	-0.33678	0.3	0.317	0.242983	11
Tbl1x	1.46E-05	-0.45197	0.267	0.328	0.243881	11
Rpl35	1.48E-05	-0.43901	0.167	0.234	0.246371	11
Sae1	1.5E-05	-0.4297	0.217	0.279	0.249694	11
Fam3a	1.51E-05	0.363502	0.167	0.05	0.251544	11
Pcif1	1.51E-05	-0.26023	0.208	0.196	0.252472	11
Kif20b	1.52E-05	-0.649	0.075	0.191	0.254088	11
Sbno1	1.54E-05	-0.6114	0.217	0.352	0.25623	11
Tspan6	1.54E-05	0.28951	0.433	0.244	0.25654	11
Zfr	1.57E-05	-0.36432	0.342	0.353	0.262139	11
Mcm5	1.59E-05	-0.53166	0.067	0.169	0.265921	11
Fut8	1.6E-05	-0.36298	0.2	0.227	0.266993	11
Ash2l	1.61E-05	-0.44201	0.158	0.215	0.269008	11
D19Bwg13	1.63E-05	-0.38036	0.217	0.273	0.272476	11
Eml3	1.64E-05	0.269238	0.133	0.04	0.274378	11
Tma7	1.65E-05	-0.25508	0.183	0.174	0.275682	11
Wdr89	1.69E-05	-0.31012	0.192	0.214	0.281295	11
Zfp637	1.69E-05	-0.35097	0.233	0.256	0.282494	11
Lgals1	1.7E-05	-0.2952	0.267	0.228	0.283938	11
Zfp207	1.7E-05	-0.33368	0.342	0.37	0.284352	11
Lasp1	1.72E-05	0.263457	0.217	0.09	0.286446	11
Atp2c1	1.75E-05	0.250805	0.2	0.075	0.291368	11
Zfp266	1.76E-05	-0.45518	0.1	0.168	0.292811	11
Polr2e	1.78E-05	-0.27449	0.333	0.324	0.29646	11
Frmd4a	1.79E-05	-0.58252	0.2	0.309	0.297797	11
Mex3b	1.79E-05	-0.48238	0.033	0.116	0.297945	11
Eif3d	1.81E-05	-0.28388	0.417	0.405	0.3021	11
Cadm3	1.82E-05	-0.66333	0.025	0.142	0.302782	11
Cox6a1	1.82E-05	0.32552	0.833	0.76	0.303913	11
Rab8a	1.82E-05	-0.31223	0.158	0.171	0.304075	11
Cplx1	1.83E-05	-0.58292	0.017	0.139	0.30518	11
Tvp23b	1.85E-05	0.258851	0.15	0.043	0.308643	11
Uhrf1	1.85E-05	-0.54399	0.025	0.144	0.30898	11
Arid4a	1.87E-05	-0.53567	0.217	0.283	0.311398	11
App	1.88E-05	0.360078	0.775	0.567	0.31312	11

Ppa1	1.93E-05	-0.33467	0.183	0.221	0.321178	11
A030009H(	1.93E-05	-0.46271	0.025	0.114	0.321511	11
Ndufb2	1.95E-05	0.255086	0.575	0.366	0.326015	11
Pigk	1.97E-05	0.31822	0.158	0.045	0.328963	11
2810004N:	1.98E-05	-0.44232	0.217	0.262	0.33047	11
Znhit6	1.99E-05	-0.49516	0.025	0.12	0.331103	11
Epb4.1l3	1.99E-05	-0.47143	0.017	0.114	0.331889	11
Echdc1	2.01E-05	0.315997	0.133	0.037	0.335489	11
Elovl4	2.04E-05	-0.25516	0.108	0.11	0.340287	11
Setd8	2.05E-05	-0.59253	0.133	0.262	0.341128	11
Rabggta	2.05E-05	0.271555	0.117	0.024	0.342517	11
Ola1	2.09E-05	-0.26909	0.242	0.228	0.348452	11
Glrx3	2.09E-05	-0.30407	0.233	0.247	0.348819	11
Luc7l	2.1E-05	-0.59793	0.208	0.361	0.350457	11
Smek2	2.12E-05	-0.35465	0.208	0.223	0.353832	11
Cox6b1	2.14E-05	0.308038	0.883	0.74	0.357315	11
Ivd	2.15E-05	0.444501	0.242	0.107	0.357985	11
Hdgfrp2	2.18E-05	-0.25909	0.225	0.214	0.36347	11
Usp34	2.2E-05	-0.3025	0.3	0.29	0.36655	11
Lyar	2.21E-05	-0.51856	0.2	0.286	0.368952	11
Smap2	2.28E-05	-0.25225	0.133	0.122	0.37953	11
Ncbp2	2.29E-05	-0.26986	0.2	0.194	0.382569	11
Zkscan1	2.29E-05	-0.43674	0.1	0.151	0.382712	11
Gnl3	2.33E-05	-0.54867	0.175	0.296	0.388551	11
Arf4	2.33E-05	-0.28421	0.4	0.403	0.389124	11
Ube3a	2.34E-05	-0.46001	0.283	0.35	0.390149	11
Mrpl24	2.35E-05	-0.32311	0.2	0.211	0.391203	11
Eif2b3	2.35E-05	-0.41701	0.117	0.159	0.392043	11
Phf3	2.51E-05	-0.38368	0.25	0.27	0.418349	11
Exosc1	2.51E-05	-0.39307	0.117	0.163	0.41935	11
Ybx3	2.55E-05	-0.5299	0.183	0.29	0.424558	11
Dzip3	2.55E-05	-0.34388	0.15	0.167	0.425154	11
Ddx50	2.55E-05	-0.43321	0.1	0.162	0.425828	11
Casp3	2.56E-05	-0.51076	0.142	0.234	0.42657	11
Tfdp2	2.6E-05	-0.51319	0.075	0.161	0.433045	11
Frg1	2.61E-05	-0.3713	0.275	0.299	0.434753	11
Ube2g2	2.63E-05	-0.3457	0.092	0.124	0.438533	11
Arxes1	2.69E-05	0.275601	0.183	0.063	0.449115	11
Stx6	2.73E-05	-0.52329	0.058	0.161	0.455036	11
Rnf220	2.76E-05	-0.48689	0.15	0.236	0.460685	11
Armc10	2.78E-05	-0.45378	0.017	0.11	0.46351	11
Eapp	2.78E-05	-0.33224	0.242	0.245	0.464338	11
Gnl3l	2.81E-05	-0.48363	0.117	0.189	0.468189	11
Whsc1l1	2.81E-05	-0.36053	0.125	0.155	0.468266	11
Slc38a1	2.84E-05	0.345781	0.467	0.268	0.472968	11
Ptrh2	2.86E-05	-0.3366	0.133	0.153	0.476507	11
Cnot1	2.87E-05	-0.40129	0.042	0.112	0.479494	11
Iws1	2.91E-05	-0.40959	0.175	0.215	0.485445	11

Ubp1	2.91E-05	-0.37946	0.133	0.169	0.485841	11
Raf1	2.94E-05	-0.40813	0.142	0.186	0.490632	11
Rfc4	2.95E-05	-0.57553	0.1	0.227	0.491253	11
Rab6b	2.96E-05	-0.50346	0.142	0.203	0.492966	11
Ube2d3	2.97E-05	-0.26499	0.275	0.267	0.494888	11
Ubn1	2.98E-05	-0.47005	0.142	0.204	0.496523	11
Polr2a	2.99E-05	-0.45754	0.108	0.176	0.499372	11
Ap3b2	3.05E-05	-0.59191	0.075	0.203	0.508254	11
Gamt	3.05E-05	-0.26632	0.183	0.169	0.509158	11
Mapk1	3.08E-05	-0.27705	0.25	0.241	0.514469	11
Dnal4	3.11E-05	-0.42361	0.033	0.11	0.519258	11
Cdc7	3.21E-05	-0.4658	0.092	0.168	0.534725	11
Eif4b	3.21E-05	-0.57247	0.15	0.276	0.535609	11
Kdm1a	3.21E-05	-0.45583	0.258	0.365	0.536042	11
Cox7a2l	3.24E-05	-0.30632	0.375	0.382	0.541137	11
Pbdc1	3.31E-05	-0.46203	0.133	0.206	0.552131	11
Cflar	3.31E-05	0.273984	0.158	0.049	0.552323	11
Rbm5	3.37E-05	-0.27855	0.408	0.401	0.562527	11
Rock1	3.42E-05	-0.37847	0.275	0.338	0.570936	11
Tex14	3.45E-05	-0.67597	0.025	0.146	0.575035	11
Sh3bgrl	3.46E-05	-0.41445	0.275	0.337	0.577571	11
Brix1	3.51E-05	-0.2705	0.208	0.205	0.584874	11
Kif15	3.51E-05	-0.5779	0.075	0.181	0.586208	11
Palm	3.57E-05	-0.52602	0.033	0.14	0.595637	11
Hprt	3.61E-05	-0.50936	0.083	0.188	0.602473	11
Mapt	3.63E-05	-0.51709	0.175	0.216	0.605263	11
Mbtd1	3.67E-05	-0.385	0.183	0.215	0.611804	11
Cyb561d2	3.72E-05	0.269368	0.117	0.03	0.619727	11
Cenpj	3.73E-05	-0.53942	0.042	0.142	0.622681	11
Tomm20	3.75E-05	-0.272	0.158	0.16	0.625341	11
Fam92a	3.77E-05	-0.32695	0.183	0.198	0.628472	11
1110038F1	3.81E-05	-0.30503	0.183	0.191	0.634805	11
Ppie	3.81E-05	-0.40315	0.075	0.134	0.635227	11
Las1l	3.82E-05	-0.52878	0.158	0.255	0.636806	11
Mtus1	3.83E-05	-0.51255	0.025	0.128	0.639411	11
Pafah1b2	3.88E-05	-0.36118	0.217	0.255	0.647954	11
Flna	3.9E-05	-0.38875	0.117	0.152	0.650441	11
Polr2i	3.91E-05	-0.25138	0.333	0.316	0.652875	11
Naa10	3.96E-05	-0.28524	0.25	0.249	0.660968	11
Ift74	4E-05	-0.34302	0.217	0.224	0.66642	11
Prpf6	4E-05	-0.4205	0.183	0.232	0.667672	11
Spag7	4E-05	-0.32322	0.267	0.276	0.667977	11
Tax1bp1	4.01E-05	-0.28615	0.6	0.578	0.669494	11
Atp5o	4.05E-05	0.321478	0.783	0.585	0.675203	11
Islr2	4.23E-05	-0.60485	0.05	0.18	0.705115	11
Mrps5	4.25E-05	-0.41149	0.225	0.272	0.70825	11
Ehbp1	4.26E-05	-0.37666	0.1	0.136	0.709781	11
Xpo7	4.26E-05	-0.35753	0.083	0.131	0.711181	11

Dync1h1	4.3E-05	-0.25294	0.175	0.159	0.716824	11
Cnot7	4.41E-05	-0.32372	0.2	0.216	0.735897	11
Pin1	4.47E-05	-0.45308	0.158	0.238	0.745767	11
Otx2	4.49E-05	-0.62274	0.083	0.217	0.749053	11
Plrg1	4.52E-05	-0.32851	0.1	0.131	0.7536	11
Fzd2	4.55E-05	-0.54046	0.042	0.161	0.75869	11
Ppp3cb	4.58E-05	-0.36904	0.283	0.31	0.764062	11
Fam57b	4.6E-05	-0.57519	0.042	0.186	0.767542	11
Fau	4.6E-05	-0.31966	0.092	0.123	0.76803	11
Epc2	4.65E-05	-0.5805	0.133	0.25	0.775374	11
Mllt4	4.72E-05	-0.48096	0.292	0.363	0.787446	11
Racgap1	4.77E-05	-0.57001	0.142	0.256	0.796225	11
Tro	4.8E-05	-0.47381	0.05	0.126	0.800441	11
Naa15	4.82E-05	-0.27761	0.383	0.391	0.803268	11
1700020I1	4.82E-05	-0.55047	0.167	0.275	0.803529	11
Bod1l	4.82E-05	-0.53431	0.258	0.379	0.804396	11
Gjc1	4.84E-05	-0.36513	0.158	0.196	0.806529	11
Cdc37	4.95E-05	-0.36656	0.258	0.291	0.825037	11
Tlk2	5.04E-05	-0.28424	0.225	0.212	0.841342	11
Prpf31	5.14E-05	-0.41295	0.125	0.2	0.857761	11
Rcn1	5.25E-05	-0.25899	0.233	0.233	0.876002	11
2810474O	5.26E-05	-0.55202	0.15	0.241	0.877772	11
Cnot3	5.27E-05	-0.35371	0.117	0.156	0.879039	11
Mpnd	5.29E-05	-0.29213	0.167	0.171	0.882899	11
Stip1	5.32E-05	-0.28101	0.283	0.303	0.887936	11
Sox11	5.35E-05	-0.46412	0.025	0.115	0.89237	11
Pip5k1a	5.38E-05	-0.25026	0.117	0.118	0.897367	11
Cdc20	5.38E-05	-0.70495	0.083	0.197	0.897459	11
Mycl	5.54E-05	-0.48069	0.042	0.137	0.923555	11
Dlgap4	5.62E-05	-0.33411	0.175	0.187	0.938089	11
Peli2	5.7E-05	-0.40213	0.125	0.165	0.950875	11
Uba52	5.84E-05	-0.30351	0.308	0.316	0.97494	11
Tacc3	5.85E-05	-0.48446	0.133	0.207	0.975067	11
Slc25a5	5.89E-05	0.3621	0.708	0.507	0.981978	11
E2f1	5.99E-05	-0.51579	0.05	0.147	0.999418	11
Ube2r2	6.04E-05	-0.43886	0.25	0.317	1	11
Timm50	6.08E-05	-0.34475	0.208	0.236	1	11
Slc39a6	6.09E-05	-0.36604	0.167	0.198	1	11
Hectd1	6.22E-05	-0.25972	0.217	0.202	1	11
Foxp1	6.3E-05	-0.57861	0.05	0.172	1	11
Zfp664	6.31E-05	-0.34788	0.2	0.229	1	11
Gpbp1	6.33E-05	-0.50804	0.242	0.357	1	11
Hsph1	6.39E-05	-0.40273	0.183	0.227	1	11
Abhd17b	6.46E-05	0.332651	0.3	0.142	1	11
Ddx27	6.46E-05	-0.50067	0.117	0.202	1	11
Mrpl35	6.47E-05	-0.30434	0.133	0.147	1	11
Gm17322	6.49E-05	-0.60133	0.025	0.132	1	11
Rrm1	6.53E-05	-0.41615	0.208	0.26	1	11

Strbp	6.7E-05	-0.5592	0.217	0.348	1	11
Ankrd10	6.71E-05	-0.46985	0.083	0.159	1	11
Lmo4	6.73E-05	-0.35456	0.292	0.335	1	11
Trp53i11	6.75E-05	-0.53705	0.1	0.202	1	11
Mphosph1(	6.76E-05	-0.48736	0.158	0.231	1	11
Mrpl52	6.99E-05	-0.29859	0.425	0.432	1	11
Klhl7	7.02E-05	-0.43518	0.1	0.165	1	11
Smchd1	7.19E-05	-0.56971	0.15	0.273	1	11
Dhx32	7.34E-05	-0.3475	0.175	0.2	1	11
Sde2	7.41E-05	-0.31816	0.125	0.146	1	11
Copa	7.46E-05	-0.34872	0.142	0.176	1	11
Txlna	7.51E-05	-0.3936	0.142	0.186	1	11
Rnf219	7.58E-05	-0.38906	0.092	0.14	1	11
Tomm70a	7.67E-05	-0.3632	0.2	0.229	1	11
Wbp4	7.75E-05	-0.27326	0.292	0.275	1	11
2310022B(	7.87E-05	-0.28872	0.242	0.248	1	11
Dusp1	7.88E-05	0.295568	0.275	0.162	1	11
Ahsa2	7.98E-05	-0.40471	0.158	0.206	1	11
Ccni	8.15E-05	-0.36092	0.183	0.222	1	11
Brd7	8.21E-05	-0.34203	0.283	0.308	1	11
Taf1d	8.26E-05	-0.54522	0.225	0.342	1	11
Dpy30	8.41E-05	-0.25211	0.3	0.298	1	11
Hes6	8.47E-05	-0.36558	0.208	0.236	1	11
Gnb1	8.54E-05	-0.3347	0.35	0.37	1	11
Atp11c	8.63E-05	-0.46568	0.033	0.12	1	11
Akap12	8.7E-05	-0.31192	0.108	0.111	1	11
Heatr6	8.75E-05	-0.26453	0.108	0.117	1	11
Ccna2	8.84E-05	-0.53145	0.1	0.222	1	11
Phactr1	8.96E-05	-0.3519	0.167	0.183	1	11
Commd3	8.98E-05	-0.29674	0.3	0.307	1	11
Sfswap	9.09E-05	-0.25955	0.25	0.239	1	11
Etf1	9.1E-05	-0.29042	0.258	0.261	1	11
Cdc26	9.2E-05	-0.3635	0.1	0.144	1	11
Nup88	9.28E-05	-0.37459	0.142	0.183	1	11
Taf15	9.4E-05	-0.49046	0.125	0.223	1	11
Pcsk2	9.47E-05	-0.45295	0.008	0.102	1	11
Ftsj3	9.58E-05	-0.48361	0.125	0.206	1	11
Atad2	9.67E-05	-0.58717	0.075	0.199	1	11
2410004N(	9.7E-05	-0.43245	0.125	0.184	1	11
Tyms	9.73E-05	-0.58561	0.1	0.219	1	11
Kti12	9.8E-05	-0.38297	0.033	0.102	1	11
Tipin	9.83E-05	-0.50708	0.167	0.255	1	11
Fam114a2	9.95E-05	-0.29916	0.142	0.155	1	11
Xrcc1	0.000101	-0.33859	0.067	0.11	1	11
Fbxo5	0.000101	-0.53672	0.05	0.16	1	11
Zfpl1	0.000103	-0.25457	0.092	0.101	1	11
Sfrs18	0.000103	-0.29614	0.733	0.778	1	11
Nefm	0.000104	-0.58558	0.008	0.117	1	11

Rbmxl1	0.000104	-0.49198	0.042	0.153	1	11
Exosc7	0.000104	-0.34167	0.15	0.18	1	11
Cdca7	0.000104	-0.58285	0.067	0.198	1	11
Zfp622	0.000105	-0.29746	0.125	0.137	1	11
4833420G:	0.000106	-0.44235	0.083	0.15	1	11
Plp1	0.00011	-0.41771	0.217	0.113	1	11
Cyp7b1	0.00011	0.317983	0.108	0.023	1	11
Cdk6	0.000113	-0.58566	0.092	0.244	1	11
Hspa9	0.000115	-0.26326	0.375	0.372	1	11
Gigyf2	0.000115	-0.39249	0.192	0.223	1	11
Cdh4	0.000116	0.407145	0.3	0.144	1	11
Dst	0.000119	-0.29635	0.275	0.266	1	11
Snhg6	0.000119	-0.25705	0.267	0.258	1	11
Etv1	0.000121	0.322518	0.117	0.027	1	11
Mroh2a	0.000121	-0.60007	0.017	0.114	1	11
D4Wsu53e	0.000121	-0.2784	0.458	0.446	1	11
Sart1	0.000123	-0.30017	0.192	0.198	1	11
Gart	0.000126	-0.39489	0.108	0.166	1	11
Kdm5a	0.00013	-0.25315	0.258	0.256	1	11
Ifitm2	0.00013	0.263186	0.242	0.113	1	11
Dnajc1	0.00013	0.399706	0.292	0.138	1	11
St3gal5	0.000134	-0.38712	0.158	0.194	1	11
1700025G(	0.000135	-0.5458	0.15	0.264	1	11
Mthfd1	0.000136	0.336746	0.258	0.114	1	11
Sf1	0.000138	-0.48567	0.142	0.237	1	11
R3hcc1	0.00014	-0.42576	0.1	0.159	1	11
Pygo1	0.00014	-0.29815	0.2	0.192	1	11
Nrd1	0.000141	-0.27551	0.2	0.221	1	11
Bub1b	0.000142	-0.38458	0.025	0.103	1	11
Brwd3	0.000145	-0.36424	0.05	0.102	1	11
Sc4mol	0.000146	0.399871	0.225	0.091	1	11
Smoc1	0.000147	-0.4654	0.025	0.135	1	11
Nol4	0.000148	-0.56615	0.058	0.172	1	11
Mis18bp1	0.00015	-0.58002	0.05	0.161	1	11
Ing3	0.000152	-0.38926	0.042	0.107	1	11
Nipsnap1	0.000152	-0.40687	0.083	0.136	1	11
BC004004	0.000152	0.292247	0.25	0.122	1	11
Apbb1	0.000154	-0.32649	0.158	0.17	1	11
Klhdc2	0.000155	-0.48871	0.208	0.327	1	11
Rnf146	0.000157	-0.30273	0.125	0.143	1	11
Sp3	0.00016	-0.39081	0.108	0.163	1	11
Spop	0.000162	-0.27171	0.308	0.315	1	11
Boc	0.000165	-0.52874	0.05	0.156	1	11
Vars	0.000166	-0.32941	0.133	0.166	1	11
Cenph	0.000166	-0.55706	0.092	0.194	1	11
Rsrc1	0.000167	-0.50246	0.158	0.251	1	11
Cdc42se2	0.000168	-0.46277	0.142	0.239	1	11
Wdr12	0.000168	-0.32613	0.15	0.175	1	11

Gm11541	0.000169	-0.49192	0.033	0.128	1	11
Mkrn1	0.000173	-0.32248	0.192	0.208	1	11
Rnf2	0.000173	-0.28108	0.1	0.12	1	11
Exosc8	0.000175	-0.40199	0.15	0.21	1	11
Nusap1	0.000175	-0.66916	0.092	0.228	1	11
Prdm2	0.000178	-0.45818	0.05	0.132	1	11
Wdr43	0.000181	-0.48761	0.133	0.242	1	11
Nol12	0.000182	-0.26662	0.117	0.128	1	11
Mapk8ip2	0.000186	-0.51394	0.025	0.143	1	11
Hmmr	0.000188	-0.68315	0.058	0.177	1	11
Atad5	0.000191	-0.51441	0.05	0.149	1	11
Ttc9c	0.000194	-0.41409	0.025	0.109	1	11
Cript	0.000194	-0.28044	0.242	0.244	1	11
Rangap1	0.000194	-0.36795	0.2	0.256	1	11
Pcf11	0.000197	-0.32267	0.158	0.179	1	11
Adam10	0.000197	-0.29567	0.192	0.203	1	11
Otud4	0.000199	-0.29621	0.092	0.115	1	11
Sowaha	0.000201	-0.65062	0.042	0.163	1	11
Ppil4	0.000202	-0.46716	0.158	0.249	1	11
Mapk8	0.000207	-0.28379	0.117	0.128	1	11
Cenpb	0.000208	-0.37608	0.167	0.209	1	11
Syne2	0.000209	-0.56181	0.108	0.22	1	11
Atp6v0a2	0.000216	0.250357	0.108	0.026	1	11
Yeats4	0.00022	-0.41666	0.2	0.263	1	11
Nfic	0.000222	-0.32826	0.242	0.261	1	11
Akap6	0.000226	-0.56034	0.1	0.219	1	11
Klf7	0.00023	-0.33218	0.242	0.262	1	11
Glyr1	0.000231	-0.50374	0.167	0.284	1	11
Ctps	0.000233	-0.43756	0.05	0.138	1	11
E330009J0	0.000236	-0.41069	0.05	0.117	1	11
Asph	0.000237	0.260215	0.233	0.113	1	11
Ankrd32	0.000242	-0.38417	0.058	0.184	1	11
Slu7	0.000243	-0.4226	0.2	0.264	1	11
Pdzrn3	0.000243	-0.53827	0.092	0.147	1	11
Ppm1a	0.000249	-0.31819	0.133	0.155	1	11
Clk1	0.00025	-0.44026	0.267	0.34	1	11
Kcnk1	0.000258	-0.25745	0.192	0.204	1	11
Pphln1	0.000259	-0.31332	0.083	0.118	1	11
Igsf3	0.000264	-0.262	0.133	0.133	1	11
Ehmt1	0.000265	-0.51793	0.133	0.252	1	11
Blmh	0.000267	-0.39971	0.225	0.317	1	11
Nme4	0.000269	-0.4103	0.033	0.118	1	11
Wwp2	0.000278	0.250841	0.117	0.035	1	11
Arrdc3	0.000279	-0.33514	0.05	0.104	1	11
Pum1	0.000282	-0.26243	0.267	0.276	1	11
Gprasp1	0.000282	-0.37998	0.167	0.206	1	11
Morf4l2	0.000283	-0.26944	0.267	0.272	1	11
Lmnb2	0.000284	-0.48194	0.042	0.159	1	11

Acot7	0.000289	-0.52989	0.067	0.195	1	11
Gnl2	0.00029	-0.41413	0.192	0.244	1	11
Grik2	0.000293	-0.52389	0.008	0.102	1	11
Pdxp	0.000294	-0.41814	0.033	0.113	1	11
Nono	0.000295	-0.33317	0.183	0.219	1	11
Celsr3	0.000309	-0.50375	0.025	0.123	1	11
Fnbp4	0.000314	-0.36973	0.225	0.276	1	11
Yy1	0.000318	-0.28404	0.192	0.204	1	11
Upf3b	0.000319	-0.40364	0.267	0.32	1	11
Gls	0.000325	-0.25613	0.117	0.123	1	11
Clvs1	0.000332	-0.52186	0.033	0.129	1	11
Cnot2	0.000332	-0.2867	0.175	0.185	1	11
Zrsr2	0.000332	-0.49355	0.108	0.236	1	11
Gps2	0.000334	-0.45581	0.158	0.236	1	11
Ptpn11	0.000338	-0.29326	0.267	0.27	1	11
Hnrnpf	0.000348	-0.26775	0.1	0.125	1	11
Eml4	0.000349	-0.28412	0.2	0.209	1	11
Ccdc59	0.000351	-0.31666	0.275	0.314	1	11
Smco4	0.000357	-0.46098	0.067	0.162	1	11
Phc2	0.000358	-0.31837	0.183	0.199	1	11
Rad50	0.000361	-0.31271	0.158	0.165	1	11
Krt10	0.000364	-0.39093	0.108	0.162	1	11
Suz12	0.000367	-0.47458	0.133	0.219	1	11
Msh2	0.000369	-0.49008	0.033	0.144	1	11
Psme4	0.000369	-0.4142	0.217	0.284	1	11
Znrd1	0.000369	-0.30231	0.192	0.212	1	11
Rpl7a	0.000369	-0.31984	0.117	0.15	1	11
Cdk7	0.000371	-0.31743	0.067	0.109	1	11
Syt13	0.000372	-0.48135	0.025	0.127	1	11
Tex30	0.00038	-0.43258	0.042	0.136	1	11
Cdca7l	0.000382	-0.41491	0.008	0.105	1	11
Fzd1	0.000384	0.345479	0.183	0.067	1	11
Erdr1	0.000396	-0.51635	0.058	0.162	1	11
Pde4dip	0.000399	-0.53022	0.108	0.221	1	11
Cux1	0.000399	-0.35062	0.158	0.202	1	11
Sdhb	0.0004	0.275174	0.592	0.399	1	11
Mcm3	0.000404	-0.53332	0.067	0.18	1	11
Tspan13	0.000407	-0.3173	0.192	0.207	1	11
Klf6	0.000414	0.473879	0.275	0.147	1	11
Zfp330	0.000417	-0.29606	0.1	0.122	1	11
Jhdm1d	0.000418	-0.49907	0.042	0.125	1	11
Ube2d1	0.000433	-0.48209	0.1	0.228	1	11
Timeless	0.000433	-0.49094	0.042	0.154	1	11
9330159F1	0.000434	-0.49241	0.083	0.169	1	11
Uqcr11	0.000437	0.352379	0.658	0.48	1	11
Grb2	0.00044	-0.25873	0.258	0.248	1	11
Plk4	0.000445	-0.42401	0.033	0.12	1	11
Jund	0.000447	-0.41057	0.3	0.351	1	11

Ppm1g	0.000448	-0.35832	0.158	0.2	1	11
Ppfia2	0.000449	-0.52571	0.033	0.112	1	11
Ssx2ip	0.000454	-0.42585	0.058	0.13	1	11
Pdcd11	0.000455	-0.29863	0.125	0.164	1	11
Mta1	0.000463	-0.2949	0.075	0.104	1	11
Rp9	0.000464	-0.36839	0.208	0.242	1	11
Prmt1	0.000467	-0.43519	0.142	0.225	1	11
1700037H(	0.000468	-0.2678	0.083	0.11	1	11
Rad51ap1	0.000473	-0.51167	0.05	0.167	1	11
Kif23	0.000473	-0.60192	0.083	0.215	1	11
Nvl	0.000475	-0.49056	0.067	0.169	1	11
Ccdc18	0.000475	-0.46131	0.042	0.115	1	11
Pcgf3	0.000476	-0.30969	0.075	0.107	1	11
Scnm1	0.000479	-0.291	0.117	0.136	1	11
Hist3h2a	0.00048	-0.25779	0.15	0.143	1	11
Srsf1	0.00048	-0.3365	0.25	0.296	1	11
Baz1a	0.000481	-0.51774	0.058	0.167	1	11
Fosb	0.000484	0.266807	0.258	0.155	1	11
Pitpnb	0.000492	-0.25399	0.208	0.213	1	11
Chd6	0.000496	-0.25764	0.292	0.304	1	11
Setx	0.000503	-0.40306	0.075	0.13	1	11
Gm11478	0.000505	-0.37825	0.158	0.215	1	11
Vopp1	0.000522	-0.37542	0.067	0.119	1	11
Ssbp1	0.000531	-0.32592	0.175	0.215	1	11
Eci2	0.000532	0.268911	0.408	0.248	1	11
Rbm22	0.000541	-0.38317	0.158	0.222	1	11
Nrn1	0.000554	-0.55693	0.1	0.222	1	11
Cecr2	0.000555	-0.45683	0.042	0.13	1	11
Flywch1	0.000556	-0.41659	0.058	0.128	1	11
Ctnnbl1	0.000557	-0.46979	0.092	0.185	1	11
Dync1li1	0.000559	-0.49394	0.042	0.144	1	11
Coro1c	0.00056	-0.48621	0.092	0.193	1	11
Smarcc2	0.000561	-0.32974	0.333	0.363	1	11
Gm10036	0.000563	-0.48623	0.1	0.176	1	11
Gmps	0.000564	-0.34039	0.175	0.21	1	11
Rab30	0.000565	0.264456	0.108	0.029	1	11
Smndc1	0.000566	-0.29294	0.158	0.179	1	11
Paxbp1	0.000567	-0.30719	0.225	0.259	1	11
Evl	0.00057	-0.51554	0.075	0.178	1	11
Ccdc25	0.00057	-0.37107	0.142	0.183	1	11
Ubfd1	0.000582	-0.31071	0.075	0.11	1	11
Xist	0.000588	-0.64212	0.183	0.3	1	11
Gpatch4	0.000606	-0.4045	0.067	0.18	1	11
Setd2	0.000607	-0.28579	0.158	0.212	1	11
Ap1s2	0.000613	-0.5051	0.058	0.16	1	11
Jam3	0.000615	-0.29585	0.108	0.135	1	11
C77370	0.000615	-0.47668	0.008	0.101	1	11
Nbea	0.000619	-0.40747	0.158	0.19	1	11

Acat2	0.000623	-0.32296	0.15	0.182	1	11
Tpt1	0.000627	-0.3463	0.217	0.256	1	11
Fxyd6	0.000628	-0.45527	0.375	0.509	1	11
H1fx	0.000635	-0.45809	0.15	0.245	1	11
Lars	0.000637	-0.4459	0.142	0.218	1	11
Zmym5	0.000637	-0.35391	0.2	0.232	1	11
Bms1	0.00064	-0.46771	0.108	0.183	1	11
Isy1	0.00065	-0.36152	0.092	0.141	1	11
Armcx4	0.000653	-0.44408	0.025	0.113	1	11
Cdt1	0.000676	-0.46731	0.025	0.125	1	11
Cep85l	0.000679	0.273146	0.125	0.039	1	11
Elavl1	0.00068	-0.31002	0.158	0.189	1	11
Xpo1	0.000682	-0.29562	0.2	0.227	1	11
Tab2	0.000684	0.256636	0.4	0.245	1	11
Smarce1	0.000684	-0.46938	0.1	0.204	1	11
Patz1	0.000693	-0.26119	0.083	0.105	1	11
Cops3	0.000706	-0.27703	0.242	0.251	1	11
Jarid2	0.000711	-0.4796	0.092	0.179	1	11
Lta4h	0.000717	-0.36006	0.192	0.242	1	11
Nedd4l	0.000726	-0.37295	0.083	0.128	1	11
Ccnb2	0.000727	-0.50878	0.117	0.187	1	11
Rps24-ps3	0.000733	-0.28989	0.15	0.179	1	11
Ncaph	0.000733	-0.38314	0.05	0.121	1	11
Ppia	0.000766	-0.29636	0.117	0.145	1	11
Ensa	0.000768	-0.36699	0.183	0.228	1	11
Stag2	0.000771	-0.41106	0.192	0.257	1	11
Ppan	0.000776	-0.43194	0.058	0.14	1	11
Snord104	0.000786	-0.46137	0.067	0.152	1	11
Rnaseh2b	0.000793	-0.35688	0.183	0.237	1	11
Atl1	0.000815	-0.37818	0.05	0.102	1	11
Ppp3r1	0.000816	-0.2541	0.125	0.137	1	11
Ddx17	0.000863	-0.25307	0.367	0.381	1	11
Satb1	0.000865	-0.46153	0.125	0.21	1	11
H2-Ke2	0.000867	-0.35	0.15	0.195	1	11
Hnrnpul1	0.00087	-0.26535	0.158	0.175	1	11
Bccip	0.000878	-0.33039	0.267	0.31	1	11
Phf6	0.000919	-0.41886	0.1	0.173	1	11
Gpc2	0.000924	-0.47167	0.033	0.14	1	11
Heatr3	0.000932	-0.35361	0.058	0.109	1	11
Peo1	0.000934	-0.41536	0.05	0.124	1	11
Tmeff1	0.000952	-0.33011	0.117	0.149	1	11
Cdc40	0.000955	-0.35513	0.133	0.169	1	11
Git2	0.000966	-0.37435	0.058	0.11	1	11
Mab21l2	0.000982	-0.46884	0.017	0.104	1	11
Ormdl3	0.000991	0.398372	0.167	0.084	1	11
Uba2	0.000993	-0.33835	0.217	0.256	1	11
Rbm6	0.001005	-0.29602	0.2	0.223	1	11
Fam64a	0.001028	-0.39748	0.05	0.118	1	11

Fbxo32	0.001038	-0.40834	0.033	0.117	1	11
G3bp1	0.001041	-0.41791	0.192	0.328	1	11
Narf	0.001043	-0.2714	0.1	0.117	1	11
9430016H(	0.001046	0.251239	0.225	0.112	1	11
Ttc33	0.001056	-0.2674	0.083	0.108	1	11
Trim59	0.00106	-0.4788	0.067	0.17	1	11
Slain2	0.001063	-0.38258	0.058	0.127	1	11
Stxbp1	0.001064	-0.48806	0.092	0.162	1	11
Krr1	0.001065	-0.37223	0.1	0.152	1	11
Ddx18	0.001075	-0.39608	0.058	0.127	1	11
Qser1	0.00109	-0.27239	0.108	0.127	1	11
Pik3r3	0.001096	-0.42061	0.083	0.136	1	11
Zbtb22	0.001105	0.304382	0.117	0.037	1	11
Ick	0.001109	-0.27271	0.133	0.147	1	11
Iqgap2	0.001133	0.302387	0.142	0.047	1	11
Cpsf3l	0.001144	-0.33958	0.058	0.11	1	11
Gid4	0.001153	-0.25133	0.1	0.115	1	11
Cnrip1	0.001173	-0.39735	0.15	0.198	1	11
Ercc1	0.00118	-0.43721	0.05	0.137	1	11
Zfp644	0.001182	-0.34281	0.175	0.207	1	11
Crebbp	0.001184	-0.42308	0.158	0.223	1	11
Tnpo3	0.001184	-0.27587	0.208	0.222	1	11
Mpped2	0.001208	-0.4142	0.058	0.127	1	11
Setbp1	0.001227	-0.43907	0.092	0.16	1	11
Hip1r	0.001231	-0.4977	0.042	0.133	1	11
Rad51	0.00124	-0.35334	0.058	0.116	1	11
Kat6b	0.001245	-0.4424	0.092	0.157	1	11
Gm10076	0.001247	-0.2691	0.108	0.134	1	11
Ints10	0.001252	-0.26261	0.108	0.128	1	11
Kat6a	0.001255	-0.32642	0.142	0.172	1	11
Abce1	0.001257	-0.27355	0.125	0.149	1	11
Msi2	0.001263	0.27694	0.325	0.186	1	11
Rundc3a	0.00127	-0.46381	0.158	0.249	1	11
Ctr9	0.001272	-0.45462	0.158	0.243	1	11
Smc5	0.001287	-0.25854	0.242	0.258	1	11
Xpa	0.001305	-0.25023	0.108	0.129	1	11
Tmem55b	0.001344	0.278923	0.233	0.111	1	11
Odf2	0.001345	-0.25963	0.167	0.17	1	11
Daam1	0.001383	-0.36391	0.142	0.183	1	11
Pnmal2	0.001415	-0.35853	0.117	0.147	1	11
Thsd7a	0.001421	-0.48153	0.05	0.127	1	11
mt-Co1	0.001436	0.251838	0.775	0.608	1	11
Uhrf2	0.001438	-0.38416	0.092	0.15	1	11
Hk2	0.001449	-0.40018	0.108	0.173	1	11
Upf3a	0.001453	-0.2599	0.217	0.219	1	11
Chchd2	0.00146	-0.25806	0.625	0.653	1	11
2700049A(	0.001471	-0.46011	0.033	0.122	1	11
Dis3	0.001478	-0.35188	0.042	0.124	1	11

Rpl15	0.00149	-0.40592	0.083	0.161	1	11
Ttc1	0.001497	-0.27513	0.175	0.185	1	11
Qrich1	0.001536	-0.32779	0.067	0.116	1	11
Exoc5	0.001549	-0.29852	0.125	0.147	1	11
Uimc1	0.001554	-0.34248	0.092	0.133	1	11
Rbbp8	0.001559	-0.41041	0.042	0.122	1	11
Ldb1	0.001567	-0.36245	0.142	0.184	1	11
Dcun1d5	0.001575	-0.36267	0.2	0.256	1	11
Ahi1	0.001601	-0.41993	0.225	0.307	1	11
Rrm2	0.001624	-0.51518	0.092	0.204	1	11
Gripap1	0.001689	-0.30876	0.167	0.188	1	11
Aurkb	0.001694	-0.41286	0.067	0.143	1	11
Ube2i	0.001703	-0.41288	0.058	0.152	1	11
Srgap2	0.001733	-0.36193	0.092	0.132	1	11
Mum1	0.00174	-0.43277	0.067	0.169	1	11
Shmt1	0.001741	-0.35321	0.033	0.103	1	11
Man1c1	0.001741	-0.3175	0.092	0.126	1	11
Gtf2e2	0.001761	-0.29555	0.15	0.178	1	11
6330403K(	0.001761	-0.38438	0.192	0.243	1	11
Trafd1	0.001762	-0.30071	0.092	0.118	1	11
Skiv2l	0.001771	-0.27413	0.15	0.162	1	11
N4bp2	0.001773	-0.44071	0.042	0.132	1	11
Mapk7	0.001789	-0.3773	0.017	0.106	1	11
Tk1	0.001805	-0.31267	0.083	0.131	1	11
Etaa1	0.001819	-0.41705	0.075	0.159	1	11
Rpl36	0.001838	-0.34255	0.05	0.108	1	11
Socs3	0.001844	0.282456	0.117	0.038	1	11
Rrnad1	0.001862	-0.31539	0.092	0.122	1	11
Pja1	0.001878	-0.25589	0.158	0.173	1	11
Mxi1	0.001878	-0.41062	0.117	0.191	1	11
Tln1	0.001891	-0.26741	0.142	0.15	1	11
BC034090	0.001903	-0.45243	0.042	0.128	1	11
Rpl3-ps1	0.001908	-0.26687	0.067	0.101	1	11
Tead1	0.00191	-0.3989	0.117	0.174	1	11
Rbbp9	0.001923	0.305897	0.167	0.067	1	11
Btg1	0.001959	-0.41552	0.108	0.183	1	11
Ctbp2	0.00198	-0.29002	0.192	0.215	1	11
Oard1	0.002	-0.29157	0.125	0.152	1	11
Zfp322a	0.002002	-0.31897	0.092	0.123	1	11
Upf2	0.002017	-0.32683	0.05	0.155	1	11
Zfp553	0.00204	-0.34451	0.1	0.144	1	11
Slc25a27	0.002045	-0.41485	0.033	0.11	1	11
Rbm18	0.002046	-0.31984	0.133	0.171	1	11
Tcof1	0.002064	-0.44245	0.083	0.168	1	11
Rab3a	0.002085	-0.5306	0.05	0.15	1	11
Rcc2	0.002105	-0.37152	0.117	0.179	1	11
Sf3b3	0.002115	-0.36126	0.067	0.126	1	11
Pknox1	0.002123	-0.37397	0.092	0.145	1	11

Fen1	0.002131	-0.40215	0.058	0.142	1	11
Cnksr2	0.002142	-0.43055	0.025	0.108	1	11
Sgol2	0.002149	-0.4941	0.067	0.157	1	11
Thoc3	0.002188	-0.32783	0.117	0.158	1	11
Usp33	0.002215	-0.43574	0.067	0.149	1	11
Elovl6	0.002234	-0.36482	0.175	0.278	1	11
Zmym2	0.002236	-0.29652	0.092	0.118	1	11
Mgea5	0.002244	-0.3378	0.2	0.231	1	11
Hist3h2ba	0.002247	-0.44851	0.042	0.138	1	11
Pnrc1	0.002255	-0.43054	0.167	0.271	1	11
Gpr85	0.002266	-0.45314	0.075	0.164	1	11
Qtrt1	0.002283	-0.34417	0.058	0.108	1	11
Ndc80	0.002307	-0.38801	0.025	0.115	1	11
Dbn1	0.002347	-0.35855	0.1	0.139	1	11
Zkscan3	0.002358	-0.27869	0.175	0.199	1	11
Parp2	0.002372	-0.39946	0.1	0.17	1	11
Tubg1	0.00243	-0.25969	0.108	0.129	1	11
Vrk1	0.002437	-0.40967	0.067	0.148	1	11
Rexo1	0.002449	-0.44369	0.125	0.212	1	11
Fam178a	0.002478	-0.4072	0.033	0.111	1	11
Naa16	0.002486	-0.34809	0.083	0.125	1	11
Gm11266	0.002511	-0.4637	0.075	0.144	1	11
Snrpa	0.002518	-0.30195	0.1	0.14	1	11
Daxx	0.002535	-0.36484	0.05	0.113	1	11
Bmi1	0.002552	-0.31821	0.058	0.147	1	11
Rps28	0.002564	-0.40398	0.117	0.203	1	11
Pik3ip1	0.002582	-0.26306	0.1	0.116	1	11
Ube2e2	0.002617	-0.25074	0.108	0.125	1	11
Ddx23	0.00262	-0.45325	0.108	0.207	1	11
Nfyb	0.002653	-0.42071	0.158	0.235	1	11
Haus1	0.002655	-0.35363	0.05	0.11	1	11
Uba1	0.002679	-0.3264	0.233	0.271	1	11
Ncapd2	0.002685	-0.38767	0.1	0.185	1	11
Tob1	0.002716	-0.31071	0.108	0.155	1	11
Knop1	0.002749	-0.26519	0.258	0.271	1	11
Ccdc66	0.002754	-0.3289	0.142	0.172	1	11
Gm17750	0.002766	-0.35535	0.333	0.367	1	11
Rpa3	0.002775	-0.35621	0.183	0.244	1	11
RP23-32A8	0.002791	-0.25019	0.142	0.187	1	11
Shoc2	0.002814	-0.3646	0.083	0.133	1	11
Kars	0.002816	-0.27039	0.175	0.195	1	11
Prpf4	0.002853	-0.35902	0.083	0.139	1	11
Ccnb1	0.002866	-0.43368	0.075	0.139	1	11
Tulp4	0.00289	-0.36789	0.158	0.205	1	11
Ddx10	0.002926	-0.44258	0.042	0.126	1	11
A330076H(	0.002926	-0.44836	0.083	0.161	1	11
Rrn3	0.002945	-0.34	0.067	0.12	1	11
Akap11	0.002966	-0.25416	0.133	0.14	1	11

R3hdm2	0.002978	-0.41333	0.092	0.168	1	11
Ints7	0.003042	-0.38472	0.05	0.117	1	11
Rnf144a	0.003083	-0.27683	0.142	0.159	1	11
Tmem183a	0.003105	-0.30293	0.142	0.173	1	11
Eif2s1	0.003168	-0.36849	0.133	0.197	1	11
Midn	0.003177	-0.27766	0.108	0.133	1	11
Brcc3	0.003188	-0.43434	0.067	0.175	1	11
Cdk5rap3	0.003218	-0.41565	0.117	0.193	1	11
Zranb1	0.003232	-0.29433	0.175	0.208	1	11
Rad23b	0.003252	-0.29377	0.175	0.202	1	11
Ythdf2	0.003269	-0.4204	0.158	0.259	1	11
C330027C(	0.003278	-0.38386	0.067	0.133	1	11
Elmo1	0.003307	-0.54137	0.033	0.126	1	11
Pbk	0.003328	-0.4687	0.117	0.223	1	11
Baz2a	0.003365	-0.28454	0.083	0.11	1	11
Nudcd3	0.003392	-0.26164	0.158	0.17	1	11
Mbp	0.003405	-0.30241	0.2	0.165	1	11
Lhfpl4	0.003443	-0.38317	0.033	0.108	1	11
Orc2	0.003446	-0.34301	0.108	0.155	1	11
Angptl2	0.00347	-0.39092	0.025	0.116	1	11
Limd2	0.003609	-0.35843	0.075	0.135	1	11
Slc24a5	0.003626	-0.4049	0.042	0.142	1	11
Zfp451	0.003678	-0.38742	0.1	0.17	1	11
Pcnt	0.003688	-0.42422	0.083	0.175	1	11
Trim24	0.003703	-0.35841	0.083	0.134	1	11
Cd3eap	0.003738	-0.29364	0.092	0.121	1	11
Vps41	0.003755	-0.32912	0.108	0.145	1	11
Rapgef6	0.003808	-0.39869	0.075	0.14	1	11
Nosip	0.003854	-0.31338	0.133	0.168	1	11
Dner	0.003887	-0.41721	0.15	0.215	1	11
Sbk1	0.00399	-0.43855	0.042	0.119	1	11
Bbx	0.004011	-0.38492	0.092	0.148	1	11
Abt1	0.00403	-0.35423	0.033	0.127	1	11
Cerk	0.004031	-0.39508	0.083	0.151	1	11
Fam192a	0.00405	-0.37879	0.142	0.199	1	11
Blm	0.004108	-0.39267	0.025	0.111	1	11
Agtpbp1	0.004174	-0.34478	0.05	0.102	1	11
Lrig3	0.004204	-0.37905	0.1	0.17	1	11
Bdp1	0.004239	-0.42807	0.092	0.155	1	11
Slc39a10	0.004312	-0.38955	0.125	0.195	1	11
Celsr2	0.004343	-0.35228	0.175	0.238	1	11
Supt5	0.004387	-0.30762	0.158	0.197	1	11
Snrnp200	0.004393	-0.33442	0.1	0.163	1	11
2700081O:	0.004408	-0.42188	0.033	0.111	1	11
Cwf19l2	0.004409	-0.26825	0.142	0.163	1	11
Zfp354c	0.004452	-0.27312	0.108	0.125	1	11
Rhobtb3	0.004492	-0.38999	0.1	0.165	1	11
Igf1r	0.004574	-0.38982	0.042	0.114	1	11

Prkacb	0.004663	-0.35695	0.142	0.187	1	11
Cspp1	0.004675	-0.31783	0.217	0.238	1	11
Tet3	0.004722	-0.25204	0.133	0.148	1	11
Bcat1	0.004734	-0.40369	0.033	0.121	1	11
Zfp512	0.004772	-0.34109	0.058	0.119	1	11
Cenpc1	0.004929	-0.36511	0.1	0.155	1	11
Pbx3	0.004984	-0.28729	0.1	0.127	1	11
Cntln	0.005086	-0.37189	0.133	0.172	1	11
Gmnn	0.005092	-0.40787	0.067	0.145	1	11
Pak7	0.005098	-0.28919	0.033	0.105	1	11
Ddx26b	0.005105	-0.33016	0.092	0.13	1	11
Ankhd1	0.005284	-0.39384	0.1	0.181	1	11
Fyttd1	0.005402	-0.26983	0.233	0.258	1	11
Snapc3	0.005448	-0.27235	0.1	0.125	1	11
Cnot4	0.005492	-0.30755	0.175	0.197	1	11
Wdr6	0.005531	-0.38613	0.083	0.145	1	11
Rev1	0.005601	-0.27512	0.092	0.117	1	11
Ncapg	0.00569	-0.42206	0.083	0.183	1	11
Clasp1	0.005843	-0.2743	0.092	0.114	1	11
Kif5a	0.005858	-0.45238	0.058	0.128	1	11
Ulk1	0.005927	-0.37206	0.042	0.105	1	11
Skiv2l2	0.005935	-0.2731	0.142	0.164	1	11
Rnf168	0.005952	-0.44436	0.117	0.208	1	11
Gm13092	0.005994	-0.3159	0.033	0.104	1	11
Tbc1d16	0.005997	-0.39862	0.1	0.196	1	11
Pcdha2	0.006113	-0.44646	0.033	0.118	1	11
Mis18a	0.00614	-0.25064	0.05	0.11	1	11
Chd2	0.00624	-0.44648	0.15	0.222	1	11
Actl6a	0.006303	-0.37301	0.142	0.227	1	11
Ddx55	0.006314	-0.27251	0.133	0.165	1	11
Ckap4	0.006426	-0.27101	0.267	0.289	1	11
Cep78	0.006676	-0.37843	0.075	0.14	1	11
Trim27	0.006693	-0.37347	0.075	0.149	1	11
Brpf1	0.006732	-0.3538	0.083	0.141	1	11
Ikbkap	0.006734	-0.32884	0.083	0.127	1	11
Rnps1	0.006833	-0.36925	0.058	0.137	1	11
Rbm10	0.006961	-0.32614	0.083	0.134	1	11
Thumpd1	0.007049	-0.39625	0.092	0.166	1	11
Akap8	0.00706	-0.30602	0.2	0.233	1	11
Dnph1	0.007125	-0.28069	0.083	0.123	1	11
Pmf1	0.007159	-0.26271	0.117	0.143	1	11
Atp11b	0.007173	-0.28386	0.067	0.102	1	11
Kif22	0.007229	-0.36284	0.075	0.163	1	11
Msl1	0.007407	-0.28279	0.2	0.223	1	11
Cd2bp2	0.007421	-0.38615	0.058	0.136	1	11
Cmip	0.007584	-0.33525	0.125	0.16	1	11
Asap1	0.007592	-0.26427	0.158	0.18	1	11
Wasf1	0.007857	-0.26778	0.083	0.11	1	11

Srpk1	0.007889	-0.39156	0.142	0.247	1	11
Cpsf1	0.00794	-0.33148	0.042	0.108	1	11
Nenf	0.008063	-0.25894	0.117	0.136	1	11
Mllt3	0.008096	-0.39391	0.15	0.226	1	11
BC005561	0.008187	-0.38395	0.208	0.29	1	11
Rpap3	0.008281	-0.39393	0.067	0.145	1	11
Cenpw	0.008445	-0.31157	0.083	0.127	1	11
Med1	0.008486	-0.34696	0.117	0.172	1	11
Gli1	0.008494	-0.25858	0.083	0.107	1	11
Fam53b	0.008521	-0.30014	0.075	0.136	1	11
0610010F(	0.008575	-0.36761	0.075	0.136	1	11
Faf1	0.008615	-0.27981	0.067	0.102	1	11
Hist1h2ak	0.008636	-0.40556	0.092	0.143	1	11
Pola1	0.008644	-0.3325	0.033	0.103	1	11
Gle1	0.008714	-0.3752	0.075	0.14	1	11
Ptch2	0.008876	-0.42068	0.058	0.153	1	11
Uri1	0.008883	-0.32808	0.158	0.209	1	11
Pom121	0.008972	-0.25576	0.158	0.179	1	11
Gpatch11	0.009079	-0.27744	0.083	0.11	1	11
Cdkn2d	0.009124	-0.32022	0.117	0.157	1	11
Brwd1	0.009399	-0.25354	0.167	0.193	1	11
G2e3	0.009452	-0.35202	0.042	0.131	1	11
Rrp15	0.009502	-0.30235	0.142	0.174	1	11
Arrb2	0.009537	-0.25801	0.058	0.119	1	11
Dck	0.009565	-0.30368	0.05	0.103	1	11
Topors	0.009878	-0.38187	0.108	0.183	1	11
Cklf	0.009991	-0.29589	0.125	0.165	1	11
Apod	0	5.195875	0.824	0.037	0	12
Ptn	0	3.212768	0.983	0.438	0	12
Col3a1	1.8E-290	4.141856	0.941	0.01	3.1E-286	12
Col4a1	7.5E-282	3.35861	0.966	0.066	1.2E-277	12
Igf2	4.5E-266	3.755193	0.815	0.03	7.5E-262	12
Col1a2	5.1E-258	3.69235	0.924	0.008	8.5E-254	12
Vtn	8.4E-252	3.633021	0.916	0.004	1.4E-247	12
Dcn	1.1E-250	4.049229	0.824	0.005	1.9E-246	12
Col4a2	4E-249	3.299603	0.933	0.034	6.7E-245	12
Itih5	5E-224	3.393441	0.84	0.009	8.3E-220	12
Sparc	2.1E-201	3.274595	0.975	0.079	3.5E-197	12
Lamb1	2.6E-194	2.573512	0.815	0.005	4.4E-190	12
Lum	3.7E-191	2.713851	0.723	0.001	6.2E-187	12
Sparcl1	2.3E-190	2.812351	0.941	0.202	3.9E-186	12
Nupr1	1.1E-186	2.713881	0.807	0.011	1.8E-182	12
Cxcl12	1.7E-181	2.906662	0.815	0.039	2.9E-177	12
Nid1	4.6E-169	2.555714	0.798	0.012	7.7E-165	12
Colec12	1.9E-167	2.169337	0.723	0.004	3.2E-163	12
Atp1a2	8.4E-166	3.037118	0.933	0.052	1.4E-161	12
Bgn	7.2E-165	2.129706	0.664	0.002	1.2E-160	12
Pcolce	2.5E-163	2.05606	0.664	0.002	4.1E-159	12

Ifitm3	8.4E-158	2.306942	0.765	0.009	1.4E-153	12
Serpinh1	7.8E-156	2.284953	0.924	0.109	1.3E-151	12
Igfbp7	1.4E-154	3.135589	0.79	0.013	2.3E-150	12
Serpinf1	8.3E-152	1.977628	0.622	0.002	1.4E-147	12
Lgals1	4.8E-151	2.29301	0.908	0.223	8E-147	12
Edn3	7.1E-148	2.448027	0.605	0.002	1.2E-143	12
Igfbp5	9.6E-143	2.83058	0.622	0.021	1.6E-138	12
Col1a1	1.5E-142	2.353612	0.571	0.002	2.6E-138	12
Col15a1	4.1E-142	2.34075	0.597	0.002	6.8E-138	12
Slc6a13	1E-139	2.010526	0.555	0.001	1.7E-135	12
Pltp	1.3E-139	2.48723	0.731	0.023	2.2E-135	12
Cp	6E-138	2.305962	0.672	0.009	1E-133	12
Igfbp2	2.7E-136	2.622625	0.739	0.015	4.5E-132	12
Col6a2	1.7E-131	1.83898	0.538	0.001	2.9E-127	12
Col6a1	1.9E-126	1.92361	0.563	0.003	3.1E-122	12
Gng11	5.8E-126	2.156862	0.672	0.01	9.6E-122	12
S100a6	1.6E-122	2.475518	0.647	0.017	2.7E-118	12
Eva1b	1.9E-122	1.769139	0.597	0.006	3.1E-118	12
Fstl1	3E-120	1.988139	0.874	0.175	4.9E-116	12
Rarres2	3.1E-119	1.76715	0.538	0.003	5.2E-115	12
Anxa5	5.4E-119	1.926346	0.681	0.015	9E-115	12
Cfh	4.5E-118	1.873068	0.546	0.003	7.6E-114	12
Tbx18	5.2E-117	1.802159	0.513	0.002	8.7E-113	12
Col18a1	2.1E-116	2.185339	0.647	0.051	3.5E-112	12
Ctsk	2.1E-116	1.711861	0.487	0.001	3.5E-112	12
Cyp1b1	6.7E-114	1.526781	0.504	0.002	1.1E-109	12
Cthrc1	4.2E-112	2.008194	0.605	0.012	7E-108	12
Laptm4a	6.5E-111	1.464596	0.924	0.551	1.1E-106	12
Igfbp4	9.4E-111	2.172961	0.622	0.014	1.6E-106	12
Cped1	1.7E-106	1.502587	0.471	0.002	2.8E-102	12
Il34	1.4E-105	1.478741	0.42	0	2.3E-101	12
Pdgfrl	6.9E-105	1.749536	0.496	0.004	1.2E-100	12
Htra3	7.4E-105	1.760055	0.487	0.003	1.2E-100	12
Sepp1	1.6E-104	2.104204	0.798	0.05	2.6E-100	12
S1pr3	2E-104	1.537161	0.462	0.002	3.4E-100	12
Enpp1	2.5E-103	1.501954	0.454	0.002	4.2E-99	12
Hsp90ab1	3.8E-102	-0.88903	0.958	0.998	6.35E-98	12
Lhfp	1.2E-101	1.958277	0.613	0.015	2.02E-97	12
Lama4	3.3E-100	1.771034	0.513	0.005	5.49E-96	12
Postn	1.2E-99	1.405128	0.403	0	2.03E-95	12
Emp3	1.2E-99	1.527272	0.538	0.006	2.08E-95	12
Il33	3.3E-99	1.649694	0.454	0.003	5.48E-95	12
Col13a1	1.04E-98	1.787062	0.429	0.001	1.73E-94	12
Fbln2	3.99E-98	1.926701	0.622	0.034	6.65E-94	12
Anxa2	4.97E-98	1.708838	0.555	0.008	8.29E-94	12
Tmem204	4.02E-96	1.627919	0.471	0.003	6.7E-92	12
Lamc1	9.74E-96	1.742274	0.588	0.016	1.62E-91	12
Col4a5	4.14E-93	1.636966	0.462	0.005	6.9E-89	12

Nid2	7.62E-92	1.456702	0.479	0.005	1.27E-87	12
Plat	3.7E-91	1.822217	0.588	0.017	6.16E-87	12
Loxl2	2.7E-90	1.284809	0.412	0.002	4.5E-86	12
Phlda1	6.85E-88	1.843166	0.597	0.022	1.14E-83	12
Trf	1.59E-87	2.248399	0.521	0.034	2.65E-83	12
Cmbl	3.81E-86	1.266675	0.353	0	6.35E-82	12
Angptl4	1.14E-85	1.624459	0.462	0.006	1.9E-81	12
Kdelr3	2.15E-85	1.45518	0.479	0.006	3.59E-81	12
Hmgcs2	2.17E-85	1.340429	0.42	0.003	3.63E-81	12
Bicc1	1.05E-83	1.477192	0.471	0.007	1.75E-79	12
Slc6a20a	1.87E-82	1.424827	0.336	0	3.12E-78	12
Pdgfrb	1.72E-81	1.436142	0.361	0.001	2.87E-77	12
Ifitm1	1.34E-80	1.524945	0.345	0.001	2.24E-76	12
Spp1	2.26E-80	2.164637	0.471	0.008	3.77E-76	12
Emp1	3.57E-80	1.469497	0.412	0.004	5.96E-76	12
Aldh1a1	5.6E-80	1.614268	0.395	0.004	9.34E-76	12
Lama1	4.97E-79	1.441196	0.437	0.006	8.29E-75	12
Rbp1	2.84E-77	1.653415	0.387	0.005	4.73E-73	12
Itga8	3.06E-77	1.241255	0.37	0.002	5.11E-73	12
Mfap2	9.04E-76	1.886366	0.597	0.042	1.51E-71	12
Tgfbi	9.95E-76	1.560464	0.361	0.002	1.66E-71	12
Fmo1	2.17E-75	1.323284	0.345	0.001	3.61E-71	12
Sdc2	2.31E-75	1.729167	0.597	0.051	3.86E-71	12
Rcn3	5.73E-75	1.681526	0.672	0.083	9.56E-71	12
Sod3	1.05E-74	1.323095	0.345	0.002	1.74E-70	12
C1qtnf2	8.77E-74	1.212675	0.319	0.001	1.46E-69	12
Ccdc80	2.92E-73	1.413296	0.42	0.006	4.87E-69	12
Adh1	3.38E-73	1.381271	0.286	0	5.64E-69	12
Apoe	8.84E-73	1.112665	0.882	0.244	1.47E-68	12
Cd302	2.04E-72	1.687606	0.613	0.049	3.4E-68	12
Mxra8	4.25E-72	1.619599	0.513	0.019	7.08E-68	12
Itm2c	5.9E-72	1.615531	0.824	0.207	9.83E-68	12
Tmem45a	7.6E-71	0.974458	0.277	0	1.27E-66	12
Ggt5	2.18E-69	0.994383	0.286	0	3.64E-65	12
Cnn2	2.76E-69	1.403636	0.387	0.005	4.6E-65	12
Vwa1	6.88E-69	1.334313	0.429	0.008	1.15E-64	12
Cpq	8.49E-69	1.163983	0.395	0.005	1.42E-64	12
Col26a1	5.86E-68	1.223047	0.328	0.002	9.77E-64	12
Lama2	1.08E-67	1.122919	0.336	0.002	1.8E-63	12
Tm4sf1	3E-67	1.299367	0.353	0.003	5E-63	12
Abca8a	2.36E-66	1.075305	0.286	0.001	3.94E-62	12
0610007N	1.21E-65	1.072342	0.294	0.001	2.02E-61	12
S100a11	4.36E-65	1.410271	0.437	0.013	7.27E-61	12
Mgp	5.55E-65	2.443985	0.294	0.002	9.26E-61	12
Itm2b	7.72E-65	1.201757	0.958	0.64	1.29E-60	12
Crip1	9.24E-65	1.686077	0.412	0.011	1.54E-60	12
Ctsl	2.74E-64	1.550506	0.815	0.256	4.56E-60	12
Foxc1	3.58E-64	1.163384	0.37	0.005	5.97E-60	12

Wls	4.08E-64	1.635068	0.63	0.083	6.81E-60	12
Id3	9.83E-64	1.804077	0.538	0.028	1.64E-59	12
Rhoj	1.92E-63	1.562445	0.496	0.029	3.21E-59	12
Gm14964	2.3E-63	1.379577	0.37	0.006	3.83E-59	12
Ctsh	4.18E-63	1.325586	0.412	0.009	6.97E-59	12
Myl9	7.4E-63	1.803131	0.353	0.01	1.23E-58	12
Col5a2	2.09E-62	1.424985	0.437	0.014	3.49E-58	12
Ppap2b	7.54E-61	1.664727	0.563	0.039	1.26E-56	12
P2ry14	9.13E-61	1.125795	0.269	0.001	1.52E-56	12
Ccl11	2.19E-60	1.076524	0.252	0	3.65E-56	12
Uaca	4.54E-60	1.663253	0.538	0.056	7.57E-56	12
Rbpms	4.54E-60	1.184324	0.311	0.003	7.58E-56	12
Msx1	2.84E-59	0.773356	0.261	0.001	4.74E-55	12
Msc	3.13E-59	1.164316	0.261	0.001	5.23E-55	12
Copz2	3.44E-59	1.143805	0.403	0.011	5.74E-55	12
Nfib	1.61E-58	-1.44685	0.605	0.935	2.69E-54	12
Hspa12a	3.08E-58	1.331883	0.387	0.011	5.14E-54	12
Rgs5	6.22E-58	3.335342	0.185	0.012	1.04E-53	12
Ifi27	4.61E-57	1.317421	0.395	0.012	7.69E-53	12
Pmp22	4.71E-57	1.280565	0.429	0.015	7.85E-53	12
Lamc3	2.45E-56	0.824315	0.235	0	4.09E-52	12
Col23a1	1.41E-55	1.28273	0.328	0.005	2.35E-51	12
Gpx8	1.05E-54	1.452113	0.521	0.05	1.75E-50	12
Mrap	1.55E-54	1.063953	0.227	0	2.58E-50	12
Bmp7	2.77E-54	1.047572	0.303	0.003	4.62E-50	12
Ggt1	1.63E-53	0.83388	0.218	0	2.71E-49	12
Islr	2.35E-53	1.497732	0.445	0.029	3.91E-49	12
Gper1	4.91E-53	1.017271	0.261	0.001	8.19E-49	12
Rasgrp2	9.2E-53	1.142479	0.328	0.006	1.54E-48	12
Cdh11	2.32E-52	1.253686	0.387	0.013	3.87E-48	12
Egflam	7.34E-52	0.768935	0.227	0	1.22E-47	12
Ece1	2.39E-51	1.472615	0.597	0.081	3.98E-47	12
Fam114a1	4.62E-51	0.874487	0.294	0.004	7.7E-47	12
Efemp1	8.03E-51	1.08137	0.303	0.005	1.34E-46	12
Pdlim2	9.54E-51	0.966767	0.261	0.002	1.59E-46	12
Heyl	1.08E-50	0.872632	0.235	0.001	1.79E-46	12
Cgnl1	2.21E-50	1.088167	0.277	0.003	3.69E-46	12
Itih2	4.09E-50	0.840833	0.202	0	6.83E-46	12
Srpx2	4.39E-50	0.814028	0.202	0	7.33E-46	12
Plxdc2	4.85E-50	1.316159	0.412	0.018	8.09E-46	12
Kcnj8	7.47E-50	1.633151	0.252	0.014	1.25E-45	12
Tubb5	8.6E-50	-1.24731	0.622	0.916	1.44E-45	12
Serinc3	1.59E-49	1.484691	0.672	0.116	2.65E-45	12
Slc16a12	7.68E-49	0.84503	0.227	0.001	1.28E-44	12
Aldh1a2	1.38E-48	1.040341	0.269	0.003	2.3E-44	12
Fcgrt	4.45E-48	1.223166	0.437	0.024	7.42E-44	12
Col6a3	6.45E-48	1.04385	0.252	0.002	1.08E-43	12
Nkd2	1.44E-47	0.963755	0.261	0.003	2.4E-43	12

Naalad2	1.67E-47	1.03664	0.218	0.001	2.79E-43	12
Cyth3	2.67E-47	1.372448	0.513	0.053	4.46E-43	12
Htra1	3.38E-47	1.073279	0.361	0.012	5.65E-43	12
Chp2	6.94E-47	1.081957	0.235	0.001	1.16E-42	12
Rcsd1	7.12E-47	0.904778	0.252	0.002	1.19E-42	12
Oaf	8.9E-47	0.879695	0.252	0.003	1.48E-42	12
Rrbp1	9.03E-47	1.412668	0.706	0.185	1.51E-42	12
Prrx1	1.58E-46	1.10273	0.277	0.004	2.63E-42	12
Csf1	1.97E-46	1.087476	0.311	0.007	3.29E-42	12
Sept11	1.87E-45	1.236716	0.807	0.318	3.11E-41	12
Klf2	2.93E-45	1.516461	0.361	0.016	4.89E-41	12
Tfpi	1.58E-44	1.092823	0.294	0.007	2.64E-40	12
Steap3	4.2E-44	0.771915	0.202	0.001	7E-40	12
Il13ra1	4.53E-44	0.794462	0.235	0.002	7.56E-40	12
Tgfbr3	4.92E-44	0.856266	0.261	0.003	8.2E-40	12
Wfdc1	9.31E-44	0.698069	0.21	0.001	1.55E-39	12
Arl4a	9.46E-44	1.397246	0.521	0.061	1.58E-39	12
Gja1	1.61E-43	1.209387	0.361	0.018	2.69E-39	12
Prelp	2.24E-43	0.689884	0.185	0	3.74E-39	12
Itga1	1.56E-42	1.04512	0.235	0.003	2.61E-38	12
C1qtnf6	1.62E-42	0.880072	0.261	0.004	2.7E-38	12
Serpine2	2.81E-42	1.529	0.513	0.054	4.69E-38	12
Rdh10	3E-42	1.32113	0.37	0.024	5E-38	12
Slc7a11	3.54E-42	1.197622	0.227	0.002	5.9E-38	12
Emilin1	5.34E-42	0.941502	0.269	0.005	8.92E-38	12
Fkbp10	1.45E-41	0.994858	0.277	0.006	2.42E-37	12
Sfrp1	1.7E-41	-1.54998	0.319	0.815	2.84E-37	12
Ifitm2	2.45E-41	1.27446	0.597	0.11	4.09E-37	12
Tagln2	5.67E-41	1.31836	0.454	0.042	9.46E-37	12
Lpar1	2.02E-40	1.043379	0.277	0.007	3.37E-36	12
Mylk	3.76E-40	1.085361	0.202	0.001	6.27E-36	12
Slc22a6	3.85E-40	0.764917	0.16	0	6.41E-36	12
Dkk3	7.11E-40	1.003889	0.277	0.008	1.19E-35	12
Abca9	1.07E-39	0.919527	0.244	0.004	1.78E-35	12
Mfge8	2.11E-39	1.275361	0.403	0.031	3.52E-35	12
Ahnak	2.81E-39	1.045171	0.252	0.005	4.69E-35	12
Slc22a8	1.16E-38	0.87747	0.21	0.002	1.93E-34	12
Clec1a	1.53E-38	0.816682	0.202	0.001	2.55E-34	12
G0s2	3.01E-38	1.098231	0.252	0.005	5.03E-34	12
Nbl1	8.92E-38	1.216392	0.37	0.025	1.49E-33	12
Myl12a	1.13E-37	1.18282	0.697	0.326	1.89E-33	12
Tnfrsf19	2.11E-37	0.756821	0.227	0.004	3.51E-33	12
Cald1	2.45E-37	1.124758	0.857	0.533	4.09E-33	12
Junb	2.52E-37	1.34122	0.387	0.032	4.21E-33	12
Cd97	2.95E-37	0.792438	0.227	0.003	4.92E-33	12
Nr1h3	3.29E-37	0.902203	0.218	0.003	5.5E-33	12
Pla2g7	3.44E-37	1.13871	0.336	0.018	5.74E-33	12
Creb3l2	9.09E-37	1.00107	0.277	0.009	1.52E-32	12

Fblim1	1.01E-36	0.968727	0.235	0.004	1.68E-32	12
Tmem37	1.15E-36	0.892414	0.252	0.006	1.92E-32	12
Cd248	1.39E-36	0.930118	0.202	0.002	2.32E-32	12
Rell1	1.48E-36	1.244791	0.546	0.093	2.46E-32	12
Vstm4	3.18E-36	0.770399	0.176	0.001	5.31E-32	12
Tcn2	6.27E-36	1.042489	0.328	0.017	1.05E-31	12
Rnase4	6.52E-36	0.790669	0.303	0.02	1.09E-31	12
Ptplad2	7.79E-36	0.807444	0.244	0.006	1.3E-31	12
Slc9a3r2	8.43E-36	1.09575	0.311	0.014	1.41E-31	12
Fkbp7	1.44E-35	1.238912	0.454	0.064	2.39E-31	12
Lbp	1.59E-35	0.606422	0.151	0	2.65E-31	12
Mmp14	1.65E-35	1.260452	0.639	0.171	2.76E-31	12
Tlr12	1.91E-35	0.553715	0.143	0	3.19E-31	12
Fam26e	2.21E-35	0.716812	0.16	0	3.68E-31	12
Mrc2	4.24E-35	0.577556	0.168	0.001	7.06E-31	12
Svil	4.3E-35	1.204745	0.37	0.031	7.17E-31	12
Tns1	4.93E-35	0.9879	0.21	0.003	8.23E-31	12
Mmp2	5.56E-35	0.957281	0.303	0.014	9.28E-31	12
Enpp2	7.97E-35	1.153462	0.378	0.029	1.33E-30	12
Pear1	1.23E-34	0.772298	0.176	0.001	2.05E-30	12
Col4a6	1.68E-34	0.706896	0.176	0.001	2.8E-30	12
Spry1	1.71E-34	0.957896	0.286	0.011	2.85E-30	12
Wnt5a	4.01E-34	0.973115	0.244	0.007	6.69E-30	12
Atp2b4	6.14E-34	0.916888	0.235	0.005	1.02E-29	12
Amica1	7.36E-34	0.668271	0.16	0.001	1.23E-29	12
Sult1a1	8.89E-34	0.949416	0.193	0.002	1.48E-29	12
Ramp2	1.13E-33	1.259505	0.521	0.116	1.89E-29	12
Tpm2	2.21E-33	1.110057	0.252	0.01	3.69E-29	12
Ptgis	2.56E-33	0.66033	0.168	0.001	4.27E-29	12
Rtn1	3.03E-33	-1.65159	0.16	0.693	5.06E-29	12
Crmp1	3.86E-33	-1.54323	0.176	0.671	6.44E-29	12
Ppic	4.2E-33	1.158745	0.681	0.236	7.01E-29	12
Pi16	4.37E-33	0.579016	0.134	0	7.29E-29	12
Rbms3	4.68E-33	0.979139	0.319	0.019	7.8E-29	12
Itm2a	6.88E-33	1.22397	0.403	0.043	1.15E-28	12
Myh9	9.58E-33	1.117162	0.328	0.025	1.6E-28	12
Samd5	1.04E-32	0.85782	0.193	0.002	1.73E-28	12
Axl	2.28E-32	0.914049	0.227	0.006	3.81E-28	12
Tmem64	3.09E-32	1.265567	0.445	0.088	5.16E-28	12
Hspg2	3.94E-32	0.867983	0.202	0.003	6.56E-28	12
Tril	4.2E-32	1.037738	0.336	0.023	7.01E-28	12
Igfbpl1	4.39E-32	-1.68234	0.16	0.65	7.33E-28	12
Cd81	4.65E-32	1.00133	0.824	0.459	7.76E-28	12
Gstt2	7.27E-32	0.726134	0.185	0.002	1.21E-27	12
CRE_RECOM	7.56E-32	-1.63049	0.311	0.771	1.26E-27	12
Hic1	1.1E-31	0.497882	0.134	0	1.83E-27	12
Ncl	1.24E-31	-0.87368	0.748	0.897	2.07E-27	12
Ctsb	1.42E-31	0.992942	0.714	0.258	2.37E-27	12

Cyr61	3.03E-31	1.339733	0.261	0.017	5.05E-27	12
Hrct1	3.8E-31	0.617034	0.151	0.001	6.34E-27	12
Filip1l	6.93E-31	1.313027	0.21	0.008	1.16E-26	12
Slc1a5	8.12E-31	1.121473	0.378	0.045	1.35E-26	12
Foxs1	8.65E-31	0.647823	0.143	0	1.44E-26	12
Fgfr1	9.92E-31	1.146272	0.471	0.078	1.65E-26	12
Hlf	1.06E-30	0.940723	0.294	0.017	1.77E-26	12
Thbd	1.19E-30	0.543506	0.143	0.001	1.99E-26	12
Mylip	1.43E-30	0.8349	0.261	0.011	2.39E-26	12
Higd1b	1.66E-30	1.440395	0.143	0.001	2.78E-26	12
Dse	1.79E-30	0.890311	0.244	0.009	2.98E-26	12
Cd24a	2.02E-30	-1.48266	0.168	0.637	3.37E-26	12
Gja4	3.47E-30	1.12348	0.143	0.001	5.79E-26	12
Tgfb1i1	4.39E-30	0.757518	0.21	0.005	7.33E-26	12
Hnrnpu	6.03E-30	-0.8412	0.706	0.866	1.01E-25	12
Myo1b	6.33E-30	1.152241	0.462	0.073	1.06E-25	12
Ckb	7.29E-30	-1.31811	0.479	0.81	1.22E-25	12
Lamb2	1.93E-29	0.848037	0.244	0.01	3.22E-25	12
Timp3	2.12E-29	1.368423	0.445	0.076	3.53E-25	12
Vamp8	2.3E-29	0.615457	0.235	0.011	3.84E-25	12
Fth1	2.36E-29	0.759734	0.908	0.707	3.94E-25	12
B3gnt9	3.06E-29	0.648536	0.168	0.002	5.1E-25	12
Rps4y2	5.14E-29	1.045659	0.345	0.032	8.57E-25	12
Foxd1	5.35E-29	0.524714	0.143	0.001	8.92E-25	12
Ajap1	6.93E-29	0.64568	0.176	0.003	1.16E-24	12
Fkbp9	7.52E-29	0.950661	0.328	0.028	1.25E-24	12
Snhg11	8.76E-29	1.025851	0.218	0.007	1.46E-24	12
Dusp6	9.67E-29	1.221106	0.42	0.09	1.61E-24	12
Acadl	1.02E-28	1.04975	0.429	0.062	1.7E-24	12
Marcksl1	1.27E-28	-1.21129	0.353	0.724	2.12E-24	12
Gnb4	2.17E-28	1.150308	0.487	0.102	3.62E-24	12
Tgm2	2.18E-28	0.649142	0.151	0.001	3.63E-24	12
Itgb1	2.35E-28	1.021514	0.731	0.36	3.93E-24	12
Scara5	2.69E-28	0.545173	0.118	0	4.48E-24	12
Tbxa2r	3.17E-28	0.478437	0.118	0	5.29E-24	12
Hnrnpa2b1	3.75E-28	-0.7237	0.84	0.934	6.26E-24	12
Unc93b1	4.15E-28	0.573613	0.218	0.011	6.92E-24	12
Anxa7	5.11E-28	0.71127	0.235	0.009	8.53E-24	12
B2m	1.2E-27	1.076387	0.647	0.228	2E-23	12
Nnat	1.55E-27	-1.39246	0.244	0.713	2.58E-23	12
Fap	1.84E-27	0.494912	0.126	0	3.08E-23	12
Sptbn1	1.94E-27	1.0372	0.739	0.351	3.23E-23	12
Tnfaip2	3.3E-27	0.693301	0.134	0.001	5.5E-23	12
Add3	3.56E-27	1.081765	0.504	0.101	5.94E-23	12
Tuba1a	3.85E-27	-0.98274	0.773	0.93	6.43E-23	12
Efemp2	5.02E-27	0.909205	0.252	0.015	8.37E-23	12
Dlc1	5.15E-27	0.703144	0.21	0.007	8.58E-23	12
Twist1	6.31E-27	0.535327	0.134	0.001	1.05E-22	12

Basp1	7.3E-27	-1.33171	0.277	0.707	1.22E-22	12
Adap2	1.19E-26	0.981498	0.16	0.003	1.98E-22	12
Cstb	1.23E-26	1.064881	0.521	0.114	2.05E-22	12
Parp3	1.26E-26	0.54286	0.151	0.002	2.09E-22	12
Maf	1.61E-26	0.963358	0.269	0.017	2.68E-22	12
Serping1	1.7E-26	1.110649	0.319	0.031	2.84E-22	12
H2-K1	1.99E-26	0.824787	0.244	0.012	3.33E-22	12
Cyba	2E-26	0.73857	0.294	0.027	3.33E-22	12
Nfia	2.29E-26	-1.16771	0.42	0.776	3.82E-22	12
Tnfrsf1a	2.4E-26	0.807166	0.294	0.023	4E-22	12
S100a10	2.65E-26	0.88992	0.311	0.027	4.42E-22	12
Vamp5	2.88E-26	0.662426	0.16	0.002	4.8E-22	12
Rhbdf1	3.02E-26	0.831455	0.185	0.005	5.04E-22	12
Ptges	3.03E-26	0.526877	0.143	0.001	5.06E-22	12
Rgs4	3.13E-26	1.122527	0.168	0.004	5.21E-22	12
Osmr	4.58E-26	0.617458	0.134	0.001	7.65E-22	12
Lpl	6.19E-26	1.166102	0.37	0.056	1.03E-21	12
Plin3	1.05E-25	0.659745	0.202	0.007	1.74E-21	12
Nfix	1.59E-25	-1.28168	0.252	0.676	2.66E-21	12
Tenc1	2.12E-25	0.763749	0.202	0.007	3.54E-21	12
Adamts12	2.17E-25	0.609595	0.168	0.003	3.61E-21	12
Ndufa4	2.27E-25	-0.99339	0.429	0.804	3.79E-21	12
Abca1	2.54E-25	0.861498	0.277	0.022	4.24E-21	12
Cebpd	2.66E-25	0.867769	0.218	0.011	4.44E-21	12
Foxq1	3.14E-25	0.681847	0.176	0.005	5.23E-21	12
BC028528	3.45E-25	0.778515	0.193	0.006	5.76E-21	12
Stard8	5.15E-25	0.697592	0.16	0.003	8.59E-21	12
Ecm1	6.27E-25	0.642991	0.168	0.003	1.05E-20	12
Tgfbr2	6.33E-25	0.747222	0.202	0.007	1.06E-20	12
Cpxm1	6.72E-25	0.950565	0.235	0.013	1.12E-20	12
Cst3	7.82E-25	0.665126	0.874	0.533	1.3E-20	12
Rab3il1	9.15E-25	0.64867	0.185	0.005	1.53E-20	12
Creb3l1	9.97E-25	0.587119	0.143	0.002	1.66E-20	12
Tbx15	1.4E-24	0.444882	0.109	0	2.33E-20	12
Barhl1	2.06E-24	-1.24625	0.076	0.524	3.43E-20	12
Ginm1	2.25E-24	1.04922	0.42	0.079	3.76E-20	12
E130114P1	2.67E-24	-1.42452	0.143	0.587	4.46E-20	12
Arpc1b	2.73E-24	0.887599	0.319	0.034	4.55E-20	12
Coro1b	2.93E-24	1.053013	0.588	0.236	4.88E-20	12
Ecm2	3.18E-24	0.743433	0.151	0.003	5.3E-20	12
H6pd	4.61E-24	0.756414	0.193	0.007	7.69E-20	12
Itpripl2	4.65E-24	0.661101	0.16	0.003	7.76E-20	12
Lox	4.91E-24	0.585486	0.118	0.001	8.19E-20	12
Nrp1	5.18E-24	1.078835	0.336	0.041	8.65E-20	12
Arhgdib	6.86E-24	0.889707	0.193	0.007	1.14E-19	12
Bex2	7.75E-24	-1.29428	0.151	0.573	1.29E-19	12
Cebpb	1.16E-23	0.625131	0.193	0.007	1.94E-19	12
Nr2f2	1.36E-23	0.960365	0.429	0.076	2.27E-19	12

Calm2	1.49E-23	-0.87843	0.63	0.875	2.49E-19	12
Pdpn	2.12E-23	0.65555	0.202	0.009	3.54E-19	12
Shisa5	2.16E-23	0.639536	0.261	0.024	3.6E-19	12
Col16a1	2.71E-23	0.481059	0.16	0.004	4.52E-19	12
Ccnd2	2.86E-23	-1.33166	0.286	0.664	4.77E-19	12
Arhgap29	3.05E-23	0.947411	0.286	0.027	5.09E-19	12
Lrrc17	3.3E-23	0.335976	0.101	0	5.51E-19	12
Anp32e	3.67E-23	-1.29506	0.252	0.615	6.13E-19	12
Kcne4	4.87E-23	0.618123	0.109	0.001	8.12E-19	12
Selenbp1	6.18E-23	0.663627	0.176	0.006	1.03E-18	12
H3f3b	7.36E-23	-0.72897	0.765	0.907	1.23E-18	12
App	9.8E-23	0.771629	0.882	0.566	1.63E-18	12
Col7a1	9.86E-23	0.619028	0.16	0.004	1.64E-18	12
Ech1	9.92E-23	0.997787	0.471	0.112	1.65E-18	12
Rapgef4	1.42E-22	0.727707	0.202	0.009	2.37E-18	12
Ubtd1	1.57E-22	0.654173	0.168	0.005	2.61E-18	12
Lamp2	1.73E-22	1.049687	0.487	0.135	2.89E-18	12
Aspn	1.87E-22	0.854738	0.109	0.001	3.12E-18	12
Ndufa4l2	2.6E-22	0.479548	0.118	0.001	4.33E-18	12
Stmn3	3.24E-22	-1.22074	0.202	0.624	5.4E-18	12
Lrp1	4.9E-22	0.925195	0.336	0.045	8.17E-18	12
Cyp2d22	5.35E-22	0.553898	0.126	0.002	8.92E-18	12
Pros1	6.52E-22	0.833869	0.252	0.022	1.09E-17	12
Frmd6	6.56E-22	0.728586	0.202	0.01	1.09E-17	12
Chd4	6.61E-22	-0.91435	0.555	0.771	1.1E-17	12
1810058I2	6.7E-22	1.027056	0.546	0.194	1.12E-17	12
Gjb6	7.75E-22	0.798624	0.151	0.004	1.29E-17	12
Flrt2	1E-21	1.013656	0.395	0.08	1.67E-17	12
Calr	1.02E-21	0.805666	0.815	0.508	1.7E-17	12
Cdh5	1.12E-21	0.491147	0.134	0.002	1.86E-17	12
Hnrnpdl	1.26E-21	-0.84784	0.513	0.757	2.1E-17	12
Gpx7	1.36E-21	0.934074	0.353	0.06	2.26E-17	12
F3	1.73E-21	0.766616	0.176	0.006	2.88E-17	12
Gucy1a3	1.96E-21	0.959749	0.16	0.009	3.27E-17	12
Malat1	2.38E-21	0.807372	0.975	0.955	3.97E-17	12
Arhgap6	2.82E-21	0.60573	0.109	0.001	4.7E-17	12
Ptrf	2.9E-21	0.599015	0.151	0.004	4.84E-17	12
Efcc1	3.1E-21	0.487784	0.101	0	5.16E-17	12
Sept7	3.19E-21	0.797056	0.782	0.587	5.33E-17	12
Cog7	3.2E-21	-1.26108	0.168	0.587	5.33E-17	12
P4hb	3.41E-21	0.90332	0.639	0.276	5.68E-17	12
Hint1	3.94E-21	-0.96947	0.403	0.705	6.56E-17	12
Wipi1	4.07E-21	0.843248	0.286	0.032	6.78E-17	12
Pax6	4.57E-21	-1.28279	0.151	0.515	7.63E-17	12
Col5a1	6.63E-21	0.505132	0.16	0.005	1.11E-16	12
Cldn11	7.24E-21	0.846225	0.277	0.032	1.21E-16	12
Mgst1	1.15E-20	0.905478	0.277	0.031	1.92E-16	12
Gap43	1.21E-20	-1.43305	0.202	0.602	2.02E-16	12

Rhob	1.64E-20	1.0089	0.454	0.136	2.73E-16	12
Slc30a10	1.92E-20	0.946337	0.353	0.06	3.2E-16	12
Slc19a1	2.29E-20	0.973344	0.252	0.033	3.83E-16	12
Sipa1	2.31E-20	0.59418	0.185	0.009	3.85E-16	12
Fxyd1	2.39E-20	0.71137	0.202	0.012	3.98E-16	12
P2ry1	3.1E-20	0.618516	0.16	0.006	5.18E-16	12
Mtch1	4.83E-20	0.921745	0.597	0.219	8.06E-16	12
Epb4.1l2	4.98E-20	0.933563	0.403	0.09	8.31E-16	12
Hnrnpab	6E-20	-0.78294	0.588	0.817	1E-15	12
Syt11	6.18E-20	-1.26653	0.109	0.507	1.03E-15	12
Rbms1	8.43E-20	0.871182	0.597	0.213	1.41E-15	12
Col5a3	9.97E-20	0.438543	0.126	0.002	1.66E-15	12
Pdxk	1.16E-19	0.733202	0.21	0.015	1.93E-15	12
Chst7	1.25E-19	0.481644	0.151	0.005	2.08E-15	12
Fbn1	1.35E-19	0.640148	0.151	0.005	2.25E-15	12
Meg3	1.4E-19	0.583441	0.387	0.093	2.33E-15	12
Gcnt2	1.48E-19	0.490927	0.109	0.001	2.48E-15	12
Gria2	1.51E-19	-1.39877	0.143	0.523	2.52E-15	12
Lmna	1.54E-19	0.752598	0.244	0.023	2.57E-15	12
Tubb2b	1.59E-19	-1.35592	0.101	0.467	2.65E-15	12
Nasp	1.83E-19	-1.06446	0.37	0.642	3.06E-15	12
Dpysl4	2.22E-19	-1.20414	0.092	0.493	3.69E-15	12
Sspn	2.22E-19	0.796091	0.193	0.013	3.71E-15	12
Clec3b	2.65E-19	0.679611	0.126	0.002	4.42E-15	12
Phactr2	3.18E-19	0.714737	0.21	0.015	5.31E-15	12
Rbm46	3.48E-19	0.357945	0.101	0.001	5.81E-15	12
Etfb	3.49E-19	1.003151	0.471	0.182	5.82E-15	12
Dock6	3.66E-19	0.693335	0.193	0.011	6.11E-15	12
Hsp90aa1	3.72E-19	-1.07577	0.235	0.619	6.2E-15	12
Rhoc	4.57E-19	0.671443	0.252	0.026	7.62E-15	12
Miat	5.45E-19	-1.45249	0.16	0.518	9.09E-15	12
Ralb	5.7E-19	0.693448	0.218	0.017	9.51E-15	12
Zcchc24	5.92E-19	0.771799	0.252	0.026	9.88E-15	12
2700094K1	6.43E-19	-1.07402	0.319	0.616	1.07E-14	12
Pdgfra	6.44E-19	0.997823	0.513	0.179	1.07E-14	12
Spats2l	7.1E-19	0.879952	0.303	0.044	1.18E-14	12
Fam46a	7.61E-19	0.647346	0.143	0.004	1.27E-14	12
Tec	8E-19	0.624168	0.16	0.006	1.33E-14	12
Ifnar2	1.02E-18	0.862535	0.311	0.048	1.7E-14	12
Cast	1.09E-18	0.766339	0.176	0.011	1.81E-14	12
Hsd11b1	1.09E-18	0.599491	0.143	0.004	1.82E-14	12
Ctdspl	1.13E-18	0.826571	0.269	0.033	1.89E-14	12
Tpm4	1.28E-18	0.872513	0.622	0.314	2.14E-14	12
Mmp19	1.32E-18	0.454825	0.126	0.002	2.21E-14	12
Tubb3	1.49E-18	-1.52319	0.101	0.476	2.48E-14	12
A230050P2	1.58E-18	0.661773	0.202	0.014	2.64E-14	12
Fkbp3	1.58E-18	-0.88976	0.487	0.746	2.64E-14	12
Rab20	1.68E-18	0.442	0.118	0.002	2.81E-14	12

Mxd4	1.7E-18	0.817355	0.58	0.195	2.84E-14	12
Lef1	1.87E-18	0.450076	0.118	0.002	3.12E-14	12
Capns1	1.99E-18	0.873229	0.588	0.225	3.31E-14	12
Draxin	2.31E-18	-1.14143	0.16	0.557	3.85E-14	12
Ier3	2.35E-18	0.877142	0.269	0.033	3.91E-14	12
Vim	2.46E-18	0.920837	0.672	0.3	4.1E-14	12
Isyna1	2.49E-18	0.61123	0.176	0.009	4.16E-14	12
Scarf2	3.46E-18	0.415206	0.109	0.002	5.77E-14	12
Anp32a	4.9E-18	-0.6603	0.689	0.837	8.18E-14	12
Nsg2	5.17E-18	-1.17905	0.109	0.489	8.62E-14	12
Wwtr1	5.34E-18	0.665813	0.176	0.01	8.91E-14	12
Scg3	5.44E-18	-1.18773	0.101	0.448	9.07E-14	12
Gm14005	5.66E-18	0.48218	0.109	0.002	9.44E-14	12
Celf2	6.18E-18	-1.09946	0.303	0.64	1.03E-13	12
Mpg	7.11E-18	0.879273	0.218	0.02	1.19E-13	12
Mmp11	7.15E-18	0.580272	0.168	0.009	1.19E-13	12
Vimp	8.15E-18	0.91688	0.538	0.187	1.36E-13	12
D430041D	9.09E-18	-1.20692	0.126	0.494	1.52E-13	12
Ywhae	1.12E-17	-0.75305	0.521	0.728	1.87E-13	12
Txnip	1.25E-17	0.981871	0.403	0.105	2.09E-13	12
Tmem59	1.27E-17	0.762261	0.739	0.389	2.11E-13	12
Lhx1	1.33E-17	-1.26451	0.126	0.499	2.22E-13	12
Gypc	1.37E-17	0.647488	0.193	0.014	2.28E-13	12
Pam	1.67E-17	0.929835	0.361	0.078	2.79E-13	12
Kdelr2	1.7E-17	0.842056	0.546	0.203	2.84E-13	12
Ppfibp2	1.74E-17	0.730689	0.193	0.014	2.91E-13	12
Ppp2r2c	1.8E-17	-1.13934	0.134	0.507	3.01E-13	12
Neurod1	2.15E-17	-1.68377	0.185	0.535	3.59E-13	12
Ybx1	2.16E-17	-0.72989	0.613	0.787	3.6E-13	12
Il6st	2.47E-17	0.682169	0.269	0.038	4.11E-13	12
Parm1	3.04E-17	0.583125	0.118	0.003	5.07E-13	12
Ccdc141	3.08E-17	0.81418	0.16	0.008	5.13E-13	12
Mcam	3.1E-17	0.73194	0.109	0.002	5.17E-13	12
Sat1	3.31E-17	0.717776	0.336	0.065	5.52E-13	12
Lrrk1	3.45E-17	0.433516	0.126	0.003	5.75E-13	12
S100a13	3.62E-17	0.435027	0.21	0.029	6.04E-13	12
Lpp	3.95E-17	0.637604	0.218	0.022	6.59E-13	12
Art3	3.97E-17	0.740958	0.101	0.002	6.63E-13	12
Ezh2	4.34E-17	-1.10305	0.269	0.612	7.25E-13	12
Maged2	6.57E-17	0.845446	0.504	0.164	1.1E-12	12
Rest	7.72E-17	0.798934	0.21	0.021	1.29E-12	12
Id2	9.19E-17	-1.09578	0.118	0.488	1.53E-12	12
Fosb	1E-16	1.197092	0.42	0.154	1.68E-12	12
Ikbip	1.08E-16	0.697696	0.261	0.035	1.8E-12	12
Ezr	1.16E-16	-1.15261	0.092	0.44	1.93E-12	12
Antxr1	1.45E-16	0.598493	0.193	0.015	2.41E-12	12
Map2	1.61E-16	-1.1385	0.126	0.494	2.69E-12	12
Gadd45b	1.62E-16	0.692772	0.176	0.012	2.7E-12	12

Gsn	1.76E-16	0.756944	0.176	0.012	2.94E-12	12
Tmem50a	2.18E-16	0.752413	0.748	0.403	3.63E-12	12
Slc41a1	2.31E-16	0.772289	0.261	0.037	3.86E-12	12
Dnajc3	2.33E-16	0.854483	0.504	0.174	3.88E-12	12
Fzd6	2.34E-16	0.50802	0.109	0.002	3.9E-12	12
Epas1	2.85E-16	0.641101	0.151	0.007	4.75E-12	12
Gprc5c	3E-16	0.373595	0.109	0.002	5.01E-12	12
Ntm	3.11E-16	0.857476	0.462	0.143	5.19E-12	12
Smc2	3.3E-16	-1.24563	0.269	0.547	5.5E-12	12
Serbp1	4.82E-16	-0.6385	0.739	0.87	8.05E-12	12
Chd7	5.32E-16	-0.9751	0.345	0.649	8.88E-12	12
Ets1	5.56E-16	0.676832	0.16	0.009	9.27E-12	12
Bmp4	5.6E-16	0.336164	0.101	0.006	9.34E-12	12
Rras	6.59E-16	0.556317	0.16	0.009	1.1E-11	12
Cd151	6.97E-16	0.784969	0.185	0.017	1.16E-11	12
Banf1	7.05E-16	-0.81587	0.479	0.695	1.18E-11	12
Dut	7.25E-16	-1.13482	0.16	0.436	1.21E-11	12
Psip1	7.93E-16	-0.86902	0.403	0.689	1.32E-11	12
Plin2	8E-16	0.813136	0.286	0.05	1.33E-11	12
Kank2	8.72E-16	0.468431	0.143	0.007	1.45E-11	12
Mex3a	8.86E-16	-1.1363	0.134	0.465	1.48E-11	12
Tacc1	8.9E-16	0.736452	0.336	0.068	1.48E-11	12
Smtn	1.1E-15	0.624783	0.244	0.032	1.84E-11	12
1500016L(	1.15E-15	-1.21481	0.067	0.385	1.92E-11	12
Cryab	1.26E-15	1.057102	0.168	0.021	2.1E-11	12
Timp2	1.26E-15	0.578681	0.202	0.025	2.11E-11	12
Apex1	1.58E-15	-1.00815	0.168	0.453	2.64E-11	12
Amotl1	1.65E-15	0.896278	0.336	0.077	2.75E-11	12
Arhgap24	1.7E-15	0.5305	0.118	0.004	2.83E-11	12
Hsp90b1	1.76E-15	0.609264	0.924	0.69	2.93E-11	12
H2afv	1.9E-15	-0.81644	0.513	0.729	3.18E-11	12
Hn1	2.4E-15	-1.03688	0.21	0.543	4E-11	12
Fos	3.18E-15	1.083262	0.571	0.302	5.3E-11	12
Plekha2	3.31E-15	0.53416	0.126	0.005	5.53E-11	12
Vcam1	3.95E-15	0.722819	0.126	0.006	6.58E-11	12
Stmn2	4.2E-15	-1.28408	0.227	0.555	7E-11	12
Txndc5	4.39E-15	0.745049	0.303	0.059	7.33E-11	12
Pde8b	4.51E-15	0.58856	0.126	0.005	7.51E-11	12
Ngfrap1	4.55E-15	-0.81136	0.437	0.659	7.59E-11	12
Jag1	5.2E-15	0.807109	0.244	0.039	8.67E-11	12
Ldlrap1	5.74E-15	0.458131	0.109	0.003	9.57E-11	12
Scg5	5.77E-15	-1.09545	0.034	0.337	9.63E-11	12
Gm9800	5.78E-15	-0.98869	0.202	0.536	9.65E-11	12
Gadd45a	6.24E-15	0.880259	0.353	0.086	1.04E-10	12
Arhgef6	6.77E-15	0.532496	0.126	0.005	1.13E-10	12
Mki67	6.86E-15	-1.47073	0.109	0.396	1.14E-10	12
Lgals9	6.97E-15	0.49778	0.143	0.008	1.16E-10	12
Itgb5	8.89E-15	0.523436	0.143	0.008	1.48E-10	12

Nhlh2	9.05E-15	-1.21946	0.092	0.425	1.51E-10	12
Sept4	9.67E-15	0.881017	0.555	0.224	1.61E-10	12
Crtap	1.13E-14	0.721094	0.227	0.033	1.88E-10	12
Ilf2	1.17E-14	-0.96893	0.202	0.543	1.96E-10	12
Mir22hg	1.18E-14	0.473312	0.126	0.005	1.97E-10	12
Surf4	1.2E-14	0.810501	0.403	0.117	2.01E-10	12
Fhl2	1.3E-14	0.617951	0.185	0.017	2.16E-10	12
Cpt1a	1.33E-14	0.745904	0.176	0.016	2.21E-10	12
Rhod	1.48E-14	0.394069	0.109	0.003	2.47E-10	12
Pabpc1	1.58E-14	-0.53817	0.807	0.879	2.64E-10	12
Pde1c	1.77E-14	-1.24511	0.076	0.389	2.96E-10	12
Ddah2	1.8E-14	-0.87012	0.454	0.687	3E-10	12
Sec24d	2.16E-14	0.500962	0.134	0.007	3.6E-10	12
Tpst1	2.19E-14	0.752956	0.345	0.081	3.65E-10	12
Gm17750	2.55E-14	-1.14787	0.059	0.369	4.25E-10	12
Aga	2.74E-14	0.779814	0.235	0.035	4.57E-10	12
Whsc1	2.89E-14	-1.04426	0.16	0.466	4.82E-10	12
Lmnb1	3.1E-14	-1.02669	0.034	0.301	5.17E-10	12
Tnfrsf21	3.13E-14	0.839088	0.294	0.062	5.22E-10	12
Rbfox3	4.1E-14	-1.08999	0.151	0.496	6.83E-10	12
Top2a	4.28E-14	-1.43754	0.143	0.429	7.13E-10	12
Kif21a	4.66E-14	-1.06474	0.059	0.347	7.77E-10	12
Fkbp11	4.84E-14	0.483104	0.151	0.01	8.07E-10	12
Eng	4.89E-14	0.44268	0.101	0.002	8.16E-10	12
Ifi30	4.93E-14	0.469946	0.16	0.015	8.23E-10	12
Oxct1	5.03E-14	-0.95303	0.252	0.554	8.4E-10	12
Hnrnph1	5.34E-14	-0.80495	0.378	0.629	8.91E-10	12
Gatm	5.36E-14	0.830245	0.227	0.033	8.94E-10	12
Leprel2	5.7E-14	0.740066	0.336	0.078	9.5E-10	12
Utrn	7.32E-14	0.690582	0.21	0.026	1.22E-09	12
Slc16a9	7.4E-14	0.347137	0.101	0.003	1.23E-09	12
Soga3	8.1E-14	-1.0608	0.143	0.459	1.35E-09	12
Tmpo	8.28E-14	-1.05562	0.134	0.454	1.38E-09	12
Rufy3	8.3E-14	-1.08726	0.092	0.373	1.38E-09	12
Myadm	8.72E-14	0.554166	0.193	0.023	1.46E-09	12
Map1b	8.9E-14	-1.00227	0.403	0.588	1.48E-09	12
Gm1673	9.13E-14	-1.02336	0.143	0.475	1.52E-09	12
Sh3pxd2a	9.36E-14	0.377135	0.101	0.002	1.56E-09	12
Nrxn2	9.51E-14	0.72022	0.353	0.088	1.59E-09	12
Mcm7	9.62E-14	-1.03826	0.092	0.39	1.6E-09	12
Arfgap3	9.94E-14	0.504385	0.202	0.026	1.66E-09	12
Gucy1b3	1.07E-13	0.863043	0.193	0.037	1.78E-09	12
Pmepa1	1.09E-13	0.806519	0.193	0.026	1.82E-09	12
Aebp1	1.31E-13	0.522858	0.134	0.008	2.19E-09	12
Scn2b	1.41E-13	0.519735	0.143	0.009	2.34E-09	12
Ina	1.51E-13	-1.11938	0.126	0.446	2.53E-09	12
Lrp10	1.52E-13	0.738718	0.244	0.04	2.54E-09	12
Tmem51	1.59E-13	0.425842	0.118	0.005	2.66E-09	12

Zfhx4	1.6E-13	0.427022	0.143	0.011	2.67E-09	12
Fbln1	1.68E-13	0.769772	0.193	0.042	2.8E-09	12
Dclk1	1.7E-13	-1.06984	0.118	0.414	2.83E-09	12
Ephx1	1.7E-13	0.461592	0.126	0.006	2.83E-09	12
Pdia3	1.8E-13	0.760278	0.563	0.294	2.99E-09	12
Degs1	1.89E-13	0.605052	0.319	0.074	3.15E-09	12
Xbp1	2.23E-13	0.743209	0.395	0.122	3.71E-09	12
Rora	2.25E-13	0.718591	0.277	0.053	3.76E-09	12
Slc9a3r1	2.64E-13	0.646817	0.143	0.013	4.4E-09	12
Snap25	2.78E-13	-1.04763	0.025	0.283	4.64E-09	12
Litaf	2.89E-13	0.729414	0.303	0.067	4.83E-09	12
Tmed3	2.99E-13	0.765062	0.319	0.081	4.98E-09	12
Gpr116	3.13E-13	0.429793	0.101	0.004	5.22E-09	12
Hes1	3.14E-13	0.818128	0.445	0.146	5.24E-09	12
Sept9	3.4E-13	0.583455	0.319	0.077	5.68E-09	12
Plxdc1	3.47E-13	0.454256	0.101	0.003	5.78E-09	12
Pkd2	3.86E-13	0.740174	0.311	0.07	6.44E-09	12
Ttyh2	3.95E-13	0.729934	0.235	0.037	6.59E-09	12
Ctsz	4.12E-13	0.565724	0.412	0.134	6.88E-09	12
H2afy	4.16E-13	-0.77802	0.395	0.593	6.94E-09	12
Pfn1	4.34E-13	0.632839	0.765	0.508	7.24E-09	12
Slc38a3	4.35E-13	0.679751	0.185	0.021	7.26E-09	12
Atpif1	4.43E-13	-0.61134	0.63	0.817	7.39E-09	12
Meis1	4.83E-13	-1.02709	0.076	0.374	8.06E-09	12
Magt1	5.29E-13	0.687958	0.277	0.055	8.82E-09	12
Gpc6	5.37E-13	0.599836	0.176	0.02	8.95E-09	12
Ptma	5.47E-13	-0.8053	0.345	0.583	9.12E-09	12
Ccnd1	6E-13	-1.04985	0.252	0.523	1E-08	12
Tmbim1	6.26E-13	0.327851	0.143	0.013	1.04E-08	12
Ppp1r14b	7.4E-13	-0.98891	0.134	0.398	1.23E-08	12
Arhgap20	7.57E-13	0.602977	0.261	0.052	1.26E-08	12
Fermt2	8.18E-13	0.826637	0.437	0.203	1.36E-08	12
Vps37b	8.38E-13	-0.95505	0.168	0.472	1.4E-08	12
Rps5	8.93E-13	-0.47667	0.933	0.954	1.49E-08	12
Dek	9.21E-13	-0.82011	0.496	0.694	1.54E-08	12
Srsf3	9.38E-13	-0.72386	0.42	0.658	1.56E-08	12
Trip6	9.68E-13	0.546349	0.193	0.024	1.61E-08	12
Nenf	1.1E-12	0.788191	0.412	0.134	1.83E-08	12
Apba2	1.11E-12	-0.97471	0.109	0.416	1.85E-08	12
Ttc3	1.17E-12	-0.65935	0.63	0.83	1.95E-08	12
Cadm1	1.19E-12	-0.97553	0.109	0.392	1.99E-08	12
Ifih1	1.23E-12	0.634525	0.109	0.005	2.06E-08	12
S1pr2	1.41E-12	0.408274	0.101	0.003	2.34E-08	12
Tsc22d3	1.47E-12	0.878982	0.261	0.09	2.46E-08	12
Ranbp1	1.64E-12	-0.74506	0.479	0.676	2.73E-08	12
Jak1	1.84E-12	0.81518	0.387	0.126	3.06E-08	12
Luc7l3	1.85E-12	-0.72461	0.504	0.749	3.08E-08	12
Sec62	1.91E-12	0.692287	0.681	0.404	3.19E-08	12

Uncx	1.98E-12	-1.06479	0.076	0.319	3.29E-08	12
Atrx	2.01E-12	-0.67701	0.496	0.742	3.35E-08	12
Grb10	2.16E-12	0.63768	0.202	0.028	3.6E-08	12
Tmem86a	2.2E-12	0.490674	0.168	0.019	3.66E-08	12
Hnrnpm	2.41E-12	-0.55542	0.647	0.735	4.02E-08	12
Rsu1	2.44E-12	0.78165	0.37	0.116	4.06E-08	12
Pth1r	2.54E-12	0.700732	0.101	0.006	4.24E-08	12
Calu	2.55E-12	0.761915	0.445	0.168	4.25E-08	12
Ecscr	2.59E-12	0.516829	0.109	0.005	4.32E-08	12
Bin1	3E-12	-1.03522	0.151	0.431	5.01E-08	12
Zfp36l1	3.08E-12	0.74951	0.429	0.149	5.14E-08	12
Slc1a3	3.12E-12	0.695053	0.42	0.151	5.21E-08	12
Pqlc1	3.83E-12	-0.96108	0.034	0.299	6.39E-08	12
Insm1	3.9E-12	-1.02154	0.042	0.294	6.5E-08	12
Pttg1ip	3.93E-12	0.714841	0.319	0.084	6.55E-08	12
Plod1	3.94E-12	0.482422	0.176	0.023	6.57E-08	12
Ebpl	4.5E-12	0.480025	0.303	0.088	7.5E-08	12
Vegfa	4.6E-12	0.687268	0.176	0.025	7.67E-08	12
Tcirg1	4.97E-12	0.543635	0.134	0.01	8.29E-08	12
Fubp1	5.12E-12	-0.83585	0.319	0.568	8.54E-08	12
Vkorc1	5.35E-12	0.706242	0.361	0.109	8.92E-08	12
Chchd10	6.83E-12	0.722705	0.21	0.032	1.14E-07	12
Matr3	7.48E-12	-0.71585	0.462	0.708	1.25E-07	12
Lims1	7.64E-12	0.720548	0.462	0.18	1.27E-07	12
Ilk	8.61E-12	0.704845	0.479	0.193	1.44E-07	12
Ivns1abp	8.79E-12	-0.90979	0.118	0.417	1.47E-07	12
Slc38a1	8.95E-12	-0.89636	0.025	0.271	1.49E-07	12
Tbata	9.34E-12	-1.12196	0.067	0.341	1.56E-07	12
Acin1	9.55E-12	-0.63099	0.58	0.724	1.59E-07	12
9430020K(	1.01E-11	0.470612	0.118	0.007	1.69E-07	12
Lamp1	1.02E-11	0.639563	0.639	0.334	1.69E-07	12
Thy1	1.03E-11	0.695712	0.134	0.014	1.72E-07	12
Hexa	1.05E-11	0.586846	0.277	0.064	1.75E-07	12
Rps9	1.08E-11	-0.47999	0.866	0.929	1.81E-07	12
Pbxip1	1.15E-11	0.668905	0.151	0.016	1.92E-07	12
Gpm6a	1.19E-11	-1.13028	0.126	0.411	1.99E-07	12
Mdk	1.23E-11	0.638803	0.697	0.377	2.04E-07	12
As3mt	1.4E-11	0.503506	0.143	0.012	2.34E-07	12
Pld1	1.6E-11	0.483034	0.109	0.006	2.68E-07	12
Hnrnpd	1.74E-11	-0.77774	0.37	0.612	2.91E-07	12
Ank3	1.76E-11	-1.01848	0.134	0.422	2.94E-07	12
Snrpf	2.09E-11	-0.90182	0.151	0.428	3.48E-07	12
Gaa	2.12E-11	0.560562	0.185	0.026	3.54E-07	12
Mycn	2.28E-11	-0.96541	0.084	0.365	3.8E-07	12
Elavl4	2.37E-11	-1.09423	0.084	0.337	3.95E-07	12
Dap	2.43E-11	0.700728	0.235	0.047	4.06E-07	12
Cacng2	2.5E-11	-0.97784	0.076	0.34	4.16E-07	12
Tspan17	2.57E-11	0.470626	0.101	0.004	4.28E-07	12

Gm10075	2.98E-11	-0.83399	0.269	0.516	4.97E-07	12
Hmgb2	3E-11	-0.98447	0.176	0.401	5.01E-07	12
Gng3	3.07E-11	-1.09475	0.076	0.318	5.11E-07	12
Comt	3.38E-11	0.707855	0.353	0.107	5.63E-07	12
Acaa2	3.4E-11	0.722045	0.193	0.034	5.67E-07	12
Arhgap42	3.47E-11	0.706283	0.134	0.02	5.78E-07	12
Dad1	3.47E-11	0.671928	0.605	0.341	5.78E-07	12
Map3k1	3.62E-11	-0.91891	0.109	0.372	6.03E-07	12
Cdc42ep2	3.65E-11	0.395589	0.109	0.006	6.09E-07	12
Nop58	3.7E-11	-0.7861	0.37	0.593	6.16E-07	12
Elavl3	3.75E-11	-0.96615	0.218	0.474	6.25E-07	12
Akap7	3.78E-11	0.568264	0.235	0.047	6.31E-07	12
Dhx9	4.13E-11	-0.86076	0.218	0.464	6.88E-07	12
Psap	4.16E-11	0.606701	0.471	0.182	6.95E-07	12
Ggact	4.4E-11	0.360411	0.101	0.005	7.35E-07	12
Kif5c	4.48E-11	-1.0214	0.134	0.4	7.47E-07	12
Cygb	5.17E-11	0.44167	0.218	0.05	8.62E-07	12
Fkbp14	5.82E-11	0.414059	0.143	0.014	9.71E-07	12
Gm2694	5.97E-11	-0.91689	0.092	0.36	9.97E-07	12
Actn4	6.57E-11	0.744734	0.42	0.174	1.1E-06	12
Rpl32	7.18E-11	-0.48698	0.815	0.889	1.2E-06	12
Dtx4	7.68E-11	0.360542	0.109	0.007	1.28E-06	12
Vwa5a	9.25E-11	0.381944	0.134	0.014	1.54E-06	12
Cyb5r3	1.05E-10	0.736199	0.462	0.197	1.76E-06	12
Sh3d19	1.09E-10	0.631642	0.202	0.034	1.82E-06	12
Cbx1	1.12E-10	-0.60057	0.521	0.686	1.87E-06	12
Hjurp	1.2E-10	-0.87526	0.227	0.435	2.01E-06	12
Leprel4	1.21E-10	0.486155	0.185	0.028	2.02E-06	12
Ncam1	1.4E-10	-0.90814	0.118	0.388	2.33E-06	12
Tox3	1.42E-10	-0.8345	0.076	0.347	2.36E-06	12
Pnn	1.43E-10	-0.64548	0.555	0.723	2.39E-06	12
Axin2	1.5E-10	0.486634	0.21	0.04	2.51E-06	12
2810417H1	1.59E-10	-1.08135	0.126	0.393	2.65E-06	12
9530068E0	1.69E-10	0.686322	0.445	0.183	2.82E-06	12
Grk5	1.81E-10	0.352486	0.118	0.01	3.03E-06	12
Ssrp1	1.83E-10	-0.72227	0.353	0.577	3.04E-06	12
Cdk4	1.94E-10	-0.68627	0.429	0.643	3.23E-06	12
Itpr2	1.95E-10	0.686298	0.185	0.028	3.26E-06	12
Cenpf	2E-10	-1.32494	0.126	0.363	3.33E-06	12
Lifr	2.07E-10	0.380068	0.101	0.005	3.45E-06	12
Fndc3b	2.07E-10	0.448681	0.126	0.011	3.45E-06	12
Zfp36	2.28E-10	0.444179	0.126	0.011	3.8E-06	12
Foxf2	2.36E-10	0.497496	0.118	0.009	3.93E-06	12
Hdgf	2.37E-10	-0.80845	0.269	0.533	3.95E-06	12
Col28a1	2.42E-10	0.628513	0.151	0.022	4.04E-06	12
Hmgn5	2.47E-10	-0.9255	0.143	0.426	4.12E-06	12
Ifngr2	2.51E-10	0.517258	0.168	0.023	4.19E-06	12
Pdia5	2.53E-10	0.569069	0.16	0.022	4.21E-06	12

Pdap1	2.55E-10	-0.64524	0.496	0.684	4.26E-06	12
Ptprd	2.94E-10	-0.89849	0.092	0.341	4.91E-06	12
Sh3pxd2b	3.4E-10	0.483496	0.151	0.02	5.66E-06	12
Tex264	3.74E-10	0.574938	0.269	0.067	6.23E-06	12
Galk1	3.9E-10	0.548621	0.227	0.049	6.51E-06	12
Engase	3.97E-10	0.509671	0.126	0.011	6.62E-06	12
Spry4	4.05E-10	0.438132	0.101	0.006	6.76E-06	12
Etv1	4.07E-10	0.63144	0.176	0.027	6.78E-06	12
Bcl7a	4.2E-10	-0.88684	0.076	0.327	7.01E-06	12
Tmem100	4.56E-10	0.467191	0.118	0.009	7.61E-06	12
Anp32b	4.82E-10	-0.66762	0.445	0.591	8.04E-06	12
Tm9sf3	4.83E-10	0.632453	0.605	0.342	8.05E-06	12
Hnrnpr	4.85E-10	-0.75157	0.311	0.546	8.1E-06	12
Akap12	4.92E-10	0.719867	0.345	0.109	8.2E-06	12
C130071C(	5.07E-10	-0.88076	0.067	0.309	8.46E-06	12
Dnajc9	5.19E-10	-0.90666	0.109	0.351	8.66E-06	12
Uap1l1	5.25E-10	0.415841	0.109	0.008	8.76E-06	12
Wasf2	5.85E-10	0.656319	0.345	0.111	9.76E-06	12
Fnbp1l	6.02E-10	-0.87347	0.218	0.455	1E-05	12
P4ha1	6.07E-10	0.610423	0.252	0.06	1.01E-05	12
Smarca5	6.43E-10	-0.85388	0.134	0.403	1.07E-05	12
Dusp3	6.78E-10	0.680185	0.168	0.028	1.13E-05	12
Hirip3	7.12E-10	-0.91261	0.134	0.384	1.19E-05	12
Sept3	7.14E-10	-0.91555	0.134	0.394	1.19E-05	12
Sntb2	7.31E-10	0.558952	0.168	0.026	1.22E-05	12
Sfpq	7.37E-10	-0.63649	0.487	0.671	1.23E-05	12
Snrpe	7.38E-10	-0.71327	0.345	0.563	1.23E-05	12
Dctpp1	7.79E-10	-0.86142	0.067	0.305	1.3E-05	12
S100a1	7.81E-10	0.308375	0.143	0.025	1.3E-05	12
Cpe	1.15E-09	-0.69704	0.168	0.457	1.92E-05	12
Klf6	1.21E-09	0.772042	0.37	0.146	2.02E-05	12
Tvp23b	1.22E-09	0.459677	0.21	0.043	2.03E-05	12
Nr3c1	1.22E-09	0.685165	0.353	0.124	2.04E-05	12
Pon2	1.27E-09	0.532196	0.202	0.038	2.11E-05	12
Eif4g2	1.29E-09	-0.59038	0.529	0.706	2.16E-05	12
Crip2	1.31E-09	0.596942	0.521	0.238	2.18E-05	12
Smc4	1.32E-09	-0.76421	0.437	0.567	2.2E-05	12
Rbm25	1.33E-09	-0.54426	0.672	0.813	2.22E-05	12
Fut9	1.39E-09	-0.83171	0.017	0.213	2.32E-05	12
Atraid	1.44E-09	0.538044	0.504	0.224	2.4E-05	12
Ccdc88a	1.62E-09	-0.82425	0.244	0.483	2.7E-05	12
Pa2g4	1.69E-09	-0.73235	0.303	0.523	2.82E-05	12
Spsb2	1.71E-09	0.411018	0.118	0.011	2.85E-05	12
Tgfb3	1.73E-09	0.412818	0.118	0.011	2.89E-05	12
Orai3	1.83E-09	0.389152	0.101	0.007	3.05E-05	12
Set	1.95E-09	-0.81075	0.151	0.388	3.25E-05	12
Rpl13a	1.99E-09	-0.34682	0.95	0.928	3.31E-05	12
Cbfa2t3	2.07E-09	-0.86535	0.143	0.404	3.45E-05	12

Npm1	2.14E-09	-0.61655	0.496	0.647	3.56E-05	12
Rps26	2.42E-09	-0.4891	0.773	0.847	4.03E-05	12
Cplx2	2.48E-09	-0.84526	0.235	0.497	4.13E-05	12
Sec31a	2.84E-09	0.557392	0.269	0.074	4.74E-05	12
Tmem110	2.96E-09	0.448417	0.16	0.024	4.93E-05	12
Smc3	2.98E-09	-0.64496	0.462	0.634	4.97E-05	12
Dcx	3E-09	-0.92114	0.126	0.377	5E-05	12
Hmgb3	3.16E-09	-0.79909	0.067	0.307	5.27E-05	12
Dnajb11	3.19E-09	0.650657	0.395	0.161	5.32E-05	12
Rnh1	3.43E-09	0.365673	0.227	0.061	5.72E-05	12
Cacna2d1	3.54E-09	-0.88431	0.126	0.376	5.91E-05	12
Epb4.1l3	3.55E-09	0.539126	0.336	0.111	5.93E-05	12
RP23-45G1	3.68E-09	-0.93353	0.092	0.328	6.14E-05	12
Grn	3.75E-09	0.459335	0.277	0.085	6.26E-05	12
Yipf3	3.77E-09	0.684067	0.353	0.137	6.3E-05	12
Jup	3.78E-09	0.419994	0.126	0.014	6.31E-05	12
Stmn1	3.85E-09	-0.81134	0.059	0.285	6.43E-05	12
Bcap31	4.07E-09	0.623503	0.42	0.172	6.8E-05	12
Cox7a2	4.07E-09	-0.48633	0.63	0.712	6.8E-05	12
Ptpn9	4.14E-09	0.678229	0.202	0.049	6.9E-05	12
Ppapdc1b	4.14E-09	0.370742	0.176	0.035	6.91E-05	12
Gse1	4.29E-09	-0.84575	0.034	0.238	7.16E-05	12
Pbk	4.52E-09	-0.80275	0.025	0.223	7.53E-05	12
Atp6v0e2	4.83E-09	-0.81811	0.05	0.261	8.06E-05	12
Stmn4	4.9E-09	-1.07093	0.118	0.342	8.18E-05	12
Imp3	4.95E-09	0.653217	0.487	0.245	8.26E-05	12
Marc2	4.97E-09	0.684692	0.269	0.082	8.28E-05	12
Kdelr1	5.19E-09	0.661941	0.37	0.145	8.66E-05	12
Rangap1	5.43E-09	-0.8189	0.067	0.257	9.05E-05	12
Anxa6	5.67E-09	0.450596	0.235	0.06	9.46E-05	12
C1ql1	5.98E-09	-0.79681	0.059	0.28	9.97E-05	12
Rnmt	6.04E-09	-0.77929	0.101	0.35	0.000101	12
Pten	6.07E-09	0.713073	0.319	0.118	0.000101	12
Cnot6	6.07E-09	-0.71955	0.202	0.392	0.000101	12
Serinc5	6.08E-09	0.613734	0.235	0.06	0.000101	12
Pid1	6.23E-09	0.563351	0.202	0.042	0.000104	12
Tspan9	6.43E-09	0.410091	0.109	0.009	0.000107	12
H1f0	6.7E-09	-0.69436	0.437	0.617	0.000112	12
Galnt18	7.03E-09	0.442021	0.109	0.01	0.000117	12
Slc50a1	7.56E-09	0.437092	0.252	0.081	0.000126	12
Elovl6	7.77E-09	-0.83323	0.076	0.279	0.00013	12
Iqgap1	7.88E-09	0.574816	0.361	0.13	0.000131	12
Matn2	8.25E-09	0.486948	0.118	0.013	0.000138	12
Ppfibp1	8.26E-09	0.604048	0.176	0.032	0.000138	12
Ran	8.42E-09	-0.64967	0.252	0.415	0.000141	12
Rcor2	8.6E-09	-0.80653	0.025	0.226	0.000144	12
Slc12a2	8.89E-09	0.724283	0.294	0.109	0.000148	12
Bcas1	8.95E-09	-0.91661	0.067	0.283	0.000149	12

Rps3	9.11E-09	-0.37331	0.891	0.922	0.000152	12
Plekhg2	9.24E-09	0.449436	0.143	0.02	0.000154	12
Cks1b	9.29E-09	-0.83378	0.101	0.346	0.000155	12
Arhgef40	9.72E-09	0.676562	0.227	0.056	0.000162	12
Tln1	9.93E-09	0.72562	0.361	0.148	0.000166	12
Mprip	1.05E-08	0.704894	0.37	0.15	0.000175	12
Cdk5r1	1.08E-08	-0.90472	0.109	0.335	0.000181	12
6330403K(	1.14E-08	-0.79946	0.059	0.244	0.00019	12
Nrn1	1.23E-08	-0.87429	0.034	0.222	0.000205	12
Bclaf1	1.26E-08	-0.63658	0.412	0.584	0.00021	12
Islr2	1.26E-08	-0.76378	0.008	0.181	0.000211	12
C530008M	1.27E-08	-0.83254	0.059	0.269	0.000212	12
Arl6ip1	1.29E-08	-0.77076	0.429	0.55	0.000215	12
Cflar	1.29E-08	0.530493	0.202	0.049	0.000216	12
Fgf9	1.33E-08	-0.8299	0.05	0.261	0.000221	12
Pdia4	1.47E-08	0.566704	0.445	0.194	0.000246	12
Ptprs	1.51E-08	-0.88252	0.21	0.422	0.000252	12
Sri	1.54E-08	0.597038	0.403	0.164	0.000256	12
Srpk2	1.61E-08	-0.68477	0.261	0.43	0.000268	12
Prmt8	1.62E-08	-0.78043	0.084	0.273	0.000271	12
Tmem150a	1.66E-08	0.485486	0.134	0.017	0.000276	12
Cyp51	1.7E-08	-0.58845	0.017	0.142	0.000283	12
Nhlh1	1.8E-08	-0.91769	0.042	0.224	0.000299	12
Thoc7	1.86E-08	-0.70166	0.269	0.523	0.000311	12
Elavl2	1.88E-08	-0.77711	0.059	0.271	0.000314	12
Srrt	1.89E-08	-0.75681	0.185	0.425	0.000315	12
Cdpf1	2.01E-08	0.251252	0.118	0.019	0.000335	12
Hnrnpc	2.35E-08	-0.53463	0.529	0.667	0.000391	12
Nfic	2.43E-08	0.637797	0.487	0.259	0.000405	12
Reep5	2.46E-08	0.280315	0.311	0.136	0.00041	12
Tpx2	2.51E-08	-1.0049	0.076	0.282	0.000418	12
Cbx5	2.6E-08	-0.55923	0.529	0.653	0.000435	12
Chgb	2.64E-08	-0.96062	0.042	0.242	0.00044	12
Zbtb18	2.67E-08	-0.80724	0.084	0.267	0.000446	12
Tmem39a	2.69E-08	0.513435	0.176	0.034	0.000448	12
Col27a1	2.74E-08	0.43713	0.118	0.013	0.000457	12
Nap1l4	2.81E-08	-0.70441	0.277	0.492	0.000469	12
Rrm1	2.82E-08	-0.76042	0.076	0.261	0.00047	12
Apc	2.84E-08	-0.8394	0.185	0.433	0.000474	12
Gjc1	2.96E-08	0.602648	0.277	0.195	0.000494	12
Gm11223	3.13E-08	-0.85422	0.025	0.214	0.000523	12
Stt3b	3.15E-08	0.653393	0.429	0.21	0.000526	12
Glul	3.4E-08	0.562174	0.429	0.199	0.000567	12
Tpm1	3.41E-08	0.71775	0.395	0.189	0.000568	12
Ccdc34	3.42E-08	-0.79347	0.218	0.416	0.00057	12
Hmgn1	3.42E-08	-0.49722	0.529	0.622	0.000571	12
Cenpa	3.56E-08	-0.97224	0.076	0.253	0.000593	12
2610017I0	3.58E-08	-0.69114	0.025	0.205	0.000597	12

Rfc4	3.63E-08	-0.74051	0.034	0.227	0.000605	12
Bet1l	3.77E-08	0.505675	0.261	0.077	0.000628	12
Gstm1	4.07E-08	0.716026	0.252	0.08	0.000678	12
Gdpd1	4.1E-08	-0.77323	0.059	0.244	0.000684	12
Slc1a2	4.15E-08	-0.84396	0.076	0.295	0.000691	12
Myo5a	4.53E-08	-0.74681	0.025	0.192	0.000756	12
St18	4.54E-08	-0.89297	0.025	0.191	0.000757	12
Ssr2	4.72E-08	0.570791	0.58	0.377	0.000788	12
Fam212b	4.9E-08	-0.76301	0.042	0.224	0.000818	12
Clip3	5.17E-08	-0.78309	0.118	0.313	0.000862	12
Sh2b3	5.43E-08	0.463268	0.126	0.016	0.000906	12
Snx18	5.46E-08	0.453654	0.185	0.039	0.000911	12
Leprot	5.49E-08	0.629246	0.319	0.116	0.000917	12
Laptm4b	5.51E-08	0.560646	0.361	0.139	0.000919	12
Prkcdbp	5.58E-08	0.591761	0.261	0.089	0.00093	12
Ddx26b	5.91E-08	0.588215	0.345	0.128	0.000985	12
Tuba1b	6.19E-08	-0.71889	0.286	0.482	0.001033	12
Hspa5	6.31E-08	0.503236	0.773	0.548	0.001052	12
H2afz	6.53E-08	-0.71791	0.042	0.232	0.00109	12
Rps19	6.59E-08	-0.45526	0.681	0.754	0.0011	12
Wipf1	6.62E-08	0.404034	0.151	0.027	0.001104	12
Smarcd1	6.64E-08	-0.76652	0.084	0.272	0.001107	12
Ahi1	6.83E-08	-0.8097	0.109	0.308	0.001139	12
Celsr2	6.95E-08	-0.77789	0.067	0.238	0.00116	12
St8sia3	7.13E-08	-0.77837	0.05	0.251	0.001189	12
Zic1	7.14E-08	-0.55271	0.647	0.785	0.001191	12
Top1	7.14E-08	-0.54729	0.546	0.663	0.001192	12
Srpr	7.3E-08	0.608859	0.286	0.095	0.001218	12
Srrm2	7.62E-08	-0.49352	0.613	0.732	0.001271	12
Klf4	7.79E-08	0.556999	0.143	0.023	0.0013	12
Racgap1	8.04E-08	-0.79145	0.059	0.256	0.00134	12
Cntn2	8.14E-08	-1.10629	0.092	0.264	0.001358	12
Serpinb6a	8.29E-08	0.611263	0.218	0.057	0.001382	12
Ddx39b	8.71E-08	-0.67637	0.252	0.445	0.001452	12
Ppp1r14c	9.05E-08	-0.90835	0.076	0.263	0.00151	12
Cenpe	9.18E-08	-0.91476	0.076	0.287	0.001532	12
C330027C(	9.29E-08	-0.51788	0.025	0.134	0.00155	12
Gpc3	9.46E-08	0.628827	0.21	0.064	0.001578	12
Ilvbl	9.7E-08	0.335156	0.109	0.012	0.001618	12
Col9a3	9.74E-08	-0.74392	0.017	0.181	0.001624	12
Pde5a	1.04E-07	0.557161	0.16	0.032	0.00173	12
Ghr	1.04E-07	0.337771	0.134	0.021	0.001733	12
Hdac2	1.04E-07	-0.628	0.319	0.516	0.001737	12
Sap30	1.05E-07	-0.56523	0	0.138	0.001744	12
Decr1	1.08E-07	0.559955	0.244	0.071	0.001795	12
Psat1	1.09E-07	-0.74889	0.151	0.368	0.001826	12
Tubb6	1.1E-07	0.438512	0.134	0.02	0.001841	12
Cltb	1.18E-07	-0.74081	0.261	0.424	0.001968	12

Bag3	1.19E-07	0.361042	0.118	0.015	0.001983	12
Alx4	1.24E-07	0.418702	0.101	0.01	0.002075	12
Rplp0	1.28E-07	-0.36674	0.857	0.872	0.00213	12
Nrxn1	1.29E-07	-0.94987	0.109	0.319	0.002148	12
Tm9sf2	1.46E-07	0.582608	0.286	0.098	0.002434	12
Stard13	1.49E-07	0.350054	0.109	0.012	0.002481	12
Efnb2	1.49E-07	0.47927	0.126	0.018	0.00249	12
Snx7	1.56E-07	0.436061	0.109	0.014	0.002595	12
Dtx1	1.57E-07	-0.67189	0.017	0.166	0.002627	12
Prox1	1.63E-07	-0.74814	0.042	0.23	0.002713	12
Cct3	1.64E-07	-0.62258	0.319	0.496	0.002729	12
Dcbld1	1.68E-07	0.40651	0.143	0.024	0.002804	12
Kif20b	1.73E-07	-0.7999	0.034	0.191	0.002885	12
Selm	1.8E-07	0.567027	0.395	0.172	0.003002	12
Rpl8	1.8E-07	-0.3223	0.866	0.897	0.003004	12
Fam101a	1.81E-07	0.622478	0.176	0.042	0.003013	12
Ppap2a	1.95E-07	0.342377	0.134	0.023	0.003256	12
Nusap1	2.03E-07	-0.8138	0.067	0.228	0.003381	12
Rcn1	2.05E-07	0.53618	0.471	0.232	0.003423	12
Rabac1	2.07E-07	0.59035	0.487	0.256	0.003455	12
Ube2c	2.07E-07	-1.11652	0.076	0.27	0.003459	12
Cdca8	2.12E-07	-0.83627	0.067	0.265	0.003541	12
Cklf	2.14E-07	-0.5817	0.025	0.166	0.003568	12
Lrpap1	2.22E-07	0.445472	0.303	0.116	0.003704	12
Notch1	2.24E-07	0.509525	0.134	0.022	0.003733	12
Lap3	2.24E-07	-0.66607	0.126	0.277	0.003743	12
Diap2	2.39E-07	0.483262	0.143	0.028	0.003993	12
Gsg1l	2.48E-07	-0.75487	0.067	0.256	0.004141	12
Adam19	2.51E-07	0.378294	0.118	0.016	0.004179	12
Tjp1	2.55E-07	0.632663	0.227	0.073	0.004253	12
Cd9	2.57E-07	-0.68421	0.294	0.486	0.004295	12
H3f3a	2.97E-07	-0.68409	0.176	0.382	0.00496	12
Nucb1	3.01E-07	0.516881	0.244	0.073	0.005019	12
Rgs16	3.03E-07	0.319601	0.101	0.011	0.005047	12
Cdc20	3.04E-07	-0.8855	0.025	0.197	0.005064	12
Cdk1	3.11E-07	-0.82052	0.092	0.247	0.005195	12
Hey1	3.29E-07	-0.7571	0.067	0.274	0.005488	12
Tsn	3.38E-07	-0.60023	0.387	0.59	0.005644	12
Homer2	3.43E-07	-0.73707	0.059	0.248	0.00572	12
Tmem167	3.44E-07	0.564077	0.487	0.271	0.005734	12
Gng12	3.46E-07	0.448743	0.311	0.119	0.005775	12
Pcm1	3.62E-07	-0.63617	0.277	0.523	0.006036	12
Rps10	3.66E-07	-0.43566	0.546	0.636	0.006106	12
Nucks1	3.72E-07	-0.57615	0.504	0.663	0.006205	12
Hnrnpk	3.98E-07	-0.50521	0.504	0.636	0.006639	12
Rap2a	4.05E-07	0.632896	0.37	0.168	0.006749	12
Mrpl34	4.12E-07	-0.70353	0.118	0.288	0.006871	12
Reln	4.31E-07	-0.75616	0.034	0.181	0.007182	12

Sgce	4.33E-07	0.576598	0.286	0.103	0.007224	12
Prpf40a	4.45E-07	-0.58764	0.378	0.572	0.007428	12
Kif23	4.48E-07	-0.53207	0.042	0.215	0.007471	12
Bex1	4.51E-07	-0.68097	0.042	0.225	0.007524	12
Tspan13	4.64E-07	-0.72078	0.034	0.208	0.007747	12
Dnajc1	4.7E-07	0.604902	0.336	0.138	0.007847	12
Khdrbs1	4.73E-07	-0.61505	0.269	0.495	0.007896	12
Srrm3	4.75E-07	-0.78319	0.05	0.241	0.007924	12
Ddx1	4.84E-07	-0.61291	0.244	0.389	0.008071	12
9330159F1	5.03E-07	-0.66064	0.025	0.169	0.008387	12
Hmgn2	5.09E-07	-0.66233	0.059	0.257	0.008492	12
Fam174a	5.12E-07	0.416225	0.218	0.063	0.008545	12
Bicd1	5.15E-07	-0.64139	0.025	0.153	0.008591	12
Rundc3a	5.21E-07	-0.75524	0.059	0.249	0.008686	12
Cct2	5.42E-07	-0.6115	0.311	0.525	0.009037	12
Pou3f2	5.51E-07	-0.76393	0.092	0.277	0.009185	12
Hpca	5.59E-07	-0.79338	0.076	0.278	0.009324	12
Pofut2	5.59E-07	0.515551	0.286	0.1	0.009331	12
Satb1	5.61E-07	-0.69234	0.034	0.211	0.009353	12
Dtymk	6.06E-07	-0.6785	0.21	0.44	0.010105	12
Ilf3	6.07E-07	-0.69702	0.168	0.377	0.010133	12
Nqo2	6.13E-07	0.401279	0.109	0.014	0.010226	12
Hnrnpa0	6.32E-07	-0.66628	0.269	0.457	0.010534	12
Ostf1	6.53E-07	0.342199	0.202	0.062	0.010899	12
Ak3	6.8E-07	0.548968	0.294	0.109	0.011344	12
Zfp704	7.01E-07	-0.70042	0.067	0.258	0.011687	12
Lmo4	7.03E-07	-0.64439	0.202	0.336	0.011731	12
Gnb2l1	7.08E-07	-0.34471	0.832	0.872	0.011815	12
Rsl1d1	7.46E-07	-0.58092	0.269	0.491	0.012449	12
Aldh7a1	7.7E-07	0.55127	0.218	0.065	0.012845	12
Caprin1	7.95E-07	-0.62523	0.235	0.394	0.013257	12
Gmds	8.2E-07	0.573778	0.143	0.039	0.013674	12
Eid1	8.3E-07	-0.64722	0.286	0.504	0.013849	12
Smad5	8.48E-07	0.46381	0.269	0.093	0.014152	12
Lig1	8.57E-07	-0.77166	0.134	0.349	0.014297	12
Rplp2	8.75E-07	-0.29616	0.849	0.84	0.014594	12
Tmem184b	8.91E-07	0.256257	0.109	0.019	0.014859	12
Rps21	9.03E-07	-0.32318	0.824	0.833	0.015059	12
Bzw2	9.13E-07	-0.61173	0.294	0.489	0.015222	12
Prdx5	9.28E-07	0.492329	0.496	0.262	0.015484	12
Rtn3	9.45E-07	-0.64037	0.261	0.478	0.015762	12
Kif1b	9.48E-07	-0.63704	0.361	0.521	0.01581	12
Pigk	9.62E-07	0.370221	0.176	0.045	0.01605	12
Sema7a	9.66E-07	-0.63448	0.008	0.156	0.016119	12
Tshz2	9.68E-07	-0.7254	0.109	0.321	0.016145	12
Cmtm3	1.01E-06	0.547149	0.303	0.116	0.016904	12
Bcat1	1.03E-06	-0.52651	0	0.121	0.017258	12
Slc3a2	1.03E-06	0.547673	0.521	0.321	0.017263	12

Sh3gl2	1.05E-06	-0.62557	0.067	0.172	0.017499	12
Amer2	1.09E-06	-0.53633	0.017	0.134	0.018174	12
Stx4a	1.14E-06	0.445175	0.353	0.153	0.018969	12
Hdlbp	1.18E-06	0.418779	0.319	0.132	0.019631	12
Tia1	1.18E-06	-0.55037	0.336	0.553	0.019647	12
Sqstm1	1.2E-06	0.431488	0.437	0.225	0.020017	12
Rpl22	1.21E-06	-0.34256	0.773	0.788	0.020252	12
Smarca4	1.22E-06	-0.6241	0.294	0.506	0.020371	12
Cdc42ep4	1.24E-06	0.565879	0.168	0.047	0.020708	12
Lrrn1	1.25E-06	-0.47877	0.017	0.113	0.020906	12
Serf2	1.27E-06	0.491121	0.403	0.186	0.021168	12
Sstr2	1.31E-06	-0.72316	0.034	0.191	0.021831	12
Chn1	1.33E-06	0.311532	0.109	0.017	0.022256	12
Bex4	1.34E-06	-0.47742	0.017	0.128	0.022319	12
Bmp1	1.36E-06	0.317412	0.235	0.085	0.022678	12
Pxdn	1.37E-06	0.410452	0.134	0.024	0.022778	12
Prrc1	1.43E-06	0.452261	0.202	0.056	0.023793	12
Rab6b	1.44E-06	-0.70375	0.034	0.203	0.024032	12
Gpr180	1.49E-06	0.494166	0.235	0.079	0.024833	12
Brca2	1.49E-06	-0.44197	0.017	0.114	0.024916	12
Slc39a13	1.49E-06	0.346332	0.151	0.034	0.024925	12
Dnmt1	1.53E-06	-0.7278	0.092	0.279	0.025478	12
Dtl	1.54E-06	-0.60907	0.008	0.146	0.025685	12
Chpf	1.57E-06	0.463141	0.151	0.032	0.026156	12
Setd8	1.58E-06	-0.63855	0.118	0.262	0.026341	12
Rpn1	1.65E-06	0.567703	0.378	0.191	0.027442	12
Nagk	1.65E-06	0.369238	0.168	0.042	0.027462	12
Ddx5	1.72E-06	-0.32959	0.765	0.816	0.028726	12
Xpc	1.73E-06	0.372021	0.193	0.056	0.028784	12
Fez1	1.75E-06	-0.69159	0.151	0.333	0.029135	12
Bmp5	1.81E-06	0.406436	0.134	0.025	0.030237	12
Smc1a	1.81E-06	-0.5443	0.471	0.631	0.030262	12
Atoh1	1.83E-06	-0.67842	0.025	0.165	0.03046	12
Knstrn	1.9E-06	-0.69856	0.042	0.186	0.031757	12
Strbp	1.92E-06	-0.69061	0.134	0.348	0.032032	12
Fam132a	1.93E-06	0.401325	0.118	0.019	0.03218	12
U2surp	1.94E-06	-0.52853	0.395	0.506	0.032383	12
Map3k3	1.95E-06	0.63761	0.185	0.068	0.032511	12
Pcna	1.97E-06	-0.75431	0.168	0.359	0.03289	12
Myh10	2E-06	-0.68307	0.168	0.325	0.03336	12
Map9	2.01E-06	-0.62885	0.008	0.148	0.03351	12
Atp5a1	2.03E-06	-0.37601	0.664	0.718	0.033787	12
Chd6	2.04E-06	-0.60498	0.176	0.305	0.034088	12
Tsg101	2.06E-06	-0.62891	0.126	0.265	0.034309	12
Slc35f5	2.06E-06	0.4953	0.143	0.031	0.034342	12
Rbms2	2.06E-06	0.435048	0.16	0.036	0.034362	12
Camk2n1	2.11E-06	0.412308	0.244	0.084	0.03525	12
Cpne3	2.15E-06	0.481842	0.412	0.195	0.035817	12

Phip	2.16E-06	-0.65346	0.286	0.46	0.035964	12
Slc25a20	2.16E-06	0.283409	0.143	0.037	0.036022	12
Rps11	2.22E-06	-0.4049	0.697	0.778	0.037016	12
Trio	2.29E-06	0.485441	0.311	0.123	0.038159	12
Fbxo6	2.29E-06	0.374634	0.126	0.022	0.038279	12
Polr3gl	2.33E-06	0.401221	0.185	0.05	0.038815	12
Cd63	2.33E-06	0.322165	0.916	0.754	0.038937	12
Zfhx3	2.42E-06	0.554035	0.168	0.043	0.040336	12
Dner	2.5E-06	-0.77922	0.05	0.216	0.041645	12
Pdgfa	2.51E-06	-0.64041	0.076	0.254	0.041928	12
Akap9	2.66E-06	-0.59316	0.403	0.501	0.044386	12
Abcg1	2.66E-06	0.407477	0.126	0.023	0.04441	12
Spc25	2.68E-06	-0.7631	0.059	0.226	0.044652	12
Snrpd1	2.68E-06	-0.5805	0.328	0.525	0.04471	12
Psmc1	2.7E-06	-0.61717	0.21	0.42	0.045005	12
Pak3	2.75E-06	-0.68898	0.05	0.207	0.045806	12
Fam43a	2.75E-06	0.400968	0.202	0.061	0.045944	12
Ccdc53	2.8E-06	0.453107	0.227	0.071	0.046686	12
Fus	2.81E-06	-0.36367	0.672	0.737	0.046849	12
Atp2b1	2.82E-06	-0.60722	0.412	0.572	0.04705	12
Purb	2.94E-06	-0.58985	0.336	0.55	0.04904	12
Tmem9	2.98E-06	0.45116	0.286	0.108	0.049765	12
Nol7	3.02E-06	-0.56251	0.395	0.57	0.050364	12
Usp1	3.09E-06	-0.65783	0.185	0.339	0.05148	12
Sf3b2	3.24E-06	-0.48224	0.487	0.626	0.053992	12
Bcl11a	3.25E-06	-0.62889	0.042	0.211	0.054229	12
Birc5	3.51E-06	-0.7715	0.109	0.274	0.058558	12
Gins2	3.65E-06	-0.60814	0.042	0.186	0.060815	12
Fkbp4	3.75E-06	-0.60218	0.252	0.415	0.062539	12
AI854517	3.75E-06	-0.64277	0.042	0.19	0.062611	12
Arfip1	3.78E-06	0.446166	0.252	0.091	0.063075	12
Smchd1	3.81E-06	-0.62054	0.143	0.273	0.063515	12
Mycl	3.84E-06	-0.50581	0.025	0.138	0.064075	12
Mab21l1	4E-06	-0.71481	0.059	0.226	0.066651	12
Sar1a	4.04E-06	0.37719	0.479	0.262	0.067433	12
Rassf4	4.06E-06	-0.68057	0.168	0.377	0.067647	12
Srrm4	4.16E-06	-0.71943	0.042	0.196	0.069348	12
Brd8	4.17E-06	-0.62612	0.202	0.393	0.06959	12
Brd3	4.21E-06	-0.50307	0.429	0.532	0.070161	12
Podxl2	4.26E-06	-0.7301	0.059	0.228	0.071115	12
Slc25a27	4.35E-06	-0.51048	0	0.11	0.072603	12
Aplp2	4.44E-06	0.427977	0.555	0.321	0.074082	12
Hsd11b2	4.46E-06	-0.73678	0.109	0.288	0.074408	12
Rpl4	4.47E-06	-0.33058	0.882	0.908	0.074492	12
Eef1b2	4.54E-06	-0.4358	0.622	0.733	0.075722	12
Kif22	4.56E-06	-0.58001	0.05	0.163	0.076004	12
Nptn	4.56E-06	0.286392	0.37	0.186	0.076074	12
Ubtf	4.67E-06	-0.5262	0.345	0.453	0.077914	12

Klhdc8b	4.72E-06	0.32078	0.118	0.022	0.078691	12
Ckap2l	4.73E-06	-0.76446	0.059	0.218	0.078894	12
Tbc1d20	4.9E-06	0.393193	0.252	0.094	0.081671	12
Arcn1	5.12E-06	0.50007	0.345	0.153	0.085471	12
Mns1	5.19E-06	-0.66873	0.067	0.204	0.086623	12
Gsk3b	5.26E-06	-0.60674	0.303	0.5	0.087794	12
Cxxc5	5.27E-06	-0.48201	0.378	0.544	0.087861	12
Pelo	5.3E-06	0.492653	0.185	0.051	0.088456	12
Tcerg1	5.36E-06	-0.55091	0.336	0.457	0.089426	12
Kdelc2	5.42E-06	0.468714	0.151	0.036	0.090329	12
Baz1b	5.52E-06	-0.56643	0.361	0.493	0.0921	12
Tprn	5.53E-06	-0.70333	0.059	0.227	0.092189	12
Prdx2	5.69E-06	-0.46651	0.546	0.68	0.094885	12
Cdk6	5.76E-06	-0.65565	0.067	0.244	0.096034	12
Eml3	5.83E-06	0.393876	0.16	0.04	0.097255	12
Hdgfrp3	5.83E-06	-0.66163	0.16	0.339	0.097304	12
Cenpm	5.9E-06	-0.58009	0.025	0.162	0.098429	12
Klhl5	6.11E-06	0.349468	0.151	0.038	0.101904	12
Tacc3	6.27E-06	-0.68061	0.05	0.207	0.104656	12
Trim62	6.35E-06	0.296316	0.16	0.046	0.105883	12
Myo6	6.39E-06	0.536583	0.176	0.048	0.106604	12
Dhx36	6.44E-06	-0.59487	0.235	0.371	0.107498	12
Eml4	6.49E-06	0.541763	0.403	0.208	0.108271	12
Kit	6.52E-06	0.305294	0.118	0.022	0.108837	12
Usp22	6.66E-06	-0.57341	0.21	0.362	0.111103	12
Pgrmc1	6.94E-06	0.487643	0.504	0.293	0.115773	12
Snrpd2	6.99E-06	-0.57685	0.261	0.442	0.116608	12
Rap1b	7.01E-06	0.424511	0.311	0.133	0.116939	12
Tex14	7.14E-06	-0.70038	0.025	0.146	0.119132	12
Rrm2	7.2E-06	-0.67119	0.059	0.204	0.120013	12
Tbrg1	7.21E-06	0.381961	0.454	0.241	0.120246	12
Hspa9	7.46E-06	-0.59284	0.218	0.373	0.124365	12
Aldh2	7.8E-06	0.338616	0.126	0.025	0.130165	12
Hells	7.89E-06	-0.62987	0.05	0.214	0.131659	12
Mmd	7.92E-06	0.442829	0.21	0.066	0.132098	12
Nme1	8.1E-06	-0.54698	0.353	0.51	0.135084	12
Myod1	8.18E-06	-0.60193	0.017	0.142	0.136435	12
Sep15	8.18E-06	0.419536	0.697	0.503	0.13648	12
Supt16	8.41E-06	-0.56967	0.345	0.494	0.140337	12
Rtf1	8.43E-06	-0.42601	0.328	0.513	0.140687	12
Camta1	8.65E-06	-0.63979	0.235	0.409	0.14432	12
Pick1	8.66E-06	-0.54278	0.017	0.128	0.144415	12
Hmgb1	8.81E-06	-0.61481	0.185	0.359	0.146896	12
Arid5b	8.87E-06	0.476132	0.16	0.04	0.147955	12
Ift20	9.13E-06	0.544683	0.387	0.193	0.152207	12
Aimp1	9.22E-06	-0.61697	0.126	0.28	0.153781	12
Nol4	9.32E-06	-0.60642	0.025	0.172	0.155536	12
Srrm1	9.48E-06	-0.45651	0.513	0.612	0.158185	12

Otx2	9.56E-06	-0.64969	0.059	0.217	0.159506	12
Rab13	9.99E-06	0.473691	0.235	0.083	0.166638	12
Msn	1.02E-05	0.529305	0.244	0.091	0.16959	12
Kif11	1.02E-05	-0.698	0.05	0.206	0.17023	12
Ostc	1.04E-05	0.485797	0.479	0.272	0.173422	12
Trp53i11	1.09E-05	-0.62809	0.067	0.202	0.180981	12
Smarcc1	1.09E-05	-0.58622	0.261	0.434	0.182291	12
Syngr2	1.11E-05	0.352051	0.118	0.021	0.184398	12
Rps14	1.11E-05	-0.31392	0.916	0.95	0.184876	12
Ltbr	1.11E-05	0.306235	0.101	0.015	0.185885	12
Syne2	1.13E-05	-0.67915	0.067	0.221	0.189011	12
Bub1	1.15E-05	-0.52252	0.017	0.131	0.191011	12
Wwc2	1.18E-05	0.307595	0.109	0.02	0.196341	12
Ctsa	1.19E-05	0.408313	0.294	0.123	0.198469	12
Nap1l1	1.2E-05	-0.50445	0.378	0.511	0.200607	12
Zmiz1	1.2E-05	-0.54496	0.261	0.375	0.200886	12
Dhrs7	1.23E-05	0.302503	0.202	0.07	0.205592	12
Tra2b	1.25E-05	-0.61152	0.227	0.406	0.208196	12
Pfdn4	1.26E-05	-0.62984	0.118	0.306	0.210088	12
Mbnl2	1.26E-05	0.513114	0.37	0.177	0.210961	12
1110038B1	1.28E-05	-0.58225	0.16	0.299	0.213277	12
Npepl1	1.28E-05	0.470123	0.143	0.033	0.213738	12
Ptov1	1.3E-05	-0.51233	0.353	0.485	0.217162	12
A330076H0	1.31E-05	-0.61779	0.05	0.161	0.218324	12
Rap1a	1.32E-05	0.519341	0.286	0.122	0.220185	12
Kif5a	1.34E-05	-0.54964	0.034	0.128	0.223269	12
Smoc1	1.34E-05	-0.5563	0.008	0.135	0.224013	12
Cenpv	1.34E-05	-0.61343	0.151	0.329	0.224226	12
Gm3764	1.37E-05	-0.57252	0.017	0.132	0.228162	12
Incenp	1.37E-05	-0.74287	0.101	0.276	0.228909	12
Sfrs18	1.37E-05	-0.43059	0.639	0.778	0.229064	12
Pdzrn3	1.4E-05	-0.35886	0.151	0.147	0.232696	12
Soat1	1.4E-05	0.263094	0.109	0.02	0.233455	12
Tram1	1.42E-05	0.450426	0.303	0.127	0.236345	12
Trip10	1.42E-05	0.280695	0.118	0.024	0.236387	12
Marcks	1.45E-05	-0.31166	0.857	0.916	0.242024	12
Atxn7l3b	1.45E-05	-0.36247	0.647	0.673	0.242267	12
Rab3a	1.46E-05	-0.49998	0.092	0.15	0.242985	12
Erdr1	1.46E-05	-0.59496	0.034	0.162	0.244283	12
H2afx	1.47E-05	-0.75547	0.168	0.331	0.245254	12
Gpr108	1.47E-05	0.470119	0.168	0.047	0.245529	12
Srebf1	1.53E-05	-0.56583	0.227	0.334	0.255558	12
Metap2	1.53E-05	-0.4916	0.445	0.597	0.255949	12
Serp1	1.54E-05	0.531047	0.395	0.216	0.257325	12
Klc1	1.56E-05	-0.65157	0.185	0.362	0.260486	12
Dbf4	1.56E-05	-0.54638	0.017	0.146	0.26073	12
Carhsp1	1.61E-05	-0.60244	0.109	0.254	0.268632	12
Oat	1.67E-05	0.401997	0.21	0.069	0.277781	12

Dixdc1	1.67E-05	-0.63627	0.118	0.235	0.278798	12
Aspm	1.69E-05	-0.63848	0.025	0.143	0.282307	12
Map4k4	1.73E-05	-0.50437	0.336	0.45	0.287974	12
Arhgap31	1.73E-05	0.36946	0.151	0.038	0.288045	12
Rbp4	1.73E-05	-0.66488	0.076	0.242	0.288763	12
Txnrd1	1.74E-05	-0.58827	0.185	0.39	0.290099	12
Ubxn1	1.77E-05	-0.54474	0.311	0.479	0.295079	12
Hbp1	1.82E-05	0.552039	0.319	0.155	0.302923	12
Tulp3	1.82E-05	0.264963	0.126	0.031	0.303106	12
Zcchc18	1.82E-05	-0.61771	0.042	0.171	0.303579	12
Lrrfip1	1.85E-05	0.304031	0.151	0.043	0.30788	12
Nell2	1.86E-05	-0.53339	0.008	0.133	0.310363	12
Celf4	1.87E-05	-0.85423	0.143	0.321	0.311325	12
Chd3	1.87E-05	0.368455	0.319	0.148	0.312685	12
Vcl	1.95E-05	0.344692	0.101	0.016	0.326041	12
Zbtb38	1.97E-05	0.391843	0.185	0.058	0.328516	12
Lsm6	1.99E-05	-0.60714	0.185	0.373	0.332748	12
Pdlim7	2.02E-05	0.373376	0.151	0.039	0.337754	12
Mllt11	2.09E-05	-0.6974	0.084	0.219	0.349272	12
Cdc5l	2.11E-05	-0.40159	0.361	0.401	0.352358	12
Lpar4	2.13E-05	0.350961	0.126	0.031	0.354542	12
Eef1a1	2.21E-05	-0.31739	0.866	0.884	0.368557	12
Uhrf1	2.31E-05	-0.49688	0.017	0.144	0.385932	12
Nop56	2.32E-05	-0.61263	0.168	0.361	0.387755	12
Tdrkh	2.35E-05	-0.40619	0.034	0.133	0.392517	12
Eif4a1	2.36E-05	-0.53487	0.303	0.485	0.39299	12
Zfp91	2.37E-05	-0.5025	0.378	0.49	0.395856	12
Ugp2	2.38E-05	0.39662	0.235	0.089	0.396601	12
Plekha1	2.39E-05	0.357153	0.185	0.06	0.398537	12
Mrps5	2.41E-05	-0.60232	0.118	0.273	0.402802	12
Ttyh3	2.44E-05	-0.49601	0.017	0.124	0.40616	12
Lamtor3	2.44E-05	0.357329	0.235	0.088	0.407383	12
Neurod6	2.54E-05	-0.53626	0.05	0.172	0.422923	12
Scarb2	2.57E-05	0.412693	0.151	0.038	0.428485	12
Rnaseh2c	2.58E-05	-0.59916	0.202	0.382	0.431001	12
Sms	2.59E-05	-0.46217	0.025	0.137	0.432479	12
Nt5dc2	2.6E-05	-0.59767	0.118	0.238	0.433755	12
Syt13	2.61E-05	-0.49814	0.017	0.127	0.434795	12
Ergic3	2.61E-05	0.348895	0.42	0.226	0.434868	12
Ap3b2	2.63E-05	-0.56722	0.05	0.203	0.437882	12
Mad2l2	2.68E-05	-0.61068	0.092	0.263	0.446387	12
2700089E2	2.7E-05	0.49899	0.387	0.197	0.450002	12
Csdc2	2.74E-05	0.491913	0.126	0.028	0.456577	12
Gins1	2.74E-05	-0.34135	0.017	0.115	0.45752	12
Cdca2	2.75E-05	-0.51838	0.008	0.129	0.458913	12
Trim56	2.77E-05	0.406634	0.126	0.027	0.461787	12
Zmat2	2.78E-05	-0.57117	0.261	0.441	0.463533	12
Vasp	2.84E-05	0.417092	0.202	0.066	0.473264	12

Rps24	2.85E-05	-0.34329	0.739	0.821	0.474879	12
Gphn	2.86E-05	-0.56	0.017	0.141	0.476789	12
Snrpn	2.91E-05	-0.53167	0.042	0.159	0.485594	12
Tgfb2	2.92E-05	-0.57831	0.042	0.192	0.487211	12
Akap13	2.94E-05	0.447231	0.21	0.071	0.489939	12
Ptch2	2.98E-05	-0.56514	0.025	0.153	0.496394	12
Ncapg	3.01E-05	-0.61979	0.042	0.184	0.502761	12
Ptk2	3.02E-05	0.541675	0.202	0.084	0.502911	12
Casc5	3.05E-05	-0.63287	0.059	0.187	0.508151	12
Cct8	3.05E-05	-0.42819	0.328	0.441	0.508354	12
BC005764	3.05E-05	-0.7058	0.059	0.186	0.508437	12
Tmx1	3.08E-05	-0.49036	0.05	0.15	0.51424	12
Frmd4a	3.09E-05	-0.63147	0.16	0.309	0.515093	12
Atad2	3.09E-05	-0.61954	0.059	0.199	0.515728	12
Tcp11l1	3.14E-05	-0.45682	0.017	0.116	0.524582	12
Rbmxl1	3.16E-05	-0.52438	0.025	0.153	0.527282	12
Sult4a1	3.17E-05	-0.61541	0.067	0.223	0.52824	12
Spc24	3.17E-05	-0.65331	0.084	0.243	0.528893	12
Phf21b	3.18E-05	-0.53376	0.008	0.128	0.530617	12
Zbtb4	3.19E-05	0.325025	0.126	0.029	0.531863	12
Mob1a	3.2E-05	0.422038	0.176	0.052	0.533496	12
Dnpep	3.23E-05	0.272073	0.244	0.109	0.539431	12
Kcnk1	3.27E-05	-0.63522	0.067	0.205	0.544959	12
Alad	3.3E-05	0.371607	0.151	0.039	0.550722	12
Mis18bp1	3.32E-05	-0.6157	0.034	0.161	0.553008	12
Pou3f3	3.33E-05	-0.56585	0.042	0.172	0.555248	12
Smpd3	3.35E-05	-0.60846	0.034	0.162	0.559081	12
Rps25	3.36E-05	-0.44029	0.395	0.48	0.559895	12
Esf1	3.4E-05	-0.63064	0.227	0.393	0.566885	12
Mtss1	3.5E-05	-0.68809	0.126	0.27	0.583859	12
Aldh9a1	3.54E-05	0.278898	0.168	0.055	0.59115	12
Ncapd2	3.66E-05	-0.49996	0.042	0.185	0.609922	12
Rab3c	3.66E-05	-0.59665	0.025	0.148	0.611102	12
Gdap1	3.7E-05	-0.62166	0.05	0.199	0.61781	12
1810043H(	3.72E-05	-0.28693	0.109	0.127	0.621186	12
Rfc2	3.74E-05	-0.53298	0.092	0.211	0.623848	12
Selk	3.74E-05	0.340246	0.605	0.394	0.624571	12
Blmh	3.78E-05	-0.51497	0.193	0.317	0.630233	12
Cct5	3.81E-05	-0.51834	0.319	0.478	0.635531	12
Cd2ap	3.82E-05	0.436662	0.336	0.157	0.637107	12
Nefm	3.87E-05	-0.61212	0.008	0.117	0.646201	12
Irf2	3.99E-05	0.258515	0.16	0.056	0.665081	12
Rif1	4.02E-05	-0.56599	0.151	0.276	0.670439	12
Fyn	4.07E-05	-0.56623	0.126	0.216	0.679138	12
Ctcf	4.15E-05	-0.5303	0.353	0.496	0.69288	12
Mcm6	4.16E-05	-0.64236	0.109	0.277	0.693872	12
Tnik	4.18E-05	-0.64399	0.025	0.147	0.696514	12
Tmeff1	4.19E-05	-0.55948	0.025	0.15	0.698482	12

Ypel1	4.2E-05	-0.54207	0.034	0.175	0.700371	12
Slc17a6	4.26E-05	-0.62028	0.034	0.148	0.711254	12
Tubb2a	4.34E-05	-0.65738	0.151	0.245	0.723413	12
Ube2e3	4.4E-05	-0.56733	0.244	0.417	0.734243	12
Chchd2	4.43E-05	-0.39639	0.555	0.653	0.738255	12
Tmem35	4.47E-05	-0.35609	0.025	0.104	0.745049	12
Cdca7	4.49E-05	-0.58767	0.059	0.198	0.748175	12
Ebf3	4.55E-05	-0.63097	0.067	0.203	0.759684	12
Rad21	4.58E-05	-0.56152	0.319	0.462	0.764085	12
Tomm7	4.67E-05	-0.38207	0.437	0.497	0.779346	12
Pde4dip	4.84E-05	-0.61059	0.067	0.222	0.806667	12
Bok	4.88E-05	-0.56598	0.084	0.252	0.813782	12
Tcp1	4.94E-05	-0.48006	0.303	0.464	0.82318	12
Lrig3	4.99E-05	-0.54678	0.042	0.17	0.832907	12
Sra1	5.16E-05	0.501403	0.286	0.124	0.859859	12
Ccnb1	5.21E-05	-0.5495	0.017	0.14	0.868924	12
Crispld1	5.23E-05	0.335225	0.101	0.018	0.872976	12
Cat	5.27E-05	0.507377	0.277	0.123	0.878952	12
D030056L2	5.31E-05	-0.50647	0.025	0.143	0.885139	12
Fam57b	5.31E-05	-0.59892	0.05	0.186	0.885341	12
Ppp1r14a	5.4E-05	0.456869	0.101	0.022	0.899952	12
Chrna3	5.42E-05	-0.57524	0.017	0.119	0.903281	12
Hes6	5.44E-05	-0.61047	0.084	0.237	0.907742	12
Cdh20	5.45E-05	-0.59986	0.059	0.203	0.909681	12
Ppp3ca	5.51E-05	-0.63274	0.235	0.379	0.919002	12
Rpn2	5.52E-05	0.379683	0.42	0.225	0.92049	12
Nans	5.56E-05	0.459465	0.261	0.106	0.927908	12
Ccna2	5.65E-05	-0.56649	0.067	0.222	0.942366	12
D630003M	5.69E-05	0.251807	0.143	0.045	0.949793	12
Dynlt3	5.77E-05	0.452874	0.16	0.045	0.962144	12
Timeless	5.8E-05	-0.54875	0.034	0.154	0.966919	12
Rpl37	5.82E-05	-0.45383	0.395	0.504	0.97135	12
Cope	5.83E-05	0.449885	0.546	0.362	0.97287	12
Prc1	5.88E-05	-0.76639	0.101	0.257	0.98059	12
Ndufa12	5.96E-05	-0.50451	0.294	0.422	0.994827	12
Gpr56	5.98E-05	-0.55413	0.067	0.223	0.996936	12
Dmd	6.01E-05	0.497764	0.109	0.025	1	12
Pgm1	6.16E-05	0.439721	0.101	0.023	1	12
Tprgl	6.24E-05	0.499357	0.277	0.123	1	12
Stk39	6.27E-05	0.452752	0.134	0.042	1	12
Mgat2	6.42E-05	0.414634	0.277	0.118	1	12
Naca	6.6E-05	-0.42647	0.378	0.465	1	12
Ddx21	6.68E-05	-0.59947	0.185	0.323	1	12
Bsg	6.7E-05	0.392614	0.597	0.423	1	12
Cnbp	6.71E-05	-0.37552	0.639	0.74	1	12
Pdzrn4	6.74E-05	-0.44837	0.025	0.147	1	12
Lhfpl2	6.75E-05	0.350649	0.118	0.027	1	12
Mtf2	6.76E-05	-0.59545	0.185	0.358	1	12

Sowaha	6.77E-05	-0.67355	0.034	0.163	1	12
Glyr1	6.82E-05	-0.48218	0.202	0.284	1	12
Fam181b	6.85E-05	-0.51303	0.025	0.143	1	12
Mapk8ip2	6.87E-05	-0.48012	0.042	0.143	1	12
Sox4	6.95E-05	-0.54277	0.412	0.583	1	12
Dpy30	7.02E-05	-0.57621	0.151	0.299	1	12
Echdc2	7.02E-05	0.381658	0.134	0.034	1	12
Spcs1	7.17E-05	0.38744	0.529	0.324	1	12
C1qbp	7.23E-05	-0.53596	0.227	0.378	1	12
Sars	7.24E-05	-0.51658	0.294	0.421	1	12
Acat2	7.31E-05	-0.49312	0.042	0.183	1	12
Prim1	7.45E-05	-0.57281	0.101	0.264	1	12
Bfar	7.51E-05	0.404559	0.37	0.19	1	12
Slc12a4	7.6E-05	0.334293	0.134	0.034	1	12
Xrn2	7.62E-05	-0.45822	0.361	0.473	1	12
Cep170	7.72E-05	-0.58218	0.168	0.306	1	12
Adamts10	7.73E-05	0.340961	0.118	0.026	1	12
Hspa4	7.79E-05	-0.56461	0.218	0.369	1	12
Sgol1	7.96E-05	-0.4918	0.017	0.128	1	12
Dpysl3	7.98E-05	-0.63634	0.134	0.22	1	12
Nol8	7.99E-05	-0.56478	0.05	0.168	1	12
Fabp5	8.14E-05	-0.52169	0.227	0.404	1	12
Mrpl42	8.29E-05	-0.57508	0.16	0.319	1	12
Zc3h15	8.29E-05	-0.44758	0.277	0.366	1	12
Rpa2	8.3E-05	-0.55462	0.067	0.2	1	12
Ctnna1	8.34E-05	0.446113	0.319	0.151	1	12
Tnfaip1	8.35E-05	0.48031	0.244	0.106	1	12
Nhp2	8.51E-05	-0.56267	0.218	0.371	1	12
Ythdf2	8.51E-05	-0.54628	0.118	0.259	1	12
Ccar1	8.59E-05	-0.50022	0.412	0.573	1	12
Pkia	8.7E-05	-0.43616	0.05	0.134	1	12
Cdc42se2	8.83E-05	-0.53826	0.118	0.239	1	12
Herpud1	8.95E-05	0.470493	0.294	0.134	1	12
Ubqln2	9E-05	-0.55237	0.092	0.215	1	12
Dcbld2	9E-05	0.334278	0.151	0.044	1	12
Creb3	9.12E-05	0.412367	0.244	0.098	1	12
Elmo1	9.16E-05	-0.6566	0.017	0.126	1	12
Ppp1r9a	9.19E-05	-0.60423	0.05	0.189	1	12
Mef2a	9.19E-05	0.543544	0.261	0.138	1	12
Bcap29	9.21E-05	-0.45676	0.034	0.123	1	12
B3gnt1	9.24E-05	0.37395	0.185	0.061	1	12
Myef2	9.31E-05	-0.56966	0.176	0.339	1	12
Trim37	9.35E-05	-0.48565	0.076	0.234	1	12
Fam118b	9.52E-05	0.45481	0.176	0.061	1	12
Ppa1	9.62E-05	-0.5275	0.109	0.222	1	12
Cadm3	9.68E-05	-0.60384	0.025	0.141	1	12
G3bp1	9.85E-05	-0.54629	0.176	0.328	1	12
Mcm2	0.000101	-0.59261	0.059	0.205	1	12

Ubc	0.000103	0.414092	0.555	0.357	1	12
Rin2	0.000103	0.305768	0.101	0.02	1	12
Myeov2	0.000106	-0.29754	0.437	0.455	1	12
Elovl1	0.000106	0.254208	0.109	0.034	1	12
Fam20c	0.000107	0.347886	0.176	0.059	1	12
Tmod2	0.000108	-0.55589	0.084	0.208	1	12
Prkcb	0.000108	-0.60646	0.092	0.257	1	12
BC005561	0.000109	-0.6196	0.126	0.29	1	12
Yipf5	0.000109	0.310108	0.269	0.121	1	12
Ssb	0.000109	-0.26109	0.613	0.704	1	12
4931406C(	0.00011	0.491974	0.151	0.044	1	12
Ndc80	0.000112	-0.30385	0.017	0.115	1	12
Apbb1	0.000114	-0.53755	0.076	0.17	1	12
Acadm	0.000114	0.276975	0.21	0.086	1	12
Prdx1	0.000115	0.342206	0.689	0.488	1	12
Mpnd	0.000116	-0.50514	0.059	0.172	1	12
Tmem57	0.000116	-0.53928	0.185	0.291	1	12
Ssh2	0.000117	0.417541	0.151	0.043	1	12
Tmem5	0.00012	0.531123	0.202	0.08	1	12
1700001O:	0.000123	-0.38624	0.008	0.11	1	12
Sema6d	0.000123	0.414363	0.126	0.031	1	12
Nes	0.000124	0.401589	0.218	0.085	1	12
Lepre1	0.000126	0.276434	0.101	0.021	1	12
Gnas	0.000126	0.275418	0.924	0.885	1	12
Sox18	0.000126	-0.47939	0.025	0.148	1	12
Las1l	0.000128	-0.59193	0.109	0.255	1	12
Mxra7	0.00013	0.278084	0.168	0.059	1	12
Eif5b	0.00013	-0.43954	0.445	0.595	1	12
Dlgap5	0.00013	-0.40531	0.042	0.112	1	12
Por	0.000134	0.426827	0.185	0.065	1	12
E2f1	0.000134	-0.52163	0.042	0.147	1	12
Nelfe	0.000135	-0.2928	0.126	0.15	1	12
Zswim7	0.000138	0.345882	0.109	0.023	1	12
Pomp	0.000144	-0.39771	0.353	0.414	1	12
Kank3	0.000146	0.294422	0.118	0.029	1	12
Frrs1l	0.000147	-0.54049	0.05	0.192	1	12
Gns	0.000148	0.400613	0.193	0.069	1	12
Cenpq	0.000149	-0.51777	0.034	0.161	1	12
Acadvl	0.00015	0.434001	0.252	0.105	1	12
Myt1l	0.000153	-0.64985	0.034	0.161	1	12
Eif4a2	0.000154	0.311157	0.345	0.179	1	12
Ssr3	0.000155	0.406433	0.58	0.424	1	12
Klf9	0.000155	0.383975	0.462	0.269	1	12
Rnf122	0.000161	-0.31118	0.042	0.102	1	12
Gpm6b	0.000161	-0.50714	0.361	0.485	1	12
Mapt	0.000162	-0.71308	0.076	0.217	1	12
Cct6a	0.000163	-0.47493	0.336	0.487	1	12
Rhog	0.000164	0.263435	0.118	0.03	1	12

Fam53b	0.000164	-0.42665	0.025	0.137	1	12
Srsf1	0.000165	-0.56134	0.143	0.297	1	12
Nuf2	0.000166	-0.44788	0.025	0.141	1	12
Ckap5	0.000166	-0.58919	0.134	0.266	1	12
Lancl2	0.000167	-0.41021	0.025	0.105	1	12
Hspe1	0.000169	-0.3845	0.269	0.341	1	12
Brd7	0.00017	-0.36189	0.168	0.308	1	12
Trim28	0.000173	-0.52344	0.227	0.362	1	12
Pik3r1	0.000174	0.515666	0.235	0.101	1	12
Cenpk	0.000175	-0.32725	0.034	0.145	1	12
Phldb2	0.000176	0.37845	0.168	0.055	1	12
Jun	0.000179	0.444465	0.765	0.654	1	12
Eef1e1	0.000179	-0.517	0.05	0.182	1	12
Prpf31	0.000182	-0.45067	0.118	0.2	1	12
Hsd17b11	0.000182	0.328178	0.176	0.062	1	12
Sept2	0.000188	0.386008	0.378	0.221	1	12
Mxd3	0.000188	-0.47155	0.017	0.127	1	12
Kdelc1	0.000188	0.453758	0.16	0.06	1	12
Fam64a	0.000189	-0.47494	0.017	0.118	1	12
Prmt5	0.000191	-0.53357	0.134	0.256	1	12
Mpped2	0.000193	-0.48471	0.017	0.127	1	12
Nfkbia	0.000198	0.474601	0.336	0.17	1	12
Fam115a	0.000198	-0.5166	0.244	0.351	1	12
Dtx3	0.0002	0.303426	0.227	0.097	1	12
Atp5j2	0.0002	-0.35091	0.513	0.615	1	12
Cers2	0.000209	0.429102	0.235	0.113	1	12
Aurkb	0.00021	-0.49111	0.025	0.143	1	12
Bola2	0.00021	-0.52538	0.227	0.398	1	12
Tk1	0.000217	-0.46616	0.042	0.131	1	12
BC005537	0.000219	-0.5034	0.227	0.333	1	12
Eprs	0.000224	-0.38379	0.437	0.491	1	12
Myt1	0.000224	-0.63482	0.034	0.153	1	12
Nsg1	0.000226	-0.55725	0.16	0.288	1	12
Ptbp2	0.000228	-0.55544	0.092	0.221	1	12
Dbp	0.000228	0.430253	0.134	0.039	1	12
Yeats4	0.000229	-0.56416	0.109	0.264	1	12
Rcc2	0.00023	-0.46863	0.084	0.179	1	12
Fam171b	0.000233	-0.58335	0.092	0.224	1	12
Aplp1	0.000235	-0.55796	0.059	0.152	1	12
Pcbp4	0.000235	-0.5411	0.168	0.325	1	12
Frmd8	0.000236	0.445238	0.101	0.026	1	12
Mgll	0.000236	-0.54125	0.034	0.133	1	12
Larp7	0.000237	-0.46745	0.16	0.312	1	12
Ttc9b	0.000239	-0.52904	0.067	0.195	1	12
Hk2	0.00024	-0.52826	0.059	0.173	1	12
Etaa1	0.000242	-0.52389	0.05	0.159	1	12
Sqle	0.000248	-0.45337	0.025	0.121	1	12
Pcdh18	0.000248	0.473615	0.176	0.077	1	12

Gm17322	0.000249	-0.56336	0.034	0.131	1	12
Zeb1	0.000252	-0.52199	0.252	0.388	1	12
Ubr1	0.000257	0.492945	0.218	0.094	1	12
Qdpr	0.000258	-0.43822	0.185	0.268	1	12
Dnajc2	0.000261	-0.52815	0.21	0.329	1	12
Ccnb2	0.000264	-0.63243	0.059	0.187	1	12
Tmem178	0.000268	-0.52653	0.067	0.187	1	12
Emc7	0.000269	0.347063	0.294	0.142	1	12
Dst	0.000279	-0.57668	0.143	0.267	1	12
Tmed2	0.000282	0.428125	0.21	0.086	1	12
Rcn2	0.000285	-0.32076	0.235	0.281	1	12
2700046A(	0.000286	0.336143	0.126	0.033	1	12
Bcas2	0.000291	-0.40477	0.261	0.406	1	12
Pold3	0.000291	-0.39723	0.084	0.149	1	12
Cacng4	0.000295	-0.59717	0.025	0.102	1	12
Rpl14	0.000296	-0.31326	0.647	0.697	1	12
Zdbf2	0.000298	-0.49184	0.034	0.134	1	12
Hsdl2	0.000299	0.431821	0.16	0.053	1	12
Ubr2	0.000299	0.290626	0.202	0.084	1	12
Rnf126	0.000302	-0.36925	0.017	0.103	1	12
Epb4.1	0.000304	-0.53851	0.084	0.23	1	12
Snrnp70	0.000308	-0.35967	0.613	0.694	1	12
Kif15	0.000308	-0.50891	0.05	0.181	1	12
Wdr33	0.000309	-0.41136	0.126	0.196	1	12
Sf3a1	0.000309	-0.37036	0.109	0.158	1	12
Ier5	0.000313	-0.46558	0.303	0.394	1	12
Fdft1	0.000317	-0.40097	0.017	0.101	1	12
E330009J0	0.000325	-0.44248	0.017	0.117	1	12
Arid2	0.000326	-0.4693	0.126	0.242	1	12
Ankrd50	0.000326	0.413299	0.118	0.032	1	12
D17H6S56I	0.000327	-0.45343	0.034	0.128	1	12
Cep78	0.000328	-0.47678	0.042	0.14	1	12
Lmnb2	0.000328	-0.48558	0.042	0.159	1	12
Mroh2a	0.000328	-0.51327	0.034	0.114	1	12
Pphln1	0.000329	-0.35152	0.059	0.119	1	12
Igsf8	0.000334	-0.5575	0.143	0.27	1	12
Cib1	0.000336	0.364278	0.168	0.056	1	12
Mcm5	0.000336	-0.48562	0.067	0.169	1	12
Olfm1	0.000341	-0.43474	0.151	0.198	1	12
Cox6c	0.000343	0.282652	0.815	0.718	1	12
Acp2	0.000345	0.340815	0.218	0.092	1	12
Tyms	0.000348	-0.57442	0.084	0.219	1	12
Scn8a	0.000349	-0.50123	0.042	0.137	1	12
Gpc2	0.000354	-0.48905	0.05	0.14	1	12
Canx	0.000358	0.340791	0.756	0.594	1	12
Rrp1	0.000359	-0.41997	0.471	0.607	1	12
Zfp608	0.000359	-0.53364	0.059	0.186	1	12
Cnpy1	0.000364	-0.52305	0.025	0.133	1	12

Aip	0.000369	0.3342	0.202	0.079	1	12
Pak7	0.000378	-0.46825	0.008	0.105	1	12
Suz12	0.00038	-0.53929	0.092	0.219	1	12
Sash1	0.000384	0.357795	0.126	0.034	1	12
Gorasp2	0.000396	0.412433	0.252	0.114	1	12
Spred1	0.000398	0.387203	0.244	0.107	1	12
Ncald	0.000399	-0.52107	0.042	0.166	1	12
Sclt1	0.000401	-0.43403	0.05	0.127	1	12
Idh3a	0.000407	-0.40267	0.084	0.163	1	12
Eif3l	0.000408	-0.42316	0.218	0.302	1	12
Scp2	0.00041	0.441173	0.185	0.072	1	12
Ndufa13	0.000412	0.325902	0.706	0.532	1	12
Spg21	0.000413	-0.4593	0.076	0.173	1	12
2700029M	0.000415	-0.4829	0.185	0.29	1	12
Cgrrf1	0.000417	0.390646	0.16	0.054	1	12
Capn2	0.000417	0.39731	0.218	0.089	1	12
AI597468	0.000418	0.432744	0.227	0.097	1	12
Mab21l2	0.000424	-0.50644	0.008	0.104	1	12
Zwint	0.000426	-0.44046	0.218	0.292	1	12
M6pr	0.000429	0.423218	0.244	0.108	1	12
Snrnp40	0.000429	-0.50906	0.143	0.271	1	12
Pcsk2	0.000429	-0.39139	0.017	0.102	1	12
Stt3a	0.000431	0.386969	0.261	0.125	1	12
Lman1	0.000441	0.445541	0.462	0.323	1	12
Ifnar1	0.000447	0.372916	0.227	0.095	1	12
Thoc1	0.000449	-0.39352	0.059	0.178	1	12
St8sia1	0.000451	0.355841	0.126	0.035	1	12
Ccp110	0.000458	-0.52857	0.101	0.249	1	12
Tnpo3	0.000465	-0.52513	0.084	0.223	1	12
Ifngr1	0.000466	0.341975	0.126	0.036	1	12
Clip1	0.000467	0.450408	0.218	0.093	1	12
Grik2	0.000467	-0.51742	0.008	0.102	1	12
Sec13	0.000472	0.435512	0.429	0.268	1	12
Eif3m	0.000477	-0.49543	0.176	0.302	1	12
H1fx	0.000482	-0.47086	0.109	0.245	1	12
Cnksr2	0.000482	-0.4454	0.017	0.108	1	12
Nxt1	0.000484	-0.33565	0.067	0.122	1	12
Gkap1	0.000488	-0.51962	0.084	0.221	1	12
Arpp21	0.000489	-0.55979	0.025	0.113	1	12
Cdk5rap2	0.000504	-0.26782	0.067	0.184	1	12
Xist	0.000509	-0.74536	0.151	0.3	1	12
Rbm8a	0.000517	-0.46408	0.319	0.466	1	12
Syncrip	0.000517	-0.38517	0.387	0.496	1	12
Rhoa	0.000524	0.385361	0.286	0.14	1	12
Emc6	0.000537	-0.51587	0.143	0.276	1	12
Nop10	0.000541	-0.43068	0.378	0.482	1	12
Ddhd2	0.000548	-0.4943	0.034	0.13	1	12
Mad2l1	0.000582	-0.42192	0.025	0.123	1	12

Cplx1	0.000583	-0.49341	0.034	0.138	1	12
Hspa8	0.000583	-0.50051	0.261	0.416	1	12
Polr3k	0.000588	-0.489	0.16	0.271	1	12
Gria4	0.000604	-0.46551	0.025	0.125	1	12
Sidt2	0.000604	0.406736	0.134	0.045	1	12
Plcb1	0.000606	-0.59174	0.034	0.141	1	12
Nudc	0.000607	-0.49825	0.126	0.281	1	12
Asf1a	0.000609	-0.4754	0.126	0.28	1	12
Esco2	0.000613	-0.59735	0.067	0.195	1	12
Mafb	0.000614	0.38652	0.151	0.05	1	12
Mrpl39	0.000615	-0.45446	0.025	0.133	1	12
Mrps10	0.000617	-0.43644	0.05	0.14	1	12
Luzp1	0.00062	0.450071	0.277	0.135	1	12
Rfc3	0.000622	-0.50574	0.084	0.205	1	12
Gm12696	0.000632	-0.43494	0.034	0.133	1	12
Dnajc24	0.000635	-0.30352	0.109	0.151	1	12
Ckap2	0.000637	-0.32712	0.05	0.164	1	12
Cpsf6	0.000647	-0.51246	0.143	0.291	1	12
Pkig	0.000648	0.441766	0.261	0.127	1	12
Wdr12	0.000652	-0.44731	0.05	0.176	1	12
Sbds	0.000654	0.483505	0.269	0.136	1	12
Dscr3	0.000663	0.32709	0.185	0.072	1	12
Dynll1	0.000674	-0.30886	0.563	0.594	1	12
Mbtd1	0.000678	-0.48894	0.126	0.215	1	12
Plscr4	0.000679	0.285096	0.109	0.028	1	12
Adh5	0.00068	-0.4229	0.303	0.405	1	12
Rrp15	0.000685	-0.4188	0.101	0.174	1	12
Pcbd2	0.000688	0.390308	0.118	0.036	1	12
Cstf2	0.000693	-0.36352	0.017	0.104	1	12
Pxk	0.000694	0.261494	0.151	0.054	1	12
Sec14l1	0.000695	0.30846	0.151	0.052	1	12
D10Wsu10	0.000699	-0.43105	0.025	0.12	1	12
Cd164	0.000702	0.313424	0.252	0.118	1	12
Eif1ax	0.000703	-0.47666	0.235	0.398	1	12
Hibadh	0.000706	0.311305	0.202	0.085	1	12
Tmem134	0.00071	0.383092	0.294	0.148	1	12
Rad51	0.000712	-0.41557	0.025	0.116	1	12
Gpr85	0.000719	-0.50899	0.05	0.164	1	12
Prr24	0.000724	0.307419	0.118	0.04	1	12
Tmem198b	0.000726	0.278348	0.118	0.034	1	12
Eif1a	0.000727	0.304975	0.227	0.114	1	12
2810025M	0.000729	-0.46257	0.05	0.167	1	12
Cdkn1b	0.000738	-0.47027	0.303	0.468	1	12
A930011O	0.000739	-0.56696	0.017	0.115	1	12
Safb	0.00074	-0.45008	0.353	0.504	1	12
Upf3b	0.000746	-0.41173	0.244	0.321	1	12
Rab3ip	0.000753	-0.40212	0.008	0.101	1	12
Arf4	0.000755	0.3308	0.588	0.402	1	12

Fasn	0.000756	-0.43056	0.059	0.138	1	12
Eif3e	0.000758	-0.48777	0.227	0.382	1	12
Gm8292	0.000762	-0.4669	0.235	0.369	1	12
Slk	0.000763	0.307869	0.328	0.179	1	12
Stat3	0.000781	0.400992	0.134	0.041	1	12
Phactr1	0.000785	-0.51986	0.059	0.184	1	12
Fbxo9	0.000803	0.300928	0.261	0.127	1	12
Sdf2	0.000804	0.351175	0.378	0.213	1	12
Slc29a1	0.000816	-0.43653	0.429	0.562	1	12
Yipf4	0.000817	0.308232	0.261	0.126	1	12
Mif	0.000818	-0.48811	0.218	0.357	1	12
Ltbp4	0.00083	0.447362	0.101	0.035	1	12
Lpcat3	0.000833	0.394498	0.134	0.043	1	12
Ppp1r7	0.000843	-0.40969	0.118	0.198	1	12
Zfp428	0.000855	-0.47796	0.05	0.167	1	12
Fam110b	0.000859	0.307028	0.118	0.034	1	12
Cox5a	0.000869	-0.38792	0.412	0.57	1	12
Rab31	0.000879	0.404923	0.143	0.054	1	12
Rpl5	0.000881	-0.41284	0.025	0.115	1	12
Stard4	0.0009	-0.36881	0.034	0.141	1	12
Mybbp1a	0.000921	-0.46634	0.109	0.25	1	12
1110004F1	0.000921	-0.39972	0.328	0.423	1	12
D17Wsu10	0.000946	0.258059	0.361	0.214	1	12
Nup88	0.000951	-0.43792	0.101	0.184	1	12
Adipor1	0.000983	0.322277	0.277	0.139	1	12
Taf1d	0.000985	-0.50976	0.193	0.342	1	12
Ddx6	0.001003	-0.42493	0.37	0.493	1	12
Sike1	0.001004	0.404343	0.193	0.085	1	12
Cep290	0.001008	-0.52262	0.034	0.143	1	12
Kras	0.001018	-0.50799	0.134	0.252	1	12
Cct7	0.00103	-0.41267	0.319	0.485	1	12
Ufsp2	0.001033	0.365256	0.252	0.12	1	12
5430416N	0.001038	-0.46079	0.05	0.141	1	12
Cacybp	0.001051	-0.44704	0.252	0.356	1	12
Prpf19	0.001055	-0.43537	0.21	0.307	1	12
Ube2e1	0.001066	-0.39911	0.16	0.228	1	12
Phf20l1	0.001079	-0.40298	0.277	0.34	1	12
Tmod3	0.001085	0.435242	0.286	0.154	1	12
Rnf187	0.001088	-0.43919	0.303	0.421	1	12
Tcf12	0.001088	0.411333	0.412	0.254	1	12
Smim14	0.001103	0.263903	0.345	0.195	1	12
Ddx46	0.001117	-0.42174	0.353	0.442	1	12
Map2k1	0.001148	0.321738	0.134	0.043	1	12
Hnrnph3	0.001163	-0.45359	0.261	0.427	1	12
Dynlrb1	0.001167	-0.39025	0.462	0.58	1	12
Srsf7	0.001179	-0.43589	0.328	0.466	1	12
Gtf2a2	0.00118	-0.41464	0.286	0.384	1	12
Snrpd3	0.001182	-0.36571	0.437	0.535	1	12

Coq7	0.001183	-0.48463	0.101	0.207	1	12
Rsrc1	0.001183	-0.52464	0.118	0.251	1	12
Cdca3	0.001227	-0.55715	0.092	0.222	1	12
Psma7	0.001229	-0.29552	0.731	0.782	1	12
Cdt1	0.001239	-0.42886	0.042	0.125	1	12
Arhgef2	0.001243	-0.30399	0.252	0.282	1	12
Alyref	0.001243	-0.40378	0.109	0.2	1	12
Htatsf1	0.001265	-0.43914	0.361	0.499	1	12
Tm7sf3	0.001268	0.311856	0.168	0.065	1	12
Acadsb	0.001278	0.438725	0.168	0.071	1	12
Sepw1	0.001298	-0.34571	0.235	0.3	1	12
Insig2	0.0013	0.371587	0.218	0.097	1	12
Ralgps2	0.001305	-0.49967	0.134	0.246	1	12
Ndufa2	0.001317	-0.27312	0.58	0.603	1	12
Fam133b	0.001335	-0.51818	0.168	0.309	1	12
Ptbp3	0.001356	0.346966	0.235	0.109	1	12
Elovl4	0.001384	-0.42	0.025	0.11	1	12
Eef2	0.001393	-0.33457	0.613	0.717	1	12
Pcdha2	0.001394	-0.4956	0.034	0.118	1	12
Gm11266	0.0014	-0.46129	0.05	0.144	1	12
Ctbp2	0.001402	-0.39773	0.084	0.215	1	12
Dynll2	0.00142	-0.49817	0.151	0.297	1	12
Hip1r	0.001425	-0.4258	0.067	0.133	1	12
Pdxp	0.001426	-0.38076	0.025	0.113	1	12
Fam160b1	0.00143	0.358813	0.193	0.081	1	12
Sltm	0.001431	-0.40246	0.42	0.532	1	12
Luc7l	0.001444	-0.46705	0.235	0.361	1	12
Fen1	0.001446	-0.36614	0.05	0.142	1	12
Msh2	0.001452	-0.44501	0.042	0.144	1	12
Naa15	0.001454	-0.4706	0.252	0.392	1	12
Tead2	0.001462	-0.49486	0.151	0.278	1	12
Tmem214	0.001464	0.34666	0.126	0.04	1	12
Tcof1	0.00148	-0.44506	0.059	0.168	1	12
Dck	0.0015	-0.39049	0.017	0.103	1	12
Cln5	0.001502	0.324261	0.118	0.035	1	12
Lsm2	0.001503	-0.4267	0.143	0.23	1	12
Atox1	0.001511	0.344856	0.462	0.291	1	12
Suv39h2	0.001511	-0.4127	0.042	0.131	1	12
Agtpbp1	0.001531	-0.39112	0.025	0.102	1	12
Trappc4	0.001551	-0.35939	0.176	0.224	1	12
Plod2	0.001554	0.407937	0.202	0.09	1	12
Klhl7	0.001555	-0.47554	0.059	0.165	1	12
Vcan	0.001555	0.365376	0.261	0.128	1	12
Spire1	0.001558	-0.45558	0.042	0.155	1	12
Slc39a6	0.001563	-0.35572	0.151	0.199	1	12
Ube4b	0.001568	-0.36775	0.05	0.11	1	12
Sox11	0.001611	-0.42608	0.025	0.115	1	12
Parp1	0.001637	-0.41326	0.176	0.314	1	12

Pard6g	0.001649	0.338403	0.227	0.104	1	12
Mphosph9	0.00166	-0.42851	0.042	0.148	1	12
Myl6	0.001661	0.326219	0.353	0.201	1	12
Nae1	0.001661	-0.49529	0.118	0.225	1	12
Kpnb1	0.001666	-0.39278	0.227	0.3	1	12
Ndufa5	0.00167	-0.37456	0.378	0.454	1	12
Rpl36al	0.001676	-0.35763	0.227	0.298	1	12
Fam3c	0.001742	0.301133	0.193	0.084	1	12
Mbp	0.001754	-0.68141	0.067	0.166	1	12
Dkc1	0.001777	-0.43721	0.176	0.333	1	12
Il11ra1	0.001783	0.292176	0.151	0.057	1	12
Elk4	0.001804	0.391969	0.134	0.047	1	12
Frmd4b	0.00181	-0.38248	0.059	0.128	1	12
Rnf5	0.001819	-0.46501	0.126	0.258	1	12
Map7d2	0.00184	-0.29637	0.059	0.101	1	12
Hmgcs1	0.001841	-0.35585	0.101	0.151	1	12
Cfl1	0.001854	-0.44511	0.227	0.378	1	12
Atp1a1	0.001871	-0.47385	0.05	0.159	1	12
Celsr3	0.001872	-0.44874	0.025	0.123	1	12
Krcc1	0.001872	0.376324	0.193	0.082	1	12
Ppfia2	0.001883	-0.46342	0.025	0.112	1	12
Mrps22	0.001901	-0.42269	0.042	0.134	1	12
Zrsr1	0.001909	0.26901	0.143	0.054	1	12
Knop1	0.001914	-0.49528	0.151	0.272	1	12
Gabrb3	0.001919	-0.38676	0.025	0.102	1	12
Gatc	0.001938	-0.37744	0.017	0.107	1	12
Snord104	0.001945	-0.43559	0.067	0.152	1	12
Usp16	0.001955	0.369749	0.395	0.24	1	12
Clspn	0.001966	-0.53576	0.076	0.19	1	12
Ctps	0.001968	-0.41642	0.042	0.138	1	12
Cenph	0.001986	-0.39072	0.076	0.194	1	12
Ints7	0.001999	-0.41301	0.034	0.117	1	12
Tubb4b	0.002009	-0.49077	0.126	0.24	1	12
Pspc1	0.002019	-0.44207	0.042	0.15	1	12
Kif4	0.002033	-0.3722	0.034	0.108	1	12
Gars	0.002044	-0.3599	0.109	0.228	1	12
Ctbp1	0.002045	-0.45135	0.252	0.394	1	12
2900011O	0.002057	-0.42357	0.042	0.104	1	12
Dhx32	0.002059	-0.46734	0.076	0.201	1	12
Txndc17	0.002067	0.357156	0.395	0.241	1	12
Aktip	0.002075	-0.30575	0.042	0.102	1	12
Lyar	0.002077	-0.47496	0.143	0.286	1	12
Skp1a	0.002118	-0.31485	0.529	0.581	1	12
Nktr	0.00213	-0.3575	0.336	0.38	1	12
Sv2b	0.002133	-0.39646	0.017	0.103	1	12
Rnf219	0.00214	-0.36898	0.067	0.14	1	12
Tardbp	0.002169	-0.40123	0.37	0.483	1	12
Kif3a	0.002198	-0.44088	0.168	0.317	1	12

Pes1	0.00221	-0.40838	0.025	0.115	1	12
Arhgef25	0.002234	0.253152	0.176	0.078	1	12
Klhl24	0.002257	-0.37005	0.084	0.139	1	12
Fjx1	0.00227	0.334526	0.118	0.037	1	12
Kif1a	0.002279	-0.44374	0.067	0.172	1	12
Prorsd1	0.002283	0.272434	0.118	0.037	1	12
Polr2j	0.002295	-0.4332	0.185	0.294	1	12
Rps8	0.002302	-0.36706	0.294	0.397	1	12
Tecr	0.002307	-0.36735	0.361	0.53	1	12
Snx5	0.002327	-0.25399	0.143	0.163	1	12
Dazap1	0.002343	-0.44843	0.16	0.273	1	12
Snx6	0.002347	0.266895	0.538	0.367	1	12
Nipsnap1	0.002371	-0.42616	0.034	0.136	1	12
Mid1ip1	0.002374	0.330199	0.143	0.05	1	12
Uchl3	0.002383	-0.42678	0.067	0.187	1	12
Zc3h13	0.002385	-0.41697	0.311	0.463	1	12
Dot1l	0.002397	-0.4426	0.168	0.28	1	12
Stim1	0.002415	0.303595	0.101	0.029	1	12
Sf3a3	0.002428	-0.38655	0.21	0.281	1	12
Zfp422	0.002434	-0.44561	0.126	0.246	1	12
Gtf2h5	0.002435	-0.41959	0.319	0.439	1	12
Gabarapl1	0.002456	0.277423	0.361	0.215	1	12
Nfe2l2	0.002458	0.333093	0.193	0.083	1	12
Rab22a	0.002468	0.380557	0.235	0.112	1	12
Tagln3	0.002474	-0.52057	0.185	0.295	1	12
Celf1	0.002476	-0.42708	0.269	0.38	1	12
Lsm3	0.002487	-0.43268	0.252	0.359	1	12
Nmt2	0.002506	-0.45746	0.067	0.181	1	12
Sdad1	0.002515	-0.40224	0.084	0.147	1	12
Glo1	0.002518	0.329314	0.227	0.107	1	12
Aamdc	0.002522	0.410276	0.176	0.091	1	12
Lsm4	0.002523	-0.36743	0.403	0.488	1	12
Rnf165	0.002534	-0.47332	0.059	0.173	1	12
Mettl21a	0.002585	0.294303	0.118	0.037	1	12
Kdm1a	0.00259	-0.46779	0.227	0.365	1	12
Gpsm1	0.002598	-0.46539	0.076	0.176	1	12
Tmem50b	0.002603	0.252501	0.101	0.028	1	12
Zdhhc1	0.002607	0.343726	0.143	0.06	1	12
Mef2c	0.002633	0.422604	0.118	0.068	1	12
4933426M	0.002649	0.33205	0.134	0.046	1	12
Timm50	0.002651	-0.44682	0.134	0.237	1	12
Tmem30a	0.002651	0.339972	0.319	0.178	1	12
Tbca	0.002658	-0.40122	0.311	0.42	1	12
Rars	0.002666	-0.36521	0.126	0.184	1	12
Akap6	0.002691	-0.41239	0.092	0.219	1	12
Phf20	0.002699	-0.48993	0.101	0.22	1	12
Commd4	0.002719	-0.36736	0.168	0.251	1	12
Rps15	0.002735	-0.26936	0.739	0.788	1	12

Scaper	0.002761	-0.32765	0.067	0.111	1	12
Srsf10	0.002786	-0.42029	0.193	0.331	1	12
Scamp2	0.002798	0.397978	0.261	0.134	1	12
Fech	0.002821	0.275106	0.109	0.035	1	12
Rrs1	0.002834	-0.31853	0.076	0.19	1	12
Atxn7l2	0.002848	-0.36091	0.017	0.103	1	12
Rgs12	0.00286	-0.42126	0.042	0.146	1	12
Polr2e	0.002877	-0.43328	0.176	0.325	1	12
Cd2bp2	0.002888	-0.38842	0.05	0.136	1	12
Pgls	0.002889	-0.33192	0.303	0.35	1	12
Capzb	0.002917	0.280434	0.513	0.349	1	12
Bcl2l13	0.002944	-0.3988	0.025	0.115	1	12
Prmt1	0.002951	-0.34103	0.118	0.225	1	12
Cenpw	0.002972	-0.39552	0.05	0.128	1	12
Zfp462	0.002973	-0.44735	0.067	0.156	1	12
Dzip3	0.002974	-0.45105	0.067	0.168	1	12
Nfyb	0.002977	-0.3726	0.168	0.235	1	12
Thsd7a	0.00298	-0.45138	0.042	0.127	1	12
Rbmx2	0.002989	-0.40831	0.025	0.115	1	12
Cenpp	0.002989	-0.36871	0.025	0.114	1	12
Rfc1	0.003048	-0.46507	0.185	0.331	1	12
Bag6	0.003056	-0.32903	0.143	0.181	1	12
Zfp326	0.003059	-0.40035	0.311	0.41	1	12
Rnaseh2b	0.003068	-0.44948	0.109	0.237	1	12
Pfn2	0.003076	-0.46901	0.143	0.241	1	12
Gucd1	0.003083	0.271376	0.126	0.044	1	12
Serf1	0.003084	-0.40211	0.21	0.281	1	12
Ccdc12	0.003093	0.400996	0.227	0.112	1	12
Mcm4	0.003097	-0.41569	0.05	0.138	1	12
Bach1	0.003098	0.303659	0.185	0.079	1	12
Tceal3	0.003186	-0.39312	0.034	0.111	1	12
Atp5k	0.003198	-0.32417	0.395	0.455	1	12
Fam213b	0.003226	-0.47952	0.05	0.158	1	12
Kmt2e	0.003226	-0.3915	0.454	0.569	1	12
Gnao1	0.003229	-0.49407	0.176	0.288	1	12
Cul7	0.003239	0.312789	0.109	0.033	1	12
Psma6	0.00324	-0.34642	0.37	0.452	1	12
Lhfpl4	0.003286	-0.35554	0.042	0.108	1	12
Limd1	0.003288	0.277259	0.168	0.07	1	12
Gstm5	0.003319	-0.45579	0.193	0.323	1	12
Xpo1	0.003324	-0.44888	0.118	0.228	1	12
Rnd2	0.003324	-0.48784	0.059	0.159	1	12
Appbp2	0.003346	-0.28907	0.168	0.199	1	12
Erp44	0.003352	0.323596	0.227	0.108	1	12
Skiv2l2	0.003366	-0.43806	0.059	0.164	1	12
Bag1	0.003367	-0.38896	0.193	0.346	1	12
Topors	0.003376	-0.37116	0.101	0.183	1	12
Mki67ip	0.003412	-0.39983	0.176	0.244	1	12

Prdx6	0.003445	-0.41682	0.235	0.326	1	12
Nrm	0.003466	-0.36436	0.067	0.17	1	12
Elf2	0.003481	-0.28573	0.168	0.198	1	12
Slc35a2	0.003491	0.306504	0.109	0.035	1	12
Camk1	0.003494	0.284458	0.143	0.055	1	12
Snap23	0.003499	0.262745	0.118	0.041	1	12
Sacs	0.003539	-0.41163	0.076	0.191	1	12
Rad51ap1	0.003545	-0.45746	0.076	0.167	1	12
2700081O	0.003625	-0.40584	0.042	0.111	1	12
Arhgef7	0.003625	-0.33625	0.101	0.149	1	12
Agrn	0.003696	0.307513	0.176	0.075	1	12
Arrb2	0.003743	-0.35609	0.042	0.119	1	12
Stim2	0.003743	0.381121	0.202	0.095	1	12
Gins4	0.003771	-0.33826	0.067	0.128	1	12
Cdk2ap2	0.003799	0.338134	0.227	0.11	1	12
Uba1	0.003848	-0.41999	0.16	0.272	1	12
Shmt1	0.003865	-0.35242	0.025	0.103	1	12
Peo1	0.003873	-0.40729	0.042	0.124	1	12
Dhx15	0.003876	-0.39416	0.277	0.417	1	12
Afg3l1	0.003891	-0.2934	0.134	0.174	1	12
Rnf130	0.003911	0.381009	0.227	0.112	1	12
Ube2g2	0.004001	-0.38652	0.042	0.125	1	12
St13	0.004006	-0.32766	0.378	0.439	1	12
Sumf1	0.004029	0.265325	0.109	0.034	1	12
Schip1	0.004047	-0.43994	0.067	0.183	1	12
Tmbim4	0.004059	0.349295	0.252	0.143	1	12
Nav2	0.004074	-0.41066	0.025	0.105	1	12
Sox9	0.004076	-0.44865	0.143	0.255	1	12
Zfp292	0.004105	-0.43964	0.218	0.342	1	12
Pard3	0.004143	0.388007	0.227	0.125	1	12
Cnpy2	0.004145	0.38296	0.471	0.342	1	12
Ptplad1	0.004159	-0.37047	0.185	0.251	1	12
Zfp644	0.00423	-0.46406	0.109	0.208	1	12
Gpr107	0.004259	0.303164	0.134	0.056	1	12
Rbm17	0.004342	-0.35925	0.294	0.39	1	12
Mrgbp	0.004359	-0.45768	0.076	0.184	1	12
Ncor1	0.004362	-0.34831	0.454	0.556	1	12
Atxn2l	0.004376	-0.44127	0.109	0.229	1	12
Clvs1	0.004419	-0.4317	0.034	0.129	1	12
BC034090	0.004446	-0.38817	0.059	0.128	1	12
H2afy2	0.004474	-0.39679	0.227	0.338	1	12
Arhgap11a	0.004494	-0.48891	0.076	0.171	1	12
Ano6	0.004537	0.418519	0.185	0.09	1	12
Tmem106b	0.004569	0.317237	0.168	0.071	1	12
Snhg5	0.004603	-0.38691	0.261	0.381	1	12
Copb2	0.004624	0.402084	0.336	0.217	1	12
Wasf1	0.004703	-0.33221	0.034	0.11	1	12
Gar1	0.004745	-0.43311	0.092	0.209	1	12

Fut11	0.004846	0.299487	0.143	0.054	1	12
Cdkn1c	0.004861	0.358583	0.151	0.062	1	12
Glce	0.004861	-0.42155	0.109	0.177	1	12
Ctnnbl1	0.004872	-0.43105	0.076	0.185	1	12
Cdk19	0.004882	-0.36076	0.05	0.154	1	12
Nolc1	0.004886	-0.40459	0.311	0.409	1	12
Ssr4	0.004894	0.273375	0.538	0.375	1	12
Nucb2	0.004933	0.304279	0.16	0.069	1	12
Magi3	0.004946	0.319192	0.151	0.059	1	12
Mrpl18	0.004964	-0.43917	0.218	0.354	1	12
Hnrnpul2	0.004999	-0.41401	0.168	0.257	1	12
Chaf1a	0.005051	-0.33684	0.067	0.169	1	12
Gm11541	0.005066	-0.38221	0.034	0.128	1	12
Dync1i2	0.005104	-0.28343	0.538	0.568	1	12
Cd63-ps	0.00511	0.339709	0.235	0.118	1	12
Tmsb10	0.005157	-0.26871	0.639	0.664	1	12
Snrpg	0.005212	-0.37874	0.244	0.335	1	12
1700025G(	0.00531	-0.46468	0.134	0.264	1	12
Shmt2	0.005327	-0.32544	0.042	0.12	1	12
Ddrgk1	0.005335	0.27731	0.303	0.187	1	12
Gart	0.00534	-0.40043	0.076	0.166	1	12
Dnajc7	0.005343	-0.43462	0.176	0.281	1	12
Mpp6	0.005346	-0.36591	0.059	0.162	1	12
Rcbtb1	0.005357	-0.3255	0.05	0.105	1	12
Psmc3ip	0.00543	-0.40713	0.042	0.132	1	12
Bcl2	0.00547	0.327321	0.101	0.032	1	12
Necap2	0.00547	0.299126	0.126	0.044	1	12
Sec16a	0.005494	0.269292	0.101	0.031	1	12
Cdc123	0.005522	-0.3834	0.235	0.313	1	12
Tro	0.005575	-0.41144	0.034	0.126	1	12
1500009L1	0.005603	0.354753	0.101	0.038	1	12
Rps18	0.005624	-0.36253	0.345	0.429	1	12
Prkar2a	0.005641	-0.30415	0.084	0.135	1	12
Scfd1	0.00574	0.267354	0.235	0.122	1	12
Prdx3	0.00575	0.357766	0.168	0.074	1	12
Hspbp1	0.005777	-0.33498	0.025	0.112	1	12
Mlf2	0.005785	-0.36605	0.21	0.28	1	12
Pkm	0.005858	-0.37425	0.277	0.43	1	12
Cep110	0.00588	-0.4435	0.059	0.163	1	12
Ccdc41	0.005906	-0.3789	0.134	0.254	1	12
Kmt2c	0.005944	-0.34322	0.218	0.296	1	12
Atat1	0.00595	-0.33432	0.042	0.104	1	12
Cbx3	0.006011	-0.41229	0.168	0.274	1	12
D19Bwg13	0.006043	-0.4211	0.151	0.274	1	12
Tmem179b	0.006044	0.314171	0.134	0.051	1	12
Ywhag	0.006059	-0.45028	0.143	0.241	1	12
Mdga1	0.00606	-0.42289	0.042	0.136	1	12
Psmc6	0.006073	-0.33356	0.277	0.34	1	12

Ubqln1	0.006098	-0.41905	0.176	0.305	1	12
Ddr1	0.006107	-0.42029	0.042	0.136	1	12
Gtf2e2	0.006135	-0.37061	0.109	0.178	1	12
Pabpn1	0.00617	-0.4349	0.084	0.194	1	12
Ddost	0.006262	0.35974	0.387	0.269	1	12
Blm	0.00628	-0.35767	0.025	0.111	1	12
Tspan7	0.006294	0.355256	0.21	0.105	1	12
Lipa	0.006295	0.287873	0.101	0.031	1	12
AU022252	0.006326	0.30047	0.143	0.06	1	12
Sdf4	0.006327	0.327322	0.361	0.221	1	12
Larp4b	0.006342	-0.39657	0.084	0.171	1	12
Uchl1	0.006351	-0.40351	0.202	0.335	1	12
Rad50	0.006379	-0.44029	0.101	0.166	1	12
Rdx	0.006413	-0.28203	0.546	0.604	1	12
1700011J1	0.006418	0.3199	0.143	0.059	1	12
Pde7a	0.006432	-0.37771	0.034	0.112	1	12
Calm3	0.006442	-0.33375	0.429	0.561	1	12
Unc50	0.006467	0.354642	0.235	0.124	1	12
Trim2	0.006508	-0.43306	0.176	0.283	1	12
Phf14	0.006543	-0.39678	0.277	0.375	1	12
Specc1	0.006592	0.368285	0.126	0.048	1	12
Rwdd4a	0.006618	-0.28795	0.059	0.109	1	12
Clasp2	0.006624	-0.42507	0.084	0.168	1	12
Hat1	0.006628	-0.40612	0.067	0.155	1	12
Rbm26	0.006638	-0.3764	0.227	0.297	1	12
Exosc8	0.006707	-0.36124	0.092	0.21	1	12
Boc	0.006736	-0.42978	0.067	0.156	1	12
Lbr	0.006747	-0.41705	0.084	0.197	1	12
Ssbp3	0.006786	-0.40306	0.101	0.167	1	12
Hspd1	0.006817	-0.35572	0.235	0.365	1	12
Siva1	0.006857	-0.42135	0.16	0.272	1	12
Cdc40	0.006991	-0.36873	0.076	0.17	1	12
Eps8	0.007011	0.347434	0.101	0.031	1	12
Cirbp	0.00705	-0.30084	0.319	0.355	1	12
Atp5o	0.007051	-0.31215	0.504	0.587	1	12
Gatad2b	0.007053	-0.34526	0.05	0.121	1	12
Pafah1b2	0.007054	-0.37324	0.185	0.255	1	12
Mrpl28	0.007101	-0.38608	0.202	0.289	1	12
Smarcad1	0.007152	-0.39096	0.084	0.168	1	12
Mak16	0.007208	-0.42118	0.109	0.225	1	12
Glud1	0.007244	0.346587	0.261	0.14	1	12
Rwdd1	0.007246	-0.32947	0.227	0.276	1	12
Jakmip2	0.007262	-0.45378	0.067	0.157	1	12
Btbd17	0.007266	-0.45265	0.076	0.16	1	12
1700037H(	0.007284	-0.31362	0.05	0.11	1	12
Sart3	0.007362	-0.41302	0.109	0.225	1	12
Cyb5	0.007368	0.38728	0.311	0.186	1	12
Fbxo5	0.007474	-0.41281	0.059	0.16	1	12

Gmppa	0.007477	0.25149	0.118	0.042	1	12
Cdca7l	0.007497	-0.34166	0.025	0.104	1	12
Setbp1	0.007501	-0.42371	0.059	0.16	1	12
Ube2b	0.007505	-0.32636	0.429	0.521	1	12
Timm13	0.007505	-0.31422	0.395	0.454	1	12
Dnph1	0.007546	-0.32629	0.034	0.123	1	12
Mvb12a	0.007617	0.365049	0.16	0.072	1	12
Hadh	0.007623	0.319023	0.168	0.073	1	12
Mphosph1(	0.00764	-0.45369	0.118	0.232	1	12
Pald1	0.007655	0.251605	0.16	0.073	1	12
Gstp1	0.007695	0.364622	0.277	0.154	1	12
Prdm8	0.007707	-0.46118	0.042	0.127	1	12
Nmral1	0.007715	-0.33964	0.092	0.188	1	12
Sec11a	0.007737	0.364998	0.387	0.251	1	12
Cox6b1	0.007821	-0.26569	0.689	0.742	1	12
Tmem123	0.007866	0.340264	0.151	0.083	1	12
Exoc2	0.007866	-0.35014	0.034	0.116	1	12
Arpc2	0.007947	0.275735	0.655	0.507	1	12
Emc3	0.008018	0.29646	0.269	0.148	1	12
Arhgap21	0.008021	-0.41959	0.084	0.164	1	12
Ncor2	0.008059	-0.25349	0.143	0.168	1	12
Tmco1	0.008109	0.316102	0.378	0.24	1	12
Prps1	0.00811	-0.35046	0.076	0.143	1	12
Vezf1	0.008167	-0.37905	0.252	0.342	1	12
Bud31	0.008168	-0.29532	0.269	0.31	1	12
Med19	0.008216	-0.43291	0.21	0.318	1	12
Ddx39	0.00823	-0.35203	0.084	0.187	1	12
Pcif1	0.008235	-0.38749	0.092	0.197	1	12
Aes	0.008396	-0.28175	0.185	0.212	1	12
Ttc14	0.008489	-0.43667	0.143	0.262	1	12
Trpc4ap	0.008507	-0.41816	0.151	0.198	1	12
Vapb	0.00854	0.345162	0.193	0.092	1	12
Psmb3	0.008627	-0.3206	0.176	0.259	1	12
Bap1	0.00864	-0.34984	0.025	0.108	1	12
Yif1a	0.008661	0.324069	0.286	0.178	1	12
Rnf181	0.008726	0.372775	0.176	0.099	1	12
Kctd10	0.008733	0.271864	0.134	0.053	1	12
Ndufaf2	0.008764	-0.42315	0.101	0.203	1	12
Tceb1	0.008771	-0.33665	0.294	0.366	1	12
Vrk1	0.008791	-0.37542	0.059	0.148	1	12
Csde1	0.008792	-0.35283	0.345	0.436	1	12
Actr6	0.00882	-0.30051	0.05	0.107	1	12
Cpsf2	0.008824	-0.43422	0.126	0.237	1	12
Snrpb	0.008826	-0.28123	0.521	0.573	1	12
Rpl18a	0.008834	-0.27489	0.37	0.4	1	12
Ost4	0.008867	0.364789	0.303	0.187	1	12
2310039H(	0.008896	0.298053	0.202	0.1	1	12
Tsr3	0.008898	-0.25236	0.076	0.102	1	12

Snhg6	0.008923	-0.39601	0.168	0.259	1	12
Gm9843	0.008952	-0.27191	0.193	0.227	1	12
Cfl2	0.009014	-0.31032	0.345	0.409	1	12
Chst15	0.009023	0.339362	0.168	0.077	1	12
Apbb2	0.009047	0.332031	0.109	0.039	1	12
Rbm34	0.009115	-0.33716	0.076	0.169	1	12
Pdcd2l	0.009126	-0.33221	0.059	0.131	1	12
Ehbp1	0.00916	-0.35241	0.05	0.137	1	12
Qsox1	0.009169	0.285322	0.168	0.076	1	12
Nxf1	0.009192	-0.30691	0.134	0.185	1	12
Fmnl2	0.009317	0.29407	0.277	0.156	1	12
Patz1	0.009319	-0.34037	0.025	0.105	1	12
Ick	0.00937	-0.3747	0.076	0.147	1	12
Actn1	0.009427	0.311714	0.151	0.063	1	12
Ankrd11	0.009496	-0.3612	0.395	0.473	1	12
Med30	0.009506	-0.39216	0.118	0.204	1	12
Ptms	0.00955	-0.33895	0.353	0.474	1	12
Ssbp4	0.00973	0.333233	0.143	0.06	1	12
Parp2	0.009751	-0.37565	0.101	0.17	1	12
Eif3c	0.009755	-0.30063	0.529	0.603	1	12
Psmg1	0.009793	-0.34522	0.042	0.118	1	12
Ccdc90b	0.009915	-0.37284	0.092	0.16	1	12
Gprasp1	0.009917	-0.30883	0.143	0.206	1	12
Rsrc2	0.009935	-0.31218	0.42	0.548	1	12
Tmx4	0.009986	-0.34356	0.126	0.177	1	12
Dnajc10	0.009989	0.315583	0.244	0.133	1	12
Meg3	7.1E-182	3.380973	0.961	0.09	1.2E-177	13
Snap25	3E-148	2.244034	0.864	0.278	4.9E-144	13
Syt4	6.2E-138	2.69085	0.699	0.016	1E-133	13
Chgb	6.4E-130	2.391751	0.845	0.237	1.1E-125	13
Gabra6	4.4E-124	2.243927	0.563	0.002	7.4E-120	13
Vsnl1	1.4E-110	2.1039	0.573	0.005	2.3E-106	13
Chn2	2.6E-109	2.382298	0.641	0.01	4.4E-105	13
Syt1	1.3E-105	2.474755	0.67	0.029	2.1E-101	13
Olfm1	3.3E-103	2.081746	0.796	0.195	5.5E-99	13
Atp1b1	6.12E-98	2.426384	0.68	0.048	1.02E-93	13
Nrxn3	1.85E-97	2.275154	0.709	0.032	3.09E-93	13
Eno2	2.09E-89	1.914055	0.524	0.007	3.49E-85	13
Calb2	8.32E-88	2.269783	0.602	0.019	1.39E-83	13
Lin7a	7.98E-86	2.109482	0.689	0.048	1.33E-81	13
Cox8a	1.95E-83	1.310372	0.913	0.699	3.25E-79	13
Camk4	9.29E-82	2.154256	0.641	0.04	1.55E-77	13
Rph3a	2.16E-81	1.956787	0.602	0.036	3.6E-77	13
Selm	3.81E-79	1.889473	0.718	0.171	6.36E-75	13
Gabra1	6.41E-68	1.84734	0.369	0.003	1.07E-63	13
Cbln1	4.67E-67	1.831269	0.65	0.086	7.79E-63	13
Calm1	7.36E-67	1.10772	0.99	0.861	1.23E-62	13
Rps5	2.57E-66	-1.52085	0.583	0.956	4.29E-62	13

Cbln3	3.67E-66	1.704428	0.35	0.002	6.13E-62	13
Dnm1	1.55E-59	1.745458	0.398	0.01	2.59E-55	13
Adcy1	2.76E-59	1.873143	0.476	0.023	4.61E-55	13
Malat1	4.21E-59	1.182062	1	0.954	7.01E-55	13
Nrep	3.52E-56	1.394048	0.883	0.567	5.86E-52	13
Etv1	5.92E-56	1.591145	0.437	0.025	9.87E-52	13
Gabrd	1.71E-55	1.575888	0.359	0.005	2.85E-51	13
Atp2b1	7.3E-55	1.299283	0.874	0.569	1.22E-50	13
Rnf112	3.32E-54	1.496717	0.35	0.005	5.54E-50	13
Atp6v1g2	4.66E-54	1.725625	0.408	0.023	7.78E-50	13
Scn2a1	1.03E-53	1.725735	0.408	0.015	1.71E-49	13
Ppp3ca	1.72E-53	1.415964	0.786	0.375	2.87E-49	13
Sncb	1.14E-51	1.592211	0.398	0.012	1.9E-47	13
Chchd10	2.93E-51	1.758663	0.447	0.031	4.89E-47	13
Cacnb4	3.55E-51	1.75603	0.427	0.031	5.92E-47	13
Rps9	8.01E-51	-1.38553	0.495	0.931	1.34E-46	13
Snca	1.31E-50	1.879441	0.534	0.057	2.18E-46	13
Kcnd2	7.6E-50	1.659234	0.466	0.023	1.27E-45	13
Stmn2	1.49E-49	1.268328	0.932	0.55	2.49E-45	13
Rps14	1.73E-49	-1.20768	0.641	0.952	2.89E-45	13
Tubb5	3.72E-49	-1.52758	0.437	0.917	6.21E-45	13
Eps8	1.46E-48	1.631554	0.437	0.029	2.43E-44	13
Sfrp1	4.44E-48	-2.06337	0.184	0.815	7.41E-44	13
Chl1	5.25E-48	1.481959	0.359	0.013	8.76E-44	13
Ntm	6.36E-48	1.700084	0.602	0.142	1.06E-43	13
Tmem59l	6.67E-48	1.429332	0.34	0.007	1.11E-43	13
Aplp2	4.01E-47	1.371826	0.699	0.32	6.69E-43	13
Actb	4.6E-47	-0.92104	0.806	0.98	7.67E-43	13
Celf4	5E-47	1.420975	0.874	0.317	8.33E-43	13
Napb	7.71E-47	1.605156	0.505	0.06	1.29E-42	13
Mgst3	2.1E-46	1.53135	0.534	0.093	3.5E-42	13
Camkk2	5.34E-46	1.569579	0.379	0.021	8.91E-42	13
Ndrg3	1.31E-45	1.488508	0.583	0.113	2.19E-41	13
Nap1l5	2.05E-44	1.659257	0.505	0.078	3.42E-40	13
Unc13c	2.18E-44	1.383708	0.291	0.005	3.63E-40	13
Gm2694	2.88E-44	1.343295	0.718	0.356	4.8E-40	13
Syn2	7.84E-44	1.682705	0.505	0.082	1.31E-39	13
Grin2c	3.71E-43	1.058516	0.233	0.002	6.18E-39	13
Rps3	4.23E-43	-1.23257	0.524	0.924	7.05E-39	13
Tspan7	2.77E-42	1.547276	0.553	0.103	4.62E-38	13
Nptxr	7.48E-42	1.554431	0.388	0.033	1.25E-37	13
Pak1	2.19E-41	1.629129	0.466	0.057	3.65E-37	13
Atp1a3	5.51E-41	1.531537	0.417	0.03	9.18E-37	13
Gpm6a	1.5E-39	1.247689	0.845	0.406	2.49E-35	13
Hsp90ab1	2.86E-39	-0.63029	0.981	0.998	4.78E-35	13
Cadm3	7.11E-39	1.555454	0.573	0.138	1.19E-34	13
Rpl13a	7.23E-39	-1.18474	0.592	0.93	1.21E-34	13
Kcnk3	1.38E-38	1.078322	0.204	0.001	2.3E-34	13

Tuba4a	2.44E-38	1.348323	0.282	0.017	4.07E-34	13
Cd24a	1.58E-37	-1.76239	0.039	0.637	2.63E-33	13
Rps26	5.47E-37	-1.4091	0.359	0.85	9.13E-33	13
Rbfox3	9.39E-37	1.19999	0.816	0.492	1.57E-32	13
Igfbpl1	2.67E-36	-1.93614	0.058	0.651	4.45E-32	13
Rpl32	8.09E-36	-1.20845	0.495	0.891	1.35E-31	13
Btbd3	8.6E-36	1.377267	0.476	0.088	1.43E-31	13
Rpl4	1.29E-35	-1.1471	0.534	0.91	2.16E-31	13
Kcnc1	2.26E-34	1.46497	0.476	0.087	3.78E-30	13
Gas7	2.55E-34	1.243782	0.223	0.005	4.25E-30	13
Camk2b	5.81E-34	1.500283	0.456	0.088	9.69E-30	13
Nrxn1	7E-34	1.281861	0.757	0.315	1.17E-29	13
Rplp1	1.04E-33	-1.21117	0.476	0.879	1.74E-29	13
Diras2	1.43E-33	1.368441	0.311	0.013	2.38E-29	13
Purb	3.27E-33	1.03087	0.796	0.547	5.45E-29	13
Grm4	4.54E-33	1.220591	0.243	0.005	7.56E-29	13
Tmsb4x	6.93E-33	-1.10076	0.641	0.95	1.16E-28	13
Ablim1	7.92E-33	1.4419	0.32	0.018	1.32E-28	13
Eef1a1	1.61E-32	-1.15899	0.466	0.886	2.68E-28	13
Pkib	1.98E-32	1.294106	0.311	0.017	3.29E-28	13
Hnrnpab	2.21E-32	-1.32435	0.34	0.818	3.68E-28	13
Gnb2l1	3.2E-32	-1.13177	0.427	0.874	5.34E-28	13
Tmod1	3.53E-32	1.272143	0.282	0.013	5.88E-28	13
Ckmt1	6.94E-32	0.988242	0.223	0.003	1.16E-27	13
Ndrg4	1.87E-31	1.496908	0.369	0.049	3.12E-27	13
Cd63	2.16E-31	-1.41419	0.214	0.758	3.61E-27	13
Atp6v1a	3.19E-31	1.130309	0.563	0.286	5.32E-27	13
Nsf	4.74E-31	1.318829	0.398	0.084	7.91E-27	13
Ccnd2	6.19E-31	-1.80535	0.117	0.665	1.03E-26	13
CRE_RECOM	1.05E-30	-1.83726	0.291	0.771	1.75E-26	13
Snhg11	1.14E-30	1.951615	0.165	0.007	1.91E-26	13
Tagln3	1.43E-30	1.217663	0.67	0.292	2.39E-26	13
Dlgap1	1.6E-30	1.463814	0.408	0.065	2.66E-26	13
Rpl8	3.29E-30	-1.01809	0.592	0.899	5.48E-26	13
Cadps2	3.64E-30	1.503228	0.408	0.059	6.08E-26	13
Tpi1	4.13E-30	1.212873	0.456	0.119	6.88E-26	13
Rps11	5.24E-30	-1.33667	0.282	0.78	8.74E-26	13
Rps24	6.74E-30	-1.20319	0.34	0.823	1.12E-25	13
Fbxw7	2.57E-29	1.362202	0.456	0.117	4.28E-25	13
Eef1a2	2.95E-29	1.148926	0.233	0.009	4.92E-25	13
Rps20	3.28E-29	-1.30619	0.301	0.78	5.48E-25	13
Kcna1	3.41E-29	1.295547	0.34	0.025	5.69E-25	13
Ndufa4	3.71E-29	0.727909	0.864	0.801	6.19E-25	13
Atp2b2	3.87E-29	1.323084	0.427	0.09	6.45E-25	13
Spock2	5.21E-29	1.293558	0.427	0.063	8.69E-25	13
Psd3	5.71E-29	1.320465	0.35	0.044	9.53E-25	13
Mdh1	6.51E-29	0.880908	0.563	0.451	1.09E-24	13
Nrxn2	6.63E-29	1.330471	0.427	0.088	1.11E-24	13

Rps19	6.96E-29	-1.34973	0.272	0.756	1.16E-24	13
Rpl22	7.79E-29	-1.25616	0.291	0.791	1.3E-24	13
Frrs1l	8.64E-29	1.256298	0.544	0.189	1.44E-24	13
Golga7b	1.12E-28	0.85568	0.165	0.001	1.88E-24	13
Cplx2	1.42E-28	1.035925	0.825	0.493	2.37E-24	13
Crtam	3E-28	0.937064	0.146	0	5E-24	13
Prdx5	3.31E-28	1.055501	0.495	0.262	5.52E-24	13
Gabrg2	4.09E-28	1.331635	0.35	0.048	6.82E-24	13
E130114P1	5.12E-28	-1.7092	0.078	0.587	8.54E-24	13
Tenm1	6.83E-28	1.305851	0.291	0.017	1.14E-23	13
Prune2	1.04E-27	1.111049	0.223	0.008	1.73E-23	13
Rims1	1.21E-27	1.248511	0.33	0.026	2.02E-23	13
Slc16a11	1.85E-27	0.887872	0.155	0.001	3.08E-23	13
Bcl2l15	2.09E-27	1.107948	0.184	0.003	3.49E-23	13
Phyhip	2.84E-27	1.025509	0.175	0.002	4.74E-23	13
Sv2a	3.45E-27	1.37017	0.408	0.07	5.76E-23	13
Cd9	7.73E-27	-1.57765	0.01	0.487	1.29E-22	13
Rplp0	9.23E-27	-1.03378	0.495	0.874	1.54E-22	13
Darc	1.33E-26	1.424498	0.301	0.033	2.22E-22	13
Aldoa	1.57E-26	1.117913	0.563	0.255	2.62E-22	13
Cox6a1	3.21E-26	0.723884	0.883	0.76	5.35E-22	13
Atp5g3	4.04E-26	0.842215	0.728	0.516	6.74E-22	13
Car4	4.71E-26	1.104216	0.204	0.007	7.85E-22	13
Cog7	5.05E-26	-1.50568	0.078	0.587	8.42E-22	13
Kcnj9	7.22E-26	0.940542	0.184	0.004	1.2E-21	13
H2afv	7.47E-26	-1.34556	0.252	0.73	1.25E-21	13
Scg2	7.68E-26	1.603123	0.311	0.05	1.28E-21	13
D3Bwg056	1.08E-25	1.305956	0.32	0.029	1.81E-21	13
Rpl41	1.2E-25	-1.1383	0.311	0.783	2E-21	13
Slc12a5	2.42E-25	0.788059	0.146	0.001	4.03E-21	13
Jph4	3.45E-25	1.316397	0.417	0.066	5.76E-21	13
Car10	5.96E-25	1.134739	0.359	0.038	9.94E-21	13
Prkce	6.29E-25	1.327153	0.33	0.063	1.05E-20	13
Cox4i1	6.49E-25	0.60705	0.893	0.877	1.08E-20	13
2010107G1	2.66E-24	1.094848	0.301	0.026	4.44E-20	13
Dnm3	3.28E-24	1.210923	0.272	0.024	5.46E-20	13
Rnf152	3.66E-24	1.22949	0.282	0.037	6.11E-20	13
Nptn	4.56E-24	1.092048	0.456	0.186	7.61E-20	13
Marcksl1	5.49E-24	-0.96733	0.223	0.725	9.16E-20	13
Stmn3	1.46E-23	0.754524	0.767	0.621	2.44E-19	13
Neurod2	1.63E-23	1.26517	0.417	0.065	2.72E-19	13
Rpl14	1.81E-23	-1.22079	0.233	0.699	3.03E-19	13
Dpp6	1.85E-23	1.232069	0.282	0.025	3.08E-19	13
Ddah2	1.94E-23	-1.25014	0.204	0.688	3.23E-19	13
Ezr	2.43E-23	-1.34365	0.029	0.44	4.05E-19	13
C1qtnf4	3.12E-23	1.153297	0.233	0.013	5.21E-19	13
Nnat	5.07E-23	-1.25368	0.233	0.712	8.46E-19	13
Rplp2	5.21E-23	-0.98164	0.456	0.842	8.69E-19	13

Cadm2	7.16E-23	1.399921	0.32	0.046	1.19E-18	13
Rps6ka1	8.38E-23	0.983935	0.184	0.007	1.4E-18	13
Slc17a7	1.12E-22	0.707472	0.117	0	1.86E-18	13
Neurod1	1.83E-22	0.664097	0.874	0.53	3.05E-18	13
Bend6	2.8E-22	1.001404	0.243	0.016	4.67E-18	13
Rps21	3.91E-22	-0.98322	0.476	0.835	6.53E-18	13
Rab3c	5.18E-22	1.130317	0.427	0.146	8.65E-18	13
Mybpc3	6.36E-22	0.983174	0.136	0.002	1.06E-17	13
Pabpc1	7.02E-22	-0.88877	0.553	0.88	1.17E-17	13
Nfia	1.85E-21	-1.13235	0.369	0.776	3.09E-17	13
Camk2d	2.84E-21	1.143578	0.369	0.072	4.73E-17	13
Cnr1	4.07E-21	1.163907	0.32	0.056	6.79E-17	13
Kcnt1	4.83E-21	0.944983	0.165	0.004	8.06E-17	13
Barhl1	7.14E-21	-1.40874	0.078	0.524	1.19E-16	13
Nasp	8.05E-21	-1.27119	0.184	0.642	1.34E-16	13
Nfib	9.39E-21	-0.86406	0.728	0.934	1.57E-16	13
Hmgn1	1.01E-20	-1.059	0.155	0.624	1.68E-16	13
Sept3	1.06E-20	0.974223	0.709	0.39	1.76E-16	13
Sybu	1.34E-20	0.773626	0.146	0.004	2.24E-16	13
Ncl	1.68E-20	-0.78844	0.65	0.897	2.8E-16	13
Cnnm1	3.16E-20	0.856277	0.155	0.007	5.28E-16	13
Grm1	3.18E-20	1.114857	0.243	0.018	5.3E-16	13
Rps15	3.68E-20	-0.97317	0.408	0.79	6.13E-16	13
Ezh2	3.83E-20	-1.2149	0.155	0.612	6.39E-16	13
Cplx1	4.08E-20	1.087654	0.437	0.136	6.81E-16	13
Rock2	4.15E-20	1.093017	0.485	0.21	6.92E-16	13
Unc80	5.78E-20	1.111887	0.233	0.032	9.64E-16	13
Kif5c	8.27E-20	0.826809	0.718	0.396	1.38E-15	13
Adamts18	8.68E-20	1.010209	0.194	0.01	1.45E-15	13
Rtn1	8.76E-20	0.675239	0.922	0.689	1.46E-15	13
Nptx1	9.23E-20	1.003098	0.223	0.032	1.54E-15	13
Pcsk1n	1.24E-19	1.171488	0.379	0.082	2.07E-15	13
Tceal5	1.4E-19	1.161008	0.252	0.031	2.34E-15	13
Ppfia4	1.69E-19	0.929891	0.204	0.011	2.82E-15	13
Stxbp1	2.15E-19	1.100061	0.485	0.16	3.58E-15	13
Tuba1a	2.61E-19	-0.85062	0.728	0.93	4.36E-15	13
Anp32b	2.63E-19	-0.93512	0.146	0.593	4.39E-15	13
Prkar1b	2.95E-19	0.975034	0.204	0.016	4.93E-15	13
Ppargc1b	3.11E-19	0.75834	0.136	0.003	5.19E-15	13
Spop	3.14E-19	0.96321	0.612	0.313	5.24E-15	13
Necab3	3.84E-19	1.131132	0.311	0.052	6.41E-15	13
Ppp1r14b	4.65E-19	-1.22053	0.019	0.399	7.75E-15	13
Nefl	5.43E-19	1.304912	0.282	0.057	9.05E-15	13
Cpe	8.48E-19	0.854589	0.641	0.454	1.42E-14	13
Cygb	8.59E-19	1.244102	0.311	0.049	1.43E-14	13
Erc1	8.82E-19	1.182205	0.388	0.109	1.47E-14	13
Npm1	1.02E-18	-1.12243	0.214	0.648	1.7E-14	13
Rit2	1.86E-18	0.796661	0.107	0.001	3.11E-14	13

Gm10075	1.95E-18	-0.99811	0.097	0.517	3.25E-14	13
Cend1	2.1E-18	0.964071	0.223	0.018	3.5E-14	13
Basp1	2.29E-18	-0.94685	0.272	0.706	3.82E-14	13
Cdk4	2.39E-18	-1.02216	0.204	0.644	3.99E-14	13
Tubb2b	2.55E-18	-1.30611	0.058	0.467	4.26E-14	13
1700020I1	2.77E-18	0.945364	0.495	0.273	4.62E-14	13
Mex3a	3.17E-18	-1.21991	0.058	0.465	5.29E-14	13
Slc4a4	3.83E-18	0.964968	0.272	0.039	6.39E-14	13
Snrpe	3.96E-18	-0.9654	0.136	0.564	6.6E-14	13
Srebf1	4.56E-18	-1.3444	0	0.335	7.61E-14	13
Atp1b2	4.58E-18	1.187659	0.262	0.046	7.64E-14	13
Dync1i1	4.98E-18	0.740435	0.136	0.003	8.31E-14	13
Hnrnpa2b1	5.74E-18	-0.63514	0.757	0.934	9.57E-14	13
Anp32e	5.79E-18	-1.2124	0.214	0.615	9.66E-14	13
Caln1	5.89E-18	0.978972	0.282	0.043	9.82E-14	13
Eef1b2	6.56E-18	-0.82992	0.311	0.735	1.09E-13	13
Tspyl4	7.67E-18	1.04446	0.398	0.125	1.28E-13	13
Ptma	9.29E-18	-1.08681	0.155	0.584	1.55E-13	13
Mdk	1.02E-17	-1.37706	0.019	0.381	1.7E-13	13
Mpp3	1.12E-17	1.108742	0.291	0.047	1.87E-13	13
Slc29a1	1.18E-17	-1.14109	0.146	0.564	1.96E-13	13
Sox4	1.44E-17	-1.08924	0.165	0.585	2.4E-13	13
Pcdh9	1.47E-17	1.064144	0.243	0.039	2.46E-13	13
Cox7a2	2.84E-17	0.527161	0.689	0.712	4.73E-13	13
Smc2	2.85E-17	-1.38044	0.136	0.547	4.76E-13	13
Cbx5	3.02E-17	-0.99777	0.243	0.655	5.03E-13	13
Itm2b	3.03E-17	0.596179	0.728	0.642	5.06E-13	13
Tshz2	3.91E-17	-1.24197	0	0.321	6.52E-13	13
Fnbp1	4.08E-17	1.064361	0.311	0.071	6.81E-13	13
Psat1	5.01E-17	-1.08171	0.019	0.369	8.35E-13	13
Ppib	5.08E-17	-0.82774	0.282	0.703	8.48E-13	13
Snrpn	5.22E-17	0.9608	0.408	0.156	8.7E-13	13
Srcin1	6.91E-17	1.072077	0.32	0.052	1.15E-12	13
AI848285	7.29E-17	0.864787	0.146	0.007	1.22E-12	13
Atp6v0e	7.5E-17	-1.23505	0.039	0.409	1.25E-12	13
Atp5o	8.01E-17	0.586691	0.67	0.586	1.34E-12	13
Sorl1	8.39E-17	1.00121	0.194	0.022	1.4E-12	13
Pnck	8.52E-17	0.864003	0.175	0.01	1.42E-12	13
Hcfc1r1	9.22E-17	0.964896	0.427	0.191	1.54E-12	13
Vps37b	1.01E-16	-1.14145	0.078	0.472	1.68E-12	13
Fabp5	1.2E-16	-1.06225	0.039	0.405	2.01E-12	13
Pcp4	1.47E-16	1.233707	0.291	0.051	2.45E-12	13
Ccnd1	1.71E-16	-1.32665	0.126	0.524	2.86E-12	13
Rps10	2.04E-16	-0.97063	0.223	0.638	3.4E-12	13
Rpl18a	2.66E-16	-1.06848	0.049	0.402	4.44E-12	13
Rps15a	3.05E-16	-0.99553	0.262	0.659	5.1E-12	13
Bmp1	3.58E-16	1.039973	0.301	0.085	5.96E-12	13
Ndufa13	4.28E-16	0.656894	0.66	0.532	7.14E-12	13

Hpcal1	4.81E-16	1.10654	0.359	0.105	8.03E-12	13
Rps3a1	5.04E-16	-0.90593	0.262	0.669	8.41E-12	13
Cald1	5.13E-16	-1.06638	0.136	0.537	8.56E-12	13
Ypel3	5.89E-16	0.745516	0.583	0.434	9.82E-12	13
Cnn3	5.99E-16	-1.0495	0.068	0.438	9.98E-12	13
Cacna1a	6.24E-16	1.01067	0.301	0.071	1.04E-11	13
Ndufb8	6.81E-16	0.597817	0.563	0.453	1.14E-11	13
Mcm7	7.86E-16	-1.19944	0.039	0.39	1.31E-11	13
Rps18	9.01E-16	-1.07215	0.058	0.43	1.5E-11	13
Rab3a	9.21E-16	1.020655	0.447	0.148	1.54E-11	13
Hn1	1.02E-15	-1.12242	0.155	0.543	1.71E-11	13
Rpl34	1.04E-15	-0.84998	0.243	0.654	1.73E-11	13
Fabp3	1.05E-15	0.96468	0.194	0.015	1.75E-11	13
Prrt1	1.14E-15	0.971711	0.214	0.021	1.9E-11	13
Ramp3	1.15E-15	0.586047	0.107	0.002	1.91E-11	13
Rbfox1	1.25E-15	1.044551	0.33	0.09	2.08E-11	13
Grina	1.27E-15	1.039765	0.33	0.108	2.11E-11	13
Camk2n1	1.31E-15	1.071258	0.33	0.084	2.18E-11	13
App	1.47E-15	0.705793	0.738	0.567	2.45E-11	13
Mapt	1.65E-15	1.049013	0.485	0.214	2.75E-11	13
Trpm3	1.69E-15	0.851561	0.146	0.011	2.82E-11	13
Draxin	1.89E-15	-0.99918	0.165	0.556	3.14E-11	13
Snap91	1.89E-15	0.987684	0.282	0.048	3.15E-11	13
Map1a	2.07E-15	1.043023	0.291	0.072	3.46E-11	13
Miat	2.17E-15	-1.26965	0.126	0.518	3.62E-11	13
Ndufa5	2.38E-15	0.694156	0.583	0.452	3.97E-11	13
Rps27l	2.42E-15	-1.05245	0.107	0.494	4.04E-11	13
Snrpb2	2.5E-15	-0.97186	0.049	0.386	4.16E-11	13
Gm9800	2.82E-15	-1.07771	0.146	0.537	4.7E-11	13
Atp6v0b	3.57E-15	0.853387	0.505	0.265	5.96E-11	13
Atp2b4	3.79E-15	0.711688	0.146	0.006	6.33E-11	13
Syt5	3.88E-15	0.894999	0.155	0.008	6.47E-11	13
Cntn1	4.38E-15	1.019164	0.301	0.05	7.3E-11	13
Zfand5	4.84E-15	0.687553	0.495	0.415	8.08E-11	13
Gnao1	4.9E-15	0.839967	0.476	0.286	8.17E-11	13
Fat2	4.93E-15	0.953176	0.175	0.013	8.22E-11	13
Hjurp	5.85E-15	-1.04801	0.087	0.436	9.76E-11	13
Atp6v1e1	7.04E-15	0.819831	0.563	0.33	1.17E-10	13
Rgs8	7.35E-15	0.680258	0.107	0.003	1.23E-10	13
Caly	7.46E-15	0.668395	0.146	0.006	1.24E-10	13
BC029214	8.2E-15	1.033848	0.311	0.091	1.37E-10	13
Atp6v0e2	8.26E-15	0.822298	0.427	0.259	1.38E-10	13
Clip1	8.64E-15	1.000761	0.311	0.092	1.44E-10	13
Fam210b	9.74E-15	-1.20543	0.01	0.315	1.62E-10	13
Rpl35a	1.02E-14	-0.90351	0.184	0.572	1.7E-10	13
Tspan17	1.17E-14	0.689935	0.117	0.004	1.95E-10	13
Rgs7bp	1.35E-14	0.862818	0.214	0.027	2.26E-10	13
C1ql1	1.48E-14	-1.1039	0	0.28	2.48E-10	13

Cox6b1	1.5E-14	0.54172	0.777	0.741	2.5E-10	13
Syt2	1.53E-14	0.984941	0.146	0.013	2.55E-10	13
Trim2	1.57E-14	0.881702	0.476	0.281	2.63E-10	13
Pbrm1	1.64E-14	-0.83663	0.155	0.532	2.74E-10	13
Fkbp3	1.72E-14	-0.80322	0.388	0.747	2.87E-10	13
Tmpo	2.11E-14	-1.11619	0.097	0.454	3.51E-10	13
Adora1	2.11E-14	0.744417	0.165	0.011	3.52E-10	13
Madd	2.13E-14	0.992454	0.262	0.052	3.56E-10	13
Npc2	2.27E-14	-1.00949	0.097	0.465	3.79E-10	13
E530001K1	2.4E-14	0.544621	0.107	0.002	4E-10	13
St3gal5	2.47E-14	0.911758	0.398	0.192	4.12E-10	13
Pura	2.72E-14	0.85412	0.466	0.285	4.54E-10	13
Snrpf	2.79E-14	-0.81224	0.087	0.429	4.65E-10	13
Eif3f	3.47E-14	-0.82665	0.243	0.632	5.78E-10	13
Uqcr11	4.1E-14	0.604965	0.524	0.481	6.84E-10	13
Fut9	4.25E-14	0.871172	0.408	0.21	7.1E-10	13
Sh3gl2	4.57E-14	0.914713	0.427	0.17	7.62E-10	13
Eif4g2	5.58E-14	-0.69931	0.33	0.707	9.31E-10	13
Serinc1	6.08E-14	0.683607	0.583	0.435	1.01E-09	13
Rpl23	6.5E-14	-0.84855	0.165	0.54	1.08E-09	13
Rora	6.81E-14	1.022585	0.243	0.054	1.14E-09	13
H2afy	7.23E-14	-0.75983	0.214	0.594	1.21E-09	13
Slc25a4	7.41E-14	0.465202	0.806	0.807	1.24E-09	13
Afap1l2	8.04E-14	0.708953	0.126	0.007	1.34E-09	13
Adam11	8.31E-14	0.823317	0.175	0.016	1.39E-09	13
Rpl39	9E-14	-0.85416	0.282	0.648	1.5E-09	13
Mbnl2	1.02E-13	0.942838	0.408	0.177	1.71E-09	13
Ppp1r1a	1.07E-13	0.988073	0.32	0.108	1.78E-09	13
6430573F1	1.14E-13	0.899119	0.126	0.013	1.91E-09	13
Pld5	1.22E-13	0.67636	0.117	0.004	2.04E-09	13
Lmnb1	1.25E-13	-1.03994	0.019	0.301	2.09E-09	13
Ranbp1	1.27E-13	-0.92492	0.32	0.677	2.12E-09	13
Tox3	1.35E-13	-1.02243	0.039	0.347	2.24E-09	13
Prpf40a	1.37E-13	-0.73337	0.194	0.573	2.28E-09	13
Schip1	1.37E-13	0.934789	0.398	0.181	2.29E-09	13
Prdx2	1.45E-13	-0.61279	0.301	0.681	2.42E-09	13
Pfkp	1.82E-13	0.918757	0.214	0.029	3.04E-09	13
Vamp2	1.84E-13	0.826842	0.282	0.071	3.06E-09	13
Gls	1.89E-13	0.915092	0.282	0.123	3.16E-09	13
Snrpg	1.93E-13	-0.96577	0.029	0.336	3.22E-09	13
Pde4a	1.98E-13	0.788822	0.165	0.016	3.31E-09	13
Mycn	2.01E-13	-1.03624	0.049	0.365	3.35E-09	13
Zfp385b	2.03E-13	0.95508	0.175	0.025	3.38E-09	13
Cyc1	2.48E-13	0.669361	0.515	0.412	4.13E-09	13
Dgkd	2.5E-13	0.929966	0.301	0.105	4.16E-09	13
Runx1t1	2.51E-13	0.982234	0.194	0.022	4.18E-09	13
Rps2	2.62E-13	-0.96079	0.097	0.451	4.38E-09	13
Pagr1a	2.65E-13	0.799556	0.301	0.17	4.42E-09	13

Slitrk4	2.83E-13	0.771569	0.107	0.005	4.71E-09	13
Atp5b	3.19E-13	0.505922	0.757	0.712	5.32E-09	13
Cdkn1a	3.33E-13	0.921502	0.311	0.091	5.55E-09	13
Hnrnpd	3.34E-13	-0.88521	0.252	0.612	5.58E-09	13
Rpl26	3.48E-13	-0.75006	0.35	0.703	5.81E-09	13
Gas5	4.47E-13	-0.76631	0.437	0.743	7.46E-09	13
Kif1b	4.49E-13	0.685987	0.66	0.52	7.49E-09	13
Matk	4.77E-13	0.768915	0.155	0.013	7.95E-09	13
Carhsp1	4.84E-13	-0.98413	0	0.255	8.07E-09	13
Dcx	4.91E-13	-0.97213	0.058	0.377	8.18E-09	13
Map3k1	5.09E-13	-0.88248	0.058	0.372	8.49E-09	13
Srsf6	5.14E-13	-0.64525	0.117	0.447	8.57E-09	13
Ndufv3	6.23E-13	0.730562	0.534	0.296	1.04E-08	13
Igfbp5	6.44E-13	0.843879	0.194	0.024	1.07E-08	13
Srsf3	7.23E-13	-0.79819	0.291	0.658	1.21E-08	13
Hsd11b2	7.55E-13	-1.1254	0.01	0.289	1.26E-08	13
Grin1	7.75E-13	0.957249	0.223	0.034	1.29E-08	13
Ier5	8.23E-13	-1.03044	0.068	0.396	1.37E-08	13
Rab6a	8.7E-13	0.723676	0.505	0.327	1.45E-08	13
Scn2b	9.05E-13	0.658191	0.146	0.009	1.51E-08	13
Zeb2	9.21E-13	-0.98574	0	0.25	1.54E-08	13
Dtymk	9.33E-13	-0.99292	0.097	0.44	1.56E-08	13
Il20rb	9.37E-13	0.655798	0.117	0.005	1.56E-08	13
Apex1	9.62E-13	-0.87171	0.107	0.453	1.6E-08	13
Vim	9.64E-13	-1.1477	0.029	0.304	1.61E-08	13
Plcxd3	1.11E-12	0.718541	0.126	0.01	1.85E-08	13
Napa	1.24E-12	0.674838	0.476	0.349	2.07E-08	13
Ryr2	1.36E-12	0.945352	0.184	0.02	2.27E-08	13
Adh5	1.38E-12	-0.98692	0.078	0.406	2.3E-08	13
Zcchc18	1.43E-12	0.863801	0.369	0.169	2.39E-08	13
Klf9	1.53E-12	0.800106	0.476	0.269	2.54E-08	13
Marcks	1.62E-12	-0.51526	0.68	0.917	2.7E-08	13
Egr1	1.67E-12	-1.14428	0.078	0.386	2.79E-08	13
Ran	1.68E-12	-0.83784	0.087	0.415	2.79E-08	13
Banf1	1.79E-12	-0.81432	0.359	0.695	2.99E-08	13
Tmem145	1.94E-12	0.900962	0.252	0.049	3.24E-08	13
Top2a	2.16E-12	-1.34503	0.097	0.429	3.61E-08	13
Scrn1	2.29E-12	0.8593	0.214	0.049	3.82E-08	13
Cox6c	2.47E-12	0.474352	0.806	0.718	4.12E-08	13
Atp1a1	2.58E-12	0.869621	0.311	0.157	4.3E-08	13
Smc4	2.73E-12	-1.06408	0.214	0.568	4.56E-08	13
Ccdc88a	2.73E-12	-0.82702	0.136	0.484	4.56E-08	13
Atp6v1b2	3.08E-12	0.729179	0.359	0.169	5.15E-08	13
Pou3f2	3.19E-12	-1.00341	0.01	0.277	5.32E-08	13
Stx1b	3.21E-12	0.860157	0.184	0.029	5.35E-08	13
Bdnf	3.21E-12	0.694906	0.117	0.007	5.36E-08	13
Zfp467	3.27E-12	0.79179	0.165	0.018	5.45E-08	13
Ppic	3.45E-12	-0.95101	0	0.24	5.75E-08	13

Trp53	3.52E-12	-0.83226	0.029	0.299	5.86E-08	13
Itgb1	3.92E-12	-0.70667	0.078	0.364	6.53E-08	13
Cacna1g	4.01E-12	0.620888	0.107	0.003	6.69E-08	13
Bsg	4.07E-12	0.566128	0.495	0.423	6.78E-08	13
Fez1	4.46E-12	0.715139	0.476	0.331	7.44E-08	13
Sfpq	4.61E-12	-0.66798	0.311	0.672	7.68E-08	13
Prim1	4.91E-12	-1.04361	0.01	0.264	8.19E-08	13
Ly6h	5.12E-12	0.803329	0.184	0.022	8.54E-08	13
Eef2	5.43E-12	-0.66698	0.369	0.718	9.06E-08	13
Birc5	5.51E-12	-1.14236	0.01	0.275	9.19E-08	13
Uqcrq	5.99E-12	0.605436	0.718	0.549	9.99E-08	13
Fnbp1l	6.18E-12	-0.77144	0.146	0.455	1.03E-07	13
Tubb3	6.47E-12	-1.22364	0.155	0.476	1.08E-07	13
Commd1	6.51E-12	-0.78912	0.039	0.299	1.09E-07	13
Atp2b3	6.97E-12	0.697657	0.126	0.006	1.16E-07	13
Dynll2	7.35E-12	0.721121	0.456	0.295	1.23E-07	13
Akap7	7.58E-12	0.889863	0.223	0.047	1.26E-07	13
Micu3	7.76E-12	0.954086	0.272	0.079	1.29E-07	13
Dctpp1	8.39E-12	-1.00378	0.029	0.305	1.4E-07	13
2210016L2	8.46E-12	0.781997	0.476	0.287	1.41E-07	13
Klc1	9.38E-12	0.695802	0.505	0.36	1.56E-07	13
Nap1l1	1.03E-11	-0.81792	0.175	0.512	1.72E-07	13
2810417H1	1.16E-11	-1.13038	0.078	0.393	1.93E-07	13
Fgf14	1.23E-11	0.702709	0.126	0.008	2.05E-07	13
Nop58	1.32E-11	-0.82815	0.252	0.593	2.2E-07	13
Tcf4	1.41E-11	-0.44814	0.709	0.926	2.35E-07	13
Syt7	1.51E-11	0.713295	0.146	0.012	2.52E-07	13
Sh3bgrl	1.56E-11	-0.82603	0.049	0.339	2.61E-07	13
Asph	1.65E-11	0.810176	0.291	0.113	2.75E-07	13
Dos	1.72E-11	0.82633	0.136	0.013	2.87E-07	13
Mcm2	1.74E-11	-0.69872	0.019	0.205	2.9E-07	13
Insm1	1.79E-11	-0.92845	0.029	0.294	2.98E-07	13
Hk1	1.85E-11	0.825758	0.175	0.022	3.09E-07	13
Uqcrb	1.89E-11	0.736509	0.369	0.219	3.14E-07	13
Hirip3	2.03E-11	-0.92802	0.078	0.384	3.38E-07	13
Spc25	2.14E-11	-1.0249	0	0.226	3.58E-07	13
Rpl37	2.24E-11	-0.85015	0.165	0.505	3.73E-07	13
Gm8292	2.31E-11	-0.78106	0.078	0.37	3.86E-07	13
Hdac2	2.5E-11	-0.79198	0.175	0.517	4.16E-07	13
Sptan1	2.53E-11	0.83012	0.34	0.168	4.22E-07	13
Gng3	2.55E-11	0.717762	0.612	0.314	4.26E-07	13
Higd2a	2.71E-11	0.70724	0.379	0.25	4.53E-07	13
Pcca	2.98E-11	0.85993	0.252	0.073	4.97E-07	13
Rassf4	2.99E-11	-0.89628	0.078	0.378	4.98E-07	13
Tspan9	3E-11	0.685951	0.126	0.009	5E-07	13
Fgf12	3E-11	0.597937	0.136	0.016	5.01E-07	13
Smarcc1	3.03E-11	-0.72202	0.126	0.435	5.05E-07	13
Got1	3.16E-11	0.744567	0.194	0.052	5.27E-07	13

Kifc2	3.23E-11	0.758578	0.155	0.016	5.38E-07	13
Fxyd7	3.5E-11	0.644424	0.117	0.007	5.84E-07	13
Rpl7	3.53E-11	-0.7186	0.184	0.511	5.88E-07	13
Tecpr1	3.58E-11	0.766606	0.262	0.076	5.98E-07	13
Ttc9b	3.74E-11	0.76945	0.32	0.193	6.23E-07	13
Tmem108	3.95E-11	0.644767	0.117	0.008	6.59E-07	13
Pgm2l1	4.09E-11	0.907914	0.301	0.096	6.83E-07	13
Adarb1	4.69E-11	0.849403	0.194	0.04	7.83E-07	13
Myl12a	4.7E-11	-0.79872	0.049	0.33	7.84E-07	13
Tead2	4.82E-11	-0.90902	0.019	0.279	8.03E-07	13
Tmem132a	4.85E-11	0.850824	0.252	0.101	8.1E-07	13
Zfyve28	5.16E-11	0.618395	0.155	0.014	8.6E-07	13
Mycbp2	5.23E-11	0.692529	0.505	0.344	8.72E-07	13
Hmgn2	5.3E-11	-0.89324	0.01	0.257	8.83E-07	13
Mcm6	5.31E-11	-0.96293	0.019	0.277	8.85E-07	13
Cenpa	5.31E-11	-1.14697	0.029	0.253	8.86E-07	13
Rbbp4	6.44E-11	-0.50983	0.194	0.485	1.07E-06	13
Abcg1	6.62E-11	0.855787	0.175	0.023	1.1E-06	13
Kdm1a	7.09E-11	-0.53754	0.097	0.365	1.18E-06	13
Calm2	7.14E-11	0.454031	0.951	0.873	1.19E-06	13
Rnaseh2c	7.25E-11	-0.70099	0.087	0.382	1.21E-06	13
Fam63b	7.39E-11	0.898585	0.301	0.086	1.23E-06	13
Plcxd2	7.7E-11	0.749904	0.117	0.01	1.29E-06	13
Hmgb3	7.72E-11	-0.83472	0.039	0.307	1.29E-06	13
Atp5j	7.9E-11	0.393728	0.699	0.722	1.32E-06	13
Il16	8.23E-11	0.753802	0.126	0.01	1.37E-06	13
Pcna	8.79E-11	-1.06047	0.068	0.359	1.47E-06	13
Hdgfrp3	8.84E-11	-0.46283	0.107	0.339	1.47E-06	13
Rabac1	9.11E-11	0.710123	0.388	0.257	1.52E-06	13
Rfc1	9.9E-11	-0.82618	0.058	0.332	1.65E-06	13
Ddx21	1.02E-10	-0.75697	0.058	0.323	1.71E-06	13
Zeb1	1.1E-10	-0.80549	0.097	0.389	1.83E-06	13
Syp	1.12E-10	0.914045	0.252	0.067	1.87E-06	13
Apba1	1.2E-10	0.73728	0.155	0.017	2E-06	13
Rcn1	1.25E-10	-0.65794	0.019	0.235	2.09E-06	13
Ybx1	1.27E-10	-0.54895	0.476	0.787	2.11E-06	13
Ssrp1	1.32E-10	-0.76397	0.243	0.578	2.21E-06	13
Nhlh2	1.44E-10	-1.05832	0.117	0.424	2.4E-06	13
Lsm3	1.45E-10	-0.91159	0.068	0.36	2.42E-06	13
Wbp2	1.47E-10	0.709167	0.311	0.194	2.45E-06	13
Atp6v1d	1.56E-10	0.740898	0.427	0.258	2.6E-06	13
Idh3a	1.6E-10	0.743202	0.32	0.161	2.67E-06	13
Trnp1	1.77E-10	0.619472	0.117	0.007	2.95E-06	13
Ptpre	1.78E-10	0.777675	0.155	0.016	2.97E-06	13
Ptgds	1.91E-10	0.676126	0.117	0.012	3.18E-06	13
Dusp5	2.09E-10	0.872899	0.301	0.087	3.49E-06	13
Bcas1	2.13E-10	-0.86861	0.049	0.283	3.56E-06	13
Apbb1	2.19E-10	0.79206	0.398	0.168	3.66E-06	13

Snrpd2	2.39E-10	-0.80639	0.136	0.442	3.98E-06	13
Id2	2.47E-10	-0.80801	0.165	0.488	4.12E-06	13
Klhdc8a	2.52E-10	0.543141	0.107	0.005	4.2E-06	13
Tcp1	2.56E-10	-0.7806	0.146	0.464	4.27E-06	13
Tpm4	2.57E-10	-0.80329	0.049	0.318	4.28E-06	13
Fbxo9	2.6E-10	0.808318	0.291	0.127	4.34E-06	13
Rab6b	2.99E-10	0.754651	0.398	0.201	4.99E-06	13
Ndufa1	2.99E-10	0.687484	0.485	0.341	4.99E-06	13
Adam22	3.02E-10	0.84419	0.194	0.056	5.04E-06	13
Cbx1	3.05E-10	-0.68362	0.369	0.687	5.08E-06	13
Gm17750	3.15E-10	-0.87014	0.078	0.368	5.25E-06	13
Hnrnpr	3.23E-10	-0.56474	0.223	0.547	5.38E-06	13
Hnrnpa0	3.31E-10	-0.67804	0.155	0.458	5.52E-06	13
Scamp1	3.37E-10	0.84889	0.233	0.071	5.62E-06	13
H1f0	3.6E-10	-0.71986	0.291	0.618	6.01E-06	13
Frmd4a	3.63E-10	-0.94554	0.049	0.31	6.05E-06	13
RP23-45G1	3.74E-10	-0.94693	0.058	0.328	6.24E-06	13
Fscn1	3.74E-10	-0.80204	0	0.204	6.24E-06	13
Pa2g4	3.75E-10	-0.6134	0.204	0.524	6.25E-06	13
Cdh20	4.02E-10	-0.82783	0	0.203	6.71E-06	13
Ntrk2	4.04E-10	0.807229	0.243	0.082	6.73E-06	13
Ankrd12	4.08E-10	0.825526	0.476	0.273	6.8E-06	13
Khdrbs1	4.48E-10	-0.7859	0.175	0.495	7.48E-06	13
Hmgb1	4.69E-10	-0.65636	0.087	0.36	7.83E-06	13
Dpf3	4.73E-10	0.59854	0.136	0.013	7.88E-06	13
Plcb4	4.89E-10	0.895684	0.301	0.138	8.15E-06	13
Ube2c	4.98E-10	-1.34285	0.029	0.27	8.31E-06	13
Ccdc92	5.01E-10	0.758908	0.146	0.022	8.36E-06	13
Ebna1bp2	5.05E-10	-0.90902	0.049	0.316	8.42E-06	13
B4galt6	5.05E-10	0.822804	0.204	0.054	8.43E-06	13
Ssr3	5.24E-10	-0.57984	0.146	0.426	8.74E-06	13
Eif3e	5.41E-10	-0.55777	0.107	0.383	9.02E-06	13
Ilf2	5.53E-10	-0.66258	0.223	0.543	9.22E-06	13
Mrpl52	5.53E-10	-0.82663	0.126	0.434	9.22E-06	13
Rbmx	5.62E-10	-0.83254	0.049	0.316	9.38E-06	13
Psmb1	6.42E-10	-0.562	0.437	0.681	1.07E-05	13
C530008M	6.62E-10	-0.80817	0.039	0.269	1.1E-05	13
Ccdc34	6.94E-10	-0.90552	0.117	0.416	1.16E-05	13
Elavl2	7.07E-10	-0.78928	0.029	0.271	1.18E-05	13
Arhgef7	7.1E-10	0.821081	0.32	0.148	1.18E-05	13
Gabbr2	7.13E-10	0.621005	0.155	0.018	1.19E-05	13
Paics	7.55E-10	-0.78495	0.117	0.415	1.26E-05	13
Ptn	8E-10	-0.82045	0.146	0.443	1.33E-05	13
Lap3	8.11E-10	-0.89015	0.029	0.277	1.35E-05	13
Lmo4	8.59E-10	-0.58555	0.078	0.337	1.43E-05	13
Rps4x	8.59E-10	-0.8473	0.039	0.296	1.43E-05	13
Tmsb10	8.67E-10	-0.6324	0.359	0.665	1.45E-05	13
Fam49b	8.69E-10	0.756013	0.223	0.084	1.45E-05	13

Oxct1	8.73E-10	0.516321	0.66	0.552	1.46E-05	13
Lsamp	8.81E-10	0.894715	0.272	0.076	1.47E-05	13
Jun	9.28E-10	-0.54454	0.34	0.656	1.55E-05	13
Casp3	9.73E-10	-0.83636	0.01	0.235	1.62E-05	13
Eef1g	9.79E-10	-0.73374	0.068	0.336	1.63E-05	13
Hmgb2	9.81E-10	-0.89201	0.117	0.401	1.64E-05	13
Cox5b	9.91E-10	0.478161	0.417	0.356	1.65E-05	13
Rdx	1.08E-09	-0.54991	0.301	0.605	1.8E-05	13
Rbm8a	1.09E-09	-0.39655	0.204	0.466	1.81E-05	13
Gnai2	1.17E-09	-0.80352	0.126	0.426	1.95E-05	13
Lig1	1.19E-09	-0.96252	0.087	0.349	1.99E-05	13
Ppip5k1	1.21E-09	0.643649	0.126	0.015	2.02E-05	13
Mpv17l	1.23E-09	0.710208	0.126	0.014	2.04E-05	13
Map7d2	1.35E-09	0.880725	0.272	0.099	2.25E-05	13
Ttc3	1.35E-09	0.432649	0.913	0.828	2.26E-05	13
Rps7	1.37E-09	-0.79092	0.107	0.401	2.29E-05	13
Arf4	1.4E-09	-0.80087	0.117	0.405	2.34E-05	13
Gria2	1.44E-09	0.662495	0.699	0.52	2.4E-05	13
Gse1	1.49E-09	-0.44538	0.058	0.238	2.49E-05	13
Cdh7	1.49E-09	0.787017	0.126	0.02	2.49E-05	13
Fam131a	1.51E-09	0.7903	0.155	0.024	2.52E-05	13
Rpl18	1.55E-09	-0.77404	0.049	0.306	2.59E-05	13
Txnrd1	1.58E-09	-0.65496	0.107	0.39	2.64E-05	13
Cdc42	1.62E-09	0.466977	0.612	0.514	2.7E-05	13
Cks1b	1.69E-09	-0.64154	0.087	0.345	2.81E-05	13
Echdc2	1.73E-09	0.756594	0.175	0.034	2.88E-05	13
Nkd1	1.79E-09	-0.71838	0.029	0.249	2.99E-05	13
H2afy2	1.82E-09	-0.67202	0.078	0.339	3.03E-05	13
Cystm1	1.83E-09	0.748232	0.194	0.037	3.05E-05	13
Nsg2	1.91E-09	-0.51995	0.194	0.489	3.18E-05	13
Sox9	1.92E-09	-0.88877	0.029	0.256	3.2E-05	13
Zcchc17	1.93E-09	0.737523	0.417	0.262	3.22E-05	13
Eny2	1.94E-09	-0.70622	0.117	0.388	3.23E-05	13
Mki67	1.98E-09	-1.12988	0.117	0.396	3.31E-05	13
Cct2	1.99E-09	-0.57852	0.214	0.525	3.31E-05	13
Ssr1	1.99E-09	-0.45169	0.068	0.29	3.33E-05	13
Snx6	2.22E-09	-0.55242	0.107	0.37	3.7E-05	13
Ebf3	2.22E-09	-0.70711	0.019	0.204	3.7E-05	13
Rad21	2.32E-09	-0.75857	0.165	0.463	3.87E-05	13
1110038B:	2.33E-09	-0.85396	0.049	0.3	3.89E-05	13
Edf1	2.4E-09	0.500022	0.476	0.406	4.01E-05	13
Pde1c	2.44E-09	-0.97406	0.107	0.388	4.07E-05	13
Lgals1	2.44E-09	-0.91692	0.019	0.229	4.08E-05	13
A930011O:	2.46E-09	0.85959	0.33	0.113	4.1E-05	13
Akap8l	2.8E-09	0.759336	0.33	0.157	4.67E-05	13
Supt16	2.83E-09	-0.72815	0.184	0.495	4.72E-05	13
Syngr1	2.85E-09	0.80679	0.282	0.092	4.75E-05	13
Pcyt2	2.85E-09	0.773663	0.165	0.044	4.76E-05	13

Dnajc5	2.9E-09	0.626323	0.379	0.247	4.84E-05	13
Cacna1d	2.96E-09	0.639775	0.165	0.044	4.94E-05	13
Cnbp	3E-09	-0.54499	0.476	0.741	5E-05	13
Klf13	3.04E-09	0.654476	0.35	0.235	5.08E-05	13
Synpr	3.35E-09	0.674637	0.204	0.041	5.58E-05	13
Cox7b	3.65E-09	0.479155	0.602	0.548	6.09E-05	13
Nhlh1	3.69E-09	-0.98726	0.01	0.224	6.16E-05	13
En2	3.72E-09	0.786512	0.214	0.059	6.2E-05	13
Syncrip	3.78E-09	-0.49345	0.214	0.497	6.31E-05	13
Pdgfa	3.86E-09	-0.73618	0.039	0.254	6.44E-05	13
Car11	4.01E-09	0.727212	0.146	0.02	6.69E-05	13
Ndufb2	4.08E-09	0.534769	0.427	0.367	6.8E-05	13
Nhp2	4.46E-09	-0.57071	0.107	0.372	7.43E-05	13
G3bp1	4.72E-09	-0.80646	0.068	0.329	7.87E-05	13
Mat2b	4.96E-09	0.633998	0.214	0.148	8.27E-05	13
Atp5k	5.05E-09	0.504829	0.524	0.454	8.42E-05	13
Ccm2	5.46E-09	-0.76588	0.019	0.226	9.12E-05	13
Tuba1b	5.54E-09	-0.74721	0.204	0.482	9.24E-05	13
Snrpd3	5.66E-09	-0.6117	0.243	0.536	9.43E-05	13
Rcor2	5.74E-09	-0.80396	0.019	0.226	9.57E-05	13
Papola	5.83E-09	-0.60765	0.126	0.403	9.72E-05	13
Megf11	5.91E-09	0.571367	0.117	0.009	9.85E-05	13
Rpl22l1	6.8E-09	-0.80187	0.087	0.352	0.000113	13
Tmem38a	7.07E-09	0.630992	0.146	0.021	0.000118	13
Eif3d	7.13E-09	-0.51422	0.136	0.407	0.000119	13
Nip7	7.14E-09	-0.42409	0.019	0.163	0.000119	13
Arxes1	7.15E-09	0.692013	0.194	0.063	0.000119	13
5730409E(	7.35E-09	0.526558	0.117	0.009	0.000123	13
Rpl14-ps1	7.68E-09	-0.6648	0.049	0.285	0.000128	13
Mdh2	8.1E-09	0.270896	0.456	0.528	0.000135	13
Napg	8.52E-09	0.700343	0.291	0.127	0.000142	13
Igsf21	8.59E-09	0.770536	0.272	0.088	0.000143	13
Aldoc	9.32E-09	0.592849	0.146	0.032	0.000155	13
Lrrc49	9.87E-09	0.724552	0.165	0.059	0.000165	13
Sep15	1.02E-08	-0.4212	0.233	0.506	0.000171	13
Tsn	1.05E-08	-0.5675	0.282	0.59	0.000175	13
Lrrtm3	1.06E-08	0.610106	0.126	0.017	0.000177	13
Tacc3	1.06E-08	-0.69332	0.019	0.207	0.000177	13
Atp5c1	1.09E-08	0.330874	0.592	0.621	0.000181	13
Gsg1l	1.1E-08	-0.83268	0.029	0.256	0.000183	13
Denr	1.11E-08	-0.66562	0.087	0.347	0.000185	13
Cenpe	1.14E-08	-1.18632	0.049	0.286	0.000189	13
Sh3glb1	1.14E-08	-0.82285	0.097	0.368	0.00019	13
Ddx42	1.16E-08	-0.38934	0.175	0.383	0.000194	13
Tceal3	1.16E-08	0.725872	0.243	0.109	0.000194	13
Phf14	1.2E-08	-0.55259	0.136	0.376	0.0002	13
Gins2	1.26E-08	-0.54125	0.019	0.186	0.000211	13
Nup85	1.31E-08	-0.5082	0.039	0.227	0.000219	13

Camta1	1.35E-08	0.607262	0.524	0.407	0.000225	13
Cct5	1.46E-08	-0.58885	0.184	0.478	0.000244	13
Prdx4	1.53E-08	-0.77017	0.097	0.367	0.000255	13
Sec62	1.56E-08	0.588098	0.553	0.405	0.000261	13
Uqcr10	1.58E-08	0.472328	0.495	0.433	0.000264	13
Rnd3	1.61E-08	-0.70193	0.146	0.432	0.000268	13
Serf1	1.66E-08	-0.52441	0.078	0.282	0.000276	13
Cyfip2	1.67E-08	0.717543	0.223	0.052	0.000278	13
Rbp4	1.67E-08	-0.83755	0.029	0.242	0.000279	13
Rps16	1.68E-08	-0.6544	0.019	0.21	0.00028	13
D4Wsu53e	1.7E-08	0.516674	0.583	0.446	0.000284	13
Esco2	1.73E-08	-0.77894	0.019	0.195	0.000288	13
Clic4	1.73E-08	-0.60031	0.039	0.228	0.000289	13
Eomes	1.75E-08	0.742483	0.107	0.01	0.000291	13
Hook1	1.86E-08	0.837647	0.194	0.061	0.00031	13
Tle1	1.86E-08	-0.81977	0.019	0.227	0.00031	13
Cdk1	1.88E-08	-0.84119	0.029	0.248	0.000314	13
1110001J0	1.88E-08	0.655196	0.34	0.237	0.000314	13
Magoh	1.9E-08	-0.56616	0.087	0.334	0.000316	13
Ccna2	1.9E-08	-0.7445	0.019	0.222	0.000317	13
Cbfa2t3	1.96E-08	-0.74103	0.126	0.404	0.000326	13
2700094K1	2.02E-08	-0.69196	0.35	0.615	0.000337	13
Bcl11a	2.04E-08	-0.79917	0.01	0.211	0.00034	13
Chka	2.08E-08	0.543953	0.223	0.158	0.000346	13
Eif3l	2.12E-08	-0.74049	0.058	0.303	0.000354	13
Rps25	2.17E-08	-0.49266	0.223	0.481	0.000361	13
Hmgn5	2.2E-08	-0.75957	0.146	0.426	0.000367	13
Crip2	2.25E-08	-0.67268	0.039	0.241	0.000375	13
Pcbp1	2.3E-08	-0.52747	0.359	0.594	0.000384	13
1110008F1	2.45E-08	0.551263	0.311	0.228	0.000408	13
Atp5j2	2.47E-08	0.328533	0.563	0.614	0.000412	13
Luc7l2	2.48E-08	0.410176	0.505	0.495	0.000414	13
Rpl37a	2.51E-08	-0.42086	0.214	0.469	0.000419	13
Bola2	2.52E-08	-0.31218	0.165	0.398	0.00042	13
Eef1d	2.78E-08	-0.60197	0.107	0.351	0.000463	13
Tra2a	2.82E-08	-0.35933	0.155	0.393	0.00047	13
Rhobtb3	2.86E-08	-0.49106	0.019	0.165	0.000477	13
Zranb2	2.87E-08	0.365175	0.398	0.406	0.000479	13
Atp6v1c1	2.9E-08	0.760522	0.252	0.108	0.000484	13
Btg2	3.01E-08	-0.76384	0.019	0.209	0.000501	13
Cacybp	3.09E-08	-0.34019	0.126	0.357	0.000516	13
Rfc3	3.12E-08	-0.43807	0.029	0.205	0.00052	13
Atp2a2	3.17E-08	0.500979	0.214	0.201	0.000529	13
Eif3i	3.18E-08	-0.74792	0.184	0.463	0.00053	13
Zfp326	3.24E-08	-0.7084	0.146	0.411	0.00054	13
Sirpa	3.24E-08	0.712716	0.175	0.037	0.000541	13
Nap1l4	3.31E-08	-0.25033	0.243	0.492	0.000553	13
Ank2	3.32E-08	0.786468	0.417	0.239	0.000553	13

Sec14l1	3.36E-08	0.691367	0.184	0.052	0.000561	13
Rpa3	3.41E-08	-0.74396	0.029	0.245	0.000569	13
Phactr3	3.47E-08	0.778149	0.214	0.073	0.000578	13
Gabbr1	3.64E-08	0.758213	0.32	0.139	0.000608	13
Syt12	3.65E-08	0.678003	0.146	0.036	0.00061	13
Rrm1	3.81E-08	-0.73901	0.039	0.261	0.000636	13
Ubxn1	3.88E-08	-0.39033	0.223	0.479	0.000648	13
Gucy1b3	3.89E-08	0.700846	0.136	0.037	0.000649	13
Usp1	3.93E-08	-0.70042	0.087	0.34	0.000655	13
Sec61b	4.03E-08	-0.27283	0.117	0.329	0.000673	13
Dek	4.08E-08	-0.70224	0.456	0.694	0.00068	13
Arpc5	4.22E-08	-0.31858	0.117	0.322	0.000704	13
Cisd1	4.31E-08	0.379136	0.379	0.342	0.000719	13
Prc1	4.36E-08	-1.07591	0.039	0.258	0.000727	13
Aifm3	4.41E-08	0.538365	0.107	0.008	0.000736	13
Zc3h14	4.48E-08	-0.44158	0.019	0.145	0.000747	13
Opcml	4.64E-08	0.650577	0.117	0.019	0.000775	13
Srsf7	4.67E-08	-0.57053	0.184	0.466	0.000779	13
Mpc2	4.68E-08	0.49569	0.379	0.297	0.000781	13
Ncdn	4.71E-08	0.718495	0.233	0.066	0.000786	13
Epb4.1l3	4.79E-08	0.65061	0.214	0.112	0.0008	13
1500012F(	4.82E-08	-0.63385	0.155	0.399	0.000803	13
Ythdf2	4.83E-08	-0.69771	0.039	0.26	0.000806	13
Cdca3	4.85E-08	-0.82112	0.019	0.222	0.000809	13
Shfm1	4.96E-08	-0.47317	0.408	0.687	0.000827	13
Nolc1	5.17E-08	-0.74307	0.136	0.41	0.000862	13
Prnp	5.31E-08	0.737871	0.388	0.205	0.000885	13
6430548M	5.32E-08	0.667834	0.146	0.02	0.000887	13
Gpr56	5.67E-08	-0.64245	0.039	0.223	0.000946	13
Psap	5.73E-08	0.598081	0.33	0.183	0.000956	13
Pfn1	5.76E-08	-0.56497	0.223	0.512	0.000961	13
Rpa2	5.82E-08	-0.74355	0.01	0.201	0.00097	13
Sept8	6.23E-08	0.705951	0.272	0.128	0.001039	13
Mapre1	6.23E-08	-0.60463	0.233	0.519	0.001039	13
Taok3	6.29E-08	0.78876	0.262	0.086	0.001049	13
Racgap1	6.36E-08	-0.72185	0.049	0.256	0.001061	13
Ypel1	6.39E-08	-0.64113	0.019	0.175	0.001066	13
Stmn1	6.4E-08	-0.63131	0.058	0.285	0.001068	13
Ccser2	6.71E-08	-0.6071	0.058	0.259	0.00112	13
Lrig3	6.98E-08	-0.5329	0.019	0.17	0.001164	13
Dock9	6.99E-08	0.561506	0.126	0.015	0.001167	13
Tcerg1l	7.18E-08	0.583352	0.117	0.017	0.001197	13
Dnajc9	7.3E-08	-0.60289	0.107	0.351	0.001218	13
Tyms	7.33E-08	-0.77274	0.019	0.22	0.001223	13
Cox7a2l	7.38E-08	-0.55303	0.136	0.383	0.001231	13
Bok	7.42E-08	-0.70009	0.039	0.252	0.001238	13
Dbi	7.72E-08	-0.71255	0.214	0.464	0.001287	13
Atp6ap2	7.97E-08	0.632531	0.301	0.183	0.001329	13

Mapre2	8.09E-08	0.640495	0.272	0.166	0.001349	13
Cdca7	8.26E-08	-0.64679	0.019	0.198	0.001378	13
Fam134a	8.35E-08	0.626168	0.175	0.086	0.001393	13
Hnrnpu	8.37E-08	-0.42657	0.65	0.866	0.001395	13
Ftsj3	8.64E-08	-0.45881	0.039	0.207	0.001441	13
Lars	8.69E-08	-0.5455	0.039	0.218	0.001449	13
Cdk2ap2	8.71E-08	0.632996	0.204	0.11	0.001453	13
Zmynd11	9.07E-08	0.440445	0.485	0.459	0.001512	13
Cfdp1	9.15E-08	-0.45243	0.223	0.485	0.001526	13
Lhx1	9.25E-08	-0.63213	0.214	0.498	0.001543	13
Grm5	9.35E-08	0.740363	0.107	0.011	0.00156	13
Ssr2	9.43E-08	-0.45822	0.155	0.38	0.001573	13
Neto2	9.44E-08	0.657134	0.194	0.073	0.001574	13
Rnaseh2b	9.46E-08	-0.56823	0.039	0.237	0.001578	13
Ktn1	9.83E-08	-0.56663	0.194	0.46	0.00164	13
Abcc8	9.85E-08	0.637215	0.117	0.018	0.001643	13
Porcn	9.87E-08	0.785386	0.252	0.082	0.001646	13
Zfr	1.1E-07	0.512138	0.388	0.353	0.001835	13
Pja2	1.11E-07	0.566428	0.388	0.233	0.001846	13
Clns1a	1.12E-07	-0.44729	0.039	0.219	0.001866	13
Zic1	1.13E-07	0.416469	0.864	0.783	0.001879	13
Gas1	1.14E-07	-0.66865	0	0.157	0.001907	13
Celf1	1.14E-07	0.506596	0.437	0.379	0.001908	13
Mpp6	1.16E-07	-0.58609	0.019	0.162	0.00193	13
Hells	1.17E-07	-0.81887	0.019	0.214	0.001947	13
Smarca2	1.19E-07	0.636441	0.398	0.284	0.001985	13
Xrn2	1.25E-07	-0.36858	0.214	0.474	0.002079	13
Tiam1	1.26E-07	0.729855	0.146	0.045	0.002105	13
Cenph	1.27E-07	-0.76258	0.01	0.194	0.002115	13
BC034090	1.28E-07	-0.37999	0.019	0.128	0.002136	13
Pkp4	1.35E-07	0.671616	0.214	0.101	0.002248	13
Pacsin1	1.35E-07	0.697931	0.175	0.035	0.00226	13
Rps8	1.39E-07	-0.69086	0.136	0.398	0.002316	13
Nol7	1.42E-07	-0.48923	0.291	0.57	0.002368	13
Kpnb1	1.46E-07	-0.27324	0.117	0.301	0.002438	13
Magee1	1.48E-07	0.650716	0.136	0.048	0.002474	13
Snrpd1	1.5E-07	-0.58347	0.252	0.525	0.002498	13
Set	1.61E-07	-0.48592	0.146	0.388	0.002692	13
Sri	1.62E-07	0.604329	0.262	0.165	0.0027	13
Pip5k1c	1.63E-07	0.692339	0.214	0.081	0.002717	13
Tgfb2	1.63E-07	-0.81903	0.01	0.192	0.002719	13
Psma6	1.63E-07	-0.44524	0.194	0.453	0.00272	13
Cdca8	1.65E-07	-0.86072	0.049	0.265	0.002754	13
Itm2c	1.67E-07	0.651488	0.311	0.211	0.002781	13
Cnot6	1.68E-07	-0.31829	0.165	0.392	0.002807	13
Ccnb2	1.72E-07	-0.77856	0.01	0.187	0.002872	13
Snhg5	1.74E-07	-0.73484	0.126	0.382	0.002898	13
Arpp21	1.8E-07	0.790729	0.311	0.111	0.003008	13

Sfrs18	1.85E-07	0.288166	0.796	0.777	0.003078	13
Gm13826	1.86E-07	-0.63877	0.078	0.313	0.003104	13
Tspan6	1.91E-07	-0.72652	0.039	0.247	0.003181	13
Cbfb	1.92E-07	-0.63207	0.01	0.184	0.003205	13
Ptch2	1.94E-07	-0.65736	0	0.153	0.003238	13
Cdipt	1.95E-07	0.679805	0.214	0.096	0.003253	13
Snord104	1.95E-07	-0.67208	0	0.153	0.00326	13
Dab1	1.96E-07	0.639284	0.155	0.032	0.00327	13
Smarca4	1.98E-07	-0.45841	0.243	0.506	0.003308	13
Hint2	2.01E-07	0.631504	0.223	0.119	0.003348	13
Sez6	2.03E-07	0.709528	0.184	0.055	0.003383	13
Pnrc1	2.11E-07	-0.67885	0.058	0.271	0.003525	13
Clspn	2.12E-07	-0.79782	0.01	0.19	0.00354	13
Fndc5	2.13E-07	0.618099	0.126	0.017	0.003546	13
Cd200	2.14E-07	0.731169	0.194	0.044	0.003567	13
Rif1	2.14E-07	0.460623	0.301	0.275	0.003567	13
Arhgef2	2.19E-07	-0.50349	0.078	0.283	0.003653	13
Trim3	2.3E-07	0.598633	0.146	0.04	0.003833	13
Nusap1	2.37E-07	-0.91968	0.039	0.228	0.003949	13
Rfc4	2.42E-07	-0.57896	0.039	0.227	0.004032	13
Clip3	2.44E-07	0.515617	0.466	0.31	0.004075	13
Pdap1	2.47E-07	-0.50544	0.408	0.684	0.004128	13
Kctd8	2.5E-07	0.647375	0.117	0.024	0.004165	13
Eif3h	2.58E-07	-0.48023	0.223	0.494	0.004295	13
Gpr153	2.63E-07	-0.75836	0.019	0.209	0.004391	13
Mrpl13	2.73E-07	-0.67507	0.078	0.309	0.00456	13
Caprin1	2.76E-07	-0.65629	0.136	0.395	0.004606	13
Ociad1	2.83E-07	0.364994	0.35	0.358	0.004713	13
Dut	2.85E-07	-0.66084	0.194	0.436	0.004753	13
Lsm4	2.93E-07	-0.61897	0.214	0.489	0.004896	13
mt-Rnr2	2.98E-07	0.349322	0.99	0.997	0.004973	13
Pou3f3	3.02E-07	-0.58201	0.01	0.172	0.005045	13
Atp5d	3.04E-07	0.334792	0.592	0.584	0.005078	13
Hmmr	3.06E-07	-0.77444	0.019	0.177	0.005111	13
Hes1	3.1E-07	-0.85664	0	0.149	0.005164	13
Nmral1	3.19E-07	-0.69019	0.01	0.188	0.005322	13
Rundc3a	3.22E-07	0.614855	0.34	0.248	0.005377	13
Ywhag	3.23E-07	0.594228	0.359	0.239	0.005388	13
Ccdc41	3.24E-07	-0.58348	0.058	0.254	0.005403	13
Pbk	3.3E-07	-0.82081	0.029	0.223	0.005509	13
Amph	3.32E-07	0.607423	0.194	0.089	0.00553	13
Knstrn	3.37E-07	-0.78602	0.01	0.186	0.00562	13
2610017I0	3.54E-07	-0.68367	0.019	0.205	0.00591	13
Magohb	3.55E-07	-0.55335	0.019	0.156	0.005928	13
Smim11	3.57E-07	-0.55285	0.068	0.277	0.00595	13
Eif4a1	3.74E-07	-0.49637	0.223	0.485	0.006233	13
Prmt8	3.92E-07	-0.72285	0.058	0.273	0.006541	13
Zfp704	3.93E-07	-0.67893	0.049	0.258	0.006552	13

Spc24	3.96E-07	-0.77055	0.039	0.243	0.006604	13
Mrpl15	3.99E-07	-0.40158	0.107	0.295	0.006651	13
Map1b	3.99E-07	0.461625	0.767	0.586	0.006654	13
Rgs12	4.01E-07	-0.64226	0	0.147	0.00669	13
Pdzrn4	4.01E-07	-0.67793	0	0.147	0.00669	13
Rpf2	4.13E-07	-0.73392	0.029	0.203	0.006884	13
Baiap2	4.15E-07	0.685645	0.194	0.05	0.006926	13
Ptp4a2	4.32E-07	-0.39745	0.126	0.343	0.007206	13
Grb2	4.32E-07	0.591579	0.35	0.248	0.007209	13
Tspan4	4.33E-07	0.734169	0.233	0.064	0.00723	13
Dstn	4.49E-07	-0.4972	0.107	0.332	0.007496	13
Tmem258	4.57E-07	-0.39844	0.233	0.452	0.007622	13
Galnt9	4.69E-07	0.518512	0.107	0.014	0.007828	13
Uqcrh	4.72E-07	0.28131	0.612	0.656	0.007881	13
Kif23	4.73E-07	-0.83474	0.029	0.215	0.007889	13
Cnpy1	4.76E-07	0.692023	0.252	0.132	0.007936	13
1110008P1	4.76E-07	0.608769	0.184	0.056	0.007946	13
Pdgfra	4.81E-07	-0.81292	0.01	0.182	0.008015	13
Fam155a	4.82E-07	0.62966	0.194	0.115	0.008041	13
Mapre3	4.89E-07	0.648977	0.146	0.026	0.008164	13
Cenpk	4.99E-07	-0.63527	0	0.145	0.008323	13
Gm10036	5.01E-07	-0.68926	0.029	0.177	0.008361	13
Uba52	5.05E-07	-0.65744	0.087	0.318	0.008419	13
Kbtbd11	5.09E-07	0.707326	0.184	0.087	0.00849	13
Snhg1	5.17E-07	-0.49826	0.136	0.358	0.008618	13
Nras	5.27E-07	-0.39099	0.058	0.221	0.008796	13
Cdc20	5.29E-07	-0.85136	0.029	0.197	0.008827	13
Pqlc1	5.4E-07	-0.63652	0.078	0.298	0.009011	13
Ndufa8	5.41E-07	0.400824	0.408	0.329	0.009027	13
Rb1cc1	5.5E-07	0.666309	0.379	0.233	0.009168	13
Abracl	5.57E-07	-0.6334	0.078	0.303	0.009297	13
Dpy30	5.59E-07	-0.43176	0.097	0.299	0.009317	13
1810009A1	5.6E-07	-0.42612	0.068	0.26	0.00934	13
Sphkap	5.73E-07	0.769598	0.175	0.047	0.009555	13
Plxnb2	5.86E-07	-0.61323	0.01	0.171	0.009776	13
Brix1	5.86E-07	-0.54017	0.029	0.206	0.009776	13
Ptprs	6.03E-07	-0.5738	0.165	0.422	0.010055	13
Cct3	6.04E-07	-0.60615	0.233	0.496	0.010067	13
Kcnk1	6.1E-07	0.719016	0.369	0.203	0.010172	13
Rrs1	6.17E-07	-0.64227	0.019	0.19	0.010284	13
Txndc9	6.18E-07	-0.58983	0.039	0.212	0.010308	13
Prdx1	6.35E-07	-0.57034	0.223	0.491	0.010592	13
Prkd3	6.41E-07	-0.57769	0	0.143	0.010696	13
Unc5c	6.44E-07	0.647254	0.126	0.022	0.010749	13
Idh2	6.58E-07	-0.56677	0.068	0.279	0.010979	13
Sel1l3	6.62E-07	0.601689	0.107	0.017	0.011045	13
Mettl9	6.81E-07	-0.6215	0.078	0.301	0.011362	13
Araf	6.84E-07	0.671095	0.262	0.136	0.011403	13

Mmp14	6.89E-07	-0.66724	0.01	0.175	0.011498	13
Tulp4	6.94E-07	0.514936	0.311	0.204	0.011572	13
Sub1	7.02E-07	-0.41305	0.32	0.59	0.011704	13
Laptm4b	7.03E-07	-0.57621	0	0.142	0.01173	13
Myod1	7.13E-07	-0.66766	0	0.142	0.011885	13
Pdxp	7.15E-07	0.656836	0.233	0.112	0.011931	13
Vdac1	7.34E-07	0.428096	0.35	0.302	0.012251	13
Ndufb11	7.48E-07	0.338916	0.456	0.462	0.012475	13
Nuf2	7.51E-07	-0.59817	0	0.141	0.012527	13
Tsc1	7.61E-07	0.285103	0.107	0.104	0.012702	13
Incenp	7.68E-07	-0.67434	0.068	0.276	0.012809	13
Ndufb9	7.8E-07	0.270164	0.485	0.539	0.013004	13
Lgi3	7.85E-07	0.540896	0.117	0.018	0.013091	13
Akr1a1	7.88E-07	-0.29874	0.359	0.587	0.013145	13
Parp1	8.14E-07	-0.42001	0.107	0.314	0.013579	13
Ccar1	8.23E-07	-0.34909	0.32	0.573	0.013734	13
Tead1	8.5E-07	-0.65163	0.01	0.174	0.01417	13
Gtpbp4	8.6E-07	-0.63784	0.068	0.283	0.014342	13
Gpatch4	8.68E-07	-0.55958	0.029	0.18	0.014482	13
Ccnb1	8.85E-07	-0.67718	0	0.14	0.014765	13
Arrb1	8.9E-07	0.562242	0.107	0.016	0.014846	13
Cnpy2	9.07E-07	-0.3575	0.155	0.344	0.015128	13
Sfxn3	9.13E-07	0.613614	0.155	0.03	0.015229	13
Tmem234	9.2E-07	0.299334	0.34	0.396	0.015351	13
Tbl1x	9.6E-07	-0.65269	0.097	0.329	0.016005	13
Sema7a	9.6E-07	-0.53322	0.019	0.156	0.016016	13
Ngdn	9.63E-07	-0.66345	0.019	0.197	0.016055	13
Gm11223	9.64E-07	-0.84019	0.049	0.213	0.016072	13
Kif11	9.7E-07	-0.52893	0.039	0.206	0.016184	13
Pcbp4	9.82E-07	-0.33111	0.126	0.325	0.016386	13
Nup62	9.91E-07	-0.57647	0.01	0.171	0.016524	13
Hspd1	9.97E-07	-0.61836	0.126	0.366	0.016638	13
Klhdc2	1.01E-06	-0.40669	0.117	0.328	0.016882	13
Bub3	1.01E-06	-0.63915	0.097	0.322	0.016915	13
Ckap4	1.03E-06	-0.29091	0.107	0.29	0.017113	13
H2afx	1.03E-06	-0.86105	0.107	0.331	0.017213	13
Ckap2	1.05E-06	-0.53185	0.019	0.164	0.017591	13
Psma7	1.1E-06	-0.45758	0.553	0.783	0.018301	13
Pdxk	1.11E-06	0.536459	0.107	0.015	0.018486	13
Cited2	1.11E-06	0.516817	0.146	0.152	0.018505	13
Map3k12	1.13E-06	0.487523	0.155	0.097	0.018885	13
Emc9	1.15E-06	0.658961	0.136	0.026	0.01923	13
Mcm3	1.17E-06	-0.56523	0.019	0.18	0.019436	13
Sbno1	1.18E-06	0.306038	0.291	0.351	0.019662	13
Whsc1l1	1.18E-06	0.3436	0.136	0.155	0.019728	13
Tsnax	1.19E-06	0.495701	0.262	0.19	0.019902	13
Setd8	1.2E-06	-0.64951	0.058	0.262	0.02007	13
Ptprr	1.2E-06	0.492203	0.107	0.015	0.020072	13

Rab7	1.2E-06	0.59036	0.301	0.183	0.020081	13
Ap3d1	1.23E-06	0.600116	0.311	0.203	0.020478	13
Naa15	1.24E-06	-0.35734	0.194	0.392	0.020685	13
Cenpf	1.24E-06	-0.89925	0.126	0.363	0.020731	13
Islr2	1.25E-06	-0.62733	0.019	0.18	0.02079	13
Snap47	1.32E-06	0.662049	0.282	0.139	0.022006	13
Ndufb10	1.33E-06	0.385097	0.485	0.423	0.022101	13
Anapc2	1.33E-06	-0.30825	0.019	0.12	0.022259	13
Ier3ip1	1.34E-06	-0.41913	0.117	0.313	0.022429	13
Gm26735	1.35E-06	-0.68344	0.019	0.181	0.022528	13
Ccdc28b	1.36E-06	0.625966	0.262	0.161	0.02275	13
Gm10260	1.39E-06	-0.57119	0.049	0.228	0.023133	13
Dnajc2	1.39E-06	-0.39529	0.126	0.329	0.023213	13
Fam168a	1.39E-06	0.537946	0.379	0.286	0.023246	13
Vldlr	1.4E-06	0.603517	0.126	0.06	0.02328	13
Mtch2	1.4E-06	0.53814	0.388	0.294	0.023394	13
Rpl38	1.42E-06	-0.58117	0.155	0.384	0.023625	13
Tomm7	1.43E-06	-0.28469	0.262	0.498	0.02379	13
Cbx6	1.43E-06	0.661275	0.223	0.087	0.023842	13
Dixdc1	1.44E-06	0.545158	0.34	0.234	0.024024	13
Acot7	1.45E-06	0.566	0.282	0.194	0.024254	13
2310022B(	1.46E-06	-0.70113	0.049	0.249	0.024349	13
Hk2	1.5E-06	-0.64793	0.01	0.173	0.025077	13
Sephs1	1.53E-06	-0.56072	0	0.135	0.025584	13
Nt5dc2	1.55E-06	-0.56785	0.049	0.238	0.02584	13
Bola1	1.57E-06	-0.44071	0.029	0.173	0.026161	13
Cebpz	1.63E-06	-0.60351	0.087	0.306	0.027163	13
Bzw2	1.63E-06	-0.57034	0.262	0.489	0.027168	13
Phf5a	1.63E-06	-0.5638	0.107	0.332	0.027243	13
Rcbtb2	1.64E-06	-0.53669	0.019	0.151	0.027383	13
Trim62	1.65E-06	0.628434	0.146	0.046	0.027525	13
Srsf2	1.67E-06	-0.52982	0.301	0.567	0.027839	13
Erbb2ip	1.7E-06	-0.45609	0.029	0.175	0.028297	13
Prdx6	1.7E-06	-0.51926	0.117	0.327	0.028333	13
Limch1	1.75E-06	0.647409	0.126	0.023	0.029239	13
Rpl13	1.77E-06	-0.4906	0.107	0.314	0.029459	13
Ilf3	1.78E-06	-0.3859	0.175	0.377	0.029752	13
Sstr2	1.79E-06	-0.65844	0.029	0.191	0.029862	13
Anapc5	1.81E-06	-0.37535	0.126	0.334	0.030214	13
Psmb6	1.81E-06	-0.35413	0.369	0.612	0.030225	13
Galnt7	1.83E-06	0.576714	0.136	0.051	0.030514	13
Dnmt1	1.85E-06	-0.70605	0.068	0.278	0.030819	13
Cltb	1.85E-06	0.373224	0.515	0.422	0.030914	13
Nbea	1.9E-06	0.621724	0.33	0.189	0.031615	13
Ppp6r1	1.9E-06	0.47651	0.107	0.049	0.031681	13
Igsf3	1.91E-06	0.481508	0.175	0.133	0.03188	13
Eif4a2	1.94E-06	0.564219	0.301	0.18	0.032414	13
Rasa3	1.94E-06	0.613463	0.136	0.038	0.032436	13

Hspa5	1.96E-06	-0.56391	0.301	0.551	0.032679	13
Brd3	1.99E-06	-0.56823	0.272	0.533	0.033259	13
Psmg4	1.99E-06	-0.64234	0.029	0.208	0.033269	13
Tpx2	2.02E-06	-0.92645	0.078	0.282	0.033712	13
2810025M	2.06E-06	-0.5895	0.01	0.168	0.03442	13
Tmem14c	2.11E-06	-0.35814	0.039	0.189	0.035205	13
Siva1	2.13E-06	-0.61893	0.068	0.273	0.035566	13
Wscd2	2.16E-06	0.532184	0.107	0.013	0.036104	13
Mns1	2.17E-06	-0.70272	0.029	0.204	0.036126	13
Tubb4a	2.17E-06	0.629591	0.204	0.066	0.036269	13
Snrk	2.21E-06	0.638903	0.165	0.048	0.036891	13
Vezf1	2.25E-06	-0.25969	0.155	0.342	0.03747	13
Meis1	2.28E-06	-0.65133	0.136	0.374	0.038033	13
Suv39h2	2.28E-06	-0.54557	0	0.132	0.038049	13
Tbata	2.33E-06	-0.72889	0.117	0.341	0.038889	13
AI413582	2.36E-06	0.527191	0.136	0.03	0.039375	13
Nucks1	2.37E-06	-0.57989	0.437	0.664	0.039538	13
Eif3a	2.39E-06	-0.47829	0.437	0.69	0.039798	13
Snrnp25	2.45E-06	-0.27352	0.019	0.126	0.040907	13
Serbp1	2.47E-06	-0.37478	0.699	0.87	0.041175	13
Hnrnpl	2.48E-06	-0.49764	0.136	0.364	0.041369	13
Cdk6	2.51E-06	-0.67302	0.049	0.244	0.041901	13
Sv2b	2.55E-06	0.605399	0.223	0.101	0.042492	13
Mrpl33	2.55E-06	-0.34011	0.117	0.283	0.042528	13
Mapk9	2.57E-06	0.605898	0.155	0.076	0.042901	13
Mef2a	2.57E-06	0.594324	0.282	0.138	0.042921	13
Jam3	2.59E-06	-0.38134	0.01	0.135	0.043161	13
Smarcad1	2.6E-06	-0.26575	0.049	0.168	0.043316	13
Cyb5	2.63E-06	-0.5989	0.029	0.188	0.043922	13
Vcan	2.68E-06	-0.61216	0	0.13	0.044746	13
Oxr1	2.69E-06	0.577088	0.184	0.108	0.044944	13
Actl6a	2.72E-06	-0.49245	0.049	0.227	0.04543	13
Polr2b	2.73E-06	-0.36762	0.029	0.178	0.045466	13
Pcm1	2.75E-06	-0.27223	0.291	0.523	0.045815	13
Cacna2d3	2.75E-06	0.579786	0.107	0.022	0.045913	13
Hspe1	2.76E-06	-0.26843	0.146	0.341	0.046119	13
Idh1	2.79E-06	-0.55287	0.01	0.157	0.046551	13
Nfix	2.79E-06	-0.43589	0.466	0.674	0.046562	13
Gpi1	2.86E-06	0.575766	0.243	0.13	0.047639	13
Baz1a	2.87E-06	-0.68527	0.01	0.167	0.047929	13
Mgll	2.89E-06	0.694832	0.262	0.132	0.048248	13
Snx1	2.91E-06	-0.32788	0.039	0.169	0.04853	13
Mcm5	2.93E-06	-0.64621	0.01	0.169	0.048851	13
D8Ertd738	2.94E-06	-0.53116	0.165	0.38	0.048998	13
Cct6a	2.98E-06	-0.50761	0.233	0.487	0.049695	13
Nr1d2	3E-06	0.586871	0.175	0.052	0.050118	13
Rpf1	3.01E-06	-0.35406	0.049	0.194	0.05013	13
Ckap2l	3.01E-06	-0.69348	0.039	0.218	0.050288	13

Hnrnpm	3.07E-06	-0.44218	0.495	0.736	0.051151	13
Hnrnpc	3.1E-06	-0.41889	0.417	0.668	0.051788	13
Gm11478	3.2E-06	-0.6681	0.039	0.215	0.053327	13
Fosb	3.2E-06	-0.59917	0.019	0.156	0.053331	13
Naa10	3.21E-06	-0.41193	0.078	0.25	0.053501	13
Sf3b5	3.22E-06	-0.56967	0.107	0.328	0.053726	13
Etf1	3.26E-06	-0.43734	0.078	0.262	0.054389	13
Thy1	3.27E-06	0.478986	0.107	0.014	0.054498	13
Dnajc15	3.28E-06	-0.53426	0	0.128	0.054684	13
Tbc1d20	3.36E-06	0.61982	0.194	0.095	0.055997	13
Ivns1abp	3.37E-06	-0.39941	0.194	0.416	0.05618	13
Oard1	3.4E-06	-0.5449	0.019	0.153	0.056641	13
Alyref	3.41E-06	-0.57403	0.029	0.2	0.056897	13
Negr1	3.43E-06	0.610796	0.155	0.046	0.057234	13
Znrd1	3.51E-06	-0.60609	0.039	0.212	0.058556	13
Elp2	3.57E-06	-0.44588	0.068	0.244	0.059468	13
Gpm6b	3.58E-06	-0.27447	0.262	0.485	0.05979	13
Cdh8	3.6E-06	0.621652	0.155	0.05	0.060053	13
Ncbp1	3.65E-06	-0.57189	0.01	0.163	0.060815	13
Trim28	3.7E-06	-0.49521	0.136	0.362	0.061746	13
Adamts1	3.72E-06	-0.50958	0.019	0.155	0.062093	13
Ctnnb1	3.74E-06	-0.40166	0.165	0.387	0.062407	13
B2m	3.8E-06	-0.79626	0.049	0.232	0.063339	13
Mxd3	3.8E-06	-0.53759	0	0.127	0.063438	13
Sh3glb2	3.86E-06	0.613004	0.204	0.083	0.06435	13
Impad1	3.86E-06	0.316596	0.184	0.223	0.064362	13
Gng5	3.91E-06	-0.61314	0.01	0.166	0.065297	13
Nsmce2	3.92E-06	-0.62841	0.039	0.222	0.065428	13
Heg1	3.95E-06	-0.5885	0.01	0.158	0.065826	13
Arxes2	4.03E-06	0.557519	0.184	0.098	0.067275	13
Tcof1	4.04E-06	-0.42638	0.029	0.168	0.067359	13
Msl3	4.14E-06	-0.46607	0.019	0.136	0.069002	13
Isg20l2	4.15E-06	-0.42849	0.019	0.118	0.069246	13
Rnd2	4.22E-06	-0.61606	0.01	0.159	0.070385	13
Slc25a22	4.23E-06	0.597166	0.165	0.037	0.070623	13
Timp2	4.26E-06	0.486801	0.117	0.025	0.071063	13
Nrm	4.26E-06	-0.48688	0.019	0.17	0.07112	13
Zfp36l1	4.35E-06	-0.53784	0.01	0.151	0.072628	13
Mical1	4.35E-06	-0.56009	0	0.126	0.072631	13
Atoh1	4.39E-06	-0.69305	0.01	0.165	0.073148	13
Efs	4.41E-06	-0.52175	0	0.126	0.073573	13
Odf2	4.42E-06	0.444636	0.214	0.17	0.073705	13
Larp7	4.52E-06	-0.61424	0.097	0.313	0.075344	13
2410006H	4.57E-06	-0.37534	0.223	0.46	0.076245	13
Lamtor4	4.61E-06	0.426021	0.155	0.127	0.076883	13
Pdia6	4.63E-06	-0.64182	0.117	0.341	0.077169	13
Pgp	4.65E-06	0.587335	0.262	0.149	0.077563	13
Adk	4.65E-06	-0.54536	0.019	0.166	0.077631	13

Sfr1	4.66E-06	-0.46231	0.165	0.395	0.07765	13
Bmi1	4.67E-06	-0.50143	0.019	0.147	0.07785	13
Brinp1	4.75E-06	0.622381	0.136	0.029	0.079263	13
Golim4	4.77E-06	-0.62442	0.049	0.233	0.079564	13
Psma2	4.8E-06	-0.38018	0.282	0.513	0.080087	13
Chd4	4.8E-06	-0.47718	0.563	0.77	0.080132	13
Polr3gl	4.85E-06	0.661343	0.126	0.051	0.080923	13
Fbxo5	4.85E-06	-0.47677	0.019	0.16	0.080938	13
Gm6472	4.97E-06	-0.50497	0.019	0.167	0.082982	13
Ska2	5.02E-06	-0.5354	0.039	0.205	0.083661	13
Slc9a3r2	5.05E-06	0.600121	0.107	0.016	0.084201	13
Txndc17	5.06E-06	-0.45528	0.068	0.243	0.084403	13
Ddx5	5.22E-06	0.262233	0.767	0.816	0.087068	13
Dkc1	5.28E-06	-0.5739	0.117	0.334	0.087991	13
Kif20b	5.33E-06	-0.57066	0.029	0.191	0.088839	13
Cnih4	5.37E-06	-0.35663	0.068	0.235	0.089514	13
Fastk	5.41E-06	0.535395	0.155	0.078	0.090205	13
Tars	5.47E-06	-0.44712	0.029	0.178	0.091252	13
Zwint	5.55E-06	0.474413	0.369	0.291	0.092623	13
Nap1l3	5.58E-06	0.498722	0.126	0.021	0.093089	13
Habp4	5.7E-06	0.589577	0.136	0.042	0.095099	13
Synj2bp	5.86E-06	0.566182	0.175	0.093	0.097677	13
Mt3	5.87E-06	0.502264	0.136	0.025	0.097863	13
St6galnac4	5.92E-06	0.607752	0.146	0.032	0.098671	13
Dnaja1	5.94E-06	-0.41069	0.165	0.375	0.099032	13
Pgls	5.96E-06	-0.46667	0.136	0.35	0.099397	13
Smco4	5.99E-06	-0.59122	0.01	0.162	0.099973	13
Eprs	6.01E-06	-0.41365	0.252	0.492	0.100176	13
Asap1	6.12E-06	-0.62057	0.019	0.181	0.102042	13
Smpd2	6.13E-06	-0.41349	0.029	0.166	0.102265	13
Fubp1	6.15E-06	-0.43556	0.32	0.567	0.102587	13
Brd7	6.18E-06	-0.48154	0.107	0.309	0.103151	13
Rac1	6.25E-06	-0.51592	0.126	0.347	0.104257	13
Topors	6.34E-06	-0.30037	0.058	0.183	0.10569	13
Rrm2	6.35E-06	-0.5733	0.039	0.204	0.105898	13
Rbbp8	6.36E-06	-0.52525	0	0.123	0.106103	13
Usmg5	6.42E-06	0.447458	0.301	0.244	0.107058	13
Rnaseh2a	6.46E-06	-0.32875	0.029	0.153	0.107741	13
Ubxn4	6.47E-06	-0.25761	0.243	0.428	0.107873	13
2900011O(	6.61E-06	0.624939	0.252	0.102	0.11023	13
Casc5	6.62E-06	-0.65964	0.029	0.187	0.110343	13
Chic2	6.62E-06	-0.46968	0.019	0.151	0.110444	13
D430041D	6.63E-06	0.36831	0.485	0.492	0.11052	13
Mad2l2	6.79E-06	-0.66434	0.068	0.263	0.113192	13
Cnksr2	6.86E-06	0.623074	0.243	0.106	0.114342	13
Cdc42se2	7.16E-06	-0.58713	0.058	0.239	0.119414	13
Dot1l	7.2E-06	0.392891	0.301	0.279	0.12007	13
Gnai3	7.27E-06	-0.33651	0.058	0.217	0.121227	13

Ncaph	7.28E-06	-0.49605	0	0.121	0.121375	13
Cenpq	7.41E-06	-0.33946	0.029	0.161	0.123579	13
Srek1	7.51E-06	-0.39744	0.165	0.384	0.12535	13
Arpp19	7.57E-06	-0.42963	0.165	0.378	0.126338	13
Lrp8	7.63E-06	0.59622	0.117	0.039	0.127204	13
Csdc2	7.81E-06	0.612242	0.117	0.028	0.130224	13
Slc35b2	7.83E-06	-0.32189	0.049	0.182	0.130527	13
Ddx39	7.83E-06	-0.59202	0.029	0.188	0.130648	13
Sptssa	7.86E-06	-0.58111	0.058	0.248	0.131025	13
Ptov1	7.89E-06	-0.3317	0.262	0.485	0.131538	13
Sugt1	7.97E-06	-0.46158	0.058	0.232	0.132912	13
Ddx39b	8.1E-06	-0.32417	0.223	0.445	0.135094	13
Polr2a	8.12E-06	-0.34544	0.049	0.176	0.13538	13
Arhgef9	8.24E-06	0.602197	0.194	0.088	0.137516	13
Smchd1	8.27E-06	-0.44154	0.087	0.274	0.137906	13
Arhgap5	8.27E-06	0.513993	0.204	0.18	0.138025	13
Lsm6	8.35E-06	-0.5786	0.146	0.373	0.139203	13
Eif3m	8.41E-06	-0.36329	0.136	0.302	0.140223	13
Sap30	8.42E-06	-0.29153	0.029	0.138	0.140421	13
Fbxo44	8.43E-06	0.63476	0.126	0.035	0.140639	13
Tcf25	8.45E-06	0.360392	0.718	0.601	0.140883	13
Sfrp2	8.45E-06	-0.58837	0.01	0.148	0.141019	13
Tmeff1	8.49E-06	-0.43219	0.029	0.15	0.14158	13
Luc7l3	8.66E-06	0.385545	0.777	0.747	0.144367	13
mt-Rnr1	8.69E-06	0.313901	0.408	0.367	0.144905	13
Pak2	8.8E-06	-0.5643	0.117	0.302	0.1468	13
Cdc5l	8.87E-06	-0.32893	0.233	0.402	0.147909	13
Flna	9.03E-06	-0.32822	0.029	0.152	0.150633	13
Sh3bp5	9.05E-06	0.678878	0.233	0.102	0.150909	13
Elf2	9.05E-06	-0.5059	0.039	0.199	0.150923	13
Peg3	9.05E-06	0.5466	0.388	0.283	0.151031	13
Dnajc6	9.07E-06	0.532495	0.107	0.016	0.151244	13
Pnrc2	9.16E-06	-0.44424	0.058	0.208	0.152723	13
Dhx9	9.38E-06	-0.38168	0.233	0.464	0.156534	13
Wdr5	9.41E-06	-0.51595	0.019	0.139	0.156897	13
Kif5a	9.59E-06	0.640205	0.291	0.127	0.159961	13
Rpl3	9.71E-06	-0.57186	0.039	0.212	0.161965	13
1500016L(	9.75E-06	-0.62568	0.165	0.384	0.162639	13
Gjc1	9.76E-06	-0.52213	0.039	0.197	0.162826	13
Chrna3	9.89E-06	-0.64371	0	0.119	0.164906	13
Afap1	9.92E-06	-0.59159	0.01	0.153	0.165394	13
Pak3	9.93E-06	-0.46046	0.058	0.206	0.165693	13
Tmod3	1E-05	-0.56865	0.01	0.155	0.167539	13
H2afz	1.01E-05	-0.3856	0.068	0.232	0.167943	13
Nme4	1.05E-05	-0.51653	0	0.118	0.174637	13
Cwc27	1.06E-05	-0.41888	0.029	0.169	0.176134	13
Btf3	1.06E-05	-0.53916	0.136	0.35	0.177285	13
Ctnna1	1.07E-05	-0.6142	0.01	0.153	0.177789	13

Sae1	1.07E-05	-0.43516	0.097	0.279	0.178057	13
Serp1	1.07E-05	-0.47474	0.049	0.218	0.178737	13
Ndufs7	1.07E-05	0.31888	0.311	0.335	0.17889	13
Smarcb1	1.08E-05	-0.38432	0.126	0.324	0.179681	13
Lphn1	1.11E-05	0.601186	0.243	0.112	0.184665	13
Ccdc50	1.11E-05	-0.26692	0.146	0.327	0.184708	13
Snrnp200	1.12E-05	-0.27683	0.049	0.163	0.186078	13
Gar1	1.13E-05	-0.55801	0.039	0.209	0.187729	13
Fxr1	1.14E-05	-0.48521	0.078	0.256	0.190034	13
0610009D(	1.14E-05	-0.51639	0.165	0.384	0.190958	13
Appl2	1.16E-05	0.593109	0.252	0.13	0.193596	13
Ift74	1.18E-05	-0.44057	0.058	0.225	0.196649	13
Cklf	1.19E-05	-0.53574	0.019	0.166	0.19813	13
Smim14	1.2E-05	0.550331	0.291	0.195	0.199837	13
Tkt	1.2E-05	-0.50831	0.039	0.204	0.20005	13
Ewsr1	1.2E-05	-0.30244	0.194	0.393	0.200594	13
Herc2	1.23E-05	0.529926	0.155	0.105	0.204955	13
Tram1	1.23E-05	-0.31745	0.019	0.129	0.204956	13
Nelfe	1.24E-05	-0.52087	0.01	0.151	0.207004	13
Abhd8	1.25E-05	0.591332	0.204	0.075	0.20834	13
Angptl2	1.27E-05	-0.51114	0	0.116	0.21135	13
Aurkb	1.27E-05	-0.48984	0.01	0.143	0.211646	13
Dhfr	1.27E-05	-0.33273	0.029	0.149	0.212563	13
Uchl3	1.28E-05	-0.55545	0.029	0.187	0.213599	13
Gmppa	1.34E-05	0.589055	0.126	0.042	0.224179	13
Cep57	1.35E-05	-0.32327	0.097	0.258	0.225401	13
Ctnnbl1	1.35E-05	-0.41053	0.039	0.185	0.225628	13
Sart1	1.37E-05	-0.60301	0.039	0.199	0.228429	13
Iws1	1.38E-05	-0.30336	0.087	0.215	0.230017	13
Ddx24	1.4E-05	0.283964	0.282	0.326	0.233171	13
Gpr107	1.4E-05	0.543214	0.126	0.056	0.234002	13
Tmem191c	1.41E-05	0.575734	0.117	0.028	0.23471	13
Ift27	1.41E-05	-0.63788	0.058	0.243	0.234732	13
Pebp1	1.43E-05	-0.43941	0.068	0.243	0.23775	13
Trim59	1.43E-05	-0.57543	0.019	0.17	0.238285	13
Prox1	1.43E-05	-0.53346	0.058	0.23	0.238828	13
Ppa1	1.44E-05	-0.34221	0.068	0.222	0.239592	13
Prkar1a	1.44E-05	0.262819	0.33	0.352	0.240548	13
Map1lc3a	1.45E-05	0.482607	0.35	0.275	0.242673	13
Lsg1	1.46E-05	-0.37474	0.019	0.147	0.243891	13
Tpm3	1.49E-05	-0.30904	0.146	0.316	0.248021	13
Rbm17	1.5E-05	-0.3354	0.184	0.39	0.250752	13
Ndc80	1.5E-05	-0.49937	0	0.115	0.250845	13
Anapc13	1.5E-05	-0.26312	0.214	0.394	0.25088	13
Ubr2	1.51E-05	0.429153	0.107	0.084	0.251617	13
Pole3	1.51E-05	-0.55729	0.068	0.251	0.252576	13
Ier2	1.51E-05	-0.51293	0.165	0.383	0.252637	13
Lman1	1.53E-05	-0.51354	0.117	0.325	0.254372	13

Rbx1	1.58E-05	-0.31675	0.272	0.496	0.26305	13
Ifitm2	1.58E-05	-0.49373	0	0.114	0.263888	13
Snx5	1.59E-05	-0.43654	0.029	0.164	0.264684	13
Ywhab	1.6E-05	0.285765	0.583	0.606	0.267458	13
Gm9843	1.62E-05	-0.34816	0.068	0.228	0.270822	13
Eml4	1.64E-05	-0.46035	0.049	0.21	0.27402	13
Gpd2	1.65E-05	0.575102	0.165	0.071	0.274802	13
Dcaf15	1.65E-05	0.255101	0.136	0.161	0.275312	13
Pfdn5	1.66E-05	-0.40654	0.282	0.521	0.276435	13
Ppp2r2d	1.68E-05	0.277415	0.155	0.203	0.279406	13
Rpn2	1.68E-05	-0.38236	0.068	0.227	0.279805	13
Phlda1	1.68E-05	0.593891	0.126	0.025	0.280652	13
2810474O	1.72E-05	-0.67183	0.058	0.241	0.286906	13
Nsmce1	1.73E-05	-0.55339	0.058	0.229	0.28827	13
Naca	1.77E-05	-0.43015	0.233	0.466	0.295267	13
Mfap4	1.78E-05	-0.68846	0.019	0.162	0.296603	13
Smc1a	1.78E-05	-0.42624	0.398	0.632	0.297641	13
Gstm5	1.79E-05	-0.41148	0.126	0.323	0.297801	13
Snrnp40	1.79E-05	-0.58334	0.078	0.271	0.299097	13
Pcdha2	1.82E-05	0.665408	0.252	0.117	0.303295	13
Camta2	1.84E-05	0.511183	0.136	0.029	0.307058	13
Csnk1e	1.84E-05	-0.27895	0.194	0.387	0.307392	13
Ppan	1.85E-05	-0.43079	0.019	0.14	0.308165	13
Chchd2	1.86E-05	-0.33493	0.427	0.654	0.309834	13
Gpr162	1.89E-05	0.479889	0.107	0.016	0.315662	13
Mlf2	1.93E-05	0.391856	0.311	0.279	0.321828	13
Fen1	1.93E-05	-0.34648	0.019	0.142	0.322223	13
Psmb4	1.93E-05	-0.36005	0.33	0.554	0.322279	13
Bcl7a	1.99E-05	-0.33287	0.155	0.326	0.331285	13
Ndufb3	1.99E-05	0.334036	0.33	0.339	0.332025	13
Trim24	2.02E-05	-0.39671	0.019	0.135	0.336916	13
Chaf1a	2.02E-05	-0.35847	0.039	0.169	0.337219	13
Osbpl1a	2.04E-05	0.587976	0.146	0.058	0.340425	13
Hbp1	2.08E-05	-0.42718	0.029	0.157	0.346192	13
Rars	2.08E-05	-0.30335	0.049	0.185	0.347604	13
Fam63a	2.11E-05	0.53927	0.165	0.087	0.35148	13
Cox5a	2.11E-05	0.257468	0.553	0.569	0.351638	13
Polr2e	2.13E-05	-0.3018	0.146	0.325	0.354951	13
Bcat1	2.13E-05	-0.38654	0.019	0.121	0.355205	13
Top1	2.16E-05	-0.26165	0.437	0.664	0.360554	13
Zbtb18	2.16E-05	0.553887	0.379	0.265	0.360761	13
Tspan3	2.19E-05	-0.42915	0.214	0.429	0.365552	13
H1fx	2.2E-05	-0.50146	0.068	0.246	0.367749	13
Lrrc58	2.24E-05	-0.4572	0	0.111	0.373569	13
Kif1a	2.24E-05	0.542059	0.282	0.17	0.373863	13
Dbf4	2.24E-05	-0.52606	0.01	0.146	0.374004	13
Synj1	2.27E-05	0.528233	0.165	0.078	0.378883	13
Hnrnph3	2.29E-05	-0.54773	0.204	0.428	0.381431	13

Cct8	2.29E-05	-0.37515	0.223	0.442	0.382777	13
Rpl29	2.31E-05	-0.48444	0.087	0.258	0.385162	13
Dpm3	2.33E-05	-0.44361	0.087	0.271	0.388574	13
Gamt	2.36E-05	-0.64261	0.029	0.17	0.394113	13
Zcrb1	2.4E-05	0.317387	0.369	0.371	0.399695	13
Atp11b	2.41E-05	0.282802	0.107	0.102	0.401353	13
Ppie	2.41E-05	-0.48147	0.019	0.135	0.402629	13
Elp5	2.42E-05	-0.29613	0.049	0.171	0.403959	13
Arid4a	2.47E-05	0.362511	0.272	0.282	0.411973	13
Copb2	2.5E-05	-0.30324	0.078	0.219	0.41676	13
Mis18a	2.52E-05	-0.44906	0	0.11	0.421072	13
Tgoln1	2.53E-05	0.559969	0.194	0.083	0.422241	13
Mcm4	2.53E-05	-0.56853	0.01	0.138	0.422275	13
Laptm4a	2.54E-05	-0.40647	0.32	0.555	0.423337	13
Prkcz	2.54E-05	0.592687	0.136	0.032	0.423384	13
Pdzrn3	2.56E-05	-0.76988	0.01	0.148	0.427229	13
Psip1	2.56E-05	-0.28567	0.466	0.688	0.427448	13
Cat	2.62E-05	-0.39173	0.019	0.124	0.437569	13
Ctbp2	2.63E-05	0.34458	0.194	0.215	0.438572	13
Utp3	2.64E-05	-0.48724	0.117	0.315	0.440676	13
Etfb	2.64E-05	-0.58949	0.029	0.185	0.440808	13
Smarcd3	2.65E-05	0.580392	0.136	0.062	0.441931	13
Hnrnpdl	2.7E-05	-0.26613	0.544	0.757	0.45039	13
Fam126b	2.73E-05	0.577925	0.136	0.063	0.455658	13
Lyar	2.74E-05	-0.53554	0.097	0.286	0.457085	13
Uqcrc1	2.75E-05	0.330969	0.301	0.294	0.459138	13
Snrpb	2.77E-05	-0.34597	0.34	0.574	0.461221	13
Robo2	2.77E-05	-0.29664	0.039	0.152	0.461856	13
Fam98a	2.77E-05	-0.30331	0.039	0.146	0.461941	13
Sin3b	2.81E-05	-0.38055	0.136	0.309	0.468661	13
Peli1	2.81E-05	-0.30045	0.068	0.202	0.46918	13
Cdv3	2.82E-05	-0.47571	0.019	0.159	0.470944	13
Arf3	2.85E-05	0.563605	0.155	0.062	0.474569	13
Tpr	2.87E-05	-0.38442	0.32	0.554	0.479463	13
Vrk1	2.91E-05	-0.52208	0.019	0.148	0.485693	13
Ubr7	3E-05	-0.43247	0.01	0.133	0.500298	13
Rabgap1l	3E-05	0.61471	0.175	0.061	0.500884	13
Rogdi	3E-05	0.571122	0.165	0.063	0.501152	13
Dip2b	3.03E-05	0.529185	0.126	0.079	0.50461	13
Sec13	3.05E-05	-0.42561	0.107	0.27	0.509044	13
Ctcf	3.09E-05	-0.26757	0.301	0.496	0.514769	13
Rufy2	3.11E-05	0.561791	0.272	0.157	0.518706	13
Sqstm1	3.11E-05	0.525672	0.311	0.226	0.518745	13
Gdi2	3.13E-05	-0.27032	0.204	0.377	0.521976	13
Mrpl36	3.14E-05	-0.38278	0.039	0.165	0.52375	13
2810004N:	3.14E-05	-0.26689	0.107	0.262	0.524564	13
Kif4	3.15E-05	-0.46367	0	0.108	0.52469	13
Spcs2	3.17E-05	-0.34254	0.223	0.425	0.529182	13

Cenpv	3.23E-05	-0.52461	0.126	0.329	0.53823	13
Ube2g2	3.23E-05	-0.33051	0.029	0.125	0.538683	13
Prpf38b	3.24E-05	0.418132	0.553	0.494	0.540767	13
Polr1c	3.25E-05	-0.27234	0.078	0.219	0.54128	13
Phf21b	3.26E-05	-0.34599	0.029	0.128	0.543908	13
Gli1	3.27E-05	-0.47212	0	0.108	0.544821	13
Nans	3.31E-05	-0.47422	0	0.108	0.5517	13
Gramd1b	3.41E-05	0.348089	0.204	0.169	0.568457	13
Ybx3	3.41E-05	-0.56773	0.107	0.291	0.568977	13
Kif2c	3.41E-05	-0.46122	0	0.107	0.569272	13
Psmd9	3.45E-05	-0.27206	0.039	0.148	0.575784	13
Hspa4	3.54E-05	-0.37732	0.165	0.369	0.589727	13
Mis18bp1	3.55E-05	-0.60947	0.019	0.161	0.592831	13
Nin	3.56E-05	-0.5173	0.019	0.146	0.594404	13
Fam98b	3.57E-05	-0.26457	0.184	0.336	0.595468	13
Helz	3.57E-05	0.397829	0.155	0.086	0.595802	13
Psma4	3.6E-05	-0.40286	0.204	0.414	0.600937	13
P4hb	3.61E-05	-0.47544	0.097	0.279	0.602314	13
Dtl	3.66E-05	-0.57701	0.01	0.146	0.609746	13
Naa38	3.67E-05	-0.39206	0.126	0.312	0.612902	13
Kmt2c	3.7E-05	0.266276	0.272	0.295	0.616784	13
Diap3	3.75E-05	-0.46214	0	0.107	0.62537	13
Pxn	3.79E-05	0.565971	0.117	0.039	0.632469	13
Eci2	3.8E-05	-0.49627	0.078	0.25	0.63331	13
Sema4g	3.82E-05	0.628207	0.136	0.037	0.637153	13
Prmt1	3.82E-05	-0.27963	0.078	0.225	0.637512	13
Fam131b	3.83E-05	0.527166	0.107	0.03	0.638514	13
C1qbp	3.83E-05	-0.30772	0.184	0.378	0.639239	13
Nudcd2	3.84E-05	-0.2972	0.078	0.224	0.640702	13
Naa50	3.86E-05	-0.37451	0.107	0.286	0.64305	13
Mrpl21	3.86E-05	-0.4911	0.097	0.281	0.643644	13
Psmd11	3.88E-05	-0.36177	0.136	0.321	0.647306	13
Cep110	3.93E-05	-0.36887	0.039	0.163	0.655896	13
Brd8	3.95E-05	-0.31621	0.194	0.393	0.658033	13
Rps27a	3.97E-05	-0.44639	0	0.106	0.661611	13
Wipi2	3.98E-05	0.340824	0.097	0.12	0.664427	13
Smarca5	4.06E-05	-0.57596	0.184	0.402	0.67754	13
Polr2j	4.06E-05	-0.34684	0.117	0.294	0.677706	13
Eif3k	4.08E-05	-0.30654	0.301	0.521	0.679908	13
Cep170	4.09E-05	-0.3799	0.126	0.306	0.682626	13
Psme4	4.15E-05	-0.4179	0.107	0.284	0.691815	13
Trip11	4.19E-05	0.541594	0.194	0.152	0.698939	13
Fam181b	4.2E-05	-0.31782	0.039	0.143	0.700796	13
Ank3	4.22E-05	0.262212	0.485	0.419	0.703634	13
Btbd17	4.24E-05	-0.54425	0.029	0.161	0.707622	13
Arhgap11a	4.3E-05	-0.50525	0.029	0.171	0.716732	13
Gdpd1	4.3E-05	-0.54672	0.068	0.244	0.717799	13
A030009H(	4.33E-05	0.602747	0.223	0.113	0.722016	13

Sod1	4.35E-05	-0.40174	0.233	0.443	0.726023	13
F2r	4.36E-05	-0.45938	0	0.105	0.726669	13
Sparcl1	4.38E-05	-0.93692	0.049	0.208	0.730945	13
Uhrf1	4.43E-05	-0.38731	0.019	0.144	0.739275	13
Ctxn1	4.51E-05	-0.3264	0.049	0.17	0.751991	13
Cadm1	4.53E-05	0.336253	0.388	0.39	0.754889	13
Clcn3	4.53E-05	0.378081	0.272	0.272	0.756329	13
Gm10076	4.53E-05	-0.51475	0.01	0.134	0.756374	13
Polr2d	4.56E-05	-0.49866	0.068	0.227	0.759889	13
Syf2	4.57E-05	0.30244	0.233	0.255	0.76237	13
Mrpl17	4.61E-05	-0.34708	0.097	0.26	0.768323	13
Gclm	4.61E-05	0.501766	0.194	0.14	0.769642	13
Tmem165	4.64E-05	-0.39346	0.019	0.11	0.774393	13
Rpl7a	4.66E-05	-0.5091	0.019	0.15	0.777608	13
Cdk5rap2	4.73E-05	-0.27071	0.058	0.184	0.789119	13
Ralgps2	4.75E-05	-0.628	0.068	0.246	0.792564	13
Spg21	4.83E-05	-0.52429	0.029	0.174	0.805785	13
Gm13092	4.84E-05	-0.43744	0	0.104	0.808051	13
BC005764	4.86E-05	-0.25718	0.078	0.186	0.810341	13
Polr3k	4.89E-05	-0.38687	0.097	0.271	0.815687	13
Ldhb	4.91E-05	0.357136	0.301	0.28	0.819167	13
Zranb1	4.99E-05	0.478391	0.243	0.208	0.832106	13
Mrpl24	5.01E-05	-0.37968	0.068	0.212	0.83617	13
Mrps17	5.02E-05	-0.43211	0.078	0.247	0.837749	13
Tipin	5.04E-05	-0.44732	0.087	0.255	0.841013	13
Itgb3bp	5.06E-05	-0.44122	0	0.104	0.844116	13
Abhd16a	5.07E-05	0.537522	0.262	0.189	0.845723	13
Pih1d1	5.16E-05	-0.3614	0.039	0.171	0.860979	13
D10Wsu10	5.18E-05	-0.323	0.019	0.12	0.863678	13
Sugp1	5.2E-05	0.4039	0.117	0.068	0.868109	13
Bnip2	5.21E-05	-0.32578	0.078	0.218	0.869658	13
Rnf220	5.3E-05	-0.35118	0.078	0.236	0.883543	13
Lphn3	5.35E-05	0.617795	0.146	0.06	0.893042	13
Sptbn1	5.36E-05	0.435736	0.417	0.353	0.894213	13
Atp5g1	5.4E-05	0.528398	0.194	0.096	0.900727	13
Hspa4l	5.43E-05	0.551449	0.136	0.084	0.904915	13
Anapc16	5.49E-05	-0.45478	0.068	0.234	0.916249	13
Tmem107	5.53E-05	-0.34429	0.019	0.127	0.921772	13
Rrp15	5.53E-05	-0.50743	0.029	0.174	0.922491	13
Stard4	5.54E-05	-0.53481	0.01	0.141	0.923379	13
Fuca1	5.55E-05	-0.36352	0.029	0.148	0.925675	13
Dnajc3	5.58E-05	-0.25729	0.078	0.177	0.931218	13
Lamp1	5.59E-05	-0.30417	0.175	0.337	0.932554	13
Ddx17	5.64E-05	0.299677	0.369	0.381	0.941413	13
Fbxl16	5.66E-05	0.414958	0.136	0.067	0.944304	13
Ostc	5.67E-05	-0.36203	0.107	0.274	0.946193	13
Lhfp	5.71E-05	0.493402	0.107	0.018	0.952054	13
Thrsp	5.75E-05	0.474341	0.117	0.032	0.959795	13

Tex30	5.8E-05	-0.45164	0.019	0.136	0.968078	13
Psmb2	5.81E-05	-0.26607	0.233	0.429	0.968842	13
Lman2	5.85E-05	-0.50876	0.01	0.14	0.975244	13
Peo1	5.86E-05	-0.31551	0.019	0.124	0.976727	13
Tk1	5.9E-05	-0.46572	0.01	0.131	0.983983	13
Abcf1	5.97E-05	-0.2952	0.252	0.458	0.99511	13
Pbdc1	6.07E-05	-0.4882	0.049	0.206	1	13
Csrp2	6.08E-05	-0.41348	0.029	0.158	1	13
Rbmxl1	6.18E-05	-0.27604	0.049	0.153	1	13
Ap1s2	6.2E-05	0.451503	0.214	0.159	1	13
Mia	6.25E-05	0.578839	0.107	0.024	1	13
Plk4	6.41E-05	-0.36578	0.01	0.12	1	13
Eif1ax	6.44E-05	-0.2795	0.204	0.398	1	13
C330027C(	6.48E-05	-0.47895	0.01	0.134	1	13
Tnpo3	6.52E-05	-0.49465	0.058	0.223	1	13
Ssbp4	6.57E-05	0.544005	0.146	0.06	1	13
Vps72	6.66E-05	-0.38067	0.029	0.154	1	13
Mapk8ip1	6.69E-05	0.508639	0.359	0.226	1	13
Rbms1	6.71E-05	-0.38024	0.068	0.217	1	13
Ube2e3	6.73E-05	-0.27757	0.223	0.417	1	13
Baz1b	6.76E-05	-0.39227	0.272	0.494	1	13
Odc1	6.82E-05	-0.42091	0	0.101	1	13
Pvrl3	6.83E-05	0.46013	0.146	0.122	1	13
Rnpc3	6.89E-05	0.589298	0.184	0.103	1	13
Bub1	6.91E-05	-0.46262	0.01	0.131	1	13
Ctsz	6.96E-05	-0.58878	0.01	0.137	1	13
Reep5	6.97E-05	0.521904	0.223	0.137	1	13
Rpl6	7.18E-05	-0.45334	0.107	0.291	1	13
Dbndd2	7.21E-05	0.474784	0.107	0.024	1	13
Mrps15	7.26E-05	-0.44271	0.068	0.23	1	13
Txlna	7.26E-05	-0.40894	0.049	0.186	1	13
Nemf	7.29E-05	0.276661	0.301	0.339	1	13
Dcakd	7.36E-05	-0.3769	0.117	0.297	1	13
Eif5a	7.39E-05	-0.34283	0.223	0.404	1	13
Psmc2	7.57E-05	-0.40865	0.136	0.3	1	13
Cct4	7.59E-05	-0.45227	0.175	0.382	1	13
Mettl16	7.65E-05	-0.30464	0.019	0.118	1	13
Gripap1	7.66E-05	0.260659	0.184	0.188	1	13
Gins1	7.68E-05	-0.34394	0.01	0.115	1	13
Atad3a	7.68E-05	0.450964	0.155	0.126	1	13
Smarcc2	7.7E-05	0.298599	0.379	0.363	1	13
Frmd4b	7.76E-05	-0.5334	0.01	0.128	1	13
Rhno1	7.78E-05	-0.52279	0.01	0.138	1	13
Cstf2t	7.79E-05	0.550057	0.155	0.088	1	13
Atad2	8.16E-05	-0.50347	0.049	0.199	1	13
Txnl1	8.19E-05	-0.48673	0.146	0.346	1	13
Stx7	8.2E-05	0.424515	0.175	0.115	1	13
Cldn25	8.31E-05	-0.46471	0.068	0.233	1	13

Snx30	8.35E-05	0.464908	0.136	0.064	1	13
Gm11266	8.36E-05	-0.51354	0.019	0.144	1	13
Psma5	8.42E-05	-0.43193	0.136	0.322	1	13
Gnptg	8.47E-05	0.348719	0.204	0.166	1	13
Rab34	8.47E-05	-0.38419	0.01	0.118	1	13
Cdk5rap3	8.55E-05	-0.43151	0.049	0.193	1	13
Adrbk2	8.58E-05	0.548582	0.204	0.099	1	13
Psmc4	8.62E-05	-0.35738	0.146	0.322	1	13
Socs7	8.71E-05	0.498081	0.194	0.095	1	13
Srpk2	8.73E-05	0.354658	0.476	0.429	1	13
Map7d1	8.74E-05	0.479811	0.359	0.241	1	13
Rad51ap1	8.75E-05	-0.52888	0.029	0.167	1	13
Tspyl5	9.06E-05	0.52543	0.107	0.028	1	13
Ccndbp1	9.07E-05	0.533584	0.233	0.116	1	13
Reep3	9.11E-05	-0.29742	0.107	0.257	1	13
Add3	9.15E-05	0.585874	0.204	0.103	1	13
Cit	9.19E-05	0.51208	0.146	0.065	1	13
Ube2r2	9.2E-05	-0.3721	0.136	0.317	1	13
Alkbh6	9.36E-05	0.510177	0.204	0.133	1	13
Ttyh1	9.4E-05	0.534979	0.126	0.031	1	13
mt-Co1	9.45E-05	-0.32741	0.388	0.611	1	13
Mphosph1(	9.79E-05	-0.48966	0.068	0.232	1	13
Mak16	9.81E-05	-0.44323	0.068	0.226	1	13
Pmf1	9.96E-05	-0.45707	0.019	0.143	1	13
Herc1	9.99E-05	0.366284	0.204	0.186	1	13
Nek6	0.000101	-0.42444	0.029	0.144	1	13
Cul7	0.000101	0.529118	0.107	0.033	1	13
Tm9sf3	0.000102	-0.43782	0.155	0.345	1	13
Taok1	0.000104	0.434412	0.262	0.224	1	13
Cdh4	0.000105	-0.51416	0.019	0.146	1	13
Cabin1	0.000108	0.495703	0.107	0.059	1	13
Psmc3ip	0.000108	-0.48493	0.01	0.132	1	13
Rell1	0.000108	0.4016	0.107	0.096	1	13
Macf1	0.000109	0.563167	0.32	0.209	1	13
Hes6	0.00011	-0.50485	0.078	0.237	1	13
Bzw1	0.000111	-0.41389	0.165	0.36	1	13
Psmd12	0.000111	-0.38851	0.175	0.374	1	13
Gpc2	0.000113	-0.4299	0.029	0.14	1	13
Polr2f	0.000113	-0.40391	0.175	0.375	1	13
Dst	0.000113	0.423714	0.301	0.266	1	13
Cecr2	0.000115	-0.48449	0.01	0.13	1	13
Emg1	0.000116	-0.49863	0.126	0.316	1	13
Blcap	0.000117	0.559544	0.165	0.078	1	13
Rhot1	0.00012	0.346326	0.136	0.087	1	13
Ncapg	0.000121	-0.55264	0.039	0.184	1	13
2510002D:	0.000121	0.621734	0.136	0.049	1	13
Dab2ip	0.000121	0.546158	0.165	0.068	1	13
Txnl4a	0.000122	-0.28124	0.068	0.194	1	13

Lta4h	0.000124	-0.51339	0.078	0.243	1	13
Smu1	0.000125	-0.28814	0.078	0.219	1	13
Samd14	0.000125	0.539222	0.155	0.076	1	13
Stip1	0.000125	-0.47937	0.126	0.304	1	13
Ube2e1	0.000125	-0.34639	0.078	0.228	1	13
Aig1	0.000126	0.571118	0.194	0.095	1	13
Ogfrl1	0.000127	0.539496	0.165	0.081	1	13
Dars	0.000129	-0.31029	0.039	0.158	1	13
Pard3	0.00013	-0.27316	0.029	0.127	1	13
Eif3g	0.00013	-0.28656	0.223	0.403	1	13
Phip	0.000132	-0.27587	0.262	0.46	1	13
Cnot2	0.000133	0.296444	0.194	0.185	1	13
Ndst3	0.000133	0.477013	0.126	0.033	1	13
Kif3b	0.000133	0.438244	0.107	0.096	1	13
Suz12	0.000134	-0.49406	0.068	0.22	1	13
Slc24a5	0.000135	-0.35108	0.029	0.142	1	13
Dusp3	0.000135	0.507505	0.107	0.028	1	13
Dnajc21	0.000136	-0.27048	0.087	0.21	1	13
N4bp2	0.000137	-0.33526	0.029	0.132	1	13
Nudt21	0.000138	-0.4815	0.01	0.134	1	13
Syngr3	0.000139	0.544786	0.136	0.036	1	13
Emc7	0.000141	0.455948	0.184	0.143	1	13
Snx32	0.000143	0.544855	0.117	0.037	1	13
Slc25a12	0.000146	0.487456	0.136	0.073	1	13
Tomm22	0.000146	-0.26562	0.136	0.291	1	13
D17H6S56I	0.000146	-0.45606	0.01	0.128	1	13
Kif15	0.000146	-0.37557	0.049	0.181	1	13
Stk11	0.000146	-0.28531	0.087	0.23	1	13
1700001O:	0.000147	-0.29146	0.019	0.11	1	13
Rps16-ps2	0.000147	-0.40091	0.019	0.14	1	13
Setd7	0.000148	0.486871	0.117	0.071	1	13
Cxx1c	0.000148	0.53472	0.146	0.054	1	13
0610012G(	0.000149	0.469227	0.204	0.146	1	13
Tpt1	0.000149	-0.35677	0.097	0.257	1	13
Map4k4	0.00015	-0.39464	0.243	0.45	1	13
Ddx3x	0.00015	-0.29223	0.282	0.467	1	13
Idh3b	0.000151	0.318269	0.262	0.246	1	13
Dctn3	0.000153	0.297204	0.35	0.368	1	13
Galt	0.000155	0.483789	0.107	0.022	1	13
Rrp1	0.000158	0.309525	0.592	0.606	1	13
Atp6ap1	0.000159	0.509259	0.214	0.138	1	13
Ndufa11	0.000159	0.297343	0.388	0.331	1	13
Bccip	0.00016	0.275273	0.301	0.31	1	13
Mmadhc	0.00016	-0.41922	0.078	0.233	1	13
Isy1	0.00016	-0.42607	0.029	0.142	1	13
Nol8	0.000165	-0.33787	0.049	0.168	1	13
Wdfy3	0.000165	0.370027	0.107	0.08	1	13
Acat1	0.000166	-0.40792	0.243	0.442	1	13

Wdr77	0.000169	-0.42764	0.01	0.124	1	13
Slc8a2	0.000169	0.492862	0.117	0.03	1	13
Rpl31	0.000171	-0.50273	0.01	0.131	1	13
Pfn2	0.000174	0.456896	0.301	0.24	1	13
Ik	0.000175	0.301902	0.476	0.464	1	13
Nktr	0.000175	0.264156	0.34	0.38	1	13
Tmem237	0.000178	-0.48148	0.01	0.13	1	13
Ppp3r1	0.000178	0.296332	0.126	0.137	1	13
Lims1	0.000179	-0.28614	0.058	0.183	1	13
Inpp5k	0.00018	0.451216	0.107	0.055	1	13
Phyhipl	0.000182	0.543998	0.146	0.061	1	13
Pola1	0.000184	-0.30538	0.019	0.103	1	13
Rgs17	0.000184	0.563718	0.136	0.046	1	13
Cenpm	0.000184	-0.45578	0.029	0.162	1	13
Sema6a	0.000185	-0.59049	0.058	0.203	1	13
Fxyd6	0.000185	-0.25519	0.359	0.509	1	13
Cmtm3	0.000186	-0.39205	0.01	0.118	1	13
Pcmtd2	0.000186	0.292389	0.136	0.143	1	13
Cst3	0.000186	-0.41878	0.427	0.536	1	13
Armcx1	0.000187	0.504932	0.155	0.083	1	13
Sdccag3	0.000189	0.362001	0.136	0.122	1	13
Dazap2	0.000191	-0.51846	0.078	0.246	1	13
Ehmt2	0.000192	-0.43578	0.068	0.209	1	13
Serp2	0.000194	0.517677	0.184	0.086	1	13
Snw1	0.000196	-0.36351	0.233	0.432	1	13
Ccng2	0.000204	-0.43224	0.078	0.212	1	13
Fzd2	0.000204	-0.51354	0.029	0.161	1	13
Serpinh1	0.000206	-0.42959	0.01	0.116	1	13
March2	0.000207	0.428817	0.136	0.069	1	13
Thsd7a	0.000208	-0.5589	0.01	0.128	1	13
Srrm3	0.000208	0.520214	0.32	0.239	1	13
Eif6	0.00021	-0.46069	0.087	0.25	1	13
Zfp422	0.000212	-0.3839	0.087	0.246	1	13
Acin1	0.000212	-0.26386	0.524	0.724	1	13
Nono	0.000212	-0.42724	0.068	0.22	1	13
Stt3b	0.000213	-0.38386	0.068	0.212	1	13
Rsl1d1	0.000214	-0.31561	0.291	0.491	1	13
Tspan31	0.000214	-0.32573	0.078	0.218	1	13
Jund	0.000217	-0.30674	0.175	0.352	1	13
Rnf6	0.000221	0.517256	0.136	0.059	1	13
Cenpc1	0.000226	-0.27299	0.039	0.155	1	13
Rspry1	0.000227	-0.27418	0.019	0.107	1	13
Gxylt1	0.000228	-0.37511	0.01	0.111	1	13
Aebp2	0.00023	0.349075	0.117	0.121	1	13
Gm11541	0.000232	-0.51975	0.01	0.128	1	13
Elavl1	0.000232	-0.45148	0.049	0.19	1	13
Lsmd1	0.000232	-0.26789	0.087	0.22	1	13
Prpf38a	0.000234	-0.39625	0.029	0.153	1	13

Dusp11	0.000235	0.416229	0.252	0.201	1	13
Dennd2a	0.000236	-0.33935	0.01	0.107	1	13
Qser1	0.000238	-0.27098	0.029	0.128	1	13
Larp4b	0.000239	0.271	0.155	0.171	1	13
Celf5	0.00024	0.436174	0.126	0.056	1	13
Scoc	0.00024	0.407079	0.117	0.078	1	13
Asf1a	0.00024	-0.37135	0.117	0.28	1	13
Srsf9	0.000242	-0.43251	0.087	0.237	1	13
Dgkz	0.000244	0.54635	0.204	0.104	1	13
Prmt5	0.000246	-0.37882	0.097	0.257	1	13
Ap2a2	0.000247	0.457053	0.165	0.121	1	13
Pkn2	0.000253	-0.33078	0.068	0.208	1	13
Fam111a	0.000253	-0.42957	0.019	0.126	1	13
Wdr60	0.000255	-0.36633	0.019	0.119	1	13
0610037L1	0.000255	-0.29484	0.058	0.183	1	13
Vgll4	0.000256	-0.43726	0.029	0.153	1	13
Aspm	0.000257	-0.5616	0.019	0.143	1	13
Yif1a	0.000257	-0.50308	0.039	0.18	1	13
Cenpj	0.000259	-0.43227	0.039	0.142	1	13
Rpl15	0.000262	-0.47518	0.029	0.162	1	13
Mki67ip	0.000262	-0.41696	0.087	0.245	1	13
Mpdz	0.000262	-0.47409	0.019	0.135	1	13
Gkap1	0.000266	-0.38142	0.078	0.221	1	13
Rsrc1	0.000266	-0.57255	0.087	0.251	1	13
Cenpp	0.000267	-0.35811	0.019	0.114	1	13
Apoe	0.000268	-1.72272	0.107	0.249	1	13
A330076H(	0.000272	0.451361	0.243	0.16	1	13
Setdb1	0.000274	-0.35087	0.01	0.109	1	13
Asnsd1	0.000278	-0.27896	0.029	0.117	1	13
Gphn	0.00028	0.492403	0.204	0.14	1	13
Pea15a	0.00028	0.368293	0.282	0.175	1	13
Strbp	0.00028	0.330385	0.359	0.347	1	13
Hpca	0.000287	-0.40492	0.117	0.278	1	13
Ccdc136	0.000288	0.56911	0.184	0.074	1	13
Cstb	0.000293	0.551545	0.233	0.116	1	13
2810055G:	0.000293	-0.45803	0.039	0.171	1	13
Pphln1	0.000293	-0.40655	0.01	0.119	1	13
Bcl2l13	0.000295	-0.40318	0.01	0.115	1	13
Cds2	0.000296	0.470363	0.117	0.044	1	13
2700029M	0.000297	-0.36755	0.126	0.29	1	13
1500011B(	0.000302	0.539419	0.194	0.106	1	13
Dnph1	0.000302	-0.43209	0.01	0.124	1	13
Tex9	0.000302	-0.39898	0.029	0.148	1	13
Bcar1	0.000306	-0.47611	0.049	0.193	1	13
Jarid2	0.000306	0.422189	0.214	0.178	1	13
U2af2	0.000307	-0.40229	0.019	0.137	1	13
Tsc22d4	0.000308	-0.49867	0.039	0.167	1	13
Slc39a10	0.000312	-0.38481	0.058	0.195	1	13

Mcmbp	0.000312	-0.31748	0.078	0.217	1	13
Anln	0.000312	-0.32211	0.01	0.104	1	13
Yeats4	0.000312	-0.29985	0.117	0.264	1	13
Ndst1	0.000313	-0.32025	0.039	0.136	1	13
Grpel2	0.000313	0.530117	0.146	0.072	1	13
Dclk1	0.000314	0.321149	0.466	0.411	1	13
Gcc2	0.000314	0.485682	0.214	0.17	1	13
Zfp612	0.00032	0.524755	0.126	0.04	1	13
Pes1	0.000322	-0.39279	0.01	0.115	1	13
Cenpw	0.000324	-0.48292	0.019	0.128	1	13
Vrk3	0.000327	-0.39702	0.01	0.115	1	13
Copg1	0.000327	0.50275	0.223	0.152	1	13
Hagh	0.000331	0.502302	0.175	0.1	1	13
Pcdhga9	0.000331	-0.3479	0.233	0.421	1	13
BC031181	0.000334	0.283404	0.32	0.261	1	13
Plekhb2	0.000335	0.483201	0.107	0.033	1	13
Tcf3	0.000335	-0.40643	0.078	0.226	1	13
Fam212b	0.000336	-0.36866	0.078	0.223	1	13
Smc6	0.000337	-0.32177	0.233	0.423	1	13
Ccl27a	0.000338	0.437636	0.146	0.043	1	13
Ssb	0.00034	-0.38043	0.515	0.704	1	13
Dync2h1	0.000341	-0.3723	0.019	0.116	1	13
Tceb1	0.000342	-0.3889	0.194	0.367	1	13
Eif4a3	0.000344	-0.33589	0.184	0.356	1	13
Cdca2	0.000344	-0.31044	0.029	0.129	1	13
Fbxw11	0.000347	0.429983	0.126	0.085	1	13
A830080D(	0.000349	-0.40699	0.01	0.114	1	13
Ppp5c	0.00035	0.421458	0.175	0.151	1	13
Park7	0.00035	-0.25134	0.35	0.547	1	13
Eif4e2	0.000361	-0.48545	0.068	0.219	1	13
Fstl1	0.000362	-0.42338	0.058	0.181	1	13
Gatc	0.000364	-0.34395	0.01	0.107	1	13
Mcl1	0.000366	-0.38397	0.068	0.212	1	13
Uhrf1bp1l	0.000367	0.343709	0.165	0.132	1	13
Orc4	0.000367	0.402338	0.117	0.09	1	13
Pafah1b1	0.000368	0.301077	0.466	0.474	1	13
Ptplb	0.000369	-0.41327	0.01	0.116	1	13
Clic1	0.000371	-0.40463	0.049	0.173	1	13
Pfdn4	0.000373	-0.29095	0.146	0.306	1	13
Pbx1	0.000375	-0.43866	0.049	0.187	1	13
Rftn2	0.000376	-0.48269	0.01	0.124	1	13
Smc5	0.000377	-0.31002	0.107	0.258	1	13
Hnrnpul1	0.00038	-0.34867	0.049	0.175	1	13
Atp1b3	0.000381	-0.30551	0.165	0.338	1	13
Mad2l1	0.000382	-0.45845	0.01	0.123	1	13
Ggh	0.000384	-0.4784	0.01	0.124	1	13
Fnbp4	0.000386	0.255003	0.272	0.276	1	13
Pnmal2	0.000389	0.546867	0.233	0.146	1	13

Wdr33	0.000389	-0.269	0.068	0.196	1	13
Ankrd46	0.000391	0.308896	0.175	0.16	1	13
Stag2	0.000395	-0.2669	0.117	0.257	1	13
Mprip	0.000397	0.273463	0.155	0.151	1	13
Isoc1	0.000397	-0.38238	0.049	0.181	1	13
Sez6l2	0.000402	0.532853	0.126	0.036	1	13
Cetn3	0.000403	-0.31848	0.291	0.487	1	13
Bdp1	0.000403	0.438089	0.184	0.154	1	13
Qdpr	0.000408	-0.4054	0.107	0.268	1	13
Tbl1xr1	0.000408	-0.437	0.01	0.12	1	13
Zfp664	0.000414	-0.25888	0.097	0.23	1	13
Pygo1	0.000419	-0.41214	0.078	0.192	1	13
Mrpl28	0.000423	-0.25074	0.146	0.289	1	13
Arhgap12	0.000427	-0.35108	0.01	0.105	1	13
Cd63-ps	0.000427	-0.42671	0.01	0.119	1	13
Pan3	0.000429	0.416331	0.136	0.078	1	13
Nek7	0.000436	-0.31807	0.019	0.117	1	13
Rps28	0.000437	-0.44189	0.058	0.204	1	13
Babam1	0.000442	-0.26707	0.049	0.151	1	13
Sec63	0.000444	-0.49321	0.068	0.211	1	13
Cdca4	0.000445	-0.3506	0.01	0.111	1	13
Tmem178	0.000445	0.327905	0.184	0.186	1	13
Ehd3	0.000448	0.506557	0.107	0.03	1	13
Ash1l	0.000454	0.334517	0.291	0.277	1	13
Mars	0.000457	-0.29667	0.039	0.151	1	13
Ywhaz	0.000472	0.291169	0.408	0.362	1	13
Hdgf	0.000475	-0.32329	0.33	0.533	1	13
Slk	0.000476	0.437015	0.223	0.18	1	13
Tex14	0.000484	-0.6396	0.029	0.146	1	13
Trim8	0.00049	-0.25424	0.049	0.14	1	13
Rabggtb	0.000495	0.387625	0.233	0.172	1	13
Eif2s2	0.000495	-0.36231	0.155	0.331	1	13
Stt3a	0.000496	-0.40078	0.019	0.126	1	13
Senp1	0.000497	-0.3206	0.029	0.136	1	13
Gnl3	0.0005	-0.42516	0.126	0.296	1	13
Cntln	0.0005	-0.40322	0.049	0.173	1	13
Fam64a	0.000501	-0.4495	0.01	0.118	1	13
Reep2	0.000503	0.523836	0.184	0.067	1	13
Pole4	0.000505	-0.40756	0.029	0.145	1	13
2310044G1	0.000505	0.491272	0.146	0.054	1	13
Exosc8	0.000511	-0.37904	0.078	0.21	1	13
Sept2	0.000514	-0.28003	0.087	0.223	1	13
Mrpl11	0.000514	-0.44216	0.107	0.238	1	13
1300002E1	0.000517	-0.3105	0.029	0.114	1	13
Thrap3	0.00052	-0.28139	0.214	0.388	1	13
Ramp2	0.000528	-0.52253	0.01	0.12	1	13
Ncor2	0.000531	-0.5461	0.049	0.169	1	13
Gcsh	0.000533	-0.39149	0.029	0.142	1	13

Rab33a	0.000535	0.482535	0.136	0.051	1	13
Polr2i	0.000535	-0.34408	0.155	0.317	1	13
Smn1	0.000537	-0.28595	0.049	0.159	1	13
Arf5	0.00054	0.288323	0.262	0.255	1	13
2210013O	0.000542	0.484785	0.204	0.117	1	13
Csnk2a1	0.000551	-0.25371	0.107	0.245	1	13
Mex3b	0.000552	-0.46089	0.01	0.116	1	13
Cntnap1	0.000559	0.484381	0.146	0.079	1	13
Atp8a1	0.000559	0.50299	0.155	0.073	1	13
Far1	0.000562	0.314298	0.136	0.104	1	13
Man2a2	0.000566	0.503215	0.107	0.044	1	13
Sox18	0.000568	-0.47751	0.029	0.148	1	13
Gm17322	0.000569	-0.58693	0.019	0.131	1	13
Mab21l2	0.000578	-0.39368	0.019	0.104	1	13
Gm20417	0.000588	0.376582	0.107	0.043	1	13
Hat1	0.000589	-0.3435	0.039	0.155	1	13
Zdhhc16	0.000591	0.349828	0.126	0.09	1	13
Actl6b	0.000591	0.393098	0.107	0.055	1	13
Rsu1	0.000592	-0.4682	0.01	0.119	1	13
Prkacb	0.000594	0.43859	0.262	0.186	1	13
Cox14	0.000596	0.259408	0.301	0.316	1	13
Gps2	0.000603	-0.2839	0.097	0.236	1	13
Tmed9	0.000608	-0.27531	0.252	0.434	1	13
Cdk7	0.000609	-0.36183	0.019	0.109	1	13
Cdk14	0.000609	-0.35806	0.01	0.104	1	13
Ywhah	0.000611	0.309172	0.184	0.162	1	13
Lima1	0.000613	-0.45901	0.019	0.122	1	13
Fam174a	0.000615	0.417178	0.136	0.064	1	13
2610203C	0.000619	-0.47651	0.019	0.121	1	13
Zkscan1	0.00062	-0.25509	0.049	0.151	1	13
Pigx	0.00062	-0.47279	0.029	0.148	1	13
Tab2	0.000623	-0.29691	0.107	0.247	1	13
Gnai1	0.000624	0.472308	0.165	0.099	1	13
Gabpb2	0.000629	0.340772	0.126	0.096	1	13
Kifap3	0.000629	0.273675	0.301	0.272	1	13
Manf	0.000632	-0.35486	0.155	0.327	1	13
Nipsnap1	0.000634	0.281108	0.126	0.136	1	13
Ctdsp2	0.00064	0.332278	0.117	0.11	1	13
Pcnt	0.000648	-0.29776	0.068	0.175	1	13
Epb4.1l1	0.00065	0.549863	0.204	0.09	1	13
Rdh5	0.000655	-0.38698	0.029	0.126	1	13
Ntrk3	0.000658	-0.28657	0.049	0.155	1	13
Mrps21	0.000659	-0.42941	0.087	0.239	1	13
2310015B	0.000674	0.46355	0.107	0.037	1	13
Ogdh	0.000676	0.372146	0.175	0.127	1	13
Tmed5	0.000679	-0.36215	0.019	0.126	1	13
Mroh2a	0.000684	-0.45678	0.029	0.114	1	13
Dpysl3	0.000685	-0.68413	0.078	0.22	1	13

Rpl36	0.000686	-0.36883	0.01	0.108	1	13
Rnf114	0.000691	-0.26836	0.058	0.164	1	13
Mrpl55	0.000694	-0.35425	0.049	0.16	1	13
Slc38a2	0.000696	-0.39896	0.078	0.221	1	13
Plxna2	0.000708	0.61002	0.155	0.069	1	13
Akap6	0.000715	-0.40117	0.078	0.219	1	13
Kat2b	0.000715	0.423561	0.126	0.047	1	13
Thoc7	0.000717	-0.38478	0.32	0.522	1	13
Hdac1	0.00072	-0.39112	0.019	0.126	1	13
Pfkm	0.000721	0.492188	0.155	0.065	1	13
Lrrc16b	0.000723	0.542623	0.126	0.052	1	13
Dcun1d5	0.000723	-0.45778	0.097	0.257	1	13
Agtpbp1	0.000729	0.472266	0.146	0.101	1	13
Mbtd1	0.000729	-0.43046	0.078	0.215	1	13
Smoc1	0.000735	-0.35862	0.029	0.135	1	13
Gm3764	0.000737	0.495906	0.184	0.131	1	13
Gprc5b	0.000743	0.433013	0.126	0.042	1	13
Atf4	0.000744	-0.28095	0.223	0.397	1	13
Fam53b	0.000746	-0.40118	0.029	0.136	1	13
Tmem242	0.000749	-0.34768	0.078	0.192	1	13
Dnttip2	0.00075	-0.30364	0.165	0.325	1	13
Rap2b	0.000756	-0.44627	0.029	0.148	1	13
Hmgcl	0.000762	0.448282	0.136	0.078	1	13
Nsun2	0.000765	-0.47263	0.039	0.169	1	13
Rps24-ps3	0.000765	-0.29377	0.058	0.18	1	13
Vars	0.000768	-0.44197	0.039	0.167	1	13
Nudt9	0.000775	-0.29551	0.029	0.116	1	13
Cfl2	0.000778	-0.32733	0.233	0.409	1	13
Kdelr2	0.000782	-0.37896	0.068	0.206	1	13
Taf1d	0.000788	-0.29837	0.175	0.342	1	13
Ankrd10	0.0008	-0.25993	0.049	0.159	1	13
Ccnl2	0.000805	0.279116	0.32	0.32	1	13
Ipo5	0.000814	-0.37223	0.029	0.139	1	13
Mycl	0.00083	-0.35277	0.029	0.137	1	13
Prr13	0.000831	0.501982	0.175	0.089	1	13
Srgap2	0.000834	0.492866	0.204	0.131	1	13
Ttc28	0.000839	-0.40826	0.019	0.123	1	13
1810022K(	0.000839	-0.31913	0.058	0.181	1	13
Satb1	0.000839	-0.38868	0.078	0.21	1	13
Mapk8ip2	0.00084	0.454969	0.223	0.142	1	13
Tmem256	0.00084	-0.3619	0.301	0.493	1	13
Cdca7l	0.000849	-0.33998	0.01	0.104	1	13
Rpl7l1	0.000856	-0.29887	0.087	0.223	1	13
Grik2	0.000856	0.420756	0.194	0.101	1	13
Ppid	0.000866	-0.34721	0.068	0.202	1	13
Mrps9	0.000869	-0.29048	0.039	0.148	1	13
Ppa2	0.000869	-0.33999	0.039	0.155	1	13
Gtf2e2	0.000876	-0.44144	0.049	0.178	1	13

AI854517	0.000879	-0.37265	0.058	0.189	1	13
Pdpk1	0.000881	0.356388	0.146	0.099	1	13
Slain2	0.000883	-0.41529	0.019	0.128	1	13
Imp3	0.000897	-0.43693	0.097	0.247	1	13
Zfp277	0.000898	-0.44695	0.019	0.132	1	13
Hif1a	0.000913	0.298596	0.117	0.126	1	13
Med15	0.000916	0.359775	0.107	0.049	1	13
Grpel1	0.00092	-0.31353	0.068	0.183	1	13
Ncapd2	0.000921	-0.30596	0.068	0.185	1	13
Thra	0.00093	0.499753	0.35	0.228	1	13
Scnm1	0.000934	-0.44064	0.029	0.136	1	13
Pttg1	0.000936	-0.55262	0.029	0.14	1	13
Clpp	0.00094	-0.4252	0.087	0.239	1	13
Smek1	0.000944	-0.34779	0.058	0.179	1	13
Wwp1	0.000952	0.483971	0.136	0.084	1	13
Lsm2	0.000953	-0.44228	0.087	0.231	1	13
Rfng	0.000956	0.379216	0.136	0.073	1	13
Chkb	0.000957	0.389234	0.204	0.156	1	13
Dcaf13	0.000976	-0.32222	0.019	0.114	1	13
Tbc1d16	0.000991	-0.35483	0.068	0.197	1	13
Akt1	0.000995	-0.44316	0.01	0.112	1	13
Pkia	0.000997	0.536666	0.233	0.133	1	13
Rcc2	0.001003	-0.34281	0.058	0.179	1	13
Pik3r3	0.001015	0.515662	0.233	0.135	1	13
Asphd1	0.001017	0.326849	0.107	0.025	1	13
Sdhc	0.001018	0.328711	0.301	0.269	1	13
Alcam	0.001018	-0.2853	0.039	0.135	1	13
Commd3	0.001022	-0.38444	0.146	0.308	1	13
Bms1	0.001035	-0.26125	0.068	0.183	1	13
Myeov2	0.00104	-0.37441	0.282	0.456	1	13
Pdzd11	0.001074	-0.35	0.039	0.154	1	13
Skp2	0.001086	-0.35688	0.029	0.12	1	13
Slc1a3	0.001102	-0.70115	0.039	0.153	1	13
Unc13a	0.001103	0.434749	0.126	0.062	1	13
1700021F(	0.001109	-0.27471	0.039	0.141	1	13
Slc22a17	0.001112	0.394035	0.32	0.25	1	13
Rnf165	0.001119	-0.44153	0.049	0.173	1	13
Lmo1	0.001121	0.415351	0.107	0.039	1	13
Cpne3	0.001131	-0.35398	0.068	0.197	1	13
Pitpnc1	0.001142	0.545147	0.155	0.074	1	13
Dcaf11	0.001145	0.44666	0.165	0.096	1	13
Ndn	0.001147	0.455962	0.32	0.222	1	13
Vps28	0.001157	0.387519	0.32	0.244	1	13
Sgpl1	0.001162	-0.3363	0.039	0.149	1	13
Zfp266	0.001185	-0.26594	0.058	0.168	1	13
Ddost	0.00119	-0.29613	0.126	0.27	1	13
Rpl10a	0.00119	-0.4354	0.01	0.112	1	13
Gria4	0.001199	0.50955	0.214	0.124	1	13

Nudt4	0.001207	-0.41881	0.078	0.218	1	13
Isca1	0.001213	0.406747	0.117	0.055	1	13
Phactr1	0.001217	0.435391	0.272	0.183	1	13
Aftph	0.001243	0.38857	0.146	0.119	1	13
Nop16	0.001243	-0.46108	0.049	0.174	1	13
Smap1	0.001246	0.29632	0.243	0.223	1	13
Yy1	0.001268	-0.4766	0.068	0.205	1	13
Ube2d1	0.001269	-0.32433	0.097	0.228	1	13
Gmnn	0.001274	-0.44208	0.029	0.145	1	13
Msantd4	0.001285	0.453692	0.175	0.121	1	13
Rsbn1	0.001293	0.299801	0.194	0.182	1	13
Lin7c	0.001295	0.34121	0.262	0.217	1	13
Acyp1	0.001303	0.395759	0.146	0.096	1	13
Rrbp1	0.001323	-0.44226	0.058	0.189	1	13
Cdh15	0.001326	0.38751	0.117	0.039	1	13
Mtus1	0.001328	0.392068	0.155	0.127	1	13
l7Rn6	0.001354	-0.26804	0.078	0.201	1	13
Tubgcp3	0.001358	-0.25265	0.019	0.103	1	13
Tmem176b	0.001381	0.460304	0.155	0.109	1	13
Pcsk2	0.001385	0.383948	0.146	0.101	1	13
Immp1l	0.001392	-0.32451	0.068	0.188	1	13
Neo1	0.001398	-0.39479	0.01	0.106	1	13
Med10	0.001408	-0.28725	0.087	0.214	1	13
Tcerg1	0.00141	-0.34113	0.272	0.458	1	13
Snx4	0.001423	0.28096	0.165	0.176	1	13
Strap	0.001427	-0.36745	0.146	0.308	1	13
Lzts1	0.001427	0.489408	0.126	0.04	1	13
Atg5	0.00143	-0.4134	0.039	0.12	1	13
Max	0.001446	0.417496	0.204	0.131	1	13
Pnkd	0.001477	0.407233	0.117	0.068	1	13
Cdh2	0.001483	-0.32257	0.049	0.153	1	13
Eif2s3y	0.001484	0.333873	0.194	0.149	1	13
Pde4b	0.001484	0.334089	0.107	0.05	1	13
Mgat2	0.001488	-0.36292	0.019	0.12	1	13
Rpain	0.001502	-0.36863	0.029	0.133	1	13
Dhx32	0.001506	-0.34833	0.078	0.2	1	13
Cdc123	0.00152	-0.26124	0.165	0.314	1	13
Sgol1	0.001523	-0.30598	0.029	0.128	1	13
Ppp3cb	0.001527	0.411502	0.408	0.309	1	13
L1cam	0.001531	0.564396	0.146	0.052	1	13
Gabrb3	0.001557	0.417238	0.146	0.101	1	13
Hist3h2a	0.001563	-0.38247	0.049	0.144	1	13
Ube2l3	0.001572	-0.38233	0.097	0.239	1	13
Gtf3c2	0.001585	-0.38158	0.029	0.137	1	13
Dbn1	0.001594	-0.47286	0.029	0.14	1	13
Psmd13	0.001594	-0.25775	0.058	0.169	1	13
Eapp	0.001598	-0.25106	0.117	0.246	1	13
Mt1	0.0016	-0.58125	0.049	0.168	1	13

Luzp1	0.0016	0.262766	0.117	0.136	1	13
Adar	0.001602	0.387744	0.126	0.07	1	13
Prkra	0.001605	-0.40071	0.019	0.122	1	13
Nr2f1	0.001608	-0.47997	0.068	0.204	1	13
Zcchc6	0.001615	0.26997	0.155	0.156	1	13
Dnm1l	0.001621	0.290793	0.33	0.291	1	13
E2f1	0.001621	-0.46814	0.039	0.147	1	13
Cyb5b	0.001625	-0.37496	0.01	0.105	1	13
Klhl13	0.001627	-0.38879	0.01	0.104	1	13
1110038F1	0.001634	-0.29391	0.078	0.192	1	13
Rpp30	0.001636	-0.37467	0.029	0.135	1	13
R3hcc1	0.001638	-0.39379	0.049	0.16	1	13
Mrps10	0.001642	-0.45959	0.029	0.14	1	13
Agpat4	0.001669	0.424588	0.175	0.117	1	13
Kars	0.00167	-0.46642	0.068	0.195	1	13
Rnf144a	0.00168	-0.40445	0.058	0.16	1	13
Cisd2	0.001694	-0.2808	0.117	0.257	1	13
Rpl30	0.001698	-0.30891	0.126	0.269	1	13
Gtpbp2	0.0017	0.444261	0.117	0.068	1	13
Msantd3	0.001705	-0.27718	0.029	0.112	1	13
Gdap1	0.001706	0.41461	0.252	0.198	1	13
Dcaf7	0.00171	0.326627	0.126	0.103	1	13
Fam178a	0.00172	0.341396	0.136	0.11	1	13
Uba5	0.001737	0.37782	0.184	0.163	1	13
Csnk1g1	0.001743	0.469716	0.117	0.077	1	13
Aff4	0.001746	0.271431	0.223	0.217	1	13
Gfer	0.001762	-0.40328	0.01	0.109	1	13
Rad23a	0.001762	0.449955	0.136	0.062	1	13
Gprasp2	0.001769	0.417553	0.107	0.069	1	13
Cox16	0.001786	-0.31214	0.029	0.129	1	13
Rbbp6	0.001819	-0.32864	0.282	0.433	1	13
Optn	0.001819	0.450946	0.146	0.056	1	13
Zmynd8	0.001822	0.38249	0.233	0.213	1	13
Slc25a23	0.00183	0.4639	0.214	0.107	1	13
Eml5	0.001842	0.523444	0.165	0.092	1	13
Shmt1	0.001843	-0.35184	0.01	0.103	1	13
Rnf11	0.001844	0.485081	0.243	0.147	1	13
Ddr1	0.001844	-0.35669	0.039	0.136	1	13
Cxxc1	0.001844	-0.36369	0.029	0.136	1	13
Plekha1	0.001848	0.492086	0.117	0.06	1	13
Abhd17b	0.001857	0.318309	0.155	0.143	1	13
Rev1	0.001859	0.296069	0.126	0.117	1	13
Ddx54	0.001887	-0.46494	0.029	0.132	1	13
Rai1	0.001901	-0.44241	0.049	0.16	1	13
Gtf2a2	0.001913	-0.2938	0.223	0.384	1	13
Rbmx2	0.001914	-0.3678	0.019	0.115	1	13
Ubac1	0.001924	0.445917	0.204	0.128	1	13
Sox11	0.001926	-0.33712	0.019	0.115	1	13

Cyb5r3	0.001934	-0.39883	0.068	0.199	1	13
Rassf3	0.001937	-0.43376	0.029	0.138	1	13
Ankrd32	0.001943	-0.29944	0.078	0.184	1	13
Mllt11	0.001945	0.391259	0.311	0.217	1	13
Eri1	0.001958	-0.33776	0.019	0.114	1	13
Akap11	0.001959	0.361357	0.165	0.139	1	13
Ahsa2	0.001964	-0.30251	0.097	0.207	1	13
Rbbp7	0.001977	-0.32536	0.165	0.32	1	13
Zcwpw1	0.001994	-0.37898	0.01	0.103	1	13
Gtf2f1	0.002	0.321274	0.35	0.343	1	13
Klc2	0.002002	0.452194	0.126	0.038	1	13
Mettl14	0.002009	-0.27203	0.029	0.123	1	13
Adam10	0.002011	0.374736	0.223	0.203	1	13
Cdc16	0.002023	-0.2594	0.146	0.283	1	13
Amz2	0.002023	-0.30223	0.049	0.157	1	13
Prpf31	0.002043	-0.47999	0.068	0.2	1	13
Pitpnb	0.002048	-0.42941	0.078	0.214	1	13
Flywch2	0.002062	0.421192	0.146	0.05	1	13
Zfp740	0.002073	-0.43962	0.01	0.107	1	13
Topbp1	0.002104	-0.32521	0.039	0.145	1	13
Exosc1	0.002108	-0.28385	0.058	0.163	1	13
Diablo	0.00211	-0.27713	0.078	0.199	1	13
Ppp2r2a	0.002124	-0.30061	0.107	0.244	1	13
2410004N(	0.002135	-0.50337	0.058	0.184	1	13
Psmb3	0.002147	-0.2526	0.136	0.259	1	13
Adss	0.00215	-0.38521	0.068	0.193	1	13
Adrbk1	0.002157	0.493214	0.126	0.054	1	13
Ccar2	0.002161	-0.32557	0.019	0.103	1	13
Trim27	0.002168	-0.2963	0.049	0.149	1	13
RP23-32A8	0.00217	0.390129	0.252	0.186	1	13
Ssna1	0.002173	-0.28509	0.126	0.267	1	13
Fbxo32	0.002215	-0.39238	0.019	0.117	1	13
Gmps	0.002231	-0.27328	0.087	0.21	1	13
Nalcn	0.002234	0.424543	0.107	0.073	1	13
Mrpl34	0.002234	-0.26483	0.155	0.288	1	13
Zfand3	0.002239	-0.37476	0.039	0.148	1	13
Rnf187	0.002248	0.26348	0.447	0.42	1	13
Atat1	0.002249	-0.40905	0.01	0.104	1	13
Msi1	0.002279	-0.29164	0.029	0.111	1	13
Nell2	0.002279	0.339202	0.155	0.132	1	13
Trappc3	0.002315	0.439582	0.175	0.1	1	13
Fabp7	0.002318	-1.10028	0.039	0.123	1	13
Chtop	0.002321	-0.28285	0.117	0.249	1	13
Yars	0.002322	0.252611	0.155	0.142	1	13
Limd2	0.002328	-0.30747	0.039	0.135	1	13
Suds3	0.002351	0.38727	0.175	0.123	1	13
Tgs1	0.00236	-0.2968	0.078	0.199	1	13
Slc25a17	0.00237	-0.46068	0.039	0.154	1	13

Eif2b2	0.002383	-0.40957	0.049	0.169	1	13
Orc2	0.002397	-0.43246	0.039	0.156	1	13
Cdc25a	0.002409	-0.41482	0.019	0.119	1	13
Glg1	0.002433	0.310863	0.233	0.219	1	13
Stmn4	0.002443	-0.61357	0.184	0.342	1	13
Mif	0.002473	-0.27054	0.214	0.357	1	13
Pknox1	0.002482	-0.36139	0.039	0.146	1	13
Nol11	0.002486	-0.38154	0.029	0.133	1	13
Etnk1	0.002505	0.379242	0.136	0.102	1	13
Nde1	0.002507	-0.39698	0.019	0.119	1	13
Wasf2	0.00251	-0.33868	0.019	0.113	1	13
Nme2	0.002511	-0.27339	0.019	0.102	1	13
Scg5	0.002526	0.32266	0.379	0.334	1	13
Dnajc24	0.002544	-0.37999	0.039	0.151	1	13
Smarce1	0.002588	-0.3246	0.078	0.204	1	13
Gpx1	0.002589	-0.39938	0.214	0.383	1	13
Trappc1	0.002603	-0.31686	0.097	0.226	1	13
Wdr3	0.002618	-0.3264	0.019	0.112	1	13
6330403K(	0.002641	-0.31376	0.117	0.243	1	13
Lsm14b	0.002642	-0.44981	0.039	0.153	1	13
Pptc7	0.002643	0.442606	0.146	0.052	1	13
Grik5	0.002656	0.355468	0.126	0.11	1	13
Ssu72	0.002677	0.383405	0.262	0.223	1	13
Ift43	0.002706	-0.31797	0.029	0.128	1	13
Unc50	0.002751	0.28918	0.146	0.125	1	13
E330009J0	0.002756	0.386368	0.175	0.116	1	13
Brca2	0.002785	-0.40079	0.019	0.114	1	13
Sgol2	0.002824	-0.49481	0.049	0.157	1	13
1110001A:	0.002845	0.377713	0.126	0.096	1	13
Slc17a6	0.002851	-0.46088	0.039	0.148	1	13
Pdcd11	0.002873	-0.40838	0.049	0.164	1	13
Kif22	0.002877	-0.337	0.058	0.163	1	13
Necap1	0.002884	0.443087	0.126	0.069	1	13
Uncx	0.002886	-0.42763	0.165	0.318	1	13
Yipf5	0.002934	0.250034	0.117	0.122	1	13
Tubb4b	0.002939	-0.41854	0.107	0.24	1	13
Dpf2	0.002954	-0.3496	0.019	0.111	1	13
Ing1	0.002977	-0.46025	0.058	0.184	1	13
Kcnc3	0.003	0.431259	0.117	0.065	1	13
Ergic3	0.003016	0.260275	0.243	0.228	1	13
Selo	0.003048	0.368028	0.117	0.053	1	13
Specc1	0.003049	0.418901	0.117	0.048	1	13
Cdk16	0.003053	0.415094	0.126	0.067	1	13
Elavl4	0.003068	0.445365	0.427	0.334	1	13
Cep68	0.00308	-0.26851	0.029	0.116	1	13
Bub1b	0.003095	-0.38818	0.01	0.103	1	13
Sec61a1	0.003113	-0.31263	0.068	0.179	1	13
Scaf11	0.003121	-0.40514	0.204	0.374	1	13

Rps6ka3	0.003126	0.419038	0.136	0.063	1	13
Tfdp2	0.003158	-0.38545	0.049	0.161	1	13
Tef	0.003179	0.452633	0.165	0.093	1	13
Tspan5	0.00321	0.336156	0.233	0.196	1	13
Fads1	0.003216	-0.33491	0.019	0.105	1	13
Chrac1	0.003289	-0.35639	0.019	0.112	1	13
Myg1	0.003299	-0.3038	0.029	0.123	1	13
Dnttip1	0.003308	0.287819	0.107	0.095	1	13
Pold2	0.003321	-0.34392	0.019	0.114	1	13
Elovl6	0.003322	-0.33578	0.136	0.278	1	13
Echs1	0.00334	-0.32832	0.078	0.187	1	13
Tmem55b	0.003402	0.294415	0.117	0.112	1	13
Kdm2a	0.003432	-0.3821	0.019	0.108	1	13
Blvrb	0.003445	0.397323	0.107	0.059	1	13
2810008D(	0.003448	0.40577	0.214	0.127	1	13
Ncapd3	0.003456	-0.35877	0.019	0.113	1	13
Tbce	0.003457	0.430213	0.126	0.078	1	13
Vps53	0.003466	0.343441	0.107	0.069	1	13
Barhl2	0.003479	0.400957	0.117	0.064	1	13
Tmed10	0.003489	-0.35389	0.078	0.204	1	13
Arhgap21	0.003495	0.404795	0.194	0.164	1	13
Mapk10	0.003502	0.482248	0.175	0.098	1	13
Xrcc5	0.003513	-0.31843	0.029	0.115	1	13
Krr1	0.003532	-0.29956	0.049	0.152	1	13
Dgke	0.003534	0.342954	0.117	0.033	1	13
Nudt19	0.003537	-0.28764	0.039	0.113	1	13
Pdha1	0.003547	0.263322	0.184	0.162	1	13
Neurod6	0.003599	-0.43128	0.068	0.172	1	13
Rbm33	0.003613	0.271212	0.126	0.13	1	13
Hivep2	0.003643	0.505655	0.126	0.054	1	13
Dlgap5	0.003654	-0.38771	0.019	0.112	1	13
Ldb1	0.003658	-0.31918	0.078	0.184	1	13
Ppil2	0.003666	-0.2577	0.029	0.118	1	13
Nop56	0.003687	-0.35007	0.204	0.361	1	13
Usp3	0.003704	0.412599	0.233	0.14	1	13
Tmem134	0.003756	-0.29194	0.049	0.149	1	13
Plgrkt	0.003756	-0.4132	0.068	0.195	1	13
Ptbp1	0.003781	-0.38424	0.01	0.101	1	13
Bfar	0.003796	0.302564	0.194	0.191	1	13
5830418K(	0.003804	-0.37024	0.058	0.168	1	13
Ctsf	0.003804	0.434163	0.175	0.098	1	13
Slc1a2	0.003811	-0.35693	0.146	0.295	1	13
Zcchc8	0.003821	-0.33117	0.039	0.143	1	13
Klhl23	0.003823	0.331344	0.126	0.089	1	13
Pop5	0.003839	0.342755	0.126	0.1	1	13
Mob4	0.003841	-0.2953	0.087	0.204	1	13
Cxx1b	0.003896	0.374775	0.184	0.12	1	13
Ypel4	0.003908	0.390355	0.126	0.039	1	13

Znhit6	0.003918	-0.35196	0.029	0.12	1	13
Smpd3	0.00392	-0.33148	0.058	0.161	1	13
Ncald	0.003931	0.353902	0.243	0.165	1	13
Rnf219	0.003933	-0.42119	0.039	0.14	1	13
Zfp830	0.003939	-0.35618	0.029	0.118	1	13
Fbxo21	0.003949	0.349621	0.117	0.09	1	13
Gramd1a	0.003952	-0.45068	0.058	0.165	1	13
Cdt1	0.003968	-0.33707	0.029	0.125	1	13
Exoc2	0.003979	-0.42077	0.029	0.116	1	13
Fam32a	0.004034	-0.39732	0.126	0.269	1	13
Slu7	0.004042	-0.44236	0.126	0.264	1	13
Fyn	0.00406	-0.44088	0.087	0.216	1	13
Eif3b	0.004061	-0.27891	0.097	0.221	1	13
Rtn2	0.004061	-0.39587	0.019	0.115	1	13
N6amt2	0.004062	0.262098	0.146	0.123	1	13
Gde1	0.004075	0.389482	0.184	0.141	1	13
Ptbp3	0.004079	-0.33644	0.019	0.11	1	13
Itfg1	0.004121	0.331217	0.117	0.086	1	13
Tacc2	0.004136	0.32952	0.165	0.141	1	13
2410004B:	0.004169	0.324852	0.126	0.113	1	13
Soga2	0.004199	0.352211	0.136	0.072	1	13
Ddt	0.004204	0.384739	0.175	0.105	1	13
Bcap29	0.004273	-0.39505	0.029	0.123	1	13
Sp3	0.004278	-0.38018	0.049	0.163	1	13
Nrip1	0.004313	0.359176	0.117	0.101	1	13
Hey1	0.004381	-0.45989	0.126	0.273	1	13
Mdga1	0.00439	-0.33848	0.039	0.136	1	13
Zhx1	0.004455	0.259439	0.146	0.129	1	13
Pitpna	0.004462	0.355675	0.175	0.131	1	13
Emc4	0.004464	0.393847	0.243	0.197	1	13
Brd9	0.00449	-0.3483	0.087	0.214	1	13
Supt6	0.004531	0.387384	0.223	0.157	1	13
Rpl5	0.004598	-0.25603	0.049	0.115	1	13
Ppp1r12c	0.004659	0.397195	0.165	0.126	1	13
Cd2ap	0.004685	-0.26294	0.058	0.159	1	13
Mettl10	0.004703	-0.28792	0.039	0.132	1	13
Sod2	0.004706	0.355113	0.223	0.175	1	13
Fbxw2	0.00478	0.300448	0.175	0.16	1	13
Fam175b	0.004794	-0.40768	0.019	0.112	1	13
Acaa1a	0.004827	0.361767	0.136	0.103	1	13
Hist1h2ak	0.004831	-0.31747	0.049	0.143	1	13
Rnf4	0.004849	-0.31181	0.029	0.124	1	13
Ezh1	0.004904	0.43979	0.107	0.061	1	13
Prdm2	0.004918	-0.3456	0.039	0.132	1	13
Dad1	0.004956	-0.26534	0.194	0.344	1	13
Shd	0.004957	0.251909	0.136	0.109	1	13
Vkorc1	0.004964	-0.34217	0.019	0.111	1	13
Sec11c	0.005018	-0.34947	0.097	0.213	1	13

Usp46	0.005032	0.288762	0.272	0.235	1	13
Tbca	0.005054	-0.25341	0.291	0.42	1	13
Fundc2	0.005055	-0.27541	0.136	0.271	1	13
Actr6	0.005073	-0.34974	0.019	0.107	1	13
Slc39a6	0.005089	-0.34131	0.078	0.199	1	13
Mia3	0.005093	0.315338	0.272	0.244	1	13
Rae1	0.005145	-0.4065	0.068	0.191	1	13
2700089E2	0.005147	0.371288	0.243	0.198	1	13
Fam133b	0.005174	0.278359	0.32	0.308	1	13
Prpf40b	0.005207	0.337231	0.165	0.146	1	13
Synrg	0.00521	-0.39141	0.039	0.135	1	13
Zfyve9	0.005271	0.325103	0.107	0.084	1	13
Bmyc	0.005275	0.329212	0.136	0.1	1	13
Exosc5	0.005307	-0.2848	0.019	0.104	1	13
Bet1l	0.005367	0.372987	0.136	0.078	1	13
Fam107b	0.00541	-0.37214	0.019	0.102	1	13
Bcas2	0.005432	-0.27345	0.252	0.406	1	13
Sac3d1	0.005509	-0.37685	0.029	0.107	1	13
Rab10	0.00552	-0.36605	0.146	0.293	1	13
Smim15	0.005528	-0.32212	0.029	0.121	1	13
Polr2h	0.00554	-0.37892	0.107	0.225	1	13
Tubgcp2	0.00554	-0.26891	0.029	0.117	1	13
Wdr43	0.005542	-0.35186	0.107	0.242	1	13
Grsf1	0.005593	-0.31651	0.068	0.176	1	13
Sirt2	0.005599	-0.42813	0.049	0.156	1	13
Zbtb7a	0.005795	0.4384	0.155	0.093	1	13
H2afj	0.005859	-0.40009	0.087	0.205	1	13
2510003E0	0.005897	0.407045	0.165	0.107	1	13
Prps1	0.005925	-0.41598	0.039	0.143	1	13
Bad	0.005947	0.334997	0.146	0.108	1	13
Psme2	0.005957	-0.36053	0.019	0.105	1	13
Ptrh2	0.005963	-0.33539	0.049	0.154	1	13
Trpc2	0.006002	-0.32545	0.039	0.135	1	13
Ergic2	0.006167	-0.25222	0.087	0.197	1	13
Stx8	0.006167	0.369373	0.117	0.083	1	13
Npdc1	0.006193	0.367658	0.311	0.261	1	13
Dnmt3a	0.006242	0.369592	0.272	0.202	1	13
Gosr1	0.006244	0.390382	0.117	0.062	1	13
Trpc4ap	0.006252	-0.28991	0.097	0.198	1	13
Bex1	0.006316	0.393341	0.282	0.223	1	13
Ctsd	0.006336	-0.79276	0.087	0.175	1	13
Glrx3	0.006416	-0.32691	0.126	0.247	1	13
Opa3	0.006447	-0.32929	0.039	0.114	1	13
Gbas	0.006453	0.380474	0.194	0.132	1	13
Cotl1	0.006456	-0.26542	0.058	0.154	1	13
Tm9sf4	0.006548	0.3212	0.165	0.116	1	13
Fam120a	0.006555	-0.29545	0.039	0.124	1	13
Tex261	0.006604	-0.33221	0.029	0.116	1	13

Lamp2	0.006609	-0.3566	0.039	0.138	1	13
Nicn1	0.006678	0.253989	0.214	0.208	1	13
Cpeb4	0.006689	0.375133	0.107	0.046	1	13
Extl2	0.006739	0.367743	0.107	0.054	1	13
5430416N(	0.006745	-0.39121	0.039	0.141	1	13
Pink1	0.00685	0.277339	0.165	0.13	1	13
Zmym2	0.00687	-0.3069	0.029	0.119	1	13
Pja1	0.00695	0.399119	0.243	0.172	1	13
Cdk19	0.006967	-0.36703	0.049	0.154	1	13
Prkrip1	0.006972	0.259969	0.175	0.145	1	13
Clvs1	0.007083	0.277158	0.146	0.128	1	13
Rev3l	0.007158	0.313404	0.175	0.097	1	13
Rap1a	0.007248	-0.28766	0.039	0.124	1	13
Ankhd1	0.007248	0.281789	0.204	0.18	1	13
Lsm1	0.007294	-0.41753	0.058	0.166	1	13
Ndufaf7	0.007303	-0.29107	0.019	0.101	1	13
Stra13	0.007325	-0.29986	0.029	0.101	1	13
Wash	0.007362	0.288787	0.107	0.102	1	13
Tsta3	0.00743	-0.25119	0.049	0.143	1	13
Aatf	0.007441	-0.39422	0.029	0.122	1	13
Etfa	0.007452	-0.27699	0.126	0.254	1	13
Rere	0.007536	0.306466	0.165	0.15	1	13
Gtf2b	0.00768	-0.2607	0.058	0.152	1	13
Blm	0.007715	-0.34874	0.029	0.111	1	13
Kat6b	0.007749	-0.42407	0.049	0.157	1	13
Daam1	0.007801	-0.37173	0.078	0.184	1	13
Mbp	0.007821	-0.47053	0.078	0.166	1	13
Surf4	0.007915	-0.29916	0.029	0.119	1	13
2410002F2	0.007944	-0.31187	0.049	0.147	1	13
Brwd3	0.007992	-0.36843	0.019	0.102	1	13
Thoc1	0.008095	-0.31418	0.068	0.178	1	13
Hars	0.008133	-0.36088	0.087	0.211	1	13
Dusp26	0.00819	0.472627	0.155	0.064	1	13
Dld	0.00822	0.308886	0.223	0.186	1	13
Exosc3	0.008234	-0.33801	0.019	0.107	1	13
Krt10	0.008267	-0.30039	0.058	0.162	1	13
Kcnj3	0.00833	0.35258	0.155	0.068	1	13
Deb1	0.008365	0.310516	0.204	0.167	1	13
Tmx3	0.0084	-0.30809	0.039	0.125	1	13
Clint1	0.008415	-0.38411	0.039	0.131	1	13
Poglut1	0.008491	-0.28763	0.039	0.13	1	13
Bcap31	0.008518	0.29839	0.214	0.173	1	13
Anapc4	0.008552	-0.30316	0.049	0.147	1	13
Tbcel	0.008651	0.402633	0.146	0.067	1	13
Cacng4	0.008654	-0.41758	0.029	0.102	1	13
Uri1	0.008692	-0.33246	0.097	0.21	1	13
5830428H2	0.008705	0.483638	0.136	0.064	1	13
Tbpl1	0.00875	-0.28458	0.078	0.187	1	13

Klf6	0.008765	-0.29052	0.068	0.148	1	13
Fam171b	0.008808	0.276523	0.243	0.223	1	13
Dynlt3	0.008818	0.407707	0.107	0.046	1	13
Rtcb	0.008838	-0.28257	0.107	0.227	1	13
Slc7a5	0.008841	-0.29581	0.029	0.115	1	13
Cpsf2	0.00886	-0.26812	0.117	0.237	1	13
Pafah1b2	0.008935	-0.25429	0.146	0.255	1	13
Epm2aip1	0.008938	0.289792	0.117	0.105	1	13
Tmed1	0.008971	-0.36326	0.019	0.105	1	13
Serpini1	0.00898	0.444479	0.146	0.064	1	13
Wbp1	0.008987	-0.33186	0.049	0.148	1	13
Cenpb	0.009052	-0.2899	0.097	0.209	1	13
Alg2	0.009134	0.367575	0.136	0.099	1	13
Xpo1	0.009137	-0.28003	0.107	0.228	1	13
Camsap2	0.00916	0.467093	0.194	0.132	1	13
Mybbp1a	0.009281	-0.31187	0.126	0.25	1	13
Poc1b	0.009466	-0.28003	0.039	0.127	1	13
Qtrt1	0.009649	-0.2797	0.029	0.108	1	13
Pard6a	0.009737	0.415425	0.175	0.091	1	13
Rhob	0.009814	-0.2604	0.049	0.139	1	13
Tubb2a	0.009854	0.29261	0.311	0.244	1	13
Anks1b	0.009878	0.399906	0.126	0.063	1	13
Atic	0.009976	-0.27346	0.029	0.11	1	13
Cd3eap	0.009998	-0.36547	0.029	0.121	1	13
Bsg	2.1E-164	2.728841	0.904	0.422	3.5E-160	14
Fth1	2.7E-151	2.505561	1	0.707	4.6E-147	14
Itm2a	8.4E-141	3.278525	0.942	0.043	1.4E-136	14
Ctla2a	4.3E-127	4.032005	0.904	0.004	7.2E-123	14
Igfbp7	6.7E-122	4.010019	1	0.016	1.1E-117	14
Slco1c1	7E-122	3.306536	0.885	0.003	1.2E-117	14
Flt1	4.7E-121	3.149466	0.885	0.003	7.9E-117	14
Esam	8.6E-119	2.798368	0.923	0.002	1.4E-114	14
Slc16a1	2.6E-114	2.919165	0.827	0.036	4.3E-110	14
Gpr116	5.4E-109	2.649378	0.846	0.002	9E-105	14
Sparc	8.7E-109	3.67872	1	0.082	1.4E-104	14
Cldn5	1.4E-107	2.592316	0.808	0.001	2.4E-103	14
Egfl7	8E-102	2.698347	0.846	0.01	1.34E-97	14
Slc2a1	1.35E-92	2.532981	0.808	0.012	2.25E-88	14
Ptprb	1.97E-92	2.519294	0.808	0.004	3.29E-88	14
Kdr	5.58E-92	2.732476	0.731	0.002	9.31E-88	14
Ly6c1	1.03E-90	3.474424	0.673	0.002	1.73E-86	14
Eltd1	1.06E-89	2.190623	0.673	0.001	1.77E-85	14
Nostrin	6E-85	2.104446	0.692	0.001	1E-80	14
Cd34	8.52E-83	2.323017	0.712	0.003	1.42E-78	14
Cd93	1.6E-78	2.2625	0.673	0.002	2.67E-74	14
Ramp2	8.42E-76	2.3797	0.846	0.117	1.4E-71	14
Vwa1	1.19E-69	2.175913	0.712	0.009	1.99E-65	14
Gsta4	1.43E-69	2.51998	0.615	0.009	2.39E-65	14

Gng11	1.96E-69	2.445859	0.75	0.013	3.27E-65	14
Ccdc141	3.99E-67	2.200312	0.673	0.007	6.66E-63	14
Sepp1	1.34E-66	3.16883	0.904	0.052	2.23E-62	14
BC028528	2.24E-65	1.886818	0.673	0.005	3.73E-61	14
Gimap6	3.45E-64	1.866636	0.538	0.001	5.76E-60	14
Col4a2	1.16E-63	2.475795	0.846	0.038	1.94E-59	14
AU021092	5.19E-63	1.951248	0.519	0.001	8.65E-59	14
Tmem252	1.33E-62	1.713059	0.5	0	2.22E-58	14
Grap	2.52E-62	1.766948	0.577	0.002	4.2E-58	14
Fn1	2.79E-62	2.164042	0.615	0.007	4.65E-58	14
Tfrc	3.31E-59	2.204395	0.75	0.167	5.53E-55	14
Slco1a4	7.17E-59	2.308063	0.5	0.002	1.2E-54	14
Erg	1.16E-57	1.467278	0.462	0	1.93E-53	14
Abcb1a	1.58E-57	1.81945	0.481	0.001	2.64E-53	14
Car4	2.79E-57	2.506705	0.577	0.006	4.66E-53	14
Sparcl1	2.63E-56	2.349609	0.942	0.205	4.38E-52	14
Nrp1	2.44E-55	2.413891	0.712	0.041	4.06E-51	14
Spock2	3.51E-55	2.462651	0.635	0.063	5.86E-51	14
Eng	1.83E-54	1.727038	0.5	0.002	3.04E-50	14
Ecscr	5.19E-54	1.845141	0.558	0.004	8.65E-50	14
Robo4	7.41E-54	1.438526	0.442	0	1.24E-49	14
Mfsd2a	1.75E-53	2.05539	0.596	0.009	2.92E-49	14
Pltp	6.76E-53	2.328366	0.673	0.026	1.13E-48	14
Tek	2.78E-51	1.449549	0.404	0	4.63E-47	14
Id3	3.34E-51	2.330638	0.75	0.03	5.56E-47	14
Col4a1	7.94E-51	2.239738	0.827	0.07	1.32E-46	14
Ly6a	9.5E-51	1.8437	0.404	0	1.58E-46	14
Vwf	2.38E-50	1.92359	0.442	0.001	3.96E-46	14
Ablim1	1.36E-49	2.113555	0.673	0.017	2.26E-45	14
Fzd6	6.28E-47	1.428127	0.442	0.001	1.05E-42	14
Rasip1	1.59E-46	1.629362	0.538	0.007	2.66E-42	14
Slc40a1	3.62E-46	1.652547	0.5	0.004	6.04E-42	14
Pecam1	4.18E-46	1.593476	0.423	0.001	6.97E-42	14
Epas1	1.63E-45	1.683157	0.538	0.007	2.72E-41	14
Sox17	5.6E-45	1.419737	0.365	0	9.35E-41	14
9430020K(	8.87E-45	1.75349	0.5	0.006	1.48E-40	14
Apln	1E-44	2.065833	0.385	0.002	1.67E-40	14
Igfbp3	3.52E-44	2.193662	0.462	0.011	5.86E-40	14
St3gal6	5.48E-44	1.584247	0.538	0.008	9.14E-40	14
C130074G:	1.22E-43	1.339263	0.385	0.001	2.04E-39	14
Fam101b	1.92E-43	2.026334	0.615	0.051	3.21E-39	14
Slc7a5	5.28E-43	1.924089	0.788	0.113	8.81E-39	14
Cgnl1	5.43E-43	1.551307	0.462	0.003	9.05E-39	14
Scgb3a1	6.62E-43	1.934721	0.346	0	1.1E-38	14
Slc38a5	6.64E-43	1.656402	0.346	0	1.11E-38	14
Slc39a8	6.88E-43	1.490826	0.442	0.004	1.15E-38	14
Wwtr1	1.34E-42	1.657978	0.519	0.01	2.23E-38	14
Ushbp1	5.51E-42	1.33087	0.365	0.001	9.2E-38	14

Ptrf	1.21E-41	1.479613	0.442	0.003	2.01E-37	14
Anxa3	1.81E-41	1.537643	0.5	0.006	3.02E-37	14
Klf2	8.39E-41	1.960074	0.577	0.017	1.4E-36	14
Tmem204	9.19E-41	1.556176	0.481	0.005	1.53E-36	14
Slc3a2	1.6E-40	1.637713	0.827	0.321	2.67E-36	14
Tmem88	1.96E-40	1.37274	0.442	0.003	3.26E-36	14
Foxq1	4.41E-40	1.794788	0.423	0.005	7.36E-36	14
Slc22a8	3.43E-39	1.347047	0.404	0.002	5.73E-35	14
Lama4	1.27E-38	1.796285	0.481	0.007	2.12E-34	14
Palmd	1.41E-38	1.907331	0.654	0.049	2.35E-34	14
Pglyrp1	1.44E-38	1.230815	0.308	0	2.4E-34	14
Slc9a3r2	6.46E-38	1.969571	0.519	0.015	1.08E-33	14
Cdh5	8.01E-38	1.157717	0.385	0.002	1.34E-33	14
Fxyd5	1.09E-36	1.510955	0.385	0.003	1.82E-32	14
Myh9	3.5E-36	1.539658	0.596	0.025	5.84E-32	14
Tdrp	3.66E-36	1.255752	0.327	0.001	6.1E-32	14
Lsr	3.69E-36	1.313203	0.346	0.001	6.15E-32	14
Fam129a	6.15E-36	1.200082	0.365	0.002	1.03E-31	14
Synm	6.43E-36	1.308702	0.404	0.003	1.07E-31	14
Ets1	1.25E-35	1.685953	0.442	0.009	2.08E-31	14
Pdgfb	1.62E-35	1.149518	0.327	0.001	2.71E-31	14
Slc38a3	2.23E-35	1.653017	0.558	0.02	3.72E-31	14
Tsc22d1	2.52E-35	1.506204	0.846	0.426	4.2E-31	14
Acvrl1	1.72E-34	1.339118	0.346	0.002	2.88E-30	14
Abcg2	1.92E-34	1.625218	0.596	0.033	3.2E-30	14
Srgn	1.34E-33	1.367393	0.404	0.004	2.23E-29	14
Ctgf	1.63E-33	1.407313	0.288	0.001	2.72E-29	14
Eogt	1.92E-33	1.409387	0.385	0.004	3.2E-29	14
Fli1	2.03E-33	1.263823	0.404	0.005	3.38E-29	14
Clec1a	2.61E-33	1.330793	0.346	0.002	4.36E-29	14
Serpinb6b	5.25E-33	1.140999	0.269	0	8.76E-29	14
Sptbn1	6.87E-33	1.58128	0.923	0.351	1.15E-28	14
Arhgap29	9.01E-33	1.662827	0.558	0.027	1.5E-28	14
Unc45b	3.38E-32	1.145734	0.346	0.002	5.64E-28	14
Atox1	4.07E-32	1.46747	0.846	0.291	6.8E-28	14
Ildr2	4.2E-32	1.263927	0.423	0.007	7.01E-28	14
Id1	2.05E-31	1.927369	0.558	0.071	3.42E-27	14
Nid1	2.62E-31	1.536016	0.5	0.016	4.38E-27	14
Tie1	4.11E-31	1.104387	0.269	0	6.86E-27	14
Gm20748	4.5E-31	1.118477	0.327	0.002	7.5E-27	14
Cd151	6.29E-31	1.405089	0.5	0.016	1.05E-26	14
Dlc1	6.3E-31	1.42054	0.404	0.007	1.05E-26	14
Rbpms	2.17E-30	1.09944	0.365	0.004	3.62E-26	14
Meox1	5.72E-30	1.058311	0.308	0.001	9.55E-26	14
Emcn	5.79E-30	1.337419	0.308	0.002	9.66E-26	14
Icam2	7.17E-30	0.806556	0.269	0	1.2E-25	14
Foxf2	8.44E-30	1.134834	0.423	0.008	1.41E-25	14
Lipa	1.35E-29	1.74575	0.462	0.03	2.25E-25	14

Fgd5	1.76E-29	0.844937	0.25	0	2.93E-25	14
Swap70	1.79E-29	1.356797	0.481	0.016	2.98E-25	14
Slc7a1	2.39E-29	1.68549	0.558	0.097	3.99E-25	14
Ahnak	2.48E-29	1.226727	0.385	0.006	4.13E-25	14
Wfdc1	2.55E-29	1.358075	0.288	0.002	4.26E-25	14
Clec14a	6.48E-29	1.038413	0.269	0.001	1.08E-24	14
Hmgcs2	6.94E-29	1.528197	0.327	0.004	1.16E-24	14
Anxa2	7.03E-29	1.533452	0.423	0.011	1.17E-24	14
St8sia4	8.65E-29	1.068321	0.308	0.002	1.44E-24	14
Serinc3	1.15E-28	1.780459	0.654	0.118	1.93E-24	14
Slc39a10	2.43E-28	1.5888	0.712	0.193	4.05E-24	14
Rasgrp3	2.66E-28	1.169841	0.346	0.004	4.44E-24	14
Sgms1	7.63E-28	1.461015	0.423	0.015	1.27E-23	14
Mmrn2	1.95E-27	0.935118	0.269	0.001	3.25E-23	14
Ddc	2.02E-27	1.06147	0.288	0.001	3.37E-23	14
Thsd1	2.52E-27	1.073058	0.288	0.001	4.21E-23	14
Nampt	3.41E-27	1.669108	0.5	0.08	5.69E-23	14
Cnn2	5.88E-27	1.371154	0.365	0.007	9.8E-23	14
Gpr4	7.59E-27	0.972075	0.25	0.001	1.27E-22	14
Vim	1.04E-26	1.459146	0.846	0.3	1.74E-22	14
Gata2	1.59E-26	1.023669	0.25	0.001	2.65E-22	14
Slco2b1	1.89E-26	1.066074	0.327	0.004	3.16E-22	14
Limch1	1.97E-26	1.62942	0.462	0.023	3.28E-22	14
Cdkn2b	2.93E-26	1.102448	0.327	0.004	4.88E-22	14
Il2rg	3.67E-26	0.94113	0.25	0.001	6.12E-22	14
Eva1b	5.35E-26	1.281117	0.385	0.008	8.93E-22	14
Zic3	5.48E-26	1.133678	0.346	0.005	9.14E-22	14
Serpinh1	7.7E-26	1.680968	0.635	0.113	1.28E-21	14
Ly6e	7.83E-26	1.443074	0.654	0.28	1.31E-21	14
Nfkbia	7.99E-26	1.598277	0.673	0.17	1.33E-21	14
Arhgap18	1.35E-25	1.312228	0.404	0.015	2.26E-21	14
Arl4a	1.64E-25	1.765511	0.462	0.063	2.73E-21	14
Nos3	2.2E-25	0.969656	0.308	0.003	3.67E-21	14
Apcdd1	2.79E-25	1.28725	0.385	0.011	4.65E-21	14
Myo10	3.15E-25	1.5649	0.615	0.12	5.25E-21	14
Itga1	5.15E-25	1.136361	0.308	0.003	8.6E-21	14
F11r	6.7E-25	0.917609	0.308	0.003	1.12E-20	14
Dock9	1.06E-24	1.253469	0.423	0.015	1.77E-20	14
Tm4sf1	1.3E-24	1.310031	0.308	0.004	2.17E-20	14
Hspg2	1.89E-24	1.100345	0.308	0.004	3.15E-20	14
Edn1	2.5E-24	1.145917	0.212	0	4.17E-20	14
Ocln	2.97E-24	0.806933	0.212	0	4.95E-20	14
Tmsb4x	3.06E-24	1.019128	1	0.948	5.1E-20	14
Paqr5	3.63E-24	1.159248	0.212	0.001	6.05E-20	14
4931406P1	4.33E-24	1.465598	0.538	0.056	7.22E-20	14
Ctla2b	5.69E-24	1.122453	0.269	0.002	9.49E-20	14
Cd59a	6.56E-24	0.998768	0.231	0.001	1.09E-19	14
Nid2	6.61E-24	1.119234	0.346	0.007	1.1E-19	14

Lamb1	8.64E-24	1.370452	0.365	0.009	1.44E-19	14
Marcks	9.39E-24	-1.32863	0.519	0.917	1.57E-19	14
Sfrp1	1.02E-23	-2.00527	0.231	0.813	1.7E-19	14
Pde2a	1.07E-23	1.097851	0.269	0.002	1.78E-19	14
Chst1	1.11E-23	1.226641	0.288	0.004	1.85E-19	14
Lmo2	1.2E-23	1.458641	0.5	0.035	2E-19	14
Abcc4	1.23E-23	1.066963	0.25	0.002	2.05E-19	14
Cdkn1a	1.4E-23	1.739379	0.596	0.091	2.34E-19	14
Angpt2	1.84E-23	1.408346	0.231	0.002	3.06E-19	14
Lef1	1.97E-23	0.890373	0.269	0.002	3.28E-19	14
Ifitm3	2.08E-23	1.222288	0.385	0.013	3.47E-19	14
Itga6	3.48E-23	1.195912	0.385	0.012	5.81E-19	14
Rgcc	3.99E-23	1.26914	0.404	0.015	6.66E-19	14
Uaca	4.83E-23	1.531628	0.538	0.058	8.06E-19	14
Egfl8	5.07E-23	1.124086	0.346	0.008	8.46E-19	14
Fcgrt	5.43E-23	1.35257	0.462	0.026	9.05E-19	14
Hsp90ab1	5.75E-23	-0.63543	1	0.997	9.6E-19	14
Kank3	7.51E-23	1.351328	0.462	0.028	1.25E-18	14
She	1.11E-22	0.815172	0.192	0	1.85E-18	14
Hspa12b	1.27E-22	0.84717	0.212	0.001	2.12E-18	14
Gimap1	1.41E-22	0.666762	0.192	0	2.36E-18	14
Plat	2.11E-22	1.541757	0.404	0.02	3.53E-18	14
Ctsh	2.18E-22	1.195929	0.365	0.011	3.64E-18	14
Plk2	2.44E-22	1.413035	0.346	0.011	4.07E-18	14
Lgals9	3.14E-22	1.221442	0.327	0.008	5.23E-18	14
Myl12a	4.52E-22	1.263681	0.788	0.327	7.54E-18	14
Nfib	5.11E-22	-1.32852	0.692	0.934	8.52E-18	14
Rrbp1	7.59E-22	1.480958	0.692	0.187	1.27E-17	14
Lamc1	9.13E-22	1.34428	0.404	0.019	1.52E-17	14
Dok4	1.94E-21	1.356603	0.346	0.012	3.24E-17	14
Ece1	2.15E-21	1.432882	0.558	0.083	3.59E-17	14
S1pr1	2.35E-21	1.097306	0.327	0.008	3.92E-17	14
Rhoj	2.66E-21	1.392815	0.442	0.031	4.43E-17	14
Rapgef5	3.82E-21	0.880173	0.269	0.003	6.38E-17	14
Car2	3.86E-21	1.365661	0.385	0.016	6.44E-17	14
Tagln2	4.01E-21	1.423472	0.5	0.043	6.69E-17	14
Parvb	4.32E-21	1.17353	0.346	0.011	7.21E-17	14
Rgs12	6.34E-21	1.485684	0.615	0.144	1.06E-16	14
Csrp2	1.27E-20	1.422423	0.615	0.156	2.12E-16	14
CRE_RECOM	1.41E-20	-2.17899	0.154	0.77	2.36E-16	14
Arpc1b	1.5E-20	1.269802	0.462	0.035	2.5E-16	14
Dusp2	1.67E-20	0.918581	0.231	0.002	2.79E-16	14
Cd63	1.74E-20	-1.7667	0.173	0.756	2.9E-16	14
Rassf9	2.08E-20	0.666253	0.192	0	3.48E-16	14
Sema3c	2.37E-20	0.810966	0.212	0.001	3.96E-16	14
Htra3	2.52E-20	1.164473	0.288	0.005	4.2E-16	14
Ly75	2.54E-20	0.857337	0.212	0.001	4.23E-16	14
Rhoc	2.77E-20	1.209281	0.423	0.026	4.62E-16	14

Notch1	5.49E-20	1.378859	0.365	0.022	9.15E-16	14
Vamp5	9.39E-20	0.809124	0.25	0.003	1.57E-15	14
Hmcn1	1.01E-19	0.939116	0.212	0.001	1.68E-15	14
Apold1	1.08E-19	1.071285	0.288	0.008	1.8E-15	14
Angptl4	1.35E-19	1.038687	0.308	0.008	2.26E-15	14
Gstm7	2.38E-19	1.171004	0.308	0.009	3.97E-15	14
Stap2	2.48E-19	0.871374	0.231	0.002	4.13E-15	14
Kcp	2.49E-19	1.044321	0.192	0.001	4.15E-15	14
Calm1	2.82E-19	0.860814	0.942	0.861	4.7E-15	14
Ralb	2.91E-19	1.09184	0.365	0.017	4.86E-15	14
Ppfibp1	3.47E-19	1.339824	0.404	0.031	5.8E-15	14
Afap1l1	3.85E-19	1.156956	0.346	0.014	6.43E-15	14
Basp1	4.9E-19	-1.825	0.115	0.705	8.17E-15	14
Zic1	5.21E-19	-1.61969	0.212	0.785	8.69E-15	14
Mcam	6.12E-19	0.792803	0.231	0.002	1.02E-14	14
Adcy4	6.19E-19	0.990068	0.173	0.001	1.03E-14	14
Fas	7.22E-19	0.785359	0.192	0.001	1.2E-14	14
Bambi	9.94E-19	1.335389	0.308	0.022	1.66E-14	14
S100a13	1.13E-18	1.314344	0.423	0.029	1.89E-14	14
B2m	1.56E-18	1.396839	0.731	0.23	2.6E-14	14
Dusp3	1.89E-18	1.222639	0.404	0.027	3.16E-14	14
Crmp1	2.36E-18	-1.78425	0.154	0.669	3.93E-14	14
Tbx3	2.56E-18	0.598397	0.192	0.001	4.26E-14	14
Anxa7	3.17E-18	0.949503	0.308	0.01	5.29E-14	14
Gpcpd1	3.23E-18	1.430945	0.519	0.082	5.38E-14	14
Aplnr	3.39E-18	0.75173	0.192	0.001	5.65E-14	14
Cyyr1	4.35E-18	0.776196	0.231	0.003	7.25E-14	14
Nes	5.37E-18	1.376808	0.558	0.085	8.96E-14	14
Scarf1	6.89E-18	0.840307	0.192	0.001	1.15E-13	14
Ttyh2	7.7E-18	1.260951	0.442	0.037	1.28E-13	14
Hnrnpa2b1	8.3E-18	-0.91091	0.731	0.934	1.38E-13	14
Smtn	8.31E-18	1.209433	0.385	0.032	1.39E-13	14
Utrn	9.02E-18	1.285457	0.385	0.026	1.5E-13	14
Slc30a1	9.03E-18	1.144781	0.346	0.018	1.51E-13	14
Ckb	1.48E-17	-1.63678	0.308	0.809	2.48E-13	14
Cst3	1.57E-17	1.041546	0.865	0.534	2.62E-13	14
Stab1	2.05E-17	1.113421	0.288	0.008	3.42E-13	14
Igfbpl1	2.48E-17	-1.93912	0.077	0.649	4.14E-13	14
Slfn5	3.3E-17	0.763739	0.173	0.001	5.5E-13	14
Marcksl1	4.16E-17	-1.55387	0.154	0.723	6.95E-13	14
Tns1	4.31E-17	1.068271	0.231	0.004	7.18E-13	14
Nfix	5.03E-17	-1.68793	0.173	0.674	8.4E-13	14
Tpm4	7.79E-17	1.083513	0.596	0.316	1.3E-12	14
Itgb1	9.16E-17	1.151776	0.692	0.361	1.53E-12	14
Lrp8	9.72E-17	1.142542	0.423	0.038	1.62E-12	14
Def6	9.97E-17	0.763011	0.192	0.001	1.66E-12	14
Tcf7	1.09E-16	0.630421	0.173	0.001	1.81E-12	14
Jag2	1.12E-16	0.596249	0.173	0.001	1.86E-12	14

Prkch	1.12E-16	0.819346	0.173	0.001	1.87E-12	14
Myo1b	1.55E-16	1.396988	0.462	0.075	2.59E-12	14
Tiam1	1.87E-16	1.138287	0.442	0.045	3.12E-12	14
Ccm2l	2.72E-16	0.698072	0.173	0.001	4.53E-12	14
Dll4	2.74E-16	0.674308	0.192	0.002	4.57E-12	14
Rtn1	2.93E-16	-1.8982	0.173	0.691	4.89E-12	14
Stmn3	3.4E-16	-1.73552	0.077	0.623	5.67E-12	14
Tspan13	3.71E-16	1.284966	0.654	0.205	6.18E-12	14
Nfia	3.98E-16	-1.4335	0.231	0.775	6.63E-12	14
Draxin	4.23E-16	-1.77581	0.058	0.556	7.05E-12	14
Map7	4.33E-16	0.86659	0.25	0.006	7.22E-12	14
Cpe	6.2E-16	1.116469	0.769	0.455	1.03E-11	14
Tuba1a	8.12E-16	-1.18205	0.712	0.93	1.35E-11	14
Tubb5	9.99E-16	-0.9757	0.712	0.915	1.67E-11	14
Ackr3	1.05E-15	0.751469	0.212	0.003	1.75E-11	14
Grrp1	1.08E-15	0.77186	0.173	0.001	1.8E-11	14
Arhgef5	1.41E-15	0.598514	0.154	0.001	2.35E-11	14
Cd24a	1.5E-15	-1.65528	0.096	0.635	2.5E-11	14
Fam212a	1.6E-15	0.768115	0.192	0.002	2.67E-11	14
Ddah2	1.65E-15	-1.66292	0.173	0.687	2.75E-11	14
Vamp8	1.85E-15	0.916324	0.288	0.012	3.08E-11	14
Slc35f2	1.92E-15	0.929971	0.173	0.002	3.21E-11	14
Notch4	2.28E-15	0.975026	0.288	0.012	3.8E-11	14
Kif26a	2.39E-15	0.929328	0.173	0.001	3.98E-11	14
Tjp1	2.8E-15	1.26325	0.462	0.072	4.67E-11	14
Fkbp10	2.96E-15	0.897024	0.25	0.007	4.93E-11	14
Igfbp4	3.04E-15	1.383778	0.308	0.018	5.07E-11	14
Hbegf	3.87E-15	1.154343	0.327	0.02	6.45E-11	14
H2-D1	4.56E-15	1.245392	0.577	0.129	7.6E-11	14
Plod1	5.07E-15	0.921081	0.346	0.023	8.46E-11	14
Timp3	5.34E-15	1.49744	0.462	0.078	8.91E-11	14
Rras	5.82E-15	0.872219	0.269	0.01	9.72E-11	14
Abhd2	5.91E-15	1.124327	0.365	0.03	9.87E-11	14
Tubb6	6.58E-15	1.036733	0.308	0.02	1.1E-10	14
Rell1	6.85E-15	1.31356	0.481	0.094	1.14E-10	14
Trim16	6.98E-15	0.576879	0.154	0.001	1.16E-10	14
Acadl	7.26E-15	1.15768	0.462	0.063	1.21E-10	14
Bex2	7.6E-15	-1.61424	0.077	0.572	1.27E-10	14
Il10rb	8.36E-15	0.694148	0.231	0.005	1.39E-10	14
Msrb3	8.42E-15	0.677902	0.173	0.001	1.41E-10	14
Ifnar2	9.66E-15	1.198201	0.404	0.049	1.61E-10	14
Cd97	1.25E-14	0.852686	0.212	0.004	2.08E-10	14
Cd200	1.53E-14	1.249022	0.365	0.044	2.55E-10	14
Rgs5	1.64E-14	1.977366	0.269	0.013	2.74E-10	14
Rnf144b	1.65E-14	0.828415	0.192	0.003	2.75E-10	14
Tgfbr2	1.93E-14	0.811608	0.25	0.008	3.21E-10	14
Actn4	2.01E-14	1.2039	0.577	0.174	3.35E-10	14
Bcl6b	2.26E-14	0.675869	0.212	0.004	3.77E-10	14

Klf6	2.6E-14	1.256282	0.596	0.147	4.34E-10	14
Dennd3	2.65E-14	0.701144	0.173	0.002	4.43E-10	14
Enpp2	2.89E-14	1.204576	0.365	0.031	4.81E-10	14
Cyr61	3.1E-14	1.305005	0.288	0.018	5.17E-10	14
Prom1	3.4E-14	1.176679	0.288	0.025	5.68E-10	14
Arhgef15	3.42E-14	0.679083	0.173	0.002	5.71E-10	14
Plxnd1	4E-14	0.907649	0.269	0.011	6.67E-10	14
Sorbs2	4.11E-14	1.012062	0.327	0.026	6.85E-10	14
Rgs3	5.15E-14	0.643751	0.173	0.002	8.59E-10	14
Itga4	5.44E-14	0.934902	0.25	0.009	9.07E-10	14
Pear1	5.56E-14	0.672674	0.173	0.002	9.28E-10	14
Gatm	5.88E-14	1.181074	0.365	0.033	9.81E-10	14
Pde8a	5.97E-14	0.587158	0.154	0.001	9.95E-10	14
Gja1	6.19E-14	1.083833	0.308	0.019	1.03E-09	14
Mpzl1	6.36E-14	1.163121	0.538	0.143	1.06E-09	14
Sult1a1	6.48E-14	0.875795	0.192	0.003	1.08E-09	14
Dcbld1	6.78E-14	1.048403	0.308	0.024	1.13E-09	14
Chp2	7.18E-14	0.527711	0.173	0.002	1.2E-09	14
Arhgap25	9.81E-14	0.609428	0.173	0.002	1.64E-09	14
Lyn	1.09E-13	0.819455	0.25	0.01	1.81E-09	14
Rapgef4	1.37E-13	0.777653	0.25	0.01	2.29E-09	14
Aqp11	1.54E-13	0.963951	0.192	0.006	2.58E-09	14
Gria2	2.02E-13	-1.84121	0.038	0.522	3.36E-09	14
Elk3	2.04E-13	0.868268	0.308	0.02	3.41E-09	14
Celf2	2.23E-13	-1.60381	0.154	0.639	3.71E-09	14
E130114P1	2.62E-13	-1.67978	0.096	0.586	4.38E-09	14
Necap2	2.63E-13	1.103631	0.385	0.044	4.38E-09	14
Agrn	3.34E-13	1.073852	0.462	0.074	5.56E-09	14
Slc31a1	4.7E-13	1.170552	0.462	0.115	7.83E-09	14
Mfsd7c	5.34E-13	0.521345	0.154	0.001	8.91E-09	14
Tgfb1i1	6.88E-13	0.731276	0.212	0.006	1.15E-08	14
Marc2	7.02E-13	1.120211	0.462	0.082	1.17E-08	14
Anxa5	7.41E-13	1.092971	0.288	0.019	1.24E-08	14
Gatsl3	7.56E-13	0.61576	0.154	0.001	1.26E-08	14
Crip2	8.01E-13	1.022366	0.654	0.238	1.34E-08	14
Hhex	9.11E-13	0.946622	0.135	0.002	1.52E-08	14
Msn	9.67E-13	1.154242	0.442	0.091	1.61E-08	14
Cast	1.01E-12	0.725582	0.25	0.011	1.68E-08	14
Nnat	1.08E-12	-1.5234	0.231	0.711	1.81E-08	14
Slc46a3	1.1E-12	0.485573	0.173	0.003	1.83E-08	14
Sipa1	1.11E-12	0.911077	0.231	0.009	1.85E-08	14
Pttg1ip	1.25E-12	1.108514	0.462	0.085	2.09E-08	14
Gm6977	1.27E-12	1.11169	0.269	0.031	2.13E-08	14
Rpl38	1.37E-12	0.982917	0.692	0.381	2.28E-08	14
Ccdc85b	1.38E-12	1.113404	0.442	0.075	2.3E-08	14
Megf6	1.49E-12	0.632404	0.192	0.004	2.48E-08	14
Luzp1	1.5E-12	1.056612	0.558	0.134	2.5E-08	14
Hnrnpab	1.57E-12	-1.05428	0.481	0.816	2.62E-08	14

Lrp10	1.71E-12	1.031438	0.365	0.04	2.86E-08	14
Adm	1.83E-12	0.60347	0.154	0.002	3.05E-08	14
Chd7	1.9E-12	-1.43701	0.173	0.648	3.16E-08	14
Rgl1	1.91E-12	1.01735	0.192	0.007	3.18E-08	14
Pcdh1	1.91E-12	0.470274	0.173	0.004	3.19E-08	14
Procr	2.03E-12	0.494513	0.115	0	3.38E-08	14
Cog7	2.16E-12	-1.51706	0.096	0.585	3.6E-08	14
Ngfrap1	2.49E-12	-1.32887	0.231	0.658	4.16E-08	14
Aplp2	2.7E-12	1.026027	0.692	0.322	4.51E-08	14
Cdh11	2.73E-12	1.036771	0.25	0.015	4.55E-08	14
Shisa5	2.98E-12	0.787313	0.308	0.024	4.96E-08	14
Cdk4	3.37E-12	-1.29283	0.25	0.643	5.62E-08	14
S100a16	3.45E-12	1.124614	0.5	0.093	5.75E-08	14
Slc16a9	3.45E-12	0.744164	0.173	0.003	5.75E-08	14
Calm2	4.06E-12	-0.90787	0.692	0.874	6.77E-08	14
Ppapdc2	4.09E-12	0.784737	0.25	0.012	6.83E-08	14
Sorbs3	4.28E-12	0.590588	0.192	0.005	7.14E-08	14
Insr	4.5E-12	1.139188	0.365	0.051	7.51E-08	14
Pon2	4.64E-12	1.08548	0.327	0.038	7.74E-08	14
Gnai2	5.44E-12	0.940396	0.712	0.423	9.07E-08	14
Sypl	5.65E-12	0.990938	0.442	0.073	9.42E-08	14
Tmsb10	5.75E-12	0.838011	0.827	0.663	9.58E-08	14
Map4k2	5.91E-12	0.886642	0.269	0.017	9.86E-08	14
Ttc3	6.72E-12	-1.01117	0.5	0.83	1.12E-07	14
Apbb2	9.78E-12	1.009568	0.346	0.038	1.63E-07	14
Slc52a2	9.85E-12	0.851303	0.308	0.026	1.64E-07	14
St6galnac2	1.06E-11	0.711409	0.135	0.002	1.77E-07	14
Arap2	1.15E-11	0.798009	0.212	0.009	1.92E-07	14
Myl6	1.2E-11	1.069533	0.558	0.201	2E-07	14
Xaf1	1.58E-11	0.493806	0.135	0.001	2.64E-07	14
Ostf1	1.61E-11	1.162602	0.385	0.062	2.69E-07	14
Ccnd2	1.98E-11	-1.38067	0.212	0.663	3.3E-07	14
Podxl	2.06E-11	0.73693	0.192	0.006	3.43E-07	14
Sdpr	2.33E-11	1.259758	0.442	0.086	3.89E-07	14
Ctnna1	2.54E-11	1.100891	0.519	0.151	4.23E-07	14
Slc16a4	2.65E-11	0.566796	0.154	0.002	4.42E-07	14
Sgk1	2.73E-11	0.91826	0.327	0.033	4.56E-07	14
Abcg1	2.74E-11	0.946518	0.288	0.023	4.57E-07	14
Ppp2r2c	2.74E-11	-1.42286	0.115	0.506	4.57E-07	14
Gnb4	2.83E-11	1.168863	0.423	0.104	4.71E-07	14
Ecm1	3.07E-11	0.697701	0.173	0.004	5.11E-07	14
Plekhg5	3.22E-11	0.605783	0.154	0.002	5.36E-07	14
Capg	3.53E-11	0.537241	0.173	0.004	5.88E-07	14
Scg3	3.62E-11	-1.49556	0.019	0.447	6.04E-07	14
Cav1	4.47E-11	0.899011	0.25	0.019	7.45E-07	14
Dpysl4	4.6E-11	-1.46783	0.096	0.492	7.67E-07	14
Tram2	4.74E-11	0.587349	0.154	0.002	7.91E-07	14
Paqr7	4.77E-11	0.80057	0.173	0.005	7.95E-07	14

Ankrd37	4.93E-11	0.990916	0.25	0.018	8.22E-07	14
Gimap8	5E-11	0.507113	0.135	0.001	8.34E-07	14
Gja4	5.11E-11	0.734086	0.135	0.001	8.52E-07	14
Fermt2	6.21E-11	1.096701	0.577	0.204	1.04E-06	14
Prcp	6.96E-11	0.993164	0.308	0.036	1.16E-06	14
Foxc1	8.53E-11	0.720959	0.192	0.007	1.42E-06	14
Rasgrp2	8.78E-11	0.847737	0.192	0.008	1.46E-06	14
2900026A(	1E-10	0.830876	0.212	0.011	1.67E-06	14
Mecom	1.03E-10	0.393393	0.115	0.001	1.71E-06	14
Tmc7	1.28E-10	0.660249	0.154	0.003	2.14E-06	14
Atpif1	1.36E-10	-0.90257	0.538	0.816	2.27E-06	14
Pcp4l1	1.45E-10	0.633294	0.173	0.005	2.42E-06	14
Miat	2.07E-10	-1.65728	0.115	0.516	3.45E-06	14
Maoa	2.17E-10	0.991403	0.385	0.067	3.63E-06	14
Ttll7	2.65E-10	0.865185	0.212	0.01	4.41E-06	14
Wwc2	2.93E-10	0.599117	0.25	0.019	4.88E-06	14
Hnrnpdl	3.08E-10	-0.81405	0.423	0.756	5.14E-06	14
Anp32a	3.32E-10	-0.79613	0.654	0.837	5.54E-06	14
Gm9917	3.47E-10	0.570479	0.154	0.003	5.79E-06	14
Hexb	3.63E-10	0.276371	0.231	0.032	6.05E-06	14
Inf2	3.81E-10	0.495841	0.154	0.004	6.36E-06	14
Anp32e	4E-10	-1.2733	0.269	0.614	6.68E-06	14
Gap43	4.01E-10	-1.50829	0.192	0.6	6.68E-06	14
Casp6	4.62E-10	0.726048	0.269	0.023	7.71E-06	14
H2afv	4.62E-10	-1.15385	0.346	0.728	7.71E-06	14
Higd1b	5.24E-10	0.901872	0.135	0.002	8.74E-06	14
Cdc42ep3	5.55E-10	1.144893	0.385	0.089	9.26E-06	14
Macf1	6.34E-10	1.092845	0.558	0.209	1.06E-05	14
Gmpr	7.43E-10	0.730066	0.212	0.012	1.24E-05	14
Hrct1	7.69E-10	0.322976	0.115	0.001	1.28E-05	14
Jak2	7.8E-10	0.946059	0.269	0.03	1.3E-05	14
Lamb2	8.15E-10	0.858288	0.192	0.011	1.36E-05	14
Gm1673	8.36E-10	-1.34159	0.077	0.474	1.39E-05	14
Cd38	8.48E-10	0.658332	0.135	0.002	1.41E-05	14
Prex2	8.61E-10	0.882737	0.212	0.013	1.44E-05	14
Prr5l	8.93E-10	0.549873	0.154	0.004	1.49E-05	14
Prkd2	9.5E-10	0.817443	0.269	0.024	1.58E-05	14
Loxl2	9.69E-10	0.494157	0.154	0.004	1.62E-05	14
Exoc3l	9.72E-10	0.802879	0.212	0.012	1.62E-05	14
Tubb2b	9.78E-10	-1.37879	0.058	0.465	1.63E-05	14
Mfng	1.02E-09	0.817206	0.269	0.028	1.7E-05	14
Filip1l	1.04E-09	0.681921	0.192	0.009	1.74E-05	14
5730508B(	1.06E-09	0.428268	0.135	0.002	1.76E-05	14
Itih5	1.06E-09	0.56673	0.192	0.014	1.77E-05	14
Slc31a2	1.07E-09	0.664413	0.212	0.014	1.79E-05	14
Arap3	1.16E-09	0.555493	0.115	0.001	1.94E-05	14
Cxcl12	1.22E-09	1.566062	0.212	0.043	2.03E-05	14
Lhx1	1.28E-09	-1.41114	0.077	0.498	2.14E-05	14

Rbfox3	1.37E-09	-1.42984	0.077	0.495	2.28E-05	14
Tpm1	1.37E-09	1.010727	0.577	0.189	2.28E-05	14
Abca1	1.63E-09	0.786756	0.25	0.023	2.72E-05	14
Tes	1.67E-09	0.777826	0.192	0.009	2.78E-05	14
Ifnar1	1.69E-09	0.911323	0.442	0.095	2.82E-05	14
Kank2	1.85E-09	0.486569	0.173	0.007	3.09E-05	14
Sat1	2.02E-09	1.127056	0.365	0.066	3.37E-05	14
Cbx5	2.15E-09	-1.15577	0.288	0.654	3.58E-05	14
Prnp	2.17E-09	0.953693	0.596	0.205	3.63E-05	14
Cd9	2.43E-09	-1.28428	0.135	0.485	4.06E-05	14
Ldlrap1	2.47E-09	0.633873	0.135	0.003	4.12E-05	14
Ptp4a3	2.49E-09	0.60212	0.192	0.009	4.15E-05	14
Myl4	2.72E-09	0.582342	0.192	0.009	4.53E-05	14
Psip1	2.98E-09	-1.04617	0.308	0.688	4.97E-05	14
Snrk	3.14E-09	1.004777	0.308	0.048	5.25E-05	14
Tnfaip1	3.47E-09	1.012512	0.423	0.106	5.79E-05	14
Piezo1	3.51E-09	0.597829	0.154	0.004	5.85E-05	14
Barhl1	3.7E-09	-1.32893	0.115	0.523	6.18E-05	14
Rps9	3.76E-09	-0.50055	0.962	0.928	6.27E-05	14
Apod	3.91E-09	1.191453	0.288	0.041	6.53E-05	14
Prdm1	4.17E-09	0.435057	0.115	0.001	6.95E-05	14
Mapk12	5E-09	0.661916	0.135	0.003	8.35E-05	14
Neurod1	5.09E-09	-1.70486	0.115	0.534	8.5E-05	14
Pmepa1	5.1E-09	0.949307	0.25	0.026	8.5E-05	14
Phactr2	5.36E-09	0.590694	0.212	0.016	8.95E-05	14
Lcp1	5.87E-09	0.359952	0.135	0.005	9.8E-05	14
Ncam1	5.92E-09	-1.28702	0.038	0.388	9.88E-05	14
Spata6	6.35E-09	0.392696	0.154	0.005	0.000106	14
Rap1b	6.37E-09	0.841529	0.5	0.133	0.000106	14
Map2	6.74E-09	-1.36916	0.115	0.492	0.000112	14
Ifi27	7.07E-09	0.668907	0.212	0.014	0.000118	14
Ccdc85a	7.08E-09	0.612933	0.154	0.005	0.000118	14
Hn1	7.12E-09	-1.221	0.192	0.542	0.000119	14
Id2	7.51E-09	-1.45227	0.096	0.487	0.000125	14
Tpx2	7.88E-09	-1.27907	0.038	0.282	0.000131	14
Arhgef12	8.23E-09	0.981481	0.462	0.124	0.000137	14
Pde1c	8.27E-09	-1.35957	0.058	0.387	0.000138	14
Syt11	8.32E-09	-1.29805	0.115	0.505	0.000139	14
Pfn1	8.46E-09	0.772028	0.75	0.51	0.000141	14
Psmb8	8.88E-09	0.768199	0.154	0.006	0.000148	14
Tacc1	8.96E-09	0.983191	0.346	0.069	0.000149	14
Tubb3	9.78E-09	-1.60226	0.096	0.475	0.000163	14
Ctsl	1.02E-08	0.908478	0.635	0.259	0.00017	14
Arhgef28	1.04E-08	0.589733	0.154	0.005	0.000173	14
Tm6sf1	1.06E-08	0.717918	0.212	0.016	0.000176	14
Klhl5	1.1E-08	0.669466	0.288	0.038	0.000183	14
Enc1	1.17E-08	0.498525	0.192	0.014	0.000196	14
Bgn	1.24E-08	0.807263	0.154	0.006	0.000207	14

Sept3	1.28E-08	-1.47061	0.038	0.393	0.000213	14
Slc38a2	1.32E-08	0.944774	0.519	0.219	0.000221	14
Snx3	1.35E-08	0.791752	0.692	0.341	0.000225	14
Apba2	1.35E-08	-1.23956	0.077	0.415	0.000226	14
Pdlim5	1.36E-08	0.503536	0.192	0.014	0.000226	14
Fkbp3	1.46E-08	-0.96884	0.423	0.746	0.000244	14
Ubtd1	1.49E-08	0.62344	0.154	0.005	0.000249	14
M6pr	1.51E-08	0.93405	0.423	0.108	0.000252	14
Cpt1a	1.58E-08	0.630108	0.212	0.017	0.000264	14
Gabarapl1	1.59E-08	0.982057	0.538	0.215	0.000265	14
Meis1	1.65E-08	-1.28254	0.019	0.373	0.000276	14
Isyna1	1.66E-08	0.636215	0.173	0.01	0.000277	14
Cstb	1.78E-08	0.960751	0.442	0.116	0.000298	14
Plekhg1	1.87E-08	0.46172	0.135	0.005	0.000312	14
Kif20b	1.91E-08	-0.53363	0.038	0.191	0.000319	14
Ezh2	1.92E-08	-0.91912	0.231	0.61	0.00032	14
Efna1	2.2E-08	0.523829	0.154	0.006	0.000366	14
Soga3	2.34E-08	-1.27274	0.096	0.458	0.00039	14
Stmn2	2.35E-08	-1.57867	0.192	0.554	0.000391	14
Gm14005	2.46E-08	0.54706	0.115	0.002	0.000411	14
Col4a3bp	2.98E-08	0.799243	0.346	0.065	0.000497	14
Ank3	3.23E-08	-1.09885	0.058	0.421	0.000538	14
Kif21a	3.26E-08	-1.3206	0.019	0.346	0.000544	14
Fam13a	3.38E-08	0.920723	0.192	0.014	0.000564	14
2810417H	3.51E-08	-1.11652	0.077	0.392	0.000585	14
Fry	3.51E-08	0.750658	0.25	0.027	0.000586	14
Mfge8	3.51E-08	0.82689	0.269	0.033	0.000586	14
Ndrg1	3.56E-08	0.439025	0.135	0.005	0.000594	14
Slc6a6	3.97E-08	1.050019	0.269	0.043	0.000662	14
Sash1	4.22E-08	0.838452	0.269	0.034	0.000703	14
Klf4	4.27E-08	0.969422	0.212	0.023	0.000712	14
Hspa1a	4.27E-08	0.583466	0.115	0.002	0.000712	14
Jam2	4.29E-08	0.970465	0.231	0.032	0.000716	14
Cdc42ep2	4.3E-08	0.588036	0.154	0.006	0.000718	14
Cyba	4.39E-08	0.774237	0.25	0.029	0.000732	14
Clip1	4.77E-08	0.898361	0.404	0.093	0.000796	14
Tcn2	4.86E-08	0.708068	0.212	0.019	0.000811	14
Alas1	4.94E-08	0.704539	0.327	0.056	0.000824	14
Ptprg	5.04E-08	0.923687	0.462	0.141	0.000841	14
Alad	5.2E-08	0.847428	0.269	0.039	0.000868	14
Sft2d1	5.21E-08	0.912568	0.192	0.021	0.000869	14
Ddx58	5.59E-08	0.351529	0.115	0.002	0.000933	14
Mal	5.6E-08	0.473691	0.115	0.003	0.000934	14
Tagln3	6.28E-08	-1.22577	0	0.295	0.001047	14
Dclk1	6.6E-08	-1.30616	0.077	0.413	0.001101	14
Zic4	6.88E-08	-1.23249	0.058	0.419	0.001148	14
H2-K1	6.93E-08	0.722412	0.192	0.013	0.001157	14
Usp6nl	7.16E-08	0.770368	0.25	0.032	0.001194	14

Cd2ap	7.66E-08	1.016509	0.423	0.158	0.001278	14
Flnb	8.24E-08	0.659175	0.192	0.014	0.001375	14
Slc7a8	8.27E-08	0.573736	0.135	0.005	0.001379	14
Rhobtb1	8.32E-08	0.634897	0.154	0.008	0.001388	14
Fxyd6	8.97E-08	-1.21193	0.135	0.51	0.001496	14
Ddx5	9.53E-08	-0.6595	0.673	0.816	0.001589	14
Hsd3b7	9.65E-08	0.672673	0.173	0.01	0.001609	14
Hdac2	9.72E-08	-1.09062	0.173	0.516	0.001621	14
Prdx2	9.83E-08	-0.77014	0.423	0.68	0.00164	14
Vasp	1E-07	0.779929	0.346	0.066	0.00167	14
Tsc22d3	1.11E-07	0.913424	0.365	0.091	0.001855	14
Extl3	1.18E-07	0.769708	0.327	0.058	0.001969	14
Mxd4	1.19E-07	0.924114	0.5	0.197	0.001985	14
Fkbp1a	1.32E-07	0.732852	0.673	0.451	0.002197	14
Serpinb9	1.42E-07	0.429521	0.115	0.002	0.002364	14
Mdk	1.42E-07	-1.33113	0.058	0.38	0.002372	14
Gpm6a	1.44E-07	-1.37132	0.058	0.41	0.002409	14
Col1a2	1.53E-07	0.517639	0.154	0.014	0.002545	14
4632428N(	1.57E-07	0.389262	0.135	0.006	0.002617	14
Nsg2	1.62E-07	-1.06796	0.115	0.488	0.002694	14
Pvrl2	1.69E-07	0.678003	0.192	0.016	0.002818	14
Wasf2	1.71E-07	0.946879	0.346	0.112	0.002844	14
Fabp5	1.73E-07	-1.00476	0.058	0.404	0.002893	14
Ddx39b	1.85E-07	-1.08009	0.135	0.445	0.003089	14
Dynlt3	1.87E-07	0.814715	0.288	0.045	0.003115	14
Ina	1.9E-07	-1.28311	0.096	0.444	0.003176	14
Grb10	2E-07	0.838203	0.231	0.028	0.003342	14
Hopx	2.11E-07	0.577484	0.154	0.008	0.003512	14
Oxct1	2.15E-07	-1.04858	0.192	0.553	0.003589	14
Tenc1	2.2E-07	0.63179	0.154	0.008	0.003672	14
Pax6	2.32E-07	-1.05753	0.135	0.514	0.003872	14
Stx3	2.35E-07	0.318604	0.115	0.004	0.003914	14
Nudt14	2.44E-07	0.652909	0.192	0.019	0.004066	14
Rab11a	2.45E-07	0.735514	0.635	0.276	0.004078	14
Pou3f2	2.46E-07	-1.091	0	0.276	0.004097	14
H2afy2	2.51E-07	-1.04858	0.019	0.338	0.004187	14
App	2.6E-07	0.685142	0.808	0.567	0.004337	14
Cacng2	2.64E-07	-1.18881	0.019	0.339	0.004407	14
Cp	2.91E-07	1.071011	0.173	0.013	0.004846	14
Cebpd	2.94E-07	0.611021	0.173	0.011	0.004902	14
Flii	3.03E-07	0.789414	0.308	0.054	0.005059	14
P4hb	3.11E-07	0.788064	0.519	0.278	0.005189	14
Cd320	3.11E-07	0.949881	0.327	0.085	0.005194	14
Kctd12b	3.15E-07	0.76235	0.212	0.025	0.005257	14
Tfpi	3.41E-07	0.519791	0.154	0.008	0.005692	14
Tprgl	3.61E-07	0.769621	0.423	0.123	0.006017	14
Slc38a1	3.62E-07	-1.01225	0	0.271	0.006044	14
RP23-45G1	3.83E-07	-1.29926	0.019	0.327	0.006395	14

Lhfpl2	4.05E-07	0.76689	0.231	0.027	0.006749	14
Cmtm6	4.47E-07	0.546937	0.212	0.023	0.00745	14
Arrdc1	4.63E-07	0.566938	0.154	0.009	0.00772	14
Hsp90aa1	4.74E-07	-0.91829	0.25	0.617	0.007905	14
Gstm1	5.07E-07	0.890557	0.346	0.081	0.008463	14
Gm2694	5.27E-07	-1.07651	0.038	0.359	0.008785	14
Cntn2	5.73E-07	-1.50338	0	0.264	0.009565	14
1700025G(	5.82E-07	-1.06368	0	0.264	0.009714	14
Rbp4	6.12E-07	-0.86361	0.038	0.242	0.0102	14
Adam10	6.67E-07	0.866606	0.5	0.202	0.011129	14
Vps37b	6.69E-07	-0.88494	0.115	0.471	0.011151	14
Itsn2	6.83E-07	0.80221	0.365	0.085	0.011388	14
Lnx2	7.05E-07	0.386772	0.115	0.004	0.011767	14
Tjp2	7.13E-07	0.695599	0.288	0.051	0.011891	14
Ptprm	7.14E-07	0.629788	0.115	0.006	0.011907	14
Fgf9	7.17E-07	-1.03385	0	0.261	0.011967	14
Apoe	7.19E-07	0.579587	0.577	0.247	0.011998	14
Mdh1	7.49E-07	-1.03806	0.154	0.453	0.012493	14
Ddah1	8.05E-07	0.623046	0.25	0.039	0.01343	14
Ccdc34	8.22E-07	-1.17678	0.115	0.415	0.013714	14
Tspan9	8.57E-07	0.647491	0.154	0.009	0.014292	14
Ptpn18	8.74E-07	0.275401	0.115	0.007	0.014575	14
Mycn	8.9E-07	-1.1358	0.058	0.364	0.014837	14
Casp8	9.12E-07	0.355941	0.115	0.003	0.01522	14
Nhlh2	9.42E-07	-1.28631	0.115	0.423	0.015716	14
Nrxn1	9.86E-07	-1.33425	0.019	0.319	0.01645	14
Trf	9.94E-07	0.8879	0.25	0.037	0.016578	14
Pcdh19	9.97E-07	0.615267	0.173	0.014	0.016622	14
Ppic	1E-06	0.833365	0.538	0.238	0.016731	14
Lphn2	1.01E-06	0.789991	0.327	0.069	0.016839	14
1500016L(	1.07E-06	-1.1324	0.058	0.383	0.017902	14
Kctd10	1.15E-06	0.778134	0.288	0.053	0.01917	14
Fcho2	1.17E-06	0.848421	0.327	0.076	0.019452	14
Creb3l2	1.19E-06	0.608252	0.154	0.011	0.019791	14
Gpx1	1.25E-06	0.721596	0.673	0.381	0.020931	14
Ltbp4	1.26E-06	0.868495	0.212	0.035	0.020949	14
Entpd1	1.3E-06	0.347692	0.115	0.005	0.021686	14
Tcf7l1	1.3E-06	0.496164	0.154	0.01	0.021734	14
Lxn	1.33E-06	0.938321	0.25	0.052	0.022235	14
Gm17750	1.38E-06	-1.18885	0.058	0.367	0.023031	14
Ezr	1.47E-06	-1.00941	0.096	0.438	0.024529	14
Smad1	1.48E-06	0.872507	0.423	0.143	0.024635	14
Gng3	1.5E-06	-1.28215	0.038	0.317	0.025078	14
Itm2b	1.52E-06	0.598874	0.885	0.641	0.025402	14
Ppp1r13b	1.53E-06	0.386897	0.173	0.023	0.025527	14
Iqgap1	1.58E-06	0.813065	0.423	0.131	0.026308	14
Ctnnb1	1.62E-06	0.653263	0.692	0.385	0.026956	14
Sfrs18	1.63E-06	-0.76751	0.577	0.778	0.027165	14

Rnd3	1.67E-06	-1.08413	0.096	0.432	0.02792	14
Grasp	1.69E-06	0.668313	0.192	0.02	0.028182	14
Ctnnbip1	1.73E-06	0.718962	0.365	0.098	0.028843	14
Dusp6	1.78E-06	0.864201	0.346	0.092	0.029726	14
Pomp	1.8E-06	0.709529	0.673	0.413	0.030106	14
Rgs4	1.82E-06	0.86037	0.115	0.005	0.030277	14
Rac1	1.93E-06	0.712813	0.673	0.345	0.03219	14
Lpar6	1.94E-06	0.861789	0.269	0.071	0.032402	14
Usp22	1.99E-06	-1.04965	0.077	0.362	0.03326	14
Prrg2	2.2E-06	0.821234	0.212	0.029	0.036719	14
Sh3bp5	2.25E-06	0.80901	0.385	0.102	0.037516	14
Prkcdbp	2.29E-06	0.830122	0.308	0.089	0.038166	14
Tecr	2.29E-06	-0.92958	0.25	0.53	0.038176	14
Ift27	2.37E-06	-0.86267	0.038	0.243	0.039579	14
Nasp	2.38E-06	-0.88295	0.327	0.641	0.03974	14
Pnn	2.41E-06	-0.81819	0.462	0.722	0.040172	14
Clmp	2.41E-06	-1.09801	0	0.243	0.04024	14
H3f3b	2.45E-06	-0.58032	0.769	0.907	0.040919	14
Maged1	2.47E-06	-0.99356	0.154	0.43	0.041156	14
Foxo1	2.48E-06	0.762615	0.192	0.026	0.041404	14
Serpine2	2.63E-06	0.896519	0.288	0.056	0.043935	14
Lpcat3	2.76E-06	0.531046	0.25	0.043	0.04608	14
Ptprs	2.78E-06	-1.19243	0.115	0.422	0.046355	14
Slc1a2	2.81E-06	-1.06344	0.058	0.294	0.046916	14
Net1	2.87E-06	0.714952	0.231	0.036	0.047886	14
Lhfp	2.95E-06	0.339499	0.154	0.019	0.049288	14
Fam43a	2.97E-06	0.79138	0.288	0.062	0.04958	14
Insm1	3.15E-06	-0.94111	0.019	0.293	0.052556	14
Junb	3.29E-06	0.755412	0.231	0.034	0.054925	14
Abhd17a	3.3E-06	0.845482	0.404	0.13	0.055006	14
D430041D	3.31E-06	-0.9682	0.154	0.493	0.055293	14
Lrrfip1	3.35E-06	0.800101	0.25	0.043	0.055818	14
Racgap1	3.39E-06	-0.95259	0.038	0.256	0.056578	14
Tbx1	3.53E-06	0.657651	0.154	0.014	0.05896	14
Cks1b	3.54E-06	-1.01367	0.115	0.345	0.059007	14
Hnrnpu	3.76E-06	-0.58282	0.731	0.865	0.062712	14
Srpr	3.77E-06	0.729777	0.365	0.096	0.062867	14
Nsg1	3.77E-06	-1.04273	0.038	0.288	0.062903	14
Uncx	4.3E-06	-1.11606	0.038	0.318	0.071727	14
Phf21a	4.37E-06	-0.67001	0.038	0.19	0.072974	14
Mkl2	4.59E-06	0.425771	0.154	0.013	0.076553	14
Lpp	4.6E-06	0.678995	0.173	0.023	0.076756	14
Gng5	4.75E-06	0.822586	0.404	0.164	0.079157	14
Serf1	4.76E-06	-1.03755	0.019	0.281	0.079322	14
Tspan5	4.76E-06	0.589655	0.519	0.195	0.079462	14
Myo1c	4.99E-06	0.396068	0.115	0.005	0.08315	14
Orai1	5.22E-06	0.539693	0.192	0.023	0.087098	14
Raph1	5.26E-06	0.563337	0.154	0.012	0.08779	14

Nrp2	5.36E-06	0.394021	0.115	0.005	0.089341	14
Cenpv	5.39E-06	-0.94541	0.038	0.329	0.089854	14
Rsrc1	5.46E-06	-0.77482	0.058	0.251	0.091129	14
Fkbp9	5.56E-06	0.688228	0.212	0.03	0.092822	14
Gpm6b	5.61E-06	-0.94283	0.154	0.485	0.093572	14
Cttnbp2nl	5.75E-06	0.837928	0.288	0.067	0.095947	14
Prox1	5.8E-06	-0.93014	0	0.229	0.0967	14
Dcaf6	5.94E-06	0.684112	0.327	0.08	0.099015	14
Litaf	5.95E-06	0.838428	0.269	0.068	0.099199	14
Slc25a4	6.15E-06	-0.60451	0.692	0.807	0.102665	14
Ppp1r16b	6.6E-06	0.588305	0.115	0.005	0.110029	14
Plcb4	7E-06	0.951779	0.365	0.138	0.116799	14
2700094K1	7E-06	-0.87974	0.308	0.614	0.116831	14
Tbata	7E-06	-1.18291	0.058	0.34	0.116831	14
Cxxc5	7.18E-06	-0.93626	0.269	0.544	0.119714	14
Rcan3	7.4E-06	0.679698	0.135	0.022	0.123431	14
Elovl6	7.43E-06	-0.97651	0.038	0.278	0.123893	14
Mex3a	7.58E-06	-0.97249	0.154	0.464	0.126365	14
mt-Rnr1	7.69E-06	0.772971	0.577	0.366	0.12825	14
Fam171b	8.03E-06	-0.92048	0	0.224	0.133999	14
Sox4	8.26E-06	-0.79022	0.462	0.583	0.137839	14
Nhlh1	8.45E-06	-1.05582	0	0.223	0.141024	14
Hnrnph1	8.46E-06	-0.80097	0.346	0.629	0.141069	14
Snrpd3	8.84E-06	-0.90031	0.25	0.535	0.147421	14
Adipor1	8.87E-06	0.825728	0.385	0.139	0.148021	14
Hsd11b2	9.11E-06	-1.05922	0.019	0.288	0.152032	14
Fez1	9.23E-06	-0.99808	0.077	0.333	0.153942	14
Kit	9.28E-06	0.707657	0.173	0.022	0.15481	14
Tmem44	9.3E-06	0.67461	0.231	0.036	0.155116	14
Pdxk	9.58E-06	0.680133	0.154	0.016	0.159827	14
Irf2	9.61E-06	0.727574	0.269	0.056	0.160282	14
Tmpo	9.63E-06	-0.79452	0.288	0.452	0.160659	14
Pitpnc1	9.65E-06	0.443864	0.288	0.074	0.16099	14
Eif4a1	9.69E-06	-0.86665	0.231	0.485	0.161653	14
Calm3	9.72E-06	-0.83415	0.327	0.561	0.162058	14
Pea15a	9.91E-06	0.733329	0.481	0.175	0.165258	14
Phldb2	9.98E-06	0.765065	0.269	0.055	0.166473	14
Hpca	1.02E-05	-0.95822	0.019	0.277	0.170783	14
Pxn	1.04E-05	0.72024	0.231	0.039	0.173293	14
Rbfox2	1.08E-05	-0.96219	0.077	0.283	0.180577	14
Fam13c	1.11E-05	0.840276	0.269	0.061	0.185091	14
Ilf2	1.14E-05	-0.84864	0.25	0.542	0.190888	14
Hmgn1	1.16E-05	-0.79665	0.365	0.622	0.194282	14
Cbx1	1.19E-05	-0.74358	0.462	0.685	0.198939	14
Rilpl1	1.22E-05	0.634666	0.25	0.046	0.203363	14
Arhgdia	1.22E-05	0.678522	0.519	0.207	0.203389	14
Lats2	1.27E-05	0.348018	0.154	0.018	0.211859	14
Ppard	1.28E-05	0.395641	0.135	0.01	0.214255	14

Ssbp3	1.32E-05	-0.67052	0.038	0.167	0.221001	14
Ltbr	1.35E-05	0.409027	0.154	0.016	0.22569	14
Ets2	1.36E-05	0.751537	0.231	0.045	0.227343	14
Tspan12	1.38E-05	0.542503	0.212	0.031	0.230663	14
Hes1	1.39E-05	0.882556	0.423	0.147	0.232455	14
Actn1	1.4E-05	0.77478	0.269	0.063	0.232796	14
Slc16a2	1.44E-05	0.793646	0.231	0.068	0.239413	14
Map3k1	1.44E-05	-0.76311	0.077	0.371	0.240409	14
H1f0	1.44E-05	-0.90086	0.327	0.617	0.240987	14
Clip3	1.47E-05	-0.95888	0.038	0.312	0.245593	14
Scg5	1.52E-05	-1.02818	0.058	0.335	0.253721	14
Elavl4	1.52E-05	-1.13146	0.058	0.336	0.254075	14
Ybx1	1.55E-05	-0.53712	0.712	0.786	0.257797	14
Msh2	1.63E-05	-0.42792	0.038	0.143	0.271265	14
Cmtm8	1.65E-05	0.587431	0.154	0.015	0.275647	14
Fut9	1.74E-05	-0.92003	0	0.212	0.289708	14
Gbp7	1.81E-05	0.451361	0.135	0.01	0.301572	14
Srebf1	1.85E-05	-0.91777	0.077	0.334	0.308736	14
Map1b	1.87E-05	-1.02951	0.327	0.588	0.312722	14
S100a11	1.94E-05	0.392724	0.154	0.016	0.323445	14
Pitpna	1.95E-05	0.725204	0.404	0.13	0.324684	14
Snap25	1.96E-05	-0.98677	0.038	0.282	0.326209	14
Degs1	2E-05	0.668715	0.308	0.075	0.334299	14
Galnt18	2.02E-05	0.459979	0.135	0.01	0.337367	14
Sf3b2	2.06E-05	-0.6728	0.327	0.626	0.342966	14
Map3k11	2.09E-05	0.500586	0.154	0.015	0.34824	14
Cdk17	2.12E-05	0.601605	0.269	0.057	0.35412	14
Cyb5r3	2.16E-05	0.729476	0.462	0.198	0.359599	14
Gpr153	2.17E-05	-0.87515	0	0.209	0.361685	14
Pcm1	2.19E-05	-0.86827	0.308	0.522	0.365564	14
Capns1	2.25E-05	0.73047	0.519	0.226	0.375312	14
Fam63b	2.27E-05	0.742838	0.327	0.087	0.378128	14
Top1	2.28E-05	-0.71362	0.481	0.663	0.380368	14
Glul	2.29E-05	0.773485	0.481	0.2	0.381396	14
Triobp	2.29E-05	0.683133	0.231	0.042	0.382266	14
Fubp1	2.33E-05	-0.83594	0.308	0.567	0.389006	14
Whsc1	2.37E-05	-0.94381	0.192	0.465	0.394608	14
Trim2	2.44E-05	-1.01737	0.038	0.283	0.407239	14
Ldlrad3	2.47E-05	0.346677	0.115	0.007	0.41255	14
Mef2a	2.49E-05	0.817883	0.385	0.138	0.415568	14
Cplx2	2.54E-05	-0.88322	0.173	0.496	0.422858	14
Slc43a2	2.58E-05	0.676813	0.173	0.023	0.429915	14
Apex1	2.72E-05	-0.88887	0.173	0.452	0.454369	14
Tnfrsf1a	2.73E-05	0.576492	0.173	0.024	0.45513	14
Pfn2	2.73E-05	-0.79306	0.038	0.241	0.455711	14
Adam15	2.82E-05	0.498316	0.115	0.008	0.470064	14
Rnmt	2.85E-05	-1.00401	0.077	0.349	0.474963	14
H1fx	2.86E-05	-0.80931	0.038	0.245	0.477145	14

Lrrc8c	2.87E-05	0.585733	0.173	0.021	0.477983	14
Tecpr1	3.03E-05	0.647571	0.308	0.077	0.505265	14
Mllt4	3.08E-05	0.549184	0.692	0.361	0.513227	14
Ebf3	3.08E-05	-0.86481	0	0.203	0.51427	14
C1ql1	3.09E-05	-0.9795	0.038	0.279	0.51465	14
Cdh20	3.12E-05	-0.8262	0	0.203	0.519761	14
Anapc13	3.13E-05	-0.86979	0.173	0.393	0.522881	14
Kitl	3.16E-05	0.743899	0.269	0.065	0.527086	14
Srsf3	3.25E-05	-0.71411	0.423	0.657	0.542391	14
Vtn	3.49E-05	0.528957	0.115	0.01	0.582277	14
Myo1e	3.56E-05	0.633324	0.135	0.016	0.593422	14
Hirip3	3.56E-05	-1.00658	0.115	0.383	0.594595	14
Mef2c	3.6E-05	0.771952	0.269	0.068	0.6008	14
Myl9	3.67E-05	0.531839	0.135	0.012	0.611358	14
Myh10	3.7E-05	0.697764	0.558	0.323	0.61759	14
H2afy	3.73E-05	-0.77269	0.308	0.592	0.621488	14
Bcas1	3.73E-05	-0.97861	0.038	0.283	0.622586	14
Mcm7	3.74E-05	-0.87214	0.115	0.389	0.623132	14
Plxna2	3.75E-05	0.719395	0.269	0.069	0.625425	14
Syngr2	3.77E-05	0.548455	0.173	0.022	0.628698	14
Celf4	3.87E-05	-1.28296	0.058	0.321	0.645356	14
Ppp1r14b	3.91E-05	-0.89503	0.173	0.397	0.652455	14
2010111I0	3.98E-05	0.529309	0.192	0.029	0.663033	14
Arhgdib	4.09E-05	0.593733	0.115	0.008	0.682011	14
Wls	4.11E-05	0.730535	0.308	0.086	0.68502	14
Metap2	4.13E-05	-0.68003	0.442	0.597	0.689029	14
Eif2ak2	4.15E-05	0.49549	0.135	0.011	0.69258	14
Maff	4.21E-05	0.376113	0.135	0.013	0.70152	14
Npm1	4.23E-05	-0.66221	0.462	0.646	0.706097	14
Cdk5r1	4.26E-05	-1.05544	0.077	0.334	0.710698	14
Ccny	4.29E-05	0.760855	0.25	0.069	0.716369	14
Pde4b	4.3E-05	0.768929	0.212	0.05	0.717583	14
Fam210b	4.33E-05	-0.87836	0.135	0.314	0.721711	14
Dek	4.34E-05	-0.72264	0.538	0.693	0.724007	14
Hspe1	4.38E-05	-0.76663	0.173	0.341	0.731333	14
Mettl14	4.42E-05	-0.45009	0.038	0.123	0.736641	14
Fus	4.46E-05	-0.42607	0.615	0.737	0.743823	14
Dtymk	4.52E-05	-0.83617	0.135	0.439	0.754258	14
Rhoa	4.54E-05	0.619038	0.404	0.141	0.756896	14
Dcx	4.56E-05	-0.83078	0.096	0.376	0.760573	14
Rassf4	4.66E-05	-0.97993	0.115	0.377	0.776654	14
Rest	4.75E-05	0.52645	0.173	0.022	0.792329	14
Cpd	4.77E-05	0.675588	0.269	0.062	0.795436	14
Rps26	4.92E-05	-0.5144	0.788	0.847	0.820582	14
Homer2	5.06E-05	-0.9205	0.019	0.247	0.844326	14
Anxa6	5.09E-05	0.742061	0.231	0.06	0.848289	14
Cdyl2	5.09E-05	0.36349	0.135	0.013	0.849574	14
Sptan1	5.11E-05	0.712311	0.442	0.168	0.851808	14

Gnao1	5.15E-05	-0.80087	0.058	0.287	0.85914	14
Srrm2	5.3E-05	-0.66494	0.5	0.732	0.883502	14
Srgap1	5.31E-05	0.622998	0.154	0.018	0.88619	14
Iqsec1	5.37E-05	0.540009	0.135	0.014	0.895789	14
Nomo1	5.49E-05	0.731375	0.212	0.04	0.915467	14
Chgb	5.59E-05	-1.11936	0.038	0.241	0.932186	14
Nipal3	5.85E-05	0.284894	0.115	0.011	0.976421	14
Rnf44	5.87E-05	-0.32898	0.077	0.103	0.978512	14
Pcbp4	6.09E-05	-0.92066	0.077	0.325	1	14
Slk	6.12E-05	0.824438	0.404	0.18	1	14
Cr1l	6.13E-05	0.659608	0.231	0.046	1	14
Myo5a	6.31E-05	-0.80984	0	0.191	1	14
Nxpe4	6.37E-05	0.531037	0.135	0.012	1	14
Pmp22	6.4E-05	0.596089	0.154	0.017	1	14
Sstr2	6.53E-05	-0.85625	0	0.191	1	14
St18	6.65E-05	-0.96358	0	0.191	1	14
Slc50a1	6.75E-05	0.801556	0.269	0.082	1	14
Epn2	6.86E-05	0.593434	0.308	0.083	1	14
Ssh2	6.96E-05	0.397411	0.212	0.044	1	14
Stt3b	7.01E-05	0.742489	0.423	0.211	1	14
Hdgf	7.02E-05	-0.72207	0.25	0.532	1	14
Ptbp3	7.11E-05	0.649699	0.346	0.109	1	14
Rundc3a	7.14E-05	-0.92511	0.038	0.249	1	14
Ech1	7.23E-05	0.719639	0.346	0.114	1	14
Snrnp70	7.24E-05	-0.58802	0.5	0.694	1	14
Pfkp	7.43E-05	0.618951	0.192	0.03	1	14
Dnajc3	7.44E-05	0.695211	0.442	0.175	1	14
Cdc42ep4	7.57E-05	0.548124	0.231	0.047	1	14
Rpl15	7.73E-05	-0.59107	0.038	0.161	1	14
Stmn4	7.9E-05	-1.10016	0.077	0.342	1	14
Serbp1	7.95E-05	-0.45277	0.769	0.869	1	14
Zdhhc18	8.14E-05	0.353696	0.135	0.014	1	14
Ube2e3	8.16E-05	-0.71283	0.25	0.417	1	14
Amotl1	8.17E-05	0.621936	0.288	0.078	1	14
Kif5c	8.18E-05	-0.87234	0.115	0.399	1	14
Sept4	8.33E-05	0.606149	0.519	0.225	1	14
Khdrbs1	8.68E-05	-0.77802	0.269	0.494	1	14
Polk	8.74E-05	0.603686	0.154	0.019	1	14
Thoc7	8.81E-05	-0.70827	0.25	0.522	1	14
6330403K(	8.88E-05	-0.97981	0.019	0.243	1	14
Dtl	9.03E-05	-0.55887	0.038	0.145	1	14
Elavl3	9.04E-05	-0.8563	0.173	0.473	1	14
Lig1	9.41E-05	-1.04581	0.096	0.348	1	14
Rpl4	9.43E-05	-0.42997	0.865	0.908	1	14
St8sia3	9.51E-05	-0.85585	0.019	0.251	1	14
Lsm2	9.75E-05	-0.65315	0.096	0.23	1	14
Akap12	9.8E-05	1.005134	0.308	0.11	1	14
Sult4a1	9.9E-05	-0.85754	0.019	0.223	1	14

Sh3bgrl3	0.0001	0.689563	0.462	0.197	1	14
Mast4	0.0001	0.378011	0.115	0.008	1	14
Mad2l2	0.000101	-0.78298	0.058	0.262	1	14
C530008M	0.000101	-0.82025	0.058	0.268	1	14
Nmi	0.000102	0.481882	0.135	0.013	1	14
Rai14	0.000103	0.660279	0.231	0.05	1	14
Pcdhga9	0.000103	-0.75138	0.25	0.42	1	14
U2surp	0.000104	-0.80343	0.231	0.506	1	14
Prdx6	0.000106	-0.8926	0.096	0.326	1	14
Idh3g	0.000107	-0.60509	0.212	0.308	1	14
As3mt	0.000107	0.490199	0.135	0.013	1	14
Bnip2	0.000108	0.697219	0.481	0.216	1	14
Rufy3	0.000108	-0.79379	0.115	0.372	1	14
Fam65a	0.000109	0.473534	0.192	0.036	1	14
Gas5	0.000109	-0.65391	0.5	0.742	1	14
Tspan4	0.00011	0.473877	0.25	0.065	1	14
Tln1	0.000114	0.779739	0.365	0.149	1	14
Pdgfra	0.000115	-0.85188	0	0.182	1	14
Bcl2	0.000117	0.537554	0.192	0.032	1	14
Hnrnpr	0.000117	-0.75683	0.288	0.545	1	14
Acat2	0.000121	-0.54605	0.058	0.182	1	14
Gng2	0.000126	-0.89838	0.154	0.396	1	14
Cacna2d1	0.000128	-0.92153	0.115	0.375	1	14
Mcf2l	0.00013	0.652524	0.192	0.032	1	14
Elovl1	0.00013	0.44873	0.192	0.035	1	14
Pomc	0.000132	0.464732	0.135	0.014	1	14
Rgl2	0.000135	0.694845	0.346	0.114	1	14
Pde4dip	0.000136	-0.76009	0.038	0.221	1	14
Bzw2	0.000144	-0.67689	0.212	0.489	1	14
Hnrnpm	0.00015	-0.56865	0.577	0.735	1	14
Supt16	0.000155	-0.77942	0.269	0.493	1	14
Fam212b	0.00016	-0.65618	0.019	0.223	1	14
Kif5b	0.00016	0.538256	0.635	0.521	1	14
Dner	0.000161	-0.96078	0.019	0.215	1	14
Rapgef6	0.000163	0.678395	0.385	0.139	1	14
Sh3bp4	0.000165	0.666546	0.154	0.024	1	14
Srsf7	0.000177	-0.79065	0.212	0.466	1	14
Shank3	0.000178	0.589436	0.154	0.02	1	14
Crem	0.000178	0.487428	0.173	0.027	1	14
Crim1	0.000181	0.57741	0.154	0.021	1	14
Hip1	0.000181	0.53444	0.346	0.116	1	14
Rps5	0.000187	-0.39554	0.923	0.954	1	14
Ano6	0.000193	0.61794	0.308	0.09	1	14
Ssrp1	0.000195	-0.70171	0.346	0.577	1	14
Nap1l1	0.000197	-0.73305	0.308	0.511	1	14
Rnf165	0.000197	-0.75612	0	0.173	1	14
Hdac7	0.000199	0.437878	0.154	0.022	1	14
Gpd2	0.000199	0.662911	0.269	0.071	1	14

Nol4	0.000201	-0.43646	0.038	0.172	1	14
Hnrnph2	0.000201	-0.43804	0.077	0.143	1	14
Bcl7a	0.000208	-0.82006	0.077	0.326	1	14
Sh3gl2	0.00021	-0.79202	0	0.172	1	14
Lactb	0.000213	0.43731	0.154	0.034	1	14
Ndufa12	0.000216	-0.76006	0.212	0.421	1	14
Polr3d	0.000217	-0.50708	0.038	0.133	1	14
Setd8	0.000219	-0.85142	0.038	0.262	1	14
Elf1	0.000219	0.493806	0.231	0.053	1	14
Wdr1	0.000219	0.645738	0.288	0.087	1	14
Tpd52	0.000221	0.516968	0.192	0.035	1	14
Idh2	0.000222	-0.76936	0.058	0.279	1	14
Zcchc18	0.000227	-0.75027	0	0.17	1	14
Impdh1	0.000231	0.674121	0.25	0.062	1	14
Hipk1	0.000234	0.373426	0.231	0.06	1	14
Sh3glb1	0.000234	0.620448	0.577	0.365	1	14
Purb	0.000239	-0.71145	0.365	0.549	1	14
Ptov1	0.000243	-0.66405	0.231	0.485	1	14
Hnrnpd	0.000243	-0.66722	0.423	0.611	1	14
Eif3i	0.000244	-0.70431	0.288	0.462	1	14
Dut	0.000251	-0.89471	0.173	0.435	1	14
Matr3	0.000259	-0.59025	0.538	0.706	1	14
Dusp23	0.00027	0.31231	0.115	0.011	1	14
Plekhg2	0.000271	0.284762	0.135	0.02	1	14
Cdipt	0.000272	0.566217	0.308	0.096	1	14
Hdgfrp3	0.000272	-0.8634	0.096	0.338	1	14
Rnf5	0.000279	-0.72824	0.096	0.258	1	14
Psat1	0.000284	-0.83396	0.115	0.368	1	14
Casp3	0.000284	-0.77947	0.019	0.234	1	14
Rnpep	0.000285	0.314838	0.192	0.044	1	14
Ide	0.000291	-0.39113	0.058	0.113	1	14
Celsr2	0.000299	-0.87375	0.038	0.238	1	14
Rsu1	0.000302	0.537266	0.346	0.117	1	14
Smc2	0.000304	-0.90235	0.288	0.546	1	14
Cds2	0.000305	0.517124	0.212	0.044	1	14
Sh2b3	0.000309	0.611547	0.115	0.016	1	14
Scarb1	0.000312	0.622669	0.173	0.036	1	14
Peli2	0.000317	-0.72303	0	0.165	1	14
Dixdc1	0.000318	-0.89187	0.038	0.235	1	14
Arpc2	0.00032	0.541135	0.712	0.507	1	14
Actb	0.000322	0.36326	0.981	0.979	1	14
Plec	0.000328	0.485213	0.115	0.011	1	14
Slc29a1	0.000332	-0.72942	0.327	0.562	1	14
Dnajb11	0.000332	0.443	0.404	0.162	1	14
Clint1	0.000333	0.672851	0.346	0.13	1	14
H2afj	0.000334	0.660396	0.442	0.204	1	14
Gm26924	0.000336	0.582092	0.442	0.182	1	14
Tuba1b	0.000337	-0.7136	0.231	0.481	1	14

Prmt8	0.000344	-0.5823	0.058	0.272	1	14
Ak3	0.000345	0.705164	0.269	0.109	1	14
Cep110	0.000348	-0.73212	0	0.163	1	14
Hmgb3	0.000351	-0.83023	0.077	0.306	1	14
Ttc9b	0.000352	-0.62452	0.038	0.195	1	14
Smarcc1	0.000354	-0.25576	0.231	0.434	1	14
Smc3	0.000357	-0.62788	0.442	0.633	1	14
Abhd5	0.000357	0.508006	0.135	0.019	1	14
Camta1	0.000366	-0.83001	0.192	0.408	1	14
Nrn1	0.000378	-0.92909	0.019	0.222	1	14
Htatsf1	0.000379	-0.79975	0.269	0.499	1	14
Mfap4	0.000382	-0.80369	0	0.162	1	14
Mrpl21	0.000384	-0.49026	0.231	0.28	1	14
Ip6k2	0.000387	-0.45626	0.058	0.121	1	14
Smpd3	0.000387	-0.72675	0	0.161	1	14
A330076H(	0.000393	-0.78342	0	0.161	1	14
Yes1	0.000396	0.528649	0.25	0.065	1	14
Ctps	0.000399	-0.44176	0.038	0.137	1	14
Smarca2	0.000399	0.659818	0.538	0.284	1	14
Podxl2	0.000402	-0.86742	0.019	0.228	1	14
Ndn	0.000403	-0.85515	0.019	0.223	1	14
Btbd17	0.000413	-0.74277	0	0.16	1	14
Zcchc11	0.000417	-0.72801	0.077	0.321	1	14
Mapk8ip1	0.000422	-0.83307	0.019	0.228	1	14
Rab11fip2	0.000422	-0.43897	0.038	0.106	1	14
Srrm3	0.000425	-0.66006	0.058	0.24	1	14
Pdap1	0.00043	-0.56452	0.538	0.683	1	14
Ddx42	0.000435	-0.73386	0.192	0.383	1	14
Gsg1l	0.000437	-0.71307	0.038	0.255	1	14
Snap23	0.000442	0.275297	0.173	0.041	1	14
Snhg5	0.000442	-0.67007	0.135	0.381	1	14
Nop58	0.000443	-0.71147	0.327	0.592	1	14
Ift74	0.000445	-0.59774	0.038	0.225	1	14
Prpf19	0.000448	-0.76147	0.115	0.307	1	14
Ppp1r14c	0.000449	-0.8969	0.058	0.262	1	14
Creg1	0.000449	0.460027	0.212	0.057	1	14
Smc4	0.000453	-0.65941	0.462	0.567	1	14
Sppl2a	0.000456	0.582214	0.327	0.118	1	14
Itpr1	0.000457	0.443675	0.135	0.016	1	14
Itm2c	0.000464	0.614585	0.442	0.211	1	14
Copz2	0.000466	0.488029	0.115	0.013	1	14
Cenpe	0.000467	-1.07485	0.058	0.286	1	14
Srpk2	0.000469	-0.79991	0.173	0.43	1	14
Tpd52l1	0.00048	0.456118	0.154	0.023	1	14
Unc93b1	0.00048	0.492457	0.115	0.012	1	14
Sema6d	0.000489	0.550098	0.173	0.031	1	14
Aplp1	0.000492	-0.58513	0.058	0.151	1	14
Rela	0.000493	0.333961	0.212	0.057	1	14

Sox9	0.000494	-0.76465	0.038	0.255	1	14
Top2a	0.000506	-0.98642	0.173	0.428	1	14
Lmo4	0.000518	-0.82608	0.115	0.336	1	14
Stat3	0.000534	0.638494	0.192	0.041	1	14
Dnajc8	0.000534	0.56893	0.654	0.433	1	14
Eif4g2	0.000535	-0.51813	0.558	0.705	1	14
Hmgb1	0.00055	-0.78229	0.135	0.359	1	14
Pabpc1	0.000553	-0.36995	0.885	0.878	1	14
Ntrk3	0.000554	-0.66751	0	0.155	1	14
Yap1	0.000556	0.401936	0.135	0.017	1	14
Cct2	0.000561	-0.69579	0.25	0.524	1	14
Smc5	0.00057	-0.67675	0.096	0.258	1	14
Pald1	0.00058	0.477577	0.25	0.073	1	14
Banf1	0.000587	-0.53499	0.558	0.694	1	14
Cenpf	0.000589	-1.15206	0.135	0.363	1	14
Dhx9	0.00059	-0.76005	0.212	0.463	1	14
Ier5	0.000592	-0.79109	0.135	0.394	1	14
Nrip1	0.000594	0.621022	0.308	0.1	1	14
Gdpd1	0.000598	-0.81361	0.038	0.243	1	14
Ehd4	0.000601	0.591944	0.135	0.02	1	14
Tra2b	0.000607	-0.71133	0.173	0.405	1	14
Cbfa2t3	0.000609	-0.80867	0.192	0.403	1	14
Otx2	0.000639	-0.83712	0.019	0.217	1	14
Myo9a	0.00064	-0.63769	0.038	0.158	1	14
Hp1bp3	0.00064	-0.68944	0.327	0.581	1	14
Gm5620	0.00065	-0.3765	0.038	0.101	1	14
Ptch2	0.000655	-0.65597	0	0.152	1	14
Park7	0.000661	-0.64244	0.346	0.546	1	14
Pcna	0.000675	-0.54529	0.115	0.358	1	14
Robo2	0.00068	-0.69869	0	0.152	1	14
Pebp1	0.000689	-0.70913	0.077	0.242	1	14
Ilf3	0.000713	-0.63028	0.25	0.376	1	14
Atp5a1	0.000718	-0.52699	0.577	0.718	1	14
Elovl7	0.000721	0.527666	0.115	0.014	1	14
Rmnd5b	0.000729	0.536193	0.231	0.059	1	14
Ginm1	0.000739	0.615657	0.269	0.081	1	14
Cdc20	0.00074	-0.80055	0.058	0.196	1	14
Rhob	0.000743	0.561949	0.365	0.138	1	14
Dab2ip	0.000744	0.584656	0.25	0.068	1	14
Kras	0.000752	-0.67054	0.096	0.252	1	14
Snrpg	0.000759	-0.69262	0.173	0.335	1	14
Asf1a	0.000759	-0.72497	0.115	0.28	1	14
Skp1a	0.000761	-0.65296	0.365	0.582	1	14
Isoc1	0.000764	-0.54026	0.058	0.181	1	14
Per1	0.000765	0.301886	0.115	0.016	1	14
Hnrnpk	0.000766	-0.49998	0.462	0.636	1	14
Myl12b	0.000772	0.537618	0.615	0.366	1	14
1110034G:	0.000772	0.366161	0.154	0.029	1	14

Cflar	0.000774	0.643785	0.192	0.05	1	14
Rbms1	0.000781	0.573992	0.462	0.215	1	14
Zbtb18	0.000788	-0.84106	0.058	0.267	1	14
Kif1b	0.000809	-0.76332	0.288	0.521	1	14
Atad5	0.000811	-0.64866	0	0.149	1	14
Olfm1	0.000832	-0.64961	0.019	0.199	1	14
Pak3	0.000834	-0.78813	0.038	0.206	1	14
Satb1	0.000836	-0.79664	0.019	0.21	1	14
Cdkn1c	0.000836	0.583888	0.212	0.062	1	14
Tmem66	0.000846	0.330853	0.423	0.198	1	14
Tfam	0.000857	-0.40784	0.115	0.154	1	14
Glud1	0.000866	0.518452	0.365	0.14	1	14
Nfkb1	0.000866	0.577671	0.135	0.024	1	14
Wipi1	0.000868	0.486731	0.173	0.033	1	14
Ahi1	0.000873	-0.80513	0.077	0.307	1	14
Ece2	0.000876	-0.39092	0.038	0.104	1	14
Acox1	0.000879	0.263283	0.192	0.056	1	14
Pdzrn3	0.00088	-0.84881	0	0.147	1	14
N4bp3	0.00093	0.668373	0.192	0.06	1	14
Cdh4	0.000951	-0.63192	0	0.146	1	14
Cdk6	0.000952	-0.69231	0.038	0.243	1	14
Arid2	0.000972	-0.75544	0.038	0.242	1	14
Igsf8	0.000973	-0.77156	0.058	0.27	1	14
Mpnd	0.000974	-0.5502	0.077	0.171	1	14
Txnip	0.000988	0.611921	0.288	0.106	1	14
Slc22a17	0.001004	-0.77811	0.077	0.251	1	14
Ranbp1	0.001008	-0.62473	0.462	0.675	1	14
Vdac1	0.001013	-0.64247	0.077	0.303	1	14
Hsp90b1	0.001025	0.474131	0.827	0.691	1	14
Tubb2a	0.001026	-0.48213	0.25	0.245	1	14
Bcl11a	0.001038	-0.76929	0.019	0.21	1	14
Gpn2	0.001038	0.574654	0.135	0.054	1	14
Hspd1	0.001038	-0.70449	0.192	0.365	1	14
Hnrnpa0	0.001041	-0.49114	0.346	0.456	1	14
Fnta	0.001042	0.631605	0.308	0.138	1	14
Atp1a2	0.00105	0.535474	0.212	0.058	1	14
Gm11266	0.001062	-0.70921	0	0.144	1	14
Lrrc59	0.001066	-0.28579	0.096	0.112	1	14
Trim56	0.00107	0.579484	0.154	0.027	1	14
Fmnl3	0.001071	0.487731	0.154	0.026	1	14
Ddx39	0.0011	-0.47955	0.115	0.187	1	14
Anp32b	0.001102	-0.6184	0.442	0.591	1	14
Pim3	0.001114	0.696827	0.212	0.087	1	14
2510003E(	0.001122	-0.41653	0.038	0.108	1	14
Psme4	0.001132	-0.78718	0.096	0.284	1	14
AI854517	0.001139	-0.74353	0.019	0.189	1	14
Trim33	0.001147	-0.48955	0.038	0.149	1	14
Prmt5	0.001162	-0.71657	0.096	0.256	1	14

Nucks1	0.001187	-0.64441	0.462	0.663	1	14
Nudcd2	0.001195	-0.54708	0.115	0.223	1	14
Dcakd	0.001205	-0.73413	0.096	0.296	1	14
Ldhb	0.001208	-0.76528	0.096	0.281	1	14
Rcor2	0.001212	-0.80219	0.038	0.225	1	14
Strbp	0.001216	-0.79815	0.115	0.347	1	14
Foxp1	0.001224	0.692422	0.346	0.171	1	14
Myod1	0.001241	-0.66625	0	0.141	1	14
Peg3	0.001254	-0.82225	0.096	0.285	1	14
D030056L2	0.00127	-0.42933	0.077	0.142	1	14
Hnrnpa3	0.001282	-0.54343	0.038	0.159	1	14
Zeb1	0.001284	-0.77959	0.154	0.388	1	14
Atxn10	0.001308	-0.55538	0.154	0.37	1	14
Cep78	0.001315	-0.6019	0	0.14	1	14
Ywhae	0.001324	-0.34007	0.615	0.726	1	14
Atp6v1b2	0.001325	-0.49097	0.096	0.17	1	14
Atl2	0.001326	0.402653	0.231	0.065	1	14
Acaa2	0.001342	0.488529	0.173	0.034	1	14
B230118H0	0.001358	-0.46199	0.038	0.122	1	14
Tmod2	0.001359	-0.75365	0.019	0.207	1	14
Zbtb38	0.001374	0.261459	0.192	0.058	1	14
Reep3	0.001376	0.553557	0.5	0.255	1	14
Phb2	0.001392	-0.69013	0.173	0.333	1	14
Nop10	0.001413	-0.6821	0.288	0.482	1	14
Slc16a6	0.001452	0.60636	0.173	0.041	1	14
Wdr77	0.001455	-0.28413	0.077	0.124	1	14
Hypk	0.001459	-0.33233	0.058	0.105	1	14
Sacs	0.001472	-0.67019	0.038	0.191	1	14
Cplx1	0.001481	-0.66501	0	0.138	1	14
Tram1	0.001482	0.459195	0.192	0.128	1	14
Ppp1r2	0.001513	0.55927	0.25	0.079	1	14
Brd3	0.001527	-0.68113	0.288	0.532	1	14
Acin1	0.00153	-0.49437	0.519	0.724	1	14
Mif	0.001562	-0.61617	0.231	0.357	1	14
Gnb2l1	0.001563	-0.41967	0.808	0.872	1	14
Epc2	0.001567	-0.7565	0.058	0.25	1	14
Cnot6l	0.001572	0.655306	0.327	0.134	1	14
Aph1a	0.001587	0.612563	0.115	0.021	1	14
Bex1	0.001595	-0.72477	0.038	0.224	1	14
Safb	0.001604	-0.66419	0.25	0.504	1	14
Cdk1	0.00161	-0.62887	0.173	0.247	1	14
Slc1a1	0.001616	0.366873	0.115	0.017	1	14
Rrm2	0.001643	-0.7529	0.019	0.204	1	14
2610017I0	0.001656	-0.73502	0.019	0.204	1	14
Srsf2	0.00167	-0.60633	0.385	0.566	1	14
Wdr33	0.001692	-0.54532	0.115	0.196	1	14
Gse1	0.001694	-0.71741	0.058	0.237	1	14
Cacybp	0.001694	-0.63004	0.212	0.356	1	14

Meaf6	0.001722	-0.5639	0	0.135	1	14
Mdga1	0.001722	-0.61044	0	0.135	1	14
Pcdh17	0.001735	0.545583	0.154	0.028	1	14
Snw1	0.001808	-0.51435	0.25	0.431	1	14
Arhgap31	0.001846	0.574251	0.173	0.039	1	14
Nrcam	0.001865	-0.65372	0	0.134	1	14
Fau	0.001876	-0.41069	0.058	0.123	1	14
Tmbim6	0.001889	0.499669	0.558	0.323	1	14
Hadha	0.001893	0.646946	0.327	0.155	1	14
Pqlc1	0.001917	-0.73985	0.135	0.297	1	14
Ccdc88a	0.001923	-0.63223	0.365	0.482	1	14
Amer2	0.001931	-0.56624	0	0.133	1	14
Ccp110	0.001944	-0.69792	0.096	0.248	1	14
Tnks1bp1	0.001968	0.334584	0.115	0.016	1	14
Cnpy1	0.001988	-0.62295	0	0.133	1	14
Vcl	0.001994	0.316549	0.115	0.016	1	14
Rab7	0.001995	0.291607	0.385	0.183	1	14
Akap6	0.002005	-0.64536	0.038	0.219	1	14
Zfp36l1	0.002012	0.51879	0.365	0.15	1	14
Chd4	0.002025	-0.50895	0.635	0.769	1	14
Tdrkh	0.002026	-0.54318	0	0.133	1	14
D19Bwg13	0.002051	-0.75363	0.077	0.274	1	14
Nell2	0.002058	-0.57279	0	0.132	1	14
Reep1	0.002064	0.41604	0.25	0.081	1	14
Dzip3	0.002091	-0.57569	0.058	0.167	1	14
2810055G	0.002093	-0.61862	0.038	0.17	1	14
BC005764	0.002105	-0.67993	0.019	0.186	1	14
Pkp4	0.002109	0.535062	0.288	0.102	1	14
Alkbh6	0.002119	-0.50373	0.038	0.134	1	14
Fam102b	0.002121	0.621657	0.173	0.043	1	14
1500012F(	0.002143	-0.6844	0.25	0.398	1	14
Cdkn1b	0.002158	-0.62836	0.288	0.467	1	14
H3f3a	0.002163	-0.65116	0.212	0.381	1	14
G2e3	0.002222	-0.54703	0	0.131	1	14
Sec62	0.002222	0.531211	0.635	0.405	1	14
Fmnl2	0.002231	-0.42425	0.077	0.157	1	14
Etfb	0.002241	0.556187	0.404	0.183	1	14
Slc35b3	0.002271	0.53322	0.154	0.032	1	14
Tcerg1	0.002293	-0.69007	0.231	0.457	1	14
2610203C	0.002306	-0.51494	0.038	0.121	1	14
Ube2c	0.002312	-1.10949	0.077	0.269	1	14
Gpr56	0.002341	-0.67475	0.038	0.223	1	14
Pygo1	0.002345	-0.80241	0.019	0.192	1	14
Cecr2	0.002367	-0.56796	0	0.13	1	14
Vcan	0.002375	-0.61083	0	0.13	1	14
4933426M	0.002395	0.523302	0.192	0.046	1	14
Fcf1	0.002413	-0.64732	0.019	0.196	1	14
Ccdc32	0.002418	0.364883	0.173	0.095	1	14

Gdpd5	0.002425	0.402007	0.154	0.033	1	14
Ralgps2	0.002444	-0.72494	0.077	0.246	1	14
2810474O:	0.002449	-0.58275	0.154	0.241	1	14
Zfp329	0.002459	-0.60294	0	0.129	1	14
Mtmr6	0.002477	0.380818	0.192	0.051	1	14
Clvs1	0.002482	-0.62085	0	0.129	1	14
Luc7l3	0.002499	-0.52357	0.538	0.748	1	14
Fam219b	0.002549	0.305396	0.154	0.044	1	14
Gsto1	0.00257	-0.56	0	0.128	1	14
Ank2	0.002586	-0.82637	0.096	0.24	1	14
Tmem123	0.002608	0.487863	0.25	0.083	1	14
Syncrip	0.002614	-0.63401	0.308	0.496	1	14
Nrbp1	0.00263	0.503983	0.269	0.091	1	14
Ccnb2	0.002636	-0.69391	0.019	0.187	1	14
Ybx3	0.002642	0.287099	0.481	0.289	1	14
Phf21b	0.002644	-0.57226	0	0.128	1	14
Clybl	0.002647	-0.47744	0.038	0.116	1	14
Srsf10	0.002649	-0.71428	0.135	0.331	1	14
Tox3	0.00265	-0.51984	0.135	0.346	1	14
Atrx	0.002663	-0.55435	0.577	0.74	1	14
Bex4	0.002686	-0.51265	0	0.128	1	14
Npdc1	0.002692	-0.55995	0.173	0.261	1	14
Qser1	0.002695	-0.54419	0	0.127	1	14
Cbx3	0.002704	-0.68185	0.077	0.274	1	14
Pdzrn4	0.002708	-0.54866	0.038	0.146	1	14
Nedd4	0.002733	-0.40951	0.615	0.683	1	14
Gmeb1	0.002736	-0.34674	0.077	0.111	1	14
Nr2c2	0.002739	-0.7272	0.019	0.186	1	14
Naa38	0.002747	-0.68724	0.096	0.311	1	14
Gm9800	0.002753	-0.57971	0.385	0.535	1	14
Pnp	0.002764	0.506089	0.115	0.019	1	14
Atf3	0.002765	0.519427	0.115	0.017	1	14
Gpatch4	0.002785	-0.51274	0.115	0.18	1	14
Tsfm	0.002786	-0.38843	0.038	0.108	1	14
Pbx3	0.00279	-0.57005	0	0.127	1	14
2900011O(	0.002792	-0.47205	0.038	0.103	1	14
Ikbkap	0.002799	-0.56042	0	0.127	1	14
Slc1a4	0.00281	0.597614	0.192	0.056	1	14
Cdc42	0.002815	0.40288	0.712	0.514	1	14
Tnpo3	0.002824	-0.61955	0.038	0.222	1	14
Bcas2	0.002865	-0.61345	0.212	0.405	1	14
Tro	0.002907	-0.58621	0	0.126	1	14
Usp10	0.002931	-0.61622	0.058	0.186	1	14
Sae1	0.002932	-0.69185	0.096	0.279	1	14
Fam63a	0.002948	0.616273	0.25	0.087	1	14
Ckap2l	0.003001	-0.73435	0.096	0.218	1	14
Calr	0.00302	0.459291	0.654	0.51	1	14
Efs	0.003028	-0.52058	0	0.125	1	14

Nop56	0.00303	-0.59305	0.154	0.361	1	14
Maged2	0.003047	-0.50026	0.096	0.167	1	14
Rpn1	0.003077	0.525975	0.404	0.191	1	14
Cpsf6	0.003085	-0.49825	0.212	0.291	1	14
Sin3b	0.003111	-0.70066	0.135	0.309	1	14
Txnrd1	0.003133	-0.50714	0.173	0.389	1	14
Mtus1	0.003168	0.432269	0.308	0.126	1	14
Fam69a	0.003177	0.456331	0.154	0.031	1	14
Tpst2	0.003183	0.372907	0.231	0.074	1	14
Erdr1	0.003194	-0.44749	0.019	0.162	1	14
Tmem50a	0.0032	0.492733	0.635	0.404	1	14
Fads1	0.003204	-0.40241	0.038	0.105	1	14
Akap13	0.003246	0.57913	0.231	0.071	1	14
Peo1	0.003255	-0.5393	0	0.124	1	14
Tmcc3	0.00327	0.658727	0.173	0.091	1	14
Tpm3	0.003287	0.54518	0.5	0.315	1	14
2810025M	0.003291	0.551452	0.346	0.166	1	14
Zfr	0.0033	-0.68505	0.154	0.354	1	14
Rnpepl1	0.003316	0.373906	0.154	0.037	1	14
Ptpn9	0.003356	0.45085	0.192	0.049	1	14
Dnph1	0.003412	-0.49588	0	0.123	1	14
Casc5	0.003412	-0.76278	0.019	0.186	1	14
Celsr3	0.003466	-0.57862	0	0.123	1	14
Tceb1	0.003498	0.34029	0.404	0.366	1	14
Rab3a	0.003541	-0.58219	0.038	0.15	1	14
Msrb2	0.00356	0.434986	0.115	0.018	1	14
Tacc3	0.003644	-0.65347	0.058	0.206	1	14
Neurod6	0.003648	-0.72925	0.019	0.172	1	14
Dnajc10	0.003651	0.548688	0.327	0.133	1	14
Gdap1	0.003665	-0.58031	0.038	0.199	1	14
Xbp1	0.003667	0.431677	0.308	0.123	1	14
Dnajc9	0.00375	-0.68686	0.192	0.35	1	14
Aldh2	0.00377	0.505942	0.135	0.025	1	14
Pop5	0.003794	0.586463	0.269	0.099	1	14
Cd81	0.003812	0.376165	0.5	0.461	1	14
Eid1	0.003841	-0.60726	0.346	0.503	1	14
Baiap2	0.003842	0.49079	0.192	0.05	1	14
Hlf	0.003867	0.2821	0.115	0.019	1	14
Lrig3	0.003913	-0.49613	0.019	0.17	1	14
Rbm28	0.003939	-0.72454	0.077	0.263	1	14
Bcat1	0.003952	-0.52496	0	0.121	1	14
H2afz	0.003997	-0.61559	0.096	0.231	1	14
Hist3h2ba	0.004032	-0.54001	0.038	0.138	1	14
Ndrg2	0.004036	-0.66097	0.019	0.185	1	14
Gprasp1	0.004037	-0.6641	0.038	0.207	1	14
Elavl2	0.004042	-0.75797	0.077	0.27	1	14
Slc10a7	0.004067	0.451742	0.115	0.02	1	14
Bin1	0.004085	-0.63155	0.192	0.43	1	14

Banp	0.004125	-0.48065	0.077	0.137	1	14
Bmp2k	0.004207	0.364921	0.135	0.025	1	14
Plekha1	0.004208	0.523819	0.212	0.06	1	14
2310036O:	0.004225	-0.55453	0.288	0.406	1	14
Shmt2	0.004233	-0.50757	0	0.119	1	14
Sox5	0.004233	-0.58013	0	0.119	1	14
Brcc3	0.004304	-0.29406	0.077	0.175	1	14
Ap3b2	0.00432	-0.68929	0.038	0.202	1	14
Wsb2	0.004345	0.430759	0.212	0.064	1	14
Irs1	0.004422	-0.528	0	0.119	1	14
Gm10075	0.004454	-0.59081	0.346	0.515	1	14
Vegfb	0.004463	-0.48412	0	0.118	1	14
Dach1	0.004496	0.499738	0.212	0.066	1	14
Nrm	0.004545	-0.55165	0.058	0.169	1	14
Rab34	0.004561	-0.52539	0	0.118	1	14
Ltbp3	0.004561	-0.55035	0	0.118	1	14
Nme4	0.004618	-0.51537	0	0.118	1	14
Ncald	0.004675	-0.69591	0.019	0.165	1	14
Rrp1b	0.004684	-0.48594	0.058	0.136	1	14
Txndc9	0.004686	-0.46448	0.077	0.212	1	14
Ppp3ca	0.00469	-0.73548	0.192	0.378	1	14
Eif4b	0.004693	-0.63873	0.135	0.276	1	14
Mybbp1a	0.004741	-0.60403	0.077	0.25	1	14
H2afx	0.004745	-0.82602	0.135	0.331	1	14
Cuedc2	0.004771	-0.64764	0.173	0.387	1	14
Ube2b	0.004828	-0.58794	0.288	0.521	1	14
Sdf2	0.004829	0.584271	0.385	0.214	1	14
Rplp0	0.004867	-0.38596	0.788	0.872	1	14
Dnmt1	0.004895	-0.72684	0.096	0.278	1	14
Pfdn2	0.004896	-0.59689	0.115	0.31	1	14
Leprel4	0.004939	0.546536	0.135	0.029	1	14
Cenpq	0.004946	-0.26361	0.058	0.16	1	14
Tspan18	0.004963	0.464676	0.192	0.051	1	14
Tm9sf3	0.004995	0.519148	0.5	0.343	1	14
Adk	0.005036	-0.47465	0.038	0.166	1	14
Grsf1	0.005047	-0.63035	0.038	0.176	1	14
Atp6v0e2	0.005086	-0.63448	0.077	0.26	1	14
Krt10	0.005138	-0.63703	0.019	0.162	1	14
Mcee	0.005145	0.364171	0.212	0.074	1	14
Nfic	0.005164	-0.68024	0.077	0.261	1	14
Fdx1l	0.005166	-0.42372	0.038	0.115	1	14
Brix1	0.005175	-0.58649	0.077	0.206	1	14
Exoc2	0.005212	-0.51092	0	0.116	1	14
Tcp11l1	0.005212	-0.52952	0	0.116	1	14
Plin2	0.005219	0.457462	0.192	0.052	1	14
Mprip	0.005234	0.616209	0.327	0.151	1	14
Esco2	0.005249	-0.61431	0.058	0.195	1	14
Nktr	0.005255	-0.71174	0.192	0.38	1	14

Cltb	0.005255	-0.6799	0.269	0.423	1	14
Ing4	0.005264	-0.65691	0.154	0.323	1	14
Fam213b	0.005333	-0.48118	0.019	0.157	1	14
Chmp6	0.005342	0.262067	0.231	0.088	1	14
Cenpm	0.005382	-0.61661	0.019	0.162	1	14
Ypel1	0.00539	-0.5915	0.058	0.174	1	14
Sox11	0.005409	-0.51187	0	0.115	1	14
A930011O	0.005442	-0.66552	0	0.115	1	14
Cgrrf1	0.005465	0.48032	0.192	0.054	1	14
Ccdc55	0.005501	-0.42246	0.135	0.267	1	14
Psma1	0.005558	-0.49335	0.327	0.444	1	14
Clasp1	0.005561	-0.52209	0	0.114	1	14
Cenpp	0.005613	-0.45782	0	0.114	1	14
Rin2	0.005614	0.379056	0.115	0.02	1	14
Clic1	0.005628	0.443491	0.365	0.172	1	14
Rrm1	0.005639	-0.62063	0.135	0.26	1	14
Mtf2	0.005647	-0.55548	0.25	0.357	1	14
Rock2	0.005667	0.634806	0.327	0.211	1	14
Rsl24d1	0.005683	-0.47852	0	0.114	1	14
Fam129b	0.005758	0.316475	0.115	0.02	1	14
Comt	0.005842	0.310173	0.269	0.109	1	14
Mapt	0.005875	-0.85826	0.058	0.216	1	14
Epb4.1l3	0.005897	-0.4974	0	0.113	1	14
Gng12	0.005898	0.587998	0.25	0.12	1	14
1810058I2	0.005981	0.434357	0.404	0.196	1	14
Reln	0.005986	-0.75341	0.038	0.18	1	14
Lrrn1	0.005989	-0.50627	0	0.113	1	14
Col9a3	0.006013	-0.69924	0.019	0.18	1	14
Pafah1b2	0.006022	-0.39332	0.173	0.255	1	14
Arpp21	0.006044	-0.67986	0	0.113	1	14
Jakmip2	0.006082	-0.34308	0.038	0.157	1	14
Ptprd	0.006103	-0.682	0.135	0.34	1	14
Arhgap11a	0.006112	-0.72202	0.019	0.17	1	14
Josd2	0.006138	-0.47459	0	0.113	1	14
Magt1	0.006189	0.365752	0.192	0.056	1	14
Stk40	0.006231	0.36259	0.154	0.036	1	14
Stk25	0.006235	0.571276	0.365	0.183	1	14
Srrm4	0.006259	-0.71091	0.038	0.195	1	14
Fez2	0.00627	0.380667	0.154	0.037	1	14
Tmem184b	0.006271	0.336515	0.115	0.019	1	14
Apbb1	0.006294	-0.60436	0.019	0.17	1	14
Mrpl40	0.006492	-0.53532	0.077	0.177	1	14
Mob1a	0.006519	0.550961	0.173	0.053	1	14
Msi1	0.006772	-0.49569	0	0.111	1	14
Nrep	0.00678	-0.27613	0.538	0.569	1	14
Nelfa	0.006785	-0.32479	0.038	0.103	1	14
Arf3	0.006817	0.302318	0.192	0.062	1	14
Rbmx	0.006867	-0.63696	0.115	0.315	1	14

Sirt7	0.006874	0.343305	0.25	0.096	1	14
Tceal3	0.006877	-0.51745	0	0.11	1	14
Pcbp2	0.006978	-0.50883	0.442	0.621	1	14
1700001O	0.007005	-0.46259	0	0.11	1	14
Pigk	0.007055	0.453975	0.154	0.045	1	14
Zfp292	0.007081	-0.71634	0.173	0.342	1	14
Elovl4	0.007113	-0.49629	0	0.11	1	14
Rab6b	0.007145	-0.68639	0.058	0.203	1	14
Eif2b3	0.007161	-0.59097	0.038	0.159	1	14
Lims1	0.007162	0.553813	0.365	0.181	1	14
Gpsm1	0.007222	-0.63902	0.038	0.176	1	14
Slc25a27	0.007223	-0.50896	0	0.11	1	14
Sgta	0.007316	-0.6284	0.096	0.241	1	14
Nhp2	0.007323	-0.51084	0.25	0.371	1	14
Abhd6	0.007323	0.446096	0.154	0.037	1	14
Hist3h2a	0.007445	-0.54756	0.058	0.143	1	14
Stat1	0.007469	0.479682	0.135	0.028	1	14
Snrpf	0.007486	-0.49703	0.327	0.427	1	14
Rabl6	0.007594	-0.49647	0.192	0.259	1	14
Cul4a	0.007599	-0.336	0.077	0.11	1	14
Dazap1	0.007711	-0.49209	0.192	0.272	1	14
Igf1r	0.007757	0.437768	0.288	0.113	1	14
Nbeal1	0.0078	0.562616	0.154	0.038	1	14
Lphn3	0.007813	0.354119	0.192	0.06	1	14
Kif4	0.007892	-0.4626	0	0.108	1	14
Cirbp	0.007929	-0.55886	0.154	0.355	1	14
Rfc5	0.007941	-0.44111	0	0.108	1	14
Wdr82	0.00801	-0.59383	0.038	0.173	1	14
Gli1	0.008038	-0.47103	0	0.108	1	14
Srgap2	0.008081	0.384435	0.308	0.131	1	14
Coq9	0.00811	-0.25686	0.115	0.118	1	14
Stag1	0.008165	-0.61077	0.077	0.207	1	14
Hmgn2	0.00823	-0.65101	0.096	0.256	1	14
Ankrd26	0.008263	-0.70849	0.038	0.176	1	14
Pou3f3	0.008267	-0.6509	0.019	0.172	1	14
Snrpb2	0.008285	-0.52652	0.173	0.384	1	14
Zfp637	0.008389	-0.64862	0.096	0.257	1	14
R3hdm1	0.008406	-0.63875	0.154	0.297	1	14
Pold3	0.008408	-0.31745	0.058	0.148	1	14
Ubqln2	0.008431	-0.59023	0.096	0.214	1	14
Dpysl3	0.008515	-0.82421	0.077	0.219	1	14
Hbs1l	0.00857	-0.46605	0	0.106	1	14
Sfpq	0.008734	-0.42617	0.519	0.67	1	14
Ccdc53	0.008764	0.518598	0.212	0.072	1	14
Fam53a	0.008779	0.534507	0.154	0.075	1	14
Fkbp7	0.008818	0.478842	0.212	0.066	1	14
Mfhas1	0.008838	0.523992	0.212	0.068	1	14
Arid5b	0.008853	0.56704	0.154	0.04	1	14

Malat1	0.008886	0.387395	0.981	0.955	1	14
Arl3	0.008961	-0.61001	0.192	0.365	1	14
Zmym2	0.008971	-0.40234	0.058	0.118	1	14
Atxn7	0.008981	0.643889	0.192	0.087	1	14
Clpp	0.009103	-0.56191	0.135	0.239	1	14
Slc25a1	0.00913	0.341624	0.192	0.06	1	14
Nckap1	0.009174	0.518094	0.308	0.13	1	14
Fhl1	0.009247	-0.46321	0	0.105	1	14
Tbc1d16	0.009271	-0.63702	0.038	0.196	1	14
Rtn4	0.0093	-0.34456	0.481	0.48	1	14
Smarca5	0.009306	-0.61127	0.192	0.402	1	14
Pls3	0.009385	0.547554	0.192	0.071	1	14
Dnaaf2	0.009388	-0.44538	0	0.105	1	14
Polr3k	0.009411	-0.63669	0.115	0.271	1	14
Zbtb7a	0.009461	0.561588	0.231	0.093	1	14
Ophn1	0.009552	0.367309	0.231	0.083	1	14
Cdca7l	0.009648	-0.43665	0	0.104	1	14
Zic5	0.009669	-0.53162	0.038	0.161	1	14
Rsrc2	0.009751	-0.45337	0.462	0.547	1	14
Yy1	0.009761	-0.47511	0.135	0.204	1	14
Uba2	0.009788	-0.61428	0.077	0.256	1	14
Mab21l2	0.009795	-0.53538	0	0.104	1	14
Xpo1	0.009815	-0.3262	0.096	0.227	1	14
Tia1	0.009856	-0.50198	0.365	0.552	1	14
Slc25a20	0.009872	0.466553	0.154	0.037	1	14
Mark3	0.009915	0.421362	0.288	0.235	1	14
Rnpc3	0.009915	-0.48963	0	0.104	1	14
Tmem98	0.009928	0.391031	0.154	0.038	1	14
Odf2	0.009987	-0.61754	0.019	0.171	1	14
