# Supplementary material for: scRNA-seq in medulloblastoma shows cellular heterogeneity and lineage expansion support resistance to SHH inhibitor therapy
Source: Nat Commun. 2019 Dec 20;10:5829. doi: 10.1038/s41467-019-13657-6 (PMC6925218; doi:10.1038/s41467-019-13657-6)
Supplement: Supplementary file 5 — Supplementary Data 2 [file 41467_2019_13657_MOESM5_ESM.pdf]

	avg_logFC	pct.within_cluster	pct.outside_cluster	p_val_adj	cluster
2810417H13Rik	1.30668484	0.838	0.308	0	0
Top2a	1.179765802	0.886	0.342	0	0
Esco2	1.087460776	0.525	0.133	0	0
Rrm2	0.969782719	0.493	0.149	0	0
Pbk	0.964207338	0.543	0.162	0	0
Mki67	0.938820317	0.788	0.321	0	0
Dut	0.925629728	0.761	0.373	0	0
Tyms	0.913277488	0.507	0.165	0	0
Lig1	0.904663159	0.673	0.287	0	0
Dek	0.89794021	0.944	0.646	0	0
Spc25	0.895991757	0.532	0.167	0	0
Smc2	0.890149564	0.888	0.481	0	0
Pcna	0.878709411	0.678	0.298	0	0
Tuba1b	0.841279452	0.789	0.423	0	0
Cdk1	0.824512075	0.583	0.183	0	0
Smc4	0.779800545	0.88	0.508	0	0
H2afx	0.771197829	0.664	0.268	0	0
Hmgb2	0.735110874	0.743	0.335	0	0
Ranbp1	0.679318456	0.882	0.636	0	0
Tubb5	0.481770189	0.982	0.902	0	0
Birc5	0.781042024	0.588	0.214	0	0
Incenp	0.818033576	0.585	0.217	0	0
Spc24	0.864215751	0.534	0.187	0	0
Hist1h2ak	0.996597898	0.384	0.098	1.014E-307	0
Prc1	0.609991955	0.545	0.202	8.185E-297	0
Nusap1	0.75381012	0.517	0.172	1.0211E-296	0
Anp32e	0.628042638	0.852	0.568	7.8293E-293	0
Clspn	0.885817954	0.455	0.14	1.4262E-290	0
Anp32b	0.630705874	0.837	0.544	1.2623E-288	0
Tk1	0.833791053	0.367	0.087	1.3103E-287	0
Nasp	0.602028773	0.865	0.598	5.698E-277	0
Atad2	0.840403018	0.456	0.15	3.1488E-276	0
Kif11	0.76285619	0.472	0.156	1.2257E-264	0
Ckb	-0.761399961	0.679	0.832	5.6582E-264	0
Cenpf	0.334115992	0.633	0.311	2.8194E-260	0
Cenph	0.826811495	0.435	0.148	5.1339E-247	0
Rtn1	-0.853930191	0.546	0.717	2.2207E-245	0
Neurod1	-1.144809506	0.443	0.549	1.6805E-244	0
H2afv	0.501325906	0.908	0.694	6.9771E-244	0
Tpx2	0.53686202	0.548	0.231	1.1749E-243	0
Tmsb4x	-0.591696467	0.917	0.954	2.5041E-243	0
Rad51ap1	0.766996586	0.397	0.123	5.2883E-235	0
Cdca8	0.675944187	0.528	0.215	2.6227E-227	0
Fbxo5	0.746311679	0.379	0.118	9.733E-220	0
Rrm1	0.7298922	0.502	0.214	1.1367E-219	0
Ncapg	0.743081872	0.409	0.14	7.0455E-214	0
Ccna2	0.650131923	0.466	0.175	2.0134E-213	0

Prim1	0.72821497	0.504	0.217	7.6996E-212	0
Tmpo	0.615618752	0.7	0.405	3.88E-211	0
Hirip3	0.672965923	0.63	0.336	1.1266E-207	0
Dnajc9	0.653753107	0.593	0.304	4.2731E-200	0
2700094K13Rik	0.517438394	0.828	0.574	1.5563E-199	0
Hnrnpab	0.415606482	0.924	0.795	9.2644E-190	0
Aurkb	0.674044008	0.338	0.106	1.757E-185	0
Dnmt1	0.649244007	0.51	0.234	7.6708E-185	0
Tcf19	0.663964938	0.268	0.066	1.9838E-183	0
Cntn2	-1.171922249	0.094	0.295	1.7652E-180	0
Hells	0.645374992	0.432	0.172	3.6663E-177	0
Stmn2	-0.876708958	0.432	0.575	2.0227E-175	0
Kif15	0.677856821	0.384	0.142	9.0779E-175	0
Ezh2	0.529046265	0.793	0.575	6.4748E-174	0
Cdca3	0.595407771	0.441	0.18	1.7241E-173	0
Tuba1a	-0.464921796	0.928	0.929	4.2249E-172	0
Ckap2l	0.562996783	0.43	0.177	3.2415E-166	0
RP23-45G16.5	0.535642762	0.561	0.283	1.9398E-165	0
Itm2b	-0.642391876	0.515	0.666	2.8407E-164	0
Neil3	0.606648989	0.231	0.055	1.6701E-163	0
Nrep	-0.729313298	0.422	0.596	5.9126E-161	0
Usp1	0.58357749	0.557	0.298	2.8711E-154	0
Dtymk	0.558123841	0.641	0.401	1.3418E-152	0
Fam111a	0.654988676	0.292	0.094	1.187E-149	0
Racgap1	0.567297779	0.463	0.216	9.7486E-146	0
Fkbp3	0.415014729	0.878	0.72	2.5058E-145	0
Tipin	0.593477626	0.455	0.217	4.307E-144	0
Mxd3	0.610979444	0.289	0.096	6.8947E-143	0
Casc5	0.595826152	0.373	0.151	2.3944E-141	0
Mns1	0.615534653	0.391	0.168	3.5444E-140	0
Ran	0.510967731	0.619	0.375	8.3172E-140	0
Paics	0.53430716	0.614	0.375	1.1298E-136	0
Nucks1	0.398256055	0.833	0.63	4.5611E-136	0
Ndc80	0.605002357	0.264	0.086	3.5891E-135	0
Mthfd2	0.598551721	0.225	0.063	6.7341E-134	0
Gm10075	0.475973365	0.707	0.478	9.948E-134	0
Gpm6a	-0.698546095	0.321	0.425	1.1393E-133	0
Tubb3	-0.788840916	0.349	0.497	8.5168E-133	0
Kif23	0.420022447	0.396	0.18	2.0872E-132	0
Rad51	0.564569336	0.266	0.088	6.227E-131	0
Smc6	0.508890509	0.625	0.384	1.2143E-130	0
Cenpm	0.582563891	0.324	0.131	3.6795E-126	0
Chaf1a	0.535012366	0.337	0.137	1.7153E-124	0
Cenpq	0.581346257	0.323	0.129	3.3071E-124	0
Gmnn	0.577850551	0.3	0.115	1.2269E-122	0
Rpa2	0.540754754	0.377	0.166	1.2231E-121	0
Map1b	-0.616593799	0.494	0.604	1.772E-121	0
Apoe	-1.675950084	0.226	0.253	2.4339E-121	0

Tacc3	0.538854008	0.384	0.173	5.4946E-120	0
Cdca5	0.507159092	0.194	0.053	9.92E-120	0
H2afy	0.435819429	0.754	0.561	1.2187E-119	0
Myod1	0.627437631	0.291	0.113	1.8795E-118	0
Dpysl3	-0.832950731	0.088	0.243	3.2304E-118	0
Rfc4	0.531447834	0.404	0.193	6.7255E-118	0
Cks1b	0.469564378	0.544	0.307	8.1979E-118	0
Nuf2	0.54637675	0.293	0.112	1.8853E-116	0
Ccdc34	0.490972028	0.609	0.378	2.8255E-116	0
CRE_RECOMBINASE	-0.597915994	0.733	0.774	1.8266E-114	0
Ncl	0.279588483	0.959	0.884	6.4151E-114	0
Serbp1	0.279686187	0.946	0.854	3.0293E-113	0
Psip1	0.386879781	0.822	0.662	2.031E-112	0
Supt16	0.460140959	0.67	0.46	2.429E-112	0
Stmn4	-0.784398543	0.217	0.364	5.6434E-112	0
H1f0	0.453694211	0.761	0.589	1.2687E-111	0
Gng3	-0.750361668	0.168	0.344	3.0099E-111	0
Bub1	0.54639526	0.275	0.103	7.6715E-111	0
Gm9800	0.440163083	0.7	0.503	2.8503E-109	0
Sfrs18	-0.440446423	0.673	0.797	1.2633E-108	0
Hnrnpd	0.411906208	0.758	0.582	1.069E-107	0
Mcm6	0.464609542	0.457	0.242	6.6496E-106	0
Kif22	0.477512545	0.318	0.133	8.6404E-106	0
Plk4	0.526317658	0.253	0.094	1.0011E-104	0
Ncapg2	0.524261874	0.195	0.057	1.5896E-104	0
Hmgn5	0.487092722	0.604	0.39	7.2972E-104	0
Id2	-0.634144581	0.346	0.512	8.6281E-104	0
Cdc45	0.49703504	0.218	0.071	2.2502E-103	0
Cenpk	0.551525565	0.285	0.118	5.4078E-102	0
Fen1	0.496710014	0.285	0.114	6.1145E-102	0
Kdm5b	-0.647638946	0.054	0.209	1.4332E-101	0
Miat	-0.599343154	0.433	0.53	7.9626E-101	0
Ccne2	0.497918274	0.179	0.049	1.4934E-100	0
Melk	0.494364927	0.212	0.072	1.31681E-98	0
Cbx5	0.345401885	0.807	0.624	2.38954E-98	0
Diap3	0.523170174	0.23	0.083	3.43949E-98	0
Whsc1	0.436166906	0.634	0.432	2.48611E-97	0
Ncapd2	0.513675997	0.335	0.156	4.12686E-97	0
Uhrf1	0.518206881	0.283	0.117	8.55769E-97	0
Cdca2	0.494869183	0.263	0.103	9.23431E-97	0
Celf4	-0.730210646	0.232	0.336	1.78021E-96	0
Rangap1	0.490526395	0.421	0.225	1.62682E-94	0
Tubb2a	-0.713169816	0.131	0.266	1.89685E-92	0
Ssrp1	0.380451071	0.735	0.546	2.12556E-92	0
Pmf1	0.498760283	0.275	0.118	2.4926E-92	0
Ptma	0.378198452	0.733	0.553	4.05989E-92	0
Gap43	-0.51654734	0.55	0.608	1.20277E-91	0
Dhfr	0.479264273	0.288	0.122	3.11216E-91	0

Prdx4	0.454201963	0.531	0.335	2.05476E-90	0
Pkmyt1	0.433672882	0.173	0.052	3.95023E-90	0
Rad21	0.374776972	0.638	0.428	1.90135E-88	0
Cst3	-0.571703656	0.515	0.539	1.62658E-87	0
Rbbp4	0.398164693	0.642	0.453	7.39965E-87	0
Dlgap4	-0.593553768	0.068	0.21	4.06309E-86	0
Sox4	-0.497757005	0.478	0.602	1.72596E-85	0
Tubb2b	-0.516659133	0.372	0.481	1.73758E-85	0
D4Wsu53e	-0.523805706	0.332	0.467	2.43291E-85	0
BC005764	-0.660295705	0.074	0.206	1.45765E-84	0
Smc3	0.367432189	0.763	0.609	1.50151E-84	0
Rad54l	0.43501651	0.201	0.071	1.5741E-84	0
Kif4	0.483286685	0.223	0.086	3.85032E-84	0
H2afz	0.439585282	0.384	0.202	6.46406E-84	0
Brca1	0.422686324	0.181	0.058	1.41317E-83	0
Hjurp	0.389576506	0.604	0.402	3.98543E-83	0
Dcx	-0.58597126	0.259	0.397	4.34257E-83	0
Lmnb1	0.425561545	0.461	0.269	2.9109E-82	0
Atad5	0.438726559	0.281	0.123	4.28968E-82	0
Elavl4	-0.616256266	0.204	0.359	5.11521E-82	0
Zic1	-0.353503639	0.749	0.79	6.75169E-82	0
Hmgb1	0.419005916	0.52	0.328	1.41771E-81	0
Rbfox3	-0.511025448	0.406	0.51	5.70467E-81	0
Hmgn2	0.444595517	0.408	0.227	8.53183E-81	0
Topbp1	0.452060758	0.274	0.121	1.63825E-80	0
Ubr7	0.457187586	0.256	0.109	2.5396E-80	0
Cdk5r1	-0.576941688	0.233	0.352	2.69737E-80	0
Pdzrn3	-0.685154062	0.048	0.165	1.02208E-79	0
Asf1b	0.394199522	0.151	0.044	2.32758E-79	0
Ccp110	0.430179208	0.4	0.219	2.67607E-79	0
D17H6S56E-5	0.437191244	0.248	0.104	3.39323E-79	0
Malat1	-0.385137649	0.919	0.961	4.05639E-79	0
Ncaph	0.462620066	0.237	0.099	4.74714E-79	0
Rfc1	0.432700671	0.489	0.3	4.86008E-79	0
Snrpd1	0.374659594	0.672	0.496	5.31911E-79	0
H1fx	0.483418874	0.389	0.218	5.73426E-79	0
Ankrd12	-0.66460035	0.158	0.296	7.74974E-79	0
Tpm4	0.430484208	0.472	0.287	3.29443E-78	0
Dtl	0.436178187	0.273	0.121	3.5125E-78	0
Ptn	-0.559088772	0.408	0.447	5.50753E-78	0
Nop58	0.352604512	0.735	0.565	1.66391E-77	0
Cltb	0.435495967	0.574	0.394	6.45424E-77	0
Rfc2	0.437500989	0.353	0.184	7.63144E-77	0
Mis18bp1	0.36515432	0.289	0.136	7.98622E-77	0
Igfbpl1	-0.382138821	0.622	0.652	9.03602E-77	0
Banf1	0.326611877	0.809	0.672	7.87313E-76	0
Calm1	-0.300964749	0.854	0.863	5.16914E-75	0
Pa2g4	0.365686699	0.668	0.495	6.28211E-75	0

Hnrnpdl	0.28785495	0.838	0.74	6.42583E-75	0
Nhlh1	-0.5930791	0.136	0.239	8.62785E-75	0
Siva1	0.435068194	0.421	0.244	1.92795E-74	0
Alyref	0.451230089	0.335	0.174	2.68071E-74	0
Smc1a	0.344776269	0.754	0.607	5.44706E-74	0
Lbr	0.422897733	0.332	0.171	8.40259E-74	0
Jhdm1d	-0.517861799	0.031	0.142	9.9508E-74	0
Cenpp	0.429457224	0.224	0.093	1.8555E-73	0
Nrxn1	-0.569125333	0.245	0.331	2.08067E-72	0
Kif20b	0.395658372	0.324	0.165	4.02305E-72	0
Timeless	0.400204582	0.279	0.13	6.08904E-72	0
Rab3a	-0.591861124	0.06	0.166	6.90782E-72	0
Thra	-0.606121308	0.121	0.248	2.47645E-71	0
Hint1	0.297666883	0.821	0.681	3.06133E-71	0
Serinc1	-0.45359806	0.346	0.453	7.77955E-71	0
Aldoa	-0.5517352	0.14	0.279	1.04413E-70	0
Mcm2	0.404403062	0.341	0.179	4.69926E-70	0
Cdc7	0.410147787	0.293	0.144	6.00746E-69	0
Ckap2	0.405197954	0.288	0.139	1.08518E-68	0
Hist1h2ag	0.374064537	0.136	0.041	1.09781E-68	0
Cenpw	0.438429119	0.238	0.106	1.19547E-68	0
Sept3	-0.487495586	0.327	0.404	1.35867E-68	0
Rbbp7	0.40098339	0.464	0.292	1.67164E-68	0
Ska1	0.416635363	0.186	0.071	7.48975E-68	0
Acat1	0.373602717	0.587	0.413	2.21675E-67	0
Dctpp1	0.408186204	0.447	0.277	2.27418E-67	0
Oxct1	0.344458825	0.685	0.527	3.87152E-67	0
Pdgfa	0.460612324	0.391	0.228	4.40622E-67	0
Rpa1	0.404889731	0.214	0.089	1.18273E-66	0
Ybx1	0.275155809	0.876	0.769	2.35572E-66	0
Mrpl18	0.401398602	0.497	0.326	2.85605E-66	0
Sae1	0.400248428	0.42	0.252	2.93977E-66	0
Zwilch	0.401447901	0.183	0.07	3.09171E-66	0
Srsf7	0.349410971	0.613	0.437	4.05166E-66	0
Ccdc25	0.416239935	0.31	0.159	4.24955E-66	0
Cdkn2c	0.425909645	0.257	0.122	6.03873E-66	0
Mcm5	0.404754111	0.292	0.145	6.20968E-66	0
Psmc3ip	0.428988613	0.242	0.111	1.55988E-65	0
Mcm10	0.394525399	0.153	0.051	1.8879E-65	0
Mlf1ip	0.401360817	0.165	0.058	2.05642E-65	0
Clic4	0.406199385	0.359	0.202	5.9176E-65	0
Ina	-0.478329641	0.357	0.459	6.15674E-65	0
Nap1l1	0.331054506	0.654	0.483	1.5755E-64	0
Zfp367	0.375667521	0.19	0.075	3.06232E-64	0
Nrm	0.406014735	0.29	0.146	6.71137E-64	0
Tsc22d1	-0.449632435	0.321	0.447	1.03672E-63	0
Srsf3	0.300188354	0.764	0.636	1.31388E-63	0
Skp2	0.398742358	0.226	0.1	2.30614E-63	0

Mapt	-0.619076609	0.119	0.234	3.6484E-63	0
Elavl3	-0.460401963	0.375	0.49	5.81966E-63	0
Nsl1	0.370525548	0.135	0.043	7.82025E-63	0
Gpm6b	-0.422408424	0.406	0.498	1.43086E-62	0
Lsm2	0.396939115	0.36	0.205	1.66298E-62	0
Mcm7	0.328996702	0.538	0.361	2.4085E-62	0
Pold3	0.378442373	0.263	0.127	3.07755E-62	0
Mtss1	-0.529282595	0.189	0.284	3.43096E-62	0
Ctsd	-0.757892935	0.155	0.179	4.32855E-62	0
Pola1	0.412747222	0.201	0.084	4.67811E-62	0
Nsmce4a	0.394751296	0.353	0.199	6.48167E-62	0
Rif1	0.379205433	0.415	0.249	7.22257E-62	0
Cdt1	0.378168872	0.233	0.105	7.61795E-62	0
Ctsb	-0.651253814	0.206	0.272	1.35033E-61	0
Ttc3	-0.299948859	0.8	0.834	2.22657E-61	0
Myt1	-0.578075654	0.061	0.169	2.71256E-61	0
Psat1	0.386439973	0.503	0.342	7.85724E-61	0
E2f7	0.339772105	0.114	0.031	1.11362E-60	0
Slbp	0.358389861	0.168	0.062	1.12861E-60	0
Syt11	-0.420807382	0.419	0.52	1.38251E-60	0
Sgol1	0.397769868	0.233	0.107	6.72144E-60	0
Tmem50a	-0.454595056	0.29	0.426	9.58881E-60	0
Ncapd3	0.385880214	0.215	0.093	1.14068E-59	0
Orc6	0.379849575	0.325	0.177	1.93032E-59	0
Cmc2	0.396934454	0.221	0.1	1.95827E-59	0
Mcm3	0.389327625	0.299	0.157	4.39908E-59	0
Blm	0.372345063	0.211	0.092	4.63921E-59	0
Ctsl	-0.498940648	0.191	0.272	7.47095E-59	0
Barhl1	-0.405211258	0.44	0.536	1.24738E-58	0
Gjc1	0.392008384	0.318	0.173	2.97521E-58	0
Rad54b	0.334300305	0.139	0.047	7.21678E-58	0
Atxn7l3b	-0.329622201	0.6	0.686	8.58761E-58	0
Cep57	0.378774577	0.389	0.232	9.11161E-58	0
Pgf	0.350790512	0.149	0.053	1.08083E-57	0
St18	-0.556857622	0.113	0.204	1.45553E-57	0
Suz12	0.391662518	0.343	0.195	2.54913E-57	0
6330403K07Rik	-0.507671297	0.143	0.261	3.01102E-57	0
Foxm1	0.328003713	0.107	0.033	3.07981E-57	0
Nhlh2	-0.45755197	0.355	0.435	1.07625E-56	0
Ttk	0.371927488	0.178	0.073	1.10776E-56	0
Lyar	0.390079664	0.418	0.261	1.18675E-56	0
Sept4	-0.560321833	0.136	0.243	1.29347E-56	0
Spag5	0.35635001	0.162	0.063	1.37151E-56	0
Snrpb	0.299697931	0.701	0.549	1.83202E-56	0
Brca2	0.377885258	0.212	0.095	2.03926E-56	0
Hmgn1	0.289576131	0.743	0.598	2.71555E-56	0
Aplp1	-0.526450139	0.07	0.166	3.24105E-56	0
Phf17	0.379317207	0.18	0.075	4.19043E-56	0

Brd3	0.328842128	0.657	0.508	8.24832E-56	0
Hpca	0.389520222	0.409	0.252	9.74834E-56	0
Mis18a	0.391663975	0.204	0.092	1.64497E-55	0
Csrp2	0.380364302	0.269	0.137	2.21996E-55	0
Nup62	0.359891503	0.284	0.15	3.21401E-55	0
Basp1	-0.309151211	0.676	0.709	9.26117E-55	0
Cpe	-0.396636264	0.377	0.47	9.81529E-55	0
Cadm3	-0.521647282	0.063	0.155	9.98706E-55	0
Prim2	0.349003756	0.161	0.062	1.09717E-54	0
A330076H08Rik	-0.507609181	0.081	0.175	1.32841E-54	0
Shcbp1	0.347628986	0.158	0.061	1.87315E-54	0
Map2	-0.394749571	0.427	0.503	5.35182E-54	0
Wdhd1	0.34278454	0.171	0.068	5.86904E-54	0
Chtf18	0.331997039	0.112	0.035	6.24163E-54	0
Elmo1	-0.554891764	0.05	0.139	1.15363E-53	0
Rbbp8	0.382318631	0.221	0.103	1.5485E-53	0
Trpc4ap	-0.521382592	0.121	0.212	2.88104E-53	0
A930011O12Rik	-0.526750335	0.042	0.128	2.98465E-53	0
Cklf	0.39503748	0.272	0.145	3.1552E-53	0
Mxd4	-0.478988896	0.103	0.215	4.68151E-53	0
Dbf4	0.332588473	0.251	0.125	5.62748E-53	0
Srsf2	0.302885933	0.686	0.543	6.13789E-53	0
Chgb	-0.561500747	0.165	0.254	6.38415E-53	0
Ptprs	-0.440539178	0.347	0.434	8.42568E-53	0
Espl1	0.347677135	0.137	0.051	1.23315E-52	0
Rfc3	0.359023532	0.323	0.182	1.28578E-52	0
Arhgap11a	0.330133078	0.28	0.149	1.86449E-52	0
Cdkn2d	0.33895262	0.264	0.137	5.16898E-52	0
Slc7a5	0.382707998	0.21	0.097	5.28436E-52	0
Mllt11	-0.502263477	0.134	0.234	1.72478E-51	0
Smarcc1	0.324289119	0.567	0.409	2.94296E-51	0
Vrk1	0.374217387	0.251	0.128	3.91111E-51	0
Baz1b	0.323108725	0.621	0.469	3.92215E-51	0
Nup85	0.345542169	0.346	0.203	4.40455E-51	0
Mad2l1	0.370767693	0.218	0.105	5.22896E-51	0
Nxt1	0.362228181	0.217	0.103	5.83256E-51	0
Nsg2	-0.359711328	0.417	0.5	6.61343E-51	0
Gria2	-0.388275696	0.469	0.53	7.32739E-51	0
Dscc1	0.331788823	0.125	0.042	1.18196E-50	0
Naa50	0.361889008	0.41	0.262	1.43714E-50	0
Fabp7	-0.930783047	0.101	0.127	1.64504E-50	0
Tex14	-0.537782185	0.067	0.159	2.1468E-50	0
Dnajc21	0.34016935	0.326	0.188	2.42254E-50	0
Apc	-0.428804903	0.351	0.446	2.43243E-50	0
Syce2	0.374539149	0.164	0.067	2.69533E-50	0
Lap3	0.380116901	0.398	0.253	2.835E-50	0
E2f2	0.297844556	0.107	0.032	3.08228E-50	0
Gins3	0.334718392	0.141	0.053	6.25901E-50	0

Sqstm1	-0.438072723	0.123	0.246	6.32913E-50	0
Ska2	0.345551535	0.32	0.183	7.68099E-50	0
Ccne1	0.276492448	0.103	0.03	1.20785E-49	0
Meg3	-0.798040512	0.033	0.106	1.31395E-49	0
Sgol2	0.29021977	0.257	0.137	1.51905E-49	0
Chd7	-0.32219224	0.592	0.657	1.56724E-49	0
Insm1	0.400848611	0.41	0.27	2.32907E-49	0
Anln	0.39012086	0.191	0.088	3.30239E-49	0
Celf2	-0.326292981	0.6	0.644	4.2419E-49	0
Cenpj	0.376022226	0.242	0.122	5.22163E-49	0
Nop56	0.328986821	0.49	0.336	5.91508E-49	0
Txn1	0.28635934	0.681	0.537	8.62848E-49	0
Cdca4	0.31280382	0.2	0.093	2.29111E-48	0
2700099C18Rik	0.343510008	0.162	0.068	5.00294E-48	0
Wbp5	0.29314414	0.643	0.495	9.74242E-48	0
Dner	-0.471640158	0.141	0.228	3.81589E-47	0
Apitd1	0.345851475	0.178	0.079	7.13823E-47	0
Cenpn	0.32071651	0.14	0.055	8.23209E-47	0
Lsm3	0.315898619	0.485	0.334	1.36598E-46	0
Rad18	0.346517875	0.172	0.075	1.85073E-46	0
Rbfox2	-0.439982936	0.195	0.299	2.07999E-46	0
Pole	0.303198109	0.107	0.034	3.09738E-46	0
Snrpe	0.27781282	0.68	0.54	3.97894E-46	0
Brip1	0.300581507	0.106	0.034	5.02481E-46	0
Dck	0.341689057	0.188	0.086	1.01592E-45	0
Ypel3	-0.371337046	0.353	0.45	1.93961E-45	0
Gsg2	0.302835855	0.11	0.038	2.49828E-45	0
Arpp21	-0.510582217	0.048	0.125	9.99628E-45	0
Sparcl1	-0.530624369	0.183	0.211	1.27019E-44	0
Hat1	0.34375996	0.253	0.136	1.37488E-44	0
Ppp1r14c	-0.37992796	0.229	0.268	1.49081E-44	0
B3galt2	-0.450055576	0.037	0.109	7.41703E-44	0
Scg5	-0.400713047	0.255	0.349	1.28597E-43	0
Calr	-0.332106929	0.444	0.523	1.32152E-43	0
Kif5a	-0.455141215	0.059	0.141	1.63334E-43	0
Bub3	0.330306797	0.441	0.298	2.01336E-43	0
Rfc5	0.331586009	0.191	0.092	3.27968E-43	0
Pde1c	-0.357654634	0.353	0.393	3.3992E-43	0
Kif1b	-0.347841586	0.454	0.533	5.10403E-43	0
Igsf21	-0.40740115	0.026	0.101	8.04576E-43	0
Ank3	-0.392451241	0.351	0.433	8.41574E-43	0
Rab6b	-0.447564367	0.121	0.217	1.07351E-42	0
Tbata	0.291122014	0.463	0.317	1.1014E-42	0
Srsf4	0.324481761	0.394	0.257	1.2613E-42	0
Gli1	0.318715489	0.192	0.091	1.51044E-42	0
Rbp4	0.321717622	0.354	0.22	1.70592E-42	0
Sema6a	-0.47107409	0.126	0.216	1.86953E-42	0
Arpp19	0.316576418	0.494	0.355	3.73418E-42	0

Prdx1	0.25784619	0.614	0.466	1.19853E-41	0
Ifrd1	0.326465292	0.278	0.158	1.75601E-41	0
Kmt2e	-0.320749454	0.504	0.58	2.40618E-41	0
Idh2	0.311914871	0.393	0.257	2.84959E-41	0
Chaf1b	0.340394195	0.157	0.069	3.58737E-41	0
Trip13	0.289346438	0.154	0.067	4.03645E-41	0
2700029M09Rik	0.314383873	0.405	0.267	4.19256E-41	0
Itsn1	-0.436382283	0.168	0.248	4.24489E-41	0
Kif5c	-0.364814861	0.343	0.408	9.47083E-41	0
Smarca5	0.294514399	0.521	0.378	9.9271E-41	0
Exo1	0.275523784	0.104	0.035	1.06957E-40	0
Prnp	-0.422936501	0.127	0.221	1.10545E-40	0
Ctsf	-0.388333526	0.034	0.11	1.41351E-40	0
Ctcf	0.290061481	0.609	0.474	1.73203E-40	0
Ybx3	0.320101623	0.403	0.268	1.86168E-40	0
Smchd1	0.343750694	0.383	0.252	2.38174E-40	0
Trim37	0.338456154	0.339	0.213	3.03227E-40	0
Pola2	0.311901351	0.168	0.078	7.24021E-40	0
Bex1	0.313280206	0.33	0.203	8.50079E-40	0
Stil	0.270420277	0.11	0.041	9.32392E-40	0
Uncx	-0.376082036	0.26	0.328	1.90988E-39	0
Rabac1	-0.404277561	0.172	0.274	2.17716E-39	0
Slfn9	0.323517891	0.142	0.06	3.34755E-39	0
Rpa3	0.330433498	0.351	0.223	3.55189E-39	0
Pold1	0.290114926	0.157	0.071	4.58631E-39	0
Cnot6	0.27945026	0.511	0.369	4.598E-39	0
Pax6	-0.301282298	0.472	0.52	6.91111E-39	0
Hist1h1e	0.317117442	0.127	0.051	7.39757E-39	0
Ank2	-0.43476186	0.183	0.25	8.56655E-39	0
Cdk2	0.301347439	0.133	0.055	8.68191E-39	0
Cdk5rap2	0.327012694	0.281	0.164	9.4259E-39	0
Snrpf	0.267192451	0.546	0.404	2.07902E-38	0
Chrna3	-0.439099538	0.053	0.13	2.32536E-38	0
1500012F01Rik	-0.360105579	0.32	0.412	2.61249E-38	0
Ndn	-0.398919706	0.146	0.237	3.06443E-38	0
Larp7	0.329214554	0.421	0.291	3.19767E-38	0
Map1lc3b	-0.364420718	0.254	0.345	3.63415E-38	0
Cdca7	0.276558594	0.298	0.179	3.64449E-38	0
Nmral1	0.326410613	0.284	0.169	6.31225E-38	0
Slc25a5	0.267529379	0.621	0.487	8.84035E-38	0
Wdr76	0.291967857	0.15	0.066	1.28685E-37	0
Trim59	0.30859921	0.264	0.152	2.53853E-37	0
Itm2c	-0.402110314	0.143	0.224	3.36593E-37	0
Dnaaf2	0.314310296	0.183	0.09	3.57369E-37	0
Fignl1	0.271762095	0.132	0.054	3.82094E-37	0
Ctps	0.295478679	0.225	0.121	5.75867E-37	0
Bub1b	0.324107269	0.176	0.088	1.01606E-36	0
Cep290	0.325722053	0.231	0.126	1.20685E-36	0

Grina	-0.387486607	0.047	0.12	2.27256E-36	0
Naa38	0.285983717	0.422	0.29	2.7378E-36	0
Ccdc41	0.32911657	0.357	0.234	2.76027E-36	0
Haus5	0.264049146	0.117	0.045	2.98597E-36	0
Dpy30	0.299268659	0.407	0.277	4.53201E-36	0
Btbd17	-0.406598678	0.092	0.172	6.67208E-36	0
Zic4	-0.340397155	0.348	0.431	9.3846E-36	0
S100a16	-0.433961296	0.04	0.105	1.0194E-35	0
Snap25	-0.389310932	0.222	0.293	1.02802E-35	0
Eif4ebp1	0.345032175	0.143	0.067	1.03335E-35	0
Iqgap2	0.25297996	0.103	0.037	1.08368E-35	0
Rnf168	0.304385639	0.307	0.189	1.55059E-35	0
Eif4a3	0.280209158	0.467	0.334	4.88736E-35	0
Nudc	0.303815503	0.386	0.261	5.95083E-35	0
Lsm6	0.257397156	0.485	0.35	6.07144E-35	0
Kpnb1	0.295384155	0.407	0.28	6.16885E-35	0
Tnik	-0.444388756	0.086	0.157	8.57072E-35	0
4930422G04Rik	0.289333731	0.139	0.062	1.51971E-34	0
Atp6v0b	-0.351988794	0.208	0.278	1.9045E-34	0
Hsd11b2	0.264704281	0.394	0.267	3.26349E-34	0
Arl6ip6	0.266855806	0.205	0.11	3.76158E-34	0
Myt1l	-0.445234897	0.101	0.172	5.32919E-34	0
Cntln	0.34711407	0.261	0.155	5.78614E-34	0
Prr11	0.262480976	0.116	0.047	6.49067E-34	0
Gng2	-0.325123273	0.334	0.407	6.96817E-34	0
Ldhb	-0.358145695	0.208	0.294	7.4533E-34	0
Slc1a3	-0.463660423	0.122	0.158	1.05156E-33	0
Os9	-0.380065646	0.176	0.264	1.06046E-33	0
Ipo5	0.299756239	0.221	0.122	1.39859E-33	0
Klf7	-0.38085116	0.195	0.274	1.43526E-33	0
Pole3	0.311430251	0.349	0.231	1.72232E-33	0
Srsf10	0.257903482	0.44	0.31	2.23931E-33	0
Rnaseh2a	0.309602725	0.238	0.137	2.7519E-33	0
Pknox1	0.318291649	0.23	0.129	3.15053E-33	0
2810008D09Rik	-0.366843523	0.067	0.138	9.84681E-33	0
Stag1	0.286315333	0.301	0.188	1.3261E-32	0
Ptms	-0.295410324	0.409	0.485	1.37109E-32	0
Gins1	0.299472398	0.191	0.1	1.41455E-32	0
Npdc1	-0.355155288	0.197	0.273	1.70544E-32	0
Gdi1	-0.333132419	0.181	0.239	1.80358E-32	0
Kcnk1	-0.384688776	0.148	0.214	1.82147E-32	0
Gins2	0.266991787	0.276	0.167	2.93405E-32	0
Casp8ap2	0.286380059	0.343	0.225	2.95415E-32	0
Gm11266	-0.39701292	0.08	0.155	6.94534E-32	0
Ube2t	0.266542966	0.141	0.065	1.03538E-31	0
Rbms1	0.286454212	0.312	0.198	1.11636E-31	0
Hprt	0.287115918	0.278	0.17	1.18424E-31	0
Zbtb18	-0.355191797	0.206	0.277	1.42937E-31	0

Nfatc2ip	0.285818219	0.133	0.061	5.1773E-31	0
Spdl1	0.293619522	0.141	0.067	6.9013E-31	0
Snrpd2	0.254941682	0.545	0.421	6.9492E-31	0
Lgmn	-0.396440571	0.074	0.127	8.32982E-31	0
Phgdh	0.284630445	0.141	0.068	8.76198E-31	0
Syncrip	0.251620248	0.595	0.477	1.0467E-30	0
Gins4	0.277958062	0.206	0.113	1.11033E-30	0
E2f1	0.262232332	0.228	0.131	1.12972E-30	0
Snrpa1	0.28532278	0.431	0.311	1.42882E-30	0
Ckap5	0.250152626	0.364	0.246	1.67549E-30	0
Tubg1	0.283187149	0.206	0.114	2.35607E-30	0
Rhno1	0.285079853	0.217	0.123	2.53879E-30	0
Kidins220	-0.391723441	0.079	0.151	2.56779E-30	0
Stub1	0.250927699	0.451	0.327	2.62196E-30	0
Mapk8ip1	-0.360822937	0.162	0.239	4.08035E-30	0
Trim28	0.254466465	0.466	0.341	4.24195E-30	0
Ect2	0.292656846	0.165	0.085	4.82624E-30	0
Aurka	0.272533346	0.167	0.085	7.22404E-30	0
Azin1	0.250180233	0.293	0.185	7.23466E-30	0
A030009H04Rik	0.2776135	0.187	0.1	8.10387E-30	0
Nsmce1	0.28746709	0.322	0.211	8.25074E-30	0
Tagln3	-0.323475699	0.251	0.302	1.02193E-29	0
Cbfb	0.284421208	0.27	0.166	1.07119E-29	0
Tex30	0.283817854	0.212	0.12	1.20942E-29	0
2610203C20Rik	-0.362404131	0.061	0.131	1.27997E-29	0
Sod1	0.263518178	0.541	0.423	1.59908E-29	0
Smco4	0.306382423	0.242	0.146	1.69754E-29	0
Gm26735	-0.370112169	0.113	0.193	1.77392E-29	0
Rcor2	-0.356138252	0.158	0.237	2.07582E-29	0
Ncaph2	0.280700995	0.259	0.157	2.1527E-29	0
Bok	0.280748973	0.347	0.233	2.18135E-29	0
Clmp	-0.374633968	0.185	0.252	2.76674E-29	0
Snrnp40	0.257646493	0.369	0.252	2.83807E-29	0
Abhd16a	-0.36058582	0.133	0.2	2.8709E-29	0
Uba2	0.281919427	0.351	0.238	3.46998E-29	0
Dnph1	0.310565612	0.191	0.11	3.62492E-29	0
Mis12	0.274344949	0.166	0.085	6.03868E-29	0
Set	0.26578533	0.487	0.368	6.16243E-29	0
Clip3	-0.317930296	0.249	0.323	1.04328E-28	0
Fam213b	-0.368043338	0.092	0.169	1.35648E-28	0
Bin1	-0.27469031	0.395	0.435	1.81976E-28	0
Mab21l1	-0.340748939	0.173	0.234	2.06521E-28	0
Soga3	-0.283024402	0.405	0.467	2.19574E-28	0
Ptges3	0.277513842	0.3	0.195	4.41496E-28	0
Nrn1	-0.380698922	0.151	0.234	1.1851E-27	0
Xbp1	-0.32042858	0.066	0.134	1.25642E-27	0
2210016L21Rik	-0.347756237	0.213	0.303	1.3078E-27	0
Vimp	-0.344862532	0.118	0.203	1.962E-27	0

Cdca7l	0.251912967	0.172	0.091	2.49376E-27	0
Mcm4	0.273392194	0.214	0.123	2.97843E-27	0
Apbb1	-0.347833835	0.121	0.179	2.99848E-27	0
Btg2	-0.373912199	0.151	0.219	3.03109E-27	0
Chd3	-0.397017939	0.087	0.16	3.17025E-27	0
Shmt1	0.265741114	0.17	0.09	4.12232E-27	0
Eid1	-0.263910385	0.451	0.512	8.66596E-27	0
Plp1	-0.754585704	0.103	0.116	1.11189E-26	0
Dbn1	-0.350360912	0.081	0.15	1.1305E-26	0
Slc3a2	0.256628012	0.42	0.304	1.54206E-26	0
Rab6a	-0.301043213	0.271	0.338	1.76591E-26	0
Pik3r3	-0.349360997	0.083	0.145	2.10144E-26	0
Ncan	-0.333926447	0.048	0.108	2.15397E-26	0
Glce	-0.347789768	0.121	0.187	2.31768E-26	0
Gnao1	-0.317135024	0.241	0.295	2.65562E-26	0
Cenpl	0.265497154	0.129	0.065	4.37694E-26	0
Podxl2	-0.325933192	0.183	0.235	5.43202E-26	0
Mbnl2	-0.336552917	0.127	0.188	5.44389E-26	0
Ddit4	-0.325154967	0.052	0.114	5.70717E-26	0
Stxbp1	-0.35517426	0.111	0.171	7.60158E-26	0
Exosc8	0.272297059	0.295	0.193	9.10106E-26	0
Zmynd8	-0.34485067	0.141	0.226	1.46124E-25	0
Prmt2	-0.307248325	0.046	0.103	1.75865E-25	0
Rtn4	-0.254154353	0.434	0.488	2.00146E-25	0
Serpinh1	-0.332614579	0.066	0.124	3.8602E-25	0
Cnrip1	-0.343766204	0.132	0.209	7.64826E-25	0
Pdlim1	0.254763642	0.118	0.056	7.72656E-25	0
Npc2	-0.262770436	0.411	0.473	9.34366E-25	0
Stag2	0.256959861	0.346	0.239	9.4791E-25	0
Gpc2	-0.314854622	0.088	0.149	1.51223E-24	0
Sh3bp5	-0.327188572	0.057	0.112	3.64784E-24	0
Tmem59	-0.252519225	0.346	0.4	4.2625E-24	0
Tspyl4	-0.330443472	0.077	0.136	5.28894E-24	0
Nicn1	-0.341094853	0.143	0.22	7.03554E-24	0
Use1	-0.303386678	0.244	0.326	1.35208E-23	0
Med30	0.25108563	0.285	0.188	1.4882E-23	0
Pbdc1	0.274877406	0.286	0.19	1.49686E-23	0
Clcn4-2	-0.314182374	0.197	0.261	1.62157E-23	0
Uchl1	-0.26290212	0.301	0.34	1.74788E-23	0
Exosc7	0.258536943	0.256	0.165	2.61805E-23	0
Clk1	-0.252173095	0.302	0.347	2.70789E-23	0
Srrm4	-0.304821681	0.156	0.202	3.72098E-23	0
Rufy3	-0.258033886	0.337	0.377	4.12825E-23	0
Cdkn1a	0.283829673	0.151	0.081	4.3559E-23	0
Kdm6b	-0.30125443	0.053	0.113	7.43724E-23	0
Sirt2	-0.360595983	0.114	0.163	9.42155E-23	0
Prkcb	-0.274876032	0.225	0.261	1.39376E-22	0
Vim	-0.298150204	0.268	0.308	6.96749E-22	0

Rnmt	-0.278362037	0.298	0.357	8.56578E-22	0
Nsg1	-0.292846599	0.235	0.297	8.95412E-22	0
Mpp6	0.279140847	0.232	0.148	9.18052E-22	0
Ogt	-0.29012	0.164	0.22	1.65543E-21	0
Pea15a	-0.329218399	0.128	0.184	1.81422E-21	0
Atp6v1e1	-0.280710871	0.277	0.341	1.99885E-21	0
Commd1	0.256884902	0.382	0.282	2.70473E-21	0
Ppil1	0.255472429	0.187	0.111	2.82749E-21	0
1500016L03Rik	-0.261688958	0.342	0.39	3.25599E-21	0
Kif20a	0.253810879	0.139	0.077	4.0642E-21	0
Sart3	0.25156336	0.304	0.21	4.67197E-21	0
Pdrg1	-0.311210523	0.156	0.23	8.9526E-21	0
Baz2b	-0.296161107	0.303	0.376	9.18536E-21	0
Akap8l	-0.311766089	0.106	0.167	1.27368E-20	0
Slc22a17	-0.293374553	0.198	0.26	1.34989E-20	0
Tgfb2	-0.326964312	0.135	0.202	1.73729E-20	0
RP23-32A8.1	-0.257085647	0.154	0.193	1.74641E-20	0
Pdcd4	-0.271165866	0.27	0.342	3.12057E-20	0
Idh1	-0.286405984	0.095	0.167	3.58807E-20	0
Gabarapl1	-0.284918937	0.168	0.225	3.84389E-20	0
H13	-0.279437933	0.19	0.246	3.91086E-20	0
Aplp2	-0.251162636	0.289	0.329	5.46275E-20	0
Gria4	-0.294390328	0.088	0.131	5.46612E-20	0
Uchl5	0.264266057	0.268	0.181	1.15896E-19	0
Hist3h2a	-0.310387886	0.095	0.152	1.2582E-19	0
2410066E13Rik	-0.283785208	0.056	0.106	1.49554E-19	0
Tmem66	-0.273941592	0.154	0.207	1.95149E-19	0
Asns	0.254111425	0.138	0.076	1.99266E-19	0
Ppfia2	-0.322168082	0.072	0.119	2.63889E-19	0
Hcfc1r1	-0.296546769	0.141	0.202	3.64771E-19	0
Sptan1	-0.278007408	0.133	0.175	4.88617E-19	0
Gabbr1	-0.262535125	0.108	0.146	5.98494E-19	0
Ccdc28b	-0.300105145	0.102	0.173	6.31898E-19	0
Tacc2	-0.311366677	0.093	0.149	7.16685E-19	0
Bcl7a	-0.253053603	0.28	0.333	7.35787E-19	0
Hpcal1	-0.302849035	0.063	0.115	7.46162E-19	0
Ing4	-0.268726878	0.265	0.333	9.61973E-19	0
St8sia3	-0.253138769	0.213	0.257	1.329E-18	0
Clvs1	-0.293890569	0.086	0.137	1.66361E-18	0
2900011O08Rik	-0.296266927	0.06	0.111	1.77035E-18	0
Ly6e	-0.255590026	0.242	0.289	2.61262E-18	0
Igsf8	-0.278506429	0.22	0.278	2.68424E-18	0
Ankra2	-0.27995321	0.077	0.128	4.03474E-18	0
Ywhag	-0.282221035	0.188	0.25	4.21941E-18	0
Psap	-0.299284854	0.141	0.192	4.53438E-18	0
Pnmal2	-0.301868904	0.1	0.155	6.18298E-18	0
Hist3h2ba	-0.289225647	0.088	0.146	7.98498E-18	0
Cacng4	-0.304271319	0.086	0.104	1.06007E-17	0

C130071C03Rik	-0.267128792	0.253	0.318	1.07787E-17	0
Flot1	-0.269384127	0.054	0.106	1.12155E-17	0
Pak7	-0.295351722	0.061	0.112	1.51224E-17	0
Grik2	-0.272328088	0.072	0.107	1.71713E-17	0
Zfp329	-0.280036922	0.086	0.137	1.72052E-17	0
Prdm8	-0.252140221	0.106	0.13	2.59599E-17	0
Pik3ip1	-0.272396866	0.073	0.124	3.36614E-17	0
Reln	-0.282856933	0.147	0.186	5.20039E-17	0
Pygo1	-0.291257846	0.146	0.2	7.4645E-17	0
Agpat4	-0.268221423	0.077	0.125	7.53464E-17	0
Lrpap1	-0.267235248	0.078	0.124	9.34275E-17	0
Klf9	-0.269757264	0.226	0.279	1.00979E-16	0
Mien1	-0.273902793	0.167	0.239	1.38708E-16	0
Bcas1	-0.302873307	0.24	0.289	1.62843E-16	0
Mkrn1	-0.268786877	0.16	0.217	3.0374E-16	0
Pcmtd2	-0.268759881	0.097	0.152	8.84804E-16	0
Wdr13	-0.261230832	0.061	0.113	1.18195E-15	0
Gm3764	-0.272665261	0.087	0.139	1.40303E-15	0
Dixdc1	-0.260939712	0.196	0.241	1.48266E-15	0
Maged2	-0.264151516	0.123	0.175	1.65882E-15	0
Sstr2	-0.278986807	0.143	0.199	1.75722E-15	0
Fbxo32	-0.269999306	0.069	0.125	2.14424E-15	0
Nt5c	-0.272010052	0.132	0.193	3.51757E-15	0
Atp6v0e2	-0.251514023	0.205	0.27	4.73767E-15	0
Gramd1b	-0.27119079	0.123	0.178	8.60812E-15	0
Rundc3a	-0.260594291	0.198	0.258	1.10858E-14	0
Kif1a	-0.267733351	0.125	0.18	2.25774E-14	0
4930402H24Rik	-0.252973993	0.075	0.119	3.37393E-14	0
Ctsa	-0.26278177	0.089	0.131	6.03887E-14	0
Tpm1	-0.269762905	0.148	0.198	6.92405E-14	0
Aprt	-0.257315649	0.095	0.149	9.60152E-14	0
Pcdha2	-0.262799999	0.082	0.124	5.46741E-13	0
Fyn	-0.260082034	0.191	0.22	8.44799E-13	0
Slc17a6	-0.26901084	0.113	0.154	1.14425E-12	0
Sox5	-0.254375263	0.081	0.126	1.80265E-12	0
Mbp	-0.335921521	0.141	0.17	3.91592E-11	0
Hes1	-0.266175896	0.126	0.152	8.91485E-11	0
Ntm	-0.259183046	0.111	0.151	2.40109E-10	0
Pcna.1	0.867280045	0.646	0.316	3.7476E-293	1
Lig1.1	0.868113677	0.649	0.304	5.981E-270	1
Mcm6.1	0.886715542	0.564	0.234	4E-268	1
Hells.1	0.940881409	0.496	0.171	7.0199E-263	1
Ung	0.848326835	0.321	0.059	2.7399E-251	1
Neurod1.1	-1.44762239	0.314	0.564	2.725E-242	1
Mcm3.1	0.83785278	0.432	0.142	3.0958E-221	1
Mcm2.1	0.779370827	0.45	0.168	9.9616E-194	1
Arl6ip1	-0.983220437	0.343	0.58	4.443E-192	1
Tuba1a.1	-0.581742929	0.888	0.935	3.031E-186	1

Stmn2.1	-0.991773403	0.384	0.577	2.9479E-170	1
Rtn1.1	-0.783624336	0.544	0.711	4.8422E-166	1
Dut.1	0.606065225	0.666	0.401	1.0642E-162	1
Rpa2.1	0.713334979	0.411	0.168	3.1922E-156	1
Ube2c	-1.221491996	0.092	0.294	2.3545E-153	1
Cntn2.1	-1.193300626	0.078	0.29	1.7978E-151	1
Ranbp1.1	0.4795246	0.819	0.654	1.6401E-150	1
Tmsb4x.1	-0.516964536	0.911	0.954	1.8414E-148	1
Nasp.1	0.484485854	0.787	0.618	3.4546E-145	1
Cenpa	-1.06604877	0.069	0.278	8.5546E-142	1
Dek.1	0.402464891	0.861	0.669	1.3064E-140	1
Cdt1.1	0.648543591	0.306	0.098	8.3775E-136	1
Mcm5.1	0.641713345	0.365	0.139	2.206E-134	1
Itm2b.1	-0.665291737	0.478	0.666	2.1729E-133	1
Calm2	-0.458321977	0.791	0.886	1.8211E-132	1
Tubb3.1	-0.870299959	0.328	0.495	9.241E-130	1
Uhrf1.1	0.639716678	0.324	0.117	1.9176E-128	1
Gap43.1	-0.748739398	0.45	0.621	6.6736E-123	1
Mcm4.1	0.617809589	0.313	0.112	1.1038E-121	1
Cdca7.1	0.633083304	0.385	0.17	7.6383E-119	1
Cenpf.1	-0.987672838	0.225	0.382	1.6659E-117	1
Ccnd2	0.480262298	0.815	0.639	2.7105E-117	1
Chaf1b.1	0.554741123	0.23	0.062	4.0984E-116	1
Rplp1	0.320913958	0.949	0.866	1.335E-114	1
Gpm6a.1	-0.818886864	0.236	0.434	1.6707E-111	1
Map1b.1	-0.689451316	0.442	0.608	5.1385E-110	1
Dhfr.1	0.610359166	0.315	0.124	4.7938E-109	1
Tbata.1	0.612509779	0.535	0.311	6.3246E-108	1
Npm1	0.406053395	0.778	0.627	5.215E-105	1
Cenpe	-0.922757813	0.137	0.307	5.6211E-105	1
Srebf1	0.583743725	0.528	0.305	1.1544E-104	1
Dtl.1	0.595804884	0.306	0.121	5.5396E-104	1
Rps5	0.250275697	0.985	0.949	1.9412E-103	1
Cdc6	0.480786527	0.157	0.031	1.0196E-102	1
Tpx2.1	-0.810123019	0.146	0.301	1.3372E-102	1
Siva1.1	0.556029844	0.456	0.245	5.0036E-99	1
Ccnd1	0.457553808	0.711	0.494	3.01921E-98	1
Anp32b.1	0.419390542	0.742	0.568	7.94243E-94	1
Nrxn1.1	-0.790381471	0.169	0.339	4.97835E-90	1
Prc1.1	-0.782229942	0.14	0.273	3.48287E-89	1
Clspn.1	0.568617055	0.354	0.165	1.5376E-88	1
Stmn4.1	-0.794907189	0.184	0.364	3.74794E-88	1
Ptn.1	-0.801824288	0.297	0.462	2.18854E-87	1
Apoe.1	-1.612921088	0.229	0.252	2.24625E-86	1
Kif23.1	-0.726010113	0.091	0.232	2.71485E-85	1
Paics.1	0.474066004	0.567	0.39	5.90238E-85	1
Cdc20	-0.775633251	0.072	0.214	1.55974E-84	1
Mcm7.1	0.47052139	0.551	0.365	3.74143E-83	1

Ccnb2	-0.68479909	0.059	0.205	8.05967E-83	1
Nrep.1	-0.547798093	0.463	0.584	5.5674E-80	1
Tubb2a.1	-0.74069734	0.103	0.265	6.41697E-80	1
Hmmr	-0.726174806	0.06	0.193	6.72759E-79	1
Rpl8	0.257434394	0.953	0.889	7.61745E-79	1
Ddah2	-0.461110152	0.582	0.701	7.97055E-79	1
Serbp1.1	0.274336774	0.922	0.861	9.0884E-79	1
Eef1b2	0.336230138	0.841	0.716	1.50221E-78	1
Tcf19.1	0.449635598	0.225	0.079	3.53611E-78	1
Rplp2	0.281808994	0.912	0.829	5.03011E-78	1
Celf4.1	-0.787111383	0.196	0.338	6.88465E-78	1
Nusap1.1	-0.705449056	0.104	0.245	8.10257E-78	1
Dpysl3.1	-0.744842005	0.088	0.238	8.34235E-78	1
H3f3b	-0.302182572	0.861	0.913	2.47695E-77	1
Ncl.1	0.265767974	0.934	0.89	4.6438E-76	1
Zic1.1	-0.397503753	0.713	0.794	1.40869E-75	1
Sfrp1	0.293831323	0.909	0.797	3.56586E-75	1
Hat1.1	0.498086312	0.295	0.134	2.17705E-74	1
Nop58.1	0.386330046	0.724	0.572	1.06949E-73	1
Cltb.1	0.478152903	0.569	0.401	4.20936E-73	1
Ccnb1	-0.596305044	0.038	0.154	7.38288E-73	1
Rps20	0.292875125	0.882	0.763	1.43498E-72	1
Mtss1.1	-0.671322565	0.13	0.289	3.06008E-72	1
Tubb2b.1	-0.56211671	0.331	0.483	1.20242E-71	1
H2afx.1	-0.514291652	0.281	0.337	3.10722E-71	1
Cdca3.1	-0.623044627	0.104	0.238	3.81218E-71	1
Prim1.1	0.469759782	0.424	0.239	7.79482E-71	1
Tyms.1	0.471121276	0.372	0.196	3.85627E-69	1
Mapt.1	-0.713541862	0.094	0.234	4.27111E-69	1
Rps21	0.278065133	0.901	0.823	8.39251E-69	1
Ckb.1	-0.381497196	0.771	0.813	1.16936E-68	1
Gng3.1	-0.651100314	0.187	0.335	2.49119E-68	1
Ckap2l.1	-0.622761805	0.106	0.233	6.39547E-67	1
E2f1.1	0.496421414	0.275	0.127	2.79294E-66	1
Rps19	0.30008233	0.851	0.739	1.84938E-65	1
Ccne2.1	0.415776511	0.171	0.055	2.79346E-65	1
Calm1.1	-0.343858044	0.799	0.87	6.89947E-65	1
St18.1	-0.657230576	0.085	0.205	4.55343E-64	1
Chaf1a.1	0.492460176	0.297	0.149	4.87236E-64	1
Hn1	-0.455090801	0.404	0.561	1.93797E-63	1
Top2a.1	-0.379096276	0.442	0.425	2.17389E-63	1
Timeless.1	0.482689341	0.28	0.135	4.84322E-63	1
Dscc1.1	0.376627613	0.146	0.042	7.15034E-63	1
Fam111a.1	0.490762734	0.247	0.108	9.0791E-63	1
Hsd11b2.1	0.478864973	0.437	0.265	1.64326E-62	1
Nhlh1.1	-0.634344359	0.109	0.239	2.19784E-62	1
Rfc3.1	0.456401942	0.344	0.183	2.45617E-62	1
Elavl4.1	-0.597084236	0.198	0.355	2.70307E-62	1

Cdk5r1.1	-0.580170831	0.196	0.353	2.48479E-61	1
Malat1.1	-0.37639112	0.937	0.957	7.36995E-61	1
Elmo1.1	-0.625580258	0.034	0.139	9.4015E-61	1
Zfp367.1	0.401023578	0.201	0.077	3.4332E-60	1
Gmnn.1	0.44839844	0.269	0.126	2.28028E-59	1
Tex14.1	-0.616072444	0.043	0.16	3.82421E-59	1
Birc5.1	-0.549391243	0.183	0.286	1.17098E-58	1
Mki67.1	-0.493074264	0.353	0.4	1.35804E-58	1
Dctpp1.1	0.430443513	0.444	0.283	1.8319E-58	1
Dnajc9.1	0.427976483	0.494	0.328	1.51727E-57	1
Slfn9.1	0.413442499	0.169	0.059	6.41285E-57	1
Nap1l1.1	0.364546712	0.627	0.493	7.71868E-57	1
Cbx5.1	0.313144287	0.764	0.636	3.48849E-56	1
Ccng2	-0.488647378	0.092	0.229	3.70111E-56	1
Dner.1	-0.574691071	0.1	0.231	7.49745E-56	1
Tipin.1	0.442859198	0.391	0.234	1.09493E-55	1
Nhlh2.1	-0.544226273	0.3	0.44	4.91634E-55	1
Spc25.1	-0.558327124	0.129	0.239	5.82203E-55	1
Rpl14	0.295113124	0.781	0.684	1.07843E-54	1
Serinc1.1	-0.470396732	0.3	0.456	1.83928E-54	1
Rfc2.1	0.439485679	0.341	0.191	2.34241E-54	1
Rpl41	0.262362335	0.859	0.769	3.05968E-54	1
Ppp1r14c.1	-0.586086223	0.163	0.276	5.90992E-54	1
Rpa1.1	0.415881518	0.215	0.093	1.39955E-53	1
Pdzrn3.1	-0.61192271	0.052	0.161	1.94121E-53	1
Mllt11.1	-0.577110268	0.107	0.234	5.32189E-53	1
Atad2.1	0.4083513	0.331	0.178	1.15393E-52	1
Aspm	-0.537860848	0.056	0.155	1.22098E-51	1
Dnmt1.1	0.416780439	0.415	0.257	1.79681E-51	1
Nhp2	0.394189262	0.502	0.351	2.32952E-51	1
Fam64a	-0.430020211	0.032	0.13	6.79351E-51	1
Cdk1.1	-0.474448158	0.185	0.255	1.68456E-50	1
Rab3a.1	-0.549283636	0.055	0.163	3.48942E-50	1
Nolc1	0.399530787	0.531	0.39	4.05273E-50	1
Sept3.1	-0.502370035	0.305	0.405	4.19494E-50	1
Gria2.1	-0.465491921	0.425	0.535	5.9237E-50	1
Rif1.1	0.426460187	0.406	0.256	1.44173E-49	1
Snhg1	0.399845143	0.485	0.338	2.72089E-49	1
Apc.1	-0.487976505	0.305	0.449	4.83406E-49	1
Cdc45.1	0.363404968	0.193	0.08	7.80359E-49	1
Cst3.1	-0.553874866	0.485	0.543	3.97577E-48	1
Cadm3.1	-0.531547499	0.055	0.153	1.19509E-47	1
Celf2.1	-0.393218518	0.549	0.65	1.34001E-47	1
Nop56.1	0.373417957	0.499	0.34	1.67516E-47	1
Alkbh2	0.288712411	0.104	0.028	1.95289E-47	1
Tagln3.1	-0.515202142	0.179	0.311	6.63281E-47	1
Cnbp	0.258314815	0.807	0.729	1.28155E-46	1
Atad5.1	0.420678126	0.258	0.132	1.55849E-46	1

Trpc4ap.1	-0.553653336	0.104	0.211	3.02252E-46	1
Sema6a.1	-0.544716216	0.107	0.215	9.99877E-46	1
Mms22l	0.339271803	0.142	0.051	2.72938E-45	1
Syt11.1	-0.421759007	0.383	0.522	2.76655E-45	1
Ckap5.1	-0.460017494	0.161	0.28	3.76272E-45	1
Shmt1.1	0.379262923	0.196	0.089	1.12372E-44	1
Aplp1.1	-0.51589178	0.063	0.164	2.6683E-44	1
Rps3a1	0.278053036	0.761	0.653	3.42123E-44	1
Gins2.1	0.406308703	0.291	0.169	1.19591E-43	1
Wdr76.1	0.35759709	0.165	0.067	2.21877E-43	1
Mif	0.372227413	0.473	0.339	2.42809E-43	1
Casp8ap2.1	0.410496305	0.365	0.226	4.09309E-43	1
Rad21.1	-0.378678208	0.388	0.472	5.27691E-43	1
Mcm10.1	0.360150441	0.146	0.055	1.04649E-42	1
Myt1.1	-0.527221109	0.061	0.165	1.08641E-42	1
Cdkn2d.1	-0.430868827	0.066	0.17	6.29199E-42	1
Ptms.1	-0.389672617	0.364	0.489	9.47668E-42	1
Wdhd1.1	0.367346019	0.17	0.072	1.55167E-41	1
Sptbn1	-0.478145065	0.246	0.369	1.67631E-41	1
Prmt8	0.39108186	0.394	0.254	2.48294E-41	1
Dtymk.1	0.338715908	0.559	0.421	4.40414E-41	1
H1f0.1	-0.356213464	0.518	0.63	5.16533E-41	1
Sparcl1.1	-0.67342804	0.143	0.216	3.86096E-40	1
Map2.1	-0.417571501	0.395	0.505	3.9665E-40	1
Itsn1.1	-0.493143597	0.141	0.249	4.06253E-40	1
Eif3a	0.270748008	0.76	0.678	5.65331E-40	1
Ssrp1.1	0.291833295	0.677	0.561	2.99103E-39	1
H2afy.1	0.284737614	0.69	0.577	4.14453E-39	1
Basp1.1	-0.306974145	0.646	0.712	5.76939E-39	1
Rad51.1	0.348522714	0.211	0.102	7.9237E-39	1
Meg3.1	-0.781410695	0.033	0.104	8.3641E-39	1
Fen1.1	0.371982281	0.237	0.127	1.08499E-38	1
Pola2.1	0.354733191	0.177	0.08	1.274E-38	1
Slc29a1	0.307479785	0.672	0.545	1.66462E-38	1
Hey1	0.387421616	0.391	0.255	1.18389E-37	1
Hist3h2a.1	-0.443278065	0.06	0.155	1.27288E-37	1
Cenph.1	0.370287445	0.304	0.177	1.74261E-37	1
Ccm2	0.388819691	0.333	0.208	1.86377E-37	1
Cdca8.1	-0.449274763	0.183	0.276	2.33766E-37	1
Fam210b	0.354301458	0.437	0.295	2.61757E-37	1
Arhgap11a.1	-0.455358842	0.087	0.182	3.7194E-37	1
RP23-45G16.5.1	-0.422819799	0.249	0.338	3.8211E-37	1
Gpm6b.1	-0.388498531	0.376	0.499	4.03317E-37	1
Tnik.1	-0.460777147	0.063	0.158	4.43552E-37	1
Srm	0.368832923	0.297	0.176	5.9435E-37	1
Arpp21.1	-0.511420408	0.045	0.122	1.00466E-36	1
Hspd1	0.338359437	0.465	0.349	3.9832E-36	1
2810417H13Rik.1	0.32205698	0.512	0.373	1.30418E-35	1

Sfrs18.1	-0.27796889	0.722	0.785	1.67507E-35	1
Rfc4.1	0.350636376	0.338	0.21	2.13317E-35	1
Ctsb.1	-0.583440281	0.209	0.27	2.23836E-35	1
Thra.1	-0.480197518	0.139	0.241	3.17433E-35	1
Tsc22d1.1	-0.363119835	0.313	0.444	6.03851E-35	1
Mis18bp1.1	-0.43879559	0.085	0.171	6.55666E-35	1
Baz1a	0.394753348	0.26	0.152	8.88763E-35	1
Rrm2.1	0.311852006	0.313	0.187	1.14502E-34	1
Dcx.1	-0.429073623	0.28	0.389	1.6455E-34	1
Kif11.1	-0.437561588	0.122	0.218	2.69724E-34	1
Rfc1.1	0.341460855	0.449	0.313	2.91516E-34	1
Ipo5.1	0.337987095	0.232	0.124	5.92667E-34	1
Chgb.1	-0.513867505	0.17	0.251	6.1127E-34	1
Kdm5b.1	-0.4413931	0.096	0.197	1.68459E-33	1
Pold1.1	0.316788668	0.162	0.073	2.22481E-33	1
Ina.1	-0.37765266	0.376	0.453	2.47329E-33	1
Apitd1.1	0.339565033	0.174	0.083	2.92287E-33	1
Hnrnpd.1	0.253599379	0.683	0.599	3.8639E-33	1
Chd3.1	-0.458809284	0.068	0.161	8.31396E-33	1
Sox9	0.360141293	0.363	0.239	1.00131E-32	1
D430041D05Rik	0.304432011	0.603	0.475	1.04352E-32	1
Msh6	0.328484079	0.166	0.077	1.05517E-32	1
Gnl3	0.345612628	0.407	0.279	1.20891E-32	1
Rrm1.1	0.327740173	0.373	0.243	1.65681E-32	1
A930011O12Rik.1	-0.466838612	0.046	0.124	3.53487E-32	1
D4Wsu53e.1	-0.365516677	0.339	0.462	3.5992E-32	1
Nop10	0.298610192	0.573	0.468	4.21661E-32	1
Cpe.1	-0.366284676	0.353	0.47	7.32727E-32	1
Chek1	0.304940631	0.144	0.062	9.84852E-32	1
Snrpd1.1	0.279972416	0.616	0.51	1.37252E-31	1
B3galt2.1	-0.417361018	0.033	0.107	4.7108E-31	1
Rbp4.1	0.375087971	0.341	0.227	6.96258E-31	1
Dync1i2	-0.310473497	0.468	0.582	7.70593E-31	1
Mdk	0.34339657	0.488	0.363	8.42362E-31	1
Cbfa2t3	0.318231714	0.515	0.386	1.22159E-30	1
Srsf7.1	0.289623502	0.557	0.451	1.22176E-30	1
Ank2.1	-0.471193132	0.157	0.252	1.27234E-30	1
Dkc1	0.328081692	0.44	0.317	2.08811E-30	1
Knstrn	-0.409560207	0.108	0.196	2.50087E-30	1
Rtn4.1	-0.336611639	0.379	0.495	3.9397E-30	1
Usp1.1	0.315533747	0.451	0.322	4.12438E-30	1
Myt1l.1	-0.472639416	0.089	0.171	4.52974E-30	1
Kcnk1.1	-0.431070733	0.119	0.216	6.23909E-30	1
Ankrd12.1	-0.468195347	0.188	0.287	7.1251E-30	1
Calm3	-0.288249209	0.462	0.574	7.12919E-30	1
Ubb	-0.307087175	0.448	0.555	8.14564E-30	1
Aurka.1	-0.306239038	0.036	0.107	1.71522E-29	1
Pkm	0.291393377	0.518	0.416	1.79554E-29	1

Msh2	0.287955535	0.233	0.13	3.07372E-29	1
4930422G04Rik.1	0.317026884	0.142	0.064	7.79671E-29	1
Idh2.1	0.326443776	0.375	0.264	7.96323E-29	1
Cdca7l.1	0.313894791	0.179	0.093	9.68022E-29	1
Tacc3.1	-0.378659737	0.127	0.218	1.02086E-28	1
Mthfd1	0.305598429	0.198	0.104	1.13861E-28	1
Syce2.1	0.295130801	0.154	0.072	1.29772E-28	1
Pdgfa.1	0.369696203	0.348	0.24	2.12154E-28	1
Btbd17.1	-0.381824691	0.081	0.171	2.48061E-28	1
Ppat	0.314682509	0.187	0.096	3.39551E-28	1
A330076H08Rik.1	-0.421036068	0.085	0.172	3.99162E-28	1
Rad54l.1	0.314827655	0.156	0.082	4.39964E-28	1
Gli1.1	0.337731774	0.175	0.097	5.85706E-28	1
Slc25a5.1	0.269868011	0.594	0.496	9.12235E-28	1
Supt16.1	0.278102189	0.577	0.48	1.04691E-27	1
Gm17322	0.31999352	0.216	0.118	1.12716E-27	1
Sgol2.1	-0.399370121	0.089	0.166	1.4681E-27	1
Incenp.1	-0.368935111	0.222	0.282	1.5813E-27	1
Baz1b.1	0.280788911	0.588	0.479	1.89239E-27	1
Rnf168.1	0.315693862	0.306	0.193	3.02213E-27	1
Prdx4.1	0.305806078	0.464	0.351	9.62903E-27	1
Pa2g4.1	0.254510911	0.613	0.509	1.30041E-26	1
Brd8	-0.333542387	0.295	0.406	2.13629E-26	1
Rps25	0.271749954	0.573	0.466	2.43632E-26	1
Rnaseh2a.1	0.297586608	0.24	0.14	3.40492E-26	1
Bola2	0.297719459	0.486	0.384	4.10222E-26	1
Gsr	0.269471711	0.16	0.08	4.95978E-26	1
Pnmal2.1	-0.350008124	0.071	0.157	5.7372E-26	1
Fabp7.1	-0.902665375	0.089	0.128	8.7779E-26	1
1110038B12Rik	0.310258081	0.391	0.285	1.48132E-25	1
Prnp.1	-0.358995108	0.12	0.219	2.05415E-25	1
Rpa3.1	0.316601862	0.336	0.23	2.75296E-25	1
Cdkn1b	-0.300029355	0.38	0.479	4.78721E-25	1
Pax6.1	-0.314991685	0.43	0.525	5.64743E-25	1
C1qbp	0.290124094	0.467	0.364	8.09327E-25	1
Rprml	0.311200404	0.147	0.071	1.54344E-24	1
Klf6	-0.356879114	0.077	0.158	1.96558E-24	1
Psmc3ip.1	0.302756183	0.208	0.12	3.82927E-24	1
Dlgap5	-0.308193115	0.053	0.12	5.5247E-24	1
Bok.1	0.30844078	0.343	0.237	8.11116E-24	1
Rad51ap1.1	0.291757018	0.253	0.154	1.1008E-23	1
Racgap1.1	-0.315123608	0.199	0.263	1.22554E-23	1
Topbp1.1	0.290595936	0.227	0.133	1.25609E-23	1
Elavl3.1	-0.323928497	0.4	0.483	1.39058E-23	1
Kif2c	-0.304074472	0.049	0.115	1.66902E-23	1
Dtx1	0.311087491	0.245	0.154	2.13342E-23	1
Akirin2	-0.311169825	0.119	0.208	2.16295E-23	1
Sept4.1	-0.414360013	0.146	0.238	2.18355E-23	1

Gins1.1	0.302233255	0.185	0.104	2.20552E-23	1
Prdm8.1	-0.388150014	0.064	0.135	3.02267E-23	1
Nsg2.1	-0.298861642	0.398	0.5	3.72996E-23	1
Kif22.1	-0.342930502	0.096	0.172	5.42653E-23	1
Kif5c.1	-0.336685245	0.328	0.408	5.52082E-23	1
Tead2	0.287301343	0.373	0.264	5.80627E-23	1
Brca2.1	0.319538773	0.184	0.103	6.15782E-23	1
Rnd3	0.264773587	0.528	0.417	6.97535E-23	1
Gpr180	0.284423175	0.143	0.071	8.31342E-23	1
Rangap1.1	-0.326751772	0.174	0.268	8.73965E-23	1
Mbnl2.1	-0.350611751	0.102	0.189	9.27204E-23	1
1700001O22Rik	0.2862688	0.18	0.099	9.49092E-23	1
Ctsd.1	-0.634394887	0.156	0.178	9.93537E-23	1
Rbfox2.1	-0.354319041	0.205	0.294	1.54958E-22	1
Abhd16a.1	-0.357556773	0.114	0.2	1.62926E-22	1
Boc	0.322327722	0.233	0.144	1.78237E-22	1
Mrpl18.1	0.283998122	0.44	0.341	2.31794E-22	1
Rab6b.1	-0.349480805	0.146	0.21	3.14956E-22	1
Rps2	0.259083072	0.522	0.438	3.50211E-22	1
Apex1	0.258911461	0.538	0.438	5.68815E-22	1
Polr2f	0.267996332	0.47	0.36	6.21234E-22	1
Lyar.1	0.312692983	0.369	0.273	6.33576E-22	1
Brca1.1	0.264701651	0.139	0.068	8.6852E-22	1
Ccna2.1	-0.316721017	0.171	0.229	1.78186E-21	1
Stxbp1.1	-0.369167927	0.096	0.172	1.85137E-21	1
Uncx.1	-0.343321122	0.236	0.329	1.99567E-21	1
Gdi1.1	-0.33828725	0.155	0.24	2.31739E-21	1
Ablim2	0.294325918	0.131	0.067	3.73651E-21	1
Kif5a.1	-0.354918091	0.067	0.137	4.34161E-21	1
Nup85.1	0.2793483	0.314	0.212	6.19124E-21	1
Id2.1	-0.327861922	0.415	0.496	7.01731E-21	1
Cacng4.1	-0.432753664	0.059	0.108	9.20842E-21	1
Sox4.1	-0.28539515	0.516	0.592	1.04595E-20	1
Cenpk.1	0.293104248	0.22	0.133	1.37616E-20	1
Atp6v0b.1	-0.332110188	0.192	0.278	3.14288E-20	1
Smc6.1	0.256804416	0.509	0.409	4.04057E-20	1
Grina.1	-0.313893399	0.056	0.117	4.27715E-20	1
Pea15a.1	-0.35912259	0.115	0.184	6.56546E-20	1
Fxyd6	-0.276578698	0.451	0.517	8.13107E-20	1
Lap3.1	0.271963853	0.369	0.262	8.49828E-20	1
Kif1b.1	-0.279091058	0.449	0.53	1.08949E-19	1
Pold3.1	0.273219023	0.224	0.137	1.26126E-19	1
Ptch2	0.294379943	0.225	0.141	1.65318E-19	1
Gsg1l	0.284255003	0.335	0.243	3.18862E-19	1
Odf2	-0.271765211	0.101	0.18	3.30209E-19	1
Set.1	0.255965546	0.47	0.375	1.09834E-18	1
Taf1d	0.269459295	0.425	0.329	1.10603E-18	1
Rbfox3.1	-0.26442715	0.459	0.499	1.32483E-18	1

Gng2.1	-0.283850183	0.322	0.406	1.77262E-18	1
Mab21l1.1	-0.322909561	0.155	0.235	1.77402E-18	1
Dbn1.1	-0.320408651	0.076	0.148	1.91558E-18	1
Casc5.1	-0.326106873	0.123	0.195	3.33338E-18	1
Mcmbp	0.282333705	0.29	0.205	4.41881E-18	1
Fnbp1l	-0.291877678	0.384	0.463	4.44893E-18	1
Ak2	0.263238292	0.289	0.202	4.92912E-18	1
Hist3h2ba.1	-0.305737898	0.08	0.145	6.26472E-18	1
Ank3.1	-0.298508965	0.367	0.427	7.04068E-18	1
Uchl1.1	-0.299260104	0.276	0.343	8.92178E-18	1
Snhg4	0.266004816	0.148	0.08	1.43703E-17	1
Klf7.1	-0.322349416	0.196	0.271	1.79415E-17	1
Nuf2.1	-0.292884367	0.087	0.148	1.92148E-17	1
Eef1d	0.254169987	0.423	0.339	2.06072E-17	1
Prkcb.1	-0.309163859	0.183	0.266	2.14102E-17	1
Srrm4.1	-0.32768182	0.132	0.204	2.72041E-17	1
Ldha	0.27166248	0.306	0.22	2.86377E-17	1
Vim.1	-0.343306921	0.233	0.312	3.46717E-17	1
Gar1	0.259706759	0.286	0.197	3.58209E-17	1
BC005764.1	-0.334853839	0.14	0.192	3.7949E-17	1
Trp53	0.255401761	0.379	0.286	4.39189E-17	1
Gnao1.1	-0.325073188	0.213	0.298	5.34769E-17	1
Fos	-0.412755716	0.245	0.313	1.38512E-16	1
Gdpd1	-0.289347814	0.172	0.253	1.51345E-16	1
Mxd4.1	-0.293603326	0.127	0.208	2.48036E-16	1
Tspyl4.1	-0.302629686	0.076	0.134	2.66591E-16	1
Nmral1.1	0.261457587	0.259	0.176	4.87683E-16	1
Pcdha2.1	-0.321902459	0.069	0.125	4.93466E-16	1
Rnf5	-0.269256707	0.181	0.269	6.4411E-16	1
Podxl2.1	-0.316891819	0.173	0.235	7.13725E-16	1
Ntrk3	0.252543999	0.224	0.144	7.16765E-16	1
Rbm5	-0.275710894	0.321	0.413	7.84669E-16	1
Rab6a.1	-0.268129796	0.263	0.337	8.75696E-16	1
Gart	0.252323928	0.233	0.156	9.84825E-16	1
Rcc2	0.263374452	0.246	0.168	1.13852E-15	1
Mrpl13	0.251277343	0.386	0.296	1.33726E-15	1
Rufy3.1	-0.283032047	0.303	0.381	1.84008E-15	1
Npdc1.1	-0.295870015	0.196	0.271	2.13999E-15	1
Zbtb18.1	-0.302190761	0.205	0.275	2.24937E-15	1
Trmt6	0.258536739	0.246	0.164	2.253E-15	1
Kif20b.1	-0.324936485	0.134	0.198	2.68231E-15	1
Nexn	0.320987115	0.127	0.069	2.69296E-15	1
Idh1.1	-0.263534775	0.101	0.164	2.9648E-15	1
Fyn.1	-0.351043729	0.155	0.224	5.42329E-15	1
Pqlc1	0.25212738	0.373	0.286	7.50469E-15	1
Ddx21	0.252758491	0.401	0.31	1.08576E-14	1
Pde1c.1	-0.285460469	0.341	0.393	1.99952E-14	1
Arhgef2	-0.277352954	0.206	0.293	2.27535E-14	1

4930402H24Rik.1	-0.268437025	0.065	0.12	2.63325E-14	1
Bin1.1	-0.265142189	0.373	0.437	2.87436E-14	1
Soga3.1	-0.252880476	0.388	0.467	3.4338E-14	1
Afap1	-0.274971028	0.093	0.16	5.02448E-14	1
Gm11266.1	-0.307409034	0.089	0.151	7.29855E-14	1
Lrpap1.1	-0.275332963	0.066	0.124	9.1258E-14	1
Apbb1.1	-0.299309127	0.112	0.178	9.47092E-14	1
Scg5.1	-0.2681194	0.272	0.343	1.0545E-13	1
Akap8l.1	-0.255868252	0.098	0.166	1.40287E-13	1
Cep170	-0.267084566	0.244	0.314	1.4109E-13	1
Os9.1	-0.278579367	0.187	0.26	1.72507E-13	1
Atp6v1e1.1	-0.262702612	0.27	0.34	2.13725E-13	1
2900011O08Rik.1	-0.284633419	0.058	0.11	2.43188E-13	1
Hpcal1.1	-0.288262728	0.06	0.113	2.59338E-13	1
Clvs1.1	-0.275883228	0.086	0.135	3.39561E-13	1
Srgap2	-0.287187461	0.082	0.139	3.54419E-13	1
Clip3.1	-0.271558735	0.246	0.321	4.30992E-13	1
Rad50	0.258892349	0.23	0.156	1.23164E-12	1
Jhdm1d.1	-0.257367227	0.072	0.132	1.26235E-12	1
Pik3r3.1	-0.270742491	0.082	0.143	1.28832E-12	1
Klc1	-0.258726001	0.301	0.37	1.59791E-12	1
Clasp2	-0.266592205	0.114	0.175	1.76134E-12	1
Ppfia2.1	-0.303956334	0.066	0.118	3.13717E-12	1
Tubb4b	-0.255606974	0.189	0.247	3.95761E-12	1
Aplp2.1	-0.263421598	0.269	0.33	6.76286E-12	1
Pkia	-0.27885227	0.086	0.14	1.36908E-11	1
Chrna3.1	-0.287974284	0.074	0.124	1.87056E-11	1
Pttg1	-0.27677817	0.089	0.146	2.11553E-11	1
Gnaq	-0.269973619	0.228	0.292	3.76423E-11	1
Clmp.1	-0.270350902	0.204	0.247	4.15397E-11	1
S100a16.1	-0.302112624	0.052	0.101	5.42522E-11	1
Nrcam	-0.263934978	0.091	0.14	6.23241E-11	1
Kidins220.1	-0.272139042	0.092	0.146	7.58371E-11	1
Plcb1	-0.287966612	0.103	0.146	9.52097E-11	1
Slc1a3.1	-0.303149335	0.142	0.154	4.27428E-10	1
Grik2.1	-0.2545242	0.067	0.106	5.79031E-10	1
Gria4.1	-0.256178908	0.084	0.13	1.99668E-09	1
Tspan7	-0.264665172	0.07	0.11	3.43893E-09	1
Snap25.1	-0.262237979	0.231	0.289	9.55546E-09	1
Itm2c.1	-0.25020043	0.164	0.219	2.00221E-08	1
Slc17a6.1	-0.2509795	0.103	0.154	6.66405E-08	1
Plp1.1	-0.657087143	0.104	0.116	9.27606E-08	1
Lgmn.1	-0.268601423	0.087	0.124	1.82392E-07	1
Gm11223	-0.280245575	0.175	0.218	4.36799E-07	1
Sirt2.1	-0.257370666	0.122	0.16	2.01818E-06	1
Ube2c.1	2.077394928	0.91	0.175	0	2
Cenpf.2	1.845794758	0.937	0.278	0	2
Cenpa.1	1.759793074	0.88	0.16	0	2

Cdc20.1	1.735096474	0.796	0.109	0	2
Prc1.2	1.631979143	0.815	0.175	0	2
Hmmr.1	1.615911126	0.73	0.096	0	2
Cenpe.1	1.590859745	0.845	0.204	0	2
Ccnb1.1	1.529882996	0.652	0.064	0	2
Tpx2.2	1.521284208	0.867	0.196	0	2
Arl6ip1.1	1.495173262	0.929	0.494	0	2
Kif23.2	1.479491911	0.758	0.135	0	2
Aspm.1	1.438697113	0.609	0.074	0	2
Nusap1.2	1.418557099	0.762	0.149	0	2
H2afx.2	1.398171954	0.809	0.261	0	2
Ckap2l.2	1.377216497	0.749	0.14	0	2
Sgol2.2	1.331622967	0.622	0.089	0	2
Ccnb2.1	1.329280016	0.683	0.114	0	2
Mki67.2	1.322523213	0.912	0.319	0	2
Birc5.2	1.321926525	0.826	0.192	0	2
Cdk1.2	1.31999443	0.75	0.173	0	2
Top2a.2	1.271942779	0.91	0.357	0	2
Mis18bp1.2	1.255988042	0.616	0.094	0	2
Cdca3.2	1.254833396	0.724	0.148	0	2
Cdca8.2	1.235544027	0.783	0.189	0	2
Kif20b.2	1.226860746	0.636	0.125	0	2
Arhgap11a.2	1.217291233	0.614	0.105	0	2
Smc4.1	1.204550122	0.965	0.508	0	2
Knstrn.1	1.200918387	0.654	0.117	0	2
Hmgb2.1	1.198756723	0.912	0.325	0	2
Ccna2.2	1.186736314	0.706	0.151	0	2
Spc25.2	1.170581444	0.703	0.155	0	2
Incenp.2	1.170565771	0.755	0.205	0	2
RP23-45G16.5.2	1.170227544	0.817	0.255	0	2
Casc5.2	1.130773627	0.621	0.122	0	2
Kif11.2	1.11828089	0.653	0.14	0	2
Fam64a.1	1.102227914	0.512	0.06	0	2
Tacc3.2	1.087764185	0.653	0.141	0	2
Dlgap5.1	1.039831988	0.469	0.06	0	2
Kif2c.1	1.030335558	0.462	0.055	0	2
Tubb4b.1	1.025804633	0.654	0.179	0	2
Ckap2.1	1.024233709	0.552	0.106	0	2
Plk1	1.0215	0.444	0.039	0	2
Kif22.2	1.019807218	0.552	0.106	0	2
Smc2.1	0.984915991	0.944	0.487	0	2
Racgap1.2	0.951242596	0.669	0.195	0	2
Cks1b.1	0.946966304	0.77	0.282	0	2
Rad21.2	0.926602963	0.859	0.403	0	2
C330027C09Rik	0.925784173	0.471	0.084	0	2
Cdca2.1	0.922644027	0.473	0.078	0	2
Ckap5.2	0.917552525	0.655	0.208	0	2
Pbk.1	0.914020991	0.621	0.164	0	2

Psrc1	0.910370243	0.385	0.034	0	2
Sgol1.1	0.902354905	0.447	0.081	0	2
Trim59.1	0.898883416	0.529	0.117	0	2
Ncapg.1	0.895890199	0.55	0.129	0	2
Tuba1c	0.887756817	0.31	0.019	0	2
Kif15.1	0.885974479	0.532	0.129	0	2
Nuf2.2	0.884831147	0.47	0.092	0	2
Aurka.2	0.88137257	0.413	0.052	0	2
Aurkb.1	0.870005845	0.479	0.094	0	2
Dbf4.1	0.867908943	0.463	0.099	0	2
Cep110	0.864581222	0.486	0.116	0	2
Gas2l3	0.85474548	0.34	0.032	0	2
Bora	0.852928832	0.385	0.05	0	2
Ect2.1	0.834426472	0.394	0.054	0	2
Anln.1	0.832890397	0.395	0.062	0	2
Nucks1.1	0.820367347	0.951	0.62	0	2
Bub1.1	0.813168371	0.441	0.085	0	2
Cep55	0.80504183	0.348	0.042	0	2
Mxd3.1	0.803887471	0.429	0.082	0	2
Bub1b.1	0.803024678	0.393	0.06	0	2
Spc24.1	0.800404151	0.613	0.188	0	2
Kif20a.1	0.789211905	0.365	0.046	0	2
Cdc25c	0.789175307	0.332	0.033	0	2
Tmpo.1	0.773671885	0.831	0.397	0	2
Rangap1.2	0.769909645	0.615	0.203	0	2
Sapcd2	0.764274326	0.347	0.04	0	2
Anp32e.1	0.760932377	0.932	0.567	0	2
H2afv.1	0.746644785	0.967	0.693	0	2
Cks2	0.73847089	0.325	0.039	0	2
Pif1	0.733355085	0.281	0.019	0	2
2700094K13Rik.1	0.654532634	0.913	0.57	0	2
2810417H13Rik.2	0.492694028	0.777	0.335	0	2
Ndc80.1	0.76093728	0.396	0.073	0	2
Troap	0.661676412	0.27	0.023	0	2
Ccdc34.1	0.714943743	0.781	0.361	0	2
Hjurp.1	0.790812294	0.78	0.383	0	2
Cenpl.1	0.682859368	0.313	0.041	1.4265E-307	2
Ckb.2	-0.803541798	0.747	0.817	2.4836E-299	2
Nek2	0.668562319	0.272	0.027	2.6688E-299	2
Kif4.1	0.741057594	0.375	0.069	3.3393E-297	2
Ska2.1	0.796515766	0.526	0.157	1.3605E-291	2
Esco2.1	0.760418116	0.51	0.149	7.3153E-290	2
Pttg1.1	0.954421451	0.418	0.098	2.1545E-283	2
Nde1	0.732744895	0.386	0.08	1.8449E-278	2
Ska1.1	0.714225298	0.331	0.054	3.0872E-278	2
Mns1.1	0.752943178	0.514	0.158	5.7506E-275	2
Kif18a	0.658277192	0.287	0.038	2.8546E-272	2
G2e3	0.668655753	0.397	0.092	5.1467E-268	2

Bub3.1	0.771226772	0.646	0.273	2.3332E-266	2
Neurod1.2	-0.956097393	0.577	0.526	2.8805E-265	2
Spdl1.1	0.674010048	0.302	0.046	1.1404E-264	2
Arhgef39	0.526344384	0.192	0.01	1.4745E-263	2
Cep89	0.77236161	0.315	0.052	2.0562E-258	2
Mad2l1.1	0.684694134	0.382	0.085	1.2033E-257	2
D17H6S56E-5.1	0.690043397	0.387	0.089	2.2142E-256	2
Cdkn2d.2	0.685947076	0.431	0.117	1.6623E-251	2
Calm2.1	0.44577485	0.979	0.859	7.8513E-251	2
Ttk.1	0.674333663	0.317	0.056	4.2764E-249	2
Lmnb1.1	0.664974753	0.622	0.253	2.0287E-248	2
Tuba1b.1	0.596295281	0.798	0.435	5.7754E-241	2
Fbxo5.1	0.687167269	0.427	0.12	1.7218E-236	2
Hirip3.1	0.588933654	0.703	0.336	2.1337E-232	2
Cdc25b	0.597142631	0.279	0.047	3.9919E-231	2
Ncapd2.1	0.566342653	0.453	0.146	1.7981E-228	2
Cdkn3	0.537636694	0.202	0.018	2.7246E-227	2
Hsp90b1	0.564873918	0.891	0.662	3.4439E-225	2
Spag5.1	0.56351855	0.28	0.049	2.6572E-224	2
Ccdc18	0.69077445	0.348	0.081	1.0467E-223	2
Fzr1	0.585391786	0.376	0.101	6.5552E-219	2
Banf1.1	0.501407448	0.917	0.661	2.3996E-216	2
2700099C18Rik.1	0.590938248	0.284	0.054	1.0468E-210	2
Melk.1	0.584961305	0.303	0.064	1.2581E-209	2
Brd8.1	0.565471633	0.696	0.347	2.5382E-209	2
Cenpc1	0.616699225	0.401	0.119	6.1164E-208	2
Ccng2.1	0.50958005	0.47	0.174	9.8441E-204	2
Miip	0.488405432	0.239	0.04	1.3843E-203	2
Hn1.1	0.541083305	0.82	0.501	7.5221E-203	2
Hmgn2.1	0.563353881	0.534	0.215	5.6554E-202	2
Hdgf	0.538310714	0.813	0.491	8.8396E-201	2
Pnrc2	0.542678291	0.464	0.17	7.1779E-194	2
Ncaph.1	0.571267042	0.339	0.089	1.0044E-193	2
Prr11.1	0.520817667	0.226	0.034	1.475E-193	2
Kifc1	0.491377373	0.218	0.031	2.7615E-193	2
Cenpp.1	0.541078069	0.326	0.083	4.5111E-193	2
Nudcd2	0.555598393	0.486	0.185	1.5318E-192	2
Ezh2.1	0.491658028	0.869	0.572	3.4802E-192	2
Rtn1.2	-0.570429818	0.724	0.685	1.3994E-190	2
Cdk5rap2.1	0.509384886	0.422	0.148	4.8892E-190	2
Ankle1	0.48897471	0.212	0.029	8.8895E-189	2
Ska3	0.506909037	0.242	0.043	1.7078E-188	2
Nrep.2	-0.654831922	0.536	0.574	2.4517E-187	2
Hyls1	0.468565595	0.213	0.033	1.81E-183	2
Usp1.2	0.478838414	0.616	0.298	2.1178E-183	2
Cdkn2c.1	0.523311899	0.364	0.111	1.4336E-182	2
Cenpm.1	0.536543764	0.392	0.128	4.0651E-182	2
Fam83d	0.366849008	0.129	0.006	2.0651E-181	2

Ran.1	0.491825817	0.698	0.372	2.0743E-181	2
Kif14	0.466266603	0.183	0.021	7.9032E-180	2
Lmnb2	0.513927323	0.383	0.125	4.603E-179	2
Tubb5.1	0.338392548	0.985	0.904	6.2131E-179	2
Gm10075.1	0.476879933	0.789	0.474	2.4596E-178	2
Hmgn5.1	0.461448455	0.705	0.383	1.1775E-177	2
Dek.2	0.319238523	0.92	0.66	1.7362E-176	2
Apoe.2	-1.691395864	0.289	0.243	1.0685E-173	2
H1fx.1	0.552518634	0.501	0.207	1.6263E-173	2
Cenpq.1	0.542583854	0.384	0.127	8.0378E-173	2
Igfbpl1.1	-0.583376192	0.616	0.652	7.4083E-171	2
Sap30	0.531626543	0.349	0.107	7.0335E-170	2
Nasp.2	0.316268099	0.884	0.604	2.3582E-169	2
Espl1.1	0.472623032	0.225	0.041	1.1729E-168	2
H2afz.1	0.506460609	0.478	0.195	2.7489E-168	2
Gpm6a.2	-0.732867357	0.355	0.417	6.218E-168	2
Rbfox3.2	-0.613904089	0.454	0.499	1.8781E-166	2
Hnrnpa2b1	0.279103732	0.99	0.925	6.0031E-166	2
Shcbp1.1	0.509503952	0.246	0.052	1.2207E-162	2
Odf2.1	0.420469445	0.378	0.14	2.6426E-162	2
Ube2t.1	0.520467885	0.246	0.052	5.8041E-161	2
Myod1.1	0.605030898	0.348	0.111	3.7666E-159	2
Cep70	0.441657986	0.239	0.057	1.4564E-158	2
Anp32b.2	0.339544895	0.837	0.555	4.1759E-153	2
Cst3.2	-0.317934392	0.689	0.513	1.8994E-152	2
Cenph.2	0.347990455	0.394	0.164	4.1552E-152	2
Nup37	0.494818168	0.267	0.067	9.8741E-152	2
Cklf.1	0.45013342	0.369	0.135	2.709E-150	2
Ddx39	0.426992893	0.397	0.156	3.3521E-149	2
Hmgb3	0.390206336	0.544	0.271	8.9174E-149	2
Pdzrn4	0.411577172	0.334	0.118	1.9893E-148	2
Rtkn2	0.424326994	0.172	0.025	2.0475E-148	2
Diap3.1	0.497858831	0.286	0.08	7.7553E-148	2
Plk4.1	0.477280521	0.304	0.092	2.25E-147	2
Celf4.2	-0.771406001	0.276	0.326	4.892E-147	2
Cntn2.2	-1.122521966	0.115	0.285	4.9866E-147	2
Gtse1	0.374732787	0.153	0.019	4.5947E-146	2
Sept3.2	-0.668369569	0.334	0.4	9.8162E-146	2
Pmf1.1	0.464572215	0.338	0.114	2.0739E-145	2
Pqlc1.1	0.371158763	0.528	0.263	1.0872E-144	2
Rnaseh2c	0.415446254	0.629	0.345	4.4642E-144	2
Fkbp2	0.381547436	0.564	0.291	7.5337E-144	2
Stmn2.2	-0.769482479	0.492	0.561	2.1369E-143	2
Hmgb1.1	0.361701793	0.597	0.324	5.8565E-143	2
Lsm6.1	0.434083964	0.621	0.335	7.1425E-143	2
Pdgfa.2	0.318794987	0.464	0.223	2.7729E-142	2
Ccnf	0.376736717	0.163	0.025	2.3945E-141	2
Tubb3.2	-0.580578246	0.486	0.472	3.2154E-141	2

Cenpw.1	0.430521361	0.307	0.101	1.5714E-140	2
Gap43.2	-0.541744989	0.613	0.597	2.729E-139	2
Gria2.2	-0.581886677	0.484	0.526	3.3845E-138	2
Dtymk.2	0.299125642	0.671	0.405	3.4657E-136	2
Vbp1	0.357750856	0.547	0.286	4.1668E-136	2
Psat1.1	0.381790438	0.606	0.332	4.5302E-136	2
Cenpk.2	0.439433726	0.328	0.117	5.7052E-135	2
Ccdc77	0.41511984	0.219	0.052	9.9764E-135	2
Miat.1	-0.664691024	0.464	0.522	1.5425E-134	2
Ccnd1.1	0.365713009	0.764	0.487	2.2366E-134	2
Reep4	0.322644534	0.179	0.039	1.1467E-133	2
Elavl3.2	-0.546990774	0.427	0.479	4.9262E-133	2
Rrm1.2	0.326277759	0.466	0.23	8.4561E-133	2
Gas1	0.442550493	0.344	0.129	2.7015E-132	2
Rad51ap1.2	0.357834271	0.348	0.14	2.955E-132	2
Zc3h7a	0.350686711	0.405	0.182	8.1688E-132	2
Barhl1.1	-0.415479245	0.526	0.521	1.2612E-131	2
Hmgn1.1	0.368728427	0.843	0.589	5.9525E-131	2
Map2.2	-0.459647706	0.49	0.491	8.4976E-131	2
Klf6.1	0.411034693	0.328	0.122	9.093E-131	2
Ctcf.1	0.349192153	0.734	0.461	3.3185E-130	2
Ubald2	0.379281533	0.255	0.077	3.9619E-130	2
Ina.2	-0.559561715	0.402	0.449	5.6004E-130	2
Syt13	0.398626566	0.295	0.102	3.8582E-127	2
Itm2b.2	-0.318132364	0.711	0.632	1.5296E-126	2
Gpsm2	0.374206646	0.179	0.036	2.9977E-126	2
Pcna.2	-0.378633133	0.377	0.355	1.5164E-125	2
Cpe.2	-0.362585804	0.475	0.452	1.1164E-124	2
Kif1b.2	-0.424682162	0.518	0.52	2.2547E-124	2
Fam110a	0.2804288	0.182	0.049	4.9142E-124	2
App	-0.406737877	0.57	0.567	1.208E-123	2
Tubb2b.2	-0.325916852	0.515	0.456	1.2533E-122	2
Cenpt	0.314177274	0.224	0.069	3.9064E-122	2
Smtn	0.304991167	0.132	0.019	7.0544E-121	2
Akirin2.1	0.364821119	0.384	0.17	1.3203E-120	2
Cep57.1	0.358692784	0.459	0.228	2.3702E-120	2
Rbmx	0.299878806	0.522	0.285	7.5642E-120	2
Lmo4	0.402555105	0.558	0.303	1.7279E-119	2
Lig1.2	-0.336663313	0.389	0.342	2.6092E-119	2
Dcx.2	-0.453434786	0.372	0.376	3.5524E-119	2
Ing1	0.303784451	0.354	0.158	7.9995E-119	2
Ptprs.1	-0.576698381	0.37	0.428	1.795E-118	2
Cenpv	0.27019733	0.527	0.299	3.5685E-118	2
Rrm2.2	0.270593587	0.375	0.179	5.0665E-118	2
Terf1	0.383240388	0.213	0.057	1.0238E-117	2
Gsg1l.1	0.273856965	0.441	0.228	1.0251E-117	2
Cit	0.391173199	0.195	0.047	1.0806E-117	2
Tgif1	0.322457023	0.24	0.079	1.5457E-117	2

Lbr.1	0.311705338	0.373	0.17	2.2155E-117	2
Depdc1b	0.31044214	0.123	0.015	3.289E-117	2
Ralgps2	0.333663851	0.438	0.217	9.6922E-117	2
Bin1.2	-0.523966965	0.381	0.436	2.4661E-116	2
Cdca5.1	0.385070671	0.212	0.055	5.3514E-116	2
Nhlh2.2	-0.430421849	0.44	0.42	2.035E-115	2
Arhgap19	0.325281216	0.134	0.019	4.4107E-115	2
Lsm4	0.314637876	0.712	0.455	2.4317E-114	2
Cenpj.1	0.415520418	0.307	0.117	1.0454E-112	2
Ppp3ca	-0.449267716	0.369	0.379	1.2702E-112	2
Cenpn.1	0.383003451	0.198	0.049	2.7354E-112	2
Cdca4.1	0.397786789	0.265	0.088	8.3655E-112	2
Ppp2r5c	0.291148082	0.396	0.193	2.0534E-111	2
Tdp1	0.338983264	0.245	0.082	2.5218E-111	2
Ank3.2	-0.385100169	0.44	0.417	4.4236E-111	2
Sec11c	0.304001057	0.389	0.187	8.3194E-111	2
Dnph1.1	0.323801929	0.272	0.101	1.685E-110	2
Lsm3.1	0.283319977	0.561	0.329	1.7569E-110	2
Chek2	0.300771718	0.157	0.033	1.1826E-109	2
Pkp4	0.353030182	0.245	0.081	2.2704E-109	2
Chd7.1	-0.321272394	0.685	0.641	3.5305E-109	2
Frmd4b	0.304458303	0.273	0.106	4.0283E-109	2
Kif18b	0.298581111	0.127	0.018	1.0855E-108	2
Basp1.2	-0.336185462	0.741	0.698	3.8272E-108	2
Vps36	0.325507044	0.501	0.272	4.148E-108	2
Clic4.1	0.263676375	0.397	0.202	1.7628E-107	2
mt-Rnr2	-0.34336829	0.996	0.997	2.1076E-107	2
Prdx1.1	0.255559304	0.698	0.459	9.0412E-107	2
Stmn1	0.261073135	0.466	0.257	1.1024E-106	2
Utp3	0.311438109	0.512	0.285	1.5176E-106	2
Fam216a	0.319017502	0.335	0.149	4.0114E-106	2
Zfp704	0.283949635	0.438	0.231	4.4778E-106	2
Ncaph2.1	0.319950638	0.339	0.149	5.1917E-106	2
Neil3.1	0.366478717	0.216	0.063	9.0934E-106	2
Celf2.2	-0.304678405	0.694	0.629	9.78E-106	2
Ctnnb1	0.274453632	0.59	0.357	9.8188E-106	2
Bok.2	0.259065913	0.424	0.225	1.1516E-105	2
Exosc8.1	0.319061161	0.384	0.184	4.582E-105	2
Efs	0.29091247	0.265	0.104	6.2701E-105	2
Ypel3.1	-0.301650421	0.46	0.431	1.0399E-104	2
Suv39h2	0.342503707	0.282	0.109	1.0967E-103	2
Ubb.1	0.308341518	0.754	0.51	1.0996E-103	2
Ccdc41.1	0.326007792	0.438	0.226	1.3841E-103	2
Smc1a.1	0.350934823	0.827	0.601	2.9108E-103	2
Sae1.1	0.312743885	0.468	0.251	2.9252E-103	2
Ctsd.2	-0.770010724	0.19	0.173	2.2825E-102	2
Wapal	0.259006997	0.445	0.244	6.1259E-102	2
Cmc2.1	0.363494777	0.267	0.097	2.4206E-101	2

Ctsb.2	-0.575609358	0.28	0.259	4.3772E-101	2
Pcf11	0.315457567	0.339	0.155	8.2383E-101	2
Cplx2	0.26517353	0.702	0.465	1.102E-100	2
Rfc4.2	0.269399458	0.393	0.202	2.8209E-100	2
Nrxn1.2	-0.507640698	0.309	0.319	2.887E-100	2
Mrpl51	0.302196999	0.326	0.146	3.1701E-100	2
Mis18a.1	0.346071922	0.251	0.089	4.1025E-100	2
Tubb2a.2	-0.605554713	0.194	0.252	4.1468E-100	2
Hist1h2ak.1	0.440499841	0.302	0.12	2.1941E-99	2
Dynll1	0.321503865	0.794	0.565	2.929E-99	2
Sephs1	0.256300807	0.27	0.114	3.7082E-99	2
Apc.2	-0.330442824	0.463	0.426	9.9092E-99	2
Rnaseh2b	0.257899643	0.401	0.212	2.79739E-98	2
Vrk1.1	0.323418879	0.299	0.126	6.20984E-98	2
Stmn4.2	-0.559285767	0.317	0.344	6.79474E-98	2
Tmem138	0.251725516	0.161	0.044	8.52105E-98	2
Smco4.1	0.306145684	0.315	0.139	1.22312E-97	2
Rhno1.1	0.283537045	0.279	0.117	1.2275E-97	2
Cdk5r1.2	-0.431453165	0.323	0.334	2.60055E-97	2
Mapt.2	-0.44609225	0.217	0.216	2.88724E-97	2
Gnao1.2	-0.364616674	0.295	0.286	3.5128E-97	2
Med30.1	0.326683923	0.372	0.179	5.15775E-97	2
Uncx.2	-0.396982478	0.312	0.318	8.99707E-97	2
Eid1.1	-0.309023932	0.522	0.5	1.28657E-96	2
Nfix	-0.260448562	0.719	0.666	2.52437E-96	2
Ddx11	0.30222153	0.157	0.038	4.39478E-96	2
Kif5c.2	-0.450600714	0.375	0.402	5.31595E-96	2
Trip13.1	0.357948393	0.207	0.062	7.81927E-96	2
Rdm1	0.337630842	0.177	0.046	7.98171E-96	2
Serinc1.2	-0.257323093	0.48	0.43	8.31869E-96	2
Taf5	0.302232099	0.202	0.065	1.14501E-95	2
Dpysl3.2	-0.682883979	0.146	0.23	2.20921E-94	2
Tmed9	-0.273926111	0.458	0.429	3.49066E-94	2
Hpca.1	0.451276549	0.469	0.249	5.87493E-94	2
Asap1	0.278175107	0.331	0.158	1.24798E-93	2
Stil.1	0.291227958	0.153	0.037	1.29985E-93	2
Ccdc61	0.258081232	0.141	0.034	2.47291E-93	2
Zic4.1	-0.253881974	0.461	0.411	1.19043E-92	2
H1f0.2	0.256153277	0.807	0.588	1.52614E-92	2
Thra.2	-0.615342943	0.155	0.239	1.83838E-92	2
Tmsb4x.2	-0.315949477	0.976	0.944	2.60443E-92	2
Hp1bp3	0.289519697	0.779	0.551	2.94333E-92	2
Pcnt	0.312999231	0.327	0.152	4.03671E-92	2
Elavl4.2	-0.367436303	0.345	0.333	6.27689E-92	2
5830418K08Rik	0.297604848	0.315	0.146	1.34216E-91	2
Lgals1	0.347888084	0.402	0.203	1.96419E-91	2
Zwilch.1	0.331318464	0.214	0.07	2.04818E-91	2
Mcm6.2	-0.348337137	0.276	0.276	2.90575E-91	2

Chgb.2	-0.736568572	0.153	0.253	3.89164E-91	2
Phf17.1	0.327028073	0.216	0.073	6.00505E-91	2
CRE_RECOMBINASE.1	-0.467382708	0.797	0.764	6.57455E-91	2
D030056L22Rik	0.324550308	0.287	0.121	8.44376E-91	2
Zcwpw1	0.329024396	0.232	0.084	8.65849E-90	2
Kmt2e.1	-0.267014132	0.609	0.562	9.43696E-90	2
Aldoa.1	-0.580068074	0.169	0.27	2.03721E-89	2
Sox4.2	-0.305030832	0.625	0.576	2.37131E-89	2
Ccp110.1	0.254107688	0.411	0.224	2.93823E-89	2
Gm1673	-0.319277916	0.478	0.472	3.3446E-89	2
Aplp2.2	-0.307524379	0.34	0.32	9.34606E-89	2
Gng3.2	-0.505904453	0.279	0.321	1.03372E-88	2
Mdc1	0.327456979	0.213	0.072	1.25271E-88	2
D4Wsu53e.2	-0.3241121	0.459	0.444	2.23138E-88	2
Ppp1r14c.2	-0.484575633	0.239	0.265	2.35285E-88	2
Hk2	0.286754291	0.318	0.151	3.5574E-88	2
2810442I21Rik	0.329614889	0.162	0.041	6.17708E-88	2
Smarcc2	-0.301620708	0.378	0.36	6.79946E-88	2
Gsg2.1	0.326732087	0.15	0.035	1.21268E-87	2
Lhx1	-0.347512598	0.509	0.495	1.32897E-87	2
Nudt4	0.254315609	0.371	0.195	4.14824E-87	2
Iqgap3	0.281549422	0.124	0.024	9.51528E-87	2
Nmral1.2	0.253900282	0.333	0.166	2.07784E-86	2
Nsg1.1	-0.471448722	0.238	0.294	2.29786E-86	2
Cntln.1	0.313388365	0.321	0.15	3.42879E-86	2
Nin	0.273845647	0.281	0.126	3.80472E-86	2
Cacna2d1	-0.313530587	0.395	0.371	3.85042E-86	2
Calm3.1	0.256163556	0.752	0.532	4.48701E-86	2
Pde1c.2	-0.265674881	0.439	0.379	4.52788E-86	2
Nsl1.1	0.334415451	0.164	0.043	4.21031E-85	2
Dclk1	0.2553529	0.597	0.385	1.0103E-84	2
Foxm1.1	0.253436048	0.133	0.031	1.34337E-84	2
Slc1a2	-0.261181791	0.329	0.288	1.79435E-84	2
Ankrd12.2	-0.621224487	0.208	0.284	3.16683E-83	2
Clmp.2	-0.382124299	0.239	0.242	1.3511E-82	2
Snap25.2	-0.362825085	0.284	0.281	6.87045E-82	2
Rbm5.1	-0.250490416	0.435	0.396	8.74648E-82	2
Ift80	0.253145341	0.164	0.05	9.97296E-82	2
Tex30.1	0.264164871	0.263	0.116	1.98769E-81	2
Npdc1.2	-0.353148055	0.25	0.263	1.12852E-80	2
Cdc27	0.254124307	0.227	0.094	1.15982E-80	2
Gen1	0.270677405	0.124	0.026	2.78812E-80	2
Atp6v0b.2	-0.365178773	0.251	0.269	3.72383E-80	2
Nhlh1.2	-0.561642337	0.16	0.232	1.22837E-78	2
Cntrob	0.289959667	0.158	0.045	2.13281E-78	2
Sptbn1.1	-0.271660701	0.388	0.348	2.4281E-78	2
Mtss1.2	-0.377512183	0.27	0.269	3.60392E-78	2
Sema6a.2	-0.459399198	0.173	0.206	3.90336E-78	2

Eri2	0.270289677	0.14	0.037	5.33773E-78	2
1500012F01Rik.1	-0.300955106	0.41	0.396	5.53594E-78	2
Rnmt.1	-0.256282044	0.375	0.344	7.70424E-78	2
Uchl1.2	-0.320601673	0.34	0.333	3.68116E-77	2
6330403K07Rik.1	-0.481452073	0.188	0.25	3.79842E-77	2
Eme1	0.271093151	0.137	0.034	1.67481E-76	2
Ank2.2	-0.4865317	0.211	0.244	2.13502E-76	2
Mum1l1	0.298383229	0.209	0.077	3.55879E-76	2
Ttc3.1	-0.283104708	0.854	0.825	1.42133E-75	2
Dner.2	-0.352883665	0.213	0.214	2.15114E-75	2
BC005764.2	-0.629505847	0.099	0.198	2.18673E-75	2
Slc22a17.1	-0.279505691	0.261	0.249	2.43696E-75	2
Csrp2.1	0.276690888	0.291	0.138	3.39671E-75	2
Zbtb18.2	-0.280538774	0.281	0.264	4.42058E-75	2
Pih1d1	0.264559239	0.304	0.15	9.21108E-75	2
Tbata.2	0.250612297	0.508	0.315	1.32965E-74	2
Ctsl.1	-0.546955917	0.201	0.268	2.23123E-74	2
Cenpi	0.251741817	0.118	0.026	2.8054E-74	2
Mis12.1	0.252662254	0.206	0.082	3.57932E-74	2
1500016L03Rik.1	-0.343195789	0.38	0.383	5.18633E-74	2
Cep135	0.253003826	0.198	0.077	1.40898E-73	2
Map1b.2	-0.272293624	0.646	0.578	7.28173E-73	2
Mllt11.2	-0.500191502	0.167	0.225	6.27124E-72	2
Tagln3.2	-0.310834701	0.304	0.293	9.12875E-71	2
Tk1.1	0.295847827	0.255	0.113	1.2005E-70	2
Oip5	0.262672524	0.123	0.029	1.51897E-70	2
Trpc4ap.2	-0.422170703	0.179	0.2	1.97154E-70	2
Map1lc3b.1	-0.280201455	0.335	0.33	2.20766E-70	2
Rundc3a.1	-0.338139484	0.237	0.25	1.68403E-69	2
Nbea	-0.300585173	0.199	0.189	8.22064E-69	2
Itsn1.2	-0.376387018	0.227	0.237	8.90517E-69	2
Rcor2.1	-0.412814883	0.184	0.23	2.05222E-68	2
Tbc1d31	0.256588764	0.152	0.047	5.04295E-68	2
Traip	0.253447781	0.129	0.034	7.71711E-68	2
Prkcb.2	-0.358488917	0.245	0.257	8.68001E-68	2
Nktr	-0.303773179	0.383	0.379	1.49427E-67	2
2810006K23Rik	0.255249913	0.237	0.106	3.34671E-67	2
Sept4.2	-0.450634105	0.199	0.23	1.9315E-66	2
Clcn4-2.1	-0.381474263	0.224	0.254	1.96226E-66	2
Atox1	-0.293963764	0.29	0.293	2.01117E-66	2
Ldhb.1	-0.339408339	0.26	0.283	2.55598E-66	2
Mycbp2	-0.277461045	0.362	0.342	1.04159E-65	2
Celsr2	-0.373856517	0.211	0.241	7.24245E-65	2
Pfn2	-0.287592105	0.242	0.241	1.18645E-64	2
Lap3.2	0.29228323	0.425	0.254	2.54112E-64	2
Itm2c.2	-0.360536607	0.192	0.214	1.25558E-63	2
Rab6b.2	-0.402022307	0.17	0.207	1.50342E-63	2
Tnik.2	-0.514553488	0.096	0.153	3.63871E-63	2

Igsf8.1	-0.377248486	0.239	0.273	1.08538E-62	2
Tmem57	-0.29096195	0.291	0.29	2.81321E-62	2
Pdzrn3.2	-0.637338689	0.067	0.159	3.49573E-62	2
Fabp7.2	-0.925548319	0.123	0.123	5.61498E-62	2
Aplp1.2	-0.527880272	0.084	0.161	6.0833E-62	2
C1ql1	-0.269109552	0.286	0.277	6.93869E-62	2
Gnl3.1	-0.294878355	0.293	0.296	8.14167E-62	2
Podxl2.2	-0.330693882	0.22	0.228	8.32213E-62	2
Mapk8ip1.1	-0.368153496	0.2	0.231	6.64534E-61	2
Cnrip1.1	-0.269039865	0.202	0.197	1.21123E-60	2
Hey1.1	-0.36273011	0.257	0.275	6.95669E-60	2
Stxbp1.2	-0.315387761	0.156	0.163	2.61474E-59	2
Cadm3.2	-0.503224875	0.083	0.149	6.01357E-59	2
Phf20l1	-0.293626698	0.345	0.339	1.492E-58	2
Ntm.1	-0.264174904	0.157	0.143	1.85166E-58	2
A330076H08Rik.2	-0.400590657	0.131	0.165	1.88397E-57	2
Psap.1	-0.339113857	0.171	0.186	1.31528E-56	2
Gdi1.2	-0.316436732	0.213	0.232	2.59981E-56	2
Ier2	-0.373973181	0.375	0.382	4.31727E-56	2
Klf7.2	-0.31465166	0.254	0.263	4.34552E-56	2
Dnajc5	-0.271074057	0.25	0.247	4.39673E-56	2
4631405J19Rik	0.265463377	0.124	0.036	9.30046E-56	2
Chrna3.2	-0.518858436	0.05	0.128	1.81549E-55	2
Sowaha	0.308589863	0.282	0.145	3.55746E-55	2
Tmem66.1	-0.253972942	0.199	0.199	4.59429E-55	2
Kdm5b.2	-0.32818838	0.169	0.187	2.13958E-54	2
Mxd4.2	-0.368884904	0.163	0.202	3.21229E-54	2
Slc1a3.2	-0.307488241	0.187	0.147	6.56085E-54	2
Myt1.2	-0.483250098	0.104	0.159	7.7693E-54	2
Hcfc1r1.1	-0.348427259	0.165	0.196	1.01721E-53	2
Apbb1.2	-0.43009219	0.123	0.176	1.82016E-53	2
Meg3.2	-0.846929002	0.034	0.104	3.01801E-53	2
Dlgap4.1	-0.340978673	0.161	0.191	8.09789E-53	2
2700089E24Rik	-0.332727786	0.169	0.202	1.18579E-52	2
Sh3gl2	-0.373630084	0.142	0.175	1.7116E-51	2
Clstn1	-0.298826156	0.195	0.203	8.22073E-51	2
Abhd16a.2	-0.310794261	0.176	0.191	1.08139E-50	2
Btg2.1	-0.265827334	0.219	0.206	2.77328E-50	2
Pygo1.1	-0.256575669	0.199	0.191	5.03368E-50	2
Aprt.1	-0.439135632	0.075	0.15	6.68223E-50	2
Reln.1	-0.274989022	0.185	0.179	2.15842E-49	2
Pdrg1.1	-0.297137202	0.203	0.221	3.21459E-49	2
Rrbp1	-0.25159275	0.195	0.187	3.58781E-49	2
Arpp21.2	-0.497323029	0.065	0.119	6.47957E-49	2
Nt5c.1	-0.380266107	0.139	0.19	1.93302E-48	2
Egr1	-0.320183282	0.405	0.381	5.05675E-48	2
Dbn1.2	-0.255096059	0.135	0.14	6.12059E-48	2
Kidins220.2	-0.386133791	0.106	0.144	7.22602E-48	2

Wdr6	-0.280159411	0.13	0.146	1.04695E-47	2
Prdm8.2	-0.29217676	0.121	0.127	2.34009E-47	2
Hells.2	-0.339153363	0.195	0.215	4.14413E-47	2
A930011O12Rik.2	-0.460986444	0.068	0.121	5.39305E-47	2
Rab3a.2	-0.505675117	0.079	0.16	5.51558E-47	2
Nrn1.1	-0.317730571	0.21	0.223	8.056E-47	2
Fam213b.1	-0.375381165	0.12	0.162	1.2156E-46	2
Ung.1	-0.411094834	0.023	0.102	1.25597E-46	2
Srrm4.2	-0.256109589	0.195	0.195	4.10566E-46	2
Elmo1.2	-0.441178578	0.09	0.131	4.32966E-46	2
Kif5a.2	-0.415865061	0.083	0.134	4.49392E-46	2
Zmynd8.1	-0.25580583	0.211	0.213	8.92632E-46	2
Fam21	-0.257592787	0.217	0.222	1.24934E-45	2
Col9a3	-0.266527443	0.175	0.18	1.41195E-45	2
Sh3bgrl3	-0.283938339	0.187	0.2	2.47262E-45	2
Plp1.2	-0.788980489	0.125	0.113	4.02479E-45	2
Pea15a.2	-0.337333489	0.156	0.178	1.14174E-44	2
Lgmn.2	-0.273403272	0.128	0.118	2.73518E-44	2
Gramd1b.1	-0.282956598	0.158	0.171	5.94109E-43	2
Mbp.1	-0.374094544	0.176	0.163	1.36602E-42	2
Glce.1	-0.28543565	0.165	0.178	1.96194E-42	2
Jhdm1d.2	-0.335540449	0.095	0.129	9.70945E-42	2
Kif1a.1	-0.270501267	0.16	0.173	1.43885E-41	2
Mcm2.2	-0.323175584	0.175	0.208	5.33482E-41	2
Cacng4.2	-0.381188535	0.09	0.103	7.83209E-40	2
Rnd2	-0.327084192	0.129	0.162	1.71246E-39	2
Cadm4	-0.293792315	0.085	0.118	7.13368E-39	2
Nrcam.1	-0.260923806	0.126	0.135	2.81862E-38	2
Pkia.1	-0.297923682	0.113	0.136	4.1164E-38	2
Ctsf.1	-0.39403115	0.042	0.106	1.80176E-37	2
Gabbr1.1	-0.320489797	0.111	0.144	1.95255E-37	2
Gria4.2	-0.319250546	0.1	0.128	5.16905E-37	2
Cnpy1	-0.282242621	0.112	0.135	8.40859E-37	2
Gm17322.1	-0.38549701	0.09	0.137	8.07493E-36	2
Ppfia2.2	-0.325908573	0.09	0.115	1.53457E-35	2
Chd3.2	-0.390243627	0.111	0.154	1.58316E-35	2
Cplx1	-0.319243565	0.112	0.142	2.07017E-35	2
Tacc2.1	-0.261500706	0.132	0.142	7.10417E-35	2
Ncan.1	-0.287130429	0.077	0.102	1.11958E-34	2
Slc17a6.2	-0.298947033	0.131	0.15	1.67076E-34	2
Gamt	-0.312384622	0.139	0.174	1.73799E-34	2
Akap12	-0.324733584	0.088	0.114	1.99848E-34	2
Mt1	-0.348154017	0.177	0.167	8.3683E-34	2
Hpcal1.2	-0.270477412	0.092	0.108	1.05184E-32	2
Malat1.2	-0.289141596	0.946	0.956	2.18341E-32	2
S100a16.2	-0.418816321	0.05	0.101	3.77408E-32	2
Sbk1	-0.252954013	0.105	0.12	4.8993E-32	2
Grina.2	-0.376791904	0.057	0.116	6.80047E-32	2

Ppp1r1a	-0.338676421	0.069	0.116	7.82643E-32	2
Sh3bp5.1	-0.303048816	0.077	0.107	2.50769E-31	2
Grik2.2	-0.300569945	0.079	0.105	2.95717E-31	2
B3galt2.2	-0.334171519	0.069	0.101	1.18236E-30	2
1500011B03Rik	-0.304886274	0.074	0.111	7.34239E-30	2
Cdt1.2	-0.341538601	0.073	0.132	1.38467E-29	2
Plcb1.1	-0.376740912	0.103	0.146	1.5645E-29	2
H2-D1	-0.296455623	0.102	0.134	2.6322E-29	2
Pmm1	-0.268457545	0.101	0.131	3.5777E-28	2
Nenf	-0.254441488	0.117	0.139	1.88954E-27	2
Fos.1	-0.285316023	0.311	0.303	2.16798E-25	2
Shd	-0.257820346	0.086	0.112	6.9155E-25	2
2900011O08Rik.2	-0.264581383	0.083	0.106	3.07602E-24	2
Xist	-0.388630333	0.244	0.307	9.07111E-23	2
Hes1.1	1.26392952	0.433	0.107	0	3
Tubb5.2	-0.737587596	0.818	0.928	0	3
Stmn2.3	-1.616189432	0.207	0.603	0	3
Neurod1.3	-1.857252384	0.203	0.58	0	3
Tuba1a.2	-0.766515807	0.851	0.94	0	3
Tubb3.3	-1.386482202	0.173	0.517	5.5908E-296	3
Rpl13a	0.438501442	0.988	0.919	2.4088E-264	3
Top2a.3	-1.392937409	0.179	0.463	2.2012E-249	3
Rps5.1	0.395649464	0.988	0.949	4.0054E-246	3
Rps14	0.405247357	0.989	0.944	1.5745E-240	3
Rps9	0.390640562	0.984	0.921	5.1156E-223	3
Gnb2l1	0.431085182	0.95	0.86	1.6377E-201	3
Rpl32	0.404383786	0.964	0.878	1.0757E-198	3
Cntn2.3	-1.334874255	0.046	0.294	1.7329E-195	3
Mki67.3	-1.199680623	0.159	0.428	1.1308E-191	3
Egr1.1	0.801092876	0.635	0.348	4.1614E-189	3
Gap43.3	-0.922554037	0.375	0.631	1.5039E-188	3
Smc4.2	-0.923135418	0.373	0.594	7.8496E-187	3
Draxin	0.61791826	0.762	0.524	3.3643E-185	3
2810417H13Rik.3	-1.061173307	0.157	0.425	2.7397E-184	3
Map1b.3	-0.879781695	0.362	0.619	5.4537E-180	3
Gas5	0.50433982	0.869	0.723	1.1975E-178	3
Birc5.3	-1.018406406	0.057	0.304	2.611E-175	3
Rps3	0.339974741	0.977	0.914	4.04E-171	3
Tpx2.3	-1.085667605	0.069	0.312	3.3731E-170	3
Hmgb2.2	-0.943578765	0.177	0.432	3.9492E-169	3
Rpl8.1	0.361230252	0.963	0.888	2.784E-167	3
Cdk1.3	-0.981523803	0.044	0.276	7.374E-167	3
Rplp2.1	0.401663422	0.93	0.827	1.8294E-166	3
Stmn4.3	-1.064879611	0.12	0.373	1.4109E-165	3
Jun	0.668096064	0.809	0.632	2.8698E-163	3
Fxyd6.1	-0.793632586	0.27	0.543	1.2398E-161	3
Rplp1.1	0.361186522	0.963	0.864	3.857E-159	3
Ube2c.2	-1.227026729	0.082	0.296	3.16E-158	3

Rps19.1	0.44477897	0.878	0.735	5.8457E-157	3
Ddah2.1	-0.653262246	0.506	0.711	5.6163E-155	3
H2afx.3	-0.973110753	0.14	0.358	1.8059E-154	3
Rpl4	0.355836143	0.96	0.9	1.3055E-153	3
Miat.2	-0.861710156	0.28	0.549	1.9265E-153	3
Prc1.3	-1.051811541	0.066	0.284	8.7246E-152	3
Rtn1.3	-0.695214137	0.552	0.71	1.3467E-151	3
Nusap1.3	-0.960368693	0.04	0.254	5.6541E-151	3
Cenpf.3	-1.14058857	0.162	0.391	3.275E-148	3
Cdca8.3	-0.883174131	0.064	0.293	4.8636E-147	3
Spc25.3	-0.888258038	0.041	0.251	1.9449E-145	3
Rps26	0.383631211	0.936	0.834	1.1814E-143	3
Ccna2.3	-0.802512341	0.04	0.247	3.4155E-142	3
Ckap2l.3	-0.876048123	0.037	0.243	1.2999E-141	3
Rplp0	0.363394324	0.948	0.861	1.3537E-139	3
Incenp.3	-0.903554585	0.076	0.303	1.9675E-139	3
Rpl22	0.413612959	0.891	0.773	8.9161E-139	3
Pbk.2	-0.82668871	0.042	0.248	1.4698E-138	3
Ina.3	-0.790937773	0.235	0.473	3.7584E-138	3
Celf4.3	-1.014589285	0.124	0.348	1.9372E-137	3
Rps21.1	0.381768294	0.923	0.82	8.8197E-135	3
Gpm6a.3	-0.831291145	0.21	0.438	1.5771E-133	3
Eef1a1	0.356778701	0.951	0.874	1.3094E-132	3
Sept3.3	-0.815271719	0.198	0.42	1.7564E-132	3
Arl6ip1.2	-0.750742688	0.402	0.571	3.4849E-131	3
Tubb2b.3	-0.719026018	0.264	0.493	6.4105E-131	3
Esco2.2	-0.803901898	0.03	0.218	6.4375E-129	3
Rps20.1	0.39817779	0.891	0.761	5.4238E-127	3
Barhl1.2	0.540299094	0.663	0.501	1.4375E-123	3
Nrxn1.3	-0.887505993	0.13	0.345	2.5539E-123	3
Cbfa2t3.1	0.592626552	0.565	0.379	7.4721E-122	3
Cenpa.2	-0.978085546	0.087	0.275	9.1979E-121	3
Sparcl1.2	0.541343558	0.382	0.181	2.3142E-117	3
Vim.2	0.644309683	0.49	0.275	2.9378E-117	3
Kif23.3	-0.820168386	0.05	0.238	6.8563E-116	3
CRE_RECOMBINASE.2	0.574839264	0.867	0.753	1.1419E-114	3
Nhlh2.3	-0.752107957	0.225	0.451	1.8677E-114	3
Apoe.3	-1.227398664	0.297	0.242	3.9533E-114	3
Spc24.2	-0.716039604	0.067	0.267	6.865E-114	3
Kif11.3	-0.743333791	0.048	0.228	6.7398E-113	3
Cdca3.3	-0.773314818	0.059	0.245	2.6997E-111	3
Rps15	0.360145528	0.877	0.775	2.2558E-109	3
Ncapg.2	-0.670594878	0.035	0.204	5.4829E-109	3
Cdc20.2	-0.85881857	0.049	0.217	7.2138E-109	3
Tubb2a.3	-0.828051875	0.081	0.268	6.6689E-108	3
RP23-45G16.5.3	-0.74920411	0.144	0.353	8.6115E-107	3
Elavl3.3	-0.654936309	0.278	0.5	2.2211E-106	3
Smc2.2	-0.658394586	0.423	0.563	6.4134E-105	3

Ccnb1.2	-0.667816327	0.014	0.157	1.0595E-104	3
Ier2.1	0.635781152	0.545	0.358	2.0467E-104	3
Ppp1r14c.3	-0.779144878	0.099	0.285	5.7543E-103	3
Ccnd1.2	0.478165909	0.709	0.495	1.7573E-102	3
Btg2.2	0.684012032	0.342	0.189	4.6464E-102	3
Efhd2	0.643090713	0.256	0.111	2.6661E-101	3
Hmmr.2	-0.775307086	0.034	0.197	2.872E-101	3
Mfap4	0.684885158	0.307	0.14	4.1095E-101	3
Mmp14	0.607650702	0.326	0.152	5.0825E-99	3
Gpr153	0.617549157	0.345	0.188	5.8446E-99	3
Racgap1.3	-0.642789638	0.089	0.279	1.21176E-97	3
St18.2	-0.762213061	0.049	0.211	1.6198E-97	3
Mis18bp1.3	-0.626463344	0.029	0.179	2.24491E-96	3
Tacc3.3	-0.630988523	0.055	0.228	1.11836E-95	3
Calm2.2	-0.380901634	0.812	0.883	1.08495E-94	3
Rpl39	0.400294015	0.77	0.628	1.09049E-94	3
Mxd3.2	-0.528559288	0.013	0.143	1.7515E-92	3
Casc5.3	-0.64654032	0.045	0.206	2.76188E-92	3
Pabpc1	0.283337406	0.942	0.869	1.30931E-91	3
Arhgap11a.3	-0.630502467	0.037	0.189	5.33672E-91	3
Cdca2.2	-0.524496882	0.015	0.145	5.34005E-90	3
Rpl26	0.364339226	0.801	0.687	7.83619E-90	3
Nuf2.3	-0.552946068	0.022	0.158	1.04126E-88	3
Kif15.2	-0.614256082	0.044	0.199	2.66984E-88	3
Cenpe.2	-0.835674668	0.14	0.306	3.82039E-88	3
Rpl23	0.415378876	0.657	0.521	4.16424E-88	3
Sgol2.3	-0.632201217	0.03	0.175	5.47746E-88	3
Sdpr	0.687133473	0.204	0.07	1.03421E-86	3
Ezr	0.487836352	0.565	0.419	1.29522E-86	3
Rangap1.3	-0.597638363	0.099	0.278	1.45736E-86	3
Rad21.3	-0.582207132	0.296	0.485	1.30209E-85	3
Cbx5.2	0.401344348	0.778	0.635	1.43563E-85	3
Rrm2.3	-0.607850943	0.062	0.224	5.03647E-85	3
Tex14.2	-0.6991475	0.026	0.162	1.12963E-84	3
Kif22.3	-0.535745963	0.036	0.181	1.60951E-84	3
Nkd1	0.534681487	0.383	0.228	3.77426E-84	3
Hes6	0.534959614	0.366	0.217	4.70038E-84	3
Chgb.3	-0.730760293	0.093	0.261	7.95315E-84	3
Rps11	0.332497886	0.875	0.763	9.16598E-84	3
Tmsb10	-0.445247145	0.526	0.683	1.14023E-83	3
Gng3.3	-0.677524379	0.16	0.339	2.42152E-83	3
Fam64a.2	-0.496823641	0.013	0.132	1.88783E-82	3
Cenph.3	-0.560127112	0.057	0.213	2.74545E-82	3
Tead2.1	0.509831859	0.408	0.259	1.04936E-81	3
Rps15a	0.357708573	0.754	0.643	2.64795E-81	3
Bub1.2	-0.506937367	0.02	0.146	3.42036E-81	3
Sowaha.1	0.624069565	0.297	0.143	1.78384E-80	3
Fstl1	0.513115983	0.312	0.161	2.56133E-80	3

1500012F01Rik.2	0.460310908	0.529	0.379	3.1333E-80	3
Aspm.2	-0.615572137	0.028	0.159	5.8299E-80	3
Rps24	0.284845184	0.89	0.81	9.08995E-80	3
Tagln3.3	-0.596254957	0.145	0.316	2.95937E-79	3
Rpl41.1	0.318987812	0.872	0.767	1.08059E-78	3
Tuba1b.2	-0.527303167	0.343	0.501	1.33952E-78	3
Zmiz1	0.475274196	0.499	0.356	1.41786E-77	3
Aurkb.2	-0.509971673	0.029	0.159	1.96748E-77	3
Basp1.3	-0.445362148	0.59	0.72	2.56786E-77	3
Tk1.2	-0.509855304	0.023	0.147	4.21533E-77	3
Mllt11.3	-0.653767298	0.086	0.237	4.30872E-77	3
Rps18	0.427725548	0.546	0.411	6.55903E-77	3
Sptbn1.2	-0.576705276	0.195	0.376	7.49497E-77	3
Rad51ap1.3	-0.503264382	0.043	0.184	1.09651E-76	3
Sept4.3	-0.681223964	0.092	0.245	2.40301E-76	3
Rpl34	0.344244716	0.745	0.639	3.16423E-76	3
Sgol1.2	-0.470389086	0.021	0.143	4.7507E-76	3
Bin1.3	-0.536051508	0.273	0.452	7.1506E-76	3
Rps3a1.1	0.336320082	0.76	0.653	1.90977E-75	3
Kif20b.3	-0.623235173	0.06	0.209	6.44891E-75	3
Mns1.2	-0.565510489	0.067	0.223	1.04909E-74	3
Dek.3	-0.444908844	0.605	0.706	6.49312E-74	3
Uchl1.3	-0.548834606	0.189	0.355	1.20562E-73	3
Zfp36l1	0.543231661	0.261	0.134	1.35224E-73	3
Ndc80.2	-0.467417561	0.016	0.128	2.32842E-73	3
Atoh1	0.55264575	0.274	0.148	2.91359E-73	3
Dlgap5.2	-0.480292374	0.014	0.126	2.94124E-73	3
Slc17a6.3	-0.599827396	0.034	0.164	3.96107E-73	3
Cdk5r1.3	-0.587253265	0.184	0.355	1.22628E-72	3
Zeb1	0.448283323	0.502	0.37	1.69763E-72	3
2410006H16Rik	0.429329605	0.579	0.441	5.0886E-72	3
Podxl2.3	-0.577072678	0.095	0.246	5.3955E-72	3
Nucks1.2	-0.431768569	0.556	0.678	5.89467E-72	3
Ckap2.2	-0.516955531	0.044	0.18	2.29453E-71	3
Dner.3	-0.599547209	0.081	0.234	4.51847E-71	3
Calm1.2	-0.357508629	0.77	0.875	5.71017E-71	3
Fbxo5.2	-0.516279328	0.044	0.176	3.18724E-69	3
Slc1a2.1	0.49544371	0.398	0.278	1.99956E-68	3
Sox9.1	0.506914822	0.375	0.237	3.46586E-68	3
Rps2.1	0.375695905	0.559	0.433	6.92992E-68	3
Hn1.2	-0.453979985	0.392	0.563	2.16152E-67	3
Prkcb.3	-0.571355798	0.116	0.276	2.21415E-67	3
Map2.3	-0.472224265	0.347	0.512	1.92227E-66	3
Kif2c.2	-0.419244933	0.016	0.12	2.26097E-66	3
Ccnb2.2	-0.579909923	0.063	0.204	2.95092E-66	3
Dut.2	-0.52049592	0.293	0.455	2.99679E-66	3
Ctsd.3	-0.498570391	0.187	0.173	3.56159E-66	3
Sox4.3	0.428501962	0.668	0.57	5.34156E-66	3

Eno1	0.436781174	0.33	0.214	5.49153E-66	3
Gsg1l.2	0.458426623	0.368	0.238	6.55601E-66	3
Rps10	0.330461481	0.727	0.623	1.01493E-65	3
Ntrk3.1	0.496324866	0.248	0.141	2.36015E-65	3
Rpl14.1	0.30225255	0.784	0.684	2.96362E-65	3
Sema6a.3	-0.585897251	0.083	0.219	3.86466E-65	3
Sfrp1.1	0.320697051	0.902	0.798	3.91133E-65	3
Serpinh1.1	0.445506826	0.222	0.099	4.1572E-65	3
Rnd3.1	0.423473514	0.541	0.415	1.93966E-64	3
Rab3a.3	-0.598190771	0.042	0.165	2.38365E-64	3
Clspn.2	-0.524700287	0.065	0.207	3.56394E-64	3
Tyms.2	-0.52958384	0.093	0.237	4.43332E-64	3
Knstrn.2	-0.545580159	0.065	0.203	6.51959E-64	3
Npm1.1	0.326861729	0.753	0.63	7.68784E-64	3
Whsc1.1	-0.438483192	0.297	0.488	1.63101E-63	3
Tmpo.2	-0.486988814	0.313	0.472	1.76619E-63	3
Ckap5.3	-0.52214243	0.128	0.285	4.13656E-63	3
Ank3.3	-0.493226954	0.278	0.44	5.01535E-63	3
Rpl35a	0.328646138	0.667	0.556	6.15922E-63	3
Ska1.2	-0.36172402	0.009	0.101	8.94049E-63	3
Rufy3.2	-0.478351506	0.223	0.392	1.37914E-62	3
Cdkn2d.3	-0.462847791	0.047	0.173	1.68328E-62	3
Dbf4.2	-0.453444273	0.039	0.16	1.87406E-62	3
D430041D05Rik.1	0.405958612	0.609	0.475	2.27843E-62	3
Pdlim3	0.514309243	0.141	0.05	5.45759E-62	3
Kif4.2	-0.408673743	0.018	0.121	7.40039E-62	3
Fos.2	0.592766645	0.431	0.286	1.13273E-61	3
Kif5c.3	-0.498347371	0.25	0.42	2.6124E-61	3
Calm3.2	-0.399428817	0.403	0.583	6.93963E-61	3
Cxcr4	0.468053415	0.178	0.078	8.59664E-61	3
Plk1.1	-0.369878058	0.01	0.102	8.76281E-61	3
Apc.3	-0.480667987	0.285	0.452	9.88223E-61	3
Celf2.3	-0.398366996	0.537	0.652	1.14283E-60	3
Rps25.1	0.368032449	0.593	0.463	1.23817E-60	3
Anp32e.2	-0.402109399	0.53	0.625	1.66142E-60	3
Nnat	-0.447211751	0.604	0.725	1.72075E-60	3
Cenpq.2	-0.452905992	0.05	0.176	3.08485E-60	3
Id2.2	0.427119092	0.609	0.468	4.7552E-60	3
Pou3f2	0.450074114	0.375	0.261	7.46193E-60	3
Tgfb2.1	0.509587735	0.293	0.177	2.00856E-59	3
Ttk.2	-0.359554615	0.011	0.101	5.08718E-59	3
Eef1b2.1	0.299956227	0.811	0.721	7.86934E-59	3
C330027C09Rik.1	-0.446841619	0.034	0.147	1.96211E-58	3
Cdh20	0.439067037	0.301	0.188	2.08419E-58	3
Gas6	0.512095528	0.171	0.067	2.77325E-58	3
Hist1h2ak.2	-0.555286442	0.043	0.157	4.0892E-58	3
Anln.2	-0.384891408	0.019	0.116	4.11343E-58	3
Eif3f	0.282760663	0.706	0.619	5.23318E-58	3

Ect2.2	-0.35537416	0.015	0.109	7.72528E-58	3
D17H6S56E-5.2	-0.386959706	0.032	0.141	9.67411E-58	3
Srrm3	-0.526650691	0.111	0.258	1.12384E-57	3
Mtss1.3	-0.543127363	0.151	0.286	6.01486E-57	3
E130114P18Rik	0.37815545	0.691	0.569	8.96857E-57	3
Cenpm.2	-0.428850399	0.054	0.177	4.21125E-56	3
Aurka.3	-0.347953753	0.017	0.11	7.51083E-56	3
Gria2.3	-0.457816751	0.384	0.54	1.12646E-55	3
Klc1.1	-0.416327194	0.219	0.382	1.23714E-55	3
Ncapd2.2	-0.41474091	0.072	0.201	2.481E-55	3
Atad2.2	-0.484201329	0.081	0.215	2.708E-55	3
Loxl1	0.432247019	0.163	0.079	5.00756E-55	3
Mapt.3	-0.589946148	0.113	0.231	8.4066E-55	3
Bub1b.2	-0.378562455	0.019	0.114	1.41014E-54	3
Myt1.3	-0.544573539	0.051	0.167	4.16684E-54	3
Lmnb1.2	-0.43848181	0.162	0.319	1.26415E-53	3
Hjurp.2	-0.448068373	0.291	0.454	1.78334E-53	3
Elmo1.3	-0.56036711	0.035	0.139	3.48577E-53	3
Angptl2	0.418708433	0.189	0.105	3.61815E-53	3
Stmn1.1	-0.424068524	0.152	0.303	6.32715E-53	3
Sema7a	0.436598094	0.246	0.142	1.27627E-52	3
Diap3.2	-0.378398081	0.022	0.118	1.53783E-52	3
Rrm1.3	-0.433745098	0.132	0.278	4.31175E-52	3
Myt1l.2	-0.5469479	0.06	0.175	4.71793E-52	3
2700094K13Rik.2	-0.384648068	0.5	0.63	5.51939E-52	3
Mad2l1.2	-0.366500763	0.033	0.136	1.10577E-51	3
Fnbp1l.1	-0.413406308	0.321	0.472	1.21685E-51	3
Nhlh1.3	-0.526995052	0.112	0.239	3.27751E-51	3
Dhx32	0.426283963	0.286	0.187	8.70071E-51	3
Ezh2.2	-0.398644469	0.485	0.627	9.19784E-51	3
Dpysl3.3	-0.581778871	0.107	0.235	1.08003E-50	3
Olfm1	-0.478206101	0.089	0.214	1.0955E-50	3
Cacng5	0.425557867	0.115	0.04	1.33146E-50	3
Eef2	0.270737484	0.789	0.705	2.27557E-50	3
Lig1.3	-0.486914443	0.215	0.367	2.82188E-50	3
Ctsb.3	-0.539921304	0.194	0.272	1.86748E-49	3
Clmp.3	-0.484726569	0.121	0.259	2.40729E-49	3
Myc	0.424082253	0.177	0.089	3.45167E-49	3
Crmp1	-0.351978655	0.547	0.685	1.50792E-48	3
Cenpk.3	-0.411024255	0.048	0.158	1.99731E-48	3
Rpl37	0.323539079	0.579	0.492	2.01253E-48	3
Melk.2	-0.322082686	0.018	0.105	2.42123E-48	3
Hirip3.2	-0.442243666	0.253	0.401	2.58397E-48	3
Ska2.2	-0.428035257	0.093	0.22	3.58041E-48	3
Mdk.1	0.391613068	0.492	0.363	9.38172E-48	3
Ank2.3	-0.534212375	0.133	0.255	9.64721E-48	3
Rpl18a	0.349925261	0.491	0.387	1.50284E-47	3
Plk4.2	-0.363044031	0.033	0.131	2.04984E-47	3

Gm1673.1	-0.368130956	0.337	0.492	2.36353E-47	3
Rpl7	0.303233518	0.583	0.498	3.93188E-47	3
Pdlim4	0.436644995	0.145	0.066	4.98311E-47	3
Fam111a.2	-0.424235007	0.039	0.138	2.31683E-46	3
Nt5dc2	-0.413298493	0.123	0.253	2.67475E-46	3
Prmt8.1	0.421984316	0.362	0.259	2.28548E-45	3
Pde1c.3	-0.463824333	0.271	0.403	2.36386E-45	3
Ccng2.2	-0.405182184	0.107	0.227	2.37748E-45	3
Ncor2	0.403812498	0.247	0.157	2.5236E-45	3
Stxbp1.3	-0.471397103	0.068	0.175	2.56191E-45	3
Myod1.2	-0.443663385	0.051	0.154	4.35462E-45	3
B3galt2.3	-0.468253543	0.022	0.108	6.91854E-45	3
H2afv.2	-0.280184419	0.703	0.731	1.21466E-44	3
Frmd4a	-0.382327644	0.184	0.326	1.62854E-44	3
Stmn3	-0.334577049	0.507	0.638	2.9092E-44	3
Gm11478	0.382849076	0.297	0.203	4.54996E-44	3
Fabp7.3	-0.851729963	0.1	0.126	6.64558E-44	3
Zic1.2	-0.29085803	0.734	0.791	1.44771E-43	3
Meg3.3	-0.79902194	0.029	0.104	2.05847E-43	3
Rfc4.3	-0.365724874	0.115	0.242	8.74129E-43	3
Dll3	0.423754745	0.136	0.055	2.61126E-42	3
Itsn1.3	-0.476250705	0.129	0.251	2.93399E-42	3
Atp6v0e	0.315126906	0.483	0.396	3.1112E-42	3
Sept11	-0.378863368	0.198	0.34	3.55734E-42	3
Ppic	0.381277176	0.331	0.226	4.73413E-42	3
Gstm5	0.344380864	0.402	0.311	8.3457E-42	3
Ppp2r2c	-0.332837994	0.374	0.523	1.88472E-41	3
Ckb.3	-0.268444238	0.779	0.812	2.06835E-41	3
Fosb	0.483434993	0.245	0.142	2.14446E-41	3
Sparc	-0.359161191	0.106	0.082	2.21053E-41	3
Pax6.2	-0.326779052	0.381	0.532	2.89342E-41	3
Ptn.2	-0.473875741	0.342	0.455	3.00667E-41	3
Uncx.3	-0.378729289	0.206	0.333	3.29676E-41	3
Plcb1.2	-0.484564328	0.053	0.153	5.50206E-41	3
Rbfox1	-0.38405717	0.022	0.102	6.87432E-41	3
Mroh2a	-0.472153352	0.039	0.124	7.05727E-41	3
Cenpp.2	-0.317789118	0.038	0.125	8.80645E-41	3
Kcnk1.2	-0.42508986	0.103	0.219	1.03569E-40	3
Nrn1.2	-0.452877025	0.115	0.237	1.06238E-40	3
Gpx8	0.34993255	0.118	0.044	1.32968E-40	3
Rpl18	0.301339387	0.37	0.295	1.34497E-40	3
Ccnd2.1	-0.350064821	0.588	0.672	1.57475E-40	3
Pdgfra	-0.414901563	0.085	0.195	1.62863E-40	3
Sfrp2	0.350926626	0.204	0.139	1.74749E-40	3
Gm10260	0.346691216	0.309	0.216	1.96109E-40	3
Ramp2	0.37273086	0.195	0.108	2.88774E-40	3
Ier5	0.360592142	0.475	0.382	4.0488E-40	3
Elavl4.3	-0.419474664	0.216	0.352	4.28687E-40	3

Ncam1	-0.310532667	0.262	0.405	7.53697E-40	3
A930011O12Rik.3	-0.473476353	0.037	0.126	1.2252E-39	3
Rad51.2	-0.303040971	0.038	0.127	3.01247E-39	3
Eef1g	0.302831759	0.403	0.325	3.6759E-39	3
Dcx.3	-0.397638483	0.264	0.391	8.4708E-39	3
Ccdc34.2	-0.387524578	0.291	0.432	1.13397E-38	3
Psip1.1	-0.292804346	0.578	0.703	2.3179E-38	3
Aplp1.3	-0.45425648	0.067	0.163	3.53711E-38	3
H2afz.2	-0.308019645	0.127	0.246	4.59571E-38	3
Cog7	-0.324221235	0.459	0.602	4.72883E-38	3
Cst3.3	-0.280081397	0.531	0.536	5.02285E-38	3
Rpl37a	0.281441078	0.547	0.457	5.41215E-38	3
Cdc45.2	-0.301129492	0.026	0.105	7.58223E-38	3
Trim59.2	-0.365920512	0.078	0.183	8.27747E-38	3
Fzd1	0.351745046	0.127	0.059	9.70828E-38	3
Pcna.3	-0.405517235	0.256	0.373	1.44912E-37	3
Gm17322.2	0.439533007	0.208	0.12	2.07272E-37	3
Nasp.3	-0.328399312	0.545	0.654	2.39067E-37	3
Tspan13	-0.366388916	0.11	0.221	3.5764E-37	3
H1fx.2	-0.368625038	0.139	0.26	5.69401E-37	3
Trpc4ap.3	-0.438989961	0.119	0.209	5.76021E-37	3
Pea15a.3	-0.424088695	0.09	0.188	1.8789E-36	3
1700025G04Rik	-0.37060885	0.158	0.278	6.606E-36	3
Ptprg	0.380711274	0.205	0.133	7.73428E-36	3
BC005764.3	-0.45824498	0.096	0.198	7.80669E-36	3
Cdc7.1	-0.270084646	0.083	0.179	8.3468E-36	3
Rprml.1	0.411236921	0.142	0.071	9.73642E-36	3
Npc2.1	0.280165142	0.535	0.453	3.19604E-35	3
Ncaph.2	-0.294382261	0.045	0.131	4.87492E-35	3
Fen1.2	-0.316567004	0.062	0.153	5.64794E-35	3
Mis18a.2	-0.291304288	0.039	0.12	5.7235E-35	3
Gnai2	0.286350742	0.508	0.412	7.99523E-35	3
Srebf1.1	0.35590825	0.42	0.321	9.77292E-35	3
Gm13826	0.279577241	0.377	0.302	1.09675E-34	3
Fabp5	-0.310580097	0.289	0.419	1.19614E-34	3
Lbr.2	-0.288739818	0.103	0.209	1.24631E-34	3
Clvs1.2	-0.385091939	0.05	0.14	1.42627E-34	3
Cenpw.2	-0.316998675	0.051	0.138	1.7505E-34	3
Rps7	0.28066284	0.461	0.39	3.51479E-34	3
Dnmt1.2	-0.331313469	0.171	0.293	5.51278E-34	3
Lhx1.1	-0.355831726	0.393	0.512	7.02539E-34	3
Cenpj.2	-0.331847604	0.061	0.153	1.03579E-33	3
Gltscr2	0.29821467	0.413	0.331	1.27402E-33	3
Hadh	0.321624428	0.129	0.065	2.7811E-33	3
Gmnn.2	-0.29680248	0.065	0.156	3.05079E-33	3
Snhg1.1	0.291445323	0.439	0.345	3.19209E-33	3
Cks1b.2	-0.378969093	0.24	0.359	4.13053E-33	3
Cdk6	0.313352003	0.3	0.235	7.00284E-33	3

2810055G20Rik	0.366163095	0.242	0.16	7.91098E-33	3
App.1	-0.279357307	0.471	0.582	9.05824E-33	3
Pmf1.2	-0.269822958	0.066	0.154	9.94007E-33	3
Ppp3ca.1	-0.318443378	0.28	0.392	1.17734E-32	3
Wdr89	0.290040463	0.268	0.206	1.28507E-32	3
Sox1	0.346536162	0.118	0.063	2.99173E-32	3
Lrig3	0.333537449	0.226	0.162	3.09373E-32	3
2410004N09Rik	0.323977395	0.247	0.175	4.01519E-32	3
Mrpl52	0.275784973	0.496	0.423	4.59407E-32	3
Vps37b	0.272903698	0.536	0.46	5.69637E-32	3
Brd8.2	-0.300467189	0.28	0.408	6.80376E-32	3
Hsd11b2.2	0.35531519	0.361	0.277	1.07099E-31	3
Smpd2	0.331662201	0.222	0.157	1.16122E-31	3
Bub3.2	-0.355129887	0.214	0.336	1.76012E-31	3
Rps28	0.289699354	0.264	0.194	2.20877E-31	3
Eif3e	0.265565834	0.439	0.373	2.58625E-31	3
Atp1b3	-0.323873818	0.229	0.352	2.81045E-31	3
Irs1	0.34512409	0.174	0.11	3.01254E-31	3
Rftn2	0.33077424	0.183	0.115	3.47418E-31	3
Dusp5	0.410726215	0.151	0.079	4.06777E-31	3
Nrep.3	-0.284695199	0.502	0.579	4.93985E-31	3
Olig2	0.344474879	0.146	0.068	5.13596E-31	3
Cadm3.3	-0.426350783	0.067	0.151	7.21653E-31	3
Dnajc9.2	-0.331346337	0.241	0.365	1.1287E-30	3
Tubb4b.2	-0.339868089	0.143	0.254	1.29264E-30	3
Cplx1.1	-0.341669613	0.062	0.149	1.3868E-30	3
Hspe1	0.254094839	0.407	0.331	1.59788E-30	3
1110038B12Rik.1	0.271437379	0.35	0.291	1.64804E-30	3
Snhg6	0.308554778	0.325	0.248	1.70021E-30	3
Usp46	-0.300275653	0.145	0.248	2.41232E-30	3
Cacng4.3	-0.471189994	0.042	0.11	3.1199E-30	3
Gm17750	-0.301447028	0.27	0.381	3.55376E-30	3
Nrcam.2	-0.362806491	0.058	0.145	4.60573E-30	3
Tceal8	0.281024563	0.296	0.238	6.33974E-30	3
2610203C20Rik.1	0.357518979	0.188	0.11	7.27344E-30	3
Mgll	-0.346814377	0.062	0.143	7.35877E-30	3
Nes	0.378073297	0.15	0.077	7.91183E-30	3
Ttc9b	-0.2906821	0.106	0.207	7.9974E-30	3
Dync1i2.1	-0.271778769	0.468	0.582	8.34433E-30	3
Use1.1	0.285646049	0.378	0.304	9.20198E-30	3
Fkbp7	0.31079766	0.115	0.06	9.24067E-30	3
Hells.3	-0.375398908	0.119	0.226	1.2692E-29	3
Rpl22l1	0.260051688	0.401	0.343	1.27866E-29	3
Rab6b.3	-0.368726764	0.114	0.215	1.40827E-29	3
Ptch2.1	0.342374931	0.205	0.144	1.45328E-29	3
Kcnip3	-0.338983315	0.05	0.13	1.61243E-29	3
A030009H04Rik.1	-0.286907284	0.047	0.123	4.36234E-29	3
Nme4	0.319255662	0.177	0.109	5.99185E-29	3

Pdzrn4.1	0.351255516	0.192	0.139	6.04351E-29	3
Rtn4.2	-0.278843715	0.376	0.495	7.85635E-29	3
Naca	0.252491123	0.527	0.455	1.22439E-28	3
Trim37.1	-0.297692828	0.141	0.246	1.2393E-28	3
Pnrc1	0.312626827	0.336	0.261	1.28405E-28	3
Smc6.2	-0.288609055	0.307	0.439	1.40191E-28	3
Fyn.2	-0.350271715	0.143	0.226	1.44847E-28	3
Tle1	0.289052272	0.281	0.217	1.6286E-28	3
Igdcc4	0.340076868	0.149	0.086	1.63876E-28	3
Prim1.2	-0.32445106	0.173	0.275	1.83487E-28	3
Rps4x	0.253810214	0.342	0.288	1.98793E-28	3
Bora.1	-0.252267678	0.032	0.101	2.378E-28	3
Srgap2.1	-0.302979885	0.061	0.142	2.47143E-28	3
Myl12a	0.267640063	0.395	0.318	4.06251E-28	3
Whrn	0.314348557	0.126	0.066	4.99772E-28	3
Kif5a.3	-0.375556841	0.061	0.137	5.38711E-28	3
Chaf1a.2	-0.284445949	0.088	0.18	6.77707E-28	3
Prdm8.3	-0.383851711	0.058	0.136	6.79936E-28	3
Pkia.2	-0.316564578	0.062	0.144	6.84273E-28	3
Odf2.2	-0.290032251	0.091	0.182	7.33532E-28	3
Hsp90aa1	-0.255758948	0.519	0.63	1.17298E-27	3
Nsg2.2	-0.275636267	0.389	0.501	1.19277E-27	3
Nav2	-0.275187634	0.043	0.114	1.35214E-27	3
Usp1.3	-0.309481235	0.238	0.353	1.41876E-27	3
Elavl2	-0.286963755	0.18	0.283	1.43874E-27	3
H2afy2	-0.267257787	0.241	0.351	1.986E-27	3
Tead1	0.336289486	0.226	0.165	2.59504E-27	3
Wdr60	0.351931555	0.177	0.11	3.69471E-27	3
Nfyb	-0.264135729	0.15	0.247	4.03103E-27	3
Apbb1.3	-0.363717443	0.096	0.18	4.64657E-27	3
Syt11.2	-0.276185592	0.405	0.518	5.44069E-27	3
Azin1.1	-0.260449703	0.118	0.215	6.33975E-27	3
Strbp	-0.272626595	0.25	0.361	6.69258E-27	3
Gm11223.1	-0.405900847	0.154	0.221	8.00865E-27	3
Snord104	0.3137062	0.217	0.143	8.62071E-27	3
Gsto1	0.324481509	0.19	0.119	1.12586E-26	3
Lmnb2.1	-0.262144526	0.085	0.169	1.15741E-26	3
Rcn3	0.310435179	0.146	0.079	1.21764E-26	3
Rbfox2.2	-0.327288109	0.197	0.295	1.3165E-26	3
Dynll2	-0.263764089	0.201	0.309	1.33031E-26	3
2810004N23Rik	0.261612372	0.31	0.255	1.66348E-26	3
Uhrf1.2	-0.293477024	0.071	0.154	1.79656E-26	3
Mab21l1.2	-0.31949971	0.142	0.237	1.82434E-26	3
Gdpd1.1	-0.257694244	0.159	0.255	4.30854E-26	3
Ap1s2	-0.284732334	0.085	0.171	4.69087E-26	3
Sh3bp5.2	-0.328085758	0.042	0.112	4.81986E-26	3
2900011O08Rik.3	-0.345528456	0.043	0.112	4.83538E-26	3
Nek7	0.297191806	0.167	0.109	1.00887E-25	3

Lpin2	0.302196595	0.22	0.174	1.0106E-25	3
Rbp4.2	0.321558397	0.304	0.232	1.04892E-25	3
Cmc2.2	-0.250224634	0.053	0.128	1.11516E-25	3
Chd3.3	-0.379853194	0.076	0.159	1.56608E-25	3
Tipin.2	-0.264098977	0.164	0.267	1.78993E-25	3
Dhfr.2	-0.264827462	0.075	0.159	2.13922E-25	3
Igsf3	-0.259312199	0.066	0.142	2.38176E-25	3
Cadm1	0.275597877	0.446	0.382	5.15935E-25	3
Lima1	0.324814054	0.182	0.113	5.33509E-25	3
Rab13	0.308873771	0.134	0.077	6.15128E-25	3
Crip2	-0.286061133	0.153	0.252	6.3265E-25	3
Actn1	0.278071488	0.106	0.058	6.71396E-25	3
Ndrg2	0.250484369	0.232	0.177	6.72619E-25	3
Epb4.1	-0.262668112	0.144	0.241	8.45618E-25	3
Akap12.1	-0.362091093	0.047	0.12	2.28904E-24	3
Nsg1.2	-0.301643192	0.209	0.298	2.55495E-24	3
Plp1.3	-0.638593288	0.111	0.115	2.84155E-24	3
Tcf19.2	-0.285661281	0.038	0.107	3.51589E-24	3
Hey1.2	0.284456159	0.335	0.263	3.56967E-24	3
Chrna3.3	-0.359224359	0.052	0.127	6.19991E-24	3
Boc.1	0.291853562	0.2	0.149	6.92864E-24	3
Arpp21.3	-0.408575641	0.054	0.121	7.79979E-24	3
Sacs	0.297056302	0.239	0.183	1.01848E-23	3
Plxnb2	-0.280883191	0.096	0.181	1.32314E-23	3
Brd7	0.258142291	0.364	0.299	2.72165E-23	3
Gnl3.2	0.272252824	0.358	0.287	4.06899E-23	3
Hip1r	-0.319667067	0.066	0.143	4.59244E-23	3
Pcdha2.2	-0.310414668	0.057	0.126	4.79875E-23	3
Tnik.3	-0.366435439	0.079	0.155	6.11701E-23	3
Mapk8ip1.2	-0.298154146	0.15	0.238	6.41063E-23	3
Dnmt3a	0.274673529	0.254	0.195	1.07814E-22	3
Cmtm3	0.257916429	0.163	0.11	1.2389E-22	3
Trps1	0.276400154	0.135	0.084	1.25718E-22	3
Stx4a	0.29095784	0.21	0.146	1.29872E-22	3
Glce.2	-0.310748865	0.106	0.186	1.80453E-22	3
Nexn.1	0.400834389	0.126	0.069	2.60628E-22	3
Adamts1	0.305942949	0.187	0.149	2.77074E-22	3
Srrm4.3	-0.278528652	0.125	0.205	7.22118E-22	3
Prnp.2	-0.27620675	0.135	0.217	1.05359E-21	3
Cep170.1	-0.272701961	0.229	0.316	1.36631E-21	3
Gm26735.1	0.324752559	0.238	0.172	3.12489E-21	3
Arhgef7	-0.265983604	0.085	0.158	3.14081E-21	3
Islr2	0.285897587	0.227	0.173	4.2117E-21	3
Tshz2	0.268135294	0.373	0.312	1.00404E-20	3
Gpatch4	0.268192425	0.232	0.172	1.13975E-20	3
Kif21a	0.269221886	0.403	0.337	1.88396E-20	3
Btbd11	0.260293523	0.105	0.063	2.31188E-20	3
Robo2	0.31025468	0.207	0.143	3.7194E-20	3

Pfn2.1	-0.265909604	0.168	0.251	5.62548E-20	3
Nbea.1	-0.312345142	0.117	0.201	5.83368E-20	3
Hmgcs1	-0.293881394	0.089	0.16	7.46752E-20	3
Sstr2.1	-0.270307286	0.124	0.2	1.05167E-19	3
Hddc2	0.266485339	0.14	0.086	1.44528E-19	3
Dusp6	0.285088509	0.136	0.086	1.68114E-19	3
Cttnbp2	0.303993337	0.134	0.076	1.80077E-19	3
Dbn1.3	-0.289771201	0.079	0.148	3.03978E-19	3
Rrp15	0.260824293	0.217	0.167	3.21419E-19	3
Cd1d1	0.258877549	0.127	0.082	4.15502E-19	3
Wwc1	0.251573785	0.113	0.063	7.51777E-19	3
Dusp1	0.251134747	0.196	0.158	1.10517E-18	3
Ifitm2	0.251963554	0.162	0.107	1.28275E-18	3
Gria4.3	-0.273824149	0.069	0.132	1.34393E-18	3
1500011K16Rik	0.252033015	0.198	0.142	1.51898E-18	3
Eif4e3	-0.269300403	0.07	0.139	2.98396E-18	3
Zfp462	0.258585507	0.197	0.15	4.71929E-18	3
C77370	-0.255829957	0.049	0.108	5.31312E-18	3
Pnmal2.2	-0.254509458	0.085	0.155	1.16359E-17	3
Tspyl4.2	-0.272341165	0.071	0.134	1.42998E-17	3
Gstm1	0.262433333	0.131	0.074	1.60888E-17	3
S100a16.3	-0.345303965	0.047	0.101	2.20101E-17	3
Mt2	0.302303355	0.144	0.084	2.47045E-17	3
Thra.3	-0.254024813	0.183	0.235	5.26135E-17	3
Pak7.1	-0.273068797	0.052	0.112	1.22005E-16	3
Abhd16a.3	-0.265970294	0.128	0.198	1.45347E-16	3
Adcyap1r1	0.278216013	0.123	0.077	3.31019E-16	3
Gria3	0.260234816	0.132	0.083	1.08025E-15	3
Lama5	0.268390205	0.116	0.075	2.81372E-15	3
Mbp.2	-0.263234039	0.129	0.17	2.97014E-12	3
Xist.1	0.256323093	0.368	0.289	1.8317E-09	3
Top2a.4	-1.320278839	0.194	0.455	8.5605E-194	4
CRE_RECOMBINASE.3	0.668830695	0.906	0.751	9.4224E-175	4
Mki67.4	-1.065347818	0.192	0.418	2.6731E-133	4
Smc4.3	-0.786387506	0.428	0.583	3.1844E-127	4
Prc1.4	-1.032445324	0.061	0.28	7.3663E-123	4
2810417H13Rik.4	-0.901697701	0.208	0.413	1.7181E-120	4
Cdk1.4	-0.884338855	0.055	0.269	6.9052E-116	4
Spc25.4	-0.851147805	0.043	0.246	1.8889E-114	4
Nusap1.4	-0.910756426	0.049	0.248	3.6749E-112	4
Birc5.4	-0.886715447	0.082	0.296	1.6318E-111	4
H2afx.4	-0.902151282	0.151	0.352	1.0812E-105	4
Smc2.3	-0.717554498	0.409	0.561	3.9741E-105	4
Pbk.3	-0.787357978	0.047	0.243	7.1584E-105	4
Neurod1.4	-0.827745465	0.483	0.538	1.1308E-104	4
Ckap2l.4	-0.81141739	0.046	0.237	1.1875E-103	4
Esco2.3	-0.795041022	0.033	0.214	2.6971E-103	4
Barhl1.3	0.507516373	0.715	0.498	6.0587E-103	4

Kif11.4	-0.736687	0.041	0.225	1.57013E-98	4
Kif23.4	-0.8248029	0.048	0.234	2.27184E-98	4
Tpx2.4	-0.892087674	0.1	0.303	4.43576E-98	4
Hmgb2.3	-0.766541022	0.223	0.42	3.43876E-97	4
Incenp.4	-0.812279533	0.089	0.297	4.19504E-97	4
Ube2c.3	-1.030158519	0.121	0.286	8.84499E-94	4
Sfrp1.2	0.373206563	0.917	0.799	6.86111E-93	4
Cdca8.4	-0.772974492	0.087	0.285	5.4911E-92	4
Arl6ip1.3	-0.669236535	0.442	0.562	6.97534E-92	4
Hmmr.3	-0.807390038	0.033	0.193	9.47611E-91	4
Ccna2.4	-0.701852432	0.056	0.241	7.63126E-90	4
Cenpf.4	-0.962236072	0.197	0.381	5.01544E-89	4
Spc24.3	-0.6818849	0.077	0.262	4.52005E-83	4
Hey1.3	0.608503857	0.437	0.253	3.37281E-80	4
Igfbpl1.2	0.378196706	0.813	0.627	1.46108E-79	4
Aurkb.3	-0.551923094	0.019	0.157	8.21058E-79	4
Casc5.4	-0.664388558	0.043	0.203	1.18227E-78	4
Apoe.4	-1.607422737	0.211	0.254	1.38941E-78	4
Cdca3.4	-0.680167754	0.068	0.239	1.5995E-76	4
Ncapg.3	-0.60292659	0.042	0.199	6.15742E-76	4
Cdc20.3	-0.785073706	0.06	0.212	4.52962E-75	4
Sgol2.4	-0.656510978	0.03	0.171	6.63425E-74	4
Dek.4	-0.462858787	0.619	0.702	9.92207E-74	4
Ptn.3	-0.746061789	0.3	0.458	2.39318E-73	4
Draxin.1	0.419696898	0.709	0.535	1.69545E-70	4
Kif22.4	-0.538370269	0.034	0.178	1.71352E-70	4
Tacc3.4	-0.607579323	0.062	0.223	7.15993E-70	4
Ccnb1.3	-0.613426044	0.024	0.153	7.52322E-70	4
Ndc80.3	-0.452437064	0.013	0.126	4.10817E-66	4
Slc29a1.1	0.420943769	0.689	0.546	2.48746E-65	4
Tubb5.3	-0.318607107	0.897	0.916	4.92571E-65	4
Kif15.3	-0.554619354	0.049	0.196	5.51385E-65	4
Srebf1.2	0.508204736	0.49	0.315	1.35049E-64	4
Rps26.1	0.275128708	0.915	0.839	3.79418E-64	4
Mis18bp1.4	-0.575053923	0.038	0.175	8.86504E-64	4
Cdca2.3	-0.459234838	0.021	0.141	1.13068E-62	4
Mxd3.3	-0.469140479	0.021	0.139	8.71377E-62	4
Tk1.3	-0.485855904	0.024	0.144	1.43211E-60	4
Gm17322.3	0.563759608	0.26	0.115	1.47803E-60	4
Fbxo5.3	-0.502311953	0.041	0.173	6.7537E-59	4
Cenph.4	-0.53118691	0.063	0.209	1.55424E-57	4
Bub1.3	-0.455675068	0.025	0.143	1.73777E-57	4
Cenpa.3	-0.721098406	0.132	0.266	3.82046E-56	4
Fam64a.3	-0.457231702	0.02	0.129	7.35436E-56	4
Lhx1.2	0.422778222	0.649	0.479	8.27924E-56	4
Tuba1b.3	-0.470723211	0.369	0.494	1.09388E-54	4
Arhgap11a.4	-0.556912541	0.052	0.184	1.23068E-54	4
Ckap2.3	-0.513634066	0.047	0.177	8.28869E-54	4

Nuf2.4	-0.453486316	0.034	0.153	2.3614E-53	4
D17H6S56E-5.3	-0.43259952	0.026	0.139	3.22118E-53	4
Cd63	0.295844802	0.856	0.742	1.55264E-52	4
Itm2b.3	-0.392511726	0.566	0.651	2.29416E-51	4
Kif2c.3	-0.406891166	0.017	0.117	1.28038E-50	4
Sgol1.3	-0.420344326	0.029	0.139	1.31119E-49	4
Cenpe.3	-0.694155038	0.158	0.3	4.31028E-49	4
Rtn1.4	-0.332646602	0.689	0.69	1.35163E-48	4
Rad51ap1.4	-0.427305499	0.055	0.18	3.163E-47	4
Bub1b.3	-0.370870237	0.017	0.112	9.88642E-47	4
C1ql1.1	0.451853955	0.409	0.263	1.3198E-46	4
Kif20b.4	-0.542347355	0.073	0.204	3.63565E-46	4
Cdkn2d.4	-0.45035438	0.05	0.17	8.17171E-46	4
Melk.3	-0.35022248	0.014	0.103	2.54717E-45	4
Aspm.3	-0.533216376	0.044	0.154	3.81489E-45	4
Miat.3	0.260013129	0.666	0.497	5.07232E-45	4
Gpm6a.4	-0.483428668	0.323	0.419	1.23371E-44	4
Rassf4	0.412814988	0.508	0.36	1.24539E-44	4
Clspn.3	-0.50988995	0.074	0.203	2.63226E-44	4
Calm2.3	-0.277040569	0.85	0.877	1.00994E-43	4
Nucks1.3	-0.350160661	0.599	0.67	1.29409E-43	4
Ncapd2.3	-0.403070835	0.071	0.198	1.46754E-43	4
Rrm2.4	-0.491210513	0.088	0.217	3.00828E-43	4
Cst3.4	-0.487858459	0.492	0.541	9.85264E-43	4
Nrxn1.4	-0.554752135	0.214	0.33	2.99205E-42	4
Meg3.4	-0.841076188	0.022	0.103	3.01824E-42	4
Kif4.3	-0.359871882	0.024	0.118	5.69851E-42	4
Celf4.4	-0.593360039	0.225	0.331	1.60467E-41	4
Crmp1.1	0.302309693	0.783	0.653	1.98692E-41	4
Dbf4.3	-0.379291294	0.047	0.157	2.87958E-41	4
Knstrn.3	-0.484187365	0.075	0.198	4.35759E-41	4
Cenpq.3	-0.413963718	0.058	0.172	2.64172E-40	4
Ccnd2.2	0.299522088	0.772	0.648	3.09953E-40	4
Fam210b.1	0.41426481	0.436	0.299	5.88888E-40	4
Hist1h2ak.3	-0.509561699	0.049	0.154	1.31764E-39	4
Islr2.1	0.428291844	0.29	0.166	2.36619E-39	4
Racgap1.4	-0.455436082	0.132	0.27	8.44484E-39	4
Cntn2.4	-0.529575948	0.214	0.269	1.70941E-37	4
Mns1.3	-0.416111491	0.091	0.216	2.41688E-37	4
1500016L03Rik.2	0.34898626	0.516	0.367	4.31356E-37	4
Ckap5.4	-0.472779819	0.149	0.279	5.7626E-37	4
Anln.3	-0.354514682	0.025	0.113	6.15007E-37	4
Rad21.4	-0.418598745	0.347	0.475	1.23489E-36	4
Cbfa2t3.2	0.341448173	0.52	0.388	2.66264E-36	4
Aurka.4	-0.304847692	0.023	0.107	5.25695E-36	4
Gamt.1	0.431243487	0.272	0.157	6.81653E-36	4
Diap3.3	-0.340151937	0.027	0.116	7.77218E-36	4
Ect2.3	-0.330751669	0.023	0.106	1.94886E-35	4

Rangap1.4	-0.412489707	0.136	0.27	2.03208E-35	4
Ccnb2.3	-0.481221123	0.083	0.199	2.89526E-35	4
Dpysl4	0.336505158	0.617	0.475	3.03085E-35	4
Dlgap5.3	-0.3680288	0.031	0.121	6.02493E-35	4
Mad2l1.3	-0.339462241	0.04	0.133	8.73749E-35	4
Ctsd.4	-0.651900951	0.152	0.178	3.2409E-34	4
RP23-45G16.5.4	-0.465454014	0.21	0.34	7.73165E-34	4
Mdk.2	0.391610662	0.485	0.366	4.11725E-33	4
Fam111a.3	-0.389081236	0.042	0.136	4.32457E-33	4
Cenpk.4	-0.400598951	0.055	0.155	4.38841E-33	4
Fabp7.4	-0.84830797	0.099	0.125	1.16416E-32	4
Lig1.4	-0.470177276	0.235	0.361	1.82115E-32	4
Gap43.4	-0.384997908	0.534	0.607	1.87207E-31	4
Rrm1.4	-0.386608017	0.146	0.273	5.34811E-31	4
Elmo1.4	-0.500492533	0.045	0.135	6.65644E-31	4
Dut.3	-0.357660506	0.369	0.442	7.85779E-31	4
Atp2b1	-0.34831582	0.462	0.584	8.18218E-30	4
C330027C09Rik.2	-0.346102082	0.05	0.143	1.68951E-29	4
Plk4.3	-0.308271383	0.042	0.128	6.60675E-29	4
Ppp1r14c.4	-0.462759602	0.163	0.274	1.03005E-28	4
Tyms.3	-0.398785665	0.117	0.231	1.23114E-28	4
Mroh2a.1	0.477098444	0.184	0.105	1.85067E-28	4
Anp32e.3	-0.251237432	0.593	0.616	5.99616E-28	4
Ska2.3	-0.335955897	0.105	0.216	8.74253E-28	4
Tmpo.3	-0.336947467	0.373	0.461	1.17845E-27	4
Cenpw.3	-0.310392527	0.051	0.136	3.6416E-27	4
Nnat.1	0.27825476	0.799	0.699	3.69224E-27	4
Dpysl3.4	-0.493477897	0.132	0.229	5.46693E-27	4
Rpl35a.1	0.255949738	0.655	0.56	7.36321E-27	4
Tubb2a.4	-0.444213616	0.166	0.254	1.59597E-26	4
Ctsb.4	-0.449252574	0.221	0.267	1.93969E-26	4
Sparcl1.3	-0.621932516	0.138	0.215	2.87388E-26	4
Trim59.3	-0.331830529	0.08	0.18	4.14291E-26	4
Sept3.4	-0.31778086	0.352	0.397	4.70437E-26	4
Mycn	0.326574747	0.471	0.35	7.49821E-26	4
Hsd11b2.3	0.323414178	0.393	0.275	1.84136E-25	4
Selm	0.346510621	0.263	0.163	2.1235E-25	4
Rcor2.2	0.331421691	0.326	0.212	3.20318E-25	4
Atad2.3	-0.372727095	0.104	0.209	3.22663E-25	4
Atoh1.1	0.346285328	0.253	0.153	4.8615E-25	4
Rad51.3	-0.306419634	0.044	0.125	6.20417E-25	4
Pde1c.4	0.289872582	0.499	0.373	6.61825E-25	4
Ncaph.3	-0.293453297	0.047	0.129	2.48345E-24	4
Slc1a2.2	0.339068121	0.386	0.282	5.7612E-24	4
Ezh2.3	-0.282343808	0.55	0.616	9.26226E-24	4
Cenpm.3	-0.318056278	0.078	0.172	1.67996E-23	4
Mapt.4	-0.447006553	0.142	0.225	3.21423E-23	4
Ccng2.3	-0.30082041	0.119	0.223	4.58252E-23	4

Malat1.3	-0.25182621	0.956	0.954	7.53607E-23	4
Hes6.1	0.316142435	0.328	0.225	3.10301E-22	4
Usp1.4	-0.33074206	0.232	0.351	8.82382E-22	4
2700094K13Rik.3	-0.25735146	0.564	0.62	1.36902E-21	4
Cacng4.4	-0.443008165	0.045	0.108	2.37477E-21	4
Arpp21.4	-0.43029268	0.052	0.12	3.84954E-21	4
Tubb4b.3	-0.340291908	0.151	0.25	5.63944E-21	4
Vim.3	-0.375380968	0.226	0.311	1.45105E-20	4
Cdkn2c.2	-0.275632122	0.069	0.152	4.32573E-20	4
Eln	0.316226537	0.125	0.062	4.93379E-20	4
Lmnb1.3	-0.325131233	0.202	0.311	6.38224E-20	4
Gmnn.3	-0.303066075	0.07	0.153	6.79412E-20	4
Plp1.4	-0.62668161	0.117	0.114	8.57846E-20	4
Tprn	0.352672688	0.309	0.216	9.76309E-20	4
Nek6	0.319236939	0.206	0.137	1.27729E-19	4
Prdm8.4	-0.375415351	0.06	0.134	1.73089E-19	4
Cxxc5	0.256502287	0.623	0.534	3.21417E-19	4
Trpc4ap.4	-0.391756523	0.125	0.206	2.52188E-18	4
Ank2.4	-0.388498588	0.157	0.25	2.63704E-18	4
Sema7a.1	0.304422252	0.227	0.146	3.64406E-18	4
Cks1b.3	-0.322393297	0.254	0.355	7.22276E-18	4
Ddit4.1	0.314774557	0.173	0.096	8.49589E-18	4
Tcf19.3	-0.279806575	0.04	0.105	1.02875E-17	4
Dnmt1.3	-0.309916466	0.186	0.288	1.07242E-17	4
Rpl18a.1	0.256949363	0.478	0.391	1.5743E-17	4
Ccm2.1	0.288006984	0.303	0.215	1.73059E-17	4
B2m	-0.275987957	0.2	0.235	1.86216E-17	4
Mtss1.4	-0.347615474	0.206	0.276	3.75262E-17	4
Cacng2	0.279113842	0.418	0.328	4.64872E-17	4
2310022B05Rik	0.289255729	0.327	0.238	6.17138E-17	4
Klf6.2	-0.34294235	0.082	0.156	9.88619E-17	4
Fxyd6.2	-0.285772112	0.433	0.518	1.58922E-16	4
Hes1.2	-0.409878484	0.089	0.155	1.61296E-16	4
Bcl7a.1	0.273415501	0.404	0.315	1.78277E-16	4
Sptbn1.3	-0.298094838	0.258	0.364	1.97431E-16	4
Add3	-0.304035955	0.046	0.11	3.24482E-16	4
Ccdc34.3	-0.307711569	0.327	0.425	3.26606E-16	4
1500012F01Rik.3	0.261875605	0.479	0.388	4.95084E-16	4
Aldoa.2	0.263844467	0.342	0.247	5.37618E-16	4
Sstr2.2	0.301186537	0.266	0.182	6.06721E-16	4
Egr1.2	-0.376156795	0.297	0.394	7.57071E-16	4
Rnd3.2	0.250130133	0.515	0.421	1.23317E-15	4
Pcna.4	-0.264353734	0.315	0.363	1.85175E-15	4
St18.3	-0.369908378	0.121	0.198	3.12287E-15	4
Atp6v0e.1	0.250354656	0.482	0.399	1.73532E-14	4
Uhrf1.3	-0.268322038	0.079	0.151	8.35863E-14	4
Stmn4.4	-0.254870062	0.322	0.343	1.44457E-13	4
G2e3.1	-0.255161769	0.07	0.138	1.92326E-13	4

Tspan7.1	-0.310394739	0.055	0.111	2.39578E-13	4
Hirip3.3	-0.283861904	0.301	0.392	3.37123E-13	4
Dnajc9.3	-0.270169685	0.266	0.359	8.91037E-13	4
Dner.4	-0.310522087	0.157	0.221	1.59365E-12	4
Heg1	0.259102791	0.225	0.149	1.87548E-12	4
Kcnk1.3	-0.28555859	0.141	0.211	1.18613E-11	4
Nsmf	0.261084864	0.131	0.079	1.21885E-11	4
Mt1.1	-0.342546458	0.131	0.173	7.22889E-11	4
Slc1a3.3	-0.326268285	0.123	0.156	1.24704E-10	4
Mfap4.1	0.307750696	0.219	0.154	1.41225E-10	4
Hells.4	-0.281819619	0.156	0.22	8.23372E-10	4
Fbxo32.1	0.273830211	0.166	0.11	1.51971E-09	4
Sema6a.4	-0.295157368	0.147	0.208	2.45375E-09	4
Gse1	0.254410123	0.297	0.229	6.59161E-09	4
Zmynd8.2	0.257240974	0.278	0.205	7.48603E-09	4
Aplp1.4	-0.252118841	0.103	0.157	1.01613E-08	4
Thra.4	-0.279305616	0.17	0.235	1.85056E-08	4
Stxbp1.4	-0.254238921	0.11	0.168	3.76639E-08	4
Rab3a.4	-0.255342997	0.099	0.156	5.49775E-07	4
Myt1l.3	-0.255719412	0.117	0.166	4.55682E-05	4
Tubb3.4	1.161993281	0.853	0.433	0	5
Igfbpl1.3	0.882645815	0.889	0.621	0	5
Stmn2.4	0.764137199	0.897	0.516	0	5
Ckb.4	0.711171111	0.95	0.792	0	5
Miat.4	0.974764068	0.818	0.482	0	5
Pde1c.5	1.046871134	0.652	0.358	0	5
Cntn2.5	1.021495584	0.651	0.221	6.1154E-288	5
Nnat.2	0.92725502	0.823	0.697	9.1376E-284	5
Tuba1a.3	0.558117971	0.986	0.923	2.9306E-282	5
Nhlh2.4	0.891511846	0.718	0.391	2.2581E-240	5
Ccnd1.3	-1.244164678	0.176	0.559	2.2443E-239	5
Neurod1.5	0.648991635	0.818	0.502	3.4852E-208	5
Smc2.4	-1.176444612	0.258	0.576	1.4011E-200	5
Ddah2.2	0.639346999	0.85	0.668	1.2981E-192	5
Dek.5	-0.883436261	0.433	0.721	1.0833E-186	5
Nhlh1.4	0.924026669	0.486	0.194	1.3823E-185	5
Elavl3.4	0.72695092	0.715	0.446	2.6236E-185	5
Lhx1.3	0.740324951	0.695	0.476	1.146E-181	5
Top2a.5	-1.379313691	0.161	0.456	1.4539E-181	5
Mki67.5	-1.332792624	0.127	0.423	6.1307E-175	5
Gap43.5	0.70388122	0.823	0.575	9.162E-175	5
Hmgb2.4	-1.055882468	0.129	0.428	4.9205E-157	5
2810417H13Rik.5	-1.090909415	0.12	0.42	1.2917E-153	5
Chrna3.4	0.88116619	0.327	0.095	2.995E-146	5
Pdzrn3.3	0.9167676	0.369	0.123	1.2399E-145	5
Birc5.5	-1.031572958	0.045	0.297	3.5696E-143	5
Anp32b.3	-0.778040986	0.336	0.618	1.4034E-141	5
Rtn1.5	0.510821316	0.862	0.671	5.7199E-140	5

Nrn1.3	0.825512913	0.427	0.199	2.0333E-138	5
Tpx2.5	-1.086286846	0.056	0.305	1.1263E-135	5
Cenpf.5	-1.246997439	0.125	0.387	8.3335E-134	5
Sept4.4	0.840848432	0.439	0.203	1.4709E-132	5
Cdk1.5	-0.980480622	0.037	0.269	1.0309E-131	5
Elavl4.4	0.729474222	0.542	0.312	4.9156E-129	5
Basp1.4	0.512117107	0.814	0.692	5.7271E-124	5
Nfix.1	0.504384664	0.796	0.659	1.0115E-123	5
Nfib	0.418782701	0.981	0.928	8.9982E-122	5
Nusap1.5	-0.972750637	0.033	0.247	2.1173E-121	5
Cdca3.5	-0.893668666	0.03	0.242	3.7835E-121	5
Bin1.4	0.643129403	0.622	0.408	4.6739E-121	5
Pbk.4	-0.867338169	0.03	0.242	1.4029E-120	5
H2afx.5	-1.020635681	0.113	0.353	5.9333E-120	5
Map2.4	0.620182672	0.662	0.473	1.0845E-118	5
Tuba1b.4	-0.765517625	0.238	0.507	2.3976E-114	5
Cdca8.5	-0.901837606	0.065	0.285	4.1703E-112	5
Smc4.4	-0.817715972	0.375	0.587	5.605E-111	5
Cbx5.3	-0.61467824	0.416	0.678	6.8834E-111	5
Ccna2.5	-0.806422832	0.036	0.241	1.3326E-110	5
Pdgfa.3	-0.852769831	0.055	0.275	4.1929E-110	5
Kif11.5	-0.821284112	0.03	0.224	2.5006E-106	5
Ina.4	0.577301286	0.62	0.424	1.9409E-105	5
Spc25.5	-0.83180291	0.04	0.245	6.3842E-105	5
Dut.4	-0.769299857	0.201	0.46	7.1353E-105	5
Spc24.4	-0.768989468	0.051	0.262	4.2698E-104	5
Map1b.4	0.524045811	0.754	0.569	2.5499E-103	5
Clmp.4	0.736769006	0.414	0.223	6.3147E-103	5
Ube2c.4	-1.120063286	0.084	0.288	1.7297E-101	5
Prc1.5	-0.978819532	0.068	0.276	1.0112E-100	5
Mllt11.4	0.679517162	0.416	0.197	7.8171E-100	5
Nasp.4	-0.617863125	0.428	0.662	1.623E-99	5
Cenpa.4	-1.016671571	0.069	0.271	2.8659E-99	5
Cenpe.4	-0.982932719	0.087	0.306	1.28496E-97	5
Incenp.5	-0.828098296	0.077	0.296	1.52937E-97	5
Dbi	-0.748993579	0.249	0.486	9.78162E-95	5
Tmpo.4	-0.680417413	0.225	0.476	3.33564E-93	5
Esco2.4	-0.762049709	0.031	0.212	7.05547E-93	5
Crmp1.2	0.419373564	0.763	0.657	2.09338E-90	5
Egr1.3	-0.808371234	0.166	0.407	5.50977E-90	5
Ranbp1.2	-0.562059744	0.499	0.694	1.23343E-89	5
Gpr153.1	-0.678630268	0.041	0.226	1.78616E-89	5
Ncapg.4	-0.665262931	0.027	0.199	3.66034E-89	5
Ckap2l.5	-0.740590534	0.047	0.235	1.2275E-88	5
Tcf4	0.344050381	0.952	0.922	9.79193E-88	5
Cks1b.4	-0.695121415	0.14	0.366	6.92123E-87	5
Pcna.5	-0.736048853	0.159	0.379	2.3376E-85	5
1500016L03Rik.3	0.581082251	0.534	0.366	3.59942E-85	5

Zic1.3	0.364026101	0.87	0.774	1.28745E-84	5
Kif23.5	-0.807273507	0.052	0.231	3.5411E-84	5
Ank3.4	0.563412553	0.558	0.405	7.39783E-84	5
Mcm6.3	-0.724379479	0.094	0.295	1.69022E-83	5
Kif15.4	-0.685971318	0.03	0.196	1.7017E-83	5
Uncx.4	0.60509558	0.48	0.3	2.96374E-83	5
Kif5c.4	0.541135778	0.558	0.381	1.77638E-82	5
Srrm3.1	0.640585532	0.382	0.224	6.02101E-82	5
Rrm2.5	-0.691044021	0.045	0.221	6.69281E-82	5
Casc5.5	-0.677972339	0.035	0.202	1.11956E-81	5
Cdc20.4	-0.832690737	0.043	0.212	8.07974E-81	5
Stmn4.5	0.569529439	0.532	0.32	9.17781E-81	5
H2afv.3	-0.506894921	0.578	0.743	3.72655E-80	5
Usp1.5	-0.639374558	0.139	0.36	5.64413E-80	5
Tex14.3	0.719196646	0.301	0.128	8.04037E-79	5
Ran.2	-0.599532904	0.21	0.435	1.50132E-78	5
Tubb2b.4	0.515115435	0.614	0.448	3.21532E-78	5
Nucks1.4	-0.524242702	0.481	0.682	9.91932E-78	5
Cxxc5.1	0.423862614	0.626	0.534	4.23961E-77	5
Abhd16a.4	0.646698133	0.327	0.175	8.49566E-77	5
Syt11.3	0.49341357	0.612	0.492	2.34856E-76	5
Ccnb2.4	-0.733233986	0.04	0.202	2.45848E-76	5
Pqlc1.2	-0.635194124	0.113	0.317	3.0108E-75	5
Knstrn.4	-0.654767636	0.038	0.201	4.53539E-75	5
Cog7.1	0.438891814	0.697	0.572	1.70861E-74	5
Ccnb1.4	-0.646163696	0.016	0.152	2.34383E-74	5
Apc.4	0.518700939	0.569	0.416	2.38934E-74	5
Nrep.4	0.385581506	0.708	0.554	2.79642E-74	5
Ckap2.4	-0.611586113	0.027	0.177	5.00763E-74	5
Tmsb4x.3	0.28804793	0.973	0.946	1.1604E-73	5
2700094K13Rik.4	-0.52086433	0.419	0.634	4.2739E-73	5
RP23-45G16.5.5	-0.690909107	0.145	0.346	1.87502E-72	5
Rplp1.2	-0.373993997	0.782	0.887	2.3391E-72	5
Ptprs.2	0.552776756	0.539	0.408	4.03485E-71	5
Clspn.4	-0.664088201	0.044	0.205	1.09273E-70	5
Cenph.5	-0.627750002	0.047	0.209	2.47524E-70	5
Myt1.4	0.667429234	0.3	0.136	5.77088E-70	5
Hmmr.4	-0.727806104	0.037	0.191	8.05315E-70	5
Anp32e.4	-0.528342864	0.432	0.632	8.1871E-70	5
Hes1.3	-0.764906924	0.024	0.161	1.13791E-69	5
Rad51ap1.5	-0.557087816	0.032	0.181	2.60989E-69	5
Aurkb.4	-0.540729023	0.02	0.156	5.58198E-69	5
Sgol2.5	-0.623042813	0.027	0.17	9.70923E-69	5
Tyms.4	-0.644739807	0.066	0.235	1.55051E-68	5
Dcx.4	0.538919301	0.517	0.36	2.15277E-68	5
Mis18bp1.5	-0.554375023	0.03	0.174	4.0586E-68	5
Kif20b.5	-0.672944861	0.047	0.205	5.40936E-68	5
Cdca2.4	-0.493487212	0.014	0.14	6.93879E-68	5

Fbxo5.4	-0.573320117	0.029	0.173	7.39489E-68	5
Cltb.2	-0.585946659	0.229	0.443	1.46649E-67	5
Zfp36l1.1	-0.563221256	0.025	0.164	8.62243E-67	5
Hirip3.4	-0.597520999	0.194	0.402	1.22593E-66	5
Tacc3.5	-0.622782547	0.058	0.222	1.38353E-66	5
Nkd1.1	-0.567578203	0.086	0.265	2.05507E-66	5
Lig1.5	-0.645518209	0.164	0.368	2.11795E-66	5
Dnajc9.4	-0.552676051	0.164	0.369	2.37002E-66	5
Dtymk.3	-0.551416512	0.246	0.459	2.76729E-66	5
Nsg2.3	0.42800577	0.584	0.476	6.27143E-66	5
Sparcl1.4	-0.937387363	0.068	0.222	9.92617E-66	5
Gas1.1	-0.547538878	0.029	0.17	1.73551E-65	5
Cenpm.4	-0.536280014	0.033	0.175	6.18177E-65	5
Npm1.2	-0.459859144	0.47	0.665	1.23442E-64	5
Tbata.3	-0.676675055	0.166	0.358	2.59447E-64	5
Cdk5r1.4	0.523820969	0.483	0.317	4.03968E-64	5
Tagln3.4	0.533887151	0.436	0.279	1.3158E-63	5
Rbp4.3	-0.614452787	0.084	0.258	1.35317E-63	5
Gnai2.1	-0.526245045	0.235	0.444	1.607E-63	5
Gng3.4	0.533513171	0.474	0.299	2.67374E-63	5
1700025G04Rik.1	0.520066249	0.36	0.252	2.78381E-63	5
Dclk1.1	-0.519023301	0.22	0.432	3.34137E-63	5
Kif22.5	-0.529892225	0.034	0.176	5.27023E-63	5
Serbp1.2	-0.340809464	0.762	0.88	6.40993E-63	5
Mxd3.4	-0.480167635	0.016	0.138	7.24746E-63	5
Tk1.4	-0.519391529	0.018	0.143	9.96472E-63	5
Nuf2.5	-0.520048592	0.023	0.153	1.86258E-62	5
Prim1.3	-0.566313936	0.102	0.28	5.06845E-61	5
Aspm.4	-0.595090848	0.025	0.155	1.35702E-60	5
Ndc80.4	-0.466688786	0.012	0.125	1.88608E-60	5
C1ql1.2	0.504923677	0.393	0.266	4.25398E-60	5
Gm10075.2	-0.459226641	0.324	0.535	4.27135E-60	5
Apoe.5	-1.584744783	0.186	0.256	4.80732E-60	5
Thsd7a	0.61581164	0.232	0.116	5.16903E-60	5
Atad2.4	-0.571560097	0.059	0.213	1.06542E-59	5
Slc17a6.4	0.614386541	0.278	0.134	1.24353E-59	5
Sgol1.4	-0.450320492	0.019	0.139	2.59736E-59	5
Rab6b.4	0.563293649	0.328	0.188	4.45052E-59	5
Tmsb10.1	0.341218228	0.717	0.658	8.09598E-59	5
Bub1.4	-0.462699217	0.021	0.142	9.12995E-59	5
Gmnn.4	-0.514268215	0.028	0.157	1.13413E-58	5
BC005764.4	0.532882852	0.341	0.169	2.0581E-58	5
Mapk8ip1.3	0.531884161	0.352	0.213	3.02933E-58	5
Ccdc34.4	-0.499581539	0.229	0.434	5.37219E-58	5
D17H6S56E-5.4	-0.45751809	0.019	0.139	6.62939E-58	5
Nop58.2	-0.446554179	0.409	0.611	6.64286E-58	5
Arhgap11a.5	-0.567804746	0.043	0.183	2.12797E-57	5
Rpa2.2	-0.546623769	0.063	0.214	2.58992E-57	5

Mns1.4	-0.587573434	0.065	0.218	5.7953E-57	5
Gsg1l.3	-0.560691641	0.102	0.271	6.35549E-57	5
Rcor2.3	0.520537765	0.349	0.211	7.54768E-57	5
Kif2c.4	-0.424904745	0.011	0.117	3.34264E-56	5
Sstr2.3	0.547601265	0.312	0.178	4.3035E-56	5
Sfrp2.1	-0.467314071	0.031	0.16	5.84612E-56	5
Cenpq.4	-0.494395942	0.038	0.173	6.5007E-56	5
Hmgn5.2	-0.566932172	0.251	0.442	1.28458E-55	5
Txn1.1	-0.426969219	0.382	0.579	2.4589E-55	5
Lap3.3	-0.548413384	0.121	0.292	3.57471E-55	5
D430041D05Rik.2	-0.453333441	0.311	0.511	1.1801E-54	5
Marcksl1	0.297611215	0.764	0.717	1.36453E-54	5
Chgb.4	0.550018992	0.388	0.224	2.27941E-54	5
Podxl2.4	0.561269454	0.348	0.214	2.75992E-54	5
Hells.5	-0.543757428	0.076	0.227	3.46387E-52	5
Kif4.4	-0.373922463	0.015	0.118	3.76347E-52	5
Prdx4.2	-0.399407742	0.199	0.384	5.23384E-52	5
C330027C09Rik.3	-0.457843516	0.026	0.144	7.22175E-52	5
Angptl2.1	-0.449166307	0.017	0.126	8.06314E-52	5
Pmf1.3	-0.453731458	0.033	0.155	1.0293E-51	5
Rrm1.5	-0.479395256	0.112	0.276	1.03009E-51	5
Elavl2.1	0.487507833	0.361	0.26	1.08343E-51	5
Mcm3.2	-0.527964553	0.054	0.193	1.20825E-51	5
Cenpk.5	-0.49027618	0.033	0.156	1.65395E-51	5
Klc1.2	0.430466534	0.453	0.351	1.93622E-51	5
CRE_RECOMBINASE.4	0.358359331	0.831	0.761	5.11804E-51	5
Prdx1.2	-0.45755939	0.325	0.507	9.31742E-51	5
Hint1.1	-0.330966429	0.536	0.721	1.17446E-50	5
Paics.2	-0.459486029	0.24	0.431	1.3337E-50	5
Dlgap5.4	-0.417946444	0.017	0.122	1.58949E-50	5
Chaf1a.3	-0.487709155	0.049	0.181	1.16818E-49	5
Bub1b.4	-0.370024758	0.013	0.111	3.0108E-49	5
Hsbp1	0.322469046	0.638	0.589	4.28149E-49	5
Gm2694	-0.458984156	0.195	0.376	1.07172E-48	5
Rab3a.5	0.500541591	0.281	0.135	1.62422E-48	5
Tipin.3	-0.46306266	0.112	0.27	1.75923E-48	5
Rufy3.3	0.412180893	0.471	0.36	2.72916E-48	5
Fam64a.4	-0.414436004	0.022	0.128	4.38421E-48	5
Arl6ip1.4	-0.507233301	0.442	0.561	1.30421E-47	5
Cnbp.1	-0.322551617	0.594	0.755	2.43759E-47	5
Trim59.4	-0.442458909	0.052	0.182	2.57368E-47	5
Gm17750.1	0.437123489	0.44	0.359	3.59929E-47	5
Ttc3.2	0.271883441	0.868	0.824	3.87375E-47	5
Cdca7.2	-0.476829933	0.071	0.211	9.05608E-47	5
Mex3a	0.368754915	0.529	0.456	9.08184E-47	5
Cenpp.3	-0.372606301	0.02	0.124	1.44069E-46	5
Mt1.2	-0.701706109	0.055	0.18	1.69189E-46	5
Ppib	0.276060145	0.725	0.698	3.67477E-46	5

Cplx2.1	-0.390757275	0.324	0.513	4.60937E-46	5
Kmt2e.2	0.33113025	0.605	0.564	5.00042E-46	5
Pax6.3	0.387806732	0.595	0.504	5.30661E-46	5
Cdkn1b.1	0.339944262	0.527	0.46	7.1966E-46	5
App.2	0.35461375	0.631	0.561	7.35648E-46	5
Plk4.4	-0.392587354	0.024	0.129	1.27963E-45	5
Lmnb1.4	-0.429166802	0.15	0.315	1.77967E-45	5
Tubb4b.4	-0.47724305	0.103	0.254	1.92292E-45	5
Nap1l1.2	-0.365204389	0.34	0.529	2.30984E-45	5
Hk2.1	-0.439066781	0.056	0.185	3.50557E-45	5
Stmn3.1	0.316549751	0.678	0.615	3.85645E-45	5
Nt5dc2.1	0.454460386	0.321	0.228	5.78522E-45	5
Rangap1.5	-0.445570902	0.116	0.271	1.06997E-44	5
Rbfox2.3	0.435389744	0.377	0.273	1.18377E-44	5
Anln.4	-0.408061393	0.016	0.113	1.19995E-44	5
Gsk3b	0.331950209	0.545	0.494	1.27909E-44	5
Hmgb1.2	-0.368736655	0.202	0.375	3.39926E-44	5
Melk.4	-0.350788082	0.012	0.103	7.54508E-44	5
Fkbp3.1	-0.346626666	0.619	0.758	8.38853E-44	5
Chd4	0.25377067	0.785	0.767	8.48046E-44	5
Barhl1.4	0.321968332	0.604	0.512	1.07565E-43	5
Dkc1.1	-0.407898419	0.18	0.349	1.2225E-43	5
Sema6a.5	0.523675509	0.304	0.191	1.24755E-43	5
Aurka.5	-0.354523871	0.015	0.107	1.50375E-43	5
Mad2l1.4	-0.377002644	0.027	0.133	1.71317E-43	5
Sptbn1.4	0.448055969	0.442	0.344	4.48151E-43	5
Igsf8.2	0.451771387	0.367	0.258	5.12512E-43	5
B2m.1	-0.578968793	0.108	0.245	5.75783E-43	5
Mcm5.2	-0.47004887	0.056	0.18	8.99718E-43	5
Hrk	0.565366846	0.134	0.052	1.08763E-42	5
Arhgef2.1	0.405999871	0.347	0.275	1.2623E-42	5
Sowaha.2	-0.573589972	0.054	0.174	1.91255E-42	5
Ncaph.4	-0.385140329	0.027	0.131	2.10008E-42	5
Ect2.4	-0.346533596	0.015	0.106	3.64721E-42	5
Sox9.2	-0.504219999	0.124	0.269	3.72681E-42	5
H2afz.3	-0.393361551	0.101	0.245	4.31251E-42	5
Dpysl4.1	0.351574599	0.554	0.484	6.65523E-42	5
Rfc4.4	-0.399876781	0.098	0.24	7.47078E-42	5
Ebf3	0.451384469	0.28	0.194	1.65775E-41	5
Ncl.2	-0.276522882	0.821	0.904	1.85813E-41	5
Celf2.4	0.288149589	0.71	0.629	2.58389E-41	5
Racgap1.5	-0.451578539	0.12	0.269	2.73407E-41	5
Dbf4.4	-0.373081165	0.044	0.156	6.81912E-41	5
Hjurp.3	-0.42281687	0.276	0.45	7.08853E-41	5
Rbbp7.1	-0.363182212	0.174	0.335	1.34545E-40	5
Itsn1.4	0.485473543	0.335	0.225	1.814E-40	5
A330076H08Rik.3	0.497314708	0.271	0.149	2.15774E-40	5
Afap1.1	0.461308573	0.236	0.143	2.47998E-40	5

Draxin.2	-0.368481717	0.398	0.571	3.48923E-40	5
Rps19.2	-0.293448453	0.621	0.768	5.56109E-40	5
E130114P18Rik.1	0.34017006	0.667	0.575	6.44048E-40	5
Brd7.1	-0.340254044	0.169	0.322	8.14455E-40	5
Supt16.2	-0.343541617	0.332	0.51	9.00636E-40	5
A930011O12Rik.4	0.520114406	0.221	0.103	2.19603E-39	5
Snrpb.1	-0.320398923	0.413	0.59	3.71707E-39	5
Slc3a2.1	-0.377161174	0.18	0.338	4.6862E-39	5
Frmd4b.1	-0.363413768	0.034	0.137	4.83014E-39	5
Rtn4.3	0.314459037	0.521	0.475	5.50235E-39	5
Rif1.2	-0.415444577	0.14	0.29	9.72147E-39	5
Cklf.2	-0.394196786	0.058	0.176	1.06829E-38	5
Mcm7.2	-0.336019892	0.24	0.404	1.79167E-38	5
Rad51.4	-0.380744066	0.027	0.126	1.89357E-38	5
Uhrf1.4	-0.413064489	0.044	0.154	2.39348E-38	5
Lrig3.1	-0.399260341	0.062	0.181	2.545E-38	5
Slc7a5.1	-0.404406739	0.027	0.124	2.76944E-38	5
Ppp2r2c.1	0.338047847	0.579	0.496	3.08729E-38	5
Hmgn2.2	-0.426392664	0.126	0.269	3.10704E-38	5
Atp2b1.1	-0.316062779	0.414	0.588	3.19559E-38	5
Siva1.2	-0.416112458	0.141	0.286	3.54054E-38	5
Gdpd1.2	0.398150633	0.313	0.235	3.81815E-38	5
Vim.4	-0.490364509	0.166	0.317	1.61915E-37	5
Nde1.1	-0.358164821	0.03	0.128	1.77728E-37	5
Banp	0.438867309	0.194	0.13	3.35104E-37	5
Rad21.5	-0.413389958	0.313	0.477	3.71117E-37	5
Fam111a.4	-0.432337706	0.034	0.135	7.79602E-37	5
Pabpc1.1	-0.267559285	0.811	0.886	8.12086E-37	5
Sept8	-0.347625237	0.039	0.139	8.36237E-37	5
Ybx1.1	-0.26757855	0.661	0.799	9.74661E-37	5
Hnrnpd.2	-0.310434353	0.464	0.626	1.46042E-36	5
Diap3.4	-0.375157993	0.023	0.115	1.46323E-36	5
Smco4.2	-0.407622945	0.058	0.172	3.54331E-36	5
Atp1b3.1	0.344248593	0.392	0.331	4.88319E-36	5
Rpl41.2	-0.26471034	0.669	0.792	4.88874E-36	5
Nop56.2	-0.382376457	0.217	0.375	5.12551E-36	5
Eef1d.1	-0.289592755	0.211	0.364	7.41867E-36	5
Fth1	-0.307475087	0.58	0.722	7.61437E-36	5
Ncapd2.4	-0.353962457	0.076	0.196	1.18269E-35	5
Gria2.4	0.325202648	0.615	0.51	1.52815E-35	5
Fos.3	-0.568578604	0.173	0.318	1.55585E-35	5
Myod1.3	-0.430546023	0.045	0.151	1.61596E-35	5
Dixdc1.1	0.414835699	0.303	0.227	2.26789E-35	5
Jun.1	-0.36011469	0.531	0.668	2.69646E-35	5
Lsm2.1	-0.368942777	0.11	0.243	3.45585E-35	5
Mif.1	-0.370202474	0.218	0.371	3.5053E-35	5
Hist1h2ak.4	-0.49815677	0.048	0.153	4.12988E-35	5
B3galt2.4	0.476636407	0.193	0.087	9.30338E-35	5

Hpca.2	-0.483908496	0.15	0.29	1.088E-34	5
Cbfa2t3.3	-0.338044544	0.269	0.417	1.64985E-34	5
Ddr1	0.431034922	0.207	0.127	2.04446E-34	5
Smc6.3	-0.288705237	0.276	0.438	2.33458E-34	5
Rplp2.2	-0.258352852	0.752	0.849	5.76782E-34	5
Banf1.2	-0.30680717	0.563	0.707	7.37188E-34	5
Hsd11b2.4	-0.45986509	0.159	0.301	8.17498E-34	5
Idh2.2	-0.34412305	0.151	0.292	9.03801E-34	5
Cd24a	0.269138658	0.678	0.629	1.05523E-33	5
Tead2.2	-0.397420799	0.153	0.291	1.39921E-33	5
Topbp1.2	-0.341603802	0.052	0.155	2.40468E-33	5
Fstl1.1	-0.426555267	0.076	0.191	2.63503E-33	5
Hat1.2	-0.365042707	0.058	0.165	6.1914E-33	5
Soga3.2	0.307440911	0.505	0.452	1.34042E-32	5
Dnmt1.4	-0.385861572	0.151	0.291	2.54368E-32	5
Klf7.3	0.403752208	0.342	0.253	3.72107E-32	5
Pard6a	0.416752615	0.155	0.085	3.94603E-32	5
Fosb.1	-0.512483004	0.059	0.166	8.42485E-32	5
Snrpd1.2	-0.308462997	0.378	0.539	1.22097E-31	5
Gli1.2	-0.363207432	0.029	0.116	1.68966E-31	5
Hip1r.1	0.44332983	0.205	0.125	1.92957E-31	5
Pkp4.1	-0.286313402	0.028	0.11	1.95656E-31	5
Chd7.2	0.266075788	0.694	0.642	2.84455E-31	5
Usp22	0.290881336	0.401	0.356	2.8798E-31	5
Rps25.2	-0.300858938	0.339	0.494	2.92169E-31	5
G3bp1	-0.292529136	0.198	0.341	3.15268E-31	5
Srsf7.2	-0.308902527	0.322	0.48	4.10174E-31	5
Clic4.2	-0.305941071	0.115	0.239	4.1544E-31	5
Snhg1.2	-0.276057678	0.231	0.37	5.10801E-31	5
Cep57.2	-0.293417977	0.141	0.27	6.03836E-31	5
Rpl22.1	-0.25709871	0.68	0.799	6.65705E-31	5
Usp46.1	0.353337663	0.287	0.23	1.02766E-30	5
Lgals1.1	-0.461904864	0.118	0.24	1.03095E-30	5
Dtl.2	-0.372276252	0.053	0.155	1.03795E-30	5
Hmgn3	-0.365021809	0.148	0.282	1.13556E-30	5
Pa2g4.2	-0.277615871	0.38	0.537	1.33269E-30	5
Snrpf.1	-0.316953166	0.289	0.441	2.18217E-30	5
Lmo4.1	-0.302117058	0.207	0.349	2.30518E-30	5
Lsm6.2	-0.300226354	0.241	0.385	3.69687E-30	5
Scg3	0.2835079	0.476	0.443	4.16879E-30	5
Isoc1	-0.317937794	0.081	0.191	4.4105E-30	5
Mcm2.3	-0.413688097	0.098	0.216	4.48978E-30	5
Rpl39.1	-0.250254221	0.512	0.66	5.44459E-30	5
Spop	-0.301242559	0.19	0.328	5.5249E-30	5
Dctpp1.2	-0.356830791	0.179	0.317	6.35494E-30	5
Aldoa.3	0.375550109	0.336	0.249	8.38867E-30	5
Dctn3	0.26984372	0.394	0.365	8.95322E-30	5
Dnph1.2	-0.322291507	0.04	0.132	1.05064E-29	5

Skp2.1	-0.282105999	0.04	0.128	1.11751E-29	5
Hdgf.1	-0.306695236	0.395	0.546	1.55315E-29	5
Nolc1.1	-0.263167852	0.275	0.422	1.67012E-29	5
Rps20.2	-0.258329716	0.679	0.788	1.8782E-29	5
Schip1	0.397434716	0.262	0.173	1.97818E-29	5
Cep110.1	-0.361685498	0.068	0.173	2.18662E-29	5
Sri	-0.361360949	0.069	0.176	2.20111E-29	5
Ccdc41.2	-0.269940242	0.144	0.265	2.36747E-29	5
Nsmce4a.1	-0.269753175	0.119	0.234	2.63487E-29	5
Celf4.5	0.283681821	0.43	0.308	2.66297E-29	5
Cdca7l.2	-0.329223483	0.029	0.112	3.5297E-29	5
Irs1.1	-0.3065311	0.039	0.127	4.10734E-29	5
6330403K07Rik.2	0.396775396	0.316	0.234	4.13653E-29	5
Ckap5.5	-0.33179636	0.148	0.278	5.18336E-29	5
Carhsp1	0.33324919	0.306	0.248	5.60621E-29	5
Rpl22l1.1	-0.294488275	0.224	0.364	9.63921E-29	5
Rundc3a.2	0.340612691	0.306	0.242	1.5287E-28	5
Lbr.3	-0.314552782	0.095	0.207	1.57945E-28	5
Serinc1.3	0.250088126	0.46	0.433	1.63073E-28	5
Cacna2d1.1	0.333717375	0.43	0.369	1.85738E-28	5
Dhfr.3	-0.382930598	0.058	0.158	1.99071E-28	5
Napa	0.26023957	0.371	0.347	2.07538E-28	5
Cenpw.4	-0.310663702	0.045	0.136	2.15277E-28	5
Nudcd2.1	-0.26317505	0.121	0.234	2.16124E-28	5
Sept3.5	0.325084376	0.502	0.38	2.33831E-28	5
Ezh2.4	-0.331336678	0.488	0.622	3.59913E-28	5
Lyar.2	-0.281794898	0.17	0.298	3.72083E-28	5
Kif1b.3	0.264670283	0.569	0.515	3.82385E-28	5
Pttg1.2	-0.381682262	0.054	0.148	4.25852E-28	5
Atp6v0e.2	0.255122422	0.444	0.404	5.11719E-28	5
H13.1	0.347131827	0.296	0.231	6.72983E-28	5
Zic4.2	0.310402262	0.47	0.412	6.99818E-28	5
Naa50.1	-0.256641623	0.171	0.297	8.93913E-28	5
Rfc2.2	-0.320422437	0.106	0.222	9.05974E-28	5
Pou3f2.1	-0.318955184	0.161	0.288	9.1178E-28	5
Mrpl42	-0.280691715	0.197	0.331	1.05464E-27	5
Cald1	0.250776493	0.573	0.531	1.11564E-27	5
Itm2b.4	0.25943581	0.686	0.638	2.01696E-27	5
Tpm4.1	-0.2648872	0.198	0.329	2.073E-27	5
Rfc1.2	-0.281029945	0.21	0.343	2.24463E-27	5
Gdi1.3	0.363347798	0.3	0.222	2.81914E-27	5
2610203C20Rik.2	0.425069609	0.192	0.113	3.65057E-27	5
BC031181	0.318599601	0.312	0.256	5.37284E-27	5
Pfn1	-0.264416085	0.38	0.524	1.1812E-26	5
Cerk	-0.30442877	0.063	0.16	1.7429E-26	5
Gng2.2	0.266730265	0.427	0.392	1.8412E-26	5
Uchl1.4	0.32924309	0.401	0.327	1.9493E-26	5
Timeless.2	-0.29649941	0.067	0.163	2.31974E-26	5

Hsp90b1.1	-0.2921509	0.578	0.703	2.32572E-26	5
Clic1	0.277777089	0.194	0.17	3.02221E-26	5
Ctnnb1.1	-0.250014464	0.261	0.4	3.25811E-26	5
Hes6.2	-0.317424195	0.13	0.247	3.78583E-26	5
Lmcd1	0.388868999	0.113	0.056	4.28137E-26	5
Fen1.3	-0.318652323	0.056	0.15	4.38608E-26	5
Cbfb.1	-0.255398646	0.092	0.193	6.00172E-26	5
Csrp2.2	-0.340480411	0.068	0.167	6.73531E-26	5
Ier2.2	-0.32442356	0.26	0.395	6.91402E-26	5
Suz12.1	-0.291053758	0.119	0.229	1.03541E-25	5
C530008M17Rik	0.322980954	0.308	0.263	1.63063E-25	5
Mab21l1.3	0.359316651	0.283	0.218	1.65467E-25	5
Psat1.2	-0.315651965	0.243	0.381	2.01086E-25	5
Mab21l2	0.402853158	0.164	0.097	2.39176E-25	5
Rnaseh2b.1	-0.257633796	0.134	0.247	3.10669E-25	5
Ctsd.5	-0.709415392	0.117	0.181	3.17633E-25	5
Add3.1	-0.284406879	0.034	0.111	3.55951E-25	5
Ptn.4	-0.326368367	0.345	0.451	4.01747E-25	5
Rnmt.2	0.30532319	0.395	0.343	4.7952E-25	5
Atoh1.2	-0.36166779	0.073	0.174	4.93667E-25	5
Cdc45.3	-0.296005024	0.027	0.102	5.86947E-25	5
Alyref.1	-0.303933554	0.102	0.21	6.96863E-25	5
Ddx39.1	-0.263344518	0.095	0.197	7.10442E-25	5
Shmt1.2	-0.27315213	0.033	0.11	7.30006E-25	5
Fbxo32.2	0.399241846	0.189	0.108	7.94362E-25	5
Nr3c1	-0.321886002	0.047	0.134	9.18474E-25	5
Sae1.2	-0.260314122	0.169	0.29	1.14102E-24	5
Acot7	-0.312014692	0.098	0.205	1.15491E-24	5
D4Wsu53e.3	0.27516717	0.491	0.441	1.19919E-24	5
Ska2.4	-0.298915916	0.107	0.215	1.23377E-24	5
Gpr56	0.334460509	0.275	0.216	1.32341E-24	5
Cdt1.3	-0.336347924	0.047	0.133	1.40752E-24	5
Syt13.1	-0.321589529	0.048	0.135	1.78896E-24	5
Ddx21.1	-0.277941402	0.21	0.334	1.90804E-24	5
Bub3.3	-0.268951546	0.207	0.333	2.81618E-24	5
Rpa1.2	-0.284897626	0.037	0.116	2.90261E-24	5
S100a16.4	0.410679755	0.163	0.087	3.91197E-24	5
Atp6v1e1.2	0.295075779	0.371	0.326	4.07023E-24	5
Cst3.5	-0.370186316	0.473	0.542	4.40693E-24	5
Fam213b.2	0.394689623	0.226	0.15	4.76922E-24	5
Cmtm3.1	-0.290818899	0.044	0.125	8.63276E-24	5
Tcf19.4	-0.32907593	0.03	0.105	8.68725E-24	5
Dlgap4.2	0.374247327	0.264	0.179	1.21023E-23	5
Btbd17.2	0.379603764	0.228	0.152	1.32919E-23	5
Stx4a.1	-0.302197643	0.069	0.163	1.44425E-23	5
Magoh	-0.253697798	0.218	0.345	1.5212E-23	5
Nrm.1	-0.284446038	0.082	0.178	1.6108E-23	5
Pik3r3.2	0.389653549	0.21	0.127	1.79637E-23	5

Map1lc3b.2	0.256157807	0.368	0.327	1.9994E-23	5
Chst15	0.370025731	0.143	0.071	2.07038E-23	5
Mphosph10	-0.267327632	0.132	0.241	2.29264E-23	5
Plk3	0.369365601	0.135	0.066	2.72849E-23	5
Smpd2.1	-0.300833491	0.079	0.175	2.79019E-23	5
St18.4	0.417078494	0.269	0.182	3.4414E-23	5
Mtap	-0.296578694	0.029	0.101	3.75242E-23	5
Gramd1a	0.328061283	0.211	0.16	4.18245E-23	5
Ube2d1	0.268499128	0.256	0.224	4.34672E-23	5
Vrk1.2	-0.269694251	0.067	0.156	4.62145E-23	5
Pola1.1	-0.295362707	0.034	0.11	4.74752E-23	5
Cadm1.1	-0.284325552	0.27	0.403	5.04414E-23	5
Smarcd1	0.281003916	0.3	0.267	5.24136E-23	5
Ntm.2	-0.380429	0.065	0.154	1.13717E-22	5
H1fx.3	-0.322496753	0.141	0.256	1.24474E-22	5
Aplp1.5	0.394459179	0.239	0.142	1.26445E-22	5
Gpc2.1	0.372620232	0.206	0.132	1.39481E-22	5
Mis12.2	-0.260400873	0.032	0.105	1.5122E-22	5
Cntln.2	-0.302797519	0.085	0.181	1.62214E-22	5
Fabp7.5	-0.8735864	0.086	0.127	2.90377E-22	5
Ccdc18.1	-0.269723625	0.045	0.122	3.0304E-22	5
Fnbp1l.2	0.258613136	0.492	0.449	3.17337E-22	5
Tprn.1	0.368883382	0.303	0.218	3.30656E-22	5
Ybx3.1	-0.252073747	0.185	0.301	3.46875E-22	5
Adamts1.1	-0.350749563	0.075	0.163	7.24187E-22	5
Clip3.2	0.305850184	0.372	0.305	7.83798E-22	5
Ccdc50	0.251871206	0.357	0.323	1.38098E-21	5
Ramp2.1	-0.334013932	0.048	0.127	1.49723E-21	5
Dda1	0.252632287	0.278	0.249	1.61293E-21	5
Id2.3	-0.290642234	0.366	0.499	1.6784E-21	5
Nudc.1	-0.270407889	0.176	0.292	1.78399E-21	5
Bcl2l1	0.347259236	0.135	0.083	2.07931E-21	5
Necab3	0.363987803	0.112	0.047	2.12812E-21	5
Boc.2	-0.307334484	0.073	0.164	2.63381E-21	5
Scg5.2	0.279099016	0.381	0.329	3.05087E-21	5
Frmd4a.1	0.304181334	0.354	0.303	3.08584E-21	5
Prkcb.4	0.337484425	0.326	0.248	3.18716E-21	5
Clvs1.3	0.373426219	0.197	0.121	3.57392E-21	5
Ldha.1	-0.280370857	0.135	0.242	3.98622E-21	5
Smc5	-0.251950628	0.159	0.268	7.66781E-21	5
Clcn4-2.2	0.303619688	0.302	0.245	1.27505E-20	5
Dbn1.4	0.364773145	0.207	0.132	1.42817E-20	5
Mmp14.1	-0.288589346	0.09	0.183	2.07833E-20	5
Maged2.1	0.329881144	0.222	0.16	2.22332E-20	5
Lpin2.1	-0.320357358	0.093	0.189	2.79591E-20	5
Mcm4.2	-0.313976296	0.061	0.146	3.53213E-20	5
Tubb2a.5	0.36465694	0.329	0.235	3.82993E-20	5
Snx5	-0.288065129	0.082	0.172	4.03533E-20	5

Ptch2.2	-0.262629599	0.074	0.16	4.35798E-20	5
Nin.1	-0.261436178	0.07	0.154	4.55004E-20	5
Apitd1.2	-0.258382224	0.033	0.101	4.96259E-20	5
Chd3.4	0.392448599	0.211	0.142	5.01594E-20	5
Strbp.1	0.266983247	0.384	0.343	7.10125E-20	5
Hist3h2a.2	0.36802048	0.204	0.137	7.61809E-20	5
Igsf21.1	0.382956296	0.155	0.082	9.38277E-20	5
Gadd45a	0.357887489	0.144	0.082	9.44389E-20	5
Srebf1.3	-0.264933949	0.231	0.345	9.49338E-20	5
Fibp	0.27759347	0.211	0.176	1.27345E-19	5
Rad50.1	-0.298556627	0.084	0.174	1.29169E-19	5
Fam107b	0.337242958	0.153	0.096	2.19511E-19	5
Rrbp1.1	-0.313874611	0.102	0.197	2.35673E-19	5
Mpp6.1	-0.276313755	0.081	0.17	2.6877E-19	5
Srgap2.2	0.293903303	0.16	0.129	2.70881E-19	5
Nsg1.3	0.277295846	0.324	0.283	2.74687E-19	5
Npdc1.3	0.284916934	0.303	0.257	3.74226E-19	5
Tmem57.1	0.27342757	0.336	0.286	4.19353E-19	5
Oraov1	-0.265836636	0.051	0.128	5.68093E-19	5
Kdm5b.3	0.344843848	0.26	0.176	6.26823E-19	5
Mien1.1	0.276473982	0.261	0.224	1.34252E-18	5
Elovl6	0.273557297	0.315	0.274	1.45731E-18	5
Atp6v0b.3	0.289184319	0.313	0.262	1.93961E-18	5
Gm11266.2	0.362701894	0.201	0.137	2.13971E-18	5
Acd	0.305967041	0.205	0.156	2.33735E-18	5
Nav2.1	0.31913609	0.143	0.101	3.12706E-18	5
Enox2	0.312033668	0.103	0.051	3.24114E-18	5
Zic5	0.288564984	0.189	0.157	3.69023E-18	5
Zfp423	0.328948996	0.137	0.077	4.77431E-18	5
Kif5a.4	0.362507494	0.198	0.12	5.33397E-18	5
Pvrl3	-0.277419779	0.054	0.129	6.15897E-18	5
Brca2.2	-0.253522785	0.049	0.12	8.42565E-18	5
Stxbp1.5	0.347882701	0.23	0.155	9.26872E-18	5
Myt1l.4	0.369362846	0.217	0.155	9.7464E-18	5
Sbk1.1	0.327222877	0.165	0.113	1.35679E-17	5
Glul	-0.276451356	0.125	0.209	1.9368E-17	5
Ldb1	0.290929754	0.225	0.179	3.17487E-17	5
Rnf165	0.311831273	0.214	0.168	3.69648E-17	5
Sox11	0.274728935	0.15	0.111	4.14122E-17	5
Map6	0.333624877	0.134	0.073	6.72494E-17	5
Ppp2r2b	0.34816326	0.134	0.079	9.47603E-17	5
Cep290.1	-0.259050062	0.072	0.15	1.05227E-16	5
Psmc3ip.2	-0.273807601	0.062	0.139	1.2188E-16	5
Dner.5	0.32122072	0.284	0.207	1.34559E-16	5
Grb2	0.255137586	0.276	0.246	1.52792E-16	5
Smpd3	0.273500748	0.189	0.158	1.67274E-16	5
Smarcd2	0.313631567	0.141	0.092	1.75754E-16	5
2810025M15Rik	-0.257121837	0.091	0.175	2.03913E-16	5

Pak7.2	0.351787911	0.162	0.098	2.90745E-16	5
Trpc4ap.5	0.351820369	0.247	0.192	3.3264E-16	5
Ncor2.1	-0.251208493	0.093	0.176	5.56821E-16	5
Scrt1	0.309889854	0.104	0.053	6.23139E-16	5
Atad5.2	-0.27973013	0.076	0.156	7.093E-16	5
Gamt.2	0.310481146	0.224	0.163	7.0976E-16	5
Tmx4	0.28332422	0.216	0.173	7.19519E-16	5
Cadm3.4	0.335020167	0.207	0.134	8.47045E-16	5
Efhd2.1	-0.273930113	0.063	0.137	1.01628E-15	5
Mical1	-0.268188591	0.059	0.132	1.44944E-15	5
Klhl7	0.250743183	0.193	0.161	1.73057E-15	5
Dcc	0.294540144	0.103	0.059	1.88677E-15	5
Pygo1.2	0.280610984	0.218	0.189	1.98588E-15	5
Bzrap1	0.336376152	0.107	0.056	2.40903E-15	5
9330159F19Rik	0.27613098	0.21	0.164	2.93807E-15	5
Rnf122	0.286369055	0.139	0.098	4.00811E-15	5
Tspan7.2	-0.302534214	0.047	0.111	4.20846E-15	5
Nek7.1	-0.263559789	0.054	0.123	4.21869E-15	5
Atl1	0.272872897	0.127	0.099	5.56898E-15	5
Nova2	0.302862142	0.103	0.053	6.37968E-15	5
Dusp8	0.315072588	0.13	0.073	7.03233E-15	5
Crip2.1	0.276960978	0.275	0.236	7.48508E-15	5
Nol4	0.269528827	0.21	0.167	1.23416E-14	5
Trak1	0.287820329	0.124	0.085	1.3935E-14	5
Slc1a3.4	-0.364694341	0.097	0.158	1.39478E-14	5
Scmh1	0.268256953	0.108	0.074	2.2556E-14	5
Myc.1	-0.277844217	0.043	0.106	3.17242E-14	5
Smoc1	-0.254548285	0.068	0.141	3.28474E-14	5
Brsk2	0.316136242	0.129	0.083	7.34104E-14	5
Arid3a	0.299282872	0.146	0.09	8.9404E-14	5
Baz1a.1	-0.28552376	0.094	0.174	8.96152E-14	5
Eif4e3.1	0.300569095	0.18	0.125	1.83147E-13	5
Srrm4.4	0.298090379	0.243	0.19	3.14793E-13	5
Prmt2.1	0.270411246	0.136	0.09	3.24001E-13	5
Tmem178b	0.283946977	0.111	0.065	5.04587E-13	5
Plcb1.3	0.323931207	0.181	0.136	6.90344E-13	5
Pea15a.4	0.278149115	0.217	0.171	8.03776E-13	5
Grina.3	0.306665443	0.157	0.104	9.33969E-13	5
Mkrn1.1	0.251508886	0.243	0.204	1.3688E-12	5
Apbb1.4	0.282853985	0.214	0.165	1.46652E-12	5
Clybl	0.282191592	0.156	0.112	2.62355E-12	5
Tmeff1	0.272319576	0.193	0.144	3.98583E-12	5
Rnasel	0.317370299	0.125	0.067	5.21076E-12	5
Pdgfra.1	0.273621135	0.237	0.175	5.26986E-12	5
2700081O15Rik	0.280324944	0.152	0.106	5.31524E-12	5
Mroh2a.2	0.317581378	0.162	0.108	5.79982E-12	5
Clstn1.1	0.257993709	0.23	0.199	6.24114E-12	5
Kif1a.2	0.254469513	0.204	0.168	1.29419E-11	5

Mapk10	0.271548356	0.144	0.094	1.58033E-11	5
Itga7	0.27737423	0.136	0.085	1.94826E-11	5
Nrcam.3	0.269217908	0.161	0.131	2.1373E-11	5
Dpysl3.5	0.312192531	0.281	0.212	3.09017E-11	5
Gramd1b.2	0.251763693	0.197	0.166	3.78028E-11	5
Lingo1	0.294675267	0.138	0.09	4.67163E-11	5
Shd.1	0.251071989	0.141	0.106	6.23215E-11	5
Tubb4a	0.261101642	0.104	0.063	9.53107E-11	5
Parp6	0.260495499	0.163	0.121	1.02963E-10	5
Atat1	0.26043479	0.144	0.099	1.33926E-10	5
Reln.2	0.263312374	0.211	0.176	1.64692E-10	5
Ncan.2	0.26728456	0.131	0.095	3.22627E-10	5
Rnd2.1	0.252985676	0.192	0.154	1.15318E-09	5
Fam57b	0.251920191	0.228	0.181	4.01126E-09	5
Eml5	0.258078252	0.127	0.089	1.71921E-08	5
Plp1.5	-0.375882354	0.107	0.115	0.451209098	5
Celf4.6	1.670783338	0.821	0.27	0	6
Neurod1.6	1.581942962	0.967	0.49	0	6
Meg3.5	1.545660771	0.42	0.063	0	6
Mapt.5	1.506170676	0.643	0.174	0	6
Arpp21.5	1.492349535	0.48	0.076	0	6
Gpm6a.5	1.477988192	0.84	0.366	0	6
Neurod2	1.289948971	0.359	0.038	0	6
Stmn2.5	1.285677509	0.949	0.513	0	6
Nrxn1.5	1.285517589	0.682	0.282	0	6
Stmn4.6	1.275609007	0.711	0.304	0	6
Tubb2a.6	1.270634153	0.616	0.208	0	6
Dpysl3.6	1.247722941	0.583	0.183	0	6
Ank2.5	1.227787162	0.57	0.207	0	6
Sept3.6	1.227093317	0.756	0.356	0	6
Thra.5	1.226266817	0.576	0.194	0	6
Rtn1.6	1.223190675	0.959	0.663	0	6
Ppp1r14c.5	1.191170672	0.604	0.228	0	6
Nrep.5	1.134537197	0.855	0.541	0	6
Mtss1.5	1.125988095	0.594	0.237	0	6
Gria2.5	0.968603053	0.783	0.495	0	6
Celf2.5	0.951401489	0.837	0.618	0	6
Atp2b1.2	0.946420858	0.749	0.553	0	6
Map1b.5	0.932599287	0.834	0.563	0	6
Zic1.4	0.844822167	0.952	0.767	0	6
Calm1.3	0.716783276	0.942	0.853	0	6
Tuba1a.4	0.684580698	0.975	0.925	0	6
Rps9.1	-0.840612004	0.715	0.95	0	6
Rpl32.1	-0.859760151	0.631	0.915	0	6
Rps5.2	-0.862416516	0.807	0.968	0	6
Rpl8.2	-0.88759759	0.642	0.922	0	6
Rps3.1	-0.956931005	0.663	0.947	0	6
Rpl13a.1	-0.986509157	0.7	0.95	0	6

Rps26.2	-0.999875869	0.521	0.879	0	6
Rps19.3	-1.062957079	0.359	0.792	0	6
Rplp1.3	-1.076960579	0.543	0.909	0	6
Sfrp1.3	-1.243693376	0.447	0.847	0	6
Pabpc1.2	-0.856996975	0.606	0.905	0	6
Rps20.3	-0.993372631	0.389	0.816	0	6
Rpl4.1	-0.78874654	0.7	0.928	0	6
Car10	1.240021458	0.278	0.017	0	6
Fxyd6.3	0.901533561	0.737	0.486	9.9389E-308	6
Gnb2l1.1	-0.83710671	0.593	0.899	6.8015E-307	6
Pcp4	1.477545837	0.299	0.028	6.4949E-306	6
Rps14.1	-0.675809111	0.826	0.962	1.6296E-302	6
Rplp2.3	-0.882734365	0.528	0.871	5.0706E-302	6
Rbfox3.3	0.91634806	0.755	0.468	6.8163E-291	6
Malat1.4	0.706772948	0.991	0.951	8.3981E-288	6
Gap43.6	0.829065406	0.848	0.574	2.3724E-287	6
Rpl22.2	-0.918257362	0.432	0.823	5.5113E-286	6
Jph4	1.243550583	0.331	0.042	9.8491E-284	6
Ccnd1.4	-1.404299526	0.119	0.561	1.6913E-282	6
Rps11.1	-0.925508689	0.418	0.813	5.5141E-282	6
L1cam	1.155726226	0.297	0.029	3.5295E-269	6
Sez6	1.165925425	0.29	0.032	9.9332E-269	6
Ttc3.3	0.645600768	0.918	0.82	4.351E-268	6
Cd63.1	-0.91864336	0.393	0.79	4.5521E-266	6
Rplp0.1	-0.778900927	0.619	0.897	1.7325E-265	6
Eef1a1.1	-0.768622354	0.662	0.906	7.643E-265	6
Ccnd2.3	-1.147440737	0.278	0.699	4.0263E-264	6
Rps21.2	-0.802773007	0.525	0.863	5.5296E-259	6
Elmo1.5	1.191353078	0.418	0.097	1.9607E-258	6
Gng3.5	1.003277844	0.611	0.287	2.1703E-253	6
Plxna2	1.141799172	0.304	0.046	2.7235E-248	6
Smc2.5	-1.352435332	0.175	0.581	3.4643E-244	6
Rpl41.3	-0.820101609	0.446	0.813	1.6101E-243	6
Npm1.3	-0.964363626	0.263	0.684	1.2843E-241	6
Prdx1.3	-1.091895417	0.119	0.526	4.5496E-235	6
Cntn2.6	0.958469305	0.614	0.228	1.2832E-234	6
Tubb2b.5	0.809222742	0.683	0.442	1.6889E-230	6
Ankrd12.3	1.053286198	0.542	0.248	4.0702E-230	6
Ranbp1.3	-0.973813836	0.318	0.71	6.3207E-230	6
H2afv.4	-0.938652688	0.408	0.759	9.9486E-228	6
Anp32b.4	-1.017230424	0.215	0.627	4.4848E-227	6
Dner.6	1.022331007	0.489	0.187	1.0268E-224	6
Cdk5r1.5	0.910360859	0.578	0.309	3.0641E-219	6
Rps15.1	-0.754396421	0.479	0.818	3.0409E-217	6
Cadps2	1.096140444	0.261	0.042	2.3334E-210	6
Tmsb4x.4	0.512334709	0.977	0.946	2.0231E-209	6
Rps3a1.2	-0.843169481	0.314	0.701	3.9385E-209	6
Cdk4	-0.845701289	0.283	0.677	6.2241E-207	6

Gnao1.3	0.914442216	0.519	0.264	6.8328E-206	6
Dusp26	1.115795234	0.28	0.044	6.8341E-206	6
Rps24.1	-0.709989643	0.55	0.847	1.5708E-205	6
Eef1b2.2	-0.783346753	0.403	0.765	2.1535E-204	6
2810417H13Rik.6	-1.345357393	0.073	0.423	2.2288E-204	6
Kcnk1.4	0.973824964	0.45	0.18	3.5918E-204	6
Rpl14.2	-0.818680617	0.362	0.729	5.2015E-204	6
Smc4.5	-1.189909594	0.236	0.599	1.2214E-201	6
Cadm2	1.083228193	0.24	0.028	1.3423E-201	6
Mdk.3	-1.214849404	0.062	0.41	5.4803E-201	6
Ina.5	0.805269236	0.664	0.421	1.0605E-199	6
Nbea.2	1.001600058	0.412	0.168	1.4501E-199	6
Basp1.5	0.629747208	0.835	0.691	3.6447E-199	6
Anp32e.5	-0.946810318	0.268	0.647	9.2782E-198	6
E130114P18Rik.2	-0.923115812	0.231	0.619	1.151E-197	6
Zfpm2	0.944383587	0.226	0.024	2.6357E-197	6
Camk2d	1.016503295	0.279	0.053	9.1267E-197	6
Srebf1.4	-1.149762737	0.039	0.363	2.0063E-194	6
Draxin.3	-0.932703581	0.204	0.588	2.7186E-194	6
Uchl1.5	0.80332006	0.52	0.316	1.1942E-191	6
Hmgb2.5	-1.195202997	0.085	0.43	1.9502E-191	6
Sv2a	1.025541212	0.286	0.051	2.5888E-189	6
Snca	1.124009806	0.241	0.042	1.237E-188	6
Dbi.1	-1.069939724	0.137	0.495	4.3311E-188	6
Rpl39.2	-0.815344172	0.309	0.679	1.4117E-187	6
Dcx.5	0.815842667	0.595	0.354	1.9298E-184	6
Mki67.6	-1.379814917	0.092	0.424	8.6615E-184	6
Zfand5	0.737188765	0.555	0.401	3.226E-183	6
Zbtb18.3	0.850703377	0.477	0.245	7.0028E-183	6
Kcna1	0.905146612	0.177	0.012	1.2811E-181	6
Kif1b.4	0.687644636	0.666	0.506	1.3693E-179	6
Phf20l1.1	0.820082698	0.512	0.323	8.394E-178	6
Psat1.3	-0.938373765	0.07	0.396	1.5985E-176	6
Tspan4	1.008455138	0.26	0.046	7.8107E-176	6
Stmn3.2	0.611666481	0.742	0.61	2.6482E-175	6
Gnai2.2	-0.881775573	0.111	0.455	2.7818E-174	6
Ppp3ca.2	0.782092787	0.557	0.36	2.4764E-173	6
Cks1b.5	-1.016531216	0.058	0.372	1.2926E-172	6
Grin2b	0.946463379	0.229	0.029	1.8611E-172	6
2900079G21Rik	0.939319742	0.224	0.031	2.1604E-172	6
Cadm3.5	0.964302854	0.368	0.118	1.3838E-171	6
Rnd3.3	-0.920665307	0.118	0.462	1.8935E-171	6
Aplp1.6	0.945219553	0.381	0.129	2.4488E-171	6
Gm12022	0.959085232	0.167	0.011	2.7463E-171	6
Camk4	0.904879409	0.212	0.027	4.325E-171	6
Sh3gl2.1	0.917467843	0.374	0.151	1.9487E-170	6
Prkcb.5	0.85475132	0.476	0.234	2.6236E-170	6
Prdm8.5	0.9790727	0.334	0.106	1.4877E-169	6

Rps15a.1	-0.740378193	0.34	0.688	1.5841E-169	6
Prkce	0.928634937	0.236	0.047	1.8673E-167	6
Rab3a.6	0.93569927	0.381	0.127	1.4183E-166	6
Tnik.4	0.990505254	0.375	0.123	2.2773E-165	6
Chd7.3	0.590469001	0.751	0.637	2.3084E-164	6
Ndst3	0.9036585	0.187	0.019	2.7326E-164	6
Top2a.6	-1.324755313	0.149	0.455	1.398E-163	6
Cygb	0.923522192	0.224	0.034	4.5377E-163	6
Nop58.3	-0.762506585	0.267	0.624	1.0538E-162	6
Anks1b	0.935984723	0.246	0.045	3.359E-160	6
Grin1	0.843676879	0.189	0.02	5.0799E-160	6
Fkbp3.2	-0.707739632	0.465	0.772	7.0448E-160	6
Hmgn5.3	-0.972280362	0.124	0.453	1.3038E-159	6
Epb4.1l1	0.947916826	0.286	0.071	2.5234E-159	6
Ryr2	0.83504123	0.148	0.008	5.9672E-159	6
Marcks	0.445898591	0.952	0.912	1.6059E-156	6
Tead2.3	-0.891517611	0.031	0.302	1.7578E-156	6
Rps27l	-0.744999706	0.182	0.522	2.8643E-155	6
Dek.6	-0.809368449	0.428	0.719	4.7151E-155	6
Mcm6.4	-0.974619053	0.031	0.3	2.8948E-154	6
Serinc1.4	0.650960843	0.577	0.422	1.5227E-153	6
Lrrtm2	0.906432457	0.215	0.035	1.7598E-153	6
Ephb1	0.859484491	0.195	0.025	1.5422E-152	6
Id2.4	0.721630482	0.629	0.472	4.2213E-150	6
D3Bwg0562e	0.897607113	0.166	0.017	1.1322E-149	6
Dut.5	-0.911615756	0.143	0.463	2.4889E-149	6
Cpe.3	0.648194954	0.599	0.441	4.8849E-149	6
Rps2.2	-0.771791455	0.151	0.479	9.7602E-149	6
Synpr	0.853278795	0.191	0.028	1.0973E-148	6
Ybx1.2	-0.612171607	0.534	0.81	1.5416E-148	6
Hnrnpab.1	-0.593517279	0.586	0.838	2.1809E-148	6
Pvrl3.1	0.853988361	0.272	0.107	4.7806E-148	6
Tbata.4	-1.057953241	0.074	0.366	6.1485E-148	6
Pcsk1n	0.892502698	0.28	0.064	5.5453E-147	6
Rpl26.1	-0.641669405	0.415	0.73	9.1342E-147	6
Pax6.4	0.618399156	0.67	0.497	5.2877E-144	6
Pcna.6	-0.976638403	0.09	0.384	6.6855E-144	6
Sema6a.6	0.847626343	0.403	0.182	1.2678E-143	6
Rbp4.4	-0.948944022	0.02	0.263	5.7466E-143	6
Nasp.5	-0.7660354	0.356	0.668	9.7974E-143	6
Nhp2.1	-0.82700313	0.096	0.397	1.0121E-142	6
C1ql1.3	-0.916437109	0.039	0.302	2.0533E-142	6
Rpl35a.2	-0.689644115	0.269	0.6	2.3036E-142	6
Stxbp1.6	0.842462118	0.347	0.144	2.1848E-141	6
Gabra2	0.696252116	0.123	0.006	6.175E-141	6
Tmpo.5	-0.821070897	0.16	0.481	1.5265E-140	6
Gsg1l.4	-0.881546561	0.029	0.277	3.0037E-139	6
Rbfox1.1	0.855003913	0.262	0.075	3.9106E-138	6

Birc5.6	-1.017401094	0.04	0.296	5.0841E-138	6
Ppp1r14b	-0.798796785	0.122	0.424	8.6473E-138	6
Ran.3	-0.780248108	0.133	0.441	9.3576E-138	6
D4Wsu53e.4	0.632193123	0.573	0.434	1.2984E-137	6
Tpx2.6	-1.072968394	0.044	0.304	2.7015E-137	6
Cplx1.2	0.84945139	0.313	0.121	1.9612E-136	6
Snrpf.2	-0.718278929	0.148	0.454	1.9636E-136	6
Porcn	0.870856044	0.256	0.066	3.7927E-136	6
Pkia.3	0.83421785	0.307	0.116	5.7092E-136	6
Tuba1b.5	-0.829196926	0.197	0.509	1.7785E-135	6
Cdk1.6	-0.989920629	0.027	0.268	3.6413E-135	6
Rps25.3	-0.700364434	0.192	0.508	3.3388E-134	6
Mllt11.5	0.809781993	0.406	0.199	1.3995E-133	6
Ncam1.1	0.623173503	0.51	0.374	7.7804E-133	6
Apbb1.5	0.831601365	0.352	0.152	1.6323E-132	6
Dtymk.4	-0.764776674	0.161	0.466	8.9423E-132	6
Grm1	0.750103657	0.131	0.008	2.0056E-131	6
Grik2.3	0.859324264	0.272	0.085	7.1733E-131	6
Hey1.4	-0.920628661	0.043	0.295	2.006E-130	6
Atp6v0e.3	-0.696402259	0.14	0.434	2.3074E-130	6
Cdca8.6	-0.951649071	0.04	0.286	4.7027E-130	6
RP23-45G16.5.6	-0.951663029	0.079	0.351	9.7623E-130	6
Rps10.1	-0.590984569	0.354	0.664	1.5445E-129	6
Fnbp1l.3	0.631295836	0.585	0.44	6.3478E-128	6
CRE_RECOMBINASE.5	-0.822728125	0.579	0.786	8.4037E-128	6
Hmgn1.2	-0.601716813	0.333	0.65	1.5011E-127	6
Prdx6	-0.788668705	0.079	0.35	3.6255E-127	6
Ier5.1	-0.728472311	0.132	0.42	2.451E-126	6
Shfm1	-0.574458709	0.411	0.713	8.2161E-126	6
Eid1.2	0.553309125	0.609	0.492	1.1625E-125	6
Rpl34.1	-0.590073729	0.372	0.68	1.6607E-125	6
Elavl3.5	0.567031559	0.64	0.455	2.1398E-125	6
Kif5c.5	0.653796717	0.552	0.383	3.067E-125	6
Syp	0.824122315	0.224	0.053	6.3458E-125	6
2700094K13Rik.5	-0.700413807	0.346	0.64	8.0242E-125	6
Tomm7	-0.64659239	0.216	0.525	1.6242E-123	6
Ube2c.5	-1.237136098	0.054	0.29	2.1603E-123	6
Hsd11b2.5	-0.841860562	0.058	0.31	6.083E-123	6
Spc24.5	-0.859816563	0.032	0.263	8.2902E-123	6
Slc25a5.2	-0.656952847	0.231	0.536	1.1798E-122	6
Cenpf.6	-1.203696858	0.121	0.385	1.0491E-121	6
Cog7.2	-0.596284465	0.303	0.612	2.5195E-121	6
Tagln3.5	0.717207657	0.469	0.277	4.0898E-121	6
Cadm1.2	-0.68656901	0.132	0.416	8.8589E-121	6
Dctpp1.3	-0.729483883	0.072	0.327	1.2939E-120	6
Cenpa.5	-1.125105305	0.043	0.272	3.222E-120	6
Rpl23.1	-0.60716758	0.261	0.566	7.3592E-120	6
Racgap1.6	-0.815119555	0.041	0.276	8.5259E-120	6

Hirip3.5	-0.809322188	0.125	0.408	1.0799E-119	6
H2afy.2	-0.606654338	0.315	0.619	4.2993E-118	6
St18.5	0.760186994	0.386	0.171	2.2587E-117	6
Myl12a.1	-0.682460013	0.092	0.351	3.5473E-117	6
Paics.3	-0.698220168	0.153	0.438	4.4141E-117	6
Pbk.5	-0.855710485	0.026	0.241	4.0779E-116	6
Lsm6.3	-0.680937865	0.122	0.396	4.493E-116	6
Itsn1.5	0.744078542	0.399	0.219	5.0463E-116	6
Nap1l1.3	-0.60655982	0.239	0.537	1.4494E-115	6
Gnaq.1	0.678647995	0.426	0.27	2.3757E-115	6
Kif5a.5	0.793413565	0.303	0.11	1.4099E-114	6
Prmt8.2	-0.795423427	0.055	0.293	1.8135E-114	6
Kcnk2	0.757793272	0.165	0.027	3.1987E-114	6
Cenpe.5	-1.085554072	0.065	0.307	4.8619E-114	6
Rufy3.4	0.620594756	0.504	0.358	5.486E-114	6
BC005764.5	0.80426905	0.372	0.167	8.8961E-114	6
Rps18.1	-0.652808304	0.169	0.454	1.0013E-113	6
Mycbp2.1	0.658316745	0.483	0.331	1.3898E-113	6
Nsg1.4	0.650005627	0.433	0.273	1.5868E-113	6
Dpysl4.2	-0.649388555	0.226	0.516	1.6494E-113	6
Hook1	0.820858084	0.196	0.049	2.672E-113	6
Sh3bp5.3	0.78309533	0.257	0.088	5.3942E-113	6
Trpc4ap.6	0.765428698	0.351	0.183	1.0371E-112	6
Sox9.3	-0.814856211	0.046	0.275	1.8591E-112	6
Pcdha2.3	0.803192549	0.272	0.102	2.3692E-112	6
Sox4.4	0.514984525	0.678	0.573	2.6369E-112	6
Ube2ql1	0.66375791	0.134	0.014	3.058E-112	6
H2afx.6	-0.963409485	0.102	0.353	1.4926E-111	6
Rpl18a.2	-0.580697863	0.155	0.424	2.2116E-111	6
Cdca3.6	-0.858943435	0.028	0.24	3.4449E-111	6
Fam63b	0.802010978	0.234	0.073	6.1347E-111	6
Siva1.3	-0.708122947	0.059	0.293	1.3407E-110	6
Sfrs18.2	0.474252366	0.848	0.77	1.656E-110	6
Mcm2.4	-0.76935014	0.021	0.222	1.9466E-110	6
Ptms.2	0.502293515	0.551	0.466	2.3444E-110	6
Ppic.1	-0.750085907	0.039	0.259	5.3252E-110	6
Gm10075.3	-0.639216506	0.25	0.54	6.7562E-110	6
Srcin1	0.774875119	0.2	0.039	1.1094E-109	6
Hnrnpm	-0.494820121	0.487	0.759	1.1971E-109	6
Fam210b.2	-0.724118784	0.086	0.336	1.271E-109	6
Apex1.1	-0.565683638	0.194	0.476	1.8304E-109	6
Itgb1	-0.57443414	0.126	0.386	2.1361E-109	6
Ntm.3	0.814878874	0.281	0.132	6.5218E-109	6
Cnbp.2	-0.519898693	0.504	0.763	1.0873E-108	6
Bola2.1	-0.61603126	0.149	0.421	1.7218E-108	6
Prdx4.3	-0.668833409	0.126	0.389	2.4304E-108	6
Prim1.4	-0.718710412	0.055	0.283	5.8491E-108	6
Banf1.3	-0.563758171	0.454	0.717	9.1261E-108	6

Scg5.3	0.643153897	0.477	0.32	1.2134E-107	6
Pqlc1.3	-0.720412902	0.078	0.318	1.7694E-107	6
Gpr153.2	-0.769437415	0.024	0.226	3.5017E-107	6
Serbp1.3	-0.454320294	0.715	0.884	1.1074E-106	6
Prc1.6	-0.976275073	0.053	0.276	1.5894E-106	6
Ncl.3	-0.445857139	0.764	0.909	3.0817E-106	6
Ccna2.6	-0.782844563	0.031	0.24	5.8859E-106	6
Ifi203	0.63268053	0.113	0.008	6.0535E-106	6
Aplp2.3	0.622027546	0.447	0.31	6.2249E-106	6
Fut9	0.699052603	0.339	0.199	6.6713E-106	6
Slc29a1.2	-0.610280899	0.305	0.586	1.3884E-105	6
Hmgn2.3	-0.698879876	0.052	0.275	1.4151E-105	6
Snrpd2.1	-0.601465672	0.188	0.465	1.6035E-105	6
Snrpe.1	-0.540723722	0.299	0.588	1.8568E-105	6
Pdgfa.4	-0.777353641	0.051	0.273	2.7019E-105	6
Eif3f.1	-0.497105255	0.37	0.656	2.832E-105	6
Trp53.1	-0.597825837	0.082	0.319	3.715E-105	6
Hdgf.2	-0.571791718	0.271	0.557	4.1233E-105	6
Apc.5	0.595985333	0.578	0.416	4.7441E-105	6
Camta1	0.546488201	0.506	0.398	6.1369E-105	6
Nnat.3	-0.640052534	0.487	0.731	1.5864E-104	6
Cdh20.1	-0.716873006	0.023	0.22	1.7841E-104	6
Lsm4.1	-0.55520057	0.229	0.513	2.2176E-104	6
Snap25.3	0.630994069	0.421	0.268	2.6602E-104	6
Itm2b.5	0.457926535	0.728	0.634	8.5441E-104	6
Gng2.3	0.531459251	0.487	0.387	1.1969E-103	6
Nefl	0.753813098	0.199	0.044	1.4516E-103	6
Atp5e	-0.50509633	0.399	0.677	1.4955E-103	6
Cbx5.4	-0.584986935	0.409	0.677	1.5932E-103	6
Nsg2.4	0.503833976	0.6	0.476	2.3116E-103	6
Scaper	0.762958715	0.247	0.097	5.2182E-103	6
Nusap1.6	-0.887829922	0.036	0.245	5.8634E-103	6
Snrpb.2	-0.51512739	0.315	0.598	7.0039E-103	6
Mbnl2.2	0.697418875	0.313	0.165	7.6852E-103	6
Nfib.1	0.387111487	0.968	0.93	8.1259E-103	6
Ndrg4	0.737576493	0.18	0.039	1.2307E-102	6
Cenpv.1	-0.59839321	0.106	0.35	5.2634E-102	6
Ccdc34.5	-0.647509491	0.17	0.438	5.8571E-102	6
Diras2	0.61438631	0.103	0.006	9.3915E-102	6
Scg2	0.782480351	0.168	0.041	1.018E-101	6
Cdca7.3	-0.714932028	0.023	0.215	1.2182E-101	6
Kcnc1	0.750286236	0.233	0.075	1.2991E-101	6
Chgb.5	0.723931391	0.434	0.221	1.8481E-101	6
Myt1l.5	0.80560979	0.329	0.144	3.2762E-101	6
5830416P10Rik	0.660944196	0.123	0.013	4.054E-101	6
Ckap2l.6	-0.839065855	0.033	0.235	7.0174E-101	6
Srsf7.3	-0.557694249	0.215	0.49	8.3241E-101	6
Map1lc3a	0.583530058	0.377	0.266	1.6869E-100	6

Galr1	0.714932559	0.182	0.034	1.7024E-100	6
Nop10.1	-0.554204237	0.232	0.506	3.422E-100	6
Ezr.1	-0.604814975	0.195	0.461	3.8601E-100	6
Rpl22l1.2	-0.637308522	0.122	0.373	1.4404E-99	6
Hes6.3	-0.664097244	0.048	0.255	1.6835E-99	6
Dnajc9.5	-0.62810922	0.122	0.372	1.7222E-99	6
Lyar.3	-0.678642453	0.076	0.306	2.2309E-99	6
Tpm4.2	-0.634810833	0.097	0.338	2.4045E-99	6
Naca.1	-0.517374637	0.219	0.489	2.6532E-99	6
Commd1.1	-0.613969606	0.087	0.319	3.7508E-99	6
Tox3	-0.628240751	0.12	0.367	4.3862E-99	6
mt-Nd1	-0.402872789	0.826	0.936	5.846E-99	6
Npdc1.4	0.629068705	0.399	0.247	5.8669E-99	6
Chd3.5	0.750890249	0.312	0.133	6.3791E-99	6
Spock2	0.749049928	0.204	0.051	1.21448E-98	6
Kif11.6	-0.740442817	0.029	0.223	1.38366E-98	6
Lig1.6	-0.801837873	0.124	0.37	1.40333E-98	6
Rrm2.6	-0.72341258	0.028	0.221	2.03895E-98	6
Eef1d.2	-0.551686666	0.13	0.371	8.06384E-98	6
Lgals1.2	-0.842199309	0.043	0.247	9.67173E-98	6
Mycn.1	-0.651549964	0.134	0.386	1.08734E-97	6
Gdi1.4	0.62993197	0.355	0.217	1.35771E-97	6
Ly6h	0.623001135	0.121	0.013	1.41652E-97	6
Arhgef7.1	0.694918032	0.285	0.136	1.44094E-97	6
Lap3.4	-0.732633734	0.072	0.296	2.53648E-97	6
Rpl7.1	-0.509693659	0.262	0.533	7.23265E-97	6
C1qbp.1	-0.511220022	0.153	0.399	7.64634E-97	6
Mcm7.3	-0.563062941	0.16	0.411	1.73042E-96	6
Zeb1.1	-0.575125942	0.157	0.409	2.01781E-96	6
Spc25.6	-0.808220663	0.041	0.243	3.79083E-96	6
Tyms.5	-0.718213214	0.038	0.236	5.24728E-96	6
Sv2b	0.705959382	0.233	0.089	6.83264E-96	6
Bok.3	-0.632221439	0.058	0.269	2.45769E-95	6
Nrxn3	0.741789015	0.149	0.025	3.13762E-95	6
Rrm1.6	-0.639975874	0.065	0.279	8.72629E-95	6
Mns1.5	-0.720984138	0.03	0.22	1.10196E-94	6
Rgs17	0.714307327	0.164	0.035	1.46656E-94	6
Tacc3.6	-0.741832765	0.032	0.223	1.93668E-94	6
Hnrnpu	-0.41279353	0.705	0.881	2.29995E-94	6
Smarcc2.1	0.535993147	0.457	0.353	2.39261E-94	6
Ssrp1.2	-0.501377849	0.323	0.601	2.62798E-94	6
Camk2b	0.744706176	0.233	0.076	2.78289E-94	6
Zwint	0.537401354	0.369	0.283	3.59098E-94	6
Dync1i2.2	0.441676167	0.629	0.561	3.83879E-94	6
Casc5.6	-0.719685703	0.022	0.202	4.21055E-94	6
Mpp3	0.699544601	0.163	0.037	9.74397E-94	6
Runx1t1	0.662405743	0.118	0.013	1.49005E-93	6
Incenp.6	-0.806039081	0.072	0.295	1.65514E-93	6

Atp1a3	0.63112429	0.141	0.022	1.69949E-93	6
Knstrn.5	-0.71375148	0.022	0.201	3.73833E-93	6
Ckb.5	0.371991336	0.861	0.803	4.78731E-93	6
Clspn.5	-0.707974872	0.025	0.205	1.6239E-92	6
Cklf.3	-0.605572406	0.014	0.18	2.64224E-92	6
B2m.2	-0.809883885	0.048	0.25	2.75628E-92	6
Pkm.1	-0.507485082	0.202	0.451	5.14427E-92	6
Tipin.4	-0.596222899	0.065	0.273	9.14463E-92	6
Btbd3	0.679716134	0.195	0.08	9.56261E-92	6
Pcbp1	-0.446116939	0.347	0.617	1.06186E-91	6
Park7	-0.4497582	0.305	0.569	1.63189E-91	6
Atp6v0b.4	0.598695718	0.386	0.255	2.55574E-91	6
Tnrc6c	0.685924038	0.303	0.166	6.39039E-91	6
Prnp.3	0.633662491	0.324	0.195	1.20077E-90	6
Hmmr.5	-0.844085608	0.019	0.191	1.36979E-90	6
Pgm2l1	0.740767408	0.229	0.084	1.75338E-90	6
Cntn1	0.673679172	0.179	0.038	3.72543E-90	6
Tshz2.1	-0.613739301	0.111	0.34	4.04181E-90	6
Sphkap	0.705536209	0.165	0.036	4.94803E-90	6
Adcy1	0.647658002	0.124	0.016	5.17416E-90	6
Rnaseh2c.1	-0.491583157	0.16	0.403	5.22099E-90	6
Pfn1.1	-0.481693487	0.271	0.534	8.64231E-90	6
Sub1	-0.386410453	0.347	0.612	9.98543E-90	6
Rpl37.1	-0.532636007	0.27	0.526	1.13195E-89	6
Pdia6	-0.551723617	0.128	0.361	1.27634E-89	6
Rps4x.1	-0.564049302	0.094	0.315	1.64877E-89	6
Calb2	0.689196959	0.114	0.013	1.69382E-89	6
Snrpd1.3	-0.45006151	0.288	0.547	2.43983E-89	6
Luc7l3	0.424295599	0.795	0.743	2.67448E-89	6
Nrxn2	0.719769156	0.23	0.076	3.81481E-89	6
Cdc20.5	-0.868853066	0.031	0.212	6.10308E-89	6
Esco2.5	-0.746163945	0.029	0.211	8.13023E-89	6
Rnasel.1	0.764739266	0.212	0.059	8.99534E-89	6
Sptan1.1	0.682392275	0.303	0.155	1.41766E-88	6
Ctnnb1.2	-0.470044584	0.168	0.408	2.50831E-88	6
Idh2.3	-0.553823336	0.085	0.297	3.72211E-88	6
Rpa2.3	-0.637980025	0.034	0.216	9.51691E-88	6
A930011O12Rik.5	0.771190856	0.268	0.099	1.37506E-87	6
BC029214	0.675578014	0.217	0.08	1.395E-87	6
2810004N23Rik.1	-0.603377229	0.074	0.28	1.50652E-87	6
Nfix.2	0.3729011	0.716	0.668	1.62485E-87	6
Zmat4	0.572279886	0.104	0.01	1.70277E-87	6
Gm1673.2	0.472914771	0.565	0.464	2.45403E-87	6
Snrpg	-0.532616911	0.126	0.355	6.32084E-87	6
Gm9800.1	-0.51581397	0.302	0.557	7.12843E-87	6
Hspe1.1	-0.517401306	0.132	0.361	7.59789E-87	6
Dkc1.2	-0.603271724	0.122	0.353	3.08434E-86	6
Atp6v1e1.3	0.516356833	0.421	0.322	3.70503E-86	6

Nmral1.3	-0.611420955	0.027	0.203	4.11354E-86	6
Adh5	-0.45954379	0.188	0.426	4.72679E-86	6
Vps37b.1	-0.432881791	0.244	0.492	5.64878E-86	6
Hells.6	-0.783016089	0.042	0.23	5.80535E-86	6
Eif3i	-0.487248542	0.232	0.484	6.52783E-86	6
Smarcc1.1	-0.515418604	0.208	0.456	1.06854E-85	6
Abracl	-0.584218128	0.101	0.321	1.29167E-85	6
Tmsb10.2	0.364557	0.705	0.659	1.74006E-85	6
Cenph.6	-0.620015963	0.032	0.209	1.94198E-85	6
Ssbp3	0.633520758	0.286	0.155	2.53753E-85	6
Uba52	-0.52766147	0.115	0.336	3.59233E-85	6
Snrpd3	-0.456930808	0.298	0.558	4.39474E-85	6
Ptprs.3	0.54555134	0.535	0.409	5.28722E-85	6
Rab6a.2	0.50313703	0.411	0.319	5.3446E-85	6
Ptma.1	-0.470090795	0.342	0.605	9.90064E-85	6
Rfc4.5	-0.538723702	0.055	0.243	1.24004E-84	6
Ccnb2.5	-0.760143798	0.028	0.202	1.32352E-84	6
Ppfia2.3	0.738020628	0.253	0.098	1.60909E-84	6
Rtn4.4	0.422865747	0.528	0.475	2.09388E-84	6
Rps8	-0.520635349	0.176	0.418	2.2967E-84	6
Mrpl52.1	-0.428582943	0.214	0.454	3.71918E-84	6
Add3.2	0.688607361	0.214	0.093	5.04574E-84	6
Usp1.6	-0.575292897	0.129	0.359	6.08162E-84	6
Rps7.1	-0.48674541	0.183	0.42	1.29448E-83	6
Csnk1e	0.461082273	0.438	0.38	1.70136E-83	6
Txn1.2	-0.415666132	0.327	0.583	1.72439E-83	6
Rsl1d1	-0.411555529	0.263	0.512	2.24042E-83	6
Reep2	0.687651206	0.183	0.056	2.95185E-83	6
Adrbk2	0.685696765	0.224	0.088	3.12863E-83	6
Ndufa2	-0.393889031	0.371	0.625	3.29819E-83	6
Rpl14-ps1	-0.488201203	0.097	0.301	5.461E-83	6
Hmgb1.3	-0.504144788	0.149	0.379	7.11773E-83	6
Mif.2	-0.543525769	0.145	0.377	1.28441E-82	6
Homer2	-0.479030251	0.073	0.263	1.59982E-82	6
Calm2.4	0.331922769	0.915	0.87	1.67257E-82	6
Kidins220.3	0.694787682	0.274	0.126	1.74281E-82	6
Pou3f2.2	-0.61873656	0.086	0.294	1.82517E-82	6
Gm8292	-0.536828867	0.158	0.389	3.13131E-82	6
Ndufa12	-0.473284153	0.205	0.442	3.1665E-82	6
Cplx2.2	0.473849932	0.55	0.489	4.20156E-82	6
Slc3a2.2	-0.486300867	0.126	0.342	4.89633E-82	6
Cct3	-0.404885643	0.273	0.517	6.26692E-82	6
Rbfox2.4	0.575585068	0.401	0.271	7.3196E-82	6
G3bp1.1	-0.472789583	0.128	0.347	7.39337E-82	6
Kif23.6	-0.756628575	0.046	0.23	9.74227E-82	6
Chchd2	-0.395485249	0.422	0.675	1.854E-81	6
Smco4.3	-0.573235108	0.018	0.175	2.15653E-81	6
Tmem145	0.658202483	0.16	0.039	2.16953E-81	6

Elavl4.5	0.559932217	0.482	0.32	2.22316E-81	6
Psma4	-0.399862879	0.203	0.434	3.29267E-81	6
Fgf13	0.61670059	0.155	0.043	3.68422E-81	6
Pnmal2.3	0.676963044	0.274	0.134	3.87416E-81	6
Ezh2.5	-0.543933412	0.384	0.632	8.23075E-81	6
Hjurp.4	-0.59972747	0.214	0.455	8.23321E-81	6
Adam10	0.563477558	0.285	0.195	1.00013E-80	6
mt-Rnr2.1	0.308684162	0.996	0.997	1.33159E-80	6
Mmp24	0.671630027	0.169	0.041	1.69399E-80	6
Unc5c	0.630283381	0.106	0.015	1.83084E-80	6
Sod1.1	-0.435793279	0.224	0.463	3.35948E-80	6
Asap1.1	-0.623133753	0.027	0.195	3.9178E-80	6
Tacc2.2	0.687529715	0.273	0.128	7.21396E-80	6
Mllt4	0.524061483	0.464	0.353	9.28744E-80	6
Srsf6	-0.428634512	0.231	0.466	9.40014E-80	6
2900011O08Rik.4	0.668396493	0.229	0.091	1.04112E-79	6
Smim11	-0.46924075	0.095	0.294	1.29432E-79	6
Supt16.3	-0.495557518	0.266	0.515	1.494E-79	6
Tcp1	-0.444573401	0.241	0.485	1.5544E-79	6
Slc1a2.3	-0.596316266	0.103	0.312	1.75861E-79	6
Gmnn.5	-0.574609908	0.011	0.157	1.85519E-79	6
Ddx39b	-0.432032957	0.227	0.465	2.35876E-79	6
Kazn	0.548715239	0.102	0.013	2.64792E-79	6
Nudt3	0.514080567	0.329	0.24	2.822E-79	6
Ncapg.5	-0.647424689	0.03	0.198	3.32494E-79	6
Mcm3.3	-0.612659207	0.028	0.194	8.67368E-79	6
Cadps	0.658339118	0.181	0.062	9.57912E-79	6
Cbfa2t3.4	-0.538887618	0.19	0.423	1.58976E-78	6
Ppib.1	-0.347960321	0.482	0.722	1.65087E-78	6
Snhg1.3	-0.521227057	0.153	0.377	1.73264E-78	6
Lrig3.2	-0.598745466	0.023	0.184	1.81476E-78	6
Nudcd2.2	-0.537060547	0.057	0.239	2.26269E-78	6
Gar1.1	-0.565640869	0.046	0.224	2.79747E-78	6
Timp3	0.735413245	0.197	0.067	3.15401E-78	6
Atoh1.3	-0.63827831	0.021	0.178	4.09696E-78	6
Ndn.1	0.529239105	0.309	0.214	1.09943E-77	6
Mrpl13.1	-0.454835473	0.12	0.326	1.98928E-77	6
1810009A15Rik	-0.534282107	0.081	0.277	2.1273E-77	6
Mtch2	0.458230371	0.354	0.288	2.58857E-77	6
Srsf3.1	-0.398356116	0.432	0.679	2.71366E-77	6
Fam115a	0.462350313	0.401	0.345	3.82792E-77	6
Chaf1a.4	-0.566463972	0.025	0.182	4.93159E-77	6
Tubb4b.5	-0.584932567	0.068	0.257	5.5878E-77	6
Smarca5.1	-0.451943459	0.193	0.421	5.60659E-77	6
Map2.5	0.43500235	0.583	0.482	5.88891E-77	6
Tspyl4.3	0.657819066	0.241	0.115	6.27008E-77	6
Cep170.2	0.533125405	0.391	0.296	1.01163E-76	6
Gm13826.1	-0.510130124	0.122	0.331	1.09679E-76	6

Larp7.1	-0.477497228	0.124	0.33	1.14258E-76	6
B3galt2.5	0.700300101	0.224	0.085	1.41477E-76	6
Reln.3	0.66791135	0.296	0.168	1.63105E-76	6
Islr2.2	-0.602971981	0.03	0.194	1.80896E-76	6
Peli2	0.583811045	0.268	0.154	2.25067E-76	6
Soga3.3	0.43393451	0.505	0.452	4.30068E-76	6
Pde1c.6	-0.608482595	0.186	0.406	5.65777E-76	6
Cnrip1.2	0.583713713	0.3	0.187	5.67271E-76	6
Cct2	-0.386300334	0.305	0.545	8.56148E-76	6
Arpp19.1	-0.384176628	0.182	0.396	1.1243E-75	6
Cald1.1	-0.394332604	0.313	0.557	1.27505E-75	6
Hpcal1.3	0.665207337	0.219	0.095	3.25425E-75	6
Klf7.4	0.579802307	0.376	0.25	4.00365E-75	6
Rpl37a.1	-0.454917081	0.253	0.489	4.81832E-75	6
Sox18	-0.596951927	0.015	0.16	5.28601E-75	6
Clcn4-2.3	0.566213182	0.357	0.24	5.66565E-75	6
Rpl6	-0.462301821	0.11	0.308	1.63381E-74	6
Rpa3.2	-0.520433478	0.073	0.26	1.92918E-74	6
Trim59.5	-0.521333819	0.028	0.183	2.0922E-74	6
Bcl11a	-0.524842399	0.052	0.225	2.22777E-74	6
Pdzrn4.2	-0.621229349	0.014	0.159	2.3237E-74	6
Syt1	0.627443492	0.13	0.023	3.20722E-74	6
Gas1.2	-0.582398318	0.019	0.17	3.25425E-74	6
Cdc42	0.332088793	0.527	0.514	4.35828E-74	6
Isoc1.1	-0.527210544	0.035	0.195	5.20894E-74	6
Hnrnpc	-0.369096874	0.452	0.688	5.36995E-74	6
Mab21l1.4	0.579713093	0.334	0.214	5.58529E-74	6
Ank3.5	0.48744406	0.503	0.411	6.51919E-74	6
Sowaha.3	-0.695211617	0.023	0.176	7.037E-74	6
Rbm8a	-0.351802937	0.259	0.485	8.30656E-74	6
Uhrf1.5	-0.544259655	0.014	0.156	8.36494E-74	6
Ckap2.5	-0.594038058	0.024	0.176	1.63992E-73	6
Grina.4	0.593977568	0.234	0.097	1.65738E-73	6
Npc2.2	-0.427313169	0.253	0.484	1.67535E-73	6
Kif15.5	-0.601090163	0.034	0.194	1.95053E-73	6
Ywhag.1	0.515467574	0.328	0.231	2.20872E-73	6
Mcm5.3	-0.567030558	0.026	0.182	2.90795E-73	6
Clcn3	0.508019636	0.346	0.265	3.68638E-73	6
App.3	0.378037486	0.631	0.561	5.32442E-73	6
Pa2g4.3	-0.393296363	0.306	0.543	6.13861E-73	6
Psmb2	-0.298764402	0.235	0.446	6.18197E-73	6
Cirbp	0.445136723	0.415	0.349	1.08378E-72	6
Srrm2	0.316265045	0.733	0.731	1.13128E-72	6
Smpd2.2	-0.549142311	0.026	0.179	1.60155E-72	6
Cnn3	-0.35667383	0.238	0.456	2.75329E-72	6
Atxn7l3b.1	0.315950977	0.678	0.672	3.12665E-72	6
Igfbpl1.4	-0.478577072	0.461	0.666	7.66689E-72	6
Sep15	-0.374556401	0.295	0.525	9.2907E-72	6

Cenpm.5	-0.548579241	0.024	0.175	1.01496E-71	6
Rpl18.1	-0.48814806	0.119	0.322	1.25683E-71	6
Gm10260.1	-0.498118482	0.067	0.243	1.61482E-71	6
Prrt1	0.536738586	0.106	0.014	1.61827E-71	6
Ccnb1.5	-0.622162556	0.013	0.151	2.0113E-71	6
Klc1.3	0.471639602	0.456	0.352	2.3887E-71	6
Sfrp2.2	-0.533429392	0.018	0.16	2.64901E-71	6
Hnrnpd.3	-0.42865607	0.398	0.631	4.54E-71	6
Nolc1.2	-0.45664667	0.203	0.428	6.51354E-71	6
Aurkb.5	-0.550818335	0.015	0.155	7.36718E-71	6
Egr1.4	0.596697086	0.477	0.375	8.40214E-71	6
Ptn.5	0.304442951	0.551	0.43	8.51767E-71	6
Nicn1.1	0.588314876	0.316	0.197	8.88796E-71	6
Rab6b.5	0.587406678	0.33	0.189	1.1002E-70	6
H2afz.4	-0.468364052	0.071	0.247	1.25366E-70	6
Tulp4	0.546511365	0.3	0.195	1.34423E-70	6
Fstl1.2	-0.58758779	0.036	0.194	1.63572E-70	6
Cd200	0.603429651	0.149	0.035	1.88504E-70	6
Mrpl17	-0.457089588	0.09	0.276	1.99371E-70	6
Pja2	0.485460475	0.302	0.227	2.80879E-70	6
Clasp2.1	0.581109189	0.268	0.157	3.17344E-70	6
Cst3.6	-0.584299107	0.356	0.553	4.32251E-70	6
Rbbp7.2	-0.408292239	0.139	0.337	5.28274E-70	6
Cct5	-0.330347828	0.274	0.496	5.53151E-70	6
Rpl38	-0.415788555	0.191	0.401	5.70173E-70	6
Cacna1a	0.62641157	0.181	0.062	8.27803E-70	6
Baz1a.2	-0.571729237	0.028	0.18	8.94256E-70	6
Rtn3	0.354688002	0.5	0.474	1.25603E-69	6
Gm11541	-0.545255328	0.01	0.139	1.29672E-69	6
Snrpb2	-0.341030629	0.202	0.402	1.47721E-69	6
Glce.3	0.59737879	0.282	0.166	2.49982E-69	6
Pmf1.4	-0.465861966	0.019	0.155	2.69003E-69	6
Chchd1	-0.344674579	0.212	0.419	2.94192E-69	6
Naa38.1	-0.369839968	0.139	0.328	3.16303E-69	6
Ddx21.2	-0.47851711	0.138	0.34	3.72559E-69	6
Gabbr1.2	0.580846853	0.246	0.13	5.21188E-69	6
Pafah1b1	0.358277269	0.497	0.472	5.82385E-69	6
Eef1g.1	-0.438507651	0.148	0.353	7.17173E-69	6
Emg1	-0.410428131	0.135	0.332	1.04644E-68	6
Polr2f.1	-0.38960815	0.184	0.393	1.20839E-68	6
Adamts1.2	-0.584353044	0.023	0.167	2.0454E-68	6
Eif5a	-0.40267604	0.208	0.422	2.17969E-68	6
Kifap3	0.51691548	0.366	0.263	2.37589E-68	6
Uqcrq	-0.351517707	0.34	0.571	2.4782E-68	6
Kmt2e.3	0.339953147	0.587	0.566	3.29124E-68	6
Rpl29	-0.463721638	0.091	0.274	4.06774E-68	6
Arhgap20	0.633589117	0.156	0.043	4.93455E-68	6
Actb	-0.250141674	0.946	0.982	7.08628E-68	6

Snrpa1.1	-0.391818385	0.15	0.348	7.2929E-68	6
Sgol2.6	-0.604149701	0.024	0.169	8.43002E-68	6
Lman1	-0.430587825	0.144	0.342	9.78108E-68	6
Apoe.6	-1.648823104	0.148	0.259	1.31009E-67	6
Lin7a	0.620711068	0.153	0.042	1.66183E-67	6
Gng5	-0.507292595	0.029	0.178	1.75909E-67	6
Kif20b.6	-0.644034979	0.043	0.205	2.23461E-67	6
Pak1	0.623754698	0.17	0.048	3.17608E-67	6
Arhgap11a.6	-0.620812689	0.031	0.184	3.91643E-67	6
Eif3k	-0.340352282	0.325	0.54	4.01853E-67	6
2210016L21Rik.1	0.486109649	0.364	0.281	4.08335E-67	6
Grb2.1	0.45382553	0.302	0.243	5.06826E-67	6
Mis18bp1.6	-0.570494526	0.027	0.173	5.70409E-67	6
Ntrk3.2	-0.579095798	0.023	0.168	6.2841E-67	6
Tceal5	0.549502511	0.119	0.023	6.39087E-67	6
Nop56.3	-0.388386591	0.175	0.378	6.46734E-67	6
Marcksl1.1	0.295377085	0.74	0.72	8.06632E-67	6
Ndufb6	-0.400393241	0.192	0.398	9.3257E-67	6
Mrpl42.1	-0.458052429	0.139	0.335	1.13163E-66	6
Pbdc1.1	-0.483779782	0.056	0.22	1.31329E-66	6
Nucks1.5	-0.451379194	0.474	0.681	1.35803E-66	6
Sez6l2	0.588998472	0.129	0.028	1.53823E-66	6
2810025M15Rik.1	-0.484786266	0.033	0.18	1.60511E-66	6
Sae1.3	-0.44610783	0.108	0.295	1.84612E-66	6
Nuf2.6	-0.513604362	0.018	0.152	2.06339E-66	6
Bub1.5	-0.514726348	0.013	0.142	2.1663E-66	6
Cenpq.5	-0.504465401	0.029	0.173	2.45941E-66	6
Tubb3.5	0.318324788	0.616	0.46	3.35448E-66	6
Ccl27a	0.579183486	0.133	0.034	4.36913E-66	6
Mt1.3	-0.800322673	0.031	0.182	4.85647E-66	6
Rpl13	-0.464008562	0.133	0.33	6.00264E-66	6
Atp5b	-0.323444597	0.511	0.732	6.26666E-66	6
Clic4.3	-0.473881173	0.07	0.242	6.8055E-66	6
Snap47	0.541921155	0.231	0.131	6.98124E-66	6
Thoc7	-0.290454239	0.325	0.54	7.94106E-66	6
Ubxn1	-0.308964937	0.284	0.497	8.14236E-66	6
Hivep2	0.595031463	0.149	0.045	1.37769E-65	6
Set.2	-0.38468286	0.2	0.405	1.49966E-65	6
Hes1.4	-0.747511423	0.021	0.16	1.8267E-65	6
Cdk6.1	-0.470035776	0.085	0.258	1.94691E-65	6
Rnmt.3	0.438743826	0.41	0.342	2.15595E-65	6
Golim4	-0.515050495	0.073	0.248	2.33214E-65	6
Eif3d	-0.299643885	0.224	0.423	3.50258E-65	6
Tcf25	0.315863763	0.606	0.601	3.67783E-65	6
Ypel3.2	0.351078513	0.464	0.432	5.22898E-65	6
Fkbp1b	0.497147383	0.101	0.015	5.26478E-65	6
Hspa5	-0.432305167	0.352	0.569	6.74658E-65	6
Atrx	0.32553764	0.748	0.739	7.21488E-65	6

Eny2	-0.379517073	0.2	0.405	7.57917E-65	6
Rps16	-0.484765138	0.059	0.223	8.33648E-65	6
Dpm3	-0.428465587	0.105	0.286	8.38182E-65	6
Alcam	-0.542002926	0.015	0.146	9.05656E-65	6
Mmp14.2	-0.540784876	0.038	0.187	1.00076E-64	6
Fbxo5.5	-0.511760117	0.029	0.172	1.02656E-64	6
Mphosph10.1	-0.476714696	0.076	0.246	1.069E-64	6
Rps6ka3	0.570802917	0.144	0.056	1.13358E-64	6
2010107G23Rik	0.532144488	0.114	0.019	1.14774E-64	6
Rbm5.2	0.3957912	0.449	0.396	1.35071E-64	6
Kcnd2	0.582659148	0.108	0.018	1.39845E-64	6
Mbd3	-0.420318394	0.122	0.307	1.50151E-64	6
Erc1	0.590596133	0.202	0.101	1.52663E-64	6
Myod1.4	-0.603600496	0.018	0.153	1.55735E-64	6
Sparcl1.5	-0.894728211	0.06	0.221	1.59388E-64	6
Sema7a.2	-0.538990107	0.026	0.168	1.87795E-64	6
Hspd1.1	-0.409906968	0.179	0.382	1.92315E-64	6
2010107E04Rik	-0.293558117	0.374	0.59	2.28726E-64	6
Gas5.1	-0.366455203	0.556	0.76	3.2713E-64	6
2310044G17Rik	0.551966503	0.134	0.047	3.73717E-64	6
Zfp36l1.2	-0.561588332	0.023	0.163	4.24792E-64	6
Polr2h	-0.418394834	0.078	0.239	4.79175E-64	6
Eif3g	-0.325167864	0.219	0.42	4.82138E-64	6
Ssr3	-0.312879912	0.242	0.443	4.93187E-64	6
D17H6S56E-5.5	-0.495589634	0.013	0.138	7.80929E-64	6
Lsamp	0.663412549	0.189	0.066	1.01505E-63	6
Cacna1b	0.611684465	0.193	0.087	1.04108E-63	6
Psmb6	-0.263037008	0.418	0.63	1.15031E-63	6
Ndufa3	-0.277020027	0.308	0.515	1.51938E-63	6
Bccip	-0.379665542	0.142	0.327	1.52032E-63	6
Sptbn1.5	0.46978807	0.462	0.342	1.65211E-63	6
Nek6.1	-0.501989106	0.021	0.156	1.79542E-63	6
Rbbp4.1	-0.349459031	0.286	0.503	2.1381E-63	6
Eif4a1	-0.381035543	0.29	0.503	2.15082E-63	6
Wbp5.1	-0.277012099	0.324	0.537	2.29457E-63	6
Uqcrh	-0.295699974	0.464	0.675	2.78675E-63	6
Optn	0.587433088	0.151	0.047	3.57952E-63	6
Scd2	-0.407508223	0.131	0.313	4.05607E-63	6
Serpini1	0.598727311	0.164	0.054	4.59925E-63	6
Acot7.1	-0.436536296	0.055	0.208	4.66174E-63	6
Uqcr11	-0.273013693	0.297	0.499	5.16873E-63	6
2410066E13Rik.1	0.586621687	0.202	0.088	5.71229E-63	6
Cnih4	-0.38976086	0.087	0.249	5.83954E-63	6
Sec11c.1	-0.495912493	0.063	0.227	6.06101E-63	6
Serping1	0.555662387	0.122	0.024	6.37E-63	6
Kcnj3	0.601796626	0.169	0.059	6.55613E-63	6
Hint1.2	-0.351628104	0.514	0.722	8.3399E-63	6
Wdr89.1	-0.429875441	0.068	0.228	8.87579E-63	6

Pole3.1	-0.386960327	0.097	0.265	8.98953E-63	6
Nktr.1	0.414375683	0.42	0.376	1.41414E-62	6
Gtpbp4	-0.462136617	0.113	0.298	1.93954E-62	6
Nup62.1	-0.4213717	0.04	0.184	2.97993E-62	6
Cacna2d1.2	0.463159645	0.448	0.367	3.63758E-62	6
Mxd3.5	-0.471003628	0.014	0.138	3.71506E-62	6
Smarca2	0.455328304	0.349	0.279	4.35514E-62	6
Srsf9	-0.392706703	0.086	0.251	4.84101E-62	6
Spcs2	-0.268544335	0.249	0.44	8.03389E-62	6
Ebna1bp2	-0.40292521	0.144	0.331	8.57473E-62	6
Lsm3.2	-0.362745153	0.182	0.376	1.16877E-61	6
Slc25a3	-0.275842119	0.383	0.591	1.31111E-61	6
Cpne3	-0.457156942	0.058	0.21	1.47027E-61	6
Irf2bpl	0.535748827	0.126	0.031	1.48757E-61	6
Dbn1.5	0.559847295	0.242	0.129	1.60509E-61	6
Dnph1.3	-0.466312701	0.012	0.134	1.78686E-61	6
Cdk14	0.533334451	0.177	0.096	1.84384E-61	6
Parp1	-0.346044086	0.149	0.329	2.81378E-61	6
Fam168a	0.460641632	0.352	0.28	4.63418E-61	6
Rad51ap1.6	-0.497558864	0.036	0.179	4.90748E-61	6
Tspan3	-0.339269788	0.246	0.446	5.38689E-61	6
mt-Cytb	-0.280466595	0.854	0.946	6.78938E-61	6
Cxcl12	0.417258833	0.131	0.035	1.02252E-60	6
Magoh.1	-0.347463519	0.163	0.349	1.17197E-60	6
Eno1.1	-0.427491335	0.081	0.244	1.4109E-60	6
Map3k12	0.553007449	0.186	0.089	1.98519E-60	6
Insm1.1	-0.517165686	0.126	0.308	2.22962E-60	6
Minos1	-0.343433601	0.372	0.588	2.65284E-60	6
Gjc1.1	-0.444618972	0.057	0.21	3.08201E-60	6
Bcas1.1	-0.542334235	0.117	0.298	3.17744E-60	6
Pitpnc1	0.568297442	0.169	0.065	3.66E-60	6
Gpm6b.2	0.360699832	0.536	0.478	4.77378E-60	6
Mak16	-0.409163988	0.079	0.239	5.53099E-60	6
2310022B05Rik.1	-0.4263027	0.094	0.263	7.15496E-60	6
Ostc	-0.392384106	0.121	0.289	7.76796E-60	6
Pdrg1.2	0.482967607	0.294	0.211	9.00692E-60	6
Akap9	0.381262902	0.528	0.497	1.00611E-59	6
Ppp3cb	0.375896655	0.339	0.307	1.02709E-59	6
Ntrk2	0.609985664	0.191	0.072	1.08772E-59	6
Btf3	-0.309078143	0.185	0.365	1.16071E-59	6
Lsm2.2	-0.373932975	0.087	0.244	1.22275E-59	6
Cct8	-0.27177954	0.266	0.458	1.41811E-59	6
Hspa9	-0.311559754	0.202	0.389	1.44611E-59	6
Actl6a	-0.358854548	0.084	0.24	1.48838E-59	6
Gm17322.4	-0.613547301	0.017	0.142	2.19771E-59	6
Ndufb9	-0.259615174	0.352	0.558	2.70015E-59	6
Lmnb1.5	-0.438354223	0.132	0.316	3.19826E-59	6
Slc4a4	0.405121702	0.122	0.032	3.64895E-59	6

Hat1.3	-0.433330804	0.034	0.166	3.9459E-59	6
Atad2.5	-0.52641753	0.056	0.212	3.95672E-59	6
Pdgfra.2	-0.534482495	0.047	0.194	4.25664E-59	6
Tcf3	-0.433393846	0.078	0.24	4.47368E-59	6
Mrpl18.2	-0.380994903	0.177	0.371	5.68962E-59	6
Serp2	0.558193546	0.178	0.078	6.63017E-59	6
Uncx.5	0.463979095	0.413	0.308	6.7766E-59	6
Cdca2.5	-0.427590571	0.019	0.139	7.67844E-59	6
Eif3e.1	-0.278851874	0.219	0.398	7.72465E-59	6
1110038B12Rik.2	-0.384788719	0.138	0.314	9.06723E-59	6
Mad2l1.5	-0.420649776	0.015	0.133	1.11821E-58	6
9330159F19Rik.1	0.527186824	0.257	0.16	1.12953E-58	6
Manf	-0.383993795	0.16	0.343	1.25095E-58	6
C330027C09Rik.4	-0.459570047	0.019	0.144	1.36441E-58	6
Ddx17	0.354345133	0.406	0.379	1.40417E-58	6
Syncrip.1	-0.298300801	0.307	0.514	1.52186E-58	6
Trim2	0.473118746	0.349	0.276	1.72369E-58	6
Sgol1.5	-0.450738936	0.017	0.138	2.13776E-58	6
Laptm4a	-0.300335123	0.364	0.572	2.29281E-58	6
Adk	-0.446712895	0.038	0.178	2.35957E-58	6
Mybbp1a	-0.358610388	0.103	0.264	2.70804E-58	6
Nsmce1.1	-0.35213985	0.088	0.242	2.81431E-58	6
Fam162a	-0.390564346	0.133	0.312	2.81853E-58	6
Fmnl2	0.501549799	0.227	0.15	2.82984E-58	6
Tk1.5	-0.471464486	0.019	0.142	2.83799E-58	6
Fundc2	-0.389258778	0.115	0.285	3.30016E-58	6
Shmt1.3	-0.390649239	0.008	0.112	3.36993E-58	6
Plcb1.4	0.61361331	0.252	0.13	3.6905E-58	6
Etfa	-0.380694357	0.102	0.268	5.2816E-58	6
Gli1.3	-0.470416187	0.008	0.117	6.65455E-58	6
AI854517	-0.446892061	0.055	0.202	1.22194E-57	6
Mrps14	-0.373085571	0.09	0.25	1.24599E-57	6
Sf3b5	-0.365916928	0.16	0.343	1.25353E-57	6
Kif2c.5	-0.442807914	0.008	0.117	1.41035E-57	6
Gria4.4	0.583692568	0.231	0.114	1.41677E-57	6
mt-Nd2	-0.26011883	0.45	0.657	1.65013E-57	6
Ramp2.2	-0.513638384	0.013	0.13	2.01197E-57	6
Macf1	0.496543096	0.268	0.204	2.386E-57	6
Ivns1abp	-0.327274374	0.241	0.432	2.83313E-57	6
Ybx3.2	-0.431525371	0.127	0.306	3.61204E-57	6
Tmem256	-0.271938307	0.318	0.509	4.08801E-57	6
Srgap2.3	0.547308454	0.214	0.124	4.74443E-57	6
Cox6c	-0.291421967	0.531	0.737	5.65069E-57	6
Gstm5.1	-0.36113342	0.158	0.338	7.60252E-57	6
Ect2.5	-0.417808368	0.004	0.107	7.87933E-57	6
Gltscr2.1	-0.276252267	0.185	0.357	8.28276E-57	6
Tmem57.2	0.407591822	0.336	0.286	8.39265E-57	6
Bub3.4	-0.424897535	0.153	0.337	1.20241E-56	6

Canx	-0.304375139	0.405	0.614	1.33691E-56	6
Ndufv3	0.365265285	0.323	0.295	1.45146E-56	6
Hmg20b	-0.401186641	0.08	0.236	1.76202E-56	6
Tle1.1	-0.405905683	0.084	0.239	1.79876E-56	6
Cox7b	-0.309196137	0.357	0.567	2.22562E-56	6
Kbtbd11	0.545298535	0.162	0.08	2.31497E-56	6
Zfp521	0.571312964	0.15	0.057	2.38488E-56	6
Cfdp1	-0.278332899	0.309	0.501	2.53172E-56	6
Actl6b	0.542738146	0.148	0.046	2.53418E-56	6
Gse1.1	-0.428240334	0.09	0.251	2.83779E-56	6
Cnpy2	-0.318006542	0.181	0.358	2.87532E-56	6
Psmb1	-0.313324518	0.491	0.698	3.07476E-56	6
1700001O22Rik.1	-0.424202689	0.01	0.12	3.5672E-56	6
Ubb.2	0.270399763	0.529	0.542	3.7734E-56	6
Cnksr2	0.552334648	0.192	0.099	3.97498E-56	6
Lrpap1.2	0.523923838	0.193	0.109	4.52424E-56	6
Dhx32.1	-0.437244476	0.063	0.213	4.94167E-56	6
Mettl9	-0.305320181	0.148	0.315	6.2849E-56	6
Zeb2	-0.402084083	0.101	0.263	6.52039E-56	6
Csrp2.3	-0.437887513	0.036	0.17	7.64359E-56	6
Igsf21.2	0.56205491	0.2	0.078	8.87855E-56	6
Psmc4	-0.301958392	0.166	0.336	9.44924E-56	6
Nde1.2	-0.429872053	0.014	0.129	1.43455E-55	6
Ndufc2	-0.321364243	0.421	0.629	1.72372E-55	6
Ppa1	-0.354385229	0.086	0.234	1.75608E-55	6
Cenpp.4	-0.402773604	0.013	0.124	1.86633E-55	6
Psma7	-0.275511709	0.611	0.798	2.01928E-55	6
Tia1	0.273130932	0.539	0.553	2.22879E-55	6
Naa10	-0.354696428	0.105	0.263	3.17479E-55	6
Tspan13.1	0.448961914	0.269	0.201	3.73321E-55	6
Srrm4.5	0.521713331	0.288	0.186	3.81581E-55	6
Ccdc88a	0.303011725	0.469	0.483	3.91278E-55	6
Eif3h	-0.276539479	0.315	0.51	5.45333E-55	6
Snap91	0.537103458	0.136	0.04	6.77053E-55	6
Kif22.6	-0.493726175	0.038	0.175	6.89283E-55	6
Aspm.5	-0.584378587	0.026	0.154	7.64128E-55	6
Vgll4	-0.372083049	0.038	0.163	9.4519E-55	6
Alyref.2	-0.369061531	0.068	0.212	9.91772E-55	6
Dnajc2	-0.32435375	0.168	0.344	1.41754E-54	6
Atxn10	0.276426338	0.369	0.37	1.45385E-54	6
Barhl1.5	-0.402765772	0.337	0.539	1.47863E-54	6
Syn2	0.580837751	0.168	0.077	1.56046E-54	6
Ccm2.2	-0.451310889	0.082	0.238	1.62465E-54	6
Gnl3.3	-0.393220809	0.136	0.311	1.64431E-54	6
Myl12b	0.293687033	0.386	0.365	1.69529E-54	6
Ncor2.2	-0.433145333	0.043	0.18	1.76056E-54	6
Rftn2.1	-0.43339814	0.018	0.134	2.63722E-54	6
Cltb.3	-0.483669477	0.246	0.44	3.00127E-54	6

2610203C20Rik.3	-0.51997495	0.015	0.131	3.04002E-54	6
Ift74	-0.41607608	0.084	0.238	3.04861E-54	6
Ccdc41.3	-0.369152131	0.107	0.268	3.09646E-54	6
Dtl.3	-0.443351848	0.03	0.156	3.2008E-54	6
2410015M20Rik	-0.300829957	0.205	0.376	3.404E-54	6
Psmg4	-0.327169269	0.079	0.22	3.40802E-54	6
Tsc22d4	-0.507138576	0.042	0.178	3.90497E-54	6
Mrpl15	-0.339430689	0.141	0.309	4.29321E-54	6
Clk1.1	0.346062159	0.366	0.337	4.47982E-54	6
P4hb	-0.2727964	0.141	0.292	6.4423E-54	6
Cnr1	0.565721013	0.143	0.049	6.48962E-54	6
Rpl35	-0.359381652	0.094	0.247	6.63124E-54	6
Hnrnpl	-0.250725757	0.207	0.378	7.3927E-54	6
Phf5a	-0.270855568	0.18	0.345	8.27721E-54	6
Luc7l2	0.271461488	0.494	0.495	9.20766E-54	6
Mad2l2	-0.404875958	0.112	0.277	1.04172E-53	6
Cbln1	0.530864688	0.167	0.082	1.52016E-53	6
Tpt1	-0.336147299	0.114	0.27	1.72568E-53	6
Polr1c	-0.351470291	0.086	0.231	1.84557E-53	6
Odf2.3	0.447001709	0.224	0.165	2.08561E-53	6
Map1lc3b.3	0.360890739	0.367	0.327	2.10707E-53	6
Dpy30.1	-0.290673772	0.151	0.312	2.24453E-53	6
RP23-32A8.1.1	0.456099549	0.243	0.181	2.49907E-53	6
Gabarapl1.1	0.432594689	0.281	0.209	2.73294E-53	6
Pttg1.3	-0.541104356	0.025	0.15	2.95201E-53	6
Cdh4	-0.478239645	0.031	0.157	2.9886E-53	6
Rad51.5	-0.420810586	0.014	0.126	3.11731E-53	6
Rb1cc1	0.465100923	0.288	0.229	3.26349E-53	6
Ncor1	0.261775771	0.548	0.556	3.36579E-53	6
Utp3.1	-0.257412998	0.167	0.328	3.63317E-53	6
Carhsp1.1	-0.329651296	0.116	0.267	4.85932E-53	6
Cep57.3	-0.274500742	0.125	0.27	5.79675E-53	6
Arl6ip1.5	-0.39702817	0.46	0.558	7.21586E-53	6
Mical1.1	-0.432302707	0.02	0.135	8.82009E-53	6
Dnajc8	-0.269060356	0.267	0.45	9.70885E-53	6
Reep5	0.462591654	0.211	0.13	1.01494E-52	6
Nek7.2	-0.436754851	0.015	0.126	1.54204E-52	6
Ptch2.3	-0.459186341	0.035	0.163	1.69545E-52	6
Itm2c.3	0.460608683	0.282	0.205	1.83386E-52	6
Nptn	0.405742663	0.235	0.183	2.22709E-52	6
Rims1	0.493121292	0.104	0.021	2.26831E-52	6
Efs.1	-0.415914134	0.02	0.135	2.4235E-52	6
Rif1.3	-0.384170699	0.124	0.291	2.605E-52	6
BC018507	0.50644025	0.189	0.115	2.62825E-52	6
Rrs1	-0.432097817	0.058	0.202	2.81984E-52	6
Syt13.2	-0.460835639	0.02	0.137	3.04842E-52	6
Gpx1	-0.356898455	0.212	0.399	3.06175E-52	6
Gm11478.1	-0.411602175	0.079	0.228	3.17074E-52	6

Smim18	0.525665835	0.134	0.04	3.33907E-52	6
Gins2.2	-0.445512924	0.055	0.197	4.00122E-52	6
Rcc2.1	-0.394362796	0.056	0.19	4.07558E-52	6
Msra	0.488807004	0.166	0.082	4.21897E-52	6
Nrcam.4	0.518253836	0.227	0.124	4.84477E-52	6
Pja1	0.460028913	0.239	0.166	5.41249E-52	6
Myt1.5	0.595075837	0.278	0.14	5.92446E-52	6
Plk4.5	-0.41895132	0.017	0.129	6.20642E-52	6
Ak2.1	-0.319803233	0.084	0.226	6.34687E-52	6
Kdelr2	-0.35845211	0.074	0.218	7.79882E-52	6
Cmtm3.2	-0.4238995	0.016	0.127	8.05319E-52	6
Eif3b	-0.315601248	0.092	0.232	8.95218E-52	6
Hsp90b1.2	-0.393285424	0.528	0.707	9.72475E-52	6
Prkacb	0.430928042	0.241	0.181	9.7994E-52	6
Fxr1	-0.268729757	0.122	0.268	1.00293E-51	6
Nptxr	0.474578848	0.106	0.027	1.01291E-51	6
Vars	-0.352085788	0.05	0.178	1.05648E-51	6
1700025G04Rik.2	-0.38961279	0.117	0.277	1.38396E-51	6
Utp14a	-0.352708007	0.099	0.245	1.58941E-51	6
Cenpw.5	-0.353319944	0.026	0.137	1.64958E-51	6
Cers4	0.510073587	0.131	0.053	2.15983E-51	6
Rpl36al	-0.354873638	0.146	0.312	2.32749E-51	6
Parp6.1	0.508894232	0.204	0.117	2.63505E-51	6
Pura	0.384083772	0.318	0.283	2.73571E-51	6
Snrnp40.1	-0.291298224	0.131	0.284	2.86332E-51	6
Akap12.2	0.579790816	0.2	0.102	3.012E-51	6
Cenpk.6	-0.463152076	0.031	0.155	3.10972E-51	6
Srsf2.1	-0.259606278	0.388	0.583	3.81379E-51	6
Gatsl2	0.550239912	0.175	0.082	3.89765E-51	6
Npepps	0.278519065	0.318	0.318	4.83857E-51	6
Tmem107	-0.380527126	0.023	0.137	5.16664E-51	6
Pcbp2	-0.265019946	0.441	0.639	5.41564E-51	6
Prmt5	-0.320532578	0.12	0.269	5.98817E-51	6
Cdc45.4	-0.372482408	0.007	0.103	6.48406E-51	6
Scrn1	0.523039101	0.126	0.042	6.94956E-51	6
Ddx39.2	-0.365493499	0.063	0.199	9.04053E-51	6
Smoc1.1	-0.452790026	0.025	0.145	9.22646E-51	6
Ndc80.5	-0.416864726	0.016	0.124	9.40631E-51	6
Ssna1	-0.343533845	0.124	0.281	1.01493E-50	6
Mroh2a.3	-0.573015303	0.014	0.123	1.07666E-50	6
E2f1.2	-0.463828861	0.033	0.158	1.14781E-50	6
Mfap4.2	-0.599898103	0.04	0.173	1.16375E-50	6
Ifitm2.1	-0.41155427	0.016	0.123	1.34858E-50	6
Fzd2	-0.4271189	0.043	0.172	1.76536E-50	6
Mrpl33	-0.297817561	0.139	0.296	1.9039E-50	6
Nol4.1	0.463484565	0.236	0.165	2.1989E-50	6
Fscn1	-0.385725615	0.074	0.215	2.2278E-50	6
Taf1d.1	-0.337559468	0.185	0.357	2.32774E-50	6

Efhd2.2	-0.488293692	0.023	0.14	2.79154E-50	6
Hist1h2ak.5	-0.550487108	0.031	0.154	3.22392E-50	6
Dad1	-0.260176407	0.195	0.358	3.58158E-50	6
Dhfr.4	-0.443990475	0.035	0.159	5.21228E-50	6
Rfc1.3	-0.269244188	0.181	0.345	5.33598E-50	6
Napb	0.528950054	0.151	0.054	6.09118E-50	6
Hmgn3.1	-0.313942471	0.131	0.283	6.17924E-50	6
Pafah1b3	0.391750873	0.238	0.185	6.69178E-50	6
Lims1	-0.37785748	0.059	0.194	7.8436E-50	6
Gm9843	-0.329429041	0.099	0.24	8.59502E-50	6
Amph	0.517023192	0.171	0.082	8.9286E-50	6
Rwdd3	0.549854696	0.156	0.068	1.07007E-49	6
Tm7sf2	0.490472783	0.104	0.026	1.17102E-49	6
Napg	0.50720057	0.2	0.121	1.37114E-49	6
Rpl3	-0.410889616	0.077	0.224	1.50227E-49	6
Exosc8.2	-0.310695596	0.085	0.221	1.74502E-49	6
Dnajc15	-0.425082581	0.023	0.138	1.85915E-49	6
Pde9a	0.474386493	0.101	0.024	2.0529E-49	6
Ppp2r3a	0.535118058	0.177	0.085	2.07373E-49	6
Polr2e	-0.258177326	0.183	0.338	2.09312E-49	6
Sh3bgrl	-0.291880546	0.183	0.352	2.26842E-49	6
Gspt1	-0.352113057	0.131	0.292	2.95169E-49	6
Slirp	-0.278462822	0.198	0.365	3.27788E-49	6
Adarb1	0.518083862	0.116	0.033	3.73684E-49	6
Lmo4.2	-0.315231677	0.183	0.35	3.75343E-49	6
Coq7	-0.349447473	0.079	0.219	4.01782E-49	6
Mrfap1	-0.269588861	0.243	0.418	4.45371E-49	6
Ube2e3	0.260602108	0.412	0.416	4.52069E-49	6
Paxbp1	0.393140266	0.31	0.253	4.95511E-49	6
Fth1.1	-0.319454459	0.55	0.724	5.33528E-49	6
2700029M09Rik.1	-0.258696063	0.151	0.303	5.47917E-49	6
Pcbp4	-0.259733384	0.18	0.338	5.88456E-49	6
Dcakd	-0.304725584	0.153	0.31	6.8817E-49	6
Pdlim7	0.46846399	0.102	0.033	6.9636E-49	6
Rrp15.1	-0.387606697	0.055	0.185	8.19799E-49	6
Rpl30	-0.356533121	0.123	0.282	8.77044E-49	6
Naa50.2	-0.366845548	0.136	0.3	8.84868E-49	6
Stxbp5l	0.544814827	0.128	0.044	8.97055E-49	6
Boc.3	-0.392589385	0.043	0.167	1.08752E-48	6
Dctn2	0.312377868	0.339	0.318	1.35164E-48	6
Romo1	-0.270600705	0.269	0.446	1.37028E-48	6
H1f0.3	-0.282026063	0.438	0.633	1.46765E-48	6
Ndufs6	-0.291116618	0.21	0.381	1.50064E-48	6
Atp6v1a	0.301111129	0.297	0.287	1.90686E-48	6
Bhlhe22	0.527829722	0.168	0.069	1.91428E-48	6
Hist3h2ba.2	0.489868869	0.216	0.129	1.93268E-48	6
Mvd	0.502335094	0.141	0.052	2.1878E-48	6
Chga	0.504721455	0.161	0.077	2.28665E-48	6

Fubp1	-0.26776979	0.398	0.583	2.4121E-48	6
Dync1li2	0.377500474	0.299	0.255	2.85136E-48	6
Ncaph.5	-0.401448945	0.02	0.13	3.6232E-48	6
Cib2	0.460128391	0.101	0.026	3.7372E-48	6
Mki67ip	-0.300224781	0.113	0.257	4.07255E-48	6
Pgrmc2	-0.349404796	0.072	0.208	4.23202E-48	6
Syngr3	0.478282375	0.101	0.03	4.6279E-48	6
Gsk3b.1	0.265529717	0.503	0.499	4.99227E-48	6
Txnrd1	-0.267173959	0.23	0.404	5.01796E-48	6
Dnajc5.1	0.381569544	0.283	0.244	5.02743E-48	6
Tsc22d1.2	0.309272845	0.459	0.424	5.51273E-48	6
Brd7.2	-0.269883036	0.17	0.321	5.60205E-48	6
Ndufs8	-0.2852978	0.197	0.363	6.32473E-48	6
Fam212b	-0.347668708	0.092	0.235	7.39126E-48	6
Gprin1	0.503677198	0.165	0.085	8.52835E-48	6
Ldha.2	-0.381686123	0.097	0.245	9.01637E-48	6
Os9.2	0.394095197	0.299	0.246	9.11081E-48	6
Nrm.2	-0.293095283	0.06	0.18	9.21228E-48	6
Gpatch4.1	-0.404982844	0.058	0.191	9.97522E-48	6
Tgif1.1	-0.383300186	0.01	0.108	1.38848E-47	6
Sec61b	-0.282365551	0.182	0.343	1.49536E-47	6
Rnaseh2b.2	-0.334086924	0.104	0.249	1.50054E-47	6
Peg3	0.37935541	0.322	0.28	1.65801E-47	6
Sobp	0.481595803	0.205	0.12	1.75486E-47	6
Mcm4.3	-0.429774915	0.031	0.148	1.88315E-47	6
Klf9.1	0.400182316	0.325	0.265	2.02487E-47	6
Rps28.1	-0.388165957	0.075	0.215	2.26605E-47	6
Casp3	0.315353234	0.249	0.232	2.38443E-47	6
Clip3.3	0.3780856	0.365	0.306	2.39906E-47	6
Rassf4.1	-0.365487923	0.221	0.391	2.76176E-47	6
Brix1	-0.30553981	0.084	0.217	2.78784E-47	6
Angptl2.2	-0.445841693	0.018	0.126	2.80519E-47	6
Pnrc2.1	-0.338470391	0.082	0.22	2.8414E-47	6
Dlgap5.5	-0.441342376	0.016	0.121	3.16099E-47	6
Cxxc5.2	-0.284784352	0.374	0.56	3.29771E-47	6
Topbp1.3	-0.341473406	0.041	0.155	3.97353E-47	6
Cdkn2c.3	-0.360113297	0.036	0.153	4.13886E-47	6
Nin.2	-0.438855167	0.035	0.157	4.60677E-47	6
Lhx1.4	-0.36361893	0.327	0.514	4.99756E-47	6
Laptm4b	-0.412221596	0.033	0.152	5.33796E-47	6
Atp6ap1	0.404756459	0.188	0.134	5.51903E-47	6
Pard3	-0.404026113	0.024	0.136	5.73637E-47	6
Cdc42se1	-0.281435633	0.06	0.175	6.45167E-47	6
Eci2	-0.313926327	0.12	0.262	6.97903E-47	6
Polr2i	-0.250920224	0.177	0.33	7.59283E-47	6
Cask	0.511835722	0.182	0.102	7.88961E-47	6
Dnmt1.5	-0.33268593	0.136	0.291	7.94273E-47	6
Mrps28	-0.379045105	0.053	0.18	8.59665E-47	6

Rab3c	0.48916206	0.222	0.14	1.06288E-46	6
Atp1b3.2	0.293162071	0.344	0.336	1.07746E-46	6
Gpr56.1	-0.359360386	0.092	0.235	1.10028E-46	6
Syt11.4	0.339265429	0.558	0.499	1.17262E-46	6
Rps24-ps3	-0.309095263	0.065	0.19	1.2407E-46	6
Snx32	0.479538782	0.109	0.03	1.51937E-46	6
Mapk8ip1.4	0.449299215	0.307	0.219	1.52815E-46	6
Nudc.2	-0.297374714	0.142	0.294	1.62762E-46	6
Atp6v1b2	0.425054347	0.222	0.165	1.74547E-46	6
Fkbp4	-0.274085629	0.256	0.429	2.23878E-46	6
Eif3a.1	-0.255761279	0.519	0.705	3.37559E-46	6
Mrpl21	-0.274793595	0.146	0.293	3.66634E-46	6
Fen1.4	-0.364007344	0.036	0.152	3.69421E-46	6
Usp22.1	0.254017397	0.359	0.361	3.70011E-46	6
Tead1.1	-0.382737931	0.057	0.185	4.2933E-46	6
Ptp4a2	-0.255842257	0.197	0.355	4.93518E-46	6
Kif4.5	-0.399857604	0.015	0.117	5.44085E-46	6
Timeless.3	-0.400074686	0.042	0.164	6.49036E-46	6
Kat6b	0.477925966	0.224	0.15	8.09358E-46	6
Tceal8.1	-0.261083061	0.124	0.257	9.95556E-46	6
Gab1	0.507288565	0.15	0.063	1.04385E-45	6
Bub1b.5	-0.365914404	0.013	0.111	1.09058E-45	6
Fam111a.5	-0.422602794	0.026	0.135	1.22463E-45	6
Clvs1.4	0.512948994	0.22	0.12	1.32228E-45	6
Lta4h	-0.314272479	0.112	0.255	1.39499E-45	6
Serp1	-0.35263876	0.09	0.23	1.47134E-45	6
Tcf19.5	-0.354788397	0.013	0.106	1.51948E-45	6
Psmc3ip.3	-0.380111692	0.03	0.142	1.52219E-45	6
Micu3	0.498751524	0.163	0.072	1.71064E-45	6
Ap3d1	0.431814198	0.266	0.198	1.7739E-45	6
Lbr.4	-0.31468832	0.077	0.208	2.76709E-45	6
Gprasp1	0.404251705	0.249	0.202	3.20425E-45	6
Taok3	0.53165223	0.176	0.079	4.16229E-45	6
Ska2.5	-0.359207484	0.082	0.216	4.56389E-45	6
Rabgap1l	0.532260415	0.141	0.054	5.5932E-45	6
Clic1.1	-0.353732973	0.06	0.184	5.99559E-45	6
Mpp6.2	-0.359145557	0.05	0.172	6.01201E-45	6
Rnaseh2a.2	-0.375446159	0.044	0.163	6.55331E-45	6
Hk2.2	-0.275560176	0.068	0.183	7.22835E-45	6
Dbf4.5	-0.370714034	0.04	0.155	8.01831E-45	6
Bin1.5	0.332300853	0.482	0.424	9.31113E-45	6
Gm10076	-0.352536168	0.033	0.144	1.08277E-44	6
Map7d1	0.338596913	0.265	0.239	2.28345E-44	6
Efr3b	0.481691085	0.118	0.042	2.34718E-44	6
Rufy2	0.41997253	0.203	0.154	3.23897E-44	6
Rpf2	-0.335030817	0.08	0.214	3.5061E-44	6
Mxd4.3	0.415232252	0.262	0.191	5.22292E-44	6
Rabac1.1	0.350751397	0.298	0.254	5.62768E-44	6

Mndal	0.482310425	0.106	0.032	6.02611E-44	6
Nop16	-0.373662603	0.058	0.185	7.38501E-44	6
Tcof1	-0.345844878	0.057	0.178	7.38541E-44	6
Imp3	-0.257906992	0.126	0.258	7.59997E-44	6
Cep110.2	-0.395421994	0.052	0.174	9.28136E-44	6
Srm.1	-0.354899827	0.074	0.203	9.46671E-44	6
Tmem151b	0.483649619	0.116	0.037	9.74317E-44	6
Smpd3.1	0.444034411	0.234	0.154	1.10048E-43	6
C530008M17Rik.1	-0.354225633	0.131	0.281	1.19367E-43	6
Zmynd8.3	0.366279351	0.239	0.21	1.56121E-43	6
Abcc5	0.412704826	0.165	0.118	1.64104E-43	6
Myc.2	-0.397802113	0.014	0.108	1.89108E-43	6
Anln.5	-0.34869547	0.018	0.112	2.16742E-43	6
Melk.5	-0.362979278	0.01	0.102	2.46051E-43	6
Cbfb.2	-0.338440672	0.069	0.194	2.4731E-43	6
Tspan6	-0.255306008	0.123	0.258	2.62108E-43	6
Phactr1	0.391654793	0.228	0.179	3.22948E-43	6
Lman2	-0.280253135	0.045	0.148	3.4637E-43	6
Cnih2	0.438722182	0.101	0.027	4.1571E-43	6
Suclg1	-0.26389149	0.142	0.285	4.57019E-43	6
Uba1	0.299424093	0.286	0.27	4.64419E-43	6
Cerk.1	-0.354739917	0.045	0.161	5.3842E-43	6
Ctnna1	-0.319334526	0.05	0.163	5.45105E-43	6
Ing4.1	0.290485477	0.338	0.321	5.65028E-43	6
Sh3glb1	-0.25318497	0.223	0.38	6.60876E-43	6
Ndrg3	0.445778037	0.188	0.108	7.04311E-43	6
Celf5	0.48815328	0.135	0.048	7.55807E-43	6
1700020I14Rik	0.284186305	0.283	0.273	7.663E-43	6
Cisd2	-0.277344589	0.128	0.269	1.04181E-42	6
Dpysl2	0.348929351	0.279	0.245	1.09985E-42	6
Ier2.3	0.322462199	0.376	0.382	1.13971E-42	6
Samm50	-0.289871573	0.09	0.214	1.40346E-42	6
Pdia4	-0.313756884	0.08	0.207	1.74641E-42	6
Prkd3	-0.407958544	0.037	0.152	1.8027E-42	6
Zic2	0.41418877	0.175	0.115	2.62649E-42	6
Klf13	-0.261749193	0.118	0.247	2.7279E-42	6
Irs1.2	-0.407245416	0.023	0.128	2.83852E-42	6
Ift27	-0.293223079	0.119	0.254	4.16104E-42	6
Ncapd2.5	-0.262254605	0.079	0.195	4.31353E-42	6
Lsmd1	-0.291305289	0.101	0.232	4.44642E-42	6
Add1	0.378112938	0.209	0.159	5.66472E-42	6
Lphn3	0.485615951	0.131	0.054	5.8494E-42	6
Cdca7l.3	-0.358320506	0.016	0.113	6.7697E-42	6
Ftsj3	-0.301684703	0.089	0.217	8.41325E-42	6
Rcn1	-0.284208627	0.11	0.245	1.0034E-41	6
Sema4g	0.481349321	0.102	0.031	1.06805E-41	6
Dnm1l	0.300779389	0.305	0.29	1.11127E-41	6
Serf1	0.273410133	0.283	0.28	1.24292E-41	6

Mrps17	-0.275032416	0.124	0.258	1.30933E-41	6
Nme2	-0.367010839	0.015	0.11	1.33579E-41	6
Suv39h2.1	-0.290340919	0.039	0.14	1.38788E-41	6
Hnrnpf	-0.317103029	0.031	0.135	1.44298E-41	6
2610017I09Rik	-0.346737136	0.086	0.215	1.45186E-41	6
Rassf3	-0.403649472	0.035	0.148	1.69043E-41	6
Kif1a.3	0.421880309	0.234	0.165	1.71904E-41	6
Mrpl12	-0.321095466	0.087	0.216	1.80545E-41	6
Tspan5	0.370583029	0.234	0.192	2.36807E-41	6
2610001J05Rik	-0.267470361	0.084	0.204	2.37213E-41	6
Ctps.1	-0.338426166	0.038	0.147	2.79832E-41	6
Gm26735.2	0.374590966	0.219	0.177	3.06599E-41	6
Rbbp8.1	-0.335745648	0.03	0.131	5.06825E-41	6
Mum1l1.1	-0.363996327	0.012	0.102	6.13942E-41	6
Csrnp3	0.470917944	0.146	0.076	6.78162E-41	6
Dclk1.2	-0.274429708	0.268	0.426	6.97112E-41	6
Strbp.2	0.265975549	0.352	0.346	7.70548E-41	6
Dlgap4.3	0.450353445	0.264	0.18	8.12125E-41	6
Eml4	-0.271590721	0.095	0.22	8.36909E-41	6
Diap3.5	-0.34442093	0.019	0.115	9.39107E-41	6
D19Bwg1357e	-0.25499066	0.147	0.285	1.46437E-40	6
Dgkd	0.432406819	0.158	0.101	1.82855E-40	6
Zfp367.2	-0.325412563	0.013	0.101	1.85933E-40	6
Serpinh1.2	-0.395317265	0.026	0.124	3.21506E-40	6
Mpdz	-0.315837078	0.04	0.144	3.53779E-40	6
Apc2	0.467031109	0.12	0.036	4.06722E-40	6
Rfc3.2	-0.316763006	0.088	0.215	4.24444E-40	6
Gabrg2	0.450631114	0.12	0.043	5.40493E-40	6
Dph3	-0.278495877	0.096	0.221	5.42469E-40	6
Vrk1.3	-0.331410132	0.048	0.157	5.80338E-40	6
Ipo5.2	-0.348747335	0.039	0.148	6.03532E-40	6
Olfm1.1	0.385508508	0.246	0.193	6.81752E-40	6
Baiap2	0.441550709	0.105	0.045	7.09231E-40	6
Pold2	-0.335627049	0.024	0.122	7.65984E-40	6
Wdr12	-0.304008157	0.068	0.186	8.02561E-40	6
Lin7c	0.333571603	0.254	0.213	8.40907E-40	6
Rars	-0.313087815	0.075	0.195	1.09303E-39	6
Rad21.6	-0.33232552	0.303	0.477	1.18593E-39	6
Tmem132a	0.42688586	0.161	0.097	1.32632E-39	6
Hpca.3	-0.428143744	0.141	0.29	1.33068E-39	6
Sqstm1.1	0.305628435	0.247	0.224	1.3662E-39	6
Hmgb3.1	-0.258213888	0.174	0.318	1.50179E-39	6
Gpatch8	0.307203037	0.3	0.278	1.77222E-39	6
Snrpn	0.358332956	0.191	0.155	1.82436E-39	6
Cdc42se2	-0.302864087	0.115	0.25	1.9529E-39	6
Nfyb.1	0.33722472	0.264	0.231	2.37172E-39	6
Ptprd	0.318530024	0.357	0.338	2.54647E-39	6
Jam3	-0.338562811	0.038	0.144	3.0076E-39	6

Eif2s1	-0.270371656	0.087	0.208	3.42491E-39	6
Aurka.6	-0.316950693	0.017	0.106	3.52198E-39	6
Man1c1	-0.37165013	0.031	0.135	3.73981E-39	6
Gm6472	-0.300903722	0.063	0.177	4.13123E-39	6
Vamp2	0.428622494	0.124	0.068	7.02088E-39	6
Fam96a	-0.318268857	0.067	0.184	7.3835E-39	6
Eef1e1	0.29497312	0.197	0.18	8.67439E-39	6
Agtpbp1	0.433132679	0.155	0.096	9.69306E-39	6
Snx5.1	-0.338070297	0.057	0.173	9.97133E-39	6
Hprt.1	-0.263443727	0.082	0.198	1.10433E-38	6
Prdx5	0.290843162	0.283	0.262	1.19695E-38	6
Mtap.1	-0.339151049	0.014	0.102	1.29133E-38	6
Yif1a	-0.258998504	0.079	0.189	1.33254E-38	6
Ankra2.1	0.411425388	0.169	0.115	1.41082E-38	6
1810037I17Rik	-0.307249467	0.086	0.207	1.75926E-38	6
Hcfc1r1.2	0.347148976	0.236	0.188	1.99284E-38	6
Plekho2	-0.35315125	0.014	0.101	2.14839E-38	6
Cd63-ps	-0.321024947	0.031	0.127	2.30655E-38	6
Zfp57	0.454420682	0.138	0.06	2.75061E-38	6
Trim27	-0.253012369	0.055	0.157	2.92374E-38	6
Pacsin1	0.434105169	0.101	0.03	3.00626E-38	6
Atp6v0e2.1	0.298046726	0.283	0.257	3.4332E-38	6
Dixdc1.2	0.357078844	0.264	0.231	3.46536E-38	6
Bod1l	0.255088102	0.367	0.379	4.02803E-38	6
Cmc2.3	-0.335494016	0.029	0.128	4.27514E-38	6
Atcay	0.445577435	0.135	0.058	4.28658E-38	6
Snx1	-0.284705607	0.068	0.178	4.38994E-38	6
Zfp148	0.262939067	0.283	0.284	5.54496E-38	6
Chchd3	-0.280733729	0.077	0.194	5.60721E-38	6
Mapre2	0.361812547	0.208	0.163	5.95649E-38	6
Exosc7.1	-0.274231763	0.075	0.19	8.07276E-38	6
Abi2	0.295174905	0.204	0.18	8.57555E-38	6
Ncdn	0.394342325	0.12	0.061	1.03281E-37	6
Gcsh	-0.312911068	0.046	0.151	1.03782E-37	6
Mest	0.43580962	0.148	0.077	1.14627E-37	6
Mapk8ip2	0.407543585	0.195	0.137	1.66648E-37	6
Ndufaf2	-0.306275794	0.091	0.213	1.76499E-37	6
Riok3	0.383068651	0.171	0.126	1.77118E-37	6
Atp6v0a1	0.455722799	0.169	0.088	1.9284E-37	6
Cd164	-0.307474204	0.031	0.128	1.95591E-37	6
Ptpra	0.251380743	0.23	0.235	2.38417E-37	6
Bcar1	-0.272737057	0.087	0.202	2.5138E-37	6
Ppa2	-0.318977979	0.054	0.165	2.62101E-37	6
Sept6	0.415842338	0.127	0.069	3.14808E-37	6
Klhl29	0.448789798	0.102	0.03	3.36908E-37	6
Gins1.2	-0.332635575	0.027	0.123	3.61798E-37	6
Fam57b.1	0.335253828	0.221	0.182	3.74866E-37	6
Gnl3l	0.330021619	0.227	0.184	3.88474E-37	6

Ptbp1	-0.336983904	0.019	0.109	3.88608E-37	6
Ip6k1	0.357345279	0.22	0.171	4.00577E-37	6
Nfasc	0.439042481	0.101	0.029	4.9504E-37	6
Etfb	-0.335542735	0.077	0.195	5.2123E-37	6
Dusp8.1	0.476094126	0.155	0.071	5.3049E-37	6
Pea15a.5	0.413383837	0.258	0.167	6.60612E-37	6
Gng12	-0.32573535	0.031	0.13	7.31441E-37	6
Zranb1	0.343226365	0.239	0.205	7.98847E-37	6
Cxadr	0.442895302	0.149	0.073	8.1101E-37	6
Wbp2	0.31433646	0.217	0.193	8.45285E-37	6
Strip1	0.369376843	0.19	0.149	8.76911E-37	6
Ptges3.1	-0.269739062	0.1	0.222	9.61093E-37	6
Ninj1	-0.349800665	0.018	0.105	9.82093E-37	6
Pom121	-0.282063448	0.075	0.189	1.04113E-36	6
Evl	0.342469937	0.219	0.174	1.05566E-36	6
C77370.1	0.467188937	0.161	0.095	1.21631E-36	6
Smc5.1	-0.273098776	0.137	0.269	1.26469E-36	6
Ptprg.1	-0.322064323	0.047	0.151	1.73746E-36	6
Ccnl2	0.259824599	0.33	0.319	2.20963E-36	6
Zfp277	-0.293166246	0.041	0.14	2.51972E-36	6
Gtf3c6	-0.33335816	0.086	0.211	2.52998E-36	6
Elavl1	-0.295525666	0.082	0.2	2.65766E-36	6
Arl6ip6.1	-0.313147275	0.036	0.134	2.70907E-36	6
Zcwpw1.1	-0.351448163	0.02	0.111	3.53991E-36	6
Tprn.2	0.364436689	0.288	0.22	3.57985E-36	6
Mlf2	0.262929843	0.294	0.278	3.59419E-36	6
Atp9a	0.470919986	0.139	0.061	3.85507E-36	6
Cntln.3	-0.339646707	0.067	0.182	4.04667E-36	6
Smap1	0.2771323	0.23	0.222	4.077E-36	6
Cdc42bpa	0.392024812	0.183	0.132	4.40047E-36	6
Plekha1	0.427509659	0.122	0.055	4.87918E-36	6
Map6.1	0.457559648	0.154	0.072	4.97318E-36	6
Arhgef2.2	0.288910707	0.31	0.279	4.98491E-36	6
Synj1	0.427458647	0.146	0.072	5.53549E-36	6
Cdt1.4	-0.362412641	0.035	0.134	7.37425E-36	6
Gart.1	-0.266434769	0.069	0.175	8.3343E-36	6
Phf3	0.276164232	0.273	0.269	8.80117E-36	6
Cdca4.2	-0.287059332	0.027	0.118	9.38784E-36	6
Gstp1	-0.251451471	0.062	0.164	1.13779E-35	6
Znrd1	-0.256218127	0.103	0.222	1.18474E-35	6
Cenpj.3	-0.343982053	0.046	0.151	1.36286E-35	6
Ppp2r2b.1	0.489820218	0.17	0.076	1.50559E-35	6
Mdga1	-0.388186842	0.04	0.145	1.76519E-35	6
Scamp1	0.451704849	0.139	0.065	1.92154E-35	6
Hn1.3	0.26135936	0.554	0.54	1.98728E-35	6
Ogt.1	0.305886408	0.235	0.209	2.01162E-35	6
Gphn	0.344397558	0.167	0.138	2.18162E-35	6
Pkn2	-0.278225118	0.099	0.218	2.21973E-35	6

Rev3l	0.422336211	0.157	0.091	2.78622E-35	6
Slc7a5.2	-0.34042444	0.031	0.123	3.03167E-35	6
Stx7	0.405943291	0.18	0.109	3.28793E-35	6
Mycl	-0.343920075	0.043	0.146	3.47211E-35	6
Zcchc18	0.394216641	0.227	0.164	3.47869E-35	6
Camsap2	0.422732212	0.187	0.127	3.48198E-35	6
Hid1	0.437629045	0.125	0.048	3.92397E-35	6
Aig1	0.424389416	0.153	0.09	4.36381E-35	6
Mlec	-0.278984859	0.065	0.171	4.68579E-35	6
Dpysl5	0.42919103	0.16	0.088	5.04256E-35	6
Zfp608	0.298376982	0.201	0.183	5.23422E-35	6
Arhgef25	0.456149355	0.146	0.072	5.40891E-35	6
Asrgl1	-0.342679612	0.043	0.142	5.62105E-35	6
Trafd1	0.396405749	0.17	0.112	6.01434E-35	6
Ypel5	0.366437379	0.16	0.11	6.49244E-35	6
Agap1	0.377040594	0.175	0.127	6.6135E-35	6
Bola1	-0.263503414	0.073	0.181	8.44751E-35	6
Ensa	0.267241398	0.229	0.227	8.56349E-35	6
Mkrn1.2	0.296483488	0.23	0.206	1.0783E-34	6
Rabep1	0.285774534	0.243	0.234	1.09232E-34	6
Mef2a	0.374635794	0.182	0.134	1.16109E-34	6
Igdcc4.1	-0.366493151	0.016	0.102	1.23107E-34	6
Fam64a.5	-0.366814995	0.03	0.126	1.28777E-34	6
Fam171b	0.327735874	0.256	0.22	1.39574E-34	6
Cnpy1.1	0.422135939	0.192	0.127	1.52755E-34	6
Prpf40b	0.293722271	0.161	0.145	1.74523E-34	6
Gm13092	-0.293663485	0.024	0.112	1.85825E-34	6
Osbpl1a	0.406301429	0.107	0.054	2.08675E-34	6
Idh1.2	0.363144457	0.2	0.151	3.13825E-34	6
Arl8a	0.410279509	0.164	0.089	3.29138E-34	6
Mgst3	0.442901907	0.166	0.088	3.37415E-34	6
Rpl7a	-0.302432022	0.055	0.159	4.12338E-34	6
Abtb1	0.414127289	0.141	0.079	4.21831E-34	6
Tspyl1	0.354182115	0.19	0.141	4.73509E-34	6
Arhgap21	0.359177862	0.198	0.16	4.84398E-34	6
Slc17a6.5	0.452473675	0.235	0.139	4.99828E-34	6
Fam171a2	0.420702478	0.114	0.05	5.15226E-34	6
Pabpn1	0.26365436	0.202	0.193	5.46767E-34	6
Dars	-0.254966389	0.065	0.167	7.0209E-34	6
Tex9	-0.302321149	0.054	0.157	7.46461E-34	6
Mrpl36	-0.290301583	0.068	0.174	8.65986E-34	6
Robo2.1	0.39395679	0.201	0.146	9.93065E-34	6
Mbd6	0.417827233	0.135	0.076	1.00535E-33	6
Fh1	-0.256041427	0.084	0.192	1.03302E-33	6
A830010M20Rik	0.44028608	0.103	0.042	1.12737E-33	6
Hist3h2a.3	0.42398652	0.213	0.136	1.70709E-33	6
Itgb3bp	-0.339919468	0.023	0.112	2.06218E-33	6
Erbb4	0.436289592	0.112	0.04	2.41688E-33	6

Mis18a.3	-0.311144691	0.027	0.118	2.49189E-33	6
Jarid2	0.310677694	0.201	0.176	2.77613E-33	6
Gdpd1.3	0.250923078	0.252	0.242	2.9218E-33	6
Apitd1.3	-0.296995755	0.019	0.102	3.26447E-33	6
Pick1	0.392191111	0.179	0.122	3.32487E-33	6
Ubr7.1	-0.276846036	0.046	0.141	3.53995E-33	6
Nudt21	-0.252932558	0.047	0.142	3.74298E-33	6
Igsf3.1	0.37513019	0.178	0.128	3.90479E-33	6
Klhdc3	0.273723821	0.196	0.184	4.2607E-33	6
Ssbp2	-0.294609545	0.04	0.134	5.85335E-33	6
Nae1	0.262013948	0.231	0.223	6.87029E-33	6
Mapk10.1	0.405320246	0.155	0.093	7.26423E-33	6
Sap30.1	-0.275356012	0.049	0.146	7.85022E-33	6
C130071C03Rik.1	0.284114838	0.334	0.305	8.0359E-33	6
Col9a3.1	0.266805226	0.189	0.178	8.38937E-33	6
Slc1a3.5	-0.506888645	0.065	0.161	1.08621E-32	6
Eftud2	-0.252256886	0.082	0.192	1.08842E-32	6
Rpa1.3	-0.282711392	0.029	0.117	1.14109E-32	6
Xrcc5	-0.324790935	0.031	0.123	1.5953E-32	6
Slc22a17.2	0.306026536	0.283	0.247	1.60949E-32	6
Prr13	0.39364508	0.143	0.084	1.85442E-32	6
Actr2	0.274332587	0.213	0.195	2.26304E-32	6
Ttc4	0.281764566	0.195	0.178	2.61715E-32	6
Dnaaf2.1	-0.306649122	0.025	0.112	2.67223E-32	6
Zfp292	0.29291756	0.357	0.339	2.98881E-32	6
Ctnnd2	0.385651997	0.141	0.094	3.16818E-32	6
Vegfb	-0.275036991	0.036	0.126	3.61704E-32	6
Rps16-ps2	-0.298564991	0.05	0.149	3.83342E-32	6
Mthfd1.1	-0.30007437	0.033	0.124	3.91486E-32	6
Rusc1	0.411930995	0.111	0.05	4.64574E-32	6
Zfp318	0.278622751	0.183	0.175	5.57119E-32	6
Pfkm	0.399652736	0.124	0.06	5.67412E-32	6
Sgip1	0.449068387	0.132	0.057	5.846E-32	6
Polb	0.325501851	0.204	0.162	6.292E-32	6
Ubl3	0.268068855	0.217	0.208	6.36263E-32	6
Rangap1.6	-0.292197205	0.141	0.267	8.95053E-32	6
Lamp2	-0.309047234	0.05	0.146	1.15928E-31	6
Pfn2.2	0.298179772	0.272	0.238	1.90012E-31	6
Ccdc58	-0.290761033	0.046	0.14	2.51434E-31	6
Ccdc18.2	-0.277833838	0.037	0.123	2.61188E-31	6
Tmem63b	0.403547651	0.136	0.075	2.65549E-31	6
Ppm1l	0.410806569	0.15	0.089	3.86359E-31	6
Snx10	0.360454223	0.188	0.142	4.2489E-31	6
Pak7.3	0.436545185	0.171	0.097	4.3755E-31	6
A630007B06Rik	0.303319145	0.229	0.203	4.74221E-31	6
Actr1a	0.270871022	0.192	0.175	5.41305E-31	6
Wdr13.1	0.408543544	0.165	0.099	6.39938E-31	6
Snord104.1	-0.281383812	0.062	0.161	6.47876E-31	6

Zc2hc1a	0.37911591	0.147	0.089	8.58985E-31	6
Ppat.1	-0.274367398	0.031	0.115	8.95283E-31	6
Nme4.1	-0.283162267	0.037	0.125	1.01715E-30	6
Jakmip2	0.380329811	0.213	0.151	1.05713E-30	6
Smarca1	0.444968439	0.111	0.051	1.11839E-30	6
Cdk5rap2.2	-0.261397307	0.085	0.192	1.43022E-30	6
Plxnb2.1	-0.257327311	0.08	0.179	1.43154E-30	6
Dis3	-0.251484299	0.045	0.131	1.56804E-30	6
Nr3c1.1	-0.264857069	0.043	0.133	1.59981E-30	6
Gamt.3	-0.348036158	0.072	0.179	1.80217E-30	6
Nhlh2.5	0.266688361	0.467	0.418	2.00376E-30	6
Ldhb.2	0.29712457	0.317	0.277	2.12518E-30	6
Pola1.2	-0.331416255	0.024	0.11	2.16339E-30	6
Syngr1	0.385836711	0.15	0.087	2.20111E-30	6
Ctsz	-0.355766947	0.049	0.145	2.24496E-30	6
Pik3r2	0.418239805	0.147	0.075	2.46881E-30	6
Ankrd46	0.314686852	0.191	0.157	3.0572E-30	6
Scamp2	-0.272538717	0.052	0.143	3.17573E-30	6
Trappc3	0.31408103	0.127	0.098	3.49785E-30	6
Lpgat1	0.310421013	0.187	0.165	3.67794E-30	6
Vcan	-0.303518824	0.045	0.138	3.89719E-30	6
Mtch1	0.253697867	0.232	0.221	7.08913E-30	6
Gsto1.1	-0.32381201	0.043	0.136	7.33095E-30	6
Sept8.1	-0.327049455	0.043	0.137	7.33243E-30	6
Rpl3-ps1	-0.265085638	0.026	0.108	7.59099E-30	6
2510002D24Rik	0.402745897	0.114	0.043	9.17984E-30	6
Mrps15	-0.253967285	0.125	0.24	1.15004E-29	6
Gpc2.2	0.361791488	0.185	0.135	1.19928E-29	6
Tspan7.3	0.370593616	0.136	0.102	1.47978E-29	6
Flywch2	0.398772354	0.101	0.046	1.71679E-29	6
Necab3.1	0.419770176	0.127	0.046	1.87393E-29	6
Akap8l.2	0.370098353	0.206	0.153	1.99342E-29	6
Grik5	0.376042974	0.153	0.105	2.29783E-29	6
Tmem30a	0.260926328	0.188	0.178	2.32029E-29	6
Cdk5rap3	-0.271995346	0.095	0.202	3.09226E-29	6
Odc1	-0.290486037	0.026	0.108	3.11742E-29	6
Samd14	0.366888666	0.116	0.072	3.5388E-29	6
Clstn1.2	0.316037037	0.242	0.198	3.74963E-29	6
BC034090	-0.307556657	0.045	0.135	3.82296E-29	6
Pcgf2	0.39627253	0.141	0.08	3.94899E-29	6
4930402H24Rik.2	0.377971708	0.161	0.108	4.05041E-29	6
Tmem237	-0.295208832	0.046	0.138	5.34777E-29	6
Brca2.3	-0.255595697	0.039	0.12	5.78783E-29	6
Alg2	0.35965716	0.146	0.095	6.28391E-29	6
Fam184a	0.405900842	0.112	0.062	6.41002E-29	6
A330076H08Rik.4	0.453533516	0.24	0.153	6.99101E-29	6
Tnrc6b	0.260035302	0.262	0.246	7.03672E-29	6
Nedd4l	0.336371084	0.166	0.124	8.05471E-29	6

Fabp7.6	-0.84966713	0.084	0.126	9.81484E-29	6
Stox2	0.269040122	0.212	0.197	1.39637E-28	6
Klhl13	-0.28394746	0.03	0.111	1.40657E-28	6
Zfp536	0.321199762	0.136	0.107	1.54639E-28	6
Ndrg2.1	-0.280099475	0.089	0.194	1.74628E-28	6
Pkp4.2	0.271755996	0.111	0.101	2.07513E-28	6
Pkig	-0.29634045	0.045	0.136	2.11304E-28	6
Araf	0.309255701	0.169	0.134	3.10527E-28	6
Ctsd.6	-0.454944994	0.144	0.178	3.24379E-28	6
Mpped2	0.339313827	0.163	0.122	4.45216E-28	6
Socs7	0.31351186	0.122	0.093	5.33326E-28	6
Prmt2.2	0.398028073	0.146	0.089	5.42448E-28	6
Tom1l1	-0.281841471	0.029	0.11	5.67019E-28	6
Lima1.1	-0.29078254	0.043	0.129	6.38406E-28	6
H1fx.4	-0.26547504	0.14	0.255	6.95438E-28	6
Rnpc3	0.329864581	0.133	0.1	9.22612E-28	6
Cspp1	0.278548364	0.256	0.236	9.78535E-28	6
Fos.4	0.444494034	0.369	0.298	1.20679E-27	6
Fbxl15	0.346382327	0.129	0.082	1.24854E-27	6
Mau2	0.318032495	0.182	0.145	1.51995E-27	6
Agpat4.1	0.356564536	0.166	0.112	1.78974E-27	6
Shd.2	0.368257668	0.162	0.104	2.09643E-27	6
Orc2	-0.272486211	0.067	0.164	2.14283E-27	6
Scn8a	0.353052815	0.175	0.133	2.34613E-27	6
Senp7	0.317464145	0.137	0.104	2.60188E-27	6
Lphn2	0.367866612	0.118	0.065	2.91367E-27	6
Rps23	-0.270873614	0.027	0.105	4.72245E-27	6
Tmem178	0.296271272	0.214	0.183	5.0799E-27	6
Nol12	-0.253362406	0.05	0.136	5.29136E-27	6
Gdap1	0.261214188	0.214	0.197	6.14802E-27	6
Ctnna2	0.338943515	0.13	0.081	6.19181E-27	6
Rps27a	-0.251227213	0.033	0.113	6.31539E-27	6
Gm5124	0.421574088	0.123	0.061	9.12843E-27	6
Fbxo11	0.309918559	0.179	0.152	1.01181E-26	6
Fbxo21	0.321449006	0.131	0.086	1.25405E-26	6
Pcmtd1	0.32100144	0.158	0.122	1.31865E-26	6
Stau2	0.316025772	0.19	0.146	1.67752E-26	6
Lpin2.2	-0.291822036	0.085	0.189	1.75948E-26	6
Rrp1b	-0.280942589	0.053	0.144	2.06174E-26	6
Plcb4	0.372522514	0.187	0.134	2.32508E-26	6
Phf21b	-0.261455019	0.05	0.135	3.16453E-26	6
Wasf2	-0.279084221	0.038	0.12	3.52297E-26	6
Tro	0.317566082	0.158	0.122	3.54427E-26	6
Tpi1	0.334114837	0.163	0.117	4.17422E-26	6
Ctxn1	0.250900518	0.189	0.168	4.2375E-26	6
Mrto4	-0.256937669	0.026	0.102	4.82507E-26	6
Hcfc2	0.371056727	0.117	0.065	5.38314E-26	6
Hmgcs1.1	0.341922717	0.19	0.147	5.75575E-26	6

Mapk9	0.378424305	0.122	0.072	6.51777E-26	6
Dennd2a	-0.269844226	0.036	0.114	7.72349E-26	6
Bdh1	0.331987654	0.117	0.067	9.77367E-26	6
Oraov1.1	-0.272355348	0.043	0.128	1.10004E-25	6
Wdr60.1	-0.288864716	0.043	0.125	1.12074E-25	6
1500011B03Rik.1	0.374610229	0.16	0.101	1.21652E-25	6
Armcx1	0.377246606	0.134	0.078	1.32505E-25	6
Rnf165.1	0.313253042	0.207	0.169	1.33595E-25	6
Kdm5b.4	0.345696637	0.23	0.18	1.73922E-25	6
Tram1l1	0.348680583	0.129	0.081	1.88485E-25	6
Cyfip2	0.356681928	0.111	0.047	1.9212E-25	6
Pip5k1c	0.362538547	0.131	0.077	2.17632E-25	6
Sox5.1	0.346338932	0.149	0.116	2.37411E-25	6
D430019H16Rik	0.376869527	0.109	0.051	2.76652E-25	6
Gabrb3	0.343080267	0.139	0.098	3.65183E-25	6
Rhebl1	0.261209132	0.122	0.104	4.84297E-25	6
Blcap	0.364103528	0.131	0.073	4.98394E-25	6
Nefm	0.399377196	0.156	0.112	5.23684E-25	6
Chd9	0.348973719	0.156	0.115	5.80538E-25	6
Elovl4	0.252485009	0.125	0.108	1.20301E-24	6
Socs2	0.306563033	0.169	0.135	1.58255E-24	6
Tsga10	0.337731681	0.106	0.058	1.87993E-24	6
Map9	0.325826589	0.181	0.144	2.36472E-24	6
Cd99l2	0.313437014	0.13	0.096	3.02385E-24	6
Tmx4.1	0.262553253	0.201	0.175	3.57287E-24	6
6330403K07Rik.3	0.277884715	0.281	0.238	3.74208E-24	6
Sqle	0.261734736	0.136	0.119	4.31105E-24	6
Podxl2.5	0.276077893	0.272	0.222	8.06392E-24	6
Atat1.1	0.256332324	0.122	0.101	9.247E-24	6
Peo1	-0.268705748	0.048	0.131	1.00733E-23	6
Gtf2f2	-0.252283575	0.044	0.124	1.11389E-23	6
Enho	0.370426357	0.106	0.044	1.56018E-23	6
Celsr2.1	0.271254491	0.269	0.234	1.74923E-23	6
Tbce	0.314002277	0.111	0.075	2.01899E-23	6
Usp33	0.288007482	0.173	0.146	2.83904E-23	6
Exosc5	-0.25798491	0.035	0.11	3.01809E-23	6
Tmem35	0.276668557	0.128	0.101	3.35277E-23	6
Tmem176b	-0.29359764	0.039	0.116	3.38577E-23	6
Slc25a27	0.306025553	0.144	0.106	3.51792E-23	6
Akap11	0.261988823	0.151	0.138	3.57806E-23	6
2810008D09Rik.1	0.301468728	0.159	0.124	4.09727E-23	6
Rps13	-0.25568459	0.029	0.102	4.24949E-23	6
Necap1	0.334533124	0.106	0.066	4.73878E-23	6
Rbm33	0.271030861	0.147	0.128	5.61093E-23	6
Hyi	0.270870003	0.101	0.073	6.38442E-23	6
Gdap1l1	0.318417525	0.119	0.079	8.28564E-23	6
Ltbp3	-0.278704956	0.045	0.125	9.7326E-23	6
Flot1.1	0.328608533	0.139	0.094	2.51941E-22	6

Fbxo9	0.285854615	0.153	0.125	3.05102E-22	6
Ddx26b	0.262610342	0.145	0.128	4.59866E-22	6
Pmvk	0.285716891	0.124	0.091	4.92117E-22	6
Nipsnap1	0.315867303	0.17	0.132	5.32826E-22	6
Ophn1	0.325283471	0.13	0.079	6.53427E-22	6
Unc13a	0.323853574	0.101	0.059	8.58845E-22	6
Kcnq2	0.351996637	0.116	0.068	1.43547E-21	6
Arhgef9	0.327704065	0.126	0.085	1.46679E-21	6
Narf	0.281541494	0.144	0.114	1.80399E-21	6
Vopp1	0.292061308	0.15	0.115	2.16494E-21	6
Reep1	0.310848656	0.117	0.078	2.78478E-21	6
Tln1	0.275550751	0.18	0.146	3.19453E-21	6
2510009E07Rik	0.281314182	0.119	0.094	4.36992E-21	6
Fnbp1	0.342685069	0.122	0.068	5.19702E-21	6
Dcaf6	0.320374852	0.114	0.077	8.07191E-21	6
Chfr	0.276862331	0.121	0.097	8.18669E-21	6
Prkx	0.296223572	0.143	0.109	1.20162E-20	6
4632415L05Rik	0.294642297	0.134	0.112	1.26346E-20	6
Phyhipl	0.316968269	0.106	0.057	1.38464E-20	6
Kif3c	0.338111356	0.114	0.058	1.41595E-20	6
Map1a	0.357793393	0.124	0.068	2.38878E-20	6
Camsap1	0.326643611	0.136	0.098	3.42619E-20	6
Zfp329.1	0.266077263	0.153	0.126	3.85027E-20	6
Ddhd2	0.252722549	0.151	0.128	5.14799E-20	6
Myo9a	0.275013925	0.18	0.155	5.97713E-20	6
Wsb2	0.307854935	0.102	0.06	6.28526E-20	6
Ncoa7	0.253702918	0.114	0.097	8.04943E-20	6
Nsf	0.260377933	0.107	0.083	1.57657E-19	6
Zdhhc17	0.289578179	0.109	0.074	1.82425E-19	6
Ctsf.2	0.329881839	0.146	0.093	1.85107E-19	6
Xpr1	0.327284914	0.132	0.088	1.89061E-19	6
Bmyc	0.267366017	0.124	0.098	1.9227E-19	6
Zfyve27	0.3323335	0.104	0.058	2.07285E-19	6
Thsd7a.1	-0.261328076	0.058	0.134	3.56043E-19	6
Asxl3	0.339635288	0.101	0.059	4.21038E-19	6
Tspyl2	0.320418737	0.106	0.065	5.94749E-19	6
Ppp1r21	0.297217493	0.101	0.061	7.25089E-19	6
Lingo1.1	0.317557555	0.138	0.09	1.47519E-18	6
Nenf.1	0.25414168	0.158	0.134	2.64216E-18	6
RP23-199B2.4	0.323050369	0.104	0.058	2.66275E-18	6
Csnk1g1	0.320214839	0.116	0.073	3.18045E-18	6
Tmem127	0.265489244	0.116	0.092	5.40386E-18	6
Clybl.1	0.261907355	0.142	0.113	1.2574E-17	6
Tsc1	0.276798608	0.138	0.1	1.4793E-17	6
Vezt	0.275293014	0.117	0.09	2.37969E-17	6
Lztr1	0.263550955	0.131	0.097	2.92894E-17	6
4933427D14Rik	0.314255256	0.114	0.074	6.56208E-17	6
Mgll.1	0.267764603	0.156	0.13	8.05843E-17	6

Eml5.1	0.308576605	0.124	0.09	1.11512E-16	6
Ppp1r1a.1	0.299795138	0.151	0.106	1.48913E-16	6
Gm10036	-0.281007543	0.101	0.183	1.57525E-16	6
Ttc28	0.253779236	0.138	0.121	1.79679E-16	6
Dmxl2	0.310489462	0.118	0.07	4.23171E-16	6
Dip2b	0.26954277	0.102	0.077	4.61628E-16	6
Dctn1	0.265989346	0.11	0.079	7.18495E-16	6
Meis3	0.263319184	0.107	0.074	9.34682E-16	6
Pik3r3.3	0.293449277	0.172	0.132	1.34566E-15	6
Egfr	0.279462621	0.112	0.08	1.69785E-15	6
Tceal3	0.264929157	0.138	0.107	1.85445E-15	6
Sbk1.2	0.309392949	0.16	0.114	2.01464E-15	6
Gm3764.1	0.273895672	0.173	0.127	3.80469E-15	6
Limk2	0.27795691	0.102	0.067	4.63137E-15	6
Ntn4	0.290311393	0.112	0.079	5.5431E-15	6
BC068157	0.29558656	0.117	0.077	9.83271E-15	6
Casd1	0.274343483	0.116	0.079	1.15402E-14	6
Mgat5b	0.28277418	0.101	0.069	2.94381E-14	6
Tecpr1	0.260458208	0.106	0.075	1.08724E-12	6
Zer1	0.290331718	0.103	0.064	1.20218E-12	6
Mmp16	0.251620487	0.114	0.085	3.96364E-12	6
Jhdm1d.3	0.251054057	0.153	0.122	1.75486E-11	6
Plp1.6	-0.506805055	0.088	0.117	9.56079E-10	6
Fosb.2	0.25106888	0.174	0.154	1.28311E-09	6
Cntn2.7	1.597898918	0.889	0.213	0	7
Neurod1.7	1.460194366	0.986	0.496	0	7
Tubb3.6	1.339017309	0.939	0.437	0	7
Gap43.7	1.175882847	0.955	0.571	0	7
Nhlh2.6	1.096764378	0.836	0.39	0	7
Stmn2.6	1.053926176	0.964	0.52	0	7
Miat.5	1.040867273	0.909	0.484	0	7
Map1b.6	1.008978727	0.932	0.56	0	7
Tuba1a.5	0.964219268	0.996	0.924	0	7
Rtn1.7	0.959724418	0.971	0.668	0	7
Ckb.6	0.86034745	0.986	0.794	0	7
Tmsb4x.5	0.729502599	0.998	0.944	0	7
Nhlh1.5	1.184748872	0.65	0.189	0	7
Tubb2b.6	0.950213918	0.825	0.435	2.5051E-297	7
Tex14.4	1.249191526	0.526	0.115	1.3826E-285	7
Basp1.6	0.795196136	0.927	0.686	4.4952E-277	7
Dpysl3.7	1.081689698	0.641	0.186	2.0317E-275	7
Ina.6	0.94823856	0.806	0.414	1.1036E-268	7
St18.6	1.201796177	0.576	0.159	2.2563E-268	7
Stmn4.7	0.995381848	0.755	0.308	2.5532E-263	7
Pdzrn3.4	1.151267468	0.529	0.117	2.1894E-259	7
Trpc4ap.7	1.121036185	0.566	0.168	9.1931E-255	7
Ccnd1.5	-1.528794516	0.114	0.554	6.5723E-249	7
Sept3.7	0.929864942	0.761	0.363	1.6649E-240	7

Rplp1.4	-0.774975802	0.731	0.888	6.775E-240	7
Map2.6	0.876826871	0.803	0.467	1.0238E-231	7
Sept4.5	1.021362133	0.602	0.196	6.0876E-228	7
Igfbpl1.5	0.748472389	0.906	0.627	2.0831E-223	7
Zic1.5	0.634144109	0.953	0.77	2.2064E-222	7
Rpl13a.2	-0.663535368	0.853	0.934	9.876E-220	7
Tubb2a.7	0.955779576	0.62	0.215	4.5695E-218	7
Podxl2.6	1.009590535	0.576	0.199	1.8507E-216	7
Ppp1r14c.6	0.949611933	0.617	0.234	3.2538E-208	7
Itm2b.6	0.713550188	0.878	0.624	8.6603E-208	7
Gpm6a.6	0.779138922	0.766	0.38	2.3724E-207	7
Ddah2.3	0.693461649	0.899	0.669	2.7558E-204	7
Ank3.6	0.869129175	0.735	0.395	9.1524E-201	7
Chgb.6	0.923418904	0.602	0.212	1.3126E-200	7
Nrep.6	0.658502157	0.86	0.546	1.3211E-199	7
Rpl8.3	-0.651250183	0.794	0.905	2.8419E-199	7
Dek.7	-1.06743748	0.418	0.715	3.5135E-199	7
Smc2.6	-1.382423837	0.223	0.571	5.6109E-199	7
BC005764.6	0.991871405	0.527	0.158	1.6645E-197	7
Mtss1.6	0.952755876	0.607	0.242	4.3467E-196	7
Elavl4.6	0.862266475	0.69	0.306	5.1617E-195	7
Draxin.4	-1.178829676	0.186	0.583	2.4669E-193	7
Cdk5r1.6	0.8557518	0.675	0.306	4.1347E-190	7
Celf4.7	0.811092723	0.679	0.291	1.8031E-189	7
Nrxn1.6	0.881609873	0.658	0.291	3.5269E-189	7
Clmp.5	0.939095357	0.569	0.216	5.3421E-183	7
2810417H13Rik.7	-1.438768438	0.063	0.417	4.4791E-182	7
Gng3.6	0.857737236	0.665	0.288	8.8534E-182	7
Dcx.6	0.810506625	0.693	0.35	1.8997E-179	7
Apc.6	0.813523174	0.728	0.407	2.65E-175	7
Myt1.6	0.908990721	0.459	0.128	1.8853E-173	7
Nfib.2	0.504663608	0.986	0.929	8.8468E-173	7
Anp32b.5	-1.042470514	0.259	0.617	4.7317E-171	7
Rab3a.7	0.864655345	0.458	0.125	2.5135E-170	7
Bin1.6	0.771804584	0.728	0.405	2.6156E-166	7
Sema6a.7	0.892426977	0.515	0.177	1.5566E-165	7
Celf2.6	0.664732563	0.856	0.62	4.9037E-164	7
H2afv.5	-0.825094928	0.538	0.742	8.9817E-162	7
Elmo1.6	0.880555044	0.401	0.104	2.0137E-154	7
Rplp2.4	-0.654099204	0.673	0.853	4.5044E-152	7
Malat1.5	0.425554063	0.995	0.951	3.8742E-151	7
Rps5.3	-0.516521604	0.925	0.956	9.9628E-151	7
Ranbp1.4	-0.869329987	0.434	0.694	4.1053E-150	7
Sfrp1.4	-0.705756347	0.72	0.819	1.9383E-149	7
Elavl3.6	0.69214587	0.757	0.449	2.915E-147	7
Myt1l.6	0.914528629	0.441	0.138	2.6185E-146	7
A930011O12Rik.6	0.893394468	0.374	0.094	4.0947E-146	7
Mki67.7	-1.398972723	0.122	0.416	1.1852E-145	7

Top2a.7	-1.38612644	0.177	0.447	7.4901E-145	7
Rpl4.2	-0.529761877	0.853	0.912	2.6122E-144	7
Pabpc1.3	-0.575356012	0.783	0.886	2.6404E-143	7
Rps19.4	-0.719084477	0.552	0.769	9.3548E-143	7
Hmgb2.6	-1.175413045	0.113	0.422	7.7403E-142	7
Itsn1.6	0.825454248	0.521	0.213	3.3423E-140	7
Npm1.4	-0.833999158	0.388	0.666	3.163E-139	7
Gria2.6	0.65167041	0.796	0.499	6.8388E-139	7
Srebf1.5	-1.104862529	0.06	0.355	6.3963E-137	7
Prdx1.4	-0.95011962	0.183	0.514	5.5628E-136	7
Mllt11.6	0.777995114	0.505	0.195	1.2287E-135	7
Dut.6	-1.064320128	0.147	0.457	4.544E-135	7
Aplp1.7	0.772777215	0.422	0.13	4.928E-135	7
Rps26.3	-0.605177338	0.736	0.856	3.9924E-134	7
Dbi.2	-1.063532602	0.17	0.486	2.0604E-131	7
Rpl32.2	-0.538683481	0.806	0.896	5.5936E-130	7
Cadm3.6	0.738459473	0.391	0.121	1.5209E-129	7
Cadm1.3	-0.975228094	0.1	0.413	2.4506E-129	7
Chrna3.5	0.804949343	0.362	0.098	1.211E-127	7
2700094K13Rik.6	-0.836746655	0.37	0.633	3.6104E-126	7
Rps20.4	-0.649873445	0.602	0.792	5.0538E-126	7
Sptbn1.6	0.768521214	0.601	0.333	5.0277E-125	7
Pax6.5	0.612595966	0.754	0.494	4.5478E-124	7
Nasp.6	-0.813543457	0.415	0.658	6.5919E-124	7
Anp32e.6	-0.849982599	0.366	0.633	7.2069E-124	7
Gnai2.3	-0.947975355	0.147	0.446	2.7195E-120	7
Kif5c.6	0.68796916	0.661	0.377	7.4666E-120	7
Tnik.5	0.776012604	0.388	0.126	1.021E-119	7
Pcna.7	-1.040484639	0.096	0.379	1.3995E-119	7
Uncx.6	0.723985189	0.583	0.296	1.2487E-117	7
B3galt2.6	0.764728525	0.317	0.08	2.0631E-116	7
Galr1.1	0.74940844	0.219	0.033	1.453E-115	7
Rbfox3.4	0.591245994	0.745	0.474	1.3703E-113	7
Birc5.7	-1.052271569	0.038	0.291	1.7412E-113	7
Tmsb10.3	0.521708311	0.837	0.65	4.3837E-113	7
Tcf4.1	0.406840802	0.977	0.921	1.5636E-112	7
Mcm6.5	-0.96694296	0.041	0.294	1.8014E-112	7
Ttc3.4	0.445230129	0.942	0.82	6.9376E-112	7
Syt11.5	0.601174206	0.741	0.485	2.1627E-111	7
Dner.7	0.743714689	0.474	0.194	2.8502E-111	7
Rps11.2	-0.595126576	0.628	0.789	6.6277E-111	7
Rpl41.4	-0.59464977	0.602	0.794	1.561E-110	7
Rpl22.3	-0.594685631	0.615	0.801	6.5106E-110	7
Tuba1b.6	-0.858191174	0.249	0.499	4.8703E-109	7
Rbp4.5	-0.937623726	0.026	0.258	3.1526E-108	7
Gsg1l.5	-0.878599866	0.032	0.273	4.2776E-107	7
Ube2c.6	-1.319390525	0.054	0.286	1.0097E-106	7
Tpx2.7	-1.122972871	0.054	0.299	2.7732E-106	7

App.4	0.568887732	0.774	0.551	5.846E-106	7
Smc4.6	-0.904121872	0.374	0.581	3.3608E-105	7
Hsd11b2.6	-0.968327893	0.055	0.306	4.8138E-105	7
Cenpf.7	-1.276090723	0.143	0.379	5.7225E-105	7
Igsf21.3	0.71939085	0.289	0.073	2.2828E-104	7
Pdgfa.5	-0.960009341	0.035	0.271	5.6092E-104	7
Btbd17.3	0.749707835	0.388	0.142	1.8265E-103	7
Srrm4.6	0.733361599	0.43	0.176	4.1182E-103	7
Rab6b.6	0.689485692	0.435	0.183	1.3435E-102	7
Ncl.4	-0.477404887	0.817	0.902	1.5746E-102	7
Cdk1.7	-0.996456472	0.035	0.263	7.6206E-102	7
Hmgn5.4	-0.902429503	0.16	0.445	1.7883E-100	7
Rufy3.5	0.637032261	0.609	0.352	1.9048E-100	7
Serbp1.4	-0.472119895	0.787	0.875	2.6518E-100	7
Pde1c.7	0.675580467	0.617	0.368	2.8625E-100	7
Gnb2l1.2	-0.475345397	0.797	0.877	1.8926E-99	7
Pnoc	0.703593979	0.123	0.008	4.2105E-99	7
Fkbp3.3	-0.620902938	0.569	0.759	7.9383E-99	7
Hist3h2a.4	0.747671216	0.352	0.126	2.76718E-98	7
Cdca8.7	-0.961278441	0.049	0.281	7.28069E-98	7
Spc24.6	-0.863236895	0.033	0.259	1.00014E-97	7
Rps3.2	-0.418336018	0.883	0.925	2.69395E-97	7
Cks1b.6	-0.872063648	0.1	0.363	2.73047E-97	7
Rps9.2	-0.423312368	0.891	0.932	3.63475E-97	7
Ptprs.4	0.626428109	0.656	0.402	4.14055E-97	7
Rbfox2.5	0.659534439	0.523	0.264	5.79619E-96	7
Ank2.6	0.696152747	0.482	0.221	3.51157E-95	7
Nfix.3	0.467940345	0.823	0.661	3.08879E-94	7
Cnbp.3	-0.558994559	0.574	0.752	7.57486E-94	7
Rplp0.2	-0.467416418	0.804	0.878	1.45646E-93	7
Prkcb.6	0.628489379	0.494	0.237	2.80286E-91	7
Pbk.6	-0.862272615	0.028	0.237	3.92811E-91	7
Epb4.1l1.1	0.732979806	0.275	0.076	4.52438E-91	7
H2afx.7	-1.00919354	0.116	0.347	1.64094E-90	7
Cenpa.6	-1.114182649	0.054	0.267	5.97066E-90	7
Tubb5.4	0.280475122	0.986	0.909	2.41926E-89	7
Pqlc1.4	-0.825280616	0.075	0.314	7.08579E-89	7
Eef1b2.3	-0.572022333	0.56	0.746	1.19108E-88	7
Plcb1.5	0.758507026	0.344	0.124	1.7823E-88	7
Kif5a.6	0.681996054	0.332	0.111	1.75066E-87	7
Gpr153.3	-0.801188094	0.024	0.223	2.13354E-87	7
Tagln3.6	0.597806512	0.53	0.276	2.49346E-87	7
Cbx5.5	-0.63972542	0.464	0.668	9.17851E-87	7
Hirip3.6	-0.81918697	0.142	0.401	2.27516E-86	7
Serinc1.5	0.547801534	0.643	0.42	1.74041E-84	7
Ccna2.7	-0.812717033	0.033	0.236	4.91741E-84	7
Stmn3.3	0.456964689	0.781	0.609	5.58813E-84	7
Stxbp1.7	0.657488634	0.37	0.145	9.27533E-84	7

Rrm2.7	-0.779403042	0.024	0.218	1.00912E-83	7
Ankrd12.4	0.656051164	0.506	0.256	1.01578E-83	7
Cdca3.7	-0.84736366	0.034	0.236	1.48728E-83	7
Kcnk1.5	0.670277578	0.417	0.187	1.83297E-83	7
Rpl14.3	-0.568398361	0.515	0.711	1.97721E-83	7
Paics.4	-0.75755661	0.177	0.431	3.44233E-83	7
Rnasel.2	0.627151373	0.238	0.059	4.31291E-83	7
Hells.7	-0.82939021	0.029	0.227	9.00988E-83	7
Prdm8.6	0.7033785	0.315	0.111	1.25452E-82	7
Ppp2r2b.2	0.694211521	0.255	0.07	2.13768E-82	7
RP23-45G16.5.7	-0.875375336	0.12	0.343	2.27522E-82	7
Eef1a1.2	-0.434207884	0.818	0.889	2.32283E-82	7
Apbb1.6	0.604158876	0.378	0.153	2.29884E-81	7
Mif.3	-0.73791843	0.13	0.374	3.2684E-81	7
Nsg1.5	0.606620447	0.505	0.27	3.53397E-81	7
Ybx1.3	-0.487089936	0.665	0.795	5.32726E-81	7
Marcksl1.2	0.424593292	0.84	0.712	1.67245E-80	7
Tmpo.6	-0.755022228	0.226	0.47	2.0491E-80	7
Ran.4	-0.728929926	0.181	0.432	2.08304E-80	7
Nusap1.7	-0.92015083	0.041	0.241	2.6843E-80	7
Glce.4	0.665420541	0.378	0.16	1.23877E-79	7
Rps15.2	-0.492308334	0.647	0.799	1.54317E-79	7
Lig1.7	-0.896625673	0.133	0.365	4.24515E-79	7
Abhd16a.5	0.609247333	0.389	0.173	1.06075E-78	7
Gdpd5	0.555593573	0.153	0.023	1.29513E-78	7
Fnbp1l.4	0.570844406	0.642	0.438	3.92304E-78	7
Incenp.7	-0.886266668	0.075	0.291	8.52796E-78	7
Tyms.6	-0.765458358	0.039	0.233	1.06343E-77	7
Cenpe.6	-1.045705258	0.087	0.301	1.19484E-77	7
Gpc2.3	0.643076584	0.328	0.124	1.39027E-77	7
Dctpp1.4	-0.728369406	0.092	0.321	2.4943E-77	7
Sox9.4	-0.777761895	0.06	0.27	4.94777E-77	7
Esco2.6	-0.823918741	0.026	0.208	7.03261E-77	7
Mcm3.4	-0.719907704	0.018	0.192	1.06061E-76	7
Kif11.7	-0.785450177	0.031	0.219	1.09771E-76	7
Nop58.4	-0.647932967	0.373	0.609	1.74718E-76	7
Elavl2.2	0.623903169	0.469	0.254	1.94717E-76	7
Hes6.4	-0.747788472	0.051	0.251	2.30188E-76	7
Klc1.4	0.560924152	0.561	0.345	3.47984E-76	7
Rps21.3	-0.460516406	0.72	0.842	4.17745E-76	7
Gdi1.5	0.549793057	0.431	0.214	5.37565E-76	7
Chd7.4	0.452272732	0.807	0.634	1.67591E-75	7
A330076H08Rik.5	0.646514924	0.358	0.145	2.19333E-75	7
Mab21l1.5	0.645412696	0.425	0.209	2.37671E-75	7
Spc25.7	-0.830368798	0.045	0.239	7.98722E-75	7
Txn1.3	-0.628377197	0.342	0.577	1.46447E-74	7
Dbn1.6	0.641817719	0.328	0.124	2.42198E-74	7
Kif1b.5	0.478531604	0.704	0.506	2.57458E-74	7

Mapk8ip1.5	0.582681492	0.436	0.21	4.64999E-74	7
Nsg2.5	0.502276755	0.672	0.472	5.7254E-74	7
Tbata.5	-0.860329789	0.135	0.356	3.17159E-73	7
Rps3a1.3	-0.537166153	0.497	0.68	1.52168E-72	7
Knstrn.6	-0.738249458	0.024	0.198	1.97558E-72	7
Usp1.7	-0.734242158	0.126	0.355	3.0578E-72	7
Apc2.1	0.547829746	0.17	0.033	4.10144E-72	7
Akap12.3	0.646542389	0.279	0.098	1.63185E-71	7
Dtymk.5	-0.696708128	0.219	0.456	2.86193E-71	7
Prim1.5	-0.71928755	0.068	0.278	2.96001E-71	7
Gng2.4	0.523986458	0.577	0.381	1.01315E-70	7
Prc1.7	-0.929445302	0.073	0.271	1.20198E-70	7
Clspn.6	-0.720143746	0.027	0.202	3.35086E-70	7
Ccnb2.6	-0.790241021	0.027	0.199	6.98032E-70	7
Kif23.7	-0.834914658	0.042	0.227	7.71695E-70	7
Ckap2l.7	-0.795177695	0.043	0.231	1.3435E-69	7
Cdca7.4	-0.682169129	0.032	0.21	2.93907E-69	7
Klf7.5	0.578077022	0.461	0.246	8.02337E-69	7
Chd3.6	0.591821231	0.338	0.134	1.93768E-68	7
Calm2.5	0.322883385	0.947	0.868	2.38544E-68	7
Nucks1.6	-0.567241457	0.506	0.675	2.79039E-68	7
Ppm1h	0.512557444	0.146	0.026	2.93728E-68	7
Hmgn2.4	-0.684184718	0.069	0.27	5.54569E-68	7
Siva1.4	-0.697744377	0.079	0.287	1.05415E-67	7
Rnmt.4	0.549813584	0.53	0.334	1.38236E-67	7
Cenph.7	-0.713721071	0.033	0.206	3.05995E-67	7
Necab3.2	0.541435216	0.186	0.043	3.25247E-67	7
Tacc3.7	-0.710182185	0.041	0.219	1.99295E-66	7
Pak7.4	0.572442589	0.262	0.091	3.49403E-66	7
D4Wsu53e.5	0.493642064	0.647	0.43	3.58119E-66	7
Gas1.3	-0.615520324	0.015	0.168	9.29488E-66	7
Nrcam.5	0.631998027	0.301	0.12	1.77736E-65	7
Ccdc34.6	-0.696185402	0.198	0.431	2.23464E-65	7
Srgap3	0.528415881	0.154	0.03	2.79408E-65	7
Clvs1.5	0.584965153	0.295	0.115	3.33955E-65	7
Mdk.4	-0.766353142	0.18	0.395	3.98079E-65	7
Cbfa2t3.5	-0.682749728	0.204	0.418	6.33097E-65	7
Atoh1.4	-0.696208631	0.021	0.175	7.65762E-65	7
Ptn.6	0.291797073	0.642	0.425	1.23418E-64	7
L1cam.1	0.510780412	0.178	0.043	1.26397E-64	7
Porcn.1	0.567964526	0.232	0.071	1.52003E-64	7
Tead2.4	-0.678511097	0.092	0.292	2.09919E-64	7
Hmmr.6	-0.785792262	0.025	0.188	2.14008E-64	7
Slc17a6.6	0.59937363	0.325	0.134	8.38234E-64	7
Cltb.4	-0.707092078	0.237	0.437	1.82962E-63	7
Pmf1.5	-0.55178699	0.011	0.153	2.10798E-63	7
Ncapg.6	-0.67750723	0.03	0.195	2.16106E-63	7
Tmem2	0.595233788	0.227	0.072	2.7845E-63	7

Lsm4.2	-0.583947328	0.277	0.504	3.80996E-63	7
Gm10075.4	-0.598888132	0.318	0.53	5.86887E-63	7
Gmnn.6	-0.575640645	0.012	0.155	8.66665E-63	7
Gadd45a.1	0.634466832	0.233	0.077	1.01333E-62	7
Uchl1.6	0.538886771	0.525	0.319	1.05397E-62	7
Npdc1.5	0.531223755	0.449	0.246	1.08969E-62	7
Cep170.3	0.566277038	0.486	0.29	1.58975E-62	7
Map6.2	0.538929366	0.223	0.068	2.08499E-62	7
Smco4.4	-0.570580238	0.019	0.172	2.24821E-62	7
Sobp.1	0.601085911	0.282	0.115	2.43939E-62	7
Igsf8.3	0.528196116	0.461	0.253	2.68657E-62	7
Soga3.4	0.477122247	0.621	0.444	3.84984E-62	7
Fam210b.3	-0.694697452	0.12	0.329	4.28465E-62	7
Aurkb.6	-0.577660721	0.012	0.153	9.10981E-62	7
Lap3.5	-0.697220815	0.091	0.291	1.00408E-61	7
Smim18.1	0.5151873	0.167	0.039	1.471E-61	7
Cenpm.6	-0.578290471	0.02	0.173	1.50428E-61	7
Slc25a5.3	-0.589080405	0.313	0.524	6.39253E-61	7
Banf1.4	-0.488640595	0.546	0.705	1.16593E-60	7
Sparcl1.6	-1.022294336	0.052	0.219	1.29348E-60	7
Psat1.4	-0.629081342	0.168	0.383	1.3219E-60	7
Rpa2.4	-0.630999526	0.041	0.212	1.34514E-60	7
Rps14.2	-0.33234827	0.922	0.952	2.83823E-60	7
mt-Nd1.1	-0.343297944	0.868	0.931	3.48451E-60	7
Rrm1.7	-0.643790223	0.083	0.274	4.59971E-60	7
Mns1.6	-0.675179243	0.044	0.216	5.7121E-60	7
B2m.3	-0.75094431	0.069	0.244	6.57534E-60	7
Gnao1.4	0.507339068	0.477	0.272	6.74001E-60	7
Nhp2.2	-0.613444744	0.167	0.386	7.20723E-60	7
Rps25.4	-0.563476996	0.28	0.495	1.1734E-59	7
Plcd1	0.455669423	0.147	0.032	1.5118E-59	7
Hes1.5	-0.778383926	0.018	0.158	1.76778E-59	7
Apoe.7	-1.739151244	0.172	0.255	2.65148E-59	7
Hpca.4	-0.743352337	0.097	0.291	4.10692E-59	7
Dnajc9.6	-0.640168896	0.15	0.365	7.13641E-59	7
Nrn1.4	0.565536624	0.404	0.207	7.9862E-59	7
Lhx1.5	0.460515198	0.679	0.482	8.0469E-59	7
Grin2b.1	0.491912935	0.163	0.038	1.07277E-58	7
Sfrp2.3	-0.553518007	0.016	0.158	1.24332E-58	7
Cdc20.6	-0.811990673	0.044	0.208	3.03078E-58	7
Ppfia2.4	0.581907671	0.267	0.099	5.09531E-58	7
Bhlhe22.1	0.569654873	0.21	0.068	6.20973E-58	7
Rnd3.4	-0.610943252	0.227	0.447	9.04799E-58	7
Zfp36l1.3	-0.59549487	0.019	0.161	1.09114E-57	7
Frmd4a.2	0.516587238	0.486	0.294	1.52131E-57	7
Casc5.7	-0.672911041	0.037	0.197	1.62951E-57	7
Nap1l1.4	-0.564498619	0.328	0.525	5.57531E-57	7
Isoc1.2	-0.57954294	0.035	0.192	2.08398E-56	7

Hey1.5	-0.722646314	0.099	0.286	3.10561E-56	7
Marcks.1	0.277272713	0.961	0.912	3.22335E-56	7
Mis18bp1.7	-0.635944946	0.024	0.171	3.36174E-56	7
Kdm5b.5	0.556954091	0.356	0.171	3.65342E-56	7
Mt1.4	-0.859599471	0.031	0.179	3.66405E-56	7
Dkc1.3	-0.584596485	0.141	0.348	5.53923E-56	7
Hist3h2ba.3	0.559342992	0.291	0.125	5.64175E-56	7
Dync1i2.3	0.435096619	0.705	0.557	1.67435E-55	7
Rtn4.5	0.43833639	0.643	0.467	1.78187E-55	7
Dpysl5.1	0.540661957	0.233	0.084	2.6377E-55	7
Serpini1.1	0.482671936	0.189	0.054	2.83364E-55	7
Fth1.2	-0.487592776	0.564	0.72	3.23244E-55	7
Ckap2.6	-0.603152137	0.026	0.174	3.27748E-55	7
Dclk1.3	-0.633461412	0.216	0.427	3.32004E-55	7
Samd12	0.446259732	0.134	0.026	4.87326E-55	7
Hint1.3	-0.450376763	0.558	0.714	5.29415E-55	7
Smarcc2.2	0.488091792	0.522	0.35	1.24201E-54	7
Hn1.4	0.427794225	0.715	0.527	1.34726E-54	7
S100a16.5	0.56189685	0.242	0.083	1.59433E-54	7
Kmt2e.4	0.417927843	0.712	0.557	1.88486E-54	7
Mcm2.5	-0.623539641	0.05	0.216	1.96494E-54	7
Cacna2d1.3	0.510452142	0.552	0.36	2.41381E-54	7
Rps24.2	-0.386958529	0.722	0.828	4.65492E-54	7
Mcm5.4	-0.587478873	0.031	0.179	6.96556E-54	7
Rad51ap1.7	-0.576156834	0.03	0.177	8.0735E-54	7
Cpe.4	0.450175591	0.629	0.442	1.96926E-53	7
Fbxo5.6	-0.565521852	0.025	0.17	1.98635E-53	7
Rpl22l1.3	-0.603476443	0.164	0.365	2.55285E-53	7
Ccnb1.6	-0.602173639	0.016	0.149	3.31052E-53	7
Afap1.2	0.570381338	0.309	0.139	3.79656E-53	7
Gm17322.5	-0.645227376	0.015	0.14	5.76009E-53	7
Kif15.6	-0.622243523	0.038	0.191	7.41806E-53	7
Jhdm1d.4	0.539333782	0.273	0.113	9.62779E-53	7
Rpl35a.3	-0.48850683	0.394	0.584	1.60519E-52	7
Cklf.4	-0.546923093	0.03	0.175	2.26659E-52	7
Uhrf1.6	-0.540108641	0.019	0.153	2.41484E-52	7
Tk1.6	-0.532289627	0.013	0.14	4.02157E-52	7
Nnat.4	0.490622034	0.807	0.702	4.49147E-52	7
Myod1.5	-0.609892139	0.018	0.151	6.44633E-52	7
Hjurp.5	-0.61604819	0.258	0.447	6.63662E-52	7
H2afy.3	-0.491198082	0.435	0.604	6.8655E-52	7
Hdgf.3	-0.526981789	0.355	0.546	9.41423E-52	7
Lsm6.4	-0.560668742	0.178	0.387	1.37498E-51	7
Acot7.2	-0.581603559	0.051	0.206	1.42308E-51	7
Sgol2.7	-0.636982683	0.026	0.167	1.82688E-51	7
Mapt.6	0.471085726	0.385	0.202	2.64767E-51	7
Aspm.6	-0.638569877	0.02	0.152	3.96233E-51	7
Kif20b.7	-0.657664964	0.047	0.201	3.97244E-51	7

Rpl39.3	-0.455663043	0.508	0.657	4.42185E-51	7
Atp1b3.3	0.491719471	0.505	0.323	4.96925E-51	7
Cenpk.7	-0.5540755	0.02	0.154	5.24964E-51	7
Bok.4	-0.590728411	0.087	0.263	5.62638E-51	7
Nmral1.4	-0.525116253	0.044	0.198	1.23914E-50	7
Hmgn1.3	-0.467823694	0.475	0.633	1.27557E-50	7
Slc3a2.3	-0.539994358	0.141	0.337	8.53539E-50	7
Cd24a.1	0.374420699	0.783	0.622	1.06541E-49	7
Nuf2.7	-0.522786525	0.019	0.15	1.09087E-49	7
Mllt4.1	0.475607739	0.525	0.35	1.25323E-49	7
Arl6ip1.6	-0.442843197	0.517	0.552	1.90166E-49	7
Thra.6	0.441470283	0.394	0.215	2.0635E-49	7
Kidins220.4	0.544407068	0.292	0.127	3.05515E-49	7
Dlgap4.4	0.501383057	0.349	0.174	3.5546E-49	7
Ebf3.1	0.502884826	0.365	0.19	4.05365E-49	7
Pa2g4.4	-0.510943624	0.335	0.537	6.05039E-49	7
D17H6S56E-5.6	-0.491287017	0.014	0.136	7.92999E-49	7
Mmp24.1	0.447151719	0.16	0.044	9.45584E-49	7
Rps2.3	-0.532000069	0.266	0.464	9.92787E-49	7
Boc.4	-0.570262038	0.028	0.166	1.12089E-48	7
Clip3.4	0.475609212	0.468	0.299	1.15633E-48	7
Gria4.5	0.492634145	0.264	0.113	1.36629E-48	7
Atad2.6	-0.584899191	0.054	0.209	1.54242E-48	7
Gsk3b.2	0.414598468	0.635	0.489	1.86614E-48	7
Rps27l.1	-0.518974533	0.305	0.507	2.86587E-48	7
Mcm7.4	-0.527828022	0.199	0.404	3.78857E-48	7
Dusp14	0.474029246	0.147	0.038	4.14411E-48	7
Kif21b	0.472415319	0.157	0.043	6.38779E-48	7
Sfrs18.3	0.338406723	0.877	0.769	8.52882E-48	7
Tipin.5	-0.554934399	0.094	0.267	1.74938E-47	7
Bub1.6	-0.47602415	0.018	0.139	2.3684E-47	7
Actl6b.1	0.432958081	0.163	0.047	2.40922E-47	7
Cdca2.6	-0.506203078	0.016	0.137	3.72485E-47	7
Sowaha.4	-0.678503182	0.037	0.172	3.73969E-47	7
Prmt8.3	-0.603916503	0.109	0.285	3.96853E-47	7
Arhgap11a.7	-0.613302851	0.038	0.18	6.87348E-47	7
Prdx4.4	-0.558949395	0.186	0.38	8.32241E-47	7
Lgals1.3	-0.708605043	0.084	0.24	1.76538E-46	7
2410066E13Rik.2	0.489610602	0.219	0.088	3.05547E-46	7
Nop10.2	-0.499733172	0.292	0.497	3.59464E-46	7
Rps15a.2	-0.412828533	0.539	0.666	4.15112E-46	7
Dusp8.2	0.447385487	0.199	0.069	6.56299E-46	7
Lrig3.3	-0.5292645	0.039	0.18	7.45474E-46	7
Scg5.4	0.469621587	0.493	0.322	8.73416E-46	7
Grina.5	0.507874179	0.246	0.098	9.15733E-46	7
Kif22.7	-0.526640545	0.035	0.173	9.61219E-46	7
Dtl.4	-0.514797985	0.025	0.154	1.36916E-45	7
Adamts1.3	-0.587717144	0.032	0.164	4.09787E-45	7

Mxd3.6	-0.491884627	0.017	0.135	4.21881E-45	7
Sv2a.1	0.444364817	0.186	0.063	4.88857E-45	7
Gli1.4	-0.463167277	0.008	0.115	5.63348E-45	7
Pou3f2.3	-0.554519123	0.116	0.288	6.39224E-45	7
Smpd2.3	-0.534554043	0.038	0.176	9.52917E-45	7
Shmt1.4	-0.411337388	0.007	0.11	1.01974E-44	7
Cenpq.6	-0.505812501	0.035	0.17	1.11316E-44	7
Nav2.2	0.506660195	0.233	0.095	1.38682E-44	7
Sox4.5	0.388708499	0.723	0.571	1.46619E-44	7
Ntrk3.3	-0.529709119	0.033	0.164	1.74298E-44	7
Homer2.1	-0.529110271	0.091	0.259	3.37507E-44	7
Prdx6.1	-0.5435424	0.157	0.339	3.42106E-44	7
Hnrnpu.1	-0.290064435	0.836	0.867	3.9996E-44	7
Stxbp5l.1	0.463235055	0.152	0.044	4.12403E-44	7
Cnih2.1	0.382776059	0.118	0.027	4.29173E-44	7
Erbb4.1	0.507028038	0.144	0.038	4.67241E-44	7
Tubb4b.6	-0.566691483	0.09	0.252	2.19466E-43	7
Serping1.1	0.419961812	0.12	0.026	2.61565E-43	7
Tomm7.1	-0.480036595	0.324	0.511	3.64102E-43	7
Snrpb.3	-0.458224034	0.415	0.586	4.64497E-43	7
Cdkn1b.2	0.386480395	0.602	0.456	5.71031E-43	7
Mex3b	0.507863893	0.243	0.105	9.1746E-43	7
Gpm6b.3	0.36322115	0.632	0.472	1.15826E-42	7
Hist1h2ak.6	-0.595201583	0.028	0.152	1.20844E-42	7
Cenpp.5	-0.405403769	0.013	0.122	1.60678E-42	7
Rps10.2	-0.406539515	0.5	0.647	1.6369E-42	7
Scd2.1	-0.56290769	0.138	0.309	3.37449E-42	7
Snrpf.3	-0.501941968	0.255	0.44	7.65927E-42	7
1500016L03Rik.4	0.395108254	0.535	0.371	1.62207E-41	7
Kif4.6	-0.422284503	0.012	0.115	1.78489E-41	7
Racgap1.7	-0.560353639	0.107	0.267	2.36118E-41	7
Zfp423.1	0.495342596	0.194	0.074	3.38091E-41	7
Rab6a.3	0.406895633	0.476	0.316	5.32553E-41	7
Mad2l1.6	-0.415658597	0.018	0.131	7.11722E-41	7
Zbtb18.4	0.452717051	0.417	0.254	7.69738E-41	7
Rps18.2	-0.479022387	0.263	0.441	1.40484E-40	7
Neurod2.1	0.437965469	0.175	0.059	1.88176E-40	7
Myl12a.2	-0.485827644	0.171	0.341	1.89933E-40	7
6330403K07Rik.4	0.454471318	0.392	0.23	3.04255E-40	7
Melk.6	-0.383668512	0.006	0.101	3.06842E-40	7
Ndc80.6	-0.435892149	0.015	0.122	3.13195E-40	7
Jph4.1	0.346366932	0.17	0.06	3.28216E-40	7
Syt13.3	-0.490066796	0.021	0.135	4.19193E-40	7
Ier5.2	-0.519983633	0.239	0.406	4.31498E-40	7
Smoc1.2	-0.494538315	0.025	0.143	4.44487E-40	7
Lingo1.2	0.48478876	0.214	0.085	4.96273E-40	7
Smpd3.2	0.496051336	0.299	0.15	5.26894E-40	7
2900079G21Rik.1	0.413038501	0.144	0.041	6.02521E-40	7

Klc2	0.4228262	0.123	0.032	8.12527E-40	7
Ect2.6	-0.397831271	0.008	0.104	8.37574E-40	7
Tspyl4.4	0.439660009	0.253	0.116	1.18963E-39	7
Chaf1a.5	-0.504579165	0.045	0.178	1.23313E-39	7
Ppp3ca.3	0.411559219	0.536	0.365	2.0895E-39	7
Cacna1b.1	0.46932429	0.218	0.087	2.78548E-39	7
Cenpw.6	-0.4221476	0.022	0.135	4.22438E-39	7
Tcf19.6	-0.429624027	0.008	0.105	4.37557E-39	7
Cnpy1.2	0.482717605	0.256	0.123	4.57774E-39	7
Rcor2.4	0.463237746	0.38	0.212	4.65644E-39	7
Nefl.1	0.49393134	0.16	0.05	5.38028E-39	7
Fmnl2.1	0.489933342	0.276	0.148	6.57068E-39	7
Lyar.4	-0.54365579	0.13	0.298	9.98497E-39	7
2900011O08Rik.5	0.433325448	0.223	0.094	1.25008E-38	7
Hsp90b1.3	-0.404632798	0.602	0.698	1.30855E-38	7
H2afz.5	-0.458101687	0.087	0.242	1.36351E-38	7
Tmem57.3	0.446017993	0.437	0.279	1.38179E-38	7
Nop56.4	-0.495712856	0.196	0.373	1.70849E-38	7
Hmgb1.4	-0.45254107	0.194	0.371	1.82655E-38	7
Arhgef2.3	0.426388359	0.424	0.271	2.1138E-38	7
Rfc4.6	-0.489344858	0.087	0.237	3.25225E-38	7
Mcm4.4	-0.462990152	0.028	0.146	4.43717E-38	7
Ramp2.3	-0.500611414	0.019	0.127	5.72353E-38	7
Cdh20.2	-0.484983287	0.07	0.213	6.22506E-38	7
Dnph1.4	-0.424828716	0.021	0.131	7.2489E-38	7
Eif5a.1	-0.450507758	0.242	0.415	1.00702E-37	7
Pkm.2	-0.457044564	0.258	0.442	1.33019E-37	7
Fstl1.3	-0.536491764	0.056	0.19	1.39864E-37	7
Clcn4-2.4	0.42279321	0.396	0.239	1.41223E-37	7
Fam213b.3	0.472353033	0.293	0.146	1.77924E-37	7
Ncam1.2	0.402028896	0.521	0.376	1.83947E-37	7
Ntm.4	-0.578638483	0.035	0.154	2.03849E-37	7
Atp6v1e1.4	0.391069892	0.47	0.32	3.87463E-37	7
Gdpd1.4	0.401845977	0.38	0.232	4.3016E-37	7
Sept8.2	-0.470909933	0.026	0.137	5.76702E-37	7
Csrp2.4	-0.457700397	0.042	0.167	6.58179E-37	7
Eef1d.3	-0.474570952	0.19	0.362	6.6682E-37	7
C330027C09Rik.5	-0.45211542	0.027	0.141	6.70771E-37	7
E130309F12Rik	0.354383389	0.116	0.028	7.39817E-37	7
Snhg1.4	-0.470806772	0.204	0.369	9.38183E-37	7
Eno1.2	-0.469869112	0.094	0.24	9.40852E-37	7
Ctsd.7	-0.648059367	0.145	0.177	1.01437E-36	7
Slc7a5.3	-0.462680716	0.018	0.122	1.28854E-36	7
Angptl2.3	-0.438888813	0.019	0.124	1.3223E-36	7
Pea15a.6	0.38320642	0.305	0.165	1.3255E-36	7
Ssrp1.3	-0.425404471	0.418	0.588	2.27694E-36	7
Pgm2l1.1	0.380101602	0.208	0.088	2.31888E-36	7
Cyfip2.1	0.398734771	0.146	0.046	3.69535E-36	7

Bola2.2	-0.465298667	0.239	0.409	3.77757E-36	7
Sgol1.6	-0.445440695	0.026	0.135	6.03061E-36	7
Timp3.1	0.459771667	0.179	0.071	7.87132E-36	7
Ptms.3	0.349921534	0.588	0.464	8.80591E-36	7
Ybx3.3	-0.495147113	0.141	0.301	8.98584E-36	7
Naa50.3	-0.468408281	0.136	0.297	9.29235E-36	7
Sox11.1	0.440915219	0.231	0.105	1.02272E-35	7
Fam111a.6	-0.474804282	0.025	0.134	2.15144E-35	7
Hk2.3	-0.468060586	0.052	0.182	2.31538E-35	7
Bub1b.6	-0.372667668	0.014	0.109	3.47107E-35	7
Hat1.4	-0.433910279	0.042	0.163	3.63535E-35	7
Slc29a1.3	-0.427106046	0.436	0.571	3.87884E-35	7
Mmp14.3	-0.522444019	0.054	0.183	4.03787E-35	7
Darc	0.355152097	0.111	0.029	4.43926E-35	7
Dnmt1.6	-0.512071899	0.13	0.289	4.8332E-35	7
Mad2l2.1	-0.46865321	0.12	0.273	5.21516E-35	7
Cmtm3.3	-0.404051778	0.021	0.124	5.23601E-35	7
Grik2.4	0.438639341	0.216	0.092	5.77701E-35	7
Ncaph.6	-0.383405838	0.023	0.128	5.79872E-35	7
Zfp521.1	0.451740287	0.163	0.058	6.05264E-35	7
Pdrg1.3	0.41797981	0.351	0.208	6.07441E-35	7
Scmh1.1	0.415572661	0.173	0.07	9.12991E-35	7
Rad51.6	-0.409170944	0.022	0.123	1.36785E-34	7
Shfm1.1	-0.36256578	0.555	0.696	1.39854E-34	7
Kif2c.6	-0.39012911	0.016	0.114	1.45041E-34	7
Ndufc2.1	-0.393885067	0.47	0.621	1.56658E-34	7
D430041D05Rik.3	-0.43922499	0.344	0.503	1.66233E-34	7
Dhfr.5	-0.476234415	0.038	0.157	1.67544E-34	7
Os9.3	0.434676757	0.387	0.239	1.77055E-34	7
Gm11223.2	0.574955476	0.327	0.203	1.9846E-34	7
Atp5e.1	-0.364650259	0.53	0.662	2.25267E-34	7
Srrm3.2	0.430778206	0.383	0.228	2.43465E-34	7
Selm.1	-0.524993138	0.057	0.183	3.15238E-34	7
Ldha.3	-0.474221651	0.098	0.242	3.27361E-34	7
Tubb4a.1	0.386535945	0.163	0.059	4.06986E-34	7
Cdc45.5	-0.36902657	0.011	0.101	6.94134E-34	7
Ttyh2	0.402706183	0.117	0.032	7.08699E-34	7
Cenpv.2	-0.453483735	0.177	0.34	7.22475E-34	7
Celf3	0.377524613	0.12	0.034	7.58686E-34	7
Gm1673.3	0.353376723	0.609	0.462	1.23228E-33	7
Pttg1.4	-0.531807747	0.036	0.147	1.48419E-33	7
Srgap2.4	0.364125096	0.243	0.123	1.63411E-33	7
Celsr2.2	0.401035866	0.373	0.227	2.15511E-33	7
Mvd.1	0.418454822	0.152	0.053	2.55916E-33	7
Snrpd3.1	-0.398683959	0.375	0.547	4.30627E-33	7
Mycbp2.2	0.415351282	0.482	0.334	4.41092E-33	7
Baz2b.1	0.421633688	0.503	0.353	4.51486E-33	7
9330159F19Rik.2	0.421687641	0.289	0.159	6.16439E-33	7

Snrpg.1	-0.439635223	0.183	0.347	6.18063E-33	7
Sphkap.1	0.356528427	0.131	0.041	8.85687E-33	7
Srsf7.4	-0.416560315	0.305	0.478	1.03198E-32	7
Chst15.1	0.409484131	0.178	0.07	1.17228E-32	7
Shf	0.351088824	0.13	0.039	1.26384E-32	7
Anln.6	-0.393447608	0.016	0.111	1.46871E-32	7
Adk.1	-0.414701438	0.051	0.174	1.66259E-32	7
Nde1.3	-0.395144067	0.024	0.126	1.67799E-32	7
Nkd1.2	-0.441596435	0.114	0.259	2.07996E-32	7
Ywhag.2	0.391253311	0.361	0.23	2.78176E-32	7
Fam115a.1	0.354581903	0.464	0.341	3.3257E-32	7
Pnmal2.4	0.436494752	0.262	0.137	4.30778E-32	7
Plk4.6	-0.391399756	0.025	0.126	4.9046E-32	7
Dixdc1.3	0.420963864	0.358	0.225	4.94395E-32	7
Trp53inp2	0.406839632	0.139	0.045	5.44979E-32	7
Hnrnpab.2	-0.288654494	0.765	0.819	7.76511E-32	7
Srcin1.1	0.358279269	0.139	0.047	1.05782E-31	7
Reep2.1	0.350244393	0.154	0.06	1.08497E-31	7
Pik3r3.4	0.44870071	0.248	0.126	1.17661E-31	7
Phf20l1.2	0.396885788	0.464	0.33	1.23289E-31	7
Dlgap5.6	-0.414612363	0.021	0.119	1.69771E-31	7
Rbm5.3	0.363341281	0.534	0.39	1.87248E-31	7
Idh2.4	-0.431786206	0.138	0.289	2.56204E-31	7
Ephb1.1	0.380605937	0.117	0.034	3.12376E-31	7
Cog7.3	0.327603409	0.714	0.574	3.19924E-31	7
Mum1l1.2	-0.373071614	0.013	0.101	3.50571E-31	7
Ddx5	0.25352573	0.893	0.809	3.76698E-31	7
Ezr.2	-0.421102251	0.297	0.449	5.0823E-31	7
Atxn10.1	0.306819381	0.472	0.361	5.18001E-31	7
Dbf4.6	-0.419025819	0.042	0.153	5.49703E-31	7
Pfn1.2	-0.395388059	0.368	0.522	5.9378E-31	7
Timeless.4	-0.458165537	0.046	0.162	5.9536E-31	7
Cdt1.5	-0.410600274	0.029	0.132	7.58123E-31	7
Pcsk1n.1	0.356526065	0.184	0.076	7.74118E-31	7
Cdca7l.4	-0.370710333	0.018	0.111	9.02626E-31	7
Diap3.6	-0.379955401	0.02	0.113	9.21229E-31	7
Syp.1	0.351417307	0.16	0.061	1.27312E-30	7
Scrt1.1	0.394437569	0.148	0.051	1.31113E-30	7
Ppp1r14b.1	-0.423715392	0.251	0.408	1.33755E-30	7
St6galnac4	0.346869683	0.103	0.027	1.49784E-30	7
Kifap3.1	0.381210212	0.394	0.262	1.56084E-30	7
2810025M15Rik.2	-0.401423161	0.057	0.175	1.62489E-30	7
Nek7.3	-0.383093566	0.025	0.124	1.69712E-30	7
Nova2.1	0.365187064	0.145	0.051	2.00836E-30	7
Atp6v0b.5	0.374453587	0.391	0.257	2.17431E-30	7
Gm11541.1	-0.450576508	0.033	0.135	2.29669E-30	7
Klhl29.1	0.365162301	0.108	0.031	2.3812E-30	7
Ccnd2.4	-0.406295955	0.559	0.67	2.39242E-30	7

Idh1.3	0.409427388	0.269	0.147	3.12048E-30	7
Pgm2	0.361395089	0.104	0.027	4.52284E-30	7
Hspe1.2	-0.418118689	0.196	0.352	5.91297E-30	7
Snrpa1.2	-0.429164826	0.189	0.341	8.81445E-30	7
Rps7.2	-0.413591908	0.258	0.41	1.19566E-29	7
Ldhb.3	0.391727804	0.403	0.271	1.23074E-29	7
Aurka.7	-0.350603706	0.016	0.105	1.27161E-29	7
Rpl26.2	-0.323325418	0.615	0.708	1.30744E-29	7
Psmc3ip.4	-0.386410336	0.035	0.139	1.50235E-29	7
Brsk2.1	0.438993243	0.183	0.08	1.67593E-29	7
Rpa3.3	-0.432869812	0.114	0.254	2.01802E-29	7
Hsbp1.1	0.263728985	0.688	0.586	3.39091E-29	7
Fam64a.6	-0.379396997	0.028	0.124	3.4224E-29	7
Rbbp7.3	-0.361885962	0.18	0.33	3.61852E-29	7
Tulp4.1	0.412407666	0.325	0.195	4.30851E-29	7
Pbdc1.2	-0.420804821	0.084	0.215	4.34133E-29	7
Atat1.2	0.400559251	0.206	0.095	4.45519E-29	7
Nt5dc2.2	0.39507335	0.356	0.227	7.6972E-29	7
Tpm4.3	-0.427712248	0.179	0.327	7.78211E-29	7
Nudcd2.3	-0.413780816	0.098	0.233	9.49756E-29	7
Bex1.1	-0.427319526	0.099	0.233	1.29968E-28	7
Nicn1.2	0.393396621	0.33	0.198	1.6521E-28	7
Sh3kbp1	0.447640017	0.191	0.089	2.08258E-28	7
Ctnna2.1	0.370142548	0.171	0.079	2.20179E-28	7
Trim59.6	-0.424195813	0.061	0.178	2.33661E-28	7
Clasp2.2	0.387506623	0.274	0.158	2.33963E-28	7
Ing4.2	0.350087501	0.432	0.314	2.47081E-28	7
Lrpap1.3	0.372358644	0.211	0.109	2.6613E-28	7
Gm11266.3	0.42874125	0.254	0.135	2.68327E-28	7
Gar1.2	-0.369494513	0.091	0.217	3.28476E-28	7
Ypel4	0.324086126	0.114	0.034	3.81266E-28	7
Csrnp3.1	0.401575253	0.177	0.075	4.2311E-28	7
Cpne3.1	-0.397414059	0.08	0.206	4.31982E-28	7
Parp6.2	0.390193288	0.226	0.117	6.24808E-28	7
Nup62.2	-0.376070947	0.063	0.179	6.34392E-28	7
Nfasc.1	0.296936607	0.103	0.03	7.59016E-28	7
Rpl23.2	-0.375391961	0.405	0.549	1.06282E-27	7
Sez6l2.1	0.336366419	0.107	0.031	1.07962E-27	7
Hmgn3.2	-0.393455008	0.137	0.28	1.17676E-27	7
Sstr2.4	0.425900957	0.309	0.181	1.30413E-27	7
Ccm2.3	-0.466906482	0.107	0.234	3.25653E-27	7
Mrpl42.2	-0.359805166	0.18	0.329	3.39415E-27	7
Wbp5.2	-0.359875917	0.378	0.529	3.64653E-27	7
Tmeff1.1	0.403992198	0.256	0.14	4.34319E-27	7
Gnaq.2	0.366186422	0.401	0.275	4.60606E-27	7
Irs1.3	-0.380316038	0.032	0.125	4.87235E-27	7
Arpp21.6	0.315819606	0.21	0.105	5.39736E-27	7
Nolc1.3	-0.403973407	0.265	0.42	5.90664E-27	7

Rnaseh2c.2	-0.404167621	0.24	0.392	5.94614E-27	7
Nktr.2	0.346998342	0.493	0.371	6.79307E-27	7
Cdk6.2	-0.405808832	0.121	0.252	7.31527E-27	7
Cct3.1	-0.36151018	0.355	0.506	1.13068E-26	7
Sbk1.3	0.375036951	0.223	0.11	1.47884E-26	7
Eid1.3	0.295993295	0.614	0.494	2.11969E-26	7
Cdh4.1	-0.408668287	0.048	0.153	2.16702E-26	7
Rad21.7	-0.414630929	0.346	0.47	3.03952E-26	7
Sae1.4	-0.380395453	0.151	0.288	4.08549E-26	7
Rltpr	0.316533591	0.108	0.034	4.84298E-26	7
Lzts1	0.325987634	0.111	0.035	5.18974E-26	7
Kcnj3.1	0.335226864	0.15	0.062	5.48083E-26	7
Rsl1d1.1	-0.363681567	0.347	0.501	5.63886E-26	7
Tacc2.3	0.403701909	0.251	0.132	6.84887E-26	7
Gnl3.4	-0.388336418	0.166	0.306	7.28516E-26	7
Cst3.7	-0.369889402	0.514	0.537	7.89353E-26	7
Ccl27a.1	0.335479088	0.113	0.038	9.17091E-26	7
Cbfb.3	-0.375616044	0.078	0.191	9.45942E-26	7
Spop.1	-0.418143096	0.183	0.325	1.2645E-25	7
Supt16.4	-0.398703502	0.36	0.503	1.29205E-25	7
Ephb2	0.39401434	0.17	0.073	1.61158E-25	7
Map1lc3a.1	0.328446822	0.382	0.267	1.61278E-25	7
Pdzrn4.3	-0.438181332	0.048	0.153	1.63742E-25	7
Cntln.4	-0.440917288	0.066	0.18	1.69947E-25	7
Topbp1.4	-0.365106743	0.048	0.152	1.75074E-25	7
Nol7	-0.337529782	0.431	0.58	1.84938E-25	7
Ndufa2.1	-0.339715881	0.486	0.612	1.86639E-25	7
1700001O22Rik.2	-0.35457087	0.026	0.116	1.9684E-25	7
Hivep2.1	0.335030975	0.128	0.049	2.37722E-25	7
Myc.3	-0.375935293	0.021	0.106	2.39908E-25	7
Naa38.2	-0.388002875	0.18	0.321	3.56887E-25	7
Strbp.3	0.34340731	0.455	0.338	3.6256E-25	7
Ubash3b	0.397792605	0.167	0.071	3.63234E-25	7
Ptch2.4	-0.39414427	0.055	0.16	3.8116E-25	7
Eny2.1	-0.394364216	0.248	0.398	3.8565E-25	7
Snrpd2.2	-0.362855088	0.316	0.45	4.29155E-25	7
Rpl18a.3	-0.379951724	0.262	0.411	4.30284E-25	7
Hmgcs1.2	0.419126925	0.245	0.143	5.6249E-25	7
Sema7a.3	-0.40461216	0.056	0.163	5.64091E-25	7
Gramd1a.1	0.364499393	0.272	0.156	5.69136E-25	7
Ubl3.1	0.3550239	0.309	0.201	5.81581E-25	7
Pcdha2.4	0.404294515	0.217	0.11	6.24465E-25	7
Ppic.2	-0.402541895	0.124	0.248	6.25976E-25	7
Islr2.3	-0.426473418	0.072	0.188	6.96675E-25	7
Clic4.4	-0.354189764	0.11	0.236	7.07727E-25	7
Rnaseh2b.3	-0.354450202	0.121	0.245	7.35787E-25	7
Ndufa12.1	-0.369061444	0.29	0.431	7.73945E-25	7
Ndrg2.2	-0.44167598	0.078	0.193	7.81535E-25	7

Vim.5	-0.48465758	0.183	0.311	8.634E-25	7
Pdia6.1	-0.370117233	0.209	0.35	1.00373E-24	7
Cplx2.3	-0.371342033	0.355	0.506	1.01897E-24	7
Alcam.1	-0.38714633	0.042	0.142	1.08949E-24	7
Baz1a.3	-0.420362275	0.063	0.174	1.25529E-24	7
Snrpd1.4	-0.358083142	0.395	0.534	1.26151E-24	7
Srsf3.2	-0.297938895	0.574	0.663	1.4861E-24	7
Gramd1b.3	0.353248255	0.269	0.161	1.70418E-24	7
Fen1.5	-0.363592904	0.047	0.149	1.78712E-24	7
Dpm3.1	-0.362319607	0.154	0.279	1.87474E-24	7
Clybl.2	0.390099236	0.213	0.108	1.97751E-24	7
Zic2.1	0.353372342	0.217	0.113	2.08273E-24	7
Ssbp3.1	0.334204786	0.27	0.159	2.46167E-24	7
Rb1cc1.1	0.378268248	0.338	0.226	2.51385E-24	7
Tshz2.2	-0.42694657	0.186	0.33	2.56497E-24	7
Trp53.2	-0.354689629	0.168	0.308	2.63757E-24	7
Akap9.1	0.327310059	0.595	0.492	2.70104E-24	7
Map1lc3b.4	0.336320436	0.447	0.321	3.02904E-24	7
Gm10260.2	-0.384549971	0.12	0.236	4.01143E-24	7
Mphosph10.2	-0.388983713	0.116	0.24	4.32341E-24	7
Cerk.2	-0.40039435	0.054	0.158	5.33347E-24	7
Nova1	0.350173832	0.16	0.076	5.63224E-24	7
Lpin2.3	-0.389292195	0.081	0.187	7.37149E-24	7
Pik3r2.1	0.351819263	0.165	0.075	7.41138E-24	7
Ube2d1.1	0.336731759	0.325	0.22	7.58955E-24	7
Ctxn1.1	0.354405779	0.262	0.162	7.92928E-24	7
Fam110a.1	0.334416414	0.132	0.061	1.06346E-23	7
Srsf2.2	-0.344823561	0.441	0.575	1.17455E-23	7
Pcbp1.1	-0.333113941	0.483	0.602	1.18144E-23	7
Larp7.2	-0.39094365	0.183	0.322	1.21375E-23	7
Exosc7.2	-0.313244567	0.078	0.187	1.31963E-23	7
Ppp2r2c.2	0.283568017	0.619	0.495	1.59433E-23	7
Cnrip1.3	0.333313214	0.307	0.189	1.80975E-23	7
Thsd7a.2	0.418852299	0.221	0.119	1.91539E-23	7
Gm20033	0.36282321	0.118	0.04	1.92351E-23	7
Ttc28.1	0.405446419	0.218	0.115	2.51927E-23	7
Lmnb1.6	-0.370675865	0.183	0.309	2.59648E-23	7
Rnaseh2a.3	-0.355163428	0.059	0.16	3.03376E-23	7
Dcps	-0.323775576	0.028	0.115	3.09185E-23	7
Nedd4l.1	0.370852025	0.22	0.121	3.38265E-23	7
Efr3b.1	0.355616744	0.121	0.043	4.23226E-23	7
Fkbp2.1	-0.347509583	0.196	0.336	5.11578E-23	7
Naca.2	-0.350902514	0.332	0.475	5.1661E-23	7
Shd.3	0.344692549	0.202	0.102	6.69572E-23	7
C1qbp.2	-0.369591394	0.245	0.388	8.01896E-23	7
Slc22a17.3	0.351450669	0.361	0.242	8.15107E-23	7
Ccp110.2	-0.372151813	0.132	0.257	8.25651E-23	7
Ncor2.3	-0.389713497	0.068	0.176	9.61816E-23	7

Rif1.4	-0.400795248	0.152	0.285	1.08621E-22	7
Rpl7.2	-0.322022931	0.382	0.519	1.14096E-22	7
Sri.1	-0.384786114	0.068	0.173	1.24749E-22	7
Meis1	0.281313278	0.48	0.364	1.34332E-22	7
Mapk8ip2.1	0.3632543	0.233	0.135	1.41546E-22	7
Mex3a.1	0.298141845	0.565	0.455	1.42391E-22	7
Dpysl4.3	-0.371617061	0.356	0.501	1.81917E-22	7
Scrn1.1	0.300211871	0.12	0.044	2.04407E-22	7
Eif3i.1	-0.349482667	0.333	0.472	2.07388E-22	7
Vegfb.1	-0.316704926	0.035	0.124	2.0975E-22	7
Asxl3.1	0.34509435	0.138	0.057	2.39405E-22	7
Fabp7.7	-0.936016991	0.08	0.126	2.4149E-22	7
Ska2.6	-0.354374204	0.104	0.212	2.90515E-22	7
Gm9800.2	-0.33078815	0.406	0.544	3.39249E-22	7
Smim11.1	-0.371056563	0.155	0.285	3.7804E-22	7
Clic1.2	0.37694843	0.259	0.166	3.9421E-22	7
Uqcrq.1	-0.317398359	0.423	0.56	3.99599E-22	7
Skp2.2	-0.329762377	0.037	0.126	4.03566E-22	7
Ap1s2.1	0.379357287	0.247	0.153	4.45E-22	7
Slco5a1	0.399890295	0.131	0.055	4.78813E-22	7
Rrs1.1	-0.36304875	0.084	0.197	5.60744E-22	7
Npepps.1	0.289695888	0.416	0.31	5.92519E-22	7
Alyref.3	-0.364362423	0.095	0.208	6.20108E-22	7
Bdh1.1	0.323744819	0.148	0.065	7.0118E-22	7
Dmxl2.1	0.355997769	0.154	0.068	8.22038E-22	7
March1	0.343800851	0.105	0.038	9.28873E-22	7
Grb2.2	0.320971782	0.345	0.241	9.38067E-22	7
Fdft1	0.342364418	0.167	0.095	1.0455E-21	7
Rusc1.1	0.278930601	0.126	0.05	1.09944E-21	7
Ninj1.1	-0.335996334	0.025	0.103	1.25266E-21	7
RP23-32A8.1.2	0.338912206	0.284	0.179	1.39814E-21	7
Peli2.1	0.343487758	0.259	0.157	1.72806E-21	7
Sod1.2	-0.357529332	0.312	0.452	1.92242E-21	7
Mical1.2	-0.367944314	0.042	0.132	2.24515E-21	7
Mroh2a.4	-0.455974896	0.041	0.119	2.39714E-21	7
Klf9.2	0.336375334	0.361	0.263	3.03272E-21	7
Mybbp1a.1	-0.368198949	0.136	0.258	3.05253E-21	7
Paxbp1.1	0.340081704	0.35	0.251	3.34939E-21	7
Celf5.1	0.351126217	0.128	0.05	3.47199E-21	7
Hnrnpd.4	-0.314180228	0.515	0.618	3.52656E-21	7
RP23-199B2.4.1	0.311793424	0.136	0.056	3.55267E-21	7
Cadps.1	0.369220294	0.15	0.066	3.96575E-21	7
Pak1.1	0.291195356	0.13	0.054	4.16653E-21	7
Rabep1.1	0.33839928	0.335	0.227	5.68682E-21	7
Tnrc6c.1	0.348931359	0.274	0.17	5.72388E-21	7
Snap91.1	0.275759847	0.111	0.044	6.22577E-21	7
Spag9	0.270680453	0.404	0.299	6.23781E-21	7
Pfn2.3	0.273188008	0.332	0.233	6.91307E-21	7

Rps4x.2	-0.37212621	0.177	0.304	8.16166E-21	7
Bicd1	0.370064613	0.245	0.145	9.10069E-21	7
Cask.1	0.369317599	0.191	0.102	9.2868E-21	7
Rps16.1	-0.327193049	0.108	0.217	9.79429E-21	7
Arid3a.1	0.364890312	0.18	0.088	9.89604E-21	7
Ddx39.3	-0.344958709	0.087	0.195	1.01068E-20	7
Wdr47	0.290021003	0.128	0.052	1.08095E-20	7
4930402H24Rik.3	0.378764375	0.2	0.106	1.24747E-20	7
Cd81	-0.332234724	0.349	0.47	1.33325E-20	7
Reln.4	0.38162638	0.285	0.171	1.34858E-20	7
Atl1.1	0.348617949	0.186	0.095	1.62218E-20	7
Mbd3.1	-0.314986062	0.17	0.3	1.6439E-20	7
Prkd3.1	-0.308301818	0.054	0.149	1.82128E-20	7
Tmem107.1	-0.336514121	0.043	0.133	2.10033E-20	7
Trafd1.1	0.32402235	0.205	0.111	2.5714E-20	7
Mapk10.2	0.294531441	0.175	0.092	2.72908E-20	7
Gng12.1	-0.312797716	0.04	0.127	3.14173E-20	7
Vgll4.1	-0.318707993	0.065	0.159	3.21358E-20	7
Dpysl2.1	0.313714626	0.348	0.24	3.27319E-20	7
Snrnp40.2	-0.296084416	0.161	0.279	3.41731E-20	7
Rtn3.1	0.270183019	0.559	0.47	3.43551E-20	7
Atp5b.1	-0.261377679	0.64	0.718	3.62195E-20	7
Rpl34.2	-0.265833676	0.563	0.659	3.70182E-20	7
Rpl18.2	-0.335693781	0.185	0.313	4.45635E-20	7
Hnrnpm.1	-0.266364976	0.645	0.742	5.0834E-20	7
Rfc5.1	-0.290982243	0.032	0.113	5.08455E-20	7
Ift27.1	-0.319277483	0.139	0.25	5.20245E-20	7
Ssr1	-0.302983951	0.181	0.297	5.55646E-20	7
Id2.5	0.331020076	0.58	0.478	5.57992E-20	7
Bccip.1	-0.333419994	0.191	0.319	6.1677E-20	7
Scg3.1	0.299026938	0.545	0.438	6.61718E-20	7
Lmnb2.2	-0.312765219	0.067	0.165	6.6288E-20	7
1810009A15Rik.1	-0.355324238	0.147	0.268	8.39089E-20	7
Eif4e3.2	0.314194334	0.211	0.124	8.61712E-20	7
Cplx1.3	0.319666768	0.229	0.131	9.17323E-20	7
Ctnnb1.3	-0.350743003	0.261	0.396	9.84299E-20	7
Asrgl1.1	-0.356826376	0.05	0.14	1.00697E-19	7
Vrk1.4	-0.335471726	0.06	0.155	1.05036E-19	7
Rfc1.4	-0.401263363	0.213	0.339	1.08777E-19	7
Tub	0.283874022	0.103	0.038	1.15423E-19	7
Hspd1.2	-0.341660113	0.25	0.373	1.16717E-19	7
Usp22.2	0.253196954	0.441	0.354	1.19287E-19	7
Srsf1	-0.308109379	0.198	0.304	1.28889E-19	7
Mthfd1.2	-0.315068463	0.038	0.122	1.37191E-19	7
Park7.1	-0.316482436	0.421	0.555	1.47796E-19	7
Atad5.3	-0.339252352	0.061	0.155	1.72905E-19	7
Mpdz.1	-0.317302358	0.051	0.141	1.81256E-19	7
Naa10.1	-0.341375087	0.142	0.257	2.30757E-19	7

Pkp4.3	-0.336492718	0.029	0.108	2.38207E-19	7
Rpl38.1	-0.314505506	0.263	0.392	2.47331E-19	7
Clk1.2	0.285640605	0.427	0.333	2.82849E-19	7
Ddx21.3	-0.36499877	0.201	0.332	2.87844E-19	7
Cyth2	0.298494513	0.361	0.265	3.11545E-19	7
Etf1	-0.301818508	0.156	0.269	3.38297E-19	7
Zfpm2.1	0.294806604	0.104	0.037	3.57136E-19	7
Gart.2	-0.322845263	0.074	0.173	3.64891E-19	7
Rpl6.1	-0.349459044	0.175	0.3	3.69135E-19	7
Cep110.3	-0.372716337	0.071	0.17	3.75466E-19	7
Sema6c	0.292179195	0.128	0.055	4.00388E-19	7
Kdm1a	0.30669377	0.466	0.356	5.0719E-19	7
Brca2.4	-0.316147193	0.037	0.119	6.12999E-19	7
Hip1r.2	0.317864949	0.226	0.125	6.30534E-19	7
Bzrap1.1	0.298770787	0.128	0.055	6.44267E-19	7
H2afy2.1	0.306539502	0.425	0.33	6.51984E-19	7
Dnmt3a.1	-0.335623492	0.101	0.211	6.67549E-19	7
Myl12b.1	0.258361911	0.45	0.36	7.56894E-19	7
Gm6472.1	-0.26090828	0.088	0.173	8.25541E-19	7
Stx7.1	0.325279001	0.191	0.11	8.34468E-19	7
Synj1.1	0.352141019	0.149	0.073	9.65654E-19	7
G3bp1.2	-0.319495896	0.206	0.337	1.17348E-18	7
Smc6.4	-0.358934205	0.299	0.432	1.29438E-18	7
Rnf165.2	0.325001983	0.271	0.164	1.36705E-18	7
Tgif1.2	-0.292668265	0.028	0.105	1.72224E-18	7
Trp53i11	0.31385763	0.286	0.194	1.7724E-18	7
Pkia.4	0.318685363	0.227	0.126	1.78868E-18	7
Ankra2.2	0.320945837	0.193	0.114	1.81804E-18	7
Vars.1	-0.331171338	0.076	0.173	2.01115E-18	7
Rpl14-ps1.1	-0.321366102	0.173	0.292	2.24685E-18	7
Mat2a	-0.29828994	0.289	0.429	2.28536E-18	7
Ccdc18.3	-0.300576555	0.04	0.121	2.3753E-18	7
Pafah1b1.1	0.277298699	0.541	0.469	2.67809E-18	7
Exosc8.3	-0.344480674	0.109	0.217	2.97824E-18	7
Etfb.1	-0.355468624	0.092	0.191	3.0795E-18	7
Cdkn2c.4	-0.286324655	0.063	0.149	3.08634E-18	7
Prmt2.3	0.341657064	0.175	0.088	3.17144E-18	7
Mak16.1	-0.301968767	0.127	0.233	3.37859E-18	7
Ipo5.3	-0.283003532	0.058	0.144	3.49881E-18	7
Arpc5	0.27398758	0.401	0.315	4.15207E-18	7
Nin.3	-0.312389981	0.061	0.152	4.32931E-18	7
Ccdc41.4	-0.349092381	0.148	0.262	5.26014E-18	7
Wsb1	0.327342784	0.341	0.239	5.535E-18	7
Tspyl1.1	0.305623966	0.227	0.139	5.54114E-18	7
Lsm3.3	-0.316844158	0.238	0.368	6.37272E-18	7
2610001J05Rik.1	-0.254643648	0.101	0.201	6.90236E-18	7
Gabarapl1.2	0.297201237	0.309	0.208	7.38595E-18	7
Rps8.1	-0.317139371	0.285	0.405	7.74527E-18	7

Eif3a.2	-0.284720977	0.594	0.696	8.07521E-18	7
Mrpl33.1	-0.320361401	0.177	0.29	8.69962E-18	7
Ankrd13a	0.306755056	0.136	0.059	9.13458E-18	7
Mpp6.3	-0.268343209	0.077	0.168	9.35461E-18	7
Riok3.1	0.324875638	0.213	0.124	1.08793E-17	7
Acd.1	0.343473829	0.246	0.154	1.23974E-17	7
Mrpl52.2	-0.26409136	0.318	0.441	1.43567E-17	7
Cep120	0.279203567	0.13	0.063	2.15845E-17	7
Prnp.4	0.278885421	0.3	0.199	2.19434E-17	7
Sfxn1	-0.349849439	0.148	0.258	2.19619E-17	7
Dst	0.333625872	0.36	0.259	2.21135E-17	7
Rpl3.1	-0.34492499	0.114	0.219	2.34221E-17	7
Rpl30.1	-0.276299006	0.167	0.276	2.52606E-17	7
Rpl37.2	-0.25563272	0.426	0.509	2.72985E-17	7
Sh3bp5.4	0.318749127	0.184	0.097	2.74593E-17	7
Nfyb.2	0.308651416	0.318	0.228	2.8054E-17	7
Dars.1	-0.2840147	0.074	0.164	3.01607E-17	7
Uqcr10	-0.289000081	0.312	0.443	3.26135E-17	7
Lsm2.3	-0.282512199	0.132	0.238	3.48025E-17	7
Nxt1.1	-0.269278381	0.046	0.127	3.80065E-17	7
Dhx32.2	-0.33339013	0.107	0.207	3.8477E-17	7
Tcp1.1	-0.305072008	0.347	0.472	4.55971E-17	7
Fundc2.1	-0.302665797	0.167	0.278	5.28578E-17	7
Pard6a.1	0.291549139	0.167	0.085	5.31752E-17	7
Laptm4b.1	-0.270125517	0.065	0.147	5.43961E-17	7
Etfa.1	-0.33108714	0.148	0.262	5.82482E-17	7
Sox18.1	-0.348458214	0.063	0.154	5.89259E-17	7
Nol4.2	0.278574304	0.257	0.164	6.72262E-17	7
Nsmce4a.2	-0.259710253	0.131	0.23	7.03964E-17	7
Pdap1	-0.262913613	0.594	0.69	7.61306E-17	7
Sap30.2	-0.253035144	0.062	0.143	8.17542E-17	7
Gm11478.2	-0.32948554	0.12	0.222	8.63619E-17	7
Brd7.3	-0.333572956	0.197	0.316	8.7016E-17	7
Cdk4.1	-0.263297438	0.572	0.647	8.7702E-17	7
Snx5.2	-0.287892654	0.076	0.17	8.82688E-17	7
Dctn2.1	0.269376702	0.407	0.313	9.92888E-17	7
Srm.2	-0.325801518	0.106	0.198	1.13174E-16	7
Tcf3.1	-0.289433733	0.137	0.232	1.21345E-16	7
Enox2.1	0.347427453	0.12	0.051	1.25846E-16	7
Jun.2	-0.337764592	0.58	0.66	1.45826E-16	7
Abracl.1	-0.333470608	0.198	0.31	1.47744E-16	7
Stau2.1	0.333458816	0.227	0.144	1.5847E-16	7
Exosc1	-0.328106636	0.081	0.169	1.63605E-16	7
Fabp5.1	0.257328229	0.488	0.396	1.64685E-16	7
Zfp57.1	0.287202199	0.131	0.062	1.98601E-16	7
Cited2	0.352307651	0.233	0.146	2.06627E-16	7
Gabbr1.3	0.324385153	0.23	0.133	2.17526E-16	7
Cox7b.1	-0.291750986	0.432	0.557	2.27884E-16	7

Pold2.1	-0.283583587	0.042	0.119	2.52093E-16	7
Snrpe.2	-0.265486985	0.468	0.569	2.72912E-16	7
Flot1.2	0.286803219	0.169	0.092	3.06782E-16	7
Arhgef7.2	0.322820847	0.236	0.142	3.38072E-16	7
Ifitm2.2	-0.309708748	0.041	0.119	3.41524E-16	7
Cnih4.1	-0.290404396	0.137	0.242	3.79133E-16	7
Ncaph2.2	-0.300448674	0.085	0.18	4.25433E-16	7
Hcfc1r1.3	0.301286767	0.276	0.186	4.3133E-16	7
Scn8a.1	0.309438124	0.22	0.13	4.57645E-16	7
Apba2	0.252473795	0.498	0.407	4.76192E-16	7
Nme2.1	-0.287051505	0.034	0.106	4.86777E-16	7
Uba52.1	-0.292581768	0.206	0.325	4.87865E-16	7
Alg2.1	0.288220818	0.167	0.094	4.95479E-16	7
Pygo1.3	0.327099865	0.28	0.185	5.61957E-16	7
Hpcal1.4	0.316596724	0.185	0.1	5.67463E-16	7
Kcnq2.1	0.281459368	0.141	0.067	5.80259E-16	7
Stx4a.2	-0.308126513	0.072	0.161	5.80295E-16	7
Sep15.1	-0.259648704	0.405	0.513	6.42269E-16	7
Ezh2.6	-0.285608644	0.543	0.615	6.60425E-16	7
Polr2i.1	-0.296539376	0.223	0.323	6.94498E-16	7
Aig1.1	0.306446099	0.166	0.09	6.98337E-16	7
Neurod6	0.319967234	0.255	0.165	7.15502E-16	7
Itgb1.1	-0.264794406	0.247	0.371	7.99322E-16	7
Atcay.1	0.293777826	0.132	0.06	8.02721E-16	7
2810004N23Rik.2	-0.335825782	0.157	0.27	8.40996E-16	7
Glo1	-0.282901919	0.041	0.113	8.54335E-16	7
Smarcd2.1	0.319617856	0.177	0.091	8.5518E-16	7
Lrrtm2.1	0.32958759	0.113	0.046	8.87089E-16	7
Tspan7.4	-0.351855582	0.048	0.11	8.91977E-16	7
Set.3	-0.294444415	0.276	0.396	8.94668E-16	7
Meis3.1	0.307114222	0.15	0.071	9.76985E-16	7
Add2	0.262361674	0.126	0.062	1.11122E-15	7
Sox5.2	0.323720368	0.193	0.113	1.13836E-15	7
Rwdd3.1	0.324706476	0.144	0.07	1.27832E-15	7
Ptma.2	-0.265790671	0.493	0.588	1.41238E-15	7
Ak2.2	-0.279427288	0.12	0.22	1.64663E-15	7
Gm5620	0.284671127	0.159	0.096	1.78372E-15	7
Ptbp1.1	-0.256501329	0.035	0.106	1.79882E-15	7
Gm17750.2	0.263186913	0.458	0.359	1.99113E-15	7
Zfp608.1	0.283755513	0.258	0.179	2.12841E-15	7
Lman1.1	-0.305917111	0.216	0.333	2.4403E-15	7
St8sia3.1	0.295325372	0.34	0.243	2.44365E-15	7
Mrfap1.1	-0.285041302	0.289	0.412	2.56942E-15	7
Cdk5rap2.3	-0.29003153	0.096	0.19	2.6806E-15	7
Sdc3	0.260905887	0.141	0.072	2.79121E-15	7
Rpl29.1	-0.284466934	0.163	0.265	2.82071E-15	7
Slc7a1	-0.289506113	0.033	0.103	2.90469E-15	7
1110038B12Rik.3	-0.300282902	0.197	0.306	2.96158E-15	7

Mrpl13.2	-0.308888397	0.2	0.316	3.00394E-15	7
BC029214.1	0.295748855	0.167	0.087	3.15736E-15	7
St7	0.288064158	0.155	0.084	3.36555E-15	7
Nrm.3	-0.307219919	0.084	0.176	3.52507E-15	7
Ppil1.1	-0.26395255	0.051	0.129	3.58478E-15	7
Gjc1.2	-0.301524012	0.107	0.203	3.67094E-15	7
Rfc2.3	-0.304979816	0.12	0.218	3.69075E-15	7
Sv2b.1	0.307545756	0.177	0.096	3.93164E-15	7
Egfr.1	0.309741423	0.147	0.078	4.08838E-15	7
Lims1.1	-0.258367354	0.098	0.189	4.156E-15	7
Ppat.2	-0.25822419	0.04	0.113	4.36702E-15	7
Ssr3.1	-0.280307909	0.312	0.433	4.52972E-15	7
Daam1	0.300572807	0.256	0.177	4.5941E-15	7
Pola1.3	-0.268900833	0.038	0.107	4.95766E-15	7
Zfp292.1	0.290971373	0.428	0.334	5.48639E-15	7
Asap1.2	-0.303073685	0.097	0.186	5.48852E-15	7
Klf13.1	-0.297729887	0.144	0.243	5.75323E-15	7
Rnf122.1	0.316562662	0.17	0.096	5.86575E-15	7
Jmjd1c	0.283585186	0.259	0.179	6.39014E-15	7
Nefm.1	0.382600805	0.188	0.11	6.48841E-15	7
Dtx1.1	-0.323234744	0.082	0.172	8.19233E-15	7
Sptan1.2	0.275664477	0.251	0.162	8.35156E-15	7
Sec11c.2	-0.33540178	0.117	0.22	8.43589E-15	7
Onecut2	0.308620175	0.111	0.046	8.7418E-15	7
Mgat5b.1	0.300579499	0.136	0.067	9.25432E-15	7
Imp3.1	-0.275699798	0.154	0.254	9.3303E-15	7
Lsmd1.1	-0.287912128	0.134	0.227	9.59728E-15	7
Sgip1.1	0.255921696	0.124	0.059	9.87723E-15	7
Mycl.1	-0.28874643	0.061	0.143	1.11153E-14	7
Tmem63b.1	0.305806519	0.148	0.075	1.18225E-14	7
Rassf3.1	-0.312563233	0.061	0.144	1.19734E-14	7
Arpp19.2	-0.273664238	0.266	0.386	1.42278E-14	7
Kif3c.1	0.253936251	0.122	0.058	1.43901E-14	7
Mkrn1.3	0.292519393	0.281	0.202	1.46962E-14	7
Ypel5.1	0.271351424	0.18	0.109	1.61476E-14	7
Sf3b5.1	-0.273173191	0.216	0.335	1.6827E-14	7
Epc1	0.291887144	0.302	0.226	2.03044E-14	7
Atp6v0a1.1	0.321547415	0.167	0.09	2.04194E-14	7
Atxn7l2	0.277364666	0.162	0.097	2.16812E-14	7
Brd3.1	-0.286285386	0.431	0.539	2.26511E-14	7
Fam162a.1	-0.295670718	0.196	0.304	2.37295E-14	7
Ncan.3	0.260633835	0.166	0.094	2.4466E-14	7
Scaper.1	0.307979165	0.177	0.105	2.44975E-14	7
Eif4a3.1	-0.257394749	0.254	0.363	2.6485E-14	7
Rbm3	-0.254913633	0.054	0.129	2.68655E-14	7
Rcn1.1	-0.265769432	0.137	0.241	2.69683E-14	7
Atp6v0e2.2	0.277814292	0.338	0.253	2.83798E-14	7
Cog1	0.271058643	0.183	0.109	3.00338E-14	7

1500011B03Rik.2	0.327613295	0.183	0.1	3.16593E-14	7
Med30.2	-0.278984071	0.114	0.21	3.17866E-14	7
Ift74.1	-0.31363747	0.131	0.232	3.27459E-14	7
Mtmr4	0.277468433	0.103	0.051	3.30474E-14	7
Nr3c1.2	-0.291703067	0.052	0.131	4.06389E-14	7
Ppa1.1	-0.26290767	0.137	0.227	4.20587E-14	7
Nudc.3	-0.274942163	0.181	0.288	4.22406E-14	7
Nbea.3	0.293920723	0.271	0.184	4.2643E-14	7
Eif3b.1	-0.271369504	0.132	0.227	4.8119E-14	7
Rftn2.2	-0.31628102	0.055	0.129	4.86493E-14	7
Blcap.1	0.304759036	0.144	0.073	4.9157E-14	7
Nop16.1	-0.281043436	0.092	0.18	5.21384E-14	7
Tpt1.1	-0.274102249	0.166	0.263	5.43239E-14	7
Apex1.2	-0.272523752	0.354	0.458	5.54369E-14	7
Jarid2.1	0.315401597	0.253	0.172	5.55788E-14	7
Wdr89.2	-0.257706576	0.132	0.22	5.61583E-14	7
Taf1d.2	-0.284934883	0.234	0.35	5.6389E-14	7
Timm13	-0.263469833	0.345	0.463	5.68828E-14	7
Hprt.2	-0.253852381	0.105	0.194	5.75103E-14	7
Kcnc1.1	0.316946964	0.161	0.083	5.86197E-14	7
Abtb1.1	0.255901861	0.152	0.079	6.45692E-14	7
Rcc2.2	-0.259807919	0.096	0.185	6.45768E-14	7
Tmx4.2	0.301014774	0.252	0.171	6.53863E-14	7
Gm8292.1	-0.255965934	0.281	0.375	7.21135E-14	7
Nipbl	0.250326614	0.561	0.486	7.30183E-14	7
H1fx.5	-0.312056892	0.16	0.251	7.33102E-14	7
1810037I17Rik.1	-0.266168351	0.114	0.203	7.42431E-14	7
Gcc2	0.272246155	0.24	0.165	7.78199E-14	7
Tmem178.1	0.311050609	0.266	0.18	7.95871E-14	7
Pole3.2	-0.251606156	0.156	0.257	8.28513E-14	7
Pard6g	0.307532902	0.169	0.099	8.81253E-14	7
Tram1	-0.251469103	0.057	0.134	8.83288E-14	7
Pold3.2	-0.263795866	0.073	0.154	9.18259E-14	7
Map7d1.1	0.277937032	0.312	0.236	9.55861E-14	7
Srsf10.1	-0.263496719	0.229	0.338	1.00112E-13	7
Aes	0.271706166	0.291	0.205	1.00587E-13	7
Nsmce1.2	-0.270939388	0.143	0.235	1.01642E-13	7
Slc37a3	0.25001772	0.121	0.057	1.01765E-13	7
Ppp2r3a.1	0.267839207	0.158	0.088	1.03181E-13	7
Mycn.2	-0.315272122	0.267	0.371	1.032E-13	7
Evl.1	0.293377241	0.257	0.171	1.12563E-13	7
Sdha	-0.265623916	0.15	0.248	1.28002E-13	7
Emg1.1	-0.271682093	0.216	0.322	1.33814E-13	7
Dirc2	0.269598396	0.153	0.09	1.39644E-13	7
Cd164.1	-0.258531659	0.05	0.125	1.4021E-13	7
Pds5b	-0.252306859	0.179	0.279	1.48929E-13	7
Rpa1.4	-0.271904165	0.043	0.114	1.61607E-13	7
Commd1.2	-0.268567183	0.204	0.305	1.79746E-13	7

Rrp15.2	-0.259090093	0.091	0.18	2.2601E-13	7
Actl6a.1	-0.279710803	0.136	0.234	2.46802E-13	7
Smc1a.2	-0.26045645	0.538	0.637	2.84744E-13	7
Cnksr2.1	0.324127919	0.173	0.102	3.21714E-13	7
Wdr13.2	0.257772503	0.167	0.1	3.24061E-13	7
Rab3c.1	0.289065711	0.228	0.141	3.30365E-13	7
Glrx5	-0.260306589	0.266	0.368	3.3818E-13	7
Peli1	0.275045034	0.272	0.196	3.62633E-13	7
Plp1.7	-0.581341899	0.114	0.114	3.71971E-13	7
Golm1	0.322934045	0.26	0.182	3.76322E-13	7
Prmt5.1	-0.273069033	0.161	0.263	4.3879E-13	7
Sqle.1	0.291283801	0.178	0.116	4.45035E-13	7
Rbm8a.1	-0.259010533	0.374	0.472	4.55076E-13	7
Brix1.1	-0.252400017	0.122	0.212	4.6209E-13	7
Jam3.1	-0.259417368	0.063	0.14	5.32496E-13	7
Tsc22d4.1	-0.297959263	0.095	0.171	5.37644E-13	7
Prkx.1	0.297564036	0.181	0.107	6.0312E-13	7
Armcx1.1	0.321190657	0.144	0.078	6.0536E-13	7
Zfp157	0.287835138	0.162	0.098	6.69383E-13	7
4933427D14Rik.1	0.270867008	0.132	0.073	6.81004E-13	7
Ftsj3.1	-0.293110449	0.124	0.212	7.48861E-13	7
Srsf9.1	-0.279042576	0.147	0.243	7.69675E-13	7
Trp53bp1	0.266551468	0.196	0.124	7.94346E-13	7
Vezt.1	0.299263775	0.157	0.087	8.12718E-13	7
Mrpl18.3	-0.263179608	0.247	0.362	8.35484E-13	7
Tmem176b.1	-0.347980694	0.05	0.113	8.39438E-13	7
Gsto1.2	-0.290828474	0.058	0.134	8.54991E-13	7
Epb4.1.1	0.285116562	0.301	0.223	9.26396E-13	7
Rai1	-0.28064525	0.082	0.165	9.73467E-13	7
Trim28.1	-0.280706372	0.257	0.369	1.00501E-12	7
1500011K16Rik.1	-0.313949397	0.075	0.155	1.04207E-12	7
Gins2.3	-0.274505197	0.108	0.191	1.05198E-12	7
Rnf168.2	-0.275008938	0.122	0.214	1.07607E-12	7
Pafah1b3.1	0.255837863	0.25	0.185	1.07965E-12	7
Eif1ax	-0.250400749	0.298	0.404	1.3342E-12	7
Psmg4.1	-0.259563813	0.12	0.214	1.52859E-12	7
Zfp462.1	-0.28598289	0.08	0.162	1.74843E-12	7
Egr1.5	-0.334984319	0.276	0.393	1.91549E-12	7
Mrps28.1	-0.259623612	0.091	0.175	2.22585E-12	7
Slc1a2.4	-0.283216761	0.211	0.3	2.37134E-12	7
Serf1.1	0.276678829	0.361	0.274	2.39776E-12	7
Gdap1l1.1	0.253472611	0.145	0.078	2.50811E-12	7
Glul.1	-0.25703338	0.139	0.206	2.5728E-12	7
Mrps14.1	-0.25969878	0.147	0.243	2.65781E-12	7
1700025G04Rik.3	0.283284325	0.331	0.257	2.72557E-12	7
Leprotl1	0.273038988	0.236	0.175	2.85948E-12	7
Aff4	0.270320212	0.287	0.211	3.00386E-12	7
Bub3.5	-0.288427535	0.233	0.328	3.4747E-12	7

Lmo4.3	-0.282497137	0.251	0.342	4.036E-12	7
Pbx1	0.255641789	0.255	0.181	4.25364E-12	7
Dnajc2.1	-0.270595662	0.239	0.335	5.03136E-12	7
Reep1.1	0.260255953	0.135	0.077	5.42796E-12	7
Gab1.1	0.274046699	0.127	0.067	5.69783E-12	7
Uchl5.1	-0.271921352	0.113	0.201	6.04466E-12	7
Ntrk2.1	0.305992144	0.146	0.078	6.08615E-12	7
Celsr3	0.254411837	0.183	0.118	6.21328E-12	7
Rfc3.3	-0.25203705	0.12	0.211	7.76211E-12	7
Banp.1	0.28636196	0.205	0.131	8.73889E-12	7
Ctsf.3	0.270906936	0.163	0.093	9.0954E-12	7
Hspa5.1	-0.280883266	0.467	0.556	9.15454E-12	7
Tln1.1	0.273913439	0.214	0.144	9.74056E-12	7
Dpy30.2	-0.255980669	0.21	0.305	1.20176E-11	7
Rad50.2	-0.260258871	0.088	0.171	1.32719E-11	7
Mtch2.1	0.251252896	0.36	0.289	1.66722E-11	7
Aldoa.4	0.253645397	0.346	0.25	1.70892E-11	7
Fam168a.1	0.263783009	0.36	0.281	1.80822E-11	7
Ppp1r1a.2	0.270250078	0.169	0.105	1.86619E-11	7
Polr1c.1	-0.261213653	0.14	0.224	1.92671E-11	7
Msl3	-0.277043561	0.066	0.14	1.98069E-11	7
Wasf2.1	-0.271414855	0.05	0.118	2.38364E-11	7
Ola1	-0.250415264	0.146	0.234	2.38747E-11	7
Chchd1.1	-0.252751601	0.299	0.408	2.44704E-11	7
Map3k12.1	0.259527659	0.157	0.093	3.16798E-11	7
Rps16-ps2.1	-0.255107066	0.072	0.145	3.36547E-11	7
Pabpn1.1	0.25666668	0.259	0.188	3.44717E-11	7
Fam57b.2	0.269966421	0.258	0.179	3.55793E-11	7
Nhsl1	0.260967303	0.106	0.052	3.74611E-11	7
Ctps.2	-0.265743983	0.068	0.143	4.37741E-11	7
Hook3	0.274850185	0.375	0.293	4.76449E-11	7
Ubr7.2	-0.269427108	0.064	0.138	4.85829E-11	7
Map9.1	0.278774285	0.214	0.142	4.99278E-11	7
Fbxo11.1	0.260454894	0.228	0.149	5.02073E-11	7
Zc2hc1a.1	0.278366168	0.157	0.089	5.06906E-11	7
2700029M09Rik.2	-0.265962014	0.198	0.296	5.77242E-11	7
Rpl36al.1	-0.25460335	0.209	0.305	5.92821E-11	7
Clcn2	0.264830119	0.116	0.057	6.15572E-11	7
Myo1b	0.256617613	0.136	0.071	6.402E-11	7
Lima1.2	-0.254979787	0.056	0.127	7.0874E-11	7
Macf1.1	0.258488389	0.269	0.205	7.14441E-11	7
Rps28.2	-0.276671644	0.121	0.209	7.28728E-11	7
Rpl10a	-0.25263662	0.05	0.116	7.4268E-11	7
Slc1a3.6	-0.326023324	0.12	0.155	7.51108E-11	7
Ly6e.1	0.26795834	0.348	0.276	8.56327E-11	7
Rexo2	-0.276772724	0.103	0.186	8.78328E-11	7
Jakmip2.1	0.274518421	0.225	0.151	8.81203E-11	7
Efhd2.3	-0.281335221	0.07	0.134	9.21737E-11	7

Gpatch8.1	0.266218323	0.348	0.275	9.44356E-11	7
Snapc3	0.259104701	0.179	0.12	9.60711E-11	7
Plk3.1	0.289694939	0.13	0.068	1.04844E-10	7
Arl8a.1	0.257282091	0.157	0.091	1.06015E-10	7
Polb.1	0.253828926	0.226	0.161	1.10848E-10	7
Oraov1.2	-0.260874541	0.056	0.126	1.53456E-10	7
Pick1.1	0.256364594	0.19	0.122	2.39962E-10	7
Mgea5	0.266723115	0.302	0.225	2.42913E-10	7
Kars	-0.252349257	0.12	0.201	2.53541E-10	7
Fnbp1.1	0.252096519	0.128	0.068	2.90979E-10	7
Gm26735.3	0.268829614	0.251	0.175	3.5179E-10	7
Mfap4.3	-0.303887121	0.087	0.167	4.0702E-10	7
Tom1l1.1	-0.253019945	0.044	0.108	4.33938E-10	7
Ccng2.4	0.255326161	0.286	0.206	6.0403E-10	7
Rsbn1	0.255983362	0.24	0.177	6.65867E-10	7
Arhgap21.1	0.25894307	0.219	0.159	7.62015E-10	7
Hid1.1	0.266957433	0.109	0.051	7.68194E-10	7
Setbp1	0.28245601	0.228	0.154	1.0012E-09	7
Jak1	0.278791488	0.182	0.123	1.00498E-09	7
Vopp1.1	0.259653418	0.175	0.114	1.02373E-09	7
Fzd2.1	-0.251621935	0.096	0.166	1.32658E-09	7
Fam107b.1	0.264483462	0.157	0.097	1.34482E-09	7
Zbtb38	0.273050944	0.105	0.055	1.59838E-09	7
Psmg2	-0.250665773	0.084	0.157	4.1438E-09	7
Nr2f2	0.252994645	0.134	0.074	4.50437E-09	7
C1ql1.4	-0.259242613	0.198	0.285	4.92424E-09	7
Mapre2.1	0.250985643	0.227	0.162	5.18257E-09	7
Maml3	0.252554109	0.161	0.095	5.63997E-09	7
Mxd4.4	0.254805377	0.276	0.191	9.21948E-09	7
Rangap1.7	-0.262289585	0.176	0.262	1.26803E-08	7
Akap8l.3	0.25187997	0.223	0.153	1.28831E-08	7
Ifrd1.1	-0.260094603	0.111	0.182	1.39203E-08	7
Myo9a.1	0.265307317	0.218	0.153	1.64033E-08	7
2410089E03Rik	0.260839222	0.177	0.126	1.97088E-08	7
Dpf2	0.263331691	0.163	0.107	2.48361E-08	7
Lmcd1.1	0.258200107	0.104	0.059	4.08975E-08	7
Zfp711	0.265677196	0.138	0.085	8.9771E-08	7
Baz2a	0.262742615	0.16	0.106	3.08994E-07	7
Ckap5.6	-0.255217154	0.196	0.271	1.6605E-06	7
Serpinh1.3	-0.260623995	0.064	0.119	2.28173E-05	7
Fosb.3	-0.256084905	0.101	0.16	0.000388388	7
Cenpa.7	1.138911783	0.784	0.23	1.2317E-197	8
Cenpe.7	1.010472014	0.724	0.268	5.0684E-132	8
Ccnb2.7	1.033226316	0.579	0.171	1.8441E-123	8
Cdc20.7	0.887002429	0.582	0.18	4.3008E-111	8
Hsp90b1.4	0.725220431	0.894	0.683	1.1858E-110	8
Tpx2.8	0.820632065	0.694	0.265	3.9734E-109	8
Cenpf.8	0.616513259	0.765	0.345	3.7232E-106	8

Mki67.8	0.579551079	0.764	0.38	1.64449E-85	8
Hmgb2.7	0.67800302	0.755	0.385	1.5293E-82	8
Knstrn.7	0.780741241	0.491	0.173	3.47482E-72	8
H2afv.6	0.483411992	0.931	0.719	9.78892E-69	8
Nucks1.7	0.545238056	0.89	0.653	3.49457E-67	8
RP23-45G16.5.8	0.64180425	0.652	0.313	3.22203E-65	8
2700094K13Rik.7	0.513727688	0.874	0.603	2.47785E-61	8
Cdca8.8	0.531866626	0.55	0.253	2.45428E-53	8
Cdca3.8	0.586154778	0.491	0.21	4.96174E-50	8
Dynll1.1	0.517309012	0.814	0.585	5.17622E-50	8
Pttg1.5	0.724235944	0.371	0.13	1.30356E-49	8
Anp32e.7	0.449542822	0.856	0.603	1.24232E-47	8
Tubb5.5	-0.43174542	0.875	0.916	4.11224E-46	8
Rad21.8	0.527130145	0.717	0.451	4.79114E-46	8
Hdgf.4	0.504048182	0.774	0.522	6.05982E-44	8
Fstl1.4	0.614779471	0.416	0.17	3.42834E-43	8
Neurod1.8	-0.875182059	0.488	0.534	4.09063E-42	8
Hnrnpa2b1.1	0.298935919	0.985	0.931	1.14178E-41	8
Gm11266.4	0.634065324	0.346	0.135	1.97363E-36	8
Cep89.1	0.556435027	0.254	0.079	8.56412E-36	8
Sowaha.5	0.719031588	0.352	0.155	3.89365E-35	8
Malat1.6	-0.463054353	0.949	0.955	4.92788E-35	8
Gria2.7	-0.728197601	0.346	0.527	1.43195E-34	8
CRE_RECOMBINASE.6	0.524031202	0.884	0.763	1.7665E-34	8
Birc5.8	0.37323737	0.499	0.264	3.1533E-34	8
Hmgn5.5	0.498362792	0.658	0.414	1.18428E-33	8
Ckb.7	-0.507546056	0.76	0.81	1.90858E-33	8
C330027C09Rik.6	0.570724496	0.32	0.125	3.59364E-33	8
Banf1.5	0.294340404	0.894	0.685	3.78496E-33	8
Miat.6	-0.756645284	0.34	0.522	6.74855E-32	8
Nrep.7	-0.518007241	0.522	0.571	3.71997E-31	8
Celf4.8	-0.834146179	0.195	0.325	1.09848E-29	8
Gm10075.5	0.400293817	0.733	0.506	2.76473E-29	8
Apoe.8	-1.581733076	0.235	0.25	1.91254E-28	8
Ccdc34.7	0.406581243	0.641	0.405	7.01829E-28	8
Racgap1.8	0.468373353	0.466	0.247	1.17941E-27	8
Lmnb1.7	0.396556641	0.516	0.291	1.7802E-27	8
Kif23.8	0.428262508	0.412	0.206	3.27628E-27	8
Rtn1.8	-0.413940869	0.691	0.69	1.10637E-26	8
Tacc3.8	0.433352887	0.4	0.198	4.9912E-26	8
Sept3.8	-0.630719618	0.287	0.396	1.47894E-25	8
Dlgap5.7	0.464661453	0.267	0.105	3.15054E-25	8
Basp1.7	-0.454567728	0.632	0.707	3.2359E-25	8
Hmgn2.5	0.46178326	0.453	0.247	1.02715E-24	8
Top1	0.371780002	0.817	0.656	1.04093E-23	8
Hes6.5	0.414778945	0.428	0.228	1.85925E-23	8
Ckap2l.8	0.262273629	0.38	0.211	4.90659E-23	8
Arhgap11a.8	0.41384311	0.339	0.163	4.37555E-22	8

Tubb4b.7	0.415019834	0.427	0.232	1.1657E-21	8
Gpm6a.7	-0.566701412	0.333	0.412	1.66798E-21	8
Hist1h2bc	0.450722394	0.135	0.034	1.69315E-21	8
Tubb2a.8	-0.637557043	0.155	0.248	1.96207E-21	8
Cdc25c.1	0.424619949	0.194	0.066	2.56795E-21	8
Cks1b.7	0.34725823	0.543	0.336	3.1263E-21	8
Stmn2.7	-0.591133137	0.491	0.555	3.82336E-21	8
Cep110.4	0.470103277	0.326	0.156	7.32533E-21	8
Bin1.7	-0.541355827	0.309	0.434	7.48735E-21	8
Vim.6	0.410116042	0.496	0.294	7.92873E-21	8
Cdkn3.1	0.357934604	0.138	0.038	1.33187E-20	8
Hmmr.7	0.29214172	0.327	0.17	1.62414E-20	8
Fabp7.8	-0.758977643	0.147	0.122	2.2527E-20	8
Zic1.6	-0.290969575	0.801	0.783	3.37519E-20	8
App.5	-0.409598238	0.506	0.57	3.6566E-20	8
Syt13.4	0.461091791	0.273	0.121	1.25041E-19	8
Shfm1.2	0.315307242	0.827	0.68	2.58895E-19	8
Rangap1.8	0.323285793	0.431	0.249	5.3516E-19	8
Gap43.8	-0.463236583	0.557	0.601	8.89412E-19	8
Pcna.8	-0.470259341	0.293	0.36	9.54579E-19	8
Hmgn1.4	0.292176961	0.792	0.614	1.37162E-18	8
Lgals1.4	0.447497448	0.4	0.221	1.38574E-18	8
Rbfox3.5	-0.508799169	0.389	0.498	3.52984E-18	8
Ezr.3	0.354776172	0.623	0.43	5.48157E-18	8
Tubb3.7	-0.487891978	0.437	0.475	5.87592E-18	8
Ctsb.5	-0.555875187	0.24	0.263	1.22248E-17	8
Hnrnpm.2	0.273780517	0.87	0.729	1.84683E-17	8
Hpca.5	0.422321157	0.45	0.27	3.75734E-17	8
Cntn2.8	-0.702798563	0.173	0.267	5.54544E-17	8
Aspm.7	0.359089865	0.282	0.137	7.65595E-17	8
Ckap5.7	0.412971712	0.435	0.258	8.37309E-17	8
Lig1.8	-0.514618433	0.271	0.351	1.59126E-16	8
Rps27l.2	0.285736631	0.673	0.485	1.92396E-16	8
Celf2.7	-0.367512642	0.6	0.639	3.03436E-16	8
Tubb2b.7	-0.384330989	0.424	0.466	4.11584E-16	8
Igfbpl1.6	-0.418541865	0.575	0.65	5.37395E-16	8
Cacna2d1.4	-0.473576592	0.289	0.378	7.09215E-16	8
Ctnnb1.4	0.336082327	0.563	0.379	1.02232E-15	8
Smim11.2	0.410092938	0.434	0.27	1.41352E-15	8
Bub1b.7	0.348885871	0.224	0.097	1.83072E-15	8
Hmgb3.2	0.33958266	0.475	0.299	2.10864E-15	8
Mad2l1.7	0.369846967	0.254	0.118	2.32692E-15	8
Mns1.7	0.355270042	0.353	0.197	2.72666E-15	8
Pde1c.8	-0.468729246	0.323	0.389	2.85464E-15	8
Vbp1.1	0.350164071	0.49	0.312	3.70582E-15	8
Ptprs.5	-0.499344234	0.324	0.425	4.00525E-15	8
Ank3.7	-0.379885748	0.383	0.421	4.1529E-15	8
Cbx1	0.276568571	0.82	0.68	4.72373E-15	8

Gas6.1	0.430843032	0.191	0.076	6.01847E-15	8
Stmn4.8	-0.516404153	0.283	0.343	9.39023E-15	8
Paip2	0.302655644	0.713	0.542	9.57717E-15	8
Gng3.7	-0.439395242	0.271	0.318	1.09786E-14	8
Dpysl3.8	-0.489383439	0.17	0.221	2.50258E-14	8
Xist.2	-0.569919926	0.214	0.302	2.71049E-14	8
Ppp1r14c.7	-0.54652425	0.174	0.265	3.63953E-14	8
Pdlim3.1	0.344203959	0.155	0.057	7.52674E-14	8
Efhd2.4	0.354215768	0.255	0.125	1.20738E-13	8
Ran.5	0.273610647	0.582	0.407	1.58232E-13	8
Nfib.3	-0.274401056	0.937	0.933	2.02734E-13	8
Gnai2.4	0.334279771	0.588	0.418	2.26415E-13	8
Nde1.4	0.385076537	0.236	0.114	2.71232E-13	8
Tra2b	0.340489212	0.551	0.399	3.36859E-13	8
Ctsd.8	-0.595748947	0.182	0.175	4.84257E-13	8
Tmpo.7	0.282141848	0.617	0.445	6.07223E-13	8
Pdia6.2	0.256422318	0.497	0.334	6.86156E-13	8
Otx2	0.359724307	0.362	0.211	8.77704E-13	8
Barhl1.6	-0.266143278	0.519	0.521	9.59233E-13	8
Sh3gl2.2	-0.511399849	0.07	0.175	1.85893E-12	8
Cwc15	0.271112634	0.56	0.391	2.37004E-12	8
Thra.7	-0.374169923	0.199	0.23	2.45846E-12	8
H2afz.6	0.315200222	0.371	0.225	2.55326E-12	8
BC005764.7	-0.51582567	0.107	0.188	2.55887E-12	8
Nrxn1.7	-0.483716048	0.26	0.32	2.76828E-12	8
Ppp1r14b.2	0.301849861	0.559	0.39	3.02307E-12	8
Map2.7	-0.36739582	0.443	0.493	3.58489E-12	8
Kif1b.6	-0.382063116	0.431	0.524	4.31317E-12	8
Lsm4.3	0.262565483	0.642	0.481	4.83847E-12	8
Hmgn3.3	0.326944387	0.416	0.264	5.8453E-12	8
Fam64a.7	0.326332059	0.232	0.113	8.67135E-12	8
Mapt.7	-0.530595026	0.145	0.219	1.03112E-11	8
Mtss1.7	-0.380976975	0.236	0.27	1.59082E-11	8
Sep15.2	0.275047143	0.658	0.499	2.17044E-11	8
Pcf11.1	0.353100624	0.309	0.173	2.30245E-11	8
Aplp1.8	-0.484859982	0.076	0.154	3.86119E-11	8
Ppp2r5c.1	0.330699513	0.353	0.214	6.97786E-11	8
Mdh1	0.270411918	0.607	0.446	7.58563E-11	8
Myt1.7	-0.451585491	0.098	0.154	9.79471E-11	8
Atp6v1e1.5	-0.395718602	0.255	0.334	1.03508E-10	8
Cct5.1	0.26627454	0.629	0.47	1.73214E-10	8
Cenpw.7	0.283772897	0.238	0.123	1.74265E-10	8
Psmc1	0.292315475	0.563	0.413	2.13378E-10	8
Srebf1.6	-0.306658887	0.318	0.334	2.23164E-10	8
Brd7.4	0.251440302	0.452	0.302	2.61112E-10	8
Sec11c.3	0.344201063	0.345	0.207	3.1883E-10	8
Chgb.7	-0.510555218	0.176	0.243	4.01036E-10	8
1700123O20Rik	0.262557773	0.251	0.135	4.06183E-10	8

Zeb1.2	-0.306167777	0.35	0.388	4.38169E-10	8
Nudcd2.4	0.338250318	0.356	0.218	6.02828E-10	8
Gadd45g	0.3540743	0.17	0.076	6.05609E-10	8
Clspn.7	-0.452732082	0.117	0.192	6.38439E-10	8
Pqlc1.5	0.290277166	0.44	0.291	6.40641E-10	8
Cks2.1	0.274417608	0.166	0.071	8.24233E-10	8
Lyar.5	0.301820748	0.427	0.28	8.4955E-10	8
Trpc4ap.8	-0.33405436	0.179	0.198	8.82381E-10	8
Mphosph10.3	0.335381603	0.364	0.226	1.15904E-09	8
Tax1bp1	0.260882593	0.714	0.572	2.14084E-09	8
Hmgb1.5	0.26555683	0.503	0.352	3.02258E-09	8
Kif20a.2	0.251864567	0.177	0.083	3.16805E-09	8
Smarcc2.3	-0.408002561	0.264	0.367	3.58525E-09	8
Ank2.7	-0.503517114	0.151	0.243	3.71142E-09	8
Cd81.1	-0.333218498	0.397	0.464	4.93202E-09	8
Kif5c.7	-0.353874716	0.35	0.4	5.73913E-09	8
Ralgps2.1	0.302985705	0.377	0.24	6.34983E-09	8
Ckap2.7	0.318561043	0.276	0.158	7.91406E-09	8
Sept4.6	-0.407032145	0.18	0.228	1.09589E-08	8
Spop.2	0.251225747	0.45	0.309	1.19838E-08	8
Cenpv.3	0.281596759	0.465	0.323	1.31844E-08	8
Kif15.7	0.289667464	0.296	0.175	1.32723E-08	8
Prkcb.7	-0.380956176	0.201	0.258	1.45244E-08	8
Taf7	0.337583202	0.239	0.129	1.54273E-08	8
Slc35b1	0.299624912	0.428	0.288	1.92053E-08	8
Ankrd12.5	-0.454320009	0.21	0.277	2.07742E-08	8
Myt1l.7	-0.459983741	0.098	0.163	2.66807E-08	8
Itsn1.7	-0.373683295	0.191	0.237	3.12302E-08	8
Rbbp6	0.297069352	0.567	0.427	3.40715E-08	8
Tnik.6	-0.483882454	0.067	0.149	3.59493E-08	8
Ect2.7	0.283651771	0.191	0.094	3.78161E-08	8
Grik2.5	-0.396372609	0.043	0.104	3.84435E-08	8
Zfc3h1	0.287692529	0.174	0.084	5.72723E-08	8
Larp7.3	0.267828538	0.446	0.306	6.20649E-08	8
Cadm3.7	-0.425906994	0.081	0.143	6.95358E-08	8
Apc.7	-0.305173956	0.4	0.432	1.19946E-07	8
Gsk3b.3	-0.327894046	0.419	0.503	1.26614E-07	8
H1fx.6	0.255240851	0.367	0.24	1.39373E-07	8
D4Wsu53e.6	-0.278887997	0.415	0.447	1.63051E-07	8
Ubb.3	-0.270035475	0.481	0.543	1.86721E-07	8
Mapk8ip1.6	-0.261777179	0.21	0.228	1.91796E-07	8
Rnaseh2c.3	0.253905119	0.516	0.375	2.12569E-07	8
Rab6b.7	-0.379153091	0.144	0.204	2.33276E-07	8
Nuf2.8	0.257814924	0.242	0.136	2.35067E-07	8
Chd3.7	-0.328723668	0.123	0.15	2.59261E-07	8
Chd7.5	-0.283570827	0.592	0.649	2.78883E-07	8
Sh3bgrl.1	0.254602083	0.469	0.332	3.11773E-07	8
Phf20l1.3	-0.384169816	0.26	0.343	4.66801E-07	8

Dnttip2	0.255073767	0.452	0.319	5.10698E-07	8
Cog7.4	-0.284291984	0.532	0.586	5.23078E-07	8
Csrp2.5	0.263878228	0.261	0.154	6.10359E-07	8
Gas1.4	0.319623834	0.26	0.152	8.13172E-07	8
Ift74.2	0.289950405	0.34	0.22	8.22826E-07	8
Akap6	-0.344202079	0.161	0.22	8.24646E-07	8
Ppp3ca.4	-0.36578027	0.302	0.381	1.14467E-06	8
Bzw1	0.25049909	0.488	0.354	1.35814E-06	8
Dcx.7	-0.264523963	0.364	0.376	1.52399E-06	8
Elavl4.7	-0.348961732	0.286	0.336	1.68287E-06	8
Odc1.1	0.309432962	0.183	0.097	1.74278E-06	8
Nbea.4	-0.410514157	0.12	0.193	1.97743E-06	8
Nsg1.6	-0.286562164	0.255	0.288	2.02805E-06	8
Rufy3.6	-0.273194834	0.343	0.372	3.1737E-06	8
Sema6a.8	-0.380672474	0.145	0.204	3.50457E-06	8
Tagln3.7	-0.385857833	0.218	0.297	3.61241E-06	8
2810474O19Rik	0.274819126	0.356	0.236	3.76735E-06	8
Gnao1.5	-0.344308223	0.235	0.289	4.52342E-06	8
A330076H08Rik.6	-0.400299982	0.094	0.163	4.90842E-06	8
Ssbp3.2	-0.266394397	0.136	0.168	5.36991E-06	8
Klc1.5	-0.297387996	0.314	0.363	5.40202E-06	8
Cnpy1.3	-0.365047393	0.067	0.135	6.01683E-06	8
Hes1.6	0.356562391	0.245	0.144	6.16896E-06	8
Gdi1.6	-0.312986706	0.182	0.231	6.74349E-06	8
Bub1.7	0.256519047	0.223	0.127	7.20933E-06	8
Jam3.2	0.271500789	0.229	0.131	7.57494E-06	8
Hist3h2a.5	-0.362706029	0.088	0.145	8.35815E-06	8
Slc1a2.5	-0.31583643	0.251	0.295	9.21687E-06	8
Aldoa.5	-0.322247571	0.207	0.259	1.04854E-05	8
Cenpp.6	0.309436448	0.195	0.11	1.23742E-05	8
Ccdc77.1	0.252751934	0.145	0.071	1.47952E-05	8
Pak7.5	-0.340799597	0.056	0.106	2.2403E-05	8
Grina.6	-0.325720716	0.062	0.111	2.51535E-05	8
Celsr2.3	-0.324317005	0.186	0.239	2.54871E-05	8
Ppp1r10	0.271282775	0.16	0.081	2.66053E-05	8
Srgap2.5	-0.300581692	0.097	0.133	2.9329E-05	8
Chrna3.6	-0.311727656	0.085	0.119	3.10083E-05	8
Ina.7	-0.281641576	0.412	0.444	3.29829E-05	8
Apbb1.7	-0.301766647	0.135	0.171	3.53991E-05	8
Cacng4.5	-0.333152285	0.084	0.102	3.93716E-05	8
Arpp21.7	-0.421618274	0.056	0.115	4.76208E-05	8
Slc17a6.7	-0.345623371	0.104	0.149	6.34922E-05	8
Zwint.1	-0.335015945	0.211	0.294	7.02521E-05	8
Mllt3	-0.333427572	0.163	0.228	7.28638E-05	8
Pou3f2.4	0.273330089	0.386	0.271	7.94988E-05	8
Atp6v0b.6	-0.325872926	0.202	0.27	9.74282E-05	8
Mycbp2.3	-0.326490689	0.276	0.347	0.00012265	8
Rab3a.8	-0.322894353	0.109	0.151	0.000124398	8

Uchl1.7	-0.253920036	0.309	0.335	0.00015259	8
Fyn.3	-0.303361967	0.188	0.217	0.000160076	8
Elmo1.7	-0.414279947	0.065	0.128	0.000174899	8
Lmnb2.3	0.270786899	0.249	0.154	0.000188393	8
Ypel3.3	-0.262790338	0.383	0.437	0.000189021	8
Cnksr2.2	-0.314997971	0.062	0.109	0.000199875	8
Kif5a.7	-0.370428932	0.066	0.13	0.000301426	8
Maml3.1	-0.305383668	0.048	0.102	0.000302931	8
Ptprd.1	-0.295398959	0.286	0.342	0.000412627	8
Gnaq.3	-0.270114093	0.249	0.286	0.000471316	8
Olig2.1	0.297735085	0.145	0.075	0.000473937	8
Mum1l1.3	0.263614487	0.167	0.091	0.000475156	8
Ntrk3.4	-0.286096572	0.109	0.156	0.000609508	8
Ppfia2.5	-0.355987204	0.059	0.114	0.000647206	8
Hells.8	-0.272765097	0.188	0.214	0.000649519	8
Zfp36l1.4	0.252522557	0.238	0.147	0.000670074	8
Pkia.5	-0.295807981	0.095	0.135	0.000774126	8
Tacc2.4	-0.317852448	0.095	0.142	0.000803966	8
Zmiz1.1	-0.270410756	0.326	0.376	0.000849171	8
Psap.2	-0.268305931	0.16	0.185	0.000859646	8
Agap1.1	-0.308885412	0.082	0.133	0.000925361	8
Fam111a.7	-0.318102502	0.072	0.128	0.000962181	8
Itm2c.4	-0.281239671	0.177	0.213	0.001070823	8
Akap9.2	-0.278254239	0.45	0.502	0.001198012	8
Dner.8	-0.290360188	0.183	0.215	0.001208743	8
Eif4g3	-0.26723106	0.327	0.386	0.001348817	8
Ppp1r1a.3	-0.297007191	0.066	0.111	0.001348827	8
Stxbp1.8	-0.32782077	0.114	0.164	0.001518207	8
Kmt2c	-0.322632823	0.216	0.298	0.001534973	8
Gria4.6	-0.326227383	0.07	0.126	0.001598375	8
Reln.5	-0.307239194	0.144	0.181	0.001734744	8
Mgll.2	-0.322080229	0.081	0.135	0.001753366	8
Rnd2.2	-0.251213322	0.13	0.159	0.001808667	8
Hcfc1r1.4	-0.301182057	0.139	0.195	0.001953195	8
Cplx1.4	-0.283343456	0.104	0.139	0.002035633	8
Erc1.1	-0.315164959	0.062	0.112	0.00236597	8
Stag2.1	-0.296334736	0.198	0.259	0.00250983	8
Clasp2.3	-0.307961045	0.113	0.169	0.002527698	8
Lphn1	-0.279050788	0.065	0.115	0.002547217	8
Trio	-0.304078919	0.067	0.127	0.002672839	8
Fut9.1	-0.317004337	0.155	0.214	0.002754574	8
Rere	-0.307625355	0.088	0.153	0.003665825	8
Kidins220.5	-0.327017711	0.089	0.141	0.004589748	8
Plcb1.6	-0.336066948	0.097	0.142	0.005108255	8
Pdzrn3.5	-0.354379987	0.103	0.149	0.005109589	8
Podxl2.7	-0.312974167	0.176	0.229	0.005374241	8
Abhd16a.6	-0.306341658	0.136	0.191	0.005830318	8
Fmnl2.2	-0.299507715	0.107	0.159	0.006288869	8

Zmynd8.4	-0.294593719	0.163	0.215	0.007755088	8
Pea15a.7	-0.30064942	0.135	0.177	0.009506148	8
Plp1.8	-0.672924328	0.111	0.114	0.011528366	8
Vcan.1	-0.263508985	0.097	0.131	0.011849437	8
Meaf6	-0.257456788	0.091	0.137	0.012232736	8
Kifap3.2	-0.277634133	0.221	0.274	0.014768488	8
Gphn.1	-0.288552855	0.087	0.143	0.015589892	8
Fam210b.4	-0.263844751	0.264	0.316	0.018805642	8
Scn8a.2	-0.275165554	0.097	0.138	0.025731828	8
Aurkb.7	-0.271781623	0.087	0.145	0.031566221	8
Snap25.4	-0.296628751	0.233	0.284	0.033537223	8
Aprt.2	-0.280415785	0.084	0.143	0.037937351	8
Ddx26b.1	-0.251215816	0.094	0.131	0.038755073	8
Klf9.3	-0.27971171	0.218	0.272	0.039556244	8
Sv2b.2	-0.259600253	0.065	0.103	0.039990499	8
Scaper.2	-0.250347762	0.084	0.111	0.064376198	8
Nrcam.6	-0.286575144	0.091	0.135	0.067871039	8
Clcn4-2.5	-0.255439185	0.214	0.252	0.073861433	8
Gamt.4	-0.26539591	0.128	0.171	0.084873771	8
6330403K07Rik.5	-0.287932771	0.185	0.244	0.106036111	8
Tspyl4.5	-0.25080789	0.084	0.128	0.144152645	8
Shd.4	-0.258010799	0.067	0.111	0.15700353	8
Adrbk2.1	-0.254305269	0.069	0.101	0.159865303	8
Stau2.2	-0.260456896	0.107	0.152	0.16713974	8
St18.7	-0.303836856	0.148	0.192	0.19468802	8
Esco2.7	-0.286042461	0.151	0.196	0.212256604	8
Rfc2.4	-0.257683193	0.157	0.213	0.251547151	8
Dnmt1.7	-0.264419995	0.216	0.28	0.256217163	8
Rabgap1	-0.270402384	0.17	0.226	0.271587883	8
Ntm.5	-0.270196669	0.11	0.146	0.296552426	8
Fam213b.4	-0.270409307	0.114	0.159	0.300661685	8
Rad51ap1.8	-0.259724185	0.109	0.169	0.471067343	8
A930011O12Rik.7	-0.250743076	0.091	0.115	0.51024293	8
Macf1.2	-0.271646914	0.151	0.212	0.537199243	8
Sox5.3	-0.25342775	0.082	0.12	0.590951458	8
Phactr1.1	-0.259499859	0.141	0.185	0.671033388	8
Hook3.1	-0.259800216	0.235	0.302	0.870931324	8
Mt1.5	-0.334867354	0.142	0.169	0.910681805	8
Olig1	2.903177018	0.937	0.059	0	9
Serpine2	2.715342148	0.844	0.038	0	9
Cspg5	2.683401309	0.693	0.013	0	9
3110035E14Rik	2.389352667	0.713	0.007	0	9
Cacng4.6	2.361277033	0.808	0.085	0	9
Fabp7.9	2.295787472	0.827	0.106	0	9
Ptprz1	2.209871928	0.698	0.021	0	9
Scrg1	2.182640109	0.703	0.009	0	9
Cntn1.1	2.163621885	0.732	0.035	0	9
Gpr17	2.022658367	0.526	0.003	0	9

Gpr37l1	1.973935391	0.684	0.015	0	9
Pllp	1.780260482	0.538	0.003	0	9
Sox10	1.68035394	0.526	0.002	0	9
Olig2.2	2.021651086	0.754	0.062	0	9
Plp1.9	3.538254237	0.326	0.109	1.3672E-305	9
Sulf2	1.741662466	0.533	0.01	4.1325E-280	9
Ptpre	1.652719821	0.496	0.005	4.497E-279	9
Bcas1.2	1.863914217	0.672	0.273	2.3412E-271	9
S100a13	2.012091919	0.535	0.019	7.3536E-267	9
Sirt2.2	2.063499527	0.55	0.146	1.3582E-264	9
Enpp2	1.804668413	0.572	0.019	1.4917E-263	9
Cnp	2.339189571	0.484	0.023	3.165E-258	9
Bcan	1.880028263	0.655	0.038	4.9274E-258	9
Ramp1	1.714919315	0.545	0.015	8.179E-253	9
Fyn.4	1.868826087	0.603	0.206	2.9751E-238	9
S100a1	1.831255061	0.482	0.016	3.9808E-235	9
Rgcc	1.551688696	0.428	0.006	2.8825E-229	9
Ugt8a	1.57139186	0.428	0.006	7.308E-229	9
Slc35f1	1.450650516	0.431	0.007	2.0159E-224	9
Ppfibp1	1.602593829	0.501	0.021	5.883E-219	9
Cspg4	1.63063144	0.433	0.01	3.5915E-210	9
Nfib.4	-1.507845392	0.54	0.942	2.087E-208	9
Pdgfra.3	1.670836497	0.62	0.171	9.4965E-197	9
Gjc3	1.458396832	0.375	0.006	1.1485E-193	9
Gatm	1.469185926	0.489	0.024	5.0855E-191	9
Ednrb	1.530134139	0.457	0.019	3.76E-184	9
Tpm1.1	1.557954069	0.674	0.179	8.4838E-182	9
Pcsk1n.2	1.52794907	0.633	0.07	1.0339E-179	9
Nap1l5	1.553548438	0.584	0.068	3.4889E-175	9
Itpr2	1.547462182	0.423	0.02	2.4382E-173	9
Epn2	1.522477717	0.572	0.072	6.631E-169	9
Tagln2	1.57173351	0.462	0.034	9.4758E-169	9
Nkx2-2	1.18013979	0.299	0.002	6.0197E-167	9
B3gat2	1.300410561	0.365	0.01	2.9875E-166	9
Plekhb1	1.189870178	0.314	0.004	3.6088E-164	9
Lsamp.1	1.501990339	0.579	0.065	5.2325E-164	9
Qpct	1.311636766	0.377	0.013	7.1506E-162	9
Sfrp1.5	-1.749242216	0.304	0.823	2.3619E-161	9
Mfsd2a	1.088448505	0.309	0.004	7.3207E-159	9
Dnm3	1.32511429	0.406	0.016	9.6592E-158	9
Omg	1.15859031	0.307	0.004	4.3622E-157	9
Gria3.1	1.523135408	0.55	0.078	7.2744E-156	9
Mbp.3	2.073127038	0.326	0.161	7.3538E-153	9
Zic1.7	-1.655142169	0.219	0.797	4.9361E-150	9
Ptn.7	1.268454632	0.818	0.432	2.3051E-149	9
Tsc22d4.2	1.461783572	0.625	0.155	2.1832E-148	9
Sox6	1.142593788	0.29	0.004	4.3875E-148	9
Phlda1	1.310544969	0.384	0.017	1.3407E-147	9

Tmem100	1.127057348	0.282	0.004	2.3755E-146	9
Asrgl1.2	1.355157661	0.608	0.122	6.418E-144	9
Spon1	1.133780244	0.311	0.007	2.0426E-140	9
Cd9	1.154669859	0.701	0.479	5.1102E-139	9
Ddah1	1.305638416	0.409	0.031	1.3827E-138	9
CRE_RECOMBINASE.7	-2.059423624	0.248	0.78	2.1263E-138	9
Cdo1	1.287858009	0.341	0.013	3.2874E-137	9
Lims2	1.020927721	0.248	0.002	2.1436E-136	9
Ncald	1.368819745	0.633	0.154	1.2412E-135	9
Hsp90ab1	-0.570900984	0.99	0.998	3.4745E-132	9
Susd4	1.012549128	0.292	0.007	3.4467E-130	9
Ascl1	0.948023641	0.234	0.002	5.0979E-130	9
Tril	1.12701058	0.363	0.017	5.597E-130	9
Ncam2	1.098274532	0.311	0.01	4.1301E-129	9
Cyp2j6	1.098736951	0.309	0.011	8.3442E-124	9
Tmem176b.2	1.363995042	0.555	0.098	8.9268E-123	9
Igfbpl1.7	-1.857745283	0.112	0.66	1.0684E-121	9
Cd24a.2	-1.622727012	0.095	0.647	1.9019E-120	9
Sox2	1.197990755	0.416	0.039	7.718E-119	9
Neu4	0.863527973	0.197	0.001	7.3022E-118	9
Kcnj10	1.128964812	0.333	0.018	8.083E-117	9
2810468N07Rik	1.115717348	0.309	0.015	1.0544E-115	9
Dbi.3	1.0943101	0.798	0.455	1.3978E-115	9
Kctd4	0.738455959	0.195	0.001	3.9768E-115	9
Ttyh1	1.160683038	0.358	0.024	5.7902E-115	9
Klhl5	1.158610348	0.382	0.031	6.5986E-115	9
S100a16.6	1.437703632	0.499	0.085	1.3223E-114	9
Cadm2.1	1.202860717	0.418	0.039	2.6949E-114	9
Dmrtb1	0.718700633	0.187	0.001	7.8755E-113	9
Pcdh17	1.213272949	0.341	0.021	5.7464E-112	9
Lrrc4c	0.958704728	0.241	0.004	1.1067E-111	9
Sox8	1.208292489	0.331	0.022	9.6056E-110	9
Cmtm5	0.904632875	0.287	0.011	9.6011E-109	9
Slc1a1	0.980568666	0.285	0.011	9.8614E-109	9
Rlbp1	1.116968929	0.241	0.006	1.193E-108	9
Afap1l2	0.828859047	0.221	0.003	2.8007E-108	9
Zcchc24	1.089565385	0.324	0.021	1.1962E-105	9
Sept7	0.927710774	0.818	0.583	2.8393E-105	9
Vcan.2	1.281117807	0.513	0.12	4.0683E-104	9
Opcml	1.017047337	0.29	0.013	4.793E-104	9
Anks1b.1	1.219028522	0.44	0.054	2.3773E-103	9
Enc1	1.012223699	0.255	0.009	8.2451E-102	9
Bmp4	1.293077849	0.187	0.002	3.2158E-101	9
Atp1a2	1.072763391	0.404	0.05	7.412E-101	9
Ostf1	1.218368773	0.411	0.055	1.9408E-100	9
Car8	0.901650861	0.219	0.005	2.5857E-99	9
S100b	1.346672715	0.358	0.033	3.9106E-99	9
Mmp15	1.008386864	0.258	0.011	3.9501E-99	9

Rprm	1.113778113	0.326	0.027	4.00311E-98	9
Nxph1	0.863248077	0.231	0.006	1.03943E-97	9
Ppap2b	1.037266795	0.365	0.035	2.27751E-97	9
Pxdc1	0.732645895	0.204	0.004	2.39814E-95	9
3632451O06Rik	1.004539507	0.333	0.028	4.42869E-93	9
Pou3f1	0.825445705	0.175	0.001	5.50129E-93	9
Gal3st1	0.576702965	0.151	0	1.61261E-92	9
Degs1	1.114262358	0.423	0.068	2.47497E-92	9
Brinp3	0.818550724	0.221	0.006	3.07502E-92	9
Gfra1	0.840531901	0.214	0.005	5.57747E-92	9
Matn4	0.792732986	0.185	0.002	7.01431E-92	9
Kcnd2.1	1.088951469	0.297	0.019	2.15682E-91	9
Tnr	0.612117329	0.165	0.001	2.16073E-91	9
Sema5a	0.796356632	0.185	0.002	2.29508E-91	9
Tspan3.1	0.925487053	0.766	0.42	1.38035E-88	9
Scd2.2	1.059209578	0.667	0.288	3.20066E-88	9
Arsb	1.001851735	0.277	0.019	2.11395E-87	9
Tmem255b	0.702148946	0.151	0.001	2.26287E-87	9
Fa2h	0.731774292	0.163	0.001	1.53673E-86	9
Tubb5.6	-0.83451973	0.766	0.918	7.42975E-86	9
Anp32a	-0.902846843	0.586	0.842	1.4978E-85	9
Sox2ot	0.77465904	0.168	0.002	7.19667E-85	9
Pcdh10	0.860702249	0.226	0.009	1.1021E-84	9
Tspan7.5	1.0934022	0.479	0.096	8.54295E-84	9
Enpp6	0.927254593	0.158	0.001	2.19412E-83	9
AW047730	1.07913264	0.265	0.018	3.38728E-83	9
Sema5b	0.774667138	0.19	0.004	4.46754E-83	9
Dscam	0.89992954	0.246	0.012	7.61881E-83	9
Timp4	0.870482397	0.238	0.011	2.08597E-82	9
Sh3d19	0.954112299	0.311	0.029	7.50836E-82	9
Cdh13	1.007250997	0.343	0.04	6.42446E-81	9
H3f3b.1	-0.750909468	0.742	0.91	1.26528E-80	9
Megf11	0.811074979	0.197	0.006	3.05292E-80	9
Barhl1.7	-1.459706256	0.085	0.532	3.11764E-80	9
Cd81.2	0.895579823	0.776	0.454	5.04606E-80	9
1810041L15Rik	0.717537414	0.18	0.003	6.55902E-80	9
Tmem132b	0.607694837	0.144	0.001	2.2798E-79	9
Il1rap	0.851585768	0.226	0.011	5.26938E-78	9
Phyhipl.1	1.151371648	0.333	0.055	5.23694E-77	9
Slc6a1	0.825782526	0.273	0.021	1.05108E-76	9
Gm2a	0.995360854	0.333	0.041	1.06753E-76	9
Grm5	0.90439797	0.202	0.007	1.36427E-76	9
Arl4a	1.100472493	0.367	0.057	1.65128E-76	9
Cldn11	1.458231684	0.168	0.031	2.54587E-76	9
Draxin.5	-1.405266343	0.134	0.564	1.82703E-75	9
Meg3.6	0.659224531	0.416	0.087	2.53742E-75	9
Resp18	0.756312695	0.165	0.003	6.27405E-75	9
Kcnip3.1	1.149367939	0.401	0.113	2.24854E-74	9

Tns3	0.75169345	0.187	0.006	6.60697E-74	9
Lypd1	0.712255712	0.156	0.002	3.70888E-73	9
Cog7.5	-1.298781113	0.165	0.594	3.97874E-73	9
Sema3d	0.66780861	0.151	0.002	7.68133E-72	9
Gltp	1.057354254	0.37	0.076	8.50803E-72	9
Crmp1.3	-1.163119281	0.268	0.677	2.49267E-71	9
Vstm2b	0.600195995	0.136	0.001	2.58146E-71	9
Scn3a	0.83943863	0.217	0.011	5.42056E-71	9
Clu	0.81585503	0.229	0.015	7.99291E-71	9
Gsn	0.959654969	0.195	0.009	6.34484E-70	9
Calm2.6	-0.798768018	0.662	0.879	7.76021E-70	9
Tspan2	0.967886887	0.207	0.011	1.02113E-69	9
Ddah2.4	-1.159180809	0.316	0.694	1.75489E-69	9
Slc22a3	0.566329897	0.117	0	2.42909E-69	9
Slc29a1.4	-1.280319689	0.151	0.571	3.02511E-69	9
Nlgn3	0.878075878	0.263	0.022	3.94682E-69	9
Luzp2	0.803925002	0.219	0.012	6.12203E-69	9
Adam9	0.94034359	0.328	0.046	2.08682E-68	9
Pik3r1	0.994563784	0.436	0.094	4.83417E-68	9
Adora1	0.763027765	0.19	0.008	8.88274E-68	9
Dab1	0.957926193	0.268	0.028	1.31637E-67	9
Stk32a	0.602182651	0.144	0.002	1.68641E-67	9
Tmem88b	0.662973307	0.148	0.003	1.90456E-66	9
Pax6.6	-1.30429638	0.114	0.522	3.28739E-66	9
Phactr3	0.982306664	0.375	0.066	8.95362E-66	9
Bricd5	0.543065636	0.127	0.001	1.15124E-65	9
Hip1	1.019536529	0.44	0.11	2.51849E-65	9
Sh3bp4	0.82476965	0.241	0.019	4.29844E-65	9
Lhx1.6	-1.396452894	0.114	0.506	4.85669E-65	9
Gnb4	0.994334752	0.411	0.097	2.13768E-64	9
Tmsb10.4	-1.0768535	0.311	0.672	3.17836E-64	9
Tgfa	0.572789077	0.146	0.003	4.20575E-64	9
Ezr.4	-1.251861014	0.061	0.446	6.00727E-64	9
Slc22a23	0.707665315	0.175	0.007	7.66117E-64	9
Lrp1	0.928571913	0.309	0.041	1.7399E-63	9
Ptprt	0.675556415	0.148	0.003	3.2909E-63	9
Dlgap1	0.964736229	0.353	0.06	4.82113E-63	9
Chst11	0.722016324	0.187	0.009	4.86492E-63	9
Nfia	-0.92778846	0.474	0.781	5.70457E-63	9
C1ql2	0.489332147	0.112	0	8.78749E-63	9
Slc22a17.4	0.92976849	0.601	0.242	1.41259E-62	9
Syt11.6	0.786683904	0.798	0.497	1.63817E-62	9
Calr.1	0.790675108	0.779	0.504	2.48234E-62	9
Alcam.2	0.990713708	0.462	0.126	2.82845E-62	9
Cd63.2	-0.912043511	0.47	0.761	3.30885E-62	9
Dcaf12l1	0.552372076	0.134	0.002	4.3653E-62	9
Rap1gap	0.650653038	0.178	0.007	1.39168E-61	9
Stmn2.8	-1.555310719	0.178	0.561	2.03844E-61	9

Zfp365	0.707745417	0.19	0.01	4.80542E-61	9
Brinp1	0.764375608	0.248	0.024	3.66412E-60	9
G0s2	0.596525651	0.148	0.004	4.32713E-60	9
Slc38a3	0.842043865	0.217	0.017	4.38254E-59	9
Rab31	0.896323851	0.311	0.049	5.54326E-59	9
Tmsb4x.6	-0.731539965	0.893	0.95	1.42462E-58	9
Gng12.2	0.942369522	0.416	0.114	3.07398E-58	9
B3gat1	0.839529267	0.217	0.02	1.04033E-57	9
Ppp1r16b	0.553661957	0.131	0.002	2.18372E-57	9
S100a6	0.837183872	0.214	0.017	2.53304E-57	9
Sstr1	0.44362285	0.107	0.001	3.25668E-57	9
Fbxo7	0.719031456	0.234	0.022	4.27929E-57	9
Neurod1.9	-1.738852191	0.182	0.541	9.82404E-57	9
Deb1	0.935912478	0.474	0.16	1.93186E-56	9
Lrrtm1	0.622305543	0.136	0.003	2.16488E-56	9
Pmp22	0.791432061	0.195	0.013	4.73484E-56	9
Rbfox3.6	-1.250327304	0.124	0.502	1.16416E-55	9
D430041D05Rik.4	-1.181191883	0.129	0.5	1.37205E-55	9
Pfn2.4	0.938523775	0.487	0.235	2.82773E-55	9
Kank1	0.786369198	0.248	0.03	4.1064E-55	9
Pcdh11x	0.621037098	0.158	0.006	4.89783E-55	9
Pcdh9	0.839325445	0.268	0.035	7.83055E-55	9
Nfasc.2	1.077835613	0.238	0.031	1.06847E-54	9
Ehd3	0.77470153	0.241	0.025	1.28907E-54	9
Emid1	0.725624693	0.153	0.006	1.54491E-54	9
Npas3	0.828489691	0.226	0.022	2.44097E-54	9
Eps8	0.783702489	0.243	0.027	3.33317E-54	9
Il18	0.625713974	0.178	0.01	5.57549E-54	9
Dock9	0.716263538	0.185	0.012	9.05686E-54	9
Zic4.3	-1.164109347	0.068	0.426	9.17263E-54	9
Nrep.8	-1.196061916	0.217	0.577	1.62083E-53	9
E130114P18Rik.3	-1.113877726	0.226	0.593	1.74291E-53	9
Pgp	0.928787949	0.445	0.143	2.08973E-53	9
4833424O15Rik	0.855479345	0.273	0.038	5.03643E-53	9
Timp2	0.678776637	0.209	0.021	4.43955E-52	9
Rnd3.5	-1.190672855	0.092	0.439	7.22883E-52	9
Trio.1	0.916290668	0.414	0.118	1.54839E-51	9
Pid1	0.817741065	0.27	0.038	1.74036E-51	9
Gpm6b.4	0.734721583	0.766	0.477	2.59358E-51	9
Sash1	0.866143133	0.234	0.03	7.30471E-51	9
Rhoc	0.757811763	0.217	0.023	9.42419E-51	9
Chn2	0.984738135	0.17	0.01	2.11776E-50	9
Tm7sf3	0.790778386	0.316	0.059	2.24909E-50	9
Igfbp3	0.654442518	0.158	0.009	7.76431E-50	9
H2afv.7	-0.885778228	0.462	0.734	9.17641E-50	9
Canx.1	0.635583119	0.766	0.591	1.13891E-49	9
Pabpc1.4	-0.583086762	0.81	0.88	1.63236E-49	9
Sox21	0.531463815	0.129	0.004	1.71533E-49	9

Spry4	0.593821192	0.124	0.003	2.43074E-49	9
2900011O08Rik.6	0.863201359	0.392	0.096	3.09716E-49	9
Nhlh2.7	-1.277112379	0.09	0.43	3.13857E-49	9
Mmd2	0.744284923	0.253	0.034	3.34233E-49	9
Wscd1	0.794846284	0.309	0.057	3.36966E-49	9
Spry1	0.694892656	0.161	0.01	4.71749E-49	9
Slitrk3	0.575874945	0.163	0.01	4.88199E-49	9
Dpp6	0.690502775	0.212	0.022	2.51073E-48	9
Mpzl1	0.937309564	0.37	0.139	3.18307E-48	9
Pde4b	0.821044737	0.275	0.045	3.62443E-48	9
Nfix.4	-0.881295978	0.336	0.681	5.30121E-48	9
Cyp2j9	0.500910864	0.124	0.003	6.25704E-48	9
Cplx2.4	-1.123059081	0.158	0.503	9.30888E-48	9
1500016L03Rik.5	-1.175123412	0.066	0.39	1.23648E-47	9
Neto1	0.486341549	0.107	0.002	1.34338E-47	9
Mmp16.1	0.917346792	0.326	0.082	2.04718E-46	9
Chadl	0.501716308	0.129	0.004	3.9216E-46	9
Epb4.1l2	0.946088263	0.26	0.089	5.3652E-46	9
Lrrfip1	0.79894207	0.253	0.039	9.05995E-46	9
Dusp26.1	0.719842055	0.299	0.059	1.88484E-45	9
Plk2	0.654664116	0.153	0.009	2.87207E-45	9
Col16a1	0.482287599	0.112	0.003	3.21922E-45	9
Flrt1	0.614289026	0.139	0.006	4.10222E-45	9
Cbr3	0.538813719	0.134	0.006	4.5811E-45	9
Ppapdc1a	0.510967321	0.122	0.004	5.9041E-45	9
Cbfa2t3.6	-1.091971113	0.083	0.41	7.49827E-45	9
Cacng2.1	-1.07212988	0.041	0.345	1.06316E-44	9
A930009A15Rik	0.699176104	0.202	0.022	2.35277E-44	9
Tbata.6	-1.198051447	0.044	0.347	3.0522E-44	9
Hnrnpab.3	-0.631770673	0.667	0.819	1.13627E-43	9
S100a4	0.522519823	0.109	0.003	1.14246E-43	9
Chpt1	0.776617176	0.285	0.054	2.36213E-43	9
Rpl13a.3	-0.529253426	0.876	0.929	2.7656E-43	9
Cbx1.1	-0.775816426	0.438	0.691	3.16365E-43	9
Sdc3.1	0.855437873	0.319	0.071	4.25448E-43	9
Mapt.8	0.720363596	0.53	0.208	4.51722E-43	9
Ntrk2.2	0.765887312	0.336	0.077	7.16434E-43	9
Arhgdig	0.642013567	0.173	0.015	8.53026E-43	9
Ptma.3	-0.876647855	0.282	0.588	1.66846E-42	9
Cadm4.1	0.769338085	0.387	0.107	1.70086E-42	9
Sgk1	0.849612736	0.209	0.03	2.41461E-42	9
Rps5.4	-0.502335002	0.92	0.954	2.66563E-42	9
Gria2.8	0.632061131	0.793	0.514	2.81471E-42	9
Cacna2d1.5	-1.115279813	0.075	0.382	2.95943E-42	9
H1f0.4	-0.898078818	0.319	0.623	9.47725E-42	9
Tmem176a	0.600261832	0.158	0.012	2.07415E-41	9
Fam3c	0.800272951	0.314	0.079	8.84799E-41	9
5730559C18Rik	0.386874404	0.102	0.002	3.10738E-40	9

Abhd12	0.722448215	0.345	0.087	4.19852E-40	9
Tubb3.8	-1.224277744	0.173	0.481	4.77463E-40	9
Zdhhc2	0.712119731	0.204	0.028	4.82874E-40	9
Rtkn	0.593957552	0.187	0.02	4.90952E-40	9
Mt3	0.643787875	0.19	0.021	6.65345E-40	9
Sh3gl3	0.690113946	0.234	0.037	1.58465E-39	9
Plekha2	0.484336698	0.107	0.003	2.28324E-39	9
Fam210b.5	-1.050775764	0.044	0.32	2.64994E-39	9
Celf2.8	-0.894241971	0.345	0.644	6.36556E-39	9
Uncx.7	-1.09977652	0.056	0.324	7.57073E-39	9
Pea15a.8	0.818662512	0.457	0.169	7.76824E-39	9
Lphn3.1	0.700028712	0.277	0.056	9.26604E-39	9
Dpysl4.4	-0.945685	0.173	0.498	1.23184E-38	9
Mycn.3	-1.001625097	0.073	0.37	1.66083E-38	9
Hsd11b2.7	-1.077814342	0.032	0.293	1.74673E-38	9
Nrxn2.1	0.763756068	0.333	0.084	1.87122E-38	9
Rplp0.3	-0.528309497	0.781	0.874	1.93733E-38	9
Cp	0.565552313	0.139	0.011	3.96522E-38	9
Ppp2r2c.3	-0.932507752	0.2	0.512	7.00216E-38	9
Acox1	0.644365345	0.265	0.051	8.04415E-38	9
Cav2	0.492688732	0.124	0.007	8.8318E-38	9
Cdh11	0.597330132	0.151	0.012	1.09454E-37	9
Cyfip2.2	0.778521588	0.255	0.048	1.31437E-37	9
Hsd17b12	0.77404089	0.46	0.181	1.88862E-37	9
Hmgcs1.3	0.834233602	0.409	0.145	2.20458E-37	9
Dusp15	0.487735378	0.134	0.008	2.87444E-37	9
Smc2.7	-1.020651663	0.304	0.551	4.79986E-37	9
Rps9.3	-0.429254388	0.915	0.929	6.68434E-37	9
Rps3.3	-0.459275569	0.876	0.923	6.83582E-37	9
Rpl4.3	-0.482447344	0.864	0.909	8.32723E-37	9
Tspan6.1	0.751643861	0.494	0.24	1.2564E-36	9
Gm2694.1	-1.0004856	0.078	0.365	1.71506E-36	9
Marc2	0.776799091	0.299	0.078	2.02198E-36	9
Scamp2.1	0.779372135	0.382	0.129	2.89733E-36	9
Kcnd3	0.623953616	0.151	0.013	7.55195E-36	9
Tead2.5	-0.887897555	0.029	0.284	1.33801E-35	9
Prkcq	0.738057726	0.255	0.058	1.44147E-35	9
Slitrk2	0.47447878	0.109	0.005	4.37204E-35	9
Lnx1	0.700622048	0.173	0.022	5.27054E-35	9
Cdh10	0.456956029	0.134	0.011	5.58611E-35	9
Rassf4.2	-0.984531898	0.1	0.382	8.71653E-35	9
Taf9b	0.472841496	0.139	0.011	9.15218E-35	9
Fbn2	0.568500024	0.117	0.007	1.45826E-34	9
Tmem255a	0.49982438	0.105	0.005	1.8416E-34	9
Nacc2	0.593524781	0.153	0.015	1.84415E-34	9
Limch1	0.627714618	0.173	0.02	2.03655E-34	9
Ier5.3	-0.916914131	0.105	0.401	2.16035E-34	9
Ccnd2.5	-0.836696671	0.421	0.667	3.01328E-34	9

Necab2	0.412957679	0.114	0.006	1.09477E-33	9
Cav1	0.570612936	0.148	0.016	1.93299E-33	9
Eid1.4	0.596747372	0.757	0.496	2.52371E-33	9
Hepacam	0.527202684	0.117	0.007	2.77768E-33	9
Dock10	0.536588947	0.144	0.013	3.16881E-33	9
Spock2.1	0.629687434	0.265	0.06	3.92907E-33	9
Srebf1.7	-1.018226196	0.078	0.34	4.76451E-33	9
Epha4	0.474740062	0.127	0.009	5.26362E-33	9
Mex3a.2	-0.886107973	0.173	0.47	6.96405E-33	9
Tmem9b	0.68094669	0.35	0.106	1.25732E-32	9
Npc1	0.650224044	0.214	0.038	1.64172E-32	9
Lrrtm3	0.48358663	0.146	0.015	1.91776E-32	9
Pde1c.9	-1.003201538	0.112	0.393	2.55678E-32	9
Wipf1	0.592930539	0.175	0.024	2.68952E-32	9
Rev3l.1	0.803466388	0.307	0.092	4.00881E-32	9
Ildr2	0.442862908	0.107	0.005	4.931E-32	9
Gabra3	0.580336829	0.144	0.015	6.76899E-32	9
Ppp2r2b.3	0.659116	0.297	0.079	8.54708E-32	9
Hbegf	0.627138423	0.156	0.017	9.61716E-32	9
Dynlt3	0.618521574	0.224	0.042	9.67328E-32	9
Ptpro	0.431418145	0.136	0.013	1.60563E-31	9
Hsp90b1.5	0.525679082	0.793	0.689	1.60582E-31	9
Insm1.2	-0.944380387	0.049	0.298	1.71936E-31	9
Camk1	0.692625392	0.241	0.052	2.16071E-31	9
Mgll.3	0.797904178	0.358	0.127	2.51368E-31	9
Lbh	0.731990438	0.27	0.069	4.87866E-31	9
Taok3.1	0.671083715	0.302	0.082	6.12174E-31	9
Sv2a.2	0.531646499	0.263	0.067	6.29742E-31	9
Pcdh7	0.465059981	0.134	0.012	6.63017E-31	9
Nasp.7	-0.704352239	0.453	0.644	8.77817E-31	9
Sapcd2.1	0.729720075	0.234	0.076	9.62166E-31	9
Gria4.7	0.734197961	0.36	0.119	2.59812E-30	9
Tln2	0.528450904	0.151	0.017	2.63113E-30	9
Kif21a.1	-0.968644716	0.102	0.351	2.78349E-30	9
Cd302	0.699127675	0.231	0.048	2.97247E-30	9
Cpne8	0.486544206	0.127	0.011	3.75873E-30	9
Kazn.1	0.512963127	0.148	0.018	4.70833E-30	9
Cryab	0.710833467	0.153	0.019	4.96693E-30	9
Ppic.3	-0.838022563	0.024	0.244	5.81853E-30	9
Mif4gd	0.551709923	0.18	0.027	6.90353E-30	9
Miat.7	-0.985969928	0.265	0.521	7.00525E-30	9
Rps14.3	-0.409736361	0.922	0.951	8.55477E-30	9
Shc4	0.641088539	0.19	0.033	1.3529E-29	9
Nim1	0.519154261	0.136	0.013	1.42605E-29	9
Hpca.6	-0.964397305	0.049	0.282	2.05913E-29	9
Lamp1	0.649471932	0.569	0.33	2.44953E-29	9
Dynll2.1	0.67823986	0.409	0.293	2.74093E-29	9
Apoe.9	-0.930516298	0.401	0.246	3.82842E-29	9

Gsg1l.6	-0.867522665	0.036	0.26	6.37284E-29	9
Lrrc4	0.370113061	0.105	0.007	6.77022E-29	9
Arxes1	0.624479692	0.253	0.06	6.88915E-29	9
Smc4.7	-0.859420827	0.372	0.571	7.99818E-29	9
Fermt2	0.642107604	0.467	0.199	1.00523E-28	9
Pdlim5	0.451822969	0.127	0.011	1.18696E-28	9
Gpt2	0.648779236	0.251	0.062	1.32934E-28	9
Shisa4	0.645948187	0.224	0.048	1.69071E-28	9
Trib2	0.691121112	0.282	0.083	1.9965E-28	9
Slc44a1	0.799649011	0.282	0.099	2.02744E-28	9
Metrn	0.522244282	0.148	0.018	2.17679E-28	9
Vimp.1	0.726505371	0.414	0.184	2.23964E-28	9
Rps24.3	-0.50333193	0.691	0.823	2.43842E-28	9
Chd5	0.523113558	0.102	0.006	2.7816E-28	9
Caskin2	0.472358585	0.105	0.007	3.15184E-28	9
Tmbim6	0.649830658	0.574	0.318	3.46052E-28	9
Rps20.5	-0.544576452	0.633	0.781	3.47869E-28	9
Hmgb2.8	-0.913626089	0.161	0.405	5.73933E-28	9
Lrch3	0.647272706	0.273	0.072	7.32067E-28	9
Meis1.1	-0.863155786	0.124	0.378	1.20313E-27	9
Aldoc	0.538621971	0.17	0.029	1.28343E-27	9
Arvcf	0.536501075	0.124	0.011	1.5648E-27	9
Cd200.1	0.618968089	0.207	0.041	1.74017E-27	9
Evi5l	0.640695302	0.197	0.037	2.93841E-27	9
Gpx3	0.493908239	0.109	0.008	3.77839E-27	9
Cdk6.3	-0.788456289	0.032	0.248	4.31928E-27	9
Gyg	0.410819018	0.105	0.007	4.76619E-27	9
Cks1b.8	-0.883823458	0.109	0.349	5.02184E-27	9
Grin3a	0.667430047	0.231	0.055	5.44497E-27	9
Tspan13.2	0.705953217	0.431	0.201	6.74461E-27	9
Syt16	0.487602503	0.119	0.011	8.2118E-27	9
Eif1b	0.621276436	0.564	0.33	1.11988E-26	9
Sec11c.4	0.69348836	0.433	0.207	1.42669E-26	9
Rps26.4	-0.464328578	0.779	0.849	1.56521E-26	9
Dbndd2	0.56937697	0.153	0.021	1.94058E-26	9
Fgf9	-0.823744683	0.044	0.265	1.97291E-26	9
Gucy1a3	0.495472337	0.105	0.008	3.0969E-26	9
Fchsd2	0.588319505	0.18	0.031	3.14393E-26	9
Tcf4.2	-0.419497529	0.876	0.926	3.38832E-26	9
Add3.3	0.633364934	0.311	0.099	1.07422E-25	9
Stmn1.2	-0.775306674	0.066	0.289	1.61062E-25	9
Spry2	0.646386088	0.202	0.045	2.23645E-25	9
Gng3.8	0.453831866	0.589	0.31	2.90146E-25	9
Cntn2.9	-1.186115539	0.061	0.268	3.04357E-25	9
Mtap.2	0.674637856	0.27	0.09	3.4687E-25	9
Chd7.6	-0.664219202	0.423	0.652	6.31285E-25	9
Rbp4.6	-0.857791459	0.039	0.246	6.4037E-25	9
Gm11223.3	-1.020180883	0.027	0.217	1.09095E-24	9

Gm17750.3	-0.835721116	0.122	0.372	1.11085E-24	9
Elovl7	0.523456483	0.117	0.012	1.22225E-24	9
Otx2.1	-0.794712794	0.034	0.221	1.27631E-24	9
Gm9800.3	-0.66762433	0.316	0.539	1.72672E-24	9
Dtd1	0.611076713	0.265	0.075	2.13986E-24	9
Clmp.6	-0.906055817	0.041	0.247	2.29254E-24	9
Arxes2	0.650583537	0.285	0.094	2.4214E-24	9
Fez1	0.61131753	0.562	0.327	2.48254E-24	9
Nenf.2	0.593214992	0.358	0.131	3.35086E-24	9
Fam49b	0.58735406	0.275	0.08	3.86479E-24	9
Mfap2	0.56059489	0.197	0.042	4.93385E-24	9
Mageh1	0.639451142	0.321	0.115	5.37309E-24	9
Kif5c.8	-0.841049937	0.173	0.404	6.82869E-24	9
Arl2bp	0.660035876	0.433	0.212	7.38768E-24	9
Lmbrd1	0.561323493	0.246	0.066	9.12448E-24	9
Itgb8	0.541440888	0.131	0.018	9.74536E-24	9
Polg	0.627434624	0.209	0.05	1.05776E-23	9
Ly6h.1	0.455287841	0.136	0.02	1.08768E-23	9
2810417H13Rik.8	-0.906508277	0.165	0.397	1.33918E-23	9
Jam2	0.524554677	0.168	0.03	1.48722E-23	9
Ggct	0.557532672	0.175	0.033	1.48941E-23	9
Cxxc5.3	-0.698193231	0.302	0.549	1.99147E-23	9
Arhgap31	0.578507472	0.18	0.036	2.24794E-23	9
Tub.1	0.582831178	0.19	0.039	2.38871E-23	9
Hnrnpu.2	-0.386989883	0.808	0.866	3.62082E-23	9
1810037I17Rik.2	0.600278544	0.418	0.191	5.15266E-23	9
Atpif1	-0.457237246	0.689	0.818	5.39035E-23	9
Limd1	0.646101652	0.234	0.067	6.40179E-23	9
Rgs7bp	0.550408062	0.153	0.025	6.81033E-23	9
Tceal3.1	0.669997714	0.302	0.106	6.83013E-23	9
Lrrn3	0.515111959	0.146	0.022	6.87568E-23	9
Scamp5	0.563210938	0.229	0.058	6.89094E-23	9
Gjc1.3	-0.696313785	0.019	0.2	7.56516E-23	9
Amz1	0.370586618	0.119	0.014	8.79527E-23	9
C1ql3	0.6206527	0.212	0.054	8.97961E-23	9
Apba2.1	-0.735578514	0.175	0.419	1.57474E-22	9
Vapa	0.573939895	0.557	0.334	1.57965E-22	9
Grid1	0.440433963	0.107	0.01	2.00303E-22	9
Nhlh1.6	-0.884461117	0.039	0.227	2.05624E-22	9
Hmgn1.5	-0.576187455	0.411	0.626	2.66277E-22	9
Rpl22.4	-0.430943791	0.713	0.79	2.81283E-22	9
2700094K13Rik.8	-0.541412639	0.448	0.618	3.64111E-22	9
A230050P20Rik	0.403332258	0.117	0.013	3.84094E-22	9
Mcm7.5	-0.717021823	0.151	0.394	4.25815E-22	9
Mmp2	0.479860836	0.114	0.014	4.65864E-22	9
Sox9.5	-0.809933322	0.056	0.259	5.02563E-22	9
Dek.8	-0.526023587	0.623	0.695	5.27434E-22	9
Fkbp3.4	-0.51542629	0.62	0.748	5.79353E-22	9

BC005764.8	-0.846675955	0.022	0.189	6.99193E-22	9
Rin2	0.50112643	0.129	0.017	8.91506E-22	9
Fam213a	0.652910158	0.263	0.084	9.71148E-22	9
Rab33a	0.682760309	0.19	0.048	1.44318E-21	9
Reep5.1	0.63177725	0.336	0.133	2.3659E-21	9
Ebf3.2	-0.746967406	0.027	0.207	2.58947E-21	9
Rap2a	0.689869341	0.353	0.165	2.76241E-21	9
Mt1.6	0.550022576	0.384	0.163	2.83617E-21	9
Nrxn1.8	0.483342478	0.572	0.312	4.5665E-21	9
Mab21l1.6	-0.756605585	0.041	0.229	7.82917E-21	9
Selk	0.524480393	0.628	0.39	1.08955E-20	9
Tle1.2	-0.721262569	0.056	0.229	1.12568E-20	9
Bcl11a.1	-0.670878757	0.034	0.214	1.83568E-20	9
Prmt8.4	-0.743987372	0.068	0.277	1.9266E-20	9
Rasl11a	0.59469683	0.131	0.027	2.14054E-20	9
Phldb1	0.583950558	0.192	0.047	2.23839E-20	9
Wasf1	0.608398831	0.294	0.105	2.92346E-20	9
Sox4.6	-0.679923332	0.389	0.587	3.68401E-20	9
Crip2.2	-0.755833733	0.051	0.244	4.61764E-20	9
Ezh2.7	-0.588170765	0.423	0.614	5.39678E-20	9
Psap.3	0.461053961	0.399	0.179	5.89902E-20	9
Abtb2	0.377298984	0.107	0.013	5.97826E-20	9
Lgi3	0.462953511	0.119	0.016	7.06436E-20	9
Hap1	0.531806579	0.158	0.032	7.49064E-20	9
Tmco3	0.425210084	0.161	0.032	7.61304E-20	9
Dhcr24	0.56573513	0.226	0.065	8.38671E-20	9
Tmem191c	0.456851348	0.144	0.025	1.11235E-19	9
Dynll1.2	0.454845354	0.691	0.591	1.13806E-19	9
Sstr2.5	-0.750957566	0.027	0.194	1.48801E-19	9
Rplp2.5	-0.383039338	0.8	0.841	1.54882E-19	9
Fnta	0.580474899	0.328	0.134	1.91405E-19	9
Ugdh	0.621592143	0.26	0.092	1.92601E-19	9
Vps37b.2	-0.670054343	0.251	0.475	2.09941E-19	9
Rab14	0.511076512	0.616	0.407	3.00439E-19	9
Ranbp1.5	-0.498159739	0.591	0.677	3.26631E-19	9
Spag9.1	0.560885446	0.526	0.301	4.36149E-19	9
Malat1.7	0.417152249	0.956	0.955	4.53308E-19	9
Dmd	0.514849509	0.136	0.023	4.54529E-19	9
Serbp1.5	-0.35174471	0.832	0.87	4.60416E-19	9
Ccnd1.6	0.544870177	0.659	0.518	5.48593E-19	9
Mllt4.2	-0.702737187	0.141	0.368	7.87893E-19	9
H2afy.4	-0.580722271	0.387	0.596	8.65616E-19	9
Rcor2.5	-0.74291742	0.051	0.229	8.82777E-19	9
Abcg1	0.40787541	0.127	0.022	8.84468E-19	9
Car11	0.441974658	0.122	0.018	1.00645E-18	9
Gnb2l1.3	-0.378362221	0.815	0.873	1.0817E-18	9
Ust	0.414286419	0.153	0.031	1.41391E-18	9
Ppt1	0.498311706	0.268	0.09	1.42905E-18	9

Fnbp1l.5	-0.712318413	0.241	0.458	1.5783E-18	9
Hk2.4	-0.636597507	0.019	0.176	1.73756E-18	9
Rps19.5	-0.446707011	0.655	0.756	2.32834E-18	9
Itm2c.5	0.545301162	0.431	0.206	2.36237E-18	9
Hey1.6	-0.791767808	0.085	0.277	2.40616E-18	9
Hirip3.7	-0.687166917	0.165	0.387	2.74376E-18	9
Fam63b.1	0.492813457	0.253	0.083	2.89596E-18	9
Snx22	0.487603543	0.185	0.045	2.91409E-18	9
Usp24	0.568247777	0.146	0.031	3.07096E-18	9
Eef2.1	-0.471543294	0.564	0.719	3.47581E-18	9
Slc17a6.8	-0.729749186	0.012	0.151	3.50141E-18	9
Map3k1	-0.694417511	0.158	0.375	3.6116E-18	9
Pqlc1.6	-0.696869447	0.097	0.302	4.30824E-18	9
Gnal	0.437775001	0.112	0.015	4.68857E-18	9
Atcay.2	0.535220922	0.217	0.062	4.85708E-18	9
Ddx5.1	-0.418081411	0.689	0.818	4.91533E-18	9
Dut.7	-0.702475339	0.217	0.44	5.34453E-18	9
Stxbp3a	0.43233708	0.117	0.018	6.61998E-18	9
Laptm4b.2	0.582311336	0.319	0.137	6.83686E-18	9
Gpm6a.8	0.331767982	0.635	0.403	7.40804E-18	9
Papss1	0.557343412	0.336	0.142	7.85773E-18	9
Taldo1	0.550700782	0.484	0.317	8.4884E-18	9
Abcd3	0.590851305	0.375	0.177	8.64014E-18	9
Irs2	0.52442014	0.209	0.06	1.25432E-17	9
Tubb2a.9	0.357754494	0.455	0.239	1.6254E-17	9
Fip1l1	0.557312671	0.509	0.303	1.65871E-17	9
Abracl.2	-0.635543714	0.1	0.306	1.72115E-17	9
Cald1.2	-0.650538348	0.348	0.54	1.72185E-17	9
Aplp2.4	0.541237787	0.54	0.317	1.79042E-17	9
Srrm4.7	-0.705429046	0.034	0.199	1.8217E-17	9
Dpysl3.9	0.385057846	0.418	0.214	2.31532E-17	9
Cxcl14	0.564397679	0.158	0.038	2.4993E-17	9
Myl12a.3	-0.682489481	0.151	0.332	2.54534E-17	9
Abhd2	0.53515595	0.136	0.029	2.56745E-17	9
Gng2.5	-0.599235015	0.173	0.401	2.84136E-17	9
Hjurp.6	-0.681234505	0.241	0.438	4.5903E-17	9
Zbtb18.5	-0.723082344	0.08	0.27	4.75065E-17	9
Nkd1.3	-0.659877846	0.068	0.252	6.55229E-17	9
Cbx5.6	-0.518668375	0.513	0.656	7.06436E-17	9
Abhd6	0.411367962	0.156	0.034	7.91538E-17	9
Acap3	0.486033894	0.178	0.044	8.84905E-17	9
Hmgcl	0.516563231	0.229	0.074	9.73363E-17	9
Itga9	0.486010228	0.109	0.017	1.16001E-16	9
Sept2	0.536985904	0.414	0.217	1.43938E-16	9
Lrrtm2.2	0.358319563	0.17	0.048	1.4942E-16	9
Scn2a1	0.361801769	0.102	0.015	1.7462E-16	9
Eef1b2.4	-0.469386205	0.584	0.736	1.89125E-16	9
Traf4	0.511361395	0.241	0.08	2.32148E-16	9

Tceal5.1	0.480142934	0.144	0.029	2.54832E-16	9
Txndc15	0.583946596	0.299	0.126	2.83043E-16	9
Atoh1.5	-0.669784123	0.024	0.167	3.3131E-16	9
Celf4.9	-0.869804308	0.146	0.324	3.38036E-16	9
Tanc2	0.41511638	0.102	0.014	4.41665E-16	9
Pcdhga9	0.484776284	0.606	0.415	4.85426E-16	9
Lcorl	0.611532739	0.248	0.092	5.14629E-16	9
Heg1.1	-0.628812947	0.022	0.16	5.2765E-16	9
Mki67.9	-0.745421393	0.2	0.399	7.33279E-16	9
Fzd2.2	-0.589045589	0.019	0.164	7.73621E-16	9
Arl6ip1.7	0.507722522	0.747	0.545	7.83312E-16	9
Reep1.2	0.418150311	0.231	0.078	8.05704E-16	9
Kcna1.1	0.312892148	0.107	0.025	9.52043E-16	9
Islr2.4	-0.629069129	0.029	0.183	9.95254E-16	9
Reep3	0.519701279	0.465	0.251	1.09984E-15	9
Ccng2.5	-0.649651283	0.054	0.215	1.13497E-15	9
Orai1	0.440454184	0.119	0.021	1.27698E-15	9
Map1b.7	-0.591328647	0.37	0.592	1.31766E-15	9
Maged1	0.473651862	0.62	0.425	1.49921E-15	9
Wdr1	0.525953458	0.238	0.084	3.09049E-15	9
Aatk	0.361012239	0.129	0.026	3.85875E-15	9
Rnmt.5	-0.675360817	0.168	0.352	4.26297E-15	9
Spred1	0.526647401	0.268	0.104	5.62731E-15	9
Rsu1	0.50937338	0.287	0.114	7.26861E-15	9
Thra.8	0.510778873	0.428	0.224	7.32504E-15	9
Anp32b.6	-0.498778261	0.457	0.594	8.04046E-15	9
Npm1.5	-0.448485701	0.53	0.649	1.02448E-14	9
Nr3c1.3	0.529766028	0.292	0.121	1.06262E-14	9
Rbms1.1	-0.594937966	0.054	0.22	1.12568E-14	9
Gaa	0.374966313	0.124	0.025	1.25723E-14	9
Ctnnd2.1	0.610690483	0.236	0.095	1.35029E-14	9
Lgals1.5	-0.75360932	0.066	0.232	1.37572E-14	9
Nipa1	0.381926492	0.127	0.025	1.42222E-14	9
Tmbim4	0.50348662	0.321	0.14	1.46698E-14	9
RP23-45G16.5.9	-0.616648179	0.131	0.331	1.54548E-14	9
Ptch2.5	-0.575685509	0.022	0.155	1.5977E-14	9
Hnrnpm.3	-0.418419818	0.625	0.737	1.62268E-14	9
Hnrnpd.5	-0.487525391	0.465	0.614	2.34564E-14	9
Mdk.5	-0.699329946	0.185	0.384	2.40416E-14	9
Ralb	0.375690446	0.105	0.017	2.89329E-14	9
Gde1	0.462591884	0.316	0.137	3.1929E-14	9
Atp6v0b.7	0.477702321	0.467	0.262	3.89112E-14	9
Fstl1.5	-0.643584519	0.039	0.183	4.41225E-14	9
Mfge8	0.446909954	0.139	0.031	4.77147E-14	9
Ndn.2	0.504564108	0.418	0.218	5.26479E-14	9
Srsf3.3	-0.434487113	0.521	0.66	6.70447E-14	9
Rpl32.3	-0.314549765	0.861	0.89	7.37381E-14	9
Serinc5	0.513261285	0.18	0.058	7.5937E-14	9

Paip2.1	-0.520706926	0.392	0.552	9.56897E-14	9
Zfand5.1	-0.620671649	0.243	0.419	9.84203E-14	9
Sept8.3	0.509628724	0.292	0.125	1.04699E-13	9
Zdbf2	-0.529767044	0.017	0.136	1.08921E-13	9
Top2a.8	-0.766296748	0.255	0.431	1.09497E-13	9
Snx25	0.43940359	0.107	0.02	1.22445E-13	9
Lima1.3	0.511113276	0.285	0.118	1.24596E-13	9
Boc.5	-0.57813808	0.024	0.159	1.49535E-13	9
Ypel3.4	-0.593266849	0.263	0.439	1.49683E-13	9
Cdkn1b.3	-0.556338924	0.282	0.471	1.66812E-13	9
Chd4.1	-0.423830339	0.647	0.772	1.72586E-13	9
Atp1b1	0.527782972	0.173	0.049	1.7532E-13	9
Tmpo.8	-0.592484005	0.263	0.456	1.80992E-13	9
Emc7	0.531985628	0.307	0.14	1.88845E-13	9
Glrb	0.455692331	0.148	0.037	2.47325E-13	9
Smc1a.3	-0.458218073	0.479	0.634	2.50517E-13	9
Celsr2.4	-0.659090951	0.083	0.241	2.51076E-13	9
Snrpf.4	-0.556392534	0.26	0.431	2.62092E-13	9
Pcna.9	-0.645807531	0.221	0.361	2.66788E-13	9
Spc24.7	-0.610167107	0.078	0.246	2.87569E-13	9
Rps21.4	-0.342828707	0.776	0.834	3.00758E-13	9
Chrna3.7	-0.615232762	0.01	0.12	3.21137E-13	9
Nckap1	0.526954845	0.292	0.127	3.28036E-13	9
Nrxn3.1	0.449402401	0.139	0.034	3.303E-13	9
Zic5.1	-0.580363765	0.027	0.163	3.53004E-13	9
Gse1.2	-0.620423622	0.073	0.24	3.54366E-13	9
Nap1l3	0.307702719	0.102	0.02	4.14596E-13	9
Hmgb3.3	-0.581930472	0.134	0.31	4.56751E-13	9
Osbpl1a.1	0.489051266	0.182	0.056	5.79918E-13	9
Gtl3	0.53422138	0.316	0.181	5.86806E-13	9
H3f3a	-0.565464691	0.207	0.385	6.24395E-13	9
Reln.6	-0.682978721	0.041	0.183	6.94913E-13	9
Nell2	0.554653919	0.265	0.129	7.79429E-13	9
BC034090.1	-0.528543482	0.012	0.13	7.9553E-13	9
Hmgn5.6	-0.637922391	0.253	0.428	8.04642E-13	9
Cuedc1	0.548170018	0.236	0.092	8.07707E-13	9
Ncl.5	-0.288804585	0.876	0.896	8.09616E-13	9
Stat3	0.422544749	0.151	0.039	9.11754E-13	9
Gm3764.2	0.54195978	0.287	0.127	9.45666E-13	9
Trp53i11.1	-0.605573516	0.056	0.204	1.07165E-12	9
Tex14.5	-0.700592916	0.024	0.148	1.20968E-12	9
Tcf7l2	0.32612188	0.114	0.023	1.3146E-12	9
Syt13.5	-0.500940219	0.012	0.129	1.34125E-12	9
Hdgf.5	-0.493159446	0.36	0.536	1.42404E-12	9
Sez6l	0.416463367	0.185	0.058	1.65435E-12	9
Frmd4b.2	-0.509842832	0.015	0.13	1.67418E-12	9
Selt	0.489031742	0.236	0.091	1.76624E-12	9
Rps11.3	-0.355328042	0.689	0.779	1.84076E-12	9

Tmem63b.2	0.408478609	0.214	0.077	2.5421E-12	9
Mfap4.4	-0.66066461	0.032	0.164	2.84021E-12	9
Ndrg2.3	0.511729401	0.355	0.18	3.09887E-12	9
8430419L09Rik	0.410302432	0.192	0.063	3.21566E-12	9
Prox1	-0.547898475	0.071	0.232	3.32624E-12	9
Dhx32.3	-0.593210466	0.063	0.203	3.62326E-12	9
Rrbp1.2	0.521922355	0.363	0.184	3.77274E-12	9
Arpc5.1	0.476819099	0.496	0.317	4.55096E-12	9
Efhd2.5	-0.523146164	0.015	0.132	4.58427E-12	9
Tuba1b.7	-0.511725653	0.348	0.484	4.62854E-12	9
Akap6.1	-0.608222434	0.071	0.222	4.74694E-12	9
Tmem30a.1	0.49060279	0.35	0.175	5.12764E-12	9
Sox18.2	-0.553530602	0.024	0.15	5.60147E-12	9
Slc35b2	0.482771863	0.353	0.177	5.75924E-12	9
Mcm6.6	-0.630973708	0.112	0.28	6.30216E-12	9
Lhfpl2	0.345541767	0.117	0.026	6.48306E-12	9
Dhrs7	0.475367389	0.197	0.068	6.61604E-12	9
Klhl13.1	0.522916148	0.241	0.1	7.16881E-12	9
Hdac2	-0.454475425	0.358	0.519	7.19963E-12	9
Dner.9	0.47970148	0.394	0.21	7.49332E-12	9
Tpm4.4	-0.581181256	0.146	0.321	7.59871E-12	9
Gpr153.4	-0.574087196	0.058	0.211	7.71314E-12	9
Cenpf.9	-0.577857108	0.197	0.366	7.91408E-12	9
Cttnbp2.1	0.488586897	0.219	0.08	7.95716E-12	9
Robo2.2	-0.574100187	0.027	0.154	8.02569E-12	9
Ppp2r2a	0.487292174	0.418	0.238	8.19281E-12	9
Abhd4	0.400214419	0.153	0.042	9.66899E-12	9
Rab10	0.434097185	0.47	0.288	9.87182E-12	9
Agrn	0.415077394	0.202	0.072	1.02603E-11	9
Btg2.3	-0.669433745	0.068	0.211	1.11067E-11	9
Zdhhc14	0.357458397	0.141	0.038	1.14987E-11	9
Coro2b	-0.566587878	0.044	0.176	1.16084E-11	9
Cyb5	0.478407724	0.36	0.183	1.21354E-11	9
Itgav	0.516728887	0.17	0.058	1.2297E-11	9
Chst2	0.455547127	0.119	0.03	1.2691E-11	9
Nova1.1	0.491176801	0.214	0.079	1.42744E-11	9
Zic2.2	-0.469936908	0.012	0.123	1.45013E-11	9
Lrig3.4	-0.517444614	0.036	0.173	1.53315E-11	9
Rest	0.386713425	0.105	0.02	1.80504E-11	9
Btbd17.4	-0.603189494	0.039	0.163	1.81945E-11	9
Gm11541.2	-0.50761551	0.017	0.13	1.85545E-11	9
Birc5.9	-0.507365394	0.114	0.277	1.91902E-11	9
Notch1	0.423230883	0.102	0.021	2.01721E-11	9
Snrpe.3	-0.462840041	0.418	0.565	2.1262E-11	9
Tsn	-0.462669688	0.414	0.593	2.26983E-11	9
Sel1l	0.392781497	0.209	0.079	2.43411E-11	9
Aplp1.9	0.49158036	0.314	0.147	2.76723E-11	9
Myod1.6	-0.572887843	0.024	0.144	2.83238E-11	9

Atxn7l3b.2	0.324050537	0.818	0.669	2.98674E-11	9
Tmem59.1	0.417476107	0.579	0.387	3.14057E-11	9
Wbp5.3	-0.497260468	0.353	0.522	3.2014E-11	9
Fubp1.1	-0.46880959	0.416	0.57	3.48757E-11	9
Atp1b2	0.414055833	0.153	0.045	3.63238E-11	9
Pigyl	0.437202543	0.304	0.14	3.97559E-11	9
Hn1.5	-0.487410451	0.392	0.545	4.16548E-11	9
Tgif1.3	-0.407392945	0.005	0.101	4.54228E-11	9
Siva1.5	-0.572376194	0.114	0.276	4.95241E-11	9
Dtna	0.350401145	0.122	0.029	4.96307E-11	9
Mdga1.1	-0.505193178	0.022	0.138	5.01663E-11	9
Neurod6.1	-0.599193309	0.046	0.175	5.40356E-11	9
Sec62	0.386887553	0.594	0.401	5.62119E-11	9
Htatsf1	-0.527605752	0.353	0.501	6.13542E-11	9
Hprt.3	-0.51912936	0.066	0.19	6.44001E-11	9
Ube2b	-0.486676499	0.358	0.524	7.12246E-11	9
Snx3	0.433871428	0.518	0.338	7.39831E-11	9
Nmnat2	0.433837245	0.122	0.029	7.49355E-11	9
Fam19a5	0.366034803	0.107	0.022	8.10833E-11	9
Pak4	0.437535416	0.127	0.036	8.54262E-11	9
Rabgap1l.1	0.43433874	0.178	0.059	8.77797E-11	9
Acsl3	0.517152318	0.277	0.143	8.83807E-11	9
Elavl2.3	-0.602585543	0.109	0.274	9.09356E-11	9
Tmem107.2	-0.456266292	0.017	0.129	9.3917E-11	9
Gm11266.5	-0.589168962	0.027	0.146	9.44421E-11	9
Baz2b.2	-0.564443158	0.182	0.369	9.57923E-11	9
Tpx2.9	-0.550444158	0.124	0.285	9.62993E-11	9
Bpgm	0.379723085	0.19	0.066	9.7029E-11	9
Nedd4	-0.369856602	0.572	0.686	1.08072E-10	9
Ccdc134	0.364006933	0.124	0.03	1.10025E-10	9
Gm17322.6	-0.575430376	0.019	0.133	1.21685E-10	9
Serpinh1.4	-0.515420768	0.015	0.117	1.28472E-10	9
Incenp.8	-0.631672607	0.117	0.278	1.31304E-10	9
Phip	-0.52468789	0.277	0.463	1.6748E-10	9
Gpc2.4	-0.541531858	0.029	0.142	1.71287E-10	9
Ccdc107	0.451622766	0.187	0.068	1.96754E-10	9
Ier3	0.419797375	0.127	0.032	2.30274E-10	9
Cdk4.2	-0.382628628	0.526	0.644	2.3777E-10	9
Specc1	0.437466963	0.153	0.046	2.41336E-10	9
Cdk1.8	-0.541311387	0.092	0.25	2.56618E-10	9
Prex1	0.457469672	0.175	0.06	2.57975E-10	9
Sept3.9	-0.607788567	0.265	0.395	2.59839E-10	9
B2m.4	0.397583556	0.399	0.228	2.65554E-10	9
Mtus1	-0.491801607	0.019	0.129	3.0101E-10	9
Ccna2.8	-0.560871745	0.075	0.225	3.37058E-10	9
Nlgn1	0.411797501	0.105	0.022	3.50755E-10	9
Tox3.1	-0.534419834	0.173	0.349	3.57697E-10	9
Hmgn2.6	-0.553620599	0.107	0.259	3.65373E-10	9

Sar1b	0.46992871	0.36	0.194	3.74479E-10	9
Cdc37l1	0.498246811	0.248	0.11	3.91143E-10	9
Gm10075.6	-0.453463726	0.392	0.517	3.92241E-10	9
Fam69b	0.356713968	0.144	0.041	4.50547E-10	9
Cdk14.1	0.342677708	0.231	0.1	4.53813E-10	9
Rpl8.4	-0.267514195	0.881	0.897	4.55689E-10	9
Sema7a.4	-0.507636879	0.034	0.158	5.41519E-10	9
Fam53b	-0.499074319	0.029	0.138	5.46897E-10	9
Ccnd3	0.474509837	0.195	0.079	5.65864E-10	9
Foxp1	-0.527338566	0.044	0.175	5.88472E-10	9
Apex1.3	-0.46625271	0.277	0.455	6.3863E-10	9
Baz1b.2	-0.4953437	0.328	0.496	6.41135E-10	9
Pbk.7	-0.527081185	0.075	0.225	6.43279E-10	9
Map1a.1	0.454805728	0.192	0.07	7.48097E-10	9
Abat	0.251158246	0.109	0.028	8.04146E-10	9
Angptl2.4	-0.444482487	0.015	0.118	8.13961E-10	9
Smco4.5	-0.483426711	0.039	0.164	8.87493E-10	9
Tspyl4.6	0.350623508	0.268	0.123	9.7038E-10	9
Ctsl.2	0.399771752	0.438	0.255	9.74436E-10	9
Bok.5	-0.531303174	0.1	0.254	1.11971E-09	9
Timp3.2	0.35485928	0.195	0.076	1.12507E-09	9
Snx18	0.351873706	0.136	0.038	1.21735E-09	9
Kif3a	0.460958211	0.477	0.313	1.22093E-09	9
Rdh5	-0.376718717	0.022	0.128	1.22502E-09	9
Aldh9a1	0.421767111	0.163	0.053	1.25772E-09	9
Ssrp1.4	-0.409636605	0.465	0.578	1.38477E-09	9
Tmem50b	0.260799393	0.109	0.027	1.44188E-09	9
Basp1.8	-0.440964764	0.577	0.707	1.44816E-09	9
Mtf2	-0.50054172	0.2	0.36	1.57164E-09	9
Banf1.6	-0.327360596	0.596	0.696	1.62732E-09	9
Prc1.8	-0.629050826	0.107	0.26	1.67927E-09	9
Prnp.5	0.426400881	0.375	0.202	1.68715E-09	9
Lmnb1.8	-0.525375808	0.144	0.303	1.7819E-09	9
Dpy19l1	0.438282666	0.178	0.063	1.78557E-09	9
Rab11a	0.422758667	0.445	0.273	1.78814E-09	9
Ina.8	-0.538555281	0.304	0.446	1.9022E-09	9
Ube2e3.1	-0.493460309	0.253	0.42	1.91463E-09	9
Slc25a4	0.28298017	0.878	0.805	2.01322E-09	9
Adcyap1r1.1	0.411341099	0.204	0.08	2.12451E-09	9
Rassf3.2	-0.483738203	0.027	0.14	2.15783E-09	9
C530008M17Rik.2	-0.543206485	0.114	0.271	2.34467E-09	9
Mroh2a.5	-0.57342437	0.017	0.115	2.41972E-09	9
Bcap29	0.455081701	0.255	0.119	2.67373E-09	9
Ifitm2.3	-0.407279157	0.015	0.116	2.71379E-09	9
Fam168a.2	0.371808795	0.462	0.283	3.08108E-09	9
Hells.9	-0.59594836	0.075	0.216	3.09761E-09	9
Smyd2	0.473052302	0.221	0.097	3.11963E-09	9
Mrps7	0.473989766	0.282	0.148	3.19264E-09	9

Spc25.8	-0.585351143	0.083	0.228	3.46607E-09	9
Lta4h.1	-0.526970754	0.114	0.245	3.6525E-09	9
Atp5j	-0.346793848	0.603	0.725	3.74033E-09	9
Rpl35a.4	-0.417859402	0.431	0.573	3.77142E-09	9
Sgcb	0.450812852	0.248	0.112	3.9917E-09	9
Tubb4a.2	0.466220091	0.178	0.064	4.13331E-09	9
Atraid	0.437617418	0.389	0.222	4.68868E-09	9
Ccdc88a.1	0.40432887	0.633	0.478	5.14549E-09	9
Map4k4	-0.501557748	0.28	0.453	5.32886E-09	9
Cdca7.5	-0.495656797	0.063	0.201	5.52849E-09	9
Ccdc34.8	-0.520912033	0.263	0.418	6.31044E-09	9
Ilf2	-0.430330965	0.409	0.544	6.44575E-09	9
Hipk2	0.309510233	0.141	0.045	6.51472E-09	9
Elf1	0.355417627	0.156	0.051	6.86125E-09	9
Ier2.4	-0.579870844	0.221	0.385	7.12144E-09	9
Lmo1	0.396495651	0.127	0.037	7.19202E-09	9
Ppp2r3a.2	0.467209767	0.219	0.091	7.75956E-09	9
Tk1.7	-0.437058895	0.024	0.133	8.9877E-09	9
Acbd5	0.489804078	0.238	0.106	9.24302E-09	9
Pdia6.3	0.401559982	0.494	0.336	9.28233E-09	9
Carhsp1.2	-0.535192154	0.109	0.257	9.35698E-09	9
Dad1.1	0.414081701	0.504	0.339	9.47915E-09	9
Fkbp15	0.431432649	0.107	0.032	9.73436E-09	9
Cyp51	0.456885433	0.282	0.137	9.82946E-09	9
Hnrnpa0	-0.486805761	0.299	0.46	1.07786E-08	9
Idh2.5	-0.523609191	0.134	0.282	1.08832E-08	9
Spcs2.1	0.392690926	0.564	0.42	1.09497E-08	9
H1fx.7	-0.50182979	0.102	0.248	1.10246E-08	9
Ebpl	0.313228592	0.207	0.086	1.10247E-08	9
Snrpg.2	-0.494606661	0.178	0.338	1.10714E-08	9
Cltb.5	-0.480499985	0.336	0.425	1.12047E-08	9
H2afz.7	-0.464432228	0.088	0.234	1.12259E-08	9
Sept11.1	-0.507241737	0.168	0.325	1.16794E-08	9
Tspan12	0.340200317	0.114	0.03	1.29537E-08	9
Ick	0.464858059	0.282	0.143	1.35161E-08	9
Pcmtd1.1	0.38063356	0.263	0.122	1.39805E-08	9
Cnpy1.4	-0.510142803	0.029	0.135	1.40257E-08	9
2310022B05Rik.2	-0.506715951	0.102	0.251	1.44998E-08	9
Ypel1	-0.46326559	0.051	0.177	1.51342E-08	9
Dhcr7	0.364981351	0.151	0.049	1.617E-08	9
Elavl3.7	-0.498435137	0.353	0.475	1.73675E-08	9
Myt1.8	0.359812303	0.294	0.149	1.74321E-08	9
Asah1	0.385993663	0.17	0.06	1.84826E-08	9
Sdf2l1	0.397813487	0.148	0.049	1.96433E-08	9
Cenph.8	-0.509796213	0.063	0.196	1.97462E-08	9
4833439L19Rik	0.387170386	0.299	0.151	2.00032E-08	9
Itgb1.2	0.395732943	0.53	0.358	2.01007E-08	9
Magee1	0.418175476	0.144	0.046	2.07379E-08	9

Pdia3	0.424762547	0.45	0.292	2.09697E-08	9
Rrm2.8	-0.47194122	0.073	0.207	2.10335E-08	9
E2f1.3	-0.490018366	0.036	0.149	2.17044E-08	9
Rbfox2.6	-0.538635021	0.131	0.286	2.23718E-08	9
Igsf21.4	0.294488939	0.197	0.086	2.24007E-08	9
Camta1.1	-0.468520267	0.243	0.412	2.36456E-08	9
Ptms.4	-0.46956747	0.341	0.476	2.52973E-08	9
Cdc42se1.1	0.417902862	0.302	0.162	2.58173E-08	9
Pcyt1b	0.451279773	0.148	0.049	2.61777E-08	9
Os9.4	0.450203582	0.406	0.247	2.83366E-08	9
Tmem9	0.413352371	0.236	0.106	2.86642E-08	9
Zeb2.1	0.458535997	0.367	0.246	2.9523E-08	9
Snx1.1	0.442317307	0.307	0.165	2.97925E-08	9
Socs2.1	-0.489486548	0.032	0.141	3.54892E-08	9
Kif23.9	-0.542936096	0.078	0.217	3.54942E-08	9
Fndc4	0.45341021	0.248	0.122	3.61193E-08	9
Cask.2	0.446273355	0.238	0.106	3.69918E-08	9
Pbrm1	-0.452157358	0.377	0.534	3.9345E-08	9
Tmcc3	0.419691108	0.207	0.088	4.03438E-08	9
Dusp6.1	0.467175345	0.192	0.09	4.13543E-08	9
Cyld	0.442986431	0.139	0.047	4.22189E-08	9
Tyms.7	-0.541336124	0.092	0.222	4.44654E-08	9
Plxnb2.2	-0.49945445	0.054	0.173	4.50259E-08	9
Pgm2.1	0.319123424	0.114	0.031	4.93327E-08	9
Cdca8.9	-0.379827126	0.122	0.267	4.96417E-08	9
Elovl1	0.318442836	0.117	0.033	5.10975E-08	9
Cdca2.7	-0.332333598	0.029	0.13	5.13495E-08	9
Iffo1	0.308921817	0.136	0.043	5.13691E-08	9
Tppp3	-0.464155244	0.044	0.16	5.28874E-08	9
Unc50	0.388628009	0.26	0.122	5.62568E-08	9
Igsf3.2	-0.48588418	0.034	0.135	5.65746E-08	9
Mcm3.5	-0.517226171	0.058	0.182	5.71487E-08	9
Jam3.3	0.397913103	0.273	0.131	5.77865E-08	9
Syncrip.2	-0.444848837	0.348	0.499	5.78483E-08	9
Ncapg.7	-0.507541541	0.061	0.186	5.84683E-08	9
Cklf.5	-0.47107696	0.051	0.167	5.86032E-08	9
Ostm1	0.302580661	0.139	0.045	6.02229E-08	9
Tgfb2.2	-0.54208836	0.066	0.194	6.08409E-08	9
Cited2.1	-0.485081221	0.044	0.155	6.08971E-08	9
Mpc2	0.426366921	0.448	0.293	6.6072E-08	9
Snrpb.4	-0.383428797	0.45	0.576	7.2035E-08	9
Fam69a	0.358208971	0.109	0.029	7.20781E-08	9
Ccdc18.4	-0.318231848	0.027	0.117	7.37258E-08	9
Dcakd.1	-0.488717831	0.153	0.299	7.41174E-08	9
Snx27	0.397280586	0.297	0.15	7.72556E-08	9
Smdt1	0.368376074	0.516	0.351	7.92212E-08	9
Sacs.1	-0.515438244	0.068	0.193	8.20867E-08	9
Racgap1.9	-0.463945289	0.109	0.258	8.45117E-08	9

A930011O12Rik.8	-0.564131519	0.027	0.116	8.67753E-08	9
Sult4a1	-0.505527167	0.092	0.225	9.17902E-08	9
Map7d2	0.411154537	0.224	0.097	9.95871E-08	9
Rnaseh2c.4	-0.455465602	0.231	0.384	1.00516E-07	9
Adrbk2.2	-0.437980083	0.015	0.102	1.01879E-07	9
Nedd4l.2	-0.468404266	0.039	0.13	1.07798E-07	9
2510003E04Rik	0.456890278	0.229	0.104	1.11628E-07	9
Psip1.2	-0.341859233	0.584	0.69	1.12016E-07	9
Fam171b.1	0.458848827	0.363	0.22	1.1954E-07	9
Ccdc28b.1	0.438647559	0.299	0.159	1.22148E-07	9
Rasa3	0.301408668	0.122	0.037	1.30277E-07	9
Pdap1.1	-0.344639786	0.572	0.685	1.50252E-07	9
Tacc2.5	0.443853485	0.273	0.137	1.50915E-07	9
Clcn3.1	0.394221788	0.433	0.269	1.54848E-07	9
Csrp2.6	-0.456235346	0.046	0.16	1.6039E-07	9
Fktn	0.399228226	0.238	0.109	1.60643E-07	9
Sv2b.3	-0.431918502	0.015	0.104	1.6209E-07	9
Clspn.8	-0.53608598	0.066	0.192	1.62715E-07	9
Dtl.5	-0.469341261	0.039	0.148	1.72387E-07	9
2810055G20Rik.1	-0.501451318	0.056	0.173	1.81583E-07	9
Ube2c.7	-0.623340974	0.131	0.272	1.84641E-07	9
Mab21l2.1	-0.445807242	0.015	0.106	1.87652E-07	9
Rad51ap1.9	-0.426467184	0.051	0.169	1.96186E-07	9
Tcf12	0.357284777	0.418	0.251	2.00024E-07	9
Rps15.3	-0.281976895	0.74	0.789	2.08116E-07	9
Dcx.8	-0.522571431	0.234	0.379	2.17625E-07	9
Smarca1.1	0.322010585	0.148	0.054	2.18553E-07	9
Chd3.8	0.473717746	0.275	0.146	2.42078E-07	9
Cenpe.8	-0.613020781	0.139	0.289	2.5153E-07	9
Tmeff2	0.42483461	0.163	0.063	2.52264E-07	9
Snhg5	-0.488425827	0.236	0.384	2.56785E-07	9
Mxd3.7	-0.410822745	0.027	0.129	2.70773E-07	9
Kif11.8	-0.29241546	0.09	0.208	2.73787E-07	9
St18.8	-0.594332569	0.095	0.192	2.86988E-07	9
Slc6a6	0.345645653	0.131	0.042	3.02906E-07	9
Arhgef2.4	-0.523668154	0.148	0.285	3.20066E-07	9
Sc4mol	0.437116873	0.202	0.089	3.37241E-07	9
Comt	0.410181748	0.231	0.106	3.4219E-07	9
Atad2.7	-0.489608306	0.075	0.201	3.45521E-07	9
Fbxo8	0.338971426	0.131	0.041	3.49179E-07	9
Gphn.2	0.342903313	0.275	0.137	3.55896E-07	9
Pdzrn3.6	-0.607575737	0.041	0.149	3.69476E-07	9
Otud7b	0.365667444	0.153	0.054	3.72099E-07	9
Ccm2.4	-0.451565213	0.097	0.227	4.14902E-07	9
Rpl14.4	-0.287170429	0.652	0.698	4.55467E-07	9
Btg1	-0.464686746	0.063	0.186	5.24345E-07	9
Lss	0.291572423	0.136	0.047	5.43991E-07	9
Setd8	-0.489671411	0.127	0.264	5.74936E-07	9

Tceal6	0.30933976	0.136	0.046	5.76489E-07	9
Tmem66.2	0.387090376	0.348	0.195	6.10017E-07	9
Gli1.5	-0.341849926	0.019	0.109	6.51448E-07	9
D17Wsu104e	0.38028246	0.367	0.212	6.72378E-07	9
Cers2	0.406977831	0.234	0.111	7.16238E-07	9
Kif1a.4	0.371168785	0.314	0.168	7.19995E-07	9
Tmem5	0.369644146	0.19	0.079	7.24106E-07	9
Evi5	0.454747667	0.26	0.139	7.33856E-07	9
Efcab14	0.378348815	0.185	0.075	7.40922E-07	9
Twf1	0.355803016	0.268	0.133	8.24584E-07	9
Mmp14.4	-0.442021274	0.058	0.177	8.60986E-07	9
BC029722	0.336686497	0.114	0.033	8.92614E-07	9
Rpl39.4	-0.323608624	0.574	0.647	9.16266E-07	9
Esco2.8	-0.551635187	0.08	0.197	9.16754E-07	9
Cdca3.9	-0.357810401	0.095	0.224	9.91359E-07	9
Serinc1.6	0.325783884	0.599	0.432	1.01763E-06	9
Myh10	-0.490685017	0.18	0.327	1.05392E-06	9
Tbc1d12	0.293520812	0.136	0.046	1.10283E-06	9
Pnrc1.1	-0.464724677	0.134	0.273	1.15954E-06	9
Ccp110.3	-0.485561718	0.117	0.251	1.21247E-06	9
Pop5	0.375898989	0.217	0.097	1.27136E-06	9
Pex2	0.385800543	0.217	0.097	1.31382E-06	9
Slc35a5	0.256297311	0.102	0.029	1.3497E-06	9
Psma7.1	-0.307285631	0.686	0.784	1.35162E-06	9
Frmd4a.3	-0.290092377	0.17	0.312	1.44943E-06	9
Hnrnph1	-0.360314601	0.501	0.631	1.52986E-06	9
Nusap1.8	-0.556902518	0.1	0.23	1.54165E-06	9
Marcksl1.3	-0.324324581	0.63	0.724	1.5711E-06	9
Tead1.2	-0.424176619	0.058	0.176	1.57984E-06	9
Atp9a.1	0.323757161	0.158	0.066	1.60991E-06	9
Vps28	0.37881275	0.397	0.24	1.68284E-06	9
Agpat4.2	0.371825011	0.238	0.114	1.68769E-06	9
Samd8	0.369950388	0.185	0.077	1.688E-06	9
Clybl.3	-0.394325595	0.024	0.118	1.80074E-06	9
Tbc1d16	-0.476096118	0.078	0.199	1.81977E-06	9
Rab8b	0.321177161	0.139	0.048	1.82799E-06	9
Stk39	0.299388053	0.122	0.041	1.8473E-06	9
Snrpb2.1	-0.444307794	0.238	0.387	1.8638E-06	9
Ncaph.7	-0.308759226	0.029	0.123	1.90178E-06	9
Snca.1	0.27022954	0.146	0.057	2.00588E-06	9
Uhrf1.7	-0.385421585	0.041	0.146	2.02235E-06	9
Cacnb4	0.347693666	0.109	0.032	2.02333E-06	9
Rap1gds1	0.276641855	0.131	0.044	2.0482E-06	9
Rps25.5	-0.415858067	0.355	0.482	2.16188E-06	9
Chchd2.1	-0.32303076	0.557	0.655	2.19907E-06	9
Kif15.8	-0.4449328	0.063	0.183	2.20794E-06	9
Nap1l1.5	-0.37141333	0.394	0.513	2.21872E-06	9
P4ha1	0.338719298	0.156	0.059	2.33946E-06	9

Itm2b.7	0.279021462	0.764	0.639	2.38395E-06	9
Rps15a.3	-0.323012807	0.577	0.659	2.50306E-06	9
Chaf1a.6	-0.40663258	0.056	0.171	2.56799E-06	9
Ccdc47	0.380681089	0.394	0.241	2.62891E-06	9
Jun.3	-0.439017148	0.54	0.657	2.96398E-06	9
Blvra	0.298086042	0.117	0.036	3.02552E-06	9
Lig1.9	-0.51459963	0.207	0.351	3.14506E-06	9
Gabbr1.4	0.430742071	0.263	0.137	3.32909E-06	9
Nt5dc2.3	-0.485681376	0.112	0.24	3.43215E-06	9
Mllt3.1	-0.480890142	0.131	0.228	3.4941E-06	9
Ap2a1	0.296351425	0.102	0.028	3.55016E-06	9
Dhx9	-0.394040161	0.333	0.466	3.55572E-06	9
Itsn1.8	-0.537506166	0.129	0.238	3.56534E-06	9
Sdc2	0.371029482	0.144	0.052	3.61683E-06	9
H2afx.8	-0.545443634	0.217	0.333	3.88179E-06	9
Tcp11l2	0.277764397	0.127	0.045	4.02337E-06	9
Cdc20.8	-0.464724225	0.078	0.199	4.03221E-06	9
Rpl26.3	-0.318169936	0.603	0.704	4.17575E-06	9
Msi1	-0.38412083	0.029	0.112	4.49469E-06	9
Vamp3	0.332445631	0.182	0.076	4.67454E-06	9
Usp1.8	-0.414136344	0.195	0.342	4.67618E-06	9
Sep15.3	0.323596373	0.65	0.501	4.76899E-06	9
Hpcal1.5	-0.455618496	0.024	0.108	5.05696E-06	9
Tmed5	0.394362152	0.236	0.123	5.23789E-06	9
Tmem147	0.397701342	0.394	0.258	5.38752E-06	9
Vldlr	0.339050297	0.153	0.058	5.82254E-06	9
Rufy3.7	-0.457966102	0.263	0.374	6.33246E-06	9
Arhgef9.1	0.399226697	0.195	0.086	6.47782E-06	9
Dirc2.1	0.342277198	0.204	0.092	6.68057E-06	9
Ssbp3.3	-0.474557837	0.068	0.169	7.64949E-06	9
Dock7	-0.406964176	0.027	0.116	7.97171E-06	9
Ltbp3.1	-0.400527486	0.032	0.12	8.49258E-06	9
Pou3f2.5	-0.457285117	0.141	0.279	8.60218E-06	9
Fam155a	0.436143874	0.221	0.113	8.69092E-06	9
Epb4.1.2	-0.446370245	0.102	0.232	9.31167E-06	9
Rad21.9	-0.359188689	0.358	0.464	9.662E-06	9
Eif4e3.3	-0.444183192	0.036	0.133	9.77332E-06	9
H2-D1.1	0.292023854	0.246	0.127	9.93864E-06	9
Casp3.1	-0.458727568	0.122	0.236	1.05244E-05	9
March1.1	0.304820094	0.122	0.041	1.11683E-05	9
Atp6v0e2.3	0.358379163	0.404	0.256	1.13142E-05	9
Dll3.1	0.402361374	0.158	0.063	1.20553E-05	9
Rbm8a.2	-0.395017748	0.336	0.468	1.21604E-05	9
Dnajc9.7	-0.439650926	0.204	0.353	1.22637E-05	9
Ppp1r18	0.28758619	0.153	0.061	1.24975E-05	9
Tshz2.3	-0.436868241	0.178	0.323	1.30812E-05	9
Cdk5r1.7	-0.505517361	0.207	0.336	1.32808E-05	9
Astn1	0.322194109	0.114	0.036	1.33764E-05	9

Tmem246	0.359807411	0.182	0.079	1.40732E-05	9
Rnf165.3	-0.445168582	0.066	0.175	1.435E-05	9
Trp53.3	-0.42587611	0.161	0.301	1.46032E-05	9
Ncan.4	0.362859061	0.207	0.096	1.57385E-05	9
Dctpp1.5	-0.428835957	0.195	0.307	1.65E-05	9
Lactb	0.306604818	0.107	0.032	1.65457E-05	9
Hadha	0.38310578	0.28	0.152	1.71152E-05	9
Etfb.2	0.353679894	0.319	0.181	1.7868E-05	9
Trim59.7	-0.391752216	0.061	0.172	1.80838E-05	9
Glud1	0.394329044	0.26	0.138	1.86608E-05	9
Ndfip1	0.35086059	0.178	0.077	1.87676E-05	9
Mrpl34	-0.448565047	0.182	0.29	1.92744E-05	9
Setbp1.1	-0.451343925	0.058	0.162	1.98238E-05	9
Sept4.7	-0.369297958	0.107	0.229	1.99732E-05	9
Ybx3.4	-0.406270422	0.163	0.293	2.18662E-05	9
Arl8a.2	0.296435107	0.2	0.093	2.30634E-05	9
Mllt11.7	-0.462588361	0.148	0.219	2.76497E-05	9
Celsr3.1	-0.411001341	0.034	0.125	2.80454E-05	9
Ccdc41.5	-0.428541112	0.129	0.256	2.87319E-05	9
Klhdc2	-0.406658686	0.19	0.33	3.10938E-05	9
Dbt	0.359557028	0.195	0.091	3.14809E-05	9
Ctsz.1	-0.473837367	0.044	0.139	3.15868E-05	9
Hmgb1.6	-0.396754572	0.229	0.361	3.24907E-05	9
Dusp1.1	-0.472316027	0.061	0.166	3.25534E-05	9
Mid1ip1	0.291789821	0.134	0.049	3.57365E-05	9
Hsp90aa1.1	-0.318985748	0.518	0.619	3.66648E-05	9
2700089E24Rik.1	0.352800331	0.331	0.195	3.99051E-05	9
Sptssa	0.371296068	0.382	0.243	3.99479E-05	9
Capns1	0.303996251	0.367	0.224	4.18439E-05	9
Fam173a	0.35465672	0.275	0.15	4.23533E-05	9
Casc4	0.360065863	0.27	0.144	4.34952E-05	9
Rhbdd2	0.334659718	0.148	0.058	4.489E-05	9
Lgalsl	0.359767774	0.195	0.09	4.67631E-05	9
Mvk	0.329669555	0.151	0.06	4.78092E-05	9
Jagn1	0.346143799	0.258	0.136	4.84801E-05	9
Nuf2.9	-0.369685491	0.044	0.143	5.07328E-05	9
Lmf1	0.283573666	0.102	0.032	5.13997E-05	9
Gabarapl1.3	0.368649184	0.348	0.213	5.17061E-05	9
Tex30.2	-0.326507063	0.041	0.137	5.19499E-05	9
Ctnna1.1	-0.404052966	0.056	0.155	5.30124E-05	9
Man2a2	0.308136655	0.122	0.042	5.37113E-05	9
Lap3.6	-0.467723118	0.151	0.279	5.38742E-05	9
Clvs1.6	-0.450232162	0.046	0.131	5.60362E-05	9
Zfp462.2	0.384184189	0.277	0.153	5.77765E-05	9
Rap2b	0.379944705	0.265	0.145	6.10279E-05	9
Nceh1	0.297913803	0.105	0.033	6.13012E-05	9
Eif3f.2	-0.314910216	0.526	0.633	6.41942E-05	9
Hmmr.8	-0.396706919	0.071	0.179	6.67673E-05	9

Sgol1.7	-0.379617961	0.036	0.129	6.67817E-05	9
Kif20b.8	-0.489002607	0.08	0.193	6.74829E-05	9
Prim1.6	-0.450538347	0.139	0.265	6.85116E-05	9
Map4k5	0.303370592	0.151	0.06	7.7113E-05	9
Mcm5.5	-0.381708218	0.066	0.17	7.88585E-05	9
Smpd3.3	-0.453589906	0.061	0.163	8.05081E-05	9
Ttc3.5	-0.270824415	0.779	0.83	8.0947E-05	9
Strbp.4	-0.43668352	0.236	0.349	8.44788E-05	9
Ctcf.2	-0.380196767	0.363	0.499	8.67787E-05	9
Mad2l1.8	-0.34190395	0.034	0.125	9.17229E-05	9
Camsap2.1	0.373583052	0.248	0.13	9.73139E-05	9
Casc5.8	-0.458757657	0.078	0.188	0.000100892	9
Ppm1l.1	0.294514002	0.195	0.092	0.000101273	9
Podxl2.8	-0.486150932	0.119	0.229	0.000101874	9
Usp16	0.386772017	0.375	0.237	0.000105611	9
Cox7a2	-0.272253724	0.65	0.714	0.000106403	9
Rph3a	0.313160428	0.107	0.037	0.000117735	9
Slc48a1	0.374603762	0.144	0.058	0.000118316	9
Abi2.1	0.369931018	0.307	0.179	0.000126162	9
Ncapd2.6	-0.364670056	0.08	0.187	0.000128172	9
Gpsm1	-0.432429324	0.073	0.178	0.000134546	9
Ddost	0.350249559	0.399	0.266	0.000140383	9
Nucks1.8	-0.266413225	0.601	0.664	0.000145012	9
Prpf40a	-0.352002794	0.467	0.573	0.000148029	9
Parp6.3	-0.41841219	0.044	0.127	0.000152646	9
Tcerg1	-0.390255372	0.338	0.459	0.00015714	9
Magoh.2	-0.402540841	0.214	0.335	0.000162914	9
Desi1	0.384375858	0.187	0.091	0.000168021	9
Grik5.1	0.333368596	0.217	0.107	0.000176071	9
Ifnar2	0.293395025	0.129	0.048	0.000184699	9
1700001O22Rik.3	-0.347258413	0.029	0.112	0.000187619	9
Glce.5	-0.459358615	0.075	0.179	0.00019399	9
Mum1	-0.411354566	0.071	0.171	0.00019973	9
Src	0.316832298	0.102	0.033	0.000201572	9
Kmt2e.5	-0.350737256	0.47	0.57	0.000202099	9
Ccnb2.8	-0.370751301	0.083	0.189	0.000206065	9
Stard3nl	0.291877411	0.224	0.114	0.000209513	9
Ank3.8	-0.374762076	0.358	0.421	0.000209747	9
Ndc80.7	-0.370053918	0.032	0.116	0.000212451	9
Pcdha2.5	0.264085073	0.221	0.115	0.000214602	9
G6pc3	0.272882912	0.112	0.038	0.000220793	9
Slc50a1	0.331540507	0.178	0.08	0.000229036	9
Sri.2	0.283704439	0.285	0.163	0.00023566	9
Gins2.4	-0.352464942	0.078	0.187	0.000236803	9
Ankrd46.1	0.374240834	0.275	0.157	0.000240747	9
Nop58.5	-0.311655242	0.53	0.593	0.000244853	9
Sqle.2	0.297013066	0.229	0.118	0.000248244	9
Dtymk.6	-0.391840426	0.326	0.441	0.000250471	9

Rpl34.3	-0.301862182	0.564	0.654	0.000257297	9
Kif22.8	-0.39207854	0.063	0.165	0.000271592	9
Impad1	0.36043458	0.348	0.22	0.000271612	9
Fam13c	0.355048429	0.144	0.06	0.000275435	9
Epb4.1l3	0.388745893	0.209	0.111	0.000277175	9
Srrt	-0.391829357	0.314	0.426	0.000277194	9
Wls	0.335764395	0.18	0.085	0.000288499	9
Tprn.3	-0.452884351	0.119	0.229	0.000290715	9
Ankrd11	0.341228558	0.586	0.47	0.000301613	9
Pmf1.6	-0.373246868	0.054	0.145	0.000302889	9
Atxn7l2.1	-0.365654106	0.024	0.104	0.000317009	9
Ufl1	0.361399464	0.209	0.104	0.000317388	9
Epdr1	0.322589654	0.141	0.057	0.000322288	9
Gnai1	0.389476263	0.192	0.097	0.000331418	9
Phf21b.1	-0.378775691	0.041	0.13	0.000332919	9
Prps1	-0.364712308	0.051	0.144	0.00033588	9
Atrx.1	-0.296079139	0.64	0.742	0.000350967	9
Rab6a.4	0.339653662	0.465	0.324	0.000351734	9
Atp8a1	0.304072643	0.163	0.071	0.000352686	9
Hist1h2ak.7	-0.425331627	0.061	0.145	0.000354415	9
Sh3bgrl.2	-0.389949893	0.207	0.34	0.000366568	9
Ankrd32	-0.437077921	0.085	0.185	0.000375269	9
Tmem106b	0.313389583	0.161	0.07	0.000377517	9
Clic1.3	-0.394403712	0.071	0.175	0.000378787	9
Ly6e.2	-0.377597163	0.156	0.285	0.000379372	9
Zfp191	0.33852771	0.345	0.214	0.00038118	9
Rpl18a.4	-0.391651001	0.275	0.403	0.000396314	9
Pcbp1.2	-0.25186752	0.557	0.594	0.000400061	9
Tmem101	0.304466607	0.165	0.074	0.000402339	9
Nrn1.5	-0.46372523	0.119	0.224	0.000407305	9
Afap1.3	-0.429909309	0.061	0.154	0.000413409	9
Usp46.2	-0.41807977	0.122	0.238	0.000417756	9
Rpn2	0.348793082	0.35	0.223	0.000423162	9
Preb	0.342216085	0.265	0.152	0.000428518	9
Tmem50a.1	0.250571756	0.538	0.402	0.000429443	9
Pknox1.1	-0.411002826	0.058	0.147	0.000432844	9
Tom1l1.2	-0.350737342	0.027	0.105	0.000445504	9
Homer2.2	-0.393257768	0.129	0.249	0.000449149	9
Zswim6	0.29232125	0.131	0.051	0.000449626	9
Cenpa.8	-0.529105329	0.134	0.254	0.000471431	9
Elovl6.1	0.374171315	0.399	0.275	0.000484797	9
Mapre2.2	0.336439744	0.287	0.164	0.000493637	9
Flna	-0.411759694	0.061	0.154	0.000510589	9
Spats2l	0.323161662	0.119	0.044	0.000530476	9
Ndufa8	0.353825093	0.443	0.327	0.000540754	9
Rabac1.2	0.290179358	0.394	0.254	0.000562272	9
Paqr4	0.304052649	0.122	0.045	0.000584652	9
Slc15a2	0.333500436	0.107	0.037	0.00060823	9

Cenpk.8	-0.337475245	0.054	0.146	0.000608882	9
Tm2d2	0.309985146	0.382	0.246	0.000625596	9
Cep290.2	-0.34822891	0.054	0.145	0.000707664	9
1500011K16Rik.2	0.341223841	0.251	0.147	0.000716746	9
Ank2.8	-0.441492243	0.182	0.241	0.000717771	9
Fjx1	0.280368436	0.105	0.035	0.000720969	9
Vmp1	0.303276336	0.221	0.114	0.000743993	9
Rpa2.5	-0.40954766	0.097	0.202	0.000749287	9
Pura.1	0.340363187	0.421	0.283	0.000772579	9
Fabp5.2	-0.344112806	0.273	0.406	0.000774291	9
Prkar2b	0.296166395	0.156	0.067	0.000783582	9
Dkc1.4	-0.290255996	0.217	0.335	0.000809766	9
Smim15	0.321665999	0.226	0.118	0.000824949	9
Napa.1	-0.390586971	0.236	0.352	0.000838872	9
Cecr2	-0.379919508	0.044	0.131	0.00083933	9
Ddx25	0.269601054	0.102	0.034	0.000844078	9
Extl2	0.296853559	0.131	0.052	0.00084421	9
Prdx4.5	-0.3838857	0.241	0.369	0.000854332	9
Larp7.4	-0.398009261	0.19	0.314	0.000854715	9
Anp32e.8	-0.29753792	0.562	0.614	0.000908392	9
Prdx5.1	0.275112785	0.399	0.261	0.000909834	9
Pgrmc2.1	0.351555569	0.302	0.193	0.00093765	9
Emc2	0.31579982	0.29	0.168	0.000957794	9
Nup62.3	-0.304980891	0.078	0.173	0.000971713	9
Ptpra.1	0.303235233	0.365	0.231	0.000990781	9
Cct2.1	-0.322768399	0.426	0.526	0.001004155	9
Dtd2	0.261172146	0.105	0.036	0.00100746	9
Cebpg	-0.387415689	0.09	0.194	0.00104704	9
Tmem167	0.358352249	0.38	0.27	0.001149662	9
Rfc1.5	-0.394844473	0.209	0.333	0.001151011	9
Tbca	-0.351135243	0.287	0.423	0.00115196	9
Eif1	-0.268798121	0.528	0.583	0.001156435	9
Tpp1	0.258164203	0.148	0.064	0.001183879	9
Ago3	0.283628137	0.165	0.075	0.001210195	9
Zfand6	-0.38727793	0.219	0.315	0.001216494	9
Eif3a.3	-0.289622762	0.618	0.69	0.001249177	9
Pepd	0.275056469	0.112	0.04	0.001322498	9
Rnf5.1	0.333200559	0.384	0.254	0.001420844	9
Kif13a	0.346517731	0.119	0.047	0.00143249	9
Ctnnal1	0.277376189	0.124	0.048	0.001441161	9
Rif1.5	-0.426511276	0.168	0.278	0.001479208	9
Lmcd1.2	0.269321657	0.141	0.06	0.00151395	9
Ankmy2	0.306810966	0.109	0.04	0.001537872	9
Aprt.3	0.260572342	0.246	0.138	0.001565979	9
Tceal1	0.316474943	0.158	0.071	0.00158143	9
0610007P14Rik	0.361918714	0.265	0.159	0.001583771	9
Krcc1	0.305735423	0.173	0.081	0.00160302	9
Uba52.2	-0.370683622	0.207	0.319	0.001605753	9

Smim13	0.262462833	0.139	0.058	0.001624371	9
Aurkb.8	-0.354949257	0.054	0.145	0.001666953	9
Smc3.1	-0.289937744	0.526	0.635	0.001683651	9
Knstrn.8	-0.375654091	0.088	0.188	0.001695241	9
Uqcrb	0.3601538	0.336	0.217	0.001695941	9
C130071C03Rik.2	-0.379321919	0.19	0.31	0.001711949	9
Znhit6	0.364098817	0.219	0.117	0.001732489	9
Lrrn1	0.371448136	0.202	0.111	0.001776739	9
Maged2.2	0.309364727	0.282	0.164	0.00177814	9
Elovl5	0.35384263	0.214	0.116	0.001799477	9
Rpl23.3	-0.322641803	0.445	0.541	0.001849289	9
Ate1	0.356278462	0.231	0.127	0.001858094	9
Samd4b	0.285946418	0.156	0.069	0.001873098	9
Midn	0.337820835	0.236	0.13	0.001960821	9
Ivns1abp.1	-0.352662113	0.29	0.418	0.001961955	9
Gm8292.2	-0.373304613	0.255	0.371	0.002017803	9
Tia1.1	-0.31412975	0.472	0.553	0.002022632	9
Pak1.2	0.293220493	0.136	0.057	0.002071493	9
Sox11.2	-0.36034432	0.036	0.116	0.002089821	9
Gpx7	0.262905402	0.141	0.061	0.002107192	9
Leprot	0.350477669	0.217	0.115	0.002110738	9
B3gnt1	0.320961548	0.141	0.06	0.002143162	9
Gmnn.7	-0.305992221	0.056	0.146	0.002189966	9
Myo6	0.2834692	0.122	0.047	0.002234762	9
Pfdn4	-0.387976268	0.204	0.307	0.002295015	9
Mboat2	0.267032074	0.119	0.046	0.002368134	9
Hmgn3.4	0.338962049	0.394	0.266	0.002369172	9
Clptm1l	0.334976721	0.221	0.12	0.002398812	9
Eif3k.1	-0.331625386	0.401	0.523	0.002429039	9
1500012F01Rik.4	-0.375913534	0.29	0.4	0.00251539	9
Snap25.5	-0.42399423	0.17	0.284	0.002589429	9
Hes6.6	-0.415082499	0.139	0.238	0.002647355	9
Ccnh	0.346693022	0.251	0.147	0.002789622	9
Cpe.5	0.296454354	0.579	0.452	0.002812106	9
Pmvk.1	0.265068102	0.185	0.092	0.002823098	9
Cd63-ps.1	-0.321949217	0.039	0.121	0.002911501	9
Nlk	0.267127656	0.178	0.086	0.002928364	9
Tmem18	0.330194179	0.112	0.044	0.00293358	9
Mtdh	0.258478017	0.667	0.546	0.003017594	9
Rnf208	0.259790907	0.161	0.075	0.00302226	9
Cdca7l.5	-0.307429316	0.029	0.106	0.003102797	9
Ramp2.4	0.279087501	0.219	0.117	0.003147062	9
Ddx26b.2	0.357902678	0.224	0.127	0.003163746	9
Myo5a	0.359225844	0.302	0.188	0.00317607	9
Syne2	-0.424461693	0.122	0.222	0.00319571	9
Ptprd.2	-0.37947686	0.214	0.343	0.003216614	9
Hdhd2	0.263665025	0.217	0.115	0.003240079	9
Sgce	0.305909175	0.2	0.101	0.003385789	9

Rab9	0.328565737	0.204	0.107	0.003451688	9
Fbxo5.7	-0.334529335	0.066	0.161	0.003464764	9
Asf1a	-0.372137232	0.163	0.282	0.003481718	9
Nsg2.6	-0.349172645	0.367	0.49	0.003505954	9
Supt16.5	-0.346251429	0.387	0.495	0.003545026	9
Baz1a.4	-0.399129279	0.088	0.168	0.003619711	9
Secisbp2l	0.378339959	0.255	0.152	0.003651474	9
Arpc1a	0.296110505	0.443	0.361	0.003691879	9
Tm9sf3	0.325115064	0.47	0.341	0.003697088	9
Cdc42se2.1	0.344298505	0.35	0.236	0.003719998	9
Chgb.8	-0.495635166	0.136	0.243	0.003800514	9
Rnf13	0.3201139	0.165	0.079	0.003885495	9
Magt1	0.301175845	0.131	0.055	0.003967682	9
Klf7.6	-0.399407729	0.192	0.263	0.004000352	9
Sbf2	0.320698541	0.168	0.08	0.00400565	9
Golm1.1	-0.401959442	0.092	0.19	0.004069609	9
Ubl3.2	0.320668873	0.328	0.206	0.004075385	9
Atp6v0e.4	0.297112923	0.506	0.405	0.004091433	9
Hsd17b4	0.298492922	0.243	0.137	0.004192998	9
Pole3.3	-0.382198553	0.141	0.252	0.004218156	9
Rab5c	0.284836168	0.19	0.095	0.004318278	9
Usp22.3	-0.351575267	0.27	0.363	0.004345376	9
Atp13a3	0.291054609	0.122	0.052	0.004352322	9
Myt1l.8	-0.474349642	0.08	0.163	0.004400116	9
Rad51.7	-0.287291571	0.039	0.118	0.004476243	9
Deaf1	0.271915476	0.175	0.085	0.004607814	9
Psph	0.340204979	0.151	0.072	0.004749798	9
Dnajb11	0.328181844	0.27	0.16	0.00485315	9
Tfdp2	-0.38958986	0.073	0.162	0.005083203	9
Soga3.5	-0.350971752	0.37	0.459	0.005491775	9
Tor1aip2	0.287793487	0.18	0.088	0.005510444	9
Sptlc1	0.301939659	0.119	0.049	0.005641143	9
Ddx3x	-0.341153983	0.353	0.469	0.005662975	9
1700025G04Rik.4	0.346397897	0.38	0.26	0.005792032	9
Rgs12	-0.372340381	0.061	0.148	0.006007506	9
Purb	-0.291774205	0.491	0.55	0.006054499	9
Atp2a2	0.303672686	0.319	0.199	0.006216528	9
Cldn25	0.296542515	0.353	0.229	0.006219847	9
Sppl2a	0.308920627	0.217	0.117	0.006481152	9
Fam184a.1	0.27651854	0.144	0.065	0.0064875	9
Rap1a	0.293768921	0.221	0.121	0.00651091	9
Rfc2.5	-0.356709939	0.114	0.213	0.006635846	9
Mis18bp1.8	-0.378635768	0.068	0.163	0.00671053	9
Ap3m1	0.279660725	0.144	0.065	0.006726144	9
Dclk1.4	-0.385709076	0.299	0.414	0.006841352	9
Ndrg3.1	0.289160228	0.212	0.113	0.006903975	9
Smarca5.2	-0.329388478	0.314	0.403	0.007115448	9
Khdrbs1	-0.307306482	0.382	0.496	0.007206751	9

Mia3	0.300867959	0.365	0.242	0.007437903	9
Rnaseh2a.4	-0.352595288	0.071	0.154	0.007617472	9
Cenpj.4	-0.304679151	0.058	0.143	0.007703119	9
Arhgap11a.9	-0.359140739	0.075	0.172	0.007876787	9
Nf1	0.295034404	0.148	0.067	0.007988159	9
Suv39h2.2	-0.327187184	0.049	0.133	0.008208142	9
Zcchc18.1	0.280026045	0.28	0.167	0.00843433	9
Mpv17	0.277469111	0.185	0.093	0.008710013	9
Ndufa13	0.26181989	0.652	0.53	0.008711601	9
Fcho2	0.320191976	0.156	0.075	0.008929813	9
Chga.1	0.309951312	0.17	0.083	0.008955817	9
Cenpw.8	-0.256934133	0.049	0.129	0.009134594	9
Snx4	0.295136634	0.287	0.173	0.009166422	9
Gpbp1	-0.353006964	0.268	0.358	0.009262683	9
Srrm3.3	-0.400390977	0.136	0.242	0.009735406	9
Cdk2ap1	-0.295141922	0.032	0.101	0.009834714	9
Mns1.8	-0.372315304	0.102	0.205	0.009870351	9
Prdm8.7	-0.426961154	0.056	0.128	0.010089081	9
Hnrnpk	-0.254000236	0.56	0.637	0.010185891	9
Smap1.1	0.280176413	0.343	0.22	0.010252161	9
Jund	-0.361253589	0.229	0.354	0.010275817	9
Sdhb	0.299941219	0.513	0.397	0.010303515	9
Cpox	0.291584908	0.151	0.071	0.010634994	9
Ehbp1	-0.376535856	0.061	0.138	0.010658561	9
Lrrc42	0.314978074	0.136	0.061	0.01115406	9
Sppl2b	0.324237903	0.114	0.055	0.011213192	9
Nfkbib	0.316078749	0.158	0.075	0.011229485	9
Arrdc3	-0.314434894	0.034	0.105	0.011457175	9
Elavl4.8	-0.401299329	0.221	0.337	0.011667553	9
Ica1	0.262998449	0.114	0.046	0.011998577	9
Npc2.3	-0.342204782	0.358	0.466	0.012513773	9
Fasn	0.303738032	0.238	0.135	0.013464312	9
Dennd5a	0.259173172	0.192	0.1	0.013465632	9
Nptn.1	0.292125804	0.299	0.185	0.014275476	9
Rbbp4.2	-0.304928093	0.377	0.486	0.014495009	9
Mbip	0.266841286	0.146	0.068	0.014532052	9
Rpl7.3	-0.297932347	0.433	0.511	0.01484396	9
Taf15	-0.367556605	0.129	0.224	0.01501341	9
Psmb10	0.287716832	0.136	0.06	0.015083254	9
Kbtbd11.1	0.318718747	0.168	0.085	0.015118018	9
Nfyb.3	-0.382198718	0.134	0.237	0.015196492	9
Agl	0.298467451	0.102	0.039	0.01547918	9
Dbf4.7	-0.304604121	0.061	0.147	0.016114303	9
Cdca4.3	-0.271771475	0.036	0.112	0.016956365	9
Limd2	-0.285760325	0.063	0.136	0.017267757	9
Pcnt.1	-0.380878549	0.085	0.176	0.017380739	9
Bub1b.8	-0.269108098	0.032	0.104	0.017749648	9
Bub1.8	-0.276557508	0.051	0.132	0.018092673	9

Timeless.5	-0.325032765	0.066	0.155	0.018672227	9
Mlf2.1	0.306305044	0.397	0.276	0.018882244	9
Maml3.2	-0.272115305	0.032	0.102	0.019465448	9
Emc3	0.266819772	0.251	0.146	0.020470184	9
Akap12.4	0.335226565	0.197	0.109	0.021409883	9
Fam107b.2	-0.304769276	0.034	0.103	0.021532401	9
Tmed10	0.294984645	0.311	0.201	0.022306312	9
Stmn4.9	-0.270103695	0.341	0.341	0.022978771	9
Fen1.6	-0.346343516	0.066	0.143	0.023814408	9
2310061I04Rik	0.310692278	0.148	0.07	0.024023026	9
Rpl37.3	-0.307976799	0.406	0.506	0.026066825	9
Chtop	0.309169175	0.343	0.246	0.027431208	9
BC004004	0.301596399	0.217	0.12	0.028162764	9
Pccb	0.316927764	0.207	0.115	0.028354643	9
Tmem33	0.297552386	0.243	0.142	0.029026741	9
Nol4.3	-0.370155624	0.083	0.173	0.030216582	9
Atp2b1.3	-0.290446398	0.533	0.572	0.03263774	9
Zfp91	-0.299213922	0.394	0.491	0.033243716	9
Glo1.1	0.304549951	0.192	0.105	0.033790846	9
Itsn2	0.296128421	0.168	0.084	0.033901488	9
Ten1	0.290475493	0.253	0.149	0.034366388	9
Arhgdia	0.276095571	0.319	0.205	0.034693756	9
Ska2.7	-0.335236815	0.139	0.206	0.035613843	9
Banp.2	-0.337519979	0.08	0.138	0.036531266	9
Tor1b	0.297218546	0.226	0.129	0.038217315	9
Golph3	0.319604941	0.139	0.072	0.038712912	9
Ubtf	-0.31433546	0.355	0.454	0.04075919	9
Palm	-0.311787365	0.08	0.14	0.040792192	9
Gm26735.4	-0.389946627	0.095	0.182	0.041237736	9
Slc3a2.4	-0.299756463	0.27	0.324	0.042286993	9
Ap1s2.2	-0.334168088	0.073	0.162	0.042710153	9
Ythdc1	-0.31034974	0.355	0.479	0.042947669	9
Whsc1.2	-0.284249701	0.401	0.465	0.045931605	9
5830418K08Rik.1	-0.297215808	0.083	0.169	0.046674226	9
Maoa	0.274070147	0.139	0.066	0.046712611	9
Cep78	-0.346725817	0.066	0.142	0.047434707	9
Lsm3.4	-0.326109224	0.268	0.36	0.049419075	9
Snhg1.5	-0.329211172	0.248	0.359	0.049893138	9
9330159F19Rik.3	-0.351377385	0.08	0.17	0.050354055	9
Frrs1l	0.3359268	0.294	0.188	0.050809897	9
Brpf1	-0.27817175	0.061	0.143	0.051094664	9
Ptbp1.2	-0.285905128	0.034	0.102	0.051477767	9
Hibadh	0.271090332	0.165	0.083	0.053870013	9
Polr2h.1	-0.293450524	0.139	0.227	0.05389283	9
Rora	0.271289884	0.119	0.053	0.0541733	9
Arhgap20.1	0.269583446	0.117	0.052	0.054212335	9
Smarcc1.2	-0.313302068	0.326	0.436	0.054348459	9
Blm.1	-0.283987742	0.039	0.112	0.054427474	9

Nfic	-0.363072077	0.158	0.263	0.054731709	9
Hmgcr	0.327111462	0.217	0.127	0.055125862	9
Fam57b.3	-0.358776318	0.095	0.187	0.056608138	9
Ndufa7	0.280952206	0.545	0.438	0.058145608	9
Slc25a1	0.299676666	0.129	0.059	0.058676668	9
Ddhd1	0.297100143	0.119	0.054	0.059690806	9
Sdf4	0.289667506	0.328	0.219	0.060363806	9
Ncdn.1	0.252764199	0.139	0.065	0.060798161	9
Ckap2l.9	-0.381169648	0.122	0.219	0.060907271	9
Bnip3l	-0.345415868	0.088	0.165	0.061007482	9
Tipin.6	-0.340076926	0.151	0.257	0.061330507	9
Lrpap1.4	0.288658442	0.207	0.115	0.062380786	9
1810009A15Rik.2	-0.366207886	0.17	0.261	0.063298955	9
Tbl1x	-0.315470014	0.212	0.33	0.06450585	9
Cadm3.8	-0.313171242	0.112	0.141	0.064810191	9
Apc.8	-0.356208659	0.35	0.433	0.065697766	9
Pbx3	-0.279109951	0.051	0.128	0.066237583	9
Zeb1.3	-0.333057532	0.273	0.39	0.066411193	9
Btbd9	0.303928629	0.195	0.107	0.071163446	9
Gpr180.1	0.270613858	0.148	0.079	0.07143545	9
Cers4.1	0.303556814	0.129	0.058	0.072179087	9
Dbn1.7	-0.371141628	0.073	0.141	0.072288582	9
Psma4.1	-0.316193628	0.324	0.415	0.073314921	9
Kifap3.3	0.308614365	0.382	0.27	0.074312348	9
Vrk1.5	-0.288320242	0.066	0.15	0.075773074	9
Cnn3.1	-0.295778035	0.331	0.439	0.079607108	9
Scp2	0.276267243	0.146	0.071	0.080990418	9
Gins1.3	-0.263231877	0.044	0.116	0.083189213	9
Eif3j1	0.269547598	0.153	0.076	0.088067944	9
Vkorc1	0.283467701	0.195	0.108	0.088462832	9
Eny2.2	-0.291161911	0.326	0.388	0.090822799	9
Rpn1	0.302342694	0.292	0.19	0.093056811	9
Cenpb	-0.327741596	0.117	0.211	0.093424508	9
Saysd1	0.323596546	0.102	0.051	0.094138715	9
Xpo1	-0.328375474	0.158	0.229	0.094397507	9
Vapb	0.256787164	0.17	0.091	0.095288629	9
Trib1	0.278685032	0.109	0.057	0.095945698	9
Rpa3.4	-0.353164259	0.153	0.246	0.097877694	9
Bag1	-0.306392141	0.229	0.347	0.098313411	9
Galnt1	0.317415153	0.18	0.102	0.099076613	9
Kif4.7	-0.259722575	0.039	0.109	0.099603587	9
Rac1	0.287452367	0.426	0.344	0.100385858	9
Stxbp1.9	-0.392975685	0.088	0.164	0.10398433	9
Tsc22d1.3	0.258911013	0.543	0.425	0.109314085	9
Rai1.1	-0.352328979	0.08	0.161	0.109944238	9
Hmg20b.1	-0.348902822	0.134	0.224	0.110362445	9
N6amt1	0.2830232	0.18	0.096	0.113322646	9
Tram1l1.1	0.257328732	0.163	0.084	0.114597747	9

Mis18a.4	-0.288702614	0.051	0.111	0.114893792	9
Fkbp2.2	0.281425982	0.436	0.323	0.11575406	9
Srp14	0.254842546	0.477	0.354	0.116049479	9
Agfg1	0.276965321	0.129	0.06	0.117660773	9
Pa2g4.5	-0.257673379	0.44	0.524	0.117894972	9
Pdgfa.6	-0.388621526	0.165	0.255	0.121463194	9
Lmnb2.4	-0.327880341	0.078	0.16	0.121879217	9
Dazap1	-0.314693966	0.17	0.274	0.129392418	9
Mier3	-0.316741307	0.051	0.121	0.129449532	9
Creb1	-0.298268114	0.131	0.224	0.131997138	9
Scfd1	0.26637358	0.212	0.121	0.132399833	9
Cdk5rap3.1	-0.327990237	0.105	0.194	0.132881908	9
Rrm1.8	-0.303211019	0.158	0.262	0.132952888	9
Mesdc2	0.318355991	0.282	0.203	0.133180349	9
Crebbp	-0.340958979	0.131	0.225	0.137335328	9
Ift27.2	-0.296915504	0.144	0.245	0.137961244	9
Pim3	0.267567058	0.165	0.085	0.139323671	9
Tdp2	-0.303389264	0.075	0.151	0.142568198	9
D030056L22Rik.1	-0.284279361	0.063	0.144	0.143079769	9
Ktn1	0.268529926	0.543	0.457	0.143113707	9
Ndufaf7	0.278314282	0.18	0.098	0.145877584	9
Pafah1b2	-0.325833256	0.158	0.257	0.148257457	9
Zfp292.2	-0.36345008	0.238	0.343	0.150842983	9
Smc6.5	-0.317151236	0.311	0.425	0.157342798	9
Arl6ip6.2	-0.303326525	0.063	0.127	0.164365161	9
Snrnp40.3	-0.288834676	0.195	0.272	0.180567795	9
Agap1.2	0.279309656	0.221	0.129	0.190089728	9
Acap2	0.294404018	0.141	0.07	0.195029263	9
Wwp1	0.28408603	0.156	0.082	0.199146572	9
Cdkn1c	0.279427837	0.129	0.061	0.199463014	9
Paics.5	-0.251840617	0.302	0.415	0.200473683	9
Cspp1.1	-0.301640749	0.153	0.24	0.216878207	9
Tkt	0.278805269	0.304	0.2	0.218375195	9
Ift46	-0.313180547	0.058	0.118	0.21858459	9
Hddc2.1	0.275704872	0.17	0.091	0.221976905	9
Grik2.6	-0.329300454	0.058	0.102	0.224250399	9
Nipbl.1	-0.310636693	0.404	0.494	0.226812195	9
Srsf7.5	-0.264780622	0.382	0.467	0.230905051	9
Set.4	-0.277030573	0.302	0.389	0.233389943	9
Dhx15	-0.27465008	0.311	0.419	0.234272633	9
Ptpla	0.26192974	0.151	0.077	0.235373857	9
Mcm2.6	-0.316937573	0.119	0.206	0.236310047	9
Cyth2.1	-0.317929291	0.204	0.274	0.239303122	9
Gnptg	0.285930293	0.26	0.164	0.243042613	9
Rbm3.1	-0.261872466	0.061	0.125	0.250169969	9
Mrpl13.3	-0.323282665	0.217	0.31	0.252371604	9
Tacc3.9	-0.344488468	0.117	0.208	0.252804712	9
Tmed9.1	-0.250099891	0.392	0.434	0.256525443	9

Sh3gl2.3	-0.355106448	0.102	0.173	0.269306219	9
Txnrd1.1	-0.306252794	0.297	0.39	0.274388302	9
Uggt2	0.3014194	0.122	0.059	0.280731923	9
Glg1	0.268952097	0.324	0.216	0.283492607	9
Litaf	0.281575497	0.131	0.067	0.286511539	9
Snx5.3	0.267470367	0.258	0.161	0.287676612	9
5830428H23Rik	0.259893623	0.129	0.063	0.28837157	9
Caprin1	-0.266541401	0.309	0.395	0.290264081	9
Nnat.5	-0.282256383	0.698	0.71	0.295612849	9
Gnao1.6	-0.311477908	0.241	0.288	0.300889838	9
Rps28.3	-0.299129168	0.129	0.204	0.301536379	9
Rfc4.7	-0.282148858	0.134	0.228	0.304254433	9
2410006H16Rik.1	-0.309183919	0.36	0.461	0.312979007	9
Fbxo32.3	-0.343261597	0.054	0.118	0.314770439	9
Commd6	0.311734472	0.263	0.177	0.326678615	9
Ggh	0.287739628	0.18	0.122	0.335882675	9
1110065P20Rik	0.271025873	0.2	0.116	0.338005716	9
2810474O19Rik.1	-0.323095958	0.144	0.243	0.348563029	9
Cdv3	0.315826175	0.231	0.157	0.348811737	9
Akap11.1	0.264772665	0.229	0.137	0.349048068	9
Pak2	0.250817193	0.409	0.298	0.363411104	9
Ccnl1	-0.277103115	0.294	0.37	0.366998093	9
Grlf1	0.271797927	0.175	0.096	0.368674591	9
Tardbp	-0.258685017	0.399	0.484	0.376914658	9
Uimc1	-0.315145117	0.061	0.134	0.382424943	9
Serf1.2	-0.313951165	0.187	0.282	0.405259347	9
Scamp3	0.289598196	0.204	0.12	0.411070337	9
Mt2.1	0.323636001	0.163	0.09	0.420283618	9
Acp2	0.261971974	0.168	0.091	0.431744908	9
Crot	0.285047414	0.156	0.087	0.463580775	9
Zc3h13	-0.316191086	0.37	0.464	0.46675792	9
Psme4	-0.302218598	0.19	0.286	0.474602123	9
Sod2	0.257300735	0.27	0.173	0.479810463	9
Hsph1	-0.329306458	0.141	0.229	0.486024875	9
Brd3.2	-0.256424466	0.455	0.533	0.491249434	9
Ap3b2	-0.27066749	0.117	0.204	0.49136893	9
Psma1	-0.270472588	0.355	0.446	0.499442157	9
Tmem57.4	-0.338589758	0.207	0.292	0.530294131	9
Stard4	-0.315293075	0.073	0.142	0.533528618	9
Pdzrn4.4	0.302032471	0.182	0.145	0.538681343	9
Hnrnph3	-0.273778175	0.338	0.428	0.540899378	9
Rps7.3	-0.289471965	0.316	0.401	0.547349865	9
Ing4.3	-0.307544366	0.226	0.324	0.548903424	9
Tbcb	0.271077963	0.416	0.308	0.565209479	9
Dnajc21.1	-0.317534822	0.136	0.211	0.568469006	9
Cep57.4	-0.313908932	0.173	0.259	0.578122001	9
Man1c1.1	-0.307074965	0.058	0.127	0.60629589	9
1110038B12Rik.4	-0.303426668	0.204	0.3	0.611064199	9

Ptp4a2.1	0.265273711	0.436	0.339	0.648309701	9
Mllt10	-0.319931316	0.202	0.288	0.651804684	9
Ensa.1	-0.322376687	0.144	0.229	0.66266796	9
Tshz1	0.29930024	0.224	0.139	0.686772875	9
Lpcat1	0.27793516	0.219	0.133	0.702720282	9
Rfc5.2	-0.272099368	0.051	0.109	0.703935543	9
Pttg1.6	-0.320669609	0.066	0.141	0.731712038	9
Ssbp2.1	-0.256375271	0.056	0.127	0.737197338	9
Arid2	-0.32519428	0.151	0.243	0.738071662	9
Rab7	0.258028242	0.277	0.181	0.767157082	9
Psmd6	0.267099655	0.397	0.306	0.784095168	9
2700049A03Rik	-0.278152088	0.056	0.122	0.789469839	9
Ldb1.1	-0.338463748	0.107	0.185	0.813853153	9
Naa38.3	-0.273185148	0.217	0.313	0.827904003	9
Vps37a	0.258803961	0.112	0.057	0.838544947	9
Zfp706	-0.287988267	0.275	0.365	0.864777811	9
Arl6ip4	-0.329386684	0.151	0.238	0.8741943	9
Phactr1.2	0.25193408	0.277	0.181	0.880610502	9
Phf20l1.4	-0.334715294	0.26	0.342	0.884469917	9
Atad5.4	-0.32026767	0.078	0.15	0.910970402	9
Med19	-0.314810763	0.238	0.319	0.964291153	9
Ccni	-0.321357414	0.139	0.224	0.966966111	9
Zmiz1.2	-0.313280882	0.285	0.377	0.975374466	9
Faim	0.307133054	0.241	0.167	0.97870454	9
Irs1.4	-0.295116426	0.058	0.12	1	9
Stk11	-0.303353638	0.151	0.231	1	9
Plcg1	-0.261544606	0.109	0.162	1	9
Klhl24	-0.315709236	0.075	0.14	1	9
Zfp827	0.261211194	0.114	0.055	1	9
Anapc5	-0.299376403	0.243	0.335	1	9
Gtf2f2.1	0.268326412	0.19	0.115	1	9
Psmc3ip.5	-0.294927717	0.066	0.133	1	9
Lbr.5	-0.297582369	0.117	0.198	1	9
Sgol2.8	-0.292413703	0.08	0.158	1	9
Myl12b.2	-0.282652203	0.29	0.369	1	9
Cirbp.1	-0.281187015	0.297	0.356	1	9
Git2	-0.290362944	0.056	0.111	1	9
Lphn1.1	0.252345641	0.19	0.111	1	9
Xpr1.1	0.253197345	0.163	0.09	1	9
Agpat6	0.308175513	0.192	0.129	1	9
Plekha1.1	0.293660004	0.112	0.059	1	9
Jarid2.2	-0.31314225	0.102	0.18	1	9
Dync2h1	-0.306585587	0.054	0.116	1	9
Sesn3	0.258981452	0.102	0.049	1	9
Bcar1.1	-0.313498952	0.117	0.194	1	9
Gtf2e2	-0.296124312	0.105	0.179	1	9
Ywhaz	-0.274147397	0.294	0.364	1	9
Shmt2	-0.265110732	0.054	0.12	1	9

Rbmx.1	-0.293058878	0.229	0.317	1 9
Tro.1	0.296407387	0.202	0.124	1 9
Slc38a1	-0.27014058	0.175	0.272	1 9
Dnajc2.2	-0.276634697	0.251	0.33	1 9
Ptprs.6	-0.312119752	0.326	0.423	1 9
Rfc3.4	-0.278843445	0.122	0.206	1 9
Armc10	-0.272525793	0.049	0.11	1 9
Mapk8ip1.7	-0.296372577	0.148	0.229	1 9
Ube2g2	-0.285252209	0.061	0.126	1 9
Evl.2	-0.298913713	0.1	0.18	1 9
Blmh	-0.27596791	0.221	0.319	1 9
Sumo3	-0.297919393	0.217	0.3	1 9
Pttg1ip	0.266342215	0.148	0.084	1 9
Cplx1.5	-0.290699713	0.068	0.139	1 9
Shmt1.5	-0.255929273	0.049	0.104	1 9
Ppp1r1a.4	-0.256219501	0.068	0.111	1 9
Rps4x.3	-0.2870137	0.209	0.297	1 9
Zfr	0.264283561	0.421	0.351	1 9
Dnajc1	0.258608504	0.219	0.138	1 9
Cdh20.3	-0.284008875	0.129	0.204	1 9
Scaf11	-0.269403036	0.292	0.375	1 9
Klc1.6	-0.28257576	0.304	0.362	1 9
Ccdc53	0.255382017	0.131	0.071	1 9
Kdm1a.1	-0.286308886	0.275	0.366	1 9
Dnmt1.8	-0.271904805	0.187	0.279	1 9
Sclt1	-0.26966876	0.063	0.128	1 9
Picalm	0.260111095	0.219	0.139	1 9
Pcbp4.1	-0.274635765	0.243	0.326	1 9
Zfp422	-0.285584695	0.161	0.247	1 9
Nsmce4a.3	-0.283766994	0.139	0.225	1 9
Wasl	0.268866419	0.311	0.234	1 9
Ctbp2	-0.279801139	0.131	0.216	1 9
D19Bwg1357e.1	-0.302171898	0.187	0.275	1 9
Ddx39b.1	-0.257789215	0.353	0.446	1 9
Prkar2a	0.260562723	0.212	0.133	1 9
Tchp	-0.286676497	0.068	0.118	1 9
Herpud1	-0.290428251	0.073	0.137	1 9
Brd7.5	-0.295775594	0.231	0.309	1 9
Scg3.2	-0.277624592	0.372	0.448	1 9
Polr1c.2	-0.269037189	0.163	0.219	1 9
Pebp1	0.267844742	0.319	0.24	1 9
Ulk1	-0.275802759	0.051	0.105	1 9
Gltscr2.2	-0.283584908	0.26	0.343	1 9
Cnksr2.3	-0.28822818	0.049	0.108	1 9
Nes.1	0.26034682	0.148	0.085	1 9
Zfp36l1.5	0.284223598	0.209	0.149	1 9
Dpy30.3	-0.280824173	0.212	0.3	1 9
Arfrp1	0.267872695	0.187	0.116	1 9

Fam64a.8	-0.26937929	0.056	0.119	1 9
Dock1	0.273607572	0.146	0.089	1 9
Pcsk2	0.25663431	0.158	0.1	1 9
Sass6	-0.259851712	0.061	0.127	1 9
Casp8ap2.2	-0.292673678	0.158	0.245	1 9
Klhl7.1	-0.280092848	0.097	0.166	1 9
Lyar.6	-0.277447846	0.195	0.288	1 9
Ankrd49	0.274627481	0.156	0.094	1 9
Ewsr1	-0.250254371	0.324	0.394	1 9
Kcnk1.6	-0.32900617	0.134	0.206	1 9
Mad2l2.2	-0.289849985	0.187	0.264	1 9
Ccdc23	0.259678914	0.285	0.196	1 9
Rnf220	-0.297244577	0.158	0.237	1 9
Rab3c.2	-0.292712165	0.08	0.149	1 9
Poc1b	-0.257339795	0.071	0.128	1 9
Nmral1.5	-0.269108775	0.112	0.189	1 9
2700081O15Rik.1	-0.292062272	0.054	0.112	1 9
Gm13826.2	-0.2704922	0.241	0.313	1 9
Tmco1	0.257697996	0.324	0.239	1 9
Gm11478.3	-0.278966582	0.136	0.216	1 9
Fkbp4.1	-0.251570295	0.333	0.416	1 9
Zdhhc20	0.272880637	0.217	0.143	1 9
Sowaha.6	-0.318382581	0.122	0.163	1 9
Ano6	0.281110262	0.146	0.089	1 9
Frg1	-0.270644975	0.238	0.3	1 9
Snord104.2	-0.285866215	0.097	0.153	1 9
Iqgap1	-0.269659773	0.068	0.133	1 9
Nfu1	0.256221035	0.204	0.13	1 9
Lin7c.1	-0.28354079	0.139	0.219	1 9
Pou3f3	0.273027593	0.238	0.17	1 9
Klf13.2	-0.280498525	0.165	0.238	1 9
Polr2c	-0.272301465	0.127	0.203	1 9
Rbbp8.2	-0.266120097	0.063	0.123	1 9
Celf1	-0.265928893	0.304	0.381	1 9
Mpp6.4	0.2584907	0.234	0.159	1 9
2610203C20Rik.4	-0.293008915	0.061	0.122	1 9
Tiparp	-0.282471639	0.097	0.148	1 9
Rpf1	-0.278160559	0.119	0.195	1 9
Mxi1	-0.283265927	0.131	0.191	1 9
Tbpl1	-0.281193784	0.119	0.188	1 9
Rundc3a.3	-0.27504882	0.19	0.25	1 9
Emd	-0.250462693	0.119	0.183	1 9
Rraga	0.259101496	0.246	0.171	1 9
Ube2d1.2	-0.250797606	0.163	0.229	1 9
Sf3a3	-0.25396842	0.204	0.282	1 9
Cotl1	0.273339137	0.217	0.152	1 9
Nkain4	0.279113984	0.192	0.127	1 9
6330403K07Rik.6	-0.295379903	0.178	0.244	1 9

R3hcc1	-0.278026127	0.102	0.16	1	9
Ndufaf2.1	0.25498247	0.28	0.2	1	9
Oraov1.3	0.259394692	0.175	0.119	1	9
Ncor2.4	-0.273008897	0.102	0.17	1	9
Nfkbia	-0.27562316	0.122	0.173	1	9
Smad4	-0.27818131	0.085	0.145	1	9
Phc2	-0.281010755	0.144	0.201	1	9
Mapk3	-0.25155761	0.063	0.113	1	9
Dgkz	-0.262538486	0.054	0.105	1	9
Pcyt1a	0.270128968	0.156	0.097	1	9
Klf9.4	-0.288449953	0.195	0.272	1	9
Rpl6.2	-0.256296831	0.212	0.292	1	9
Aspm.8	-0.298107152	0.08	0.144	1	9
Suz12.2	-0.253670749	0.144	0.22	1	9
Slc12a2	0.264856902	0.168	0.109	1	9
Cyb5r3	-0.271264089	0.151	0.199	1	9
Elp3	-0.255282287	0.083	0.138	1	9
Mrpl48	0.255203711	0.192	0.136	1	9
1700020I14Rik.1	-0.256415643	0.212	0.275	1	9
Smchd1.1	-0.260272171	0.204	0.274	1	9
A330076H08Rik.7	-0.296124285	0.1	0.162	1	9
Zfp704.1	-0.259111582	0.202	0.258	1	9
Tagln3.8	-0.2688369	0.234	0.296	1	9
Nemf	-0.259361789	0.268	0.34	1	9
Pnpla8	-0.256070009	0.178	0.232	1	9
Srgap2.6	-0.262342637	0.085	0.133	1	9
Esf1	-0.258067961	0.328	0.393	1	9
Atp1b3.4	-0.251872607	0.263	0.338	1	9
Acd.2	-0.25515901	0.109	0.162	1	9
Rab6b.8	-0.251578911	0.161	0.203	1	9
Hes1.7	-0.298818067	0.107	0.149	1	9
Kdm5a	-0.254713678	0.202	0.257	1	9
A030009H04Rik.2	-0.251379115	0.066	0.115	1	9
Pde4dip	-0.251271037	0.158	0.222	1	9
Plcb4.1	-0.253040599	0.088	0.14	1	9
Apoe.10	4.754507745	0.984	0.241	0	10
Ctsd.9	4.288821601	0.957	0.167	0	10
C1qb	4.143669221	0.946	0.009	0	10
Lyz2	4.126398341	0.924	0.009	0	10
C1qc	3.83865028	0.946	0.007	0	10
Sepp1	3.781710177	0.957	0.046	0	10
Hexb	3.717133298	0.886	0.024	0	10
Tyrobp	3.683428741	0.957	0.008	0	10
Ctsb.6	3.615737244	0.962	0.255	0	10
Ctss	3.536774357	0.897	0.004	0	10
Cst3.8	3.248175531	0.989	0.531	0	10
Fcrls	3.212615988	0.87	0.004	0	10
Fcer1g	3.126130303	0.876	0.004	0	10

C1qa	2.97252039	0.854	0.003	0	10
Aif1	2.959846314	0.87	0.003	0	10
Grn	2.843422276	0.822	0.078	0	10
B2m.5	2.82339197	0.941	0.224	0	10
Lgmn.3	2.794107802	0.876	0.111	0	10
Ctsz.2	2.59995032	0.886	0.129	0	10
Trem2	3.029375626	0.822	0.003	0	10
Laptm5	2.855579211	0.822	0.003	0	10
Ly86	2.634753428	0.816	0.004	1.8845E-295	10
Csf1r	2.59660831	0.746	0.001	1.7043E-290	10
Cx3cr1	2.671047448	0.735	0.003	2.8507E-269	10
Cd68	2.518763391	0.762	0.006	9.4599E-264	10
Hsp90ab1.1	-1.032551105	0.935	0.998	1.1419E-253	10
Fcgr3	2.224377694	0.676	0.001	2.453E-250	10
Fth1.3	1.694028259	0.989	0.705	2.0596E-247	10
Igf1	2.237240319	0.708	0.003	1.2841E-244	10
Ctsa.1	2.36196583	0.773	0.117	2.0778E-234	10
Rnase4	2.52328173	0.719	0.014	9.3328E-234	10
Timp2.1	2.687213447	0.762	0.018	3.6452E-228	10
Cyba	2.350316809	0.751	0.022	2.7036E-226	10
Mpeg1	2.221837322	0.638	0.002	6.8572E-226	10
Hexa	2.393966891	0.778	0.058	6.7547E-223	10
Creg1	2.430485037	0.778	0.051	7.297E-214	10
Rgs10	2.122829879	0.714	0.01	1.0751E-212	10
Ctsl.3	2.249459625	0.789	0.254	3.1458E-205	10
AF251705	1.701074389	0.551	0.001	2.1178E-198	10
P2ry12	2.054767686	0.546	0.001	6.839E-195	10
Fyb	1.796592367	0.584	0.002	1.0994E-193	10
Emr1	1.64077606	0.519	0	4.1443E-192	10
Unc93b1	2.020386608	0.622	0.006	1.6129E-191	10
Hpgds	1.753281188	0.568	0.002	1.8745E-191	10
Ptpn18	1.821549307	0.562	0.002	6.1146E-190	10
Maf	2.295062746	0.649	0.013	7.0704E-182	10
Cd53	1.753324753	0.514	0.001	5.2181E-181	10
Lamp1.1	1.806257441	0.854	0.33	5.7448E-179	10
Gpr34	1.751237825	0.508	0.001	1.4253E-178	10
C3ar1	1.672629254	0.486	0.001	2.2191E-169	10
Psap.4	2.043269464	0.773	0.178	1.368E-168	10
Cyth4	1.537006842	0.492	0.001	1.1544E-167	10
Vamp8	1.795268931	0.573	0.007	2.0126E-167	10
Sat1	2.128460554	0.746	0.06	4.6626E-166	10
Cd52	1.860648433	0.459	0.001	1.3791E-162	10
Pld4	1.489703063	0.454	0.001	3.0057E-159	10
Tmsb4x.7	1.409223102	1	0.948	1.774E-158	10
Ctsc	1.790088575	0.508	0.003	8.5928E-158	10
Arhgdib	1.649696483	0.514	0.003	1.4328E-157	10
Arpc1b	1.901181997	0.676	0.03	1.1892E-156	10
Rac2	1.394312794	0.465	0.001	1.3332E-155	10

Npc2.4	1.555492545	0.935	0.459	2.2396E-154	10
Abca1	2.041642992	0.616	0.017	5.1934E-154	10
Ctsh	1.812446815	0.551	0.006	7.7466E-153	10
Ltc4s	1.517644818	0.432	0.001	1.934E-149	10
Man2b1	1.900302355	0.643	0.027	5.6622E-148	10
Sirpa	1.95997929	0.654	0.031	2.5162E-147	10
Stab1	1.94600942	0.497	0.004	1.0757E-145	10
Pycard	1.759068248	0.514	0.006	7.5021E-145	10
Apbb1ip	1.505889956	0.492	0.004	1.4893E-143	10
Itgb5	1.520005955	0.486	0.004	3.1035E-143	10
Cd84	1.150913402	0.4	0	1.1544E-142	10
Lpcat2	1.43284087	0.459	0.002	3.551E-141	10
Ccl3	2.274054043	0.405	0.001	1.6678E-140	10
Evi2a	1.454700348	0.454	0.002	5.1835E-140	10
Ms4a7	2.296434931	0.411	0.001	5.1856E-139	10
Ms4a6c	1.548861845	0.4	0.001	3.3063E-137	10
Apoc1	2.322374208	0.432	0.003	1.3852E-136	10
H2-K1	1.733247496	0.524	0.009	2.833E-135	10
Ncf1	1.270018118	0.4	0.001	7.3292E-132	10
Irf8	1.415136026	0.481	0.006	4.737E-131	10
Bst2	1.407111721	0.4	0.001	1.2182E-129	10
Serinc3	1.967577056	0.73	0.113	4.2815E-129	10
Itm2b.8	1.437156205	0.957	0.639	1.6582E-126	10
Anxa3	1.422885937	0.432	0.003	3.5801E-126	10
Lair1	1.278885584	0.368	0.001	2.5598E-125	10
Fam105a	1.456467669	0.492	0.008	2.9693E-123	10
Pf4	2.590723509	0.373	0.002	2.8932E-122	10
Cd300a	1.227598739	0.373	0.001	4.9694E-122	10
Abhd12.1	1.78782206	0.681	0.086	7.2248E-121	10
Mertk	1.479799462	0.411	0.003	1.8393E-119	10
4632428N05Rik	1.322448855	0.395	0.002	3.2875E-118	10
Mt1.7	1.944901605	0.827	0.161	5.5894E-118	10
Spi1	1.167856644	0.346	0	9.8549E-118	10
Abcg1.1	1.722133804	0.551	0.018	2.0759E-117	10
Rgs2	1.871260545	0.605	0.046	2.6134E-116	10
Npl	1.211870953	0.4	0.003	1.1976E-114	10
Tnfaip8l2	1.056735614	0.335	0	1.9391E-114	10
Fcgr1	1.068791094	0.335	0	2.2799E-113	10
Lcp1	1.253191274	0.362	0.002	5.9581E-112	10
Cd86	1.012189426	0.324	0	1.9357E-111	10
Mrc1	1.93342006	0.351	0.002	1.1671E-109	10
Bin2	1.154352966	0.319	0	5.2435E-109	10
Sh3bgrl3.1	1.667333322	0.751	0.193	1.2536E-108	10
Cotl1.1	1.660350157	0.746	0.147	3.8694E-108	10
Selplg	1.204835444	0.308	0	7.8093E-108	10
Syngr1.1	1.806695819	0.659	0.087	8.0861E-108	10
Nfib.5	-1.570808828	0.476	0.938	3.9201E-106	10
Cd37	1.095629483	0.33	0.001	2.2902E-105	10

Psmb8	1.214193918	0.373	0.003	8.0928E-105	10
Fgd2	1.001742244	0.308	0	4.9083E-104	10
Ptpn6	0.958680157	0.314	0.001	2.8453E-103	10
Anxa5	1.566499479	0.486	0.015	1.758E-102	10
Plek	1.379078212	0.422	0.007	1.9443E-102	10
Cryba4	1.168384814	0.308	0.001	5.4863E-102	10
Tuba1a.6	-1.626256053	0.438	0.934	6.726E-102	10
Gpx3.1	1.265804525	0.416	0.006	7.093E-102	10
Spp1	2.805588122	0.395	0.007	8.3994E-102	10
Gns	1.662358271	0.6	0.064	4.3512E-99	10
Clec7a	1.297406537	0.308	0.001	2.69491E-97	10
Ptgs1	1.178721578	0.292	0.001	5.65148E-96	10
Ms4a6d	0.990671247	0.292	0.001	1.59364E-95	10
Plin2	1.628693394	0.53	0.047	1.08211E-93	10
Ccl4	2.259233002	0.286	0.001	1.37905E-93	10
Hmha1	0.937243335	0.286	0.001	3.90398E-93	10
Tbxas1	0.93088952	0.281	0	6.50975E-93	10
Il6ra	1.106399577	0.319	0.002	9.3711E-93	10
Inpp5d	1.002764847	0.281	0	1.5357E-92	10
Ms4a6b	1.180279736	0.265	0	8.31852E-91	10
P2ry13	0.931099651	0.265	0	1.168E-90	10
Rasgrp3	1.056087527	0.319	0.002	3.4816E-90	10
Scamp2.2	1.600738918	0.638	0.13	3.70426E-90	10
Zfp36	1.615345403	0.362	0.009	2.01688E-89	10
Ccr5	0.893846317	0.254	0	5.51184E-89	10
Wfdc17	1.311865698	0.276	0.001	1.75948E-88	10
Ccl12	1.533799203	0.27	0	1.77567E-88	10
Pld3	1.56879502	0.568	0.052	5.80489E-88	10
Cst7	0.959338227	0.259	0	2.70734E-87	10
Gusb	1.529397357	0.627	0.075	5.94237E-87	10
Fermt3	0.950062112	0.303	0.002	1.05785E-86	10
Tmem86a	1.33686765	0.438	0.015	2.18909E-85	10
H2-D1.2	1.580032763	0.665	0.124	2.1496E-84	10
Sparc.1	2.177046139	0.622	0.079	2.22462E-83	10
Plxdc2	1.287458753	0.443	0.016	2.88907E-83	10
Lgals9	1.169652041	0.346	0.005	3.12443E-83	10
Cybb	1.210355355	0.27	0.001	1.34805E-82	10
Cstb	1.580029216	0.632	0.111	1.51122E-82	10
Slc11a1	0.973788412	0.265	0.001	2.1882E-82	10
Rgs1	1.07349366	0.243	0	2.32073E-82	10
Arl4c	1.504839814	0.503	0.036	3.78618E-82	10
Nckap1l	0.897949018	0.265	0.001	3.89242E-81	10
Trf	1.453481385	0.514	0.032	5.72512E-81	10
Mylip	1.200241921	0.373	0.009	6.8587E-81	10
Myo1f	0.788499036	0.243	0	2.78889E-80	10
Hpgd	1.216000523	0.286	0.002	3.39777E-80	10
Olfml3	1.002275458	0.341	0.006	5.52974E-80	10
Snx5.4	1.428601268	0.703	0.157	1.16029E-79	10

Syk	0.809556854	0.259	0.001	1.21885E-79	10
Abcc3	0.890617109	0.249	0	1.94475E-79	10
Tpp1.1	1.470687784	0.557	0.061	3.40991E-79	10
Slc7a7	0.903834567	0.286	0.002	7.70843E-79	10
Cd83	1.145404795	0.303	0.003	9.6661E-79	10
Tlr7	0.82792778	0.227	0	1.68632E-78	10
Clta	1.11721389	0.897	0.561	1.84275E-78	10
Serpine2.1	1.710628545	0.568	0.051	1.97803E-78	10
Siglech	1.119812305	0.238	0	2.83545E-78	10
Rab3il1	1.007307452	0.308	0.003	4.09622E-78	10
Lpl	1.697435098	0.449	0.054	6.14164E-78	10
Ucp2	0.86987915	0.286	0.002	1.80715E-77	10
Dcxr	1.128945857	0.368	0.009	5.016E-77	10
Ehd4	1.281560285	0.411	0.016	6.59747E-77	10
Bcl2a1b	0.744298995	0.222	0	9.83841E-77	10
Ccl6	1.00001993	0.243	0.001	1.92239E-76	10
Ctse	0.866709123	0.227	0	2.35138E-75	10
Lgals3	1.51981578	0.281	0.003	5.24228E-75	10
Entpd1	0.936751519	0.276	0.002	2.65023E-74	10
Nrp1	1.59770849	0.497	0.039	2.85906E-74	10
Mef2c	1.468050189	0.551	0.063	9.4993E-74	10
Mafb	1.437177691	0.508	0.046	1.02598E-73	10
Blnk	0.796552379	0.232	0	1.05577E-73	10
Il10ra	0.751771459	0.227	0	3.15182E-73	10
Tcn2	1.154193186	0.4	0.015	5.34261E-73	10
Lyn	1.039973978	0.33	0.007	1.42628E-72	10
Adap2	0.817942612	0.254	0.002	1.68325E-72	10
Sfrp1.6	-1.6650176	0.33	0.816	1.78579E-72	10
Tcf4.3	-1.10024783	0.611	0.928	2.73124E-72	10
Tmem37	0.942726568	0.303	0.004	5.33186E-72	10
H2-DMa	1.016627777	0.281	0.003	7.43287E-72	10
Srgn	1.066922652	0.276	0.003	9.1904E-72	10
Ighm	0.814245816	0.222	0	2.17913E-71	10
Uap1l1	1.131101388	0.314	0.006	2.46824E-71	10
Gngt2	0.748301447	0.238	0.001	2.65704E-71	10
Havcr2	0.767981448	0.222	0	3.76891E-71	10
Arhgap30	0.745016952	0.216	0	8.47681E-71	10
Klhl6	0.754186557	0.222	0.001	2.41662E-69	10
Lrp1.1	1.427927573	0.497	0.042	3.3E-69	10
Slc6a6.1	1.17740017	0.497	0.039	7.56052E-69	10
Il10rb	0.899667805	0.276	0.003	8.04472E-69	10
Gpx1.1	1.218920858	0.822	0.377	9.45635E-69	10
Lyl1	0.706710977	0.222	0.001	1.02807E-68	10
Slco2b1	0.807549655	0.254	0.002	2.13595E-68	10
Hcls1	0.798371465	0.216	0	4.72438E-68	10
Lipa	1.225621115	0.438	0.027	1.95632E-67	10
Emp3	1.146589516	0.314	0.007	2.26017E-67	10
Slc15a3	0.974436338	0.281	0.004	6.38562E-67	10

Ptprc	0.68433114	0.211	0	1.42015E-66	10
Lgals3bp	0.724572511	0.254	0.002	1.45862E-66	10
Itgb2	0.696967511	0.222	0.001	4.23978E-66	10
Dhrs3	1.068637061	0.389	0.018	5.77229E-66	10
F11r	0.761132437	0.243	0.002	7.5028E-66	10
Renbp	0.888865292	0.276	0.004	9.28229E-66	10
Lamp2.1	1.36058842	0.638	0.132	1.29912E-65	10
Hnrnpa2b1.2	-0.859134199	0.784	0.935	1.40823E-65	10
Slc40a1	0.958340789	0.265	0.003	2.94194E-65	10
Cd180	0.72409681	0.205	0	3.05484E-65	10
Tmem176a.1	1.213518503	0.346	0.012	5.17437E-65	10
Tlr13	0.730531045	0.195	0	6.60807E-65	10
Ifi30	1.183229267	0.346	0.012	6.62242E-65	10
Dnase2a	1.04220527	0.341	0.011	1.0686E-64	10
Folr2	0.864030889	0.189	0	1.28351E-64	10
Actb.1	0.757989781	0.989	0.979	1.48825E-64	10
Ccl2	1.646801717	0.211	0.001	4.74177E-64	10
Cd36	1.113962443	0.205	0.001	4.48533E-63	10
Rhog	1.17295097	0.411	0.026	7.02579E-63	10
Fcgr2b	0.885259055	0.189	0	8.93054E-63	10
Vav1	0.748144478	0.189	0	1.89693E-62	10
Ctla2b	0.804196853	0.211	0.001	2.83254E-61	10
Lst1	0.898600529	0.205	0.001	4.3485E-61	10
Arsb.1	1.176043519	0.384	0.021	1.17822E-60	10
Lcp2	0.76993457	0.227	0.002	7.48734E-60	10
Sgk1.1	1.369021184	0.4	0.03	9.62739E-60	10
Apobec1	1.216369987	0.319	0.015	1.37515E-59	10
Ifi27	1.055403846	0.324	0.012	4.76005E-59	10
Zic1.8	-1.550140718	0.265	0.789	6.33041E-59	10
Prkcd	0.876877745	0.259	0.004	9.0897E-59	10
Gna15	0.61605996	0.173	0	1.01569E-57	10
Ncf2	0.66065271	0.184	0	2.12571E-57	10
Cxcl16	1.043150647	0.232	0.003	3.83676E-57	10
Arhgap25	0.656948552	0.195	0.001	4.01365E-57	10
Ostf1.1	1.2373527	0.503	0.058	4.45857E-57	10
Dock2	0.546242468	0.178	0	4.6402E-57	10
Axl	0.87746811	0.259	0.005	1.47257E-56	10
Rps6ka1	0.812903378	0.259	0.005	1.63742E-56	10
Sla	0.621237154	0.178	0	1.6955E-56	10
Tcirg1	1.04990763	0.286	0.008	2.8129E-56	10
Camk1.1	1.18824813	0.476	0.052	1.10321E-55	10
Gmfg	0.657920273	0.168	0	1.38637E-55	10
Nrros	0.817539405	0.216	0.002	2.22185E-54	10
Lgals1.6	1.411783078	0.67	0.224	2.22702E-54	10
Gpr183	0.690433132	0.162	0	6.44829E-54	10
Msr1	0.772254732	0.168	0	7.74307E-54	10
Itga6	0.895626416	0.297	0.01	1.63067E-53	10
Fli1	0.767241135	0.232	0.003	5.09446E-53	10

Pla2g15	0.919013682	0.308	0.012	5.57929E-53	10
CRE_RECOMBINASE.8	-1.868933722	0.292	0.773	6.40307E-53	10
Cfh	0.769458346	0.232	0.005	7.48708E-53	10
Arpc2	1.030855757	0.854	0.504	8.72173E-53	10
Ang	0.809148194	0.205	0.002	2.00464E-52	10
Susd3	0.606301155	0.184	0.001	2.01172E-52	10
Igsf6	0.700993675	0.184	0.001	2.04929E-52	10
KCTD12	1.173004853	0.314	0.015	2.51812E-52	10
Cd14	0.887079094	0.168	0	3.10142E-52	10
Ptplad2	0.822806616	0.243	0.005	8.92404E-52	10
P2ry6	0.569527023	0.157	0	1.07798E-51	10
Pfn1.3	0.985894547	0.827	0.507	1.09673E-51	10
Gcnt1	0.68239025	0.178	0.001	1.61653E-51	10
Rrbp1.3	1.244515882	0.67	0.183	4.34414E-51	10
Rin2.1	1.079467594	0.324	0.017	4.72069E-51	10
Tpd52	1.116100849	0.384	0.032	5.79863E-51	10
Pon3	0.80311648	0.227	0.004	8.16246E-51	10
Dock8	0.565543939	0.168	0	1.17758E-50	10
Tmem106a	0.767098936	0.184	0.001	2.40468E-50	10
Pmepa1	1.146116003	0.346	0.024	7.48064E-50	10
Fuca2	0.63930972	0.254	0.007	8.12964E-50	10
H2-DMb1	0.572112925	0.146	0	9.94197E-50	10
Ecscr	0.825662195	0.216	0.003	1.09084E-49	10
Lilrb4	0.615814764	0.162	0	2.28702E-49	10
Efhd2.6	1.193235442	0.6	0.125	3.80357E-49	10
Abi3	0.584050272	0.157	0	3.84013E-49	10
Slc37a2	0.697696967	0.189	0.001	5.28278E-49	10
Hk3	0.591638081	0.151	0	7.985E-49	10
Tlr2	0.690620132	0.184	0.001	8.97516E-49	10
Runx1	0.607789947	0.178	0.001	1.05668E-48	10
Tifab	0.531074457	0.151	0	1.53711E-48	10
Klf2	1.480869548	0.286	0.016	5.97352E-48	10
Rab32	0.765711166	0.211	0.003	1.06882E-47	10
Atf3	1.368025642	0.243	0.015	1.61227E-47	10
Tnfrsf1b	0.577920659	0.151	0	2.01327E-47	10
Ikzf1	0.522554111	0.151	0	2.74979E-47	10
Kcnk6	0.541161834	0.151	0	2.76533E-47	10
Slc7a8	0.618030304	0.205	0.003	1.26425E-46	10
Slc9a9	0.723260529	0.227	0.005	1.49212E-46	10
Tubb5.7	-0.933942906	0.692	0.917	2.07589E-46	10
Erp29	1.086200857	0.719	0.275	3.71161E-46	10
Pbxip1	0.934058687	0.292	0.014	3.88102E-46	10
Csf2rb	0.478996322	0.151	0	4.52991E-46	10
Parvg	0.606561044	0.178	0.001	5.42789E-46	10
Tmem176b.3	1.321711902	0.508	0.105	6.47178E-46	10
Tspo	0.851317587	0.286	0.013	9.54182E-46	10
Fes	0.689144186	0.178	0.001	7.48337E-45	10
Soat1	0.847351397	0.308	0.018	7.93666E-45	10

Tnfrsf11a	0.557839166	0.151	0	1.02558E-44	10
A630001G21Rik	0.485757391	0.146	0	4.09973E-44	10
Skap2	0.67179195	0.2	0.003	4.63207E-44	10
Gpr65	0.655384307	0.146	0	5.9099E-44	10
Ntpcr	0.922800897	0.324	0.022	6.29738E-44	10
Asah1.1	1.128834216	0.427	0.059	1.06778E-43	10
Csf3r	0.526394733	0.13	0	1.28477E-43	10
Pmp22.1	0.961411688	0.286	0.015	1.92035E-43	10
Plcg2	0.475553563	0.151	0.001	2.37566E-43	10
Anp32a.1	-0.956802428	0.551	0.839	2.84299E-43	10
Scarb2	0.910630545	0.373	0.036	5.40454E-43	10
Hhex	0.458097902	0.146	0.001	6.20379E-43	10
Rasal3	0.426515792	0.135	0	8.00986E-43	10
Stxbp2	0.546089943	0.168	0.002	2.90085E-42	10
Neat1	0.965476167	0.27	0.013	3.50445E-42	10
Epb4.1l2.1	1.111106358	0.492	0.088	7.02858E-42	10
Ebi3	0.548001091	0.146	0	1.07794E-41	10
Igfbpl1.8	-1.570667849	0.157	0.652	1.23819E-41	10
Pnpla7	0.777876252	0.243	0.009	1.39983E-41	10
Ddah2.5	-1.415275435	0.227	0.69	1.80042E-41	10
Tgfbr2	0.677214307	0.222	0.006	3.4189E-41	10
Crmp1.4	-1.297433036	0.162	0.673	3.54355E-41	10
Rtn1.9	-1.595529251	0.227	0.695	4.79985E-41	10
Ppfia4	0.796661059	0.238	0.01	6.51531E-41	10
Ttc3.6	-1.085561287	0.481	0.832	8.66375E-41	10
Cd24a.3	-1.366539685	0.141	0.639	1.04182E-40	10
Hck	0.737607896	0.205	0.006	1.52743E-40	10
Alox5ap	0.681922382	0.151	0.001	2.29814E-40	10
Fblim1	0.571552346	0.184	0.004	2.34694E-40	10
Lmo2	0.926613159	0.351	0.033	2.82919E-40	10
Fxyd5	0.675905922	0.173	0.002	3.94804E-40	10
Ncl.6	-0.745963917	0.768	0.897	5.98258E-40	10
Ccl9	0.87655996	0.13	0	7.44189E-40	10
Nfix.5	-1.284464563	0.178	0.678	7.45449E-40	10
Npc1.1	0.912814207	0.368	0.038	7.85396E-40	10
Gas6.2	1.198075783	0.454	0.077	9.34247E-40	10
Tnf	0.499543145	0.119	0	1.47182E-39	10
Plin3	0.729408371	0.211	0.006	2.06644E-39	10
AB124611	0.577291714	0.135	0	3.27554E-39	10
Ccl7	1.12860426	0.13	0	6.27769E-39	10
Glul.2	1.141638395	0.605	0.196	7.42656E-39	10
Cd4	0.709430098	0.146	0.001	1.59705E-38	10
Marcksl1.4	-1.137780304	0.276	0.727	1.65116E-38	10
Hvcn1	0.531762856	0.141	0.001	2.08121E-38	10
Gpr157	0.444957906	0.141	0.001	2.42165E-38	10
Daglb	0.898937093	0.351	0.036	3.2092E-38	10
Icam1	0.5054308	0.157	0.001	5.30414E-38	10
Syngr2	0.838791085	0.286	0.019	5.3242E-38	10

Shisa5	0.790050524	0.297	0.022	6.46542E-38	10
Fam49b.1	1.121605508	0.443	0.081	1.04552E-37	10
Ifngr1	0.969265993	0.335	0.034	1.21727E-37	10
Glipr1	0.509944655	0.151	0.001	1.25837E-37	10
Pik3cg	0.446444779	0.124	0	1.79676E-37	10
Tgfbr1	1.053169528	0.395	0.058	2.35298E-37	10
Nfia.1	-1.172047912	0.438	0.777	2.802E-37	10
Nnat.6	-1.442556081	0.27	0.714	3.04455E-37	10
Cd48	0.430104379	0.119	0	5.68938E-37	10
Atp13a2	0.943251378	0.395	0.053	1.20683E-36	10
Trim30a	0.517938909	0.124	0	1.65716E-36	10
Nedd4.1	-1.096477442	0.238	0.688	1.86969E-36	10
Ncf4	0.35413869	0.108	0	2.11107E-36	10
Npnt	0.51422002	0.157	0.002	2.27936E-36	10
Psmb9	0.501840001	0.135	0.001	2.95484E-36	10
Slc43a2	0.931991085	0.286	0.021	3.08636E-36	10
Cd33	0.470904006	0.13	0	3.17224E-36	10
0610031J06Rik	0.851265011	0.405	0.058	3.35384E-36	10
Tmem140	0.418567447	0.151	0.002	5.67076E-36	10
Arhgap9	0.429810511	0.124	0	6.57921E-36	10
Aldh2	0.822762972	0.292	0.023	8.64125E-36	10
Nfam1	0.435420677	0.108	0	1.68047E-35	10
Slfn2	0.670734538	0.13	0.001	2.1771E-35	10
H3f3b.2	-0.688182898	0.784	0.907	2.38195E-35	10
Slamf9	0.431195257	0.114	0	3.04413E-35	10
Gba	0.943204375	0.389	0.054	4.18354E-35	10
Slc29a3	0.677858109	0.189	0.005	5.20577E-35	10
P2rx4	0.739864695	0.227	0.011	7.4746E-35	10
Ccnd2.6	-1.368054801	0.243	0.666	7.67978E-35	10
Litaf.1	0.93357549	0.411	0.065	9.30709E-35	10
Ptafr	0.402257132	0.114	0	1.05307E-34	10
Clec5a	0.387647469	0.103	0	1.70024E-34	10
Tapbp	0.702653376	0.249	0.015	1.83524E-34	10
Tnfaip3	0.564724157	0.141	0.001	2.34599E-34	10
Tmem119	0.620728934	0.141	0.001	2.53884E-34	10
Hnrnpu.3	-0.691847215	0.751	0.866	2.72928E-34	10
Coro1a	0.697970651	0.211	0.009	4.15508E-34	10
Hebp1	0.663838295	0.184	0.005	9.88735E-34	10
Itgal	0.398243378	0.114	0	1.20753E-33	10
Man1a	0.548272652	0.178	0.004	1.30593E-33	10
C5ar1	0.537921131	0.108	0	1.60019E-33	10
Gpr137b	0.84912671	0.232	0.014	2.74364E-33	10
Hpse	0.344124534	0.124	0.001	3.14566E-33	10
Sipa1	0.577286978	0.2	0.008	4.1997E-33	10
Ifngr2	0.790492612	0.27	0.021	7.60309E-33	10
Stard8	0.690325827	0.151	0.002	1.62362E-32	10
Fam26f	0.489300916	0.119	0.001	1.92167E-32	10
Gap43.9	-1.535059451	0.162	0.603	1.94927E-32	10

Stmn3.4	-1.19371191	0.189	0.626	3.66942E-32	10
Psip1.3	-1.108315985	0.265	0.692	5.99157E-32	10
Chst1	0.45870246	0.151	0.003	6.13762E-32	10
Csf2ra	0.769209642	0.254	0.018	1.17702E-31	10
Sdcbp	1.032521207	0.551	0.187	1.24513E-31	10
Atox1.1	0.927074196	0.697	0.288	1.37123E-31	10
Nagpa	0.769746907	0.281	0.025	2.00319E-31	10
Anxa2	0.868413806	0.211	0.01	2.85421E-31	10
Fam212a	0.526640117	0.135	0.001	3.58331E-31	10
H2afv.8	-0.954387508	0.492	0.73	3.90741E-31	10
Tnfrsf13b	0.372512353	0.119	0.001	3.95299E-31	10
Adipor1	0.981047456	0.519	0.136	4.41202E-31	10
Gatm.1	1.019102987	0.297	0.032	4.71907E-31	10
Cmtm6	0.768583001	0.259	0.021	7.31357E-31	10
S100a1.1	0.794408415	0.265	0.024	8.07476E-31	10
Fkbp3.5	-0.918269571	0.443	0.748	9.55872E-31	10
Rassf5	0.355020872	0.114	0.001	1.49836E-30	10
Tmsb10.5	-1.156909572	0.249	0.668	1.69075E-30	10
Rnh1	0.884400584	0.373	0.059	1.72372E-30	10
Cyfip1	0.979636375	0.481	0.117	2.10731E-30	10
Eva1a	0.540378223	0.168	0.004	2.90162E-30	10
Rbm47	0.395688435	0.108	0	2.95155E-30	10
Gaa.1	0.634359423	0.259	0.024	3.20814E-30	10
Itgam	0.884288642	0.232	0.017	3.3172E-30	10
Hnrnpab.4	-0.743103317	0.573	0.818	3.41213E-30	10
Fcgrt	1.001378361	0.243	0.025	4.28418E-30	10
Msn	0.997440686	0.422	0.089	4.89138E-30	10
Ralb.1	0.729690914	0.238	0.016	6.0576E-30	10
E130114P18Rik.4	-1.286241958	0.168	0.589	9.21225E-30	10
Cbx5.7	-1.077996442	0.238	0.657	9.30913E-30	10
Wwp2	0.909495285	0.297	0.033	1.49355E-29	10
Junb	1.099419036	0.27	0.032	1.49564E-29	10
Ifnar2.1	0.8373389	0.341	0.047	1.50755E-29	10
Bex2	-1.196727544	0.146	0.575	1.86756E-29	10
Luc7l3.1	-0.988695975	0.427	0.751	1.96426E-29	10
Capzb	0.879971882	0.697	0.347	2.7395E-29	10
Adssl1	0.378706434	0.119	0.001	3.58196E-29	10
Tm6sf1	0.775424494	0.222	0.015	4.60501E-29	10
Lhfpl2.1	0.733516141	0.265	0.025	5.60937E-29	10
Colec12	0.677193862	0.173	0.007	1.07633E-28	10
Slc25a45	0.405043002	0.108	0	1.11264E-28	10
Smagp	0.45832125	0.114	0.001	1.45987E-28	10
Cd38	0.602962585	0.124	0.001	1.57021E-28	10
Cpe.6	-1.334917279	0.065	0.459	2.76244E-28	10
Gdi2	0.850380006	0.697	0.373	3.70836E-28	10
Pros1	0.734607376	0.249	0.021	6.21168E-28	10
Paqr7	0.532640508	0.157	0.004	6.90389E-28	10
Sdc4	0.628537186	0.178	0.007	7.1461E-28	10

Gpnmb	1.048242129	0.13	0.003	8.65079E-28	10
Pla2g7	0.787899851	0.227	0.018	1.06615E-27	10
Gm26532	0.658743836	0.108	0.001	2.34894E-27	10
Cd302.1	0.890826345	0.335	0.05	2.87637E-27	10
Gm2a.1	0.820515241	0.319	0.045	3.22587E-27	10
Lhx1.7	-1.4073599	0.114	0.501	4.25465E-27	10
Nceh1.1	0.81084254	0.281	0.032	4.423E-27	10
Acss1	0.421822416	0.146	0.004	4.88873E-27	10
Cela1	0.612142387	0.141	0.003	5.35704E-27	10
Fbxw4	0.624799405	0.238	0.021	6.15772E-27	10
Snx20	0.345691383	0.124	0.002	6.52767E-27	10
Msrb1	0.968215595	0.33	0.058	7.41702E-27	10
Cog7.6	-1.182581439	0.205	0.588	8.3514E-27	10
Nrp2	0.459649038	0.146	0.004	8.83575E-27	10
Cebpa	0.493052098	0.157	0.005	2.32055E-26	10
Necap2	0.722636952	0.308	0.042	2.46578E-26	10
Barhl1.8	-1.268065573	0.124	0.525	2.85669E-26	10
Plod1	0.711898449	0.243	0.022	3.67101E-26	10
Serbp1.6	-0.576399285	0.784	0.87	3.97185E-26	10
6330416G13Rik	0.525895626	0.173	0.007	3.981E-26	10
Ccrl2	0.485564608	0.108	0.001	4.23945E-26	10
Stmn2.9	-1.610030822	0.2	0.556	4.57639E-26	10
Stat6	0.367053598	0.114	0.001	4.97821E-26	10
Slc38a6	0.617615383	0.2	0.013	5.41676E-26	10
Hnrnpdl.1	-0.765426876	0.514	0.758	8.04245E-26	10
Soga3.6	-1.246928062	0.081	0.461	8.82986E-26	10
Fam46c	0.674198022	0.151	0.005	9.91545E-26	10
Il4ra	0.693030647	0.205	0.014	1.00679E-25	10
Matr3	-0.908881527	0.432	0.709	1.19664E-25	10
Akr1a1	0.719285156	0.832	0.583	1.41576E-25	10
Cndp2	0.871638639	0.357	0.065	2.02087E-25	10
Htatip2	0.456899764	0.124	0.002	2.04015E-25	10
S1pr1	0.581293956	0.168	0.007	2.73562E-25	10
Rtn4rl1	0.446625588	0.119	0.002	3.51917E-25	10
Blvrb	0.930970377	0.319	0.057	4.1372E-25	10
Gria2.9	-1.356466487	0.141	0.524	4.25395E-25	10
Map1b.8	-1.343187985	0.216	0.591	4.52093E-25	10
Pax6.7	-1.252182511	0.13	0.517	4.63875E-25	10
Xlr	0.358453016	0.103	0.001	4.65417E-25	10
Adam15	0.601747556	0.162	0.006	4.7183E-25	10
Cryl1	0.644091642	0.173	0.008	5.08945E-25	10
Banf1.7	-0.820397477	0.476	0.696	6.85776E-25	10
Tnfrsf1a	0.51562438	0.227	0.023	1.25978E-24	10
Adcy7	0.474837361	0.151	0.005	1.48584E-24	10
Edem1	0.847099277	0.238	0.028	1.69332E-24	10
Bmp2k	0.696367153	0.238	0.023	1.83861E-24	10
Cnn3.2	-1.17141266	0.07	0.44	2.83872E-24	10
Pon2	0.738587613	0.281	0.036	2.85086E-24	10

Tubb3.9	-1.528196411	0.108	0.477	2.93646E-24	10
Rcsd1	0.583682852	0.124	0.003	5.39326E-24	10
Miat.8	-1.347793968	0.135	0.519	5.48388E-24	10
Arap1	0.28879488	0.114	0.002	5.93021E-24	10
Mex3a.3	-1.21233749	0.092	0.467	6.49302E-24	10
Smim1	0.513722972	0.135	0.003	7.12605E-24	10
Tmem50a.2	0.745923487	0.746	0.401	8.12808E-24	10
Ier3.1	1.048664805	0.243	0.032	8.1808E-24	10
Grap	0.41336353	0.119	0.003	8.43594E-24	10
Draxin.6	-1.175511438	0.173	0.558	8.63385E-24	10
Naglu	0.50321445	0.162	0.007	1.01602E-23	10
Ngfrap1	-0.893316405	0.314	0.661	1.07203E-23	10
Plbd2	0.658572663	0.27	0.035	1.43777E-23	10
Cd9.1	0.765131726	0.719	0.482	2.35235E-23	10
Klf6.3	1.078303948	0.384	0.145	2.61611E-23	10
Vcam1	0.786934724	0.141	0.006	2.6398E-23	10
Cbx1.2	-0.875598159	0.4	0.688	2.71812E-23	10
Sfrs18.4	-0.814452524	0.546	0.78	3.97356E-23	10
Elavl3.8	-1.258535565	0.103	0.476	4.34672E-23	10
Vwa5a	0.631856018	0.189	0.013	4.40992E-23	10
Dpp7	0.49895943	0.2	0.019	5.14752E-23	10
Cald1.3	-1.139069143	0.157	0.539	5.8232E-23	10
C2	0.475280496	0.103	0.001	6.10215E-23	10
Ftl1	0.57865181	0.276	0.04	8.60742E-23	10
P2rx7	0.604285866	0.151	0.006	8.6462E-23	10
S100a13.1	0.669310529	0.238	0.028	1.04045E-22	10
Slc46a3	0.42822935	0.119	0.002	1.05566E-22	10
Gabarap	0.758160253	0.724	0.441	1.28187E-22	10
Tubb2b.8	-1.257364921	0.124	0.468	1.30887E-22	10
Nsg2.7	-1.153951881	0.119	0.491	1.36859E-22	10
Nrep.9	-1.185902036	0.189	0.573	1.46052E-22	10
Akap13	0.863780309	0.346	0.069	1.5992E-22	10
Myh9	0.733115983	0.232	0.025	1.72098E-22	10
Tspan4.1	0.525445878	0.303	0.063	1.81732E-22	10
Hnrnph1.1	-0.870552098	0.357	0.631	2.07734E-22	10
Kcnk13	0.332860545	0.135	0.006	2.46014E-22	10
Map2.8	-1.20979107	0.124	0.495	2.998E-22	10
Ptbp3	0.88374107	0.411	0.107	3.77611E-22	10
Rapsn	0.476554977	0.103	0.001	3.79809E-22	10
Irf5	0.564822512	0.151	0.006	4.95286E-22	10
Calm2.7	-0.618076563	0.768	0.875	5.67698E-22	10
Cpd	0.750597097	0.33	0.06	6.04165E-22	10
Nhlh2.8	-1.306095749	0.07	0.426	6.15584E-22	10
Scg3.3	-1.164783341	0.097	0.45	6.72438E-22	10
1700017B05Rik	0.510532289	0.146	0.006	6.99132E-22	10
Ppp2r2c.4	-1.096647375	0.135	0.508	7.39784E-22	10
P4ha1.1	0.620498715	0.319	0.058	8.83485E-22	10
H1f0.5	-1.018676446	0.281	0.619	9.05453E-22	10

Sft2d2	0.446057845	0.124	0.003	1.06243E-21	10
Capza2	0.770481066	0.686	0.357	1.1554E-21	10
AI413582	0.604203166	0.238	0.029	1.26525E-21	10
Chd4.2	-0.754945252	0.497	0.772	1.73588E-21	10
Atrx.2	-0.718707006	0.476	0.743	2.04324E-21	10
Acin1	-0.840992635	0.454	0.726	2.26378E-21	10
Nfkbid	0.606950041	0.13	0.004	2.51366E-21	10
Fam46a	0.580526338	0.13	0.004	4.42887E-21	10
Hist1h1c	0.816524202	0.232	0.031	6.3597E-21	10
Ank3.9	-1.247832768	0.081	0.423	7.59904E-21	10
0610040J01Rik	0.400405659	0.114	0.002	7.7954E-21	10
Cebpb	0.604972785	0.146	0.007	8.33624E-21	10
Lpcat3	0.633491513	0.27	0.041	8.77735E-21	10
Tmem141	0.454292172	0.13	0.004	8.94043E-21	10
Ilf2.1	-0.992018422	0.173	0.545	9.90046E-21	10
Elovl1.1	0.615311169	0.249	0.033	1.05527E-20	10
Ezh2.8	-0.988984946	0.286	0.613	1.13619E-20	10
Ppt1.1	0.80572795	0.384	0.091	1.23733E-20	10
H2-T23	0.650690788	0.227	0.026	1.38559E-20	10
Pik3cd	0.492540347	0.13	0.005	1.66907E-20	10
Speg	0.294771474	0.103	0.002	1.95356E-20	10
Rnf130	0.853338193	0.4	0.109	2.19876E-20	10
Pcm1	-1.040030942	0.184	0.525	2.37371E-20	10
Atxn7l3b.3	-0.882213046	0.384	0.675	2.46462E-20	10
Cap1	0.786830272	0.384	0.093	2.92514E-20	10
Man2b2	0.319990945	0.108	0.002	2.93325E-20	10
Abca9	0.519163227	0.13	0.004	3.32858E-20	10
Smc3.2	-0.961650181	0.308	0.636	3.52507E-20	10
Hfe	0.44723483	0.114	0.003	3.59546E-20	10
Srsf3.4	-0.781569002	0.411	0.659	3.62794E-20	10
Sfpq	-0.735519762	0.405	0.673	4.7714E-20	10
Rps5.5	-0.462296525	0.957	0.954	5.1835E-20	10
Tor3a	0.49646362	0.146	0.007	5.70912E-20	10
BC028528	0.484686367	0.135	0.006	6.33616E-20	10
Gpsm3	0.401178329	0.103	0.002	6.59967E-20	10
Gm13476	0.672067935	0.222	0.026	8.74785E-20	10
Fxyd6.4	-1.098060929	0.157	0.512	9.23966E-20	10
Gm17750.4	-1.176180671	0.049	0.37	1.09152E-19	10
Prdx2	-0.774123487	0.405	0.682	1.24855E-19	10
Clic1.4	0.785169231	0.503	0.169	1.27512E-19	10
Tfpi	0.355796388	0.135	0.007	1.64645E-19	10
Syt11.7	-1.102330981	0.157	0.508	1.68983E-19	10
Ly6e.3	0.834694878	0.595	0.278	1.83653E-19	10
Rnd3.6	-1.107683107	0.097	0.434	1.94027E-19	10
Chd7.7	-0.967603009	0.341	0.65	2.27944E-19	10
Ramp1.1	0.712119177	0.216	0.025	2.32213E-19	10
Crip1	0.938679385	0.162	0.012	2.39557E-19	10
D430041D05Rik.5	-1.049523535	0.141	0.495	2.56036E-19	10

Gpm6a.9	-1.297957907	0.081	0.412	2.66946E-19	10
Picalm.1	0.815509657	0.449	0.138	2.80132E-19	10
Cltc	0.808942038	0.465	0.147	3.6341E-19	10
Gmpr	0.431583563	0.157	0.011	3.68585E-19	10
Ninj1.2	0.787049768	0.378	0.094	3.86501E-19	10
Trim47	0.422895399	0.103	0.002	4.16696E-19	10
Dpysl4.5	-1.07863671	0.146	0.494	4.61818E-19	10
Sept3.10	-1.2677656	0.076	0.395	5.3106E-19	10
Tia1.2	-0.943161723	0.232	0.555	6.12181E-19	10
Rab11fip5	0.545933936	0.184	0.017	7.075E-19	10
Wdr1.1	0.748473771	0.351	0.085	7.30988E-19	10
Ndufa4	-0.571594055	0.643	0.803	7.35086E-19	10
Slc29a1.5	-0.972574438	0.27	0.564	7.55814E-19	10
Ubtd1	0.592846485	0.124	0.004	7.90946E-19	10
Pnn	-0.661314855	0.492	0.724	9.67169E-19	10
Neurod1.10	-1.587733468	0.243	0.535	1.62436E-18	10
Ckb.8	-0.846864008	0.627	0.81	1.6281E-18	10
Twf2	0.6292038	0.292	0.054	1.69508E-18	10
Gpm6b.5	-1.059274962	0.141	0.487	1.91978E-18	10
Mfsd1	0.735063124	0.4	0.108	2.23104E-18	10
Casp8	0.343741681	0.108	0.003	2.45619E-18	10
Golm1.2	0.867563165	0.476	0.185	2.69333E-18	10
Dse	0.502351665	0.151	0.009	2.8999E-18	10
Dtx4	0.371365109	0.13	0.006	2.96888E-18	10
Zic4.4	-1.034257117	0.092	0.421	3.15891E-18	10
Dcx.9	-1.186888689	0.065	0.379	3.74409E-18	10
Hdac2.1	-0.96204513	0.195	0.518	3.93462E-18	10
Serpinb6a	0.782905633	0.286	0.056	4.74176E-18	10
Smc1a.4	-0.787676935	0.341	0.633	5.01699E-18	10
Tmed3	0.719677333	0.341	0.08	6.34183E-18	10
Nasp.8	-0.765761633	0.395	0.642	7.5516E-18	10
Tec	0.416846264	0.13	0.006	7.99703E-18	10
Itpripl2	0.333906265	0.108	0.003	8.47005E-18	10
Hsp90aa1.2	-0.781456397	0.292	0.62	9.54819E-18	10
Ranbp1.6	-0.757109705	0.481	0.677	1.02498E-17	10
Scoc	0.679176587	0.335	0.076	1.15654E-17	10
Prune2	0.52748212	0.141	0.008	1.17248E-17	10
Gm6977	0.489892665	0.216	0.03	1.34494E-17	10
Pnp	0.539697948	0.184	0.018	1.37096E-17	10
Tnfrsf21	0.640327901	0.297	0.061	1.61549E-17	10
Pfkfb3	0.613759781	0.189	0.019	2.09264E-17	10
Snrnp70	-0.716317938	0.492	0.695	2.21425E-17	10
Pde1c.10	-1.227974556	0.092	0.39	2.47352E-17	10
Ncam1.3	-0.972328112	0.076	0.39	2.51784E-17	10
Arpc5.2	0.784102841	0.605	0.318	2.52495E-17	10
Itm2c.6	0.799921197	0.53	0.208	2.81168E-17	10
Rbfox3.7	-1.125136275	0.178	0.497	2.82902E-17	10
Il18.1	0.516225269	0.162	0.013	2.91981E-17	10

Limd2.1	0.828595335	0.4	0.132	3.47923E-17	10
Hmgn1.6	-0.620383463	0.459	0.623	3.5467E-17	10
Cplx2.5	-1.016503816	0.173	0.498	3.57773E-17	10
Hnrnpm.4	-0.559214328	0.595	0.736	3.75673E-17	10
Cpt1a	0.459014424	0.168	0.016	3.98749E-17	10
Cnbp.4	-0.628267728	0.573	0.741	4.58214E-17	10
Mfsd12	0.490976294	0.168	0.016	4.8611E-17	10
Celf4.10	-1.390660508	0.038	0.323	5.11321E-17	10
Apba2.2	-1.011474823	0.097	0.417	7.18467E-17	10
Comt.1	0.79699241	0.378	0.106	7.4008E-17	10
Hnrnpd.6	-0.853672903	0.351	0.613	7.93937E-17	10
Usp2	0.406729204	0.146	0.011	8.25128E-17	10
1500016L03Rik.6	-1.040328508	0.076	0.386	9.06194E-17	10
Cln3	0.680108351	0.227	0.034	9.4121E-17	10
Pcdhga9.1	-0.981010143	0.114	0.423	1.19412E-16	10
Pdk1	0.450963046	0.124	0.006	1.20162E-16	10
Abhd6.1	0.521798299	0.227	0.035	1.31396E-16	10
Slc46a1	0.287114728	0.124	0.009	1.3773E-16	10
Parp9	0.444456177	0.135	0.008	1.49434E-16	10
Scg5.5	-1.065212124	0.049	0.337	1.74105E-16	10
Slc16a6	0.643429377	0.243	0.039	1.83768E-16	10
Ina.9	-1.122999293	0.141	0.446	1.90304E-16	10
Dynlt3.1	0.612965853	0.254	0.044	1.96654E-16	10
Commd9	0.749600438	0.33	0.081	2.03398E-16	10
Anp32e.9	-0.808064498	0.357	0.616	2.0418E-16	10
Tanc2.1	0.431805897	0.151	0.014	2.19444E-16	10
Adam17	0.715091076	0.254	0.047	2.98782E-16	10
Vps37b.3	-0.977356411	0.168	0.473	3.05923E-16	10
Uncx.8	-1.098939006	0.038	0.32	3.29126E-16	10
Dek.9	-0.610581045	0.616	0.694	3.68714E-16	10
Srrm2.1	-0.673324799	0.508	0.734	4.68355E-16	10
Fubp1.2	-0.828278673	0.292	0.569	4.79518E-16	10
Mycn.4	-1.025520526	0.07	0.366	5.11683E-16	10
Gnao1.7	-1.021214384	0.027	0.289	5.72651E-16	10
Rnf128	0.341940864	0.114	0.005	7.40625E-16	10
1110001A16Rik	0.792754034	0.346	0.094	7.47879E-16	10
Edem2	0.5702824	0.249	0.044	7.62426E-16	10
Kif5c.9	-1.1145026	0.114	0.401	8.28792E-16	10
Plekha2.1	0.405312617	0.114	0.005	8.46211E-16	10
Isyna1	0.356653369	0.13	0.009	8.48163E-16	10
Mt2.2	0.747133288	0.346	0.089	9.84616E-16	10
Atp6v0b.8	0.738726047	0.573	0.264	1.16391E-15	10
Map3k8	0.43573753	0.108	0.004	1.19006E-15	10
Galns	0.377178994	0.114	0.005	1.2429E-15	10
Tns3.1	0.540784016	0.135	0.008	1.41245E-15	10
Sall1	0.409586091	0.141	0.01	1.41923E-15	10
Nop58.6	-0.860554374	0.319	0.595	1.4484E-15	10
Bhlhe41	0.571511252	0.141	0.011	1.48472E-15	10

Ssb	-0.666051015	0.497	0.705	1.56324E-15	10
Leprot.1	0.57399346	0.384	0.115	1.66938E-15	10
Nfkbia.1	0.906763727	0.357	0.169	1.82227E-15	10
Smpdl3a	0.390977665	0.135	0.01	1.82655E-15	10
Tagln3.9	-1.083568903	0.032	0.297	1.89754E-15	10
Manba	0.353522435	0.108	0.004	1.9948E-15	10
Arpc4	0.706430757	0.443	0.152	2.68728E-15	10
Sulf2.1	0.523942238	0.173	0.02	2.77301E-15	10
Cdkn1b.4	-0.921154665	0.162	0.47	3.10251E-15	10
Gas5.2	-0.657347074	0.535	0.744	3.16403E-15	10
Tmem135	0.510876911	0.205	0.03	3.47967E-15	10
Prdx5.2	0.722623468	0.573	0.261	3.52214E-15	10
Plekho1	0.569621844	0.259	0.05	3.60717E-15	10
Capg	0.500221685	0.103	0.004	3.71705E-15	10
As3mt	0.463166104	0.146	0.012	3.77144E-15	10
Mef2a.1	0.671305964	0.422	0.135	3.87708E-15	10
Nfe2l2	0.726708861	0.319	0.081	4.95507E-15	10
Pepd.1	0.692336437	0.232	0.04	5.2105E-15	10
Hnrnpr	-0.802895825	0.281	0.547	5.40037E-15	10
Pkib	0.369723932	0.146	0.018	5.42611E-15	10
Nap1l1.6	-0.820566278	0.227	0.513	5.89766E-15	10
Anxa4	0.627817545	0.227	0.039	6.1758E-15	10
Orai1.1	0.485831119	0.178	0.021	7.53141E-15	10
Cd81.3	0.627862528	0.562	0.46	7.996E-15	10
Top2b	-0.96750916	0.168	0.416	8.27153E-15	10
Ptma.4	-0.745680194	0.308	0.584	8.44662E-15	10
Sec14l1	0.557470291	0.254	0.051	9.66931E-15	10
Blvra.1	0.678449712	0.222	0.036	9.91838E-15	10
M6pr	0.69535976	0.368	0.106	1.00071E-14	10
Rnf13.1	0.593523661	0.314	0.078	1.18618E-14	10
Csf1	0.591526701	0.124	0.008	1.33224E-14	10
Rasa4	0.316227862	0.103	0.006	1.35759E-14	10
Ddx5.2	-0.533255768	0.676	0.817	1.58192E-14	10
Aga	0.485758875	0.211	0.034	1.64894E-14	10
Gm10075.7	-0.870441649	0.227	0.517	2.05981E-14	10
Hist1h2bc.1	0.751295995	0.216	0.036	2.101E-14	10
Fuca1	0.735590264	0.422	0.144	2.19519E-14	10
Ptn.8	-1.222661816	0.157	0.444	2.19598E-14	10
Hnrnpc.1	-0.625697978	0.47	0.669	2.5115E-14	10
Nacc2.1	0.413020013	0.157	0.017	2.55541E-14	10
Pla2g16	0.562640353	0.119	0.007	2.67355E-14	10
Sgpl1	0.693971438	0.427	0.146	2.77614E-14	10
Dnajb14	0.641267862	0.292	0.067	3.32308E-14	10
Elk3	0.459317736	0.168	0.019	3.39893E-14	10
Cib1	0.669590905	0.265	0.055	3.89356E-14	10
Celf2.9	-0.812792705	0.465	0.639	4.05623E-14	10
Rassf2	0.447667044	0.151	0.014	4.48851E-14	10
Skp1a	-0.787117328	0.297	0.584	5.03841E-14	10

Gm9800.4	-0.729451877	0.27	0.537	5.81845E-14	10
Mpp1	0.61663011	0.211	0.033	6.34407E-14	10
Bclaf1	-0.76610115	0.368	0.585	6.35711E-14	10
Htatsf1.1	-0.900815488	0.205	0.501	6.51983E-14	10
Amdhd2	0.62628165	0.232	0.042	6.83536E-14	10
Arhgap17	0.593389651	0.319	0.083	7.73027E-14	10
Rufy3.8	-1.002938343	0.114	0.374	8.73939E-14	10
Hdgfrp3	-0.828428646	0.07	0.34	1.02944E-13	10
Gyg.1	0.496808412	0.124	0.008	1.07277E-13	10
Krcc1.1	0.7032939	0.308	0.081	1.21401E-13	10
Thoc7.1	-0.769193174	0.286	0.523	1.24559E-13	10
Rps26.5	-0.434329872	0.827	0.847	1.33187E-13	10
Ubc	0.697728037	0.557	0.357	1.37412E-13	10
Atp6ap2	0.68744587	0.47	0.181	1.49384E-13	10
Rap2a.1	0.66277484	0.454	0.166	1.5041E-13	10
Gla	0.459884802	0.178	0.023	1.5375E-13	10
Fez1.1	-0.933044713	0.065	0.335	1.54148E-13	10
Khk	0.272944085	0.119	0.01	1.74396E-13	10
Bin1.8	0.636338788	0.708	0.426	1.74707E-13	10
Cacna2d1.6	-0.998578965	0.092	0.377	1.80997E-13	10
Snrpd1.5	-0.808282503	0.27	0.526	1.8855E-13	10
Cdk4.3	-0.632641582	0.449	0.644	2.13657E-13	10
Slc25a4.1	-0.498879137	0.724	0.808	2.17129E-13	10
Pdap1.2	-0.651167815	0.465	0.685	2.24801E-13	10
Supt16.6	-0.766886865	0.292	0.495	2.33996E-13	10
Acox3	0.558597483	0.157	0.017	3.00331E-13	10
Stmn4.10	-1.206687916	0.076	0.344	3.05581E-13	10
Fnbp1l.6	-0.959075825	0.173	0.456	3.07977E-13	10
Fam134b	0.431396631	0.195	0.032	3.20756E-13	10
Kif1b.7	-0.91931987	0.243	0.523	3.27084E-13	10
Tox3.2	-0.848891369	0.07	0.348	3.38806E-13	10
Atp6v1c1	0.614300463	0.357	0.106	3.55285E-13	10
Nfkbiz	0.551037992	0.13	0.011	3.80682E-13	10
Gmip	0.419237452	0.141	0.015	4.1576E-13	10
Rragc	0.586644718	0.249	0.051	4.5572E-13	10
Insm1.3	-0.979143671	0.043	0.295	4.64004E-13	10
Ppp1r18.1	0.598585785	0.27	0.061	4.76892E-13	10
Glb1	0.592658078	0.189	0.028	5.09479E-13	10
Mgat4a	0.402532773	0.124	0.009	5.35785E-13	10
Dynll1.3	-0.736331727	0.346	0.596	5.56402E-13	10
H2afj	0.625969651	0.497	0.202	5.89518E-13	10
Arhgdia.1	0.732617116	0.459	0.205	5.9714E-13	10
Brd3.3	-0.809619783	0.292	0.534	8.00384E-13	10
Elavl4.9	-1.079970446	0.086	0.337	8.01924E-13	10
Mdk.6	-1.073121183	0.114	0.382	8.87462E-13	10
Gsdmd	0.343622539	0.157	0.023	9.11835E-13	10
Zfhx3	0.643681517	0.222	0.042	9.93331E-13	10
Smc4.8	-0.939507771	0.351	0.568	1.12718E-12	10

Akap9.3	-0.959914306	0.249	0.503	1.1563E-12	10
Srsf11	-0.571359108	0.481	0.691	1.22816E-12	10
Pip4k2a	0.549669633	0.227	0.044	1.24841E-12	10
Camta1.2	-0.899592994	0.141	0.411	1.35357E-12	10
Tsc22d1.4	-0.90293742	0.146	0.43	1.37542E-12	10
Tmem9b.1	0.614164004	0.357	0.109	1.53786E-12	10
Basp1.9	-0.721832522	0.535	0.705	1.55007E-12	10
Mvp	0.307791353	0.103	0.007	1.75626E-12	10
Rnpep	0.560030561	0.227	0.043	1.80551E-12	10
Sash1.1	0.444671578	0.195	0.033	1.90448E-12	10
Cacng2.2	-0.938165293	0.076	0.34	1.92709E-12	10
Dtnbp1	0.607591401	0.33	0.094	1.99401E-12	10
Snx3.1	0.646291135	0.611	0.339	2.17181E-12	10
Ezr.5	-0.906106973	0.189	0.44	2.52839E-12	10
Hn1.6	-0.768329174	0.292	0.544	3.05665E-12	10
Nhlrc3	0.379268845	0.13	0.012	3.3605E-12	10
Il18bp	0.597727585	0.114	0.008	3.54301E-12	10
Ssrp1.5	-0.74829292	0.314	0.579	3.61824E-12	10
Tpi1.1	0.622307425	0.368	0.118	3.934E-12	10
H2afy2.2	-0.888982357	0.076	0.34	3.97437E-12	10
Kmt2e.6	-0.776030714	0.324	0.571	4.01239E-12	10
Prcp	0.546784193	0.205	0.035	4.71776E-12	10
Uchl1.8	-0.885844122	0.07	0.337	4.98706E-12	10
Sox4.7	-0.836625533	0.346	0.585	5.23664E-12	10
Wasf2.2	0.654671488	0.346	0.11	5.38845E-12	10
Sdf2l1.1	0.591319273	0.238	0.049	6.43047E-12	10
Cltb.6	-0.939807754	0.178	0.425	6.45241E-12	10
Mllt4.3	-0.888797922	0.097	0.365	6.48108E-12	10
Irf9	0.368288526	0.135	0.015	6.76099E-12	10
Capns1.1	0.639358823	0.514	0.224	6.86366E-12	10
Trim2.1	-0.934835414	0.043	0.285	7.09258E-12	10
Nucks1.9	-0.61028195	0.53	0.664	8.05183E-12	10
Rap1b	0.645481241	0.384	0.131	8.92362E-12	10
Zeb1.4	-0.919492319	0.124	0.39	9.09868E-12	10
Hdgf.6	-0.77294223	0.286	0.534	1.15532E-11	10
Pde3b	0.616848247	0.297	0.082	1.35347E-11	10
Ktn1.1	-0.801247512	0.195	0.462	1.4067E-11	10
Tep1	0.37558049	0.103	0.006	1.62571E-11	10
Ptov1	-0.779549509	0.249	0.486	1.67744E-11	10
Fus	-0.475172393	0.584	0.738	1.75797E-11	10
Smc2.8	-0.80839753	0.314	0.547	1.7755E-11	10
Tpst2	0.613041313	0.281	0.072	1.78134E-11	10
Cd164.2	0.653554518	0.357	0.117	1.81712E-11	10
Hnrnph3.1	-0.824510172	0.168	0.429	2.09553E-11	10
Galc	0.538954517	0.211	0.039	2.18911E-11	10
Eif4g2	-0.495132915	0.535	0.707	2.35254E-11	10
Cyb5r1	0.462707915	0.168	0.024	2.40883E-11	10
Tram1.1	0.590035759	0.373	0.125	2.49707E-11	10

Rad21.10	-0.858058828	0.216	0.464	2.62643E-11	10
Nrxn1.9	-1.110054233	0.086	0.32	2.69797E-11	10
Bri3	0.6557909	0.292	0.085	2.98452E-11	10
Gnai2.5	0.54424108	0.714	0.421	3.01513E-11	10
Dusp3	0.445855077	0.173	0.027	3.08947E-11	10
Ddx26b.3	0.781148963	0.341	0.127	3.12177E-11	10
Dram2	0.53896958	0.292	0.085	3.26143E-11	10
Hsd11b2.8	-0.887242328	0.049	0.29	4.21431E-11	10
Myh10.1	-0.886233482	0.081	0.326	4.86591E-11	10
Kif21a.2	-0.951434744	0.097	0.348	4.87437E-11	10
Magt1.1	0.564318359	0.243	0.055	5.11405E-11	10
Dclk1.5	-0.864317042	0.146	0.414	5.1417E-11	10
6330403K07Rik.7	-0.91801779	0.038	0.244	5.2738E-11	10
Rpl4.4	-0.438796162	0.832	0.909	5.59691E-11	10
Fnip2	0.452249851	0.141	0.015	5.80008E-11	10
Pcbp2.1	-0.532589771	0.47	0.623	5.84364E-11	10
Ptpre.1	0.250251821	0.114	0.016	5.94789E-11	10
Cfdp1.1	-0.755070254	0.276	0.486	6.03385E-11	10
Atpif1.1	-0.469982555	0.724	0.816	6.2645E-11	10
Fam115a.2	-0.900992005	0.108	0.353	6.73134E-11	10
Rdx	-0.685305961	0.4	0.605	7.68277E-11	10
Gsn.1	0.444282346	0.124	0.012	8.99161E-11	10
Crlf2	0.41442643	0.135	0.014	9.80169E-11	10
Clip3.5	-0.829401608	0.07	0.314	1.10253E-10	10
Sh3glb1.1	0.605136863	0.627	0.363	1.15479E-10	10
Rrp1	-0.633205924	0.449	0.607	1.24606E-10	10
Ttyh2.1	0.417799469	0.184	0.037	1.26071E-10	10
Elf1.1	0.571138266	0.232	0.051	1.26389E-10	10
Pdlim4.1	0.567370568	0.276	0.074	1.28433E-10	10
Tor1aip1	0.528374611	0.303	0.089	1.29303E-10	10
Pfn2.5	-0.85909281	0.032	0.243	1.36759E-10	10
Ptgr2	0.411050926	0.151	0.021	1.44103E-10	10
Anp32b.7	-0.520493274	0.449	0.592	1.4612E-10	10
Hmgb3.4	-0.8249368	0.07	0.308	1.52038E-10	10
Snhg5.1	-0.68108443	0.119	0.384	1.54573E-10	10
Pcbp4.2	-0.815666205	0.076	0.327	1.57171E-10	10
Vps18	0.391977347	0.168	0.028	1.6789E-10	10
Dpysl3.10	-1.030324469	0.022	0.221	1.73627E-10	10
Hnrnpa0.1	-0.759188758	0.249	0.458	1.77417E-10	10
Top1.1	-0.626975472	0.481	0.665	1.93337E-10	10
Hmgn5.7	-0.824009629	0.168	0.427	2.10502E-10	10
Meis1.2	-0.862570425	0.119	0.375	2.20663E-10	10
Tshz2.4	-0.880487159	0.081	0.322	2.25842E-10	10
C1ql1.5	-0.861505148	0.049	0.281	2.36341E-10	10
Whsc1.3	-0.810578506	0.222	0.466	2.4202E-10	10
Slc36a1	0.402307884	0.13	0.015	2.54013E-10	10
Abcd2	0.44868551	0.297	0.092	2.58188E-10	10
Cox7a2.1	-0.379029349	0.643	0.713	2.76921E-10	10

Ccnd1.7	-0.789905983	0.324	0.524	2.8196E-10	10
Ppp1r14c.8	-0.996456901	0.049	0.264	2.83102E-10	10
Eif2ak2	0.491088094	0.114	0.01	2.8585E-10	10
Strbp.5	-0.859451032	0.097	0.349	2.88332E-10	10
Snrpf.5	-0.778437187	0.195	0.429	2.90172E-10	10
Metap2	-0.664130454	0.357	0.598	3.28255E-10	10
mt-Cytb.1	-0.367493282	0.897	0.938	3.63215E-10	10
Mfsd11	0.554300451	0.227	0.05	3.78782E-10	10
Tmem179b	0.625934259	0.222	0.05	4.10632E-10	10
Aldoa.6	0.618336061	0.524	0.254	4.3599E-10	10
Ncald.1	-0.71668494	0.011	0.167	4.59649E-10	10
Rab31.1	0.471127786	0.227	0.053	4.89607E-10	10
Arrdc1	0.390544939	0.108	0.009	5.28225E-10	10
Bzw2	-0.778970983	0.232	0.491	5.31581E-10	10
Nap1l4	-0.711411584	0.308	0.492	5.46034E-10	10
Tmbim6.1	0.593785932	0.584	0.321	5.66463E-10	10
Rbfox2.7	-0.86373363	0.054	0.285	5.9382E-10	10
Gng3.9	-0.967751203	0.076	0.319	6.51059E-10	10
Lamtor1	0.636440495	0.449	0.196	6.81257E-10	10
Slc12a2.1	0.599179187	0.33	0.108	6.89193E-10	10
Nhlh1.7	-0.954510063	0.032	0.225	7.20511E-10	10
Nsg1.7	-0.878737374	0.07	0.289	8.58894E-10	10
Tacc1	0.590121887	0.259	0.068	8.63962E-10	10
Lactb.1	0.513098289	0.178	0.033	9.28673E-10	10
Podxl2.9	-0.876403448	0.027	0.229	9.31427E-10	10
Tmem219	0.477176137	0.265	0.073	9.39365E-10	10
Ahi1	-0.858911112	0.07	0.309	9.58387E-10	10
Apc.9	-0.900903379	0.195	0.433	1.06354E-09	10
Fam210b.6	-0.880168751	0.081	0.316	1.08184E-09	10
Arid4b	-0.787739957	0.184	0.442	1.24739E-09	10
H2afy.5	-0.566494497	0.443	0.593	1.73184E-09	10
Cdk5rap2.4	-0.726592964	0.011	0.185	1.81231E-09	10
Serp1.1	0.558635173	0.47	0.215	1.87838E-09	10
Gm5617	0.553944889	0.238	0.058	1.89596E-09	10
Rundc3a.4	-0.841269933	0.038	0.25	1.98792E-09	10
Pald1	0.484661728	0.259	0.072	1.9987E-09	10
Gm2694.2	-0.864007848	0.119	0.361	2.01288E-09	10
Map4k4.1	-0.773933152	0.243	0.451	2.06428E-09	10
Safb	-0.72744523	0.303	0.505	2.07195E-09	10
Tmem173	0.495549374	0.124	0.015	2.15405E-09	10
Tsn.1	-0.619876572	0.389	0.591	2.37569E-09	10
Hpca.7	-0.697190849	0.059	0.279	2.37995E-09	10
Tecr	-0.64893771	0.357	0.531	2.92311E-09	10
Ptprs.7	-0.895100146	0.178	0.423	3.24201E-09	10
Tcerg1.1	-0.745879947	0.243	0.459	3.58163E-09	10
Smap2	0.648559689	0.341	0.12	3.66697E-09	10
Ppib.2	-0.456229605	0.649	0.701	3.7839E-09	10
Mab21l1.7	-0.809392625	0.027	0.227	3.82658E-09	10

Luc7l	-0.791791198	0.162	0.362	4.08137E-09	10
Wipf1.1	0.466521426	0.162	0.027	4.40199E-09	10
Tbata.7	-0.956163206	0.108	0.342	4.62194E-09	10
Cdk5r1.8	-0.908830331	0.119	0.335	5.36543E-09	10
Cyp4f13	0.345643747	0.114	0.011	5.47374E-09	10
Thra.9	-0.886771076	0.059	0.23	6.02522E-09	10
Bcas1.3	-0.912345931	0.065	0.284	6.2026E-09	10
Ccdc88a.2	-0.799151616	0.265	0.484	6.57583E-09	10
Mcfd2	0.549597978	0.276	0.081	6.87966E-09	10
Ctcf.3	-0.734252984	0.286	0.498	7.09217E-09	10
Dixdc1.4	-0.821900182	0.065	0.236	7.91377E-09	10
Flcn	0.483215781	0.184	0.036	8.29268E-09	10
Itpripl1	0.304534158	0.108	0.011	8.36646E-09	10
Ncor1.1	-0.664739949	0.362	0.557	8.62008E-09	10
Nagk	0.564684154	0.195	0.041	8.78184E-09	10
Gng12.3	0.646787322	0.324	0.119	8.98132E-09	10
Acp2.1	0.316498529	0.259	0.091	9.14423E-09	10
1110007C09Rik	0.405463145	0.2	0.049	9.34633E-09	10
Cfl2	-0.712039662	0.162	0.411	1.07648E-08	10
Sppl2a.1	0.582690817	0.324	0.117	1.17448E-08	10
St3gal6	0.373099768	0.103	0.009	1.2696E-08	10
Arrb2	0.6150594	0.319	0.116	1.3759E-08	10
Srebf1.8	-0.876123647	0.108	0.336	1.38344E-08	10
Fam171b.2	-0.776904963	0.043	0.225	1.42086E-08	10
Txnrd1.2	-0.761977844	0.146	0.391	1.53716E-08	10
Atp6v0e2.4	-0.777437107	0.054	0.262	1.73476E-08	10
Tmed5.1	0.542195997	0.341	0.124	1.81945E-08	10
Snrpe.4	-0.584856966	0.368	0.564	1.86518E-08	10
Prpf4b	-0.72415466	0.276	0.473	1.87406E-08	10
Psma7.2	-0.451134174	0.659	0.783	1.88075E-08	10
Hey1.7	-0.882746225	0.076	0.275	2.008E-08	10
Degs1.1	0.554862726	0.259	0.074	2.23091E-08	10
Lrp10	0.36526484	0.178	0.04	2.29901E-08	10
Chgb.9	-0.996040979	0.043	0.242	2.37085E-08	10
Nop56.5	-0.768479271	0.124	0.363	2.44838E-08	10
Hmgb2.9	-0.836815099	0.184	0.402	2.52212E-08	10
Bex1.2	-0.71585929	0.032	0.225	2.88802E-08	10
Cntn2.10	-1.132256667	0.081	0.265	2.88853E-08	10
Adcy3	0.278385835	0.124	0.019	3.11013E-08	10
Atp6v1f	0.56318487	0.551	0.298	3.17258E-08	10
Scpep1	0.406834002	0.211	0.052	3.29897E-08	10
Elavl2.4	-0.821700271	0.059	0.272	3.30119E-08	10
Hmgn3.5	-0.651601877	0.059	0.272	3.54056E-08	10
Slc1a2.6	-0.858314971	0.086	0.296	3.76283E-08	10
Wwp1.1	0.659568547	0.265	0.082	3.79179E-08	10
Pou2f2	0.410972225	0.146	0.027	3.855E-08	10
Zfp710	0.332482856	0.103	0.01	4.04166E-08	10
Atp6ap1.1	0.436423817	0.351	0.137	4.09812E-08	10

Dnaja1	-0.715041072	0.168	0.376	4.20648E-08	10
Tuba1c.1	0.514991115	0.216	0.055	4.26099E-08	10
2700094K13Rik.9	-0.534991336	0.449	0.615	4.66113E-08	10
Cd63.3	0.411481883	0.849	0.754	4.97783E-08	10
Grina.7	0.266172796	0.265	0.107	5.04759E-08	10
AI854517.1	-0.651564149	0.016	0.19	5.15236E-08	10
Sord	0.408610111	0.151	0.025	5.27113E-08	10
Fgf9.1	-0.778220706	0.054	0.262	5.70371E-08	10
Srrm4.8	-0.803023871	0.022	0.197	5.86698E-08	10
Sf3b2	-0.541259816	0.481	0.626	5.8995E-08	10
Akap6.2	-0.747725781	0.032	0.22	5.93426E-08	10
Snx18.1	0.453243706	0.184	0.039	6.10403E-08	10
Mki67.10	-0.896848653	0.2	0.396	6.3343E-08	10
Psmb10.1	0.47764265	0.227	0.06	6.5161E-08	10
Tmem66.3	0.459413852	0.438	0.196	7.08379E-08	10
Rcn1.2	-0.702680043	0.038	0.235	7.15128E-08	10
1110004F10Rik	-0.618872178	0.2	0.425	7.57866E-08	10
Itfg3	0.440517221	0.119	0.016	7.61289E-08	10
Zc3h13.1	-0.7639875	0.259	0.464	8.016E-08	10
Smarca4	-0.681093932	0.281	0.507	8.43811E-08	10
Tmpo.9	-0.680783336	0.249	0.454	8.95883E-08	10
Mapk14	0.479794799	0.232	0.068	9.11032E-08	10
Lmnb1.9	-0.750765091	0.086	0.302	9.30236E-08	10
Arhgap19.1	0.471014691	0.162	0.033	9.97176E-08	10
Akr1b10	0.450940247	0.168	0.033	9.98016E-08	10
Map3k1.1	-0.750898566	0.178	0.372	1.05156E-07	10
Apex1.4	-0.595840608	0.211	0.453	1.05784E-07	10
Tln1.2	0.603457305	0.368	0.147	1.09224E-07	10
Prmt8.5	-0.771941295	0.065	0.274	1.17035E-07	10
Bcl11a.2	-0.700210231	0.027	0.211	1.17114E-07	10
Clmp.7	-0.856922813	0.049	0.244	1.17422E-07	10
Dynlrb1	-0.544199674	0.454	0.58	1.19221E-07	10
Klhdc2.1	-0.746605464	0.124	0.329	1.30106E-07	10
Rnmt.6	-0.789315325	0.124	0.35	1.36877E-07	10
P4hb.1	0.564477168	0.519	0.276	1.38012E-07	10
Adam9.1	0.528941549	0.205	0.051	1.46186E-07	10
Srrt.1	-0.734988185	0.205	0.426	1.54846E-07	10
Btbd17.5	-0.709395548	0.005	0.161	1.58162E-07	10
Set.5	-0.672785921	0.189	0.389	1.60315E-07	10
Tsc22d4.3	0.494554156	0.389	0.163	1.63522E-07	10
Elovl6.2	-0.782729476	0.081	0.28	1.66833E-07	10
Ywhae	-0.406167192	0.654	0.727	1.77506E-07	10
Scarb1	0.322816896	0.162	0.035	1.82469E-07	10
Fut9.2	-0.787433177	0.032	0.213	1.84753E-07	10
Kif3a.1	-0.754097944	0.135	0.318	1.88441E-07	10
Pde1b	0.363665469	0.124	0.019	1.92132E-07	10
Cbfa2t3.7	-0.76109767	0.173	0.405	2.15105E-07	10
Fkbp4.2	-0.571897269	0.211	0.416	2.15507E-07	10

Rab8b.1	0.483465903	0.2	0.048	2.29811E-07	10
Sacs.2	-0.723956335	0.032	0.192	2.47674E-07	10
Maged1.1	-0.712454072	0.216	0.431	2.59131E-07	10
Pcbp1.3	-0.464405209	0.486	0.594	2.6193E-07	10
Cirbp.2	-0.749042843	0.157	0.357	2.6345E-07	10
Bcl7a.2	-0.770276388	0.119	0.327	2.72124E-07	10
Prim1.7	-0.784488434	0.081	0.264	2.77998E-07	10
Hirip3.8	-0.757556813	0.173	0.384	2.83349E-07	10
Cnot6.1	-0.695914178	0.173	0.393	2.91926E-07	10
Dap	0.333342668	0.184	0.047	2.94171E-07	10
Khdrbs1.1	-0.636005413	0.308	0.495	3.02276E-07	10
Npdc1.6	-0.758137095	0.059	0.263	3.09467E-07	10
Dner.10	-0.838695913	0.038	0.216	3.16915E-07	10
Actr3	0.557778561	0.319	0.126	3.23879E-07	10
Hjurp.7	-0.738666518	0.238	0.436	3.47515E-07	10
Mllt3.2	-0.75981149	0.043	0.227	3.51048E-07	10
Mast3	0.344167362	0.103	0.011	3.63001E-07	10
Cct6a	-0.636637233	0.292	0.488	3.74982E-07	10
Gstm5.2	-0.724789948	0.146	0.324	3.75052E-07	10
Slc45a4	0.471459761	0.13	0.021	4.00467E-07	10
Snx2	0.588410488	0.265	0.094	4.00836E-07	10
Kdm1a.2	-0.659924674	0.135	0.366	4.35053E-07	10
Tpm3	0.522449081	0.562	0.313	4.35219E-07	10
1700025G04Rik.5	-0.761123591	0.07	0.265	4.36833E-07	10
Ap3b2.1	-0.65598169	0.027	0.204	4.37297E-07	10
Rplp1.5	0.316808755	0.962	0.876	4.45061E-07	10
Mapk8ip2.2	-0.631715565	0.011	0.144	4.56577E-07	10
Nkd1.4	-0.754732104	0.059	0.25	4.62299E-07	10
Rsrc2	-0.532674829	0.335	0.549	4.80827E-07	10
Dbnl	0.538283255	0.292	0.108	5.10689E-07	10
Pigk	0.467056453	0.189	0.044	5.352E-07	10
Sf3b1	-0.468836698	0.557	0.669	5.52389E-07	10
Mcm7.6	-0.66723399	0.162	0.391	5.86286E-07	10
March1.2	0.29762962	0.162	0.042	6.33505E-07	10
Ptprd.3	-0.702616687	0.114	0.342	6.44883E-07	10
Wls.1	0.49028537	0.265	0.085	6.62047E-07	10
Hnrnpk.1	-0.511878565	0.476	0.637	6.95192E-07	10
Dusp6.2	0.58310753	0.27	0.091	7.52853E-07	10
Mcur1	0.471207357	0.184	0.042	7.5954E-07	10
Dgkz.1	0.397160464	0.281	0.102	7.93457E-07	10
Neu1	0.482976778	0.184	0.045	8.10094E-07	10
Tle1.3	-0.706215825	0.043	0.227	8.13848E-07	10
Hp1bp3.1	-0.482309388	0.449	0.582	8.19443E-07	10
Ptpn1	0.542557993	0.373	0.16	8.36203E-07	10
Nek6.2	0.542578359	0.341	0.142	8.73127E-07	10
Aldh9a1.1	0.449930558	0.205	0.054	9.05853E-07	10
Gng5.1	0.56423596	0.373	0.162	9.37481E-07	10
Rps3a1.4	-0.38341699	0.6	0.667	9.39962E-07	10

Aim2	0.366278397	0.103	0.012	9.52703E-07	10
Stx7.2	0.43989494	0.303	0.114	9.65831E-07	10
Pbrm1.1	-0.603870262	0.373	0.532	9.75554E-07	10
Slc35c2	0.444269504	0.168	0.036	9.8976E-07	10
Rap2b.1	0.614847703	0.324	0.146	1.05055E-06	10
Pou3f2.6	-0.755658745	0.076	0.278	1.07463E-06	10
Usp22.4	-0.708167553	0.146	0.363	1.09377E-06	10
Apbb1.8	-0.694361834	0.032	0.171	1.15511E-06	10
Paip2.2	-0.596345852	0.346	0.551	1.15836E-06	10
Syncrip.3	-0.599175537	0.308	0.497	1.23391E-06	10
Kif2a	-0.711004765	0.092	0.252	1.29935E-06	10
Mid1ip1.1	0.407730941	0.195	0.05	1.29958E-06	10
Heg1.2	-0.630377991	0.027	0.158	1.30341E-06	10
Ostm1.1	0.510971144	0.189	0.046	1.36706E-06	10
Myo9b	0.356940917	0.178	0.046	1.39268E-06	10
Sypl	0.500176082	0.238	0.072	1.41818E-06	10
Ccar1	-0.496144895	0.422	0.573	1.51919E-06	10
Slc35f6	0.277559632	0.114	0.018	1.53042E-06	10
Ddx46	-0.706773907	0.232	0.444	1.59291E-06	10
Cln5	0.39066543	0.162	0.034	1.5956E-06	10
Dock10.1	0.381484412	0.114	0.015	1.64893E-06	10
Sdf4.1	0.492022243	0.454	0.22	1.68756E-06	10
Cflar	0.502936721	0.195	0.049	1.68969E-06	10
Col27a1	0.302600707	0.103	0.013	1.75404E-06	10
Npm1.6	-0.422748571	0.551	0.647	1.77106E-06	10
Otx2.2	-0.738706748	0.043	0.218	1.85681E-06	10
Atp6v1g1	0.516652066	0.427	0.209	1.90363E-06	10
Dkc1.5	-0.680834916	0.119	0.335	1.97949E-06	10
Purb.1	-0.620657012	0.368	0.551	1.98567E-06	10
St8sia3.2	-0.72978837	0.07	0.252	2.00013E-06	10
Rbm39	-0.368626619	0.714	0.793	2.01133E-06	10
Gm8292.3	-0.687650978	0.173	0.371	2.02412E-06	10
Chd6	-0.741000875	0.108	0.306	2.0495E-06	10
Srrm3.4	-0.790735792	0.054	0.241	2.12838E-06	10
Tead2.6	-0.684093272	0.076	0.28	2.1777E-06	10
BC005764.9	-0.777278348	0.027	0.187	2.20673E-06	10
B4galt1	0.400078032	0.151	0.029	2.25306E-06	10
Rogdi	0.543080148	0.2	0.062	2.28905E-06	10
Eny2.3	-0.494130337	0.184	0.389	2.40948E-06	10
Ldhb.4	0.587244238	0.432	0.279	2.47425E-06	10
Pdgfra.4	-0.761278607	0.027	0.183	2.57128E-06	10
Ubtf.1	-0.651545298	0.249	0.454	2.83664E-06	10
Pak3	-0.74657908	0.038	0.207	2.94281E-06	10
Plod3	0.315757338	0.205	0.064	3.03164E-06	10
2310022B05Rik.3	-0.697927845	0.065	0.25	3.06461E-06	10
Ehbp1l1	0.35910019	0.124	0.021	3.25456E-06	10
Ube2e3.2	-0.647489844	0.238	0.418	3.30358E-06	10
Tex14.6	-0.756051053	0.022	0.146	3.44856E-06	10

Ank	0.517690949	0.2	0.054	3.44897E-06	10
Zc3hav1	0.342612944	0.103	0.012	3.45514E-06	10
Rps19.6	-0.295837007	0.762	0.753	3.47356E-06	10
Eif1ax.1	-0.603020347	0.232	0.398	3.547E-06	10
Nol7.1	-0.529076328	0.405	0.571	3.59236E-06	10
Cdh20.4	-0.628863945	0.032	0.204	3.63092E-06	10
Rab6b.9	-0.750795786	0.049	0.204	3.63595E-06	10
Sept4.8	-0.859826348	0.054	0.228	3.80571E-06	10
Ubb.4	-0.527486848	0.422	0.542	4.08163E-06	10
Rhob	0.609444103	0.297	0.137	4.27686E-06	10
Fbxo6	0.408917306	0.13	0.022	4.34397E-06	10
Prr13.1	0.473508353	0.259	0.088	4.35256E-06	10
Ythdc1.1	-0.628871774	0.249	0.479	4.6151E-06	10
Dnm2	0.406622754	0.222	0.069	4.6679E-06	10
Ltbr	0.31303719	0.108	0.015	4.73366E-06	10
Cenpv.4	-0.577517065	0.119	0.33	4.83833E-06	10
Actr2.1	0.570705826	0.405	0.195	5.00547E-06	10
Prrc2c	-0.533412497	0.384	0.554	5.24231E-06	10
Cxxc5.4	-0.603410658	0.373	0.545	5.33132E-06	10
Rsf1	-0.755116988	0.151	0.34	5.44436E-06	10
Tspan6.2	-0.582876077	0.059	0.247	5.55075E-06	10
Tmem178.2	-0.672826774	0.027	0.188	5.8897E-06	10
Atoh1.6	-0.685942539	0.022	0.165	5.90397E-06	10
Txnip	0.567018921	0.286	0.105	6.49002E-06	10
Csnk1a1	-0.44397548	0.53	0.668	6.56512E-06	10
Gna12	0.296057522	0.227	0.081	6.60618E-06	10
Rbmx.2	-0.663862039	0.114	0.317	6.61167E-06	10
Kcnk1.7	-0.760761808	0.038	0.206	6.6171E-06	10
Srrm1	-0.4874533	0.47	0.612	6.82674E-06	10
Cln8	0.350849395	0.178	0.047	6.8591E-06	10
Rhoc.1	0.347611106	0.135	0.026	6.92765E-06	10
Klc1.7	-0.703522544	0.146	0.363	7.00992E-06	10
Naga	0.357781575	0.151	0.032	7.01129E-06	10
Satb1	-0.65965265	0.038	0.211	7.55469E-06	10
Thoc2	-0.608373591	0.319	0.449	7.56656E-06	10
Pdcd4.1	-0.723290372	0.135	0.332	7.70891E-06	10
Sptbn1.7	-0.755598971	0.151	0.355	7.87918E-06	10
Pgd	0.511146261	0.243	0.081	8.18577E-06	10
Dnajc9.8	-0.69682152	0.178	0.351	8.78534E-06	10
Mapt.9	-0.859103025	0.054	0.217	9.14806E-06	10
Dut.8	-0.581920601	0.227	0.437	9.30692E-06	10
Prdx1.5	0.480674721	0.676	0.487	9.31971E-06	10
Snrnp40.4	-0.592562554	0.081	0.272	9.50387E-06	10
Tcp1.2	-0.482188836	0.357	0.464	9.5136E-06	10
Sept7.1	-0.486344139	0.465	0.59	9.5984E-06	10
Chrna3.8	-0.622471416	0.011	0.119	9.60849E-06	10
Rbp4.7	-0.677609564	0.059	0.243	1.03824E-05	10
Coa5	0.468584624	0.189	0.05	1.06991E-05	10

Plcb4.2	-0.646532685	0.005	0.14	1.07169E-05	10
Elovl4.1	-0.45659661	0.011	0.111	1.07224E-05	10
Derl1	0.363337312	0.249	0.093	1.07838E-05	10
Clstn1.3	-0.58342716	0.119	0.203	1.08249E-05	10
Ppp3cb.1	-0.695667864	0.135	0.311	1.12959E-05	10
Sult4a1.1	-0.591024244	0.049	0.224	1.1533E-05	10
Rbm25	-0.410252563	0.692	0.813	1.18895E-05	10
Trp53i11.2	-0.588183808	0.038	0.203	1.19383E-05	10
Sh3gl2.4	-0.676634397	0.038	0.172	1.19476E-05	10
Coro2b.1	-0.635286463	0.022	0.175	1.19538E-05	10
Sirt2.3	0.385969802	0.351	0.153	1.2277E-05	10
Tgfb2.3	-0.681027848	0.032	0.193	1.25907E-05	10
Aplp1.10	-0.672303526	0.038	0.152	1.26135E-05	10
Ilf3	-0.585178085	0.189	0.378	1.30699E-05	10
Colgalt1	0.346880746	0.292	0.119	1.31616E-05	10
St18.9	-0.801466224	0.032	0.192	1.31968E-05	10
Pkn1	0.41061632	0.173	0.043	1.34305E-05	10
Homer2.3	-0.683963999	0.07	0.248	1.3485E-05	10
Fam57b.4	-0.649058427	0.049	0.187	1.3703E-05	10
Ubl3.3	0.442026659	0.422	0.206	1.3796E-05	10
Gltp.1	0.293513609	0.227	0.082	1.38647E-05	10
Crip2.3	-0.732811421	0.059	0.242	1.38797E-05	10
C130071C03Rik.3	-0.580302196	0.103	0.31	1.39105E-05	10
Brk1	0.491726331	0.589	0.374	1.40032E-05	10
Sept11.2	-0.700053598	0.119	0.324	1.40034E-05	10
Sema6a.9	-0.742736878	0.038	0.203	1.43063E-05	10
Reep5.2	0.406852354	0.324	0.136	1.44577E-05	10
Nfkb1	0.291317165	0.124	0.024	1.47454E-05	10
Sstr2.6	-0.712283261	0.038	0.192	1.51089E-05	10
Vasp	0.4325967	0.216	0.065	1.6175E-05	10
Atp5b.2	-0.344228264	0.676	0.713	1.62158E-05	10
Ppcdc	0.323771353	0.135	0.028	1.65961E-05	10
Tmed10.1	0.520314654	0.411	0.202	1.69857E-05	10
Nucb1	0.411582748	0.227	0.073	1.75687E-05	10
Islr2.5	-0.662843776	0.027	0.181	1.80889E-05	10
Pa2g4.6	-0.499632898	0.373	0.523	1.85814E-05	10
Peg3.1	-0.744200671	0.097	0.286	1.87501E-05	10
Ttc9b.1	-0.626073608	0.038	0.196	1.98485E-05	10
Ube2b.1	-0.538207497	0.368	0.522	2.08576E-05	10
Zcchc18.2	-0.634177107	0.022	0.171	2.08992E-05	10
Itgav.1	0.297112111	0.189	0.059	2.10213E-05	10
Ophn1.1	0.450912012	0.243	0.082	2.14376E-05	10
Stmn1.3	-0.660478796	0.097	0.286	2.17382E-05	10
Tmod2	-0.684980852	0.043	0.209	2.31633E-05	10
Nell2.1	-0.564203967	0.005	0.133	2.32207E-05	10
Arl3	-0.576939037	0.151	0.367	2.32954E-05	10
Zbtb18.6	-0.721565338	0.108	0.268	2.35078E-05	10
Ssh2	0.482491709	0.173	0.043	2.37317E-05	10

Zfp326	-0.638660303	0.205	0.411	2.5272E-05	10
Ppic.4	-0.666918795	0.065	0.241	2.5522E-05	10
Wbp5.4	-0.550631653	0.362	0.52	2.56607E-05	10
Rtf1	-0.603370853	0.346	0.513	2.58048E-05	10
RP23-45G16.5.10	-0.748753007	0.13	0.329	2.6138E-05	10
Baz1b.3	-0.575397067	0.324	0.494	2.72309E-05	10
Rcor2.6	-0.705235738	0.065	0.226	2.77026E-05	10
Taok3.2	0.416223727	0.243	0.086	2.79875E-05	10
Acat1.1	-0.617412944	0.254	0.442	2.82022E-05	10
Kifap3.4	-0.718881495	0.097	0.274	2.84018E-05	10
Cyc1	-0.562262792	0.27	0.414	3.15952E-05	10
Klf9.5	-0.680276169	0.081	0.272	3.25841E-05	10
Tro.2	-0.546005833	0.022	0.127	3.33818E-05	10
Prc1.9	-0.800949015	0.119	0.258	3.37216E-05	10
Dhx36	-0.669040364	0.162	0.372	3.54273E-05	10
App.6	-0.563838298	0.427	0.569	3.54499E-05	10
Rab5c.1	0.501739138	0.265	0.095	3.55099E-05	10
Cetn3	-0.573396178	0.335	0.488	3.5741E-05	10
Pdzrn3.7	-0.789057284	0.016	0.148	3.78375E-05	10
Gm11266.6	-0.657290175	0.022	0.145	3.82529E-05	10
Rbbp6.1	-0.415174914	0.33	0.433	3.9626E-05	10
Rplp0.4	-0.274983271	0.881	0.872	4.01706E-05	10
Rnf144a	-0.496799724	0.022	0.161	4.03524E-05	10
H1fx.8	-0.644466374	0.065	0.247	4.15646E-05	10
Arpc3	0.469037221	0.324	0.14	4.27689E-05	10
Cct2.2	-0.530074354	0.368	0.525	4.38905E-05	10
H2afx.9	-0.751630354	0.173	0.332	4.42479E-05	10
Cr1l	0.276267539	0.162	0.045	4.49449E-05	10
Dusp1.2	0.616730817	0.33	0.161	4.56553E-05	10
Adrbk1	0.356363854	0.178	0.053	4.66E-05	10
Ubash3b.1	0.358800881	0.222	0.076	4.6978E-05	10
Myef2	-0.508895698	0.146	0.34	4.73429E-05	10
Fam168a.3	-0.647938148	0.157	0.288	4.88478E-05	10
Gsk3b.4	-0.565304029	0.281	0.502	4.96971E-05	10
Dhrs1	0.363457039	0.249	0.091	5.06576E-05	10
Slc23a2	0.257601163	0.108	0.019	5.18336E-05	10
Prkcb.8	-0.74003882	0.097	0.257	5.23084E-05	10
Vezf1	-0.63953657	0.168	0.343	5.56158E-05	10
Cdkn1a.1	0.738102189	0.173	0.091	5.79342E-05	10
Lpp	0.367718545	0.119	0.022	5.93248E-05	10
Pacsin2	0.427514867	0.119	0.022	5.98725E-05	10
Mllt11.8	-0.682656083	0.049	0.22	5.99793E-05	10
Fnip1	0.257298248	0.2	0.071	6.06641E-05	10
Igsf3.3	-0.569945641	0.011	0.134	6.15936E-05	10
St6galnac4.1	0.297606843	0.13	0.032	6.22251E-05	10
Myt1.9	-0.734789621	0.016	0.154	6.35451E-05	10
Rgl2	0.376768709	0.281	0.113	6.39483E-05	10
Rab7.1	0.505072475	0.384	0.181	6.52776E-05	10

Fermt2.1	-0.643386549	0.043	0.206	6.5715E-05	10
Ebf3.3	-0.671365023	0.043	0.204	6.96406E-05	10
Gm3764.3	-0.476589905	0.011	0.132	6.99725E-05	10
Zfp462.3	-0.57207775	0.022	0.157	7.13403E-05	10
Ccp110.4	-0.63379985	0.07	0.249	7.2451E-05	10
Igfbp4	0.594522504	0.108	0.018	7.35697E-05	10
Cdc5l	-0.636235101	0.211	0.402	7.61808E-05	10
Spc25.9	-0.713044389	0.086	0.226	7.84408E-05	10
Aprt.4	0.551677429	0.319	0.139	8.22349E-05	10
Med19.1	-0.608631029	0.146	0.319	8.52159E-05	10
Ddx42	-0.605654197	0.232	0.384	8.67755E-05	10
Sec11c.5	0.469094229	0.422	0.21	8.74441E-05	10
Efs.2	-0.481367459	0.016	0.126	8.77889E-05	10
Top2a.9	-0.763315833	0.259	0.429	8.91648E-05	10
Amz1.1	0.324696874	0.103	0.015	9.21198E-05	10
Epc1.1	-0.603812907	0.114	0.233	9.59249E-05	10
Eid1.5	-0.580949139	0.346	0.504	9.81017E-05	10
Rhoa	0.443818256	0.324	0.14	0.000103202	10
Smc6.6	-0.607263747	0.232	0.424	0.000104784	10
Slc17a6.9	-0.681935744	0.016	0.149	0.000107643	10
Nudt14	0.283502061	0.108	0.018	0.000107904	10
Srpk2	-0.537714395	0.276	0.43	0.000108675	10
Stat3.1	0.389317463	0.157	0.04	0.000108991	10
Nrn1.6	-0.707044339	0.076	0.223	0.00011103	10
Sox9.6	-0.663906908	0.076	0.256	0.000111554	10
Tmeff1.2	-0.5270648	0.016	0.15	0.000114284	10
Fbxo32.4	-0.540088799	0.011	0.117	0.00011694	10
Bach1	0.297569629	0.216	0.079	0.000123048	10
Rbm8a.3	-0.532889203	0.319	0.467	0.000124458	10
Arglu1	-0.47172203	0.384	0.559	0.000125178	10
Snrpn.1	-0.592110054	0.022	0.159	0.000125551	10
Fez2	0.349633432	0.151	0.036	0.000128135	10
Nr2f1	-0.596355745	0.043	0.205	0.000128167	10
Ptprg.2	-0.460586679	0.016	0.143	0.000129184	10
Setbp1.2	-0.614391932	0.022	0.161	0.000134196	10
Cenpe.9	-0.889564147	0.114	0.287	0.00013462	10
Ssr4	0.483834802	0.573	0.374	0.000134866	10
Bnip2	0.479845399	0.395	0.215	0.000135423	10
Tspyl4.7	-0.579422699	0.005	0.128	0.000138687	10
2810417H13Rik.9	-0.718942774	0.227	0.393	0.000148716	10
Sema7a.5	-0.484814619	0.022	0.157	0.000150024	10
Snap25.6	-0.618308434	0.103	0.284	0.000151032	10
Polr2h.2	-0.598685924	0.065	0.226	0.000151577	10
Tnik.7	-0.715545465	0.022	0.147	0.000162945	10
Fmnl3	0.317952799	0.124	0.025	0.000170134	10
Camk1d	0.341377705	0.108	0.022	0.000173569	10
Ldha.4	0.326146747	0.416	0.229	0.000181653	10
Fmr1	-0.543081147	0.108	0.301	0.000182552	10

9330159F19Rik.4	-0.643816766	0.032	0.17	0.000199729	10
Dtx1.2	-0.611186627	0.027	0.167	0.000204084	10
Mtss1.8	-0.756596919	0.119	0.27	0.000208159	10
Marc2.1	0.318997762	0.222	0.082	0.000213323	10
Mfap4.5	-0.680048845	0.027	0.163	0.000222799	10
Extl3	0.380999399	0.189	0.058	0.000230301	10
Smpd3.4	-0.620801558	0.032	0.162	0.000240813	10
Rbm5.4	-0.58039333	0.276	0.402	0.000248813	10
Bnip3	0.349594937	0.124	0.025	0.000254847	10
Fstl1.6	-0.624141469	0.032	0.181	0.000265839	10
Tcf25.1	-0.445333691	0.497	0.603	0.000270409	10
Rnd2.3	-0.630259401	0.027	0.159	0.000274797	10
Gpr153.5	-0.63002183	0.049	0.21	0.000281691	10
Slc12a9	0.423146614	0.157	0.042	0.000285074	10
1110001J03Rik	-0.628658728	0.07	0.239	0.000291349	10
Myo5a.1	0.499469095	0.384	0.189	0.000302958	10
Snrpb.5	-0.439542291	0.438	0.574	0.000304527	10
Sltm	-0.565151106	0.362	0.533	0.000312522	10
Tppp3.1	-0.515560595	0.027	0.159	0.000321631	10
Cmtm3.4	0.420699281	0.276	0.115	0.000323803	10
Cttnbp2nl	0.428611275	0.2	0.066	0.000326452	10
Coro1b	0.418808597	0.443	0.236	0.0003383	10
Armcx4	-0.475730743	0.016	0.114	0.000341546	10
Zfp704.2	-0.662127738	0.103	0.259	0.0003432	10
Sft2d1	0.312592591	0.114	0.021	0.000344063	10
Pcbd2	0.35110653	0.146	0.035	0.000356481	10
Tardbp.1	-0.548117297	0.308	0.484	0.000363409	10
Fut8	-0.613731169	0.065	0.229	0.000382811	10
Chtop.1	-0.57277108	0.119	0.25	0.000406494	10
Fkbp15.1	0.365794207	0.135	0.032	0.000414436	10
Tbc1d12.1	0.372688463	0.168	0.047	0.000416281	10
Smox	0.336682754	0.195	0.064	0.000420179	10
Mphosph8	-0.595821036	0.238	0.366	0.000425693	10
Hmgn2.7	-0.62445237	0.092	0.257	0.000426261	10
Calm3.3	-0.442030094	0.47	0.561	0.000426443	10
Snx6	0.421894844	0.557	0.366	0.000448063	10
Usp1.9	-0.50771172	0.151	0.34	0.000460903	10
Bicd1.1	-0.624299172	0.022	0.154	0.000461893	10
Ccser2	-0.652574634	0.097	0.26	0.000470043	10
Ten1.1	0.399110351	0.33	0.15	0.000470449	10
Kmt2a	-0.540061004	0.227	0.331	0.00047184	10
Pltp	0.363557733	0.124	0.026	0.000472544	10
Bok.6	-0.609397702	0.081	0.252	0.000475015	10
Zfp422.1	-0.598203202	0.092	0.247	0.000487291	10
Siva1.6	-0.642481066	0.108	0.274	0.000489236	10
Gde1.1	0.399119574	0.314	0.14	0.000493312	10
Cadm3.9	-0.666581184	0.022	0.142	0.0004955	10
Atp5o	-0.386267773	0.465	0.588	0.000500538	10

Rab2a	-0.528931119	0.308	0.432	0.000507674	10
Hist3h2ba.4	-0.472273649	0.016	0.138	0.000514489	10
Fam133b	-0.652334463	0.151	0.309	0.000517405	10
Nemf.1	-0.598154681	0.222	0.34	0.000525962	10
Tpm4.5	-0.577216588	0.178	0.318	0.000574222	10
Fam212b.1	-0.630370898	0.065	0.224	0.000574756	10
Carhsp1.3	-0.563829268	0.092	0.255	0.000577468	10
Mknk1	0.354463914	0.13	0.033	0.000586629	10
Rcn2	-0.597942764	0.124	0.282	0.000587311	10
Slc25a23	-0.450733519	0.027	0.108	0.000593906	10
Rbfa	0.291458906	0.151	0.042	0.000597369	10
Sowaha.7	-0.651821982	0.027	0.164	0.000602662	10
Sub1.1	-0.395691508	0.508	0.589	0.000607383	10
Rsl1d1.2	-0.529767764	0.308	0.491	0.000612572	10
Eprs	-0.549844897	0.319	0.492	0.000618454	10
Thrap3	-0.581430136	0.222	0.389	0.00062502	10
Pold2.2	-0.427496447	0.016	0.114	0.000629264	10
Zwint.2	-0.637493658	0.13	0.293	0.000630953	10
Cdh2	-0.51793439	0.022	0.154	0.000653257	10
Gpbp1.1	-0.59675626	0.184	0.358	0.000654022	10
Ddx17.1	-0.565225044	0.259	0.382	0.000678838	10
Rnf187	-0.519343757	0.243	0.422	0.000698854	10
Cttn	-0.420915245	0.011	0.115	0.000706781	10
Ank2.9	-0.762662182	0.081	0.242	0.00070847	10
Cks1b.9	-0.582800911	0.162	0.346	0.00072832	10
Zfp637	-0.52765367	0.086	0.258	0.000736753	10
Bcap31	0.414936768	0.357	0.172	0.00076211	10
Rnf20	-0.609889854	0.13	0.29	0.000764575	10
Ndufa12.2	-0.504682684	0.243	0.423	0.00076876	10
Akirin1	0.344388822	0.135	0.031	0.000779654	10
Os9.5	0.305629677	0.443	0.248	0.000784533	10
Cdc42.1	0.338895518	0.73	0.513	0.000801606	10
Cnot7	-0.594351147	0.081	0.217	0.000823622	10
Gsg1l.7	-0.596946318	0.086	0.257	0.000827311	10
Mfng	0.287067911	0.119	0.028	0.000835836	10
Snx8	0.376752773	0.119	0.024	0.000854319	10
Stox2.1	-0.618323286	0.049	0.2	0.000855347	10
Gabrb3.1	-0.420628056	0.011	0.103	0.000856337	10
Cenpf.10	-0.794656737	0.205	0.363	0.000865854	10
Cdk2ap1.1	-0.394328351	0.011	0.101	0.000869663	10
Gprasp1.1	-0.623832573	0.054	0.208	0.000874216	10
A630007B06Rik.1	-0.673763528	0.076	0.206	0.000881199	10
Syne2.1	-0.595424574	0.059	0.221	0.000939225	10
Csnk1e.1	-0.402123138	0.346	0.386	0.000942506	10
Itsn2.1	0.445243472	0.227	0.084	0.000973273	10
Sars	-0.56994994	0.254	0.422	0.000981292	10
Snrpd3.2	-0.444386638	0.378	0.536	0.000995875	10
Man1c1.2	0.359337482	0.286	0.124	0.001006394	10

Cpeb4	0.273081185	0.151	0.045	0.001028803	10
Smarcc1.3	-0.57689018	0.259	0.435	0.001041918	10
Bub3.6	-0.505490883	0.222	0.322	0.001093807	10
Scly	0.277274045	0.114	0.023	0.00110832	10
2610017I09Rik.1	-0.595342089	0.054	0.205	0.00111877	10
R3hdm1	-0.570217724	0.135	0.299	0.001154266	10
Ndn.3	-0.566155542	0.07	0.224	0.001165893	10
Odf2.4	-0.452961705	0.103	0.171	0.001217153	10
Atp1b3.5	0.388425985	0.546	0.334	0.001225932	10
BC005561	-0.643637668	0.146	0.291	0.001228531	10
Rab11a.1	0.447610221	0.476	0.275	0.001230157	10
Tubb2a.10	-0.658596216	0.151	0.245	0.001231082	10
Cox20	-0.503504745	0.2	0.286	0.001238818	10
Nolc1.4	-0.553502031	0.249	0.41	0.001264404	10
Smarcd1.1	-0.47300156	0.097	0.272	0.001277526	10
Orc6.1	-0.565711844	0.049	0.202	0.0013237	10
Phip.1	-0.554273878	0.292	0.461	0.001324518	10
Cdk6.4	-0.619583386	0.086	0.244	0.001324918	10
Zfp91.1	-0.483472082	0.357	0.49	0.001368666	10
Celsr2.5	-0.646667011	0.081	0.239	0.001369963	10
Nptn.2	0.425054418	0.373	0.186	0.001375125	10
Rp2h	0.433506103	0.157	0.045	0.00152787	10
Wdr26	0.379227176	0.551	0.337	0.00163016	10
Vat1	0.274542519	0.151	0.046	0.001666782	10
Snap23	0.29097813	0.146	0.04	0.001688119	10
Arf3	0.383788719	0.189	0.061	0.001699457	10
Casc4.1	-0.530561126	0.022	0.149	0.001714342	10
Epb4.1l3.1	0.309598292	0.259	0.112	0.001716332	10
Snhg1.6	-0.566361646	0.189	0.359	0.001719901	10
C530008M17Rik.3	-0.617726888	0.097	0.27	0.001740656	10
Gjc1.4	-0.54398437	0.049	0.197	0.001753798	10
Pou3f3.1	-0.581358415	0.038	0.173	0.001773784	10
Lamtor4	0.40902466	0.286	0.126	0.001817009	10
Ewsr1.1	-0.424268121	0.314	0.393	0.001829059	10
Etfb.3	0.414482149	0.368	0.182	0.001830994	10
Kat6b.1	-0.596963319	0.027	0.158	0.001847334	10
A330076H08Rik.8	-0.645316818	0.032	0.162	0.001872724	10
Cct5.2	-0.392777843	0.378	0.478	0.001907789	10
Mpdz.2	-0.526558996	0.016	0.136	0.001927905	10
Plcb1.7	-0.642968082	0.027	0.142	0.00193288	10
Ppfia2.6	-0.596964288	0.005	0.113	0.001948864	10
Frrs1l.1	-0.626525125	0.054	0.192	0.002088612	10
Dtymk.7	-0.565731771	0.259	0.441	0.002110962	10
Cecr2.1	-0.520075059	0.016	0.131	0.002114482	10
Lrrn1.1	-0.479712222	0.011	0.114	0.002115557	10
Daam1.1	-0.544338589	0.043	0.185	0.00212464	10
Olfm1.2	-0.654544747	0.054	0.2	0.00212735	10
Ift43	-0.47011295	0.016	0.129	0.002168106	10

Nkain4.1	-0.549926992	0.016	0.13	0.002207915	10
Eif1.1	-0.420496646	0.476	0.583	0.002358535	10
Serf1.3	-0.610342182	0.135	0.282	0.002361576	10
Fndc4.1	-0.479690466	0.022	0.126	0.002410436	10
Col9a3.2	-0.613876023	0.043	0.181	0.002438659	10
Cdca3.10	-0.627085169	0.076	0.223	0.002456813	10
Tmem57.5	-0.624262659	0.13	0.292	0.002532797	10
Tspan14	0.270725716	0.146	0.043	0.002545055	10
Ralgps2.2	-0.617068778	0.086	0.247	0.002577394	10
1700001O22Rik.4	-0.451656562	0.005	0.111	0.002600879	10
Son	-0.325137631	0.751	0.788	0.002628219	10
Eef1a1.3	-0.259963197	0.903	0.884	0.002717761	10
Cct7	-0.49925138	0.314	0.486	0.00274516	10
Fam98b	-0.587668368	0.157	0.337	0.002757961	10
Gas1.5	-0.50624615	0.027	0.158	0.002828077	10
Amer2	-0.455761764	0.032	0.134	0.002888643	10
Slc22a17.5	-0.626891173	0.097	0.252	0.002891021	10
Phf14	-0.577911279	0.232	0.376	0.002903106	10
Mpped2.1	-0.495696982	0.016	0.127	0.002978787	10
Pygo1.4	-0.596564952	0.049	0.193	0.003015509	10
Hip1r.3	-0.537408705	0.016	0.134	0.00305038	10
Map7d2.1	-0.496092153	0.016	0.101	0.003064397	10
Stxbp1.10	-0.635221828	0.038	0.163	0.003102501	10
Reln.7	-0.660504327	0.043	0.181	0.003143314	10
Rap1a.1	0.411868635	0.281	0.122	0.003205104	10
Trappc6a	0.300289338	0.141	0.04	0.003293731	10
Smarca5.3	-0.513745773	0.243	0.402	0.003304846	10
Smarcb1	-0.511348468	0.216	0.324	0.003374711	10
Snrpa1.3	-0.557107446	0.2	0.332	0.003429791	10
Zfp292.3	-0.632508536	0.184	0.343	0.003438609	10
Rab3ip	-0.43445116	0.011	0.101	0.00346164	10
Gm11223.4	-0.743401226	0.07	0.214	0.003513481	10
Ybx3.5	-0.540312109	0.119	0.291	0.003651476	10
Zfp428	-0.51474753	0.032	0.168	0.003659874	10
Senp6	-0.585275041	0.227	0.39	0.003678521	10
Bmyc.1	0.308163855	0.238	0.099	0.003778428	10
Hgsnat	0.407081999	0.151	0.043	0.003826141	10
Mbnl1	0.395898783	0.319	0.149	0.003832938	10
Ppp2r5e	-0.56375734	0.103	0.221	0.003886285	10
Bod1	-0.563459205	0.049	0.178	0.003897989	10
Trove2	-0.600898507	0.054	0.187	0.003900717	10
Spcs2.2	0.42342939	0.6	0.421	0.003961205	10
Tmem87b	0.263381261	0.227	0.097	0.004022128	10
Rap1gds1.1	0.351231508	0.151	0.045	0.004036374	10
Wasf1.1	-0.456710845	0.011	0.111	0.004211399	10
Tfrc	-0.569575392	0.049	0.17	0.004246698	10
Casp3.2	-0.57749062	0.086	0.235	0.004314415	10
Ran.6	-0.523510653	0.265	0.415	0.004431556	10

Upf3b	-0.560423743	0.189	0.322	0.004439657	10
Prpf40a.1	-0.47350728	0.405	0.573	0.004520258	10
Spats2	-0.557472129	0.043	0.183	0.004545327	10
Neurod6.2	-0.567625376	0.038	0.173	0.004579019	10
Bcar1.2	-0.526007539	0.049	0.193	0.004579638	10
Cox6c.1	-0.257072535	0.703	0.719	0.004658881	10
Sh3kbp1.1	0.333482229	0.232	0.095	0.004679218	10
Specc1.1	0.403247125	0.162	0.048	0.004683419	10
Tprn.4	-0.606272428	0.07	0.228	0.00474706	10
Serpinh1.5	-0.506333812	0.011	0.116	0.004771137	10
Ccdc34.9	-0.568044437	0.249	0.416	0.004789033	10
Pnmal2.5	-0.533921104	0.027	0.147	0.004811441	10
Eif3c	-0.390242862	0.492	0.603	0.004862104	10
Usp46.3	-0.603848248	0.097	0.237	0.004921091	10
Jakmip2.2	-0.586725524	0.032	0.158	0.004976361	10
Tfdp2.1	-0.556160722	0.032	0.162	0.004993329	10
Fam3c.1	0.303933291	0.211	0.083	0.005028104	10
H3f3a.1	-0.513401529	0.249	0.382	0.00507485	10
Pitpnc1.1	0.342960656	0.2	0.073	0.00520799	10
Nrcam.7	-0.554771902	0.027	0.135	0.005383137	10
Srsf2.3	-0.361522326	0.443	0.567	0.005582598	10
Rgs19	0.317139563	0.157	0.047	0.005597195	10
Asf1a.1	-0.571598374	0.119	0.281	0.005699577	10
Gm1673.4	-0.482473675	0.373	0.474	0.005726744	10
Srsf10.2	-0.543500966	0.205	0.331	0.005735599	10
Fkbp1a	-0.494650629	0.286	0.453	0.005757908	10
Grb2.3	0.360529789	0.432	0.247	0.005805649	10
Gse1.3	-0.594888754	0.081	0.238	0.005806284	10
U2surp	-0.450248253	0.346	0.507	0.005824524	10
Sfrp2.4	-0.508089946	0.027	0.149	0.00584912	10
Hsd17b11	0.284784458	0.173	0.061	0.005995298	10
Clspn.9	-0.538707297	0.049	0.191	0.006143516	10
Atp6v1b2.1	0.26289788	0.324	0.169	0.00636916	10
Kif11.9	-0.577300637	0.059	0.207	0.006379655	10
Smarcc2.4	-0.546856264	0.205	0.364	0.006428208	10
Mrps5	-0.591746209	0.114	0.274	0.006447468	10
Rassf4.3	-0.516992255	0.259	0.377	0.006586473	10
Ckap2l.10	-0.529867574	0.076	0.218	0.006661129	10
Eif3h.1	-0.42198319	0.389	0.494	0.006687482	10
Fam111a.8	0.346116028	0.276	0.124	0.006730077	10
Vav2	0.341731288	0.146	0.042	0.006981224	10
Tet1	-0.482460012	0.022	0.141	0.007326997	10
Calr.2	0.398553721	0.676	0.509	0.007643038	10
Rcbtb2	0.451853437	0.314	0.148	0.0076882	10
Mtf2.1	-0.575502955	0.184	0.359	0.007893912	10
Ccdc104	-0.592696833	0.146	0.315	0.007916669	10
Il11ra1	0.274416733	0.162	0.056	0.00816497	10
Magoh.3	-0.521213288	0.178	0.334	0.008244695	10

Srsf7.6	-0.44272675	0.324	0.466	0.008304149	10
Mum1.1	-0.501815759	0.038	0.17	0.008370288	10
Ssx2ip	-0.461815955	0.043	0.131	0.008681318	10
Ptpn11	-0.559097135	0.146	0.271	0.008739996	10
Trim37.2	-0.447581133	0.097	0.234	0.008880532	10
Fbxo5.8	-0.446697575	0.038	0.16	0.008905618	10
Tex9.1	-0.515011444	0.027	0.149	0.009196161	10
Prpf38b	-0.494848188	0.373	0.496	0.009513115	10
Ndufc2.2	-0.37048898	0.519	0.611	0.009616024	10
Sf3a3.1	-0.546801049	0.157	0.282	0.009923645	10
Prpf6	-0.585211169	0.097	0.233	0.009941669	10
G3bp2	-0.366911423	0.476	0.552	0.010119321	10
Chmp6	0.376547563	0.222	0.087	0.010668036	10
Rac1.1	0.372712677	0.546	0.344	0.010770221	10
Lmo4.4	-0.53128506	0.162	0.337	0.010791406	10
Tmem160	0.334391639	0.416	0.232	0.01081393	10
Atp5a1	-0.277082841	0.681	0.718	0.01103634	10
Naa38.4	-0.45518717	0.135	0.313	0.011267428	10
Luc7l2.1	-0.479633738	0.341	0.496	0.011374463	10
Zfp664	-0.558860359	0.086	0.23	0.011488036	10
Cwc27	-0.572918848	0.043	0.17	0.01173256	10
Atl1.2	-0.482854888	0.005	0.103	0.011982041	10
Lman2.1	0.284495686	0.286	0.137	0.01222566	10
Egr2	0.479327758	0.124	0.046	0.012232454	10
Ccng1	0.388468201	0.141	0.038	0.012250659	10
Ndufa5	-0.475753223	0.335	0.454	0.012285791	10
Schip1.1	-0.569543621	0.054	0.183	0.012413466	10
Tbc1d20	0.257197513	0.216	0.094	0.012582367	10
Mapk8ip1.8	-0.570369981	0.076	0.228	0.012857311	10
Setd8.1	-0.55780696	0.103	0.263	0.013044667	10
Kif1a.5	-0.566112755	0.043	0.173	0.013275859	10
Gtf2i	-0.484912149	0.249	0.389	0.013712513	10
Eef2.2	-0.344700494	0.605	0.717	0.013726754	10
Mns1.9	-0.596442258	0.065	0.204	0.013822142	10
Zc3h15	-0.522416175	0.222	0.367	0.013851421	10
Nfyb.4	-0.567201636	0.108	0.236	0.014159923	10
Rnf166	0.390558456	0.184	0.066	0.014688068	10
Fam174a	0.392744587	0.178	0.063	0.014716934	10
Rab3c.3	-0.531523594	0.027	0.149	0.014961447	10
Naa10.2	-0.547393712	0.108	0.25	0.01501085	10
Ift27.3	-0.526843737	0.086	0.244	0.015355479	10
Pde4dip.1	-0.529547806	0.086	0.222	0.01536801	10
Tcf3.2	-0.480349032	0.081	0.227	0.015432318	10
Ulk2	0.279844846	0.195	0.077	0.015605017	10
Plgrkt	0.366792145	0.368	0.193	0.015827923	10
Ap1b1	0.369861288	0.151	0.045	0.015840056	10
Ppp3ca.5	-0.557059204	0.27	0.379	0.015873431	10
2900011O08Rik.7	-0.398695924	0.011	0.104	0.01611905	10

Epc2	-0.56434967	0.119	0.251	0.016533625	10
Gdpd1.5	-0.565146162	0.092	0.244	0.016755171	10
Atp6v1a.1	0.425227551	0.47	0.286	0.017122983	10
Rtn3.2	-0.4131016	0.395	0.477	0.017159426	10
Cyth2.2	-0.560381472	0.135	0.274	0.017290723	10
Vps26a	0.41581124	0.276	0.13	0.017597005	10
Brd8.3	-0.526294592	0.254	0.393	0.018051221	10
Eps15	0.329749637	0.173	0.059	0.018160977	10
Hscb	0.461738489	0.151	0.059	0.01820599	10
Arpc1a.1	-0.467611705	0.265	0.364	0.019138828	10
Prox1.1	-0.471076612	0.119	0.23	0.019140731	10
Tubb4b.8	-0.536925075	0.13	0.241	0.019526742	10
Xrn2	-0.471429956	0.303	0.474	0.019698118	10
Morc3	0.257453591	0.222	0.096	0.019713299	10
Nbea.5	-0.629048356	0.065	0.191	0.019740236	10
Hes6.7	-0.558590681	0.124	0.237	0.02012443	10
Dcakd.2	-0.510745705	0.13	0.298	0.020230645	10
Tgoln1	0.290813907	0.2	0.082	0.020437578	10
Cacng4.7	-0.638827569	0.011	0.102	0.02103909	10
Rhobtb3	-0.529871145	0.043	0.165	0.021240981	10
Ehmt1	-0.48067643	0.119	0.253	0.022290973	10
Plk4.7	-0.434031878	0.016	0.12	0.022301041	10
Surf4	0.303832271	0.249	0.117	0.022823127	10
Gpatch8.2	-0.571269822	0.151	0.282	0.023454652	10
Nedd4l.3	-0.491464755	0.027	0.129	0.023648798	10
Gtf2h5	-0.369010282	0.286	0.44	0.024809213	10
Pafah1b3.2	-0.530627611	0.065	0.191	0.025215616	10
Ctr9	-0.556601737	0.108	0.243	0.02529205	10
Rab6a.5	-0.488219186	0.168	0.329	0.025344313	10
Tmem14c	0.36670142	0.357	0.186	0.025627958	10
Chchd2.2	-0.304936692	0.611	0.653	0.025773621	10
Gnl3l.1	-0.54526509	0.059	0.189	0.025961532	10
Maf1	-0.48412606	0.227	0.332	0.026390251	10
Ccdc41.6	-0.588565166	0.119	0.255	0.027043546	10
Mad2l2.3	-0.523948929	0.119	0.263	0.027123021	10
Nob1	-0.486271008	0.043	0.171	0.027218457	10
2210018M11Rik	-0.3850532	0.043	0.105	0.027403205	10
Dnajc8.1	-0.409356783	0.324	0.435	0.027885253	10
Pcyox1	0.286580269	0.2	0.082	0.028042643	10
Appl1	-0.552722861	0.076	0.21	0.028272487	10
Dnajc3	0.375315388	0.335	0.174	0.028555779	10
Afap1.4	-0.528723311	0.032	0.153	0.029311197	10
Ddx39b.2	-0.359620447	0.341	0.445	0.029363411	10
Brd2	-0.493788165	0.254	0.396	0.029906679	10
Atp5j.1	-0.351888223	0.627	0.723	0.030126987	10
Rfc4.8	-0.534329172	0.081	0.227	0.030681144	10
Ptpn12	-0.440394202	0.022	0.124	0.030777721	10
Ddx6	-0.40194678	0.373	0.493	0.031378655	10

Hmox2	0.380044393	0.297	0.142	0.032148048	10
Atp8a1.1	0.379664039	0.195	0.072	0.032671541	10
Samhd1	0.418259002	0.151	0.052	0.032867703	10
Rsbn1l	-0.584383496	0.168	0.313	0.034028399	10
Pgrmc1	-0.549753524	0.146	0.296	0.034608236	10
Tex264	0.360232284	0.184	0.067	0.035072691	10
Dbn1.8	-0.542196426	0.032	0.14	0.035339156	10
Ankrd12.6	-0.309283061	0.297	0.274	0.035417208	10
Tpx2.10	-0.658357238	0.135	0.283	0.035428104	10
Npepl1	0.288262737	0.119	0.033	0.035644261	10
Ska2.8	-0.520095183	0.065	0.206	0.036336127	10
Aagab	0.278341622	0.173	0.063	0.036476278	10
Polr2i.2	-0.545573076	0.168	0.317	0.036525589	10
Tacc2.6	-0.558077552	0.038	0.142	0.037669538	10
Mdga1.2	-0.474118199	0.032	0.136	0.038541163	10
Rps15a.4	-0.259129322	0.622	0.657	0.038878204	10
Stk4	0.370480668	0.205	0.085	0.039039871	10
Usp34	-0.551111408	0.168	0.291	0.039613555	10
Nuak1	0.291110996	0.124	0.034	0.04009019	10
Mrpl20	-0.3974691	0.243	0.311	0.040095464	10
Hsp90b1.6	0.283317631	0.849	0.69	0.040228838	10
Pip5k1a	-0.420461932	0.043	0.118	0.040381499	10
Esf1.1	-0.514607141	0.238	0.393	0.041083148	10
Polr2m	-0.409693702	0.135	0.245	0.041295768	10
Lyar.7	-0.565728937	0.146	0.287	0.041302707	10
Myt1l.9	-0.645926024	0.043	0.162	0.041378307	10
Mdh2	-0.413364655	0.4	0.529	0.041866488	10
Scn8a.3	-0.503967098	0.027	0.138	0.042373642	10
Jak1.1	0.288904969	0.259	0.126	0.043262117	10
Snrpd2.3	-0.43338343	0.297	0.442	0.043272163	10
Kcnip3.2	-0.485683605	0.016	0.121	0.044108721	10
Ckap5.8	-0.380828378	0.141	0.266	0.045149729	10
Pttg1ip.1	0.385700268	0.2	0.084	0.047685225	10
Rpn2.1	0.379218135	0.395	0.225	0.047849542	10
Pqlc1.7	-0.533948965	0.135	0.299	0.048029781	10
Cdc16	-0.516151848	0.157	0.283	0.048133792	10
Tuba1b.8	-0.289306571	0.427	0.481	0.049029971	10
Nicn1.3	-0.586860037	0.076	0.209	0.049114583	10
Mcl1	0.449766009	0.351	0.21	0.049641128	10
Rnps1	-0.443601258	0.027	0.138	0.052284811	10
Pbx1.1	-0.50047667	0.054	0.188	0.05345888	10
Phf21b.2	-0.476148997	0.022	0.129	0.053801983	10
Tax1bp1.1	-0.411730052	0.411	0.579	0.054039888	10
Kdm5b.6	-0.593798514	0.07	0.186	0.054045151	10
Timeless.6	-0.500421377	0.043	0.154	0.054062388	10
Ndrg2.4	-0.559427298	0.059	0.185	0.056520379	10
Wdr82	-0.462395016	0.086	0.174	0.057734907	10
Racgap1.10	-0.525283635	0.103	0.257	0.058389204	10

Creb1.1	-0.489226508	0.135	0.223	0.059008381	10
Slc39a6	-0.532561998	0.086	0.199	0.059395272	10
Arl8b	0.260404006	0.314	0.166	0.059781396	10
Zmynd11	-0.468708754	0.324	0.461	0.059866367	10
Nans	0.334800459	0.238	0.106	0.061071021	10
Ski	0.340698919	0.184	0.068	0.061192516	10
Rnf7	0.328180455	0.368	0.207	0.061500004	10
Rbx1	-0.32437591	0.368	0.496	0.06199906	10
Prps2	0.302578226	0.157	0.052	0.06225721	10
Slc15a4	0.314223795	0.119	0.035	0.062267588	10
Ube2k	-0.448752091	0.341	0.452	0.06245464	10
Gdap1.1	-0.539517937	0.065	0.2	0.062554093	10
Cdc7.2	-0.482242064	0.043	0.168	0.063038362	10
Sox11.3	-0.438322616	0.016	0.115	0.06328316	10
Rufy2.1	-0.471483185	0.038	0.159	0.063677118	10
Sumf1	0.375947072	0.124	0.034	0.063836642	10
Adam10.1	0.345859344	0.368	0.201	0.064444039	10
Atp6v0a1.2	0.364797497	0.222	0.094	0.064789795	10
Selm.2	-0.564356543	0.059	0.175	0.06982174	10
Cdca8.10	-0.56937294	0.146	0.265	0.071561112	10
Clic4.5	-0.503160004	0.086	0.228	0.071614111	10
Hes1.8	-0.670327695	0.049	0.149	0.07190758	10
Lrpap1.5	0.373571279	0.254	0.115	0.071928626	10
Eif3d.1	-0.437706931	0.297	0.406	0.072854009	10
2610203C20Rik.5	-0.498746052	0.022	0.121	0.073593913	10
Rnpc3.1	-0.435746369	0.022	0.104	0.073822916	10
Wdr6.1	-0.508093831	0.032	0.145	0.07424295	10
Pabpn1.2	-0.520608001	0.081	0.195	0.074497833	10
Magohb	-0.451615646	0.038	0.156	0.074596071	10
Fos.5	0.568584663	0.443	0.303	0.074841604	10
Mgst1	0.359444356	0.119	0.032	0.074940048	10
Clip1	0.378750253	0.222	0.092	0.07562923	10
Gpr85	-0.509094191	0.043	0.165	0.076210285	10
Pfdn4.1	-0.50689322	0.151	0.306	0.077144795	10
Cdk11b	-0.340387445	0.368	0.512	0.077235916	10
Vbp1.2	-0.423956106	0.157	0.321	0.079033581	10
Ndufb2	-0.292941105	0.351	0.368	0.079879582	10
Acot7.3	-0.485173503	0.065	0.196	0.079985882	10
Morf4l2	-0.508645243	0.135	0.273	0.080307573	10
Ckap4	-0.49159111	0.162	0.291	0.080460911	10
Clptm1l.1	0.344553017	0.259	0.121	0.080549203	10
Tmem256.1	0.362513179	0.654	0.49	0.081452785	10
Mical1.3	-0.340284431	0.086	0.126	0.08199839	10
Trp53.4	-0.485349987	0.168	0.299	0.082792549	10
Ebna1bp2.1	-0.505876412	0.162	0.316	0.083386344	10
Tead1.3	-0.53596316	0.059	0.174	0.083728947	10
Eif3e.2	-0.403472535	0.254	0.383	0.085057644	10
Hint1.4	-0.32107713	0.627	0.704	0.085360743	10

Pdzrn4.5	-0.48703157	0.032	0.147	0.085725366	10
Rnaset2a	0.273028254	0.146	0.048	0.088071781	10
Ddx1	-0.395888459	0.292	0.389	0.088135444	10
Caprin1.1	-0.392346009	0.308	0.394	0.088221487	10
Ercc1	-0.457790182	0.043	0.137	0.089771669	10
Atad5.5	-0.482401001	0.054	0.149	0.0915522	10
Spc24.8	-0.428283421	0.103	0.243	0.091657439	10
Pbk.8	-0.577573688	0.092	0.223	0.0925394	10
Cct3.2	-0.399188454	0.341	0.496	0.093613328	10
Prdx6.2	-0.511309425	0.184	0.327	0.093613983	10
Csrp2.7	-0.365619583	0.043	0.159	0.095903077	10
Stard3nl.1	0.382817445	0.254	0.115	0.097004802	10
Nsd1	-0.484546841	0.189	0.341	0.098294241	10
Cuta	0.384516815	0.459	0.294	0.099798825	10
Clpp	-0.513957669	0.124	0.24	0.100095624	10
Cdc20.9	-0.656895077	0.07	0.197	0.102252714	10
Glce.6	-0.559091133	0.065	0.177	0.104194384	10
Ddr1.1	-0.506251537	0.038	0.136	0.105506576	10
Xist.3	-0.739841409	0.168	0.3	0.107065573	10
Cnksr2.4	-0.453065775	0.016	0.108	0.10717641	10
Cfl1	0.400419979	0.524	0.376	0.107476305	10
Cct8.1	-0.329978917	0.335	0.442	0.108415331	10
Nipsnap1.1	-0.466917841	0.027	0.137	0.108512931	10
Ntm.6	-0.579351484	0.043	0.146	0.108742562	10
Fam181b	-0.463362796	0.032	0.143	0.112736287	10
Cc2d1b	0.300887841	0.13	0.04	0.112769237	10
Ggh.1	0.308978966	0.254	0.122	0.113155345	10
Cdh4.2	-0.473218116	0.032	0.147	0.113193784	10
Celsr3.2	-0.485638742	0.022	0.124	0.113817036	10
Ptbp2	-0.525812943	0.097	0.221	0.114056015	10
Cdca7.6	-0.465922168	0.065	0.199	0.114241326	10
Tmem35.1	-0.419222468	0.022	0.104	0.115343326	10
Dnajc2.3	-0.450252955	0.238	0.329	0.116734011	10
Arpp21.8	-0.584444853	0.016	0.113	0.117174068	10
Hmgb1.7	-0.480232247	0.216	0.36	0.117381698	10
Ndufs4	-0.398926026	0.292	0.366	0.118205873	10
Nol4.4	-0.511403178	0.049	0.172	0.118858324	10
Cdc123	-0.507610743	0.178	0.314	0.11939578	10
Prdm8.8	-0.550827891	0.032	0.127	0.120953733	10
Ube3a	-0.501049899	0.232	0.351	0.121652305	10
Zfp266	-0.488639166	0.059	0.168	0.122645714	10
Eif4a1.1	-0.383754382	0.346	0.485	0.122801068	10
Shmt2.1	-0.390493577	0.022	0.12	0.123603794	10
Elp3.1	-0.422079048	0.059	0.137	0.12372824	10
Abcf1	-0.457275387	0.341	0.458	0.123757858	10
Appbp2	-0.466577532	0.103	0.2	0.125558071	10
Ppp1r14b.3	-0.454153198	0.243	0.398	0.127025361	10
Zfp580	-0.423033524	0.022	0.123	0.127033291	10

Zfp36l1.6	0.452352646	0.292	0.149	0.128162862	10
Tiprl	-0.489874567	0.076	0.205	0.128979645	10
Dis3.1	-0.399816785	0.022	0.125	0.129618993	10
Bptf	-0.435010508	0.303	0.374	0.130280895	10
Tiparp.1	-0.45517555	0.07	0.147	0.131099825	10
Smarcad1	-0.491818059	0.07	0.169	0.13291851	10
Tceal8.2	-0.49180946	0.13	0.246	0.133207351	10
Zcchc11	-0.50394814	0.162	0.322	0.137620363	10
Cisd1	-0.473948048	0.232	0.343	0.137949509	10
Mllt10.1	-0.527554504	0.157	0.287	0.13975006	10
Anapc5.1	-0.44313847	0.232	0.334	0.143091883	10
Rpa3.5	-0.506054357	0.103	0.245	0.143956759	10
Ppp1cb	-0.431715265	0.205	0.292	0.144345616	10
Zic5.2	-0.506191251	0.043	0.161	0.145877736	10
Ptch2.6	-0.482299241	0.038	0.153	0.146949637	10
Sox5.4	-0.492081728	0.022	0.12	0.147971345	10
Pik3r3.5	-0.517731225	0.043	0.136	0.150776899	10
Pdcd6ip	0.381936457	0.222	0.095	0.152481786	10
Ywhag.3	-0.472616516	0.157	0.241	0.155485838	10
Sbk1.4	-0.469025403	0.022	0.119	0.155770916	10
Map1lc3a.2	-0.455247944	0.195	0.276	0.157004949	10
Got1	0.300731083	0.151	0.052	0.157585225	10
Srsf5	-0.337959756	0.373	0.427	0.159084374	10
Pdia3.1	0.343231982	0.465	0.294	0.163332511	10
C77370.2	-0.453950134	0.011	0.102	0.166210701	10
Cacybp	-0.376641793	0.232	0.357	0.167304115	10
Zfml	-0.517019349	0.195	0.313	0.167373898	10
Ifnar1	0.37353712	0.216	0.095	0.16800727	10
Tmem222	-0.258677805	0.135	0.143	0.171472345	10
Ykt6	0.406392027	0.243	0.111	0.174558122	10
Nono	-0.462715213	0.119	0.22	0.175417881	10
Naca.3	-0.395562224	0.357	0.466	0.176174928	10
Gpsm1.1	-0.503058776	0.054	0.177	0.181862075	10
Fam92a	-0.363193426	0.07	0.199	0.183138594	10
Ankrd11.1	-0.442248263	0.362	0.474	0.18447467	10
Tmed7	0.261136039	0.238	0.123	0.184841844	10
Lhfpl4	-0.41679756	0.016	0.108	0.185059093	10
Gpatch4.2	-0.467693142	0.103	0.18	0.187617124	10
Aspm.9	-0.537390386	0.043	0.143	0.187638787	10
Slc25a5.4	0.294658089	0.681	0.507	0.187933231	10
Txn1.4	-0.361627996	0.47	0.561	0.193199785	10
Rab14.1	0.313388622	0.595	0.41	0.193382675	10
Rnf165.4	-0.520427862	0.054	0.173	0.194003196	10
Phpt1	-0.465753145	0.108	0.203	0.194920354	10
Casp8ap2.3	-0.560995691	0.114	0.245	0.197769763	10
Smco4.6	-0.345407662	0.049	0.162	0.202291927	10
2210016L21Rik.2	-0.532186366	0.168	0.29	0.206854005	10
Tmem30a.2	0.323377999	0.33	0.177	0.207056583	10

Cbx3	-0.403429325	0.135	0.275	0.207277867	10
Yipf1	0.278480965	0.195	0.082	0.209000546	10
Srek1	-0.476108799	0.254	0.384	0.216445192	10
Agpat4.3	-0.465957827	0.027	0.118	0.220566749	10
Nhp2.3	-0.404097834	0.27	0.371	0.222135704	10
Mob1a	0.314638561	0.151	0.052	0.223562046	10
Sqle.3	-0.452239374	0.027	0.121	0.223890412	10
Parp6.4	-0.474348683	0.027	0.126	0.224314073	10
Taldo1.1	0.323568632	0.492	0.319	0.226474295	10
Lpar6	0.296790637	0.173	0.071	0.229386086	10
Zdbf2.1	-0.361400316	0.032	0.134	0.232737146	10
Map9.2	-0.507551355	0.038	0.148	0.237764068	10
Lmnb2.5	-0.434247389	0.043	0.159	0.238687929	10
Pitpna	0.34538459	0.265	0.13	0.239229847	10
Rexo2.1	0.449037086	0.303	0.179	0.240970185	10
Mycbp2.4	-0.53151083	0.227	0.346	0.244102576	10
Tbl1x.1	-0.348752539	0.286	0.328	0.244219421	10
Eif3m	-0.452554646	0.2	0.302	0.250103336	10
Smchd1.2	-0.513801291	0.157	0.274	0.250866338	10
Ube2e2	-0.405249862	0.027	0.126	0.255512788	10
Polr3k	-0.498469446	0.13	0.272	0.257001554	10
Clcn4-2.6	-0.511300261	0.146	0.252	0.261954264	10
Mageh1.1	-0.402693461	0.022	0.12	0.262188676	10
Birc5.10	-0.57745343	0.151	0.274	0.2633757	10
Oat	0.354989118	0.178	0.069	0.265130933	10
Phf3.1	-0.527422801	0.13	0.271	0.271880926	10
Zranb2	-0.434623253	0.281	0.407	0.272793817	10
Fabp7.10	-0.732478319	0.151	0.122	0.275461967	10
Prnp.6	-0.514918384	0.076	0.208	0.280727505	10
Emg1.2	-0.406495391	0.2	0.316	0.28221115	10
Id2.6	-0.506016879	0.357	0.487	0.286996178	10
D19Bwg1357e.2	-0.469643356	0.173	0.274	0.287713655	10
Klhl7.2	-0.481118948	0.059	0.165	0.289889563	10
E330009J07Rik	-0.43917871	0.027	0.117	0.292460497	10
Vopp1.2	-0.396950541	0.022	0.12	0.293312033	10
Dzip3	-0.485860988	0.059	0.168	0.293923422	10
Vps36.1	-0.377660018	0.216	0.302	0.296352074	10
Dync1li2.1	-0.500360942	0.151	0.26	0.298366348	10
Pafah1b1.2	-0.3439872	0.422	0.475	0.30064195	10
Tulp4.2	-0.514186275	0.097	0.205	0.307823478	10
Arf4	-0.421625373	0.297	0.405	0.30799747	10
Dync2h1.1	-0.457547213	0.022	0.116	0.308386821	10
Gtl3.1	-0.453848179	0.092	0.185	0.311780966	10
Lrig3.5	-0.457448059	0.059	0.171	0.315477891	10
Kif5a.8	-0.480762842	0.027	0.129	0.315724804	10
Ube2e1	-0.42201275	0.146	0.228	0.319009181	10
Csde1	-0.325696364	0.368	0.437	0.322471606	10
Trp53bp1.1	-0.435048511	0.027	0.13	0.322605112	10

Papola	-0.412162563	0.297	0.402	0.328368864	10
Vcp	-0.396025698	0.238	0.306	0.330112821	10
Paics.6	-0.424004554	0.303	0.414	0.330222231	10
Nudt3.1	-0.414372171	0.178	0.249	0.334526065	10
Vcan.3	-0.45376644	0.027	0.131	0.335236264	10
Zfp451	-0.487856271	0.059	0.171	0.335834014	10
Usp10	-0.468428532	0.086	0.187	0.340204014	10
Clybl.4	-0.461789637	0.027	0.117	0.34711059	10
Ryk	-0.464462214	0.038	0.144	0.348601105	10
Twf1.1	0.385596352	0.27	0.135	0.356232488	10
Cul3	-0.427383221	0.092	0.163	0.356424303	10
Eif5a.2	-0.305564947	0.335	0.403	0.363973415	10
Clvs1.7	-0.461729971	0.027	0.13	0.371445433	10
Ndufv2	-0.292615378	0.4	0.443	0.372013946	10
Npepps.2	-0.407843301	0.243	0.318	0.373580783	10
Srp19	-0.326215774	0.319	0.354	0.37745603	10
Spag9.2	-0.505722128	0.168	0.308	0.377703004	10
Cdca7l.6	-0.32531696	0.016	0.105	0.378551963	10
Nipa2	0.316937242	0.173	0.067	0.378851149	10
Smarce1	-0.483708513	0.081	0.205	0.384888325	10
Ywhaq	-0.408409746	0.205	0.361	0.38580556	10
Ech1	0.377136513	0.238	0.113	0.386269764	10
Robo2.3	-0.504332842	0.043	0.152	0.388969469	10
Brwd1	-0.488274082	0.092	0.194	0.398506925	10
Mfsd5	0.311867959	0.119	0.035	0.398863628	10
Chmp3	0.305861314	0.249	0.127	0.4005825	10
Ctbp1	-0.434279621	0.249	0.395	0.401200589	10
Kif22.9	-0.363051578	0.054	0.164	0.402067493	10
Klf13.3	-0.454603395	0.108	0.237	0.403024749	10
Snw1	-0.375066472	0.351	0.432	0.403795917	10
Mycl.2	-0.436225878	0.032	0.138	0.413385668	10
Cenpa.9	-0.64873522	0.135	0.252	0.413707692	10
Thsd7a.3	-0.48983553	0.027	0.128	0.41561148	10
Prmt5.2	-0.489394384	0.135	0.257	0.418142882	10
Igsf8.4	-0.501362622	0.13	0.27	0.419997766	10
Flywch1	-0.426663236	0.027	0.129	0.4219351	10
Rrm1.9	-0.476697303	0.157	0.261	0.423344394	10
Ilkap	-0.412142007	0.124	0.231	0.428919386	10
Gtf2a2	-0.363296045	0.286	0.384	0.432454196	10
Ptrhd1	0.417617974	0.178	0.074	0.43371187	10
Dync1i2.4	-0.32615181	0.519	0.568	0.439166046	10
Dctpp1.6	-0.478350844	0.162	0.306	0.440197054	10
Tipin.7	-0.498469458	0.141	0.256	0.446271909	10
Psmc6	-0.440487425	0.216	0.341	0.446675796	10
Zfp354c	-0.434630567	0.032	0.126	0.449778984	10
Tpm1.2	-0.53296934	0.092	0.191	0.452903438	10
Cdkn2c.5	-0.426159908	0.038	0.144	0.453849499	10
Srsf1.1	-0.482491844	0.168	0.297	0.461902935	10

Ift74.3	-0.503757911	0.124	0.225	0.465427278	10
Pdcd11	-0.474873069	0.054	0.165	0.46694487	10
Nckap1.1	-0.461675422	0.038	0.132	0.472563454	10
Nktr.3	-0.495681668	0.259	0.381	0.484759442	10
Tmem107.3	-0.397064894	0.027	0.127	0.508269998	10
Acot13	0.411922339	0.238	0.112	0.510139625	10
Prmt1	-0.457223608	0.092	0.225	0.514065065	10
Las1l	-0.466225338	0.124	0.256	0.524629485	10
Nt5dc2.4	-0.507029578	0.108	0.238	0.528146204	10
Akap8	-0.366254514	0.178	0.233	0.528499067	10
Tomm70a	-0.400272288	0.168	0.229	0.529098879	10
Trpc2	-0.421438598	0.032	0.135	0.532601098	10
Hspa5.2	0.333309204	0.708	0.548	0.543685659	10
Abi1	0.365608889	0.281	0.143	0.547823884	10
Nedd8	-0.276169697	0.519	0.542	0.548051804	10
Rnf168.3	-0.457997268	0.124	0.208	0.550977528	10
Ubqln2	-0.497861566	0.097	0.215	0.55341843	10
Bcat1	-0.427616996	0.027	0.121	0.572031036	10
Mrps30	-0.251723214	0.081	0.108	0.572175736	10
Zranb1.1	-0.4923946	0.092	0.209	0.580731932	10
Slc3a2.5	0.300690687	0.492	0.321	0.586502366	10
Pard3.1	-0.400073864	0.027	0.127	0.586996534	10
Stip1	-0.415091021	0.168	0.304	0.589769784	10
Sec62.1	-0.309718458	0.378	0.406	0.592583918	10
Bzw1.1	-0.338098557	0.27	0.36	0.614471637	10
Tceal3.2	-0.437747907	0.022	0.111	0.622482242	10
Ube2d1.3	-0.476533954	0.114	0.229	0.624230738	10
Sfswap	-0.422571938	0.168	0.24	0.634033869	10
Lsm6.5	-0.394841304	0.276	0.372	0.6345444	10
Cadm4.2	-0.415317679	0.022	0.114	0.664597914	10
Fasn.1	-0.392431146	0.038	0.138	0.665096306	10
Golga4	-0.433498658	0.265	0.325	0.668330998	10
Mgea5.1	-0.47207793	0.141	0.232	0.669025429	10
Rab3a.9	-0.546482872	0.049	0.151	0.68955858	10
Nop10.3	-0.295299392	0.378	0.483	0.694898348	10
Fyn.5	-0.536091027	0.13	0.216	0.697532883	10
Knstrn.9	-0.514033208	0.07	0.186	0.699522216	10
Polr2b	-0.46235902	0.065	0.179	0.71455866	10
A930011O12Rik.9	-0.545662585	0.027	0.115	0.717144198	10
Ap2s1	0.266826968	0.427	0.274	0.720638405	10
Strn3	-0.447619415	0.162	0.314	0.72583156	10
Tada2a	-0.372679098	0.016	0.101	0.730326753	10
Rbmxl1	-0.327273544	0.103	0.152	0.73936246	10
Lta4h.2	-0.438152036	0.141	0.243	0.752303298	10
Rbbp4.3	-0.308009612	0.422	0.484	0.752584967	10
Osbpl11	0.313525849	0.151	0.055	0.770289194	10
Hsbp1.2	-0.34884797	0.492	0.595	0.77835156	10
Ctxn1.2	-0.456763232	0.059	0.171	0.784413133	10

Snap47.1	-0.4257556	0.065	0.141	0.791307829	10
Fbxl5	-0.294523419	0.086	0.118	0.79763362	10
Lphn1.2	-0.407661102	0.022	0.114	0.812090901	10
Smim15.1	0.42548673	0.232	0.119	0.817404304	10
Hmgcl.1	0.389979695	0.184	0.077	0.829639656	10
Psma5	-0.428086171	0.216	0.322	0.834866479	10
Gm17322.7	-0.508781351	0.038	0.132	0.837952803	10
Nusap1.9	-0.5613591	0.097	0.228	0.846602982	10
Prpf4	-0.363865883	0.086	0.14	0.855753016	10
Mxd3.8	-0.373542007	0.032	0.127	0.856223366	10
Asrgl1.3	-0.454316916	0.038	0.134	0.871237656	10
Ube2j1	0.341009856	0.189	0.079	0.886506268	10
Ssu72	0.309661141	0.373	0.221	0.909038943	10
Zmiz1.3	-0.403480805	0.303	0.375	0.910886465	10
1110037F02Rik	-0.387537867	0.027	0.105	0.917385769	10
Ankrd32.1	-0.473300772	0.081	0.184	0.918965509	10
Pds5b.1	-0.328007405	0.146	0.273	0.929295815	10
Sri.3	0.36345383	0.303	0.164	0.932680521	10
Ctdspl2	-0.416383513	0.043	0.141	0.941115572	10
1110051M20Rik	-0.386756111	0.022	0.101	0.944071961	10
Ash1l	-0.513349744	0.157	0.278	0.949971582	10
Deb1.1	-0.425602742	0.065	0.168	0.953621494	10
Mroh2a.6	-0.531527777	0.027	0.114	0.965830101	10
Ccdc28b.2	-0.480052025	0.059	0.163	0.969753371	10
Jarid2.3	-0.470500812	0.065	0.18	0.974535363	10
Sh3bp2	0.286199846	0.124	0.041	0.974698154	10
Adk.2	-0.401176641	0.054	0.166	0.978278086	10
Phactr1.3	-0.50730721	0.076	0.184	0.982215056	10
Tmem251	0.390918327	0.146	0.057	0.986793077	10
Hnrnpl.1	-0.328318838	0.319	0.363	1	10
Acat2	-0.455058654	0.076	0.183	1	10
Ier5.4	-0.446748439	0.27	0.395	1	10
Pja1.1	-0.459922951	0.059	0.174	1	10
Itpa	-0.406577299	0.13	0.211	1	10
2410089E03Rik.1	-0.442095859	0.038	0.131	1	10
Ndufa10	-0.336938821	0.324	0.372	1	10
Lsm14b	-0.447686433	0.054	0.154	1	10
Phf6	-0.414766319	0.059	0.174	1	10
Stx6	-0.396981088	0.092	0.161	1	10
Dctn2.2	-0.300844013	0.297	0.321	1	10
Spop.3	-0.450630516	0.216	0.316	1	10
G2e3.2	-0.401738845	0.032	0.132	1	10
Smdt1.1	0.26842339	0.514	0.353	1	10
Mis18bp1.9	-0.318491847	0.07	0.161	1	10
Dph3.1	-0.454105814	0.108	0.211	1	10
Nefm.2	-0.459781829	0.027	0.117	1	10
Mapre1	-0.296943123	0.422	0.518	1	10
Ap3b1	0.32003171	0.243	0.121	1	10

Ypel1.1	-0.423205263	0.059	0.175	1	10
Fam155a.1	-0.377492524	0.027	0.116	1	10
Klf7.7	-0.481612601	0.151	0.263	1	10
Fnbp1.2	0.286941813	0.168	0.072	1	10
Tspan5.1	-0.473978119	0.081	0.197	1	10
Abi2.2	-0.456699622	0.081	0.183	1	10
Ift81	-0.415781369	0.043	0.147	1	10
Pole3.4	-0.436524506	0.162	0.251	1	10
Mga	-0.462032768	0.097	0.191	1	10
Palm.1	-0.40078588	0.038	0.14	1	10
Paxbp1.2	-0.439887758	0.151	0.26	1	10
Cep57.5	-0.461339451	0.124	0.258	1	10
Rdh5.1	-0.413408032	0.032	0.126	1	10
Erc1.2	-0.442970097	0.038	0.111	1	10
Tubg1.1	-0.39730891	0.049	0.13	1	10
Msi2	-0.412107611	0.07	0.188	1	10
Scd2.3	0.321838294	0.449	0.295	1	10
Pura.2	-0.430542264	0.195	0.287	1	10
Ttc4.1	-0.461497789	0.081	0.181	1	10
Spen	-0.464556404	0.119	0.203	1	10
Stard3	0.324050003	0.119	0.045	1	10
Mpzl1.1	-0.432430696	0.054	0.145	1	10
Rbm6	-0.417504406	0.146	0.224	1	10
Pcdha2.6	-0.424347372	0.027	0.119	1	10
Utp3.2	-0.363306972	0.189	0.315	1	10
Pdrg1.4	-0.453301091	0.135	0.22	1	10
Ppp1r1a.5	-0.408029523	0.022	0.111	1	10
Gdi1.7	-0.490163764	0.114	0.231	1	10
Ppp2ca	-0.255311075	0.357	0.376	1	10
Atxn7l2.2	-0.389373634	0.032	0.103	1	10
Mocs2	-0.308242833	0.168	0.195	1	10
Rsu1.1	0.330072611	0.227	0.117	1	10
Ankrd46.2	-0.432411938	0.076	0.161	1	10
Tpr	-0.317912653	0.465	0.553	1	10
Rbm26	-0.471963346	0.178	0.298	1	10
Smarca2.1	-0.486804734	0.178	0.286	1	10
Stoml2	-0.440972893	0.157	0.263	1	10
Cep290.3	-0.472749381	0.065	0.143	1	10
Ick.1	-0.401484958	0.043	0.147	1	10
Kif15.9	-0.451068042	0.065	0.181	1	10
Irs1.5	-0.369316959	0.027	0.119	1	10
Agtpbp1.1	-0.410938347	0.022	0.102	1	10
Rpf2.1	-0.460930637	0.097	0.203	1	10
Gnb4.1	-0.390788563	0.027	0.105	1	10
Dnaja2	-0.298831241	0.243	0.334	1	10
Pdxp	-0.405891913	0.027	0.114	1	10
Psmc3ip.6	-0.416751932	0.043	0.132	1	10
Amer1	-0.404510434	0.022	0.108	1	10

Ntrk3.5	-0.399390941	0.049	0.156	1	10
Vdac2	0.275934066	0.508	0.347	1	10
Gna13	0.273699652	0.243	0.127	1	10
Stard4.1	-0.374730305	0.043	0.141	1	10
Cask.3	-0.442122067	0.032	0.11	1	10
Dnajb11.1	0.282811685	0.292	0.161	1	10
Pnrc2.2	-0.369126715	0.146	0.208	1	10
BC034090.2	-0.441083921	0.038	0.128	1	10
Ncapd2.7	-0.431169037	0.07	0.186	1	10
Gpr56.2	-0.413287196	0.151	0.223	1	10
Bex4	-0.357134027	0.032	0.128	1	10
Bod1l.1	-0.404790829	0.319	0.379	1	10
Csnk2b	-0.390783684	0.124	0.2	1	10
Slc38a1.1	-0.461147036	0.146	0.271	1	10
5031439G07Rik	0.257068075	0.119	0.041	1	10
Pkia.6	-0.469720386	0.043	0.134	1	10
Rnf220.1	-0.446842949	0.141	0.236	1	10
U2af1l4	-0.414578881	0.119	0.196	1	10
Ccdc25.1	-0.328214124	0.076	0.184	1	10
Fbxw7	-0.395173183	0.054	0.12	1	10
Nsmce4a.4	-0.452506779	0.103	0.224	1	10
0610009D07Rik	-0.262249456	0.303	0.384	1	10
Copg1	-0.364504857	0.092	0.153	1	10
Pik3ip1.1	-0.3331217	0.032	0.117	1	10
Esco2.9	-0.522108853	0.081	0.196	1	10
2700049A03Rik.1	-0.428587563	0.038	0.122	1	10
Gm11541.3	-0.400490756	0.032	0.128	1	10
Tecpr1.1	0.311974828	0.178	0.076	1	10
Slc39a10	-0.450997895	0.092	0.195	1	10
Cd63-ps.2	0.286036369	0.232	0.117	1	10
Dhx15.1	-0.371038078	0.324	0.417	1	10
Ube2c.8	-0.642657713	0.162	0.27	1	10
Tex261	0.257371092	0.227	0.114	1	10
Ddx55	-0.447407361	0.065	0.166	1	10
Prpf19	-0.411087177	0.2	0.307	1	10
Cct4	-0.306250564	0.319	0.382	1	10
R3hdm2	-0.40268079	0.086	0.168	1	10
Rab1	0.281336878	0.514	0.36	1	10
Prdm2	-0.422713885	0.043	0.133	1	10
Ctbp2.1	-0.43245724	0.124	0.215	1	10
Nisch	-0.409847724	0.216	0.301	1	10
Polr2f.2	-0.400619844	0.265	0.376	1	10
Qdpr	-0.364770691	0.211	0.268	1	10
Wdr61	-0.404413117	0.103	0.181	1	10
Cpsf6	-0.404550776	0.157	0.292	1	10
Ccnb2.9	-0.449477218	0.076	0.188	1	10
Tnrc6a	-0.437169303	0.211	0.3	1	10
Nt5c3	-0.416122371	0.049	0.15	1	10

Prps1.1	-0.394549803	0.043	0.143	1	10
Golim4.1	-0.485244875	0.119	0.233	1	10
Myl12a.4	0.32350823	0.47	0.327	1	10
Cdk14.2	-0.385044661	0.022	0.104	1	10
4921524J17Rik	-0.357029113	0.049	0.15	1	10
Cplx1.6	-0.470955519	0.043	0.139	1	10
Btf3.1	-0.372943144	0.265	0.35	1	10
Rufy1	0.332398735	0.141	0.052	1	10
Tob1	-0.438732884	0.054	0.155	1	10
Snrpb2.2	-0.286093749	0.341	0.384	1	10
Srsf6.1	-0.281487442	0.384	0.446	1	10
Sobp.2	-0.451843132	0.038	0.128	1	10
Cfp	0.415902549	0.103	0.042	1	10
Psmc1.1	-0.402951534	0.276	0.42	1	10
Jund.1	-0.423291723	0.211	0.353	1	10
Ywhaz.1	-0.325870068	0.303	0.363	1	10
Eif4g1	-0.423964228	0.249	0.353	1	10
Hnrnpul2	-0.420254332	0.168	0.258	1	10
Usp12	0.258529991	0.103	0.031	1	10
Sv2b.4	-0.414664741	0.022	0.103	1	10
Zfp131	-0.452523818	0.097	0.202	1	10
Sfr1	-0.359150015	0.276	0.395	1	10
5830418K08Rik.2	-0.456004069	0.07	0.168	1	10
Rps7.4	-0.381325195	0.297	0.4	1	10
Rnf146	-0.398727412	0.043	0.144	1	10
Gar1.3	-0.419297165	0.092	0.209	1	10
Lig1.10	-0.421348098	0.281	0.349	1	10
Celf1.1	-0.385628822	0.281	0.38	1	10
Sptssa.1	0.312093447	0.346	0.246	1	10
Acaa2	0.306532934	0.108	0.034	1	10
Mab21l2.2	-0.43314286	0.022	0.104	1	10
Fhl1	-0.372185065	0.022	0.106	1	10
Bcap29.1	0.365813534	0.232	0.121	1	10
Srpk1	-0.426246709	0.157	0.247	1	10
Tra2a	-0.414112499	0.27	0.393	1	10
Zfp148.1	-0.458464066	0.184	0.285	1	10
Scaper.3	-0.450068928	0.032	0.111	1	10
Chkb	-0.331261692	0.108	0.157	1	10
Scp2.1	0.284213752	0.168	0.072	1	10
Ahsa2	-0.419483411	0.114	0.207	1	10
2810004N23Rik.3	-0.455885367	0.157	0.263	1	10
Gli1.6	-0.376360148	0.032	0.108	1	10
Rcor3	-0.352527007	0.043	0.107	1	10
Eif3f.3	-0.279658505	0.578	0.631	1	10
Cdc42bpa.1	-0.447124837	0.054	0.137	1	10
Uhrf2	-0.390342119	0.076	0.151	1	10
Cenpj.5	-0.360433413	0.049	0.142	1	10
Jmy	-0.46460339	0.081	0.187	1	10

Krr1	-0.40013105	0.059	0.152	1	10
Cspp1.2	-0.487365064	0.119	0.239	1	10
Dhx9.1	-0.31115504	0.384	0.464	1	10
Uhrf1.8	-0.382840279	0.049	0.144	1	10
Vamp3.1	0.275218328	0.173	0.078	1	10
Fzd2.3	-0.410625455	0.059	0.161	1	10
Eef1g.2	-0.363617999	0.259	0.335	1	10
Park7.2	-0.323262628	0.47	0.546	1	10
Zic2.3	-0.376413338	0.032	0.121	1	10
Uqcrfs1	-0.36386176	0.281	0.388	1	10
Psmd14	-0.280182569	0.276	0.351	1	10
Cyp51.1	-0.400744034	0.043	0.142	1	10
Gtf2f1	-0.416942497	0.232	0.344	1	10
Irf2bp2	0.347998161	0.2	0.093	1	10
Cnpy1.5	-0.406220383	0.038	0.134	1	10
Iws1	-0.451678551	0.114	0.216	1	10
Dnmt1.9	-0.360253302	0.157	0.279	1	10
Nav2.3	-0.331115854	0.059	0.105	1	10
Nek7.4	0.2617605	0.227	0.115	1	10
Usp11	-0.30561871	0.022	0.101	1	10
Hprt.4	0.27275419	0.319	0.186	1	10
Tcf12.1	-0.442904396	0.157	0.256	1	10
Mff	-0.394797494	0.184	0.285	1	10
1700021F05Rik	-0.363421669	0.081	0.141	1	10
Fdft1.1	-0.355355532	0.022	0.101	1	10
Gna11	-0.411759391	0.043	0.138	1	10
Zmym5	-0.460024607	0.13	0.233	1	10
Rab11b	-0.344445356	0.097	0.153	1	10
Tm9sf3.1	0.330184789	0.492	0.342	1	10
Hsph1.1	-0.447014503	0.114	0.228	1	10
Grik5.2	-0.391358797	0.027	0.11	1	10
Rangap1.9	-0.420331181	0.162	0.257	1	10
Cox7c	-0.29278108	0.232	0.284	1	10
Fam216a.1	-0.380833947	0.065	0.174	1	10
5430416N02Rik	-0.395618089	0.043	0.141	1	10
Sipa1l2	0.269902729	0.141	0.056	1	10
Maged2.3	-0.429212729	0.081	0.167	1	10
Cenpm.7	-0.382178634	0.059	0.163	1	10
Acadl	0.277405032	0.151	0.063	1	10
Tbrg1	-0.447374759	0.124	0.244	1	10
Bmpr1a	-0.334015398	0.022	0.103	1	10
Rbmx2	-0.39496196	0.032	0.115	1	10
Zcrb1	-0.397846691	0.254	0.372	1	10
Chmp4b	0.307377257	0.286	0.213	1	10
Arl2bp.1	-0.410528123	0.097	0.218	1	10
Zeb2.2	0.377929339	0.357	0.247	1	10
Hist3h2a.6	-0.429357768	0.076	0.144	1	10
Vma21	-0.38379211	0.043	0.137	1	10

Gphn.3	-0.426617611	0.054	0.142	1	10
Bcas2	-0.302034197	0.33	0.406	1	10
Rad51ap1.10	-0.310178326	0.065	0.167	1	10
Igf1r	-0.375686529	0.038	0.114	1	10
Psmd7	-0.316650655	0.4	0.48	1	10
Ndufb11	-0.259559783	0.427	0.462	1	10
Trim28.2	-0.364577896	0.286	0.362	1	10
Frmd4a.4	-0.349126232	0.259	0.309	1	10
Txndc17	0.283605136	0.357	0.241	1	10
Grik2.7	-0.414743234	0.022	0.102	1	10
Aimp1	-0.3856341	0.2	0.28	1	10
Gamt.5	-0.454506771	0.081	0.17	1	10
Boc.6	-0.410609758	0.054	0.157	1	10
2810008D09Rik.2	-0.287284356	0.103	0.127	1	10
Arl6	-0.36470201	0.032	0.115	1	10
Kpnb1.1	-0.29293187	0.265	0.3	1	10
Sox18.3	-0.413416168	0.049	0.148	1	10
Fcho2.1	0.312780408	0.173	0.076	1	10
Tmem70	-0.304632495	0.07	0.119	1	10
Phf20	-0.451114205	0.119	0.221	1	10
Dnm1l.1	-0.414758691	0.173	0.293	1	10
Brd9	-0.432993582	0.114	0.214	1	10
Rrs1.2	-0.408005433	0.092	0.19	1	10
Dnajc7	-0.307275	0.243	0.281	1	10
Gm5914	-0.354207661	0.043	0.112	1	10
Dhx40	-0.402795394	0.065	0.148	1	10
Lbr.6	-0.323402256	0.092	0.197	1	10
Taok1	-0.318871961	0.189	0.225	1	10
Gabbr1.5	-0.419303647	0.059	0.141	1	10
Hmmr.9	-0.481642107	0.07	0.177	1	10
Ddit4.2	-0.381794838	0.043	0.105	1	10
Psme4.1	-0.433169405	0.157	0.285	1	10
Rnf122.2	-0.35922014	0.022	0.103	1	10
Alas1	0.265681408	0.141	0.056	1	10
Atad3a	-0.305799752	0.086	0.127	1	10
Rps8.2	-0.314389909	0.314	0.397	1	10
Dtl.6	-0.380906256	0.049	0.146	1	10
Kidins220.6	-0.325071568	0.114	0.14	1	10
Lrrc45	-0.337475561	0.032	0.102	1	10
Kif2c.7	-0.357557194	0.027	0.108	1	10
Nup62.4	-0.340286495	0.114	0.171	1	10
Jam3.4	-0.318469781	0.043	0.136	1	10
Nipbl.2	-0.313293973	0.449	0.492	1	10
Mrfap1.2	-0.328256991	0.286	0.404	1	10
Srm.3	-0.406406983	0.114	0.192	1	10
Dscr3	0.258587621	0.162	0.072	1	10
Acd.3	-0.432016888	0.065	0.162	1	10
Tdp2.1	-0.296459921	0.054	0.151	1	10

G3bp1.3	-0.352312432	0.243	0.328	1	10
Phb2	-0.326933607	0.281	0.333	1	10
Dot1l	-0.431050444	0.157	0.28	1	10
Cox5b	-0.287925103	0.308	0.357	1	10
0610011F06Rik	-0.376423776	0.059	0.127	1	10
Gria4.8	-0.440074441	0.049	0.125	1	10
Epb4.1.3	-0.402346279	0.108	0.23	1	10
Rheb	-0.297741027	0.222	0.274	1	10
Cnep1r1	-0.254157019	0.108	0.132	1	10
Tmed9.2	-0.292514204	0.389	0.433	1	10
Tex30.3	-0.368360262	0.043	0.136	1	10
A830080D01Rik	-0.383966637	0.032	0.114	1	10
Ikzf5	-0.340439939	0.049	0.145	1	10
Pebp1.1	-0.373404909	0.141	0.243	1	10
Nudcd3	-0.340643975	0.124	0.17	1	10
Bms1	-0.275002057	0.097	0.183	1	10
Ccna2.9	-0.385483692	0.103	0.222	1	10
Upf2	-0.438418297	0.07	0.155	1	10
Mcm2.7	-0.340908606	0.092	0.205	1	10
Mettl9.1	-0.377493531	0.211	0.301	1	10
Zfr.1	-0.391980341	0.259	0.354	1	10
Hspa9.1	-0.349449747	0.254	0.373	1	10
Trip11	-0.450868717	0.07	0.153	1	10
Tra2b.1	-0.335722098	0.324	0.405	1	10
Zfp536.1	-0.363249239	0.027	0.111	1	10
Ubap2l	-0.267323423	0.286	0.317	1	10
Rtn2	-0.37697445	0.032	0.116	1	10
Bfar	-0.421119201	0.114	0.192	1	10
D030056L22Rik.2	-0.345919941	0.049	0.143	1	10
Rpap3	-0.421398581	0.054	0.145	1	10
Wnk1	0.340689881	0.308	0.182	1	10
Nae1.1	-0.430386041	0.108	0.225	1	10
Snrpg.3	-0.392295235	0.227	0.336	1	10
Ctnnbl1	-0.415384875	0.092	0.185	1	10
Stx4a.3	0.30103058	0.259	0.153	1	10
Ip6k2	-0.408133564	0.038	0.122	1	10
Ltbp3.2	-0.389644045	0.032	0.118	1	10
Zkscan1	-0.415510505	0.054	0.152	1	10
Ndnl2	-0.35674944	0.054	0.13	1	10
Syt13.6	-0.358253358	0.038	0.128	1	10
Vps16	-0.252996142	0.07	0.101	1	10
Mcm6.7	-0.437756634	0.151	0.277	1	10
Ep400	-0.411835084	0.081	0.181	1	10
Ccnb1.7	-0.447513223	0.049	0.14	1	10
Odc1.2	-0.334267053	0.027	0.101	1	10
Txndc9	-0.407986333	0.119	0.212	1	10
Sae1.5	-0.370588814	0.189	0.279	1	10
Ankhd1	-0.358363186	0.086	0.181	1	10

Tom1l1.3	-0.343962328	0.038	0.104	1	10
Cul5	-0.373600879	0.184	0.316	1	10
Cep78.1	-0.356227691	0.049	0.141	1	10
Mex3b.1	-0.390586067	0.032	0.116	1	10
Ppil1.2	-0.297747211	0.043	0.124	1	10
Acap2.1	0.299876088	0.162	0.071	1	10
Timm50	-0.367810691	0.146	0.237	1	10
Tceb2	-0.285732524	0.454	0.514	1	10
Fam98a	-0.392667337	0.065	0.146	1	10
Lsm3.5	-0.344479107	0.259	0.359	1	10
Eif3g.1	-0.33287886	0.324	0.403	1	10
Pomp	0.267823983	0.557	0.412	1	10
Ppid	-0.400334207	0.097	0.202	1	10
Ccdc66	-0.364482405	0.07	0.173	1	10
Hmgxb4	-0.399946573	0.059	0.155	1	10
Cntln.5	-0.421452175	0.07	0.173	1	10
Epm2aip1	-0.347940687	0.027	0.106	1	10
Nup85.2	-0.377829638	0.108	0.227	1	10
Pick1.2	-0.381519123	0.059	0.128	1	10
Sgol1.8	-0.374426191	0.043	0.128	1	10
Emc6	-0.269060461	0.232	0.276	1	10
Cenpc1.1	-0.401369444	0.059	0.156	1	10
Kif23.10	-0.457205353	0.103	0.215	1	10
Neo1	-0.358921298	0.032	0.106	1	10
Tbc1d16.1	-0.27900363	0.173	0.196	1	10
Rhno1.2	-0.371800469	0.054	0.139	1	10
Rabgap1.1	-0.441527958	0.13	0.225	1	10
Gnb2	-0.296148037	0.427	0.484	1	10
Ddx3x.1	-0.261400489	0.432	0.466	1	10
Cdc27.1	-0.303332989	0.065	0.112	1	10
Ist1	-0.31361463	0.151	0.201	1	10
Galk2	0.273882953	0.13	0.052	1	10
Mta1	-0.333799453	0.049	0.104	1	10
Myod1.7	-0.406048009	0.049	0.142	1	10
Impad1.1	-0.429133113	0.124	0.224	1	10
Pafah1b2.1	-0.374476827	0.157	0.255	1	10
Anln.7	-0.295048853	0.027	0.105	1	10
Vmp1.1	0.258339347	0.216	0.115	1	10
Twistnb	-0.392684091	0.065	0.151	1	10
Erdr1	-0.416632862	0.07	0.162	1	10
Tbca.1	-0.266222403	0.373	0.42	1	10
Vps33a	0.265669775	0.124	0.048	1	10
Sec61a2	-0.371936562	0.038	0.116	1	10
Abt1	-0.328741304	0.049	0.127	1	10
Psmc4.1	-0.380036706	0.211	0.322	1	10
Poglut1	-0.378310097	0.054	0.131	1	10
Taf1	-0.398525568	0.108	0.202	1	10
Hs6st1	0.318476832	0.108	0.039	1	10

Trappc4	-0.377052051	0.157	0.225	1	10
Zfp207	-0.359682491	0.286	0.37	1	10
Gmps	-0.273518834	0.103	0.211	1	10
1500012F01Rik.5	-0.350181296	0.319	0.399	1	10
Zfp260	-0.417111207	0.103	0.206	1	10
Pard6g.1	-0.355650858	0.027	0.105	1	10
4930402H24Rik.4	-0.315319172	0.032	0.113	1	10
Psme1	0.288360991	0.286	0.172	1	10
Sparcl1.7	-0.701283366	0.108	0.208	1	10
Alcam.3	-0.2810839	0.049	0.135	1	10
Mki67ip.1	-0.349270826	0.124	0.245	1	10
Rbm4b	-0.389216664	0.054	0.141	1	10
Zcwpw1.2	-0.285005756	0.027	0.104	1	10
Fip1l1.1	-0.367111277	0.205	0.309	1	10
Rftn1	0.322612108	0.184	0.088	1	10
Gm26735.5	-0.428774352	0.081	0.181	1	10
Cep170.4	-0.408357677	0.216	0.306	1	10
Bcl2l13	-0.324173732	0.065	0.115	1	10
Prpf40b.1	-0.337481187	0.054	0.147	1	10
Sarnp	-0.401382081	0.13	0.239	1	10
Dcaf15	-0.270661194	0.135	0.161	1	10
C1qbp.3	-0.364680527	0.281	0.378	1	10
Ccndbp1	-0.299564061	0.076	0.117	1	10
Inpp5f	-0.350128604	0.054	0.113	1	10
Tdp1.1	-0.312419937	0.027	0.103	1	10
Gga1	0.2609167	0.114	0.044	1	10
Mnat1	-0.278038099	0.059	0.104	1	10
Vimp.2	0.309875798	0.314	0.188	1	10
Tsnax	-0.384130927	0.114	0.191	1	10
Baz2b.3	-0.37154215	0.286	0.365	1	10
Socs2.2	-0.387416443	0.049	0.139	1	10
Chic2	-0.26326319	0.124	0.15	1	10
Snap29	0.250544235	0.157	0.072	1	10
Mak16.2	-0.415812817	0.119	0.226	1	10
Ddx18	-0.263840214	0.097	0.127	1	10
Rgs12.1	-0.335112338	0.059	0.147	1	10
Pvrl3.2	-0.382404688	0.038	0.123	1	10
N4bp2	-0.379422457	0.065	0.132	1	10
Nabp2	-0.336695086	0.081	0.162	1	10
Acsl3.1	-0.366909013	0.054	0.147	1	10
Trim59.8	-0.322555127	0.07	0.17	1	10
Ubqln1	-0.293325779	0.265	0.304	1	10
Adamts1.4	-0.417545436	0.065	0.155	1	10
Dlgap5.8	-0.301295084	0.032	0.112	1	10
Knop1	-0.406559038	0.184	0.272	1	10
Mau2.1	-0.290670196	0.119	0.149	1	10
Cdca2.8	-0.358806771	0.043	0.129	1	10
Tnrc6b.1	-0.322960481	0.168	0.248	1	10

Fam168b	-0.319385631	0.184	0.241	1	10
Actl6a.2	-0.381170729	0.13	0.227	1	10
Imp3.2	-0.397360741	0.141	0.248	1	10
Ift57	-0.333012893	0.027	0.103	1	10
Sms	-0.380333963	0.059	0.137	1	10
Fam107b.3	-0.347462834	0.038	0.102	1	10
Rps6kb1	-0.371961651	0.184	0.279	1	10
Kif20b.9	-0.464195825	0.097	0.191	1	10
Sbno1	-0.386082878	0.243	0.352	1	10
0610007P14Rik.1	-0.359301441	0.07	0.163	1	10
Gsto1.3	0.306421192	0.232	0.127	1	10
Rps18.3	-0.331094565	0.319	0.429	1	10
Cenpp.7	-0.323438071	0.038	0.115	1	10
2700081O15Rik.2	-0.37939443	0.049	0.111	1	10
Smc5.2	-0.404665991	0.141	0.259	1	10
Rbm22	-0.338539537	0.141	0.223	1	10
Snrnp27	-0.364698136	0.222	0.311	1	10
Cenpq.7	-0.358015127	0.081	0.161	1	10
Exosc1.1	-0.365460375	0.092	0.163	1	10
Ensa.2	-0.367664752	0.151	0.228	1	10
Sepw1	-0.38504017	0.195	0.301	1	10
Uchl3	-0.37869717	0.092	0.187	1	10
Slc7a5.4	-0.369844498	0.038	0.115	1	10
Supt6	-0.399024151	0.086	0.158	1	10
Hnrnpll	-0.356228649	0.103	0.171	1	10
Rnaseh2c.5	-0.381564509	0.265	0.382	1	10
Psma6	-0.26827103	0.395	0.452	1	10
Stub1.1	-0.28017902	0.286	0.347	1	10
Zmat2	-0.300445431	0.33	0.441	1	10
Bbip1	-0.274954727	0.2	0.231	1	10
Ddx23	-0.299758581	0.114	0.207	1	10
Emc10	-0.295196694	0.254	0.316	1	10
Uba1.1	-0.343888321	0.211	0.272	1	10
Dazap1.1	-0.394099643	0.173	0.273	1	10
Uri1	-0.302997666	0.124	0.21	1	10
Phf20l1.5	-0.413881969	0.238	0.341	1	10
Tcea1	-0.340760358	0.054	0.137	1	10
Phax	-0.353613838	0.276	0.348	1	10
Wbp1	-0.374475524	0.059	0.149	1	10
Kdm5a.1	-0.404202405	0.173	0.257	1	10
Ttc14	-0.369975687	0.162	0.262	1	10
Gripap1	-0.400288023	0.086	0.189	1	10
Tmem132a.1	-0.314971166	0.027	0.103	1	10
Clcn3.2	-0.304285059	0.211	0.273	1	10
Ubp1	-0.394023858	0.081	0.17	1	10
Eif2a	-0.39652406	0.13	0.224	1	10
Shd.5	-0.374285878	0.032	0.11	1	10
Gabarapl1.4	-0.409725657	0.114	0.217	1	10

Mat2a.1	-0.345306374	0.303	0.42	1	10
Zfand5.2	-0.325354093	0.308	0.416	1	10
Ccnt2	-0.332175824	0.086	0.135	1	10
Rexo1	-0.324336472	0.151	0.212	1	10
March7	-0.294418318	0.2	0.239	1	10
Ndufaf2.2	-0.308872229	0.097	0.203	1	10
Rpf1.1	-0.384923873	0.114	0.194	1	10
Pmpcb	-0.332179928	0.157	0.218	1	10
Ndufa1	0.31285743	0.47	0.341	1	10
Ndufv1	-0.384215919	0.141	0.237	1	10
Senp2	-0.323791086	0.049	0.115	1	10
Tmem55b	0.28397525	0.211	0.111	1	10
Srsf4.1	-0.320351974	0.162	0.28	1	10
Arid4a	-0.368404879	0.162	0.284	1	10
Meis3.2	0.279567026	0.162	0.076	1	10
Ccar2	-0.340126793	0.032	0.103	1	10
Ube2g1	-0.288675163	0.059	0.144	1	10
Ptges3.2	-0.353963901	0.135	0.212	1	10
Ercc5	-0.350389699	0.059	0.112	1	10
Ntan1	-0.388466709	0.103	0.187	1	10
Mark3	-0.342054635	0.13	0.236	1	10
Lsm4.4	-0.3102517	0.411	0.488	1	10
Mphosph6	-0.323133674	0.086	0.141	1	10
Arid1b	-0.277044655	0.07	0.103	1	10
Zfp68	-0.364366698	0.065	0.14	1	10
Eif4b	-0.349076461	0.178	0.276	1	10
Mrps6	-0.333861745	0.059	0.152	1	10
Hspa8	-0.263543524	0.357	0.416	1	10
Lancl2	-0.317966324	0.032	0.105	1	10
Rrm2.9	-0.316131281	0.103	0.205	1	10
Mapk7	-0.327724636	0.032	0.106	1	10
Erbb2ip	0.276990588	0.259	0.174	1	10
Tshz1.1	-0.32547422	0.076	0.142	1	10
Mettl14	-0.352834772	0.054	0.123	1	10
1110038F14Rik	-0.372992195	0.114	0.192	1	10
Pfdn5	0.27560865	0.632	0.519	1	10
Sugt1	-0.364181918	0.157	0.232	1	10
Cnot2	-0.391738271	0.097	0.186	1	10
Mbtd1	-0.385152682	0.13	0.215	1	10
Psme2	0.299919369	0.2	0.103	1	10
Zfp512	-0.337729839	0.038	0.119	1	10
Sf1	-0.344979894	0.162	0.237	1	10
Ing1.1	-0.373232495	0.103	0.184	1	10
R3hcc1.1	-0.347021365	0.076	0.16	1	10
H2-Ke2	-0.328629826	0.114	0.196	1	10
Ppa1.2	-0.361596954	0.124	0.222	1	10
Sart3.1	-0.391997251	0.135	0.225	1	10
Ncor2.5	-0.393333713	0.081	0.169	1	10

Smoc1.3	-0.353804479	0.049	0.135	1	10
Usp7	-0.383822065	0.168	0.265	1	10
Tsen34	-0.382801646	0.135	0.234	1	10
Mrpl38	-0.274187707	0.032	0.106	1	10
Casc5.9	-0.394495434	0.086	0.187	1	10
Stau2.3	-0.397629067	0.076	0.151	1	10
Cops5	-0.376710531	0.13	0.223	1	10
Lin7c.2	-0.364775087	0.141	0.218	1	10
Stk16	-0.315962607	0.092	0.169	1	10
Mzt1	-0.356218844	0.065	0.151	1	10
Bap1	-0.331817742	0.032	0.109	1	10
Snord104.3	-0.387509642	0.076	0.153	1	10
Cbr1	-0.339535872	0.076	0.149	1	10
Anapc11	-0.29401488	0.249	0.329	1	10
Ranbp3	-0.339732134	0.103	0.172	1	10
Hmgcs1.4	-0.406787112	0.065	0.152	1	10
Elp2	-0.351527925	0.173	0.243	1	10
Wdr12.1	-0.311000045	0.114	0.176	1	10
Akap12.5	-0.401877632	0.038	0.112	1	10
Setd5	-0.387412186	0.141	0.248	1	10
F2r	-0.326135598	0.038	0.105	1	10
Aurkb.9	-0.26559457	0.059	0.143	1	10
Gng2.6	-0.315477401	0.319	0.397	1	10
Rbbp8.3	-0.315555828	0.049	0.123	1	10
Zfp322a	-0.351146928	0.065	0.123	1	10
Rpl36	-0.316591815	0.038	0.109	1	10
Rb1cc1.2	-0.40628093	0.157	0.235	1	10
Man2a1	0.284735422	0.108	0.041	1	10
Ppil4	-0.40030423	0.146	0.25	1	10
Casc3	-0.331541096	0.059	0.129	1	10
1500011B03Rik.3	-0.357696313	0.043	0.107	1	10
Chchd1.2	-0.284823563	0.357	0.401	1	10
Ccdc127	-0.307237961	0.114	0.208	1	10
Cpsf7	-0.369218712	0.086	0.184	1	10
l7Rn6	-0.341264692	0.119	0.201	1	10
Fbxw2	-0.356461588	0.086	0.16	1	10
Cdc26	-0.349356342	0.07	0.144	1	10
Pnpla8.1	-0.363099937	0.146	0.232	1	10
Med11	0.329591188	0.114	0.048	1	10
Slc35b1.1	-0.299139199	0.243	0.294	1	10
Kdm6b.1	-0.351587027	0.038	0.105	1	10
Ppp1r12a	-0.374484258	0.173	0.251	1	10
Paf1	-0.334987746	0.103	0.171	1	10
Lrrfip1.1	0.258006978	0.108	0.043	1	10
Rsbn1.1	-0.377764748	0.114	0.183	1	10
Zc3h7b	-0.289277714	0.086	0.128	1	10
Rpa2.6	-0.37936305	0.124	0.2	1	10
Chmp7	-0.350514179	0.059	0.129	1	10

Copb2	-0.316775153	0.157	0.218	1 10
Ccnl2.1	-0.279071054	0.286	0.321	1 10
Rwdd1	-0.357883285	0.162	0.277	1 10
Foxp1.1	-0.389899663	0.092	0.172	1 10
Gkap1	-0.337351422	0.124	0.221	1 10
Qser1	-0.346760818	0.049	0.128	1 10
Raf1	-0.345971851	0.092	0.187	1 10
Palld	0.295944488	0.103	0.04	1 10
Tmem183a	-0.355528392	0.092	0.174	1 10
Vps4a	-0.294848391	0.086	0.137	1 10
Cstf3	-0.351389364	0.059	0.14	1 10
Bccip.2	-0.272198014	0.243	0.311	1 10
Zfp608.2	-0.400035618	0.097	0.186	1 10
Ssbp2.2	-0.341594991	0.049	0.126	1 10
H2afz.8	-0.343171058	0.135	0.232	1 10
Cenpk.9	-0.374913124	0.059	0.145	1 10
Rnf11	-0.35833739	0.081	0.148	1 10
Ckap2.8	-0.387228946	0.081	0.164	1 10
Fnbp4	-0.360476128	0.168	0.277	1 10
Ccdc18.5	-0.264892008	0.043	0.116	1 10
Mpnd	-0.361831392	0.097	0.172	1 10
Kmt2c.1	-0.348060446	0.205	0.296	1 10
Pum1	-0.381390512	0.168	0.277	1 10
Psma4.2	-0.280879306	0.319	0.414	1 10
Scaf11.1	-0.280074053	0.314	0.373	1 10
Eif4e3.4	-0.350560898	0.049	0.131	1 10
Ogt.2	-0.280135698	0.178	0.211	1 10
Parl	-0.335207017	0.059	0.124	1 10
Naa15	-0.334668748	0.286	0.392	1 10
Tmem184c	-0.332946503	0.043	0.123	1 10
Sod1.3	-0.259540179	0.378	0.442	1 10
Xrcc5.1	-0.307583308	0.038	0.115	1 10
Usp3	-0.342283026	0.081	0.142	1 10
A430005L14Rik	-0.291328619	0.054	0.116	1 10
Adprh	-0.270438126	0.249	0.339	1 10
Ralgds	-0.346254427	0.032	0.103	1 10
Bhlhb9	-0.314908513	0.043	0.117	1 10
Wbp11	-0.324489153	0.232	0.297	1 10
Dnttip2.1	-0.385549013	0.227	0.325	1 10
Zfp191.1	-0.357097021	0.124	0.218	1 10
Lrrcc1	-0.346355479	0.097	0.152	1 10
Dck.1	-0.301437006	0.032	0.103	1 10
Gars	-0.369370507	0.135	0.228	1 10
5730455P16Rik	-0.356425435	0.054	0.128	1 10
Isoc1.3	-0.36309604	0.103	0.181	1 10
Gzf1	-0.259563357	0.043	0.115	1 10
Ccdc59	-0.328522471	0.232	0.314	1 10
Dhx32.4	-0.371533707	0.108	0.201	1 10

Fopnl	-0.333196326	0.103	0.203	1	10
Psmb2.1	-0.25197871	0.351	0.428	1	10
Ssbp3.4	-0.347242694	0.076	0.168	1	10
Dhx30	-0.337299326	0.049	0.124	1	10
Frg1.1	-0.309241594	0.211	0.299	1	10
4632415L05Rik.1	-0.327824684	0.043	0.115	1	10
Ssr3.2	-0.275711297	0.373	0.425	1	10
Pbdc1.3	-0.307551272	0.157	0.206	1	10
Hmgcr.1	-0.329500098	0.049	0.13	1	10
Napg.1	-0.341915854	0.076	0.129	1	10
Midn.1	-0.317396384	0.081	0.133	1	10
Ntmt1	-0.330440748	0.059	0.144	1	10
Uba2.1	-0.334279156	0.162	0.256	1	10
Topors	-0.355062284	0.108	0.183	1	10
Mrpl47	-0.345583309	0.081	0.147	1	10
Suv420h1	-0.335038815	0.092	0.143	1	10
Ppp2r1a	-0.2788097	0.173	0.213	1	10
Itsn1.9	-0.413401709	0.151	0.236	1	10
Brca2.5	-0.325998172	0.059	0.114	1	10
Sfxn1.1	-0.32547313	0.189	0.25	1	10
Ubfd1	-0.275194724	0.07	0.11	1	10
Brcc3	-0.310738036	0.081	0.176	1	10
Gatad2b	-0.318392493	0.043	0.121	1	10
Fosb.4	0.436264592	0.249	0.154	1	10
Actr6	-0.32118643	0.043	0.108	1	10
Rbm17	-0.284195428	0.319	0.39	1	10
Ubxn6	-0.315441262	0.114	0.168	1	10
Senp1	-0.347908858	0.065	0.136	1	10
Krt10	-0.344471956	0.076	0.162	1	10
Slc25a27.1	-0.335895049	0.038	0.11	1	10
Pmm1.1	-0.334916071	0.065	0.128	1	10
Aff4.1	-0.371228075	0.151	0.217	1	10
Rfk	-0.278697023	0.097	0.139	1	10
Carkd	-0.30118388	0.178	0.225	1	10
Mysm1	-0.360896098	0.081	0.145	1	10
Mrpl13.4	-0.250084417	0.232	0.308	1	10
Gm13092.1	-0.269181642	0.054	0.104	1	10
Hdac3	-0.330573922	0.086	0.153	1	10
Tomm40	-0.276719884	0.124	0.166	1	10
Phf5a.1	-0.305090472	0.227	0.332	1	10
Papss1.1	-0.340479068	0.065	0.147	1	10
Pop4	-0.311911721	0.076	0.162	1	10
Ing4.4	-0.320037297	0.259	0.323	1	10
Cherp	-0.299047729	0.065	0.121	1	10
Tor2a	0.260145569	0.141	0.069	1	10
Ammecr1l	-0.297084501	0.076	0.123	1	10
Mecp2	-0.347354384	0.049	0.12	1	10
Mrpl11	-0.356917238	0.151	0.238	1	10

Pja2.1	-0.374546464	0.151	0.235	1 10
Eftud2.1	-0.335509053	0.114	0.183	1 10
Rcc2.3	-0.321339011	0.114	0.179	1 10
Eif4h	-0.261946459	0.4	0.471	1 10
H13.2	-0.286424772	0.2	0.238	1 10
Ppan	-0.330031472	0.076	0.139	1 10
St3gal5	-0.309836381	0.119	0.194	1 10
Mmadhc	-0.326496656	0.168	0.233	1 10
Arid2.1	-0.355002284	0.151	0.242	1 10
Dohh	-0.332350841	0.097	0.169	1 10
Lrp11	-0.286497095	0.032	0.101	1 10
Rtca	-0.338860058	0.059	0.129	1 10
Xrn1	-0.344797878	0.043	0.111	1 10
Smek2	-0.282006171	0.189	0.223	1 10
1110038B12Rik.5	-0.345774169	0.216	0.299	1 10
Sumo1	-0.352419433	0.13	0.218	1 10
Lars	-0.339358141	0.119	0.218	1 10
Gnl3.5	-0.364771198	0.205	0.297	1 10
Nvl	-0.348596915	0.081	0.169	1 10
Taf2	-0.311234944	0.054	0.106	1 10
Ap4s1	-0.279301349	0.059	0.104	1 10
Cog1.1	-0.250281187	0.043	0.115	1 10
Nme1	-0.298084067	0.422	0.51	1 10
2410006H16Rik.2	-0.327746672	0.362	0.46	1 10
Cdk1.9	-0.366568234	0.146	0.247	1 10
Bola1.1	-0.347950014	0.086	0.173	1 10
Nip7	-0.326988837	0.092	0.163	1 10
Cenph.9	-0.260252991	0.108	0.194	1 10
Exosc7.3	-0.315556036	0.086	0.18	1 10
Rab24	-0.273348787	0.178	0.215	1 10
Nin.4	-0.358665387	0.076	0.146	1 10
Blmh.1	-0.282146397	0.254	0.317	1 10
Slc35a1	-0.306172134	0.07	0.124	1 10
Gle1	-0.273020257	0.059	0.14	1 10
Lcmt1	-0.33573584	0.086	0.169	1 10
Arhgef7.3	-0.296736052	0.114	0.149	1 10
Nudc.4	-0.305794235	0.173	0.282	1 10
Commd1.3	-0.284916023	0.222	0.299	1 10
Mprip	-0.35176177	0.086	0.152	1 10
Irak1bp1	-0.315606083	0.059	0.14	1 10
Eif2ak1	-0.314513323	0.054	0.122	1 10
Polr3f	-0.284530636	0.032	0.102	1 10
Dnal4	-0.310609258	0.049	0.11	1 10
Elp4	-0.295072629	0.049	0.103	1 10
Incenp.9	-0.335925376	0.195	0.276	1 10
Mrpl30	-0.289944068	0.238	0.309	1 10
Cby1	-0.317191203	0.059	0.127	1 10
Yars	-0.28672561	0.076	0.142	1 10

2610524H06Rik	-0.317116955	0.07	0.127	1	10
Hook3.2	-0.373887774	0.2	0.3	1	10
1700123O20Rik.1	-0.301975936	0.081	0.141	1	10
Stk25	-0.344743361	0.114	0.185	1	10
Slc25a17	-0.319229057	0.092	0.154	1	10
Thumpd1	-0.34066045	0.097	0.166	1	10
Oxsr1	-0.28802529	0.076	0.122	1	10
Armc10.1	-0.299068344	0.038	0.11	1	10
Vps29	0.270893546	0.319	0.225	1	10
Yme1l1	-0.267540673	0.178	0.222	1	10
Suv39h2.3	-0.322211449	0.054	0.132	1	10
Usp5	-0.305771224	0.086	0.14	1	10
Wdr43	-0.345422852	0.151	0.242	1	10
Wasl.1	-0.364971553	0.141	0.237	1	10
Smek1	-0.292470175	0.13	0.179	1	10
Csnk2a1	-0.326604821	0.141	0.245	1	10
Cxx1b	-0.328608572	0.049	0.121	1	10
Nosip	-0.293294103	0.124	0.168	1	10
Sssca1	-0.325367161	0.135	0.203	1	10
Ndufs7	-0.284870575	0.276	0.335	1	10
Agap1.3	-0.338434972	0.054	0.132	1	10
Pias2	-0.311203138	0.054	0.131	1	10
Drg1	-0.32937983	0.108	0.201	1	10
Zfp329.2	-0.358071578	0.059	0.129	1	10
Chka	-0.334057188	0.097	0.159	1	10
Ppdpf	-0.313428926	0.103	0.175	1	10
Pqbp1	-0.345730645	0.178	0.264	1	10
Fam53b.1	-0.264870458	0.103	0.136	1	10
Aatf	-0.322763108	0.049	0.122	1	10
Hsf2	-0.306420172	0.043	0.113	1	10
Cul4a	-0.313206494	0.049	0.111	1	10
Fen1.7	-0.295954853	0.092	0.142	1	10
Rfc1.6	-0.262011806	0.249	0.331	1	10
Stk11.1	-0.257424389	0.151	0.23	1	10
Trnt1	-0.278478953	0.076	0.153	1	10
Zfp30	-0.34276243	0.07	0.124	1	10
Zfp386	-0.332149011	0.059	0.139	1	10
Eif5	-0.259975315	0.486	0.544	1	10
Mphosph9	-0.314491069	0.065	0.148	1	10
Hspd1.3	-0.30943394	0.27	0.365	1	10
Hells.10	-0.272490937	0.13	0.214	1	10
Mrpl15.1	-0.295964116	0.238	0.294	1	10
Thoc3	-0.291884046	0.076	0.159	1	10
Nop14	-0.286959029	0.146	0.218	1	10
Rad23b	-0.272730582	0.157	0.203	1	10
Med1	-0.335034709	0.097	0.173	1	10
Sclt1.1	-0.328924285	0.054	0.127	1	10
Bmi1	-0.279344345	0.065	0.147	1	10

Pcnt.2	-0.338149296	0.108	0.175	1	10
Sf3b3	-0.298097749	0.076	0.126	1	10
1300002E11Rik	-0.30398087	0.065	0.113	1	10
Prcc	-0.272635784	0.07	0.118	1	10
Vars.2	-0.291209966	0.092	0.167	1	10
Polr1c.3	-0.256443092	0.146	0.219	1	10
Fyttd1	-0.250128826	0.189	0.258	1	10
Ifrd1.2	-0.302712851	0.124	0.178	1	10
Rab12	-0.297846099	0.146	0.193	1	10
Asun	-0.297002069	0.038	0.107	1	10
Eif3b.2	-0.288971948	0.173	0.22	1	10
Chd1	-0.309316056	0.114	0.21	1	10
Emd.1	-0.310338015	0.119	0.182	1	10
Ftsj3.2	-0.331199539	0.119	0.207	1	10
Mphosph10.4	-0.349425871	0.141	0.232	1	10
Rbm28	-0.287441976	0.205	0.263	1	10
Zfp397	-0.304225831	0.049	0.104	1	10
C330027C09Rik.7	-0.330227632	0.065	0.134	1	10
Tsg101	-0.328628574	0.189	0.265	1	10
Wbp4	-0.291744839	0.232	0.276	1	10
Mrgbp	-0.341466855	0.108	0.184	1	10
Rai1.2	-0.332612623	0.081	0.16	1	10
Zfp318.1	-0.362233881	0.108	0.176	1	10
Znhit6.1	-0.330207828	0.054	0.12	1	10
Grsf1	-0.325806735	0.114	0.176	1	10
Snx17	0.262042588	0.222	0.13	1	10
Smim11.3	-0.327077113	0.178	0.277	1	10
Pdha1	-0.315516356	0.097	0.163	1	10
Med4	-0.29731041	0.103	0.155	1	10
Chd2	-0.381586288	0.141	0.222	1	10
Cdc40	-0.309734814	0.108	0.17	1	10
Rps19bp1	-0.334081761	0.092	0.175	1	10
Rps27a.1	-0.277943169	0.054	0.106	1	10
Atf4	-0.277811962	0.314	0.397	1	10
Parp1.1	-0.32782431	0.216	0.314	1	10
Lias	-0.281157039	0.108	0.151	1	10
Trim33	-0.33185193	0.07	0.15	1	10
Orc2.1	-0.302596027	0.097	0.156	1	10
Snapc3.1	-0.315568564	0.065	0.125	1	10
Ubxn2a	-0.356257828	0.086	0.163	1	10
Mcmbp.1	-0.340483297	0.135	0.217	1	10
Ubr7.3	-0.306809073	0.07	0.133	1	10
Hbs1l	-0.301446111	0.043	0.107	1	10
Taf3	-0.355642445	0.081	0.156	1	10
Ash2l	-0.301021366	0.146	0.216	1	10
Mtch2.2	-0.300100521	0.232	0.295	1	10
Ccdc174	-0.325759271	0.13	0.205	1	10
Cops8	-0.313648021	0.162	0.241	1	10

Cops3	-0.310885318	0.189	0.251	1	10
Safb2	-0.268301914	0.276	0.313	1	10
Crebbp.1	-0.331070297	0.13	0.224	1	10
Snx27.1	-0.327097288	0.086	0.154	1	10
Cuedc2	-0.314603588	0.303	0.388	1	10
Mbd3.2	-0.266918906	0.227	0.291	1	10
Arhgap21.2	-0.343271496	0.081	0.165	1	10
Spin1	-0.294708198	0.059	0.119	1	10
Rab11fip2	-0.314857392	0.054	0.107	1	10
Surf2	-0.299648713	0.043	0.101	1	10
Fabp7.11	4.487435605	0.992	0.117	0	11
Slc1a3.7	3.662214228	0.992	0.146	0	11
Dbi.4	2.747454896	1	0.459	0	11
Apoe.11	3.599922742	1	0.244	0	11
Aqp4	3.971284743	0.967	0.019	7.5425E-305	11
Cst3.9	2.720436276	1	0.533	1.2387E-295	11
Sparcl1.8	3.172003634	1	0.201	2.6556E-264	11
Slc4a4.1	3.342358033	0.95	0.034	3.7672E-257	11
Mt1.8	3.10200897	0.933	0.163	7.61E-239	11
Aldoc.1	3.162592765	0.933	0.026	6.1364E-233	11
Atp1a2.1	3.35279902	1	0.052	1.7933E-227	11
Glul.3	2.490068101	0.933	0.196	1.3333E-224	11
Ttyh1.1	2.889073579	0.958	0.025	1.4223E-217	11
Pla2g7.1	2.958266753	0.883	0.014	1.4498E-215	11
Ednrb.1	2.995422259	0.95	0.023	1.3801E-204	11
Mlc1	2.232767102	0.833	0.004	5.825E-194	11
Ppap2b.1	2.780775899	0.908	0.037	5.9596E-184	11
Clu.1	2.675126603	0.833	0.014	3.9786E-178	11
Id3	2.983473726	0.858	0.026	5.5836E-174	11
Car2	2.626796719	0.767	0.012	3.2605E-171	11
Agt	2.425091363	0.792	0.01	2.5912E-167	11
Gpr37l1.1	2.597927517	0.892	0.025	3.4084E-163	11
Sparc.2	2.945330789	0.975	0.079	2.7181E-162	11
Acsbg1	2.018016443	0.742	0.005	1.433E-160	11
Timp4.1	2.138913416	0.8	0.011	4.3982E-157	11
Gpm6b.6	1.952342348	0.95	0.48	7.834E-153	11
Aldh1l1	1.937880214	0.675	0.004	1.0948E-149	11
Ptn.9	2.074900101	0.975	0.437	3.3151E-144	11
Tril.1	2.168765874	0.8	0.02	1.131E-142	11
Kcnj10.1	2.5082552	0.767	0.02	6.1153E-142	11
Mt2.3	2.542996183	0.858	0.086	2.0072E-141	11
Mt3.1	2.18615285	0.783	0.02	1.0335E-139	11
Slc25a18	1.679454649	0.658	0.004	9.8026E-138	11
Igfbp2	2.590472636	0.758	0.015	2.9666E-137	11
Cd81.4	1.709122222	0.975	0.458	1.2829E-131	11
Mmd2.1	2.055826181	0.833	0.034	8.4508E-131	11
Tnc	1.870539642	0.567	0.002	2.6233E-130	11
Serpine2.2	2.388124328	0.908	0.051	9.1161E-130	11

Hsp90ab1.2	-1.018943128	0.975	0.998	3.0803E-129	11
Ramp1.2	2.052174204	0.8	0.022	5.9633E-128	11
Bcan.1	2.224166131	0.892	0.046	3.865E-127	11
Hepacam.1	1.628504529	0.633	0.005	1.1841E-124	11
Cmtm5.1	1.857121138	0.717	0.013	1.9808E-124	11
Scd2.4	1.916660249	0.908	0.293	1.5533E-123	11
Id4	1.80110291	0.667	0.008	4.2638E-123	11
Ptprz1.1	2.270488348	0.825	0.031	1.6081E-121	11
Atp1b2.1	2.089659148	0.825	0.042	2.1265E-121	11
Gria1	1.677459721	0.625	0.007	4.7341E-117	11
Tspan7.6	2.10392258	0.9	0.1	3.4375E-114	11
Lcat	1.660314012	0.608	0.007	1.7212E-111	11
Npy	1.95427703	0.492	0.002	2.4071E-107	11
S100b.1	2.125999341	0.767	0.035	3.0425E-105	11
Pea15a.9	1.9894622	0.875	0.171	1.094E-103	11
Pla2g16.1	1.358459034	0.542	0.005	1.5596E-102	11
Nid1	1.739883553	0.642	0.014	1.6188E-101	11
Chchd10	1.923660394	0.717	0.029	1.1473E-100	11
Hopx	1.636674019	0.525	0.005	9.6525E-100	11
S1pr1.1	1.459879296	0.533	0.005	5.6411E-99	11
Sepp1.1	1.140566197	0.758	0.051	1.59946E-98	11
Cspg5.1	1.555799779	0.667	0.024	1.08683E-97	11
Paqr8	1.399807929	0.542	0.007	1.65164E-95	11
Gja1	1.865887277	0.583	0.016	5.04085E-93	11
Htra1	1.658987468	0.558	0.011	7.84595E-93	11
Gstm1.1	1.907377408	0.717	0.077	4.8018E-89	11
Mfge8.1	1.780406243	0.65	0.029	2.19121E-88	11
Lxn	1.79491209	0.708	0.048	8.72725E-87	11
Adora1.1	1.368031132	0.525	0.008	2.35305E-86	11
Tmem176a.2	1.498087206	0.558	0.012	5.0913E-86	11
Ncan.5	1.81753108	0.8	0.094	2.65106E-84	11
Lsamp.2	1.77482144	0.808	0.072	3.26763E-84	11
Sdc4.1	1.273831084	0.483	0.006	4.69968E-84	11
Cryab.1	1.58617618	0.6	0.018	2.37593E-83	11
Emid1.1	1.485959696	0.483	0.006	2.43759E-82	11
Gfap	1.844027933	0.558	0.026	5.75413E-82	11
Ntrk2.3	1.746380883	0.8	0.078	4.58425E-81	11
Ifitm3	1.431684062	0.517	0.01	7.02308E-81	11
Casp12	1.074625596	0.375	0.001	1.75878E-80	11
Scrg1.1	1.524143829	0.6	0.021	3.10372E-80	11
F3	1.348047896	0.45	0.005	6.30722E-80	11
AW047730.1	1.573503253	0.592	0.02	2.3258E-79	11
Tsc22d4.4	1.731538888	0.8	0.161	6.16468E-79	11
Tmem176b.4	1.726772851	0.817	0.104	8.57172E-79	11
Lrig1	1.366953621	0.533	0.014	1.49054E-76	11
Metrn.1	1.313571037	0.558	0.017	3.5277E-75	11
Plat	1.412497772	0.558	0.018	6.26152E-75	11
S100a1.2	1.442643636	0.575	0.023	8.83743E-74	11

Slc9a3r1	1.119122363	0.492	0.01	1.63258E-73	11
Hsd11b1	1.608872981	0.383	0.003	2.26233E-73	11
Glud1.1	1.603171589	0.792	0.137	3.81301E-72	11
Ccdc80	1.299342554	0.433	0.006	5.32982E-72	11
Luzp2.1	1.35595928	0.517	0.014	5.91302E-72	11
Gabrb1	0.839923645	0.342	0.001	6.72483E-72	11
Hes5	1.366502765	0.408	0.004	1.98912E-71	11
Slc6a1.1	1.486620432	0.575	0.023	2.27532E-71	11
Prss23	1.181308133	0.367	0.002	9.69817E-71	11
Atp1b1.1	1.781254311	0.658	0.047	2.36813E-70	11
Chl1	1.237694109	0.483	0.012	5.21788E-70	11
Fgfr3	0.956575617	0.342	0.001	4.96775E-69	11
Kcnj16	1.093082897	0.408	0.005	6.43578E-69	11
Cyp26b1	1.141285763	0.35	0.002	3.63877E-68	11
Rgs5	1.838227382	0.442	0.01	3.94827E-68	11
Gjb6	1.279196359	0.35	0.002	1.26555E-66	11
Rps5.6	-1.076494635	0.9	0.954	3.30997E-66	11
Limch1.1	1.322585159	0.533	0.02	4.25337E-66	11
Lpar1	1.065784254	0.408	0.006	6.63772E-66	11
Sfxn5	1.172667794	0.483	0.014	4.19325E-65	11
Lamp1.2	1.380649515	0.908	0.332	4.82709E-65	11
Npas3.1	1.32877035	0.542	0.023	2.67218E-64	11
Slc39a12	0.929573661	0.267	0	3.50286E-64	11
Tubb5.8	-1.506480264	0.583	0.916	5.3909E-64	11
Vcam1.1	0.852284395	0.367	0.005	7.29448E-64	11
Ndrg2.5	1.611367075	0.783	0.18	2.59685E-63	11
Cnp.1	1.025316596	0.525	0.03	1.56542E-62	11
Sfrp1.7	-1.984855323	0.367	0.814	3.15765E-62	11
Prex2	1.231396472	0.442	0.01	3.68426E-62	11
Emp2	1.067369884	0.35	0.003	1.6347E-61	11
Vim.7	1.565710646	0.883	0.298	1.8278E-61	11
Fxyd1	1.133942415	0.433	0.01	2.05598E-61	11
Gpnmb.1	1.007803827	0.325	0.002	2.3081E-61	11
Slc38a3.1	1.259391158	0.483	0.018	5.56431E-61	11
Sox2.1	1.286044004	0.617	0.044	5.60002E-61	11
Pbxip1.1	1.11280466	0.458	0.014	6.68602E-61	11
Ddah1.1	1.524711738	0.567	0.036	1.77656E-60	11
Tlcd1	1.396380591	0.5	0.024	6.51059E-60	11
Thbs3	0.925954452	0.383	0.007	1.06408E-59	11
Tspan3.2	1.273291469	0.908	0.425	5.29399E-59	11
Gm3764.4	1.540476387	0.75	0.127	4.45802E-58	11
Cacng4.8	1.550239669	0.733	0.097	1.14244E-57	11
Slc7a10	1.145424287	0.325	0.003	1.96932E-57	11
Nfib.6	-1.382781801	0.75	0.934	3.00906E-57	11
Tmem47	1.443509149	0.617	0.06	4.05302E-57	11
Atp13a4	0.954864365	0.258	0	7.71959E-57	11
Smpdl3a.1	0.97568664	0.392	0.008	8.99019E-57	11
Shisa9	1.103311418	0.35	0.004	9.71174E-57	11

Gsta4	0.83600147	0.367	0.009	3.25092E-56	11
Ckb.9	1.04749604	0.975	0.807	3.43743E-56	11
Pdpn	0.999792341	0.383	0.008	8.07182E-56	11
S100a13.2	1.151872559	0.5	0.027	8.96688E-56	11
H2-K1.1	1.206408328	0.417	0.011	1.54295E-55	11
Dkk3	0.891669033	0.367	0.007	3.46037E-55	11
Gjc3.1	1.300557675	0.417	0.012	2.17667E-54	11
Slc13a3	1.010428959	0.283	0.001	5.94205E-54	11
Mgst1.1	1.245983497	0.517	0.03	1.32134E-53	11
Itm2b.9	1.136270212	0.95	0.64	2.95454E-53	11
Myo6.1	1.386172512	0.575	0.046	3.03978E-53	11
Daam2	0.833183077	0.283	0.002	9.07598E-53	11
S100a10	1.300678119	0.483	0.026	2.82031E-52	11
Cp.1	0.803143451	0.358	0.011	9.12862E-52	11
Slc1a2.7	1.414776745	0.842	0.29	5.53461E-51	11
Cd44	0.922623246	0.425	0.017	3.74684E-50	11
Asrgl1.4	1.478670287	0.692	0.129	4.05727E-50	11
Cybrd1	0.727126425	0.267	0.002	4.7644E-50	11
Spon1.1	0.896230277	0.375	0.012	6.13126E-50	11
Gucy1a3.1	0.705345652	0.333	0.008	8.84709E-50	11
Eps8.1	1.073677169	0.475	0.029	2.41613E-49	11
Omg.1	1.017437841	0.358	0.009	3.15278E-49	11
Gmpr.1	0.996054295	0.367	0.01	1.29801E-48	11
Gabrg1	0.725329202	0.283	0.003	1.50536E-48	11
Acadl.1	1.293413623	0.592	0.06	1.59909E-48	11
Ncl.7	-1.147477775	0.692	0.897	2.8052E-48	11
Itga6.1	1.03244704	0.375	0.011	4.90661E-48	11
Acot1	1.316586976	0.508	0.037	5.52886E-48	11
S100a16.7	1.326731441	0.667	0.09	1.07832E-47	11
Cyp2j9.1	0.811865912	0.3	0.004	2.11588E-47	11
Psap.5	1.286506798	0.808	0.18	5.12533E-47	11
Slc6a11	1.39739189	0.375	0.016	7.94126E-47	11
S100a6.1	1.23277085	0.417	0.019	3.2263E-46	11
Fjx1.1	1.077633977	0.492	0.034	4.06155E-46	11
Cd302.2	1.10091668	0.542	0.049	1.00406E-45	11
Itm2c.7	1.314086668	0.808	0.207	1.22947E-45	11
Rps9.4	-1.018259717	0.808	0.929	1.26626E-45	11
Me1	0.727772375	0.3	0.006	1.90541E-45	11
Ctsl.4	1.281506416	0.808	0.256	2.24172E-45	11
Arhgef26	0.932919152	0.392	0.016	4.27691E-45	11
Gpd1	0.964055302	0.325	0.007	5.97982E-45	11
Hnrnpa2b1.3	-0.782368169	0.917	0.933	9.68686E-45	11
B2m.6	1.353274814	0.783	0.228	2.70439E-44	11
Tjp2	1.083243362	0.533	0.048	2.83241E-44	11
Sat1.1	1.475023513	0.558	0.063	3.27561E-44	11
Pcp4l1	1.009823086	0.275	0.003	3.50441E-44	11
Chst2.1	1.031812615	0.458	0.029	5.59351E-44	11
Rfx4	0.656157908	0.242	0.002	6.87559E-44	11

Wnt7a	0.744755013	0.233	0.002	7.23651E-44	11
Zcchc24.1	0.954482562	0.425	0.025	1.34541E-43	11
Slc6a9	0.899456816	0.317	0.007	1.37248E-43	11
Rpl13a.4	-0.911701647	0.9	0.928	2.16577E-43	11
Pcdh10.1	0.751482131	0.342	0.012	2.97897E-43	11
Tuba1a.7	-1.248325262	0.8	0.93	5.87303E-43	11
Cldn10	0.948553042	0.242	0.002	8.15284E-43	11
Timp3.3	1.083889172	0.592	0.075	1.15643E-42	11
1700084C01Rik	0.565969161	0.208	0.001	2.0435E-42	11
Pcx	1.145814291	0.542	0.057	1.17726E-41	11
Vamp8.1	0.631934444	0.3	0.011	1.41943E-41	11
Degs1.2	1.143524081	0.583	0.072	1.6878E-41	11
Abhd3	0.843997366	0.275	0.004	2.14787E-41	11
Cntn1.2	0.756957594	0.442	0.048	4.62874E-41	11
Adcyap1r1.2	1.125016524	0.6	0.079	6.83445E-41	11
Jam2.1	1.013695335	0.442	0.03	9.79695E-41	11
Cntnap2	0.838175708	0.3	0.007	1.21149E-40	11
H3f3b.3	-1.014882082	0.692	0.908	1.2754E-40	11
Rps26.6	-1.128355363	0.65	0.848	1.3457E-40	11
Abca1.1	0.842213542	0.367	0.021	1.47853E-40	11
Abat.1	0.911609554	0.417	0.027	1.85083E-40	11
Sorl1	1.025986286	0.392	0.02	5.36718E-40	11
Timp1	0.641720136	0.242	0.002	7.26111E-40	11
Igfbp4.1	1.198100302	0.367	0.016	8.50192E-40	11
Cpq	0.786443165	0.283	0.006	1.21493E-39	11
Rps3.4	-0.857344999	0.858	0.922	2.16414E-39	11
Pamr1	0.57993951	0.208	0.001	2.63074E-39	11
Gabra4	0.689227005	0.325	0.012	2.83002E-39	11
Naaa	0.792790459	0.258	0.004	4.09554E-39	11
Dbx2	0.898930181	0.25	0.003	4.67518E-39	11
Nrarp	0.818334464	0.283	0.006	4.76964E-39	11
Adrb1	0.549838979	0.2	0.001	4.7942E-39	11
Phyhipl.2	0.935768463	0.517	0.058	5.31465E-39	11
Lyn.1	0.609145056	0.283	0.009	6.64496E-39	11
Sox21.1	0.636780763	0.258	0.005	8.35517E-39	11
1810037I17Rik.3	1.211040328	0.75	0.192	8.87572E-39	11
Rpl4.5	-0.915066221	0.817	0.908	2.10457E-38	11
Slc14a1	0.582644084	0.192	0.001	3.03918E-38	11
Rplp0.5	-1.063561987	0.7	0.873	4.2848E-38	11
Adcy8	0.560656061	0.2	0.001	4.78891E-38	11
Gpx8.1	0.970845652	0.492	0.05	7.40009E-38	11
Plcd4	0.69972929	0.242	0.003	8.73828E-38	11
Slc35f1.1	0.699607917	0.317	0.015	1.09457E-37	11
CRE_RECOMBINASE.9	-1.973926561	0.408	0.77	1.17796E-37	11
S100a11	0.689193552	0.325	0.014	3.83328E-37	11
Hnrnpab.5	-1.073212822	0.608	0.817	5.88525E-37	11
Calm2.8	-1.032173269	0.7	0.875	1.13245E-36	11
Rhod	0.674868552	0.225	0.002	1.2097E-36	11

Pax3	0.681809329	0.267	0.008	1.4398E-36	11
Anp32a.2	-0.943803729	0.65	0.837	1.6019E-36	11
Cryl1.1	0.920215046	0.292	0.008	2.04379E-36	11
Gatm.2	0.895682286	0.408	0.032	2.14369E-36	11
Ifi27.1	0.776767079	0.317	0.013	4.15643E-36	11
Epha4.1	0.943845817	0.3	0.01	4.27552E-36	11
Rpl32.4	-0.890113236	0.792	0.89	5.31484E-36	11
Paqr7.1	0.776249194	0.25	0.004	7.0788E-36	11
Igsf11	0.673501648	0.242	0.004	1.85182E-35	11
Prdx6.3	1.121163259	0.792	0.322	7.30879E-35	11
Trpm3	0.730702111	0.283	0.01	1.5075E-34	11
Aldh2.1	0.839131107	0.367	0.023	1.61629E-34	11
Dhrs1.1	1.111643658	0.575	0.089	1.65204E-34	11
Gdpd2	0.476480397	0.175	0.001	1.75051E-34	11
Slc1a4	1.152933774	0.475	0.054	1.82632E-34	11
Rps14.4	-0.80255813	0.875	0.95	2.00541E-34	11
Gpr126	0.532877083	0.225	0.004	2.01761E-34	11
Slc13a5	0.654282149	0.225	0.003	2.65322E-34	11
Grm3	0.484999047	0.192	0.001	4.32377E-34	11
Il33	0.509001458	0.217	0.004	4.61915E-34	11
Fam213a.1	1.042641529	0.558	0.085	6.80092E-34	11
Hnrnpu.4	-0.84350654	0.767	0.866	8.21114E-34	11
Ctso	0.714543158	0.308	0.014	1.33353E-33	11
Rab31.2	0.996978835	0.475	0.052	1.44711E-33	11
Bmpr1b	0.62182506	0.233	0.004	1.95996E-33	11
Angpt1	1.051166834	0.45	0.044	1.99373E-33	11
Rgl1	0.738495897	0.258	0.006	2.0256E-33	11
Efhd1	0.693523906	0.358	0.028	6.24666E-33	11
Kcnn2	0.870904842	0.283	0.01	7.59203E-33	11
Nacc2.2	0.838438528	0.325	0.016	9.37064E-33	11
Arap2	0.727177526	0.267	0.007	9.69522E-33	11
Hbegf.1	0.806469091	0.325	0.018	1.06286E-32	11
Sash1.2	0.899430724	0.392	0.033	1.27827E-32	11
BC064078	0.499768477	0.167	0.001	2.071E-32	11
2810459M11Rik	0.497499586	0.192	0.002	2.10049E-32	11
Abhd4.1	0.915290314	0.433	0.042	2.13117E-32	11
Cml1	0.824560254	0.292	0.011	2.64588E-32	11
Igfbpl1.9	-1.773073592	0.133	0.651	4.25131E-32	11
Mro	0.503907596	0.2	0.003	6.77984E-32	11
Basp1.10	-1.614444748	0.233	0.707	1.05599E-31	11
Add3.4	0.897024774	0.567	0.1	1.30572E-31	11
Tmem37.1	0.727038491	0.242	0.006	2.15076E-31	11
Itih5	0.297986492	0.217	0.013	3.47717E-31	11
Rarres2	0.722685298	0.225	0.005	3.52173E-31	11
Fgfr1	1.015263011	0.525	0.077	4.32545E-31	11
Pon2.1	0.848449958	0.4	0.036	4.81754E-31	11
Gria4.9	1.066582526	0.617	0.121	5.52988E-31	11
Sncg	0.774430856	0.158	0	6.18293E-31	11

Sirpa.1	0.894753543	0.392	0.035	6.9909E-31	11
Nbl1	0.76901171	0.342	0.025	7.38131E-31	11
Rplp2.6	-0.959184107	0.675	0.841	3.44076E-30	11
Rrbp1.4	1.113170756	0.7	0.185	4.67678E-30	11
Rps21.5	-0.900915419	0.692	0.834	4.94541E-30	11
Serbp1.7	-0.774078934	0.8	0.869	5.2072E-30	11
Nwd1	0.431022175	0.158	0.001	6.27592E-30	11
Paqr4.1	0.900168984	0.417	0.045	7.85908E-30	11
0610040J01Rik.1	0.41100597	0.183	0.002	8.59661E-30	11
Dtna.1	0.677557434	0.333	0.029	1.51315E-29	11
Slc12a4	0.821905324	0.375	0.033	2.78882E-29	11
Hnrnpm.5	-0.98967814	0.55	0.736	2.91969E-29	11
Epas1	0.635345439	0.233	0.007	3.84986E-29	11
Agrn.1	0.850274677	0.483	0.073	4.34081E-29	11
Angptl4	0.635940037	0.233	0.007	6.39611E-29	11
Aldh1a1	0.580045226	0.208	0.005	7.43593E-29	11
Fam20a	0.551393121	0.167	0.001	7.71314E-29	11
Tpp1.2	0.861221141	0.467	0.064	9.06261E-29	11
Rhoc.2	0.855457652	0.342	0.025	1.59723E-28	11
Id1	1.135346557	0.483	0.07	1.86824E-28	11
Gnb2l1.4	-0.857818984	0.742	0.872	1.87802E-28	11
Clrn1	0.385925807	0.167	0.003	1.87935E-28	11
Crmp1.5	-1.432256931	0.242	0.67	2.35474E-28	11
Chpt1.1	0.998207296	0.45	0.057	3.20728E-28	11
Adam11	0.459868878	0.242	0.016	3.38619E-28	11
Ephx1	0.663133558	0.225	0.006	4.4325E-28	11
Pabpc1.5	-0.842060547	0.792	0.879	4.81053E-28	11
Tmsb10.6	-1.461308493	0.217	0.666	6.00291E-28	11
Btbd17.6	1.102238959	0.642	0.156	7.7778E-28	11
Slc27a1	0.862894864	0.408	0.045	8.39005E-28	11
Elovl5.1	1.022028816	0.575	0.116	8.78735E-28	11
Nfasc.3	0.915679216	0.358	0.033	9.62888E-28	11
Itih3	1.067360585	0.208	0.007	9.80112E-28	11
Slc7a2	0.542351134	0.217	0.005	1.01859E-27	11
Cbr3.1	0.567127857	0.225	0.007	1.30559E-27	11
Egfl6	0.590240879	0.167	0.001	1.35168E-27	11
Acox1.1	0.644770191	0.4	0.054	1.59692E-27	11
Gnb4.2	0.9276551	0.55	0.102	1.69955E-27	11
Stat3.2	0.701969786	0.367	0.039	1.8249E-27	11
Pmm1.2	0.9016243	0.592	0.124	1.83607E-27	11
Sfpq.1	-1.090715364	0.425	0.672	1.85388E-27	11
Aspa	0.45863534	0.158	0.001	1.87516E-27	11
Asah1.2	0.864900891	0.45	0.06	2.19003E-27	11
Cog7.7	-1.585871741	0.15	0.587	2.24603E-27	11
Cd9.2	0.92423555	0.867	0.482	3.34682E-27	11
Tmem56	0.448239162	0.217	0.009	3.40941E-27	11
Tmem198b	0.876754496	0.358	0.033	4.27638E-27	11
Prrx1	0.558512954	0.2	0.005	4.29384E-27	11

Sema6d	0.640994844	0.325	0.03	4.71364E-27	11
Tmem9b.2	0.827348274	0.55	0.109	5.35861E-27	11
Slc25a33	0.770170514	0.392	0.044	5.41735E-27	11
Fam181a	0.337538986	0.125	0	6.10867E-27	11
Tspan12.1	0.716645642	0.333	0.03	7.71221E-27	11
Abi3bp	0.400554867	0.15	0.001	7.75816E-27	11
Emc7.1	0.787434259	0.608	0.14	8.07157E-27	11
Nfia.2	-1.162257331	0.533	0.775	1.30442E-26	11
Dlgap1.1	0.73489396	0.425	0.064	1.71413E-26	11
Pld2	0.423957849	0.158	0.001	2.47028E-26	11
Gpld1	0.760845114	0.183	0.003	2.61631E-26	11
Slc22a4	0.487236396	0.192	0.005	2.68885E-26	11
Grid2	0.676728258	0.258	0.012	2.83237E-26	11
Ctsd.10	0.414261166	0.633	0.172	2.89118E-26	11
Ddx5.3	-0.88905533	0.625	0.817	3.1296E-26	11
Gpr123	0.498096876	0.175	0.002	3.15335E-26	11
Scrn1.2	0.664255253	0.367	0.047	3.76781E-26	11
Tpi1.2	0.890086217	0.567	0.118	3.85494E-26	11
Gde1.2	0.908831418	0.608	0.138	3.90681E-26	11
Hnrnpc.2	-0.789716351	0.583	0.667	5.10352E-26	11
AI464131	0.494006286	0.167	0.002	5.12997E-26	11
Mrps6.1	1.055429713	0.608	0.148	5.33575E-26	11
Cd24a.4	-1.237404892	0.167	0.637	6.51802E-26	11
Cyp2d22	0.428127334	0.158	0.001	7.47524E-26	11
Scg3.4	0.943806959	0.858	0.443	8.62814E-26	11
Stk32a.1	0.620756496	0.2	0.004	1.05625E-25	11
Ptpra.2	0.940036273	0.733	0.231	1.14464E-25	11
Cpxm1	0.553279158	0.242	0.013	1.15465E-25	11
Rnf13.2	0.687413906	0.458	0.078	1.25855E-25	11
Clec3b	0.479736099	0.167	0.002	2.05606E-25	11
Pmp22.2	0.722502065	0.275	0.016	2.14536E-25	11
Rps24.4	-0.79607428	0.725	0.821	2.69874E-25	11
Wnt3	0.68482606	0.217	0.007	3.12693E-25	11
Pth1r	0.533647722	0.192	0.005	3.45908E-25	11
Slc43a3	0.559274174	0.2	0.004	3.79798E-25	11
Irx2	0.597030604	0.225	0.008	3.89819E-25	11
Rnf182	0.540401901	0.217	0.009	4.20563E-25	11
Ranbp1.7	-0.941699624	0.575	0.676	4.49641E-25	11
Rxrg	0.404902538	0.133	0.001	4.57157E-25	11
Rps19.7	-0.899705827	0.617	0.754	4.92498E-25	11
Dtx4.1	0.332856136	0.183	0.006	5.04686E-25	11
Atpif1.2	-0.824458773	0.683	0.816	5.15853E-25	11
Vwa1	0.409263473	0.2	0.01	5.20716E-25	11
Fbxo2	0.788149993	0.258	0.012	5.81409E-25	11
Elmo2	0.720238118	0.375	0.045	6.84741E-25	11
Cdh13.1	0.754569982	0.375	0.044	6.99305E-25	11
Itpr2.1	0.639644211	0.3	0.028	7.71191E-25	11
Hnrnph1.2	-1.100365577	0.358	0.63	7.8544E-25	11

Mcc	0.511785443	0.175	0.002	8.13153E-25	11
Ampd3	0.539833611	0.167	0.002	1.0528E-24	11
Hsdl2	0.826927906	0.4	0.051	1.0619E-24	11
Eps15.1	0.906663939	0.425	0.057	1.0945E-24	11
Msx2	0.326730443	0.117	0	1.54455E-24	11
Akr1b10.1	0.692933469	0.333	0.032	1.90226E-24	11
Lix1l	0.417871638	0.192	0.006	2.01651E-24	11
Ppp1r1a.6	0.926702465	0.533	0.107	2.52264E-24	11
Gng12.4	1.024780926	0.542	0.118	2.6743E-24	11
Gabbr2	0.663288841	0.25	0.017	3.25291E-24	11
Rps15.4	-0.774928444	0.7	0.788	3.35273E-24	11
Ppargc1a	0.537762886	0.192	0.005	3.68859E-24	11
Osbpl1a.2	0.757449189	0.4	0.056	3.7516E-24	11
Sept4.9	1.138542158	0.667	0.223	5.68347E-24	11
Mertk.1	0.518176813	0.192	0.006	6.18069E-24	11
Npm1.7	-1.050224247	0.433	0.647	9.96376E-24	11
Cpne2	0.545629207	0.2	0.006	1.91117E-23	11
Axl.1	0.545060813	0.2	0.006	2.0234E-23	11
Spry2.1	0.876790752	0.383	0.046	2.02676E-23	11
Tst	0.541232282	0.25	0.015	2.58078E-23	11
Tnfrsf19	0.442324141	0.175	0.004	2.60613E-23	11
Sez6l.1	0.811553484	0.4	0.059	2.94456E-23	11
Gaa.2	0.551073171	0.275	0.025	3.42689E-23	11
Rgs7bp.1	0.63481563	0.283	0.026	3.47061E-23	11
H2afv.9	-0.907811346	0.617	0.728	4.00292E-23	11
Cystm1	0.620691244	0.317	0.036	4.0161E-23	11
Grina.8	0.530118466	0.458	0.107	4.40286E-23	11
AI413582.1	0.526503987	0.283	0.029	5.50233E-23	11
Frem2	0.439356703	0.15	0.002	6.55658E-23	11
Cbx1.3	-0.993145056	0.442	0.687	7.96868E-23	11
Amot	0.596935453	0.25	0.014	9.96818E-23	11
Serpina3n	0.354481025	0.117	0	1.11757E-22	11
Pltp.1	0.597704759	0.283	0.026	1.17259E-22	11
Chd7.8	-1.225817269	0.35	0.649	1.27901E-22	11
Gramd3	0.513622779	0.192	0.005	1.34468E-22	11
Marc2.2	0.850270073	0.458	0.081	1.82258E-22	11
Gm2a.2	0.706727184	0.358	0.046	2.19666E-22	11
Fuca2.1	0.416489658	0.2	0.008	2.23771E-22	11
Rps20.6	-0.827644334	0.658	0.779	2.30802E-22	11
Timp2.2	0.492023868	0.25	0.024	2.3227E-22	11
Trim9	0.619233979	0.242	0.015	2.4207E-22	11
Zic1.9	-0.901235476	0.658	0.785	2.42308E-22	11
Arxes2.1	0.925703371	0.475	0.096	2.47719E-22	11
Cgrrf1	0.592310733	0.358	0.052	2.53774E-22	11
Hist1h2bc.2	0.840507542	0.333	0.036	2.63113E-22	11
Bhlhe40	0.339032252	0.15	0.002	2.63468E-22	11
Pfkm.1	0.822662087	0.417	0.064	3.02832E-22	11
Fth1.4	0.691380801	0.917	0.707	3.26883E-22	11

Hrsp12	0.606530732	0.333	0.041	3.35365E-22	11
Ddah2.6	-1.155270973	0.425	0.687	3.37093E-22	11
Tmbim6.2	0.930956668	0.767	0.321	3.57354E-22	11
Ctsb.7	0.563885708	0.725	0.259	3.93999E-22	11
Ubtd1.1	0.372374752	0.167	0.005	4.05406E-22	11
Lrrc4c.1	0.404657058	0.183	0.009	4.2262E-22	11
Cyr61	0.891189763	0.267	0.017	4.8483E-22	11
Nfix.6	-1.071503641	0.45	0.674	4.96868E-22	11
Nop58.7	-1.187239751	0.308	0.594	5.56246E-22	11
Tmem229a	0.893899917	0.383	0.053	5.91263E-22	11
Slc29a1.6	-1.423984387	0.142	0.564	6.75529E-22	11
Mfn1	0.564598874	0.308	0.036	6.95471E-22	11
C030005K06Rik	0.524527337	0.125	0.001	8.2397E-22	11
Nebl	0.491478197	0.175	0.006	9.62977E-22	11
Elovl2	0.82375917	0.333	0.036	9.65315E-22	11
Trf.1	0.78539561	0.325	0.035	1.14976E-21	11
Wwc1.1	0.690886835	0.408	0.067	1.15571E-21	11
Cgref1	0.285599901	0.125	0.001	1.81645E-21	11
Plce1	0.537612938	0.192	0.006	1.86124E-21	11
Ecm2	0.515327637	0.158	0.002	2.02695E-21	11
Oat.1	0.80378865	0.417	0.068	2.05561E-21	11
Cadm2.2	0.37389096	0.275	0.046	2.15087E-21	11
Mpp6.5	0.94279231	0.592	0.158	2.19652E-21	11
Phgdh.1	0.908253683	0.442	0.077	2.34232E-21	11
Wls.2	0.98034084	0.458	0.084	2.73053E-21	11
Psip1.4	-0.785247278	0.558	0.688	2.73648E-21	11
Arpp21.9	0.286170468	0.383	0.111	2.88493E-21	11
Agtrap	0.533131789	0.183	0.006	3.76577E-21	11
Lmbrd1.1	0.61547391	0.392	0.068	5.68246E-21	11
Fkbp10	0.418452656	0.175	0.007	5.97601E-21	11
Snrpd1.6	-0.716663449	0.475	0.524	6.24021E-21	11
Sec14l2	0.428451343	0.158	0.003	6.38867E-21	11
Prr5l	0.492390873	0.158	0.003	6.96312E-21	11
Map1b.9	-1.522075777	0.258	0.589	6.967E-21	11
Dek.10	-1.222029249	0.4	0.695	7.1383E-21	11
Ldha.5	0.941535519	0.667	0.228	8.06184E-21	11
Acsl6	0.76742068	0.3	0.028	8.23573E-21	11
Hn1.7	-1.132315704	0.3	0.543	8.56654E-21	11
Slc15a2.1	0.60371005	0.308	0.037	9.06227E-21	11
Dbndd2.1	0.581032946	0.258	0.023	9.50035E-21	11
Rps11.4	-0.806660923	0.625	0.778	1.00713E-20	11
Notch1.1	0.585997804	0.258	0.021	1.0722E-20	11
Acss1.1	0.567145333	0.175	0.004	1.17712E-20	11
Cd151	0.600058424	0.242	0.016	1.40656E-20	11
Npl.1	0.443648419	0.175	0.006	1.76946E-20	11
Col1a2	0.329858612	0.183	0.013	1.85551E-20	11
Anxa5.1	0.465512888	0.225	0.018	1.87186E-20	11
Smad9	0.339026727	0.142	0.003	1.96911E-20	11

Itgb8.1	0.589297719	0.25	0.019	3.33092E-20	11
Tmie	0.461943079	0.133	0.001	3.7065E-20	11
Sned1	0.869689662	0.267	0.022	3.76622E-20	11
Gas5.3	-0.861488547	0.592	0.743	4.1241E-20	11
Bmpr1a.1	0.772680443	0.475	0.1	4.12723E-20	11
Fus.1	-0.742199788	0.617	0.737	4.22152E-20	11
Draxin.7	-1.278952938	0.233	0.556	4.46993E-20	11
Rarres1	0.34352321	0.117	0.001	4.60738E-20	11
Matr3.1	-0.799857421	0.6	0.707	4.70656E-20	11
Stmn3.5	-1.239572117	0.258	0.624	5.24584E-20	11
Spry1.1	0.502141642	0.208	0.012	5.37852E-20	11
Oaf	0.303575823	0.142	0.003	5.48337E-20	11
Cxcl14.1	0.916657702	0.333	0.039	5.9692E-20	11
Itgb5.1	0.420719974	0.175	0.008	6.65628E-20	11
Cbs	0.5159502	0.142	0.002	8.42679E-20	11
Eif3a.4	-0.822553763	0.542	0.689	9.94431E-20	11
4931406C07Rik	0.459749047	0.292	0.044	1.17677E-19	11
Smc2.9	-1.340830203	0.308	0.547	1.19584E-19	11
Banf1.8	-0.708400109	0.633	0.694	1.4466E-19	11
Dbp	0.653467832	0.317	0.038	1.61423E-19	11
Rgma	0.652096893	0.333	0.044	1.67654E-19	11
Entpd2	0.452988461	0.133	0.001	1.8618E-19	11
Lysmd2	0.697935632	0.4	0.069	2.08569E-19	11
Appl2	0.784846139	0.525	0.128	2.1536E-19	11
Tex264.1	0.623327162	0.375	0.066	2.15366E-19	11
4930402H24Rik.5	0.652552201	0.467	0.11	2.23467E-19	11
Cck	0.771531044	0.133	0.001	2.49195E-19	11
Olfm2	0.328125867	0.133	0.003	2.53029E-19	11
Reep5.3	0.824444102	0.533	0.135	2.57027E-19	11
Chd4.3	-0.783058448	0.675	0.77	3.05339E-19	11
Pgm2.2	0.571861479	0.275	0.031	3.16991E-19	11
Anp32b.8	-1.098481266	0.342	0.592	4.02469E-19	11
Eef1a1.4	-0.732059945	0.783	0.884	4.1107E-19	11
Hnrnpdl.2	-0.831468439	0.575	0.757	4.75615E-19	11
Cdh22	0.56335926	0.192	0.008	4.95724E-19	11
Capns1.2	0.86624903	0.65	0.224	5.20835E-19	11
Klhl5.1	0.674302158	0.308	0.037	5.21325E-19	11
Cdk4.4	-0.904828199	0.425	0.643	5.22256E-19	11
Ptma.5	-1.09628918	0.258	0.583	6.39492E-19	11
Mras	0.706441137	0.35	0.051	6.40848E-19	11
Usp24.1	0.39757733	0.25	0.032	6.42448E-19	11
Rpl23.4	-0.82558947	0.425	0.539	6.52673E-19	11
Etv4	0.435816959	0.158	0.005	6.76082E-19	11
Mmp14.5	0.813172961	0.592	0.171	7.34989E-19	11
Slc7a11	0.690107528	0.15	0.003	8.66555E-19	11
Il6st	0.50289616	0.292	0.038	8.75022E-19	11
Thrsp	0.968651132	0.292	0.031	1.02382E-18	11
Aldoa.7	0.847301142	0.7	0.254	1.0971E-18	11

Clip1.1	0.721581021	0.442	0.091	1.11919E-18	11
Abcd3.1	0.854790265	0.6	0.179	1.69453E-18	11
Smc3.3	-0.838025407	0.517	0.634	1.73205E-18	11
Them4	0.795772579	0.342	0.049	1.73853E-18	11
Hsd17b12.1	0.894378276	0.6	0.185	1.90622E-18	11
Plec	0.391823758	0.183	0.01	2.00323E-18	11
Neurod1.11	-2.022331255	0.175	0.535	2.18579E-18	11
Lamb2	0.640707891	0.2	0.01	2.28987E-18	11
Cyp4v3	0.322296481	0.133	0.002	2.42983E-18	11
Sybu	0.338288488	0.142	0.004	2.44039E-18	11
Serpinh1.6	0.669895626	0.475	0.112	2.46565E-18	11
Eef2.3	-0.791983305	0.558	0.717	2.64007E-18	11
Srsf3.5	-0.82527652	0.483	0.658	2.66033E-18	11
St3gal6.1	0.455931406	0.183	0.009	2.75897E-18	11
Mtss1l	0.538547466	0.25	0.025	2.99562E-18	11
Rpl8.5	-0.615213526	0.875	0.897	3.18335E-18	11
Lfng	0.666850605	0.283	0.029	3.21003E-18	11
Phyh	0.745218438	0.375	0.062	3.25495E-18	11
Kcne1l	0.821834631	0.342	0.047	3.34992E-18	11
Epdr1.1	0.581132591	0.342	0.057	3.48102E-18	11
Stom	0.427220936	0.158	0.004	3.87381E-18	11
Cib1.1	0.709450783	0.358	0.055	3.90893E-18	11
Kank1.1	0.589181421	0.283	0.033	4.09837E-18	11
Ldhb.5	0.767724417	0.725	0.277	4.24352E-18	11
Nasp.9	-1.070856101	0.392	0.641	5.1179E-18	11
Slc30a10	0.695608254	0.367	0.06	5.45426E-18	11
Gsap	0.376212833	0.167	0.007	5.56151E-18	11
Ccnd2.7	-1.137167707	0.442	0.663	5.99325E-18	11
Atp6v0b.9	0.507860841	0.683	0.264	6.40673E-18	11
Ccdc24	0.408910242	0.142	0.003	8.00043E-18	11
Syt15	0.309182943	0.108	0.001	8.13157E-18	11
Hip1.1	0.67543533	0.475	0.115	8.91737E-18	11
Pgpep1	0.330938501	0.167	0.009	1.03629E-17	11
Ttyh3	0.451372308	0.442	0.121	1.0414E-17	11
Ttc3.7	-0.800401527	0.717	0.829	1.10214E-17	11
Hdhd2.1	0.568004917	0.458	0.115	1.31157E-17	11
Tapbp.1	0.29882391	0.183	0.016	1.35566E-17	11
Ybx1.4	-0.704195524	0.675	0.786	1.42797E-17	11
Sox4.8	-1.150719059	0.35	0.584	1.76078E-17	11
Rpl34.4	-0.762495311	0.517	0.653	1.80373E-17	11
Hexa.1	0.422238208	0.325	0.064	1.8116E-17	11
Dmd.1	0.558299846	0.25	0.024	1.93656E-17	11
Adamts5	0.407568871	0.15	0.004	2.00005E-17	11
Tagln2.1	0.585288954	0.3	0.043	2.01454E-17	11
Parp3	0.390530532	0.125	0.002	2.53672E-17	11
Lrp1.2	0.718196628	0.317	0.045	2.65882E-17	11
Dynlt3.2	0.735454172	0.325	0.044	2.82427E-17	11
Pdap1.3	-0.7220358	0.567	0.683	2.89828E-17	11

Necap2.1	0.549327443	0.3	0.043	3.11963E-17	11
Mex3a.4	-1.322428511	0.108	0.466	3.82059E-17	11
Smc1a.5	-0.840274417	0.483	0.631	3.98238E-17	11
Acin1.1	-0.782982335	0.542	0.724	4.19841E-17	11
Slc20a2	0.663318248	0.317	0.045	4.20052E-17	11
C4b	0.475917701	0.117	0.001	4.26252E-17	11
Ech1.1	0.781903717	0.467	0.112	4.29832E-17	11
Elavl3.9	-1.393911333	0.075	0.475	4.47254E-17	11
Colgalt2	0.281569463	0.108	0.001	5.53469E-17	11
Rasl11a.1	0.860210364	0.267	0.028	5.86051E-17	11
Nnat.7	-0.946325176	0.442	0.711	6.11627E-17	11
Rbm25.1	-0.737027274	0.692	0.813	6.8125E-17	11
Hdac2.2	-1.04535971	0.267	0.517	7.18519E-17	11
Tns3.2	0.330880863	0.158	0.009	8.6205E-17	11
Il18.2	0.597431775	0.208	0.013	1.01211E-16	11
Gm9800.5	-1.058453152	0.267	0.536	1.15702E-16	11
Barhl1.9	-1.326066892	0.15	0.524	1.16769E-16	11
Mboat2.1	0.620737738	0.317	0.046	1.3916E-16	11
Fgfr2	0.45303086	0.192	0.011	1.43719E-16	11
Fkbp11	0.560248541	0.192	0.01	1.56014E-16	11
Phlda1.1	0.609577741	0.242	0.024	1.69409E-16	11
Marcks.2	-0.586268195	0.858	0.916	1.75622E-16	11
Prex1.1	0.735101173	0.358	0.061	1.93029E-16	11
Proca1	0.37727546	0.117	0.001	2.27934E-16	11
Rpl22.5	-0.724102086	0.667	0.789	2.86722E-16	11
Pygb	0.587336946	0.225	0.017	2.87591E-16	11
Ezh2.9	-1.108915926	0.325	0.611	3.17541E-16	11
Rplp1.6	-0.544114761	0.892	0.876	4.05445E-16	11
Plvap	0.288839312	0.117	0.002	4.28166E-16	11
Pdgfrl	0.380399869	0.15	0.007	4.30889E-16	11
Plin3.1	0.366534897	0.158	0.007	4.57634E-16	11
Nkain4.2	0.834390206	0.492	0.126	4.61716E-16	11
Celf2.10	-0.940957248	0.5	0.638	4.81549E-16	11
Hpgd.1	0.441447926	0.142	0.004	4.83004E-16	11
Col5a3	0.390504133	0.125	0.002	5.39318E-16	11
Fkbp3.6	-0.708554832	0.683	0.745	5.68447E-16	11
Shisa4.1	0.488090672	0.292	0.051	5.91222E-16	11
Lpl.1	0.710009842	0.342	0.056	6.00463E-16	11
Crot.1	0.661574111	0.4	0.086	6.37005E-16	11
Fubp1.3	-0.900950285	0.383	0.567	8.05479E-16	11
Rpl14.5	-0.633492627	0.617	0.697	9.2974E-16	11
Smc4.9	-1.063678788	0.425	0.567	9.31954E-16	11
Orai1.2	0.51888175	0.225	0.022	9.39852E-16	11
Pdlim5.1	0.456660771	0.192	0.013	9.44959E-16	11
Fgf1	0.257404263	0.108	0.001	9.55348E-16	11
Gstk1	0.456663561	0.167	0.007	1.13085E-15	11
Rpl26.4	-0.679933365	0.6	0.702	1.22734E-15	11
Spred1.1	0.50546733	0.417	0.106	1.28163E-15	11

Trib2.1	0.713901604	0.408	0.086	1.32163E-15	11
Fads2	0.533242581	0.217	0.017	1.40348E-15	11
Nrep.10	-1.171387583	0.342	0.57	1.48357E-15	11
Megf10	0.490411018	0.167	0.007	1.48507E-15	11
Kdelr3	0.346711727	0.15	0.008	1.66279E-15	11
Spock3	0.288948831	0.117	0.002	1.91022E-15	11
Tcf4.4	-0.652367457	0.85	0.925	1.9146E-15	11
Lamp2.2	0.812483811	0.5	0.135	2.09448E-15	11
Cdh10.1	0.265769549	0.15	0.013	2.17593E-15	11
Mfap3l	0.315744365	0.133	0.004	2.38302E-15	11
Rbfox3.8	-1.349255584	0.125	0.496	2.44148E-15	11
Cpeb4.1	0.409939116	0.267	0.045	2.57398E-15	11
Rora.1	0.454866659	0.292	0.053	2.81713E-15	11
Grm5.1	0.689254837	0.183	0.01	3.04933E-15	11
Gm10075.8	-0.818323259	0.367	0.515	3.09569E-15	11
Cap2	0.363248449	0.158	0.009	3.24743E-15	11
Tmem33.1	0.612456571	0.492	0.142	3.26512E-15	11
Lhx1.8	-1.335452314	0.158	0.499	3.33046E-15	11
Hsp90aa1.3	-0.819863009	0.433	0.617	3.90477E-15	11
Grin3a.1	0.773233875	0.342	0.058	3.95934E-15	11
Ccnd1.8	-1.1321565	0.308	0.523	4.77694E-15	11
Ptchd4	0.349533663	0.158	0.011	5.79192E-15	11
Abhd6.2	0.56932079	0.267	0.035	6.22658E-15	11
Nrcam.8	0.721825085	0.483	0.131	7.9047E-15	11
KCTD12.1	0.484649929	0.2	0.017	8.73763E-15	11
Bclaf1.1	-0.994638397	0.317	0.584	8.85535E-15	11
Sirt2.4	0.488484813	0.492	0.153	8.92955E-15	11
Ahcyl1	0.673189025	0.525	0.157	9.09268E-15	11
Sdc3.2	0.437505659	0.325	0.075	1.01316E-14	11
Gas7	0.257937872	0.125	0.006	1.04415E-14	11
Plekhb1.1	0.49012956	0.167	0.01	1.10359E-14	11
Pigs	0.578447135	0.233	0.027	1.11639E-14	11
Sgcb.1	0.315507202	0.367	0.113	1.15776E-14	11
Golph3.1	0.606187795	0.358	0.071	1.29895E-14	11
Lrp4	0.391317141	0.15	0.006	1.30136E-14	11
Rpl39.5	-0.735009847	0.533	0.646	1.34838E-14	11
Stmn2.10	-1.402701145	0.325	0.554	1.36107E-14	11
Cdo1.1	0.458332384	0.2	0.02	1.38686E-14	11
Fads1	0.56791867	0.408	0.103	1.43401E-14	11
Galc.1	0.525521486	0.275	0.04	1.45558E-14	11
Vimp.3	0.78002415	0.567	0.187	1.4668E-14	11
Fam195a	0.435951514	0.167	0.01	1.52421E-14	11
Gm266	0.339086952	0.125	0.003	1.61665E-14	11
Fuca1.1	0.666774598	0.5	0.145	1.6297E-14	11
Srsf11.1	-0.737165053	0.5	0.69	1.96923E-14	11
Aco2	0.719254239	0.742	0.335	1.97966E-14	11
Rasa3.1	0.524622653	0.258	0.037	2.03035E-14	11
Tcn2.1	0.309939895	0.175	0.018	2.0319E-14	11

Hnrnpd.7	-0.857801292	0.375	0.612	2.0856E-14	11
Rps3a1.5	-0.75358737	0.525	0.667	2.26976E-14	11
Tmem106b.1	0.602095036	0.35	0.07	2.39323E-14	11
Slc35e4	0.479068037	0.2	0.016	2.49514E-14	11
Rab7l1	0.501282654	0.192	0.013	2.52064E-14	11
Sypl.1	0.590282519	0.358	0.072	2.59816E-14	11
Hdgf.7	-0.764091918	0.408	0.532	2.60133E-14	11
Nfkbiz.1	0.444092789	0.175	0.011	2.80863E-14	11
Atrx.3	-0.776995183	0.608	0.741	3.23926E-14	11
Srrm2.2	-0.507479633	0.717	0.732	3.3146E-14	11
Efemp2	0.475412356	0.192	0.015	4.06635E-14	11
Tnfaip6	0.286137141	0.125	0.004	4.18411E-14	11
Laptm4b.3	0.623849021	0.483	0.139	4.44459E-14	11
Nucks1.10	-0.648756732	0.617	0.663	4.53423E-14	11
4933407L21Rik	0.393565685	0.125	0.003	5.03953E-14	11
Miat.9	-1.415434918	0.192	0.517	5.5529E-14	11
Pnn.1	-0.736107712	0.508	0.723	5.73232E-14	11
Decr1	0.538265261	0.342	0.07	5.99136E-14	11
Fabp5.3	0.797304781	0.733	0.4	6.11665E-14	11
Igfbp3.1	0.664336924	0.183	0.011	6.33905E-14	11
Enho.1	0.341522794	0.242	0.048	6.54243E-14	11
Suclg2	0.382028195	0.208	0.025	6.7447E-14	11
Camk1.2	0.359069974	0.267	0.055	6.78723E-14	11
Fermt2.2	0.712370876	0.583	0.202	7.02983E-14	11
Tubb3.10	-1.489226605	0.117	0.476	7.17969E-14	11
Rgcc.1	0.521614209	0.183	0.015	7.30213E-14	11
Uqcr10.1	0.703911347	0.783	0.431	7.59951E-14	11
Ctnnd2.2	0.55201105	0.392	0.096	7.72536E-14	11
D430041D05Rik.6	-1.219963254	0.142	0.494	7.76821E-14	11
Rhoj	0.525163346	0.242	0.03	7.87438E-14	11
Cox14	0.712290978	0.717	0.314	8.59121E-14	11
Samd4	0.55529673	0.258	0.036	8.66022E-14	11
Itgav.2	0.504694021	0.308	0.058	8.6628E-14	11
Eya1	0.371722978	0.125	0.003	9.65431E-14	11
Cdh11.1	0.5430236	0.192	0.014	9.83513E-14	11
Mid1ip1.2	0.65151364	0.308	0.049	1.14545E-13	11
BC026585	0.322278038	0.117	0.002	1.15312E-13	11
Rlbp1.1	0.458080467	0.158	0.01	1.21894E-13	11
Fuom	0.490127527	0.233	0.03	1.22662E-13	11
Adcy2	0.539342485	0.133	0.004	1.53727E-13	11
Renbp.1	0.374414554	0.142	0.006	1.60527E-13	11
Nckap5	0.278199518	0.108	0.002	1.68391E-13	11
Snrpb.6	-0.653406543	0.5	0.574	1.70904E-13	11
Cct6a.1	-0.776442093	0.35	0.487	1.7573E-13	11
Lpcat1.1	0.568959648	0.458	0.133	1.90959E-13	11
Lrp10.1	0.544584012	0.258	0.039	1.98384E-13	11
Marcksl1.5	-0.717975559	0.6	0.723	2.00313E-13	11
Cacng5.1	0.599245478	0.292	0.048	2.2859E-13	11

Agpat5	0.70617945	0.558	0.197	2.29028E-13	11
Car10.1	0.472759874	0.233	0.039	2.68873E-13	11
Usp2.1	0.325760451	0.158	0.011	2.7037E-13	11
Hp1bp3.2	-0.678023997	0.517	0.581	2.77421E-13	11
Ilf2.2	-0.91259259	0.325	0.542	2.83056E-13	11
Rps15a.5	-0.707958707	0.533	0.658	2.83875E-13	11
Itpkb	0.477199741	0.158	0.008	2.94115E-13	11
Zfyve21	0.509379404	0.292	0.051	3.1654E-13	11
Gng11	0.352519169	0.158	0.014	3.21323E-13	11
Acadm	0.463824569	0.35	0.085	3.42226E-13	11
Gm15417	0.361341882	0.133	0.006	3.46042E-13	11
Atraid.1	0.531405301	0.592	0.224	3.55779E-13	11
H1f0.6	-1.068880689	0.317	0.618	3.59361E-13	11
Cbfa2t3.8	-1.216336082	0.1	0.404	3.63432E-13	11
Rhbdf1	0.294526593	0.125	0.005	3.98421E-13	11
Tmed5.2	0.581955485	0.442	0.124	4.02599E-13	11
Tmem66.4	0.657609779	0.567	0.196	4.10344E-13	11
Mfhas1	0.592567851	0.333	0.067	4.11788E-13	11
Rnh1.1	0.545062123	0.308	0.061	4.30398E-13	11
Safb.1	-0.839455844	0.342	0.504	4.43571E-13	11
Pde4b.1	0.577217864	0.292	0.049	4.60014E-13	11
Luc7l3.2	-0.615682325	0.642	0.748	4.76884E-13	11
Ppp1r14b.4	-0.910658798	0.225	0.398	4.91454E-13	11
Uap1l1.1	0.393469974	0.15	0.008	5.61816E-13	11
Ank3.10	-1.237201642	0.142	0.422	6.26098E-13	11
Tspan15	0.332269324	0.15	0.009	6.28957E-13	11
Slc5a3	0.419842572	0.175	0.014	6.44349E-13	11
Rabac1.3	0.622440984	0.642	0.255	6.85879E-13	11
Bzw2.1	-0.92077217	0.283	0.489	7.15336E-13	11
Anp32e.10	-0.675061835	0.525	0.614	7.15664E-13	11
Soat1.1	0.574081893	0.208	0.019	7.35708E-13	11
Nudt19	0.440901773	0.392	0.11	7.58859E-13	11
Gap43.10	-1.183273429	0.375	0.6	7.8645E-13	11
Ptplb	0.771907277	0.433	0.113	8.04344E-13	11
Malat1.8	0.561602047	0.958	0.955	8.13753E-13	11
Ina.10	-1.2604318	0.117	0.445	8.50524E-13	11
Cyp4f13.1	0.354389127	0.158	0.012	8.62634E-13	11
Ssbp4	0.416171468	0.283	0.058	8.84264E-13	11
Eva1a.1	0.486208791	0.142	0.005	8.88737E-13	11
Ilvbl	0.292817432	0.15	0.012	8.92638E-13	11
Acadvl	0.564386386	0.4	0.104	8.95892E-13	11
Ptplad1	0.653350165	0.633	0.248	9.65311E-13	11
Sdf2	0.479659827	0.558	0.212	9.83845E-13	11
Bex2.1	-0.917801526	0.308	0.573	1.01368E-12	11
Vamp5	0.377508996	0.117	0.003	1.01974E-12	11
Pcdh17.1	0.497767991	0.217	0.027	1.02478E-12	11
Enpp5	0.319489863	0.183	0.026	1.09809E-12	11
Mxra8	0.415056031	0.192	0.021	1.11399E-12	11

Slc35c2.1	0.652905183	0.258	0.036	1.12974E-12	11
Ngfrap1.1	-0.687206872	0.525	0.658	1.18515E-12	11
Fam181b.1	0.716211244	0.475	0.14	1.19963E-12	11
S1pr3	0.476799652	0.125	0.004	1.20553E-12	11
Cplx2.6	-1.192390866	0.183	0.497	1.3255E-12	11
1110065P20Rik.1	0.325734966	0.367	0.117	1.33463E-12	11
Syt12	0.511168089	0.25	0.035	1.38703E-12	11
Npc1.2	0.416311867	0.242	0.04	1.55116E-12	11
Rbbp4.4	-0.621867537	0.408	0.484	1.57049E-12	11
Hmgn1.7	-0.693660324	0.475	0.622	1.5768E-12	11
Cnbp.5	-0.535721244	0.717	0.739	1.69005E-12	11
Rps10.3	-0.722936215	0.5	0.637	1.7045E-12	11
Mamdc2	0.405208455	0.2	0.02	1.81939E-12	11
Gpd2	0.726845126	0.342	0.07	1.8541E-12	11
Cyp2j6.1	0.351380323	0.167	0.017	1.9053E-12	11
Iqsec1	0.371469696	0.167	0.014	1.93973E-12	11
Eno1.3	0.611874941	0.6	0.226	1.9471E-12	11
Rasa2	0.568233897	0.325	0.071	2.05483E-12	11
Ctsc.1	0.27843206	0.125	0.008	2.05948E-12	11
Arhgef12	0.406439289	0.4	0.123	2.23345E-12	11
Syngr1.2	0.482134906	0.358	0.091	2.29449E-12	11
Galnt18	0.276845045	0.142	0.01	2.37063E-12	11
Magt1.2	0.490183846	0.292	0.055	2.3804E-12	11
Rdh14	0.355816062	0.208	0.029	2.44959E-12	11
Slitrk2.1	0.324642521	0.133	0.007	2.48808E-12	11
Kcnip3.3	0.551725581	0.417	0.118	2.52135E-12	11
Rhoq	0.530038318	0.283	0.049	2.61369E-12	11
1110001A16Rik.1	0.2749222	0.317	0.095	2.91455E-12	11
Acot13.1	0.590014004	0.408	0.111	3.0396E-12	11
Gstt1	0.503515759	0.258	0.042	3.17664E-12	11
Haghl	0.519774337	0.358	0.088	3.28252E-12	11
Skap2.1	0.461028233	0.125	0.004	3.59303E-12	11
Ugp2	0.464727431	0.35	0.088	3.80423E-12	11
Chst7	0.260950867	0.117	0.005	3.94144E-12	11
Nhlh2.9	-1.299611641	0.133	0.424	4.27038E-12	11
Gabbr1.6	0.462099913	0.425	0.138	4.34055E-12	11
Cyba.1	0.329178173	0.192	0.028	4.38249E-12	11
Baz1b.4	-0.895516915	0.317	0.494	4.66279E-12	11
Hnrnpa0.2	-0.870251931	0.292	0.457	4.7231E-12	11
Hirip3.9	-1.124730815	0.133	0.384	4.78093E-12	11
Hmgb2.10	-1.03464737	0.208	0.401	5.20571E-12	11
Ddt	0.568652031	0.392	0.103	5.22962E-12	11
Tprgl	0.421463812	0.4	0.122	5.35332E-12	11
Tuba1b.9	-0.798359746	0.342	0.482	5.44738E-12	11
Cdkn1b.5	-0.703585143	0.375	0.467	5.67661E-12	11
Fzd9	0.450929901	0.108	0.002	5.82574E-12	11
As3mt.1	0.425475392	0.167	0.012	5.83915E-12	11
Naga.1	0.376625345	0.217	0.032	5.83931E-12	11

Cacng2.3	-1.151517279	0.05	0.34	6.07777E-12	11
mt-Nd2.1	0.642750243	0.867	0.637	6.09136E-12	11
Alpl	0.373242836	0.108	0.002	6.10141E-12	11
Srrm1.1	-0.703257491	0.458	0.612	6.15998E-12	11
Ccar1.1	-0.862991109	0.358	0.573	6.66063E-12	11
Metap2.1	-0.712494946	0.408	0.597	6.90107E-12	11
Vcl	0.40340719	0.175	0.016	7.02263E-12	11
H2afy.6	-0.598106129	0.542	0.592	7.18162E-12	11
Fcgrt.1	0.481172414	0.208	0.026	7.38229E-12	11
Glo1.2	0.649699856	0.408	0.105	7.79198E-12	11
2700094K13Rik.10	-0.688254292	0.517	0.614	8.67027E-12	11
Ctsh.1	0.280128119	0.133	0.011	9.21286E-12	11
Phlpp1	0.2856915	0.175	0.022	9.66189E-12	11
Ccdc107.1	0.589452961	0.325	0.069	9.96234E-12	11
Pttg1ip.2	0.607335958	0.358	0.084	1.04174E-11	11
Epn2.1	0.318433075	0.3	0.082	1.06396E-11	11
Nsg2.8	-1.041115825	0.167	0.489	1.13694E-11	11
Eif3c.1	-0.570485367	0.533	0.603	1.28642E-11	11
Dclk1.6	0.700447041	0.775	0.409	1.33647E-11	11
Dpysl4.6	-1.031886466	0.217	0.492	1.57795E-11	11
Nim1.1	0.452459176	0.175	0.015	1.90514E-11	11
Stk25.1	-0.278355157	0.25	0.184	1.92823E-11	11
Sf3b1.1	-0.651074465	0.542	0.669	2.13901E-11	11
Ppp2r2c.5	-0.987942306	0.242	0.506	2.49833E-11	11
Sec11c.6	0.603618285	0.55	0.21	2.5072E-11	11
Dag1	0.442138991	0.233	0.036	2.56659E-11	11
Junb.1	0.739823202	0.242	0.033	2.66599E-11	11
Eif4g2.1	-0.597235705	0.6	0.706	2.6904E-11	11
Creg1.1	0.328816958	0.258	0.057	2.71377E-11	11
Ap3m2	0.284517745	0.225	0.049	2.71888E-11	11
Cpt1a.1	0.446001399	0.175	0.016	3.06941E-11	11
Tulp3	0.403892575	0.208	0.03	3.08408E-11	11
Aldh6a1	0.367040806	0.175	0.02	3.36239E-11	11
Cnn3.3	0.651390661	0.758	0.434	3.44731E-11	11
Extl3.1	0.268211471	0.242	0.058	3.53387E-11	11
Csf1.1	0.329239714	0.133	0.008	3.67307E-11	11
Hibadh.1	0.464984878	0.333	0.084	3.80635E-11	11
Acyp2	0.517443643	0.3	0.062	3.94283E-11	11
Usp6nl	0.377459489	0.208	0.031	4.01582E-11	11
Tmem147.1	0.305567056	0.567	0.259	4.10669E-11	11
Tmem50b.1	0.404135023	0.2	0.027	4.199E-11	11
Luc7l2.2	-0.766258463	0.35	0.496	4.22996E-11	11
Lgalsl.1	0.351124001	0.317	0.091	4.26981E-11	11
Klf15	0.344407173	0.108	0.003	4.34035E-11	11
Nap1l1.7	-0.560979807	0.475	0.511	4.34175E-11	11
Cyfip1.1	0.496751818	0.4	0.118	4.39374E-11	11
Ppt1.2	0.611335703	0.367	0.092	4.41355E-11	11
Dut.9	-1.08422239	0.192	0.436	4.66146E-11	11

Rnd3.7	-1.114047951	0.133	0.433	4.9024E-11	11
Aebp1	0.329955704	0.133	0.008	5.31928E-11	11
Rcn2.1	0.626968319	0.625	0.278	5.46943E-11	11
Etfb.4	0.576642824	0.517	0.182	6.0264E-11	11
Sft2d2.1	0.267407106	0.108	0.004	6.13379E-11	11
Tmem38a	0.335188366	0.175	0.02	6.44287E-11	11
Lrpap1.6	0.280972866	0.342	0.115	6.65041E-11	11
Cald1.4	-0.954351873	0.325	0.537	7.20836E-11	11
Nr3c1.4	0.561097535	0.408	0.123	7.4305E-11	11
Ncor1.2	-0.693516602	0.467	0.556	7.48886E-11	11
Dab1.1	0.448872808	0.217	0.032	8.08396E-11	11
Trip6	0.327489194	0.183	0.024	8.11068E-11	11
Ctsa.2	0.291431191	0.367	0.122	8.14934E-11	11
Mtss1.9	-0.900758587	0.15	0.27	8.43952E-11	11
2410006H16Rik.3	-0.653115219	0.4	0.459	9.00084E-11	11
H2afx.10	-0.959861762	0.217	0.331	9.51359E-11	11
Vegfa	0.480028399	0.208	0.025	9.76229E-11	11
Ralb.2	0.407862966	0.175	0.018	1.0173E-10	11
Erp29.1	0.367598744	0.6	0.278	1.05681E-10	11
Fam173a.1	0.534140497	0.45	0.151	1.09865E-10	11
Fam69a.1	0.402583264	0.208	0.03	1.11538E-10	11
Zfp36l1.7	0.341070318	0.408	0.149	1.13731E-10	11
Txndc15.1	0.39004889	0.392	0.129	1.1518E-10	11
Fam167a	0.287043533	0.117	0.005	1.15304E-10	11
Gna13.1	0.509267833	0.408	0.126	1.17492E-10	11
Slc33a1	0.343647842	0.192	0.028	1.19545E-10	11
Tnfrsf21.1	0.334325334	0.258	0.062	1.2017E-10	11
Nedd4.2	-0.618670584	0.583	0.684	1.267E-10	11
Gadd45g.1	0.670204649	0.342	0.078	1.32015E-10	11
Shisa5.1	0.461571545	0.2	0.024	1.42E-10	11
Cd200.2	0.431039781	0.225	0.044	1.42353E-10	11
Prpf40a.2	-0.679416646	0.45	0.572	1.51533E-10	11
Capn2	0.490540853	0.342	0.088	1.5264E-10	11
Eif3f.4	-0.557103699	0.583	0.631	1.63077E-10	11
Fam210b.7	-0.920644832	0.15	0.315	1.65051E-10	11
Pex11a	0.339437887	0.133	0.008	1.65283E-10	11
Klhl13.2	0.584525431	0.375	0.102	1.7583E-10	11
Tmem30a.3	0.391375777	0.467	0.177	1.76664E-10	11
Gpm6a.10	0.451092877	0.758	0.406	1.80721E-10	11
Rnf215	0.422114462	0.275	0.061	1.82131E-10	11
Tgfb2.4	0.334667448	0.467	0.19	1.83801E-10	11
Tmpo.10	-0.980410466	0.208	0.453	1.91886E-10	11
Srsf2.4	-0.61157795	0.467	0.566	1.92747E-10	11
Crip1.1	0.376498344	0.15	0.012	1.93486E-10	11
Hmgcs1.5	0.619244325	0.45	0.149	1.9572E-10	11
Naprt1	0.367787537	0.125	0.006	2.03723E-10	11
Gas2l1	0.37295958	0.125	0.006	2.07544E-10	11
Rgs20	0.472369006	0.342	0.089	2.14039E-10	11

1110054M08Rik	0.511432115	0.142	0.011	2.19375E-10	11
Sept9	0.482602033	0.317	0.077	2.22491E-10	11
Mxd4.5	0.286701111	0.45	0.196	2.31E-10	11
0610031J06Rik.1	0.539687056	0.292	0.06	2.35412E-10	11
Scd1	0.570009581	0.133	0.009	2.36122E-10	11
Dcxr.1	0.461928951	0.158	0.012	2.42489E-10	11
Top2a.10	-1.308774513	0.217	0.429	2.45361E-10	11
Olig1.1	0.93520709	0.333	0.078	2.46549E-10	11
Ggh.2	0.551703746	0.408	0.121	2.51997E-10	11
Anxa7	0.297237414	0.133	0.01	2.58854E-10	11
Hsd17b10	0.300914466	0.417	0.163	2.65943E-10	11
Pcdhga9.2	0.505813528	0.783	0.417	2.76396E-10	11
Fam49a	0.411319087	0.233	0.043	2.77976E-10	11
Eva1b	0.382279033	0.133	0.009	2.80917E-10	11
Wipi1	0.522149109	0.225	0.032	2.84271E-10	11
Pcbp1.4	-0.510675242	0.567	0.593	3.04379E-10	11
1500009L16Rik	0.437817341	0.225	0.037	3.12335E-10	11
Dcx.10	-1.197535505	0.083	0.377	3.15477E-10	11
Ntsr2	0.609567342	0.125	0.007	3.18699E-10	11
Sept3.11	-1.232886154	0.117	0.394	3.3227E-10	11
Gm5607	0.562158271	0.133	0.007	3.52267E-10	11
Arl6ip1.8	0.593730566	0.858	0.547	3.61895E-10	11
Tmbim4.1	0.455787555	0.417	0.142	3.66818E-10	11
Stt3b	0.503594061	0.533	0.209	3.70544E-10	11
Sltm.1	-0.648802777	0.467	0.532	3.78083E-10	11
Mcm7.7	-0.973380539	0.142	0.39	4.04445E-10	11
Eef1b2.5	-0.54104428	0.692	0.732	4.46944E-10	11
Tacc1.1	0.434937715	0.292	0.068	4.52876E-10	11
Unc50.1	0.454577431	0.392	0.123	4.64601E-10	11
Fnbp1l.7	-1.017454912	0.225	0.455	5.38517E-10	11
Ptpmt1	0.390161612	0.358	0.11	5.58446E-10	11
Pcm1.1	-0.733754646	0.417	0.522	6.42709E-10	11
G3bp2.1	-0.676952618	0.45	0.552	6.50702E-10	11
Agpat3	0.534655861	0.408	0.128	6.84477E-10	11
Ergic3	0.302303578	0.508	0.226	6.97343E-10	11
Syncrip.4	-0.592823015	0.425	0.496	7.19786E-10	11
Tia1.3	-0.810682985	0.358	0.553	7.59243E-10	11
Smox.1	0.509679595	0.292	0.064	8.19406E-10	11
Prnp.7	0.618948006	0.525	0.204	8.29009E-10	11
2700060E02Rik	-0.410271713	0.383	0.352	8.29409E-10	11
Ssrp1.6	-0.70224972	0.45	0.577	8.40151E-10	11
Acsl3.2	0.649316652	0.442	0.144	8.52549E-10	11
Vcan.4	0.527563404	0.408	0.128	9.27655E-10	11
Dnajc3.1	0.596478063	0.492	0.174	9.34186E-10	11
Fat1	0.254663054	0.167	0.025	9.35314E-10	11
H3f3a.2	-0.821280478	0.2	0.382	9.37459E-10	11
Plk2.1	0.504382282	0.15	0.012	1.04797E-09	11
Sox9.7	0.599027768	0.6	0.252	1.0518E-09	11

Eny2.4	-0.592454342	0.333	0.387	1.05407E-09	11
Rpl37a.2	-0.589482738	0.4	0.468	1.06317E-09	11
Fam63b.2	0.319898543	0.292	0.086	1.16064E-09	11
Ufl1.1	0.496951306	0.367	0.105	1.1836E-09	11
Cenpf.11	-1.307224829	0.2	0.363	1.22581E-09	11
Syt11.8	0.528146044	0.842	0.502	1.31664E-09	11
Sf3b2.1	-0.472024815	0.6	0.625	1.40061E-09	11
Hnrnpr.1	-0.695177207	0.383	0.546	1.4897E-09	11
Lhfp	0.341714529	0.15	0.018	1.50452E-09	11
Irx1	0.294694492	0.108	0.005	1.52033E-09	11
Ndufc2.3	0.598152687	0.8	0.609	1.61182E-09	11
Hadha.1	0.551113466	0.442	0.153	1.64755E-09	11
Ppp2r5a	0.477051195	0.258	0.049	1.74517E-09	11
Zc3h13.2	-0.913373781	0.283	0.463	1.95726E-09	11
Ier5.5	-0.975774596	0.125	0.396	1.96674E-09	11
Kif5c.10	-1.097711419	0.133	0.4	1.98287E-09	11
Snta1	0.301837146	0.133	0.01	2.09077E-09	11
Cxxc5.5	-0.838170967	0.292	0.545	2.0994E-09	11
H2afj.1	0.387291267	0.492	0.203	2.26208E-09	11
Phip.2	-0.930658655	0.242	0.461	2.26515E-09	11
Zfp36.1	0.312754421	0.133	0.011	2.39216E-09	11
Rpl41.5	-0.597298022	0.65	0.781	2.46518E-09	11
Abca3	0.33348111	0.2	0.037	2.57294E-09	11
Grn.1	0.328698225	0.3	0.085	2.59497E-09	11
Gng2.7	-1.031109122	0.133	0.397	2.63705E-09	11
Etfa.2	0.556696179	0.592	0.251	2.64277E-09	11
Os9.6	0.357217616	0.55	0.248	2.70449E-09	11
Cyb5.1	0.553817679	0.5	0.185	2.72949E-09	11
Tm7sf2.1	0.274918146	0.175	0.032	2.87626E-09	11
Maf.1	0.301618538	0.15	0.018	3.00007E-09	11
Top1.2	-0.571279458	0.583	0.663	3.06713E-09	11
Ikbip	0.480075409	0.217	0.035	3.14581E-09	11
Btbd6	0.353910871	0.167	0.02	3.16232E-09	11
Lifr	0.364247557	0.117	0.005	3.35823E-09	11
Gyg.2	0.280086834	0.125	0.009	3.37039E-09	11
Lgmn.4	0.368551304	0.367	0.117	3.39488E-09	11
Neat1.1	0.565118453	0.167	0.015	3.42474E-09	11
8430419L09Rik.1	0.348117209	0.258	0.065	3.54919E-09	11
Eif4a1.2	-0.703100601	0.333	0.485	3.67546E-09	11
2810417H13Rik.10	-1.104899087	0.183	0.393	3.69188E-09	11
Efemp1	0.25131902	0.108	0.006	3.73728E-09	11
Smpd1	0.447793344	0.217	0.038	3.80874E-09	11
Mir22hg	0.295309861	0.108	0.005	4.09762E-09	11
Anxa2.1	0.447004422	0.142	0.011	4.30891E-09	11
Akap9.4	-0.774764215	0.408	0.501	4.50436E-09	11
Col9a3.3	0.549511367	0.475	0.177	4.53475E-09	11
Camta1.3	-0.858582847	0.233	0.409	4.56694E-09	11
Dolk	0.300830423	0.192	0.034	4.75123E-09	11

Gpr56.3	0.586321972	0.525	0.22	4.8355E-09	11
Galk1	0.281084921	0.217	0.049	4.96492E-09	11
Gpi1	0.370871088	0.375	0.129	5.01636E-09	11
Tox3.3	-0.994440058	0.083	0.347	5.31617E-09	11
Cpped1	0.537232229	0.275	0.059	5.4506E-09	11
Snrpf.6	-0.788805775	0.242	0.428	5.4775E-09	11
Surf4.1	0.518062039	0.375	0.117	5.81189E-09	11
Xrn2.1	-0.809026969	0.308	0.474	5.94711E-09	11
Psma7.3	-0.492688246	0.725	0.782	6.47121E-09	11
Emc3.1	0.525613927	0.425	0.147	6.5911E-09	11
Rfc1.7	-0.808754959	0.2	0.331	6.77366E-09	11
Pnp.1	0.319444936	0.158	0.019	6.87319E-09	11
Kifc3	0.332485213	0.108	0.005	6.88851E-09	11
Rps2.4	-0.542968068	0.408	0.449	7.09484E-09	11
Acaa2.1	0.490246261	0.217	0.034	7.19362E-09	11
Cct3.3	-0.493757099	0.475	0.495	7.22623E-09	11
Ptgr2.1	0.445984114	0.175	0.021	7.71585E-09	11
Serhl	0.338653056	0.108	0.004	8.32003E-09	11
C230037L18Rik	0.315853219	0.108	0.006	8.33976E-09	11
D3Bwg0562e.1	0.317070052	0.167	0.029	9.08547E-09	11
Htatsf1.2	-0.822496988	0.317	0.499	9.27064E-09	11
Tmtc2	0.331976253	0.183	0.032	9.96753E-09	11
Scamp2.3	0.509996298	0.408	0.133	1.05223E-08	11
Cox7a1	0.312971853	0.108	0.005	1.07983E-08	11
Hjurp.8	-1.01682324	0.175	0.435	1.10919E-08	11
Rab10.1	0.313867422	0.592	0.291	1.21246E-08	11
Ginm1	0.395864026	0.292	0.08	1.23869E-08	11
Rsl1d1.3	-0.565073538	0.433	0.49	1.23884E-08	11
Irgm1	0.263433057	0.15	0.02	1.35784E-08	11
Gsk3b.5	-0.444009023	0.508	0.499	1.3675E-08	11
Cyhr1	0.530752632	0.333	0.093	1.38027E-08	11
Srsf6.2	-0.70180574	0.292	0.446	1.40556E-08	11
Ddrgk1	0.400243157	0.45	0.186	1.44755E-08	11
Mki67.11	-1.226643383	0.175	0.396	1.46029E-08	11
Cenpe.10	-1.28778113	0.075	0.287	1.46244E-08	11
Gm2694.3	-1.053052231	0.083	0.36	1.50407E-08	11
Cbx5.8	-0.551191786	0.55	0.653	1.50843E-08	11
Ccdc141	0.413721336	0.125	0.008	1.54366E-08	11
Cables1	0.319664027	0.117	0.008	1.56536E-08	11
Srsf7.7	-0.807454965	0.242	0.466	1.62658E-08	11
Cotl1.2	0.452440305	0.425	0.151	1.6271E-08	11
Prkcdbp	0.546442498	0.333	0.088	1.70067E-08	11
Tubb2a.11	0.338015574	0.525	0.242	1.70909E-08	11
Orai3	0.25725887	0.108	0.007	1.83311E-08	11
Tmem205	0.337245801	0.25	0.064	1.85763E-08	11
Uqcrb.1	0.466406317	0.525	0.218	2.01497E-08	11
Lrrc8a	0.28746698	0.167	0.027	2.0423E-08	11
Ctsf.4	0.27736499	0.267	0.097	2.07164E-08	11

Desi1.1	0.259489581	0.283	0.092	2.08309E-08	11
Naca.4	-0.614362163	0.367	0.465	2.08998E-08	11
Pa2g4.7	-0.617368898	0.408	0.523	2.11313E-08	11
Preb.1	0.392903667	0.408	0.153	2.16533E-08	11
Specc1.2	0.44994404	0.242	0.047	2.2304E-08	11
Ntm.7	0.439700666	0.408	0.143	2.245E-08	11
Sccpdh	0.298742787	0.275	0.082	2.24614E-08	11
Efhd2.7	0.358655419	0.367	0.128	2.27516E-08	11
Laptm4a.1	0.521142475	0.85	0.552	2.29556E-08	11
Slc41a1	0.550800473	0.225	0.038	2.31186E-08	11
Tpr.1	-0.585587408	0.467	0.553	2.38821E-08	11
Echs1	0.523674084	0.483	0.185	2.39082E-08	11
Ctcf.4	-0.761608794	0.342	0.496	2.47293E-08	11
Tshz2.5	-0.811662723	0.183	0.32	2.48447E-08	11
Lrrc1	0.412060002	0.175	0.022	2.48777E-08	11
Soga3.7	-0.885921148	0.258	0.458	2.63348E-08	11
Casc4.2	0.275719757	0.375	0.146	2.65883E-08	11
Snrpd3.3	-0.475478232	0.5	0.535	2.66847E-08	11
Sec14l1.1	0.390317166	0.233	0.051	2.73106E-08	11
Khdrbs1.2	-0.630205677	0.383	0.494	2.88514E-08	11
E130114P18Rik.5	-0.685584981	0.467	0.585	2.89974E-08	11
Ndufa1.1	0.423263277	0.667	0.34	2.94344E-08	11
Eif2ak2.1	0.259435587	0.117	0.011	3.01841E-08	11
Pbrm1.2	-0.663197333	0.425	0.531	3.0656E-08	11
Rpl35a.5	-0.545995423	0.483	0.57	3.14946E-08	11
Cyth2.3	-0.611635602	0.2	0.273	3.36208E-08	11
Hcfc1r1.5	0.291257853	0.442	0.191	3.41691E-08	11
Nckap1.2	0.254200467	0.342	0.129	3.56372E-08	11
Tmod2.1	0.596390559	0.517	0.205	3.57729E-08	11
Rnase4.1	0.331618985	0.15	0.021	3.60027E-08	11
Rufy3.9	-0.913343908	0.167	0.372	3.76745E-08	11
Ssb.1	-0.520370244	0.642	0.704	3.79236E-08	11
Pigyl.1	0.288103313	0.367	0.142	3.8253E-08	11
Ptms.5	-0.598002195	0.4	0.474	3.85676E-08	11
Slc7a4	0.285217565	0.108	0.007	3.97579E-08	11
St8sia3.3	-0.950303321	0.017	0.252	4.15637E-08	11
Ypel3.5	-0.769266228	0.292	0.436	4.51624E-08	11
Bcl2	0.263180508	0.175	0.031	4.68699E-08	11
Snrpe.5	-0.716716938	0.383	0.563	4.72387E-08	11
Slc25a4.2	0.425577301	0.933	0.806	4.96245E-08	11
Fmn2	0.425378385	0.217	0.04	5.0672E-08	11
Dhx9.2	-0.753227858	0.292	0.464	5.14979E-08	11
Snrnp27.1	-0.37527647	0.35	0.31	5.15999E-08	11
Gng5.2	0.348616047	0.417	0.163	5.16971E-08	11
Snx5.5	0.401791086	0.425	0.161	5.17128E-08	11
Tmem100.1	0.388919906	0.125	0.009	5.19798E-08	11
Rps7.5	-0.669161286	0.283	0.4	5.31099E-08	11
Rorb	0.3447592	0.158	0.02	5.38576E-08	11

Impact	0.363153064	0.225	0.05	5.4226E-08	11
Pccb.1	0.47898903	0.367	0.116	5.7514E-08	11
Klhdc8b	0.294220043	0.15	0.022	6.38458E-08	11
D17Wsu104e.1	0.556402137	0.508	0.213	6.50723E-08	11
Pmvk.2	0.272375064	0.275	0.093	6.63949E-08	11
Tsn.2	-0.581044189	0.483	0.589	6.68728E-08	11
Vldlr.1	0.303317633	0.233	0.059	6.70606E-08	11
Fahd2a	0.26914006	0.208	0.051	6.8891E-08	11
Ak1	0.324010115	0.117	0.009	7.34179E-08	11
Itfg1	0.358847847	0.292	0.084	7.37385E-08	11
Tmem9.1	0.440797809	0.342	0.108	7.49411E-08	11
Pam	0.428168695	0.283	0.078	7.50289E-08	11
Sdhc	0.542938683	0.592	0.267	7.50319E-08	11
Stard3.1	0.254500749	0.192	0.045	7.73665E-08	11
Rbm8a.4	-0.484232901	0.408	0.465	7.90842E-08	11
Fbxo44	0.45130673	0.2	0.035	8.02298E-08	11
Plekhh2	0.303194727	0.125	0.011	8.06355E-08	11
Surf1	0.344828477	0.317	0.103	8.44815E-08	11
Adam17.1	0.364826306	0.225	0.048	8.9159E-08	11
Srp72	-0.600535788	0.258	0.322	9.15877E-08	11
Tardbp.2	-0.574681727	0.408	0.483	9.68819E-08	11
Rdh10	0.449054066	0.183	0.026	9.76128E-08	11
Uncx.9	-1.037570624	0.092	0.319	9.81345E-08	11
Wwtr1	0.31888628	0.125	0.01	9.85711E-08	11
Nipa1.1	0.318455488	0.167	0.026	1.02625E-07	11
Nucb1.1	0.417444278	0.275	0.073	1.04114E-07	11
Tbata.8	-1.140470118	0.075	0.341	1.06693E-07	11
Trp53.5	-0.63070172	0.2	0.299	1.06846E-07	11
Efr3a	0.260575169	0.283	0.093	1.12318E-07	11
mt-Rnr2.2	-0.348386643	1	0.997	1.14989E-07	11
Hint1.5	-0.438487496	0.7	0.703	1.1743E-07	11
Rps25.6	-0.574402857	0.392	0.48	1.21052E-07	11
Smarca5.4	-0.826701739	0.217	0.402	1.21207E-07	11
Smc6.7	-0.61913118	0.342	0.423	1.23172E-07	11
Psmb10.2	0.410391907	0.25	0.061	1.24359E-07	11
Lmnb1.10	-0.918532901	0.075	0.301	1.3592E-07	11
Serf1.4	-0.518310789	0.258	0.28	1.36935E-07	11
Eif5b	-0.633230354	0.508	0.595	1.37326E-07	11
Wnt5a	0.253129822	0.108	0.008	1.39386E-07	11
Kpnb1.2	-0.437526837	0.3	0.3	1.40431E-07	11
Pqbp1.1	-0.30830008	0.317	0.263	1.42117E-07	11
Ccm2.5	-0.620655443	0.15	0.225	1.42464E-07	11
Ube2b.2	-0.555394426	0.442	0.521	1.43654E-07	11
Hsd11b2.9	-0.983286069	0.033	0.289	1.4506E-07	11
Bphl	0.366490164	0.233	0.058	1.4734E-07	11
Smarca4.1	-0.705732454	0.342	0.506	1.51907E-07	11
Polr3h	0.360989267	0.308	0.098	1.63642E-07	11
Tnfrsf1a.1	0.341838169	0.167	0.024	1.64071E-07	11

Anxa6	0.273389664	0.225	0.06	1.72544E-07	11
Ptprg.3	0.382354988	0.383	0.14	1.74436E-07	11
Adam9.2	0.341608971	0.225	0.051	1.76691E-07	11
Hpca.8	-1.061089145	0.042	0.278	1.81921E-07	11
Commd6.1	0.282715324	0.4	0.178	1.82384E-07	11
Vps36.2	-0.529543682	0.275	0.302	1.8253E-07	11
G0s2.1	0.324806757	0.108	0.006	1.9565E-07	11
Pxdc1.1	0.339728081	0.108	0.007	1.96032E-07	11
Selk.1	0.445631721	0.7	0.394	2.0008E-07	11
Zfand5.3	-0.537562911	0.392	0.415	2.05799E-07	11
Nrbp2	0.488685836	0.175	0.028	2.06299E-07	11
Rgs2.1	0.482042457	0.233	0.05	2.06833E-07	11
Rap2a.2	0.264889205	0.4	0.168	2.13721E-07	11
Gas6.3	0.286800094	0.258	0.079	2.17727E-07	11
Mycn.5	-0.963825361	0.125	0.365	2.18461E-07	11
Hnrnph3.2	-0.723216442	0.267	0.427	2.22839E-07	11
Psmc1.2	-0.367896051	0.458	0.418	2.23181E-07	11
Ccnd3.1	0.380693961	0.283	0.081	2.25506E-07	11
Gabarapl1.5	0.283428477	0.467	0.214	2.29749E-07	11
0610011F06Rik.1	0.341832347	0.342	0.125	2.3291E-07	11
Klhl25	0.260320476	0.117	0.01	2.3378E-07	11
Ptprf	0.435311064	0.317	0.096	2.48088E-07	11
Tusc3	0.299644529	0.367	0.146	2.54533E-07	11
Bhlhe41.1	0.268420499	0.125	0.011	2.58666E-07	11
Ly6e.4	-0.956334611	0.042	0.283	2.62332E-07	11
Fkbp1a.1	-0.501060544	0.392	0.452	2.6748E-07	11
Hnrnpk.2	-0.525702341	0.533	0.636	2.80393E-07	11
Aard	0.299321275	0.158	0.023	2.84116E-07	11
Trappc3.1	0.3470459	0.308	0.099	2.93659E-07	11
Eif3h.2	-0.391124799	0.483	0.493	2.94199E-07	11
Mbd3.3	-0.264011604	0.358	0.29	2.97238E-07	11
Rest.1	0.268263789	0.15	0.022	3.032E-07	11
Ddhd1.1	0.416114708	0.242	0.055	3.05993E-07	11
Ahcyl2	0.309358246	0.158	0.025	3.0912E-07	11
Slc25a29	0.411841492	0.125	0.009	3.17747E-07	11
Pigc	0.269991727	0.167	0.031	3.20379E-07	11
Cacna2d1.7	-1.011203695	0.1	0.376	3.37773E-07	11
Amer2.1	0.335338794	0.35	0.132	3.67007E-07	11
Tcerg1.2	-0.802108595	0.267	0.458	3.80369E-07	11
Usp22.5	-0.708190111	0.233	0.362	3.93389E-07	11
Rab7.2	0.467441183	0.458	0.181	4.03916E-07	11
Hlf	0.45152466	0.158	0.018	4.0453E-07	11
Pitpnc1.2	0.336800889	0.258	0.073	4.15423E-07	11
Pls3	0.513477282	0.275	0.07	4.46656E-07	11
Acbd5.1	0.552439249	0.342	0.107	4.54907E-07	11
Ppp2r1a.1	-0.255721825	0.275	0.213	4.59645E-07	11
Hmgn3.6	0.430110107	0.567	0.267	4.76055E-07	11
Camk2n1	0.362137627	0.275	0.084	4.76853E-07	11

Csad	0.453169265	0.267	0.067	4.92615E-07	11
Csde1.1	-0.572785722	0.358	0.436	5.1015E-07	11
Shfm1.3	-0.471132039	0.633	0.686	5.29513E-07	11
Rpl37.4	-0.549551815	0.408	0.504	5.32495E-07	11
Paip2.3	-0.652194571	0.417	0.549	5.41061E-07	11
Pax6.8	-0.791951437	0.342	0.514	5.51611E-07	11
Brd3.4	-0.806019946	0.317	0.533	5.54852E-07	11
Ddx39b.3	-0.63887721	0.317	0.445	5.58768E-07	11
Rdx.1	-0.322261753	0.667	0.603	5.67742E-07	11
Gpc4	0.337274358	0.117	0.008	5.71868E-07	11
Sod2.1	0.398977249	0.433	0.174	5.79999E-07	11
Nup93	0.313114836	0.233	0.059	5.8725E-07	11
Prim1.8	-0.84128914	0.1	0.264	6.0544E-07	11
Prmt8.6	-0.955997688	0.05	0.273	6.56727E-07	11
Bicc1	0.260240251	0.108	0.009	6.57436E-07	11
mt-Nd4	0.467680414	0.925	0.775	6.62688E-07	11
Derl2	0.342377268	0.25	0.069	6.66565E-07	11
Fam102b	0.381589789	0.208	0.042	6.76177E-07	11
Comt.2	0.416831059	0.333	0.108	7.05683E-07	11
Fos.6	0.990509397	0.525	0.303	7.30634E-07	11
Gab2	0.34949587	0.125	0.013	7.42141E-07	11
Ncam2.1	0.303628639	0.133	0.016	7.63102E-07	11
Erlec1	0.363765039	0.375	0.143	7.77555E-07	11
Zfp326.1	-0.794957319	0.233	0.41	8.17333E-07	11
Mtf2.2	-0.84737875	0.183	0.358	8.42284E-07	11
E2f5	0.474045029	0.275	0.068	8.56959E-07	11
Cfdp1.2	-0.594367785	0.392	0.484	8.91894E-07	11
Cct7.1	-0.424804842	0.467	0.484	9.01429E-07	11
Vhl	0.301656768	0.217	0.055	9.0751E-07	11
Arl8a.3	0.38959741	0.3	0.094	9.14504E-07	11
Lactb.2	0.268956336	0.167	0.033	9.16251E-07	11
C1galt1c1	0.323747689	0.175	0.031	9.23703E-07	11
Sfxn1.2	0.528683878	0.542	0.248	9.23814E-07	11
Smarcc1.4	-0.846868231	0.217	0.435	9.25534E-07	11
Eif1.2	-0.417837058	0.558	0.582	9.74448E-07	11
Plxnb1	0.375344069	0.233	0.058	9.90038E-07	11
Sstr2.7	-0.82989882	0.017	0.192	9.90848E-07	11
1500012F01Rik.6	-0.850309057	0.217	0.399	9.90889E-07	11
Rbp4.8	-0.936860812	0.042	0.243	9.9758E-07	11
Apba2.3	-0.552841643	0.358	0.414	1.00875E-06	11
Snw1.1	-0.532278701	0.375	0.431	1.01396E-06	11
Aldh7a1	0.440507599	0.25	0.065	1.04664E-06	11
Aplp2.5	0.461629559	0.633	0.32	1.04932E-06	11
Nudt2	0.318306175	0.242	0.068	1.05484E-06	11
Sema6a.10	0.262982171	0.433	0.2	1.07694E-06	11
Klc1.8	-0.31054823	0.433	0.36	1.08891E-06	11
Stmn4.11	-1.143249706	0.108	0.342	1.11042E-06	11
Tmem184c.1	0.250802869	0.317	0.121	1.12915E-06	11

Tmem14c.1	0.327516877	0.433	0.186	1.14467E-06	11
Macf1.3	0.485149247	0.483	0.208	1.1511E-06	11
Ddx46.1	-0.779259672	0.267	0.443	1.15447E-06	11
Gnptg.1	0.308275004	0.392	0.165	1.1668E-06	11
C530008M17Rik.4	-0.859622545	0.092	0.269	1.18367E-06	11
Hspa5.3	0.331545058	0.85	0.548	1.19474E-06	11
Usp1.10	-0.818569772	0.175	0.34	1.19861E-06	11
Anapc5.2	-0.571415175	0.258	0.334	1.21251E-06	11
Rprm.1	0.316581578	0.175	0.033	1.22096E-06	11
Scp2.2	0.359371656	0.258	0.072	1.2246E-06	11
Trps1.1	0.491061908	0.308	0.089	1.22946E-06	11
Pigt	0.262186341	0.15	0.024	1.239E-06	11
Kmt2e.7	-0.728821122	0.408	0.569	1.25539E-06	11
Ube2e3.3	-0.74160945	0.233	0.417	1.2689E-06	11
Slc25a1.1	0.275531897	0.217	0.059	1.29692E-06	11
Sec62.2	0.525239623	0.717	0.403	1.31218E-06	11
B4galt4	0.368429562	0.167	0.025	1.37527E-06	11
Cltb.7	-0.822061418	0.267	0.424	1.46199E-06	11
Arsk	0.328232753	0.167	0.027	1.47292E-06	11
Ddx42.1	-0.668455804	0.275	0.383	1.48469E-06	11
Tmem246.1	0.318806934	0.267	0.08	1.50501E-06	11
Nhp2.4	-0.466055773	0.358	0.37	1.5378E-06	11
Gusb.1	0.390168843	0.275	0.079	1.54207E-06	11
Etv5	0.26075563	0.158	0.03	1.54448E-06	11
Celf4.11	-1.206610959	0.067	0.322	1.60073E-06	11
Prdx5.3	0.426331359	0.55	0.262	1.61709E-06	11
Uqcrc1	0.408004806	0.592	0.292	1.68893E-06	11
Map3k1.2	-0.889937208	0.142	0.372	1.74305E-06	11
Gpt2.1	0.546862676	0.267	0.065	1.78301E-06	11
Sall2	0.366364391	0.208	0.048	1.85904E-06	11
Mmp15.1	0.252046482	0.125	0.016	1.89916E-06	11
Sspn	0.270577146	0.125	0.014	1.90949E-06	11
Incenp.10	-1.000328924	0.083	0.276	1.95111E-06	11
Ndufa11	0.458737104	0.625	0.329	2.00757E-06	11
Tagln3.10	0.445778316	0.592	0.292	2.11553E-06	11
Rad21.11	-0.625498638	0.342	0.462	2.16656E-06	11
Cetn3.1	-0.559146787	0.417	0.487	2.16724E-06	11
Caprin1.2	-0.589523416	0.308	0.394	2.17714E-06	11
Apex1.5	-0.793841654	0.242	0.452	2.21782E-06	11
Snrpg.4	-0.644407207	0.208	0.335	2.21803E-06	11
Sumo3.1	-0.635110626	0.2	0.298	2.22532E-06	11
Nolc1.5	-0.760602207	0.25	0.409	2.2419E-06	11
Nol7.2	-0.44900593	0.55	0.569	2.2708E-06	11
Rbmx.3	-0.747503521	0.158	0.316	2.34136E-06	11
Smn1	-0.377765077	0.15	0.159	2.35869E-06	11
Ghr	0.261018997	0.142	0.021	2.46564E-06	11
Itgb1bp1	0.279126223	0.225	0.062	2.47166E-06	11
Rtn3.3	0.423401329	0.783	0.474	2.48213E-06	11

Mboat7	0.553184001	0.2	0.038	2.542E-06	11
Polr3k.1	-0.595422146	0.192	0.271	2.5959E-06	11
Sox18.4	-0.630948113	0.017	0.148	2.60761E-06	11
BC031181.1	0.374760083	0.542	0.259	2.62419E-06	11
Rbfox2.8	-0.932029724	0.075	0.284	2.72365E-06	11
Srp68	-0.257866078	0.125	0.108	2.76195E-06	11
Smim13.1	0.264003923	0.217	0.059	2.82772E-06	11
Pcdh9.1	0.42779843	0.2	0.039	2.83044E-06	11
Pdlim3.2	0.398355969	0.233	0.06	2.88271E-06	11
Rassf4.4	-0.902637235	0.142	0.377	2.91719E-06	11
Tmed1	0.385044935	0.317	0.103	2.9244E-06	11
Rpl7.4	-0.33352082	0.55	0.509	3.00666E-06	11
Sys1	0.447720629	0.442	0.195	3.11858E-06	11
Pcbp4.3	-0.888574377	0.092	0.326	3.18843E-06	11
Metrnl	0.288175345	0.108	0.009	3.24277E-06	11
Trib1.1	0.252423524	0.208	0.057	3.28457E-06	11
Jtb	0.388145342	0.483	0.217	3.30544E-06	11
Ndp	0.352935376	0.133	0.014	3.43879E-06	11
Snhg5.2	-0.889381623	0.133	0.382	3.58105E-06	11
Wbp5.5	-0.377469451	0.517	0.518	3.68608E-06	11
Sub1.2	-0.395268572	0.567	0.589	3.7082E-06	11
Dhrs4	0.365648548	0.2	0.045	3.82772E-06	11
Epha5	0.355795859	0.133	0.015	3.90386E-06	11
Ide	0.410696641	0.325	0.111	3.91085E-06	11
Tcp1.3	-0.527575237	0.392	0.463	4.03894E-06	11
Stk17b	0.264117018	0.225	0.064	4.26764E-06	11
Elavl4.10	-0.991913583	0.133	0.336	4.38588E-06	11
Zmiz1.4	-0.590245884	0.308	0.375	4.46383E-06	11
Wscd1.1	0.352517459	0.233	0.062	4.4771E-06	11
Snn	0.468934083	0.35	0.126	4.66366E-06	11
Cox8a	0.448935139	0.833	0.699	4.68593E-06	11
Ankrd11.2	-0.623248148	0.392	0.473	4.77659E-06	11
Pex2.1	0.463086281	0.3	0.099	4.82097E-06	11
Hsd17b4.1	0.484182982	0.383	0.138	4.83391E-06	11
Prpsap1	0.346909695	0.408	0.172	5.03792E-06	11
Thrap3.1	-0.607556122	0.267	0.388	5.30822E-06	11
Krcc1.2	0.34298288	0.267	0.082	5.40162E-06	11
Snap25.7	-0.962521662	0.067	0.283	5.54229E-06	11
Gm8292.4	-0.607116382	0.258	0.369	5.54327E-06	11
Slc50a1.1	0.315191085	0.258	0.081	5.58904E-06	11
Cirbp.3	-0.646417946	0.258	0.355	5.79558E-06	11
Maoa.1	0.347695944	0.242	0.066	5.88817E-06	11
Cdk1.10	-0.74313706	0.15	0.247	5.9878E-06	11
Pcbp2.2	-0.370261943	0.6	0.621	6.1078E-06	11
Mlf2.2	0.271111972	0.533	0.277	6.32286E-06	11
Nkd1.5	-0.860625406	0.042	0.249	6.3529E-06	11
Erp44	0.285499991	0.3	0.107	6.40464E-06	11
Vmp1.2	0.368965691	0.317	0.115	6.47363E-06	11

Leprel4	0.354866442	0.167	0.028	6.60174E-06	11
Bai1	0.456936628	0.15	0.021	6.6076E-06	11
Cox6b2	0.425627976	0.133	0.014	6.62551E-06	11
Evi5.1	0.542441859	0.392	0.14	7.09231E-06	11
Rnmt.7	-0.830052847	0.167	0.349	7.16734E-06	11
Hmgb1.8	-0.704667706	0.217	0.359	7.29337E-06	11
Clptm1	0.267369167	0.208	0.059	7.51559E-06	11
Fndc5	0.285059837	0.125	0.017	7.51789E-06	11
Zmat2.1	-0.606444847	0.35	0.44	7.58863E-06	11
Fkbp2.3	0.261312779	0.575	0.324	7.69158E-06	11
Dscr3.1	0.363182161	0.25	0.071	7.70057E-06	11
Phf5a.2	-0.401940963	0.325	0.331	7.78918E-06	11
Zeb1.5	-0.770823737	0.217	0.388	8.29096E-06	11
Zwint.3	-0.267238035	0.358	0.29	8.43499E-06	11
Eif4h.1	-0.408870607	0.45	0.471	8.56508E-06	11
Eif3k.2	-0.459552312	0.492	0.521	8.61507E-06	11
Kctd5	0.353637439	0.308	0.106	8.7748E-06	11
Abracl.3	-0.791154255	0.125	0.303	9.29179E-06	11
Hspa8.1	-0.524683988	0.342	0.415	9.30966E-06	11
Lig1.11	-1.029440333	0.125	0.349	9.53969E-06	11
Clcn3.3	0.414412731	0.55	0.27	9.84597E-06	11
Tm2d2.1	0.263807949	0.492	0.248	1.01433E-05	11
Grhpr	0.41068292	0.133	0.015	1.02849E-05	11
1500016L03Rik.7	-0.909417224	0.142	0.384	1.04496E-05	11
Hdgfrp3.1	-0.785073672	0.167	0.339	1.0754E-05	11
Ubtf.2	-0.703286132	0.3	0.453	1.10591E-05	11
Dpy19l3	0.296968611	0.142	0.023	1.14039E-05	11
Cpe.7	0.589719908	0.7	0.454	1.14143E-05	11
Gpx1.2	-0.379098109	0.408	0.382	1.14701E-05	11
Rab9.1	0.294810087	0.3	0.108	1.1519E-05	11
Samd8.1	0.381535854	0.25	0.078	1.19318E-05	11
Ran.7	-0.531888337	0.308	0.414	1.22232E-05	11
Slc35f5	0.28117297	0.158	0.031	1.24076E-05	11
Dhrs7.1	0.303027942	0.233	0.07	1.24228E-05	11
Fut9.3	0.51280131	0.483	0.21	1.28833E-05	11
Rtn1.10	-0.710343412	0.617	0.69	1.30911E-05	11
Cks1b.10	-0.862258805	0.142	0.345	1.32415E-05	11
Scg5.6	-0.851194088	0.108	0.336	1.34327E-05	11
Psmd4	-0.351945315	0.425	0.392	1.35649E-05	11
Txnrd1.3	-0.650050801	0.267	0.389	1.35965E-05	11
Aldh9a1.2	0.379933585	0.217	0.055	1.37114E-05	11
Ddx6.1	-0.635718564	0.358	0.493	1.41047E-05	11
Nop56.6	-0.814566358	0.158	0.361	1.42297E-05	11
Mrpl13.5	-0.271192279	0.367	0.307	1.4365E-05	11
Rps8.3	-0.622379444	0.275	0.397	1.45416E-05	11
Lamtor4.1	0.263888623	0.317	0.126	1.54138E-05	11
Aga.1	0.273829053	0.167	0.035	1.60018E-05	11
Smim15.2	0.338361763	0.317	0.119	1.60854E-05	11

Vdac1	0.377507658	0.575	0.3	1.61616E-05	11
Whsc1.4	-0.644628724	0.342	0.465	1.71396E-05	11
Cldn25.1	0.274336436	0.45	0.23	1.72357E-05	11
Eif3e.3	-0.664396425	0.25	0.382	1.72525E-05	11
Tmem59.2	0.430437079	0.692	0.39	1.76122E-05	11
Mcm6.8	-0.9023416	0.075	0.277	1.89742E-05	11
Nme1.1	-0.482619668	0.442	0.509	1.91245E-05	11
Yif1a.1	0.291326251	0.392	0.177	1.91362E-05	11
Bmyc.2	0.262583443	0.275	0.099	1.97902E-05	11
Sh3glb2	0.348982622	0.258	0.082	2.04848E-05	11
Pcna.10	-0.686596342	0.267	0.358	2.08967E-05	11
Vegfb.2	0.367083215	0.325	0.117	2.11163E-05	11
Sbds	0.274089419	0.333	0.135	2.12565E-05	11
Rhoa.1	0.37113917	0.358	0.14	2.17057E-05	11
Sugt1.1	-0.39934296	0.225	0.231	2.22857E-05	11
Vps37b.4	-0.578817363	0.383	0.47	2.24742E-05	11
Map2.9	-0.41494386	0.533	0.491	2.28106E-05	11
Hmgb3.5	-0.832573508	0.092	0.307	2.28524E-05	11
Cit.1	0.275665835	0.225	0.064	2.33052E-05	11
Rpe	0.25720422	0.192	0.05	2.38589E-05	11
Cct5.3	-0.38241257	0.475	0.477	2.46897E-05	11
Tmx2	0.288973775	0.292	0.106	2.5286E-05	11
Fam212b.2	-0.482278129	0.192	0.223	2.54183E-05	11
Gbp7	0.297230306	0.108	0.009	2.72652E-05	11
Pde1c.11	-0.888497546	0.225	0.388	2.745E-05	11
2610507B11Rik	0.375141841	0.417	0.179	2.75919E-05	11
U2surp.1	-0.431329024	0.492	0.505	2.76936E-05	11
C1ql1.6	-0.867433323	0.083	0.28	2.78062E-05	11
Acaa1a	0.423356148	0.308	0.102	2.81324E-05	11
Sdc2.1	0.423718641	0.225	0.053	2.90513E-05	11
Supt16.7	-0.594341237	0.375	0.494	2.9249E-05	11
Sept2.1	0.261304676	0.45	0.22	2.98241E-05	11
Eprs.1	-0.525224674	0.433	0.491	3.04295E-05	11
Rpl18a.5	-0.649871566	0.25	0.401	3.05369E-05	11
Slc35f6.1	0.334565492	0.133	0.018	3.09354E-05	11
Suv39h2.4	-0.488135113	0.042	0.131	3.25562E-05	11
Ik	-0.422474285	0.425	0.465	3.42358E-05	11
Arl2	0.252607541	0.275	0.102	3.43118E-05	11
Per3	0.269039085	0.117	0.015	3.47986E-05	11
Suclg1.1	0.33176795	0.533	0.27	3.48989E-05	11
Cs	0.47638755	0.5	0.227	3.56976E-05	11
Birc5.11	-0.880667599	0.117	0.274	3.5775E-05	11
Adh5.1	-0.386128507	0.417	0.404	3.64503E-05	11
Ssfa2	0.270526373	0.133	0.02	3.81009E-05	11
Pros1.1	0.289170235	0.142	0.023	3.81875E-05	11
Thoc7.2	-0.539830185	0.417	0.522	3.84213E-05	11
Cachd1	0.29811819	0.15	0.026	3.87682E-05	11
Gse1.4	-0.8459618	0.042	0.238	3.9028E-05	11

Gng3.10	-0.993591739	0.1	0.318	3.95696E-05	11
Slc48a1.1	0.325727611	0.217	0.059	3.96095E-05	11
Hadh.1	0.406197939	0.258	0.072	3.96412E-05	11
Cdca8.11	-0.868435559	0.1	0.265	4.06099E-05	11
Cdip1	0.275590155	0.267	0.101	4.07104E-05	11
Neurod6.3	-0.73463602	0.025	0.173	4.25034E-05	11
2700029M09Rik.3	-0.466388174	0.258	0.289	4.2776E-05	11
Dusp6.3	0.500090886	0.3	0.091	4.28519E-05	11
Skp1a.1	-0.374842009	0.592	0.581	4.30505E-05	11
Ubc.1	0.289939693	0.625	0.357	4.48909E-05	11
Fip1l1.2	-0.644503544	0.208	0.309	4.55504E-05	11
Smim20	0.404152528	0.208	0.048	4.6292E-05	11
Phb2.1	-0.283118125	0.383	0.332	4.68884E-05	11
Rpl14-ps1.2	-0.583805193	0.183	0.284	5.12323E-05	11
Actb.2	-0.270342448	1	0.978	5.28368E-05	11
Hspb6	0.294144201	0.108	0.011	5.69733E-05	11
Snrnp70.1	-0.437328014	0.617	0.694	5.73579E-05	11
Nars	-0.363386991	0.475	0.441	5.73581E-05	11
D10Jhu81e	0.298523488	0.242	0.074	5.73683E-05	11
Arpc1a.2	-0.397883391	0.367	0.363	5.78879E-05	11
Tm7sf3.1	0.335898592	0.225	0.064	5.92423E-05	11
Rsrc2.1	-0.557004044	0.458	0.548	6.15043E-05	11
H2afy2.3	-0.830757236	0.108	0.339	6.53699E-05	11
Prpf38b.1	-0.481418451	0.467	0.495	6.64166E-05	11
Sptssa.2	0.28350606	0.475	0.245	6.8479E-05	11
Vbp1.3	-0.403952369	0.308	0.319	6.9738E-05	11
Ncald.2	0.32252728	0.375	0.164	6.9838E-05	11
Dirc2.2	0.376538947	0.258	0.094	7.05118E-05	11
Ophn1.2	0.300681002	0.25	0.082	7.14195E-05	11
Gnai2.6	0.300157834	0.7	0.422	7.1596E-05	11
Srsf10.3	-0.656887374	0.2	0.331	7.16758E-05	11
Uqcc2	0.311739113	0.575	0.31	7.29281E-05	11
Arfip1	0.295778517	0.267	0.091	7.508E-05	11
Tra2b.2	-0.697196597	0.25	0.406	7.60998E-05	11
Emg1.3	-0.414263019	0.3	0.315	7.77381E-05	11
Gpx7.1	0.42265087	0.225	0.061	7.92323E-05	11
Mad2l2.4	-0.548203737	0.192	0.262	8.0773E-05	11
Meis1.3	-0.609510292	0.283	0.373	8.18968E-05	11
Nudt4.1	0.512611227	0.458	0.216	8.30417E-05	11
Gdpd1.6	-0.651334835	0.133	0.244	8.33942E-05	11
Kazn.2	0.340872029	0.133	0.02	8.52796E-05	11
Lbh.1	0.410210755	0.242	0.072	8.53542E-05	11
Pigp	0.252427167	0.308	0.127	8.80126E-05	11
Eif3b.3	-0.44298468	0.192	0.22	8.81792E-05	11
Ghitm	0.301658197	0.533	0.282	8.87878E-05	11
Lsm6.6	0.339093616	0.642	0.37	9.168E-05	11
Prr13.2	0.260846356	0.25	0.088	9.67971E-05	11
Scaf11.2	-0.516219672	0.317	0.373	9.73772E-05	11

Spag9.3	0.261576344	0.542	0.305	9.76879E-05	11
Kif11.10	-0.729591267	0.083	0.206	0.000100134	11
Neto2	0.329727906	0.242	0.072	0.000100398	11
Asf1a.2	-0.441496768	0.233	0.28	0.000100925	11
Gm11223.5	-1.039303413	0.025	0.214	0.000101276	11
Lactb2	0.301081128	0.117	0.013	0.000101675	11
Ing4.5	-0.52753725	0.267	0.323	0.000104206	11
Krtcap2	0.352514344	0.75	0.466	0.000104222	11
1110008F13Rik	0.500139657	0.483	0.227	0.000107729	11
Thoc2.1	-0.701007653	0.3	0.449	0.000109693	11
Cdc42se1.2	0.304317081	0.375	0.164	0.000110674	11
Csnk1e.2	-0.671772352	0.258	0.386	0.000112725	11
Srrm4.9	-0.805596005	0.033	0.196	0.000116635	11
Myt1.10	-0.75519657	0.033	0.153	0.000119586	11
Rpn2.2	0.368575437	0.475	0.225	0.000121844	11
Tead2.7	-0.650592849	0.158	0.278	0.000122978	11
Esf1.2	-0.550919195	0.35	0.392	0.000127436	11
Ythdc1.2	-0.639770803	0.325	0.478	0.00013395	11
Mdk.7	-0.387408334	0.425	0.379	0.000135042	11
Hsp90b1.7	0.387318789	0.917	0.69	0.000136797	11
Pfkl	0.336924261	0.117	0.013	0.000138607	11
Cluh	0.256500193	0.175	0.046	0.000138975	11
Nipbl.3	-0.44929498	0.458	0.492	0.000141476	11
Tmem5.1	0.321046821	0.25	0.08	0.000144205	11
Pak2.1	-0.302187377	0.342	0.3	0.000146958	11
Sar1b.1	0.418072646	0.442	0.196	0.000148336	11
Ano6.1	0.362684905	0.267	0.089	0.00014956	11
Pnkd	0.259789426	0.217	0.067	0.000153383	11
Id2.7	0.362749916	0.733	0.484	0.000153774	11
Rbm17.1	-0.548902097	0.308	0.39	0.000154136	11
Cntn2.11	-1.153534321	0.092	0.264	0.000155555	11
0610012G03Rik	0.250691174	0.325	0.145	0.000161136	11
Slc35b2.1	0.284267401	0.392	0.18	0.000162051	11
Insm1.4	-0.879713899	0.075	0.293	0.000162222	11
Cct2.3	-0.550409232	0.392	0.524	0.000162488	11
Srgap1	0.403515664	0.133	0.017	0.000163413	11
Tmed9.3	-0.277352644	0.475	0.432	0.000163717	11
Prrc2b	-0.422529675	0.208	0.222	0.00016388	11
0610009L18Rik	0.309761421	0.117	0.015	0.000170302	11
Atp1b3.6	-0.25531986	0.408	0.336	0.000172095	11
N4bp2l2	-0.538749954	0.2	0.261	0.000174944	11
Arl8b.1	0.250431237	0.35	0.166	0.000180424	11
Psat1.5	-0.305857314	0.4	0.367	0.000183985	11
Brd2.1	-0.410278611	0.375	0.395	0.000186745	11
Srsf5.1	-0.423605104	0.4	0.426	0.000186901	11
Arsb.2	0.398261866	0.15	0.024	0.00018977	11
Tle1.4	-0.462274819	0.192	0.226	0.00019649	11
H2-D1.3	0.40771918	0.333	0.129	0.000197704	11

Cadm4.3	0.252284372	0.275	0.112	0.000198549	11
Bcl7a.3	-0.773804277	0.142	0.326	0.000199011	11
Abcf1.1	-0.314388645	0.4	0.457	0.000201048	11
Brd8.4	-0.658948218	0.258	0.392	0.000202869	11
Cdc5l.1	-0.678172603	0.25	0.401	0.000205651	11
Pdgfra.5	-0.830991903	0.008	0.182	0.00020781	11
Lrrc42.1	0.273068163	0.208	0.062	0.000212594	11
Klf4	0.504749559	0.142	0.023	0.00021743	11
Set.6	-0.734682613	0.192	0.388	0.000218156	11
Celf1.2	-0.676080195	0.242	0.38	0.000227877	11
Rpn1.1	0.48366279	0.425	0.19	0.000228267	11
Pde6d	0.316871462	0.325	0.128	0.000230407	11
Dkc1.6	-0.756898815	0.125	0.334	0.000237226	11
Akr1e1	0.338748587	0.267	0.092	0.000241369	11
Ube2q1	-0.319308131	0.208	0.19	0.000251375	11
Ctage5	-0.587234224	0.192	0.267	0.000257816	11
Fam115a.3	-0.378182717	0.367	0.35	0.000262169	11
Clmp.8	-0.900733875	0.042	0.243	0.000264231	11
Pou3f3.2	0.311273511	0.383	0.17	0.000265978	11
Arhgap31.1	0.254365199	0.158	0.038	0.000274557	11
Tmbim1	0.272832294	0.108	0.014	0.000275109	11
Hells.11	-0.749923898	0.092	0.214	0.000276869	11
Prkcb.9	-0.87384201	0.067	0.257	0.000284193	11
Sars.1	-0.383463499	0.425	0.42	0.000291594	11
Sc5d	0.510611312	0.233	0.059	0.000299291	11
Rdh11	0.317605667	0.233	0.079	0.000304174	11
Bola2.3	-0.448388874	0.358	0.397	0.000305863	11
Fadd	0.297876272	0.108	0.012	0.000306953	11
Dixdc1.5	-0.817659119	0.042	0.236	0.000315239	11
Dync1i2.5	-0.28930858	0.617	0.567	0.000315569	11
Fam98b.1	-0.347040497	0.35	0.335	0.000319709	11
Cdc42ep4	0.325568914	0.192	0.047	0.000320302	11
Selt.1	0.305995198	0.258	0.094	0.000321388	11
Srrt.2	-0.737200128	0.225	0.425	0.000326099	11
Fkbp4.3	-0.530417261	0.333	0.414	0.00033004	11
Ndufa9	0.288115358	0.425	0.208	0.000332056	11
Klf13.4	-0.730477638	0.058	0.237	0.000332765	11
Dzip1l	0.370845326	0.142	0.023	0.000334305	11
Gm13826.3	-0.538432544	0.233	0.312	0.000336321	11
Stmn1.4	-0.743416399	0.108	0.285	0.000338158	11
Ctbp1.1	-0.430812047	0.35	0.394	0.000339262	11
Gm6472.2	-0.344375631	0.158	0.166	0.000348827	11
Pja2.2	-0.267237189	0.283	0.234	0.000359118	11
Ier3.2	0.255855793	0.142	0.033	0.000361912	11
Eif2s2	-0.440784378	0.308	0.33	0.000362669	11
Rab2a.1	-0.300433667	0.475	0.43	0.000362969	11
Cenpa.10	-0.721888326	0.175	0.252	0.000363418	11
Iars2	0.359044139	0.258	0.083	0.000365338	11

Snhg1.7	-0.55413766	0.283	0.357	0.000371261	11
Derl1.1	0.393594085	0.275	0.093	0.000372147	11
Rhbdd2.1	0.264892594	0.2	0.059	0.000373324	11
Slc16a1	0.260255175	0.167	0.037	0.00037886	11
Plscr4	0.300944527	0.15	0.027	0.000381828	11
Stxbp3a.1	0.258693496	0.125	0.019	0.00038755	11
Sox8.1	0.276267725	0.142	0.029	0.000388026	11
Rbm39.1	-0.426573484	0.717	0.793	0.000396268	11
Cyp51.2	0.456667907	0.35	0.139	0.00039861	11
Ncam1.4	-0.392151637	0.392	0.387	0.000408192	11
Tprn.5	-0.829458879	0.05	0.227	0.000408263	11
Atp2b1.4	-0.38475585	0.617	0.571	0.000416412	11
Cox6c.2	0.433543788	0.867	0.717	0.000443124	11
Zc3h15.1	-0.484514304	0.317	0.366	0.000445908	11
Zbtb18.7	-0.830601795	0.067	0.267	0.000458233	11
Uqcrq.2	0.428240806	0.8	0.548	0.000467482	11
Siva1.7	-0.66714243	0.142	0.273	0.000490197	11
Nop10.4	-0.446169934	0.45	0.482	0.000490761	11
Ogfr	-0.351006306	0.167	0.17	0.00050501	11
Chchd1.3	-0.334111059	0.425	0.4	0.000516217	11
Arhgap11a.10	-0.765054445	0.008	0.171	0.000516484	11
Bin1.9	-0.803414833	0.225	0.43	0.000523444	11
Lsm4.5	-0.432192574	0.442	0.488	0.000526116	11
mt-Nd1.2	0.379110694	0.992	0.926	0.000531013	11
Prmt5.3	-0.364917489	0.258	0.256	0.00055712	11
Slc39a1	0.356418047	0.167	0.034	0.00056167	11
Nap1l5.1	0.392241204	0.25	0.079	0.000570889	11
Lbr.7	-0.667622587	0.05	0.197	0.000591648	11
Csnk1a1.1	-0.305058534	0.7	0.666	0.000591812	11
Adamts1.5	0.654718074	0.342	0.153	0.00059436	11
Gdap1.2	-0.734450458	0.05	0.199	0.000630501	11
Atp5g3	0.436557937	0.775	0.515	0.000653216	11
Stk40	0.378214291	0.15	0.035	0.000655076	11
Idh1.4	0.30861561	0.35	0.154	0.000656472	11
Ppp1ca	-0.369529744	0.475	0.483	0.000662758	11
Blvrb.1	0.326782017	0.208	0.058	0.000670048	11
Gm2000	-0.424728399	0.058	0.129	0.000673879	11
Zfp292.4	-0.832514499	0.15	0.342	0.000677872	11
Eid1.6	-0.424447075	0.492	0.503	0.00067835	11
Cenpv.5	-0.639040982	0.192	0.329	0.000694534	11
Mphosph8.1	-0.374393313	0.375	0.365	0.00070961	11
Ndufa13.1	0.49263284	0.75	0.531	0.000740593	11
Pfdn4.2	-0.594441023	0.208	0.305	0.000792148	11
Ctnnb1.5	0.341059486	0.633	0.384	0.000792565	11
Dnajc9.9	-0.644863982	0.225	0.35	0.000798468	11
Erlin2	0.337892459	0.183	0.045	0.000810372	11
Papola.1	-0.302895409	0.442	0.401	0.000816069	11
Mfsd1.1	0.267646576	0.275	0.11	0.000817454	11

Fmr1.1	-0.359929552	0.267	0.299	0.000819541	11
Rnft1	0.262936611	0.158	0.036	0.000823586	11
Napa.2	-0.318350943	0.367	0.349	0.000827842	11
Rnf20.1	-0.701829685	0.15	0.289	0.00082893	11
Rtf1.1	-0.658906614	0.358	0.513	0.000834239	11
Tpx2.11	-0.755372708	0.175	0.282	0.000834287	11
Cdk5r1.9	-0.83553678	0.133	0.334	0.000840665	11
Ccdc55	-0.508061113	0.233	0.267	0.000851881	11
Fkbp9	0.31626478	0.15	0.03	0.000860795	11
Ralbp1	-0.440683991	0.3	0.322	0.000864068	11
Canx.2	0.433744954	0.817	0.594	0.00087881	11
Gtf3a	-0.444918903	0.15	0.194	0.000895694	11
Polr2m.1	-0.35947172	0.242	0.244	0.000899511	11
Prpf4b.1	-0.552637907	0.383	0.471	0.000909699	11
Cox20.1	-0.38113166	0.283	0.285	0.000921844	11
Ash1l.1	-0.259752144	0.333	0.277	0.00093274	11
Klf2.1	0.401943007	0.125	0.018	0.000954972	11
Hnrnpul2.1	-0.533903639	0.192	0.257	0.000968692	11
Ywhaz.2	-0.451056609	0.308	0.362	0.000996147	11
Arhgef2.5	-0.690169506	0.158	0.283	0.001015392	11
Fgf9.2	-0.780997319	0.075	0.261	0.001026149	11
Rbbp7.4	-0.290428375	0.35	0.319	0.001031559	11
Aes.1	-0.402360383	0.192	0.212	0.001041159	11
Amfr	0.47269153	0.242	0.073	0.001049744	11
Parp1.2	-0.66192349	0.167	0.314	0.001069762	11
Ddx21.4	-0.713599126	0.183	0.323	0.001088892	11
Atp6v0e2.5	0.272090735	0.483	0.258	0.001093156	11
Bcas2.1	-0.444840938	0.367	0.405	0.001096806	11
Srebf1.9	-0.703832824	0.225	0.334	0.001116086	11
Gbas	0.383877098	0.333	0.131	0.00112872	11
Tpm4.6	-0.624302625	0.2	0.317	0.001152378	11
Kif2a.1	-0.534721175	0.183	0.251	0.001154435	11
Fam32a	-0.59626301	0.175	0.268	0.001163948	11
Nudc.5	-0.542361437	0.183	0.281	0.001186096	11
Apc.10	-0.483859981	0.425	0.431	0.001200792	11
Mrfap1.3	-0.339753915	0.408	0.403	0.001209643	11
Cct8.2	-0.433958256	0.383	0.441	0.00123302	11
Myef2.1	-0.515605856	0.267	0.338	0.001236778	11
Dad1.2	0.389131256	0.608	0.341	0.001264437	11
Vamp3.2	0.320407449	0.233	0.078	0.001277382	11
Arsa	0.295479438	0.117	0.017	0.001303401	11
Top2b.1	-0.570773665	0.325	0.414	0.001370064	11
Nktr.4	-0.491778304	0.35	0.38	0.001379076	11
Ccdc50.1	-0.532653773	0.242	0.327	0.001414569	11
Spc25.10	-0.540853	0.175	0.225	0.001466013	11
Ncln	0.254339335	0.233	0.1	0.001487164	11
Rab24.1	-0.328251001	0.225	0.214	0.00149273	11
Ubb.5	-0.402294877	0.517	0.541	0.00160694	11

Fam168a.4	0.335224176	0.525	0.285	0.001612288	11
Tceal3.3	0.553619777	0.3	0.109	0.001626618	11
Lpgat1.1	-0.635479379	0.008	0.168	0.001626679	11
Mia3.1	-0.279984417	0.283	0.244	0.00162867	11
Atoh1.7	-0.631649216	0.058	0.165	0.001645894	11
Kif21a.3	-0.315069563	0.383	0.345	0.001674492	11
Dusp3.1	0.313295523	0.142	0.028	0.001685811	11
Gar1.4	-0.678283728	0.05	0.209	0.001691954	11
Fam213b.5	-0.452869615	0.125	0.157	0.001693802	11
Mocs2.1	0.280455083	0.4	0.193	0.001724498	11
Golga4.1	-0.691586558	0.217	0.325	0.001730915	11
Pak3.1	-0.68801104	0.092	0.206	0.001763978	11
Mfsd11.1	0.283029069	0.175	0.051	0.001791113	11
Casc5.10	-0.725929248	0.05	0.187	0.001797287	11
Dennd5a.1	0.282001479	0.267	0.101	0.001807038	11
Gucy1b3	0.340719869	0.167	0.037	0.001853362	11
Zranb2.1	-0.336195289	0.425	0.406	0.001995551	11
Cfl2.1	-0.323862027	0.417	0.408	0.002023292	11
Dctpp1.7	-0.621283149	0.183	0.305	0.002057383	11
Ppp3ca.6	-0.409727968	0.383	0.378	0.002062911	11
C1qbp.4	-0.320786751	0.392	0.377	0.002115472	11
Sac3d1	-0.352254304	0.067	0.107	0.002123302	11
Rpl6.3	-0.635597487	0.158	0.291	0.00216944	11
Idh3a	0.299467615	0.358	0.161	0.002171172	11
Smim11.4	-0.290663549	0.308	0.276	0.002180846	11
Rab34	0.37260998	0.3	0.116	0.00220741	11
Rexo2.2	0.384445277	0.4	0.179	0.002208472	11
Gpr153.6	-0.704629479	0.075	0.209	0.002214204	11
Aimp1.1	-0.515684962	0.217	0.28	0.002239395	11
March7.1	-0.353216489	0.233	0.238	0.002245888	11
Supt4a	-0.26953208	0.167	0.154	0.002294899	11
Cwc15.1	-0.273768999	0.425	0.398	0.002309091	11
Itsn1.10	-0.416375142	0.242	0.235	0.002387194	11
Kdm5b.7	-0.440628015	0.167	0.185	0.002401992	11
Mut	0.314934904	0.192	0.053	0.002435327	11
Zic4.5	-0.677198992	0.25	0.419	0.002436362	11
Pqlc1.8	-0.73429074	0.142	0.298	0.002448497	11
Zcchc11.1	-0.751026086	0.15	0.322	0.002515697	11
Ddx3x.2	-0.427583258	0.408	0.467	0.002579424	11
Prdx1.6	0.333877284	0.75	0.488	0.002588535	11
Chgb.10	-0.966842384	0.067	0.241	0.0026095	11
Ndufa12.3	0.33564392	0.65	0.419	0.002642641	11
Elavl2.5	-0.79072109	0.067	0.271	0.002663281	11
Bex1.3	-0.645898745	0.108	0.224	0.002692509	11
A630007B06Rik.2	-0.603343029	0.133	0.206	0.00271435	11
C130071C03Rik.4	-0.506625374	0.25	0.308	0.002723301	11
Kif3a.2	-0.458157925	0.275	0.317	0.002728368	11
Setd5.1	-0.413425058	0.225	0.247	0.002738212	11

Gapvd1	-0.338227802	0.242	0.23	0.002799271	11
Cyth3	0.493626227	0.208	0.055	0.002822526	11
Tmem150a	0.336262412	0.117	0.017	0.002861233	11
Zfp422.2	-0.55890415	0.15	0.246	0.002869326	11
Rps4x.4	-0.488283704	0.233	0.295	0.002900413	11
Pdgfa.7	-0.808623516	0.1	0.254	0.003096323	11
Cited2.2	-0.556659098	0.075	0.153	0.003111849	11
RP23-45G16.5.11	-0.555832793	0.25	0.327	0.003167379	11
Ebf3.4	-0.716553801	0.058	0.204	0.00319452	11
Podxl2.10	-0.800336209	0.05	0.228	0.003235889	11
Myl12b.3	-0.391519796	0.342	0.367	0.003255033	11
Rrp1.1	-0.49411515	0.517	0.606	0.003270082	11
Mns1.10	-0.735779249	0.05	0.204	0.003311069	11
Gtf2a2.1	-0.325601748	0.367	0.383	0.003371546	11
Ndufs8.1	0.406331294	0.608	0.346	0.00346874	11
Ncor2.6	-0.627578649	0.042	0.169	0.003469126	11
Rnf122.3	-0.326805191	0.075	0.102	0.003511309	11
Magoh.4	-0.533322955	0.25	0.333	0.003586291	11
Sfr1.1	-0.302538878	0.408	0.394	0.003623773	11
Paics.7	-0.324913119	0.433	0.413	0.003698561	11
Ccp110.5	-0.675945998	0.108	0.249	0.00373807	11
1110038B12Rik.6	-0.640643545	0.167	0.299	0.003753803	11
Gpr19	0.287608555	0.208	0.066	0.003790613	11
P4ha1.2	0.345934941	0.208	0.06	0.003921376	11
Alcam.4	0.365743015	0.317	0.133	0.003927475	11
Cebpz	-0.450571209	0.267	0.305	0.003992085	11
Ckap2l.11	-0.529394383	0.175	0.217	0.004036955	11
Hmgxb4.1	-0.484668994	0.092	0.155	0.004079614	11
Psmd7.1	-0.389230902	0.458	0.479	0.004113051	11
Dctn3.1	-0.371115784	0.367	0.368	0.004215289	11
Esco2.10	-0.761732166	0.058	0.196	0.004252734	11
Gcsh.1	0.397528451	0.342	0.14	0.004264273	11
Rcor2.7	-0.740917997	0.067	0.226	0.004539558	11
Cacng7	0.278777095	0.183	0.051	0.004590181	11
Fam58b	0.286296063	0.158	0.04	0.004620303	11
Ctxn1.3	-0.616197943	0.042	0.171	0.004631273	11
Txnl4a	-0.332631846	0.192	0.194	0.004678871	11
Dazap1.2	-0.637143534	0.142	0.273	0.004689368	11
Lgr4	0.259858507	0.108	0.017	0.004744013	11
Ewsr1.2	-0.476314647	0.325	0.392	0.004747324	11
Itpa.1	-0.278564503	0.233	0.21	0.004755673	11
Smarcd1.2	-0.519772739	0.2	0.271	0.004836875	11
Lsm3.6	-0.655798967	0.2	0.359	0.004943105	11
Srrm3.5	-0.726333593	0.117	0.24	0.004970655	11
Txn1.5	-0.383766835	0.533	0.56	0.004986364	11
Bcar1.3	-0.619083228	0.075	0.193	0.004996242	11
Acat1.2	-0.314825758	0.45	0.44	0.005008011	11
Phf14.1	-0.554660296	0.258	0.375	0.005011686	11

Hmgn2.8	-0.664116963	0.108	0.256	0.00506284	11
Gsg1l.8	-0.732779219	0.058	0.256	0.005111983	11
Btf3.2	-0.288383325	0.375	0.349	0.005151527	11
Casc3.1	-0.27792229	0.133	0.129	0.005235583	11
Nap1l4.1	-0.353825101	0.475	0.491	0.005402301	11
Actr1a.1	-0.28924009	0.192	0.177	0.005434848	11
Gltscr2.3	-0.547089761	0.25	0.342	0.005455724	11
Dnmt1.10	-0.738168373	0.1	0.278	0.005561589	11
BC005764.10	-0.799403213	0.042	0.186	0.005654	11
Srpk2.1	-0.35237544	0.433	0.429	0.005719136	11
Pkm.3	-0.281658797	0.458	0.429	0.00577323	11
Rpl13.1	-0.366927992	0.292	0.313	0.005845291	11
Mllt11.9	-0.753392964	0.092	0.219	0.005898681	11
Ndufa3.1	0.328078128	0.75	0.495	0.005941359	11
Polr2h.3	-0.359570675	0.217	0.225	0.006004294	11
Zcrb1.1	-0.400633664	0.342	0.371	0.006022566	11
Med19.2	-0.532675682	0.242	0.317	0.006025548	11
Frrs1l.2	0.362311895	0.4	0.189	0.00607887	11
St18.10	-0.793484842	0.058	0.191	0.006730943	11
Strn3.1	-0.336057514	0.325	0.312	0.006928704	11
Ahsa1	-0.263762055	0.275	0.249	0.007113188	11
Bcl11a.3	-0.669790814	0.033	0.211	0.007150009	11
Psma1.1	-0.429220001	0.392	0.445	0.007166707	11
Fam214b	0.274137379	0.125	0.025	0.007175098	11
Snrpb2.3	-0.554127559	0.267	0.385	0.007224152	11
Utp14a.1	-0.559513466	0.158	0.233	0.00725801	11
Bptf.1	-0.530480808	0.308	0.374	0.007608562	11
Slc16a2	0.282664232	0.2	0.068	0.00761957	11
Sumo1.1	-0.412790412	0.175	0.218	0.00772729	11
Flcn.1	0.333272213	0.158	0.036	0.007729128	11
Ssna1.1	-0.298467548	0.283	0.266	0.007857208	11
Gars.1	-0.430440361	0.192	0.227	0.008054717	11
Pfdn1	0.281401076	0.508	0.297	0.008376448	11
Pds5b.2	-0.505084015	0.208	0.272	0.008437907	11
Hmgn5.8	-0.690156719	0.258	0.425	0.008506856	11
Atp2a2.1	0.275724659	0.392	0.2	0.008799772	11
Nsmce4a.5	-0.535206181	0.133	0.224	0.008878519	11
Purb.2	-0.282623407	0.592	0.549	0.008972436	11
Rpl38.2	-0.430506858	0.342	0.383	0.00901792	11
Mab21l1.8	-0.761675586	0.067	0.226	0.009022884	11
Arid4b.1	-0.676333498	0.275	0.44	0.009030843	11
Calr.3	0.330594369	0.75	0.509	0.009131446	11
Bcap31.1	0.40361393	0.383	0.172	0.009163543	11
Glce.7	-0.723433347	0.025	0.177	0.009291481	11
Psmd12	-0.257746937	0.417	0.372	0.009501161	11
Apoa1bp	0.25569213	0.367	0.196	0.009980132	11
Tmco3.1	0.300201416	0.15	0.034	0.009988504	11
Tmem18.1	0.27413455	0.167	0.045	0.009991697	11

Trpc4ap.9	-0.271513459	0.25	0.197	0.010041162	11
Ank.1	0.508532664	0.2	0.054	0.010106018	11
Tpm3.1	-0.305670048	0.317	0.315	0.010109302	11
Zmynd8.5	-0.650062788	0.1	0.213	0.010190988	11
Acer3	0.422170694	0.192	0.051	0.010215454	11
Denr	-0.50465231	0.258	0.346	0.010485368	11
2410089E03Rik.2	-0.409112456	0.092	0.13	0.010556676	11
Idh2.6	-0.282197376	0.3	0.278	0.010853417	11
Spag5.2	0.44686504	0.225	0.078	0.010866332	11
Snrnp40.5	-0.533524926	0.175	0.271	0.010921922	11
Bpnt1	0.336856305	0.167	0.051	0.010932679	11
Rnaseh2c.6	-0.254248726	0.408	0.381	0.011073176	11
Cep57.6	-0.606837036	0.15	0.258	0.011135035	11
Lpp.1	0.316703725	0.125	0.022	0.011147356	11
Igsf8.5	-0.451393748	0.233	0.269	0.011195853	11
Hes1.9	0.439185012	0.342	0.147	0.011206426	11
Akirin2.2	-0.341746588	0.192	0.197	0.011319087	11
Fam168b.1	-0.427650683	0.208	0.241	0.011328655	11
Fgd6	0.254037564	0.2	0.067	0.011741476	11
Dpysl2.2	-0.373358227	0.242	0.248	0.012052792	11
Dtymk.8	-0.460641648	0.375	0.439	0.012324859	11
Fmnl2.3	0.307321968	0.342	0.156	0.012445971	11
Myod1.8	-0.657427512	0.008	0.142	0.012491331	11
Eef1e1.1	-0.381069799	0.158	0.181	0.01293611	11
Ubap2l.1	-0.544304251	0.217	0.317	0.013078468	11
Bok.7	-0.664786549	0.083	0.252	0.013243191	11
Clip3.6	-0.543343455	0.233	0.312	0.013594356	11
Pdcd4.2	-0.390131828	0.317	0.331	0.013855962	11
Rpl3.2	-0.437736118	0.158	0.211	0.013999949	11
mt-Cytb.2	0.335027026	0.967	0.938	0.014068529	11
Dhx15.2	-0.442592676	0.358	0.417	0.014151773	11
Nmral1.6	-0.612959744	0.05	0.188	0.014324409	11
Cnot6.2	-0.533729796	0.3	0.392	0.014489175	11
Ppic.5	-0.385054556	0.225	0.239	0.01459798	11
Atg3	0.371278593	0.383	0.178	0.015091245	11
Eif3g.2	-0.402941551	0.367	0.402	0.015117145	11
Ten1.2	0.301141221	0.325	0.151	0.015180256	11
Btd	0.327540863	0.117	0.018	0.01539355	11
Rbm4b.1	-0.524717767	0.042	0.141	0.015424296	11
Cyc1.1	0.403257834	0.658	0.41	0.015501539	11
Kras	-0.324459619	0.258	0.251	0.015823935	11
Ift27.4	-0.39377471	0.217	0.242	0.015834858	11
Mrpl21.1	-0.321943569	0.292	0.28	0.015951264	11
4933426M11Rik	0.359416546	0.175	0.046	0.016039249	11
Actr6.1	-0.303545458	0.092	0.107	0.016176307	11
Sidt2	0.334732883	0.167	0.045	0.016527978	11
Baz2b.4	-0.466017639	0.333	0.364	0.016657194	11
Cuedc2.1	-0.350551049	0.367	0.387	0.017614589	11

Psph.1	0.372678931	0.225	0.073	0.017696969	11
Tnrc6a.1	-0.291515589	0.325	0.299	0.017884737	11
Sucla2	0.329289927	0.317	0.146	0.018212442	11
Ivns1abp.2	-0.421854905	0.367	0.415	0.018251326	11
Ppig	-0.444633637	0.442	0.485	0.018368164	11
1110004F10Rik.1	-0.473732246	0.35	0.423	0.018628635	11
Spcs2.3	0.312920177	0.667	0.422	0.018685715	11
Naa38.5	-0.381926632	0.275	0.311	0.018926287	11
Mphosph9.1	-0.595328168	0.025	0.148	0.019125689	11
Chd8	-0.464174506	0.15	0.201	0.019285336	11
Uchl1.9	-0.577813006	0.242	0.335	0.019531087	11
Dtl.7	-0.595163879	0.017	0.146	0.019761783	11
Cadm1.4	-0.599698119	0.225	0.391	0.019902299	11
Sult4a1.2	-0.686289107	0.067	0.223	0.019993571	11
Olfm1.3	-0.623043755	0.108	0.199	0.020341689	11
Rnf165.5	-0.670029721	0.033	0.173	0.020365313	11
Ift81.1	-0.342707747	0.133	0.146	0.02037375	11
Adipor1.1	0.297487683	0.308	0.139	0.020485379	11
Slc22a17.6	0.337045925	0.45	0.249	0.020732206	11
Epb4.1.4	-0.684705409	0.083	0.23	0.020909048	11
Cdk11b.1	-0.413388597	0.433	0.511	0.021016181	11
Nptn.3	0.372922423	0.392	0.186	0.021378418	11
Il11ra1.1	0.312290109	0.183	0.057	0.021770417	11
Lsm2.4	-0.378638011	0.175	0.23	0.021869537	11
Tacc2.7	-0.671455245	0.008	0.141	0.021966638	11
Ylpm1	-0.40149733	0.225	0.241	0.022084704	11
Ppp1r14c.9	-0.425374229	0.267	0.262	0.022108003	11
Uqcrfs1.1	0.264384073	0.608	0.386	0.022591537	11
Larp7.5	-0.633447378	0.175	0.312	0.022925791	11
Snx13	0.257312223	0.158	0.044	0.02308936	11
Gm10260.3	-0.427147118	0.183	0.228	0.023689401	11
Nhsl1.1	0.358521999	0.183	0.056	0.023861187	11
Cpsf2	-0.326019369	0.242	0.236	0.024138275	11
Rpl29.2	-0.309557094	0.225	0.258	0.024456591	11
Ypel1.2	-0.629700406	0.042	0.175	0.024608349	11
Hook3.3	-0.332709402	0.317	0.299	0.024897396	11
Nsd1.1	-0.396161732	0.325	0.339	0.025352464	11
Rab33b	0.262756377	0.15	0.038	0.025795562	11
Kmt2a.1	-0.569340768	0.242	0.33	0.026190999	11
Tnrc6c.2	-0.503776163	0.125	0.178	0.026206879	11
Fhl1.1	0.253675837	0.25	0.104	0.02654968	11
Myl12a.5	-0.279195799	0.358	0.328	0.027018181	11
Myt1l.10	-0.771759744	0.017	0.162	0.027631479	11
Arl6ip4.1	-0.251964006	0.275	0.236	0.027849122	11
Sept7.2	0.284739023	0.817	0.587	0.028048529	11
Ilf3.1	-0.616858884	0.208	0.377	0.028137057	11
Eif4g1.1	-0.291171132	0.367	0.351	0.02823484	11
Smpd3.5	-0.619899565	0.05	0.162	0.029195516	11

Rif1.6	-0.555956172	0.192	0.276	0.029301884	11
Carhsp1.4	-0.495820655	0.192	0.254	0.029502345	11
Rbm26.1	-0.502585279	0.225	0.297	0.02995493	11
Tprkb	0.273517839	0.283	0.125	0.031147141	11
Nup85.3	-0.563590995	0.117	0.226	0.031239663	11
Bzw1.2	-0.312659299	0.367	0.359	0.031679122	11
Nup62.5	-0.577322542	0.042	0.172	0.031933447	11
Ttc9b.2	-0.648420583	0.033	0.195	0.032171833	11
Rb1cc1.3	-0.333142764	0.25	0.234	0.032334386	11
Trim28.3	-0.644013466	0.183	0.362	0.032410026	11
Ezr.6	-0.35667774	0.442	0.437	0.032745193	11
Atp5d	0.394869131	0.783	0.583	0.032812448	11
Ska2.9	-0.591755825	0.092	0.205	0.032902817	11
A930011O12Rik.10	-0.667145979	0	0.115	0.032964411	11
Sh3gl2.5	-0.441354457	0.142	0.171	0.0339592	11
Cbx3.1	-0.535907784	0.167	0.274	0.03418363	11
Tmem57.6	-0.669098093	0.125	0.292	0.034604073	11
Csrp2.8	-0.550353673	0.042	0.158	0.03517516	11
Gtpbp4.1	-0.544783578	0.2	0.282	0.03560633	11
Smdt1.2	0.38197934	0.592	0.353	0.037014337	11
Senp6.1	-0.380196905	0.383	0.389	0.037128692	11
Cd320	0.257682877	0.225	0.085	0.037192996	11
Mki67ip.2	-0.473343362	0.192	0.244	0.037694718	11
Ube2c.9	-0.857755064	0.183	0.269	0.038320775	11
Pfdn2	-0.582020335	0.183	0.31	0.039881273	11
Mpv17l2	0.34691295	0.208	0.071	0.040184455	11
Rpf2.2	-0.560948952	0.1	0.202	0.040534775	11
Tra2a.1	-0.406400195	0.317	0.392	0.040612965	11
Sobp.3	-0.402359615	0.1	0.128	0.040951358	11
Mpc2.1	0.256410621	0.508	0.296	0.040999858	11
Sf3a3.2	-0.6802059	0.108	0.282	0.04141576	11
Golim4.2	-0.476229807	0.183	0.232	0.041426556	11
Hspa4	-0.347871762	0.35	0.368	0.042549083	11
Orc6.2	-0.548552211	0.108	0.201	0.043464939	11
5430416N02Rik.1	-0.599612172	0.008	0.141	0.043517368	11
Ube2a	0.397575217	0.35	0.161	0.045293672	11
Sema7a.6	-0.610330678	0.017	0.156	0.045770678	11
Clspn.10	-0.728411151	0.042	0.19	0.045854171	11
Utp3.3	-0.527006021	0.225	0.315	0.045858422	11
Usp3.1	-0.552728634	0.033	0.142	0.047134247	11
B230219D22Rik	-0.373032858	0.3	0.321	0.047378837	11
Dcakd.3	-0.5868178	0.167	0.297	0.047476296	11
Zic5.3	-0.449876441	0.117	0.161	0.0479579	11
Gspt1.1	-0.282134001	0.292	0.277	0.048115316	11
Dhrs3.1	0.253048204	0.108	0.021	0.048604204	11
Crnkl1	-0.475397472	0.117	0.186	0.050745383	11
Myh10.2	-0.681358078	0.133	0.325	0.0508701	11
Ank2.10	0.280933183	0.442	0.238	0.051279772	11

Bub3.7	-0.380900495	0.25	0.321	0.051323568	11
Tbpl1.1	-0.520043467	0.1	0.187	0.051408923	11
Srsf9.2	-0.257151067	0.258	0.236	0.0516775	11
Slc17a6.10	-0.665590193	0.042	0.148	0.052141839	11
Rfc3.5	-0.588693547	0.092	0.205	0.052762053	11
Pole3.5	-0.404069309	0.217	0.25	0.053179227	11
Dnajc21.2	-0.657994723	0.092	0.21	0.053419948	11
Rpl18.3	-0.517350217	0.2	0.305	0.054103517	11
Dnajc2.4	-0.598326064	0.208	0.329	0.05416385	11
Wapal.1	-0.433772661	0.225	0.27	0.054653376	11
Usp7.1	-0.480155406	0.2	0.264	0.055445103	11
Cenpm.8	-0.514113543	0.067	0.162	0.055577923	11
Prdx2.1	-0.277663638	0.708	0.679	0.055587382	11
Mdga1.3	-0.596371244	0.008	0.136	0.056412611	11
Fam50a	-0.298374973	0.25	0.239	0.057254136	11
Trappc4.1	-0.265401499	0.25	0.224	0.05741081	11
Nemf.2	-0.515231915	0.275	0.339	0.05851116	11
Gm561	0.2512162	0.242	0.112	0.059638947	11
Peg3.2	-0.666774697	0.158	0.285	0.060000224	11
Fez1.2	0.270302916	0.533	0.331	0.060788017	11
Wbp11.1	-0.396437043	0.25	0.296	0.06120057	11
Cln3.1	0.337908026	0.142	0.035	0.062681085	11
Plscr1	0.284449596	0.125	0.024	0.06327579	11
Hnrnpa3	-0.469477734	0.075	0.159	0.063891149	11
Smarca2.2	-0.368745738	0.267	0.285	0.064102373	11
Twf1.2	0.251387724	0.283	0.135	0.064476423	11
Mllt10.2	-0.337987779	0.292	0.286	0.064557129	11
Eif5a.3	-0.319651169	0.392	0.403	0.066852348	11
Rps18.4	-0.485600596	0.333	0.429	0.066886625	11
Fopnl.1	-0.355454621	0.183	0.202	0.067010131	11
Nop14.1	-0.309740984	0.233	0.217	0.067034594	11
Mfap4.6	-0.717722534	0.033	0.162	0.067721482	11
Snrnp25	-0.273670298	0.125	0.126	0.068031686	11
Ep400.1	-0.441460133	0.125	0.18	0.069462165	11
Slc3a2.6	0.260740004	0.533	0.321	0.070177386	11
Mycbp2.5	-0.392979216	0.342	0.345	0.071651547	11
Ppp1r7	-0.437755649	0.15	0.198	0.073391788	11
Rnf187.1	-0.416963698	0.333	0.421	0.073688502	11
Cep170.5	-0.679007453	0.158	0.306	0.074041363	11
Jakmip2.3	-0.303045609	0.167	0.156	0.074431137	11
Wwp1.2	0.316626863	0.225	0.083	0.075626416	11
Zfp318.2	-0.342326877	0.175	0.175	0.075976183	11
Polr2c.1	-0.418513784	0.15	0.202	0.076147226	11
Nfkbia.2	0.317384024	0.35	0.17	0.077821791	11
Rsbn1l.1	-0.413175474	0.283	0.312	0.078124469	11
Psmd14.1	-0.285703826	0.35	0.35	0.078586371	11
Dnajc8.2	-0.259882338	0.458	0.434	0.079239968	11
Coro2b.2	-0.364927274	0.15	0.173	0.080371833	11

Lap3.7	-0.47421332	0.225	0.276	0.080601648	11
Calm1.4	-0.300327124	0.883	0.861	0.08372537	11
Safb2.1	-0.562098698	0.217	0.314	0.083806216	11
Prdm8.9	-0.605748939	0.025	0.127	0.084137036	11
Dhx36.1	-0.366456162	0.358	0.37	0.085218417	11
Polr2f.3	-0.326348713	0.358	0.375	0.085233703	11
Myo10	0.464330764	0.275	0.12	0.086096053	11
Tsg101.1	-0.325227166	0.258	0.264	0.086659691	11
Cops2	-0.333817112	0.217	0.226	0.088235789	11
Rps16.2	-0.408667996	0.167	0.209	0.08922314	11
Abr	0.327936841	0.242	0.098	0.089721808	11
Gtf2f1.1	-0.529517307	0.25	0.344	0.090314528	11
Stau2.4	-0.402256208	0.117	0.15	0.090650578	11
Nop16.2	-0.467301945	0.1	0.174	0.090965186	11
Zfml.1	-0.440961291	0.275	0.312	0.091644793	11
Rpa2.7	-0.583637157	0.092	0.2	0.091682569	11
Tmem178.3	-0.641577262	0.033	0.187	0.092972981	11
Ikzf5.1	-0.342906666	0.117	0.144	0.09352893	11
Gm26735.6	-0.567752559	0.092	0.181	0.094355543	11
Tubgcp2	-0.296774571	0.092	0.117	0.094961073	11
Mrpl30.1	-0.373270804	0.283	0.308	0.095462028	11
Nhlh1.8	-0.781144588	0.067	0.224	0.096434747	11
Mcm2.8	-0.589245708	0.1	0.205	0.099411035	11
H2afz.9	-0.442614403	0.15	0.231	0.101313856	11
Dstn	-0.40896432	0.275	0.331	0.102689679	11
Zfp704.3	-0.565785558	0.15	0.258	0.10403707	11
Eif3l	-0.326867245	0.283	0.302	0.104603278	11
Pde7a	-0.379249773	0.067	0.112	0.105111096	11
Tcf3.3	-0.570039176	0.108	0.226	0.106289154	11
Tubb2b.9	-0.365701432	0.467	0.464	0.108581611	11
Tmem192	0.269215508	0.125	0.031	0.111524259	11
2610001J05Rik.2	0.257239057	0.367	0.192	0.111669357	11
Mapk10.3	0.349405043	0.242	0.097	0.117707319	11
Gm5617.1	0.461331794	0.183	0.059	0.119691941	11
Mcl1.1	-0.253564578	0.233	0.211	0.120054427	11
Sacs.3	-0.59872396	0.083	0.191	0.120770431	11
Mgrn1	0.307764667	0.175	0.053	0.120833683	11
Rbbp6.2	-0.505130278	0.342	0.433	0.121580202	11
Dnaja1.1	-0.331363303	0.367	0.374	0.123870561	11
Pdzrn4.6	-0.390140671	0.125	0.146	0.124766142	11
Smap1.2	0.254648606	0.4	0.222	0.126368207	11
Ccng2.6	-0.613916725	0.083	0.212	0.129349311	11
Nt5dc2.5	-0.635056089	0.1	0.238	0.130269193	11
Ptprs.8	-0.33285677	0.458	0.42	0.130775215	11
Mapk8ip1.9	-0.453402081	0.183	0.227	0.131388585	11
Phf20l1.6	-0.596904877	0.225	0.34	0.131402019	11
Mak16.3	-0.561480408	0.125	0.225	0.133177458	11
Nsg1.8	-0.646619448	0.142	0.288	0.135448372	11

Chrna3.9	-0.606147553	0.017	0.119	0.141545437	11
2900011O08Rik.8	-0.545370729	0	0.104	0.143639396	11
Sep15.4	0.374069986	0.725	0.503	0.147059458	11
Smarcd3	0.27659505	0.183	0.061	0.147858088	11
Nt5c3.1	-0.361482622	0.117	0.149	0.149342115	11
Fam155a.2	0.375638884	0.275	0.114	0.149531613	11
Trim2.2	-0.401847972	0.267	0.282	0.149645983	11
Nadk2	0.341489976	0.142	0.033	0.151154969	11
Rpl30.2	-0.401314459	0.217	0.269	0.151955965	11
Prc1.10	-0.780978528	0.133	0.257	0.158868975	11
Chd1.1	-0.53868544	0.125	0.209	0.158970436	11
Eif4e3.5	-0.54310103	0.033	0.131	0.158992825	11
Smek1.1	-0.401943348	0.133	0.179	0.160477235	11
Cpsf6.1	-0.389290711	0.258	0.291	0.160963095	11
Snrpa1.4	-0.471705715	0.258	0.331	0.161926046	11
Hibch	0.322733585	0.142	0.033	0.162895174	11
Txndc9.1	-0.282513417	0.217	0.211	0.163577758	11
Reln.8	-0.700149654	0.042	0.181	0.164283104	11
Nin.5	-0.501094655	0.017	0.147	0.166647798	11
Hars	-0.371342847	0.183	0.211	0.167773633	11
Ralgps2.3	-0.62813445	0.117	0.246	0.168563364	11
R3hdm1.1	-0.641891415	0.142	0.298	0.16892999	11
Bcas1.4	-0.715492354	0.15	0.283	0.169143279	11
Mapre1.1	-0.289990574	0.525	0.517	0.169789683	11
Srsf4.2	-0.366213743	0.233	0.279	0.173234311	11
Pgrmc1.1	0.319102904	0.508	0.293	0.178469271	11
Msantd3	-0.250890518	0.108	0.111	0.178484117	11
Nrm.4	-0.501233914	0.075	0.17	0.181545977	11
Birc6	-0.25958611	0.183	0.168	0.181632095	11
Stag1.1	-0.566388583	0.1	0.207	0.182100356	11
Nicn1.4	-0.395009173	0.183	0.208	0.18300649	11
Rbms1.2	-0.483981017	0.15	0.216	0.191781006	11
Imp3.3	-0.447372866	0.183	0.247	0.196381566	11
Prox1.2	-0.66287721	0.075	0.23	0.197228991	11
Lpcat3.1	0.389921364	0.158	0.042	0.197597283	11
Ercc5.1	-0.286937863	0.108	0.112	0.201169778	11
Smg6	-0.454505103	0.058	0.126	0.201628709	11
Vps29.1	0.360622923	0.425	0.224	0.202331452	11
Tsc22d1.5	-0.52058232	0.308	0.428	0.208933971	11
Spc24.9	-0.672212164	0.092	0.243	0.211560612	11
Kcnd2.2	0.284603157	0.108	0.025	0.217037825	11
Dnttip2.2	-0.484138609	0.258	0.325	0.223299975	11
Bnip3l.1	-0.331020102	0.15	0.163	0.224932405	11
Polr1c.4	-0.443358386	0.158	0.218	0.226910113	11
Ccnl1.1	-0.413526942	0.317	0.368	0.230467705	11
Homer2.4	-0.56817354	0.108	0.247	0.230654258	11
Psmc6.1	-0.386906912	0.3	0.34	0.23129595	11
Vps4b	-0.315011908	0.158	0.171	0.236589283	11

Tbl1x.2	-0.451749986	0.267	0.328	0.243691037	11
Arf1	-0.336462472	0.3	0.317	0.245991122	11
Rpl35.1	-0.438968332	0.167	0.234	0.246123302	11
Sae1.6	-0.429670284	0.217	0.279	0.247531994	11
Pcif1	-0.260298159	0.208	0.196	0.249490616	11
Kif20b.10	-0.649339698	0.075	0.191	0.250877439	11
Fam3a	0.363410555	0.167	0.05	0.253417065	11
Tspan6.3	0.289400181	0.433	0.244	0.253589673	11
Sbno1.1	-0.611435083	0.217	0.352	0.25479515	11
Mcm5.6	-0.531740339	0.067	0.169	0.264173577	11
Zfr.2	-0.363888457	0.342	0.353	0.265179212	11
Ash2l.1	-0.442393648	0.158	0.215	0.267857771	11
Fut8.1	-0.362617617	0.2	0.227	0.270868128	11
Tma7	-0.255570376	0.183	0.174	0.272271768	11
D19Bwg1357e.3	-0.380248418	0.217	0.273	0.274223694	11
Wdr89.3	-0.310186795	0.192	0.214	0.280101276	11
Zfp637.1	-0.350843335	0.233	0.256	0.281762756	11
Lgals1.7	-0.295558481	0.267	0.228	0.282099279	11
Lasp1	0.263457283	0.217	0.09	0.283979758	11
Zfp207.1	-0.333311475	0.342	0.37	0.284296036	11
Eml3	0.26884106	0.133	0.04	0.285243025	11
Atp2c1	0.251166671	0.2	0.075	0.285762878	11
Zfp266.1	-0.455331253	0.1	0.168	0.291017928	11
Mex3b.2	-0.482537432	0.033	0.116	0.295448812	11
Polr2e.1	-0.274617918	0.333	0.324	0.296181916	11
Frmd4a.5	-0.582357474	0.2	0.309	0.296994374	11
App.7	0.360730706	0.775	0.566	0.298309343	11
Eif3d.2	-0.284087069	0.417	0.405	0.299926486	11
Cplx1.7	-0.583439984	0.017	0.139	0.301351117	11
Rab8a	-0.312066316	0.158	0.171	0.304317328	11
Cadm3.10	-0.662964512	0.025	0.141	0.30494107	11
Cox6a1	0.325407118	0.833	0.76	0.306301227	11
Uhrf1.9	-0.543866185	0.025	0.144	0.309512668	11
Tvp23b	0.258893927	0.15	0.043	0.310054193	11
Arid4a.1	-0.535537446	0.217	0.283	0.311544267	11
Ppa1.3	-0.334809652	0.183	0.221	0.315549016	11
A030009H04Rik.3	-0.46303884	0.025	0.114	0.318883478	11
Epb4.1l3.2	-0.472269466	0.017	0.114	0.324445263	11
Echdc1	0.316200214	0.133	0.037	0.330728787	11
2810004N23Rik.4	-0.44219785	0.217	0.262	0.332623182	11
Znhit6.2	-0.494826571	0.025	0.12	0.333514758	11
Pigk.1	0.317800707	0.158	0.045	0.334762883	11
Ndufb2.1	0.254743058	0.575	0.366	0.336354495	11
Elovl4.2	-0.255416485	0.108	0.11	0.340315408	11
Setd8.2	-0.592463205	0.133	0.262	0.341630052	11
Ola1.1	-0.269261867	0.242	0.228	0.342190168	11
Rabggta	0.271501318	0.117	0.025	0.34754791	11
Glrx3	-0.303813494	0.233	0.247	0.351090039	11

Smek2.1	-0.354566441	0.208	0.222	0.351116031	11
Ivd	0.444850021	0.242	0.107	0.352449002	11
Luc7l.1	-0.597691221	0.208	0.361	0.352699169	11
Hdgfrp2	-0.259210839	0.225	0.214	0.357576617	11
Cox6b1	0.308035131	0.883	0.74	0.358737777	11
Lyar.8	-0.518757014	0.2	0.286	0.36499312	11
Usp34.1	-0.302459093	0.3	0.29	0.365073954	11
Smap2.1	-0.252370477	0.133	0.122	0.373595459	11
Gnl3.6	-0.548944059	0.175	0.296	0.382579326	11
Ube3a.1	-0.460146168	0.283	0.35	0.384413054	11
Ncbp2	-0.270177007	0.2	0.194	0.385054424	11
Mrpl24	-0.322891133	0.2	0.211	0.388502361	11
Zkscan1.1	-0.436280105	0.1	0.151	0.38952272	11
Eif2b3	-0.417232808	0.117	0.159	0.39038724	11
Arf4.1	-0.284060285	0.4	0.403	0.395027809	11
Ybx3.6	-0.530436947	0.183	0.29	0.416317618	11
Exosc1.2	-0.392870398	0.117	0.163	0.420383404	11
Ddx50	-0.433777862	0.1	0.162	0.423082939	11
Dzip3.1	-0.343503721	0.15	0.167	0.425686328	11
Phf3.2	-0.383382181	0.25	0.27	0.425752912	11
Casp3.3	-0.51069519	0.142	0.234	0.430776015	11
Frg1.2	-0.371465957	0.275	0.298	0.432070688	11
Tfdp2.2	-0.513083214	0.075	0.161	0.437680774	11
Ube2g2.1	-0.345547618	0.092	0.124	0.443571954	11
Arxes1.1	0.275706801	0.183	0.063	0.445831984	11
Stx6.1	-0.523338915	0.058	0.161	0.450864338	11
Eapp	-0.332320004	0.242	0.245	0.45992509	11
Armc10.2	-0.453678025	0.017	0.11	0.464945032	11
Slc38a1.2	0.346042511	0.467	0.268	0.466629984	11
Rnf220.2	-0.486443351	0.15	0.235	0.466673578	11
Whsc1l1	-0.360049972	0.125	0.155	0.47012547	11
Gnl3l.2	-0.483191369	0.117	0.189	0.47626066	11
Ptrh2	-0.336498767	0.133	0.153	0.47648617	11
Iws1.1	-0.409518554	0.175	0.215	0.48116118	11
Rfc4.9	-0.576168457	0.1	0.227	0.483424108	11
Ubp1.1	-0.379316285	0.133	0.169	0.484336473	11
Cnot1	-0.400815807	0.042	0.111	0.484908757	11
Raf1.1	-0.408087446	0.142	0.186	0.487498606	11
Polr2a	-0.457740145	0.108	0.176	0.492969118	11
Rab6b.10	-0.502818108	0.142	0.202	0.498207773	11
Ube2d3	-0.264440452	0.275	0.267	0.499604157	11
Ubn1	-0.469644034	0.142	0.204	0.501269923	11
Gamt.6	-0.266625928	0.183	0.169	0.510701792	11
Ap3b2.2	-0.591693466	0.075	0.203	0.510931832	11
Mapk1	-0.277101948	0.25	0.241	0.512144299	11
Dnal4.1	-0.423445991	0.033	0.11	0.520715641	11
Kdm1a.3	-0.45581072	0.258	0.365	0.529432148	11
Cdc7.3	-0.465628688	0.092	0.168	0.5357415	11

Eif4b.1	-0.572810028	0.15	0.276	0.53722765	11
Cox7a2l	-0.305824989	0.375	0.382	0.539030258	11
Cflar.1	0.274244607	0.158	0.049	0.543342528	11
Pbdc1.4	-0.461740818	0.133	0.206	0.557792952	11
Rbm5.5	-0.278078864	0.408	0.401	0.564137371	11
Rock1	-0.37945442	0.275	0.338	0.566159156	11
Sh3bgrl.3	-0.414459623	0.275	0.337	0.569121068	11
Tex14.7	-0.675489851	0.025	0.146	0.582404102	11
Brix1.2	-0.270458176	0.208	0.205	0.585411279	11
Kif15.10	-0.577690177	0.075	0.181	0.586613746	11
Hprt.5	-0.509949518	0.083	0.188	0.598307602	11
Palm.2	-0.525704006	0.033	0.14	0.601423931	11
Mapt.10	-0.516604339	0.175	0.216	0.607499298	11
Mbtd1.1	-0.384932621	0.183	0.215	0.609166582	11
Cyb561d2	0.269553388	0.117	0.03	0.61186356	11
Tomm20	-0.272023869	0.158	0.161	0.620714111	11
Fam92a.1	-0.326636494	0.183	0.198	0.624282846	11
Cenpj.6	-0.539296182	0.042	0.142	0.625159566	11
1110038F14Rik.1	-0.305042642	0.183	0.191	0.63777051	11
Las1l.1	-0.528397449	0.158	0.255	0.639271017	11
Pafah1b2.2	-0.361189332	0.217	0.255	0.640364771	11
Ppie	-0.40269515	0.075	0.134	0.642766155	11
Polr2i.3	-0.250976509	0.333	0.316	0.645059267	11
Mtus1.1	-0.512002777	0.025	0.127	0.650225997	11
Flna.1	-0.388337996	0.117	0.152	0.657100169	11
Ift74.4	-0.343106107	0.217	0.224	0.660612869	11
Spag7	-0.323011783	0.267	0.276	0.661753021	11
Tax1bp1.2	-0.285768198	0.6	0.577	0.667060921	11
Naa10.3	-0.285237956	0.25	0.249	0.673102533	11
Prpf6.1	-0.419963102	0.183	0.232	0.673723565	11
Atp5o.1	0.321138034	0.783	0.585	0.686179484	11
Ehbp1.1	-0.376552696	0.1	0.136	0.702698594	11
Mrps5.1	-0.411198817	0.225	0.272	0.704595588	11
Dync1h1	-0.252697046	0.175	0.158	0.708805414	11
Islr2.6	-0.604626775	0.05	0.18	0.708924184	11
Xpo7	-0.357129053	0.083	0.131	0.715570342	11
Pin1	-0.453643808	0.158	0.238	0.731511317	11
Cnot7.1	-0.32365182	0.2	0.216	0.738024754	11
Otx2.3	-0.622687767	0.083	0.217	0.751226032	11
Plrg1	-0.328301647	0.1	0.131	0.755233136	11
Fzd2.4	-0.540612074	0.042	0.161	0.756041165	11
Fau	-0.320200441	0.092	0.123	0.760685834	11
Ppp3cb.2	-0.368670694	0.283	0.31	0.76321483	11
Fam57b.5	-0.574907682	0.042	0.186	0.768623161	11
Epc2.1	-0.580668238	0.133	0.25	0.769524037	11
Naa15.1	-0.277700474	0.383	0.391	0.78273546	11
Mllt4.4	-0.48097143	0.292	0.363	0.792483746	11
1700020I14Rik.2	-0.550828352	0.167	0.275	0.794092937	11

Gjc1.5	-0.365477201	0.158	0.196	0.797591036	11
Bod1l.2	-0.534177827	0.258	0.379	0.800665587	11
Racgap1.11	-0.569547455	0.142	0.256	0.805686702	11
Tro.3	-0.473252356	0.05	0.126	0.81046764	11
Cdc37	-0.366859183	0.258	0.291	0.82426199	11
Tlk2	-0.284153199	0.225	0.212	0.831405642	11
Prpf31	-0.413075209	0.125	0.2	0.85469659	11
Rcn1.3	-0.259023132	0.233	0.233	0.864401791	11
2810474O19Rik.2	-0.552154839	0.15	0.241	0.871848591	11
Cnot3	-0.35401054	0.117	0.156	0.873969043	11
Mpnd.1	-0.291956091	0.167	0.171	0.891856579	11
Sox11.4	-0.463869958	0.025	0.115	0.898463491	11
Stip1.1	-0.280851299	0.283	0.303	0.904740335	11
Cdc20.10	-0.704385991	0.083	0.197	0.907385968	11
Mycl.3	-0.480610884	0.042	0.137	0.927771819	11
Dlgap4.5	-0.334136644	0.175	0.187	0.939470619	11
Uba52.3	-0.303584022	0.308	0.316	0.962639596	11
Peli2.2	-0.401875778	0.125	0.165	0.962814238	11
Tacc3.10	-0.48468061	0.133	0.207	0.971451016	11
Slc25a5.5	0.361816689	0.708	0.507	1	11
E2f1.4	-0.515401125	0.05	0.147	1	11
Slc39a6.1	-0.366034993	0.167	0.199	1	11
Ube2r2	-0.438637111	0.25	0.317	1	11
Hectd1	-0.260049158	0.217	0.202	1	11
Timm50.1	-0.344219838	0.208	0.236	1	11
Foxp1.2	-0.578338347	0.05	0.172	1	11
Hsph1.2	-0.402875386	0.183	0.227	1	11
Zfp664.1	-0.347497499	0.2	0.229	1	11
Gpbp1.2	-0.507669374	0.242	0.357	1	11
Mrpl35	-0.304277142	0.133	0.147	1	11
Ddx27	-0.500491382	0.117	0.202	1	11
Rrm1.10	-0.41623016	0.208	0.26	1	11
Abhd17b	0.332088393	0.3	0.142	1	11
Gm17322.8	-0.601152199	0.025	0.132	1	11
Ankrd10	-0.469996957	0.083	0.159	1	11
Strbp.6	-0.559319483	0.217	0.348	1	11
Trp53i11.3	-0.536824892	0.1	0.202	1	11
Mphosph10.5	-0.487413332	0.158	0.231	1	11
Lmo4.5	-0.354458801	0.292	0.335	1	11
Klhl7.3	-0.435084428	0.1	0.165	1	11
Mrpl52.3	-0.29867204	0.425	0.432	1	11
Smchd1.3	-0.570062819	0.15	0.274	1	11
Dhx32.5	-0.3476914	0.175	0.2	1	11
Sde2	-0.318054107	0.125	0.146	1	11
Copa	-0.34819589	0.142	0.176	1	11
Txlna	-0.393838436	0.142	0.186	1	11
Rnf219	-0.389406115	0.092	0.14	1	11
Wbp4.1	-0.27335109	0.292	0.275	1	11

Dusp1.3	0.29586324	0.275	0.162	1 11
Tomm70a.1	-0.362853826	0.2	0.229	1 11
Ahsa2.1	-0.404859251	0.158	0.207	1 11
2310022B05Rik.4	-0.289215127	0.242	0.248	1 11
Ccni.1	-0.361591591	0.183	0.222	1 11
Brd7.6	-0.342631627	0.283	0.308	1 11
Taf1d.3	-0.545741867	0.225	0.342	1 11
Dpy30.4	-0.252679296	0.3	0.298	1 11
Hes6.8	-0.365398595	0.208	0.236	1 11
Atp11c	-0.466245507	0.033	0.12	1 11
Gnb1	-0.334512758	0.35	0.37	1 11
Ccna2.10	-0.531818656	0.1	0.222	1 11
Akap12.6	-0.311430196	0.108	0.111	1 11
Heatr6	-0.264335101	0.108	0.117	1 11
Commd3	-0.296432667	0.3	0.307	1 11
Phactr1.4	-0.351638438	0.167	0.183	1 11
Etf1.1	-0.290497291	0.258	0.261	1 11
Cdc26.1	-0.36326864	0.1	0.144	1 11
Sfswap.1	-0.259323193	0.25	0.239	1 11
Nup88	-0.374452546	0.142	0.183	1 11
Taf15.1	-0.490536562	0.125	0.223	1 11
Pcsk2.1	-0.452876064	0.008	0.102	1 11
Ftsj3.3	-0.483970094	0.125	0.206	1 11
Atad2.8	-0.587171459	0.075	0.199	1 11
2410004N09Rik.1	-0.432301773	0.125	0.184	1 11
Tyms.8	-0.585710012	0.1	0.219	1 11
Tipin.8	-0.506967114	0.167	0.255	1 11
Kti12	-0.382681765	0.033	0.102	1 11
Xrcc1	-0.338747959	0.067	0.11	1 11
Fam114a2	-0.29881849	0.142	0.155	1 11
Zfpl1	-0.254875107	0.092	0.101	1 11
Fbxo5.9	-0.536372088	0.05	0.16	1 11
Exosc7.4	-0.341626834	0.15	0.18	1 11
Nefm.3	-0.5852728	0.008	0.117	1 11
Cdca7.7	-0.58301471	0.067	0.198	1 11
Rbmxl1.1	-0.491765777	0.042	0.153	1 11
Zfp622	-0.297690521	0.125	0.137	1 11
Sfrs18.5	-0.295662697	0.733	0.777	1 11
4833420G17Rik	-0.442023478	0.083	0.15	1 11
Plp1.10	-0.417424429	0.217	0.113	1 11
Cyp7b1	0.31795179	0.108	0.023	1 11
Cdk6.5	-0.58572526	0.092	0.244	1 11
Cdh4.3	0.407331663	0.3	0.144	1 11
Hspa9.2	-0.263754294	0.375	0.372	1 11
Gigyf2	-0.392180576	0.192	0.223	1 11
Snhg6.1	-0.25721888	0.267	0.258	1 11
Dst.1	-0.296469213	0.275	0.266	1 11
Sart1	-0.300199462	0.192	0.198	1 11

Mroh2a.7	-0.599719133	0.017	0.114	1	11
Etv1	0.322487505	0.117	0.027	1	11
D4Wsu53e.7	-0.277767275	0.458	0.446	1	11
Gart.3	-0.394935294	0.108	0.166	1	11
Dnajc1.1	0.399693398	0.292	0.138	1	11
Ifitm2.4	0.263362858	0.242	0.113	1	11
Kdm5a.2	-0.252983101	0.258	0.256	1	11
St3gal5.1	-0.386650316	0.158	0.194	1	11
1700025G04Rik.6	-0.545693574	0.15	0.264	1	11
Sf1.1	-0.485720273	0.142	0.237	1	11
R3hcc1.2	-0.425791651	0.1	0.16	1	11
Mthfd1.3	0.33601221	0.258	0.115	1	11
Nrd1	-0.275151155	0.2	0.221	1	11
Bub1b.9	-0.384516985	0.025	0.103	1	11
Pygo1.5	-0.298302747	0.2	0.192	1	11
Brwd3	-0.363763749	0.05	0.101	1	11
Smoc1.4	-0.465472885	0.025	0.135	1	11
Sc4mol.1	0.399893237	0.225	0.091	1	11
BC004004.1	0.292758663	0.25	0.121	1	11
Nol4.5	-0.565704155	0.058	0.172	1	11
Mis18bp1.10	-0.580071885	0.05	0.161	1	11
Ing3	-0.389170472	0.042	0.107	1	11
Nipsnap1.2	-0.406559407	0.083	0.136	1	11
Klhdc2.2	-0.48877438	0.208	0.327	1	11
Apbb1.9	-0.325974857	0.158	0.17	1	11
Rnf146.1	-0.302160299	0.125	0.143	1	11
Sp3	-0.390847346	0.108	0.163	1	11
Boc.7	-0.529550586	0.05	0.156	1	11
Spop.4	-0.272090879	0.308	0.315	1	11
Vars.3	-0.330373491	0.133	0.166	1	11
Wdr12.2	-0.326747291	0.15	0.175	1	11
Cenph.10	-0.556852488	0.092	0.194	1	11
Gm11541.4	-0.492201919	0.033	0.128	1	11
Rsrc1	-0.502438965	0.158	0.251	1	11
Cdc42se2.2	-0.462468836	0.142	0.239	1	11
Rnf2	-0.281096237	0.1	0.12	1	11
Exosc8.4	-0.401803392	0.15	0.21	1	11
Mkrn1.4	-0.322013338	0.192	0.208	1	11
Nusap1.10	-0.668799096	0.092	0.228	1	11
Prdm2.1	-0.457837488	0.05	0.132	1	11
Wdr43.1	-0.487521713	0.133	0.242	1	11
Nol12.1	-0.266227343	0.117	0.128	1	11
Hmmr.10	-0.683857486	0.058	0.177	1	11
Mapk8ip2.3	-0.513533026	0.025	0.143	1	11
Atad5.6	-0.514051744	0.05	0.149	1	11
Otud4	-0.297316736	0.092	0.115	1	11
Ttc9c	-0.413798849	0.025	0.109	1	11
Pcf11.2	-0.322736737	0.158	0.179	1	11

Cript	-0.279935126	0.242	0.244	1	11
Rangap1.10	-0.36739583	0.2	0.256	1	11
Ppil4.1	-0.467581781	0.158	0.25	1	11
Adam10.2	-0.295453013	0.192	0.203	1	11
Sowaha.8	-0.650939549	0.042	0.163	1	11
Mapk8	-0.283529889	0.117	0.128	1	11
Syne2.2	-0.561964362	0.108	0.22	1	11
Cenpb.1	-0.376206014	0.167	0.209	1	11
Atp6v0a2	0.250139054	0.108	0.026	1	11
Yeats4	-0.416923217	0.2	0.264	1	11
Nfic.1	-0.32852717	0.242	0.261	1	11
Glyr1	-0.504019594	0.167	0.284	1	11
Akap6.3	-0.559764182	0.1	0.219	1	11
Ctps.3	-0.437846296	0.05	0.138	1	11
Klf7.8	-0.331603553	0.242	0.262	1	11
Asph	0.260220141	0.233	0.113	1	11
E330009J07Rik.1	-0.41043179	0.05	0.117	1	11
Ankrd32.2	-0.384040338	0.058	0.184	1	11
Slu7	-0.422671281	0.2	0.264	1	11
Ppm1a	-0.318511017	0.133	0.156	1	11
Pdzrn3.8	-0.5380119	0.092	0.147	1	11
Clk1.3	-0.439915854	0.267	0.34	1	11
Pphln1	-0.313801474	0.083	0.119	1	11
Kcnk1.8	-0.256890211	0.192	0.204	1	11
Igsf3.4	-0.261965815	0.133	0.133	1	11
Ehmt1.1	-0.518086296	0.133	0.252	1	11
Blmh.2	-0.399344609	0.225	0.317	1	11
Nme4.2	-0.410265774	0.033	0.118	1	11
Wwp2.1	0.251056266	0.117	0.035	1	11
Arrdc3.1	-0.335265856	0.05	0.104	1	11
Pum1.1	-0.262325428	0.267	0.276	1	11
Morf4l2.1	-0.269319149	0.267	0.272	1	11
Gprasp1.2	-0.379549976	0.167	0.206	1	11
Lmnb2.6	-0.482061854	0.042	0.159	1	11
Gnl2	-0.414214873	0.192	0.244	1	11
Acot7.4	-0.529714353	0.067	0.195	1	11
Grik2.8	-0.523852495	0.008	0.102	1	11
Nono.1	-0.332939818	0.183	0.219	1	11
Pdxp.1	-0.417773551	0.033	0.113	1	11
Celsr3.3	-0.503821726	0.025	0.123	1	11
Upf3b.1	-0.403582291	0.267	0.32	1	11
Fnbp4.1	-0.369491711	0.225	0.276	1	11
Yy1	-0.283686506	0.192	0.204	1	11
Gls	-0.255748747	0.117	0.123	1	11
Clvs1.8	-0.522186212	0.033	0.129	1	11
Zrsr2	-0.493879946	0.108	0.236	1	11
Cnot2.1	-0.286600428	0.175	0.185	1	11
Gps2	-0.45580519	0.158	0.236	1	11

Ptpn11.1	-0.292828543	0.267	0.27	1	11
Eml4.1	-0.284359784	0.2	0.209	1	11
Hnrnpf.1	-0.267488105	0.1	0.125	1	11
Ccdc59.1	-0.316629923	0.275	0.314	1	11
Smco4.7	-0.461122002	0.067	0.162	1	11
Rad50.3	-0.313595989	0.158	0.165	1	11
Phc2.1	-0.318390844	0.183	0.199	1	11
Suz12.3	-0.474971658	0.133	0.219	1	11
Psme4.2	-0.41427052	0.217	0.284	1	11
Znrd1.1	-0.301960531	0.192	0.211	1	11
Krt10.1	-0.390392793	0.108	0.162	1	11
Rpl7a.1	-0.320362751	0.117	0.15	1	11
Msh2.1	-0.489865469	0.033	0.144	1	11
Syt13.7	-0.481188933	0.025	0.127	1	11
Cdk7	-0.317040298	0.067	0.109	1	11
Tex30.4	-0.43266018	0.042	0.136	1	11
Cdca7l.7	-0.415180052	0.008	0.105	1	11
Fzd1.1	0.345319503	0.183	0.067	1	11
Erdr1.1	-0.516535768	0.058	0.162	1	11
Mcm3.6	-0.533470812	0.067	0.18	1	11
Cux1	-0.350405475	0.158	0.202	1	11
Sdhb.1	0.275253848	0.592	0.399	1	11
Pde4dip.2	-0.529767109	0.108	0.221	1	11
Tspan13.3	-0.317558079	0.192	0.207	1	11
Zfp330	-0.296562839	0.1	0.122	1	11
Klf6.4	0.473974293	0.275	0.147	1	11
Jhdm1d.5	-0.498493937	0.042	0.125	1	11
Timeless.7	-0.49154915	0.042	0.154	1	11
Ube2d1.4	-0.482005518	0.1	0.228	1	11
9330159F19Rik.5	-0.492256027	0.083	0.169	1	11
Jund.2	-0.411176084	0.3	0.352	1	11
Grb2.4	-0.258721104	0.258	0.248	1	11
Uqcr11.1	0.352035832	0.658	0.48	1	11
Pdcd11.1	-0.298878819	0.125	0.164	1	11
Ppfia2.7	-0.525754786	0.033	0.112	1	11
Ppm1g	-0.358186723	0.158	0.199	1	11
Plk4.8	-0.423516614	0.033	0.12	1	11
Ssx2ip.1	-0.425516784	0.058	0.13	1	11
Mta1.1	-0.294573139	0.075	0.104	1	11
Prmt1.1	-0.43539351	0.142	0.225	1	11
1700037H04Rik	-0.267484202	0.083	0.109	1	11
Rp9	-0.367974308	0.208	0.242	1	11
Rad51ap1.11	-0.511635261	0.05	0.167	1	11
Ccdc18.6	-0.461958751	0.042	0.115	1	11
Kif23.11	-0.601821785	0.083	0.215	1	11
Hist3h2a.7	-0.257805346	0.15	0.143	1	11
Baz1a.5	-0.518293076	0.058	0.167	1	11
Pcgf3	-0.309724632	0.075	0.107	1	11

Scnm1	-0.291351154	0.117	0.136	1	11
Srsf1.2	-0.336384858	0.25	0.296	1	11
Nvl.1	-0.489932764	0.067	0.169	1	11
Fosb.5	0.266251687	0.258	0.155	1	11
Pitpnb	-0.254249097	0.208	0.213	1	11
Chd6.1	-0.257086156	0.292	0.304	1	11
Gm11478.4	-0.379277	0.158	0.215	1	11
Setx	-0.402911636	0.075	0.13	1	11
Ssbp1	-0.327341336	0.175	0.215	1	11
Vopp1.3	-0.375147048	0.067	0.119	1	11
Eci2.1	0.268991163	0.408	0.248	1	11
Rbm22.1	-0.383311674	0.158	0.222	1	11
Nrn1.7	-0.556915403	0.1	0.222	1	11
Smndc1	-0.293753567	0.158	0.18	1	11
Flywch1.1	-0.416627848	0.058	0.128	1	11
Gmps.1	-0.340766921	0.175	0.21	1	11
Cecr2.2	-0.456557911	0.042	0.13	1	11
Ctnnbl1.1	-0.470141672	0.092	0.185	1	11
Rab30	0.264623998	0.108	0.029	1	11
Coro1c	-0.486180112	0.092	0.193	1	11
Smarcc2.5	-0.329136806	0.333	0.363	1	11
Ccdc25.2	-0.371144015	0.142	0.183	1	11
Dync1li1	-0.493560205	0.042	0.144	1	11
Gm10036.1	-0.486276803	0.1	0.177	1	11
Evl.3	-0.515514941	0.075	0.178	1	11
Paxbp1.3	-0.306832638	0.225	0.259	1	11
Ubfd1.1	-0.311071432	0.075	0.11	1	11
Xist.4	-0.641917103	0.183	0.3	1	11
Setd2	-0.285875896	0.158	0.212	1	11
Gpatch4.3	-0.40439471	0.067	0.18	1	11
Jam3.5	-0.295645424	0.108	0.135	1	11
Ap1s2.3	-0.504863483	0.058	0.16	1	11
C77370.3	-0.476375404	0.008	0.101	1	11
Tpt1.2	-0.346507865	0.217	0.256	1	11
Nbea.6	-0.407073812	0.158	0.19	1	11
Fxyd6.5	-0.45517576	0.375	0.509	1	11
Acat2.1	-0.322740462	0.15	0.182	1	11
Lars.1	-0.446057744	0.142	0.218	1	11
H1fx.9	-0.458386417	0.15	0.245	1	11
Zmym5.1	-0.353902922	0.2	0.232	1	11
Bms1.1	-0.467449146	0.108	0.183	1	11
Isy1	-0.361670236	0.092	0.141	1	11
Armcx4.1	-0.444086145	0.025	0.113	1	11
Cdt1.6	-0.467557028	0.025	0.125	1	11
Xpo1.1	-0.296091632	0.2	0.227	1	11
Tab2	0.256426594	0.4	0.245	1	11
Cep85l	0.27324465	0.125	0.039	1	11
Patz1	-0.261282583	0.083	0.105	1	11

Elavl1.1	-0.309564805	0.158	0.189	1	11
Smarce1.1	-0.469294794	0.1	0.204	1	11
Cops3.1	-0.277443983	0.242	0.251	1	11
Lta4h.3	-0.360367398	0.192	0.243	1	11
Jarid2.4	-0.479418387	0.092	0.179	1	11
Ccnb2.10	-0.508919477	0.117	0.187	1	11
Rps24-ps3.1	-0.290083831	0.15	0.179	1	11
Nedd4l.4	-0.372478948	0.083	0.128	1	11
Ncaph.8	-0.382793038	0.05	0.121	1	11
Ppia	-0.297289789	0.117	0.145	1	11
Ensa.3	-0.366562261	0.183	0.228	1	11
Stag2.2	-0.410880057	0.192	0.257	1	11
Snord104.4	-0.461622951	0.067	0.153	1	11
Ppan.1	-0.431382243	0.058	0.139	1	11
Rnaseh2b.4	-0.356683743	0.183	0.237	1	11
Ppp3r1	-0.253942464	0.125	0.137	1	11
Atl1.3	-0.377771488	0.05	0.102	1	11
Hnrnpul1	-0.265698292	0.158	0.175	1	11
Ddx17.2	-0.25262373	0.367	0.381	1	11
H2-Ke2.1	-0.349965562	0.15	0.195	1	11
Satb1.1	-0.461143702	0.125	0.21	1	11
Bccip.3	-0.330307114	0.267	0.31	1	11
Phf6.1	-0.419051904	0.1	0.173	1	11
Heatr3	-0.35360501	0.058	0.109	1	11
Gpc2.5	-0.471406694	0.033	0.14	1	11
Peo1.1	-0.415130448	0.05	0.124	1	11
Tmeff1.3	-0.329955832	0.117	0.149	1	11
Cdc40.1	-0.355316257	0.133	0.169	1	11
Git2.1	-0.374212725	0.058	0.11	1	11
Mab21l2.3	-0.46874419	0.017	0.104	1	11
Uba2.2	-0.338446483	0.217	0.256	1	11
Ormdl3	0.398318851	0.167	0.084	1	11
Rbm6.1	-0.29592243	0.2	0.223	1	11
Trim59.9	-0.479991797	0.067	0.17	1	11
Narf.1	-0.271770407	0.1	0.117	1	11
G3bp1.4	-0.417939103	0.192	0.328	1	11
Fam64a.9	-0.397205937	0.05	0.118	1	11
9430016H08Rik	0.251629599	0.225	0.112	1	11
Fbxo32.5	-0.407881297	0.033	0.117	1	11
Ttc33	-0.267226255	0.083	0.108	1	11
Krr1.1	-0.372119483	0.1	0.152	1	11
Slain2	-0.382634237	0.058	0.127	1	11
Ddx18.1	-0.396239286	0.058	0.127	1	11
Stxbp1.11	-0.487485265	0.092	0.162	1	11
Qser1.1	-0.272253453	0.108	0.127	1	11
Zbtb22	0.304589507	0.117	0.037	1	11
Pik3r3.6	-0.420082345	0.083	0.136	1	11
Ick.2	-0.272377344	0.133	0.146	1	11

Iqgap2.1	0.302642347	0.142	0.047	1	11
Cpsf3l	-0.339386574	0.058	0.11	1	11
Gid4	-0.251214683	0.1	0.115	1	11
Cnrip1.4	-0.397143025	0.15	0.198	1	11
Zfp644	-0.342815013	0.175	0.207	1	11
Tnpo3	-0.276108501	0.208	0.222	1	11
Crebbp.2	-0.422645539	0.158	0.223	1	11
Ercc1.1	-0.436745792	0.05	0.137	1	11
Gm10076.1	-0.270751249	0.108	0.134	1	11
Mpped2.2	-0.413656472	0.058	0.126	1	11
Rad51.8	-0.353830018	0.058	0.116	1	11
Hip1r.4	-0.497668108	0.042	0.133	1	11
Setbp1.3	-0.438727719	0.092	0.16	1	11
Msi2.1	0.277624906	0.325	0.185	1	11
Ints10	-0.262620433	0.108	0.128	1	11
Rundc3a.5	-0.464216456	0.158	0.249	1	11
Kat6b.2	-0.442390692	0.092	0.157	1	11
Abce1	-0.273816297	0.125	0.149	1	11
Kat6a	-0.326572941	0.142	0.172	1	11
Smc5.3	-0.25875555	0.242	0.258	1	11
Ctr9.1	-0.454364406	0.158	0.242	1	11
Xpa	-0.250219904	0.108	0.129	1	11
Tmem55b.1	0.279268725	0.233	0.111	1	11
Odf2.5	-0.259061672	0.167	0.17	1	11
Daam1.2	-0.363847205	0.142	0.183	1	11
Pnmal2.6	-0.358198817	0.117	0.146	1	11
Thsd7a.4	-0.481434741	0.05	0.127	1	11
Uhrf2.1	-0.384087491	0.092	0.15	1	11
Hk2.5	-0.400057121	0.108	0.173	1	11
mt-Co1	0.251698122	0.775	0.609	1	11
Upf3a	-0.25992442	0.217	0.219	1	11
Chchd2.3	-0.257907026	0.625	0.653	1	11
2700049A03Rik.2	-0.459776747	0.033	0.122	1	11
Ttc1	-0.27506759	0.175	0.185	1	11
Dis3.2	-0.351356628	0.042	0.124	1	11
Rpl15	-0.405392058	0.083	0.161	1	11
Qrich1	-0.327785564	0.067	0.116	1	11
Exoc5	-0.298458418	0.125	0.147	1	11
Uimc1.1	-0.342755545	0.092	0.133	1	11
Rbbp8.4	-0.410937823	0.042	0.122	1	11
Dcun1d5	-0.362955825	0.2	0.256	1	11
Ldb1.2	-0.362169239	0.142	0.184	1	11
Ahi1.1	-0.419791448	0.225	0.307	1	11
Rrm2.10	-0.515921084	0.092	0.204	1	11
Aurkb.10	-0.41314384	0.067	0.143	1	11
Gripap1.1	-0.308925342	0.167	0.189	1	11
Mum1.2	-0.433440434	0.067	0.17	1	11
Ube2i	-0.413244415	0.058	0.152	1	11

Gtf2e2.1	-0.296001751	0.15	0.178	1	11
Shmt1.6	-0.35314692	0.033	0.103	1	11
Srgap2.7	-0.361480586	0.092	0.132	1	11
Man1c1.3	-0.316971018	0.092	0.126	1	11
Trafd1.2	-0.300565358	0.092	0.118	1	11
Tk1.8	-0.313450043	0.083	0.131	1	11
N4bp2.1	-0.440652658	0.042	0.132	1	11
6330403K07Rik.8	-0.383655429	0.192	0.243	1	11
Skiv2l	-0.273878013	0.15	0.162	1	11
Mapk7.1	-0.376826636	0.017	0.105	1	11
Etaa1	-0.417209428	0.075	0.159	1	11
Socs3	0.282661888	0.117	0.038	1	11
Rpl36.1	-0.342481714	0.05	0.108	1	11
Rrnad1	-0.315561639	0.092	0.122	1	11
Pja1.2	-0.255671556	0.158	0.173	1	11
Rpl3-ps1.1	-0.267108813	0.067	0.101	1	11
Tln1.3	-0.266893396	0.142	0.149	1	11
BC034090.3	-0.452391432	0.042	0.128	1	11
Mxi1.1	-0.41000651	0.117	0.191	1	11
Tead1.4	-0.398795937	0.117	0.174	1	11
Rbbp9	0.306045993	0.167	0.067	1	11
Btg1.1	-0.414858523	0.108	0.183	1	11
Ctbp2.2	-0.289688335	0.192	0.215	1	11
Oard1	-0.291750423	0.125	0.152	1	11
Slc25a27.2	-0.415228836	0.033	0.11	1	11
Zfp322a.1	-0.318520198	0.092	0.123	1	11
Upf2.1	-0.326510906	0.05	0.154	1	11
Rbm18	-0.319756299	0.133	0.171	1	11
Zfp553	-0.344541605	0.1	0.144	1	11
Tcof1.1	-0.442527147	0.083	0.168	1	11
Rcc2.4	-0.372080407	0.117	0.179	1	11
Rab3a.10	-0.530091013	0.05	0.15	1	11
Sf3b3.1	-0.361197536	0.067	0.126	1	11
Pknox1.2	-0.373908424	0.092	0.145	1	11
Sgol2.9	-0.494445815	0.067	0.157	1	11
Cnksr2.5	-0.430819641	0.025	0.108	1	11
Fen1.8	-0.403127907	0.058	0.142	1	11
Thoc3.1	-0.327545184	0.117	0.158	1	11
Usp33.1	-0.435692763	0.067	0.149	1	11
Pnrc1.2	-0.430641395	0.167	0.271	1	11
Elovl6.3	-0.364702049	0.175	0.278	1	11
Zmym2	-0.296105181	0.092	0.118	1	11
Mgea5.2	-0.337580915	0.2	0.231	1	11
Gpr85.1	-0.453111063	0.075	0.164	1	11
Hist3h2ba.5	-0.448024313	0.042	0.138	1	11
Qtrt1	-0.343828112	0.058	0.108	1	11
Ndc80.8	-0.387921369	0.025	0.115	1	11
Dbn1.9	-0.358069487	0.1	0.139	1	11

Parp2	-0.399554444	0.1	0.17	1	11
Zkscan3	-0.27807115	0.175	0.199	1	11
Tubg1.2	-0.259997671	0.108	0.129	1	11
Vrk1.6	-0.410167654	0.067	0.148	1	11
Rexo1.1	-0.443597538	0.125	0.212	1	11
Naa16	-0.348126867	0.083	0.125	1	11
Fam178a	-0.407176757	0.033	0.111	1	11
Snrpa	-0.302271598	0.1	0.14	1	11
Gm11266.7	-0.463179238	0.075	0.144	1	11
Daxx	-0.364358732	0.05	0.113	1	11
Bmi1.1	-0.318122081	0.058	0.147	1	11
Rps28.4	-0.403722271	0.117	0.203	1	11
Pik3ip1.2	-0.262535489	0.1	0.116	1	11
Ddx23.1	-0.453626244	0.108	0.207	1	11
Haus1	-0.354335871	0.05	0.11	1	11
Ube2e2.1	-0.250862057	0.108	0.125	1	11
Nfyb.5	-0.420638183	0.158	0.235	1	11
Ncapd2.8	-0.387828378	0.1	0.185	1	11
Uba1.2	-0.326842804	0.233	0.272	1	11
Ccdc66.1	-0.329082151	0.142	0.172	1	11
Tob1.1	-0.310409581	0.108	0.155	1	11
Gm17750.5	-0.355555218	0.333	0.367	1	11
Knop1.1	-0.264717165	0.258	0.271	1	11
Rpa3.6	-0.356163465	0.183	0.244	1	11
RP23-32A8.1.3	-0.25030103	0.142	0.187	1	11
Kars.1	-0.270783624	0.175	0.195	1	11
Shoc2	-0.36445555	0.083	0.133	1	11
Prpf4.1	-0.359035117	0.083	0.139	1	11
Ccnb1.8	-0.433303371	0.075	0.139	1	11
Ddx10	-0.442926909	0.042	0.126	1	11
Tulp4.3	-0.36726768	0.158	0.205	1	11
A330076H08Rik.9	-0.448164736	0.083	0.161	1	11
Rrn3	-0.340229179	0.067	0.12	1	11
Akap11.2	-0.254084123	0.133	0.14	1	11
R3hdm2.1	-0.412709773	0.092	0.168	1	11
Ints7	-0.384785065	0.05	0.117	1	11
Tmem183a.1	-0.303095844	0.142	0.173	1	11
Rnf144a.1	-0.276217696	0.142	0.159	1	11
Eif2s1.1	-0.368686884	0.133	0.197	1	11
Brcc3.1	-0.435072212	0.067	0.176	1	11
Midn.2	-0.277852829	0.108	0.133	1	11
Zranb1.2	-0.294058323	0.175	0.208	1	11
Ythdf2	-0.420435557	0.158	0.259	1	11
Cdk5rap3.2	-0.415249024	0.117	0.193	1	11
Rad23b.1	-0.293767548	0.175	0.202	1	11
C330027C09Rik.8	-0.383677155	0.067	0.133	1	11
Elmo1.8	-0.541261648	0.033	0.126	1	11
Mbp.4	-0.302370518	0.2	0.165	1	11

Baz2a.1	-0.284493851	0.083	0.11	1	11
Pbk.9	-0.468094475	0.117	0.223	1	11
Orc2.2	-0.343891324	0.108	0.156	1	11
Angptl2.5	-0.391389119	0.025	0.117	1	11
Nudcd3.1	-0.261651386	0.158	0.17	1	11
Lhfpl4.1	-0.382818306	0.033	0.108	1	11
Limd2.2	-0.358917111	0.075	0.135	1	11
Slc24a5	-0.404864405	0.042	0.141	1	11
Pcnt.3	-0.424333111	0.083	0.175	1	11
Trim24	-0.358166517	0.083	0.134	1	11
Zfp451.1	-0.386945718	0.1	0.17	1	11
Cd3eap	-0.293418774	0.092	0.121	1	11
Vps41	-0.32899778	0.108	0.145	1	11
Rapgef6	-0.398237156	0.075	0.14	1	11
Nosip.1	-0.312959675	0.133	0.168	1	11
Dner.11	-0.416572993	0.15	0.215	1	11
Sbk1.5	-0.438612455	0.042	0.119	1	11
Cerk.3	-0.395718633	0.083	0.151	1	11
Bbx	-0.384778215	0.092	0.148	1	11
Abt1.1	-0.354099798	0.033	0.127	1	11
Fam192a	-0.37874559	0.142	0.199	1	11
Blm.2	-0.393064968	0.025	0.111	1	11
Lrig3.6	-0.379934865	0.1	0.17	1	11
Agtpbp1.2	-0.344298298	0.05	0.102	1	11
Bdp1	-0.427568755	0.092	0.154	1	11
Supt5	-0.307760516	0.158	0.197	1	11
Slc39a10.1	-0.389212288	0.125	0.195	1	11
Celsr2.6	-0.351935069	0.175	0.238	1	11
Snrnp200	-0.334300252	0.1	0.163	1	11
Cwf19l2	-0.268279718	0.142	0.163	1	11
2700081O15Rik.3	-0.421482771	0.033	0.111	1	11
Zfp354c.1	-0.273295194	0.108	0.126	1	11
Rhobtb3.1	-0.389983075	0.1	0.164	1	11
Igf1r.1	-0.389455335	0.042	0.114	1	11
Prkacb.1	-0.356898021	0.142	0.187	1	11
Cspp1.3	-0.317705341	0.217	0.238	1	11
Tet3	-0.25170136	0.133	0.148	1	11
Bcat1.1	-0.403732174	0.033	0.121	1	11
Zfp512.1	-0.341327424	0.058	0.119	1	11
Cenpc1.2	-0.365363706	0.1	0.155	1	11
Pbx3.1	-0.286955867	0.1	0.127	1	11
Gmnn.8	-0.408315161	0.067	0.145	1	11
Cntln.6	-0.371848942	0.133	0.172	1	11
Ddx26b.4	-0.330003721	0.092	0.13	1	11
Pak7.6	-0.288869171	0.033	0.105	1	11
Ankhd1.1	-0.393476262	0.1	0.181	1	11
Snapc3.2	-0.272447922	0.1	0.125	1	11
Fyttd1.1	-0.269102064	0.233	0.257	1	11

Rev1	-0.275662225	0.092	0.117	1	11
Wdr6.2	-0.386181459	0.083	0.145	1	11
Cnot4	-0.307151411	0.175	0.197	1	11
Ncapg.8	-0.421715369	0.083	0.183	1	11
Clasp1	-0.27478805	0.092	0.114	1	11
Kif5a.9	-0.452123826	0.058	0.128	1	11
Skiv2l2	-0.273370533	0.142	0.164	1	11
Gm13092.2	-0.316047568	0.033	0.104	1	11
Ulk1.1	-0.371719521	0.042	0.105	1	11
Tbc1d16.2	-0.398664628	0.1	0.197	1	11
Rnf168.4	-0.444191473	0.117	0.208	1	11
Pcdha2.7	-0.446140309	0.033	0.118	1	11
Mis18a.5	-0.251211079	0.05	0.11	1	11
Actl6a.3	-0.37336309	0.142	0.227	1	11
Chd2.1	-0.445968812	0.15	0.222	1	11
Ddx55.1	-0.271957116	0.133	0.165	1	11
Ckap4.1	-0.271091499	0.267	0.289	1	11
Cep78.2	-0.379049751	0.075	0.14	1	11
Brpf1.1	-0.354087785	0.083	0.141	1	11
Trim27.1	-0.373098359	0.075	0.149	1	11
Ikbkap	-0.328639221	0.083	0.127	1	11
Rnps1.1	-0.368715479	0.058	0.137	1	11
Rbm10	-0.326346861	0.083	0.134	1	11
Thumpd1.1	-0.396229864	0.092	0.166	1	11
Akap8.1	-0.306019153	0.2	0.233	1	11
Dnph1.5	-0.281869319	0.083	0.123	1	11
Pmf1.7	-0.262696766	0.117	0.143	1	11
Atp11b	-0.283810648	0.067	0.102	1	11
Kif22.10	-0.362979018	0.075	0.163	1	11
Msl1	-0.283498083	0.2	0.223	1	11
Cd2bp2	-0.386488885	0.058	0.136	1	11
Cmip	-0.335316939	0.125	0.16	1	11
Asap1.3	-0.264268058	0.158	0.18	1	11
Srpk1.1	-0.39163858	0.142	0.247	1	11
Wasf1.2	-0.267296671	0.083	0.11	1	11
Cpsf1	-0.331150277	0.042	0.108	1	11
Nenf.3	-0.258484655	0.117	0.136	1	11
Mllt3.3	-0.393724964	0.15	0.226	1	11
BC005561.1	-0.383578152	0.208	0.29	1	11
Rpap3.1	-0.393533752	0.067	0.145	1	11
Cenpw.9	-0.312115262	0.083	0.127	1	11
Fam53b.2	-0.30023624	0.075	0.136	1	11
Hist1h2ak.8	-0.406748692	0.092	0.143	1	11
Med1.1	-0.346567249	0.117	0.172	1	11
Faf1	-0.280281429	0.067	0.102	1	11
Gli1.7	-0.258312071	0.083	0.107	1	11
Pola1.4	-0.332982332	0.033	0.103	1	11
0610010F05Rik	-0.367250405	0.075	0.136	1	11

Gle1.1	-0.374926115	0.075	0.139	1	11
Uri1.1	-0.328069722	0.158	0.209	1	11
Ptch2.7	-0.420442845	0.058	0.153	1	11
Pom121.1	-0.255485735	0.158	0.179	1	11
Gpatch11	-0.277466501	0.083	0.11	1	11
Cdkn2d.5	-0.31994374	0.117	0.157	1	11
Brwd1.1	-0.253955185	0.167	0.193	1	11
Dck.2	-0.303863139	0.05	0.103	1	11
G2e3.3	-0.35180202	0.042	0.131	1	11
Rrp15.3	-0.302124691	0.142	0.174	1	11
Arrb2.1	-0.257653369	0.058	0.118	1	11
Topors.1	-0.381885471	0.108	0.183	1	11
Apod	5.196128317	0.824	0.037	0	12
Ptn.10	3.213414212	0.983	0.437	0	12
Col3a1	4.141714635	0.941	0.01	1.3008E-286	12
Col4a1	3.358682168	0.966	0.066	9.7311E-278	12
Igf2	3.754831938	0.815	0.03	1.049E-261	12
Col1a2.1	3.692406366	0.924	0.008	7.4356E-254	12
Vtn	3.633053415	0.916	0.004	1.2254E-247	12
Dcn	4.049268128	0.824	0.005	1.6414E-246	12
Col4a2	3.299821434	0.933	0.034	5.8478E-245	12
Itih5.1	3.393501346	0.84	0.009	7.3088E-220	12
Sparc.3	3.273672521	0.975	0.079	3.0581E-197	12
Lamb1	2.573550195	0.815	0.005	3.8875E-190	12
Lum	2.713860199	0.723	0.001	5.598E-187	12
Sparcl1.9	2.812451609	0.941	0.202	3.0801E-186	12
Nupr1	2.713957927	0.807	0.011	1.5635E-182	12
Cxcl12.1	2.906768982	0.815	0.039	2.2072E-177	12
Nid1.1	2.555813967	0.798	0.012	6.8117E-165	12
Colec12.1	2.169364328	0.723	0.004	2.8479E-163	12
Atp1a2.2	3.037597859	0.933	0.052	1.226E-161	12
Bgn	2.1297172	0.664	0.002	1.0947E-160	12
Pcolce	2.056072574	0.664	0.002	3.7349E-159	12
Ifitm3.1	2.306645992	0.765	0.009	1.8404E-153	12
Serpinh1.7	2.285145172	0.924	0.109	1.1116E-151	12
Igfbp7	3.135847379	0.79	0.013	2.0465E-150	12
Serpinf1	1.977638242	0.622	0.002	1.2702E-147	12
Lgals1.8	2.292549831	0.908	0.224	5.691E-147	12
Edn3	2.44804173	0.605	0.002	1.087E-143	12
Igfbp5	2.830728393	0.622	0.021	1.4571E-138	12
Col1a1	2.353623933	0.571	0.002	2.3616E-138	12
Col15a1	2.340766072	0.597	0.002	6.2426E-138	12
Slc6a13	2.010531449	0.555	0.001	1.5689E-135	12
Pltp.2	2.487244001	0.731	0.023	1.8272E-135	12
Cp.2	2.306028787	0.672	0.009	9.0707E-134	12
Igfbp2.1	2.622794703	0.739	0.015	4.0139E-132	12
Col6a2	1.8389862	0.538	0.001	2.6953E-127	12
Col6a1	1.923629057	0.563	0.003	2.8869E-122	12

Gng11.1	2.156949763	0.672	0.01	8.6895E-122	12
S100a6.2	2.475382526	0.647	0.018	2.5284E-118	12
Eva1b.1	1.76917439	0.597	0.006	2.8428E-118	12
Fstl1.7	1.987913348	0.874	0.175	5.2561E-116	12
Rarres2.1	1.767167405	0.538	0.003	4.8123E-115	12
Anxa5.2	1.925998429	0.681	0.015	1.2001E-114	12
Cfh.1	1.873091634	0.546	0.003	7.0008E-114	12
Tbx18	1.802173069	0.513	0.002	8.1111E-113	12
Col18a1	2.185019787	0.647	0.051	2.7811E-112	12
Ctsk	1.711868444	0.487	0.001	3.3014E-112	12
Cyp1b1	1.526792672	0.504	0.002	1.0416E-109	12
Cthrc1	2.008275022	0.605	0.012	6.3796E-108	12
Laptm4a.2	1.464541515	0.924	0.551	9.6291E-107	12
Igfbp4.2	2.173068619	0.622	0.014	1.4297E-106	12
Cped1	1.502105159	0.471	0.002	1.0482E-101	12
Il34	1.478743655	0.42	0	2.1429E-101	12
Htra3	1.760073759	0.487	0.003	1.1486E-100	12
Pdgfrl.1	1.749173906	0.496	0.004	2.2341E-100	12
S1pr3.1	1.537171992	0.462	0.002	3.1441E-100	12
Sepp1.2	2.101735094	0.798	0.051	9.2507E-100	12
Enpp1	1.501964198	0.454	0.002	3.8923E-99	12
Hsp90ab1.3	-0.888941182	0.958	0.998	5.95904E-98	12
Lhfp.1	1.95806742	0.613	0.015	2.20292E-97	12
Lama4	1.771076042	0.513	0.005	5.09188E-96	12
Postn	1.405131711	0.403	0	1.91106E-95	12
Emp3.1	1.527096779	0.538	0.006	3.01758E-95	12
Il33.1	1.649708164	0.454	0.003	5.12447E-95	12
Col13a1	1.787072301	0.429	0.001	1.62259E-94	12
Fbln2	1.926506195	0.622	0.034	7.59655E-94	12
Anxa2.2	1.708076863	0.555	0.009	1.98464E-93	12
Tmem204	1.627945101	0.471	0.003	6.2516E-92	12
Lamc1	1.742372089	0.588	0.016	1.49133E-91	12
Col4a5	1.636992138	0.462	0.005	6.45059E-89	12
Nid2	1.456733761	0.479	0.005	1.18539E-87	12
Plat.1	1.822337967	0.588	0.017	5.6572E-87	12
Loxl2	1.284818064	0.412	0.002	4.23231E-86	12
Phlda1.2	1.843318649	0.597	0.022	1.04765E-83	12
Trf.2	2.248256663	0.521	0.034	2.04058E-83	12
Cmbl	1.266677993	0.353	0	6.02606E-82	12
Angptl4.1	1.624495666	0.462	0.006	1.77394E-81	12
Kdelr3.1	1.455223049	0.479	0.006	3.34704E-81	12
Hmgcs2	1.340450214	0.42	0.003	3.4088E-81	12
Bicc1.1	1.477235659	0.471	0.007	1.62938E-79	12
Slc6a20a	1.424829786	0.336	0	2.96854E-78	12
Pdgfrb	1.436149142	0.361	0.001	2.72262E-77	12
Ifitm1	1.524950319	0.345	0.001	2.12515E-76	12
Spp1.1	2.164043803	0.471	0.008	7.351E-76	12
Aldh1a1.1	1.614290095	0.395	0.004	8.81483E-76	12

Emp1	1.469034772	0.412	0.004	1.27479E-75	12
Lama1	1.441093911	0.437	0.006	1.00382E-74	12
Rbp1	1.653440796	0.387	0.005	4.47052E-73	12
Itga8	1.241269022	0.37	0.002	4.84003E-73	12
Tgfbi	1.560479113	0.361	0.002	1.57442E-71	12
Mfap2.1	1.886074527	0.597	0.042	1.76012E-71	12
Fmo1	1.323292086	0.345	0.001	3.43645E-71	12
Sdc2.2	1.729422766	0.597	0.051	3.54213E-71	12
Rcn3.1	1.68146965	0.672	0.083	9.81155E-71	12
Sod3	1.323104209	0.345	0.002	1.65766E-70	12
C1qtnf2	1.212680002	0.319	0.001	1.39603E-69	12
Ccdc80.1	1.413338596	0.42	0.006	4.57702E-69	12
Adh1	1.381271281	0.286	0	5.40475E-69	12
Apoe.12	1.10959726	0.882	0.245	3.31464E-68	12
Cd302.3	1.686918909	0.613	0.049	4.40933E-68	12
Mxra8.1	1.619725199	0.513	0.019	6.57832E-68	12
Itm2c.8	1.61507349	0.824	0.207	1.07654E-67	12
Tmem45a	0.974458914	0.277	0	1.21761E-66	12
Ggt5	0.994384686	0.286	0	3.48846E-65	12
Cnn2	1.403669434	0.387	0.005	4.34843E-65	12
Vwa1.1	1.334381867	0.429	0.008	1.07751E-64	12
Cpq.1	1.164017363	0.395	0.005	1.3365E-64	12
Col26a1	1.22305733	0.328	0.002	9.31518E-64	12
Lama2	1.122932307	0.336	0.002	1.70996E-63	12
Tm4sf1	1.299389308	0.353	0.003	4.752E-63	12
Abca8a	1.07530862	0.286	0.001	3.77498E-62	12
S100a11.1	1.410345799	0.437	0.013	6.82818E-61	12
0610007N19Rik	1.071990401	0.294	0.001	7.49301E-61	12
Mgp	2.44400584	0.294	0.002	8.86498E-61	12
Itm2b.10	1.201543235	0.958	0.64	1.31749E-60	12
Crip1.2	1.686155813	0.412	0.01	1.45193E-60	12
Ctsl.5	1.550696295	0.815	0.256	4.3451E-60	12
Foxc1	1.163415417	0.37	0.005	5.65349E-60	12
Wls.3	1.634386343	0.63	0.083	8.31494E-60	12
Id3.1	1.804212045	0.538	0.028	1.67397E-59	12
Rhoj.1	1.56239111	0.496	0.029	3.12786E-59	12
Gm14964	1.379619754	0.37	0.006	3.62812E-59	12
Ctsh.2	1.325673807	0.412	0.009	6.56723E-59	12
Myl9	1.802723694	0.353	0.01	1.67458E-58	12
Col5a2	1.425071019	0.437	0.014	3.27249E-58	12
Ppap2b.2	1.665040945	0.563	0.039	1.16042E-56	12
P2ry14	1.125798765	0.269	0.001	1.46452E-56	12
Ccl11	1.076525399	0.252	0	3.51344E-56	12
Uaca	1.663550989	0.538	0.056	7.01859E-56	12
Rbpms	1.184340489	0.311	0.003	7.24031E-56	12
Msx1	0.773358628	0.261	0.001	4.56217E-55	12
Msc	1.164321428	0.261	0.001	5.03055E-55	12
Copz2	1.143866853	0.403	0.011	5.41769E-55	12

Nfib.7	-1.446447039	0.605	0.935	2.89989E-54	12
Hspa12a	1.331947125	0.387	0.011	4.86047E-54	12
Rgs5.1	3.335474124	0.185	0.012	1.01133E-53	12
Pmp22.3	1.280665575	0.429	0.015	7.37894E-53	12
Ifi27.2	1.317139045	0.395	0.012	8.91752E-53	12
Lamc3	0.824316518	0.235	0	3.95324E-52	12
Col23a1	1.282765476	0.328	0.005	2.23712E-51	12
Gpx8.2	1.451942301	0.521	0.05	1.80293E-50	12
Mrap	1.063955366	0.227	0	2.4984E-50	12
Bmp7	1.047594142	0.303	0.003	4.42011E-50	12
Ggt1	0.833881705	0.218	0	2.6255E-49	12
Islr	1.497903037	0.445	0.029	3.67286E-49	12
Gper1	1.01728031	0.261	0.001	7.87842E-49	12
Rasgrp2	1.142517249	0.328	0.006	1.46369E-48	12
Cdh11.2	1.253769282	0.387	0.013	3.6609E-48	12
Egflam	0.768938699	0.227	0	1.18445E-47	12
Ece1	1.472855082	0.597	0.081	3.72819E-47	12
Fam114a1	0.874508396	0.294	0.004	7.37468E-47	12
Efemp1.1	1.081398023	0.303	0.005	1.28142E-46	12
Pdlim2	0.966781273	0.261	0.002	1.53158E-46	12
Heyl	0.872636718	0.235	0.001	1.73344E-46	12
Cgnl1	1.088187852	0.277	0.003	3.54751E-46	12
Itih2	0.840834008	0.202	0	6.62621E-46	12
Srpx2	0.814028836	0.202	0	7.11494E-46	12
Plxdc2.1	1.316286323	0.412	0.018	7.62804E-46	12
Kcnj8	1.633227025	0.252	0.014	1.20192E-45	12
Tubb5.9	-1.247271379	0.622	0.916	1.33802E-45	12
Serinc3.1	1.483311478	0.672	0.116	3.34054E-45	12
Slc16a12	0.845035558	0.227	0.001	1.23845E-44	12
Aldh1a2	1.040363074	0.269	0.003	2.21559E-44	12
Fcgrt.2	1.222990706	0.437	0.024	7.87653E-44	12
Col6a3	1.04386418	0.252	0.002	1.03757E-43	12
Nkd2	0.963770277	0.261	0.003	2.31288E-43	12
Naalad2	1.036644791	0.218	0.001	2.69848E-43	12
Cyth3.1	1.372381431	0.513	0.053	4.4741E-43	12
Htra1.1	1.073365232	0.361	0.012	5.35925E-43	12
Chp2	1.081967617	0.235	0.001	1.11911E-42	12
Rcsd1.1	0.904795538	0.252	0.002	1.14491E-42	12
Oaf.1	0.879707994	0.252	0.003	1.43122E-42	12
Rrbp1.5	1.41269412	0.706	0.185	1.44369E-42	12
Prrx1.1	1.102757893	0.277	0.004	2.52364E-42	12
Csf1.2	1.087521899	0.311	0.007	3.14565E-42	12
Sept11.3	1.236455697	0.807	0.318	3.20788E-41	12
Klf2.2	1.515921521	0.361	0.016	6.12435E-41	12
Tfpi.1	1.092863694	0.294	0.007	2.52583E-40	12
Steap3	0.771918751	0.202	0.001	6.79972E-40	12
Il13ra1	0.794473738	0.235	0.002	7.30795E-40	12
Tgfbr3	0.856287815	0.261	0.003	7.89374E-40	12

Wfdc1	0.698082569	0.21	0.001	1.506E-39	12
Arl4a.1	1.396922711	0.521	0.061	1.66746E-39	12
Gja1.1	1.209514888	0.361	0.018	2.55419E-39	12
Prelp	0.689885541	0.185	0	3.64009E-39	12
Itga1	1.045137914	0.235	0.003	2.51718E-38	12
C1qtnf6	0.880094827	0.261	0.004	2.59742E-38	12
Serpine2.3	1.529440399	0.513	0.054	4.62102E-38	12
Rdh10.1	1.321272083	0.37	0.024	4.74987E-38	12
Slc7a11.1	1.197639118	0.227	0.002	5.70958E-38	12
Emilin1	0.941532849	0.269	0.005	8.57424E-38	12
Fkbp10.1	0.994896906	0.277	0.006	2.32916E-37	12
Sfrp1.8	-1.5496902	0.319	0.815	2.93727E-37	12
Ifitm2.5	1.274621077	0.597	0.11	3.94993E-37	12
Tagln2.2	1.318631439	0.454	0.042	8.87793E-37	12
Lpar1.1	1.043423048	0.277	0.007	3.23326E-36	12
Mylk	1.085371002	0.202	0.001	6.08898E-36	12
Slc22a6	0.764917245	0.16	0	6.26585E-36	12
Dkk3.1	1.003934028	0.277	0.008	1.1401E-35	12
Abca9.1	0.919549294	0.244	0.004	1.71606E-35	12
Mfge8.2	1.275557735	0.403	0.031	3.32972E-35	12
Ahnak	1.045204689	0.252	0.005	4.52187E-35	12
Slc22a8	0.87748481	0.21	0.002	1.8686E-34	12
Clec1a	0.816695519	0.202	0.001	2.47723E-34	12
G0s2.2	1.098268195	0.252	0.005	4.84761E-34	12
Nbl1.1	1.216400018	0.37	0.025	1.49715E-33	12
Myl12a.6	1.182442048	0.697	0.326	1.95045E-33	12
Tnfrsf19.1	0.756840941	0.227	0.004	3.40028E-33	12
Cald1.5	1.124685356	0.857	0.533	4.14285E-33	12
Junb.2	1.340843977	0.387	0.033	4.50784E-33	12
Cd97	0.792461522	0.227	0.003	4.75831E-33	12
Pla2g7.2	1.138922541	0.336	0.018	5.46858E-33	12
Nr1h3	0.90208392	0.218	0.003	6.99984E-33	12
Creb3l2	1.001125235	0.277	0.009	1.45658E-32	12
Tmem37.2	0.892450743	0.252	0.006	1.84703E-32	12
Cd248	0.930133674	0.202	0.002	2.25525E-32	12
Rell1	1.245230267	0.546	0.092	2.28663E-32	12
Fblim1.1	0.968275546	0.235	0.004	2.33973E-32	12
Vstm4	0.770404487	0.176	0.001	5.17085E-32	12
Tcn2.2	1.042598998	0.328	0.017	9.98623E-32	12
Slc9a3r2	1.095866395	0.311	0.014	1.34593E-31	12
Rnase4.2	0.790208552	0.303	0.02	1.54257E-31	12
Ptplad2.1	0.807085779	0.244	0.006	1.6872E-31	12
Lbp	0.606423541	0.151	0	2.59571E-31	12
Mmp14.6	1.260520817	0.639	0.171	2.68996E-31	12
Fkbp7.1	1.23833768	0.454	0.064	2.69722E-31	12
Tlr12	0.553715579	0.143	0	3.1203E-31	12
Fam26e	0.716815867	0.16	0	3.59629E-31	12
Mrc2	0.577561328	0.168	0.001	6.89269E-31	12

Svil	1.204424106	0.37	0.031	7.62648E-31	12
Tns1	0.987922995	0.21	0.003	7.98275E-31	12
Mmp2.1	0.957364604	0.303	0.014	8.88714E-31	12
Enpp2.1	1.153706351	0.378	0.029	1.26154E-30	12
Pear1	0.772305162	0.176	0.001	1.99858E-30	12
Col4a6	0.706903702	0.176	0.001	2.72581E-30	12
Spry1.2	0.957970385	0.286	0.011	2.73579E-30	12
Wnt5a.1	0.973159292	0.244	0.007	6.45897E-30	12
Atp2b4	0.91692882	0.235	0.005	9.90036E-30	12
Amica1	0.668273777	0.16	0.001	1.19852E-29	12
Sult1a1	0.949432051	0.193	0.002	1.44247E-29	12
Ramp2.5	1.25875104	0.521	0.116	2.12976E-29	12
Tpm2	1.110120231	0.252	0.01	3.55661E-29	12
Ptgis	0.66033625	0.168	0.001	4.16978E-29	12
Rtn1.11	-1.651114556	0.16	0.693	5.19203E-29	12
Crmp1.6	-1.543048486	0.176	0.671	6.57699E-29	12
Pi16	0.579016237	0.134	0	7.14972E-29	12
Ppic.6	1.158410826	0.681	0.236	7.18478E-29	12
Rbms3	0.979043875	0.319	0.019	8.19631E-29	12
Itm2a	1.223983735	0.403	0.043	1.17752E-28	12
Myh9.1	1.11730933	0.328	0.025	1.52607E-28	12
Samd5	0.85783848	0.193	0.002	1.68317E-28	12
Axl.2	0.914085774	0.227	0.006	3.68316E-28	12
Tmem64	1.265814688	0.445	0.088	4.87192E-28	12
Hspg2	0.868005189	0.202	0.003	6.37487E-28	12
Tril.2	1.037915369	0.336	0.023	6.69118E-28	12
Igfbpl1.10	-1.682088908	0.16	0.65	7.37399E-28	12
Cd81.5	1.001168958	0.824	0.459	8.42789E-28	12
Gstt2	0.726145926	0.185	0.002	1.18018E-27	12
CRE_RECOMBINASE.10	-1.630240962	0.311	0.771	1.25026E-27	12
Hic1	0.497882956	0.134	0	1.79116E-27	12
Ncl.8	-0.873711361	0.748	0.897	2.06654E-27	12
Ctsb.8	0.991236934	0.714	0.259	3.00449E-27	12
Cyr61.1	1.339535723	0.261	0.018	5.1383E-27	12
Hrct1	0.617038156	0.151	0.001	6.19702E-27	12
Filip1l	1.313089052	0.21	0.008	1.12248E-26	12
Slc1a5	1.1214183	0.378	0.045	1.36134E-26	12
Foxs1	0.647825644	0.143	0	1.41259E-26	12
Fgfr1.1	1.146148596	0.471	0.078	1.69034E-26	12
Hlf.1	0.940535562	0.294	0.017	1.89999E-26	12
Thbd	0.543511232	0.143	0.001	1.94819E-26	12
Mylip.1	0.834972258	0.261	0.011	2.30247E-26	12
Higd1b	1.440405799	0.143	0.001	2.71986E-26	12
Dse.1	0.890364246	0.244	0.009	2.88106E-26	12
Cd24a.5	-1.483068369	0.168	0.637	3.20907E-26	12
Gja4	1.123486115	0.143	0.001	5.6696E-26	12
Tgfb1i1	0.757547519	0.21	0.005	7.1121E-26	12
Myo1b.1	1.152629589	0.462	0.073	9.93363E-26	12

Hnrnpu.5	-0.841028487	0.706	0.866	1.02921E-25	12
Ckb.10	-1.317925062	0.479	0.81	1.21783E-25	12
Lamb2.1	0.848102079	0.244	0.01	3.1088E-25	12
Timp3.4	1.36860703	0.445	0.076	3.4236E-25	12
Vamp8.2	0.615550382	0.235	0.011	3.71606E-25	12
Fth1.5	0.758635815	0.908	0.707	4.7925E-25	12
B3gnt9	0.648547539	0.168	0.002	4.97392E-25	12
Foxd1	0.524719573	0.143	0.001	8.73628E-25	12
Rps4y2	1.045576326	0.345	0.032	8.80666E-25	12
Ajap1	0.64570192	0.176	0.003	1.1274E-24	12
Fkbp9.1	0.950381148	0.328	0.028	1.32676E-24	12
Snhg11	1.025937424	0.218	0.007	1.41582E-24	12
Dusp6.4	1.220981905	0.42	0.09	1.56257E-24	12
Acadl.2	1.049741417	0.429	0.062	1.6898E-24	12
Marcksl1.6	-1.21135715	0.353	0.724	2.08231E-24	12
Tgm2	0.649149797	0.151	0.001	3.55508E-24	12
Gnb4.3	1.149820198	0.487	0.102	3.87757E-24	12
Itgb1.3	1.021600756	0.731	0.36	3.96311E-24	12
Scara5	0.545173123	0.118	0	4.4073E-24	12
Tbxa2r	0.478439507	0.118	0	5.19759E-24	12
Hnrnpa2b1.4	-0.723527296	0.84	0.934	6.82302E-24	12
Unc93b1.1	0.573458589	0.218	0.011	7.86309E-24	12
Anxa7.1	0.711322035	0.235	0.009	8.24755E-24	12
Nnat.8	-1.392373011	0.244	0.713	2.59051E-23	12
B2m.7	1.073988925	0.647	0.229	2.63596E-23	12
Fap	0.494914033	0.126	0	3.02122E-23	12
Sptbn1.8	1.03741433	0.739	0.35	3.15046E-23	12
Tnfaip2	0.693305376	0.134	0.001	5.38959E-23	12
Add3.5	1.082262249	0.504	0.101	5.56075E-23	12
Tuba1a.8	-0.98273745	0.773	0.93	6.13267E-23	12
Dlc1	0.703192071	0.21	0.007	8.32877E-23	12
Twist1	0.535332595	0.134	0.001	1.03202E-22	12
Efemp2.1	0.908445795	0.252	0.015	1.12499E-22	12
Basp1.11	-1.331237765	0.277	0.707	1.22379E-22	12
Adap2.1	0.981518286	0.16	0.003	1.93299E-22	12
Cstb.1	1.063840102	0.521	0.114	2.26869E-22	12
Serping1.2	1.110689554	0.319	0.031	2.84373E-22	12
Parp3.1	0.542353032	0.151	0.002	3.5302E-22	12
Nfia.3	-1.167661492	0.42	0.776	3.81705E-22	12
Maf.2	0.962225501	0.269	0.018	3.97293E-22	12
H2-K1.2	0.824019856	0.244	0.013	4.0312E-22	12
Tnfrsf1a.2	0.80717484	0.294	0.023	4.15117E-22	12
S100a10.1	0.890085349	0.311	0.027	4.23626E-22	12
Vamp5.1	0.662438907	0.16	0.002	4.69035E-22	12
Rhbdf1.1	0.831486259	0.185	0.005	4.90678E-22	12
Ptges	0.526884025	0.143	0.001	4.95594E-22	12
Cyba.2	0.737031855	0.294	0.028	4.97737E-22	12
Rgs4	1.122112708	0.168	0.004	6.43004E-22	12

Osmr	0.617463301	0.134	0.001	7.49832E-22	12
Lpl.2	1.164560568	0.37	0.056	1.31087E-21	12
Plin3.2	0.659381202	0.202	0.007	2.07085E-21	12
Nfix.7	-1.281513251	0.252	0.676	2.67114E-21	12
Tenc1	0.763797527	0.202	0.007	3.43916E-21	12
Adamts12	0.609613277	0.168	0.003	3.52495E-21	12
Ndufa4.1	-0.993449793	0.429	0.804	3.71827E-21	12
Cebpd	0.867832684	0.218	0.01	4.30246E-21	12
Foxq1	0.681892302	0.176	0.005	5.09937E-21	12
Abca1.2	0.860569176	0.277	0.022	5.12932E-21	12
BC028528.1	0.778559431	0.193	0.006	5.60004E-21	12
Ecm1	0.643013295	0.168	0.003	1.02119E-20	12
Tgfbr2.1	0.747265081	0.202	0.007	1.02657E-20	12
Cpxm1.1	0.950655068	0.235	0.013	1.08462E-20	12
Stard8.1	0.697202973	0.16	0.003	1.25219E-20	12
Cst3.10	0.663741457	0.874	0.533	1.55119E-20	12
Creb3l1	0.587129199	0.143	0.002	1.62874E-20	12
Rab3il1.1	0.648225623	0.185	0.005	1.79976E-20	12
Tbx15	0.444884275	0.109	0	2.29257E-20	12
Barhl1.10	-1.24625743	0.076	0.524	3.50698E-20	12
Ginm1.1	1.049079268	0.42	0.079	3.83019E-20	12
E130114P18Rik.6	-1.424296789	0.143	0.587	4.61261E-20	12
Coro1b.1	1.052718848	0.588	0.236	4.88513E-20	12
Arpc1b.1	0.886734505	0.319	0.034	5.16859E-20	12
Ecm2.1	0.743447979	0.151	0.003	5.18268E-20	12
H6pd	0.756458268	0.193	0.007	7.47824E-20	12
Itpripl2.1	0.661119996	0.16	0.003	7.58512E-20	12
Lox	0.585489004	0.118	0.001	8.05146E-20	12
Nrp1.1	1.077788341	0.336	0.042	1.04156E-19	12
Bex2.2	-1.294457548	0.151	0.574	1.24741E-19	12
Arhgdib.1	0.889552523	0.193	0.007	1.31085E-19	12
Cebpb.1	0.625174937	0.193	0.007	1.88762E-19	12
Nr2f2.1	0.960418399	0.429	0.076	2.28535E-19	12
Calm2.9	-0.878489881	0.63	0.876	2.41847E-19	12
Pdpn.1	0.655604643	0.202	0.009	3.44471E-19	12
Shisa5.2	0.639675471	0.261	0.024	3.46975E-19	12
Col16a1.1	0.481087626	0.16	0.004	4.41835E-19	12
Ccnd2.8	-1.331401521	0.286	0.664	4.76962E-19	12
Arhgap29	0.94758239	0.286	0.027	4.90014E-19	12
Lrrc17	0.335977223	0.101	0	5.42803E-19	12
Anp32e.11	-1.295643272	0.252	0.616	5.84638E-19	12
Kcne4	0.618125461	0.109	0.001	7.99226E-19	12
Selenbp1	0.663659857	0.176	0.006	1.00468E-18	12
H3f3b.4	-0.728902744	0.765	0.907	1.22363E-18	12
App.8	0.772279074	0.882	0.565	1.54209E-18	12
Col7a1	0.619056597	0.16	0.004	1.60773E-18	12
Ech1.2	0.997904458	0.471	0.112	1.62559E-18	12
Rapgef4	0.727769488	0.202	0.009	2.30468E-18	12

Ubtd1.2	0.654203758	0.168	0.005	2.55085E-18	12
Lamp2.3	1.049720802	0.487	0.135	2.82253E-18	12
Aspn	0.854744294	0.109	0.001	3.07291E-18	12
Ndufa4l2	0.479553873	0.118	0.001	4.25796E-18	12
Stmn3.6	-1.220845281	0.202	0.624	5.24553E-18	12
Lrp1.3	0.925106097	0.336	0.045	8.17266E-18	12
Cyp2d22.1	0.553906014	0.126	0.002	8.75889E-18	12
Frmd6	0.728648793	0.202	0.01	1.06324E-17	12
1810058I24Rik	1.027113679	0.546	0.194	1.08737E-17	12
Chd4.4	-0.914384253	0.555	0.771	1.09175E-17	12
Pros1.2	0.833268931	0.252	0.022	1.25714E-17	12
Gjb6.1	0.798651933	0.151	0.004	1.26506E-17	12
Calr.4	0.805512912	0.815	0.508	1.75745E-17	12
Flrt2	1.013237318	0.395	0.08	1.76775E-17	12
Cdh5	0.491160748	0.134	0.002	1.82702E-17	12
Hnrnpdl.3	-0.848204743	0.513	0.757	2.05318E-17	12
Gpx7.2	0.934047409	0.353	0.06	2.27324E-17	12
F3.1	0.766664533	0.176	0.006	2.81015E-17	12
Gucy1a3.2	0.959805106	0.16	0.009	3.1936E-17	12
Malat1.9	0.807635849	0.975	0.955	3.85534E-17	12
Arhgap6	0.605734942	0.109	0.001	4.62443E-17	12
Ptrf	0.599040996	0.151	0.004	4.73643E-17	12
Efcc1	0.487787095	0.101	0	5.08731E-17	12
Cog7.8	-1.261002098	0.168	0.587	5.23071E-17	12
Sept7.3	0.797401929	0.782	0.587	5.25762E-17	12
P4hb.2	0.902590564	0.639	0.276	6.22355E-17	12
Wipi1.1	0.843431557	0.286	0.031	6.52878E-17	12
Hint1.6	-0.969352952	0.403	0.705	6.61524E-17	12
Pax6.9	-1.282995223	0.151	0.515	7.44268E-17	12
Col5a1	0.505160156	0.16	0.005	1.08053E-16	12
Cldn11.1	0.846457532	0.277	0.032	1.16339E-16	12
Mgst1.2	0.905332128	0.277	0.031	1.968E-16	12
Gap43.11	-1.432783074	0.202	0.602	2.01243E-16	12
Rhob.1	1.008892976	0.454	0.136	2.68589E-16	12
Slc30a10.1	0.946375401	0.353	0.06	3.20365E-16	12
Slc19a1	0.973533374	0.252	0.033	3.70208E-16	12
Fxyd1.1	0.711446971	0.202	0.012	3.87191E-16	12
Sipa1.1	0.594097372	0.185	0.009	4.3334E-16	12
P2ry1	0.618546319	0.16	0.006	5.05992E-16	12
Mtch1.1	0.921555509	0.597	0.219	8.13193E-16	12
Epb4.1l2.2	0.933311075	0.403	0.09	8.6062E-16	12
Hnrnpab.6	-0.783117892	0.588	0.817	9.85515E-16	12
Syt11.9	-1.266297013	0.109	0.507	1.05494E-15	12
Rbms1.3	0.871360381	0.597	0.213	1.3883E-15	12
Col5a3.1	0.438557073	0.126	0.002	1.63281E-15	12
Pdxk	0.733291261	0.21	0.015	1.87287E-15	12
Chst7.1	0.481677412	0.151	0.005	2.03386E-15	12
Fbn1	0.640177397	0.151	0.005	2.20318E-15	12

Meg3.7	0.584126316	0.387	0.093	2.29294E-15	12
Gcnt2	0.490933701	0.109	0.001	2.43659E-15	12
Gria2.10	-1.398101813	0.143	0.523	2.62857E-15	12
Tubb2b.10	-1.355155356	0.101	0.466	2.79161E-15	12
Lmna	0.752024331	0.244	0.023	2.87019E-15	12
Nasp.10	-1.064620459	0.37	0.642	2.98966E-15	12
Sspn.1	0.796168024	0.193	0.013	3.6116E-15	12
Dpysl4.7	-1.203807784	0.092	0.493	3.73435E-15	12
Clec3b.1	0.679625808	0.126	0.002	4.34106E-15	12
Etfb.5	1.003623681	0.471	0.182	5.53244E-15	12
Phactr2	0.714704285	0.21	0.015	5.6103E-15	12
Rbm46	0.357950394	0.101	0.001	5.72791E-15	12
Dock6	0.693408932	0.193	0.011	5.94968E-15	12
Hsp90aa1.4	-1.076049692	0.235	0.619	6.15927E-15	12
Rhoc.3	0.671594542	0.252	0.026	7.36847E-15	12
Miat.10	-1.451677886	0.16	0.517	9.63812E-15	12
Zcchc24.2	0.771610969	0.252	0.026	1.01534E-14	12
Pdgfra.6	0.998374479	0.513	0.179	1.0212E-14	12
2700094K13Rik.11	-1.074190905	0.319	0.616	1.0481E-14	12
Ralb.3	0.69293947	0.218	0.017	1.08329E-14	12
Spats2l.1	0.880202287	0.303	0.044	1.13901E-14	12
Fam46a.1	0.647376206	0.143	0.004	1.24302E-14	12
Tec.1	0.624206611	0.16	0.006	1.30519E-14	12
Ifnar2.2	0.862464947	0.311	0.048	1.71124E-14	12
Cast	0.76640217	0.176	0.011	1.76904E-14	12
Hsd11b1.1	0.599538808	0.143	0.004	1.78539E-14	12
Ctdspl	0.826767059	0.269	0.033	1.81918E-14	12
Tpm4.7	0.872640542	0.622	0.314	2.07728E-14	12
Mmp19	0.454838989	0.126	0.002	2.16653E-14	12
A230050P20Rik.1	0.661852633	0.202	0.014	2.57033E-14	12
Tubb3.11	-1.522751553	0.101	0.476	2.59315E-14	12
Mxd4.6	0.81806917	0.58	0.195	2.65048E-14	12
Fkbp3.7	-0.889646433	0.487	0.746	2.70806E-14	12
Rab20	0.442010024	0.118	0.002	2.75808E-14	12
Lef1	0.450089495	0.118	0.002	3.07197E-14	12
Capns1.3	0.87259917	0.588	0.225	3.48748E-14	12
Ier3.3	0.87737801	0.269	0.033	3.77642E-14	12
Vim.8	0.921216903	0.672	0.299	3.97156E-14	12
Isyna1.1	0.611289552	0.176	0.009	4.05637E-14	12
Draxin.8	-1.14073895	0.16	0.556	4.09264E-14	12
Scarf2	0.415215824	0.109	0.002	5.68419E-14	12
Anp32a.3	-0.660344763	0.689	0.837	7.96049E-14	12
Wwtr1.1	0.665874132	0.176	0.01	8.69472E-14	12
Nsg2.9	-1.178622853	0.109	0.489	8.87845E-14	12
Scg3.5	-1.187583396	0.101	0.448	9.08518E-14	12
Gm14005	0.482190215	0.109	0.002	9.29606E-14	12
Celf2.11	-1.098841816	0.303	0.64	1.07804E-13	12
Mpg	0.879414953	0.218	0.02	1.15197E-13	12

Mmp11	0.579856322	0.168	0.009	1.29622E-13	12
Vimp.4	0.916425298	0.538	0.187	1.40777E-13	12
D430041D05Rik.7	-1.206737124	0.126	0.494	1.50414E-13	12
Ywhae.1	-0.753232517	0.521	0.728	1.85024E-13	12
Tmem59.3	0.762144828	0.739	0.389	2.13568E-13	12
Txnip.1	0.981359438	0.403	0.105	2.17955E-13	12
Gypc	0.647570907	0.193	0.014	2.22369E-13	12
Lhx1.9	-1.264093574	0.126	0.499	2.24896E-13	12
Ppfibp2	0.730787479	0.193	0.014	2.83286E-13	12
Kdelr2.1	0.842005794	0.546	0.203	2.83427E-13	12
Pam.1	0.929541234	0.361	0.078	2.86862E-13	12
Ppp2r2c.6	-1.139109663	0.134	0.507	3.01609E-13	12
Ybx1.5	-0.72988521	0.613	0.787	3.54749E-13	12
Neurod1.12	-1.683426969	0.185	0.535	3.5949E-13	12
Il6st.1	0.682372807	0.269	0.038	3.97299E-13	12
Parm1	0.583140929	0.118	0.003	4.9817E-13	12
Ccdc141.1	0.814247312	0.16	0.008	5.02076E-13	12
Mcam	0.73195799	0.109	0.002	5.08985E-13	12
Lrrk1	0.433533518	0.126	0.003	5.64472E-13	12
Sat1.2	0.717019464	0.336	0.065	6.2397E-13	12
S100a13.3	0.435118995	0.21	0.029	6.27494E-13	12
Lpp.2	0.63774615	0.218	0.022	6.39462E-13	12
Art3	0.740974184	0.101	0.002	6.53089E-13	12
Ezh2.10	-1.102855096	0.269	0.612	7.25571E-13	12
Maged2.4	0.84564819	0.504	0.164	1.08519E-12	12
Rest.2	0.799058084	0.21	0.021	1.2518E-12	12
Id2.8	-1.095418086	0.118	0.488	1.54425E-12	12
Ikbip.1	0.697894235	0.261	0.035	1.7394E-12	12
Fosb.6	1.196512772	0.42	0.154	1.77517E-12	12
Ezr.7	-1.152615601	0.092	0.44	1.90901E-12	12
Antxr1	0.598452001	0.193	0.015	2.54257E-12	12
Gadd45b	0.692853294	0.176	0.012	2.63329E-12	12
Map2.10	-1.138300575	0.126	0.494	2.71645E-12	12
Gsn.2	0.757039673	0.176	0.012	2.87025E-12	12
Tmem50a.3	0.752095946	0.748	0.403	3.725E-12	12
Fzd6	0.508035027	0.109	0.002	3.83551E-12	12
Slc41a1.1	0.77229259	0.261	0.037	3.85569E-12	12
Dnajc3.2	0.854476407	0.504	0.174	3.88426E-12	12
Epas1.1	0.641155737	0.151	0.007	4.65079E-12	12
Gprc5c	0.37361199	0.109	0.002	4.93135E-12	12
Ntm.8	0.85756826	0.462	0.143	5.23212E-12	12
Smc2.10	-1.245917883	0.269	0.547	5.38747E-12	12
Serbp1.8	-0.638823634	0.739	0.87	7.74209E-12	12
Ets1	0.676895323	0.16	0.009	9.07111E-12	12
Chd7.9	-0.974668321	0.345	0.649	9.11885E-12	12
Bmp4.1	0.336247875	0.101	0.006	9.21362E-12	12
Rras	0.556374597	0.16	0.009	1.07477E-11	12
Banf1.9	-0.815911546	0.479	0.695	1.17054E-11	12

Dut.10	-1.135428916	0.16	0.436	1.17779E-11	12
Psip1.5	-0.86926462	0.403	0.689	1.25352E-11	12
Cd151.1	0.784612435	0.185	0.017	1.26038E-11	12
Kank2	0.468469126	0.143	0.007	1.42586E-11	12
Plin2.1	0.812605039	0.286	0.051	1.42715E-11	12
Mex3a.5	-1.136006985	0.134	0.465	1.47341E-11	12
Tacc1.2	0.73626847	0.336	0.068	1.50441E-11	12
Smtn.1	0.624943596	0.244	0.032	1.78302E-11	12
1500016L03Rik.8	-1.214906589	0.067	0.385	1.89374E-11	12
Cryab.2	1.057257031	0.168	0.021	2.05757E-11	12
Timp2.3	0.578308795	0.202	0.025	2.30576E-11	12
Apex1.6	-1.008338399	0.168	0.453	2.60939E-11	12
Amotl1	0.896505454	0.336	0.076	2.67335E-11	12
Arhgap24	0.530522427	0.118	0.004	2.78685E-11	12
Hsp90b1.8	0.609447698	0.924	0.69	2.8865E-11	12
H2afv.10	-0.816547335	0.513	0.729	3.07639E-11	12
Hn1.8	-1.037185794	0.21	0.543	3.89682E-11	12
Plekha2.2	0.534191462	0.126	0.005	5.43207E-11	12
Fos.7	1.082225224	0.571	0.302	5.73288E-11	12
Vcam1.2	0.722859568	0.126	0.006	6.46708E-11	12
Stmn2.11	-1.283631519	0.227	0.555	7.01208E-11	12
Ngfrap1.2	-0.811853954	0.437	0.659	7.24268E-11	12
Pde8b	0.588590785	0.126	0.005	7.38295E-11	12
Txndc5	0.744621011	0.303	0.059	7.76809E-11	12
Jag1	0.807316942	0.244	0.039	8.40188E-11	12
Ldlrap1	0.458146067	0.109	0.003	9.42468E-11	12
Gm9800.6	-0.988680912	0.202	0.537	9.64539E-11	12
Scg5.7	-1.095059588	0.034	0.336	9.92798E-11	12
Gadd45a.2	0.880567608	0.353	0.086	1.01342E-10	12
Mki67.12	-1.470298339	0.109	0.396	1.14673E-10	12
Arhgef6	0.532220058	0.126	0.005	1.30298E-10	12
Lgals9.1	0.497277828	0.143	0.008	1.43399E-10	12
Itgb5.2	0.523497452	0.143	0.008	1.45374E-10	12
Sept4.10	0.881749861	0.555	0.224	1.52744E-10	12
Nhlh2.10	-1.218860455	0.092	0.425	1.56112E-10	12
Mir22hg.1	0.473346656	0.126	0.005	1.93062E-10	12
Ilf2.3	-0.968852217	0.202	0.543	1.97272E-10	12
Surf4.2	0.810689423	0.403	0.117	1.97324E-10	12
Crtap	0.720861414	0.227	0.033	1.9781E-10	12
Cpt1a.2	0.746010612	0.176	0.016	2.16214E-10	12
Fhl2	0.617842475	0.185	0.017	2.23177E-10	12
Rhod.1	0.394085574	0.109	0.003	2.43393E-10	12
Pabpc1.6	-0.538374261	0.807	0.879	2.64274E-10	12
Pde1c.12	-1.245515571	0.076	0.389	2.94006E-10	12
Ddah2.7	-0.869819411	0.454	0.687	3.09497E-10	12
Tpst1	0.752731972	0.345	0.081	3.69906E-10	12
Sec24d	0.500654482	0.134	0.007	4.06438E-10	12
Gm17750.6	-1.148068861	0.059	0.369	4.17328E-10	12

Aga.2	0.779825791	0.235	0.035	4.56689E-10	12
Whsc1.5	-1.044152539	0.16	0.466	4.83757E-10	12
Tnfrsf21.2	0.839418722	0.294	0.062	5.03827E-10	12
Lmnb1.11	-1.026291917	0.034	0.301	5.28699E-10	12
Rbfox3.9	-1.089637352	0.151	0.496	7.02044E-10	12
Top2a.11	-1.437700728	0.143	0.429	7.09595E-10	12
Kif21a.4	-1.06509844	0.059	0.347	7.55074E-10	12
Fkbp11.1	0.483164993	0.151	0.01	7.90635E-10	12
Ifi30.1	0.469329737	0.16	0.015	8.0833E-10	12
Oxct1.1	-0.952905948	0.252	0.554	8.50178E-10	12
Hnrnph1.3	-0.80494767	0.378	0.629	8.91874E-10	12
Leprel2	0.740464206	0.336	0.078	9.12437E-10	12
Eng	0.442353731	0.101	0.003	9.91109E-10	12
Gatm.3	0.829640599	0.227	0.033	9.97877E-10	12
Utrn	0.690752803	0.21	0.026	1.18859E-09	12
Slc16a9	0.347157718	0.101	0.003	1.21713E-09	12
Soga3.8	-1.06054568	0.143	0.459	1.35599E-09	12
Tmpo.11	-1.055703983	0.134	0.454	1.36039E-09	12
Myadm	0.554307479	0.193	0.023	1.41859E-09	12
Rufy3.10	-1.086659324	0.092	0.373	1.42446E-09	12
Map1b.10	-1.001813826	0.403	0.588	1.51606E-09	12
Sh3pxd2a	0.377150512	0.101	0.002	1.53887E-09	12
Nrxn2.2	0.720291212	0.353	0.088	1.54896E-09	12
Gm1673.5	-1.02322532	0.143	0.475	1.55423E-09	12
Mcm7.8	-1.038569459	0.092	0.391	1.56563E-09	12
Arfgap3	0.504196655	0.202	0.026	1.6744E-09	12
Gucy1b3.1	0.86305144	0.193	0.037	1.73589E-09	12
Pmepa1.1	0.806679088	0.193	0.026	1.7786E-09	12
Aebp1.1	0.522906225	0.134	0.008	2.14708E-09	12
Scn2b	0.519799776	0.143	0.009	2.29873E-09	12
Lrp10.2	0.738838223	0.244	0.04	2.54093E-09	12
Tmem51	0.425873783	0.118	0.005	2.612E-09	12
Zfhx4	0.42709378	0.143	0.011	2.62081E-09	12
Ina.11	-1.118550032	0.126	0.445	2.66426E-09	12
Fbln1	0.769630632	0.193	0.042	2.76877E-09	12
Ephx1.1	0.461636408	0.126	0.006	2.78227E-09	12
Dclk1.7	-1.069425043	0.118	0.414	2.90247E-09	12
Pdia3.2	0.759820506	0.563	0.294	3.14402E-09	12
Degs1.3	0.605081606	0.319	0.074	3.19368E-09	12
Rora.2	0.718917036	0.277	0.053	3.62801E-09	12
Xbp1.1	0.743125387	0.395	0.122	3.7898E-09	12
Slc9a3r1.1	0.646890762	0.143	0.013	4.32075E-09	12
Snap25.8	-1.04761809	0.025	0.283	4.684E-09	12
Litaf.2	0.729055622	0.303	0.067	4.95061E-09	12
Gpr116	0.42984855	0.101	0.004	5.14755E-09	12
Hes1.10	0.818310661	0.445	0.146	5.28772E-09	12
Tmed3.1	0.764509669	0.319	0.081	5.28806E-09	12
Sept9.1	0.583132593	0.319	0.077	5.63932E-09	12

Ttyh2.2	0.730181242	0.235	0.037	6.39177E-09	12
Pkd2	0.740123206	0.311	0.07	6.49371E-09	12
H2afy.7	-0.777978001	0.395	0.593	6.97282E-09	12
Slc38a3.2	0.679895736	0.185	0.021	7.08487E-09	12
Plxdc1	0.453922202	0.101	0.003	7.17751E-09	12
Pfn1.4	0.632499506	0.765	0.509	7.44009E-09	12
Ctsz.3	0.564148797	0.412	0.135	7.47571E-09	12
Atpif1.3	-0.611184094	0.63	0.817	7.55906E-09	12
Meis1.4	-1.02700006	0.076	0.374	8.01527E-09	12
Gpc6	0.599936245	0.176	0.02	8.7485E-09	12
Ptma.6	-0.805556526	0.345	0.583	8.99713E-09	12
Magt1.3	0.687648518	0.277	0.055	9.21306E-09	12
Ccnd1.9	-1.049755932	0.252	0.524	9.913E-09	12
Tmbim1.1	0.327937009	0.143	0.013	1.02394E-08	12
Ppp1r14b.5	-0.988794413	0.134	0.398	1.22769E-08	12
Arhgap20.2	0.603150085	0.261	0.052	1.2673E-08	12
Fermt2.3	0.826729055	0.437	0.203	1.33594E-08	12
Vps37b.5	-0.954837118	0.168	0.472	1.41372E-08	12
Rps5.7	-0.476912055	0.933	0.954	1.42304E-08	12
Dek.11	-0.820692203	0.496	0.695	1.49142E-08	12
Srsf3.6	-0.724210826	0.42	0.658	1.52231E-08	12
Trip6.1	0.546177228	0.193	0.024	1.64915E-08	12
Nenf.4	0.788641284	0.412	0.134	1.76448E-08	12
Apba2.4	-0.974277572	0.109	0.416	1.89124E-08	12
Cadm1.5	-0.975872046	0.109	0.392	1.95255E-08	12
Ttc3.8	-0.658870872	0.63	0.83	2.03826E-08	12
S1pr2	0.408293132	0.101	0.003	2.31099E-08	12
Ifih1	0.634215347	0.109	0.005	2.31488E-08	12
Ranbp1.8	-0.745461941	0.479	0.676	2.62313E-08	12
Tsc22d3	0.878392048	0.261	0.09	2.82205E-08	12
Sec62.3	0.692450934	0.681	0.404	3.13063E-08	12
Jak1.2	0.814820772	0.387	0.126	3.13997E-08	12
Luc7l3.3	-0.724373455	0.504	0.749	3.14997E-08	12
Atrx.4	-0.67713751	0.496	0.742	3.35719E-08	12
Uncx.10	-1.064075691	0.076	0.319	3.42037E-08	12
Grb10	0.637847536	0.202	0.028	3.51237E-08	12
Tmem86a.1	0.490804597	0.168	0.019	3.58198E-08	12
Hnrnpm.6	-0.555524719	0.647	0.735	3.89474E-08	12
Rsu1.2	0.781542407	0.37	0.117	4.08314E-08	12
Calu	0.761878894	0.445	0.168	4.25109E-08	12
Bin1.10	-1.035280579	0.151	0.431	4.95869E-08	12
Ecscr.1	0.516652168	0.109	0.005	4.96058E-08	12
Pth1r.1	0.700290526	0.101	0.006	4.99285E-08	12
Slc1a3.8	0.695650525	0.42	0.15	5.05626E-08	12
Zfp36l1.8	0.749622801	0.429	0.149	5.10343E-08	12
Pqlc1.9	-0.961401487	0.034	0.299	6.25904E-08	12
Insm1.5	-1.021214793	0.042	0.294	6.51962E-08	12
Pttg1ip.3	0.71475101	0.319	0.084	6.61137E-08	12

Plod1.1	0.482191917	0.176	0.023	6.72424E-08	12
Ebpl.1	0.4804552	0.303	0.088	7.24955E-08	12
Vegfa.1	0.68740218	0.176	0.025	7.49792E-08	12
Fubp1.4	-0.836392973	0.319	0.568	8.25102E-08	12
Tcirg1.1	0.543363238	0.134	0.01	8.96409E-08	12
Vkorc1.1	0.705460439	0.361	0.109	9.49816E-08	12
Chchd10.1	0.72293671	0.21	0.032	1.10883E-07	12
Matr3.2	-0.715663502	0.462	0.708	1.25397E-07	12
Lims1.2	0.720291724	0.462	0.18	1.29069E-07	12
Ivns1abp.3	-0.909806399	0.118	0.417	1.44809E-07	12
Ilk	0.704689136	0.479	0.193	1.44872E-07	12
Tbata.9	-1.122241246	0.067	0.341	1.51506E-07	12
Slc38a1.3	-0.896089469	0.025	0.271	1.51669E-07	12
Acin1.2	-0.630821065	0.58	0.724	1.60663E-07	12
Lamp1.3	0.639963242	0.639	0.333	1.65591E-07	12
9430020K01Rik	0.470662668	0.118	0.007	1.66296E-07	12
Thy1	0.695798689	0.134	0.014	1.69196E-07	12
Rps9.5	-0.480314725	0.866	0.929	1.731E-07	12
Hexa.2	0.586029907	0.277	0.064	1.91243E-07	12
Pbxip1.2	0.668792835	0.151	0.016	1.95313E-07	12
Gpm6a.11	-1.129926868	0.126	0.411	1.98332E-07	12
Mdk.8	0.638820473	0.697	0.377	2.05383E-07	12
As3mt.2	0.503579341	0.143	0.012	2.29417E-07	12
Pld1	0.483068508	0.109	0.006	2.63616E-07	12
Hnrnpd.8	-0.777650044	0.37	0.612	2.93777E-07	12
Ank3.11	-1.0181453	0.134	0.422	2.99107E-07	12
Gaa.3	0.560732924	0.185	0.026	3.4581E-07	12
Snrpf.7	-0.901861189	0.151	0.429	3.46316E-07	12
Mycn.6	-0.965290844	0.084	0.365	3.85719E-07	12
Dap.1	0.700977898	0.235	0.047	3.94522E-07	12
Elavl4.11	-1.093503211	0.084	0.336	4.1021E-07	12
Tspan17	0.47065551	0.101	0.004	4.22414E-07	12
Cacng2.4	-0.977217901	0.076	0.339	4.288E-07	12
Hmgb2.11	-0.984430873	0.176	0.401	4.96051E-07	12
Gm10075.9	-0.833872677	0.269	0.516	4.98898E-07	12
Gng3.11	-1.094474969	0.076	0.318	5.1565E-07	12
Acaa2.2	0.722038299	0.193	0.034	5.62133E-07	12
Comt.3	0.70799982	0.353	0.107	5.62997E-07	12
Arhgap42	0.706393091	0.134	0.02	5.68405E-07	12
Dad1.3	0.671959886	0.605	0.341	5.75655E-07	12
Map3k1.3	-0.918813668	0.109	0.372	5.98252E-07	12
Cdc42ep2	0.395631942	0.109	0.006	6.00215E-07	12
Nop58.8	-0.786256276	0.37	0.593	6.07175E-07	12
Akap7	0.568557409	0.235	0.047	6.13236E-07	12
Elavl3.10	-0.965777149	0.218	0.474	6.37079E-07	12
Dhx9.3	-0.86108235	0.218	0.464	6.75635E-07	12
Ggact	0.360442556	0.101	0.005	7.24358E-07	12
Psap.6	0.605584403	0.471	0.182	7.39825E-07	12

Kif5c.11	-1.021089197	0.134	0.4	7.4344E-07	12
Cygb.1	0.442028433	0.218	0.05	8.39773E-07	12
Gm2694.4	-0.91730336	0.092	0.36	9.85769E-07	12
Fkbp14	0.414007366	0.143	0.014	1.02194E-06	12
Actn4	0.744845477	0.42	0.174	1.08088E-06	12
Rpl32.5	-0.487406118	0.815	0.89	1.1573E-06	12
Dtx4.2	0.360235105	0.109	0.007	1.39691E-06	12
Vwa5a.1	0.382026722	0.134	0.014	1.51598E-06	12
Cyb5r3.1	0.736150224	0.462	0.197	1.75676E-06	12
Sh3d19.1	0.631844789	0.202	0.034	1.77116E-06	12
Cbx1.4	-0.60063036	0.521	0.686	1.8461E-06	12
Hjurp.9	-0.875256502	0.227	0.435	1.99436E-06	12
Leprel4.1	0.486167791	0.185	0.028	2.06002E-06	12
Tox3.4	-0.834620485	0.076	0.347	2.33357E-06	12
Ncam1.5	-0.907997953	0.118	0.388	2.33372E-06	12
Pnn.2	-0.645430137	0.555	0.723	2.34307E-06	12
Axin2	0.486862654	0.21	0.04	2.44371E-06	12
2810417H13Rik.11	-1.0816094	0.126	0.393	2.60408E-06	12
9530068E07Rik	0.686189781	0.445	0.183	2.84816E-06	12
Grk5	0.352549507	0.118	0.01	2.97856E-06	12
Ssrp1.7	-0.721995284	0.353	0.577	3.06875E-06	12
Cdk4.5	-0.686478663	0.429	0.643	3.19009E-06	12
Lifr.1	0.380098264	0.101	0.005	3.39955E-06	12
Itpr2.2	0.686206734	0.185	0.029	3.43974E-06	12
Cenpf.12	-1.324187532	0.126	0.363	3.44653E-06	12
Fndc3b	0.44861389	0.126	0.011	3.59943E-06	12
Foxf2	0.497557309	0.118	0.009	3.86929E-06	12
Hdgf.8	-0.808406247	0.269	0.533	3.94074E-06	12
Col28a1	0.628644977	0.151	0.022	3.96791E-06	12
Zfp36.2	0.444067467	0.126	0.012	4.04965E-06	12
Hmgn5.9	-0.925533684	0.143	0.426	4.12559E-06	12
Pdia5	0.56918868	0.16	0.022	4.12959E-06	12
Pdap1.4	-0.645324454	0.496	0.684	4.23505E-06	12
Ifngr2.1	0.51720153	0.168	0.023	4.28642E-06	12
Ptprd.4	-0.898583583	0.092	0.341	4.8907E-06	12
Sh3pxd2b	0.483609787	0.151	0.02	5.55514E-06	12
Tex264.2	0.575187054	0.269	0.067	6.19258E-06	12
Engase	0.509743175	0.126	0.011	6.50838E-06	12
Galk1.1	0.548589119	0.227	0.049	6.5262E-06	12
Spry4.1	0.438170253	0.101	0.006	6.66337E-06	12
Etv1.1	0.631405146	0.176	0.027	6.85148E-06	12
Bcl7a.4	-0.886292011	0.076	0.326	7.14805E-06	12
Tmem100.2	0.467266903	0.118	0.009	7.48638E-06	12
Tm9sf3.2	0.632854919	0.605	0.342	7.8241E-06	12
Anp32b.9	-0.668209924	0.445	0.591	7.94545E-06	12
Hnrnpr.2	-0.751931459	0.311	0.546	7.97322E-06	12
Akap12.7	0.720349341	0.345	0.109	7.99074E-06	12
C130071C03Rik.5	-0.880813331	0.067	0.309	8.39299E-06	12

Dnajc9.10	-0.907065469	0.109	0.351	8.45962E-06	12
Wasf2.3	0.656802694	0.345	0.111	9.39406E-06	12
Uap1l1.2	0.415513088	0.109	0.008	9.46462E-06	12
Fnbp1l.8	-0.873836488	0.218	0.455	9.9106E-06	12
P4ha1.3	0.610324726	0.252	0.06	1.04043E-05	12
Smarca5.5	-0.853519384	0.134	0.403	1.09662E-05	12
Dusp3.2	0.679824069	0.168	0.028	1.16912E-05	12
Hirip3.10	-0.91262702	0.134	0.384	1.17725E-05	12
Sfpq.2	-0.636857115	0.487	0.671	1.20913E-05	12
Snrpe.6	-0.713391271	0.345	0.563	1.22113E-05	12
Sept3.12	-0.914958845	0.134	0.394	1.23903E-05	12
Dctpp1.8	-0.862247865	0.067	0.306	1.26255E-05	12
Sntb2	0.55824136	0.168	0.026	1.29347E-05	12
S100a1.3	0.307779149	0.143	0.026	1.44463E-05	12
Cpe.8	-0.696545763	0.168	0.457	1.96282E-05	12
Klf6.5	0.772133481	0.37	0.146	2.01221E-05	12
Tvp23b.1	0.459716918	0.21	0.043	2.03891E-05	12
Nr3c1.5	0.685212436	0.353	0.124	2.04395E-05	12
Pon2.2	0.532408003	0.202	0.038	2.05897E-05	12
Eif4g2.2	-0.590439087	0.529	0.706	2.17412E-05	12
Crip2.4	0.597175876	0.521	0.238	2.19404E-05	12
Smc4.10	-0.763866764	0.437	0.567	2.20499E-05	12
Rbm25.2	-0.544341603	0.672	0.813	2.21819E-05	12
Fut9.4	-0.831172208	0.017	0.213	2.36824E-05	12
Atraid.2	0.538123852	0.504	0.224	2.42606E-05	12
Ccdc88a.3	-0.823865531	0.244	0.483	2.73576E-05	12
Pa2g4.8	-0.732341927	0.303	0.523	2.81668E-05	12
Tgfb3	0.412886316	0.118	0.011	2.84696E-05	12
Spsb2	0.410863376	0.118	0.011	2.95342E-05	12
Orai3.1	0.389193167	0.101	0.007	3.00759E-05	12
Set.7	-0.810613977	0.151	0.388	3.25461E-05	12
Rpl13a.5	-0.346966378	0.95	0.928	3.31478E-05	12
Cbfa2t3.9	-0.865300826	0.143	0.404	3.41623E-05	12
Npm1.8	-0.616574644	0.496	0.647	3.5256E-05	12
Rps26.7	-0.489430703	0.773	0.848	3.94179E-05	12
Cplx2.7	-0.844878695	0.235	0.496	4.23545E-05	12
Sec31a	0.557590269	0.269	0.074	4.6936E-05	12
Smc3.4	-0.644654728	0.462	0.634	5.00838E-05	12
Dcx.11	-0.920630478	0.126	0.377	5.09481E-05	12
Dnajb11.2	0.651008484	0.395	0.161	5.18127E-05	12
Hmgb3.6	-0.798616106	0.067	0.307	5.38186E-05	12
Tmem110	0.448026673	0.16	0.024	5.49659E-05	12
Rnh1.2	0.365442981	0.227	0.061	5.97882E-05	12
Cacna2d1.8	-0.883956697	0.126	0.376	6.00344E-05	12
RP23-45G16.5.12	-0.933795794	0.092	0.328	6.09449E-05	12
Yipf3	0.684349551	0.353	0.137	6.16273E-05	12
Jup	0.420093263	0.126	0.014	6.21072E-05	12
Epb4.1l3.3	0.538268327	0.336	0.112	6.22907E-05	12

Stmn1.5	-0.811551416	0.059	0.285	6.38083E-05	12
Grn.2	0.458926377	0.277	0.085	6.51492E-05	12
Bcap31.2	0.623843839	0.42	0.172	6.65352E-05	12
Ptpn9	0.678302054	0.202	0.049	6.80604E-05	12
Cox7a2.2	-0.48630988	0.63	0.713	6.91408E-05	12
Gse1.5	-0.845853644	0.034	0.238	7.07809E-05	12
Ppapdc1b	0.370377194	0.176	0.035	7.10046E-05	12
Pbk.10	-0.802152907	0.025	0.223	7.70081E-05	12
Imp3.4	0.653641609	0.487	0.245	8.03414E-05	12
Atp6v0e2.6	-0.8177675	0.05	0.261	8.16351E-05	12
Marc2.3	0.684618389	0.269	0.082	8.26151E-05	12
Stmn4.12	-1.070493835	0.118	0.342	8.28124E-05	12
Kdelr1	0.662055351	0.37	0.145	8.58367E-05	12
Rangap1.11	-0.818338961	0.067	0.257	9.27976E-05	12
Anxa6.1	0.450779611	0.235	0.06	9.44353E-05	12
C1ql1.7	-0.796800676	0.059	0.28	9.92589E-05	12
Pten	0.713269638	0.319	0.118	9.9785E-05	12
Cnot6.3	-0.71991253	0.202	0.392	9.99966E-05	12
Serinc5.1	0.613906037	0.235	0.06	0.000100016	12
Pid1.1	0.563616409	0.202	0.042	0.000101427	12
Rnmt.8	-0.778946185	0.101	0.35	0.000101996	12
Tspan9	0.410154205	0.109	0.009	0.000105765	12
H1f0.7	-0.694282239	0.437	0.617	0.000112529	12
Galnt18.1	0.442075467	0.109	0.01	0.00011554	12
Slc50a1.2	0.437369016	0.252	0.081	0.000125168	12
Iqgap1.1	0.575128166	0.361	0.13	0.000128206	12
Elovl6.4	-0.833111849	0.076	0.279	0.000129351	12
Ppfibp1.1	0.604266293	0.176	0.031	0.0001348	12
Matn2	0.487019471	0.118	0.013	0.000135439	12
Ran.8	-0.6497852	0.252	0.415	0.000139628	12
Rcor2.8	-0.806413736	0.025	0.226	0.00014351	12
Rps3.5	-0.373494537	0.891	0.922	0.000147732	12
Slc12a2.2	0.724294208	0.294	0.109	0.000147937	12
Bcas1.5	-0.916397932	0.067	0.283	0.000152236	12
Cks1b.11	-0.833747641	0.101	0.346	0.000154548	12
Plekhg2	0.449078582	0.143	0.02	0.000157248	12
Tln1.4	0.72613413	0.361	0.148	0.00016062	12
Arhgef40	0.676097129	0.227	0.056	0.000166105	12
Mprip.1	0.705188465	0.37	0.15	0.000172411	12
Cdk5r1.10	-0.904366224	0.109	0.334	0.000183631	12
6330403K07Rik.9	-0.798727087	0.059	0.243	0.000195188	12
Nrn1.8	-0.874265719	0.034	0.222	0.00020255	12
Cflar.2	0.530750548	0.202	0.049	0.000211036	12
Islr2.7	-0.76355544	0.008	0.181	0.000212305	12
Bclaf1.2	-0.636365336	0.412	0.584	0.000213057	12
C530008M17Rik.5	-0.832163099	0.059	0.269	0.00021503	12
Arl6ip1.9	-0.770540026	0.429	0.55	0.000216823	12
Fgf9.3	-0.829955829	0.05	0.261	0.000219332	12

Pdia4.1	0.566528725	0.445	0.194	0.000249635	12
Sri.4	0.5971193	0.403	0.164	0.000254964	12
Ptprs.9	-0.882137355	0.21	0.422	0.000256145	12
Prmt8.7	-0.780686608	0.084	0.273	0.000267677	12
Srpk2.2	-0.684620527	0.261	0.43	0.000271298	12
Tmem150a.1	0.485608498	0.134	0.017	0.000271375	12
Cyp51.3	-0.588188771	0.017	0.142	0.000285704	12
Nhlh1.9	-0.917492612	0.042	0.224	0.000301052	12
Elavl2.6	-0.777019791	0.059	0.271	0.000311283	12
Srrt.3	-0.756722755	0.185	0.425	0.000312669	12
Thoc7.3	-0.701212637	0.269	0.523	0.00031799	12
Cdpf1	0.250731157	0.118	0.019	0.000350027	12
Hnrnpc.3	-0.534962548	0.529	0.668	0.000384357	12
Nfic.2	0.63752192	0.487	0.259	0.000411117	12
Tpx2.12	-1.005164914	0.076	0.282	0.000414541	12
Reep5.4	0.280588113	0.311	0.136	0.000416263	12
Cbx5.9	-0.559673173	0.529	0.653	0.000417248	12
Tmem39a	0.51361498	0.176	0.034	0.000439071	12
Chgb.11	-0.960246065	0.042	0.242	0.000444286	12
Zbtb18.8	-0.807181912	0.084	0.267	0.000445648	12
Rrm1.11	-0.760493149	0.076	0.261	0.000467073	12
Nap1l4.2	-0.704267706	0.277	0.492	0.000470429	12
Col27a1.1	0.437080898	0.118	0.013	0.000472042	12
Apc.11	-0.838669411	0.185	0.433	0.000492918	12
Gjc1.6	0.60228447	0.277	0.195	0.000513817	12
Stt3b.1	0.653645389	0.429	0.21	0.00051548	12
Gm11223.6	-0.853866874	0.025	0.214	0.000520621	12
Tpm1.3	0.718341	0.395	0.189	0.000549695	12
Ccdc34.10	-0.793408675	0.218	0.415	0.000568306	12
Hmgn1.8	-0.496915731	0.529	0.622	0.000579914	12
Glul.4	0.561672114	0.429	0.199	0.000580126	12
Rfc4.10	-0.741143507	0.034	0.227	0.000595413	12
2610017I09Rik.2	-0.691210539	0.025	0.205	0.000595534	12
Cenpa.11	-0.971509454	0.076	0.252	0.000607228	12
Bet1l	0.506067969	0.261	0.077	0.000610604	12
Gstm1.2	0.716013055	0.252	0.081	0.000677179	12
Gdpd1.7	-0.773135534	0.059	0.244	0.000682093	12
Slc1a2.8	-0.843612376	0.076	0.295	0.000708109	12
Myo5a.2	-0.746659658	0.025	0.192	0.000753001	12
St18.11	-0.892781478	0.025	0.191	0.000761675	12
Ssr2	0.57041812	0.58	0.377	0.000811183	12
Fam212b.3	-0.763140319	0.042	0.224	0.000811683	12
Clip3.7	-0.783218601	0.118	0.313	0.000854271	12
Snx18.2	0.453870877	0.185	0.039	0.000891056	12
Sh2b3	0.463372772	0.126	0.016	0.000891835	12
Prkcdbp.1	0.592151944	0.261	0.089	0.000905559	12
Laptm4b.4	0.560158058	0.361	0.139	0.000924552	12
Leprot.2	0.628735283	0.319	0.116	0.000941485	12

Ddx26b.5	0.588362322	0.345	0.128	0.000975196	12
Tuba1b.10	-0.718945639	0.286	0.482	0.001025965	12
Hspa5.4	0.502683729	0.773	0.548	0.001077165	12
Wipf1.2	0.404188696	0.151	0.027	0.001083869	12
Rps19.8	-0.455381787	0.681	0.754	0.001091535	12
H2afz.10	-0.717917694	0.042	0.232	0.001097097	12
Smarcd1.3	-0.766249799	0.084	0.272	0.001116426	12
Ahi1.2	-0.809566021	0.109	0.308	0.001148062	12
Top1.3	-0.547635318	0.546	0.663	0.001164456	12
Celsr2.7	-0.777539979	0.067	0.238	0.001168295	12
St8sia3.4	-0.777903966	0.05	0.251	0.001203376	12
Zic1.10	-0.552433614	0.647	0.785	0.001213135	12
Klf4.1	0.557146114	0.143	0.023	0.00127726	12
Srpr	0.60805574	0.286	0.095	0.001280263	12
Srrm2.3	-0.493231507	0.613	0.732	0.001289783	12
Serpinb6a.1	0.61156483	0.218	0.057	0.001348475	12
Racgap1.12	-0.790992238	0.059	0.256	0.001365458	12
Cntn2.12	-1.105750055	0.092	0.264	0.001379418	12
Ddx39b.4	-0.676580846	0.252	0.445	0.001440119	12
Ppp1r14c.10	-0.908151338	0.076	0.263	0.00151377	12
Cenpe.11	-0.914684388	0.076	0.287	0.001514092	12
Gpc3	0.629135314	0.21	0.064	0.001542917	12
C330027C09Rik.9	-0.517701782	0.025	0.134	0.001559305	12
Ilvbl.1	0.335234575	0.109	0.012	0.001595333	12
Col9a3.4	-0.743525672	0.017	0.181	0.001647722	12
Ghr.1	0.33789667	0.134	0.021	0.001704311	12
Pde5a	0.557140088	0.16	0.032	0.001729239	12
Hdac2.3	-0.627789193	0.319	0.516	0.001740875	12
Sap30.3	-0.565046922	0	0.138	0.001744444	12
Psat1.6	-0.74909858	0.151	0.369	0.001791064	12
Decr1.1	0.559275628	0.244	0.071	0.001860002	12
Cltb.8	-0.74098113	0.261	0.424	0.001950466	12
Bag3	0.361124828	0.118	0.015	0.00195388	12
Tubb6	0.4379646	0.134	0.02	0.001968522	12
Rplp0.6	-0.367283227	0.857	0.872	0.002028136	12
Alx4	0.418761706	0.101	0.01	0.002048225	12
Nrxn1.10	-0.949310596	0.109	0.319	0.002192724	12
Tm9sf2	0.582767055	0.286	0.098	0.002403599	12
Stard13	0.350131993	0.109	0.012	0.002445981	12
Efnb2	0.479382446	0.126	0.018	0.002451075	12
Snx7	0.436139319	0.109	0.014	0.002559367	12
Dtx1.3	-0.671682378	0.017	0.166	0.002624192	12
Cct3.4	-0.623108088	0.319	0.496	0.002656165	12
Prox1.3	-0.748001868	0.042	0.23	0.002714465	12
Dcbld1	0.406441786	0.143	0.024	0.00285752	12
Kif20b.11	-0.800238193	0.034	0.191	0.002859769	12
Rpl8.6	-0.322530343	0.866	0.897	0.002920526	12
Selm.3	0.567116226	0.395	0.172	0.003004782	12

Fam101a	0.622303195	0.176	0.042	0.003055611	12
Ppap2a	0.342513367	0.134	0.023	0.003202806	12
Rcn1.4	0.5361422	0.471	0.232	0.003408384	12
Nusap1.11	-0.813446121	0.067	0.228	0.003422893	12
Rabac1.4	0.590304207	0.487	0.256	0.003455752	12
Ube2c.10	-1.116003697	0.076	0.27	0.003487018	12
Cdca8.12	-0.83607542	0.067	0.265	0.00355874	12
Lrpap1.7	0.445999948	0.303	0.115	0.003592903	12
Cklf.6	-0.581284955	0.025	0.166	0.00361088	12
Notch1.2	0.509662102	0.134	0.022	0.00367192	12
Lap3.8	-0.665546503	0.126	0.277	0.003796293	12
Diap2	0.483416503	0.143	0.028	0.00392556	12
Adam19	0.378389363	0.118	0.016	0.00411734	12
Gsg1l.9	-0.754965127	0.067	0.256	0.00412564	12
Tjp1	0.632611736	0.227	0.073	0.004261713	12
Cd9.3	-0.683954893	0.294	0.486	0.004329782	12
H3f3a.3	-0.684209567	0.176	0.382	0.004888873	12
Rgs16	0.319676301	0.101	0.011	0.004981689	12
Nucb1.2	0.516851533	0.244	0.073	0.005051767	12
Cdc20.11	-0.88494219	0.025	0.197	0.00514953	12
Cdk1.11	-0.820370226	0.092	0.247	0.005201866	12
Hey1.8	-0.757170552	0.067	0.274	0.005415422	12
Tsn.3	-0.600055575	0.387	0.59	0.005709531	12
Tmem167.1	0.564156382	0.487	0.271	0.005728747	12
Homer2.5	-0.736885258	0.059	0.247	0.005767389	12
Rps10.4	-0.436360041	0.546	0.637	0.005841905	12
Gng12.5	0.447856048	0.311	0.119	0.00601115	12
Pcm1.2	-0.635605998	0.277	0.523	0.006289744	12
Nucks1.11	-0.575649268	0.504	0.663	0.006374486	12
Rap2a.3	0.633206479	0.37	0.168	0.006644427	12
Hnrnpk.3	-0.504932261	0.504	0.636	0.006752013	12
Mrpl34.1	-0.703294581	0.118	0.288	0.006929789	12
Sgce.1	0.576375857	0.286	0.103	0.007275367	12
Reln.9	-0.755492209	0.034	0.181	0.00733141	12
Prpf40a.3	-0.587622066	0.378	0.572	0.007376962	12
Kif23.12	-0.531967305	0.042	0.215	0.007441748	12
Bex1.4	-0.68066332	0.042	0.225	0.007628953	12
Tspan13.4	-0.72104509	0.034	0.208	0.007722502	12
Dnajc1.2	0.604886657	0.336	0.138	0.007882257	12
Khdrbs1.3	-0.61478022	0.269	0.495	0.00795221	12
Srrm3.6	-0.782939319	0.05	0.241	0.007993172	12
Ddx1.1	-0.613013253	0.244	0.389	0.008072676	12
9330159F19Rik.6	-0.660488341	0.025	0.169	0.008384146	12
Fam174a.1	0.416360914	0.218	0.063	0.008499409	12
Rundc3a.6	-0.755648616	0.059	0.25	0.008585268	12
Hmgn2.9	-0.661891437	0.059	0.257	0.008645032	12
Bicd1.2	-0.641079482	0.025	0.153	0.008668276	12
Cct2.4	-0.611533417	0.311	0.525	0.00901974	12

Pou3f2.7	-0.763924136	0.092	0.277	0.009159475	12
Hpca.9	-0.793053335	0.076	0.278	0.009396709	12
Satb1.2	-0.691954996	0.034	0.21	0.009585579	12
Pofut2	0.51519693	0.286	0.101	0.009692091	12
Dtymk.9	-0.678955509	0.21	0.44	0.009748041	12
Ilf3.2	-0.697181726	0.168	0.377	0.010130128	12
Nqo2	0.401223955	0.109	0.014	0.010464102	12
Hnrnpa0.3	-0.666232936	0.269	0.457	0.010479796	12
Ostf1.2	0.341493429	0.202	0.062	0.011347684	12
Ak3	0.548741918	0.294	0.109	0.011508259	12
Zfp704.4	-0.70032768	0.067	0.258	0.011670811	12
Lmo4.6	-0.644289414	0.202	0.336	0.01179001	12
Gnb2l1.5	-0.344796227	0.832	0.872	0.011792991	12
Rsl1d1.4	-0.581060147	0.269	0.491	0.012368041	12
Aldh7a1.1	0.550655498	0.218	0.066	0.013320074	12
Gmds	0.573977809	0.143	0.039	0.013463766	12
Caprin1.3	-0.624749775	0.235	0.394	0.013519818	12
Smad5	0.464248631	0.269	0.093	0.013764262	12
Eid1.7	-0.647204743	0.286	0.504	0.013808279	12
Lig1.12	-0.772011111	0.134	0.349	0.014031119	12
Rplp2.7	-0.296609048	0.849	0.84	0.0142776	12
Tmem184b	0.256369726	0.109	0.019	0.014663934	12
Rps21.6	-0.323246808	0.824	0.833	0.015100218	12
Bzw2.2	-0.611594333	0.294	0.489	0.015259716	12
Tshz2.6	-0.725827595	0.109	0.321	0.015839607	12
Prdx5.4	0.49215708	0.496	0.262	0.015852946	12
Rtn3.4	-0.64018589	0.261	0.478	0.015935339	12
Pigk.2	0.369800717	0.176	0.045	0.016170396	12
Sema7a.7	-0.634139965	0.008	0.156	0.016292111	12
Kif1b.8	-0.636258081	0.361	0.521	0.016456512	12
Cmtm3.5	0.547394197	0.303	0.116	0.016622856	12
Bcat1.2	-0.526548899	0	0.121	0.017341967	12
Slc3a2.7	0.547337141	0.521	0.321	0.017373305	12
Sh3gl2.6	-0.62515546	0.067	0.172	0.01768903	12
Amer2.2	-0.536257556	0.017	0.134	0.018170773	12
Stx4a.4	0.445218558	0.353	0.153	0.018996026	12
Hdlbp	0.418838056	0.319	0.132	0.019386005	12
Tia1.4	-0.550353283	0.336	0.553	0.019536814	12
Rpl22.6	-0.343009518	0.773	0.788	0.019671001	12
Sqstm1.2	0.43178865	0.437	0.225	0.019829842	12
Smarca4.2	-0.624086838	0.294	0.506	0.020314054	12
Cdc42ep4.1	0.566118953	0.168	0.047	0.020335314	12
Lrrn1.2	-0.479075499	0.017	0.113	0.020768366	12
Sstr2.8	-0.723120945	0.034	0.192	0.021544606	12
Serf2	0.490259767	0.403	0.186	0.021807725	12
Chn1	0.311630791	0.109	0.017	0.021958301	12
Bex4.1	-0.477590079	0.017	0.128	0.022271896	12
Pxdn	0.410597053	0.134	0.024	0.022411827	12

Bmp1	0.316933755	0.235	0.085	0.022775273	12
Prrc1	0.452132382	0.202	0.056	0.024152226	12
Slc39a13	0.346536772	0.151	0.034	0.024495841	12
Rab6b.11	-0.70310467	0.034	0.203	0.024609217	12
Gpr180.2	0.494206482	0.235	0.079	0.024689742	12
Brca2.6	-0.442215509	0.017	0.114	0.024690807	12
Dnmt1.11	-0.728200621	0.092	0.278	0.025466914	12
Dtl.8	-0.608756503	0.008	0.146	0.025907491	12
Setd8.3	-0.638480917	0.118	0.262	0.026376778	12
Rpn1.2	0.568018126	0.378	0.191	0.026865213	12
Chpf	0.462818189	0.151	0.032	0.027016096	12
Nagk.1	0.369205636	0.168	0.042	0.027434238	12
Ddx5.4	-0.3296434	0.765	0.816	0.028635282	12
Xpc	0.372237097	0.193	0.056	0.028866352	12
Fez1.3	-0.691289786	0.151	0.333	0.02949029	12
Smc1a.6	-0.544399963	0.471	0.631	0.030079874	12
Atoh1.8	-0.678647118	0.025	0.165	0.0301825	12
Bmp5	0.406432562	0.134	0.025	0.030739268	12
Strbp.7	-0.690736087	0.134	0.348	0.031619327	12
Fam132a	0.401435077	0.118	0.018	0.031718559	12
Knstrn.10	-0.698453772	0.042	0.186	0.031769333	12
Pcna.11	-0.754762517	0.168	0.359	0.031843368	12
Map3k3	0.637847422	0.185	0.068	0.03204798	12
U2surp.2	-0.528301601	0.395	0.506	0.032725571	12
Atp5a1.1	-0.376227856	0.664	0.718	0.033249023	12
Map9.3	-0.628713686	0.008	0.148	0.033562744	12
Myh10.3	-0.682587489	0.168	0.325	0.03388116	12
Chd6.2	-0.604423602	0.176	0.305	0.034672262	12
Cpne3.2	0.482459554	0.412	0.195	0.034775999	12
Tsg101.2	-0.628396971	0.126	0.265	0.034897859	12
Rbms2	0.434752271	0.16	0.036	0.035420845	12
Slc25a20	0.283618277	0.143	0.037	0.035461276	12
Phip.3	-0.653664041	0.286	0.46	0.035735461	12
Slc35f5.1	0.494738778	0.143	0.031	0.035800806	12
Camk2n1.1	0.412171427	0.244	0.084	0.03591827	12
Rps11.5	-0.405069667	0.697	0.778	0.036618828	12
Trio.2	0.485222006	0.311	0.123	0.038212906	12
Cd63.4	0.322327499	0.916	0.754	0.038370913	12
Polr3gl	0.400354971	0.185	0.05	0.038471923	12
Fbxo6.1	0.374012523	0.126	0.022	0.040144276	12
Pdgfa.8	-0.642622436	0.076	0.255	0.040168243	12
Zfhx3.1	0.554089183	0.168	0.043	0.040184227	12
Dner.12	-0.778582307	0.05	0.215	0.042634605	12
Snrpd1.7	-0.580465521	0.328	0.525	0.0446724	12
Psmc1.3	-0.61713633	0.21	0.42	0.044740147	12
Abcg1.2	0.407287616	0.126	0.023	0.04491623	12
Fam43a	0.40128202	0.202	0.061	0.044972639	12
Akap9.5	-0.592693829	0.403	0.501	0.045283853	12

Spc25.11	-0.762297129	0.059	0.226	0.046049049	12
Pak3.2	-0.688639544	0.05	0.207	0.046068383	12
Ccdc53.1	0.452968524	0.227	0.071	0.047012807	12
Fus.2	-0.363714193	0.672	0.737	0.047148077	12
Atp2b1.5	-0.607064609	0.412	0.572	0.047480832	12
Purb.3	-0.58972993	0.336	0.55	0.048718188	12
Tmem9.2	0.451486103	0.286	0.108	0.049008496	12
Nol7.3	-0.56260152	0.395	0.57	0.05001272	12
Usp1.11	-0.658935495	0.185	0.339	0.050191818	12
Sf3b2.2	-0.482116526	0.487	0.626	0.053859108	12
Bcl11a.4	-0.628479601	0.042	0.211	0.054818592	12
Birc5.12	-0.771108071	0.109	0.274	0.059290785	12
Gins2.5	-0.60783703	0.042	0.186	0.061302968	12
Fkbp4.4	-0.602648072	0.252	0.415	0.061907395	12
Smchd1.4	-0.620887502	0.143	0.274	0.062650928	12
AI854517.2	-0.642751792	0.042	0.19	0.06265754	12
Mycl.4	-0.505732184	0.025	0.137	0.064405621	12
Arfip1.1	0.445621267	0.252	0.091	0.064645399	12
Sar1a	0.377342466	0.479	0.262	0.066824183	12
Mab21l1.9	-0.71425827	0.059	0.226	0.067941186	12
Rassf4.5	-0.680477413	0.168	0.377	0.068109845	12
Brd3.5	-0.50347511	0.429	0.532	0.06886202	12
Brd8.5	-0.626082618	0.202	0.393	0.069627039	12
Srrm4.10	-0.719254009	0.042	0.196	0.069828725	12
Slc25a27.3	-0.51086035	0	0.11	0.071243581	12
Podxl2.11	-0.729638347	0.059	0.228	0.072044793	12
Rpl4.6	-0.330836031	0.882	0.908	0.072418062	12
Aplp2.6	0.428295735	0.555	0.321	0.073532775	12
Hsd11b2.10	-0.737280404	0.109	0.288	0.074011272	12
Nptn.4	0.286817483	0.37	0.186	0.075062758	12
Kif22.11	-0.580148335	0.05	0.163	0.075900887	12
Eef1b2.6	-0.435599523	0.622	0.733	0.076693125	12
Ubtf.3	-0.526142996	0.345	0.453	0.077758458	12
Ckap2l.12	-0.76383601	0.059	0.218	0.080663007	12
Tbc1d20.1	0.392922307	0.252	0.094	0.082539343	12
Klhdc8b.1	0.320436925	0.118	0.022	0.082840834	12
Mns1.11	-0.669616859	0.067	0.204	0.084188387	12
Arcn1	0.500393304	0.345	0.153	0.08430667	12
Cxxc5.6	-0.482272726	0.378	0.544	0.086807226	12
Gsk3b.6	-0.606940327	0.303	0.501	0.086931907	12
Pelo	0.492774521	0.185	0.051	0.088012717	12
Tcerg1.3	-0.550888712	0.336	0.457	0.089831303	12
Baz1b.5	-0.566635778	0.361	0.493	0.090795728	12
Tprn.6	-0.703283775	0.059	0.227	0.091756921	12
Kdelc2	0.46846496	0.151	0.036	0.091867606	12
Prdx2.2	-0.466694732	0.546	0.68	0.094804833	12
Cdk6.6	-0.655715289	0.067	0.244	0.096144707	12
Hdgfrp3.2	-0.6617738	0.16	0.339	0.097915075	12

Cenpm.9	-0.58022294	0.025	0.162	0.09801325	12
Eml3.1	0.393477379	0.16	0.04	0.102006822	12
Tacc3.11	-0.680828564	0.05	0.207	0.104253359	12
Kit	0.305427645	0.118	0.022	0.107327111	12
Dhx36.2	-0.59483431	0.235	0.371	0.107522164	12
Trim62	0.296120345	0.16	0.046	0.107835323	12
Klhl5.2	0.349147354	0.151	0.038	0.107849953	12
Myo6.2	0.5360621	0.176	0.048	0.109461931	12
Eml4.2	0.541512825	0.403	0.208	0.109954003	12
Usp22.6	-0.573471458	0.21	0.362	0.110894512	12
Pgrmc1.2	0.488273928	0.504	0.293	0.112348696	12
Snrpd2.4	-0.576977734	0.261	0.442	0.115371443	12
Rrm2.11	-0.671921405	0.059	0.205	0.11794325	12
Rap1b.1	0.424581674	0.311	0.133	0.118784037	12
Tex14.8	-0.699899849	0.025	0.146	0.120791296	12
Hspa9.3	-0.593332705	0.218	0.373	0.122347157	12
Tbrg1.1	0.382001716	0.454	0.241	0.122463576	12
Mmd	0.443155765	0.21	0.066	0.129225707	12
Hells.12	-0.630827661	0.05	0.214	0.129702875	12
Aldh2.2	0.3385569	0.126	0.025	0.132363099	12
Nme1.2	-0.547309353	0.353	0.51	0.13306477	12
Myod1.9	-0.602114232	0.017	0.142	0.136434945	12
Rtf1.2	-0.42626665	0.328	0.513	0.138147457	12
Sep15.5	0.419157746	0.697	0.503	0.139366024	12
Supt16.8	-0.569623834	0.345	0.494	0.140858535	12
Camta1.4	-0.639817103	0.235	0.409	0.14375364	12
Pick1.3	-0.542624108	0.017	0.128	0.143787414	12
Hmgb1.9	-0.615033196	0.185	0.359	0.144615953	12
Arid5b	0.475650309	0.16	0.04	0.151359423	12
Ift20	0.544860847	0.387	0.193	0.151731726	12
Aimp1.2	-0.616928655	0.126	0.28	0.153651327	12
Nol4.6	-0.605971312	0.025	0.172	0.157405592	12
Srrm1.2	-0.456331722	0.513	0.611	0.15888766	12
Otx2.4	-0.649639668	0.059	0.217	0.160190803	12
Rab13.1	0.473880711	0.235	0.083	0.167199145	12
Kif11.11	-0.698285775	0.05	0.207	0.169335654	12
Msn.1	0.528739206	0.244	0.092	0.174757557	12
Ostc.1	0.485251558	0.479	0.272	0.177500204	12
Smarcc1.5	-0.586234929	0.261	0.435	0.180993056	12
Rps14.5	-0.314290763	0.916	0.95	0.181282036	12
Trp53i11.4	-0.627867757	0.067	0.202	0.181461394	12
Ltbr.1	0.30632301	0.101	0.015	0.183603172	12
Syne2.3	-0.679298775	0.067	0.221	0.187528843	12
Bub1.9	-0.522417895	0.017	0.131	0.190962862	12
Wwc2	0.307704396	0.109	0.02	0.193800847	12
Syngr2.1	0.351357884	0.118	0.022	0.198725911	12
Zmiz1.5	-0.54469935	0.261	0.375	0.202269252	12
Nap1l1.8	-0.504128626	0.378	0.511	0.203681502	12

Tra2b.3	-0.612032671	0.227	0.406	0.204043134	12
Dhrs7.2	0.302753365	0.202	0.07	0.205487815	12
Ctsa.3	0.40810815	0.294	0.123	0.206792861	12
Mbnl2.3	0.513604025	0.37	0.177	0.207300453	12
1110038B12Rik.7	-0.582686453	0.16	0.299	0.209882686	12
Npepl1.1	0.470317183	0.143	0.033	0.210321316	12
Pfdn4.3	-0.629814609	0.118	0.306	0.211666684	12
Ptov1.1	-0.512649791	0.353	0.485	0.213829975	12
A330076H08Rik.10	-0.617594482	0.05	0.161	0.21914137	12
Smoc1.5	-0.556376555	0.008	0.135	0.222474653	12
Cenpv.6	-0.613778415	0.151	0.329	0.223152881	12
Rap1a.2	0.518942369	0.286	0.123	0.224274267	12
Kif5a.10	-0.549380119	0.034	0.128	0.224434226	12
Gm3764.5	-0.572277399	0.017	0.132	0.228470987	12
Incenp.11	-0.742910979	0.101	0.276	0.228499035	12
Soat1.2	0.263203802	0.109	0.02	0.230455663	12
Trip10	0.280838773	0.118	0.024	0.233195438	12
Sfrs18.6	-0.430105649	0.639	0.778	0.236301391	12
Tram1.2	0.450452542	0.303	0.127	0.236892748	12
Pdzrn3.9	-0.358602761	0.151	0.147	0.237084284	12
Erdr1.2	-0.595146907	0.034	0.162	0.240299047	12
H2afx.11	-0.755526868	0.168	0.331	0.244008793	12
Rab3a.11	-0.499465758	0.092	0.15	0.244527429	12
Marcks.3	-0.311456018	0.857	0.916	0.245890069	12
Gpr108	0.470056544	0.168	0.047	0.246394775	12
Atxn7l3b.4	-0.362077092	0.647	0.672	0.246494157	12
Srebf1.10	-0.565970632	0.227	0.334	0.254920753	12
Metap2.2	-0.491312849	0.445	0.597	0.25935941	12
Klc1.9	-0.651535829	0.185	0.362	0.259786263	12
Dbf4.8	-0.54616233	0.017	0.146	0.262831199	12
Carhsp1.5	-0.602713208	0.109	0.254	0.267340363	12
Oat.2	0.40209977	0.21	0.069	0.275928596	12
Serp1.2	0.529569925	0.395	0.216	0.27629538	12
Dixdc1.6	-0.635911017	0.118	0.235	0.28227863	12
Arhgap31.2	0.369681216	0.151	0.038	0.28327223	12
Rbp4.9	-0.666013065	0.076	0.242	0.28562855	12
Aspm.10	-0.63791308	0.025	0.143	0.286494316	12
Map4k4.2	-0.504646727	0.336	0.45	0.287566407	12
Txnrd1.4	-0.588189618	0.185	0.39	0.289420479	12
Ubxn1.1	-0.544862495	0.311	0.479	0.295397646	12
Hbp1	0.552552107	0.319	0.155	0.295991528	12
Zcchc18.3	-0.617258519	0.042	0.171	0.307235522	12
Lrrfip1.2	0.304111423	0.151	0.043	0.309980123	12
Tulp3.1	0.264601949	0.126	0.031	0.311357444	12
Chd3.9	0.368111441	0.319	0.148	0.313180723	12
Nell2.2	-0.53313847	0.008	0.133	0.314527692	12
Celf4.12	-0.85364682	0.143	0.321	0.31635148	12
Vcl.1	0.344788956	0.101	0.016	0.322073989	12

Zbtb38.1	0.392002141	0.185	0.058	0.327987083	12
Pdlim7.1	0.373614793	0.151	0.039	0.332191564	12
Lsm6.7	-0.606964857	0.185	0.373	0.334176571	12
Lpar4	0.35112475	0.126	0.031	0.34961773	12
Mllt11.10	-0.696986784	0.084	0.219	0.353195076	12
Cdc5l.2	-0.401004222	0.361	0.401	0.354306258	12
Eef1a1.5	-0.317456814	0.866	0.884	0.367599799	12
Uhrf1.10	-0.496748496	0.017	0.144	0.386280383	12
Plekha1.2	0.35749278	0.185	0.06	0.391077496	12
Nop56.7	-0.612416497	0.168	0.361	0.391636183	12
Ugp2.1	0.396334934	0.235	0.089	0.392544974	12
Tdrkh	-0.406039555	0.034	0.133	0.394683475	12
Eif4a1.3	-0.53464764	0.303	0.485	0.396120968	12
Zfp91.2	-0.502295495	0.378	0.49	0.396607152	12
Ttyh3.1	-0.496207898	0.017	0.124	0.403179396	12
Mrps5.2	-0.602020519	0.118	0.273	0.407341425	12
Lamtor3	0.35657904	0.235	0.089	0.413144607	12
Neurod6.4	-0.537441821	0.05	0.172	0.415980294	12
Rnaseh2c.7	-0.59925353	0.202	0.382	0.426537412	12
Scarb2.1	0.412758378	0.151	0.038	0.429881341	12
Ergic3.1	0.349102588	0.42	0.226	0.431802947	12
Syt13.8	-0.49797823	0.017	0.127	0.436999601	12
Sms.1	-0.461738668	0.025	0.137	0.438007643	12
Nt5dc2.6	-0.597383262	0.118	0.237	0.438187838	12
Mad2l2.5	-0.611283604	0.092	0.263	0.441401523	12
Ap3b2.3	-0.566998953	0.05	0.203	0.44211818	12
2700089E24Rik.2	0.499265174	0.387	0.197	0.446662323	12
Csdc2	0.492106802	0.126	0.028	0.450061813	12
Trim56	0.406812112	0.126	0.027	0.45515733	12
Cdca2.9	-0.518244362	0.008	0.129	0.458658816	12
Gins1.4	-0.341094528	0.017	0.115	0.462093798	12
Zmat2.2	-0.571190502	0.261	0.441	0.463412275	12
Rps24.5	-0.343361505	0.739	0.821	0.465462826	12
Vasp.1	0.41695512	0.202	0.066	0.468931948	12
Gphn.4	-0.559802613	0.017	0.141	0.480065342	12
Tgfb2.5	-0.578593306	0.042	0.192	0.485098004	12
Snrpn.2	-0.531512379	0.042	0.159	0.487381107	12
Ptk2	0.541919817	0.202	0.084	0.494707763	12
Ptch2.8	-0.56490141	0.025	0.153	0.495486669	12
Cct8.3	-0.429001914	0.328	0.442	0.496201845	12
Akap13.1	0.446373616	0.21	0.071	0.499310543	12
Ncapg.9	-0.619444139	0.042	0.184	0.506259576	12
Casc5.11	-0.632676878	0.059	0.186	0.512391107	12
BC005764.11	-0.705397397	0.059	0.186	0.514099256	12
Atad2.9	-0.61954706	0.059	0.199	0.514466116	12
Frmd4a.6	-0.63131026	0.16	0.309	0.515273612	12
Tmx1	-0.490177875	0.05	0.15	0.516679862	12
Spc24.10	-0.654211095	0.084	0.243	0.519873527	12

Mob1a.1	0.422303214	0.176	0.052	0.523661635	12
Zbtb4	0.325218638	0.126	0.029	0.524395219	12
Tcp11l1	-0.456842523	0.017	0.116	0.526396127	12
Sult4a1.3	-0.615657788	0.067	0.223	0.527169547	12
Rbmxl1.2	-0.524166579	0.025	0.153	0.528999821	12
Phf21b.3	-0.533833037	0.008	0.128	0.53013339	12
Alad	0.371817457	0.151	0.039	0.541683154	12
Dnpep	0.271939405	0.244	0.109	0.542401941	12
Rps25.7	-0.440417661	0.395	0.48	0.555263702	12
Kcnk1.9	-0.634659768	0.067	0.205	0.555493283	12
Mis18bp1.11	-0.615745335	0.034	0.161	0.556998625	12
Pou3f3.3	-0.565522455	0.042	0.172	0.561303065	12
Esf1.3	-0.630629194	0.227	0.393	0.561951341	12
Smpd3.6	-0.608138425	0.034	0.162	0.561953962	12
Aldh9a1.3	0.27891985	0.168	0.055	0.588260246	12
Mtss1.10	-0.687581424	0.126	0.27	0.591621039	12
Rfc2.6	-0.534032158	0.092	0.211	0.606227898	12
Ncapd2.9	-0.500116239	0.042	0.186	0.607220013	12
Rab3c.4	-0.596883745	0.025	0.148	0.607383495	12
Gdap1.3	-0.62205108	0.05	0.199	0.613984364	12
1810043H04Rik	-0.287020751	0.109	0.127	0.62254185	12
Selk.2	0.340203452	0.605	0.394	0.625190482	12
Blmh.3	-0.51460263	0.193	0.317	0.63777679	12
Cct5.4	-0.51815448	0.319	0.478	0.640959697	12
Cd2ap	0.436352535	0.336	0.157	0.644636332	12
Nefm.4	-0.61181292	0.008	0.117	0.645895611	12
Rif1.7	-0.566708652	0.151	0.276	0.657107449	12
Irf2	0.258530558	0.16	0.056	0.663834631	12
Mcm6.9	-0.643223192	0.109	0.277	0.674685788	12
Fyn.6	-0.566369097	0.126	0.216	0.676964878	12
Ypel1.3	-0.542620892	0.034	0.175	0.690858004	12
Ctcf.5	-0.53031624	0.353	0.496	0.692451365	12
Tmeff1.4	-0.559329196	0.025	0.15	0.703805084	12
Tnik.8	-0.643310026	0.025	0.147	0.710689208	12
Slc17a6.11	-0.619908033	0.034	0.148	0.717158938	12
Ube2e3.4	-0.567214098	0.244	0.417	0.736677374	12
Tubb2a.12	-0.656573449	0.151	0.245	0.736950649	12
Chchd2.4	-0.396241314	0.555	0.653	0.743831945	12
Cdca7.8	-0.587834295	0.059	0.198	0.745045688	12
Tmem35.2	-0.356108543	0.025	0.104	0.745272061	12
Ebf3.5	-0.631030786	0.067	0.203	0.754153652	12
Rad21.12	-0.56122362	0.319	0.462	0.771280524	12
Tomm7.2	-0.381949484	0.437	0.497	0.779591051	12
Bok.8	-0.566417769	0.084	0.252	0.809979247	12
Lrig3.7	-0.547667851	0.042	0.171	0.811179629	12
Pde4dip.3	-0.610137221	0.067	0.222	0.813540181	12
Tcp1.4	-0.480137147	0.303	0.464	0.815746748	12
Crispld1	0.335329582	0.101	0.018	0.862582727	12

D030056L22Rik.3	-0.507079045	0.025	0.143	0.873587067	12
Sra1	0.50112116	0.286	0.125	0.875159711	12
Ccnb1.9	-0.549119802	0.017	0.14	0.880923478	12
Cat	0.507271734	0.277	0.123	0.882605708	12
Fam57b.6	-0.598628936	0.05	0.186	0.888980088	12
Ppp1r14a	0.457001212	0.101	0.022	0.889747709	12
Rpn2.3	0.379577796	0.42	0.225	0.904399542	12
Cdh20.5	-0.599921554	0.059	0.203	0.90495175	12
Hes6.9	-0.610289999	0.084	0.237	0.910984354	12
Chrna3.10	-0.574771344	0.017	0.119	0.912939579	12
Ppp3ca.7	-0.632385902	0.235	0.379	0.92700496	12
Ccna2.11	-0.56686243	0.067	0.222	0.933263364	12
D630003M21Rik	0.252042655	0.143	0.045	0.936143892	12
Dynlt3.3	0.453128455	0.16	0.045	0.945957551	12
Nans.1	0.459055722	0.261	0.106	0.953387233	12
Timeless.8	-0.549355062	0.034	0.154	0.95588296	12
Cope	0.44993876	0.546	0.362	0.968103707	12
Rpl37.5	-0.453838393	0.395	0.504	0.97338275	12
Dmd.2	0.497920829	0.109	0.025	0.990546746	12
Prc1.11	-0.765826177	0.101	0.257	0.992149606	12
Ndufa12.4	-0.504495955	0.294	0.422	0.992551358	12
Gpr56.4	-0.553946885	0.067	0.223	1	12
Pgm1	0.439852205	0.101	0.023	1	12
Tprgl.1	0.499790471	0.277	0.123	1	12
Stk39.1	0.45281925	0.134	0.042	1	12
Mgat2	0.414796167	0.277	0.118	1	12
Ddx21.5	-0.599865876	0.185	0.323	1	12
Bsg	0.39299994	0.597	0.422	1	12
Naca.5	-0.426401499	0.378	0.465	1	12
Pdzrn4.7	-0.44837369	0.025	0.147	1	12
Sowaha.9	-0.673864958	0.034	0.163	1	12
Cnbp.6	-0.375358166	0.639	0.74	1	12
Glyr1.1	-0.482456942	0.202	0.284	1	12
Mtf2.3	-0.595062876	0.185	0.358	1	12
Fam181b.2	-0.512750791	0.025	0.143	1	12
Dpy30.5	-0.5767685	0.151	0.299	1	12
Mapk8ip2.4	-0.479711198	0.042	0.143	1	12
Echdc2	0.38187538	0.134	0.034	1	12
Sox4.9	-0.542586737	0.412	0.583	1	12
Spcs1	0.387665455	0.529	0.324	1	12
C1qbp.5	-0.536285049	0.227	0.378	1	12
Acat2.2	-0.492902658	0.042	0.183	1	12
Sars.2	-0.516238941	0.294	0.421	1	12
Prim1.9	-0.572772364	0.101	0.264	1	12
Slc12a4.1	0.334488526	0.134	0.034	1	12
Lhfpl2.2	0.349651536	0.118	0.027	1	12
Bfar.1	0.40475567	0.37	0.19	1	12
Xrn2.2	-0.458147645	0.361	0.473	1	12

Adamts10	0.341103096	0.118	0.026	1	12
Hspa4.1	-0.564628292	0.218	0.369	1	12
Cep170.6	-0.581692896	0.168	0.306	1	12
Nol8	-0.564833886	0.05	0.168	1	12
Dpysl3.11	-0.635859302	0.134	0.22	1	12
Sgol1.9	-0.49135806	0.017	0.128	1	12
Fabp5.4	-0.521679397	0.227	0.404	1	12
Rpa2.8	-0.55468789	0.067	0.2	1	12
Zc3h15.2	-0.447859707	0.277	0.366	1	12
Mrpl42.3	-0.574979891	0.16	0.319	1	12
Ctnna1.2	0.445982986	0.319	0.151	1	12
Ythdf2.1	-0.546315743	0.118	0.259	1	12
Tnfaip1	0.479749655	0.244	0.106	1	12
Nhp2.5	-0.562405074	0.218	0.371	1	12
Ccar1.2	-0.499925584	0.412	0.573	1	12
Pkia.7	-0.435818628	0.05	0.134	1	12
Cdc42se2.3	-0.53795734	0.118	0.239	1	12
Creb3	0.412377917	0.244	0.098	1	12
Ubqln2.1	-0.552276088	0.092	0.215	1	12
Herpud1.1	0.470525282	0.294	0.134	1	12
Dcbld2	0.334388474	0.151	0.044	1	12
Mef2a.2	0.543768953	0.261	0.138	1	12
Ppp1r9a	-0.60421323	0.05	0.189	1	12
Elmo1.9	-0.656490096	0.017	0.126	1	12
B3gnt1.1	0.37415697	0.185	0.061	1	12
Trim37.3	-0.486493723	0.076	0.234	1	12
Bcap29.2	-0.456240674	0.034	0.123	1	12
Fam118b	0.45511694	0.176	0.061	1	12
Myef2.2	-0.569350588	0.176	0.339	1	12
Ppa1.4	-0.527636175	0.109	0.222	1	12
Cadm3.11	-0.603469402	0.025	0.141	1	12
G3bp1.5	-0.546324028	0.176	0.329	1	12
Mcm2.9	-0.59323065	0.059	0.205	1	12
Rin2.2	0.305892884	0.101	0.02	1	12
Ubc.2	0.413974475	0.555	0.357	1	12
Elovl1.2	0.254395279	0.109	0.034	1	12
Myeov2	-0.297571903	0.437	0.455	1	12
Fam20c	0.348107031	0.176	0.059	1	12
Tmod2.2	-0.555829912	0.084	0.208	1	12
Yipf5	0.310507209	0.269	0.121	1	12
Ssb.2	-0.261119054	0.613	0.704	1	12
Prkcb.10	-0.605940216	0.092	0.257	1	12
BC005561.2	-0.619234028	0.126	0.29	1	12
Ndc80.9	-0.303763813	0.017	0.115	1	12
Acadm.1	0.276980504	0.21	0.086	1	12
Apbb1.10	-0.537030907	0.076	0.17	1	12
4931406C07Rik.1	0.491037306	0.151	0.045	1	12
Ssh2.1	0.41778394	0.151	0.043	1	12

Tmem57.7	-0.539264406	0.185	0.291	1	12
Prdx1.7	0.341948777	0.689	0.488	1	12
Mpnd.2	-0.504959937	0.059	0.171	1	12
Tmem5.2	0.531520462	0.202	0.08	1	12
1700001O22Rik.5	-0.386241691	0.008	0.111	1	12
Sema6d.1	0.41411092	0.126	0.031	1	12
Lepre1	0.276557055	0.101	0.021	1	12
Gnas	0.275530553	0.924	0.885	1	12
Nes.2	0.401280898	0.218	0.085	1	12
Sox18.5	-0.48015315	0.025	0.148	1	12
Las1l.2	-0.591553969	0.109	0.255	1	12
Dlgap5.9	-0.405140096	0.042	0.112	1	12
Eif5b.1	-0.439330186	0.445	0.595	1	12
Por	0.427156684	0.185	0.065	1	12
Mxra7	0.278115442	0.168	0.059	1	12
E2f1.5	-0.521246245	0.042	0.147	1	12
Zswim7	0.346014821	0.109	0.023	1	12
Nelfe	-0.292496798	0.126	0.15	1	12
Kank3	0.294595414	0.118	0.029	1	12
Pomp.1	-0.397271508	0.353	0.414	1	12
Cenpq.8	-0.517910558	0.034	0.161	1	12
Frrs1l.3	-0.540203207	0.05	0.191	1	12
Gns.1	0.400391322	0.193	0.069	1	12
Acadvl.1	0.433982924	0.252	0.105	1	12
Myt1l.11	-0.649663164	0.034	0.161	1	12
Klf9.6	0.384163127	0.462	0.269	1	12
Eif4a2	0.311329198	0.345	0.179	1	12
Ssr3.3	0.406342096	0.58	0.424	1	12
Rnf122.4	-0.311516953	0.042	0.102	1	12
Rhog.1	0.263604698	0.118	0.03	1	12
Fam53b.3	-0.426738212	0.025	0.137	1	12
Cct6a.2	-0.474770935	0.336	0.487	1	12
Gpm6b.7	-0.506805585	0.361	0.484	1	12
Mapt.11	-0.712600794	0.076	0.217	1	12
Srsf1.3	-0.561217096	0.143	0.297	1	12
Nuf2.10	-0.448176653	0.025	0.141	1	12
Lancl2.1	-0.410459487	0.025	0.105	1	12
Hspe1.3	-0.384868764	0.269	0.341	1	12
Ckap5.9	-0.588674626	0.134	0.266	1	12
Brd7.7	-0.362485588	0.168	0.309	1	12
Cenpk.10	-0.327342251	0.034	0.145	1	12
Trim28.4	-0.523469631	0.227	0.362	1	12
Phldb2	0.378742046	0.168	0.055	1	12
Pik3r1.1	0.515100073	0.235	0.101	1	12
Hsd17b11.1	0.328518935	0.176	0.061	1	12
Eef1e1.2	-0.516761292	0.05	0.182	1	12
Prpf31.1	-0.450790001	0.118	0.2	1	12
Jun.4	0.443874339	0.765	0.654	1	12

Mxd3.9	-0.472034962	0.017	0.127	1 12
Kdelc1	0.453834757	0.16	0.06	1 12
Sept2.2	0.386125585	0.378	0.221	1 12
Fam64a.10	-0.474666154	0.017	0.118	1 12
Prmt5.4	-0.533430231	0.134	0.256	1 12
Dtx3	0.303865441	0.227	0.096	1 12
Mpped2.3	-0.484162667	0.017	0.127	1 12
Atp5j2	-0.351207381	0.513	0.615	1 12
Fam115a.4	-0.516540011	0.244	0.351	1 12
Nfkbia.3	0.473344471	0.336	0.17	1 12
Bola2.4	-0.525579714	0.227	0.398	1 12
Aurkb.11	-0.491393023	0.025	0.143	1 12
Cers2.1	0.428940007	0.235	0.113	1 12
Tk1.9	-0.466939115	0.042	0.132	1 12
BC005537	-0.503269271	0.227	0.333	1 12
Yeats4.1	-0.56442817	0.109	0.264	1 12
Eprs.2	-0.383345161	0.437	0.491	1 12
Myt1.11	-0.634339721	0.034	0.153	1 12
Rcc2.5	-0.469189194	0.084	0.179	1 12
Ptbp2.1	-0.555511196	0.092	0.221	1 12
Dbp.1	0.43034846	0.134	0.039	1 12
Nsg1.9	-0.556764348	0.16	0.288	1 12
Frmd8	0.4453881	0.101	0.026	1 12
Mgll.4	-0.541401727	0.034	0.133	1 12
Aplp1.11	-0.557885397	0.059	0.152	1 12
Fam171b.3	-0.583051387	0.092	0.224	1 12
Larp7.6	-0.467986118	0.16	0.313	1 12
Pcbp4.4	-0.540710068	0.168	0.325	1 12
Hk2.6	-0.528131155	0.059	0.173	1 12
Etaa1.1	-0.524052399	0.05	0.159	1 12
Ttc9b.3	-0.528648503	0.067	0.195	1 12
Sqle.4	-0.453066985	0.025	0.121	1 12
Pcdh18	0.473601882	0.176	0.077	1 12
Gm17322.9	-0.563186435	0.034	0.131	1 12
Zeb1.6	-0.521530984	0.252	0.388	1 12
Qdpr.1	-0.437967071	0.185	0.268	1 12
Ubr1	0.49266678	0.218	0.094	1 12
Dnajc2.5	-0.528109315	0.21	0.329	1 12
Ccnb2.11	-0.632572729	0.059	0.187	1 12
Tmem178.4	-0.52647338	0.067	0.187	1 12
Emc7.2	0.347129332	0.294	0.142	1 12
Dst.2	-0.576799465	0.143	0.267	1 12
Tmed2	0.427987159	0.21	0.086	1 12
Bcas2.2	-0.405225271	0.261	0.406	1 12
2700046A07Rik	0.336209058	0.126	0.033	1 12
Rcn2.2	-0.320120341	0.235	0.281	1 12
Pold3.3	-0.396980043	0.084	0.149	1 12
Ubr2	0.291021748	0.202	0.084	1 12

Cacng4.9	-0.597019226	0.025	0.102	1	12
Hsdl2.1	0.431921137	0.16	0.053	1	12
Zdbf2.2	-0.491772723	0.034	0.134	1	12
Rnf126	-0.369419033	0.017	0.103	1	12
Rpl14.6	-0.313085082	0.647	0.697	1	12
Epb4.1.5	-0.538132574	0.084	0.23	1	12
Ier5.6	-0.466047898	0.303	0.394	1	12
Sf3a1	-0.370161158	0.109	0.158	1	12
Snrnp70.2	-0.359600342	0.613	0.694	1	12
Kif15.11	-0.508697123	0.05	0.181	1	12
Wdr33	-0.411258229	0.126	0.196	1	12
Fdft1.2	-0.40051918	0.017	0.101	1	12
Ankrd50	0.413485573	0.118	0.032	1	12
Cep78.3	-0.477396063	0.042	0.141	1	12
Pphln1.1	-0.352004266	0.059	0.119	1	12
Arid2.2	-0.469256979	0.126	0.242	1	12
D17H6S56E-5.7	-0.453646411	0.034	0.128	1	12
E330009J07Rik.2	-0.442219011	0.017	0.117	1	12
Lmnb2.7	-0.485695824	0.042	0.159	1	12
Mroh2a.8	-0.512923612	0.034	0.114	1	12
Mcm5.7	-0.485694024	0.067	0.169	1	12
Acp2.2	0.341256398	0.218	0.092	1	12
Igsf8.6	-0.557045665	0.143	0.27	1	12
Cib1.2	0.364318082	0.168	0.057	1	12
Olfm1.4	-0.434598126	0.151	0.198	1	12
Tyms.9	-0.574524267	0.084	0.219	1	12
Cox6c.3	0.282199434	0.815	0.718	1	12
Scn8a.4	-0.501245817	0.042	0.137	1	12
Canx.3	0.341263647	0.756	0.594	1	12
Gpc2.6	-0.488786532	0.05	0.14	1	12
Zfp608.3	-0.533632866	0.059	0.186	1	12
Rrp1.2	-0.419665889	0.471	0.607	1	12
Cnpy1.6	-0.52316271	0.025	0.133	1	12
Aip	0.334376335	0.202	0.079	1	12
Suz12.4	-0.539683294	0.092	0.219	1	12
Pak7.7	-0.467937329	0.008	0.105	1	12
Gorasp2	0.412535095	0.252	0.114	1	12
Sash1.3	0.356800618	0.126	0.034	1	12
Ncald.3	-0.521500383	0.042	0.166	1	12
Sclt1.2	-0.434129994	0.05	0.127	1	12
Eif3l.1	-0.423497958	0.218	0.302	1	12
Spred1.2	0.386957822	0.244	0.107	1	12
Scp2.3	0.441352141	0.185	0.072	1	12
Idh3a.1	-0.402665013	0.084	0.163	1	12
AI597468	0.432935561	0.227	0.097	1	12
Spg21	-0.458985301	0.076	0.173	1	12
2700029M09Rik.4	-0.482749375	0.185	0.29	1	12
Ndufa13.2	0.325379413	0.706	0.532	1	12

Cgrrf1.1	0.390601039	0.16	0.054	1	12
Capn2.1	0.397162096	0.218	0.089	1	12
Snrnp40.6	-0.509301032	0.143	0.271	1	12
Mab21l2.4	-0.506343464	0.008	0.104	1	12
Stt3a	0.387161327	0.261	0.124	1	12
Pcsk2.2	-0.391310478	0.017	0.102	1	12
Zwint.4	-0.440004168	0.218	0.291	1	12
M6pr.1	0.422776954	0.244	0.108	1	12
Lman1.2	0.445331519	0.462	0.323	1	12
Thoc1	-0.393659461	0.059	0.178	1	12
Ifnar1.1	0.371972673	0.227	0.095	1	12
St8sia1	0.355849993	0.126	0.035	1	12
Ccp110.6	-0.528396859	0.101	0.249	1	12
Tnpo3.1	-0.525358322	0.084	0.223	1	12
Grik2.9	-0.517378936	0.008	0.102	1	12
Sec13	0.435555562	0.429	0.268	1	12
Eif3m.1	-0.495735603	0.176	0.302	1	12
Clip1.2	0.44995976	0.218	0.093	1	12
Gkap1.1	-0.520527446	0.084	0.221	1	12
H1fx.10	-0.471157261	0.109	0.246	1	12
Cnksr2.6	-0.445668117	0.017	0.108	1	12
Nxt1.2	-0.335519494	0.067	0.122	1	12
Arpp21.10	-0.559352134	0.025	0.113	1	12
Ifngr1.1	0.340919324	0.126	0.036	1	12
Rbm8a.5	-0.464455146	0.319	0.466	1	12
Xist.5	-0.745158061	0.151	0.3	1	12
Cdk5rap2.5	-0.267861025	0.067	0.184	1	12
Syncrip.5	-0.38495689	0.387	0.496	1	12
Nop10.5	-0.43193176	0.378	0.482	1	12
Rhoa.2	0.385402547	0.286	0.14	1	12
Emc6.1	-0.516001777	0.143	0.276	1	12
Ddhd2.1	-0.494550534	0.034	0.13	1	12
Cplx1.8	-0.49392773	0.034	0.139	1	12
Mad2l1.9	-0.42209842	0.025	0.124	1	12
Hspa8.2	-0.500258551	0.261	0.416	1	12
Polr3k.2	-0.488922242	0.16	0.271	1	12
Sidt2.1	0.406998962	0.134	0.045	1	12
Asf1a.3	-0.475645778	0.126	0.281	1	12
Nudc.6	-0.498886724	0.126	0.281	1	12
Esco2.11	-0.597450805	0.067	0.195	1	12
Gria4.10	-0.465182322	0.025	0.125	1	12
Plcb1.8	-0.591262155	0.034	0.141	1	12
Mrpl39	-0.45420513	0.025	0.133	1	12
Rfc3.6	-0.50572157	0.084	0.205	1	12
Mrps10	-0.43600051	0.05	0.14	1	12
Luzp1	0.449892801	0.277	0.135	1	12
Gm12696	-0.435171855	0.034	0.133	1	12
Dnajc24	-0.303371277	0.109	0.151	1	12

Pkig.1	0.442071587	0.261	0.127	1	12
Mafb.1	0.38543582	0.151	0.05	1	12
Wdr12.3	-0.447921357	0.05	0.176	1	12
Ckap2.9	-0.326926543	0.05	0.164	1	12
Cpsf6.2	-0.512354699	0.143	0.291	1	12
Sbds.1	0.483137577	0.269	0.136	1	12
Adh5.2	-0.423307039	0.303	0.405	1	12
Plscr4.1	0.285244414	0.109	0.028	1	12
Dscr3.2	0.326653644	0.185	0.072	1	12
Dynll1.4	-0.308912919	0.563	0.594	1	12
Mbtd1.2	-0.48886585	0.126	0.215	1	12
Pcbd2.1	0.390496676	0.118	0.035	1	12
Rrp15.4	-0.41857895	0.101	0.174	1	12
D10Wsu102e	-0.431435653	0.025	0.12	1	12
Pxk	0.261632499	0.151	0.054	1	12
Hibadh.2	0.310891337	0.202	0.085	1	12
Rad51.9	-0.416052335	0.025	0.117	1	12
Cstf2	-0.363165714	0.017	0.104	1	12
Cd164.3	0.313317137	0.252	0.119	1	12
Sec14l1.2	0.307783109	0.151	0.052	1	12
Eif1ax.2	-0.476260912	0.235	0.397	1	12
Tmem134	0.383143858	0.294	0.148	1	12
Prr24	0.307668572	0.118	0.04	1	12
Tmem198b.1	0.278548243	0.118	0.034	1	12
Eif1a	0.305373796	0.227	0.114	1	12
Gpr85.2	-0.508954203	0.05	0.164	1	12
2810025M15Rik.3	-0.462555582	0.05	0.167	1	12
Eif3e.4	-0.488941953	0.227	0.383	1	12
A930011O12Rik.11	-0.566707244	0.017	0.115	1	12
Safb.2	-0.449705887	0.353	0.504	1	12
Upf3b.2	-0.411668991	0.244	0.321	1	12
Slk	0.308125583	0.328	0.179	1	12
Cdkn1b.6	-0.469723557	0.303	0.468	1	12
Fasn.2	-0.43084673	0.059	0.138	1	12
Arf4.2	0.3309419	0.588	0.402	1	12
Gm8292.5	-0.467176824	0.235	0.369	1	12
Rab3ip.1	-0.401947115	0.008	0.101	1	12
Stat3.3	0.401241003	0.134	0.041	1	12
Phactr1.5	-0.519605212	0.059	0.184	1	12
Fbxo9.1	0.301057428	0.261	0.127	1	12
Mif.4	-0.488332088	0.218	0.357	1	12
Sdf2.1	0.350585785	0.378	0.213	1	12
Ltbp4	0.447572344	0.101	0.035	1	12
Slc29a1.7	-0.436197038	0.429	0.562	1	12
Yipf4	0.308132971	0.261	0.126	1	12
Lpcat3.2	0.394415976	0.134	0.043	1	12
Cox5a	-0.388912054	0.412	0.571	1	12
Ppp1r7.1	-0.409455433	0.118	0.198	1	12

Fam110b	0.307242207	0.118	0.034	1	12
Zfp428.1	-0.477452592	0.05	0.167	1	12
Rab31.3	0.405215062	0.143	0.054	1	12
Rpl5	-0.412642764	0.025	0.115	1	12
Stard4.2	-0.368747209	0.034	0.141	1	12
Mybbp1a.2	-0.466315535	0.109	0.25	1	12
1110004F10Rik.2	-0.399179859	0.328	0.423	1	12
Nup88.1	-0.437778574	0.101	0.184	1	12
D17Wsu104e.2	0.258163427	0.361	0.214	1	12
Taf1d.4	-0.510283894	0.193	0.343	1	12
Sike1	0.404650192	0.193	0.085	1	12
Ddx6.2	-0.425062926	0.37	0.493	1	12
Adipor1.2	0.321713461	0.277	0.139	1	12
Cep290.4	-0.522528252	0.034	0.143	1	12
Kras.1	-0.507907166	0.134	0.252	1	12
Cct7.2	-0.412640761	0.319	0.485	1	12
Ufsp2	0.365073523	0.252	0.12	1	12
5430416N02Rik.2	-0.460413794	0.05	0.141	1	12
Cacybp.1	-0.447159262	0.252	0.356	1	12
Prpf19.1	-0.435242452	0.21	0.307	1	12
Tmod3	0.435827043	0.286	0.153	1	12
Ube2e1.1	-0.399087035	0.16	0.228	1	12
Rnf187.2	-0.43952456	0.303	0.421	1	12
Phf20l1.7	-0.402706507	0.277	0.34	1	12
Smim14	0.26415692	0.345	0.195	1	12
Tcf12.2	0.410575959	0.412	0.254	1	12
Ddx46.2	-0.421809173	0.353	0.442	1	12
Map2k1	0.321960335	0.134	0.043	1	12
Hnrnph3.3	-0.453409525	0.261	0.427	1	12
Dynlrb1.1	-0.390188012	0.462	0.579	1	12
Gtf2a2.2	-0.414765997	0.286	0.384	1	12
Srsf7.8	-0.436017767	0.328	0.466	1	12
Snrpd3.4	-0.36582881	0.437	0.535	1	12
Rsrc1.1	-0.52461517	0.118	0.251	1	12
Coq7.1	-0.484110835	0.101	0.207	1	12
Alyref.4	-0.404800729	0.109	0.2	1	12
Arhgef2.6	-0.304647198	0.252	0.282	1	12
Psma7.4	-0.2956938	0.731	0.782	1	12
Cdt1.7	-0.429108169	0.042	0.125	1	12
Cdca3.11	-0.556655943	0.092	0.222	1	12
Tm7sf3.2	0.312077597	0.168	0.065	1	12
Acadsb	0.438559889	0.168	0.071	1	12
Htatsf1.3	-0.438407454	0.361	0.499	1	12
Sepw1.1	-0.345715966	0.235	0.3	1	12
Ndufa2.2	-0.273370487	0.58	0.603	1	12
Ralgps2.4	-0.499553033	0.134	0.246	1	12
Insig2	0.371706534	0.218	0.097	1	12
Fam133b.1	-0.517625873	0.168	0.308	1	12

Ptbp3.1	0.346937903	0.235	0.109	1	12
Elovl4.3	-0.420261577	0.025	0.11	1	12
Eef2.4	-0.33453883	0.613	0.717	1	12
Pcdha2.8	-0.495281183	0.034	0.118	1	12
Ctbp2.3	-0.397401973	0.084	0.215	1	12
Fam160b1	0.35922141	0.193	0.08	1	12
Dynll2.2	-0.498225837	0.151	0.297	1	12
Gm11266.8	-0.460770688	0.05	0.144	1	12
Fen1.9	-0.367116144	0.05	0.142	1	12
Hip1r.5	-0.425768418	0.067	0.133	1	12
Sltm.2	-0.402423474	0.42	0.532	1	12
Pdxp.2	-0.38039571	0.025	0.113	1	12
Tead2.8	-0.495341677	0.151	0.278	1	12
Tmem214	0.346888947	0.126	0.04	1	12
Luc7l.2	-0.46680786	0.235	0.361	1	12
Naa15.2	-0.47068918	0.252	0.392	1	12
Msh2.2	-0.444798693	0.042	0.144	1	12
Tcof1.2	-0.445134387	0.059	0.168	1	12
Lsm2.5	-0.427394382	0.143	0.23	1	12
Dck.3	-0.390669023	0.017	0.103	1	12
Cln5.1	0.324148259	0.118	0.035	1	12
Suv39h2.5	-0.412545962	0.042	0.131	1	12
Atox1.2	0.344807229	0.462	0.291	1	12
Trappc4.2	-0.360177933	0.176	0.224	1	12
Plod2	0.408095346	0.202	0.09	1	12
Agtpbp1.3	-0.390641773	0.025	0.102	1	12
Klhl7.4	-0.47544751	0.059	0.165	1	12
Spire1	-0.45546864	0.042	0.155	1	12
Slc39a6.2	-0.355714388	0.151	0.199	1	12
Vcan.5	0.364960137	0.261	0.129	1	12
Ube4b	-0.367510712	0.05	0.11	1	12
Parp1.3	-0.413407979	0.176	0.314	1	12
Sox11.5	-0.42582573	0.025	0.115	1	12
Myl6	0.326604248	0.353	0.201	1	12
Rpl36al.2	-0.358342578	0.227	0.298	1	12
Pard6g.2	0.33838474	0.227	0.104	1	12
Ndufa5.1	-0.374734475	0.378	0.454	1	12
Kpnb1.3	-0.392816904	0.227	0.3	1	12
Mphosph9.2	-0.428540032	0.042	0.148	1	12
Nae1.2	-0.494825591	0.118	0.225	1	12
Dkc1.7	-0.43753523	0.176	0.333	1	12
Mbp.5	-0.681367479	0.067	0.166	1	12
Fam3c.2	0.300340514	0.193	0.084	1	12
Elk4	0.392221633	0.134	0.047	1	12
Frmd4b.3	-0.382344387	0.059	0.127	1	12
Rnf5.2	-0.464883676	0.126	0.258	1	12
Il11ra1.2	0.291652436	0.151	0.057	1	12
Hmgcs1.6	-0.355691127	0.101	0.151	1	12

Cfl1.1	-0.445162501	0.227	0.378	1	12
Atp1a1	-0.473842095	0.05	0.159	1	12
Celsr3.4	-0.448807202	0.025	0.123	1	12
Ppfia2.8	-0.46346203	0.025	0.112	1	12
Mrps22	-0.423142016	0.042	0.134	1	12
Zrsr1	0.269319247	0.143	0.054	1	12
Krcc1.3	0.375793812	0.193	0.082	1	12
Gabrb3.2	-0.386532821	0.025	0.102	1	12
Snord104.5	-0.435839647	0.067	0.152	1	12
Usp16.1	0.370024518	0.395	0.24	1	12
Knop1.2	-0.494806296	0.151	0.272	1	12
Gatc	-0.377381434	0.017	0.107	1	12
Clspn.11	-0.536063841	0.076	0.19	1	12
Ctps.4	-0.416707572	0.042	0.138	1	12
Ints7.1	-0.413067755	0.034	0.117	1	12
Tubb4b.9	-0.490929362	0.126	0.24	1	12
Cenph.11	-0.390505947	0.076	0.194	1	12
Kif4.8	-0.37222449	0.034	0.108	1	12
Ctbp1.2	-0.451477134	0.252	0.395	1	12
Dhx32.6	-0.467529679	0.076	0.201	1	12
Pspc1	-0.441490846	0.042	0.15	1	12
Lyar.9	-0.47516003	0.143	0.286	1	12
Gars.2	-0.361161385	0.109	0.228	1	12
Aktip	-0.305764445	0.042	0.102	1	12
2900011O08Rik.9	-0.423131336	0.042	0.104	1	12
Txndc17.1	0.356619096	0.395	0.241	1	12
Rnf219.1	-0.369324761	0.067	0.14	1	12
Kif3a.3	-0.441211074	0.168	0.317	1	12
Skp1a.2	-0.314520045	0.529	0.581	1	12
Nktr.5	-0.357462841	0.336	0.38	1	12
Sv2b.5	-0.39610812	0.017	0.103	1	12
Tardbp.3	-0.401472258	0.37	0.483	1	12
Pes1	-0.408298245	0.025	0.115	1	12
Arhgef25.1	0.25340951	0.176	0.078	1	12
Fjx1.2	0.334729672	0.118	0.037	1	12
Klhl24.1	-0.370331878	0.084	0.139	1	12
Prorsd1	0.272639561	0.118	0.036	1	12
Kif1a.6	-0.444007634	0.067	0.172	1	12
Rps8.4	-0.367371988	0.294	0.397	1	12
Snx5.6	-0.254751147	0.143	0.163	1	12
Polr2j	-0.432814785	0.185	0.293	1	12
Tecr.1	-0.366995854	0.361	0.53	1	12
Dazap1.3	-0.448298128	0.16	0.273	1	12
Snx6.1	0.266503325	0.538	0.367	1	12
Nipsnap1.3	-0.425847331	0.034	0.136	1	12
Dot1l.1	-0.442760565	0.168	0.279	1	12
Stim1	0.303750703	0.101	0.029	1	12
Zc3h13.3	-0.416880886	0.311	0.462	1	12

Uchl3.1	-0.426633635	0.067	0.187	1	12
Sf3a3.3	-0.386727182	0.21	0.281	1	12
Mid1ip1.3	0.32983337	0.143	0.05	1	12
Nfe2l2.1	0.333388302	0.193	0.083	1	12
Zfp422.3	-0.445357456	0.126	0.246	1	12
Gtf2h5.1	-0.41942512	0.319	0.439	1	12
Gabarapl1.6	0.277418692	0.361	0.215	1	12
Lsm3.7	-0.433293388	0.252	0.359	1	12
Celf1.3	-0.426983149	0.269	0.38	1	12
Rab22a	0.380382388	0.235	0.112	1	12
Aamdc	0.410702449	0.176	0.091	1	12
Nmt2	-0.457405679	0.067	0.181	1	12
Sdad1	-0.402274717	0.084	0.147	1	12
Tagln3.11	-0.519908252	0.185	0.295	1	12
Glo1.3	0.329509653	0.227	0.106	1	12
Lsm4.6	-0.367259102	0.403	0.488	1	12
Rnf165.6	-0.47297631	0.059	0.173	1	12
Kdm1a.4	-0.467772849	0.227	0.365	1	12
Gpsm1.2	-0.465492483	0.076	0.176	1	12
Mettl21a	0.294315606	0.118	0.037	1	12
4933426M11Rik.1	0.332310623	0.134	0.046	1	12
Tmem50b.2	0.252534115	0.101	0.028	1	12
Tmem30a.4	0.340086627	0.319	0.178	1	12
Zdhhc1	0.343281431	0.143	0.061	1	12
Mef2c.1	0.422600755	0.118	0.068	1	12
Tbca.2	-0.401280127	0.311	0.421	1	12
Rars.1	-0.365452306	0.126	0.184	1	12
Timm50.2	-0.446288262	0.134	0.237	1	12
Rps15.5	-0.269652368	0.739	0.788	1	12
Phf20.1	-0.489628313	0.101	0.22	1	12
Akap6.4	-0.411821179	0.092	0.219	1	12
Commd4	-0.367204135	0.168	0.251	1	12
Srsf10.4	-0.420598681	0.193	0.331	1	12
Scaper.4	-0.327105724	0.067	0.11	1	12
Fech	0.275311685	0.109	0.035	1	12
Rrs1.3	-0.318149028	0.076	0.19	1	12
Polr2e.2	-0.433413745	0.176	0.325	1	12
Atxn7l2.3	-0.361314271	0.017	0.103	1	12
Rgs12.2	-0.421612545	0.042	0.146	1	12
Cd2bp2.1	-0.388755325	0.05	0.136	1	12
Scamp2.4	0.396769559	0.261	0.134	1	12
Bcl2l13.1	-0.399300854	0.025	0.115	1	12
Pgls	-0.331827288	0.303	0.35	1	12
Prmt1.2	-0.341240435	0.118	0.225	1	12
Cenpw.10	-0.396064572	0.05	0.128	1	12
Zfp462.4	-0.447183214	0.067	0.156	1	12
Nfyb.6	-0.372522934	0.168	0.235	1	12
Capzb.1	0.280021895	0.513	0.349	1	12

Thsd7a.5	-0.451279849	0.042	0.127	1	12
Rbmx2.1	-0.40817836	0.025	0.115	1	12
Dzip3.2	-0.45067139	0.067	0.167	1	12
Cenpp.8	-0.3682525	0.025	0.114	1	12
Bag6	-0.328941128	0.143	0.181	1	12
Rfc1.8	-0.464740733	0.185	0.331	1	12
Zfp326.2	-0.400342388	0.311	0.41	1	12
Rnaseh2b.5	-0.449284873	0.109	0.237	1	12
Gucd1	0.271206433	0.126	0.044	1	12
Mcm4.5	-0.415918014	0.05	0.138	1	12
Pfn2.6	-0.468670728	0.143	0.241	1	12
Serf1.5	-0.40181092	0.21	0.281	1	12
Ccdc12	0.400849388	0.227	0.112	1	12
Bach1.1	0.303385964	0.185	0.08	1	12
Atp5k	-0.324350623	0.395	0.455	1	12
Tceal3.4	-0.392838653	0.034	0.111	1	12
Cul7	0.312988235	0.109	0.033	1	12
Gnao1.8	-0.493838063	0.176	0.287	1	12
Psma6.1	-0.346241773	0.37	0.452	1	12
Fam213b.6	-0.479017764	0.05	0.158	1	12
Kmt2e.8	-0.391104867	0.454	0.569	1	12
Xpo1.2	-0.44934977	0.118	0.228	1	12
Lhfpl4.2	-0.355187571	0.042	0.108	1	12
Skiv2l2.1	-0.438329107	0.059	0.164	1	12
Limd1.1	0.27708146	0.168	0.07	1	12
Appbp2.1	-0.289246499	0.168	0.199	1	12
Gstm5.3	-0.455426647	0.193	0.323	1	12
Rnd2.4	-0.487501213	0.059	0.159	1	12
Bag1.1	-0.388792607	0.193	0.346	1	12
Topors.2	-0.371173603	0.101	0.183	1	12
Mki67ip.3	-0.399836666	0.176	0.244	1	12
Nrm.5	-0.36552635	0.067	0.17	1	12
Erp44.1	0.323336794	0.227	0.108	1	12
Prdx6.4	-0.416804662	0.235	0.326	1	12
Elf2	-0.28546948	0.168	0.198	1	12
Snap23.1	0.262844768	0.118	0.041	1	12
Camk1.3	0.284210832	0.143	0.055	1	12
Slc35a2	0.306223701	0.109	0.035	1	12
Rad51ap1.12	-0.457420608	0.076	0.167	1	12
Sacs.4	-0.411215719	0.076	0.191	1	12
Arhgef7.4	-0.33600938	0.101	0.149	1	12
2700081O15Rik.4	-0.405446339	0.042	0.111	1	12
Agrn.2	0.307354705	0.176	0.075	1	12
Cdk2ap2	0.338473213	0.227	0.109	1	12
Gins4.1	-0.338212153	0.067	0.128	1	12
Arrb2.2	-0.355733283	0.042	0.119	1	12
Uba1.3	-0.420435061	0.16	0.272	1	12
Stim2	0.380837421	0.202	0.095	1	12

Afg3l1	-0.29425493	0.134	0.174	1	12
Dhx15.3	-0.394154692	0.277	0.417	1	12
Shmt1.7	-0.352358215	0.025	0.103	1	12
Rnf130.1	0.381287337	0.227	0.112	1	12
Peo1.2	-0.407061244	0.042	0.124	1	12
Ube2g2.2	-0.386370129	0.042	0.125	1	12
Sumf1.1	0.265191146	0.109	0.034	1	12
St13	-0.327569184	0.378	0.44	1	12
Tmbim4.2	0.349479864	0.252	0.143	1	12
Nav2.4	-0.410576979	0.025	0.105	1	12
Schip1.2	-0.439481774	0.067	0.183	1	12
Pard3.2	0.388292721	0.227	0.125	1	12
Cnpy2.1	0.383193432	0.471	0.342	1	12
Sox9.8	-0.448182782	0.143	0.255	1	12
Ptplad1.1	-0.370707157	0.185	0.251	1	12
Zfp292.5	-0.43897585	0.218	0.342	1	12
Gpr107	0.303461226	0.134	0.056	1	12
Zfp644.1	-0.464060114	0.109	0.208	1	12
Rbm17.2	-0.359249559	0.294	0.39	1	12
Atxn2l	-0.44128342	0.109	0.229	1	12
Ncor1.3	-0.348163334	0.454	0.556	1	12
Clvs1.9	-0.432021434	0.034	0.129	1	12
Mrgbp.1	-0.45725796	0.076	0.184	1	12
BC034090.4	-0.388130314	0.059	0.128	1	12
H2afy2.4	-0.396579275	0.227	0.338	1	12
Arhgap11a.11	-0.488837458	0.076	0.171	1	12
Ano6.2	0.418158535	0.185	0.09	1	12
Snhg5.3	-0.387352273	0.261	0.382	1	12
Copb2.1	0.402363677	0.336	0.217	1	12
Tmem106b.2	0.317067138	0.168	0.071	1	12
Gar1.5	-0.433542465	0.092	0.209	1	12
Wasf1.3	-0.331730729	0.034	0.11	1	12
Cdkn1c.1	0.358948042	0.151	0.061	1	12
Nolc1.6	-0.405379059	0.311	0.409	1	12
Cdk19	-0.361309734	0.05	0.155	1	12
Fut11	0.299591807	0.143	0.054	1	12
Ctnnbl1.2	-0.431409613	0.076	0.185	1	12
Glce.8	-0.421677273	0.109	0.177	1	12
Ssr4.1	0.273744382	0.538	0.375	1	12
Nucb2	0.304644864	0.16	0.069	1	12
Magi3	0.319509001	0.151	0.059	1	12
Mrpl18.4	-0.439474597	0.218	0.354	1	12
Chaf1a.7	-0.337457127	0.067	0.169	1	12
Hnrnpul2.2	-0.414003414	0.168	0.257	1	12
Gm11541.5	-0.382485323	0.034	0.128	1	12
Cd63-ps.3	0.33926219	0.235	0.118	1	12
Dync1i2.6	-0.282939091	0.538	0.568	1	12
Tmsb10.7	-0.268648824	0.639	0.664	1	12

Snrpg.5	-0.3788321	0.244	0.335	1	12
1700025G04Rik.7	-0.464575179	0.134	0.264	1	12
Mpp6.6	-0.365780297	0.059	0.162	1	12
Dnajc7.1	-0.434729806	0.176	0.281	1	12
Gart.4	-0.400472095	0.076	0.166	1	12
Ddrgk1.1	0.277403247	0.303	0.187	1	12
Shmt2.2	-0.32509743	0.042	0.119	1	12
Psmc3ip.7	-0.407809504	0.042	0.132	1	12
Rcbtb1	-0.325036258	0.05	0.105	1	12
Bcl2.1	0.327496956	0.101	0.032	1	12
Cdc123.1	-0.383439117	0.235	0.313	1	12
1500009L16Rik.1	0.35495191	0.101	0.037	1	12
Rps18.5	-0.362678887	0.345	0.429	1	12
Necap2.2	0.298416636	0.126	0.045	1	12
Sec16a	0.269003984	0.101	0.031	1	12
Tro.4	-0.410880674	0.034	0.126	1	12
Prkar2a.1	-0.303894567	0.084	0.135	1	12
Scfd1.1	0.267580622	0.235	0.122	1	12
Hspbp1	-0.335475362	0.025	0.112	1	12
Prdx3	0.357681703	0.168	0.074	1	12
Mlf2.3	-0.365844051	0.21	0.279	1	12
Pkm.4	-0.374108034	0.277	0.43	1	12
Cep110.5	-0.443752383	0.059	0.163	1	12
Ccdc41.7	-0.378689511	0.134	0.254	1	12
Atat1.3	-0.334173404	0.042	0.104	1	12
Kmt2c.2	-0.34293895	0.218	0.296	1	12
Mdga1.4	-0.423136723	0.042	0.136	1	12
Gtf2e2.2	-0.371069119	0.109	0.178	1	12
Psmc6.2	-0.333251234	0.277	0.34	1	12
Cbx3.2	-0.411767099	0.168	0.274	1	12
D19Bwg1357e.4	-0.420985685	0.151	0.274	1	12
Ubqln1.1	-0.418931989	0.176	0.305	1	12
Ywhag.4	-0.449761465	0.143	0.241	1	12
Tmem179b.1	0.31400504	0.134	0.051	1	12
Ddr1.2	-0.419841238	0.042	0.135	1	12
Ddost.1	0.359795434	0.387	0.269	1	12
Pabpn1.3	-0.43443885	0.084	0.194	1	12
Tspan7.7	0.35553014	0.21	0.104	1	12
Rad50.4	-0.441170951	0.101	0.166	1	12
Blm.3	-0.35806869	0.025	0.111	1	12
Uchl1.10	-0.40348625	0.202	0.335	1	12
Sdf4.2	0.327509164	0.361	0.221	1	12
AU022252	0.300340108	0.143	0.06	1	12
Larp4b	-0.396268225	0.084	0.171	1	12
Lipa.1	0.287743185	0.101	0.031	1	12
Rdx.2	-0.281917828	0.546	0.604	1	12
Pde7a.1	-0.377529211	0.034	0.112	1	12
1700011J10Rik	0.319913173	0.143	0.059	1	12

Trim2.3	-0.432891502	0.176	0.283	1	12
Calm3.4	-0.333515265	0.429	0.561	1	12
Specc1.3	0.368548922	0.126	0.048	1	12
Phf14.2	-0.396913435	0.277	0.375	1	12
Unc50.2	0.354163143	0.235	0.124	1	12
Boc.8	-0.430585696	0.067	0.156	1	12
Rwdd4a	-0.287950584	0.059	0.109	1	12
Clasp2.4	-0.424913045	0.084	0.168	1	12
Rbm26.2	-0.37636697	0.227	0.297	1	12
Hat1.5	-0.405803558	0.067	0.155	1	12
Siva1.8	-0.422304754	0.16	0.273	1	12
Lbr.8	-0.417277962	0.084	0.197	1	12
Exosc8.5	-0.361045641	0.092	0.21	1	12
Ssbp3.5	-0.403232571	0.101	0.167	1	12
Hspd1.4	-0.355718665	0.235	0.365	1	12
Atp5o.2	-0.312481249	0.504	0.587	1	12
Cirbp.4	-0.301011461	0.319	0.355	1	12
Mrpl28	-0.38661178	0.202	0.289	1	12
Cdc40.2	-0.368923875	0.076	0.17	1	12
Pafah1b2.3	-0.373247017	0.185	0.255	1	12
Eps8.2	0.347472443	0.101	0.031	1	12
Gatad2b.1	-0.345026538	0.05	0.121	1	12
Mak16.4	-0.421324519	0.109	0.226	1	12
Smarcad1.1	-0.390917575	0.084	0.168	1	12
Rwdd1.1	-0.329426763	0.227	0.276	1	12
Glud1.2	0.346582953	0.261	0.14	1	12
Btbd17.7	-0.45229779	0.076	0.16	1	12
Jakmip2.4	-0.453341516	0.067	0.157	1	12
1700037H04Rik.1	-0.31330245	0.05	0.11	1	12
Cyb5.2	0.387343873	0.311	0.186	1	12
Sart3.2	-0.41329311	0.109	0.225	1	12
Gmppa	0.251728939	0.118	0.042	1	12
Dnph1.6	-0.327465209	0.034	0.124	1	12
Cdca7l.8	-0.341927762	0.025	0.104	1	12
Timm13.1	-0.314406692	0.395	0.454	1	12
Fbxo5.10	-0.41245673	0.059	0.16	1	12
Ube2b.3	-0.326043856	0.429	0.521	1	12
Setbp1.4	-0.423358688	0.059	0.16	1	12
Mvb12a	0.365118734	0.16	0.072	1	12
Nmral1.7	-0.340429022	0.092	0.188	1	12
Hadh.2	0.318806402	0.168	0.073	1	12
Mphosph10.6	-0.453742558	0.118	0.232	1	12
Sec11a	0.365232038	0.387	0.251	1	12
Pald1.1	0.25153993	0.16	0.073	1	12
Gstp1.1	0.364289382	0.277	0.154	1	12
Prdm8.10	-0.460764572	0.042	0.127	1	12
Cox6b1.1	-0.265682719	0.689	0.742	1	12
Tmem123	0.340362732	0.151	0.083	1	12

Exoc2	-0.349987714	0.034	0.116	1	12
Emc3.2	0.296318061	0.269	0.148	1	12
Arpc2.1	0.275377138	0.655	0.507	1	12
Tmco1.1	0.316401698	0.378	0.24	1	12
Ncor2.7	-0.253417583	0.143	0.168	1	12
Arhgap21.3	-0.419285293	0.084	0.164	1	12
Bud31	-0.295596357	0.269	0.31	1	12
Prps1.2	-0.350239755	0.076	0.142	1	12
Ddx39.4	-0.352269268	0.084	0.188	1	12
Pcif1.1	-0.387559486	0.092	0.197	1	12
Med19.3	-0.432659007	0.21	0.318	1	12
Vezf1.1	-0.378568931	0.252	0.342	1	12
Aes.2	-0.281463417	0.185	0.212	1	12
Ttc14.1	-0.437050298	0.143	0.262	1	12
Trpc4ap.10	-0.417755497	0.151	0.198	1	12
Yif1a.2	0.324369397	0.286	0.178	1	12
Psmb3	-0.321029268	0.176	0.259	1	12
Vapb.1	0.345155667	0.193	0.092	1	12
Bap1.1	-0.350153176	0.025	0.108	1	12
Rnf181	0.372971125	0.176	0.099	1	12
Rpl18a.6	-0.275653334	0.37	0.4	1	12
Vrk1.7	-0.37592669	0.059	0.148	1	12
Cpsf2.1	-0.43464761	0.126	0.237	1	12
Snrpb.7	-0.281557533	0.521	0.573	1	12
Tceb1	-0.336565345	0.294	0.366	1	12
Csde1.2	-0.352825851	0.345	0.436	1	12
Ndufaf2.3	-0.423064549	0.101	0.203	1	12
Gm9843.1	-0.272677576	0.193	0.227	1	12
Actr6.2	-0.300494773	0.05	0.107	1	12
Ost4	0.364792516	0.303	0.187	1	12
Snhg6.2	-0.396179316	0.168	0.259	1	12
2310039H08Rik	0.298298176	0.202	0.1	1	12
Kctd10	0.271609498	0.134	0.053	1	12
Tsr3	-0.251898285	0.076	0.102	1	12
Apbb2	0.33225232	0.109	0.039	1	12
Chst15.2	0.339328814	0.168	0.077	1	12
Nxf1	-0.307955561	0.134	0.185	1	12
Cfl2.2	-0.310168373	0.345	0.409	1	12
Rbm34	-0.337276019	0.076	0.169	1	12
Qsox1	0.284982489	0.168	0.076	1	12
Pdcd2l	-0.331893204	0.059	0.131	1	12
Ehbp1.2	-0.35230728	0.05	0.137	1	12
Actn1.1	0.312024303	0.151	0.063	1	12
Patz1.1	-0.34046399	0.025	0.105	1	12
Fmnl2.4	0.29392322	0.277	0.156	1	12
Ick.3	-0.374361431	0.076	0.147	1	12
Ankrd11.3	-0.361307114	0.395	0.473	1	12
Med30.3	-0.39174536	0.118	0.204	1	12

Ptms.6	-0.338641871	0.353	0.474	1	12
Ssbp4.1	0.333554404	0.143	0.059	1	12
Psmg1	-0.345893987	0.042	0.118	1	12
Parp2.1	-0.375739557	0.101	0.17	1	12
Rsrc2.2	-0.312453174	0.42	0.548	1	12
Eif3c.2	-0.300376336	0.529	0.603	1	12
Dcakd.4	-0.292496115	0.176	0.297	1	12
Dnajc10	0.315908557	0.244	0.133	1	12
Ccdc90b	-0.372595502	0.092	0.16	1	12
Gprasp1.3	-0.308407832	0.143	0.206	1	12
Meg3.8	3.370066536	0.954	0.089	1.3424E-183	13
Snap25.9	2.225637245	0.861	0.278	2.1056E-145	13
Syt4	2.656734449	0.676	0.016	4.0888E-134	13
Chgb.12	2.3744119	0.833	0.237	3.4208E-127	13
Gabra6	2.213667594	0.546	0.002	1.986E-121	13
Chn2.1	2.349281141	0.63	0.01	9.9436E-107	13
Vsnl1	2.067963982	0.556	0.005	1.5244E-106	13
Olfm1.5	2.054003152	0.787	0.195	1.5604E-99	13
Syt1.1	2.430687476	0.639	0.029	3.1301E-99	13
Atp1b1.2	2.414687711	0.676	0.048	9.17405E-98	13
Nrxn3.2	2.255388265	0.694	0.032	1.22909E-94	13
Eno2	1.880225328	0.509	0.007	2.01571E-85	13
Lin7a.1	2.092672482	0.685	0.048	3.29827E-84	13
Calb2.1	2.231185598	0.583	0.019	4.40455E-83	13
Cox8a.1	1.302944389	0.898	0.699	1.342E-80	13
Camk4.1	2.127822874	0.63	0.04	1.07535E-78	13
Rph3a.1	1.924250587	0.583	0.036	6.02865E-77	13
Selm.4	1.86775096	0.713	0.171	2.84248E-76	13
Calm1.5	1.113480461	0.991	0.861	2.1525E-67	13
Cbln1.1	1.818964153	0.648	0.086	2.37887E-65	13
Rps5.8	-1.504801798	0.583	0.956	4.48408E-64	13
Gabra1	1.807432352	0.352	0.003	9.30317E-63	13
Cbln3	1.665697467	0.333	0.002	4.6433E-61	13
Malat1.10	1.181243122	1	0.954	3.27857E-58	13
Adcy1.1	1.862382793	0.481	0.023	4.50813E-58	13
Nrep.11	1.407508839	0.889	0.567	1.15929E-56	13
Dnm1	1.713516535	0.389	0.01	1.51129E-55	13
Atp2b1.6	1.30423302	0.87	0.569	2.02639E-54	13
Atp6v1g2	1.716607483	0.407	0.023	3.10299E-52	13
Ppp3ca.8	1.414844131	0.778	0.375	5.88405E-52	13
Scn2a1.1	1.716502363	0.407	0.015	6.05847E-52	13
Rnf112	1.469911461	0.343	0.005	7.10485E-51	13
Etv1.2	1.552332319	0.417	0.025	1.32008E-50	13
Gabrd	1.538133067	0.343	0.005	2.27814E-50	13
Snca.2	1.900855908	0.537	0.057	3.19125E-50	13
Cacnb4.1	1.750716702	0.426	0.031	2.21014E-49	13
Stmn2.12	1.292365877	0.935	0.55	3.89859E-49	13
Rps14.6	-1.203252946	0.648	0.952	1.79599E-47	13

Kcnd2.3	1.645188245	0.463	0.023	2.19684E-47	13
Tubb5.10	-1.526529817	0.444	0.917	2.5215E-47	13
Ntm.9	1.698432942	0.611	0.142	1.1445E-46	13
Sncb	1.553944709	0.38	0.012	1.97508E-46	13
Chchd10.2	1.718348801	0.426	0.031	6.86375E-46	13
Celf4.13	1.433296683	0.88	0.316	7.52751E-46	13
Rps9.6	-1.316470775	0.519	0.931	1.38875E-45	13
Sfrp1.9	-2.020070856	0.194	0.815	4.82037E-45	13
Chl1.1	1.468052597	0.352	0.013	5.64387E-45	13
Camkk2	1.577192672	0.38	0.021	8.02567E-45	13
Tmem59l	1.403617096	0.333	0.007	2.03946E-44	13
Aplp2.7	1.364316341	0.694	0.32	2.28551E-44	13
Unc13c	1.398633413	0.296	0.005	5.34732E-44	13
Actb.3	-0.906273935	0.815	0.98	9.8773E-44	13
Mgst3.1	1.517570453	0.528	0.092	1.40591E-43	13
Eps8.3	1.592040562	0.417	0.029	3.21227E-43	13
Napb.1	1.575145247	0.491	0.06	1.98876E-42	13
Rps3.6	-1.262953739	0.509	0.924	2.18632E-42	13
Ndrg3.2	1.472310345	0.574	0.112	2.28804E-42	13
Syn2.1	1.677418623	0.509	0.082	9.81846E-42	13
Nap1l5.2	1.64341651	0.5	0.078	3.71347E-41	13
Tspan7.8	1.544843691	0.556	0.102	3.88327E-40	13
Pak1.3	1.628230364	0.472	0.057	1.07631E-39	13
Nptxr.1	1.545719537	0.389	0.032	1.54276E-39	13
Gm2694.5	1.310553608	0.704	0.356	3.14342E-39	13
Grin2c	1.027627786	0.222	0.002	2.15017E-38	13
Atp1a3.1	1.510974804	0.407	0.03	3.67522E-37	13
Rpl13a.6	-1.171784623	0.611	0.93	3.29269E-36	13
Gpm6a.12	1.241994938	0.833	0.406	1.27356E-35	13
Cadm3.12	1.537626662	0.574	0.138	1.65126E-35	13
Rps26.8	-1.417299316	0.352	0.85	6.81762E-35	13
Tuba4a	1.329205232	0.278	0.017	7.21814E-35	13
Hsp90ab1.4	-0.604933174	0.981	0.998	4.17316E-34	13
Cd24a.6	-1.727735155	0.046	0.637	4.44784E-34	13
Rbfox3.10	1.191587523	0.806	0.492	5.5935E-34	13
Kcnk3	1.047227379	0.194	0.001	6.71608E-34	13
Rpl32.6	-1.200517184	0.5	0.891	4.30669E-33	13
Rpl4.7	-1.135407058	0.546	0.91	9.49108E-33	13
Btbd3.1	1.36835049	0.472	0.088	1.61693E-32	13
Nrxn1.11	1.282355254	0.769	0.315	2.95493E-32	13
Igfbpl1.11	-1.877169612	0.074	0.651	7.05596E-32	13
Kcnc1.2	1.450795801	0.472	0.087	3.58131E-31	13
Hnrnpab.7	-1.342813405	0.333	0.818	2.04797E-30	13
Rplp1.7	-1.189612215	0.491	0.879	2.36284E-30	13
Diras2.1	1.348319658	0.306	0.013	4.66335E-30	13
Camk2b.1	1.47664419	0.454	0.088	5.35221E-30	13
Gas7.1	1.20979334	0.213	0.005	1.3214E-29	13
Purb.4	1.018605819	0.796	0.547	1.47479E-29	13

Ckmt1	0.985786915	0.222	0.003	4.35943E-29	13
Ablim1	1.41433664	0.315	0.017	5.35235E-29	13
Eef1a1.6	-1.120659624	0.481	0.886	1.58585E-28	13
Ndrg4.1	1.486124204	0.37	0.049	1.70736E-28	13
Rpl8.7	-1.036515408	0.583	0.899	2.27771E-28	13
Grm4	1.186942412	0.231	0.005	2.7397E-28	13
CRE_RECOMBINASE.11	-1.828931521	0.296	0.771	6.68669E-28	13
Ccnd2.9	-1.789815567	0.12	0.665	7.31481E-28	13
Gnb2l1.6	-1.098051635	0.444	0.874	7.35763E-28	13
Tmsb4x.8	-1.052577784	0.657	0.95	9.06898E-28	13
Pkib.1	1.258574463	0.296	0.017	1.75202E-27	13
Spock2.2	1.30279133	0.435	0.063	2.13265E-27	13
Tmod1	1.237383177	0.269	0.013	2.63199E-27	13
Rps11.6	-1.337704217	0.287	0.78	3.36309E-27	13
Cadps2.1	1.48595959	0.407	0.059	6.66924E-27	13
Nsf.1	1.292965888	0.389	0.084	7.42638E-27	13
Rps24.6	-1.194265317	0.343	0.823	8.54834E-27	13
Rpl22.7	-1.279899441	0.287	0.791	1.04983E-26	13
Rps20.7	-1.315483978	0.296	0.781	1.17561E-26	13
Sv2a.3	1.419468576	0.426	0.07	1.27446E-26	13
Cd63.5	-1.329822484	0.231	0.758	2.01297E-26	13
Rps19.9	-1.353914702	0.269	0.756	2.8968E-26	13
Dlgap1.2	1.43572852	0.398	0.065	3.01058E-26	13
Tagln3.12	1.192235822	0.657	0.292	3.07128E-26	13
Tpi1.3	1.192778911	0.444	0.119	3.35809E-26	13
Snhg11.1	1.910803659	0.157	0.007	4.31779E-26	13
Atp6v1a.2	1.101189722	0.556	0.286	4.35702E-26	13
Nrxn2.3	1.322976914	0.435	0.087	6.94718E-26	13
Psd3	1.315537627	0.343	0.044	1.09913E-25	13
Cplx2.8	1.039112803	0.815	0.493	1.15553E-25	13
E130114P18Rik.7	-1.724261698	0.074	0.587	1.62032E-25	13
Mdh1.1	0.866738837	0.556	0.451	1.65271E-25	13
Kcna1.2	1.269827404	0.333	0.025	2.81825E-25	13
Atp2b2	1.303651594	0.417	0.089	3.14843E-25	13
Fbxw7.1	1.333273651	0.444	0.117	6.78709E-25	13
Tenm1	1.306405379	0.287	0.017	1.14828E-24	13
Frrs1l.4	1.23431809	0.528	0.188	1.16437E-24	13
Rplp0.7	-1.049721497	0.491	0.875	1.75936E-24	13
Eef1a2	1.115612598	0.222	0.009	2.09044E-24	13
Golga7b	0.828563029	0.157	0.001	4.36646E-24	13
Crtam	0.908449609	0.139	0	1.04812E-23	13
Darc.1	1.417040631	0.306	0.033	1.10824E-23	13
Car10.2	1.166067908	0.37	0.038	1.36093E-23	13
Cog7.9	-1.524703898	0.074	0.587	1.51219E-23	13
Prdx5.5	1.032626256	0.491	0.263	1.66002E-23	13
Ndufa4.2	0.700472518	0.861	0.801	1.66649E-23	13
Cd9.4	-1.494831124	0.019	0.487	1.76662E-23	13
Car4	1.105416962	0.204	0.007	2.62777E-23	13

Gabrg2.1	1.294325635	0.333	0.048	4.07993E-23	13
D3Bwg0562e.2	1.301663143	0.324	0.028	5.14965E-23	13
Aldoa.8	1.10919664	0.556	0.255	5.16966E-23	13
Prune2.1	1.078864056	0.213	0.008	5.46878E-23	13
Slc16a11	0.860145797	0.148	0.001	6.80747E-23	13
Bcl2l15	1.076083257	0.176	0.003	8.99642E-23	13
Phyhip	0.995139213	0.167	0.002	1.16E-22	13
Rims1.1	1.213001956	0.315	0.026	1.17392E-22	13
Scg2.1	1.585775264	0.306	0.05	2.95353E-22	13
Atp5g3.1	0.830737367	0.722	0.516	3.71263E-22	13
H2afv.11	-1.30762935	0.259	0.73	4.77722E-22	13
Cox6a1.1	0.709093343	0.88	0.76	6.95724E-22	13
Rpl41.6	-1.118681999	0.324	0.783	8.25442E-22	13
Neurod2.2	1.307150057	0.426	0.065	9.53439E-22	13
Jph4.2	1.298970642	0.417	0.066	1.05223E-21	13
Prkce.1	1.316686454	0.333	0.062	1.72106E-21	13
Rpl14.7	-1.251177668	0.222	0.699	2.82064E-21	13
Kcnj9	0.911292624	0.176	0.004	3.37268E-21	13
C1qtnf4	1.164350495	0.241	0.013	3.62542E-21	13
Dpp6.1	1.233433467	0.287	0.025	6.36553E-21	13
Slc12a5	0.762363811	0.139	0.001	8.41838E-21	13
Stmn3.7	0.75289588	0.759	0.621	1.03727E-20	13
Dnm3.1	1.194994812	0.269	0.024	1.15381E-20	13
Bend6	1.038194261	0.25	0.016	1.30251E-20	13
Ddah2.8	-1.26567515	0.204	0.688	1.84853E-20	13
Ezr.8	-1.352827305	0.028	0.44	2.60792E-20	13
Cox4i1	0.581139936	0.88	0.877	3.40487E-20	13
Nptn.5	1.07521418	0.454	0.186	3.8059E-20	13
Neurod1.13	0.666562042	0.88	0.53	1.36785E-19	13
Mybpc3	0.993360682	0.139	0.002	1.6208E-19	13
Cnr1.1	1.20626134	0.333	0.056	1.76919E-19	13
2010107G23Rik.1	1.061256697	0.287	0.026	2.11093E-19	13
Rnf152	1.193459469	0.269	0.037	2.41269E-19	13
Rab3c.5	1.138827551	0.435	0.146	2.50351E-19	13
Cadm2.3	1.388310408	0.315	0.046	2.76878E-19	13
Marcksl1.7	-0.92281194	0.241	0.725	2.77658E-19	13
Rps21.7	-0.986600071	0.481	0.835	4.5988E-19	13
Nnat.9	-1.236026045	0.25	0.712	1.21435E-18	13
Rplp2.8	-0.946644508	0.472	0.842	1.30023E-18	13
Camk2d.1	1.162828216	0.37	0.072	2.16344E-18	13
Slc17a7	0.683671497	0.111	0	3.33178E-18	13
Rps6ka1.1	0.953892474	0.176	0.007	3.53306E-18	13
Sybu.1	0.778627388	0.148	0.004	4.97293E-18	13
Ppfia4.1	0.963202444	0.213	0.011	7.0733E-18	13
Nasp.11	-1.285679672	0.185	0.643	9.09067E-18	13
Pabpc1.7	-0.850884559	0.556	0.88	9.11119E-18	13
Kif5c.12	0.866350415	0.731	0.396	9.66383E-18	13
Sept3.13	0.977657102	0.704	0.39	1.65575E-17	13

Erc1.3	1.225588562	0.398	0.109	1.78945E-17	13
Rtn1.12	0.68552641	0.926	0.688	1.79865E-17	13
Hmgn1.9	-1.070482328	0.157	0.624	2.81991E-17	13
Rock2	1.095661348	0.481	0.21	3.7733E-17	13
Unc80	1.116158615	0.231	0.032	6.08805E-17	13
Pcsk1n.3	1.193010796	0.38	0.082	6.86737E-17	13
Ezh2.11	-1.218885257	0.157	0.612	8.22181E-17	13
Grm1.1	1.102921286	0.241	0.018	9.3573E-17	13
Cplx1.9	1.07937823	0.435	0.136	1.02206E-16	13
Kcnt1	0.915769832	0.157	0.004	1.85008E-16	13
Caln1	1.01318661	0.296	0.043	2.18113E-16	13
Ncl.9	-0.75474448	0.657	0.897	5.1304E-16	13
Rps15.6	-0.94434909	0.417	0.79	5.45529E-16	13
Rit2	0.801171656	0.111	0.001	6.78542E-16	13
Ppp1r14b.6	-1.228497225	0.019	0.399	6.79574E-16	13
Tuba1a.9	-0.842630806	0.722	0.93	7.43697E-16	13
Tceal5.2	1.14604127	0.25	0.031	7.45036E-16	13
Nfib.8	-0.824146311	0.741	0.934	8.74765E-16	13
Slc4a4.2	0.974441322	0.278	0.039	9.48701E-16	13
Nfia.4	-1.034376339	0.389	0.776	1.02334E-15	13
Cnnm1	0.828684798	0.148	0.007	1.13145E-15	13
Npm1.9	-1.137277743	0.213	0.649	1.26693E-15	13
Barhl1.11	-1.267053044	0.093	0.524	1.41116E-15	13
Gm10075.10	-1.022874013	0.093	0.517	1.46268E-15	13
Cpe.9	0.852607956	0.648	0.454	2.0816E-15	13
Snrpe.7	-0.992938548	0.13	0.565	2.2017E-15	13
Adamts18	0.97902979	0.185	0.01	3.80645E-15	13
Kirrel3	0.641988015	0.102	0.001	4.19826E-15	13
Nptx1	0.970967395	0.213	0.032	4.32087E-15	13
Stxbp1.12	1.076238878	0.472	0.16	4.70548E-15	13
Anp32b.10	-0.90436114	0.157	0.593	7.69729E-15	13
Pcp4.1	1.260205975	0.306	0.05	8.63024E-15	13
Cbx5.10	-1.031588858	0.231	0.655	9.57596E-15	13
Ppib.3	-0.863409244	0.269	0.703	9.77874E-15	13
Ppargc1b	0.733115619	0.13	0.003	1.01645E-14	13
Cygb.2	1.221484887	0.306	0.049	1.04835E-14	13
Prkar1b	0.944014479	0.194	0.016	1.43713E-14	13
1700020I14Rik.3	0.933346338	0.491	0.273	1.48807E-14	13
Mdk.9	-1.385676118	0.019	0.381	1.73462E-14	13
Spop.5	0.932680509	0.593	0.313	1.78473E-14	13
Hnrnpa2b1.5	-0.633880135	0.769	0.934	2.09396E-14	13
Eef1b2.7	-0.84156831	0.315	0.735	2.44E-14	13
Necab3.3	1.095250372	0.296	0.052	2.83832E-14	13
Nefl.2	1.266853826	0.269	0.057	3.23E-14	13
Tubb2b.11	-1.276462968	0.065	0.466	3.46373E-14	13
Tspyl4.8	1.035986233	0.398	0.125	3.6939E-14	13
AI848285	0.879527101	0.148	0.007	3.90519E-14	13
Cdk4.6	-0.966527321	0.213	0.644	4.22892E-14	13

Sox4.10	-1.068694699	0.167	0.585	4.55418E-14	13
Anp32e.12	-1.179449038	0.222	0.615	4.92848E-14	13
Atp5o.3	0.594476251	0.676	0.586	5.82309E-14	13
Rps10.5	-1.002617484	0.213	0.639	7.11068E-14	13
Mpp3.1	1.092933461	0.287	0.047	8.67652E-14	13
Psat1.7	-1.091520457	0.019	0.369	9.09536E-14	13
Tshz2.7	-1.242602699	0	0.321	9.37116E-14	13
Smc2.11	-1.371489996	0.139	0.547	9.58872E-14	13
Cend1	0.933305563	0.213	0.018	1.04876E-13	13
Vps37b.6	-1.157977713	0.074	0.472	1.07036E-13	13
Atp6v0e.5	-1.24403127	0.037	0.41	1.10179E-13	13
Pnck	0.865297344	0.176	0.01	1.13297E-13	13
Dync1i1	0.715532893	0.13	0.003	1.62476E-13	13
Slc29a1.8	-1.094966911	0.157	0.564	1.66867E-13	13
Fabp5.5	-1.07568945	0.037	0.405	1.87021E-13	13
Fnbp1.3	1.054620901	0.306	0.071	2.12501E-13	13
Ptma.7	-1.030323116	0.167	0.584	2.36415E-13	13
Atp1b2.2	1.151704931	0.25	0.046	2.53783E-13	13
Syt5	0.930102981	0.167	0.008	2.55097E-13	13
Fat2	0.993574689	0.185	0.013	2.58735E-13	13
Rpl34.5	-0.884301607	0.231	0.655	3.28528E-13	13
Mapt.12	1.062314064	0.5	0.214	4.26645E-13	13
Rpl18a.7	-1.08164873	0.046	0.402	4.36425E-13	13
Rps15a.6	-1.003253249	0.259	0.659	4.86093E-13	13
Itm2b.11	0.589135071	0.731	0.642	4.93336E-13	13
Cntn1.3	1.02929057	0.315	0.049	4.96096E-13	13
Snrpn.3	0.943105138	0.398	0.156	5.36418E-13	13
Cox7a2.3	0.497580567	0.676	0.712	5.62145E-13	13
Rps3a1.6	-0.926929353	0.259	0.669	7.17895E-13	13
Pcdh9.2	1.030468693	0.231	0.039	7.48405E-13	13
Basp1.12	-0.813826308	0.296	0.706	9.37821E-13	13
BC029214.2	1.058706544	0.324	0.091	1.59879E-12	13
Gabrb2	0.564510419	0.102	0.002	1.83455E-12	13
Srebf1.11	-1.252048571	0.009	0.336	1.94496E-12	13
Atp6v0b.10	0.862886041	0.519	0.265	1.96745E-12	13
Bmp1.1	1.031615314	0.306	0.085	1.98566E-12	13
Fabp3	0.970888229	0.194	0.015	2.02669E-12	13
Rps27l.3	-1.073885273	0.102	0.494	2.30606E-12	13
Mex3a.6	-1.086974775	0.083	0.465	2.50852E-12	13
Snap91.2	0.986863832	0.287	0.048	2.54009E-12	13
Ypel3.6	0.735396605	0.574	0.434	3.13188E-12	13
Sorl1.1	0.969770161	0.185	0.021	3.47199E-12	13
Gnao1.9	0.850469064	0.481	0.286	4.85722E-12	13
Srcin1.2	1.036730326	0.306	0.052	5.50633E-12	13
Hcfc1r1.6	0.933395429	0.417	0.191	6.13808E-12	13
Caly	0.681231301	0.148	0.006	6.70467E-12	13
Atp6v0e2.7	0.833447442	0.426	0.259	6.86155E-12	13
Prrt1.1	0.955406327	0.213	0.021	7.06157E-12	13

Ccnd1.10	-1.251748776	0.139	0.524	7.7556E-12	13
E530001K10Rik	0.641480079	0.111	0.002	9.54274E-12	13
Cnn3.4	-1.037633753	0.074	0.438	9.60429E-12	13
App.9	0.697813437	0.731	0.567	1.04466E-11	13
St3gal5.2	0.936962557	0.407	0.192	1.11295E-11	13
Ndufa13.3	0.630490173	0.648	0.532	1.3587E-11	13
Gm9800.7	-1.067775105	0.148	0.537	1.39234E-11	13
Rps18.6	-1.05716339	0.065	0.43	1.4203E-11	13
Rbfox1.2	1.02462035	0.324	0.09	1.48179E-11	13
Mcm7.9	-1.17058472	0.046	0.391	1.50551E-11	13
Camk2n1.2	1.049770068	0.324	0.084	1.89974E-11	13
Snrpb2.4	-0.91942514	0.056	0.386	1.94255E-11	13
Zfand5.4	0.68361116	0.5	0.415	2.10184E-11	13
Cald1.6	-0.978985508	0.148	0.537	2.17881E-11	13
Fam210b.8	-1.207468285	0.009	0.315	2.56207E-11	13
Hjurp.10	-1.026756422	0.093	0.436	2.97213E-11	13
Ndufb8	0.56462674	0.546	0.453	3.00607E-11	13
Ramp3	0.565091659	0.102	0.002	3.22161E-11	13
Rpl35a.6	-0.914663954	0.185	0.572	3.84615E-11	13
Cacna1a.1	0.975391228	0.287	0.071	3.9274E-11	13
Hpcal1.6	1.068315467	0.343	0.105	4.06819E-11	13
Snrpf.8	-0.834450856	0.083	0.429	4.50311E-11	13
Runx1t1.1	1.019302288	0.204	0.022	4.66604E-11	13
C1ql1.8	-1.104076536	0	0.28	4.86401E-11	13
Atp6v1e1.6	0.81231499	0.565	0.329	4.92651E-11	13
Ranbp1.9	-0.96156208	0.306	0.677	4.95102E-11	13
Rpl23.5	-0.877826415	0.157	0.541	5.33496E-11	13
Trpm3.1	0.823450548	0.139	0.011	5.58364E-11	13
Eif4g2.3	-0.725908428	0.324	0.707	7.90025E-11	13
Ndufa5.2	0.683809114	0.593	0.452	7.96968E-11	13
Grina.9	1.002108598	0.315	0.108	8.02767E-11	13
Miat.11	-1.21522883	0.139	0.517	8.24585E-11	13
Eif3f.5	-0.838410655	0.241	0.633	8.44377E-11	13
Serinc1.7	0.697047868	0.583	0.435	8.64714E-11	13
Adam11.1	0.84888451	0.176	0.016	1.08117E-10	13
Rab3a.12	0.981696019	0.426	0.148	1.13809E-10	13
Clip1.3	0.980662992	0.306	0.092	1.1969E-10	13
Hn1.9	-1.035358695	0.176	0.543	1.20234E-10	13
Map1a.2	1.007569834	0.278	0.071	1.21292E-10	13
Ly6h.2	0.846474887	0.204	0.021	1.25634E-10	13
Atp2b4.1	0.687018582	0.139	0.006	1.2856E-10	13
Tmpo.12	-1.092811343	0.102	0.454	1.37642E-10	13
Draxin.9	-0.932411421	0.176	0.556	1.4423E-10	13
Fkbp3.8	-0.811857395	0.398	0.747	1.6678E-10	13
Rgs8	0.656778974	0.102	0.003	2.05634E-10	13
Npc2.5	-0.925397556	0.102	0.466	2.28029E-10	13
Rora.3	1.017287743	0.241	0.053	2.73095E-10	13
Grin1.1	0.978143529	0.231	0.034	3.12657E-10	13

Tspan17.1	0.666098853	0.111	0.004	3.40797E-10	13
Trim2.4	0.861797774	0.472	0.281	3.44156E-10	13
Pura.3	0.837009446	0.463	0.285	3.60913E-10	13
Rps2.5	-0.980954654	0.093	0.451	3.65662E-10	13
Zfyve28	0.696795034	0.176	0.014	3.95542E-10	13
Lmnb1.12	-1.04311573	0.019	0.301	4.01121E-10	13
Rpl26.5	-0.763768176	0.343	0.704	4.02664E-10	13
Mbnl2.4	0.936169256	0.407	0.177	4.40129E-10	13
Mycn.7	-1.049209395	0.046	0.365	4.61719E-10	13
Snrpg.6	-0.974622518	0.028	0.336	4.90008E-10	13
Syt2	0.954090901	0.139	0.013	5.01438E-10	13
Pfkp	0.92135176	0.213	0.029	5.43873E-10	13
Plcxd3	0.742639104	0.13	0.01	5.7255E-10	13
Rgs7bp.2	0.832828072	0.204	0.026	6.10867E-10	13
Cox6b1.2	0.525242723	0.778	0.741	6.25664E-10	13
Fut9.5	0.85702299	0.407	0.21	6.65778E-10	13
Ryr2.1	0.951824173	0.194	0.019	6.82873E-10	13
Hnrnpd.9	-0.902801137	0.25	0.612	7.28101E-10	13
Atp1a1.1	0.901324652	0.324	0.157	7.30196E-10	13
Adora1.2	0.71842853	0.157	0.011	7.78373E-10	13
Rpl39.6	-0.830215069	0.287	0.648	7.93623E-10	13
Srsf3.7	-0.814553597	0.287	0.659	1.20024E-09	13
Madd	0.958226402	0.25	0.052	1.20776E-09	13
Vamp2.1	0.815258835	0.278	0.071	1.2333E-09	13
Dtymk.10	-1.013485123	0.093	0.441	1.39595E-09	13
H2afy.8	-0.758130441	0.222	0.594	1.46149E-09	13
Pbrm1.3	-0.72265367	0.176	0.533	1.68815E-09	13
Gls.1	0.901078162	0.278	0.122	1.72321E-09	13
Uqcr11.2	0.579833086	0.519	0.481	1.75209E-09	13
Carhsp1.6	-0.984579059	0	0.255	1.90507E-09	13
Sptan1.3	0.88326523	0.352	0.167	2.21913E-09	13
Afap1l2.1	0.684287943	0.12	0.007	2.43269E-09	13
Hsd11b2.11	-1.129598327	0.009	0.289	2.47181E-09	13
Slc25a4.3	0.440371961	0.796	0.807	3.02878E-09	13
Ran.9	-0.859033133	0.083	0.416	3.0674E-09	13
Ppp1r1a.7	0.96154001	0.315	0.108	3.35003E-09	13
6430573F11Rik	0.869916686	0.12	0.013	3.37572E-09	13
Pld5	0.652835543	0.111	0.004	3.58166E-09	13
Top2a.12	-1.371501079	0.093	0.429	3.71496E-09	13
Zeb2.3	-0.985808716	0	0.25	3.77056E-09	13
Tox3.5	-0.998611042	0.046	0.347	3.87682E-09	13
Sh3gl2.7	0.876422313	0.407	0.17	4.23805E-09	13
Bsg.1	0.59361075	0.509	0.423	4.26798E-09	13
Rab6a.6	0.713815373	0.5	0.327	5.15597E-09	13
Schip1.3	0.905052045	0.389	0.181	6.09508E-09	13
Prdx2.3	-0.59213626	0.315	0.681	6.83976E-09	13
Pde4a	0.761583348	0.157	0.016	7.11305E-09	13
Zfp385b	0.923672058	0.167	0.025	7.33209E-09	13

Marcks.4	-0.506742794	0.676	0.917	7.3764E-09	13
Apex1.7	-0.848405132	0.111	0.453	7.46689E-09	13
Slitrk4	0.745821418	0.102	0.005	7.81075E-09	13
Zcchc18.4	0.856783723	0.361	0.169	8.41115E-09	13
2210016L21Rik.3	0.799202964	0.491	0.287	8.61424E-09	13
Pagr1a	0.763160707	0.287	0.17	9.43687E-09	13
Pcca	0.922084527	0.25	0.073	9.62581E-09	13
Ndufv3.1	0.714646609	0.528	0.296	1.05276E-08	13
Itgb1.4	-0.727587837	0.074	0.364	1.05639E-08	13
Banf1.10	-0.803918278	0.361	0.695	1.114E-08	13
Pou3f2.8	-1.007549558	0.009	0.277	1.12265E-08	13
Klf9.7	0.799874654	0.472	0.269	1.15056E-08	13
Trp53.6	-0.842477163	0.028	0.3	1.17014E-08	13
Kif1b.9	0.665181054	0.657	0.519	1.23411E-08	13
Egr1.6	-1.121069155	0.083	0.386	1.2728E-08	13
Nap1l1.9	-0.845329484	0.167	0.512	1.2764E-08	13
Dgkd.1	0.894431449	0.287	0.105	1.28841E-08	13
Gas5.4	-0.74042477	0.435	0.743	1.33031E-08	13
Prpf40a.4	-0.674239418	0.213	0.573	1.40683E-08	13
Ppic.7	-0.951485095	0	0.24	1.46848E-08	13
Stx1b	0.854837923	0.185	0.029	1.49314E-08	13
Vim.9	-1.095132549	0.037	0.304	1.57737E-08	13
Matk	0.7423769	0.148	0.013	1.64778E-08	13
Cyc1.2	0.634130846	0.5	0.411	1.65399E-08	13
Prim1.10	-1.044556624	0.009	0.264	1.90036E-08	13
Map3k1.4	-0.871209993	0.065	0.372	1.96772E-08	13
Atp5b.3	0.473235655	0.741	0.712	1.99466E-08	13
Birc5.13	-1.145969484	0.009	0.274	2.02023E-08	13
Syt7	0.736024073	0.148	0.012	2.03157E-08	13
Cdkn1a.2	0.884429144	0.296	0.091	2.2395E-08	13
Commd1.4	-0.801676066	0.037	0.3	2.27788E-08	13
Fxyd7	0.674692531	0.12	0.007	2.33959E-08	13
Tmem145.1	0.881923669	0.25	0.049	2.3713E-08	13
Dctpp1.9	-1.00962793	0.028	0.306	2.61352E-08	13
Igfbp5.1	0.813264415	0.185	0.024	2.62531E-08	13
Il20rb	0.63256612	0.111	0.005	2.7193E-08	13
Scn2b.1	0.634402246	0.139	0.009	3.01228E-08	13
Smc4.11	-1.048712693	0.222	0.568	3.41165E-08	13
Eef2.5	-0.654362851	0.37	0.718	3.64197E-08	13
Srsf6.3	-0.610725498	0.13	0.447	3.70955E-08	13
Dcx.12	-0.896975066	0.074	0.377	3.99252E-08	13
Atp6v1b2.2	0.70941971	0.352	0.169	4.25006E-08	13
Rpl7.5	-0.747569556	0.176	0.511	4.60235E-08	13
Plcb4.3	0.94842525	0.315	0.138	5.27843E-08	13
Fez1.4	0.699581069	0.472	0.331	5.89888E-08	13
Syp.2	0.972124084	0.259	0.067	6.19467E-08	13
Insm1.6	-0.938690731	0.028	0.294	6.256E-08	13
D10Bwg1379e	0.572031308	0.102	0.004	6.48556E-08	13

Ier5.7	-0.957925863	0.083	0.396	7.4573E-08	13
Uqcrq.3	0.589309638	0.713	0.549	7.98166E-08	13
Fnbp1l.9	-0.736844925	0.157	0.455	8.16121E-08	13
Plcxd2	0.768177783	0.12	0.01	8.47911E-08	13
Nop58.9	-0.804074056	0.25	0.594	9.12072E-08	13
Napa.3	0.641486577	0.463	0.349	9.226E-08	13
Bdnf	0.670651496	0.111	0.007	9.25122E-08	13
Scrn1.3	0.827452781	0.204	0.049	9.28824E-08	13
Adarb1.1	0.884847418	0.194	0.04	9.83883E-08	13
Mcm2.10	-0.706513147	0.019	0.205	1.02835E-07	13
Spc25.12	-1.024279753	0	0.226	1.05361E-07	13
Ccdc88a.4	-0.778731781	0.148	0.484	1.10239E-07	13
Cacna1g	0.598668374	0.102	0.003	1.11749E-07	13
Zfp467	0.764224837	0.157	0.018	1.16185E-07	13
Casp3.4	-0.779140452	0.019	0.235	1.3045E-07	13
2810417H13Rik.12	-1.111530787	0.083	0.393	1.36392E-07	13
Rpl37.6	-0.830369891	0.167	0.505	1.44976E-07	13
Tead2.9	-0.915748611	0.019	0.279	1.73291E-07	13
Tecpr1.2	0.766638844	0.259	0.076	1.81105E-07	13
Mcm6.10	-0.97084307	0.019	0.277	1.90515E-07	13
Kdm1a.5	-0.563204722	0.093	0.366	2.04846E-07	13
Atp2b3	0.673147635	0.12	0.006	2.11981E-07	13
Hirip3.11	-0.887480143	0.083	0.384	2.12499E-07	13
Rabac1.5	0.71347214	0.389	0.257	2.17859E-07	13
Hmgn2.10	-0.896337744	0.009	0.257	2.19892E-07	13
Klc1.10	0.673809547	0.5	0.36	2.34621E-07	13
Adh5.3	-0.83080248	0.093	0.406	2.36291E-07	13
Gng3.12	0.739512613	0.602	0.314	2.45732E-07	13
Cenpa.12	-1.154853311	0.028	0.253	2.50587E-07	13
Tubb3.12	-1.159830586	0.176	0.475	2.62496E-07	13
Dusp5.1	0.893738705	0.306	0.086	2.6252E-07	13
Mycbp2.6	0.684137722	0.5	0.344	2.63259E-07	13
Zeb1.7	-0.826368317	0.093	0.389	2.67591E-07	13
Nhlh2.11	-1.080336833	0.111	0.424	2.96911E-07	13
Rfc1.9	-0.842622973	0.056	0.332	3.04627E-07	13
Akap7.1	0.857864194	0.213	0.047	3.2627E-07	13
Ddx21.6	-0.775496397	0.056	0.324	3.30852E-07	13
Hdgfrp3.3	-0.488496286	0.102	0.339	3.39209E-07	13
Fgf14	0.678024582	0.12	0.008	3.69972E-07	13
Lsm3.8	-0.926413101	0.065	0.36	3.77169E-07	13
Micu3.1	0.919153385	0.259	0.079	3.80837E-07	13
Dynll2.3	0.681201943	0.435	0.295	3.96436E-07	13
Ankrd12.7	0.842679049	0.491	0.273	4.04401E-07	13
Il16	0.747324069	0.13	0.01	4.11187E-07	13
Snrpd2.5	-0.829320198	0.13	0.442	4.53103E-07	13
Smarcc1.6	-0.727728539	0.13	0.435	5.24347E-07	13
Dos	0.798293199	0.13	0.013	5.43152E-07	13
Rassf4.6	-0.888314035	0.083	0.378	5.66078E-07	13

Tmem132a.2	0.836497624	0.25	0.101	5.68014E-07	13
Hnrnpa0.4	-0.706111661	0.148	0.458	6.10779E-07	13
Rcn1.5	-0.667765217	0.019	0.235	6.23971E-07	13
Sfpq.3	-0.621039915	0.333	0.672	6.3066E-07	13
Ttc9b.4	0.748415448	0.315	0.193	6.58042E-07	13
Ilf2.4	-0.693940065	0.213	0.543	6.69194E-07	13
Hk1	0.796810043	0.167	0.022	6.83994E-07	13
Myl12a.7	-0.743602951	0.056	0.33	6.962E-07	13
Apbb1.11	0.792867385	0.398	0.168	7.16281E-07	13
Asph.1	0.775713466	0.278	0.113	7.82892E-07	13
Atp5j.2	0.369880517	0.685	0.722	8.09375E-07	13
Fam63b.3	0.882652488	0.296	0.086	8.11837E-07	13
Tpm4.8	-0.81701558	0.046	0.318	8.31367E-07	13
Lsamp.3	0.934051273	0.278	0.076	8.43243E-07	13
Uqcrb.2	0.698382018	0.352	0.219	8.51801E-07	13
Tcf4.5	-0.427101274	0.722	0.926	8.74715E-07	13
Ccdc92	0.77259189	0.148	0.022	8.97772E-07	13
Tspan9.1	0.661591485	0.12	0.009	8.98173E-07	13
H1f0.8	-0.739549093	0.287	0.618	9.18313E-07	13
Fgf12	0.574902311	0.13	0.016	9.54608E-07	13
Syngr1.3	0.842937436	0.296	0.092	9.90215E-07	13
Wbp2.1	0.697276948	0.306	0.194	1.02459E-06	13
Higd2a	0.668476449	0.361	0.251	1.05746E-06	13
Cox6c.4	0.448560864	0.806	0.718	1.06671E-06	13
Pcna.12	-1.045851751	0.074	0.36	1.07184E-06	13
Kifc2	0.731715	0.148	0.016	1.09646E-06	13
Hnrnpr.3	-0.558379843	0.222	0.547	1.12328E-06	13
Got1.1	0.715280291	0.185	0.052	1.12438E-06	13
Tmem108	0.621557729	0.111	0.008	1.12791E-06	13
Atp6v1d	0.732603385	0.426	0.258	1.14476E-06	13
RP23-45G16.5.13	-0.963536084	0.056	0.328	1.17925E-06	13
Ssr3.4	-0.607103843	0.139	0.426	1.21181E-06	13
Hmgb3.7	-0.748589338	0.046	0.307	1.21471E-06	13
Rnaseh2c.8	-0.688089418	0.093	0.383	1.24373E-06	13
Frmd4a.7	-0.956134485	0.046	0.31	1.2553E-06	13
Gria2.11	0.681393284	0.713	0.519	1.34199E-06	13
Calm2.10	0.438299971	0.954	0.873	1.42342E-06	13
Ccdc34.11	-0.928685408	0.111	0.416	1.49961E-06	13
Id2.9	-0.765264528	0.167	0.488	1.60817E-06	13
Ebna1bp2.2	-0.918839106	0.046	0.316	1.69349E-06	13
Rbmx.4	-0.845430914	0.046	0.316	1.79423E-06	13
Tcp1.5	-0.784238753	0.148	0.465	1.93321E-06	13
Ube2c.11	-1.351013225	0.028	0.27	2.06243E-06	13
Fscn1.1	-0.801849131	0	0.204	2.06311E-06	13
Gm8292.6	-0.715995712	0.093	0.37	2.09923E-06	13
Rab6b.12	0.744916435	0.398	0.201	2.13596E-06	13
Cdh20.6	-0.828049495	0	0.204	2.19087E-06	13
Pgm2l1.2	0.872652272	0.287	0.096	2.23102E-06	13

Hmgb2.12	-0.916976252	0.111	0.401	2.40481E-06	13
Gnai2.7	-0.827336611	0.12	0.426	2.45451E-06	13
Abcg1.3	0.825652891	0.167	0.023	2.52045E-06	13
Sh3bgrl.4	-0.750826724	0.065	0.339	2.70989E-06	13
Cdh7	0.799116933	0.13	0.02	2.8671E-06	13
C530008M17Rik.6	-0.818378823	0.037	0.269	2.91248E-06	13
Rps4x.5	-0.857436712	0.037	0.297	3.04065E-06	13
Cdc42.2	0.480098326	0.62	0.514	3.12374E-06	13
Lap3.9	-0.898752883	0.028	0.277	3.19583E-06	13
Ttc3.9	0.450987032	0.917	0.828	3.28595E-06	13
Fam49b.2	0.757892221	0.222	0.084	3.30116E-06	13
Hmgb1.10	-0.62083953	0.093	0.36	3.46511E-06	13
Cbx1.5	-0.680519468	0.38	0.687	3.63944E-06	13
Lig1.13	-0.982397577	0.083	0.35	3.67362E-06	13
Apba1	0.710848974	0.148	0.017	4.0583E-06	13
Ybx1.6	-0.539618559	0.491	0.787	4.11828E-06	13
Eif3e.5	-0.537795618	0.111	0.383	4.66143E-06	13
Psmb1.1	-0.552644112	0.435	0.681	4.77135E-06	13
Mki67.13	-1.155771337	0.111	0.396	5.03739E-06	13
Trnp1	0.596725401	0.111	0.007	5.07902E-06	13
Ssrp1.8	-0.721185898	0.259	0.578	5.21101E-06	13
Mrpl52.4	-0.825269381	0.13	0.434	5.22601E-06	13
Ptgds	0.650442687	0.111	0.012	5.34419E-06	13
Cks1b.12	-0.664843201	0.083	0.346	5.44933E-06	13
Ndufa1.2	0.662648124	0.472	0.341	5.66813E-06	13
Pa2g4.9	-0.588555921	0.213	0.524	6.03281E-06	13
Ptpre.2	0.749619821	0.148	0.016	6.39798E-06	13
Klhdc8a	0.522732896	0.102	0.005	6.93908E-06	13
Idh3a.2	0.70781724	0.306	0.161	7.03903E-06	13
Syncrip.6	-0.525702865	0.204	0.497	7.0859E-06	13
Rbbp4.5	-0.470773024	0.213	0.485	7.22015E-06	13
Paics.8	-0.774096921	0.12	0.415	7.26265E-06	13
Cnbp.7	-0.555133303	0.463	0.741	7.26794E-06	13
Cct2.5	-0.562833873	0.213	0.525	7.87399E-06	13
1110038B12Rik.8	-0.864942472	0.046	0.3	8.43083E-06	13
Ssr1.1	-0.473355018	0.065	0.29	8.49308E-06	13
Gse1.6	-0.464576117	0.056	0.238	8.66328E-06	13
Sox9.9	-0.895802184	0.028	0.256	8.76235E-06	13
Atp2a2.2	0.553038435	0.213	0.201	9.63065E-06	13
Adam22	0.812558211	0.185	0.056	1.04229E-05	13
Tuba1b.11	-0.777192418	0.194	0.483	1.08386E-05	13
Fbxo9.2	0.773125271	0.278	0.127	1.12635E-05	13
Hook1.1	0.885508085	0.204	0.061	1.13632E-05	13
Khdrbs1.4	-0.750390954	0.185	0.495	1.17196E-05	13
Lgals1.9	-0.924345416	0.019	0.23	1.21885E-05	13
Cox5b.1	0.465166002	0.417	0.356	1.31293E-05	13
Nhp2.6	-0.595283041	0.102	0.372	1.31888E-05	13
Ebf3.6	-0.714156227	0.019	0.204	1.31922E-05	13

Arhgef7.5	0.800160262	0.315	0.148	1.34342E-05	13
Scamp1.1	0.816047743	0.222	0.071	1.35478E-05	13
Tmsb10.8	-0.59026777	0.361	0.665	1.35505E-05	13
Camta1.5	0.631335148	0.528	0.407	1.42388E-05	13
En2	0.792517165	0.213	0.059	1.44387E-05	13
Rdx.3	-0.549940472	0.306	0.605	1.45116E-05	13
Synpr.1	0.675790757	0.204	0.041	1.54547E-05	13
G3bp1.6	-0.819960288	0.065	0.329	1.54693E-05	13
Echdc2.1	0.745771219	0.176	0.034	1.61336E-05	13
Ntrk2.4	0.773745659	0.231	0.082	1.62824E-05	13
Akap8l.4	0.757584616	0.324	0.157	1.71953E-05	13
Dpf3	0.574226173	0.13	0.013	1.75989E-05	13
Pdgfa.9	-0.752902948	0.037	0.255	1.79271E-05	13
B4galt6	0.791320847	0.194	0.054	1.97969E-05	13
Rpl22l1.4	-0.818952368	0.083	0.352	2.08793E-05	13
Nsg2.10	-0.537694268	0.194	0.488	2.14585E-05	13
Lmo4.7	-0.574043081	0.083	0.337	2.17733E-05	13
Jun.5	-0.536474445	0.352	0.656	2.24131E-05	13
H2afy2.5	-0.649073531	0.083	0.339	2.28787E-05	13
Gabbr2.1	0.59651978	0.148	0.018	2.38927E-05	13
Rbm8a.6	-0.414877781	0.204	0.467	2.39367E-05	13
Cct5.5	-0.617856121	0.176	0.478	2.66753E-05	13
Hdac2.4	-0.672490389	0.213	0.517	2.81539E-05	13
Rcor2.9	-0.809993164	0.019	0.226	3.00594E-05	13
Sirpa.2	0.731586647	0.185	0.036	3.13605E-05	13
Rpl14-ps1.3	-0.678552227	0.046	0.285	3.19213E-05	13
A930011O12Rik.12	0.843073582	0.324	0.113	3.32938E-05	13
Gm17750.7	-0.80347292	0.093	0.368	3.40824E-05	13
Epb4.1l3.4	0.68279974	0.222	0.112	3.45921E-05	13
Rnd3.8	-0.729437578	0.139	0.433	3.53004E-05	13
Denr.1	-0.686321016	0.083	0.347	3.55493E-05	13
Mpv17l	0.684524777	0.12	0.014	3.57769E-05	13
Edf1	0.489567326	0.472	0.406	3.6475E-05	13
Igsf21.5	0.769526777	0.278	0.088	3.83777E-05	13
D4Wsu53e.8	0.541913945	0.583	0.445	3.84951E-05	13
Ppip5k1	0.619279834	0.12	0.015	3.91301E-05	13
Tsn.4	-0.572888047	0.278	0.59	4.03301E-05	13
Sec62.4	0.59532355	0.546	0.405	4.12095E-05	13
Elavl2.7	-0.769343797	0.037	0.271	4.47988E-05	13
Papola.2	-0.588393514	0.13	0.403	4.76132E-05	13
Fam131a	0.762065623	0.148	0.024	4.91382E-05	13
Cbfa2t3.10	-0.765783536	0.12	0.404	4.92433E-05	13
Gsg1l.10	-0.840440748	0.028	0.256	4.95573E-05	13
Zcchc17	0.718642264	0.417	0.262	4.95923E-05	13
Rpl18.4	-0.736589812	0.056	0.306	4.96995E-05	13
Atp5f1	0.250321848	0.556	0.611	5.27671E-05	13
Nip7.1	-0.433679434	0.019	0.163	5.5463E-05	13
Cacna1d	0.631189142	0.167	0.044	5.65463E-05	13

Map7d2.2	0.845607862	0.259	0.099	5.94335E-05	13
Lrrtm3.1	0.608235132	0.13	0.017	6.14504E-05	13
Lrrc49	0.721995644	0.167	0.059	6.31685E-05	13
Pde1c.13	-0.892836264	0.111	0.388	6.33136E-05	13
Klf13.5	0.631514385	0.343	0.235	6.4823E-05	13
Magoh.5	-0.588552311	0.083	0.334	6.56599E-05	13
Eny2.5	-0.671161731	0.13	0.389	6.70696E-05	13
Mpc2.2	0.532786284	0.389	0.296	6.7278E-05	13
Cystm1.1	0.719610526	0.185	0.037	6.95213E-05	13
Pja2.3	0.60784599	0.398	0.233	7.00646E-05	13
Nhlh1.10	-0.949904213	0.019	0.224	7.16078E-05	13
Arf4.3	-0.725334154	0.13	0.405	7.2867E-05	13
Nup85.4	-0.522398283	0.037	0.227	7.56734E-05	13
Snx6.2	-0.503777696	0.12	0.37	7.72419E-05	13
Supt16.9	-0.705266866	0.194	0.495	7.79351E-05	13
Eif3l.2	-0.754180976	0.056	0.303	7.95961E-05	13
Rbp4.10	-0.846940487	0.028	0.243	8.17344E-05	13
Serf1.6	-0.544137558	0.074	0.281	8.19413E-05	13
Eif3d.3	-0.427657608	0.148	0.407	8.1995E-05	13
Sep15.6	-0.39714607	0.241	0.506	8.47609E-05	13
Gins2.6	-0.549399506	0.019	0.186	8.53055E-05	13
Napg.2	0.687437809	0.287	0.127	8.64963E-05	13
Snrpd3.5	-0.608313068	0.25	0.536	8.76264E-05	13
Cdk1.12	-0.852925801	0.028	0.248	9.09357E-05	13
Eef1g.3	-0.680343113	0.083	0.336	9.1017E-05	13
Rps25.8	-0.494522705	0.222	0.481	9.19157E-05	13
Pcyt2	0.743732723	0.157	0.044	9.27536E-05	13
Atp6v1c1.1	0.76973463	0.25	0.108	9.35088E-05	13
Srsf7.9	-0.600272078	0.176	0.467	9.45365E-05	13
Phf14.3	-0.521209079	0.139	0.376	9.46414E-05	13
Oxct1.2	0.477730346	0.639	0.552	9.71056E-05	13
Zic1.11	0.4418965	0.87	0.783	9.79168E-05	13
Ndufb2.2	0.49473846	0.407	0.367	9.9738E-05	13
Ccna2.12	-0.753414084	0.019	0.222	0.000100691	13
Txnrd1.5	-0.609836102	0.12	0.39	0.00010347	13
Esco2.12	-0.7850555	0.019	0.196	0.000107393	13
Pcbp1.5	-0.551691007	0.352	0.595	0.000107554	13
Cacybp.2	-0.368656973	0.12	0.357	0.000112733	13
Bcl11a.5	-0.800831535	0.009	0.211	0.000113566	13
Crip2.5	-0.68644014	0.037	0.241	0.000117151	13
Kbtbd11.2	0.778954223	0.204	0.087	0.000119099	13
Mat2b	0.600709897	0.204	0.148	0.000119631	13
Car11.1	0.700525643	0.139	0.02	0.000126223	13
Chka.1	0.538155414	0.222	0.158	0.00012779	13
Nolc1.7	-0.768527274	0.13	0.41	0.00013015	13
Cox7b.2	0.443565072	0.583	0.548	0.0001307	13
Clic4.6	-0.554148498	0.046	0.228	0.000134148	13
Atp5k.1	0.463539336	0.5	0.454	0.000146217	13

Dnajc5.2	0.597554383	0.37	0.247	0.000148445	13
Atp5j2.1	0.314919777	0.556	0.615	0.000150184	13
1110008F13Rik.1	0.542540311	0.306	0.228	0.000162749	13
Rpa3.7	-0.752033818	0.028	0.245	0.000167035	13
Megf11.1	0.549397628	0.111	0.009	0.000167408	13
Lhx1.10	-0.665392119	0.204	0.499	0.000167854	13
Psap.7	0.601202235	0.333	0.183	0.000175711	13
Sh3glb1.2	-0.795435949	0.102	0.368	0.000176377	13
Hmgn5.10	-0.757487737	0.148	0.426	0.000184926	13
Dbi.5	-0.743405148	0.204	0.465	0.000187725	13
Taok3.3	0.796307636	0.269	0.086	0.000194946	13
Rfc3.7	-0.452935604	0.028	0.205	0.000196105	13
2700094K13Rik.12	-0.684833429	0.361	0.615	0.000197029	13
Sec14l1.3	0.687923851	0.185	0.052	0.000198879	13
Mdh2.1	0.268886336	0.463	0.528	0.000200233	13
6430548M08Rik	0.672623656	0.148	0.02	0.000203721	13
Nkd1.6	-0.626332217	0.046	0.249	0.000207602	13
Prc1.12	-1.085115865	0.037	0.258	0.000207879	13
5730409E04Rik	0.506068964	0.111	0.009	0.000208638	13
Dek.12	-0.70919903	0.463	0.695	0.00021213	13
Rhobtb3.2	-0.499511412	0.019	0.165	0.000218405	13
Ythdf2.2	-0.709019803	0.037	0.26	0.000222727	13
Tmem38a.1	0.606485904	0.139	0.021	0.000227757	13
Arxes1.2	0.662539943	0.185	0.063	0.000239959	13
Arpp21.11	0.80408727	0.324	0.111	0.000241881	13
Dnajc9.11	-0.627151888	0.102	0.351	0.000248729	13
Shfm1.4	-0.486897457	0.407	0.688	0.000262005	13
Cdca3.12	-0.82798583	0.019	0.222	0.000263788	13
Atp6ap2.1	0.641171693	0.306	0.183	0.000264626	13
Uqcr10.2	0.445019879	0.481	0.433	0.000265053	13
Snrpd1.8	-0.615683714	0.241	0.525	0.000266334	13
Ubxn1.2	-0.402958888	0.222	0.479	0.000268848	13
Rps16.3	-0.580687013	0.028	0.21	0.000268966	13
Stmn1.6	-0.647393066	0.056	0.285	0.000275884	13
Luc7l2.3	0.424994654	0.528	0.495	0.000280172	13
Grm5.2	0.743948351	0.111	0.011	0.000285235	13
Sphkap.2	0.828880436	0.185	0.046	0.000300724	13
Zfp326.3	-0.711734676	0.148	0.411	0.000302552	13
Bola2.5	-0.320082318	0.167	0.398	0.000306027	13
Mapre3	0.686447685	0.157	0.026	0.00030981	13
Sfrs18.7	0.302700252	0.796	0.777	0.000314189	13
Rps7.6	-0.659516737	0.13	0.401	0.000320003	13
Racgap1.13	-0.733683631	0.046	0.256	0.00032274	13
Btg2.4	-0.688595707	0.028	0.209	0.000323872	13
Gpr56.5	-0.652707481	0.037	0.223	0.00033545	13
Rpa2.9	-0.747101009	0.009	0.201	0.000337535	13
Ank2.11	0.77672927	0.407	0.239	0.00033882	13
Phactr3.1	0.767466148	0.213	0.073	0.000339492	13

Zfr2	0.511887644	0.111	0.01	0.000344077	13
Rpl37a.3	-0.438865682	0.213	0.47	0.00035737	13
Bok.9	-0.711552873	0.037	0.252	0.000363254	13
Zc3h14	-0.449529459	0.019	0.145	0.00037788	13
Ptn.11	-0.712152769	0.176	0.443	0.00037909	13
Mapre1.2	-0.587228523	0.231	0.519	0.000383379	13
Rps8.5	-0.714075127	0.13	0.398	0.000383947	13
Psma6.2	-0.476049263	0.185	0.453	0.000399236	13
Tceal3.5	0.692638212	0.231	0.109	0.000403087	13
Cisd1.1	0.359440983	0.37	0.342	0.000420899	13
Cfdp1.3	-0.453324255	0.222	0.485	0.000432771	13
Ypel1.4	-0.645591323	0.019	0.175	0.000452152	13
Eomes	0.716401305	0.102	0.01	0.000468644	13
Rif1.8	0.476923046	0.306	0.275	0.000472859	13
Gucy1b3.2	0.692205711	0.139	0.037	0.000491742	13
Lrig3.8	-0.540935067	0.019	0.171	0.000500748	13
Cdca7.9	-0.654328972	0.019	0.198	0.00051302	13
Atp5c1	0.311495052	0.593	0.621	0.000515681	13
Eif3h.3	-0.512236651	0.213	0.494	0.000518841	13
Snhg5.4	-0.757845305	0.12	0.382	0.000520795	13
Lars.2	-0.558840441	0.037	0.219	0.000526977	13
Neto2.1	0.655998271	0.194	0.073	0.000536	13
Eif3i.2	-0.724145774	0.194	0.463	0.000551114	13
Ftsj3.4	-0.47422854	0.037	0.207	0.000554354	13
Zranb2.2	0.358200905	0.398	0.406	0.000569284	13
Eef1d.4	-0.600456036	0.111	0.351	0.000585968	13
1110001J03Rik.1	0.617313838	0.324	0.237	0.000605965	13
Rad21.13	-0.609972277	0.185	0.463	0.000618872	13
Ktn1.2	-0.571437556	0.194	0.46	0.000626593	13
mt-Rnr2.3	0.353768752	0.991	0.997	0.000628909	13
Hells.13	-0.826294106	0.019	0.214	0.000652563	13
Ccser2.1	-0.558953094	0.065	0.259	0.000667212	13
Ccm2.6	-0.734839943	0.028	0.226	0.000693201	13
Cyfip2.3	0.687815346	0.213	0.052	0.000696313	13
Cenph.12	-0.765765338	0.009	0.194	0.000781562	13
Rrm1.12	-0.678004206	0.046	0.261	0.000798849	13
Usp1.12	-0.697203722	0.093	0.34	0.000801179	13
Set.8	-0.430995272	0.148	0.388	0.000815354	13
Eif4a1.4	-0.52765566	0.213	0.486	0.000816501	13
Caprin1.4	-0.679401599	0.13	0.395	0.000819538	13
Gas1.6	-0.66892454	0	0.157	0.000831551	13
Prdx4.6	-0.694876526	0.111	0.367	0.000867541	13
Pkp4.4	0.677194789	0.222	0.101	0.000877715	13
Mpp6.7	-0.590008764	0.019	0.162	0.000895757	13
Kpnb1.4	-0.255997062	0.12	0.301	0.000948052	13
Tspan6.4	-0.735406501	0.037	0.247	0.000969811	13
Tle1.5	-0.782229599	0.028	0.226	0.001000592	13
Ssr2.1	-0.419556228	0.167	0.38	0.001029419	13

Map1b.11	0.467102267	0.769	0.586	0.001059119	13
Porcn.2	0.773543068	0.25	0.082	0.00107463	13
Pfn1.5	-0.541682381	0.231	0.512	0.001077572	13
Syt12.1	0.650957369	0.139	0.036	0.001080702	13
Ccnb2.12	-0.783902302	0.009	0.187	0.001089	13
Aifm3	0.517491786	0.102	0.008	0.001194972	13
BC034090.5	-0.388507929	0.019	0.128	0.00120292	13
Mapre2.3	0.623680431	0.269	0.166	0.001226232	13
Cbfb.4	-0.637097185	0.009	0.184	0.00123281	13
Cox7a2l.1	-0.557142896	0.139	0.383	0.001241755	13
Opcml.1	0.625461348	0.111	0.019	0.00125557	13
Prdx1.8	-0.602140522	0.213	0.491	0.001306366	13
Clspn.12	-0.801379479	0.009	0.19	0.001320693	13
Fam155a.3	0.644696965	0.194	0.115	0.001324913	13
Prnp.8	0.713852196	0.38	0.205	0.001332728	13
Cenpe.12	-1.099571624	0.065	0.287	0.001339666	13
Nusap1.12	-0.927116049	0.037	0.228	0.001373145	13
Rfc4.11	-0.591616077	0.037	0.227	0.001376736	13
1500012F01Rik.7	-0.546578375	0.176	0.399	0.001415201	13
Ptch2.9	-0.657259568	0	0.153	0.001439103	13
Snord104.6	-0.672470259	0	0.153	0.001439103	13
Arrb1	0.590560901	0.111	0.016	0.001447339	13
Pdap1.5	-0.522851755	0.407	0.684	0.001463795	13
Zmynd11.1	0.411714062	0.472	0.459	0.00151025	13
Clip3.8	0.509302444	0.463	0.311	0.0015159	13
Gpr153.7	-0.763404984	0.019	0.209	0.00155658	13
Zfr.3	0.501552008	0.389	0.353	0.001559022	13
Bcas1.6	-0.710029822	0.065	0.283	0.00161088	13
Dut.11	-0.657783535	0.194	0.436	0.001691874	13
Ccdc41.8	-0.599925885	0.056	0.254	0.001741821	13
Gabbr1.7	0.722681443	0.306	0.139	0.001799066	13
Prmt8.8	-0.737662915	0.056	0.273	0.001823781	13
Arpc5.3	-0.326583055	0.12	0.322	0.001889841	13
Pbk.11	-0.826889234	0.028	0.223	0.00189509	13
1110008P14Rik	0.625128573	0.185	0.056	0.001923524	13
Ncdn.2	0.688394791	0.222	0.066	0.001942612	13
Tcerg1l	0.560482027	0.111	0.017	0.001956967	13
Nmral1.8	-0.69291021	0.009	0.188	0.001985073	13
Lsm4.7	-0.632837328	0.213	0.489	0.001996486	13
Dock9.1	0.539326901	0.12	0.015	0.002024018	13
Tra2a.2	-0.347476977	0.167	0.393	0.002035235	13
Spc24.11	-0.781315129	0.037	0.243	0.002035873	13
Mrpl15.2	-0.424754246	0.102	0.295	0.002089615	13
2610017I09Rik.3	-0.689845051	0.019	0.205	0.002123819	13
Knstrn.11	-0.787982659	0.009	0.186	0.002163162	13
Hmmr.11	-0.78160303	0.019	0.177	0.002171289	13
Ctsz.4	-0.52770262	0.019	0.137	0.002252345	13
Itm2c.9	0.646200348	0.315	0.211	0.002279876	13

Fam134a	0.595864928	0.167	0.086	0.002294	13
Hnrnpu.6	-0.418244256	0.667	0.866	0.002312062	13
Pqlc1.10	-0.656042389	0.074	0.298	0.00233322	13
Rnaseh2b.6	-0.52972598	0.046	0.237	0.002339085	13
Abracl.4	-0.651643985	0.074	0.303	0.002343287	13
Celf1.4	0.489524053	0.435	0.379	0.002347509	13
Hes1.11	-0.856612302	0	0.149	0.002347724	13
Smarca4.3	-0.443401203	0.25	0.506	0.002357216	13
Sept8.4	0.671927863	0.259	0.128	0.002384325	13
Cdk2ap2.1	0.601559759	0.194	0.11	0.002393167	13
Unc5c.1	0.652007393	0.13	0.022	0.002408878	13
Gm13826.4	-0.592432405	0.083	0.313	0.002557053	13
Rpf2.3	-0.737559226	0.028	0.203	0.002643186	13
Psma7.5	-0.483882886	0.546	0.783	0.002670325	13
Abcc8	0.612766405	0.111	0.018	0.002676964	13
Dpy30.6	-0.454872911	0.093	0.299	0.002690822	13
Arhgef2.7	-0.37239264	0.093	0.283	0.002795384	13
Kif23.13	-0.842864422	0.028	0.215	0.002798117	13
Magohb.1	-0.557302784	0.019	0.156	0.002854483	13
Aldoc.2	0.730937318	0.148	0.032	0.002857876	13
Mettl9.2	-0.638658943	0.074	0.301	0.002877969	13
Ociad1	0.345377965	0.343	0.358	0.002964067	13
Pip5k1c.1	0.676628648	0.213	0.081	0.003062735	13
Idh2.7	-0.584268218	0.065	0.28	0.003078363	13
Rgs12.3	-0.642748225	0	0.147	0.003101641	13
Pdzrn4.8	-0.678074807	0	0.147	0.003101641	13
Atp5d.1	0.34495084	0.593	0.584	0.003127851	13
Plxnb2.3	-0.535801886	0.019	0.171	0.003193235	13
Pdgfra.7	-0.814364195	0.009	0.182	0.003204802	13
Ddx42.2	-0.328293994	0.185	0.383	0.003318797	13
Ywhag.5	0.590349101	0.361	0.239	0.003341031	13
Hspd1.5	-0.641052406	0.12	0.366	0.003445368	13
Nras	-0.408228027	0.056	0.221	0.003496225	13
Slc25a22	0.647689996	0.176	0.037	0.00354454	13
Cdc20.12	-0.85859352	0.028	0.197	0.003554957	13
Tiam1	0.700995031	0.139	0.045	0.003569012	13
Hspa5.5	-0.600257817	0.287	0.551	0.003620256	13
Cdca8.13	-0.839788442	0.056	0.265	0.003623939	13
Parp1.4	-0.444197412	0.102	0.314	0.003637837	13
Gm10036.2	-0.694660619	0.028	0.177	0.003716024	13
Incenp.12	-0.694946574	0.065	0.276	0.003733675	13
Rundc3a.7	0.619426885	0.352	0.248	0.003744426	13
Snhg1.8	-0.479650246	0.139	0.358	0.003779859	13
Magee1.1	0.641632594	0.139	0.048	0.00384242	13
Tspan4.2	0.726188361	0.231	0.064	0.003844064	13
Cenpk.11	-0.63549158	0	0.145	0.003846501	13
Txndc9.2	-0.599406947	0.037	0.212	0.003950007	13
Map3k12.2	0.498901251	0.157	0.098	0.003961309	13

Ccar1.3	-0.371042779	0.315	0.573	0.003967936	13
Tyms.10	-0.741143273	0.028	0.22	0.004040034	13
Rrs1.4	-0.647265588	0.019	0.19	0.004089677	13
H2afx.12	-0.883696309	0.102	0.332	0.004129453	13
Dixdc1.7	0.559531665	0.343	0.234	0.004186418	13
Bub3.8	-0.658901644	0.093	0.322	0.004237604	13
Smarca2.3	0.596773988	0.38	0.285	0.004272296	13
Cenpf.13	-0.929126723	0.12	0.363	0.004529923	13
Nbea.7	0.639555766	0.333	0.189	0.004553698	13
Tgfb2.6	-0.753818493	0.019	0.192	0.004559485	13
Pacsin1.1	0.67030772	0.167	0.035	0.004631798	13
Pcbp4.5	-0.357624257	0.12	0.325	0.004632108	13
Pnrc1.3	-0.66118183	0.065	0.271	0.004655314	13
Sri.5	0.570254395	0.25	0.165	0.004721809	13
Mmp14.7	-0.671898167	0.009	0.175	0.004727972	13
Sec61b.1	-0.255362129	0.13	0.33	0.004825531	13
Grb2.5	0.593448597	0.361	0.248	0.00501471	13
Vldlr.2	0.616468444	0.13	0.06	0.005047377	13
Prkd3.2	-0.577638132	0	0.143	0.005109145	13
Sel1l3	0.600295339	0.111	0.017	0.005120673	13
Kcnk1.10	0.713887227	0.37	0.203	0.005122955	13
Nol7.4	-0.46146595	0.306	0.571	0.005423559	13
Psmb6.1	-0.370155943	0.361	0.612	0.005488547	13
Ckap4.2	-0.316596652	0.102	0.29	0.005516995	13
Laptm4b.5	-0.576807894	0	0.142	0.005589279	13
Araf.1	0.664936709	0.259	0.136	0.005655269	13
Myod1.10	-0.667982346	0	0.142	0.005667003	13
Tead1.5	-0.655845839	0.009	0.174	0.005897033	13
Hint2	0.599064746	0.213	0.119	0.005904548	13
Ngdn	-0.669290276	0.019	0.198	0.005953447	13
Nuf2.11	-0.598591846	0	0.141	0.005988729	13
Setd8.4	-0.663294143	0.056	0.262	0.006084156	13
Cdipt	0.647961057	0.204	0.096	0.006108033	13
Dab1.2	0.613117477	0.148	0.032	0.006144692	13
Tulp4.4	0.532269501	0.306	0.204	0.006316604	13
Gm11223.7	-0.851819357	0.046	0.213	0.00635174	13
Kif11.12	-0.544428577	0.037	0.206	0.006359318	13
Gpatch4.4	-0.569054396	0.028	0.18	0.006393238	13
Phf5a.3	-0.585738407	0.102	0.332	0.00648099	13
Fndc5.1	0.593448137	0.12	0.017	0.006490771	13
Mrpl13.6	-0.663895663	0.083	0.309	0.006490782	13
Trim3	0.572302882	0.139	0.04	0.006584853	13
Emc9	0.663119549	0.139	0.026	0.006587742	13
Sez6.1	0.679017602	0.176	0.054	0.006648958	13
Igsf3.5	0.506726907	0.185	0.132	0.006685096	13
Ptprs.10	-0.588589413	0.167	0.422	0.006737211	13
Nup62.6	-0.581268551	0.009	0.172	0.006750277	13
Gtpbp4.2	-0.537646383	0.074	0.283	0.007164534	13

Kctd8	0.621255016	0.111	0.024	0.007179697	13
Ilf3.3	-0.414837923	0.167	0.377	0.007464425	13
Prdx6.5	-0.542620013	0.111	0.327	0.007478399	13
Sema7a.8	-0.539080833	0.019	0.156	0.007786746	13
Meis1.5	-0.675083145	0.13	0.374	0.007826867	13
Tacc3.12	-0.66517213	0.028	0.207	0.007930284	13
Mcm3.7	-0.574119105	0.019	0.18	0.00796154	13
Rpl13.2	-0.512597306	0.102	0.314	0.007993552	13
Cnpy1.7	0.676030774	0.25	0.132	0.008004845	13
Cd200.3	0.701111895	0.185	0.044	0.00806192	13
Ddx5.5	0.283810271	0.769	0.816	0.00812751	13
Gm10260.4	-0.583754403	0.046	0.228	0.008177328	13
Ckap2.10	-0.539379959	0.019	0.164	0.008196036	13
Uba52.4	-0.647660496	0.093	0.318	0.008479275	13
Pdxp.3	0.646181013	0.231	0.112	0.008537576	13
Islr2.8	-0.633853047	0.019	0.18	0.008750001	13
Dnmt1.12	-0.721514297	0.065	0.279	0.008853631	13
Nt5dc2.7	-0.58286244	0.046	0.238	0.00889058	13
Tbata.10	-0.7538167	0.111	0.341	0.009087236	13
AI413582.2	0.555402167	0.139	0.03	0.009198983	13
Bola1.2	-0.373562012	0.037	0.173	0.009245312	13
Amph.1	0.592770665	0.194	0.089	0.009527879	13
Tpx2.13	-0.943295957	0.074	0.282	0.009819827	13
Dstn.1	-0.497909037	0.111	0.332	0.010043779	13
Pcm1.3	-0.267434085	0.287	0.523	0.010126978	13
Vdac1.1	0.407729789	0.343	0.302	0.010241802	13
Siva1.9	-0.636265723	0.065	0.273	0.010293921	13
Hk2.7	-0.650841336	0.009	0.173	0.010415242	13
Xrn2.3	-0.332289966	0.231	0.474	0.010533942	13
Ndufb11.1	0.298253707	0.435	0.462	0.010827314	13
Cnot6.4	-0.309442052	0.176	0.392	0.011082867	13
Snap47.2	0.660329306	0.287	0.139	0.011141113	13
Clns1a	-0.43215826	0.046	0.219	0.011338492	13
Ivns1abp.4	-0.429604649	0.185	0.416	0.011356706	13
Nucks1.12	-0.589766109	0.435	0.664	0.011942315	13
Sbno1.2	0.26807646	0.278	0.351	0.012017427	13
Sstr2.9	-0.666949413	0.028	0.192	0.012040787	13
Hspe1.4	-0.297408307	0.139	0.342	0.012558672	13
Klhdc2.3	-0.397768672	0.12	0.328	0.012645712	13
Erbb2ip.1	-0.467808749	0.028	0.175	0.012654072	13
D19Ertd737e	-0.253461691	0.046	0.191	0.012659942	13
Galnt9	0.497240468	0.102	0.014	0.012697211	13
Sephs1.1	-0.560539539	0	0.135	0.012839044	13
Gpm6b.8	-0.25877824	0.259	0.485	0.012854047	13
Smim11.5	-0.543925207	0.074	0.277	0.012877458	13
Anapc2	-0.317503472	0.019	0.12	0.013098072	13
Sf3b5.2	-0.591181796	0.102	0.328	0.013323274	13
Mns1.12	-0.711071222	0.028	0.204	0.013482324	13

Cited2.3	0.513498314	0.148	0.152	0.013531502	13
Rcbtb2.1	-0.540489142	0.019	0.151	0.013565568	13
Rpl38.3	-0.584638913	0.157	0.384	0.013612478	13
Cdk6.7	-0.6845555	0.046	0.244	0.013714742	13
Tbl1x.3	-0.616145503	0.102	0.329	0.013862201	13
Tmem234	0.274642695	0.333	0.396	0.014545697	13
Baiap2.1	0.65707124	0.185	0.05	0.014717055	13
2810025M15Rik.4	-0.593206754	0.009	0.168	0.014895435	13
Eif4a2.1	0.558245585	0.296	0.18	0.015513087	13
Tmem14c.2	-0.372482093	0.037	0.189	0.015536647	13
Ndufa8.1	0.362332683	0.389	0.329	0.015657504	13
Sfr1.2	-0.490730453	0.157	0.395	0.015984457	13
Tmem258	-0.341190474	0.241	0.452	0.016124521	13
Tcf25.2	0.381916582	0.731	0.601	0.016475801	13
Whsc1l1.1	0.314375075	0.13	0.155	0.017331503	13
Etf1.2	-0.457086391	0.074	0.262	0.01815615	13
Cyb5.3	-0.605995273	0.028	0.188	0.018364412	13
Pdia6.4	-0.662385226	0.111	0.341	0.018423587	13
Fam168a.5	0.518761065	0.37	0.286	0.018521535	13
Lamtor4.2	0.437112183	0.157	0.127	0.018757385	13
Naa15.3	-0.301119943	0.213	0.392	0.018899049	13
Serbp1.9	-0.390872813	0.704	0.87	0.018948368	13
Negr1	0.613009706	0.157	0.046	0.018958757	13
Cct6a.3	-0.505710639	0.231	0.488	0.019220805	13
Nap1l3.1	0.51227064	0.13	0.021	0.019242066	13
Ap3d1.1	0.582040729	0.306	0.203	0.019328596	13
Suv39h2.6	-0.545535647	0	0.132	0.019335755	13
Gm11478.5	-0.676563888	0.037	0.216	0.019398011	13
Larp7.7	-0.635021874	0.093	0.313	0.01964064	13
Cltb.9	0.357570833	0.509	0.422	0.019773499	13
Polr2b.1	-0.381006446	0.028	0.178	0.020266432	13
Baz1a.6	-0.687595815	0.009	0.167	0.020522215	13
Ier3ip1	-0.36093132	0.13	0.313	0.020564617	13
Mcm5.8	-0.648505388	0.009	0.169	0.020596755	13
Sugt1.2	-0.34336095	0.065	0.232	0.02126914	13
Pou3f3.4	-0.531305162	0.019	0.172	0.021410458	13
mt-Nd6	0.266215047	0.083	0.113	0.021548733	13
Alyref.5	-0.583812181	0.028	0.2	0.021594388	13
Atoh1.9	-0.654823709	0.019	0.165	0.021685265	13
Zwint.5	0.504234252	0.38	0.29	0.021706539	13
Dkc1.8	-0.596930774	0.111	0.334	0.021848181	13
Lgi3.1	0.518534372	0.111	0.018	0.021870906	13
Cct3.5	-0.549836629	0.241	0.496	0.021880907	13
Rb1cc1.4	0.636201116	0.37	0.233	0.022049361	13
Psma2	-0.385911759	0.278	0.513	0.022052566	13
Smarcad1.2	-0.282550458	0.046	0.168	0.022339246	13
Mrpl33.2	-0.283034133	0.13	0.283	0.022382842	13
Jam3.6	-0.389236996	0.009	0.135	0.02244199	13

Rpf1.2	-0.369901103	0.046	0.194	0.022486509	13
Znrd1.2	-0.614535263	0.037	0.212	0.022606549	13
Vcan.6	-0.612691533	0	0.13	0.022610276	13
Cebpz.1	-0.568051084	0.093	0.306	0.022767497	13
Nsmce2	-0.638060838	0.037	0.222	0.023399556	13
Acot7.5	0.56155546	0.287	0.194	0.02346435	13
Snx1.2	-0.341694463	0.037	0.169	0.023557255	13
Slc9a3r2.1	0.598767559	0.111	0.015	0.023827531	13
Sub1.3	-0.386902378	0.333	0.59	0.024705625	13
2410006H16Rik.4	-0.365094219	0.222	0.46	0.025250107	13
Fosb.7	-0.611913555	0.019	0.156	0.025700652	13
Fubp1.5	-0.448072882	0.315	0.568	0.025956421	13
Smc1a.7	-0.4654458	0.38	0.632	0.025969964	13
Mgll.5	0.688619464	0.259	0.132	0.026539729	13
Uqcrh.1	0.260420331	0.611	0.657	0.026602551	13
Chd4.5	-0.481644691	0.556	0.77	0.026754408	13
Ncbp1	-0.57529922	0.009	0.163	0.027084	13
Ubxn4	-0.291195588	0.231	0.428	0.027157617	13
Gpi1.1	0.568028325	0.241	0.13	0.027625589	13
Idh1.5	-0.48216549	0.019	0.157	0.028012354	13
Golim4.3	-0.637601005	0.046	0.233	0.028030833	13
Dnajc15.1	-0.534024651	0	0.128	0.028286286	13
Sfxn3	0.588263222	0.148	0.03	0.028652659	13
Pdxk.1	0.515065703	0.102	0.015	0.028895096	13
Brd7.8	-0.505525646	0.102	0.309	0.029210059	13
Cacna2d3	0.578082472	0.111	0.022	0.029650372	13
Tsnax.1	0.461846077	0.25	0.19	0.030022957	13
Txndc17.2	-0.473721279	0.065	0.243	0.03028071	13
Adamts1.6	-0.517929858	0.019	0.155	0.030382019	13
Heg1.3	-0.592829207	0.009	0.158	0.030497984	13
Nrm.6	-0.496118215	0.019	0.17	0.031299403	13
Tcof1.3	-0.437430795	0.028	0.168	0.031474324	13
Ptprr	0.471746498	0.102	0.015	0.031475168	13
Mtch2.3	0.51348984	0.38	0.294	0.031688463	13
Mxd3.10	-0.538187043	0	0.127	0.032389453	13
Atp11b.1	0.339555287	0.12	0.102	0.032974505	13
Cnpy2.2	-0.322152841	0.167	0.344	0.033107432	13
Ska2.10	-0.546832376	0.037	0.205	0.033505901	13
Naa10.4	-0.387061389	0.083	0.25	0.03411446	13
Zfp36l1.9	-0.543674999	0.009	0.151	0.034468786	13
Cnih4.2	-0.375591858	0.065	0.235	0.034624745	13
Ndufb10	0.345358068	0.463	0.423	0.035058074	13
Elp2.1	-0.405355779	0.074	0.244	0.035627736	13
1500016L03Rik.9	-0.652503444	0.157	0.384	0.036300968	13
Kif20b.12	-0.584175951	0.028	0.191	0.036481799	13
Dnajc2.6	-0.402199204	0.13	0.33	0.037012986	13
Zbtb18.9	0.581162927	0.389	0.265	0.037276611	13
Msl3.1	-0.47096828	0.019	0.136	0.03743494	13

Brinp1.1	0.617347067	0.139	0.029	0.037932565	13
Gm6472.3	-0.512579357	0.019	0.167	0.038035293	13
Mical1.4	-0.559891188	0	0.126	0.038095472	13
Fbxo5.11	-0.485249495	0.019	0.16	0.038574433	13
Efs.3	-0.52135967	0	0.126	0.039138594	13
Actl6a.4	-0.441289227	0.056	0.227	0.039216092	13
Gm26735.7	-0.613704905	0.028	0.181	0.039608484	13
Bmi1.2	-0.506441396	0.019	0.147	0.039943937	13
Tars	-0.458368166	0.028	0.179	0.04023669	13
Rab7.3	0.555868257	0.287	0.183	0.040628028	13
1810009A15Rik.3	-0.349183178	0.083	0.26	0.040806877	13
Atad5.7	-0.257223188	0.037	0.149	0.041423029	13
2310022B05Rik.5	-0.661489694	0.056	0.249	0.041454443	13
Dnaja1.2	-0.359031555	0.167	0.375	0.042328326	13
Cdc42se2.4	-0.599626764	0.056	0.239	0.042541095	13
0610009D07Rik.1	-0.542557758	0.157	0.384	0.042782014	13
Asap1.4	-0.6255628	0.019	0.181	0.043022969	13
Btf3.3	-0.562544859	0.13	0.35	0.043146017	13
Sptssa.3	-0.595033068	0.056	0.248	0.043896164	13
Brix1.3	-0.516762469	0.037	0.206	0.044180601	13
Smco4.8	-0.593995425	0.009	0.162	0.044446353	13
Smchd1.5	-0.463839625	0.083	0.274	0.044833866	13
Ppp6r1	0.451681075	0.102	0.049	0.045093611	13
Ccdc28b.3	0.603113609	0.259	0.161	0.045240037	13
Trim62.1	0.60071816	0.139	0.046	0.046201664	13
Galnt7	0.550486905	0.13	0.051	0.046284703	13
Casc5.12	-0.667066473	0.028	0.187	0.047367611	13
Rtn3.5	0.284290892	0.491	0.476	0.047405318	13
Smpd2.4	-0.424904441	0.028	0.166	0.04814041	13
Impad1.2	0.283034084	0.176	0.223	0.048518164	13
Limch1.2	0.621909929	0.12	0.023	0.04865722	13
Gnai3	-0.354709304	0.056	0.217	0.049006265	13
Nfix.8	-0.424201483	0.481	0.674	0.050396233	13
Cbx6	0.630118576	0.213	0.087	0.050579012	13
Smarcb1.1	-0.409327143	0.12	0.324	0.051146864	13
Zfp704.5	-0.595883966	0.065	0.258	0.051518635	13
Akr1a1.1	-0.251197898	0.38	0.587	0.05172779	13
Topors.3	-0.317120744	0.056	0.183	0.052537032	13
Rasa3.2	0.587452125	0.13	0.038	0.052817272	13
Smarcd3.1	0.617920607	0.148	0.062	0.052910666	13
Ddx39.5	-0.598600141	0.028	0.188	0.054398939	13
Chic2.1	-0.476696696	0.019	0.151	0.054651673	13
Rnaseh2a.5	-0.340561255	0.028	0.153	0.055143902	13
Ptp4a2.2	-0.330773066	0.148	0.342	0.055195127	13
Rbbp8.5	-0.525888658	0	0.123	0.055936928	13
Hnrnpc.4	-0.419049808	0.426	0.668	0.056004824	13
Ccdc50.2	-0.296047279	0.139	0.327	0.05602722	13
Ier2.5	-0.545332495	0.157	0.383	0.05656241	13

Wscd2	0.510653059	0.102	0.013	0.057013328	13
Ctnnb1.6	-0.413306546	0.167	0.388	0.057826518	13
Luc7l3.4	0.382171529	0.778	0.747	0.058761956	13
Cenpq.9	-0.352842039	0.028	0.161	0.059690425	13
Sae1.7	-0.455928863	0.093	0.279	0.059707751	13
Rbm17.3	-0.365397384	0.176	0.39	0.05993811	13
Clmp.9	-0.254152442	0.083	0.243	0.061550102	13
Srsf2.5	-0.504320135	0.315	0.567	0.062206719	13
Rpl3.3	-0.581262937	0.037	0.212	0.062338676	13
Peg3.3	0.54649046	0.389	0.283	0.063563541	13
Top1.4	-0.283352158	0.426	0.664	0.064228977	13
Mapk9.1	0.576470311	0.148	0.076	0.064363589	13
Rnd2.5	-0.556385458	0.019	0.159	0.06441358	13
Psmg4.2	-0.612498733	0.037	0.208	0.064501471	13
Herc2	0.538235847	0.157	0.105	0.065171664	13
Ncaph.9	-0.49580948	0	0.121	0.065722336	13
H2afz.11	-0.403459719	0.065	0.232	0.066066604	13
Arhgef9.2	0.597702701	0.194	0.088	0.06676498	13
Oxr1	0.546982143	0.176	0.108	0.066893822	13
Serp1.3	-0.490107905	0.046	0.218	0.066975424	13
Cnksr2.7	0.614322745	0.241	0.106	0.067196521	13
Polr2a.1	-0.360175496	0.046	0.176	0.067341402	13
Sh3bp5.5	0.678541257	0.231	0.102	0.068425368	13
Snrk	0.611379819	0.157	0.048	0.068432816	13
Sfrp2.5	-0.58880566	0.009	0.148	0.069038046	13
Gar1.6	-0.568680207	0.037	0.209	0.07147058	13
Pak3.3	-0.47686947	0.056	0.206	0.072077057	13
Ywhab	0.28967482	0.583	0.607	0.074200985	13
Tmeff1.5	-0.441458081	0.028	0.15	0.074958901	13
Ift74.5	-0.45889505	0.056	0.225	0.075502212	13
Tubb4a.3	0.600437837	0.194	0.066	0.076134692	13
Sap30.4	-0.30256607	0.028	0.138	0.07848067	13
Tmod3.1	-0.571338142	0.009	0.155	0.079430614	13
Afap1.5	-0.595220575	0.009	0.153	0.079552096	13
Rufy2.2	0.588846492	0.287	0.157	0.080374627	13
Sv2b.6	0.574214629	0.213	0.101	0.082067213	13
Ctnna1.3	-0.615706727	0.009	0.153	0.083522285	13
Wdr5	-0.51842184	0.019	0.139	0.083770786	13
Tpm3.2	-0.335111685	0.139	0.316	0.084068718	13
2410015M20Rik.1	-0.273811571	0.176	0.362	0.084384691	13
Gstm5.4	-0.436346067	0.12	0.323	0.084960858	13
Thy1.1	0.459164628	0.102	0.014	0.08557941	13
Lphn1.3	0.600170117	0.241	0.112	0.086020235	13
Cep57.7	-0.346303746	0.093	0.258	0.086902493	13
Pole3.6	-0.572776517	0.065	0.251	0.087251885	13
Arxes2.2	0.545225573	0.185	0.098	0.088593776	13
Srek1.1	-0.3989222	0.167	0.384	0.088813438	13
Chrna3.11	-0.643378533	0	0.119	0.090643354	13

Uchl3.2	-0.563262238	0.028	0.187	0.090946539	13
Cklf.7	-0.541076888	0.019	0.166	0.091591856	13
Odf2.6	0.412846376	0.204	0.17	0.091606224	13
Snrnp40.7	-0.599323369	0.074	0.272	0.09388577	13
Nme4.3	-0.516615766	0	0.118	0.095621112	13
Pak2.2	-0.558386555	0.12	0.302	0.096088966	13
Psmb4	-0.37922089	0.324	0.554	0.096205024	13
Tbc1d20.2	0.588763609	0.185	0.095	0.096715255	13
Nr1d2	0.559933396	0.167	0.052	0.09768383	13
Nelfe.1	-0.524230643	0.009	0.151	0.098525769	13
Mef2a.3	0.561465153	0.269	0.138	0.098622137	13
Mlf2.4	0.392555219	0.306	0.279	0.098703007	13
Snrnp200.1	-0.292124869	0.046	0.163	0.099065066	13
Ppa1.5	-0.360992047	0.065	0.222	0.099829384	13
Rrm2.12	-0.515062579	0.046	0.205	0.099925461	13
Ctnnbl1.3	-0.424142596	0.037	0.185	0.101265675	13
Lsm6.8	-0.581214155	0.148	0.373	0.101805999	13
Pcdha2.9	0.672977209	0.259	0.117	0.101923844	13
Pgls.1	-0.400073487	0.148	0.351	0.102885339	13
Iws1.2	-0.268304268	0.093	0.215	0.103094518	13
Cdh8	0.594263096	0.148	0.05	0.103302854	13
Trim59.10	-0.58208123	0.019	0.17	0.103708064	13
Aurkb.12	-0.495223901	0.009	0.143	0.104605872	13
Eprs.3	-0.377143972	0.259	0.492	0.107176101	13
Nsmce1.3	-0.566169361	0.056	0.229	0.108484461	13
Gm9843.2	-0.367597884	0.065	0.228	0.108517421	13
Eml4.3	-0.475573388	0.046	0.21	0.109503618	13
Polr2e.3	-0.329144395	0.139	0.325	0.110485291	13
Dhfr.6	-0.345814841	0.028	0.149	0.110854138	13
Ndufs7.1	0.320514501	0.315	0.335	0.111163459	13
Angptl2.6	-0.51171793	0	0.117	0.113738527	13
Chchd2.5	-0.338451498	0.426	0.654	0.114591007	13
B2m.8	-0.732248433	0.056	0.233	0.115103163	13
Tram1.3	-0.326330307	0.019	0.129	0.115413029	13
Timp2.4	0.46205507	0.111	0.025	0.115840893	13
Ddx39b.5	-0.291092796	0.231	0.445	0.117893832	13
Bzw2.3	-0.506473112	0.269	0.489	0.119906882	13
Naca.6	-0.429001897	0.231	0.466	0.121276118	13
Anapc13	-0.270475071	0.213	0.394	0.121293343	13
Samd14.1	0.594658557	0.167	0.076	0.122262935	13
Sh3glb2.1	0.583176438	0.194	0.083	0.12521022	13
Hnrnpm.7	-0.419486941	0.509	0.736	0.125720736	13
Lsg1	-0.385060912	0.019	0.146	0.126657016	13
Laptm4a.3	-0.423451242	0.315	0.555	0.12667804	13
Snx5.7	-0.446839342	0.028	0.164	0.127116396	13
Dpm3.2	-0.464385958	0.083	0.271	0.127884297	13
H1fx.11	-0.519830482	0.065	0.246	0.130595957	13
Psma4.3	-0.432959254	0.194	0.415	0.13230187	13

Polr3gl.1	0.632286627	0.12	0.051	0.133730253	13
Fastk	0.507909605	0.148	0.078	0.13474111	13
Mfap4.7	-0.694292693	0.019	0.162	0.136018654	13
Rpl29.3	-0.502364614	0.083	0.258	0.136644826	13
Dot1l.2	0.356474198	0.287	0.279	0.136799505	13
Tpr.2	-0.392846431	0.315	0.554	0.137384476	13
Eif3a.5	-0.416100019	0.454	0.69	0.137560409	13
Rp9.1	0.34393439	0.25	0.242	0.138812435	13
D8Ertd738e	-0.476452039	0.176	0.38	0.139786588	13
Ndc80.10	-0.499397467	0	0.115	0.1398119	13
Fxr1.1	-0.458615872	0.083	0.256	0.141821803	13
Lyar.10	-0.556353751	0.093	0.287	0.14339894	13
Ckap2l.13	-0.673339841	0.046	0.218	0.143921834	13
Tmem191c.1	0.57283785	0.12	0.028	0.14412013	13
Bcl7a.5	-0.318698141	0.157	0.326	0.145200605	13
Usmg5	0.412583665	0.287	0.244	0.146190201	13
Mad2l2.6	-0.62995642	0.074	0.263	0.147333361	13
Ifitm2.6	-0.493658242	0	0.114	0.14744814	13
Pgp.1	0.553971098	0.25	0.15	0.14770415	13
Cenpv.7	-0.547363521	0.12	0.329	0.148190443	13
Taok1.1	0.506876319	0.287	0.224	0.148413355	13
Smarca5.6	-0.602174645	0.176	0.402	0.150513113	13
Map1lc3a.3	0.46909262	0.343	0.275	0.150593445	13
Synj2bp	0.536111991	0.167	0.093	0.153747751	13
Habp4	0.563485722	0.13	0.042	0.155183115	13
Arhgap5	0.480272003	0.194	0.18	0.155219329	13
Gjc1.7	-0.48238066	0.046	0.197	0.156133348	13
Rbx1.1	-0.287544893	0.278	0.496	0.161380163	13
Chaf1a.8	-0.372744537	0.037	0.169	0.163974425	13
Rpn2.4	-0.340547582	0.074	0.227	0.166462087	13
Ppan.2	-0.437079408	0.019	0.14	0.166730412	13
Rars.2	-0.320310754	0.046	0.185	0.167082905	13
C1qbp.6	-0.338066095	0.176	0.379	0.167888	13
Mt3.2	0.479794707	0.13	0.025	0.170076079	13
Hnrnpdl.4	-0.276589185	0.537	0.757	0.170389688	13
Fen1.10	-0.357407104	0.019	0.142	0.171359172	13
Psmd11	-0.289339173	0.139	0.321	0.173811887	13
Gng5.3	-0.518898464	0.019	0.166	0.173996568	13
St6galnac4.2	0.582155856	0.139	0.032	0.174056193	13
Ptov1.2	-0.331874541	0.269	0.485	0.174675503	13
Cct8.4	-0.381946204	0.222	0.442	0.175263668	13
Fam63a	0.539327757	0.167	0.087	0.175682042	13
Hbp1.1	-0.43689189	0.028	0.157	0.177168867	13
Hspa4l	0.602840708	0.157	0.084	0.179924414	13
Gamt.7	-0.64806498	0.028	0.17	0.181653267	13
Ybx3.7	-0.586830267	0.102	0.291	0.182641313	13
Eif3m.2	-0.366128868	0.139	0.302	0.184317764	13
Dbf4.9	-0.529547599	0.009	0.146	0.184681925	13

Trim24.1	-0.405340281	0.019	0.135	0.184959195	13
Hnrnph3.4	-0.546601983	0.204	0.428	0.185571543	13
Lrp8	0.570685509	0.111	0.039	0.186113923	13
Sec13.1	-0.445635345	0.102	0.27	0.19039959	13
Pebp1.2	-0.390754683	0.074	0.243	0.190872503	13
Etfb.6	-0.59614277	0.028	0.185	0.191583655	13
Arid4a.2	0.354307897	0.269	0.282	0.191603277	13
Cdc5l.3	-0.322184602	0.241	0.401	0.19410333	13
Prkcz	0.58788826	0.139	0.032	0.199102113	13
P4hb.3	-0.495951751	0.093	0.28	0.199961291	13
Lhfp.2	0.507339387	0.111	0.018	0.200295281	13
Csdc2.1	0.586996802	0.111	0.028	0.201282884	13
Prox1.4	-0.479423447	0.065	0.23	0.202352815	13
2810004N23Rik.5	-0.292354066	0.102	0.263	0.203119087	13
Elp5	-0.312330892	0.046	0.172	0.203255592	13
Spcs2.4	-0.339003627	0.222	0.425	0.204926934	13
Fam131b	0.538703231	0.111	0.029	0.204958397	13
Pdzrn3.10	-0.773359645	0.009	0.148	0.206353671	13
Asphd1	0.491376169	0.13	0.025	0.206660187	13
Bcat1.3	-0.392721446	0.019	0.121	0.210528381	13
Fam98b.2	-0.293259336	0.176	0.336	0.213983537	13
Mcm4.6	-0.569686183	0.009	0.138	0.214707806	13
Peli1.1	-0.319557506	0.065	0.203	0.217373313	13
Naa50.4	-0.397459481	0.102	0.286	0.220357207	13
Fbxo44.1	0.608384933	0.12	0.035	0.220535964	13
Cdv3.1	-0.483880598	0.019	0.159	0.221642009	13
Ppie.1	-0.484801309	0.019	0.135	0.222701984	13
Rabgap1l.2	0.614259635	0.176	0.061	0.22290084	13
Sqstm1.3	0.540588807	0.324	0.226	0.225429893	13
Rogdi.1	0.572243403	0.167	0.063	0.225759671	13
Prpf38b.2	0.422031697	0.556	0.494	0.226088318	13
Ldhb.6	0.368409287	0.306	0.28	0.228163789	13
Eci2.2	-0.513649148	0.074	0.25	0.231220539	13
Dnajc6	0.510670606	0.102	0.016	0.235551726	13
Mis18a.6	-0.449739646	0	0.11	0.239156519	13
Vrk1.8	-0.526928078	0.019	0.148	0.243265368	13
2900011O08Rik.10	0.592913815	0.241	0.102	0.251180511	13
Cat.1	-0.397800499	0.019	0.124	0.253164958	13
Sin3b	-0.354432719	0.139	0.309	0.253840005	13
Fam98a.1	-0.31616111	0.037	0.146	0.258191901	13
Mrpl36.1	-0.395158815	0.037	0.166	0.260996756	13
Gdi2.1	-0.27573563	0.204	0.377	0.26303863	13
Fbxl16	0.439735486	0.139	0.067	0.263559387	13
Ubr7.4	-0.437338771	0.009	0.133	0.264530558	13
Brd3.6	-0.473127689	0.296	0.533	0.267485202	13
Ssbp4.2	0.565883958	0.157	0.059	0.269118313	13
Arpp19.3	-0.28723045	0.185	0.378	0.271502001	13
Lman1.3	-0.515494994	0.12	0.325	0.273335091	13

Phf21b.4	-0.298401241	0.037	0.128	0.274938218	13
Nudcd2.5	-0.317258106	0.074	0.224	0.27842715	13
Ralgps2.5	-0.641962543	0.065	0.246	0.284339394	13
Polr3k.3	-0.408543469	0.093	0.271	0.286944736	13
D430041D05Rik.8	0.341926385	0.481	0.492	0.289202745	13
Mrpl17.1	-0.369097472	0.093	0.26	0.289481848	13
Srgap3.1	0.540129186	0.111	0.039	0.290808043	13
Tkt.1	-0.465224085	0.046	0.204	0.294356757	13
Ndufb3	0.296573634	0.315	0.339	0.297417251	13
Plekha1.3	0.622262921	0.139	0.06	0.297885244	13
Ctcf.6	-0.254521168	0.306	0.497	0.298217113	13
Eif3k.3	-0.25365101	0.306	0.522	0.300989415	13
Kif4.9	-0.463802923	0	0.108	0.301257903	13
Dtl.9	-0.579096342	0.009	0.146	0.301567999	13
Nin.6	-0.522669309	0.019	0.146	0.306793706	13
Cct4.1	-0.479112528	0.167	0.383	0.307650266	13
Rac1.2	-0.480846494	0.139	0.348	0.310062629	13
Nans.2	-0.47472628	0	0.108	0.313397757	13
Polr2d	-0.512295422	0.065	0.227	0.313539474	13
Gli1.8	-0.471956454	0	0.108	0.315467497	13
Psmd9	-0.286146478	0.037	0.148	0.319995349	13
Lamp1.4	-0.33245626	0.167	0.337	0.320479831	13
Ube2g2.3	-0.339545902	0.028	0.125	0.324846944	13
Gpr107.1	0.517327278	0.12	0.056	0.330630302	13
Kif2c.8	-0.460866531	0	0.107	0.334712927	13
Hspa4.2	-0.36462609	0.167	0.369	0.335655388	13
Ppp2r2d	0.270952537	0.157	0.203	0.337244075	13
Arhgap11a.12	-0.516485092	0.028	0.171	0.338834516	13
Gmppa.1	0.563432579	0.12	0.042	0.339832818	13
Cep110.6	-0.383483605	0.037	0.163	0.340277905	13
Ostc.2	-0.384685549	0.102	0.275	0.341845683	13
Pitpnc1.3	0.643688237	0.176	0.074	0.343452729	13
Gramd1b.4	0.340975904	0.204	0.169	0.343753754	13
Naa38.6	-0.369503849	0.13	0.312	0.347325917	13
Smim14.1	0.515694345	0.278	0.195	0.347935378	13
Anapc16	-0.472594749	0.065	0.234	0.349301596	13
Trim28.5	-0.405806567	0.157	0.362	0.350129573	13
Mia	0.586797319	0.111	0.024	0.353282179	13
Dhx9.4	-0.334564433	0.25	0.464	0.355672864	13
Herc1	0.390835997	0.204	0.186	0.358409486	13
Diap3.7	-0.462591108	0	0.107	0.359814874	13
Aig1.2	0.608337257	0.204	0.095	0.362783216	13
Ewsr1.3	-0.290681009	0.204	0.393	0.366650114	13
Pea15a.10	0.409918239	0.296	0.175	0.367589572	13
Zcrb1.2	0.278963532	0.352	0.371	0.368069418	13
Ctbp2.4	0.312168508	0.185	0.215	0.370147067	13
Spg21.1	-0.531407447	0.028	0.174	0.372078602	13
Mrpl24.1	-0.396530575	0.065	0.212	0.373871753	13

Gm10076.2	-0.51731087	0.009	0.135	0.377882441	13
Appl2.1	0.561166773	0.241	0.13	0.379768514	13
Rps27a.2	-0.447057487	0	0.106	0.381727883	13
Rpl7a.2	-0.514080775	0.019	0.151	0.382938633	13
Uhrf1.11	-0.396562387	0.019	0.144	0.383494331	13
Rpl6.4	-0.474632882	0.102	0.292	0.385278511	13
Bnip2.1	-0.345061003	0.074	0.218	0.385544469	13
Pnrc2.3	-0.42711312	0.065	0.208	0.396258915	13
Dcakd.5	-0.401348723	0.111	0.297	0.396584608	13
Gripap1.2	0.302662568	0.185	0.188	0.398040985	13
Fam181b.3	-0.329021598	0.037	0.143	0.401456571	13
Cdk5rap2.6	-0.288966777	0.056	0.184	0.401781447	13
Abhd8	0.562988348	0.194	0.075	0.410349469	13
Kif5a.11	0.606080942	0.278	0.127	0.413644765	13
Pbdc1.5	-0.500818809	0.046	0.206	0.421956783	13
Rrp15.5	-0.516497472	0.028	0.174	0.423952204	13
Pih1d1.1	-0.373713754	0.037	0.171	0.427835689	13
F2r.1	-0.459144403	0	0.105	0.429581069	13
Blcap.2	0.582738483	0.167	0.078	0.438415998	13
Snx30	0.473267582	0.139	0.064	0.441259171	13
Uqcrc1.1	0.295486823	0.287	0.294	0.4505932	13
Ccndbp1.1	0.575951162	0.231	0.116	0.45076882	13
Elf2.1	-0.484163593	0.046	0.199	0.45339942	13
Gpd2.1	0.546658083	0.157	0.071	0.454116355	13
2810474O19Rik.3	-0.62283637	0.065	0.242	0.454943219	13
Psmc2	-0.431537566	0.13	0.3	0.455966778	13
Phlda1.3	0.569195958	0.12	0.025	0.461567969	13
Stard4.3	-0.537627846	0.009	0.141	0.462992327	13
Dgkz.2	0.591269046	0.204	0.103	0.467875012	13
Mrps15.1	-0.460479929	0.065	0.231	0.475970917	13
Rbms1.4	-0.398386081	0.065	0.217	0.483319742	13
Itgb3bp.1	-0.442341552	0	0.104	0.48334643	13
Gpr162	0.459298565	0.102	0.016	0.489681218	13
Fuca1.2	-0.374551664	0.028	0.148	0.492064837	13
Hnrnpl.2	-0.38063184	0.167	0.364	0.493610547	13
Adk.3	-0.465329125	0.028	0.166	0.495221816	13
Csrp2.9	-0.424357533	0.028	0.158	0.501683204	13
mt-Rnr1	0.401522666	0.398	0.367	0.503304614	13
Map7d1.2	0.484811903	0.361	0.24	0.508471448	13
Ndufb6.1	0.282075613	0.352	0.379	0.509897464	13
Tk1.10	-0.471280416	0.009	0.132	0.514178617	13
Eif5a.4	-0.34566616	0.222	0.404	0.516933308	13
Lman2.2	-0.424154458	0.019	0.14	0.51748193	13
Tmem107.4	-0.35231085	0.019	0.127	0.522954213	13
Tex30.5	-0.456083625	0.019	0.136	0.527152478	13
Ccnb1.10	-0.633649887	0.009	0.14	0.528057754	13
Pfdn5.1	-0.347769011	0.296	0.521	0.529635555	13
Inpp5k	0.478204929	0.111	0.055	0.532181685	13

Osbpl1a.3	0.560093609	0.139	0.058	0.535451014	13
Dnajc3.3	-0.274964618	0.074	0.177	0.535522699	13
Camta2	0.487281407	0.13	0.029	0.537515146	13
Psme4.3	-0.403532493	0.111	0.284	0.541135523	13
Cldn25.2	-0.480622915	0.065	0.233	0.542251653	13
Psip1.6	-0.293836733	0.472	0.689	0.546236446	13
Zfp207.2	-0.252706818	0.204	0.37	0.548472233	13
Adrbk2.3	0.55459531	0.204	0.099	0.562739353	13
Psmb2.2	-0.277340492	0.231	0.429	0.564103796	13
Txlna.1	-0.422755949	0.046	0.186	0.566287709	13
C330027C09Rik.10	-0.482949538	0.009	0.134	0.571591116	13
Peo1.3	-0.324738856	0.019	0.124	0.572672598	13
Wipi2	0.312923549	0.093	0.12	0.578368647	13
Rbmxl1.3	-0.289005579	0.046	0.153	0.587906172	13
Atad2.10	-0.516812581	0.046	0.199	0.591233051	13
Synj1.2	0.499965969	0.157	0.078	0.5970811	13
Bub1.10	-0.46699855	0.009	0.131	0.614080725	13
Plk4.9	-0.37129497	0.009	0.12	0.616331399	13
Polr2j.1	-0.275079802	0.13	0.294	0.617222095	13
Ndst3.1	0.501089488	0.13	0.033	0.617415351	13
Kif1a.7	0.520057054	0.278	0.171	0.624604384	13
Sart1.1	-0.534691427	0.046	0.199	0.635365013	13
Gclm	0.493749441	0.194	0.14	0.636297681	13
A330076H08Rik.11	0.491782806	0.25	0.16	0.638762769	13
Cdk5rap3.3	-0.445318321	0.046	0.193	0.641018242	13
Mphosph10.7	-0.506630236	0.065	0.232	0.64565445	13
Rhno1.3	-0.524811042	0.009	0.139	0.652687	13
Fam126b	0.550292837	0.13	0.063	0.65420286	13
Tmem127.1	0.28556768	0.102	0.094	0.654714119	13
Mak16.5	-0.46040599	0.065	0.226	0.655426398	13
Rnpc3.2	0.599585027	0.194	0.103	0.663838492	13
Dab2ip	0.55661156	0.167	0.068	0.678257283	13
Ogfrl1	0.550447594	0.167	0.081	0.67972034	13
2510002D24Rik.1	0.633493452	0.139	0.049	0.679900566	13
Abcf1.2	-0.317286699	0.25	0.458	0.681220838	13
Odc1.3	-0.420673302	0	0.101	0.687748425	13
Stip1.2	-0.501056236	0.12	0.304	0.688885903	13
Rsl1d1.5	-0.351090129	0.278	0.491	0.696231937	13
Rad51ap1.13	-0.536883329	0.028	0.167	0.696735458	13
Frmd4b.4	-0.534177728	0.009	0.128	0.706866802	13
Ubr2.1	0.423568451	0.111	0.084	0.707010501	13
Clcn3.4	0.342169416	0.259	0.272	0.715327658	13
Gltscr2.4	-0.265447362	0.167	0.342	0.719538645	13
Psmc4.2	-0.335038591	0.148	0.322	0.732600705	13
Gm11266.9	-0.521458597	0.019	0.144	0.733696585	13
Hes6.10	-0.522010888	0.074	0.237	0.734037205	13
Snw1.2	-0.395770105	0.222	0.432	0.745297482	13
Eif1ax.3	-0.286993391	0.204	0.398	0.749417366	13

Gins1.5	-0.34988797	0.009	0.115	0.752288522	13
Tgoln1.1	0.531376897	0.185	0.083	0.756149221	13
Ptms.7	0.253675579	0.454	0.473	0.756982709	13
Syf2	0.287441644	0.231	0.255	0.757893262	13
Cnot2.2	0.303640512	0.194	0.185	0.762577359	13
Helz	0.391318535	0.157	0.086	0.76613853	13
Ank3.12	0.274254845	0.491	0.419	0.772801533	13
Mettl16	-0.313060657	0.019	0.118	0.773503128	13
Arf3.1	0.536231493	0.148	0.062	0.774374156	13
Mrpl21.2	-0.486662564	0.102	0.281	0.778007644	13
Ddx17.3	0.261668916	0.352	0.381	0.79972141	13
Sptbn1.9	0.421395185	0.417	0.353	0.804107939	13
Rab34.1	-0.390357319	0.009	0.118	0.811490247	13
Reep3.1	-0.271215857	0.111	0.257	0.837134328	13
Cep170.7	-0.313969765	0.139	0.306	0.842285891	13
Tnpo3.2	-0.421598591	0.065	0.223	0.843582418	13
Reep5.5	0.512958809	0.222	0.137	0.851572432	13
Puf60	-0.265417126	0.111	0.244	0.853193072	13
Rgs17.1	0.579667574	0.139	0.046	0.856745248	13
Cwc27.1	-0.400361821	0.037	0.169	0.860144591	13
Trip11.1	0.508010303	0.185	0.152	0.860287684	13
Pmf1.8	-0.46203248	0.019	0.143	0.868626355	13
Macf1.4	0.569228784	0.324	0.209	0.869026454	13
Eif3g.3	-0.28195492	0.222	0.403	0.875523164	13
Nek6.3	-0.434279778	0.028	0.145	0.885105311	13
Add3.6	0.579765402	0.204	0.103	0.886264468	13
Ube2e3.5	-0.294976309	0.222	0.417	0.905406478	13
Sod1.4	-0.396011398	0.241	0.443	0.912851541	13
Tspan3.3	-0.395111576	0.231	0.429	0.913780146	13
Tomm22	-0.291048186	0.13	0.291	0.923133088	13
Ncapg.10	-0.562255367	0.037	0.184	0.923390217	13
Smu1	-0.30776359	0.074	0.219	0.925410514	13
Dip2b.1	0.519486485	0.13	0.079	0.935627808	13
Tipin.9	-0.358695589	0.102	0.255	0.936327709	13
Psma5.1	-0.422612774	0.139	0.322	0.939100025	13
Txnl1	-0.476770592	0.148	0.346	0.939144961	13
Suz12.5	-0.509044575	0.065	0.22	0.948578794	13
Psmc3ip.8	-0.489087174	0.009	0.132	0.957048842	13
Tpt1.3	-0.378105816	0.093	0.257	0.9571878	13
N4bp2.2	-0.272220627	0.037	0.132	0.957954217	13
Pxn	0.540639352	0.111	0.039	0.959638654	13
Sparcl1.10	-0.919779154	0.056	0.208	0.975309564	13
Park7.3	-0.288085631	0.333	0.547	0.981046787	13
Txnl4a.1	-0.299252012	0.065	0.195	0.997336883	13
Slc35b2.2	-0.307830318	0.056	0.182	1	13
Ssb.3	-0.409386516	0.5	0.704	1	13
Ift27.5	-0.483762399	0.074	0.243	1	13
Cecr2.3	-0.488097843	0.009	0.13	1	13

Sema4g.1	0.60142344	0.13	0.037	1	13
Alkbh6	0.504094363	0.204	0.133	1	13
Snrpb.8	-0.34205363	0.352	0.574	1	13
Gpc2.7	-0.438463677	0.028	0.14	1	13
Gdpd1.8	-0.53362186	0.074	0.244	1	13
Tm9sf3.3	-0.397740221	0.157	0.345	1	13
Sugp1	0.37958379	0.111	0.068	1	13
Mmadhc.1	-0.436796014	0.074	0.233	1	13
Ddx3x.3	-0.271138088	0.278	0.467	1	13
Mapk8ip1.10	0.490287839	0.352	0.226	1	13
Dars.2	-0.323306906	0.037	0.158	1	13
Utp3.4	-0.438799607	0.13	0.315	1	13
Nemf.3	0.279050603	0.306	0.339	1	13
Tsc1.1	0.279795298	0.111	0.104	1	13
Kif15.12	-0.391335564	0.046	0.181	1	13
Nudt21.1	-0.483606346	0.009	0.134	1	13
Dazap2	-0.53444828	0.074	0.246	1	13
Brd8.6	-0.29974687	0.204	0.393	1	13
Rfc2.7	-0.256518266	0.083	0.211	1	13
Pvrl3.3	0.429936106	0.139	0.122	1	13
Jarid2.5	0.46309082	0.222	0.178	1	13
Rps16-ps2.2	-0.409114656	0.019	0.141	1	13
Polr2f.4	-0.402025864	0.176	0.376	1	13
Pard3.3	-0.284178693	0.028	0.127	1	13
Gphn.5	0.529899368	0.222	0.14	1	13
D17H6S56E-5.8	-0.459975071	0.009	0.128	1	13
A030009H04Rik.4	0.570168981	0.213	0.113	1	13
Eif6	-0.47893491	0.083	0.25	1	13
Smarcc2.6	0.261032541	0.361	0.363	1	13
Ap1s2.4	0.419691213	0.204	0.159	1	13
Lphn3.2	0.589558392	0.139	0.06	1	13
Baz1b.6	-0.396146275	0.278	0.494	1	13
Pcdhga9.3	-0.379985825	0.222	0.421	1	13
Nktr.6	0.250672151	0.333	0.38	1	13
Sema6a.11	-0.604547968	0.056	0.202	1	13
Zfp422.4	-0.402912322	0.083	0.246	1	13
Cadm1.6	0.304783066	0.38	0.39	1	13
Nol8.1	-0.353166844	0.046	0.168	1	13
Polr1c.5	-0.267695785	0.083	0.219	1	13
Thrsp.1	0.451585999	0.111	0.032	1	13
Atad3a.1	0.421633985	0.148	0.126	1	13
Prmt1.3	-0.274762893	0.083	0.225	1	13
Mrps17.1	-0.42357167	0.083	0.247	1	13
Asf1a.4	-0.394348086	0.111	0.281	1	13
Isy1.1	-0.433124201	0.028	0.142	1	13
Emg1.4	-0.464455672	0.13	0.316	1	13
Rpl31	-0.504654664	0.009	0.131	1	13
Plxna2.1	0.6560305	0.167	0.069	1	13

Cenpm.10	-0.464331227	0.028	0.162	1	13
Nono.2	-0.442484296	0.065	0.22	1	13
Mis18bp1.12	-0.576963299	0.028	0.161	1	13
1700001O22Rik.6	-0.298898908	0.019	0.11	1	13
Lims1.3	-0.302789427	0.056	0.183	1	13
Atp5g1	0.499755076	0.185	0.096	1	13
Tmem237.1	-0.484526081	0.009	0.131	1	13
Ccng2.7	-0.44921237	0.074	0.212	1	13
Wdr77	-0.431369003	0.009	0.124	1	13
Stt3b.2	-0.400178171	0.065	0.212	1	13
Tspan31	-0.343938135	0.074	0.218	1	13
Rnf220.3	-0.274273377	0.093	0.236	1	13
Zranb1.3	0.455387695	0.241	0.208	1	13
Abhd16a.7	0.514172141	0.259	0.189	1	13
Plekhb2	0.499335297	0.111	0.033	1	13
Spag9.4	0.310723885	0.278	0.307	1	13
Robo2.4	-0.267963752	0.046	0.152	1	13
Fzd2.5	-0.520883509	0.028	0.161	1	13
Gnptg.2	0.318441805	0.194	0.166	1	13
Ndufa11.1	0.285249571	0.38	0.331	1	13
Rsrc1.2	-0.588909072	0.083	0.251	1	13
Lsmd1.2	-0.288965628	0.083	0.221	1	13
Tceb1.1	-0.416730336	0.185	0.367	1	13
Eif4a3.2	-0.364590609	0.176	0.356	1	13
Shank3	0.422796275	0.102	0.02	1	13
2700029M09Rik.5	-0.391488591	0.12	0.29	1	13
Cmtm3.6	-0.396656174	0.009	0.118	1	13
Elavl1.2	-0.462373872	0.046	0.19	1	13
Cstf2t	0.521366233	0.148	0.088	1	13
Tsen34.1	-0.26138602	0.093	0.234	1	13
Dbndd2.2	0.453440321	0.102	0.024	1	13
Stx7.3	0.395685111	0.167	0.115	1	13
Rell1.1	0.375111457	0.102	0.095	1	13
Thsd7a.6	-0.562080139	0.009	0.128	1	13
Pkn2.1	-0.349196464	0.065	0.209	1	13
Gkap1.2	-0.400736214	0.074	0.221	1	13
Pola1.5	-0.312332228	0.019	0.103	1	13
Ckap5.10	-0.252034997	0.12	0.266	1	13
Srpk2.3	0.325559206	0.463	0.429	1	13
Serpinh1.8	-0.435273173	0.009	0.116	1	13
Cenpc1.3	-0.288317596	0.037	0.155	1	13
Yif1a.3	-0.511854167	0.037	0.18	1	13
Prpf38a	-0.405545785	0.028	0.153	1	13
Atp1b3.7	-0.333883898	0.157	0.338	1	13
Rrp1.3	0.317213873	0.602	0.606	1	13
Slc25a23.1	0.555286196	0.222	0.107	1	13
Gm11541.6	-0.522577759	0.009	0.128	1	13
2310015B20Rik	0.496070809	0.111	0.037	1	13

0610037L13Rik	-0.311207939	0.056	0.183	1	13
Gcc2.1	0.490966579	0.213	0.17	1	13
Rpl15.1	-0.482351166	0.028	0.162	1	13
Acyp1	0.447858346	0.157	0.096	1	13
Tspyl5	0.50223342	0.102	0.028	1	13
Dnajc21.3	-0.257576431	0.093	0.21	1	13
Cabin1	0.470505538	0.102	0.059	1	13
Vgll4.2	-0.444992524	0.028	0.153	1	13
Lta4h.4	-0.499965614	0.083	0.243	1	13
Aspm.11	-0.567896157	0.019	0.143	1	13
Nck1	0.473605031	0.102	0.059	1	13
Cit.2	0.486412277	0.139	0.065	1	13
Ank.2	0.449150546	0.111	0.055	1	13
Dst.3	0.386752465	0.287	0.266	1	13
Pfdn4.4	-0.317536165	0.139	0.306	1	13
Gxylt1	-0.380665563	0.009	0.111	1	13
Kif3b	0.410689482	0.102	0.096	1	13
Bcar1.4	-0.488406552	0.046	0.193	1	13
Mcmbp.2	-0.336490317	0.074	0.217	1	13
Mpdz.3	-0.479447619	0.019	0.135	1	13
2810055G20Rik.2	-0.470702226	0.037	0.171	1	13
Dennd2a.1	-0.345362772	0.009	0.107	1	13
Cul7.1	0.505651491	0.102	0.033	1	13
Fam212b.4	-0.388058392	0.074	0.223	1	13
Rspry1	-0.282514264	0.019	0.107	1	13
Socs7.1	0.469623561	0.185	0.095	1	13
Ndn.4	0.507709368	0.333	0.222	1	13
Ik.1	0.297811375	0.481	0.464	1	13
Ttyh1.2	0.50927332	0.12	0.031	1	13
Pafah1b1.3	0.339078453	0.491	0.474	1	13
Rhot1	0.321903559	0.13	0.087	1	13
Sez6l2.2	0.539199329	0.13	0.036	1	13
Prkacb.2	0.4667262	0.278	0.186	1	13
Thrap3.2	-0.312402065	0.204	0.388	1	13
Wdr60.2	-0.376002072	0.019	0.119	1	13
Eif4e2	-0.499783771	0.065	0.219	1	13
Smc5.4	-0.334128864	0.102	0.258	1	13
Tsc22d4.5	-0.509315881	0.037	0.167	1	13
Gprc5b	0.467128076	0.13	0.042	1	13
Cox14.1	0.270135733	0.296	0.317	1	13
2310044G17Rik.1	0.508054585	0.148	0.054	1	13
Acin1.3	-0.27092036	0.528	0.724	1	13
Tex9.2	-0.409158927	0.028	0.148	1	13
Idh3b	0.28450065	0.25	0.246	1	13
Gnai1.1	0.504102726	0.176	0.099	1	13
Mcl1.2	-0.400688543	0.065	0.212	1	13
Ppp3r1.1	0.269097073	0.12	0.137	1	13
Setdb1	-0.356202961	0.009	0.109	1	13

Cenpp.9	-0.362189833	0.019	0.114	1	13
Dnph1.7	-0.43628355	0.009	0.124	1	13
Pphln1.2	-0.410989968	0.009	0.119	1	13
Bcl2l13.2	-0.408452421	0.009	0.115	1	13
Stag2.3	-0.291103635	0.111	0.257	1	13
Mki67ip.4	-0.360857593	0.093	0.245	1	13
Pan3	0.42394102	0.139	0.078	1	13
Phip.4	-0.275073329	0.269	0.46	1	13
Emc7.3	0.425169039	0.176	0.143	1	13
Dnaaf2.2	-0.251493815	0.019	0.105	1	13
Pcmtd2.1	0.264323736	0.13	0.143	1	13
Ehd3.1	0.518091164	0.111	0.03	1	13
Pbx1.2	-0.452411714	0.046	0.187	1	13
Asnsd1	-0.288225978	0.028	0.117	1	13
Acat1.3	-0.399836794	0.25	0.442	1	13
Dusp11	0.405100271	0.25	0.201	1	13
Agtpbp1.4	0.495551896	0.148	0.101	1	13
Larp4b.1	0.270058649	0.157	0.17	1	13
Smc6.8	-0.328735392	0.231	0.423	1	13
Fstl1.8	-0.438188805	0.056	0.181	1	13
Uhrf1bp1l	0.344309205	0.167	0.132	1	13
Wdfy3	0.345606278	0.102	0.08	1	13
Strbp.8	0.326408018	0.361	0.347	1	13
Bzw1.3	-0.379303982	0.176	0.36	1	13
Clic1.5	-0.417206252	0.046	0.173	1	13
Ndst1	-0.331321037	0.037	0.136	1	13
Wdr33.1	-0.28823255	0.065	0.196	1	13
Pes1.1	-0.397285051	0.009	0.115	1	13
Hnrnpul1.1	-0.36274724	0.046	0.175	1	13
Oard1.1	-0.451250334	0.037	0.153	1	13
Hdgf.9	-0.344049222	0.324	0.533	1	13
Vrk3	-0.400988675	0.009	0.115	1	13
Isoc1.4	-0.395701035	0.046	0.181	1	13
Wdr89.4	-0.255874078	0.083	0.214	1	13
Anln.8	-0.327941463	0.009	0.104	1	13
Polr2i.4	-0.370077968	0.148	0.317	1	13
Rps28.5	-0.455061371	0.056	0.204	1	13
Serp2.1	0.506428437	0.185	0.086	1	13
Sec63	-0.506964328	0.065	0.21	1	13
0610012G03Rik.1	0.437741593	0.194	0.146	1	13
Dusp3.3	0.484599482	0.102	0.028	1	13
Cdca2.10	-0.32024952	0.028	0.129	1	13
Slc25a12	0.460343296	0.13	0.073	1	13
Manf.1	-0.381734695	0.148	0.328	1	13
Dync2h1.2	-0.379448106	0.019	0.116	1	13
Snx32.1	0.520208238	0.111	0.037	1	13
Setd7	0.480349722	0.12	0.071	1	13
Mad2l1.10	-0.46036203	0.009	0.124	1	13

Ptplb.1	-0.417739933	0.009	0.116	1	13
Atf4.1	-0.312810944	0.213	0.398	1	13
Ywhaz.3	0.289005534	0.407	0.362	1	13
Pygo1.6	-0.429695545	0.074	0.193	1	13
Aebp2	0.322052127	0.111	0.121	1	13
Zfp612	0.520401965	0.13	0.04	1	13
Syngr3.1	0.520293718	0.13	0.036	1	13
2210013O21Rik	0.491249022	0.204	0.117	1	13
Atp6ap1.2	0.492768146	0.213	0.138	1	13
Tbl1xr1	-0.440286284	0.009	0.12	1	13
Cdh4.4	-0.429476181	0.028	0.146	1	13
Galt	0.462618504	0.102	0.022	1	13
Cxx1c	0.508803704	0.139	0.054	1	13
Ube2e1.2	-0.339086834	0.083	0.228	1	13
Ehmt2	-0.420014388	0.074	0.209	1	13
Cd63-ps.4	-0.430502362	0.009	0.119	1	13
Ube2r2.1	-0.34482752	0.148	0.317	1	13
Exosc8.6	-0.395467717	0.074	0.21	1	13
Pfn2.7	0.421339968	0.287	0.24	1	13
Slc39a10.2	-0.332464224	0.065	0.195	1	13
Psmd12.1	-0.372406369	0.185	0.374	1	13
Srrm3.7	0.498494379	0.315	0.239	1	13
Mars	-0.30920495	0.037	0.151	1	13
Cntln.7	-0.418006218	0.046	0.173	1	13
Ankrd46.3	0.333175076	0.185	0.16	1	13
Armcx1.2	0.494151754	0.157	0.083	1	13
Nek7.5	-0.326139486	0.019	0.117	1	13
Qdpr.2	-0.354892845	0.111	0.268	1	13
Atp8a1.2	0.508506209	0.157	0.072	1	13
Slc8a2	0.470163742	0.111	0.03	1	13
Tex14.9	-0.646329331	0.028	0.146	1	13
Rsu1.3	-0.403826517	0.019	0.119	1	13
Tmem261	-0.251940086	0.093	0.22	1	13
Arhgap12	-0.35664987	0.009	0.105	1	13
Cdca4.4	-0.354546641	0.009	0.111	1	13
Btbd17.8	-0.464804013	0.046	0.16	1	13
Grik2.10	0.49445472	0.204	0.101	1	13
Mrps21	-0.447770257	0.083	0.239	1	13
Bccip.4	0.250784159	0.296	0.31	1	13
Pole4	-0.415242498	0.028	0.145	1	13
Phyhipl.3	0.517284809	0.139	0.061	1	13
Ncor2.8	-0.553203477	0.046	0.169	1	13
Ctxn1.4	-0.310643487	0.056	0.17	1	13
Stk11.2	-0.281157129	0.093	0.23	1	13
March2	0.403602997	0.13	0.069	1	13
Senp1.1	-0.331418815	0.028	0.136	1	13
Scoc.1	0.382230007	0.111	0.078	1	13
Stt3a.1	-0.405503109	0.019	0.126	1	13

Cenpj.7	-0.405458473	0.046	0.142	1	13
Gcsh.2	-0.400081119	0.028	0.142	1	13
Tcf3.4	-0.377022168	0.083	0.226	1	13
Fam64a.11	-0.452624733	0.009	0.118	1	13
Smn1.1	-0.300325104	0.046	0.159	1	13
Lmo1.1	0.433480969	0.111	0.039	1	13
Ramp2.6	-0.525516425	0.009	0.12	1	13
Sox18.6	-0.486003807	0.028	0.148	1	13
Dctn3.2	0.282099138	0.352	0.368	1	13
Ap2a2	0.427269896	0.157	0.121	1	13
Slc38a2	-0.417872895	0.074	0.221	1	13
Gnl3.7	-0.384597843	0.13	0.297	1	13
Gm17322.10	-0.590699302	0.019	0.131	1	13
Mex3b.3	-0.464889312	0.009	0.116	1	13
Cetn3.2	-0.306680065	0.296	0.488	1	13
Hat1.6	-0.354942189	0.037	0.155	1	13
Rnf6	0.491519845	0.13	0.059	1	13
Kifap3.5	0.277373864	0.296	0.272	1	13
Pigx	-0.479798754	0.028	0.148	1	13
1300002E11Rik.1	-0.319381343	0.028	0.114	1	13
Mbtd1.3	-0.447483059	0.074	0.215	1	13
Celf5.2	0.411661912	0.12	0.056	1	13
Pfkm.2	0.496041886	0.157	0.065	1	13
Epb4.1l1.2	0.550512535	0.204	0.09	1	13
Zmynd8.6	0.424556717	0.241	0.212	1	13
Pcnt.4	-0.314730712	0.065	0.175	1	13
Mapk8ip2.5	0.471386833	0.231	0.142	1	13
Kdelr2.2	-0.39422642	0.065	0.206	1	13
Ash1l.2	0.341308782	0.296	0.277	1	13
Lima1.4	-0.46316561	0.019	0.122	1	13
Sdccag3	0.352719407	0.139	0.122	1	13
2610203C20Rik.6	-0.481110665	0.019	0.121	1	13
Ube2d3.1	-0.258268687	0.12	0.268	1	13
Pkia.8	0.548670522	0.241	0.133	1	13
Pde4b.2	0.354566906	0.111	0.05	1	13
Mab21l2.5	-0.399859701	0.019	0.104	1	13
Ntrk3.6	-0.30129863	0.046	0.155	1	13
Mrpl55	-0.367376526	0.046	0.16	1	13
Arf5	0.29871562	0.269	0.255	1	13
Nsun2	-0.481080564	0.037	0.169	1	13
Vars.4	-0.452089861	0.037	0.167	1	13
Prmt5.5	-0.306580182	0.111	0.256	1	13
Sidt2.2	0.447919797	0.102	0.045	1	13
Ppp5c	0.391169954	0.167	0.151	1	13
Cdk7.1	-0.36678535	0.019	0.109	1	13
Clpp.1	-0.444397556	0.083	0.239	1	13
Tmed5.3	-0.370394095	0.019	0.126	1	13
Dgke	0.425368653	0.13	0.033	1	13

Rps24-ps3.2	-0.30918425	0.056	0.18	1	13
Wdr12.4	-0.250249538	0.065	0.176	1	13
Rpl7l1	-0.319143523	0.083	0.223	1	13
Lin7c.3	0.358308855	0.269	0.217	1	13
Rdh5.2	-0.394033443	0.028	0.126	1	13
Rap2b.2	-0.453947321	0.028	0.149	1	13
Tmem242	-0.362954967	0.074	0.192	1	13
Dcaf11	0.460248867	0.167	0.096	1	13
Satb1.3	-0.405905242	0.074	0.21	1	13
Dnm1l.2	0.316375416	0.333	0.291	1	13
Thra.10	0.50611996	0.352	0.228	1	13
Hdac1	-0.397037045	0.019	0.126	1	13
Kat2b	0.42384443	0.13	0.047	1	13
Lsm2.6	-0.459283537	0.083	0.231	1	13
Rpl36.2	-0.373030007	0.009	0.108	1	13
Ppid.1	-0.362998611	0.065	0.202	1	13
Dclk1.8	0.291973829	0.454	0.411	1	13
Fam53b.4	-0.409271391	0.028	0.137	1	13
Smoc1.6	-0.368335442	0.028	0.135	1	13
Dusp26.2	0.552599034	0.176	0.064	1	13
Orc4	0.376309746	0.111	0.09	1	13
Fbxw11	0.403143869	0.12	0.085	1	13
1810022K09Rik	-0.334307288	0.056	0.181	1	13
AI854517.3	-0.388363711	0.056	0.189	1	13
Gtf2e2.3	-0.452735157	0.046	0.178	1	13
Cds2	0.446787873	0.111	0.044	1	13
Hpca.10	-0.409893116	0.12	0.278	1	13
Ppp3cb.3	0.442594408	0.417	0.309	1	13
Mroh2a.9	-0.464440134	0.028	0.114	1	13
Grpel2	0.502771668	0.139	0.073	1	13
Ipo5.4	-0.381039963	0.028	0.139	1	13
Ccnl2.2	0.283131895	0.324	0.32	1	13
Bdp1.1	0.406325536	0.176	0.154	1	13
Ppa2.1	-0.352519234	0.037	0.155	1	13
Hivep2.2	0.561151925	0.148	0.054	1	13
1500011B03Rik.4	0.508939895	0.185	0.106	1	13
Mycl.5	-0.362527774	0.028	0.137	1	13
Copg1.1	0.47031478	0.213	0.152	1	13
Tbc1d16.3	-0.372134244	0.065	0.197	1	13
Ncapd2.10	-0.322547889	0.065	0.185	1	13
Mrps9	-0.304566922	0.037	0.148	1	13
Hagh	0.473764	0.167	0.1	1	13
Gps2.1	-0.254516726	0.102	0.236	1	13
Tubgcp5	0.326540533	0.102	0.06	1	13
Ttc28.2	-0.416283362	0.019	0.123	1	13
Ecsit	0.260678152	0.102	0.094	1	13
Grpel1	-0.328692564	0.065	0.183	1	13
Nudt9	-0.303301209	0.028	0.115	1	13

Slain2.1	-0.421512602	0.019	0.128	1	13
Ccdc136	0.540543193	0.176	0.074	1	13
Lrrc58	-0.386421687	0.009	0.111	1	13
Tab2.1	-0.281627501	0.111	0.247	1	13
A830080D01Rik.1	-0.353274877	0.019	0.114	1	13
Rcc2.6	-0.358449561	0.056	0.179	1	13
Cdca7l.9	-0.344656169	0.009	0.104	1	13
Pttg1.7	-0.558384151	0.028	0.14	1	13
Dnttip2.3	-0.302913427	0.167	0.325	1	13
Taf1d.5	-0.294001904	0.176	0.343	1	13
Gm13092.3	-0.344766952	0.009	0.104	1	13
Uba5	0.393302915	0.185	0.163	1	13
Bms1.2	-0.280169398	0.065	0.183	1	13
Ccl27a.2	0.414540152	0.139	0.043	1	13
Cstb.2	0.519168037	0.222	0.116	1	13
Pdzd11	-0.361428326	0.037	0.155	1	13
Ddt.1	0.431133961	0.185	0.105	1	13
Ctdsp2	0.33529857	0.12	0.11	1	13
Rftn2.3	-0.389681156	0.019	0.124	1	13
Bscl2	0.262450862	0.185	0.155	1	13
Akt1	-0.446124688	0.009	0.112	1	13
Slk.1	0.403591942	0.213	0.18	1	13
Dcaf13	-0.329856683	0.019	0.114	1	13
Srgap2.8	0.486533757	0.204	0.131	1	13
Nell2.3	0.36534933	0.157	0.132	1	13
Slc1a3.9	-0.709722458	0.037	0.153	1	13
Srsf9.3	-0.362400411	0.102	0.237	1	13
Pnmal2.7	0.513692384	0.222	0.146	1	13
Dpysl3.12	-0.671427153	0.083	0.22	1	13
Cfl2.3	-0.296413343	0.241	0.409	1	13
Nop16.3	-0.470438675	0.046	0.174	1	13
Ankhd1.2	0.352737375	0.222	0.18	1	13
Rrbp1.6	-0.457903165	0.056	0.189	1	13
Tmem256.2	-0.353268042	0.306	0.493	1	13
Sgpl1.1	-0.348110918	0.037	0.149	1	13
Adrbk1.1	0.513665988	0.13	0.054	1	13
Ggh.3	-0.412364885	0.019	0.124	1	13
Yme1l1.1	-0.255269848	0.102	0.222	1	13
Nipsnap1.4	0.281204722	0.13	0.136	1	13
Rabggtb	0.371912819	0.231	0.173	1	13
Dcun1d5.1	-0.413470032	0.102	0.257	1	13
Ywhah	0.280507926	0.176	0.162	1	13
l7Rn6.1	-0.287323656	0.074	0.201	1	13
Skp2.3	-0.363473546	0.028	0.12	1	13
Gmnn.9	-0.449917517	0.028	0.145	1	13
Med10	-0.307223422	0.083	0.214	1	13
Rere.1	0.384816147	0.176	0.15	1	13
Myeov2.1	-0.360323151	0.278	0.456	1	13

Ube2l3	-0.40102978	0.093	0.239	1	13
Aff4.2	0.280223308	0.222	0.217	1	13
Zdhhc16	0.325298957	0.12	0.09	1	13
Immp1l	-0.340205872	0.065	0.188	1	13
Rpl10a.1	-0.436991788	0.009	0.112	1	13
Rpl30.3	-0.332114309	0.12	0.269	1	13
Gabpb2	0.315033585	0.12	0.096	1	13
Cisd2.1	-0.304134583	0.111	0.258	1	13
Vps72	-0.306494376	0.046	0.154	1	13
Map4k4.3	-0.33821907	0.269	0.45	1	13
Smek1.2	-0.290080178	0.065	0.179	1	13
Tmed9.4	-0.262431589	0.259	0.434	1	13
Commd3.1	-0.376359311	0.148	0.308	1	13
Actl6b.2	0.369887427	0.102	0.055	1	13
Dhx32.7	-0.365926185	0.074	0.201	1	13
Man2a2.1	0.479110201	0.102	0.043	1	13
Far1	0.310274599	0.139	0.104	1	13
Optn.1	0.457919141	0.148	0.056	1	13
Cntnap1	0.457245383	0.139	0.08	1	13
Eml5.2	0.533455984	0.167	0.092	1	13
Gm20417	0.356963765	0.102	0.043	1	13
Mt1.9	-0.599067828	0.046	0.169	1	13
Kars.2	-0.478628428	0.065	0.196	1	13
Rab33a.1	0.457423055	0.13	0.051	1	13
Tmem178.5	0.333578771	0.194	0.186	1	13
Slc24a5.1	-0.326088498	0.037	0.141	1	13
Hif1a	0.272065177	0.111	0.126	1	13
Cdh2.1	-0.335053278	0.046	0.153	1	13
Neo1.1	-0.398325808	0.009	0.106	1	13
Rpain	-0.377161819	0.028	0.134	1	13
Psmd13	-0.273863883	0.056	0.169	1	13
Cdc16.1	-0.28443212	0.139	0.283	1	13
Fam174a.2	0.393252844	0.13	0.064	1	13
Prr13.3	0.490716377	0.176	0.089	1	13
Tubgcp3	-0.260130238	0.019	0.103	1	13
Mgat2.1	-0.369102155	0.019	0.12	1	13
Imp3.5	-0.391163721	0.102	0.247	1	13
Gtf2f1.2	0.315242366	0.343	0.343	1	13
Gtf3c2	-0.390078112	0.028	0.137	1	13
Sgol1.10	-0.315518812	0.028	0.128	1	13
Reep2.2	0.496311493	0.176	0.067	1	13
Dcaf7	0.335117844	0.13	0.103	1	13
E2f1.6	-0.47529959	0.037	0.147	1	13
Soga2	0.396428047	0.148	0.071	1	13
Mrps10.1	-0.465441928	0.028	0.14	1	13
Sept2.3	-0.275036883	0.093	0.223	1	13
Ddost.2	-0.285044758	0.13	0.27	1	13
Ogdh	0.361528849	0.176	0.127	1	13

Ssna1.2	-0.309052026	0.12	0.267	1	13
Akap11.3	0.368727438	0.167	0.139	1	13
Cyb5r3.2	-0.415545463	0.065	0.199	1	13
Ube2d1.5	-0.302422485	0.102	0.228	1	13
Gm3764.6	0.464570235	0.176	0.131	1	13
Pitpnb.1	-0.445394997	0.074	0.214	1	13
Prkra	-0.405890781	0.019	0.122	1	13
Rpp30	-0.38195261	0.028	0.135	1	13
Gria4.11	0.500305007	0.213	0.124	1	13
Jund.3	-0.263228939	0.194	0.352	1	13
Cebpg.1	-0.253424346	0.074	0.193	1	13
Cib2.1	0.451217369	0.111	0.032	1	13
Amer1.1	0.261047397	0.111	0.107	1	13
U2af2	-0.370175959	0.028	0.137	1	13
Cyb5b	-0.378719264	0.009	0.106	1	13
Rnf144a.2	-0.414926584	0.056	0.16	1	13
Strap	-0.345143708	0.148	0.308	1	13
Mtus1.2	0.392169444	0.157	0.126	1	13
Nudt4.2	-0.351711911	0.083	0.218	1	13
Klhl13.3	-0.392653043	0.009	0.104	1	13
Hmgcl.2	0.422597478	0.13	0.078	1	13
Eif2s2.1	-0.338200804	0.167	0.331	1	13
Selo	0.382531288	0.12	0.053	1	13
Lrrc16b	0.516577894	0.12	0.052	1	13
Rai1.3	-0.452454622	0.046	0.16	1	13
Cxxc1	-0.372111321	0.028	0.136	1	13
Elavl4.12	0.459374619	0.426	0.334	1	13
Agpat4.4	0.423703768	0.176	0.117	1	13
Adss	-0.400479805	0.065	0.193	1	13
Gfer	-0.405584111	0.009	0.109	1	13
Chtop.2	-0.30461512	0.111	0.249	1	13
2410004N09Rik.2	-0.514079028	0.056	0.184	1	13
Diablo	-0.295508438	0.074	0.198	1	13
Rassf3.3	-0.440889084	0.028	0.138	1	13
Rbbp7.5	-0.293842942	0.167	0.32	1	13
Gtf2a2.3	-0.29968171	0.222	0.384	1	13
Rbbp6.3	-0.315672926	0.287	0.433	1	13
Abca2	0.520622302	0.102	0.032	1	13
Pdpk1	0.330577707	0.139	0.099	1	13
Eef2k	0.386858304	0.102	0.057	1	13
Gmps.2	-0.29355052	0.083	0.211	1	13
Ddx54	-0.469389919	0.028	0.132	1	13
Amz2	-0.316608981	0.046	0.157	1	13
Klhl23	0.35731934	0.13	0.089	1	13
Akap6.5	-0.39155551	0.083	0.219	1	13
Shmt1.8	-0.355134548	0.009	0.103	1	13
Csnk1g1.1	0.474728533	0.12	0.076	1	13
Sox11.6	-0.344453113	0.019	0.115	1	13

Topbp1.5	-0.33709563	0.037	0.145	1	13
Tmem176b.5	0.454611409	0.157	0.109	1	13
Sdhc.1	0.29400339	0.287	0.269	1	13
Exosc1.3	-0.298486199	0.056	0.163	1	13
Med15	0.338898966	0.102	0.049	1	13
Chkb.1	0.35832074	0.194	0.156	1	13
Tgs1	-0.315489586	0.074	0.198	1	13
Wwp1.3	0.45639393	0.13	0.084	1	13
RP23-32A8.1.4	0.388182754	0.25	0.186	1	13
Gdap1.4	0.421333563	0.259	0.198	1	13
Glg1.1	0.310550719	0.231	0.219	1	13
Gars.3	-0.268209944	0.102	0.228	1	13
Eif2b2	-0.420421963	0.046	0.169	1	13
Zfand3	-0.384556678	0.037	0.148	1	13
Zcwpw1.3	-0.382118194	0.009	0.103	1	13
Ppp1r12c	0.436930948	0.176	0.126	1	13
Trim27.2	-0.308452978	0.046	0.149	1	13
Anks1b.2	0.44893458	0.139	0.063	1	13
Rfng	0.355361018	0.13	0.073	1	13
Abhd17b.1	0.31873765	0.157	0.143	1	13
Slc25a17.1	-0.467479726	0.037	0.154	1	13
Thoc7.4	-0.32520555	0.333	0.522	1	13
Snx4.1	0.250756225	0.157	0.175	1	13
Rnf165.7	-0.401285705	0.056	0.173	1	13
Fam101b	0.480473189	0.102	0.052	1	13
Mllt11.11	0.389287415	0.306	0.217	1	13
Tubb4b.10	-0.43833375	0.102	0.241	1	13
Fbxo32.6	-0.399529173	0.019	0.117	1	13
Cenpw.11	-0.444660181	0.028	0.128	1	13
Aftph	0.360680854	0.139	0.119	1	13
Atat1.4	-0.412164215	0.009	0.104	1	13
Dnajc24.1	-0.390003467	0.037	0.151	1	13
Pknox1.3	-0.372379492	0.037	0.146	1	13
Rad23a	0.447179126	0.139	0.062	1	13
Ccar2.1	-0.331071161	0.019	0.103	1	13
2810008D09Rik.3	0.417489013	0.222	0.126	1	13
Arhgap21.4	0.416512969	0.194	0.164	1	13
Safb2.2	0.323354275	0.324	0.313	1	13
Cdc25a	-0.420836453	0.019	0.119	1	13
Lsm14b.1	-0.458306135	0.037	0.153	1	13
Gm2000.1	-0.252706859	0.037	0.129	1	13
Fbxo21.1	0.389757678	0.13	0.09	1	13
Vps53	0.357295722	0.111	0.069	1	13
Unc13a.1	0.41021849	0.12	0.062	1	13
Ing1.2	-0.471937705	0.056	0.184	1	13
Nde1.5	-0.400412239	0.019	0.119	1	13
Smap1.3	0.296888113	0.25	0.223	1	13
Frg1.3	-0.25135967	0.157	0.299	1	13

Pdcd11.2	-0.420844882	0.046	0.164	1	13
Slc1a2.9	-0.383317991	0.139	0.294	1	13
Suds3	0.386652209	0.176	0.123	1	13
Wasf2.4	-0.345123982	0.019	0.113	1	13
Fam111a.9	-0.400244062	0.028	0.126	1	13
Ift43.1	-0.328577686	0.028	0.128	1	13
D10Wsu102e.1	-0.294630798	0.028	0.12	1	13
Ypel4.1	0.40453124	0.13	0.039	1	13
Kif22.12	-0.351222304	0.056	0.163	1	13
Wdr3	-0.333392766	0.019	0.112	1	13
Nme2.2	-0.280779273	0.019	0.102	1	13
Isca1	0.383581201	0.111	0.055	1	13
Pdha1.1	0.294142272	0.194	0.162	1	13
Msantd4	0.423884032	0.167	0.121	1	13
Rsbn1.2	0.307115754	0.204	0.182	1	13
Pcsk2.3	0.357208241	0.139	0.101	1	13
Pik3r3.7	0.483648842	0.222	0.135	1	13
Camsap1.1	0.269018279	0.093	0.101	1	13
Brca2.7	-0.4063484	0.019	0.114	1	13
R3hcc1.3	-0.34092119	0.056	0.16	1	13
Tfdp2.3	-0.397819524	0.046	0.161	1	13
Gde1.3	0.399964818	0.185	0.141	1	13
Vps28.1	0.365080443	0.315	0.244	1	13
Zmym4	-0.253892814	0.028	0.108	1	13
Hey1.9	-0.483453337	0.12	0.273	1	13
Ensa.4	0.278268653	0.259	0.227	1	13
Ddr1.3	-0.289380465	0.046	0.135	1	13
Rnf187.3	0.25875486	0.444	0.42	1	13
Cpeb4.2	0.410073314	0.111	0.046	1	13
Echs1.1	-0.343534852	0.074	0.187	1	13
Eif2s3y	0.305120226	0.185	0.149	1	13
Slc22a17.7	0.369954693	0.315	0.25	1	13
Dpf2.1	-0.356134516	0.019	0.111	1	13
Tbca.3	-0.286127499	0.278	0.421	1	13
Plgrkt.1	-0.427487968	0.065	0.195	1	13
Pnkd.1	0.382950143	0.111	0.068	1	13
Mrpl11.1	-0.394178382	0.111	0.238	1	13
Gabrb3.3	0.389705289	0.139	0.101	1	13
Tacc2.8	0.338777913	0.167	0.14	1	13
Fam178a.1	0.314668635	0.13	0.11	1	13
Nr2f1.1	-0.449287295	0.074	0.205	1	13
Agap3	0.362964923	0.13	0.111	1	13
Bub1b.10	-0.390286881	0.009	0.103	1	13
Rev1.1	0.269162251	0.12	0.117	1	13
Cep68	-0.277515146	0.028	0.116	1	13
Cdh15	0.366849231	0.111	0.04	1	13
Ergic3.2	0.276260069	0.25	0.227	1	13
Yy1.1	-0.421132062	0.074	0.205	1	13

Phactr1.6	0.402114683	0.259	0.183	1	13
Neurod6.5	-0.446099822	0.065	0.172	1	13
Myg1	-0.312829906	0.028	0.123	1	13
Eif3b.4	-0.299454165	0.093	0.221	1	13
Chrac1	-0.362514204	0.019	0.112	1	13
Ldb1.3	-0.336466751	0.074	0.184	1	13
Pold2.3	-0.348809167	0.019	0.114	1	13
Bfar.2	0.304029761	0.194	0.191	1	13
Max	0.38791297	0.194	0.131	1	13
Adar	0.363095142	0.12	0.07	1	13
Gprasp2	0.392898387	0.102	0.069	1	13
5830418K08Rik.3	-0.383744826	0.056	0.168	1	13
Adam10.3	0.341725325	0.213	0.203	1	13
Lzts1.1	0.465350537	0.12	0.04	1	13
Gtpbp2	0.418527182	0.111	0.068	1	13
Kdm2a	-0.386753448	0.019	0.108	1	13
Zcchc8	-0.341691806	0.037	0.143	1	13
Tmem134.1	-0.304577842	0.046	0.149	1	13
Gramd1a.2	-0.461414228	0.056	0.165	1	13
Fundc2.2	-0.300439037	0.13	0.271	1	13
Tcerg1.4	-0.292315904	0.287	0.458	1	13
Xrcc5.2	-0.325721383	0.028	0.115	1	13
Dlgap5.10	-0.39290231	0.019	0.112	1	13
Sp3.1	-0.391424572	0.046	0.163	1	13
Ahsa2.2	-0.295579252	0.102	0.207	1	13
Ppil2	-0.267955059	0.028	0.118	1	13
Ptbp1.3	-0.387208932	0.009	0.101	1	13
Cdt1.8	-0.34653601	0.028	0.125	1	13
Uncx.11	-0.392955082	0.167	0.318	1	13
L1cam.2	0.53615861	0.139	0.052	1	13
Nudt19.1	-0.295848773	0.037	0.112	1	13
Gatc.1	-0.310704825	0.019	0.107	1	13
Znhit6.3	-0.359845641	0.028	0.12	1	13
Prpf31.2	-0.459491328	0.074	0.2	1	13
Mrpl34.2	-0.271330063	0.157	0.288	1	13
Tubb2a.13	0.322774013	0.333	0.244	1	13
Zfp830	-0.362840418	0.028	0.118	1	13
Scnm1.1	-0.384612069	0.037	0.136	1	13
Scg5.8	0.30071552	0.37	0.334	1	13
Slc39a6.3	-0.359140459	0.074	0.199	1	13
Nalcn	0.399406253	0.102	0.073	1	13
Sec11c.7	-0.368590705	0.093	0.213	1	13
Mdga1.5	-0.349949185	0.037	0.136	1	13
Pax6.10	-0.253423993	0.352	0.514	1	13
Rae1	-0.421387488	0.065	0.191	1	13
Med13l	0.259766224	0.111	0.113	1	13
Sgip1.2	0.510329426	0.148	0.063	1	13
Mif.5	-0.255988503	0.222	0.357	1	13

Polr2h.4	-0.397595572	0.102	0.225	1	13
Cnrip1.5	0.25954302	0.185	0.197	1	13
Pitpna.1	0.356142423	0.176	0.131	1	13
Eri1	-0.288478389	0.028	0.114	1	13
Zrsr2.1	-0.259742282	0.111	0.236	1	13
Mob4	-0.255256131	0.093	0.204	1	13
Grik5.3	0.328530277	0.12	0.109	1	13
Zfp740	-0.35335583	0.019	0.107	1	13
Ubac1	0.415752049	0.194	0.128	1	13
1200014J11Rik	-0.250197125	0.028	0.101	1	13
Fads1.1	-0.278667612	0.028	0.105	1	13
Mettl10	-0.299263649	0.037	0.132	1	13
Gpx1.3	-0.367193105	0.222	0.383	1	13
Ndrg2.6	-0.25775418	0.093	0.185	1	13
Fam175b	-0.41198424	0.019	0.112	1	13
Etnk1	0.35253771	0.13	0.102	1	13
Usp33.2	0.303909781	0.194	0.148	1	13
Rnf4	-0.320128199	0.028	0.124	1	13
Zhx1	0.261022801	0.148	0.129	1	13
H2afj.2	-0.415792131	0.083	0.205	1	13
Prdm2.2	-0.355281557	0.037	0.132	1	13
Pja1.3	0.412992579	0.25	0.172	1	13
Klc2.1	0.429452302	0.12	0.038	1	13
Unc50.3	0.262414938	0.139	0.125	1	13
Rnf11.1	0.453907991	0.231	0.147	1	13
Vkorc1.2	-0.347892776	0.019	0.111	1	13
Grsf1.1	-0.331865513	0.065	0.176	1	13
Sirt2.5	-0.441947794	0.046	0.156	1	13
Glrx3.1	-0.347877794	0.12	0.247	1	13
Sgol2.10	-0.45555244	0.056	0.157	1	13
Synrg	-0.400348811	0.037	0.135	1	13
Actr6.3	-0.355240381	0.019	0.108	1	13
Apoe.13	-1.435046084	0.12	0.25	1	13
1110001A16Rik.2	0.350770277	0.12	0.096	1	13
Ssu72.1	0.349663746	0.25	0.223	1	13
Dbn1.10	-0.444246106	0.037	0.14	1	13
Hist3h2a.8	-0.253361389	0.065	0.143	1	13
Trappc3.2	0.411689552	0.167	0.1	1	13
Tmem55b.2	0.269053874	0.111	0.112	1	13
Dnttip1	0.264056803	0.102	0.095	1	13
Ptrh2.1	-0.348189066	0.046	0.154	1	13
Exosc5.1	-0.292144232	0.019	0.104	1	13
Flywch2.1	0.396691439	0.139	0.05	1	13
Smim15.3	-0.330465706	0.028	0.121	1	13
Prps1.3	-0.422814278	0.037	0.143	1	13
Nol11	-0.334886085	0.037	0.133	1	13
Ezh1	0.446726521	0.111	0.061	1	13
Trpc4ap.11	-0.312205076	0.093	0.198	1	13

Fam107b.4	-0.376208797	0.019	0.102	1	13
Trappc1	-0.312103468	0.102	0.226	1	13
1700021F05Rik.1	-0.255903087	0.046	0.141	1	13
Dad1.4	-0.265528159	0.194	0.344	1	13
Sac3d1.1	-0.381177322	0.028	0.107	1	13
E330009J07Rik.3	0.358571924	0.167	0.116	1	13
Ankrd32.3	-0.291559237	0.083	0.184	1	13
Ctsd.11	-0.812903255	0.083	0.175	1	13
Necap1.1	0.417413342	0.12	0.069	1	13
Nop56.8	-0.324471754	0.213	0.361	1	13
Kcnc3	0.406269346	0.111	0.065	1	13
Psme2.1	-0.36558685	0.019	0.105	1	13
Tspan5.2	0.303690024	0.222	0.196	1	13
Bex1.5	0.404594223	0.287	0.223	1	13
Fam133b.2	0.293490199	0.333	0.307	1	13
Lamp2.4	-0.366064859	0.037	0.138	1	13
Mapk10.4	0.470823726	0.176	0.098	1	13
5430416N02Rik.3	-0.400397264	0.037	0.141	1	13
Cdk16	0.391231527	0.12	0.067	1	13
Lsm1	-0.428683414	0.056	0.166	1	13
Gsr.1	0.261716178	0.102	0.09	1	13
Epm2aip1.1	0.31443838	0.12	0.105	1	13
Dld	0.31347979	0.222	0.186	1	13
Smarce1.2	-0.314602773	0.083	0.204	1	13
Specc1.4	0.396580489	0.111	0.048	1	13
Cxx1b.1	0.365350552	0.185	0.12	1	13
Pptc7	0.418543973	0.139	0.052	1	13
Tex261.1	-0.339158274	0.028	0.116	1	13
Fam120a	-0.30545516	0.037	0.124	1	13
Emc4	0.381635303	0.241	0.197	1	13
Fbxw2.1	0.317195344	0.185	0.159	1	13
Ncapd3.1	-0.282654791	0.028	0.113	1	13
Blvrb.2	0.37446683	0.102	0.059	1	13
Slc2a3	0.353009134	0.102	0.032	1	13
Cpne3.3	-0.298945712	0.083	0.197	1	13
Opa3	-0.336638838	0.037	0.115	1	13
2410004B18Rik	0.298482741	0.12	0.113	1	13
Zmym2.1	-0.316076145	0.028	0.119	1	13
Tbce.1	0.403678208	0.12	0.078	1	13
Pop5.1	0.316538185	0.12	0.1	1	13
Kat6b.3	-0.435910938	0.046	0.157	1	13
Rps6ka3.1	0.39457406	0.13	0.063	1	13
Thoc1.1	-0.330652653	0.065	0.178	1	13
Barhl2	0.376955513	0.111	0.064	1	13
Rtcb	-0.303162311	0.102	0.227	1	13
Tsta3	-0.265234543	0.046	0.143	1	13
Tef	0.424613386	0.157	0.093	1	13
Xpo1.3	-0.301232462	0.102	0.228	1	13

Scaper.5	0.319164642	0.12	0.11	1	13
Nrip1	0.333310489	0.111	0.101	1	13
Fhl1.2	0.256395762	0.12	0.105	1	13
Gtf2b	-0.275365674	0.056	0.153	1	13
Rnf219.2	-0.393995048	0.046	0.14	1	13
Nap1l2	0.399355043	0.102	0.033	1	13
Rap1a.3	-0.297431525	0.037	0.124	1	13
Smg1	0.346704417	0.157	0.125	1	13
Aatf.1	-0.399646966	0.028	0.122	1	13
Kcnj3.2	0.37976668	0.157	0.068	1	13
Bcas2.3	-0.258782996	0.259	0.406	1	13
Cenpb.2	-0.309429285	0.093	0.21	1	13
Tbpl1.2	-0.301769393	0.074	0.187	1	13
Fyn.7	-0.434116927	0.093	0.216	1	13
Tmed10.2	-0.327363625	0.083	0.205	1	13
Prpf40b.2	0.332568419	0.167	0.146	1	13
2700089E24Rik.3	0.359794363	0.241	0.198	1	13
Rab10.2	-0.354436182	0.148	0.293	1	13
Sec61a1	-0.299718748	0.074	0.179	1	13
Orc2.3	-0.406742837	0.046	0.156	1	13
Mia3.2	0.300403644	0.269	0.244	1	13
Stra13	-0.305612013	0.028	0.102	1	13
Anapc4	-0.315990742	0.046	0.147	1	13
Limd2.3	-0.284598204	0.046	0.135	1	13
Itfg1.1	0.329821445	0.12	0.086	1	13
Rbm33.1	0.294093259	0.139	0.13	1	13
Exosc3	-0.343987358	0.019	0.107	1	13
Poglut1.1	-0.298634452	0.037	0.13	1	13
Brwd3.1	-0.372888328	0.019	0.102	1	13
Tmx3	-0.317426516	0.037	0.125	1	13
Acaa1a.1	0.334979534	0.13	0.103	1	13
4930402H24Rik.6	0.254936766	0.111	0.113	1	13
Mmp24.2	0.446161514	0.13	0.052	1	13
Atp6v0a1.3	0.438055235	0.194	0.095	1	13
Ctsf.5	0.40524905	0.167	0.098	1	13
Slc7a5.5	-0.30615961	0.028	0.115	1	13
Zfyve9	0.300801461	0.102	0.084	1	13
5830428H23Rik.1	0.486454125	0.139	0.064	1	13
Tmed1.1	-0.366968364	0.019	0.105	1	13
Cox16	-0.288289854	0.037	0.129	1	13
Poc1b.1	-0.290750486	0.037	0.127	1	13
Slc48a1.2	0.402535739	0.12	0.06	1	13
Cacng4.10	-0.427772414	0.028	0.102	1	13
Sod2.2	0.323620105	0.213	0.175	1	13
Usp3.2	0.382557772	0.222	0.14	1	13
Ncald.4	0.322191049	0.231	0.165	1	13
Usp46.4	0.255070673	0.259	0.235	1	13
Rhob.2	-0.273794621	0.046	0.139	1	13

Cd3eap.1	-0.372798428	0.028	0.121	1	13
Slu7.1	-0.382886264	0.13	0.264	1	13
Supt6.1	0.356354736	0.213	0.157	1	13
Ogfr.1	-0.293737964	0.065	0.17	1	13
Gnl3l.3	0.342909105	0.25	0.188	1	13
Bmyc.3	0.324106949	0.139	0.1	1	13
Bcap29.3	-0.344858223	0.037	0.123	1	13
Npdc1.7	0.350795627	0.306	0.261	1	13
Dck.4	-0.343711612	0.019	0.103	1	13
Atic	-0.281839369	0.028	0.11	1	13
Pdcd4.3	-0.27890368	0.194	0.331	1	13
Slc17a6.12	-0.440181783	0.046	0.148	1	13
Deb1.2	0.321991152	0.213	0.167	1	13
Eif2b3.1	-0.334281517	0.056	0.16	1	13
Taf2.1	-0.264963964	0.028	0.105	1	13
Tnik.9	-0.399001968	0.065	0.146	1	13
Dph3.2	-0.311811718	0.093	0.211	1	13
Tpp2	-0.341347367	0.065	0.173	1	13
Isg20l2	-0.369730394	0.028	0.118	1	13
2810006K23Rik.1	-0.304269894	0.037	0.123	1	13
Fam32a.1	-0.36077141	0.139	0.269	1	13
Bet1l.1	0.348491687	0.13	0.078	1	13
Znhit1	-0.292208142	0.037	0.12	1	13
Stx8	0.344851833	0.111	0.083	1	13
Bcl2l1.1	0.379543114	0.12	0.088	1	13
Bad	0.329276002	0.148	0.108	1	13
Wdr43.2	-0.347818948	0.111	0.242	1	13
Mybbp1a.3	-0.264754427	0.13	0.25	1	13
Blm.4	-0.317219754	0.037	0.111	1	13
Trpc2.1	-0.285629245	0.046	0.135	1	13
Wash	0.264324214	0.102	0.102	1	13
Smpd3.7	-0.316716011	0.065	0.161	1	13
Zfp277.1	-0.325153275	0.037	0.132	1	13
Ccdc55.1	-0.286274595	0.139	0.268	1	13
2510003E04Rik.1	0.379479515	0.157	0.107	1	13
Pdlim7.2	0.380847131	0.111	0.039	1	13
Atp11c.1	-0.258232881	0.037	0.12	1	13
Zbtb7a	0.410577262	0.148	0.093	1	13
Rtn2.1	-0.316681068	0.028	0.115	1	13
Pink1	0.250306568	0.157	0.13	1	13
Ptbp3.2	-0.286890917	0.028	0.11	1	13
Daam1.3	-0.357869762	0.083	0.184	1	13
Gtf3c6.1	-0.263816238	0.093	0.201	1	13
Gosr1	0.367075414	0.111	0.062	1	13
Ip6k2.1	0.294098823	0.148	0.121	1	13
Mrps18a	-0.353152851	0.074	0.17	1	13
Tm9sf4	0.294278865	0.157	0.116	1	13
Prpf4.2	-0.350607706	0.046	0.14	1	13

Pard6a.2	0.407868245	0.176	0.091	1	13
Extl2.1	0.345795268	0.102	0.054	1	13
Exoc2.1	-0.38498359	0.037	0.116	1	13
Gbas.1	0.351751561	0.185	0.132	1	13
Fam171a2.1	0.413003026	0.102	0.055	1	13
Dnmt3a.2	0.336771646	0.259	0.202	1	13
Ncoa7.1	0.424019807	0.176	0.098	1	13
Myt1l.12	0.469618841	0.25	0.16	1	13
Camsap2.2	0.458913648	0.194	0.132	1	13
Scaf11.3	-0.350901278	0.222	0.374	1	13
Clint1	-0.355182823	0.046	0.131	1	13
Pdcd2l.1	-0.337275335	0.037	0.131	1	13
Pik3ip1.3	0.312130951	0.139	0.116	1	13
9930021J03Rik	-0.258710742	0.046	0.13	1	13
Cst3.11	-0.293829507	0.435	0.536	1	13
Ip6k1.1	0.385194053	0.231	0.175	1	13
Uri1.2	-0.326219279	0.102	0.21	1	13
Lgmn.5	-0.470666519	0.037	0.119	1	13
Acd.4	-0.252074712	0.065	0.162	1	13
Pold3.4	-0.256822963	0.056	0.149	1	13
Bsg.2	2.729192797	0.904	0.422	2.9063E-160	14
Fth1.6	2.504407897	1	0.708	7.3039E-147	14
Itm2a.1	3.278467338	0.942	0.043	9.7739E-137	14
Ctla2a	4.03203715	0.904	0.004	6.7739E-123	14
Igfbp7.1	4.010266913	1	0.016	1.0498E-117	14
Slco1c1	3.306555033	0.885	0.003	1.106E-117	14
Flt1	3.149487364	0.885	0.003	7.4205E-117	14
Esam	2.798389438	0.923	0.002	1.3547E-114	14
Slc16a1.1	2.918646822	0.827	0.036	3.639E-110	14
Gpr116.1	2.649391766	0.846	0.002	8.5442E-105	14
Sparc.4	3.67790733	1	0.082	1.1565E-104	14
Cldn5	2.592324965	0.808	0.001	2.2388E-103	14
Egfl7	2.698404047	0.846	0.01	1.26856E-97	14
Slc2a1	2.533049596	0.808	0.012	2.13931E-88	14
Ptprb	2.519325836	0.808	0.004	3.12652E-88	14
Kdr	2.732491857	0.731	0.002	8.88508E-88	14
Ly6c1	3.474436285	0.673	0.002	1.65206E-86	14
Eltd1	2.190625421	0.673	0.001	1.69734E-85	14
Nostrin	2.104455483	0.692	0.001	9.56441E-81	14
Cd34	2.323036676	0.712	0.003	1.35808E-78	14
Cd93	2.262514675	0.673	0.002	2.56146E-74	14
Ramp2.7	2.378925611	0.846	0.117	2.04464E-71	14
Vwa1.2	2.175976487	0.712	0.009	1.90346E-65	14
Gsta4.1	2.520039602	0.615	0.009	2.29959E-65	14
Gng11.2	2.445969364	0.75	0.013	3.11492E-65	14
Ccdc141.2	2.200361109	0.673	0.007	6.37898E-63	14
Sepp1.3	3.166305326	0.904	0.053	2.58419E-62	14
BC028528.2	1.886852904	0.673	0.005	3.57543E-61	14

Gimap6	1.866641742	0.538	0.001	5.56402E-60	14
Col4a2.1	2.476137338	0.846	0.038	1.8376E-59	14
AU021092	1.951252412	0.519	0.001	8.36868E-59	14
Tmem252	1.713062083	0.5	0	2.15439E-58	14
Grap.1	1.76696263	0.577	0.002	4.05161E-58	14
Fn1	2.16408578	0.615	0.007	4.4704E-58	14
Tfrc.1	2.204620776	0.75	0.167	5.42558E-55	14
Slco1a4	2.308075537	0.5	0.002	1.15777E-54	14
Erg	1.467280703	0.462	0	1.87308E-53	14
Abcb1a	1.819455261	0.481	0.001	2.55683E-53	14
Car4.1	2.506762139	0.577	0.006	4.48887E-53	14
Sparcl1.11	2.349788577	0.942	0.205	4.15672E-52	14
Nrp1.2	2.412810058	0.712	0.042	4.30113E-51	14
Spock2.3	2.4630348	0.635	0.063	5.63411E-51	14
Eng.1	1.72669796	0.5	0.002	5.74568E-50	14
Ecscr.2	1.844949602	0.558	0.004	1.00895E-49	14
Robo4	1.438528166	0.442	0	1.20117E-49	14
Mfsd2a.1	2.055457649	0.596	0.009	2.81019E-49	14
Pltp.3	2.328432792	0.673	0.026	1.06021E-48	14
Tek	1.449550496	0.404	0	4.51312E-47	14
Id3.2	2.330779866	0.75	0.03	5.48398E-47	14
Col4a1.1	2.239969556	0.827	0.07	1.29133E-46	14
Ly6a	1.843701506	0.404	0	1.54396E-46	14
Vwf	1.923598118	0.442	0.001	3.85177E-46	14
Ablim1.1	2.11370414	0.673	0.017	2.16903E-45	14
Fzd6.1	1.428136194	0.442	0.001	1.01856E-42	14
Rasip1	1.629403947	0.538	0.007	2.56995E-42	14
Pecam1	1.593483763	0.423	0.001	6.78515E-42	14
Slc40a1.1	1.652271175	0.5	0.004	7.79655E-42	14
Epas1.2	1.683203174	0.538	0.007	2.63214E-41	14
Sox17	1.41973874	0.365	0	9.12938E-41	14
9430020K01Rik.1	1.753529026	0.5	0.006	1.43322E-40	14
Apln	2.06584269	0.385	0.002	1.63236E-40	14
Igfbp3.2	2.193738414	0.462	0.011	5.69411E-40	14
St3gal6.2	1.583995202	0.538	0.008	1.04637E-39	14
C130074G19Rik	1.339268456	0.385	0.001	1.98747E-39	14
Fam101b.1	2.026514842	0.615	0.051	3.03219E-39	14
Cgnl1.1	1.551331211	0.462	0.003	8.79116E-39	14
Slc7a5.6	1.923806041	0.788	0.113	9.77049E-39	14
Scgb3a1	1.934722201	0.346	0	1.07963E-38	14
Slc38a5	1.656404489	0.346	0	1.08363E-38	14
Slc39a8	1.490845153	0.442	0.004	1.11589E-38	14
Wwtr1.2	1.658031117	0.519	0.01	2.15965E-38	14
Ushbp1	1.330873807	0.365	0.001	8.98515E-38	14
Ptrf.1	1.479632997	0.442	0.003	1.95499E-37	14
Anxa3.1	1.536670197	0.5	0.006	6.61348E-37	14
Tmem204.1	1.556221692	0.481	0.005	1.48581E-36	14
Klf2.3	1.959547166	0.577	0.017	1.58488E-36	14

Slc3a2.8	1.63736776	0.827	0.321	2.53996E-36	14
Tmem88	1.372760565	0.442	0.003	3.17247E-36	14
Foxq1.1	1.794822831	0.423	0.005	7.16057E-36	14
Slc22a8.1	1.347063202	0.404	0.002	5.58057E-35	14
Lama4.1	1.796347457	0.481	0.007	2.05259E-34	14
Palmd	1.907474912	0.654	0.049	2.23836E-34	14
Pglyrp1	1.230816067	0.308	0	2.35038E-34	14
Slc9a3r2.2	1.969681082	0.519	0.015	1.04194E-33	14
Cdh5.1	1.157727959	0.385	0.002	1.30402E-33	14
Fxyd5.1	1.510534667	0.385	0.003	2.93096E-32	14
Myh9.2	1.539808136	0.596	0.025	5.62935E-32	14
Tdrp	1.255757021	0.327	0.001	5.97367E-32	14
Lsr	1.313211009	0.346	0.001	6.01365E-32	14
Fam129a	1.200093036	0.365	0.002	1.00244E-31	14
Synm	1.308722582	0.404	0.003	1.04455E-31	14
Ets1.1	1.68600835	0.442	0.009	2.02547E-31	14
Pdgfb	1.149522296	0.327	0.001	2.65136E-31	14
Slc38a3.3	1.653154877	0.558	0.02	3.59034E-31	14
Tsc22d1.6	1.506630027	0.846	0.426	3.90806E-31	14
Acvrl1	1.339129093	0.346	0.002	2.81225E-30	14
Abcg2	1.625051289	0.596	0.033	3.3098E-30	14
Srgn.1	1.36742573	0.404	0.004	2.1715E-29	14
Ctgf	1.407318684	0.288	0.001	2.67143E-29	14
Eogt	1.409413149	0.385	0.004	3.1177E-29	14
Fli1.1	1.263850003	0.404	0.005	3.29301E-29	14
Clec1a.1	1.330807085	0.346	0.002	4.26619E-29	14
Serpinb6b	1.140999681	0.269	0	8.60782E-29	14
Sptbn1.10	1.58149501	0.923	0.351	1.12014E-28	14
Arhgap29.1	1.662995859	0.558	0.027	1.45132E-28	14
Unc45b	1.145751706	0.346	0.002	5.51564E-28	14
Atox1.3	1.467411847	0.846	0.291	6.67303E-28	14
Ildr2.1	1.263968241	0.423	0.006	6.82195E-28	14
Id1.1	1.927761889	0.558	0.071	3.30868E-27	14
Nid1.2	1.53618922	0.5	0.016	4.24097E-27	14
Tie1	1.104389977	0.269	0	6.74078E-27	14
Gm20748	1.118489439	0.327	0.002	7.34786E-27	14
Dlc1.1	1.420585562	0.404	0.007	1.02353E-26	14
Cd151.2	1.404732033	0.5	0.016	1.14191E-26	14
Rbpms.1	1.099467582	0.365	0.004	3.5343E-26	14
Meox1	1.058323065	0.308	0.001	9.36233E-26	14
Emcn	1.337431397	0.308	0.002	9.47483E-26	14
Icam2	0.80655885	0.269	0	1.17537E-25	14
Foxf2.1	1.134892754	0.423	0.008	1.37132E-25	14
Lipa.2	1.745604371	0.462	0.03	2.12152E-25	14
Fgd5	0.844938588	0.25	0	2.88236E-25	14
Swap70	1.356898895	0.481	0.016	2.89183E-25	14
Slc7a1.1	1.685506813	0.558	0.097	3.73141E-25	14
Ahnak.1	1.226766572	0.385	0.006	4.03469E-25	14

Wfdc1.1	1.358086634	0.288	0.002	4.1796E-25	14
Clec14a	1.038417102	0.269	0.001	1.06172E-24	14
Hmgcs2.1	1.528228681	0.327	0.004	1.13451E-24	14
St8sia4	1.068332251	0.308	0.002	1.41547E-24	14
Anxa2.3	1.532731257	0.423	0.011	1.484E-24	14
Serinc3.2	1.779106272	0.654	0.118	2.21559E-24	14
Slc39a10.3	1.589128599	0.712	0.193	3.82574E-24	14
Rasgrp3.1	1.169476344	0.346	0.004	5.74693E-24	14
Sgms1	1.461101452	0.423	0.015	1.23992E-23	14
Mmrn2	0.935123312	0.269	0.001	3.19601E-23	14
Ddc	1.061479973	0.288	0.001	3.30382E-23	14
Thsd1	1.073069726	0.288	0.001	4.13174E-23	14
Nampt	1.669022278	0.5	0.08	5.79516E-23	14
Cnn2.1	1.371201201	0.365	0.007	9.57952E-23	14
Gpr4	0.972078379	0.25	0.001	1.24675E-22	14
Vim.10	1.459524823	0.846	0.3	1.68787E-22	14
Gata2	1.023673716	0.25	0.001	2.60992E-22	14
Slco2b1.1	1.066096843	0.327	0.004	3.09068E-22	14
Limch1.3	1.629579098	0.462	0.023	3.18505E-22	14
Cdkn2b	1.102472352	0.327	0.004	4.78084E-22	14
Il2rg	0.941133649	0.25	0.001	6.02358E-22	14
Eva1b.2	1.281182183	0.385	0.008	8.71323E-22	14
Zic3	1.1337136	0.346	0.005	8.94233E-22	14
Ly6e.5	1.442762722	0.654	0.281	1.22467E-21	14
Serpinh1.9	1.681205678	0.635	0.113	1.24574E-21	14
Nfkbia.4	1.597006151	0.673	0.17	1.61124E-21	14
Arhgap18	1.312307583	0.404	0.015	2.20207E-21	14
Arl4a.2	1.765198631	0.462	0.063	2.81896E-21	14
Nos3	0.969676896	0.308	0.003	3.60216E-21	14
Apcdd1	1.287319305	0.385	0.011	4.53586E-21	14
Myo10.1	1.564329626	0.615	0.12	5.40544E-21	14
Itga1.1	1.136385997	0.308	0.003	8.42962E-21	14
F11r.1	0.917630669	0.308	0.003	1.0961E-20	14
Dock9.2	1.25356513	0.423	0.015	1.71899E-20	14
Tm4sf1.1	1.31006524	0.308	0.004	2.12586E-20	14
Hspg2.1	1.100371274	0.308	0.004	3.086E-20	14
Edn1	1.145919792	0.212	0	4.1121E-20	14
Ocln	0.806934882	0.212	0	4.88009E-20	14
Tmsb4x.9	1.018709346	1	0.948	5.34904E-20	14
Paqr5	1.159251359	0.212	0.001	5.9704E-20	14
4931406P16Rik	1.465782405	0.538	0.056	7.01423E-20	14
Ctla2b.1	1.122469151	0.269	0.002	9.32762E-20	14
Nid2.1	1.119283887	0.346	0.007	1.07798E-19	14
Cd59a	0.99877425	0.231	0.001	1.07799E-19	14
Lamb1.1	1.370571209	0.365	0.009	1.40798E-19	14
Marcks.5	-1.328430603	0.519	0.917	1.5927E-19	14
Sfrp1.10	-2.004982963	0.231	0.813	1.72319E-19	14
Pde2a	1.097865462	0.269	0.002	1.74761E-19	14

Chst1.1	1.226665601	0.288	0.004	1.81619E-19	14
Abcc4	1.066973138	0.25	0.002	2.01971E-19	14
Lmo2.1	1.458491952	0.5	0.035	2.02398E-19	14
Cdkn1a.3	1.738279136	0.596	0.091	2.52159E-19	14
Angpt2	1.408357582	0.231	0.002	3.01799E-19	14
Lef1.1	0.890386036	0.269	0.002	3.22337E-19	14
Ifitm3.2	1.222071438	0.385	0.013	3.75549E-19	14
Itga6.2	1.195854212	0.385	0.012	6.06014E-19	14
Rgcc.2	1.269268061	0.404	0.015	6.4927E-19	14
Uaca.1	1.531941786	0.538	0.058	7.79954E-19	14
Egfl8	1.124137202	0.346	0.008	8.28131E-19	14
Fcgrt.3	1.352406388	0.462	0.026	9.2957E-19	14
Hsp90ab1.5	-0.635343981	1	0.997	9.4798E-19	14
Kank3.1	1.351494211	0.462	0.028	1.2182E-18	14
She	0.815173866	0.192	0	1.82529E-18	14
Hspa12b	0.847172525	0.212	0.001	2.08682E-18	14
Gimap1	0.666763436	0.192	0	2.33097E-18	14
Plat.2	1.541903952	0.404	0.02	3.43953E-18	14
Ctsh.3	1.196029903	0.365	0.011	3.56077E-18	14
Plk2.2	1.413115814	0.346	0.011	3.97927E-18	14
Lgals9.2	1.220936672	0.327	0.008	6.48196E-18	14
Myl12a.8	1.263316319	0.788	0.327	7.66998E-18	14
Nfib.9	-1.328118164	0.692	0.934	8.7974E-18	14
Rrbp1.7	1.480996806	0.692	0.187	1.23225E-17	14
Lamc1.1	1.34440366	0.404	0.019	1.48452E-17	14
Dok4	1.356695504	0.346	0.012	3.1717E-17	14
Ece1.1	1.433134475	0.558	0.083	3.49387E-17	14
S1pr1.2	1.09735944	0.327	0.008	3.84027E-17	14
Rhoj.2	1.392781524	0.442	0.031	4.40131E-17	14
Tagln2.3	1.423751483	0.5	0.043	6.48485E-17	14
Car2.1	1.365479676	0.385	0.016	6.80057E-17	14
Parvb	1.173600564	0.346	0.011	7.05834E-17	14
Rapgef5	0.879847793	0.269	0.003	7.59166E-17	14
Rgs12.4	1.48530972	0.615	0.144	1.13318E-16	14
Csrp2.10	1.421416154	0.615	0.156	2.33635E-16	14
CRE_RECOMBINASE.12	-2.178744422	0.154	0.77	2.35572E-16	14
Dusp2	0.918593104	0.231	0.002	2.74662E-16	14
Arpc1b.2	1.26894541	0.462	0.035	2.75306E-16	14
Cd63.6	-1.766531867	0.173	0.756	2.9819E-16	14
Rassf9	0.66625601	0.192	0	3.43414E-16	14
Sema3c	0.810971459	0.212	0.001	3.90766E-16	14
Htra3.1	1.164522794	0.288	0.005	4.12858E-16	14
Ly75	0.857343198	0.212	0.001	4.17787E-16	14
Rhoc.4	1.209431177	0.423	0.026	4.49914E-16	14
Notch1.3	1.378990509	0.365	0.022	8.94874E-16	14
Vamp5.2	0.80914009	0.25	0.003	1.54219E-15	14
Hmcn1	0.939124282	0.212	0.001	1.66135E-15	14
Apold1	1.07132622	0.288	0.008	1.7656E-15	14

Angptl4.2	1.038750028	0.308	0.008	2.21433E-15	14
Gstm7	1.171065183	0.308	0.009	3.89396E-15	14
Stap2	0.871388779	0.231	0.002	4.07019E-15	14
Kcp	1.044326943	0.192	0.001	4.10009E-15	14
Calm1.6	0.861452039	0.942	0.861	4.44805E-15	14
Ralb.4	1.091335717	0.365	0.018	5.43249E-15	14
Ppfibp1.2	1.340038907	0.404	0.031	5.65512E-15	14
Afap1l1	1.156833123	0.346	0.014	6.63591E-15	14
Basp1.13	-1.824536354	0.115	0.705	8.1146E-15	14
Zic1.12	-1.619416151	0.212	0.785	8.67801E-15	14
Mcam.1	0.792825231	0.231	0.002	1.00522E-14	14
Adcy4	0.990071956	0.173	0.001	1.02054E-14	14
Fas	0.785363187	0.192	0.001	1.18969E-14	14
Bambi	1.335506236	0.308	0.022	1.62784E-14	14
S100a13.4	1.31443063	0.423	0.029	1.91831E-14	14
B2m.9	1.394463799	0.731	0.23	3.01467E-14	14
Dusp3.4	1.222278488	0.404	0.028	3.2909E-14	14
Crmp1.7	-1.784078889	0.154	0.669	3.97114E-14	14
Tbx3	0.598403611	0.192	0.001	4.21232E-14	14
Anxa7.2	0.949557541	0.308	0.01	5.19262E-14	14
Gpcpd1	1.431355101	0.519	0.082	5.22304E-14	14
Aplnr	0.751737	0.192	0.001	5.58507E-14	14
Cyyr1	0.776213538	0.231	0.003	7.14564E-14	14
Nes.3	1.376492995	0.558	0.085	9.16916E-14	14
Scarf1	0.840313855	0.192	0.001	1.13485E-13	14
Ttyh2.3	1.261196418	0.442	0.037	1.25069E-13	14
Smtn.2	1.209591824	0.385	0.032	1.35459E-13	14
Hnrnpa2b1.6	-0.910740728	0.731	0.934	1.4493E-13	14
Utrn.1	1.285625347	0.385	0.026	1.46891E-13	14
Slc30a1	1.14489017	0.346	0.018	1.47442E-13	14
Ckb.11	-1.636600134	0.308	0.809	2.45973E-13	14
Cst3.12	1.040170082	0.865	0.535	2.99714E-13	14
Stab1.1	1.113060186	0.288	0.009	3.62993E-13	14
Igfbpl1.12	-1.938872132	0.077	0.649	4.14113E-13	14
Slfn5	0.763744005	0.173	0.001	5.43865E-13	14
Marcksl1.8	-1.553937846	0.154	0.724	6.78568E-13	14
Tns1.1	1.068300847	0.231	0.004	7.07854E-13	14
Nfix.9	-1.687767728	0.173	0.674	8.40649E-13	14
Tpm4.9	1.083643251	0.596	0.316	1.26778E-12	14
Itgb1.5	1.151869334	0.692	0.361	1.54635E-12	14
Lrp8.1	1.142764483	0.423	0.038	1.58084E-12	14
Def6	0.763020054	0.192	0.001	1.64224E-12	14
Tcf7	0.630426577	0.173	0.001	1.79395E-12	14
Jag2	0.596253929	0.173	0.001	1.84261E-12	14
Myo1b.2	1.397380182	0.462	0.075	2.51782E-12	14
Prkch	0.819127129	0.173	0.001	2.95117E-12	14
Tiam1.1	1.138202166	0.442	0.045	3.13005E-12	14
Ccm2l	0.698079473	0.173	0.001	4.48008E-12	14

Dll4	0.67432472	0.192	0.002	4.51421E-12	14
Rtn1.13	-1.897727227	0.173	0.691	4.95628E-12	14
Stmn3.8	-1.735627239	0.077	0.623	5.5789E-12	14
Tspan13.5	1.284689337	0.654	0.205	6.39261E-12	14
Nfia.5	-1.433453164	0.231	0.775	6.58636E-12	14
Map7	0.866638138	0.25	0.006	7.10927E-12	14
Draxin.10	-1.775121421	0.058	0.555	7.28516E-12	14
Cpe.10	1.116957453	0.769	0.454	9.93157E-12	14
Tuba1a.10	-1.182047813	0.712	0.93	1.31847E-11	14
Tubb5.11	-0.975658438	0.712	0.915	1.62556E-11	14
Ackr3	0.751486217	0.212	0.003	1.72834E-11	14
Grrp1	0.771867267	0.173	0.001	1.77617E-11	14
Arhgef5	0.598517032	0.154	0.001	2.33097E-11	14
Cd24a.7	-1.655691153	0.096	0.635	2.432E-11	14
Fam212a.1	0.768129618	0.192	0.002	2.63557E-11	14
Ddah2.9	-1.66262659	0.173	0.687	2.79491E-11	14
Vamp8.3	0.916417872	0.288	0.012	3.02609E-11	14
Slc35f2	0.929982074	0.173	0.002	3.1716E-11	14
Notch4	0.975100185	0.288	0.012	3.73177E-11	14
Kif26a	0.929338129	0.173	0.001	3.93927E-11	14
Tjp1.1	1.26319693	0.462	0.072	4.67224E-11	14
Fkbp10.2	0.897070527	0.25	0.007	4.85493E-11	14
Igfbp4.3	1.383932497	0.308	0.018	4.97192E-11	14
Hbegf.2	1.154484707	0.327	0.02	6.32369E-11	14
H2-D1.4	1.245019065	0.577	0.129	7.69705E-11	14
Plod1.2	0.920851466	0.346	0.023	8.61116E-11	14
Timp3.5	1.497633352	0.462	0.078	8.77454E-11	14
Rras.1	0.872276759	0.269	0.01	9.55463E-11	14
Abhd2.1	1.124503254	0.365	0.03	9.65067E-11	14
Rell1.2	1.314004611	0.481	0.094	1.11147E-10	14
Trim16	0.576882265	0.154	0.001	1.15327E-10	14
Acadl.3	1.157678553	0.462	0.063	1.20709E-10	14
Tubb6.1	1.036183846	0.308	0.02	1.21614E-10	14
Bex2.3	-1.614422901	0.077	0.572	1.24372E-10	14
Il10rb.1	0.694179506	0.231	0.005	1.37441E-10	14
Msrb3	0.677909918	0.173	0.001	1.38981E-10	14
Ifnar2.3	1.198134655	0.404	0.049	1.61715E-10	14
Cd97.1	0.852714032	0.212	0.004	2.05347E-10	14
Cd200.4	1.248805622	0.365	0.044	2.58048E-10	14
Rgs5.2	1.977651058	0.269	0.013	2.69826E-10	14
Rnf144b	0.828254773	0.192	0.003	3.05153E-10	14
Tgfbr2.2	0.811655711	0.25	0.008	3.16424E-10	14
Actn4.1	1.204011438	0.577	0.174	3.28526E-10	14
Bcl6b	0.675896488	0.212	0.004	3.72523E-10	14
Klf6.6	1.256373864	0.596	0.147	4.31192E-10	14
Dennd3	0.701153267	0.173	0.002	4.37757E-10	14
Enpp2.2	1.204826625	0.365	0.031	4.70936E-10	14
Cyr61.2	1.304824463	0.288	0.018	5.2494E-10	14

Prom1	1.176818711	0.288	0.025	5.5785E-10	14
Arhgef15	0.679097949	0.173	0.002	5.64839E-10	14
Plxnd1	0.907723194	0.269	0.011	6.5639E-10	14
Sorbs2	1.012220282	0.327	0.026	6.71383E-10	14
Rgs3	0.643761391	0.173	0.002	8.49428E-10	14
Itga4	0.934963554	0.25	0.009	8.92949E-10	14
Pear1.1	0.672687402	0.173	0.002	9.17819E-10	14
Pde8a	0.587164705	0.154	0.001	9.8565E-10	14
Gja1.2	1.08397114	0.308	0.019	1.01317E-09	14
Gatm.4	1.180475803	0.365	0.034	1.06445E-09	14
Sult1a1.1	0.875819845	0.192	0.003	1.06803E-09	14
Mpzl1.2	1.163051023	0.538	0.143	1.0694E-09	14
Dcbld1.1	1.048332912	0.308	0.024	1.13664E-09	14
Chp2.1	0.527734945	0.173	0.002	1.18547E-09	14
Arhgap25.1	0.609217683	0.173	0.002	2.09616E-09	14
Lyn.2	0.818778022	0.25	0.01	2.12197E-09	14
Rapgef4.1	0.777719608	0.25	0.01	2.25336E-09	14
Aqp11	0.963986713	0.192	0.006	2.5452E-09	14
Elk3.1	0.868387133	0.308	0.02	3.34496E-09	14
Gria2.12	-1.840543062	0.038	0.522	3.43282E-09	14
Celf2.12	-1.603192556	0.154	0.639	3.79846E-09	14
E130114P18Rik.8	-1.679553396	0.096	0.586	4.44829E-09	14
Necap2.3	1.102916963	0.385	0.044	4.64422E-09	14
Agrn.3	1.073690007	0.462	0.074	5.64197E-09	14
Slc31a1	1.170335483	0.462	0.115	7.95028E-09	14
Mfsd7c	0.521352194	0.154	0.001	8.82649E-09	14
Tgfb1i1.1	0.731310033	0.212	0.006	1.13195E-08	14
Marc2.4	1.120138751	0.462	0.082	1.17228E-08	14
Gatsl3	0.615770818	0.154	0.001	1.24807E-08	14
Anxa5.3	1.092675985	0.288	0.019	1.27972E-08	14
Crip2.6	1.02259962	0.654	0.238	1.31599E-08	14
Cast.1	0.725650573	0.25	0.011	1.65189E-08	14
Msn.2	1.153675172	0.442	0.092	1.68772E-08	14
Hhex.1	0.946276766	0.135	0.002	1.79805E-08	14
Slc46a3.1	0.485592945	0.173	0.003	1.8111E-08	14
Nnat.10	-1.523318981	0.231	0.711	1.81268E-08	14
Sipa1.2	0.910996542	0.231	0.009	1.89686E-08	14
Gm6977.1	1.111855209	0.269	0.031	2.0938E-08	14
Pttg1ip.4	1.108426017	0.462	0.085	2.10239E-08	14
Ccdc85b	1.113252992	0.442	0.075	2.3186E-08	14
Rpl38.4	0.982337582	0.692	0.381	2.33031E-08	14
Megf6	0.632428244	0.192	0.004	2.45422E-08	14
Luzp1.1	1.056431878	0.558	0.134	2.54427E-08	14
Hnrnpab.8	-1.054456907	0.481	0.816	2.60166E-08	14
Lrp10.3	1.031559752	0.365	0.04	2.84338E-08	14
Adm	0.603479076	0.154	0.002	3.01754E-08	14
Rgl1.1	1.017394082	0.192	0.007	3.14092E-08	14
Pcdh1	0.470298692	0.173	0.004	3.15165E-08	14

Chd7.10	-1.436580759	0.173	0.648	3.19838E-08	14
Procr	0.494515168	0.115	0	3.35921E-08	14
Cog7.10	-1.51698382	0.096	0.585	3.563E-08	14
Ngfrap1.3	-1.329361771	0.231	0.659	4.05168E-08	14
Aplp2.8	1.026342916	0.692	0.322	4.41764E-08	14
Cdh11.3	1.036867619	0.25	0.015	4.48609E-08	14
Shisa5.3	0.787453939	0.308	0.024	4.87396E-08	14
Cdk4.7	-1.293038499	0.25	0.643	5.58196E-08	14
Slc16a9.1	0.74418428	0.173	0.003	5.68913E-08	14
S100a16.8	1.124771496	0.5	0.093	5.73185E-08	14
Calm2.11	-0.907928339	0.692	0.874	6.70292E-08	14
Ppapdc2	0.784810689	0.25	0.012	6.72572E-08	14
Sorbs3	0.590616267	0.192	0.005	7.05287E-08	14
Insr	1.139472426	0.365	0.051	7.35356E-08	14
Pon2.3	1.085690131	0.327	0.038	7.59096E-08	14
Gnai2.8	0.94040648	0.712	0.423	9.08385E-08	14
Sypl.2	0.991178096	0.442	0.073	9.27511E-08	14
Tmsb10.9	0.838063015	0.827	0.663	9.50864E-08	14
Map4k2	0.886353217	0.269	0.017	1.00675E-07	14
Ttc3.10	-1.01069707	0.5	0.83	1.14917E-07	14
Apbb2.1	1.00978662	0.346	0.038	1.59876E-07	14
Slc52a2	0.851455982	0.308	0.026	1.61253E-07	14
St6galnac2	0.711418141	0.135	0.002	1.75629E-07	14
Arap2.1	0.798061931	0.212	0.009	1.89205E-07	14
Myl6.1	1.069914454	0.558	0.201	1.96431E-07	14
Xaf1	0.493811745	0.135	0.001	2.61491E-07	14
Ostf1.3	1.16189095	0.385	0.062	2.80588E-07	14
Ccnd2.10	-1.38040638	0.212	0.663	3.28852E-07	14
Podxl	0.736746804	0.192	0.006	3.60703E-07	14
Sdpr.1	1.25951911	0.442	0.086	3.92941E-07	14
Ctnna1.4	1.100758247	0.519	0.151	4.2821E-07	14
Slc16a4	0.566809622	0.154	0.002	4.3766E-07	14
Sgk1.2	0.918475299	0.327	0.033	4.47476E-07	14
Ppp2r2c.7	-1.422640155	0.115	0.506	4.57508E-07	14
Abcg1.4	0.946327306	0.288	0.023	4.61558E-07	14
Gnb4.4	1.168390532	0.423	0.104	4.84453E-07	14
Ecm1.1	0.697726709	0.173	0.004	5.05879E-07	14
Plekhg5	0.605800514	0.154	0.002	5.31248E-07	14
Capg.1	0.537269245	0.173	0.004	5.81999E-07	14
Scg3.6	-1.495416811	0.019	0.447	6.03499E-07	14
Cav1.1	0.899109276	0.25	0.019	7.34367E-07	14
Dpysl4.8	-1.467503621	0.096	0.491	7.71833E-07	14
Tram2	0.587362624	0.154	0.002	7.83758E-07	14
Paqr7.2	0.800601486	0.173	0.005	7.86887E-07	14
Ankrd37	0.991032806	0.25	0.018	8.09873E-07	14
Gimap8	0.507123646	0.135	0.001	8.27155E-07	14
Gja4.1	0.734104937	0.135	0.001	8.44375E-07	14
Fermt2.4	1.09679566	0.577	0.204	1.02365E-06	14

Prcp.1	0.992895076	0.308	0.036	1.18838E-06	14
Foxc1.1	0.721004393	0.192	0.007	1.40685E-06	14
Rasgrp2.1	0.847787735	0.192	0.008	1.44758E-06	14
2900026A02Rik	0.830940194	0.212	0.011	1.65279E-06	14
Mecom	0.393402714	0.115	0.001	1.70027E-06	14
Tmc7	0.660269938	0.154	0.003	2.11484E-06	14
Atpif1.4	-0.902415509	0.538	0.816	2.29551E-06	14
Pcp4l1.1	0.633325271	0.173	0.005	2.39887E-06	14
Miat.12	-1.656472359	0.115	0.516	3.548E-06	14
Maoa.2	0.991557502	0.385	0.067	3.58659E-06	14
Ttll7	0.865262827	0.212	0.01	4.35706E-06	14
Wwc2.1	0.599225895	0.25	0.019	4.80877E-06	14
Hnrnpdl.5	-0.814420608	0.423	0.756	5.10394E-06	14
Anp32a.4	-0.796170596	0.654	0.837	5.46464E-06	14
Gm9917	0.570498308	0.154	0.003	5.73372E-06	14
Inf2	0.495867226	0.154	0.004	6.2957E-06	14
Anp32e.13	-1.273876882	0.269	0.614	6.55653E-06	14
Gap43.12	-1.50802654	0.192	0.6	6.67427E-06	14
H2afv.12	-1.153960619	0.346	0.729	7.5709E-06	14
Hexb.1	0.274228379	0.231	0.032	7.71072E-06	14
Casp6	0.725523197	0.269	0.023	8.35441E-06	14
Higd1b.1	0.90190368	0.135	0.002	8.66387E-06	14
Cdc42ep3	1.145341934	0.385	0.089	9.07077E-06	14
Macf1.5	1.092796118	0.558	0.209	1.05909E-05	14
Hrct1.1	0.322985942	0.115	0.001	1.2728E-05	14
Jak2	0.946238575	0.269	0.03	1.28168E-05	14
Gmpr.2	0.729669104	0.212	0.012	1.30039E-05	14
Lamb2.2	0.858358541	0.192	0.011	1.34404E-05	14
Cd38.1	0.658347364	0.135	0.002	1.40249E-05	14
Gm1673.6	-1.341464606	0.077	0.474	1.40924E-05	14
Prex2.1	0.882820063	0.212	0.013	1.41877E-05	14
Prr5l.1	0.549904779	0.154	0.004	1.47602E-05	14
Prkd2	0.817591568	0.269	0.024	1.55894E-05	14
Loxl2.1	0.494185421	0.154	0.004	1.60114E-05	14
Tubb2b.12	-1.378030481	0.058	0.465	1.6653E-05	14
Exoc3l	0.802476854	0.212	0.012	1.69258E-05	14
Filip1l.1	0.681999832	0.192	0.009	1.71593E-05	14
5730508B09Rik	0.428283834	0.135	0.002	1.74733E-05	14
Mfng.1	0.817020024	0.269	0.028	1.74955E-05	14
Itih5.2	0.566979763	0.192	0.014	1.75245E-05	14
Slc31a2	0.664498646	0.212	0.014	1.76693E-05	14
Arap3	0.555499494	0.115	0.001	1.92787E-05	14
Cxcl12.2	1.566275325	0.212	0.043	1.98262E-05	14
Lhx1.11	-1.410725079	0.077	0.498	2.15192E-05	14
Tpm1.4	1.011317405	0.577	0.189	2.22555E-05	14
Rbfox3.11	-1.429493729	0.077	0.495	2.31226E-05	14
Tes	0.77789411	0.192	0.009	2.74983E-05	14
Abca1.3	0.785842715	0.25	0.023	2.91655E-05	14

Ifnar1.2	0.910380508	0.442	0.095	2.92287E-05	14
Kank2.1	0.486609616	0.173	0.007	3.05936E-05	14
Sat1.3	1.126303701	0.365	0.066	3.4621E-05	14
Cbx5.11	-1.156206264	0.288	0.654	3.4907E-05	14
Prnp.9	0.953904005	0.596	0.205	3.58856E-05	14
Cd9.5	-1.284028009	0.135	0.485	4.06804E-05	14
Ldlrap1.1	0.633889595	0.135	0.003	4.09061E-05	14
Ptp4a3	0.602174097	0.192	0.009	4.09772E-05	14
Myl4	0.582393957	0.192	0.009	4.48066E-05	14
Psip1.7	-1.046421301	0.308	0.688	4.82411E-05	14
Snrk.1	1.004714999	0.308	0.048	5.26613E-05	14
Piezo1	0.597858437	0.154	0.004	5.79282E-05	14
Tnfaip1.1	1.011952097	0.423	0.106	6.03807E-05	14
Rps9.7	-0.500877952	0.962	0.929	6.15268E-05	14
Barhl1.12	-1.328944612	0.115	0.522	6.22067E-05	14
Apod.1	1.192243855	0.288	0.041	6.42015E-05	14
Prdm1	0.435063178	0.115	0.001	6.89842E-05	14
Mapk12	0.661936627	0.135	0.003	8.2772E-05	14
Pmepa1.2	0.949469717	0.25	0.026	8.37976E-05	14
Neurod1.14	-1.704516054	0.115	0.534	8.48918E-05	14
Phactr2.1	0.590667486	0.212	0.016	9.32841E-05	14
Ncam1.6	-1.286877656	0.038	0.388	9.87451E-05	14
Spata6	0.392724555	0.154	0.005	0.000104992	14
Rap1b.2	0.841598739	0.5	0.133	0.000106812	14
Lcp1.1	0.359778565	0.135	0.005	0.000108332	14
Map2.11	-1.368965018	0.115	0.492	0.000112801	14
Ccdc85a	0.612962333	0.154	0.005	0.000116909	14
Hn1.10	-1.221301859	0.192	0.542	0.000117097	14
Id2.10	-1.451908679	0.096	0.487	0.000125905	14
Ifi27.3	0.668649516	0.212	0.014	0.000126806	14
Tpx2.14	-1.279337958	0.038	0.282	0.000131006	14
Arhgef12.1	0.981870265	0.462	0.124	0.000134686	14
Pde1c.14	-1.35997911	0.058	0.387	0.000137611	14
Syt11.10	-1.297820059	0.115	0.505	0.000140016	14
Pfn1.6	0.771693314	0.75	0.51	0.000143024	14
Tacc1.3	0.983011692	0.346	0.069	0.000150882	14
Tubb3.13	-1.601824503	0.096	0.475	0.000166237	14
Ctsl.6	0.908688633	0.635	0.259	0.000169297	14
Psmb8.1	0.767569038	0.154	0.006	0.000169877	14
Arhgef28	0.589767543	0.154	0.005	0.000171056	14
Tm6sf1.1	0.718008068	0.212	0.016	0.000174262	14
Klhl5.3	0.669146914	0.288	0.038	0.000193155	14
Enc1.1	0.498627132	0.192	0.014	0.000193384	14
Bgn.1	0.807328724	0.154	0.006	0.000205455	14
Sept3.14	-1.470021248	0.038	0.393	0.000218279	14
Snx3.2	0.792295096	0.692	0.341	0.000219512	14
Pdlim5.2	0.503618045	0.192	0.014	0.000223868	14
Apba2.5	-1.239122739	0.077	0.415	0.000228341	14

Slc38a2.1	0.943943034	0.519	0.219	0.000232552	14
Fkbp3.9	-0.968724815	0.423	0.746	0.000245136	14
Ubtd1.3	0.623475122	0.154	0.005	0.000246708	14
M6pr.2	0.933608527	0.423	0.108	0.000257016	14
Cpt1a.3	0.630220017	0.212	0.017	0.000261085	14
Gabarapl1.7	0.982049042	0.538	0.215	0.000262412	14
Isyna1.2	0.636278596	0.173	0.01	0.000273833	14
Meis1.6	-1.282445746	0.019	0.373	0.000274684	14
Plekhg1	0.461749048	0.135	0.005	0.000309372	14
Cstb.3	0.959732085	0.442	0.116	0.000310995	14
Kif20b.13	-0.533972249	0.038	0.191	0.000318536	14
Ezh2.12	-0.918922942	0.231	0.61	0.000318726	14
Efna1	0.523871093	0.154	0.006	0.000362736	14
Soga3.9	-1.272492148	0.096	0.458	0.000389803	14
Stmn2.13	-1.578219142	0.192	0.554	0.000391602	14
Gm14005.1	0.547072406	0.115	0.002	0.000407773	14
Col4a3bp	0.799582351	0.346	0.065	0.00048774	14
Kif21a.5	-1.32096465	0.019	0.346	0.000536315	14
Ank3.13	-1.098523417	0.058	0.421	0.000542429	14
Fam13a	0.920830973	0.192	0.014	0.000557588	14
Mfge8.3	0.827100609	0.269	0.033	0.000577017	14
Fry	0.750833894	0.25	0.027	0.00057721	14
2810417H13Rik.13	-1.116780782	0.077	0.392	0.000580266	14
Ndrg1	0.439058591	0.135	0.005	0.000588867	14
Slc6a6.2	1.050092416	0.269	0.044	0.000670705	14
Klf4.2	0.969568288	0.212	0.023	0.000703614	14
Jam2.2	0.970646195	0.231	0.032	0.000707155	14
Hspa1a	0.583484182	0.115	0.002	0.000707216	14
Cdc42ep2.1	0.58807997	0.154	0.006	0.000711347	14
Sash1.4	0.837458675	0.269	0.034	0.000746839	14
Tcn2.3	0.708187733	0.212	0.019	0.00080089	14
Clip1.4	0.89791302	0.404	0.093	0.000807827	14
Cyba.3	0.772715402	0.25	0.029	0.000819851	14
Ptprg.4	0.924189688	0.462	0.141	0.000826936	14
Alas1.1	0.704266764	0.327	0.056	0.000833556	14
Alad.1	0.847636917	0.269	0.039	0.000854987	14
Sft2d1.1	0.912306401	0.192	0.022	0.000865827	14
Ddx58	0.351543908	0.115	0.002	0.000926445	14
Mal	0.473738786	0.115	0.003	0.000927053	14
Tagln3.13	-1.225117013	0	0.295	0.001062114	14
Dclk1.9	-1.305743803	0.077	0.413	0.001113248	14
Zic4.6	-1.232147328	0.058	0.419	0.001158816	14
Usp6nl.1	0.770550866	0.25	0.032	0.001177585	14
H2-K1.3	0.721658444	0.192	0.014	0.001231766	14
Cd2ap.1	1.016198211	0.423	0.158	0.001286593	14
Flnb	0.659265544	0.192	0.014	0.001359732	14
Slc7a8.1	0.573770357	0.135	0.005	0.001367394	14
Rhobtb1	0.634948657	0.154	0.008	0.001375639	14

Fxyd6.6	-1.211834274	0.135	0.51	0.001490732	14
Ddx5.6	-0.659555225	0.673	0.816	0.00158369	14
Hsd3b7	0.672738063	0.173	0.01	0.001592634	14
Hdac2.5	-1.090409391	0.173	0.516	0.001621465	14
Prdx2.4	-0.770324257	0.423	0.68	0.001648682	14
Vasp.2	0.779792483	0.346	0.066	0.001663797	14
Tsc22d3.1	0.912845576	0.365	0.091	0.001937614	14
Mxd4.7	0.924828252	0.5	0.197	0.001938592	14
Extl3.2	0.769491997	0.327	0.058	0.002014914	14
Fkbp1a.2	0.73296253	0.673	0.451	0.002183952	14
Serpinb9	0.429535936	0.115	0.002	0.002347664	14
Mdk.10	-1.331100084	0.058	0.38	0.002366491	14
Gpm6a.13	-1.3709689	0.058	0.41	0.002400864	14
Col1a2.2	0.517940469	0.154	0.014	0.00252259	14
Nsg2.11	-1.06753353	0.115	0.488	0.002726992	14
Pvrl2	0.678097375	0.192	0.016	0.002786117	14
Wasf2.5	0.947362009	0.346	0.112	0.00279769	14
Fabp5.6	-1.00474834	0.058	0.404	0.00288823	14
4632428N05Rik.1	0.388825488	0.135	0.006	0.003023525	14
Dynlt3.4	0.814968357	0.288	0.045	0.003065351	14
Ddx39b.6	-1.080295452	0.135	0.445	0.003079809	14
Ina.12	-1.282291214	0.096	0.444	0.003255593	14
Grb10.1	0.838371649	0.231	0.028	0.003299087	14
Hopx.1	0.577547704	0.154	0.008	0.003479477	14
Oxct1.3	-1.048455457	0.192	0.553	0.003615217	14
Tenc1.1	0.631844495	0.154	0.008	0.003638462	14
Pax6.11	-1.057741171	0.135	0.514	0.003827758	14
Stx3	0.318627817	0.115	0.004	0.003886254	14
Rab11a.2	0.735790455	0.635	0.276	0.004023712	14
Nudt14.1	0.652809851	0.192	0.019	0.004068276	14
Pou3f2.9	-1.090993446	0	0.276	0.004082223	14
H2afy2.6	-1.048370236	0.019	0.338	0.004212366	14
App.10	0.685792493	0.808	0.567	0.004261944	14
Cacng2.5	-1.188193078	0.019	0.339	0.004470682	14
Cp.3	1.071138592	0.173	0.013	0.004797349	14
Cebpd.1	0.611092788	0.173	0.011	0.004852341	14
Flii	0.789526044	0.308	0.054	0.005037496	14
Cd320.1	0.949836859	0.327	0.085	0.005169292	14
Kctd12b	0.762496096	0.212	0.025	0.00519422	14
P4hb.4	0.787349613	0.519	0.278	0.005386724	14
Tfpi.2	0.519844816	0.154	0.008	0.0056398	14
Tprgl.2	0.770053432	0.423	0.123	0.00591848	14
Slc38a1.4	-1.011982984	0	0.27	0.006089829	14
RP23-45G16.5.14	-1.299523333	0.019	0.327	0.00637182	14
Lhfpl2.3	0.765894716	0.231	0.027	0.007401246	14
Cmtm6.1	0.546902316	0.212	0.023	0.007579393	14
Arrdc1.1	0.567007062	0.154	0.009	0.007649997	14
Hsp90aa1.5	-0.918573821	0.25	0.617	0.007894485	14

Gstm1.3	0.890547331	0.346	0.081	0.008462829	14
Gm2694.6	-1.076923882	0.038	0.359	0.008748972	14
Cntn2.13	-1.502852265	0	0.264	0.009631844	14
1700025G04Rik.8	-1.063581726	0	0.264	0.00974419	14
Rbp4.11	-0.864743762	0.038	0.242	0.010113675	14
Adam10.4	0.866813995	0.5	0.202	0.011009873	14
Vps37b.7	-0.884729227	0.115	0.471	0.011198833	14
Itsn2.2	0.802387429	0.365	0.085	0.011298067	14
Lnx2	0.386796273	0.115	0.004	0.011684176	14
Ptprm	0.629821502	0.115	0.006	0.011825673	14
Fgf9.4	-1.033907633	0	0.261	0.011908058	14
Tjp2.1	0.695394881	0.288	0.051	0.011980981	14
Mdh1.2	-1.037856892	0.154	0.453	0.012550351	14
Ddah1.2	0.622741952	0.25	0.039	0.0133641	14
Apoe.14	0.576579424	0.577	0.248	0.013671611	14
Ccdc34.12	-1.17672691	0.115	0.415	0.013692275	14
Tspan9.2	0.6475547	0.154	0.009	0.014162731	14
Mycn.8	-1.135685364	0.058	0.364	0.014963858	14
Ptpn18.1	0.275302285	0.115	0.007	0.015675878	14
Nhlh2.12	-1.285711452	0.115	0.423	0.015965983	14
Casp8.1	0.355608472	0.115	0.003	0.016337707	14
Pcdh19	0.615357228	0.173	0.014	0.016454639	14
Nrxn1.12	-1.333695978	0.019	0.318	0.01664599	14
Trf.3	0.887827783	0.25	0.037	0.016846736	14
Lphn2.1	0.789983719	0.327	0.069	0.01686687	14
Ppic.8	0.833048591	0.538	0.238	0.016887312	14
1500016L03Rik.10	-1.132499101	0.058	0.384	0.017799892	14
Fcho2.2	0.848343016	0.327	0.076	0.019502053	14
Kctd10.1	0.777877659	0.288	0.053	0.019528438	14
Creb3l2.1	0.608317995	0.154	0.011	0.019612519	14
Ltbp4.1	0.868704281	0.212	0.035	0.020710679	14
Gpx1.4	0.721433989	0.673	0.381	0.021066659	14
Lxn.1	0.938223254	0.25	0.052	0.022080065	14
Tcf7l1	0.496090572	0.154	0.01	0.022560997	14
Gm17750.8	-1.18905175	0.058	0.368	0.022847985	14
Entpd1.1	0.347580307	0.115	0.005	0.024042748	14
Ezr.9	-1.009416985	0.096	0.439	0.02440234	14
Smad1	0.872150434	0.423	0.143	0.025051668	14
Gng3.13	-1.281871747	0.038	0.317	0.025157406	14
Ppp1r13b	0.387053563	0.173	0.023	0.025282435	14
Itm2b.12	0.598679426	0.885	0.642	0.025640925	14
Iqgap1.2	0.813378155	0.423	0.13	0.025999711	14
Ctnnb1.7	0.653093494	0.692	0.385	0.027232872	14
Sfrs18.8	-0.767029879	0.577	0.778	0.027736091	14
Rnd3.9	-1.08420852	0.096	0.432	0.027776862	14
Grasp	0.6684358	0.192	0.02	0.027874235	14
Ctnnbip1	0.719027243	0.365	0.098	0.029058851	14
Pomp.2	0.709963621	0.673	0.413	0.029601358	14

Dusp6.5	0.86409349	0.346	0.092	0.029813228	14
Rgs4.1	0.859974289	0.115	0.005	0.031205431	14
Rac1.3	0.712789741	0.673	0.345	0.032223663	14
Lpar6.1	0.861819628	0.269	0.071	0.03249496	14
Usp22.7	-1.049711357	0.077	0.362	0.033194295	14
Prrg2	0.821404654	0.212	0.029	0.036289641	14
Sh3bp5.6	0.80924438	0.385	0.102	0.037129115	14
Prkcdbp.2	0.830513403	0.308	0.089	0.037598449	14
Tecr.2	-0.929222577	0.25	0.53	0.038610891	14
Nasp.12	-0.883117492	0.327	0.641	0.039377776	14
Ift27.6	-0.862805392	0.038	0.243	0.039649843	14
Pnn.3	-0.818144334	0.462	0.722	0.039871376	14
Clmp.10	-1.097679418	0	0.243	0.040441354	14
Maged1.2	-0.993768001	0.154	0.43	0.040737492	14
H3f3b.5	-0.580261679	0.769	0.907	0.040947234	14
Foxo1	0.762762131	0.192	0.026	0.04096502	14
Serpine2.4	0.896973479	0.288	0.056	0.043869746	14
Ptprs.11	-1.192049269	0.115	0.422	0.046672766	14
Lpcat3.3	0.530965317	0.25	0.043	0.047129005	14
Net1	0.715135391	0.231	0.036	0.047287677	14
Slc1a2.10	-1.063100424	0.058	0.294	0.047463048	14
Fam43a.1	0.791691771	0.288	0.062	0.048837503	14
Lhfp.3	0.339345124	0.154	0.019	0.049772821	14
Insm1.7	-0.940783411	0.019	0.293	0.052597718	14
Abhd17a	0.846011634	0.404	0.13	0.053937284	14
D430041D05Rik.9	-0.968024394	0.154	0.493	0.055149508	14
Lrrfip1.3	0.800179754	0.25	0.043	0.055575297	14
Junb.3	0.755062502	0.231	0.034	0.055830021	14
Racgap1.14	-0.952128903	0.038	0.256	0.057073311	14
Cks1b.13	-1.013638375	0.115	0.345	0.058972705	14
Tbx1	0.657314109	0.154	0.014	0.060895874	14
Hnrnpu.7	-0.582653583	0.731	0.865	0.063133862	14
Nsg1.10	-1.042245571	0.038	0.288	0.063619209	14
Srpr.1	0.72898177	0.365	0.096	0.064627715	14
Phf21a	-0.670590212	0.038	0.19	0.072357631	14
Uncx.12	-1.115343728	0.038	0.318	0.073063214	14
Mkl2	0.425856852	0.154	0.013	0.075875135	14
Lpp.3	0.679140308	0.173	0.022	0.076022805	14
Serf1.7	-1.037253345	0.019	0.281	0.079957278	14
Tspan5.3	0.589388927	0.519	0.195	0.080866749	14
Myo1c	0.396100121	0.115	0.005	0.082576116	14
Gng5.4	0.82161007	0.404	0.164	0.084704372	14
Raph1	0.563407381	0.154	0.012	0.087007978	14
Orai1.3	0.539700816	0.192	0.023	0.088542856	14
Nrp2.1	0.3940604	0.115	0.005	0.088727443	14
Cenpv.8	-0.945750818	0.038	0.329	0.08988329	14
Rsrc1.3	-0.774803212	0.058	0.251	0.090853672	14
Fkbp9.2	0.687960758	0.212	0.03	0.094266643	14

Gpm6b.9	-0.942497531	0.154	0.485	0.094611745	14
Cttnbp2nl.1	0.837647502	0.288	0.067	0.096591555	14
Prox1.5	-0.93000791	0	0.229	0.096712908	14
Dcaf6.1	0.684509693	0.327	0.08	0.097370734	14
Litaf.3	0.838076889	0.269	0.068	0.101405268	14
Slc25a4.4	-0.604448556	0.692	0.807	0.102430884	14
Ppp1r16b.1	0.588343258	0.115	0.005	0.109272085	14
Tbata.11	-1.183190613	0.058	0.34	0.115433859	14
Plcb4.4	0.952109434	0.365	0.138	0.115551814	14
2700094K13Rik.13	-0.879910855	0.308	0.615	0.11584737	14
Cxxc5.7	-0.93652334	0.269	0.544	0.118733828	14
Rcan3	0.679846236	0.135	0.022	0.122551102	14
Elovl6.5	-0.976389331	0.038	0.278	0.123664557	14
Mex3a.7	-0.97219593	0.154	0.464	0.126124417	14
mt-Rnr1.1	0.772768394	0.577	0.366	0.128564422	14
Fam171b.4	-0.920190064	0	0.224	0.134451572	14
Sox4.11	-0.790044882	0.462	0.583	0.138493311	14
Hnrnph1.4	-0.800962981	0.346	0.629	0.141084157	14
Nhlh1.11	-1.055633935	0	0.223	0.14149061	14
Snrpd3.6	-0.900431047	0.25	0.535	0.14730408	14
Adipor1.3	0.825164992	0.385	0.139	0.150281312	14
Hsd11b2.12	-1.059718327	0.019	0.288	0.152212801	14
Tmem44	0.67483745	0.231	0.036	0.153181706	14
Kit.1	0.707788636	0.173	0.022	0.15332953	14
Fez1.5	-0.997776836	0.077	0.333	0.154935237	14
Pdxk.2	0.680226829	0.154	0.016	0.158436207	14
Irf2.1	0.72758735	0.269	0.056	0.159556648	14
Tmpo.13	-0.794609207	0.288	0.452	0.160514755	14
Eif4a1.5	-0.866425968	0.231	0.485	0.16215218	14
Pitpnc1.4	0.443447547	0.288	0.074	0.162321232	14
Pea15a.11	0.733609811	0.481	0.175	0.163434786	14
Calm3.5	-0.833912524	0.327	0.561	0.163495904	14
Phldb2.1	0.765356112	0.269	0.055	0.16414517	14
Hpca.11	-0.957899047	0.019	0.277	0.171407264	14
Pxn.1	0.719792197	0.231	0.039	0.17675577	14
Rbfox2.9	-0.961740213	0.077	0.283	0.182261549	14
Fam13c.1	0.840603388	0.269	0.061	0.18255745	14
Ilf2.5	-0.848560381	0.25	0.542	0.191549195	14
Hmgn1.10	-0.796350477	0.365	0.622	0.195427955	14
Cbx1.6	-0.74363629	0.462	0.686	0.197750601	14
Arhgdia.2	0.678251161	0.519	0.207	0.20295865	14
Rilpl1	0.634795288	0.25	0.046	0.203511803	14
Lats2	0.348128674	0.154	0.018	0.210044381	14
Ppard	0.39570264	0.135	0.01	0.212582847	14
Ssbp3.6	-0.670695417	0.038	0.167	0.220333242	14
Ltbr.2	0.40911587	0.154	0.016	0.223728065	14
Ets2	0.751477219	0.231	0.045	0.227430913	14
Actn1.2	0.77508849	0.269	0.063	0.229634763	14

Tspan12.2	0.542105434	0.212	0.031	0.230627808	14
Hes1.12	0.882742068	0.423	0.147	0.232196088	14
Slc16a2.1	0.793668525	0.231	0.068	0.235673357	14
Map3k1.5	-0.763018771	0.077	0.371	0.238456545	14
H1f0.9	-0.900783274	0.327	0.617	0.241247197	14
Clip3.9	-0.959012046	0.038	0.312	0.243700818	14
Ybx1.7	-0.5371157	0.712	0.786	0.256524263	14
Scg5.9	-1.027799879	0.058	0.335	0.256663286	14
Elavl4.13	-1.130737638	0.058	0.335	0.2589233	14
Msh2.3	-0.427710636	0.038	0.143	0.27220187	14
Cmtm8	0.58752205	0.154	0.015	0.273249473	14
Fut9.6	-0.919498464	0	0.212	0.2924834	14
Gbp7.1	0.451424148	0.135	0.01	0.29921911	14
Srebf1.12	-0.917905528	0.077	0.334	0.306518904	14
Map1b.12	-1.029054738	0.327	0.588	0.316096847	14
S100a11.2	0.392820833	0.154	0.016	0.320642237	14
Snap25.10	-0.986759491	0.038	0.282	0.327308162	14
Pitpna.2	0.724603446	0.404	0.131	0.330319072	14
Degs1.4	0.668747623	0.308	0.075	0.334514391	14
Galnt18.2	0.460035181	0.135	0.01	0.334742114	14
Sf3b2.3	-0.672676275	0.327	0.626	0.343772356	14
Map3k11	0.500476995	0.154	0.015	0.353621311	14
Cdk17	0.600963103	0.269	0.057	0.359617879	14
Cyb5r3.3	0.729432387	0.462	0.198	0.360470198	14
Gpr153.8	-0.874855791	0	0.209	0.363732394	14
Pcm1.4	-0.86770662	0.308	0.522	0.371118121	14
Top1.5	-0.713967593	0.481	0.663	0.375331854	14
Fam63b.4	0.742916841	0.327	0.087	0.378502604	14
Triobp	0.683175594	0.231	0.042	0.381064858	14
Capns1.4	0.729854009	0.519	0.227	0.382346024	14
Fubp1.6	-0.836484075	0.308	0.567	0.383662833	14
Glul.5	0.772988022	0.481	0.2	0.386842573	14
Whsc1.6	-0.943709009	0.192	0.465	0.39525399	14
Trim2.5	-1.017202925	0.038	0.283	0.407469259	14
Ldlrad3	0.346718085	0.115	0.007	0.409773927	14
Mef2a.4	0.818108189	0.385	0.138	0.411910753	14
Slc43a2.1	0.676965314	0.173	0.023	0.425846915	14
Cplx2.9	-0.882838367	0.173	0.496	0.428861287	14
Apex1.8	-0.889063383	0.173	0.452	0.452314009	14
Pfn2.8	-0.792719958	0.038	0.241	0.459092204	14
Tnfrsf1a.3	0.576508083	0.173	0.024	0.45977392	14
Adam15.1	0.498369248	0.115	0.008	0.466909918	14
H1fx.12	-0.809607903	0.038	0.245	0.474617284	14
Rnmt.9	-1.003668918	0.077	0.349	0.47794756	14
Lrrc8c	0.585242427	0.173	0.021	0.491500508	14
Tecpr1.3	0.647965294	0.308	0.077	0.49748765	14
Ebf3.7	-0.86486597	0	0.203	0.511476606	14
C1ql1.9	-0.97949649	0.038	0.279	0.513192243	14

Mllt4.5	0.549162565	0.692	0.362	0.518705547	14
Cdh20.7	-0.826265743	0	0.203	0.518762811	14
Anapc13.1	-0.869826773	0.173	0.393	0.521330482	14
Kitl	0.743949373	0.269	0.065	0.525889989	14
Srsf3.8	-0.714470168	0.423	0.657	0.53546493	14
Vtn.1	0.52922802	0.115	0.01	0.578444051	14
Myo1e	0.63341162	0.135	0.016	0.589005139	14
Hirip3.12	-1.006599032	0.115	0.383	0.590577043	14
Mef2c.2	0.7719485	0.269	0.068	0.59930637	14
Myh10.4	0.698239741	0.558	0.323	0.607863254	14
Mcm7.10	-0.872457447	0.115	0.389	0.618647248	14
H2afy.9	-0.772643576	0.308	0.592	0.620922343	14
Plxna2.2	0.719679453	0.269	0.069	0.622378427	14
Myl9.1	0.531484612	0.135	0.012	0.628192814	14
Bcas1.7	-0.978403481	0.038	0.282	0.629243646	14
Ppp1r14b.7	-0.894911258	0.173	0.397	0.651682684	14
Celf4.14	-1.282384569	0.058	0.321	0.652002792	14
Syngr2.2	0.547766387	0.173	0.022	0.661122193	14
2010111I01Rik	0.529285897	0.192	0.029	0.666002888	14
Eif2ak2.2	0.495563625	0.135	0.011	0.687243145	14
Metap2.3	-0.679742016	0.442	0.596	0.693616786	14
Maff	0.3761966	0.135	0.013	0.696188115	14
Wls.4	0.729901559	0.308	0.086	0.70014683	14
Npm1.10	-0.662236719	0.462	0.646	0.701780295	14
Arhgdib.2	0.593589917	0.115	0.008	0.703931651	14
Ccny	0.761189114	0.25	0.069	0.70778386	14
Fam210b.9	-0.878946713	0.135	0.314	0.712811376	14
Pde4b.3	0.768942984	0.212	0.05	0.713162693	14
Cdk5r1.11	-1.055093024	0.077	0.334	0.716140635	14
Dek.13	-0.723221434	0.538	0.694	0.716276108	14
Hspe1.5	-0.766995621	0.173	0.341	0.72435669	14
Mettl14.1	-0.449922773	0.038	0.123	0.737358299	14
Dtymk.11	-0.836630171	0.135	0.44	0.739394397	14
Fus.3	-0.426119908	0.615	0.737	0.747017903	14
Rhoa.3	0.619079441	0.404	0.141	0.761507009	14
Dcx.13	-0.830267766	0.096	0.376	0.766134724	14
Rassf4.7	-0.979837507	0.115	0.377	0.779732202	14
Rest.3	0.526580412	0.173	0.022	0.78478929	14
Cpd.1	0.67556603	0.269	0.062	0.794108728	14
Rps26.9	-0.514733062	0.788	0.847	0.813427652	14
Anxa6.2	0.742243773	0.231	0.06	0.839406536	14
Cdyl2	0.363569263	0.135	0.013	0.843105872	14
Homer2.6	-0.920317527	0.019	0.247	0.847893819	14
Sptan1.4	0.712435305	0.442	0.168	0.849869327	14
Gnao1.10	-0.80063998	0.058	0.287	0.863385206	14
Srgap1.1	0.623110165	0.154	0.018	0.878615662	14
Iqsec1.1	0.54010216	0.135	0.014	0.889053014	14
Srrm2.4	-0.664653529	0.5	0.732	0.893240408	14

Nomo1	0.731594959	0.212	0.04	0.905379913	14
Chgb.13	-1.118991783	0.038	0.241	0.936707364	14
Nipal3	0.284968847	0.115	0.011	0.970042149	14
Rnf44	-0.328771357	0.077	0.103	0.976818477	14
Slk.2	0.824692143	0.404	0.18	1	14
Cr1l.1	0.659730697	0.231	0.046	1	14
Pcbp4.6	-0.920274522	0.077	0.325	1	14
Myo5a.3	-0.80969393	0	0.192	1	14
Pmp22.4	0.596205314	0.154	0.017	1	14
Nxpe4	0.530974926	0.135	0.012	1	14
Sstr2.10	-0.856218642	0	0.191	1	14
Slc50a1.3	0.80183288	0.269	0.082	1	14
St18.12	-0.96339634	0	0.191	1	14
Epn2.2	0.593682359	0.308	0.083	1	14
Ssh2.2	0.397655738	0.212	0.044	1	14
Stt3b.3	0.742742886	0.423	0.211	1	14
Hdgf.10	-0.722029283	0.25	0.532	1	14
Rundc3a.8	-0.925519922	0.038	0.249	1	14
Ptbp3.3	0.649670793	0.346	0.109	1	14
Ech1.3	0.719765157	0.346	0.114	1	14
Snrnp70.3	-0.587950563	0.5	0.694	1	14
Pfkp.1	0.619151688	0.192	0.03	1	14
Dnajc3.4	0.69521254	0.442	0.175	1	14
Cdc42ep4.2	0.548365937	0.231	0.047	1	14
Rpl15.2	-0.590551442	0.038	0.161	1	14
Serbp1.10	-0.453087478	0.769	0.869	1	14
Stmn4.13	-1.099726561	0.077	0.342	1	14
Zdhhc18	0.35379295	0.135	0.014	1	14
Amotl1.1	0.622169857	0.288	0.078	1	14
Kif5c.13	-0.872033291	0.115	0.399	1	14
Sept4.11	0.606882091	0.519	0.225	1	14
Ube2e3.6	-0.712715622	0.25	0.417	1	14
Khdrbs1.5	-0.777753603	0.269	0.494	1	14
Polk	0.603669812	0.154	0.019	1	14
Thoc7.5	-0.707825896	0.25	0.522	1	14
6330403K07Rik.10	-0.979083803	0.019	0.243	1	14
Dtl.10	-0.558558421	0.038	0.145	1	14
Elavl3.11	-0.855925259	0.173	0.473	1	14
Rpl4.8	-0.430225883	0.865	0.908	1	14
Lig1.14	-1.046166462	0.096	0.349	1	14
St8sia3.5	-0.855383368	0.019	0.251	1	14
Lsm2.7	-0.653851534	0.096	0.23	1	14
Akap12.8	1.005616015	0.308	0.11	1	14
Sult4a1.4	-0.857795388	0.019	0.223	1	14
Mast4	0.378059007	0.115	0.008	1	14
Mad2l2.7	-0.783585835	0.058	0.263	1	14
Nmi	0.481961226	0.135	0.013	1	14
Sh3bgrl3.2	0.689225327	0.462	0.198	1	14

C530008M17Rik.7	-0.819881054	0.058	0.268	1	14
Pcdhga9.4	-0.751800416	0.25	0.42	1	14
Rai14	0.659959172	0.231	0.05	1	14
U2surp.3	-0.803206332	0.231	0.506	1	14
Prdx6.6	-0.892587542	0.096	0.326	1	14
As3mt.3	0.4902755	0.135	0.013	1	14
Idh3g	-0.605306606	0.212	0.308	1	14
Bnip2.2	0.697338528	0.481	0.217	1	14
Fam65a	0.473755004	0.192	0.036	1	14
Tspan4.3	0.474303206	0.25	0.065	1	14
Rufy3.11	-0.793190587	0.115	0.372	1	14
Gas5.5	-0.653706683	0.5	0.742	1	14
Tln1.5	0.780254084	0.365	0.149	1	14
Bcl2.2	0.537730657	0.192	0.032	1	14
Pdgfra.8	-0.851325974	0	0.182	1	14
Hnrnpr.4	-0.757189908	0.288	0.545	1	14
Acat2.3	-0.545832265	0.058	0.182	1	14
Gng2.8	-0.898336727	0.154	0.396	1	14
Mcf2l	0.652733511	0.192	0.032	1	14
Cacna2d1.9	-0.921184805	0.115	0.375	1	14
Elovl1.3	0.448917323	0.192	0.034	1	14
Pomc	0.464491692	0.135	0.014	1	14
Rgl2.1	0.694529921	0.346	0.114	1	14
Pde4dip.4	-0.75964414	0.038	0.221	1	14
Bzw2.4	-0.676753665	0.212	0.489	1	14
Hnrnpm.8	-0.56876152	0.577	0.735	1	14
Supt16.10	-0.779375015	0.269	0.494	1	14
Fam212b.5	-0.656313815	0.019	0.223	1	14
Rapgef6.1	0.6788414	0.385	0.139	1	14
Kif5b	0.53757636	0.635	0.521	1	14
Dner.13	-0.960141934	0.019	0.215	1	14
Sh3bp4.1	0.666701585	0.154	0.024	1	14
Srsf7.10	-0.790776309	0.212	0.466	1	14
Shank3.1	0.589566143	0.154	0.02	1	14
Crim1	0.577404785	0.154	0.021	1	14
Crem	0.487202568	0.173	0.027	1	14
Rps5.9	-0.395776928	0.923	0.954	1	14
Hip1.2	0.534194138	0.346	0.117	1	14
Ssrp1.9	-0.701429996	0.346	0.576	1	14
Ano6.3	0.617582092	0.308	0.09	1	14
Hdac7	0.438024241	0.154	0.022	1	14
Nap1l1.10	-0.732732608	0.308	0.511	1	14
Rnf165.8	-0.755780518	0	0.173	1	14
Gpd2.2	0.662728113	0.269	0.071	1	14
Hnrnph2	-0.438338496	0.077	0.143	1	14
Nol4.7	-0.436017845	0.038	0.171	1	14
Bcl7a.6	-0.819521741	0.077	0.326	1	14
Sh3gl2.8	-0.791611649	0	0.172	1	14

Lactb.3	0.437334944	0.154	0.034	1	14
Ndufa12.5	-0.760049689	0.212	0.421	1	14
Polr3d	-0.507230467	0.038	0.133	1	14
Wdr1.2	0.645982866	0.288	0.087	1	14
Setd8.5	-0.851356208	0.038	0.262	1	14
Elf1.2	0.49398233	0.231	0.053	1	14
Idh2.8	-0.769446047	0.058	0.279	1	14
Tpd52.1	0.516594096	0.192	0.035	1	14
Zcchc18.5	-0.749816769	0	0.17	1	14
Hipk1	0.373469915	0.231	0.06	1	14
Impdh1	0.673670828	0.25	0.062	1	14
Sh3glb1.3	0.620448868	0.577	0.365	1	14
Ptov1.3	-0.664373998	0.231	0.485	1	14
Purb.5	-0.711339668	0.365	0.549	1	14
Eif3i.3	-0.704362149	0.288	0.462	1	14
Hnrnpd.10	-0.667127245	0.423	0.611	1	14
Dut.12	-0.895325686	0.173	0.435	1	14
Matr3.3	-0.590066442	0.538	0.706	1	14
Dusp23	0.312374282	0.115	0.011	1	14
Plekhg2.1	0.28440989	0.135	0.02	1	14
Hdgfrp3.4	-0.863551387	0.096	0.338	1	14
Cdipt.1	0.566038534	0.308	0.096	1	14
Rnf5.3	-0.728116037	0.096	0.258	1	14
Psat1.8	-0.834162542	0.115	0.368	1	14
Rnpep.1	0.31486966	0.192	0.044	1	14
Casp3.5	-0.779403775	0.019	0.234	1	14
Ide.1	-0.390984453	0.058	0.113	1	14
Celsr2.8	-0.873405589	0.038	0.238	1	14
Smc2.12	-0.902644908	0.288	0.546	1	14
Cds2.1	0.516975499	0.212	0.044	1	14
Rsu1.4	0.53716753	0.346	0.118	1	14
Sh2b3.1	0.61165265	0.115	0.016	1	14
Scarb1.1	0.622512493	0.173	0.036	1	14
Peli2.3	-0.722785765	0	0.165	1	14
Dixdc1.8	-0.891513692	0.038	0.235	1	14
Arpc2.2	0.540778699	0.712	0.507	1	14
Actb.4	0.363047685	0.981	0.979	1	14
Plec.1	0.485279886	0.115	0.011	1	14
Dnajb11.3	0.443353726	0.404	0.162	1	14
Clint1.1	0.672903847	0.346	0.13	1	14
H2afj.3	0.660441254	0.442	0.204	1	14
Slc29a1.9	-0.729091403	0.327	0.562	1	14
Tuba1b.12	-0.713663436	0.231	0.482	1	14
Gm26924	0.582282487	0.442	0.182	1	14
Prmt8.9	-0.582560566	0.058	0.272	1	14
Ak3.1	0.704941609	0.269	0.109	1	14
Smarcc1.7	-0.255775418	0.231	0.434	1	14
Cep110.7	-0.732380061	0	0.163	1	14

Hmgb3.8	-0.829760299	0.077	0.306	1	14
Abhd5	0.508115213	0.135	0.019	1	14
Ttc9b.5	-0.624129689	0.038	0.195	1	14
Smc3.5	-0.627582349	0.442	0.633	1	14
Camta1.6	-0.830042353	0.192	0.408	1	14
Nrn1.9	-0.929074752	0.019	0.222	1	14
Mfap4.8	-0.804262404	0	0.162	1	14
Mrpl21.3	-0.490939339	0.231	0.28	1	14
Htatsf1.4	-0.799015855	0.269	0.499	1	14
Smpd3.8	-0.726439216	0	0.161	1	14
Ip6k2.2	-0.455718788	0.058	0.121	1	14
A330076H08Rik.12	-0.783229277	0	0.161	1	14
Ctps.5	-0.442045543	0.038	0.137	1	14
Yes1	0.528632041	0.25	0.065	1	14
Smarca2.4	0.659513438	0.538	0.285	1	14
Ndn.5	-0.855291156	0.019	0.223	1	14
Podxl2.12	-0.866962719	0.019	0.227	1	14
Btbd17.9	-0.742421735	0	0.16	1	14
Zcchc11.2	-0.727926559	0.077	0.321	1	14
Rab11fip2.1	-0.43911682	0.038	0.106	1	14
Mapk8ip1.11	-0.832792065	0.019	0.227	1	14
Srrm3.8	-0.659820274	0.058	0.24	1	14
Pdap1.6	-0.564604752	0.538	0.683	1	14
Ddx42.3	-0.734046366	0.192	0.383	1	14
Gsg1l.11	-0.713160616	0.038	0.256	1	14
Nop58.10	-0.711635568	0.327	0.592	1	14
Snhg5.5	-0.67050998	0.135	0.381	1	14
Ift74.6	-0.597821534	0.038	0.225	1	14
Snap23.2	0.275396677	0.173	0.041	1	14
Prpf19.2	-0.761340414	0.115	0.307	1	14
Ppp1r14c.11	-0.896705976	0.058	0.262	1	14
Smc4.12	-0.659069954	0.462	0.567	1	14
Itpr1	0.443773102	0.135	0.016	1	14
Sppl2a.2	0.581951738	0.327	0.118	1	14
Copz2.1	0.488105532	0.115	0.013	1	14
Cenpe.13	-1.074780522	0.058	0.286	1	14
Itm2c.10	0.614170344	0.442	0.211	1	14
Creg1.2	0.459286136	0.212	0.058	1	14
Srpk2.4	-0.799761112	0.173	0.43	1	14
Tpd52l1	0.456082311	0.154	0.023	1	14
Sema6d.2	0.549848929	0.173	0.031	1	14
Unc93b1.2	0.492308339	0.115	0.012	1	14
Aplp1.12	-0.585057227	0.058	0.151	1	14
Sox9.10	-0.764185579	0.038	0.255	1	14
Rela	0.334013837	0.212	0.057	1	14
Top2a.13	-0.986590588	0.173	0.428	1	14
Lmo4.8	-0.825974652	0.115	0.336	1	14
Stat3.4	0.638742253	0.192	0.041	1	14

Dnajc8.3	0.569101106	0.654	0.433	1	14
Eif4g2.4	-0.518192928	0.558	0.705	1	14
Hmgb1.11	-0.782510057	0.135	0.359	1	14
Yap1	0.402035239	0.135	0.017	1	14
Pabpc1.8	-0.370156826	0.885	0.878	1	14
Ntrk3.7	-0.667332172	0	0.155	1	14
Cct2.6	-0.695824433	0.25	0.524	1	14
Smc5.5	-0.67695713	0.096	0.258	1	14
Pald1.2	0.477511374	0.25	0.073	1	14
Nrip1.1	0.621479792	0.308	0.1	1	14
Ier5.8	-0.791561264	0.135	0.395	1	14
Dhx9.5	-0.76037479	0.212	0.463	1	14
Banf1.11	-0.535038491	0.558	0.694	1	14
Gdpd1.9	-0.813524562	0.038	0.243	1	14
Cenpf.14	-1.151307434	0.135	0.362	1	14
Tra2b.4	-0.711843955	0.173	0.405	1	14
Pwp1	-0.355169462	0.038	0.101	1	14
Cbfa2t3.11	-0.808623139	0.192	0.403	1	14
Ehd4.1	0.591067781	0.135	0.02	1	14
Hp1bp3.3	-0.689846353	0.327	0.581	1	14
Myo9a.2	-0.637782396	0.038	0.158	1	14
Otx2.5	-0.837075139	0.019	0.217	1	14
Gm5620.1	-0.377231198	0.038	0.101	1	14
Ptch2.10	-0.65573377	0	0.152	1	14
Pcna.13	-0.545753498	0.115	0.359	1	14
Park7.4	-0.642070529	0.346	0.546	1	14
Robo2.5	-0.698728685	0	0.152	1	14
Pebp1.3	-0.708958598	0.077	0.242	1	14
Ilf3.4	-0.630448244	0.25	0.376	1	14
Atp5a1.2	-0.527209627	0.577	0.718	1	14
Elovl7.1	0.527759971	0.115	0.014	1	14
Rmnd5b	0.536093669	0.231	0.059	1	14
Dab2ip.1	0.585004952	0.25	0.068	1	14
Ginm1.2	0.615530607	0.269	0.081	1	14
Cdc20.13	-0.799994715	0.058	0.196	1	14
Rhob.3	0.561953664	0.365	0.138	1	14
Kras.2	-0.670452745	0.096	0.252	1	14
Asf1a.5	-0.725217474	0.115	0.28	1	14
Snrpg.7	-0.692717835	0.173	0.335	1	14
Per1	0.301998913	0.115	0.016	1	14
1110034G24Rik	0.366342354	0.154	0.029	1	14
Isoc1.5	-0.540024373	0.058	0.181	1	14
Cflar.3	0.644043824	0.192	0.05	1	14
Skp1a.3	-0.65262851	0.365	0.582	1	14
Hnrnpk.4	-0.499707565	0.462	0.636	1	14
Myl12b.4	0.537404115	0.615	0.366	1	14
Rbms1.5	0.574177147	0.462	0.215	1	14
Zbtb18.10	-0.841005891	0.058	0.267	1	14

Atad5.8	-0.648305187	0	0.149	1	14
Kif1b.10	-0.76254819	0.288	0.521	1	14
Cdkn1c.2	0.58425256	0.212	0.062	1	14
Olfm1.6	-0.64947175	0.019	0.199	1	14
Pak3.4	-0.787797673	0.038	0.206	1	14
Satb1.4	-0.796257152	0.019	0.21	1	14
Tfam	-0.408032435	0.115	0.154	1	14
Tmem66.5	0.330703866	0.423	0.198	1	14
Nfkb1.1	0.577811374	0.135	0.024	1	14
Wipi1.2	0.486921894	0.173	0.033	1	14
Glud1.3	0.51844895	0.365	0.14	1	14
Acox1.2	0.263582368	0.192	0.056	1	14
Ece2	-0.390726624	0.038	0.104	1	14
Ahi1.3	-0.804995771	0.077	0.307	1	14
Pdzrn3.11	-0.848549645	0	0.147	1	14
N4bp3	0.668357189	0.192	0.06	1	14
Cdk6.8	-0.69237856	0.038	0.243	1	14
Cdh4.5	-0.631725877	0	0.146	1	14
Arid2.3	-0.755390009	0.038	0.242	1	14
Mpnd.3	-0.550026138	0.077	0.171	1	14
Igsf8.7	-0.771111468	0.058	0.269	1	14
Txnip.2	0.611426302	0.288	0.106	1	14
Ranbp1.10	-0.625134435	0.462	0.676	1	14
Vdac1.2	-0.642649452	0.077	0.303	1	14
Slc22a17.8	-0.777802907	0.077	0.251	1	14
Hsp90b1.9	0.474317661	0.827	0.691	1	14
Tubb2a.14	-0.481323406	0.25	0.244	1	14
Gpn2	0.574913683	0.135	0.054	1	14
Hspd1.6	-0.704485573	0.192	0.365	1	14
Hnrnpa0.5	-0.491090593	0.346	0.457	1	14
Atp1a2.3	0.536041075	0.212	0.058	1	14
Fnta.1	0.631689112	0.308	0.138	1	14
Bcl11a.6	-0.768886608	0.019	0.21	1	14
Trim56.1	0.579662236	0.154	0.027	1	14
Fmnl3.1	0.487881133	0.154	0.026	1	14
Lrrc59	-0.285522881	0.096	0.112	1	14
Gm11266.10	-0.70869475	0	0.144	1	14
Anp32b.11	-0.618987394	0.442	0.591	1	14
Ddx39.6	-0.479787055	0.115	0.187	1	14
Pim3.1	0.696816483	0.212	0.087	1	14
Psme4.4	-0.787254112	0.096	0.284	1	14
2510003E04Rik.2	-0.416270536	0.038	0.108	1	14
Trim33.1	-0.490516842	0.038	0.149	1	14
AI854517.4	-0.743507946	0.019	0.189	1	14
Prmt5.6	-0.716427047	0.096	0.256	1	14
Dcakd.6	-0.734899413	0.096	0.297	1	14
Nucks1.13	-0.643905741	0.462	0.663	1	14
Nudcd2.6	-0.546662013	0.115	0.223	1	14

Strbp.9	-0.798272405	0.115	0.347	1 14
Ldhb.7	-0.765060943	0.096	0.281	1 14
Foxp1.3	0.692691256	0.346	0.171	1 14
Rcor2.10	-0.802080954	0.038	0.225	1 14
Myod1.11	-0.666439026	0	0.141	1 14
Peg3.4	-0.822665316	0.096	0.285	1 14
D030056L22Rik.4	-0.429942316	0.077	0.142	1 14
Hnrnpa3.1	-0.543800933	0.038	0.159	1 14
Atxn10.2	-0.555806347	0.154	0.37	1 14
Zeb1.8	-0.779134283	0.154	0.387	1 14
Cep78.4	-0.602517627	0	0.14	1 14
Ywhae.2	-0.340254515	0.615	0.727	1 14
Atl2	0.40278958	0.231	0.065	1 14
Atp6v1b2.3	-0.490783559	0.096	0.17	1 14
Acaa2.3	0.488528758	0.173	0.034	1 14
Reep3.2	0.554185171	0.5	0.255	1 14
Tmod2.3	-0.753586957	0.019	0.207	1 14
B230118H07Rik	-0.461856143	0.038	0.122	1 14
Zbtb38.2	0.261620152	0.192	0.058	1 14
Phb2.2	-0.69044326	0.173	0.333	1 14
Nop10.6	-0.683347922	0.288	0.482	1 14
Slc16a6.1	0.606582082	0.173	0.041	1 14
Hypk	-0.33249073	0.058	0.105	1 14
Wdr77.1	-0.283820382	0.077	0.123	1 14
Cplx1.10	-0.665532309	0	0.138	1 14
Tram1.4	0.459224219	0.192	0.128	1 14
Sacs.5	-0.669780482	0.038	0.191	1 14
Ppp1r2	0.559081306	0.25	0.079	1 14
Brd3.7	-0.681539367	0.288	0.532	1 14
Acin1.4	-0.494203845	0.519	0.724	1 14
Mif.6	-0.616396499	0.231	0.357	1 14
Gnb2l1.7	-0.41975922	0.808	0.872	1 14
Epc2.2	-0.756669676	0.058	0.25	1 14
Aph1a	0.612694962	0.115	0.021	1 14
Cnot6l	0.654949633	0.327	0.134	1 14
Cdk1.13	-0.628730855	0.173	0.247	1 14
Bex1.6	-0.724469993	0.038	0.224	1 14
Slc1a1.1	0.366987573	0.115	0.017	1 14
Safb.3	-0.663825373	0.25	0.504	1 14
Rrm2.13	-0.753639443	0.019	0.204	1 14
2610017I09Rik.4	-0.735093572	0.019	0.204	1 14
Srsf2.6	-0.606323844	0.385	0.566	1 14
Gse1.7	-0.717517307	0.058	0.237	1 14
Cacybp.3	-0.63016507	0.212	0.356	1 14
Wdr33.2	-0.545212437	0.115	0.196	1 14
Mdga1.6	-0.610683905	0	0.136	1 14
Pcdh17.2	0.545793936	0.154	0.028	1 14
Meaf6.1	-0.56370131	0	0.135	1 14

Snw1.3	-0.514543199	0.25	0.432	1	14
Arhgap31.3	0.574472223	0.173	0.039	1	14
Fau.1	-0.411225412	0.058	0.123	1	14
Tmbim6.3	0.499948494	0.558	0.323	1	14
Nrcam.9	-0.653360561	0	0.134	1	14
Hadha.2	0.647300526	0.327	0.155	1	14
Pqlc1.11	-0.74017629	0.135	0.297	1	14
Amer2.3	-0.566169282	0	0.133	1	14
Ccdc88a.5	-0.631845782	0.365	0.482	1	14
Ccp110.7	-0.697749627	0.096	0.248	1	14
Tnks1bp1	0.334682066	0.115	0.016	1	14
Vcl.2	0.316647975	0.115	0.016	1	14
Cnpy1.8	-0.623064786	0	0.133	1	14
Rab7.4	0.291795841	0.385	0.183	1	14
Zfp36l1.10	0.518908814	0.365	0.15	1	14
Chd4.6	-0.50899351	0.635	0.77	1	14
Akap6.6	-0.644787291	0.038	0.219	1	14
Tdrkh.1	-0.543032113	0	0.133	1	14
Reep1.3	0.416452498	0.25	0.081	1	14
D19Bwg1357e.5	-0.753518013	0.077	0.273	1	14
Nell2.4	-0.572539316	0	0.132	1	14
2810055G20Rik.3	-0.618711711	0.038	0.171	1	14
Pkp4.5	0.53536788	0.288	0.102	1	14
Dzip3.3	-0.575309461	0.058	0.167	1	14
Alkbh6.1	-0.503880919	0.038	0.134	1	14
BC005764.12	-0.679529843	0.019	0.186	1	14
Fam102b.1	0.621580844	0.173	0.043	1	14
1500012F01Rik.8	-0.684476444	0.25	0.398	1	14
H3f3a.4	-0.651275655	0.212	0.381	1	14
Cdkn1b.7	-0.627818361	0.288	0.467	1	14
Sec62.5	0.531378997	0.635	0.405	1	14
Etfb.7	0.556664666	0.404	0.183	1	14
G2e3.4	-0.546813146	0	0.131	1	14
Fmnl2.5	-0.424391815	0.077	0.157	1	14
Slc35b3	0.533410899	0.154	0.032	1	14
Tcerg1.5	-0.690052925	0.231	0.457	1	14
2610203C20Rik.7	-0.514492113	0.038	0.12	1	14
Ube2c.12	-1.108981861	0.077	0.269	1	14
Pygo1.7	-0.802554472	0.019	0.192	1	14
Gpr56.6	-0.674561874	0.038	0.223	1	14
Vcan.7	-0.611240603	0	0.13	1	14
4933426M11Rik.2	0.523561731	0.192	0.046	1	14
Cecr2.4	-0.567693249	0	0.13	1	14
Fcf1	-0.64731492	0.019	0.196	1	14
Ccdc32	0.365243771	0.173	0.095	1	14
Gdpd5.1	0.40204583	0.154	0.033	1	14
2810474O19Rik.4	-0.582883371	0.154	0.241	1	14
Ralgps2.6	-0.724822629	0.077	0.246	1	14

Zfp329.3	-0.602803432	0	0.129	1	14
Clvs1.10	-0.621176743	0	0.129	1	14
Mtmr6	0.380322681	0.192	0.051	1	14
Luc7l3.5	-0.52333078	0.538	0.748	1	14
Fam219b	0.305639573	0.154	0.044	1	14
Gsto1.4	-0.560469628	0	0.128	1	14
Ank2.12	-0.82601896	0.096	0.24	1	14
Tmem123.1	0.487962032	0.25	0.083	1	14
Syncrip.7	-0.633795602	0.308	0.496	1	14
Ccnb2.13	-0.694053505	0.019	0.187	1	14
Srsf10.5	-0.714583252	0.135	0.331	1	14
Phf21b.5	-0.572335145	0	0.128	1	14
Tox3.6	-0.519962037	0.135	0.346	1	14
Clybl.5	-0.477416899	0.038	0.116	1	14
Ybx3.8	0.286556927	0.481	0.289	1	14
Atrx.5	-0.554476373	0.577	0.74	1	14
Bex4.2	-0.512819632	0	0.128	1	14
Nrbp1	0.502722014	0.269	0.091	1	14
Qser1.2	-0.54405032	0	0.127	1	14
Npdc1.8	-0.559527946	0.173	0.261	1	14
Pdzrn4.9	-0.548659181	0.038	0.146	1	14
Cbx3.3	-0.681329836	0.077	0.274	1	14
Nr2c2	-0.727114996	0.019	0.186	1	14
Gmeb1	-0.346339326	0.077	0.111	1	14
Pnp.2	0.50619695	0.115	0.019	1	14
Naa38.7	-0.687329489	0.096	0.311	1	14
Nedd4.3	-0.409455372	0.615	0.683	1	14
Gm9800.8	-0.579706349	0.385	0.535	1	14
Tsfm	-0.388515426	0.038	0.108	1	14
Gpatch4.5	-0.512632571	0.115	0.179	1	14
Slc1a4.1	0.597722633	0.192	0.056	1	14
Ikbkap.1	-0.56022457	0	0.127	1	14
Pbx3.2	-0.569725176	0	0.127	1	14
2900011O08Rik.11	-0.471609648	0.038	0.103	1	14
Atf3.1	0.518894735	0.115	0.017	1	14
Cdc42.3	0.402961474	0.712	0.514	1	14
Tnpo3.3	-0.619781253	0.038	0.223	1	14
Bcas2.4	-0.613905992	0.212	0.406	1	14
Usp10.1	-0.616334511	0.058	0.186	1	14
Tro.5	-0.585657936	0	0.126	1	14
Sae1.8	-0.691829534	0.096	0.279	1	14
Fam63a.1	0.615502766	0.25	0.087	1	14
Ckap2l.14	-0.733726473	0.096	0.217	1	14
Calr.5	0.459147483	0.654	0.51	1	14
Nop56.9	-0.592830602	0.154	0.361	1	14
Rpn1.3	0.526291316	0.404	0.191	1	14
Efs.4	-0.520072978	0	0.125	1	14
Maged2.5	-0.500046128	0.096	0.167	1	14

Cpsf6.3	-0.49814957	0.212	0.291	1	14
Txnrd1.6	-0.507062617	0.173	0.389	1	14
Mtus1.3	0.432806649	0.308	0.126	1	14
Sin3b.1	-0.700132635	0.135	0.309	1	14
Fam69a.2	0.456506142	0.154	0.03	1	14
Erdr1.3	-0.447685341	0.019	0.162	1	14
Tpst2.1	0.372894992	0.231	0.074	1	14
Fads1.2	-0.402202274	0.038	0.105	1	14
Tmem50a.4	0.492425495	0.635	0.404	1	14
Tmcc3.1	0.659143313	0.173	0.091	1	14
Tpm3.3	0.545646641	0.5	0.315	1	14
Peo1.4	-0.539067929	0	0.124	1	14
2810025M15Rik.5	0.551464482	0.346	0.166	1	14
Akap13.2	0.578278622	0.231	0.071	1	14
Rnpepl1	0.373922898	0.154	0.037	1	14
Zfr.4	-0.684620198	0.154	0.354	1	14
Ptpn9.1	0.450928737	0.192	0.049	1	14
Dnph1.8	-0.497054857	0	0.123	1	14
Casc5.13	-0.76258987	0.019	0.186	1	14
Celsr3.5	-0.57869223	0	0.123	1	14
Tceb1.2	0.340372027	0.404	0.366	1	14
Rab3a.13	-0.58167696	0.038	0.15	1	14
Msrb2	0.434762171	0.115	0.018	1	14
Dnajc10.1	0.549012916	0.327	0.133	1	14
Neurod6.6	-0.730427307	0.019	0.172	1	14
Tacc3.13	-0.65369573	0.058	0.207	1	14
Gdap1.5	-0.580696393	0.038	0.199	1	14
Xbp1.2	0.431601688	0.308	0.123	1	14
Dnajc9.12	-0.687262103	0.192	0.35	1	14
Aldh2.3	0.505884085	0.135	0.025	1	14
Baiap2.2	0.491081804	0.192	0.05	1	14
Pop5.2	0.58599785	0.269	0.099	1	14
Eid1.8	-0.607245611	0.346	0.503	1	14
Cd81.6	0.376019044	0.5	0.461	1	14
Lrig3.9	-0.497016068	0.019	0.17	1	14
Hlf.2	0.28192638	0.115	0.019	1	14
Rbm28.1	-0.724127847	0.077	0.263	1	14
Bcat1.4	-0.524997616	0	0.121	1	14
H2afz.12	-0.615594518	0.096	0.231	1	14
Elavl2.8	-0.75787907	0.077	0.27	1	14
Slc10a7	0.45185128	0.115	0.02	1	14
Ndrg2.7	-0.660579006	0.019	0.185	1	14
Hist3h2ba.6	-0.539519334	0.038	0.137	1	14
Gprasp1.4	-0.663680673	0.038	0.206	1	14
Bin1.11	-0.631612802	0.192	0.43	1	14
Banp.3	-0.480427186	0.077	0.137	1	14
Plekha1.4	0.524158168	0.212	0.06	1	14
Bmp2k.1	0.365064662	0.135	0.025	1	14

2310036O22Rik	-0.55475033	0.288	0.406	1	14
Shmt2.3	-0.507222266	0	0.119	1	14
Sox5.5	-0.579837142	0	0.119	1	14
Brcc3.2	-0.294791499	0.077	0.175	1	14
Wsb2.1	0.431101719	0.212	0.063	1	14
Ap3b2.4	-0.689076062	0.038	0.202	1	14
Irs1.6	-0.527932914	0	0.119	1	14
Dach1	0.500079225	0.212	0.066	1	14
Gm10075.11	-0.590689003	0.346	0.515	1	14
Vegfb.3	-0.483734151	0	0.118	1	14
Nrm.7	-0.552811101	0.058	0.169	1	14
Rab34.2	-0.525303231	0	0.118	1	14
Ltbp3.3	-0.549902554	0	0.118	1	14
Nme4.4	-0.515338021	0	0.118	1	14
Ncald.5	-0.696339667	0.019	0.165	1	14
Rrp1b.1	-0.485835917	0.058	0.136	1	14
Eif4b.2	-0.639080364	0.135	0.276	1	14
Txndc9.3	-0.464180997	0.077	0.211	1	14
Ppp3ca.9	-0.735131769	0.192	0.378	1	14
H2afx.13	-0.826079154	0.135	0.331	1	14
Mybbp1a.4	-0.604002951	0.077	0.25	1	14
Cuedc2.2	-0.647576071	0.173	0.387	1	14
Rplp0.8	-0.386510755	0.788	0.872	1	14
Sdf2.2	0.583685115	0.385	0.214	1	14
Ube2b.4	-0.58762005	0.288	0.521	1	14
Pfdn2.1	-0.596970723	0.115	0.31	1	14
Dnmt1.13	-0.727240576	0.096	0.278	1	14
Cenpq.10	-0.263758475	0.058	0.16	1	14
Tspan18	0.464941174	0.192	0.051	1	14
Leprel4.2	0.546551894	0.135	0.029	1	14
Tm9sf3.4	0.51955156	0.5	0.343	1	14
Adk.4	-0.474482189	0.038	0.166	1	14
Grsf1.2	-0.630007235	0.038	0.176	1	14
Atp6v0e2.8	-0.634144672	0.077	0.26	1	14
Fdx1l	-0.424247826	0.038	0.115	1	14
Nfic.3	-0.680504247	0.077	0.261	1	14
Brix1.4	-0.586451061	0.077	0.206	1	14
Krt10.2	-0.636496071	0.019	0.162	1	14
Mprip.2	0.616506729	0.327	0.151	1	14
Mcee	0.363801832	0.212	0.075	1	14
Exoc2.2	-0.510771087	0	0.116	1	14
Tcp11l1.1	-0.529538401	0	0.116	1	14
Esco2.13	-0.614413056	0.058	0.195	1	14
Cltb.10	-0.680071249	0.269	0.423	1	14
Nktr.7	-0.711704958	0.192	0.38	1	14
Ing4.6	-0.656613906	0.154	0.323	1	14
Plin2.2	0.456945373	0.192	0.052	1	14
Chmp6.1	0.261951843	0.231	0.088	1	14

Ypel1.5	-0.592050312	0.058	0.174	1	14
Fam213b.7	-0.480676485	0.019	0.157	1	14
Cenpm.11	-0.616740686	0.019	0.162	1	14
Sox11.7	-0.511619607	0	0.115	1	14
A930011O12Rik.13	-0.665273289	0	0.115	1	14
Ccdc55.2	-0.422427949	0.135	0.267	1	14
Cgrrf1.2	0.480277021	0.192	0.054	1	14
Psma1.2	-0.493830341	0.327	0.445	1	14
Clasp1.1	-0.522573734	0	0.115	1	14
Rin2.3	0.379182018	0.115	0.02	1	14
Rrm1.13	-0.620707181	0.135	0.26	1	14
Rock2.1	0.634901389	0.327	0.211	1	14
Clic1.6	0.443521669	0.365	0.172	1	14
Cenpp.10	-0.457365229	0	0.114	1	14
Mtf2.4	-0.555097728	0.25	0.357	1	14
Fam129b	0.316600253	0.115	0.02	1	14
Rsl24d1	-0.478048028	0	0.114	1	14
Epb4.1l3.5	-0.498241411	0	0.113	1	14
Comt.4	0.310324247	0.269	0.109	1	14
Mapt.13	-0.857784444	0.058	0.216	1	14
Lrrn1.3	-0.506580853	0	0.113	1	14
1810058I24Rik.1	0.434426762	0.404	0.196	1	14
Gng12.6	0.587116835	0.25	0.12	1	14
Pafah1b2.4	-0.393335411	0.173	0.255	1	14
Reln.10	-0.752746223	0.038	0.18	1	14
Col9a3.5	-0.698848508	0.019	0.18	1	14
Arpp21.12	-0.67943081	0	0.113	1	14
Ptprd.5	-0.68209959	0.135	0.34	1	14
Jakmip2.5	-0.342636	0.038	0.157	1	14
Josd2	-0.475653932	0	0.113	1	14
Arhgap11a.13	-0.721950531	0.019	0.17	1	14
Fez2.1	0.380870621	0.154	0.037	1	14
Tmem184b.1	0.336628384	0.115	0.019	1	14
Srrm4.11	-0.710738423	0.038	0.195	1	14
Magt1.4	0.36545117	0.192	0.056	1	14
Stk25.2	0.570796415	0.365	0.183	1	14
Apbb1.12	-0.603851469	0.019	0.17	1	14
Stk40.1	0.362281487	0.154	0.036	1	14
Mob1a.2	0.551226509	0.173	0.053	1	14
Mrpl40	-0.535389358	0.077	0.177	1	14
Msi1.1	-0.495886225	0	0.111	1	14
Nelfa	-0.325260248	0.038	0.103	1	14
Arf3.2	0.302285231	0.192	0.062	1	14
Rbmx.5	-0.637317984	0.115	0.315	1	14
Sirt7	0.343754252	0.25	0.096	1	14
Nrep.12	-0.275715531	0.538	0.569	1	14
Tceal3.6	-0.517172429	0	0.11	1	14
Pcbp2.3	-0.508935068	0.442	0.621	1	14

1700001O22Rik.7	-0.462593172	0	0.11	1	14
Elovl4.4	-0.496551832	0	0.11	1	14
Eif2b3.2	-0.591199395	0.038	0.159	1	14
Zfp292.6	-0.715679095	0.173	0.342	1	14
Slc25a27.4	-0.509344485	0	0.11	1	14
Pigk.3	0.453557959	0.154	0.045	1	14
Lims1.4	0.553565624	0.365	0.181	1	14
Rab6b.13	-0.685746679	0.058	0.202	1	14
Gpsm1.3	-0.63912521	0.038	0.176	1	14
Sgta	-0.628422557	0.096	0.241	1	14
Nhp2.7	-0.510578272	0.25	0.371	1	14
Abhd6.3	0.446111579	0.154	0.037	1	14
Hist3h2a.9	-0.54757937	0.058	0.143	1	14
Stat1	0.479626259	0.135	0.028	1	14
Snrpf.9	-0.497077616	0.327	0.427	1	14
Rabl6	-0.496463204	0.192	0.259	1	14
Cul4a.1	-0.336276597	0.077	0.11	1	14
Igf1r.2	0.438124367	0.288	0.113	1	14
Dazap1.4	-0.491957425	0.192	0.272	1	14
Nbeal1	0.562845869	0.154	0.038	1	14
Lphn3.3	0.354459114	0.192	0.06	1	14
Kif4.10	-0.462627694	0	0.108	1	14
Cirbp.5	-0.559029675	0.154	0.355	1	14
Rfc5.3	-0.441441942	0	0.108	1	14
Wdr82.1	-0.593692236	0.038	0.173	1	14
Srgap2.9	0.384881778	0.308	0.131	1	14
Gli1.9	-0.470765042	0	0.108	1	14
Coq9	-0.256913928	0.115	0.118	1	14
Stag1.2	-0.610125509	0.077	0.207	1	14
Hmgn2.11	-0.650570639	0.096	0.256	1	14
Ankrd26	-0.708401612	0.038	0.176	1	14
Snrpb2.5	-0.526471369	0.173	0.384	1	14
Pou3f3.5	-0.650577566	0.019	0.172	1	14
R3hdm1.2	-0.638693599	0.154	0.297	1	14
Pold3.5	-0.31720101	0.058	0.148	1	14
Ubqln2.2	-0.590146551	0.096	0.214	1	14
Zfp637.2	-0.648492925	0.096	0.256	1	14
Dpysl3.13	-0.823733421	0.077	0.219	1	14
Hbs1l.1	-0.46572719	0	0.106	1	14
Sfpq.4	-0.426543049	0.519	0.67	1	14
Fam53a	0.534531489	0.154	0.075	1	14
Mfhas1.1	0.524343518	0.212	0.068	1	14
Ccdc53.2	0.518463106	0.212	0.072	1	14
Malat1.11	0.387664227	0.981	0.955	1	14
Fkbp7.2	0.47829541	0.212	0.066	1	14
Arl3.1	-0.61004311	0.192	0.365	1	14
Atxn7	0.644087028	0.192	0.087	1	14
Arid5b.1	0.566563204	0.154	0.04	1	14

Zmym2.2	-0.401928435	0.058	0.118	1	14
Clpp.2	-0.562675849	0.135	0.239	1	14
Nckap1.3	0.518339992	0.308	0.13	1	14
Slc25a1.2	0.341206765	0.192	0.06	1	14
Tbc1d16.4	-0.637072659	0.038	0.196	1	14
Fhl1.3	-0.463327107	0	0.105	1	14
Rtn4.6	-0.344190641	0.481	0.48	1	14
Dnaaf2.3	-0.445915402	0	0.105	1	14
Smarca5.7	-0.610918578	0.192	0.401	1	14
Pls3.1	0.547456407	0.192	0.071	1	14
Polr3k.4	-0.636614208	0.115	0.271	1	14
Ophn1.3	0.367734764	0.231	0.083	1	14
Zbtb7a.1	0.561306781	0.231	0.093	1	14
Cdca7l.10	-0.436921901	0	0.104	1	14
Zic5.4	-0.531550011	0.038	0.161	1	14
Rsrc2.3	-0.453649393	0.462	0.547	1	14
Xpo1.4	-0.32667132	0.096	0.228	1	14
Yy1.2	-0.474760253	0.135	0.204	1	14
Uba2.3	-0.614375522	0.077	0.256	1	14
Slc25a20.1	0.46676211	0.154	0.037	1	14
Mab21l2.6	-0.535279556	0	0.104	1	14
Tia1.5	-0.501964835	0.365	0.552	1	14
Tmem98	0.391095047	0.154	0.038	1	14
Cpt2	0.381699472	0.115	0.021	1	14
Rnpc3.3	-0.489150303	0	0.104	1	14

gene
2810417H13Rik
Top2a
Esco2
Rrm2
Pbk
Mki67
Dut
Tyms
Lig1
Dek
Spc25
Smc2
Pcna
Tuba1b
Cdk1
Smc4
H2afx
Hmgb2
Ranbp1
Tubb5
Birc5
Incenp
Spc24
Hist1h2ak
Prc1
Nusap1
Anp32e
Clspn
Anp32b
Tk1
Nasp
Atad2
Kif11
Ckb
Cenpf
Cenph
Rtn1
Neurod1
H2afv
Tpx2
Tmsb4x
Rad51ap1
Cdca8
Fbxo5
Rrm1
Ncapg
Ccna2

Prim1
Tmpo
Hirip3
Dnajc9
2700094K13Rik
Hnrnpab
Aurkb
Dnmt1
Tcf19
Cntn2
Hells
Stmn2
Kif15
Ezh2
Cdca3
Tuba1a
Ckap2l
RP23-45G16.5
Itm2b
Neil3
Nrep
Usp1
Dtymk
Fam111a
Racgap1
Fkbp3
Tipin
Mxd3
Casc5
Mns1
Ran
Paics
Nucks1
Ndc80
Mthfd2
Gm10075
Gpm6a
Tubb3
Kif23
Rad51
Smc6
Cenpm
Chaf1a
Cenpq
Gmnn
Rpa2
Map1b
Apoe

Tacc3
Cdca5
H2afy
Myod1
Dpysl3
Rfc4
Cks1b
Nuf2
Ccdc34
CRE_RECOMBINASE
Ncl
Serbp1
Psip1
Supt16
Stmn4
H1f0
Gng3
Bub1
Gm9800
Sfrs18
Hnrnpd
Mcm6
Kif22
Plk4
Ncapg2
Hmgn5
Id2
Cdc45
Cenpk
Fen1
Kdm5b
Miat
Ccne2
Melk
Cbx5
Diap3
Whsc1
Ncapd2
Uhrf1
Cdca2
Celf4
Rangap1
Tubb2a
Ssrp1
Pmf1
Ptma
Gap43
Dhfr

Prdx4
Pkmyt1
Rad21
Cst3
Rbbp4
Dlgap4
Sox4
Tubb2b
D4Wsu53e
BC005764
Smc3
Rad54l
Kif4
H2afz
Brca1
Hjurp
Dcx
Lmnb1
Atad5
Elavl4
Zic1
Hmgb1
Rbfox3
Hmgn2
Topbp1
Ubr7
Cdk5r1
Pdzrn3
Asf1b
Ccp110
D17H6S56E-5
Malat1
Ncaph
Rfc1
Snrpd1
H1fx
Ankrd12
Tpm4
Dtl
Ptn
Nop58
Cltb
Rfc2
Mis18bp1
Igfbpl1
Banf1
Calm1
Pa2g4

Hnrnpdl
Nhlh1
Siva1
Alyref
Smc1a
Lbr
Jhdm1d
Cenpp
Nrxn1
Kif20b
Timeless
Rab3a
Thra
Hint1
Serinc1
Aldoa
Mcm2
Cdc7
Ckap2
Hist1h2ag
Cenpw
Sept3
Rbbp7
Ska1
Acat1
Dctpp1
Oxct1
Pdgfa
Rpa1
Ybx1
Mrpl18
Sae1
Zwilch
Srsf7
Ccdc25
Cdkn2c
Mcm5
Psmc3ip
Mcm10
Mlf1ip
Clic4
Ina
Nap1l1
Zfp367
Nrm
Tsc22d1
Srsf3
Skp2

Mapt
Elavl3
Nsl1
Gpm6b
Lsm2
Mcm7
Pold3
Mtss1
Ctsd
Pola1
Nsmce4a
Rif1
Cdt1
Ctsb
Ttc3
Myt1
Psat1
E2f7
Slbp
Syt11
Sgol1
Tmem50a
Ncapd3
Orc6
Cmc2
Mcm3
Blm
Ctsl
Barhl1
Gjc1
Rad54b
Atxn7l3b
Cep57
Pgf
St18
Suz12
6330403K07Rik
Foxm1
Nhlh2
Ttk
Lyar
Sept4
Spag5
Snrpb
Brca2
Hmgn1
Aplp1
Phf17

Brd3
Hpca
Mis18a
Csrp2
Nup62
Basp1
Cpe
Cadm3
Prim2
A330076H08Rik
Shcbp1
Map2
Wdhd1
Chtf18
Elmo1
Rbbp8
Trpc4ap
A930011O12Rik
Cklf
Mxd4
Dbf4
Srsf2
Chgb
Ptprs
Espl1
Rfc3
Arhgap11a
Cdkn2d
Slc7a5
Mllt11
Smarcc1
Vrk1
Baz1b
Nup85
Mad2l1
Nxt1
Nsg2
Gria2
Dscc1
Naa50
Fabp7
Tex14
Dnajc21
Apc
Syce2
Lap3
E2f2
Gins3

Sqstm1
Ska2
Ccne1
Meg3
Sgol2
Chd7
Insm1
Anln
Celf2
Cenpj
Nop56
Txn1
Cdca4
2700099C18Rik
Wbp5
Dner
Apitd1
Cenpn
Lsm3
Rad18
Rbfox2
Pole
Snrpe
Brip1
Dck
Ypel3
Gsg2
Arpp21
Sparcl1
Hat1
Ppp1r14c
B3galt2
Scg5
Calr
Kif5a
Bub3
Rfc5
Pde1c
Kif1b
Igsf21
Ank3
Rab6b
Tbata
Srsf4
Gli1
Rbp4
Sema6a
Arpp19

Prdx1
Ifrd1
Kmt2e
Idh2
Chaf1b
Trip13
2700029M09Rik
Itsn1
Kif5c
Smarca5
Exo1
Prnp
Ctsf
Ctcf
Ybx3
Smchd1
Trim37
Pola2
Bex1
Stil
Uncx
Rabac1
Slfn9
Rpa3
Pold1
Cnot6
Pax6
Hist1h1e
Ank2
Cdk2
Cdk5rap2
Snrpf
Chrna3
1500012F01Rik
Ndn
Larp7
Map1lc3b
Cdca7
Nmral1
Slc25a5
Wdr76
Trim59
Itm2c
Dnaaf2
Fignl1
Ctps
Bub1b
Cep290

Grina
Naa38
Ccdc41
Haus5
Dpy30
Btbd17
Zic4
S100a16
Snap25
Eif4ebp1
Iqgap2
Rnf168
Eif4a3
Nudc
Lsm6
Kpnb1
Tnik
4930422G04Rik
Atp6v0b
Hsd11b2
Arl6ip6
Myt1l
Cntln
Prr11
Gng2
Ldhb
Slc1a3
Os9
Ipo5
Klf7
Pole3
Srsf10
Rnaseh2a
Pknox1
2810008D09Rik
Stag1
Ptms
Gins1
Npdc1
Gdi1
Kcnk1
Gins2
Casp8ap2
Gm11266
Ube2t
Rbms1
Hprt
Zbtb18

Nfatc2ip
Spdl1
Snrpd2
Lgmn
Phgdh
Syncrip
Gins4
E2f1
Snrpa1
Ckap5
Tubg1
Rhno1
Kidins220
Stub1
Mapk8ip1
Trim28
Ect2
Aurka
Azin1
A030009H04Rik
Nsmce1
Tagln3
Cbfb
Tex30
2610203C20Rik
Sod1
Smco4
Gm26735
Rcor2
Ncaph2
Bok
Clmp
Snrnp40
Abhd16a
Uba2
Dnph1
Mis12
Set
Clip3
Fam213b
Bin1
Mab21l1
Soga3
Ptges3
Nrn1
Xbp1
2210016L21Rik
Vimp

Cdca7l
Mcm4
Apbb1
Btg2
Chd3
Shmt1
Eid1
Plp1
Dbn1
Slc3a2
Rab6a
Pik3r3
Ncan
Glce
Gnao1
Cenpl
Podxl2
Mbnl2
Ddit4
Stxbp1
Exosc8
Zmynd8
Prmt2
Rtn4
Serpinh1
Cnrip1
Pdlim1
Npc2
Stag2
Gpc2
Sh3bp5
Tmem59
Tspyl4
Nicn1
Use1
Med30
Pbdc1
Clcn4-2
Uchl1
Exosc7
Clk1
Srrm4
Rufy3
Cdkn1a
Kdm6b
Sirt2
Prkcb
Vim

Rnmt
Nsg1
Mpp6
Ogt
Pea15a
Atp6v1e1
Commd1
Ppil1
1500016L03Rik
Kif20a
Sart3
Pdrg1
Baz2b
Akap8l
Slc22a17
Tgfb2
RP23-32A8.1
Pdcd4
Idh1
Gabarapl1
H13
Aplp2
Gria4
Uchl5
Hist3h2a
2410066E13Rik
Tmem66
Asns
Ppfia2
Hcfc1r1
Sptan1
Gabbr1
Ccdc28b
Tacc2
Bcl7a
Hpcal1
Ing4
St8sia3
Clvs1
2900011O08Rik
Ly6e
Igsf8
Ankra2
Ywhag
Psap
Pnmal2
Hist3h2ba
Cacng4

C130071C03Rik
Flot1
Pak7
Grik2
Zfp329
Prdm8
Pik3ip1
Reln
Pygo1
Agpat4
Lrpap1
Klf9
Mien1
Bcas1
Mkrn1
Pcmtd2
Wdr13
Gm3764
Dixdc1
Maged2
Sstr2
Fbxo32
Nt5c
Atp6v0e2
Gramd1b
Rundc3a
Kif1a
4930402H24Rik
Ctsa
Tpm1
Aprt
Pcdha2
Fyn
Slc17a6
Sox5
Mbp
Hes1
Ntm
Pcna
Lig1
Mcm6
Hells
Ung
Neurod1
Mcm3
Mcm2
Arl6ip1
Tuba1a

Stmn2
Rtn1
Dut
Rpa2
Ube2c
Cntn2
Ranbp1
Tmsb4x
Nasp
Cenpa
Dek
Cdt1
Mcm5
Itm2b
Calm2
Tubb3
Uhrf1
Gap43
Mcm4
Cdca7
Cenpf
Ccnd2
Chaf1b
Rplp1
Gpm6a
Map1b
Dhfr
Tbata
Npm1
Cenpe
Srebf1
Dtl
Rps5
Cdc6
Tpx2
Siva1
Ccnd1
Anp32b
Nrxn1
Prc1
Clspn
Stmn4
Ptn
Apoe
Kif23
Paics
Cdc20
Mcm7

Ccnb2
Nrep
Tubb2a
Hmmr
Rpl8
Ddah2
Serbp1
Eef1b2
Tcf19
Rplp2
Celf4
Nusap1
Dpysl3
H3f3b
Ncl
Zic1
Sfrp1
Hat1
Nop58
Cltb
Ccnb1
Rps20
Mtss1
Tubb2b
H2afx
Cdca3
Prim1
Tyms
Mapt
Rps21
Ckb
Gng3
Ckap2l
E2f1
Rps19
Ccne2
Calm1
St18
Chaf1a
Hn1
Top2a
Timeless
Dscc1
Fam111a
Hsd11b2
Nhlh1
Rfc3
Elavl4

Cdk5r1
Malat1
Elmo1
Zfp367
Gmnn
Tex14
Birc5
Mki67
Dctpp1
Dnajc9
Slfn9
Nap1l1
Cbx5
Ccng2
Dner
Tipin
Nhlh2
Spc25
Rpl14
Serinc1
Rfc2
Rpl41
Ppp1r14c
Rpa1
Pdzrn3
Mllt11
Atad2
Aspm
Dnmt1
Nhp2
Fam64a
Cdk1
Rab3a
Nolc1
Sept3
Gria2
Rif1
Snhg1
Apc
Cdc45
Cst3
Cadm3
Celf2
Nop56
Alkbh2
Tagln3
Cnbp
Atad5

Trpc4ap
Sema6a
Mms22l
Syt11
Ckap5
Shmt1
Aplp1
Rps3a1
Gins2
Wdr76
Mif
Casp8ap2
Rad21
Mcm10
Myt1
Cdkn2d
Ptms
Wdhd1
Sptbn1
Prmt8
Dtymk
H1f0
Sparcl1
Map2
Itsn1
Eif3a
Ssrp1
H2afy
Basp1
Rad51
Meg3
Fen1
Pola2
Slc29a1
Hey1
Hist3h2a
Cenph
Ccm2
Cdca8
Fam210b
Arhgap11a
RP23-45G16.5
Gpm6b
Tnik
Srm
Arpp21
Hspd1
2810417H13Rik

Sfrs18
Rfc4
Ctsb
Thra
Tsc22d1
Mis18bp1
Baz1a
Rrm2
Dcx
Kif11
Rfc1
Ipo5
Chgb
Kdm5b
Pold1
Ina
Apitd1
Hnrnpd
Chd3
Sox9
D430041D05Rik
Msh6
Gnl3
Rrm1
A930011O12Rik
D4Wsu53e
Nop10
Cpe
Chek1
Snrpd1
B3galt2
Rbp4
Dync1i2
Mdk
Cbfa2t3
Srsf7
Ank2
Dkc1
Knstrn
Rtn4
Usp1
Myt1l
Kcnk1
Ankrd12
Calm3
Ubb
Aurka
Pkm

Msh2
4930422G04Rik
Idh2
Cdca7l
Tacc3
Mthfd1
Syce2
Pdgfa
Btbd17
Ppat
A330076H08Rik
Rad54l
Gli1
Slc25a5
Supt16
Gm17322
Sgol2
Incenp
Baz1b
Rnf168
Prdx4
Pa2g4
Brd8
Rps25
Rnaseh2a
Bola2
Gsr
Pnmal2
Fabp7
1110038B12Rik
Prnp
Rpa3
Cdkn1b
Pax6
C1qbp
Rprml
Klf6
Psmc3ip
Dlgap5
Bok
Rad51ap1
Racgap1
Topbp1
Elavl3
Kif2c
Dtx1
Akirin2
Sept4

Gins1
Prdm8
Nsg2
Kif22
Kif5c
Tead2
Brca2
Rnd3
Gpr180
Rangap1
Mbnl2
1700001O22Rik
Ctsd
Rbfox2
Abhd16a
Boc
Mrpl18
Rab6b
Rps2
Apex1
Polr2f
Lyar
Brca1
Ccna2
Stxbp1
Uncx
Gdi1
Ablim2
Kif5a
Nup85
Id2
Cacng4
Sox4
Cenpk
Atp6v0b
Smc6
Grina
Pea15a
Fxyd6
Lap3
Kif1b
Pold3
Ptch2
Gsg1l
Odf2
Set
Taf1d
Rbfox3

Gng2
Mab21l1
Dbn1
Casc5
Mcmbp
Fnbp1l
Ak2
Hist3h2ba
Ank3
Uchl1
Snhg4
Klf7
Nuf2
Eef1d
Prkcb
Srrm4
Ldha
Vim
Gar1
BC005764
Trp53
Gnao1
Fos
Gdpd1
Mxd4
Tspyl4
Nmral1
Pcdha2
Rnf5
Podxl2
Ntrk3
Rbm5
Rab6a
Gart
Rcc2
Mrpl13
Rufy3
Npdc1
Zbtb18
Trmt6
Kif20b
Nexn
Idh1
Fyn
Pqlc1
Ddx21
Pde1c
Arhgef2

4930402H24Rik
Bin1
Soga3
Afap1
Gm11266
Lrpap1
Apbb1
Scg5
Akap8l
Cep170
Os9
Atp6v1e1
2900011O08Rik
Hpcal1
Clvs1
Srgap2
Clip3
Rad50
Jhdm1d
Pik3r3
Klc1
Clasp2
Ppfia2
Tubb4b
Aplp2
Pkia
Chrna3
Pttg1
Gnaq
Clmp
S100a16
Nrcam
Kidins220
Plcb1
Slc1a3
Grik2
Gria4
Tspan7
Snap25
Itm2c
Slc17a6
Plp1
Lgmn
Gm11223
Sirt2
Ube2c
Cenpf
Cenpa

Cdc20
Prc1
Hmmr
Cenpe
Ccnb1
Tpx2
Arl6ip1
Kif23
Aspm
Nusap1
H2afx
Ckap2l
Sgol2
Ccnb2
Mki67
Birc5
Cdk1
Top2a
Mis18bp1
Cdca3
Cdca8
Kif20b
Arhgap11a
Smc4
Knstrn
Hmgb2
Ccna2
Spc25
Incenp
RP23-45G16.5
Casc5
Kif11
Fam64a
Tacc3
Dlgap5
Kif2c
Tubb4b
Ckap2
Plk1
Kif22
Smc2
Racgap1
Cks1b
Rad21
C330027C09Rik
Cdca2
Ckap5
Pbk

Psrc1
Sgol1
Trim59
Ncapg
Tuba1c
Kif15
Nuf2
Aurka
Aurkb
Dbf4
Cep110
Gas2l3
Bora
Ect2
Anln
Nucks1
Bub1
Cep55
Mxd3
Bub1b
Spc24
Kif20a
Cdc25c
Tmpo
Rangap1
Sapcd2
Anp32e
H2afv
Cks2
Pif1
2700094K13Rik
2810417H13Rik
Ndc80
Troap
Ccdc34
Hjurp
Cenpl
Ckb
Nek2
Kif4
Ska2
Esco2
Pttg1
Nde1
Ska1
Mns1
Kif18a
G2e3

Bub3
Neurod1
Spdl1
Arhgef39
Cep89
Mad2l1
D17H6S56E-5
Cdkn2d
Calm2
Ttk
Lmnb1
Tuba1b
Fbxo5
Hirip3
Cdc25b
Ncapd2
Cdkn3
Hsp90b1
Spag5
Ccdc18
Fzr1
Banf1
2700099C18Rik
Melk
Brd8
Cenpc1
Ccng2
Miip
Hn1
Hmgn2
Hdgf
Pnrc2
Ncaph
Prr11
Kifc1
Cenpp
Nudcd2
Ezh2
Rtn1
Cdk5rap2
Ankle1
Ska3
Nrep
Hyls1
Usp1
Cdkn2c
Cenpm
Fam83d

Ran
Kif14
Lmnb2
Tubb5
Gm10075
Hmgn5
Dek
Apoe
H1fx
Cenpq
Igfbpl1
Sap30
Nasp
Espl1
H2afz
Gpm6a
Rbfox3
Hnrnpa2b1
Shcbp1
Odf2
Ube2t
Myod1
Cep70
Anp32b
Cst3
Cenph
Nup37
Cklf
Ddx39
Hmgb3
Pdzrn4
Rtkn2
Diap3
Plk4
Celf4
Cntn2
Gtse1
Sept3
Pmf1
Pqlc1
Rnaseh2c
Fkbp2
Stmn2
Hmgb1
Lsm6
Pdgfa
Ccnf
Tubb3

Cenpw
Gap43
Gria2
Dtymk
Vbp1
Psat1
Cenpk
Ccdc77
Miat
Ccnd1
Reep4
Elavl3
Rrm1
Gas1
Rad51ap1
Zc3h7a
Barhl1
Hmgn1
Map2
Klf6
Ctcf
Ubald2
Ina
Syt13
Itm2b
Gpsm2
Pcna
Cpe
Kif1b
Fam110a
App
Tubb2b
Cenpt
Smtn
Akirin2
Cep57
Rbmx
Lmo4
Lig1
Dcx
Ing1
Ptprs
Cenpv
Rrm2
Terf1
Gsg1l
Cit
Tgif1

Lbr
Depdc1b
Ralgps2
Bin1
Cdca5
Nhlh2
Arhgap19
Lsm4
Cenpj
Ppp3ca
Cenpn
Cdca4
Ppp2r5c
Tdp1
Ank3
Sec11c
Dnph1
Lsm3
Chek2
Pkp4
Chd7
Frmd4b
Kif18b
Basp1
Vps36
Clic4
mt-Rnr2
Prdx1
Stmn1
Utp3
Fam216a
Zfp704
Ncaph2
Neil3
Celf2
Ctnnb1
Bok
Exosc8
Efs
Ypel3
Suv39h2
Ubb
Ccdc41
Smc1a
Sae1
Ctsd
Wapal
Cmc2

Ctsb
Pcf11
Cplx2
Rfc4
Nrxn1
Mrpl51
Mis18a
Tubb2a
Hist1h2ak
Dynll1
Sephs1
Apc
Rnaseh2b
Vrk1
Stmn4
Tmem138
Smco4
Rhno1
Cdk5r1
Mapt
Gnao1
Med30
Uncx
Eid1
Nfix
Ddx11
Kif5c
Trip13
Rdm1
Serinc1
Taf5
Dpysl3
Tmed9
Hpca
Asap1
Stil
Ccdc61
Zic4
H1f0
Thra
Tmsb4x
Hp1bp3
Pcnt
Elavl4
5830418K08Rik
Lgals1
Zwilch
Mcm6

Chgb
Phf17
CRE_RECOMBINASE
D030056L22Rik
Zcwpw1
Kmt2e
Aldoa
Sox4
Ccp110
Gm1673
Aplp2
Gng3
Mdc1
D4Wsu53e
Ppp1r14c
Hk2
2810442I21Rik
Smarcc2
Gsg2
Lhx1
Nudt4
Iqgap3
Nmral1
Nsg1
Cntln
Nin
Cacna2d1
Calm3
Pde1c
Nsl1
Dclk1
Foxm1
Slc1a2
Ankrd12
Clmp
Snap25
Rbm5
Ift80
Tex30
Npdc1
Cdc27
Gen1
Atp6v0b
Nhlh1
Cntrob
Sptbn1
Mtss1
Sema6a

Eri2
1500012F01Rik
Rnmt
Uchl1
6330403K07Rik
Eme1
Ank2
Mum1l1
Ttc3
Dner
BC005764
Slc22a17
Csrp2
Zbtb18
Pih1d1
Tbata
Ctsl
Cenpi
Mis12
1500016L03Rik
Cep135
Map1b
Mllt11
Tagln3
Tk1
Oip5
Trpc4ap
Map1lc3b
Rundc3a
Nbea
Itsn1
Rcor2
Tbc1d31
Traip
Prkcb
Nktr
2810006K23Rik
Sept4
Clcn4-2
Atox1
Ldhb
Mycbp2
Celsr2
Pfn2
Lap3
Itm2c
Rab6b
Tnik

Igsf8
Tmem57
Pdzrn3
Fabp7
Aplp1
C1ql1
Gnl3
Podxl2
Mapk8ip1
Cnrip1
Hey1
Stxbp1
Cadm3
Phf20l1
Ntm
A330076H08Rik
Psap
Gdi1
Ier2
Klf7
Dnajc5
4631405J19Rik
Chrna3
Sowaha
Tmem66
Kdm5b
Mxd4
Slc1a3
Myt1
Hcfc1r1
Apbb1
Meg3
Dlgap4
2700089E24Rik
Sh3gl2
Clstn1
Abhd16a
Btg2
Pygo1
Aprt
Reln
Pdrg1
Rrbp1
Arpp21
Nt5c
Egr1
Dbn1
Kidins220

Wdr6
Prdm8
Hells
A930011O12Rik
Rab3a
Nrn1
Fam213b
Ung
Srrm4
Elmo1
Kif5a
Zmynd8
Fam21
Col9a3
Sh3bgrl3
Plp1
Pea15a
Lgmn
Gramd1b
Mbp
Glce
Jhdm1d
Kif1a
Mcm2
Cacng4
Rnd2
Cadm4
Nrcam
Pkia
Ctsf
Gabbr1
Gria4
Cnpy1
Gm17322
Ppfia2
Chd3
Cplx1
Tacc2
Ncan
Slc17a6
Gamt
Akap12
Mt1
Hpcal1
Malat1
S100a16
Sbk1
Grina

Ppp1r1a
Sh3bp5
Grik2
B3galt2
1500011B03Rik
Cdt1
Plcb1
H2-D1
Pmm1
Nenf
Fos
Shd
2900011O08Rik
Xist
Hes1
Tubb5
Stmn2
Neurod1
Tuba1a
Tubb3
Rpl13a
Top2a
Rps5
Rps14
Rps9
Gnb2l1
Rpl32
Cntn2
Mki67
Egr1
Gap43
Smc4
Draxin
2810417H13Rik
Map1b
Gas5
Birc5
Rps3
Tpx2
Hmgb2
Rpl8
Cdk1
Rplp2
Stmn4
Jun
Fxyd6
Rplp1
Ube2c

Rps19
Ddah2
H2afx
Rpl4
Miat
Prc1
Rtn1
Nusap1
Cenpf
Cdca8
Spc25
Rps26
Ccna2
Ckap2l
Rplp0
Incenp
Rpl22
Pbk
Ina
Celf4
Rps21
Gpm6a
Eef1a1
Sept3
Arl6ip1
Tubb2b
Esco2
Rps20
Barhl1
Nrxn1
Cbfa2t3
Cenpa
Sparcl1
Vim
Kif23
CRE_RECOMBINASE
Nhlh2
Apoe
Spc24
Kif11
Cdca3
Rps15
Ncapg
Cdc20
Tubb2a
RP23-45G16.5
Elavl3
Smc2

Ccnb1
Ier2
Ppp1r14c
Ccnd1
Btg2
Efhd2
Hmmr
Mfap4
Mmp14
Gpr153
Racgap1
St18
Mis18bp1
Tacc3
Calm2
Rpl39
Mxd3
Casc5
Pabpc1
Arhgap11a
Cdca2
Rpl26
Nuf2
Kif15
Cenpe
Rpl23
Sgol2
Sdpr
Ezr
Rangap1
Rad21
Cbx5
Rrm2
Tex14
Kif22
Nkd1
Hes6
Chgb
Rps11
Tmsb10
Gng3
Fam64a
Cenph
Tead2
Rps15a
Bub1
Sowaha
Fstl1

1500012F01Rik
Aspm
Rps24
Tagln3
Rpl41
Tuba1b
Zmiz1
Aurkb
Basp1
Tk1
Mllt11
Rps18
Sptbn1
Rad51ap1
Sept4
Rpl34
Sgol1
Bin1
Rps3a1
Kif20b
Mns1
Dek
Uchl1
Zfp36l1
Ndc80
Atoh1
Dlgap5
Slc17a6
Cdk5r1
Zeb1
2410006H16Rik
Podxl2
Nucks1
Ckap2
Dner
Calm1
Fbxo5
Slc1a2
Sox9
Rps2
Hn1
Prkcb
Map2
Kif2c
Ccnb2
Dut
Ctsd
Sox4

Eno1
Gsg1l
Rps10
Ntrk3
Rpl14
Sema6a
Sfrp1
Serpinh1
Rnd3
Rab3a
Clspn
Tyms
Knstrn
Npm1
Whsc1
Tmpo
Ckap5
Ank3
Rpl35a
Ska1
Rufy3
Cdkn2d
Dbf4
D430041D05Rik
Pdlim3
Kif4
Fos
Kif5c
Calm3
Cxcr4
Plk1
Apc
Celf2
Rps25
Anp32e
Nnat
Cenpq
Id2
Pou3f2
Tgfb2
Ttk
Eef1b2
C330027C09Rik
Cdh20
Gas6
Hist1h2ak
Anln
Eif3f

Ect2
D17H6S56E-5
Srrm3
Mtss1
E130114P18Rik
Cenpm
Aurka
Gria2
Klc1
Ncapd2
Atad2
Loxl1
Mapt
Bub1b
Myt1
Lmnb1
Hjurp
Elmo1
Angptl2
Stmn1
Sema7a
Diap3
Rrm1
Myt1l
2700094K13Rik
Mad2l1
Fnbp1l
Nhlh1
Dhx32
Ezh2
Dpysl3
Olfm1
Cacng5
Eef2
Lig1
Ctsb
Clmp
Myc
Crmp1
Cenpk
Rpl37
Melk
Hirip3
Ska2
Mdk
Ank2
Rpl18a
Plk4

Gm1673
Rpl7
Pdlim4
Fam111a
Nt5dc2
Prmt8
Pde1c
Ccng2
Ncor2
Stxbp1
Myod1
B3galt2
H2afv
Frmd4a
Stmn3
Gm11478
Fabp7
Zic1
Meg3
Rfc4
Dll3
Itsn1
Atp6v0e
Sept11
Ppic
Gstm5
Ppp2r2c
Ckb
Fosb
Sparc
Pax6
Ptn
Uncx
Plcb1
Rbfox1
Mroh2a
Cenpp
Kcnk1
Nrn1
Gpx8
Rpl18
Ccnd2
Pdgfra
Sfrp2
Gm10260
Ramp2
Ier5
Elavl4

Ncam1
A930011O12Rik
Rad51
Eef1g
Dcx
Ccdc34
Psip1
Aplp1
H2afz
Cog7
Cst3
Rpl37a
Cdc45
Trim59
Fzd1
Pcna
Gm17322
Nasp
Tspan13
H1fx
Trpc4ap
Pea15a
1700025G04Rik
Ptprg
BC005764
Cdc7
Rprml
Npc2
Ncaph
Fen1
Mis18a
Gnai2
Srebf1
Gm13826
Fabp5
Lbr
Clvs1
Cenpw
Rps7
Dnmt1
Lhx1
Cenpj
Gltscr2
Hadh
Gmnn
Snhg1
Cks1b
Cdk6

2810055G20Rik
App
Pmf1
Ppp3ca
Wdr89
Sox1
Lrig3
2410004N09Rik
Mrpl52
Vps37b
Brd8
Hsd11b2
Smpd2
Bub3
Rps28
Eif3e
Atp1b3
Irs1
Rftn2
Dusp5
Nrep
Olig2
Cadm3
Dnajc9
Tubb4b
Cplx1
Hspe1
1110038B12Rik
Snhg6
Usp46
Cacng4
Gm17750
Nrcam
Tceal8
2610203C20Rik
Mgll
Nes
Ttc9b
Dync1i2
Use1
Fkbp7
Hells
Rpl22l1
Rab6b
Ptch2
Kcnip3
A030009H04Rik
Nme4

Pdzrn4
Rtn4
Naca
Trim37
Pnrc1
Smc6
Fyn
Tle1
Igdcc4
Prim1
Rps4x
Bora
Srgap2
Myl12a
Whrn
Kif5a
Chaf1a
Prdm8
Pkia
Odf2
Hsp90aa1
Nsg2
Nav2
Usp1
Elavl2
H2afy2
Tead1
Wdr60
Nfyb
Apbb1
Syt11
Azin1
Strbp
Gm11223
Snord104
Gsto1
Lmnb2
Rcn3
Rbfox2
Dynll2
2810004N23Rik
Uhrf1
Mab21l1
Gdpd1
Ap1s2
Sh3bp5
2900011O08Rik
Nek7

Lpin2
Rbp4
Cmc2
Chd3
Tipin
Dhfr
Igsf3
Cadm1
Lima1
Rab13
Crip2
Actn1
Ndrg2
Epb4.1
Akap12
Nsg1
Plp1
Tcf19
Hey1
Chrna3
Boc
Arpp21
Sacs
Plxnb2
Brd7
Gnl3
Hip1r
Pcdha2
Tnik
Mapk8ip1
Dnmt3a
Cmtm3
Trps1
Stx4a
Glce
Nexn
Adamts1
Srrm4
Prnp
Cep170
Gm26735
Arhgef7
Islr2
Tshz2
Gpatch4
Kif21a
Btbd11
Robo2

Pfn2
Nbea
Hmgcs1
Sstr2
Hddc2
Dusp6
Cttnbp2
Dbn1
Rrp15
Cd1d1
Wwc1
Dusp1
Ifitm2
Gria4
1500011K16Rik
Eif4e3
Zfp462
C77370
Pnmal2
Tspyl4
Gstm1
S100a16
Mt2
Thra
Pak7
Abhd16a
Adcyap1r1
Gria3
Lama5
Mbp
Xist
Top2a
CRE_RECOMBINASE
Mki67
Smc4
Prc1
2810417H13Rik
Cdk1
Spc25
Nusap1
Birc5
H2afx
Smc2
Pbk
Neurod1
Ckap2l
Esco2
Barhl1

Kif11
Kif23
Tpx2
Hmgb2
Incenp
Ube2c
Sfrp1
Cdca8
Arl6ip1
Hmmr
Ccna2
Cenpf
Spc24
Hey1
Igfbpl1
Aurkb
Casc5
Apoe
Cdca3
Ncapg
Cdc20
Sgol2
Dek
Ptn
Draxin
Kif22
Tacc3
Ccnb1
Ndc80
Slc29a1
Tubb5
Kif15
Srebf1
Rps26
Mis18bp1
Cdca2
Mxd3
Tk1
Gm17322
Fbxo5
Cenph
Bub1
Cenpa
Fam64a
Lhx1
Tuba1b
Arhgap11a
Ckap2

Nuf2
D17H6S56E-5
Cd63
Itm2b
Kif2c
Sgol1
Cenpe
Rtn1
Rad51ap1
Bub1b
C1ql1
Kif20b
Cdkn2d
Melk
Aspm
Miat
Gpm6a
Rassf4
Clspn
Calm2
Nucks1
Ncapd2
Rrm2
Cst3
Nrxn1
Meg3
Kif4
Celf4
Crmp1
Dbf4
Knstrn
Cenpq
Ccnd2
Fam210b
Hist1h2ak
Islr2
Racgap1
Cntn2
Mns1
1500016L03Rik
Ckap5
Anln
Rad21
Cbfa2t3
Aurka
Gamt
Diap3
Ect2

Rangap1
Ccnb2
Dpysl4
Dlgap5
Mad2l1
Ctsd
RP23-45G16.5
Mdk
Fam111a
Cenpk
Fabp7
Lig1
Gap43
Rrm1
Elmo1
Dut
Atp2b1
C330027C09Rik
Plk4
Ppp1r14c
Tyms
Mroh2a
Anp32e
Ska2
Tmpo
Cenpw
Nnat
Dpysl3
Rpl35a
Tubb2a
Ctsb
Sparcl1
Trim59
Sept3
Mycn
Hsd11b2
Selm
Rcor2
Atad2
Atoh1
Rad51
Pde1c
Ncaph
Slc1a2
Ezh2
Cenpm
Mapt
Ccng2

Malat1
Hes6
Usp1
2700094K13Rik
Cacng4
Arpp21
Tubb4b
Vim
Cdkn2c
Eln
Lmnb1
Gmnn
Plp1
Tprn
Nek6
Prdm8
Cxxc5
Trpc4ap
Ank2
Sema7a
Cks1b
Ddit4
Tcf19
Dnmt1
Rpl18a
Ccm2
B2m
Mtss1
Cacng2
2310022B05Rik
Klf6
Fxyd6
Hes1
Bcl7a
Sptbn1
Add3
Ccdc34
1500012F01Rik
Aldoa
Sstr2
Egr1
Rnd3
Pcna
St18
Atp6v0e
Uhrf1
Stmn4
G2e3

Tspan7
Hirip3
Dnajc9
Dner
Heg1
Kcnk1
Nsmf
Mt1
Slc1a3
Mfap4
Hells
Fbxo32
Sema6a
Gse1
Zmynd8
Aplp1
Thra
Stxbp1
Rab3a
Myt1l
Tubb3
Igfbpl1
Stmn2
Ckb
Miat
Pde1c
Cntn2
Nnat
Tuba1a
Nhlh2
Ccnd1
Neurod1
Smc2
Ddah2
Dek
Nhlh1
Elavl3
Lhx1
Top2a
Mki67
Gap43
Hmgb2
2810417H13Rik
Chrna3
Pdzrn3
Birc5
Anp32b
Rtn1

Nrn1
Tpx2
Cenpf
Sept4
Cdk1
Elavl4
Basp1
Nfix
Nfib
Nusap1
Cdca3
Bin1
Pbk
H2afx
Map2
Tuba1b
Cdca8
Smc4
Cbx5
Ccna2
Pdgfa
Kif11
Ina
Spc25
Dut
Spc24
Map1b
Clmp
Ube2c
Prc1
Mllt11
Nasp
Cenpa
Cenpe
Incenp
Dbi
Tmpo
Esco2
Crmp1
Egr1
Ranbp1
Gpr153
Ncapg
Ckap2l
Tcf4
Cks1b
Pcna
1500016L03Rik

Zic1
Kif23
Ank3
Mcm6
Kif15
Uncx
Kif5c
Srrm3
Rrm2
Casc5
Cdc20
Stmn4
H2afv
Usp1
Tex14
Ran
Tubb2b
Nucks1
Cxxc5
Abhd16a
Syt11
Ccnb2
Pqlc1
Knstrn
Cog7
Ccnb1
Apc
Nrep
Ckap2
Tmsb4x
2700094K13Rik
RP23-45G16.5
Rplp1
Ptprs
Clspn
Cenph
Myt1
Hmmr
Anp32e
Hes1
Rad51ap1
Aurkb
Sgol2
Tyms
Dcx
Mis18bp1
Kif20b
Cdca2

Fbxo5
Cltb
Zfp36l1
Hirip3
Tacc3
Nkd1
Lig1
Dnajc9
Dtymk
Nsg2
Sparcl1
Gas1
Cenpm
Npm1
Tbata
Cdk5r1
Tagln3
Rbp4
Gnai2
Gng3
1700025G04Rik
Dclk1
Kif22
Serbp1
Mxd3
Tk1
Nuf2
Prim1
Aspm
Ndc80
C1ql1
Gm10075
Apoe
Thsd7a
Atad2
Slc17a6
Sgol1
Rab6b
Tmsb10
Bub1
Gmnn
BC005764
Mapk8ip1
Ccdc34
D17H6S56E-5
Nop58
Arhgap11a
Rpa2

Mns1
Gsg1l
Rcor2
Kif2c
Sstr2
Sfrp2
Cenpq
Hmgn5
Txn1
Lap3
D430041D05Rik
Marcksl1
Chgb
Podxl2
Hells
Kif4
Prdx4
C330027C09Rik
Angptl2
Pmf1
Rrm1
Elavl2
Mcm3
Cenpk
Klc1
CRE_RECOMBINASE
Prdx1
Hint1
Paics
Dlgap5
Chaf1a
Bub1b
Hsbp1
Gm2694
Rab3a
Tipin
Rufy3
Fam64a
Arl6ip1
Cnbp
Trim59
Gm17750
Ttc3
Cdca7
Mex3a
Cenpp
Mt1
Ppib

Cplx2
Kmt2e
Pax6
Cdkn1b
App
Plk4
Lmnb1
Tubb4b
Nap1l1
Hk2
Stmn3
Nt5dc2
Rangap1
Rbfox2
Anln
Gsk3b
Hmgb1
Melk
Fkbp3
Chd4
Barhl1
Dkc1
Sema6a
Aurka
Mad2l1
Sptbn1
Igsf8
B2m
Mcm5
Hrk
Arhgef2
Sowaha
Ncaph
Ect2
Sox9
H2afz
Dpysl4
Rfc4
Ebf3
Ncl
Celf2
Racgap1
Dbf4
Hjurp
Rbbp7
Itsn1
A330076H08Rik
Afap1

Draxin
Rps19
E130114P18Rik
Brd7
Supt16
A930011O12Rik
Snrpb
Slc3a2
Frmd4b
Rtn4
Rif1
Cklf
Mcm7
Rad51
Uhrf1
Lrig3
Slc7a5
Ppp2r2c
Hmgn2
Atp2b1
Siva1
Gdpd1
Vim
Nde1
Banp
Rad21
Fam111a
Pabpc1
Sept8
Ybx1
Hnrnpd
Diap3
Smco4
Atp1b3
Rpl41
Nop56
Eef1d
Fth1
Ncapd2
Gria2
Fos
Myod1
Dixdc1
Jun
Lsm2
Mif
Hist1h2ak
B3galt2

Hpca
Cbfa2t3
Ddr1
Smc6
Rplp2
Banf1
Hsd11b2
Idh2
Cd24a
Tead2
Topbp1
Fstl1
Hat1
Soga3
Dnmt1
Klf7
Pard6a
Fosb
Snrpd1
Gli1
Hip1r
Pkp4
Chd7
Usp22
Rps25
G3bp1
Srsf7
Clic4
Snhg1
Cep57
Rpl22
Usp46
Lgals1
Dtl
Hmgn3
Pa2g4
Snrpf
Lmo4
Lsm6
Scg3
Isoc1
Mcm2
Rpl39
Spop
Dctpp1
Aldoa
Dctn3
Dnph1

Skp2
Hdgf
Nolc1
Rps20
Schip1
Cep110
Sri
Ccdc41
Nsmce4a
Celf4
Cdca7l
Irs1
6330403K07Rik
Ckap5
Carhsp1
Rpl22l1
Rundc3a
Lbr
Serinc1
Cacna2d1
Dhfr
Napa
Cenpw
Nudcd2
Sept3
Ezh2
Lyar
Kif1b
Pttg1
Atp6v0e
H13
Zic4
Naa50
Rfc2
Pou3f2
Mrpl42
Cald1
Itm2b
Tpm4
Rfc1
Gdi1
2610203C20Rik
BC031181
Pfn1
Cerk
Gng2
Uchl1
Timeless

Hsp90b1
Clic1
Ctnnb1
Hes6
Lmcd1
Fen1
Cbfb
Csrp2
Ier2
Suz12
C530008M17Rik
Mab21l1
Psat1
Mab21l2
Rnaseh2b
Ctsd
Add3
Ptn
Rnmt
Atoh1
Cdc45
Alyref
Ddx39
Shmt1
Fbxo32
Nr3c1
Sae1
Acot7
D4Wsu53e
Ska2
Gpr56
Cdt1
Syt13
Ddx21
Bub3
Rpa1
S100a16
Atp6v1e1
Cst3
Fam213b
Cmtm3
Tcf19
Dlgap4
Btbd17
Stx4a
Magoh
Nrm
Pik3r3

Map1lc3b
Chst15
Mphosph10
Plk3
Smpd2
St18
Mtap
Gramd1a
Ube2d1
Vrk1
Pola1
Cadm1
Smarcd1
Ntm
H1fx
Aplp1
Gpc2
Mis12
Cntln
Fabp7
Ccdc18
Fnbp1l
Tprn
Ybx3
Adamts1
Clip3
Ccdc50
Ramp2
Dda1
Id2
Nudc
Bcl2l1
Necab3
Boc
Scg5
Frmd4a
Prkcb
Clvs1
Ldha
Smc5
Clcn4-2
Dbn1
Mmp14
Maged2
Lpin2
Mcm4
Tubb2a
Snx5

Ptch2
Nin
Apitd1
Chd3
Strbp
Hist3h2a
Igsf21
Gadd45a
Srebf1
Fibp
Rad50
Fam107b
Rrbp1
Mpp6
Srgap2
Nsg1
Npdc1
Tmem57
Oraov1
Kdm5b
Mien1
Elovl6
Atp6v0b
Gm11266
Acd
Nav2
Enox2
Zic5
Zfp423
Kif5a
Pvrl3
Brca2
Stxbp1
Myt1l
Sbk1
Glul
Ldb1
Rnf165
Sox11
Map6
Ppp2r2b
Cep290
Psmc3ip
Dner
Grb2
Smpd3
Smarcd2
2810025M15Rik

Pak7
Trpc4ap
Ncor2
Scrt1
Atad5
Gamt
Tmx4
Cadm3
Efhd2
Mical1
Klhl7
Dcc
Pygo1
Bzrap1
9330159F19Rik
Rnf122
Tspan7
Nek7
Atl1
Nova2
Dusp8
Crip2
Nol4
Trak1
Slc1a3
Scmh1
Myc
Smoc1
Brsk2
Arid3a
Baz1a
Eif4e3
Srrm4
Prmt2
Tmem178b
Plcb1
Pea15a
Grina
Mkrn1
Apbb1
Clybl
Tmeff1
Rnasel
Pdgfra
2700081O15Rik
Mroh2a
Clstn1
Kif1a

Mapk10
Itga7
Nrcam
Dpysl3
Gramd1b
Lingo1
Shd
Tubb4a
Parp6
Atat1
Reln
Ncan
Rnd2
Fam57b
Eml5
Plp1
Celf4
Neurod1
Meg3
Mapt
Arpp21
Gpm6a
Neurod2
Stmn2
Nrxn1
Stmn4
Tubb2a
Dpysl3
Ank2
Sept3
Thra
Rtn1
Ppp1r14c
Nrep
Mtss1
Gria2
Celf2
Atp2b1
Map1b
Zic1
Calm1
Tuba1a
Rps9
Rpl32
Rps5
Rpl8
Rps3
Rpl13a

Rps26
Rps19
Rplp1
Sfrp1
Pabpc1
Rps20
Rpl4
Car10
Fxyd6
Gnb2l1
Pcp4
Rps14
Rplp2
Rbfox3
Malat1
Gap43
Rpl22
Jph4
Ccnd1
Rps11
L1cam
Sez6
Ttc3
Cd63
Rplp0
Eef1a1
Ccnd2
Rps21
Elmo1
Gng3
Plxna2
Smc2
Rpl41
Npm1
Prdx1
Cntn2
Tubb2b
Ankrd12
Ranbp1
H2afv
Anp32b
Dner
Cdk5r1
Rps15
Cadps2
Tmsb4x
Rps3a1
Cdk4

Gnao1
Dusp26
Rps24
Eef1b2
2810417H13Rik
Kcnk1
Rpl14
Smc4
Cadm2
Mdk
Ina
Nbea
Basp1
Anp32e
E130114P18Rik
Zfpm2
Camk2d
Srebf1
Draxin
Uchl1
Hmgb2
Sv2a
Snca
Dbi
Rpl39
Dcx
Mki67
Zfand5
Zbtb18
Kcna1
Kif1b
Phf20l1
Psat1
Tspan4
Stmn3
Gnai2
Ppp3ca
Cks1b
Grin2b
2900079G21Rik
Cadm3
Rnd3
Aplp1
Gm12022
Camk4
Sh3gl2
Prkcb
Prdm8

Rps15a
Prkce
Rab3a
Tnik
Chd7
Ndst3
Top2a
Cygb
Nop58
Anks1b
Grin1
Fkbp3
Hmgn5
Epb4.1l1
Ryr2
Marcks
Tead2
Rps27l
Dek
Mcm6
Serinc1
Lrrtm2
Ephb1
Id2
D3Bwg0562e
Dut
Cpe
Rps2
Synpr
Ybx1
Hnrnpab
Pvrl3
Tbata
Pcsk1n
Rpl26
Pax6
Pcna
Sema6a
Rbp4
Nasp
Nhp2
C1ql1
Rpl35a
Stxbp1
Gabra2
Tmpo
Gsg1l
Rbfox1

Birc5
Ppp1r14b
Ran
D4Wsu53e
Tpx2
Cplx1
Snrpf
Porcn
Pkia
Tuba1b
Cdk1
Rps25
Mllt11
Ncam1
Apbb1
Dtymk
Grm1
Grik2
Hey1
Atp6v0e
Cdca8
RP23-45G16.5
Rps10
Fnbp1l
CRE_RECOMBINASE
Hmgn1
Prdx6
Ier5
Shfm1
Eid1
Rpl34
Elavl3
Kif5c
Syp
2700094K13Rik
Tomm7
Ube2c
Hsd11b2
Spc24
Slc25a5
Cenpf
Cog7
Tagln3
Cadm1
Dctpp1
Cenpa
Rpl23
Racgap1

Hirip3
H2afy
St18
Myl12a
Paics
Pbk
Lsm6
Itsn1
Nap1l1
Gnaq
Kif5a
Prmt8
Kcnk2
Cenpe
Rufy3
BC005764
Rps18
Mycbp2
Nsg1
Dpysl4
Hook1
Sh3bp5
Trpc4ap
Sox9
Pcdha2
Sox4
Ube2ql1
H2afx
Rpl18a
Cdca3
Fam63b
Siva1
Sfrs18
Mcm2
Ptms
Ppic
Gm10075
Srcin1
Hnrnpm
Fam210b
Apex1
Itgb1
Ntm
Cnbp
Bola2
Prdx4
Prim1
Banf1

Scg5
Pqlc1
Gpr153
Serbp1
Prc1
Ncl
Ccna2
Ifi203
Aplp2
Fut9
Slc29a1
Hmgn2
Snrpd2
Snrpe
Pdgfa
Eif3f
Trp53
Hdgf
Apc
Camta1
Nnat
Cdh20
Lsm4
Snap25
Itm2b
Gng2
Nefl
Atp5e
Cbx5
Nsg2
Scaper
Nusap1
Snrpb
Mbnl2
Nfib
Ndrg4
Cenpv
Ccdc34
Diras2
Scg2
Cdca7
Kcnc1
Chgb
Myt1l
5830416P10Rik
Ckap2l
Srsf7
Map1lc3a

Galr1
Nop10
Ezr
Rpl22l1
Hes6
Dnajc9
Lyar
Tpm4
Naca
Commd1
Tox3
mt-Nd1
Npdc1
Chd3
Spock2
Kif11
Lig1
Rrm2
Eef1d
Lgals1
Mycn
Gdi1
Ly6h
Arhgef7
Lap3
Rpl7
C1qbp
Mcm7
Zeb1
Spc25
Tyms
Sv2b
Bok
Nrxn3
Rrm1
Mns1
Rgs17
Tacc3
Hnrnpu
Smarcc2
Ssrp1
Camk2b
Zwint
Dync1i2
Casc5
Mpp3
Runx1t1
Incenp

Atp1a3
Knstrn
Ckb
Clspn
Cklf
B2m
Pkm
Tipin
Btbd3
Pcbp1
Park7
Atp6v0b
Tnrc6c
Prnp
Hmmr
Pgm2l1
Cntn1
Tshz2
Sphkap
Adcy1
Rnaseh2c
Pfn1
Sub1
Rpl37
Pdia6
Rps4x
Calb2
Snrpd1
Luc7l3
Nrxn2
Cdc20
Esco2
Rnasel
Sptan1
Ctnnb1
Idh2
Rpa2
A930011O12Rik
BC029214
2810004N23Rik
Nfix
Zmat4
Gm1673
Snrpg
Gm9800
Hspe1
Dkc1
Atp6v1e1

Nmral1
Adh5
Vps37b
Hells
Eif3i
Smarcc1
Abracl
Tmsb10
Cenph
Ssbp3
Uba52
Snrpd3
Ptprs
Rab6a
Ptma
Rfc4
Ccnb2
Ppfia2
Rtn4
Rps8
Mrpl52
Add3
Usp1
Rps7
Csnk1e
Txn1
Rsl1d1
Reep2
Adrbk2
Ndufa2
Rpl14-ps1
Hmgb1
Mif
Homer2
Calm2
Kidins220
Pou3f2
Gm8292
Ndufa12
Cplx2
Slc3a2
Cct3
Rbfox2
G3bp1
Kif23
Chchd2
Smco4
Tmem145

Elavl4
Psma4
Fgf13
Pnmal2
Ezh2
Hjurp
Adam10
mt-Rnr2
Mmp24
Unc5c
Sod1
Asap1
Tacc2
Mllt4
Srsf6
2900011O08Rik
Smim11
Supt16
Tcp1
Slc1a2
Gmnn
Ddx39b
Kazn
Nudt3
Ncapg
Mcm3
Cadps
Cbfa2t3
Ppib
Snhg1
Lrig3
Nudcd2
Gar1
Timp3
Atoh1
Ndn
Mrpl13
1810009A15Rik
Mtch2
Srsf3
Fam115a
Chaf1a
Tubb4b
Smarca5
Map2
Tspyl4
Cep170
Gm13826

Larp7
B3galt2
Reln
Islr2
Peli2
Soga3
Pde1c
Cnrip1
Cct2
Arpp19
Cald1
Hpcal1
Klf7
Rpl37a
Sox18
Clcn4-2
Rpl6
Rpa3
Trim59
Bcl11a
Pdzrn4
Syt1
Gas1
Cdc42
Isoc1
Hnrnpc
Mab21l1
Ank3
Sowaha
Rbm8a
Uhrf1
Ckap2
Grina
Npc2
Kif15
Ywhag
Mcm5
Clcn3
App
Pa2g4
Psmb2
Cirbp
Srrm2
Smpd2
Cnn3
Atxn7l3b
Igfbpl1
Sep15

Cenpm
Rpl18
Gm10260
Prrt1
Ccnb1
Klc1
Sfrp2
Hnrnpd
Nolc1
Aurkb
Egr1
Ptn
Nicn1
Rab6b
H2afz
Tulp4
Fstl1
Cd200
Mrpl17
Pja2
Clasp2
Cst3
Rbbp7
Cct5
Rpl38
Cacna1a
Baz1a
Rtn3
Gm11541
Snrpb2
Glce
Pmf1
Chchd1
Naa38
Ddx21
Gabbr1
Pafah1b1
Eef1g
Emg1
Polr2f
Adamts1
Eif5a
Kifap3
Uqcrq
Kmt2e
Rpl29
Arhgap20
Actb

Snrpa1
Sgol2
Lman1
Apoe
Lin7a
Gng5
Kif20b
Pak1
Arhgap11a
Eif3k
2210016L21Rik
Grb2
Mis18bp1
Ntrk3
Tceal5
Nop56
Marcksl1
Ndufb6
Mrpl42
Pbdc1
Nucks1
Sez6l2
2810025M15Rik
Sae1
Nuf2
Bub1
Cenpq
Tubb3
Ccl27a
Mt1
Rpl13
Atp5b
Clic4
Snap47
Thoc7
Ubxn1
Hivep2
Set
Hes1
Cdk6
Rnmt
Golim4
Eif3d
Tcf25
Ypel3
Fkbp1b
Hspa5
Atrx

Eny2
Rps16
Dpm3
Alcam
Mmp14
Fbxo5
Mphosph10
Rps6ka3
2010107G23Rik
Rbm5
Kcnd2
Mbd3
Erc1
Myod1
Sparcl1
Sema7a
Hspd1
2010107E04Rik
Gas5
2310044G17Rik
Zfp36l1
Polr2h
Eif3g
Ssr3
D17H6S56E-5
Lsamp
Cacna1b
Psmb6
Ndufa3
Bccip
Sptbn1
Nek6
Rbbp4
Eif4a1
Wbp5
Uqcrh
Optn
Scd2
Serpini1
Acot7
Uqcr11
2410066E13Rik
Cnih4
Sec11c
Serping1
Kcnj3
Hint1
Wdr89

Pole3
Nktr
Gtpbp4
Nup62
Cacna2d1
Mxd3
Smarca2
Srsf9
Spcs2
Ebna1bp2
Lsm3
Slc25a3
Cpne3
Irf2bpl
Dbn1
Dnph1
Cdk14
Parp1
Fam168a
Rad51ap1
Tspan3
mt-Cytb
Cxcl12
Magoh
Eno1
Map3k12
Insm1
Minos1
Gjc1
Bcas1
Pitpnc1
Gpm6b
Mak16
2310022B05Rik
Ostc
Pdrg1
Akap9
Ppp3cb
Ntrk2
Btf3
Lsm2
Cct8
Hspa9
Actl6a
Gm17322
Ndufb9
Lmnb1
Slc4a4

Hat1
Atad2
Pdgfra
Tcf3
Mrpl18
Serp2
Uncx
Cdca2
Eif3e
1110038B12Rik
Mad2l1
9330159F19Rik
Manf
C330027C09Rik
Ddx17
Syncrip
Trim2
Sgol1
Laptm4a
Adk
Mybbp1a
Nsmce1
Fam162a
Fmnl2
Tk1
Fundc2
Shmt1
Plcb1
Etfa
Gli1
AI854517
Mrps14
Sf3b5
Kif2c
Gria4
mt-Nd2
Ramp2
Macf1
Ivns1abp
Ybx3
Tmem256
Srgap2
Cox6c
Gstm5
Ect2
Gltscr2
Tmem57
Bub3

Canx
Ndufv3
Hmg20b
Tle1
Cox7b
Kbtbd11
Zfp521
Cfdp1
Actl6b
Gse1
Cnpy2
Psmb1
1700001O22Rik
Ubb
Cnksr2
Lrpap1
Dhx32
Mettl9
Zeb2
Csrp2
Igsf21
Psmc4
Nde1
Ndufc2
Ppa1
Cenpp
Psma7
Tia1
Naa10
Tspan13
Srrm4
Ccdc88a
Eif3h
Snap91
Kif22
Aspm
Vgll4
Alyref
Dnajc2
Atxn10
Barhl1
Syn2
Ccm2
Gnl3
Myl12b
Ncor2
Rftn2
Cltb

2610203C20Rik
Ift74
Ccdc41
Dtl
2410015M20Rik
Psmg4
Tsc22d4
Mrpl15
Clk1
P4hb
Cnr1
Rpl35
Hnrnpl
Phf5a
Luc7l2
Mad2l2
Cbln1
Tpt1
Polr1c
Odf2
Map1lc3b
Dpy30
RP23-32A8.1
Gabarapl1
Pttg1
Cdh4
Rad51
Rb1cc1
Ncor1
Utp3
Carhsp1
Cep57
Arl6ip1
Mical1
Dnajc8
Reep5
Nek7
Ptch2
Itm2c
Nptn
Rims1
Efs
Rif1
BC018507
Rrs1
Syt13
Gpx1
Gm11478

Smim18
Gins2
Rcc2
Msra
Nrcam
Pja1
Myt1
Plk4
Ak2
Kdelr2
Cmtm3
Eif3b
Hsp90b1
Prkacb
Fxr1
Nptxr
Vars
1700025G04Rik
Utp14a
Cenpw
Cers4
Rpl36al
Parp6
Pura
Snrnp40
Akap12
Cenpk
Srsf2
Gatsl2
Npepps
Tmem107
Pcbp2
Prmt5
Cdc45
Scrn1
Ddx39
Smoc1
Ndc80
Ssna1
Mroh2a
E2f1
Mfap4
Ifitm2
Fzd2
Mrpl33
Nol4
Fscn1
Taf1d

Efhd2
Hist1h2ak
Dad1
Dhfr
Rfc1
Napb
Hmgn3
Pafah1b3
Lims1
Gm9843
Amph
Rwdd3
Tm7sf2
Napg
Rpl3
Exosc8
Dnajc15
Pde9a
Ppp2r3a
Polr2e
Sh3bgrl
Gspt1
Slirp
Adarb1
Lmo4
Coq7
Mrfap1
Ube2e3
Paxbp1
Fth1
2700029M09Rik
Pcbp4
Dcakd
Pdlim7
Rrp15
Rpl30
Naa50
Stxbp5l
Boc
Dctn2
Romo1
H1f0
Ndufs6
Atp6v1a
Bhlhe22
Hist3h2ba
Mvd
Chga

Fubp1
Dync1li2
Ncaph
Cib2
Mki67ip
Pgrmc2
Syngr3
Gsk3b
Txnrd1
Dnajc5
Tsc22d1
Brd7
Ndufs8
Fam212b
Gprin1
Ldha
Os9
Nrm
Gpatch4
Tgif1
Sec61b
Rnaseh2b
Peg3
Sobp
Mcm4
Klf9
Rps28
Casp3
Clip3
Rassf4
Brix1
Angptl2
Pnrc2
Dlgap5
Cxxc5
Topbp1
Cdkn2c
Nin
Lhx1
Laptm4b
Atp6ap1
Pard3
Cdc42se1
Eci2
Polr2i
Cask
Dnmt1
Mrps28

Rab3c
Atp1b3
Gpr56
Syt11
Rps24-ps3
Snx32
Mapk8ip1
Nudc
Atp6v1b2
Fkbp4
Eif3a
Mrpl21
Fen1
Usp22
Tead1
Ptp4a2
Kif4
Timeless
Kat6b
Tceal8
Gab1
Bub1b
Fam111a
Clvs1
Lta4h
Serp1
Tcf19
Psmc3ip
Micu3
Ap3d1
Lbr
Gprasp1
Taok3
Ska2
Rabgap1l
Clic1
Mpp6
Rnaseh2a
Hk2
Dbf4
Bin1
Gm10076
Map7d1
Efr3b
Rufy2
Rpf2
Mxd4
Rabac1

Mndal
Nop16
Tcof1
Imp3
Cep110
Srm
Tmem151b
Smpd3
C530008M17Rik
Zmynd8
Abcc5
Myc
Anln
Melk
Cbfb
Tspan6
Phactr1
Lman2
Cnih2
Suclg1
Uba1
Cerk
Ctnna1
Ing4
Sh3glb1
Ndrg3
Celf5
1700020I14Rik
Cisd2
Dpysl2
Ier2
Samm50
Pdia4
Prkd3
Zic2
Klf13
Irs1
Ift27
Ncapd2
Lsmd1
Add1
Lphn3
Cdca7l
Ftsj3
Rcn1
Sema4g
Dnm1l
Serf1

Mrps17
Nme2
Suv39h2
Hnrnpf
2610017I09Rik
Rassf3
Kif1a
Mrpl12
Tspan5
2610001J05Rik
Ctps
Gm26735
Rbbp8
Mum1l1
Csrnp3
Dclk1
Strbp
Dlgap4
Eml4
Diap3
D19Bwg1357e
Dgkd
Zfp367
Serpinh1
Mpdz
Apc2
Rfc3
Gabrg2
Dph3
Vrk1
Ipo5
Olfm1
Baiap2
Pold2
Wdr12
Lin7c
Rars
Rad21
Tmem132a
Hpca
Sqstm1
Hmgb3
Gpatch8
Snrpn
Cdc42se2
Nfyb
Ptprd
Jam3

Eif2s1
Aurka
Man1c1
Gm6472
Vamp2
Fam96a
Eef1e1
Agtpbp1
Snx5
Hprt
Prdx5
Mtap
Yif1a
Ankra2
1810037I17Rik
Hcfc1r1
Plekho2
Cd63-ps
Zfp57
Trim27
Pacsin1
Atp6v0e2
Dixdc1
Bod1l
Cmc2
Atcay
Snx1
Zfp148
Chchd3
Mapre2
Exosc7
Abi2
Ncdn
Gcsh
Mest
Mapk8ip2
Ndufaf2
Riok3
Atp6v0a1
Cd164
Ptpra
Bcar1
Ppa2
Sept6
Klhl29
Gins1
Fam57b
Gnl3l

Ptbp1
Ip6k1
Nfasc
Etfb
Dusp8
Pea15a
Gng12
Zranb1
Cxadr
Wbp2
Strip1
Ptges3
Ninj1
Pom121
Evl
C77370
Smc5
Ptprg
Ccnl2
Zfp277
Gtf3c6
Elavl1
Arl6ip6
Zcwpw1
Tprn
Mlf2
Atp9a
Cntln
Smap1
Cdc42bpa
Plekha1
Map6
Arhgef2
Synj1
Cdt1
Gart
Phf3
Cdca4
Gstp1
Znrd1
Cenpj
Ppp2r2b
Mdga1
Scamp1
Hn1
Ogt
Gphn
Pkn2

Rev3l
Slc7a5
Stx7
Mycl
Zcchc18
Camsap2
Hid1
Aig1
Mlec
Dpysl5
Zfp608
Arhgef25
Asrgl1
Trafd1
Ypel5
Agap1
Bola1
Ensa
Mkrn1
Rabep1
Mef2a
Igdcc4
Fam64a
Fam171b
Cnpy1
Prpf40b
Gm13092
Osbpl1a
Idh1
Arl8a
Mgst3
Rpl7a
Abtb1
Tspyl1
Arhgap21
Slc17a6
Fam171a2
Pabpn1
Dars
Tex9
Mrpl36
Robo2
Mbd6
Fh1
A830010M20Rik
Hist3h2a
Itgb3bp
Erbb4

Mis18a
Jarid2
Gdpd1
Apitd1
Pick1
Ubr7
Nudt21
Igsf3
Klhdc3
Ssbp2
Nae1
Mapk10
Sap30
C130071C03Rik
Col9a3
Slc1a3
Eftud2
Rpa1
Xrcc5
Slc22a17
Prr13
Actr2
Ttc4
Dnaaf2
Zfp292
Ctnnd2
Vegfb
Rps16-ps2
Mthfd1
Rusc1
Zfp318
Pfkm
Sgip1
Polb
Ubl3
Rangap1
Lamp2
Pfn2
Ccdc58
Ccdc18
Tmem63b
Ppm1l
Snx10
Pak7
A630007B06Rik
Actr1a
Wdr13
Snord104

Zc2hc1a
Ppat
Nme4
Jakmip2
Smarca1
Cdk5rap2
Plxnb2
Dis3
Nr3c1
Gamt
Nhlh2
Ldhb
Pola1
Syngr1
Ctsz
Pik3r2
Ankrd46
Scamp2
Trappc3
Lpgat1
Vcan
Mtch1
Gsto1
Sept8
Rpl3-ps1
2510002D24Rik
Mrps15
Gpc2
Tspan7
Flywch2
Necab3
Akap8l
Grik5
Tmem30a
Cdk5rap3
Odc1
Samd14
Clstn1
BC034090
Pcgf2
4930402H24Rik
Tmem237
Brca2
Alg2
Fam184a
A330076H08Rik
Tnrc6b
Nedd4l

Fabp7
Stox2
Klhl13
Zfp536
Ndrg2
Pkp4
Pkig
Araf
Ctsd
Mpped2
Socs7
Prmt2
Tom1l1
Lima1
H1fx
Rnpc3
Cspp1
Fos
Fbxl15
Mau2
Agpat4
Shd
Orc2
Scn8a
Senp7
Lphn2
Rps23
Tmem178
Nol12
Gdap1
Ctnna2
Rps27a
Gm5124
Fbxo11
Fbxo21
Pcmtd1
Stau2
Lpin2
Rrp1b
Plcb4
Phf21b
Wasf2
Tro
Tpi1
Ctxn1
Mrto4
Hcfc2
Hmgcs1

Mapk9
Dennd2a
Bdh1
Oraov1
Wdr60
1500011B03Rik
Armcx1
Rnf165
Kdm5b
Tram1l1
Cyfip2
Pip5k1c
Sox5
D430019H16Rik
Gabrb3
Rhebl1
Blcap
Nefm
Chd9
Elovl4
Socs2
Tsga10
Map9
Cd99l2
Tmx4
6330403K07Rik
Sqle
Podxl2
Atat1
Peo1
Gtf2f2
Enho
Celsr2
Tbce
Usp33
Exosc5
Tmem35
Tmem176b
Slc25a27
Akap11
2810008D09Rik
Rps13
Necap1
Rbm33
Hyi
Gdap1l1
Ltbp3
Flot1

Fbxo9
Ddx26b
Pmvk
Nipsnap1
Ophn1
Unc13a
Kcnq2
Arhgef9
Narf
Vopp1
Reep1
Tln1
2510009E07Rik
Fnbp1
Dcaf6
Chfr
Prkx
4632415L05Rik
Phyhipl
Kif3c
Map1a
Camsap1
Zfp329
Ddhd2
Myo9a
Wsb2
Ncoa7
Nsf
Zdhhc17
Ctsf
Xpr1
Bmyc
Zfyve27
Thsd7a
Asxl3
Tspyl2
Ppp1r21
Lingo1
Nenf
RP23-199B2.4
Csnk1g1
Tmem127
Clybl
Tsc1
Vezt
Lztr1
4933427D14Rik
Mgll

Eml5
Ppp1r1a
Gm10036
Ttc28
Dmxl2
Dip2b
Dctn1
Meis3
Pik3r3
Egfr
Tceal3
Sbk1
Gm3764
Limk2
Ntn4
BC068157
Casd1
Mgat5b
Tecpr1
Zer1
Mmp16
Jhdm1d
Plp1
Fosb
Cntn2
Neurod1
Tubb3
Gap43
Nhlh2
Stmn2
Miat
Map1b
Tuba1a
Rtn1
Ckb
Tmsb4x
Nhlh1
Tubb2b
Tex14
Basp1
Dpysl3
Ina
St18
Stmn4
Pdzrn3
Trpc4ap
Ccnd1
Sept3

Rplp1
Map2
Sept4
Igfbpl1
Zic1
Rpl13a
Tubb2a
Podxl2
Ppp1r14c
Itm2b
Gpm6a
Ddah2
Ank3
Chgb
Nrep
Rpl8
Dek
Smc2
BC005764
Mtss1
Elavl4
Draxin
Cdk5r1
Celf4
Nrxn1
Clmp
2810417H13Rik
Gng3
Dcx
Apc
Myt1
Nfib
Anp32b
Rab3a
Bin1
Sema6a
Celf2
H2afv
Elmo1
Rplp2
Malat1
Rps5
Ranbp1
Sfrp1
Elavl3
Myt1l
A930011O12Rik
Mki67

Top2a
Rpl4
Pabpc1
Rps19
Hmgb2
Itsn1
Npm1
Gria2
Srebf1
Prdx1
Mllt11
Dut
Aplp1
Rps26
Dbi
Rpl32
Cadm3
Cadm1
Chrna3
2700094K13Rik
Rps20
Sptbn1
Pax6
Nasp
Anp32e
Gnai2
Kif5c
Tnik
Pcna
Uncx
B3galt2
Galr1
Rbfox3
Birc5
Tmsb10
Tcf4
Mcm6
Ttc3
Syt11
Dner
Rps11
Rpl41
Rpl22
Tuba1b
Rbp4
Gsg1l
Ube2c
Tpx2

App
Smc4
Hsd11b2
Cenpf
Igsf21
Pdgfa
Btbd17
Srrm4
Rab6b
Ncl
Cdk1
Hmgn5
Rufy3
Serbp1
Pde1c
Gnb2l1
Pnoc
Fkbp3
Hist3h2a
Cdca8
Spc24
Rps3
Cks1b
Rps9
Ptprs
Rbfox2
Ank2
Nfix
Cnbp
Rplp0
Prkcb
Pbk
Epb4.1l1
H2afx
Cenpa
Tubb5
Pqlc1
Eef1b2
Plcb1
Kif5a
Gpr153
Tagln3
Cbx5
Hirip3
Serinc1
Ccna2
Stmn3
Stxbp1

Rrm2
Ankrd12
Cdca3
Kcnk1
Rpl14
Paics
Rnasel
Hells
Prdm8
Ppp2r2b
RP23-45G16.5
Eef1a1
Apbb1
Mif
Nsg1
Ybx1
Marcksl1
Tmpo
Ran
Nusap1
Glce
Rps15
Lig1
Abhd16a
Gdpd5
Fnbp1l
Incenp
Tyms
Cenpe
Gpc2
Dctpp1
Sox9
Esco2
Mcm3
Kif11
Nop58
Elavl2
Hes6
Klc1
Rps21
Gdi1
Chd7
A330076H08Rik
Mab21l1
Spc25
Txn1
Dbn1
Kif1b

Mapk8ip1
Nsg2
Tbata
Rps3a1
Knstrn
Usp1
Apc2
Akap12
Dtymk
Prim1
Gng2
Prc1
Clspn
Ccnb2
Kif23
Ckap2l
Cdca7
Klf7
Chd3
Calm2
Nucks1
Ppm1h
Hmgn2
Siva1
Rnmt
Cenph
Necab3
Tacc3
Pak7
D4Wsu53e
Gas1
Nrcam
Ccdc34
Srgap3
Clvs1
Mdk
Cbfa2t3
Atoh1
Ptn
L1cam
Porcn
Tead2
Hmmr
Slc17a6
Cltb
Pmf1
Ncapg
Tmem2

Lsm4
Gm10075
Gmnn
Gadd45a
Uchl1
Npdc1
Cep170
Map6
Smco4
Sobp
Igsf8
Soga3
Fam210b
Aurkb
Lap3
Smim18
Cenpm
Slc25a5
Banf1
Sparcl1
Psat1
Rpa2
Rps14
mt-Nd1
Rrm1
Mns1
B2m
Gnao1
Nhp2
Rps25
Plcd1
Hes1
Apoe
Hpca
Dnajc9
Nrn1
Lhx1
Grin2b
Sfrp2
Cdc20
Ppfia2
Bhlhe22
Rnd3
Zfp36l1
Frmd4a
Casc5
Nap1l1
Isoc1

Hey1
Marcks
Mis18bp1
Kdm5b
Mt1
Dkc1
Hist3h2ba
Dync1i2
Rtn4
Dpysl5
Serpini1
Fth1
Ckap2
Dclk1
Samd12
Hint1
Smarcc2
Hn1
S100a16
Kmt2e
Mcm2
Cacna2d1
Rps24
Mcm5
Rad51ap1
Cpe
Fbxo5
Rpl22l1
Ccnb1
Afap1
Gm17322
Kif15
Jhdm1d
Rpl35a
Cklf
Uhrf1
Tk1
Nnat
Myod1
Hjurp
H2afy
Hdgf
Lsm6
Acot7
Sgol2
Mapt
Aspm
Kif20b

Rpl39
Atp1b3
Cenpk
Bok
Nmral1
Hmgn1
Slc3a2
Cd24a
Nuf2
Mllt4
Arl6ip1
Thra
Kidins220
Dlgap4
Ebf3
Pa2g4
D17H6S56E-5
Mmp24
Rps2
Boc
Clip3
Gria4
Atad2
Gsk3b
Rps27l
Mcm7
Dusp14
Kif21b
Sfrs18
Tipin
Bub1
Actl6b
Cdca2
Sowaha
Prmt8
Arhgap11a
Prdx4
Lgals1
2410066E13Rik
Nop10
Rps15a
Dusp8
Lrig3
Scg5
Grina
Kif22
Dtl
Adamts1

Mxd3
Sv2a
Gli1
Pou3f2
Smpd2
Shmt1
Cenpq
Nav2
Sox4
Ntrk3
Homer2
Prdx6
Hnrnpu
Stxbp5l
Cnih2
Erbb4
Tubb4b
Serping1
Tomm7
Snrpb
Cdkn1b
Mex3b
Gpm6b
Hist1h2ak
Cenpp
Rps10
Scd2
Snrpf
1500016L03Rik
Kif4
Racgap1
Zfp423
Rab6a
Mad2l1
Zbtb18
Rps18
Neurod2
Myl12a
6330403K07Rik
Melk
Ndc80
Jph4
Syt13
Ier5
Smoc1
Lingo1
Smpd3
2900079G21Rik

Klc2
Ect2
Tspyl4
Chaf1a
Ppp3ca
Cacna1b
Cenpw
Tcf19
Cnpy1
Rcor2
Nefl
Fmnl2
Lyar
2900011O08Rik
Hsp90b1
H2afz
Tmem57
Nop56
Hmgb1
Arhgef2
Rfc4
Mcm4
Ramp2
Cdh20
Dnph1
Eif5a
Pkm
Fstl1
Clcn4-2
Fam213b
Ncam1
Ntm
Atp6v1e1
Gdpd1
Sept8
Csrp2
Eef1d
C330027C09Rik
E130309F12Rik
Snhg1
Eno1
Ctsd
Slc7a5
Angptl2
Pea15a
Ssrp1
Pgm2l1
Cyfip2

Bola2
Sgol1
Timp3
Ptms
Ybx3
Naa50
Sox11
Fam111a
Hk2
Bub1b
Hat1
Slc29a1
Mmp14
Darc
Dnmt1
Mad2l2
Cmtm3
Grik2
Ncaph
Zfp521
Pdrg1
Scmh1
Rad51
Shfm1
Kif2c
Ndufc2
D430041D05Rik
Dhfr
Os9
Gm11223
Atp5e
Srrm3
Selm
Ldha
Tubb4a
Cdc45
Ttyh2
Cenpv
Celf3
Gm1673
Pttg1
Srgap2
Celsr2
Mvd
Snrpd3
Mycbp2
Baz2b
9330159F19Rik

Snrpg
Sphkap
Srsf7
Chst15
Shf
Anln
Adk
Nde1
Nkd1
Ywhag
Fam115a
Pnmal2
Plk4
Dixdc1
Trp53inp2
Hnrnpab
Srcin1
Reep2
Pik3r3
Phf20l1
Dlgap5
Rbm5
Idh2
Ephb1
Cog7
Mum1l1
Ddx5
Ezr
Atxn10
Dbf4
Pfn1
Timeless
Cdt1
Pcsk1n
Cdca7l
Diap3
Syp
Scrt1
Ppp1r14b
St6galnac4
Kifap3
2810025M15Rik
Nek7
Nova2
Atp6v0b
Gm11541
Klhl29
Ccnd2

Idh1
Pgm2
Hspe1
Snrpa1
Rps7
Ldhb
Aurka
Rpl26
Psmc3ip
Brsk2
Rpa3
Hsbp1
Fam64a
Rbbp7
Tulp4
Pbdc1
Atat1
Nt5dc2
Tpm4
Nudcd2
Bex1
Nicn1
Sh3kbp1
Ctnna2
Trim59
Clasp2
Ing4
Lrpap1
Gm11266
Gar1
Ypel4
Csrnp3
Cpne3
Parp6
Nup62
Nfasc
Rpl23
Sez6l2
Hmgn3
Sstr2
Ccm2
Mrpl42
Wbp5
Tmeff1
Gnaq
Irs1
Arpp21
Nolc1

Rnaseh2c
Nktr
Cdk6
Cct3
Sbk1
Eid1
Cdh4
Rad21
Sae1
Rltpr
Lzts1
Kcnj3
Rsl1d1
Tacc2
Gnl3
Cst3
Ccl27a
Cbfb
Spop
Supt16
Ephb2
Map1lc3a
Pdzrn4
Cntln
Topbp1
Nol7
Ndufa2
1700001O22Rik
Hivep2
Myc
Naa38
Strbp
Ubash3b
Ptch2
Eny2
Snrpd2
Rpl18a
Hmgcs1
Sema7a
Gramd1a
Ubl3
Pcdha2
Ppic
Islr2
Clic4
Rnaseh2b
Ndufa12
Ndrg2

Vim
Pdia6
Cplx2
Alcam
Baz1a
Snrpd1
Srsf3
Gramd1b
Fen1
Dpm3
Clybl
Zic2
Ssbp3
Rb1cc1
Tshz2
Trp53
Akap9
Map1lc3b
Gm10260
Mphosph10
Cerk
Nova1
Lpin2
Pik3r2
Ube2d1
Ctxn1
Fam110a
Srsf2
Pcbp1
Larp7
Exosc7
Ppp2r2c
Cnrip1
Thsd7a
Gm20033
Ttc28
Lmnb1
Rnaseh2a
Dcps
Nedd4l
Efr3b
Fkbp2
Naca
Shd
C1qbp
Slc22a17
Ccp110
Ncor2

Rif1
Rpl7
Sri
Meis1
Mapk8ip2
Mex3a
Dpysl4
Scrn1
Eif3i
Vegfb
Asxl3
Fabp7
Ska2
Gm9800
Smim11
Clic1
Uqcrq
Skp2
Ap1s2
Slco5a1
Rrs1
Npepps
Alyref
Bdh1
Dmxl2
March1
Grb2
Fdft1
Rusc1
Ninj1
RP23-32A8.1
Peli2
Sod1
Mical1
Mroh2a
Klf9
Mybbp1a
Paxbp1
Celf5
Hnrnpd
RP23-199B2.4
Cadps
Pak1
Rabep1
Tnrc6c
Snap91
Spag9
Pfn2

Rps4x
Bicd1
Cask
Rps16
Arid3a
Ddx39
Wdr47
4930402H24Rik
Cd81
Reln
Atl1
Mbd3
Prkd3
Tmem107
Trafd1
Mapk10
Gng12
Vgll4
Dpysl2
Snrnp40
Rtn3
Atp5b
Rpl34
Rpl18
Hnrnpm
Rfc5
Ift27
Ssr1
Id2
Bccip
Scg3
Lmnb2
1810009A15Rik
Eif4e3
Cplx1
Ctnnb1
Asrgl1
Vrk1
Rfc1
Tub
Hspd1
Usp22
Srsf1
Mthfd1
Park7
Atad5
Mpdz
Naa10

Pkp4
Rpl38
Clk1
Ddx21
Cyth2
Etf1
Zfpm2
Gart
Rpl6
Cep110
Sema6c
Kdm1a
Brca2
Hip1r
Bzrap1
H2afy2
Dnmt3a
Myl12b
Gm6472
Stx7
Synj1
G3bp1
Smc6
Rnf165
Tgif1
Trp53i11
Pkia
Ankra2
Vars
Rpl14-ps1
Mat2a
Ccdc18
Pafah1b1
Exosc8
Etfb
Cdkn2c
Prmt2
Mak16
Ipo5
Arpc5
Nin
Ccdc41
Wsb1
Tspyl1
Lsm3
2610001J05Rik
Gabarapl1
Rps8

Eif3a
Mrpl33
Ankrd13a
Mpp6
Riok3
Acd
Mrpl52
Cep120
Prnp
Sfxn1
Dst
Rpl3
Rpl30
Rpl37
Sh3bp5
Nfyb
Dars
Uqcr10
Lsm2
Nxt1
Dhx32
Tcp1
Fundc2
Pard6a
Laptm4b
Etfa
Sox18
Nol4
Nsmce4a
Pdap1
Sap30
Gm11478
Brd7
Cdk4
Snx5
Dctn2
Srm
Tcf3
Enox2
Jun
Abracl
Stau2
Exosc1
Fabp5
Zfp57
Cited2
Gabbr1
Cox7b

Pold2
Snrpe
Flot1
Arhgef7
Ifitm2
Cnih4
Ncaph2
Hcfc1r1
Scn8a
Apba2
Nme2
Uba52
Alg2
Pygo1
Hpcal1
Kcnq2
Stx4a
Sep15
Ezh2
Polr2i
Aig1
Neurod6
Itgb1
Atcay
2810004N23Rik
Glo1
Smarcd2
Lrrtm2
Tspan7
Set
Meis3
Add2
Sox5
Rwdd3
Ptma
Ak2
Gm5620
Ptbp1
Gm17750
Zfp608
Lman1
St8sia3
Mrfap1
Cdk5rap2
Sdc3
Rpl29
Slc7a1
1110038B12Rik

Mrpl13
BC029214
St7
Nrm
Ppil1
Gjc1
Rfc2
Sv2b
Egfr
Lims1
Ppat
Ssr3
Daam1
Pola1
Zfp292
Asap1
Klf13
Rnf122
Jmjd1c
Nefm
Dtx1
Sptan1
Sec11c
Onecut2
Mgat5b
Imp3
Lsmd1
Sgip1
Mycl
Tmem63b
Rassf3
Arpp19
Kif3c
Mkrn1
Ypel5
Sf3b5
Epc1
Atp6v0a1
Atxn7l2
Brd3
Fam162a
Ncan
Scaper
Eif4a3
Rbm3
Rcn1
Atp6v0e2
Cog1

1500011B03Rik
Med30
Ift74
Mtmr4
Nr3c1
Ppa1
Nudc
Nbea
Eif3b
Rftn2
Blcap
Nop16
Tpt1
Apex1
Jarid2
Wdr89
Taf1d
Timm13
Hprt
Kcnc1
Abtb1
Rcc2
Tmx4
Gm8292
Nipbl
H1fx
1810037I17Rik
Gcc2
Tmem178
Pole3
Pard6g
Tram1
Pold3
Map7d1
Srsf10
Aes
Nsmce1
Slc37a3
Ppp2r3a
Mycn
Evl
Sdha
Emg1
Dirc2
Cd164
Pds5b
Rpa1
Commd1

Rrp15
Actl6a
Smc1a
Cnksr2
Wdr13
Rab3c
Glrx5
Peli1
Plp1
Golm1
Prmt5
Sqle
Rbm8a
Brix1
Jam3
Tsc22d4
Prkx
Armcx1
Zfp157
4933427D14Rik
Ftsj3
Srsf9
Trp53bp1
Vezt
Mrpl18
Tmem176b
Gsto1
Epb4.1
Rai1
Trim28
1500011K16Rik
Gins2
Rnf168
Pafah1b3
Eif1ax
Psmg4
Zfp462
Egr1
Mrps28
Slc1a2
Serf1
Gdap1l1
Glul
Mrps14
1700025G04Rik
Leprotl1
Aff4
Bub3

Lmo4
Pbx1
Dnajc2
Reep1
Gab1
Uchl5
Ntrk2
Celsr3
Rfc3
Banp
Ctsf
Hspa5
Tln1
Dpy30
Rad50
Mtch2
Aldoa
Fam168a
Ppp1r1a
Polr1c
Msl3
Wasf2
Ola1
Chchd1
Map3k12
Rps16-ps2
Pabpn1
Fam57b
Nhsl1
Ctps
Hook3
Ubr7
Map9
Fbxo11
Zc2hc1a
2700029M09Rik
Rpl36al
Clcn2
Myo1b
Lima1
Macf1
Rps28
Rpl10a
Slc1a3
Ly6e
Rexo2
Jakmip2
Efhd2

Gpatch8
Snapc3
Plk3
Arl8a
Polb
Oraov1
Pick1
Mgea5
Kars
Fnbp1
Gm26735
Mfap4
Tom1l1
Ccng2
Rsbn1
Arhgap21
Hid1
Setbp1
Jak1
Vopp1
Fzd2
Fam107b
Zbtb38
Psmg2
Nr2f2
C1ql1
Mapre2
Maml3
Mxd4
Rangap1
Akap8l
Ifrd1
Myo9a
2410089E03Rik
Dpf2
Lmcd1
Zfp711
Baz2a
Ckap5
Serpinh1
Fosb
Cenpa
Cenpe
Ccnb2
Cdc20
Hsp90b1
Tpx2
Cenpf

Mki67
Hmgb2
Knstrn
H2afv
Nucks1
RP23-45G16.5
2700094K13Rik
Cdca8
Cdca3
Dynll1
Pttg1
Anp32e
Tubb5
Rad21
Hdgf
Fstl1
Neurod1
Hnrnpa2b1
Gm11266
Cep89
Sowaha
Malat1
Gria2
CRE_RECOMBINASE
Birc5
Hmgn5
Ckb
C330027C09Rik
Banf1
Miat
Nrep
Celf4
Gm10075
Apoe
Ccdc34
Racgap1
Lmnb1
Kif23
Rtn1
Tacc3
Sept3
Dlgap5
Basp1
Hmgn2
Top1
Hes6
Ckap2l
Arhgap11a

Tubb4b
Gpm6a
Hist1h2bc
Tubb2a
Cdc25c
Cks1b
Stmn2
Cep110
Bin1
Vim
Cdkn3
Hmmr
Fabp7
Zic1
App
Syt13
Shfm1
Rangap1
Gap43
Pcna
Hmgn1
Lgals1
Rbfox3
Ezr
Tubb3
Ctsb
Hnrnpm
Hpca
Cntn2
Aspm
Ckap5
Lig1
Rps27l
Celf2
Tubb2b
Igfbpl1
Cacna2d1
Ctnnb1
Smim11
Bub1b
Hmgb3
Mad2l1
Mns1
Pde1c
Vbp1
Ptprs
Ank3
Cbx1

Gas6
Stmn4
Paip2
Gng3
Dpysl3
Xist
Ppp1r14c
Pdlim3
Efhd2
Ran
Nfib
Gnai2
Nde1
Tra2b
Ctsd
Tmpo
Pdia6
Otx2
Barhl1
Sh3gl2
Cwc15
Thra
H2afz
BC005764
Nrxn1
Ppp1r14b
Map2
Kif1b
Lsm4
Hmgn3
Fam64a
Mapt
Mtss1
Sep15
Pcf11
Aplp1
Ppp2r5c
Mdh1
Myt1
Atp6v1e1
Cct5
Cenpw
Psmc1
Srebf1
Brd7
Sec11c
Chgb
1700123O20Rik

Zeb1
Nudcd2
Gadd45g
Clspn
Pqlc1
Cks2
Lyar
Trpc4ap
Mphosph10
Tax1bp1
Hmgb1
Kif20a
Smarcc2
Ank2
Cd81
Kif5c
Ralgps2
Ckap2
Sept4
Spop
Cenpv
Kif15
Prkcb
Taf7
Slc35b1
Ankrd12
Myt1l
Itsn1
Rbbp6
Tnik
Ect2
Grik2
Zfc3h1
Larp7
Cadm3
Apc
Gsk3b
H1fx
D4Wsu53e
Ubb
Mapk8ip1
Rnaseh2c
Rab6b
Nuf2
Chd3
Chd7
Sh3bgrl
Phf20l1

Dnttip2
Cog7
Csrp2
Gas1
Ift74
Akap6
Ppp3ca
Bzw1
Dcx
Elavl4
Odc1
Nbea
Nsg1
Rufy3
Sema6a
Tagln3
2810474O19Rik
Gnao1
A330076H08Rik
Ssbp3
Klc1
Cnpy1
Hes1
Gdi1
Bub1
Jam3
Hist3h2a
Slc1a2
Aldoa
Cenpp
Ccdc77
Pak7
Grina
Celsr2
Ppp1r10
Srgap2
Chrna3
Ina
Apbb1
Cacng4
Arpp21
Slc17a6
Zwint
Mllt3
Pou3f2
Atp6v0b
Mycbp2
Rab3a

Uchl1
Fyn
Elmo1
Lmnb2
Ypel3
Cnksr2
Kif5a
Maml3
Ptprd
Gnaq
Olig2
Mum1l1
Ntrk3
Ppfia2
Hells
Zfp36l1
Pkia
Tacc2
Zmiz1
Psap
Agap1
Fam111a
Itm2c
Akap9
Dner
Eif4g3
Ppp1r1a
Stxbp1
Kmt2c
Gria4
Reln
Mgll
Rnd2
Hcfc1r1
Cplx1
Erc1
Stag2
Clasp2
Lphn1
Trio
Fut9
Rere
Kidins220
Plcb1
Pdzrn3
Podxl2
Abhd16a
Fmnl2

Zmynd8
Pea15a
Plp1
Vcan
Meaf6
Kifap3
Gphn
Fam210b
Scn8a
Aurkb
Snap25
Aprt
Ddx26b
Klf9
Sv2b
Scaper
Nrcam
Clcn4-2
Gamt
6330403K07Rik
Tspyl4
Shd
Adrbk2
Stau2
St18
Esco2
Rfc2
Dnmt1
Rabgap1
Ntm
Fam213b
Rad51ap1
A930011O12Rik
Macf1
Sox5
Phactr1
Hook3
Mt1
Olig1
Serpine2
Cspg5
3110035E14Rik
Cacng4
Fabp7
Ptprz1
Scrg1
Cntn1
Gpr17

Gpr37l1
Pllp
Sox10
Olig2
Plp1
Sulf2
Ptpre
Bcas1
S100a13
Sirt2
Enpp2
Cnp
Bcan
Ramp1
Fyn
S100a1
Rgcc
Ugt8a
Slc35f1
Ppfibp1
Cspg4
Nfib
Pdgfra
Gjc3
Gatm
Ednrb
Tpm1
Pcsk1n
Nap1l5
Itpr2
Epn2
Tagln2
Nkx2-2
B3gat2
Plekhb1
Lsamp
Qpct
Sfrp1
Mfsd2a
Dnm3
Omg
Gria3
Mbp
Zic1
Ptn
Tsc22d4
Sox6
Phlda1

Tmem100
Asrgl1
Spon1
Cd9
Ddah1
CRE_RECOMBINASE
Cdo1
Lims2
Ncald
Hsp90ab1
Susd4
Ascl1
Tril
Ncam2
Cyp2j6
Tmem176b
Igfbpl1
Cd24a
Sox2
Neu4
Kcnj10
2810468N07Rik
Dbi
Kctd4
Ttyh1
Klhl5
S100a16
Cadm2
Dmrtb1
Pcdh17
Lrrc4c
Sox8
Cmtm5
Slc1a1
Rlbp1
Afap1l2
Zcchc24
Sept7
Vcan
Opcml
Anks1b
Enc1
Bmp4
Atp1a2
Ostf1
Car8
S100b
Mmp15

Rprm
Nxph1
Ppap2b
Pxdc1
3632451O06Rik
Pou3f1
Gal3st1
Degs1
Brinp3
Gfra1
Matn4
Kcnd2
Tnr
Sema5a
Tspan3
Scd2
Arsb
Tmem255b
Fa2h
Tubb5
Anp32a
Sox2ot
Pcdh10
Tspan7
Enpp6
AW047730
Sema5b
Dscam
Timp4
Sh3d19
Cdh13
H3f3b
Megf11
Barhl1
Cd81
1810041L15Rik
Tmem132b
Il1rap
Phyhipl
Slc6a1
Gm2a
Grm5
Arl4a
Cldn11
Draxin
Meg3
Resp18
Kcnip3

Tns3
Lypd1
Cog7
Sema3d
Gltp
Crmp1
Vstm2b
Scn3a
Clu
Gsn
Calm2
Tspan2
Ddah2
Slc22a3
Slc29a1
Nlgn3
Luzp2
Adam9
Pik3r1
Adora1
Dab1
Stk32a
Tmem88b
Pax6
Phactr3
Bricd5
Hip1
Sh3bp4
Lhx1
Gnb4
Tmsb10
Tgfa
Ezr
Slc22a23
Lrp1
Ptprt
Dlgap1
Chst11
Nfia
C1ql2
Slc22a17
Syt11
Calr
Alcam
Cd63
Dcaf12l1
Rap1gap
Stmn2

Zfp365
Brinp1
G0s2
Slc38a3
Rab31
Tmsb4x
Gng12
B3gat1
Ppp1r16b
S100a6
Sstr1
Fbxo7
Neurod1
Deb1
Lrrtm1
Pmp22
Rbfox3
D430041D05Rik
Pfn2
Kank1
Pcdh11x
Pcdh9
Nfasc
Ehd3
Emid1
Npas3
Eps8
Il18
Dock9
Zic4
Nrep
E130114P18Rik
Pgp
4833424O15Rik
Timp2
Rnd3
Trio
Pid1
Gpm6b
Sash1
Rhoc
Chn2
Tm7sf3
Igfbp3
H2afv
Canx
Pabpc1
Sox21

Spry4
2900011O08Rik
Nhlh2
Mmd2
Wscd1
Spry1
Slitrk3
Dpp6
Mpzl1
Pde4b
Nfix
Cyp2j9
Cplx2
1500016L03Rik
Neto1
Mmp16
Chadl
Epb4.1l2
Lrrfip1
Dusp26
Plk2
Col16a1
Flrt1
Cbr3
Ppapdc1a
Cbfa2t3
Cacng2
A930009A15Rik
Tbata
Hnrnpab
S100a4
Chpt1
Rpl13a
Cbx1
Sdc3
Mapt
Ntrk2
Arhgdig
Ptma
Cadm4
Sgk1
Rps5
Gria2
Cacna2d1
H1f0
Tmem176a
Fam3c
5730559C18Rik

Abhd12
Tubb3
Zdhhc2
Rtkn
Mt3
Sh3gl3
Plekha2
Fam210b
Celf2
Uncx
Pea15a
Lphn3
Dpysl4
Mycn
Hsd11b2
Nrxn2
Rplp0
Cp
Ppp2r2c
Acox1
Cav2
Cdh11
Cyfip2
Hsd17b12
Hmgcs1
Dusp15
Smc2
Rps9
Rps3
Rpl4
Tspan6
Gm2694
Marc2
Scamp2
Kcnd3
Tead2
Prkcq
Slitrk2
Lnx1
Cdh10
Rassf4
Taf9b
Fbn2
Tmem255a
Nacc2
Limch1
Ier5
Ccnd2

Necab2
Cav1
Eid1
Hepacam
Dock10
Spock2
Srebf1
Epha4
Mex3a
Tmem9b
Npc1
Lrrtm3
Pde1c
Wipf1
Rev3l
Ildr2
Gabra3
Ppp2r2b
Hbegf
Dynlt3
Ptpro
Hsp90b1
Insm1
Camk1
Mgll
Lbh
Taok3
Sv2a
Pcdh7
Nasp
Sapcd2
Gria4
Tln2
Kif21a
Cd302
Cpne8
Kazn
Cryab
Ppic
Mif4gd
Miat
Rps14
Shc4
Nim1
Hpca
Lamp1
Dynll2
Apoe

Gsg1l
Lrrc4
Arxes1
Smc4
Fermt2
Pdlim5
Gpt2
Shisa4
Trib2
Slc44a1
Metrn
Vimp
Rps24
Chd5
Caskin2
Tmbim6
Rps20
Hmgb2
Lrch3
Meis1
Aldoc
Arvcf
Cd200
Evi5l
Gpx3
Cdk6
Gyg
Cks1b
Grin3a
Tspan13
Syt16
Eif1b
Sec11c
Rps26
Dbndd2
Fgf9
Gucy1a3
Fchsd2
Tcf4
Add3
Stmn1
Spry2
Gng3
Cntn2
Mtap
Chd7
Rbp4
Gm11223

Gm17750
Elovl7
Otx2
Gm9800
Dtd1
Clmp
Arxes2
Fez1
Nenf
Fam49b
Mfap2
Mageh1
Kif5c
Arl2bp
Lmbrd1
Itgb8
Polg
Ly6h
2810417H13Rik
Jam2
Ggct
Cxxc5
Arhgap31
Tub
Hnrnpu
1810037I17Rik
Atpif1
Limd1
Rgs7bp
Tceal3
Lrrn3
Scamp5
Gjc1
Amz1
C1ql3
Apba2
Vapa
Grid1
Nhlh1
Hmgn1
Rpl22
2700094K13Rik
A230050P20Rik
Mcm7
Mmp2
Sox9
Dek
Fkbp3

BC005764
Rin2
Fam213a
Rab33a
Reep5
Ebf3
Rap2a
Mt1
Nrxn1
Mab21l1
Selk
Tle1
Bcl11a
Prmt8
Rasl11a
Phldb1
Wasf1
Sox4
Crip2
Ezh2
Psap
Abtb2
Lgi3
Hap1
Tmco3
Dhcr24
Tmem191c
Dynll1
Sstr2
Rplp2
Fnta
Ugdh
Vps37b
Rab14
Ranbp1
Spag9
Malat1
Dmd
Serbp1
Ccnd1
Mllt4
H2afy
Rcor2
Abcg1
Car11
Gnb2l1
Ust
Ppt1

Fnbp1l
Hk2
Rps19
Itm2c
Hey1
Hirip3
Fam63b
Snx22
Usp24
Eef2
Slc17a6
Map3k1
Pqlc1
Gnal
Atcay
Ddx5
Dut
Stxbp3a
Laptm4b
Gpm6a
Papss1
Taldo1
Abcd3
Irs2
Tubb2a
Fip1l1
Abracl
Cald1
Aplp2
Srrm4
Dpysl3
Cxcl14
Myl12a
Abhd2
Gng2
Hjurp
Zbtb18
Nkd1
Cbx5
Abhd6
Acap3
Hmgcl
Itga9
Sept2
Lrrtm2
Scn2a1
Eef1b2
Traf4

Tceal5
Txndc15
Atoh1
Celf4
Tanc2
Pcdhga9
Lcorl
Heg1
Mki67
Fzd2
Arl6ip1
Reep1
Kcna1
Islr2
Reep3
Ccng2
Orai1
Map1b
Maged1
Wdr1
Aatk
Rnmt
Spred1
Rsu1
Thra
Anp32b
Npm1
Nr3c1
Rbms1
Gaa
Ctnnd2
Lgals1
Nipa1
Tmbim4
RP23-45G16.5
Ptch2
Hnrnpm
Hnrnpd
Mdk
Ralb
Gde1
Atp6v0b
Fstl1
Mfge8
Ndn
Srsf3
Rpl32
Serinc5

Paip2
Zfand5
Sept8
Zdbf2
Top2a
Snx25
Lima1
Boc
Ypel3
Cdkn1b
Chd4
Atp1b1
Tmpo
Emc7
Glrb
Smc1a
Celsr2
Snrpf
Pcna
Spc24
Rps21
Chrna3
Nckap1
Nrxn3
Zic5
Gse1
Nap1l3
Hmgb3
Osbpl1a
Gtl3
H3f3a
Reln
Nell2
BC034090
Hmgn5
Cuedc1
Ncl
Stat3
Gm3764
Trp53i11
Tex14
Tcf7l2
Syt13
Hdgf
Sez6l
Frmd4b
Selt
Rps11

Tmem63b
Mfap4
Ndrg2
8430419L09Rik
Prox1
Dhx32
Rrbp1
Arpc5
Efhd2
Tuba1b
Akap6
Tmem30a
Sox18
Slc35b2
Mcm6
Lhfpl2
Dhrs7
Klhl13
Hdac2
Dner
Tpm4
Gpr153
Cenpf
Cttnbp2
Robo2
Ppp2r2a
Abhd4
Rab10
Agrn
Btg2
Zdhhc14
Coro2b
Cyb5
Itgav
Chst2
Nova1
Zic2
Lrig3
Rest
Btbd17
Gm11541
Birc5
Notch1
Snrpe
Tsn
Sel1l
Aplp1
Myod1

Atxn7l3b
Tmem59
Wbp5
Fubp1
Atp1b2
Pigyl
Hn1
Tgif1
Siva1
Dtna
Mdga1
Neurod6
Sec62
Htatsf1
Hprt
Ube2b
Snx3
Nmnat2
Fam19a5
Pak4
Rabgap1l
Acsl3
Elavl2
Tmem107
Gm11266
Baz2b
Tpx2
Bpgm
Nedd4
Ccdc134
Gm17322
Serpinh1
Incenp
Phip
Gpc2
Ccdc107
Ier3
Cdk4
Specc1
Cdk1
Prex1
Sept3
B2m
Mtus1
Ccna2
Nlgn1
Tox3
Hmgn2

Sar1b
Cdc37l1
Gm10075
Fam69b
Cdk14
Rpl8
Sema7a
Fam53b
Ccnd3
Foxp1
Apex1
Baz1b
Pbk
Map1a
Abat
Angptl2
Smco4
Tspyl4
Ctsl
Bok
Timp3
Snx18
Kif3a
Rdh5
Aldh9a1
Ssrp1
Tmem50b
Basp1
Mtf2
Banf1
Prc1
Prnp
Lmnb1
Dpy19l1
Rab11a
Ina
Ube2e3
Slc25a4
Adcyap1r1
Rassf3
C530008M17Rik
Mroh2a
Bcap29
Ifitm2
Fam168a
Hells
Smyd2
Mrps7

Spc25
Lta4h
Atp5j
Rpl35a
Sgcb
Tubb4a
Atraid
Ccdc88a
Map4k4
Cdca7
Ccdc34
Ilf2
Hipk2
Elf1
Ier2
Lmo1
Ppp2r3a
Tk1
Acbd5
Pdia6
Carhsp1
Dad1
Fkbp15
Cyp51
Hnrnpa0
Idh2
Spcs2
H1fx
Ebpl
Snrpg
Cltb
H2afz
Sept11
Tspan12
Ick
Pcmtd1
Cnpy1
2310022B05Rik
Ypel1
Dhcr7
Elavl3
Myt1
Asah1
Sdf2l1
Cenph
4833439L19Rik
Itgb1
Magee1

Pdia3
Rrm2
E2f1
Rbfox2
Igsf21
Camta1
Ptms
Cdc42se1
Pcyt1b
Os9
Tmem9
Zeb2
Snx1
Socs2
Kif23
Fndc4
Cask
Pbrm1
Tmcc3
Dusp6
Cyld
Tyms
Plxnb2
Pgm2
Cdca8
Elovl1
Cdca2
Iffo1
Tppp3
Unc50
Igsf3
Mcm3
Jam3
Syncrip
Ncapg
Cklf
Ostm1
Tgfb2
Cited2
Mpc2
Snrpb
Fam69a
Ccdc18
Dcakd
Snx27
Smdt1
Sacs
Racgap1

A930011O12Rik
Sult4a1
Map7d2
Rnaseh2c
Adrbk2
Nedd4l
2510003E04Rik
Psip1
Fam171b
Ccdc28b
Rasa3
Pdap1
Tacc2
Clcn3
Csrp2
Fktn
Sv2b
Clspn
Dtl
2810055G20Rik
Ube2c
Mab21l2
Rad51ap1
Tcf12
Rps15
Dcx
Smarca1
Chd3
Cenpe
Tmeff2
Snhg5
Mxd3
Kif11
St18
Slc6a6
Arhgef2
Sc4mol
Comt
Atad2
Fbxo8
Gphn
Pdzrn3
Otud7b
Ccm2
Rpl14
Btg1
Lss
Setd8

Tceal6
Tmem66
Gli1
D17Wsu104e
Cers2
Kif1a
Tmem5
Evi5
Efcab14
Twf1
Mmp14
BC029722
Rpl39
Esco2
Cdca3
Serinc1
Myh10
Tbc1d12
Pnrc1
Ccp110
Pop5
Pex2
Slc35a5
Psma7
Frmd4a
Hnrnph1
Nusap1
Marcksl1
Tead1
Atp9a
Vps28
Agpat4
Samd8
Clybl
Tbc1d16
Rab8b
Stk39
Snrpb2
Ncaph
Snca
Uhrf1
Cacnb4
Rap1gds1
Rps25
Chchd2
Kif15
Nap1l1
P4ha1

Itm2b
Rps15a
Chaf1a
Ccdc47
Jun
Blvra
Lig1
Gabbr1
Nt5dc2
Mllt3
Ap2a1
Dhx9
Itsn1
Sdc2
H2afx
Tcp11l2
Cdc20
Rpl26
Msi1
Vamp3
Usp1
Sep15
Hpcal1
Tmed5
Tmem147
Vldlr
Rufy3
Arhgef9
Dirc2
Ssbp3
Dock7
Ltbp3
Pou3f2
Fam155a
Epb4.1
Rad21
Eif4e3
H2-D1
Casp3
March1
Atp6v0e2
Dll3
Rbm8a
Dnajc9
Ppp1r18
Tshz2
Cdk5r1
Astn1

Tmem246
Rnf165
Trp53
Ncan
Dctpp1
Lactb
Hadha
Etfb
Trim59
Glud1
Ndfip1
Mrpl34
Setbp1
Sept4
Ybx3
Arl8a
Mllt11
Celsr3
Ccdc41
Klhdc2
Dbt
Ctsz
Hmgb1
Dusp1
Mid1ip1
Hsp90aa1
2700089E24Rik
Sptssa
Capns1
Fam173a
Casc4
Rhbdd2
Lgalsl
Mvk
Jagn1
Nuf2
Lmf1
Gabarapl1
Tex30
Ctnna1
Man2a2
Lap3
Clvs1
Zfp462
Rap2b
Nceh1
Eif3f
Hmmr

Sgol1
Kif20b
Prim1
Map4k5
Mcm5
Smpd3
Ttc3
Strbp
Ctcf
Mad2l1
Camsap2
Casc5
Ppm1l
Podxl2
Usp16
Cox7a2
Rph3a
Slc48a1
Abi2
Ncapd2
Gpsm1
Ddost
Nucks1
Prpf40a
Parp6
Tcerg1
Magoh
Desi1
Grik5
Ifnar2
1700001O22Rik
Glce
Mum1
Src
Kmt2e
Ccnb2
Stard3nl
Ank3
Ndc80
Pcdha2
G6pc3
Slc50a1
Sri
Gins2
Ankrd46
Nop58
Sqle
Dtymk

Rpl34
Kif22
Impad1
Fam13c
Epb4.1l3
Srrt
Wls
Tprn
Ankrd11
Pmf1
Atxn7l2
Ufl1
Epdr1
Gnai1
Phf21b
Prps1
Atrx
Rab6a
Atp8a1
Hist1h2ak
Sh3bgrl
Ankrd32
Tmem106b
Clic1
Ly6e
Zfp191
Rpl18a
Pcbp1
Tmem101
Nrn1
Afap1
Usp46
Rpn2
Preb
Tmem50a
Pknox1
Tom1l1
Homer2
Zswim6
Cenpa
Elovl6
Mapre2
Flna
Spats2l
Ndufa8
Rabac1
Paqr4
Slc15a2

Cenpk
Tm2d2
Cep290
1500011K16Rik
Ank2
Fjx1
Vmp1
Rpa2
Pura
Fabp5
Prkar2b
Dkc1
Smim15
Napa
Cecr2
Ddx25
Extl2
Prdx4
Larp7
Anp32e
Prdx5
Pgrmc2
Emc2
Nup62
Ptpra
Cct2
Dtd2
Cebpg
Tmem167
Rfc1
Tbca
Eif1
Tpp1
Ago3
Zfand6
Eif3a
Pepd
Rnf5
Kif13a
Ctnnal1
Rif1
Lmcd1
Ankmy2
Aprt
Tceal1
0610007P14Rik
Krcc1
Uba52

Smim13
Aurkb
Smc3
Knstrn
Uqcrb
C130071C03Rik
Znhit6
Lrrn1
Maged2
Elovl5
Rpl23
Ate1
Samd4b
Midn
Ivns1abp
Gm8292
Tia1
Pak1
Sox11
Gpx7
Leprot
B3gnt1
Gmnn
Myo6
Pfdn4
Mboat2
Hmgn3
Clptm1l
Eif3k
1500012F01Rik
Snap25
Hes6
Ccnh
Cpe
Pmvk
Cd63-ps
Nlk
Tmem18
Mtdh
Rnf208
Cdca7l
Ramp2
Ddx26b
Myo5a
Syne2
Ptprd
Hdhd2
Sgce

Rab9
Fbxo5
Asf1a
Nsg2
Supt16
Baz1a
Secisbp2l
Arpc1a
Tm9sf3
Cdc42se2
Chgb
Rnf13
Magt1
Klf7
Sbf2
Golm1
Ubl3
Atp6v0e
Hsd17b4
Pole3
Rab5c
Usp22
Atp13a3
Myt1l
Rad51
Deaf1
Psph
Dnajb11
Tfdp2
Soga3
Tor1aip2
Sptlc1
Ddx3x
1700025G04Rik
Rgs12
Purb
Atp2a2
Cldn25
Sppl2a
Fam184a
Rap1a
Rfc2
Mis18bp1
Ap3m1
Dclk1
Ndrg3
Smarca5
Khdrbs1

Mia3
Rnaseh2a
Cenpj
Arhgap11a
Nf1
Suv39h2
Zcchc18
Mpv17
Ndufa13
Fcho2
Chga
Cenpw
Snx4
Gpbp1
Srrm3
Cdk2ap1
Mns1
Prdm8
Hnrnpk
Smap1
Jund
Sdhb
Cpox
Ehbp1
Lrrc42
Sppl2b
Nfkbib
Arrdc3
Elavl4
Ica1
Npc2
Fasn
Dennd5a
Nptn
Rbbp4
Mbip
Rpl7
Taf15
Psmb10
Kbtbd11
Nfyb
Agl
Dbf4
Cdca4
Limd2
Pcnt
Bub1b
Bub1

Timeless
Mlf2
Maml3
Emc3
Akap12
Fam107b
Tmed10
Stmn4
Fen1
2310061I04Rik
Rpl37
Chtop
BC004004
Pccb
Tmem33
Nol4
Atp2b1
Zfp91
Glo1
Itsn2
Ten1
Arhgdia
Ska2
Banp
Tor1b
Golph3
Ubtf
Palm
Gm26735
Slc3a2
Ap1s2
Ythdc1
Whsc1
5830418K08Rik
Maoa
Cep78
Lsm3
Snhg1
9330159F19Rik
Frrs1l
Brpf1
Ptbp1
Hibadh
Polr2h
Rora
Arhgap20
Smarcc1
Blm

Nfic
Hmgcr
Fam57b
Ndufa7
Slc25a1
Ddhd1
Sdf4
Ncdn
Ckap2l
Bnip3l
Tipin
Lrpap1
1810009A15Rik
Tbl1x
Cadm3
Apc
Pbx3
Zeb1
Btbd9
Gpr180
Cers4
Dbn1
Psma4
Kifap3
Vrk1
Cnn3
Scp2
Gins1
Eif3j1
Vkorc1
Eny2
Rpn1
Cenpb
Saysd1
Xpo1
Vapb
Trib1
Rpa3
Bag1
Galnt1
Kif4
Rac1
Stxbp1
Tsc22d1
Rai1
Hmg20b
N6amt1
Tram1l1

Mis18a
Fkbp2
Srp14
Agfg1
Pa2g4
Pdgfa
Lmnb2
Dazap1
Mier3
Creb1
Scfd1
Cdk5rap3
Rrm1
Mesdc2
Crebbp
Ift27
Pim3
Tdp2
D030056L22Rik
Ktn1
Ndufaf7
Pafah1b2
Zfp292
Smc6
Arl6ip6
Snrnp40
Agap1
Acap2
Wwp1
Cdkn1c
Paics
Cspp1
Tkt
Ift46
Hddc2
Grik2
Nipbl
Srsf7
Set
Dhx15
Ptpla
Mcm2
Cyth2
Gnptg
Rbm3
Mrpl13
Tacc3
Tmed9

Sh3gl2
Txnrd1
Uggt2
Glg1
Litaf
Snx5
5830428H23Rik
Caprin1
Nnat
Gnao1
Rps28
Rfc4
2410006H16Rik
Fbxo32
Commd6
Ggh
1110065P20Rik
2810474O19Rik
Cdv3
Akap11
Pak2
Ccnl1
Grlf1
Tardbp
Uimc1
Serf1
Scamp3
Mt2
Acp2
Crot
Zc3h13
Psme4
Sod2
Hsph1
Brd3
Ap3b2
Psma1
Tmem57
Stard4
Pdzrn4
Hnrnph3
Rps7
Ing4
Tbcb
Dnajc21
Cep57
Man1c1
1110038B12Rik

Ptp4a2
Mllt10
Ensa
Tshz1
Lpcat1
Rfc5
Pttg1
Ssbp2
Arid2
Rab7
Psmd6
2700049A03Rik
Ldb1
Naa38
Vps37a
Zfp706
Arl6ip4
Phactr1
Phf20l1
Atad5
Med19
Ccni
Zmiz1
Faim
Irs1
Stk11
Plcg1
Klhl24
Zfp827
Anapc5
Gtf2f2
Psmc3ip
Lbr
Sgol2
Myl12b
Cirbp
Git2
Lphn1
Xpr1
Agpat6
Plekha1
Jarid2
Dync2h1
Sesn3
Bcar1
Gtf2e2
Ywhaz
Shmt2

Rbmx
Tro
Slc38a1
Dnajc2
Ptprs
Rfc3
Armc10
Mapk8ip1
Ube2g2
Evl
Blmh
Sumo3
Pttg1ip
Cplx1
Shmt1
Ppp1r1a
Rps4x
Zfr
Dnajc1
Cdh20
Scaf11
Klc1
Ccdc53
Kdm1a
Dnmt1
Sclt1
Picalm
Pcbp4
Zfp422
Nsmce4a
Wasl
Ctbp2
D19Bwg1357e
Ddx39b
Prkar2a
Tchp
Herpud1
Brd7
Scg3
Polr1c
Pebp1
Ulk1
Gltscr2
Cnksr2
Nes
Zfp36l1
Dpy30
Arfrp1

Fam64a
Dock1
Pcsk2
Sass6
Casp8ap2
Klhl7
Lyar
Ankrd49
Ewsr1
Kcnk1
Mad2l2
Ccdc23
Rnf220
Rab3c
Poc1b
Nmral1
2700081O15Rik
Gm13826
Tmco1
Gm11478
Fkbp4
Zdhhc20
Sowaha
Ano6
Frg1
Snord104
Iqgap1
Nfu1
Lin7c
Pou3f3
Klf13
Polr2c
Rbbp8
Celf1
Mpp6
2610203C20Rik
Tiparp
Rpf1
Mxi1
Tbpl1
Rundc3a
Emd
Rraga
Ube2d1
Sf3a3
Cotl1
Nkain4
6330403K07Rik

R3hcc1
Ndufaf2
Oraov1
Ncor2
Nfkbia
Smad4
Phc2
Mapk3
Dgkz
Pcyt1a
Klf9
Rpl6
Aspm
Suz12
Slc12a2
Cyb5r3
Elp3
Mrpl48
1700020I14Rik
Smchd1
A330076H08Rik
Zfp704
Tagln3
Nemf
Pnpla8
Srgap2
Esf1
Atp1b3
Acd
Rab6b
Hes1
Kdm5a
A030009H04Rik
Pde4dip
Plcb4
Apoe
Ctsd
C1qb
Lyz2
C1qc
Sepp1
Hexb
Tyrobp
Ctsb
Ctss
Cst3
Fcrls
Fcer1g

C1qa
Aif1
Grn
B2m
Lgmn
Ctsz
Trem2
Laptm5
Ly86
Csf1r
Cx3cr1
Cd68
Hsp90ab1
Fcgr3
Fth1
Igf1
Ctsa
Rnase4
Timp2
Cyba
Mpeg1
Hexa
Creg1
Rgs10
Ctsl
AF251705
P2ry12
Fyb
Emr1
Unc93b1
Hpgds
Ptpn18
Maf
Cd53
Lamp1
Gpr34
C3ar1
Psap
Cyth4
Vamp8
Sat1
Cd52
Pld4
Tmsb4x
Ctsc
Arhgdib
Arpc1b
Rac2

Npc2
Abca1
Ctsh
Ltc4s
Man2b1
Sirpa
Stab1
Pycard
Apbb1ip
Itgb5
Cd84
Lpcat2
Ccl3
Evi2a
Ms4a7
Ms4a6c
Apoc1
H2-K1
Ncf1
Irf8
Bst2
Serinc3
Itm2b
Anxa3
Lair1
Fam105a
Pf4
Cd300a
Abhd12
Mertk
4632428N05Rik
Mt1
Spi1
Abcg1
Rgs2
Npl
Tnfaip8l2
Fcgr1
Lcp1
Cd86
Mrc1
Bin2
Sh3bgrl3
Cotl1
Selplg
Syngr1
Nfib
Cd37

Psmb8
Fgd2
Ptpn6
Anxa5
Plek
Cryba4
Tuba1a
Gpx3
Spp1
Gns
Clec7a
Ptgs1
Ms4a6d
Plin2
Ccl4
Hmha1
Tbxas1
Il6ra
Inpp5d
Ms4a6b
P2ry13
Rasgrp3
Scamp2
Zfp36
Ccr5
Wfdc17
Ccl12
Pld3
Cst7
Gusb
Fermt3
Tmem86a
H2-D1
Sparc
Plxdc2
Lgals9
Cybb
Cstb
Slc11a1
Rgs1
Arl4c
Nckap1l
Trf
Mylip
Myo1f
Hpgd
Olfml3
Snx5

Syk
Abcc3
Tpp1
Slc7a7
Cd83
Tlr7
Clta
Serpine2
Siglech
Rab3il1
Lpl
Ucp2
Dcxr
Ehd4
Bcl2a1b
Ccl6
Ctse
Lgals3
Entpd1
Nrp1
Mef2c
Mafb
Blnk
Il10ra
Tcn2
Lyn
Adap2
Sfrp1
Tcf4
Tmem37
H2-DMa
Srgn
Ighm
Uap1l1
Gngt2
Havcr2
Arhgap30
Klhl6
Lrp1
Slc6a6
Il10rb
Gpx1
Lyl1
Slco2b1
Hcls1
Lipa
Emp3
Slc15a3

Ptprc
Lgals3bp
Itgb2
Dhrs3
F11r
Renbp
Lamp2
Hnrnpa2b1
Slc40a1
Cd180
Tmem176a
Tlr13
Ifi30
Dnase2a
Folr2
Actb
Ccl2
Cd36
Rhog
Fcgr2b
Vav1
Ctla2b
Lst1
Arsb
Lcp2
Sgk1
Apobec1
Ifi27
Zic1
Prkcd
Gna15
Ncf2
Cxcl16
Arhgap25
Ostf1
Dock2
Axl
Rps6ka1
Sla
Tcirg1
Camk1
Gmfg
Nrros
Lgals1
Gpr183
Msr1
Itga6
Fli1

Pla2g15
CRE_RECOMBINASE
Cfh
Arpc2
Ang
Susd3
Igsf6
KCTD12
Cd14
Ptplad2
P2ry6
Pfn1
Gcnt1
Rrbp1
Rin2
Tpd52
Pon3
Dock8
Tmem106a
Pmepa1
Fuca2
H2-DMb1
Ecscr
Lilrb4
Efhd2
Abi3
Slc37a2
Hk3
Tlr2
Runx1
Tifab
Klf2
Rab32
Atf3
Tnfrsf1b
Ikzf1
Kcnk6
Slc7a8
Slc9a9
Tubb5
Erp29
Pbxip1
Csf2rb
Parvg
Tmem176b
Tspo
Fes
Soat1

Tnfrsf11a
A630001G21Rik
Skap2
Gpr65
Ntpcr
Asah1
Csf3r
Pmp22
Plcg2
Anp32a
Scarb2
Hhex
Rasal3
Stxbp2
Neat1
Epb4.1l2
Ebi3
Igfbpl1
Pnpla7
Ddah2
Tgfbr2
Crmp1
Rtn1
Ppfia4
Ttc3
Cd24a
Hck
Alox5ap
Fblim1
Lmo2
Fxyd5
Ncl
Ccl9
Nfix
Npc1
Gas6
Tnf
Plin3
AB124611
Ccl7
Glul
Cd4
Marcksl1
Hvcn1
Gpr157
Daglb
Icam1
Syngr2

Shisa5
Fam49b
Ifngr1
Glipr1
Pik3cg
Tgfbr1
Nfia
Nnat
Cd48
Atp13a2
Trim30a
Nedd4
Ncf4
Npnt
Psmb9
Slc43a2
Cd33
0610031J06Rik
Tmem140
Arhgap9
Aldh2
Nfam1
Slfn2
H3f3b
Slamf9
Gba
Slc29a3
P2rx4
Ccnd2
Litaf
Ptafr
Clec5a
Tapbp
Tnfaip3
Tmem119
Hnrnpu
Coro1a
Hebp1
Itgal
Man1a
C5ar1
Gpr137b
Hpse
Sipa1
Ifngr2
Stard8
Fam26f
Gap43

Stmn3
Psip1
Chst1
Csf2ra
Sdcbp
Atox1
Nagpa
Anxa2
Fam212a
H2afv
Tnfrsf13b
Adipor1
Gatm
Cmtm6
S100a1
Fkbp3
Rassf5
Tmsb10
Rnh1
Cyfip1
Eva1a
Rbm47
Gaa
Itgam
Hnrnpab
Fcgrt
Msn
Ralb
E130114P18Rik
Cbx5
Wwp2
Junb
Ifnar2
Bex2
Luc7l3
Capzb
Adssl1
Tm6sf1
Lhfpl2
Colec12
Slc25a45
Smagp
Cd38
Cpe
Gdi2
Pros1
Paqr7
Sdc4

Gpnmb
Pla2g7
Gm26532
Cd302
Gm2a
Lhx1
Nceh1
Acss1
Cela1
Fbxw4
Snx20
Msrb1
Cog7
Nrp2
Cebpa
Necap2
Barhl1
Plod1
Serbp1
6330416G13Rik
Ccrl2
Stmn2
Stat6
Slc38a6
Hnrnpdl
Soga3
Fam46c
Il4ra
Matr3
Akr1a1
Cndp2
Htatip2
S1pr1
Rtn4rl1
Blvrb
Gria2
Map1b
Pax6
Xlr
Adam15
Cryl1
Banf1
Tnfrsf1a
Adcy7
Edem1
Bmp2k
Cnn3
Pon2

Tubb3
Rcsd1
Miat
Arap1
Mex3a
Smim1
Tmem50a
Ier3
Grap
Draxin
Naglu
Ngfrap1
Plbd2
Cd9
Klf6
Vcam1
Cbx1
Sfrs18
Elavl3
Vwa5a
Dpp7
Cald1
C2
Ftl1
P2rx7
S100a13
Slc46a3
Gabarap
Tubb2b
Nsg2
Nrep
Akap13
Myh9
Tspan4
Hnrnph1
Kcnk13
Map2
Ptbp3
Rapsn
Irf5
Calm2
Cpd
Nhlh2
Scg3
1700017B05Rik
Ppp2r2c
P4ha1
H1f0

Sft2d2
Capza2
AI413582
Chd4
Atrx
Acin1
Nfkbid
Fam46a
Hist1h1c
Ank3
0610040J01Rik
Cebpb
Lpcat3
Tmem141
Ilf2
Elovl1
Ezh2
Ppt1
H2-T23
Pik3cd
Speg
Rnf130
Pcm1
Atxn7l3b
Cap1
Man2b2
Abca9
Smc3
Hfe
Srsf3
Sfpq
Rps5
Tor3a
BC028528
Gpsm3
Gm13476
Fxyd6
Gm17750
Prdx2
Clic1
Tfpi
Syt11
Ly6e
Rnd3
Chd7
Ramp1
Crip1
D430041D05Rik

Gpm6a
Picalm
Cltc
Gmpr
Ninj1
Trim47
Dpysl4
Sept3
Tia1
Rab11fip5
Wdr1
Ndufa4
Slc29a1
Ubtd1
Pnn
Neurod1
Ckb
Twf2
Gpm6b
Mfsd1
Casp8
Golm1
Dse
Dtx4
Zic4
Dcx
Hdac2
Serpinb6a
Smc1a
Tmed3
Nasp
Tec
Itpripl2
Hsp90aa1
Ranbp1
Scoc
Prune2
Gm6977
Pnp
Tnfrsf21
Pfkfb3
Snrnp70
Pde1c
Ncam1
Arpc5
Itm2c
Rbfox3
Il18

Limd2
Hmgn1
Cplx2
Hnrnpm
Cpt1a
Cnbp
Mfsd12
Celf4
Apba2
Comt
Hnrnpd
Usp2
1500016L03Rik
Cln3
Pcdhga9
Pdk1
Abhd6
Slc46a1
Parp9
Scg5
Slc16a6
Ina
Dynlt3
Commd9
Anp32e
Tanc2
Adam17
Vps37b
Uncx
Dek
Srrm2
Fubp1
Mycn
Gnao1
Rnf128
1110001A16Rik
Edem2
Kif5c
Plekha2
Isyna1
Mt2
Atp6v0b
Map3k8
Galns
Tns3
Sall1
Nop58
Bhlhe41

Ssb
Leprot
Nfkbia
Smpdl3a
Tagln3
Manba
Arpc4
Sulf2
Cdkn1b
Gas5
Tmem135
Prdx5
Plekho1
Capg
As3mt
Mef2a
Nfe2l2
Pepd
Hnrnpr
Pkib
Nap1l1
Anxa4
Orai1
Cd81
Top2b
Ptma
Sec14l1
Blvra
M6pr
Rnf13
Csf1
Rasa4
Ddx5
Aga
Gm10075
Hist1h2bc
Fuca1
Ptn
Hnrnpc
Nacc2
Pla2g16
Sgpl1
Dnajb14
Elk3
Cib1
Celf2
Rassf2
Skp1a

Gm9800
Mpp1
Bclaf1
Htatsf1
Amdhd2
Arhgap17
Rufy3
Hdgfrp3
Gyg
Krcc1
Thoc7
Rps26
Ubc
Atp6ap2
Rap2a
Gla
Fez1
Khk
Bin1
Cacna2d1
Snrpd1
Cdk4
Slc25a4
Pdap1
Supt16
Acox3
Stmn4
Fnbp1l
Fam134b
Kif1b
Tox3
Atp6v1c1
Nfkbiz
Gmip
Rragc
Insm1
Ppp1r18
Glb1
Mgat4a
Dynll1
H2afj
Arhgdia
Brd3
Elavl4
Mdk
Gsdmd
Zfhx3
Smc4

Akap9
Srsf11
Pip4k2a
Camta1
Tsc22d1
Tmem9b
Basp1
Mvp
Rnpep
Sash1
Cacng2
Dtnbp1
Snx3
Ezr
Hn1
Nhlrc3
Il18bp
Ssrp1
Tpi1
H2afy2
Kmt2e
Prcp
Uchl1
Sox4
Wasf2
Sdf2l1
Cltb
Mllt4
Irf9
Capns1
Trim2
Nucks1
Rap1b
Zeb1
Hdgf
Pde3b
Ktn1
Tep1
Ptov1
Fus
Smc2
Tpst2
Cd164
Hnrnph3
Galc
Eif4g2
Cyb5r1
Tram1

Rad21
Nrxn1
Bri3
Gnai2
Dusp3
Ddx26b
Dram2
Hsd11b2
Myh10
Kif21a
Magt1
Dclk1
6330403K07Rik
Rpl4
Fnip2
Pcbp2
Ptpre
Cfdp1
Atpif1
Fam115a
Rdx
Gsn
Crlf2
Clip3
Sh3glb1
Rrp1
Ttyh2
Elf1
Pdlim4
Tor1aip1
Pfn2
Ptgr2
Anp32b
Hmgb3
Snhg5
Pcbp4
Vps18
Dpysl3
Hnrnpa0
Top1
Hmgn5
Meis1
Tshz2
C1ql1
Whsc1
Slc36a1
Abcd2
Cox7a2

Ccnd1
Ppp1r14c
Eif2ak2
Strbp
Snrpf
Metap2
mt-Cytb
Mfsd11
Tmem179b
Aldoa
Ncald
Rab31
Arrdc1
Bzw2
Nap1l4
Tmbim6
Rbfox2
Gng3
Lamtor1
Slc12a2
Nhlh1
Nsg1
Tacc1
Lactb
Podxl2
Tmem219
Ahi1
Apc
Fam210b
Arid4b
H2afy
Cdk5rap2
Serp1
Gm5617
Rundc3a
Pald1
Gm2694
Map4k4
Safb
Tmem173
Tsn
Hpca
Tecr
Ptprs
Tcerg1
Smap2
Ppib
Mab21l1

Luc7l
Wipf1
Tbata
Cdk5r1
Cyp4f13
Thra
Bcas1
Ccdc88a
Mcfd2
Ctcf
Dixdc1
Flcn
Itpripl1
Ncor1
Nagk
Gng12
Acp2
1110007C09Rik
Cfl2
Sppl2a
St3gal6
Arrb2
Srebf1
Fam171b
Txnrd1
Atp6v0e2
Tmed5
Snrpe
Prpf4b
Psma7
Hey1
Degs1
Lrp10
Chgb
Nop56
Hmgb2
Bex1
Cntn2
Adcy3
Atp6v1f
Scpep1
Elavl2
Hmgn3
Slc1a2
Wwp1
Pou2f2
Zfp710
Atp6ap1

Dnaja1
Tuba1c
2700094K13Rik
Cd63
Grina
AI854517
Sord
Fgf9
Srrm4
Sf3b2
Akap6
Snx18
Mki67
Psmb10
Tmem66
Rcn1
1110004F10Rik
Itfg3
Zc3h13
Smarca4
Tmpo
Mapk14
Lmnb1
Arhgap19
Akr1b10
Map3k1
Apex1
Tln1
Prmt8
Bcl11a
Clmp
Dynlrb1
Klhdc2
Rnmt
P4hb
Adam9
Srrt
Btbd17
Set
Tsc22d4
Elovl6
Ywhae
Scarb1
Fut9
Kif3a
Pde1b
Cbfa2t3
Fkbp4

Rab8b
Sacs
Maged1
Pcbp1
Cirbp
Bcl7a
Prim1
Hirip3
Cnot6
Dap
Khdrbs1
Npdc1
Dner
Actr3
Hjurp
Mllt3
Mast3
Cct6a
Gstm5
Slc45a4
Snx2
Kdm1a
Tpm3
1700025G04Rik
Ap3b2
Rplp1
Mapk8ip2
Nkd1
Rsrc2
Dbnl
Pigk
Sf3b1
Mcm7
March1
Ptprd
Wls
Hnrnpk
Dusp6
Mcur1
Dgkz
Neu1
Tle1
Hp1bp3
Ptpn1
Nek6
Aldh9a1
Gng5
Rps3a1

Aim2
Stx7
Pbrm1
Slc35c2
Rap2b
Pou3f2
Usp22
Apbb1
Paip2
Syncrip
Kif2a
Mid1ip1
Heg1
Ostm1
Myo9b
Sypl
Ccar1
Slc35f6
Ddx46
Cln5
Dock10
Sdf4
Cflar
Col27a1
Npm1
Otx2
Atp6v1g1
Dkc1
Purb
St8sia3
Rbm39
Gm8292
Chd6
Srrm3
Tead2
BC005764
B4galt1
Rogdi
Eny2
Ldhb
Pdgfra
Ubtf
Pak3
Plod3
2310022B05Rik
Ehbp1l1
Ube2e3
Tex14

Ank
Zc3hav1
Rps19
Eif1ax
Nol7
Cdh20
Rab6b
Sept4
Ubb
Rhob
Fbxo6
Prr13
Ythdc1
Dnm2
Ltbr
Cenpv
Actr2
Prrc2c
Cxxc5
Rsf1
Tspan6
Tmem178
Atoh1
Txnip
Csnk1a1
Gna12
Rbmx
Kcnk1
Srrm1
Cln8
Rhoc
Klc1
Naga
Satb1
Thoc2
Pdcd4
Sptbn1
Pgd
Dnajc9
Mapt
Dut
Prdx1
Snrnp40
Tcp1
Sept7
Chrna3
Rbp4
Coa5

Plcb4
Elovl4
Derl1
Clstn1
Ppp3cb
Sult4a1
Rbm25
Trp53i11
Sh3gl2
Coro2b
Sirt2
Tgfb2
Aplp1
Ilf3
Colgalt1
St18
Pkn1
Homer2
Fam57b
Ubl3
Gltp
Crip2
C130071C03Rik
Brk1
Sept11
Sema6a
Reep5
Nfkb1
Sstr2
Vasp
Atp5b
Ppcdc
Tmed10
Nucb1
Islr2
Pa2g4
Peg3
Ttc9b
Ube2b
Zcchc18
Itgav
Ophn1
Stmn1
Tmod2
Nell2
Arl3
Zbtb18
Ssh2

Zfp326
Ppic
Wbp5
Rtf1
RP23-45G16.5
Baz1b
Rcor2
Taok3
Acat1
Kifap3
Cyc1
Klf9
Tro
Prc1
Dhx36
App
Rab5c
Cetn3
Pdzrn3
Gm11266
Rbbp6
Rplp0
Rnf144a
H1fx
Arpc3
Cct2
H2afx
Cr1l
Dusp1
Adrbk1
Ubash3b
Myef2
Fam168a
Gsk3b
Dhrs1
Slc23a2
Prkcb
Vezf1
Cdkn1a
Lpp
Pacsin2
Mllt11
Fnip1
Igsf3
St6galnac4
Myt1
Rgl2
Rab7

Fermt2
Ebf3
Gm3764
Zfp462
Ccp110
Igfbp4
Cdc5l
Spc25
Aprt
Med19
Ddx42
Sec11c
Efs
Top2a
Amz1
Epc1
Eid1
Rhoa
Smc6
Slc17a6
Nudt14
Srpk2
Stat3
Nrn1
Sox9
Tmeff1
Fbxo32
Bach1
Rbm8a
Arglu1
Snrpn
Fez2
Nr2f1
Ptprg
Setbp1
Cenpe
Ssr4
Bnip2
Tspyl4
2810417H13Rik
Sema7a
Snap25
Polr2h
Tnik
Fmnl3
Camk1d
Ldha
Fmr1

9330159F19Rik
Dtx1
Mtss1
Marc2
Mfap4
Extl3
Smpd3
Rbm5
Bnip3
Fstl1
Tcf25
Rnd2
Gpr153
Slc12a9
1110001J03Rik
Myo5a
Snrpb
Sltm
Tppp3
Cmtm3
Cttnbp2nl
Coro1b
Armcx4
Zfp704
Sft2d1
Pcbd2
Tardbp
Fut8
Chtop
Fkbp15
Tbc1d12
Smox
Mphosph8
Hmgn2
Calm3
Snx6
Usp1
Bicd1
Ccser2
Ten1
Kmt2a
Pltp
Bok
Zfp422
Siva1
Gde1
Cadm3
Atp5o

Rab2a
Hist3h2ba
Fam133b
Nemf
Tpm4
Fam212b
Carhsp1
Mknk1
Rcn2
Slc25a23
Rbfa
Sowaha
Sub1
Rsl1d1
Eprs
Thrap3
Pold2
Zwint
Cdh2
Gpbp1
Ddx17
Rnf187
Cttn
Ank2
Cks1b
Zfp637
Bcap31
Rnf20
Ndufa12
Akirin1
Os9
Cdc42
Cnot7
Gsg1l
Mfng
Snx8
Stox2
Gabrb3
Cenpf
Cdk2ap1
Gprasp1
A630007B06Rik
Syne2
Csnk1e
Itsn2
Sars
Snrpd3
Man1c1

Cpeb4
Smarcc1
Bub3
Scly
2610017I09Rik
R3hdm1
Ndn
Odf2
Atp1b3
BC005561
Rab11a
Tubb2a
Cox20
Nolc1
Smarcd1
Orc6
Phip
Cdk6
Zfp91
Celsr2
Nptn
Rp2h
Wdr26
Vat1
Snap23
Arf3
Casc4
Epb4.1l3
Snhg1
C530008M17Rik
Gjc1
Pou3f3
Lamtor4
Ewsr1
Etfb
Kat6b
A330076H08Rik
Cct5
Mpdz
Plcb1
Ppfia2
Frrs1l
Dtymk
Cecr2
Lrrn1
Daam1
Olfm1
Ift43

Nkain4
Eif1
Serf1
Fndc4
Col9a3
Cdca3
Tmem57
Tspan14
Ralgps2
1700001O22Rik
Son
Eef1a1
Cct7
Fam98b
Gas1
Amer2
Slc22a17
Phf14
Mpped2
Pygo1
Hip1r
Map7d2
Stxbp1
Reln
Rap1a
Trappc6a
Smarca5
Smarcb1
Snrpa1
Zfp292
Rab3ip
Gm11223
Ybx3
Zfp428
Senp6
Bmyc
Hgsnat
Mbnl1
Ppp2r5e
Bod1
Trove2
Spcs2
Tmem87b
Rap1gds1
Wasf1
Tfrc
Casp3
Ran

Upf3b
Prpf40a
Spats2
Neurod6
Bcar1
Cox6c
Sh3kbp1
Specc1
Tprn
Serpinh1
Ccdc34
Pnmal2
Eif3c
Usp46
Jakmip2
Tfdp2
Fam3c
H3f3a
Pitpnc1
Nrcam
Srsf2
Rgs19
Asf1a
Gm1673
Srsf10
Fkbp1a
Grb2
Gse1
U2surp
Sfrp2
Hsd17b11
Clspn
Atp6v1b2
Kif11
Smarcc2
Mrps5
Rassf4
Ckap2l
Eif3h
Fam111a
Vav2
Tet1
Calr
Rcbtb2
Mtf2
Ccdc104
Il11ra1
Magoh

Srsf7
Mum1
Ssx2ip
Ptpn11
Trim37
Fbxo5
Tex9
Prpf38b
Ndufc2
Sf3a3
Prpf6
G3bp2
Chmp6
Rac1
Lmo4
Tmem160
Atp5a1
Naa38
Luc7l2
Zfp664
Cwc27
Atl1
Lman2
Egr2
Ccng1
Ndufa5
Schip1
Tbc1d20
Mapk8ip1
Setd8
Kif1a
Gtf2i
Eef2
Mns1
Zc3h15
Nfyb
Rnf166
Fam174a
Rab3c
Naa10
Ift27
Pde4dip
Tcf3
Ulk2
Plgrkt
Ap1b1
Ppp3ca
2900011O08Rik

Epc2
Gdpd1
Atp6v1a
Rtn3
Cyth2
Vps26a
Brd8
Eps15
Hscb
Arpc1a
Prox1
Tubb4b
Xrn2
Morc3
Nbea
Hes6
Dcakd
Tgoln1
Cacng4
Rhobtb3
Ehmt1
Plk4
Surf4
Gpatch8
Nedd4l
Gtf2h5
Pafah1b3
Ctr9
Rab6a
Tmem14c
Chchd2
Gnl3l
Maf1
Ccdc41
Mad2l2
Nob1
2210018M11Rik
Dnajc8
Pcyox1
Appl1
Dnajc3
Afap1
Ddx39b
Brd2
Atp5j
Rfc4
Ptpn12
Ddx6

Hmox2
Atp8a1
Samhd1
Rsbn1l
Pgrmc1
Tex264
Dbn1
Ankrd12
Tpx2
Npepl1
Ska2
Aagab
Polr2i
Tacc2
Mdga1
Rps15a
Stk4
Usp34
Nuak1
Mrpl20
Hsp90b1
Pip5k1a
Esf1
Polr2m
Lyar
Myt1l
Mdh2
Scn8a
Jak1
Snrpd2
Kcnip3
Ckap5
Pttg1ip
Rpn2
Pqlc1
Cdc16
Tuba1b
Nicn1
Mcl1
Rnps1
Pbx1
Phf21b
Tax1bp1
Kdm5b
Timeless
Ndrg2
Wdr82
Racgap1

Creb1
Slc39a6
Arl8b
Zmynd11
Nans
Ski
Rnf7
Rbx1
Prps2
Slc15a4
Ube2k
Gdap1
Cdc7
Sox11
Rufy2
Sumf1
Adam10
Atp6v0a1
Selm
Cdca8
Clic4
Hes1
Lrpap1
Eif3d
2610203C20Rik
Rnpc3
Wdr6
Pabpn1
Magohb
Fos
Mgst1
Clip1
Gpr85
Pfdn4
Cdk11b
Vbp1
Ndufb2
Acot7
Morf4l2
Ckap4
Clptm1l
Tmem256
Mical1
Trp53
Ebna1bp2
Tead1
Eif3e
Hint1

Pdzrn4
Rnaset2a
Ddx1
Caprin1
Ercc1
Atad5
Spc24
Pbk
Cct3
Prdx6
Csrp2
Stard3nl
Nsd1
Cuta
Clpp
Cdc20
Glce
Ddr1
Xist
Cnksr2
Cfl1
Cct8
Nipsnap1
Ntm
Fam181b
Cc2d1b
Ggh
Cdh4
Celsr3
Ptbp2
Cdca7
Tmem35
Dnajc2
Arpp21
Hmgb1
Ndufs4
Nol4
Cdc123
Prdm8
Ube3a
Zfp266
Eif4a1
Shmt2
Elp3
Abcf1
Appbp2
Ppp1r14b
Zfp580

Zfp36l1
Tiprl
Dis3
Bptf
Tiparp
Smarcad1
Tceal8
Zcchc11
Cisd1
Mllt10
Anapc5
Rpa3
Ppp1cb
Zic5
Ptch2
Sox5
Pik3r3
Pdcd6ip
Ywhag
Sbk1
Map1lc3a
Got1
Srsf5
Pdia3
C77370
Cacybp
Zfml
Ifnar1
Tmem222
Ykt6
Nono
Naca
Gpsm1
Fam92a
Ankrd11
Tmed7
Lhfpl4
Gpatch4
Aspm
Slc25a5
Txn1
Rab14
Rnf165
Phpt1
Casp8ap2
Smco4
2210016L21Rik
Tmem30a

Cbx3
Yipf1
Srek1
Agpat4
Nhp2
Mob1a
Sqle
Parp6
Taldo1
Lpar6
Zdbf2
Map9
Lmnb2
Pitpna
Rexo2
Mycbp2
Tbl1x
Eif3m
Smchd1
Ube2e2
Polr3k
Clcn4-2
Mageh1
Birc5
Oat
Phf3
Zranb2
Fabp7
Prnp
Emg1
Id2
D19Bwg1357e
Klhl7
E330009J07Rik
Vopp1
Dzip3
Vps36
Dync1li2
Pafah1b1
Tulp4
Arf4
Dync2h1
Gtl3
Lrig3
Kif5a
Ube2e1
Csde1
Trp53bp1

Papola
Vcp
Paics
Nudt3
Vcan
Zfp451
Usp10
Clybl
Ryk
Twf1
Cul3
Eif5a
Clvs1
Ndufv2
Npepps
Srp19
Spag9
Cdca7l
Nipa2
Smarce1
Ywhaq
Ech1
Robo2
Brwd1
Mfsd5
Chmp3
Ctbp1
Kif22
Klf13
Snw1
Mycl
Cenpa
Thsd7a
Prmt5
Igsf8
Flywch1
Rrm1
Ilkap
Gtf2a2
Ptrhd1
Dync1i2
Dctpp1
Tipin
Psmc6
Zfp354c
Tpm1
Cdkn2c
Srsf1

Ift74
Pdcd11
Nckap1
Nktr
Tmem107
Acot13
Prmt1
Las1l
Nt5dc2
Akap8
Tomm70a
Trpc2
Hspa5
Abi1
Nedd8
Rnf168
Ubqln2
Bcat1
Mrps30
Zranb1
Slc3a2
Pard3
Stip1
Sec62
Bzw1
Tceal3
Ube2d1
Sfswap
Lsm6
Cadm4
Fasn
Golga4
Mgea5
Rab3a
Nop10
Fyn
Knstrn
Polr2b
A930011O12Rik
Ap2s1
Strn3
Tada2a
Rbmxl1
Lta4h
Rbbp4
Osbpl11
Hsbp1
Ctxn1

Snap47
Fbxl5
Lphn1
Smim15
Hmgcl
Psma5
Gm17322
Nusap1
Prpf4
Mxd3
Asrgl1
Ube2j1
Ssu72
Zmiz1
1110037F02Rik
Ankrd32
Pds5b
Sri
Ctdspl2
1110051M20Rik
Ash1l
Deb1
Mroh2a
Ccdc28b
Jarid2
Sh3bp2
Adk
Phactr1
Tmem251
Hnrnpl
Acat2
Ier5
Pja1
Itpa
2410089E03Rik
Ndufa10
Lsm14b
Phf6
Stx6
Dctn2
Spop
G2e3
Smdt1
Mis18bp1
Dph3
Nefm
Mapre1
Ap3b1

Ypel1
Fam155a
Klf7
Fnbp1
Tspan5
Abi2
Ift81
Pole3
Mga
Palm
Paxbp1
Cep57
Rdh5
Erc1
Tubg1
Msi2
Scd2
Pura
Ttc4
Spen
Stard3
Mpzl1
Rbm6
Pcdha2
Utp3
Pdrg1
Ppp1r1a
Gdi1
Ppp2ca
Atxn7l2
Mocs2
Rsu1
Ankrd46
Tpr
Rbm26
Smarca2
Stoml2
Cep290
Ick
Kif15
Irs1
Agtpbp1
Rpf2
Gnb4
Dnaja2
Pdxp
Psmc3ip
Amer1

Ntrk3
Vdac2
Gna13
Stard4
Cask
Dnajb11
Pnrc2
BC034090
Ncapd2
Gpr56
Bex4
Bod1l
Csnk2b
Slc38a1
5031439G07Rik
Pkia
Rnf220
U2af1l4
Ccdc25
Fbxw7
Nsmce4a
0610009D07Rik
Copg1
Pik3ip1
Esco2
2700049A03Rik
Gm11541
Tecpr1
Slc39a10
Cd63-ps
Dhx15
Ube2c
Tex261
Ddx55
Prpf19
Cct4
R3hdm2
Rab1
Prdm2
Ctbp2
Nisch
Polr2f
Qdpr
Wdr61
Cpsf6
Ccnb2
Tnrc6a
Nt5c3

Prps1
Golim4
Myl12a
Cdk14
4921524J17Rik
Cplx1
Btf3
Rufy1
Tob1
Snrpb2
Srsf6
Sobp
Cfp
Psmc1
Jund
Ywhaz
Eif4g1
Hnrnpul2
Usp12
Sv2b
Zfp131
Sfr1
5830418K08Rik
Rps7
Rnf146
Gar1
Lig1
Celf1
Sptssa
Acaa2
Mab21l2
Fhl1
Bcap29
Srpk1
Tra2a
Zfp148
Scaper
Chkb
Scp2
Ahsa2
2810004N23Rik
Gli1
Rcor3
Eif3f
Cdc42bpa
Uhrf2
Cenpj
Jmy

Krr1
Cspp1
Dhx9
Uhrf1
Vamp3
Fzd2
Eef1g
Park7
Zic2
Uqcrfs1
Psmd14
Cyp51
Gtf2f1
Irf2bp2
Cnpy1
Iws1
Dnmt1
Nav2
Nek7
Usp11
Hprt
Tcf12
Mff
1700021F05Rik
Fdft1
Gna11
Zmym5
Rab11b
Tm9sf3
Hsph1
Grik5
Rangap1
Cox7c
Fam216a
5430416N02Rik
Sipa1l2
Maged2
Cenpm
Acadl
Tbrg1
Bmpr1a
Rbmx2
Zcrb1
Chmp4b
Arl2bp
Zeb2
Hist3h2a
Vma21

Gphn
Bcas2
Rad51ap1
Igf1r
Psmd7
Ndufb11
Trim28
Frmd4a
Txndc17
Grik2
Aimp1
Gamt
Boc
2810008D09Rik
Arl6
Kpnb1
Sox18
Fcho2
Tmem70
Phf20
Dnm1l
Brd9
Rrs1
Dnajc7
Gm5914
Dhx40
Lbr
Taok1
Gabbr1
Hmmr
Ddit4
Psme4
Rnf122
Alas1
Atad3a
Rps8
Dtl
Kidins220
Lrrc45
Kif2c
Nup62
Jam3
Nipbl
Mrfap1
Srm
Dscr3
Acd
Tdp2

G3bp1
Phb2
Dot1l
Cox5b
0610011F06Rik
Gria4
Epb4.1
Rheb
Cnep1r1
Tmed9
Tex30
A830080D01Rik
Ikzf5
Pebp1
Nudcd3
Bms1
Ccna2
Upf2
Mcm2
Mettl9
Zfr
Hspa9
Trip11
Tra2b
Zfp536
Ubap2l
Rtn2
Bfar
D030056L22Rik
Rpap3
Wnk1
Nae1
Snrpg
Ctnnbl1
Stx4a
Ip6k2
Ltbp3
Zkscan1
Ndnl2
Syt13
Vps16
Mcm6
Ep400
Ccnb1
Odc1
Txndc9
Sae1
Ankhd1

Tom1l1
Cul5
Cep78
Mex3b
Ppil1
Acap2
Timm50
Tceb2
Fam98a
Lsm3
Eif3g
Pomp
Ppid
Ccdc66
Hmgxb4
Cntln
Epm2aip1
Nup85
Pick1
Sgol1
Emc6
Cenpc1
Kif23
Neo1
Tbc1d16
Rhno1
Rabgap1
Gnb2
Ddx3x
Cdc27
Ist1
Galk2
Mta1
Myod1
Impad1
Pafah1b2
Anln
Vmp1
Twistnb
Erdr1
Tbca
Vps33a
Sec61a2
Abt1
Psmc4
Poglut1
Taf1
Hs6st1

Trappc4
Zfp207
Gmps
1500012F01Rik
Zfp260
Pard6g
4930402H24Rik
Psme1
Sparcl1
Alcam
Mki67ip
Rbm4b
Zcwpw1
Fip1l1
Rftn1
Gm26735
Cep170
Bcl2l13
Prpf40b
Sarnp
Dcaf15
C1qbp
Ccndbp1
Inpp5f
Tdp1
Gga1
Mnat1
Vimp
Tsnax
Baz2b
Socs2
Chic2
Snap29
Mak16
Ddx18
Rgs12
Pvrl3
N4bp2
Nabp2
Acsl3
Trim59
Ubqln1
Adamts1
Dlgap5
Knop1
Mau2
Cdca2
Tnrc6b

Fam168b
Actl6a
Imp3
Ift57
Sms
Fam107b
Rps6kb1
Kif20b
Sbno1
0610007P14Rik
Gsto1
Rps18
Cenpp
2700081O15Rik
Smc5
Rbm22
Snrnp27
Cenpq
Exosc1
Ensa
Sepw1
Uchl3
Slc7a5
Supt6
Hnrnpll
Rnaseh2c
Psma6
Stub1
Zmat2
Bbip1
Ddx23
Emc10
Uba1
Dazap1
Uri1
Phf20l1
Tcea1
Phax
Wbp1
Kdm5a
Ttc14
Gripap1
Tmem132a
Clcn3
Ubp1
Eif2a
Shd
Gabarapl1

Mat2a
Zfand5
Ccnt2
Rexo1
March7
Ndufaf2
Rpf1
Pmpcb
Ndufa1
Ndufv1
Senp2
Tmem55b
Srsf4
Arid4a
Meis3
Ccar2
Ube2g1
Ptges3
Ercc5
Ntan1
Mark3
Lsm4
Mphosph6
Arid1b
Zfp68
Eif4b
Mrps6
Hspa8
Lancl2
Rrm2
Mapk7
Erbb2ip
Tshz1
Mettl14
1110038F14Rik
Pfdn5
Sugt1
Cnot2
Mbtd1
Psme2
Zfp512
Sf1
Ing1
R3hcc1
H2-Ke2
Ppa1
Sart3
Ncor2

Smoc1
Usp7
Tsen34
Mrpl38
Casc5
Stau2
Cops5
Lin7c
Stk16
Mzt1
Bap1
Snord104
Cbr1
Anapc11
Ranbp3
Hmgcs1
Elp2
Wdr12
Akap12
Setd5
F2r
Aurkb
Gng2
Rbbp8
Zfp322a
Rpl36
Rb1cc1
Man2a1
Ppil4
Casc3
1500011B03Rik
Chchd1
Ccdc127
Cpsf7
l7Rn6
Fbxw2
Cdc26
Pnpla8
Med11
Slc35b1
Kdm6b
Ppp1r12a
Paf1
Lrrfip1
Rsbn1
Zc3h7b
Rpa2
Chmp7

Copb2
Ccnl2
Rwdd1
Foxp1
Gkap1
Qser1
Raf1
Palld
Tmem183a
Vps4a
Cstf3
Bccip
Zfp608
Ssbp2
H2afz
Cenpk
Rnf11
Ckap2
Fnbp4
Ccdc18
Mpnd
Kmt2c
Pum1
Psma4
Scaf11
Eif4e3
Ogt
Parl
Naa15
Tmem184c
Sod1
Xrcc5
Usp3
A430005L14Rik
Adprh
Ralgds
Bhlhb9
Wbp11
Dnttip2
Zfp191
Lrrcc1
Dck
Gars
5730455P16Rik
Isoc1
Gzf1
Ccdc59
Dhx32

Fopnl
Psmb2
Ssbp3
Dhx30
Frg1
4632415L05Rik
Ssr3
Pbdc1
Hmgcr
Napg
Midn
Ntmt1
Uba2
Topors
Mrpl47
Suv420h1
Ppp2r1a
Itsn1
Brca2
Sfxn1
Ubfd1
Brcc3
Gatad2b
Fosb
Actr6
Rbm17
Ubxn6
Senp1
Krt10
Slc25a27
Pmm1
Aff4
Rfk
Carkd
Mysm1
Mrpl13
Gm13092
Hdac3
Tomm40
Phf5a
Papss1
Pop4
Ing4
Cherp
Tor2a
Ammecr1l
Mecp2
Mrpl11

Pja2
Eftud2
Rcc2
Eif4h
H13
Ppan
St3gal5
Mmadhc
Arid2
Dohh
Lrp11
Rtca
Xrn1
Smek2
1110038B12Rik
Sumo1
Lars
Gnl3
Nvl
Taf2
Ap4s1
Cog1
Nme1
2410006H16Rik
Cdk1
Bola1
Nip7
Cenph
Exosc7
Rab24
Nin
Blmh
Slc35a1
Gle1
Lcmt1
Arhgef7
Nudc
Commd1
Mprip
Irak1bp1
Eif2ak1
Polr3f
Dnal4
Elp4
Incenp
Mrpl30
Cby1
Yars

2610524H06Rik
Hook3
1700123O20Rik
Stk25
Slc25a17
Thumpd1
Oxsr1
Armc10
Vps29
Yme1l1
Suv39h2
Usp5
Wdr43
Wasl
Smek1
Csnk2a1
Cxx1b
Nosip
Sssca1
Ndufs7
Agap1
Pias2
Drg1
Zfp329
Chka
Ppdpf
Pqbp1
Fam53b
Aatf
Hsf2
Cul4a
Fen1
Rfc1
Stk11
Trnt1
Zfp30
Zfp386
Eif5
Mphosph9
Hspd1
Hells
Mrpl15
Thoc3
Nop14
Rad23b
Med1
Sclt1
Bmi1

Pcnt
Sf3b3
1300002E11Rik
Prcc
Vars
Polr1c
Fyttd1
Ifrd1
Rab12
Asun
Eif3b
Chd1
Emd
Ftsj3
Mphosph10
Rbm28
Zfp397
C330027C09Rik
Tsg101
Wbp4
Mrgbp
Rai1
Zfp318
Znhit6
Grsf1
Snx17
Smim11
Pdha1
Med4
Chd2
Cdc40
Rps19bp1
Rps27a
Atf4
Parp1
Lias
Trim33
Orc2
Snapc3
Ubxn2a
Mcmbp
Ubr7
Hbs1l
Taf3
Ash2l
Mtch2
Ccdc174
Cops8

Cops3
Safb2
Crebbp
Snx27
Cuedc2
Mbd3
Arhgap21
Spin1
Rab11fip2
Surf2
Fabp7
Slc1a3
Dbi
Apoe
Aqp4
Cst3
Sparcl1
Slc4a4
Mt1
Aldoc
Atp1a2
Glul
Ttyh1
Pla2g7
Ednrb
Mlc1
Ppap2b
Clu
Id3
Car2
Agt
Gpr37l1
Sparc
Acsbg1
Timp4
Gpm6b
Aldh1l1
Ptn
Tril
Kcnj10
Mt2
Mt3
Slc25a18
Igfbp2
Cd81
Mmd2
Tnc
Serpine2

Hsp90ab1
Ramp1
Bcan
Hepacam
Cmtm5
Scd2
Id4
Ptprz1
Atp1b2
Gria1
Tspan7
Lcat
Npy
S100b
Pea15a
Pla2g16
Nid1
Chchd10
Hopx
S1pr1
Sepp1
Cspg5
Paqr8
Gja1
Htra1
Gstm1
Mfge8
Lxn
Adora1
Tmem176a
Ncan
Lsamp
Sdc4
Cryab
Emid1
Gfap
Ntrk2
Ifitm3
Casp12
Scrg1
F3
AW047730
Tsc22d4
Tmem176b
Lrig1
Metrn
Plat
S100a1

Slc9a3r1
Hsd11b1
Glud1
Ccdc80
Luzp2
Gabrb1
Hes5
Slc6a1
Prss23
Atp1b1
Chl1
Fgfr3
Kcnj16
Cyp26b1
Rgs5
Gjb6
Rps5
Limch1
Lpar1
Sfxn5
Lamp1
Npas3
Slc39a12
Tubb5
Vcam1
Ndrg2
Cnp
Sfrp1
Prex2
Emp2
Vim
Fxyd1
Gpnmb
Slc38a3
Sox2
Pbxip1
Ddah1
Tlcd1
Thbs3
Tspan3
Gm3764
Cacng4
Slc7a10
Nfib
Tmem47
Atp13a4
Smpdl3a
Shisa9

Gsta4
Ckb
Pdpn
S100a13
H2-K1
Dkk3
Gjc3
Slc13a3
Mgst1
Itm2b
Myo6
Daam2
S100a10
Cp
Slc1a2
Cd44
Asrgl1
Cybrd1
Spon1
Gucy1a3
Eps8
Omg
Gmpr
Gabrg1
Acadl
Ncl
Itga6
Acot1
S100a16
Cyp2j9
Psap
Slc6a11
S100a6
Fjx1
Cd302
Itm2c
Rps9
Me1
Ctsl
Arhgef26
Gpd1
Hnrnpa2b1
B2m
Tjp2
Sat1
Pcp4l1
Chst2
Rfx4

Wnt7a
Zcchc24
Slc6a9
Rpl13a
Pcdh10
Tuba1a
Cldn10
Timp3
1700084C01Rik
Pcx
Vamp8
Degs1
Abhd3
Cntn1
Adcyap1r1
Jam2
Cntnap2
H3f3b
Rps26
Abca1
Abat
Sorl1
Timp1
Igfbp4
Cpq
Rps3
Pamr1
Gabra4
Naaa
Dbx2
Nrarp
Adrb1
Phyhipl
Lyn
Sox21
1810037I17Rik
Rpl4
Slc14a1
Rplp0
Adcy8
Gpx8
Plcd4
Slc35f1
CRE_RECOMBINASE
S100a11
Hnrnpab
Calm2
Rhod

Pax3
Anp32a
Cryl1
Gatm
Ifi27
Epha4
Rpl32
Paqr7
Igsf11
Prdx6
Trpm3
Aldh2
Dhrs1
Gdpd2
Slc1a4
Rps14
Gpr126
Slc13a5
Grm3
Il33
Fam213a
Hnrnpu
Ctso
Rab31
Bmpr1b
Angpt1
Rgl1
Efhd1
Kcnn2
Nacc2
Arap2
Hbegf
Sash1
BC064078
2810459M11Rik
Abhd4
Cml1
Igfbpl1
Mro
Basp1
Add3
Tmem37
Itih5
Rarres2
Fgfr1
Pon2
Gria4
Sncg

Sirpa
Nbl1
Rplp2
Rrbp1
Rps21
Serbp1
Nwd1
Paqr4
0610040J01Rik
Dtna
Slc12a4
Hnrnpm
Epas1
Agrn
Angptl4
Aldh1a1
Fam20a
Tpp1
Rhoc
Id1
Gnb2l1
Clrn1
Crmp1
Chpt1
Adam11
Ephx1
Pabpc1
Tmsb10
Btbd17
Slc27a1
Elovl5
Nfasc
Itih3
Slc7a2
Cbr3
Egfl6
Acox1
Gnb4
Stat3
Pmm1
Sfpq
Aspa
Asah1
Cog7
Cd9
Tmem56
Tmem198b
Prrx1

Sema6d
Tmem9b
Slc25a33
Fam181a
Tspan12
Abi3bp
Emc7
Nfia
Dlgap1
Pld2
Gpld1
Slc22a4
Grid2
Ctsd
Ddx5
Gpr123
Scrn1
Tpi1
Gde1
Hnrnpc
AI464131
Mrps6
Cd24a
Cyp2d22
Scg3
Stk32a
Ptpra
Cpxm1
Rnf13
Clec3b
Pmp22
Rps24
Wnt3
Pth1r
Slc43a3
Irx2
Rnf182
Ranbp1
Rxrg
Rps19
Dtx4
Atpif1
Vwa1
Fbxo2
Elmo2
Cdh13
Itpr2
Hnrnph1

Mcc
Ampd3
Hsdl2
Eps15
Msx2
Akr1b10
Lix1l
Ppp1r1a
Gng12
Gabbr2
Rps15
Ppargc1a
Osbpl1a
Sept4
Mertk
Npm1
Cpne2
Axl
Spry2
Tst
Tnfrsf19
Sez6l
Gaa
Rgs7bp
H2afv
Cystm1
Grina
AI413582
Frem2
Cbx1
Amot
Serpina3n
Pltp
Chd7
Gramd3
Marc2
Gm2a
Fuca2
Rps20
Timp2
Trim9
Zic1
Arxes2
Cgrrf1
Hist1h2bc
Bhlhe40
Pfkm
Fth1

Hrsp12
Ddah2
Tmbim6
Ctsb
Ubtd1
Lrrc4c
Cyr61
Nfix
Nop58
Tmem229a
Slc29a1
Mfn1
C030005K06Rik
Nebl
Elovl2
Trf
Wwc1
Cgref1
Plce1
Ecm2
Oat
Cadm2
Mpp6
Phgdh
Wls
Psip1
Arpp21
Agtrap
Lmbrd1
Fkbp10
Snrpd1
Sec14l2
Prr5l
Map1b
Dek
Ldha
Acsl6
Hn1
Slc15a2
Dbndd2
Rps11
Notch1
Acss1
Cd151
Npl
Col1a2
Anxa5
Smad9

Itgb8
Tmie
Sned1
Gas5
Bmpr1a
Fus
Draxin
Rarres1
Matr3
Stmn3
Spry1
Oaf
Cxcl14
Itgb5
Cbs
Eif3a
4931406C07Rik
Smc2
Banf1
Dbp
Rgma
Entpd2
Lysmd2
Appl2
Tex264
4930402H24Rik
Cck
Olfm2
Reep5
Chd4
Pgm2
Anp32b
Eef1a1
Hnrnpdl
Cdh22
Capns1
Klhl5
Cdk4
Ptma
Mras
Usp24
Rpl23
Etv4
Mmp14
Slc7a11
Il6st
Thrsp
Aldoa

Clip1
Abcd3
Smc3
Them4
Hsd17b12
Plec
Neurod1
Lamb2
Cyp4v3
Sybu
Serpinh1
Eef2
Srsf3
St3gal6
Mtss1l
Rpl8
Lfng
Phyh
Kcne1l
Epdr1
Stom
Cib1
Kank1
Ldhb
Nasp
Slc30a10
Gsap
Ccnd2
Atp6v0b
Ccdc24
Syt15
Hip1
Pgpep1
Ttyh3
Ttc3
Hdhd2
Tapbp
Ybx1
Sox4
Rpl34
Hexa
Dmd
Adamts5
Tagln2
Parp3
Lrp1
Dynlt3
Pdap1

Necap2
Mex3a
Smc1a
Acin1
Slc20a2
C4b
Ech1
Elavl3
Colgalt2
Rasl11a
Nnat
Rbm25
Hdac2
Tns3
Il18
Gm9800
Barhl1
Mboat2
Fgfr2
Fkbp11
Phlda1
Marcks
Prex1
Proca1
Rpl22
Pygb
Ezh2
Rplp1
Plvap
Pdgfrl
Plin3
Nkain4
Celf2
Hpgd
Col5a3
Fkbp3
Shisa4
Lpl
Crot
Fubp1
Rpl14
Smc4
Orai1
Pdlim5
Fgf1
Gstk1
Rpl26
Spred1

Trib2
Fads2
Nrep
Megf10
Kdelr3
Spock3
Tcf4
Lamp2
Cdh10
Mfap3l
Rbfox3
Cpeb4
Rora
Grm5
Gm10075
Cap2
Tmem33
Lhx1
Hsp90aa1
Grin3a
Ccnd1
Ptchd4
Abhd6
Nrcam
KCTD12
Bclaf1
Sirt2
Ahcyl1
Sdc3
Gas7
Plekhb1
Pigs
Sgcb
Golph3
Lrp4
Rpl39
Stmn2
Cdo1
Fads1
Galc
Vimp
Fam195a
Gm266
Fuca1
Srsf11
Aco2
Rasa3
Tcn2

Hnrnpd
Rps3a1
Tmem106b
Slc35e4
Rab7l1
Sypl
Hdgf
Nfkbiz
Atrx
Srrm2
Efemp2
Tnfaip6
Laptm4b
Nucks1
4933407L21Rik
Miat
Pnn
Decr1
Fabp5
Igfbp3
Enho
Suclg2
Camk1
Fermt2
Tubb3
Rgcc
Uqcr10
Ctnnd2
D430041D05Rik
Rhoj
Cox14
Samd4
Itgav
Eya1
Cdh11
Mid1ip1
BC026585
Rlbp1
Fuom
Adcy2
Renbp
Nckap5
Snrpb
Cct6a
Lpcat1
Lrp10
Marcksl1
Cacng5

Agpat5
Car10
Usp2
Hp1bp3
Ilf2
Rps15a
Itpkb
Zfyve21
Gng11
Acadm
Gm15417
Atraid
H1f0
Cbfa2t3
Rhbdf1
Tmed5
Tmem66
Mfhas1
Rnh1
Safb
Pde4b
Luc7l3
Ppp1r14b
Uap1l1
Ank3
Tspan15
Slc5a3
Rabac1
Bzw2
Anp32e
Soat1
Nudt19
Gap43
Ptplb
Malat1
Ina
Cyp4f13
Ssbp4
Eva1a
Ilvbl
Acadvl
Ptplad1
Sdf2
Bex2
Vamp5
Pcdh17
Enpp5
Mxra8

Slc35c2
Ngfrap1
Fam181b
S1pr3
Cplx2
1110065P20Rik
Syt12
Npc1
Rbbp4
Hmgn1
Cnbp
Rps10
Mamdc2
Gpd2
Cyp2j6
Iqsec1
Eno1
Rasa2
Ctsc
Arhgef12
Syngr1
Galnt18
Magt1
Rdh14
Slitrk2
Kcnip3
Rhoq
1110001A16Rik
Acot13
Gstt1
Haghl
Skap2
Ugp2
Chst7
Nhlh2
Gabbr1
Cyba
Baz1b
Hnrnpa0
Hirip3
Hmgb2
Ddt
Tprgl
Tuba1b
Cdkn1b
Fzd9
As3mt
Naga

Cacng2
mt-Nd2
Alpl
Srrm1
Ccar1
Metap2
Vcl
H2afy
Fcgrt
Glo1
2700094K13Rik
Ctsh
Phlpp1
Ccdc107
Pttg1ip
Epn2
Nsg2
Eif3c
Dclk1
Dpysl4
Nim1
Stk25
Sf3b1
Ppp2r2c
Sec11c
Dag1
Junb
Eif4g2
Creg1
Ap3m2
Cpt1a
Tulp3
Aldh6a1
Cnn3
Extl3
Csf1
Hibadh
Acyp2
Usp6nl
Tmem147
Tmem50b
Luc7l2
Lgalsl
Klf15
Nap1l1
Cyfip1
Ppt1
Dut

Rnd3
Aebp1
Rcn2
Etfb
Sft2d2
Tmem38a
Lrpap1
Cald1
Nr3c1
Ncor1
Dab1
Trip6
Ctsa
Mtss1
2410006H16Rik
H2afx
Vegfa
Ralb
Erp29
Fam173a
Fam69a
Zfp36l1
Txndc15
Fam167a
Gna13
Slc33a1
Tnfrsf21
Nedd4
Gadd45g
Shisa5
Cd200
Prpf40a
Capn2
Eif3f
Fam210b
Pex11a
Klhl13
Tmem30a
Gpm6a
Rnf215
Tgfb2
Tmpo
Srsf2
Crip1
Hmgcs1
Naprt1
Gas2l1
Rgs20

1110054M08Rik
Sept9
Mxd4
0610031J06Rik
Scd1
Dcxr
Top2a
Olig1
Ggh
Anxa7
Hsd17b10
Pcdhga9
Fam49a
Eva1b
Wipi1
Pcbp1
1500009L16Rik
Dcx
Ntsr2
Sept3
Gm5607
Arl6ip1
Tmbim4
Stt3b
Sltm
Mcm7
Eef1b2
Tacc1
Unc50
Fnbp1l
Ptpmt1
Pcm1
G3bp2
Agpat3
Ergic3
Syncrip
Tia1
Smox
Prnp
2700060E02Rik
Ssrp1
Acsl3
Vcan
Dnajc3
Fat1
H3f3a
Plk2
Sox9

Eny2
Rpl37a
Fam63b
Ufl1
Cenpf
Syt11
Sf3b2
Hnrnpr
Lhfp
Irx1
Ndufc2
Hadha
Ppp2r5a
Zc3h13
Ier5
Kif5c
Snta1
Cxxc5
H2afj
Phip
Zfp36
Rpl41
Abca3
Grn
Gng2
Etfa
Os9
Cyb5
Tm7sf2
Maf
Top1
Ikbip
Btbd6
Lifr
Gyg
Lgmn
Neat1
8430419L09Rik
Eif4a1
2810417H13Rik
Efemp1
Smpd1
Mir22hg
Anxa2
Akap9
Col9a3
Camta1
Dolk

Gpr56
Galk1
Gpi1
Tox3
Cpped1
Snrpf
Surf4
Xrn2
Psma7
Emc3
Rfc1
Pnp
Kifc3
Rps2
Acaa2
Cct3
Ptgr2
Serhl
C230037L18Rik
D3Bwg0562e
Htatsf1
Tmtc2
Scamp2
Cox7a1
Hjurp
Rab10
Ginm1
Rsl1d1
Irgm1
Gsk3b
Cyhr1
Srsf6
Ddrgk1
Mki67
Cenpe
Gm2694
Cbx5
Ccdc141
Cables1
Srsf7
Cotl1
Prkcdbp
Tubb2a
Orai3
Tmem205
Uqcrb
Lrrc8a
Ctsf

Desi1
Naca
Pa2g4
Preb
Specc1
Ntm
Sccpdh
Efhd2
Laptm4a
Slc41a1
Tpr
Echs1
Ctcf
Tshz2
Lrrc1
Soga3
Casc4
Snrpd3
Sec14l1
Khdrbs1
E130114P18Rik
Ndufa1
Eif2ak2
Pbrm1
Rpl35a
Cyth2
Hcfc1r1
Nckap1
Tmod2
Rnase4
Rufy3
Ssb
Pigyl
Ptms
Slc7a4
St8sia3
Ypel3
Bcl2
Snrpe
Slc25a4
Fmn2
Dhx9
Snrnp27
Gng5
Snx5
Tmem100
Rps7
Rorb

Impact
Pccb
Klhdc8b
D17Wsu104e
Pmvk
Tsn
Vldlr
Fahd2a
Ak1
Itfg1
Tmem9
Pam
Sdhc
Stard3
Rbm8a
Fbxo44
Plekhh2
Surf1
Adam17
Srp72
Tardbp
Rdh10
Uncx
Wwtr1
Nipa1
Nucb1
Tbata
Trp53
Efr3a
mt-Rnr2
Hint1
Rps25
Smarca5
Smc6
Psmb10
Lmnb1
Serf1
Eif5b
Wnt5a
Kpnb1
Pqbp1
Ccm2
Ube2b
Hsd11b2
Bphl
Smarca4
Polr3h
Tnfrsf1a

Anxa6
Ptprg
Adam9
Hpca
Commd6
Vps36
G0s2
Pxdc1
Selk
Zfand5
Nrbp2
Rgs2
Rap2a
Gas6
Mycn
Hnrnph3
Psmc1
Ccnd3
Gabarapl1
0610011F06Rik
Klhl25
Ptprf
Tusc3
Bhlhe41
Ly6e
Fkbp1a
Hnrnpk
Aard
Trappc3
Eif3h
Mbd3
Rest
Ddhd1
Ahcyl2
Slc25a29
Pigc
Cacna2d1
Amer2
Tcerg1
Usp22
Rab7
Hlf
Pitpnc1
Pls3
Acbd5
Ppp2r1a
Hmgn3
Camk2n1

Csad
Csde1
Shfm1
Rpl37
Paip2
Pax6
Brd3
Ddx39b
Rdx
Gpc4
Sod2
Nup93
Prim1
Prmt8
Bicc1
mt-Nd4
Derl2
Fam102b
Comt
Fos
Gab2
Ncam2
Erlec1
Zfp326
Mtf2
E2f5
Cfdp1
Cct7
Vhl
Arl8a
Lactb
C1galt1c1
Sfxn1
Smarcc1
Eif1
Plxnb1
Sstr2
1500012F01Rik
Rbp4
Apba2
Snw1
Aldh7a1
Aplp2
Nudt2
Sema6a
Klc1
Stmn4
Tmem184c

Tmem14c
Macf1
Ddx46
Gnptg
C530008M17Rik
Hspa5
Usp1
Anapc5
Rprm
Scp2
Trps1
Pigt
Kmt2e
Ube2e3
Slc25a1
Sec62
B4galt4
Cltb
Arsk
Ddx42
Tmem246
Nhp2
Gusb
Etv5
Celf4
Prdx5
Uqcrc1
Map3k1
Gpt2
Sall2
Mmp15
Sspn
Incenp
Ndufa11
Tagln3
Rad21
Cetn3
Caprin1
Apex1
Snrpg
Sumo3
Nolc1
Nol7
Rbmx
Smn1
Ghr
Itgb1bp1
Rtn3

Mboat7
Polr3k
Sox18
BC031181
Rbfox2
Srp68
Smim13
Pcdh9
Pdlim3
Rassf4
Tmed1
Rpl7
Sys1
Pcbp4
Metrnl
Trib1
Jtb
Ndp
Snhg5
Wbp5
Sub1
Dhrs4
Epha5
Ide
Tcp1
Stk17b
Elavl4
Zmiz1
Wscd1
Snn
Cox8a
Ankrd11
Pex2
Hsd17b4
Prpsap1
Thrap3
Krcc1
Snap25
Gm8292
Slc50a1
Cirbp
Maoa
Cdk1
Pcbp2
Mlf2
Nkd1
Erp44
Vmp1

Leprel4
Bai1
Cox6b2
Evi5
Rnmt
Hmgb1
Clptm1
Fndc5
Zmat2
Fkbp2
Dscr3
Phf5a
Zeb1
Zwint
Eif4h
Eif3k
Kctd5
Abracl
Hspa8
Lig1
Clcn3
Tm2d2
Grhpr
1500016L03Rik
Hdgfrp3
Ubtf
Dpy19l3
Cpe
Gpx1
Rab9
Samd8
Ran
Slc35f5
Dhrs7
Fut9
Rtn1
Cks1b
Scg5
Psmd4
Txnrd1
Aldh9a1
Ddx6
Nop56
Mrpl13
Rps8
Lamtor4
Aga
Smim15

Vdac1
Whsc1
Cldn25
Eif3e
Tmem59
Mcm6
Nme1
Yif1a
Bmyc
Sh3glb2
Pcna
Vegfb
Sbds
Rhoa
Sugt1
Vps37b
Map2
Hmgb3
Cit
Rpe
Cct5
Tmx2
Fam212b
Gbp7
Pde1c
2610507B11Rik
U2surp
C1ql1
Acaa1a
Sdc2
Supt16
Sept2
Eprs
Rpl18a
Slc35f6
Suv39h2
Ik
Arl2
Per3
Suclg1
Cs
Birc5
Adh5
Ssfa2
Pros1
Thoc7
Cachd1
Gse1

Gng3
Slc48a1
Hadh
Cdca8
Cdip1
Neurod6
2700029M09Rik
Dusp6
Skp1a
Ubc
Fip1l1
Smim20
Phb2
Rpl14-ps1
Actb
Hspb6
Snrnp70
Nars
D10Jhu81e
Arpc1a
Tm7sf3
Rsrc2
H2afy2
Prpf38b
Sptssa
Vbp1
Ncald
Dirc2
Ophn1
Gnai2
Srsf10
Uqcc2
Arfip1
Tra2b
Emg1
Gpx7
Mad2l2
Meis1
Nudt4
Gdpd1
Kazn
Lbh
Pigp
Eif3b
Ghitm
Lsm6
Prr13
Scaf11

Spag9
Kif11
Neto2
Asf1a
Gm11223
Lactb2
Ing4
Krtcap2
1110008F13Rik
Thoc2
Cdc42se1
Csnk1e
Srrm4
Myt1
Rpn2
Tead2
Esf1
Ythdc1
Mdk
Hsp90b1
Pfkl
Cluh
Nipbl
Tmem5
Pak2
Sar1b
Ano6
Pnkd
Id2
Rbm17
Cntn2
0610012G03Rik
Slc35b2
Insm1
Cct2
Srgap1
Tmed9
Prrc2b
0610009L18Rik
Atp1b3
N4bp2l2
Arl8b
Psat1
Brd2
Srsf5
Arsb
Tle1
H2-D1

Cadm4
Bcl7a
Abcf1
Brd8
Cdc5l
Pdgfra
Lrrc42
Klf4
Set
Celf1
Rpn1
Pde6d
Dkc1
Akr1e1
Ube2q1
Ctage5
Fam115a
Clmp
Pou3f3
Arhgap31
Tmbim1
Hells
Prkcb
Sars
Sc5d
Rdh11
Bola2
Fadd
Dixdc1
Dync1i2
Fam98b
Cdc42ep4
Selt
Srrt
Fkbp4
Ndufa9
Klf13
Dzip1l
Gm13826
Stmn1
Ctbp1
Gm6472
Pja2
Ier3
Eif2s2
Rab2a
Cenpa
Iars2

Snhg1
Derl1
Rhbdd2
Slc16a1
Plscr4
Stxbp3a
Sox8
Rbm39
Cyp51
Ncam1
Tprn
Atp2b1
Cox6c
Zc3h15
Zbtb18
Uqcrq
Siva1
Nop10
Ogfr
Chchd1
Arhgap11a
Bin1
Lsm4
mt-Nd1
Prmt5
Slc39a1
Nap1l5
Lbr
Csnk1a1
Adamts1
Gdap1
Atp5g3
Stk40
Idh1
Ppp1ca
Blvrb
Gm2000
Zfp292
Eid1
Cenpv
Mphosph8
Ndufa13
Pfdn4
Ctnnb1
Dnajc9
Erlin2
Papola
Mfsd1

Fmr1
Rnft1
Napa
Rnf20
Rtf1
Tpx2
Cdk5r1
Ccdc55
Fkbp9
Ralbp1
Canx
Gtf3a
Polr2m
Prpf4b
Cox20
Ash1l
Klf2
Hnrnpul2
Ywhaz
Arhgef2
Fgf9
Rbbp7
Aes
Amfr
Parp1
Ddx21
Atp6v0e2
Bcas2
Srebf1
Gbas
Tpm4
Kif2a
Fam32a
Nudc
Apc
Mrfap1
Cct8
Myef2
Dad1
Vamp3
Arsa
Top2b
Nktr
Ccdc50
Spc25
Ncln
Rab24
Ubb

Fam168a
Tceal3
Lpgat1
Mia3
Atoh1
Kif21a
Dusp3
Gar1
Fam213b
Mocs2
Golga4
Pak3
Mfsd11
Casc5
Dennd5a
Gucy1b3
Zranb2
Cfl2
Dctpp1
Ppp3ca
C1qbp
Sac3d1
Rpl6
Idh3a
Smim11
Rab34
Rexo2
Gpr153
Aimp1
March7
Supt4a
Cwc15
Itsn1
Kdm5b
Mut
Zic4
Pqlc1
Zcchc11
Ddx3x
Prdx1
Chgb
Ndufa12
Elavl2
Bex1
A630007B06Rik
C130071C03Rik
Kif3a
Setd5

Gapvd1
Cyth3
Tmem150a
Zfp422
Rps4x
Pdgfa
Cited2
RP23-45G16.5
Ebf3
Podxl2
Myl12b
Rrp1
Mns1
Gtf2a2
Ndufs8
Ncor2
Rnf122
Magoh
Sfr1
Paics
Ccp110
1110038B12Rik
Gpr19
P4ha1
Alcam
Cebpz
Ckap2l
Hmgxb4
Psmd7
Dctn3
Esco2
Gcsh
Rcor2
Cacng7
Fam58b
Ctxn1
Txnl4a
Dazap1
Lgr4
Ewsr1
Itpa
Smarcd1
Lsm3
Srrm3
Txn1
Bcar1
Acat1
Phf14

Hmgn2
Gsg1l
Btf3
Casc3
Nap1l4
Actr1a
Gltscr2
Dnmt1
BC005764
Srpk2
Pkm
Rpl13
Mllt11
Ndufa3
Polr2h
Zcrb1
Med19
Frrs1l
St18
Strn3
Ahsa1
Bcl11a
Psma1
Fam214b
Snrpb2
Utp14a
Bptf
Slc16a2
Sumo1
Flcn
Ssna1
Gars
Pfdn1
Pds5b
Hmgn5
Atp2a2
Nsmce4a
Purb
Rpl38
Mab21l1
Arid4b
Calr
Bcap31
Glce
Psmd12
Apoa1bp
Tmco3
Tmem18

Trpc4ap
Ank
Tpm3
Zmynd8
Acer3
Denr
2410089E03Rik
Idh2
Spag5
Snrnp40
Bpnt1
Rnaseh2c
Cep57
Lpp
Igsf8
Hes1
Akirin2
Fam168b
Fgd6
Dpysl2
Dtymk
Fmnl2
Myod1
Eef1e1
Ubap2l
Bok
Clip3
Pdcd4
Rpl3
mt-Cytb
Dhx15
Nmral1
Cnot6
Ppic
Atg3
Eif3g
Ten1
Btd
Rbm4b
Cyc1
Kras
Ift27
Mrpl21
4933426M11Rik
Actr6
Sidt2
Baz2b
Cuedc2

Psph
Tnrc6a
Sucla2
Ivns1abp
Ppig
1110004F10Rik
Spcs2
Naa38
Mphosph9
Chd8
Uchl1
Dtl
Cadm1
Sult4a1
Olfm1
Rnf165
Ift81
Adipor1
Slc22a17
Epb4.1
Cdk11b
Nptn
Il11ra1
Lsm2
Tacc2
Ylpm1
Ppp1r14c
Uqcrfs1
Larp7
Snx13
Gm10260
Nhsl1
Cpsf2
Rpl29
Ypel1
Hook3
Nsd1
Rab33b
Kmt2a
Tnrc6c
Fhl1
Myl12a
Myt1l
Arl6ip4
Sept7
Ilf3
Eif4g1
Smpd3

Rif1
Carhsp1
Rbm26
Tprkb
Nup85
Bzw1
Nup62
Ttc9b
Rb1cc1
Trim28
Ezr
Atp5d
Ska2
A930011O12Rik
Sh3gl2
Cbx3
Tmem57
Csrp2
Gtpbp4
Smdt1
Senp6
Cd320
Mki67ip
Ube2c
Pfdn2
Mpv17l2
Rpf2
Tra2a
Sobp
Mpc2
Sf3a3
Golim4
Hspa4
Orc6
5430416N02Rik
Ube2a
Sema7a
Clspn
Utp3
Usp3
B230219D22Rik
Dcakd
Zic5
Gspt1
Dhrs3
Crnkl1
Myh10
Ank2

Bub3
Tbpl1
Srsf9
Slc17a6
Rfc3
Pole3
Dnajc21
Rpl18
Dnajc2
Wapal
Usp7
Cenpm
Prdx2
Mdga1
Fam50a
Trappc4
Nemf
Gm561
Peg3
Fez1
Wbp11
Cln3
Plscr1
Hnrnpa3
Smarca2
Twf1
Mllt10
Eif5a
Rps18
Fopnl
Nop14
Mfap4
Snrnp25
Ep400
Slc3a2
Mycbp2
Ppp1r7
Rnf187
Cep170
Jakmip2
Wwp1
Zfp318
Polr2c
Nfkbia
Rsbn1l
Psmd14
Dnajc8
Coro2b

Lap3
Calm1
Safb2
Prdm8
Dhx36
Polr2f
Myo10
Tsg101
Cops2
Rps16
Abr
Gtf2f1
Stau2
Nop16
Zfml
Rpa2
Tmem178
Ikzf5
Gm26735
Tubgcp2
Mrpl30
Nhlh1
Mcm2
H2afz
Dstn
Zfp704
Eif3l
Pde7a
Tcf3
Tubb2b
Tmem192
2610001J05Rik
Mapk10
Gm5617
Mcl1
Sacs
Mgrn1
Rbbp6
Dnaja1
Pdzrn4
Smap1
Ccng2
Nt5dc2
Ptprs
Mapk8ip1
Phf20l1
Mak16
Nsg1

Chrna3
2900011O08Rik
Sep15
Smarcd3
Nt5c3
Fam155a
Trim2
Nadk2
Rpl30
Prc1
Chd1
Eif4e3
Smek1
Cpsf6
Snrpa1
Hibch
Txndc9
Reln
Nin
Hars
Ralgps2
R3hdm1
Bcas1
Mapre1
Srsf4
Pgrmc1
Msantd3
Nrm
Birc6
Stag1
Nicn1
Rbms1
Imp3
Prox1
Lpcat3
Ercc5
Smg6
Vps29
Tsc22d1
Spc24
Kcnd2
Dnttip2
Bnip3l
Polr1c
Ccnl1
Homer2
Psmc6
Vps4b

Tbl1x
Arf1
Rpl35
Sae1
Pcif1
Kif20b
Fam3a
Tspan6
Sbno1
Mcm5
Zfr
Ash2l
Fut8
Tma7
D19Bwg1357e
Wdr89
Zfp637
Lgals1
Lasp1
Zfp207
Eml3
Atp2c1
Zfp266
Mex3b
Polr2e
Frmd4a
App
Eif3d
Cplx1
Rab8a
Cadm3
Cox6a1
Uhrf1
Tvp23b
Arid4a
Ppa1
A030009H04Rik
Epb4.1l3
Echdc1
2810004N23Rik
Znhit6
Pigk
Ndufb2
Elovl4
Setd8
Ola1
Rabggta
Glrx3

Smek2
Ivd
Luc7l
Hdgfrp2
Cox6b1
Lyar
Usp34
Smap2
Gnl3
Ube3a
Ncbp2
Mrpl24
Zkscan1
Eif2b3
Arf4
Ybx3
Exosc1
Ddx50
Dzip3
Phf3
Casp3
Frg1
Tfdp2
Ube2g2
Arxes1
Stx6
Eapp
Armc10
Slc38a1
Rnf220
Whsc1l1
Gnl3l
Ptrh2
Iws1
Rfc4
Ubp1
Cnot1
Raf1
Polr2a
Rab6b
Ube2d3
Ubn1
Gamt
Ap3b2
Mapk1
Dnal4
Kdm1a
Cdc7

Eif4b
Cox7a2l
Cflar
Pbdc1
Rbm5
Rock1
Sh3bgrl
Tex14
Brix1
Kif15
Hprt
Palm
Mapt
Mbtd1
Cyb561d2
Tomm20
Fam92a
Cenpj
1110038F14Rik
Las1l
Pafah1b2
Ppie
Polr2i
Mtus1
Flna
Ift74
Spag7
Tax1bp1
Naa10
Prpf6
Atp5o
Ehbp1
Mrps5
Dync1h1
Islr2
Xpo7
Pin1
Cnot7
Otx2
Plrg1
Fzd2
Fau
Ppp3cb
Fam57b
Epc2
Naa15
Mllt4
1700020I14Rik

Gjc1
Bod1l
Racgap1
Tro
Cdc37
Tlk2
Prpf31
Rcn1
2810474O19Rik
Cnot3
Mpnd
Sox11
Stip1
Cdc20
Mycl
Dlgap4
Uba52
Peli2
Tacc3
Slc25a5
E2f1
Slc39a6
Ube2r2
Hectd1
Timm50
Foxp1
Hsph1
Zfp664
Gpbp1
Mrpl35
Ddx27
Rrm1
Abhd17b
Gm17322
Ankrd10
Strbp
Trp53i11
Mphosph10
Lmo4
Klhl7
Mrpl52
Smchd1
Dhx32
Sde2
Copa
Txlna
Rnf219
Wbp4

Dusp1
Tomm70a
Ahsa2
2310022B05Rik
Ccni
Brd7
Taf1d
Dpy30
Hes6
Atp11c
Gnb1
Ccna2
Akap12
Heatr6
Commd3
Phactr1
Etf1
Cdc26
Sfswap
Nup88
Taf15
Pcsk2
Ftsj3
Atad2
2410004N09Rik
Tyms
Tipin
Kti12
Xrcc1
Fam114a2
Zfpl1
Fbxo5
Exosc7
Nefm
Cdca7
Rbmxl1
Zfp622
Sfrs18
4833420G17Rik
Plp1
Cyp7b1
Cdk6
Cdh4
Hspa9
Gigyf2
Snhg6
Dst
Sart1

Mroh2a
Etv1
D4Wsu53e
Gart
Dnajc1
Ifitm2
Kdm5a
St3gal5
1700025G04Rik
Sf1
R3hcc1
Mthfd1
Nrd1
Bub1b
Pygo1
Brwd3
Smoc1
Sc4mol
BC004004
Nol4
Mis18bp1
Ing3
Nipsnap1
Klhdc2
Apbb1
Rnf146
Sp3
Boc
Spop
Vars
Wdr12
Cenph
Gm11541
Rsrc1
Cdc42se2
Rnf2
Exosc8
Mkrn1
Nusap1
Prdm2
Wdr43
Nol12
Hmmr
Mapk8ip2
Atad5
Otud4
Ttc9c
Pcf11

Cript
Rangap1
Ppil4
Adam10
Sowaha
Mapk8
Syne2
Cenpb
Atp6v0a2
Yeats4
Nfic
Glyr1
Akap6
Ctps
Klf7
Asph
E330009J07Rik
Ankrd32
Slu7
Ppm1a
Pdzrn3
Clk1
Pphln1
Kcnk1
Igsf3
Ehmt1
Blmh
Nme4
Wwp2
Arrdc3
Pum1
Morf4l2
Gprasp1
Lmnb2
Gnl2
Acot7
Grik2
Nono
Pdxp
Celsr3
Upf3b
Fnbp4
Yy1
Gls
Clvs1
Zrsr2
Cnot2
Gps2

Ptpn11
Eml4
Hnrnpf
Ccdc59
Smco4
Rad50
Phc2
Suz12
Psme4
Znrd1
Krt10
Rpl7a
Msh2
Syt13
Cdk7
Tex30
Cdca7l
Fzd1
Erdr1
Mcm3
Cux1
Sdhb
Pde4dip
Tspan13
Zfp330
Klf6
Jhdm1d
Timeless
Ube2d1
9330159F19Rik
Jund
Grb2
Uqcr11
Pdcd11
Ppfia2
Ppm1g
Plk4
Ssx2ip
Mta1
Prmt1
1700037H04Rik
Rp9
Rad51ap1
Ccdc18
Kif23
Hist3h2a
Baz1a
Pcgf3

Scnm1
Srsf1
Nvl
Fosb
Pitpnb
Chd6
Gm11478
Setx
Ssbp1
Vopp1
Eci2
Rbm22
Nrn1
Smndc1
Flywch1
Gmps
Cecr2
Ctnnbl1
Rab30
Coro1c
Smarcc2
Ccdc25
Dync1li1
Gm10036
Evl
Paxbp1
Ubfd1
Xist
Setd2
Gpatch4
Jam3
Ap1s2
C77370
Tpt1
Nbea
Fxyd6
Acat2
Lars
H1fx
Zmym5
Bms1
Isy1
Armcx4
Cdt1
Xpo1
Tab2
Cep85l
Patz1

Elavl1
Smarce1
Cops3
Lta4h
Jarid2
Ccnb2
Rps24-ps3
Nedd4l
Ncaph
Ppia
Ensa
Stag2
Snord104
Ppan
Rnaseh2b
Ppp3r1
Atl1
Hnrnpul1
Ddx17
H2-Ke2
Satb1
Bccip
Phf6
Heatr3
Gpc2
Peo1
Tmeff1
Cdc40
Git2
Mab21l2
Uba2
Ormdl3
Rbm6
Trim59
Narf
G3bp1
Fam64a
9430016H08Rik
Fbxo32
Ttc33
Krr1
Slain2
Ddx18
Stxbp1
Qser1
Zbtb22
Pik3r3
Ick

Iqgap2
Cpsf3l
Gid4
Cnrip1
Zfp644
Tnpo3
Crebbp
Ercc1
Gm10076
Mpped2
Rad51
Hip1r
Setbp1
Msi2
Ints10
Rundc3a
Kat6b
Abce1
Kat6a
Smc5
Ctr9
Xpa
Tmem55b
Odf2
Daam1
Pnmal2
Thsd7a
Uhrf2
Hk2
mt-Co1
Upf3a
Chchd2
2700049A03Rik
Ttc1
Dis3
Rpl15
Qrich1
Exoc5
Uimc1
Rbbp8
Dcun1d5
Ldb1
Ahi1
Rrm2
Aurkb
Gripap1
Mum1
Ube2i

Gtf2e2
Shmt1
Srgap2
Man1c1
Trafd1
Tk1
N4bp2
6330403K07Rik
Skiv2l
Mapk7
Etaa1
Socs3
Rpl36
Rrnad1
Pja1
Rpl3-ps1
Tln1
BC034090
Mxi1
Tead1
Rbbp9
Btg1
Ctbp2
Oard1
Slc25a27
Zfp322a
Upf2
Rbm18
Zfp553
Tcof1
Rcc2
Rab3a
Sf3b3
Pknox1
Sgol2
Cnksr2
Fen1
Thoc3
Usp33
Pnrc1
Elovl6
Zmym2
Mgea5
Gpr85
Hist3h2ba
Qtrt1
Ndc80
Dbn1

Parp2
Zkscan3
Tubg1
Vrk1
Rexo1
Naa16
Fam178a
Snrpa
Gm11266
Daxx
Bmi1
Rps28
Pik3ip1
Ddx23
Haus1
Ube2e2
Nfyb
Ncapd2
Uba1
Ccdc66
Tob1
Gm17750
Knop1
Rpa3
RP23-32A8.1
Kars
Shoc2
Prpf4
Ccnb1
Ddx10
Tulp4
A330076H08Rik
Rrn3
Akap11
R3hdm2
Ints7
Tmem183a
Rnf144a
Eif2s1
Brcc3
Midn
Zranb1
Ythdf2
Cdk5rap3
Rad23b
C330027C09Rik
Elmo1
Mbp

Baz2a
Pbk
Orc2
Angptl2
Nudcd3
Lhfpl4
Limd2
Slc24a5
Pcnt
Trim24
Zfp451
Cd3eap
Vps41
Rapgef6
Nosip
Dner
Sbk1
Cerk
Bbx
Abt1
Fam192a
Blm
Lrig3
Agtpbp1
Bdp1
Supt5
Slc39a10
Celsr2
Snrnp200
Cwf19l2
2700081O15Rik
Zfp354c
Rhobtb3
Igf1r
Prkacb
Cspp1
Tet3
Bcat1
Zfp512
Cenpc1
Pbx3
Gmnn
Cntln
Ddx26b
Pak7
Ankhd1
Snapc3
Fyttd1

Rev1
Wdr6
Cnot4
Ncapg
Clasp1
Kif5a
Skiv2l2
Gm13092
Ulk1
Tbc1d16
Rnf168
Pcdha2
Mis18a
Actl6a
Chd2
Ddx55
Ckap4
Cep78
Brpf1
Trim27
Ikbkap
Rnps1
Rbm10
Thumpd1
Akap8
Dnph1
Pmf1
Atp11b
Kif22
Msl1
Cd2bp2
Cmip
Asap1
Srpk1
Wasf1
Cpsf1
Nenf
Mllt3
BC005561
Rpap3
Cenpw
Fam53b
Hist1h2ak
Med1
Faf1
Gli1
Pola1
0610010F05Rik

Gle1
Uri1
Ptch2
Pom121
Gpatch11
Cdkn2d
Brwd1
Dck
G2e3
Rrp15
Arrb2
Topors
Apod
Ptn
Col3a1
Col4a1
Igf2
Col1a2
Vtn
Dcn
Col4a2
Itih5
Sparc
Lamb1
Lum
Sparcl1
Nupr1
Cxcl12
Nid1
Colec12
Atp1a2
Bgn
Pcolce
Ifitm3
Serpinh1
Igfbp7
Serpinf1
Lgals1
Edn3
Igfbp5
Col1a1
Col15a1
Slc6a13
Pltp
Cp
Igfbp2
Col6a2
Col6a1

Gng11
S100a6
Eva1b
Fstl1
Rarres2
Anxa5
Cfh
Tbx18
Col18a1
Ctsk
Cyp1b1
Cthrc1
Laptm4a
Igfbp4
Cped1
Il34
Htra3
Pdgfrl
S1pr3
Sepp1
Enpp1
Hsp90ab1
Lhfp
Lama4
Postn
Emp3
Il33
Col13a1
Fbln2
Anxa2
Tmem204
Lamc1
Col4a5
Nid2
Plat
Loxl2
Phlda1
Trf
Cmbl
Angptl4
Kdelr3
Hmgcs2
Bicc1
Slc6a20a
Pdgfrb
Ifitm1
Spp1
Aldh1a1

Emp1
Lama1
Rbp1
Itga8
Tgfbi
Mfap2
Fmo1
Sdc2
Rcn3
Sod3
C1qtnf2
Ccdc80
Adh1
Apoe
Cd302
Mxra8
Itm2c
Tmem45a
Ggt5
Cnn2
Vwa1
Cpq
Col26a1
Lama2
Tm4sf1
Abca8a
S100a11
0610007N19Rik
Mgp
Itm2b
Crip1
Ctsl
Foxc1
Wls
Id3
Rhoj
Gm14964
Ctsh
Myl9
Col5a2
Ppap2b
P2ry14
Ccl11
Uaca
Rbpms
Msx1
Msc
Copz2

Nfib
Hspa12a
Rgs5
Pmp22
Ifi27
Lamc3
Col23a1
Gpx8
Mrap
Bmp7
Ggt1
Islr
Gper1
Rasgrp2
Cdh11
Egflam
Ece1
Fam114a1
Efemp1
Pdlim2
Heyl
Cgnl1
Itih2
Srpx2
Plxdc2
Kcnj8
Tubb5
Serinc3
Slc16a12
Aldh1a2
Fcgrt
Col6a3
Nkd2
Naalad2
Cyth3
Htra1
Chp2
Rcsd1
Oaf
Rrbp1
Prrx1
Csf1
Sept11
Klf2
Tfpi
Steap3
Il13ra1
Tgfbr3

Wfdc1
Arl4a
Gja1
Prelp
Itga1
C1qtnf6
Serpine2
Rdh10
Slc7a11
Emilin1
Fkbp10
Sfrp1
Ifitm2
Tagln2
Lpar1
Mylk
Slc22a6
Dkk3
Abca9
Mfge8
Ahnak
Slc22a8
Clec1a
G0s2
Nbl1
Myl12a
Tnfrsf19
Cald1
Junb
Cd97
Pla2g7
Nr1h3
Creb3l2
Tmem37
Cd248
Rell1
Fblim1
Vstm4
Tcn2
Slc9a3r2
Rnase4
Ptplad2
Lbp
Mmp14
Fkbp7
Tlr12
Fam26e
Mrc2

Svil
Tns1
Mmp2
Enpp2
Pear1
Col4a6
Spry1
Wnt5a
Atp2b4
Amica1
Sult1a1
Ramp2
Tpm2
Ptgis
Rtn1
Crmp1
Pi16
Ppic
Rbms3
Itm2a
Myh9
Samd5
Axl
Tmem64
Hspg2
Tril
Igfbpl1
Cd81
Gstt2
CRE_RECOMBINASE
Hic1
Ncl
Ctsb
Cyr61
Hrct1
Filip1l
Slc1a5
Foxs1
Fgfr1
Hlf
Thbd
Mylip
Higd1b
Dse
Cd24a
Gja4
Tgfb1i1
Myo1b

Hnrnpu
Ckb
Lamb2
Timp3
Vamp8
Fth1
B3gnt9
Foxd1
Rps4y2
Ajap1
Fkbp9
Snhg11
Dusp6
Acadl
Marcksl1
Tgm2
Gnb4
Itgb1
Scara5
Tbxa2r
Hnrnpa2b1
Unc93b1
Anxa7
Nnat
B2m
Fap
Sptbn1
Tnfaip2
Add3
Tuba1a
Dlc1
Twist1
Efemp2
Basp1
Adap2
Cstb
Serping1
Parp3
Nfia
Maf
H2-K1
Tnfrsf1a
S100a10
Vamp5
Rhbdf1
Ptges
Cyba
Rgs4

Osmr
Lpl
Plin3
Nfix
Tenc1
Adamts12
Ndufa4
Cebpd
Foxq1
Abca1
BC028528
Ecm1
Tgfbr2
Cpxm1
Stard8
Cst3
Creb3l1
Rab3il1
Tbx15
Barhl1
Ginm1
E130114P18Rik
Coro1b
Arpc1b
Ecm2
H6pd
Itpripl2
Lox
Nrp1
Bex2
Arhgdib
Cebpb
Nr2f2
Calm2
Pdpn
Shisa5
Col16a1
Ccnd2
Arhgap29
Lrrc17
Anp32e
Kcne4
Selenbp1
H3f3b
App
Col7a1
Ech1
Rapgef4

Ubtd1
Lamp2
Aspn
Ndufa4l2
Stmn3
Lrp1
Cyp2d22
Frmd6
1810058I24Rik
Chd4
Pros1
Gjb6
Calr
Flrt2
Cdh5
Hnrnpdl
Gpx7
F3
Gucy1a3
Malat1
Arhgap6
Ptrf
Efcc1
Cog7
Sept7
P4hb
Wipi1
Hint1
Pax6
Col5a1
Cldn11
Mgst1
Gap43
Rhob
Slc30a10
Slc19a1
Fxyd1
Sipa1
P2ry1
Mtch1
Epb4.1l2
Hnrnpab
Syt11
Rbms1
Col5a3
Pdxk
Chst7
Fbn1

Meg3
Gcnt2
Gria2
Tubb2b
Lmna
Nasp
Sspn
Dpysl4
Clec3b
Etfb
Phactr2
Rbm46
Dock6
Hsp90aa1
Rhoc
Miat
Zcchc24
Pdgfra
2700094K13Rik
Ralb
Spats2l
Fam46a
Tec
Ifnar2
Cast
Hsd11b1
Ctdspl
Tpm4
Mmp19
A230050P20Rik
Tubb3
Mxd4
Fkbp3
Rab20
Lef1
Capns1
Ier3
Vim
Isyna1
Draxin
Scarf2
Anp32a
Wwtr1
Nsg2
Scg3
Gm14005
Celf2
Mpg

Mmp11
Vimp
D430041D05Rik
Ywhae
Tmem59
Txnip
Gypc
Lhx1
Ppfibp2
Kdelr2
Pam
Ppp2r2c
Ybx1
Neurod1
Il6st
Parm1
Ccdc141
Mcam
Lrrk1
Sat1
S100a13
Lpp
Art3
Ezh2
Maged2
Rest
Id2
Ikbip
Fosb
Ezr
Antxr1
Gadd45b
Map2
Gsn
Tmem50a
Fzd6
Slc41a1
Dnajc3
Epas1
Gprc5c
Ntm
Smc2
Serbp1
Ets1
Chd7
Bmp4
Rras
Banf1

Dut
Psip1
Cd151
Kank2
Plin2
Mex3a
Tacc1
Smtn
1500016L03Rik
Cryab
Timp2
Apex1
Amotl1
Arhgap24
Hsp90b1
H2afv
Hn1
Plekha2
Fos
Vcam1
Stmn2
Ngfrap1
Pde8b
Txndc5
Jag1
Ldlrap1
Gm9800
Scg5
Gadd45a
Mki67
Arhgef6
Lgals9
Itgb5
Sept4
Nhlh2
Mir22hg
Ilf2
Surf4
Crtap
Cpt1a
Fhl2
Rhod
Pabpc1
Pde1c
Ddah2
Tpst1
Sec24d
Gm17750

Aga
Whsc1
Tnfrsf21
Lmnb1
Rbfox3
Top2a
Kif21a
Fkbp11
Ifi30
Oxct1
Hnrnph1
Leprel2
Eng
Gatm
Utrn
Slc16a9
Soga3
Tmpo
Myadm
Rufy3
Map1b
Sh3pxd2a
Nrxn2
Gm1673
Mcm7
Arfgap3
Gucy1b3
Pmepa1
Aebp1
Scn2b
Lrp10
Tmem51
Zfhx4
Ina
Fbln1
Ephx1
Dclk1
Pdia3
Degs1
Rora
Xbp1
Slc9a3r1
Snap25
Litaf
Gpr116
Hes1
Tmed3
Sept9

Ttyh2
Pkd2
H2afy
Slc38a3
Plxdc1
Pfn1
Ctsz
Atpif1
Meis1
Gpc6
Ptma
Magt1
Ccnd1
Tmbim1
Ppp1r14b
Arhgap20
Fermt2
Vps37b
Rps5
Dek
Srsf3
Trip6
Nenf
Apba2
Cadm1
Ttc3
S1pr2
Ifih1
Ranbp1
Tsc22d3
Sec62
Jak1
Luc7l3
Atrx
Uncx
Grb10
Tmem86a
Hnrnpm
Rsu1
Calu
Bin1
Ecscr
Pth1r
Slc1a3
Zfp36l1
Pqlc1
Insm1
Pttg1ip

Plod1
Ebpl
Vegfa
Fubp1
Tcirg1
Vkorc1
Chchd10
Matr3
Lims1
Ivns1abp
Ilk
Tbata
Slc38a1
Acin1
Lamp1
9430020K01Rik
Thy1
Rps9
Hexa
Pbxip1
Gpm6a
Mdk
As3mt
Pld1
Hnrnpd
Ank3
Gaa
Snrpf
Mycn
Dap
Elavl4
Tspan17
Cacng2
Hmgb2
Gm10075
Gng3
Acaa2
Comt
Arhgap42
Dad1
Map3k1
Cdc42ep2
Nop58
Akap7
Elavl3
Dhx9
Ggact
Psap

Kif5c
Cygb
Gm2694
Fkbp14
Actn4
Rpl32
Dtx4
Vwa5a
Cyb5r3
Sh3d19
Cbx1
Hjurp
Leprel4
Tox3
Ncam1
Pnn
Axin2
2810417H13Rik
9530068E07Rik
Grk5
Ssrp1
Cdk4
Lifr
Itpr2
Cenpf
Fndc3b
Foxf2
Hdgf
Col28a1
Zfp36
Hmgn5
Pdia5
Pdap1
Ifngr2
Ptprd
Sh3pxd2b
Tex264
Engase
Galk1
Spry4
Etv1
Bcl7a
Tmem100
Tm9sf3
Anp32b
Hnrnpr
Akap12
C130071C03Rik

Dnajc9
Wasf2
Uap1l1
Fnbp1l
P4ha1
Smarca5
Dusp3
Hirip3
Sfpq
Snrpe
Sept3
Dctpp1
Sntb2
S100a1
Cpe
Klf6
Tvp23b
Nr3c1
Pon2
Eif4g2
Crip2
Smc4
Rbm25
Fut9
Atraid
Ccdc88a
Pa2g4
Tgfb3
Spsb2
Orai3
Set
Rpl13a
Cbfa2t3
Npm1
Rps26
Cplx2
Sec31a
Smc3
Dcx
Dnajb11
Hmgb3
Tmem110
Rnh1
Cacna2d1
RP23-45G16.5
Yipf3
Jup
Epb4.1l3

Stmn1
Grn
Bcap31
Ptpn9
Cox7a2
Gse1
Ppapdc1b
Pbk
Imp3
Atp6v0e2
Marc2
Stmn4
Kdelr1
Rangap1
Anxa6
C1ql1
Pten
Cnot6
Serinc5
Pid1
Rnmt
Tspan9
H1f0
Galnt18
Slc50a1
Iqgap1
Elovl6
Ppfibp1
Matn2
Ran
Rcor2
Rps3
Slc12a2
Bcas1
Cks1b
Plekhg2
Tln1
Arhgef40
Mprip
Cdk5r1
6330403K07Rik
Nrn1
Cflar
Islr2
Bclaf1
C530008M17Rik
Arl6ip1
Fgf9

Pdia4
Sri
Ptprs
Prmt8
Srpk2
Tmem150a
Cyp51
Nhlh1
Elavl2
Srrt
Thoc7
Cdpf1
Hnrnpc
Nfic
Tpx2
Reep5
Cbx5
Tmem39a
Chgb
Zbtb18
Rrm1
Nap1l4
Col27a1
Apc
Gjc1
Stt3b
Gm11223
Tpm1
Ccdc34
Hmgn1
Glul
Rfc4
2610017I09Rik
Cenpa
Bet1l
Gstm1
Gdpd1
Slc1a2
Myo5a
St18
Ssr2
Fam212b
Clip3
Snx18
Sh2b3
Prkcdbp
Laptm4b
Leprot

Ddx26b
Tuba1b
Hspa5
Wipf1
Rps19
H2afz
Smarcd1
Ahi1
Top1
Celsr2
St8sia3
Zic1
Klf4
Srpr
Srrm2
Serpinb6a
Racgap1
Cntn2
Ddx39b
Ppp1r14c
Cenpe
Gpc3
C330027C09Rik
Ilvbl
Col9a3
Ghr
Pde5a
Hdac2
Sap30
Psat1
Decr1
Cltb
Bag3
Tubb6
Rplp0
Alx4
Nrxn1
Tm9sf2
Stard13
Efnb2
Snx7
Dtx1
Cct3
Prox1
Dcbld1
Kif20b
Rpl8
Selm

Fam101a
Ppap2a
Rcn1
Nusap1
Rabac1
Ube2c
Cdca8
Lrpap1
Cklf
Notch1
Lap3
Diap2
Adam19
Gsg1l
Tjp1
Cd9
H3f3a
Rgs16
Nucb1
Cdc20
Cdk1
Hey1
Tsn
Tmem167
Homer2
Rps10
Gng12
Pcm1
Nucks1
Rap2a
Hnrnpk
Mrpl34
Sgce
Reln
Prpf40a
Kif23
Bex1
Tspan13
Dnajc1
Khdrbs1
Srrm3
Ddx1
9330159F19Rik
Fam174a
Rundc3a
Hmgn2
Bicd1
Cct2

Pou3f2
Hpca
Satb1
Pofut2
Dtymk
Ilf3
Nqo2
Hnrnpa0
Ostf1
Ak3
Zfp704
Lmo4
Gnb2l1
Rsl1d1
Aldh7a1
Gmds
Caprin1
Smad5
Eid1
Lig1
Rplp2
Tmem184b
Rps21
Bzw2
Tshz2
Prdx5
Rtn3
Pigk
Sema7a
Kif1b
Cmtm3
Bcat1
Slc3a2
Sh3gl2
Amer2
Stx4a
Hdlbp
Tia1
Rpl22
Sqstm1
Smarca4
Cdc42ep4
Lrrn1
Sstr2
Serf2
Chn1
Bex4
Pxdn

Bmp1
Prrc1
Slc39a13
Rab6b
Gpr180
Brca2
Dnmt1
Dtl
Setd8
Rpn1
Chpf
Nagk
Ddx5
Xpc
Fez1
Smc1a
Atoh1
Bmp5
Strbp
Fam132a
Knstrn
Pcna
Map3k3
U2surp
Atp5a1
Map9
Myh10
Chd6
Cpne3
Tsg101
Rbms2
Slc25a20
Phip
Slc35f5
Camk2n1
Rps11
Trio
Cd63
Polr3gl
Fbxo6
Pdgfa
Zfhx3
Dner
Snrpd1
Psmc1
Abcg1
Fam43a
Akap9

Spc25
Pak3
Ccdc53
Fus
Atp2b1
Purb
Tmem9
Nol7
Usp1
Sf3b2
Bcl11a
Birc5
Gins2
Fkbp4
Smchd1
AI854517
Mycl
Arfip1
Sar1a
Mab21l1
Rassf4
Brd3
Brd8
Srrm4
Slc25a27
Podxl2
Rpl4
Aplp2
Hsd11b2
Nptn
Kif22
Eef1b2
Ubtf
Ckap2l
Tbc1d20
Klhdc8b
Mns1
Arcn1
Cxxc5
Gsk3b
Pelo
Tcerg1
Baz1b
Tprn
Kdelc2
Prdx2
Cdk6
Hdgfrp3

Cenpm
Eml3
Tacc3
Kit
Dhx36
Trim62
Klhl5
Myo6
Eml4
Usp22
Pgrmc1
Snrpd2
Rrm2
Rap1b
Tex14
Hspa9
Tbrg1
Mmd
Hells
Aldh2
Nme1
Myod1
Rtf1
Sep15
Supt16
Camta1
Pick1
Hmgb1
Arid5b
Ift20
Aimp1
Nol4
Srrm1
Otx2
Rab13
Kif11
Msn
Ostc
Smarcc1
Rps14
Trp53i11
Ltbr
Syne2
Bub1
Wwc2
Syngr2
Zmiz1
Nap1l1

Tra2b
Dhrs7
Ctsa
Mbnl2
1110038B12Rik
Npepl1
Pfdn4
Ptov1
A330076H08Rik
Smoc1
Cenpv
Rap1a
Kif5a
Gm3764
Incenp
Soat1
Trip10
Sfrs18
Tram1
Pdzrn3
Erdr1
H2afx
Rab3a
Marcks
Gpr108
Atxn7l3b
Srebf1
Metap2
Klc1
Dbf4
Carhsp1
Oat
Serp1
Dixdc1
Arhgap31
Rbp4
Aspm
Map4k4
Txnrd1
Ubxn1
Hbp1
Zcchc18
Lrrfip1
Tulp3
Chd3
Nell2
Celf4
Vcl

Zbtb38
Pdlim7
Lsm6
Lpar4
Mllt11
Cdc5l
Eef1a1
Uhrf1
Plekha1
Nop56
Ugp2
Tdrkh
Eif4a1
Zfp91
Ttyh3
Mrps5
Lamtor3
Neurod6
Rnaseh2c
Scarb2
Ergic3
Syt13
Sms
Nt5dc2
Mad2l2
Ap3b2
2700089E24Rik
Csdc2
Trim56
Cdca2
Gins1
Zmat2
Rps24
Vasp
Gphn
Tgfb2
Snrpn
Ptk2
Ptch2
Cct8
Akap13
Ncapg
Casc5
BC005764
Atad2
Frmd4a
Tmx1
Spc24

Mob1a
Zbtb4
Tcp11l1
Sult4a1
Rbmxl1
Phf21b
Alad
Dnpep
Rps25
Kcnk1
Mis18bp1
Pou3f3
Esf1
Smpd3
Aldh9a1
Mtss1
Rfc2
Ncapd2
Rab3c
Gdap1
1810043H04Rik
Selk
Blmh
Cct5
Cd2ap
Nefm
Rif1
Irf2
Mcm6
Fyn
Ypel1
Ctcf
Tmeff1
Tnik
Slc17a6
Ube2e3
Tubb2a
Chchd2
Cdca7
Tmem35
Ebf3
Rad21
Tomm7
Bok
Lrig3
Pde4dip
Tcp1
Crispld1

D030056L22Rik
Sra1
Ccnb1
Cat
Fam57b
Ppp1r14a
Rpn2
Cdh20
Hes6
Chrna3
Ppp3ca
Ccna2
D630003M21Rik
Dynlt3
Nans
Timeless
Cope
Rpl37
Dmd
Prc1
Ndufa12
Gpr56
Pgm1
Tprgl
Stk39
Mgat2
Ddx21
Bsg
Naca
Pdzrn4
Sowaha
Cnbp
Glyr1
Mtf2
Fam181b
Dpy30
Mapk8ip2
Echdc2
Sox4
Spcs1
C1qbp
Acat2
Sars
Prim1
Slc12a4
Lhfpl2
Bfar
Xrn2

Adamts10
Hspa4
Cep170
Nol8
Dpysl3
Sgol1
Fabp5
Rpa2
Zc3h15
Mrpl42
Ctnna1
Ythdf2
Tnfaip1
Nhp2
Ccar1
Pkia
Cdc42se2
Creb3
Ubqln2
Herpud1
Dcbld2
Mef2a
Ppp1r9a
Elmo1
B3gnt1
Trim37
Bcap29
Fam118b
Myef2
Ppa1
Cadm3
G3bp1
Mcm2
Rin2
Ubc
Elovl1
Myeov2
Fam20c
Tmod2
Yipf5
Ssb
Prkcb
BC005561
Ndc80
Acadm
Apbb1
4931406C07Rik
Ssh2

Tmem57
Prdx1
Mpnd
Tmem5
1700001O22Rik
Sema6d
Lepre1
Gnas
Nes
Sox18
Las1l
Dlgap5
Eif5b
Por
Mxra7
E2f1
Zswim7
Nelfe
Kank3
Pomp
Cenpq
Frrs1l
Gns
Acadvl
Myt1l
Klf9
Eif4a2
Ssr3
Rnf122
Rhog
Fam53b
Cct6a
Gpm6b
Mapt
Srsf1
Nuf2
Lancl2
Hspe1
Ckap5
Brd7
Cenpk
Trim28
Phldb2
Pik3r1
Hsd17b11
Eef1e1
Prpf31
Jun

Mxd3
Kdelc1
Sept2
Fam64a
Prmt5
Dtx3
Mpped2
Atp5j2
Fam115a
Nfkbia
Bola2
Aurkb
Cers2
Tk1
BC005537
Yeats4
Eprs
Myt1
Rcc2
Ptbp2
Dbp
Nsg1
Frmd8
Mgll
Aplp1
Fam171b
Larp7
Pcbp4
Hk2
Etaa1
Ttc9b
Sqle
Pcdh18
Gm17322
Zeb1
Qdpr
Ubr1
Dnajc2
Ccnb2
Tmem178
Emc7
Dst
Tmed2
Bcas2
2700046A07Rik
Rcn2
Pold3
Ubr2

Cacng4
Hsdl2
Zdbf2
Rnf126
Rpl14
Epb4.1
Ier5
Sf3a1
Snrnp70
Kif15
Wdr33
Fdft1
Ankrd50
Cep78
Pphln1
Arid2
D17H6S56E-5
E330009J07Rik
Lmnb2
Mroh2a
Mcm5
Acp2
Igsf8
Cib1
Olfm1
Tyms
Cox6c
Scn8a
Canx
Gpc2
Zfp608
Rrp1
Cnpy1
Aip
Suz12
Pak7
Gorasp2
Sash1
Ncald
Sclt1
Eif3l
Spred1
Scp2
Idh3a
AI597468
Spg21
2700029M09Rik
Ndufa13

Cgrrf1
Capn2
Snrnp40
Mab21l2
Stt3a
Pcsk2
Zwint
M6pr
Lman1
Thoc1
Ifnar1
St8sia1
Ccp110
Tnpo3
Grik2
Sec13
Eif3m
Clip1
Gkap1
H1fx
Cnksr2
Nxt1
Arpp21
Ifngr1
Rbm8a
Xist
Cdk5rap2
Syncrip
Nop10
Rhoa
Emc6
Ddhd2
Cplx1
Mad2l1
Hspa8
Polr3k
Sidt2
Asf1a
Nudc
Esco2
Gria4
Plcb1
Mrpl39
Rfc3
Mrps10
Luzp1
Gm12696
Dnajc24

Pkig
Mafb
Wdr12
Ckap2
Cpsf6
Sbds
Adh5
Plscr4
Dscr3
Dynll1
Mbtd1
Pcbd2
Rrp15
D10Wsu102e
Pxk
Hibadh
Rad51
Cstf2
Cd164
Sec14l1
Eif1ax
Tmem134
Prr24
Tmem198b
Eif1a
Gpr85
2810025M15Rik
Eif3e
A930011O12Rik
Safb
Upf3b
Slk
Cdkn1b
Fasn
Arf4
Gm8292
Rab3ip
Stat3
Phactr1
Fbxo9
Mif
Sdf2
Ltbp4
Slc29a1
Yipf4
Lpcat3
Cox5a
Ppp1r7

Fam110b
Zfp428
Rab31
Rpl5
Stard4
Mybbp1a
1110004F10Rik
Nup88
D17Wsu104e
Taf1d
Sike1
Ddx6
Adipor1
Cep290
Kras
Cct7
Ufsp2
5430416N02Rik
Cacybp
Prpf19
Tmod3
Ube2e1
Rnf187
Phf20l1
Smim14
Tcf12
Ddx46
Map2k1
Hnrnph3
Dynlrb1
Gtf2a2
Srsf7
Snrpd3
Rsrc1
Coq7
Alyref
Arhgef2
Psma7
Cdt1
Cdca3
Tm7sf3
Acadsb
Htatsf1
Sepw1
Ndufa2
Ralgps2
Insig2
Fam133b

Ptbp3
Elovl4
Eef2
Pcdha2
Ctbp2
Fam160b1
Dynll2
Gm11266
Fen1
Hip1r
Sltm
Pdxp
Tead2
Tmem214
Luc7l
Naa15
Msh2
Tcof1
Lsm2
Dck
Cln5
Suv39h2
Atox1
Trappc4
Plod2
Agtpbp1
Klhl7
Spire1
Slc39a6
Vcan
Ube4b
Parp1
Sox11
Myl6
Rpl36al
Pard6g
Ndufa5
Kpnb1
Mphosph9
Nae1
Dkc1
Mbp
Fam3c
Elk4
Frmd4b
Rnf5
Il11ra1
Hmgcs1

Cfl1
Atp1a1
Celsr3
Ppfia2
Mrps22
Zrsr1
Krcc1
Gabrb3
Snord104
Usp16
Knop1
Gatc
Clspn
Ctps
Ints7
Tubb4b
Cenph
Kif4
Ctbp1
Dhx32
Pspc1
Lyar
Gars
Aktip
2900011O08Rik
Txndc17
Rnf219
Kif3a
Skp1a
Nktr
Sv2b
Tardbp
Pes1
Arhgef25
Fjx1
Klhl24
Prorsd1
Kif1a
Rps8
Snx5
Polr2j
Tecr
Dazap1
Snx6
Nipsnap1
Dot1l
Stim1
Zc3h13

Uchl3
Sf3a3
Mid1ip1
Nfe2l2
Zfp422
Gtf2h5
Gabarapl1
Lsm3
Celf1
Rab22a
Aamdc
Nmt2
Sdad1
Tagln3
Glo1
Lsm4
Rnf165
Kdm1a
Gpsm1
Mettl21a
4933426M11Rik
Tmem50b
Tmem30a
Zdhhc1
Mef2c
Tbca
Rars
Timm50
Rps15
Phf20
Akap6
Commd4
Srsf10
Scaper
Fech
Rrs1
Polr2e
Atxn7l2
Rgs12
Cd2bp2
Scamp2
Bcl2l13
Pgls
Prmt1
Cenpw
Zfp462
Nfyb
Capzb

Thsd7a
Rbmx2
Dzip3
Cenpp
Bag6
Rfc1
Zfp326
Rnaseh2b
Gucd1
Mcm4
Pfn2
Serf1
Ccdc12
Bach1
Atp5k
Tceal3
Cul7
Gnao1
Psma6
Fam213b
Kmt2e
Xpo1
Lhfpl4
Skiv2l2
Limd1
Appbp2
Gstm5
Rnd2
Bag1
Topors
Mki67ip
Nrm
Erp44
Prdx6
Elf2
Snap23
Camk1
Slc35a2
Rad51ap1
Sacs
Arhgef7
2700081O15Rik
Agrn
Cdk2ap2
Gins4
Arrb2
Uba1
Stim2

Afg3l1
Dhx15
Shmt1
Rnf130
Peo1
Ube2g2
Sumf1
St13
Tmbim4
Nav2
Schip1
Pard3
Cnpy2
Sox9
Ptplad1
Zfp292
Gpr107
Zfp644
Rbm17
Atxn2l
Ncor1
Clvs1
Mrgbp
BC034090
H2afy2
Arhgap11a
Ano6
Snhg5
Copb2
Tmem106b
Gar1
Wasf1
Cdkn1c
Nolc1
Cdk19
Fut11
Ctnnbl1
Glce
Ssr4
Nucb2
Magi3
Mrpl18
Chaf1a
Hnrnpul2
Gm11541
Cd63-ps
Dync1i2
Tmsb10

Snrpg
1700025G04Rik
Mpp6
Dnajc7
Gart
Ddrgk1
Shmt2
Psmc3ip
Rcbtb1
Bcl2
Cdc123
1500009L16Rik
Rps18
Necap2
Sec16a
Tro
Prkar2a
Scfd1
Hspbp1
Prdx3
Mlf2
Pkm
Cep110
Ccdc41
Atat1
Kmt2c
Mdga1
Gtf2e2
Psmc6
Cbx3
D19Bwg1357e
Ubqln1
Ywhag
Tmem179b
Ddr1
Ddost
Pabpn1
Tspan7
Rad50
Blm
Uchl1
Sdf4
AU022252
Larp4b
Lipa
Rdx
Pde7a
1700011J10Rik

Trim2
Calm3
Specc1
Phf14
Unc50
Boc
Rwdd4a
Clasp2
Rbm26
Hat1
Siva1
Lbr
Exosc8
Ssbp3
Hspd1
Atp5o
Cirbp
Mrpl28
Cdc40
Pafah1b2
Eps8
Gatad2b
Mak16
Smarcad1
Rwdd1
Glud1
Btbd17
Jakmip2
1700037H04Rik
Cyb5
Sart3
Gmppa
Dnph1
Cdca7l
Timm13
Fbxo5
Ube2b
Setbp1
Mvb12a
Nmral1
Hadh
Mphosph10
Sec11a
Pald1
Gstp1
Prdm8
Cox6b1
Tmem123

Exoc2
Emc3
Arpc2
Tmco1
Ncor2
Arhgap21
Bud31
Prps1
Ddx39
Pcif1
Med19
Vezf1
Aes
Ttc14
Trpc4ap
Yif1a
Psmb3
Vapb
Bap1
Rnf181
Rpl18a
Vrk1
Cpsf2
Snrpb
Tceb1
Csde1
Ndufaf2
Gm9843
Actr6
Ost4
Snhg6
2310039H08Rik
Kctd10
Tsr3
Apbb2
Chst15
Nxf1
Cfl2
Rbm34
Qsox1
Pdcd2l
Ehbp1
Actn1
Patz1
Fmnl2
Ick
Ankrd11
Med30

Ptms
Ssbp4
Psmg1
Parp2
Rsrc2
Eif3c
Dcakd
Dnajc10
Ccdc90b
Gprasp1
Meg3
Snap25
Syt4
Chgb
Gabra6
Chn2
Vsnl1
Olfm1
Syt1
Atp1b1
Nrxn3
Eno2
Lin7a
Calb2
Cox8a
Camk4
Rph3a
Selm
Calm1
Cbln1
Rps5
Gabra1
Cbln3
Malat1
Adcy1
Nrep
Dnm1
Atp2b1
Atp6v1g2
Ppp3ca
Scn2a1
Rnf112
Etv1
Gabrd
Snca
Cacnb4
Stmn2
Rps14

Kcnd2
Tubb5
Ntm
Sncb
Chchd10
Celf4
Rps9
Sfrp1
Chl1
Camkk2
Tmem59l
Aplp2
Unc13c
Actb
Mgst3
Eps8
Napb
Rps3
Ndrg3
Syn2
Nap1l5
Tspan7
Pak1
Nptxr
Gm2694
Grin2c
Atp1a3
Rpl13a
Gpm6a
Cadm3
Rps26
Tuba4a
Hsp90ab1
Cd24a
Rbfox3
Kcnk3
Rpl32
Rpl4
Btbd3
Nrxn1
Igfbpl1
Kcnc1
Hnrnpab
Rplp1
Diras2
Camk2b
Gas7
Purb

Ckmt1
Ablim1
Eef1a1
Ndrg4
Rpl8
Grm4
CRE_RECOMBINASE
Ccnd2
Gnb2l1
Tmsb4x
Pkib
Spock2
Tmod1
Rps11
Cadps2
Nsf
Rps24
Rpl22
Rps20
Sv2a
Cd63
Rps19
Dlgap1
Tagln3
Tpi1
Snhg11
Atp6v1a
Nrxn2
Psd3
Cplx2
E130114P18Rik
Mdh1
Kcna1
Atp2b2
Fbxw7
Tenm1
Frrs1l
Rplp0
Eef1a2
Golga7b
Crtam
Darc
Car10
Cog7
Prdx5
Ndufa4
Cd9
Car4

Gabrg2
D3Bwg0562e
Aldoa
Prune2
Slc16a11
Bcl2l15
Phyhip
Rims1
Scg2
Atp5g3
H2afv
Cox6a1
Rpl41
Neurod2
Jph4
Prkce
Rpl14
Kcnj9
C1qtnf4
Dpp6
Slc12a5
Stmn3
Dnm3
Bend6
Ddah2
Ezr
Cox4i1
Nptn
Neurod1
Mybpc3
Cnr1
2010107G23Rik
Rnf152
Rab3c
Cadm2
Marcksl1
Rps21
Nnat
Rplp2
Camk2d
Slc17a7
Rps6ka1
Sybu
Ppfia4
Nasp
Pabpc1
Kif5c
Sept3

Erc1
Rtn1
Hmgn1
Rock2
Unc80
Pcsk1n
Ezh2
Grm1
Cplx1
Kcnt1
Caln1
Ncl
Rps15
Rit2
Ppp1r14b
Tuba1a
Tceal5
Nfib
Slc4a4
Nfia
Cnnm1
Npm1
Barhl1
Gm10075
Cpe
Snrpe
Adamts18
Kirrel3
Nptx1
Stxbp1
Anp32b
Pcp4
Cbx5
Ppib
Ppargc1b
Cygb
Prkar1b
1700020I14Rik
Mdk
Spop
Hnrnpa2b1
Eef1b2
Necab3
Nefl
Tubb2b
Tspyl4
AI848285
Cdk4

Sox4
Anp32e
Atp5o
Rps10
Mpp3
Psat1
Tshz2
Smc2
Cend1
Vps37b
Atp6v0e
Pnck
Dync1i1
Slc29a1
Fabp5
Fnbp1
Ptma
Atp1b2
Syt5
Fat2
Rpl34
Mapt
Rpl18a
Rps15a
Itm2b
Cntn1
Snrpn
Cox7a2
Rps3a1
Pcdh9
Basp1
BC029214
Gabrb2
Srebf1
Atp6v0b
Bmp1
Fabp3
Rps27l
Mex3a
Snap91
Ypel3
Sorl1
Gnao1
Srcin1
Hcfc1r1
Caly
Atp6v0e2
Prrt1

Ccnd1
E530001K10Rik
Cnn3
App
St3gal5
Ndufa13
Gm9800
Rps18
Rbfox1
Mcm7
Camk2n1
Snrpb2
Zfand5
Cald1
Fam210b
Hjurp
Ndufb8
Ramp3
Rpl35a
Cacna1a
Hpcal1
Snrpf
Runx1t1
C1ql1
Atp6v1e1
Ranbp1
Rpl23
Trpm3
Eif4g2
Ndufa5
Grina
Miat
Eif3f
Serinc1
Adam11
Rab3a
Clip1
Hn1
Map1a
Ly6h
Atp2b4
Tmpo
Draxin
Fkbp3
Rgs8
Npc2
Rora
Grin1

Tspan17
Trim2
Pura
Rps2
Zfyve28
Lmnb1
Rpl26
Mbnl2
Mycn
Snrpg
Syt2
Pfkp
Plcxd3
Rgs7bp
Cox6b1
Fut9
Ryr2
Hnrnpd
Atp1a1
Adora1
Rpl39
Srsf3
Madd
Vamp2
Dtymk
H2afy
Pbrm1
Gls
Uqcr11
Carhsp1
Sptan1
Afap1l2
Hsd11b2
Slc25a4
Ran
Ppp1r1a
6430573F11Rik
Pld5
Top2a
Zeb2
Tox3
Sh3gl2
Bsg
Rab6a
Schip1
Prdx2
Pde4a
Zfp385b

Marcks
Apex1
Slitrk4
Zcchc18
2210016L21Rik
Pagr1a
Pcca
Ndufv3
Itgb1
Banf1
Pou3f2
Klf9
Trp53
Kif1b
Egr1
Nap1l1
Dgkd
Gas5
Prpf40a
Ppic
Stx1b
Vim
Matk
Cyc1
Prim1
Map3k1
Atp5b
Birc5
Syt7
Cdkn1a
Commd1
Fxyd7
Tmem145
Dctpp1
Igfbp5
Il20rb
Scn2b
Smc4
Eef2
Srsf6
Dcx
Atp6v1b2
Rpl7
Plcb4
Fez1
Syp
Insm1
D10Bwg1379e

Ier5
Uqcrq
Fnbp1l
Plcxd2
Nop58
Napa
Bdnf
Scrn1
Adarb1
Mcm2
Spc25
Ccdc88a
Cacna1g
Zfp467
Casp3
2810417H13Rik
Rpl37
Tead2
Tecpr1
Mcm6
Kdm1a
Atp2b3
Hirip3
Rabac1
Hmgn2
Klc1
Adh5
Gng3
Cenpa
Tubb3
Dusp5
Mycbp2
Zeb1
Nhlh2
Rfc1
Akap7
Ddx21
Hdgfrp3
Fgf14
Lsm3
Micu3
Dynll2
Ankrd12
Il16
Snrpd2
Smarcc1
Dos
Rassf4

Tmem132a
Hnrnpa0
Rcn1
Sfpq
Ttc9b
Ilf2
Hk1
Myl12a
Apbb1
Asph
Atp5j
Fam63b
Tpm4
Lsamp
Uqcrb
Tcf4
Ccdc92
Tspan9
H1f0
Fgf12
Syngr1
Wbp2
Higd2a
Cox6c
Pcna
Kifc2
Hnrnpr
Got1
Tmem108
Atp6v1d
RP23-45G16.5
Ssr3
Hmgb3
Rnaseh2c
Frmd4a
Gria2
Calm2
Ccdc34
Id2
Ebna1bp2
Rbmx
Tcp1
Ube2c
Fscn1
Gm8292
Rab6b
Cdh20
Pgm2l1

Hmgb2
Gnai2
Abcg1
Sh3bgrl
Cdh7
C530008M17Rik
Rps4x
Cdc42
Lap3
Ttc3
Fam49b
Hmgb1
Cbx1
Lig1
Apba1
Ybx1
Eif3e
Psmb1
Mki67
Trnp1
Ssrp1
Mrpl52
Ptgds
Cks1b
Ndufa1
Pa2g4
Ptpre
Klhdc8a
Idh3a
Syncrip
Rbbp4
Paics
Cnbp
Cct2
1110038B12Rik
Ssr1
Gse1
Sox9
Atp2a2
Adam22
Tuba1b
Fbxo9
Hook1
Khdrbs1
Lgals1
Cox5b
Nhp2
Ebf3

Arhgef7
Scamp1
Tmsb10
Camta1
En2
Rdx
Synpr
G3bp1
Echdc2
Ntrk2
Akap8l
Dpf3
Pdgfa
B4galt6
Rpl22l1
Nsg2
Lmo4
Jun
H2afy2
Gabbr2
Rbm8a
Cct5
Hdac2
Rcor2
Sirpa
Rpl14-ps1
A930011O12Rik
Gm17750
Epb4.1l3
Rnd3
Denr
Mpv17l
Edf1
Igsf21
D4Wsu53e
Ppip5k1
Tsn
Sec62
Elavl2
Papola
Fam131a
Cbfa2t3
Gsg1l
Zcchc17
Rpl18
Atp5f1
Nip7
Cacna1d

Map7d2
Lrrtm3
Lrrc49
Pde1c
Klf13
Magoh
Eny2
Mpc2
Cystm1
Pja2
Nhlh1
Arf4
Nup85
Snx6
Supt16
Eif3l
Rbp4
Serf1
Eif3d
Sep15
Gins2
Napg
Snrpd3
Cdk1
Eef1g
Rps25
Pcyt2
Atp6v1c1
Srsf7
Phf14
Oxct1
Zic1
Ndufb2
Ccna2
Txnrd1
Esco2
Pcbp1
Cacybp
Bcl11a
Crip2
Kbtbd11
Mat2b
Car11
Chka
Nolc1
Cox7b
Clic4
Atp5k

Dnajc5
Atp5j2
1110008F13Rik
Rpa3
Megf11
Lhx1
Psap
Sh3glb1
Hmgn5
Dbi
Taok3
Rfc3
2700094K13Rik
Sec14l1
Mdh2
6430548M08Rik
Nkd1
Prc1
5730409E04Rik
Dek
Rhobtb3
Ythdf2
Tmem38a
Arxes1
Arpp21
Dnajc9
Shfm1
Cdca3
Atp6ap2
Uqcr10
Snrpd1
Ubxn1
Rps16
Stmn1
Luc7l2
Grm5
Sphkap
Zfp326
Bola2
Mapre3
Sfrs18
Rps7
Racgap1
Btg2
Gpr56
Rpa2
Ank2
Phactr3

Zfr2
Rpl37a
Bok
Zc3h14
Ptn
Mapre1
Rps8
Psma6
Tceal3
Cisd1
Cfdp1
Ypel1
Eomes
Rif1
Gucy1b3
Lrig3
Cdca7
Atp5c1
Eif3h
Snhg5
Lars
Neto2
Eif3i
Ftsj3
Zranb2
Eef1d
1110001J03Rik
Rad21
Ktn1
mt-Rnr2
Hells
Ccser2
Ccm2
Cyfip2
Cenph
Rrm1
Usp1
Set
Eif4a1
Caprin1
Gas1
Prdx4
Pkp4
Mpp6
Kpnb1
Tspan6
Tle1
Ssr2

Map1b
Porcn
Pfn1
Syt12
Ccnb2
Aifm3
BC034090
Mapre2
Cbfb
Cox7a2l
Opcml
Prdx1
Clspn
Fam155a
Prnp
Cenpe
Nusap1
Rfc4
1500012F01Rik
Ptch2
Snord104
Arrb1
Pdap1
Zmynd11
Clip3
Gpr153
Zfr
Bcas1
Dut
Ccdc41
Gabbr1
Prmt8
Arpc5
Pbk
1110008P14Rik
Ncdn
Tcerg1l
Nmral1
Lsm4
Dock9
Tra2a
Spc24
Mrpl15
2610017I09Rik
Knstrn
Hmmr
Ctsz
Itm2c

Fam134a
Hnrnpu
Pqlc1
Rnaseh2b
Abracl
Celf1
Hes1
Smarca4
Sept8
Cdk2ap2
Unc5c
Gm13826
Rpf2
Psma7
Abcc8
Dpy30
Arhgef2
Kif23
Magohb
Aldoc
Mettl9
Ociad1
Pip5k1c
Idh2
Rgs12
Pdzrn4
Atp5d
Plxnb2
Pdgfra
Ddx42
Ywhag
Hspd1
Nras
Slc25a22
Cdc20
Tiam1
Hspa5
Cdca8
Parp1
Gm10036
Incenp
Rundc3a
Snhg1
Magee1
Tspan4
Cenpk
Txndc9
Map3k12

Ccar1
Tyms
Rrs1
H2afx
Dixdc1
Bub3
Smarca2
Cenpf
Nbea
Tgfb2
Pacsin1
Pcbp4
Pnrc1
Sri
Mmp14
Sec61b
Grb2
Vldlr
Prkd3
Sel1l3
Kcnk1
Nol7
Psmb6
Ckap4
Laptm4b
Araf
Myod1
Tead1
Hint2
Ngdn
Nuf2
Setd8
Cdipt
Dab1
Tulp4
Gm11223
Kif11
Gpatch4
Phf5a
Fndc5
Mrpl13
Trim3
Emc9
Sez6
Igsf3
Ptprs
Nup62
Gtpbp4

Kctd8
Ilf3
Prdx6
Sema7a
Meis1
Tacc3
Mcm3
Rpl13
Cnpy1
Cd200
Ddx5
Gm10260
Ckap2
Uba52
Pdxp
Islr2
Dnmt1
Nt5dc2
Tbata
AI413582
Bola1
Amph
Tpx2
Dstn
Pcm1
Vdac1
Siva1
Hk2
Xrn2
Ndufb11
Cnot6
Snap47
Clns1a
Ivns1abp
Nucks1
Sbno1
Sstr2
Hspe1
Klhdc2
Erbb2ip
D19Ertd737e
Galnt9
Sephs1
Gpm6b
Smim11
Anapc2
Sf3b5
Mns1

Cited2
Rcbtb2
Rpl38
Cdk6
Tbl1x
Tmem234
Baiap2
2810025M15Rik
Eif4a2
Tmem14c
Ndufa8
Sfr1
Tmem258
Tcf25
Whsc1l1
Etf1
Cyb5
Pdia6
Fam168a
Lamtor4
Naa15
Serbp1
Negr1
Cct6a
Nap1l3
Ap3d1
Suv39h2
Gm11478
Larp7
Cltb
Polr2b
Baz1a
Ier3ip1
Mcm5
Sugt1
Pou3f3
mt-Nd6
Alyref
Atoh1
Zwint
Dkc1
Lgi3
Cct3
Rb1cc1
Psma2
Smarcad1
Mrpl33
Jam3

Rpf1
Znrd1
Vcan
Cebpz
Nsmce2
Acot7
Snx1
Slc9a3r2
Sub1
2410006H16Rik
Fosb
Fubp1
Smc1a
Mgll
Uqcrh
Chd4
Ncbp1
Ubxn4
Gpi1
Idh1
Golim4
Dnajc15
Sfxn3
Pdxk
Brd7
Cacna2d3
Tsnax
Txndc17
Adamts1
Heg1
Nrm
Tcof1
Ptprr
Mtch2
Mxd3
Atp11b
Cnpy2
Ska2
Naa10
Zfp36l1
Cnih4
Ndufb10
Elp2
1500016L03Rik
Kif20b
Dnajc2
Zbtb18
Msl3

Brinp1
Gm6472
Mical1
Fbxo5
Efs
Actl6a
Gm26735
Bmi1
Tars
Rab7
1810009A15Rik
Atad5
2310022B05Rik
Dnaja1
Cdc42se2
0610009D07Rik
Asap1
Btf3
Sptssa
Brix1
Smco4
Smchd1
Ppp6r1
Ccdc28b
Trim62
Galnt7
Casc5
Rtn3
Smpd2
Impad1
Limch1
Gnai3
Nfix
Cbx6
Smarcb1
Zfp704
Akr1a1
Topors
Rasa3
Smarcd3
Ddx39
Chic2
Rnaseh2a
Ptp4a2
Rbbp8
Hnrnpc
Ccdc50
Ier2

Wscd2
Ctnnb1
Luc7l3
Cenpq
Sae1
Rbm17
Clmp
Srsf2
Rpl3
Peg3
Top1
Mapk9
Rnd2
Psmg4
Herc2
Ncaph
H2afz
Arhgef9
Oxr1
Serp1
Cnksr2
Polr2a
Sh3bp5
Snrk
Sfrp2
Gar1
Pak3
Ywhab
Tmeff1
Ift74
Tubb4a
Sap30
Tmod3
Afap1
Rufy2
Sv2b
Ctnna1
Wdr5
Tpm3
2410015M20Rik
Gstm5
Thy1
Lphn1
Cep57
Pole3
Arxes2
Srek1
Chrna3

Uchl3
Cklf
Odf2
Snrnp40
Nme4
Pak2
Psmb4
Tbc1d20
Nr1d2
Nelfe
Mef2a
Mlf2
Snrnp200
Ppa1
Rrm2
Ctnnbl1
Lsm6
Pcdha2
Pgls
Iws1
Cdh8
Trim59
Aurkb
Eprs
Nsmce1
Gm9843
Eml4
Polr2e
Dhfr
Ndufs7
Angptl2
Chchd2
B2m
Tram1
Timp2
Ddx39b
Bzw2
Naca
Anapc13
Samd14
Sh3glb2
Hnrnpm
Lsg1
Laptm4a
Snx5
Dpm3
H1fx
Psma4

Polr3gl
Fastk
Mfap4
Rpl29
Dot1l
Tpr
Eif3a
Rp9
D8Ertd738e
Ndc80
Fxr1
Lyar
Ckap2l
Tmem191c
Bcl7a
Usmg5
Mad2l2
Ifitm2
Pgp
Cenpv
Taok1
Smarca5
Map1lc3a
Synj2bp
Habp4
Arhgap5
Gjc1
Rbx1
Chaf1a
Rpn2
Ppan
Rars
C1qbp
Mt3
Hnrnpdl
Fen1
Psmd11
Gng5
St6galnac4
Ptov1
Cct8
Fam63a
Hbp1
Hspa4l
Gamt
Ybx3
Eif3m
Dbf4

Trim24
Hnrnph3
Lrp8
Sec13
Pebp1
Etfb
Arid4a
Cdc5l
Prkcz
P4hb
Lhfp
Csdc2
Prox1
2810004N23Rik
Elp5
Spcs2
Fam131b
Pdzrn3
Asphd1
Bcat1
Fam98b
Mcm4
Peli1
Naa50
Fbxo44
Cdv3
Ppie
Rabgap1l
Sqstm1
Rogdi
Prpf38b
Ldhb
Eci2
Dnajc6
Mis18a
Vrk1
2900011O08Rik
Cat
Sin3b
Fam98a
Mrpl36
Gdi2
Fbxl16
Ubr7
Brd3
Ssbp4
Arpp19
Lman1

Phf21b
Nudcd2
Ralgps2
Polr3k
D430041D05Rik
Mrpl17
Srgap3
Tkt
Ndufb3
Plekha1
Ctcf
Eif3k
Kif4
Dtl
Nin
Cct4
Rac1
Nans
Polr2d
Gli1
Psmd9
Lamp1
Ube2g2
Gpr107
Kif2c
Hspa4
Ppp2r2d
Arhgap11a
Gmppa
Cep110
Ostc
Pitpnc1
Gramd1b
Naa38
Smim14
Anapc16
Trim28
Mia
Dhx9
Herc1
Diap3
Aig1
Ewsr1
Pea15a
Zcrb1
Ctbp2
Spg21
Mrpl24

Gm10076
Appl2
Rps27a
Rpl7a
Uhrf1
Rpl6
Bnip2
Pnrc2
Dcakd
Gripap1
Fam181b
Cdk5rap2
Abhd8
Kif5a
Pbdc1
Rrp15
Pih1d1
F2r
Blcap
Snx30
Uqcrc1
Ccndbp1
Elf2
Gpd2
2810474O19Rik
Psmc2
Phlda1
Stard4
Dgkz
Mrps15
Rbms1
Itgb3bp
Gpr162
Fuca1
Hnrnpl
Adk
Csrp2
mt-Rnr1
Map7d1
Ndufb6
Tk1
Eif5a
Lman2
Tmem107
Tex30
Ccnb1
Pfdn5
Inpp5k

Osbpl1a
Dnajc3
Camta2
Psme4
Cldn25
Psip1
Zfp207
Adrbk2
Psmb2
Txlna
C330027C09Rik
Peo1
Wipi2
Rbmxl1
Atad2
Synj1
Bub1
Plk4
Polr2j
Ndst3
Kif1a
Sart1
Gclm
A330076H08Rik
Cdk5rap3
Mphosph10
Rhno1
Fam126b
Tmem127
Mak16
Rnpc3
Dab2ip
Ogfrl1
2510002D24Rik
Abcf1
Odc1
Stip1
Rsl1d1
Rad51ap1
Frmd4b
Ubr2
Clcn3
Gltscr2
Psmc4
Gm11266
Hes6
Snw1
Eif1ax

Gins1
Tgoln1
Ptms
Syf2
Cnot2
Helz
Ank3
Mettl16
Arf3
Mrpl21
Ddx17
Sptbn1
Rab34
Reep3
Cep170
Tnpo3
Reep5
Puf60
Rgs17
Cwc27
Trip11
Pmf1
Macf1
Eif3g
Nek6
Add3
Ube2e3
Sod1
Tspan3
Tomm22
Ncapg
Smu1
Dip2b
Tipin
Psma5
Txnl1
Suz12
Psmc3ip
Tpt1
N4bp2
Pxn
Sparcl1
Park7
Txnl4a
Slc35b2
Ssb
Ift27
Cecr2

Sema4g
Alkbh6
Snrpb
Gpc2
Gdpd1
Tm9sf3
Sugp1
Mmadhc
Ddx3x
Mapk8ip1
Dars
Utp3
Nemf
Tsc1
Kif15
Nudt21
Dazap2
Brd8
Rfc2
Pvrl3
Jarid2
Rps16-ps2
Polr2f
Pard3
Gphn
D17H6S56E-5
A030009H04Rik
Eif6
Smarcc2
Ap1s2
Lphn3
Baz1b
Pcdhga9
Nktr
Sema6a
Zfp422
Cadm1
Nol8
Polr1c
Thrsp
Atad3a
Prmt1
Mrps17
Asf1a
Isy1
Emg1
Rpl31
Plxna2

Cenpm
Nono
Mis18bp1
1700001O22Rik
Lims1
Atp5g1
Tmem237
Ccng2
Wdr77
Stt3b
Tspan31
Rnf220
Zranb1
Abhd16a
Plekhb2
Spag9
Robo2
Fzd2
Gnptg
Ndufa11
Rsrc1
Lsmd1
Tceb1
Eif4a3
Shank3
2700029M09Rik
Cmtm3
Elavl1
Cstf2t
Tsen34
Dbndd2
Stx7
Rell1
Thsd7a
Pkn2
Gkap1
Pola1
Ckap5
Srpk2
Serpinh1
Cenpc1
Yif1a
Prpf38a
Atp1b3
Rrp1
Slc25a23
Gm11541
2310015B20Rik

0610037L13Rik
Gcc2
Rpl15
Acyp1
Tspyl5
Dnajc21
Cabin1
Vgll4
Lta4h
Aspm
Nck1
Cit
Ank
Dst
Pfdn4
Gxylt1
Kif3b
Bcar1
Mcmbp
Mpdz
2810055G20Rik
Dennd2a
Cul7
Fam212b
Rspry1
Socs7
Ndn
Ik
Ttyh1
Pafah1b1
Rhot1
Sez6l2
Prkacb
Thrap3
Wdr60
Eif4e2
Smc5
Tsc22d4
Gprc5b
Cox14
2310044G17Rik
Acin1
Tex9
Idh3b
Gnai1
Mcl1
Ppp3r1
Setdb1

Cenpp
Dnph1
Pphln1
Bcl2l13
Stag2
Mki67ip
Pan3
Phip
Emc7
Dnaaf2
Pcmtd2
Ehd3
Pbx1
Asnsd1
Acat1
Dusp11
Agtpbp1
Larp4b
Smc6
Fstl1
Uhrf1bp1l
Wdfy3
Strbp
Bzw1
Clic1
Ndst1
Wdr33
Pes1
Hnrnpul1
Oard1
Hdgf
Vrk3
Isoc1
Wdr89
Anln
Polr2i
Rps28
Serp2
Sec63
0610012G03Rik
Dusp3
Cdca2
Slc25a12
Manf
Dync2h1
Snx32
Setd7
Mad2l1

Ptplb
Atf4
Ywhaz
Pygo1
Aebp2
Zfp612
Syngr3
2210013O21Rik
Atp6ap1
Tbl1xr1
Cdh4
Galt
Cxx1c
Ube2e1
Ehmt2
Cd63-ps
Ube2r2
Exosc8
Pfn2
Slc39a10
Psmd12
Srrm3
Mars
Cntln
Ankrd46
Armcx1
Nek7
Qdpr
Atp8a1
Slc8a2
Tex14
Rsu1
Tmem261
Arhgap12
Cdca4
Btbd17
Grik2
Mrps21
Bccip
Pole4
Phyhipl
Ncor2
Ctxn1
Stk11
March2
Senp1
Scoc
Stt3a

Cenpj
Gcsh
Tcf3
Fam64a
Smn1
Lmo1
Ramp2
Sox18
Dctn3
Ap2a2
Slc38a2
Gnl3
Gm17322
Mex3b
Cetn3
Hat1
Rnf6
Kifap3
Pigx
1300002E11Rik
Mbtd1
Celf5
Pfkm
Epb4.1l1
Zmynd8
Pcnt
Mapk8ip2
Kdelr2
Ash1l
Lima1
Sdccag3
2610203C20Rik
Ube2d3
Pkia
Pde4b
Mab21l2
Ntrk3
Mrpl55
Arf5
Nsun2
Vars
Prmt5
Sidt2
Ppp5c
Cdk7
Clpp
Tmed5
Dgke

Rps24-ps3
Wdr12
Rpl7l1
Lin7c
Rdh5
Rap2b
Tmem242
Dcaf11
Satb1
Dnm1l
Thra
Hdac1
Kat2b
Lsm2
Rpl36
Ppid
Dclk1
Fam53b
Smoc1
Dusp26
Orc4
Fbxw11
1810022K09Rik
AI854517
Gtf2e2
Cds2
Hpca
Ppp3cb
Mroh2a
Grpel2
Ipo5
Ccnl2
Bdp1
Ppa2
Hivep2
1500011B03Rik
Mycl
Copg1
Tbc1d16
Ncapd2
Mrps9
Hagh
Gps2
Tubgcp5
Ttc28
Ecsit
Grpel1
Nudt9

Slain2
Ccdc136
Lrrc58
Tab2
A830080D01Rik
Rcc2
Cdca7l
Pttg1
Dnttip2
Taf1d
Gm13092
Uba5
Bms1
Ccl27a
Cstb
Pdzd11
Ddt
Ctdsp2
Rftn2
Bscl2
Akt1
Slk
Dcaf13
Srgap2
Nell2
Slc1a3
Srsf9
Pnmal2
Dpysl3
Cfl2
Nop16
Ankhd1
Rrbp1
Tmem256
Sgpl1
Adrbk1
Ggh
Yme1l1
Nipsnap1
Rabggtb
Dcun1d5
Ywhah
l7Rn6
Skp2
Gmnn
Med10
Rere
Myeov2

Ube2l3
Aff4
Zdhhc16
Immp1l
Rpl10a
Rpl30
Gabpb2
Cisd2
Vps72
Map4k4
Smek1
Tmed9
Commd3
Actl6b
Dhx32
Man2a2
Far1
Optn
Cntnap1
Eml5
Gm20417
Mt1
Kars
Rab33a
Tmem178
Slc24a5
Hif1a
Cdh2
Neo1
Rpain
Psmd13
Cdc16
Fam174a
Prr13
Tubgcp3
Mgat2
Imp3
Gtf2f1
Gtf3c2
Sgol1
Reep2
Dcaf7
E2f1
Soga2
Mrps10
Sept2
Ddost
Ogdh

Ssna1
Akap11
Cyb5r3
Ube2d1
Gm3764
Pitpnb
Prkra
Rpp30
Gria4
Jund
Cebpg
Cib2
Amer1
U2af2
Cyb5b
Rnf144a
Strap
Mtus1
Nudt4
Klhl13
Hmgcl
Eif2s2
Selo
Lrrc16b
Rai1
Cxxc1
Elavl4
Agpat4
Adss
Gfer
Chtop
2410004N09Rik
Diablo
Rassf3
Rbbp7
Gtf2a2
Rbbp6
Abca2
Pdpk1
Eef2k
Gmps
Ddx54
Amz2
Klhl23
Akap6
Shmt1
Csnk1g1
Sox11

Topbp1
Tmem176b
Sdhc
Exosc1
Med15
Chkb
Tgs1
Wwp1
RP23-32A8.1
Gdap1
Glg1
Gars
Eif2b2
Zfand3
Zcwpw1
Ppp1r12c
Trim27
Anks1b
Rfng
Abhd17b
Slc25a17
Thoc7
Snx4
Rnf165
Fam101b
Mllt11
Tubb4b
Fbxo32
Cenpw
Aftph
Atat1
Dnajc24
Pknox1
Rad23a
Ccar2
2810008D09Rik
Arhgap21
Safb2
Cdc25a
Lsm14b
Gm2000
Fbxo21
Vps53
Unc13a
Ing1
Nde1
Smap1
Frg1

Pdcd11
Slc1a2
Suds3
Wasf2
Fam111a
Ift43
D10Wsu102e
Ypel4
Kif22
Wdr3
Nme2
Isca1
Pdha1
Msantd4
Rsbn1
Pcsk2
Pik3r3
Camsap1
Brca2
R3hcc1
Tfdp2
Gde1
Vps28
Zmym4
Hey1
Ensa
Ddr1
Rnf187
Cpeb4
Echs1
Eif2s3y
Slc22a17
Dpf2
Tbca
Plgrkt
Pnkd
Mrpl11
Gabrb3
Tacc2
Fam178a
Nr2f1
Agap3
Bub1b
Rev1
Cep68
Cdh15
Ergic3
Yy1

Phactr1
Neurod6
Myg1
Eif3b
Chrac1
Ldb1
Pold2
Bfar
Max
Adar
Gprasp2
5830418K08Rik
Adam10
Lzts1
Gtpbp2
Kdm2a
Zcchc8
Tmem134
Gramd1a
Fundc2
Tcerg1
Xrcc5
Dlgap5
Sp3
Ahsa2
Ppil2
Ptbp1
Cdt1
Uncx
L1cam
Nudt19
Gatc
Znhit6
Prpf31
Mrpl34
Tubb2a
Zfp830
Scnm1
Scg5
Slc39a6
Nalcn
Sec11c
Mdga1
Pax6
Rae1
Med13l
Sgip1
Mif

Polr2h
Cnrip1
Pitpna
Eri1
Zrsr2
Mob4
Grik5
Zfp740
Ubac1
1200014J11Rik
Fads1
Mettl10
Gpx1
Ndrg2
Fam175b
Etnk1
Usp33
Rnf4
Zhx1
H2afj
Prdm2
Pja1
Klc2
Unc50
Rnf11
Vkorc1
Grsf1
Sirt2
Glrx3
Sgol2
Synrg
Actr6
Apoe
1110001A16Rik
Ssu72
Dbn1
Hist3h2a
Trappc3
Tmem55b
Dnttip1
Ptrh2
Exosc5
Flywch2
Smim15
Prps1
Nol11
Ezh1
Trpc4ap

Fam107b
Trappc1
1700021F05Rik
Dad1
Sac3d1
E330009J07Rik
Ankrd32
Ctsd
Necap1
Nop56
Kcnc3
Psme2
Tspan5
Bex1
Fam133b
Lamp2
Mapk10
5430416N02Rik
Cdk16
Lsm1
Gsr
Epm2aip1
Dld
Smarce1
Specc1
Cxx1b
Pptc7
Tex261
Fam120a
Emc4
Fbxw2
Ncapd3
Blvrb
Slc2a3
Cpne3
Opa3
2410004B18Rik
Zmym2
Tbce
Pop5
Kat6b
Rps6ka3
Thoc1
Barhl2
Rtcb
Tsta3
Tef
Xpo1

Scaper
Nrip1
Fhl1
Gtf2b
Rnf219
Nap1l2
Rap1a
Smg1
Aatf
Kcnj3
Bcas2
Cenpb
Tbpl1
Fyn
Tmed10
Prpf40b
2700089E24Rik
Rab10
Sec61a1
Orc2
Mia3
Stra13
Anapc4
Limd2
Itfg1
Rbm33
Exosc3
Poglut1
Brwd3
Tmx3
Acaa1a
4930402H24Rik
Mmp24
Atp6v0a1
Ctsf
Slc7a5
Zfyve9
5830428H23Rik
Tmed1
Cox16
Poc1b
Slc48a1
Cacng4
Sod2
Usp3
Ncald
Usp46
Rhob

Cd3eap
Slu7
Supt6
Ogfr
Gnl3l
Bmyc
Bcap29
Npdc1
Dck
Atic
Pdcd4
Slc17a6
Deb1
Eif2b3
Taf2
Tnik
Dph3
Tpp2
Isg20l2
2810006K23Rik
Fam32a
Bet1l
Znhit1
Stx8
Bcl2l1
Bad
Wdr43
Mybbp1a
Blm
Trpc2
Wash
Smpd3
Zfp277
Ccdc55
2510003E04Rik
Pdlim7
Atp11c
Zbtb7a
Rtn2
Pink1
Ptbp3
Daam1
Gtf3c6
Gosr1
Ip6k2
Mrps18a
Tm9sf4
Prpf4

Pard6a
Extl2
Exoc2
Gbas
Fam171a2
Dnmt3a
Ncoa7
Myt1l
Camsap2
Scaf11
Clint1
Pdcd2l
Pik3ip1
9930021J03Rik
Cst3
Ip6k1
Uri1
Lgmn
Acd
Pold3
Bsg
Fth1
Itm2a
Ctla2a
Igfbp7
Slco1c1
Flt1
Esam
Slc16a1
Gpr116
Sparc
Cldn5
Egfl7
Slc2a1
Ptprb
Kdr
Ly6c1
Eltd1
Nostrin
Cd34
Cd93
Ramp2
Vwa1
Gsta4
Gng11
Ccdc141
Sepp1
BC028528

Gimap6
Col4a2
AU021092
Tmem252
Grap
Fn1
Tfrc
Slco1a4
Erg
Abcb1a
Car4
Sparcl1
Nrp1
Spock2
Eng
Ecscr
Robo4
Mfsd2a
Pltp
Tek
Id3
Col4a1
Ly6a
Vwf
Ablim1
Fzd6
Rasip1
Pecam1
Slc40a1
Epas1
Sox17
9430020K01Rik
Apln
Igfbp3
St3gal6
C130074G19Rik
Fam101b
Cgnl1
Slc7a5
Scgb3a1
Slc38a5
Slc39a8
Wwtr1
Ushbp1
Ptrf
Anxa3
Tmem204
Klf2

Slc3a2
Tmem88
Foxq1
Slc22a8
Lama4
Palmd
Pglyrp1
Slc9a3r2
Cdh5
Fxyd5
Myh9
Tdrp
Lsr
Fam129a
Synm
Ets1
Pdgfb
Slc38a3
Tsc22d1
Acvrl1
Abcg2
Srgn
Ctgf
Eogt
Fli1
Clec1a
Serpinb6b
Sptbn1
Arhgap29
Unc45b
Atox1
Ildr2
Id1
Nid1
Tie1
Gm20748
Dlc1
Cd151
Rbpms
Meox1
Emcn
Icam2
Foxf2
Lipa
Fgd5
Swap70
Slc7a1
Ahnak

Wfdc1
Clec14a
Hmgcs2
St8sia4
Anxa2
Serinc3
Slc39a10
Rasgrp3
Sgms1
Mmrn2
Ddc
Thsd1
Nampt
Cnn2
Gpr4
Vim
Gata2
Slco2b1
Limch1
Cdkn2b
Il2rg
Eva1b
Zic3
Ly6e
Serpinh1
Nfkbia
Arhgap18
Arl4a
Nos3
Apcdd1
Myo10
Itga1
F11r
Dock9
Tm4sf1
Hspg2
Edn1
Ocln
Tmsb4x
Paqr5
4931406P16Rik
Ctla2b
Nid2
Cd59a
Lamb1
Marcks
Sfrp1
Pde2a

Chst1
Abcc4
Lmo2
Cdkn1a
Angpt2
Lef1
Ifitm3
Itga6
Rgcc
Uaca
Egfl8
Fcgrt
Hsp90ab1
Kank3
She
Hspa12b
Gimap1
Plat
Ctsh
Plk2
Lgals9
Myl12a
Nfib
Rrbp1
Lamc1
Dok4
Ece1
S1pr1
Rhoj
Tagln2
Car2
Parvb
Rapgef5
Rgs12
Csrp2
CRE_RECOMBINASE
Dusp2
Arpc1b
Cd63
Rassf9
Sema3c
Htra3
Ly75
Rhoc
Notch1
Vamp5
Hmcn1
Apold1

Angptl4
Gstm7
Stap2
Kcp
Calm1
Ralb
Ppfibp1
Afap1l1
Basp1
Zic1
Mcam
Adcy4
Fas
Bambi
S100a13
B2m
Dusp3
Crmp1
Tbx3
Anxa7
Gpcpd1
Aplnr
Cyyr1
Nes
Scarf1
Ttyh2
Smtn
Hnrnpa2b1
Utrn
Slc30a1
Ckb
Cst3
Stab1
Igfbpl1
Slfn5
Marcksl1
Tns1
Nfix
Tpm4
Itgb1
Lrp8
Def6
Tcf7
Jag2
Myo1b
Prkch
Tiam1
Ccm2l

Dll4
Rtn1
Stmn3
Tspan13
Nfia
Map7
Draxin
Cpe
Tuba1a
Tubb5
Ackr3
Grrp1
Arhgef5
Cd24a
Fam212a
Ddah2
Vamp8
Slc35f2
Notch4
Kif26a
Tjp1
Fkbp10
Igfbp4
Hbegf
H2-D1
Plod1
Timp3
Rras
Abhd2
Rell1
Trim16
Acadl
Tubb6
Bex2
Il10rb
Msrb3
Ifnar2
Cd97
Cd200
Rgs5
Rnf144b
Tgfbr2
Actn4
Bcl6b
Klf6
Dennd3
Enpp2
Cyr61

Prom1
Arhgef15
Plxnd1
Sorbs2
Rgs3
Itga4
Pear1
Pde8a
Gja1
Gatm
Sult1a1
Mpzl1
Dcbld1
Chp2
Arhgap25
Lyn
Rapgef4
Aqp11
Elk3
Gria2
Celf2
E130114P18Rik
Necap2
Agrn
Slc31a1
Mfsd7c
Tgfb1i1
Marc2
Gatsl3
Anxa5
Crip2
Cast
Msn
Hhex
Slc46a3
Nnat
Sipa1
Gm6977
Pttg1ip
Ccdc85b
Rpl38
Megf6
Luzp1
Hnrnpab
Lrp10
Adm
Rgl1
Pcdh1

Chd7
Procr
Cog7
Ngfrap1
Aplp2
Cdh11
Shisa5
Cdk4
Slc16a9
S100a16
Calm2
Ppapdc2
Sorbs3
Insr
Pon2
Gnai2
Sypl
Tmsb10
Map4k2
Ttc3
Apbb2
Slc52a2
St6galnac2
Arap2
Myl6
Xaf1
Ostf1
Ccnd2
Podxl
Sdpr
Ctnna1
Slc16a4
Sgk1
Ppp2r2c
Abcg1
Gnb4
Ecm1
Plekhg5
Capg
Scg3
Cav1
Dpysl4
Tram2
Paqr7
Ankrd37
Gimap8
Gja4
Fermt2

Prcp
Foxc1
Rasgrp2
2900026A02Rik
Mecom
Tmc7
Atpif1
Pcp4l1
Miat
Maoa
Ttll7
Wwc2
Hnrnpdl
Anp32a
Gm9917
Inf2
Anp32e
Gap43
H2afv
Hexb
Casp6
Higd1b
Cdc42ep3
Macf1
Hrct1
Jak2
Gmpr
Lamb2
Cd38
Gm1673
Prex2
Prr5l
Prkd2
Loxl2
Tubb2b
Exoc3l
Filip1l
5730508B09Rik
Mfng
Itih5
Slc31a2
Arap3
Cxcl12
Lhx1
Tpm1
Rbfox3
Tes
Abca1

Ifnar1
Kank2
Sat1
Cbx5
Prnp
Cd9
Ldlrap1
Ptp4a3
Myl4
Psip1
Snrk
Piezo1
Tnfaip1
Rps9
Barhl1
Apod
Prdm1
Mapk12
Pmepa1
Neurod1
Phactr2
Ncam1
Spata6
Rap1b
Lcp1
Map2
Ccdc85a
Hn1
Id2
Ifi27
Tpx2
Arhgef12
Pde1c
Syt11
Pfn1
Tacc1
Tubb3
Ctsl
Psmb8
Arhgef28
Tm6sf1
Klhl5
Enc1
Bgn
Sept3
Snx3
Pdlim5
Apba2

Slc38a2
Fkbp3
Ubtd1
M6pr
Cpt1a
Gabarapl1
Isyna1
Meis1
Plekhg1
Cstb
Kif20b
Ezh2
Efna1
Soga3
Stmn2
Gm14005
Col4a3bp
Kif21a
Ank3
Fam13a
Mfge8
Fry
2810417H13Rik
Ndrg1
Slc6a6
Klf4
Jam2
Hspa1a
Cdc42ep2
Sash1
Tcn2
Clip1
Cyba
Ptprg
Alas1
Alad
Sft2d1
Ddx58
Mal
Tagln3
Dclk1
Zic4
Usp6nl
H2-K1
Cd2ap
Flnb
Slc7a8
Rhobtb1

Fxyd6
Ddx5
Hsd3b7
Hdac2
Prdx2
Vasp
Tsc22d3
Mxd4
Extl3
Fkbp1a
Serpinb9
Mdk
Gpm6a
Col1a2
Nsg2
Pvrl2
Wasf2
Fabp5
4632428N05Rik
Dynlt3
Ddx39b
Ina
Grb10
Hopx
Oxct1
Tenc1
Pax6
Stx3
Rab11a
Nudt14
Pou3f2
H2afy2
App
Cacng2
Cp
Cebpd
Flii
Cd320
Kctd12b
P4hb
Tfpi
Tprgl
Slc38a1
RP23-45G16.5
Lhfpl2
Cmtm6
Arrdc1
Hsp90aa1

Gstm1
Gm2694
Cntn2
1700025G04Rik
Rbp4
Adam10
Vps37b
Itsn2
Lnx2
Ptprm
Fgf9
Tjp2
Mdh1
Ddah1
Apoe
Ccdc34
Tspan9
Mycn
Ptpn18
Nhlh2
Casp8
Pcdh19
Nrxn1
Trf
Lphn2
Ppic
1500016L03Rik
Fcho2
Kctd10
Creb3l2
Ltbp4
Gpx1
Lxn
Tcf7l1
Gm17750
Entpd1
Ezr
Smad1
Gng3
Ppp1r13b
Itm2b
Iqgap1
Ctnnb1
Sfrs18
Rnd3
Grasp
Ctnnbip1
Pomp

Dusp6
Rgs4
Rac1
Lpar6
Usp22
Prrg2
Sh3bp5
Prkcdbp
Tecr
Nasp
Ift27
Pnn
Clmp
Maged1
H3f3b
Foxo1
Serpine2
Ptprs
Lpcat3
Net1
Slc1a2
Fam43a
Lhfp
Insm1
Abhd17a
D430041D05Rik
Lrrfip1
Junb
Racgap1
Cks1b
Tbx1
Hnrnpu
Nsg1
Srpr
Phf21a
Uncx
Mkl2
Lpp
Serf1
Tspan5
Myo1c
Gng5
Raph1
Orai1
Nrp2
Cenpv
Rsrc1
Fkbp9

Gpm6b
Cttnbp2nl
Prox1
Dcaf6
Litaf
Slc25a4
Ppp1r16b
Tbata
Plcb4
2700094K13Rik
Cxxc5
Rcan3
Elovl6
Mex3a
mt-Rnr1
Fam171b
Sox4
Hnrnph1
Nhlh1
Snrpd3
Adipor1
Hsd11b2
Tmem44
Kit
Fez1
Pdxk
Irf2
Tmpo
Eif4a1
Pitpnc1
Pea15a
Calm3
Phldb2
Hpca
Pxn
Rbfox2
Fam13c
Ilf2
Hmgn1
Cbx1
Arhgdia
Rilpl1
Lats2
Ppard
Ssbp3
Ltbr
Ets2
Actn1

Tspan12
Hes1
Slc16a2
Map3k1
H1f0
Clip3
Ybx1
Scg5
Elavl4
Msh2
Cmtm8
Fut9
Gbp7
Srebf1
Map1b
S100a11
Snap25
Pitpna
Degs1
Galnt18
Sf3b2
Map3k11
Cdk17
Cyb5r3
Gpr153
Pcm1
Top1
Fam63b
Triobp
Capns1
Fubp1
Glul
Whsc1
Trim2
Ldlrad3
Mef2a
Slc43a2
Cplx2
Apex1
Pfn2
Tnfrsf1a
Adam15
H1fx
Rnmt
Lrrc8c
Tecpr1
Ebf3
C1ql1

Mllt4
Cdh20
Anapc13
Kitl
Srsf3
Vtn
Myo1e
Hirip3
Mef2c
Myh10
Mcm7
H2afy
Plxna2
Myl9
Bcas1
Ppp1r14b
Celf4
Syngr2
2010111I01Rik
Eif2ak2
Metap2
Maff
Wls
Npm1
Arhgdib
Ccny
Fam210b
Pde4b
Cdk5r1
Dek
Hspe1
Mettl14
Dtymk
Fus
Rhoa
Dcx
Rassf4
Rest
Cpd
Rps26
Anxa6
Cdyl2
Homer2
Sptan1
Gnao1
Srgap1
Iqsec1
Srrm2

Nomo1
Chgb
Nipal3
Rnf44
Slk
Cr1l
Pcbp4
Myo5a
Pmp22
Nxpe4
Sstr2
Slc50a1
St18
Epn2
Ssh2
Stt3b
Hdgf
Rundc3a
Ptbp3
Ech1
Snrnp70
Pfkp
Dnajc3
Cdc42ep4
Rpl15
Serbp1
Stmn4
Zdhhc18
Amotl1
Kif5c
Sept4
Ube2e3
Khdrbs1
Polk
Thoc7
6330403K07Rik
Dtl
Elavl3
Rpl4
Lig1
St8sia3
Lsm2
Akap12
Sult4a1
Mast4
Mad2l2
Nmi
Sh3bgrl3

C530008M17Rik
Pcdhga9
Rai14
U2surp
Prdx6
As3mt
Idh3g
Bnip2
Fam65a
Tspan4
Rufy3
Gas5
Tln1
Bcl2
Pdgfra
Hnrnpr
Acat2
Gng2
Mcf2l
Cacna2d1
Elovl1
Pomc
Rgl2
Pde4dip
Bzw2
Hnrnpm
Supt16
Fam212b
Rapgef6
Kif5b
Dner
Sh3bp4
Srsf7
Shank3
Crim1
Crem
Rps5
Hip1
Ssrp1
Ano6
Hdac7
Nap1l1
Rnf165
Gpd2
Hnrnph2
Nol4
Bcl7a
Sh3gl2

Lactb
Ndufa12
Polr3d
Wdr1
Setd8
Elf1
Idh2
Tpd52
Zcchc18
Hipk1
Impdh1
Sh3glb1
Ptov1
Purb
Eif3i
Hnrnpd
Dut
Matr3
Dusp23
Plekhg2
Hdgfrp3
Cdipt
Rnf5
Psat1
Rnpep
Casp3
Ide
Celsr2
Smc2
Cds2
Rsu1
Sh2b3
Scarb1
Peli2
Dixdc1
Arpc2
Actb
Plec
Dnajb11
Clint1
H2afj
Slc29a1
Tuba1b
Gm26924
Prmt8
Ak3
Smarcc1
Cep110

Hmgb3
Abhd5
Ttc9b
Smc3
Camta1
Nrn1
Mfap4
Mrpl21
Htatsf1
Smpd3
Ip6k2
A330076H08Rik
Ctps
Yes1
Smarca2
Ndn
Podxl2
Btbd17
Zcchc11
Rab11fip2
Mapk8ip1
Srrm3
Pdap1
Ddx42
Gsg1l
Nop58
Snhg5
Ift74
Snap23
Prpf19
Ppp1r14c
Smc4
Itpr1
Sppl2a
Copz2
Cenpe
Itm2c
Creg1
Srpk2
Tpd52l1
Sema6d
Unc93b1
Aplp1
Sox9
Rela
Top2a
Lmo4
Stat3

Dnajc8
Eif4g2
Hmgb1
Yap1
Pabpc1
Ntrk3
Cct2
Smc5
Pald1
Nrip1
Ier5
Dhx9
Banf1
Gdpd1
Cenpf
Tra2b
Pwp1
Cbfa2t3
Ehd4
Hp1bp3
Myo9a
Otx2
Gm5620
Ptch2
Pcna
Park7
Robo2
Pebp1
Ilf3
Atp5a1
Elovl7
Rmnd5b
Dab2ip
Ginm1
Cdc20
Rhob
Kras
Asf1a
Snrpg
Per1
1110034G24Rik
Isoc1
Cflar
Skp1a
Hnrnpk
Myl12b
Rbms1
Zbtb18

Atad5
Kif1b
Cdkn1c
Olfm1
Pak3
Satb1
Tfam
Tmem66
Nfkb1
Wipi1
Glud1
Acox1
Ece2
Ahi1
Pdzrn3
N4bp3
Cdk6
Cdh4
Arid2
Mpnd
Igsf8
Txnip
Ranbp1
Vdac1
Slc22a17
Hsp90b1
Tubb2a
Gpn2
Hspd1
Hnrnpa0
Atp1a2
Fnta
Bcl11a
Trim56
Fmnl3
Lrrc59
Gm11266
Anp32b
Ddx39
Pim3
Psme4
2510003E04Rik
Trim33
AI854517
Prmt5
Dcakd
Nucks1
Nudcd2

Strbp
Ldhb
Foxp1
Rcor2
Myod1
Peg3
D030056L22Rik
Hnrnpa3
Atxn10
Zeb1
Cep78
Ywhae
Atl2
Atp6v1b2
Acaa2
Reep3
Tmod2
B230118H07Rik
Zbtb38
Phb2
Nop10
Slc16a6
Hypk
Wdr77
Cplx1
Tram1
Sacs
Ppp1r2
Brd3
Acin1
Mif
Gnb2l1
Epc2
Aph1a
Cnot6l
Cdk1
Bex1
Slc1a1
Safb
Rrm2
2610017I09Rik
Srsf2
Gse1
Cacybp
Wdr33
Mdga1
Pcdh17
Meaf6

Snw1
Arhgap31
Fau
Tmbim6
Nrcam
Hadha
Pqlc1
Amer2
Ccdc88a
Ccp110
Tnks1bp1
Vcl
Cnpy1
Rab7
Zfp36l1
Chd4
Akap6
Tdrkh
Reep1
D19Bwg1357e
Nell2
2810055G20Rik
Pkp4
Dzip3
Alkbh6
BC005764
Fam102b
1500012F01Rik
H3f3a
Cdkn1b
Sec62
Etfb
G2e3
Fmnl2
Slc35b3
Tcerg1
2610203C20Rik
Ube2c
Pygo1
Gpr56
Vcan
4933426M11Rik
Cecr2
Fcf1
Ccdc32
Gdpd5
2810474O19Rik
Ralgps2

Zfp329
Clvs1
Mtmr6
Luc7l3
Fam219b
Gsto1
Ank2
Tmem123
Syncrip
Ccnb2
Srsf10
Phf21b
Tox3
Clybl
Ybx3
Atrx
Bex4
Nrbp1
Qser1
Npdc1
Pdzrn4
Cbx3
Nr2c2
Gmeb1
Pnp
Naa38
Nedd4
Gm9800
Tsfm
Gpatch4
Slc1a4
Ikbkap
Pbx3
2900011O08Rik
Atf3
Cdc42
Tnpo3
Bcas2
Usp10
Tro
Sae1
Fam63a
Ckap2l
Calr
Nop56
Rpn1
Efs
Maged2

Cpsf6
Txnrd1
Mtus1
Sin3b
Fam69a
Erdr1
Tpst2
Fads1
Tmem50a
Tmcc3
Tpm3
Peo1
2810025M15Rik
Akap13
Rnpepl1
Zfr
Ptpn9
Dnph1
Casc5
Celsr3
Tceb1
Rab3a
Msrb2
Dnajc10
Neurod6
Tacc3
Gdap1
Xbp1
Dnajc9
Aldh2
Baiap2
Pop5
Eid1
Cd81
Lrig3
Hlf
Rbm28
Bcat1
H2afz
Elavl2
Slc10a7
Ndrg2
Hist3h2ba
Gprasp1
Bin1
Banp
Plekha1
Bmp2k

2310036O22Rik
Shmt2
Sox5
Brcc3
Wsb2
Ap3b2
Irs1
Dach1
Gm10075
Vegfb
Nrm
Rab34
Ltbp3
Nme4
Ncald
Rrp1b
Eif4b
Txndc9
Ppp3ca
H2afx
Mybbp1a
Cuedc2
Rplp0
Sdf2
Ube2b
Pfdn2
Dnmt1
Cenpq
Tspan18
Leprel4
Tm9sf3
Adk
Grsf1
Atp6v0e2
Fdx1l
Nfic
Brix1
Krt10
Mprip
Mcee
Exoc2
Tcp11l1
Esco2
Cltb
Nktr
Ing4
Plin2
Chmp6

Ypel1
Fam213b
Cenpm
Sox11
A930011O12Rik
Ccdc55
Cgrrf1
Psma1
Clasp1
Rin2
Rrm1
Rock2
Clic1
Cenpp
Mtf2
Fam129b
Rsl24d1
Epb4.1l3
Comt
Mapt
Lrrn1
1810058I24Rik
Gng12
Pafah1b2
Reln
Col9a3
Arpp21
Ptprd
Jakmip2
Josd2
Arhgap11a
Fez2
Tmem184b
Srrm4
Magt1
Stk25
Apbb1
Stk40
Mob1a
Mrpl40
Msi1
Nelfa
Arf3
Rbmx
Sirt7
Nrep
Tceal3
Pcbp2

1700001O22Rik
Elovl4
Eif2b3
Zfp292
Slc25a27
Pigk
Lims1
Rab6b
Gpsm1
Sgta
Nhp2
Abhd6
Hist3h2a
Stat1
Snrpf
Rabl6
Cul4a
Igf1r
Dazap1
Nbeal1
Lphn3
Kif4
Cirbp
Rfc5
Wdr82
Srgap2
Gli1
Coq9
Stag1
Hmgn2
Ankrd26
Snrpb2
Pou3f3
R3hdm1
Pold3
Ubqln2
Zfp637
Dpysl3
Hbs1l
Sfpq
Fam53a
Mfhas1
Ccdc53
Malat1
Fkbp7
Arl3
Atxn7
Arid5b

Zmym2
Clpp
Nckap1
Slc25a1
Tbc1d16
Fhl1
Rtn4
Dnaaf2
Smarca5
Pls3
Polr3k
Ophn1
Zbtb7a
Cdca7l
Zic5
Rsrc2
Xpo1
Yy1
Uba2
Slc25a20
Mab21l2
Tia1
Tmem98
Cpt2
Rnpc3
