# Supplementary material for: scRNA-seq in medulloblastoma shows cellular heterogeneity and lineage expansion support resistance to SHH inhibitor therapy
Source: Nat Commun. 2019 Dec 20;10:5829. doi: 10.1038/s41467-019-13657-6 (PMC6925218; doi:10.1038/s41467-019-13657-6)
Supplement: Supplementary file 6 — Supplementary Data 3 [file 41467_2019_13657_MOESM6_ESM.pdf]

Gene_ID	Gene_Loading	IC	Gene_ID	Gene_Loading	IC	Gene_ID	Gene_Loading
Mki67	-11971.43172	1	1700025G(	-2444.5426	2	Tubb3	-9617.152996
Top2a	-11879.54736	1	Clspn	-2477.19826	2	Stmn2	-8694.390497
Tpx2	-11053.14459	1	Mki67	-2493.07427	2	Neurod1	-8288.089934
Nusap1	-10911.50315	1	Birc5	-2557.191186	2	Tuba1a	-8286.856037
Prc1	-10881.22739	1	Hmgn5	-2624.092627	2	Gap43	-6915.397672
Birc5	-10843.48041	1	Ezh2	-2646.166671	2	Tex14	-6755.009882
Cdk1	-10677.59391	1	H2afx	-2667.345046	2	Cntn2	-6530.976424
Ube2c	-10658.19716	1	Cdk1	-2688.798551	2	Map1b	-6516.570926
Cenpf	-10597.69863	1	Top2a	-2721.804482	2	Tmsb4x	-6341.147279
Hmgb2	-10170.3448	1	Pbk	-2739.312725	2	Rtn1	-5979.214788
Cdca8	-10153.52037	1	RP23-45G1	-2745.280926	2	Calm2	-5871.551944
Kif11	-10091.31406	1	Spc24	-2793.879656	2	Tubb2b	-5848.247894
Smc2	-10019.16705	1	Rrm2	-2819.054878	2	Nhlh2	-5832.732372
Kif23	-9836.102543	1	Hells	-2823.580392	2	Nhlh1	-5812.681903
Spc25	-9816.479626	1	Myod1	-2888.118415	2	Ube2c	-5588.542887
Incenp	-9795.308835	1	Sox18	-2934.5016	2	Ina	-5257.805444
Smc4	-9769.673857	1	Lig1	-2961.175864	2	Cdc20	-5223.263287
Cdca3	-9768.681575	1	Rassf4	-2973.732622	2	Stmn4	-5202.93228
Ckap2l	-9753.00463	1	Prmt8	-3074.785958	2	Cenpa	-5178.132105
H2afx	-9732.164282	1	Gm17322	-3084.687932	2	St18	-5020.828302
Cenpa	-9431.504957	1	Lhx1	-3123.356911	2	Gng3	-5010.175744
Hmmr	-9382.727136	1	Rbp4	-3124.40703	2	Tubb5	-4975.678838
Pbk	-9363.787653	1	Hmgb2	-3174.623007	2	Ccnb1	-4936.49695
2810417H1	-9345.19343	1	Dek	-3294.941125	2	sept4	-4927.753895
Cenpe	-9196.207901	1	Pde1c	-3331.50318	2	Mllt11	-4904.766292
Casc5	-8870.808036	1	Ccnd1	-3354.152429	2	Elavl4	-4885.157873
Mis18bp1	-8855.663222	1	Smc4	-3385.326944	2	Tagln3	-4817.296395
Cdc20	-8853.130489	1	Igfbpl1	-3466.232609	2	Miat	-4811.309139
Esco2	-8843.820683	1	Mcm6	-3524.8178	2	Arl6ip1	-4781.089559
Tacc3	-8806.398984	1	E130114P1	-3545.046756	2	Ckb	-4710.431208
Ncapg	-8762.032736	1	Hsd11b2	-3650.044021	2	Cdk5r1	-4674.643612
Ccnb1	-8546.663514	1	Tbata	-3653.299635	2	Gpm6a	-4507.086413
Spc24	-8476.614771	1	Nnat	-3722.462187	2	Tubb2a	-4439.43296
Sgol2	-8451.346116	1	Smc2	-3863.266426	2	Elavl3	-4415.190764
Aspm	-8192.915424	1	Fam210b	-3897.703897	2	Nrxn1	-4356.19469
RP23-45G1	-8033.934784	1	Pcna	-3899.226964	2	Calm1	-4321.947964
Kif15	-7963.022105	1	Mroh2a	-3951.379556	2	Pdzrn3	-4317.214535
Kif20b	-7890.635047	1	Anp32e	-3985.42846	2	Nnat	-4282.814265
Ccnb2	-7849.077038	1	2810417H1	-3999.940787	2	Hmmr	-4204.708395
Arhgap11a	-7745.326076	1	Hey1	-4027.406285	2	Ppp1r14c	-4204.382698
Knstrn	-7696.144042	1	CRE_RECON	-4030.468115	2	Sept3	-4189.021459
Dek	-7320.5881	1	Eef1a1	-4101.307641	2	Pde1c	-4177.621637
Dlgap5	-6825.790013	1	Dut	-4106.828342	2	Rab3a	-4129.598382
Arl6ip1	-6593.878835	1	Mdk	-4345.422263	2	Ckap2l	-4005.851736
Anp32e	-6592.063146	1	Srebf1	-4503.601022	2	Fxyd6	-3989.549054
Mns1	-6195.93851	1	Pabpc1	-4633.23606	2	Trpc4ap	-3964.65779
Cenph	-6151.660822	1	Rps26	-4910.314743	2	Uncx	-3893.203496

Ccdc34	-6118.950214	1	Rplp1	-5306.026882	2	Celf4	-3891.205532	
Rrm2	-5854.586018	1	Sfrp1	-5536.223589	2	Apc	-3869.469518	
Ckap5	-5642.061088	1	Ccnd2	-5630.297843	2	Podxl2	-3859.699508	
Rbfox3	2629.899218	1	Celf4	6872.872473	2	Fosb	2868.08776	
Myt1	2632.295495	1	Neurod1	6870.87533	2	Hey1	2892.932362	
Dcx	2640.280455	1	Gpm6a	6683.712419	2	Rps26	2893.424828	
Jhdm1d	2643.527088	1	Mapt	6307.141973	2	Fam210b	2906.190485	
Stmn4	2686.338304	1	Rtn1	6018.027347	2	Nexn	2909.001474	
Kif5c	2689.762142	1	Arpp21	5827.164329	2	Dek	2933.100696	
Cdk5r1	2736.741577	1	Dpysl3	5815.992159	2	Ier2	2945.680855	
Gap43	2744.266129	1	Nrxn1	5670.581019	2	Lama5	2987.95228	
Chrnb4	2755.581401	1	Mtss1	5522.716974	2	Rif1	3022.462048	
Chgb	2763.656666	1	Tubb2a	5518.484332	2	Adamts1	3037.491771	
Igsf8	2767.117561	1	Ppp1r14c	5439.416221	2	Efhd2	3040.358565	
Rab3a	2832.985062	1	Sept3	5419.356752	2	Wnt4	3048.79197	
Bin1	2881.838236	1	Thra	5402.620111	2	Olig2	3111.490414	
Uncx	2897.392541	1	Ank2	5269.370377	2	Sparcl1	3179.000114	
1500016L(	2900.535026	1	Rbfox3	5225.918537	2	Xist	3228.372247	
S100a16	2910.820819	1	Nrep	5160.435885	2	Rbp4	3242.803509	
Nhlh2	2956.013681	1	Elmo1	5150.980132	2	Ier5	3255.444817	
Tubb2b	2966.054323	1	Meg3	5126.403489	2	Pdlim4	3298.318791	
Nrn1	3008.032668	1	Stmn4	5007.257421	2	Tbata	3340.408267	
Dlgap4	3010.8637	1	Stmn2	4927.474449	2	Sox1	3371.828955	
Tex14	3123.706538	1	Atp2b1	4830.405693	2	Sept8	3422.769308	
A330076H(	3145.242031	1	Gria2	4814.383155	2	Gas6	3532.67052	
Gm17322	3158.942561	1	Plxna2	4688.439917	2	Nes	3556.604519	
Tuba1a	3159.503149	1	Neurod2	4651.094625	2	Irs1	3589.094076	
Kdm5b	3188.058868	1	Jph4	4636.819048	2	Cacng5	3592.646033	
Neurod1	3205.597897	1	L1cam	4581.476147	2	Dclk1	3728.239186	
Lhx1	3237.662761	1	Celf2	4569.727375	2	Sdpr	3754.911541	
Gng3	3330.935489	1	Dusp26	4550.010291	2	Jun	3834.903284	
Elavl3	3337.864733	1	Sez6	4549.444727	2	Pou3f2	3927.254622	
Rtn1	3366.59368	1	Car10	4477.095808	2	Lap3	3932.285133	
BC005764	3403.626107	1	Ankrd12	4462.984559	2	Pcna	3935.02244	
Sept4	3475.165774	1	Sv2a	4383.374922	2	Pdlim3	3964.199009	
Mllt11	3489.351125	1	Gnao1	4298.035368	2	Hells	4009.978124	
Elavl4	3491.129864	1	Dner	4252.838741	2	Lig1	4025.358241	
Pde1c	3532.28799	1	Map1b	4248.692664	2	Ung	4096.155273	
Ina	3550.54752	1	Zfpm2	4243.701113	2	Prmt8	4147.113655	
Nrep	3829.461351	1	Cntn2	4190.519432	2	Rprml	4252.00122	
Miat	4005.801096	1	Tnik	4181.346587	2	Pdgfa	4258.616532	
6330403K(	4040.352137	1	Kcnk1	4139.20094	2	Cltb	4441.79928	
Aldoa	4159.978333	1	Epb4.1l1	4116.962245	2	Hsd11b2	4500.21898	
CRE_RECON	4352.807726	1	Cadm2	4110.492323	2	Rplp1	4610.131426	
Chrna3	4406.603627	1	Gap43	4078.324717	2	Zfp36l1	4655.080902	
Cntn2	4502.59998	1	Calm1	4045.430444	2	Sowaha	4709.12134	
Nhlh1	4557.76745	1	2900079G:	4029.323441	2	Egr1	4821.754465	
Stmn2	4754.216413	1	Ndst3	4016.286984	2	Srebf1	5088.222944	

Pdzrn3	4904.244313	1	Cygb	4016.190549	2	Gpr153	5223.538593
Tmsb4x	5191.281738	1	Cadm3	4014.083398	2	Mcm6	5289.30825
Igfbpl1	5288.386884	1	Cadps2	3980.735545	2	Sox9	5677.462661
Tubb3	5797.964089	1	Pcsk1n	3976.300404	2	Ccnd1	6850.528738
Ckb	7014.042823	1	Gng3	3968.646474	2	Hes1	7027.729924

IC	Gene_ID	Gene_Loading	IC
3	Cenpa	-5473.991796	4
3	Ccnb2	-5031.499298	4
3	Pttg1	-4988.211177	4
3	Cdc20	-4782.242217	4
3	Ube2c	-4753.859159	4
3	Eef1a1	-4399.750713	4
3	Vim	-4358.502268	4
3	Pabpc1	-3934.815331	4
3	Rps26	-3899.481461	4
3	Knstrn	-3829.493632	4
3	Ccnb1	-3790.376639	4
3	Cdca3	-3650.886841	4
3	Hes1	-3478.534186	4
3	Sparcl1	-3219.243954	4
3	Lgals1	-3210.856365	4
3	Rplp1	-3111.194245	4
3	Pdlim4	-2976.668132	4
3	Pdlim3	-2974.846498	4
3	Tmsb4x	-2903.822338	4
3	Cenpe	-2869.637596	4
3	Hpca	-2856.88711	4
3	H2afx	-2837.946498	4
3	Sowaha	-2772.633003	4
3	Rbp4	-2771.445866	4
3	Birc5	-2723.976573	4
3	Ccnd1	-2694.677378	4
3	Cenpf	-2676.058254	4
3	RP23-45G1	-2660.408504	4
3	Tbata	-2649.203255	4
3	Ckap2l	-2607.668093	4
3	Tpx2	-2600.519183	4
3	Hmmr	-2586.411714	4
3	Gm2694	-2551.933993	4
3	Dlgap5	-2511.407147	4
3	Id2	-2463.828071	4
3	Hist1h2ak	-2458.351599	4
3	Olig2	-2452.013593	4
3	Calm1	-2431.13799	4
3	Arl6ip1	-2381.236835	4
3	Gas6	-2332.633946	4
3	Pdzrn4	-2214.462586	4
3	Lap3	-2182.782517	4
3	Cep89	-2182.132784	4
3	Sdpr	-2161.278571	4
3	Olig1	-2129.267724	4
3	Kif23	-2044.21792	4
3	Hmgb2	-1993.849734	4

3	Cdca8	-1993.826633	4
3	Efhd2	-1962.939708	4
3	Cdk1	-1953.456075	4
3	Bzrap1	3199.914889	4
3	Srrm3	3205.801362	4
3	Pdzrn3	3206.470204	4
3	Gap43	3211.910927	4
3	Chrna3	3243.972974	4
3	mt-Rnr1	3314.381603	4
3	Ppp3ca	3314.620983	4
3	A930011O	3328.25625	4
3	BC005764	3343.969142	4
3	Sept4	3345.881205	4
3	Frmd4a	3365.649617	4
3	Ptprd	3372.974653	4
3	Pax6	3376.107415	4
3	2700081O	3423.49447	4
3	Nktr	3460.9905	4
3	Nrn1	3467.136101	4
3	Slc17a6	3479.669817	4
3	Map1b	3479.678073	4
3	Celsr2	3491.548478	4
3	Sema6a	3529.643713	4
3	Ankrd11	3564.167678	4
3	Dcx	3581.984767	4
3	Sptbn1	3595.344856	4
3	Apc	3606.0844	4
3	Ebf3	3622.342122	4
3	Chd7	3640.835884	4
3	Elavl2	3670.72644	4
3	Syt11	3717.685781	4
3	Neurod1	3790.900393	4
3	Myt1	3793.00858	4
3	Gse1	3806.240465	4
3	Podxl2	3815.271058	4
3	Reln	3860.730872	4
3	Lhx1	4049.374128	4
3	Clmp	4147.440945	4
3	Pde1c	4276.910275	4
3	Insm1	4440.489788	4
3	Elavl3	4450.023101	4
3	Celf2	4466.598147	4
3	Bin1	4474.053116	4
3	Igfbpl1	4507.409701	4
3	Gria2	4571.22659	4
3	Cacna2d1	4648.139359	4
3	Ank3	4691.475296	4
3	Map2	4723.782726	4

3	Xist	5059.270044	4
3	Cntn2	5150.154853	4
3	Nhlh2	5655.175992	4
3	Ptprs	6301.521569	4
3	Miat	7691.477154	4
