# Supplementary material for: scRNA-seq in medulloblastoma shows cellular heterogeneity and lineage expansion support resistance to SHH inhibitor therapy
Source: Nat Commun. 2019 Dec 20;10:5829. doi: 10.1038/s41467-019-13657-6 (PMC6925218; doi:10.1038/s41467-019-13657-6)
Supplement: Supplementary file 7 — Supplementary Data 4 [file 41467_2019_13657_MOESM7_ESM.pdf]

gene	p_val	avg_logFC	pct.In_Sox2_Pop	pct.AllOthers	p_val_adj
Sox2	0	1.930036	1	0	0
Fabp7	5.6E-188	2.283031	0.374	0.11	9.3E-184
Dbi	1.7E-157	1.049306	0.708	0.45	2.9E-153
Slc1a3	5.2E-137	1.421355	0.327	0.144	8.7E-133
Olig1	8.9E-131	1.360579	0.352	0.066	1.5E-126
Gpr37l1	8.5E-127	1.113887	0.235	0.02	1.4E-122
Ptprz1	1.7E-118	1.154132	0.245	0.026	2.8E-114
Scrg1	1.4E-117	0.932002	0.209	0.016	2.3E-113
Serpine2	1.4E-112	1.420294	0.279	0.046	2.3E-108
Bcan	2E-111	1.022319	0.279	0.041	3.3E-107
Cspg5	8.5E-103	1.24908	0.206	0.02	1.42E-98
Ramp1	2.6E-100	0.852691	0.2	0.019	4.4E-96
Ednrb	5.6E-100	1.240174	0.195	0.021	9.35E-96
Olig2	1E-98	0.968456	0.316	0.066	1.66E-94
Sparcl1	1.84E-90	1.206161	0.439	0.195	3.06E-86
Neurod1	9.78E-90	-1.37984	0.334	0.542	1.63E-85
Sirt2	2.44E-89	1.018936	0.287	0.149	4.07E-85
Ppap2b	4.66E-89	0.999632	0.224	0.034	7.78E-85
Gjc3	4.53E-85	0.736946	0.139	0.008	7.56E-81
Stmn2	8.3E-85	-1.1098	0.32	0.564	1.38E-80
3110035E1	1.11E-84	1.010172	0.169	0.016	1.85E-80
Timp4	1.71E-83	0.679285	0.146	0.01	2.85E-79
Bcas1	4.97E-82	0.804233	0.422	0.275	8.29E-78
S100a1	9.15E-82	0.831888	0.174	0.019	1.53E-77
Kcnj10	5.56E-81	0.876115	0.168	0.018	9.28E-77
Slc35f1	2.49E-80	0.596622	0.144	0.011	4.15E-76
Plp1	2.67E-80	1.431722	0.211	0.109	4.45E-76
Tril	2.87E-80	0.735137	0.171	0.018	4.79E-76
Cnp	1.96E-79	1.132199	0.184	0.026	3.26E-75
Cst3	1.64E-78	0.785195	0.724	0.526	2.73E-74
Mt1	5.19E-78	1.12706	0.349	0.159	8.66E-74
Omg	2.42E-76	0.440862	0.114	0.006	4.04E-72
S100b	3.87E-73	0.876174	0.201	0.032	6.46E-69
Fyn	4.72E-73	0.815644	0.338	0.209	7.87E-69
S100a13	3.88E-72	0.854515	0.175	0.023	6.48E-68
Sox10	8.07E-72	0.636146	0.125	0.009	1.35E-67
Nfib	5.98E-71	-0.47242	0.862	0.937	9.97E-67
Cacng4	6.09E-69	0.968544	0.296	0.092	1.02E-64
Cmtm5	5.33E-67	0.486631	0.132	0.012	8.9E-63
Ttyh1	7.07E-67	0.945421	0.165	0.025	1.18E-62
Tsc22d4	9.87E-66	0.784298	0.368	0.156	1.65E-61
Pllp	8.28E-65	0.6281	0.122	0.01	1.38E-60
Spon1	1.55E-63	0.429457	0.116	0.009	2.59E-59
Gpr17	3.32E-63	0.788403	0.12	0.01	5.53E-59
Cntn1	4.55E-63	0.814974	0.205	0.043	7.58E-59
Tubb3	4.03E-62	-0.96387	0.272	0.484	6.72E-58
Aqp4	4.56E-62	1.628615	0.102	0.021	7.6E-58

Apoe	8.8E-62	1.15054	0.385	0.241	1.47E-57
Scd2	1.06E-61	0.673057	0.515	0.286	1.76E-57
Rgcc	4.54E-61	0.558433	0.12	0.011	7.58E-57
Atp1a2	4.83E-61	1.198687	0.207	0.051	8.05E-57
Clu	5.33E-60	0.76301	0.131	0.014	8.9E-56
Miat	2.47E-59	-0.86873	0.321	0.525	4.12E-55
Gatm	2.67E-59	0.537779	0.169	0.028	4.45E-55
Ptpre	3.57E-59	0.622464	0.121	0.011	5.96E-55
Lsamp	3.74E-58	0.776192	0.248	0.069	6.24E-54
Vim	5.35E-58	0.645699	0.551	0.29	8.92E-54
Cd9	9.56E-58	0.53772	0.672	0.475	1.59E-53
Slc4a4	5.89E-57	1.169035	0.119	0.036	9.83E-53
Sulf2	2.01E-56	0.590939	0.134	0.016	3.36E-52
Ptn	3.57E-56	0.661534	0.608	0.433	5.96E-52
Luzp2	4.37E-56	0.387668	0.116	0.012	7.28E-52
Basp1	1.49E-55	-0.51332	0.646	0.707	2.48E-51
Aldoc	2.39E-54	1.040833	0.128	0.027	3.98E-50
Zic1	6.31E-54	-0.50041	0.706	0.788	1.05E-49
Mt3	8.18E-54	0.596474	0.139	0.02	1.36E-49
Tmem176b	1.37E-52	0.672733	0.291	0.1	2.28E-48
Zcchc24	1.97E-52	0.449114	0.141	0.022	3.28E-48
Pde4b	2.58E-52	0.397951	0.188	0.044	4.31E-48
Cd81	3.46E-52	0.517257	0.655	0.452	5.77E-48
Itpr2	3.62E-52	0.628099	0.147	0.024	6.04E-48
Igfbpl1	3.72E-52	-0.60654	0.493	0.655	6.21E-48
Asrgl1	1.04E-51	0.648901	0.324	0.124	1.73E-47
Nhlh2	1.62E-51	-0.79539	0.258	0.431	2.7E-47
Glul	2.4E-51	0.627785	0.313	0.195	4.01E-47
Ncam2	5.75E-51	0.391809	0.111	0.012	9.59E-47
Ddah1	2.1E-50	0.5513	0.166	0.033	3.5E-46
Tspan7	3.39E-50	0.705577	0.278	0.097	5.66E-46
Ugt8a	5.25E-50	0.495295	0.108	0.012	8.75E-46
Map1b	8.81E-50	-0.67396	0.461	0.593	1.47E-45
Nfix	2.35E-49	-0.45415	0.614	0.676	3.91E-45
Crmp1	5.91E-49	-0.50527	0.542	0.674	9.86E-45
Enpp2	1.65E-48	0.664862	0.146	0.026	2.75E-44
Id3	1.7E-48	1.070847	0.124	0.027	2.83E-44
Ppfibp1	3.01E-48	0.551675	0.149	0.027	5.02E-44
Ina	7.12E-48	-0.72914	0.288	0.451	1.19E-43
Celf2	9.31E-48	-0.51985	0.574	0.641	1.55E-43
Tagln2	9.5E-47	0.520468	0.172	0.038	1.58E-42
Tmem176a	5.03E-46	0.389055	0.101	0.011	8.39E-42
Gng12	1.26E-44	0.493486	0.3	0.111	2.11E-40
Phyhipl	1.81E-44	0.555871	0.192	0.055	3.03E-40
Tmsb10	2.61E-44	-0.45632	0.578	0.668	4.36E-40
Chd7	2.62E-44	-0.4566	0.586	0.65	4.38E-40
Arl4a	3.48E-44	0.412502	0.202	0.057	5.8E-40
Slc6a1	1.32E-43	0.369404	0.125	0.022	2.2E-39

Nrep	2.93E-43	-0.57062	0.486	0.573	4.9E-39
AW04773C	3.87E-43	0.523434	0.12	0.019	6.46E-39
Gpm6b	6.25E-43	0.47094	0.625	0.477	1.04E-38
Mbp	7.38E-43	0.847035	0.222	0.162	1.23E-38
Phlda1	1.07E-42	0.43268	0.125	0.021	1.78E-38
Tspan3	2.44E-42	0.446616	0.639	0.417	4.06E-38
Npas3	9.48E-42	0.441303	0.127	0.022	1.58E-37
Rtn1	2.29E-41	-0.4532	0.696	0.69	3.82E-37
Elavl3	4.87E-41	-0.55224	0.379	0.477	8.12E-37
Hes1	5.94E-41	0.484319	0.327	0.139	9.9E-37
Cntn2	6.12E-41	-0.9437	0.109	0.271	1.02E-36
Sox8	8.08E-41	0.451463	0.129	0.025	1.35E-36
Gap43	1.4E-40	-0.60706	0.516	0.603	2.34E-36
Hip1	1.55E-40	0.41018	0.28	0.109	2.58E-36
Degs1	2.21E-40	0.507742	0.22	0.069	3.69E-36
Metrn	3.48E-40	0.316129	0.109	0.017	5.8E-36
Sept3	3.58E-40	-0.63492	0.296	0.397	5.98E-36
Klhl5	6.1E-39	0.43492	0.149	0.033	1.02E-34
Gm2a	8.25E-39	0.36518	0.162	0.042	1.38E-34
Mt2	1.04E-38	0.763552	0.202	0.086	1.73E-34
Mgst1	2.1E-38	0.349025	0.132	0.028	3.5E-34
Ddah2	2.28E-38	-0.45027	0.612	0.689	3.8E-34
Sept7	5.43E-38	0.377288	0.766	0.58	9.05E-34
Mmd2	1.04E-37	0.518268	0.146	0.034	1.74E-33
Rhoc	1.18E-37	0.316405	0.12	0.023	1.96E-33
Eps8	1.36E-37	0.291554	0.121	0.027	2.27E-33
Ank3	1.58E-36	-0.47759	0.378	0.422	2.63E-32
Calm2	2.54E-36	-0.29914	0.855	0.875	4.23E-32
Gnb4	3.89E-36	0.459742	0.255	0.097	6.49E-32
S100a6	6.6E-36	0.366612	0.102	0.018	1.1E-31
Sash1	6.69E-36	0.354279	0.133	0.03	1.12E-31
Lhx1	6.94E-36	-0.57632	0.358	0.504	1.16E-31
Cspg4	7.16E-36	0.527226	0.101	0.016	1.19E-31
Ppp2r2c	9.1E-36	-0.49997	0.394	0.51	1.52E-31
Sparc	9.59E-36	0.784304	0.224	0.078	1.6E-31
Rprm	1.16E-35	0.355434	0.131	0.029	1.93E-31
Qpct	1.29E-35	0.374749	0.105	0.017	2.15E-31
Map2	1.58E-35	-0.37641	0.487	0.492	2.64E-31
Celf4	3.34E-35	-0.79433	0.192	0.326	5.57E-31
1810037I1	4.4E-35	0.402201	0.378	0.187	7.33E-31
Limch1	9.94E-35	0.35413	0.108	0.02	1.66E-30
Atp1b2	1.24E-34	0.555869	0.151	0.042	2.07E-30
Cryab	1.3E-34	0.386087	0.105	0.018	2.16E-30
Tcf4	2.54E-34	-0.28675	0.929	0.924	4.23E-30
Tuba1a	2.76E-34	-0.36183	0.924	0.929	4.6E-30
Dnm3	2.97E-34	0.42033	0.112	0.021	4.95E-30
Cog7	5.03E-34	-0.44837	0.473	0.589	8.39E-30
Gria3	5.73E-34	0.457246	0.226	0.082	9.56E-30

Cd24a	1.24E-33	-0.41898	0.516	0.64	2.07E-29
Acot1	1.26E-33	0.357795	0.141	0.035	2.11E-29
Nr3c1	2.03E-33	0.298458	0.267	0.118	3.38E-29
Rufy3	4.55E-33	-0.47634	0.302	0.375	7.59E-29
Ccnd2	6.2E-33	-0.39431	0.628	0.663	1.03E-28
Pax6	6.74E-33	-0.4697	0.432	0.517	1.12E-28
Chpt1	6.88E-33	0.333723	0.172	0.054	1.15E-28
Ttc3	8.63E-33	-0.3111	0.827	0.829	1.44E-28
Kif5c	1.3E-32	-0.52144	0.315	0.402	2.16E-28
Apc	2.81E-32	-0.39949	0.412	0.432	4.68E-28
Cdh13	1.2E-31	0.374498	0.149	0.041	2.01E-27
B2m	2.82E-31	0.332843	0.408	0.222	4.71E-27
Kank1	4.71E-31	0.289182	0.125	0.03	7.86E-27
Mfge8	2.76E-30	0.44287	0.122	0.029	4.61E-26
Tmbim6	3.4E-30	0.287997	0.508	0.315	5.67E-26
Cdkn1b	4.15E-30	-0.25488	0.475	0.466	6.92E-26
Pcdh17	7.27E-30	0.336517	0.109	0.024	1.21E-25
Stmn4	7.57E-30	-0.65433	0.228	0.347	1.26E-25
Uchl1	1.69E-29	-0.41055	0.294	0.336	2.81E-25
Epn2	2.63E-29	0.457268	0.206	0.078	4.38E-25
Soga3	3.44E-29	-0.29031	0.464	0.457	5.74E-25
Tspan6	3.73E-29	0.317734	0.412	0.237	6.22E-25
CRE_RECOM	5.78E-29	-0.37526	0.661	0.773	9.63E-25
Jam2	6.99E-29	0.253953	0.115	0.029	1.17E-24
Cdk5r1	9.45E-29	-0.55332	0.24	0.338	1.58E-24
Dlgap1	9.61E-29	0.265813	0.167	0.062	1.6E-24
Wls	1.2E-28	0.357276	0.209	0.081	2E-24
Slc29a1	1.38E-28	-0.40705	0.469	0.566	2.3E-24
Lamp1	1.52E-28	0.388774	0.514	0.327	2.53E-24
Ndrg2	1.59E-28	0.427932	0.335	0.177	2.65E-24
Tpm1	1.74E-28	0.439935	0.334	0.183	2.9E-24
Rab31	2.3E-28	0.316379	0.154	0.05	3.83E-24
Ncald	2.47E-28	0.383019	0.313	0.158	4.11E-24
Nap1l5	3.61E-28	0.501442	0.199	0.075	6.03E-24
Scamp2	5.01E-28	0.316997	0.273	0.128	8.36E-24
Spry2	4.12E-27	0.369553	0.146	0.044	6.87E-23
Add3	4.99E-27	0.294892	0.224	0.098	8.32E-23
Pde1c	6.32E-27	-0.47982	0.326	0.389	1.05E-22
Zfand5	7.61E-27	-0.31115	0.405	0.416	1.27E-22
D4Wsu53e	8.05E-27	-0.26448	0.462	0.446	1.34E-22
Ypel3	9.62E-27	-0.27104	0.434	0.435	1.6E-22
Dcx	1.18E-26	-0.49688	0.284	0.38	1.97E-22
Trib2	1.54E-26	0.273143	0.199	0.082	2.56E-22
Klc1	1.91E-26	-0.30242	0.358	0.361	3.19E-22
1500016L0	2.33E-26	-0.52432	0.261	0.389	3.89E-22
Apba2	3.56E-26	-0.32913	0.379	0.416	5.94E-22
Cadm2	4.23E-26	0.317856	0.132	0.043	7.05E-22
Ptprs	4.98E-26	-0.4078	0.388	0.422	8.31E-22

Mtss1	5.9E-26	-0.45934	0.222	0.271	9.84E-22
Dab1	7.12E-26	0.296712	0.112	0.029	1.19E-21
Tlcd1	7.68E-26	0.305893	0.101	0.023	1.28E-21
Stmn3	9.51E-26	-0.3302	0.585	0.623	1.59E-21
Sh3d19	1.93E-25	0.316227	0.118	0.031	3.23E-21
Camta1	2.17E-25	-0.3129	0.389	0.408	3.62E-21
Fxyd6	3.8E-25	-0.26602	0.524	0.508	6.34E-21
S100a16	5.14E-25	0.519419	0.212	0.088	8.57E-21
Pcsk1n	5.41E-25	0.406288	0.189	0.078	9.02E-21
Cd302	6.54E-25	0.289444	0.144	0.048	1.09E-20
Ccnd1	6.77E-25	0.275829	0.695	0.513	1.13E-20
Pdlim3	7.13E-25	0.336597	0.16	0.056	1.19E-20
St18	7.65E-25	-0.57077	0.112	0.194	1.28E-20
Marc2	9.59E-25	0.297069	0.191	0.078	1.6E-20
Hsd17b12	9.87E-25	0.316062	0.333	0.18	1.65E-20
Abcd3	1.86E-24	0.267649	0.319	0.174	3.11E-20
Tubb2b	2.1E-24	-0.3172	0.462	0.464	3.51E-20
Uncx	2.38E-24	-0.5263	0.212	0.323	3.98E-20
Adam9	4.02E-24	0.294651	0.144	0.048	6.71E-20
Mllt11	4.25E-24	-0.49561	0.151	0.221	7.08E-20
Epb4.1l2	5.06E-24	0.420458	0.195	0.087	8.45E-20
Gas6	5.42E-24	0.274843	0.181	0.076	9.04E-20
Bin1	5.55E-24	-0.38725	0.38	0.431	9.26E-20
Nes	6.73E-24	0.287382	0.191	0.081	1.12E-19
Hsp90b1	7.35E-24	0.301275	0.819	0.685	1.23E-19
Acadl	7.39E-24	0.355029	0.165	0.059	1.23E-19
Sec11c	7.66E-24	0.277063	0.355	0.205	1.28E-19
Nnat	1.07E-23	-0.34326	0.7	0.71	1.78E-19
BC005764	2.75E-23	-0.58008	0.087	0.19	4.59E-19
Glud1	4.23E-23	0.380093	0.264	0.135	7.06E-19
Rrbp1	4.89E-23	0.324843	0.326	0.181	8.15E-19
Angpt1	5.11E-23	0.266099	0.129	0.043	8.53E-19
Mab21l1	5.64E-23	-0.47377	0.151	0.229	9.41E-19
Laptm4b	7.33E-23	0.303361	0.267	0.135	1.22E-18
Dusp6	1.06E-22	0.311148	0.202	0.087	1.76E-18
Adcyap1r1	1.22E-22	0.256164	0.181	0.078	2.03E-18
Chgb	1.5E-22	-0.63987	0.132	0.246	2.5E-18
Zeb2	1.64E-22	0.262283	0.395	0.241	2.74E-18
Itsn1	3.4E-22	-0.41808	0.193	0.238	5.67E-18
Rnmt	3.75E-22	-0.3829	0.295	0.351	6.26E-18
Ppp3ca	4.24E-22	-0.28885	0.379	0.378	7.08E-18
Rap2a	4.31E-22	0.310931	0.301	0.163	7.19E-18
Vcan	4.82E-22	0.385213	0.254	0.123	8.04E-18
Cxxc5	6.14E-22	-0.26602	0.528	0.544	1.02E-17
Atp1b3	6.47E-22	-0.25431	0.339	0.336	1.08E-17
Podxl2	6.84E-22	-0.48153	0.151	0.231	1.14E-17
Gm17750	8.67E-22	-0.41044	0.311	0.369	1.45E-17
Mex3a	1E-21	-0.31761	0.431	0.465	1.67E-17

Pea15a	1.12E-21	0.463037	0.275	0.171	1.86E-17
Mtap	1.17E-21	0.256921	0.199	0.089	1.95E-17
Rbfox2	1.19E-21	-0.36624	0.245	0.285	1.98E-17
Nsg2	1.32E-21	-0.30775	0.441	0.489	2.21E-17
Nfasc	1.45E-21	0.384378	0.104	0.032	2.41E-17
Tex14	2.41E-21	-0.57536	0.055	0.15	4.02E-17
Arl6ip1	2.73E-21	0.352564	0.692	0.542	4.55E-17
Mpzl1	2.91E-21	0.360212	0.238	0.139	4.86E-17
H2afy2	3.35E-21	-0.25512	0.332	0.338	5.58E-17
Ank2	3.62E-21	-0.39907	0.216	0.241	6.04E-17
Canx	4.19E-21	0.252005	0.749	0.588	6.99E-17
Pdgfra	6.1E-21	0.43008	0.228	0.179	1.02E-16
Rab3a	1.07E-20	-0.46577	0.089	0.153	1.79E-16
Alcam	1.11E-20	0.302467	0.253	0.128	1.85E-16
Fnbp1l	2.38E-20	-0.25769	0.46	0.453	3.97E-16
Lmo4	2.41E-20	0.282519	0.478	0.328	4.03E-16
Srrm3	2.41E-20	-0.48956	0.156	0.244	4.03E-16
Id1	2.6E-20	0.35081	0.167	0.068	4.33E-16
Nhlh1	3.25E-20	-0.52187	0.133	0.227	5.41E-16
Gdi1	4.56E-20	-0.25031	0.227	0.23	7.61E-16
Celsr2	4.81E-20	-0.33187	0.208	0.239	8.03E-16
Gm3764	5.09E-20	0.358984	0.249	0.125	8.5E-16
Klf7	5.77E-20	-0.28435	0.254	0.262	9.63E-16
Cacna2d1	7.2E-20	-0.38602	0.318	0.377	1.2E-15
Chchd10	8.02E-20	0.378688	0.102	0.03	1.34E-15
Trpc4ap	9.11E-20	-0.2614	0.208	0.197	1.52E-15
Pgp	1.3E-19	0.25051	0.264	0.144	2.17E-15
Fos	1.36E-19	0.376159	0.441	0.297	2.26E-15
Deb1	1.81E-19	0.299587	0.282	0.161	3.02E-15
Elavl4	1.94E-19	-0.46678	0.239	0.34	3.24E-15
Sema6a	1.95E-19	-0.40107	0.162	0.204	3.26E-15
Mapk8ip1	2.02E-19	-0.38084	0.176	0.229	3.36E-15
Vimp	2.35E-19	0.281587	0.318	0.183	3.93E-15
Tmem47	5.05E-19	0.343135	0.148	0.059	8.42E-15
Ntrk2	5.99E-19	0.365605	0.18	0.078	1E-14
3632451O	7.67E-19	0.294893	0.102	0.032	1.28E-14
Clmp	7.67E-19	-0.48665	0.161	0.246	1.28E-14
Myt1l	1.12E-18	-0.52564	0.076	0.165	1.88E-14
Rassf4	1.19E-18	-0.37381	0.306	0.379	1.99E-14
Ppp1r14c	1.55E-18	-0.39644	0.224	0.264	2.58E-14
Kcnk1	1.69E-18	-0.4217	0.148	0.207	2.82E-14
Nsg1	2.09E-18	-0.27413	0.275	0.288	3.48E-14
Meg3	2.34E-18	-0.29821	0.128	0.093	3.9E-14
Gng2	3.66E-18	-0.29434	0.368	0.397	6.1E-14
Ebf3	6.25E-18	-0.37349	0.153	0.205	1.04E-13
Carhsp1	9.64E-18	-0.3192	0.218	0.255	1.61E-13
Lap3	1.09E-17	0.266655	0.414	0.269	1.81E-13
Abhd16a	1.24E-17	-0.25045	0.184	0.19	2.07E-13

A930011O	1.63E-17	-0.49314	0.036	0.118	2.72E-13
Rab6b	1.82E-17	-0.35145	0.166	0.204	3.04E-13
Atp1b1	1.82E-17	0.440487	0.125	0.048	3.04E-13
Igsf8	1.94E-17	-0.28143	0.247	0.27	3.24E-13
Scg5	2.1E-17	-0.26994	0.321	0.335	3.51E-13
Gstm1	2.23E-17	0.382215	0.168	0.077	3.72E-13
Dpysl4	2.3E-17	-0.29722	0.451	0.492	3.84E-13
Cacng2	2.92E-17	-0.36041	0.275	0.341	4.88E-13
Arhgef2	3.95E-17	-0.29495	0.252	0.283	6.59E-13
Rcor2	4.47E-17	-0.39368	0.158	0.228	7.46E-13
Ctsl	5.62E-17	0.27318	0.392	0.253	9.38E-13
Slc17a6	7.21E-17	-0.469	0.075	0.151	1.2E-12
Rbfox3	1.02E-16	-0.33872	0.445	0.496	1.7E-12
Chrna3	1.05E-16	-0.45354	0.046	0.122	1.76E-12
Gas1	1.24E-16	0.251962	0.264	0.151	2.07E-12
Klf6	1.56E-16	0.332632	0.252	0.143	2.61E-12
Itm2c	1.61E-16	0.268366	0.326	0.206	2.68E-12
Elavl2	1.96E-16	-0.36704	0.219	0.272	3.28E-12
Ncan	1.97E-16	0.369488	0.189	0.094	3.29E-12
Rundc3a	1.98E-16	-0.30545	0.219	0.25	3.31E-12
Stxbp1	2.74E-16	-0.43119	0.098	0.165	4.57E-12
Fam210b	3.94E-16	-0.2899	0.287	0.315	6.58E-12
Srrm4	7.12E-16	-0.43195	0.12	0.199	1.19E-11
Tnik	1.16E-15	-0.3317	0.121	0.147	1.93E-11
Cadm3	2.16E-15	-0.40736	0.091	0.143	3.6E-11
Sstr2	3.43E-15	-0.38472	0.134	0.193	5.72E-11
Glce	5.09E-15	-0.30235	0.149	0.178	8.49E-11
Gng3	5.44E-15	-0.27469	0.312	0.316	9.07E-11
Reln	6.22E-15	-0.41315	0.125	0.183	1.04E-10
Ly6e	8.49E-15	-0.29967	0.246	0.283	1.42E-10
Nrn1	8.61E-15	-0.40716	0.156	0.224	1.44E-10
Barhl1	1.1E-14	-0.27858	0.462	0.524	1.83E-10
Gm11223	1.83E-14	-0.40766	0.178	0.214	3.05E-10
Zbtb18	1.99E-14	-0.32306	0.226	0.268	3.31E-10
Mycn	2.25E-14	-0.26952	0.322	0.365	3.75E-10
Ostf1	2.46E-14	0.300166	0.136	0.059	4.1E-10
Hbb-bs	2.75E-14	-0.43318	0.102	0.064	4.59E-10
Nt5dc2	3.16E-14	-0.31714	0.196	0.239	5.28E-10
Prkcb	3.34E-14	-0.37116	0.201	0.258	5.58E-10
Fam3c	4.81E-14	0.261241	0.166	0.08	8.02E-10
Dbn1	6.69E-14	-0.30522	0.107	0.141	1.12E-09
Tagln3	8.75E-14	-0.32183	0.256	0.297	1.46E-09
Zic4	1.14E-13	-0.25213	0.389	0.419	1.89E-09
Trio	1.44E-13	0.25172	0.213	0.12	2.4E-09
Gpm6a	2.05E-13	-0.28411	0.408	0.409	3.42E-09
Ttc9b	5.54E-13	-0.31546	0.147	0.197	9.25E-09
Rab3c	6.01E-13	-0.30236	0.114	0.149	1E-08
Lxn	6.11E-13	0.330418	0.111	0.049	1.02E-08

Gltp	6.41E-13	0.267813	0.161	0.079	1.07E-08
Zic5	1.23E-12	-0.25512	0.141	0.161	2.05E-08
Tprn	1.51E-12	-0.3735	0.16	0.229	2.52E-08
Crip2	2.94E-12	-0.3008	0.205	0.241	4.91E-08
Prdm8	6.44E-12	-0.32043	0.095	0.128	1.07E-07
Fam53b	9.19E-12	-0.25377	0.107	0.137	1.53E-07
Gpc2	1.33E-11	-0.30483	0.098	0.141	2.21E-07
Elmo1	5.39E-11	-0.31244	0.101	0.127	8.99E-07
Sult4a1	1.23E-10	-0.25072	0.189	0.224	2.06E-06
9330159F1	1.27E-10	-0.29618	0.126	0.17	2.12E-06
Clvs1	1.32E-10	-0.33929	0.061	0.132	2.21E-06
Grik2	1.47E-10	-0.28981	0.072	0.103	2.45E-06
Pdzrn3	1.49E-10	-0.39395	0.098	0.149	2.49E-06
Kcnip3	1.5E-10	0.29454	0.198	0.116	2.5E-06
Celsr3	1.65E-10	-0.28738	0.085	0.125	2.75E-06
Kif5a	2.84E-10	-0.32242	0.086	0.13	4.74E-06
Rnf165	9.3E-10	-0.29302	0.124	0.175	1.55E-05
St8sia3	9.36E-10	-0.25042	0.219	0.252	1.56E-05
Sv2b	9.78E-10	-0.25806	0.072	0.104	1.63E-05
Eif4e3	1.96E-09	-0.27657	0.094	0.132	3.27E-05
Snap25	2.54E-09	-0.31133	0.229	0.284	4.23E-05
Gm11266	2.77E-09	-0.29485	0.107	0.145	4.63E-05
Neurod6	2.81E-09	-0.29659	0.128	0.174	4.69E-05
Dixdc1	3.01E-09	-0.28828	0.186	0.237	5.03E-05
Myt1	3.95E-09	-0.3502	0.1	0.155	6.59E-05
Dner	1.25E-08	-0.27132	0.187	0.216	0.000209
A330076H(	1.78E-08	-0.26613	0.133	0.162	0.000297
Fam57b	2.8E-08	-0.27545	0.139	0.188	0.000467
Cplx1	3.09E-07	-0.28207	0.095	0.14	0.00516
Mroh2a	3.13E-07	-0.31969	0.071	0.115	0.005215
