# Supplementary material for: scRNA-seq in medulloblastoma shows cellular heterogeneity and lineage expansion support resistance to SHH inhibitor therapy
Source: Nat Commun. 2019 Dec 20;10:5829. doi: 10.1038/s41467-019-13657-6 (PMC6925218; doi:10.1038/s41467-019-13657-6)
Supplement: Supplementary file 8 — Supplementary Data 5 [file 41467_2019_13657_MOESM8_ESM.pdf]

	p_val	avg_logFC	pct.popula	pct.Other	p_val_adj	Population
Serpine2	2.3E-140	-2.8026	0.035	0.885	3.9E-136	CGNP-Like
Gpr37l1	3.2E-126	-2.27261	0.015	0.783	5.3E-122	CGNP-Like
Ptprz1	1.1E-121	-2.29213	0.026	0.787	1.9E-117	CGNP-Like
Fabp7	3.9E-120	-3.85587	0.155	0.918	6.5E-116	CGNP-Like
Cacng4	1.1E-114	-2.31234	0.081	0.832	1.9E-110	CGNP-Like
Scrg1	1.4E-113	-2.02294	0.008	0.709	2.3E-109	CGNP-Like
Lsamp	1.3E-110	-2.01863	0.043	0.758	2.2E-106	CGNP-Like
Cspg5	1.4E-109	-2.5258	0.01	0.693	2.4E-105	CGNP-Like
Bcan	6.1E-106	-1.89505	0.068	0.803	1E-101	CGNP-Like
Ramp1	1.27E-96	-1.8091	0.018	0.652	2.12E-92	CGNP-Like
Cntn1	1.72E-92	-1.99993	0.026	0.648	2.86E-88	CGNP-Like
S100a1	1.08E-87	-1.83489	0.01	0.582	1.8E-83	CGNP-Like
Ednrb	3.37E-84	-2.29974	0.03	0.607	5.63E-80	CGNP-Like
3110035E1	5.6E-82	-2.08375	0.013	0.557	9.34E-78	CGNP-Like
Pcsk1n	2.38E-78	-1.58736	0.03	0.586	3.97E-74	CGNP-Like
S100a13	3.63E-78	-1.88152	0.021	0.557	6.06E-74	CGNP-Like
Sfrp1	1.93E-76	1.890114	0.914	0.352	3.22E-72	CGNP-Like
Olig1	6.66E-76	-2.15524	0.185	0.766	1.11E-71	CGNP-Like
S100a16	2.15E-75	-1.69382	0.053	0.607	3.59E-71	CGNP-Like
Gatm	3.71E-75	-1.40047	0.02	0.541	6.18E-71	CGNP-Like
Gjc3	5.95E-74	-1.64951	0.002	0.48	9.93E-70	CGNP-Like
Tril	3.16E-73	-1.59479	0.023	0.537	5.27E-69	CGNP-Like
Asrgl1	9.47E-73	-1.48392	0.157	0.738	1.58E-68	CGNP-Like
Kcnj10	1.05E-70	-1.83684	0.025	0.525	1.75E-66	CGNP-Like
Cnp	2.46E-70	-2.20494	0.036	0.549	4.11E-66	CGNP-Like
Atp1a2	9.34E-70	-2.57577	0.058	0.578	1.56E-65	CGNP-Like
Slc35f1	9.89E-70	-1.37422	0.008	0.48	1.65E-65	CGNP-Like
Tsc22d4	1.32E-69	-1.68411	0.211	0.758	2.2E-65	CGNP-Like
Ppap2b	1.93E-69	-1.90571	0.069	0.607	3.23E-65	CGNP-Like
Enpp2	7.24E-69	-1.67418	0.012	0.48	1.21E-64	CGNP-Like
Timp4	8.05E-69	-1.52263	0.012	0.48	1.34E-64	CGNP-Like
Nfib	1.06E-65	1.326535	0.954	0.635	1.77E-61	CGNP-Like
Gpr17	3.03E-64	-1.79263	0	0.418	5.06E-60	CGNP-Like
Sox10	6.96E-64	-1.49695	0.003	0.426	1.16E-59	CGNP-Like
Cmtm5	2.67E-63	-1.25515	0.008	0.439	4.46E-59	CGNP-Like
Tspan7	3.77E-63	-1.55961	0.124	0.66	6.29E-59	CGNP-Like
Ttyh1	8.79E-62	-1.92755	0.033	0.492	1.47E-57	CGNP-Like
Tmem176b	9.42E-62	-1.46584	0.137	0.672	1.57E-57	CGNP-Like
Ptpre	1.29E-61	-1.48926	0.003	0.414	2.15E-57	CGNP-Like
Rps5	2.87E-61	0.779279	0.998	0.934	4.8E-57	CGNP-Like
Cadm2	3.45E-61	-1.33534	0.01	0.434	5.75E-57	CGNP-Like
Pllp	4.47E-61	-1.50608	0.005	0.414	7.46E-57	CGNP-Like
Rpl13a	1.55E-60	0.819698	0.99	0.922	2.59E-56	CGNP-Like
Hsp90ab1	3.99E-60	0.665646	0.998	0.988	6.66E-56	CGNP-Like
Ddah1	1.4E-59	-1.34836	0.036	0.488	2.34E-55	CGNP-Like
Rgcc	2.59E-59	-1.37993	0.005	0.406	4.32E-55	CGNP-Like
Omg	3.08E-59	-1.14851	0.002	0.393	5.14E-55	CGNP-Like

Draxin	6.53E-59	1.684104	0.771	0.176	1.09E-54	CGNP-Like
Aldoc	6.83E-57	-2.18274	0.013	0.414	1.14E-52	CGNP-Like
Sulf2	7.64E-57	-1.40683	0.016	0.426	1.27E-52	CGNP-Like
Ptn	3.12E-56	-1.74742	0.514	0.84	5.21E-52	CGNP-Like
Spon1	9.22E-56	-1.12403	0.007	0.389	1.54E-51	CGNP-Like
Ntrk2	1.54E-55	-1.37101	0.051	0.5	2.57E-51	CGNP-Like
Pabpc1	1.62E-55	0.831631	0.964	0.857	2.7E-51	CGNP-Like
CRE_RECON	1.07E-53	2.014977	0.806	0.303	1.79E-49	CGNP-Like
Clu	1.99E-53	-1.64793	0.018	0.41	3.31E-49	CGNP-Like
Cd81	2.59E-53	-1.23734	0.565	0.877	4.32E-49	CGNP-Like
Epn2	1.16E-52	-1.33475	0.074	0.533	1.93E-48	CGNP-Like
Nap1l5	1.65E-52	-1.36931	0.069	0.52	2.75E-48	CGNP-Like
S100b	7.03E-52	-1.43668	0.072	0.52	1.17E-47	CGNP-Like
Rps9	1.16E-51	0.686808	0.992	0.939	1.93E-47	CGNP-Like
Mmd2	1.76E-51	-1.3409	0.031	0.43	2.94E-47	CGNP-Like
Ugt8a	5.27E-51	-1.26226	0.007	0.361	8.8E-47	CGNP-Like
Rpl4	9.39E-51	0.75001	0.974	0.906	1.57E-46	CGNP-Like
AW04773C	3.19E-50	-1.3058	0.015	0.381	5.32E-46	CGNP-Like
Syt11	2.37E-49	-1.01702	0.486	0.873	3.95E-45	CGNP-Like
H3f3b	2.57E-49	0.928829	0.949	0.754	4.28E-45	CGNP-Like
Phyhipl	1.41E-48	-1.36084	0.069	0.496	2.35E-44	CGNP-Like
Dnm3	1.9E-48	-1.21697	0.012	0.361	3.17E-44	CGNP-Like
Itpr2	1.99E-48	-1.33296	0.035	0.426	3.32E-44	CGNP-Like
Zic1	7.9E-48	1.161364	0.84	0.369	1.32E-43	CGNP-Like
Cd24a	8.24E-48	1.480676	0.677	0.119	1.38E-43	CGNP-Like
Tspan3	1.62E-47	-1.00338	0.544	0.873	2.7E-43	CGNP-Like
Mt3	3.67E-47	-1.32402	0.031	0.406	6.12E-43	CGNP-Like
Ppfibp1	4.18E-47	-1.28367	0.04	0.422	6.97E-43	CGNP-Like
Pea15a	4.39E-47	-1.54356	0.148	0.59	7.33E-43	CGNP-Like
Anp32a	5.66E-47	0.964512	0.908	0.664	9.44E-43	CGNP-Like
Plekhb1	1.57E-46	-1.14791	0.007	0.332	2.63E-42	CGNP-Like
Cdh13	2.28E-46	-1.11179	0.04	0.422	3.8E-42	CGNP-Like
Dlgap1	2.51E-46	-1.04082	0.053	0.455	4.19E-42	CGNP-Like
Scd2	2.6E-46	-1.34084	0.409	0.779	4.34E-42	CGNP-Like
Rplp0	3.22E-46	0.803318	0.957	0.807	5.37E-42	CGNP-Like
Anks1b	4.56E-46	-1.17651	0.023	0.381	7.61E-42	CGNP-Like
D430041D	4.71E-46	1.361888	0.687	0.143	7.86E-42	CGNP-Like
Adora1	5.14E-46	-1.02782	0.002	0.311	8.57E-42	CGNP-Like
Hepacam	1.03E-45	-1.01797	0.007	0.328	1.73E-41	CGNP-Like
Slc6a1	1.22E-45	-1.03643	0.023	0.377	2.03E-41	CGNP-Like
Tmem176a	2.35E-45	-1.03158	0.008	0.332	3.91E-41	CGNP-Like
Degs1	2.66E-45	-1.17089	0.097	0.525	4.43E-41	CGNP-Like
Luzp2	6.13E-45	-0.97204	0.018	0.361	1.02E-40	CGNP-Like
Rps3	1.55E-44	0.662595	0.977	0.902	2.58E-40	CGNP-Like
Olig2	3.99E-44	-1.3258	0.189	0.631	6.65E-40	CGNP-Like
Klhl5	5.43E-44	-1.09601	0.043	0.414	9.05E-40	CGNP-Like
Rps26	1.34E-43	0.803963	0.952	0.762	2.23E-39	CGNP-Like
Tubb5	2.11E-43	0.949236	0.932	0.77	3.53E-39	CGNP-Like

Dbi	4.21E-43	-1.58523	0.643	0.873	7.03E-39	CGNP-Like
Mfsd2a	8.72E-43	-1.02518	0.002	0.291	1.45E-38	CGNP-Like
H2afv	1.58E-42	1.029357	0.862	0.586	2.64E-38	CGNP-Like
Eps8	2.48E-42	-0.96796	0.025	0.361	4.13E-38	CGNP-Like
Rps20	2.56E-42	0.83206	0.931	0.693	4.28E-38	CGNP-Like
Nfasc	3.78E-42	-1.23701	0.013	0.328	6.3E-38	CGNP-Like
Tagln2	7.42E-42	-1.25441	0.063	0.443	1.24E-37	CGNP-Like
Sirt2	9.34E-42	-2.11841	0.165	0.59	1.56E-37	CGNP-Like
Igfbpl1	1.37E-41	1.572049	0.639	0.127	2.29E-37	CGNP-Like
Phlda1	5.28E-41	-1.13619	0.03	0.361	8.8E-37	CGNP-Like
Cplx2	7.23E-41	1.38266	0.656	0.156	1.21E-36	CGNP-Like
Cyp2j6	9.52E-41	-1.00963	0.013	0.316	1.59E-36	CGNP-Like
Gria4	1.34E-40	-1.10502	0.102	0.508	2.24E-36	CGNP-Like
Nfia	7.27E-40	1.057138	0.857	0.545	1.21E-35	CGNP-Like
Slc38a3	2.63E-39	-0.98707	0.015	0.311	4.39E-35	CGNP-Like
Gpm6b	2.64E-39	-1.34575	0.539	0.836	4.41E-35	CGNP-Like
Slc4a4	3.9E-39	-2.36729	0.03	0.344	6.5E-35	CGNP-Like
Cryab	4.73E-39	-1.0858	0.018	0.32	7.89E-35	CGNP-Like
Gnb4	6.36E-39	-1.0974	0.137	0.549	1.06E-34	CGNP-Like
Hnrnpab	6.69E-39	0.819906	0.908	0.705	1.12E-34	CGNP-Like
Cspg4	9.47E-39	-1.31592	0.016	0.311	1.58E-34	CGNP-Like
Nkx2-2	1.04E-38	-0.89908	0.005	0.279	1.73E-34	CGNP-Like
Lrrc4c	1.29E-38	-0.8739	0.005	0.279	2.16E-34	CGNP-Like
B3gat2	1.36E-38	-1.09908	0.008	0.287	2.27E-34	CGNP-Like
Npas3	2.08E-38	-0.97283	0.033	0.361	3.47E-34	CGNP-Like
Pcdh10	3.21E-38	-0.77148	0.002	0.262	5.36E-34	CGNP-Like
Ncam2	4.81E-38	-0.96799	0.023	0.328	8.03E-34	CGNP-Like
Limch1	8.68E-38	-0.97384	0.021	0.324	1.45E-33	CGNP-Like
Atp1b1	1.28E-37	-1.30403	0.033	0.352	2.13E-33	CGNP-Like
Cdo1	1.65E-37	-0.99889	0.012	0.291	2.76E-33	CGNP-Like
Sox6	2E-37	-0.97178	0.007	0.275	3.34E-33	CGNP-Like
Barhl1	2.53E-37	1.369506	0.603	0.115	4.23E-33	CGNP-Like
Igfbp2	3.02E-37	-1.71868	0.012	0.287	5.03E-33	CGNP-Like
Rplp2	3.34E-37	0.681883	0.951	0.811	5.57E-33	CGNP-Like
Aqp4	3.66E-37	-2.90985	0.02	0.307	6.11E-33	CGNP-Like
Acsbg1	4.46E-37	-1.14529	0.005	0.266	7.43E-33	CGNP-Like
Qpct	2.24E-36	-1.00072	0.021	0.311	3.74E-32	CGNP-Like
Car2	2.94E-36	-1.65957	0.018	0.299	4.91E-32	CGNP-Like
Ier5	3.34E-36	1.243021	0.578	0.094	5.56E-32	CGNP-Like
Fyn	3.98E-36	-1.9647	0.226	0.615	6.64E-32	CGNP-Like
Gnb2l1	1.8E-35	0.651981	0.954	0.84	3.01E-31	CGNP-Like
Smc2	1.9E-35	1.382705	0.717	0.357	3.17E-31	CGNP-Like
Emid1	1.92E-35	-1.00325	0.003	0.25	3.21E-31	CGNP-Like
Afap1l2	2.08E-35	-0.81418	0.003	0.25	3.46E-31	CGNP-Like
Dek	5.5E-35	0.999336	0.843	0.57	9.18E-31	CGNP-Like
Npm1	5.85E-35	0.883902	0.839	0.541	9.76E-31	CGNP-Like
Tmem100	6.55E-35	-0.84826	0.003	0.246	1.09E-30	CGNP-Like
E130114P1	7.1E-35	1.101826	0.738	0.311	1.19E-30	CGNP-Like

Atp1b2	1.01E-34	-1.31359	0.058	0.381	1.68E-30	CGNP-Like
Ptma	1.06E-34	1.010916	0.72	0.32	1.77E-30	CGNP-Like
S100a6	1.26E-34	-0.94225	0.021	0.303	2.1E-30	CGNP-Like
Hnrnpu	1.32E-34	0.644379	0.947	0.857	2.21E-30	CGNP-Like
Slc22a17	1.36E-34	-1.05875	0.226	0.623	2.28E-30	CGNP-Like
Rps14	2.91E-34	0.545295	0.985	0.951	4.85E-30	CGNP-Like
Sox8	3E-34	-1.04342	0.041	0.348	5.01E-30	CGNP-Like
Itm2c	3.38E-34	-1.01742	0.213	0.607	5.65E-30	CGNP-Like
Cbfa2t3	4.71E-34	1.228937	0.544	0.09	7.86E-30	CGNP-Like
Serbp1	1.26E-33	0.616696	0.949	0.865	2.1E-29	CGNP-Like
Agt	1.67E-33	-1.37826	0.008	0.254	2.79E-29	CGNP-Like
Ncald	1.72E-33	-1.08465	0.198	0.598	2.87E-29	CGNP-Like
Calm2	2.47E-33	0.768458	0.908	0.725	4.13E-29	CGNP-Like
Kcnj16	3.02E-33	-0.70396	0.002	0.23	5.04E-29	CGNP-Like
Lrp1	3.06E-33	-0.87816	0.031	0.32	5.11E-29	CGNP-Like
Pla2g7	3.14E-33	-1.89784	0.021	0.287	5.24E-29	CGNP-Like
Gria1	4E-33	-0.87288	0.005	0.242	6.68E-29	CGNP-Like
Cbx1	4.27E-33	0.875144	0.811	0.512	7.12E-29	CGNP-Like
Rpl32	4.98E-33	0.593811	0.964	0.881	8.31E-29	CGNP-Like
Gm2a	5.04E-33	-0.91001	0.068	0.398	8.41E-29	CGNP-Like
Opcml	5.58E-33	-0.87941	0.007	0.246	9.31E-29	CGNP-Like
Acox1	5.98E-33	-0.72928	0.056	0.381	9.97E-29	CGNP-Like
Rps24	1.08E-32	0.71476	0.921	0.742	1.8E-28	CGNP-Like
Cyp2j9	1.7E-32	-0.58239	0.002	0.225	2.84E-28	CGNP-Like
Tbata	3.36E-32	1.505599	0.494	0.057	5.61E-28	CGNP-Like
Gpm6a	3.59E-32	-0.90713	0.292	0.697	5.98E-28	CGNP-Like
Slc1a1	3.65E-32	-0.86095	0.015	0.266	6.09E-28	CGNP-Like
Susd4	9.12E-32	-0.81738	0.003	0.225	1.52E-27	CGNP-Like
Nasp	9.12E-32	0.976383	0.783	0.467	1.52E-27	CGNP-Like
Lims2	9.73E-32	-0.89542	0	0.213	1.62E-27	CGNP-Like
Pfn2	1.02E-31	-1.17114	0.214	0.582	1.71E-27	CGNP-Like
Cbx5	1.75E-31	0.826171	0.847	0.607	2.91E-27	CGNP-Like
Zcchc24	2.53E-31	-0.83623	0.053	0.361	4.22E-27	CGNP-Like
Ezr	2.7E-31	1.128308	0.616	0.189	4.5E-27	CGNP-Like
Slc25a18	3.46E-31	-0.80977	0.007	0.234	5.77E-27	CGNP-Like
Rbfox3	3.89E-31	1.227954	0.57	0.135	6.5E-27	CGNP-Like
Tmem9b	4.41E-31	-0.75339	0.109	0.475	7.35E-27	CGNP-Like
Aldh1l1	8.75E-31	-1.05136	0.005	0.225	1.46E-26	CGNP-Like
Arsb	1.05E-30	-0.90674	0.023	0.279	1.76E-26	CGNP-Like
Crmp1	1.08E-30	1.106141	0.659	0.25	1.8E-26	CGNP-Like
Tmsb10	1.1E-30	1.013891	0.694	0.291	1.84E-26	CGNP-Like
Cd9	1.42E-30	-1.17487	0.613	0.816	2.37E-26	CGNP-Like
Rnd3	1.43E-30	1.209489	0.552	0.131	2.39E-26	CGNP-Like
Pxdc1	1.63E-30	-0.67026	0	0.205	2.73E-26	CGNP-Like
Bmp4	1.63E-30	-1.43971	0	0.205	2.73E-26	CGNP-Like
Rlbp1	1.71E-30	-0.9138	0.008	0.234	2.85E-26	CGNP-Like
Mlc1	1.84E-30	-1.17864	0.016	0.258	3.06E-26	CGNP-Like
Hbegf	1.93E-30	-0.68864	0.01	0.242	3.22E-26	CGNP-Like

Pmp22	2.23E-30	-0.89425	0.015	0.254	3.73E-26	CGNP-Like
2810468N(	2.26E-30	-0.85488	0.016	0.262	3.77E-26	CGNP-Like
Cp	2.39E-30	-0.69754	0.003	0.217	3.99E-26	CGNP-Like
Gfra1	3.19E-30	-0.77398	0.002	0.209	5.32E-26	CGNP-Like
Rps19	3.57E-30	0.686247	0.898	0.713	5.95E-26	CGNP-Like
Mapt	3.64E-30	-0.99222	0.147	0.5	6.08E-26	CGNP-Like
Rpl8	4.38E-30	0.523604	0.974	0.918	7.3E-26	CGNP-Like
Psap	5.01E-30	-0.88149	0.198	0.561	8.36E-26	CGNP-Like
H1f0	5.27E-30	1.060525	0.725	0.332	8.78E-26	CGNP-Like
Lamp1	5.39E-30	-0.94215	0.428	0.73	8.99E-26	CGNP-Like
Ckb	6.29E-30	-0.91535	0.794	0.918	1.05E-25	CGNP-Like
Nid1	6.59E-30	-1.00278	0.007	0.225	1.1E-25	CGNP-Like
Cog7	7.07E-30	1.198045	0.59	0.18	1.18E-25	CGNP-Like
Kank1	7.12E-30	-0.74251	0.043	0.328	1.19E-25	CGNP-Like
Enc1	7.17E-30	-0.90792	0.008	0.23	1.2E-25	CGNP-Like
Tubb2a	8.51E-30	-0.86105	0.166	0.533	1.42E-25	CGNP-Like
Pcdh17	1.28E-29	-0.90171	0.033	0.299	2.13E-25	CGNP-Like
F3	1.35E-29	-0.80355	0.005	0.217	2.24E-25	CGNP-Like
Ncl	1.63E-29	0.583249	0.964	0.869	2.72E-25	CGNP-Like
Tead2	2.07E-29	1.116905	0.451	0.049	3.45E-25	CGNP-Like
Ncan	3.22E-29	-1.17584	0.099	0.414	5.37E-25	CGNP-Like
Tm7sf3	3.34E-29	-0.79897	0.072	0.377	5.57E-25	CGNP-Like
Gsg1l	3.56E-29	1.130117	0.445	0.041	5.93E-25	CGNP-Like
Gria3	3.67E-29	-1.06761	0.129	0.467	6.12E-25	CGNP-Like
Tns3	3.9E-29	-0.64522	0.003	0.209	6.51E-25	CGNP-Like
Rpl22	4.19E-29	0.659994	0.916	0.75	6.99E-25	CGNP-Like
Grm5	5.08E-29	-0.91319	0.005	0.213	8.48E-25	CGNP-Like
Tpm1	5.09E-29	-1.25765	0.236	0.578	8.49E-25	CGNP-Like
Sv2a	5.93E-29	-0.7454	0.026	0.279	9.9E-25	CGNP-Like
Sparc	6.22E-29	-1.82921	0.132	0.455	1.04E-24	CGNP-Like
Dscam	6.3E-29	-0.77206	0.002	0.201	1.05E-24	CGNP-Like
Id4	6.4E-29	-0.98513	0.012	0.234	1.07E-24	CGNP-Like
Dbndd2	6.43E-29	-0.73944	0.02	0.262	1.07E-24	CGNP-Like
Id3	1E-28	-1.87152	0.046	0.316	1.67E-24	CGNP-Like
Eef1a1	1.39E-28	0.562405	0.951	0.889	2.32E-24	CGNP-Like
Nrxn2	1.44E-28	-0.71375	0.051	0.34	2.4E-24	CGNP-Like
Cd302	1.71E-28	-0.79055	0.059	0.352	2.85E-24	CGNP-Like
Ctsl	1.75E-28	-0.94566	0.293	0.635	2.92E-24	CGNP-Like
Chpt1	1.79E-28	-0.78907	0.082	0.393	2.98E-24	CGNP-Like
3632451O(	1.8E-28	-0.88689	0.03	0.283	3.01E-24	CGNP-Like
Metrn	1.85E-28	-0.76185	0.035	0.295	3.09E-24	CGNP-Like
Kcnd2	1.95E-28	-0.98261	0.013	0.234	3.25E-24	CGNP-Like
Mmp15	2.4E-28	-0.83674	0.01	0.225	4E-24	CGNP-Like
Igfbp3	2.78E-28	-0.84319	0.01	0.225	4.63E-24	CGNP-Like
Sash1	3.25E-28	-0.83801	0.053	0.336	5.42E-24	CGNP-Like
Rps21	3.76E-28	0.623555	0.939	0.807	6.27E-24	CGNP-Like
Vcan	4.26E-28	-0.99855	0.157	0.496	7.11E-24	CGNP-Like
Sox21	5.02E-28	-0.54773	0.005	0.209	8.38E-24	CGNP-Like

Paqr8	6.33E-28	-0.6808	0.008	0.217	1.06E-23	CGNP-Like
2900011O(	6.59E-28	-0.95892	0.056	0.336	1.1E-23	CGNP-Like
Nlgn3	8.61E-28	-0.79129	0.021	0.258	1.44E-23	CGNP-Like
Meg3	1.67E-27	-0.88784	0.049	0.324	2.78E-23	CGNP-Like
Brinp3	1.79E-27	-0.64209	0	0.184	2.99E-23	CGNP-Like
Smc4	2.03E-27	1.133448	0.72	0.426	3.39E-23	CGNP-Like
Slc29a1	2.05E-27	1.079336	0.586	0.176	3.42E-23	CGNP-Like
Pik3r1	2.31E-27	-0.91223	0.107	0.426	3.85E-23	CGNP-Like
Reep5	2.54E-27	-0.84232	0.117	0.447	4.23E-23	CGNP-Like
Rab31	2.95E-27	-0.85373	0.071	0.361	4.93E-23	CGNP-Like
Hirip3	3.98E-27	1.061617	0.56	0.172	6.63E-23	CGNP-Like
Plat	5.72E-27	-0.79021	0.016	0.238	9.55E-23	CGNP-Like
Spry1	6.07E-27	-0.73858	0.012	0.221	1.01E-22	CGNP-Like
Ehd3	7.08E-27	-0.77476	0.015	0.23	1.18E-22	CGNP-Like
Kctd4	7.21E-27	-0.6406	0	0.18	1.2E-22	CGNP-Like
Gria2	7.49E-27	-0.94106	0.465	0.75	1.25E-22	CGNP-Like
Adam9	7.93E-27	-0.81942	0.063	0.344	1.32E-22	CGNP-Like
Hsd11b2	9.5E-27	1.228709	0.39	0.02	1.59E-22	CGNP-Like
Gm3764	9.84E-27	-1.02133	0.157	0.48	1.64E-22	CGNP-Like
2810417H:	1.28E-26	1.270177	0.572	0.209	2.14E-22	CGNP-Like
Alcam	1.56E-26	-0.88735	0.157	0.492	2.61E-22	CGNP-Like
Hmgb2	1.59E-26	1.215058	0.568	0.209	2.66E-22	CGNP-Like
Timp2	1.96E-26	-0.68242	0.021	0.25	3.28E-22	CGNP-Like
Hmgn1	2.07E-26	0.76731	0.778	0.467	3.46E-22	CGNP-Like
S1pr1	2.89E-26	-0.76482	0	0.176	4.82E-22	CGNP-Like
Hopx	3.35E-26	-0.87554	0.003	0.189	5.6E-22	CGNP-Like
Abat	3.48E-26	-0.63899	0.025	0.258	5.81E-22	CGNP-Like
Rprm	3.74E-26	-0.87485	0.054	0.32	6.23E-22	CGNP-Like
Hnrnpm	3.98E-26	0.665953	0.86	0.668	6.63E-22	CGNP-Like
Dpp6	4.45E-26	-0.63453	0.008	0.205	7.41E-22	CGNP-Like
Rpl41	4.5E-26	0.570287	0.921	0.758	7.5E-22	CGNP-Like
Sfxn5	4.69E-26	-0.56379	0.008	0.205	7.83E-22	CGNP-Like
Shisa9	5.92E-26	-0.58626	0.002	0.18	9.87E-22	CGNP-Like
Enpp6	6.07E-26	-1.06874	0.002	0.18	1.01E-21	CGNP-Like
Rgs7bp	7.4E-26	-0.70551	0.013	0.221	1.23E-21	CGNP-Like
Tnc	1.12E-25	-1.0359	0.003	0.184	1.87E-21	CGNP-Like
Stk32a	1.15E-25	-0.53721	0	0.172	1.92E-21	CGNP-Like
Chchd10	1.52E-25	-0.99294	0.035	0.27	2.53E-21	CGNP-Like
Fkbp3	1.75E-25	0.679692	0.878	0.705	2.91E-21	CGNP-Like
Scn3a	3.01E-25	-0.74282	0.002	0.176	5.02E-21	CGNP-Like
Chl1	3.39E-25	-0.71008	0.01	0.205	5.66E-21	CGNP-Like
Ascl1	4.59E-25	-0.72841	0	0.168	7.66E-21	CGNP-Like
Deb1	5.19E-25	-0.93625	0.189	0.516	8.66E-21	CGNP-Like
Tmpo	6.28E-25	0.911897	0.624	0.254	1.05E-20	CGNP-Like
Phactr3	7.2E-25	-0.76814	0.036	0.275	1.2E-20	CGNP-Like
Brinp1	7.32E-25	-0.71858	0.015	0.217	1.22E-20	CGNP-Like
Ranbp1	9.55E-25	0.810443	0.807	0.672	1.59E-20	CGNP-Like
Car8	1.02E-24	-0.77423	0.005	0.184	1.7E-20	CGNP-Like

Gm2694	1.16E-24	1.073449	0.45	0.082	1.93E-20	CGNP-Like
Arhgef26	1.27E-24	-0.61086	0.016	0.221	2.11E-20	CGNP-Like
Mro	1.82E-24	-0.55213	0	0.164	3.04E-20	CGNP-Like
Eef1b2	2.46E-24	0.653866	0.875	0.668	4.1E-20	CGNP-Like
Kcnip3	3.02E-24	-0.99828	0.114	0.406	5.04E-20	CGNP-Like
Lpar1	3.74E-24	-0.57364	0.002	0.168	6.23E-20	CGNP-Like
Ostf1	3.91E-24	-0.86233	0.061	0.324	6.53E-20	CGNP-Like
Neu4	4.17E-24	-0.6355	0.002	0.168	6.95E-20	CGNP-Like
Shisa4	4.32E-24	-0.71407	0.04	0.275	7.2E-20	CGNP-Like
Ddah2	7.87E-24	0.874502	0.7	0.389	1.31E-19	CGNP-Like
Anp32b	9.47E-24	0.76745	0.771	0.516	1.58E-19	CGNP-Like
Hsd17b12	1.17E-23	-0.80648	0.244	0.557	1.94E-19	CGNP-Like
Pla2g16	1.22E-23	-0.61845	0.008	0.189	2.04E-19	CGNP-Like
Npy	1.4E-23	-1.07992	0.005	0.176	2.34E-19	CGNP-Like
Gltp	1.48E-23	-0.85996	0.084	0.352	2.47E-19	CGNP-Like
G0s2	1.83E-23	-0.60244	0.005	0.176	3.06E-19	CGNP-Like
Gm9800	2.44E-23	0.841969	0.659	0.344	4.06E-19	CGNP-Like
Dbx2	2.85E-23	-0.58421	0	0.156	4.76E-19	CGNP-Like
Cpq	2.85E-23	-0.60404	0	0.156	4.76E-19	CGNP-Like
Osbpl1a	3.29E-23	-0.721	0.056	0.307	5.48E-19	CGNP-Like
Mfge8	3.3E-23	-0.91886	0.053	0.295	5.51E-19	CGNP-Like
Nrxn1	3.82E-23	-0.9156	0.257	0.586	6.38E-19	CGNP-Like
Lcat	5.51E-23	-0.76129	0.018	0.213	9.2E-19	CGNP-Like
Il18	5.81E-23	-0.58816	0.015	0.205	9.69E-19	CGNP-Like
Dut	6.06E-23	0.956523	0.6	0.258	1.01E-18	CGNP-Like
Gabrg1	6.7E-23	-0.50128	0.002	0.16	1.12E-18	CGNP-Like
Atp6v0b	7.24E-23	-0.72913	0.247	0.574	1.21E-18	CGNP-Like
Map3k1	7.49E-23	0.874958	0.522	0.16	1.25E-18	CGNP-Like
Tspan2	7.76E-23	-0.66954	0.012	0.193	1.29E-18	CGNP-Like
Cdh10	7.76E-23	-0.46543	0.005	0.172	1.29E-18	CGNP-Like
Pdgfra	8.74E-23	-1.53039	0.152	0.418	1.46E-18	CGNP-Like
Nxph1	1.06E-22	-0.66035	0.007	0.176	1.77E-18	CGNP-Like
Nkd1	1.07E-22	0.914538	0.39	0.053	1.78E-18	CGNP-Like
Lypd1	1.12E-22	-0.59274	0	0.152	1.87E-18	CGNP-Like
Htra1	1.23E-22	-0.78595	0.015	0.201	2.05E-18	CGNP-Like
Rps11	1.3E-22	0.550966	0.881	0.734	2.17E-18	CGNP-Like
Me1	1.48E-22	-0.48399	0.003	0.164	2.46E-18	CGNP-Like
1810037I1	1.66E-22	-0.7874	0.29	0.598	2.78E-18	CGNP-Like
Plk2	1.73E-22	-0.68268	0.008	0.18	2.89E-18	CGNP-Like
Cks1b	1.9E-22	0.970363	0.501	0.148	3.17E-18	CGNP-Like
Chn2	1.92E-22	-1.21609	0.005	0.168	3.21E-18	CGNP-Like
Prss23	2.4E-22	-0.66606	0.002	0.156	4E-18	CGNP-Like
Ccdc80	2.51E-22	-0.70295	0.005	0.168	4.19E-18	CGNP-Like
Ezh2	2.66E-22	0.866225	0.728	0.475	4.43E-18	CGNP-Like
Ifitm3	2.97E-22	-0.72931	0.01	0.184	4.95E-18	CGNP-Like
Il1rap	3.1E-22	-0.72287	0.02	0.213	5.17E-18	CGNP-Like
Ppp2r2b	3.29E-22	-0.71141	0.044	0.275	5.49E-18	CGNP-Like
Hnrnpa2b1	3.47E-22	0.41812	0.97	0.943	5.79E-18	CGNP-Like

Fam213a	3.86E-22	-0.72047	0.099	0.377	6.44E-18	CGNP-Like
Slc22a23	3.87E-22	-0.65765	0.012	0.189	6.46E-18	CGNP-Like
Igfbp4	3.93E-22	-0.68739	0.007	0.172	6.56E-18	CGNP-Like
Dusp26	3.97E-22	-0.7295	0.028	0.234	6.62E-18	CGNP-Like
Cadm4	4.43E-22	-0.67358	0.119	0.414	7.39E-18	CGNP-Like
Top2a	4.77E-22	1.126351	0.583	0.258	7.96E-18	CGNP-Like
Fermt2	5.28E-22	-0.73121	0.275	0.59	8.8E-18	CGNP-Like
Pid1	5.7E-22	-0.75493	0.021	0.213	9.51E-18	CGNP-Like
Mcm7	6.06E-22	0.874524	0.509	0.16	1.01E-17	CGNP-Like
Zfp365	7.43E-22	-0.69653	0.016	0.201	1.24E-17	CGNP-Like
Rpl35a	7.93E-22	0.686915	0.746	0.467	1.32E-17	CGNP-Like
Tmem108	9.62E-22	-0.51474	0.002	0.152	1.6E-17	CGNP-Like
Timp3	1.04E-21	-0.69594	0.063	0.307	1.73E-17	CGNP-Like
2700094K:	1.11E-21	0.71327	0.761	0.516	1.85E-17	CGNP-Like
Rhoc	1.13E-21	-0.70163	0.053	0.287	1.89E-17	CGNP-Like
Jam2	1.2E-21	-0.64768	0.049	0.279	2E-17	CGNP-Like
Ctsb	1.3E-21	-0.52523	0.285	0.602	2.18E-17	CGNP-Like
Lrig1	1.39E-21	-0.63821	0.018	0.205	2.31E-17	CGNP-Like
Thra	1.62E-21	-0.83379	0.194	0.508	2.71E-17	CGNP-Like
Aplp1	1.74E-21	-0.78087	0.066	0.311	2.91E-17	CGNP-Like
Cacng2	2.38E-21	0.95097	0.367	0.045	3.96E-17	CGNP-Like
Fa2h	2.41E-21	-0.55931	0.003	0.156	4.01E-17	CGNP-Like
Cbr3	2.76E-21	-0.48143	0.008	0.172	4.6E-17	CGNP-Like
Trio	3.22E-21	-0.88113	0.133	0.41	5.37E-17	CGNP-Like
Dmrtb1	4.54E-21	-0.55711	0.002	0.148	7.58E-17	CGNP-Like
Hes5	5.15E-21	-0.75179	0.01	0.176	8.59E-17	CGNP-Like
Sdc3	5.18E-21	-0.73553	0.071	0.32	8.63E-17	CGNP-Like
Ptprt	5.27E-21	-0.54967	0.005	0.16	8.78E-17	CGNP-Like
Cyfip2	5.41E-21	-0.75711	0.033	0.234	9.02E-17	CGNP-Like
Stat3	6.89E-21	-0.57662	0.04	0.254	1.15E-16	CGNP-Like
Lmbrd1	8.6E-21	-0.63044	0.079	0.332	1.44E-16	CGNP-Like
Dynlt3	8.87E-21	-0.566	0.056	0.291	1.48E-16	CGNP-Like
Gucy1a3	9.03E-21	-0.52435	0.021	0.209	1.51E-16	CGNP-Like
Cdk6	9.36E-21	0.929767	0.372	0.049	1.56E-16	CGNP-Like
Chst11	9.93E-21	-0.52695	0.007	0.164	1.66E-16	CGNP-Like
Cenpe	1.04E-20	1.074503	0.471	0.127	1.73E-16	CGNP-Like
Zic4	1.15E-20	0.909151	0.486	0.152	1.92E-16	CGNP-Like
Gaa	1.24E-20	-0.41446	0.018	0.201	2.06E-16	CGNP-Like
Abhd6	1.35E-20	-0.54273	0.033	0.238	2.25E-16	CGNP-Like
Tnr	1.63E-20	-0.47468	0.002	0.143	2.71E-16	CGNP-Like
Sema5b	1.86E-20	-0.55718	0.005	0.156	3.1E-16	CGNP-Like
Tuba1b	2.15E-20	0.807852	0.657	0.381	3.59E-16	CGNP-Like
Chst2	2.22E-20	-0.62009	0.035	0.238	3.71E-16	CGNP-Like
Hdgf	2.27E-20	0.691413	0.708	0.439	3.78E-16	CGNP-Like
Cst3	2.39E-20	-1.528	0.685	0.82	3.99E-16	CGNP-Like
Kazn	2.46E-20	-0.58654	0.012	0.176	4.1E-16	CGNP-Like
Dusp15	2.57E-20	-0.55205	0.003	0.148	4.29E-16	CGNP-Like
Gal3st1	2.63E-20	-0.43989	0	0.135	4.38E-16	CGNP-Like

Cyp26b1	2.63E-20	-0.52643	0	0.135	4.38E-16	CGNP-Like
Mki67	2.64E-20	0.930463	0.578	0.246	4.4E-16	CGNP-Like
Hpca	3.02E-20	1.050281	0.343	0.037	5.03E-16	CGNP-Like
Sema5a	3.12E-20	-0.51691	0.003	0.148	5.21E-16	CGNP-Like
Fjx1	3.39E-20	-0.62186	0.056	0.279	5.66E-16	CGNP-Like
Sept7	3.73E-20	-0.73833	0.728	0.857	6.21E-16	CGNP-Like
Sh3d19	3.83E-20	-0.77283	0.054	0.275	6.38E-16	CGNP-Like
H2-K1	4.78E-20	-0.56727	0.008	0.164	7.98E-16	CGNP-Like
Hjurp	4.9E-20	0.840887	0.563	0.234	8.18E-16	CGNP-Like
Gng12	5.27E-20	-0.79597	0.217	0.504	8.79E-16	CGNP-Like
Rap1gap	5.75E-20	-0.54737	0.008	0.164	9.59E-16	CGNP-Like
Sh3bp4	6.28E-20	-0.58277	0.023	0.205	1.05E-15	CGNP-Like
Hnrnpd	6.49E-20	0.673309	0.759	0.504	1.08E-15	CGNP-Like
Pcdhga9	7.34E-20	-0.64151	0.451	0.717	1.22E-15	CGNP-Like
Smc1a	7.64E-20	0.706748	0.755	0.52	1.27E-15	CGNP-Like
Mgll	7.8E-20	-0.83872	0.094	0.34	1.3E-15	CGNP-Like
Rpl26	7.88E-20	0.581462	0.825	0.639	1.31E-15	CGNP-Like
Hmgcs1	8.98E-20	-0.8698	0.171	0.447	1.5E-15	CGNP-Like
Pax6	9.31E-20	0.88412	0.524	0.205	1.55E-15	CGNP-Like
1810041L1	1.01E-19	-0.55677	0.007	0.156	1.68E-15	CGNP-Like
Ppp1r16b	1.02E-19	-0.47897	0	0.131	1.7E-15	CGNP-Like
Asah1	1.18E-19	-0.61464	0.066	0.299	1.96E-15	CGNP-Like
Thbs3	1.2E-19	-0.45193	0.003	0.143	2.01E-15	CGNP-Like
Myo6	1.28E-19	-0.69912	0.049	0.262	2.14E-15	CGNP-Like
Marc2	1.33E-19	-0.75331	0.115	0.377	2.22E-15	CGNP-Like
Cd200	1.38E-19	-0.59065	0.04	0.246	2.31E-15	CGNP-Like
Tmbim6	1.39E-19	-0.63511	0.428	0.705	2.31E-15	CGNP-Like
Orai1	1.58E-19	-0.56411	0.015	0.18	2.64E-15	CGNP-Like
Tmem63b	1.59E-19	-0.52786	0.058	0.287	2.65E-15	CGNP-Like
Dtna	1.77E-19	-0.538	0.023	0.205	2.95E-15	CGNP-Like
4833424O1	2.14E-19	-0.65524	0.043	0.25	3.57E-15	CGNP-Like
Rgs5	2.29E-19	-1.19788	0.013	0.172	3.83E-15	CGNP-Like
Fgf9	2.37E-19	0.904372	0.356	0.057	3.95E-15	CGNP-Like
Tox3	2.62E-19	0.814533	0.461	0.135	4.38E-15	CGNP-Like
Pqlc1	2.65E-19	0.916697	0.443	0.127	4.41E-15	CGNP-Like
Dpysl4	2.65E-19	0.82631	0.545	0.213	4.43E-15	CGNP-Like
Add3	2.68E-19	-0.68498	0.143	0.422	4.47E-15	CGNP-Like
Nacc2	3.91E-19	-0.51268	0.023	0.201	6.52E-15	CGNP-Like
Gabrb1	3.95E-19	-0.36732	0	0.127	6.59E-15	CGNP-Like
Sema3d	3.95E-19	-0.47626	0	0.127	6.59E-15	CGNP-Like
Rplp1	4.15E-19	0.406945	0.969	0.91	6.93E-15	CGNP-Like
Dpysl3	4.17E-19	-0.75147	0.157	0.439	6.96E-15	CGNP-Like
Apoe	4.37E-19	-2.72531	0.318	0.553	7.29E-15	CGNP-Like
Gde1	4.37E-19	-0.64115	0.189	0.48	7.3E-15	CGNP-Like
Mt1	4.72E-19	-1.69687	0.278	0.529	7.88E-15	CGNP-Like
Calr	4.79E-19	-0.71506	0.598	0.799	7.98E-15	CGNP-Like
Mcm6	5.49E-19	0.938051	0.44	0.123	9.16E-15	CGNP-Like
Cenpf	6.24E-19	1.006378	0.53	0.221	1.04E-14	CGNP-Like

Dab1	6.78E-19	-0.70067	0.051	0.262	1.13E-14	CGNP-Like
Arl4a	7.32E-19	-0.8142	0.129	0.385	1.22E-14	CGNP-Like
Tmem88b	7.37E-19	-0.5254	0.005	0.143	1.23E-14	CGNP-Like
Lap3	7.72E-19	0.915577	0.501	0.197	1.29E-14	CGNP-Like
Epha4	8.27E-19	-0.58987	0.021	0.193	1.38E-14	CGNP-Like
Itgb8	8.51E-19	-0.55648	0.026	0.205	1.42E-14	CGNP-Like
Npc1	8.91E-19	-0.55744	0.041	0.242	1.49E-14	CGNP-Like
Nrep	8.92E-19	0.864957	0.577	0.262	1.49E-14	CGNP-Like
Fam63b	9.76E-19	-0.58014	0.064	0.291	1.63E-14	CGNP-Like
Gpr153	1.01E-18	0.838344	0.348	0.057	1.69E-14	CGNP-Like
Pcdh7	1.13E-18	-0.49994	0.016	0.18	1.88E-14	CGNP-Like
Mex3a	1.23E-18	0.759645	0.526	0.193	2.06E-14	CGNP-Like
Sox4	1.25E-18	0.837463	0.685	0.406	2.09E-14	CGNP-Like
Glud1	1.32E-18	-0.92554	0.189	0.447	2.2E-14	CGNP-Like
Hnrnph1	1.44E-18	0.612693	0.758	0.504	2.41E-14	CGNP-Like
Fgfr3	1.52E-18	-0.36009	0	0.123	2.54E-14	CGNP-Like
Prmt8	1.73E-18	0.90808	0.381	0.078	2.88E-14	CGNP-Like
Rpl14	1.78E-18	0.491923	0.865	0.705	2.97E-14	CGNP-Like
Fam3c	1.84E-18	-0.73914	0.097	0.34	3.06E-14	CGNP-Like
Scamp2	2.33E-18	-0.72484	0.194	0.467	3.88E-14	CGNP-Like
Enpp5	2.35E-18	-0.43969	0.013	0.168	3.91E-14	CGNP-Like
Chd4	2.45E-18	0.571347	0.857	0.725	4.09E-14	CGNP-Like
Rps15	2.65E-18	0.483108	0.914	0.807	4.42E-14	CGNP-Like
Gm10075	2.66E-18	0.660704	0.692	0.439	4.43E-14	CGNP-Like
Cacna2d1	2.8E-18	0.921585	0.405	0.102	4.68E-14	CGNP-Like
Gng3	3.09E-18	-0.73781	0.232	0.512	5.16E-14	CGNP-Like
Rbp4	3.6E-18	0.909107	0.344	0.053	6.01E-14	CGNP-Like
Abhd4	3.84E-18	-0.62687	0.066	0.283	6.4E-14	CGNP-Like
Mycn	3.93E-18	0.876082	0.41	0.102	6.56E-14	CGNP-Like
Cdk4	4.1E-18	0.55379	0.792	0.586	6.84E-14	CGNP-Like
Snrpf	4.13E-18	0.80082	0.557	0.275	6.88E-14	CGNP-Like
Lmnb1	4.37E-18	0.759104	0.458	0.143	7.3E-14	CGNP-Like
Cdh11	4.47E-18	-0.54395	0.02	0.184	7.45E-14	CGNP-Like
RP23-45G1	5.23E-18	0.921177	0.465	0.156	8.73E-14	CGNP-Like
Pde4b	5.75E-18	-0.69079	0.117	0.365	9.59E-14	CGNP-Like
Tmem255b	5.87E-18	-0.59636	0	0.119	9.8E-14	CGNP-Like
Pou3f1	5.87E-18	-0.60964	0	0.119	9.8E-14	CGNP-Like
Arxes2	6.84E-18	-0.76424	0.11	0.352	1.14E-13	CGNP-Like
Lgi3	7.39E-18	-0.54286	0.008	0.148	1.23E-13	CGNP-Like
Hmgn5	7.58E-18	0.82133	0.595	0.295	1.26E-13	CGNP-Like
Birc5	8.01E-18	0.815836	0.433	0.127	1.34E-13	CGNP-Like
Rpl39	8.04E-18	0.586307	0.811	0.619	1.34E-13	CGNP-Like
Cryl1	8.12E-18	-0.4311	0.008	0.148	1.35E-13	CGNP-Like
Mt2	8.62E-18	-1.39133	0.135	0.369	1.44E-13	CGNP-Like
Ppt1	8.9E-18	-0.59232	0.097	0.34	1.48E-13	CGNP-Like
Gpt2	8.92E-18	-0.68019	0.071	0.291	1.49E-13	CGNP-Like
Spock2	9.67E-18	-0.62385	0.048	0.246	1.61E-13	CGNP-Like
Dmd	1.02E-17	-0.60311	0.03	0.205	1.7E-13	CGNP-Like

Prrx1	1.31E-17	-0.42386	0.002	0.123	2.19E-13	CGNP-Like
Gjb6	1.41E-17	-0.41616	0.002	0.123	2.36E-13	CGNP-Like
Tspan17	1.44E-17	-0.3025	0.002	0.123	2.39E-13	CGNP-Like
Slc7a10	1.45E-17	-0.46235	0.005	0.135	2.42E-13	CGNP-Like
Cdk1	1.77E-17	0.937813	0.399	0.111	2.95E-13	CGNP-Like
Pcdh9	1.79E-17	-0.63615	0.051	0.246	2.99E-13	CGNP-Like
Atpif1	1.8E-17	0.52257	0.858	0.758	3E-13	CGNP-Like
Dkk3	1.87E-17	-0.45986	0.01	0.152	3.11E-13	CGNP-Like
Nedd4	1.89E-17	0.525331	0.815	0.635	3.15E-13	CGNP-Like
Lhx1	2.04E-17	0.976695	0.443	0.143	3.4E-13	CGNP-Like
Ccnd2	2.08E-17	0.743137	0.71	0.426	3.46E-13	CGNP-Like
Sox2ot	2.08E-17	-0.54628	0.007	0.139	3.47E-13	CGNP-Like
Nfix	2.16E-17	0.621542	0.694	0.414	3.61E-13	CGNP-Like
Lxn	2.17E-17	-0.93746	0.054	0.25	3.62E-13	CGNP-Like
Gpnmb	2.26E-17	-0.35018	0	0.115	3.77E-13	CGNP-Like
Casp12	2.26E-17	-0.43448	0	0.115	3.77E-13	CGNP-Like
Ptpro	2.31E-17	-0.40486	0.007	0.139	3.85E-13	CGNP-Like
Gsn	2.32E-17	-0.41955	0.012	0.156	3.87E-13	CGNP-Like
Slc25a4	2.37E-17	-0.43315	0.881	0.939	3.96E-13	CGNP-Like
Anp32e	2.48E-17	0.605295	0.781	0.59	4.14E-13	CGNP-Like
Nusap1	2.64E-17	0.916975	0.389	0.094	4.4E-13	CGNP-Like
Nrarp	2.78E-17	-0.46428	0.007	0.139	4.63E-13	CGNP-Like
Abhd3	2.97E-17	-0.36079	0.003	0.127	4.95E-13	CGNP-Like
Arpp21	3.49E-17	-0.63099	0.077	0.303	5.83E-13	CGNP-Like
Grina	3.91E-17	-0.55644	0.074	0.291	6.52E-13	CGNP-Like
Nim1	4.57E-17	-0.49801	0.01	0.148	7.62E-13	CGNP-Like
Banf1	5.15E-17	0.540173	0.825	0.684	8.59E-13	CGNP-Like
Fxyd1	5.26E-17	-0.43853	0.005	0.131	8.78E-13	CGNP-Like
Chadl	5.4E-17	-0.40422	0.002	0.119	9.01E-13	CGNP-Like
Atraid	5.47E-17	-0.62557	0.259	0.541	9.12E-13	CGNP-Like
Emp2	5.72E-17	-0.44196	0.002	0.119	9.54E-13	CGNP-Like
Tubb2b	5.98E-17	-0.64879	0.389	0.643	9.97E-13	CGNP-Like
Scrn1	6.31E-17	-0.51047	0.048	0.242	1.05E-12	CGNP-Like
Fbxo7	6.46E-17	-0.48543	0.026	0.193	1.08E-12	CGNP-Like
Hip1	6.51E-17	-0.68803	0.201	0.48	1.09E-12	CGNP-Like
Arl8a	6.6E-17	-0.56314	0.079	0.299	1.1E-12	CGNP-Like
H2afy	6.63E-17	0.642365	0.718	0.492	1.11E-12	CGNP-Like
Snrpb	7.02E-17	0.62352	0.715	0.512	1.17E-12	CGNP-Like
H2afx	7.07E-17	1.027343	0.506	0.242	1.18E-12	CGNP-Like
Usp24	7.53E-17	-0.57804	0.028	0.197	1.26E-12	CGNP-Like
Sdc4	7.7E-17	-0.49597	0.013	0.156	1.28E-12	CGNP-Like
Lrrfip1	7.72E-17	-0.58748	0.04	0.221	1.29E-12	CGNP-Like
Pou3f2	8.3E-17	0.68752	0.484	0.172	1.38E-12	CGNP-Like
Wnt7a	8.65E-17	-0.42051	0	0.111	1.44E-12	CGNP-Like
Cybrd1	8.65E-17	-0.34298	0	0.111	1.44E-12	CGNP-Like
Dcaf12l1	8.65E-17	-0.36181	0	0.111	1.44E-12	CGNP-Like
Gpr123	8.65E-17	-0.36453	0	0.111	1.44E-12	CGNP-Like
Cldn10	8.65E-17	-0.48562	0	0.111	1.44E-12	CGNP-Like

Abhd12	9.36E-17	-0.5907	0.109	0.348	1.56E-12	CGNP-Like
Hmgb3	9.57E-17	0.691617	0.451	0.152	1.6E-12	CGNP-Like
Slc44a1	1.01E-16	-0.85524	0.096	0.32	1.69E-12	CGNP-Like
Slitrk3	1.04E-16	-0.42072	0.007	0.135	1.74E-12	CGNP-Like
Tgfa	1.05E-16	-0.36985	0.003	0.123	1.75E-12	CGNP-Like
Jun	1.14E-16	0.670354	0.815	0.574	1.9E-12	CGNP-Like
Hmgn2	1.21E-16	0.793566	0.41	0.127	2.01E-12	CGNP-Like
Nop58	1.21E-16	0.590422	0.773	0.533	2.02E-12	CGNP-Like
Eef2	1.29E-16	0.522005	0.83	0.656	2.15E-12	CGNP-Like
Mif4gd	1.34E-16	-0.53433	0.033	0.205	2.24E-12	CGNP-Like
Nipa1	1.36E-16	-0.4767	0.025	0.184	2.26E-12	CGNP-Like
Selk	1.36E-16	-0.62679	0.456	0.709	2.27E-12	CGNP-Like
Prc1	1.43E-16	1.006927	0.42	0.139	2.39E-12	CGNP-Like
Tpm4	1.47E-16	0.712062	0.479	0.189	2.45E-12	CGNP-Like
Dirc2	1.56E-16	-0.58475	0.077	0.295	2.61E-12	CGNP-Like
Pdlim5	1.61E-16	-0.49014	0.025	0.184	2.68E-12	CGNP-Like
Specc1	1.75E-16	-0.62925	0.054	0.25	2.92E-12	CGNP-Like
Epcam	1.95E-16	-0.48638	0.002	0.115	3.25E-12	CGNP-Like
Trim9	2.1E-16	-0.41742	0.01	0.143	3.51E-12	CGNP-Like
Snrpg	2.14E-16	0.717819	0.484	0.201	3.57E-12	CGNP-Like
Spry2	2.21E-16	-0.68931	0.084	0.299	3.69E-12	CGNP-Like
Cnbp	2.22E-16	0.446357	0.876	0.795	3.7E-12	CGNP-Like
Spc24	2.28E-16	0.870068	0.367	0.094	3.8E-12	CGNP-Like
Sar1b	2.33E-16	-0.58715	0.221	0.492	3.88E-12	CGNP-Like
Otx2	2.74E-16	0.785605	0.3	0.041	4.57E-12	CGNP-Like
Paqr4	2.8E-16	-0.67025	0.054	0.246	4.67E-12	CGNP-Like
Lrrtm3	2.91E-16	-0.49161	0.013	0.152	4.85E-12	CGNP-Like
Fam210b	3.24E-16	0.854927	0.364	0.094	5.4E-12	CGNP-Like
Epas1	3.7E-16	-0.36831	0.007	0.131	6.17E-12	CGNP-Like
Acss1	3.7E-16	-0.37049	0.003	0.119	6.17E-12	CGNP-Like
Srsf3	3.75E-16	0.533667	0.778	0.586	6.26E-12	CGNP-Like
Vcam1	4.04E-16	-0.33645	0.012	0.148	6.73E-12	CGNP-Like
Gpx3	4.2E-16	-0.51583	0.003	0.119	7E-12	CGNP-Like
Sybu	4.54E-16	-0.36679	0.003	0.119	7.57E-12	CGNP-Like
Arvcf	4.65E-16	-0.51106	0.008	0.135	7.75E-12	CGNP-Like
Irx2	4.79E-16	-0.46649	0.015	0.156	7.98E-12	CGNP-Like
Itm2b	4.8E-16	-0.66953	0.727	0.807	8.01E-12	CGNP-Like
Ptch2	4.84E-16	0.742684	0.267	0.02	8.07E-12	CGNP-Like
Slc1a3	5.19E-16	-2.33688	0.269	0.471	8.66E-12	CGNP-Like
Tspan15	5.4E-16	-0.37012	0.008	0.135	9E-12	CGNP-Like
Srebf1	5.75E-16	0.918988	0.417	0.143	9.59E-12	CGNP-Like
Sec62	6.18E-16	-0.54365	0.456	0.697	1.03E-11	CGNP-Like
Insm1	6.23E-16	0.81546	0.339	0.07	1.04E-11	CGNP-Like
Gabbr1	6.94E-16	-0.67612	0.112	0.34	1.16E-11	CGNP-Like
Taok3	6.96E-16	-0.54781	0.084	0.303	1.16E-11	CGNP-Like
Gpd1	7.01E-16	-0.46187	0.008	0.135	1.17E-11	CGNP-Like
Matn4	7.29E-16	-0.51371	0.005	0.123	1.22E-11	CGNP-Like
Serinc1	7.92E-16	-0.61173	0.435	0.684	1.32E-11	CGNP-Like

Smpdl3a	8.43E-16	-0.44712	0.01	0.139	1.41E-11	CGNP-Like
Gas5	8.49E-16	0.50727	0.867	0.746	1.42E-11	CGNP-Like
Sez6l	8.86E-16	-0.51386	0.077	0.291	1.48E-11	CGNP-Like
Wscd1	9.66E-16	-0.56348	0.081	0.291	1.61E-11	CGNP-Like
Rpl34	9.66E-16	0.491699	0.839	0.648	1.61E-11	CGNP-Like
Ccna2	1.14E-15	0.81003	0.364	0.094	1.9E-11	CGNP-Like
Spag9	1.2E-15	-0.69412	0.369	0.623	1.99E-11	CGNP-Like
Ifi27	1.21E-15	-0.41452	0.015	0.152	2.03E-11	CGNP-Like
Camk1	1.22E-15	-0.60746	0.064	0.262	2.03E-11	CGNP-Like
Pld2	1.26E-15	-0.28598	0	0.102	2.1E-11	CGNP-Like
Gpr146	1.26E-15	-0.30719	0	0.102	2.1E-11	CGNP-Like
Resp18	1.26E-15	-0.55583	0	0.102	2.1E-11	CGNP-Like
Cml1	1.3E-15	-0.49713	0.016	0.156	2.16E-11	CGNP-Like
Dner	1.3E-15	-0.71835	0.122	0.348	2.16E-11	CGNP-Like
Pgm2	1.43E-15	-0.43656	0.018	0.16	2.39E-11	CGNP-Like
Slitrk2	1.47E-15	-0.45383	0.007	0.127	2.45E-11	CGNP-Like
Nucks1	1.52E-15	0.603337	0.799	0.684	2.54E-11	CGNP-Like
Tlcd1	1.54E-15	-0.66752	0.049	0.23	2.56E-11	CGNP-Like
Tpi1	1.7E-15	-0.58087	0.115	0.344	2.84E-11	CGNP-Like
Rps25	1.71E-15	0.597783	0.689	0.443	2.85E-11	CGNP-Like
Meis1	1.87E-15	0.801748	0.466	0.193	3.11E-11	CGNP-Like
S100a4	2.02E-15	-0.45121	0.008	0.131	3.37E-11	CGNP-Like
Cpne2	2.22E-15	-0.40169	0.008	0.131	3.7E-11	CGNP-Like
Slc9a3r1	2.27E-15	-0.39666	0.028	0.184	3.78E-11	CGNP-Like
Dock9	2.34E-15	-0.47139	0.021	0.168	3.91E-11	CGNP-Like
Ndrg2	2.46E-15	-0.88014	0.265	0.508	4.11E-11	CGNP-Like
Hsd11b1	2.72E-15	-0.69743	0.005	0.119	4.53E-11	CGNP-Like
Incenp	2.72E-15	0.84111	0.402	0.123	4.54E-11	CGNP-Like
Lrrtm1	2.86E-15	-0.50768	0.002	0.107	4.77E-11	CGNP-Like
1500016L(	3.06E-15	0.86387	0.334	0.078	5.1E-11	CGNP-Like
Vwa1	3.15E-15	-0.30376	0.002	0.107	5.26E-11	CGNP-Like
Tmem132b	3.18E-15	-0.43543	0.002	0.107	5.3E-11	CGNP-Like
Stmn1	3.34E-15	0.712009	0.349	0.086	5.57E-11	CGNP-Like
Aatk	3.38E-15	-0.38107	0.02	0.164	5.63E-11	CGNP-Like
Taf9b	3.39E-15	-0.36004	0.012	0.139	5.65E-11	CGNP-Like
Gabbr2	3.59E-15	-0.45446	0.01	0.135	5.99E-11	CGNP-Like
B3gat1	3.61E-15	-0.69116	0.03	0.184	6.02E-11	CGNP-Like
Cltb	4.16E-15	0.837041	0.601	0.373	6.93E-11	CGNP-Like
Pcdh11x	4.51E-15	-0.45949	0.007	0.123	7.52E-11	CGNP-Like
Sh3bp5	4.58E-15	-0.58893	0.063	0.254	7.64E-11	CGNP-Like
Bcas1	4.62E-15	-1.69286	0.371	0.549	7.71E-11	CGNP-Like
Amz1	4.64E-15	-0.40301	0.013	0.143	7.73E-11	CGNP-Like
Prex2	4.86E-15	-0.56754	0.02	0.16	8.1E-11	CGNP-Like
Prkcq	5.72E-15	-0.68332	0.068	0.254	9.53E-11	CGNP-Like
Kifc3	6.24E-15	-0.41966	0.003	0.111	1.04E-10	CGNP-Like
Aldoa	6.32E-15	-0.64855	0.219	0.48	1.05E-10	CGNP-Like
Ctcf	6.75E-15	0.603186	0.636	0.385	1.13E-10	CGNP-Like
Sh3gl3	6.76E-15	-0.5069	0.026	0.176	1.13E-10	CGNP-Like

Snrpe	6.77E-15	0.59704	0.68	0.439	1.13E-10	CGNP-Like
Emc7	7.31E-15	-0.59498	0.196	0.447	1.22E-10	CGNP-Like
Cdca8	7.39E-15	0.772077	0.399	0.123	1.23E-10	CGNP-Like
Rrbp1	7.52E-15	-0.6964	0.252	0.512	1.25E-10	CGNP-Like
Ppic	7.64E-15	0.807444	0.357	0.102	1.27E-10	CGNP-Like
Hells	7.7E-15	0.887136	0.328	0.078	1.28E-10	CGNP-Like
Rassf4	8.45E-15	0.856196	0.381	0.123	1.41E-10	CGNP-Like
Trp53	9.42E-15	0.7479	0.451	0.197	1.57E-10	CGNP-Like
Syncrip	9.91E-15	0.602247	0.666	0.434	1.65E-10	CGNP-Like
Adcyap1r1	9.92E-15	-0.57787	0.117	0.34	1.65E-10	CGNP-Like
Slc6a9	1.04E-14	-0.36573	0.01	0.131	1.73E-10	CGNP-Like
1500009C(	1.12E-14	-0.3132	0.002	0.102	1.87E-10	CGNP-Like
Arhgdig	1.15E-14	-0.50867	0.012	0.135	1.92E-10	CGNP-Like
2810459M	1.19E-14	-0.30739	0.002	0.102	1.99E-10	CGNP-Like
Abracl	1.21E-14	0.719367	0.356	0.094	2.02E-10	CGNP-Like
Sat1	1.24E-14	-0.6994	0.063	0.25	2.06E-10	CGNP-Like
Cntnap2	1.26E-14	-0.34984	0.002	0.102	2.11E-10	CGNP-Like
Zeb1	1.45E-14	0.581592	0.549	0.266	2.42E-10	CGNP-Like
Tpp1	1.53E-14	-0.5184	0.087	0.295	2.56E-10	CGNP-Like
Mdk	1.54E-14	0.765739	0.539	0.279	2.57E-10	CGNP-Like
Rasl11a	1.64E-14	-0.69828	0.035	0.189	2.73E-10	CGNP-Like
Gmpr	1.67E-14	-0.40722	0.013	0.139	2.79E-10	CGNP-Like
Nrxn3	1.74E-14	-0.58528	0.015	0.143	2.91E-10	CGNP-Like
Rps15a	1.79E-14	0.505501	0.799	0.635	2.99E-10	CGNP-Like
Megf11	1.87E-14	-0.53511	0.013	0.139	3.11E-10	CGNP-Like
Bcl11a	1.93E-14	0.654499	0.29	0.049	3.23E-10	CGNP-Like
Tln2	1.97E-14	-0.4595	0.028	0.176	3.28E-10	CGNP-Like
Prox1	2.07E-14	0.7229	0.32	0.07	3.45E-10	CGNP-Like
Bricd5	2.13E-14	-0.4291	0.003	0.107	3.55E-10	CGNP-Like
Ust	2.14E-14	-0.47451	0.054	0.234	3.57E-10	CGNP-Like
Rpl23	2.25E-14	0.538609	0.723	0.516	3.76E-10	CGNP-Like
Grid1	2.34E-14	-0.37261	0.008	0.123	3.9E-10	CGNP-Like
Rps3a1	2.39E-14	0.498743	0.829	0.66	3.99E-10	CGNP-Like
Fstl1	2.58E-14	0.813538	0.348	0.098	4.3E-10	CGNP-Like
Agrn	2.64E-14	-0.56852	0.105	0.32	4.4E-10	CGNP-Like
Vimp	3.16E-14	-0.67074	0.247	0.492	5.27E-10	CGNP-Like
Gramd3	3.26E-14	-0.31376	0.008	0.123	5.44E-10	CGNP-Like
Htatsf1	3.27E-14	0.637911	0.623	0.365	5.45E-10	CGNP-Like
Kif21a	3.32E-14	0.757908	0.491	0.23	5.54E-10	CGNP-Like
Pdap1	3.49E-14	0.505734	0.787	0.631	5.82E-10	CGNP-Like
Tpx2	3.78E-14	0.800154	0.433	0.172	6.31E-10	CGNP-Like
Hmgb1	3.88E-14	0.617001	0.498	0.242	6.47E-10	CGNP-Like
Cxcl14	3.96E-14	-0.66964	0.048	0.213	6.61E-10	CGNP-Like
Fubp1	3.98E-14	0.574696	0.702	0.5	6.65E-10	CGNP-Like
Dkc1	4.02E-14	0.574457	0.521	0.246	6.71E-10	CGNP-Like
S100a10	4.31E-14	-0.7292	0.048	0.213	7.19E-10	CGNP-Like
5730559C:	4.34E-14	-0.33457	0.005	0.111	7.24E-10	CGNP-Like
Ncapg	5.36E-14	0.708747	0.323	0.074	8.93E-10	CGNP-Like

App	5.37E-14	-0.58449	0.593	0.758	8.95E-10	CGNP-Like
Arap2	5.65E-14	-0.367	0.007	0.115	9.43E-10	CGNP-Like
Prnp	5.67E-14	-0.55987	0.198	0.447	9.46E-10	CGNP-Like
Rad51ap1	6E-14	0.705677	0.27	0.041	1E-09	CGNP-Like
Fnta	6.32E-14	-0.5899	0.15	0.373	1.05E-09	CGNP-Like
Dock10	6.35E-14	-0.52793	0.018	0.148	1.06E-09	CGNP-Like
Mfap4	6.66E-14	0.845642	0.259	0.037	1.11E-09	CGNP-Like
Nckap1	7.55E-14	-0.57192	0.137	0.361	1.26E-09	CGNP-Like
Wbp5	7.61E-14	0.57124	0.677	0.451	1.27E-09	CGNP-Like
Lix1l	8.14E-14	-0.29484	0.013	0.135	1.36E-09	CGNP-Like
Rps10	8.3E-14	0.497518	0.789	0.631	1.38E-09	CGNP-Like
Mtss1l	8.3E-14	-0.50463	0.035	0.184	1.38E-09	CGNP-Like
Gyg	8.37E-14	-0.38737	0.016	0.143	1.4E-09	CGNP-Like
Rbm8a	1.02E-13	0.567407	0.618	0.385	1.69E-09	CGNP-Like
Prim1	1.06E-13	0.712837	0.41	0.148	1.76E-09	CGNP-Like
Elovl5	1.09E-13	-0.64572	0.15	0.369	1.82E-09	CGNP-Like
Clspn	1.22E-13	0.793277	0.303	0.066	2.03E-09	CGNP-Like
Canx	1.24E-13	-0.48959	0.728	0.799	2.07E-09	CGNP-Like
Fads2	1.36E-13	-0.34714	0.01	0.123	2.27E-09	CGNP-Like
Cpxm1	1.45E-13	-0.34735	0.012	0.127	2.42E-09	CGNP-Like
Cklf	1.5E-13	0.701729	0.308	0.074	2.5E-09	CGNP-Like
Ier2	1.59E-13	0.664325	0.539	0.266	2.65E-09	CGNP-Like
Pth1r	1.61E-13	-0.33795	0.005	0.107	2.69E-09	CGNP-Like
Myh10	1.66E-13	0.651479	0.455	0.18	2.76E-09	CGNP-Like
Sema7a	1.66E-13	0.707841	0.217	0.012	2.77E-09	CGNP-Like
Csf1	1.72E-13	-0.27855	0.01	0.123	2.87E-09	CGNP-Like
Smco4	1.73E-13	0.669656	0.285	0.053	2.89E-09	CGNP-Like
Flrt1	1.85E-13	-0.48431	0.005	0.107	3.09E-09	CGNP-Like
Itga6	1.86E-13	-0.42869	0.012	0.127	3.1E-09	CGNP-Like
Acin1	1.88E-13	0.452577	0.84	0.68	3.14E-09	CGNP-Like
Cdca7	1.95E-13	0.686721	0.303	0.066	3.26E-09	CGNP-Like
Rrm2	1.96E-13	0.711069	0.336	0.09	3.27E-09	CGNP-Like
Trib2	2.01E-13	-0.55545	0.137	0.352	3.35E-09	CGNP-Like
Cav2	2.04E-13	-0.4009	0.007	0.111	3.4E-09	CGNP-Like
Pgp	2.17E-13	-0.73249	0.199	0.422	3.62E-09	CGNP-Like
Pbk	2.18E-13	0.623441	0.353	0.098	3.64E-09	CGNP-Like
Tshz2	2.2E-13	0.709381	0.433	0.18	3.67E-09	CGNP-Like
Pdpn	2.42E-13	-0.47955	0.021	0.152	4.04E-09	CGNP-Like
4930402H	2.48E-13	-0.50785	0.13	0.352	4.13E-09	CGNP-Like
Myl12a	2.51E-13	0.712277	0.502	0.27	4.19E-09	CGNP-Like
Glrb	2.57E-13	-0.46738	0.04	0.193	4.29E-09	CGNP-Like
Tmem106b	3.06E-13	-0.49558	0.071	0.254	5.11E-09	CGNP-Like
Kif23	3.14E-13	0.687763	0.341	0.09	5.24E-09	CGNP-Like
Fam49b	3.27E-13	-0.52849	0.1	0.303	5.45E-09	CGNP-Like
Tsn	3.37E-13	0.49716	0.712	0.48	5.63E-09	CGNP-Like
Foxp1	3.38E-13	0.669417	0.247	0.033	5.64E-09	CGNP-Like
Necab2	3.5E-13	-0.33944	0.008	0.115	5.84E-09	CGNP-Like
Hnrnpa0	3.62E-13	0.650809	0.537	0.303	6.04E-09	CGNP-Like

Fus	3.94E-13	0.387613	0.847	0.746	6.57E-09	CGNP-Like
Arl2bp	4.12E-13	-0.57813	0.232	0.484	6.87E-09	CGNP-Like
Lgals1	4.39E-13	0.789119	0.394	0.148	7.32E-09	CGNP-Like
Lnx1	4.5E-13	-0.51689	0.013	0.127	7.51E-09	CGNP-Like
Eif3a	4.67E-13	0.473512	0.812	0.66	7.78E-09	CGNP-Like
Siva1	4.83E-13	0.738561	0.386	0.143	8.05E-09	CGNP-Like
Lyn	5.36E-13	-0.34413	0.01	0.119	8.93E-09	CGNP-Like
Spry4	5.41E-13	-0.46817	0.012	0.123	9.02E-09	CGNP-Like
Lrig3	5.43E-13	0.667683	0.287	0.061	9.06E-09	CGNP-Like
Galnt10	5.54E-13	-0.36385	0.005	0.102	9.24E-09	CGNP-Like
Plcd4	5.7E-13	-0.32342	0.005	0.102	9.51E-09	CGNP-Like
Lig1	5.72E-13	0.735428	0.448	0.184	9.54E-09	CGNP-Like
Cox8a	5.91E-13	-0.38278	0.819	0.869	9.86E-09	CGNP-Like
Mbp	6.21E-13	-2.03814	0.163	0.369	1.04E-08	CGNP-Like
Pgpep1	6.43E-13	-0.32017	0.012	0.123	1.07E-08	CGNP-Like
Arxes1	6.52E-13	-0.43457	0.086	0.275	1.09E-08	CGNP-Like
Dnajc9	6.69E-13	0.597207	0.507	0.246	1.12E-08	CGNP-Like
Abcd3	6.82E-13	-0.62676	0.254	0.48	1.14E-08	CGNP-Like
Ube2c	6.94E-13	1.012956	0.397	0.156	1.16E-08	CGNP-Like
Cask	7.15E-13	-0.54699	0.084	0.266	1.19E-08	CGNP-Like
Ppp2r2c	7.47E-13	0.69183	0.465	0.217	1.25E-08	CGNP-Like
Vps37b	7.66E-13	0.685499	0.567	0.328	1.28E-08	CGNP-Like
Shfm1	7.67E-13	0.427712	0.837	0.693	1.28E-08	CGNP-Like
Ildr2	7.93E-13	-0.42233	0.007	0.107	1.32E-08	CGNP-Like
Tub	8.03E-13	-0.51958	0.02	0.143	1.34E-08	CGNP-Like
Chd3	8.53E-13	-0.64209	0.097	0.291	1.42E-08	CGNP-Like
Pbrm1	8.69E-13	0.543236	0.651	0.418	1.45E-08	CGNP-Like
Esco2	8.9E-13	0.85984	0.318	0.09	1.48E-08	CGNP-Like
Car11	8.94E-13	-0.39626	0.015	0.131	1.49E-08	CGNP-Like
Usp1	8.98E-13	0.566494	0.488	0.23	1.5E-08	CGNP-Like
Angptl2	9.67E-13	0.63214	0.204	0.012	1.61E-08	CGNP-Like
Ube2e3	9.69E-13	0.621693	0.489	0.246	1.62E-08	CGNP-Like
Hk2	9.88E-13	0.676656	0.259	0.049	1.65E-08	CGNP-Like
Akap6	1.04E-12	0.699346	0.328	0.094	1.73E-08	CGNP-Like
Plp1	1.07E-12	-2.97185	0.155	0.348	1.78E-08	CGNP-Like
Fbxo5	1.09E-12	0.579038	0.28	0.057	1.81E-08	CGNP-Like
Atad2	1.09E-12	0.7177	0.323	0.09	1.82E-08	CGNP-Like
Hmmr	1.21E-12	0.765818	0.295	0.066	2.02E-08	CGNP-Like
Larp7	1.22E-12	0.632351	0.473	0.213	2.04E-08	CGNP-Like
Sowaha	1.55E-12	0.846426	0.336	0.102	2.58E-08	CGNP-Like
Zdhhc2	1.56E-12	-0.57919	0.044	0.193	2.61E-08	CGNP-Like
Grin3a	1.57E-12	-0.59286	0.082	0.262	2.62E-08	CGNP-Like
Rtkn	1.63E-12	-0.40072	0.033	0.172	2.72E-08	CGNP-Like
Grhpr	1.65E-12	-0.42656	0.01	0.115	2.76E-08	CGNP-Like
Racgap1	1.67E-12	0.589975	0.392	0.139	2.78E-08	CGNP-Like
Ddx5	1.71E-12	0.38839	0.871	0.746	2.85E-08	CGNP-Like
Mpzl1	1.74E-12	-0.95734	0.18	0.381	2.9E-08	CGNP-Like
Kif15	1.8E-12	0.757054	0.298	0.074	3E-08	CGNP-Like

Kcnd3	1.96E-12	-0.48971	0.013	0.123	3.27E-08	CGNP-Like
Nhlh2	2E-12	0.711812	0.323	0.094	3.33E-08	CGNP-Like
Ptpra	2E-12	-0.56867	0.306	0.537	3.33E-08	CGNP-Like
Laptm4b	2.12E-12	-0.49546	0.201	0.434	3.54E-08	CGNP-Like
Fgf12	2.14E-12	-0.41487	0.013	0.123	3.57E-08	CGNP-Like
Atoh1	2.17E-12	0.724674	0.25	0.045	3.62E-08	CGNP-Like
Lmo4	2.33E-12	0.690781	0.544	0.316	3.89E-08	CGNP-Like
Rev3l	2.38E-12	-0.73637	0.102	0.283	3.97E-08	CGNP-Like
Adam11	2.72E-12	-0.26363	0.012	0.119	4.54E-08	CGNP-Like
Baz1b	2.79E-12	0.603853	0.638	0.426	4.65E-08	CGNP-Like
Celf2	2.82E-12	0.635214	0.638	0.418	4.7E-08	CGNP-Like
Abcg1	2.82E-12	-0.38407	0.013	0.123	4.71E-08	CGNP-Like
Gabra4	2.9E-12	-0.27371	0.016	0.131	4.84E-08	CGNP-Like
Car10	2.93E-12	-0.38075	0.012	0.119	4.89E-08	CGNP-Like
Snx32	2.96E-12	-0.28209	0.013	0.123	4.94E-08	CGNP-Like
Tcf4	3.06E-12	0.341624	0.944	0.893	5.1E-08	CGNP-Like
Frmd4b	3.08E-12	0.592037	0.234	0.033	5.13E-08	CGNP-Like
Cald1	3.08E-12	0.664401	0.613	0.393	5.14E-08	CGNP-Like
Arhgap11a	3.11E-12	0.645169	0.285	0.061	5.18E-08	CGNP-Like
Iqsec1	3.17E-12	-0.47749	0.015	0.127	5.28E-08	CGNP-Like
H3f3a	3.22E-12	0.591274	0.479	0.254	5.38E-08	CGNP-Like
Apex1	3.37E-12	0.603774	0.547	0.311	5.63E-08	CGNP-Like
Gja1	3.41E-12	-0.75573	0.046	0.193	5.7E-08	CGNP-Like
Ssrp1	3.43E-12	0.469034	0.71	0.537	5.72E-08	CGNP-Like
Slc15a2	3.59E-12	-0.41895	0.049	0.205	5.99E-08	CGNP-Like
Itgav	3.63E-12	-0.53792	0.079	0.254	6.06E-08	CGNP-Like
Syt13	3.65E-12	0.600545	0.206	0.016	6.08E-08	CGNP-Like
H2afz	3.99E-12	0.591701	0.341	0.111	6.65E-08	CGNP-Like
Pax3	4.04E-12	-0.43994	0.02	0.139	6.74E-08	CGNP-Like
Lhfpl2	4.13E-12	-0.34523	0.023	0.148	6.89E-08	CGNP-Like
Sgk1	4.15E-12	-0.65587	0.036	0.172	6.92E-08	CGNP-Like
Cacnb4	4.45E-12	-0.38405	0.023	0.148	7.43E-08	CGNP-Like
Homer2	4.52E-12	0.599777	0.361	0.127	7.54E-08	CGNP-Like
Tnfrsf19	4.55E-12	-0.31037	0.008	0.107	7.59E-08	CGNP-Like
Lbh	4.57E-12	-0.5725	0.097	0.283	7.62E-08	CGNP-Like
Abca1	4.65E-12	-0.38285	0.021	0.143	7.75E-08	CGNP-Like
Snrpd1	4.94E-12	0.525822	0.677	0.537	8.24E-08	CGNP-Like
Pcp4l1	5.06E-12	-0.39186	0.008	0.107	8.45E-08	CGNP-Like
Rab33a	5.2E-12	-0.66149	0.043	0.184	8.68E-08	CGNP-Like
Tjp2	5.3E-12	-0.42974	0.087	0.27	8.84E-08	CGNP-Like
Ybx3	6.07E-12	0.576866	0.45	0.205	1.01E-07	CGNP-Like
Phip	6.14E-12	0.515768	0.555	0.303	1.02E-07	CGNP-Like
Gabra3	6.2E-12	-0.39861	0.01	0.111	1.03E-07	CGNP-Like
Glul	6.36E-12	-1.38792	0.26	0.443	1.06E-07	CGNP-Like
Eif1b	6.76E-12	-0.59798	0.412	0.615	1.13E-07	CGNP-Like
Tmco3	7.02E-12	-0.39061	0.043	0.189	1.17E-07	CGNP-Like
Nap1l1	7.23E-12	0.530592	0.672	0.508	1.21E-07	CGNP-Like
Fnbp1l	7.41E-12	0.581892	0.534	0.275	1.24E-07	CGNP-Like

Bok	7.74E-12	0.580536	0.318	0.094	1.29E-07	CGNP-Like
Hnrnpdl	7.78E-12	0.378423	0.86	0.721	1.3E-07	CGNP-Like
Elovl7	8.05E-12	-0.5172	0.021	0.139	1.34E-07	CGNP-Like
A230050P2	8.65E-12	-0.34297	0.016	0.127	1.44E-07	CGNP-Like
Cenpv	8.94E-12	0.541963	0.471	0.225	1.49E-07	CGNP-Like
Lphn3	9.97E-12	-0.49588	0.077	0.25	1.66E-07	CGNP-Like
Gng2	1E-11	0.548568	0.44	0.189	1.67E-07	CGNP-Like
Tipin	1.01E-11	0.654746	0.407	0.176	1.68E-07	CGNP-Like
Cdc42se1	1.02E-11	-0.50193	0.219	0.443	1.7E-07	CGNP-Like
Sacs	1.06E-11	0.630384	0.275	0.066	1.76E-07	CGNP-Like
Cxxc5	1.08E-11	0.562236	0.603	0.344	1.8E-07	CGNP-Like
Trim59	1.09E-11	0.603358	0.288	0.074	1.81E-07	CGNP-Like
Snrpb2	1.09E-11	0.538099	0.496	0.254	1.82E-07	CGNP-Like
Hmgcl	1.1E-11	-0.46652	0.104	0.291	1.83E-07	CGNP-Like
Mns1	1.1E-11	0.584103	0.338	0.107	1.83E-07	CGNP-Like
Capns1	1.1E-11	-0.54496	0.29	0.508	1.83E-07	CGNP-Like
Kif21b	1.12E-11	-0.37125	0.016	0.127	1.87E-07	CGNP-Like
Mmp16	1.14E-11	-0.64167	0.086	0.254	1.91E-07	CGNP-Like
Gsta4	1.2E-11	-0.28225	0.013	0.119	2E-07	CGNP-Like
Man2a2	1.23E-11	-0.31362	0.04	0.18	2.05E-07	CGNP-Like
Cenph	1.29E-11	0.691329	0.293	0.078	2.15E-07	CGNP-Like
Lrch3	1.33E-11	-0.52587	0.097	0.275	2.21E-07	CGNP-Like
A930009A1	1.34E-11	-0.47918	0.038	0.172	2.23E-07	CGNP-Like
Rabac1	1.45E-11	-0.46344	0.292	0.529	2.41E-07	CGNP-Like
Rap2a	1.59E-11	-0.68728	0.241	0.451	2.65E-07	CGNP-Like
Loxl1	1.64E-11	0.616895	0.208	0.025	2.74E-07	CGNP-Like
Tmem30a	1.68E-11	-0.51775	0.214	0.43	2.81E-07	CGNP-Like
Elavl2	1.71E-11	0.652435	0.278	0.07	2.84E-07	CGNP-Like
H1fx	1.72E-11	0.671519	0.369	0.148	2.87E-07	CGNP-Like
Spc25	1.79E-11	0.667622	0.367	0.139	2.98E-07	CGNP-Like
Sparcl1	1.83E-11	-1.60458	0.395	0.549	3.05E-07	CGNP-Like
Rad21	1.88E-11	0.557541	0.586	0.389	3.14E-07	CGNP-Like
Tmem50b	1.89E-11	-0.36919	0.038	0.176	3.16E-07	CGNP-Like
Il6st	2.05E-11	-0.35326	0.041	0.18	3.43E-07	CGNP-Like
Nrcam	2.26E-11	-0.49109	0.12	0.311	3.77E-07	CGNP-Like
Acsl3	2.26E-11	-0.62235	0.196	0.402	3.77E-07	CGNP-Like
Spred1	2.35E-11	-0.44753	0.163	0.377	3.91E-07	CGNP-Like
Eid1	2.37E-11	-0.51157	0.55	0.746	3.95E-07	CGNP-Like
Tgif1	2.42E-11	0.544615	0.18	0.008	4.04E-07	CGNP-Like
Mageh1	2.52E-11	-0.55299	0.157	0.352	4.2E-07	CGNP-Like
Gabarapl1	2.65E-11	-0.49685	0.217	0.443	4.42E-07	CGNP-Like
Txndc15	2.66E-11	-0.55793	0.168	0.385	4.43E-07	CGNP-Like
Cdca3	2.81E-11	0.574796	0.361	0.131	4.7E-07	CGNP-Like
Pfkm	2.82E-11	-0.47241	0.071	0.234	4.7E-07	CGNP-Like
Atcay	2.93E-11	-0.52186	0.048	0.189	4.89E-07	CGNP-Like
Fam168a	2.95E-11	-0.52457	0.325	0.541	4.92E-07	CGNP-Like
Ndc80	2.98E-11	0.566121	0.231	0.037	4.98E-07	CGNP-Like
Ckap2l	3.09E-11	0.714077	0.366	0.148	5.15E-07	CGNP-Like

Papss1	3.3E-11	-0.54485	0.183	0.389	5.51E-07	CGNP-Like
Reep1	3.34E-11	-0.42034	0.059	0.213	5.57E-07	CGNP-Like
Atrx	3.38E-11	0.472661	0.802	0.66	5.64E-07	CGNP-Like
Tmem5	3.46E-11	-0.48461	0.099	0.275	5.76E-07	CGNP-Like
Cdca2	3.51E-11	0.602367	0.236	0.041	5.85E-07	CGNP-Like
Ccp110	3.53E-11	0.595289	0.353	0.131	5.88E-07	CGNP-Like
Ybx1	3.56E-11	0.355065	0.895	0.82	5.94E-07	CGNP-Like
Arhgef9	3.59E-11	-0.50293	0.096	0.275	5.98E-07	CGNP-Like
Paip2	3.59E-11	0.522792	0.666	0.484	5.99E-07	CGNP-Like
Acsl6	3.63E-11	-0.4052	0.016	0.123	6.06E-07	CGNP-Like
Tle1	3.7E-11	0.697663	0.328	0.127	6.18E-07	CGNP-Like
Supt16	3.95E-11	0.479101	0.652	0.447	6.59E-07	CGNP-Like
Dctpp1	3.98E-11	0.624751	0.455	0.246	6.64E-07	CGNP-Like
Timeless	4.03E-11	0.577611	0.254	0.053	6.73E-07	CGNP-Like
Atp6ap1	4.06E-11	-0.40098	0.12	0.311	6.77E-07	CGNP-Like
Trpm3	4.15E-11	-0.31045	0.015	0.119	6.93E-07	CGNP-Like
Pcnt	4.21E-11	0.604948	0.272	0.07	7.02E-07	CGNP-Like
Ccdc107	4.49E-11	-0.51699	0.091	0.262	7.49E-07	CGNP-Like
Hdac2	4.85E-11	0.483525	0.596	0.393	8.08E-07	CGNP-Like
Ccdc34	4.92E-11	0.560344	0.585	0.377	8.21E-07	CGNP-Like
Plcb1	5.27E-11	-0.56194	0.1	0.279	8.78E-07	CGNP-Like
Mid1ip1	5.4E-11	-0.43995	0.072	0.234	9.01E-07	CGNP-Like
Chd7	5.71E-11	0.542306	0.644	0.443	9.52E-07	CGNP-Like
Lyar	5.83E-11	0.544581	0.446	0.209	9.72E-07	CGNP-Like
Arpc5	6.15E-11	-0.49174	0.356	0.582	1.03E-06	CGNP-Like
Nkain4	6.17E-11	-0.63293	0.135	0.32	1.03E-06	CGNP-Like
Hnrnpc	6.2E-11	0.406299	0.794	0.672	1.03E-06	CGNP-Like
Dtymk	6.29E-11	0.565944	0.577	0.373	1.05E-06	CGNP-Like
Kif11	6.35E-11	0.438335	0.343	0.115	1.06E-06	CGNP-Like
Zbtb18	6.58E-11	0.58706	0.285	0.078	1.1E-06	CGNP-Like
Srsf7	7.54E-11	0.491091	0.585	0.361	1.26E-06	CGNP-Like
Cenpa	7.81E-11	0.845197	0.372	0.156	1.3E-06	CGNP-Like
Nova1	8.12E-11	-0.4719	0.063	0.213	1.35E-06	CGNP-Like
Gfap	8.13E-11	-0.89768	0.046	0.18	1.36E-06	CGNP-Like
Tmem59	8.17E-11	-0.46086	0.461	0.66	1.36E-06	CGNP-Like
Rbms1	8.19E-11	0.599643	0.293	0.09	1.37E-06	CGNP-Like
8430419L(	8.26E-11	-0.41096	0.076	0.238	1.38E-06	CGNP-Like
Cenpk	8.38E-11	0.551034	0.239	0.045	1.4E-06	CGNP-Like
B2m	8.4E-11	-0.5735	0.354	0.545	1.4E-06	CGNP-Like
Tk1	8.47E-11	0.548252	0.245	0.053	1.41E-06	CGNP-Like
Gse1	8.54E-11	0.558563	0.28	0.074	1.42E-06	CGNP-Like
Sgol2	8.6E-11	0.697356	0.273	0.07	1.43E-06	CGNP-Like
Gm11223	8.78E-11	0.730764	0.231	0.045	1.46E-06	CGNP-Like
Rpa2	8.86E-11	0.577305	0.328	0.111	1.48E-06	CGNP-Like
Tnfrsf21	8.88E-11	-0.39306	0.058	0.205	1.48E-06	CGNP-Like
Tceal5	9.16E-11	-0.45599	0.021	0.131	1.53E-06	CGNP-Like
Prdx4	9.19E-11	0.533618	0.56	0.34	1.53E-06	CGNP-Like
Fchsd2	9.63E-11	-0.39541	0.036	0.164	1.61E-06	CGNP-Like

Casc5	9.74E-11	0.617217	0.305	0.094	1.62E-06	CGNP-Like
Reep3	9.87E-11	-0.43671	0.323	0.561	1.65E-06	CGNP-Like
Rbbp4	1E-10	0.512659	0.606	0.418	1.67E-06	CGNP-Like
Cyb5	1.03E-10	-0.4988	0.259	0.471	1.72E-06	CGNP-Like
Kif20b	1.04E-10	0.666934	0.298	0.09	1.74E-06	CGNP-Like
AI413582	1.05E-10	-0.32473	0.025	0.139	1.76E-06	CGNP-Like
Sstr2	1.05E-10	0.595852	0.181	0.016	1.76E-06	CGNP-Like
Fkbp15	1.06E-10	-0.4715	0.025	0.139	1.77E-06	CGNP-Like
Tmem66	1.15E-10	-0.52289	0.239	0.447	1.92E-06	CGNP-Like
Pa2g4	1.16E-10	0.51675	0.667	0.488	1.94E-06	CGNP-Like
Desi1	1.18E-10	-0.51075	0.092	0.258	1.97E-06	CGNP-Like
Dnmt1	1.23E-10	0.524424	0.4	0.172	2.05E-06	CGNP-Like
Ccnb2	1.24E-10	0.626914	0.333	0.111	2.06E-06	CGNP-Like
Aurkb	1.26E-10	0.622159	0.262	0.066	2.1E-06	CGNP-Like
Tspan12	1.26E-10	-0.36704	0.058	0.205	2.11E-06	CGNP-Like
Cd63	1.27E-10	0.375066	0.862	0.672	2.11E-06	CGNP-Like
Pbxip1	1.29E-10	-0.34771	0.036	0.164	2.16E-06	CGNP-Like
Rif1	1.32E-10	0.643505	0.405	0.193	2.2E-06	CGNP-Like
Mxd3	1.35E-10	0.574166	0.217	0.037	2.25E-06	CGNP-Like
Rabgap1l	1.45E-10	-0.51769	0.048	0.184	2.42E-06	CGNP-Like
Dtl	1.54E-10	0.551555	0.219	0.037	2.57E-06	CGNP-Like
Stxbp3a	1.58E-10	-0.33185	0.033	0.156	2.64E-06	CGNP-Like
Kif1a	1.67E-10	-0.39183	0.14	0.34	2.78E-06	CGNP-Like
Zfp91	1.76E-10	0.521809	0.619	0.455	2.93E-06	CGNP-Like
Mtus1	1.9E-10	0.504756	0.165	0.008	3.17E-06	CGNP-Like
Sepp1	1.96E-10	-0.25598	0.086	0.246	3.27E-06	CGNP-Like
Ncor2	2E-10	0.56792	0.295	0.094	3.34E-06	CGNP-Like
Lbr	2.03E-10	0.541205	0.29	0.086	3.38E-06	CGNP-Like
Jagn1	2.04E-10	-0.4534	0.132	0.316	3.4E-06	CGNP-Like
Tcp1	2.07E-10	0.505142	0.616	0.418	3.45E-06	CGNP-Like
Gjc1	2.08E-10	0.560792	0.269	0.074	3.48E-06	CGNP-Like
Uncx	2.12E-10	0.729918	0.265	0.078	3.54E-06	CGNP-Like
Tnfrsf1a	2.16E-10	-0.43877	0.026	0.139	3.61E-06	CGNP-Like
Psip1	2.3E-10	0.438253	0.766	0.652	3.83E-06	CGNP-Like
Mlf2	2.34E-10	-0.49781	0.323	0.516	3.91E-06	CGNP-Like
Knstrn	2.38E-10	0.58443	0.311	0.102	3.98E-06	CGNP-Like
Klhl13	2.48E-10	-0.56111	0.15	0.336	4.14E-06	CGNP-Like
4833439L1	2.54E-10	-0.39032	0.18	0.393	4.24E-06	CGNP-Like
Mad2l1	2.74E-10	0.518224	0.231	0.049	4.57E-06	CGNP-Like
Ypel3	2.75E-10	0.618842	0.493	0.291	4.59E-06	CGNP-Like
Stambpl1	2.76E-10	-0.33993	0.012	0.102	4.61E-06	CGNP-Like
Sh3rf1	2.78E-10	-0.27112	0.012	0.102	4.63E-06	CGNP-Like
Kif22	2.96E-10	0.591218	0.262	0.07	4.94E-06	CGNP-Like
Srrt	3.02E-10	0.54557	0.534	0.311	5.03E-06	CGNP-Like
Hsp90b1	3.02E-10	-0.41783	0.802	0.861	5.03E-06	CGNP-Like
Brinp2	3.04E-10	-0.32104	0.012	0.102	5.07E-06	CGNP-Like
Fam19a5	3.04E-10	-0.3282	0.025	0.135	5.08E-06	CGNP-Like
Ssbp4	3.08E-10	-0.41739	0.058	0.201	5.13E-06	CGNP-Like

Snhg5	3.14E-10	0.544494	0.484	0.258	5.23E-06	CGNP-Like
Csrp2	3.14E-10	0.537155	0.249	0.061	5.24E-06	CGNP-Like
Basp1	3.16E-10	0.390091	0.71	0.488	5.28E-06	CGNP-Like
Hist1h2bc	3.32E-10	-0.49756	0.049	0.184	5.55E-06	CGNP-Like
Tmem9	3.37E-10	-0.47118	0.13	0.311	5.61E-06	CGNP-Like
Klf13	3.42E-10	0.609368	0.356	0.148	5.7E-06	CGNP-Like
Boc	3.72E-10	0.637808	0.199	0.033	6.21E-06	CGNP-Like
Sox18	3.79E-10	0.529007	0.204	0.033	6.33E-06	CGNP-Like
Ctnnd2	3.79E-10	-0.6637	0.125	0.291	6.33E-06	CGNP-Like
Dad1	4.19E-10	-0.47631	0.422	0.611	6.99E-06	CGNP-Like
Rsu1	4.29E-10	-0.45912	0.155	0.348	7.16E-06	CGNP-Like
Kidins220	4.3E-10	-0.39998	0.087	0.25	7.17E-06	CGNP-Like
Pde1c	4.46E-10	0.570848	0.387	0.172	7.43E-06	CGNP-Like
Nr3c1	4.5E-10	-0.49005	0.209	0.41	7.51E-06	CGNP-Like
Asf1a	4.52E-10	0.513327	0.443	0.234	7.54E-06	CGNP-Like
Tmbim4	4.77E-10	-0.42148	0.173	0.373	7.95E-06	CGNP-Like
Fam69b	4.87E-10	-0.35466	0.03	0.143	8.12E-06	CGNP-Like
Tead1	5.06E-10	0.568792	0.29	0.09	8.44E-06	CGNP-Like
Eif4a1	5.23E-10	0.464364	0.643	0.439	8.72E-06	CGNP-Like
Prex1	5.43E-10	-0.42501	0.091	0.254	9.05E-06	CGNP-Like
Slc35b2	5.53E-10	-0.48698	0.226	0.426	9.23E-06	CGNP-Like
Prdx5	5.98E-10	-0.45437	0.293	0.508	9.98E-06	CGNP-Like
Mis18bp1	6.05E-10	0.584861	0.264	0.07	1.01E-05	CGNP-Like
Cpe	6.46E-10	-0.50844	0.481	0.676	1.08E-05	CGNP-Like
Clcn3	6.65E-10	-0.49709	0.325	0.541	1.11E-05	CGNP-Like
Mcm3	6.89E-10	0.627939	0.26	0.074	1.15E-05	CGNP-Like
Lrrtm2	6.9E-10	-0.35691	0.03	0.143	1.15E-05	CGNP-Like
Smarcc1	6.98E-10	0.498332	0.56	0.344	1.16E-05	CGNP-Like
Rps4x	7.07E-10	0.521104	0.455	0.242	1.18E-05	CGNP-Like
Smc6	7.24E-10	0.545698	0.562	0.352	1.21E-05	CGNP-Like
Samd4	7.29E-10	-0.41111	0.056	0.193	1.22E-05	CGNP-Like
Dbf4	7.42E-10	0.500794	0.262	0.07	1.24E-05	CGNP-Like
Tm7sf2	7.7E-10	-0.2898	0.028	0.139	1.28E-05	CGNP-Like
1500012F(	7.71E-10	0.566586	0.517	0.303	1.29E-05	CGNP-Like
Myt1	7.85E-10	-0.47717	0.059	0.201	1.31E-05	CGNP-Like
Ccar1	7.88E-10	0.35547	0.667	0.467	1.31E-05	CGNP-Like
Rassf3	7.9E-10	0.600909	0.204	0.037	1.32E-05	CGNP-Like
Dusp3	8.14E-10	-0.33584	0.026	0.135	1.36E-05	CGNP-Like
Slc12a4	8.2E-10	-0.43402	0.069	0.213	1.37E-05	CGNP-Like
Eif3f	8.31E-10	0.390803	0.786	0.672	1.39E-05	CGNP-Like
Cpeb4	8.51E-10	-0.31964	0.046	0.176	1.42E-05	CGNP-Like
Rab10	8.71E-10	-0.43196	0.39	0.602	1.45E-05	CGNP-Like
Acadl	8.78E-10	-0.56967	0.117	0.283	1.47E-05	CGNP-Like
Fbn2	8.94E-10	-0.46396	0.013	0.102	1.49E-05	CGNP-Like
Dcakd	8.97E-10	0.622119	0.371	0.176	1.5E-05	CGNP-Like
Ndn	9.29E-10	-0.51493	0.231	0.43	1.55E-05	CGNP-Like
Gsap	9.79E-10	-0.34797	0.021	0.123	1.63E-05	CGNP-Like
Etfb	9.92E-10	-0.43079	0.264	0.475	1.65E-05	CGNP-Like

Naca	1E-09	0.454058	0.656	0.488	1.67E-05	CGNP-Like
Slc33a1	1.05E-09	-0.29026	0.035	0.152	1.75E-05	CGNP-Like
Tmem147	1.25E-09	-0.43792	0.3	0.512	2.08E-05	CGNP-Like
Sirpa	1.27E-09	-0.40439	0.046	0.172	2.11E-05	CGNP-Like
Ube2b	1.28E-09	0.461128	0.621	0.443	2.13E-05	CGNP-Like
Tspan13	1.31E-09	-0.65341	0.194	0.373	2.18E-05	CGNP-Like
Lsm3	1.32E-09	0.501657	0.493	0.283	2.21E-05	CGNP-Like
Rdx	1.33E-09	0.378165	0.755	0.623	2.22E-05	CGNP-Like
Uhrf1	1.37E-09	0.484174	0.227	0.049	2.29E-05	CGNP-Like
Haghl	1.39E-09	-0.36713	0.082	0.234	2.32E-05	CGNP-Like
Ilf2	1.41E-09	0.470468	0.61	0.426	2.35E-05	CGNP-Like
Tpr	1.45E-09	0.473548	0.687	0.525	2.42E-05	CGNP-Like
Slain1	1.48E-09	-0.27434	0.02	0.119	2.46E-05	CGNP-Like
Lmnb2	1.48E-09	0.526172	0.232	0.057	2.47E-05	CGNP-Like
Rab6a	1.49E-09	-0.45695	0.333	0.541	2.49E-05	CGNP-Like
Rpl37	1.49E-09	0.478424	0.649	0.471	2.49E-05	CGNP-Like
Fzd2	1.5E-09	0.532525	0.194	0.033	2.51E-05	CGNP-Like
Ccdc41	1.55E-09	0.503638	0.384	0.176	2.59E-05	CGNP-Like
Ctso	1.63E-09	-0.29055	0.031	0.143	2.72E-05	CGNP-Like
Tmem198b	1.7E-09	-0.37901	0.046	0.172	2.84E-05	CGNP-Like
Ccng2	1.71E-09	0.541954	0.264	0.082	2.85E-05	CGNP-Like
Ufl1	1.75E-09	-0.4909	0.135	0.307	2.93E-05	CGNP-Like
Polg	1.77E-09	-0.51389	0.064	0.201	2.96E-05	CGNP-Like
Lta4h	1.78E-09	0.547159	0.362	0.168	2.97E-05	CGNP-Like
Abca3	1.81E-09	-0.30426	0.03	0.139	3.03E-05	CGNP-Like
Btd	1.82E-09	-0.3615	0.028	0.135	3.03E-05	CGNP-Like
Scarb2	1.83E-09	-0.30016	0.03	0.139	3.06E-05	CGNP-Like
Them4	1.88E-09	-0.40423	0.044	0.168	3.14E-05	CGNP-Like
D17Wsu10	1.96E-09	-0.47586	0.288	0.496	3.26E-05	CGNP-Like
Nenf	2E-09	-0.48693	0.15	0.328	3.33E-05	CGNP-Like
Comt	2.04E-09	-0.46915	0.127	0.295	3.41E-05	CGNP-Like
Hsp90aa1	2.06E-09	0.441902	0.723	0.578	3.43E-05	CGNP-Like
Tubb4a	2.07E-09	-0.57493	0.044	0.164	3.45E-05	CGNP-Like
Kcnk2	2.09E-09	-0.35842	0.026	0.131	3.49E-05	CGNP-Like
Pcbp1	2.16E-09	0.453531	0.728	0.615	3.6E-05	CGNP-Like
Ggct	2.21E-09	-0.37959	0.043	0.164	3.69E-05	CGNP-Like
Mcm5	2.22E-09	0.551614	0.272	0.082	3.69E-05	CGNP-Like
Cct2	2.24E-09	0.445229	0.647	0.467	3.73E-05	CGNP-Like
Ap3m2	2.24E-09	-0.33984	0.043	0.164	3.74E-05	CGNP-Like
Cdc20	2.24E-09	0.746003	0.283	0.094	3.74E-05	CGNP-Like
Bpgm	2.26E-09	-0.32668	0.086	0.242	3.76E-05	CGNP-Like
Vps28	2.29E-09	-0.45132	0.259	0.455	3.82E-05	CGNP-Like
Tmem191c	2.36E-09	-0.36772	0.02	0.115	3.94E-05	CGNP-Like
Ralb	2.49E-09	-0.34432	0.023	0.123	4.15E-05	CGNP-Like
Os9	2.49E-09	-0.45618	0.285	0.492	4.15E-05	CGNP-Like
Dhcr24	2.52E-09	-0.43185	0.084	0.234	4.2E-05	CGNP-Like
Mllt4	2.67E-09	0.543121	0.414	0.201	4.45E-05	CGNP-Like
Rab11a	2.71E-09	-0.45891	0.331	0.529	4.52E-05	CGNP-Like

Rest	2.73E-09	-0.32727	0.035	0.148	4.55E-05	CGNP-Like
Unc50	2.74E-09	-0.38692	0.161	0.348	4.57E-05	CGNP-Like
Tyms	2.78E-09	0.569548	0.321	0.123	4.64E-05	CGNP-Like
Tmsb4x	2.79E-09	0.395682	0.959	0.943	4.65E-05	CGNP-Like
Phldb1	2.84E-09	-0.49313	0.043	0.16	4.73E-05	CGNP-Like
Dtd1	2.91E-09	-0.47481	0.102	0.258	4.85E-05	CGNP-Like
Cystm1	3.01E-09	-0.34498	0.035	0.148	5.02E-05	CGNP-Like
Pcna	3.13E-09	0.731269	0.491	0.324	5.23E-05	CGNP-Like
Grid2	3.17E-09	-0.2793	0.026	0.131	5.29E-05	CGNP-Like
Lrrc42	3.2E-09	-0.47291	0.086	0.234	5.33E-05	CGNP-Like
Lrrn3	3.23E-09	-0.37769	0.02	0.115	5.39E-05	CGNP-Like
Rfc4	3.31E-09	0.465531	0.346	0.143	5.52E-05	CGNP-Like
Idh2	3.4E-09	0.634465	0.392	0.209	5.67E-05	CGNP-Like
Samd8	3.43E-09	-0.43169	0.087	0.238	5.73E-05	CGNP-Like
Itfg3	3.47E-09	-0.28708	0.015	0.102	5.78E-05	CGNP-Like
Aldh2	3.7E-09	-0.29089	0.044	0.168	6.17E-05	CGNP-Like
Lrp10	3.7E-09	-0.29468	0.041	0.16	6.17E-05	CGNP-Like
Paics	3.76E-09	0.45926	0.586	0.402	6.27E-05	CGNP-Like
Kcnn2	3.93E-09	-0.30447	0.016	0.107	6.56E-05	CGNP-Like
Cldn11	3.96E-09	-0.92531	0.053	0.176	6.61E-05	CGNP-Like
Rab8b	4.03E-09	-0.36741	0.058	0.189	6.72E-05	CGNP-Like
Blmh	4.05E-09	0.481534	0.45	0.246	6.75E-05	CGNP-Like
Apba2	4.11E-09	0.558682	0.432	0.246	6.86E-05	CGNP-Like
Bclaf1	4.37E-09	0.416126	0.712	0.529	7.29E-05	CGNP-Like
Fam49a	4.37E-09	-0.36327	0.044	0.164	7.29E-05	CGNP-Like
Eif3k	4.41E-09	0.429027	0.661	0.48	7.35E-05	CGNP-Like
Gm8292	4.45E-09	0.514761	0.506	0.324	7.42E-05	CGNP-Like
Clmp	4.79E-09	0.585089	0.208	0.045	7.98E-05	CGNP-Like
Nup62	4.8E-09	0.376431	0.267	0.082	8E-05	CGNP-Like
C530008M	4.85E-09	0.583803	0.336	0.143	8.09E-05	CGNP-Like
Dhrs1	4.9E-09	-0.47331	0.12	0.283	8.18E-05	CGNP-Like
Agpat4	5.04E-09	-0.45854	0.115	0.275	8.4E-05	CGNP-Like
Ccdc18	5.04E-09	0.514244	0.206	0.041	8.4E-05	CGNP-Like
Rps18	5.22E-09	0.442819	0.585	0.381	8.71E-05	CGNP-Like
Pcyt2	5.27E-09	-0.34927	0.044	0.164	8.79E-05	CGNP-Like
Chaf1a	5.37E-09	0.444898	0.255	0.074	8.96E-05	CGNP-Like
Rtn1	5.4E-09	-0.35475	0.661	0.787	9.01E-05	CGNP-Like
Ddhd1	5.61E-09	-0.402	0.054	0.18	9.36E-05	CGNP-Like
Camta1	6.32E-09	0.470176	0.448	0.246	0.000105	CGNP-Like
Ebf3	6.48E-09	0.504072	0.198	0.041	0.000108	CGNP-Like
Rpl18a	6.66E-09	0.514032	0.516	0.324	0.000111	CGNP-Like
Lsm2	6.69E-09	0.486995	0.372	0.176	0.000112	CGNP-Like
Hhip	6.82E-09	0.425938	0.165	0.02	0.000114	CGNP-Like
Sec11c	7.06E-09	-0.533	0.3	0.492	0.000118	CGNP-Like
Rpa3	7.1E-09	0.539102	0.381	0.184	0.000118	CGNP-Like
Sh3bgrl	7.2E-09	0.469341	0.458	0.25	0.00012	CGNP-Like
Atad5	7.27E-09	0.518187	0.229	0.061	0.000121	CGNP-Like
Sgcb	7.43E-09	-0.41889	0.138	0.316	0.000124	CGNP-Like

G3bp1	7.51E-09	0.533444	0.468	0.283	0.000125	CGNP-Like
Rhbdd2	7.69E-09	-0.31267	0.061	0.193	0.000128	CGNP-Like
Taf15	7.99E-09	0.484173	0.334	0.143	0.000133	CGNP-Like
Bub1	8.05E-09	0.360902	0.232	0.057	0.000134	CGNP-Like
Acbd5	8.16E-09	-0.50427	0.137	0.303	0.000136	CGNP-Like
Ppp2r3a	8.28E-09	-0.41109	0.107	0.262	0.000138	CGNP-Like
Nptn	8.35E-09	-0.49133	0.206	0.393	0.000139	CGNP-Like
Rfc1	8.44E-09	0.495871	0.455	0.262	0.000141	CGNP-Like
Mmp2	8.66E-09	-0.38526	0.016	0.102	0.000145	CGNP-Like
Elf1	8.68E-09	-0.33985	0.069	0.205	0.000145	CGNP-Like
Sdpr	8.74E-09	0.742679	0.199	0.045	0.000146	CGNP-Like
2700099C:	9E-09	0.425157	0.166	0.02	0.00015	CGNP-Like
Matr3	9.19E-09	0.33802	0.804	0.68	0.000153	CGNP-Like
Dnajc2	9.2E-09	0.464271	0.481	0.283	0.000153	CGNP-Like
Dbp	9.4E-09	-0.34749	0.04	0.152	0.000157	CGNP-Like
Map1a	9.43E-09	-0.34369	0.053	0.176	0.000157	CGNP-Like
Rprml	9.44E-09	0.571391	0.132	0.004	0.000157	CGNP-Like
Ccdc47	1.01E-08	-0.48864	0.292	0.48	0.000169	CGNP-Like
Pnn	1.02E-08	0.347685	0.84	0.705	0.00017	CGNP-Like
Myod1	1.02E-08	0.569737	0.183	0.033	0.000171	CGNP-Like
Wdr89	1.06E-08	0.493215	0.356	0.168	0.000177	CGNP-Like
Lgmn	1.07E-08	-0.28983	0.099	0.254	0.000179	CGNP-Like
Eif3e	1.09E-08	0.418179	0.545	0.332	0.000182	CGNP-Like
Islr2	1.1E-08	0.543826	0.216	0.053	0.000184	CGNP-Like
Snx18	1.15E-08	-0.32901	0.038	0.148	0.000191	CGNP-Like
Cdca7l	1.17E-08	0.448975	0.183	0.033	0.000195	CGNP-Like
Shisa2	1.18E-08	0.466895	0.14	0.008	0.000196	CGNP-Like
Pole3	1.19E-08	0.502233	0.372	0.184	0.000199	CGNP-Like
4931406C(	1.19E-08	-0.26474	0.049	0.172	0.000199	CGNP-Like
Setd8	1.22E-08	0.487917	0.326	0.135	0.000203	CGNP-Like
Mtf2	1.34E-08	0.54065	0.425	0.238	0.000223	CGNP-Like
Lamtor4	1.34E-08	-0.40573	0.132	0.299	0.000224	CGNP-Like
Sf3b2	1.35E-08	0.368871	0.73	0.594	0.000225	CGNP-Like
Sel1l	1.37E-08	-0.37857	0.099	0.246	0.000228	CGNP-Like
Neurod1	1.37E-08	0.654655	0.39	0.193	0.000229	CGNP-Like
Malat1	1.42E-08	-0.54866	0.982	0.98	0.000236	CGNP-Like
Rrm1	1.43E-08	0.504126	0.407	0.209	0.000239	CGNP-Like
Magee1	1.44E-08	-0.31725	0.044	0.16	0.00024	CGNP-Like
Snx22	1.47E-08	-0.37479	0.051	0.172	0.000245	CGNP-Like
Ift27	1.5E-08	0.558189	0.354	0.172	0.00025	CGNP-Like
Lcorl	1.5E-08	-0.563	0.135	0.295	0.000251	CGNP-Like
Dhx9	1.51E-08	0.497172	0.55	0.385	0.000252	CGNP-Like
Ran	1.51E-08	0.484886	0.583	0.439	0.000252	CGNP-Like
Zmiz1	1.56E-08	0.475806	0.516	0.324	0.000261	CGNP-Like
Smc3	1.57E-08	0.40158	0.728	0.611	0.000261	CGNP-Like
Tlx3	1.64E-08	0.481223	0.152	0.016	0.000273	CGNP-Like
Lrpap1	1.66E-08	-0.39427	0.11	0.266	0.000276	CGNP-Like
Mfap2	1.66E-08	-0.44043	0.058	0.18	0.000277	CGNP-Like

Ly6e	1.67E-08	0.455829	0.3	0.115	0.000278	CGNP-Like
BC034090	1.67E-08	0.469656	0.171	0.029	0.000279	CGNP-Like
Gmnn	1.7E-08	0.466538	0.231	0.066	0.000283	CGNP-Like
Arhgap31	1.75E-08	-0.35592	0.066	0.197	0.000291	CGNP-Like
Casc4	1.77E-08	-0.35142	0.189	0.377	0.000295	CGNP-Like
Eif4g2	1.78E-08	0.334739	0.802	0.693	0.000297	CGNP-Like
Bub1b	1.81E-08	0.423896	0.175	0.029	0.000302	CGNP-Like
Fam173a	1.91E-08	-0.45831	0.199	0.381	0.000318	CGNP-Like
Enho	2.03E-08	-0.27346	0.036	0.143	0.000339	CGNP-Like
Hsdl2	2.05E-08	-0.29581	0.069	0.205	0.000343	CGNP-Like
Cenpm	2.06E-08	0.506405	0.262	0.09	0.000344	CGNP-Like
Cyb561d2	2.11E-08	-0.2862	0.025	0.119	0.000352	CGNP-Like
Brd3	2.12E-08	0.454173	0.634	0.447	0.000354	CGNP-Like
Rps7	2.13E-08	0.440169	0.535	0.361	0.000355	CGNP-Like
Prpf40a	2.16E-08	0.4004	0.694	0.541	0.000359	CGNP-Like
Ldhb	2.16E-08	-0.44876	0.344	0.512	0.00036	CGNP-Like
Luc7l2	2.23E-08	0.469728	0.593	0.422	0.000372	CGNP-Like
Egr1	2.29E-08	0.407153	0.563	0.344	0.000383	CGNP-Like
Rps2	2.32E-08	0.408753	0.621	0.484	0.000387	CGNP-Like
Cyp51	2.33E-08	-0.52196	0.171	0.336	0.000389	CGNP-Like
Rap1gds1	2.33E-08	-0.32378	0.046	0.16	0.000389	CGNP-Like
D17H6S56I	2.41E-08	0.430405	0.227	0.066	0.000401	CGNP-Like
Ppp1r1a	2.47E-08	-0.45648	0.099	0.242	0.000412	CGNP-Like
Ncaph	2.57E-08	0.439355	0.191	0.041	0.000429	CGNP-Like
Nuf2	2.58E-08	0.408687	0.231	0.066	0.000431	CGNP-Like
Magoh	2.59E-08	0.489061	0.461	0.287	0.000432	CGNP-Like
Pnrc1	2.61E-08	0.563383	0.351	0.168	0.000436	CGNP-Like
Magt1	2.62E-08	-0.34118	0.096	0.242	0.000436	CGNP-Like
Utp3	2.62E-08	0.519641	0.46	0.275	0.000437	CGNP-Like
Gm2000	2.64E-08	0.460824	0.199	0.049	0.00044	CGNP-Like
Shc4	2.64E-08	-0.50349	0.051	0.164	0.00044	CGNP-Like
Gm11541	2.66E-08	0.499637	0.189	0.041	0.000444	CGNP-Like
Sept11	2.7E-08	0.465045	0.405	0.23	0.00045	CGNP-Like
Cfdp1	2.71E-08	0.406564	0.641	0.475	0.000453	CGNP-Like
Sema6d	2.73E-08	-0.31498	0.049	0.168	0.000455	CGNP-Like
Whrn	2.73E-08	0.424519	0.157	0.02	0.000456	CGNP-Like
Gltscr2	2.79E-08	0.482598	0.458	0.279	0.000465	CGNP-Like
Sbds	2.83E-08	-0.39102	0.147	0.311	0.000473	CGNP-Like
Schip1	2.84E-08	-0.35899	0.153	0.324	0.000474	CGNP-Like
Iffo1	2.87E-08	-0.34653	0.044	0.156	0.000478	CGNP-Like
Tceal3	2.9E-08	-0.53021	0.138	0.295	0.000484	CGNP-Like
Bccip	2.93E-08	0.449911	0.458	0.258	0.000489	CGNP-Like
Gm11478	3.02E-08	0.566634	0.326	0.152	0.000503	CGNP-Like
Rasa3	3.03E-08	-0.35084	0.063	0.189	0.000505	CGNP-Like
Rin2	3.1E-08	-0.41381	0.038	0.143	0.000516	CGNP-Like
Heg1	3.13E-08	0.420068	0.198	0.045	0.000522	CGNP-Like
Ect2	3.13E-08	0.380832	0.188	0.037	0.000522	CGNP-Like
Rbfox2	3.23E-08	0.382183	0.298	0.111	0.000538	CGNP-Like

Rnaseh2c	3.27E-08	0.449976	0.519	0.34	0.000546	CGNP-Like
Rnf13	3.3E-08	-0.36712	0.12	0.275	0.00055	CGNP-Like
Hdhd2	3.4E-08	-0.29795	0.165	0.344	0.000567	CGNP-Like
Golph3	3.42E-08	-0.42728	0.105	0.25	0.000571	CGNP-Like
Psmb10	3.48E-08	-0.41449	0.071	0.201	0.00058	CGNP-Like
Kif20a	3.51E-08	0.365976	0.163	0.025	0.000586	CGNP-Like
Neu1	3.68E-08	-0.31572	0.04	0.148	0.000613	CGNP-Like
Rad51	3.89E-08	0.404507	0.203	0.049	0.000648	CGNP-Like
Wasf1	3.9E-08	-0.42391	0.13	0.287	0.00065	CGNP-Like
Kif5c	4.07E-08	0.54233	0.369	0.18	0.000679	CGNP-Like
Tspyl4	4.28E-08	-0.40785	0.107	0.254	0.000715	CGNP-Like
Aurka	4.29E-08	0.358104	0.178	0.033	0.000716	CGNP-Like
Mcm2	4.31E-08	0.515766	0.3	0.123	0.000718	CGNP-Like
Smchd1	4.36E-08	0.530764	0.372	0.193	0.000727	CGNP-Like
Rpl7	4.37E-08	0.449389	0.644	0.508	0.000729	CGNP-Like
Zdbf2	4.38E-08	0.528172	0.178	0.037	0.00073	CGNP-Like
Baz1a	4.42E-08	0.513123	0.259	0.094	0.000738	CGNP-Like
Phyh	4.71E-08	-0.35389	0.064	0.189	0.000786	CGNP-Like
Ncapd2	4.99E-08	0.402603	0.27	0.098	0.000833	CGNP-Like
Btg2	5.15E-08	0.542932	0.267	0.098	0.000859	CGNP-Like
Set	5.19E-08	0.49493	0.493	0.328	0.000866	CGNP-Like
Tacc3	5.25E-08	0.458123	0.323	0.139	0.000876	CGNP-Like
Hadha	5.31E-08	-0.4329	0.231	0.41	0.000885	CGNP-Like
Ifnar2	5.31E-08	-0.38258	0.054	0.172	0.000886	CGNP-Like
Rora	5.37E-08	-0.30525	0.061	0.184	0.000896	CGNP-Like
Kif4	5.58E-08	0.388517	0.181	0.037	0.00093	CGNP-Like
Cdc45	5.61E-08	0.411968	0.165	0.029	0.000935	CGNP-Like
Hes6	5.67E-08	0.591227	0.372	0.201	0.000946	CGNP-Like
2810055G	5.85E-08	0.561771	0.264	0.098	0.000976	CGNP-Like
Ypel1	6.13E-08	0.439718	0.209	0.057	0.001022	CGNP-Like
Synj1	6.2E-08	-0.40269	0.074	0.205	0.001035	CGNP-Like
Tcf7l2	6.25E-08	-0.32998	0.058	0.176	0.001043	CGNP-Like
Tfdp2	6.39E-08	0.480033	0.229	0.074	0.001066	CGNP-Like
Aco2	6.45E-08	-0.41575	0.417	0.586	0.001076	CGNP-Like
Melk	6.52E-08	0.40355	0.152	0.02	0.001088	CGNP-Like
Hprt	6.67E-08	0.487813	0.239	0.082	0.001112	CGNP-Like
Mab21l1	6.82E-08	0.419129	0.193	0.045	0.001137	CGNP-Like
Sdc2	7.01E-08	-0.39233	0.072	0.201	0.001169	CGNP-Like
Tcerg1	7.01E-08	0.448869	0.562	0.373	0.00117	CGNP-Like
Eif3h	7.08E-08	0.389489	0.633	0.504	0.001182	CGNP-Like
Pbdc1	7.13E-08	0.433466	0.346	0.164	0.001189	CGNP-Like
Mblac2	7.25E-08	-0.26672	0.025	0.115	0.00121	CGNP-Like
Tlk1	7.28E-08	0.455061	0.219	0.061	0.001215	CGNP-Like
Chchd2	7.35E-08	0.347961	0.773	0.648	0.001226	CGNP-Like
Hn1	7.38E-08	0.471075	0.598	0.434	0.00123	CGNP-Like
Fktn	7.4E-08	-0.42139	0.137	0.295	0.001234	CGNP-Like
Gm17750	7.6E-08	0.462665	0.366	0.176	0.001268	CGNP-Like
Txn1	7.7E-08	0.370507	0.725	0.586	0.001284	CGNP-Like

Nmral1	7.7E-08	0.432083	0.265	0.094	0.001285	CGNP-Like
Csmd2	7.87E-08	-0.38994	0.02	0.102	0.001313	CGNP-Like
Ssb	7.95E-08	0.308129	0.822	0.734	0.001327	CGNP-Like
Acap3	8.07E-08	-0.28912	0.031	0.127	0.001345	CGNP-Like
Snhg1	8.14E-08	0.503274	0.483	0.303	0.001358	CGNP-Like
Vhl	8.2E-08	-0.27027	0.059	0.18	0.001368	CGNP-Like
Cenpw	8.39E-08	0.42472	0.241	0.078	0.001399	CGNP-Like
Otud7b	8.41E-08	-0.3573	0.071	0.197	0.001403	CGNP-Like
Ndfip1	8.53E-08	-0.39515	0.084	0.217	0.001423	CGNP-Like
Tmem50a	8.65E-08	-0.37594	0.445	0.631	0.001442	CGNP-Like
Fndc4	9.1E-08	-0.46596	0.152	0.311	0.001518	CGNP-Like
Sema6a	9.14E-08	-0.40523	0.119	0.27	0.001524	CGNP-Like
Cdca4	9.14E-08	0.367057	0.198	0.049	0.001524	CGNP-Like
Tmem246	9.27E-08	-0.35532	0.081	0.213	0.001546	CGNP-Like
Epb4.1l2	9.38E-08	-0.81956	0.155	0.295	0.001564	CGNP-Like
Lgalsl	9.48E-08	-0.39628	0.122	0.275	0.001581	CGNP-Like
Pam	9.65E-08	-0.39174	0.091	0.225	0.00161	CGNP-Like
Sult4a1	9.66E-08	0.507058	0.234	0.078	0.001612	CGNP-Like
Commd9	9.76E-08	-0.35845	0.091	0.225	0.001629	CGNP-Like
1500009L1	9.83E-08	-0.29557	0.038	0.139	0.00164	CGNP-Like
Tia1	9.87E-08	0.396196	0.657	0.5	0.001647	CGNP-Like
Tspan6	9.89E-08	-0.50911	0.366	0.525	0.00165	CGNP-Like
Nlgn1	9.94E-08	-0.34113	0.03	0.123	0.001658	CGNP-Like
Tbc1d16	1.01E-07	0.475352	0.257	0.094	0.001678	CGNP-Like
Mboat2	1.01E-07	-0.29255	0.063	0.184	0.001692	CGNP-Like
Tmem107	1.02E-07	0.434447	0.209	0.061	0.001706	CGNP-Like
Pdgfa	1.03E-07	0.670164	0.331	0.164	0.001711	CGNP-Like
Hlf	1.04E-07	-0.33575	0.028	0.119	0.001729	CGNP-Like
Tex30	1.05E-07	0.415724	0.194	0.049	0.001752	CGNP-Like
Rab7	1.07E-07	-0.43003	0.231	0.398	0.001782	CGNP-Like
Ago3	1.08E-07	-0.34597	0.096	0.234	0.001802	CGNP-Like
Anapc5	1.08E-07	0.414395	0.463	0.283	0.001802	CGNP-Like
Mapre2	1.08E-07	-0.41697	0.183	0.352	0.001805	CGNP-Like
Hes1	1.08E-07	0.523444	0.381	0.197	0.001809	CGNP-Like
Swap70	1.09E-07	-0.34955	0.03	0.123	0.001812	CGNP-Like
Eif5a	1.1E-07	0.442317	0.555	0.406	0.001842	CGNP-Like
Hist1h2ak	1.12E-07	0.572507	0.244	0.086	0.001861	CGNP-Like
Nxph3	1.17E-07	0.404938	0.124	0.008	0.001951	CGNP-Like
Lmf1	1.18E-07	-0.36019	0.033	0.127	0.001969	CGNP-Like
Taf1d	1.19E-07	0.420138	0.488	0.287	0.001991	CGNP-Like
Robo2	1.2E-07	0.516593	0.213	0.066	0.001996	CGNP-Like
4631405J1	1.2E-07	0.43268	0.115	0.004	0.002003	CGNP-Like
Pik3r2	1.21E-07	-0.2896	0.068	0.193	0.002024	CGNP-Like
Fam69a	1.28E-07	-0.33169	0.041	0.143	0.002139	CGNP-Like
Naa38	1.29E-07	0.424861	0.446	0.27	0.002148	CGNP-Like
Syngr1	1.29E-07	-0.34266	0.102	0.246	0.002159	CGNP-Like
Pmm1	1.31E-07	-0.39414	0.138	0.291	0.00218	CGNP-Like
Dlgap5	1.31E-07	0.465214	0.193	0.049	0.002188	CGNP-Like

Aspm	1.33E-07	0.594303	0.237	0.082	0.002225	CGNP-Like
Ppig	1.34E-07	0.340729	0.643	0.447	0.002241	CGNP-Like
Taldo1	1.34E-07	-0.48654	0.379	0.533	0.002241	CGNP-Like
Slc25a33	1.36E-07	-0.29513	0.066	0.189	0.002275	CGNP-Like
Sgol1	1.38E-07	0.433585	0.198	0.053	0.002308	CGNP-Like
Tnik	1.39E-07	-0.47733	0.084	0.213	0.002322	CGNP-Like
Brca2	1.44E-07	0.423026	0.199	0.053	0.002403	CGNP-Like
Trip13	1.49E-07	0.332178	0.152	0.025	0.00249	CGNP-Like
Snrnp40	1.52E-07	0.47839	0.404	0.238	0.002534	CGNP-Like
Ppm1l	1.55E-07	-0.47935	0.082	0.209	0.002591	CGNP-Like
Ddrgk1	1.59E-07	-0.34811	0.226	0.41	0.002654	CGNP-Like
Slc50a1	1.62E-07	-0.38284	0.094	0.23	0.002706	CGNP-Like
Rdh11	1.63E-07	-0.36744	0.097	0.234	0.002717	CGNP-Like
Mdga1	1.68E-07	0.437839	0.143	0.02	0.0028	CGNP-Like
Ift74	1.71E-07	0.444808	0.362	0.184	0.002858	CGNP-Like
Slc48a1	1.73E-07	-0.31108	0.063	0.18	0.002882	CGNP-Like
Gli1	1.74E-07	0.411424	0.189	0.049	0.002897	CGNP-Like
Vapa	1.75E-07	-0.43319	0.423	0.598	0.002912	CGNP-Like
Bahcc1	1.77E-07	0.375583	0.165	0.033	0.00295	CGNP-Like
Pigs	1.84E-07	-0.25554	0.035	0.131	0.003065	CGNP-Like
Baz2b	1.86E-07	0.53514	0.443	0.258	0.003104	CGNP-Like
Abi2	1.87E-07	-0.43775	0.221	0.398	0.003126	CGNP-Like
Ilf3	1.88E-07	0.388053	0.496	0.307	0.003135	CGNP-Like
Yipf5	1.89E-07	-0.31613	0.135	0.287	0.003152	CGNP-Like
Fut9	1.9E-07	-0.4974	0.214	0.377	0.003169	CGNP-Like
Cacng7	1.91E-07	-0.32297	0.048	0.152	0.003178	CGNP-Like
Ndrg3	1.93E-07	-0.37033	0.115	0.262	0.003214	CGNP-Like
Wls	1.98E-07	-0.42688	0.166	0.316	0.003311	CGNP-Like
Slc12a9	2.06E-07	-0.27974	0.035	0.131	0.003438	CGNP-Like
Nsmce4a	2.11E-07	0.409121	0.323	0.152	0.003523	CGNP-Like
Vrk1	2.17E-07	0.400962	0.226	0.074	0.003624	CGNP-Like
Map2	2.3E-07	-0.33317	0.433	0.623	0.003831	CGNP-Like
Ptgr2	2.33E-07	-0.26764	0.023	0.107	0.003882	CGNP-Like
Atp6v0e2	2.33E-07	-0.40751	0.295	0.471	0.003892	CGNP-Like
Dpy19l3	2.45E-07	-0.25195	0.021	0.102	0.004092	CGNP-Like
Plk1	2.48E-07	0.413985	0.163	0.033	0.004136	CGNP-Like
C130071C(	2.51E-07	0.502264	0.386	0.209	0.004183	CGNP-Like
Vldlr	2.55E-07	-0.29073	0.084	0.213	0.004249	CGNP-Like
Smdt1	2.56E-07	-0.35302	0.465	0.623	0.004271	CGNP-Like
Nme4	2.59E-07	0.439227	0.226	0.074	0.004323	CGNP-Like
Ythdc1	2.62E-07	0.349721	0.578	0.389	0.004365	CGNP-Like
Ebpl	2.62E-07	-0.32101	0.099	0.234	0.004377	CGNP-Like
Acp2	2.63E-07	-0.37615	0.082	0.209	0.004383	CGNP-Like
C330027C(	2.64E-07	0.358785	0.221	0.07	0.0044	CGNP-Like
Mis18a	2.65E-07	0.434538	0.191	0.053	0.004415	CGNP-Like
Scp2	2.65E-07	-0.3456	0.089	0.221	0.004415	CGNP-Like
Nudc	2.68E-07	0.519512	0.392	0.23	0.004463	CGNP-Like
Flna	2.73E-07	0.494859	0.206	0.066	0.004547	CGNP-Like

Wwc2	2.73E-07	-0.35025	0.03	0.119	0.004548	CGNP-Like
Atg9a	2.82E-07	-0.28118	0.021	0.102	0.004708	CGNP-Like
Pde4dip	2.83E-07	0.416764	0.31	0.139	0.004721	CGNP-Like
Fen1	2.85E-07	0.474402	0.217	0.074	0.004756	CGNP-Like
Tmem33	2.93E-07	-0.36762	0.196	0.365	0.004886	CGNP-Like
Stx7	3E-07	-0.345	0.11	0.25	0.004997	CGNP-Like
Tmed5	3.02E-07	-0.35324	0.18	0.348	0.005045	CGNP-Like
Pop5	3.19E-07	-0.33959	0.092	0.221	0.005323	CGNP-Like
Abhd2	3.31E-07	-0.45763	0.033	0.123	0.005529	CGNP-Like
Brd7	3.35E-07	0.438524	0.473	0.307	0.005583	CGNP-Like
Wipf1	3.4E-07	-0.39301	0.048	0.152	0.005676	CGNP-Like
Sc4mol	3.45E-07	-0.53014	0.104	0.234	0.005762	CGNP-Like
Ddx39b	3.46E-07	0.40781	0.554	0.381	0.005771	CGNP-Like
Epb4.1	3.54E-07	0.472318	0.264	0.111	0.00591	CGNP-Like
Khdrbs1	3.55E-07	0.366754	0.586	0.43	0.00592	CGNP-Like
Sept4	3.57E-07	-0.74053	0.142	0.283	0.005958	CGNP-Like
Sf3b1	3.62E-07	0.326527	0.778	0.668	0.006039	CGNP-Like
Snx30	3.67E-07	-0.30695	0.046	0.148	0.006128	CGNP-Like
Bai1	3.74E-07	-0.35533	0.033	0.123	0.006233	CGNP-Like
Cdk14	3.74E-07	-0.39782	0.124	0.258	0.00624	CGNP-Like
Coro2b	3.88E-07	0.518697	0.239	0.094	0.006466	CGNP-Like
Pttg1ip	3.92E-07	-0.38467	0.112	0.25	0.006537	CGNP-Like
Tbc1d12	3.95E-07	-0.29688	0.051	0.156	0.006584	CGNP-Like
Rpl14-ps1	3.97E-07	0.43595	0.422	0.254	0.006618	CGNP-Like
Ubl3	4.31E-07	-0.38437	0.264	0.434	0.007195	CGNP-Like
Wdr1	4.33E-07	-0.39947	0.112	0.246	0.007215	CGNP-Like
Arl6ip1	4.38E-07	-0.32928	0.643	0.816	0.007308	CGNP-Like
Rab3a	4.43E-07	-0.28754	0.058	0.168	0.00739	CGNP-Like
Ttk	4.44E-07	0.339118	0.16	0.033	0.007407	CGNP-Like
Ccnb1	4.47E-07	0.323093	0.249	0.09	0.007452	CGNP-Like
Map4k4	4.51E-07	0.49182	0.55	0.398	0.00752	CGNP-Like
Wdhd1	4.51E-07	0.383391	0.153	0.029	0.007526	CGNP-Like
Thrap3	4.66E-07	0.352615	0.527	0.352	0.007774	CGNP-Like
Rgs17	4.78E-07	-0.28873	0.033	0.123	0.007971	CGNP-Like
Skp2	4.9E-07	0.360967	0.196	0.057	0.008179	CGNP-Like
Csad	5.02E-07	-0.32184	0.1	0.234	0.008367	CGNP-Like
Slc20a2	5.06E-07	-0.34087	0.053	0.156	0.008444	CGNP-Like
Sfpq	5.18E-07	0.286774	0.774	0.676	0.008642	CGNP-Like
Tagln3	5.2E-07	-0.43082	0.213	0.365	0.008674	CGNP-Like
Cxcr4	5.3E-07	0.510122	0.181	0.053	0.008843	CGNP-Like
2510003E0	5.37E-07	-0.56426	0.122	0.254	0.008962	CGNP-Like
Maged2	5.38E-07	-0.35415	0.163	0.32	0.008967	CGNP-Like
Top1	5.38E-07	0.363447	0.792	0.684	0.008973	CGNP-Like
Dhx15	5.45E-07	0.356794	0.534	0.365	0.009099	CGNP-Like
D030056L2	5.48E-07	0.39968	0.229	0.082	0.009132	CGNP-Like
Rfc3	5.52E-07	0.403604	0.288	0.127	0.009215	CGNP-Like
Lysmd2	5.58E-07	-0.3341	0.084	0.205	0.009301	CGNP-Like
Marcksl1	5.62E-07	0.3054	0.773	0.672	0.009373	CGNP-Like

Plk4	5.66E-07	0.417329	0.194	0.057	0.009433	CGNP-Like
Tbl1x	5.76E-07	0.36447	0.397	0.213	0.009612	CGNP-Like
Reln	5.97E-07	0.470902	0.16	0.037	0.009957	CGNP-Like
Prss23	2.1E-99	1.667073	0.812	0.016	3.47E-95	Astrocytic
Hopx	2.58E-94	2.046628	0.875	0.024	4.3E-90	Astrocytic
Id4	5.92E-92	1.979821	1	0.039	9.87E-88	Astrocytic
Acsbg1	6.22E-85	1.925892	1	0.044	1.04E-80	Astrocytic
Nid1	7.72E-82	1.980698	0.906	0.037	1.29E-77	Astrocytic
Gpnmb	1.91E-81	1.150858	0.625	0.01	3.19E-77	Astrocytic
Rgs5	1.99E-78	2.483813	0.812	0.029	3.31E-74	Astrocytic
Npy	9.75E-78	2.164797	0.781	0.026	1.63E-73	Astrocytic
Ifitm3	9.7E-76	1.774252	0.812	0.031	1.62E-71	Astrocytic
Tnc	2.18E-75	1.914476	0.781	0.027	3.64E-71	Astrocytic
Cntnap2	1.07E-70	1.18076	0.562	0.01	1.79E-66	Astrocytic
Aqp4	5.17E-70	3.095774	1	0.067	8.63E-66	Astrocytic
Pla2g16	4.53E-69	1.415937	0.781	0.032	7.55E-65	Astrocytic
Rhod	6.88E-68	1.150082	0.469	0.005	1.15E-63	Astrocytic
Igfbp2	7.77E-68	2.282606	0.938	0.057	1.3E-63	Astrocytic
Agt	8.35E-67	2.082201	0.875	0.048	1.39E-62	Astrocytic
Pcp4l1	2.81E-66	1.347991	0.594	0.015	4.68E-62	Astrocytic
Aldh1l1	1.54E-65	1.801593	0.812	0.039	2.57E-61	Astrocytic
Chchd10	2.11E-65	2.080497	0.969	0.068	3.52E-61	Astrocytic
Emid1	1.3E-64	1.637149	0.844	0.044	2.16E-60	Astrocytic
Fxyd1	1.71E-64	1.284785	0.625	0.018	2.85E-60	Astrocytic
Car2	4.52E-64	2.498761	0.938	0.066	7.53E-60	Astrocytic
Timp1	5.39E-63	0.962047	0.5	0.009	8.99E-59	Astrocytic
Wnt3	5.89E-63	1.00993	0.5	0.009	9.82E-59	Astrocytic
Pla2g7	5.98E-63	2.461342	0.938	0.065	9.98E-59	Astrocytic
Ccdc80	7.58E-62	1.542546	0.688	0.027	1.26E-57	Astrocytic
Slc25a18	4.33E-61	1.449568	0.812	0.043	7.22E-57	Astrocytic
Naaa	4.5E-61	1.215147	0.531	0.012	7.51E-57	Astrocytic
Gabra4	8.31E-59	1.026461	0.656	0.026	1.39E-54	Astrocytic
Casp12	3.24E-58	1.183592	0.531	0.013	5.41E-54	Astrocytic
Slc4a4	3.45E-57	2.332793	1	0.085	5.75E-53	Astrocytic
Chl1	6.88E-57	1.450545	0.75	0.039	1.15E-52	Astrocytic
Hsd11b1	1.07E-56	1.388928	0.562	0.017	1.78E-52	Astrocytic
Gfap	1.58E-56	1.971445	0.844	0.055	2.64E-52	Astrocytic
Dkk3	5.88E-56	1.149849	0.656	0.027	9.81E-52	Astrocytic
Tmem37	2.48E-55	0.871154	0.469	0.01	4.14E-51	Astrocytic
Mlc1	2.49E-54	1.631621	0.844	0.056	4.15E-50	Astrocytic
Id3	7.02E-52	2.184395	0.969	0.09	1.17E-47	Astrocytic
Gabrb1	1.77E-51	0.822766	0.531	0.017	2.95E-47	Astrocytic
Aldoc	2.03E-51	2.276152	0.969	0.095	3.39E-47	Astrocytic
Paqr8	4.42E-50	1.263281	0.719	0.043	7.38E-46	Astrocytic
Abi3bp	1.31E-49	0.694174	0.344	0.004	2.18E-45	Astrocytic
Clrn1	1.59E-49	0.610021	0.344	0.004	2.66E-45	Astrocytic
H2-K1	3.79E-49	1.296567	0.625	0.031	6.32E-45	Astrocytic
Gria1	4.21E-49	1.16297	0.75	0.046	7.03E-45	Astrocytic

Cyp26b1	2.14E-48	1.048874	0.531	0.02	3.57E-44	Astrocytic
Lxn	3.71E-48	1.915312	0.875	0.081	6.19E-44	Astrocytic
Gpd1	4.11E-48	1.340366	0.562	0.024	6.86E-44	Astrocytic
Timp4	1.04E-47	1.824263	1	0.112	1.73E-43	Astrocytic
Atp1b1	2.36E-47	2.264217	0.906	0.094	3.93E-43	Astrocytic
Vamp8	5.22E-47	0.800209	0.5	0.017	8.7E-43	Astrocytic
Spock3	6.08E-46	0.62511	0.344	0.005	1.01E-41	Astrocytic
Cybrd1	7.57E-46	0.726372	0.469	0.015	1.26E-41	Astrocytic
Lyn	1.49E-45	0.944683	0.531	0.022	2.49E-41	Astrocytic
Cryab	1.52E-44	1.482363	0.844	0.076	2.54E-40	Astrocytic
Parp3	4.23E-44	0.557098	0.312	0.004	7.05E-40	Astrocytic
S100a11	4.77E-44	1.063007	0.594	0.032	7.95E-40	Astrocytic
Prex2	7.81E-43	1.269471	0.625	0.038	1.3E-38	Astrocytic
Fjx1	1.64E-42	1.349829	0.875	0.09	2.74E-38	Astrocytic
Lpar1	2.65E-42	1.08335	0.562	0.029	4.42E-38	Astrocytic
Slc9a3r1	3.64E-42	1.057594	0.688	0.049	6.07E-38	Astrocytic
Cd44	5.09E-42	1.250947	0.719	0.056	8.49E-38	Astrocytic
Metrn	7.17E-42	1.304977	0.844	0.081	1.2E-37	Astrocytic
Trpm3	1.35E-41	1.095945	0.531	0.026	2.25E-37	Astrocytic
Lcat	1.7E-41	1.346309	0.688	0.05	2.84E-37	Astrocytic
Emp2	1.83E-41	1.007497	0.469	0.018	3.05E-37	Astrocytic
Plat	3.15E-41	1.375759	0.719	0.055	5.25E-37	Astrocytic
Vcam1	3.75E-41	0.973201	0.562	0.031	6.26E-37	Astrocytic
Syt15	5.27E-41	0.662645	0.312	0.005	8.79E-37	Astrocytic
Htra1	6.9E-41	1.334904	0.656	0.045	1.15E-36	Astrocytic
Ecm2	7.21E-41	0.73621	0.312	0.005	1.2E-36	Astrocytic
Vwa1	1.53E-40	0.874906	0.438	0.016	2.55E-36	Astrocytic
Tmem176a	2.39E-40	1.240968	0.812	0.073	3.99E-36	Astrocytic
Chst2	8.02E-40	1.237049	0.75	0.067	1.34E-35	Astrocytic
Adora1	1.6E-39	1.304686	0.75	0.065	2.66E-35	Astrocytic
Mfge8	3.03E-39	1.710288	0.844	0.094	5.05E-35	Astrocytic
Ttyh1	4.45E-39	1.811404	0.969	0.133	7.42E-35	Astrocytic
Igfbp4	7.22E-39	1.473846	0.562	0.034	1.2E-34	Astrocytic
Sfxn5	7.63E-39	1.002539	0.625	0.043	1.27E-34	Astrocytic
Aspa	3.56E-38	0.651262	0.312	0.006	5.93E-34	Astrocytic
Renbp	4.08E-38	0.753063	0.344	0.009	6.8E-34	Astrocytic
Mcc	4.57E-38	0.778405	0.312	0.006	7.62E-34	Astrocytic
Sdc4	4.84E-38	1.051324	0.562	0.034	8.08E-34	Astrocytic
Pamr1	5.12E-38	0.71071	0.344	0.009	8.55E-34	Astrocytic
Mt2	6.41E-38	2.303231	1	0.171	1.07E-33	Astrocytic
Sparc	1.35E-37	2.73169	1	0.194	2.25E-33	Astrocytic
Mmd2	1.38E-37	1.627109	0.906	0.116	2.3E-33	Astrocytic
Gstm1	2.08E-37	1.673484	0.938	0.138	3.47E-33	Astrocytic
Cck	3.6E-37	1.072221	0.25	0.002	6E-33	Astrocytic
Atp1a2	2.4E-36	2.154277	1	0.176	4.01E-32	Astrocytic
Plvap	7.94E-36	0.682685	0.281	0.005	1.32E-31	Astrocytic
Mt3	8.84E-36	1.495996	0.875	0.11	1.47E-31	Astrocytic
Gucy1a3	1.15E-35	0.953707	0.656	0.053	1.92E-31	Astrocytic

Emp3	1.28E-35	0.497772	0.281	0.005	2.13E-31	Astrocytic
Sncg	1.52E-35	0.548765	0.281	0.005	2.54E-31	Astrocytic
Ubtd1	2.15E-35	0.454645	0.312	0.007	3.59E-31	Astrocytic
Hepacam	3.08E-35	1.233352	0.75	0.073	5.13E-31	Astrocytic
Atp1b2	3.88E-35	1.461377	0.906	0.121	6.47E-31	Astrocytic
S100a10	4.29E-35	1.398149	0.719	0.071	7.16E-31	Astrocytic
Shisa9	4.36E-35	1.224944	0.531	0.034	7.27E-31	Astrocytic
Gjb6	2.17E-34	0.762579	0.438	0.021	3.63E-30	Astrocytic
Trf	2.24E-34	1.234852	0.562	0.039	3.74E-30	Astrocytic
Eps15	4.77E-34	1.323894	0.719	0.077	7.95E-30	Astrocytic
F3	6.44E-34	1.139273	0.594	0.045	1.07E-29	Astrocytic
Rxrg	1.38E-33	0.568651	0.25	0.004	2.3E-29	Astrocytic
Ttpa	1.38E-33	0.404438	0.25	0.004	2.3E-29	Astrocytic
Npnt	1.44E-33	0.457372	0.25	0.004	2.4E-29	Astrocytic
Plec	2.94E-33	0.649968	0.375	0.015	4.9E-29	Astrocytic
Gas7	3.4E-33	0.578188	0.312	0.009	5.67E-29	Astrocytic
Cmtm5	3.51E-33	1.541477	0.812	0.105	5.86E-29	Astrocytic
Prr5l	3.88E-33	0.802064	0.281	0.006	6.46E-29	Astrocytic
Tmem56	9.92E-33	0.586982	0.406	0.018	1.66E-28	Astrocytic
Pax3	1.17E-32	1.11882	0.531	0.035	1.95E-28	Astrocytic
Ddah1	1.41E-32	1.410683	0.906	0.137	2.35E-28	Astrocytic
Cml1	2.26E-32	1.05014	0.531	0.038	3.77E-28	Astrocytic
Abca1	6.87E-32	1.021415	0.531	0.038	1.15E-27	Astrocytic
Rfx4	7.97E-32	0.754031	0.375	0.016	1.33E-27	Astrocytic
Lrrk2	8.75E-32	0.514476	0.219	0.002	1.46E-27	Astrocytic
Vamp5	1.04E-31	0.378061	0.219	0.002	1.73E-27	Astrocytic
Pea15a	1.47E-31	2.164311	1	0.247	2.45E-27	Astrocytic
Ednrb	4.27E-31	1.587461	0.969	0.165	7.12E-27	Astrocytic
Sepp1	5.37E-31	1.094413	0.812	0.105	8.96E-27	Astrocytic
Tjp2	6.51E-31	1.148213	0.844	0.112	1.09E-26	Astrocytic
Smad9	7.15E-31	0.509792	0.281	0.007	1.19E-26	Astrocytic
Ntrk2	9.04E-31	1.561965	0.906	0.151	1.51E-26	Astrocytic
Cgref1	9.64E-31	0.480108	0.281	0.007	1.61E-26	Astrocytic
1700084C(	1.09E-30	0.91648	0.344	0.013	1.82E-26	Astrocytic
Car13	1.28E-30	0.391797	0.25	0.005	2.13E-26	Astrocytic
Lrig1	2.14E-30	0.953829	0.594	0.051	3.56E-26	Astrocytic
Nebl	2.23E-30	0.696366	0.375	0.017	3.71E-26	Astrocytic
Slc22a4	2.36E-30	0.624018	0.375	0.017	3.93E-26	Astrocytic
Slc25a34	3.25E-30	0.28594	0.188	0.001	5.42E-26	Astrocytic
Slc27a1	4.82E-30	1.19195	0.656	0.068	8.03E-26	Astrocytic
Glul	8.65E-30	2.337334	1	0.286	1.44E-25	Astrocytic
Nr1h4	8.68E-30	0.393209	0.156	0	1.45E-25	Astrocytic
Myo6	9.43E-30	1.270652	0.719	0.087	1.57E-25	Astrocytic
Ppp1r1a	2.07E-29	1.208447	0.812	0.114	3.45E-25	Astrocytic
Gmpr	4.74E-29	1.02959	0.469	0.033	7.9E-25	Astrocytic
Acadl	4.81E-29	1.46555	0.844	0.138	8.02E-25	Astrocytic
Igsf11	5.27E-29	0.49429	0.344	0.015	8.78E-25	Astrocytic
Adcyap1r1	1.38E-28	1.251311	0.906	0.153	2.3E-24	Astrocytic

Unc93b1	1.53E-28	0.590273	0.281	0.009	2.56E-24	Astrocytic
Timp3	1.87E-28	1.168603	0.781	0.107	3.11E-24	Astrocytic
Slc1a3	2.13E-28	2.620344	1	0.3	3.56E-24	Astrocytic
Thbs3	2.16E-28	0.857449	0.438	0.028	3.61E-24	Astrocytic
Ltbp1	2.39E-28	0.312643	0.219	0.004	3.99E-24	Astrocytic
Ncan	2.74E-28	1.515574	0.906	0.161	4.58E-24	Astrocytic
Gsta4	3.51E-28	0.756737	0.438	0.028	5.86E-24	Astrocytic
Rnf182	4.49E-28	0.715183	0.344	0.016	7.48E-24	Astrocytic
Oaf	4.85E-28	0.367791	0.25	0.006	8.09E-24	Astrocytic
Crip1	7.55E-28	0.486027	0.312	0.012	1.26E-23	Astrocytic
Ephx1	8.12E-28	0.712693	0.344	0.016	1.35E-23	Astrocytic
0610040J0	1.02E-27	0.576719	0.344	0.016	1.7E-23	Astrocytic
AI413582	1.25E-27	0.848165	0.5	0.04	2.09E-23	Astrocytic
Luzp2	1.78E-27	1.189525	0.719	0.093	2.97E-23	Astrocytic
Thrsp	1.79E-27	1.349435	0.594	0.062	2.98E-23	Astrocytic
Fabp7	2.03E-27	2.8286	1	0.349	3.38E-23	Astrocytic
Mt1	2.17E-27	2.522367	1	0.325	3.62E-23	Astrocytic
Hes5	2.72E-27	1.021718	0.5	0.04	4.54E-23	Astrocytic
Sat1	3.45E-27	1.326469	0.719	0.093	5.76E-23	Astrocytic
Pbxip1	3.87E-27	1.00838	0.562	0.054	6.46E-23	Astrocytic
S1pr1	4.26E-27	0.72167	0.469	0.034	7.1E-23	Astrocytic
Pdpn	4.43E-27	1.074532	0.5	0.042	7.39E-23	Astrocytic
Ppap2b	5.93E-27	1.459277	0.969	0.194	9.89E-23	Astrocytic
Hpgd	6.77E-27	0.690286	0.281	0.01	1.13E-22	Astrocytic
Adamts5	1.18E-26	0.750135	0.312	0.013	1.96E-22	Astrocytic
Gprc5c	2.28E-26	0.438672	0.188	0.002	3.81E-22	Astrocytic
Npr1	2.51E-26	0.400591	0.188	0.002	4.19E-22	Astrocytic
P2ry14	2.51E-26	0.355218	0.188	0.002	4.19E-22	Astrocytic
Plin3	3.09E-26	0.515417	0.312	0.013	5.16E-22	Astrocytic
BC064078	3.36E-26	0.736536	0.25	0.007	5.61E-22	Astrocytic
Ramp1	4.89E-26	1.369636	0.906	0.172	8.16E-22	Astrocytic
Smpdl3a	5.79E-26	0.721876	0.438	0.032	9.65E-22	Astrocytic
Ctsh	6.36E-26	0.466625	0.25	0.007	1.06E-21	Astrocytic
Tril	6.77E-26	1.498935	0.844	0.144	1.13E-21	Astrocytic
Gpr37l1	8.44E-26	1.413545	0.969	0.206	1.41E-21	Astrocytic
Entpd2	9.46E-26	0.544867	0.219	0.005	1.58E-21	Astrocytic
S100b	9.77E-26	1.391579	0.906	0.173	1.63E-21	Astrocytic
Slc7a2	1.08E-25	0.696402	0.344	0.018	1.81E-21	Astrocytic
Abat	1.15E-25	0.97901	0.625	0.071	1.93E-21	Astrocytic
Acot1	1.33E-25	1.330397	0.75	0.117	2.22E-21	Astrocytic
Apoe	1.5E-25	2.764975	1	0.361	2.51E-21	Astrocytic
Creb3l1	1.52E-25	0.34465	0.219	0.005	2.54E-21	Astrocytic
2610305D1	1.52E-25	0.279671	0.219	0.005	2.54E-21	Astrocytic
Daam2	1.92E-25	0.848602	0.344	0.018	3.2E-21	Astrocytic
Cyp2d22	2.36E-25	0.597293	0.281	0.011	3.94E-21	Astrocytic
Angptl4	4.14E-25	0.605448	0.312	0.015	6.9E-21	Astrocytic
Agtrap	5.32E-25	0.461896	0.312	0.015	8.87E-21	Astrocytic
Ptx3	9.02E-25	0.409506	0.156	0.001	1.51E-20	Astrocytic

Efhd1	1.01E-24	0.875466	0.531	0.053	1.68E-20	Astrocytic
Slc13a5	1.26E-24	0.795559	0.344	0.02	2.1E-20	Astrocytic
Rhoj	1.4E-24	0.710049	0.531	0.051	2.34E-20	Astrocytic
Rab7l1	1.45E-24	0.784278	0.406	0.029	2.41E-20	Astrocytic
Fgfr1	1.79E-24	1.213175	0.812	0.143	2.98E-20	Astrocytic
Elovl2	1.85E-24	0.955882	0.531	0.055	3.09E-20	Astrocytic
Klhdc8b	2.79E-24	0.555823	0.344	0.02	4.65E-20	Astrocytic
Dbp	3.07E-24	0.850393	0.531	0.054	5.11E-20	Astrocytic
Rarres2	3.24E-24	0.614526	0.25	0.009	5.41E-20	Astrocytic
Rarres1	3.82E-24	0.546485	0.25	0.009	6.38E-20	Astrocytic
Col4a6	3.95E-24	0.327127	0.125	0	6.59E-20	Astrocytic
Gja1	4.17E-24	1.192242	0.594	0.068	6.95E-20	Astrocytic
Cp	4.41E-24	0.944985	0.5	0.048	7.35E-20	Astrocytic
Me1	5.06E-24	0.587198	0.438	0.034	8.44E-20	Astrocytic
Clu	5.31E-24	1.277937	0.719	0.107	8.85E-20	Astrocytic
Ptchd4	6.6E-24	0.541237	0.312	0.016	1.1E-19	Astrocytic
Limch1	6.67E-24	1.126004	0.656	0.087	1.11E-19	Astrocytic
Pgm2	7.1E-24	0.850709	0.469	0.043	1.18E-19	Astrocytic
Angpt1	1.52E-23	1.004868	0.719	0.106	2.54E-19	Astrocytic
Itih5	1.63E-23	0.612224	0.344	0.021	2.72E-19	Astrocytic
Hmgcs2	1.72E-23	0.51734	0.219	0.006	2.87E-19	Astrocytic
Efemp1	2.06E-23	0.457796	0.219	0.006	3.44E-19	Astrocytic
S100a1	2.45E-23	0.938538	0.875	0.147	4.08E-19	Astrocytic
Btbd17	2.59E-23	1.468068	0.812	0.158	4.33E-19	Astrocytic
Plce1	2.78E-23	0.671113	0.375	0.026	4.64E-19	Astrocytic
Prrx2	3.15E-23	0.284832	0.188	0.004	5.25E-19	Astrocytic
Cpq	3.41E-23	0.719923	0.406	0.031	5.69E-19	Astrocytic
Glud1	3.56E-23	1.345365	0.938	0.237	5.94E-19	Astrocytic
Arhgef26	6.35E-23	0.960541	0.531	0.057	1.06E-18	Astrocytic
Dhrs1	7.28E-23	1.18767	0.781	0.143	1.22E-18	Astrocytic
Slc38a3	8.28E-23	0.960296	0.625	0.079	1.38E-18	Astrocytic
Grm3	1.18E-22	0.483312	0.25	0.01	1.97E-18	Astrocytic
Dtna	1.34E-22	0.876792	0.531	0.057	2.24E-18	Astrocytic
Ifi27	1.66E-22	0.753529	0.438	0.039	2.77E-18	Astrocytic
Etv4	2.8E-22	0.50987	0.344	0.022	4.68E-18	Astrocytic
Cryl1	2.83E-22	1.02041	0.406	0.034	4.72E-18	Astrocytic
Gdpd2	2.91E-22	0.394589	0.281	0.013	4.85E-18	Astrocytic
Cib1	6.49E-22	0.954508	0.656	0.096	1.08E-17	Astrocytic
Rdh10	8.09E-22	0.771005	0.406	0.034	1.35E-17	Astrocytic
Gabrg1	8.13E-22	0.515479	0.406	0.033	1.36E-17	Astrocytic
Rgl1	9.75E-22	0.587099	0.312	0.018	1.63E-17	Astrocytic
Gpm6b	1.02E-21	1.970318	1	0.609	1.7E-17	Astrocytic
Mgst1	1.06E-21	1.149708	0.688	0.111	1.77E-17	Astrocytic
Clec3b	1.19E-21	0.378213	0.219	0.007	1.99E-17	Astrocytic
Il33	1.3E-21	0.376326	0.312	0.018	2.17E-17	Astrocytic
Sparcl1	1.87E-21	1.707467	1	0.418	3.12E-17	Astrocytic
Epha4	2.02E-21	0.838764	0.5	0.054	3.37E-17	Astrocytic
Stk32a	2.32E-21	0.900235	0.406	0.035	3.87E-17	Astrocytic

Slc13a3	2.84E-21	0.54197	0.281	0.015	4.73E-17	Astrocytic
Lsamp	3.29E-21	1.143111	0.969	0.22	5.49E-17	Astrocytic
Adam11	3.52E-21	0.62113	0.375	0.029	5.88E-17	Astrocytic
Cd151	3.57E-21	0.804726	0.406	0.035	5.96E-17	Astrocytic
KCTD12	3.7E-21	0.744188	0.312	0.02	6.16E-17	Astrocytic
AI464131	3.89E-21	0.564171	0.25	0.011	6.48E-17	Astrocytic
Fam167a	4.17E-21	0.326441	0.156	0.002	6.96E-17	Astrocytic
Thrb	4.17E-21	0.268302	0.156	0.002	6.96E-17	Astrocytic
Fam20a	4.36E-21	0.469473	0.25	0.011	7.28E-17	Astrocytic
Sned1	4.52E-21	1.300074	0.469	0.05	7.53E-17	Astrocytic
Fam198b	4.56E-21	0.264811	0.156	0.002	7.61E-17	Astrocytic
Sphk1	6.55E-21	0.364948	0.188	0.005	1.09E-16	Astrocytic
Tspan7	6.59E-21	1.133234	0.969	0.25	1.1E-16	Astrocytic
Bcan	6.7E-21	1.081193	1	0.25	1.12E-16	Astrocytic
Fgf1	7.6E-21	0.270739	0.188	0.005	1.27E-16	Astrocytic
Sod3	8.19E-21	0.287919	0.188	0.005	1.37E-16	Astrocytic
Tmem9b	8.81E-21	0.969745	0.906	0.187	1.47E-16	Astrocytic
Aldh2	1.22E-20	0.809708	0.531	0.062	2.03E-16	Astrocytic
Ctso	1.71E-20	0.572039	0.469	0.048	2.84E-16	Astrocytic
Snta1	2.96E-20	0.553667	0.281	0.016	4.94E-16	Astrocytic
Dbi	3.03E-20	2.076135	1	0.697	5.06E-16	Astrocytic
Gm3764	3.21E-20	1.117884	0.938	0.222	5.35E-16	Astrocytic
Pgpep1	3.36E-20	0.482885	0.375	0.031	5.61E-16	Astrocytic
Pmp22	4.34E-20	0.865517	0.531	0.066	7.24E-16	Astrocytic
Scrn1	5.05E-20	0.885001	0.594	0.084	8.43E-16	Astrocytic
Kcnj10	5.2E-20	1.062241	0.781	0.144	8.67E-16	Astrocytic
F730043M	5.98E-20	0.50349	0.219	0.009	9.97E-16	Astrocytic
Psap	6.87E-20	1.124625	0.969	0.276	1.15E-15	Astrocytic
Cystm1	7.39E-20	0.785773	0.469	0.051	1.23E-15	Astrocytic
Aldh1a1	8.43E-20	0.482519	0.25	0.012	1.41E-15	Astrocytic
Oat	1.15E-19	1.025538	0.656	0.109	1.91E-15	Astrocytic
Npas3	1.44E-19	1.056614	0.656	0.106	2.4E-15	Astrocytic
Cpxm1	1.55E-19	0.491307	0.375	0.032	2.59E-15	Astrocytic
Kcnj16	1.56E-19	0.688272	0.469	0.051	2.6E-15	Astrocytic
S100a16	1.91E-19	0.987603	0.875	0.186	3.19E-15	Astrocytic
Tpi1	2.33E-19	1.094605	0.781	0.158	3.89E-15	Astrocytic
Tsc22d4	2.45E-19	1.415307	0.938	0.346	4.09E-15	Astrocytic
Plcd4	2.48E-19	0.618074	0.312	0.022	4.14E-15	Astrocytic
Isg15	2.85E-19	0.251577	0.125	0.001	4.76E-15	Astrocytic
Lpl	2.95E-19	0.892098	0.562	0.079	4.93E-15	Astrocytic
Fbxo2	4.84E-19	0.912296	0.406	0.042	8.07E-15	Astrocytic
Gpr126	6.37E-19	0.391021	0.312	0.022	1.06E-14	Astrocytic
A330048O	6.79E-19	0.324444	0.188	0.006	1.13E-14	Astrocytic
Slc14a1	8.33E-19	0.509886	0.25	0.013	1.39E-14	Astrocytic
Hbegf	8.52E-19	0.628746	0.5	0.06	1.42E-14	Astrocytic
Pcx	1E-18	0.758634	0.688	0.116	1.67E-14	Astrocytic
Pth1r	1.44E-18	0.666586	0.312	0.023	2.4E-14	Astrocytic
Fkbp11	1.63E-18	0.62652	0.312	0.023	2.72E-14	Astrocytic

Uap1l1	1.72E-18	0.656082	0.281	0.018	2.87E-14	Astrocytic
Dtx4	1.85E-18	0.441623	0.312	0.023	3.08E-14	Astrocytic
Syt12	2.07E-18	0.615487	0.469	0.055	3.46E-14	Astrocytic
Mertk	2.11E-18	0.555107	0.281	0.018	3.52E-14	Astrocytic
Cgrrf1	2.35E-18	0.680717	0.562	0.079	3.92E-14	Astrocytic
Hsdl2	2.39E-18	0.759736	0.594	0.089	3.99E-14	Astrocytic
Add3	2.42E-18	0.98185	0.844	0.199	4.04E-14	Astrocytic
Rab20	2.45E-18	0.273425	0.156	0.004	4.08E-14	Astrocytic
AW04773(	3.21E-18	0.85328	0.625	0.1	5.35E-14	Astrocytic
Them4	3.23E-18	0.840326	0.5	0.063	5.39E-14	Astrocytic
Tulp3	4.18E-18	0.68788	0.406	0.043	6.97E-14	Astrocytic
Cd302	5.1E-18	0.877697	0.688	0.122	8.51E-14	Astrocytic
Degs1	5.19E-18	1.063845	0.812	0.197	8.65E-14	Astrocytic
Tmem198b	5.94E-18	0.846638	0.5	0.066	9.91E-14	Astrocytic
St3gal6	5.96E-18	0.615294	0.312	0.024	9.95E-14	Astrocytic
Cst3	6.38E-18	1.496219	1	0.713	1.06E-13	Astrocytic
Tapbp	7.44E-18	0.466289	0.344	0.029	1.24E-13	Astrocytic
Tmem176b	7.57E-18	1.304992	0.875	0.267	1.26E-13	Astrocytic
Rgs7bp	8.14E-18	0.751034	0.469	0.057	1.36E-13	Astrocytic
Epas1	9.16E-18	0.644519	0.344	0.031	1.53E-13	Astrocytic
B2m	9.54E-18	1.235634	0.938	0.388	1.59E-13	Astrocytic
Tmie	1.04E-17	0.508766	0.25	0.015	1.74E-13	Astrocytic
Wls	1.13E-17	1.116988	0.781	0.187	1.88E-13	Astrocytic
Ptn	1.19E-17	1.437597	1	0.592	1.98E-13	Astrocytic
Sirpa	1.24E-17	0.762266	0.5	0.066	2.07E-13	Astrocytic
Axl	1.26E-17	0.45077	0.25	0.015	2.11E-13	Astrocytic
Cyr61	1.42E-17	1.260603	0.469	0.062	2.37E-13	Astrocytic
Acss1	1.91E-17	0.683413	0.312	0.026	3.19E-13	Astrocytic
Ptges	2.54E-17	0.528656	0.188	0.007	4.24E-13	Astrocytic
Eva1b	2.71E-17	0.442908	0.219	0.011	4.53E-13	Astrocytic
Tmem47	2.8E-17	0.970381	0.688	0.127	4.67E-13	Astrocytic
Mir22hg	2.94E-17	0.519058	0.219	0.011	4.9E-13	Astrocytic
Paqr7	3.03E-17	0.322449	0.281	0.02	5.05E-13	Astrocytic
C4b	3.15E-17	0.519505	0.188	0.007	5.26E-13	Astrocytic
Lrp10	3.81E-17	0.632662	0.469	0.06	6.35E-13	Astrocytic
Elmo2	4.11E-17	0.550128	0.5	0.066	6.85E-13	Astrocytic
Pfkm	4.35E-17	0.897998	0.594	0.099	7.26E-13	Astrocytic
Omg	4.54E-17	0.794487	0.594	0.095	7.57E-13	Astrocytic
Sybu	5.8E-17	0.511736	0.312	0.026	9.68E-13	Astrocytic
4931406C(	6E-17	0.621408	0.5	0.068	1E-12	Astrocytic
Cd81	7.07E-17	1.20126	1	0.641	1.18E-12	Astrocytic
Kcne1l	8.33E-17	1.029176	0.438	0.055	1.39E-12	Astrocytic
Sept9	1.13E-16	0.853639	0.562	0.094	1.89E-12	Astrocytic
Bmpr1b	1.15E-16	0.483404	0.281	0.021	1.93E-12	Astrocytic
Pmm1	1.27E-16	0.959275	0.719	0.161	2.11E-12	Astrocytic
Necap2	1.48E-16	0.60511	0.469	0.061	2.46E-12	Astrocytic
Acox1	1.55E-16	0.67899	0.688	0.128	2.58E-12	Astrocytic
Slc25a33	1.55E-16	0.788534	0.531	0.084	2.59E-12	Astrocytic

Hrsp12	1.64E-16	0.618461	0.531	0.078	2.73E-12	Astrocytic
Grina	1.64E-16	0.839418	0.625	0.117	2.74E-12	Astrocytic
Phgdh	1.93E-16	0.894005	0.688	0.143	3.22E-12	Astrocytic
Hiatl1	2.07E-16	0.44531	0.312	0.027	3.46E-12	Astrocytic
Msx2	2.09E-16	0.39924	0.156	0.005	3.48E-12	Astrocytic
Tpp1	2.31E-16	0.912551	0.656	0.127	3.86E-12	Astrocytic
Clip1	2.8E-16	0.879703	0.719	0.149	4.67E-12	Astrocytic
Dusp22	3.77E-16	0.385186	0.219	0.012	6.3E-12	Astrocytic
Eva1c	3.9E-16	0.332209	0.125	0.002	6.51E-12	Astrocytic
Fam213a	4.12E-16	0.732262	0.75	0.156	6.87E-12	Astrocytic
Icam1	4.42E-16	0.301448	0.125	0.002	7.38E-12	Astrocytic
Itm2b	4.57E-16	1.106766	1	0.74	7.63E-12	Astrocytic
Tnfaip6	4.59E-16	0.509726	0.281	0.022	7.65E-12	Astrocytic
Slc39a12	4.6E-16	0.585721	0.25	0.017	7.67E-12	Astrocytic
Kirrel3	4.81E-16	0.292655	0.125	0.002	8.02E-12	Astrocytic
Trmt2b	4.87E-16	0.501737	0.281	0.022	8.13E-12	Astrocytic
Pdgfrl	5.22E-16	0.309367	0.219	0.012	8.71E-12	Astrocytic
Tiam2	5.45E-16	0.26092	0.125	0.002	9.09E-12	Astrocytic
Ctsl	5.49E-16	1.188488	0.938	0.37	9.15E-12	Astrocytic
Usp24	5.68E-16	0.439733	0.469	0.061	9.47E-12	Astrocytic
Sft2d2	6.48E-16	0.33476	0.188	0.009	1.08E-11	Astrocytic
Slc35e4	6.56E-16	0.505216	0.312	0.028	1.09E-11	Astrocytic
Ly96	6.86E-16	0.383845	0.188	0.009	1.14E-11	Astrocytic
Colgalt2	7.06E-16	0.40827	0.188	0.009	1.18E-11	Astrocytic
Nwd1	7.47E-16	0.446407	0.188	0.009	1.25E-11	Astrocytic
Serpine2	7.8E-16	0.958899	0.938	0.253	1.3E-11	Astrocytic
Gm15417	7.9E-16	0.398769	0.188	0.009	1.32E-11	Astrocytic
Olfm2	9.67E-16	0.382176	0.25	0.017	1.61E-11	Astrocytic
Rab31	1.08E-15	0.864185	0.656	0.134	1.79E-11	Astrocytic
Ahcyl2	1.24E-15	0.378117	0.312	0.028	2.06E-11	Astrocytic
Cacng5	1.34E-15	0.752946	0.594	0.106	2.23E-11	Astrocytic
1810037I1	1.73E-15	1.177162	0.906	0.358	2.89E-11	Astrocytic
Lysmd2	1.78E-15	0.862033	0.562	0.101	2.97E-11	Astrocytic
Spry1	1.8E-15	0.627163	0.438	0.057	3E-11	Astrocytic
Marc2	4.2E-15	0.821412	0.75	0.168	7E-11	Astrocytic
Btbd3	4.65E-15	0.714102	0.531	0.089	7.75E-11	Astrocytic
Gpx8	5.02E-15	0.880874	0.625	0.125	8.37E-11	Astrocytic
Me3	5.97E-15	0.283579	0.219	0.013	9.96E-11	Astrocytic
Grid2	6.51E-15	0.541292	0.375	0.044	1.09E-10	Astrocytic
Rnf215	6.54E-15	0.71965	0.5	0.079	1.09E-10	Astrocytic
Npl	6.64E-15	0.3168	0.25	0.018	1.11E-10	Astrocytic
Mrps6	6.87E-15	1.124944	0.812	0.25	1.15E-10	Astrocytic
Fcgrt	7.59E-15	0.57	0.344	0.037	1.27E-10	Astrocytic
Ckb	9.55E-15	1.111909	0.969	0.824	1.59E-10	Astrocytic
Ech1	9.73E-15	0.916288	0.719	0.171	1.62E-10	Astrocytic
Hist1h2bc	9.89E-15	0.922941	0.469	0.073	1.65E-10	Astrocytic
St5	1.13E-14	0.397044	0.188	0.01	1.88E-10	Astrocytic
Eya1	1.16E-14	0.398491	0.188	0.01	1.93E-10	Astrocytic

Frem2	1.35E-14	0.298639	0.188	0.01	2.26E-10	Astrocytic
Asah1	1.48E-14	0.760744	0.594	0.115	2.46E-10	Astrocytic
Kcnn2	1.73E-14	0.575769	0.312	0.032	2.88E-10	Astrocytic
Hsp90ab1	1.77E-14	-0.9349	0.969	0.996	2.95E-10	Astrocytic
Paqr4	1.84E-14	0.795978	0.531	0.093	3.07E-10	Astrocytic
Pcdh10	1.85E-14	0.650021	0.438	0.062	3.08E-10	Astrocytic
Tex264	1.97E-14	0.604696	0.5	0.079	3.28E-10	Astrocytic
Cap2	2.27E-14	0.40797	0.25	0.02	3.79E-10	Astrocytic
Lamp1	2.33E-14	1.062883	0.969	0.497	3.88E-10	Astrocytic
Maf	2.36E-14	0.362801	0.25	0.02	3.94E-10	Astrocytic
Ldha	2.36E-14	1.047709	0.906	0.344	3.94E-10	Astrocytic
Gadd45b	2.49E-14	0.524795	0.219	0.015	4.15E-10	Astrocytic
Ilvbl	2.77E-14	0.395149	0.312	0.032	4.62E-10	Astrocytic
Fgfr3	2.95E-14	0.446161	0.281	0.026	4.91E-10	Astrocytic
Anxa7	3.11E-14	0.372651	0.219	0.015	5.18E-10	Astrocytic
Nbl1	3.55E-14	0.830396	0.438	0.065	5.92E-10	Astrocytic
Pygb	3.8E-14	0.554519	0.312	0.033	6.34E-10	Astrocytic
Cpne2	4.39E-14	0.629856	0.312	0.033	7.32E-10	Astrocytic
Timp2	4.49E-14	0.549781	0.469	0.072	7.49E-10	Astrocytic
Nkain4	4.64E-14	0.953565	0.688	0.168	7.74E-10	Astrocytic
Prdx6	5.17E-14	1.233719	0.906	0.403	8.62E-10	Astrocytic
Itm2c	6.01E-14	1.013817	0.875	0.304	1E-09	Astrocytic
Vim	6.17E-14	1.096945	0.969	0.534	1.03E-09	Astrocytic
Gpm6a	6.26E-14	0.964443	0.969	0.386	1.04E-09	Astrocytic
Pld2	6.66E-14	0.443437	0.25	0.021	1.11E-09	Astrocytic
S100a13	7.12E-14	0.558568	0.719	0.154	1.19E-09	Astrocytic
Itga6	9.68E-14	0.699161	0.312	0.034	1.61E-09	Astrocytic
Cmya5	9.8E-14	0.307586	0.125	0.004	1.63E-09	Astrocytic
Ptprz1	1.01E-13	0.978818	0.812	0.222	1.69E-09	Astrocytic
Gper1	1.01E-13	0.267032	0.125	0.004	1.69E-09	Astrocytic
Map3k5	1.05E-13	0.261603	0.125	0.004	1.75E-09	Astrocytic
Eps8	1.08E-13	0.676108	0.562	0.104	1.81E-09	Astrocytic
Gstk1	1.1E-13	0.472794	0.281	0.027	1.83E-09	Astrocytic
Gm12022	1.13E-13	0.507438	0.188	0.011	1.88E-09	Astrocytic
Mfn1	1.15E-13	0.60522	0.438	0.066	1.92E-09	Astrocytic
Slc26a6	1.16E-13	0.391933	0.188	0.011	1.93E-09	Astrocytic
Gaa	1.16E-13	0.659016	0.406	0.057	1.94E-09	Astrocytic
Ncl	1.21E-13	-1.29122	0.812	0.941	2.02E-09	Astrocytic
Col9a3	1.27E-13	0.858494	0.781	0.215	2.12E-09	Astrocytic
Rps5	1.33E-13	-1.09212	0.969	0.98	2.23E-09	Astrocytic
Arpp21	1.36E-13	0.660637	0.625	0.123	2.26E-09	Astrocytic
Cdh4	1.38E-13	0.97537	0.688	0.172	2.3E-09	Astrocytic
Chst7	1.41E-13	0.334377	0.188	0.011	2.34E-09	Astrocytic
Phyh	1.52E-13	0.598963	0.5	0.084	2.54E-09	Astrocytic
Skap2	1.53E-13	0.407987	0.219	0.016	2.54E-09	Astrocytic
Ndrg2	1.54E-13	1.15921	0.875	0.314	2.57E-09	Astrocytic
D3Bwg056	1.67E-13	0.741373	0.281	0.028	2.79E-09	Astrocytic
Gde1	1.81E-13	0.879198	0.812	0.252	3.01E-09	Astrocytic

Abcc4	1.96E-13	0.301072	0.219	0.016	3.27E-09	Astrocytic
Slc35c2	1.96E-13	0.88311	0.438	0.072	3.27E-09	Astrocytic
Tlcd1	1.97E-13	0.656071	0.5	0.085	3.29E-09	Astrocytic
Fbxo44	2.24E-13	0.719415	0.406	0.06	3.74E-09	Astrocytic
Cdip1	2.38E-13	0.720606	0.625	0.125	3.97E-09	Astrocytic
Ugp2	2.56E-13	0.67803	0.625	0.133	4.27E-09	Astrocytic
Fam129a	2.59E-13	0.359177	0.156	0.007	4.33E-09	Astrocytic
Ptpra	2.87E-13	0.978116	0.906	0.352	4.79E-09	Astrocytic
Vcl	2.9E-13	0.547955	0.312	0.035	4.84E-09	Astrocytic
Stard8	3.07E-13	0.288503	0.156	0.007	5.11E-09	Astrocytic
Frmpd1	3.15E-13	0.331504	0.156	0.007	5.26E-09	Astrocytic
Notch3	3.43E-13	0.258189	0.156	0.007	5.71E-09	Astrocytic
Pltp	3.68E-13	0.747692	0.406	0.06	6.14E-09	Astrocytic
Pam	3.73E-13	0.839454	0.562	0.112	6.22E-09	Astrocytic
Rgs2	4.48E-13	0.763509	0.469	0.084	7.47E-09	Astrocytic
Dlgap1	4.64E-13	0.516708	0.688	0.148	7.73E-09	Astrocytic
Trappc3	4.71E-13	0.681157	0.562	0.114	7.85E-09	Astrocytic
Sept4	5.97E-13	1.104164	0.656	0.164	9.96E-09	Astrocytic
Anxa5	6.33E-13	0.54023	0.281	0.029	1.06E-08	Astrocytic
Stom	7.8E-13	0.503417	0.25	0.023	1.3E-08	Astrocytic
Cspg5	7.87E-13	0.272589	0.812	0.182	1.31E-08	Astrocytic
Cacng4	8.4E-13	0.745282	0.906	0.272	1.4E-08	Astrocytic
Acadvl	9.36E-13	0.800148	0.625	0.145	1.56E-08	Astrocytic
Sorbs2	9.68E-13	0.329459	0.312	0.035	1.61E-08	Astrocytic
Ptgr2	1.05E-12	0.63522	0.312	0.037	1.76E-08	Astrocytic
Gbp7	1.09E-12	0.458152	0.188	0.012	1.82E-08	Astrocytic
Scd2	1.1E-12	1.022964	0.938	0.498	1.84E-08	Astrocytic
Lgi4	1.12E-12	0.313403	0.188	0.012	1.86E-08	Astrocytic
Mfap3l	1.22E-12	0.275467	0.219	0.017	2.03E-08	Astrocytic
Gng12	1.24E-12	1.008778	0.812	0.28	2.06E-08	Astrocytic
Hexb	1.26E-12	0.252588	0.375	0.053	2.11E-08	Astrocytic
Rps9	1.34E-12	-0.93543	0.938	0.978	2.23E-08	Astrocytic
Syn3	1.43E-12	0.254002	0.188	0.012	2.39E-08	Astrocytic
Acvr1	1.49E-12	0.340896	0.25	0.023	2.49E-08	Astrocytic
Irx2	1.54E-12	0.554762	0.344	0.044	2.58E-08	Astrocytic
Dgkz	1.57E-12	0.573991	0.5	0.09	2.62E-08	Astrocytic
Pfkfb3	1.57E-12	0.325889	0.25	0.023	2.62E-08	Astrocytic
Sema6d	1.61E-12	0.561943	0.438	0.07	2.69E-08	Astrocytic
Cyp2j9	1.65E-12	0.578231	0.375	0.054	2.76E-08	Astrocytic
Rlbp1	1.78E-12	0.504742	0.406	0.06	2.98E-08	Astrocytic
Spon1	1.85E-12	0.624998	0.531	0.1	3.08E-08	Astrocytic
Id1	1.92E-12	0.894818	0.625	0.149	3.2E-08	Astrocytic
Rasl11a	1.96E-12	0.840965	0.406	0.066	3.27E-08	Astrocytic
Hexa	2.01E-12	0.558071	0.531	0.103	3.35E-08	Astrocytic
Hnrnpa2b1	2.89E-12	-0.97358	0.938	0.963	4.81E-08	Astrocytic
Slc6a1	2.94E-12	0.734979	0.531	0.109	4.9E-08	Astrocytic
Tmod1	3.26E-12	0.539361	0.25	0.024	5.43E-08	Astrocytic
Wwc1	3.36E-12	0.560372	0.625	0.138	5.61E-08	Astrocytic

Tcirg1	3.36E-12	0.336317	0.219	0.018	5.61E-08	Astrocytic
Slain1	3.42E-12	0.455053	0.312	0.038	5.71E-08	Astrocytic
Abhd4	3.48E-12	0.873704	0.531	0.112	5.81E-08	Astrocytic
Ldhb	3.8E-12	0.879842	0.906	0.372	6.34E-08	Astrocytic
Slc2a1	4.19E-12	0.417459	0.25	0.024	6.99E-08	Astrocytic
Aldoa	4.53E-12	0.923191	0.812	0.274	7.55E-08	Astrocytic
Ap3m2	4.69E-12	0.495122	0.406	0.065	7.82E-08	Astrocytic
Nrcam	4.98E-12	0.871689	0.625	0.158	8.31E-08	Astrocytic
Anxa2	5.17E-12	0.566125	0.188	0.013	8.62E-08	Astrocytic
Ephx2	5.49E-12	0.321697	0.125	0.005	9.15E-08	Astrocytic
Adrb1	5.49E-12	0.250888	0.219	0.018	9.16E-08	Astrocytic
Pxmp2	5.6E-12	0.257477	0.219	0.018	9.33E-08	Astrocytic
Appl2	6.74E-12	0.79274	0.625	0.158	1.12E-07	Astrocytic
Ctsc	6.81E-12	0.357064	0.188	0.013	1.14E-07	Astrocytic
Tuba1a	6.98E-12	-1.29624	0.812	0.928	1.16E-07	Astrocytic
Usp2	7.03E-12	0.261805	0.25	0.024	1.17E-07	Astrocytic
Gatm	8.14E-12	0.648602	0.656	0.15	1.36E-07	Astrocytic
Ampd3	8.36E-12	0.576949	0.219	0.02	1.4E-07	Astrocytic
Ssbp4	9.98E-12	0.54971	0.469	0.084	1.66E-07	Astrocytic
Lrp4	1.06E-11	0.457716	0.25	0.026	1.77E-07	Astrocytic
Fads2	1.16E-11	0.609935	0.281	0.033	1.93E-07	Astrocytic
S100a6	1.21E-11	0.681927	0.469	0.088	2.01E-07	Astrocytic
Rpl13a	1.38E-11	-0.98628	0.969	0.971	2.3E-07	Astrocytic
Mpp6	1.57E-11	1.004422	0.75	0.242	2.62E-07	Astrocytic
Akr1b10	1.6E-11	0.751006	0.406	0.07	2.66E-07	Astrocytic
Rhbdf1	1.69E-11	0.340766	0.219	0.02	2.82E-07	Astrocytic
Jam2	1.74E-11	0.676159	0.5	0.1	2.91E-07	Astrocytic
Tspan3	1.97E-11	0.914804	0.969	0.625	3.28E-07	Astrocytic
Ctsa	2.11E-11	0.613182	0.656	0.162	3.51E-07	Astrocytic
Aard	2.24E-11	0.50172	0.312	0.042	3.73E-07	Astrocytic
Junb	2.29E-11	0.649361	0.344	0.05	3.81E-07	Astrocytic
Acaa2	2.31E-11	0.513828	0.375	0.059	3.85E-07	Astrocytic
Slc7a10	2.47E-11	0.381632	0.281	0.033	4.12E-07	Astrocytic
Pon2	2.48E-11	0.687213	0.438	0.082	4.14E-07	Astrocytic
Rpl4	2.51E-11	-0.96525	0.906	0.956	4.18E-07	Astrocytic
Naga	3.01E-11	0.505651	0.375	0.06	5.01E-07	Astrocytic
Fam69a	3.18E-11	0.472995	0.375	0.059	5.3E-07	Astrocytic
Slc26a11	3.2E-11	0.395827	0.188	0.015	5.34E-07	Astrocytic
Slc35f1	3.68E-11	0.385583	0.594	0.126	6.15E-07	Astrocytic
Golph3	3.94E-11	0.735076	0.562	0.131	6.58E-07	Astrocytic
Aebp1	3.98E-11	0.46864	0.25	0.027	6.64E-07	Astrocytic
Orai1	4.31E-11	0.529163	0.344	0.051	7.19E-07	Astrocytic
Sorl1	4.45E-11	0.680834	0.375	0.061	7.42E-07	Astrocytic
Ppap2a	4.55E-11	0.600419	0.281	0.035	7.59E-07	Astrocytic
Cpt1a	4.62E-11	0.383035	0.312	0.042	7.7E-07	Astrocytic
C1galt1c1	4.63E-11	0.487442	0.344	0.051	7.72E-07	Astrocytic
Pdzd2	4.8E-11	0.286157	0.156	0.01	8E-07	Astrocytic
Nacc2	4.91E-11	0.685967	0.375	0.062	8.19E-07	Astrocytic

Tagln3	4.95E-11	0.852902	0.719	0.238	8.25E-07	Astrocytic
Hfe	5.14E-11	0.276906	0.156	0.01	8.58E-07	Astrocytic
Rnh1	5.53E-11	0.462514	0.5	0.099	9.22E-07	Astrocytic
Dhrs4	5.8E-11	0.443931	0.406	0.068	9.67E-07	Astrocytic
Plscr4	6.18E-11	0.549569	0.312	0.044	1.03E-06	Astrocytic
Slc30a10	6.25E-11	0.604243	0.438	0.082	1.04E-06	Astrocytic
Vhl	6.35E-11	0.542645	0.438	0.081	1.06E-06	Astrocytic
Slc1a2	6.53E-11	0.988229	0.875	0.4	1.09E-06	Astrocytic
Tubb5	6.77E-11	-1.42414	0.656	0.895	1.13E-06	Astrocytic
Gria4	7.19E-11	0.812686	0.688	0.2	1.2E-06	Astrocytic
1110001A1	8.07E-11	0.447996	0.594	0.132	1.35E-06	Astrocytic
Hsd17b11	8.26E-11	0.484104	0.406	0.071	1.38E-06	Astrocytic
Zcchc24	8.5E-11	0.51333	0.562	0.125	1.42E-06	Astrocytic
Cyba	8.87E-11	0.485109	0.312	0.044	1.48E-06	Astrocytic
Lgals3	9.09E-11	0.44435	0.125	0.006	1.52E-06	Astrocytic
Fabp5	9.15E-11	0.974618	0.844	0.43	1.53E-06	Astrocytic
Ndufc2	1.03E-10	0.830618	0.969	0.753	1.73E-06	Astrocytic
Amot	1.05E-10	0.579615	0.281	0.037	1.74E-06	Astrocytic
Tnfrsf19	1.07E-10	0.349207	0.25	0.028	1.79E-06	Astrocytic
Tspan15	1.12E-10	0.358848	0.281	0.035	1.87E-06	Astrocytic
Serbp1	1.19E-10	-0.97546	0.875	0.927	1.98E-06	Astrocytic
Clptm1	1.26E-10	0.503547	0.438	0.081	2.1E-06	Astrocytic
Bmpr1a	1.47E-10	0.80505	0.625	0.165	2.45E-06	Astrocytic
Dscr3	1.49E-10	0.589619	0.469	0.098	2.48E-06	Astrocytic
Slc41a1	1.5E-10	0.582505	0.344	0.055	2.49E-06	Astrocytic
Fat1	1.5E-10	0.435741	0.344	0.054	2.5E-06	Astrocytic
Tns3	1.59E-10	0.288153	0.344	0.051	2.64E-06	Astrocytic
Glo1	1.63E-10	0.7942	0.625	0.182	2.72E-06	Astrocytic
Magt1	1.66E-10	0.631993	0.531	0.122	2.77E-06	Astrocytic
Col5a3	1.67E-10	0.370952	0.188	0.016	2.78E-06	Astrocytic
Nrarp	1.75E-10	0.305035	0.281	0.035	2.91E-06	Astrocytic
Mapk4	1.76E-10	0.303393	0.188	0.016	2.94E-06	Astrocytic
Asrgl1	1.84E-10	0.7902	0.844	0.303	3.07E-06	Astrocytic
Rpl32	1.93E-10	-0.94583	0.875	0.943	3.21E-06	Astrocytic
Usp6nl	1.97E-10	0.441982	0.312	0.045	3.28E-06	Astrocytic
Gpr146	2.11E-10	0.285591	0.219	0.022	3.52E-06	Astrocytic
Hnrnpu	2.21E-10	-1.0031	0.875	0.923	3.69E-06	Astrocytic
1500009L1	2.28E-10	0.563028	0.344	0.056	3.8E-06	Astrocytic
Sspn	2.31E-10	0.349023	0.281	0.037	3.86E-06	Astrocytic
Lamtor4	2.44E-10	0.672191	0.625	0.162	4.07E-06	Astrocytic
Etnk1	2.67E-10	0.589869	0.594	0.15	4.46E-06	Astrocytic
As3mt	2.85E-10	0.508821	0.25	0.031	4.76E-06	Astrocytic
Cox7a1	3.04E-10	0.268357	0.156	0.011	5.07E-06	Astrocytic
Fam81a	3.11E-10	0.322711	0.156	0.011	5.18E-06	Astrocytic
Proca1	3.17E-10	0.278155	0.156	0.011	5.29E-06	Astrocytic
Itgb8	3.33E-10	0.549578	0.375	0.066	5.56E-06	Astrocytic
Abhd3	3.48E-10	0.450747	0.25	0.031	5.8E-06	Astrocytic
H3f3b	3.48E-10	-1.1805	0.719	0.9	5.8E-06	Astrocytic

Tspan12	3.76E-10	0.574509	0.438	0.087	6.28E-06	Astrocytic
2810459M	4.03E-10	0.546148	0.219	0.023	6.72E-06	Astrocytic
Lamb2	4.6E-10	0.450958	0.25	0.031	7.68E-06	Astrocytic
Rps3	4.9E-10	-0.82608	0.938	0.956	8.17E-06	Astrocytic
Efhd2	4.96E-10	0.552328	0.688	0.189	8.28E-06	Astrocytic
Ndp	5E-10	0.576112	0.25	0.032	8.35E-06	Astrocytic
Extl3	5.04E-10	0.460571	0.406	0.076	8.41E-06	Astrocytic
Arf3	5.06E-10	0.522287	0.375	0.067	8.44E-06	Astrocytic
Pdlim7	5.44E-10	0.41958	0.312	0.046	9.08E-06	Astrocytic
Il11ra1	5.45E-10	0.557726	0.281	0.039	9.09E-06	Astrocytic
Ppp2r5a	5.53E-10	0.587875	0.469	0.1	9.23E-06	Astrocytic
Dcxr	5.73E-10	0.670193	0.219	0.024	9.56E-06	Astrocytic
Acadm	5.76E-10	0.599898	0.562	0.139	9.6E-06	Astrocytic
Phlda1	5.83E-10	0.535702	0.5	0.11	9.73E-06	Astrocytic
Stk40	5.87E-10	0.278997	0.281	0.038	9.79E-06	Astrocytic
BC026585	6.05E-10	0.27855	0.188	0.017	1.01E-05	Astrocytic
Fam49a	6.24E-10	0.59529	0.375	0.067	1.04E-05	Astrocytic
Fgfr2	6.33E-10	0.427604	0.312	0.048	1.06E-05	Astrocytic
Epdr1	6.66E-10	0.673691	0.5	0.115	1.11E-05	Astrocytic
Gadd45g	6.87E-10	0.74016	0.469	0.105	1.15E-05	Astrocytic
Pnn	7.22E-10	-1.35767	0.5	0.813	1.2E-05	Astrocytic
Cnn3	7.25E-10	0.744813	0.969	0.57	1.21E-05	Astrocytic
Cox14	7.61E-10	0.870695	0.906	0.438	1.27E-05	Astrocytic
Smpd1	7.72E-10	0.512567	0.312	0.049	1.29E-05	Astrocytic
Prex1	7.75E-10	0.48178	0.531	0.122	1.29E-05	Astrocytic
Bdh1	8.2E-10	0.457435	0.438	0.088	1.37E-05	Astrocytic
Scrg1	8.74E-10	0.648719	0.688	0.19	1.46E-05	Astrocytic
Dbndd2	9.03E-10	0.47798	0.406	0.077	1.51E-05	Astrocytic
Lix1l	9.15E-10	0.347083	0.281	0.039	1.53E-05	Astrocytic
Lpcat1	9.61E-10	0.679701	0.688	0.206	1.6E-05	Astrocytic
Grhpr	9.9E-10	0.482221	0.25	0.032	1.65E-05	Astrocytic
Mxra8	1.06E-09	0.353841	0.281	0.039	1.76E-05	Astrocytic
Sh3bp5	1.07E-09	0.610745	0.469	0.104	1.78E-05	Astrocytic
Wnt7a	1.09E-09	0.479141	0.219	0.024	1.82E-05	Astrocytic
Fth1	1.15E-09	0.626451	0.969	0.846	1.92E-05	Astrocytic
Ahcyl1	1.29E-09	0.745936	0.656	0.206	2.15E-05	Astrocytic
Ptplb	1.3E-09	0.755781	0.562	0.153	2.16E-05	Astrocytic
S1pr3	1.31E-09	0.452891	0.125	0.007	2.19E-05	Astrocytic
Sec14l2	1.34E-09	0.326559	0.219	0.024	2.24E-05	Astrocytic
Rorb	1.35E-09	0.355844	0.25	0.032	2.25E-05	Astrocytic
Gpr123	1.36E-09	0.365069	0.219	0.024	2.27E-05	Astrocytic
Rrbp1	1.37E-09	0.667057	0.812	0.308	2.29E-05	Astrocytic
Col4a5	1.42E-09	0.385148	0.156	0.012	2.37E-05	Astrocytic
Mamdc2	1.45E-09	0.359095	0.281	0.04	2.42E-05	Astrocytic
Galnt18	1.46E-09	0.378871	0.188	0.018	2.43E-05	Astrocytic
Nudt7	1.48E-09	0.314751	0.156	0.012	2.47E-05	Astrocytic
Rps26	1.48E-09	-1.04185	0.812	0.901	2.47E-05	Astrocytic
Rnf13	1.51E-09	0.599015	0.562	0.149	2.52E-05	Astrocytic

Tm7sf2	1.56E-09	0.470791	0.312	0.05	2.6E-05	Astrocytic
Rgs17	1.6E-09	0.402797	0.312	0.049	2.67E-05	Astrocytic
Slc20a2	1.61E-09	0.566455	0.375	0.071	2.69E-05	Astrocytic
Rhoq	1.65E-09	0.575053	0.469	0.105	2.76E-05	Astrocytic
Spry2	1.66E-09	0.556781	0.531	0.131	2.77E-05	Astrocytic
Scp2	1.78E-09	0.418576	0.5	0.112	2.97E-05	Astrocytic
Itgb5	1.84E-09	0.368971	0.188	0.018	3.08E-05	Astrocytic
Cyhr1	1.92E-09	0.56721	0.531	0.133	3.2E-05	Astrocytic
Naglu	2.15E-09	0.285407	0.188	0.018	3.58E-05	Astrocytic
Aldh6a1	2.26E-09	0.385427	0.312	0.049	3.77E-05	Astrocytic
Fuca2	2.29E-09	0.367988	0.25	0.033	3.83E-05	Astrocytic
Abca3	2.43E-09	0.473135	0.312	0.051	4.06E-05	Astrocytic
Cacnb4	2.44E-09	0.251414	0.312	0.049	4.07E-05	Astrocytic
Oxld1	2.54E-09	0.29128	0.188	0.018	4.23E-05	Astrocytic
Tcn2	2.58E-09	0.366083	0.25	0.033	4.3E-05	Astrocytic
Nrbp2	2.61E-09	0.779874	0.25	0.034	4.36E-05	Astrocytic
Dek	2.68E-09	-1.58269	0.469	0.777	4.46E-05	Astrocytic
Sfrp1	2.72E-09	-1.97505	0.469	0.764	4.54E-05	Astrocytic
Samd4	2.76E-09	0.591158	0.406	0.083	4.61E-05	Astrocytic
Cotl1	2.77E-09	0.695997	0.656	0.204	4.62E-05	Astrocytic
Acot13	3E-09	0.625694	0.625	0.181	5E-05	Astrocytic
St6galnac4	3.04E-09	0.352234	0.219	0.026	5.07E-05	Astrocytic
Uqcrc1	3.15E-09	0.646276	0.844	0.353	5.25E-05	Astrocytic
Cdh22	3.17E-09	0.360457	0.219	0.026	5.29E-05	Astrocytic
Tesk1	3.31E-09	0.3676	0.219	0.026	5.53E-05	Astrocytic
Klhl25	3.31E-09	0.341735	0.219	0.026	5.53E-05	Astrocytic
Rps4y2	3.81E-09	0.323811	0.312	0.05	6.35E-05	Astrocytic
2510009E(	4.16E-09	0.477546	0.469	0.107	6.94E-05	Astrocytic
Eno1	4.25E-09	0.715387	0.844	0.352	7.09E-05	Astrocytic
Marcks	4.47E-09	-0.80786	0.875	0.952	7.46E-05	Astrocytic
Ctnnd2	4.74E-09	0.643117	0.562	0.158	7.91E-05	Astrocytic
Rhoc	4.84E-09	0.516167	0.469	0.106	8.07E-05	Astrocytic
Slc7a4	4.85E-09	0.414581	0.188	0.02	8.09E-05	Astrocytic
Nfkbiz	5.17E-09	0.348138	0.219	0.027	8.63E-05	Astrocytic
Gm12892	5.42E-09	0.382034	0.281	0.043	9.04E-05	Astrocytic
Ppargc1a	5.43E-09	0.331382	0.188	0.02	9.05E-05	Astrocytic
Pnp	5.67E-09	0.414649	0.219	0.027	9.45E-05	Astrocytic
Megf10	6.47E-09	0.270156	0.188	0.02	0.000108	Astrocytic
Kdelr3	6.49E-09	0.278949	0.156	0.013	0.000108	Astrocytic
Cd200	6.64E-09	0.697233	0.406	0.087	0.000111	Astrocytic
Slc15a2	6.65E-09	0.474527	0.406	0.082	0.000111	Astrocytic
Snhg11	7E-09	0.289209	0.156	0.013	0.000117	Astrocytic
Capns1	7.45E-09	0.832277	0.75	0.337	0.000124	Astrocytic
Fkbp10	7.46E-09	0.264747	0.188	0.02	0.000124	Astrocytic
Sypl	7.74E-09	0.64737	0.5	0.131	0.000129	Astrocytic
Acyp2	7.76E-09	0.638536	0.469	0.116	0.000129	Astrocytic
Lmbrd1	8.98E-09	0.479486	0.531	0.137	0.00015	Astrocytic
Pigt	9.11E-09	0.404082	0.281	0.044	0.000152	Astrocytic

Dusp18	9.31E-09	0.37394	0.281	0.044	0.000155	Astrocytic
Ssfa2	9.7E-09	0.412391	0.281	0.045	0.000162	Astrocytic
Elovl5	1.03E-08	0.649709	0.625	0.197	0.000172	Astrocytic
Fam149a	1.08E-08	0.375767	0.312	0.054	0.00018	Astrocytic
Hdhd2	1.09E-08	0.590822	0.656	0.199	0.000183	Astrocytic
Rasa2	1.12E-08	0.650712	0.5	0.129	0.000187	Astrocytic
Nr4a1	1.16E-08	0.439254	0.219	0.028	0.000194	Astrocytic
Gm15794	1.17E-08	0.281025	0.125	0.009	0.000194	Astrocytic
Dio2	1.17E-08	0.277881	0.125	0.009	0.000194	Astrocytic
Rplp2	1.19E-08	-0.87469	0.844	0.913	0.000198	Astrocytic
Spag4	1.19E-08	0.291776	0.125	0.009	0.000199	Astrocytic
Arntl	1.22E-08	0.301839	0.125	0.009	0.000203	Astrocytic
Cachd1	1.24E-08	0.468067	0.25	0.037	0.000207	Astrocytic
E530001K1	1.3E-08	0.291119	0.125	0.009	0.000217	Astrocytic
Tspo	1.31E-08	0.415853	0.188	0.021	0.000218	Astrocytic
Lifr	1.35E-08	0.385919	0.188	0.021	0.000225	Astrocytic
Pitpna	1.38E-08	0.612123	0.562	0.161	0.000231	Astrocytic
Enox2	1.39E-08	0.383691	0.281	0.045	0.000231	Astrocytic
Pabpc1	1.4E-08	-0.80801	0.906	0.934	0.000234	Astrocytic
Gm6166	1.49E-08	0.452849	0.469	0.112	0.000249	Astrocytic
mt-Rnr2	1.58E-08	-0.73283	1	0.996	0.000263	Astrocytic
Emc7	1.69E-08	0.576447	0.719	0.25	0.000282	Astrocytic
Dpy19l3	1.91E-08	0.333999	0.25	0.037	0.000318	Astrocytic
Pacsin1	1.97E-08	0.288284	0.188	0.021	0.000328	Astrocytic
Ablim1	1.97E-08	0.269195	0.188	0.021	0.000328	Astrocytic
Grin3a	1.98E-08	0.659175	0.469	0.121	0.00033	Astrocytic
Glod4	2.01E-08	0.584774	0.531	0.14	0.000335	Astrocytic
Fuca1	2.06E-08	0.478145	0.688	0.212	0.000343	Astrocytic
Hsd3b7	2.08E-08	0.364274	0.156	0.015	0.000348	Astrocytic
Sema6a	2.11E-08	0.656198	0.531	0.148	0.000353	Astrocytic
Prrx1	2.16E-08	0.529384	0.219	0.029	0.00036	Astrocytic
Trib1	2.17E-08	0.443087	0.375	0.077	0.000363	Astrocytic
Osbpl1a	2.2E-08	0.515061	0.469	0.115	0.000367	Astrocytic
Crip2	2.23E-08	0.555553	0.625	0.188	0.000372	Astrocytic
Mfhas1	2.49E-08	0.516053	0.438	0.103	0.000415	Astrocytic
Slc38a7	2.52E-08	0.289645	0.156	0.015	0.000421	Astrocytic
Rgs20	2.54E-08	0.547403	0.531	0.143	0.000423	Astrocytic
Acaa1a	2.64E-08	0.550703	0.562	0.164	0.000441	Astrocytic
Dip2b	2.93E-08	0.474403	0.375	0.079	0.000488	Astrocytic
Suclg2	2.98E-08	0.402088	0.312	0.056	0.000497	Astrocytic
Nucb1	2.98E-08	0.561631	0.469	0.116	0.000497	Astrocytic
Cbr3	3.03E-08	0.32397	0.281	0.046	0.000505	Astrocytic
Prkcdbp	3.08E-08	0.610672	0.562	0.165	0.000514	Astrocytic
Slc33a1	3.08E-08	0.46545	0.312	0.059	0.000514	Astrocytic
Arsa	3.43E-08	0.36778	0.188	0.022	0.000573	Astrocytic
Arxes2	3.5E-08	0.582959	0.562	0.165	0.000584	Astrocytic
Abhd6	3.82E-08	0.519794	0.375	0.081	0.000638	Astrocytic
Gusb	3.91E-08	0.530663	0.469	0.118	0.000653	Astrocytic

B3galt1	3.93E-08	0.389582	0.25	0.038	0.000655	Astrocytic
Fam195a	3.95E-08	0.279085	0.219	0.029	0.00066	Astrocytic
P2rx4	3.97E-08	0.271557	0.25	0.038	0.000662	Astrocytic
Adcy8	3.97E-08	0.290582	0.188	0.022	0.000662	Astrocytic
Tmbim6	4E-08	0.651901	0.906	0.492	0.000667	Astrocytic
Hnrnpab	4.16E-08	-0.96437	0.781	0.852	0.000694	Astrocytic
Serpinb6a	4.34E-08	0.385806	0.375	0.079	0.000723	Astrocytic
Cluh	4.37E-08	0.354124	0.312	0.057	0.000729	Astrocytic
Nop58	4.5E-08	-1.36245	0.406	0.716	0.000751	Astrocytic
Reep5	4.83E-08	0.659071	0.625	0.195	0.000805	Astrocytic
Hnrnph1	5.01E-08	-1.30473	0.375	0.697	0.000836	Astrocytic
Gpr137b	5.07E-08	0.257968	0.188	0.022	0.000846	Astrocytic
Acin1	5.09E-08	-1.10056	0.531	0.805	0.000849	Astrocytic
Acsl6	5.14E-08	0.507991	0.25	0.039	0.000858	Astrocytic
Atraid	5.16E-08	0.636828	0.812	0.321	0.000861	Astrocytic
Tmem66	5.28E-08	0.690382	0.719	0.282	0.00088	Astrocytic
Cdh13	5.43E-08	0.285936	0.531	0.134	0.000905	Astrocytic
Stt3b	5.54E-08	0.619701	0.75	0.28	0.000924	Astrocytic
Fam19a5	5.57E-08	0.277387	0.281	0.048	0.00093	Astrocytic
March2	5.64E-08	0.502254	0.406	0.094	0.000941	Astrocytic
Hnrnpm	5.66E-08	-1.05648	0.688	0.81	0.000944	Astrocytic
Chst3	5.66E-08	0.529491	0.125	0.01	0.000944	Astrocytic
Gnb2l1	5.7E-08	-0.79121	0.906	0.922	0.000951	Astrocytic
Rgma	5.84E-08	0.453737	0.438	0.106	0.000973	Astrocytic
Slc1a4	5.87E-08	0.567021	0.438	0.109	0.000979	Astrocytic
Enho	5.92E-08	0.31879	0.312	0.057	0.000988	Astrocytic
Trp53inp2	6.43E-08	0.35178	0.25	0.039	0.001073	Astrocytic
Scg3	6.67E-08	0.919955	0.844	0.501	0.001112	Astrocytic
Cox6c	6.7E-08	0.678254	0.969	0.802	0.001117	Astrocytic
Nnat	6.81E-08	-1.6168	0.406	0.712	0.001137	Astrocytic
Serhl	6.83E-08	0.302066	0.156	0.016	0.001138	Astrocytic
Tst	6.95E-08	0.260082	0.281	0.048	0.001159	Astrocytic
Rab7	6.95E-08	0.631216	0.688	0.263	0.00116	Astrocytic
Sys1	7.02E-08	0.828005	0.719	0.263	0.001172	Astrocytic
Rrad	7.41E-08	0.389525	0.156	0.016	0.001236	Astrocytic
Fnbp1	7.44E-08	0.394543	0.375	0.081	0.001241	Astrocytic
Bmyc	7.47E-08	0.483541	0.5	0.134	0.001245	Astrocytic
Basp1	7.55E-08	-1.41384	0.25	0.662	0.001259	Astrocytic
Anp32b	7.58E-08	-1.33853	0.375	0.711	0.001264	Astrocytic
Nasp	7.59E-08	-1.52022	0.375	0.705	0.001266	Astrocytic
Il6st	7.63E-08	0.430092	0.344	0.071	0.001273	Astrocytic
Sh3pxd2b	7.85E-08	0.349254	0.219	0.031	0.001309	Astrocytic
Nfasc	7.96E-08	0.267861	0.406	0.092	0.001328	Astrocytic
C1qtnf6	8.04E-08	0.274115	0.156	0.016	0.001341	Astrocytic
Tmem51	8.3E-08	0.270161	0.156	0.016	0.001385	Astrocytic
Insig2	8.5E-08	0.30917	0.469	0.114	0.001417	Astrocytic
Rplp0	8.66E-08	-0.80715	0.906	0.915	0.001444	Astrocytic
Dusp3	9.04E-08	0.329565	0.281	0.049	0.001508	Astrocytic

Dynlt3	9.12E-08	0.529555	0.438	0.111	0.001522	Astrocytic
Dclk1	9.36E-08	0.644456	0.938	0.524	0.001561	Astrocytic
Enpp5	9.36E-08	0.355597	0.281	0.049	0.001562	Astrocytic
Tmem246	9.55E-08	0.393498	0.438	0.106	0.001594	Astrocytic
Snn	9.94E-08	0.449425	0.531	0.15	0.001658	Astrocytic
Shisa5	1.05E-07	0.338597	0.25	0.04	0.001755	Astrocytic
D430041D	1.07E-07	-1.51591	0.031	0.551	0.001786	Astrocytic
Sorcs1	1.12E-07	0.282729	0.188	0.023	0.001862	Astrocytic
Smox	1.12E-07	0.499602	0.406	0.1	0.001871	Astrocytic
Cdk4	1.17E-07	-1.10967	0.469	0.744	0.001954	Astrocytic
Sfpq	1.19E-07	-1.13995	0.594	0.752	0.001986	Astrocytic
Sorbs1	1.21E-07	0.365894	0.281	0.05	0.002011	Astrocytic
Camkk2	1.23E-07	0.312378	0.219	0.032	0.002057	Astrocytic
Rps19	1.24E-07	-0.93816	0.719	0.85	0.002062	Astrocytic
Trim9	1.29E-07	0.316828	0.25	0.04	0.002155	Astrocytic
Eef1a1	1.35E-07	-0.7246	0.938	0.933	0.00225	Astrocytic
Hnrnpd	1.43E-07	-1.28262	0.344	0.7	0.002393	Astrocytic
Mras	1.48E-07	0.494988	0.375	0.087	0.00246	Astrocytic
Aga	1.54E-07	0.328395	0.344	0.071	0.002577	Astrocytic
Abhd8	1.57E-07	0.423283	0.312	0.061	0.002624	Astrocytic
Mpv17l2	1.68E-07	0.417993	0.312	0.062	0.002802	Astrocytic
1110008P1	1.71E-07	0.46885	0.312	0.063	0.002855	Astrocytic
Gba	1.81E-07	0.524157	0.344	0.077	0.00302	Astrocytic
Ctsb	1.84E-07	0.678897	0.781	0.36	0.003068	Astrocytic
Naprt1	1.91E-07	0.307208	0.156	0.017	0.00319	Astrocytic
Frrs1l	1.92E-07	0.539038	0.719	0.26	0.003207	Astrocytic
Sec14l1	2.01E-07	0.425048	0.375	0.084	0.003351	Astrocytic
Leprel4	2.08E-07	0.391128	0.312	0.062	0.003464	Astrocytic
1110065P2	2.1E-07	0.439032	0.594	0.183	0.003506	Astrocytic
Fuom	2.17E-07	0.302453	0.281	0.051	0.003624	Astrocytic
Hsd17b12	2.17E-07	0.71823	0.75	0.317	0.003627	Astrocytic
Col1a2	2.19E-07	0.291818	0.188	0.024	0.003659	Astrocytic
Ezh2	2.22E-07	-1.44062	0.281	0.67	0.003699	Astrocytic
Fus	2.22E-07	-0.88394	0.719	0.822	0.003711	Astrocytic
Pacrgl	2.28E-07	0.540679	0.188	0.026	0.003798	Astrocytic
Mboat2	2.32E-07	0.43058	0.375	0.087	0.003871	Astrocytic
Nat8l	2.38E-07	0.297441	0.219	0.033	0.003972	Astrocytic
Mpc1	2.48E-07	0.447095	0.281	0.054	0.004129	Astrocytic
H1f0	2.48E-07	-1.58079	0.219	0.628	0.004143	Astrocytic
Rabac1	2.64E-07	0.650993	0.781	0.343	0.004412	Astrocytic
Cd24a	2.71E-07	-1.60056	0.062	0.535	0.004512	Astrocytic
Rbm25	2.81E-07	-0.87989	0.719	0.856	0.004688	Astrocytic
Rnase4	2.82E-07	0.388158	0.219	0.034	0.004708	Astrocytic
Anp32a	2.82E-07	-0.98845	0.781	0.84	0.00471	Astrocytic
Pttg1ip	2.85E-07	0.619369	0.469	0.139	0.004751	Astrocytic
Txndc15	2.91E-07	0.417069	0.656	0.214	0.004858	Astrocytic
Tmem38a	3.09E-07	0.378162	0.219	0.034	0.005154	Astrocytic
Jak1	3.25E-07	0.732788	0.531	0.167	0.005419	Astrocytic

Ctsd	3.29E-07	0.339333	0.719	0.272	0.00549	Astrocytic
Pdha1	3.44E-07	0.466742	0.625	0.197	0.005739	Astrocytic
Cox6b2	3.46E-07	0.454893	0.219	0.034	0.005769	Astrocytic
Rps24	3.51E-07	-0.85373	0.812	0.872	0.005848	Astrocytic
Cchcr1	3.59E-07	0.371402	0.281	0.054	0.005987	Astrocytic
Sh3glb2	3.79E-07	0.470462	0.406	0.101	0.006324	Astrocytic
Ddt	3.88E-07	0.552772	0.531	0.164	0.006465	Astrocytic
Ccdc107	4.26E-07	0.370562	0.469	0.127	0.007103	Astrocytic
Rpl14	4.28E-07	-0.86128	0.781	0.821	0.007139	Astrocytic
Taf13	4.36E-07	0.304023	0.312	0.063	0.007274	Astrocytic
Cflar	4.54E-07	0.362265	0.312	0.066	0.007579	Astrocytic
Erlin2	4.76E-07	0.456542	0.312	0.067	0.007939	Astrocytic
Slc16a2	4.92E-07	0.417205	0.375	0.087	0.008215	Astrocytic
Fam214b	5.14E-07	0.490445	0.188	0.027	0.008579	Astrocytic
Tmbim4	5.3E-07	0.488843	0.625	0.215	0.008836	Astrocytic
Pigs	5.37E-07	0.33295	0.281	0.054	0.00896	Astrocytic
Ybx1	5.45E-07	-0.76712	0.844	0.874	0.009084	Astrocytic
Tnfrsf21	5.5E-07	0.38178	0.375	0.089	0.009167	Astrocytic
Sdhc	5.6E-07	0.620822	0.781	0.375	0.009348	Astrocytic
Ppp2r2b	5.61E-07	0.343553	0.406	0.099	0.009364	Astrocytic
Chpt1	5.67E-07	0.624849	0.5	0.159	0.009451	Astrocytic
Slc16a1	5.91E-07	0.318924	0.312	0.065	0.009852	Astrocytic
Trip6	5.94E-07	0.274398	0.281	0.054	0.009902	Astrocytic
Gpi1	5.98E-07	0.411479	0.594	0.19	0.009973	Astrocytic
S1pr1	1.91E-75	1.792318	0.659	0.02	3.18E-71	AP-Like
Mlc1	1.6E-74	2.061884	0.854	0.047	2.67E-70	AP-Like
Aqp4	3.09E-72	2.504482	0.927	0.06	5.15E-68	AP-Like
Pla2g7	3.42E-72	2.319065	0.902	0.057	5.7E-68	AP-Like
Agt	8.92E-69	1.974747	0.805	0.042	1.49E-64	AP-Like
Slc4a4	4.16E-66	2.801291	0.927	0.079	6.93E-62	AP-Like
Aldh1l1	1.28E-61	1.701644	0.707	0.036	2.14E-57	AP-Like
Aldoc	1.68E-59	2.450285	0.927	0.088	2.8E-55	AP-Like
Slc6a11	4.47E-59	1.797242	0.683	0.038	7.45E-55	AP-Like
Clu	1.15E-58	2.484669	0.902	0.091	1.91E-54	AP-Like
Ttyh1	1.17E-58	2.36939	1	0.122	1.95E-54	AP-Like
Lcat	1.25E-57	1.524847	0.707	0.042	2.09E-53	AP-Like
Acsbg1	1.26E-56	1.682677	0.732	0.047	2.1E-52	AP-Like
Id3	2.1E-56	2.576496	0.878	0.085	3.5E-52	AP-Like
Lrig1	7.48E-56	1.491258	0.683	0.041	1.25E-51	AP-Like
Dbx2	2.6E-55	1.303881	0.537	0.02	4.34E-51	AP-Like
Slc25a18	4.37E-55	1.441271	0.683	0.041	7.29E-51	AP-Like
Fgfr3	8.28E-53	1.010789	0.463	0.014	1.38E-48	AP-Like
Ednrb	1.36E-50	2.447465	1	0.154	2.26E-46	AP-Like
Htra1	1.87E-50	1.541602	0.634	0.04	3.13E-46	AP-Like
Itga6	8.38E-50	1.202418	0.512	0.021	1.4E-45	AP-Like
C030005K(	8.87E-50	0.993414	0.293	0.001	1.48E-45	AP-Like
Atp1a2	6.56E-49	2.630373	1	0.167	1.09E-44	AP-Like
Nid1	3.44E-47	1.415651	0.634	0.041	5.73E-43	AP-Like

Thbs3	9.53E-47	1.042175	0.488	0.021	1.59E-42	AP-Like
Atp1b2	1.95E-46	2.05117	0.878	0.114	3.26E-42	AP-Like
Slc7a11	3.29E-46	1.171292	0.293	0.002	5.49E-42	AP-Like
Egfl6	5.71E-46	0.694552	0.293	0.002	9.52E-42	AP-Like
Slc6a9	8.4E-46	1.102877	0.488	0.022	1.4E-41	AP-Like
Gja1	1.02E-45	1.737407	0.683	0.058	1.7E-41	AP-Like
Atp13a4	2.66E-45	1.144521	0.366	0.009	4.43E-41	AP-Like
Hepacam	5.24E-45	1.413278	0.732	0.067	8.73E-41	AP-Like
Car2	1.51E-44	1.855193	0.732	0.067	2.51E-40	AP-Like
Ppap2b	6.54E-44	2.334235	0.976	0.185	1.09E-39	AP-Like
Sepp1	3.87E-43	1.182421	0.829	0.096	6.46E-39	AP-Like
Gria1	7.84E-43	1.516956	0.61	0.046	1.31E-38	AP-Like
Mmd2	7.95E-43	1.62751	0.854	0.11	1.33E-38	AP-Like
Lpar1	1.87E-40	1.147403	0.488	0.027	3.12E-36	AP-Like
Id4	2.53E-40	1.38339	0.61	0.048	4.23E-36	AP-Like
Mt3	2.7E-40	1.827766	0.805	0.105	4.5E-36	AP-Like
Igfbp2	5.97E-40	2.152676	0.659	0.062	9.96E-36	AP-Like
Slc39a12	5.81E-39	1.125881	0.341	0.01	9.69E-35	AP-Like
Kcnj10	6.94E-39	2.391071	0.829	0.135	1.16E-34	AP-Like
Sfxn5	3.56E-37	1.076377	0.537	0.041	5.95E-33	AP-Like
Sdc4	7.21E-37	1.120433	0.488	0.032	1.2E-32	AP-Like
Sparc	7.57E-37	1.858104	0.951	0.188	1.26E-32	AP-Like
Chchd10	1.99E-36	1.26844	0.683	0.073	3.32E-32	AP-Like
Lxn	2.53E-36	1.403753	0.707	0.08	4.23E-32	AP-Like
Gjb6	1.01E-35	1.060014	0.39	0.019	1.69E-31	AP-Like
Tlcd1	1.33E-35	1.523398	0.659	0.073	2.22E-31	AP-Like
Tril	1.34E-35	1.564759	0.854	0.136	2.24E-31	AP-Like
Tnc	2.29E-35	1.536771	0.488	0.033	3.81E-31	AP-Like
Slc1a3	1.14E-34	2.573368	1	0.293	1.91E-30	AP-Like
Plcd4	1.18E-34	0.90836	0.366	0.016	1.97E-30	AP-Like
Paqr7	1.57E-34	1.027251	0.341	0.014	2.61E-30	AP-Like
Slc7a10	2.08E-34	1.124295	0.415	0.023	3.48E-30	AP-Like
Gm266	2.37E-34	0.585388	0.244	0.004	3.96E-30	AP-Like
Timp4	2.99E-34	1.494075	0.78	0.114	4.98E-30	AP-Like
Limch1	6.33E-34	1.335726	0.683	0.079	1.06E-29	AP-Like
Abhd3	2.52E-33	0.913879	0.39	0.021	4.21E-29	AP-Like
Tspan7	4.03E-33	1.882259	0.976	0.242	6.72E-29	AP-Like
Slc6a1	5.23E-33	1.463818	0.707	0.095	8.73E-29	AP-Like
Cmtm5	9.92E-33	1.353546	0.732	0.101	1.66E-28	AP-Like
Plat	7.17E-32	1.251598	0.561	0.056	1.2E-27	AP-Like
Fam181a	8.98E-32	0.547103	0.244	0.005	1.5E-27	AP-Like
Gpld1	1.74E-31	0.75878	0.268	0.007	2.9E-27	AP-Like
AW04773C	3.92E-31	1.424748	0.683	0.091	6.54E-27	AP-Like
Sparcl1	4.11E-31	2.639639	1	0.411	6.85E-27	AP-Like
Wnt7a	5.49E-31	0.900738	0.341	0.016	9.16E-27	AP-Like
Ncan	5.88E-31	1.680896	0.829	0.157	9.81E-27	AP-Like
Apoe	1.9E-30	2.792425	1	0.354	3.18E-26	AP-Like
Itih3	1.99E-30	1.204962	0.317	0.014	3.32E-26	AP-Like

Mt2	2.09E-30	1.709439	0.854	0.169	3.49E-26	AP-Like
Fabp7	2.19E-30	2.211136	1	0.342	3.65E-26	AP-Like
Ccdc80	3.5E-30	1.126951	0.439	0.032	5.84E-26	AP-Like
Arap2	4.26E-30	0.909632	0.366	0.021	7.1E-26	AP-Like
Kcnn2	6.18E-30	0.978278	0.39	0.025	1.03E-25	AP-Like
Ramp1	1.74E-29	1.399025	0.854	0.167	2.91E-25	AP-Like
Cyp26b1	4.08E-29	1.127126	0.366	0.022	6.81E-25	AP-Like
Ano1	6.19E-29	0.692214	0.195	0.002	1.03E-24	AP-Like
Ntrk2	6.52E-29	1.411942	0.805	0.148	1.09E-24	AP-Like
Adrb1	1.59E-28	0.773748	0.293	0.012	2.66E-24	AP-Like
Sept4	1.6E-28	1.676493	0.805	0.151	2.67E-24	AP-Like
Lfng	2.08E-28	1.036293	0.512	0.052	3.47E-24	AP-Like
Paqr8	2.12E-28	1.141543	0.488	0.047	3.54E-24	AP-Like
Ppp1r3c	5.82E-28	0.457255	0.171	0.001	9.72E-24	AP-Like
Ntsr2	8.3E-28	0.724573	0.22	0.005	1.38E-23	AP-Like
Bmpr1b	8.34E-28	0.788291	0.317	0.016	1.39E-23	AP-Like
Emp2	9E-28	0.953563	0.341	0.02	1.5E-23	AP-Like
Alpl	1.36E-27	0.705775	0.268	0.01	2.27E-23	AP-Like
Gabrb1	4.08E-27	0.864846	0.341	0.021	6.8E-23	AP-Like
Gdpd2	1.2E-26	0.704997	0.268	0.011	2E-22	AP-Like
Pdpn	1.34E-26	1.022407	0.439	0.04	2.23E-22	AP-Like
Glul	1.67E-26	1.803376	0.902	0.283	2.78E-22	AP-Like
Sorl1	3.41E-26	0.965394	0.488	0.052	5.68E-22	AP-Like
Cst3	4.26E-26	2.593034	1	0.71	7.11E-22	AP-Like
Kcnj16	8.41E-26	1.072903	0.463	0.047	1.4E-21	AP-Like
Pbxip1	1.6E-25	0.872643	0.488	0.052	2.67E-21	AP-Like
Slc13a3	3.55E-25	0.881945	0.268	0.012	5.92E-21	AP-Like
Slc43a3	4.81E-25	0.682749	0.268	0.012	8.02E-21	AP-Like
Il33	7.83E-25	0.722507	0.293	0.016	1.31E-20	AP-Like
Tst	9.79E-25	0.915958	0.415	0.038	1.63E-20	AP-Like
Nrarp	1.28E-24	0.873182	0.366	0.028	2.13E-20	AP-Like
Luzp2	1.3E-24	1.131198	0.61	0.091	2.16E-20	AP-Like
Tmem176a	1.78E-24	1.238886	0.561	0.078	2.97E-20	AP-Like
Myo6	2.18E-24	1.210412	0.585	0.086	3.63E-20	AP-Like
Lsamp	3.43E-24	1.366401	0.878	0.216	5.72E-20	AP-Like
Adora1	3.53E-24	1.027637	0.537	0.068	5.9E-20	AP-Like
Serpine2	3.68E-24	1.279785	0.951	0.244	6.13E-20	AP-Like
Cables1	4.47E-24	0.543936	0.244	0.01	7.45E-20	AP-Like
Cbs	4.91E-24	0.651063	0.22	0.007	8.2E-20	AP-Like
Mt1	5.25E-24	1.940578	0.902	0.322	8.75E-20	AP-Like
4933407L2	7.72E-24	0.603553	0.22	0.007	1.29E-19	AP-Like
Dbi	1.88E-23	1.670898	1	0.694	3.14E-19	AP-Like
2810459M	2.42E-23	0.534896	0.293	0.017	4.04E-19	AP-Like
Cryab	2.56E-23	1.252929	0.561	0.081	4.27E-19	AP-Like
Gm3764	3.58E-23	1.43926	0.829	0.22	5.97E-19	AP-Like
Chl1	3.92E-23	1.07543	0.439	0.047	6.53E-19	AP-Like
Acsl6	3.98E-23	0.852539	0.366	0.031	6.63E-19	AP-Like
Cybrd1	4.24E-23	0.861936	0.293	0.019	7.07E-19	AP-Like

Bcan	4.37E-23	1.412381	0.902	0.247	7.29E-19	AP-Like
Prex2	4.38E-23	1.079553	0.415	0.042	7.3E-19	AP-Like
Slc14a1	6.83E-23	0.583531	0.244	0.011	1.14E-18	AP-Like
Smpdl3a	7.52E-23	0.885015	0.366	0.031	1.25E-18	AP-Like
Psap	7.88E-23	1.193831	0.902	0.272	1.32E-18	AP-Like
Pla2g16	1.23E-22	0.954003	0.415	0.042	2.05E-18	AP-Like
Tmem47	1.27E-22	1.417576	0.634	0.123	2.11E-18	AP-Like
Trim9	2.3E-22	0.782035	0.366	0.032	3.84E-18	AP-Like
Npy	2.83E-22	1.283477	0.39	0.037	4.73E-18	AP-Like
Car10	3.18E-22	0.785922	0.341	0.027	5.3E-18	AP-Like
Ifitm3	3.8E-22	0.904476	0.415	0.042	6.33E-18	AP-Like
Npas3	4.01E-22	1.117956	0.61	0.102	6.7E-18	AP-Like
Ptprz1	5.46E-22	1.50729	0.829	0.215	9.11E-18	AP-Like
Gmpr	6.14E-22	0.816037	0.366	0.033	1.02E-17	AP-Like
Ckb	6.15E-22	1.358052	1	0.821	1.03E-17	AP-Like
Scd2	1.4E-21	1.588454	0.951	0.493	2.34E-17	AP-Like
Gpr37l1	2.62E-21	1.519813	0.78	0.207	4.37E-17	AP-Like
Pcx	3.76E-21	1.095875	0.61	0.114	6.27E-17	AP-Like
Ptn	4.15E-21	1.750392	0.976	0.589	6.91E-17	AP-Like
Gstm1	6.14E-21	1.588531	0.659	0.143	1.02E-16	AP-Like
Atp1b1	6.31E-21	0.936303	0.61	0.1	1.05E-16	AP-Like
AI464131	6.33E-21	0.628835	0.22	0.01	1.06E-16	AP-Like
Vcam1	9.1E-21	0.603975	0.366	0.035	1.52E-16	AP-Like
Sirpa	1.04E-20	1.036417	0.463	0.063	1.74E-16	AP-Like
Slc5a3	1.63E-20	0.647505	0.317	0.026	2.71E-16	AP-Like
Gabbr2	2.3E-20	0.846312	0.341	0.031	3.84E-16	AP-Like
Slc9a3r1	3.02E-20	0.75111	0.439	0.054	5.04E-16	AP-Like
Cd81	4.12E-20	1.367204	0.976	0.638	6.87E-16	AP-Like
Gabrg1	4.53E-20	0.750865	0.341	0.032	7.55E-16	AP-Like
Plscr2	4.65E-20	0.435825	0.171	0.005	7.76E-16	AP-Like
Tmem176b	6.58E-20	1.165948	0.878	0.26	1.1E-15	AP-Like
Gramd3	8.46E-20	0.707308	0.317	0.027	1.41E-15	AP-Like
Aldh1a1	1.18E-19	0.659053	0.22	0.011	1.98E-15	AP-Like
Angptl4	1.33E-19	0.666343	0.244	0.015	2.22E-15	AP-Like
Metrn	1.39E-19	0.976893	0.537	0.088	2.32E-15	AP-Like
Arhgef26	1.46E-19	0.850944	0.439	0.057	2.44E-15	AP-Like
Glud1	1.5E-19	1.389936	0.78	0.237	2.5E-15	AP-Like
Itm2c	1.51E-19	1.264071	0.878	0.298	2.51E-15	AP-Like
Slc38a3	1.68E-19	0.977536	0.512	0.079	2.8E-15	AP-Like
Dennd3	1.94E-19	0.27059	0.122	0.001	3.24E-15	AP-Like
1700084C(	2.01E-19	0.460304	0.244	0.015	3.35E-15	AP-Like
Paqr6	2.11E-19	0.426734	0.122	0.001	3.52E-15	AP-Like
Rps5	2.35E-19	-1.26368	0.951	0.981	3.92E-15	AP-Like
Eps8	4.43E-19	0.933416	0.561	0.099	7.38E-15	AP-Like
Bhlhe40	7.8E-19	0.416059	0.195	0.009	1.3E-14	AP-Like
Cldn10	9.41E-19	0.844404	0.268	0.02	1.57E-14	AP-Like
Cxcl14	1.23E-18	1.07297	0.488	0.075	2.06E-14	AP-Like
Npl	1.26E-18	0.604514	0.244	0.016	2.1E-14	AP-Like

Pon2	1.66E-18	0.801587	0.488	0.075	2.77E-14	AP-Like
Gsta4	1.7E-18	0.672234	0.317	0.03	2.83E-14	AP-Like
Dkk3	1.92E-18	0.793772	0.341	0.036	3.2E-14	AP-Like
Pea15a	2.3E-18	1.105772	0.829	0.247	3.84E-14	AP-Like
Ccdc37	2.87E-18	0.659552	0.171	0.006	4.79E-14	AP-Like
Pdgfd	3.32E-18	0.339977	0.146	0.004	5.54E-14	AP-Like
Hopx	3.4E-18	0.853549	0.366	0.041	5.67E-14	AP-Like
Casp12	4.62E-18	0.740975	0.268	0.021	7.7E-14	AP-Like
Slc1a2	5.61E-18	1.35265	0.902	0.394	9.36E-14	AP-Like
F3	6.12E-18	1.030626	0.39	0.049	1.02E-13	AP-Like
Gm5607	9.84E-18	0.884955	0.244	0.017	1.64E-13	AP-Like
Shisa9	1.09E-17	0.787504	0.341	0.038	1.81E-13	AP-Like
Cyp2j9	1.09E-17	0.711419	0.39	0.049	1.82E-13	AP-Like
Acadl	1.1E-17	1.027598	0.634	0.141	1.84E-13	AP-Like
Paqr4	1.21E-17	1.007616	0.512	0.089	2.01E-13	AP-Like
Hsp90ab1	1.39E-17	-0.94594	0.976	0.996	2.32E-13	AP-Like
Grm3	1.54E-17	0.423883	0.195	0.01	2.57E-13	AP-Like
Adcy8	1.71E-17	0.517587	0.244	0.017	2.85E-13	AP-Like
Kdelr3	1.91E-17	0.469732	0.195	0.01	3.18E-13	AP-Like
Ctsl	2.17E-17	1.147404	0.878	0.367	3.61E-13	AP-Like
Rps9	2.29E-17	-1.08557	0.878	0.981	3.82E-13	AP-Like
Sardh	5.75E-17	0.691955	0.171	0.007	9.59E-13	AP-Like
Jam2	6.85E-17	0.903708	0.512	0.095	1.14E-12	AP-Like
Rgl1	7.17E-17	0.607546	0.244	0.019	1.2E-12	AP-Like
Rps26	1E-16	-1.46984	0.561	0.915	1.67E-12	AP-Like
Fxyd1	1.06E-16	0.673902	0.293	0.028	1.77E-12	AP-Like
Mro	1.27E-16	0.569951	0.317	0.033	2.12E-12	AP-Like
Nlrx1	1.3E-16	0.311074	0.122	0.002	2.17E-12	AP-Like
Baalc	1.6E-16	0.263184	0.122	0.002	2.68E-12	AP-Like
Rps3	1.7E-16	-1.0977	0.878	0.959	2.84E-12	AP-Like
Fam20a	1.79E-16	0.57586	0.195	0.011	2.98E-12	AP-Like
Prss35	2.06E-16	0.436122	0.146	0.005	3.44E-12	AP-Like
Itgb5	2.52E-16	0.446249	0.22	0.015	4.2E-12	AP-Like
Cyp4v3	2.53E-16	0.314366	0.146	0.005	4.22E-12	AP-Like
Fam189a2	2.84E-16	0.370795	0.146	0.005	4.74E-12	AP-Like
Fads2	3.04E-16	0.647369	0.293	0.03	5.07E-12	AP-Like
Daam2	3.36E-16	0.593853	0.244	0.02	5.61E-12	AP-Like
Gria4	3.52E-16	1.031761	0.707	0.194	5.87E-12	AP-Like
Ncl	3.87E-16	-1.25399	0.707	0.948	6.46E-12	AP-Like
Me1	4E-16	0.683996	0.317	0.036	6.68E-12	AP-Like
Elovl5	4.51E-16	1.11154	0.683	0.189	7.53E-12	AP-Like
Rpl4	4.52E-16	-1.12858	0.829	0.96	7.53E-12	AP-Like
Hes5	5.24E-16	1.208271	0.341	0.043	8.73E-12	AP-Like
Megf10	6.25E-16	0.707481	0.22	0.016	1.04E-11	AP-Like
Abca1	6.66E-16	0.754839	0.341	0.042	1.11E-11	AP-Like
Ndrg2	6.86E-16	1.403607	0.805	0.311	1.14E-11	AP-Like
Fkbp10	8.05E-16	0.543423	0.22	0.016	1.34E-11	AP-Like
Ppargc1a	8.37E-16	0.627959	0.22	0.016	1.4E-11	AP-Like

Tsc22d4	8.56E-16	1.002813	0.878	0.342	1.43E-11	AP-Like
Abhd4	9.7E-16	0.772281	0.537	0.107	1.62E-11	AP-Like
Hsd11b1	1.25E-15	1.318142	0.268	0.026	2.08E-11	AP-Like
Grm5	1.25E-15	0.873205	0.366	0.049	2.08E-11	AP-Like
Frem2	1.28E-15	0.51726	0.171	0.009	2.13E-11	AP-Like
Gjc3	1.49E-15	1.093881	0.561	0.117	2.49E-11	AP-Like
Btbd17	1.62E-15	1.089895	0.634	0.159	2.71E-11	AP-Like
Rplp0	1.79E-15	-1.28498	0.683	0.926	2.99E-11	AP-Like
Gfap	1.8E-15	1.328875	0.415	0.068	2.99E-11	AP-Like
Mfge8	1.85E-15	1.027658	0.512	0.102	3.08E-11	AP-Like
Slc35f6	1.92E-15	0.566828	0.268	0.026	3.2E-11	AP-Like
Gpr56	2.16E-15	1.039919	0.707	0.209	3.6E-11	AP-Like
Cyp2d22	2.22E-15	0.447306	0.195	0.012	3.69E-11	AP-Like
Mgst1	2.6E-15	0.997303	0.537	0.112	4.34E-11	AP-Like
Gpr126	3.92E-15	0.730192	0.244	0.022	6.54E-11	AP-Like
Cp	4.91E-15	0.548524	0.366	0.049	8.2E-11	AP-Like
S100b	5.48E-15	0.970501	0.683	0.177	9.14E-11	AP-Like
Fndc5	5.97E-15	0.563015	0.22	0.017	9.96E-11	AP-Like
Rplp2	6.9E-15	-1.20359	0.634	0.925	1.15E-10	AP-Like
Rpl13a	7.12E-15	-1.02353	0.927	0.973	1.19E-10	AP-Like
Ccdc24	8.8E-15	0.423377	0.146	0.006	1.47E-10	AP-Like
H2-K1	1.1E-14	0.810814	0.317	0.04	1.84E-10	AP-Like
Sat1	1.24E-14	1.184168	0.488	0.098	2.07E-10	AP-Like
Pcdh10	1.83E-14	0.677882	0.39	0.06	3.05E-10	AP-Like
Naprt1	1.98E-14	0.446575	0.195	0.014	3.31E-10	AP-Like
Ak1	2.2E-14	0.502047	0.171	0.01	3.66E-10	AP-Like
Ephx1	2.37E-14	0.588946	0.22	0.019	3.95E-10	AP-Like
Nfkbiz	2.71E-14	0.63402	0.244	0.023	4.52E-10	AP-Like
Rgma	4E-14	0.819332	0.488	0.1	6.68E-10	AP-Like
Tmem229a	4.01E-14	0.87433	0.488	0.099	6.68E-10	AP-Like
Itm2b	5.79E-14	1.063377	0.927	0.741	9.65E-10	AP-Like
Lamp1	6.57E-14	0.990771	0.902	0.495	1.1E-09	AP-Like
Rplp1	7.41E-14	-0.91378	0.878	0.956	1.24E-09	AP-Like
Slc12a4	7.76E-14	0.822407	0.463	0.093	1.29E-09	AP-Like
Gpm6b	9.38E-14	1.281779	0.927	0.609	1.57E-09	AP-Like
Rps14	9.7E-14	-0.86401	0.951	0.977	1.62E-09	AP-Like
Sox21	1E-13	0.590803	0.341	0.049	1.68E-09	AP-Like
Axl	1.05E-13	0.510814	0.195	0.015	1.76E-09	AP-Like
Abat	1.4E-13	0.820409	0.415	0.075	2.34E-09	AP-Like
Chi3l1	1.66E-13	0.392698	0.146	0.007	2.76E-09	AP-Like
Asrgl1	1.79E-13	1.020742	0.78	0.3	2.98E-09	AP-Like
Ttyh3	1.88E-13	0.791617	0.585	0.147	3.13E-09	AP-Like
Gpc4	2.1E-13	0.402649	0.171	0.011	3.5E-09	AP-Like
Ndrg4	2.56E-13	0.477441	0.268	0.031	4.27E-09	AP-Like
Aldoa	3.09E-13	1.153428	0.732	0.272	5.15E-09	AP-Like
Slc27a1	3.1E-13	0.614981	0.415	0.074	5.17E-09	AP-Like
Slc1a4	3.55E-13	0.902862	0.463	0.104	5.93E-09	AP-Like
Tnfrsf19	4.58E-13	0.535395	0.244	0.026	7.65E-09	AP-Like

Cspg5	4.59E-13	0.556075	0.707	0.18	7.66E-09	AP-Like
Slc7a2	4.86E-13	0.515891	0.22	0.021	8.11E-09	AP-Like
Gnb2l1	4.94E-13	-1.0333	0.707	0.932	8.24E-09	AP-Like
Prrx1	5.08E-13	0.58239	0.244	0.026	8.48E-09	AP-Like
Ank2	5.33E-13	1.053395	0.634	0.195	8.89E-09	AP-Like
Stat3	5.71E-13	0.628783	0.439	0.084	9.52E-09	AP-Like
Enho	6.22E-13	0.609125	0.341	0.053	1.04E-08	AP-Like
Tjp2	7.57E-13	0.758362	0.512	0.121	1.26E-08	AP-Like
Tubb5	7.99E-13	-1.31627	0.61	0.9	1.33E-08	AP-Like
Rps21	8.06E-13	-1.07811	0.707	0.911	1.34E-08	AP-Like
Nckap5	8.16E-13	0.410861	0.195	0.016	1.36E-08	AP-Like
Sez6l	8.6E-13	0.849898	0.512	0.12	1.43E-08	AP-Like
Timp3	1.24E-12	0.881536	0.488	0.115	2.08E-08	AP-Like
Trpm3	1.32E-12	0.532747	0.268	0.033	2.2E-08	AP-Like
Hnrnpu	1.59E-12	-1.00905	0.756	0.93	2.66E-08	AP-Like
Pabpc1	1.82E-12	-0.93642	0.805	0.94	3.04E-08	AP-Like
Rpl32	1.87E-12	-0.91352	0.829	0.946	3.11E-08	AP-Like
Adcy2	2.08E-12	0.550013	0.146	0.009	3.47E-08	AP-Like
Adra1b	2.22E-12	0.36615	0.146	0.009	3.7E-08	AP-Like
Nwd1	2.22E-12	0.253529	0.146	0.009	3.7E-08	AP-Like
Ccdc141	2.24E-12	0.558536	0.22	0.022	3.73E-08	AP-Like
Mras	2.29E-12	0.746601	0.415	0.081	3.82E-08	AP-Like
Rpl8	2.34E-12	-0.79552	0.927	0.959	3.91E-08	AP-Like
Cd9	2.65E-12	0.698684	1	0.654	4.42E-08	AP-Like
Rps20	2.69E-12	-1.14585	0.659	0.873	4.49E-08	AP-Like
Rrbp1	2.89E-12	1.095101	0.756	0.305	4.82E-08	AP-Like
Sfrp1	3.4E-12	-1.91106	0.341	0.774	5.67E-08	AP-Like
Gpd1	3.41E-12	0.60939	0.268	0.033	5.69E-08	AP-Like
Wipi1	3.63E-12	0.782042	0.317	0.051	6.05E-08	AP-Like
Tpp1	3.8E-12	0.832424	0.512	0.128	6.35E-08	AP-Like
Prdx6	4.22E-12	1.052889	0.805	0.402	7.04E-08	AP-Like
Acot1	4.93E-12	1.047489	0.488	0.123	8.23E-08	AP-Like
Ctso	5.34E-12	0.681508	0.317	0.051	8.91E-08	AP-Like
Adcyap1r1	5.55E-12	0.821801	0.585	0.16	9.26E-08	AP-Like
Ppp1r1a	7.52E-12	0.939378	0.488	0.122	1.25E-07	AP-Like
Tspan12	7.94E-12	0.655379	0.415	0.084	1.32E-07	AP-Like
Il6st	8E-12	0.610821	0.366	0.067	1.33E-07	AP-Like
Scg3	8.43E-12	0.966975	0.878	0.495	1.41E-07	AP-Like
Ctsb	8.66E-12	0.738929	0.805	0.354	1.45E-07	AP-Like
Cbr3	1.11E-11	0.584704	0.293	0.043	1.85E-07	AP-Like
Eef1a1	1.19E-11	-0.90075	0.756	0.942	1.98E-07	AP-Like
S100a16	1.3E-11	0.805231	0.634	0.19	2.18E-07	AP-Like
Phyhipl	1.32E-11	0.646899	0.61	0.17	2.2E-07	AP-Like
Proca1	1.64E-11	0.396537	0.146	0.01	2.74E-07	AP-Like
Osbpl1a	1.92E-11	0.778378	0.463	0.111	3.2E-07	AP-Like
4930402H	2.1E-11	0.794	0.585	0.174	3.51E-07	AP-Like
S1pr3	2.18E-11	0.306792	0.122	0.006	3.63E-07	AP-Like
Foxj1	2.18E-11	0.27399	0.122	0.006	3.63E-07	AP-Like

Nfasc	2.21E-11	0.744585	0.415	0.088	3.69E-07	AP-Like
BC064078	2.27E-11	0.294864	0.146	0.01	3.79E-07	AP-Like
Fuca2	2.37E-11	0.46675	0.244	0.031	3.95E-07	AP-Like
Dtx4	2.37E-11	0.332121	0.22	0.025	3.96E-07	AP-Like
Pmm1	2.66E-11	0.843328	0.561	0.163	4.44E-07	AP-Like
Hnrnpab	2.72E-11	-1.0888	0.61	0.862	4.54E-07	AP-Like
Agrn	2.84E-11	0.857927	0.537	0.148	4.73E-07	AP-Like
Nacc2	2.91E-11	0.583736	0.341	0.06	4.86E-07	AP-Like
B2m	2.97E-11	0.932727	0.829	0.388	4.95E-07	AP-Like
Rpl22	3.18E-11	-1.0174	0.585	0.883	5.3E-07	AP-Like
Dtna	3.22E-11	0.72111	0.341	0.062	5.37E-07	AP-Like
Mertk	3.69E-11	0.494376	0.195	0.02	6.15E-07	AP-Like
Gabbr1	4.26E-11	0.698157	0.561	0.158	7.1E-07	AP-Like
Mapk4	4.65E-11	0.417224	0.171	0.015	7.75E-07	AP-Like
Rnf182	4.71E-11	0.374746	0.195	0.02	7.86E-07	AP-Like
St3gal6	5.22E-11	0.524579	0.22	0.026	8.71E-07	AP-Like
S100a10	5.39E-11	0.985605	0.39	0.08	8.99E-07	AP-Like
Adrbk2	6.47E-11	0.780426	0.39	0.081	1.08E-06	AP-Like
Epas1	6.77E-11	0.454662	0.244	0.032	1.13E-06	AP-Like
Them4	6.8E-11	0.888249	0.341	0.067	1.13E-06	AP-Like
Bcl2	7.18E-11	0.494557	0.293	0.047	1.2E-06	AP-Like
Lrrc16a	7.44E-11	0.409215	0.22	0.026	1.24E-06	AP-Like
Rps15	7.58E-11	-0.93681	0.707	0.893	1.26E-06	AP-Like
Scrg1	7.97E-11	0.710923	0.634	0.188	1.33E-06	AP-Like
Cd302	8.9E-11	0.824448	0.488	0.126	1.48E-06	AP-Like
Rhbdl2	9.71E-11	0.578414	0.146	0.011	1.62E-06	AP-Like
Lgmn	1.04E-10	0.754784	0.488	0.126	1.74E-06	AP-Like
Hnrnpa2b1	1.05E-10	-0.72309	0.951	0.963	1.75E-06	AP-Like
Soat1	1.08E-10	0.723836	0.268	0.042	1.79E-06	AP-Like
Cyp4f13	1.14E-10	0.504626	0.22	0.027	1.9E-06	AP-Like
Itgav	1.2E-10	0.616737	0.463	0.112	1.99E-06	AP-Like
Rarres2	1.39E-10	0.4908	0.146	0.011	2.32E-06	AP-Like
Sdc3	1.54E-10	0.714305	0.488	0.125	2.56E-06	AP-Like
Dhrs1	1.62E-10	0.867921	0.512	0.149	2.7E-06	AP-Like
Gpd2	1.82E-10	0.900719	0.463	0.12	3.03E-06	AP-Like
Specc1	1.86E-10	0.650682	0.415	0.095	3.11E-06	AP-Like
Lhfp	1.87E-10	0.373243	0.171	0.016	3.12E-06	AP-Like
Macf1	1.92E-10	0.882557	0.683	0.269	3.21E-06	AP-Like
Gpr162	2.16E-10	0.462628	0.122	0.007	3.61E-06	AP-Like
Serbp1	2.21E-10	-0.83359	0.829	0.93	3.69E-06	AP-Like
Serpina3n	2.26E-10	0.365299	0.122	0.007	3.77E-06	AP-Like
Trim47	2.31E-10	0.320111	0.122	0.007	3.85E-06	AP-Like
Dio2	2.41E-10	0.376237	0.122	0.007	4.02E-06	AP-Like
Frmpd1	2.41E-10	0.323594	0.122	0.007	4.02E-06	AP-Like
Sash1	2.43E-10	0.765044	0.463	0.117	4.05E-06	AP-Like
Rps24	2.45E-10	-0.97217	0.707	0.878	4.09E-06	AP-Like
Gm12892	2.51E-10	0.416971	0.268	0.041	4.19E-06	AP-Like
Gm12222	2.52E-10	0.267071	0.122	0.007	4.2E-06	AP-Like

Angpt1	2.71E-10	0.883917	0.439	0.114	4.51E-06	AP-Like
Atp2b4	2.74E-10	0.306805	0.122	0.007	4.58E-06	AP-Like
Npm1	3.4E-10	-1.19175	0.439	0.769	5.67E-06	AP-Like
Efhd1	3.66E-10	0.511035	0.317	0.058	6.1E-06	AP-Like
S100a6	3.66E-10	0.82547	0.39	0.088	6.11E-06	AP-Like
Cyfip1	3.85E-10	0.675545	0.561	0.167	6.42E-06	AP-Like
Ddhd1	3.91E-10	0.544722	0.366	0.077	6.52E-06	AP-Like
Cyth3	3.95E-10	0.758553	0.366	0.078	6.58E-06	AP-Like
Dmd	3.99E-10	0.519885	0.341	0.067	6.66E-06	AP-Like
Pltp	4.1E-10	0.685244	0.317	0.06	6.84E-06	AP-Like
Col1a2	4.15E-10	0.385531	0.195	0.022	6.92E-06	AP-Like
Rps19	4.97E-10	-0.97577	0.659	0.854	8.28E-06	AP-Like
H3f3b	5.22E-10	-0.95532	0.659	0.905	8.71E-06	AP-Like
Rab31	5.62E-10	0.864055	0.488	0.137	9.37E-06	AP-Like
Amot	5.68E-10	0.482254	0.244	0.036	9.47E-06	AP-Like
Nop58	6.1E-10	-1.2746	0.268	0.726	1.02E-05	AP-Like
Slc15a2	6.21E-10	0.724351	0.366	0.08	1.04E-05	AP-Like
Tpi1	6.43E-10	0.712668	0.537	0.163	1.07E-05	AP-Like
Rpl41	7.04E-10	-0.88298	0.683	0.884	1.17E-05	AP-Like
Id1	7.46E-10	1.028741	0.512	0.149	1.24E-05	AP-Like
Tmtc2	7.9E-10	0.529815	0.244	0.037	1.32E-05	AP-Like
Cpeb4	8.08E-10	0.584084	0.341	0.07	1.35E-05	AP-Like
Rnf13	8.12E-10	0.595343	0.512	0.147	1.35E-05	AP-Like
Slc35e4	8.52E-10	0.574761	0.22	0.03	1.42E-05	AP-Like
Reep5	8.7E-10	0.704374	0.585	0.193	1.45E-05	AP-Like
Ccnd1	9.11E-10	-1.43617	0.293	0.715	1.52E-05	AP-Like
Ptplb	9.46E-10	0.846201	0.512	0.151	1.58E-05	AP-Like
Sema6d	9.63E-10	0.616041	0.341	0.07	1.61E-05	AP-Like
Mrps6	1.05E-09	0.942368	0.634	0.253	1.76E-05	AP-Like
Ptp4a3	1.09E-09	0.367914	0.195	0.023	1.82E-05	AP-Like
Capns1	1.12E-09	0.774507	0.732	0.333	1.87E-05	AP-Like
Tspan3	1.19E-09	0.779905	0.902	0.625	1.98E-05	AP-Like
Notch1	1.28E-09	0.590177	0.366	0.08	2.14E-05	AP-Like
Gpr123	1.28E-09	0.383215	0.195	0.023	2.14E-05	AP-Like
Ptpmt1	1.57E-09	0.582182	0.512	0.147	2.61E-05	AP-Like
Hbegf	1.59E-09	0.792942	0.317	0.064	2.66E-05	AP-Like
C4b	1.62E-09	0.556031	0.122	0.009	2.71E-05	AP-Like
Rora	1.7E-09	0.664373	0.366	0.083	2.83E-05	AP-Like
Agtrap	1.74E-09	0.403674	0.171	0.019	2.89E-05	AP-Like
Apoc1	1.79E-09	0.313063	0.122	0.009	2.99E-05	AP-Like
Spon1	2.03E-09	0.660271	0.415	0.101	3.39E-05	AP-Like
Cdh22	2.15E-09	0.571609	0.195	0.025	3.59E-05	AP-Like
Emid1	2.17E-09	0.811531	0.317	0.062	3.62E-05	AP-Like
Akr1b10	2.31E-09	0.419579	0.341	0.069	3.86E-05	AP-Like
Gpnmb	2.35E-09	0.456262	0.195	0.025	3.93E-05	AP-Like
Lix1l	2.64E-09	0.385494	0.244	0.038	4.41E-05	AP-Like
Mamdc2	2.73E-09	0.551761	0.244	0.04	4.56E-05	AP-Like
Itih5	2.78E-09	0.373336	0.195	0.025	4.64E-05	AP-Like

Pdgfrl	2.93E-09	0.327806	0.146	0.014	4.88E-05	AP-Like
Suclg2	2.97E-09	0.53391	0.293	0.054	4.96E-05	AP-Like
Asah1	2.99E-09	0.775495	0.439	0.117	4.99E-05	AP-Like
Gstt1	3.03E-09	0.594277	0.317	0.064	5.05E-05	AP-Like
Ybx1	3.09E-09	-0.83912	0.707	0.881	5.15E-05	AP-Like
Kcne1l	3.25E-09	0.688093	0.293	0.058	5.42E-05	AP-Like
Vim	3.63E-09	0.887921	0.902	0.532	6.06E-05	AP-Like
Prnp	3.7E-09	0.807078	0.659	0.249	6.18E-05	AP-Like
Hsdl2	3.93E-09	0.563557	0.39	0.094	6.56E-05	AP-Like
Lyn	4.32E-09	0.438774	0.22	0.032	7.21E-05	AP-Like
Gpx8	4.36E-09	0.594822	0.463	0.127	7.27E-05	AP-Like
Dek	4.41E-09	-1.23211	0.439	0.781	7.36E-05	AP-Like
Timp1	4.71E-09	0.606425	0.171	0.02	7.85E-05	AP-Like
Rgs5	4.95E-09	0.847959	0.268	0.048	8.25E-05	AP-Like
Itgb8	5.15E-09	0.533931	0.317	0.065	8.6E-05	AP-Like
Nfib	5.25E-09	-1.07015	0.659	0.873	8.76E-05	AP-Like
Smpd1	5.55E-09	0.456813	0.268	0.048	9.26E-05	AP-Like
Metrnl	5.72E-09	0.454239	0.171	0.02	9.54E-05	AP-Like
Zfyve21	5.76E-09	0.564006	0.366	0.085	9.62E-05	AP-Like
Apba1	6.1E-09	0.481917	0.195	0.026	0.000102	AP-Like
Rps3a1	6.31E-09	-0.99587	0.512	0.794	0.000105	AP-Like
Pnp	6.65E-09	0.387683	0.195	0.026	0.000111	AP-Like
Trib2	6.83E-09	0.797542	0.537	0.181	0.000114	AP-Like
Appl2	7.42E-09	0.6439	0.512	0.158	0.000124	AP-Like
Hsd17b12	7.42E-09	0.773554	0.683	0.316	0.000124	AP-Like
Tmsb10	7.66E-09	-1.352	0.122	0.601	0.000128	AP-Like
Klhl13	8.13E-09	0.652153	0.561	0.185	0.000136	AP-Like
Efemp2	8.14E-09	0.481022	0.22	0.033	0.000136	AP-Like
Hnrnpdl	8.53E-09	-0.89249	0.512	0.836	0.000142	AP-Like
Acsl3	8.78E-09	0.8005	0.61	0.237	0.000147	AP-Like
Ttc3	9.61E-09	-0.96541	0.61	0.837	0.00016	AP-Like
Rps11	9.83E-09	-0.88433	0.683	0.847	0.000164	AP-Like
Pamr1	9.97E-09	0.377771	0.146	0.015	0.000166	AP-Like
Hist1h2bc	1E-08	0.602727	0.341	0.075	0.000167	AP-Like
Eif3a	1.02E-08	-1.04462	0.61	0.777	0.00017	AP-Like
Rasgrp2	1.13E-08	0.337903	0.122	0.01	0.000189	AP-Like
Serpinh1	1.14E-08	0.664504	0.537	0.178	0.000189	AP-Like
Cacng4	1.2E-08	0.457709	0.732	0.274	0.0002	AP-Like
Slc25a33	1.2E-08	0.536961	0.366	0.088	0.0002	AP-Like
Tns1	1.22E-08	0.310267	0.122	0.01	0.000203	AP-Like
Vamp8	1.29E-08	0.394918	0.195	0.027	0.000214	AP-Like
Wfs1	1.33E-08	0.367379	0.122	0.01	0.000222	AP-Like
Gaa	1.34E-08	0.452557	0.293	0.059	0.000223	AP-Like
Draxin	1.36E-08	-1.42269	0.171	0.622	0.000228	AP-Like
Cbx1	1.38E-08	-1.03541	0.415	0.741	0.000231	AP-Like
Rpl39	1.4E-08	-1.03054	0.488	0.769	0.000234	AP-Like
Sfpq	1.43E-08	-1.00629	0.439	0.762	0.000238	AP-Like
Nbl1	1.49E-08	0.444804	0.317	0.067	0.000248	AP-Like

Bzw2	1.5E-08	-1.26526	0.146	0.601	0.000251	AP-Like
Neat1	1.59E-08	0.470517	0.22	0.035	0.000265	AP-Like
Slc20a2	1.61E-08	0.59247	0.317	0.07	0.000269	AP-Like
Ptpra	1.76E-08	0.72371	0.756	0.353	0.000293	AP-Like
Rpl34	1.76E-08	-0.91156	0.488	0.799	0.000294	AP-Like
0610040J0	1.8E-08	0.334685	0.171	0.021	0.0003	AP-Like
Fkbp3	1.82E-08	-0.94792	0.61	0.84	0.000303	AP-Like
Aig1	1.84E-08	0.558138	0.39	0.101	0.000307	AP-Like
Hnrnpm	2.17E-08	-0.91028	0.585	0.816	0.000362	AP-Like
Grina	2.37E-08	0.48286	0.439	0.121	0.000395	AP-Like
Tmem198b	2.43E-08	0.581793	0.317	0.07	0.000405	AP-Like
Pfkm	2.62E-08	0.656074	0.39	0.104	0.000438	AP-Like
Nrxn3	2.68E-08	0.359727	0.244	0.042	0.000448	AP-Like
Pttg1ip	2.77E-08	0.661588	0.463	0.136	0.000462	AP-Like
Cd44	2.89E-08	0.447774	0.317	0.069	0.000482	AP-Like
Atpif1	3.06E-08	-0.84166	0.683	0.837	0.00051	AP-Like
Chst2	3.2E-08	0.542304	0.341	0.08	0.000534	AP-Like
Pld2	3.35E-08	0.347902	0.171	0.022	0.000558	AP-Like
Col5a3	3.45E-08	0.440112	0.146	0.016	0.000576	AP-Like
Slc9a3r2	3.76E-08	0.274335	0.146	0.016	0.000628	AP-Like
Gng11	4.04E-08	0.504352	0.171	0.022	0.000675	AP-Like
Thy1	4.15E-08	0.44818	0.171	0.022	0.000692	AP-Like
Atp6v0b	4.19E-08	0.612266	0.732	0.321	0.000699	AP-Like
Wnt7b	4.35E-08	0.313066	0.146	0.016	0.000725	AP-Like
Tubb2a	4.42E-08	0.71728	0.634	0.253	0.000738	AP-Like
Kank2	4.51E-08	0.4266	0.122	0.011	0.000753	AP-Like
Cox7a1	4.67E-08	0.319862	0.122	0.011	0.000778	AP-Like
Degs1	4.69E-08	0.540069	0.585	0.201	0.000783	AP-Like
Rfx4	4.7E-08	0.369042	0.171	0.022	0.000784	AP-Like
Gnb4	5E-08	0.657343	0.61	0.237	0.000834	AP-Like
Necap2	5.5E-08	0.63349	0.293	0.065	0.000917	AP-Like
Crot	5.5E-08	0.582758	0.463	0.143	0.000918	AP-Like
Maff	5.6E-08	0.281775	0.122	0.011	0.000934	AP-Like
Cdc42ep4	5.94E-08	0.494919	0.244	0.046	0.000991	AP-Like
Emc7	6.21E-08	0.611822	0.634	0.249	0.001035	AP-Like
Phyh	6.41E-08	0.611476	0.341	0.088	0.001068	AP-Like
Srsf3	6.86E-08	-0.95605	0.366	0.741	0.001145	AP-Like
Fut9	6.91E-08	0.834414	0.585	0.244	0.001152	AP-Like
Eva1a	7.18E-08	0.520427	0.146	0.017	0.001198	AP-Like
Cml1	7.61E-08	0.657332	0.244	0.047	0.00127	AP-Like
Gucy1a3	7.9E-08	0.487119	0.293	0.064	0.001318	AP-Like
Ech1	7.9E-08	0.770376	0.512	0.175	0.001318	AP-Like
Cgrrf1	8.55E-08	0.518678	0.341	0.085	0.001426	AP-Like
Cdk4	8.82E-08	-0.92557	0.439	0.748	0.001472	AP-Like
Rps10	9E-08	-0.87226	0.415	0.76	0.001501	AP-Like
Anxa5	9.07E-08	0.504638	0.195	0.031	0.001513	AP-Like
Cntnap2	9.09E-08	0.448253	0.171	0.023	0.001516	AP-Like
Anp32b	9.5E-08	-1.09135	0.415	0.712	0.001585	AP-Like

Tmie	9.59E-08	0.427789	0.146	0.017	0.001599	AP-Like
Zcchc24	9.95E-08	0.449983	0.439	0.126	0.00166	AP-Like
Basp1	1.01E-07	-1.10108	0.244	0.667	0.001677	AP-Like
Grid2	1.02E-07	0.44376	0.244	0.047	0.001694	AP-Like
Lamb2	1.06E-07	0.47499	0.195	0.031	0.001767	AP-Like
Nrxn1	1.11E-07	0.718338	0.707	0.333	0.001845	AP-Like
Acin1	1.12E-07	-0.8834	0.561	0.806	0.001873	AP-Like
Plk2	1.14E-07	0.77186	0.244	0.048	0.001907	AP-Like
Hdhd2	1.32E-07	0.514732	0.561	0.199	0.002199	AP-Like
Nrcam	1.44E-07	0.732227	0.463	0.16	0.002403	AP-Like
Rpl23	1.44E-07	-1.01269	0.39	0.678	0.002405	AP-Like
Fam213a	1.44E-07	0.593921	0.488	0.163	0.00241	AP-Like
Rorb	1.47E-07	0.493245	0.195	0.032	0.002457	AP-Like
Hnrnph1	1.53E-07	-0.99713	0.439	0.698	0.002548	AP-Like
Pfkfb3	1.62E-07	0.411142	0.171	0.025	0.002698	AP-Like
Spry2	1.64E-07	0.84908	0.415	0.132	0.002739	AP-Like
C230037L1	1.72E-07	0.347554	0.122	0.012	0.002871	AP-Like
Pitpnc1	1.75E-07	0.547354	0.341	0.088	0.002915	AP-Like
Cycs	1.75E-07	0.373707	0.171	0.025	0.002926	AP-Like
Dlgap1	1.76E-07	0.762922	0.463	0.153	0.002933	AP-Like
Oat	1.86E-07	0.702737	0.39	0.116	0.003096	AP-Like
Omg	1.86E-07	0.549321	0.366	0.101	0.003106	AP-Like
Chst7	1.89E-07	0.305559	0.122	0.012	0.00315	AP-Like
Slc13a5	2.09E-07	0.41096	0.171	0.025	0.00348	AP-Like
Tuba1a	2.1E-07	-0.78288	0.854	0.927	0.003498	AP-Like
H2afv	2.13E-07	-0.95515	0.585	0.793	0.003551	AP-Like
AI836003	2.27E-07	0.323286	0.122	0.012	0.003787	AP-Like
Wwc1	2.3E-07	0.650575	0.439	0.142	0.003844	AP-Like
Fuca1	2.32E-07	0.749136	0.537	0.215	0.003878	AP-Like
Hmgn1	2.37E-07	-0.95519	0.366	0.705	0.003953	AP-Like
Eif2ak2	2.38E-07	0.362506	0.146	0.019	0.003978	AP-Like
Kazn	2.53E-07	0.505308	0.244	0.049	0.004219	AP-Like
S100a1	2.66E-07	0.561496	0.488	0.158	0.004432	AP-Like
Myl9	2.75E-07	0.317229	0.146	0.019	0.004586	AP-Like
Anp32a	2.77E-07	-0.79582	0.683	0.846	0.004613	AP-Like
Hdgf	2.96E-07	-1.05927	0.317	0.647	0.004945	AP-Like
Fabp5	2.98E-07	0.828138	0.78	0.428	0.004971	AP-Like
Arl8a	2.98E-07	0.591032	0.415	0.128	0.004979	AP-Like
Rdh14	3.06E-07	0.382427	0.268	0.058	0.0051	AP-Like
Tmbim6	3.07E-07	0.670877	0.805	0.493	0.005113	AP-Like
Igfbpl1	3.19E-07	-1.55001	0.122	0.511	0.005313	AP-Like
1500009L1	3.19E-07	0.377596	0.268	0.057	0.005321	AP-Like
Pex11a	3.34E-07	0.390754	0.171	0.026	0.005575	AP-Like
Gcdh	3.38E-07	0.394225	0.244	0.049	0.005631	AP-Like
Bclaf1	3.44E-07	-0.96474	0.317	0.677	0.005731	AP-Like
Aldh2	3.46E-07	0.462491	0.293	0.069	0.005777	AP-Like
Srsf11	3.65E-07	-0.83793	0.439	0.767	0.006083	AP-Like
Fth1	3.71E-07	0.558679	0.951	0.846	0.006191	AP-Like

Grin3a	3.77E-07	0.833219	0.39	0.121	0.00629	AP-Like
Galnt10	3.78E-07	0.420384	0.171	0.026	0.0063	AP-Like
Igfbp3	3.8E-07	0.889254	0.268	0.062	0.006343	AP-Like
Tnfrsf1a	3.86E-07	0.380655	0.244	0.049	0.006443	AP-Like
Pdlim5	4.45E-07	0.459561	0.268	0.06	0.007421	AP-Like
Ptplad1	4.83E-07	0.61792	0.683	0.317	0.008062	AP-Like
Ddx5	4.85E-07	-0.74397	0.585	0.848	0.008087	AP-Like
Lpp	5.03E-07	0.331684	0.195	0.033	0.008384	AP-Like
Hnrnpc	5.17E-07	-0.82534	0.561	0.769	0.008615	AP-Like
Tnk2	5.19E-07	0.528444	0.268	0.06	0.00865	AP-Like
Decr1	5.23E-07	0.578726	0.39	0.119	0.008726	AP-Like
Ubtd1	5.25E-07	0.361742	0.122	0.014	0.008749	AP-Like
Entpd6	5.25E-07	0.305826	0.22	0.041	0.008754	AP-Like
Rnf215	5.26E-07	0.598569	0.317	0.084	0.008777	AP-Like
Klhl32	5.32E-07	0.406087	0.171	0.026	0.008867	AP-Like
Calm2	5.6E-07	-0.77468	0.756	0.86	0.009343	AP-Like
Bmpr1a	5.62E-07	0.495558	0.488	0.167	0.00938	AP-Like
Pde4b	5.65E-07	0.636306	0.488	0.173	0.009422	AP-Like
Palld	5.7E-07	0.37997	0.293	0.07	0.009509	AP-Like
Mid1ip1	5.73E-07	0.590861	0.366	0.106	0.009552	AP-Like
0610031J0	5.74E-07	0.55474	0.317	0.085	0.00958	AP-Like
Ier5	5.77E-07	-1.30248	0.049	0.459	0.009616	AP-Like
Fjx1	5.82E-07	0.54551	0.366	0.107	0.009705	AP-Like
Bmp4	5.8E-172	2.768665	0.942	0.001	9.6E-168	Oligodendrocytic
Enpp6	2.8E-153	2.303395	0.846	0.001	4.6E-149	Oligodendrocytic
Neu4	1.7E-101	1.463828	0.673	0.009	2.9E-97	Oligodendrocytic
Lims2	1.04E-96	1.793156	0.731	0.018	1.73E-92	Oligodendrocytic
Epcam	4.53E-95	1.375768	0.538	0.001	7.56E-91	Oligodendrocytic
Tmem88b	1.91E-94	1.394594	0.615	0.008	3.18E-90	Oligodendrocytic
Chn2	1.93E-92	2.406228	0.654	0.013	3.22E-88	Oligodendrocytic
Slc1a1	7.91E-89	1.502095	0.846	0.038	1.32E-84	Oligodendrocytic
Lgi3	6.71E-87	1.405298	0.615	0.011	1.12E-82	Oligodendrocytic
Gpr17	3.09E-77	2.327066	0.904	0.069	5.16E-73	Oligodendrocytic
Gp1bb	7.67E-74	0.989282	0.442	0.003	1.28E-69	Oligodendrocytic
Ptpre	4.51E-72	1.708743	0.904	0.07	7.52E-68	Oligodendrocytic
Fa2h	1.57E-71	1.2402	0.558	0.014	2.61E-67	Oligodendrocytic
Zfp365	1.66E-70	1.443237	0.673	0.03	2.77E-66	Oligodendrocytic
Ugt8a	9.53E-68	1.685289	0.827	0.061	1.59E-63	Oligodendrocytic
Ppp1r16b	6.06E-67	1.061089	0.481	0.009	1.01E-62	Oligodendrocytic
Cbln2	1.37E-66	1.017792	0.385	0.001	2.29E-62	Oligodendrocytic
Cnp	2.21E-65	3.025833	0.962	0.133	3.68E-61	Oligodendrocytic
S100a13	1.08E-64	2.214263	0.981	0.123	1.8E-60	Oligodendrocytic
Pllp	2.99E-64	1.696213	0.865	0.074	4.99E-60	Oligodendrocytic
Myrf	2.57E-63	0.763609	0.346	0	4.28E-59	Oligodendrocytic
Cldn11	8.23E-63	2.164271	0.712	0.048	1.37E-58	Oligodendrocytic
Tubb4a	1.39E-62	1.582081	0.673	0.04	2.31E-58	Oligodendrocytic
Slc22a23	4.75E-61	1.338207	0.596	0.028	7.92E-57	Oligodendrocytic
Dbndd2	6.88E-61	1.429569	0.712	0.049	1.15E-56	Oligodendrocytic

Itpr2	3.91E-60	1.865284	0.904	0.098	6.53E-56	Oligodendrocytic
Nkx2-2	7.41E-60	1.319475	0.692	0.044	1.24E-55	Oligodendrocytic
Cyfip2	1.4E-59	1.465504	0.712	0.05	2.33E-55	Oligodendrocytic
Enpp2	1.59E-59	1.90853	0.885	0.098	2.65E-55	Oligodendrocytic
Tnr	1.89E-58	1.004182	0.481	0.014	3.15E-54	Oligodendrocytic
Mag	4.12E-58	1.778038	0.385	0.005	6.87E-54	Oligodendrocytic
2810468N(	5.84E-58	1.451651	0.692	0.048	9.74E-54	Oligodendrocytic
S100a1	9.41E-58	1.953724	0.942	0.124	1.57E-53	Oligodendrocytic
Bfsp2	9.96E-58	0.994322	0.385	0.005	1.66E-53	Oligodendrocytic
Nfasc	1.4E-57	1.946067	0.75	0.061	2.34E-53	Oligodendrocytic
9530059O:	2.36E-56	0.721974	0.308	0	3.93E-52	Oligodendrocytic
Tspan2	4.05E-55	1.249117	0.577	0.03	6.75E-51	Oligodendrocytic
Tns3	1.04E-53	1.246352	0.558	0.03	1.73E-49	Oligodendrocytic
1810041L1	2.52E-53	1.019794	0.5	0.02	4.2E-49	Oligodendrocytic
Tspan17	3.14E-53	0.781473	0.423	0.011	5.24E-49	Oligodendrocytic
Elovl7	2.28E-52	1.39119	0.519	0.025	3.81E-48	Oligodendrocytic
Epb4.1l2	7.04E-52	2.017193	0.904	0.149	1.17E-47	Oligodendrocytic
Rab33a	2.2E-51	1.50431	0.635	0.048	3.67E-47	Oligodendrocytic
Sirt2	2.47E-51	3.186744	1	0.24	4.13E-47	Oligodendrocytic
3110035E1	3.51E-51	2.03339	0.885	0.123	5.86E-47	Oligodendrocytic
Sox8	1.81E-49	1.388288	0.788	0.086	3.03E-45	Oligodendrocytic
Plp1	1.62E-48	4.42866	0.885	0.166	2.7E-44	Oligodendrocytic
Plxnb3	1.65E-48	0.921998	0.346	0.006	2.75E-44	Oligodendrocytic
Phyhipl	2.25E-48	2.01869	0.885	0.146	3.75E-44	Oligodendrocytic
Mfsd2a	8.22E-48	1.159709	0.635	0.049	1.37E-43	Oligodendrocytic
Slc44a1	1.11E-47	1.803515	0.827	0.116	1.85E-43	Oligodendrocytic
Tmem108	6.01E-47	1.081875	0.442	0.019	1E-42	Oligodendrocytic
Dusp15	1.11E-46	1.125661	0.442	0.019	1.85E-42	Oligodendrocytic
Fyn	1.97E-46	2.972438	1	0.294	3.29E-42	Oligodendrocytic
Sgk1	2.26E-46	1.381977	0.577	0.043	3.77E-42	Oligodendrocytic
Gng4	3.27E-46	0.811798	0.288	0.003	5.45E-42	Oligodendrocytic
Srpk3	5.78E-46	0.544766	0.25	0	9.65E-42	Oligodendrocytic
Fbxo7	2.26E-45	1.009725	0.577	0.041	3.77E-41	Oligodendrocytic
5730559C:	6.14E-45	0.829031	0.385	0.013	1.02E-40	Oligodendrocytic
Ehd3	1.74E-44	1.179613	0.577	0.044	2.9E-40	Oligodendrocytic
Cpm	3.23E-44	0.801535	0.308	0.005	5.38E-40	Oligodendrocytic
Sox10	1.33E-43	1.233971	0.75	0.084	2.22E-39	Oligodendrocytic
Dock9	1.66E-43	1.02206	0.519	0.034	2.76E-39	Oligodendrocytic
Mbp	5.99E-43	3.371459	0.904	0.178	9.99E-39	Oligodendrocytic
Mpzl1	7.27E-43	2.017837	0.904	0.194	1.21E-38	Oligodendrocytic
S100b	1.93E-42	1.724728	0.885	0.156	3.22E-38	Oligodendrocytic
Gjc3	3.92E-42	1.677749	0.75	0.099	6.54E-38	Oligodendrocytic
Dusp26	4.57E-42	1.267417	0.596	0.054	7.62E-38	Oligodendrocytic
Cdc37l1	8.94E-42	1.393563	0.808	0.118	1.49E-37	Oligodendrocytic
Cyp2j6	3.83E-41	1.310048	0.635	0.065	6.38E-37	Oligodendrocytic
Kazn	7.13E-41	1.185101	0.481	0.031	1.19E-36	Oligodendrocytic
Bcas1	8.16E-41	2.719461	1	0.384	1.36E-36	Oligodendrocytic
Pxdc1	1.29E-40	0.958596	0.481	0.031	2.16E-36	Oligodendrocytic

Pak4	1.43E-40	1.231353	0.519	0.04	2.39E-36	Oligodendrocytic
Gal3st1	2.38E-40	0.890972	0.385	0.016	3.97E-36	Oligodendrocytic
Arsb	2.88E-40	1.372204	0.615	0.063	4.81E-36	Oligodendrocytic
Ppfibp1	4.09E-40	1.321575	0.788	0.108	6.82E-36	Oligodendrocytic
Dnm3	6.92E-39	1.182079	0.673	0.075	1.15E-34	Oligodendrocytic
Kctd4	1.17E-38	0.909253	0.442	0.026	1.95E-34	Oligodendrocytic
Gltp	3.98E-38	1.407552	0.769	0.121	6.65E-34	Oligodendrocytic
Pfn2	5.17E-38	1.835339	0.942	0.279	8.62E-34	Oligodendrocytic
Ust	8.22E-38	1.079854	0.635	0.071	1.37E-33	Oligodendrocytic
Tm7sf3	3.31E-36	1.114053	0.769	0.12	5.52E-32	Oligodendrocytic
Klhl5	6.19E-36	1.291353	0.731	0.111	1.03E-31	Oligodendrocytic
3632451O(	8.93E-36	1.10946	0.615	0.069	1.49E-31	Oligodendrocytic
Kif26a	1.53E-35	0.611842	0.212	0.001	2.56E-31	Oligodendrocytic
Sv2a	2E-35	1.08807	0.596	0.066	3.33E-31	Oligodendrocytic
Opcml	2.13E-35	1.047438	0.519	0.046	3.55E-31	Oligodendrocytic
Sh3bp4	3.8E-35	0.817303	0.519	0.046	6.35E-31	Oligodendrocytic
Fam163a	4.73E-35	0.495582	0.269	0.006	7.89E-31	Oligodendrocytic
Ptpro	3E-34	0.736028	0.385	0.023	5E-30	Oligodendrocytic
Sox6	3.86E-34	1.07853	0.538	0.054	6.43E-30	Oligodendrocytic
Ncam2	1.23E-33	1.163876	0.615	0.078	2.05E-29	Oligodendrocytic
Prkcq	1.5E-33	1.154117	0.635	0.088	2.51E-29	Oligodendrocytic
Spsb1	4.11E-33	0.661231	0.308	0.013	6.86E-29	Oligodendrocytic
2900011O(	4.24E-33	1.197073	0.692	0.1	7.07E-29	Oligodendrocytic
Snx30	8.08E-33	0.918031	0.5	0.048	1.35E-28	Oligodendrocytic
Scrg1	1.13E-32	1.360612	0.846	0.168	1.89E-28	Oligodendrocytic
Kank1	1.34E-32	1.004838	0.654	0.09	2.23E-28	Oligodendrocytic
Rasgef1b	2.23E-32	0.789888	0.25	0.006	3.72E-28	Oligodendrocytic
Plekhb1	1.06E-31	1.098849	0.577	0.069	1.77E-27	Oligodendrocytic
Ap2a1	1.16E-31	0.809119	0.385	0.026	1.94E-27	Oligodendrocytic
Sulf2	2.63E-31	0.923086	0.692	0.098	4.38E-27	Oligodendrocytic
Pim3	2.68E-31	1.091067	0.577	0.076	4.48E-27	Oligodendrocytic
Brinp1	3.42E-31	0.961017	0.481	0.046	5.71E-27	Oligodendrocytic
Thra	3.72E-31	1.437171	0.904	0.244	6.21E-27	Oligodendrocytic
Mapt	4.03E-31	1.439596	0.846	0.209	6.72E-27	Oligodendrocytic
Susd4	4.25E-31	0.918486	0.462	0.041	7.1E-27	Oligodendrocytic
Pcdh7	9.64E-31	0.921765	0.442	0.039	1.61E-26	Oligodendrocytic
Rab3a	9.66E-31	0.846404	0.538	0.06	1.61E-26	Oligodendrocytic
Trio	1.76E-30	1.426688	0.788	0.175	2.94E-26	Oligodendrocytic
Map1a	2.53E-30	1.037943	0.519	0.06	4.22E-26	Oligodendrocytic
Mog	2.95E-30	0.696212	0.212	0.004	4.93E-26	Oligodendrocytic
S100a16	5.83E-30	1.502934	0.788	0.174	9.72E-26	Oligodendrocytic
AC163993.	5.94E-30	0.440683	0.212	0.004	9.9E-26	Oligodendrocytic
Iffo1	6.56E-30	0.90919	0.481	0.05	1.09E-25	Oligodendrocytic
Fam3c	7.54E-30	1.229705	0.712	0.131	1.26E-25	Oligodendrocytic
S100a6	8.74E-30	1.282832	0.558	0.073	1.46E-25	Oligodendrocytic
Afap1l2	9.1E-30	0.818567	0.481	0.048	1.52E-25	Oligodendrocytic
Sh3rf1	2.49E-29	0.592982	0.327	0.019	4.15E-25	Oligodendrocytic
Chd3	2.62E-29	1.248934	0.692	0.118	4.37E-25	Oligodendrocytic

Cd9	3.13E-29	1.910416	0.962	0.652	5.22E-25	Oligodendrocytic
Rnf128	4.27E-29	0.650552	0.231	0.006	7.12E-25	Oligodendrocytic
Cdk14	8.57E-29	1.115091	0.692	0.128	1.43E-24	Oligodendrocytic
Tmbim1	1.3E-28	0.735372	0.327	0.02	2.17E-24	Oligodendrocytic
Susd5	1.5E-28	0.634874	0.25	0.009	2.51E-24	Oligodendrocytic
Gpr37l1	1.81E-28	0.988425	0.923	0.19	3.03E-24	Oligodendrocytic
Mgll	2.15E-28	1.27117	0.692	0.13	3.59E-24	Oligodendrocytic
Mpv17l	2.3E-28	0.493669	0.288	0.014	3.84E-24	Oligodendrocytic
Gnb4	2.89E-28	1.348867	0.846	0.217	4.81E-24	Oligodendrocytic
Ctnnal1	2.96E-28	0.942546	0.538	0.07	4.93E-24	Oligodendrocytic
Glrb	5.61E-28	1.066701	0.481	0.058	9.35E-24	Oligodendrocytic
Rtkn	6.57E-28	0.623516	0.462	0.048	1.1E-23	Oligodendrocytic
Tmem132b	3.18E-27	0.610535	0.288	0.015	5.31E-23	Oligodendrocytic
8430427H1	3.51E-27	0.561473	0.231	0.008	5.86E-23	Oligodendrocytic
A930009A1	3.74E-27	0.918924	0.462	0.051	6.23E-23	Oligodendrocytic
Sema5a	3.74E-27	0.781908	0.346	0.025	6.23E-23	Oligodendrocytic
Lsamp	4.17E-27	1.181227	0.865	0.208	6.95E-23	Oligodendrocytic
Tsc22d4	5.64E-27	1.495422	0.923	0.332	9.41E-23	Oligodendrocytic
Atcay	6.5E-27	0.872048	0.5	0.061	1.08E-22	Oligodendrocytic
Srcin1	6.98E-27	0.558784	0.269	0.013	1.16E-22	Oligodendrocytic
Col9a1	8.46E-27	0.534431	0.308	0.019	1.41E-22	Oligodendrocytic
Sema3d	1.48E-26	0.625084	0.308	0.019	2.47E-22	Oligodendrocytic
Usp20	1.57E-26	0.65202	0.423	0.041	2.63E-22	Oligodendrocytic
Deb1	2.81E-26	1.215353	0.865	0.245	4.68E-22	Oligodendrocytic
Gpr146	3.25E-26	0.659939	0.269	0.014	5.42E-22	Oligodendrocytic
Aatk	3.54E-26	0.688532	0.404	0.039	5.9E-22	Oligodendrocytic
Fcor	5.29E-26	0.564871	0.212	0.006	8.82E-22	Oligodendrocytic
Plekhf1	5.87E-26	0.529826	0.212	0.006	9.8E-22	Oligodendrocytic
Fnta	6.17E-26	1.089798	0.769	0.178	1.03E-21	Oligodendrocytic
Arhgap24	7.06E-26	0.725698	0.25	0.011	1.18E-21	Oligodendrocytic
Fkbp15	8.01E-26	1.171167	0.385	0.036	1.34E-21	Oligodendrocytic
Ldlrad3	1.03E-25	0.619028	0.288	0.018	1.73E-21	Oligodendrocytic
Tuba1a	2.88E-25	1.140422	1	0.919	4.8E-21	Oligodendrocytic
Elf1	3.34E-25	0.890783	0.538	0.08	5.57E-21	Oligodendrocytic
Lcorl	5.01E-25	1.174414	0.692	0.148	8.36E-21	Oligodendrocytic
Dynll1	5.15E-25	1.134689	1	0.696	8.59E-21	Oligodendrocytic
Zdhhc12	5.23E-25	0.692631	0.346	0.029	8.72E-21	Oligodendrocytic
Shisa4	6.9E-25	1.106799	0.519	0.08	1.15E-20	Oligodendrocytic
Arl4a	1.13E-24	1.162389	0.731	0.168	1.89E-20	Oligodendrocytic
Camsap2	1.36E-24	0.963087	0.712	0.146	2.26E-20	Oligodendrocytic
Prickle1	1.75E-24	0.513668	0.25	0.013	2.92E-20	Oligodendrocytic
Sept7	2.28E-24	1.315497	0.981	0.751	3.8E-20	Oligodendrocytic
Agpat4	3.4E-24	0.996483	0.654	0.129	5.66E-20	Oligodendrocytic
Dynll2	3.5E-24	1.61197	0.846	0.348	5.84E-20	Oligodendrocytic
Vcan	3.58E-24	1.227058	0.788	0.219	5.97E-20	Oligodendrocytic
Sh3gl3	3.95E-24	0.678736	0.423	0.046	6.59E-20	Oligodendrocytic
Gm4876	4.08E-24	0.497293	0.231	0.01	6.8E-20	Oligodendrocytic
Rap2a	4.74E-24	1.403688	0.827	0.267	7.91E-20	Oligodendrocytic

Sh3d19	5.14E-24	1.028667	0.558	0.089	8.58E-20	Oligodendrocytic
Rnd2	6.03E-24	1.339979	0.712	0.163	1.01E-19	Oligodendrocytic
Zdhhc14	2.03E-23	0.81306	0.481	0.068	3.39E-19	Oligodendrocytic
Serinc5	2.52E-23	0.894642	0.538	0.089	4.21E-19	Oligodendrocytic
Stxbp3a	2.6E-23	0.787031	0.404	0.046	4.33E-19	Oligodendrocytic
Amz1	2.98E-23	0.681079	0.346	0.031	4.96E-19	Oligodendrocytic
Gatm	3.56E-23	1.016733	0.673	0.136	5.95E-19	Oligodendrocytic
Nfkbib	3.88E-23	0.899919	0.519	0.081	6.47E-19	Oligodendrocytic
2510003E0	9.59E-23	1.375908	0.615	0.13	1.6E-18	Oligodendrocytic
Tmcc2	1.74E-22	0.635578	0.288	0.021	2.9E-18	Oligodendrocytic
Lrrc42	2E-22	1.070501	0.558	0.1	3.34E-18	Oligodendrocytic
Ago3	3.52E-22	0.883317	0.577	0.106	5.87E-18	Oligodendrocytic
Cadm2	3.92E-22	0.976001	0.577	0.103	6.54E-18	Oligodendrocytic
Arhgef28	4.57E-22	0.491717	0.25	0.015	7.63E-18	Oligodendrocytic
Scamp2	5.17E-22	1.259005	0.769	0.24	8.62E-18	Oligodendrocytic
Zfp488	1.39E-21	0.26907	0.173	0.005	2.32E-17	Oligodendrocytic
Pcyt2	2.06E-21	0.851139	0.423	0.056	3.43E-17	Oligodendrocytic
Arhgap23	2.23E-21	0.548376	0.212	0.01	3.72E-17	Oligodendrocytic
Rgcc	3.71E-21	0.900766	0.538	0.093	6.18E-17	Oligodendrocytic
Degs1	5.81E-21	1.07185	0.731	0.186	9.69E-17	Oligodendrocytic
Map1b	6.82E-21	1.23072	0.923	0.431	1.14E-16	Oligodendrocytic
Map6d1	7.27E-21	0.496839	0.269	0.02	1.21E-16	Oligodendrocytic
Matn2	1.1E-20	0.630569	0.308	0.029	1.84E-16	Oligodendrocytic
Osbpl1a	1.3E-20	1.062842	0.538	0.101	2.17E-16	Oligodendrocytic
Ick	1.34E-20	0.951267	0.75	0.193	2.24E-16	Oligodendrocytic
Acox1	1.45E-20	0.772254	0.596	0.12	2.41E-16	Oligodendrocytic
Fam102a	1.52E-20	0.416355	0.269	0.02	2.54E-16	Oligodendrocytic
Dynlt3	1.75E-20	0.795663	0.538	0.096	2.92E-16	Oligodendrocytic
5430435G1	2.17E-20	0.298466	0.154	0.004	3.62E-16	Oligodendrocytic
Wasf1	3.31E-20	0.902245	0.654	0.144	5.52E-16	Oligodendrocytic
Aldh3b1	4.81E-20	0.380772	0.173	0.006	8.02E-16	Oligodendrocytic
Creb3l2	5.37E-20	0.429838	0.25	0.018	8.96E-16	Oligodendrocytic
Pcsk1n	9.95E-20	0.845791	0.692	0.156	1.66E-15	Oligodendrocytic
1110007C0	1.11E-19	0.893579	0.365	0.046	1.86E-15	Oligodendrocytic
Sept5	2.18E-19	0.367489	0.135	0.003	3.63E-15	Oligodendrocytic
Lhfpl2	2.4E-19	0.640365	0.346	0.04	3.99E-15	Oligodendrocytic
Asrgl1	2.48E-19	0.955667	0.846	0.289	4.14E-15	Oligodendrocytic
Cadm4	3.88E-19	0.808631	0.692	0.171	6.48E-15	Oligodendrocytic
Dscam	7.85E-19	0.547176	0.346	0.04	1.31E-14	Oligodendrocytic
Fam13c	8E-19	1.095703	0.462	0.08	1.33E-14	Oligodendrocytic
Evi2a	8.58E-19	0.631757	0.192	0.01	1.43E-14	Oligodendrocytic
Mtap	1E-18	0.93887	0.654	0.169	1.67E-14	Oligodendrocytic
Tspan15	1.48E-18	0.622153	0.288	0.029	2.47E-14	Oligodendrocytic
Calr	2.57E-18	1.015531	0.962	0.636	4.28E-14	Oligodendrocytic
Olig2	3.48E-18	0.962407	0.846	0.282	5.8E-14	Oligodendrocytic
Cdv3	3.79E-18	1.100114	0.712	0.218	6.33E-14	Oligodendrocytic
Mif4gd	4.49E-18	0.708781	0.404	0.061	7.5E-14	Oligodendrocytic
Rictor	5.77E-18	0.550773	0.346	0.044	9.63E-14	Oligodendrocytic

Vimp	6.51E-18	1.119503	0.788	0.287	1.09E-13	Oligodendrocytic
Zeb2	7.88E-18	1.199795	0.827	0.367	1.31E-13	Oligodendrocytic
Ccdc134	1.22E-17	0.639898	0.327	0.04	2.04E-13	Oligodendrocytic
Npc1	1.27E-17	0.704596	0.442	0.076	2.13E-13	Oligodendrocytic
Chadl	1.4E-17	0.391271	0.25	0.021	2.34E-13	Oligodendrocytic
Aplp1	1.63E-17	1.040856	0.519	0.111	2.72E-13	Oligodendrocytic
Nfib	1.9E-17	-1.58554	0.538	0.884	3.16E-13	Oligodendrocytic
Parvb	5.64E-17	0.656111	0.192	0.013	9.41E-13	Oligodendrocytic
Zic1	6.29E-17	-1.80697	0.173	0.74	1.05E-12	Oligodendrocytic
Ppp2r3a	7.37E-17	0.957646	0.538	0.126	1.23E-12	Oligodendrocytic
Taldo1	7.63E-17	1.126717	0.846	0.395	1.27E-12	Oligodendrocytic
Olig1	1.07E-16	0.673667	0.904	0.315	1.79E-12	Oligodendrocytic
Lmf1	1.73E-16	0.546881	0.327	0.043	2.89E-12	Oligodendrocytic
Scarb2	1.74E-16	0.601992	0.327	0.044	2.9E-12	Oligodendrocytic
Timp4	1.74E-16	0.739597	0.558	0.119	2.9E-12	Oligodendrocytic
Jakmip1	1.76E-16	0.415299	0.192	0.013	2.94E-12	Oligodendrocytic
Tgfa	1.88E-16	0.492478	0.25	0.024	3.13E-12	Oligodendrocytic
Tmcc3	2.08E-16	0.864427	0.558	0.136	3.47E-12	Oligodendrocytic
Vldlr	2.19E-16	0.731789	0.481	0.098	3.65E-12	Oligodendrocytic
Sema5b	2.31E-16	0.54201	0.288	0.033	3.85E-12	Oligodendrocytic
Rap1gap	2.32E-16	0.378527	0.308	0.036	3.88E-12	Oligodendrocytic
Kcna1	2.63E-16	0.47337	0.231	0.02	4.38E-12	Oligodendrocytic
Slc4a8	2.64E-16	0.495822	0.269	0.029	4.4E-12	Oligodendrocytic
Col11a2	3.56E-16	0.34745	0.115	0.003	5.93E-12	Oligodendrocytic
Arhgap20	4.67E-16	0.613634	0.327	0.044	7.79E-12	Oligodendrocytic
Arhgap5	4.75E-16	1.066816	0.692	0.245	7.91E-12	Oligodendrocytic
Ptprt	5.16E-16	0.588145	0.288	0.034	8.61E-12	Oligodendrocytic
Rin2	8.24E-16	0.528849	0.346	0.05	1.37E-11	Oligodendrocytic
Abtb2	9.76E-16	0.499405	0.269	0.03	1.63E-11	Oligodendrocytic
Gm15446	1.03E-15	0.510673	0.212	0.018	1.72E-11	Oligodendrocytic
Pld1	1.12E-15	0.500965	0.212	0.018	1.86E-11	Oligodendrocytic
Phactr3	1.33E-15	0.61792	0.442	0.083	2.22E-11	Oligodendrocytic
Map4k5	1.49E-15	0.791158	0.442	0.088	2.48E-11	Oligodendrocytic
Cdh10	2.59E-15	0.620466	0.288	0.038	4.33E-11	Oligodendrocytic
Sema4a	4.48E-15	0.34847	0.173	0.011	7.47E-11	Oligodendrocytic
Sapcd1	4.82E-15	0.412058	0.173	0.011	8.05E-11	Oligodendrocytic
Dirc2	7.24E-15	0.921452	0.5	0.116	1.21E-10	Oligodendrocytic
Dgat2	7.4E-15	0.516976	0.212	0.019	1.23E-10	Oligodendrocytic
Pdcd4	8.03E-15	1.231245	0.808	0.36	1.34E-10	Oligodendrocytic
Grid1	8.13E-15	0.535546	0.25	0.028	1.36E-10	Oligodendrocytic
Tbc1d24	8.64E-15	0.5615	0.308	0.043	1.44E-10	Oligodendrocytic
Gramd3	9.04E-15	0.399934	0.25	0.028	1.51E-10	Oligodendrocytic
Btd	9.04E-15	0.631261	0.308	0.043	1.51E-10	Oligodendrocytic
Snx22	9.23E-15	0.662018	0.385	0.066	1.54E-10	Oligodendrocytic
Csf1	1.28E-14	0.563011	0.25	0.029	2.14E-10	Oligodendrocytic
Sash1	2.21E-14	0.912683	0.481	0.111	3.69E-10	Oligodendrocytic
Rhbdl2	2.23E-14	0.25875	0.154	0.009	3.72E-10	Oligodendrocytic
Rapgef1	2.24E-14	0.630255	0.423	0.084	3.74E-10	Oligodendrocytic

Pik3r1	2.51E-14	0.697836	0.615	0.171	4.19E-10	Oligodendrocytic
Slc5a7	2.89E-14	0.588756	0.173	0.013	4.82E-10	Oligodendrocytic
H2afv	2.99E-14	-1.40979	0.442	0.805	5E-10	Oligodendrocytic
Slc27a3	3.12E-14	0.765996	0.192	0.016	5.2E-10	Oligodendrocytic
Zfp276	3.24E-14	0.479334	0.231	0.025	5.4E-10	Oligodendrocytic
A230050P2	3.59E-14	0.446313	0.269	0.034	6E-10	Oligodendrocytic
Alad	3.69E-14	0.578213	0.308	0.045	6.16E-10	Oligodendrocytic
Kif21b	4.15E-14	0.494629	0.269	0.034	6.93E-10	Oligodendrocytic
Shroom2	4.31E-14	0.428706	0.212	0.02	7.19E-10	Oligodendrocytic
Lrch3	4.45E-14	0.668436	0.519	0.124	7.42E-10	Oligodendrocytic
Kbtbd3	4.77E-14	0.631365	0.385	0.073	7.95E-10	Oligodendrocytic
Fktn	6.33E-14	0.827966	0.577	0.156	1.06E-09	Oligodendrocytic
Tmco3	8.62E-14	0.66384	0.365	0.066	1.44E-09	Oligodendrocytic
Gtf2h1	9.58E-14	0.647764	0.538	0.143	1.6E-09	Oligodendrocytic
Syt11	1.07E-13	0.732743	0.923	0.576	1.78E-09	Oligodendrocytic
Csgalnact1	1.18E-13	0.644188	0.308	0.048	1.96E-09	Oligodendrocytic
Ube2e2	1.3E-13	0.699877	0.519	0.135	2.17E-09	Oligodendrocytic
Nipa1	1.34E-13	0.592051	0.327	0.054	2.23E-09	Oligodendrocytic
Gfra1	1.48E-13	0.405447	0.308	0.045	2.47E-09	Oligodendrocytic
Gng12	1.7E-13	0.84974	0.731	0.272	2.84E-09	Oligodendrocytic
Ppm1l	1.77E-13	0.753335	0.442	0.098	2.95E-09	Oligodendrocytic
Impa1	1.93E-13	0.565557	0.519	0.126	3.22E-09	Oligodendrocytic
Capn5	1.98E-13	0.44835	0.192	0.018	3.29E-09	Oligodendrocytic
Sfrp1	2.13E-13	-1.66249	0.385	0.777	3.55E-09	Oligodendrocytic
Kcnj10	2.43E-13	0.272513	0.577	0.141	4.05E-09	Oligodendrocytic
Ncald	2.68E-13	0.70679	0.788	0.282	4.47E-09	Oligodendrocytic
Slc35b2	2.76E-13	0.756156	0.712	0.255	4.61E-09	Oligodendrocytic
1700021K1	2.81E-13	0.54356	0.288	0.043	4.68E-09	Oligodendrocytic
Eid1	3.19E-13	0.715314	0.981	0.582	5.32E-09	Oligodendrocytic
Lima1	3.24E-13	0.679814	0.635	0.189	5.41E-09	Oligodendrocytic
Dpp6	4.49E-13	0.574211	0.308	0.049	7.48E-09	Oligodendrocytic
Cdh13	4.79E-13	0.797206	0.5	0.126	7.98E-09	Oligodendrocytic
Phactr1	5.36E-13	0.735709	0.615	0.186	8.93E-09	Oligodendrocytic
Tmem9b	5.98E-13	0.707516	0.615	0.188	9.97E-09	Oligodendrocytic
Tcf7l2	6.41E-13	0.729418	0.365	0.074	1.07E-08	Oligodendrocytic
Dlgap1	6.48E-13	0.698141	0.538	0.144	1.08E-08	Oligodendrocytic
Scd2	6.85E-13	0.782013	0.885	0.491	1.14E-08	Oligodendrocytic
Tubb2b	8.01E-13	0.839786	0.865	0.436	1.34E-08	Oligodendrocytic
Ostm1	8.04E-13	0.559373	0.327	0.056	1.34E-08	Oligodendrocytic
Ifnar2	8.05E-13	0.554169	0.365	0.07	1.34E-08	Oligodendrocytic
Eif1b	9.15E-13	0.899107	0.827	0.447	1.53E-08	Oligodendrocytic
Adam9	9.15E-13	0.646699	0.481	0.121	1.53E-08	Oligodendrocytic
Efcab14	9.21E-13	0.704988	0.481	0.123	1.54E-08	Oligodendrocytic
Pcdh11x	1.25E-12	0.40416	0.231	0.028	2.09E-08	Oligodendrocytic
Mtmr10	1.28E-12	0.48098	0.231	0.029	2.14E-08	Oligodendrocytic
Dusp19	1.53E-12	0.36935	0.212	0.024	2.55E-08	Oligodendrocytic
Plcb1	1.81E-12	0.753926	0.5	0.129	3.02E-08	Oligodendrocytic
Anp32a	2.23E-12	-1.05835	0.673	0.849	3.72E-08	Oligodendrocytic

Rtn4	2.25E-12	0.838199	0.904	0.541	3.75E-08	Oligodendrocytic
Tmem63b	2.25E-12	0.563368	0.442	0.103	3.75E-08	Oligodendrocytic
Mycl	2.32E-12	0.608062	0.538	0.148	3.87E-08	Oligodendrocytic
E130114P1	2.56E-12	-1.56686	0.154	0.646	4.27E-08	Oligodendrocytic
Rab4a	2.59E-12	0.552129	0.192	0.02	4.32E-08	Oligodendrocytic
Rap1gds1	2.64E-12	0.635025	0.327	0.063	4.41E-08	Oligodendrocytic
Nfia	2.67E-12	-1.30894	0.481	0.786	4.45E-08	Oligodendrocytic
Hmgcs1	2.69E-12	1.039851	0.615	0.227	4.48E-08	Oligodendrocytic
Ppp1r15b	2.99E-12	0.641161	0.442	0.109	4.99E-08	Oligodendrocytic
Lrrc4c	3.53E-12	0.583996	0.346	0.066	5.89E-08	Oligodendrocytic
Bcan	3.56E-12	0.600561	0.769	0.247	5.93E-08	Oligodendrocytic
Ccnd1	3.56E-12	-1.45125	0.308	0.72	5.94E-08	Oligodendrocytic
Tpd52	3.6E-12	0.583268	0.327	0.063	6.01E-08	Oligodendrocytic
Lss	3.64E-12	0.656742	0.327	0.061	6.07E-08	Oligodendrocytic
G0s2	3.77E-12	0.471797	0.269	0.04	6.29E-08	Oligodendrocytic
Jagn1	4.12E-12	0.697219	0.558	0.16	6.87E-08	Oligodendrocytic
Bin1	5.32E-12	0.995813	0.788	0.353	8.87E-08	Oligodendrocytic
Ppapdc2	5.62E-12	0.334883	0.192	0.02	9.38E-08	Oligodendrocytic
Atp6v0b	5.76E-12	0.798002	0.731	0.315	9.6E-08	Oligodendrocytic
Zadh2	5.8E-12	0.57518	0.385	0.085	9.67E-08	Oligodendrocytic
Cask	6.92E-12	0.814565	0.442	0.116	1.15E-07	Oligodendrocytic
Stx7	6.95E-12	0.667224	0.481	0.129	1.16E-07	Oligodendrocytic
Lrrtm2	7.94E-12	0.483176	0.288	0.048	1.32E-07	Oligodendrocytic
Actb	9.29E-12	0.478358	1	0.989	1.55E-07	Oligodendrocytic
Tln2	1.16E-11	0.435591	0.308	0.055	1.94E-07	Oligodendrocytic
Yipf1	1.19E-11	0.577513	0.462	0.12	1.98E-07	Oligodendrocytic
Draxin	1.2E-11	-1.56261	0.154	0.63	2E-07	Oligodendrocytic
Slc35f1	1.45E-11	0.601629	0.481	0.121	2.42E-07	Oligodendrocytic
Atp9a	1.51E-11	0.403617	0.288	0.048	2.51E-07	Oligodendrocytic
Tspan3	1.61E-11	0.672259	0.942	0.618	2.68E-07	Oligodendrocytic
Sox2ot	1.66E-11	0.735946	0.231	0.033	2.77E-07	Oligodendrocytic
Slc24a3	1.9E-11	0.454113	0.173	0.018	3.17E-07	Oligodendrocytic
Bpgm	1.91E-11	0.522975	0.442	0.11	3.19E-07	Oligodendrocytic
Pgp	1.94E-11	0.782539	0.654	0.238	3.24E-07	Oligodendrocytic
Pdzd8	2.21E-11	0.617719	0.404	0.094	3.69E-07	Oligodendrocytic
Cnn2	3.11E-11	0.31201	0.135	0.01	5.18E-07	Oligodendrocytic
Cobll1	3.35E-11	0.303645	0.173	0.018	5.58E-07	Oligodendrocytic
Vat1	3.68E-11	0.516909	0.25	0.039	6.13E-07	Oligodendrocytic
Strn	4.09E-11	0.492279	0.269	0.045	6.83E-07	Oligodendrocytic
Cpox	4.38E-11	0.636082	0.423	0.105	7.3E-07	Oligodendrocytic
Bricd5	4.41E-11	0.442003	0.192	0.023	7.35E-07	Oligodendrocytic
Reep1	4.69E-11	0.504053	0.385	0.085	7.82E-07	Oligodendrocytic
Prkar2b	5.46E-11	0.557453	0.385	0.089	9.11E-07	Oligodendrocytic
Egr1	5.57E-11	-1.95408	0.038	0.531	9.3E-07	Oligodendrocytic
Epn2	5.88E-11	0.529636	0.596	0.18	9.81E-07	Oligodendrocytic
App	7.52E-11	0.852221	0.923	0.622	1.25E-06	Oligodendrocytic
4930402H:	1.09E-10	0.74301	0.538	0.171	1.81E-06	Oligodendrocytic
Canx	1.2E-10	0.726443	0.885	0.74	2.01E-06	Oligodendrocytic

Qpct	1.24E-10	0.570763	0.385	0.086	2.07E-06	Oligodendrocytic
Trit1	1.28E-10	0.463365	0.365	0.083	2.13E-06	Oligodendrocytic
Bdh1	1.31E-10	0.480017	0.365	0.084	2.19E-06	Oligodendrocytic
Ephb1	1.43E-10	0.30726	0.192	0.024	2.39E-06	Oligodendrocytic
Znrf1	1.57E-10	0.604912	0.481	0.14	2.62E-06	Oligodendrocytic
H3f3b	1.68E-10	-0.84685	0.865	0.895	2.81E-06	Oligodendrocytic
Phldb1	1.73E-10	0.460842	0.308	0.061	2.88E-06	Oligodendrocytic
Pcdhga9	1.78E-10	0.667503	0.885	0.504	2.97E-06	Oligodendrocytic
Ptprj	1.84E-10	0.315217	0.135	0.011	3.08E-06	Oligodendrocytic
Chst3	1.96E-10	0.280289	0.115	0.008	3.27E-06	Oligodendrocytic
Slain1	1.97E-10	0.499142	0.231	0.036	3.29E-06	Oligodendrocytic
Wls	1.98E-10	0.781247	0.538	0.188	3.3E-06	Oligodendrocytic
Arpc1a	2.04E-10	0.750321	0.769	0.412	3.41E-06	Oligodendrocytic
C430049B(	2.1E-10	0.397304	0.135	0.011	3.5E-06	Oligodendrocytic
Gria3	2.61E-10	0.674181	0.596	0.202	4.35E-06	Oligodendrocytic
Mvd	2.79E-10	0.49721	0.231	0.036	4.65E-06	Oligodendrocytic
Slc22a17	2.86E-10	0.750597	0.712	0.315	4.77E-06	Oligodendrocytic
Pld2	2.87E-10	0.430631	0.173	0.02	4.79E-06	Oligodendrocytic
Il18	3.15E-10	0.519541	0.288	0.055	5.26E-06	Oligodendrocytic
Fam73a	3.5E-10	0.519877	0.404	0.101	5.83E-06	Oligodendrocytic
Dek	3.74E-10	-1.19143	0.577	0.777	6.24E-06	Oligodendrocytic
Vps36	3.92E-10	0.760181	0.75	0.372	6.54E-06	Oligodendrocytic
Acsl3	4.92E-10	0.822906	0.596	0.233	8.21E-06	Oligodendrocytic
Frmd5	5.1E-10	0.476271	0.25	0.044	8.5E-06	Oligodendrocytic
Abhd12	5.14E-10	0.524607	0.5	0.156	8.58E-06	Oligodendrocytic
Col9a3	5.63E-10	0.899626	0.558	0.215	9.38E-06	Oligodendrocytic
Cdc42se2	6.79E-10	0.683005	0.712	0.307	1.13E-05	Oligodendrocytic
Irs2	7.59E-10	0.590478	0.423	0.119	1.27E-05	Oligodendrocytic
Hipk2	7.73E-10	0.510496	0.327	0.074	1.29E-05	Oligodendrocytic
Fchsd2	8.47E-10	0.552847	0.288	0.059	1.41E-05	Oligodendrocytic
Ctsl	8.53E-10	0.641676	0.808	0.364	1.42E-05	Oligodendrocytic
Hsp90b1	9.34E-10	0.541232	0.962	0.81	1.56E-05	Oligodendrocytic
Omg	9.44E-10	0.557084	0.385	0.096	1.57E-05	Oligodendrocytic
Man2a2	1E-09	0.390519	0.308	0.065	1.67E-05	Oligodendrocytic
Klhl18	1.01E-09	0.358912	0.173	0.021	1.69E-05	Oligodendrocytic
Rexo2	1.05E-09	0.508388	0.712	0.275	1.76E-05	Oligodendrocytic
Traf4	1.07E-09	0.531572	0.481	0.14	1.78E-05	Oligodendrocytic
Tecr	1.08E-09	0.613429	0.904	0.602	1.81E-05	Oligodendrocytic
Selk	1.11E-09	0.575246	0.885	0.506	1.84E-05	Oligodendrocytic
Celf2	1.12E-09	-1.22731	0.173	0.601	1.86E-05	Oligodendrocytic
Ccnd2	1.13E-09	-1.32991	0.269	0.652	1.89E-05	Oligodendrocytic
Nlk	1.22E-09	0.437972	0.423	0.114	2.04E-05	Oligodendrocytic
Cdyl	1.32E-09	0.403291	0.231	0.039	2.2E-05	Oligodendrocytic
Ebpl	1.33E-09	0.542767	0.423	0.119	2.22E-05	Oligodendrocytic
Dmrtb1	1.34E-09	0.598383	0.212	0.033	2.24E-05	Oligodendrocytic
Slc15a4	1.38E-09	0.404367	0.25	0.045	2.31E-05	Oligodendrocytic
Dnajb14	1.4E-09	0.501079	0.327	0.074	2.34E-05	Oligodendrocytic
Klhl17	1.43E-09	0.370118	0.212	0.033	2.38E-05	Oligodendrocytic

Atp6v0a2	1.47E-09	0.360252	0.212	0.033	2.45E-05	Oligodendrocytic
Lamp1	1.51E-09	0.772958	0.808	0.496	2.52E-05	Oligodendrocytic
S100a4	1.53E-09	0.354065	0.212	0.033	2.56E-05	Oligodendrocytic
Ccny	1.68E-09	0.441677	0.346	0.081	2.81E-05	Oligodendrocytic
Pacs2	1.72E-09	0.527687	0.25	0.046	2.87E-05	Oligodendrocytic
Ppp1r18	1.81E-09	0.401603	0.308	0.065	3.02E-05	Oligodendrocytic
Scamp5	1.85E-09	0.566535	0.365	0.093	3.09E-05	Oligodendrocytic
Jun	2.09E-09	-1.27934	0.404	0.768	3.49E-05	Oligodendrocytic
Cnksr3	2.22E-09	0.355229	0.192	0.028	3.7E-05	Oligodendrocytic
Slc25a44	2.27E-09	0.383196	0.269	0.054	3.79E-05	Oligodendrocytic
Nipal3	2.35E-09	0.267245	0.154	0.018	3.92E-05	Oligodendrocytic
Tor1aip2	2.8E-09	0.566682	0.462	0.144	4.66E-05	Oligodendrocytic
Tmem41b	2.8E-09	0.38211	0.288	0.06	4.67E-05	Oligodendrocytic
Mitf	3.05E-09	0.308075	0.173	0.023	5.08E-05	Oligodendrocytic
Casc4	3.06E-09	0.518496	0.596	0.22	5.1E-05	Oligodendrocytic
Slc35a5	3.72E-09	0.357826	0.25	0.046	6.2E-05	Oligodendrocytic
Acap3	3.78E-09	0.348554	0.25	0.046	6.31E-05	Oligodendrocytic
Polr3h	3.94E-09	0.628758	0.5	0.164	6.58E-05	Oligodendrocytic
Apbb1	3.97E-09	0.524386	0.462	0.144	6.63E-05	Oligodendrocytic
Gtf2f2	3.98E-09	0.555985	0.5	0.159	6.64E-05	Oligodendrocytic
Csad	4.03E-09	0.560999	0.423	0.12	6.73E-05	Oligodendrocytic
Crmp1	4.73E-09	-1.30836	0.154	0.567	7.88E-05	Oligodendrocytic
Tm7sf2	4.76E-09	0.369595	0.25	0.048	7.95E-05	Oligodendrocytic
Rnf5	5.05E-09	0.591611	0.731	0.333	8.43E-05	Oligodendrocytic
Abat	5.22E-09	0.414434	0.327	0.076	8.71E-05	Oligodendrocytic
Akirin1	5.23E-09	0.286017	0.231	0.04	8.72E-05	Oligodendrocytic
Vps28	5.26E-09	0.650236	0.654	0.293	8.78E-05	Oligodendrocytic
Etv6	5.41E-09	0.353319	0.173	0.024	9.02E-05	Oligodendrocytic
Tmem167	5.48E-09	0.706219	0.731	0.349	9.14E-05	Oligodendrocytic
3222401L1	5.48E-09	0.359954	0.135	0.014	9.14E-05	Oligodendrocytic
Faah	5.72E-09	0.322143	0.135	0.014	9.54E-05	Oligodendrocytic
Nenf	5.98E-09	0.484537	0.538	0.179	9.98E-05	Oligodendrocytic
Cryab	6.64E-09	0.767898	0.346	0.089	0.000111	Oligodendrocytic
Tmem30a	7.35E-09	0.549963	0.635	0.253	0.000123	Oligodendrocytic
Samd4b	7.55E-09	0.572484	0.385	0.106	0.000126	Oligodendrocytic
Rhog	7.58E-09	0.392349	0.25	0.049	0.000126	Oligodendrocytic
Smc2	7.87E-09	-1.53435	0.327	0.632	0.000131	Oligodendrocytic
Hmg20a	8.05E-09	0.501016	0.404	0.113	0.000134	Oligodendrocytic
Arhgdig	9.02E-09	0.437069	0.212	0.036	0.000151	Oligodendrocytic
Echdc1	9.41E-09	0.379382	0.25	0.049	0.000157	Oligodendrocytic
Nnat	9.72E-09	-1.26159	0.481	0.715	0.000162	Oligodendrocytic
Ggct	1.02E-08	0.464005	0.288	0.064	0.000171	Oligodendrocytic
Elovl1	1.08E-08	0.291517	0.269	0.055	0.000181	Oligodendrocytic
Mpi	1.13E-08	0.276235	0.269	0.055	0.000188	Oligodendrocytic
Ilk	1.17E-08	0.582175	0.635	0.26	0.000195	Oligodendrocytic
Ppp1r21	1.22E-08	0.489953	0.327	0.08	0.000203	Oligodendrocytic
Jam3	1.24E-08	0.580189	0.558	0.2	0.000207	Oligodendrocytic
Sar1b	1.28E-08	0.504628	0.673	0.274	0.000214	Oligodendrocytic

Bcar1	1.29E-08	0.549716	0.5	0.178	0.000215	Oligodendrocytic
Vim	1.36E-08	-1.39232	0.192	0.573	0.000227	Oligodendrocytic
Wipf1	1.47E-08	0.451282	0.288	0.064	0.000245	Oligodendrocytic
Gphn	1.49E-08	0.395293	0.577	0.202	0.000248	Oligodendrocytic
Cldn25	1.51E-08	0.561639	0.712	0.33	0.000252	Oligodendrocytic
Rac1	1.55E-08	0.642843	0.788	0.434	0.000258	Oligodendrocytic
Calm2	1.57E-08	-0.78574	0.808	0.859	0.000263	Oligodendrocytic
A430035B1	1.59E-08	0.458583	0.154	0.02	0.000266	Oligodendrocytic
Cd81	1.67E-08	0.513408	0.923	0.637	0.000278	Oligodendrocytic
BC002163	1.87E-08	0.272807	0.135	0.015	0.000311	Oligodendrocytic
2610035D1	1.97E-08	0.255865	0.173	0.025	0.000328	Oligodendrocytic
CRE_RECOM	2E-08	-1.55222	0.365	0.681	0.000334	Oligodendrocytic
Mki67	2.07E-08	-1.74061	0.135	0.506	0.000346	Oligodendrocytic
Smim15	2.18E-08	0.467946	0.519	0.178	0.000363	Oligodendrocytic
Mien1	2.22E-08	0.468478	0.673	0.277	0.00037	Oligodendrocytic
Tmbim4	2.25E-08	0.524994	0.558	0.209	0.000375	Oligodendrocytic
Taf9b	2.28E-08	0.310771	0.212	0.038	0.000381	Oligodendrocytic
Dtd1	2.47E-08	0.491432	0.423	0.129	0.000412	Oligodendrocytic
D630045J1	2.5E-08	0.379231	0.212	0.038	0.000416	Oligodendrocytic
Taok3	2.52E-08	0.504581	0.423	0.129	0.00042	Oligodendrocytic
Gaa	2.59E-08	0.299139	0.269	0.058	0.000432	Oligodendrocytic
Cyp51	2.62E-08	0.636531	0.519	0.199	0.000437	Oligodendrocytic
Tubb2a	2.69E-08	0.558835	0.615	0.249	0.000448	Oligodendrocytic
Fermt2	2.82E-08	0.489445	0.769	0.339	0.000471	Oligodendrocytic
Id2	3.04E-08	-1.28358	0.231	0.583	0.000507	Oligodendrocytic
Rbfox3	3.07E-08	-1.37327	0.077	0.469	0.000512	Oligodendrocytic
Selt	3.09E-08	0.523716	0.462	0.153	0.000516	Oligodendrocytic
Tmf1	3.36E-08	0.514372	0.404	0.126	0.00056	Oligodendrocytic
Rimkla	3.41E-08	0.296083	0.115	0.011	0.000569	Oligodendrocytic
Ten1	3.44E-08	0.523014	0.577	0.219	0.000574	Oligodendrocytic
Sema4d	3.74E-08	0.281193	0.173	0.026	0.000624	Oligodendrocytic
Sc4mol	3.88E-08	0.68149	0.404	0.124	0.000647	Oligodendrocytic
5031439G0	4.13E-08	0.400748	0.231	0.046	0.000688	Oligodendrocytic
Fam168a	4.4E-08	0.593979	0.75	0.363	0.000733	Oligodendrocytic
B3gat1	4.47E-08	0.447956	0.269	0.061	0.000746	Oligodendrocytic
B3gat2	4.56E-08	0.364539	0.308	0.074	0.000761	Oligodendrocytic
Slc6a1	4.63E-08	0.374574	0.385	0.108	0.000772	Oligodendrocytic
Grlf1	4.68E-08	0.515048	0.404	0.126	0.000781	Oligodendrocytic
Barhl1	4.79E-08	-1.31068	0.096	0.487	0.000799	Oligodendrocytic
Igfbpl1	4.83E-08	-1.3946	0.135	0.516	0.000805	Oligodendrocytic
Arxes1	4.85E-08	0.4327	0.404	0.123	0.00081	Oligodendrocytic
Pabpc1	4.91E-08	-0.55452	0.923	0.934	0.000819	Oligodendrocytic
Klhl2	5.07E-08	0.288735	0.154	0.021	0.000846	Oligodendrocytic
Map7d1	5.09E-08	0.616418	0.615	0.275	0.000849	Oligodendrocytic
Ncdn	5.13E-08	0.456319	0.269	0.061	0.000856	Oligodendrocytic
Smc4	5.58E-08	-1.48576	0.442	0.648	0.000931	Oligodendrocytic
Arl8a	5.62E-08	0.549412	0.404	0.125	0.000938	Oligodendrocytic
Ezr	5.65E-08	-1.20353	0.154	0.516	0.000943	Oligodendrocytic

Ralgds	5.69E-08	0.544986	0.346	0.1	0.00095	Oligodendrocytic
Gria2	5.75E-08	0.651493	0.827	0.528	0.000959	Oligodendrocytic
Smad7	6.13E-08	0.288088	0.135	0.016	0.001023	Oligodendrocytic
Dscaml1	6.34E-08	0.280466	0.154	0.021	0.001058	Oligodendrocytic
Abi1	6.48E-08	0.565883	0.538	0.214	0.00108	Oligodendrocytic
Tmpo	6.69E-08	-1.19779	0.192	0.539	0.001116	Oligodendrocytic
Rab5a	7.53E-08	0.538385	0.269	0.063	0.001257	Oligodendrocytic
Spag9	7.65E-08	0.536526	0.788	0.419	0.001276	Oligodendrocytic
Dnajb11	7.68E-08	0.634594	0.577	0.243	0.001281	Oligodendrocytic
Mdk	7.9E-08	-1.12958	0.096	0.488	0.001318	Oligodendrocytic
Serinc1	8.09E-08	0.629132	0.788	0.488	0.00135	Oligodendrocytic
Pus7	8.14E-08	0.450668	0.365	0.108	0.001359	Oligodendrocytic
Dhcr24	8.35E-08	0.557258	0.365	0.111	0.001392	Oligodendrocytic
Klhl42	8.75E-08	0.26329	0.173	0.028	0.001459	Oligodendrocytic
Nckap1	8.93E-08	0.513025	0.5	0.181	0.00149	Oligodendrocytic
Aprt	9.1E-08	0.508038	0.519	0.19	0.001519	Oligodendrocytic
Plekha1	9.3E-08	0.694494	0.269	0.065	0.001551	Oligodendrocytic
Sdf2l1	9.85E-08	0.50758	0.327	0.09	0.001643	Oligodendrocytic
Ltv1	9.98E-08	0.490061	0.365	0.109	0.001665	Oligodendrocytic
Meg3	1.01E-07	0.359601	0.385	0.111	0.001683	Oligodendrocytic
Ier5	1.01E-07	-1.19917	0.077	0.463	0.00169	Oligodendrocytic
Pdia3	1.11E-07	0.601918	0.75	0.395	0.001851	Oligodendrocytic
Tbc1d8	1.13E-07	0.424434	0.115	0.013	0.001883	Oligodendrocytic
Cbx1	1.14E-07	-0.84724	0.558	0.736	0.001906	Oligodendrocytic
Pou3f2	1.14E-07	-1.15411	0.038	0.418	0.001908	Oligodendrocytic
Atp6ap1	1.17E-07	0.526642	0.442	0.158	0.001955	Oligodendrocytic
Col27a1	1.2E-07	0.280497	0.154	0.023	0.002003	Oligodendrocytic
Cnn3	1.21E-07	-0.9736	0.308	0.603	0.002017	Oligodendrocytic
Arvcf	1.23E-07	0.446302	0.192	0.035	0.002058	Oligodendrocytic
Hexdc	1.27E-07	0.418367	0.173	0.029	0.002119	Oligodendrocytic
Dbt	1.33E-07	0.514352	0.442	0.153	0.002211	Oligodendrocytic
Cbx5	1.33E-07	-0.81583	0.692	0.783	0.002216	Oligodendrocytic
St6gal2	1.42E-07	0.254639	0.115	0.013	0.002369	Oligodendrocytic
Kif1a	1.46E-07	0.472201	0.5	0.178	0.002433	Oligodendrocytic
Tmem86a	1.58E-07	0.324395	0.135	0.018	0.002634	Oligodendrocytic
Ier2	1.58E-07	-1.39216	0.135	0.482	0.002634	Oligodendrocytic
Utp14b	1.58E-07	0.277103	0.173	0.029	0.002643	Oligodendrocytic
Mrps7	1.59E-07	0.588445	0.558	0.228	0.002644	Oligodendrocytic
Usp16	1.75E-07	0.529134	0.635	0.289	0.002911	Oligodendrocytic
Nap1l5	1.77E-07	0.322484	0.519	0.178	0.002952	Oligodendrocytic
Fdps	1.83E-07	0.473749	0.25	0.059	0.003056	Oligodendrocytic
Tril	1.86E-07	0.366221	0.462	0.151	0.003099	Oligodendrocytic
Pcdh9	1.93E-07	0.456107	0.327	0.093	0.003221	Oligodendrocytic
Arpc5	1.93E-07	0.514032	0.769	0.398	0.003222	Oligodendrocytic
Urm1	1.95E-07	0.525142	0.365	0.116	0.003254	Oligodendrocytic
Sept8	1.98E-07	0.494785	0.538	0.218	0.003306	Oligodendrocytic
Arpc1b	2E-07	0.285311	0.25	0.056	0.003328	Oligodendrocytic
Atl1	2.04E-07	0.377693	0.308	0.083	0.003406	Oligodendrocytic

Dhrs7	2.07E-07	0.395021	0.423	0.138	0.00346	Oligodendrocytic
Ophn1	2.11E-07	0.397057	0.365	0.11	0.003515	Oligodendrocytic
Prtg	2.17E-07	0.496184	0.154	0.024	0.003621	Oligodendrocytic
Zfp579	2.2E-07	0.347915	0.308	0.083	0.003665	Oligodendrocytic
Ttll12	2.32E-07	0.322378	0.231	0.05	0.003869	Oligodendrocytic
Abhd5	2.33E-07	0.263175	0.212	0.043	0.003892	Oligodendrocytic
Ctsb	2.37E-07	0.354767	0.731	0.353	0.003955	Oligodendrocytic
Twf1	2.38E-07	0.455648	0.538	0.21	0.003962	Oligodendrocytic
Snx18	2.38E-07	0.342018	0.25	0.058	0.003963	Oligodendrocytic
Tmeff2	2.38E-07	0.702138	0.346	0.106	0.003966	Oligodendrocytic
Dpysl2	2.59E-07	0.732284	0.577	0.283	0.004323	Oligodendrocytic
Tead2	2.64E-07	-1.10694	0	0.358	0.004405	Oligodendrocytic
Cmtm5	2.66E-07	0.304987	0.385	0.115	0.004438	Oligodendrocytic
Lactb	2.75E-07	0.418747	0.231	0.051	0.004591	Oligodendrocytic
Tmem126a	2.87E-07	0.517855	0.538	0.217	0.004791	Oligodendrocytic
Rpl13a	3.01E-07	-0.51414	0.942	0.972	0.005013	Oligodendrocytic
Fam49b	3.03E-07	0.53175	0.423	0.141	0.005052	Oligodendrocytic
Arpp21	3.13E-07	0.475344	0.385	0.126	0.00522	Oligodendrocytic
Peli1	3.15E-07	0.745872	0.519	0.232	0.005258	Oligodendrocytic
Nfix	3.57E-07	-0.85231	0.308	0.633	0.005952	Oligodendrocytic
Gsn	3.65E-07	0.257451	0.212	0.043	0.006083	Oligodendrocytic
Fsd1l	3.83E-07	0.501011	0.327	0.096	0.006381	Oligodendrocytic
Itfg3	3.84E-07	0.437231	0.173	0.031	0.006412	Oligodendrocytic
Cd24a	4.01E-07	-1.04481	0.192	0.538	0.006684	Oligodendrocytic
Cmip	4.06E-07	0.532533	0.5	0.193	0.006777	Oligodendrocytic
Tubb5	4.08E-07	-0.75055	0.942	0.882	0.006798	Oligodendrocytic
2700094K1	4.47E-07	-0.9298	0.558	0.7	0.007451	Oligodendrocytic
Nkiras1	4.48E-07	0.312628	0.269	0.066	0.007466	Oligodendrocytic
Ece1	4.55E-07	0.500538	0.346	0.109	0.007589	Oligodendrocytic
Hmgn1	4.59E-07	-0.806	0.481	0.702	0.00765	Oligodendrocytic
Atmin	4.63E-07	0.412689	0.308	0.086	0.007716	Oligodendrocytic
Hjurp	4.68E-07	-1.20874	0.173	0.488	0.007804	Oligodendrocytic
Pax6	4.78E-07	-1.11941	0.115	0.453	0.00798	Oligodendrocytic
Stmn3	4.79E-07	-0.96426	0.288	0.603	0.007988	Oligodendrocytic
Dzip1	4.86E-07	0.492482	0.346	0.108	0.008109	Oligodendrocytic
Papss1	4.94E-07	0.553448	0.538	0.223	0.008246	Oligodendrocytic
1810037I1	5.21E-07	0.574778	0.712	0.357	0.008694	Oligodendrocytic
Josd2	5.32E-07	0.442552	0.423	0.151	0.008872	Oligodendrocytic
Rnf181	5.68E-07	0.382073	0.385	0.126	0.009471	Oligodendrocytic
Dixdc1	5.88E-07	0.419639	0.462	0.168	0.009812	Oligodendrocytic
Tub	5.89E-07	0.353064	0.212	0.045	0.009822	Oligodendrocytic
Cspg5	2.1E-108	2.579409	0.916	0.09	3.6E-104	OPC-Like
Cspg4	1.72E-85	1.867276	0.597	0.02	2.87E-81	OPC-Like
Serpine2	1.76E-85	2.15234	0.958	0.168	2.94E-81	OPC-Like
Cacng4	1.78E-80	2.068577	0.941	0.191	2.97E-76	OPC-Like
Cntn1	1.02E-78	2.237482	0.79	0.109	1.71E-74	OPC-Like
Pdgfra	7.34E-76	2.231377	0.815	0.133	1.22E-71	OPC-Like
Olig1	1.13E-71	2.205983	0.933	0.257	1.89E-67	OPC-Like

3110035E1	7.65E-68	1.422364	0.739	0.077	1.28E-63	OPC-Like
Ptprz1	9.45E-68	1.649893	0.849	0.146	1.58E-63	OPC-Like
Ascl1	1.86E-59	1.162409	0.345	0	3.11E-55	OPC-Like
Pcsk1n	3.56E-57	1.662943	0.681	0.109	5.94E-53	OPC-Like
Nap1l5	1.14E-55	1.627466	0.689	0.119	1.9E-51	OPC-Like
Sox10	7.37E-52	1.481943	0.546	0.056	1.23E-47	OPC-Like
B3gat2	2.06E-51	1.441385	0.445	0.03	3.43E-47	OPC-Like
Sulf2	8.44E-51	1.55914	0.555	0.066	1.41E-46	OPC-Like
Anks1b	9.54E-51	1.558613	0.529	0.06	1.59E-46	OPC-Like
Enc1	2.21E-50	1.321877	0.395	0.019	3.68E-46	OPC-Like
Fabp7	9.95E-50	0.556854	0.941	0.281	1.66E-45	OPC-Like
Cdo1	1.47E-48	1.294903	0.445	0.034	2.44E-44	OPC-Like
Mmp15	6.15E-48	1.250285	0.387	0.02	1.03E-43	OPC-Like
Olig2	8.41E-48	1.463231	0.815	0.235	1.4E-43	OPC-Like
Slc35f1	3.57E-47	1.44516	0.555	0.077	5.95E-43	OPC-Like
Kcnd2	1.73E-46	1.324457	0.395	0.025	2.88E-42	OPC-Like
Scrg1	3.81E-44	1.357029	0.681	0.133	6.36E-40	OPC-Like
Tpm1	7.83E-44	1.734841	0.756	0.265	1.31E-39	OPC-Like
Nxph1	1.71E-42	1.016423	0.319	0.012	2.85E-38	OPC-Like
Qpct	9.03E-42	1.242786	0.445	0.049	1.51E-37	OPC-Like
Ptn	2.13E-41	1.039588	0.975	0.548	3.55E-37	OPC-Like
Scn3a	9.53E-41	1.095176	0.303	0.011	1.59E-36	OPC-Like
Phlda1	3.99E-40	1.388986	0.479	0.067	6.65E-36	OPC-Like
Tagln2	4.1E-39	1.566386	0.555	0.109	6.84E-35	OPC-Like
Ddah1	4.91E-39	1.221243	0.563	0.101	8.19E-35	OPC-Like
Epn2	9.5E-39	1.503515	0.605	0.141	1.58E-34	OPC-Like
Rlbp1	2.89E-38	1.238424	0.353	0.027	4.82E-34	OPC-Like
Pou3f1	6.47E-36	0.966782	0.227	0.003	1.08E-31	OPC-Like
Tmem255b	7.13E-36	0.946713	0.227	0.003	1.19E-31	OPC-Like
Bcan	1.42E-35	1.181831	0.731	0.205	2.38E-31	OPC-Like
Pllp	2.16E-34	1.151089	0.462	0.067	3.6E-30	OPC-Like
Pid1	3.39E-34	1.127948	0.345	0.033	5.65E-30	OPC-Like
Rgcc	1.32E-33	1.319734	0.445	0.067	2.21E-29	OPC-Like
Resp18	3.3E-33	0.908976	0.202	0.001	5.51E-29	OPC-Like
Gpr37l1	1.1E-32	1.066685	0.672	0.164	1.83E-28	OPC-Like
Gpr17	1.63E-32	0.830944	0.462	0.064	2.71E-28	OPC-Like
Ptpre	1.77E-32	1.126241	0.454	0.067	2.96E-28	OPC-Like
Enpp2	2.83E-32	1.063865	0.504	0.087	4.73E-28	OPC-Like
Ostf1	5.79E-32	1.263919	0.462	0.083	9.66E-28	OPC-Like
Ppfibp1	1.81E-31	1.188533	0.487	0.094	3.03E-27	OPC-Like
Ednrb	2.3E-31	0.738706	0.613	0.127	3.83E-27	OPC-Like
Ramp1	2.69E-31	1.123322	0.597	0.135	4.48E-27	OPC-Like
Ajap1	1.32E-30	0.763761	0.176	0	2.21E-26	OPC-Like
Sfrp1	1.35E-30	-1.58552	0.311	0.825	2.26E-26	OPC-Like
Dner	4.2E-30	1.143424	0.538	0.13	7E-26	OPC-Like
Tmem100	5.92E-30	0.984229	0.319	0.033	9.87E-26	OPC-Like
Ildr2	5.52E-29	0.730499	0.21	0.007	9.2E-25	OPC-Like
Neto1	6.94E-29	0.691294	0.176	0.001	1.16E-24	OPC-Like

Gng3	8.27E-29	1.095464	0.689	0.251	1.38E-24	OPC-Like
Sstr1	8.73E-29	0.570306	0.185	0.003	1.46E-24	OPC-Like
Spock2	1.03E-28	0.947559	0.387	0.059	1.72E-24	OPC-Like
Cadm2	1.08E-28	1.259196	0.437	0.082	1.81E-24	OPC-Like
Mmp16	2.28E-28	1.121978	0.437	0.085	3.8E-24	OPC-Like
Brinp3	2.29E-28	0.857461	0.261	0.019	3.81E-24	OPC-Like
Kcnip3	3.18E-28	1.333069	0.529	0.143	5.3E-24	OPC-Like
Meg3	3.23E-28	1.15923	0.429	0.079	5.4E-24	OPC-Like
Car8	7.02E-28	0.98142	0.269	0.022	1.17E-23	OPC-Like
C1ql2	8.71E-28	0.546044	0.16	0	1.45E-23	OPC-Like
Gatm	1.62E-27	1.106665	0.496	0.116	2.71E-23	OPC-Like
Dnm3	1.82E-27	1.156252	0.395	0.066	3.04E-23	OPC-Like
Gria3	2.24E-27	1.281131	0.563	0.171	3.74E-23	OPC-Like
Gfra1	3.02E-27	0.985832	0.277	0.026	5.03E-23	OPC-Like
CRE_RECON	8.93E-27	-1.93292	0.193	0.738	1.49E-22	OPC-Like
Zic1	1.07E-26	-1.2728	0.244	0.78	1.78E-22	OPC-Like
Igfbp3	3.06E-26	0.892697	0.303	0.034	5.11E-22	OPC-Like
Rprm	6.29E-26	1.151004	0.412	0.085	1.05E-21	OPC-Like
Alcam	7.05E-26	1.110515	0.588	0.198	1.18E-21	OPC-Like
Draxin	1.25E-25	-1.50836	0.134	0.676	2.08E-21	OPC-Like
Pcdh17	1.53E-25	1.091566	0.378	0.066	2.55E-21	OPC-Like
Lsamp	1.32E-24	0.978298	0.613	0.189	2.21E-20	OPC-Like
Dscam	1.4E-24	0.970739	0.261	0.026	2.34E-20	OPC-Like
Tmem176b	2.1E-24	1.021146	0.655	0.231	3.5E-20	OPC-Like
Sall3	2.25E-24	0.568479	0.168	0.004	3.76E-20	OPC-Like
Lrrtm3	2.89E-24	0.769163	0.244	0.022	4.82E-20	OPC-Like
Nfib	4.7E-24	-1.06794	0.613	0.903	7.83E-20	OPC-Like
Spon1	8.96E-24	1.024635	0.378	0.074	1.5E-19	OPC-Like
Cd24a	1.64E-23	-1.47397	0.067	0.59	2.73E-19	OPC-Like
Rev3l	1.82E-23	1.153565	0.437	0.108	3.03E-19	OPC-Like
Cobl	2.62E-23	0.554968	0.16	0.004	4.38E-19	OPC-Like
Grm5	4.67E-23	1.023635	0.269	0.031	7.79E-19	OPC-Like
Pmp22	5.86E-23	1.019212	0.311	0.046	9.77E-19	OPC-Like
Lrp1	6.14E-23	0.994207	0.37	0.072	1.02E-18	OPC-Like
Ezr	6.25E-23	-1.39821	0.042	0.567	1.04E-18	OPC-Like
Ugt8a	1.16E-22	0.861831	0.37	0.066	1.93E-18	OPC-Like
D430041D	1.52E-22	-1.27938	0.084	0.604	2.54E-18	OPC-Like
Lypd1	3.26E-22	0.800019	0.21	0.016	5.44E-18	OPC-Like
Slc22a3	3.51E-22	0.506004	0.126	0	5.86E-18	OPC-Like
Megf11	4.18E-22	0.828815	0.227	0.02	6.97E-18	OPC-Like
Spry4	4.4E-22	0.755953	0.21	0.016	7.34E-18	OPC-Like
Dmrtb1	5.87E-22	0.644977	0.21	0.016	9.8E-18	OPC-Like
Il1rap	5.97E-22	0.964357	0.286	0.041	9.97E-18	OPC-Like
Dpysl3	8.86E-22	1.084494	0.546	0.187	1.48E-17	OPC-Like
Gria2	1.04E-21	0.952107	0.832	0.5	1.73E-17	OPC-Like
Gap43	1.47E-21	0.971498	0.765	0.475	2.45E-17	OPC-Like
Syt11	2.34E-21	0.885476	0.84	0.557	3.91E-17	OPC-Like
Ncald	3.11E-21	1.194526	0.613	0.264	5.19E-17	OPC-Like

Gabra3	5.14E-21	0.629767	0.193	0.014	8.57E-17	OPC-Like
Rpl13a	8.82E-21	-0.61535	0.899	0.982	1.47E-16	OPC-Like
Nlgn3	1.05E-20	0.94499	0.311	0.053	1.76E-16	OPC-Like
Lnx1	1.15E-20	0.765062	0.21	0.019	1.91E-16	OPC-Like
Scn1a	1.49E-20	0.595	0.151	0.005	2.49E-16	OPC-Like
Cd63	1.74E-20	-0.8869	0.462	0.863	2.91E-16	OPC-Like
Asrgl1	1.85E-20	0.931728	0.647	0.27	3.08E-16	OPC-Like
Vstm2b	1.9E-20	0.581337	0.134	0.003	3.17E-16	OPC-Like
Spry1	2.51E-20	0.922036	0.269	0.04	4.19E-16	OPC-Like
Pabpc1	2.57E-20	-0.67669	0.832	0.949	4.29E-16	OPC-Like
Tspan13	2.86E-20	1.117684	0.529	0.199	4.77E-16	OPC-Like
Slitrk3	3.66E-20	0.622143	0.202	0.018	6.11E-16	OPC-Like
Hsp90ab1	4.39E-20	-0.49433	0.992	0.996	7.32E-16	OPC-Like
Lrrtm1	4.58E-20	0.702963	0.168	0.01	7.64E-16	OPC-Like
Nrxn2	4.83E-20	0.858561	0.387	0.093	8.05E-16	OPC-Like
Rps5	7.62E-20	-0.56007	0.908	0.992	1.27E-15	OPC-Like
Omg	8.12E-20	0.954049	0.353	0.075	1.35E-15	OPC-Like
Matn4	2.35E-19	0.7755	0.185	0.015	3.91E-15	OPC-Like
Chst11	2.72E-19	0.753207	0.218	0.025	4.54E-15	OPC-Like
Tmsb4x	3.7E-19	-0.79378	0.882	0.966	6.16E-15	OPC-Like
Cplx2	4.08E-19	-1.24262	0.109	0.578	6.81E-15	OPC-Like
Gpx3	7.1E-19	0.778452	0.176	0.014	1.18E-14	OPC-Like
Plekhb1	8.19E-19	0.994534	0.319	0.064	1.37E-14	OPC-Like
2900011O(	8.3E-19	0.911868	0.387	0.096	1.38E-14	OPC-Like
AW04773C	9.73E-19	0.992219	0.361	0.081	1.62E-14	OPC-Like
Susd4	1.36E-18	0.803703	0.252	0.037	2.27E-14	OPC-Like
Polg	2.88E-18	0.90374	0.319	0.068	4.8E-14	OPC-Like
Vwc2	2.96E-18	0.511086	0.143	0.007	4.94E-14	OPC-Like
Tacc2	3.16E-18	0.921969	0.403	0.112	5.28E-14	OPC-Like
Barhl1	4.14E-18	-1.31016	0.076	0.526	6.9E-14	OPC-Like
Mapt	4.9E-18	0.759834	0.555	0.198	8.18E-14	OPC-Like
Plk2	5.72E-18	0.729112	0.227	0.03	9.54E-14	OPC-Like
Scn2a1	9.45E-18	0.560274	0.126	0.004	1.58E-13	OPC-Like
Gsx1	9.71E-18	0.543172	0.118	0.003	1.62E-13	OPC-Like
Rps3	9.81E-18	-0.5632	0.882	0.967	1.64E-13	OPC-Like
Ptma	1.04E-17	-0.98853	0.252	0.663	1.73E-13	OPC-Like
Kcnd3	1.08E-17	0.754345	0.193	0.02	1.81E-13	OPC-Like
Elfn1	1.09E-17	0.583522	0.118	0.003	1.82E-13	OPC-Like
Lrrc4c	1.26E-17	0.950112	0.277	0.052	2.09E-13	OPC-Like
Nt5e	1.75E-17	0.558027	0.151	0.01	2.93E-13	OPC-Like
Lrrfip1	1.98E-17	0.79956	0.294	0.059	3.3E-13	OPC-Like
Sapcd2	2.14E-17	0.971215	0.378	0.102	3.57E-13	OPC-Like
Creg2	2.93E-17	0.600577	0.143	0.008	4.89E-13	OPC-Like
Necab2	3.08E-17	0.50798	0.176	0.016	5.14E-13	OPC-Like
Myt1	3.1E-17	0.868489	0.311	0.066	5.17E-13	OPC-Like
4833424O:	4.06E-17	0.881545	0.311	0.068	6.77E-13	OPC-Like
Slc22a17	5.19E-17	0.953829	0.605	0.296	8.66E-13	OPC-Like
Mfsd2a	8.2E-17	0.905255	0.277	0.053	1.37E-12	OPC-Like

Grik1	1.11E-16	0.560024	0.126	0.005	1.86E-12	OPC-Like
E130114P1	1.26E-16	-0.99907	0.252	0.675	2.1E-12	OPC-Like
Ptpn5	1.81E-16	0.455412	0.118	0.004	3.02E-12	OPC-Like
Afap1l2	1.83E-16	0.826719	0.252	0.045	3.05E-12	OPC-Like
Kif21a	2.06E-16	-1.18024	0.059	0.474	3.44E-12	OPC-Like
Syt6	2.17E-16	0.443585	0.118	0.004	3.61E-12	OPC-Like
Gm2a	2.35E-16	0.967764	0.395	0.124	3.92E-12	OPC-Like
Dab1	2.36E-16	0.966954	0.319	0.078	3.94E-12	OPC-Like
Igfbpl1	2.67E-16	-1.29028	0.126	0.552	4.46E-12	OPC-Like
Cdh13	3.06E-16	0.960417	0.378	0.112	5.11E-12	OPC-Like
Cbfa2t3	6.34E-16	-1.11483	0.059	0.471	1.06E-11	OPC-Like
Npm1	7.81E-16	-0.77991	0.462	0.801	1.3E-11	OPC-Like
Rpl4	8.95E-16	-0.53843	0.899	0.963	1.49E-11	OPC-Like
Ppapdc1a	8.99E-16	0.594052	0.151	0.012	1.5E-11	OPC-Like
Rcan2	1.4E-15	0.502584	0.126	0.007	2.33E-11	OPC-Like
Rps26	1.47E-15	-0.63963	0.756	0.921	2.46E-11	OPC-Like
Anp32a	2.17E-15	-0.70846	0.622	0.873	3.63E-11	OPC-Like
Rhoc	2.66E-15	0.835567	0.328	0.086	4.44E-11	OPC-Like
Prkg2	2.69E-15	0.460416	0.118	0.005	4.49E-11	OPC-Like
Rps9	2.82E-15	-0.47435	0.941	0.982	4.71E-11	OPC-Like
Rplp0	3.01E-15	-0.59797	0.79	0.934	5.02E-11	OPC-Like
Ppp2r2b	3.12E-15	0.858708	0.311	0.078	5.21E-11	OPC-Like
Dbi	3.46E-15	0.334413	0.857	0.684	5.77E-11	OPC-Like
Chd5	4.66E-15	0.598611	0.134	0.01	7.77E-11	OPC-Like
Nova1	4.88E-15	0.763409	0.303	0.074	8.15E-11	OPC-Like
Tmem255a	4.93E-15	0.575312	0.134	0.01	8.23E-11	OPC-Like
Nrxn1	5.34E-15	1.024948	0.605	0.31	8.9E-11	OPC-Like
Gabra1	6.23E-15	0.473965	0.101	0.003	1.04E-10	OPC-Like
Timp2	6.29E-15	0.803453	0.269	0.057	1.05E-10	OPC-Like
Ncam2	8.55E-15	0.806216	0.311	0.078	1.43E-10	OPC-Like
Ppap2b	1.03E-14	0.451983	0.504	0.178	1.72E-10	OPC-Like
Sox9	1.11E-14	-1.01732	0.059	0.451	1.85E-10	OPC-Like
Arhgdig	1.37E-14	0.666317	0.185	0.025	2.29E-10	OPC-Like
Rtn1	1.39E-14	0.606292	0.857	0.671	2.32E-10	OPC-Like
Hnrnpab	1.48E-14	-0.65808	0.655	0.881	2.47E-10	OPC-Like
Zcchc24	1.91E-14	0.903773	0.353	0.107	3.18E-10	OPC-Like
Opcml	2.09E-14	0.803878	0.244	0.048	3.48E-10	OPC-Like
Tead2	2.21E-14	-0.9926	0.017	0.388	3.69E-10	OPC-Like
Tub	2.36E-14	0.730057	0.202	0.031	3.93E-10	OPC-Like
Dock10	2.51E-14	0.70097	0.202	0.031	4.19E-10	OPC-Like
Tbata	2.89E-14	-1.35031	0.05	0.421	4.82E-10	OPC-Like
Flrt1	2.95E-14	0.648136	0.151	0.015	4.92E-10	OPC-Like
Sox4	2.98E-14	-0.95109	0.277	0.658	4.96E-10	OPC-Like
Zdhhc2	3.14E-14	0.84072	0.261	0.059	5.24E-10	OPC-Like
Sorcs3	3.64E-14	0.491517	0.118	0.007	6.07E-10	OPC-Like
H3f3b	4.07E-14	-0.6404	0.748	0.917	6.79E-10	OPC-Like
Pik3r1	4.56E-14	0.998842	0.42	0.163	7.6E-10	OPC-Like
Pea15a	4.86E-14	0.628013	0.538	0.232	8.1E-10	OPC-Like

Rap1gap	4.91E-14	0.737944	0.193	0.03	8.18E-10	OPC-Like
Gpm6a	5.07E-14	0.839645	0.655	0.367	8.46E-10	OPC-Like
Ier5	5.44E-14	-0.93684	0.101	0.495	9.08E-10	OPC-Like
Myl12a	7.16E-14	-0.93343	0.109	0.489	1.19E-09	OPC-Like
Rps20	7.81E-14	-0.59348	0.639	0.899	1.3E-09	OPC-Like
Creb5	1.28E-13	0.458991	0.151	0.016	2.13E-09	OPC-Like
Ntrk2	1.51E-13	0.673958	0.42	0.141	2.51E-09	OPC-Like
B3gat1	1.52E-13	0.878173	0.235	0.048	2.53E-09	OPC-Like
Sox6	1.55E-13	0.877231	0.252	0.056	2.59E-09	OPC-Like
Nfia	1.67E-13	-0.75612	0.513	0.809	2.79E-09	OPC-Like
Grik3	1.98E-13	0.519724	0.118	0.008	3.31E-09	OPC-Like
Rps21	3.3E-13	-0.57624	0.773	0.922	5.51E-09	OPC-Like
Cog7	3.77E-13	-0.99864	0.151	0.525	6.29E-09	OPC-Like
Camk1	4.21E-13	0.835252	0.311	0.09	7.03E-09	OPC-Like
Sox3	4.55E-13	0.467998	0.109	0.007	7.59E-09	OPC-Like
Zic4	4.77E-13	-0.90656	0.076	0.441	7.96E-09	OPC-Like
Sdc3	4.97E-13	0.854155	0.345	0.109	8.29E-09	OPC-Like
H1f0	6.35E-13	-0.88265	0.311	0.661	1.06E-08	OPC-Like
Gjc3	6.37E-13	0.643032	0.353	0.104	1.06E-08	OPC-Like
Rbfox3	6.43E-13	-1.05032	0.134	0.496	1.07E-08	OPC-Like
Slc38a3	8.3E-13	0.760209	0.277	0.071	1.39E-08	OPC-Like
Shc4	8.52E-13	0.810562	0.244	0.057	1.42E-08	OPC-Like
Olfm1	9.23E-13	0.758078	0.395	0.142	1.54E-08	OPC-Like
Rpl41	9.53E-13	-0.52986	0.723	0.899	1.59E-08	OPC-Like
Csmd3	1.08E-12	0.545897	0.101	0.005	1.81E-08	OPC-Like
Slitrk2	1.1E-12	0.639112	0.16	0.022	1.84E-08	OPC-Like
Smc2	1.16E-12	-1.01774	0.353	0.656	1.94E-08	OPC-Like
Nrep	1.45E-12	-0.86531	0.16	0.54	2.43E-08	OPC-Like
Nim1	1.79E-12	0.66852	0.176	0.029	2.99E-08	OPC-Like
H2afv	1.86E-12	-0.69342	0.588	0.814	3.11E-08	OPC-Like
Rnd3	1.89E-12	-0.97448	0.126	0.481	3.15E-08	OPC-Like
Gsg1l	2.11E-12	-0.96101	0.042	0.376	3.52E-08	OPC-Like
Col16a1	2.51E-12	0.447251	0.126	0.012	4.18E-08	OPC-Like
Clgn	2.69E-12	0.592509	0.126	0.012	4.48E-08	OPC-Like
Malat1	2.7E-12	0.603752	0.966	0.984	4.51E-08	OPC-Like
S100a16	2.99E-12	0.787374	0.445	0.173	4.99E-08	OPC-Like
Akap12	3.32E-12	0.912677	0.286	0.082	5.54E-08	OPC-Like
Ptprt	3.84E-12	0.561306	0.176	0.029	6.41E-08	OPC-Like
G0s2	4.27E-12	0.659834	0.185	0.033	7.12E-08	OPC-Like
Phactr3	5.4E-12	0.795142	0.277	0.077	9.01E-08	OPC-Like
Eef1b2	5.48E-12	-0.60433	0.555	0.858	9.13E-08	OPC-Like
Ckb	6.33E-12	0.417411	0.908	0.817	1.06E-07	OPC-Like
C1ql3	7.05E-12	0.735291	0.277	0.078	1.18E-07	OPC-Like
Mgat4a	9.39E-12	0.435857	0.101	0.007	1.57E-07	OPC-Like
1700086L1	9.66E-12	0.373183	0.101	0.007	1.61E-07	OPC-Like
Tspan3	1.1E-11	0.560463	0.807	0.611	1.84E-07	OPC-Like
Pcdh11x	1.12E-11	0.598895	0.151	0.022	1.87E-07	OPC-Like
Syt16	1.15E-11	0.468952	0.126	0.014	1.92E-07	OPC-Like

Cav2	1.24E-11	0.538171	0.143	0.019	2.06E-07	OPC-Like
Rps24	1.38E-11	-0.56059	0.681	0.9	2.3E-07	OPC-Like
Bricd5	1.55E-11	0.528638	0.134	0.016	2.58E-07	OPC-Like
3632451O(	1.59E-11	0.811122	0.269	0.075	2.65E-07	OPC-Like
Adora1	1.99E-11	0.658573	0.252	0.064	3.32E-07	OPC-Like
Gm9800	2.12E-11	-0.7101	0.269	0.617	3.54E-07	OPC-Like
Kctd4	2.14E-11	0.582219	0.176	0.031	3.57E-07	OPC-Like
Ppic	2.16E-11	-0.92213	0.025	0.327	3.61E-07	OPC-Like
Hsd11b2	2.37E-11	-1.06901	0.025	0.327	3.96E-07	OPC-Like
S100a13	2.77E-11	0.682412	0.387	0.141	4.62E-07	OPC-Like
Srebf1	3.33E-11	-0.97555	0.067	0.383	5.56E-07	OPC-Like
Rpl35a	3.5E-11	-0.65337	0.378	0.713	5.84E-07	OPC-Like
Mmp2	3.81E-11	0.572988	0.151	0.023	6.35E-07	OPC-Like
Sox2	3.87E-11	0.270612	1	1	6.46E-07	OPC-Like
Pax6	4.5E-11	-0.86836	0.151	0.478	7.51E-07	OPC-Like
Orai1	4.69E-11	0.654123	0.193	0.041	7.83E-07	OPC-Like
Tgfa	5.3E-11	0.40643	0.143	0.02	8.84E-07	OPC-Like
Hmgb2	5.37E-11	-0.94973	0.193	0.51	8.95E-07	OPC-Like
Tril	6.16E-11	0.636078	0.378	0.137	1.03E-06	OPC-Like
Anp32b	6.81E-11	-0.64347	0.454	0.738	1.14E-06	OPC-Like
Tubb5	7.42E-11	-0.59728	0.782	0.903	1.24E-06	OPC-Like
Nasp	8.1E-11	-0.66482	0.429	0.735	1.35E-06	OPC-Like
Cdk6	8.42E-11	-0.90036	0.025	0.321	1.41E-06	OPC-Like
Slc29a1	9.21E-11	-0.83978	0.176	0.516	1.54E-06	OPC-Like
Dlgap1	9.58E-11	0.756289	0.353	0.138	1.6E-06	OPC-Like
Klhl5	9.97E-11	0.724427	0.336	0.119	1.66E-06	OPC-Like
Dpysl4	1.21E-10	-0.71381	0.151	0.499	2.01E-06	OPC-Like
Ttll7	1.28E-10	0.503876	0.118	0.014	2.13E-06	OPC-Like
Dcaf12l1	1.5E-10	0.473639	0.126	0.016	2.51E-06	OPC-Like
Cbx5	1.7E-10	-0.59939	0.563	0.813	2.83E-06	OPC-Like
Calm2	1.74E-10	-0.52785	0.647	0.889	2.9E-06	OPC-Like
Rps14	1.78E-10	-0.36781	0.958	0.978	2.98E-06	OPC-Like
Tubb2b	2.02E-10	0.609861	0.63	0.434	3.37E-06	OPC-Like
Brinp1	2.04E-10	0.664816	0.21	0.051	3.41E-06	OPC-Like
Arl2bp	2.33E-10	0.801272	0.504	0.272	3.88E-06	OPC-Like
Eid1	2.53E-10	0.626263	0.765	0.581	4.22E-06	OPC-Like
Rgs7bp	2.58E-10	0.686062	0.21	0.051	4.3E-06	OPC-Like
Stmn4	2.67E-10	0.691539	0.429	0.195	4.46E-06	OPC-Like
Lgals1	2.8E-10	-0.95316	0.067	0.365	4.67E-06	OPC-Like
Gria4	2.8E-10	0.790658	0.42	0.186	4.67E-06	OPC-Like
Nrxn3	3.04E-10	0.773176	0.168	0.033	5.07E-06	OPC-Like
Vstm2a	3.06E-10	0.393761	0.101	0.01	5.1E-06	OPC-Like
Cd200	3.88E-10	0.63738	0.252	0.074	6.47E-06	OPC-Like
Nkd1	4.19E-10	-0.78886	0.05	0.333	6.98E-06	OPC-Like
Gm2694	4.2E-10	-0.84527	0.084	0.387	7E-06	OPC-Like
Sema5b	4.37E-10	0.623256	0.16	0.03	7.28E-06	OPC-Like
Ccdc34	5.12E-10	-0.62439	0.244	0.571	8.53E-06	OPC-Like
Tmsb10	5.6E-10	-0.70072	0.303	0.623	9.34E-06	OPC-Like

Cnih2	6.14E-10	0.365457	0.109	0.012	1.02E-05	OPC-Like
S100a1	6.26E-10	0.60141	0.378	0.141	1.04E-05	OPC-Like
Prrg1	6.34E-10	0.431191	0.118	0.015	1.06E-05	OPC-Like
Cyp2j6	7.12E-10	0.676189	0.252	0.075	1.19E-05	OPC-Like
Pqlc1	8.07E-10	-0.78795	0.092	0.395	1.35E-05	OPC-Like
Cacng2	8.21E-10	-0.79807	0.034	0.314	1.37E-05	OPC-Like
Ugdh	8.49E-10	0.815884	0.328	0.128	1.42E-05	OPC-Like
Mageh1	9.3E-10	0.747447	0.395	0.183	1.55E-05	OPC-Like
Vps37b	9.41E-10	-0.72562	0.21	0.545	1.57E-05	OPC-Like
Tspan7	9.49E-10	0.538757	0.496	0.242	1.58E-05	OPC-Like
Rpl26	1.04E-09	-0.49289	0.521	0.813	1.74E-05	OPC-Like
Dpf1	1.09E-09	0.585662	0.168	0.036	1.82E-05	OPC-Like
Rplp2	1.12E-09	-0.43015	0.807	0.928	1.88E-05	OPC-Like
Fstl1	1.13E-09	-0.86361	0.042	0.314	1.88E-05	OPC-Like
Ddah2	1.15E-09	-0.67948	0.353	0.653	1.92E-05	OPC-Like
Rabgap1l	1.16E-09	0.770038	0.227	0.064	1.93E-05	OPC-Like
Smc4	1.19E-09	-0.7677	0.387	0.676	1.99E-05	OPC-Like
Abhd2	1.3E-09	0.74806	0.176	0.04	2.17E-05	OPC-Like
Rpl22	1.82E-09	-0.4839	0.731	0.891	3.04E-05	OPC-Like
Mfap2	1.96E-09	0.624575	0.235	0.07	3.27E-05	OPC-Like
Gnb2l1	2.07E-09	-0.41068	0.832	0.936	3.46E-05	OPC-Like
Bag1	2.09E-09	-0.62061	0.176	0.497	3.48E-05	OPC-Like
Cbx1	2.16E-09	-0.57058	0.513	0.76	3.61E-05	OPC-Like
Npas3	2.26E-09	0.614798	0.294	0.1	3.77E-05	OPC-Like
Fkbp3	2.67E-09	-0.48958	0.664	0.855	4.46E-05	OPC-Like
Ranbp1	2.79E-09	-0.63446	0.605	0.795	4.66E-05	OPC-Like
Tmem191c	2.89E-09	0.530953	0.151	0.03	4.81E-05	OPC-Like
Ccnd1	2.89E-09	0.609047	0.815	0.675	4.82E-05	OPC-Like
Gpt2	2.98E-09	0.739034	0.294	0.108	4.96E-05	OPC-Like
Fbn2	3.21E-09	0.647267	0.134	0.023	5.35E-05	OPC-Like
Dpp6	3.3E-09	0.566406	0.185	0.045	5.51E-05	OPC-Like
Lap3	3.64E-09	-0.73399	0.151	0.456	6.08E-05	OPC-Like
Cd81	3.75E-09	0.442946	0.79	0.633	6.26E-05	OPC-Like
Map3k1	3.79E-09	-0.65434	0.16	0.46	6.32E-05	OPC-Like
Cthrc1	4.15E-09	0.512441	0.126	0.02	6.92E-05	OPC-Like
Lrrn3	4.16E-09	0.556641	0.151	0.03	6.94E-05	OPC-Like
Chpt1	4.58E-09	0.630044	0.345	0.143	7.64E-05	OPC-Like
Hdgf	4.85E-09	-0.51136	0.361	0.675	8.09E-05	OPC-Like
Caskin2	4.93E-09	0.534109	0.118	0.018	8.23E-05	OPC-Like
Dmd	4.99E-09	0.693905	0.21	0.059	8.32E-05	OPC-Like
Snrpf	5.26E-09	-0.65543	0.218	0.518	8.78E-05	OPC-Like
Hirip3	5.31E-09	-0.68874	0.193	0.49	8.85E-05	OPC-Like
Galnt13	5.4E-09	0.426156	0.126	0.02	9.02E-05	OPC-Like
Rps11	5.67E-09	-0.4383	0.664	0.867	9.46E-05	OPC-Like
1500016L0	5.76E-09	-0.87678	0.042	0.296	9.61E-05	OPC-Like
Rplp1	6.11E-09	-0.36998	0.882	0.963	0.000102	OPC-Like
Cpne8	6.16E-09	0.369908	0.118	0.018	0.000103	OPC-Like
Fam210b	6.32E-09	-0.77684	0.059	0.324	0.000105	OPC-Like

Kcnip1	6.65E-09	0.451755	0.101	0.012	0.000111	OPC-Like
Nceh1	6.85E-09	0.456867	0.168	0.038	0.000114	OPC-Like
Eef1a1	7.07E-09	-0.38566	0.874	0.943	0.000118	OPC-Like
Unc5c	7.16E-09	0.450807	0.118	0.018	0.000119	OPC-Like
Tceal5	7.7E-09	0.661477	0.16	0.036	0.000128	OPC-Like
Deb1	8.02E-09	0.72772	0.471	0.253	0.000134	OPC-Like
Midn	8.1E-09	0.619007	0.336	0.139	0.000135	OPC-Like
Cks1b	8.92E-09	-0.76483	0.16	0.439	0.000149	OPC-Like
Fgf12	9.4E-09	0.57072	0.143	0.029	0.000157	OPC-Like
Pcdh10	9.66E-09	0.593032	0.202	0.056	0.000161	OPC-Like
2810417H	9.73E-09	-0.87239	0.235	0.505	0.000162	OPC-Like
Rps19	9.86E-09	-0.45638	0.664	0.874	0.000164	OPC-Like
Snrpg	1.03E-08	-0.60996	0.151	0.444	0.000172	OPC-Like
Crmp1	1.04E-08	-0.69616	0.294	0.582	0.000173	OPC-Like
Fgf9	1.22E-08	-0.72082	0.05	0.306	0.000203	OPC-Like
Gpr153	1.3E-08	-0.71308	0.05	0.299	0.000218	OPC-Like
Cacna2d1	1.43E-08	-0.81402	0.092	0.355	0.000238	OPC-Like
Tsc22d4	1.44E-08	0.425747	0.597	0.331	0.000241	OPC-Like
Sema5a	1.49E-08	0.466323	0.143	0.029	0.000249	OPC-Like
Igsf21	1.5E-08	0.482149	0.202	0.055	0.000249	OPC-Like
Abcg1	1.79E-08	0.467457	0.143	0.029	0.000299	OPC-Like
Hey1	1.88E-08	-0.80496	0.084	0.348	0.000313	OPC-Like
Csmd1	1.91E-08	0.634397	0.109	0.016	0.000318	OPC-Like
Pcyt1b	1.98E-08	0.689665	0.193	0.053	0.00033	OPC-Like
Cdk1	1.99E-08	-0.68713	0.084	0.354	0.000333	OPC-Like
Pcdh9	2E-08	0.680402	0.244	0.085	0.000333	OPC-Like
Rpl8	2.03E-08	-0.35863	0.882	0.97	0.000339	OPC-Like
Sox2ot	2.05E-08	0.433366	0.143	0.029	0.000342	OPC-Like
Rpl32	2.09E-08	-0.36995	0.866	0.952	0.000348	OPC-Like
Aplp1	2.15E-08	0.597977	0.294	0.111	0.000358	OPC-Like
Cltb	2.17E-08	-0.73916	0.303	0.574	0.000362	OPC-Like
Reep1	2.28E-08	0.518028	0.244	0.081	0.00038	OPC-Like
Hk2	2.67E-08	-0.67611	0.008	0.23	0.000445	OPC-Like
Tcp1	2.7E-08	-0.54264	0.303	0.601	0.00045	OPC-Like
Map4k4	2.73E-08	-0.58688	0.244	0.549	0.000456	OPC-Like
Sh3d19	2.79E-08	0.650753	0.261	0.094	0.000465	OPC-Like
Pcdh19	2.83E-08	0.373049	0.134	0.026	0.000473	OPC-Like
Dtd1	2.84E-08	0.640425	0.303	0.122	0.000473	OPC-Like
March1	2.87E-08	0.503397	0.151	0.034	0.000478	OPC-Like
Adam9	2.88E-08	0.773462	0.294	0.119	0.00048	OPC-Like
Cuedc1	3E-08	0.66602	0.286	0.109	0.0005	OPC-Like
Taok3	3.06E-08	0.631326	0.303	0.122	0.000511	OPC-Like
Gm3764	3.22E-08	0.554661	0.437	0.219	0.000537	OPC-Like
Rbp4	3.32E-08	-0.79668	0.05	0.295	0.000554	OPC-Like
Reep5	3.4E-08	0.656615	0.387	0.183	0.000567	OPC-Like
Hip1	3.95E-08	0.761759	0.445	0.254	0.000658	OPC-Like
Mcm7	4.03E-08	-0.60004	0.168	0.448	0.000673	OPC-Like
Cp	4.27E-08	0.572138	0.176	0.046	0.000712	OPC-Like

Usp24	4.37E-08	0.769826	0.193	0.057	0.00073	OPC-Like
Hpca	4.39E-08	-0.83961	0.05	0.288	0.000733	OPC-Like
Epb4.1l3	4.66E-08	0.673068	0.319	0.137	0.000778	OPC-Like
Hes1	5.4E-08	-0.88719	0.109	0.363	0.0009	OPC-Like
Evi5l	5.47E-08	0.668996	0.193	0.056	0.000913	OPC-Like
Aplp2	5.48E-08	0.698902	0.588	0.432	0.000914	OPC-Like
Elmo1	5.92E-08	0.620632	0.235	0.079	0.000988	OPC-Like
Ly6h	6.15E-08	0.481292	0.109	0.018	0.001025	OPC-Like
Gpm6b	6.33E-08	0.269444	0.748	0.604	0.001056	OPC-Like
Nap1l3	7.05E-08	0.449755	0.126	0.025	0.001176	OPC-Like
Zfp91	7.27E-08	-0.44244	0.328	0.612	0.001212	OPC-Like
Meis1	7.42E-08	-0.66173	0.16	0.425	0.001238	OPC-Like
Cav1	7.54E-08	0.62167	0.176	0.049	0.001258	OPC-Like
Eps8	7.89E-08	0.690092	0.261	0.098	0.001316	OPC-Like
Pak1	8.25E-08	0.595909	0.176	0.048	0.001376	OPC-Like
Nenf	8.66E-08	0.655655	0.361	0.175	0.001444	OPC-Like
Map2	8.79E-08	0.543522	0.622	0.466	0.001467	OPC-Like
Otx2	8.95E-08	-0.68948	0.034	0.257	0.001494	OPC-Like
Nell2	9.3E-08	0.815915	0.353	0.173	0.001551	OPC-Like
Lphn3	1E-07	0.657596	0.269	0.104	0.001668	OPC-Like
Ndn	1.01E-07	0.631665	0.454	0.261	0.001689	OPC-Like
H2afx	1.02E-07	-0.73293	0.202	0.467	0.001706	OPC-Like
Cadm4	1.02E-07	0.593397	0.37	0.176	0.001707	OPC-Like
Abhd6	1.06E-07	0.481793	0.218	0.071	0.00176	OPC-Like
Gsn	1.1E-07	0.442081	0.151	0.037	0.001836	OPC-Like
Hsd17b12	1.17E-07	0.622788	0.496	0.307	0.001949	OPC-Like
Car11	1.19E-07	0.470799	0.143	0.033	0.001981	OPC-Like
Hnrnpm	1.19E-07	-0.43937	0.63	0.833	0.001982	OPC-Like
Arsb	1.2E-07	0.520238	0.227	0.075	0.001995	OPC-Like
Tpm4	1.21E-07	-0.51935	0.16	0.434	0.002014	OPC-Like
Svil	1.23E-07	0.638305	0.16	0.041	0.002047	OPC-Like
Efhd2	1.29E-07	-0.7332	0.025	0.238	0.002155	OPC-Like
Itgb8	1.3E-07	0.551471	0.193	0.059	0.002168	OPC-Like
Gjc1	1.31E-07	-0.60354	0.025	0.243	0.002188	OPC-Like
Mmp14	1.42E-07	-0.51774	0.076	0.327	0.00237	OPC-Like
Cnn3	1.48E-07	-0.52855	0.345	0.624	0.002464	OPC-Like
Arhgef2	1.5E-07	-0.64473	0.05	0.284	0.002501	OPC-Like
Rpl39	1.55E-07	-0.47434	0.563	0.787	0.002585	OPC-Like
Fam69b	1.55E-07	0.442007	0.168	0.045	0.002587	OPC-Like
H2afy	1.62E-07	-0.47672	0.42	0.691	0.002706	OPC-Like
Serpinh1	1.69E-07	-0.67888	0.017	0.224	0.002821	OPC-Like
Ptch2	1.72E-07	-0.64376	0.017	0.225	0.002874	OPC-Like
Ppp1r14c	1.74E-07	0.700295	0.378	0.198	0.002902	OPC-Like
Trp53	1.86E-07	-0.57464	0.151	0.415	0.003095	OPC-Like
Cdh11	1.92E-07	0.51573	0.176	0.049	0.0032	OPC-Like
Psma7	1.99E-07	-0.4046	0.672	0.863	0.003325	OPC-Like
Tubb2a	2.1E-07	0.656839	0.429	0.246	0.003496	OPC-Like
Tle1	2.11E-07	-0.61889	0.076	0.302	0.003526	OPC-Like

Abracl	2.15E-07	-0.60244	0.076	0.314	0.003579	OPC-Like
Cpq	2.15E-07	0.52403	0.134	0.03	0.003582	OPC-Like
Dut	2.21E-07	-0.63845	0.277	0.538	0.003686	OPC-Like
Pnmal2	2.45E-07	0.658902	0.311	0.134	0.004084	OPC-Like
Matk	2.46E-07	0.502374	0.118	0.023	0.004095	OPC-Like
Nkx2-2	2.49E-07	0.584935	0.202	0.064	0.004158	OPC-Like
Cxcl14	2.52E-07	0.617123	0.218	0.075	0.004199	OPC-Like
Chga	2.52E-07	0.579	0.227	0.079	0.004209	OPC-Like
Psat1	2.67E-07	-0.52955	0.269	0.54	0.004454	OPC-Like
Hnrnpa0	2.73E-07	-0.54127	0.235	0.508	0.004546	OPC-Like
Hnrnpu	2.73E-07	-0.35238	0.849	0.933	0.004559	OPC-Like
Trio	2.76E-07	0.544754	0.378	0.186	0.004608	OPC-Like
Smarca1	2.79E-07	0.50901	0.185	0.056	0.004659	OPC-Like
Etfa	2.8E-07	-0.47801	0.168	0.455	0.004669	OPC-Like
Fip1l1	2.85E-07	0.629341	0.563	0.415	0.004755	OPC-Like
Mdk	2.94E-07	-0.59847	0.235	0.501	0.004911	OPC-Like
Mycn	3.01E-07	-0.62875	0.109	0.357	0.005015	OPC-Like
Stat3	3.03E-07	0.532683	0.227	0.081	0.005061	OPC-Like
Rassf4	3.25E-07	-0.74834	0.109	0.339	0.005419	OPC-Like
Stmn1	3.3E-07	-0.56566	0.076	0.306	0.005503	OPC-Like
Crip2	3.4E-07	-0.6264	0.025	0.234	0.005677	OPC-Like
Galnt10	3.44E-07	0.431271	0.109	0.02	0.00574	OPC-Like
Pter	3.46E-07	0.413773	0.109	0.02	0.005779	OPC-Like
Serbp1	3.49E-07	-0.35982	0.849	0.937	0.005818	OPC-Like
Foxp1	3.54E-07	-0.63711	0.017	0.213	0.005909	OPC-Like
Lhx1	3.57E-07	-0.75309	0.143	0.392	0.005951	OPC-Like
Itpr2	3.69E-07	0.577101	0.294	0.123	0.006147	OPC-Like
Tsc22d1	3.75E-07	0.55866	0.613	0.448	0.006261	OPC-Like
Hmgn1	3.91E-07	-0.45603	0.462	0.725	0.006526	OPC-Like
Pdap1	3.93E-07	-0.42078	0.529	0.777	0.006554	OPC-Like
Top2a	3.93E-07	-0.64871	0.277	0.525	0.006563	OPC-Like
Pbrm1	4.37E-07	-0.49813	0.345	0.623	0.007296	OPC-Like
Dclk1	4.4E-07	-0.57074	0.319	0.575	0.007342	OPC-Like
Kcnj10	4.53E-07	0.298824	0.328	0.142	0.007554	OPC-Like
RP23-45G1	4.56E-07	-0.61269	0.151	0.413	0.007611	OPC-Like
Ezh2	4.68E-07	-0.58009	0.471	0.686	0.007799	OPC-Like
Insm1	4.68E-07	-0.64743	0.067	0.294	0.007799	OPC-Like
Ctnnd2	4.81E-07	0.814779	0.311	0.15	0.008025	OPC-Like
Kpnb1	4.97E-07	-0.36547	0.176	0.445	0.008293	OPC-Like
Lrig3	5.06E-07	-0.61504	0.042	0.251	0.008441	OPC-Like
Dll3	5.17E-07	0.469959	0.21	0.071	0.008615	OPC-Like
Loxl1	5.37E-07	-0.59959	0	0.18	0.008953	OPC-Like
Apba2	5.4E-07	-0.52802	0.16	0.414	0.009	OPC-Like
Lrrc4	5.51E-07	0.270838	0.101	0.018	0.009184	OPC-Like
Smc1a	5.59E-07	-0.45479	0.471	0.723	0.009316	OPC-Like
Lmcd1	5.69E-07	0.482075	0.202	0.067	0.009496	OPC-Like
Pdgfa	5.78E-07	-0.76508	0.092	0.314	0.009636	OPC-Like
