# Supplementary material for: scRNA-seq in medulloblastoma shows cellular heterogeneity and lineage expansion support resistance to SHH inhibitor therapy
Source: Nat Commun. 2019 Dec 20;10:5829. doi: 10.1038/s41467-019-13657-6 (PMC6925218; doi:10.1038/s41467-019-13657-6)
Supplement: Supplementary file 9 — Supplementary Data 6 [file 41467_2019_13657_MOESM9_ESM.pdf]

IC1 Gene	Gene Loading IC1	IC2 Gene	Gene Loading IC2	IC3 Gene
Pcp4	17249	Ckb	12408	Neurod1
Ntm	16404	Tubb3	11077	Stmn2
Snca	15593	Cntn2	11009	Gap43
Meg3	15384	Tmsb4x	10178	Cntn2
Car10	14647	Stmn2	10068	Rtn1
Mapt	14402	Rtn1	9080	Map1b
Jph4	13552	Neurod1	9042	Tubb3
Cadm2	13465	Nhlh1	8584	Stmn4
Calb2	13380	Pdzrn3	8529	Celf4
D3Bwg0562e	13167	Gng3	8180	Gpm6a
Celf4	13114	Nrep	7904	Nrxn1
Neurod2	13110	Gap43	7875	Tubb2a
Arpp21	13021	Ina	7731	Dpysl3
Atp2b1	12874	Dpysl3	7689	Tmsb4x
Cntn1	12758	Stmn4	7424	Sept3
Camk4	12740	Mllt11	7305	Gng3
Pvrl3	12376	BC005764	7290	Mtss1
Sez6	12343	Sept3	7267	St18
Kcnd2	12125	Tubb2a	7222	Ppp1r14c
Gpm6a	12125	Miat	7077	Mapt
Prkce	12123	Chrna3	7031	Nrep
Grm1	11789	Igfbpl1	6977	Ina
Spock2	11728	Sept4	6974	Cdk5r1
Camk2d	11722	Dcx	6930	Elmo1
Btbd3	11611	Elavl4	6825	Dner
Lin7a	11566	Trpc4ap	6761	Rab3a
Tspan4	11511	Gpm6a	6759	Dcx
Ablim1	11507	Elavl3	6726	Trpc4ap
Ryr2	11399	Chgb	6589	Mllt11
Tenm1	11236	Map1b	6570	Tex14
Dpp6	11231	Rab3a	6557	Nhlh2
Adamts18	11199	Rbfox3	6440	Elavl3
Grin1	11180	Cdk5r1	6367	Aplp1
Nrep	10918	Aplp1	6308	Elavl4
Fat2	10820	6330403K07Rik	6295	Sema6a
Nrxn3	10770	Tex14	6261	Fxyd6
Thra	10756	Map2	6260	Apc
Cadps2	10645	Myt1	6232	Ank2
Anks1b	10623	Celf4	6048	Kcnk1
Nrxn2	10556	A330076H08Rik	6034	Map2
Synpr	10515	Bin1	6031	Arpp21
Tspan7	10477	Ppp1r14c	5988	Chgb
Runx1t1	10422	Apc	5918	Myt1l
Diras2	10409	Kif5c	5692	Nhlh1
Scg2	10392	Nhlh2	5684	L1cam
Kcna1	10258	Rab6b	5668	Tagln3
L1cam	10212	Mtss1	5663	Arl6ip1

Ank2	10199	St18	5590	Tnik
Gm12022	10076	Nrxn1	5564	B3galt2
Ly6h	9949	Itsn1	5494	Sptbn1
Tacc3	-5082	Hmgn5	-9256	Clspn
Knstrn	-5084	Arl6ip1	-9354	Hmgn5
Nusap1	-5091	Rrm2	-9503	Mycn
Ckb	-5113	Hjurp	-9835	Jun
Nasp	-5134	Ccdc34	-9962	CRE_RECOMBINASE
Hmmr	-5183	Nasp	-10133	Islr2
Chrna3	-5189	Hirip3	-10211	Sdpr
Prc1	-5290	Dlgap5	-10313	Ptch2
Kif23	-5315	Nucks1	-10395	Rprml
Spc25	-5324	Tuba1b	-10854	Pou3f2
H2afx	-5326	Cks1b	-10872	Ccnd2
Ckap2l	-5364	Ckap2	-11923	Sowaha
Pdzrn3	-5421	Ccnb2	-12046	Pqlc1
Cdk1	-5481	Arhgap11a	-12154	Tshz2
Ranbp1	-5496	Knstrn	-12177	C1ql1
Cks1b	-5597	Aspm	-12386	Baz1a
Cdca8	-5690	Kif20b	-12401	Nasp
Cdc20	-5731	Dek	-12443	Zfp36l1
2810417H13Rik	-5800	Esco2	-12558	Cltb
Mycn	-5825	Sgol2	-12597	Boc
Cenpe	-5927	Kif15	-12717	Ier5
Mdk	-5954	Ncapg	-12841	Mmp14
Insm1	-5963	Casc5	-13014	Pdgfa
Birc5	-5993	Ccnb1	-13080	Dek
Sept4	-6029	Mis18bp1	-13081	Dut
C1ql1	-6117	RP23-45G16.5	-13385	Ntrk3
Miat	-6201	Tacc3	-13808	Atoh1
Cenpa	-6226	Cdc20	-13892	Rbp4
Cenpf	-6232	Hmmr	-14344	Gpr153
Top2a	-6246	Cenpa	-14542	Gm17322
Ube2c	-6292	H2afx	-14562	Nop58
Tpx2	-6438	Ckap2l	-14567	Hes1
Mki67	-6443	2810417H13Rik	-14813	Gnai2
RP23-45G16.5	-6466	Incenp	-15096	Tbata
Tex14	-6604	Spc25	-15116	Hey1
Smc2	-6714	Cenpe	-15128	Ranbp1
Nhlh1	-6863	Kif23	-15225	Fam210b
Hmgb2	-6928	Cdk1	-15536	Cbfa2t3
Elavl2	-7098	Smc4	-15899	Sox9
Nhlh2	-7116	Cdca8	-15920	Hells
Smc4	-7157	Nusap1	-16169	Mdk
CRE_RECOMBINASE	-7177	Smc2	-16196	Lig1
Nnat	-7341	Ube2c	-16458	Mcm3
Lhx1	-7475	Hmgb2	-16459	Ung
Pde1c	-8167	Birc5	-16578	Hsd11b2

E130114P18Rik	-9635	Prc1	-16628	Pcna
Cog7	-10111	Cenpf	-17060	Sfrp1
Ccnd2	-10165	Tpx2	-17133	Srebf1
Sfrp1	-10762	Mki67	-18741	Mcm6
Igfbpl1	-12652	Top2a	-18846	Ccnd1

Gene Loading IC3	IC4 Gene	Gene Loading IC4
17176	Ptprs	11791
15188	Miat	10235
14408	Xist	9795
14174	Nfib	8684
13535	Cntn2	7721
13289	Reln	7151
12571	Ank3	6987
12486	Cacna2d1	6662
11902	Insm1	6623
11706	Gm26924	6596
11436	Nhlh2	6513
10982	Ankrd11	6306
10951	Gria2	6193
10940	Dync1h1	6019
10777	Ppp3ca	5943
10621	Clmp	5811
10211	mt-Rnr1	5719
10117	Ccdc88a	5618
10057	Phf20l1	5578
9402	Plxnb2	5574
9286	Ankrd12	5544
9217	Brsk2	5514
9206	Sema6a	5513
9069	Elavl3	5507
8770	Map2	5482
8618	Podxl2	5467
8579	Flna	5407
8531	Celsr2	5331
8462	Nktr	5316
8414	Mdga1	5057
8333	Dcx	5051
8294	2700081O15Rik	5046
8291	Sptbn1	5032
8283	Elavl2	5024
8247	A930011O12Rik	4941
8104	Scn8a	4921
8071	Mgat5b	4874
8061	Zc3h13	4838
7969	Prdm8	4820
7814	Srcin1	4786
7801	Kif1b	4784
7719	Tmem259	4762
7693	Kidins220	4743
7682	Map1b	4705
7673	Tnik	4688
7658	Sema6c	4662
7624	Zfp292	4634

7577	Tnrc6a	4601
7511	Atp9a	4563
7484	BC005764	4554
-5219	Prc1	-2744
-5239	Nucks1	-2822
-5321	Hes1	-2948
-5471	Tax1bp1	-2976
-5473	Kif23	-2983
-5485	Nusap1	-3002
-5491	Aspm	-3074
-5672	Cltb	-3079
-5673	Atoh1	-3088
-5689	Hmgb2	-3092
-5772	Arhgap11a	-3189
-5818	Sparcl1	-3200
-5864	Pdzrn4	-3241
-5874	Cdca8	-3248
-5875	Cenpe	-3254
-5910	Ccnd1	-3428
-6009	Sowaha	-3454
-6156	Cenpf	-3606
-6190	Id2	-3671
-6242	Dlgap5	-3750
-6366	Sfrp1	-3827
-6404	Gm2694	-3895
-6461	Tpx2	-3926
-6477	Lgals1	-3952
-6601	Hmmr	-3955
-6644	RP23-45G16.5	-3960
-6817	Cdk1	-3996
-7095	Vim	-4109
-7127	Hpca	-4198
-7477	Arl6ip1	-4198
-7534	Birc5	-4208
-7727	Tbata	-4455
-7762	Rbp4	-4480
-7817	Ckap2l	-4502
-7900	E130114P18Rik	-4641
-8000	Ranbp1	-4734
-8094	Tmsb4x	-4763
-8180	CRE_RECOMBINASE	-4825
-8510	H2afx	-4990
-8519	Hist1h2ak	-5015
-8529	Knstrn	-5155
-8618	mt-Nd5	-5173
-8703	Cks1b	-5712
-9012	Ccnb1	-5718
-9027	Ccnb2	-6638

-9777	Cdc20	-6888
-10415	Pttg1	-7489
-10754	Ube2c	-7670
-11203	Cenpa	-8021
-12162	mt-Nd1	-8702
