# Supplementary material for: scRNA-seq in medulloblastoma shows cellular heterogeneity and lineage expansion support resistance to SHH inhibitor therapy
Source: Nat Commun. 2019 Dec 20;10:5829. doi: 10.1038/s41467-019-13657-6 (PMC6925218; doi:10.1038/s41467-019-13657-6)
Supplement: Supplementary file 10 — Supplementary Data 7 [file 41467_2019_13657_MOESM10_ESM.pdf]

	p_val	avg_logFC	pct.Cluster0	pct.AllOthers	p_val_adj
Rpl13a	0	0.437042593	0.977	0.913	0
Rps5	0	0.430126209	0.985	0.944	0
Rps14	0	0.403282137	0.982	0.94	0
Tuba1a	0	-0.741963024	0.858	0.95	0
Rtn1	0	-0.781283764	0.518	0.74	0
Map1b	0	-0.884520861	0.358	0.653	0
Gap43	0	-0.952991478	0.367	0.666	0
Tubb3	0	-1.172262378	0.223	0.546	0
Stmn2	0	-1.341248288	0.271	0.634	0
Cntn2	0	-1.386584341	0.047	0.326	0
Neurod1	0	-1.780023473	0.226	0.621	0
Gnb2l1	0	0.441826047	0.935	0.853	4.1003E-305
Rpl8	7.0433E-307	0.412225533	0.953	0.881	1.1748E-302
Rps9	1.2475E-304	0.392851621	0.974	0.915	2.0809E-300
Gpm6a	2.173E-302	-0.926823803	0.19	0.472	3.6246E-298
Arl6ip1	3.1651E-299	-0.880699347	0.36	0.604	5.2794E-295
Ube2c	3.9319E-297	-1.276097579	0.08	0.323	6.5584E-293
Tpx2	1.2565E-293	-1.069658238	0.081	0.339	2.0958E-289
Rps3	1.7825E-289	0.382436424	0.969	0.908	2.9732E-285
Rplp1	7.4285E-278	0.400456256	0.951	0.855	1.2391E-273
Top2a	1.3275E-276	-1.059705631	0.252	0.478	2.2142E-272
Stmn4	1.2E-275	-1.005199309	0.134	0.401	2.0016E-271
Nrxn1	1.5383E-270	-0.965380192	0.116	0.376	2.5658E-266
Tubb5	3.1447E-267	-0.478753376	0.851	0.933	5.2453E-263
Rpl32	3.5565E-267	0.395474175	0.948	0.872	5.9322E-263
Celf4	3.503E-256	-1.013004692	0.128	0.376	5.8431E-252
Prc1	1.1501E-254	-1.035594565	0.078	0.308	1.9184E-250
Cenpf	1.1583E-254	-1.119429622	0.173	0.416	1.932E-250
Rplp2	2.3212E-253	0.4107213	0.916	0.818	3.8718E-249
Nusap1	2.4021E-253	-0.939868949	0.052	0.277	4.0067E-249
Rps19	5.4498E-253	0.464460648	0.858	0.723	9.0902E-249
Rpl4	4.2219E-251	0.379081229	0.952	0.895	7.0422E-247
Rplp0	1.3584E-249	0.398554785	0.932	0.855	2.2659E-245
Mki67	2.8339E-247	-0.998216686	0.211	0.448	4.727E-243
Birc5	2.2598E-246	-0.901182018	0.087	0.327	3.7693E-242
Ckap2l	2.8315E-243	-0.869121106	0.048	0.266	4.723E-239
Spc25	7.9248E-243	-0.866107822	0.054	0.275	1.3219E-238
Ddah2	2.8571E-242	-0.607137506	0.508	0.737	4.7657E-238
Kif23	1.0364E-236	-0.886369094	0.049	0.262	1.7288E-232
Nhlh2	1.9103E-233	-0.76741019	0.221	0.481	3.1863E-229
Tubb2b	2.1545E-233	-0.691192939	0.263	0.522	3.5937E-229
Draxin	4.3124E-231	0.573203554	0.708	0.509	7.1931E-227
Hes1	2.6653E-223	0.899432748	0.286	0.108	4.4456E-219
Sept3	1.5857E-221	-0.756627714	0.211	0.444	2.645E-217
Ina	8.6672E-217	-0.703517018	0.253	0.498	1.4457E-212
Cdk1	6.8344E-216	-0.839456806	0.079	0.295	1.14E-211
Cenpa	1.0959E-215	-0.971616032	0.087	0.299	1.828E-211

Tubb2a	7.5835E-215	-0.855273614	0.08	0.292	1.2649E-210
Cdca8	2.752E-214	-0.802452065	0.091	0.314	4.5903E-210
Rps20	3.2535E-214	0.41722107	0.879	0.748	5.4268E-210
H2afx	6.4487E-214	-0.852112999	0.171	0.376	1.0756E-209
Ccnd1	6.8592E-214	0.56034816	0.69	0.473	1.1441E-209
Rps26	5.5225E-213	0.384634192	0.921	0.825	9.2116E-209
Calm2	7.251E-213	-0.447528711	0.785	0.9	1.2095E-208
Apoe	1.5744E-211	-1.421613477	0.265	0.245	2.6261E-207
Smc4	3.7287E-207	-0.706289827	0.436	0.604	6.2194E-203
Hmmr	3.8258E-207	-0.84216831	0.034	0.218	6.3814E-203
Rps21	3.1142E-204	0.387817381	0.9	0.813	5.1945E-200
Gas5	3.6088E-203	0.430357344	0.819	0.719	6.0195E-199
Eef1a1	1.0429E-201	0.367255891	0.933	0.869	1.7396E-197
Rpl22	1.64E-201	0.405220592	0.867	0.765	2.7356E-197
Kif11	2.4246E-198	-0.714650081	0.055	0.249	4.0442E-194
St18	8.7562E-196	-0.806413086	0.047	0.232	1.4605E-191
Cdc20	1.9531E-193	-0.859182039	0.055	0.237	3.2578E-189
Cdca3	7.9515E-191	-0.746472024	0.069	0.266	1.3263E-186
Fxyd6	1.0516E-188	-0.577482398	0.322	0.563	1.7541E-184
Elavl3	6.6866E-186	-0.586706994	0.288	0.526	1.1153E-181
Ccnb1	6.7941E-184	-0.684049992	0.02	0.173	1.1333E-179
Miat	2.0117E-183	-0.645511702	0.33	0.569	3.3555E-179
Rps15	1.2773E-182	0.372739989	0.855	0.768	2.1305E-178
Ppp1r14c	1.4781E-182	-0.754892534	0.108	0.307	2.4655E-178
Incenp	5.6348E-180	-0.757698904	0.112	0.322	9.3989E-176
Map2	8.3181E-177	-0.553670734	0.318	0.542	1.3875E-172
Ccna2	1.6985E-175	-0.656756255	0.073	0.264	2.8332E-171
Rps11	6.8692E-175	0.379559601	0.853	0.755	1.1458E-170
Cenpe	1.1171E-173	-0.867524303	0.13	0.33	1.8634E-169
Cbfa2t3	3.7517E-169	0.535493915	0.501	0.374	6.2578E-165
Sptbn1	1.706E-168	-0.611032913	0.189	0.401	2.8456E-164
Gng3	3.286E-168	-0.706488246	0.157	0.362	5.481E-164
Cdk5r1	1.6374E-167	-0.620674236	0.17	0.38	2.7311E-163
Tagln3	3.1885E-167	-0.612775824	0.137	0.34	5.3184E-163
Hn1	5.8048E-166	-0.5023608	0.367	0.592	9.6825E-162
Eef1b2	1.385E-164	0.384095066	0.805	0.711	2.3101E-160
Apc	1.0246E-163	-0.552708992	0.26	0.481	1.7091E-159
Npm1	6.5704E-163	0.411556466	0.742	0.618	1.0959E-158
Pabpc1	1.7897E-159	0.317760648	0.93	0.863	2.9853E-155
Sfrp1	3.4108E-159	0.397815627	0.897	0.786	5.6893E-155
Aspm	7.9516E-159	-0.649626919	0.028	0.175	1.3263E-154
Hmgb2	1.3767E-157	-0.659018922	0.247	0.444	2.2963E-153
Mtss1	1.5308E-157	-0.669312723	0.124	0.311	2.5533E-153
Pbk	1.4349E-156	-0.630232419	0.081	0.263	2.3933E-152
Fam64a	8.1772E-156	-0.525648209	0.016	0.147	1.364E-151
Arhgap11a	1.622E-155	-0.620595546	0.047	0.206	2.7055E-151
Chgb	3.4524E-155	-0.725290645	0.101	0.281	5.7585E-151
Basp1	6.5799E-155	-0.464008684	0.577	0.741	1.0975E-150

Gria2	1.5894E-153	-0.533091085	0.355	0.568	2.651E-149
Esco2	3.2345E-153	-0.637283671	0.062	0.233	5.3952E-149
Mis18bp1	1.1253E-152	-0.600339769	0.042	0.195	1.8771E-148
Tacc3	1.082E-151	-0.572684747	0.071	0.245	1.8048E-147
Sema6a	6.2705E-151	-0.66232876	0.075	0.238	1.0459E-146
Tex14	1.1021E-150	-0.69910757	0.032	0.178	1.8383E-146
Ckap5	1.3955E-150	-0.570332648	0.117	0.308	2.3278E-146
Tead2	4.8206E-150	0.507564649	0.37	0.251	8.0408E-146
Casc5	4.0507E-149	-0.592652492	0.059	0.223	6.7566E-145
Dpysl3	3.8583E-147	-0.715464944	0.086	0.258	6.4356E-143
Rps2	5.7245E-147	0.403616754	0.518	0.429	9.5485E-143
Mllt11	5.6878E-146	-0.658137621	0.086	0.256	9.4872E-142
Celf2	9.3536E-146	-0.459169724	0.499	0.677	1.5602E-141
Rpl41	1.35E-145	0.343934471	0.853	0.759	2.2519E-141
Sgol2	4.3661E-144	-0.607545061	0.041	0.19	7.2826E-140
Ccnb2	1.3989E-143	-0.615505267	0.062	0.223	2.3334E-139
Kif22	4.8909E-143	-0.52167582	0.046	0.196	8.158E-139
Dner	1.3074E-142	-0.619749638	0.082	0.253	2.1807E-138
Srebf1	1.3945E-141	0.537956743	0.441	0.302	2.326E-137
Gpr153	8.1124E-141	0.542107251	0.286	0.185	1.3531E-136
Mapt	1.8159E-140	-0.698678508	0.094	0.251	3.029E-136
Ccng2	3.5197E-140	-0.520272016	0.084	0.249	5.8709E-136
Rpl14	2.2842E-139	0.352480642	0.763	0.677	3.81E-135
Nuf2	6.1435E-139	-0.522245112	0.033	0.172	1.0247E-134
Ank3	7.1654E-139	-0.503647974	0.267	0.464	1.1952E-134
Ncapg	2.6456E-138	-0.539230668	0.061	0.218	4.4128E-134
Cdkn2d	3.2042E-138	-0.503656452	0.045	0.189	5.3447E-134
RP23-45G16.5	3.2536E-138	-0.601594891	0.175	0.37	5.427E-134
Rps18	1.3494E-137	0.409936159	0.496	0.409	2.2507E-133
Racgap1	1.4191E-137	-0.53583405	0.116	0.296	2.3671E-133
Rpl39	3.8248E-137	0.372212455	0.723	0.623	6.3797E-133
Rad21	5.5928E-137	-0.519747355	0.304	0.507	9.3288E-133
Ptn	8.7687E-137	-0.639858473	0.305	0.481	1.4626E-132
Zic1	4.6228E-136	-0.394099089	0.687	0.812	7.7109E-132
Rangap1	1.3723E-135	-0.505490996	0.116	0.296	2.2891E-131
Kif15	4.4542E-135	-0.546856079	0.06	0.215	7.4297E-131
Rps25	9.0975E-135	0.404838663	0.558	0.456	1.5175E-130
Podxl2	4.1454E-134	-0.555367645	0.1	0.264	6.9145E-130
Cdca2	1.8813E-133	-0.474206359	0.028	0.157	3.138E-129
Kif5c	4.9042E-133	-0.493115671	0.247	0.442	8.1802E-129
Rps24	3.3643E-131	0.3041914	0.874	0.805	5.6117E-127
Bin1	9.2589E-131	-0.465333328	0.282	0.472	1.5444E-126
Cbx5	1.4164E-130	0.387104671	0.736	0.629	2.3625E-126
Elmo1	2.1937E-130	-0.658844278	0.028	0.154	3.659E-126
Nrep	6.2788E-130	-0.448842482	0.452	0.603	1.0473E-125
Mmp14	1.7008E-129	0.539276826	0.27	0.146	2.8369E-125
Bub1	2.3772E-129	-0.458858285	0.031	0.159	3.9651E-125
Rps3a1	3.0913E-129	0.348180659	0.735	0.647	5.1563E-125

Itm2b	6.4574E-129	-0.404295104	0.53	0.675	1.0771E-124
Rab3a	1.479E-128	-0.605505847	0.043	0.18	2.4669E-124
Nhlh1	2.1662E-128	-0.62542023	0.101	0.258	3.6132E-124
Ezr	2.4225E-128	0.425627893	0.509	0.417	4.0407E-124
Gsg1l	9.5808E-128	0.473170388	0.331	0.233	1.5981E-123
Slc17a6	1.2791E-127	-0.573805987	0.042	0.178	2.1336E-123
Rufy3	3.1244E-127	-0.455216204	0.225	0.413	5.2116E-123
Rpl23	5.0879E-127	0.360936182	0.596	0.522	8.4866E-123
Barhl1	5.2844E-127	0.416837082	0.597	0.499	8.8144E-123
Elavl4	1.28E-126	-0.532081308	0.191	0.376	2.135E-122
Uchl1	6.4168E-126	-0.48344453	0.199	0.374	1.0703E-121
Prmt8	8.0458E-126	0.501575854	0.355	0.248	1.342E-121
Rpl26	1.4575E-123	0.334974172	0.762	0.684	2.4311E-119
Kif20b	6.226E-123	-0.570725708	0.072	0.225	1.0385E-118
Jun	7.8066E-123	0.476903948	0.731	0.632	1.3021E-118
Calm1	8.7849E-123	-0.339590503	0.768	0.888	1.4653E-118
Dcx	2.4553E-122	-0.471782062	0.236	0.416	4.0954E-118
Mxd3	1.4947E-121	-0.430948598	0.031	0.154	2.4931E-117
Rps15a	1.731E-121	0.339272719	0.717	0.639	2.8874E-117
Hsd11b2	1.9194E-121	0.501079064	0.373	0.262	3.2016E-117
Plk1	4.6953E-121	-0.404435575	0.011	0.113	7.8317E-117
Tmsb10	5.3839E-121	-0.382013048	0.527	0.703	8.9804E-117
Hes6	5.8808E-120	0.456251303	0.304	0.216	9.8093E-116
Mcm6	7.7111E-120	0.471658373	0.348	0.255	1.2862E-115
Rps10	3.9722E-119	0.322935549	0.699	0.618	6.6257E-115
Rnd3	6.2489E-118	0.409471802	0.504	0.41	1.0423E-113
Dlgap5	9.9646E-117	-0.465126962	0.023	0.137	1.6621E-112
Fnbp1l	2.2669E-116	-0.410711256	0.31	0.495	3.7813E-112
Nhp2	4.7591E-116	0.377801124	0.428	0.353	7.9381E-112
Myt1	2.2748E-115	-0.582426632	0.051	0.182	3.7944E-111
Ank2	7.3777E-115	-0.57811952	0.121	0.274	1.2306E-110
Nkd1	1.5666E-114	0.44782349	0.318	0.228	2.613E-110
Rpl34	2.1434E-114	0.317784362	0.702	0.638	3.5752E-110
Tbata	5.7208E-114	0.505245912	0.414	0.318	9.5423E-110
Prkcb	1.141E-113	-0.485733186	0.127	0.293	1.9032E-109
Myt1l	1.7157E-113	-0.588172122	0.058	0.19	2.8618E-109
Ndc80	1.9452E-113	-0.430881609	0.026	0.14	3.2446E-109
Calm3	2.3929E-113	-0.337537439	0.407	0.604	3.9913E-109
Aurka	2.7868E-113	-0.3694774	0.018	0.121	4.6484E-109
Rpl35a	4.912E-113	0.328796634	0.623	0.554	8.1933E-109
Ctsb	4.7434E-112	-0.601780386	0.182	0.285	7.9119E-108
Aurkb	5.71E-112	-0.428286492	0.045	0.171	9.5244E-108
Kif2c	8.5684E-112	-0.419589222	0.023	0.131	1.4292E-107
Sept4	9.1773E-112	-0.577835215	0.11	0.26	1.5308E-107
D430041D05Rik	2.02E-111	0.388803443	0.558	0.472	3.3694E-107
CRE_RECOMBINASE	3.4127E-111	0.441252087	0.838	0.747	5.6923E-107
Pax6	3.5128E-111	-0.358652033	0.369	0.555	5.8594E-107
Ckap2	4.6297E-111	-0.453189763	0.059	0.193	7.7223E-107

Itsn1	3.1697E-110	-0.518182133	0.115	0.271	5.2871E-106
Anln	8.6165E-110	-0.389986938	0.022	0.128	1.4372E-105
Knstrn	9.9731E-110	-0.503881807	0.075	0.217	1.6635E-105
Ntrk3	6.361E-109	0.44424756	0.209	0.139	1.061E-104
Rpl7	8.1575E-108	0.323797653	0.554	0.496	1.3607E-103
1500012F01Rik	1.3276E-107	0.387035174	0.459	0.38	2.2144E-103
Srrm3	1.5009E-107	-0.419422825	0.121	0.274	2.5035E-103
Brd8	5.9139E-107	-0.354529416	0.255	0.431	9.8644E-103
Eno1	6.0844E-107	0.386079918	0.284	0.213	1.0149E-102
Eif3f	2.6888E-106	0.286837836	0.673	0.618	4.485E-102
Cdca7	3.955E-106	0.426743794	0.253	0.181	6.597E-102
Bub1b	1.0592E-105	-0.35919372	0.023	0.125	1.7668E-101
Mdk	1.6948E-105	0.456762843	0.477	0.351	2.827E-101
Rpl18a	1.7132E-105	0.342307712	0.451	0.386	2.8577E-101
Sox9	4.1842E-105	0.471067878	0.33	0.233	6.9792E-101
Atoh1	5.2016E-105	0.479997317	0.233	0.144	8.6763E-101
Egr1	1.2304E-104	0.510389482	0.494	0.352	2.0524E-100
Syt11	1.6628E-104	-0.368413675	0.37	0.543	2.7736E-100
Klc1	7.2259E-104	-0.376892112	0.232	0.399	1.2053E-99
Kif4	5.9385E-103	-0.392335654	0.026	0.131	9.9054E-99
Ctsd	4.5814E-102	-0.594954806	0.162	0.179	7.6417E-98
Dbf4	4.7509E-102	-0.40472985	0.051	0.172	7.92445E-98
Aplp1	1.2978E-101	-0.54139839	0.056	0.179	2.16475E-97
2410006H16Rik	2.5811E-101	0.360008093	0.512	0.443	4.30526E-97
Trpc4ap	9.1778E-101	-0.535485126	0.099	0.226	1.53086E-96
B3galt2	9.4332E-101	-0.504119223	0.021	0.12	1.57346E-96
Ung	4.5905E-100	0.476293833	0.158	0.073	7.65692E-96
Snhg1	1.5059E-99	0.374689928	0.42	0.339	2.5119E-95
Cltb	1.6572E-99	0.370334034	0.479	0.406	2.76423E-95
Serinc1	3.9601E-99	-0.34894766	0.31	0.473	6.60545E-95
Pde1c	5.2733E-99	-0.464806481	0.262	0.423	8.7959E-95
Ect2	1.17432E-98	-0.339444502	0.022	0.119	1.95876E-94
Mfap4	9.48063E-98	0.532507983	0.237	0.139	1.58137E-93
Hey1	1.13344E-97	0.428535275	0.338	0.254	1.89058E-93
Rbp4	2.76227E-97	0.452811505	0.308	0.222	4.60747E-93
Rtn4	3.07622E-97	-0.32349319	0.348	0.518	5.13113E-93
Ncam1	1.95758E-96	-0.331888838	0.26	0.424	3.26525E-92
Ckb	4.19239E-96	-0.321740923	0.755	0.823	6.99291E-92
1110038B12Rik	4.3941E-96	0.331931936	0.337	0.287	7.32937E-92
Ppic	6.54107E-96	0.398105725	0.302	0.221	1.09105E-91
Spc24	7.85279E-96	-0.425770406	0.127	0.276	1.30984E-91
Kif20a	1.03899E-95	-0.318865795	0.017	0.107	1.73303E-91
Meg3	1.6288E-95	-0.86680898	0.026	0.115	2.71684E-91
Stxbp1	3.40398E-95	-0.483241253	0.067	0.189	5.67783E-91
Cst3	1.15627E-94	-0.402500837	0.494	0.548	1.92865E-90
Ier5	2.21792E-94	0.369774013	0.444	0.379	3.69949E-90
Frmd4a	2.2756E-94	-0.315669665	0.192	0.342	3.7957E-90
Dync1i2	4.5161E-94	-0.319379707	0.435	0.606	7.53285E-90

Rpl18	8.72271E-94	0.271955014	0.325	0.298	1.45495E-89
Uncx	1.02729E-93	-0.390415206	0.203	0.351	1.71351E-89
Sgol1	4.17944E-93	-0.377245716	0.043	0.152	6.9713E-89
Kcnk1	4.21365E-93	-0.441027466	0.103	0.234	7.02837E-89
Rps7	1.31468E-92	0.292273503	0.434	0.389	2.19288E-88
Dhx32	2.07205E-92	0.373274885	0.247	0.186	3.45618E-88
C330027C09Rik	2.29519E-92	-0.360374231	0.047	0.158	3.82838E-88
Fbxo5	6.29032E-92	-0.388046945	0.065	0.186	1.04923E-87
Cadm3	6.50417E-92	-0.522979917	0.054	0.166	1.0849E-87
Stmn1	8.31258E-92	-0.323357433	0.168	0.317	1.38654E-87
A930011O12Rik	1.37156E-91	-0.524207082	0.035	0.137	2.28776E-87
Ncapd2	1.77549E-91	-0.352890798	0.087	0.213	2.96152E-87
Ttk	3.62193E-91	-0.305278856	0.021	0.109	6.04137E-87
Ptms	3.71714E-91	-0.314957438	0.351	0.509	6.20019E-87
Gstm5	3.81469E-91	0.344165561	0.37	0.308	6.3629E-87
Gm10260	9.8366E-90	0.32973986	0.268	0.216	1.64075E-85
Zfp36l1	1.0844E-88	0.422251462	0.205	0.135	1.80877E-84
Nucks1	1.99008E-88	-0.357141554	0.569	0.689	3.31945E-84
Clmp	3.56503E-88	-0.470205326	0.135	0.273	5.94647E-84
Mif	1.01436E-87	0.304737573	0.392	0.346	1.69195E-83
Atp6v0e	1.13298E-87	0.288124697	0.437	0.399	1.88981E-83
Sema7a	1.37018E-87	0.39903433	0.206	0.14	2.28545E-83
Btg2	2.41127E-87	0.462560525	0.259	0.193	4.02201E-83
Ier2	2.44324E-87	0.439875322	0.454	0.36	4.07532E-83
Angptl2	3.08675E-87	0.393912457	0.165	0.102	5.14869E-83
Siva1	5.75713E-87	0.343845129	0.318	0.259	9.60289E-83
Lmnb1	7.09983E-87	-0.294133245	0.186	0.332	1.18425E-82
Eef1d	7.62164E-87	0.302867769	0.382	0.34	1.27129E-82
Ppp3ca	8.08065E-87	-0.31660818	0.271	0.409	1.34785E-82
Pcsk1n	1.10033E-86	-0.490242259	0.018	0.103	1.83536E-82
Zeb1	1.25498E-86	0.312443999	0.424	0.376	2.0933E-82
Ska2	2.69903E-86	-0.352633039	0.105	0.233	4.50198E-82
Nnat	7.8779E-86	-0.369766295	0.625	0.734	1.31403E-81
Ska1	8.69079E-86	-0.308223371	0.022	0.109	1.44962E-81
Ppp2r2b	1.0936E-85	-0.447469431	0.018	0.103	1.82412E-81
Loxl1	1.23331E-85	0.361665153	0.13	0.078	2.05717E-81
Mns1	1.24599E-85	-0.344848212	0.104	0.232	2.07831E-81
Rps4x	1.99105E-85	0.294369202	0.322	0.287	3.32107E-81
Pou3f2	3.92561E-85	0.351127418	0.313	0.264	6.54792E-81
Sdpr	4.51259E-85	0.54095045	0.154	0.068	7.527E-81
Tomm7	2.44808E-84	0.260740896	0.528	0.488	4.0834E-80
Slc1a2	4.39829E-84	0.353733258	0.328	0.283	7.33636E-80
Nsg2	4.66034E-84	-0.291455552	0.37	0.521	7.77345E-80
Cdh20	4.84998E-84	0.372917409	0.255	0.187	8.08977E-80
Hspe1	7.42925E-84	0.296453943	0.38	0.329	1.2392E-79
C1qbp	8.19163E-83	0.292060852	0.411	0.367	1.36636E-78
BC005764	1.36046E-82	-0.476561192	0.094	0.212	2.26925E-78
Zmiz1	3.02383E-82	0.317418775	0.41	0.364	5.04374E-78

Klf7	9.32668E-82	-0.345182088	0.159	0.291	1.55569E-77
Cnbp	1.17475E-81	0.259916546	0.783	0.727	1.95948E-77
Rpl37	1.30441E-81	0.267847558	0.532	0.495	2.17576E-77
Eef1g	1.58019E-81	0.271788449	0.363	0.326	2.63575E-77
H1fx	1.65047E-81	-0.323010871	0.143	0.274	2.75298E-77
Gnai2	7.2128E-81	0.296213885	0.469	0.411	1.2031E-76
Cxcr4	7.78319E-81	0.384417205	0.141	0.076	1.29824E-76
Atp1b3	8.17657E-81	-0.318690394	0.224	0.369	1.36385E-76
App	1.22514E-80	-0.279451852	0.456	0.6	2.04353E-76
Elavl2	1.89504E-80	-0.305219163	0.171	0.299	3.16092E-76
Trim59	2.44519E-80	-0.330518131	0.081	0.195	4.07858E-76
Smpd2	2.67952E-80	0.323521556	0.194	0.157	4.46945E-76
Plcb1	2.93433E-80	-0.464588515	0.059	0.164	4.89446E-76
Odf2	3.15109E-80	-0.316198038	0.084	0.195	5.25602E-76
Gm17322	4.55566E-80	0.452661648	0.194	0.112	7.59884E-76
Tnik	4.9985E-80	-0.465023693	0.062	0.17	8.33749E-76
Gdpd1	7.37019E-80	-0.299407231	0.145	0.271	1.22935E-75
Stmn3	7.63899E-80	-0.270470405	0.511	0.654	1.27418E-75
Gnl3	2.73411E-79	0.352339453	0.343	0.282	4.5605E-75
Gm8292	2.97685E-79	0.267542351	0.397	0.36	4.96538E-75
Chd3	5.5971E-79	-0.459795507	0.065	0.173	9.33596E-75
Fabp7	7.32003E-79	-0.884331803	0.096	0.13	1.22098E-74
Rbfox2	1.14105E-78	-0.369050636	0.18	0.313	1.90327E-74
Wdr89	1.71795E-78	0.282828233	0.242	0.206	2.86554E-74
Rpl37a	3.92375E-78	0.263601614	0.499	0.459	6.54481E-74
Mrpl52	4.82763E-78	0.258333188	0.456	0.425	8.05249E-74
Nrn1	5.51596E-78	-0.397956831	0.124	0.249	9.20062E-74
Gm13826	8.25605E-78	0.266223052	0.337	0.305	1.37711E-73
Prdm8	1.30684E-77	-0.45071617	0.05	0.148	2.1798E-73
Rab6a	2.6406E-77	-0.267816825	0.228	0.357	4.40451E-73
Whsc1	3.58308E-77	-0.274006771	0.34	0.5	5.97659E-73
Arhgef2	5.36885E-77	-0.301402465	0.182	0.311	8.95523E-73
Arpp21	5.65688E-77	-0.509252282	0.041	0.133	9.43567E-73
Atp6v0b	5.89029E-77	-0.309810199	0.172	0.295	9.825E-73
Dkc1	1.19775E-76	0.312223789	0.375	0.32	1.99784E-72
Rab6b	1.29823E-76	-0.397668338	0.109	0.229	2.16544E-72
Lrig3	6.88969E-76	0.343979627	0.208	0.159	1.1492E-71
Nt5dc2	7.14012E-76	-0.303326892	0.145	0.263	1.19097E-71
Ncor2	8.30266E-76	0.335994568	0.203	0.158	1.38488E-71
Rprml	1.1395E-75	0.424825803	0.128	0.067	1.90069E-71
Efhd2	1.48759E-75	0.396517184	0.177	0.116	2.4813E-71
Melk	2.02599E-75	-0.277503374	0.029	0.113	3.37935E-71
Ldha	3.05346E-75	0.311837996	0.27	0.22	5.09317E-71
Fyn	3.13184E-75	-0.36778104	0.135	0.239	5.22391E-71
Tspan13	5.31896E-75	-0.304316208	0.118	0.233	8.87202E-71
Olfm1	1.25308E-74	-0.335846706	0.114	0.223	2.09014E-70
Hist1h2ak	1.26049E-74	-0.458815808	0.062	0.166	2.1025E-70
Boc	1.33988E-74	0.338845077	0.191	0.145	2.23492E-70

Pdlim4	1.62715E-74	0.396153364	0.126	0.061	2.71408E-70
Ttc9b	2.10567E-74	-0.287127463	0.107	0.219	3.51226E-70
Gm11478	2.5307E-74	0.29320684	0.246	0.205	4.2212E-70
Clvs1	5.246E-74	-0.390796632	0.053	0.151	8.75032E-70
Polr2h	5.83625E-74	0.296583854	0.258	0.215	9.73487E-70
Gnaq	7.93357E-74	-0.291988083	0.189	0.312	1.32332E-69
Tmsb4x	8.78923E-74	-0.280238651	0.918	0.957	1.46604E-69
Trp53	2.08417E-73	0.264814055	0.331	0.288	3.47639E-69
Tubb4b	3.97711E-73	-0.327277713	0.146	0.267	6.63382E-69
Fam162a	4.08589E-73	0.251716823	0.323	0.288	6.81527E-69
Rpl14-ps1	4.43353E-73	0.255073754	0.304	0.277	7.39514E-69
Gdi1	4.64182E-73	-0.324006295	0.138	0.256	7.74256E-69
Diap3	5.17255E-73	-0.288910542	0.038	0.126	8.62782E-69
Ptch2	6.72151E-73	0.361727898	0.193	0.14	1.12115E-68
Cdkn1b	7.78305E-73	-0.274019175	0.349	0.501	1.29821E-68
Srgap2	1.06384E-72	-0.315598025	0.06	0.153	1.77449E-68
Pea15a	1.84661E-72	-0.41726236	0.09	0.2	3.08015E-68
Anp32e	4.92313E-72	-0.328314548	0.541	0.634	8.21178E-68
Fam210b	9.00646E-72	0.334550333	0.369	0.297	1.50228E-67
Sfrp2	1.71757E-71	0.30117505	0.176	0.139	2.8649E-67
Sept11	1.82531E-71	-0.273077199	0.217	0.352	3.04461E-67
1700025G04Rik	3.23555E-71	-0.316681152	0.168	0.291	5.39689E-67
Prdx6	1.51947E-70	0.269268048	0.355	0.317	2.53448E-66
Bola2	2.16073E-70	0.271555576	0.424	0.389	3.6041E-66
Fstl1	2.33988E-70	0.344517341	0.234	0.164	3.90292E-66
Mab21l1	5.07056E-70	-0.335585123	0.134	0.251	8.45769E-66
Afap1	7.27631E-70	-0.31167205	0.075	0.174	1.21369E-65
2810417H13Rik	7.49925E-70	-0.41582094	0.278	0.424	1.25087E-65
Mcm3	2.11003E-69	0.31873209	0.212	0.17	3.51953E-65
Sowaha	3.04175E-69	0.457031252	0.229	0.143	5.07363E-65
Ankrd12	4.12649E-69	-0.362900831	0.181	0.301	6.88299E-65
Nfyb	1.32982E-68	-0.258384278	0.148	0.26	2.21814E-64
Bub3	1.58747E-68	-0.320701216	0.22	0.35	2.6479E-64
Pqlc1	1.67199E-68	0.310434786	0.333	0.286	2.78888E-64
Srm	2.58649E-68	0.304938282	0.226	0.181	4.31427E-64
Pdgfa	3.409E-68	0.357769964	0.292	0.242	5.68622E-64
Cadm1	3.82363E-68	0.267080381	0.416	0.383	6.37782E-64
Mapk8ip1	4.56449E-68	-0.297657814	0.139	0.253	7.61358E-64
Smc2	6.91857E-68	-0.369899806	0.495	0.56	1.15402E-63
2810004N23Rik	6.98915E-68	0.281649685	0.29	0.254	1.16579E-63
Mycn	8.50553E-68	0.312458005	0.4	0.352	1.41872E-63
Cep170	1.90323E-67	-0.311355464	0.209	0.333	3.17459E-63
Gnao1	2.08787E-67	-0.306961319	0.195	0.313	3.48257E-63
Chrna3	6.36914E-67	-0.391347651	0.048	0.138	1.06237E-62
Nol4	1.6615E-66	-0.253231394	0.096	0.193	2.77138E-62
Myc	4.64673E-66	0.345476158	0.139	0.088	7.75075E-62
Mad2l1	5.81062E-66	-0.255538165	0.056	0.142	9.69211E-62
Mybbp1a	5.88315E-66	0.264388542	0.275	0.242	9.81309E-62

Irs1	7.29041E-66	0.326857454	0.152	0.108	1.21604E-61
Isoc1	1.06846E-65	0.278268307	0.208	0.172	1.78218E-61
Thra	1.40979E-65	-0.382833969	0.149	0.252	2.35154E-61
Nbea	1.54084E-65	-0.339103596	0.108	0.214	2.57012E-61
Vim	2.10808E-65	0.36692503	0.371	0.282	3.51628E-61
Pkia	3.35533E-65	-0.355337691	0.062	0.154	5.59669E-61
Baz1a	4.26992E-65	0.30613014	0.192	0.159	7.12223E-61
Cdk6	6.31582E-65	0.266482731	0.263	0.237	1.05348E-60
Nrcam	6.99061E-65	-0.35224131	0.061	0.155	1.16603E-60
Ccm2	1.16674E-64	0.275394498	0.246	0.218	1.94613E-60
Gli1	1.34792E-64	0.28245211	0.127	0.101	2.24834E-60
Mcm2	1.35106E-64	0.315019325	0.24	0.194	2.25356E-60
Prnp	2.76642E-64	-0.3135947	0.124	0.23	4.61439E-60
Etfb	4.97496E-64	0.252129901	0.203	0.179	8.29824E-60
Pdgfra	5.85565E-64	-0.29827107	0.106	0.203	9.76723E-60
Tgfb2	7.5032E-64	0.346102728	0.232	0.18	1.25153E-59
Rps16	9.77572E-64	0.251512535	0.229	0.203	1.63059E-59
Npdc1	1.05867E-63	-0.273030284	0.178	0.285	1.76587E-59
Igsf21	3.07831E-63	-0.381354068	0.03	0.106	5.13463E-59
Cenpc1	1.08612E-62	-0.251289628	0.083	0.175	1.81165E-58
Cenpq	1.44879E-62	-0.271580783	0.085	0.182	2.41658E-58
Gsto1	3.73243E-62	0.334186258	0.171	0.116	6.2257E-58
Cacng4	6.99007E-62	-0.500355976	0.044	0.118	1.16594E-57
2900011O08Rik	7.43049E-62	-0.366649474	0.04	0.121	1.23941E-57
Lpin2	1.30668E-61	0.296219387	0.202	0.173	2.17954E-57
Bora	2.83238E-61	-0.253547644	0.035	0.109	4.72441E-57
Whrn	3.49627E-61	0.310170301	0.11	0.064	5.83178E-57
Srrm4	3.77879E-61	-0.305692781	0.119	0.217	6.30301E-57
Rbfox1	1.06107E-60	-0.338008908	0.033	0.109	1.76987E-56
Rpa2	1.0794E-60	0.25379072	0.217	0.194	1.80044E-56
Ap1s2	1.52292E-60	-0.266125082	0.088	0.181	2.54022E-56
Kif5a	1.70926E-60	-0.384056709	0.06	0.147	2.85105E-56
Abhd16a	2.35076E-60	-0.305563185	0.113	0.211	3.92107E-56
Dixdc1	3.94028E-60	-0.26865654	0.156	0.257	6.57239E-56
Mcm4	5.24922E-60	0.293938122	0.168	0.129	8.75569E-56
Clasp2	6.67423E-60	-0.278028748	0.095	0.188	1.11326E-55
Nolc1	2.7941E-59	0.267347739	0.438	0.4	4.66055E-55
Pnmal2	3.37932E-59	-0.31234338	0.075	0.167	5.63671E-55
Rftn2	4.18082E-59	0.273717811	0.149	0.116	6.97361E-55
Nme4	1.20423E-58	0.281864794	0.149	0.108	2.00865E-54
Apbb1	1.56146E-58	-0.347605956	0.095	0.191	2.60451E-54
Gria4	2.3491E-58	-0.321038559	0.061	0.143	3.9183E-54
Nsg1	2.43E-58	-0.261012068	0.207	0.31	4.05324E-54
Pcdha2	3.46826E-58	-0.343321304	0.055	0.136	5.78506E-54
Malat1	3.74694E-58	-0.277828617	0.95	0.956	6.24989E-54
Tshz2	4.78745E-58	0.268182148	0.35	0.311	7.98547E-54
Serpinh1	1.5018E-57	0.299020643	0.168	0.099	2.50501E-53
Dbn1	2.21393E-57	-0.315014926	0.073	0.158	3.69284E-53

Snord104	3.6845E-57	0.331386225	0.195	0.14	6.14575E-53
Lhx1	4.6283E-57	-0.280772422	0.402	0.524	7.72001E-53
Glce	6.24702E-57	-0.295026956	0.105	0.197	1.042E-52
Ramp2	1.74693E-56	0.307515424	0.164	0.106	2.91389E-52
Rrp15	2.41813E-56	0.266211868	0.201	0.166	4.03344E-52
Hadh	4.06528E-55	0.295114434	0.107	0.064	6.78089E-51
Sh3bp5	1.03047E-54	-0.274721759	0.046	0.119	1.71882E-50
Klf6	1.6261E-54	-0.251184915	0.082	0.167	2.71233E-50
Tspyl4	3.86884E-54	-0.306522292	0.065	0.144	6.45322E-50
Hist3h2a	9.33212E-54	-0.321728066	0.076	0.163	1.5566E-49
Arhgef7	6.52807E-53	-0.277522943	0.085	0.167	1.08888E-48
Akap12	1.44064E-52	-0.359113371	0.05	0.129	2.40298E-48
Hells	1.16584E-51	0.285457242	0.234	0.207	1.94462E-47
Hist3h2ba	1.21136E-51	-0.294437818	0.072	0.156	2.02056E-47
Hmgcs1	1.37125E-51	-0.305822276	0.087	0.17	2.28724E-47
Pdzrn3	2.45012E-51	-0.412444817	0.079	0.167	4.0868E-47
Lima1	5.01936E-51	0.299619944	0.155	0.112	8.37228E-47
Nek7	6.08429E-51	0.267709249	0.144	0.108	1.01486E-46
Ptprg	5.03368E-50	0.25772841	0.163	0.136	8.39618E-46
Tnrc6c	8.01556E-50	-0.250652858	0.112	0.197	1.337E-45
Myod1	1.04456E-49	-0.276086479	0.08	0.159	1.74233E-45
Thsd7a	1.45148E-49	-0.269393358	0.067	0.144	2.42107E-45
Igdcc4	1.91912E-49	0.276241004	0.121	0.086	3.20108E-45
Hddc2	3.31988E-49	0.274414723	0.122	0.084	5.53755E-45
Cplx1	3.91826E-49	-0.285702982	0.077	0.156	6.53566E-45
Smoc1	7.31246E-49	0.252757742	0.155	0.128	1.21972E-44
Gpatch4	1.35901E-48	0.266204822	0.212	0.17	2.26683E-44
Epb4.1l1	3.07723E-48	-0.342605451	0.039	0.106	5.13283E-44
Pak7	3.97538E-48	-0.278825913	0.049	0.12	6.63093E-44
Adamts1	1.85798E-47	0.269308839	0.169	0.15	3.09911E-43
Lrpap1	4.32168E-47	-0.260956306	0.061	0.133	7.20857E-43
Kcnip3	1.30452E-46	-0.268106139	0.062	0.137	2.17594E-42
Plp1	2.2539E-46	-0.701696178	0.1	0.118	3.7595E-42
Ifitm2	7.30504E-46	0.2590106	0.146	0.104	1.21848E-41
Nexn	1.43462E-45	0.395268752	0.113	0.065	2.39295E-41
Grina	2.30184E-45	-0.298034856	0.053	0.125	3.83948E-41
Tmpo	3.3466E-45	-0.25831542	0.367	0.476	5.58212E-41
Snhg4	6.99546E-45	0.270848366	0.123	0.079	1.16684E-40
Kidins220	1.18777E-44	-0.261191825	0.08	0.157	1.9812E-40
Gm11223	4.34462E-44	-0.344074942	0.156	0.229	7.24683E-40
Gas6	1.01483E-43	0.324513192	0.121	0.069	1.69274E-39
Sobp	8.94806E-43	-0.251486171	0.071	0.144	1.49254E-38
Rab13	9.52715E-42	0.26804961	0.113	0.075	1.58913E-37
Grik2	4.27987E-41	-0.276994822	0.052	0.116	7.13883E-37
Nrxn2	6.48877E-41	-0.272614672	0.042	0.103	1.08233E-36
S100a16	9.10018E-41	-0.342359813	0.046	0.109	1.51791E-36
Add3	5.56102E-40	-0.252662718	0.055	0.118	9.27579E-36
Nes	9.39883E-40	0.323361237	0.122	0.076	1.56772E-35

Wdr60	1.19301E-36	0.283278637	0.148	0.11	1.98994E-32
Ppfia2	1.41645E-36	-0.27155335	0.062	0.126	2.36264E-32
Fos	5.46633E-29	0.289556147	0.341	0.293	9.11783E-25
Dusp5	2.95671E-28	0.253159518	0.111	0.081	4.9318E-24
Xist	4.25717E-19	0.268066758	0.351	0.284	7.10095E-15
