# Supplementary material for: scRNA-seq in medulloblastoma shows cellular heterogeneity and lineage expansion support resistance to SHH inhibitor therapy
Source: Nat Commun. 2019 Dec 20;10:5829. doi: 10.1038/s41467-019-13657-6 (PMC6925218; doi:10.1038/s41467-019-13657-6)
Supplement: Supplementary file 11 — Supplementary Data 8 [file 41467_2019_13657_MOESM11_ESM.pdf]

	p_val	avg_logFC	pct.Vismo_	pct.Veh_Nc	p_val_adj
Top2a	0	2.096832	0.881	0.249	0
Cenpf	0	1.893585	0.774	0.18	0
Mki67	0	1.844122	0.853	0.21	0
Ube2c	0	1.701852	0.586	0.089	0
Prc1	0	1.689608	0.594	0.077	0
Tpx2	0	1.630632	0.669	0.085	0
Cenpe	0	1.563214	0.654	0.135	0
Spc25	0	1.482867	0.553	0.056	0
Hmgb2	0	1.463203	0.812	0.25	0
Nusap1	0	1.452931	0.528	0.052	0
Smc4	0	1.431319	0.914	0.452	0
Cdca8	0	1.427112	0.629	0.091	0
Birc5	0	1.41048	0.626	0.087	0
Kif23	0	1.394968	0.511	0.051	0
Incenp	0	1.380446	0.624	0.111	0
Cenpa	0	1.351657	0.498	0.09	0
Hmmr	0	1.341213	0.432	0.034	0
Ckap2l	0	1.276544	0.487	0.053	0
Ccna2	0	1.263635	0.555	0.072	0
Kif11	0	1.263456	0.502	0.056	0
Cdc20	0	1.257126	0.431	0.056	0
RP23-45G1	0	1.253883	0.696	0.189	0
Cdk1	0	1.229088	0.509	0.077	0
H2afx	0	1.205755	0.618	0.176	0
Kif15	0	1.19237	0.491	0.059	0
Pbk	0	1.144159	0.5	0.076	0
2810417H	0	1.13713	0.761	0.267	0
Tacc3	0	1.135167	0.494	0.072	0
Cdca3	0	1.131043	0.492	0.065	0
Arhgap11a	0	1.112936	0.412	0.051	0
Arl6ip1	0	1.079468	0.697	0.388	0
Sgol2	0	1.076652	0.372	0.041	0
Ccnb1	0	1.05952	0.347	0.021	0
Rps3a3	0	1.043953	0.335	0.024	0
Rangap1	0	1.025322	0.545	0.127	0
Mns1	0	1.018056	0.517	0.111	0
Lmnb1	0	0.944209	0.64	0.211	0
Smc2	0	0.90764	0.865	0.478	0
Dek	0	0.73661	0.933	0.676	0
Casc5	0	0.994632	0.415	0.061	0
Kif20b	0	1.112887	0.44	0.076	0
Mis18bp1	0	0.979993	0.371	0.043	0
Ncapg	0	0.959381	0.409	0.062	0
Esco2	3.9E-307	0.970344	0.408	0.063	6.5E-303
Nuf2	5.1E-307	0.915352	0.343	0.036	8.6E-303
Nucks1	1.2E-305	0.758037	0.867	0.591	1.9E-301
Kif22	7.6E-305	0.889678	0.372	0.048	1.3E-300

Spc24	1.3E-304	0.930405	0.513	0.123	2.1E-300
Racgap1	1.3E-303	0.921473	0.522	0.131	2.2E-299
H1fx	1.3E-303	0.982209	0.558	0.162	2.2E-299
Aspm	1.2E-301	1.017828	0.333	0.035	2E-297
Bub1	3.2E-290	0.867546	0.319	0.031	5.4E-286
Nasp	8E-290	0.706296	0.904	0.629	1.3E-285
Ccnb2	2.9E-282	0.920581	0.381	0.06	4.9E-278
Knstrn	2.6E-280	0.925691	0.408	0.074	4.4E-276
Sgol1	1.6E-277	0.899715	0.342	0.044	2.7E-273
Mxd3	3.6E-276	0.813072	0.313	0.032	5.9E-272
Anp32e	6.7E-275	0.723496	0.848	0.573	1.1E-270
Anp32b	4E-273	0.682858	0.897	0.61	6.7E-269
Fam64a	1.2E-272	0.811687	0.267	0.018	1.9E-268
Ndc80	2.9E-267	0.78581	0.284	0.024	4.9E-263
Bub1b	1.6E-265	0.74286	0.279	0.023	2.6E-261
Ckap2	9.1E-263	0.867613	0.366	0.06	1.5E-258
Cdca2	3.2E-261	0.756175	0.294	0.029	5.3E-257
Kif4	3.9E-255	0.797023	0.285	0.028	6.6E-251
C330027C(	5.4E-253	0.834923	0.337	0.049	9E-249
Ect2	3.1E-248	0.757209	0.27	0.024	5.1E-244
Ckap5	2.2E-247	0.915176	0.479	0.135	3.7E-243
Plk1	9E-244	0.686163	0.227	0.012	1.5E-239
Fbxo5	4.1E-241	0.8016	0.359	0.065	6.8E-237
Aurkb	2.7E-235	0.730627	0.301	0.04	4.5E-231
Ncapd2	2.7E-234	0.765558	0.399	0.092	4.6E-230
Tmpo	1.1E-233	0.733444	0.738	0.383	1.8E-229
Dbf4	1.1E-232	0.76904	0.338	0.057	1.8E-228
Rrm2	4.4E-226	0.767846	0.463	0.133	7.4E-222
Gm10075	5.5E-223	0.660353	0.804	0.483	9.2E-219
Kif2c	5.2E-221	0.664772	0.241	0.022	8.6E-217
Tuba1b	2.2E-220	0.748737	0.717	0.416	3.7E-216
Dlgap5	7.6E-217	0.716154	0.254	0.027	1.3E-212
Myod1	2.7E-214	0.821603	0.381	0.09	4.5E-210
Rad21	5.4E-209	0.740619	0.674	0.336	8.9E-205
Hnrnpu	8.3E-208	0.420587	0.97	0.876	1.4E-203
Aurka	2.8E-207	0.634736	0.233	0.022	4.7E-203
Kif20a	4.5E-206	0.561231	0.212	0.015	7.6E-202
Melk	1.1E-202	0.611007	0.251	0.03	1.9E-198
Spag5	2.4E-200	0.602384	0.21	0.016	4.1E-196
Ttk	4.9E-198	0.596823	0.228	0.023	8.2E-194
Diap3	3.8E-195	0.66604	0.272	0.041	6.4E-191
Ska1	1.7E-193	0.586851	0.217	0.02	2.8E-189
Anln	1.1E-192	0.622573	0.234	0.026	1.8E-188
Ccnd2	4.9E-189	0.53492	0.925	0.723	8.2E-185
Cep55	1.3E-188	0.573723	0.205	0.017	2.2E-184
Prr11	1.6E-188	0.500139	0.18	0.01	2.7E-184
Trim59	6.2E-187	0.701633	0.357	0.088	1E-182
Tubb5	1.8E-181	0.434601	0.963	0.881	3E-177

Gas2l3	7.5E-181	0.596699	0.186	0.013	1.2E-176
Cks2	8.6E-178	0.531468	0.199	0.018	1.4E-173
Insm1	2.4E-174	0.614307	0.579	0.266	4.1E-170
Tubb4b	2.5E-174	0.668516	0.437	0.152	4.1E-170
Kifc1	1.9E-173	0.465789	0.17	0.01	3.2E-169
Ran	2E-173	0.647851	0.676	0.371	3.3E-169
Gm11223	3.5E-171	0.68486	0.471	0.179	5.9E-167
Hirip3	1.2E-168	0.64428	0.65	0.332	2.1E-164
Rad51ap1	9.8E-168	0.586596	0.356	0.102	1.6E-163
Cdkn2d	1.2E-167	0.605122	0.268	0.052	2E-163
Usp1	1.6E-167	0.612409	0.609	0.29	2.7E-163
Neurod1	1.8E-165	0.662263	0.664	0.347	2.9E-161
Sapcd2	4E-165	0.489773	0.164	0.01	6.7E-161
Hmgn5	9.5E-165	0.632845	0.715	0.412	1.6E-160
Lbr	1.7E-160	0.606083	0.407	0.14	2.8E-156
Pif1	3E-160	0.487839	0.158	0.01	5E-156
D17H6S56I	5E-160	0.564582	0.269	0.055	8.3E-156
Pmf1	4.3E-159	0.590624	0.34	0.096	7.2E-155
Cenpm	5.1E-159	0.5505	0.348	0.102	8.4E-155
Nek2	1.9E-158	0.467652	0.165	0.012	3.2E-154
Espl1	1.4E-157	0.531137	0.183	0.017	2.3E-153
Ska2	6.3E-154	0.633653	0.371	0.118	1.1E-149
Nt5dc2	3.8E-153	0.582414	0.478	0.196	6.3E-149
Plxnb2	8E-153	0.529274	0.414	0.156	1.3E-148
Kif14	5.5E-152	0.461697	0.147	0.008	9.2E-148
Psrc1	4E-151	0.502674	0.174	0.017	6.6E-147
Shcbp1	6.5E-151	0.557152	0.194	0.024	1.1E-146
Ttr	7.6E-150	0.803784	0.225	0.039	1.3E-145
Ccdc34	1.1E-149	0.548554	0.643	0.337	1.8E-145
Foxm1	5.4E-149	0.522274	0.158	0.012	9E-145
Mad2l1	6.5E-149	0.506365	0.254	0.055	1.1E-144
Tuba1c	1.1E-148	0.580864	0.154	0.011	1.8E-144
Zfp704	2.8E-148	0.514366	0.49	0.212	4.7E-144
Ankle1	4.7E-147	0.473096	0.148	0.009	7.8E-143
2700094K1	3.3E-145	0.518739	0.817	0.573	5.6E-141
Cdc25c	9.8E-144	0.47739	0.162	0.015	1.6E-139
Xpo1	2.1E-143	0.540281	0.464	0.2	3.6E-139
Erdr1	4.1E-142	0.648238	0.414	0.168	6.8E-138
Hnrnpa2b1	4.6E-141	0.342009	0.972	0.925	7.7E-137
Whsc1	4.3E-140	0.553454	0.673	0.382	7.3E-136
2700099C1	7.5E-139	0.478296	0.191	0.028	1.3E-134
Cenpl	1E-138	0.414457	0.159	0.016	1.7E-134
Nde1	1.4E-138	0.549538	0.269	0.067	2.3E-134
Hdgf	4.2E-138	0.533215	0.77	0.521	7E-134
Tyms	1.1E-137	0.579728	0.427	0.173	1.8E-133
Hmgn1	1.8E-136	0.472761	0.828	0.631	3.1E-132
Bora	2.1E-136	0.55484	0.208	0.036	3.5E-132
Ccdc18	1.3E-135	0.661813	0.27	0.066	2.2E-131

Rrm1	4.5E-135	0.513504	0.462	0.201	7.4E-131
Troap	3.6E-134	0.414952	0.146	0.012	5.9E-130
Cdk5rap2	3.6E-133	0.512599	0.345	0.119	6E-129
Spdl1	4E-132	0.482225	0.175	0.023	6.7E-128
Cdca5	4.3E-131	0.454041	0.175	0.023	7.1E-127
Hjurp	5.1E-131	0.591369	0.655	0.387	8.5E-127
Cenpk	1.7E-130	0.578098	0.317	0.098	2.9E-126
Cmc2	3.1E-129	0.470516	0.262	0.07	5.2E-125
G2e3	9.5E-129	0.494287	0.259	0.068	1.6E-124
Pkp4	9.8E-129	0.536259	0.239	0.055	1.6E-124
Purb	1.9E-128	0.484199	0.778	0.519	3.1E-124
Neil3	6.2E-128	0.405119	0.172	0.024	1E-123
Cit	7.6E-128	0.452191	0.169	0.024	1.3E-123
Hmgb1	1.2E-127	0.564903	0.598	0.326	2E-123
H2afz	1.4E-127	0.495035	0.416	0.171	2.3E-123
Kif18a	1.1E-126	0.408783	0.155	0.019	1.9E-122
Trim37	1.3E-126	0.502302	0.432	0.184	2.2E-122
Ccnf	8.2E-126	0.369982	0.133	0.011	1.4E-121
Rtkn2	1.4E-125	0.399419	0.135	0.01	2.4E-121
Cenph	5.8E-125	0.502527	0.353	0.128	9.7E-121
Ptn	1.9E-123	0.554457	0.579	0.304	3.2E-119
Fxyd6	3.5E-123	0.564222	0.647	0.381	5.8E-119
Cenpw	1.2E-122	0.422866	0.258	0.074	2E-118
Selm	2.6E-121	0.482575	0.458	0.211	4.4E-117
Hist1h2ak	6.1E-121	0.714794	0.241	0.06	1E-116
Mthfd2	2.2E-120	0.533811	0.235	0.058	3.6E-116
Stmn1	2.5E-120	0.465151	0.45	0.205	4.2E-116
Sae1	3.3E-120	0.466914	0.487	0.237	5.6E-116
Ptma	4.1E-120	0.453015	0.808	0.588	6.9E-116
H2afv	6.1E-120	0.393674	0.887	0.735	1E-115
Cdkn2c	1.4E-119	0.442284	0.27	0.08	2.3E-115
Plk4	1.4E-119	0.451531	0.239	0.062	2.4E-115
Psmc1	1E-118	0.470895	0.646	0.381	1.7E-114
Gpc1	2.6E-117	0.506684	0.268	0.081	4.3E-113
Clspn	5.7E-117	0.536134	0.366	0.146	9.5E-113
Cenpq	1.2E-116	0.476087	0.28	0.087	2E-112
Ptms	2.5E-116	0.470999	0.652	0.409	4.1E-112
Psip1	6.4E-116	0.433	0.854	0.656	1.1E-111
Suz12	1.5E-114	0.420649	0.391	0.171	2.5E-110
Tk1	1.5E-113	0.437936	0.239	0.065	2.4E-109
Map1b	2.1E-113	0.500286	0.709	0.469	3.5E-109
Lmnb2	2.5E-111	0.451868	0.301	0.106	4.1E-107
Ybx3	9.3E-110	0.437932	0.54	0.295	1.5E-105
Dut	1.1E-109	0.428615	0.683	0.42	1.9E-105
Smc6	4.8E-109	0.458101	0.626	0.37	8E-105
Ezh2	1.7E-108	0.466104	0.784	0.563	2.9E-104
Zic5	1.6E-107	0.40241	0.346	0.142	2.6E-103
Cenpn	5.1E-107	0.38466	0.167	0.03	8.6E-103

Ndc1	6.4E-107	0.425539	0.216	0.056	1.1E-102
Hnrnpd	4.1E-106	0.416463	0.817	0.605	6.9E-102
Drap1	1.2E-105	0.4424	0.489	0.255	2E-101
Gm9800	1.5E-104	0.455386	0.752	0.528	2.6E-100
Kpnb1	2.5E-104	0.428501	0.507	0.276	4.2E-100
Ncaph	5.8E-104	0.424435	0.227	0.064	9.7E-100
Odf2	7.8E-104	0.409387	0.28	0.099	1.3E-99
Srsf1	1.7E-103	0.421884	0.52	0.286	2.8E-99
Eif2s2	5.4E-103	0.450264	0.559	0.312	9E-99
Neurl1b	1E-102	0.361529	0.108	0.007	1.73E-98
Hnrnpa0	1.3E-102	0.339451	0.694	0.453	2.13E-98
Nudc	1.8E-102	0.439195	0.492	0.258	3.04E-98
Sptbn1	1.5E-101	0.433949	0.457	0.233	2.43E-97
Ppp1r14c	2.5E-101	0.365436	0.332	0.139	4.15E-97
Pttg1	7.3E-101	0.488978	0.267	0.092	1.22E-96
Cenpp	1.9E-100	0.394734	0.21	0.056	3.09E-96
Cks1b	8.1E-100	0.46225	0.515	0.277	1.35E-95
Ncapg2	8.7E-100	0.382898	0.193	0.048	1.45E-95
Trim28	1.6E-99	0.327404	0.562	0.343	2.67E-95
Rfc1	2.7E-99	0.431533	0.569	0.331	4.46E-95
Alyref	4.2E-99	0.352884	0.374	0.178	6.93E-95
mt-Rnr1	4.5E-99	0.388761	0.594	0.352	7.56E-95
Vps36	1.1E-98	0.360338	0.47	0.251	1.83E-94
Ncaph2	1.25E-98	0.325886	0.309	0.13	2.09E-94
Mdc1	1.41E-98	0.464886	0.227	0.066	2.35E-94
Lyar	2.45E-98	0.39543	0.503	0.276	4.09E-94
Cenpc1	2.03E-97	0.429904	0.27	0.095	3.38E-93
Cenpj	6.96E-97	0.366171	0.268	0.098	1.16E-92
Elavl2	2.42E-96	0.402493	0.442	0.229	4.04E-92
Ska3	2.72E-96	0.320906	0.148	0.027	4.54E-92
Ywhaz	6.07E-96	0.344956	0.548	0.325	1.01E-91
Cep89	1.09E-95	0.485342	0.183	0.041	1.82E-91
Naa50	1.41E-94	0.407762	0.49	0.271	2.35E-90
Set	2.45E-94	0.393103	0.627	0.397	4.09E-90
Ubald2	3.49E-94	0.435629	0.202	0.055	5.83E-90
Gtse1	4.71E-94	0.305886	0.104	0.009	7.86E-90
Uhrf1	1.87E-93	0.323689	0.313	0.14	3.12E-89
Hmgb3	1.88E-93	0.415033	0.488	0.261	3.14E-89
Atp5k	2.4E-93	0.39188	0.667	0.441	4E-89
Ccdc25	2.45E-93	0.41278	0.335	0.145	4.09E-89
Fzr1	2.91E-93	0.401954	0.235	0.078	4.86E-89
Sept11	3.04E-93	0.367522	0.467	0.248	5.06E-89
Larp7	3.5E-93	0.411797	0.522	0.291	5.84E-89
Ehd1	4.98E-93	0.385471	0.241	0.085	8.31E-89
Lsm2	9.44E-93	0.333486	0.39	0.198	1.57E-88
Cited1	1.52E-92	0.404857	0.178	0.042	2.54E-88
Ccng2	2.53E-92	0.338567	0.267	0.106	4.22E-88
Clic4	3.72E-92	0.379264	0.388	0.187	6.2E-88

Asf1b	3.92E-92	0.373789	0.156	0.03	6.54E-88
Gsg2	7.93E-92	0.324592	0.128	0.018	1.32E-87
Pknox1	8.31E-92	0.352722	0.254	0.092	1.39E-87
Dnmt1	1.11E-91	0.380743	0.455	0.243	1.86E-87
Fkbp2	1.74E-91	0.368834	0.489	0.274	2.9E-87
Hnrnpul1	1.93E-91	0.317553	0.319	0.142	3.21E-87
Plp1	2.3E-91	0.424842	0.278	0.107	3.84E-87
Lsm6	3.21E-91	0.352035	0.58	0.355	5.35E-87
Smarcd3	1.07E-90	0.419874	0.196	0.054	1.78E-86
Nfyb	1.5E-90	0.359918	0.356	0.171	2.5E-86
E2f8	1.74E-90	0.336025	0.107	0.01	2.89E-86
Bub3	2.73E-90	0.447635	0.461	0.246	4.55E-86
Cdk11b	7.17E-90	0.393108	0.716	0.484	1.2E-85
Brd9	1.11E-89	0.309074	0.349	0.171	1.86E-85
Atad2	1.41E-89	0.447059	0.344	0.152	2.36E-85
Cdca4	1.69E-89	0.278735	0.197	0.064	2.82E-85
Ppp2r2c	1.75E-89	0.332399	0.733	0.503	2.92E-85
Rnf187	2.11E-89	0.37878	0.603	0.382	3.52E-85
Hnrnpl	3.63E-89	0.324447	0.581	0.36	6.06E-85
Hsp90aa1	3.65E-89	0.393662	0.777	0.567	6.09E-85
Sephs1	4.67E-89	0.381334	0.264	0.104	7.79E-85
Ctcf	2.78E-88	0.392034	0.673	0.436	4.64E-84
Dnajc9	3.54E-88	0.387609	0.545	0.316	5.91E-84
Hnrnpul2	7.14E-88	0.403113	0.422	0.219	1.19E-83
Cep110	1.11E-87	0.453321	0.285	0.11	1.85E-83
Azin1	1.11E-87	0.337288	0.328	0.154	1.85E-83
Trip13	4.27E-87	0.361634	0.175	0.047	7.12E-83
Peg3	9.98E-87	0.315076	0.442	0.243	1.67E-82
Fen1	1.42E-86	0.334927	0.28	0.117	2.36E-82
Stag1	2.98E-86	0.351696	0.341	0.161	4.97E-82
Slc17a6	3.38E-86	0.259857	0.259	0.111	5.64E-82
Ywhae	4.26E-86	0.345007	0.868	0.702	7.11E-82
Rfc4	1.26E-85	0.336706	0.382	0.193	2.1E-81
Phf17	2.54E-85	0.3609	0.19	0.054	4.23E-81
Psmd13	2.68E-85	0.275494	0.311	0.149	4.46E-81
5830418K(	3.29E-85	0.306623	0.289	0.13	5.48E-81
Cbx3	4.34E-85	0.426019	0.451	0.243	7.24E-81
U2af2	5.48E-85	0.350254	0.301	0.138	9.14E-81
Nudcd2	8.76E-85	0.357181	0.359	0.177	1.46E-80
Ank3	1.16E-84	0.309692	0.568	0.352	1.94E-80
Otx2	1.64E-84	0.326407	0.374	0.187	2.73E-80
Cul3	2.59E-84	0.358981	0.307	0.137	4.32E-80
Pvalb	3.39E-84	0.435181	0.223	0.072	5.65E-80
Ncl	6.28E-84	0.263668	0.973	0.921	1.05E-79
Sap30	7.02E-84	0.281661	0.23	0.09	1.17E-79
Neo1	1.21E-83	0.345857	0.251	0.1	2.01E-79
Anapc11	9.89E-83	0.35692	0.491	0.283	1.65E-78
Cux1	1.54E-82	0.355595	0.356	0.175	2.57E-78

Hist1h1e	1.72E-82	0.441094	0.152	0.036	2.87E-78
Rbbp7	4.03E-82	0.319897	0.497	0.293	6.71E-78
Dynll2	4.58E-82	0.312591	0.428	0.244	7.63E-78
Wnk1	5.62E-82	0.269642	0.308	0.152	9.37E-78
Hsph1	6.03E-82	0.319669	0.424	0.242	1.01E-77
Ddah2	1.16E-81	0.376314	0.79	0.602	1.93E-77
Itgb3bp	1.34E-81	0.333628	0.236	0.089	2.23E-77
Ptges3	2.03E-81	0.303551	0.373	0.199	3.39E-77
Mphosph9	2.34E-81	0.288584	0.274	0.12	3.9E-77
Zcwpw1	2.42E-81	0.385611	0.224	0.078	4.03E-77
Frmd4a	3.45E-81	0.272767	0.437	0.254	5.75E-77
Blm	4.09E-81	0.35488	0.228	0.082	6.82E-77
2700029M	4.65E-81	0.325803	0.454	0.261	7.76E-77
BC005537	5.02E-81	0.264868	0.471	0.288	8.38E-77
Cntln	7.19E-81	0.327301	0.311	0.144	1.2E-76
E2f2	7.27E-81	0.37026	0.154	0.035	1.21E-76
Slc7a5	9.33E-81	0.280205	0.245	0.102	1.56E-76
St18	1.06E-80	0.348983	0.235	0.088	1.77E-76
Rad51	1.16E-80	0.350172	0.231	0.085	1.93E-76
Nmt1	2.14E-80	0.325733	0.312	0.15	3.57E-76
Gm26924	6.63E-80	0.307285	0.354	0.186	1.11E-75
Gnb1	1.34E-79	0.265392	0.512	0.334	2.23E-75
Gen1	2.33E-79	0.282049	0.107	0.014	3.88E-75
Aars	3.06E-79	0.310575	0.291	0.133	5.1E-75
Brd8	4.76E-79	0.370783	0.496	0.287	7.94E-75
Prmt1	6.14E-78	0.265992	0.408	0.236	1.02E-73
Klf6	1.15E-77	0.284204	0.223	0.086	1.92E-73
Mprip	1.77E-77	0.280582	0.282	0.13	2.95E-73
Nhlh2	2.84E-77	0.298109	0.542	0.338	4.74E-73
Ywhah	5.08E-77	0.30362	0.265	0.115	8.47E-73
Rcc2	1.43E-76	0.299003	0.349	0.186	2.39E-72
Hyls1	8.76E-76	0.322095	0.11	0.017	1.46E-71
Cdc25b	1.34E-75	0.358804	0.167	0.047	2.23E-71
Pum2	2.02E-75	0.26331	0.312	0.163	3.36E-71
Sf1	2.15E-75	0.253007	0.376	0.221	3.59E-71
Calm3	2.34E-75	0.342063	0.678	0.461	3.91E-71
Gm10036	2.74E-75	0.304378	0.371	0.197	4.57E-71
mt-Rnr2	5.39E-75	0.313034	0.998	0.996	9E-71
Clic1	7.9E-75	0.273399	0.271	0.127	1.32E-70
Podxl2	1.79E-74	0.278773	0.333	0.171	2.98E-70
Gmnn	2.38E-74	0.329402	0.268	0.118	3.97E-70
Dnajc21	4.84E-74	0.333082	0.366	0.192	8.07E-70
Celf2	5.1E-74	0.313042	0.769	0.567	8.51E-70
Naa15	7.75E-74	0.266473	0.588	0.394	1.29E-69
Taf1	9.41E-74	0.267077	0.323	0.172	1.57E-69
Pkdcc	1.11E-73	0.284422	0.151	0.042	1.86E-69
Myh10	1.84E-73	0.279144	0.474	0.293	3.06E-69
Smarca5	4.21E-73	0.335798	0.602	0.393	7.03E-69

Nsl1	9.35E-73	0.281191	0.125	0.025	1.56E-68
Exosc8	1.15E-72	0.254543	0.335	0.178	1.92E-68
Nrxn1	1.19E-72	0.327917	0.337	0.17	1.99E-68
Rock2	1.7E-72	0.253038	0.334	0.181	2.83E-68
Dnph1	1.79E-72	0.307914	0.243	0.104	2.98E-68
Calm2	1.91E-72	0.318335	0.9	0.819	3.18E-68
Mrfap1	3.11E-72	0.28094	0.588	0.407	5.19E-68
E2f7	1.41E-71	0.300084	0.12	0.024	2.36E-67
Taf5	1.91E-71	0.270521	0.163	0.051	3.18E-67
Abcf1	2.26E-71	0.299348	0.644	0.445	3.78E-67
Ppp2r2b	3.51E-71	0.303389	0.139	0.037	5.86E-67
Syne2	5.95E-71	0.256812	0.344	0.189	9.92E-67
Zc3h7a	7.75E-71	0.264445	0.293	0.151	1.29E-66
Pcf11	1.52E-70	0.295446	0.298	0.15	2.53E-66
Hdgfrp3	2.55E-70	0.254246	0.47	0.291	4.25E-66
Fut9	4.6E-70	0.287	0.331	0.174	7.67E-66
Syncrip	5.67E-70	0.348126	0.684	0.48	9.47E-66
Hn1	5.79E-70	0.363761	0.63	0.425	9.66E-66
Stil	1.36E-69	0.301278	0.12	0.023	2.27E-65
G3bp1	1.97E-69	0.278236	0.51	0.332	3.28E-65
Mis12	2.24E-69	0.288037	0.188	0.068	3.73E-65
Suv39h2	6.5E-69	0.283524	0.241	0.107	1.08E-64
Ccp110	7.78E-69	0.26921	0.368	0.21	1.3E-64
Smchd1	1.01E-68	0.281306	0.412	0.242	1.68E-64
Traip	1.01E-68	0.27973	0.11	0.021	1.69E-64
Smarcc1	1.33E-68	0.328193	0.637	0.433	2.22E-64
Tpm4	1.47E-68	0.305913	0.486	0.297	2.45E-64
Rb1	2.48E-68	0.295418	0.176	0.059	4.14E-64
Gap43	3.46E-68	0.398195	0.641	0.459	5.78E-64
Cenpb	1.41E-67	0.283565	0.376	0.209	2.35E-63
Miip	1.6E-67	0.274942	0.125	0.03	2.66E-63
Smc1a	1.7E-67	0.363023	0.768	0.598	2.84E-63
Ubtf	1.83E-67	0.262765	0.61	0.417	3.06E-63
Iws1	3.92E-67	0.251632	0.343	0.191	6.54E-63
Pbrm1	4.53E-67	0.305617	0.7	0.499	7.56E-63
Mis18a	5.88E-67	0.288277	0.189	0.069	9.81E-63
Ctps	6.61E-67	0.256271	0.272	0.136	1.1E-62
Chaf1a	9.9E-67	0.283514	0.296	0.152	1.65E-62
Gpsm2	1.18E-66	0.250859	0.12	0.029	1.97E-62
Hmg20b	3.83E-66	0.256974	0.382	0.23	6.39E-62
Mtss1	4.33E-66	0.25748	0.306	0.163	7.22E-62
Tbcb	5.63E-66	0.265831	0.458	0.283	9.4E-62
2810442I2	7.39E-66	0.287687	0.127	0.03	1.23E-61
Basp1	1.44E-65	0.3218	0.814	0.649	2.41E-61
Hsp90b1	1.46E-65	0.365887	0.797	0.655	2.43E-61
Zwilch	1.57E-65	0.272558	0.156	0.048	2.61E-61
Ryk	2.09E-65	0.252739	0.275	0.142	3.48E-61
Supt16	2.43E-65	0.335854	0.679	0.478	4.06E-61

Numa1	5.01E-65	0.269117	0.222	0.096	8.36E-61
Zc3h15	5.41E-65	0.266147	0.534	0.351	9.02E-61
Mgat5b	6.49E-65	0.318999	0.168	0.055	1.08E-60
Sox11	7.37E-65	0.274143	0.204	0.089	1.23E-60
Terf1	1.26E-64	0.270307	0.15	0.047	2.11E-60
Arpp19	1.47E-64	0.266699	0.533	0.356	2.46E-60
Mphosph8	1.61E-64	0.252633	0.517	0.339	2.68E-60
Hmgn2	1.64E-64	0.319848	0.38	0.209	2.74E-60
Rock1	1.89E-64	0.257943	0.469	0.302	3.16E-60
Hist1h2ag	1.46E-63	0.250323	0.115	0.025	2.43E-59
Shf	1.48E-63	0.285766	0.118	0.028	2.48E-59
Cse1l	1.51E-63	0.301731	0.215	0.091	2.52E-59
Rpa1	1.6E-63	0.307812	0.231	0.103	2.66E-59
Ppig	1.63E-63	0.257348	0.655	0.467	2.72E-59
Mybl1	3.08E-63	0.262546	0.106	0.021	5.13E-59
Rbbp8	6.97E-63	0.25335	0.228	0.105	1.16E-58
Mdm1	8.93E-63	0.250198	0.141	0.042	1.49E-58
Cdc42bpa	1.34E-62	0.258599	0.222	0.1	2.23E-58
Zfp91	1.45E-62	0.259471	0.662	0.471	2.42E-58
Oxct1	2.68E-62	0.281627	0.681	0.486	4.47E-58
Cars	5.1E-62	0.33198	0.164	0.053	8.51E-58
H2afy	9.47E-62	0.279471	0.782	0.6	1.58E-57
Chst2	7.04E-61	0.286209	0.112	0.023	1.17E-56
Phldb1	8.4E-61	0.25908	0.131	0.04	1.4E-56
Ctnnb1	1.53E-60	0.252246	0.527	0.365	2.55E-56
Prrc2a	1.67E-60	0.346561	0.162	0.051	2.78E-56
Ccdc88a	3.23E-60	0.322125	0.616	0.429	5.38E-56
Baz1b	8.89E-60	0.282081	0.664	0.482	1.48E-55
Neto2	1.95E-59	0.299586	0.157	0.053	3.25E-55
Pdap1	2.51E-59	0.281924	0.848	0.696	4.18E-55
Eif5b	2.74E-59	0.277349	0.766	0.592	4.57E-55
Ccdc77	5.5E-59	0.254679	0.143	0.047	9.17E-55
Cadps2	8.85E-59	0.294174	0.112	0.027	1.48E-54
Ttf2	2.81E-58	0.268728	0.113	0.026	4.69E-54
Calm1	1.65E-57	0.255912	0.914	0.802	2.75E-53
Cbx1	1.1E-56	0.297652	0.818	0.672	1.84E-52
Metap2	2.19E-56	0.281437	0.772	0.604	3.65E-52
Nup37	6.69E-56	0.27416	0.163	0.06	1.12E-51
E2f1	1.11E-55	0.264475	0.304	0.168	1.85E-51
C77370	5.29E-55	0.261971	0.171	0.067	8.82E-51
Nup210	7.93E-55	0.279362	0.147	0.051	1.32E-50
Ipo5	1.51E-54	0.257276	0.263	0.139	2.52E-50
Ccne2	8.26E-54	0.35159	0.178	0.066	1.38E-49
Nars	1.23E-53	0.306324	0.579	0.407	2.06E-49
Rdx	2.73E-53	0.269159	0.75	0.576	4.55E-49
Ssrp1	7.57E-53	0.254725	0.745	0.576	1.26E-48
Ddx11	1.73E-52	0.272532	0.12	0.033	2.89E-48
Pou4f1	1.8E-51	0.267518	0.151	0.056	2.99E-47

Fkbp5	1.13E-50	0.252655	0.161	0.061	1.89E-46
Rad54l	4.92E-50	0.295425	0.17	0.065	8.2E-46
Cdh15	1.03E-49	0.324709	0.131	0.042	1.73E-45
Sltm	1.06E-48	0.253639	0.682	0.518	1.77E-44
Fam111a	7.75E-48	0.262702	0.207	0.097	1.29E-43
Maz	1.13E-47	0.279939	0.16	0.062	1.89E-43
Mest	1.39E-47	0.26026	0.147	0.056	2.33E-43
Acat1	7.63E-47	0.262623	0.568	0.405	1.27E-42
Eif4e3	1.04E-45	0.253937	0.22	0.108	1.73E-41
Chtf18	2.35E-45	0.253315	0.109	0.031	3.92E-41
Khdrbs3	1.45E-44	0.255082	0.121	0.04	2.42E-40
Cep290	1.26E-41	0.267639	0.25	0.139	2.09E-37
Nefm	5.9E-40	0.256578	0.19	0.095	9.85E-36
Tmsb10	3.48E-38	0.266372	0.74	0.605	5.8E-34
Cntn2	1.95E-36	0.256506	0.225	0.136	3.25E-32
Dynll1	1.31E-35	0.267059	0.682	0.552	2.19E-31
