# Supplementary material for: scRNA-seq in medulloblastoma shows cellular heterogeneity and lineage expansion support resistance to SHH inhibitor therapy
Source: Nat Commun. 2019 Dec 20;10:5829. doi: 10.1038/s41467-019-13657-6 (PMC6925218; doi:10.1038/s41467-019-13657-6)
Supplement: Supplementary file 12 — Supplementary Data 9 [file 41467_2019_13657_MOESM12_ESM.pdf]

Figure	Gene	Expression Threshold (scaled nUMI)
2b	Col3a1	3
2b	C1qb	3
2b	Meg3	4
2b	Sox10	1
2b	Aqp4	3
2b	Pecam1	2
3a	Col3a1	3
3a	C1qb	3
3a	Meg3	4
3a	Sox10	1
3a	Aqp4	3
3a	Pecam1	1
3b	Pax2	1
3b	Pax3	1
3b	Sox2	2
3b	Ascl1	2
3c	Ccnd2	3
3c	Barhl1	2
3c	Cntn2	1
3c	Rbfox3	2
3c	Grin2b	2
3c	Gli1	1
4c	Ccnd1	2
4c	Ccne2	1
4c	Ccna2	2
4c	Ccnb2	2
5a	Sox2	1
5a	SmoM2-EYFP	1
5d	Aqp4	1
5d	Sox10	1
5e	Mbp	1
5e	Pdgfra	1
5e	Mog	1
5e	SmoM2-EYFP	1
5f	Olig1	1
5f	SmoM2-EYFP	1
6a	Col3a1	3
6a	C1qb	3
6a	Meg3	4
6a	Sox10	1
6a	Aqp4	3
6a	Pecam1	2
6e	SmoM2-EYFP	1
7a	Gli1	1

7c	Hes1	1
7e	Myod1	1
8a	Ezh2	1
8a	Suz12	1
8a	Eed	1
8d	Fabp7	1
8d	Hes1	1
8e	Nes	1
8e	Vim	3
8e	Yfp	1
Supplemental Figure 1	Slc17a1	1
Supplemental Figure 1	Calb2	1
Supplemental Figure 1	Gad1	1
Supplemental Figure 1	Calb1	1
Supplemental Figure 1	Eomes	1
Supplemental Figure 4	Ccnd1	2
Supplemental Figure 4	Ccne2	1
Supplemental Figure 4	Ccna2	2
Supplemental Figure 4	Ccnb2	2
Supplemental Figure 4	Mki67	1
Supplemental Figure 4	Pcna	1
Supplemental Figure 4	Ccnd1	1
Supplemental Figure 4	Ccnd2	1
Supplemental Figure 4	Gli1	1
Supplemental Figure 4	Atoh1	1
Supplemental Figure 4	Barhl1	1
Supplemental Figure 4	Cntn2	1
Supplemental Figure 4	Rbfox3	1
Supplemental Figure 6	Ptch1	1
Supplemental Figure 5	Hhip	1
Supplemental Figure 5	Sfrp1	1
