# Supplementary material for: scRNA-seq in medulloblastoma shows cellular heterogeneity and lineage expansion support resistance to SHH inhibitor therapy
Source: Nat Commun. 2019 Dec 20;10:5829. doi: 10.1038/s41467-019-13657-6 (PMC6925218; doi:10.1038/s41467-019-13657-6)
Supplement: Supplementary file 13 — Reporting Summary [file 41467_2019_13657_MOESM13_ESM.pdf]

nature research

Corresponding author(s): Timothy Gershon
Kirk Wilhelmsen

Last updated by author(s): Nov 12, 2019

Reporting Summary

Nature Research wishes to improve the reproducibility of the work that we publish. This form provides structure for consistency and transparency in reporting. For further information on Nature Research policies, see Authors & Referees and the Editorial Policy Checklist.

Statistics

For all statistical analyses, confirm that the following items are present in the figure legend, table legend, main text, or Methods section.

n/a	Confirmed	
☐	☒	The exact sample size (*n*) for each experimental group/condition, given as a discrete number and unit of measurement
☐	☒	A statement on whether measurements were taken from distinct samples or whether the same sample was measured repeatedly
☐	☒	The statistical test(s) used AND whether they are one- or two-sided<br>*Only common tests should be described solely by name; describe more complex techniques in the Methods section.*
☐	☒	A description of all covariates tested
☐	☒	A description of any assumptions or corrections, such as tests of normality and adjustment for multiple comparisons
☐	☒	A full description of the statistical parameters including central tendency (e.g. means) or other basic estimates (e.g. regression coefficient) AND variation (e.g. standard deviation) or associated estimates of uncertainty (e.g. confidence intervals)
☐	☒	For null hypothesis testing, the test statistic (e.g. *F*, *t*, *r*) with confidence intervals, effect sizes, degrees of freedom and *P* value noted<br>*Give P values as exact values whenever suitable.*
☒	☐	For Bayesian analysis, information on the choice of priors and Markov chain Monte Carlo settings
☐	☒	For hierarchical and complex designs, identification of the appropriate level for tests and full reporting of outcomes
☐	☒	Estimates of effect sizes (e.g. Cohen's *d*, Pearson's *r*), indicating how they were calculated

Our web collection on statistics for biologists contains articles on many of the points above.

Software and code

Policy information about availability of computer code

Data collection	Sequencing Data was collected on an Illumina HiSeq 4000 and pre-processed using bcl2fastq v. 2.18 pipeline software.
Data analysis	Raw fastq files produced by bcl2fastq are concatenated to produce sample-specific paired-end files. Reads were tagged with cell & molecular barcodes, and trimmed with Drop-Seq Tools 1.13. Reads were aligned to a mixed-species mm10/hg19 reference using STAR 5.4b. Mouse cells were identified from the HEK cell spike-in by proportion of mm10-aligned reads, and were re-aligned only against mm10 using STAR 5.4b. A Digital Expression matrix (DGE) was generated with Drop-Seq Tools 1.13. Data were filtered using the Seurat 2.3.0 package for R to include only robust cells. Cell-type assignment, clustering, tSNE visualization and differential gene expression was performed with a mixture of Seurat v. 2.3.0 and custom code, which has been provided.

For manuscripts utilizing custom algorithms or software that are central to the research but not yet described in published literature, software must be made available to editors/reviewers. We strongly encourage code deposition in a community repository (e.g. GitHub). See the Nature Research guidelines for submitting code & software for further information.

Data

Policy information about availability of data

All manuscripts must include a data availability statement. This statement should provide the following information, where applicable:

- Accession codes, unique identifiers, or web links for publicly available datasets
- A list of figures that have associated raw data
- A description of any restrictions on data availability

Single-Cell RNA-Seq data that support the findings of this study have been deposited in GEO with the accession codes GSE129730

Field-specific reporting

Please select the one below that is the best fit for your research. If you are not sure, read the appropriate sections before making your selection.

☒ Life sciences ☐ Behavioural & social sciences ☐ Ecological, evolutionary & environmental sciences

For a reference copy of the document with all sections, see nature.com/documents/nr-reporting-summary-flat.pdf

Life sciences study design

All studies must disclose on these points even when the disclosure is negative.

Sample size	For the study on the effect of vismodegib treatment on survival, the minimum sample size on 9 was determined based on prior published studies by our group in which this sample size was sufficient to detect clinically relevant treatment effects.<br>For the single cell-transcriptomic studies, we considered 2 interrelated parameters: the number of replicate mice per treatment group, and the total number of cells.<br>Justification of 5 mice per treatment group: As the degree of variability and normality in the data was not knowable in advance, we could not prospectively determine the ideal number of mice. We therefore set the number of replicate mice per treatment group to 5 in order achieve a reasonable level of precision in sampling around the mean.<br>Justification of >10,000 cells per treatment group: Pilot studies indicated that at least 10,000 total cells were needed to identify rare cell types in a group. As we increased the number of cells sequenced, we found that we were able to subdivide cell types with progressively greater granularity. We noted that when sequencing >10,000 cells we were able to identify a sufficient number of cells within the vascular cell type that we could differentiate vascular fibroblasts from endothelial cells, which were both about 0.1% of the total population. We considered that ability to detect cells as rare as 0.1% to be a reasonable level of sensitivity.
Data exclusions	No mice were excluded from the analysis. As described in the paper, we restricted analysis to cells with > 500 detected genes, and removed outlier cells with > 4 SD above the median number of genes, UMIs and mitochondrial content per cell to address the common problems of gene drop out, unintentional cell-cell multiplexing and premature cell lysis.
Replication	For a representative sample of genes, we used immunohistochemistry to confirm changes in protein expression suggested by our transcriptomic analysis. In all cases tested, we were able to confirm the transcriptomic data.
Randomization	Mice were randomly allocated to treatment groups
Blinding	Software was blind to treatment co-variate during PCA analysis

Reporting for specific materials, systems and methods

We require information from authors about some types of materials, experimental systems and methods used in many studies. Here, indicate whether each material, system or method listed is relevant to your study. If you are not sure if a list item applies to your research, read the appropriate section before selecting a response.

Materials & experimental systems

n/a	Involved in the study
☐	☒ Antibodies
☒	☐ Eukaryotic cell lines
☒	☐ Palaeontology
☐	☒ Animals and other organisms
☒	☐ Human research participants
☒	☐ Clinical data

Methods

n/a	Involved in the study
☒	☐ ChIP-seq
☐	☒ Flow cytometry
☒	☐ MRI-based neuroimaging

Antibodies

Antibodies used	SOX2 Cell Signaling Technology Catalog #4900<br>HES1 Cell Signaling Technology Catalog #11988<br>MyoD1 Novus Catalog #NBP2-32882<br>MyoD1 Cell Signaling Technology Catalog #13812<br>phospho-RB (Ser807/811) Cell Signaling Technology Catalog #8516<br>GFAP (GA5) Mouse mAb (Alexa Fluor® 647 Conjugate) Cell Signaling Technology Catalog #3657<br>NeuN Millipore Catalog #MAB377<br>Calbindin Cell Signaling Technology Catalog #2173<br>Goat anti-rabbit Alexa Fluor 488 ThermoFisher Scientific Catalog #A-11034<br>Goat anti-mouse Alexa Fluor 555 ThermoFisher Scientific Catalog #A-21424<br>ImmPRESS™ HRP Anti-Rabbit IgG Vector Laboratories Catalog #MP-7401

	ImmPRESS™ HRP Anti-Mouse IgG Vector Laboratories Catalog #MP-7402
Validation	All antibodies used were validated by the manufacturers, as described on their websites. For the GFAP antibody, the use of the antibody for flow cytometry was not specifically documented by the manufacturer. However, data presented in the paper address the validity of the antibody for this purpose by showing an appropriately sized GFAP+ population detected in dissociated normal cerebella.

Animals and other organisms

Policy information about studies involving animals; ARRIVE guidelines recommended for reporting animal research

Laboratory animals	All mice were bred onto the C57BL/6 mice background for at least 5 generations. Male and female mice were included in roughly equal numbers.
Wild animals	The study did not involve wild animals.
Field-collected samples	The study did not involve field-collected samples.
Ethics oversight	All procedures were approved by the UNC IACUC, under protocol 16-099

Note that full information on the approval of the study protocol must also be provided in the manuscript.

Flow Cytometry

Plots

Confirm that:

☒ The axis labels state the marker and fluorochrome used (e.g. CD4-FITC).

☒ The axis scales are clearly visible. Include numbers along axes only for bottom left plot of group (a 'group' is an analysis of identical markers).

☒ All plots are contour plots with outliers or pseudocolor plots.

☒ A numerical value for number of cells or percentage (with statistics) is provided.

Methodology

Sample preparation	Cells were obtained by dissociating cerebella and medulloblastomas from freshly harvested mice, as described. Cells were subjected to papain dissociation, fixation and permeabiliztion as described.
Instrument	Becton Dickinson LSR Fortressa
Software	FlowJo V10
Cell population abundance	The Cre-activated Dendra2 fluorescent marker was used to define the Math1-Cre lineage, which was ~90% of the population.
Gating strategy	Cells were gated to exclude debris and dead cells, identified by low FSC and SSC and by <G1 DNA content, and to exclude doublets, identified by H:A ratio of FxCycle Violet DNA dye.

☒ Tick this box to confirm that a figure exemplifying the gating strategy is provided in the Supplementary Information.
